# Footprints of a Singular 22-Nucleotide RNA Ring at the Origin of Life

**DOI:** 10.3390/biology9050088

**Published:** 2020-04-25

**Authors:** Jacques Demongeot, Alexandra Henrion-Caude

**Affiliations:** 1 Laboratory AGEIS EA 7407, Faculty of Medicine, University of Grenoble Alpes, 38700 La Tronche, France; 2 Future of Research Team, SimplissimA International Research Institute, 39 rue saint Louis, 11324 Port-Louis, Mauritius; a.caude@simplissima.org

**Keywords:** origin of life, evolution, amino acid RNA interaction

## Abstract

(1) Background: Previous experimental observations and theoretical hypotheses have been providing insight into a hypothetical world where an RNA hairpin or ring may have debuted as the primary informational and functional molecule. We propose a model revisiting the architecture of RNA-peptide interactions at the origin of life through the evolutionary dynamics of RNA populations. (2) Methods: By performing a step-by-step computation of the smallest possible hairpin/ring RNA sequences compatible with building up a variety of peptides of the primitive network, we inferred the sequence of a singular docosameric RNA molecule, we call the ALPHA sequence. Then, we searched for any relics of the peptides made from ALPHA in sequences deposited in the different public databases. (3) Results: Sequence matching between ALPHA and sequences from organisms among the earliest forms of life on Earth were found at high statistical relevance. We hypothesize that the frequency of appearance of relics from ALPHA sequence in present genomes has a functional necessity. (4) Conclusions: Given the fitness of ALPHA as a supportive sequence of the framework of all existing theories, and the evolution of Archaea and giant viruses, it is anticipated that the unique properties of this singular archetypal ALPHA sequence should prove useful as a model matrix for future applications, ranging from synthetic biology to DNA computing.

## 1. Introduction

Elucidating the prebiotic ingredients from which life arose on Earth is one key focus of the origin-of-life research. Previous theoretical hypotheses have been providing insight into a hypothetical world where hairpin or ring of RNA molecules may have debuted as the primary informational and functional molecule [1,2,3,4]. Our model starts with revisiting the architecture of RNA–peptide interactions at the origin of life through the evolutionary dynamics of molecular evolution of RNA populations. By performing a step-by-step computation of the smallest possible hairpin/ring RNA sequences compatible with building up a variety of peptides of the primitive network, we inferred the sequence of a singular docosameric RNA molecule, which we refer to as the ALPHA sequence. In compliance with the stereochemical constraints of prebiotic conditions, we searched for any relics of the peptides made from ALPHA in sequences deposited in the different public databases. Sequence matching between ALPHA and sequences from organisms among the earliest forms of life on Earth were found at high statistical relevance.

### 1.1. Miller’s Experiment as a Bridge between the Old towards the New Theories of the Origin of Life

In 1953, Stanley Miller, who was looking for the origin of the first biomolecules, made the most remarkable breakthrough by successfully generating five amino acids out of a chamber simply containing a mixture of water and gas, and submitted to an electrical discharge [5]. To represent an environment with the four basic atoms of life (oxygen, hydrogen, nitrogen, and carbon), he had used gas made of methane (CH_4_), ammonia (NH_3_) and hydrogen, and found in the resulting solution the unexpected presence of: glycine, α- and β-alanine, aspartic acid, and α-aminobutyric acid. Later on, another set of amino acids were to be found by Jeffrey Bada, upon analyzing the products of another old experiment by Miller, dating back to 1958, and having assessed a hydrogen sulphide (H_2_S)-containing mixture [6]. In 1961, Joan Oró used a solution of hydrogen cyanide (HCN) and ammonia solution, and found that adenine had been synthesized, in addition to amino acids [7]. Two years ago, an adaptation from Miller’s experiment was conducted with a simple mixture of NH_3_ + CO and H_2_O, and resulted in all RNA nucleotides [8], concluding this extraordinary series of experiments that proved that the biomolecules, which are necessary to begin life, could be obtained from simple atmosphere exposed to electrical sparks. “For many years, researchers have asked themselves what preceded what—the proteins, the nucleic acids, or vice versa?” [1]. However, substituting ‘protein’ by ‘function’, and ‘nucleic acids’ by ‘information’, as proposed by Eigen [2,3], leads to rephrase the question *ad absurdum*, because “function” cannot occur in an organized manner unless “information” is present, and this “information” only acquires its meaning via the “function” for which it is coding. Hence, viewed by Eigen [3] as a “function”, proteogenesis enabled to deduce a tRNA structure, which could be the ancestor of all the present tRNAs.

To attain the adequate concentration of the products, which would be an absolute necessity for evolution, it was suggested in 1951 by Bernal that adsorption could take place on very fine clay deposits. As a “polymerization catalyst”, montmorillonite would result in decreasing the free amino acid content consequently to polymerization [4], and after a certain time, obtaining polypeptides at the same molecular weight distribution pattern as those produced if amino acid adenylates were polymerized. In fact, Ponnamperuma and his collaborators described the formation of ATP [9], and in 1995, the interactions between amino acids and nucleotides as a possible physicochemical basis for the origin of the genetic code [10]. Altogether, those observations actually form the experimental corpus of the stereochemical theory of the origin of life [11,12], and this was the origin of a long debate.

If Shapiro [13] admitted that “life began within a mixture of simple organic molecules, with possible participation by minerals” and that “a triple stem-loop (RNA) structure, containing 40–60 nucleotides, offered a reasonable hope of functioning as a replicase ribozyme”, he and his colleague Bernhardt [14] were critical to the montmorillonite hypothesis and the stereochemical approach. Conversely, in line with Ponnamperuma, Yarus recently defended the idea of a catalytic role of RNA rings favoring the peptidic bonds between amino acids [15,16]. Accordingly, an illustration of his proposal was that: “a ready rationale exists for smaller individual amino acid sites, still side chain specific. These can be extreme single-ended sites, forced to be small because of the crowding of two sites produced by the short single covalent peptide bond between His and Phe” [15].

At last, a number of recent models have pointed to the role of a lipid component in the very early stages of the origin of RNA structures [17,18,19,20,21], which secondarily could have functioned independently [22]. Another possibility is that those lipids may result from the apparition of proteins catalyzing their synthesis, as in Archaea where a number of enzymes contribute to the biosynthesis of lipids [23]. In fact, Forterre and his coworkers have proposed that Archaea and Bacteria may have a common ancestor, referred to as LUCA, and that the co-evolution of Archaea, Bacteria and Eukarya may be driven in part by viruses (their phages and giant viruses) [24,25]. Accordingly, the complexity of Eukarya may have taken the advantage from retroviruses and large DNA viruses, whilst similar selection pressure could explain the somewhat similar evolution of the archaeal and bacterial mobilomes [25]. Coining the name of “Domain Cell Theory”, Staley supported the independent evolution of Archaea, Bacteria and Eukarya, based on an evolution from three distinct cellular lineages [26].

Since the pioneer work by H.J. Muller in 1922, who claimed 20 years before the Beadle and Tatum’s “one gene—one enzyme” hypothesis, that life began not as an enzyme but as a gene, the scientific community has been divided as to the origin of life along two hypotheses: the “genetics first” and the “metabolism first”, as accurately pointed out by Fontecilla-Camps [27]. The “genetics first” view, which is based on Miller’s experiments and organic components from meteorites, proposes the “RNA World” concept as the origin of life. The “metabolism first” hypothesis posits that life began autotrophically on minerals and/or hydrothermal vents. While lending solid support to either hypothesis is still impossible, the “metabolism first” option may be further explored assuming a “continuous geochemical, catalytically dynamic process” [27]. In this context, the nucleotide synthesis that may have originated on a mineral surface, could have been later replaced by ATP.

In the same “metabolism first” view, Aguirre et al. [28] proposed that large neutral networks of genotypes formed mapping into sets of phenotypes having the same fitness, but several strong connected components. Despite the fact that an actual set of genotypes visited by an evolving population is rarely neutral, Aguirre suggests that nearly neutral mutations often increase the adaptive ability of finite populations, similarly to the model of Eigen and Schuster of quasi-species [2,3]. Because this old model of quasi-species offers the view of a large group of related genotypes in an environment with high mutation rate that may lead to hypercycles [2,3], it is now commonly assumed that the evolution of the first genome is based on the physicochemical properties of the amino acids that compose their proteins (i.e., their inertness [29], structural complexity [30,31] and stereochemical affinities between peptides and small RNAs [15,32,33]), that may have co-evolved with metabolic pathways [34] (e.g., N-fixation [35]) to parallel the metabolism of amino acids [36,37].

### 1.2. Stereo-Chemical Theory of Singular Docosameric Sequences

Forterre and his coworkers [24,25] have emitted the idea that Archaea and Bacteria have a common ancestor he called LUCA (for Last Universal Common Ancestor) and that the co-evolution of Archaea, Bacteria and Eukarya have been driven in part by Viruses (their phages and the giant viruses): “selection of different parts of the ancestral virosphere at the onset of the three domains played a critical role in shaping their respective biology. Eukarya probably evolved toward complexity with the help of retroviruses and large DNA viruses, whereas similar selection pressure (thermo-reduction) could explain why the archaeal and bacterial mobilomes somehow resemble each other”. Staley [26] proposed in the framework of his Domain Cell Theory about the evolution of Archaea, Bacteria and Eukarya, that “when the domains of life evolved, each of the three domains evolved from separate and unique cellular lineages”.

A recent model [38] in the spirit of the Eigen-Schuster’s hypercycles [2,3] presents a plausible co-evolution of two types of replicating molecules, denoted P and Q: P can represent the primitive RNA molecules and Q the primitive DNA molecules. The dynamical evolution of P and Q leads to a stable stationary state, in which coexist the functional species P (ancestor of enzymes) and the informative species Q (ancestor of DNA). We will search in the following two analogues of P and Q, from the same primitive nucleotide sequences, one P in ring form able to catalyze the peptide formation from amino acids present among the early life components, and the second Q in hairpin form with the maximal thermo-dynamical stability able to store the memory of sequence P and being in chemical equilibrium with it.

**Figure 1:**
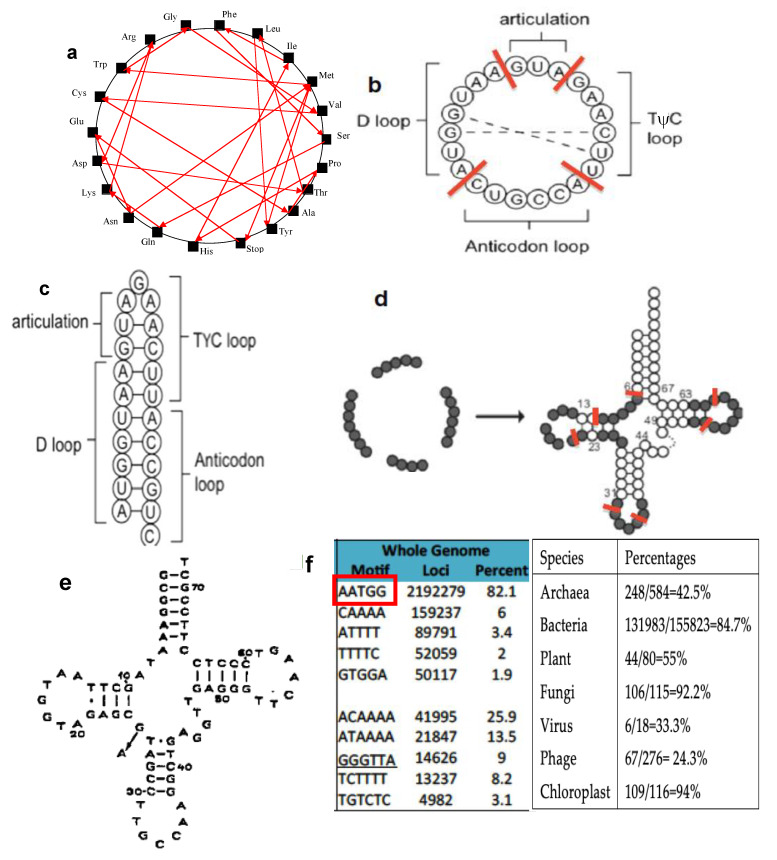
Computation of the Archetypal Loop AL. (**a**) Hamiltonian path in the graph having amino acids as vertices; (**b**) AL ring AUGGUACUGCCAUUCAAGAUG; (**c**) Optimal hairpin form of AL; (**d**) Urancestral tRNA-Gly [6], where nucleotides common with AL are indicated in black. (**e**) GlytRNA^GCC^ of *Œnothera coquimbensis* [53], whose loops (D-, anti-codon, articulation and T_Ψ_-loops) fit quasi-perfectly AL; (**f**) pentamer frequencies in whole human genome [42] and percentages of tRNAs containing TGGTA and TTCNA in their D- and T_Ψ_-loops, among tRNAs having NTGCCAN as an anticodon loop in different species of the tRNADB-CE database [51].

All the previous theoretical hypotheses and experimental observations made it possible to develop present theories on the origin of protein translation in which the central role of hairpin or ring RNAs [39,40,41] was postulated and then supported by a large amount of research on the available genomic databases. In the present paper, dealing with the genomes (notably tRNAs) of plants, human, Archae and giant viruses, we will focus on the construction of an ancestral hairpin/ring called AL (for Ancestral Loop or ALPHA structure), which could have played an important role at the origin of the protein translation and we show the existence of relics of AL in present genomes, in a hypothetical framework consistent with the theories previously mentioned. The lack in our current knowledge of satisfactory models prompted us to find what kind of rationale could enable to reconcile a common ancestry to all present genomes. The bioinformatics tools for analyzing RNA, DNA and protein sequences, coupled with the availability of complete genome sequences [42,43,44,45,46,47,48,49,50,51,52], and dedicated high-throughput databases, enabled us to postulate and decipher a sequence candidate, which could have played a role at the start of evolution. By performing a step-by-step computation of the smallest possible RNA sequence compatible with building up a variety of peptides, a small RNA model was identified called AL (ALPHA ring) thanks to a constraint propagation algorithm [39,40] searching a Hamiltonian path in a graph (Figure 1a) with the following properties:

- All dinucleotides should appear at least once (apart CG, because of CG suppression).
- Among the rings satisfying the principle “to be as short as possible and containing at least one codon of each amino acid”, there is no solution for a length below 22 nucleotides. For the length 22, 29, 520 solutions (i.e., about 10^−9^ of the possible solutions) contain only one repeated codon AUN, N being G for 52% of the solutions.
- From these 29,520 solutions, 25 rings only satisfy with the formation of a hairpin 9-nucleotide-long or greater.
- From these 25 rings, 19 encompass both a start and a stop codon.
- Through the calculation of several distances (e.g., circular Hamming distance, permutation distance and edit distance), one singular ring (ALPHA ring) exhibits a minimum average distance as compared to the others. Only this sequence is thus acting as the barycenter of the set of the 18 others (Figure 1b).

The AL sequence is the following:

                     5′-AUGGUACUGCCAUUCAAGAUGA-3′

Using the Kinefold® algorithm [46], it is possible to exhibit the most thermodynamically stable hairpin formed from AL ring (Figure 1c) and an analysis of the primary structure of tRNAs like GlytRNA^GCC^ reveals that three well-characterized oligomers: a hexamer CUGCCA that is usually known as an anticodon loop, and two heptamers UUCAAGA and AAUGGUA, are respectively known as the Tψ-loop and the D-loop (cf. Figure 1d,e) of a high percentage of tRNAs (cf. the supplementary material 1).

## 2. Materials and Methods

Searching the genomes of plants [52,53], human [54], Archaea [55,56,57], and giant viruses [58,59], we further searched the fitness of this ALPHA sequence, as a prototype, which could have played an important role at the origin of the protein translation. The recent discovery by Claverie and Abergel of three new strains of pandoraviruses solved the unresolved question on a common ancestor to the *Pandoraviridae* family [58]. Spontaneous neogenesis partially alleviates this new mystery but yet does not explain the diversity of those neogenes of unknown functions. Complying with previous theories including that built on Eigen and Schuster’s model, we made the hypothesis that the proximity to a putative archetypal genome at the origin of life could explain the specificity of the Pandora family with respect to the rest of the whole giant virus set and the classification of Archaea [55,56,57], and measured the singular proximity of ALPHA by assessing the presence of oligomers derived from ALPHA. This approach offered the possibility to confront the optimal properties of the ALPHA ring and to search for the existence of relics of ALPHA in present genomes.

**Figure 2:**
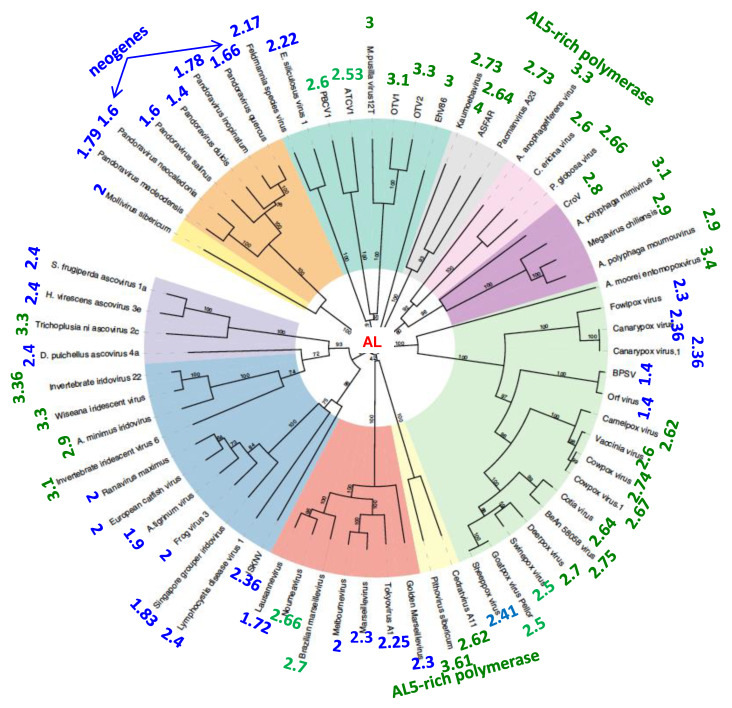
Giant viruses classification tree. The numbers at the periphery of the circular tree (from [58]) indicate the ALPHA pentamer-proximity P_22_ of the Giant viruses genomes to the ALPHA sequence.

We calculated the probability P_22_ of occurrence of the 22 pentamers derived from ALPHA (100xP_22_ is called the ALPHA pentamer-proximity noted P_22_) in the genomes of giant viruses, and compared it to 0.0215 + 0.0024 * as being the probability of random occurrences of a pentamer from ALPHA sequence (denoted AL5) for a genome of size 1,000,000 nucleotides-long (* indicating the upper limit of the 95%-confidence interval of the right-tailed test of significance of an empirical frequency). As shown in Figure 2, we observed two groups. In one group, which figures are noted in blue, the probability P_22_ was similar to those of a random occurrence (P_22_ ≤ 0.0222). The group contained in particular the large family of Pandora viruses having a tendency to create neogenes (93% of their genes differ from all other known viral or cellular genes [58]). In the second group, in which the figures are noted in green, P_22_ was above 0.025 (significantly different from the purely random probability with *p* < 0.5 × 10^−124^).

## 3. Results

The search for ALPHA relics consisted in identifying in present genomes from different databases [42,43,44,45,46,47,48,49,50,51,52], the footprints of some ALPHA sub-sequences. By doing so, some remarkable facts arose such as the high similarity of ALPHA-derived sequences with the consensus sequences at exon/intron boundaries in complex eukaryotes, typically GGTAAGT and TTCAAG. Moreover, the footprints often matched with RNA genes, typically tRNAs, rRNAs, miRNAs, and circRNAs (as assessed using NCBI sources, and GtRNAdb, miRBase and circBase, see Supplementary material 4).

Further sequence analysis of the primary structure of ALPHA revealed three well-characterized oligomers: the hexamer CUGCCA, which is usually known as the anticodon loop of tRNA-Gly’s, and the two heptamers UUCAAGA and AAUGGUA, that are respectively known as the Tψ-loop and the D-loop of most tRNAs. This observation fitted nicely with the possibility of encountering the ALPHA sequence as a possible RNA present at origin of life. Indeed, the distribution of the pentamers frequencies within the genomes of Rfam database [43] shows the greatest survival probabilities for both pentamers coming from the most stable part of ALPHA, which correspond to the parts of the D-loop and Tψ-loop of many present tRNAs. We present in Figure 1e the example casted for *Œnothera coquimbensis* [52] and in Supplementary material 2, matches found with all known tRNAs.

### 3.1. ALPHA Remnants in the Genome of Archaea

**Figure 3:**
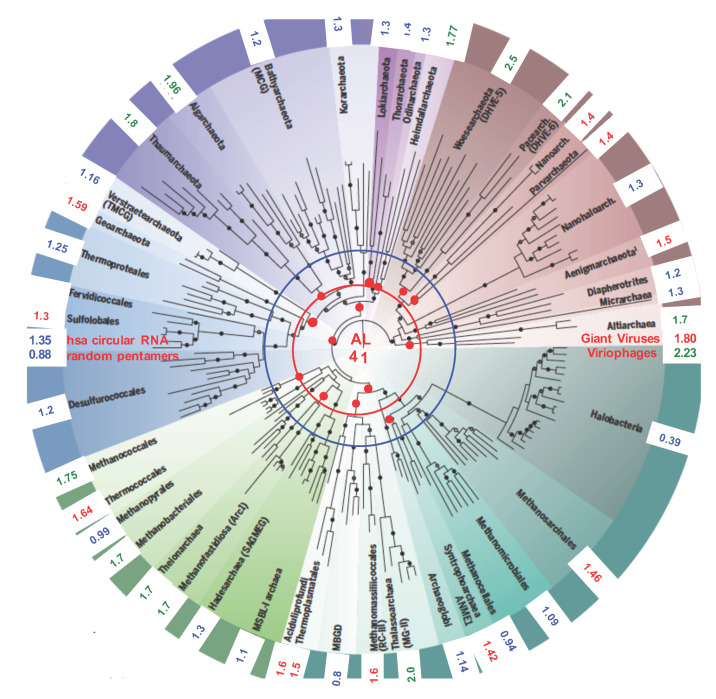
Archaea classification tree. The numbers at the periphery of the circular tree (from [55,56]) indicate the ALPHA pentamer-proximity P_9_ of the Archaea genomes to the subset of nine pentamers from ALPHA: {ATTCA, TTCAA, TCAAG, CAAGA, AAGAT, AGATG, GATGA, ATGAA, TGAAT}.

Like with giant viruses, we assessed any proximity of the ALPHA with the well-known Archaea realm (Figure 3). Assessing their ALPHA pentamer-proximity P_9_ (equal to the percentage of occurrence in their genomes of nine pentamers issued from ALPHA: ATTCA, TTCAA, TCAAG, CAAGA, AAGAT, AGATG, GATGA, ATGAA, TGAAT), Archaea fell into three categories. The first group (in blue) corresponded to 0.0109 ≤ P_9_ ≤ 0.014 (that differs significantly from the purely random probability with *p* < 4 × 10^−22^, with a genome of about 160,000 nucleotides-long), the second (in red) with 1.4 < P_9_ ≤ 1.8 (*p* < 10^−122^), and the third group (in green) with P_9_ > 1.8 (*p* ≈ 1.5 × 10^−382^).

### 3.2. ALPHA Remnants in the Genome of Bacteria

**Figure 4:**
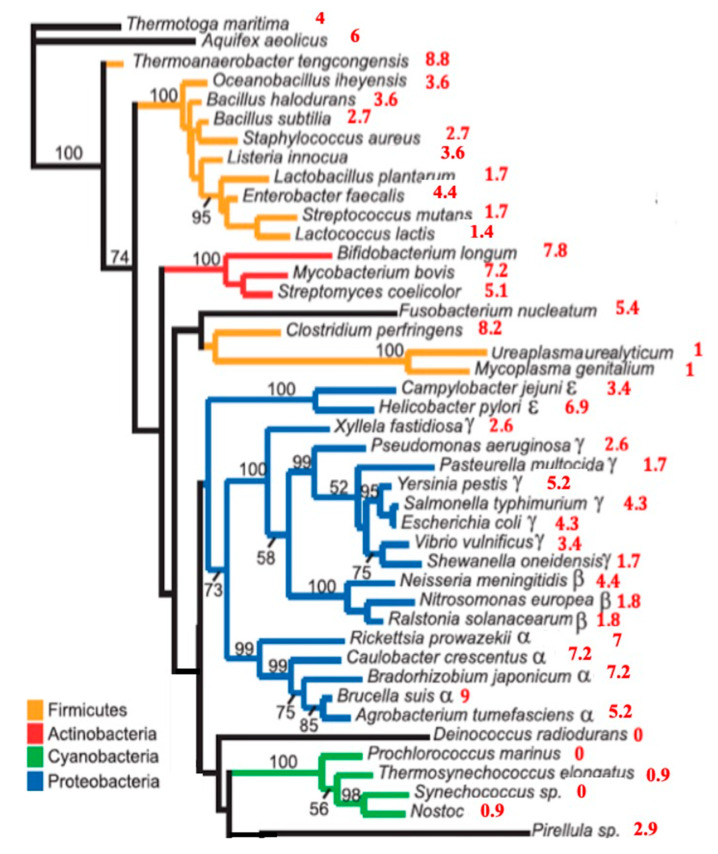
Bacteria phylogeny (from [60]) based on the sequences of the 16S ribosomal RNA genes. The numbers at the periphery of the phylogeny indicate the ALPHA pentamer-proximity P_22_ of the 5S ribosomal RNAs (in red).

By using the ALPHA pentamer-proximity P_22_ values for the 5S ribosomal RNAs (in red), Figure 4 (see also Supplementary material 3) shows that P_22_ explains partly a bacteria phylogeny based on the sequences of the 16S ribosomal RNA genes [59] with the following descending order: actinobacteria (mean P_22_ = 6.7), proteobacteria (mean P_22_ = 4.4), firmicutes without mycoplasmataceae (mean P_22_ = 3.9), mycoplasmataceae (mean P_22_ = 1), and cyanobacteria (mean P_22_ = 0.45), which corresponds to the classical order based on morphology and Gram stain. The upper limit U of significance of the observed frequencies of ALPHA pentamers for a 5S sequence of length 115 is equal to the upper bound of the 95% confidence interval of the right tailed test with H0 as pure random frequency f = 22/1024 ≈ 0.0214; then, we have: U = f + 1.64[f(1 − f)/115]^1/2^ ≈ 0.043, corresponding to P_22_ = 4.3.

### 3.3. ALPHA Remnants in Different Living Realms

**Figure 5:**
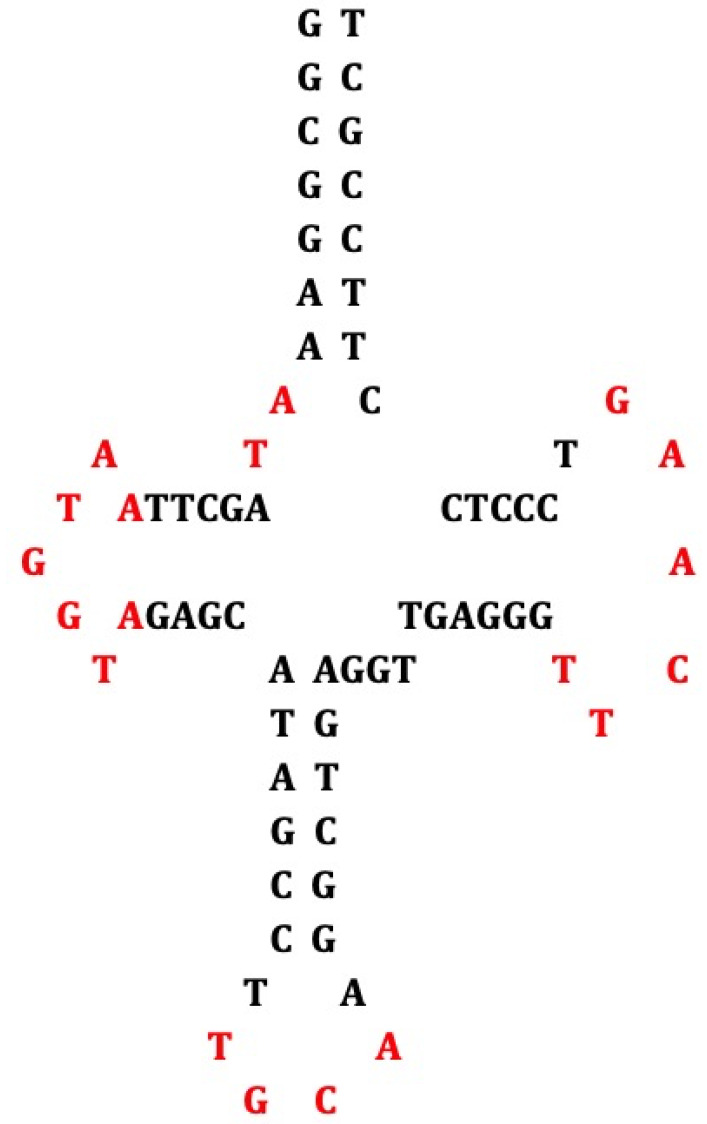
Lupine mitochondrial tRNA-Gly [61].

**Figure 6:**
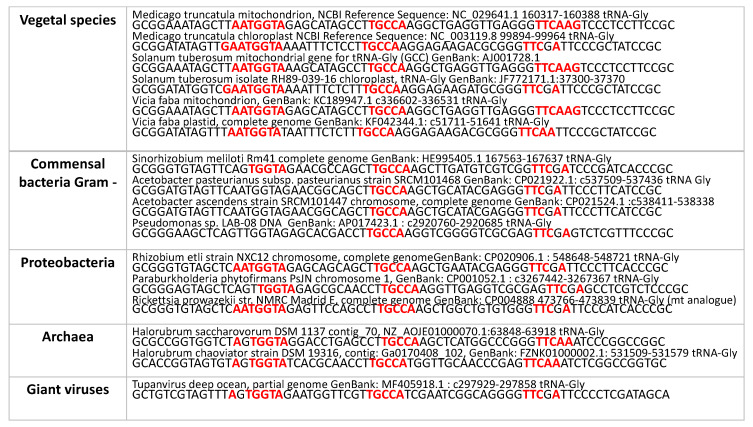
tRNA-Gly sequences from different living realms [42,43,44,45,46,47,48,49,50,51,52].

Other remnants from the ring ALPHA were found by exploring the tRNA-Gly’s of different living realms. As shown in Figure 5, the whole sequence of ALPHA, but with one distinct nucleotide, is encompassed within the lupine mt tRNA-Gly [60], and, as further presented in Figure 6, sequence similarity is found with 242 other vegetal species [42] and their commensal bacteria [61], and in particular the *Rickettsia prowazekii* genome (close to mitochondrial genomes), the Archae-like Halorubrum [62], and the giant-virus-like Tupanvirus, described as very ancient genomes [63], with the possibility of horizontal gene transfer between plants and bacteria [64]. Of note, the ALPHA-derived pentamer UGGUA (in bold) were also found twice within the 50 nucleotides-long sequence of the RNA catalytic domain harbored by the satellite RNA sequence of tobacco ringspot virus [65]:

5′-AAACAGAGAAGUCAACCAGAGAAACACACGUUG**UGGUA**UAUUACC**UGGUA**-3′

Likewise, a minimal RNA hairpin ribozyme, chain D, discovered 18 years later showed similarity with one ALPHA-derived hexamer and one pentamer spanning 19 bases: 5′-UCG**UGGUAC**AUUAC**CUGCC**-3′ [65]. Interestingly, the ALPHA-derived tetramer motif UGGU is a motif that is known to generally not be cleavable by ribozymes [66], and which could explain its ongoing presence in present ribozymes. Likewise, those ALPHA-derived pentamers were present in the D Chain of many hairpin ribozymes. This remnant presence of motifs could have been used to build simple RNA “cells”, consisting of two ribozymes with concerted activity allowing RNA replication [67,68,69,70,71,72].

## 4. Discussion: The Proximity to ALPHA as Criterion of Primitivity

From our observations described above, ALPHA could well be the ancestor of tRNAs. Moreover, by searching the ALPHA proximity with additional molecules, as presented in Table 1, we found the interesting trend according to which the greatest pentameric proximity with ALPHA was encountered for those molecules that are considered as essential to the process of protein translation. Searching for primitive mechanisms of proteogenesis, Agmon [72] proposed a scenario describing the emergence of “life as we know it”, i.e., “based on nucleic-acid and amino acid polymers that must include a proto-ribosome, which would have catalyzed the formation of a peptide bond between two amino acids and produced simple peptides.” Aligned with this scenario and based on proximities defined throughout this work, we propose as proto-ribosome the ring/hairpin ALPHA as featured above, because it has with ribozymes, tRNAs and rRNAs a greater pentamer proximity than with other RNAs (see Table 1 and Supplementary material 2).

Other theories concerning the origin of life and ancestors of tRNA structure and function exist.

First, the circular code discovered by Arquès and Michel [73,74] contains a remarkable set X_1_ of 20 highly frequent codons in RNAs involved in the protein translation:

X_1_ = {**AAT (ATT), TAC (GTA), GAA (TTC), GAT** **(ATC)**, **GCC** **(GGC)**, **GGT** **(ACC)**, **CTG** **(CAG)**, GAC (GTC), GAG (CTC), GTT (AAC)}

Among the codons of X_1_, 10 belong to ALPHA (in red) plus 4 to anti-ALPHA (in blue), and 12 of these 14 codons code all the 12 amino acids coded by X_1_, which shows the close connection between ALPHA and the circular code.

Second, the tRNAs obtained from repeats like UAGCC [75] present loops close to ALPHA, with the following hexamer motifs:

- D-loop hexamer motif is CTGGTC for types I and II tRNA, and ATGGTA for ALPHA
- Anticodon-loop hexamer motif is CTanticodonA for type I tRNA and for ALPHA
- T-loop hexamer motif is TTCAAA for types I and II tRNA, and TTCAAG for ALPHA.

Hence, these hexamer motifs represent 18/22 bases in ALPHA and present similarities with types I and II evolving tRNAs of [75].

**Figure 7:**
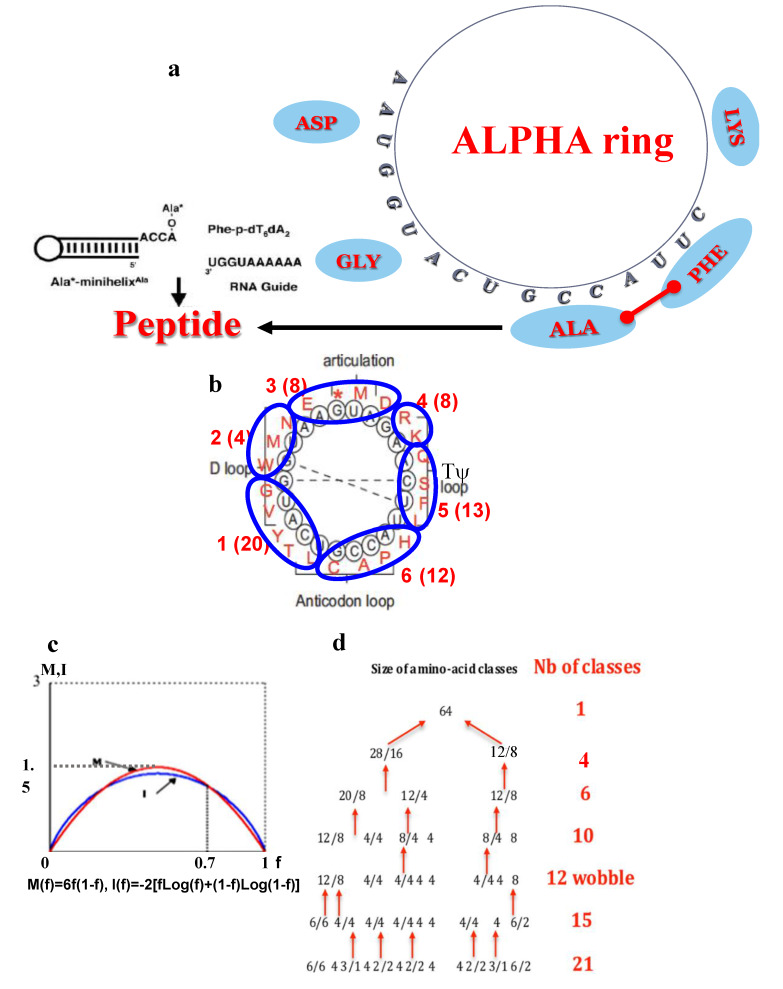
ALPHA ring as ribosome ancestor. (**a**) Primitive translation machinery centered on ALPHA ring with synthesis of Ala-Phe peptide on UGGUAA RNA guide [78] or on its complement in ALPHA GCCAUU. (**b**) ALPHA can be divided into six sub-sequences corresponding to the codons classes candidates for the second step of the descending partition, which follows (**c**) the min-max principle: “mean mutation error M equals information I” [79] and gives at step 4 (**d**) the “wobble” partition coding for the 11 early assigned amino acids plus a group of codons assigned to late amino acids [80,81,82].

Third, in [76], the authors claimed that the protein translation mechanism emerged when the genetic code started to evolve due to a stabilizing effect on RNA–peptide complexes with bridge peptides present in polymerase motifs, like the QLSLF amino acid motif whose nucleotide sequence contains twice the ALPHA tetramer ACTG: CA(ACTG)TC(ACTG)TTC. The hybridization-induced proximity peptides of short amino-acetylated RNAs could have favored the emergence of random peptides [77,78], initiating the Darwinian transformation of the genetic code (Figure 7) in a harmonious co-evolution of the biological information and function. The present RNA ring theory is compatible with an RNA–peptides scenario in which peptides were synthesized thanks to ALPHA sequence.

Eventually, in [60], it is shown that ALPHA ring belongs to a family of ancient RNAs made from diverse RNA types, including replication origins (OL) and OL-like structure, riboswitches, ribozymes, rRNAs, and tRNAs, molecules that are presumably close to ancestral RNAs.

## 5. Conclusions

To support the origin of life, a network view, as discussed by Aguirre at al. [28] and priorly by Seligmann and Raoult [83] can be proposed and centered on the ALPHA ring, as a key in the primitive machinery building peptides (Figure 7a,b). In this model, which could be viewed as a Mother Goose model, we hypothesize that the boundary of this first functional « machine », which was able to build peptides, could be defined as a peptidic gradient boundary, centered on the “proto-nucleus” ALPHA. The amino acids confinement around this protonucleus ALPHA could favor the occurrence of peptide bounds. This “organ” functioned as a “proto-ribosome” into a “proto-membrane”, and thus as a “proto-cell” with a circular organization. In fact, this model stands as a solution of a variational problem that is that peptide synthesis favored by ALPHA was necessary to repair the proto-cell membrane made of hydrophobic peptides and lipids, which reciprocally ensured the integrity of the proto-nucleus, and so-protected it against denaturation.

This mechanism is supported by different works, theoretical as well as experimental:

- In 1926, H.J. Muller already suggested that life began not as an enzyme but as a gene [84]
- The four amino acids: glycine, aspartic acid, asparagine, and serine have been claimed to have been coded by the first four triplets of the early, evolving genetic code [2], constituting the first class of amino acids (Figure 7d) selected following the min-max principle: “mean mutation error M equals information I” (Figure 7c), which uses the notion of information as proposed by Eigen [85]
- In the theory of autopoiesis [86,87], the first living system is self-reproducing [88,89] and “continuously generates and specifies its own organization through its operation as a system of production of its own components, and does this in an endless turnover of components”
- Experimental evidence of direct RNA–amino acid interactions has been shown for Arginine by Yarus et al. [33] and Alanine by Tamura and Schimmel [90,91,92]
- Statistical and theoretical arguments about the role of the primitive RNAs in the progressive constitution of the genetic code are given in [93,94,95,96,97].

Strikingly, the unique properties exhibited by the ALPHA ring address a possible role at the origins of life, giving birth to footprints as molecular relics in the present structures involved in the ribosomal translation. As a theoretical singular prototype, this ALPHA sequence should be useful to assess as a model matrix of future applications, ranging from synthetic biology to DNA computing. The sequence ALPHA and pentamers extracted from ALPHA are indeed frequently retrieved as remnants in many genomes [40], notably in proteins essential for the protein translation and maintenance of the cell integrity (tRNA synthetases, polymerases, tRNA nucleotidyl transferases, lipids synthetases, CRISPR Cas 9, etc.), which are considered as essential building blocks for cell survival.

Further studies should experimentally investigate the ring ALPHA as a potential catalyzer of peptide synthesis and search for its role in building after the RNA world, the protein and cell worlds and its role in consolidation of the genetic code, in accordance with previous knowledge in the field [98,99,100,101,102,103,104,105,106,107,108].

## Figures and Tables

**Table 1:** ALPHA proximity. The more the molecules considered are essential for protein translation, the greater is their ALPHA pentameric proximity P_22_ (calculated on the 22 pentamers of AL).

Molecular Family	5S RNA	SASP	Deaminase	Polymerase	cl. II Synthetase cl. I	Rprotein L7
Nb of species	100	100	50	30	3	30
Mean observed AL-proximity	4.45	4.5	4.5	3.5	3.5–2.9	3.4
Mean expected AL-proximity	2.1 ± 2 *	2.1 ± 2 *	2.1 ± 1.2 *	2.1 ± 0.9 *	2.1 ± 0.5 *	2.1 ± 1.25 *
% over max 0.90-confidence upper threshold *	42%	100%	48%	100%	96–53%	50%

* indicates the max (among species) of the 0.90-confidence upper threshold of expected P22.

## Supplementary Materials

The following are available online at https://www.mdpi.com/2079-7737/9/5/88/s1, S1: 1000#tRNAs#extracted#from#GtRNAdb; S2: All tRNAs from the GtRNAdb data base; S3: P22 proximity to AL of Bacteria 5S ribosomal RNAs; S4: Sample of size 200 from sequences of human circular RNAs from starbase.
