# Supplementary material for: Footprints of a Singular 22-Nucleotide RNA Ring at the Origin of Life
Source: Biology (Basel). 2020 Apr 25;9(5):88. doi: 10.3390/biology9050088 (PMC7285048; doi:10.3390/biology9050088)
Supplement: Supplementary file 1 [file biology-09-00088-s001.pdf]

1000 tRNAs extracted from GtRNAdb

>Acaryochloris_marina_MBIC11017_chr.trna68-GlyCCC (671119-671048) Gly (CCC) 72 bp Sc: 81.70 18 (Nb matches with AL)
GCGGATGTAGTTCAGTGGTAGAACGTCAGCTTCCCAAGCTGAATGTCGTCGGTTCGAGTCCGATCATCCGCT
Articulation D-loop Anticodon-loop Tψ-loop
>Acaryochloris_marina_MBIC11017_chr.trna39-GlyGCC (3821193-3821263) Gly (GCC) 71 bp Sc: 41.08 15
GCTCGATTAGCTCAGAGGGAGAGCACCGAGGGGCCATCTCGGGGGGCGTAGGTTCGAATC
CTACATCGACA
>Acaryochloris_marina_MBIC11017_chr.trna67-GlyGCC (978217-978146) Gly (GCC) 72 bp Sc: 77.98 16
GCGGGTATAGCTCAGTGGTAGAGCGTCACCTTGCCAAGGTGAATGTCGCGCGTTCGAATC
GCGTTACCCGCT
>Acaryochloris_marina_MBIC11017_chr.trna44-GlyTCC (4814711-4814781) Gly (TCC) 71 bp Sc: 68.07 15
GCGGGTGTAGTTTAGTGGTAAAACCTTAGCCTTCCAAGCTAATGATGGGGGTTCGATTCC
CCCCACCCGCT
>Acaryochloris_marina_MBIC11017_chr.trna20-GlyGCC (297174-297244) Gly (GCC) 71 bp Sc: 27.15 16
GCGAGTGTGATGTAGCAGTAGCATCTGGCGGTGCCAACGCCAGCGCGTGAGTGCGAATCT
CACCACTCGCT
>Anolis_carolinensis_chr2.trna284-GlyCCC (197419617-197419687) Gly (CCC) 71 bp Sc: 62.44 15
GCACCAGTGGTGTAGAGGCATCATGCGAGGTTCCCAATCTTGTGACCCGGGTTCGAGTCC
CGGCTGGTGCA
>Anolis_carolinensis_chr2.trna289-GlyCCC (197433343-197433413) Gly (CCC) 71 bp Sc: 62.44 15
GCACCAGTGGTGTAGAGGCATCATGCGAGGTTCCCAATCTTGTGACCCGGGTTCGAGTCC
CGGCTGGTGCA
>Anolis_carolinensis_chrUn_GL343784.trna1-GlyGCC (117103-117173) Gly (GCC) 71 bp Sc: 74.27 19
GCATTGGTGGTTCAGTGGTAGAATTCTCGCCTGCCACGCAGGAGGCCCAGGTTCGATTCC
TGGCCAATGCA
>Anolis_carolinensis_chrUn_GL343400.trna10-GlyGCC (556460-556390) Gly (GCC) 71 bp Sc: 81.62 19
GCATTGGTGGTTCAGTGGTAGAATTCTCGCCTGCCACGCGGGAGGCCCGGGTTCGATTCC
CGGCCAATGCA
>Anolis_carolinensis_chrUn_GL343400.trna12-GlyGCC (524602-524532) Gly (GCC) 71 bp Sc: 81.62 19
GCATTGGTGGTTCAGTGGTAGAATTCTCGCCTGCCACGCGGGAGGCCCGGGTTCGATTCC
CGGCCAATGCA
>Anolis_carolinensis_chrUn_GL343400.trna14-GlyGCC (398937-398867) Gly (GCC) 71 bp Sc: 81.62 19
GCATTGGTGGTTCAGTGGTAGAATTCTCGCCTGCCACGCGGGAGGCCCGGGTTCGATTCC
CGGCCAATGCA
>Anolis_carolinensis_chrUn_GL343400.trna16-GlyGCC (355340-355270) Gly (GCC) 71 bp Sc: 81.62 19
GCATTGGTGGTTCAGTGGTAGAATTCTCGCCTGCCACGCGGGAGGCCCGGGTTCGATTCC
CGGCCAATGCA
>Anolis_carolinensis_chrUn_GL343400.trna2-GlyGCC (399073-399143) Gly (GCC) 71 bp Sc: 81.62 19
GCATTGGTGGTTCAGTGGTAGAATTCTCGCCTGCCACGCGGGAGGCCCGGGTTCGATTCC
CGGCCAATGCA
>Anolis_carolinensis_chrUn_GL343400.trna9-GlyGCC (559474-559404) Gly (GCC) 71 bp Sc: 81.62 19
GCATTGGTGGTTCAGTGGTAGAATTCTCGCCTGCCACGCGGGAGGCCCGGGTTCGATTCC
CGGCCAATGCA
>Anolis_carolinensis_chrUn_GL343930.trna1-GlyTCC (85174-85103) Gly (TCC) 72 bp Sc: 66.22 17
GCGTTGGTGGTATAGTGGTTAGCATAGCTGCCTTCCAAGCAGTTGACCCAGGTTCGATTC
CCGGTCAACGCA
>Anolis_carolinensis_chrUn_GL343466.trna13-GlyTCC (263636-263707) Gly (TCC) 72 bp Sc: 73.26 17
GCGTTGGTGGTATAGTGGTGAGCATAGCTGCCTTCCAAGCAGTTGACCCGGGTTCGATTC
CCGGCCAACGCA
>Anolis_carolinensis_chrUn_GL343466.trna41-GlyTCC (246495-246424) Gly (TCC) 72 bp Sc: 73.26 17
GCGTTGGTGGTATAGTGGTGAGCATAGCTGCCTTCCAAGCAGTTGACCCGGGTTCGATTC
CCGGCCAACGCA
>Anolis_carolinensis_chrUn_GL343466.trna6-GlyTCC (251232-251303) Gly (TCC) 72 bp Sc: 73.26 17
GCGTTGGTGGTATAGTGGTGAGCATAGCTGCCTTCCAAGCAGTTGACCCGGGTTCGATTC
CCGGCCAACGCA
>Anolis_carolinensis_chrUn_GL343660.trna2-GlyTCC (87039-87110) Gly (TCC) 72 bp Sc: 73.26 17
GCGTTGGTGGTATAGTGGTGAGCATAGCTGCCTTCCAAGCAGTTGACCCGGGTTCGATTC
CCGGCCAACGCA
>Anolis_carolinensis_chr2.trna294-GlyTCC (197649527-197649598) Gly (TCC) 72 bp Sc: 76.83 17
GCGTTGGTGGTATAGTGGTTAGCATAGCTGCCTTCCAAGCAGTTGACCCGGGTTCGATTC
CCGGCCAACGCA
>Anolis_carolinensis_chr2.trna307-GlyTCC (197655656-197655585) Gly (TCC) 72 bp Sc: 76.83 17
GCGTTGGTGGTATAGTGGTTAGCATAGCTGCCTTCCAAGCAGTTGACCCGGGTTCGATTC
CCGGCCAACGCA
>Anolis_carolinensis_chrUn_GL343194.trna26-GlyTCC (589968-589897) Gly (TCC) 72 bp Sc: 76.83 17
GCGTTGGTGGTATAGTGGTTAGCATAGCTGCCTTCCAAGCAGTTGACCCGGGTTCGATTC
CCGGCCAACGCA
>Acholeplasma_laidlawii_PG_8A_chr.trna36-GlyGCC (301013-300939) Gly (GCC) 75 bp Sc: 55.52 16
GTCGGTGTCGTATAATGGTTATTACAAGGCCTTGCCAAGGCTTAGACGGCAGTTCGATCC
TGCTCACCGGCTCCA
>Acholeplasma_laidlawii_PG_8A_chr.trna31-GlyTCC (1196659-1196585) Gly (TCC) 75 bp Sc: 64.35 16
GCGGGTGTCGTATAATGGTTATTACCCCAGCCTTCCAAGCTGAAGACGGCGGTTCGATTC
CGCTCACCCGCTCCA

>Acidovorax_JS42_chr.trna47-GlyCCC (2077544-2077471) Gly (CCC) 74 bp Sc: 60.87 17
GCGGGCGTCGTTCAATGGTAGGACCTGAGCTTCCCAAGCTCAAGACGTGGGTTCGATTCC
CATCACCCGCTCCA
>Acidovorax_JS42_chr.trna10-GlyGCC (1408638-1408713) Gly (GCC) 76 bp Sc: 88.82 17
GCGGGAATAGCTCAGTTGGTAGAGCGCAACCTTGCCAAGGTTGAGGTCGTCGGTTCGAGA
CCGATTTCCCGCTCCA
>Acidovorax_JS42_chr.trna8-GlyGCC (1287848-1287923) Gly (GCC) 76 bp Sc: 88.82 17
GCGGGAATAGCTCAGTTGGTAGAGCGCAACCTTGCCAAGGTTGAGGTCGTCGGTTCGAGA
CCGATTTCCCGCTCCA
>Acidovorax_JS42_chr.trna31-GlyTCC (4151676-4151603) Gly (TCC) 74 bp Sc: 77.70 18
GCGGGAGTAGTTCAATGGTAGAACCCTAGCCTTCCAAGCTAATGACGCGGGTTCGATTCC
CGTCTCCCGCTCCA
>Acidobacteria_bacterium_Ellin345_chr.trna41-GlyCCC (2354670-2354596) Gly (CCC) 75 bp Sc: 83.59 16
GCGGGAGTAACTCAGTGGTAGAGTCACGGCTTCCCAAGCCGTTGGTCGCGGGTTCGATCC
CCGTCTCCCGCTCCA
>Acidobacteria_bacterium_Ellin345_chr.trna6-GlyGCC (1015247-1015321) Gly (GCC) 75 bp Sc: 84.50 18
GCGGGAGTAACTCAGTGGTAGAGTGCGACCTTGCCAAGGTCGAAGTCGCGGGTTCAAATC
CCGTCTCCCGCTCCA
>Acidobacteria_bacterium_Ellin345_chr.trna21-GlyTCC (5545681-5545607) Gly (TCC) 75 bp Sc: 86.70 17
GCGGGAGTAACTCAGTGGTAGAGTCACAGCCTTCCAAGCTGTTGGTCGCGGGTTCGATTC
CCGTCTCCCGCTCCA
>Acidothermus_cellulolyticus_11B_chr.trna32-GlyCCC (2389324-2389254) Gly (CCC) 71 bp Sc: 70.87 17
GCGGGTGTAGTTCAATGGCAGAACGTCAGCTTCCCAAGCTGATAATGCGGGTTCGATTCC
CGTCACCCGCT
>Acidothermus_cellulolyticus_11B_chr.trna19-GlyGCC (1524762-1524834) Gly (GCC) 73 bp Sc: 88.54 17
GCGGACGTGGCTCAGTTGGTAGAGCATCACCTTGCCAAGGTGAGGGTCGCGGGTTCGAGT
CCCGTCGTCCGCT
>Acidothermus_cellulolyticus_11B_chr.trna22-GlyTCC (1797034-1797104) Gly (TCC) 71 bp Sc: 77.75 18
GCGGGTGTAGCTCAATGGCAGAGCTCCAGCCTTCCAAGCTGGCGGTGCGGGTTCGATTCC
CGTCACCCGCT
>Acinetobacter_baumannii_chr.trna27-GlyGCC (2542259-2542334) Gly (GCC) 76 bp Sc: 90.38 17
GCGGGAATAGCTCAGTTGGTAGAGCATAACCTTGCCAAGGTTGGGGTCGCGAGTTCGAGT
CTCGTTTCCCGCTCCA
>Acinetobacter_baumannii_chr.trna35-GlyGCC (3776785-3776860) Gly (GCC) 76 bp Sc: 90.38 17
GCGGGAATAGCTCAGTTGGTAGAGCATAACCTTGCCAAGGTTGGGGTCGCGAGTTCGAGT
CTCGTTTCCCGCTCCA
>Acinetobacter_baumannii_chr.trna36-GlyGCC (3776906-3776981) Gly (GCC) 76 bp Sc: 90.38 17
GCGGGAATAGCTCAGTTGGTAGAGCATAACCTTGCCAAGGTTGGGGTCGCGAGTTCGAGT
CTCGTTTCCCGCTCCA
>Acinetobacter_baumannii_chr.trna43-GlyTCC (3553417-3553342) Gly (TCC) 76 bp Sc: 91.76 16
GCGGGAGTAGCTCAGTTGGTAGAGCGGCAGCCTTCCAAGCTGCATGTCGCGAGTTCGATC
CTCGTCTCCCGCTCCA
>Acinetobacter_baumannii_ATCC_17978_chr.trna57-GlyGCC (1446506-1446431) Gly (GCC) 76 bp Sc: 90.38 17
GCGGGAATAGCTCAGTTGGTAGAGCATAACCTTGCCAAGGTTGGGGTCGCGAGTTCGAGT
CTCGTTTCCCGCTCCA
>Acinetobacter_baumannii_ATCC_17978_chr.trna69-GlyGCC (149356-149281) Gly (GCC) 76 bp Sc: 90.38 17
GCGGGAATAGCTCAGTTGGTAGAGCATAACCTTGCCAAGGTTGGGGTCGCGAGTTCGAGT
CTCGTTTCCCGCTCCA
>Acinetobacter_baumannii_ATCC_17978_chr.trna70-GlyGCC (149235-149160) Gly (GCC) 76 bp Sc: 90.38 17
GCGGGAATAGCTCAGTTGGTAGAGCATAACCTTGCCAAGGTTGGGGTCGCGAGTTCGAGT
CTCGTTTCCCGCTCCA
>Acinetobacter_baumannii_ATCC_17978_chr.trna5-GlyTCC (300127-300202) Gly (TCC) 76 bp Sc: 91.76 16
GCGGGAGTAGCTCAGTTGGTAGAGCGGCAGCCTTCCAAGCTGCATGTCGCGAGTTCGATC
CTCGTCTCCCGCTCCA
>Acinetobacter_baumannii_AYE_chr.trna27-GlyGCC (2542259-2542334) Gly (GCC) 76 bp Sc: 90.38 16
GCGGGAATAGCTCAGTTGGTAGAGCATAACCTTGCCAAGGTTGGGGTCGCGAGTTCGAGT
CTCGTTTCCCGCTCCA
>Acinetobacter_baumannii_AYE_chr.trna35-GlyGCC (3776785-3776860) Gly (GCC) 76 bp Sc: 90.38 16
GCGGGAATAGCTCAGTTGGTAGAGCATAACCTTGCCAAGGTTGGGGTCGCGAGTTCGAGT
CTCGTTTCCCGCTCCA
>Acinetobacter_baumannii_AYE_chr.trna36-GlyGCC (3776906-3776981) Gly (GCC) 76 bp Sc: 90.38 16
GCGGGAATAGCTCAGTTGGTAGAGCATAACCTTGCCAAGGTTGGGGTCGCGAGTTCGAGT
CTCGTTTCCCGCTCCA
>Acinetobacter_baumannii_AYE_chr.trna43-GlyTCC (3553417-3553342) Gly (TCC) 76 bp Sc: 91.76 16
GCGGGAGTAGCTCAGTTGGTAGAGCGGCAGCCTTCCAAGCTGCATGTCGCGAGTTCGATC
CTCGTCTCCCGCTCCA
>Acinetobacter_baumannii_SDF_NC_010400.trna23-GlyGCC (2157296-2157371) Gly (GCC) 76 bp Sc: 90.38 16
GCGGGAATAGCTCAGTTGGTAGAGCATAACCTTGCCAAGGTTGGGGTCGCGAGTTCGAGT
CTCGTTTCCCGCTCCA
>Acinetobacter_baumannii_SDF_NC_010400.trna63-GlyGCC (133032-132957) Gly (GCC) 76 bp Sc: 90.38 16
GCGGGAATAGCTCAGTTGGTAGAGCATAACCTTGCCAAGGTTGGGGTCGCGAGTTCGAGT
CTCGTTTCCCGCTCCA
>Acinetobacter_baumannii_SDF_NC_010400.trna64-GlyGCC (132911-132836) Gly (GCC) 76 bp Sc: 90.38 16
GCGGGAATAGCTCAGTTGGTAGAGCATAACCTTGCCAAGGTTGGGGTCGCGAGTTCGAGT

CTCGTTTCCCGCTCCA
>Acinetobacter_baumannii_SDF_NC_010400.trna32-GlyTCC (3005698-3005623) Gly (TCC) 76 bp Sc: 87.92 16
GCGGGAGTAGCTCAGTTGGTAGAGCGGCAGCCTTCCAAGCTGCATGTCGCGAGTTCGATC
CTCGTCTTCCGCTCCA
>Actinobacillus_pleuropneumoniae_serovar_3_JL03_chr.trna43-GlyGCC (1492267-1492192) Gly (GCC) 76 bp Sc: 91.37 16
GCGGGAATAGCTCAGTTGGTAGAGCACGACCTTGCCAAGGTCGGGGTCGCGAGTTCGAGC
CTCGTTTCCCGCTCCA
>Actinobacillus_pleuropneumoniae_serovar_3_JL03_chr.trna45-GlyGCC (1492067-1491992) Gly (GCC) 76 bp Sc: 91.37 16
GCGGGAATAGCTCAGTTGGTAGAGCACGACCTTGCCAAGGTCGGGGTCGCGAGTTCGAGC
CTCGTTTCCCGCTCCA
>Actinobacillus_pleuropneumoniae_serovar_3_JL03_chr.trna48-GlyGCC (1491717-1491642) Gly (GCC) 76 bp Sc: 91.37 16
GCGGGAATAGCTCAGTTGGTAGAGCACGACCTTGCCAAGGTCGGGGTCGCGAGTTCGAAC
CTCGTTTCCCGCTCCA
>Actinobacillus_pleuropneumoniae_serovar_3_JL03_chr.trna61-GlyGCC (847198-847123) Gly (GCC) 76 bp Sc: 91.37 16
GCGGGAATAGCTCAGTTGGTAGAGCACGACCTTGCCAAGGTCGGGGTCGCGAGTTCGAGC
CTCGTTTCCCGCTCCA
>Actinobacillus_pleuropneumoniae_serovar_3_JL03_chr.trna36-GlyTCC (1690474-1690400) Gly (TCC) 75 bp Sc: 63.54 16
GCGGGCATCGTATAATGGCTATTACCTTAGCCTTCCAAGCTAATGATGCGGGTTCGATTC
CCGCTGCCCGCTCCA
>Actinobacillus_succinogenes_130Z_chr.trna28-GlyGCC (1922674-1922749) Gly (GCC) 76 bp Sc: 92.69 16
GCGGGAATAGCTCAGTTGGTAGAGCACAACCTTGCCAAGGTTGGGGTCGCGAGTTCGAGC
CTCGTTTCCCGCTCCA
>Actinobacillus_succinogenes_130Z_chr.trna30-GlyGCC (2030325-2030400) Gly (GCC) 76 bp Sc: 92.69 16
GCGGGAATAGCTCAGTTGGTAGAGCACAACCTTGCCAAGGTTGGGGTCGCGAGTTCGAGC
CTCGTTTCCCGCTCCA
>Actinobacillus_succinogenes_130Z_chr.trna41-GlyGCC (1071701-1071626) Gly (GCC) 76 bp Sc: 92.69 16
GCGGGAATAGCTCAGTTGGTAGAGCACAACCTTGCCAAGGTTGGGGTCGCGAGTTCGAGC
CTCGTTTCCCGCTCCA
>Actinobacillus_succinogenes_130Z_chr.trna55-GlyTCC (112045-111971) Gly (TCC) 75 bp Sc: 63.54 16
GCGGGCATCGTATAATGGCTATTACCTTAGCCTTCCAAGCTAATGATGCGGGTTCGATTC
CCGCTGCCCGCTCCA
>Aeromonas_hydrophila_ATCC_7966_chr.trna97-GlyCCC (3414662-3414589) Gly (CCC) 74 bp Sc: 74.92 18
GCGGGTGTAGTTCAATGGTAGAACGGTAGCTTCCCAAGCTGCATACGTGGGTTCGATTCC
CATCACCCGCTCCA
>Aeromonas_hydrophila_ATCC_7966_chr.trna80-GlyGCC (3882630-3882555) Gly (GCC) 76 bp Sc: 93.74 17
GCGGGAATAGCTCAGTTGGTAGAGCACGACCTTGCCAAGGTCGGGGTCGCGAGTTCGAGT
CTCGTTTCCCGCTCCA
>Aeromonas_hydrophila_ATCC_7966_chr.trna81-GlyGCC (3882529-3882454) Gly (GCC) 76 bp Sc: 93.74 17
GCGGGAATAGCTCAGTTGGTAGAGCACGACCTTGCCAAGGTCGGGGTCGCGAGTTCGAGT
CTCGTTTCCCGCTCCA
>Aeromonas_hydrophila_ATCC_7966_chr.trna82-GlyGCC (3882420-3882345) Gly (GCC) 76 bp Sc: 93.74 17
GCGGGAATAGCTCAGTTGGTAGAGCACGACCTTGCCAAGGTCGGGGTCGCGAGTTCGAGT
CTCGTTTCCCGCTCCA
>Aeromonas_hydrophila_ATCC_7966_chr.trna83-GlyGCC (3882320-3882245) Gly (GCC) 76 bp Sc: 93.74 17
GCGGGAATAGCTCAGTTGGTAGAGCACGACCTTGCCAAGGTCGGGGTCGCGAGTTCGAGT
CTCGTTTCCCGCTCCA
>Aeromonas_hydrophila_ATCC_7966_chr.trna84-GlyGCC (3882220-3882145) Gly (GCC) 76 bp Sc: 93.74 17
GCGGGAATAGCTCAGTTGGTAGAGCACGACCTTGCCAAGGTCGGGGTCGCGAGTTCGAGT
CTCGTTTCCCGCTCCA
>Aeromonas_hydrophila_ATCC_7966_chr.trna85-GlyGCC (3882120-3882045) Gly (GCC) 76 bp Sc: 93.74 17
GCGGGAATAGCTCAGTTGGTAGAGCACGACCTTGCCAAGGTCGGGGTCGCGAGTTCGAGT
CTCGTTTCCCGCTCCA
>Aeromonas_hydrophila_ATCC_7966_chr.trna86-GlyGCC (3882020-3881945) Gly (GCC) 76 bp Sc: 93.74 17
GCGGGAATAGCTCAGTTGGTAGAGCACGACCTTGCCAAGGTCGGGGTCGCGAGTTCGAGT
CTCGTTTCCCGCTCCA
>Aeromonas_hydrophila_ATCC_7966_chr.trna95-GlyGCC (3487654-3487579) Gly (GCC) 76 bp Sc: 93.74 17
GCGGGAATAGCTCAGTTGGTAGAGCACGACCTTGCCAAGGTCGGGGTCGCGAGTTCGAGT
CTCGTTTCCCGCTCCA
>Aeromonas_hydrophila_ATCC_7966_chr.trna99-GlyGCC (3111461-3111386) Gly (GCC) 76 bp Sc: 93.74 17
GCGGGAATAGCTCAGTTGGTAGAGCACGACCTTGCCAAGGTCGGGGTCGCGAGTTCGAGT
CTCGTTTCCCGCTCCA
>Aeromonas_hydrophila_ATCC_7966_chr.trna47-GlyTCC (4476138-4476064) Gly (TCC) 75 bp Sc: 64.85 16
GCGGGCATCGTATAATGGCTATTACCTCAGCCTTCCAAGCTGATGATGCGGGTTCGATTC
CCGCTGCCCGCTCCA
>Aeromonas_hydrophila_ATCC_7966_chr.trna98-GlyTCC (3414494-3414420) Gly (TCC) 75 bp Sc: 64.85 16
GCGGGCATCGTATAATGGCTATTACCTCAGCCTTCCAAGCTGATGATGCGGGTTCGATTC
CCGCTGCCCGCTCCA
>Aeromonas_hydrophila_ATCC_7966_chr.trna87-GlyGCC (3881881-3881802) Gly (GCC) 80 bp Sc: 30.09
GCTGCCATATTCAGTTGGTAGAGCATGATCCGCGCTTGCCAAGGCTGGGGTCGCGAGTTC
GAGTCTCGTTTCCCGCTCCA
>Aeromonas_salmonicida_A449_chr.trna79-GlyCCC (3283584-3283511) Gly (CCC) 74 bp Sc: 74.92 18
GCGGGTGTAGTTCAATGGTAGAACGGTAGCTTCCCAAGCTGCATACGTGGGTTCGATTCC
CATCACCCGCTCCA
>Aeromonas_salmonicida_A449_chr.trna28-GlyGCC (890391-890466) Gly (GCC) 76 bp Sc: 93.74 17

GCGGGAATAGCTCAGTTGGTAGAGCACGACCTTGCCAAGGTCGGGGTCGCGAGTTCGAGT
CTCGTTTCCCGCTCCA
>Aeromonas_salmonicida_A449_chr.trna29-GlyGCC (890503-890578) Gly (GCC) 76 bp Sc: 93.74 17
GCGGGAATAGCTCAGTTGGTAGAGCACGACCTTGCCAAGGTCGGGGTCGCGAGTTCGAGT
CTCGTTTCCCGCTCCA
>Aeromonas_salmonicida_A449_chr.trna30-GlyGCC (890614-890689) Gly (GCC) 76 bp Sc: 93.74 17
GCGGGAATAGCTCAGTTGGTAGAGCACGACCTTGCCAAGGTCGGGGTCGCGAGTTCGAGT
CTCGTTTCCCGCTCCA
>Aeromonas_salmonicida_A449_chr.trna31-GlyGCC (890726-890801) Gly (GCC) 76 bp Sc: 93.74 17
GCGGGAATAGCTCAGTTGGTAGAGCACGACCTTGCCAAGGTCGGGGTCGCGAGTTCGAGT
CTCGTTTCCCGCTCCA
>Aeromonas_salmonicida_A449_chr.trna32-GlyGCC (890828-890903) Gly (GCC) 76 bp Sc: 93.74 17
GCGGGAATAGCTCAGTTGGTAGAGCACGACCTTGCCAAGGTCGGGGTCGCGAGTTCGAGT
CTCGTTTCCCGCTCCA
>Aeromonas_salmonicida_A449_chr.trna40-GlyGCC (1635316-1635391) Gly (GCC) 76 bp Sc: 93.74 17
GCGGGAATAGCTCAGTTGGTAGAGCACGACCTTGCCAAGGTCGGGGTCGCGAGTTCGAGT
CTCGTTTCCCGCTCCA
>Aeromonas_salmonicida_A449_chr.trna77-GlyGCC (3340716-3340641) Gly (GCC) 76 bp Sc: 93.74 17
GCGGGAATAGCTCAGTTGGTAGAGCACGACCTTGCCAAGGTCGGGGTCGCGAGTTCGAGT
CTCGTTTCCCGCTCCA
>Aeromonas_salmonicida_A449_chr.trna10-GlyTCC (291554-291628) Gly (TCC) 75 bp Sc: 61.33 16
GCGGGCATCGTATAATGGCTATTACCTCAGCCTTCCAAGCTGATGATGCGGGTTCGATTC
CCGCTGCCCGTTCCA
>Aeromonas_salmonicida_A449_chr.trna80-GlyTCC (3283410-3283336) Gly (TCC) 75 bp Sc: 64.85 16
GCGGGCATCGTATAATGGCTATTACCTCAGCCTTCCAAGCTGATGATGCGGGTTCGATTC
CCGCTGCCCGCTCCA
>Anopheles_gambiae_chr2L.trna130-GlyGCC (15482432-15482362) Gly (GCC) 71 bp Sc: 82.25 19
GCATCGGTGGTTCAGTGGTAGAATGCTCGCCTGCCACGCGGGCGGCCCGGGTTCGATTCC
CGGCCGATGCA
>Anopheles_gambiae_chr2L.trna131-GlyGCC (15480024-15479954) Gly (GCC) 71 bp Sc: 82.25 19
GCATCGGTGGTTCAGTGGTAGAATGCTCGCCTGCCACGCGGGCGGCCCGGGTTCGATTCC
CGGCCGATGCA
>Anopheles_gambiae_chr2L.trna132-GlyGCC (15473270-15473200) Gly (GCC) 71 bp Sc: 82.25 19
GCATCGGTGGTTCAGTGGTAGAATGCTCGCCTGCCACGCGGGCGGCCCGGGTTCGATTCC
CGGCCGATGCA
>Anopheles_gambiae_chr2L.trna7-GlyGCC (14668048-14668118) Gly (GCC) 71 bp Sc: 82.25 19
GCATCGGTGGTTCAGTGGTAGAATGCTCGCCTGCCACGCGGGCGGCCCGGGTTCGATTCC
CGGCCGATGCA
>Anopheles_gambiae_chr2R.trna61-GlyGCC (55542894-55542964) Gly (GCC) 71 bp Sc: 82.25 19
GCATCGGTGGTTCAGTGGTAGAATGCTCGCCTGCCACGCGGGCGGCCCGGGTTCGATTCC
CGGCCGATGCA
>Anopheles_gambiae_chr2R.trna66-GlyGCC (57612141-57612211) Gly (GCC) 71 bp Sc: 82.25 19
GCATCGGTGGTTCAGTGGTAGAATGCTCGCCTGCCACGCGGGCGGCCCGGGTTCGATTCC
CGGCCGATGCA
>Anopheles_gambiae_chr2R.trna72-GlyGCC (51448701-51448631) Gly (GCC) 71 bp Sc: 82.25 19
GCATCGGTGGTTCAGTGGTAGAATGCTCGCCTGCCACGCGGGCGGCCCGGGTTCGATTCC
CGGCCGATGCA
>Anopheles_gambiae_chr2R.trna73-GlyGCC (51401819-51401749) Gly (GCC) 71 bp Sc: 82.25 19
GCATCGGTGGTTCAGTGGTAGAATGCTCGCCTGCCACGCGGGCGGCCCGGGTTCGATTCC
CGGCCGATGCA
>Anopheles_gambiae_chr2R.trna96-GlyGCC (32435252-32435182) Gly (GCC) 71 bp Sc: 82.25 19
GCATCGGTGGTTCAGTGGTAGAATGCTCGCCTGCCACGCGGGCGGCCCGGGTTCGATTCC
CGGCCGATGCA
>Anopheles_gambiae_chr2R.trna99-GlyGCC (32429500-32429430) Gly (GCC) 71 bp Sc: 82.25 19
GCATCGGTGGTTCAGTGGTAGAATGCTCGCCTGCCACGCGGGCGGCCCGGGTTCGATTCC
CGGCCGATGCA
>Anopheles_gambiae_chr3L.trna10-GlyGCC (8129363-8129433) Gly (GCC) 71 bp Sc: 82.25 19
GCATCGGTGGTTCAGTGGTAGAATGCTCGCCTGCCACGCGGGCGGCCCGGGTTCGATTCC
CGGCCGATGCA
>Anopheles_gambiae_chr3L.trna87-GlyGCC (5637949-5637879) Gly (GCC) 71 bp Sc: 82.25 19
GCATCGGTGGTTCAGTGGTAGAATGCTCGCCTGCCACGCGGGCGGCCCGGGTTCGATTCC
CGGCCGATGCA
>Anopheles_gambiae_chr3R.trna23-GlyGCC (32850262-32850332) Gly (GCC) 71 bp Sc: 82.25 19
GCATCGGTGGTTCAGTGGTAGAATGCTCGCCTGCCACGCGGGCGGCCCGGGTTCGATTCC
CGGCCGATGCA
>Anopheles_gambiae_chr3R.trna24-GlyGCC (33021487-33021557) Gly (GCC) 71 bp Sc: 82.25 19
GCATCGGTGGTTCAGTGGTAGAATGCTCGCCTGCCACGCGGGCGGCCCGGGTTCGATTCC
CGGCCGATGCA
>Anopheles_gambiae_chr3R.trna32-GlyGCC (33706356-33706286) Gly (GCC) 71 bp Sc: 82.25 19
GCATCGGTGGTTCAGTGGTAGAATGCTCGCCTGCCACGCGGGCGGCCCGGGTTCGATTCC
CGGCCGATGCA
>Anopheles_gambiae_chr2L.trna71-GlyTCC (44792453-44792382) Gly (TCC) 72 bp Sc: 72.21 18
GCGTCGGTGGTGTAATGGTCAGCATAGTTGCCTTCCAAGCAGTTGATCCGGGTTCGATTC
CCGGCCGACGCA

>Anopheles_gambiae_chr2R.trna27-GlyTCC (27030814-27030885) Gly (TCC) 72 bp Sc: 72.21 18
GCGTCGGTGGTGTAATGGTCAGCATAGTTGCCTTCCAAGCAGTTGATCCGGGTTCGATTC
CCGGCCGACGCA
>Anopheles_gambiae_chr2R.trna37-GlyTCC (37856478-37856549) Gly (TCC) 72 bp Sc: 72.21 18
GCGTCGGTGGTGTAATGGTCAGCATAGTTGCCTTCCAAGCAGTTGATCCGGGTTCGATTC
CCGGCCGACGCA
>Anopheles_gambiae_chr2R.trna40-GlyTCC (37884513-37884584) Gly (TCC) 72 bp Sc: 72.21 18
GCGTCGGTGGTGTAATGGTCAGCATAGTTGCCTTCCAAGCAGTTGATCCGGGTTCGATTC
CCGGCCGACGCA
>Anopheles_gambiae_chr2R.trna47-GlyTCC (46945357-46945428) Gly (TCC) 72 bp Sc: 72.21 18
GCGTCGGTGGTGTAATGGTCAGCATAGTTGCCTTCCAAGCAGTTGATCCGGGTTCGATTC
CCGGCCGACGCA
>Anopheles_gambiae_chr2R.trna59-GlyTCC (55074547-55074618) Gly (TCC) 72 bp Sc: 72.21 18
GCGTCGGTGGTGTAATGGTCAGCATAGTTGCCTTCCAAGCAGTTGATCCGGGTTCGATTC
CCGGCCGACGCA
>Anopheles_gambiae_chr2R.trna65-GlyTCC (57467215-57467286) Gly (TCC) 72 bp Sc: 72.21 18
GCGTCGGTGGTGTAATGGTCAGCATAGTTGCCTTCCAAGCAGTTGATCCGGGTTCGATTC
CCGGCCGACGCA
>Anopheles_gambiae_chr3R.trna54-GlyTCC (9550053-9549982) Gly (TCC) 72 bp Sc: 72.21 18
GCGTCGGTGGTGTAATGGTCAGCATAGTTGCCTTCCAAGCAGTTGATCCGGGTTCGATTC
CCGGCCGACGCA
>Agrobacterium_tumefaciens_C58_UWash_chrcircular.trna5-GlyCCC (430676-430749) Gly (CCC) 74 bp Sc: 80.72 18
GCGGGTGTAGCTCAATGGTAGAGCAGCAGCTTCCCAAGCTGAATACGAGGGTTCGATTCC
CTTCACCCGCTCCA
>Agrobacterium_tumefaciens_C58_UWash_chrcircular.trna20-GlyGCC (2446476-2446550) Gly (GCC) 75 bp Sc: 87.04 18
GCGGGTGTAGCTCAGGGGTAGAGCACAACCTTGCCAAGGTTGGGGTCGAGGGTTCAAATC
CCTTCGCCCGCTCCA
>Agrobacterium_tumefaciens_C58_UWash_chrlinear.trna2-GlyGCC (779527-779601) Gly (GCC) 75 bp Sc: 87.04 18
GCGGGTGTAGCTCAGGGGTAGAGCACAACCTTGCCAAGGTTGGGGTCGAGGGTTCAAATC
CCTTCGCCCGCTCCA
>Agrobacterium_tumefaciens_C58_UWash_chrcircular.trna27-GlyTCC (1956756-1956683) Gly (TCC) 74 bp Sc: 83.28 18
GCGGGTATAGCTCAATGGTAGAGCAGCAGCCTTCCAAGCTGAATACGCGGGTTCGATTCC
CGCTACCCGCTCCA
>Alcanivorax_borkumensis_SK2_chr.trna37-GlyCCC (1807972-1807899) Gly (CCC) 74 bp Sc: 77.93 18
GCGGGTGTAGTTCAATGGTAGAACGGCAGCTTCCCAAGCTGCATACGAGAGTTCGATTCT
CTTCACCCGCTCCA
>Alcanivorax_borkumensis_SK2_chr.trna16-GlyGCC (972692-972767) Gly (GCC) 76 bp Sc: 93.74 16
GCGGGAATAGCTCAGTTGGTAGAGCACGACCTTGCCAAGGTCGGGGTCGCGAGTTCGAAT
CTCGTTTCCCGCTCCA
>Alcanivorax_borkumensis_SK2_chr.trna24-GlyGCC (1481751-1481826) Gly (GCC) 76 bp Sc: 95.06 16
GCGGGAATAGCTCAGTTGGTAGAGCACAACCTTGCCAAGGTTGGGGTCGCGAGTTCGAAT
CTCGTTTCCCGCTCCA
>Alcanivorax_borkumensis_SK2_chr.trna7-GlyTCC (413664-413737) Gly (TCC) 74 bp Sc: 83.13 17
GCGGGTATAGTTCAACGGTAGAACCTCAGCCTTCCAAGCTGATGGTGCGGGTTCGATTCC
CGCTACCCGCTCCA
>Alkaliphilus_metalliredigens_QYMF_chr.trna26-GlyGCC (1843694-1843768) Gly (GCC) 75 bp Sc: 91.02 18
GCGGAAGTGGCTCAGTGGTAGAGCATCGCCTTGCCAAGGCGAGGGTCGCGAGTTCAAATC
TCGTCTTCCGCTCCA
>Alkaliphilus_metalliredigens_QYMF_chr.trna54-GlyGCC (4446065-4445991) Gly (GCC) 75 bp Sc: 91.02 18
GCGGAAGTGGCTCAGTGGTAGAGCATCGCCTTGCCAAGGCGAGGGTCGCGAGTTCAAATC
TCGTCTTCCGCTCCA
>Alkaliphilus_metalliredigens_QYMF_chr.trna98-GlyGCC (4429559-4429485) Gly (GCC) 75 bp Sc: 91.02 18
GCGGAAGTGGCTCAGTGGTAGAGCATCGCCTTGCCAAGGCGAGGGTCGCGAGTTCAAATC
TCGTCTTCCGCTCCA
>Alkaliphilus_metalliredigens_QYMF_chr.trna38-GlyTCC (4453023-4452950) Gly (TCC) 74 bp Sc: 75.29 18
GCGGGTGTAGTTTAGTGGTAGAACTTCAGCCTTCCAAGCTGATCGCGAGGGTTCGATTCC
CTCTACCCGCTCCA
>Alkaliphilus_metalliredigens_QYMF_chr.trna5-GlyTCC (996643-996716) Gly (TCC) 74 bp Sc: 75.29 18
GCGGGTGTAGTTTAGTGGTAGAACTTCAGCCTTCCAAGCTGATCGCGAGGGTTCGATTCC
CTCTACCCGCTCCA
>Alkaliphilus_metalliredigens_QYMF_chr.trna66-GlyTCC (4444833-4444760) Gly (TCC) 74 bp Sc: 75.29 18
GCGGGTGTAGTTTAGTGGTAGAACTTCAGCCTTCCAAGCTGATCGCGAGGGTTCGATTCC
CTCTACCCGCTCCA
>Alkaliphilus_metalliredigens_QYMF_chr.trna72-GlyTCC (4437637-4437564) Gly (TCC) 74 bp Sc: 75.29 18
GCGGGTGTAGTTTAGTGGTAGAACTTCAGCCTTCCAAGCTGATCGCGAGGGTTCGATTCC
CTCTACCCGCTCCA
>Alkaliphilus_metalliredigens_QYMF_chr.trna88-GlyTCC (4436089-4436016) Gly (TCC) 74 bp Sc: 75.29 18
GCGGGTGTAGTTTAGTGGTAGAACTTCAGCCTTCCAAGCTGATCGCGAGGGTTCGATTCC
CTCTACCCGCTCCA
>Alkaliphilus_oremlandii_OhILAs_chr.trna50-GlyGCC (2604016-2603942) Gly (GCC) 75 bp Sc: 94.96 18
GCGGAAGTGGCTCAGTGGTAGAGCATCGCCTTGCCAAGGCGAGGGTCGCGGGTTCGAGTC
CCGTCTTCCGCTCCA
>Alkaliphilus_oremlandii_OhILAs_chr.trna76-GlyGCC (1916013-1915939) Gly (GCC) 75 bp Sc: 94.96 18
GCGGAAGTGGCTCAGTGGTAGAGCATCGCCTTGCCAAGGCGAGGGTCGCGGGTTCGAGTC

CCGTCTTCCGCTCCA
>Alkaliphilus_oremlandii_OhILAs_chr.trna9-GlyGCC (617874-617948) Gly (GCC) 75 bp Sc: 94.96 18
GCGGAAGTGGCTCAGTGGTAGAGCATCGCCTTGCCAAGGCGAGGGTCGCGGGTTCGAGTC
CCGTCTTCCGCTCCA
>Alkaliphilus_oremlandii_OhILAs_chr.trna21-GlyTCC (2612075-2612002) Gly (TCC) 74 bp Sc: 83.64 19
GCGGGTGTAGTTCAATGGTAGAACGTCAGCCTTCCAAGCTGATCGCGAGGGTTCGATTCC
CTTCACCCGCTCCA
>Alkaliphilus_oremlandii_OhILAs_chr.trna38-GlyTCC (2610381-2610308) Gly (TCC) 74 bp Sc: 83.64 19
GCGGGTGTAGTTCAATGGTAGAACGTCAGCCTTCCAAGCTGATCGCGAGGGTTCGATTCC
CTTCACCCGCTCCA
>Alkaliphilus_oremlandii_OhILAs_chr.trna4-GlyTCC (617432-617505) Gly (TCC) 74 bp Sc: 83.64 19
GCGGGTGTAGTTCAATGGTAGAACGTCAGCCTTCCAAGCTGATCGCGAGGGTTCGATTCC
CTTCACCCGCTCCA
>Alkaliphilus_oremlandii_OhILAs_chr.trna70-GlyTCC (1923704-1923631) Gly (TCC) 74 bp Sc: 83.64 19
GCGGGTGTAGTTCAATGGTAGAACGTCAGCCTTCCAAGCTGATCGCGAGGGTTCGATTCC
CTTCACCCGCTCCA
>Ailuropoda_melanoleuca_GL193488.1.trna37-GlyCCC (585553-585481) Gly (CCC) 73 bp Sc: 44.89 16
ACCCAGCTGGCTCAGCTGGAAGAGCATGCGACTCCCTATCTCAGGGTTGTGAGTTCAAGC
CCCATGTTGGGTG
>Ailuropoda_melanoleuca_GL194257.1.trna6-GlyCCC (231134-231204) Gly (CCC) 71 bp Sc: 48.80 17
GCATTGGTGGTTCAGTGGTAGAATTCTTGCCTCCCACGTGGGAGAGCTGTGTTTGATTCC
CAGCCAATGCA
>Ailuropoda_melanoleuca_GL192781.1.trna53-GlyCCC (1229045-1228973) Gly (CCC) 73 bp Sc: 50.08 15
GCCTGGCTGGCTCAGTCAGAAAAGCGTGTGACTCCCGATCTCGGGGTCATGGGTTCAAGT
CCCACGTTGGGCA
>Ailuropoda_melanoleuca_GL192406.1.trna56-GlyCCC (2450118-2450188) Gly (CCC) 71 bp Sc: 76.98 16
GCGCCGCTGGTGTAGTGGTATCATGCAAGATTCCCATTCTTGCGACCCGGGTTCGATTCC
CGGGCGGCGCA
>Ailuropoda_melanoleuca_GL192733.1.trna61-GlyCCC (507191-507121) Gly (CCC) 71 bp Sc: 76.98 19
GCGCCGCTGGTGTAGTGGTATCATGCAAGATTCCCATTCTTGCGACCCGGGTTCGATTCC
CGGGCGGCGCA
>Ailuropoda_melanoleuca_GL193160.1.trna47-GlyCCC (916532-916462) Gly (CCC) 71 bp Sc: 80.05 19
GCATTGGTGGTTCAGTGGTAGAATTCTCGCCTCCCACGCGGGAGACCCGGGTTCGATTCC
CGGCCAATGCA
>Ailuropoda_melanoleuca_GL195218.1.trna5-GlyCCC (4083-4013) Gly (CCC) 71 bp Sc: 80.05 18
GCATTGGTGGTTCAGTGGTAGAATTCTCGCCTCCCACGCGGGAGACCCGGGTTCGATTCC
CGGCCAATGCA
>Ailuropoda_melanoleuca_GL193347.1.trna4-GlyGCC (157229-157299) Gly (GCC) 71 bp Sc: 66.28 17
GCATTGGTGGTTCAGTGGTAGAATTCTCGCCTGCCATGCGGGAGGCCCAGGTTTGATTTC
TGGCCAATGCA
>Ailuropoda_melanoleuca_GL192795.1.trna12-GlyGCC (370472-370542) Gly (GCC) 71 bp Sc: 81.62 18
GCATTGGTGGTTCAGTGGTAGAATTCTCGCCTGCCACGCGGGAGGCCCGGGTTCGATTCC
CGGCCAATGCA
>Ailuropoda_melanoleuca_GL192795.1.trna13-GlyGCC (371132-371202) Gly (GCC) 71 bp Sc: 81.62 18
GCATTGGTGGTTCAGTGGTAGAATTCTCGCCTGCCACGCGGGAGGCCCGGGTTCGATTCC
CGGCCAATGCA
>Ailuropoda_melanoleuca_GL192795.1.trna96-GlyGCC (362025-361955) Gly (GCC) 71 bp Sc: 81.62 18
GCATTGGTGGTTCAGTGGTAGAATTCTCGCCTGCCACGCGGGAGGCCCGGGTTCGATTCC
CGGCCAATGCA
>Ailuropoda_melanoleuca_GL192808.1.trna79-GlyGCC (1199023-1198953) Gly (GCC) 71 bp Sc: 81.62 18
GCATTGGTGGTTCAGTGGTAGAATTCTCGCCTGCCACGCGGGAGGCCCGGGTTCGATTCC
CGGCCAATGCA
>Ailuropoda_melanoleuca_GL193406.1.trna25-GlyGCC (116810-116740) Gly (GCC) 71 bp Sc: 81.62 18
GCATTGGTGGTTCAGTGGTAGAATTCTCGCCTGCCACGCGGGAGGCCCGGGTTCGATTCC
CGGCCAATGCA
>Ailuropoda_melanoleuca_GL193552.1.trna5-GlyGCC (104498-104568) Gly (GCC) 71 bp Sc: 81.62 18
GCATTGGTGGTTCAGTGGTAGAATTCTCGCCTGCCACGCGGGAGGCCCGGGTTCGATTCC
CGGCCAATGCA
>Ailuropoda_melanoleuca_GL193874.1.trna17-GlyGCC (379275-379205) Gly (GCC) 71 bp Sc: 81.62 18
GCATTGGTGGTTCAGTGGTAGAATTCTCGCCTGCCACGCGGGAGGCCCGGGTTCGATTCC
CGGCCAATGCA
>Ailuropoda_melanoleuca_GL194913.1.trna5-GlyGCC (100377-100447) Gly (GCC) 71 bp Sc: 81.62 18
GCATTGGTGGTTCAGTGGTAGAATTCTCGCCTGCCACGCGGGAGGCCCGGGTTCGATTCC
CGGCCAATGCA
>Ailuropoda_melanoleuca_GL195041.1.trna9-GlyGCC (40417-40347) Gly (GCC) 71 bp Sc: 81.62 18
GCATTGGTGGTTCAGTGGTAGAATTCTCGCCTGCCACGCGGGAGGCCCGGGTTCGATTCC
CGGCCAATGCA
>Ailuropoda_melanoleuca_GL192559.1.trna24-GlyTCC (1788749-1788821) Gly (TCC) 73 bp Sc: 32.28 15
ACCTGGCTGGCTCAGTCAGTAGAGCATGCGACTTCCGATCTTGGGTTTGTGAGTTCAAAG
CCCACGTTGGGTG
>Ailuropoda_melanoleuca_GL193376.1.trna15-GlyTCC (527758-527841) Gly (TCC) 84 bp Sc: 37.22 14
GCCTGACTGGCTCAGCGGGTTGAGCGTCTGCCTTCCACTCAGGTCATGGTCCCAGGGTCC
TAGGATCGAGTCCTGCGTCAGGCT
>Ailuropoda_melanoleuca_GL192808.1.trna85-GlyTCC (1127539-1127468) Gly (TCC) 72 bp Sc: 73.26 17

GCGTTGGTGGTATAGTGGTGAGCATAGCTGCCTTCCAAGCAGTTGACCCGGGTTCGATTC
CCGGCCAACGCA
>Ailuropoda_melanoleuca_GL193160.1.trna35-GlyTCC (833047-833118) Gly (TCC) 72 bp Sc: 73.26 17
GCGTTGGTGGTATAGTGGTGAGCATAGCTGCCTTCCAAGCAGTTGACCCGGGTTCGATTC
CCGGCCAACGCA
>Ailuropoda_melanoleuca_GL194913.1.trna8-GlyTCC (111712-111641) Gly (TCC) 72 bp Sc: 73.26 17
GCGTTGGTGGTATAGTGGTGAGCATAGCTGCCTTCCAAGCAGTTGACCCGGGTTCGATTC
CCGGCCAACGCA
>Ailuropoda_melanoleuca_GL194913.1.trna9-GlyTCC (91941-91870) Gly (TCC) 72 bp Sc: 73.26 17
GCGTTGGTGGTATAGTGGTGAGCATAGCTGCCTTCCAAGCAGTTGACCCGGGTTCGATTC
CCGGCCAACGCA
>Ailuropoda_melanoleuca_GL194164.1.trna5-GlyTCC (84198-84269) Gly (TCC) 72 bp Sc: 76.83 17
GCGTTGGTGGTATAGTGGTTAGCATAGCTGCCTTCCAAGCAGTTGACCCGGGTTCGATTC
CCGGCCAACGCA
>Anabaena_variabilis_ATCC_29413_chr.trna28-GlyCCC (5209484-5209413) Gly (CCC) 72 bp Sc: 72.00 16
GCGGGCGTAATTCAGTGGTAGAATGTCACCTTCCCAAGGTGAACGTCGTGGGTTCGAGTC
CCATCGCCCGCT
>Anabaena_variabilis_ATCC_29413_chr.trna40-GlyGCC (2133971-2133900) Gly (GCC) 72 bp Sc: 77.98 17
GCGGGTATAGCTCAGTGGTAGAGCGTCACCTTGCCAAGGTGAATGTCGCGCGTTCGAATC
GCGTTACCCGCT
>Anabaena_variabilis_ATCC_29413_chr.trna13-GlyTCC (4023869-4023939) Gly (TCC) 71 bp Sc: 66.79 16
GCGGGCGTAGTTTAGTGGTAAAACTATAGCCTTCCAAGCTATTAATGCGGGTTCGATTCC
CGCCGCCCGCT
>Anaplasma_phagocytophilum_HZ_chr.trna29-GlyGCC (920897-920826) Gly (GCC) 72 bp Sc: 78.14 17
GCGGGAGTAGCTCAGTGGTAGAGTATAACCTTGCCAAGGTTAGGGTCGAGGGTTCGAACC
CCTTCTCCCGCT
>Anaplasma_phagocytophilum_HZ_chr.trna7-GlyTCC (273587-273657) Gly (TCC) 71 bp Sc: 71.64 18
GCGGGTATAGCTCAATGGTAGAGTAGCAGCCTTCCAAGCTGCCTGTGTGGGTTCGATTCC
CATTACCCGCT
>Arcobacter_butzleri_RM4018_chr.trna21-GlyTCC (1876218-1876294) Gly (TCC) 77 bp Sc: 91.96 15
GCGGGAGTAGCTCAGTTGGCTAGAGCTTCTGCCTTCCAAGCAGACTGTCGCGAGTTCGAG
TCTCGTCTCCCGCTCCA
>Arcobacter_butzleri_RM4018_chr.trna39-GlyTCC (1898064-1897988) Gly (TCC) 77 bp Sc: 91.96 15
GCGGGAGTAGCTCAGTTGGCTAGAGCTTCTGCCTTCCAAGCAGACTGTCGCGAGTTCGAG
TCTCGTCTCCCGCTCCA
>Arcobacter_butzleri_RM4018_chr.trna5-GlyTCC (169789-169865) Gly (TCC) 77 bp Sc: 91.96 15
GCGGGAGTAGCTCAGTTGGCTAGAGCTTCTGCCTTCCAAGCAGACTGTCGCGAGTTCGAG
TCTCGTCTCCCGCTCCA
>Arcobacter_butzleri_RM4018_chr.trna7-GlyTCC (170017-170093) Gly (TCC) 77 bp Sc: 91.96 15
GCGGGAGTAGCTCAGTTGGCTAGAGCTTCTGCCTTCCAAGCAGACTGTCGCGAGTTCGAG
TCTCGTCTCCCGCTCCA
>Arthrobacter_FB24_chr1.trna26-GlyCCC (4364506-4364579) Gly (CCC) 74 bp Sc: 81.46 18
GCGGCTGTAGCTCAATGGTAGAGCGCTAGCTTCCCAAGCTTGATACGCGGGTTCGATTCC
CGTCAGCCGCTCCA
>Arthrobacter_FB24_chr1.trna41-GlyGCC (1863537-1863465) Gly (GCC) 73 bp Sc: 85.80 17
GCGGACGTAGCTCAGCTGGTAGAGCACCACCTTGCCAAGGTGGATGTCGCGAGTTCGAAT
CTCGTCGTCCGCT
>Arthrobacter_FB24_chr1.trna44-GlyGCC (1863175-1863103) Gly (GCC) 73 bp Sc: 85.80 17
GCGGACGTAGCTCAGCTGGTAGAGCACCACCTTGCCAAGGTGGATGTCGCGAGTTCGAAT
CTCGTCGTCCGCT
>Arthrobacter_FB24_chr1.trna19-GlyTCC (2701975-2702048) Gly (TCC) 74 bp Sc: 75.34 19
GGGGACGTAGCTTAATGGTAAAGCCTCAGTCTTCCAAACTGATTACGCGGGTTCGATTCC
CGTCGTCCCCTCCA
>Arthrobacter_aurescens_TC1_chr.trna25-GlyCCC (4045671-4045744) Gly (CCC) 74 bp Sc: 79.70 17
GCGGGCGTAGCTCAATGGTAGAGCGCTAGCTTCCCAAGCTCGATACGCGGGTTCGATTCC
CGTCGCCCGCTCCA
>Arthrobacter_aurescens_TC1_chr.trna42-GlyGCC (1987872-1987800) Gly (GCC) 73 bp Sc: 85.80 18
GCGGACGTAGCTCAGCTGGTAGAGCACCACCTTGCCAAGGTGGATGTCGCGAGTTCGAAT
CTCGTCGTCCGCT
>Arthrobacter_aurescens_TC1_chr.trna45-GlyGCC (1987512-1987440) Gly (GCC) 73 bp Sc: 85.80 18
GCGGACGTAGCTCAGCTGGTAGAGCACCACCTTGCCAAGGTGGATGTCGCGAGTTCGAAT
CTCGTCGTCCGCT
>Arthrobacter_aurescens_TC1_chr.trna47-GlyGCC (1987283-1987208) Gly (GCC) 76 bp Sc: 94.10 17
GCGGACGTAGCTCAGCTGGTAGAGCACCACCTTGCCAAGGTGGATGTCGCGAGTTCGAAT
CTCGTCGTCCGCTCCA
>Arthrobacter_aurescens_TC1_chr.trna18-GlyTCC (2625965-2626038) Gly (TCC) 74 bp Sc: 75.34 19
GGGGACGTAGCTTAATGGTAAAGCCTCAGTCTTCCAAACTGATTACGCGGGTTCGATTCC
CGTCGTCCCCTCCA
>Aspergillus_fumigatus_chr4.trna9-GlyCCC (2307984-2307898) Gly (CCC) 87 bp Sc: 60.39 17
GCGCCAATGGTCTAGTGGTTAGGATGCGGTCCTCCCATTCCCCTACTGGGCAAGACCGAG
ACACGAGTTCGAATCCCGTTTGGCGCA
>Aspergillus_fumigatus_chr1.trna13-GlyGCC (2907410-2907480) Gly (GCC) 71 bp Sc: 66.83 17
GCATCATTGGTCTAGTGGTAGAATTCGTCGTTGCCATCGACGAGGCCCGTGTTCGATTCA
CGGATGATGCA

>Aspergillus_fumigatus_chr1.trna7-GlyGCC (1651256-1651326) Gly (GCC) 71 bp Sc: 66.83 17
GCATCATTGGTCTAGTGGTAGAATTCGTCGTTGCCATCGACGAGGCCCGTGTTCGATTCA
CGGATGATGCA
>Aspergillus_fumigatus_chr2.trna23-GlyGCC (465305-465235) Gly (GCC) 71 bp Sc: 66.83 17
GCATCATTGGTCTAGTGGTAGAATTCGTCGTTGCCATCGACGAGGCCCGTGTTCGATTCA
CGGATGATGCA
>Aspergillus_fumigatus_chr3.trna13-GlyGCC (3556767-3556837) Gly (GCC) 71 bp Sc: 66.83 17
GCATCATTGGTCTAGTGGTAGAATTCGTCGTTGCCATCGACGAGGCCCGTGTTCGATTCA
CGGATGATGCA
>Aspergillus_fumigatus_chr4.trna6-GlyGCC (3650153-3650223) Gly (GCC) 71 bp Sc: 66.83 17
GCATCATTGGTCTAGTGGTAGAATTCGTCGTTGCCATCGACGAGGCCCGTGTTCGATTCA
CGGATGATGCA
>Aspergillus_fumigatus_chr4.trna8-GlyGCC (2409096-2409026) Gly (GCC) 71 bp Sc: 66.83 17
GCATCATTGGTCTAGTGGTAGAATTCGTCGTTGCCATCGACGAGGCCCGTGTTCGATTCA
CGGATGATGCA
>Aspergillus_fumigatus_chr5.trna33-GlyGCC (1903658-1903588) Gly (GCC) 71 bp Sc: 66.83 17
GCATCATTGGTCTAGTGGTAGAATTCGTCGTTGCCATCGACGAGGCCCGTGTTCGATTCA
CGGATGATGCA
>Aspergillus_fumigatus_chr5.trna35-GlyGCC (321680-321610) Gly (GCC) 71 bp Sc: 66.83 17
GCATCATTGGTCTAGTGGTAGAATTCGTCGTTGCCATCGACGAGGCCCGTGTTCGATTCA
CGGATGATGCA
>Aspergillus_fumigatus_chr5.trna9-GlyGCC (3086241-3086311) Gly (GCC) 71 bp Sc: 66.83 17
GCATCATTGGTCTAGTGGTAGAATTCGTCGTTGCCATCGACGAGGCCCGTGTTCGATTCA
CGGATGATGCA
>Aspergillus_fumigatus_chr6.trna9-GlyGCC (3477652-3477722) Gly (GCC) 71 bp Sc: 66.83 17
GCATCATTGGTCTAGTGGTAGAATTCGTCGTTGCCATCGACGAGGCCCGTGTTCGATTCA
CGGATGATGCA
>Aspergillus_fumigatus_chr8.trna16-GlyGCC (665850-665780) Gly (GCC) 71 bp Sc: 66.83 17
GCATCATTGGTCTAGTGGTAGAATTCGTCGTTGCCATCGACGAGGCCCGTGTTCGATTCA
CGGATGATGCA
>Aspergillus_fumigatus_chr1.trna19-GlyTCC (4157779-4157850) Gly (TCC) 72 bp Sc: 71.91 16
GCGTTAGTGGTGTAGCGGTTAGCATTGTTGCCTTCCAAGCAATAGATCTGGGTTCGACTC
CCAGCTAACGCA
>Aspergillus_fumigatus_chr5.trna30-GlyTCC (2561769-2561698) Gly (TCC) 72 bp Sc: 71.91 16
GCGTTAGTGGTGTAGCGGTTAGCATTGTTGCCTTCCAAGCAATAGATCTGGGTTCGACTC
CCAGCTAACGCA
>Aspergillus_fumigatus_chr7.trna4-GlyTCC (220338-220409) Gly (TCC) 72 bp Sc: 71.91 16
GCGTTAGTGGTGTAGCGGTTAGCATTGTTGCCTTCCAAGCAATAGATCTGGGTTCGACTC
CCAGCTAACGCA
>Arabidopsis_thaliana_chr1.trna215-GlyACC (12097327-12097259) Gly (ACC) 69 bp Sc: 20.99 18
GCACCAATGGTTCAGTGGTAGATTAATACTCTACCACTATCTAACCCGGGTTCGATCCCA
GACGGTGTA
>Arabidopsis_thaliana_chr2.trna60-GlyCCC (19566995-19566925) Gly (CCC) 71 bp Sc: 68.50 17
GCGCATCTGGTGTAGTGGTATCATAGTACCCTCCCACGGTACTGACCAGGGTTCGATTCC
CTGGATGCGCA
>Arabidopsis_thaliana_chr3.trna60-GlyCCC (23221683-23221753) Gly (CCC) 71 bp Sc: 68.50 17
GCGCATCTGGTGTAGTGGTATCATAGTACCCTCCCACGGTACTGACCAGGGTTCGATTCC
CTGGATGCGCA
>Arabidopsis_thaliana_chr4.trna59-GlyCCC (12486282-12486212) Gly (CCC) 71 bp Sc: 68.50 17
GCGCATCTGGTGTAGTGGTATCATAGTACCCTCCCACGGTACTGACCAGGGTTCGATTCC
CTGGATGCGCA
>Arabidopsis_thaliana_chr4.trna6-GlyCCC (6646426-6646496) Gly (CCC) 71 bp Sc: 68.50 17
GCGCATCTGGTGTAGTGGTATCATAGTACCCTCCCACGGTACTGACCAGGGTTCGATTCC
CTGGATGCGCA
>Arabidopsis_thaliana_chr5.trna78-GlyCCC (21740410-21740340) Gly (CCC) 71 bp Sc: 68.50 17
GCGCATCTGGTGTAGTGGTATCATAGTACCCTCCCACGGTACTGACCAGGGTTCGATTCC
CTGGATGCGCA
>Arabidopsis_thaliana_chr1.trna7-GlyGCC (1159022-1159092) Gly (GCC) 71 bp Sc: 57.43 18
TAACCAGTGGTCTAGTGGTAGAATAGTACCCTGCCACGGTACAGACCCGGGTTCGATTCC
CGGCTGGTGCA
>Arabidopsis_thaliana_chr2.trna88-GlyGCC (3444431-3444360) Gly (GCC) 72 bp Sc: 62.60 20
GCGGAAATAGCTTAATGGTAGAGCATAGCCTTGCCAAGGCTAAGGTTGAGGGTTCAAGTC
CCTCCTTCCGCT
>Arabidopsis_thaliana_chr3.trna91-GlyGCC (1843400-1843330) Gly (GCC) 71 bp Sc: 65.04 18
GCACCAGTGGTCTAGTGGTAGAATAGAACCCTGCCACGGTACAGACCCGGGTTCGATTCC
CGGCTGGTGCA
>Arabidopsis_thaliana_chr5.trna122-GlyGCC (389960-389890) Gly (GCC) 71 bp Sc: 66.11 18
GCACCAGTGGTCTAGTGGCATGATAGTACCCTGCCACGGTACATACCCGGGTTCAATTCC
CGGCTGGTGCA
>Arabidopsis_thaliana_chr1.trna201-GlyGCC (22401327-22401257) Gly (GCC) 71 bp Sc: 67.59 18
GCACCAGTGGTCTAGTGGTAGAATAGTACTCTGCCACGGTACAGACCCGGGTTCGATTCC
CGGCTGGTGCA
>Arabidopsis_thaliana_chr1.trna161-GlyGCC (22431832-22431902) Gly (GCC) 71 bp Sc: 69.82 18
GCACCAGTGGTCTAGTGGTAGAATAGTACCCTGCCACGGTACAGACCCGGGTTCAATTCC

CGGCTGGTGCA
>Arabidopsis_thaliana_chr2.trna54-GlyGCC (17667856-17667926) Gly (GCC) 71 bp Sc: 69.82 18
GCACCAGTGGTCTAGTGGTAGAATAGTACCCTGCCACGGTACAGACCCGGGTTCAATTCC
CGGCTGGTGCA
>Arabidopsis_thaliana_chr1.trna171-GlyGCC (26977051-26977121) Gly (GCC) 71 bp Sc: 70.74 18
GCACCAGTGGTCTAGTGGCATGATAGTACCCTGCCACGGTACAGACCCGGGTTCAATTCC
CGGCTGGTGCA
>Arabidopsis_thaliana_chr5.trna87-GlyGCC (17512839-17512769) Gly (GCC) 71 bp Sc: 70.74 18
GCACCAGTGGTCTAGTGGCATGATAGTACCCTGCCACGGTACAGACCCGGGTTCAATTCC
CGGCTGGTGCA
>Arabidopsis_thaliana_chr1.trna202-GlyGCC (22398372-22398302) Gly (GCC) 71 bp Sc: 71.56 18
GCACCAGTGGTCTAGTGGTAGAATAGTACCCTGCCACGGTACAGACCCGGGTTCGATTCC
CGGCTGGTGCA
>Arabidopsis_thaliana_chr1.trna203-GlyGCC (22317149-22317079) Gly (GCC) 71 bp Sc: 71.56 18
GCACCAGTGGTCTAGTGGTAGAATAGTACCCTGCCACGGTACAGACCCGGGTTCGATTCC
CGGCTGGTGCA
>Arabidopsis_thaliana_chr1.trna240-GlyGCC (2111217-2111147) Gly (GCC) 71 bp Sc: 71.56 18
GCACCAGTGGTCTAGTGGTAGAATAGTACCCTGCCACGGTACAGACCCGGGTTCGATTCC
CGGCTGGTGCA
>Arabidopsis_thaliana_chr1.trna241-GlyGCC (2107467-2107397) Gly (GCC) 71 bp Sc: 71.56 18
GCACCAGTGGTCTAGTGGTAGAATAGTACCCTGCCACGGTACAGACCCGGGTTCGATTCC
CGGCTGGTGCA
>Arabidopsis_thaliana_chr2.trna32-GlyGCC (12888668-12888738) Gly (GCC) 71 bp Sc: 71.56 18
GCACCAGTGGTCTAGTGGTAGAATAGTACCCTGCCACGGTACAGACCCGGGTTCGATTCC
CGGCTGGTGCA
>Arabidopsis_thaliana_chr2.trna33-GlyGCC (12889811-12889881) Gly (GCC) 71 bp Sc: 71.56 18
GCACCAGTGGTCTAGTGGTAGAATAGTACCCTGCCACGGTACAGACCCGGGTTCGATTCC
CGGCTGGTGCA
>Arabidopsis_thaliana_chr2.trna72-GlyGCC (12983095-12983025) Gly (GCC) 71 bp Sc: 71.56 18
GCACCAGTGGTCTAGTGGTAGAATAGTACCCTGCCACGGTACAGACCCGGGTTCGATTCC
CGGCTGGTGCA
>Arabidopsis_thaliana_chr3.trna47-GlyGCC (19995919-19995989) Gly (GCC) 71 bp Sc: 71.56 18
GCACCAGTGGTCTAGTGGTAGAATAGTACCCTGCCACGGTACAGACCCGGGTTCGATTCC
CGGCTGGTGCA
>Arabidopsis_thaliana_chr3.trna51-GlyGCC (21553259-21553329) Gly (GCC) 71 bp Sc: 71.56 18
GCACCAGTGGTCTAGTGGTAGAATAGTACCCTGCCACGGTACAGACCCGGGTTCGATTCC
CGGCTGGTGCA
>Arabidopsis_thaliana_chr3.trna56-GlyGCC (22853497-22853567) Gly (GCC) 71 bp Sc: 71.56 18
GCACCAGTGGTCTAGTGGTAGAATAGTACCCTGCCACGGTACAGACCCGGGTTCGATTCC
CGGCTGGTGCA
>Arabidopsis_thaliana_chr4.trna33-GlyGCC (15671399-15671469) Gly (GCC) 71 bp Sc: 71.56 18
GCACCAGTGGTCTAGTGGTAGAATAGTACCCTGCCACGGTACAGACCCGGGTTCGATTCC
CGGCTGGTGCA
>Arabidopsis_thaliana_chr5.trna113-GlyGCC (2212749-2212679) Gly (GCC) 71 bp Sc: 71.56 18
GCACCAGTGGTCTAGTGGTAGAATAGTACCCTGCCACGGTACAGACCCGGGTTCGATTCC
CGGCTGGTGCA
>Arabidopsis_thaliana_chr5.trna23-GlyGCC (6401241-6401311) Gly (GCC) 71 bp Sc: 71.56 18
GCACCAGTGGTCTAGTGGTAGAATAGTACCCTGCCACGGTACAGACCCGGGTTCGATTCC
CGGCTGGTGCA
>Arabidopsis_thaliana_chr5.trna65-GlyGCC (26700004-26699934) Gly (GCC) 71 bp Sc: 71.56 18
GCACCAGTGGTCTAGTGGTAGAATAGTACCCTGCCACGGTACAGACCCGGGTTCGATTCC
CGGCTGGTGCA
>Arabidopsis_thaliana_chr1.trna14-GlyTCC (2588522-2588593) Gly (TCC) 72 bp Sc: 75.78 15
GCGTCTGTAGTCCAACGGTTAGGATAATTGCCTTCCAAGCAATAGACCCGGGTTCGACTC
CCGGCAGACGCA
>Arabidopsis_thaliana_chr1.trna232-GlyTCC (4285956-4285885) Gly (TCC) 72 bp Sc: 75.78 15
GCGTCTGTAGTCCAACGGTTAGGATAATTGCCTTCCAAGCAATAGACCCGGGTTCGACTC
CCGGCAGACGCA
>Arabidopsis_thaliana_chr2.trna74-GlyTCC (12478921-12478850) Gly (TCC) 72 bp Sc: 75.78 15
GCGTCTGTAGTCCAACGGTTAGGATAATTGCCTTCCAAGCAATAGACCCGGGTTCGACTC
CCGGCAGACGCA
>Arabidopsis_thaliana_chr2.trna75-GlyTCC (11871302-11871231) Gly (TCC) 72 bp Sc: 75.78 15
GCGTCTGTAGTCCAACGGTTAGGATAATTGCCTTCCAAGCAATAGACCCGGGTTCGACTC
CCGGCAGACGCA
>Arabidopsis_thaliana_chr3.trna35-GlyTCC (17160737-17160808) Gly (TCC) 72 bp Sc: 75.78 15
GCGTCTGTAGTCCAACGGTTAGGATAATTGCCTTCCAAGCAATAGACCCGGGTTCGACTC
CCGGCAGACGCA
>Arabidopsis_thaliana_chr3.trna74-GlyTCC (16438097-16438026) Gly (TCC) 72 bp Sc: 75.78 15
GCGTCTGTAGTCCAACGGTTAGGATAATTGCCTTCCAAGCAATAGACCCGGGTTCGACTC
CCGGCAGACGCA
>Arabidopsis_thaliana_chr4.trna63-GlyTCC (10326047-10325976) Gly (TCC) 72 bp Sc: 75.78 15
GCGTCTGTAGTCCAACGGTTAGGATAATTGCCTTCCAAGCAATAGACCCGGGTTCGACTC
CCGGCAGACGCA
>Arabidopsis_thaliana_chr5.trna105-GlyTCC (3578367-3578296) Gly (TCC) 72 bp Sc: 75.78 15

GCGTCTGTAGTCCAACGGTTAGGATAATTGCCTTCCAAGCAATAGACCCGGGTTCGACTC
CCGGCAGACGCA
>Arabidopsis_thaliana_chr5.trna13-GlyTCC (3617059-3617130) Gly (TCC) 72 bp Sc: 75.78 15
GCGTCTGTAGTCCAACGGTTAGGATAATTGCCTTCCAAGCAATAGACCCGGGTTCGACTC
CCGGCAGACGCA
>Arabidopsis_thaliana_chr5.trna34-GlyTCC (18576072-18576143) Gly (TCC) 72 bp Sc: 75.78 15
GCGTCTGTAGTCCAACGGTTAGGATAATTGCCTTCCAAGCAATAGACCCGGGTTCGACTC
CCGGCAGACGCA
>Arabidopsis_thaliana_chr5.trna50-GlyTCC (23971168-23971239) Gly (TCC) 72 bp Sc: 75.78 15
GCGTCTGTAGTCCAACGGTTAGGATAATTGCCTTCCAAGCAATAGACCCGGGTTCGACTC
CCGGCAGACGCA
>Arabidopsis_thaliana_chr5.trna51-GlyTCC (23974656-23974727) Gly (TCC) 72 bp Sc: 75.78 15
GCGTCTGTAGTCCAACGGTTAGGATAATTGCCTTCCAAGCAATAGACCCGGGTTCGACTC
CCGGCAGACGCA
>Arabidopsis_thaliana_chr1.trna63-GlyGCC (12083875-12083945) Gly (GCC) 71 bp Sc: 27.76 16
GCATTAGTCGTCTAGTGGTTGTATTGTAGCCTGCCACTTGACAGTCCAGGGTTCGATTCC
CGGATGGTGCA
>Azoarcus_BH72_chr.trna28-GlyCCC (4144876-4144950) Gly (CCC) 75 bp Sc: 60.99 14
GCGGGCGTCGTATAACGGCTATTACCTCAGCTTCCCAAGCTGATGACGAGGGTTCGACTC
CCTTCGCCCGCTCCA
>Azoarcus_BH72_chr.trna23-GlyGCC (1809899-1809974) Gly (GCC) 76 bp Sc: 88.26 16
GCGGGAATAGCTCAGTTGGTAGAGCGCAACCTTGCCAAGGTTGAGGTCGCGAGTTCGAGA
CTCGTTTCCCGCTCCA
>Azoarcus_BH72_chr.trna25-GlyGCC (1810132-1810207) Gly (GCC) 76 bp Sc: 88.26 16
GCGGGAATAGCTCAGTTGGTAGAGCGCAACCTTGCCAAGGTTGAGGTCGCGAGTTCGAGA
CTCGTTTCCCGCTCCA
>Azoarcus_BH72_chr.trna30-GlyTCC (3760855-3760782) Gly (TCC) 74 bp Sc: 82.17 19
GCGGGTGTAGCTCAATGGTAGAGCAGAAGCCTTCCAAGCTTACGACGAGGGTTCGATTCC
CTTCACCCGCTCCA
>Azoarcus_sp_EbN1_chr.trna8-GlyCCC (1383723-1383796) Gly (CCC) 74 bp Sc: 65.01 17
GCGGGCGTCGTATAATGGTAATACCCTAGCTTCCCAAGCTAGAGCCGTGGGTTCGATTCC
CATCGCCCGCTCCA
>Azoarcus_sp_EbN1_chr.trna32-GlyGCC (3304938-3305013) Gly (GCC) 76 bp Sc: 88.26 16
GCGGGAATAGCTCAGTTGGTAGAGCGCAACCTTGCCAAGGTTGAGGTCGCGAGTTCGAGA
CTCGTTTCCCGCTCCA
>Azoarcus_sp_EbN1_chr.trna35-GlyGCC (3400810-3400885) Gly (GCC) 76 bp Sc: 88.26 16
GCGGGAATAGCTCAGTTGGTAGAGCGCAACCTTGCCAAGGTTGAGGTCGCGAGTTCGAGA
CTCGTTTCCCGCTCCA
>Azoarcus_sp_EbN1_chr.trna16-GlyTCC (2242786-2242859) Gly (TCC) 74 bp Sc: 82.26 19
GCGGGTGTAGCTCAATGGTAGAGCAGAAGCCTTCCAAGCTTATGACGAGGGTTCGATTCC
CTTCACCCGCTCCA
>Azorhizobium_caulinodans_ORS_571_chr.trna53-GlyCCC (260304-260230) Gly (CCC) 75 bp Sc: 90.16 16
GCGGGCGTAGTTCAGTGGTAGAACGACAGCTTCCCAAGCTGTATGTCGTGGGTTCGATTC
CCATCGCCCGCTCCA
>Azorhizobium_caulinodans_ORS_571_chr.trna26-GlyGCC (4346061-4346135) Gly (GCC) 75 bp Sc: 88.17 17
GCGGGTGTAGCTCAGGGGTAGAGCACAACCTTGCCAAGGTTGGGGTCGAGGGTTCGAATC
CCTTCGCCCGCTCCA
>Azorhizobium_caulinodans_ORS_571_chr.trna7-GlyTCC (981811-981884) Gly (TCC) 74 bp Sc: 82.42 19
GCGGGTGTAGCTCAATGGTAGAGCAACAGCCTTCCAAGCTGATGACGAGGGTTCGATTCC
CTTCACCCGCTCCA
>Bacillus_amyloliquefaciens_FZB42_chr.trna15-GlyGCC (167072-167146) Gly (GCC) 75 bp Sc: 90.25 18
GCGGAAGTAGTTCAGTGGTAGAACATCACCTTGCCAAGGTGGGGGTCGCGGGTTCGAATC
CCGTCTTCCGCTCCA
>Bacillus_amyloliquefaciens_FZB42_chr.trna51-GlyGCC (882172-882246) Gly (GCC) 75 bp Sc: 90.25 17
GCGGAAGTAGTTCAGTGGTAGAACATCACCTTGCCAAGGTGGGGGTCGCGGGTTCGAATC
CCGTCTTCCGCTCCA
>Bacillus_amyloliquefaciens_FZB42_chr.trna70-GlyGCC (2922228-2922154) Gly (GCC) 75 bp Sc: 90.25 17
GCGGAAGTAGTTCAGTGGTAGAACATCACCTTGCCAAGGTGGGGGTCGCGGGTTCGAATC
CCGTCTTCCGCTCCA
>Bacillus_amyloliquefaciens_FZB42_chr.trna8-GlyGCC (96464-96538) Gly (GCC) 75 bp Sc: 94.93 17
GCGGAAGTAGTTCAGTGGTAGAACACCACCTTGCCAAGGTGGGGGTCGCGGGTTCGAATC
CCGTCTTCCGCTCCA
>Bacillus_amyloliquefaciens_FZB42_chr.trna36-GlyTCC (604884-604957) Gly (TCC) 74 bp Sc: 78.55 18
GCGGGTGTAGTTTAGTGGTAAAACCTCAGCCTTCCAAGCTGATGTCGTGAGTTCGATTCT
CATCACCCGCTCCA
>Bacillus_amyloliquefaciens_FZB42_chr.trna55-GlyTCC (897337-897410) Gly (TCC) 74 bp Sc: 78.55 18
GCGGGTGTAGTTTAGTGGTAAAACCTCAGCCTTCCAAGCTGATGTCGTGAGTTCGATTCT
CATCACCCGCTCCA
>Bacillus_amyloliquefaciens_FZB42_chr.trna82-GlyTCC (2921146-2921073) Gly (TCC) 74 bp Sc: 81.36 18
GCGGGTGTAGTTTAGTGGTAAAACCTCAGCCTTCCAAGCTGATGTCGTGGGTTCGATTCC
CATCACCCGCTCCA
>Bacillus_anthracis_A2012_,_unfinished_sequence.trna15-GlyGCC (1049307-1049381) Gly (GCC) 75 bp Sc: 88.86 18
GCGGAAGTAGTTCAGTGGTAGAATACAACCTTGCCAAGGTTGGGGTCGCGGGTTCGAATC
CCGTCTTCCGCTCCA

>Bacillus_anthracis_A2012_,_unfinished_sequence.trna18-GlyTCC (1053988-1054061) Gly (TCC) 74 bp Sc: 60.03 17
TCCGGAGTAGTTTAGTGGTAAAACAAGAGCCTTCCAAGCTCTGGTCGAGAGTTCGATTCT
CTTCACCCGCTCCA
>Bacillus_anthracis_Ames_chr.trna15-GlyGCC (150978-151049) Gly (GCC) 72 bp Sc: 80.56 18
GCGGAAGTAGTTCAGTGGTAGAATACAACCTTGCCAAGGTTGGGGTCGCGGGTTCGAATC
CCGTCTTCCGCT
>Bacillus_anthracis_Ames_chr.trna42-GlyGCC (538386-538460) Gly (GCC) 75 bp Sc: 88.86 18
GCGGAAGTAGTTCAGTGGTAGAATACAACCTTGCCAAGGTTGGGGTCGCGGGTTCGAATC
CCGTCTTCCGCTCCA
>Bacillus_anthracis_Ames_chr.trna50-GlyGCC (746745-746819) Gly (GCC) 75 bp Sc: 88.86 18
GCGGAAGTAGTTCAGTGGTAGAATACAACCTTGCCAAGGTTGGGGTCGCGGGTTCGAATC
CCGTCTTCCGCTCCA
>Bacillus_anthracis_Ames_chr.trna74-GlyGCC (4651530-4651456) Gly (GCC) 75 bp Sc: 88.86 19
GCGGAAGTAGTTCAGTGGTAGAATACAACCTTGCCAAGGTTGGGGTCGCGGGTTCGAATC
CCGTCTTCCGCTCCA
>Bacillus_anthracis_Ames_chr.trna27-GlyTCC (246061-246131) Gly (TCC) 71 bp Sc: 71.78 18
GCGGGTGTAGTTTAGTGGTAAAACAAGAGCCTTCCAAGCTCTGGTCGAGAGTTCGATTCT
CTTCACCCGCT
>Bacillus_anthracis_Ames_chr.trna87-GlyTCC (4650354-4650284) Gly (TCC) 71 bp Sc: 71.78 16
GCGGGTGTAGTTTAGTGGTAAAACAAGAGCCTTCCAAGCTCTGGTCGAGAGTTCGATTCT
CTTCACCCGCT
>Bacillus_anthracis_Ames_chr.trna45-GlyTCC (543071-543144) Gly (TCC) 74 bp Sc: 80.08 18
GCGGGTGTAGTTTAGTGGTAAAACAAGAGCCTTCCAAGCTCTGGTCGAGAGTTCGATTCT
CTTCACCCGCTCCA
>Bacillus_anthracis_Ames_chr.trna92-GlyTCC (4287652-4287579) Gly (TCC) 74 bp Sc: 80.08 18
GCGGGTGTAGTTTAGTGGTAAAACAAGAGCCTTCCAAGCTCTGGTCGAGAGTTCGATTCT
CTTCACCCGCTCCA
>Bacillus_cereus_ATCC_10987_chr.trna15-GlyGCC (150984-151055) Gly (GCC) 72 bp Sc: 80.56 19
GCGGAAGTAGTTCAGTGGTAGAATACAACCTTGCCAAGGTTGGGGTCGCGGGTTCGAATC
CCGTCTTCCGCT
>Bacillus_cereus_ATCC_10987_chr.trna44-GlyGCC (610888-610962) Gly (GCC) 75 bp Sc: 88.86 19
GCGGAAGTAGTTCAGTGGTAGAATACAACCTTGCCAAGGTTGGGGTCGCGGGTTCGAATC
CCGTCTTCCGCTCCA
>Bacillus_cereus_ATCC_10987_chr.trna53-GlyGCC (823438-823512) Gly (GCC) 75 bp Sc: 88.86 19
GCGGAAGTAGTTCAGTGGTAGAATACAACCTTGCCAAGGTTGGGGTCGCGGGTTCGAATC
CCGTCTTCCGCTCCA
>Bacillus_cereus_ATCC_10987_chr.trna77-GlyGCC (4650508-4650434) Gly (GCC) 75 bp Sc: 88.86 19
GCGGAAGTAGTTCAGTGGTAGAATACAACCTTGCCAAGGTTGGGGTCGCGGGTTCGAATC
CCGTCTTCCGCTCCA
>Bacillus_cereus_ATCC_10987_chr.trna27-GlyTCC (279452-279522) Gly (TCC) 71 bp Sc: 71.78 18
GCGGGTGTAGTTTAGTGGTAAAACAAGAGCCTTCCAAGCTCTGGTCGAGAGTTCGATTCT
CTTCACCCGCT
>Bacillus_cereus_ATCC_10987_chr.trna90-GlyTCC (4649333-4649263) Gly (TCC) 71 bp Sc: 71.78 18
GCGGGTGTAGTTTAGTGGTAAAACAAGAGCCTTCCAAGCTCTGGTCGAGAGTTCGATTCT
CTTCACCCGCT
>Bacillus_cereus_ATCC_10987_chr.trna47-GlyTCC (615179-615252) Gly (TCC) 74 bp Sc: 80.08 18
GCGGGTGTAGTTTAGTGGTAAAACAAGAGCCTTCCAAGCTCTGGTCGAGAGTTCGATTCT
CTTCACCCGCTCCA
>Bacillus_cereus_ATCC_10987_chr.trna95-GlyTCC (4248374-4248301) Gly (TCC) 74 bp Sc: 80.08 18
GCGGGTGTAGTTTAGTGGTAAAACAAGAGCCTTCCAAGCTCTGGTCGAGAGTTCGATTCT
CTTCACCCGCTCCA
>Bacillus_licheniformis_ATCC_14580_chr.trna55-GlyGCC (3116926-3116855) Gly (GCC) 72 bp Sc: 86.63 18
GCGGAAGTAGTTCAGTGGTAGAACACCACCTTGCCAAGGTGGGGGTCGCGGGTTCGAATC
CCGTCTTCCGCT
>Bacillus_licheniformis_ATCC_14580_chr.trna38-GlyGCC (926641-926715) Gly (GCC) 75 bp Sc: 94.93 18
GCGGAAGTAGTTCAGTGGTAGAACACCACCTTGCCAAGGTGGGGGTCGCGGGTTCGAATC
CCGTCTTCCGCTCCA
>Bacillus_licheniformis_ATCC_14580_chr.trna23-GlyTCC (611775-611848) Gly (TCC) 74 bp Sc: 78.55 18
GCGGGTGTAGTTTAGTGGTAAAACCTCAGCCTTCCAAGCTGATGTCGTGAGTTCGATTCT
CATCACCCGCTCCA
>Bacillus_licheniformis_ATCC_14580_chr.trna42-GlyTCC (953171-953244) Gly (TCC) 74 bp Sc: 81.36 18
GCGGGTGTAGTTTAGTGGTAAAACCTCAGCCTTCCAAGCTGATGTCGTGGGTTCGATTCC
CATCACCCGCTCCA
>Bacillus_licheniformis_ATCC_14580_chr.trna66-GlyTCC (3115907-3115834) Gly (TCC) 74 bp Sc: 81.36 18
GCGGGTGTAGTTTAGTGGTAAAACCTCAGCCTTCCAAGCTGATGTCGTGGGTTCGATTCC
CATCACCCGCTCCA
>Bacillus_licheniformis_DSM_13_chr.trna55-GlyGCC (3117103-3117032) Gly (GCC) 72 bp Sc: 86.63 18
GCGGAAGTAGTTCAGTGGTAGAACACCACCTTGCCAAGGTGGGGGTCGCGGGTTCGAATC
CCGTCTTCCGCT
>Bacillus_licheniformis_DSM_13_chr.trna38-GlyGCC (926934-927008) Gly (GCC) 75 bp Sc: 94.93 18
GCGGAAGTAGTTCAGTGGTAGAACACCACCTTGCCAAGGTGGGGGTCGCGGGTTCGAATC
CCGTCTTCCGCTCCA
>Bacillus_licheniformis_DSM_13_chr.trna23-GlyTCC (611567-611640) Gly (TCC) 74 bp Sc: 78.55 18
GCGGGTGTAGTTTAGTGGTAAAACCTCAGCCTTCCAAGCTGATGTCGTGAGTTCGATTCT

CATCACCCGCTCCA
>Bacillus_licheniformis_DSM_13_chr.trna42-GlyTCC (953465-953538) Gly (TCC) 74 bp Sc: 81.36 18
GCGGGTGTAGTTTAGTGGTAAAACCTCAGCCTTCCAAGCTGATGTCGTGGGTTCGATTCC
CATCACCCGCTCCA
>Bacillus_licheniformis_DSM_13_chr.trna66-GlyTCC (3116084-3116011) Gly (TCC) 74 bp Sc: 81.36 18
GCGGGTGTAGTTTAGTGGTAAAACCTCAGCCTTCCAAGCTGATGTCGTGGGTTCGATTCC
CATCACCCGCTCCA
>Bacillus_thuringiensis_Al_Hakam_chr.trna24-GlyGCC (156705-156776) Gly (GCC) 72 bp Sc: 80.56 18
GCGGAAGTAGTTCAGTGGTAGAATACAACCTTGCCAAGGTTGGGGTCGCGGGTTCGAATC
CCGTCTTCCGCT
>Bacillus_thuringiensis_Al_Hakam_chr.trna12-GlyGCC (87647-87721) Gly (GCC) 75 bp Sc: 88.86 18
GCGGAAGTAGTTCAGTGGTAGAATACAACCTTGCCAAGGTTGGGGTCGCGGGTTCGAATC
CCGTCTTCCGCTCCA
>Bacillus_thuringiensis_Al_Hakam_chr.trna51-GlyGCC (540414-540488) Gly (GCC) 75 bp Sc: 88.86 16
GCGGAAGTAGTTCAGTGGTAGAATACAACCTTGCCAAGGTTGGGGTCGCGGGTTCGAATC
CCGTCTTCCGCTCCA
>Bacillus_thuringiensis_Al_Hakam_chr.trna60-GlyGCC (767283-767357) Gly (GCC) 75 bp Sc: 88.86 18
GCGGAAGTAGTTCAGTGGTAGAATACAACCTTGCCAAGGTTGGGGTCGCGGGTTCGAATC
CCGTCTTCCGCTCCA
>Bacillus_thuringiensis_Al_Hakam_chr.trna84-GlyGCC (4667815-4667741) Gly (GCC) 75 bp Sc: 88.86 18
GCGGAAGTAGTTCAGTGGTAGAATACAACCTTGCCAAGGTTGGGGTCGCGGGTTCGAATC
CCGTCTTCCGCTCCA
>Bacillus_thuringiensis_Al_Hakam_chr.trna36-GlyTCC (258057-258127) Gly (TCC) 71 bp Sc: 71.78 18
GCGGGTGTAGTTTAGTGGTAAAACAAGAGCCTTCCAAGCTCTGGTCGAGAGTTCGATTCT
CTTCACCCGCT
>Bacillus_thuringiensis_Al_Hakam_chr.trna97-GlyTCC (4666638-4666568) Gly (TCC) 71 bp Sc: 71.78 18
GCGGGTGTAGTTTAGTGGTAAAACAAGAGCCTTCCAAGCTCTGGTCGAGAGTTCGATTCT
CTTCACCCGCT
>Bacillus_thuringiensis_Al_Hakam_chr.trna102-GlyTCC (4294741-4294668) Gly (TCC) 74 bp Sc: 80.08 16
GCGGGTGTAGTTTAGTGGTAAAACAAGAGCCTTCCAAGCTCTGGTCGAGAGTTCGATTCT
CTTCACCCGCTCCA
>Bacillus_thuringiensis_Al_Hakam_chr.trna54-GlyTCC (552778-552851) Gly (TCC) 74 bp Sc: 80.08 18
GCGGGTGTAGTTTAGTGGTAAAACAAGAGCCTTCCAAGCTCTGGTCGAGAGTTCGATTCT
CTTCACCCGCTCCA
>Bacillus_weihenstephanensis_KBAB4_chr.trna24-GlyGCC (156592-156663) Gly (GCC) 72 bp Sc: 80.56 18
GCGGAAGTAGTTCAGTGGTAGAATACAACCTTGCCAAGGTTGGGGTCGCGGGTTCGAATC
CCGTCTTCCGCT
>Bacillus_weihenstephanensis_KBAB4_chr.trna12-GlyGCC (87492-87566) Gly (GCC) 75 bp Sc: 88.86 18
GCGGAAGTAGTTCAGTGGTAGAATACAACCTTGCCAAGGTTGGGGTCGCGGGTTCGAATC
CCGTCTTCCGCTCCA
>Bacillus_weihenstephanensis_KBAB4_chr.trna53-GlyGCC (522040-522114) Gly (GCC) 75 bp Sc: 88.86 18
GCGGAAGTAGTTCAGTGGTAGAATACAACCTTGCCAAGGTTGGGGTCGCGGGTTCGAATC
CCGTCTTCCGCTCCA
>Bacillus_weihenstephanensis_KBAB4_chr.trna62-GlyGCC (733628-733702) Gly (GCC) 75 bp Sc: 88.86 18
GCGGAAGTAGTTCAGTGGTAGAATACAACCTTGCCAAGGTTGGGGTCGCGGGTTCGAATC
CCGTCTTCCGCTCCA
>Bacillus_weihenstephanensis_KBAB4_chr.trna86-GlyGCC (4694979-4694905) Gly (GCC) 75 bp Sc: 88.86 18
GCGGAAGTAGTTCAGTGGTAGAATACAACCTTGCCAAGGTTGGGGTCGCGGGTTCGAATC
CCGTCTTCCGCTCCA
>Bacillus_weihenstephanensis_KBAB4_chr.trna37-GlyTCC (247654-247724) Gly (TCC) 71 bp Sc: 71.78 18
GCGGGTGTAGTTTAGTGGTAAAACAAGAGCCTTCCAAGCTCTGGTCGAGAGTTCGATTCT
CTTCACCCGCT
>Bacillus_weihenstephanensis_KBAB4_chr.trna99-GlyTCC (4693800-4693730) Gly (TCC) 71 bp Sc: 71.78 18
GCGGGTGTAGTTTAGTGGTAAAACAAGAGCCTTCCAAGCTCTGGTCGAGAGTTCGATTCT
CTTCACCCGCT
>Bacillus_weihenstephanensis_KBAB4_chr.trna104-GlyTCC (4322849-4322776) Gly (TCC) 74 bp Sc: 80.08 18
GCGGGTGTAGTTTAGTGGTAAAACAAGAGCCTTCCAAGCTCTGGTCGAGAGTTCGATTCT
CTTCACCCGCTCCA
>Bacillus_weihenstephanensis_KBAB4_chr.trna56-GlyTCC (527074-527147) Gly (TCC) 74 bp Sc: 80.08 18
GCGGGTGTAGTTTAGTGGTAAAACAAGAGCCTTCCAAGCTCTGGTCGAGAGTTCGATTCT
CTTCACCCGCTCCA
>Bacteroides_fragilis_YCH46_chr.trna55-GlyCCC (2858492-2858417) Gly (CCC) 76 bp Sc: 72.47 14
GCGGTAGTAGCTCAGTTGGCAGAGCGGCGGCTTCCCAAGCCGCAGGTCACGAGTTCGACC
CTCGCCTACCGCTCTA
>Bacteroides_fragilis_YCH46_chr.trna2-GlyGCC (589029-589101) Gly (GCC) 73 bp Sc: 84.53 17
GCGGAAATAGCTCAGTTGGTAGAGCATAACCTTGCCAAGGTTAGGGTCGCGAGTTCGAGT
CTCGTTTTCCGCT
>Bacteroides_fragilis_YCH46_chr.trna49-GlyGCC (2904824-2904752) Gly (GCC) 73 bp Sc: 84.53 17
GCGGAAATAGCTCAGTTGGTAGAGCATAACCTTGCCAAGGTTAGGGTCGCGAGTTCGAGT
CTCGTTTTCCGCT
>Bacteroides_fragilis_YCH46_chr.trna54-GlyGCC (2904274-2904202) Gly (GCC) 73 bp Sc: 84.53 17
GCGGAAATAGCTCAGTTGGTAGAGCATAACCTTGCCAAGGTTAGGGTCGCGAGTTCGAGT
CTCGTTTTCCGCT
>Bacteroides_fragilis_YCH46_chr.trna52-GlyGCC (2904501-2904426) Gly (GCC) 76 bp Sc: 85.15 16

GCGGAAATAGCTCAGTTGGTAGAGCATAACCTTGCCAAGGTTAGGGTCGCGAGTTCGAGT
CTCGTTTTCCGCTCAA
>Bacteroides_fragilis_YCH46_chr.trna31-GlyTCC (4795253-4795181) Gly (TCC) 73 bp Sc: 68.88 15
GCGGAAGTAGCTCAGTTGATAGAGCATTAGCCTTCCAAGCTGAGGGTCGCGGGTTTGAGC
CCCGTCTTCCGCT
>Bacteroides_fragilis_YCH46_chr.trna67-GlyTCC (1157452-1157377) Gly (TCC) 76 bp Sc: 69.50 15
GCGGAAGTAGCTCAGTTGATAGAGCATTAGCCTTCCAAGCTGAGGGTCGCGGGTTTGAGC
CCCGTCTTCCGCTCTA
>Bacteroides_vulgatus_ATCC_8482_chr.trna30-GlyCCC (3958542-3958639) Gly (CCC) 98 bp Sc: 53.14 14
GCGGCAGTAGCTCAGTTGGTCACTTTTAATCCGCAGGATTAAAAGGCATCAGCTTCCCAA
GCTGAGGGTCGCGGGTTCGAATCCCGTTTGCCGCTCCA
>Bacteroides_vulgatus_ATCC_8482_chr.trna29-GlyCCC (3943164-3943236) Gly (CCC) 73 bp Sc: 76.85 15
GCGGCAGTAGCTCAGTTGGTAGAGCATCAGCTTCCCAAGCTGAGGGTCACGAGTTCGAAC
CTCGCTTGCCGCT
>Bacteroides_vulgatus_ATCC_8482_chr.trna59-GlyCCC (3633774-3633700) Gly (CCC) 75 bp Sc: 83.20 15
GGACGATTAGCTCAGAGGCAGAGCATCAGCTTCCCAAGCTGAGGGTCGCGGGTTCAAGTC
CCGTATCGTCCACGA
>Bacteroides_vulgatus_ATCC_8482_chr.trna45-GlyGCC (4390009-4389937) Gly (GCC) 73 bp Sc: 84.53 17
GCGGAAATAGCTCAGTTGGTAGAGCATAACCTTGCCAAGGTTAGGGTCGCGAGTTCGAGT
CTCGTTTTCCGCT
>Bacteroides_vulgatus_ATCC_8482_chr.trna48-GlyGCC (4389685-4389613) Gly (GCC) 73 bp Sc: 84.53 17
GCGGAAATAGCTCAGTTGGTAGAGCATAACCTTGCCAAGGTTAGGGTCGCGAGTTCGAGT
CTCGTTTTCCGCT
>Bacteroides_vulgatus_ATCC_8482_chr.trna69-GlyGCC (1160616-1160544) Gly (GCC) 73 bp Sc: 84.53 17
GCGGAAATAGCTCAGTTGGTAGAGCATAACCTTGCCAAGGTTAGGGTCGCGAGTTCGAGT
CTCGTTTTCCGCT
>Bacteroides_vulgatus_ATCC_8482_chr.trna50-GlyGCC (4389469-4389394) Gly (GCC) 76 bp Sc: 85.15 17
GCGGAAATAGCTCAGTTGGTAGAGCATAACCTTGCCAAGGTTAGGGTCGCGAGTTCGAGT
CTCGTTTTCCGCTCAA
>Bacteroides_vulgatus_ATCC_8482_chr.trna73-GlyTCC (1120213-1120141) Gly (TCC) 73 bp Sc: 68.88 15
GCGGAAGTAGCTCAGTTGATAGAGCATTAGCCTTCCAAGCTGAGGGTCGCGGGTTTGAGC
CCCGTCTTCCGCT
>Bacteroides_vulgatus_ATCC_8482_chr.trna62-GlyTCC (3328976-3328901) Gly (TCC) 76 bp Sc: 69.50 15
GCGGAAGTAGCTCAGTTGATAGAGCATTAGCCTTCCAAGCTGAGGGTCGCGGGTTTGAGC
CCCGTCTTCCGCTCTA
>Bartonella_bacilliformis_KC583_chr.trna44-GlyGCC (50872-50798) Gly (GCC) 75 bp Sc: 87.83 15
GCGGGTGTAGCTCAGGGGTAGAGCACAACCTTGCCAAGGTTGGGGTCGTGGGTTCGAATC
CCATCGCCCGCTCCA
>Bartonella_bacilliformis_KC583_chr.trna10-GlyTCC (572451-572524) Gly (TCC) 74 bp Sc: 81.40 18
GCGGGTATAGCTCAATGGTAGAGCAGCAGCCTTCCAAGCTGAATATGCGGGTTCGATTCC
CGCTACCCGCTCCA
>Bartonella_quintana_Toulouse_chr.trna16-GlyGCC (1535763-1535837) Gly (GCC) 75 bp Sc: 87.83 15
GCGGGTGTAGCTCAGGGGTAGAGCACAACCTTGCCAAGGTTGGGGTCGTGGGTTCGAATC
CCATCGCCCGCTCCA
>Bartonella_quintana_Toulouse_chr.trna31-GlyTCC (855787-855714) Gly (TCC) 74 bp Sc: 81.40 18
GCGGGTATAGCTCAATGGTAGAGCAGCAGCCTTCCAAGCTGAATATGCGGGTTCGATTCC
CGCTACCCGCTCCA
>Bartonella_tribocorum_CIP_105476_chr.trna21-GlyGCC (2530958-2531032) Gly (GCC) 75 bp Sc: 87.83 15
GCGGGTGTAGCTCAGGGGTAGAGCACAACCTTGCCAAGGTTGGGGTCGTGGGTTCGAATC
CCATCGCCCGCTCCA
>Bartonella_tribocorum_CIP_105476_chr.trna6-GlyTCC (949166-949239) Gly (TCC) 74 bp Sc: 81.40 18
GCGGGTATAGCTCAATGGTAGAGCAGCAGCCTTCCAAGCTGAATATGCGGGTTCGATTCC
CGCTACCCGCTCCA
>Brachypodium_distachyon_chr1.trna109-GlyCCC (67836802-67836732) Gly (CCC) 71 bp Sc: 68.50 17
GCGCATCTGGTGTAGTGGTATCATAGTACCCTCCCACGGTACTGACCAGGGTTCGATTCC
CTGGATGCGCA
>Brachypodium_distachyon_chr1.trna161-GlyCCC (14510054-14509984) Gly (CCC) 71 bp Sc: 68.50 17
GCGCATCTGGTGTAGTGGTATCATAGTACCCTCCCACGGTACTGACCAGGGTTCGATTCC
CTGGATGCGCA
>Brachypodium_distachyon_chr1.trna29-GlyCCC (14682083-14682153) Gly (CCC) 71 bp Sc: 68.50 17
GCGCATCTGGTGTAGTGGTATCATAGTACCCTCCCACGGTACTGACCAGGGTTCGATTCC
CTGGATGCGCA
>Brachypodium_distachyon_chr1.trna80-GlyCCC (62214183-62214253) Gly (CCC) 71 bp Sc: 68.50 17
GCGCATCTGGTGTAGTGGTATCATAGTACCCTCCCACGGTACTGACCAGGGTTCGATTCC
CTGGATGCGCA
>Brachypodium_distachyon_chr2.trna93-GlyCCC (48559532-48559462) Gly (CCC) 71 bp Sc: 68.50 17
GCGCATCTGGTGTAGTGGTATCATAGTACCCTCCCACGGTACTGACCAGGGTTCGATTCC
CTGGATGCGCA
>Brachypodium_distachyon_chr3.trna7-GlyCCC (5483812-5483882) Gly (CCC) 71 bp Sc: 68.50 17
GCGCATCTGGTGTAGTGGTATCATAGTACCCTCCCACGGTACTGACCAGGGTTCGATTCC
CTGGATGCGCA
>Brachypodium_distachyon_chr3.trna85-GlyCCC (38548253-38548183) Gly (CCC) 71 bp Sc: 68.50 17
GCGCATCTGGTGTAGTGGTATCATAGTACCCTCCCACGGTACTGACCAGGGTTCGATTCC
CTGGATGCGCA

>Brachypodium_distachyon_chr4.trna104-GlyCCC (4667360-4667290) Gly (CCC) 71 bp Sc: 68.50 17
GCGCATCTGGTGTAGTGGTATCATAGTACCCTCCCACGGTACTGACCAGGGTTCGATTCC
CTGGATGCGCA
>Brachypodium_distachyon_chr4.trna72-GlyCCC (35285672-35285602) Gly (CCC) 71 bp Sc: 68.50 17
GCGCATCTGGTGTAGTGGTATCATAGTACCCTCCCACGGTACTGACCAGGGTTCGATTCC
CTGGATGCGCA
>Brachypodium_distachyon_chr4.trna113-GlyGCC (407298-407228) Gly (GCC) 71 bp Sc: 54.35 17
GCGAGTGTAGTTCAATGGTAAACCATCTCCTTGCCAAGGAGAAGATACGGGTTCGATTCC
CGCCGCTCGCC
>Brachypodium_distachyon_chr5.trna58-GlyGCC (9608389-9608319) Gly (GCC) 71 bp Sc: 55.65 17
GCGAGCGTAGTTCAATGGTAAAACATCTCCTTGCCAAGGAGAAGATACGGGTTTGATTCC
CGCCGCTCGCC
>Brachypodium_distachyon_chr1.trna175-GlyGCC (778570-778500) Gly (GCC) 71 bp Sc: 55.92 17
GCAAGTGTAGTTCAATGGTAAAACATCTCCTTGCCAAGGAGAAGATACGGGTTCGATTCC
CGCCGCTCGCC
>Brachypodium_distachyon_chr3.trna98-GlyGCC (28296252-28296182) Gly (GCC) 71 bp Sc: 61.61 17
GCGAGCGTAGTTCAATGGTAAAACATCTCCTTGCCAAGGAGAAGATACGGGTTCGATTCC
CGCCGCTTGCC
>Brachypodium_distachyon_chr1.trna130-GlyGCC (54727850-54727780) Gly (GCC) 71 bp Sc: 62.56 17
GCGAGCGTAGTTCAATGGTAAAACATCTCCTTGCCAAGGAGAAGATACGGGTTCGATTCC
CGCCGCTCGCC
>Brachypodium_distachyon_chr1.trna92-GlyGCC (74163058-74163128) Gly (GCC) 71 bp Sc: 62.56 17
GCGAGCGTAGTTCAATGGTAAAACATCTCCTTGCCAAGGAGAAGATACGGGTTCGATTCC
CGCCGCTCGCC
>Brachypodium_distachyon_chr2.trna116-GlyGCC (16388847-16388777) Gly (GCC) 71 bp Sc: 62.56 17
GCGAGCGTAGTTCAATGGTAAAACATCTCCTTGCCAAGGAGAAGATACGGGTTCGATTCC
CGCCGCTCGCC
>Brachypodium_distachyon_chr3.trna27-GlyGCC (35358440-35358510) Gly (GCC) 71 bp Sc: 65.59 17
GCACCAGTGGTCTAGTGGTAGAATAGTACCCTGCCACTGTACAGACCCGGGTTCGATTCC
CGGCTGGTGCA
>Brachypodium_distachyon_chr5.trna45-GlyGCC (18805152-18805082) Gly (GCC) 71 bp Sc: 67.64 17
GCACCAGTGGTCTAGTGGCAGAATAGTACCCTGCCACGGTACAGACCCGGGTTCGATTCC
CGGCTGGTGCA
>Brachypodium_distachyon_chr1.trna101-GlyGCC (72602209-72602139) Gly (GCC) 71 bp Sc: 71.56 17
GCACCAGTGGTCTAGTGGTAGAATAGTACCCTGCCACGGTACAGACCCGGGTTCGATTCC
CGGCTGGTGCA
>Brachypodium_distachyon_chr1.trna102-GlyGCC (72597467-72597397) Gly (GCC) 71 bp Sc: 71.56 17
GCACCAGTGGTCTAGTGGTAGAATAGTACCCTGCCACGGTACAGACCCGGGTTCGATTCC
CGGCTGGTGCA
>Brachypodium_distachyon_chr1.trna117-GlyGCC (64237306-64237236) Gly (GCC) 71 bp Sc: 71.56 17
GCACCAGTGGTCTAGTGGTAGAATAGTACCCTGCCACGGTACAGACCCGGGTTCGATTCC
CGGCTGGTGCA
>Brachypodium_distachyon_chr1.trna129-GlyGCC (54918233-54918163) Gly (GCC) 71 bp Sc: 71.56 17
GCACCAGTGGTCTAGTGGTAGAATAGTACCCTGCCACGGTACAGACCCGGGTTCGATTCC
CGGCTGGTGCA
>Brachypodium_distachyon_chr1.trna163-GlyGCC (10042289-10042219) Gly (GCC) 71 bp Sc: 71.56 17
GCACCAGTGGTCTAGTGGTAGAATAGTACCCTGCCACGGTACAGACCCGGGTTCGATTCC
CGGCTGGTGCA
>Brachypodium_distachyon_chr1.trna169-GlyGCC (4075009-4074939) Gly (GCC) 71 bp Sc: 71.56 17
GCACCAGTGGTCTAGTGGTAGAATAGTACCCTGCCACGGTACAGACCCGGGTTCGATTCC
CGGCTGGTGCA
>Brachypodium_distachyon_chr1.trna47-GlyGCC (32957780-32957850) Gly (GCC) 71 bp Sc: 71.56 17
GCACCAGTGGTCTAGTGGTAGAATAGTACCCTGCCACGGTACAGACCCGGGTTCGATTCC
CGGCTGGTGCA
>Brachypodium_distachyon_chr1.trna52-GlyGCC (42995888-42995958) Gly (GCC) 71 bp Sc: 71.56 17
GCACCAGTGGTCTAGTGGTAGAATAGTACCCTGCCACGGTACAGACCCGGGTTCGATTCC
CGGCTGGTGCA
>Brachypodium_distachyon_chr1.trna59-GlyGCC (49670122-49670192) Gly (GCC) 71 bp Sc: 71.56 17
GCACCAGTGGTCTAGTGGTAGAATAGTACCCTGCCACGGTACAGACCCGGGTTCGATTCC
CGGCTGGTGCA
>Brachypodium_distachyon_chr1.trna88-GlyGCC (72190585-72190655) Gly (GCC) 71 bp Sc: 71.56 17
GCACCAGTGGTCTAGTGGTAGAATAGTACCCTGCCACGGTACAGACCCGGGTTCGATTCC
CGGCTGGTGCA
>Brachypodium_distachyon_chr2.trna128-GlyGCC (3857235-3857165) Gly (GCC) 71 bp Sc: 71.56 17
GCACCAGTGGTCTAGTGGTAGAATAGTACCCTGCCACGGTACAGACCCGGGTTCGATTCC
CGGCTGGTGCA
>Brachypodium_distachyon_chr3.trna124-GlyGCC (6185863-6185793) Gly (GCC) 71 bp Sc: 71.56 17
GCACCAGTGGTCTAGTGGTAGAATAGTACCCTGCCACGGTACAGACCCGGGTTCGATTCC
CGGCTGGTGCA
>Brachypodium_distachyon_chr3.trna31-GlyGCC (36642001-36642071) Gly (GCC) 71 bp Sc: 71.56 17
GCACCAGTGGTCTAGTGGTAGAATAGTACCCTGCCACGGTACAGACCCGGGTTCGATTCC
CGGCTGGTGCA
>Brachypodium_distachyon_chr3.trna33-GlyGCC (37560538-37560608) Gly (GCC) 71 bp Sc: 71.56 17
GCACCAGTGGTCTAGTGGTAGAATAGTACCCTGCCACGGTACAGACCCGGGTTCGATTCC

CGGCTGGTGCA
>Brachypodium_distachyon_chr3.trna94-GlyGCC (29683449-29683379) Gly (GCC) 71 bp Sc: 71.56 17
GCACCAGTGGTCTAGTGGTAGAATAGTACCCTGCCACGGTACAGACCCGGGTTCGATTCC
CGGCTGGTGCA
>Brachypodium_distachyon_chr4.trna105-GlyGCC (3885550-3885480) Gly (GCC) 71 bp Sc: 71.56 17
GCACCAGTGGTCTAGTGGTAGAATAGTACCCTGCCACGGTACAGACCCGGGTTCGATTCC
CGGCTGGTGCA
>Brachypodium_distachyon_chr4.trna111-GlyGCC (752951-752881) Gly (GCC) 71 bp Sc: 71.56 17
GCACCAGTGGTCTAGTGGTAGAATAGTACCCTGCCACGGTACAGACCCGGGTTCGATTCC
CGGCTGGTGCA
>Brachypodium_distachyon_chr4.trna59-GlyGCC (44583073-44583003) Gly (GCC) 71 bp Sc: 71.56 17
GCACCAGTGGTCTAGTGGTAGAATAGTACCCTGCCACGGTACAGACCCGGGTTCGATTCC
CGGCTGGTGCA
>Brachypodium_distachyon_chr1.trna82-GlyTCC (63387217-63387287) Gly (TCC) 71 bp Sc: 49.97 15
GTGTCTGTAGTCCAACGGTTAGGATAATTGCCTTCCAAGCAATAGACCCTGTTCGACTCC
CGGCAGACGCA
>Brachypodium_distachyon_chr1.trna34-GlyTCC (20063451-20063522) Gly (TCC) 72 bp Sc: 73.14 15
GCGTTTGTAGTCCAACGGTTAGGATAATTGCCTTCCAAGCAATAGACCCGGGTTCGACTC
CCGGCAGACGCA
>Brachypodium_distachyon_chr2.trna31-GlyTCC (20084762-20084833) Gly (TCC) 72 bp Sc: 73.14 15
GCGTTTGTAGTCCAACGGTTAGGATAATTGCCTTCCAAGCAATAGACCCGGGTTCGACTC
CCGGCAGACGCA
>Brachypodium_distachyon_chr2.trna60-GlyTCC (52940374-52940445) Gly (TCC) 72 bp Sc: 73.14 15
GCGTTTGTAGTCCAACGGTTAGGATAATTGCCTTCCAAGCAATAGACCCGGGTTCGACTC
CCGGCAGACGCA
>Brachypodium_distachyon_chr2.trna96-GlyTCC (42096168-42096097) Gly (TCC) 72 bp Sc: 73.14 15
GCGTTTGTAGTCCAACGGTTAGGATAATTGCCTTCCAAGCAATAGACCCGGGTTCGACTC
CCGGCAGACGCA
>Brachypodium_distachyon_chr1.trna69-GlyTCC (55523598-55523669) Gly (TCC) 72 bp Sc: 75.78 15
GCGTCTGTAGTCCAACGGTTAGGATAATTGCCTTCCAAGCAATAGACCCGGGTTCGACTC
CCGGCAGACGCA
>Brachypodium_distachyon_chr3.trna11-GlyTCC (12376076-12376147) Gly (TCC) 72 bp Sc: 75.78 15
GCGTCTGTAGTCCAACGGTTAGGATAATTGCCTTCCAAGCAATAGACCCGGGTTCGACTC
CCGGCAGACGCA
>Brachypodium_distachyon_chr4.trna107-GlyTCC (3767461-3767390) Gly (TCC) 72 bp Sc: 75.78 15
GCGTCTGTAGTCCAACGGTTAGGATAATTGCCTTCCAAGCAATAGACCCGGGTTCGACTC
CCGGCAGACGCA
>Brachypodium_distachyon_chr5.trna10-GlyTCC (10925504-10925575) Gly (TCC) 72 bp Sc: 75.78 15
GCGTCTGTAGTCCAACGGTTAGGATAATTGCCTTCCAAGCAATAGACCCGGGTTCGACTC
CCGGCAGACGCA
>Brachypodium_distachyon_chr1.trna26-GlyGCC (13487392-13487459) Gly (GCC) 68 bp Sc: 28.93 18
GCACCAGTGGTCTTATGGTAGAATAGTACCCTGCCACGGTACAGACCCGGGTTCGATTCC
GGGGTACA
>Beijerinckia_indica_ATCC_9039_chr.trna1-GlyCCC (431977-432050) Gly (CCC) 74 bp Sc: 80.62 16
GCGGGCGTAGTTCAATGGTAGAACGGCAGCTTCCCAAGCTGCATACGAGGGTTCGATTCC
CTTCGCCCGCTCCA
>Beijerinckia_indica_ATCC_9039_chr.trna34-GlyGCC (3178840-3178766) Gly (GCC) 75 bp Sc: 88.17 15
GCGGGTGTAGCTCAGGGGTAGAGCACAACCTTGCCAAGGTTGGGGTCGAGGGTTCGAATC
CCTTCGCCCGCTCCA
>Beijerinckia_indica_ATCC_9039_chr.trna13-GlyTCC (1547422-1547495) Gly (TCC) 74 bp Sc: 82.33 17
GCGGGTGTAGCTCAATGGTAGAGCAGCAGCCTTCCAAGCTGAATACGAGGGTTCGATTCC
CTTCACCCGCTCCA
>Bifidobacterium_adolescentis_ATCC_15703_chr.trna1-GlyCCC (81167-81240) Gly (CCC) 74 bp Sc: 67.14 14
GCGGATGTAGTTCATCGGTAGAACGAAAGCTTCCCAAGCTTTAAAGGCGGGTTCGACTCC
CGTCATCCGCTCCA
>Bifidobacterium_adolescentis_ATCC_15703_chr.trna35-GlyGCC (1060616-1060541) Gly (GCC) 76 bp Sc: 76.41 15
GCGGACATAGCTTAGTTGGTAAAGCGCAACCTTGCCAAGGTTGAGACCGCGGGTCCGAGT
CCCGTTGTCCGCTCCA
>Bifidobacterium_adolescentis_ATCC_15703_chr.trna45-GlyGCC (628805-628730) Gly (GCC) 76 bp Sc: 76.41 15
GCGGACATAGCTTAGTTGGTAAAGCGCAACCTTGCCAAGGTTGAGACCGCGGGTCCGAGT
CCCGTTGTCCGCTCCA
>Bifidobacterium_adolescentis_ATCC_15703_chr.trna36-GlyGCC (1029147-1029072) Gly (GCC) 76 bp Sc: 76.69 15
GCGGACATAGCTTAGTTGGTAAAGCGCAACCTTGCCAAGGTTGAGACCGCGGGTTCGAGT
CCCGTTGTCCGCTCTA
>Bifidobacterium_adolescentis_ATCC_15703_chr.trna7-GlyTCC (342497-342570) Gly (TCC) 74 bp Sc: 79.76 17
GCGGATGTAGCTCAATGGTAGAGCCTCAGTCTTCCAAACTGATTACGCGGGTTCGATTCC
CGTCATCCGCTCCA
>Blochmannia_floridanus_chr.trna2-GlyGCC (82596-82669) Gly (GCC) 74 bp Sc: 79.17 16
GCGGGAATAGCTCAGATGGTTAGAGTGCAACCTTGCCAAGGTTGAGGTCGCGAGTTCGAA
TCTCGTTTCCCGCT
>Blochmannia_floridanus_chr.trna9-GlyTCC (220138-220209) Gly (TCC) 72 bp Sc: 42.59 17
GTGGGCATCGTATAATGGTTATTACCTCAACCTTCCAAGTTGATGATGTGGGTTCGATTC
CCACTGTCCGCT
>Bordetella_avium_197N_chr.trna6-GlyCCC (455847-455920) Gly (CCC) 74 bp Sc: 77.64 16

GCGGGTGTAGTTCAATGGTAGAACGGCGGCTTCCCAAGCCTCAAGCGTGGGTTCGATTCC
CATCACCCGCTCCA
>Bordetella_avium_197N_chr.trna21-GlyGCC (2953742-2953817) Gly (GCC) 76 bp Sc: 89.05 16
GCGGGAGTAGCTCAGTTGGTAGAGCGCAACCTTGCCAAGGTTGAGGTCGCGAGTTCGAGA
CTCGTCTCCCGCTCCA
>Bordetella_avium_197N_chr.trna22-GlyGCC (2962130-2962205) Gly (GCC) 76 bp Sc: 89.05 16
GCGGGAGTAGCTCAGTTGGTAGAGCGCAACCTTGCCAAGGTTGAGGTCGCGAGTTCGAGA
CTCGTCTCCCGCTCCA
>Bordetella_avium_197N_chr.trna2-GlyTCC (5642-5715) Gly (TCC) 74 bp Sc: 82.17 17
GCGGGTGTAGCTCAATGGTAGAGCAGAAGCCTTCCAAGCTTACGACGAGGGTTCGATTCC
CTTCACCCGCTCCA
>Borrelia_afzelii_PKo_chr.trna15-GlyGCC (640242-640313) Gly (GCC) 72 bp Sc: 66.76 16
GCGAAAGTAACTCAGGGGTAGAGTGTCACCTTGCCAAGGTGGAAGTCGCGGGTTCAAATC
CCGTCTTTCGCT
>Borrelia_afzelii_PKo_chr.trna1-GlyTCC (46313-46384) Gly (TCC) 72 bp Sc: 50.44 15
GCGTCCTTCGTATAATGGCTATTACCTTAGCCTTCCAAGCTAATGATGTCGGTTCGATTC
CGATAGGACGCT
>Borrelia_garinii_PBi_chrlinear.trna15-GlyGCC (638415-638486) Gly (GCC) 72 bp Sc: 66.76 16
GCGAAAGTAACTCAGGGGTAGAGTGTCACCTTGCCAAGGTGGAAGTCGCGGGTTCAAATC
CCGTCTTTCGCT
>Borrelia_garinii_PBi_chrlinear.trna1-GlyTCC (46379-46450) Gly (TCC) 72 bp Sc: 50.44 15
GCGTCCTTCGTATAATGGCTATTACCTTAGCCTTCCAAGCTAATGATGTCGGTTCGATTC
CGATAGGACGCT
>Bradyrhizobium_BTAi1_chr.trna7-GlyCCC (799661-799734) Gly (CCC) 74 bp Sc: 80.62 16
GCGGGCGTAGTTCAATGGTAGAACGGCAGCTTCCCAAGCTGCATACGAGGGTTCGATTCC
CTTCGCCCGCTCCA
>Bradyrhizobium_BTAi1_chr.trna4-GlyGCC (310428-310502) Gly (GCC) 75 bp Sc: 88.70 16
GCGGGTGTAGCTCAGTGGTAGAGCACGACCTTGCCAAGGTCGGGGTCGAGGGTTCGAACC
CCTTCGCCCGCTCCA
>Bradyrhizobium_BTAi1_chr.trna37-GlyTCC (5356535-5356462) Gly (TCC) 74 bp Sc: 82.33 15
GCGGGTGTAGCTCAATGGTAGAGCAGCAGCCTTCCAAGCTGAATACGAGGGTTCGATTCC
CTTCACCCGCTCCA
>Brucella_canis_ATCC_23365_chrI.trna33-GlyCCC (936171-936098) Gly (CCC) 74 bp Sc: 65.12 17
GCGGGTATGATGTAATGGTAGCCTGTCAGCTTCCCAAGCTGAACGCGCGGGTTCGATTCC
CGCTACCCGCTCCA
>Brucella_canis_ATCC_23365_chrII.trna3-GlyGCC (877831-877905) Gly (GCC) 75 bp Sc: 88.17 17
GCGGGTGTAGCTCAGGGGTAGAGCACAACCTTGCCAAGGTTGGGGTCGAGGGTTCGAATC
CCTTCGCCCGCTCCA
>Brucella_canis_ATCC_23365_chrII.trna4-GlyGCC (1006981-1007055) Gly (GCC) 75 bp Sc: 88.17 17
GCGGGTGTAGCTCAGGGGTAGAGCACAACCTTGCCAAGGTTGGGGTCGAGGGTTCGAATC
CCTTCGCCCGCTCCA
>Brucella_canis_ATCC_23365_chrI.trna31-GlyTCC (1219037-1218964) Gly (TCC) 74 bp Sc: 83.28 18
GCGGGTATAGCTCAATGGTAGAGCAGCAGCCTTCCAAGCTGAATACGCGGGTTCGATTCC
CGCTACCCGCTCCA
>Brucella_melitensis_chrI.trna13-GlyCCC (1050587-1050660) Gly (CCC) 74 bp Sc: 65.12 17
GCGGGTATGATGTAATGGTAGCCTGTCAGCTTCCCAAGCTGAACGCGCGGGTTCGATTCC
CGCTACCCGCTCCA
>Brucella_melitensis_chrII.trna11-GlyGCC (419834-419760) Gly (GCC) 75 bp Sc: 88.17 15
GCGGGTGTAGCTCAGGGGTAGAGCACAACCTTGCCAAGGTTGGGGTCGAGGGTTCGAATC
CCTTCGCCCGCTCCA
>Brucella_melitensis_chrII.trna12-GlyGCC (291445-291371) Gly (GCC) 75 bp Sc: 88.17 15
GCGGGTGTAGCTCAGGGGTAGAGCACAACCTTGCCAAGGTTGGGGTCGAGGGTTCGAATC
CCTTCGCCCGCTCCA
>Brucella_melitensis_chrI.trna11-GlyTCC (767877-767950) Gly (TCC) 74 bp Sc: 83.28 18
GCGGGTATAGCTCAATGGTAGAGCAGCAGCCTTCCAAGCTGAATACGCGGGTTCGATTCC
CGCTACCCGCTCCA
>Brucella_suis_1330_chrI.trna33-GlyCCC (937721-937648) Gly (CCC) 74 bp Sc: 65.12 18
GCGGGTATGATGTAATGGTAGCCTGTCAGCTTCCCAAGCTGAACGCGCGGGTTCGATTCC
CGCTACCCGCTCCA
>Brucella_suis_1330_chrII.trna3-GlyGCC (878530-878604) Gly (GCC) 75 bp Sc: 88.17 15
GCGGGTGTAGCTCAGGGGTAGAGCACAACCTTGCCAAGGTTGGGGTCGAGGGTTCGAATC
CCTTCGCCCGCTCCA
>Brucella_suis_1330_chrII.trna4-GlyGCC (1007558-1007632) Gly (GCC) 75 bp Sc: 88.17 15
GCGGGTGTAGCTCAGGGGTAGAGCACAACCTTGCCAAGGTTGGGGTCGAGGGTTCGAATC
CCTTCGCCCGCTCCA
>Brucella_suis_1330_chrI.trna31-GlyTCC (1220146-1220073) Gly (TCC) 74 bp Sc: 83.28 18
GCGGGTATAGCTCAATGGTAGAGCAGCAGCCTTCCAAGCTGAATACGCGGGTTCGATTCC
CGCTACCCGCTCCA
>Brucella_suis_ATCC_23445_chrI.trna27-GlyCCC (957474-957401) Gly (CCC) 74 bp Sc: 65.12 18
GCGGGTATGATGTAATGGTAGCCTGTCAGCTTCCCAAGCTGAACGCGCGGGTTCGATTCC
CGCTACCCGCTCCA
>Brucella_suis_ATCC_23445_chrII.trna3-GlyGCC (866152-866226) Gly (GCC) 75 bp Sc: 88.17 15
GCGGGTGTAGCTCAGGGGTAGAGCACAACCTTGCCAAGGTTGGGGTCGAGGGTTCGAATC
CCTTCGCCCGCTCCA

>Brucella_suis_ATCC_23445_chrII.trna4-GlyGCC (996011-996085) Gly (GCC) 75 bp Sc: 88.17 15
GCGGGTGTAGCTCAGGGGTAGAGCACAACCTTGCCAAGGTTGGGGTCGAGGGTTCGAATC
CCTTCGCCCGCTCCA
>Brucella_suis_ATCC_23445_chrI.trna25-GlyTCC (1240896-1240823) Gly (TCC) 74 bp Sc: 83.28 18
GCGGGTATAGCTCAATGGTAGAGCAGCAGCCTTCCAAGCTGAATACGCGGGTTCGATTCC
CGCTACCCGCTCCA
>Bos_taurus_chr10.trna865-GlyACC (20039325-20039397) Gly (ACC) 73 bp Sc: 30.58 15
TCTCTGATGGTCCAGGGCTTAAGACGATGTATTACCAGTACAGGAGGCCCAGGTTAGATC
CCTGGTCAGGGAA
>Bos_taurus_chr9.trna3118-GlyACC (90597498-90597570) Gly (ACC) 73 bp Sc: 33.61 16
TCCCTGGTGGTCCAGTGGCTGAGATCCTGAGCTACCAATCCAGGGACCTCAGGTTAGATC
CCTGATTAGGGAA
>Bos_taurus_chr25.trna630-GlyACC (11264063-11264135) Gly (ACC) 73 bp Sc: 33.84 16
TCCCTGGTGGTCCAGTGGTTAAGATTCTGTGCTACCAGTACAGGGGGTCTAGGTTCCATC
CTTGGTCAGGGAA
>Bos_taurus_chr22.trna1672-GlyACC (47319352-47319424) Gly (ACC) 73 bp Sc: 36.83 16
TCCCTGGTGGTCCAGCAGCTAAGACTCTGCACCACCAATGCAGGGGGCCTGGGTTTGATC
CCTGGTCAGGGAA
>Bos_taurus_chr19.trna2490-GlyACC (49326425-49326496) Gly (ACC) 72 bp Sc: 37.80 17
TCCCTGGTGGTCCAGTGGCTGAGACTCTGCGCTACCAATGCAGGGGTCTGGGTTCCATCC
CTGGTCAGGGAA
>Bos_taurus_chr5.trna5618-GlyACC (114430852-114430780) Gly (ACC) 73 bp Sc: 39.12 16
TCCCTGGTGGTCCAGTGGCTAAGTCTCTGTGCTACCAATGCAGGGTGCTCAGGTTTGAAC
CCTGGTCAGGGAA
>Bos_taurus_chr22.trna113-GlyACC (3385873-3385945) Gly (ACC) 73 bp Sc: 39.64 15
TCCCTCATGGTCCAGTGGCTAAGATTCTGTGCTACCAATGTAGGGGGCTCAGGTTTGATT
CCTGGTCAGGGAA
>Bos_taurus_chr15.trna5149-GlyACC (30970782-30970710) Gly (ACC) 73 bp Sc: 42.71 17
TCCTTAGTGGTTCAATGGCTAAGACTCTGTGCTACCAATGCAGATGGCCCAGGTTCAATC
CCTGGTCAGGGAA
>Bos_taurus_chr20.trna1184-GlyACC (33508899-33508971) Gly (ACC) 73 bp Sc: 43.40 17
TCCTTGATGGTCCAGTGGCTAAGATTCTGTGCCACCACTGCAGGGGGCCCGGGTTCAAGC
CCTGGTTGGGGAA
>Bos_taurus_chr14.trna289-GlyACC (7423277-7423349) Gly (ACC) 73 bp Sc: 44.88 17
TCCCTGGTGGTGCAATGGCTAAGACTCTGCACTACCAGTGCAGAGGGCTCGGGTTCGATC
CCTGGTCAGGGAA
>Bos_taurus_chrX.trna2724-GlyACC (73152721-73152793) Gly (ACC) 73 bp Sc: 45.65 15
TCCCTGGTGGTCCAGTGGCTAAAACTCTGAGCTACCAATGCAGAAGTCCTAGGTTTGATC
CCTGGTCAGGGAG
>Bos_taurus_chr11.trna4048-GlyACC (99239103-99239175) Gly (ACC) 73 bp Sc: 47.06 17
TCTCTGGTGGTCCAGTGGCTAAGACTCTGTGCTACCAATGCAGGGGGCCTGGGTTCAATC
CCTGGCCAGGGAA
>Bos_taurus_chr13.trna6530-GlyACC (25688027-25687955) Gly (ACC) 73 bp Sc: 47.54 15
TCCCTGGTGGTCCAGTGGCCAAGACTCCATGTTACCAATGTAGGGGACCCAGGTTCGATC
CCTGGTCAGGGAA
>Bos_taurus_chr4.trna4061-GlyACC (117003365-117003437) Gly (ACC) 73 bp Sc: 50.18 16
TCCCTGGTGGTCTAGTGGTTAAGACTCTGCCCTACCACTGCAGGGGACATAGGTTCAATC
CCTGGTCAGGGAA
>Bos_taurus_chr22.trna4309-GlyACC (6815948-6815876) Gly (ACC) 73 bp Sc: 52.52 16
TCCTAGGTGGTCTAGAGGCTAAGACTCTGCACTACCAAGGCAGGGGGCCTGGGTTCAATC
CCCGGTCTGGGAA
>Bos_taurus_chrX.trna1341-GlyACC (34808900-34808972) Gly (ACC) 73 bp Sc: 53.22 16
TCCCTGATTGTCCAGTGGCTAAGACTCTGTGCTACCAATGCAGGGGGCCTAGGTTCGATC
CCTGGTCAGGGAA
>Bos_taurus_chr12.trna3870-GlyACC (74898435-74898363) Gly (ACC) 73 bp Sc: 55.45 14
GGGCCAGTGGCACAATGGGTAATGCATCTGACTACCCATCAGAAGATTCCAAGTTCAACT
CCTGGCTGGCTCA
>Bos_taurus_chrUn.004.891.trna9-GlyACC (51647-51719) Gly (ACC) 73 bp Sc: 55.74 16
TCCCTGGTGGTCCAGTGGCTACGACTCTGTGCTACCAATGCAGGGGGCCTGGGTTCGATT
CCTAGCCAGGGAA
>Bos_taurus_chr4.trna4265-GlyACC (120631359-120631439) Gly (ACC) 81 bp Sc: 49.30 18
TCCCAGGTGGCTTACTGGTAAAGCATCTGCCTACCAATGTAGAAGATGCAGGAGACGCAG
GTTCGATCCCTGGTTTGGGAA
>Bos_taurus_chr15.trna1863-GlyACC (53760639-53760727) Gly (ACC) 89 bp Sc: 36.15 18
TTCCAGTTGGCTCAGTGGTAAAGAATCTGTCTACCAGTGGTTAAAGAATTGAACCAAAGG
AGATGCAGGTTCGATCCCTGTGTTGGAAG
>Bos_taurus_chr28.trna1008-GlyCCC (27712875-27712948) Gly (CCC) 74 bp Sc: 21.36 16
TCCCTGATGGTCCAAGTGGCTAAGGCTCTGCTGTCCCATAGAGGGGGGTACAGGTTAGAT
CCCTGTTCAGGCAA
>Bos_taurus_chr11.trna7020-GlyCCC (51357939-51357868) Gly (CCC) 72 bp Sc: 21.83 15
TCCCTGGTGGTCCAGTGGCTGAGGCTCTGTGCTCCCTATACAGGGAGCCCGGTTCCATCC
CTGGTCAGGGAA
>Bos_taurus_chr14.trna1278-GlyCCC (29536419-29536491) Gly (CCC) 73 bp Sc: 22.39 14
TCTCTGGTGGTCCAGTGCCTAAGAGTCTGTGCTCCCGATGCAGGGGGGTCAGGTTCCATC

CCTGGTCAGGGAA
>Bos_taurus_chr7.trna8147-GlyCCC (10678868-10678798) Gly (CCC) 71 bp Sc: 23.21 15
TCCTTGGTGGTTTAGTGGCTGAGACTCTGTGCTCCCAGTGCAAGGGCCTGGTTTGATCCC
TGGCCAGGGAA
>Bos_taurus_chr5.trna4914-GlyCCC (121374003-121374074) Gly (CCC) 72 bp Sc: 24.04 15
TCCCTGGTGGTTCAGTGGAGAAGACTCTGCACTCCCAATGCACGGGGCTGGGTCTAGTCC
CTGGTCAGGGAA
>Bos_taurus_chr5.trna5246-GlyCCC (121258068-121257997) Gly (CCC) 72 bp Sc: 24.04 15
TCCCTGGTGGTTCAGTGGAGAAGACTCTGCACTCCCAATGCACGGGGCTGGGTCTAGTCC
CTGGTCAGGGAA
>Bos_taurus_chr1.trna781-GlyCCC (19050095-19050167) Gly (CCC) 73 bp Sc: 24.07 14
TCCCTGGTTGTCCAGTGGCTGAGACTCTGCATTCCCAGTGTAAGGGGTCCAGGTTTGATC
CCTGGTCAGGGAA
>Bos_taurus_chr1.trna2695-GlyCCC (79185717-79185790) Gly (CCC) 74 bp Sc: 24.18 17
TCCCTGGTGGTCCAATGGCTAAGACTCTGTGCTCCCAATGCAGGGGACTTTAAGTTTAAA
CCCTGGTCAGGGAA
>Bos_taurus_chr19.trna5941-GlyCCC (18790877-18790805) Gly (CCC) 73 bp Sc: 24.94 14
TCTCTGGTGGTCCAGTGACCAAGACTCTGAACTCCCCATGCAGGCAGTCCAGGTTCCATC
CCTGGTCAGGGAA
>Bos_taurus_chr12.trna6294-GlyCCC (16048422-16048350) Gly (CCC) 73 bp Sc: 25.37 16
TCCCTGGTGGTCCAGTGGCTGAGACTCTGCGCTCCCAACGCAGGGGGGCTGGGTTCCATC
TCTGATCAGGGAA
>Bos_taurus_chr7.trna8290-GlyCCC (9341356-9341284) Gly (CCC) 73 bp Sc: 25.43 14
TCTCTGGTGGTCCAGTAGCTAAGACTCTGTGCTCCCAATACAGGGTGCCCAGGTTTGATT
CCTAGTCAGGGGA
>Bos_taurus_chr2.trna4315-GlyCCC (125661715-125661787) Gly (CCC) 73 bp Sc: 25.46 15
TCCCTGGTGGTCCACTGACTATGTCTCTGTGCTCCCAATGCAGATAGCCTGGGTTCGATC
CCTAGTCAGGGAA
>Bos_taurus_chr29.trna2770-GlyCCC (33718462-33718390) Gly (CCC) 73 bp Sc: 25.50 14
TCTCTGGTGGTCCAGTGGCTAAGATTCCATGGTCCCAGCGCGGGGGCCCTTGGTTTGATC
CCCAGTCAGGGAA
>Bos_taurus_chr3.trna8832-GlyCCC (16840182-16840109) Gly (CCC) 74 bp Sc: 25.57 15
TCCCTGGTAGTCCAGTGGCTAAGACTCTGTGCTCCCAGGGCAGGGGGCCTCAGGATAGAT
CCCTGGTCAGGAAA
>Bos_taurus_chr12.trna6683-GlyCCC (9933513-9933441) Gly (CCC) 73 bp Sc: 25.65 14
TCCCTGGCAGTCCAGTGGCTAAGACTCTGTGTTCCCAATGCAGGGGAGCCAGGCTCAATC
CTTGGTTGGGGAA
>Bos_taurus_chr7.trna3254-GlyCCC (81131023-81131095) Gly (CCC) 73 bp Sc: 25.80 15
TTCCTGGTGGTCCAGCGGCTGAGACTCTGCATTCCCAGTACAGGGGGCTCAAGTTCAATC
CCTGGTCAGGGAA
>Bos_taurus_chr6.trna5669-GlyCCC (93704615-93704542) Gly (CCC) 74 bp Sc: 25.86 15
TCTCCTGATGGTCCAGTGACTAAGACCCCGTACTCCCAATGCTGCAGGCCCGGGTTTGAT
CCCTGGTCAGGGAA
>Bos_taurus_chr7.trna7971-GlyCCC (13335281-13335208) Gly (CCC) 74 bp Sc: 25.95 16
TCCCTGGTGGTTCAGTGACTAAGACTCTGTGCTCCCAATGCAGGGGGCCTCACGTTCTAT
CCTTGGTCAGGGAA
>Bos_taurus_chr29.trna3340-GlyCCC (19448852-19448780) Gly (CCC) 73 bp Sc: 26.05 16
TCCCTGATGGTCCAGTGGCTCGGACTATGTACTCCCAATGCAAGTAATCTGGGTTTGATT
CCTGGTCAGGGGC
>Bos_taurus_chr29.trna3345-GlyCCC (19389163-19389091) Gly (CCC) 73 bp Sc: 26.05 16
TCCCTGATGGTCCAGTGGCTCGGACTATGTACTCCCAATGCAAGTAATCTGGGTTTGATT
CCTGGTCAGGGGC
>Bos_taurus_chr1.trna3337-GlyCCC (97482414-97482486) Gly (CCC) 73 bp Sc: 26.15 14
TCCCTGGTGGTCCGGCAGGCGGGACTCTGAGCTCCCGATGCAGGGTGTCCGGGTTCGATC
CCTGGTCAGGGAA
>Bos_taurus_chr5.trna9084-GlyCCC (31663926-31663854) Gly (CCC) 73 bp Sc: 26.39 16
TCCCTGGTGGTCCAGTGGCTGGGCCTCTATGCTCCCAATGCAGGGAACCCAGGTTCCATC
TCTGGTCAGGGAA
>Bos_taurus_chr11.trna3030-GlyCCC (75469807-75469880) Gly (CCC) 74 bp Sc: 26.92 16
TCCCTGATGGTCCAGTGACTGAGATTCTGAGCTCCCAATGCAGGGAGCCCAGGGTTCGAT
CCTTGGTCAGGGAA
>Bos_taurus_chr7.trna8139-GlyCCC (10855599-10855527) Gly (CCC) 73 bp Sc: 27.00 15
TCCCTGGTGGTCCAGTGGCTGAGTCTCTGTGCTCCCAGTGCAGGGGGCCCAGGTTTGATC
CCTGGTCAGGGGA
>Bos_taurus_chr9.trna1675-GlyCCC (52124825-52124899) Gly (CCC) 75 bp Sc: 27.03 17
TCCCTGGTGGTCCAAGTGGCTGAGACTCCGTGCTCCCAACACAGGGGGTGCAGGGTTCGA
TCCCTAGTCAGGGAA
>Bos_taurus_chr19.trna1623-GlyCCC (31984601-31984673) Gly (CCC) 73 bp Sc: 27.15 15
TCCCTGGTGGTCCACAGGCTAAGACTCTGTGCTCCCACTGCAGGGGGCCAGGGTTTGATC
CCTCGTCAGGGAA
>Bos_taurus_chrX.trna4259-GlyCCC (72988999-72988928) Gly (CCC) 72 bp Sc: 27.16 14
TCCCTGGTTGTCCAGTGACTAAGACTCCGCATTCCCAGTGCAGGGGTCCGGGTTCCATCC
CTGGTCAGGGAA
>Bos_taurus_chrX.trna4835-GlyCCC (59392720-59392648) Gly (CCC) 73 bp Sc: 36.85 16

TCCCTGATGGTCCAGTGTCTAAGACTCTGTTTTCCCAATGCAGGGGGCACGGGTTCAACC
CCTGGTTGGGGAA
>Bos_taurus_chr19.trna4121-GlyCCC (51161989-51161917) Gly (CCC) 73 bp Sc: 36.87 14
TCCCTGGTGGTCCAGTGAGTAAGATTCTGAGCTCCCAATGCAGGGGGCCCAGGTTTGATC
CCTGATCAGGGAA
>Bos_taurus_chrUn.004.1096.trna13-GlyCCC (5691-5619) Gly (CCC) 73 bp Sc: 36.89 14
TTCCTGGTGGTCTGGTGGCTAAGACTCTGCACTCCCAATGCAGGGGCCCTGGGTTGGATG
CCTGGTCAGGGAA
>Bos_taurus_chrUn.004.1030.trna5-GlyCCC (49046-48974) Gly (CCC) 73 bp Sc: 36.89 15
TCCCTGGTGGTCCAGTGGCTAAGATTCTGCCCTCCCAATGCAGGAGGCCTGGGTTTGATC
TCTAGTCAGGGAA
>Bos_taurus_chrUn.004.2470.trna3-GlyCCC (12273-12345) Gly (CCC) 73 bp Sc: 36.89 15
TTCTTGGTGGTCCAGTGGCTTAGACTCTGCACTCCCAATGCAGAGGGCCTAGGTTCCATC
CTTGGTCACGGAA
>Bos_taurus_chr21.trna4290-GlyCCC (31049276-31049204) Gly (CCC) 73 bp Sc: 36.90 15
TCCCTGGTGGTCCAGTGGCTGAGACTCTGCAGTCCCAATGCAGGGGGCCTGAGTTCGATC
CCTGGTCAGGGAA
>Bos_taurus_chr22.trna4208-GlyCCC (8252936-8252864) Gly (CCC) 73 bp Sc: 36.91 13
TCCCTGGTGGTCCTGTGGCTAGGACTCTGTGTTCCCAACGCAAGGGGCCTGGGTTTGATC
CCTGGTCAGGGAA
>Bos_taurus_chr19.trna5536-GlyCCC (25025075-25025002) Gly (CCC) 74 bp Sc: 36.92 13
TCCCCGGTAGTCCAGTGAGTAGGTCTCCACACTCCCAATGGAGGGGGCCTCAGGTTTGAT
CCCTGGCTGGGGAA
>Bos_taurus_chr11.trna41-GlyCCC (896975-897046) Gly (CCC) 72 bp Sc: 36.93 16
TCCCTGGTGGTCCAGTGGTTGGGACTCTGTGCTCCCAATGCAGGGGGCCTGGGTCCATTC
CTGGTCAGGGAC
>Bos_taurus_chr14.trna1426-GlyCCC (32678831-32678902) Gly (CCC) 72 bp Sc: 36.93 15
TCCCTGGTGGTCCAGTGGCTGAGACTCTGTGCTCCCAATGCAGGGACCTGGGTTTGATCC
CTGGTCAGGGAA
>Bos_taurus_chr2.trna3537-GlyCCC (108668029-108668101) Gly (CCC) 73 bp Sc: 36.96 15
TCCCTGGTGGTCCAGTGGCTAAGACCCTATGCTCCCAATGCAGGGGGCCCAGGTTTGATC
CCTGGTCAGGAAC
>Bos_taurus_chr17.trna4437-GlyCCC (57364912-57364829) Gly (CCC) 84 bp Sc: 36.98 15
TCCCTGGTGGTCCAGTGGCTGGGACGCCACGCTCCCAAAGTGGAATGGGGTGGAGAGCCC
CAGGCTCGATCCCTGGTCGGGGAA
>Bos_taurus_chr14.trna2679-GlyCCC (64323388-64323459) Gly (CCC) 72 bp Sc: 37.00 17
TCCCTGGTGGTCTGGTGGTAAGACTCAGTGCTCCCACTGCAGGGGGCCTAGGTTCCATCC
CTGGTCAGGGAA
>Bos_taurus_chr4.trna6212-GlyCCC (82053861-82053789) Gly (CCC) 73 bp Sc: 37.03 15
TCCCTGGTGGTCCAATGACTAAGACTCTGCACTCCCAGTGCAGAGGGTCCAGGTTTGATC
CCTAGTCAGGGAA
>Bos_taurus_chr21.trna4139-GlyCCC (34243037-34242965) Gly (CCC) 73 bp Sc: 37.03 17
TCTCTGGTGGTCCAGTGGCTAAGACTCGGCACTCCCAGTGCAGGGGGCCTGGGTTCAATC
CGTAGTCAGAGAA
>Bos_taurus_chr7.trna8273-GlyCCC (9549043-9548971) Gly (CCC) 73 bp Sc: 37.05 15
TCCCTGATGGTCCAGTGGCTAAGATTCTGTGTTCCCAATGCAGGGGCCCTGGGTTTGTTC
CCTGGTCAGGGAA
>Bos_taurus_chr16.trna4816-GlyCCC (39145437-39145365) Gly (CCC) 73 bp Sc: 37.08 14
CCCCTAGTGGTCTACTGTTTAAGACTCTGTGCTCCCAATGCAGGAGGCGCAGGTTCGATC
CCTGGTTGGGGGA
>Bos_taurus_chr23.trna3958-GlyCCC (18591476-18591404) Gly (CCC) 73 bp Sc: 37.08 15
TCCCTGGCAGTCCAGTGGCTAAGACTCTGCGCTCCCAACTCAGGGGGCCTGGGTTTGAAC
CCTGGTCGGGGAA
>Bos_taurus_chr3.trna2457-GlyCCC (70925298-70925370) Gly (CCC) 73 bp Sc: 37.09 15
TCCCTGGTAGTCCAATAGGTAAGACTCTGCATTCCCAATGCAGAGAGCTTGGGATCAATG
CCTGGTTAGGGAA
>Bos_taurus_chr25.trna2912-GlyCCC (35521296-35521223) Gly (CCC) 74 bp Sc: 37.10 17
TACCTGGTGGTCCAGTGGCGAAGACTCTGTGCTCCCAAGACAGGGGTCCCCGGGTTCAAT
CCCTGGTCAGGGAG
>Bos_taurus_chr17.trna4522-GlyCCC (56192424-56192352) Gly (CCC) 73 bp Sc: 37.10 14
TCCCTGGTGGTCCAGTAGCTAAGACTCTGTGCTCCCAATGCAGGGGCCCTGGGTTTGATC
CCTGGTCAGGGAA
>Bos_taurus_chr15.trna1860-GlyCCC (53694854-53694926) Gly (CCC) 73 bp Sc: 37.13 15
TCCCTGGTGGTCCAGTGACTGAGACTCCGCATTCCCAATGCTGGGGACCTAGGTTCAATC
CCTGGTCAGGGAA
>Bos_taurus_chr10.trna3813-GlyCCC (97744140-97744211) Gly (CCC) 72 bp Sc: 37.14 16
TCCCTGGTGGTTCAGTGGCTAAGACTCCATGCTCCCATTGTGGGGGCTTGGGTTTAATCC
CAAGTCAGGGAA
>Bos_taurus_chr17.trna6344-GlyCCC (11726350-11726277) Gly (CCC) 74 bp Sc: 37.17 15
TCTCTGGTGGCCCAGTGGCTAAGACTCCGTGCTCCCCATGCAGGGAGGGCCAGGTTCAAT
CCCTGGTCAGGGAA
>Bos_taurus_chr13.trna3070-GlyCCC (72607861-72607933) Gly (CCC) 73 bp Sc: 37.18 17
TCCCTGATGGTCCAGTGGGTCAGACTCTGCTTTCCCAATGCAGGGGGCCTGGATTCAATC
CCTGGTCAGGGAC

>Bos_taurus_chr25.trna907-GlyCCC (15278634-15278706) Gly (CCC) 73 bp Sc: 37.22 15
TCCCTGGTGGTCCAGTGGCTGAGACTCTGAGCTCCCAATGCAGGGGGGCCAGGTTCAATC
CCAGGTCAGGGAA
>Bos_taurus_chr19.trna3934-GlyCCC (55560283-55560211) Gly (CCC) 73 bp Sc: 37.24 14
TTCCTGGTGGTCCAGTGGCCAAGAGTCTGTGCTCCCAATGAAGGGGGCCCAGGTTTGATC
CCTGGTCAGGAAG
>Bos_taurus_chr11.trna4524-GlyCCC (107561178-107561250) Gly (CCC) 73 bp Sc: 37.24 16
TCCCTGGTGGTCCAGTGGGTGAGACTCTTCACTCCCAGTGCAGGGGGCCCGGGTTCAACC
CCTGGTCAGGGAA
>Bos_taurus_chr10.trna6934-GlyCCC (34223146-34223074) Gly (CCC) 73 bp Sc: 37.29 14
TCCCTGGTGGTCCAGTGGCTAAGACTCTGCGCTCCCCATGCAGGGGGCCTGGGTACAATC
CCTGGTCAGGGAA
>Bos_taurus_chr5.trna9801-GlyCCC (10461926-10461854) Gly (CCC) 73 bp Sc: 37.39 14
TCCCTGGTGGTCCAGTGGCTCAGACTCTGTGCTCCCAATTCAGGGGGCCCGGGTTTGATC
CCTGGTTAGGGAA
>Bos_taurus_chr9.trna1178-GlyCCC (36950579-36950651) Gly (CCC) 73 bp Sc: 37.40 17
TCTCTGGTGGTCTAGTGGCTGAGACTCTGCACTCCCAATGCAGGGCACCCCGGTTCAATC
CTTGGTCAGGGAA
>Bos_taurus_chr13.trna4950-GlyCCC (62951606-62951534) Gly (CCC) 73 bp Sc: 37.45 14
TTCCTGGTGGTCCAGTGGCTAAGACTCTGCACTCCCAATGCAGGGGGCCTGGGTCCGCTC
CCTGGTCAGGGAA
>Bos_taurus_chr2.trna7147-GlyCCC (101660663-101660591) Gly (CCC) 73 bp Sc: 37.50 15
TCCCTGGTGGTCCAGTGGCTAAGACTCTGAGCTCCCAGTGCAGGGGCCCCAGGTTTGATC
CTTGGTTGGGGAA
>Bos_taurus_chr25.trna1190-GlyCCC (21314396-21314468) Gly (CCC) 73 bp Sc: 37.50 15
TCCCTGGTGGTCCATTGGCTAAGACTCTGTGCTCCCAATGCAGGGGGCCTGGGTTTGATC
CCTGGTCAGGGAA
>Bos_taurus_chr2.trna7421-GlyCCC (95391307-95391236) Gly (CCC) 72 bp Sc: 37.51 16
TCCTTGGTGGTCCAATGGCTAGGACTCTGTGCTCCCAGTGCAGGGGCACAGGTTCGTTTC
TTGGTCAGGGAA
>Bos_taurus_chr29.trna2437-GlyCCC (42679436-42679364) Gly (CCC) 73 bp Sc: 37.60 17
TCCCTGGTGATCCAGTGGCTAAGATTCTGAGCTCCCAATGCAGGGGGCCTGAGTTCAATC
CTTGGTCAGGGAA
>Bos_taurus_chr27.trna2309-GlyCCC (38672371-38672299) Gly (CCC) 73 bp Sc: 37.62   15
TCCCTGGTGGTCCAGGGGCTGAGACTTTGAGCTCCCAATGCAGGGGGCTCTGGTTCGATC
CCAGGTCAGGGAG
>Bos_taurus_chr11.trna7077-GlyCCC (50759049-50758977) Gly (CCC) 73 bp Sc: 37.66 15
TCCATGGTGGTCCAGGGGCTAAGACTCTGTGCTCCCAATGCAGGAGGCCCAGGGTCAATC
CCTGGTCAGGGAA
>Bos_taurus_chr28.trna304-GlyCCC (7149380-7149451) Gly (CCC) 72 bp Sc: 37.67 16
TCCCTGGTTGTCCAGTGGCTAAGACTCTGTGCTCCCAATGCATACGGCCAGGTTCGATCC
CTGGTCAGGAAA
>Bos_taurus_chr21.trna4879-GlyCCC (20915733-20915661) Gly (CCC) 73 bp Sc: 37.70 17
TCCCTGGAGGTCCAGTAGCTAAGACTCTATGCTCCCAATGCAGAGGACCCAGGTTCAATC
CCTGGTCAGGGAA
>Bos_taurus_chr25.trna862-GlyCCC (14569352-14569426) Gly (CCC) 75 bp Sc: 37.70 15
TCCCTGGTAGTCCAGTGTCTAAGACTCTGCACTCCCAGTGCAGGGGGGCCCTGGGTTCAA
TTCCTGGTCAGGGAA
>Bos_taurus_chr18.trna4238-GlyCCC (45422792-45422720) Gly (CCC) 73 bp Sc: 37.79 15
ACCCTGGTGGTCCAGTGGCTAAGACTCTTTGCTCCCGATACAGGGGGCCCAGGTTCACTT
CCTGGTCAGGGAG
>Bos_taurus_chr12.trna3546-GlyCCC (80717644-80717572) Gly (CCC) 73 bp Sc: 37.81 15
TCCCTGGTGGTCTGGTGGCTAAGATTCTGCACTCCCAGTGCAAGAGGTCTGGGTTCGATC
TCCAGTCAGGGAT
>Bos_taurus_chr28.trna1151-GlyCCC (32651715-32651788) Gly (CCC) 74 bp Sc: 37.84 13
TCCCTGGTGGTCAGGTGGCTAAGACTCTGAGTTCCCAATGCAGGGGGCCCCAGGTTTGAT
CCCTGGTCAGGGAA
>Bos_taurus_chr25.trna3100-GlyCCC (33195984-33195912) Gly (CCC) 73 bp Sc: 37.87 16
TCCCTGGTGGTCTTGTGACTAAGACTCAGCACTCCCAATGCAGGGGGTCCGGGTTCAATC
CCTGGTCAGGGAA
>Bos_taurus_chr23.trna3102-GlyCCC (38738678-38738606) Gly (CCC) 73 bp Sc: 37.88 13
TCCCTGGTGGTTAAGTGACTAAGACTTTACATTCCCAATGCAGTGGGCCCAGGTTTAATC
CCTGGTCGGGGAA
>Bos_taurus_chr17.trna536-GlyCCC (14616526-14616598) Gly (CCC) 73 bp Sc: 37.89 16
TCCCTGATGGTCCAGTGGCTGGGACCCTGTGCTCCCAGTGCAGGGGGCCCAGGTTTGATC
CCTGGTCAGGGGA
>Bos_taurus_chr25.trna987-GlyCCC (16747021-16747092) Gly (CCC) 72 bp Sc: 37.91 15
TCCCTGGTGGTCCAATGACTAAGACTCTGAGCTCCCAGTGCAGGGGCCCAGGTTTGATTC
CTGGTCAGGGAA
>Bos_taurus_chr9.trna5689-GlyCCC (65942917-65942845) Gly (CCC) 73 bp Sc: 37.97 15
TCCTTGGTGGTCCAGGGGTCAAGACTCTGAACTCCCAATACAGGGGGCCTGAGTTCGATC
TCTGGTCAGGGAA
>Bos_taurus_chr9.trna5692-GlyCCC (65893767-65893695) Gly (CCC) 73 bp Sc: 37.97 15
TCCTTGGTGGTCCAGGGGTCAAGACTCTGAACTCCCAATACAGGGGGCCTGAGTTCGATC

TCTGGTCAGGGAA
>Bos_taurus_chrUn.004.3863.trna1-GlyCCC (3779-3707) Gly (CCC) 73 bp Sc: 37.97 17
TCCTTGGTGGTCCAGGGGTCAAGACTCTGAACTCCCAATACAGGGGGCCTGAGTTCGATC
TCTGGTCAGGGAA
>Bos_taurus_chr27.trna333-GlyCCC (13042916-13042988) Gly (CCC) 73 bp Sc: 38.08 16
TCCCTGGTGGTCCAGTGGTCACGACTCTGTGCTCCCAGTGTAGGGAGCCCAGGTTTGATC
CCTGGTCAGGGAA
>Bos_taurus_chr19.trna5695-GlyCCC (22418383-22418311) Gly (CCC) 73 bp Sc: 38.09 16
TCCCTGATGGTCCAGTGGCTAAGACACTGTGCTCCCGAGGCAGGTGGCCCGAGTTCGATC
TCTGGTCAGGGAA
>Bos_taurus_chr13.trna4157-GlyCCC (76326016-76325944) Gly (CCC) 73 bp Sc: 38.11 14
TCCCTGGTGGTCCAGTGGCTAAGACTCTGCATTCCCAATGAAGGGGGCCCGGGTTTGACC
CGTGGTCAGGGAA
>Bos_taurus_chr9.trna3835-GlyCCC (105129960-105130034) Gly (CCC) 75 bp Sc: 38.12 15
TCCATGGTGGTCCAGTGGCTAAGACTCTGCACTCCCAATGCAGGGAGCCCCCAGGGTTGA
TCCCTGGTCAGGGAA
>Bos_taurus_chr10.trna1815-GlyCCC (47203318-47203390) Gly (CCC) 73 bp Sc: 38.13 14
TCCCTGGTGGTCCAGTGGCTAAGATTCTGTGGTCCCAATGCAGAGGGCTCAGGTTTGATT
CCTGGTGAGGGAA
>Bos_taurus_chr21.trna1525-GlyCCC (33789299-33789371) Gly (CCC) 73 bp Sc: 38.18 15
TCCCTGGTGGTCCAGTGGTTAAGACTCTGTTCTCCCAATGCAGGGGGCCCGGGCTTGATC
CCTGGTTAGGTAA
>Bos_taurus_chr4.trna5508-GlyCCC (98684374-98684304) Gly (CCC) 71 bp Sc: 38.19 17
TCCCTGGTGGTCCAGTGGCTAAGACTCTGAGCTCCCAGTGCAGGGTCCAGGTTCAATCTC
TGGTCGGGGAA
>Bos_taurus_chr18.trna3407-GlyCCC (56252354-56252282) Gly (CCC) 73 bp Sc: 38.20 16
TCCCTGGTTGTCCAGCGGCAAAGACTCTACGCTCCCAATGCAGGGGCCCCAGGTTCCATC
CCTGGTCAGGGAA
>Bos_taurus_chr25.trna2098-GlyCCC (35458262-35458335) Gly (CCC) 74 bp Sc: 38.26 15
TCCCTGGTGGTCTAGCGGCCAAGATTCTGAGCTCCCAGTGCAGGGGGGCCCAAGGTTCGAT
CCCTGGCCAGGGAA
>Bos_taurus_chr27.trna2413-GlyCCC (36317371-36317299) Gly (CCC) 73 bp Sc: 38.27 14
TCCCTGGTCGTCTAGTGGCTAAGACTCTGCACTCCCATTGCATGGGGCTCAGGTTTGATC
CTTGGTCAGGGAA
>Bos_taurus_chr12.trna3661-GlyCCC (78437324-78437252) Gly (CCC) 73 bp Sc: 38.27 14
TCCCTGGTAGTCCAGTTACTAAGATTTTGCATTCCCAATGCAGGGGGCCTGGGTTCAATT
CCTGGTCAGGGAA
>Bos_taurus_chr17.trna1924-GlyCCC (51809202-51809274) Gly (CCC) 73 bp Sc: 38.34 15
TCCCTGGTGGTCCAGTGGCTAAGACTCTGCATTCCCAATGCAGGGGACTTGGGTTCCATC
CCTAGTCAGGAAC
>Bos_taurus_chrX.trna830-GlyCCC (19611789-19611861) Gly (CCC) 73 bp Sc: 38.35 13
TCCCTAGTGGTCCAGCAGCTAAGACTCTGCACTCCCAAAGCAGGGGGCTCAGGTTTGATC
CCTGGCCAGGGAA
>Bos_taurus_chr10.trna3535-GlyCCC (91250286-91250358) Gly (CCC) 73 bp Sc: 38.35 14
TCCCTGGTAGTCCAGAGGCTAAGACTCTGTGCTCCCAATGCAGGGGGCCCAGGTTCCATC
CCTGGTTGGAGAA
>Bos_taurus_chr1.trna7266-GlyCCC (120764378-120764306) Gly (CCC) 73 bp Sc: 38.36 14
TCCCTGGTGGTCCAGTGGCTAAGACTCTGTGCTCCCAATGCAGGTGACCCAGGTTAGCTC
CCTGGTCAGGGAA
>Bos_taurus_chrUn.004.6187.trna1-GlyCCC (3957-3885) Gly (CCC) 73 bp Sc: 38.36 14
TCCCTGGTGGTCCAGTGGCTAAGACTCTGTGCTCCCAATGCAGGTGACCCAGGTTAGCTC
CCTGGTCAGGGAA
>Bos_taurus_chr13.trna2976-GlyCCC (70654643-70654715) Gly (CCC) 73 bp Sc: 38.36 14
TCCCTGGTGGTCTAGTGGCTAAGACTCTGCACTCCCAGTGCAGGGGTCTTGGGTCTGATC
CCTGGTCAGGGAA
>Bos_taurus_chr7.trna7527-GlyCCC (18535047-18534975) Gly (CCC) 73 bp Sc: 38.43 15
TCCCTAGTGGTCCAGTGGCTAAGACTCTGAGCTCCCAATGCAGGCCAGCCAGGTTCGATC
CCTGGTCAGGGAA
>Bos_taurus_chr1.trna4771-GlyCCC (137110519-137110590) Gly (CCC) 72 bp Sc: 38.44 14
TCTCTGGTGGTCCCGTGGCTAAGACTCTGAGCTCCCAATGCAGGGATCCAGGTTTGATTC
CTGGTCAGGGAA
>Bos_taurus_chr29.trna1641-GlyCCC (45118969-45119041) Gly (CCC) 73 bp Sc: 38.66 14
TCCCTGGTGGTCCAGTGGCTAAGACTCCGTATTCCCAATGCAGGTGGCCTGGGTTCAGTC
CCTGGTCAGGGAA
>Bos_taurus_chr11.trna4951-GlyCCC (102296396-102296324) Gly (CCC) 73 bp Sc: 38.71 14
TCCCTGATGGTCCAGGGGCTAAGACTTCATATTCCCAATGTAGGAGGCCTGGGTTTGATC
CCTGGTCAGGGAA
>Bos_taurus_chr10.trna715-GlyCCC (16979762-16979834) Gly (CCC) 73 bp Sc: 38.99 17
TCCCTGATGGTTTAGTGGTTAAGACTCTGTTCTCCCAATGCAGGGAGCTGGGGTTTGATC
CCTGGTCAGGGAA

>Bos_taurus_chr28.trna2513-GlyCCC (16271730-16271658) Gly (CCC) 73 bp Sc: 39.17 17
TCCCCGATGGTCCAGTGACTAAGATTCTGTCCTCCCAGTGCAGGGGGCCCAGGTTCAATC
CCTGGTCAGGGAA

>Bos_taurus_chr11.trna1300-GlyCCC (30526576-30526648) Gly (CCC) 73 bp Sc: 39.30 15
TTCCTGATGGTCCAGAGTCTAAGACTCTGCACTCCCAATGCAGGAGGCTCAGGTTTGATC
CCTGGTTAGGGAA
>Bos_taurus_chr11.trna3929-GlyCCC (96088850-96088922) Gly (CCC) 73 bp Sc: 39.85 18
TCCCTGATGGTCCAATGGCTAAGATTCTGAGCTCCCAATGCAGGGGGCCTGGGTTCCATC
CCTGGTCAGGGAA
>Bos_taurus_chr8.trna2570-GlyCCC (76928327-76928399) Gly (CCC) 73 bp Sc: 39.87 16
TCCCTGGTGGTCCAGTGGCTGAGATTCTGAGCTCCCAATGCAGGGGGCCCAGGTTCCATC
CCTGGTTAGGGAA
>Bos_taurus_chr11.trna307-GlyCCC (5263737-5263809) Gly (CCC) 73 bp Sc: 39.90 16
TCCCTAGTGGTCCAGTGGCTAAGACTCCGCACTCCCAGTGCAGGGAGGCCAGGTTCAATC
CCTGGTCAGGGAA
>Bos_taurus_chr11.trna8477-GlyCCC (15393232-15393158) Gly (CCC) 75 bp Sc: 39.95 16
CCCCTGGTGGTCCACAGTGGTTAAGACTCTGAGCTCCCAATTCAGGGGGCCCAGGTTTGA
TCCCTGGTCAGGGAA
>Bos_taurus_chr2.trna5775-GlyCCC (131049705-131049633) Gly (CCC) 73 bp Sc: 40.02 14
TCTCTGGTCATCCAGTGGCTAAGATGCTGAGCTCCCAATACAGGGGGCCCAGGTTTGATC
CCTGGTCAGAGAA
>Bos_taurus_chr13.trna878-GlyCCC (23469716-23469788) Gly (CCC) 73 bp Sc: 40.08 18
TTGCTGATGGTCCAGTGGCTAAGATTCTGAGCTCCCAATACAGTAGACCCAGGTTCAATC
CCTGGTTGGGGAA
>Bos_taurus_chr3.trna4153-GlyCCC (114514328-114514400) Gly (CCC) 73 bp Sc: 40.09 16
TCCCTGGTGGTCCAAGGGGTGAGACTCTGCACTCCCACTGCGGGTGGGCCAGGTTCGATC
CCTGGTCAGGGAA
>Bos_taurus_chr2.trna4264-GlyCCC (124878460-124878532) Gly (CCC) 73 bp Sc: 40.10 13
TCCCTGGTAGTCCAGTGGCTAAGAAGCTGCATTCCCGATGCAGGGGGCCTGGGTCCAATC
CCTGGTCAGGGAA
>Bos_taurus_chr19.trna6491-GlyCCC (9485905-9485833) Gly (CCC) 73 bp Sc: 40.10 14
GCCCTGGTGGTCCAGTGGCTAAGACTCTGTGTTCCCAATGCAGGGCACCCAGGTTTGATC
CCTGGTCAGGGAA
>Bos_taurus_chr24.trna4009-GlyCCC (35979696-35979624) Gly (CCC) 73 bp Sc: 40.12 15
TCCCTGGTGGTCCAGTGGCTAAGACTCTGTAGTCCCAATACAGAGGGCCTGGGTACAATC
CCTGGTCAGGGAA
>Bos_taurus_chr5.trna2900-GlyCCC (78072613-78072685) Gly (CCC) 73 bp Sc: 40.19 16
TCCCTGGTGGTCCAGTGGCTAAGACTCTGTGGTCCCAATTCAGGGAGCACAGGTTCAATC
CCTGGTCAGGGAA
>Bos_taurus_chr5.trna7271-GlyCCC (78101166-78101094) Gly (CCC) 73 bp Sc: 40.19 16
TCCCTGGTGGTCCAGTGGCTAAGACTCTGTGGTCCCAATTCAGGGAGCACAGGTTCAATC
CCTGGTCAGGGAA
>Bos_taurus_chr9.trna7392-GlyCCC (14803269-14803197) Gly (CCC) 73 bp Sc: 40.20 14
TCCCTGGTGGTCCAGTGGCTAAGATTTTGAGCTCCCCATGCAGGGGGCCTGGGTTCGATT
ACCAGTCAGGGAA
>Bos_taurus_chr2.trna3758-GlyCCC (115078803-115078875) Gly (CCC) 73 bp Sc: 40.21 14
TCCCTGGTGGTCCAGTGGCTAAGACTCTGAATTCCCAATGCAGAGGATCCAGGTTTGTTC
CCTGGTCAAGGAA
>Bos_taurus_chr6.trna5628-GlyCCC (94345971-94345899) Gly (CCC) 73 bp Sc: 40.30 17
TCCCTGGTGGTCTAATGGCTAAGACTCTGAGCTCCCAATTCAGGGGGCCTTGGTTTAATC
CCTGGTCAGGGAA
>Bos_taurus_chr16.trna813-GlyCCC (24705874-24705946) Gly (CCC) 73 bp Sc: 40.34 13
TTCCTGGTGGTCCAGCAGCTAAGACTCTGCACCCCCAATGCAGGGGGTCCAGGTTTGATT
CCTGGTCAGGGAA
>Bos_taurus_chr6.trna1317-GlyCCC (46863999-46864071) Gly (CCC) 73 bp Sc: 40.34 18
TCCTTGATGGTCCAGTGGCTAAGACTCCAAGCTCCCAACTCGGGGGGTCTAGGTTCAATC
CCTGATCAGGGGA
>Bos_taurus_chr25.trna2203-GlyCCC (36902193-36902264) Gly (CCC) 72 bp Sc: 40.37 17
CCCCTGGTGGTCCAATGGTTAAGACTCTGTGCTCCCAGTGCAGGGGGCCAGGTTCGATCC
TTGGTCGGGGAA
>Bos_taurus_chr9.trna7011-GlyCCC (25192943-25192871) Gly (CCC) 73 bp Sc: 40.39 14
TCCCTGGTGGTCCAGGGACTAAGGCTTTGCACTCCCAATGCAGGGGCCCCTGGTTCGACG
CCTGGTCAGGGAA
>Bos_taurus_chr25.trna743-GlyCCC (12770093-12770165) Gly (CCC) 73 bp Sc: 40.39 16
TCCCTGGTGGTCCAGTGGCTAAGACTCCGCACTCCCAATGCAGGGCACCTGGATTCAATC
CCTGGTCAGGGAA
>Bos_taurus_chrUn.004.175.trna3-GlyCCC (126299-126371) Gly (CCC) 73 bp Sc: 40.40 15
TCCCTGGTGGTCCAGTGGCTAAGACTCTGTGCTCCCGATACAGGGGGGCTGGGTTCAATC
CCTGGTCAGGGAA
>Bos_taurus_chr12.trna3794-GlyCCC (75824740-75824668) Gly (CCC) 73 bp Sc: 40.92 17
TCCCTGCTGGTCCAGTGGTAAAGACTCTGAGTTCCCAGTGCAGGGGGCTCAGGTTCGATC
CCTGATCAGGGAA
>Bos_taurus_chr10.trna693-GlyCCC (16145820-16145892) Gly (CCC) 73 bp Sc: 40.94 14
TCCCTGGTGGCCCAGTGGCTAAGACTCTGTACTCCCAGTGCCGGGGGTCCAGGTTTGATC
CCTGGCCAGGGAA
>Bos_taurus_chr17.trna4400-GlyCCC (58209195-58209123) Gly (CCC) 73 bp Sc: 40.95 17
TCCCTGGTTGTCCAGTGGCTAAGACTCTGTGCTCCCAATGCAGGGGGTGCACGTTCAATC

CCTGTTCAGGGAA
>Bos_taurus_chr13.trna6150-GlyCCC (32836285-32836213) Gly (CCC) 73 bp Sc: 40.95 17
TCCTTGGTGGTCTAGTGGCTGAGACTCCATACTCCCAATGCGGAGGACCTGGGTTCAATC
CCTGGTCAAGGAA
>Bos_taurus_chr5.trna5262-GlyCCC (121025927-121025855) Gly (CCC) 73 bp Sc: 40.96 16
TCCCTGGCGGTCCAGTGGCTAAGGCTCTGCACTCCCAGTGCAAGGGGCCCAGGTTCAATC
CCTGGTCAGGGAA
>Bos_taurus_chrX.trna2108-GlyCCC (59367170-59367242) Gly (CCC) 73 bp Sc: 47.78 19
TCCATGGTGGTCCAGTGGTTAAGACTATGTGCTCCCAATGCAGGGGACCCAGGTTCAAGC
CCTGGTCAGGGAA
>Bos_taurus_chr13.trna6584-GlyCCC (24871216-24871144) Gly (CCC) 73 bp Sc: 47.79 16
TCCCTGGTGGTCCAGTAGCTAAGACACTGTGCTCCCAATGCAGGGGTCCTGGGTTCAATT
CCTGGTCAGGGAA
>Bos_taurus_chr13.trna3172-GlyCCC (73947299-73947371) Gly (CCC) 73 bp Sc: 47.81 16
CCCCTGGTGGTCCAGTGGCTAAGACTCTGTGCTCCCAATGCAGGGGGCCCAGGTTCCATC
CCTGGTCAGGGAA
>Bos_taurus_chr2.trna4263-GlyCCC (124867110-124867182) Gly (CCC) 73 bp Sc: 52.05 15
TCCCTGGTGGTCCAGTGGCTAAGACACTGCACTCCCAGTGCAGGGGACCCAGGTTCCATC
CCTGGTCAGGGAG
>Bos_taurus_chr21.trna3345-GlyCCC (58803621-58803549) Gly (CCC) 73 bp Sc: 52.06 14
TCCCTGGTGGTCCAGTGTCTAAGACACTGCACTCCCAATGCAGGGGACCCAGGATGGATT
CCTGGTCAGGGAA
>Bos_taurus_chr28.trna2500-GlyGCC (16682481-16682409) Gly (GCC) 73 bp Sc: 36.64 14
TCCTTAGTGGTCCCGTGGCTAAGACTATGCATTGCCAATGCAGGTGACCCAGGTTTGATC
CCTGGTTGGGGAA
>Bos_taurus_chr11.trna5460-GlyGCC (90787823-90787751) Gly (GCC) 73 bp Sc: 37.06 16
TCCCTGGTGGTCCAGGGGTTAAGACTCTGAGCTGCCACTGCAGGGAATGCAGGTTTGATC
CCTGGTCAGGGAA
>Bos_taurus_chr5.trna5024-GlyGCC (123381315-123381387) Gly (GCC) 73 bp Sc: 38.82 18
TCCCTGGTGGCCCAGTGGTTAGGATTTGGTTCTGCCACTGCCAAGGACCTGGGTTCAGTC
CCTGGTCAGGGAG
>Bos_taurus_chr11.trna8811-GlyGCC (7196046-7195974) Gly (GCC) 73 bp Sc: 38.90 16
TCCCTGGTGGCTCAGTCGGTAAAGATCCCGCCTGCCAATGCAGGAGACTCAGGTTTGATC
CCTGGGTAGGGAA
>Bos_taurus_chr17.trna4142-GlyGCC (62738694-62738621) Gly (GCC) 74 bp Sc: 39.37 18
TCCCTGGTGGTCCACTGGCTAAAGACTCTGAGCTGCCAATGCGGGGGGCCCAGGTTCAAT
CCCTGGTCAGGGAA
>Bos_taurus_chr13.trna4340-GlyGCC (73383939-73383865) Gly (GCC) 75 bp Sc: 39.63 17
TCCCTGGTGGTCCAGCGGCTAAGGCTCTGTGCTGCCAATGCAGATTGGTATCAGGTTCAA
CACCTGGTCAGGGAA
>Bos_taurus_chr2.trna8333-GlyGCC (67079038-67078964) Gly (GCC) 75 bp Sc: 40.37 16
TCCCTGGTGGTCCAGCGGCTAAAAGACTCAGCAATGCCAATGCAGGGGGCCCAGGTTCAA
TCCCTGGTCAGGGAA
>Bos_taurus_chr13.trna3017-GlyGCC (71262409-71262480) Gly (GCC) 72 bp Sc: 40.38 17
TCCCTGGTGGTCCAGTGGTTAAGACTCTGTGCTGCCAGTGCAGGGGTCTGGGTTTGATCC
CTGGTCAGGGAA
>Bos_taurus_chr8.trna310-GlyGCC (8342043-8342114) Gly (GCC) 72 bp Sc: 45.36 18
TTTCTACTGGCTCAGACGGTAAAGAATCTGCCTGCCATGCAGGAGACCCAGGTTCAATCC
CTGGGTGGGGAA
>Bos_taurus_chrUn.004.75.trna10-GlyGCC (162402-162474) Gly (GCC) 73 bp Sc: 45.37 17
TCCCTTGTTGTCCAGTGGTTAAGATTCTGTGCTGCCAATGCAAAGGGCCCAGGTTTGATC
CCTGGCTGGGGAA
>Bos_taurus_chr18.trna2862-GlyGCC (62991276-62991349) Gly (GCC) 74 bp Sc: 45.40 17
TCCCTGGTGGTCCAGGGGCTAAGACTCCGTGCTGCCACTGCAGGGGGGCCCAGGTTCAAT
TCCTGGTCAGGGAA
>Bos_taurus_chr15.trna1211-GlyGCC (35991918-35991996) Gly (GCC) 79 bp Sc: 45.64 17
TCCCCAGTGGTCCAGTTGTTAAGACTTTGCACTGCCACTGCAGGGGGGCACGGGTTAGGT
TCAATCCCTAATTGGGGAA
>Bos_taurus_chrX.trna719-GlyGCC (16213473-16213545) Gly (GCC) 73 bp Sc: 46.12 17
TCCCTGGTGGTCAAGTGGTTAAGACTCTGCACTGCCAATGCATGGGGCACAGGTTGGATA
CCTGGTCAGGGGA
>Homo_sapiens_chr1.trna2-GlyCCC (17053780-17053850) Gly (CCC) 71 bp Sc: 63.06 19
GCCTTGGTGGTGCAGTGGTAGAATTCTCGCCTCCCACGTGGGAGACCCGGGTTCAATTCC
CGGCCAATGCA
>Homo_sapiens_chr1.trna131-GlyCCC (17004836-17004766) Gly (CCC) 71 bp Sc: 69.75 19
GCGTTGGTGGTTTAGTGGTAGAATTCTCGCCTCCCATGCGGGAGACCCGGGTTCAATTCC
CGGCCACTGCA
>Homo_sapiens_chr17.trna13-GlyCCC (19764175-19764245) Gly (CCC) 71 bp Sc: 70.13 19
GCATTGGTGGTTCAATGGTAGAATTCTCGCCTCCCACGCAGGAGACCCAGGTTCGATTCC
TGGCCAATGCA
>Homo_sapiens_chr16.trna34-GlyCCC (686806-686736) Gly (CCC) 71 bp Sc: 76.98 17
GCGCCGCTGGTGTAGTGGTATCATGCAAGATTCCCATTCTTGCGACCCGGGTTCGATTCC
CGGGCGGCGCA
>Homo_sapiens_chr2.trna27-GlyCCC (70476193-70476123) Gly (CCC) 71 bp Sc: 76.98 17

GCGCCGCTGGTGTAGTGGTATCATGCAAGATTCCCATTCTTGCGACCCGGGTTCGATTCC
CGGGCGGCGCA
>Homo_sapiens_chr1.trna133-GlyCCC (16872504-16872434) Gly (CCC) 71 bp Sc: 78.31 19
GCATTGGTGGTTCAGTGGTAGAATTCTCGCCTCCCACGCGGGAGACCCGGGTTCAATTCC
CGGCCAATGCA
>Homo_sapiens_chr1.trna4-GlyCCC (17188416-17188486) Gly (CCC) 71 bp Sc: 78.31 19
GCATTGGTGGTTCAGTGGTAGAATTCTCGCCTCCCACGCGGGAGACCCGGGTTCAATTCC
CGGCCAATGCA
>Homo_sapiens_chr6.trna82-GlyGCC (142578776-142578846) Gly (GCC) 71 bp Sc: 46.73 19
GCATGGGTGATTCAGTGGTAGAATTTTCACCTGCCATGCAGGAGGTCCAGGTTCATTTCC
TGGCCTATGCA
>Homo_sapiens_chr16.trna18-GlyGCC (70822597-70822667) Gly (GCC) 71 bp Sc: 56.35 19
GCATTGGTGGTTCAGTGGTAGAATTCTCGCCTGCCATGCGGGCGGCCGGGCTTCGATTCC
TGGCCAATGCA
>Homo_sapiens_chr1.trna43-GlyGCC (161450356-161450426) Gly (GCC) 71 bp Sc: 69.02 18
GCATAGGTGGTTCAGTGGTAGAATTCTTGCCTGCCACGCAGGAGGCCCAGGTTTGATTCC
TGGCCCATGCA
>Homo_sapiens_chr16.trna25-GlyGCC (70812184-70812114) Gly (GCC) 71 bp Sc: 73.97 18
GCATTGGTGGTTCAGTGGTAGAATTCTCGCCTGCCACGCGGGAGGCCCGGGTTTGATTCC
CGGCCAGTGCA
>Homo_sapiens_chr1.trna68-GlyGCC (161493707-161493637) Gly (GCC) 71 bp Sc: 81.62 19
GCATTGGTGGTTCAGTGGTAGAATTCTCGCCTGCCACGCGGGAGGCCCGGGTTCGATTCC
CGGCCAATGCA
>Homo_sapiens_chr16.trna19-GlyGCC (70823410-70823480) Gly (GCC) 71 bp Sc: 81.62 19
GCATTGGTGGTTCAGTGGTAGAATTCTCGCCTGCCACGCGGGAGGCCCGGGTTCGATTCC
CGGCCAATGCA
>Homo_sapiens_chr16.trna24-GlyGCC (70813012-70812942) Gly (GCC) 71 bp Sc: 81.62 19
GCATTGGTGGTTCAGTGGTAGAATTCTCGCCTGCCACGCGGGAGGCCCGGGTTCGATTCC
CGGCCAATGCA
>Homo_sapiens_chr17.trna5-GlyGCC (8029064-8029134) Gly (GCC) 71 bp Sc: 81.62 19
GCATTGGTGGTTCAGTGGTAGAATTCTCGCCTGCCACGCGGGAGGCCCGGGTTCGATTCC
CGGCCAATGCA
>Homo_sapiens_chr2.trna19-GlyGCC (157257729-157257659) Gly (GCC) 71 bp Sc: 81.62 19
GCATTGGTGGTTCAGTGGTAGAATTCTCGCCTGCCACGCGGGAGGCCCGGGTTCGATTCC
CGGCCAATGCA
>Homo_sapiens_chr6.trna128-GlyGCC (27870756-27870686) Gly (GCC) 71 bp Sc: 81.62 19
GCATTGGTGGTTCAGTGGTAGAATTCTCGCCTGCCACGCGGGAGGCCCGGGTTCGATTCC
CGGCCAATGCA
>Homo_sapiens_chr1.trna35-GlyGCC (161413094-161413164) Gly (GCC) 71 bp Sc: 82.15 19
GCATGGGTGGTTCAGTGGTAGAATTCTCGCCTGCCACGCGGGAGGCCCGGGTTCGATTCC
CGGCCCATGCA
>Homo_sapiens_chr1.trna37-GlyGCC (161420467-161420537) Gly (GCC) 71 bp Sc: 82.15 19
GCATGGGTGGTTCAGTGGTAGAATTCTCGCCTGCCACGCGGGAGGCCCGGGTTCGATTCC
CGGCCCATGCA
>Homo_sapiens_chr1.trna39-GlyGCC (161427898-161427968) Gly (GCC) 71 bp Sc: 82.15 19
GCATGGGTGGTTCAGTGGTAGAATTCTCGCCTGCCACGCGGGAGGCCCGGGTTCGATTCC
CGGCCCATGCA
>Homo_sapiens_chr1.trna41-GlyGCC (161435258-161435328) Gly (GCC) 71 bp Sc: 82.15 19
GCATGGGTGGTTCAGTGGTAGAATTCTCGCCTGCCACGCGGGAGGCCCGGGTTCGATTCC
CGGCCCATGCA
>Homo_sapiens_chr21.trna2-GlyGCC (18827177-18827107) Gly (GCC) 71 bp Sc: 82.15 19
GCATGGGTGGTTCAGTGGTAGAATTCTCGCCTGCCACGCGGGAGGCCCGGGTTCGATTCC
CGGCCCATGCA
>Homo_sapiens_chr1.trna82-GlyTCC (161410032-161409961) Gly (TCC) 72 bp Sc: 55.96 16
GCGTTGGTGGTATAGTGGTGAGCATAGTTGCCTTCCAAGCAGTTGACCCGGGCTCGATTC
CCGCCCAACGCA
>Homo_sapiens_chr17.trna10-GlyTCC (8124866-8124937) Gly (TCC) 72 bp Sc: 71.94 18
GCGTTGGTGGTATAGTGGTAAGCATAGCTGCCTTCCAAGCAGTTGACCCGGGTTCGATTC
CCGGCCAACGCA
>Homo_sapiens_chr1.trna117-GlyTCC (145397935-145397864) Gly (TCC) 72 bp Sc: 73.26 17
GCGTTGGTGGTATAGTGGTGAGCATAGCTGCCTTCCAAGCAGTTGACCCGGGTTCGATTC
CCGGCCAACGCA
>Homo_sapiens_chr1.trna45-GlyTCC (161500903-161500974) Gly (TCC) 72 bp Sc: 73.26v 17
GCGTTGGTGGTATAGTGGTGAGCATAGCTGCCTTCCAAGCAGTTGACCCGGGTTCGATTC
CCGGCCAACGCA
>Homo_sapiens_chr1.trna70-GlyTCC (161439618-161439547) Gly (TCC) 72 bp Sc: 73.26 17
GCGTTGGTGGTATAGTGGTGAGCATAGCTGCCTTCCAAGCAGTTGACCCGGGTTCGATTC
CCGGCCAACGCA
>Homo_sapiens_chr1.trna73-GlyTCC (161432237-161432166) Gly (TCC) 72 bp Sc: 73.26 17
GCGTTGGTGGTATAGTGGTGAGCATAGCTGCCTTCCAAGCAGTTGACCCGGGTTCGATTC
CCGGCCAACGCA
>Homo_sapiens_chr1.trna76-GlyTCC (161424827-161424756) Gly (TCC) 72 bp Sc: 73.26 17
GCGTTGGTGGTATAGTGGTGAGCATAGCTGCCTTCCAAGCAGTTGACCCGGGTTCGATTC
CCGGCCAACGCA

>Homo_sapiens_chr1.trna79-GlyTCC (161417446-161417375) Gly (TCC) 72 bp Sc: 73.26 17
GCGTTGGTGGTATAGTGGTGAGCATAGCTGCCTTCCAAGCAGTTGACCCGGGTTCGATTC
CCGGCCAACGCA
>Homo_sapiens_chr19.trna2-GlyTCC (4724082-4724153) Gly (TCC) 72 bp Sc: 76.83 17
GCGTTGGTGGTATAGTGGTTAGCATAGCTGCCTTCCAAGCAGTTGACCCGGGTTCGATTC
CCGGCCAACGCA
>Bos_taurus_chr26.trna1853-GlyGCC (47012730-47012802) Gly (GCC) 73 bp Sc: 46.21 18
TTCCTGGTGGCCCAGTGGTTAAGGCTCTGCTCTGCCAAAGCAGGGAGCACAGGTTCAGGC
CCTGGCTGGGGAA
>Bos_taurus_chr12.trna479-GlyGCC (14072328-14072400) Gly (GCC) 73 bp Sc: 46.62 18
TCCCTGGTGGTCGAGTGGTTAAGACTCTGTGCTGCCAACACAGGGGGCATGGGTTTGAAC
CCTGGTCAGGGAA
>Bos_taurus_chr10.trna227-GlyGCC (6490906-6490978) Gly (GCC) 73 bp Sc: 47.55 16
TTCCTGGTTGTCCAGTGGCTAAGACTCTGCACTGCCAATGCAGGGGGCCTGGGTTTGATC
CCCGGTCAGGGAA
>Bos_taurus_chr17.trna3577-GlyGCC (71988247-71988176) Gly (GCC) 72 bp Sc: 47.59 16
TCCCTGGTGGTCCAGTGGCTAAGAGTCTGTACTGCCAATGCAGGGGGCCAGGTTTAATCC
CTGGTCAGGGAG
>Bos_taurus_chrUn.004.428.trna9-GlyGCC (89168-89239) Gly (GCC) 72 bp Sc: 47.59 16
TCCCTGGTGGTCCAGTGGCTAAGAGTCTGTACTGCCAATGCAGGGGGCCAGGTTTAATCC
CTGGTCAGGGAG
>Bos_taurus_chr22.trna3485-GlyGCC (25395275-25395203) Gly (GCC) 73 bp Sc: 47.90 17
TCTCTGATGGTCTGCTGGCTAAGACTCTGCACTGCCAATGCAGGGGGGCCAGGTTCGATC
CCTGGTCAGGGAA
>Bos_taurus_chr1.trna8456-GlyGCC (83350963-83350891) Gly (GCC) 73 bp Sc: 48.09 17
TCCCTGGTGGTCCAGTGGCTAAGACTCTGTGCTGCCAAAGCAGGGGGCCTAGGTTCGATC
CCTGCTCAGGGAA
>Bos_taurus_chr2.trna181-GlyGCC (6289102-6289172) Gly (GCC) 71 bp Sc: 48.23 17
GCATAGGTGGTTCAGTGGTAGAATTCTTGCTTGCCACATGGGGGGCCTGGGTTTGATTCC
CAGCCCATGCA
>Bos_taurus_chr19.trna4069-GlyGCC (52507144-52507072) Gly (GCC) 73 bp Sc: 48.31 19
TCCCTGGTGGTCCAGTGGTTAAGATTCTGAGCTGCCAATGCAGGGGGAACAGGTTCAATT
CCTGGTCAGGGAA
>Bos_taurus_chr16.trna3234-GlyGCC (76151092-76151020) Gly (GCC) 73 bp Sc: 49.72 20
TCCCTGATGGTCCAGTGGTTAGGACTTGGCACTGCCACTGCAGGGGGCATGGGTTCAATC
CCTGATCAGGGAA
>Bos_taurus_chr24.trna2043-GlyGCC (49624304-49624374) Gly (GCC) 71 bp Sc: 49.99 18
TCCCAGCTGGCTCAGTGGTTAAGAATCTGCCTGCCAAGCAGGAGACTCGGGTTCGATCCC
TGGGTTGGGAA
>Bos_taurus_chr23.trna4570-GlyGCC (7869087-7869015) Gly (GCC) 73 bp Sc: 50.21 18
TCCCTGGTGGTCCAGTGGTTAAGATTCCGTGCTGCCACTGCAGGGGGCTCAGGTTCGATC
CCTGGTCAGGGAA
>Bos_taurus_chr18.trna2120-GlyGCC (49962265-49962337) Gly (GCC) 73 bp Sc: 50.38 17
ACCCTGGTGGTCCAGTGGCTAAGACTCTGCGCTGCCAATGCAGGGGGCCCAGGTTCCATC
CCTGGTCAGGGAA
>Bos_taurus_chr21.trna974-GlyGCC (22893829-22893901) Gly (GCC) 73 bp Sc: 50.48 17
TCCCTGGTGGTCCAGTGGCTAAGACTCTGTGCTGCCAATGCAGGGGACCCAGGTTCCATC
CCTGGTCAGGGAG
>Bos_taurus_chr18.trna3165-GlyGCC (61943024-61942953) Gly (GCC) 72 bp Sc: 50.97 17
TTCCTGCTGGCTCAGTTGGTAAAGAATCTGCCTGCCGTGCAGGAGACCCAGGTTTGATTC
CTGGGTAGGGAA
>Bos_taurus_chr23.trna3986-GlyGCC (18113158-18113086) Gly (GCC) 73 bp Sc: 52.48 16
TCCCTGGTGGTCGAGTGGGTAAGACTCTGCGCTGCCAATGCAGGGGGCCCAGGTTTGATC
CTTGGTCAGGGAA
>Bos_taurus_chr29.trna28-GlyGCC (1022300-1022372) Gly (GCC) 73 bp Sc: 54.37 18
TCCCTGGTGGTCCAGTGGTTAAGACTCTGCGCTGCCAATGCAGGGGACACAGGTTCAATC
CCTGGTTGGGGAA
>Bos_taurus_chr29.trna39-GlyGCC (1429324-1429396) Gly (GCC) 73 bp Sc: 54.37 18
TCCCTGGTGGTCCAGTGGTTAAGACTCTGCGCTGCCAATGCAGGGGACACAGGTTCAATC
CCTGGTTGGGGAA
>Bos_taurus_chr1.trna7467-GlyGCC (114410195-114410123) Gly (GCC) 73 bp Sc: 55.34 18
TCCCTGGTGGTCCAGTGGCTAAGACTCTGGACTGCCAATGCAGAAGGCCTGGGTTCAATC
CCCAGTCAAGGAA
>Bos_taurus_chr4.trna2020-GlyGCC (63774130-63774202) Gly (GCC) 73 bp Sc: 55.74 18
TCTCTGGTGGTCCAGTGGTTAAGATTCTGCACTGCCAATGCATGGGTTACAGGTTCGATC
CCTGGTCAGGGAA
>Bos_taurus_chr5.trna2122-GlyGCC (58785546-58785617) Gly (GCC) 72 bp Sc: 56.86 19
TCCCTGGTGGCTCAGACGGTAAAGCGTCTGCCTGCCATGCAGGAGACCCAGGTTCAATCC
CTGGGTCGGGAA
>Bos_taurus_chr17.trna4080-GlyGCC (63453252-63453180) Gly (GCC) 73 bp Sc: 57.78 18
TTCCTGGTGGTCCAGTGGTCAAGACTCTCCACTGCCAATGCAGAGGGCTCAGGTTCGATC
CCTGGTCAGGGAA
>Bos_taurus_chr27.trna1901-GlyGCC (48285783-48285712) Gly (GCC) 72 bp Sc: 57.82 17
TCCCTGCTGGCTCAGACGGTAAAGCGTCTGCCTGCCATGCAGGAGACCCGGGTTCGATCC

CTGGGTTGGGAA
>Bos_taurus_chr28.trna3102-GlyGCC (3150077-3150006) Gly (GCC) 72 bp Sc: 59.49 16
TCCCTGGTGGTCCAGTGGCTAGGACTCTGCACTGCCAATGCAGTGACTCTGGTTTGATCC
CTGGTCAGGGAG
>Bos_taurus_chr27.trna2093-GlyGCC (43147196-43147126) Gly (GCC) 71 bp Sc: 59.70 20
GCATGGGTGCTTCAGTGGTAGGATTCCCGCCTGCCACGCGGGAGGCCCGGGTTCAATTTC
TGGCCCGTGCA
>Bos_taurus_chr3.trna2984-GlyGCC (86322865-86322938) Gly (GCC) 74 bp Sc: 60.75 19
TCCCTGGTGGTCTAGTGGTTTAAGACTCTGCACTGCCAATACAGCAGGCCCAGGTTCAAT
CCCTGGTCAGGGAG
>Bos_taurus_chr1.trna8310-GlyGCC (87549283-87549213) Gly (GCC) 71 bp Sc: 66.42 20
GCATTGGTGGTTCAGTGGTAGAATTCTCACCTGCCACTCGGGAGGCCTGGGTTCAATTCC
CAGCCAATGCA
>Bos_taurus_chr6.trna6754-GlyGCC (61649573-61649503) Gly (GCC) 71 bp Sc: 67.09 19
GCATTGGTGGTTCAGTGGTAGAATTCTTGCCTGCCACGTGGGAGGCCTGGGTTCGATTCC
CAGCTGATGCA
>Bos_taurus_chr18.trna27-GlyGCC (774792-774862) Gly (GCC) 71 bp Sc: 81.62 19
GCATTGGTGGTTCAGTGGTAGAATTCTCGCCTGCCACGCGGGAGGCCCGGGTTCGATTCC
CGGCCAATGCA
>Bos_taurus_chr18.trna37-GlyGCC (1170238-1170308) Gly (GCC) 71 bp Sc: 81.62 19
GCATTGGTGGTTCAGTGGTAGAATTCTCGCCTGCCACGCGGGAGGCCCGGGTTCGATTCC
CGGCCAATGCA
>Bos_taurus_chr18.trna5998-GlyGCC (767760-767690) Gly (GCC) 71 bp Sc: 81.62 19
GCATTGGTGGTTCAGTGGTAGAATTCTCGCCTGCCACGCGGGAGGCCCGGGTTCGATTCC
CGGCCAATGCA
>Bos_taurus_chr18.trna5999-GlyGCC (767120-767050) Gly (GCC) 71 bp Sc: 81.62 19
GCATTGGTGGTTCAGTGGTAGAATTCTCGCCTGCCACGCGGGAGGCCCGGGTTCGATTCC
CGGCCAATGCA
>Bos_taurus_chr19.trna1409-GlyGCC (28311388-28311458) Gly (GCC) 71 bp Sc: 81.62 19
GCATTGGTGGTTCAGTGGTAGAATTCTCGCCTGCCACGCGGGAGGCCCGGGTTCGATTCC
CGGCCAATGCA
>Bos_taurus_chr2.trna1341-GlyGCC (41255098-41255168) Gly (GCC) 71 bp Sc: 81.62 19
GCATTGGTGGTTCAGTGGTAGAATTCTCGCCTGCCACGCGGGAGGCCCGGGTTCGATTCC
CGGCCAATGCA
>Bos_taurus_chr23.trna1359-GlyGCC (31005308-31005378) Gly (GCC) 71 bp Sc: 81.62 19
GCATTGGTGGTTCAGTGGTAGAATTCTCGCCTGCCACGCGGGAGGCCCGGGTTCGATTCC
CGGCCAATGCA
>Bos_taurus_chr23.trna1423-GlyGCC (31703264-31703334) Gly (GCC) 71 bp Sc: 81.62 19
GCATTGGTGGTTCAGTGGTAGAATTCTCGCCTGCCACGCGGGAGGCCCGGGTTCGATTCC
CGGCCAATGCA
>Bos_taurus_chr3.trna274-GlyGCC (8751409-8751479) Gly (GCC) 71 bp Sc: 81.62 19
GCATTGGTGGTTCAGTGGTAGAATTCTCGCCTGCCACGCGGGAGGCCCGGGTTCGATTCC
CGGCCAATGCA
>Bos_taurus_chr3.trna272-GlyGCC (8631775-8631845) Gly (GCC) 71 bp Sc: 82.15 19
GCATGGGTGGTTCAGTGGTAGAATTCTCGCCTGCCACGCGGGAGGCCCGGGTTCGATTCC
CGGCCCATGCA
>Bos_taurus_chr3.trna276-GlyGCC (8817913-8817983) Gly (GCC) 71 bp Sc: 82.15 19
GCATGGGTGGTTCAGTGGTAGAATTCTCGCCTGCCACGCGGGAGGCCCGGGTTCGATTCC
CGGCCCATGCA
>Bos_taurus_chr3.trna278-GlyGCC (8941221-8941291) Gly (GCC) 71 bp Sc: 82.15 19
GCATGGGTGGTTCAGTGGTAGAATTCTCGCCTGCCACGCGGGAGGCCCGGGTTCGATTCC
CGGCCCATGCA
>Bos_taurus_chr21.trna3271-GlyGCC (60062795-60062673) Gly (GCC) 123 bp Sc: 54.61 17
TCCCTGGTGGTCCAGTGGCTAAGACTCTGCACTGCCAAGACCAGGGGACATTCCTGGTGG
ACTAGTTGCTAAGACTCCACACTGCCAATGCAGGGGGCCCAGGTTCGATCCCTGGTCAGG
GAA
>Bos_taurus_chr14.trna192-GlyTCC (5693404-5693474) Gly (TCC) 71 bp Sc: 23.47 14
TCCGTGGTAGTCCAGTGGCTAAGACTCTGTGCTTCCAGTGCAGAAGCTGGGTTTGATGCC
TGGCCAGGGAA
>Bos_taurus_chr16.trna2301-GlyTCC (58903532-58903603) Gly (TCC) 72 bp Sc: 38.37 15
TCCCTGGTAGTCCAGTGGTTAGGACTTGGCACTTCCACTGCCAGGGATTGGGTTCAGCCC
CTTGTTGGGGAA
>Bos_taurus_chr13.trna2165-GlyTCC (55195749-55195819) Gly (TCC) 71 bp Sc: 38.53 16
TCCCTGGTGGTCCAGTGGTTAAGACTCCTCGCTTCCTCAGAGGAGTTTGGGTTCAATCCC
CGATCGGTGAA
>Bos_taurus_chr14.trna5433-GlyTCC (27762521-27762449) Gly (TCC) 73 bp Sc: 38.53 15
TCCCTGGGGGTTCAGTGGTTATGACTCTGAGCTTCCAATGCAGGGGGTCCAGGTTTGATC
CCTGGTCAGGGAA
>Bos_taurus_chr15.trna1557-GlyTCC (44445149-44445224) Gly (TCC) 76 bp Sc: 38.58 16
TCCCTGGTGGTCTAGTGGCTAAGACTCTACACTTCCAATGTGAGATTAAATCCAGGTTCG
ACCCTTAGTCAGGGAA
>Bos_taurus_chr3.trna5161-GlyTCC (117921628-117921556) Gly (TCC) 73 bp Sc: 38.74 15
TCCCTGGTAGTCCAGTGAGTAAGACTCTGTGCTTCCAATGCAGGGGGCACAGGTTCAATC
CTTGGTCAGGGAA

>Bos_taurus_chr27.trna1060-GlyTCC (30311833-30311905) Gly (TCC) 73 bp Sc: 39.26 17
TCCCTGGTGGTCCAAGTGTTAAGACTCTTTACTTCCATTGCAGGGGACACAGGTTCGATC
CCTGGTCAGGGAA
>Bos_taurus_chrUn.004.41.trna23-GlyTCC (266271-266343) Gly (TCC) 73 bp Sc: 39.28 16
TCCCTGGCAGTCCAGTGGTTAAGACTCTATGCTTCCACTGCAGAGAGCCCAGGTTTGAGC
CCTGGTCGGGGAA
>Bos_taurus_chr14.trna6475-GlyTCC (6141482-6141410) Gly (TCC) 73 bp Sc: 39.34 16
TCCCTGGTGGTCCAGTGGCTAGGATTCTGTGTTTCCACTGCAGGGTACCAGGGTTCAATC
CCTAGTCAGGGAA
>Bos_taurus_chr5.trna4418-GlyTCC (112311776-112311848) Gly (TCC) 73 bp Sc: 39.34 18
TCTCTGATGGTCCAGTGGCTGAGACTCTGCACTTCCAGTTCAGGGGGCCCGGGTTCAATC
CCTGGTCAGGGAA
>Bos_taurus_chr17.trna4687-GlyTCC (54606519-54606447) Gly (TCC) 73 bp Sc: 39.43 16
TCCCTGATGGTCCAGGTGTTAAGACTCTGTGCTTCCAATACAGGGATCTCAGGTTTGATC
CTTGATTGGGGAA
>Bos_taurus_chr20.trna1698-GlyTCC (44974552-44974623) Gly (TCC) 72 bp Sc: 39.43 16
TCCCTGGTAGTCTAGTGGTTAAGACTCTGTGCTTCCACTGCAGGGGCATAAGGTCAATCC
TTGGTTGGGGAA
>Bos_taurus_chr13.trna5381-GlyTCC (51762459-51762387) Gly (TCC) 73 bp Sc: 39.49 18
TCCCTGATGGTCCAGTGGTAAGGACTTTGTTCTTCCATTGCAGGAGGCATAGGTTTGATC
CCTGGTCAGGGAA
>Bos_taurus_chr10.trna327-GlyTCC (8464872-8464944) Gly (TCC) 73 bp Sc: 39.50 16
TCCCTGGTGGTCCAGTGGTTACGACTCTGGGCTTCCAATGCAGGGCACACAGGTTTGATC
CCTGATCAGGGAA
>Bos_taurus_chr16.trna5511-GlyTCC (23311561-23311489) Gly (TCC) 73 bp Sc: 43.65 15
TCTCTGGTGGTCCAATGGCTAAGACTCTGCACTTCCAATTCAGGGGGTCCAGGTTGGATC
CCTGGTAAGAGAA
>Bos_taurus_chr11.trna3633-GlyTCC (89660999-89661071) Gly (TCC) 73 bp Sc: 43.69 15
TCCCTGGTGGTCCAGTGACGAGGACTCCCTGCTTCCAATGTGGTGGGCCCTGGTTCGATC
CCTGGTCAGGGAA
>Bos_taurus_chr21.trna4179-GlyTCC (33691758-33691686) Gly (TCC) 73 bp Sc: 43.7 17
TCCCTGGAAGTCCAATGGTTAAGACTCTATACTTCCACTGCAGGGAGTCCAGGTTCAATT
CCTGGTTGGGGAA
>Bos_taurus_chr20.trna5520-GlyTCC (6168442-6168371) Gly (TCC) 72 bp Sc: 43.77 17
TCCTTGGTGGTCTAGTGGTTAAGACTCTGTGCTTCCAATGCAGGAGCACAGGCTCAATCC
CTGGTTGGGGAA
>Bos_taurus_chr17.trna5152-GlyTCC (47385623-47385551) Gly (TCC) 73 bp Sc: 43.79 14
TCCCTGGTGGTCCAGTGGCTAAGACTCTGCACTTCCAATGCAGGGGGCCTGGGTTTGGTC
CCTGGTCAGGGAA
>Bos_taurus_chr5.trna4591-GlyTCC (115947496-115947569) Gly (TCC) 74 bp Sc: 43.81 16
TTCCTTGTGGTCCAGTGGTTAGGACTCTGTGCTTCCACTGCTGAGAGCACAGGTTCGATC
CTTGTTCAGGGAAC
>Bos_taurus_chr20.trna2598-GlyTCC (68252569-68252641) Gly (TCC) 73 bp Sc: 43.86 17
TCCCTGGTGGTTCAGTGGTTAAGACTCTGCACTTCCACTACAGGGGACACAGGTTCCATC
CCTGGTTGGGGAA
>Bos_taurus_chr1.trna5274-GlyTCC (151622382-151622454) Gly (TCC) 73 bp Sc: 43.86 17
TCCCTGGTGGTCCAGTGGTTAAGACTCTGTGCTTCCACTGCAGGGGGCATGGGTTCTATC
CCTGTTCAGGGAA
>Bos_taurus_chr5.trna2751-GlyTCC (73789445-73789517) Gly (TCC) 73 bp Sc: 43.86 17
TCCCTGGTGGTCCAGTGGTTAAGACTCTGTGCTTCCACTGCAGGGGGCATGGGTTCTATC
CCTGTTCAGGGAA
>Bos_taurus_chr7.trna413-GlyTCC (9174406-9174478) Gly (TCC) 73 bp Sc: 45.02 16
TTCCTGGTGGTTCAGTGGCTAGGACTCTGCCCTTCCAGTGCAGAGGGCCCAGGTTCTATC
CCTGGTCAGGGAA
>Bos_taurus_chr13.trna6602-GlyTCC (24692773-24692701) Gly (TCC) 73 bp Sc: 45.09 20
TCCCTGATGGTCCAATGGTTAAGACTCTGTGCTTCCAATGCAGTGGGTGTGGGTTCAATC
CCTGATCAGGGAA
>Bos_taurus_chrX.trna6685-GlyTCC (3862467-3862395) Gly (TCC) 73 bp Sc: 45.10 17
TCCCTGGAAGTCCAGTGGTTAGGACTCTGTGCTTCCACTACAGGGGGTCTGGGTTCGATC
CCTGGCTGGGGAA
>Bos_taurus_chr4.trna2306-GlyTCC (72326941-72327013) Gly (TCC) 73 bp Sc: 45.30 18
TCCCTGGTGGTCCAGTGGTTGGGACTCTGTGCTTCCACTGCAGGGAGTGCAGGTTCAATC
CCTGGTCAGGGAA
>Bos_taurus_chr16.trna2138-GlyTCC (54926605-54926677) Gly (TCC) 73 bp Sc: 45.35 16
TCCCTGGTGGTCCAGTGGTTAAGACTCTGAGCTTCCACTGCAGAGACCTCAGGTTGGATT
CCTGGTCAGAGAA
>Bos_taurus_chr15.trna4440-GlyTCC (50393774-50393702) Gly (TCC) 73 bp Sc: 45.40 15
TCCCTGGTGGTCCAGTGGCTAAGACTCTGAGCTTCCAATGCAGGGGGCCCAGGTTTGACT
CCTGGTCTGGGAA
>Bos_taurus_chr14.trna6588-GlyTCC (3844636-3844564) Gly (TCC) 73 bp Sc: 45.43 14
TCCCTGGTGGTCCAGTGGCTAAGATGCTGCACTTCCTATGCAGGGAGCCCAGGTTTGATC
CCTAGTCAGGGAA
>Bos_taurus_chr6.trna5225-GlyTCC (101384748-101384676) Gly (TCC) 73 bp Sc: 45.47 15
TCCCTGGTGGTCCAGCAGTTAAGACTCTGCACTTCCAGTGCGGAAGCCACAGGTTCGATT

CCTGGTTAGGGAA
>Bos_taurus_chr12.trna449-GlyTCC (13814048-13814120) Gly (TCC) 73 bp Sc: 45.51 16
TCCCTGGTGGTCCAGTGGCTAAGACTCTGTGCTTCCAATGCAGGGGGCCTGGGTGCAATT
CCTGGCCAGGGAA
>Bos_taurus_chrX.trna2211-GlyTCC (61993047-61993119) Gly (TCC) 73 bp Sc: 45.55 16
TCCCTGGTGGTCCAGTGATTAAGACTCTGCACTTCCACTGCAGGGTGCACAGGTTCCATC
CCTGGTCAGGGAA
>Bos_taurus_chrUn.004.36.trna8-GlyTCC (546664-546592) Gly (TCC) 73 bp Sc: 45.74 16
TCCCTGGTGGTCCAGTGGCTAAGACTCTGTGCTTCCAATACAGAGGGGCTGGGTTCGATC
CCTGGTCAGGGAA
>Bos_taurus_chr15.trna4439-GlyTCC (50497624-50497553) Gly (TCC) 72 bp Sc: 45.75 16
TCCCTGGTGGTCCAGTGGTTAGGACTCTGTGCTTCCACTGCAGGGTCCTGGGTTTGATCC
CTTGTTGGGGAA
>Bos_taurus_chr12.trna1446-GlyTCC (31704040-31704112) Gly (TCC) 73 bp Sc: 45.79 18
TCCCTGGTGGTCCAGTGGTTAAGACTCTGTGCTTCCAACGCAGGGGGCATGGGTTCAATG
CCTGGTCAGGGAA
>Bos_taurus_chr1.trna2838-GlyTCC (82934266-82934338) Gly (TCC) 73 bp Sc: 45.81 14
TCCCTGGTGGTCCAGTGGCTAAGATTCTGTGTTTCCAATGCAGGGGTTTCAGGTTTGATC
CCTGGTTGGGGAA
>Bos_taurus_chr17.trna2045-GlyTCC (53552239-53552311) Gly (TCC) 73 bp Sc: 45.93 15
TCCTTGGCAGTCCAGCGGCTAAGACTCTGCACTTCCAGTGCAGGGGACCCAGGTTCAATC
CCTGGTCAGGGAA
>Bos_taurus_chr10.trna7778-GlyTCC (11756994-11756922) Gly (TCC) 73 bp Sc: 46.13 16
TCCCTGGCAGTCCAGTGGTTAGGGTTCTGTGCTTCCACCACAGGGGGCCCAGGTTCAGTC
CCTGGTCAGGGAA
>Bos_taurus_chr17.trna3119-GlyTCC (69312903-69312975) Gly (TCC) 73 bp Sc: 46.21 16
TCCCTGGTAGTCTAGAGGTTAGGATTCTGCGCTTCCACTTCAGGGGCCACAGGTTCAACC
CCTGGTCAGGGAA
>Bos_taurus_chr9.trna4969-GlyTCC (84456389-84456316) Gly (TCC) 74 bp Sc: 47.63 17
TCCCTGGTGGTCCAGTGGCTAAGACTCTGCACTTCCAAGGCAGGAGGCCCAGGGTTCAAT
CCCTGGTCAGGGAA
>Bos_taurus_chr3.trna2384-GlyTCC (68084172-68084244) Gly (TCC) 73 bp Sc: 47.75 17
TCCCTGGTAGTCTAGTGGTTAAGACTCTGTGCTTCCACTGCAGGGGGCTCAGGTTCAAAC
TCTGGTTGGGGAA
>Bos_taurus_chr10.trna3988-GlyTCC (103228335-103228407) Gly (TCC) 73 bp Sc: 47.77 16
TCCCTGGTGGTCCAGTGGTTAGGATTCTGCTCTTCCAGTGCAGGGGACTCAGGTTTGATC
CCAGACCAGGGAA
>Bos_taurus_chr10.trna3995-GlyTCC (103311664-103311736) Gly (TCC) 73 bp Sc: 47.77 16
TCCCTGGTGGTCCAGTGGTTAGGATTCTGCTCTTCCAGTGCAGGGGACTCAGGTTTGATC
CCAGACCAGGGAA
>Bos_taurus_chr18.trna4284-GlyTCC (44467032-44466960) Gly (TCC) 73 bp Sc: 47.81 14
TCCCTGGTGGTTTGGTGGCTAAAACTTTGTGTTTCCAATGCAAAGGTCCCAGGTTTTATT
CCTGGTCAGGGAA
>Bos_taurus_chr11.trna2791-GlyTCC (70242206-70242278) Gly (TCC) 73 bp Sc: 47.82 17
TCCCTGGTGGTCCAGTAGTTAAGACTCTGCGCTTCCAATGCAGGGGGTGCAGGTTCAATC
CCTGGTCAGGGAA
>Bos_taurus_chr2.trna4176-GlyTCC (123377674-123377746) Gly (TCC) 73 bp Sc: 47.86 16
TCCCTGGTGGTCCAGTGGTTAAGACTCTGTGCTTCCATCGCAGGGGGCATGGGTTGGATC
CCTGTTCAGGGAA
>Bos_taurus_chr22.trna4476-GlyTCC (3914163-3914091) Gly (TCC) 73 bp Sc: 47.98 13
TCCCCGGTGGTCCAGTGACTAAGACTCTGCACCTCCAATGCAGGGGTCCTGGGTTTGATC
CCTAGTTGGGGAA
>Bos_taurus_chr27.trna3782-GlyTCC (5092807-5092736) Gly (TCC) 72 bp Sc: 48.03 18
TCTCTGGTGGTCCAGTGGTTAAGACTCTGCACTTCCAATGCAGGGGGCATGGGTTCAATC
CCTGTCGGAGAA
>Bos_taurus_chr7.trna2284-GlyTCC (51480482-51480554) Gly (TCC) 73 bp Sc: 48.13 16
TCCCTGGTCGTCCAGTGGTTAGGATGCTGTGCTTCCATTTCAGAGGGCCCAGGTTCATCC
CCTGGTCAGGGAA
>Bos_taurus_chr8.trna932-GlyTCC (26612068-26612140) Gly (TCC) 73 bp Sc: 48.66 17
TTCCTGGTGGTCCAGTGATTAGGACTCTGTGCTTCCACTGCTGGGGGCCCAGGTTCAATT
CCTGGTTGGGAAA
>Bos_taurus_chr26.trna2586-GlyTCC (38281852-38281781) Gly (TCC) 72 bp Sc: 48.66 15
TCCCTGGTGGTTCAGGGGTTAAGATGCTGCACTTCCAGTGCAGGAGCACAGGTTCGATCC
CTGGTTGGGGAA
>Bos_taurus_chr7.trna3327-GlyTCC (82985078-82985150) Gly (TCC) 73 bp Sc: 48.77 15
TCCTTGGTGGTCCAGTGGTTAAGACTCTGCGTTTCCACTGCAGGGGGCACAGGTTGGATC
CCTGGTCAGGGAA
>Bos_taurus_chr6.trna5176-GlyTCC (101965807-101965735) Gly (TCC) 73 bp Sc: 48.87 16
TCCCTGGTGGTCCAGTGGTTAAGACTCTGTGCTTCCAATGCAGGGGGTGTGGGTTTGATC
CCCGCTTGGGGAA
>Bos_taurus_chr21.trna929-GlyTCC (22307748-22307820) Gly (TCC) 73 bp Sc: 48.90 16
TCCCTGGTGGTCCAGTGGTTAAGACTCTGCACTTCCAGTGCAGGGGGTGCAGGTTTGATC
CCTGGTCAGGGAA
>Bos_taurus_chr18.trna3992-GlyTCC (48470361-48470288) Gly (TCC) 74 bp Sc: 48.91 19

TCCCTGGTGGTCCAATGGTGAAGATGCCATGCTTCCAACGTGGGGGTGTGCGGGTTCAAT
CCCTGCTCGGGGAA
>Bos_taurus_chr11.trna1937-GlyTCC (48300437-48300509) Gly (TCC) 73 bp Sc: 48.91 17
TCCCAGGTGGTCCAGTGGTTAGGACTCTGCACTTCCACTGCAGGGAGCCTGGGTTCCATC
CCTGGTTGGGGAA
>Bos_taurus_chr20.trna3067-GlyTCC (69945000-69944928) Gly (TCC) 73 bp Sc: 48.93 17
TCCCTGGTGGTCCAGTGGTTAGGATTCTGTGCTTCCACTGGAGGGGGCCCAGGTTCAGTC
CCTGGTCAGGGAA
>Bos_taurus_chr17.trna6367-GlyTCC (11242440-11242369) Gly (TCC) 72 bp Sc: 48.97 17
TCCCTGGTGGTCCAGTGATTTGGACTCTGCCCTTCCACTGCAGGGTCACAGGTTCAATCC
CTGACTGGGGAA
>Bos_taurus_chr6.trna4516-GlyTCC (115339366-115339294) Gly (TCC) 73 bp Sc: 49.07 20
TCCCTGGTGGTTCAGTGGGTATGATTCTGCACTTCCAATACAGGGGGCCCAGGTTCAATC
CCTGGTCAGGGGA
>Bos_taurus_chr11.trna7896-GlyTCC (30955110-30955038) Gly (TCC) 73 bp Sc: 49.12 17
TCCCCAGTAGTCCAGTGGTTAAGACTCTGAGCTTCCACCACAGAGGGCTTGGGTTCAATC
CCTAGCTAGGGAT
>Bos_taurus_chr9.trna571-GlyTCC (20120981-20121053) Gly (TCC) 73 bp Sc: 49.34 18
TCCCTGATGGCCCAGTGGTTAGGGTGCTGAGCTTCCACTGCAGGAGACATGGGTTCGATC
CCTAGTCAGGGAA
>Bos_taurus_chr2.trna767-GlyTCC (24804843-24804913) Gly (TCC) 71 bp Sc: 49.40 17
TCCTTGGTGGTCCAGTGGCTAGGACTCTGTGCTTCCAATGCAGGGACCCAGGTTCAATAC
CTGGTCAGGAG
>Bos_taurus_chr18.trna5003-GlyTCC (25826099-25826028) Gly (TCC) 72 bp Sc: 49.41 17
TCCCTGGTGGTCCAGTGGTTAGCATGTGGTATTTCCACTGCTGTGACCCAGGTTCAATCC
TTGGTTAGGGAA
>Bos_taurus_chr1.trna6453-GlyTCC (144251698-144251626) Gly (TCC) 73 bp Sc: 49.56 18
TCCCTGGTGGTTCAGTGGTTAAGACTTTGCACTTCCATTGCAGGGGGAGTGGGTTCAATC
CCTACTCAGGGAA
>Bos_taurus_chr21.trna1320-GlyTCC (28127754-28127825) Gly (TCC) 72 bp Sc: 49.59 18
TCCCTGGTGGTCCAGTGGTTAAGACCCTGTGCTTCCACAGCAGGGGCATAGGTTCAATCC
CTGGTCAGGGAA
>Bos_taurus_chrUn.004.2.trna168-GlyTCC (1030665-1030593) Gly (TCC) 73 bp Sc: 49.60 15
TCCCTGGTGGTCCAGTGGTTAAGACTCTGCATTTCCACTACAGTTGGCCTAGGTTTGATC
CCTGGTCAGGGAA
>Bos_taurus_chr18.trna3012-GlyTCC (65597076-65597005) Gly (TCC) 72 bp Sc: 49.66 17
TTCCTGATGGTCCAGTGGTTAAGACTCTGCTCTTCCAATGCAGGGGCTCAGGTTTGATCC
CTGGTGGGGGAA
>Bos_taurus_chr8.trna1367-GlyTCC (41852184-41852256) Gly (TCC) 73 bp Sc: 49.76 15
TCCCTGGTAGTCCAGTGGTTAAGGCTCTGCACTTCCACTGCAGGGGGCATAGGTTTGATT
CCTGGTCAGGGAA
>Bos_taurus_chr11.trna1285-GlyTCC (30091494-30091566) Gly (TCC) 73 bp Sc: 49.85 16
TTCTTGGTGGTCCAGTGGCTAAGACTCTGGGCCTCCAACGCAGGGGGCCCAGGTTCAATC
CCTGGTCAGGGAA
>Bos_taurus_chr17.trna446-GlyTCC (13261272-13261344) Gly (TCC) 73 bp Sc: 49.92 16
TCCCTGGTGGTCCAGTGGTTAGGATGCTGTGCTTCCACTGCAGGGGGCACAGGTTTTATC
CCTGGCCAGGGAA
>Bos_taurus_chr18.trna669-GlyTCC (17594232-17594304) Gly (TCC) 73 bp Sc: 50.08 16
TTCCTGGTGGTCCAGTGGCTAAGACTCTGTGCTTCCAACGCAGGGATCCCAGCTTCGATC
CCTGGTCAGGGGA
>Bos_taurus_chr1.trna11015-GlyTCC (2375663-2375591) Gly (TCC) 73 bp Sc: 50.10 15
TCCCTGGTGGTTCAGTGGCTAAGACTCTGTTCTTCCAATGCAGGGGGCCCAGGTTGGATC
CCTGGTCAGGGAA
>Bos_taurus_chr11.trna3678-GlyTCC (90429261-90429332) Gly (TCC) 72 bp Sc: 50.11 18
TCCCTGCTGGTCCAATGGTTAAGACTCTGAGCTTCCAATTCAGGGGCCCAGGTTCCATCC
CTGGCTGGGGAA
>Bos_taurus_chr3.trna9288-GlyTCC (3596604-3596532) Gly (TCC) 73 bp Sc: 50.16 15
TCCCTGGTGGTCCAGTAGTTAAGACTCTGCACTTCCGATGCAGGGGGCATGGGTTCGATC
CCTGGCCAGGGAA
>Bos_taurus_chr19.trna3744-GlyTCC (58181285-58181213) Gly (TCC) 73 bp Sc: 50.22 14
TCCCTGGTAGTCCAGGGGTGAAGACCCTGCGCTTCCAATGCAGGGGGCCCAGGTTTGATC
CCTGGTCAGGGAA
>Bos_taurus_chr4.trna1541-GlyTCC (48707072-48707144) Gly (TCC) 73 bp Sc: 50.29 15
TCCCTGGTGGTCCAGTGGCTAAGACTCTGCACTTCCACTGCAGGGGGCCTGGGTTTGATC
CCTGGTCAGGGAA
>Bos_taurus_chr2.trna8973-GlyTCC (47020760-47020688) Gly (TCC) 73 bp Sc: 50.38 15
TCCCTGGCAGTCCAGTGGTTAGGACTCGGCACTTCCACTGCAGGGGGCCCAGGTTTGATC
CCTGGTTGGGGAA
>Bos_taurus_chr22.trna653-GlyTCC (14831746-14831818) Gly (TCC) 73 bp Sc: 50.40 17
TCCCTGGTGGTTCAGTGGTTAAGACTCTGCGCTTCCAATGCAGGGGTCTCAGGATCAATC
TCTGGTTGGGGAA
>Bos_taurus_chr6.trna640-GlyTCC (23651667-23651739) Gly (TCC) 73 bp Sc: 50.54 16
TCCCTGGTGGTCCAGTGGTTAGGACTCTACACTTCCACTGTAGAGGATACAGGTTCCGTC
CCTGGTCAGGGAA

>Bos_taurus_chrUn.004.2284.trna1-GlyTCC (17168-17096) Gly (TCC) 73 bp Sc: 50.54 16
TCCCTGGTGGTCCAGTGGTTAGGACTCTACACTTCCACTGTAGAGGATACAGGTTCCGTC
CCTGGTCAGGGAA
>Bos_taurus_chrUn.004.5644.trna1-GlyTCC (7435-7363) Gly (TCC) 73 bp Sc: 50.54 16
TCCCTGGTGGTCCAGTGGTTAGGACTCTACACTTCCACTGTAGAGGATACAGGTTCCGTC
CCTGGTCAGGGAA
>Bos_taurus_chrUn.004.6077.trna1-GlyTCC (6524-6452) Gly (TCC) 73 bp Sc: 50.54 16
TCCCTGGTGGTCCAGTGGTTAGGACTCTACACTTCCACTGTAGAGGATACAGGTTCCGTC
CCTGGTCAGGGAA
>Bos_taurus_chr26.trna3485-GlyTCC (14647620-14647548) Gly (TCC) 73 bp Sc: 50.63 14
TCCCTGGCAGTCCAGTGGTTAAGACTCTGCATTTCCACTGCAGGGGCCACAGGTTTGATC
CCTGTTTGGGGAA
>Bos_taurus_chrUn.004.125.trna1-GlyTCC (61294-61366) Gly (TCC) 73 bp Sc: 50.77 18
TCCCTGGTGGTCCAGTGGTTAGACTCCACACTTCCAATGTAGGCAGCACAGGTTCAATC
CCTGGCCAGGGAA
>Bos_taurus_chr22.trna1848-GlyTCC (51956972-51957044) Gly (TCC) 73 bp Sc: 50.77 16
TCCCTGGTGGTCCAGTGGTTAGGGCTCTGTGCTTCCACTGCAGGGGCCACAGGTTTGATC
CCTGGCTGGGGAA
>Bos_taurus_chr11.trna1286-GlyTCC (30102233-30102305) Gly (TCC) 73 bp Sc: 50.89 16
TCCCTGGTGGTTCAGTGGTTTGGATTCTGCACTTCCAATGCAGGGGGCCCAGGTTTGATC
CCTGGTTGAGGAA
>Bos_taurus_chr7.trna6176-GlyTCC (57889845-57889773) Gly (TCC) 73 bp Sc: 51.02 17
TCCCTGGTGGTCCAGTGGTGAAGACGCTGCACTTCCACTGCAGGGAGCATGGGTTCGATC
CCTAGTTGGGGAA
>Bos_taurus_chrUn.004.6.trna22-GlyTCC (513296-513368) Gly (TCC) 73 bp Sc: 51.06 16
TCCCTGGTGGTCCAGTGGTTAAGACGCTGCGCTTCCACTGCAGGGGGCACAGGTTTGATC
CCTGGTTGGGGAA
>Bos_taurus_chr9.trna6653-GlyTCC (35959930-35959858) Gly (TCC) 73 bp Sc: 51.08 16
TCCCTGGTGGTCCAGTGGTTAGGATTCTGTGCTTCCACTGCAGGGGGCACAGGTTTGATC
CCTGGTCAGGGAA
>Bos_taurus_chr14.trna5777-GlyTCC (20910450-20910378) Gly (TCC) 73 bp Sc: 51.25 17
TCCCCAATGGTCTAGTGGTTAAGACTCTGCACTTCCAATGCAGAAGGCATGGGTTCCATC
CCTGGTTGGGGAA
>Bos_taurus_chr21.trna2608-GlyTCC (62628622-62628694) Gly (TCC) 73 bp Sc: 51.26 17
TCCCTGGTGGTCCAGTGGCTAAGACTCTGCACTTCCAATGTAGGGGGCACAGGTTCAACC
CCTGGTCAGGGAA
>Bos_taurus_chr11.trna4880-GlyTCC (103448059-103447987) Gly (TCC) 73 bp Sc: 51.31 16
TCCCTGGTGGTCCAGTGGTTAAGACTCTGTGCTTCCAATGCAGGGGGTGCAGGTTTGATC
CCTGGCCAGGGAA
>Bos_taurus_chr11.trna3980-GlyTCC (97295437-97295509) Gly (TCC) 73 bp Sc: 51.34 15
TCCTTGGTGGTCCAGCTGTTAGGATGCTGTTCTTCCAATGCAGGGGGCCTGGGTTCGATT
CCTAGTCAGGGAA
>Bos_taurus_chr17.trna802-GlyTCC (19391859-19391931) Gly (TCC) 73 bp Sc: 51.39 17
TCCCTGGCAGTCCAGTGGTTAAGGCTTTGCACTTCCACTGCAGGGAGCCCAGGTTCAATC
CCTGGTCAGGGAA
>Bos_taurus_chr8.trna5106-GlyTCC (89913094-89913022) Gly (TCC) 73 bp Sc: 51.45 17
TCCCTGGTGGTCCAGTGGCTAGGACTCCACACTTCCAATGTAGGCTGCCCAAGTTCAATC
CCTGGTCAGGGAA
>Bos_taurus_chr8.trna1392-GlyTCC (42791127-42791199) Gly (TCC) 73 bp Sc: 51.58 18
TCCCTGGTGGTCCAGTGGTTAAGGCTCTGCACTTCCACTGCATGGGGCACAGGTTCAATC
CCTGGTCAGGGAA
>Bos_taurus_chr13.trna6646-GlyTCC (24146783-24146712) Gly (TCC) 72 bp Sc: 51.80 15
TCCCTGCTGGTCCAGTGGCTAAGACTCTGCACTTCCAATGCAGGGGCCTGGGTTGGATCC
CTGGTCAGGGAA
>Bos_taurus_chrUn.004.2057.trna1-GlyTCC (15073-15001) Gly (TCC) 73 bp Sc: 51.90 18
TCTCTGATGGTCCAGTGGTGAAGACTCTGCGCTTCCACTGCAGGGGGCACAGGTTCAATC
CCTGGTCAGGGAA
>Bos_taurus_chr17.trna5087-GlyTCC (48808049-48807977) Gly (TCC) 73 bp Sc: 51.95 17
TCCCTGGTGGTGCAGTGGTTAATATTCTGCACTTCCACTGCAGGGGTTGCAGGTTCCATC
CCTGGTCAGGGAA
>Bos_taurus_chr10.trna4968-GlyTCC (86620416-86620344) Gly (TCC) 73 bp Sc: 52.01 16
TCTCTGGTGGTCCAGTGGTTAAGACTCTGTGCTTCCACTGCAGGGGTCTCAGGTTTGATC
CCTGGTCAGGGAA
>Bos_taurus_chr13.trna808-GlyTCC (22071497-22071568) Gly (TCC) 72 bp Sc: 52.02 19
TCCCTGGTGGTCCAATGGTTAAGACTTGGCACTTCCACTGCAGTGGCCCATGTTCAACCC
CTGGTCAGGGAA
>Bos_taurus_chr23.trna3898-GlyTCC (19482018-19481946) Gly (TCC) 73 bp Sc: 52.13 18
TCCCTGGTGGTCTAGTGGTTAAGACTCTGTGCTTCCAATGCAGGGGGCCCAGGTTCAATC
ACTGGTTGGGGAA
>Bos_taurus_chr2.trna2108-GlyTCC (67270832-67270903) Gly (TCC) 72 bp Sc: 52.14 18
TCCCTGATGGTCTAGTCGTTAAGACAGTGCACTTCCACTGCAGGGGCACAGGTTCAATCC
CTGGTTGGGGAA
>Bos_taurus_chr22.trna3237-GlyTCC (33010416-33010345) Gly (TCC) 72 bp Sc: 52.20 17
TCCCTGGTGGTCCAGTGCTTAGGACTCAGCACTTCCACTGCTGTGGTCCAGGTTCAATCT

CTGGTCAGAGAA
>Bos_taurus_chr4.trna3669-GlyTCC (107629935-107630007) Gly (TCC) 73 bp Sc: 52.32 18
TCCCCGGTGGTCCAATGGTTAGGACTCCACACTTCCACTGTGGGGACATGGGTTCGATA
CCTGGTTGGGGAA
>Bos_taurus_chr11.trna2834-GlyTCC (70869324-70869396) Gly (TCC) 73 bp Sc: 52.34 17
TCCCTGGTGGTCCAGTGGTTAAGACTCTGCACTTCCACTGCAGGGTCCGTGGGTTCGATC
CCTGGTCAGGGAA
>Bos_taurus_chr18.trna4654-GlyTCC (34985649-34985577) Gly (TCC) 73 bp Sc: 52.35 15
TCCCTGGTGGTCCAGTGGCTAAGACGCTGCACTTCCAATTCAGGGGGCCTGGGTTTGATC
CCCAATCAGGGAA
>Bos_taurus_chr11.trna8837-GlyTCC (6654582-6654510) Gly (TCC) 73 bp Sc: 52.42 16
TCCCTGGTGGTCCAGTGGCTAAGACTTTGCGCTTCCAACGCAGAGGGCCTGGGTTCGATG
CCTGGTCAGGGAA
>Bos_taurus_chr13.trna6650-GlyTCC (24050821-24050749) Gly (TCC) 73 bp Sc: 52.43 16
TCCTTGGTGGTCCAGTGGTTAAGACTCTGCACTTCCAATGCAGGGGGTGCAGGTTTGATC
CCTGGTTGGGGAA
>Bos_taurus_chrUn.004.21.trna79-GlyTCC (322528-322457) Gly (TCC) 72 bp Sc: 52.53 15
TCCCTGGTGGTCCAGTAGCTAAGACTCTGTGCTTCCAATGCAGGGGTCCAGGTTCGATCC
CTGGTCAGGGAA
>Bos_taurus_chr13.trna1846-GlyTCC (44051177-44051249) Gly (TCC) 73 bp Sc: 52.56 17
TCCCTGGTAGTCCAGTGGTTAAGACTCTGCACTTCCATTGCAGAGGGCGTAGGTTCAAAG
CCTGGTTGGGGAA
>Bos_taurus_chr11.trna4989-GlyTCC (101514014-101513942) Gly (TCC) 73 bp Sc: 52.57 15
TCCCTGGTGGTCTAGTGGTTAGGACTCTGTGCTTCCAATGCAGGGGCCACAGGTTTGATC
CCTGGTTAGGGAA
>Bos_taurus_chr24.trna3418-GlyTCC (49945762-49945690) Gly (TCC) 73 bp Sc: 52.62 16
TCCCTGGTGGTCTAGTCGCTAGGACTCTGTGCTTCCAATGCAGGGGGCCCGGGTTCAATT
CCTGGCCGGGGAA
>Bos_taurus_chr12.trna6139-GlyTCC (19075796-19075725) Gly (TCC) 72 bp Sc: 52.67 16
TCCCTGGTGGTCCAGGGTTAGGACTCTGCACTTCCAGTGCAGGGGCCCAGGGTTCAATTC
CTGGTCAGGGAA
>Bos_taurus_chr13.trna4196-GlyTCC (75659455-75659383) Gly (TCC) 73 bp Sc: 53.07 18
TCCCTGCTGGTCCAGTGGTTAAGACTCTGCGCTTCCACTGCAGGAGGCACGGGTTCAATC
CCCGAGTGGGGAA
>Bos_taurus_chr22.trna720-GlyTCC (16573962-16574034) Gly (TCC) 73 bp Sc: 53.10 16
TCCCTGGTGGTCAAGTGGTTGGGACTCTGCACTTCCACTGCAGGGGGCCCAGGTTTGATT
CCTGGTTGGGGAA
>Bos_taurus_chr23.trna820-GlyTCC (18685036-18685108) Gly (TCC) 73 bp Sc: 53.10 17
CCCTTGGTGGTCCAGTGGTTAAGACTCTGCTCTTCCAAAGCAGGGGGCCTGGGTTCGATC
CCTGGGCGGGGGA
>Bos_taurus_chr3.trna6121-GlyTCC (93693139-93693067) Gly (TCC) 73 bp Sc: 53.29 16
ACCCTGGTGGTCCAGTGGTTAGGACTCTGCTCTTCCACTGCAGAGGGTCTAGGTTTGATC
CCTGGTCAGGGAA
>Bos_taurus_chr20.trna1413-GlyTCC (39125012-39125083) Gly (TCC) 72 bp Sc: 53.33 17
TCCCTGGTGGTCCAGTGGTTAAGACTCTGCACTTCCTCTGCAGAGGCCTGGGTTCAATCC
CTAGTCAGAGAA
>Bos_taurus_chr1.trna8836-GlyTCC (70538849-70538777) Gly (TCC) 73 bp Sc: 53.34 16
TCCCTGGTGGTCCAGTGGCTAAGACTCTGTGCTTCCAATGCAGGGGGCGCAGGTTCGATC
CCTGGTCAGGGAA
>Bos_taurus_chr13.trna1699-GlyTCC (40257852-40257924) Gly (TCC) 73 bp Sc: 53.41 17
TCCCTGGTGGTCCAGTGGTGAAGACTCTGCACTTCCAATGCAGGGGGCCCAGATTCCATC
CCTGGTCAGGGAA
>Bos_taurus_chr18.trna5070-GlyTCC (24099824-24099752) Gly (TCC) 73 bp Sc: 53.41 17
TCCCTGGTGGTCCAGTGGTTAGGACTCTGTGCTTCCAATGCTGGGGGCCCAGGTTTAATC
CCTGGTCAGGGAA
>Bos_taurus_chr24.trna4587-GlyTCC (24859388-24859317) Gly (TCC) 72 bp Sc: 53.44 17
GCGTTGGTGGTGTAGTGGTGAGTATAGCTGCCTTCCAAGCACTTGATCCAGGTTCTATTC
CTGGCCAATGCA
>Bos_taurus_chr11.trna6395-GlyTCC (69182616-69182544) Gly (TCC) 73 bp Sc: 53.52 17
TCCCTGGTGGTTCAGTGGCTAAGGCTCTGTACTTCCAATGCAGGGGGTCCAGGTTCAAAC
CCTGGTCAGGGAA
>Bos_taurus_chr24.trna5142-GlyTCC (7201194-7201122) Gly (TCC) 73 bp Sc: 53.55 17
TCCCTGGTGGTCCAGTGGTTAAGACCCTGTGCTTCCACTGCAGGGGGGCGCAGGTTCGATC
CCTGGTCAGGGAA
>Bos_taurus_chr12.trna1587-GlyTCC (35792927-35792999) Gly (TCC) 73 bp Sc: 53.63 16
TCCCTGGTGGTCCAGTGGCTAAGACTCTGCACTTCCAATGCAGGGGGCCCAGGTTCTATC
CTTGGACAGGGAA
>Bos_taurus_chr18.trna3346-GlyTCC (57016681-57016609) Gly (TCC) 73 bp Sc: 53.83 16
TCCCTGGTGGTCCAGTGGTTAAGACTCTGTGCTTCCAACGCAGGGGACGCAGGTTTGATC
CCTGGTCAGGGAA
>Bos_taurus_chr5.trna1675-GlyTCC (47362529-47362601) Gly (TCC) 73 bp Sc: 53.92 16
TCCCTGGCAGTCCAGTGGTTAAGACTCTGCATTTCCACTGCAGGAGGCTCGGGTTCAATC
CCCGACTGGGGAA
>Bos_taurus_chr5.trna1117-GlyTCC (32140816-32140888) Gly (TCC) 73 bp Sc: 53.92 16

TCCCTGGTAGTCCAGTGGTTAAGACTCTGTGCTTCCACTGCAGAGGACACAGGTTCGATC
CCTGGTTGGGGAA
>Bos_taurus_chr5.trna5736-GlyTCC (112552075-112552003) Gly (TCC) 73 bp Sc: 53.93 19
TCCCTGATGGTCCAGTGGTTAGGACTCTGCACTTCCACTGCTGAGGGTGTGGGTTCAATC
CCTGGTCAGGGAA
>Bos_taurus_chr18.trna947-GlyTCC (23285845-23285918) Gly (TCC) 74 bp Sc: 54.19 17
TCCTTGGTGGGTCCAGTGGCTAAGACTCTGTGCTTCCAATGCAGGGGCCCCAGGTTCAAT
CCCTGGTCAGGGAA
>Bos_taurus_chr23.trna299-GlyTCC (8777674-8777746) Gly (TCC) 73 bp Sc: 54.25 17
TCCCTGGTGGTCCAGCGGTTAAGACTCTACACTTCCAATGCAGGGGGCACAGGTTCAATC
CCTGGTCGGGGAA
>Bos_taurus_chr11.trna7180-GlyTCC (49426379-49426307) Gly (TCC) 73 bp Sc: 54.34 17
TCCCTGGTGGTCCAGTGGCTAAGACTCTGCACTTCCAATGTAGGGGGCCCAGGTTCAATC
TCTGGTCAGGGAA
>Bos_taurus_chr17.trna3802-GlyTCC (67340975-67340903) Gly (TCC) 73 bp Sc: 54.39 17
TCCCTGGTGGTCCAGTGGTTAAGACTCTGTGCTTCCAATGCAGGGGGCCCAGGTTCGATC
CCTGGTTGGAGAA
>Bos_taurus_chr28.trna508-GlyTCC (12687269-12687341) Gly (TCC) 73 bp Sc: 54.72 18
TCCCTGGTGGTCCAGTGGTTAAGACTCTGCACTTCCACTGCAGGGAGTGTGGGTTCAATC
CCTGCTCAGGGAA
>Bos_taurus_chr27.trna832-GlyTCC (25851512-25851584) Gly (TCC) 73 bp Sc: 54.99 18
TCTCTGGTTGTCCAGTGGTTAAGACTCTGCGCTTCCAATGCAGGGGGCACAGGTTCAATT
CCTGGCTAGAGAA
>Bos_taurus_chr14.trna6666-GlyTCC (628117-628045) Gly (TCC) 73 bp Sc: 55.04 18
TCCTTGGTGGTCCAATGGTTAAGACTTTGCATTTCCAATGCAGGGGGCACAGGTTCAATC
CCTGGTCAGGGAA
>Bos_taurus_chrUn.004.2377.trna1-GlyTCC (20202-20130) Gly (TCC) 73 bp Sc: 55.04 18
TCCTTGGTGGTCCAATGGTTAAGACTTTGCATTTCCAATGCAGGGGGCACAGGTTCAATC
CCTGGTCAGGGAA
>Bos_taurus_chr17.trna6215-GlyTCC (14993290-14993218) Gly (TCC) 73 bp Sc: 55.08 17
TCCCTAGTGGTCCAGTGGTTAAGACTCTGCACTTCCACTGCAGGGGACGCAGGTTCAATC
CCTGGTCAGGGAA
>Bos_taurus_chr13.trna4038-GlyTCC (78412038-78411966) Gly (TCC) 73 bp Sc: 55.14 16
TCCTTGGCAGTCCAGTGGTTAAGTCTCTGCACTTCCACTGCAGGGGGGCCCAGGTTCGATC
CCTGGTCAGGGAA
>Bos_taurus_chrX.trna1886-GlyTCC (54061538-54061610) Gly (TCC) 73 bp Sc: 55.40 16
TCCCTGATGGTCCAGTGGTTAGGACTCTGTGCTTCCACTGCAGGGGGCCTGGGTTTGATC
CCTAGTCAGGGAA
>Bos_taurus_chr18.trna1531-GlyTCC (38344919-38344991) Gly (TCC) 73 bp Sc: 55.42 19
TCCCTGGTGGTCCAATGGTTAGGACTCTGCACTTCCACTACAGGGGGCTCAGGTTCAATC
CTTGGTCAGGGAA
>Bos_taurus_chrUn.004.437.trna6-GlyTCC (57269-57341) Gly (TCC) 73 bp Sc: 55.46 15
TCCGTGGTGGTCCAGTGGCTAAGACTCTGCACTTCCAATGCAGGGGGCCCAGGTTCGCTC
CCTGGTCAGGGAA
>Bos_taurus_chr11.trna7884-GlyTCC (31122496-31122424) Gly (TCC) 73 bp Sc: 55.48 18
TCCCTGGTGGTCCAGTGGTTAAGACTCTGAGCTTCCACTGCAGGGGGCCCAGGTTCAATC
CCTGGACAGGGAA
>Bos_taurus_chr27.trna3306-GlyTCC (18985647-18985575) Gly (TCC) 73 bp Sc: 55.52 17
TCCCTGGTGGTCCAGTGGTTAGGACTCTGTGTTTCCACTGCAGGGGGCCCAGGTTCAATC
CCTGGTGGGGGAA
>Bos_taurus_chr2.trna4688-GlyTCC (132056903-132056974) Gly (TCC) 72 bp Sc: 55.71 18
TCCCTGGTGGCCCAGTGGTTAGGACTCTGCACTTCCACTTCAGCGTCGCAGGTTCAATCC
CTGGTCGGGGAG
>Bos_taurus_chr15.trna4729-GlyTCC (41072021-41071949) Gly (TCC) 73 bp Sc: 55.71
TCCCTGGTGGTCCAGTGGCTAAGACTCTGGGTTTCCAATGCAGGGGTCCCAGGTTCGATC
CCTGATCAGGGAA
>Bos_taurus_chr7.trna8144-GlyTCC (10706427-10706355) Gly (TCC) 73 bp Sc: 55.84
TCCCTGGTGGTCCAGTGGTTAAGACTCTGCACTTCCACTGCAGGGGGCCTGGGTTTGATC
CCTAGTCGGGGAA
>Bos_taurus_chr1.trna4425-GlyTCC (128541245-128541317) Gly (TCC) 73 bp Sc: 56.09
TCCCTGGTGGTCCAGTGGTTAAGACTCTACGCTTCCAACACAGGGGACCCAGGTTCAATC
CCTGGTCGGGGAA
>Bos_taurus_chr9.trna1800-GlyTCC (55747909-55747981) Gly (TCC) 73 bp Sc: 56.10
TCCCTGGTGGTCCAGTGGTTAGGACTCTGCATTTCCATTGCTGATGGCACAGGTTCAATC
CCTGATCAGGGAA
>Bos_taurus_chrUn.004.1.trna321-GlyTCC (1750083-1750011) Gly (TCC) 73 bp Sc: 56.12
TCCCTGGTGGCCCAGTGGTTAAGGTGCCACACTTCCACTGTAGGGGGTGCAGGTTCGATC
CCTGGTCAGGGAA
>Bos_taurus_chr19.trna5696-GlyTCC (22408349-22408277) Gly (TCC) 73 bp Sc: 56.14
TCCTCGGTGGTCCAGTGGTTAAGACTCTGCACTTCCACTGCAGGGGGCATGGGTTCGATC
CCTAGTTGGGGAA
>Bos_taurus_chr15.trna3226-GlyTCC (80849237-80849166) Gly (TCC) 72 bp Sc: 56.19
TCCCTGGTGGTCCAGTGGTTAAGACTCCACACTTCCAATGCAGGGGCACAGGTTCGATCC
CTGATCAGGGAA

>Bos_taurus_chrUn.004.4759.trna3-GlyTCC (5826-5755) Gly (TCC) 72 bp Sc: 56.19
TCCCTGGTGGTCCAGTGGTTAAGACTCCACACTTCCAATGCAGGGGCACAGGTTCGATCC
CTGATCAGGGAA
>Bos_taurus_chr15.trna4768-GlyTCC (39961784-39961712) Gly (TCC) 73 bp Sc: 56.21
TCCCTGGTGGTTCGGTGGTTAGGACTCTGCACTTCCACTGCAAGGGGCCTGGGTTCAATC
CCCAGTCAGGGAA
>Bos_taurus_chr1.trna1031-GlyTCC (25808955-25809026) Gly (TCC) 72 bp Sc: 56.43
TCCCTGGTGGTCCAGTGGTTAAGACTCTGAGTTTCCACTTCAGGGGCTCAGGTTCAATTC
CTGGTTAGGGAA
>Bos_taurus_chr12.trna4612-GlyTCC (55880584-55880513) Gly (TCC) 72 bp Sc: 56.44
TCCCTGGTGGTCCAGTGGTTAAGACTCTGAACTTCCAATGCAGGGGGCCAGGTTCAAACC
CTGTTCAGGGAA
>Bos_taurus_chr15.trna4031-GlyTCC (60868740-60868667) Gly (TCC) 74 bp Sc: 56.45
TCCCTGATGGTCTAGTGGCTAGGATTCTGGCACTTCCAATGCAGGGGCCCCAGGTTTGAT
TCCTGGTTAGGGAA
>Bos_taurus_chr29.trna3333-GlyTCC (19555058-19554986) Gly (TCC) 73 bp Sc: 56.60
TCCCTGGTGGTCCAGTGGTTAGGACTCTGTGCTTCCACTGCAGGGAGCCCAGGTTAGAAC
CCTGGTCAGGGAA
>Bos_taurus_chr19.trna5804-GlyTCC (20924919-20924847) Gly (TCC) 73 bp Sc: 56.70
TCCCTAGTGGTCCAGTGGCTAAGACTCTGTGCTTCCAATGCAGAGGGCCCAGGTTCGATC
CCTGGTCAGGGAA
>Bos_taurus_chr11.trna895-GlyTCC (20983490-20983562) Gly (TCC) 73 bp Sc: 56.82 17
TCCCTGGTGGTCCAATGGCTAAGACTCTGCACTTCCACTGCAGGGGTCACAGGTTCGATT
GCTGGTCAGGGAA
>Bos_taurus_chr15.trna2875-GlyTCC (80804829-80804901) Gly (TCC) 73 bp Sc: 56.92 18
TCCCTGGTGGCCCAGTGGTTAGGACTCTGTGCTTCCACAGCAGGGGTCACAGGTTCAATC
CCTGGTCAGGGAA
>Bos_taurus_chr11.trna51-GlyTCC (1022099-1022171) Gly (TCC) 73 bp Sc: 56.96 17
TCCCTGGTGGTCCAGTGGTTAGGACTCTGTGCTTCCACTGCAGGGGGCACAGGTTCGATC
CCTGGTTGGGGAA
>Bos_taurus_chr19.trna2521-GlyTCC (49866781-49866853) Gly (TCC) 73 bp Sc: 56.97 18
TCCCTGGTGGTCCAGTGGTTAGGACTCTGTGCTTCCACTGAAGGGGACCCAGGTTCAAAC
CCTGGTCAGGGAA
>Bos_taurus_chrUn.004.1196.trna1-GlyTCC (26447-26519) Gly (TCC) 73 bp Sc: 56.98 19
TCCCTGATGGTCTAATGGATAAGACTCTGTGCTTCCAATGCAGGGTGCCCAGGTTCAATC
CCTGGTCAGGGAA
>Bos_taurus_chr27.trna871-GlyTCC (26880606-26880678) Gly (TCC) 73 bp Sc: 57.20 18
TCCCTGGTGGTCTAGTGGTTAAGATTCTGCACTTCCACTGCACGGGGCACAGGTTCAATC
CCTGGCCGGGGAA
>Bos_taurus_chr22.trna2827-GlyTCC (45670205-45670133) Gly (TCC) 73 bp Sc: 57.25 19
TCCTTGATGGTCCAGTGGTTAGGACTTTGCACTTCCACTACAGGGGTCACAGGTTCAATC
ACTGGTCAGGGAA
>Bos_taurus_chr5.trna5347-GlyTCC (119598805-119598733) Gly (TCC) 73 bp Sc: 57.30 17
TCCCTGGTGGTCCAGTGGTTAAGACTCTGTACTTCCAATGCAGGGGGTGCAGGTTCGATC
CTTGGCCGGGGAA
>Bos_taurus_chr22.trna287-GlyTCC (6825085-6825157) Gly (TCC) 73 bp Sc: 57.33 17
TCCCTGGTGGTCCAGTGGTTAAGACTCTGTGCTTCCAATGCAGAGGGCACAGGTTCGATC
CCTGGTTGGGGAA
>Bos_taurus_chr25.trna3519-GlyTCC (26949218-26949146) Gly (TCC) 73 bp Sc: 57.42 17
TCCCTGGTGATCCAGTGGTTAGGATTCTGTGCTTCCAATGCAGGGGGCTCAGGTTCGATC
CCTGGTCAGGGAA
>Bos_taurus_chr13.trna631-GlyTCC (17717452-17717524) Gly (TCC) 73 bp Sc: 57.45 18
TTCCTGGTGGTCCAGTGGTTAGGACTCTGAACTTCCAATGCAGGGGACTCAGGTTCAATC
CCTGGTCAGGGAA
>Bos_taurus_chr7.trna2170-GlyTCC (48750970-48751042) Gly (TCC) 73 bp Sc: 57.77 16
TCCCCAGTGGTCCAGTGGTTAGGACTCTGCATTTCCACTGCAGGGGGCACAGGTTCAATC
CCTGGTTGGAGAA
>Bos_taurus_chr1.trna3103-GlyTCC (90425742-90425814) Gly (TCC) 73 bp Sc: 57.86 15
TCCCTGGTGGTCCAGTGGGTAAGATTCTGCACTTCCAATGCAGGGAGCCCAGGTTTGATC
CCTGGTCAGGGAA
>Bos_taurus_chr7.trna521-GlyTCC (10560503-10560575) Gly (TCC) 73 bp Sc: 58.06 16
CCCCTGGTGGTACAGTGGTTAAGACTCTGCATTTCCAATGCAGAGGCCACAGGTTCGATT
CCTGGTCAGGGAA
>Bos_taurus_chr29.trna1541-GlyTCC (42619822-42619894) Gly (TCC) 73 bp Sc: 58.11 16
TCCCTGGTGGTCCAGTGGTTAGGACTCTGCACTTCCACTGCAGGGGGCATAGGTTTGATT
CCTGGTCAGGGAA
>Bos_taurus_chr12.trna229-GlyTCC (9083078-9083150) Gly (TCC) 73 bp Sc: 58.55 18
TCCCAGGTGGTCCAGTGGTTAAGACTCTGCACTTCCAATGCAGGGGGTGCAGGTTCAATC
CCTGGCTGGGGAA
>Bos_taurus_chr19.trna924-GlyTCC (20121272-20121344) Gly (TCC) 73 bp Sc: 58.59 16
TCCTTGGTGGTTCAGTGGCTAAGATTCTGCACTTCCAATGCAGGAGGCCCAGGTTCGATC
CCTGGTCAGGGAA
>Bos_taurus_chr18.trna4022-GlyTCC (48229702-48229630) Gly (TCC) 73 bp Sc: 58.78 19
TCCCTGATGGTTCAGTGGTTAAGACGTTGCACTTCCAATGCAGAGGGCCAGGGTTCAATC

CCTGGTCAGGGAA
>Bos_taurus_chr28.trna105-GlyTCC (3513103-3513175) Gly (TCC) 73 bp Sc: 58.92 16
TCCCTGGTGGTCCAGTGGTTAGGACTCTGAACGTCCAATGCAGAGGGCCCAAGTTCGATC
CCTGGTCAGGGAA
>Bos_taurus_chr28.trna94-GlyTCC (3401682-3401754) Gly (TCC) 73 bp Sc: 58.92 16
TCCCTGGTGGTCCAGTGGTTAGGACTCTGAACGTCCAATGCAGAGGGCCCAAGTTCGATC
CCTGGTCAGGGAA
>Bos_taurus_chr7.trna498-GlyTCC (10300164-10300236) Gly (TCC) 73 bp Sc: 59.07 19
TCCCTGATGGTCCAATGGTTAAGACTCTGCACTTCCAATGCAGGGGGCACGGGTTCAATC
CCTGGTCAGGGAA
>Bos_taurus_chr18.trna1415-GlyTCC (35174892-35174964) Gly (TCC) 73 bp Sc: 59.30 18
TCCCTGGTGGTCCAGTGGTTAGGACTCTGTACTTCCAATGTAGGGGGCTCAGGTTCAATC
CCTGGTCGGGGAG
>Bos_taurus_chr7.trna5819-GlyTCC (68170077-68170005) Gly (TCC) 73 bp Sc: 59.35 19
TCCCTGATGATCCAGTGGTTAAGACTCTGCACTTCCAATGCAGAGTGCACAGGTTCAAAC
CCTGGTCAGGGAA
>Bos_taurus_chr14.trna4007-GlyTCC (63644842-63644770) Gly (TCC) 73 bp Sc: 59.72 18
CCTCTGGTGGTCCAGTGGTTAAAACTCTGCACTTCCACTGCAGGGGTCCTAGGTTCAATC
CCTGGCCAGGGAA
>Bos_taurus_chr19.trna3166-GlyTCC (61016359-61016431) Gly (TCC) 73 bp Sc: 59.73 16
TCCCTAGTGGTCCAGTGGTTAAGACTCTGCACTTCCAATGCAGAGGGTGCAGGTTCGATC
CCTGGTCAGGGAA
>Bos_taurus_chr8.trna2311-GlyTCC (72216033-72216105) Gly (TCC) 73 bp Sc: 59.76 19
TCCCTGATGGTCCAGTGGTTACGACTCTGCACTTCCACTGCAGGAGGCCCAGGTTCAATC
CCTAGTCAGGGAA
>Bos_taurus_chrUn.004.74.trna50-GlyTCC (42667-42595) Gly (TCC) 73 bp Sc: 59.97 16
TCCCTGGTGGTCCAGTGGTTAAGACTCTGCACTTCCACTGCAGAGGGCACAGGTTTGATC
CCTGGTCAGGGAA
>Bos_taurus_chr14.trna5722-GlyTCC (21992232-21992160) Gly (TCC) 73 bp Sc: 60.09 15
TCCCCAGTGGTCCAGTGGTTAAGACTCTGCATTTCCAATGCAGGGGTTACATGTTCGATC
CCTGGTTGGGGAG
>Bos_taurus_chr12.trna1361-GlyTCC (30458042-30458114) Gly (TCC) 73 bp Sc: 60.29 17
TCCCTGGTGGTCTAGTGGTTATGAGTCTGCACTTCCACTGCAGGGGGCACAGGTTCGATC
CCTGGTCAGGGAA
>Bos_taurus_chr22.trna1727-GlyTCC (48837345-48837417) Gly (TCC) 73 bp Sc: 60.39 17
TCCCTGGTGGTCCAGTGGCTAAGAGTCTGCACTTCCAATGCAGGGGGCCCAGGTTCAATC
CCTGGTCAGGGAA
>Bos_taurus_chr25.trna3509-GlyTCC (27013244-27013172) Gly (TCC) 73 bp Sc: 60.50 17
TCCGTAGTGGTCCAGTGGTTAGGACTCTGCACTTCCACTGCAGGGGTCCCGGGTTCAATT
CCTGGTCAGGGAT
>Bos_taurus_chr28.trna2582-GlyTCC (15045122-15045052) Gly (TCC) 71 bp Sc: 60.80 17
TCCCAGGTGGTCCAGTGGTTAGGACTCAGCACTTCCACTGCTGGGGCCTGGGTTCAGTCC
CAGGTTGGGAA
>Bos_taurus_chr20.trna2287-GlyTCC (61951815-61951887) Gly (TCC) 73 bp Sc: 61.54 19
TCCCTGATGGTCCAGTGGTTAGGACTCTACCCTTCCATTGCAGGGGTCACAGGTTCAATC
CCTGATCAGGGAA
>Bos_taurus_chr5.trna7781-GlyTCC (66118341-66118270) Gly (TCC) 72 bp Sc: 61.60 19
TCCCTGATGGTCCAGTGGTTAAGACTCTGCACTTCCACTGCAGAAGCACAGGTTCAATCC
CTGGTCGGGGGA
>Bos_taurus_chr5.trna7787-GlyTCC (66008243-66008172) Gly (TCC) 72 bp Sc: 61.60 19
TCCCTGATGGTCCAGTGGTTAAGACTCTGCACTTCCACTGCAGAAGCACAGGTTCAATCC
CTGGTCGGGGGA
>Bos_taurus_chr2.trna10132-GlyTCC (10394475-10394403) Gly (TCC) 73 bp Sc: 62.52 18
TCCCTGGTGGTCCAGTGGTTAAGACTCTGCACTTCCATTGGAGAGGCCCCAGGTTCAAGC
CCTGGTTGGGGAA
>Bos_taurus_chr25.trna2401-GlyTCC (40597711-40597783) Gly (TCC) 73 bp Sc: 63.74 17
TCCCTGATGGTCCAGTGGTCAGGACTCAGCGATTCCACCGCCGAGGGCCCAGGTTCGATC
CCTGGTCAGGGAA
>Bos_taurus_chr9.trna4189-GlyTCC (100778605-100778534) Gly (TCC) 72 bp Sc: 64.88 16
TTCCTGGTAGTCCAGTGGCTAAGACTCTGCACTTCCAATGCAGGGGCCCAGGTTCAATCC
CTGGCTGGGGAA
>Bos_taurus_chr27.trna1366-GlyTCC (36069172-36069244) Gly (TCC) 73 bp Sc: 65.41 18
TCCCTGGTGGTCCAGTGGTTAAGACTCTGCACTTCCACTGCAGGGGTCACAGGTTCAATC
CCTGACCGGGAAA
>Bos_taurus_chr6.trna3436-GlyTCC (105499867-105499939) Gly (TCC) 73 bp Sc: 68.51 16
TCCCTGGTGGTCCAGTGGCTAAGACTCTGCACTTCCAATGCAGGGGGCCCAGGTTCGATC
CCTGGTCAGGGAA
>Bos_taurus_chr6.trna2313-GlyTCC (76394728-76394799) Gly (TCC) 72 bp Sc: 71.29 18
GCATTGGTGGTATAGTGGTGAGCATAGCTGCCTTCCAAGCAGTTGACCCGGGTTCAATTC
CCGGCCAATGCA
>Bos_taurus_chr19.trna1416-GlyTCC (28390273-28390344) Gly (TCC) 72 bp Sc: 73.26 17
GCGTTGGTGGTATAGTGGTGAGCATAGCTGCCTTCCAAGCAGTTGACCCGGGTTCGATTC
CCGGCCAACGCA
>Bos_taurus_chr3.trna8535-GlyTCC (23222187-23222116) Gly (TCC) 72 bp Sc: 73.26 17

GCGTTGGTGGTATAGTGGTGAGCATAGCTGCCTTCCAAGCAGTTGACCCGGGTTCGATTC
CCGGCCAACGCA
>Bos_taurus_chr3.trna9106-GlyTCC (8948000-8947929) Gly (TCC) 72 bp Sc: 73.26 17
GCGTTGGTGGTATAGTGGTGAGCATAGCTGCCTTCCAAGCAGTTGACCCGGGTTCGATTC
CCGGCCAACGCA
>Bos_taurus_chr3.trna9109-GlyTCC (8824689-8824618) Gly (TCC) 72 bp Sc: 73.26 17
GCGTTGGTGGTATAGTGGTGAGCATAGCTGCCTTCCAAGCAGTTGACCCGGGTTCGATTC
CCGGCCAACGCA
>Bos_taurus_chr3.trna9112-GlyTCC (8815222-8815151) Gly (TCC) 72 bp Sc: 73.26 17
GCGTTGGTGGTATAGTGGTGAGCATAGCTGCCTTCCAAGCAGTTGACCCGGGTTCGATTC
CCGGCCAACGCA
>Bos_taurus_chr3.trna9118-GlyTCC (8741938-8741867) Gly (TCC) 72 bp Sc: 73.26 17
GCGTTGGTGGTATAGTGGTGAGCATAGCTGCCTTCCAAGCAGTTGACCCGGGTTCGATTC
CCGGCCAACGCA
>Bos_taurus_chr3.trna9124-GlyTCC (8629023-8628952) Gly (TCC) 72 bp Sc: 73.26 17
GCGTTGGTGGTATAGTGGTGAGCATAGCTGCCTTCCAAGCAGTTGACCCGGGTTCGATTC
CCGGCCAACGCA
>Bos_taurus_chrUn.004.4089.trna1-GlyTCC (148-219) Gly (TCC) 72 bp Sc: 73.26 17
GCGTTGGTGGTATAGTGGTGAGCATAGCTGCCTTCCAAGCAGTTGACCCGGGTTCGATTC
CCGGCCAACGCA
>Bos_taurus_chr15.trna1363-GlyTCC (39230675-39230747) Gly (TCC) 73 bp Sc: 74.45 17
TCGCTGGTGGTCCAGTGGTTAAGACTCTGCACTTCCAATGCAGAGGTCCCAGGTTCGATC
CCTGGTCAGGGAA
>Bos_taurus_chr7.trna7571-GlyTCC (17992863-17992792) Gly (TCC) 72 bp Sc: 76.83 17
GCGTTGGTGGTATAGTGGTTAGCATAGCTGCCTTCCAAGCAGTTGACCCGGGTTCGATTC
CCGGCCAACGCA
>Bos_taurus_chr11.trna7542-GlyTCC (42055486-42055405) Gly (TCC) 82 bp Sc: 37.33 17
TTCCTGGTGGTCCAGTGGTTAAGACTTCATGCTTCCAGTGCAGGTGGTGGTGATGGGATG
GGTTCGATCCCCAGTCAGGGAA
>Bos_taurus_chr22.trna4443-GlyTCC (4540645-4540565) Gly (TCC) 81 bp Sc: 38.10 16
TCCCGGGTGGTTCAGTCATTAAGACTCTGCACTTCCAATGCAAGGATGCAGAGGGTGCAG
GTTCAATCCCTGGTCCGGGAA
>Bos_taurus_chr5.trna3095-GlyTCC (82986884-82986970) Gly (TCC) 87 bp Sc: 28.08 18
TCCTTGGCTGTCCAGTGGTTAAGACCCCATGCTTCCACTGAAGGGGAATCATGGTTCAAT
CACCAGGTTCAATCCCTGGTCAGGGAA
>Danio_rerio_Zv9_scaffold3521.trna40-GlyGCC (36994-36924) Gly (GCC) 71 bp Sc: 71.81 18
GCATTGGTGGTTCAGTGGTAGAATTCTCGCCTGCCACGCGGGAGACCCGGGTCCGATTCC
CGGCCAATGCA
>Danio_rerio_Zv9_scaffold3521.trna46-GlyGCC (35882-35812) Gly (GCC) 71 bp Sc: 71.81 18
GCATTGGTGGTTCAGTGGTAGAATTCTCGCCTGCCACGCGGGAGACCCGGGTCCGATTCC
CGGCCAATGCA
>Danio_rerio_Zv9_scaffold3521.trna47-GlyGCC (35564-35494) Gly (GCC) 71 bp Sc: 71.81 18
GCATTGGTGGTTCAGTGGTAGAATTCTCGCCTGCCACGCGGGAGACCCGGGTCCGATTCC
CGGCCAATGCA
>Danio_rerio_Zv9_scaffold3530.trna134-GlyGCC (539251-539321) Gly (GCC) 71 bp Sc: 71.81 18
GCATTGGTGGTTCAGTGGTAGAATTCTCGCCTGCCACGCGGGAGACCCGGGTCCGATTCC
CGGCCAATGCA
>Danio_rerio_Zv9_scaffold3530.trna139-GlyGCC (540046-540116) Gly (GCC) 71 bp Sc: 71.81 18
GCATTGGTGGTTCAGTGGTAGAATTCTCGCCTGCCACGCGGGAGACCCGGGTCCGATTCC
CGGCCAATGCA
>Danio_rerio_Zv9_scaffold3530.trna150-GlyGCC (541954-542024) Gly (GCC) 71 bp Sc: 71.81 18
GCATTGGTGGTTCAGTGGTAGAATTCTCGCCTGCCACGCGGGAGACCCGGGTCCGATTCC
CGGCCAATGCA
>Danio_rerio_Zv9_scaffold3530.trna290-GlyGCC (871866-871796) Gly (GCC) 71 bp Sc: 71.81 18
GCATTGGTGGTTCAGTGGTAGAATTCTCGCCTGCCACGCGGGAGACCCGGGTCCGATTCC
CGGCCAATGCA
>Danio_rerio_Zv9_scaffold3530.trna303-GlyGCC (869003-868933) Gly (GCC) 71 bp Sc: 71.81 18
GCATTGGTGGTTCAGTGGTAGAATTCTCGCCTGCCACGCGGGAGACCCGGGTCCGATTCC
CGGCCAATGCA
>Danio_rerio_Zv9_scaffold3530.trna397-GlyGCC (410090-410020) Gly (GCC) 71 bp Sc: 71.81 18
GCATTGGTGGTTCAGTGGTAGAATTCTCGCCTGCCACGCGGGAGACCCGGGTCCGATTCC
CGGCCAATGCA
>Danio_rerio_Zv9_scaffold3530.trna470-GlyGCC (397141-397071) Gly (GCC) 71 bp Sc: 71.81 18
GCATTGGTGGTTCAGTGGTAGAATTCTCGCCTGCCACGCGGGAGACCCGGGTCCGATTCC
CGGCCAATGCA
>Danio_rerio_Zv9_scaffold3538.trna69-GlyGCC (36750-36680) Gly (GCC) 71 bp Sc: 71.81 18
GCATTGGTGGTTCAGTGGTAGAATTCTCGCCTGCCACGCGGGAGACCCGGGTCCGATTCC
CGGCCAATGCA
>Danio_rerio_chr4.trna179-GlyGCC (29752775-29752845) Gly (GCC) 71 bp Sc: 71.83 18
GCATTGGTGGTTCAGTGGTAGAATTCTCGCCTGCCACGCGGGAGACCCGGATTCGATTCC
CGGCCAATGCA
>Danio_rerio_chr8.trna769-GlyGCC (40291565-40291495) Gly (GCC) 71 bp Sc: 71.83 18
GCATTGGTGGTTCAGTGGTAGAATTCTCGCCTGCCACGCGGGAGACCCGGATTCGATTCC
CGGCCAATGCA

>Danio_rerio_Zv9_scaffold3473.trna150-GlyGCC (2577-2507) Gly (GCC) 71 bp Sc: 71.83 19
GCATTGGTGGTTCAGTGGTAGAATTCTCGCCTGCCACGCGGGAGACCCGGATTCGATTCC
CGGCCAATGCA
>Danio_rerio_Zv9_scaffold3506.trna141-GlyGCC (78402-78332) Gly (GCC) 71 bp Sc: 72.05 19
GCATTGGTGGTTCAGTGGTAGAATTCTCGCCTGCCATGCGGGAGACCCGGGTTCGATTCC
TGGCCAATGCA
>Danio_rerio_Zv9_scaffold3530.trna122-GlyGCC (537184-537254) Gly (GCC) 71 bp Sc: 73.56 19
GCATTGGTGGTTCAGTGGTAGAATGCTCGCCTGCCACGCGGGAGACCCGGGTCCGATTCC
CGGCCAATGCA
>Danio_rerio_Zv9_scaffold3506.trna139-GlyGCC (78711-78641) Gly (GCC) 71 bp Sc: 74.88 18
GCATTGGTGGTTCAGTGGTAGAATTCTCGCCTGCCACGCGAGAGACCCGGGTCCGATTCC
CGGCCAATGCA
>Danio_rerio_Zv9_scaffold3506.trna162-GlyGCC (74913-74843) Gly (GCC) 71 bp Sc: 74.88 18
GCATTGGTGGTTCAGTGGTAGAATTCTCGCCTGCCACGCGAGAGACCCGGGTCCGATTCC
CGGCCAATGCA
>Danio_rerio_chr4.trna7874-GlyGCC (33255689-33255619) Gly (GCC) 71 bp Sc: 76.67 20
GCATTGGTGGTTCAGTGGTAGAATTCTCGCCTGCCACGCGGGAGACCCGGGTTCAATTCC
CGGCTAATGCA
>Danio_rerio_Zv9_scaffold3530.trna385-GlyGCC (412278-412208) Gly (GCC) 71 bp Sc: 77.65 19
GCATTGGTGGTTCAGTGGTAGAATTCTCGCCTGCCATGCGGGAGACCCGGGTTCGATCCC
CGGCCAATGCA
>Danio_rerio_chr4.trna7876-GlyGCC (33255371-33255301) Gly (GCC) 71 bp Sc: 78.07 18
GCATTGGTGGTTCAGTGGTAGAACTCTCGCTTGCCACGCGGGAGACCCGGGTTCGATTCC
CGGCTAATGCA
>Danio_rerio_Zv9_scaffold3506.trna109-GlyGCC (83949-83879) Gly (GCC) 71 bp Sc: 79.03 19
GCATTGGTGGTTCAGTGGTAGAATTCTCGCCTGCCATGCGGGAGACCCGGGTTCGATTCC
CGGCCAATGCA
>Danio_rerio_Zv9_scaffold3506.trna77-GlyGCC (89496-89426) Gly (GCC) 71 bp Sc: 79.03 19
GCATTGGTGGTTCAGTGGTAGAATTCTCGCCTGCCATGCGGGAGACCCGGGTTCGATTCC
CGGCCAATGCA
>Danio_rerio_chr4.trna1980-GlyGCC (41623818-41623888) Gly (GCC) 71 bp Sc: 79.74 19
GCATTGGTGGTTCAGTGGTAGAATTCTCGCCTGCCACGCGGGAGACCCGGGTTCGATTCC
CGGCCAATGCA
>Danio_rerio_chr10.trna196-GlyGCC (43105916-43105846) Gly (GCC) 71 bp Sc: 81.62 19
GCATTGGTGGTTCAGTGGTAGAATTCTCGCCTGCCACGCGGGAGGCCCGGGTTCGATTCC
CGGCCAATGCA
>Danio_rerio_chr10.trna199-GlyGCC (43100762-43100692) Gly (GCC) 71 bp Sc: 81.62 19
GCATTGGTGGTTCAGTGGTAGAATTCTCGCCTGCCACGCGGGAGGCCCGGGTTCGATTCC
CGGCCAATGCA
>Danio_rerio_chr10.trna203-GlyGCC (43095594-43095524) Gly (GCC) 71 bp Sc: 81.62 19
GCATTGGTGGTTCAGTGGTAGAATTCTCGCCTGCCACGCGGGAGGCCCGGGTTCGATTCC
CGGCCAATGCA
>Danio_rerio_chr10.trna207-GlyGCC (43094467-43094397) Gly (GCC) 71 bp Sc: 81.62 19
GCATTGGTGGTTCAGTGGTAGAATTCTCGCCTGCCACGCGGGAGGCCCGGGTTCGATTCC
CGGCCAATGCA
>Danio_rerio_chr13.trna292-GlyGCC (52603308-52603378) Gly (GCC) 71 bp Sc: 81.62 19
GCATTGGTGGTTCAGTGGTAGAATTCTCGCCTGCCACGCGGGAGGCCCGGGTTCGATTCC
CGGCCAATGCA
>Danio_rerio_chr13.trna472-GlyGCC (20102754-20102684) Gly (GCC) 71 bp Sc: 81.62 19
GCATTGGTGGTTCAGTGGTAGAATTCTCGCCTGCCACGCGGGAGGCCCGGGTTCGATTCC
CGGCCAATGCA
>Danio_rerio_chr13.trna473-GlyGCC (20099996-20099926) Gly (GCC) 71 bp Sc: 81.62 19
GCATTGGTGGTTCAGTGGTAGAATTCTCGCCTGCCACGCGGGAGGCCCGGGTTCGATTCC
CGGCCAATGCA
>Danio_rerio_chr13.trna474-GlyGCC (20097756-20097686) Gly (GCC) 71 bp Sc: 81.62 19
GCATTGGTGGTTCAGTGGTAGAATTCTCGCCTGCCACGCGGGAGGCCCGGGTTCGATTCC
CGGCCAATGCA
>Danio_rerio_chr22.trna998-GlyGCC (2134445-2134375) Gly (GCC) 71 bp Sc: 81.62 19
GCATTGGTGGTTCAGTGGTAGAATTCTCGCCTGCCACGCGGGAGGCCCGGGTTCGATTCC
CGGCCAATGCA
>Danio_rerio_chr22.trna999-GlyGCC (2133633-2133563) Gly (GCC) 71 bp Sc: 81.62 19
GCATTGGTGGTTCAGTGGTAGAATTCTCGCCTGCCACGCGGGAGGCCCGGGTTCGATTCC
CGGCCAATGCA
>Danio_rerio_chr25.trna215-GlyGCC (17958477-17958407) Gly (GCC) 71 bp Sc: 81.62 19
GCATTGGTGGTTCAGTGGTAGAATTCTCGCCTGCCACGCGGGAGGCCCGGGTTCGATTCC
CGGCCAATGCA
>Danio_rerio_chr25.trna62-GlyGCC (17958672-17958742) Gly (GCC) 71 bp Sc: 81.62 19
GCATTGGTGGTTCAGTGGTAGAATTCTCGCCTGCCACGCGGGAGGCCCGGGTTCGATTCC
CGGCCAATGCA
>Danio_rerio_chr23.trna25-GlyTCC (4942719-4942790) Gly (TCC) 72 bp Sc: 45.92 15
GCGTTGGTGGTATAGTGTTGAGCATAGCTGCCTTCCAAGCAATTGACCTGGGTTTGATAC
CCGGCCAATGCA
>Equus_caballus_chr5.trna101-GlyCCC (47579367-47579297) Gly (CCC) 71 bp Sc: 60.59 17
GCATTGGTCGTTCAGTGGTAGAATTCTCACCTCCCACGCGGGTGACCCGGGTTCGATTCC

CTGCCAGTGCA
>Equus_caballus_chr5.trna104-GlyCCC (47520173-47520103) Gly (CCC) 71 bp Sc: 71.94 18
GCATTGGTGGTTCAGTGGTAGAATTCTCGCCTCCCACGCGGGTGACCCGGGTTCGATTCC
AGGCCAATGCA
>Equus_caballus_chr5.trna41-GlyCCC (47518661-47518731) Gly (CCC) 71 bp Sc: 71.94 18
GCATTGGTGGTTCAGTGGTAGAATTCTCGCCTCCCACGCGGGTGACCCGGGTTCGATTCC
AGGCCAATGCA
>Equus_caballus_chr13.trna22-GlyCCC (42006827-42006897) Gly (CCC) 71 bp Sc: 76.98 16
GCGCCGCTGGTGTAGTGGTATCATGCAAGATTCCCATTCTTGCGACCCGGGTTCGATTCC
CGGGCGGCGCA
>Equus_caballus_chr15.trna19-GlyCCC (32296879-32296949) Gly (CCC) 71 bp Sc: 76.98 16
GCGCCGCTGGTGTAGTGGTATCATGCAAGATTCCCATTCTTGCGACCCGGGTTCGATTCC
CGGGCGGCGCA
>Equus_caballus_chr5.trna107-GlyCCC (47362426-47362356) Gly (CCC) 71 bp Sc: 77.49 19
GCATTGGTGGTTCAATGGTAGAATTCTCGCCTCCCACGCGGGAGACCCGGGTTCGATTCC
CGGCCAATGCA
>Equus_caballus_chr5.trna114-GlyCCC (47196901-47196831) Gly (CCC) 71 bp Sc: 78.93 18
GCATTGGTGGTTCAGTGGTAGAATTCTCGCCTCCCACGCGGGTGACCCGGGTTCGATTCC
CGGCCAATGCA
>Equus_caballus_chr5.trna32-GlyCCC (47192293-47192363) Gly (CCC) 71 bp Sc: 78.93 18
GCATTGGTGGTTCAGTGGTAGAATTCTCGCCTCCCACGCGGGTGACCCGGGTTCGATTCC
CGGCCAATGCA
>Equus_caballus_chr11.trna41-GlyGCC (51012631-51012701) Gly (GCC) 71 bp Sc: 81.62 18
GCATTGGTGGTTCAGTGGTAGAATTCTCGCCTGCCACGCGGGAGGCCCGGGTTCGATTCC
CGGCCAATGCA
>Equus_caballus_chr18.trna63-GlyGCC (37609841-37609771) Gly (GCC) 71 bp Sc: 81.62 18
GCATTGGTGGTTCAGTGGTAGAATTCTCGCCTGCCACGCGGGAGGCCCGGGTTCGATTCC
CGGCCAATGCA
>Equus_caballus_chr20.trna167-GlyGCC (26139947-26139877) Gly (GCC) 71 bp Sc: 81.62 18
GCATTGGTGGTTCAGTGGTAGAATTCTCGCCTGCCACGCGGGAGGCCCGGGTTCGATTCC
CGGCCAATGCA
>Equus_caballus_chr20.trna230-GlyGCC (24189907-24189837) Gly (GCC) 71 bp Sc: 81.62 18
GCATTGGTGGTTCAGTGGTAGAATTCTCGCCTGCCACGCGGGAGGCCCGGGTTCGATTCC
CGGCCAATGCA
>Equus_caballus_chr3.trna10-GlyGCC (23358461-23358531) Gly (GCC) 71 bp Sc: 81.62 18
GCATTGGTGGTTCAGTGGTAGAATTCTCGCCTGCCACGCGGGAGGCCCGGGTTCGATTCC
CGGCCAATGCA
>Equus_caballus_chr3.trna7-GlyGCC (22928699-22928769) Gly (GCC) 71 bp Sc: 81.62 18
GCATTGGTGGTTCAGTGGTAGAATTCTCGCCTGCCACGCGGGAGGCCCGGGTTCGATTCC
CGGCCAATGCA
>Equus_caballus_chr3.trna8-GlyGCC (22929646-22929716) Gly (GCC) 71 bp Sc: 81.62 18
GCATTGGTGGTTCAGTGGTAGAATTCTCGCCTGCCACGCGGGAGGCCCGGGTTCGATTCC
CGGCCAATGCA
>Equus_caballus_chr3.trna91-GlyGCC (22920248-22920178) Gly (GCC) 71 bp Sc: 81.62 18
GCATTGGTGGTTCAGTGGTAGAATTCTCGCCTGCCACGCGGGAGGCCCGGGTTCGATTCC
CGGCCAATGCA
>Equus_caballus_chr3.trna92-GlyGCC (22919636-22919566) Gly (GCC) 71 bp Sc: 81.62 18
GCATTGGTGGTTCAGTGGTAGAATTCTCGCCTGCCACGCGGGAGGCCCGGGTTCGATTCC
CGGCCAATGCA
>Equus_caballus_chr5.trna122-GlyGCC (36205793-36205723) Gly (GCC) 71 bp Sc: 81.62 19
GCATTGGTGGTTCAGTGGTAGAATTCTCGCCTGCCACGCGGGAGGCCCGGGTTCGATTCC
CGGCCAATGCA
>Equus_caballus_chr11.trna46-GlyTCC (51097308-51097379) Gly (TCC) 72 bp Sc: 73.26 17
GCGTTGGTGGTATAGTGGTGAGCATAGCTGCCTTCCAAGCAGTTGACCCGGGTTCGATTC
CCGGCCAACGCA
>Equus_caballus_chr5.trna18-GlyTCC (36146122-36146193) Gly (TCC) 72 bp Sc: 73.26 17
GCGTTGGTGGTATAGTGGTGAGCATAGCTGCCTTCCAAGCAGTTGACCCGGGTTCGATTC
CCGGCCAACGCA
>Equus_caballus_chr5.trna20-GlyTCC (36213682-36213753) Gly (TCC) 72 bp Sc: 73.26 17
GCGTTGGTGGTATAGTGGTGAGCATAGCTGCCTTCCAAGCAGTTGACCCGGGTTCGATTC
CCGGCCAACGCA
>Equus_caballus_chr7.trna3-GlyTCC (2872560-2872631) Gly (TCC) 72 bp Sc: 76.83 17
GCGTTGGTGGTATAGTGGTTAGCATAGCTGCCTTCCAAGCAGTTGACCCGGGTTCGATTC
CCGGCCAACGCA
>Encephalitozoon_cuniculi_chrXI.trna2-GlyCCC (158598-158669) Gly (CCC) 72 bp Sc: 77.77 15
GCATTGGTAGTATAGTGGTTATTATGTGAGCTTCCCAAGCTCATGGCCCGGGTTCGATTC
CCGGTCAATGCA
>Encephalitozoon_cuniculi_chrVI.trna4-GlyGCC (54370-54300) Gly (GCC) 71 bp Sc: 66.16 17
GCATCATTGGTTTAGTGGTATAATGGCCGGTTGCCATCTGGCCGACCCGAGTTCGATTCT
CGGATGATGCA
>Encephalitozoon_cuniculi_chrI.trna1-GlyTCC (55599-55670) Gly (TCC) 72 bp Sc: 72.96 16
GCGTTGTTGGTCTAGTGGATAGGATAACAGCCTTCCAAGCTGTTGGCCCGGGTTCGATTC
CCGGACATCGCA
>Enterococcus_faecalis_V583_chr.trna5-GlyGCC (253721-253792) Gly (GCC) 72 bp Sc: 85.49 17

GCGGAAATAGCTCAGTGGTAGAGCACCACCTTGCCAAGGTGGGGGTCGCGGGTTCGAACC
CCGTTTTCCGCT
>Enterococcus_faecalis_V583_chr.trna54-GlyGCC (3164782-3164711) Gly (GCC) 72 bp Sc: 85.49 17
GCGGAAATAGCTCAGTGGTAGAGCACCACCTTGCCAAGGTGGGGGTCGCGGGTTCGAACC
CCGTTTTCCGCT
>Enterococcus_faecalis_V583_chr.trna15-GlyTCC (254690-254760) Gly (TCC) 71 bp Sc: 71.75 18
GCGGGTGTAGTTTAGTGGTAAAACCACAGCCTTCCAAGCTGTTGTCGCGAGTTCGATTCT
CGTCACCCGCT
>Enterococcus_faecalis_V583_chr.trna22-GlyTCC (255353-255423) Gly (TCC) 71 bp Sc: 71.75 18
GCGGGTGTAGTTTAGTGGTAAAACCACAGCCTTCCAAGCTGTTGTCGCGAGTTCGATTCT
CGTCACCCGCT
>Escherichia_coli_APEC_O1_chr.trna66-GlyCCC (3194999-3194926) Gly (CCC) 74 bp Sc: 78.75 18
GCGGGCGTAGTTCAATGGTAGAACGAGAGCTTCCCAAGCTCTATACGAGGGTTCGATTCC
CTTCGCCCGCTCCA
>Escherichia_coli_APEC_O1_chr.trna52-GlyGCC (4799896-4799971) Gly (GCC) 76 bp Sc: 93.74 17
GCGGGAATAGCTCAGTTGGTAGAGCACGACCTTGCCAAGGTCGGGGTCGCGAGTTCGAGT
CTCGTTTCCCGCTCCA
>Escherichia_coli_APEC_O1_chr.trna53-GlyGCC (4800008-4800083) Gly (GCC) 76 bp Sc: 93.74 17
GCGGGAATAGCTCAGTTGGTAGAGCACGACCTTGCCAAGGTCGGGGTCGCGAGTTCGAGT
CTCGTTTCCCGCTCCA
>Escherichia_coli_APEC_O1_chr.trna54-GlyGCC (4800119-4800194) Gly (GCC) 76 bp Sc: 93.74 17
GCGGGAATAGCTCAGTTGGTAGAGCACGACCTTGCCAAGGTCGGGGTCGCGAGTTCGAGT
CTCGTTTCCCGCTCCA
>Escherichia_coli_APEC_O1_chr.trna82-GlyGCC (1997090-1997015) Gly (GCC) 76 bp Sc: 93.74 17
GCGGGAATAGCTCAGTTGGTAGAGCACGACCTTGCCAAGGTCGGGGTCGCGAGTTCGAGT
CTCGTTTCCCGCTCCA
>Escherichia_coli_APEC_O1_chr.trna49-GlyTCC (4497254-4497328) Gly (TCC) 75 bp Sc: 64.85 16
GCGGGCATCGTATAATGGCTATTACCTCAGCCTTCCAAGCTGATGATGCGGGTTCGATTC
CCGCTGCCCGCTCCA
>Escherichia_coli_APEC_O1_chr.trna81-GlyTCC (2074679-2074605) Gly (TCC) 75 bp Sc: 64.85 16
GCGGGCATCGTATAATGGCTATTACCTCAGCCTTCCAAGCTGATGATGCGGGTTCGATTC
CCGCTGCCCGCTCCA
>Escherichia_coli_APEC_O1_chr.trna18-GlyTCC (1483347-1483421) Gly (TCC) 75 bp Sc: 64.97 16
GCGGGTATCGTATAATGGCTATTACCTCAGCCTTCCAAGCTGATGATGCGGGTTCGATTC
CCGCTACCCGCTCCA
>Felis_catus_scaffold_213975.trna4-GlyACC (72962-73046) Gly (ACC) 85 bp Sc: 47.59 14
GCCTGGGTGGCTCAGTCGGTTGGGCGTCTGACTACCGCTCAGGTCACGATCTCACGGCTC
GTGGGTTCGAGCCCCACATCGGGCT
>Felis_catus_scaffold_149452.trna6-GlyCCC (241599-241529) Gly (CCC) 71 bp Sc: 76.98 17
GCGCCGCTGGTGTAGTGGTATCATGCAAGATTCCCATTCTTGCGACCCGGGTTCGATTCC
CGGGCGGCGCA
>Felis_catus_scaffold_101630.trna1-GlyCCC (2816-2886) Gly (CCC) 71 bp Sc: 80.05 18
GCATTGGTGGTTCAGTGGTAGAATTCTCGCCTCCCACGCGGGAGACCCGGGTTCGATTCC
CGGCCAATGCA
>Felis_catus_scaffold_150864.trna1-GlyCCC (2876-2786) Gly (CCC) 91 bp Sc: 31.77 14
GCCTGGGTGGCTCAGCTGGTTAAGCCTCAGACTCCCGATTTCGGCTCAAGCCTTGATCTC
AGGTTTGTGGGATCGAGTCCCATGTCGGGCT
>Felis_catus_scaffold_100746.trna1-GlyCCC (42809-42899) Gly (CCC) 91 bp Sc: 31.77 14
GCCTGGGTGGCTCAGCTGGTTAAGCCTCAGACTCCCGATTTCGGCTCAAGCCTTGATCTC
AGGTTTGTGGGATCGAGTCCCATGTCGGGCT
>Felis_catus_scaffold_150629.trna7-GlyGCC (77823-77753) Gly (GCC) 71 bp Sc: 80.92 19
GCATTGGTGGTTCAGTGGTAGAATTCTCGCCTGCCATGCGGGAGGCCCGGGTTCGATTCC
CGGCCAATGCA
>Felis_catus_scaffold_150638.trna1-GlyGCC (11505-11575) Gly (GCC) 71 bp Sc: 81.62 19
GCATTGGTGGTTCAGTGGTAGAATTCTCGCCTGCCACGCGGGAGGCCCGGGTTCGATTCC
CGGCCAATGCA
>Felis_catus_scaffold_150638.trna2-GlyGCC (12186-12256) Gly (GCC) 71 bp Sc: 81.62 19
GCATTGGTGGTTCAGTGGTAGAATTCTCGCCTGCCACGCGGGAGGCCCGGGTTCGATTCC
CGGCCAATGCA
>Felis_catus_scaffold_150638.trna3-GlyGCC (3634-3564) Gly (GCC) 71 bp Sc: 81.62 19
GCATTGGTGGTTCAGTGGTAGAATTCTCGCCTGCCACGCGGGAGGCCCGGGTTCGATTCC
CGGCCAATGCA
>Felis_catus_scaffold_151171.trna7-GlyGCC (159227-159297) Gly (GCC) 71 bp Sc: 81.62 19
GCATTGGTGGTTCAGTGGTAGAATTCTCGCCTGCCACGCGGGAGGCCCGGGTTCGATTCC
CGGCCAATGCA
>Felis_catus_scaffold_185924.trna2-GlyGCC (11677-11607) Gly (GCC) 71 bp Sc: 81.62 19
GCATTGGTGGTTCAGTGGTAGAATTCTCGCCTGCCACGCGGGAGGCCCGGGTTCGATTCC
CGGCCAATGCA
>Felis_catus_scaffold_7734.trna7-GlyGCC (12254-12184) Gly (GCC) 71 bp Sc: 81.62 19
GCATTGGTGGTTCAGTGGTAGAATTCTCGCCTGCCACGCGGGAGGCCCGGGTTCGATTCC
CGGCCAATGCA
>Felis_catus_scaffold_91493.trna1-GlyGCC (534-604) Gly (GCC) 71 bp Sc: 82.15 19
GCATGGGTGGTTCAGTGGTAGAATTCTCGCCTGCCACGCGGGAGGCCCGGGTTCGATTCC
CGGCCCATGCA

>Felis_catus_scaffold_91497.trna1-GlyGCC (494-564) Gly (GCC) 71 bp Sc: 82.15 19
GCATGGGTGGTTCAGTGGTAGAATTCTCGCCTGCCACGCGGGAGGCCCGGGTTCGATTCC
CGGCCCATGCA
>Felis_catus_scaffold_172619.trna8-GlyTCC (218098-218014) Gly (TCC) 85 bp Sc: 24.94
GCCTGGGTGGCTCAGCCGGTTAAGTGTCTGACTTCCGTACAGGTCCTGATTTCACGGTTC
TTGGGTTTGAGCCCCGAGTCGGGCT
>Felis_catus_scaffold_202635.trna5-GlyTCC (49456-49372) Gly (TCC) 85 bp Sc: 27.62 14
GCCTGGGTGGCTCAGTCAGTTAAGCATCCGACTTCCGCTCAGGTCATGATATTGTGGTTT
GTGGGTTCGAGCCCCACGTCAGGCT
>Felis_catus_scaffold_202636.trna2-GlyTCC (492-408) Gly (TCC) 85 bp Sc: 27.62 14
GCCTGGGTGGCTCAGTCAGTTAAGCATCCGACTTCCGCTCAGGTCATGATATTGTGGTTT
GTGGGTTCGAGCCCCACGTCAGGCT
>Felis_catus_scaffold_144093.trna4-GlyTCC (129614-129530) Gly (TCC) 85 bp Sc: 30.42 14
GCCTGGGTGGCTGAGTCAGTTAAGCATCTGATTTCCACTCAGGTCATGATCTCACAGCTC
GTGGGTTCGAGCCCTGCGTCGGGCT
>Felis_catus_scaffold_160030.trna2-GlyTCC (93714-93798) Gly (TCC) 85 bp Sc: 32.01 16
GCCTGGGTGGCTCAGTTGGTTAAGCATCTGACTTCCACTCAGGTCATGATCTCACAGTTT
GTGGGTTTAAGCCCCATGTTGGGCT
>Felis_catus_scaffold_148307.trna1-GlyTCC (142104-142188) Gly (TCC) 85 bp Sc: 32.04 15
GCCTGGGTGGCTCAGTCGGTTAAGTGTCTGACTTCCGCTCAGGTCATGATCTCACAGTCT
GTGAGTTCAAGCCTCGCGTCAGGCT
>Felis_catus_scaffold_212875.trna27-GlyTCC (43108-43024) Gly (TCC) 85 bp Sc: 32.62 15
GCCTGGGTGGCTCAGTGGGTTAAGCATCCGACTTCCGCTCAGGTCATGATTTCATGGTTC
GTGGGTTCCAGCCCCACATCGGGCT
>Felis_catus_scaffold_100856.trna2-GlyTCC (85275-85359) Gly (TCC) 85 bp Sc: 34.84 15
GCCTGGGTGGCTCAGTTGGTTAAGCATCCGACTTCCACTCGGGTCATGATCTCGCGATTT
GTGGGTTTGAGCCCCATGTCAGGCT
>Felis_catus_scaffold_141020.trna22-GlyTCC (44681-44597) Gly (TCC) 85 bp Sc: 35.89 15
GCCTGAGTGGCTTAGTTGGTTAGGTGTCTGACTTCCGCTCAGGTCATGATCTCACAGTTT
GTGGGTTCGAGCCCCGTGTCAGGCT
>Felis_catus_scaffold_200406.trna2-GlyTCC (39167-39251) Gly (TCC) 85 bp Sc: 38.20 17
GCCTGGATGGCTCAGTTGGTTAAGCATCTGACTTCCGCTCAGGTCACTATCTCATAGTTC
GTGGGTTCAAGGCCCACGTCAGGCT
>Felis_catus_scaffold_193187.trna2-GlyTCC (4686-4602) Gly (TCC) 85 bp Sc: 39.17 15
ACCTGGGTGGCTCAGTTGGTTAAGCATCTGACTTCCGCTCAGATCATAATCTCACAGGTG
GTGGGTTTGAGCCCCACCTCAGATG
>Felis_catus_scaffold_104634.trna1-GlyTCC (396-312) Gly (TCC) 85 bp Sc: 39.37 15
GCCTGAGTGGCTCAGTCGGTTAAGCATCTGACTTCCGCTCAGGTCATGATCTTACGGTTC
GTGGGTTCAAGCCCCACATCAGGTT
>Felis_catus_scaffold_195526.trna18-GlyTCC (171136-171052) Gly (TCC) 85 bp Sc: 39.91 15
GCCTGGGTGGCTCAGTCGGATGAGTGTCTGACTTCCGCTCAGGTCAAGATCTTGCAGTCC
GTGGGTTCGAGCCCCACGTCAGGCT
>Felis_catus_scaffold_216256.trna20-GlyTCC (130732-130648) Gly (TCC) 85 bp Sc: 39.99 14
GCCTGGGTGGCTCAGTTGGTGAAGCGTCTGACTTCCGCTCAGGTCATGATCTCACAGTTC
GTGGGATCGAGCCCCGCATCAGGCT
>Felis_catus_scaffold_149208.trna5-GlyTCC (90962-91046) Gly (TCC) 85 bp Sc: 41.90 14
GCCTGGGTGGCTTAGTCGGTTAAGCATCTGACTTCCGCTCAGGTCTTAATCTCACGGCTT
GTGGGTTCGAGCCCCGCTTCGGGCT
>Felis_catus_scaffold_154326.trna1-GlyTCC (5896-5812) Gly (TCC) 85 bp Sc: 41.93 14
GCCTGGGTGGCTCAGGCGGTTGAGCGTCCGACTTCCGCTCAGGTCAGTATCTCACGGTTT
GTGGGTTCGAGCCCCACATCAGGCT
>Felis_catus_scaffold_49848.trna1-GlyTCC (971-887) Gly (TCC) 85 bp Sc: 42.03 15
GCCTGGGTGGCTCAGTGGGTTGAGCATCTGACTTCCGCTCAGGTCATGATCTCGCGGTTT
GTGGGTTCGAGCCCCGCGTCGGGCT
>Felis_catus_scaffold_93499.trna1-GlyTCC (475-559) Gly (TCC) 85 bp Sc: 44.73 15
GCCTGGGTGGCTCAGTTGGTTAAGCATCTGACTTCCGCTCAGATCATGATCTCGCGGTTC
ATGGGTTCGAGCCCCGTGCTGGGCT
>Felis_catus_scaffold_110340.trna2-GlyTCC (247-163) Gly (TCC) 85 bp Sc: 45.78 14
GCCTGGGTGGCTCAGTCGGTTAAGCGTCCGACTTCCGCTCAGGGCGTGATCTCACTGTTC
GTGGGTTCGAGCCCCGCCCCGGGCT
>Felis_catus_scaffold_101101.trna1-GlyTCC (820-904) Gly (TCC) 85 bp Sc: 47.19 14
GCCTGGGTGGCTCAGTCGGTTGAGCATCTGACTTCCGCTCAGGTCACGATCTCGCGGTTC
ATGGGTTCGAGCCCCGTGTCGGGCT
>Felis_catus_scaffold_205476.trna4-GlyTCC (157281-157365) Gly (TCC) 85 bp Sc: 47.19 14
GCCTGGGTGGCTCAGTCGGTTGAGCATCTGACTTCCGCTCAGGTCACGATCTCGCGGTTC
ATGGGTTCGAGCCCCGTGTCGGGCT
>Felis_catus_scaffold_778.trna1-GlyTCC (6552-6468) Gly (TCC) 85 bp Sc: 47.19 14
GCCTGGGTGGCTCAGTCGGTTGAGCATCTGACTTCCGCTCAGGTCACGATCTCGCGGTTC
ATGGGTTCGAGCCCCGTGTCGGGCT
>Felis_catus_scaffold_145142.trna1-GlyTCC (9817-9733) Gly (TCC) 85 bp Sc: 50.01 15
GCCTGGGTAGCTCAGTCGGTTGAGCGTCCGACTTCCACTCAGGTCATGATCTCACGGTTC
GTGGGTTCGAGCCCCACTTCAGGCT
>Felis_catus_scaffold_166258.trna6-GlyTCC (111031-111115) Gly (TCC) 85 bp Sc: 55.73 15
GCCTGGGTGGCTCAGTTGGTTGAGCGCCCGACTTCCGCTCGGGTTGTGATTTTGTAGGTC

GTGGGTTCGAGCCCCGCGTCGGGCT
>Felis_catus_scaffold_127375.trna4-GlyTCC (46238-46167) Gly (TCC) 72 bp Sc: 73.26 17
GCGTTGGTGGTATAGTGGTGAGCATAGCTGCCTTCCAAGCAGTTGACCCGGGTTCGATTC
CCGGCCAACGCA
>Felis_catus_scaffold_151173.trna2-GlyTCC (22131-22202) Gly (TCC) 72 bp Sc: 73.26 17
GCGTTGGTGGTATAGTGGTGAGCATAGCTGCCTTCCAAGCAGTTGACCCGGGTTCGATTC
CCGGCCAACGCA
>Felis_catus_scaffold_534.trna1-GlyTCC (24787-24858) Gly (TCC) 72 bp Sc: 73.26 17
GCGTTGGTGGTATAGTGGTGAGCATAGCTGCCTTCCAAGCAGTTGACCCGGGTTCGATTC
CCGGCCAACGCA
>Ferroplasma_acidarmanus_Fer1_Contig90.trna17-GlyCCC (1-73) Gly (CCC) 73 bp Sc: 66.64 14
GCGATTGTGGTGTAGCCTGGTATCACAGTGGCTTCCCAAGCCACTAACCCGGGTCCAAAT
CCCGGCAATCGCA
>Ferroplasma_acidarmanus_Fer1_Contig118.trna43-GlyGCC (1-73) Gly (GCC) 73 bp Sc: 66.00 14
GCGAGTGTGGTGTAGCCTGGCAACACGCGAGCTTGCCAAGCTCGTGCCCCGGGTCCAAAT
CCCGGCACTCGCA
>Ferroplasma_acidarmanus_Fer1_Contig106.trna5-GlyTCC (1-73) Gly (TCC) 73 bp Sc: 71.55 18
GCAGGTGTGGTGTAGCCTGGTAGCACGGAAGCCTTCCAAGCTTTCGACTCGGGTTCAAAT
CCCGGCACCTGCA
>Flavobacterium_psychrophilum_JIP02_86_chr.trna44-GlyGCC (480868-480793) Gly (GCC) 76 bp Sc: 81.94 17
GCGAAAATAGCTCAGCTGGTAGAGCGCGACCTTGCCAAGGTCGAGGTCGCGGGTTCGAGC
CCCGTTTTTCGCTCTA
>Flavobacterium_psychrophilum_JIP02_86_chr.trna24-GlyTCC (2404859-2404787) Gly (TCC) 73 bp Sc: 79.52 18
GCGAAAGTAGCTCAGTTGGTAGAGCTCCAGCCTTCCAAGCTGGTTGTCGCGAGTTCGAGC
CTCGTCTTTCGCT
>Homo_sapiens_chr1.trna2-GlyCCC (17053780-17053850) Gly (CCC) 71 bp Sc: 63.06 19
GCCTTGGTGGTGCAGTGGTAGAATTCTCGCCTCCCACGTGGGAGACCCGGGTTCAATTCC
CGGCCAATGCA
>Homo_sapiens_chr1.trna131-GlyCCC (17004836-17004766) Gly (CCC) 71 bp Sc: 69.75 19
GCGTTGGTGGTTTAGTGGTAGAATTCTCGCCTCCCATGCGGGAGACCCGGGTTCAATTCC
CGGCCACTGCA
>Homo_sapiens_chr17.trna13-GlyCCC (19764175-19764245) Gly (CCC) 71 bp Sc: 70.13 18
GCATTGGTGGTTCAATGGTAGAATTCTCGCCTCCCACGCAGGAGACCCAGGTTCGATTCC
TGGCCAATGCA
>Homo_sapiens_chr16.trna34-GlyCCC (686806-686736) Gly (CCC) 71 bp Sc: 76.98 18
GCGCCGCTGGTGTAGTGGTATCATGCAAGATTCCCATTCTTGCGACCCGGGTTCGATTCC
CGGGCGGCGCA
>Homo_sapiens_chr2.trna27-GlyCCC (70476193-70476123) Gly (CCC) 71 bp Sc: 76.98 18
GCGCCGCTGGTGTAGTGGTATCATGCAAGATTCCCATTCTTGCGACCCGGGTTCGATTCC
CGGGCGGCGCA
>Homo_sapiens_chr1.trna133-GlyCCC (16872504-16872434) Gly (CCC) 71 bp Sc: 78.31 19
GCATTGGTGGTTCAGTGGTAGAATTCTCGCCTCCCACGCGGGAGACCCGGGTTCAATTCC
CGGCCAATGCA
>Homo_sapiens_chr1.trna4-GlyCCC (17188416-17188486) Gly (CCC) 71 bp Sc: 78.31 19
GCATTGGTGGTTCAGTGGTAGAATTCTCGCCTCCCACGCGGGAGACCCGGGTTCAATTCC
CGGCCAATGCA
>Homo_sapiens_chr6.trna82-GlyGCC (142578776-142578846) Gly (GCC) 71 bp Sc: 46.73 19
GCATGGGTGATTCAGTGGTAGAATTTTCACCTGCCATGCAGGAGGTCCAGGTTCATTTCC
TGGCCTATGCA
>Homo_sapiens_chr16.trna18-GlyGCC (70822597-70822667) Gly (GCC) 71 bp Sc: 56.35 18
GCATTGGTGGTTCAGTGGTAGAATTCTCGCCTGCCATGCGGGCGGCCGGGCTTCGATTCC
TGGCCAATGCA
>Homo_sapiens_chr1.trna43-GlyGCC (161450356-161450426) Gly (GCC) 71 bp Sc: 69.02 18
GCATAGGTGGTTCAGTGGTAGAATTCTTGCCTGCCACGCAGGAGGCCCAGGTTTGATTCC
TGGCCCATGCA
>Homo_sapiens_chr16.trna25-GlyGCC (70812184-70812114) Gly (GCC) 71 bp Sc: 73.97 18
GCATTGGTGGTTCAGTGGTAGAATTCTCGCCTGCCACGCGGGAGGCCCGGGTTTGATTCC
CGGCCAGTGCA
>Homo_sapiens_chr1.trna68-GlyGCC (161493707-161493637) Gly (GCC) 71 bp Sc: 81.62 19
GCATTGGTGGTTCAGTGGTAGAATTCTCGCCTGCCACGCGGGAGGCCCGGGTTCGATTCC
CGGCCAATGCA
>Homo_sapiens_chr16.trna19-GlyGCC (70823410-70823480) Gly (GCC) 71 bp Sc: 81.62 19
GCATTGGTGGTTCAGTGGTAGAATTCTCGCCTGCCACGCGGGAGGCCCGGGTTCGATTCC
CGGCCAATGCA
>Homo_sapiens_chr16.trna24-GlyGCC (70813012-70812942) Gly (GCC) 71 bp Sc: 81.62 19
GCATTGGTGGTTCAGTGGTAGAATTCTCGCCTGCCACGCGGGAGGCCCGGGTTCGATTCC
CGGCCAATGCA
>Homo_sapiens_chr17.trna5-GlyGCC (8029064-8029134) Gly (GCC) 71 bp Sc: 81.62 19
GCATTGGTGGTTCAGTGGTAGAATTCTCGCCTGCCACGCGGGAGGCCCGGGTTCGATTCC
CGGCCAATGCA
>Homo_sapiens_chr2.trna19-GlyGCC (157257729-157257659) Gly (GCC) 71 bp Sc: 81.62 19
GCATTGGTGGTTCAGTGGTAGAATTCTCGCCTGCCACGCGGGAGGCCCGGGTTCGATTCC
CGGCCAATGCA
>Homo_sapiens_chr6.trna128-GlyGCC (27870756-27870686) Gly (GCC) 71 bp Sc: 81.62 19

GCATTGGTGGTTCAGTGGTAGAATTCTCGCCTGCCACGCGGGAGGCCCGGGTTCGATTCC
CGGCCAATGCA
>Homo_sapiens_chr1.trna35-GlyGCC (161413094-161413164) Gly (GCC) 71 bp Sc: 82.15 19
GCATGGGTGGTTCAGTGGTAGAATTCTCGCCTGCCACGCGGGAGGCCCGGGTTCGATTCC
CGGCCCATGCA
>Homo_sapiens_chr1.trna37-GlyGCC (161420467-161420537) Gly (GCC) 71 bp Sc: 82.15 19
GCATGGGTGGTTCAGTGGTAGAATTCTCGCCTGCCACGCGGGAGGCCCGGGTTCGATTCC
CGGCCCATGCA
>Homo_sapiens_chr1.trna39-GlyGCC (161427898-161427968) Gly (GCC) 71 bp Sc: 82.15 19
GCATGGGTGGTTCAGTGGTAGAATTCTCGCCTGCCACGCGGGAGGCCCGGGTTCGATTCC
CGGCCCATGCA
>Homo_sapiens_chr1.trna41-GlyGCC (161435258-161435328) Gly (GCC) 71 bp Sc: 82.15 19
GCATGGGTGGTTCAGTGGTAGAATTCTCGCCTGCCACGCGGGAGGCCCGGGTTCGATTCC
CGGCCCATGCA
>Homo_sapiens_chr21.trna2-GlyGCC (18827177-18827107) Gly (GCC) 71 bp Sc: 82.15 19
GCATGGGTGGTTCAGTGGTAGAATTCTCGCCTGCCACGCGGGAGGCCCGGGTTCGATTCC
CGGCCCATGCA
>Homo_sapiens_chr1.trna82-GlyTCC (161410032-161409961) Gly (TCC) 72 bp Sc: 55.96 17
GCGTTGGTGGTATAGTGGTGAGCATAGTTGCCTTCCAAGCAGTTGACCCGGGCTCGATTC
CCGCCCAACGCA
>Homo_sapiens_chr17.trna10-GlyTCC (8124866-8124937) Gly (TCC) 72 bp Sc: 71.94 17
GCGTTGGTGGTATAGTGGTAAGCATAGCTGCCTTCCAAGCAGTTGACCCGGGTTCGATTC
CCGGCCAACGCA
>Homo_sapiens_chr1.trna117-GlyTCC (145397935-145397864) Gly (TCC) 72 bp Sc: 73.26 17
GCGTTGGTGGTATAGTGGTGAGCATAGCTGCCTTCCAAGCAGTTGACCCGGGTTCGATTC
CCGGCCAACGCA
>Homo_sapiens_chr1.trna45-GlyTCC (161500903-161500974) Gly (TCC) 72 bp Sc: 73.26 17
GCGTTGGTGGTATAGTGGTGAGCATAGCTGCCTTCCAAGCAGTTGACCCGGGTTCGATTC
CCGGCCAACGCA
>Homo_sapiens_chr1.trna70-GlyTCC (161439618-161439547) Gly (TCC) 72 bp Sc: 73.26 17
GCGTTGGTGGTATAGTGGTGAGCATAGCTGCCTTCCAAGCAGTTGACCCGGGTTCGATTC
CCGGCCAACGCA
>Homo_sapiens_chr1.trna73-GlyTCC (161432237-161432166) Gly (TCC) 72 bp Sc: 73.26 17
GCGTTGGTGGTATAGTGGTGAGCATAGCTGCCTTCCAAGCAGTTGACCCGGGTTCGATTC
CCGGCCAACGCA
>Homo_sapiens_chr1.trna76-GlyTCC (161424827-161424756) Gly (TCC) 72 bp Sc: 73.26 17
GCGTTGGTGGTATAGTGGTGAGCATAGCTGCCTTCCAAGCAGTTGACCCGGGTTCGATTC
CCGGCCAACGCA
>Homo_sapiens_chr1.trna79-GlyTCC (161417446-161417375) Gly (TCC) 72 bp Sc: 73.26 17
GCGTTGGTGGTATAGTGGTGAGCATAGCTGCCTTCCAAGCAGTTGACCCGGGTTCGATTC
CCGGCCAACGCA
>Homo_sapiens_chr19.trna2-GlyTCC (4724082-4724153) Gly (TCC) 72 bp Sc: 76.83 17
GCGTTGGTGGTATAGTGGTTAGCATAGCTGCCTTCCAAGCAGTTGACCCGGGTTCGATTC
CCGGCCAACGCA

All tRNAs from the GtRNAdb data base

Number of TGGTA…TTCGA occurrences: 11182
Number of TGGTA… TTCAA occurrences: 7014
Total number of tRNAs: 111 385

TGGTA TTC A 18 196 observed and 111 385 / 10^9 = 0.42 ± 1*
>Acaryochloris_marina_MBIC11017_chr.trna26-AlaCGC (603738-603810) Ala (CGC) 73 bp Sc: 77.50
GGGGAATTAGCTCAGCTGGTAGAGTGCTGCGATCGCACCGCAGAGGTCAGGGGTTCGAAT
CCCCTATTCTCCA
>Acaryochloris_marina_MBIC11017_chr.trna43-AlaGGC (4592246-4592318) Ala (GGC) 73 bp Sc: 86.53
GGGGCTATAGCTCAGTTGGTAGAGCACCTGCATGGCATGCAGGGGGTCAGCGGTTCGAAT
CCGCTTAGCTCCA
>Acaryochloris_marina_MBIC11017_chr.trna49-AlaTGC (5637934-5638009) Ala (TGC) 76 bp Sc: 99.24
GGGGGTATAGCTCAGTTGGTAGAGCGCCTGCTTTGCAAGCAGGATGTCAGCGGTTCGAGT
CCGCTTACCTCCACCA
>Acaryochloris_marina_MBIC11017_chr.trna66-AlaTGC (1408891-1408816) Ala (TGC) 76 bp Sc: 99.24
GGGGGTATAGCTCAGTTGGTAGAGCGCCTGCTTTGCAAGCAGGATGTCAGCGGTTCGAGT
CCGCTTACCTCCACCA
>Acaryochloris_marina_MBIC11017_chr.trna8-ArgACG (291610-291683) Arg (ACG) 74 bp Sc: 44.55
GAGAACGTGGTGTAACTGGATAACATCTCAGGCTACGAACTTGAAGATTGAGGGTTCAAG
TCCTTCCGTTCTCG
>Acaryochloris_marina_MBIC11017_chr.trna69-ArgACG (119056-118983) Arg (ACG) 74 bp Sc: 64.84
GGGCTTGTAGCTCAGTGGACTAGAGCACGTGGCTACGGACCACGGTGTCGGGGGTTCGAA
TCCCTCCTAGCCCG
>Acaryochloris_marina_MBIC11017_chr.trna50-ArgCCG (5884305-5884377) Arg (CCG) 73 bp Sc: 75.78
GGGCATGTAGCTCAGTGGATAGAGCATCAGATTCCGGTTCTGAGGGTCGGGGGTTCGAAT
CCCTCCATGCTCG
>Acaryochloris_marina_MBIC11017_chr.trna51-ArgCCT (6020270-6020197) Arg (CCT) 74 bp Sc: 82.76
GGGGCTGTGGCTCAGTTGGATAGAGCAAGCGCCTCCTAAGCGCTAGGTCGGCGGTTCGAG
TCCGCCCAGTCCCG
>Acaryochloris_marina_MBIC11017_chr.trna33-ArgTCG (1924427-1924500) Arg (TCG) 74 bp Sc: 67.64
GGGCTTGTAGCTCAGTGGACTAGAGCACGTGGCTTCGGACCACGGTGTCGGGGGTTCGAA
TCCCTCCTGGCCCG
>Acaryochloris_marina_MBIC11017_chr.trna22-ArgTCT (297423-297498) Arg (TCT) 76 bp Sc: 50.66
GGGAGTGTCGCCTAAGGGATGGGGCACCGATCTTCTAAATCGGTCTGTGTAGGTTCGAAT
CCTACCACTCCTGCCA
>Acaryochloris_marina_MBIC11017_chr.trna1-ArgTCT (29248-29320) Arg (TCT) 73 bp Sc: 82.00
GCGCTCGTAGCTCAGCGGATAGAGCAGTTGCCTTCTAAGCAATTGGTCGCAGGTTCGAGT
CCTGCCGAGCGCG
>Acaryochloris_marina_MBIC11017_chr.trna10-AsnGTT (291790-291865) Asn (GTT) 76 bp Sc: 73.87
TGCCCTGTAGCTCAGTGGTAGTAGCGGTCGCCTGTTAAGCGAACGGTCGCAGGTTCAATC
CCTGCCGGGGCAGCCA
>Acaryochloris_marina_MBIC11017_chr.trna56-AsnGTT (4581964-4581893) Asn (GTT) 72 bp Sc: 77.99
TCCTCAGTAGCTCAGTGGTAGAGCGGTCGACTGTTAATCGATTGGTCGTAGGTTCGAATC
CTACCTGGGGAG
>Acaryochloris_marina_MBIC11017_chr.trna2-AspGTC (290954-291028) Asp (GTC) 75 bp Sc: 76.28
GGGGGTGTAGCTCAGTCGGTCTAGAGCAGTCGCCTGTCGAGCGAAAGGTCGCGGGTTCAA
ATCCCGTCTCCCCCG
>Acaryochloris_marina_MBIC11017_chr.trna53-AspGTC (5189191-5189118) Asp (GTC) 74 bp Sc: 79.90
GGGACTGTAGTTCAACTGGTTAGAGCACCGCCCTGTCACGGCGGAAGTTGCGGGTTCGAA
TCCCGTCAGTCCCG
>Acaryochloris_marina_MBIC11017_chr.trna3-CysGCA (291114-291189) Cys (GCA) 76 bp Sc: 56.02
GTCCAGGTCGCCAAGGGGTCGAAGGCGTCGGTCTGCAAAATCGATATCGTGGGTTCAACT
CCCACCCTGGACTCCA
>Acaryochloris_marina_MBIC11017_chr.trna61-CysGCA (2965157-2965087) Cys (GCA) 71 bp Sc: 67.58
GGCGGCATAGCCAAGTGGTAAGGCAGGGGCCTGCAAAGCCTTTATCCCCAGTTCGAATCT
GGGTGCCGCCT
>Acaryochloris_marina_MBIC11017_chr.trna11-GlnCTG (291872-291943) Gln (CTG) 72 bp Sc: 55.44
TGCCGAGTGGACGAATAGCAAGTCGCCTGACTCTGAATCAGGAGGTTGTAGGTGCGAATC
CTACCTCGGCAT
>Acaryochloris_marina_MBIC11017_chr.trna12-GlnTTG (291955-292026) Gln (TTG) 72 bp Sc: 52.48
TGTCCTGTGGTGTAACTGGCAACATTTCGCTCTTTGAAAGCGAAGTCCTAGGTTCGAATC
CTGGCGGGACAG
>Acaryochloris_marina_MBIC11017_chr.trna41-GlnTTG (4092442-4092513) Gln (TTG) 72 bp Sc: 68.24
TGGGGCGTCGCCAAGTGGTAAGGCACCTGGTTTGGTCCAGGCATTCGGAGGTTCGAATC
CTTCCGCCCCAG

>Acaryochloris_marina_MBIC11017_chr.trna30-GluTTC (1347891-1347963) Glu (TTC) 73 bp Sc: 50.51
GCCCCCATCGTCTAGAGGCCTAGGACACCTCCCTTTCACGGAGGCGACAGGGATTCGAAT
TCCCTTGGGGGTA
>Acaryochloris_marina_MBIC11017_chr.trna5-GluTTC (291300-291376) Glu (TTC) 77 bp Sc: 63.44
AGTGGTGTACGCAAATGGTTTAAGCGACTTGATTTTCAACCAAGTGATTGCGGGTTCAAA
TCCCGTCACCACTCCCA
>Acaryochloris_marina_MBIC11017_chr.trna68-GlyCCC (671119-671048) Gly (CCC) 72 bp Sc: 81.70
GCGGATGTAGTTCAGTGGTAGAACGTCAGCTTCCCAAGCTGAATGTCGTCGGTTCGAGTC
CGATCATCCGCT
>Acaryochloris_marina_MBIC11017_chr.trna39-GlyGCC (3821193-3821263) Gly (GCC) 71 bp Sc: 41.08
GCTCGATTAGCTCAGAGGGAGAGCACCGAGGGGCCATCTCGGGGGGCGTAGGTTCGAATC
CTACATCGACA
>Acaryochloris_marina_MBIC11017_chr.trna67-GlyGCC (978217-978146) Gly (GCC) 72 bp Sc: 77.98
GCGGGTATAGCTCAGTGGTAGAGCGTCACCTTGCCAAGGTGAATGTCGCGCGTTCGAATC
GCGTTACCCGCT
>Acaryochloris_marina_MBIC11017_chr.trna44-GlyTCC (4814711-4814781) Gly (TCC) 71 bp Sc: 68.07
GCGGGTGTAGTTTAGTGGTAAAACCTTAGCCTTCCAAGCTAATGATGGGGGTTCGATTCC
CCCCACCCGCT
>Acaryochloris_marina_MBIC11017_chr.trna23-HisGTG (297605-297681) His (GTG) 77 bp Sc: 34.16
TTGGCCGAATCCAAAGCGGACGAGGAGCTAGATTGTGATCCTAGTGTTAGCGGGTTCAAG
TCCCGTCGGCCACCCCA
>Acaryochloris_marina_MBIC11017_chr.trna59-HisGTG (4050489-4050417) His (GTG) 73 bp Sc: 65.94
GCGGGCGTAGCCAAGTGGTTAAGGCAGTGGATTGTGGTTCCACCATTCGTGGGTTCGATT
CCCATCGTCCGCC
>Acaryochloris_marina_MBIC11017_chr.trna48-IleGAT (5637846-5637919) Ile (GAT) 74 bp Sc: 83.56
GGGCTATTAGCTCAGGTGGTTAGAGCGCACCCCTGATAAGGGTGAGGTCCCTGGTTCGAG
TCCAGGATGGCCCA
>Acaryochloris_marina_MBIC11017_chr.trna65-IleGAT (1408979-1408906) Ile (GAT) 74 bp Sc: 83.56
GGGCTATTAGCTCAGGTGGTTAGAGCGCACCCCTGATAAGGGTGAGGTCCCTGGTTCGAG
TCCAGGATGGCCCA
>Acaryochloris_marina_MBIC11017_chr.trna14-LeuCAA (292238-292311) Leu (CAA) 74 bp Sc: 53.70
ACTCCTGTAGCCCAATGGGAAGAGGCGCTGGTTTCAAACTCCAGAGGTTGTGGGTTCGAC
TCCCACCAGGAGTA
>Acaryochloris_marina_MBIC11017_chr.trna52-LeuCAA (5521633-5521553) Leu (CAA) 81 bp Sc: 61.17
GGGCGGGTGGCGAAATGGTAGACGCACCACACTCAAAATGTGGCGACTTCGGTCATGAGA
GTTCGAGTCTCTCCCTGCCCA
>Acaryochloris_marina_MBIC11017_chr.trna31-LeuCAG (1478678-1478761) Leu (CAG) 84 bp Sc: 61.76
GCGGAACTGGCGGAATTGGTAGACGCGCTAGGTTCAGGTCCTAGTGTCTGCAAAGACATC
CGGGTTCAAGTCCCGGGTTCCGCA
>Acaryochloris_marina_MBIC11017_chr.trna16-LeuGAG (292427-292504) Leu (GAG) 78 bp Sc: 57.94
GCTCCCGTGGCGAAATTGGCAAACGCCTCTGTTTGAGAGACAGAGTTTTTACAGGTTCAA
CTCCTGTCGGGAGTACCA
>Acaryochloris_marina_MBIC11017_chr.trna28-LeuGAG (1034783-1034864) Leu (GAG) 82 bp Sc: 61.61
GCGGATGTGGTGGAATTGGTAGACACGCACGCTTGAGGGGCGTGTGGCTTATGCCTTGCG
AGTTCGATTCTCGCCATCCGCA
>Acaryochloris_marina_MBIC11017_chr.trna13-LeuTAA (292083-292156) Leu (TAA) 74 bp Sc: 60.96
ACTCCTGTGGCGCAATTGGCAGACGCAACTGTCTTAAACACAGTttgttgtgGGTTCGAC
TCCCACCAGGAGTA
>Acaryochloris_marina_MBIC11017_chr.trna15-LeuTAG (292349-292422) Leu (TAG) 74 bp Sc: 63.64
ACTCCTGTAGCCCAACTGGAAGAGGCGCTGGTCTTAGAAACCAGAGGTTGTGGGTTCGAC
TCCCACCAGGAGTA
>Acaryochloris_marina_MBIC11017_chr.trna27-LeuTAG (638687-638768) Leu (TAG) 82 bp Sc: 66.00
GCGGACGTGGCGGAATTGGTAGACGCGCTAGATTTAGGTTCTAGTACTGCAAGGTGTGAA
GGTTCAAGTCCTTTCGTCCGCA
>Acaryochloris_marina_MBIC11017_chr.trna18-LysCTT (293109-293184) Lys (CTT) 76 bp Sc: 66.91
GGGTTGTTAGCTCAGTTGGTAGAGCAGCCGCCTCTTAAGCGGAAGCGCGTAGGTTCGAAG
CCTACACAGCCCATCA
>Acaryochloris_marina_MBIC11017_chr.trna32-LysTTT (1493820-1493891) Lys (TTT) 72 bp Sc: 66.30
GGGTCGCTAACTCAACGGTAGAGTACTCGGCTTTTAACCGATTAGTTCCGGGTTCGAATC
CCGGGCGACCCA
>Acaryochloris_marina_MBIC11017_chr.trna62-MetCAT (2953445-2953373) Met (CAT) 73 bp Sc: 51.62
CCAGGGTTGGCCGAGCGGTTGAGGCAGCGAACTCATAATTCGCCTTAGGCAGGTTCAACT
CCTGCACCCTGGA
>Acaryochloris_marina_MBIC11017_chr.trna40-MetCAT (3821268-3821344) Met (CAT) 77 bp Sc: 71.11
TGAAAGCTAGCTCAGTTGGTTAGAGCATCAGACTCATAATCTGACGGACACAGGTTCAAA
TCCTGTACTTTCCACCG
>Acaryochloris_marina_MBIC11017_chr.trna45-MetCAT (5145506-5145578) Met (CAT) 73 bp Sc: 71.75

GGCTCAGTAGCTCAGTGGTTAGAGCAGGGGACTCATAAGCCCAAGGTCGCAAGTTCAAAT
CTCGCCTGAGCCA
>Acaryochloris_marina_MBIC11017_chr.trna64-MetCAT (1812833-1812757) Met (CAT) 77 bp Sc: 80.01
CGCGGGGTAGAGCAGTCTGGTAGCTCGTCGGGCTCATAACCCGAAGGTCCATGGTTCAAA
TCCGTGCCCCGCCACCA
>Acaryochloris_marina_MBIC11017_chr.trna9-PheGAA (291711-291783) Phe (GAA) 73 bp Sc: 44.21
CCCCCGGTAGCTCAGAGGTAGTAGCGCTTGTCTGAAGAGCAAGAGGTCGCCAGCTCAAAA
CTGGTTCGGGGGG
>Acaryochloris_marina_MBIC11017_chr.trna55-PheGAA (5014172-5014100) Phe (GAA) 73 bp Sc: 78.53
GCCGGGATAGCTCAGTCGGTAGAGCAGAGGACTGAAAATCCTCGTGTCGCCAGTTCAATT
CTGGCTCCTGGCA
>Acaryochloris_marina_MBIC11017_chr.trna25-ProCGG (407567-407640) Pro (CGG) 74 bp Sc: 77.78
CGGGATGTAGCGCAGTTTGGTAGCGCACTTCGTTCGGGACGAAGGGGCCGGAGGTTCAAA
TCCTCTCATCCCGA
>Acaryochloris_marina_MBIC11017_chr.trna36-ProGGG (2268029-2268102) Pro (GGG) 74 bp Sc: 73.77
CGGGGCGTAGCGCAGCTTGGTAGCGCACCACTTTGGGGTAGTGGGGGTCGTGGGTTCAAA
TCCCGCCGCTCCGA
>Acaryochloris_marina_MBIC11017_chr.trna17-ProTGG (292510-292583) Pro (TGG) 74 bp Sc: 80.25
CCGGGTGTAGCGCAGTTTGGTAGCGCGCCTGGTTTGGGACCAGGATGTCGCAGGTTCAAA
TCCTGCCACTCGGA
>Acaryochloris_marina_MBIC11017_chr.trna35-ProTGG (2187899-2187972) Pro (TGG) 74 bp Sc: 86.50
CGGGATGTAGCGCAGTTTGGTAGCGCGCCTGCTTTGGGAGCAGGATGTCGCAGGTTCGAA
TCCTGTCATCCCGA
>Acaryochloris_marina_MBIC11017_chr.trna20-GlyGCC (297174-297244) Gly (GCC) 71 bp Sc: 27.15
GCGAGTGTGATGTAGCAGTAGCATCTGGCGGTGCCAACGCCAGCGCGTGAGTGCGAATCT
CACCACTCGCT
>Acaryochloris_marina_MBIC11017_chr.trna24-ThrTGT (299739-299814) Thr (TGT) 76 bp Sc: 30.18
TACATACTCGCCAAATGGTAAGGCACTTGATTTGTAGATCAAGCACATACAGGTTCAACT
CCTGTGTATGACACAA
>Acaryochloris_marina_MBIC11017_chr.trna34-SerCGA (2136825-2136911) Ser (CGA) 87 bp Sc: 65.07
GGAGAGGTGGCAGAGTGGTTGAATGCGGCAGACTCGAAATCTGTTTTAGGGTCAAACCTA
ACGGGGGTTCGAATCCCCCCCTCTCCG
>Acaryochloris_marina_MBIC11017_chr.trna7-SerGCT (291520-291605) Ser (GCT) 86 bp Sc: 62.80
GGAGAGGTGGCTGAGTGGGGAAAAGCGCTCCCCTGCTAAGGGAGTACGGTTTCTGACCGT
TGCAGGTTCAAATCCTGTCCTCTCCG
>Acaryochloris_marina_MBIC11017_chr.trna37-SerGCT (2727847-2727935) Ser (GCT) 89 bp Sc: 71.14
GGAGAGGTGGCTGAGTGGTCGAAAGCGGCAGATTGCTAATCtgttgtACGGTGAACTCCG
TACCGAGGGTTCGAATCCCTCCCTCTCCG
>Acaryochloris_marina_MBIC11017_chr.trna60-SerGGA (4036402-4036316) Ser (GGA) 87 bp Sc: 67.05
GGAGAGGTGGCCGAGTGGTTGAAGGCGCAGCACTGGAAATGCTGTTTAGGGGTAACTTTA
ACGAGGGTTCGAATCCCTCCCTCTCCG
>Acaryochloris_marina_MBIC11017_chr.trna58-SerTGA (4085504-4085417) Ser (TGA) 88 bp Sc: 65.72
GGAGAGGTGGCAGAGTTGGTCGATTGCGTCTGACTTGAAATCAGATGAGCCCTCACGGGC
TCCGGAGGTTCGAATCCTCTCCTCTCCG
>Acaryochloris_marina_MBIC11017_chr.trna29-ThrCGT (1278907-1278978) Thr (CGT) 72 bp Sc: 74.70
GCCGATGTAGCTCAGTGGTAGAGCACTCGATTCGTAATCGAGCGGCCGTGCGTTCAAATC
GCATCATCGGCT
>Acaryochloris_marina_MBIC11017_chr.trna47-ThrGGT (5410007-5410078) Thr (GGT) 72 bp Sc: 80.70
GCCCATGTGGCTCAGTGGTAGAGCACACCCTTGGTAAGGGTGAGGTCACCGGTTCAATCC
CGGTCATGGGCT
>Acaryochloris_marina_MBIC11017_chr.trna19-ThrTGT (296371-296443) Thr (TGT) 73 bp Sc: 63.06
GCTGGTGTAGCTCAATGGCCAGAGCAATTGTCCTGTAAACAATAGGCTGCAGGTTCGACT
CCTGTCACCAGCT
>Acaryochloris_marina_MBIC11017_chr.trna63-ThrTGT (2715788-2715716) Thr (TGT) 73 bp Sc: 86.74
GCTGGTGTAGCTCAGTTGGTAGAGCAGCTGATTTGTAATCAGCCGGTCGCAAGTTCGAGT
CTTGTCACCAGCT
>Acaryochloris_marina_MBIC11017_chr.trna4-TrpCCA (291196-291266) Trp (CCA) 71 bp Sc: 49.77
GCGGGGATGGTGTAATGGCAACACCTCAGTCTCCAAAGCTGATGTTCTGGGTTCAAATCC
TAGTCTCCGCG
>Acaryochloris_marina_MBIC11017_chr.trna38-TrpCCA (2904038-2904110) Trp (CCA) 73 bp Sc: 80.45
GCGCCTTTAGTTCAGTTGGTAGAACGCAGGTCTCCAAAACCTGATGTCGGGGGTTCGAGT
CCTCCAGGGCGCG
>Acaryochloris_marina_MBIC11017_chr.trna6-TyrGTA (291382-291466) Tyr (GTA) 85 bp Sc: 41.92
AGGTCGGCTCGCCTACTGGCGTGGGCAACTGCCTGTAAAGCAGCCGCTTAATGCGTTGCA
GGTTCAATTCCTGCCCGGCCTACCA
>Acaryochloris_marina_MBIC11017_chr.trna46-TyrGTA (5409912-5409993) Tyr (GTA) 82 bp Sc: 55.66
GGGTCGGTGCCCGAGTGGTTAATGGGGGCGGACTGTAAATCCGCTGGCTACGCCTACGCT

GGTTCAAATCCAGCCCGGCCCA
>Acaryochloris_marina_MBIC11017_chr.trna54-ValCAC (5124932-5124861) Val (CAC) 72 bp Sc: 74.29
GGGCGATTAGCACAGGGGTAGCGCGCTTCCTTCACACGGAAGAGGTCACTGGTTCAAATC
CAGTATCGCCCA
>Acaryochloris_marina_MBIC11017_chr.trna57-ValGAC (4535157-4535084) Val (GAC) 74 bp Sc: 74.35
GGACGCATAGCTCAGTTGGTTAGAGCACCACGTTGACATCGTGGGGGTCTCCCGTTCGAG
TCGGGATGTGTCCA
>Acaryochloris_marina_MBIC11017_chr.trna21-ValTAC (297249-297325) Val (TAC) 77 bp Sc: 64.66
GGTCCTGTAACTCAGCCTGGGAGAGTGCCTTTCTTACAAAAAGGTGGCCGTAGGTTCGAA
TCCTACCGGGACCATCA
>Acaryochloris_marina_MBIC11017_chr.trna42-ValTAC (4267478-4267550) Val (TAC) 73 bp Sc: 84.52
GGGCGATTAGCTCAGTTGGTAGAGCGCTTCGTTTACACCGAAGATGTCGGCGGTTCGAGC
CCGTCATCGCCCA
>Anolis_carolinensis_chrUn_GL343590.trna2-AlaAGC (218800-218872) Ala (AGC) 73 bp Sc: 49.55
TGGGAATTAGCTCAAATGGTAGAGCGCTCGCTTAGCATGTGAGAGGTAGTGGGATCGATG
CCCACATTCTCCA
>Anolis_carolinensis_chrUn_GL343207.trna3-AlaAGC (1513626-1513698) Ala (AGC) 73 bp Sc: 56.15
GGGGAATTAGCTCAAATGGTAGAGCGCTCGCTTAGCATGCGAGAGGTAGCGGGATTGATG
CCCGCATTCTCCA
>Anolis_carolinensis_chrUn_GL343590.trna5-AlaAGC (252093-252021) Ala (AGC) 73 bp Sc: 56.98
GGGGAATTAGCTCAAATGGTAGAGCGCTCGCTTAGCATGTGAGAGGTAGTGGGATCGATG
CCCACATTCTCCA
>Anolis_carolinensis_chrUn_GL343207.trna10-AlaAGC (1511147-1511075) Ala (AGC) 73 bp Sc: 58.48
GGGGAATTAGCTCAAATGGTAGAGCGCTCGCTTAGCATGTGAGAGGTAGCGGGATCGATG
CCCGCATTCTCCA
>Anolis_carolinensis_chrUn_GL343590.trna1-AlaAGC (216966-217038) Ala (AGC) 73 bp Sc: 61.57
GGGGAATTAGCTCAAATGGTAGAGCGCTCGCTTAGCATGCGAGAGGTAGTGGGATCGATG
CCCACATTCTCCA
>Anolis_carolinensis_chr1.trna549-AlaAGC (110254275-110254203) Ala (AGC) 73 bp Sc: 63.06
GGGGAATTAGCTCAAATGGTAGAGCGCTCGCTTAGCATGCGAGAGGTAGCGGGATCGATG
CCCGCATTCTCCA
>Anolis_carolinensis_chr1.trna606-AlaAGC (75558738-75558666) Ala (AGC) 73 bp Sc: 63.06
GGGGAATTAGCTCAAATGGTAGAGCGCTCGCTTAGCATGCGAGAGGTAGCGGGATCGATG
CCCGCATTCTCCA
>Anolis_carolinensis_chr1.trna97-AlaAGC (74941454-74941526) Ala (AGC) 73 bp Sc: 63.06
GGGGAATTAGCTCAAATGGTAGAGCGCTCGCTTAGCATGCGAGAGGTAGCGGGATCGATG
CCCGCATTCTCCA
>Anolis_carolinensis_chrUn_GL343207.trna2-AlaAGC (1513243-1513315) Ala (AGC) 73 bp Sc: 63.06
GGGGAATTAGCTCAAATGGTAGAGCGCTCGCTTAGCATGCGAGAGGTAGCGGGATCGATG
CCCGCATTCTCCA
>Anolis_carolinensis_chrUn_GL343207.trna4-AlaAGC (1513908-1513980) Ala (AGC) 73 bp Sc: 63.06
GGGGAATTAGCTCAAATGGTAGAGCGCTCGCTTAGCATGCGAGAGGTAGCGGGATCGATG
CCCGCATTCTCCA
>Anolis_carolinensis_chrUn_GL343207.trna5-AlaAGC (1515011-1515083) Ala (AGC) 73 bp Sc: 63.06
GGGGAATTAGCTCAAATGGTAGAGCGCTCGCTTAGCATGCGAGAGGTAGCGGGATCGATG
CCCGCATTCTCCA
>Anolis_carolinensis_chrUn_GL343207.trna6-AlaAGC (1515644-1515716) Ala (AGC) 73 bp Sc: 63.06
GGGGAATTAGCTCAAATGGTAGAGCGCTCGCTTAGCATGCGAGAGGTAGCGGGATCGATG
CCCGCATTCTCCA
>Anolis_carolinensis_chrUn_GL343400.trna13-AlaCGC (523424-523353) Ala (CGC) 72 bp Sc: 73.07
GGGGATGTAGCTCAGTGGTAGAGCGCATGCTTCGCATGTATGAGGCCCCGGGTTCAATCC
CCGGCATCTCCA
>Anolis_carolinensis_chr6.trna101-AlaCGC (53478293-53478364) Ala (CGC) 72 bp Sc: 73.12
GGGGATGTAGCTCAGTGGTAGAGCGCGCGCTTCGCATGTGTGAGGTCCCGGGTTCAATCC
CCGGCATCTCCA
>Anolis_carolinensis_chrUn_GL343466.trna45-AlaCGC (175722-175651) Ala (CGC) 72 bp Sc: 75.68
GGGGATGTAGCTCAGTGGTAGAGCGCATGCTTCGCATGTATGAGGTCCTGGGTTCAATCC
CCAGCATCTCCA
>Anolis_carolinensis_chrUn_GL343590.trna4-AlaTGC (255276-255348) Ala (TGC) 73 bp Sc: 61.38
GGGGAATTAGCTCAAATGGTAGAGCACTTGCTTTGCATGCAAGAGGTAGCGGGATCGATG
CCCGCATTCTCCA
>Anolis_carolinensis_chrLGb.trna4-AlaTGC (1332527-1332598) Ala (TGC) 72 bp Sc: 72.71
GGGGATGTAGCTCAGTGGTAGAGCGCATGCTTTGCATGTATGAGGCCCCGGGTTCAATCC
CCGGCATCTCCA
>Anolis_carolinensis_chrUn_GL343466.trna46-AlaTGC (174509-174438) Ala (TGC) 72 bp Sc: 72.95
GGGGATGTAGCTCAGTGGTAGAGCGCATGCTTTGCATGTATGAGGCCCTGGGTTCGATCC
CCAGCATCTCCA

>Anolis_carolinensis_chrUn_GL343400.trna1-AlaTGC (343994-344065) Ala (TGC) 72 bp Sc: 74.45
GGGGATGTAGCTCAGTGGTAGAGCGCATGCTTTGCATGTATGAGGCCCCGGGTTCGATCC
CCGGCATCTCCA
>Anolis_carolinensis_chrUn_GL343466.trna47-AlaTGC (173950-173879) Ala (TGC) 72 bp Sc: 76.65
GGGGATATAGCTCAGTGGTAGAGCGCATGCTTTGCATGTATGAGGTCCTGGGTTCGATCC
CCAGTATCTCCA
>Anolis_carolinensis_chrUn_GL343466.trna44-AlaTGC (177072-177001) Ala (TGC) 72 bp Sc: 77.05
GGGGATGTAGCTCAGTGGTAGAGCGCATGCTTTGCATGTATGAGGTCCTGGGTTCGATCC
CCAGCATCTCCA
>Anolis_carolinensis_chr4.trna386-ArgACG (30677960-30677888) Arg (ACG) 73 bp Sc: 67.96
GGGCCAGTGGCGCAATGGATAACGCGTCTGACTACGGATCAGAAGATTGTAGGTTCGACT
CCTGCCTGGCTCG
>Anolis_carolinensis_chr4.trna45-ArgACG (30674809-30674881) Arg (ACG) 73 bp Sc: 67.96
GGGCCAGTGGCGCAATGGATAACGCGTCTGACTACGGATCAGAAGATTGTAGGTTCGACT
CCTGCCTGGCTCG
>Anolis_carolinensis_chrUn_GL343324.trna1-ArgACG (212215-212287) Arg (ACG) 73 bp Sc: 68.07
GGGCCAGTGGCGCAATGGATAACGCGTCTGACTACGGATCAGAAGATTCTAGGTTCGACT
CCTGGCTGGCTCG
>Anolis_carolinensis_chrUn_GL343879.trna1-ArgACG (76258-76330) Arg (ACG) 73 bp Sc: 68.07
GGGCCAGTGGCGCAATGGATAACGCGTCTGACTACGGATCAGAAGATTCTAGGTTCGACT
CCTGGCTGGCTCG
>Anolis_carolinensis_chrUn_GL343250.trna18-ArgCCG (1112312-1112240) Arg (CCG) 73 bp Sc: 65.49
GACCCAGTGGCCTAATGGATAAGGCATCAGCCTCCGGAGCTGGGGATTGTGGGTTCGAGT
CCCATCTGGGTCG
>Anolis_carolinensis_chrUn_GL343614.trna2-ArgCCT (265404-265332) Arg (CCT) 73 bp Sc: 67.28
GCCCCAGTGGCCTAATGGATAAGGCATTGGCCTCCTAAGCCAGGGATTGTGGGTTCGAGT
CCCATCTGGGGTG
>Anolis_carolinensis_chr2.trna417-ArgCCT (114946158-114946086) Arg (CCT) 73 bp Sc: 68.55
GCCCCAGTGGCCTAATGGACAAGGCACTGGCCTCCTAAGCCAGGGATTGTGGGTTCGAGT
CCCATCTGGGGTG
>Anolis_carolinensis_chr2.trna167-ArgCCT (114949614-114949686) Arg (CCT) 73 bp Sc: 70.63
GCCCCAGTGGCCTAATGGATAAGGCACTGGCCTCCTAAGCCAGGGATTGTGGGTTCGAGT
CCCATCTGGGGTG
>Anolis_carolinensis_chr2.trna166-ArgTCG (114913607-114913679) Arg (TCG) 73 bp Sc: 67.43
GACCACGTGGCCTAATGGATAAGGCGTCTGACTTCGGATCAGAAGATTGAGGGTTCGAGT
CCCTTCGTGGTTG
>Anolis_carolinensis_chr2.trna416-ArgTCG (114947183-114947111) Arg (TCG) 73 bp Sc: 70.52
GACCGCGTGGCCTAATGGATAAGGCGTCTGACTTCGGATCAGAAGATTGAGGGTTCGAGT
CCCTTCGTGGTCG
>Anolis_carolinensis_chrUn_GL343444.trna1-ArgTCG (327474-327546) Arg (TCG) 73 bp Sc: 76.93
GGCCGCGTGGCCTAATGGATAAGGCGTCTGACTTCGGATCAGAAGATTGCAGGTTCGAGT
CCTGCCGCGGTCG
>Anolis_carolinensis_chrUn_GL344271.trna1-ArgTCT (36009-35936) Arg (TCT) 74 bp Sc: 76.29
GTCTCTGTGGCGCAATGGACGAGCGCGCTGGACTTCTAATCCAGAGGTTCCGGGTTCGAG
TCCCGGCAGAGATG
>Anolis_carolinensis_chr4.trna127-ArgTCT (88622018-88622102) Arg (TCT) 85 bp Sc: 72.09
GGCTCCGTGGCGCAATGGATAGCGCATTGGACTTCTAGAGGGTGAAGAGATTCAAAGGTT
CCGGGTTCGAGTCCCGGCGGAGTCG
>Anolis_carolinensis_chr1.trna121-ArgTCT (91048895-91048981) Arg (TCT) 87 bp Sc: 69.04
GGCTCTGTGGCGCAATGGATAGCGCATTGGACTTCTAGTCGAGTTGAAGCGATTCAAAGG
TTGTGGGTTCGAGTCCCACCAGAGTCA
>Anolis_carolinensis_chr1.trna261-AsnGTT (210585843-210585916) Asn (GTT) 74 bp Sc: 67.07
ATCTCTGTGGCGCAATTGGTTAGCGTGTTCGGCTGTTAACCGAAAGGTTGGTGGTTTGAG
CCCACCCAGGGACG
>Anolis_carolinensis_chr2.trna310-AsnGTT (197432403-197432330) Asn (GTT) 74 bp Sc: 71.78
GTCTCTGTGGCGCAATGGGTTAGCGCGTCTGGCTGTTAACCAGAAGGATGGTGGTTCGAG
CCCACCCAGGGACG
>Anolis_carolinensis_chr2.trna312-AsnGTT (197424875-197424802) Asn (GTT) 74 bp Sc: 71.78
GTCTCTGTGGCGCAATGGGTTAGCGCGTCTGGCTGTTAACCAGAAGGATGGTGGTTCGAG
CCCACCCAGGGACG
>Anolis_carolinensis_chr2.trna290-AsnGTT (197436069-197436142) Asn (GTT) 74 bp Sc: 76.79
GTCTCTGTGGCGCAATGGGTCAGCGCGTCTGGCTGTTAACCAGAAGGTTGGTGGTTCGAG
CCCACCCAGGGACG
>Anolis_carolinensis_chr3.trna253-AsnGTT (184514323-184514396) Asn (GTT) 74 bp Sc: 82.24
GTCTCTGTGGCGCAATCGGTTAGCGCGTTCGGCTGTTAACCGAAAGGTTGGTGGTTCGAG
CCCACCCAGGGACG
>Anolis_carolinensis_chr6.trna112-AsnGTT (63087249-63087322) Asn (GTT) 74 bp Sc: 82.24

GTCTCTGTGGCGCAATCGGTTAGCGCGTTCGGCTGTTAACCGAAAGGTTGGTGGTTCGAG
CCCACCCAGGGACG
>Anolis_carolinensis_chr6.trna30-AsnGTT (16405952-16406025) Asn (GTT) 74 bp Sc: 82.24
GTCTCTGTGGCGCAATCGGTTAGCGCGTTCGGCTGTTAACCGAAAGGTTGGTGGTTCGAG
CCCACCCAGGGACG
>Anolis_carolinensis_chrUn_GL343466.trna18-AsnGTT (280687-280760) Asn (GTT) 74 bp Sc: 82.24
GTCTCTGTGGCGCAATCGGTTAGCGCGTTCGGCTGTTAACCGAAAGGTTGGTGGTTCGAG
CCCACCCAGGGACG
>Anolis_carolinensis_chrUn_GL343466.trna20-AsnGTT (289703-289776) Asn (GTT) 74 bp Sc: 82.24
GTCTCTGTGGCGCAATCGGTTAGCGCGTTCGGCTGTTAACCGAAAGGTTGGTGGTTCGAG
CCCACCCAGGGACG
>Anolis_carolinensis_chrUn_GL343466.trna37-AsnGTT (257477-257404) Asn (GTT) 74 bp Sc: 82.24
GTCTCTGTGGCGCAATCGGTTAGCGCGTTCGGCTGTTAACCGAAAGGTTGGTGGTTCGAG
CCCACCCAGGGACG
>Anolis_carolinensis_chr2.trna308-AspGTC (197652736-197652665) Asp (GTC) 72 bp Sc: 71.60
TCCTCGTTAGTATAGTGGTAAGTATCCCCGCCTGTCACGCGGGAGACCGGGGTTCGATTC
CCCGACGGGGAG
>Anolis_carolinensis_chr5.trna47-AspGTC (29377281-29377352) Asp (GTC) 72 bp Sc: 71.60
TCCTCGTTAGTATAGTGGTAAGTATCCCCGCCTGTCACGCGGGAGACCGGGGTTCGATTC
CCCGACGGGGAG
>Anolis_carolinensis_chrLGb.trna3-AspGTC (1332253-1332324) Asp (GTC) 72 bp Sc: 71.60
TCCTCGTTAGTATAGTGGTAAGTATCCCCGCCTGTCACGCGGGAGACCGGGGTTCGATTC
CCCGACGGGGAG
>Anolis_carolinensis_chrUn_GL343466.trna19-AspGTC (289439-289510) Asp (GTC) 72 bp Sc: 71.60
TCCTCGTTAGTATAGTGGTAAGTATCCCCGCCTGTCACGCGGGAGACCGGGGTTCGATTC
CCCGACGGGGAG
>Anolis_carolinensis_chrUn_GL343466.trna30-AspGTC (278556-278485) Asp (GTC) 72 bp Sc: 71.60
TCCTCGTTAGTATAGTGGTAAGTATCCCCGCCTGTCACGCGGGAGACCGGGGTTCGATTC
CCCGACGGGGAG
>Anolis_carolinensis_chrUn_GL343466.trna32-AspGTC (270851-270780) Asp (GTC) 72 bp Sc: 71.60
TCCTCGTTAGTATAGTGGTAAGTATCCCCGCCTGTCACGCGGGAGACCGGGGTTCGATTC
CCCGACGGGGAG
>Anolis_carolinensis_chrUn_GL343466.trna7-AspGTC (252001-252072) Asp (GTC) 72 bp Sc: 71.60
TCCTCGTTAGTATAGTGGTAAGTATCCCCGCCTGTCACGCGGGAGACCGGGGTTCGATTC
CCCGACGGGGAG
>Anolis_carolinensis_chr5.trna454-AspGTC (30548157-30548086) Asp (GTC) 72 bp Sc: 72.92
TCCTCGTTAGTATAGTGGTGAGTATCCCCGCCTGTCACGCGGGAGACCGGGGTTCGATTC
CCCGACGGGGAG
>Anolis_carolinensis_chr2.trna271-CysGCA (194672081-194672152) Cys (GCA) 72 bp Sc: 71.79
GGGGGTATAGCTCAGCGGTAGAGCATTTGACTGCAGATCAAGAGGTCCCTGGTTCAACTC
CGGGTGCCCCCT
>Anolis_carolinensis_chr2.trna272-CysGCA (194675336-194675407) Cys (GCA) 72 bp Sc: 71.79
GGGGGTATAGCTCAGCGGTAGAGCATTTGACTGCAGATCAAGAGGTCCCTGGTTCAACTC
CGGGTGCCCCCT
>Anolis_carolinensis_chr2.trna273-CysGCA (194680399-194680470) Cys (GCA) 72 bp Sc: 71.79
GGGGGTATAGCTCAGCGGTAGAGCATTTGACTGCAGATCAAGAGGTCCCTGGTTCAACTC
CGGGTGCCCCCT
>Anolis_carolinensis_chr2.trna274-CysGCA (194680730-194680801) Cys (GCA) 72 bp Sc: 71.79
GGGGGTATAGCTCAGCGGTAGAGCATTTGACTGCAGATCAAGAGGTCCCTGGTTCAACTC
CGGGTGCCCCCT
>Anolis_carolinensis_chr2.trna275-CysGCA (194684855-194684926) Cys (GCA) 72 bp Sc: 71.79
GGGGGTATAGCTCAGCGGTAGAGCATTTGACTGCAGATCAAGAGGTCCCTGGTTCAACTC
CGGGTGCCCCCT
>Anolis_carolinensis_chr2.trna276-CysGCA (194695175-194695246) Cys (GCA) 72 bp Sc: 71.79
GGGGGTATAGCTCAGCGGTAGAGCATTTGACTGCAGATCAAGAGGTCCCTGGTTCAACTC
CGGGTGCCCCCT
>Anolis_carolinensis_chr2.trna277-CysGCA (194696706-194696777) Cys (GCA) 72 bp Sc: 71.79
GGGGGTATAGCTCAGCGGTAGAGCATTTGACTGCAGATCAAGAGGTCCCTGGTTCAACTC
CGGGTGCCCCCT
>Anolis_carolinensis_chr2.trna270-CysGCA (194669519-194669590) Cys (GCA) 72 bp Sc: 73.07
GGGGGTATAGCTCAGCGGTAGAGCATTTGACTGCAGATCAAGAGGTCCCTGGTTCAAGTC
CGGGTGCCCCCT
>Anolis_carolinensis_chr6.trna113-CysGCA (63176702-63176773) Cys (GCA) 72 bp Sc: 77.18
GGGGGTATAGCTCAGTGGTAGAGCATTTGACTGCAGATCAAGAGGTCCCTGGTTCAAATC
CGGGTGCCCCCT
>Anolis_carolinensis_chr6.trna144-CysGCA (78603289-78603218) Cys (GCA) 72 bp Sc: 77.18
GGGGGTATAGCTCAGTGGTAGAGCATTTGACTGCAGATCAAGAGGTCCCTGGTTCAAATC

CGGGTGCCCCCT
>Anolis_carolinensis_chr2.trna54-CysGCA (40845728-40845799) Cys (GCA) 72 bp Sc: 77.65
GGGGGTATAGCTCAGTGGTAGAGCATTTGACTGCAGATCAAGAGGTCCCTGGTTCAAATC
CAGGTGCCCCCT
>Anolis_carolinensis_chr6.trna138-CysGCA (78617140-78617211) Cys (GCA) 72 bp Sc: 77.65
GGGGGTATAGCTCAGTGGTAGAGCATTTGACTGCAGATCAAGAGGTCCCTGGTTCAAATC
CAGGTGCCCCCT
>Anolis_carolinensis_chr2.trna156-GlnCTG (109470426-109470497) Gln (CTG) 72 bp Sc: 73.65
GGTTCCATGGTGTAATGGTTAGCACTCTGGACTCTGAATCCAGCGATCCGAGTTCAAATC
TCGGTGGAACCT
>Anolis_carolinensis_chrUn_GL343466.trna16-GlnCTG (274307-274378) Gln (CTG) 72 bp Sc: 73.65
GGTTCCATGGTGTAATGGTTAGCACTCTGGACTCTGAATCCAGCGATCCGAGTTCAAATC
TCGGTGGAACCT
>Anolis_carolinensis_chrUn_GL343503.trna2-GlnCTG (381803-381732) Gln (CTG) 72 bp Sc: 73.65
GGTTCCATGGTGTAATGGTTAGCACTCTGGACTCTGAATCCAGCGATCCGAGTTCAAATC
TCGGTGGAACCT
>Anolis_carolinensis_chrUn_GL343400.trna6-GlnCTG (586061-586132) Gln (CTG) 72 bp Sc: 73.66
GGTTCCGTGGTGTAATGGTTAGCACTTTGGACTCTGAATCCAACGATCCGAGTTCAAATC
TCGGCGGAACCT
>Anolis_carolinensis_chrUn_GL343466.trna43-GlnCTG (201031-200960) Gln (CTG) 72 bp Sc: 74.71
GGTTCCATGGTGTAATGGTTAGCACTTTGGACTCTGAATCCAATGATCCGAGTTCAAATC
TCGGTGGAACCT
>Anolis_carolinensis_chr2.trna157-GlnTTG (109470811-109470882) Gln (TTG) 72 bp Sc: 72.03
GGTTCTATGGTGTAATGGTTAGCACTCTGGACTTTGAATCCAGCGATCCGAGTTCAAATC
TCGGTAGAACCT
>Anolis_carolinensis_chrUn_GL343400.trna15-GlnTTG (356008-355937) Gln (TTG) 72 bp Sc: 73.28
GGTTCCATGGTGTAATGGTTAGCACTCTGGACTTTGAATCCAGCGATCCGAGTTCAAATC
TCGGTGGAACCT
>Anolis_carolinensis_chr6.trna134-GlnTTG (76176912-76176983) Gln (TTG) 72 bp Sc: 73.77
GGTCCCATGGTGTAATGGTTAGCACTCTGGACTTTGAATCCAGCGATCCGAGTTCAAATC
TCGGTGGGACCT
>Anolis_carolinensis_chr1.trna159-GluCTC (116139938-116140009) Glu (CTC) 72 bp Sc: 69.93
TCCCTGGTGGTCTAGTGGTTAGGATTCGGTGCTCTCACCGCTGTGGCCTGGGTTCGATTC
CCAGTCAGGGAA
>Anolis_carolinensis_chr2.trna337-GluCTC (189094442-189094371) Glu (CTC) 72 bp Sc: 74.53
TCCCTGGTGGTCTAGTGGTCAGGATTCGGCGCTCTCACCGCCGCGGCCCGGGTTCGATTC
CCGGTCAGGGAA
>Anolis_carolinensis_chr2.trna287-GluCTC (197429818-197429889) Glu (CTC) 72 bp Sc: 76.23
TCCCTGGTGGTCTAGTGGTTAGGATTTGGCGCTCTCACCGCCACGGCCCGGGTTCGATTC
CCGGTCAGGGAA
>Anolis_carolinensis_chr2.trna309-GluCTC (197437539-197437468) Glu (CTC) 72 bp Sc: 76.23
TCCCTGGTGGTCTAGTGGTTAGGATTTGGCGCTCTCACCGCCACGGCCCGGGTTCGATTC
CCGGTCAGGGAA
>Anolis_carolinensis_chr2.trna336-GluCTC (189107265-189107194) Glu (CTC) 72 bp Sc: 76.61
TCCCTGGTGGTCTAGTGGTTAGGATTCGGCGCTCTCACCGCCGCGGCCCGGGTTCGATTC
CCGGTCAGGGAA
>Anolis_carolinensis_chr2.trna511-GluTTC (68983472-68983401) Glu (TTC) 72 bp Sc: 75.00
TCCCATATGGTCTAGCGGTTAGGATTCCTGGTTTTCACCCAGGCGGCCCGGGTTCGACTC
CCGGTATGGGAA
>Anolis_carolinensis_chr2.trna512-GluTTC (68982916-68982845) Glu (TTC) 72 bp Sc: 75.00
TCCCATATGGTCTAGCGGTTAGGATTCCTGGTTTTCACCCAGGCGGCCCGGGTTCGACTC
CCGGTATGGGAA
>Anolis_carolinensis_chr2.trna513-GluTTC (68982443-68982372) Glu (TTC) 72 bp Sc: 75.00
TCCCATATGGTCTAGCGGTTAGGATTCCTGGTTTTCACCCAGGCGGCCCGGGTTCGACTC
CCGGTATGGGAA
>Anolis_carolinensis_chr2.trna515-GluTTC (68672125-68672054) Glu (TTC) 72 bp Sc: 75.00
TCCCATATGGTCTAGCGGTTAGGATTCCTGGTTTTCACCCAGGCGGCCCGGGTTCGACTC
CCGGTATGGGAA
>Anolis_carolinensis_chr2.trna95-GluTTC (68981470-68981541) Glu (TTC) 72 bp Sc: 75.00
TCCCATATGGTCTAGCGGTTAGGATTCCTGGTTTTCACCCAGGCGGCCCGGGTTCGACTC
CCGGTATGGGAA
>Anolis_carolinensis_chrUn_GL343577.trna5-GluTTC (234023-233952) Glu (TTC) 72 bp Sc: 75.00
TCCCATATGGTCTAGCGGTTAGGATTCCTGGTTTTCACCCAGGCGGCCCGGGTTCGACTC
CCGGTATGGGAA
>Anolis_carolinensis_chr2.trna292-GluTTC (197441892-197441963) Glu (TTC) 72 bp Sc: 77.41
TCCCTGGTGGTCTAGTGGTCAGGATTTGGCGCTTTCACCGCCACGGCCCGGGTTCGATTC
CCGGCCAGGGAA

>Anolis_carolinensis_chr2.trna284-GlyCCC (197419617-197419687) Gly (CCC) 71 bp Sc: 62.44
GCACCAGTGGTGTAGAGGCATCATGCGAGGTTCCCAATCTTGTGACCCGGGTTCGAGTCC
CGGCTGGTGCA
>Anolis_carolinensis_chr2.trna289-GlyCCC (197433343-197433413) Gly (CCC) 71 bp Sc: 62.44
GCACCAGTGGTGTAGAGGCATCATGCGAGGTTCCCAATCTTGTGACCCGGGTTCGAGTCC
CGGCTGGTGCA
>Anolis_carolinensis_chrUn_GL343784.trna1-GlyGCC (117103-117173) Gly (GCC) 71 bp Sc: 74.27
GCATTGGTGGTTCAGTGGTAGAATTCTCGCCTGCCACGCAGGAGGCCCAGGTTCGATTCC
TGGCCAATGCA
>Anolis_carolinensis_chrUn_GL343400.trna10-GlyGCC (556460-556390) Gly (GCC) 71 bp Sc: 81.62
GCATTGGTGGTTCAGTGGTAGAATTCTCGCCTGCCACGCGGGAGGCCCGGGTTCGATTCC
CGGCCAATGCA
>Anolis_carolinensis_chrUn_GL343400.trna12-GlyGCC (524602-524532) Gly (GCC) 71 bp Sc: 81.62
GCATTGGTGGTTCAGTGGTAGAATTCTCGCCTGCCACGCGGGAGGCCCGGGTTCGATTCC
CGGCCAATGCA
>Anolis_carolinensis_chrUn_GL343400.trna14-GlyGCC (398937-398867) Gly (GCC) 71 bp Sc: 81.62
GCATTGGTGGTTCAGTGGTAGAATTCTCGCCTGCCACGCGGGAGGCCCGGGTTCGATTCC
CGGCCAATGCA
>Anolis_carolinensis_chrUn_GL343400.trna16-GlyGCC (355340-355270) Gly (GCC) 71 bp Sc: 81.62
GCATTGGTGGTTCAGTGGTAGAATTCTCGCCTGCCACGCGGGAGGCCCGGGTTCGATTCC
CGGCCAATGCA
>Anolis_carolinensis_chrUn_GL343400.trna2-GlyGCC (399073-399143) Gly (GCC) 71 bp Sc: 81.62
GCATTGGTGGTTCAGTGGTAGAATTCTCGCCTGCCACGCGGGAGGCCCGGGTTCGATTCC
CGGCCAATGCA
>Anolis_carolinensis_chrUn_GL343400.trna9-GlyGCC (559474-559404) Gly (GCC) 71 bp Sc: 81.62
GCATTGGTGGTTCAGTGGTAGAATTCTCGCCTGCCACGCGGGAGGCCCGGGTTCGATTCC
CGGCCAATGCA
>Anolis_carolinensis_chrUn_GL343930.trna1-GlyTCC (85174-85103) Gly (TCC) 72 bp Sc: 66.22
GCGTTGGTGGTATAGTGGTTAGCATAGCTGCCTTCCAAGCAGTTGACCCAGGTTCGATTC
CCGGTCAACGCA
>Anolis_carolinensis_chrUn_GL343466.trna13-GlyTCC (263636-263707) Gly (TCC) 72 bp Sc: 73.26
GCGTTGGTGGTATAGTGGTGAGCATAGCTGCCTTCCAAGCAGTTGACCCGGGTTCGATTC
CCGGCCAACGCA
>Anolis_carolinensis_chrUn_GL343466.trna41-GlyTCC (246495-246424) Gly (TCC) 72 bp Sc: 73.26
GCGTTGGTGGTATAGTGGTGAGCATAGCTGCCTTCCAAGCAGTTGACCCGGGTTCGATTC
CCGGCCAACGCA
>Anolis_carolinensis_chrUn_GL343466.trna6-GlyTCC (251232-251303) Gly (TCC) 72 bp Sc: 73.26
GCGTTGGTGGTATAGTGGTGAGCATAGCTGCCTTCCAAGCAGTTGACCCGGGTTCGATTC
CCGGCCAACGCA
>Anolis_carolinensis_chrUn_GL343660.trna2-GlyTCC (87039-87110) Gly (TCC) 72 bp Sc: 73.26
GCGTTGGTGGTATAGTGGTGAGCATAGCTGCCTTCCAAGCAGTTGACCCGGGTTCGATTC
CCGGCCAACGCA
>Anolis_carolinensis_chr2.trna294-GlyTCC (197649527-197649598) Gly (TCC) 72 bp Sc: 76.83
GCGTTGGTGGTATAGTGGTTAGCATAGCTGCCTTCCAAGCAGTTGACCCGGGTTCGATTC
CCGGCCAACGCA
>Anolis_carolinensis_chr2.trna307-GlyTCC (197655656-197655585) Gly (TCC) 72 bp Sc: 76.83
GCGTTGGTGGTATAGTGGTTAGCATAGCTGCCTTCCAAGCAGTTGACCCGGGTTCGATTC
CCGGCCAACGCA
>Anolis_carolinensis_chrUn_GL343194.trna26-GlyTCC (589968-589897) Gly (TCC) 72 bp Sc: 76.83
GCGTTGGTGGTATAGTGGTTAGCATAGCTGCCTTCCAAGCAGTTGACCCGGGTTCGATTC
CCGGCCAACGCA
>Anolis_carolinensis_chr3.trna94-HisGTG (61550949-61551020) His (GTG) 72 bp Sc: 49.46
GCCGTGATCGTATAGTGGTTAGTACTTTGCGTTGTGGCCGCAGCAACCTCAGTTCAAATC
TGAGTCATGACA
>Anolis_carolinensis_chr2.trna43-HisGTG (34423791-34423862) His (GTG) 72 bp Sc: 64.63
GCCGTGATCGTATAGTGGTTAGTACTCTGCGTTGTGGCCGCAGCAACCTCGGTTCGAATC
CGAGTCACGGCA
>Anolis_carolinensis_chr2.trna575-HisGTG (34425966-34425895) His (GTG) 72 bp Sc: 64.63
GCCGTGATCGTATAGTGGTTAGTACTCTGCGTTGTGGCCGCAGCAACCTCGGTTCGAATC
CGAGTCACGGCA
>Anolis_carolinensis_chr3.trna239-HisGTG (175022718-175022789) His (GTG) 72 bp Sc: 64.63
GCCGTGATCGTATAGTGGTTAGTACTCTGCGTTGTGGCCGCAGCAACCTCGGTTCGAATC
CGAGTCACGGCA
>Anolis_carolinensis_chrUn_GL343220.trna5-HisGTG (92985-93056) His (GTG) 72 bp Sc: 64.63
GCCGTGATCGTATAGTGGTTAGTACTCTGCGTTGTGGCCGCAGCAACCTCGGTTCGAATC
CGAGTCACGGCA
>Anolis_carolinensis_chrUn_GL343199.trna8-IleAAT (3985415-3985488) Ile (AAT) 74 bp Sc: 77.09

GGCCAGTTAGCTCAGTTGGTTAGAGCGTGGTGCTAATAACGCCAAGGTCGCAGGTTCGAT
CCCCGTACTGGCCA
>Anolis_carolinensis_chr3.trna331-IleAAT (175023208-175023135) Ile (AAT) 74 bp Sc: 80.66
GGCCGGTTAGCTCAGTTGGTTAGAGCGTGGTGCTAATAACGCCAAGGTCGCGGGTTCGAT
CCCCGTACGGGCCA
>Anolis_carolinensis_chrUn_GL343232.trna5-IleAAT (1810787-1810714) Ile (AAT) 74 bp Sc: 80.66
GGCCGGTTAGCTCAGTTGGTTAGAGCGTGGTGCTAATAACGCCAAGGTCGCGGGTTCGAT
CCCCGTACGGGCCA
>Anolis_carolinensis_chrUn_GL343466.trna1-IleAAT (235105-235178) Ile (AAT) 74 bp Sc: 80.66
GGCCGGTTAGCTCAGTTGGTTAGAGCGTGGTGCTAATAACGCCAAGGTCGCGGGTTCGAT
CCCCGTACGGGCCA
>Anolis_carolinensis_chrUn_GL343466.trna34-IleAAT (265655-265582) Ile (AAT) 74 bp Sc: 80.66
GGCCGGTTAGCTCAGTTGGTTAGAGCGTGGTGCTAATAACGCCAAGGTCGCGGGTTCGAT
CCCCGTACGGGCCA
>Anolis_carolinensis_chrUn_GL343466.trna5-IleAAT (245844-245917) Ile (AAT) 74 bp Sc: 80.66
GGCCGGTTAGCTCAGTTGGTTAGAGCGTGGTGCTAATAACGCCAAGGTCGCGGGTTCGAT
CCCCGTACGGGCCA
>Anolis_carolinensis_chrUn_GL343466.trna40-IleTAT (247375-247282) Ile (TAT) 94 bp Sc: 69.02
GCTCCAGTGGCGCAATCGGTTAGCGCGCGGTACTTATACAGCAGTACTAGTGAAAGCAAT
GCCGAGGTTGTGAGTTCGAGCCTCACCTGGAGCA
>Anolis_carolinensis_chrUn_GL343466.trna36-LeuAAG (258276-258195) Leu (AAG) 82 bp Sc: 69.34
GGTAGCGTGGCCGAGCGGTCTAAGGCGCTGGATTAAGGCTCCAGTCTCTCCGGGGGCGTG
GGTTCGAATCCCACCGCTGCCA
>Anolis_carolinensis_chr2.trna340-LeuAAG (188948631-188948550) Leu (AAG) 82 bp Sc: 69.84
GGTAGCGTGGCCGAGCGGTCTAAGGCGCTGGATTAAGGCTCCAGTCTCTTCGGGGGCGTG
GGTTCGAATCCCACCGCTGCCA
>Anolis_carolinensis_chrUn_GL343466.trna11-LeuAAG (257035-257116) Leu (AAG) 82 bp Sc: 69.84
GGTAGCGTGGCCGAGCGGTCTAAGGCGCTGGATTAAGGCTCCAGTCTCTTCGGGGGCGTG
GGTTCGAATCCCACCGCTGCCA
>Anolis_carolinensis_chrUn_GL343846.trna2-LeuAAG (51297-51216) Leu (AAG) 82 bp Sc: 69.84
GGTAGCGTGGCCGAGCGGTCTAAGGCGCTGGATTAAGGCTCCAGTCTCTTCGGGGGCGTG
GGTTCGAATCCCACCGCTGCCA
>Anolis_carolinensis_chr2.trna260-LeuCAA (189052090-189052199) Leu (CAA) 110 bp Sc: 62.78
GTCAGGATGGCCGAGTGGTCTAAGGCGCCAGACTCAAGGCTTCTTCCCTTCCCAGCTCTG
GGTGTTCTGGTCTCCGAATGGAGGCGTGGGTTCAAATCCCACTTCTGACA
>Anolis_carolinensis_chr2.trna335-LeuCAA (189107696-189107587) Leu (CAA) 110 bp Sc: 62.78
GTCAGGATGGCCGAGTGGTCTAAGGCGCCAGACTCAAGGCTTCTTCCCTTCCCAGCTCTG
GGTGTTCTGGTCTCCGAATGGAGGCGTGGGTTCAAATCCCACTTCTGACA
>Anolis_carolinensis_chr3.trna330-LeuCAG (175024320-175024238) Leu (CAG) 83 bp Sc: 75.90
GTCAGGATGGCCGAGCGGTCTAAGGCGCTGCGTTCAGGTCGCAGTCTCCCCTGGAGGCGT
GGGTTCGAATCCCACTTCTGACA
>Anolis_carolinensis_chrUn_GL343466.trna4-LeuCAG (237248-237330) Leu (CAG) 83 bp Sc: 75.90
GTCAGGATGGCCGAGCGGTCTAAGGCGCTGCGTTCAGGTCGCAGTCTCCCCTGGAGGCGT
GGGTTCGAATCCCACTTCTGACA
>Anolis_carolinensis_chrUn_GL343677.trna1-LeuCAG (206362-206444) Leu (CAG) 83 bp Sc: 75.90
GTCAGGATGGCCGAGCGGTCTAAGGCGCTGCGTTCAGGTCGCAGTCTCCCCTGGAGGCGT
GGGTTCGAATCCCACTTCTGACA
>Anolis_carolinensis_chrUn_GL343466.trna39-LeuCAG (249799-249717) Leu (CAG) 83 bp Sc: 79.14
GTCAGGATGGCCGAGCGGTCTAAGGCGCTGCGTTCAGGTCGCAGTCTCCTCTGGAGGCGT
GGGTTCGAATCCCACTCCTGACA
>Anolis_carolinensis_chr1.trna122-LeuTAA (91051930-91052012) Leu (TAA) 83 bp Sc: 74.15
ACCAGAATGGCCGAGTGGTTAAGGCGTTGGACTTAAGTTCCAATGGACGTATGTCTGCGT
GGGTTCGAACCCCACTTCTGGTA
>Anolis_carolinensis_chr1.trna256-LeuTAA (206107715-206107797) Leu (TAA) 83 bp Sc: 80.55
ACCAGGATGGCCGAGTGGTTAAGGCGTTGGACTTAAGATCCAATGGACGCATGTCCGCGT
GGGTTCGAACCCCACTCCTGGTA
>Anolis_carolinensis_chrUn_GL343660.trna1-LeuTAG (86778-86859) Leu (TAG) 82 bp Sc: 67.69
GGTAGCGTGGCCGAGCGGTCTAAGGCGCTGGATTTAGGCTCCAGTCTCTCCGGGGACGTG
GGTTCGAATCCCACCGCTGCCA
>Anolis_carolinensis_chrUn_AAWZ02039051.trna1-LeuTAG (8372-8291) Leu (TAG) 82 bp Sc: 68.14
GGTAGCGTGGCCGAGTGGTCTAAGGCGCTGGATTTAGGCTCCAGTCATTTCGATGGCGTG
GGTTCGAATCCCACCGCTGCCA
>Anolis_carolinensis_chrUn_GL343466.trna9-LeuTAG (254481-254562) Leu (TAG) 82 bp Sc: 69.80
GGTAGTGTGGCCGAGCGGTCTAAGGCGCTGGATTTAGGCTCCAGTCTCTCCGGAGGCGTG
GGTTCGAATCCCACCGCTGCCA
>Anolis_carolinensis_chr3.trna222-LysCTT (160886900-160886972) Lys (CTT) 73 bp Sc: 58.41
GCCTGGCTAGCTCAGTCGGTAGAGCATAAGACTCTTAATCTCAGGGTCGTAGGTTCGAGC

TCCATGTTGGGCG
>Anolis_carolinensis_chr2.trna255-LysCTT (188811754-188811826) Lys (CTT) 73 bp Sc: 80.47
GCCCGGCTAGCTCAGTCGGTAGAGCATGAGACTCTTAATCTCAGGGTCGTGGGTTCGAGC
CCCACGTTGGGCG
>Anolis_carolinensis_chrUn_GL343220.trna4-LysCTT (92251-92323) Lys (CTT) 73 bp Sc: 80.47
GCCCGGCTAGCTCAGTCGGTAGAGCATGAGACTCTTAATCTCAGGGTCGTGGGTTCGAGC
CCCACGTTGGGCG
>Anolis_carolinensis_chrUn_GL343466.trna10-LysCTT (255015-255087) Lys (CTT) 73 bp Sc: 80.47
GCCCGGCTAGCTCAGTCGGTAGAGCATGAGACTCTTAATCTCAGGGTCGTGGGTTCGAGC
CCCACGTTGGGCG
>Anolis_carolinensis_chrUn_GL343466.trna28-LysCTT (291804-291732) Lys (CTT) 73 bp Sc: 80.47
GCCCGGCTAGCTCAGTCGGTAGAGCATGAGACTCTTAATCTCAGGGTCGTGGGTTCGAGC
CCCACGTTGGGCG
>Anolis_carolinensis_chrUn_GL343459.trna5-LysCTT (303220-303148) Lys (CTT) 73 bp Sc: 80.72
GCCCGGCTAGCTCAGTCGGTAGAGCATGGGACTCTTAATCCCAGGGTCGTGGGTTCGAGC
CCCACGTTGGGCG
>Anolis_carolinensis_chrUn_GL343466.trna14-LysTTT (268858-268931) Lys (TTT) 74 bp Sc: 75.16
GCCCGGGTAGCTCAGTCGGTAGAGCATCAGACTTTTAATCTGAGGGTCCAGGGTTCATAG
TCCCTGCTCGGGCA
>Anolis_carolinensis_chrUn_GL343466.trna8-LysTTT (253384-253456) Lys (TTT) 73 bp Sc: 83.31
GCCTGGATAGCTCAGTCGGTAGAGCATCAGACTTTTAATCTGAGGGTCCAGGGTTCAAGT
CCCTGTTCAGGCG
>Anolis_carolinensis_chr4.trna158-LysTTT (117415304-117415376) Lys (TTT) 73 bp Sc: 83.80
GCCCGGATAGCTCAGTCGGTAGAGCATCAGACTTTTAATCTGAGGGTCCAGGGTTCAAGT
CCCTGTTCGGGCG
>Anolis_carolinensis_chr4.trna258-LysTTT (117415695-117415623) Lys (TTT) 73 bp Sc: 83.80
GCCCGGATAGCTCAGTCGGTAGAGCATCAGACTTTTAATCTGAGGGTCCAGGGTTCAAGT
CCCTGTTCGGGCG
>Anolis_carolinensis_chrUn_GL343220.trna15-LysTTT (83915-83843) Lys (TTT) 73 bp Sc: 83.80
GCCCGGATAGCTCAGTCGGTAGAGCATCAGACTTTTAATCTGAGGGTCCAGGGTTCAAGT
CCCTGTTCGGGCG
>Anolis_carolinensis_chr2.trna288-MetCAT (197432511-197432582) Met (CAT) 72 bp Sc: 60.41
ATCAGAGTGGCGCAGCGGAAGCGTGCTGGGCCCATAACCCAGAGGTTGATGGATCGAAAC
CATCCTCTGCTA
>Anolis_carolinensis_chr2.trna314-MetCAT (197416297-197416226) Met (CAT) 72 bp Sc: 61.97
AGCAGAATGGCGCAGCGGGAGCGTGCTGGGCCCATAACCCAGAGGTCGATGGATCGAAAC
CATCCTCTGCTA
>Anolis_carolinensis_chr2.trna283-MetCAT (197419240-197419311) Met (CAT) 72 bp Sc: 65.51
AGCAGAGTGGCGCAGAGGGAGCGTGCTGGGCCCATAACCCAGAGGTCGATGGATCGAAAC
CATCCTCTGCTA
>Anolis_carolinensis_chrUn_GL343466.trna27-MetCAT (310214-310143) Met (CAT) 72 bp Sc: 66.20
AGCAGAGTGGCGCAGCGGAAGCGTGCTGGGCCCATAACCCAGAGGTCGATGGATCGAAAC
CATTCTCTGCTA
>Anolis_carolinensis_chrUn_GL343220.trna14-MetCAT (92684-92613) Met (CAT) 72 bp Sc: 68.98
AGCAGAGTGGCGCAGCGGAAGCGTGCTGGGCCCATAACCCAGAGGTCGATGGATCGAAAC
CATCCTCTGCTA
>Anolis_carolinensis_chrUn_GL343220.trna1-MetCAT (83325-83396) Met (CAT) 72 bp Sc: 68.98
AGCAGAGTGGCGCAGCGGAAGCGTGCTGGGCCCATAACCCAGAGGTCGATGGATCGAAAC
CATCCTCTGCTA
>Anolis_carolinensis_chrUn_GL343466.trna21-MetCAT (309734-309805) Met (CAT) 72 bp Sc: 68.98
AGCAGAGTGGCGCAGCGGAAGCGTGCTGGGCCCATAACCCAGAGGTCGATGGATCGAAAC
CATCCTCTGCTA
>Anolis_carolinensis_chrUn_GL343466.trna3-MetCAT (236657-236728) Met (CAT) 72 bp Sc: 68.98
AGCAGAGTGGCGCAGCGGAAGCGTGCTGGGCCCATAACCCAGAGGTCGATGGATCGAAAC
CATCCTCTGCTA
>Anolis_carolinensis_chrUn_GL343220.trna2-MetCAT (86053-86125) Met (CAT) 73 bp Sc: 73.48
GCCTTCTTAGCGCAGTAGGCAGCGCGTCAGTCTCATAATCTGAAGGTCCTGAGTTCGAGC
CTCAGAGAGGGCA
>Anolis_carolinensis_chrUn_GL343220.trna3-MetCAT (91879-91951) Met (CAT) 73 bp Sc: 73.48
GCCTTCTTAGCGCAGTAGGCAGCGCGTCAGTCTCATAATCTGAAGGTCCTGAGTTCGAGC
CTCAGAGAGGGCA
>Anolis_carolinensis_chrUn_GL343581.trna2-MetCAT (438586-438514) Met (CAT) 73 bp Sc: 74.51
GCCTCGTTAGCGCAGTAGGCAGCGCGTCAGTCTCATAATCTGAAGGTCGTGAGTTCGAGC
CTCACACGGGGCA
>Anolis_carolinensis_chrUn_GL343957.trna1-MetCAT (67154-67082) Met (CAT) 73 bp Sc: 74.91
GCCTCGTTGGCGCAGTAGGCAGCGCGTCAGTCTCATAATCTGAAGGTCGTGAGTTCGAGC
CTCACACGGGGCA

>Anolis_carolinensis_chr3.trna412-PheGAA (84756409-84756337) Phe (GAA) 73 bp Sc: 84.19
GCCGAAATAGCTCAGTTGGGAGAGCGTTAGACTGAAGATCTAAAGGTCCCTGGTTCGATC
CCGGGTTTCGGCA
>Anolis_carolinensis_chrLGb.trna2-PheGAA (1331842-1331914) Phe (GAA) 73 bp Sc: 84.19
GCCGAAATAGCTCAGTTGGGAGAGCGTTAGACTGAAGATCTAAAGGTCCCTGGTTCGATC
CCGGGTTTCGGCA
>Anolis_carolinensis_chrUn_GL343400.trna17-PheGAA (355065-354993) Phe (GAA) 73 bp Sc: 84.19
GCCGAAATAGCTCAGTTGGGAGAGCGTTAGACTGAAGATCTAAAGGTCCCTGGTTCGATC
CCGGGTTTCGGCA
>Anolis_carolinensis_chrUn_GL343466.trna22-PheGAA (315845-315917) Phe (GAA) 73 bp Sc: 84.19
GCCGAAATAGCTCAGTTGGGAGAGCGTTAGACTGAAGATCTAAAGGTCCCTGGTTCGATC
CCGGGTTTCGGCA
>Anolis_carolinensis_chrUn_GL343466.trna29-PheGAA (283278-283206) Phe (GAA) 73 bp Sc: 84.19
GCCGAAATAGCTCAGTTGGGAGAGCGTTAGACTGAAGATCTAAAGGTCCCTGGTTCGATC
CCGGGTTTCGGCA
>Anolis_carolinensis_chr3.trna345-ProAGG (163842384-163842313) Pro (AGG) 72 bp Sc: 75.92
GGCTCGTTGGTCTAGGGGTATGATTCTCGCTTAGGGTGCGAGAGGTCCCGGGTTCAAATC
CCGGACGAGCCC
>Anolis_carolinensis_chrUn_GL343220.trna6-ProAGG (1267132-1267203) Pro (AGG) 72 bp Sc: 75.92
GGCTCGTTGGTCTAGGGGTATGATTCTCGCTTAGGGTGCGAGAGGTCCCGGGTTCAAATC
CCGGACGAGCCC
>Anolis_carolinensis_chrUn_GL343220.trna9-ProAGG (1277635-1277706) Pro (AGG) 72 bp Sc: 75.92
GGCTCGTTGGTCTAGGGGTATGATTCTCGCTTAGGGTGCGAGAGGTCCCGGGTTCAAATC
CCGGACGAGCCC
>Anolis_carolinensis_chr3.trna226-ProCGG (163841764-163841835) Pro (CGG) 72 bp Sc: 76.52
GGCTCGTTGGTCTAGGGGTATGATTCTCGCTTCGGGTGCGAGAGGTCCCGGGTTCAAATC
CCGGACGAGCCC
>Anolis_carolinensis_chrUn_GL343466.trna17-ProCGG (280209-280280) Pro (CGG) 72 bp Sc: 76.52
GGCTCGTTGGTCTAGGGGTATGATTCTCGCTTCGGGTGCGAGAGGTCCCGGGTTCAAATC
CCGGACGAGCCC
>Anolis_carolinensis_chr3.trna93-ProGGG (61544223-61544294) Pro (GGG) 72 bp Sc: 58.12
GCCGTGGTAGTATAGTGGTTAATACTCTGCCTTGGGGCTGCAGCAACCTCGGTTCGAATC
CGAGTCACGGCA
>Anolis_carolinensis_chr2.trna258-ProTGG (188947534-188947605) Pro (TGG) 72 bp Sc: 76.15
GGCTCGTTGGTCTAGGGGTATGATTCTCGCTTTGGGTGCGAGAGGTCCCGGGTTCAAATC
CCGGACGAGCCC
>Anolis_carolinensis_chrUn_GL343220.trna7-ProTGG (1268285-1268356) Pro (TGG) 72 bp Sc: 76.15
GGCTCGTTGGTCTAGGGGTATGATTCTCGCTTTGGGTGCGAGAGGTCCCGGGTTCAAATC
CCGGACGAGCCC
>Anolis_carolinensis_chrUn_GL343466.trna35-ProTGG (262609-262538) Pro (TGG) 72 bp Sc: 76.15
GGCTCGTTGGTCTAGGGGTATGATTCTCGCTTTGGGTGCGAGAGGTCCCGGGTTCAAATC
CCGGACGAGCCC
>Anolis_carolinensis_chr2.trna175-SeC(e)TCA (125400547-125400632) SeC(e) (TCA) 86 bp Sc: 75.67
GCCCGGATGATCCTCAGTGGTCTGGGGTGCAGGCTTCAAACCTGTAGCTGTTTAGCGACA
GAGTGGTTCAATTCCACCTTTCGGGC
>Anolis_carolinensis_chr1.trna492-SerAGA (155487259-155487178) Ser (AGA) 82 bp Sc: 88.01
GTAGTCGTGGCCGAGTGGTTAAGGCGATGGACTAGAAATCCATTGGGGTCTCCCCGCGCA
GGTTCGAATCCTGCCGACTACG
>Anolis_carolinensis_chr4.trna401-SerAGA (19517037-19516956) Ser (AGA) 82 bp Sc: 88.01
GTAGTCGTGGCCGAGTGGTTAAGGCGATGGACTAGAAATCCATTGGGGTCTCCCCGCGCA
GGTTCGAATCCTGCCGACTACG
>Anolis_carolinensis_chrUn_GL343466.trna12-SerAGA (262900-262981) Ser (AGA) 82 bp Sc: 88.01
GTAGTCGTGGCCGAGTGGTTAAGGCGATGGACTAGAAATCCATTGGGGTCTCCCCGCGCA
GGTTCGAATCCTGCCGACTACG
>Anolis_carolinensis_chrUn_GL343466.trna31-SerAGA (271267-271186) Ser (AGA) 82 bp Sc: 88.01
GTAGTCGTGGCCGAGTGGTTAAGGCGATGGACTAGAAATCCATTGGGGTCTCCCCGCGCA
GGTTCGAATCCTGCCGACTACG
>Anolis_carolinensis_chrUn_GL343466.trna38-SerAGA (250541-250460) Ser (AGA) 82 bp Sc: 88.01
GTAGTCGTGGCCGAGTGGTTAAGGCGATGGACTAGAAATCCATTGGGGTCTCCCCGCGCA
GGTTCGAATCCTGCCGACTACG
>Anolis_carolinensis_chrUn_GL343400.trna11-SerCGA (549466-549385) Ser (CGA) 82 bp Sc: 88.82
GCTGTGATGGCCGAGTGGTTAAGGCGTTGGACTCGAAATCCAATAGGGTTTCCCTGCGCA
GGTTCGAATCCTGCTCACAGCG
>Anolis_carolinensis_chr2.trna437-SerCGA (93757405-93757324) Ser (CGA) 82 bp Sc: 89.14
GTCACGGTGGCCGAGTGGTTAAGGCGTTGGACTCGAAATCCAATGGGGTTTCCCCGCACA
GGTTCGAATCCTGTTCGTGACG
>Anolis_carolinensis_chrUn_GL343400.trna18-SerGCT (354532-354451) Ser (GCT) 82 bp Sc: 85.34

GACGAGGTGGCCGAGTGGTTAAGGCGATGGACTGCTAATCCATTGTGCTCTGCACGCGTG
GGTTCGAATCCCATCCTCGTCG
>Anolis_carolinensis_chrUn_GL343890.trna1-SerGCT (321-240) Ser (GCT) 82 bp Sc: 85.34
GACGAGGTGGCCGAGTGGTTAAGGCGATGGACTGCTAATCCATTGTGCTCTGCACGCGTG
GGTTCGAATCCCATCCTCGTCG
>Anolis_carolinensis_chrUn_GL343466.trna2-SerGCT (236124-236205) Ser (GCT) 82 bp Sc: 86.80
GACGAGGTGGCCGAGTGGTTAAGGCGATGGACTGCTAATCCATTGTGCTCTGCACGCGTG
GGTTCGAATCCCACCTTCGTCG
>Anolis_carolinensis_chrUn_GL343466.trna33-SerGCT (270195-270114) Ser (GCT) 82 bp Sc: 86.80
GACGAGGTGGCCGAGTGGTTAAGGCGATGGACTGCTAATCCATTGTGCTCTGCACGCGTG
GGTTCGAATCCCACCTTCGTCG
>Anolis_carolinensis_chr2.trna291-SerGCT (197436163-197436244) Ser (GCT) 82 bp Sc: 88.19
GACAAGGTGGCCGAGTGGTTAAGGCGATGGACTGCTAATCCATTGTGCTCTGCACGCGTG
GGTTCGAATCCCACCCTTGTCG
>Anolis_carolinensis_chrUn_GL343400.trna4-SerTGA (522609-522690) Ser (TGA) 82 bp Sc: 80.91
GCTGTGATGGCCGAGTGGTTAAGGCGTTGGACTTGAAATCCAATGGGGACTCCCTGCGTA
GGTTCGAATCCTGCTCACAGCG
>Anolis_carolinensis_chr3.trna448-ThrAGT (61545526-61545453) Thr (AGT) 74 bp Sc: 76.88
GGCACCATGGCTAAGTTGGCTAAAGCGCCTGTCTAGTAAACAGGAGATCCTGGGTTCAAC
TCCCAGTGGTGCCT
>Anolis_carolinensis_chrUn_GL343466.trna15-ThrAGT (273706-273779) Thr (AGT) 74 bp Sc: 81.57
GGCGCCATGGCTTAGTTGGTCAAAGCGCCTGTCTAGTAAACAGGAGATCCTGGGTTCGAA
TCCCAGTGGTGCCT
>Anolis_carolinensis_chr3.trna240-ThrAGT (175023645-175023718) Thr (AGT) 74 bp Sc: 84.06
GGCGCCGTGGCTTAGTTGGTTAAAGCGCCTGTCTAGTAAACAGGAGATCCTGGGTTCGAA
TCCCAGCGGTGCCT
>Anolis_carolinensis_chrUn_GL343400.trna5-ThrAGT (524139-524212) Thr (AGT) 74 bp Sc: 84.06
GGCGCCGTGGCTTAGTTGGTTAAAGCGCCTGTCTAGTAAACAGGAGATCCTGGGTTCGAA
TCCCAGCGGTGCCT
>Anolis_carolinensis_chrUn_GL343198.trna4-ThrCGT (991360-991431) Thr (CGT) 72 bp Sc: 79.77
GGCGCGGTGGCCAAGTGGTAAGGCGTCGGTCTCGTAAACCGAAGATCGCGGGTTCGAACC
CCGTCCGTGCCT
>Anolis_carolinensis_chr2.trna257-ThrTGT (188944926-188944998) Thr (TGT) 73 bp Sc: 79.46
GGCTCCATAGCTCAGGGGTTAGAGCACTGGTCTTGTAAACCAGGGGTCGCGAGTTCAAAT
CTCGCTGGGGCCT
>Anolis_carolinensis_chrUn_GL343220.trna8-ThrTGT (1275703-1275775) Thr (TGT) 73 bp Sc: 79.46
GGCTCCATAGCTCAGGGGTTAGAGCACTGGTCTTGTAAACCAGGGGTCGCGAGTTCAAAT
CTCGCTGGGGCCT
>Anolis_carolinensis_chr1.trna301-ThrTGT (244354538-244354610) Thr (TGT) 73 bp Sc: 83.03
GGCTCCATAGCTCAGTGGTTAGAGCACTGGTCTTGTAAACCAGGGGTCGCGAGTTCGATC
CTCGCTGGGGCCT
>Anolis_carolinensis_chr5.trna447-TrpCCA (35172915-35172844) Trp (CCA) 72 bp Sc: 62.47
AGCCCTGTGGCACAATGGCAGTGCATCTGACTCCAGATCAGAAGGTTGTGTGTTCAAGCC
ACACCAGGGTCA
>Anolis_carolinensis_chrUn_AAWZ02038165.trna1-TrpCCA (11912-11841) Trp (CCA) 72 bp Sc: 62.47
AGCCCTGTGGCACAATGGCAGTGCATCTGACTCCAGATCAGAAGGTTGTGTGTTCAAGCC
ACACCAGGGTCA
>Anolis_carolinensis_chr5.trna449-TrpCCA (35102271-35102200) Trp (CCA) 72 bp Sc: 70.92
GACCTTGTGGCGCAATGGTAGCGTGTCTGACTCCAGATCAGAAGGCTGTGTGTTCAAATC
ACACCAGGGTCA
>Anolis_carolinensis_chr5.trna455-TrpCCA (29376780-29376709) Trp (CCA) 72 bp Sc: 71.65
GACCTCGTGGCGCAACGGTAGCGCGTCTGACTCCAGATCAGAAGGCTGCGTGTTCGAATC
ACGTCGGGGTCA
>Anolis_carolinensis_chrUn_GL343228.trna5-TrpCCA (22768-22697) Trp (CCA) 72 bp Sc: 71.65
GACCTCGTGGCGCAACGGTAGCGCGTCTGACTCCAGATCAGAAGGCTGCGTGTTCGAATC
ACGTCGGGGTCA
>Anolis_carolinensis_chr5.trna448-TrpCCA (35171570-35171499) Trp (CCA) 72 bp Sc: 75.88
GGCCCTGTGGCACAATGGTAGCGCGTCTGACTCCAGATCAGAAGGTTGTGTGTTCAAATC
ACACCAGGGTCA
>Anolis_carolinensis_chrUn_AAWZ02038165.trna2-TrpCCA (10542-10471) Trp (CCA) 72 bp Sc: 75.88
GGCCCTGTGGCACAATGGTAGCGCGTCTGACTCCAGATCAGAAGGTTGTGTGTTCAAATC
ACACCAGGGTCA
>Anolis_carolinensis_chr2.trna325-TyrGTA (192633426-192633338) Tyr (GTA) 89 bp Sc: 76.09
CCTTCGATAGCTCAGTTGGTAGAGCGGAGGACTGTAGTAGTGCTACTAAAGTAATCCTTA
GGTCGCTGGTTCGACTCCGGCTCGAAGGA
>Anolis_carolinensis_chr2.trna324-TyrGTA (192663290-192663203) Tyr (GTA) 88 bp Sc: 72.74
CCTTCGATAGCTCAGCTGGTAGAGCGGAGGACTGTAGTGTGCTAACAAAGTCATCCTTAG

GTCGCTGGTTCGACTCCGGCTCGAAGGA
>Anolis_carolinensis_chr2.trna267-TyrGTA (192836103-192836194) Tyr (GTA) 92 bp Sc: 75.72
CCTTCGATAGCTCAGCTGGTAGAGCGGAGGACTGTAGAGTGTGTAATGCTAAAGTCATCC
TTAGGTCGCTGGTTCGATTCCGGCTCGAAGGA
>Anolis_carolinensis_chr4.trna387-TyrGTA (30674446-30674357) Tyr (GTA) 90 bp Sc: 75.12
CCTTCGATAGCTCAGCTGGTAGAGCGGAGGACTGTAGGACGTACAAAAGCAGGAATCCTT
AGGTCGCTGGTTCGATTCCGGCTCGAAGGA
>Anolis_carolinensis_chr4.trna46-TyrGTA (30678406-30678499) Tyr (GTA) 94 bp Sc: 72.80
CCTTCGATAGCTCAGCTGGTAGAGCGGAGGACTGTAGAGGCGAGCTTCCCTTGTAGGAAT
CCTTAGGTCGCTGGTTCGATTCCGGCTCGAAGGA
>Anolis_carolinensis_chr4.trna385-TyrGTA (30902345-30902252) Tyr (GTA) 94 bp Sc: 71.16
CCTTCGATAGCTCAGCTGGTAGAGCGGAGGACTGTAGAGGCAGCGGCTGCTTGCGGGAAT
CCTTAGGTCGCTGGTTCGATTCCGGCTCGAAGGA
>Anolis_carolinensis_chr2.trna293-ValAAC (197445434-197445506) Val (AAC) 73 bp Sc: 70.52
GTTTCTGTAGTGTAGTGGATATCACATTTGCCTAACACGCAAAAGGTCCCCAGATCGAAG
CTGGGCAGAAACA
>Anolis_carolinensis_chr2.trna259-ValAAC (188948109-188948181) Val (AAC) 73 bp Sc: 85.53
GTTTCTGTAGTGTAGTGGTTATCACGTTCGCCTAACACGCGAAAGGTCCCCGGTTCGAAA
CCGGGCAGAAACA
>Anolis_carolinensis_chr3.trna254-ValAAC (184514616-184514688) Val (AAC) 73 bp Sc: 86.79
GTTTCCGTAGTGTAGTGGTTATCACGTTCGCCTAACACGCGAAAGGTCCCCGGTTCGAAA
CCGGGCGGAAACA
>Anolis_carolinensis_chr3.trna515-ValAAC (7998105-7998033) Val (AAC) 73 bp Sc: 86.79
GTTTCCGTAGTGTAGTGGTTATCACGTTCGCCTAACACGCGAAAGGTCCCCGGTTCGAAA
CCGGGCGGAAACA
>Anolis_carolinensis_chr3.trna516-ValAAC (7997547-7997475) Val (AAC) 73 bp Sc: 86.79
GTTTCCGTAGTGTAGTGGTTATCACGTTCGCCTAACACGCGAAAGGTCCCCGGTTCGAAA
CCGGGCGGAAACA
>Anolis_carolinensis_chr2.trna254-ValCAC (188811173-188811245) Val (CAC) 73 bp Sc: 86.14
GTTTCTGTAGTGTAGTGGTTATCACGTTCGCCTCACACGCGAAAGGTCCCCGGTTCGAAA
CCGGGCAGAAACA
>Anolis_carolinensis_chr2.trna315-ValCAC (197146724-197146652) Val (CAC) 73 bp Sc: 86.14
GTTTCTGTAGTGTAGTGGTTATCACGTTCGCCTCACACGCGAAAGGTCCCCGGTTCGAAA
CCGGGCAGAAACA
>Anolis_carolinensis_chr2.trna341-ValCAC (188772097-188772025) Val (CAC) 73 bp Sc: 86.14
GTTTCTGTAGTGTAGTGGTTATCACGTTCGCCTCACACGCGAAAGGTCCCCGGTTCGAAA
CCGGGCAGAAACA
>Anolis_carolinensis_chr3.trna377-ValTAC (123065684-123065612) Val (TAC) 73 bp Sc: 78.02
GGTTCCATAGTGTAGTGGTTATCACGTCTGCTTTACACGCAGAAGGACCTGGGTTCGAGC
CCCAGTGGAACCA
>Anolis_carolinensis_chrUn_GL343466.trna42-ValTAC (235784-235712) Val (TAC) 73 bp Sc: 85.12
GGTTCCATAGTGTAGTGGTTATCACGTCTGCTTTACACGCAGAAGGTCCTGGGTTCGAGC
CCCAGTGGAACCA
>Acholeplasma_laidlawii_PG_8A_chr.trna26-AlaTGC (1203405-1203330) Ala (TGC) 76 bp Sc: 93.92
GGGGCCTTAGCTCAGCTGGGAGAGCGCCTGCCTTGCACGCAGGAGGTCAGCGGTTCGATC
CCGCTAGGCTCCACCA
>Acholeplasma_laidlawii_PG_8A_chr.trna6-AlaTGC (78593-78668) Ala (TGC) 76 bp Sc: 94.04
GGGGCTTTAGCTCAGCTGGGAGAGCGCCTGCCTTGCACGCAGGAGGTCAGCGGTTCGATC
CCGCTAAGCTCCACCA
>Acholeplasma_laidlawii_PG_8A_chr.trna29-ArgACG (1196879-1196803) Arg (ACG) 77 bp Sc: 81.60
GCACCCATAGCTCAATTGGATAGAGCGTTTGACTACGGATCAAAAGGTTAGGGGTTCAAG
TCCTCTTGGGTGCGCCA
>Acholeplasma_laidlawii_PG_8A_chr.trna14-ArgCCG (429859-429935) Arg (CCG) 77 bp Sc: 65.64
GTATCCGTAGCTTAACTGGATAAAGCATTCCCCTCCGAAGGGAAAGAGTGTCAGTTCAAA
CCTGATCGGATACACCA
>Acholeplasma_laidlawii_PG_8A_chr.trna33-ArgTCT (965090-965014) Arg (TCT) 77 bp Sc: 91.20
GTCCGAATAGCTCAGCTGGATAGAGCAATAGCCTTCTAAGCTATCGGTCGGGGGTTCGAA
TCCCTCTTCGGACGCCA
>Acholeplasma_laidlawii_PG_8A_chr.trna27-AsnGTT (1200174-1200098) Asn (GTT) 77 bp Sc: 98.72
GCCTACTTAGCTCAGTTGGTTAGAGCACCTGACTGTTAATCAGGGGGTCGCTGGTTCGAG
TCCAGCAGTGGGCGCCA
>Acholeplasma_laidlawii_PG_8A_chr.trna11-AspGTC (79115-79190) Asp (GTC) 76 bp Sc: 77.06
GGTCCGGTGGTGTAGGGGTTAACATGCCTGCCTGTCACGCAGGAGATCGCGGGTTCAAAT
CCCGTCCGGACCGCCA
>Acholeplasma_laidlawii_PG_8A_chr.trna32-CysGCA (1185530-1185456) Cys (GCA) 75 bp Sc: 67.72
GGCCCCGTAGCCAAGCGGTAAGGCATGGGTCTGCAAAACCTTGACGCGTCGGTTCAAATC
CGATCGGGGCCTCCA

>Acholeplasma_laidlawii_PG_8A_chr.trna23-GlnTTG (1333645-1333571) Gln (TTG) 75 bp Sc: 77.26
AGGCCCATAGCCAAGCGGTAAGGCAACGGACTTTGACTCCGTCACTCGTAGGTTCAAATC
CTGCTGGGCCTGCCA
>Acholeplasma_laidlawii_PG_8A_chr.trna28-GluTTC (1196963-1196888) Glu (TTC) 76 bp Sc: 77.52
GGCCCGTTGGAGAAACGGTTAACTCACATGCCTTTCACGCATGCACTCACGGGTTCGAAT
CCCGTACGGGTCACCA
>Acholeplasma_laidlawii_PG_8A_chr.trna21-GluTTC (1396762-1396687) Glu (TTC) 76 bp Sc: 79.52
GGCCCGTTGGAGAAACGGTTAACTCACATGCCTTTCACGCATGCATTCACGGGTTCGAAT
CCCGTACGGGTCACCA
>Acholeplasma_laidlawii_PG_8A_chr.trna36-GlyGCC (301013-300939) Gly (GCC) 75 bp Sc: 55.52
GTCGGTGTCGTATAATGGTTATTACAAGGCCTTGCCAAGGCTTAGACGGCAGTTCGATCC
TGCTCACCGGCTCCA
>Acholeplasma_laidlawii_PG_8A_chr.trna31-GlyTCC (1196659-1196585) Gly (TCC) 75 bp Sc: 64.35
GCGGGTGTCGTATAATGGTTATTACCCCAGCCTTCCAAGCTGAAGACGGCGGTTCGATTC
CGCTCACCCGCTCCA
>Acholeplasma_laidlawii_PG_8A_chr.trna22-HisGTG (1333744-1333669) His (GTG) 76 bp Sc: 73.22
GCGGTTGTGGCGAAGTGGTTAACGCATCGGCTTGTGGCGCCGACACTCGGGGGTTCAATT
CCCCTCGGCCGCCCCA
>Acholeplasma_laidlawii_PG_8A_chr.trna25-IleGAT (1203504-1203428) Ile (GAT) 77 bp Sc: 101.29
GGGCCTGTAGCTCAGTTGGTTAGAGCACTCGCTTGATAAGCGAGGGGTCGATGGTTCAAG
TCCATTCAGGCCCACCA
>Acholeplasma_laidlawii_PG_8A_chr.trna17-LeuCAA (512174-512257) Leu (CAA) 84 bp Sc: 71.26
CCCCGTGTGGCGAAATGGTAGACGCGCTTGACTCAAAATCAAGTAGTGAAGACTGTGCTG
GTTCGAGTCCGGTCACGGGGACCA
>Acholeplasma_laidlawii_PG_8A_chr.trna13-LeuGAG (257049-257134) Leu (GAG) 86 bp Sc: 63.71
GCTCGGGTGGTGAAACGGTATACACGCTACCTTGAGGTGGTAGTGGGAGAAATCCTGTGA
GGGTTCAAATCCCTTCCCGAGCACCA
>Acholeplasma_laidlawii_PG_8A_chr.trna5-LeuTAA (78484-78570) Leu (TAA) 87 bp Sc: 75.97
GCCCGGGTGGTGAAATCGGTAGACACGCAGGACTTAAAATCCTGTGGCATAAAAGCCATG
TCGGTTCAAGTCCGACCCCGGGCACCA
>Acholeplasma_laidlawii_PG_8A_chr.trna24-LeuTAG (1333543-1333459) Leu (TAG) 85 bp Sc: 76.21
GCGGGTGTGGCGAAATTGGCAGACGCACTAGACTTAGGATCTAGCGCTTTACGGCATGCA
GGTTCAAGTCCTGTCACCCGCACCA
>Acholeplasma_laidlawii_PG_8A_chr.trna18-LysCTT (892220-892295) Lys (CTT) 76 bp Sc: 87.81
GCATCCATAGCTCAGTTGGTAGAGCAACAGACTCTTAATCTGTGGGTCCACGGTTCGAGC
CCGTGTGGGTGTACCA
>Acholeplasma_laidlawii_PG_8A_chr.trna4-LysTTT (78393-78468) Lys (TTT) 76 bp Sc: 91.65
GTCCCGTTAGCTCAGGTGGTAGAGCACTTGACTTTTAATCAAGGTGTCGATGGTTCGAGT
CCATCACGGGACACCA
>Acholeplasma_laidlawii_PG_8A_chr.trna7-MetCAT (78714-78790) Met (CAT) 77 bp Sc: 84.33
GGCGGTGTAGCTCAGCTGGCTAGAGCGTGCGGTTCATACCCGCAAGGTCGAGGGTTCAAG
TCCCCCCGCCGCTACCA
>Acholeplasma_laidlawii_PG_8A_chr.trna10-MetCAT (79023-79099) Met (CAT) 77 bp Sc: 87.76
CGCGGGATAGAGCAGTCTGGTAGCTCGTCGGGCTCATAACCCGAAGGTCGATGGTTCAAA
TCCATCTCCCGCAACCA
>Acholeplasma_laidlawii_PG_8A_chr.trna8-MetCAT (78795-78871) Met (CAT) 77 bp Sc: 96.08
GGACCCGTAGCTCAGTTGGTTAGAGCTACCGGCTCATAACCGGTCGGTCGTTGGTTCGAG
TCCAACCGGGTCCACCA
>Acholeplasma_laidlawii_PG_8A_chr.trna12-PheGAA (79198-79273) Phe (GAA) 76 bp Sc: 81.41
GGCTCTGTAGCTCAGTCGGTAGAGCAGTGGCCTGAAGAGCCTCGTGTCAGCGGTTCGATT
CCGCTTGGAGCCACCA
>Acholeplasma_laidlawii_PG_8A_chr.trna30-ProTGG (1196744-1196668) Pro (TGG) 77 bp Sc: 86.41
CGGGAAGTAGCTCAGCTTGGTGGAGCACTTGGTTTGGGACCAAGGGGCCGCAGGTTCAAA
TCCTGTCTTCCCGACCA
>Acholeplasma_laidlawii_PG_8A_chr.trna20-SerGCT (1396855-1396765) Ser (GCT) 91 bp Sc: 56.67
GGAGCGATACTCAAGAGGCCGAAGAGGGCGCACTGCTAACGCGTTAGACGGTTAACCCCG
TGCGAGGGTTCAAATCCCTCTCGCTCCGCCA
>Acholeplasma_laidlawii_PG_8A_chr.trna15-SerGGA (459789-459877) Ser (GGA) 89 bp Sc: 66.78
GGAACAATACTCAAGAGGCTGAAGAGGCACGCTTGGAAAGCGTGTAGGGTGTAATAGCCG
CGAGGGTTCAAATCCCTCTTGTTCCGCCA
>Acholeplasma_laidlawii_PG_8A_chr.trna34-SerTGA (809453-809362) Ser (TGA) 92 bp Sc: 27.73
GGAGAAGTAGCTAAGTTGTACTGAAGCGTCTAGCCTTGAAAACTAGTAAGACAAGAGATC
GTCGTGTGGGTTCAAATCCCTCTTTCCCGCCA
>Acholeplasma_laidlawii_PG_8A_chr.trna9-SerTGA (78894-78982) Ser (TGA) 89 bp Sc: 72.22
GGAGGAATACCCAAGAGGCTGAAGGGATCGGTCTTGAAAACCGACAGGGTGTAAAAGCCG
CGGGGGTTCAAATCCCTCTTCCTCCGCCA
>Acholeplasma_laidlawii_PG_8A_chr.trna35-ThrGGT (303160-303087) Thr (GGT) 74 bp Sc: 64.99

GCCCAATTGGTGAAGTGGTATCACGCATCCATGGTAAGGATGAATCGCAAGTCCGATTCT
TGCATTGGGCACCA
>Acholeplasma_laidlawii_PG_8A_chr.trna3-ThrTGT (78310-78385) Thr (TGT) 76 bp Sc: 92.23
GCCGAAATAGCTCAATCGGTAGAGCAACTGATTTGTAATCAGTAGGTTGCGGGTTCAATT
CCTGTTTTCGGCACCA
>Acholeplasma_laidlawii_PG_8A_chr.trna19-TrpCCA (1335875-1335949) Trp (CCA) 75 bp Sc: 56.46
GGGGGCATGGTGTCAACGGTAGCACACAGGTCTCCAAAACCTTTAGTGTGGGTTCGAATC
CTGCTGCCCTCGCCA
>Acholeplasma_laidlawii_PG_8A_chr.trna1-TyrGTA (73228-73311) Tyr (GTA) 84 bp Sc: 74.68
GGAGGGGTAGCGAAGTGGCTAAACGCGGCAGACTGTAAATCTGTTCCTAACGGTTCGGCA
GTTCGAATCTGCCCCCCTCCACCA
>Acholeplasma_laidlawii_PG_8A_chr.trna16-ValGAC (477127-477201) Val (GAC) 75 bp Sc: 83.22
GCACAATTAGCTCAGCGGGAGAGCACCTGCTTGACGTGCAGGGGGTCACAAGTTCAATCC
TTGTATTGTGCACCA
>Acholeplasma_laidlawii_PG_8A_chr.trna2-ValTAC (78226-78301) Val (TAC) 76 bp Sc: 97.70
GGAGGATTAGCTCAGTTGGGAGAGCACCTGCCTTACAAGCAGGGGGTCGGCGGTTCAAGC
CCGTCATCCTCCACCA
>Acidovorax_JS42_chr.trna11-AlaGGC (1659919-1659994) Ala (GGC) 76 bp Sc: 86.46
GGGGGTATAGCTCAGCTGGGAGAGCGCTTGCATGGCATGCAAGAGGTCAGCGGTTCGATC
CCGCTTACCTCCACCA
>Acidovorax_JS42_chr.trna39-AlaGGC (2895519-2895444) Ala (GGC) 76 bp Sc: 86.46
GGGGGTATAGCTCAGCTGGGAGAGCGCTTGCATGGCATGCAAGAGGTCAGCGGTTCGATC
CCGCTTACCTCCACCA
>Acidovorax_JS42_chr.trna29-AlaTGC (4157018-4156943) Ala (TGC) 76 bp Sc: 90.93
GGGGGATTAGCTCAGCTGGGAGAGCACCTGCTTTGCAAGCAGGGGGTCGTCGGTTCGATC
CCGTCATCCTCCACCA
>Acidovorax_JS42_chr.trna36-AlaTGC (4019290-4019215) Ala (TGC) 76 bp Sc: 90.93
GGGGGATTAGCTCAGCTGGGAGAGCACCTGCTTTGCAAGCAGGGGGTCGTCGGTTCGATC
CCGTCATCCTCCACCA
>Acidovorax_JS42_chr.trna6-AlaTGC (1250292-1250367) Ala (TGC) 76 bp Sc: 90.93
GGGGGATTAGCTCAGCTGGGAGAGCACCTGCTTTGCAAGCAGGGGGTCGTCGGTTCGATC
CCGTCATCCTCCACCA
>Acidovorax_JS42_chr.trna49-ArgACG (992994-992918) Arg (ACG) 77 bp Sc: 86.82
GCGGCTGTAGCTCAGCTGGATAGAGTACTTGGCTACGAACCAAGGGGTCGTGGGTTCGAT
TCCTGCCAGCCGCACCA
>Acidovorax_JS42_chr.trna1-ArgCCG (333762-333837) Arg (CCG) 76 bp Sc: 87.79
GCGGCTGTAGCTCAGTGGATAGAGTATTGGCCTCCGAAGCCAAGGGTCGTGGGTTCGATC
CCCGCCAGCCGCACCA
>Acidovorax_JS42_chr.trna25-ArgCCT (3754391-3754465) Arg (CCT) 75 bp Sc: 70.31
CTCTCCGTAGTTCAATGGATAGAACGAGCGCCTCCTAAGCGCTAGATACAGGTTCGATTC
CTGTCGGAGGGACCA
>Acidovorax_JS42_chr.trna22-ArgTCT (2779715-2779791) Arg (TCT) 77 bp Sc: 86.67
CTGCCCGTAGCTCAACCGGATAGAGCAATTGCCTTCTAAGCAATAGGTCGGGGGTTCGAG
TCCCTCCGGGCAGGCCA
>Acidovorax_JS42_chr.trna27-ArgTCT (4301818-4301742) Arg (TCT) 77 bp Sc: 86.67
CTGCCCGTAGCTCAACCGGATAGAGCAATTGCCTTCTAAGCAATAGGTCGGGGGTTCGAG
TCCCTCCGGGCAGGCCA
>Acidovorax_JS42_chr.trna18-AsnGTT (2435001-2435076) Asn (GTT) 76 bp Sc: 83.40
TCCTCGATAGCTCAGTCGGTAGAGCGACGGACTGTTAATCCGCAGGTCCCTGGTTCGAGC
CCAGGTCGAGGAGCCA
>Acidovorax_JS42_chr.trna19-AsnGTT (2435129-2435204) Asn (GTT) 76 bp Sc: 84.28
TCCTCAATAGCTCAGTCGGTAGAGCGCCGGACTGTTAATCCGTAGGTCCCTGGTTCGAGC
CCAGGTTGAGGAGCCA
>Acidovorax_JS42_chr.trna13-AspGTC (1660159-1660235) Asp (GTC) 77 bp Sc: 92.46
GGAGTGGTAGTTCAGTCGGTTAGAATACCGGCCTGTCACGCCGGGGGTCGCGGGTTCGAG
TCCCGTCCACTCCGCCA
>Acidovorax_JS42_chr.trna41-AspGTC (2895289-2895213) Asp (GTC) 77 bp Sc: 92.46
GGAGTGGTAGTTCAGTCGGTTAGAATACCGGCCTGTCACGCCGGGGGTCGCGGGTTCGAG
TCCCGTCCACTCCGCCA
>Acidovorax_JS42_chr.trna9-CysGCA (1408450-1408523) Cys (GCA) 74 bp Sc: 59.35
GGCGCGGTAGCAAAGCGGTTATGCACCGGATTGCAAATCCGTTTAGCCCGGTTCGACTCC
GGGCCGCGCCTCCA
>Acidovorax_JS42_chr.trna50-GlnTTG (944945-944869) Gln (TTG) 77 bp Sc: 75.81
AGGGGAGTCGCCAAGTTGGTTAAGGCACCGGATTTTGATTCCGGCATGCGAGGGTTCGAG
TCCTTCCTCCCCTGCCA
>Acidovorax_JS42_chr.trna12-GluTTC (1660026-1660101) Glu (TTC) 76 bp Sc: 58.09
GACCCTATCGTCTAGAGGCCTAGGACATCACCCTTTCACGGTGAGTACCGGGGTTCGAAT

CCCCGTAGGGTCGCCA
>Acidovorax_JS42_chr.trna40-GluTTC (2895411-2895336) Glu (TTC) 76 bp Sc: 58.09
GACCCTATCGTCTAGAGGCCTAGGACATCACCCTTTCACGGTGAGTACCGGGGTTCGAAT
CCCCGTAGGGTCGCCA
>Acidovorax_JS42_chr.trna47-GlyCCC (2077544-2077471) Gly (CCC) 74 bp Sc: 60.87
GCGGGCGTCGTTCAATGGTAGGACCTGAGCTTCCCAAGCTCAAGACGTGGGTTCGATTCC
CATCACCCGCTCCA
>Acidovorax_JS42_chr.trna10-GlyGCC (1408638-1408713) Gly (GCC) 76 bp Sc: 88.82
GCGGGAATAGCTCAGTTGGTAGAGCGCAACCTTGCCAAGGTTGAGGTCGTCGGTTCGAGA
CCGATTTCCCGCTCCA
>Acidovorax_JS42_chr.trna8-GlyGCC (1287848-1287923) Gly (GCC) 76 bp Sc: 88.82
GCGGGAATAGCTCAGTTGGTAGAGCGCAACCTTGCCAAGGTTGAGGTCGTCGGTTCGAGA
CCGATTTCCCGCTCCA
>Acidovorax_JS42_chr.trna31-GlyTCC (4151676-4151603) Gly (TCC) 74 bp Sc: 77.70
GCGGGAGTAGTTCAATGGTAGAACCCTAGCCTTCCAAGCTAATGACGCGGGTTCGATTCC
CGTCTCCCGCTCCA
>Acidovorax_JS42_chr.trna43-HisGTG (2791230-2791155) His (GTG) 76 bp Sc: 78.40
GTGGCTGTAGCTCAGTTGGTAGAGTCCAGGATTGTGATTCCTGTCGTCGTGGGTTCGAGT
CCCATCAGCCACCCCA
>Acidovorax_JS42_chr.trna28-IleGAT (4157129-4157053) Ile (GAT) 77 bp Sc: 88.56
GGGTCTGTAGCTCAGCTGGTTAGAGCACCGTCTTGATAAGGCGGGGGTCGTTGGTTCGAG
CCCAACTAGACCCACCA
>Acidovorax_JS42_chr.trna35-IleGAT (4019401-4019325) Ile (GAT) 77 bp Sc: 88.56
GGGTCTGTAGCTCAGCTGGTTAGAGCACCGTCTTGATAAGGCGGGGGTCGTTGGTTCGAG
CCCAACTAGACCCACCA
>Acidovorax_JS42_chr.trna5-IleGAT (1250181-1250257) Ile (GAT) 77 bp Sc: 88.56
GGGTCTGTAGCTCAGCTGGTTAGAGCACCGTCTTGATAAGGCGGGGGTCGTTGGTTCGAG
CCCAACTAGACCCACCA
>Acidovorax_JS42_chr.trna20-LeuCAG (2734229-2734313) Leu (CAG) 85 bp Sc: 67.85
GCCCAGGTGGCGGAATTGGTAGACGCACTAGTTTCAGGTACTAGCGAGTAACATCGTGGA
GGTTCGAGTCCTCTCCTGGGCACCA
>Acidovorax_JS42_chr.trna21-LeuCAG (2734494-2734578) Leu (CAG) 85 bp Sc: 67.85
GCCCAGGTGGCGGAATTGGTAGACGCACTAGTTTCAGGTACTAGCGAGTAACATCGTGGA
GGTTCGAGTCCTCTCCTGGGCACCA
>Acidovorax_JS42_chr.trna4-LeuGAG (998729-998813) Leu (GAG) 85 bp Sc: 61.56
GCCGTCGTGGTGAAATTGGTAGACACGCTATCTTGAGGGGGTAGTGGCGAAAGCTGTGCG
AGTTCGAGTCTCGCCGACGGCACCA
>Acidovorax_JS42_chr.trna7-LeuTAG (1281620-1281706) Leu (TAG) 87 bp Sc: 74.97
GCGCGGGTGGCGAAATTGGTAGACGCACCAGGTTTAGGTCCTGACGCCAGCAATGGTGTG
GGGGTTCGAGTCCCCCCCCGCGCACCA
>Acidovorax_JS42_chr.trna24-LysCTT (3523853-3523928) Lys (CTT) 76 bp Sc: 89.48
GGGCTCTTAGCTCAGTTGGTAGAGCAGCGGACTCTTAATCCGTTGGTCGAGTGTTCGAGT
CACTCAGGGCCCACCA
>Acidovorax_JS42_chr.trna16-LysTTT (1795778-1795853) Lys (TTT) 76 bp Sc: 92.97
GGGACGTTAGCTCAGTTGGTAGAGCAGCGGACTTTTAATCCGTTGGTCACAAGTTCGAAT
CTTGTACGTCCTACCA
>Acidovorax_JS42_chr.trna2-MetCAT (414117-414193) Met (CAT) 77 bp Sc: 85.64
CGCGCGATGGAGCAGTCTGGTAGCTCGTTGGGCTCATAACCCAAAGGTCGGAGGTTCAAA
TCCTTCTCGCGCAACCA
>Acidovorax_JS42_chr.trna26-MetCAT (3828784-3828860) Met (CAT) 77 bp Sc: 90.60
GGTGGTATAGCTCAGTTGGTTAGAGCGCAGCATTCATAATGCTGATGTCCCAGGTTCAAG
TCCCGGTACCACCACCA
>Acidovorax_JS42_chr.trna34-PheGAA (4132101-4132026) Phe (GAA) 76 bp Sc: 88.27
GGCCCGGTAGCTCAGTTGGTAGAGCAGAGGATTGAAAATCCTTGTGTCGGCGGTTCGATT
CCGTCCCAGGCCACCA
>Acidovorax_JS42_chr.trna51-ProCGG (887085-887009) Pro (CGG) 77 bp Sc: 81.43
CGGGGTGTAGCTTAGCCTGGTAGAGCGCTACGTTCGGGACGTAGAGGCCGGAGGTTCGAA
TCCTCTCACCCCGACCA
>Acidovorax_JS42_chr.trna44-ProGGG (2545856-2545780) Pro (GGG) 77 bp Sc: 80.41
CGGCGTGTAGCGCAGCCTGGTAGCGCACTTGCATGGGGTGCAAGGGGTCGCGAGTTCGAA
TCCCGCCACGCCGACCA
>Acidovorax_JS42_chr.trna15-ProTGG (1735478-1735554) Pro (TGG) 77 bp Sc: 84.89
CGGAGCGTAGCGCAGCCTGGTAGCGCATCTGCTTTGGGAGCAGAGGGTCGCGAGTTCGAA
TCCCGCCGCTCCGACCA
>Acidovorax_JS42_chr.trna37-SerCGA (3633117-3633028) Ser (CGA) 90 bp Sc: 71.98
GGAGAGGTGGCAGAGTGGTCGAATGTACCTGACTCGAAATCAGGCGTAGTGGCAACACTA
CCGTGGGTTCGAATCCCACCCTCTCCGCCA

>Acidovorax_JS42_chr.trna14-SerGCT (1684418-1684510) Ser (GCT) 93 bp Sc: 66.42
GGAAACGTGACCGAGTGGCCGAAGGTGCTCCCCTGCTAAGGGAGTATGGGGTGTAGAGCC
TCATCGAGGGTTCGAATCCCTCCGTTTCCGCCA
>Acidovorax_JS42_chr.trna17-SerGGA (1870836-1870925) Ser (GGA) 90 bp Sc: 73.88
GGAGAGGTGGATGAGCGGTTTAAGTCGCACGCCTGGAAAGCGTGTGTGGGCTAATCCCCA
CCGCGGGTTCGAATCCCGCCCTCTCCGCCA
>Acidovorax_JS42_chr.trna48-SerTGA (1158831-1158744) Ser (TGA) 88 bp Sc: 72.92
GGAGACTTGGGTGAGTGGTTTAAACCAGCAGTCTTGAAAACTGCCGACGGGCAACCGTCC
GTGAGTTCGAATCTCACAGTCTCCGCCA
>Acidovorax_JS42_chr.trna23-ThrCGT (2923874-2923949) Thr (CGT) 76 bp Sc: 84.83
GCCGGAGTAGCTCAGTCGGTAGAGCAGCTCATTCGTAATGAGAAGGTCGCGTGTTCGATT
CATGTCTCCGGCACCA
>Acidovorax_JS42_chr.trna32-ThrGGT (4151561-4151487) Thr (GGT) 75 bp Sc: 88.86
GCCCATGTGGCTCAGTGGTAGAGCACTCCCTTGGTAAGGGAGAGGTCGCGGGTCCGATTC
CCGCCATGGGCACCA
>Acidovorax_JS42_chr.trna3-ThrTGT (836662-836737) Thr (TGT) 76 bp Sc: 90.94
GCCGATTTAGCTCAGTTGGTAGAGCACCCGCCTTGTAAGCGGTAGGTCGTCAGTTCGAAT
CCGACAATCGGCACCA
>Acidovorax_JS42_chr.trna33-TrpCCA (4150205-4150130) Trp (CCA) 76 bp Sc: 85.26
AGGGGTATAGCTCAATTGGCAGAGCGTCGGTCTCCAAAACCGAAGGTTGTAGGTTCGATT
CCTACTGCCCCTGCCA
>Acidovorax_JS42_chr.trna30-TyrGTA (4151810-4151725) Tyr (GTA) 86 bp Sc: 73.25
GGAGGGGTGCCCGAGTGGCTAAAGGGGGCAGACTGTAAATCTGTTGGCTTACGCCTACAC
TGGTTCGAATCCAGTCCCCTCCACCA
>Acidovorax_JS42_chr.trna45-ValCAC (2261396-2261322) Val (CAC) 75 bp Sc: 82.83
GGGTGATTAGCTCAGCGGTAGAGCACTGCCTTCACACGGCAGGGGTCACATGTTCGATCC
ATGTATCACCCACCA
>Acidovorax_JS42_chr.trna46-ValCAC (2261282-2261208) Val (CAC) 75 bp Sc: 82.83
GGGTGATTAGCTCAGCGGTAGAGCACTGCCTTCACACGGCAGGGGTCACATGTTCGATCC
ATGTATCACCCACCA
>Acidovorax_JS42_chr.trna38-ValGAC (3328325-3328249) Val (GAC) 77 bp Sc: 93.06
AGGCGCGTAGCTCAGCTGGTTAGAGCACCACCTTGACATGGTGGGGGTCGTTGGTTCGAG
TCCAATCGCGCCTACCA
>Acidovorax_JS42_chr.trna42-ValTAC (2793337-2793262) Val (TAC) 76 bp Sc: 91.12
GGGTGCTTAGCTCAGTTGGTAGAGCGGCGCCCTTACAAGGCGTAGGTCAGCGGTTCGACC
CCGTTAGCACCCACCA
>Acidobacteria_bacterium_Ellin345_chr.trna11-AlaCGC (2568155-2568230) Ala (CGC) 76 bp Sc: 84.13
GGGGCTATAGCTCAGTTGGGAGAGCGCTGCAATCGCACTGCAGAGGTCGTCAGTTCGAAC
CTGACTAGCTCCACCA
>Acidobacteria_bacterium_Ellin345_chr.trna34-AlaGGC (4551819-4551744) Ala (GGC) 76 bp Sc: 82.47
GGGCCTGTGGCGCAGCTGGGAGCGCGCTTCCATGGCATGGAAGAGGTCGTCGGTTCGATC
CCGACCAGGTCCACCA
>Acidobacteria_bacterium_Ellin345_chr.trna27-AlaTGC (5259742-5259667) Ala (TGC) 76 bp Sc: 92.82
GGGGCTGTAGCTCAGTTGGGAGAGCGCGTGCTTTGCAAGCATGAGGTCACCGGTTCGATC
CCGGTCAGCTCCACCA
>Acidobacteria_bacterium_Ellin345_chr.trna18-ArgACG (4776758-4776834) Arg (ACG) 77 bp Sc: 92.03
GCGCCCTTAGCTCAGCTGGATAGAGCGTCTGACTACGAATCAGAAGGTCGGAAGTTCGAA
TCTTCCAGGGCGCACCA
>Acidobacteria_bacterium_Ellin345_chr.trna44-ArgCCG (2183459-2183383) Arg (CCG) 77 bp Sc: 91.68
GCGCCCGTAGCTCAGCTGGATAGAGCGAATGCCTCCGGAGCATTAGGTCGGGAGTTCGAA
TCTCTCCGGGCGCACCA
>Acidobacteria_bacterium_Ellin345_chr.trna43-ArgCCT (2275787-2275711) Arg (CCT) 77 bp Sc: 82.95
GGCCCTGTAGCTCAGATGGATAGAGCAGCGGTTTCCTAAACCGTTGGTCGGCAGTTCGAC
TCTGCCCAGGGCCTCCA
>Acidobacteria_bacterium_Ellin345_chr.trna47-ArgTCG (226325-226249) Arg (TCG) 77 bp Sc: 94.97
GCGCCCTTAGCTCAGTTGGATAGAGCGACTGACTTCGAATCAGTAGGTCGGGGGTTCGAG
CCCCTCAGGGCGCGCCA
>Acidobacteria_bacterium_Ellin345_chr.trna14-ArgTCT (3783475-3783551) Arg (TCT) 77 bp Sc: 96.37
GGGGCTGTAGCTCAGGTGGATAGAGCATTTGCCTTCTAAGCAAAGGGTCGCAGGTTCGAG
TCCTGCCAGCCCCGCCA
>Acidobacteria_bacterium_Ellin345_chr.trna10-AsnGTT (2308256-2308330) Asn (GTT) 75 bp Sc: 86.18
TCCTCAGTAGCTCAATGGCAGAGCATCCGGCTGTTAACCGGAGGGTTGTAGGTTCGAGTC
CTACCTGAGGAGCCA
>Acidobacteria_bacterium_Ellin345_chr.trna31-AspGTC (4763200-4763124) Asp (GTC) 77 bp Sc: 99.01
GGGGACGTAGTTCAGTTGGTTAGAACGCTGCCCTGTCACGGCAGAGGTCGCGGGTTCGAG
TCCCGTCGTCCCCGCCA
>Acidobacteria_bacterium_Ellin345_chr.trna7-CysGCA (1015412-1015486) Cys (GCA) 75 bp Sc: 73.18

GGCGCGGTACCCAAGTGGTAAGGGAGAGGTCTGCAAAACCTTTATGCGTCGGTTCGATTC
CGACCCGCGCCTCCA
>Acidobacteria_bacterium_Ellin345_chr.trna45-GlnCTG (1343372-1343299) Gln (CTG) 74 bp Sc: 59.82
TGCCCCCTCGTTCAATGGTAGGACAGCTGACTCTGGATCAGCATATCGGGGTTCGAATCC
CTGGGGGGCAGCCA
>Acidobacteria_bacterium_Ellin345_chr.trna24-GlnTTG (5385160-5385087) Gln (TTG) 74 bp Sc: 63.87
TGGGCGATCGACTAATGGTAGGTCAAGTGCCTTTGGAGCACTTTGTCTAGGTTCGAATCC
TAGTCGCCCAGCCA
>Acidobacteria_bacterium_Ellin345_chr.trna33-GluCTC (4614336-4614262) Glu (CTC) 75 bp Sc: 64.12
GGCGCTATCGTCTAGGGGTTAGGACGTGAGATTCTCAATCTTAAAACCCGGGTTCGATTC
CCGGTAGCGCTACCA
>Acidobacteria_bacterium_Ellin345_chr.trna39-GluTTC (2481062-2480985) Glu (TTC) 78 bp Sc: 59.80
GTCCCCGTCGTCTAGCCCGGCCTAGGACACCGCCCTTTCACGGCGATAACACGGGTTCGA
ATCCCGTCGGGGACGCCA
>Acidobacteria_bacterium_Ellin345_chr.trna41-GlyCCC (2354670-2354596) Gly (CCC) 75 bp Sc: 83.59
GCGGGAGTAACTCAGTGGTAGAGTCACGGCTTCCCAAGCCGTTGGTCGCGGGTTCGATCC
CCGTCTCCCGCTCCA
>Acidobacteria_bacterium_Ellin345_chr.trna6-GlyGCC (1015247-1015321) Gly (GCC) 75 bp Sc: 84.50
GCGGGAGTAACTCAGTGGTAGAGTGCGACCTTGCCAAGGTCGAAGTCGCGGGTTCAAATC
CCGTCTCCCGCTCCA
>Acidobacteria_bacterium_Ellin345_chr.trna21-GlyTCC (5545681-5545607) Gly (TCC) 75 bp Sc: 86.70
GCGGGAGTAACTCAGTGGTAGAGTCACAGCCTTCCAAGCTGTTGGTCGCGGGTTCGATTC
CCGTCTCCCGCTCCA
>Acidobacteria_bacterium_Ellin345_chr.trna16-HisGTG (4616518-4616592) His (GTG) 75 bp Sc: 81.74
GTGGGTATAGTTCAGTGGTAGAACTCCGGACTGTGGCTCCGGCTGTCGTGGGTTCGAATC
CCACTACCCACCCCA
>Acidobacteria_bacterium_Ellin345_chr.trna26-IleGAT (5259826-5259750) Ile (GAT) 77 bp Sc: 93.22
GGGCCTGTAGCTCAGCTGGCTAGAGCGCACCCCTGATAAGGGTGAGGTCGGTAGTTCGAC
TCTACCCAGGCCCACCA
>Acidobacteria_bacterium_Ellin345_chr.trna2-LeuCAA (478049-478137) Leu (CAA) 89 bp Sc: 72.54
GCCGGGATGGCGGAACTGGCAGACGCAGCGGACTCAAAATCCGCCGAGGTTTACCCCTCG
TGGGGGTTCGACCCCCCCTCCCGGCACCA
>Acidobacteria_bacterium_Ellin345_chr.trna1-LeuCAG (327098-327184) Leu (CAG) 87 bp Sc: 76.33
GCCGAAGTGGCGGAATTGGCAGACGCGCATGGTTCAGGTCCATGTACCGGCAACGGTGTG
GGGGTTCGAGTCCCTTCTTCGGCACCA
>Acidobacteria_bacterium_Ellin345_chr.trna42-LeuGAG (2284838-2284754) Leu (GAG) 85 bp Sc: 63.22
GCGGAAGTGGTGAAATTGGCAGACACACCATCTTGAGGGGGTGGCGCCGAAAGGCATGGG
GGTTCAAGTCCCCCCTTCCGCACCA
>Acidobacteria_bacterium_Ellin345_chr.trna37-LeuTAA (3720719-3720631) Leu (TAA) 89 bp Sc: 79.93
GCCGGGATGGCGAAACTGGCAGACGCAGCGGACTTAAAATCCGCAGGTCTTAACCGACCG
TGGGGGTTCAAGTCCCCCTCCCGGCACCA
>Acidobacteria_bacterium_Ellin345_chr.trna12-LeuTAG (2891224-2891308) Leu (TAG) 85 bp Sc: 74.77
GCGAGAGTGGCGGAATTGGCAGACGCACCAGACTTAGGATCTGGCGGGTAAAACCGTGGG
GGTTCGAGTCCCCCCTTTCGCACCA
>Acidobacteria_bacterium_Ellin345_chr.trna13-LysCTT (3278034-3278109) Lys (CTT) 76 bp Sc: 92.83
GGGCGCGTAGCTCAATTGGCAGAGCAACTGACTCTTAATCAGTAGGTTGAAGGTTCGATT
CCTTCCGCGCTCACCA
>Acidobacteria_bacterium_Ellin345_chr.trna4-LysTTT (782919-782994) Lys (TTT) 76 bp Sc: 96.77
GAGCGCGTAGCTCAGTTGGTAGAGCACCTGACTTTTAATCAGGTGGTCATGGGTTCGATC
CCCATCGCGCTCACCA
>Acidobacteria_bacterium_Ellin345_chr.trna36-MetCAT (3907713-3907637) Met (CAT) 77 bp Sc: 86.92
TGCGGGGTGGAGCAGCCTGGTAGCTCGTTGGGCTCATAACCCAAAGGTCGGAGGTTCAAA
TCCTCTCCCCGCAACCA
>Acidobacteria_bacterium_Ellin345_chr.trna15-MetCAT (4130979-4131054) Met (CAT) 76 bp Sc: 90.96
GGGCCTGTAGCTCAACGGTTAGAGCAGCGGACTCATAATCCGTTGGTTGTAGGTTCAAAT
CCTACCGGGCCCACCA
>Acidobacteria_bacterium_Ellin345_chr.trna29-MetCAT (5023126-5023050) Met (CAT) 77 bp Sc: 94.36
GGCGGCGTAGCTCAGCTGGTTAGAGCGACGGTCTCATAATCCGTAGGTCCGTGGTTCGAG
TCCACGCGCCGCCACCA
>Acidobacteria_bacterium_Ellin345_chr.trna8-PheGAA (1319335-1319410) Phe (GAA) 76 bp Sc: 95.43
GGCCAGGTAGCTCAGCTGGTAGAGCAGCGGACTGAAAATCCGCGTGTCGGCGGTTCAATT
CCGTCCCTGGCCACCA
>Acidobacteria_bacterium_Ellin345_chr.trna3-ProCGG (623241-623317) Pro (CGG) 77 bp Sc: 91.08
CGGGGAGTGGCTCAGCCTGGTAGAGCACCTGGTTCGGGACCAGGGGGTCGGAGGTTCAAA
TCCTCTCTCCCCGACCA
>Acidobacteria_bacterium_Ellin345_chr.trna32-ProGGG (4689914-4689838) Pro (GGG) 77 bp Sc: 84.91
CGGGACGTGGCTCAGCCTGGTAGAGCACTCGCTTGGGGTGCGAGGGGTCGGCAGTTCAAA

TCTGCCCGTCCCGACCA
>Acidobacteria_bacterium_Ellin345_chr.trna46-ProTGG (322040-321964) Pro (TGG) 77 bp Sc: 93.25
CGGGGCGTAGCGCAGCCTGGTAGCGCACCTGCCTTGGGCGCAGGGGGTCGGAGGTTCGAA
TCCTCTCGCCCCGACCA
>Acidobacteria_bacterium_Ellin345_chr.trna38-Undet??? (3458821-3458746) Undet (???) 76 bp Sc: 21.86
CGATCGGTGGCGAAGACATCGGTTACTTCGACTCGTAATCGGGAGGTCGCAGGTTCGAAT
CCTGCCGGGTCCATCA
>Acidobacteria_bacterium_Ellin345_chr.trna40-SerCGA (2379997-2379908) Ser (CGA) 90 bp Sc: 68.65
GGAGAGGTGCGTGAGTGGTTGAAACGAGCCGCCTCGAAAGCGGCCATACCTGAAAGGGTA
TCGGGGGTTCAAATCCCTCCCTCTCCGCCA
>Acidobacteria_bacterium_Ellin345_chr.trna28-SerGCT (5080294-5080202) Ser (GCT) 93 bp Sc: 75.80
GGAGAGGTGGCCGAGTGGCTGAAGGCGGCGGTTTGCTAAACCGTTATACGGTCAAAAGCT
GTATCGGGGGTTCGAATCCCCCCCTCTCCGCCA
>Acidobacteria_bacterium_Ellin345_chr.trna35-SerGGA (4447637-4447548) Ser (GGA) 90 bp Sc: 75.72
GGAGAGGTGGCCGAGTGGCTGAAGGCGCACGCTTGGAAAGCGTGTTTAGGGGAAACTCTA
ACGTGGGTTCGAATCCCACCCTCTCCGCCA
>Acidobacteria_bacterium_Ellin345_chr.trna25-SerTGA (5276776-5276687) Ser (TGA) 90 bp Sc: 66.86
GGAGGGATGTGTGAGCGGTTGAAACAGGCGGTCTTGAAAACCGCTTTACGGGAAACCGTA
ACGGGGGTTCGAATCCCTCTCCCTCCGCCA
>Acidobacteria_bacterium_Ellin345_chr.trna9-ThrCGT (1411021-1411096) Thr (CGT) 76 bp Sc: 94.03
GCCGACGTAGCTCAGTTGGTAGAGCAGCCGATTCGTAATCGGCAGGTCACCAGTTCAAGT
CTGGTCGTCGGCTCCA
>Acidobacteria_bacterium_Ellin345_chr.trna22-ThrGGT (5545586-5545511) Thr (GGT) 76 bp Sc: 91.44
GCCGATGTAGCTCAGTTGGTAGAGCACTCCCTTGGTAAGGGAGAGGTCACCAGTTCAATC
CTGGTCATCGGCTCCA
>Acidobacteria_bacterium_Ellin345_chr.trna19-ThrTGT (5546397-5546322) Thr (TGT) 76 bp Sc: 96.24
GCTGGCGTAGCTCAGCTGGTAGAGCATCTGATTTGTAATCAGAGGGTCGGGGGTTCAAAT
CCCTTCGCCAGCTCCA
>Acidobacteria_bacterium_Ellin345_chr.trna23-TrpCCA (5543588-5543513) Trp (CCA) 76 bp Sc: 52.51
AGGGGCGTAAGCTCAACGGCTAAACTGCCGGTCTCCAAAACCGGACTTGGGGGTTCGAAT
CCCTCCGCCCCTGCCA
>Acidobacteria_bacterium_Ellin345_chr.trna20-TyrGTA (5546057-5545970) Tyr (GTA) 88 bp Sc: 71.62
GCACAGGTGGCCGAGTGGTTAATGGCAGCAGACTGTAAATCTGCCGCTCCCAGGAGCTAC
GGAGGTTCGAATCCTCCCCTGTGCACCA
>Acidobacteria_bacterium_Ellin345_chr.trna5-ValCAC (965656-965730) Val (CAC) 75 bp Sc: 85.23
GGGCGCTTAACTCAGCGGTAGAGTGCCACCTTCACACGGTGGAAGTCATAGGTTCGAATC
CTATAGCGCCCACCA
>Acidobacteria_bacterium_Ellin345_chr.trna17-ValGAC (4748291-4748368) Val (GAC) 78 bp Sc: 91.55
AGGCCCGTAGCTCAGTCTGGTTAGAGCGCTTCCTTGACACGGAAGAGGTCGATGGTTCGA
GTCCATTCGGGCCTACCA
>Acidobacteria_bacterium_Ellin345_chr.trna30-ValTAC (4763302-4763226) Val (TAC) 77 bp Sc: 100.75
GGGCGGTTAGCTCAGTTGGTTAGAGCGCCTGCCTTACAAGCAGGAGGTCGCAGGTTCGAG
CCCTGCACTGCCCACCA
>Acidothermus_cellulolyticus_11B_chr.trna45-AlaCGC (179235-179159) Ala (CGC) 77 bp Sc: 91.06
GGGGCTGTAGCGCAGTCCGGTAGCGCACCTCGTTCGCATCGAGGGGGTCACGGGTTCAAA
TCCCGTCAGCTCCACCA
>Acidothermus_cellulolyticus_11B_chr.trna14-AlaGGC (842997-843072) Ala (GGC) 76 bp Sc: 84.62
GGGGCTATAGCTCAGCTGGTAGAGCGCTTGTCTGGCAGACAAGAGGTCAGGGGTTCGAGT
CCCCTTAGCTCCACAA
>Acidothermus_cellulolyticus_11B_chr.trna2-AlaTGC (10372-10447) Ala (TGC) 76 bp Sc: 88.19
GGGGCCATAGCTCAGCTGGTAGAGCACCGCCTTTGCAAGGCGGGTGTCCGGGGTTCGAGT
CCCCGTGGCTCCACAA
>Acidothermus_cellulolyticus_11B_chr.trna5-ArgACG (41078-41153) Arg (ACG) 76 bp Sc: 72.39
GCGCTCGTAGCTCAACGGACAGAGCATCTGACTACGGATCAGAAGGTTGGGGGTTCGAAT
CCCTCCGAGCGCGCTA
>Acidothermus_cellulolyticus_11B_chr.trna44-ArgCCG (677195-677124) Arg (CCG) 72 bp Sc: 75.65
GCCCCCGTAGCTCAGGGGAGAGAGCACTGCCCTCCGGAGGCAGGGGCGCAGGTTCGAATC
CTGCCGGGGGCA
>Acidothermus_cellulolyticus_11B_chr.trna46-ArgCCT (160793-160721) Arg (CCT) 73 bp Sc: 74.19
GCCCTCGTAGCTCAGGGGATAGAGCAGCGGTTTCCTAAACCGCGTGTCGCAGGTTCGATT
CCTGCCGGGGGCA
>Acidothermus_cellulolyticus_11B_chr.trna23-ArgTCT (1824619-1824691) Arg (TCT) 73 bp Sc: 83.15
GCCCCCGTAGCTCAGCGGACAGAGCAGCTGCCTTCTAAGCAGTCGGTCGCAGGTTCGAGT
CCTGCCGGGGGCG
>Acidothermus_cellulolyticus_11B_chr.trna15-AsnGTT (893343-893418) Asn (GTT) 76 bp Sc: 87.39
TCCCGCGTAGCTCAATTGGCAGAGCGTCCGGCTGTTAACCGGTAGGTTGCAGGTTCGAGT
CCTGCCGCGGGAGCCA

>Acidothermus_cellulolyticus_11B_chr.trna29-AspGTC (2339683-2339760) Asp (GTC) 78 bp Sc: 76.71
GGTCCCGTGGTGAAGCCTGGAGTTCACGCCGCCCTGTCAAGGCGGAGGTCGCGGGTTCGA
ATCCCGTCGGGACCGCCA
>Acidothermus_cellulolyticus_11B_chr.trna20-CysGCA (1524883-1524954) Cys (GCA) 72 bp Sc: 65.70
GGCGGAGTGGCCGAGTGGCTTAGGCAAGGGCCTGCAAAGCCCTGTACGCGGGTTCGATTC
CCGCCTCCGCCT
>Acidothermus_cellulolyticus_11B_chr.trna12-GlnCTG (783342-783416) Gln (CTG) 75 bp Sc: 62.70
TGGGGTATGGGGTAATTGGCAGCCCGGCGGATTCTGGCTCCGCTAGTCTAGGTTCGAGTC
CTGGTACCCCAGCCA
>Acidothermus_cellulolyticus_11B_chr.trna37-GlnTTG (2206822-2206752) Gln (TTG) 71 bp Sc: 43.26
TCCCGGGTCGTCTAAGGGCAGGACAGTGGGTTTTGGTCCCATTAATGGGGGTTCGAATCC
TCCCCCGGGAG
>Acidothermus_cellulolyticus_11B_chr.trna13-GluCTC (783484-783559) Glu (CTC) 76 bp Sc: 77.05
GGCCCCGTAGTCTAGCGGCCTAGGACGCCGCCCTCTCAAGGCGGTAGCGCCGGTTCGAAT
CCGGTCGGGGCTACCA
>Acidothermus_cellulolyticus_11B_chr.trna28-GluTTC (2339526-2339598) Glu (TTC) 73 bp Sc: 68.03
GCCCTCATCGTCTAGCGGTCTAGGACACCGCCCTTTCAAGGCGGCGGCACGGGTTCGAAT
CCCGTTGGGGGCA
>Acidothermus_cellulolyticus_11B_chr.trna32-GlyCCC (2389324-2389254) Gly (CCC) 71 bp Sc: 70.87
GCGGGTGTAGTTCAATGGCAGAACGTCAGCTTCCCAAGCTGATAATGCGGGTTCGATTCC
CGTCACCCGCT
>Acidothermus_cellulolyticus_11B_chr.trna19-GlyGCC (1524762-1524834) Gly (GCC) 73 bp Sc: 88.54
GCGGACGTGGCTCAGTTGGTAGAGCATCACCTTGCCAAGGTGAGGGTCGCGGGTTCGAGT
CCCGTCGTCCGCT
>Acidothermus_cellulolyticus_11B_chr.trna22-GlyTCC (1797034-1797104) Gly (TCC) 71 bp Sc: 77.75
GCGGGTGTAGCTCAATGGCAGAGCTCCAGCCTTCCAAGCTGGCGGTGCGGGTTCGATTCC
CGTCACCCGCT
>Acidothermus_cellulolyticus_11B_chr.trna24-HisGTG (1855719-1855794) His (GTG) 76 bp Sc: 80.21
GTGGGTGTAGCTCAGTTGGCAGAGCACTGGGTTGTGGTCCCAGGTGTCATGGGTTCGAGT
CCCATCACTCACCCCA
>Acidothermus_cellulolyticus_11B_chr.trna1-IleGAT (10111-10184) Ile (GAT) 74 bp Sc: 87.35
GGGCCTGTAGCTCAGGCGGTTAGAGCGCACCCCTGATAAGGGTGAGGTCGGAGGTTCAAG
TCCTCCCAGGCCCA
>Acidothermus_cellulolyticus_11B_chr.trna43-LeuCAA (1195870-1195798) Leu (CAA) 73 bp Sc: 52.25
GCCCCGGTAGCCCAACGGTAGAGGCAATGGTCTCAAACACCATCCAGTGTCGGTTCGAAT
CCGACCCGGGGTA
>Acidothermus_cellulolyticus_11B_chr.trna3-LeuCAG (24698-24783) Leu (CAG) 86 bp Sc: 70.36
GGGCGAGTGGCGGAATGGCAGACGCGCACGGTTCAGGTCCGTGTGTCCGGAAGGACGTGG
GGGTTCAACTCCCCCCTCGCCCACCA
>Acidothermus_cellulolyticus_11B_chr.trna42-LeuGAG (1294313-1294229) Leu (GAG) 85 bp Sc: 54.88
GTCCGGGTGGCGGAATAGGCAGACGCGCTAGCTTGAGGTGCTAGTGCCCGTTGAGGGCGT
GGGGGTTCAAGTCCCCCCTCGGACA
>Acidothermus_cellulolyticus_11B_chr.trna38-LeuTAA (2147532-2147460) Leu (TAA) 73 bp Sc: 56.03
GCCCCCGTAGCCCAACGGCAGAGGCGAACCCCTTAAAAGGGTTACAGTGTCGGTTCGAAT
CCGACCGGGGGCA
>Acidothermus_cellulolyticus_11B_chr.trna26-LeuTAG (1894564-1894636) Leu (TAG) 73 bp Sc: 45.23
GGGGCCGTAGCCCAATGGCAGAGGCACGCGGTTTAGGTCCGCGACAGTGTGAGTTCGACT
CTCACCGGCCCTA
>Acidothermus_cellulolyticus_11B_chr.trna25-LysCTT (1861376-1861451) Lys (CTT) 76 bp Sc: 81.01
GCGCCGCTAGCTCAACTGGCAGAGCAGCGGACTCTTAATCCGCGGGTTCGGGGTTCGAGT
CCCTGGCGGCGCACGA
>Acidothermus_cellulolyticus_11B_chr.trna33-LysTTT (2336318-2336246) Lys (TTT) 73 bp Sc: 77.41
GGGCCGTTAGCTCAACTGGCAGAGCAGCGGACTTTTAATCCGCGGGTTCCGGGTTCGATC
CCCGGGCGGCCCA
>Acidothermus_cellulolyticus_11B_chr.trna9-MetCAT (308433-308509) Met (CAT) 77 bp Sc: 77.25
GGCGGAGTAGCTCAGCCTGGTAGAGCAAGCGGCTCATAATCGCTGTGTCACGGGTTCAAA
TCCCGTCTCCGCTACGA
>Acidothermus_cellulolyticus_11B_chr.trna11-MetCAT (509883-509959) Met (CAT) 77 bp Sc: 80.42
CGCGGGGTGGAGCAGCTCGGTAGCTCGCTGGGCTCATAACCCAGAGGTCGCAGGTTCAAA
TCCTGCCCCCGCTACGA
>Acidothermus_cellulolyticus_11B_chr.trna17-MetCAT (921140-921213) Met (CAT) 74 bp Sc: 89.71
GGGGCGGTAGCTCAGCCGGTTAGAGCTGCGGACTCATAATCCGCTGGTCGCAGGTTCGAG
TCCTGCCCGCCCCA
>Acidothermus_cellulolyticus_11B_chr.trna30-PheGAA (2339803-2339879) Phe (GAA) 77 bp Sc: 92.35
GGCCAGGTAGCTCAGTTGGTACGAGCGCCCGCCTGAAAAGCGGGAGGTCGGCGGTTCGAT
CCCGCCCCTGGCCACCA
>Acidothermus_cellulolyticus_11B_chr.trna35-ProCGG (2281657-2281581) Pro (CGG) 77 bp Sc: 83.85

CGGGGTGTGGCGCAGCTTGGTAGCGCGCTTCGTTCGGGACGAAGAGGCCGTGGGTTCAAA
TCCCGCCACCCCGACCA
>Acidothermus_cellulolyticus_11B_chr.trna41-ProGGG (1372838-1372762) Pro (GGG) 77 bp Sc: 85.13
CGGGACGTGGCGCAGCTTGGTAGCGCACTTGACTGGGGGTCAAGGGGTCGTGGGTTCAAA
TCCCGCCGTCCCGACCA
>Acidothermus_cellulolyticus_11B_chr.trna39-ProTGG (1796952-1796877) Pro (TGG) 76 bp Sc: 75.20
CGGGGCGTAGCGTAGTGGCCAGCGCGCCTGCTTTGGGAGCAGGAGATCGGGAGTTCGAGT
CTCCCCGCCCCGACGA
>Acidothermus_cellulolyticus_11B_chr.trna34-SerCGA (2293483-2293394) Ser (CGA) 90 bp Sc: 67.59
GGTGGAGTCGCCTAGTGGCCGATGGCGCACGCCTCGAAAGCGTGTGAGGGTGCAAGCCCT
CCGTGGGTTCAAATCCCACCTCCACCGCCA
>Acidothermus_cellulolyticus_11B_chr.trna4-SerGCT (40835-40924) Ser (GCT) 90 bp Sc: 59.29
GGAGGCTTCGCCTAGTCCGGTCTATGGCGCCGCACTGCTAATGCGGTTGGGGCTTACCGC
CCCTCCCGGGTTCAAATCCCGGAGCCTCCG
>Acidothermus_cellulolyticus_11B_chr.trna27-SerGGA (2292585-2292671) Ser (GGA) 87 bp Sc: 63.12
GGAGGATTCGCCTAGCGGCCGAGGGCGCACGCTTGGAAAGCGTGTAGAGGGGCAACCCTC
TCGCGGGTTCAAATCCCGCATCCTCCG
>Acidothermus_cellulolyticus_11B_chr.trna31-SerTGA (2374948-2375032) Ser (TGA) 85 bp Sc: 62.73
GGAGGCGTCGCCTAGCGGCCGATGGCGCCGGTCTTGAAAACCGGTAGGGCGCAAGCCCTC
GTGGGTTCAAATCCCACCGCCTCCG
>Acidothermus_cellulolyticus_11B_chr.trna36-ThrCGT (2230630-2230555) Thr (CGT) 76 bp Sc: 92.47
GCCGCCTTAGCTCAGCCGGCAGAGCAACGCACTCGTAATGCGTAGGTCCGGGGTTCGAAT
CCCCGAGGCGGCTCCA
>Acidothermus_cellulolyticus_11B_chr.trna8-ThrGGT (308349-308424) Thr (GGT) 76 bp Sc: 83.24
GCCCCCTTAGCTCAGTCGGCAGAGCGTCTCCATGGTAAGGAGAAGGTCTACGGTTCGATT
CCGTAAGGGGGCTCCA
>Acidothermus_cellulolyticus_11B_chr.trna6-ThrTGT (89585-89660) Thr (TGT) 76 bp Sc: 84.52
GCCGGCGTGGCGCAATTGGCAGCGCATCCGACTTGTAATCGGAAGGTTAGGGGTTCGATT
CCCCTCGCCGGCTCGA
>Acidothermus_cellulolyticus_11B_chr.trna10-TrpCCA (313372-313447) Trp (CCA) 76 bp Sc: 74.95
AGGGCAGTAGCTCAATTGGCAGAGTCCCGGTCTCCAAAACCGGTGGTTGGGGGTTCGAGT
CCCTCCTGCCCTGCAA
>Acidothermus_cellulolyticus_11B_chr.trna7-TyrGTA (307544-307629) Tyr (GTA) 86 bp Sc: 66.37
GGCAGGTTGCCCGAGTGGCCAAAGGGAGCGGTCTGTAAAACCGTCGGCGAACGCCTACCC
AGGTTCGAATCCTGGACCTGCCACCA
>Acidothermus_cellulolyticus_11B_chr.trna16-Undet??? (895201-895275) Undet (???) 75 bp Sc: 55.62
TCCCGCGTAGCTCAATTGGCAGAGCGTCCGACTGTTAACGGTAGGTTGCAGATTCGAGTC
CTGCCCCGGAAGCCA
>Acidothermus_cellulolyticus_11B_chr.trna40-ValCAC (1523720-1523649) Val (CAC) 72 bp Sc: 80.49
GGGCGTATAGCTCAGCGGGAGAGCGCTGCCTTCACACGGCAGAGGTCCATGGTTCGAATC
CATGTACGCCCA
>Acidothermus_cellulolyticus_11B_chr.trna21-ValGAC (1525000-1525074) Val (GAC) 75 bp Sc: 85.75
GGGCGCTTAGCTCAGTGGGAGAGCGCTACCTTGACACGGTAGAGGTCGGCAGTTCAATCC
TGCCAGCGCCCACCA
>Acidothermus_cellulolyticus_11B_chr.trna18-ValTAC (974815-974886) Val (TAC) 72 bp Sc: 83.91
GGGCGCGTAGCTCAGTGGAAGAGCACCCGGCTTACAACCGGGGGGTCACAGGTTCGAACC
CTGTCGTGCCCA
>Acinetobacter_baumannii_chr.trna67-AlaGGC (296406-296331) Ala (GGC) 76 bp Sc: 83.49
GGGGTCATAGCTCAGTTGGTAGAGCGCTACAATGGCATTGTAGAGGTCAGCGGTTCGATC
CCGCTTGGCTCCACCA
>Acinetobacter_baumannii_chr.trna10-AlaTGC (489162-489237) Ala (TGC) 76 bp Sc: 89.91
GGGGACTTAGCTTAGTTGGTAGAGCGCCTGCTTTGCACGCAGGAGGTCAGGAGTTCGACT
CTCCTAGTCTCCACCA
>Acinetobacter_baumannii_chr.trna12-AlaTGC (521395-521470) Ala (TGC) 76 bp Sc: 89.91
GGGGACTTAGCTTAGTTGGTAGAGCGCCTGCTTTGCACGCAGGAGGTCAGGAGTTCGACT
CTCCTAGTCTCCACCA
>Acinetobacter_baumannii_chr.trna2-AlaTGC (20043-20118) Ala (TGC) 76 bp Sc: 89.91
GGGGACTTAGCTTAGTTGGTAGAGCGCCTGCTTTGCACGCAGGAGGTCAGGAGTTCGACT
CTCCTAGTCTCCACCA
>Acinetobacter_baumannii_chr.trna38-AlaTGC (3897509-3897434) Ala (TGC) 76 bp Sc: 89.91
GGGGACTTAGCTTAGTTGGTAGAGCGCCTGCTTTGCACGCAGGAGGTCAGGAGTTCGACT
CTCCTAGTCTCCACCA
>Acinetobacter_baumannii_chr.trna40-AlaTGC (3731654-3731579) Ala (TGC) 76 bp Sc: 89.91
GGGGACTTAGCTTAGTTGGTAGAGCGCCTGCTTTGCACGCAGGAGGTCAGGAGTTCGACT
CTCCTAGTCTCCACCA
>Acinetobacter_baumannii_chr.trna50-AlaTGC (3175824-3175749) Ala (TGC) 76 bp Sc: 89.91
GGGGACTTAGCTTAGTTGGTAGAGCGCCTGCTTTGCACGCAGGAGGTCAGGAGTTCGACT

CTCCTAGTCTCCACCA
>Acinetobacter_baumannii_chr.trna6-ArgACG (461415-461491) Arg (ACG) 77 bp Sc: 83.63
GCGCTCATAGCTCAGCTGGATAGAGCACTTGGCTACGAACTAAGGGGTCGGGAGTTCGAA
TCTCTCTGAGCGCACCA
>Acinetobacter_baumannii_chr.trna7-ArgACG (461531-461607) Arg (ACG) 77 bp Sc: 83.63
GCGCTCATAGCTCAGCTGGATAGAGCACTTGGCTACGAACTAAGGGGTCGGGAGTTCGAA
TCTCTCTGAGCGCACCA
>Acinetobacter_baumannii_chr.trna8-ArgACG (465383-465459) Arg (ACG) 77 bp Sc: 83.63
GCGCTCATAGCTCAGCTGGATAGAGCACTTGGCTACGAACTAAGGGGTCGGGAGTTCGAA
TCTCTCTGAGCGCACCA
>Acinetobacter_baumannii_chr.trna59-ArgCCG (1250422-1250346) Arg (CCG) 77 bp Sc: 81.26
GCGCTCGTAGCTCAGTTGGATAGAGTACAGGTTTCCGAAGCCTGGGGTCGTGGGTTCGAT
CCCCGCCGAGCGCACCA
>Acinetobacter_baumannii_chr.trna66-ArgCCT (738086-738014) Arg (CCT) 73 bp Sc: 67.72
GCGCCCTTAGCTTAACTGGATAGAGCAGTTGCCTCCTAAGCGACCGACGTGGGTTCGAGT
CCCGCAGGGCGCA
>Acinetobacter_baumannii_chr.trna52-ArgTCT (3138974-3138898) Arg (TCT) 77 bp Sc: 98.82
GCGCCTGTAGCTCAGTTGGATAGAGCATCCGCCTTCTAAGCGGATGGTCACAGGTTCGAA
TCCTGTCAGGCGCGCCA
>Acinetobacter_baumannii_chr.trna60-AsnGTT (1213070-1212995) Asn (GTT) 76 bp Sc: 77.17
TCTCCAATAGCTCAGTCGGTAGAGCGACGGACTGTTAATCCGCAGGTCCCTGGTTCGAGC
CCAGGTGGGAGAGCCA
>Acinetobacter_baumannii_chr.trna61-AsnGTT (1211624-1211549) Asn (GTT) 76 bp Sc: 83.03
TCTCCCATAGCTCAGTCGGTAGAGCGACGGACTGTTAATCCGCAGGTCCCTGGTTCGAGC
CCAGGTGGGAGAGCCA
>Acinetobacter_baumannii_chr.trna62-AsnGTT (1004960-1004885) Asn (GTT) 76 bp Sc: 83.03
TCTCCCATAGCTCAGTCGGTAGAGCGACGGACTGTTAATCCGCAGGTCCCTGGTTCGAGC
CCAGGTGGGAGAGCCA
>Acinetobacter_baumannii_chr.trna15-AspGTC (696987-697063) Asp (GTC) 77 bp Sc: 94.40
GCAGCGGTAGTTCAGTTGGTTAGAATACCGGCCTGTCACGCCGGGGGTCGCGGGTTCGAG
CCCCGTCCGCTGCGCCA
>Acinetobacter_baumannii_chr.trna16-AspGTC (697111-697187) Asp (GTC) 77 bp Sc: 94.40
GCAGCGGTAGTTCAGTTGGTTAGAATACCGGCCTGTCACGCCGGGGGTCGCGGGTTCGAG
CCCCGTCCGCTGCGCCA
>Acinetobacter_baumannii_chr.trna18-AspGTC (697324-697400) Asp (GTC) 77 bp Sc: 94.40
GCAGCGGTAGTTCAGTTGGTTAGAATACCGGCCTGTCACGCCGGGGGTCGCGGGTTCGAG
CCCCGTCCGCTGCGCCA
>Acinetobacter_baumannii_chr.trna63-CysGCA (857440-857367) Cys (GCA) 74 bp Sc: 59.57
GGCGGGGTGGCAGAGTGGTCATGCAGCGGACTGCAACTCCGTGGACGCCGGTTCGATTCC
GACCTCCGCCTCCA
>Acinetobacter_baumannii_chr.trna29-GlnTTG (3009677-3009751) Gln (TTG) 75 bp Sc: 68.77
AGGGGCGTCGCCAAGTGGTAAGGCAGCGGGTTTTGATCCCGCCATCCGTTGGTTCGAATC
CAGCCGCCCCTGCCA
>Acinetobacter_baumannii_chr.trna30-GlnTTG (3009779-3009853) Gln (TTG) 75 bp Sc: 68.77
AGGGGCGTCGCCAAGTGGTAAGGCAGCGGGTTTTGATCCCGCCATCCGTTGGTTCGAATC
CAGCCGCCCCTGCCA
>Acinetobacter_baumannii_chr.trna31-GlnTTG (3009875-3009949) Gln (TTG) 75 bp Sc: 68.77
AGGGGCGTCGCCAAGTGGTAAGGCAGCGGGTTTTGATCCCGCCATCCGTTGGTTCGAATC
CAGCCGCCCCTGCCA
>Acinetobacter_baumannii_chr.trna32-GlnTTG (3010041-3010115) Gln (TTG) 75 bp Sc: 68.77
AGGGGCGTCGCCAAGTGGTAAGGCAGCGGGTTTTGATCCCGCCATCCGTTGGTTCGAATC
CAGCCGCCCCTGCCA
>Acinetobacter_baumannii_chr.trna28-GluTTC (2684257-2684332) Glu (TTC) 76 bp Sc: 56.39
GTCCCTATCGTCTAGAGGCCTAGGACATCGCCCTTTCACGGCGGTAACCGGGGTTCGAAT
CCCCGTAGGGACGCCA
>Acinetobacter_baumannii_chr.trna68-GluTTC (296305-296230) Glu (TTC) 76 bp Sc: 56.39
GTCCCTATCGTCTAGAGGCCTAGGACATCGCCCTTTCACGGCGGTAACCGGGGTTCGAAT
CCCCGTAGGGACGCCA
>Acinetobacter_baumannii_chr.trna69-GluTTC (293543-293468) Glu (TTC) 76 bp Sc: 56.39
GTCCCTATCGTCTAGAGGCCTAGGACATCGCCCTTTCACGGCGGTAACCGGGGTTCGAAT
CCCCGTAGGGACGCCA
>Acinetobacter_baumannii_chr.trna70-GluTTC (293439-293364) Glu (TTC) 76 bp Sc: 56.39
GTCCCTATCGTCTAGAGGCCTAGGACATCGCCCTTTCACGGCGGTAACCGGGGTTCGAAT
CCCCGTAGGGACGCCA
>Acinetobacter_baumannii_chr.trna27-GlyGCC (2542259-2542334) Gly (GCC) 76 bp Sc: 90.38
GCGGGAATAGCTCAGTTGGTAGAGCATAACCTTGCCAAGGTTGGGGTCGCGAGTTCGAGT
CTCGTTTCCCGCTCCA

>Acinetobacter_baumannii_chr.trna35-GlyGCC (3776785-3776860) Gly (GCC) 76 bp Sc: 90.38
GCGGGAATAGCTCAGTTGGTAGAGCATAACCTTGCCAAGGTTGGGGTCGCGAGTTCGAGT
CTCGTTTCCCGCTCCA
>Acinetobacter_baumannii_chr.trna36-GlyGCC (3776906-3776981) Gly (GCC) 76 bp Sc: 90.38
GCGGGAATAGCTCAGTTGGTAGAGCATAACCTTGCCAAGGTTGGGGTCGCGAGTTCGAGT
CTCGTTTCCCGCTCCA
>Acinetobacter_baumannii_chr.trna43-GlyTCC (3553417-3553342) Gly (TCC) 76 bp Sc: 91.76
GCGGGAGTAGCTCAGTTGGTAGAGCGGCAGCCTTCCAAGCTGCATGTCGCGAGTTCGATC
CTCGTCTCCCGCTCCA
>Acinetobacter_baumannii_chr.trna53-HisGTG (3138864-3138789) His (GTG) 76 bp Sc: 81.86
GTGGATGTAGCTCAGTTGGTAGAGCCCTGGATTGTGATTCCAGTTGTCGCGGGTTCGAAT
CCCGTCATTCACCCCA
>Acinetobacter_baumannii_chr.trna1-IleGAT (19911-19987) Ile (GAT) 77 bp Sc: 98.53
GGGTCTGTAGCTCAGTTGGTTAGAGCACACGCTTGATAAGCGTGGGGTCACAAGTTCAAG
TCTTGTCAGACCCACCA
>Acinetobacter_baumannii_chr.trna11-IleGAT (521263-521339) Ile (GAT) 77 bp Sc: 98.53
GGGTCTGTAGCTCAGTTGGTTAGAGCACACGCTTGATAAGCGTGGGGTCACAAGTTCAAG
TCTTGTCAGACCCACCA
>Acinetobacter_baumannii_chr.trna37-IleGAT (3897641-3897565) Ile (GAT) 77 bp Sc: 98.53
GGGTCTGTAGCTCAGTTGGTTAGAGCACACGCTTGATAAGCGTGGGGTCACAAGTTCAAG
TCTTGTCAGACCCACCA
>Acinetobacter_baumannii_chr.trna39-IleGAT (3731786-3731710) Ile (GAT) 77 bp Sc: 98.53
GGGTCTGTAGCTCAGTTGGTTAGAGCACACGCTTGATAAGCGTGGGGTCACAAGTTCAAG
TCTTGTCAGACCCACCA
>Acinetobacter_baumannii_chr.trna49-IleGAT (3175956-3175880) Ile (GAT) 77 bp Sc: 98.53
GGGTCTGTAGCTCAGTTGGTTAGAGCACACGCTTGATAAGCGTGGGGTCACAAGTTCAAG
TCTTGTCAGACCCACCA
>Acinetobacter_baumannii_chr.trna9-IleGAT (489030-489106) Ile (GAT) 77 bp Sc: 98.53
GGGTCTGTAGCTCAGTTGGTTAGAGCACACGCTTGATAAGCGTGGGGTCACAAGTTCAAG
TCTTGTCAGACCCACCA
>Acinetobacter_baumannii_chr.trna13-LeuCAA (548277-548363) Leu (CAA) 87 bp Sc: 71.58
CCCGAGGTGGTGAAATTGGTAGACGCGGCGGACTCAAAATCCGCTGTCAGAGATGACGTG
TCGGTTCGAGTCCGACCCTCGGGACCA
>Acinetobacter_baumannii_chr.trna46-LeuGAG (3496945-3496861) Leu (GAG) 85 bp Sc: 59.44
GCGGTGGTGGTGGAATTGGTAGACACGCTACCTTGAGGTGGTAGTGCTTTCGGGCGTGGG
GGTTCAAGTCCCCCCTTCCGCACCA
>Acinetobacter_baumannii_chr.trna64-LeuTAA (857355-857270) Leu (TAA) 86 bp Sc: 70.97
GCCCGGGTGGTGAAATAGGTAGACACAGGGGATTTAAAATCCCCCGCCCTCAAAGCGTGC
CGGTTCGAGTCCGGCCCCGGGCACCA
>Acinetobacter_baumannii_chr.trna65-LeuTAA (855210-855125) Leu (TAA) 86 bp Sc: 70.97
GCCCGGGTGGTGAAATAGGTAGACACAGGGGATTTAAAATCCCCCGCCCTCAAAGCGTGC
CGGTTCGAGTCCGGCCCCGGGCACCA
>Acinetobacter_baumannii_chr.trna22-LeuTAG (1358479-1358563) Leu (TAG) 85 bp Sc: 77.55
GGGGGCGTGGCGAAATTGGTAGACGCACTGGATTTAGGTTCCAGCGCCGCAAGGTGTAAG
AGTTCGAGTCTCTTCGCCCCCACCA
>Acinetobacter_baumannii_chr.trna24-LeuTAG (1358719-1358803) Leu (TAG) 85 bp Sc: 77.55
GGGGGCGTGGCGAAATTGGTAGACGCACTGGATTTAGGTTCCAGCGCCGCAAGGTGTAAG
AGTTCGAGTCTCTTCGCCCCCACCA
>Acinetobacter_baumannii_chr.trna33-LysTTT (3423128-3423203) Lys (TTT) 76 bp Sc: 92.37
GGGTCGTTAGCTCAGTTGGTAGAGCAGCGGACTTTTAATCCGTTGGTCCCGCGTTCGAGT
CGCGGACGACCCACCA
>Acinetobacter_baumannii_chr.trna34-LysTTT (3423226-3423301) Lys (TTT) 76 bp Sc: 92.37
GGGTCGTTAGCTCAGTTGGTAGAGCAGCGGACTTTTAATCCGTTGGTCCCGCGTTCGAGT
CGCGGACGACCCACCA
>Acinetobacter_baumannii_chr.trna47-MetCAT (3496818-3496742) Met (CAT) 77 bp Sc: 82.06
TGCGGGATGGAGCAGTCTGGTAGCTCGTCGGGCTCATAACCCGAAGGTCGTTGGTTCAAA
TCCAGCTCCCGCTACCA
>Acinetobacter_baumannii_chr.trna48-MetCAT (3496652-3496576) Met (CAT) 77 bp Sc: 82.06
TGCGGGATGGAGCAGTCTGGTAGCTCGTCGGGCTCATAACCCGAAGGTCGTTGGTTCAAA
TCCAGCTCCCGCTACCA
>Acinetobacter_baumannii_chr.trna58-MetCAT (2575189-2575113) Met (CAT) 77 bp Sc: 82.10
TGCGGGGTGGAGCAGTCTGGTAGCTCGTCGGGCTCATAACCCGAAGGTCGTTGGTTCAAA
TCCGACCCCCGCATCCA
>Acinetobacter_baumannii_chr.trna26-MetCAT (2197426-2197502) Met (CAT) 77 bp Sc: 91.80
GGGCCTATAGCTCAGTCGGTTAGAGCAGCGGACTCATAATCCGTTGGTCCACAGTTCGAG
TCTGTGTGGGCCCACCA
>Acinetobacter_baumannii_chr.trna55-MetCAT (2997482-2997406) Met (CAT) 77 bp Sc: 94.07

GGCTATGTAGCTCAGTTGGTTAGAGCACCGCACTCATAATGCGGGGGTCACAAGTTCAAG
TCTCGTCATAGCCACCA
>Acinetobacter_baumannii_chr.trna56-MetCAT (2985991-2985915) Met (CAT) 77 bp Sc: 94.07
GGCTATGTAGCTCAGTTGGTTAGAGCACCGCACTCATAATGCGGGGGTCACAAGTTCAAG
TCTCGTCATAGCCACCA
>Acinetobacter_baumannii_chr.trna3-PheGAA (267736-267811) Phe (GAA) 76 bp Sc: 86.19
GGCGTGATAGCTCAGTTGGTAGAGCAACGGATTGAAAATCCGTGTGTCCCCAGTTCGATC
CTGGGTCTCGCCACCA
>Acinetobacter_baumannii_chr.trna4-PheGAA (267938-268013) Phe (GAA) 76 bp Sc: 86.19
GGCGTGATAGCTCAGTTGGTAGAGCAACGGATTGAAAATCCGTGTGTCCCCAGTTCGATC
CTGGGTCTCGCCACCA
>Acinetobacter_baumannii_chr.trna51-ProTGG (3139107-3139031) Pro (TGG) 77 bp Sc: 85.67
CGGAGCATAGCACAGCCTGGTAGTGCACCTGGTTTGGGACCAGGGGGTCGTAGGTTCGAA
TCCTACTGCTCCGACCA
>Acinetobacter_baumannii_chr.trna54-ProTGG (3138784-3138708) Pro (TGG) 77 bp Sc: 85.67
CGGAGCATAGCACAGCCTGGTAGTGCACCTGGTTTGGGACCAGGGGGTCGTAGGTTCGAA
TCCTACTGCTCCGACCA
>Acinetobacter_baumannii_chr.trna5-SerGCT (461314-461404) Ser (GCT) 91 bp Sc: 67.25
GGTGAGGTGGATGAGTGGCTGAAATCACTTCCCTGCTAAGGAAGCATACCTATTACTGGT
ATCGAGGGTTCGAATCCCTCTCTCACCGCCA
>Acinetobacter_baumannii_chr.trna71-SerGGA (251490-251401) Ser (GGA) 90 bp Sc: 64.72
GGTGAGGTGTCCGAGTGGCTGAAGGAGCACGCCTGGAAAGTGTGTATACGTTTACGCGTA
TCGAGGGTTCGAATCCCTCTCTCACCGCCA
>Acinetobacter_baumannii_chr.trna20-SerTGA (894406-894495) Ser (TGA) 90 bp Sc: 65.75
GGAAGCGTGGCAGAGCGGTTTAATGCACCGGTCTTGAAAACCGACGAGGGTGTGAGTCCT
CCGTGAGTTCGAATCTCACCGCTTCCGCCA
>Acinetobacter_baumannii_chr.trna57-SerTGA (2676155-2676066) Ser (TGA) 90 bp Sc: 65.75
GGAAGCGTGGCAGAGCGGTTTAATGCACCGGTCTTGAAAACCGACGAGGGTGTGAGTCCT
CCGTGAGTTCGAATCTCACCGCTTCCGCCA
>Acinetobacter_baumannii_chr.trna44-ThrGGT (3553328-3553254) Thr (GGT) 75 bp Sc: 83.04
GCTCTTATAGCTCAGTGGTAGAGCACTCCCTTGGTAAGGGAGAGGTCTCGAGTTCAAATC
TCGATAAGAGCTCCA
>Acinetobacter_baumannii_chr.trna41-ThrTGT (3553670-3553595) Thr (TGT) 76 bp Sc: 94.17
GCCGGCATAGCTCAGTTGGTAGAGCAACTGACTTGTAATCAGTAGGTCCACAGTTCGAAT
CCGTGTGCCGGCACCA
>Acinetobacter_baumannii_chr.trna72-ThrTGT (238466-238391) Thr (TGT) 76 bp Sc: 94.17
GCCGGCATAGCTCAGTTGGTAGAGCAACTGACTTGTAATCAGTAGGTCCACAGTTCGAAT
CCGTGTGCCGGCACCA
>Acinetobacter_baumannii_chr.trna23-TrpCCA (1358618-1358691) Trp (CCA) 74 bp Sc: 62.59
AGAGGATTGGTGTAATGGTAGCATGACGGTCTCCAAAACCGTTCGTCAAGGTTCGAGTCC
TTGATCCTCTGCCA
>Acinetobacter_baumannii_chr.trna25-TrpCCA (1358869-1358942) Trp (CCA) 74 bp Sc: 62.59
AGAGGATTGGTGTAATGGTAGCATGACGGTCTCCAAAACCGTTCGTCAAGGTTCGAGTCC
TTGATCCTCTGCCA
>Acinetobacter_baumannii_chr.trna45-TrpCCA (3551839-3551764) Trp (CCA) 76 bp Sc: 81.08
AGGTCAGTAGTTCAATTGGTAGAGCGTCGGTCTCCAAAACCGAATGTTGGGGGTTCGAGT
CCCTCCTGACCTGCCA
>Acinetobacter_baumannii_chr.trna42-TyrGTA (3553538-3553455) Tyr (GTA) 84 bp Sc: 62.89
GGTGAGATTCCCGAGCGGCCAAAGGGGGCAGACTGTAACTCTGTTGCGAAAGCTTCGAAG
GTTCGAATCCTTCTCTCACCACCA
>Acinetobacter_baumannii_chr.trna21-ValGAC (1127257-1127333) Val (GAC) 77 bp Sc: 80.80
AGGTGTATAGCTCAGTTGGTTAGAGCGCTACGTTGACATCGTAGAGGTCTCCAGTTCGAG
TCTGGATATACCTACCA
>Acinetobacter_baumannii_chr.trna14-ValTAC (696887-696962) Val (TAC) 76 bp Sc: 97.11
GGGCGCTTAGCTCAGTTGGTAGAGCGTCTGCCTTACAAGCAGAATGTCGGCGGTTCGATC
CCGTCAGCGCCCACCA
>Acinetobacter_baumannii_chr.trna17-ValTAC (697209-697284) Val (TAC) 76 bp Sc: 97.11
GGGCGCTTAGCTCAGTTGGTAGAGCGTCTGCCTTACAAGCAGAATGTCGGCGGTTCGATC
CCGTCAGCGCCCACCA
>Acinetobacter_baumannii_chr.trna19-ValTAC (697467-697542) Val (TAC) 76 bp Sc: 97.11
GGGCGCTTAGCTCAGTTGGTAGAGCGTCTGCCTTACAAGCAGAATGTCGGCGGTTCGATC
CCGTCAGCGCCCACCA
>Acinetobacter_baumannii_ATCC_17978_chr.trna27-AlaGGC (3697006-3697081) Ala (GGC) 76 bp Sc: 83.49
GGGGTCATAGCTCAGTTGGTAGAGCGCTACAATGGCATTGTAGAGGTCAGCGGTTCGATC
CCGCTTGGCTCCACCA
>Acinetobacter_baumannii_ATCC_17978_chr.trna2-AlaTGC (195048-195123) Ala (TGC) 76 bp Sc: 89.91
GGGGACTTAGCTTAGTTGGTAGAGCGCCTGCTTTGCACGCAGGAGGTCAGGAGTTCGACT

CTCCTAGTCTCCACCA
>Acinetobacter_baumannii_ATCC_17978_chr.trna40-AlaTGC (3503931-3503856) Ala (TGC) 76 bp Sc: 89.91
GGGGACTTAGCTTAGTTGGTAGAGCGCCTGCTTTGCACGCAGGAGGTCAGGAGTTCGACT
CTCCTAGTCTCCACCA
>Acinetobacter_baumannii_ATCC_17978_chr.trna42-AlaTGC (3439549-3439474) Ala (TGC) 76 bp Sc: 89.91
GGGGACTTAGCTTAGTTGGTAGAGCGCCTGCTTTGCACGCAGGAGGTCAGGAGTTCGACT
CTCCTAGTCTCCACCA
>Acinetobacter_baumannii_ATCC_17978_chr.trna64-AlaTGC (810449-810374) Ala (TGC) 76 bp Sc: 89.91
GGGGACTTAGCTTAGTTGGTAGAGCGCCTGCTTTGCACGCAGGAGGTCAGGAGTTCGACT
CTCCTAGTCTCCACCA
>Acinetobacter_baumannii_ATCC_17978_chr.trna66-AlaTGC (804625-804550) Ala (TGC) 76 bp Sc: 89.91
GGGGACTTAGCTTAGTTGGTAGAGCGCCTGCTTTGCACGCAGGAGGTCAGGAGTTCGACT
CTCCTAGTCTCCACCA
>Acinetobacter_baumannii_ATCC_17978_chr.trna36-ArgACG (3531614-3531538) Arg (ACG) 77 bp Sc: 83.63
GCGCTCATAGCTCAGCTGGATAGAGCACTTGGCTACGAACTAAGGGGTCGGGAGTTCGAA
TCTCTCTGAGCGCACCA
>Acinetobacter_baumannii_ATCC_17978_chr.trna37-ArgACG (3531498-3531422) Arg (ACG) 77 bp Sc: 83.63
GCGCTCATAGCTCAGCTGGATAGAGCACTTGGCTACGAACTAAGGGGTCGGGAGTTCGAA
TCTCTCTGAGCGCACCA
>Acinetobacter_baumannii_ATCC_17978_chr.trna38-ArgACG (3527704-3527628) Arg (ACG) 77 bp Sc: 83.63
GCGCTCATAGCTCAGCTGGATAGAGCACTTGGCTACGAACTAAGGGGTCGGGAGTTCGAA
TCTCTCTGAGCGCACCA
>Acinetobacter_baumannii_ATCC_17978_chr.trna18-ArgCCG (2678380-2678456) Arg (CCG) 77 bp Sc: 81.26
GCGCTCGTAGCTCAGTTGGATAGAGTACAGGTTTCCGAAGCCTGGGGTCGTGGGTTCGAT
CCCCGCCGAGCGCACCA
>Acinetobacter_baumannii_ATCC_17978_chr.trna26-ArgCCT (3204641-3204713) Arg (CCT) 73 bp Sc: 67.72
GCGCCCTTAGCTTAACTGGATAGAGCAGTTGCCTCCTAAGCGACCGACGTGGGTTCGAGT
CCCGCAGGGCGCA
>Acinetobacter_baumannii_ATCC_17978_chr.trna11-ArgTCT (845536-845612) Arg (TCT) 77 bp Sc: 98.82
GCGCCTGTAGCTCAGTTGGATAGAGCATCCGCCTTCTAAGCGGATGGTCACAGGTTCGAA
TCCTGTCAGGCGCGCCA
>Acinetobacter_baumannii_ATCC_17978_chr.trna19-AsnGTT (2715729-2715804) Asn (GTT) 76 bp Sc: 83.01
TCTCCAATAGCTCAGTCGGTAGAGCGACGGACTGTTAATCCGCAGGTCCCTGGTTCGAGC
CCAGGTTGGAGAGCCA
>Acinetobacter_baumannii_ATCC_17978_chr.trna20-AsnGTT (2715813-2715888) Asn (GTT) 76 bp Sc: 83.03
TCTCCCATAGCTCAGTCGGTAGAGCGACGGACTGTTAATCCGCAGGTCCCTGGTTCGAGC
CCAGGTGGGAGAGCCA
>Acinetobacter_baumannii_ATCC_17978_chr.trna21-AsnGTT (2716067-2716142) Asn (GTT) 76 bp Sc: 83.03
TCTCCCATAGCTCAGTCGGTAGAGCGACGGACTGTTAATCCGCAGGTCCCTGGTTCGAGC
CCAGGTGGGAGAGCCA
>Acinetobacter_baumannii_ATCC_17978_chr.trna22-AsnGTT (2914390-2914465) Asn (GTT) 76 bp Sc: 83.03
TCTCCCATAGCTCAGTCGGTAGAGCGACGGACTGTTAATCCGCAGGTCCCTGGTTCGAGC
CCAGGTGGGAGAGCCA
>Acinetobacter_baumannii_ATCC_17978_chr.trna45-AspGTC (3248135-3248059) Asp (GTC) 77 bp Sc: 94.40
GCAGCGGTAGTTCAGTTGGTTAGAATACCGGCCTGTCACGCCGGGGGTCGCGGGTTCGAG
CCCCGTCCGCTGCGCCA
>Acinetobacter_baumannii_ATCC_17978_chr.trna46-AspGTC (3248011-3247935) Asp (GTC) 77 bp Sc: 94.40
GCAGCGGTAGTTCAGTTGGTTAGAATACCGGCCTGTCACGCCGGGGGTCGCGGGTTCGAG
CCCCGTCCGCTGCGCCA
>Acinetobacter_baumannii_ATCC_17978_chr.trna48-AspGTC (3247798-3247722) Asp (GTC) 77 bp Sc: 94.40
GCAGCGGTAGTTCAGTTGGTTAGAATACCGGCCTGTCACGCCGGGGGTCGCGGGTTCGAG
CCCCGTCCGCTGCGCCA
>Acinetobacter_baumannii_ATCC_17978_chr.trna23-CysGCA (3101818-3101891) Cys (GCA) 74 bp Sc: 59.57
GGCGGGGTGGCAGAGTGGTCATGCAGCGGACTGCAACTCCGTGGACGCCGGTTCGATTCC
GACCTCCGCCTCCA
>Acinetobacter_baumannii_ATCC_17978_chr.trna59-GlnTTG (976105-976031) Gln (TTG) 75 bp Sc: 68.77
AGGGGCGTCGCCAAGTGGTAAGGCAGCGGGTTTTGATCCCGCCATCCGTTGGTTCGAATC
CAGCCGCCCCTGCCA
>Acinetobacter_baumannii_ATCC_17978_chr.trna60-GlnTTG (976003-975929) Gln (TTG) 75 bp Sc: 68.77
AGGGGCGTCGCCAAGTGGTAAGGCAGCGGGTTTTGATCCCGCCATCCGTTGGTTCGAATC
CAGCCGCCCCTGCCA
>Acinetobacter_baumannii_ATCC_17978_chr.trna61-GlnTTG (975907-975833) Gln (TTG) 75 bp Sc: 68.77
AGGGGCGTCGCCAAGTGGTAAGGCAGCGGGTTTTGATCCCGCCATCCGTTGGTTCGAATC
CAGCCGCCCCTGCCA
>Acinetobacter_baumannii_ATCC_17978_chr.trna62-GlnTTG (975740-975666) Gln (TTG) 75 bp Sc: 68.77
AGGGGCGTCGCCAAGTGGTAAGGCAGCGGGTTTTGATCCCGCCATCCGTTGGTTCGAATC
CAGCCGCCCCTGCCA

>Acinetobacter_baumannii_ATCC_17978_chr.trna28-GluTTC (3697107-3697182) Glu (TTC) 76 bp Sc: 56.39
GTCCCTATCGTCTAGAGGCCTAGGACATCGCCCTTTCACGGCGGTAACCGGGGTTCGAAT
CCCCGTAGGGACGCCA
>Acinetobacter_baumannii_ATCC_17978_chr.trna29-GluTTC (3699871-3699946) Glu (TTC) 76 bp Sc: 56.39
GTCCCTATCGTCTAGAGGCCTAGGACATCGCCCTTTCACGGCGGTAACCGGGGTTCGAAT
CCCCGTAGGGACGCCA
>Acinetobacter_baumannii_ATCC_17978_chr.trna30-GluTTC (3699975-3700050) Glu (TTC) 76 bp Sc: 56.39
GTCCCTATCGTCTAGAGGCCTAGGACATCGCCCTTTCACGGCGGTAACCGGGGTTCGAAT
CCCCGTAGGGACGCCA
>Acinetobacter_baumannii_ATCC_17978_chr.trna58-GluTTC (1229719-1229644) Glu (TTC) 76 bp Sc: 56.39
GTCCCTATCGTCTAGAGGCCTAGGACATCGCCCTTTCACGGCGGTAACCGGGGTTCGAAT
CCCCGTAGGGACGCCA
>Acinetobacter_baumannii_ATCC_17978_chr.trna57-GlyGCC (1446506-1446431) Gly (GCC) 76 bp Sc: 90.38
GCGGGAATAGCTCAGTTGGTAGAGCATAACCTTGCCAAGGTTGGGGTCGCGAGTTCGAGT
CTCGTTTCCCGCTCCA
>Acinetobacter_baumannii_ATCC_17978_chr.trna69-GlyGCC (149356-149281) Gly (GCC) 76 bp Sc: 90.38
GCGGGAATAGCTCAGTTGGTAGAGCATAACCTTGCCAAGGTTGGGGTCGCGAGTTCGAGT
CTCGTTTCCCGCTCCA
>Acinetobacter_baumannii_ATCC_17978_chr.trna70-GlyGCC (149235-149160) Gly (GCC) 76 bp Sc: 90.38
GCGGGAATAGCTCAGTTGGTAGAGCATAACCTTGCCAAGGTTGGGGTCGCGAGTTCGAGT
CTCGTTTCCCGCTCCA
>Acinetobacter_baumannii_ATCC_17978_chr.trna5-GlyTCC (300127-300202) Gly (TCC) 76 bp Sc: 91.76
GCGGGAGTAGCTCAGTTGGTAGAGCGGCAGCCTTCCAAGCTGCATGTCGCGAGTTCGATC
CTCGTCTCCCGCTCCA
>Acinetobacter_baumannii_ATCC_17978_chr.trna12-HisGTG (845646-845721) His (GTG) 76 bp Sc: 81.86
GTGGATGTAGCTCAGTTGGTAGAGCCCTGGATTGTGATTCCAGTTGTCGCGGGTTCGAAT
CCCGTCATTCACCCCA
>Acinetobacter_baumannii_ATCC_17978_chr.trna1-IleGAT (194916-194992) Ile (GAT) 77 bp Sc: 98.53
GGGTCTGTAGCTCAGTTGGTTAGAGCACACGCTTGATAAGCGTGGGGTCACAAGTTCAAG
TCTTGTCAGACCCACCA
>Acinetobacter_baumannii_ATCC_17978_chr.trna39-IleGAT (3504063-3503987) Ile (GAT) 77 bp Sc: 98.53
GGGTCTGTAGCTCAGTTGGTTAGAGCACACGCTTGATAAGCGTGGGGTCACAAGTTCAAG
TCTTGTCAGACCCACCA
>Acinetobacter_baumannii_ATCC_17978_chr.trna41-IleGAT (3439681-3439605) Ile (GAT) 77 bp Sc: 98.53
GGGTCTGTAGCTCAGTTGGTTAGAGCACACGCTTGATAAGCGTGGGGTCACAAGTTCAAG
TCTTGTCAGACCCACCA
>Acinetobacter_baumannii_ATCC_17978_chr.trna63-IleGAT (810581-810505) Ile (GAT) 77 bp Sc: 98.53
GGGTCTGTAGCTCAGTTGGTTAGAGCACACGCTTGATAAGCGTGGGGTCACAAGTTCAAG
TCTTGTCAGACCCACCA
>Acinetobacter_baumannii_ATCC_17978_chr.trna65-IleGAT (804757-804681) Ile (GAT) 77 bp Sc: 98.53
GGGTCTGTAGCTCAGTTGGTTAGAGCACACGCTTGATAAGCGTGGGGTCACAAGTTCAAG
TCTTGTCAGACCCACCA
>Acinetobacter_baumannii_ATCC_17978_chr.trna43-LeuCAA (3371509-3371423) Leu (CAA) 87 bp Sc: 71.58
CCCGAGGTGGTGAAATTGGTAGACGCGGCGGACTCAAAATCCGCTGTCAGAGATGACGTG
TCGGTTCGAGTCCGACCCTCGGGACCA
>Acinetobacter_baumannii_ATCC_17978_chr.trna8-LeuGAG (359440-359524) Leu (GAG) 85 bp Sc: 59.44
GCGGTGGTGGTGGAATTGGTAGACACGCTACCTTGAGGTGGTAGTGCTTTCGGGCGTGGG
GGTTCAAGTCCCCCCTTCCGCACCA
>Acinetobacter_baumannii_ATCC_17978_chr.trna24-LeuTAA (3101903-3101988) Leu (TAA) 86 bp Sc: 70.97
GCCCGGGTGGTGAAATAGGTAGACACAGGGGATTTAAAATCCCCCGCCCTCAAAGCGTGC
CGGTTCGAGTCCGGCCCCGGGCACCA
>Acinetobacter_baumannii_ATCC_17978_chr.trna25-LeuTAA (3104049-3104134) Leu (TAA) 86 bp Sc: 70.97
GCCCGGGTGGTGAAATAGGTAGACACAGGGGATTTAAAATCCCCCGCCCTCAAAGCGTGC
CGGTTCGAGTCCGGCCCCGGGCACCA
>Acinetobacter_baumannii_ATCC_17978_chr.trna52-LeuTAG (2601327-2601243) Leu (TAG) 85 bp Sc: 77.55
GGGGGCGTGGCGAAATTGGTAGACGCACTGGATTTAGGTTCCAGCGCCGCAAGGTGTAAG
AGTTCGAGTCTCTTCGCCCCCACCA
>Acinetobacter_baumannii_ATCC_17978_chr.trna54-LeuTAG (2601085-2601001) Leu (TAG) 85 bp Sc: 77.55
GGGGGCGTGGCGAAATTGGTAGACGCACTGGATTTAGGTTCCAGCGCCGCAAGGTGTAAG
AGTTCGAGTCTCTTCGCCCCCACCA
>Acinetobacter_baumannii_ATCC_17978_chr.trna67-LysTTT (433078-433003) Lys (TTT) 76 bp Sc: 92.37
GGGTCGTTAGCTCAGTTGGTAGAGCAGCGGACTTTTAATCCGTTGGTCCCGCGTTCGAGT
CGCGGACGACCCACCA
>Acinetobacter_baumannii_ATCC_17978_chr.trna68-LysTTT (432980-432905) Lys (TTT) 76 bp Sc: 92.37
GGGTCGTTAGCTCAGTTGGTAGAGCAGCGGACTTTTAATCCGTTGGTCCCGCGTTCGAGT
CGCGGACGACCCACCA
>Acinetobacter_baumannii_ATCC_17978_chr.trna9-MetCAT (359567-359643) Met (CAT) 77 bp Sc: 82.06

TGCGGGATGGAGCAGTCTGGTAGCTCGTCGGGCTCATAACCCGAAGGTCGTTGGTTCAAA
TCCAGCTCCCGCTACCA
>Acinetobacter_baumannii_ATCC_17978_chr.trna17-MetCAT (1432695-1432771) Met (CAT) 77 bp Sc: 82.10
TGCGGGGTGGAGCAGTCTGGTAGCTCGTCGGGCTCATAACCCGAAGGTCGTTGGTTCAAA
TCCGACCCCCGCATCCA
>Acinetobacter_baumannii_ATCC_17978_chr.trna56-MetCAT (1745378-1745302) Met (CAT) 77 bp Sc: 91.80
GGGCCTATAGCTCAGTCGGTTAGAGCAGCGGACTCATAATCCGTTGGTCCACAGTTCGAG
TCTGTGTGGGCCCACCA
>Acinetobacter_baumannii_ATCC_17978_chr.trna14-MetCAT (988300-988376) Met (CAT) 77 bp Sc: 94.07
GGCTATGTAGCTCAGTTGGTTAGAGCACCGCACTCATAATGCGGGGGTCACAAGTTCAAG
TCTCGTCATAGCCACCA
>Acinetobacter_baumannii_ATCC_17978_chr.trna15-MetCAT (998453-998529) Met (CAT) 77 bp Sc: 94.07
GGCTATGTAGCTCAGTTGGTTAGAGCACCGCACTCATAATGCGGGGGTCACAAGTTCAAG
TCTCGTCATAGCCACCA
>Acinetobacter_baumannii_ATCC_17978_chr.trna33-PheGAA (3727708-3727633) Phe (GAA) 76 bp Sc: 86.19
GGCGTGATAGCTCAGTTGGTAGAGCAACGGATTGAAAATCCGTGTGTCCCCAGTTCGATC
CTGGGTCTCGCCACCA
>Acinetobacter_baumannii_ATCC_17978_chr.trna34-PheGAA (3727507-3727432) Phe (GAA) 76 bp Sc: 86.19
GGCGTGATAGCTCAGTTGGTAGAGCAACGGATTGAAAATCCGTGTGTCCCCAGTTCGATC
CTGGGTCTCGCCACCA
>Acinetobacter_baumannii_ATCC_17978_chr.trna10-ProTGG (845403-845479) Pro (TGG) 77 bp Sc: 85.67
CGGAGCATAGCACAGCCTGGTAGTGCACCTGGTTTGGGACCAGGGGGTCGTAGGTTCGAA
TCCTACTGCTCCGACCA
>Acinetobacter_baumannii_ATCC_17978_chr.trna13-ProTGG (845726-845802) Pro (TGG) 77 bp Sc: 85.67
CGGAGCATAGCACAGCCTGGTAGTGCACCTGGTTTGGGACCAGGGGGTCGTAGGTTCGAA
TCCTACTGCTCCGACCA
>Acinetobacter_baumannii_ATCC_17978_chr.trna35-SerGCT (3531715-3531625) Ser (GCT) 91 bp Sc: 67.25
GGTGAGGTGGATGAGTGGCTGAAATCACTTCCCTGCTAAGGAAGCATACCTATTACTGGT
ATCGAGGGTTCGAATCCCTCTCTCACCGCCA
>Acinetobacter_baumannii_ATCC_17978_chr.trna31-SerGGA (3744227-3744316) Ser (GGA) 90 bp Sc: 64.72
GGTGAGGTGTCCGAGTGGCTGAAGGAGCACGCCTGGAAAGTGTGTATACGTTTACGCGTA
TCGAGGGTTCGAATCCCTCTCTCACCGCCA
>Acinetobacter_baumannii_ATCC_17978_chr.trna16-SerTGA (1237135-1237224) Ser (TGA) 90 bp Sc: 65.75
GGAAGCGTGGCAGAGCGGTTTAATGCACCGGTCTTGAAAACCGACGAGGGTGTGAGTCCT
CCGTGAGTTCGAATCTCACCGCTTCCGCCA
>Acinetobacter_baumannii_ATCC_17978_chr.trna50-SerTGA (3075300-3075211) Ser (TGA) 90 bp Sc: 65.75
GGAAGCGTGGCAGAGCGGTTTAATGCACCGGTCTTGAAAACCGACGAGGGTGTGAGTCCT
CCGTGAGTTCGAATCTCACCGCTTCCGCCA
>Acinetobacter_baumannii_ATCC_17978_chr.trna6-ThrGGT (300216-300290) Thr (GGT) 75 bp Sc: 83.04
GCTCTTATAGCTCAGTGGTAGAGCACTCCCTTGGTAAGGGAGAGGTCTCGAGTTCAAATC
TCGATAAGAGCTCCA
>Acinetobacter_baumannii_ATCC_17978_chr.trna3-ThrTGT (299874-299949) Thr (TGT) 76 bp Sc: 94.17
GCCGGCATAGCTCAGTTGGTAGAGCAACTGACTTGTAATCAGTAGGTCCACAGTTCGAAT
CCGTGTGCCGGCACCA
>Acinetobacter_baumannii_ATCC_17978_chr.trna32-ThrTGT (3762438-3762513) Thr (TGT) 76 bp Sc: 94.17
GCCGGCATAGCTCAGTTGGTAGAGCAACTGACTTGTAATCAGTAGGTCCACAGTTCGAAT
CCGTGTGCCGGCACCA
>Acinetobacter_baumannii_ATCC_17978_chr.trna53-TrpCCA (2601186-2601113) Trp (CCA) 74 bp Sc: 62.59
AGAGGATTGGTGTAATGGTAGCATGACGGTCTCCAAAACCGTTCGTCAAGGTTCGAGTCC
TTGATCCTCTGCCA
>Acinetobacter_baumannii_ATCC_17978_chr.trna55-TrpCCA (2600935-2600862) Trp (CCA) 74 bp Sc: 62.59
AGAGGATTGGTGTAATGGTAGCATGACGGTCTCCAAAACCGTTCGTCAAGGTTCGAGTCC
TTGATCCTCTGCCA
>Acinetobacter_baumannii_ATCC_17978_chr.trna7-TrpCCA (301704-301779) Trp (CCA) 76 bp Sc: 81.08
AGGTCAGTAGTTCAATTGGTAGAGCGTCGGTCTCCAAAACCGAATGTTGGGGGTTCGAGT
CCCTCCTGACCTGCCA
>Acinetobacter_baumannii_ATCC_17978_chr.trna4-TyrGTA (300006-300089) Tyr (GTA) 84 bp Sc: 62.89
GGTGAGATTCCCGAGCGGCCAAAGGGGGCAGACTGTAACTCTGTTGCGAAAGCTTCGAAG
GTTCGAATCCTTCTCTCACCACCA
>Acinetobacter_baumannii_ATCC_17978_chr.trna51-ValGAC (2800473-2800397) Val (GAC) 77 bp Sc: 82.28
AGGCGTATAGCTCAGTTGGTTAGAGCGCTACGTTGACATCGTAGAGGTCTCCAGTTCGAG
TCTGGATATGCCTACCA
>Acinetobacter_baumannii_ATCC_17978_chr.trna44-ValTAC (3248235-3248160) Val (TAC) 76 bp Sc: 97.11
GGGCGCTTAGCTCAGTTGGTAGAGCGTCTGCCTTACAAGCAGAATGTCGGCGGTTCGATC
CCGTCAGCGCCCACCA
>Acinetobacter_baumannii_ATCC_17978_chr.trna47-ValTAC (3247913-3247838) Val (TAC) 76 bp Sc: 97.11
GGGCGCTTAGCTCAGTTGGTAGAGCGTCTGCCTTACAAGCAGAATGTCGGCGGTTCGATC

CCGTCAGCGCCCACCA
>Acinetobacter_baumannii_ATCC_17978_chr.trna49-ValTAC (3247655-3247580) Val (TAC) 76 bp Sc: 97.11
GGGCGCTTAGCTCAGTTGGTAGAGCGTCTGCCTTACAAGCAGAATGTCGGCGGTTCGATC
CCGTCAGCGCCCACCA
>Acinetobacter_baumannii_AYE_chr.trna67-AlaGGC (296406-296331) Ala (GGC) 76 bp Sc: 83.49
GGGGTCATAGCTCAGTTGGTAGAGCGCTACAATGGCATTGTAGAGGTCAGCGGTTCGATC
CCGCTTGGCTCCACCA
>Acinetobacter_baumannii_AYE_chr.trna10-AlaTGC (489162-489237) Ala (TGC) 76 bp Sc: 89.91
GGGGACTTAGCTTAGTTGGTAGAGCGCCTGCTTTGCACGCAGGAGGTCAGGAGTTCGACT
CTCCTAGTCTCCACCA
>Acinetobacter_baumannii_AYE_chr.trna12-AlaTGC (521395-521470) Ala (TGC) 76 bp Sc: 89.91
GGGGACTTAGCTTAGTTGGTAGAGCGCCTGCTTTGCACGCAGGAGGTCAGGAGTTCGACT
CTCCTAGTCTCCACCA
>Acinetobacter_baumannii_AYE_chr.trna2-AlaTGC (20043-20118) Ala (TGC) 76 bp Sc: 89.91
GGGGACTTAGCTTAGTTGGTAGAGCGCCTGCTTTGCACGCAGGAGGTCAGGAGTTCGACT
CTCCTAGTCTCCACCA
>Acinetobacter_baumannii_AYE_chr.trna38-AlaTGC (3897509-3897434) Ala (TGC) 76 bp Sc: 89.91
GGGGACTTAGCTTAGTTGGTAGAGCGCCTGCTTTGCACGCAGGAGGTCAGGAGTTCGACT
CTCCTAGTCTCCACCA
>Acinetobacter_baumannii_AYE_chr.trna40-AlaTGC (3731654-3731579) Ala (TGC) 76 bp Sc: 89.91
GGGGACTTAGCTTAGTTGGTAGAGCGCCTGCTTTGCACGCAGGAGGTCAGGAGTTCGACT
CTCCTAGTCTCCACCA
>Acinetobacter_baumannii_AYE_chr.trna50-AlaTGC (3175824-3175749) Ala (TGC) 76 bp Sc: 89.91
GGGGACTTAGCTTAGTTGGTAGAGCGCCTGCTTTGCACGCAGGAGGTCAGGAGTTCGACT
CTCCTAGTCTCCACCA
>Acinetobacter_baumannii_AYE_chr.trna6-ArgACG (461415-461491) Arg (ACG) 77 bp Sc: 83.63
GCGCTCATAGCTCAGCTGGATAGAGCACTTGGCTACGAACTAAGGGGTCGGGAGTTCGAA
TCTCTCTGAGCGCACCA
>Acinetobacter_baumannii_AYE_chr.trna7-ArgACG (461531-461607) Arg (ACG) 77 bp Sc: 83.63
GCGCTCATAGCTCAGCTGGATAGAGCACTTGGCTACGAACTAAGGGGTCGGGAGTTCGAA
TCTCTCTGAGCGCACCA
>Acinetobacter_baumannii_AYE_chr.trna8-ArgACG (465383-465459) Arg (ACG) 77 bp Sc: 83.63
GCGCTCATAGCTCAGCTGGATAGAGCACTTGGCTACGAACTAAGGGGTCGGGAGTTCGAA
TCTCTCTGAGCGCACCA
>Acinetobacter_baumannii_AYE_chr.trna59-ArgCCG (1250422-1250346) Arg (CCG) 77 bp Sc: 81.26
GCGCTCGTAGCTCAGTTGGATAGAGTACAGGTTTCCGAAGCCTGGGGTCGTGGGTTCGAT
CCCCGCCGAGCGCACCA
>Acinetobacter_baumannii_AYE_chr.trna66-ArgCCT (738086-738014) Arg (CCT) 73 bp Sc: 67.72
GCGCCCTTAGCTTAACTGGATAGAGCAGTTGCCTCCTAAGCGACCGACGTGGGTTCGAGT
CCCGCAGGGCGCA
>Acinetobacter_baumannii_AYE_chr.trna52-ArgTCT (3138974-3138898) Arg (TCT) 77 bp Sc: 98.82
GCGCCTGTAGCTCAGTTGGATAGAGCATCCGCCTTCTAAGCGGATGGTCACAGGTTCGAA
TCCTGTCAGGCGCGCCA
>Acinetobacter_baumannii_AYE_chr.trna60-AsnGTT (1213070-1212995) Asn (GTT) 76 bp Sc: 77.17
TCTCCAATAGCTCAGTCGGTAGAGCGACGGACTGTTAATCCGCAGGTCCCTGGTTCGAGC
CCAGGTGGGAGAGCCA
>Acinetobacter_baumannii_AYE_chr.trna61-AsnGTT (1211624-1211549) Asn (GTT) 76 bp Sc: 83.03
TCTCCCATAGCTCAGTCGGTAGAGCGACGGACTGTTAATCCGCAGGTCCCTGGTTCGAGC
CCAGGTGGGAGAGCCA
>Acinetobacter_baumannii_AYE_chr.trna62-AsnGTT (1004960-1004885) Asn (GTT) 76 bp Sc: 83.03
TCTCCCATAGCTCAGTCGGTAGAGCGACGGACTGTTAATCCGCAGGTCCCTGGTTCGAGC
CCAGGTGGGAGAGCCA
>Acinetobacter_baumannii_AYE_chr.trna15-AspGTC (696987-697063) Asp (GTC) 77 bp Sc: 94.40
GCAGCGGTAGTTCAGTTGGTTAGAATACCGGCCTGTCACGCCGGGGGTCGCGGGTTCGAG
CCCCGTCCGCTGCGCCA
>Acinetobacter_baumannii_AYE_chr.trna16-AspGTC (697111-697187) Asp (GTC) 77 bp Sc: 94.40
GCAGCGGTAGTTCAGTTGGTTAGAATACCGGCCTGTCACGCCGGGGGTCGCGGGTTCGAG
CCCCGTCCGCTGCGCCA
>Acinetobacter_baumannii_AYE_chr.trna18-AspGTC (697324-697400) Asp (GTC) 77 bp Sc: 94.40
GCAGCGGTAGTTCAGTTGGTTAGAATACCGGCCTGTCACGCCGGGGGTCGCGGGTTCGAG
CCCCGTCCGCTGCGCCA
>Acinetobacter_baumannii_AYE_chr.trna63-CysGCA (857440-857367) Cys (GCA) 74 bp Sc: 59.57
GGCGGGGTGGCAGAGTGGTCATGCAGCGGACTGCAACTCCGTGGACGCCGGTTCGATTCC
GACCTCCGCCTCCA
>Acinetobacter_baumannii_AYE_chr.trna29-GlnTTG (3009677-3009751) Gln (TTG) 75 bp Sc: 68.77
AGGGGCGTCGCCAAGTGGTAAGGCAGCGGGTTTTGATCCCGCCATCCGTTGGTTCGAATC
CAGCCGCCCCTGCCA

>Acinetobacter_baumannii_AYE_chr.trna30-GlnTTG (3009779-3009853) Gln (TTG) 75 bp Sc: 68.77
AGGGGCGTCGCCAAGTGGTAAGGCAGCGGGTTTTGATCCCGCCATCCGTTGGTTCGAATC
CAGCCGCCCCTGCCA
>Acinetobacter_baumannii_AYE_chr.trna31-GlnTTG (3009875-3009949) Gln (TTG) 75 bp Sc: 68.77
AGGGGCGTCGCCAAGTGGTAAGGCAGCGGGTTTTGATCCCGCCATCCGTTGGTTCGAATC
CAGCCGCCCCTGCCA
>Acinetobacter_baumannii_AYE_chr.trna32-GlnTTG (3010041-3010115) Gln (TTG) 75 bp Sc: 68.77
AGGGGCGTCGCCAAGTGGTAAGGCAGCGGGTTTTGATCCCGCCATCCGTTGGTTCGAATC
CAGCCGCCCCTGCCA
>Acinetobacter_baumannii_AYE_chr.trna28-GluTTC (2684257-2684332) Glu (TTC) 76 bp Sc: 56.39
GTCCCTATCGTCTAGAGGCCTAGGACATCGCCCTTTCACGGCGGTAACCGGGGTTCGAAT
CCCCGTAGGGACGCCA
>Acinetobacter_baumannii_AYE_chr.trna68-GluTTC (296305-296230) Glu (TTC) 76 bp Sc: 56.39
GTCCCTATCGTCTAGAGGCCTAGGACATCGCCCTTTCACGGCGGTAACCGGGGTTCGAAT
CCCCGTAGGGACGCCA
>Acinetobacter_baumannii_AYE_chr.trna69-GluTTC (293543-293468) Glu (TTC) 76 bp Sc: 56.39
GTCCCTATCGTCTAGAGGCCTAGGACATCGCCCTTTCACGGCGGTAACCGGGGTTCGAAT
CCCCGTAGGGACGCCA
>Acinetobacter_baumannii_AYE_chr.trna70-GluTTC (293439-293364) Glu (TTC) 76 bp Sc: 56.39
GTCCCTATCGTCTAGAGGCCTAGGACATCGCCCTTTCACGGCGGTAACCGGGGTTCGAAT
CCCCGTAGGGACGCCA
>Acinetobacter_baumannii_AYE_chr.trna27-GlyGCC (2542259-2542334) Gly (GCC) 76 bp Sc: 90.38
GCGGGAATAGCTCAGTTGGTAGAGCATAACCTTGCCAAGGTTGGGGTCGCGAGTTCGAGT
CTCGTTTCCCGCTCCA
>Acinetobacter_baumannii_AYE_chr.trna35-GlyGCC (3776785-3776860) Gly (GCC) 76 bp Sc: 90.38
GCGGGAATAGCTCAGTTGGTAGAGCATAACCTTGCCAAGGTTGGGGTCGCGAGTTCGAGT
CTCGTTTCCCGCTCCA
>Acinetobacter_baumannii_AYE_chr.trna36-GlyGCC (3776906-3776981) Gly (GCC) 76 bp Sc: 90.38
GCGGGAATAGCTCAGTTGGTAGAGCATAACCTTGCCAAGGTTGGGGTCGCGAGTTCGAGT
CTCGTTTCCCGCTCCA
>Acinetobacter_baumannii_AYE_chr.trna43-GlyTCC (3553417-3553342) Gly (TCC) 76 bp Sc: 91.76
GCGGGAGTAGCTCAGTTGGTAGAGCGGCAGCCTTCCAAGCTGCATGTCGCGAGTTCGATC
CTCGTCTCCCGCTCCA
>Acinetobacter_baumannii_AYE_chr.trna53-HisGTG (3138864-3138789) His (GTG) 76 bp Sc: 81.86
GTGGATGTAGCTCAGTTGGTAGAGCCCTGGATTGTGATTCCAGTTGTCGCGGGTTCGAAT
CCCGTCATTCACCCCA
>Acinetobacter_baumannii_AYE_chr.trna1-IleGAT (19911-19987) Ile (GAT) 77 bp Sc: 98.53
GGGTCTGTAGCTCAGTTGGTTAGAGCACACGCTTGATAAGCGTGGGGTCACAAGTTCAAG
TCTTGTCAGACCCACCA
>Acinetobacter_baumannii_AYE_chr.trna11-IleGAT (521263-521339) Ile (GAT) 77 bp Sc: 98.53
GGGTCTGTAGCTCAGTTGGTTAGAGCACACGCTTGATAAGCGTGGGGTCACAAGTTCAAG
TCTTGTCAGACCCACCA
>Acinetobacter_baumannii_AYE_chr.trna37-IleGAT (3897641-3897565) Ile (GAT) 77 bp Sc: 98.53
GGGTCTGTAGCTCAGTTGGTTAGAGCACACGCTTGATAAGCGTGGGGTCACAAGTTCAAG
TCTTGTCAGACCCACCA
>Acinetobacter_baumannii_AYE_chr.trna39-IleGAT (3731786-3731710) Ile (GAT) 77 bp Sc: 98.53
GGGTCTGTAGCTCAGTTGGTTAGAGCACACGCTTGATAAGCGTGGGGTCACAAGTTCAAG
TCTTGTCAGACCCACCA
>Acinetobacter_baumannii_AYE_chr.trna49-IleGAT (3175956-3175880) Ile (GAT) 77 bp Sc: 98.53
GGGTCTGTAGCTCAGTTGGTTAGAGCACACGCTTGATAAGCGTGGGGTCACAAGTTCAAG
TCTTGTCAGACCCACCA
>Acinetobacter_baumannii_AYE_chr.trna9-IleGAT (489030-489106) Ile (GAT) 77 bp Sc: 98.53
GGGTCTGTAGCTCAGTTGGTTAGAGCACACGCTTGATAAGCGTGGGGTCACAAGTTCAAG
TCTTGTCAGACCCACCA
>Acinetobacter_baumannii_AYE_chr.trna13-LeuCAA (548277-548363) Leu (CAA) 87 bp Sc: 71.58
CCCGAGGTGGTGAAATTGGTAGACGCGGCGGACTCAAAATCCGCTGTCAGAGATGACGTG
TCGGTTCGAGTCCGACCCTCGGGACCA
>Acinetobacter_baumannii_AYE_chr.trna46-LeuGAG (3496945-3496861) Leu (GAG) 85 bp Sc: 59.44
GCGGTGGTGGTGGAATTGGTAGACACGCTACCTTGAGGTGGTAGTGCTTTCGGGCGTGGG
GGTTCAAGTCCCCCCTTCCGCACCA
>Acinetobacter_baumannii_AYE_chr.trna64-LeuTAA (857355-857270) Leu (TAA) 86 bp Sc: 70.97
GCCCGGGTGGTGAAATAGGTAGACACAGGGGATTTAAAATCCCCCGCCCTCAAAGCGTGC
CGGTTCGAGTCCGGCCCCGGGCACCA
>Acinetobacter_baumannii_AYE_chr.trna65-LeuTAA (855210-855125) Leu (TAA) 86 bp Sc: 70.97
GCCCGGGTGGTGAAATAGGTAGACACAGGGGATTTAAAATCCCCCGCCCTCAAAGCGTGC
CGGTTCGAGTCCGGCCCCGGGCACCA
>Acinetobacter_baumannii_AYE_chr.trna22-LeuTAG (1358479-1358563) Leu (TAG) 85 bp Sc: 77.55

GGGGGCGTGGCGAAATTGGTAGACGCACTGGATTTAGGTTCCAGCGCCGCAAGGTGTAAG
AGTTCGAGTCTCTTCGCCCCCACCA
>Acinetobacter_baumannii_AYE_chr.trna24-LeuTAG (1358719-1358803) Leu (TAG) 85 bp Sc: 77.55
GGGGGCGTGGCGAAATTGGTAGACGCACTGGATTTAGGTTCCAGCGCCGCAAGGTGTAAG
AGTTCGAGTCTCTTCGCCCCCACCA
>Acinetobacter_baumannii_AYE_chr.trna33-LysTTT (3423128-3423203) Lys (TTT) 76 bp Sc: 92.37
GGGTCGTTAGCTCAGTTGGTAGAGCAGCGGACTTTTAATCCGTTGGTCCCGCGTTCGAGT
CGCGGACGACCCACCA
>Acinetobacter_baumannii_AYE_chr.trna34-LysTTT (3423226-3423301) Lys (TTT) 76 bp Sc: 92.37
GGGTCGTTAGCTCAGTTGGTAGAGCAGCGGACTTTTAATCCGTTGGTCCCGCGTTCGAGT
CGCGGACGACCCACCA
>Acinetobacter_baumannii_AYE_chr.trna47-MetCAT (3496818-3496742) Met (CAT) 77 bp Sc: 82.06
TGCGGGATGGAGCAGTCTGGTAGCTCGTCGGGCTCATAACCCGAAGGTCGTTGGTTCAAA
TCCAGCTCCCGCTACCA
>Acinetobacter_baumannii_AYE_chr.trna48-MetCAT (3496652-3496576) Met (CAT) 77 bp Sc: 82.06
TGCGGGATGGAGCAGTCTGGTAGCTCGTCGGGCTCATAACCCGAAGGTCGTTGGTTCAAA
TCCAGCTCCCGCTACCA
>Acinetobacter_baumannii_AYE_chr.trna58-MetCAT (2575189-2575113) Met (CAT) 77 bp Sc: 82.10
TGCGGGGTGGAGCAGTCTGGTAGCTCGTCGGGCTCATAACCCGAAGGTCGTTGGTTCAAA
TCCGACCCCCGCATCCA
>Acinetobacter_baumannii_AYE_chr.trna26-MetCAT (2197426-2197502) Met (CAT) 77 bp Sc: 91.80
GGGCCTATAGCTCAGTCGGTTAGAGCAGCGGACTCATAATCCGTTGGTCCACAGTTCGAG
TCTGTGTGGGCCCACCA
>Acinetobacter_baumannii_AYE_chr.trna55-MetCAT (2997482-2997406) Met (CAT) 77 bp Sc: 94.07
GGCTATGTAGCTCAGTTGGTTAGAGCACCGCACTCATAATGCGGGGGTCACAAGTTCAAG
TCTCGTCATAGCCACCA
>Acinetobacter_baumannii_AYE_chr.trna56-MetCAT (2985991-2985915) Met (CAT) 77 bp Sc: 94.07
GGCTATGTAGCTCAGTTGGTTAGAGCACCGCACTCATAATGCGGGGGTCACAAGTTCAAG
TCTCGTCATAGCCACCA
>Acinetobacter_baumannii_AYE_chr.trna3-PheGAA (267736-267811) Phe (GAA) 76 bp Sc: 86.19
GGCGTGATAGCTCAGTTGGTAGAGCAACGGATTGAAAATCCGTGTGTCCCCAGTTCGATC
CTGGGTCTCGCCACCA
>Acinetobacter_baumannii_AYE_chr.trna4-PheGAA (267938-268013) Phe (GAA) 76 bp Sc: 86.19
GGCGTGATAGCTCAGTTGGTAGAGCAACGGATTGAAAATCCGTGTGTCCCCAGTTCGATC
CTGGGTCTCGCCACCA
>Acinetobacter_baumannii_AYE_chr.trna51-ProTGG (3139107-3139031) Pro (TGG) 77 bp Sc: 85.67
CGGAGCATAGCACAGCCTGGTAGTGCACCTGGTTTGGGACCAGGGGGTCGTAGGTTCGAA
TCCTACTGCTCCGACCA
>Acinetobacter_baumannii_AYE_chr.trna54-ProTGG (3138784-3138708) Pro (TGG) 77 bp Sc: 85.67
CGGAGCATAGCACAGCCTGGTAGTGCACCTGGTTTGGGACCAGGGGGTCGTAGGTTCGAA
TCCTACTGCTCCGACCA
>Acinetobacter_baumannii_AYE_chr.trna5-SerGCT (461314-461404) Ser (GCT) 91 bp Sc: 67.25
GGTGAGGTGGATGAGTGGCTGAAATCACTTCCCTGCTAAGGAAGCATACCTATTACTGGT
ATCGAGGGTTCGAATCCCTCTCTCACCGCCA
>Acinetobacter_baumannii_AYE_chr.trna71-SerGGA (251490-251401) Ser (GGA) 90 bp Sc: 64.72
GGTGAGGTGTCCGAGTGGCTGAAGGAGCACGCCTGGAAAGTGTGTATACGTTTACGCGTA
TCGAGGGTTCGAATCCCTCTCTCACCGCCA
>Acinetobacter_baumannii_AYE_chr.trna20-SerTGA (894406-894495) Ser (TGA) 90 bp Sc: 65.75
GGAAGCGTGGCAGAGCGGTTTAATGCACCGGTCTTGAAAACCGACGAGGGTGTGAGTCCT
CCGTGAGTTCGAATCTCACCGCTTCCGCCA
>Acinetobacter_baumannii_AYE_chr.trna57-SerTGA (2676155-2676066) Ser (TGA) 90 bp Sc: 65.75
GGAAGCGTGGCAGAGCGGTTTAATGCACCGGTCTTGAAAACCGACGAGGGTGTGAGTCCT
CCGTGAGTTCGAATCTCACCGCTTCCGCCA
>Acinetobacter_baumannii_AYE_chr.trna44-ThrGGT (3553328-3553254) Thr (GGT) 75 bp Sc: 83.04
GCTCTTATAGCTCAGTGGTAGAGCACTCCCTTGGTAAGGGAGAGGTCTCGAGTTCAAATC
TCGATAAGAGCTCCA
>Acinetobacter_baumannii_AYE_chr.trna41-ThrTGT (3553670-3553595) Thr (TGT) 76 bp Sc: 94.17
GCCGGCATAGCTCAGTTGGTAGAGCAACTGACTTGTAATCAGTAGGTCCACAGTTCGAAT
CCGTGTGCCGGCACCA
>Acinetobacter_baumannii_AYE_chr.trna72-ThrTGT (238466-238391) Thr (TGT) 76 bp Sc: 94.17
GCCGGCATAGCTCAGTTGGTAGAGCAACTGACTTGTAATCAGTAGGTCCACAGTTCGAAT
CCGTGTGCCGGCACCA
>Acinetobacter_baumannii_AYE_chr.trna23-TrpCCA (1358618-1358691) Trp (CCA) 74 bp Sc: 62.59
AGAGGATTGGTGTAATGGTAGCATGACGGTCTCCAAAACCGTTCGTCAAGGTTCGAGTCC
TTGATCCTCTGCCA
>Acinetobacter_baumannii_AYE_chr.trna25-TrpCCA (1358869-1358942) Trp (CCA) 74 bp Sc: 62.59
AGAGGATTGGTGTAATGGTAGCATGACGGTCTCCAAAACCGTTCGTCAAGGTTCGAGTCC

TTGATCCTCTGCCA
>Acinetobacter_baumannii_AYE_chr.trna45-TrpCCA (3551839-3551764) Trp (CCA) 76 bp Sc: 81.08
AGGTCAGTAGTTCAATTGGTAGAGCGTCGGTCTCCAAAACCGAATGTTGGGGGTTCGAGT
CCCTCCTGACCTGCCA
>Acinetobacter_baumannii_AYE_chr.trna42-TyrGTA (3553538-3553455) Tyr (GTA) 84 bp Sc: 62.89
GGTGAGATTCCCGAGCGGCCAAAGGGGGCAGACTGTAACTCTGTTGCGAAAGCTTCGAAG
GTTCGAATCCTTCTCTCACCACCA
>Acinetobacter_baumannii_AYE_chr.trna21-ValGAC (1127257-1127333) Val (GAC) 77 bp Sc: 80.80
AGGTGTATAGCTCAGTTGGTTAGAGCGCTACGTTGACATCGTAGAGGTCTCCAGTTCGAG
TCTGGATATACCTACCA
>Acinetobacter_baumannii_AYE_chr.trna14-ValTAC (696887-696962) Val (TAC) 76 bp Sc: 97.11
GGGCGCTTAGCTCAGTTGGTAGAGCGTCTGCCTTACAAGCAGAATGTCGGCGGTTCGATC
CCGTCAGCGCCCACCA
>Acinetobacter_baumannii_AYE_chr.trna17-ValTAC (697209-697284) Val (TAC) 76 bp Sc: 97.11
GGGCGCTTAGCTCAGTTGGTAGAGCGTCTGCCTTACAAGCAGAATGTCGGCGGTTCGATC
CCGTCAGCGCCCACCA
>Acinetobacter_baumannii_AYE_chr.trna19-ValTAC (697467-697542) Val (TAC) 76 bp Sc: 97.11
GGGCGCTTAGCTCAGTTGGTAGAGCGTCTGCCTTACAAGCAGAATGTCGGCGGTTCGATC
CCGTCAGCGCCCACCA
>Acinetobacter_baumannii_SDF_NC_010400.trna59-AlaGGC (260374-260299) Ala (GGC) 76 bp Sc: 83.49
GGGGTCATAGCTCAGTTGGTAGAGCGCTACAATGGCATTGTAGAGGTCAGCGGTTCGATC
CCGCTTGGCTCCACCA
>Acinetobacter_baumannii_SDF_NC_010400.trna11-AlaTGC (452148-452223) Ala (TGC) 76 bp Sc: 89.91
GGGGACTTAGCTTAGTTGGTAGAGCGCCTGCTTTGCACGCAGGAGGTCAGGAGTTCGACT
CTCCTAGTCTCCACCA
>Acinetobacter_baumannii_SDF_NC_010400.trna2-AlaTGC (23290-23365) Ala (TGC) 76 bp Sc: 89.91
GGGGACTTAGCTTAGTTGGTAGAGCGCCTGCTTTGCACGCAGGAGGTCAGGAGTTCGACT
CTCCTAGTCTCCACCA
>Acinetobacter_baumannii_SDF_NC_010400.trna39-AlaTGC (2647651-2647576) Ala (TGC) 76 bp Sc: 89.91
GGGGACTTAGCTTAGTTGGTAGAGCGCCTGCTTTGCACGCAGGAGGTCAGGAGTTCGACT
CTCCTAGTCTCCACCA
>Acinetobacter_baumannii_SDF_NC_010400.trna4-AlaTGC (179597-179672) Ala (TGC) 76 bp Sc: 89.91
GGGGACTTAGCTTAGTTGGTAGAGCGCCTGCTTTGCACGCAGGAGGTCAGGAGTTCGACT
CTCCTAGTCTCCACCA
>Acinetobacter_baumannii_SDF_NC_010400.trna41-AlaTGC (2612405-2612330) Ala (TGC) 76 bp Sc: 89.91
GGGGACTTAGCTTAGTTGGTAGAGCGCCTGCTTTGCACGCAGGAGGTCAGGAGTTCGACT
CTCCTAGTCTCCACCA
>Acinetobacter_baumannii_SDF_NC_010400.trna8-ArgACG (425622-425698) Arg (ACG) 77 bp Sc: 83.63
GCGCTCATAGCTCAGCTGGATAGAGCACTTGGCTACGAACTAAGGGGTCGGGAGTTCGAA
TCTCTCTGAGCGCACCA
>Acinetobacter_baumannii_SDF_NC_010400.trna9-ArgACG (425738-425814) Arg (ACG) 77 bp Sc: 83.63
GCGCTCATAGCTCAGCTGGATAGAGCACTTGGCTACGAACTAAGGGGTCGGGAGTTCGAA
TCTCTCTGAGCGCACCA
>Acinetobacter_baumannii_SDF_NC_010400.trna50-ArgCCG (1359894-1359818) Arg (CCG) 77 bp Sc: 81.26
GCGCTCGTAGCTCAGTTGGATAGAGTACAGGTTTCCGAAGCCTGGGGTCGTGGGTTCGAT
CCCCGCCGAGCGCACCA
>Acinetobacter_baumannii_SDF_NC_010400.trna58-ArgCCT (655191-655116) Arg (CCT) 76 bp Sc: 74.51
GCGCCCTTAGCTTAACTGGATAGAGCAGTTGCCTCCTAAGCGACCGACGTGGGTTCGAGT
CCCGCAGGGCGCGCCA
>Acinetobacter_baumannii_SDF_NC_010400.trna43-ArgTCT (2573769-2573693) Arg (TCT) 77 bp Sc: 98.82
GCGCCTGTAGCTCAGTTGGATAGAGCATCCGCCTTCTAAGCGGATGGTCACAGGTTCGAA
TCCTGTCAGGCGCGCCA
>Acinetobacter_baumannii_SDF_NC_010400.trna52-AsnGTT (1092322-1092247) Asn (GTT) 76 bp Sc: 83.01
TCTCCAATAGCTCAGTCGGTAGAGCGACGGACTGTTAATCCGCAGGTCCCTGGTTCGAGC
CCAGGTTGGAGAGCCA
>Acinetobacter_baumannii_SDF_NC_010400.trna53-AsnGTT (1092238-1092163) Asn (GTT) 76 bp Sc: 83.03
TCTCCCATAGCTCAGTCGGTAGAGCGACGGACTGTTAATCCGCAGGTCCCTGGTTCGAGC
CCAGGTGGGAGAGCCA
>Acinetobacter_baumannii_SDF_NC_010400.trna54-AsnGTT (888728-888653) Asn (GTT) 76 bp Sc: 83.03
TCTCCCATAGCTCAGTCGGTAGAGCGACGGACTGTTAATCCGCAGGTCCCTGGTTCGAGC
CCAGGTGGGAGAGCCA
>Acinetobacter_baumannii_SDF_NC_010400.trna14-AspGTC (602326-602402) Asp (GTC) 77 bp Sc: 94.40
GCAGCGGTAGTTCAGTTGGTTAGAATACCGGCCTGTCACGCCGGGGGTCGCGGGTTCGAG
CCCCGTCCGCTGCGCCA
>Acinetobacter_baumannii_SDF_NC_010400.trna55-CysGCA (746785-746712) Cys (GCA) 74 bp Sc: 59.57
GGCGGGGTGGCAGAGTGGTCATGCAGCGGACTGCAACTCCGTGGACGCCGGTTCGATTCC
GACCTCCGCCTCCA

>Acinetobacter_baumannii_SDF_NC_010400.trna24-GlnTTG (2409753-2409827) Gln (TTG) 75 bp Sc: 68.77
AGGGGCGTCGCCAAGTGGTAAGGCAGCGGGTTTTGATCCCGCCATCCGTTGGTTCGAATC
CAGCCGCCCCTGCCA
>Acinetobacter_baumannii_SDF_NC_010400.trna25-GlnTTG (2409919-2409993) Gln (TTG) 75 bp Sc: 68.77
AGGGGCGTCGCCAAGTGGTAAGGCAGCGGGTTTTGATCCCGCCATCCGTTGGTTCGAATC
CAGCCGCCCCTGCCA
>Acinetobacter_baumannii_SDF_NC_010400.trna51-GluTTC (1271471-1271396) Glu (TTC) 76 bp Sc: 56.39
GTCCCTATCGTCTAGAGGCCTAGGACATCGCCCTTTCACGGCGGTAACCGGGGTTCGAAT
CCCCGTAGGGACGCCA
>Acinetobacter_baumannii_SDF_NC_010400.trna60-GluTTC (260273-260198) Glu (TTC) 76 bp Sc: 56.39
GTCCCTATCGTCTAGAGGCCTAGGACATCGCCCTTTCACGGCGGTAACCGGGGTTCGAAT
CCCCGTAGGGACGCCA
>Acinetobacter_baumannii_SDF_NC_010400.trna61-GluTTC (257510-257435) Glu (TTC) 76 bp Sc: 56.39
GTCCCTATCGTCTAGAGGCCTAGGACATCGCCCTTTCACGGCGGTAACCGGGGTTCGAAT
CCCCGTAGGGACGCCA
>Acinetobacter_baumannii_SDF_NC_010400.trna62-GluTTC (257406-257331) Glu (TTC) 76 bp Sc: 56.39
GTCCCTATCGTCTAGAGGCCTAGGACATCGCCCTTTCACGGCGGTAACCGGGGTTCGAAT
CCCCGTAGGGACGCCA
>Acinetobacter_baumannii_SDF_NC_010400.trna23-GlyGCC (2157296-2157371) Gly (GCC) 76 bp Sc: 90.38
GCGGGAATAGCTCAGTTGGTAGAGCATAACCTTGCCAAGGTTGGGGTCGCGAGTTCGAGT
CTCGTTTCCCGCTCCA
>Acinetobacter_baumannii_SDF_NC_010400.trna63-GlyGCC (133032-132957) Gly (GCC) 76 bp Sc: 90.38
GCGGGAATAGCTCAGTTGGTAGAGCATAACCTTGCCAAGGTTGGGGTCGCGAGTTCGAGT
CTCGTTTCCCGCTCCA
>Acinetobacter_baumannii_SDF_NC_010400.trna64-GlyGCC (132911-132836) Gly (GCC) 76 bp Sc: 90.38
GCGGGAATAGCTCAGTTGGTAGAGCATAACCTTGCCAAGGTTGGGGTCGCGAGTTCGAGT
CTCGTTTCCCGCTCCA
>Acinetobacter_baumannii_SDF_NC_010400.trna32-GlyTCC (3005698-3005623) Gly (TCC) 76 bp Sc: 87.92
GCGGGAGTAGCTCAGTTGGTAGAGCGGCAGCCTTCCAAGCTGCATGTCGCGAGTTCGATC
CTCGTCTTCCGCTCCA
>Acinetobacter_baumannii_SDF_NC_010400.trna44-HisGTG (2573659-2573584) His (GTG) 76 bp Sc: 81.86
GTGGATGTAGCTCAGTTGGTAGAGCCCTGGATTGTGATTCCAGTTGTCGCGGGTTCGAAT
CCCGTCATTCACCCCA
>Acinetobacter_baumannii_SDF_NC_010400.trna1-IleGAT (23158-23234) Ile (GAT) 77 bp Sc: 98.53
GGGTCTGTAGCTCAGTTGGTTAGAGCACACGCTTGATAAGCGTGGGGTCACAAGTTCAAG
TCTTGTCAGACCCACCA
>Acinetobacter_baumannii_SDF_NC_010400.trna10-IleGAT (452016-452092) Ile (GAT) 77 bp Sc: 98.53
GGGTCTGTAGCTCAGTTGGTTAGAGCACACGCTTGATAAGCGTGGGGTCACAAGTTCAAG
TCTTGTCAGACCCACCA
>Acinetobacter_baumannii_SDF_NC_010400.trna3-IleGAT (179465-179541) Ile (GAT) 77 bp Sc: 98.53
GGGTCTGTAGCTCAGTTGGTTAGAGCACACGCTTGATAAGCGTGGGGTCACAAGTTCAAG
TCTTGTCAGACCCACCA
>Acinetobacter_baumannii_SDF_NC_010400.trna38-IleGAT (2647783-2647707) Ile (GAT) 77 bp Sc: 98.53
GGGTCTGTAGCTCAGTTGGTTAGAGCACACGCTTGATAAGCGTGGGGTCACAAGTTCAAG
TCTTGTCAGACCCACCA
>Acinetobacter_baumannii_SDF_NC_010400.trna40-IleGAT (2612537-2612461) Ile (GAT) 77 bp Sc: 98.53
GGGTCTGTAGCTCAGTTGGTTAGAGCACACGCTTGATAAGCGTGGGGTCACAAGTTCAAG
TCTTGTCAGACCCACCA
>Acinetobacter_baumannii_SDF_NC_010400.trna12-LeuCAA (478657-478743) Leu (CAA) 87 bp Sc: 71.58
CCCGAGGTGGTGAAATTGGTAGACGCGGCGGACTCAAAATCCGCTGTCAGAGATGACGTG
TCGGTTCGAGTCCGACCCTCGGGACCA
>Acinetobacter_baumannii_SDF_NC_010400.trna35-LeuGAG (2946230-2946146) Leu (GAG) 85 bp Sc: 59.44
GCGGTGGTGGTGGAATTGGTAGACACGCTACCTTGAGGTGGTAGTGCTTTCGGGCGTGGG
GGTTCAAGTCCCCCCTTCCGCACCA
>Acinetobacter_baumannii_SDF_NC_010400.trna56-LeuTAA (746700-746615) Leu (TAA) 86 bp Sc: 70.97
GCCCGGGTGGTGAAATAGGTAGACACAGGGGATTTAAAATCCCCCGCCCTCAAAGCGTGC
CGGTTCGAGTCCGGCCCCGGGCACCA
>Acinetobacter_baumannii_SDF_NC_010400.trna57-LeuTAA (744555-744470) Leu (TAA) 86 bp Sc: 70.97
GCCCGGGTGGTGAAATAGGTAGACACAGGGGATTTAAAATCCCCCGCCCTCAAAGCGTGC
CGGTTCGAGTCCGGCCCCGGGCACCA
>Acinetobacter_baumannii_SDF_NC_010400.trna17-LeuTAG (1195182-1195266) Leu (TAG) 85 bp Sc: 77.55
GGGGGCGTGGCGAAATTGGTAGACGCACTGGATTTAGGTTCCAGCGCCGCAAGGTGTAAG
AGTTCGAGTCTCTTCGCCCCCACCA
>Acinetobacter_baumannii_SDF_NC_010400.trna19-LeuTAG (1195422-1195506) Leu (TAG) 85 bp Sc: 77.55
GGGGGCGTGGCGAAATTGGTAGACGCACTGGATTTAGGTTCCAGCGCCGCAAGGTGTAAG
AGTTCGAGTCTCTTCGCCCCCACCA
>Acinetobacter_baumannii_SDF_NC_010400.trna26-LysTTT (2868370-2868445) Lys (TTT) 76 bp Sc: 92.37

GGGTCGTTAGCTCAGTTGGTAGAGCAGCGGACTTTTAATCCGTTGGTCCCGCGTTCGAGT
CGCGGACGACCCACCA
>Acinetobacter_baumannii_SDF_NC_010400.trna27-LysTTT (2868468-2868543) Lys (TTT) 76 bp Sc: 92.37
GGGTCGTTAGCTCAGTTGGTAGAGCAGCGGACTTTTAATCCGTTGGTCCCGCGTTCGAGT
CGCGGACGACCCACCA
>Acinetobacter_baumannii_SDF_NC_010400.trna36-MetCAT (2946103-2946027) Met (CAT) 77 bp Sc: 82.06
TGCGGGATGGAGCAGTCTGGTAGCTCGTCGGGCTCATAACCCGAAGGTCGTTGGTTCAAA
TCCAGCTCCCGCTACCA
>Acinetobacter_baumannii_SDF_NC_010400.trna37-MetCAT (2945937-2945861) Met (CAT) 77 bp Sc: 82.06
TGCGGGATGGAGCAGTCTGGTAGCTCGTCGGGCTCATAACCCGAAGGTCGTTGGTTCAAA
TCCAGCTCCCGCTACCA
>Acinetobacter_baumannii_SDF_NC_010400.trna49-MetCAT (2161475-2161399) Met (CAT) 77 bp Sc: 82.10
TGCGGGGTGGAGCAGTCTGGTAGCTCGTCGGGCTCATAACCCGAAGGTCGTTGGTTCAAA
TCCGACCCCCGCATCCA
>Acinetobacter_baumannii_SDF_NC_010400.trna22-MetCAT (1976784-1976860) Met (CAT) 77 bp Sc: 91.80
GGGCCTATAGCTCAGTCGGTTAGAGCAGCGGACTCATAATCCGTTGGTCCACAGTTCGAG
TCTGTGTGGGCCCACCA
>Acinetobacter_baumannii_SDF_NC_010400.trna47-MetCAT (2376531-2376455) Met (CAT) 77 bp Sc: 94.07
GGCTATGTAGCTCAGTTGGTTAGAGCACCGCACTCATAATGCGGGGGTCACAAGTTCAAG
TCTCGTCATAGCCACCA
>Acinetobacter_baumannii_SDF_NC_010400.trna48-MetCAT (2373845-2373769) Met (CAT) 77 bp Sc: 94.07
GGCTATGTAGCTCAGTTGGTTAGAGCACCGCACTCATAATGCGGGGGTCACAAGTTCAAG
TCTCGTCATAGCCACCA
>Acinetobacter_baumannii_SDF_NC_010400.trna5-PheGAA (224675-224750) Phe (GAA) 76 bp Sc: 86.19
GGCGTGATAGCTCAGTTGGTAGAGCAACGGATTGAAAATCCGTGTGTCCCCAGTTCGATC
CTGGGTCTCGCCACCA
>Acinetobacter_baumannii_SDF_NC_010400.trna6-PheGAA (224878-224953) Phe (GAA) 76 bp Sc: 86.19
GGCGTGATAGCTCAGTTGGTAGAGCAACGGATTGAAAATCCGTGTGTCCCCAGTTCGATC
CTGGGTCTCGCCACCA
>Acinetobacter_baumannii_SDF_NC_010400.trna42-ProTGG (2573902-2573826) Pro (TGG) 77 bp Sc: 85.67
CGGAGCATAGCACAGCCTGGTAGTGCACCTGGTTTGGGACCAGGGGGTCGTAGGTTCGAA
TCCTACTGCTCCGACCA
>Acinetobacter_baumannii_SDF_NC_010400.trna45-ProTGG (2573579-2573503) Pro (TGG) 77 bp Sc: 85.67
CGGAGCATAGCACAGCCTGGTAGTGCACCTGGTTTGGGACCAGGGGGTCGTAGGTTCGAA
TCCTACTGCTCCGACCA
>Acinetobacter_baumannii_SDF_NC_010400.trna46-ProTGG (2531166-2531090) Pro (TGG) 77 bp Sc: 85.67
CGGAGCATAGCACAGCCTGGTAGTGCACCTGGTTTGGGACCAGGGGGTCGTAGGTTCGAA
TCCTACTGCTCCGACCA
>Acinetobacter_baumannii_SDF_NC_010400.trna7-SerGCT (425521-425611) Ser (GCT) 91 bp Sc: 67.25
GGTGAGGTGGATGAGTGGCTGAAATCACTTCCCTGCTAAGGAAGCATACCTATTACTGGT
ATCGAGGGTTCGAATCCCTCTCTCACCGCCA
>Acinetobacter_baumannii_SDF_NC_010400.trna28-SerGGA (3095851-3095940) Ser (GGA) 90 bp Sc: 64.72
GGTGAGGTGTCCGAGTGGCTGAAGGAGCACGCCTGGAAAGTGTGTATACGTTTACGCGTA
TCGAGGGTTCGAATCCCTCTCTCACCGCCA
>Acinetobacter_baumannii_SDF_NC_010400.trna21-SerTGA (1280438-1280527) Ser (TGA) 90 bp Sc: 65.75
GGAAGCGTGGCAGAGCGGTTTAATGCACCGGTCTTGAAAACCGACGAGGGTGTGAGTCCT
CCGTGAGTTCGAATCTCACCGCTTCCGCCA
>Acinetobacter_baumannii_SDF_NC_010400.trna33-ThrGGT (3005609-3005535) Thr (GGT) 75 bp Sc: 83.04
GCTCTTATAGCTCAGTGGTAGAGCACTCCCTTGGTAAGGGAGAGGTCTCGAGTTCAAATC
TCGATAAGAGCTCCA
>Acinetobacter_baumannii_SDF_NC_010400.trna29-ThrTGT (3111646-3111721) Thr (TGT) 76 bp Sc: 94.17
GCCGGCATAGCTCAGTTGGTAGAGCAACTGACTTGTAATCAGTAGGTCCACAGTTCGAAT
CCGTGTGCCGGCACCA
>Acinetobacter_baumannii_SDF_NC_010400.trna30-ThrTGT (3005951-3005876) Thr (TGT) 76 bp Sc: 94.17
GCCGGCATAGCTCAGTTGGTAGAGCAACTGACTTGTAATCAGTAGGTCCACAGTTCGAAT
CCGTGTGCCGGCACCA
>Acinetobacter_baumannii_SDF_NC_010400.trna18-TrpCCA (1195321-1195394) Trp (CCA) 74 bp Sc: 62.59
AGAGGATTGGTGTAATGGTAGCATGACGGTCTCCAAAACCGTTCGTCAAGGTTCGAGTCC
TTGATCCTCTGCCA
>Acinetobacter_baumannii_SDF_NC_010400.trna20-TrpCCA (1195572-1195645) Trp (CCA) 74 bp Sc: 62.59
AGAGGATTGGTGTAATGGTAGCATGACGGTCTCCAAAACCGTTCGTCAAGGTTCGAGTCC
TTGATCCTCTGCCA
>Acinetobacter_baumannii_SDF_NC_010400.trna34-TrpCCA (3004122-3004047) Trp (CCA) 76 bp Sc: 81.08
AGGTCAGTAGTTCAATTGGTAGAGCGTCGGTCTCCAAAACCGAATGTTGGGGGTTCGAGT
CCCTCCTGACCTGCCA
>Acinetobacter_baumannii_SDF_NC_010400.trna31-TyrGTA (3005819-3005736) Tyr (GTA) 84 bp Sc: 62.89
GGTGAGATTCCCGAGCGGCCAAAGGGGGCAGACTGTAACTCTGTTGCGAAAGCTTCGAAG

GTTCGAATCCTTCTCTCACCACCA
>Acinetobacter_baumannii_SDF_NC_010400.trna16-ValGAC (1058319-1058395) Val (GAC) 77 bp Sc: 82.28
AGGCGTATAGCTCAGTTGGTTAGAGCGCTACGTTGACATCGTAGAGGTCTCCAGTTCGAG
TCTGGATATGCCTACCA
>Acinetobacter_baumannii_SDF_NC_010400.trna13-ValTAC (602211-602286) Val (TAC) 76 bp Sc: 97.11
GGGCGCTTAGCTCAGTTGGTAGAGCGTCTGCCTTACAAGCAGAATGTCGGCGGTTCGATC
CCGTCAGCGCCCACCA
>Acinetobacter_baumannii_SDF_NC_010400.trna15-ValTAC (602469-602544) Val (TAC) 76 bp Sc: 97.11
GGGCGCTTAGCTCAGTTGGTAGAGCGTCTGCCTTACAAGCAGAATGTCGGCGGTTCGATC
CCGTCAGCGCCCACCA
>Actinobacillus_pleuropneumoniae_serovar_3_JL03_chr.trna12-AlaGGC (1417314-1417389) Ala (GGC) 76 bp Sc: 85.98
GGGGATATAGCTCAGTTGGGAGAGCGCTTGAATGGCATTCAAGAGGTCGTCGGTTCGATC
CCGATTATCTCCACCA
>Actinobacillus_pleuropneumoniae_serovar_3_JL03_chr.trna2-AlaTGC (69678-69753) Ala (TGC) 76 bp Sc: 93.20
GGGGATATAGCTCAGCTGGGAGAGCGCCTGCCTTGCACGCAGGAGGTCAGCGGTTCGATC
CCGCTTATCTCCACCA
>Actinobacillus_pleuropneumoniae_serovar_3_JL03_chr.trna20-AlaTGC (2028848-2028923) Ala (TGC) 76 bp Sc: 93.20
GGGGATATAGCTCAGCTGGGAGAGCGCCTGCCTTGCACGCAGGAGGTCAGCGGTTCGATC
CCGCTTATCTCCACCA
>Actinobacillus_pleuropneumoniae_serovar_3_JL03_chr.trna27-AlaTGC (1834087-1834012) Ala (TGC) 76 bp Sc: 93.20
GGGGATATAGCTCAGCTGGGAGAGCGCCTGCCTTGCACGCAGGAGGTCAGCGGTTCGATC
CCGCTTATCTCCACCA
>Actinobacillus_pleuropneumoniae_serovar_3_JL03_chr.trna39-AlaTGC (1531068-1530993) Ala (TGC) 76 bp Sc: 93.20
GGGGATATAGCTCAGCTGGGAGAGCGCCTGCCTTGCACGCAGGAGGTCAGCGGTTCGATC
CCGCTTATCTCCACCA
>Actinobacillus_pleuropneumoniae_serovar_3_JL03_chr.trna41-ArgACG (1521096-1521020) Arg (ACG) 77 bp Sc: 86.34
GCACCCGTAGCTCAGCTGGATAGAGTACTCGGCTACGAACCGAGCGGTCAGAGGTTCGAA
TCCTCTCGGGTGCGCCA
>Actinobacillus_pleuropneumoniae_serovar_3_JL03_chr.trna42-ArgACG (1520947-1520871) Arg (ACG) 77 bp Sc: 86.34
GCACCCGTAGCTCAGCTGGATAGAGTACTCGGCTACGAACCGAGCGGTCAGAGGTTCGAA
TCCTCTCGGGTGCGCCA
>Actinobacillus_pleuropneumoniae_serovar_3_JL03_chr.trna28-ArgCCG (1724602-1724526) Arg (CCG) 77 bp Sc: 85.75
GCGTTCGTAGCTCAGTTGGATAGAGCGTTGGCCTCCGGAGCCAAAGGTCGCAAGTTCGAA
TCTTGTCGAGCGCGCCA
>Actinobacillus_pleuropneumoniae_serovar_3_JL03_chr.trna60-ArgTCT (1004801-1004725) Arg (TCT) 77 bp Sc: 91.33
GCGCCCTTAGCTCAGCTGGATAGAGCAACGCCCTTCTAAGGCGTGGGTCAAAGGTTCGAA
TCCTTTAGGGCGTGCCA
>Actinobacillus_pleuropneumoniae_serovar_3_JL03_chr.trna16-AsnGTT (1938754-1938829) Asn (GTT) 76 bp Sc: 82.59
TCCTCCTTAGTTCAGTCGGTAGAACGGTGGACTGTTAATCCATATGTCGCAGGTTCGAGT
CCCGCAGGAGGAGCCA
>Actinobacillus_pleuropneumoniae_serovar_3_JL03_chr.trna18-AsnGTT (1939043-1939118) Asn (GTT) 76 bp Sc: 82.59
TCCTCCTTAGTTCAGTCGGTAGAACGGTGGACTGTTAATCCATATGTCGCAGGTTCGAGT
CCCGCAGGAGGAGCCA
>Actinobacillus_pleuropneumoniae_serovar_3_JL03_chr.trna6-AsnGTT (350988-351063) Asn (GTT) 76 bp Sc: 82.59
TCCTCCTTAGTTCAGTCGGTAGAACGGTGGACTGTTAATCCATATGTCGCAGGTTCGAGT
CCCGCAGGAGGAGCCA
>Actinobacillus_pleuropneumoniae_serovar_3_JL03_chr.trna49-AspGTC (1422132-1422056) Asp (GTC) 77 bp Sc: 89.09
GGAGTGGTAGTTCAGCTGGTTAGAATACCTGCCTGTCACGCAGGGGGTCGCGGGTTCGAG
TCCCGTCCATTCCGCCA
>Actinobacillus_pleuropneumoniae_serovar_3_JL03_chr.trna32-AspGTC (1718815-1718739) Asp (GTC) 77 bp Sc: 91.98
GGAGCGGTAGTTCAGCTGGTTAGAATACCTGCCTGTCACGCAGGGGGTCGCGGGTTCGAG
TCCCGTCCGTTCCGCCA
>Actinobacillus_pleuropneumoniae_serovar_3_JL03_chr.trna50-AspGTC (1422014-1421938) Asp (GTC) 77 bp Sc: 91.98
GGAGCGGTAGTTCAGCTGGTTAGAATACCTGCCTGTCACGCAGGGGGTCGCGGGTTCGAG
TCCCGTCCGTTCCGCCA
>Actinobacillus_pleuropneumoniae_serovar_3_JL03_chr.trna62-CysGCA (847105-847032) Cys (GCA) 74 bp Sc: 53.55
GGCGTGTTAGCAAAGCGGTTATGCACTGGATTGCAAATCCATGTAGATCGGTTCGACTCC
GGTACACGCCTCCA
>Actinobacillus_pleuropneumoniae_serovar_3_JL03_chr.trna57-GlnTTG (1280880-1280806) Gln (TTG) 75 bp Sc: 65.69
TGGGGTATCGCCAAGCGGTAAGGCACCGGGTTTTGATCTCGGCATCCCTAGGTTCGAATC
CTAGTACCCCAGCCA
>Actinobacillus_pleuropneumoniae_serovar_3_JL03_chr.trna58-GlnTTG (1280757-1280683) Gln (TTG) 75 bp Sc: 65.69
TGGGGTATCGCCAAGCGGTAAGGCACCGGGTTTTGATCTCGGCATCCCTAGGTTCGAATC
CTAGTACCCCAGCCA
>Actinobacillus_pleuropneumoniae_serovar_3_JL03_chr.trna5-GluTTC (283371-283447) Glu (TTC) 77 bp Sc: 32.17
GTCCATCGTCTAGCTCAGGCCTGGGACATCGCCCTTTCACGGCGGTAACCGGGGTTCGAA
TCCCCGTGGGGACGCCA

>Actinobacillus_pleuropneumoniae_serovar_3_JL03_chr.trna31-GluTTC (1722422-1722347) Glu (TTC) 76 bp Sc: 56.27
GTCCCCATCGTCTAGAGGCCTAGGACATCGCCCTTTCACGGCGGTAACCGGGGTTCGAAT
CCCCGTGGGGACGCCA
>Actinobacillus_pleuropneumoniae_serovar_3_JL03_chr.trna43-GlyGCC (1492267-1492192) Gly (GCC) 76 bp Sc: 91.37
GCGGGAATAGCTCAGTTGGTAGAGCACGACCTTGCCAAGGTCGGGGTCGCGAGTTCGAGC
CTCGTTTCCCGCTCCA
>Actinobacillus_pleuropneumoniae_serovar_3_JL03_chr.trna45-GlyGCC (1492067-1491992) Gly (GCC) 76 bp Sc: 91.37
GCGGGAATAGCTCAGTTGGTAGAGCACGACCTTGCCAAGGTCGGGGTCGCGAGTTCGAGC
CTCGTTTCCCGCTCCA
>Actinobacillus_pleuropneumoniae_serovar_3_JL03_chr.trna48-GlyGCC (1491717-1491642) Gly (GCC) 76 bp Sc: 91.37
GCGGGAATAGCTCAGTTGGTAGAGCACGACCTTGCCAAGGTCGGGGTCGCGAGTTCGAAC
CTCGTTTCCCGCTCCA
>Actinobacillus_pleuropneumoniae_serovar_3_JL03_chr.trna61-GlyGCC (847198-847123) Gly (GCC) 76 bp Sc: 91.37
GCGGGAATAGCTCAGTTGGTAGAGCACGACCTTGCCAAGGTCGGGGTCGCGAGTTCGAGC
CTCGTTTCCCGCTCCA
>Actinobacillus_pleuropneumoniae_serovar_3_JL03_chr.trna36-GlyTCC (1690474-1690400) Gly (TCC) 75 bp Sc: 63.54
GCGGGCATCGTATAATGGCTATTACCTTAGCCTTCCAAGCTAATGATGCGGGTTCGATTC
CCGCTGCCCGCTCCA
>Actinobacillus_pleuropneumoniae_serovar_3_JL03_chr.trna29-HisGTG (1724495-1724420) His (GTG) 76 bp Sc: 83.36
GTGGCTATAGCTCAGTTGGTAGAGCCCCGGATTGTGATTCCGGTTGTCGTGAGTTCGAGT
CTCATTAGCCACCCCA
>Actinobacillus_pleuropneumoniae_serovar_3_JL03_chr.trna38-IleGAT (1531196-1531120) Ile (GAT) 77 bp Sc: 89.12
GGGTCTGTAGCTCAGGTGGTTAGAGCGCACCCCTGATAAGGGTGAGGTCGGTGGTTCAAG
TCCAATCAGACCCACCA
>Actinobacillus_pleuropneumoniae_serovar_3_JL03_chr.trna1-IleGAT (69550-69626) Ile (GAT) 77 bp Sc: 94.77
GGGTCTGTAGCTCAGGTGGTTAGAGCGCACCCCTGATAAGGGTGAGGTCGGTGGTTCAAG
TCCACTCAGACCCACCA
>Actinobacillus_pleuropneumoniae_serovar_3_JL03_chr.trna19-IleGAT (2028720-2028796) Ile (GAT) 77 bp Sc: 94.77
GGGTCTGTAGCTCAGGTGGTTAGAGCGCACCCCTGATAAGGGTGAGGTCGGTGGTTCAAG
TCCACTCAGACCCACCA
>Actinobacillus_pleuropneumoniae_serovar_3_JL03_chr.trna26-IleGAT (1834215-1834139) Ile (GAT) 77 bp Sc: 94.77
GGGTCTGTAGCTCAGGTGGTTAGAGCGCACCCCTGATAAGGGTGAGGTCGGTGGTTCAAG
TCCACTCAGACCCACCA
>Actinobacillus_pleuropneumoniae_serovar_3_JL03_chr.trna4-IleGAT (283247-283323) Ile (GAT) 77 bp Sc: 94.77
GGGTCTGTAGCTCAGGTGGTTAGAGCGCACCCCTGATAAGGGTGAGGTCGGTGGTTCAAG
TCCACTCAGACCCACCA
>Actinobacillus_pleuropneumoniae_serovar_3_JL03_chr.trna11-LeuCAA (907849-907934) Leu (CAA) 86 bp Sc: 73.70
GCCAGTGTGGCGAAATCGGTAGACGCAGCGGATTCAAAATCCGCCGTTGAATAAACGTGT
CGGTTCGAGTCCGACCACTGGCACCA
>Actinobacillus_pleuropneumoniae_serovar_3_JL03_chr.trna63-LeuGAG (813142-813057) Leu (GAG) 86 bp Sc: 56.99
GCTCTGGTGGTGGAATTGGTAGACACGCTATCTTGAGGGGGTAGTGTCCATAGGATGTGC
GAGTTCGAGTCTCGCCCAGAGCACCA
>Actinobacillus_pleuropneumoniae_serovar_3_JL03_chr.trna44-LeuTAA (1492180-1492094) Leu (TAA) 87 bp Sc: 73.83
GCCCGAGTGGCGGAATCGGTAGACGCAAGGGATTTAAAATCCCTCGCCTTTCGGGGCGTG
CCAGTTCAAGTCTGGCCTCGGGCACCA
>Actinobacillus_pleuropneumoniae_serovar_3_JL03_chr.trna46-LeuTAA (1491969-1491883) Leu (TAA) 87 bp Sc: 73.83
GCCCGAGTGGCGGAATCGGTAGACGCAAGGGATTTAAAATCCCTCGCCTTTCGGGGCGTG
CCAGTTCAAGTCTGGCCTCGGGCACCA
>Actinobacillus_pleuropneumoniae_serovar_3_JL03_chr.trna47-LeuTAA (1491850-1491764) Leu (TAA) 87 bp Sc: 73.83
GCCCGAGTGGCGGAATCGGTAGACGCAAGGGATTTAAAATCCCTCGCCTTTCGGGGCGTG
CCAGTTCAAGTCTGGCCTCGGGCACCA
>Actinobacillus_pleuropneumoniae_serovar_3_JL03_chr.trna56-LeuTAG (1280990-1280906) Leu (TAG) 85 bp Sc: 72.50
GCGGGAATGGCGAAATTGGTAGACGCACCAGATTTAGGTTCTGGCGCCGTGAGGTGTGTG
GGTTCAAGTCCCTCTTCCCGCACCA
>Actinobacillus_pleuropneumoniae_serovar_3_JL03_chr.trna23-LysTTT (2003452-2003377) Lys (TTT) 76 bp Sc: 83.10
GGGTCGTTAGCTCAGTCGGTAGAGCAGCGGTCTTTTAATCCGTTGGTCGAAGGTTAGAAT
CCTTCACGACCCACCA
>Actinobacillus_pleuropneumoniae_serovar_3_JL03_chr.trna24-LysTTT (2003353-2003278) Lys (TTT) 76 bp Sc: 97.43
GGGTCGTTAGCTCAGTCGGTAGAGCAGCGGACTTTTAATCCGTTGGTCGAAGGTTCGAAT
CCTTCACGACCCACCA
>Actinobacillus_pleuropneumoniae_serovar_3_JL03_chr.trna25-LysTTT (2003243-2003168) Lys (TTT) 76 bp Sc: 97.43
GGGTCGTTAGCTCAGTCGGTAGAGCAGCGGACTTTTAATCCGTTGGTCGAAGGTTCGAAT
CCTTCACGACCCACCA
>Actinobacillus_pleuropneumoniae_serovar_3_JL03_chr.trna10-MetCAT (905935-906011) Met (CAT) 77 bp Sc: 83.87
CGCGGGGTGGAGCAGCTTGGTAGCTCGTCGGGCTCATAACCCGAAGGTCGTTGGTTCAAA
TCCGGCCCCCGCAACCA
>Actinobacillus_pleuropneumoniae_serovar_3_JL03_chr.trna8-MetCAT (681139-681215) Met (CAT) 77 bp Sc: 83.87

CGCGGGGTGGAGCAGCTTGGTAGCTCGTCGGGCTCATAACCCGAAGGTCGTTGGTTCAAA
TCCGGCCCCCGCAACCA
>Actinobacillus_pleuropneumoniae_serovar_3_JL03_chr.trna7-MetCAT (495149-495225) Met (CAT) 77 bp Sc: 86.41
CCCCCCTTAGCTCAGTCGGTTAGAGCAGGCGACTCATAATCGCTTGGTCACTGGTTCAAG
TCCGGTAGGGGGGACCA
>Actinobacillus_pleuropneumoniae_serovar_3_JL03_chr.trna55-MetCAT (1281070-1280994) Met (CAT) 77 bp Sc: 91.65
GGCTACATAGCTCAGCTGGTTAGAGCACAACACTCATAATGTTGGGGTCGCAAGTTCGAA
TCTCGCTGTAGCCACCA
>Actinobacillus_pleuropneumoniae_serovar_3_JL03_chr.trna17-PheGAA (1938962-1939037) Phe (GAA) 76 bp Sc: 80.10
GCCTCGATAGCTCAGTCGGTAGAGCAGGGGATTGAAAATCCCCGTGTCGTTGGTTCGATT
CCGCCTCGAGGCACCA
>Actinobacillus_pleuropneumoniae_serovar_3_JL03_chr.trna15-PheGAA (1938673-1938748) Phe (GAA) 76 bp Sc: 85.75
GCCTCGATAGCTCAGTCGGTAGAGCAGGGGATTGAAAATCCCCGTGTCGGTGGTTCGATT
CCGCCTCGAGGCACCA
>Actinobacillus_pleuropneumoniae_serovar_3_JL03_chr.trna30-ProTGG (1724391-1724315) Pro (TGG) 77 bp Sc: 90.87
CGGCGAGTAGCGCAGCTTGGTAGCGCAACTGGTTTGGGACCAGTGGGTCGTAGGTTCAAA
TCCTATCTCGCCGACCA
>Actinobacillus_pleuropneumoniae_serovar_3_JL03_chr.trna59-ProTGG (1004921-1004845) Pro (TGG) 77 bp Sc: 90.87
CGGCGAGTAGCGCAGCTTGGTAGCGCAACTGGTTTGGGACCAGTGGGTCGTAGGTTCAAA
TCCTATCTCGCCGACCA
>Actinobacillus_pleuropneumoniae_serovar_3_JL03_chr.trna14-SeC(p)TCA (1749469-1749559) SeC(p) (TCA) 91 bp Sc: 68.95
GGAAGCGAGTCATCTCCGGTGAGGTGGCAGGACTTCAAATCCTGTTGAGGACGCCAGCGT
TCTTGGGTGGGTTCGACTCCCATTCGCTTCC
>Actinobacillus_pleuropneumoniae_serovar_3_JL03_chr.trna40-SerGCT (1521204-1521110) Ser (GCT) 95 bp Sc: 74.07
GGTGAGATGGCCGAGCAGGCTGAAGGCGCTCCCCTGCTAAGGGAGTATAGGGTTCAAAAG
CTCTATCGAGGGTTCGAATCCCTCTCTCACCGCCA
>Actinobacillus_pleuropneumoniae_serovar_3_JL03_chr.trna3-SerGGA (256948-257037) Ser (GGA) 90 bp Sc: 69.67
GGTGAGATGTCCGAGTGGTTGAAGGAGCACGCCTGGAAAGCGTGTATATGCGAAAGTGTA
TCGGGGGTTCGAATCCCCCTCTCACCGCCA
>Actinobacillus_pleuropneumoniae_serovar_3_JL03_chr.trna21-SerTGA (2175149-2175238) Ser (TGA) 90 bp Sc: 71.65
GGAGGAATGGTCGAGTGGTTGAAGGCACCGGTCTTGAAAACCGGCGAGGGTTTACGCCCT
CCGTGAGTTCGAATCTCACTTCCTCCGCCA
>Actinobacillus_pleuropneumoniae_serovar_3_JL03_chr.trna22-SerTGA (2175322-2175411) Ser (TGA) 90 bp Sc: 71.65
GGAGGAATGGTCGAGTGGTTGAAGGCACCGGTCTTGAAAACCGGCGAGGGTTTACGCCCT
CCGTGAGTTCGAATCTCACTTCCTCCGCCA
>Actinobacillus_pleuropneumoniae_serovar_3_JL03_chr.trna37-ThrGGT (1690396-1690321) Thr (GGT) 76 bp Sc: 96.44
GCTGATATAGCTCAGTTGGTAGAGCGCACCCTTGGTAAGGGTGAGGTCGGCGGTTCAAAT
CCGCCTATCAGCACCA
>Actinobacillus_pleuropneumoniae_serovar_3_JL03_chr.trna13-ThrTGT (1661939-1662014) Thr (TGT) 76 bp Sc: 91.83
GCCGACTTAGCTCAGTAGGTAGAGCAACTGACTTGTAATCAGTAGGTCACCAGTTCGATT
CCGGTAGTCGGCACCA
>Actinobacillus_pleuropneumoniae_serovar_3_JL03_chr.trna34-ThrTGT (1690715-1690640) Thr (TGT) 76 bp Sc: 91.83
GCCGACTTAGCTCAGTAGGTAGAGCAACTGACTTGTAATCAGTAGGTCACCAGTTCGATT
CCGGTAGTCGGCACCA
>Actinobacillus_pleuropneumoniae_serovar_3_JL03_chr.trna33-TrpCCA (1718689-1718614) Trp (CCA) 76 bp Sc: 79.69
AGGGGCGTAGTTCAATTGGTAGAGCACCGGTCTCCAAAACCGGGTGTTGGGAGTTCGAGC
CTCTCCGCCCCTGCCA
>Actinobacillus_pleuropneumoniae_serovar_3_JL03_chr.trna35-TyrGTA (1690603-1690519) Tyr (GTA) 85 bp Sc: 68.41
GGAGGGATTCCCGAGCGGCCAAAGGGGGCAGACTGTAAATCTGTTGGCTCAGCCTTCGAA
GGTTCGAATCCTTCTCCCTCCACCA
>Actinobacillus_pleuropneumoniae_serovar_3_JL03_chr.trna9-ValGAC (835398-835474) Val (GAC) 77 bp Sc: 90.13
GCGACTATAGCTCAGTTGGTTAGAGCACCACCTTGACATGGTGGGGGTCACTGGTTCGAG
TCCAGCTAGTCGCACCA
>Actinobacillus_pleuropneumoniae_serovar_3_JL03_chr.trna51-ValTAC (1415621-1415546) Val (TAC) 76 bp Sc: 90.00
GGGCGATTAGCTCAGTTGGGAGAGCACCTCCCTTACAAGGAGGGGGTCACTGGTTCGAGA
CCGGTATCGCCCACCA
>Actinobacillus_pleuropneumoniae_serovar_3_JL03_chr.trna52-ValTAC (1415497-1415422) Val (TAC) 76 bp Sc: 90.00
GGGCGATTAGCTCAGTTGGGAGAGCACCTCCCTTACAAGGAGGGGGTCACTGGTTCGAGA
CCGGTATCGCCCACCA
>Actinobacillus_pleuropneumoniae_serovar_3_JL03_chr.trna53-ValTAC (1415371-1415296) Val (TAC) 76 bp Sc: 90.00
GGGCGATTAGCTCAGTTGGGAGAGCACCTCCCTTACAAGGAGGGGGTCACTGGTTCGAGA
CCGGTATCGCCCACCA
>Actinobacillus_pleuropneumoniae_serovar_3_JL03_chr.trna54-ValTAC (1415245-1415170) Val (TAC) 76 bp Sc: 90.00
GGGCGATTAGCTCAGTTGGGAGAGCACCTCCCTTACAAGGAGGGGGTCACTGGTTCGAGA
CCGGTATCGCCCACCA
>Actinobacillus_succinogenes_130Z_chr.trna49-AlaGGC (613607-613532) Ala (GGC) 76 bp Sc: 85.98

GGGGATATAGCTCAGTTGGGAGAGCGCTTGAATGGCATTCAAGAGGTCGTCGGTTCGATC
CCGATTATCTCCACCA
>Actinobacillus_succinogenes_130Z_chr.trna17-AlaTGC (1132316-1132391) Ala (TGC) 76 bp Sc: 93.20
GGGGATATAGCTCAGCTGGGAGAGCGCCTGCCTTGCACGCAGGAGGTCAGCGGTTCGATC
CCGCTTATCTCCACCA
>Actinobacillus_succinogenes_130Z_chr.trna52-AlaTGC (322736-322661) Ala (TGC) 76 bp Sc: 93.20
GGGGATATAGCTCAGCTGGGAGAGCGCCTGCCTTGCACGCAGGAGGTCAGCGGTTCGATC
CCGCTTATCTCCACCA
>Actinobacillus_succinogenes_130Z_chr.trna58-AlaTGC (37440-37365) Ala (TGC) 76 bp Sc: 93.20
GGGGATATAGCTCAGCTGGGAGAGCGCCTGCCTTGCACGCAGGAGGTCAGCGGTTCGATC
CCGCTTATCTCCACCA
>Actinobacillus_succinogenes_130Z_chr.trna9-ArgACG (742943-743019) Arg (ACG) 77 bp Sc: 85.94
GCACCCGTAGCTCAGCTGGATAGAGTACTCGGCTACGAACCGAGCGGTCAAAGGTTCGAA
TCCTTTCGGGTGCGCCA
>Actinobacillus_succinogenes_130Z_chr.trna8-ArgACG (742800-742876) Arg (ACG) 77 bp Sc: 86.34
GCACCCGTAGCTCAGCTGGATAGAGTACTCGGCTACGAACCGAGCGGTCAGAGGTTCGAA
TCCTCTCGGGTGCGCCA
>Actinobacillus_succinogenes_130Z_chr.trna13-ArgCCG (1130125-1130201) Arg (CCG) 77 bp Sc: 89.40
GCGCTCGTAGCTCAGTTGGATAGAGCGTTGGCCTCCGGAGCCAAAGGTCGCAAGTTCGAA
TCTTGTCGAGCGCGCCA
>Actinobacillus_succinogenes_130Z_chr.trna47-ArgTCT (821230-821154) Arg (TCT) 77 bp Sc: 91.33
GCGCCCTTAGCTCAGCTGGATAGAGCAACGCCCTTCTAAGGCGTGGGTCAAAGGTTCGAA
TCCTTTAGGGCGTGCCA
>Actinobacillus_succinogenes_130Z_chr.trna24-AsnGTT (1206949-1207024) Asn (GTT) 76 bp Sc: 82.59
TCCTCCTTAGTTCAGTCGGTAGAACGGTGGACTGTTAATCCATATGTCGCAGGTTCGAGT
CCCGCAGGAGGAGCCA
>Actinobacillus_succinogenes_130Z_chr.trna3-AsnGTT (596506-596581) Asn (GTT) 76 bp Sc: 82.59
TCCTCCTTAGTTCAGTCGGTAGAACGGTGGACTGTTAATCCATATGTCGCAGGTTCGAGT
CCCGCAGGAGGAGCCA
>Actinobacillus_succinogenes_130Z_chr.trna44-AspGTC (938374-938298) Asp (GTC) 77 bp Sc: 89.09
GGAGTGGTAGTTCAGCTGGTTAGAATACCTGCCTGTCACGCAGGGGGTCGCGGGTTCGAG
TCCCGTCCATTCCGCCA
>Actinobacillus_succinogenes_130Z_chr.trna45-AspGTC (938263-938187) Asp (GTC) 77 bp Sc: 89.09
GGAGTGGTAGTTCAGCTGGTTAGAATACCTGCCTGTCACGCAGGGGGTCGCGGGTTCGAG
TCCCGTCCATTCCGCCA
>Actinobacillus_succinogenes_130Z_chr.trna59-AspGTC (33678-33602) Asp (GTC) 77 bp Sc: 89.09
GGAGTGGTAGTTCAGCTGGTTAGAATACCTGCCTGTCACGCAGGGGGTCGCGGGTTCGAG
TCCCGTCCATTCCGCCA
>Actinobacillus_succinogenes_130Z_chr.trna32-CysGCA (2030564-2030637) Cys (GCA) 74 bp Sc: 52.06
GGCGTGTTAGCAAAGCGGTTATGCACTGGATTGCAAATCCATGTAGCTCGGTTCGACTCC
GGGACACGCCTCCA
>Actinobacillus_succinogenes_130Z_chr.trna23-GlnCTG (1202915-1202989) Gln (CTG) 75 bp Sc: 73.78
TGGGGTATCGCCAAGCGGTAAGGCACTGGATTCTGATTCCAGCATTCCTAGGTTCGAATC
CTAGTACCCCAGCCA
>Actinobacillus_succinogenes_130Z_chr.trna21-GlnTTG (1202681-1202755) Gln (TTG) 75 bp Sc: 68.96
TGGGGTATCGCCAAGCGGTAAGGCACTGGGTTTTGATCTCAGCATTCCTAGGTTCGAATC
CTAGTACCCCAGCCA
>Actinobacillus_succinogenes_130Z_chr.trna22-GlnTTG (1202798-1202872) Gln (TTG) 75 bp Sc: 68.96
TGGGGTATCGCCAAGCGGTAAGGCACTGGGTTTTGATCTCAGCATTCCTAGGTTCGAATC
CTAGTACCCCAGCCA
>Actinobacillus_succinogenes_130Z_chr.trna1-GluTTC (564543-564618) Glu (TTC) 76 bp Sc: 56.27
GTCCCCATCGTCTAGAGGCCTAGGACATCGCCCTTTCACGGCGGTAACCGGGGTTCGAAT
CCCCGTGGGGACGCCA
>Actinobacillus_succinogenes_130Z_chr.trna10-GluTTC (913168-913243) Glu (TTC) 76 bp Sc: 56.27
GTCCCCATCGTCTAGAGGCCTAGGACATCGCCCTTTCACGGCGGTAACCGGGGTTCGAAT
CCCCGTGGGGACGCCA
>Actinobacillus_succinogenes_130Z_chr.trna18-GluTTC (1182183-1182258) Glu (TTC) 76 bp Sc: 56.27
GTCCCCATCGTCTAGAGGCCTAGGACATCGCCCTTTCACGGCGGTAACCGGGGTTCGAAT
CCCCGTGGGGACGCCA
>Actinobacillus_succinogenes_130Z_chr.trna28-GlyGCC (1922674-1922749) Gly (GCC) 76 bp Sc: 92.69
GCGGGAATAGCTCAGTTGGTAGAGCACAACCTTGCCAAGGTTGGGGTCGCGAGTTCGAGC
CTCGTTTCCCGCTCCA
>Actinobacillus_succinogenes_130Z_chr.trna30-GlyGCC (2030325-2030400) Gly (GCC) 76 bp Sc: 92.69
GCGGGAATAGCTCAGTTGGTAGAGCACAACCTTGCCAAGGTTGGGGTCGCGAGTTCGAGC
CTCGTTTCCCGCTCCA
>Actinobacillus_succinogenes_130Z_chr.trna41-GlyGCC (1071701-1071626) Gly (GCC) 76 bp Sc: 92.69
GCGGGAATAGCTCAGTTGGTAGAGCACAACCTTGCCAAGGTTGGGGTCGCGAGTTCGAGC

CTCGTTTCCCGCTCCA
>Actinobacillus_succinogenes_130Z_chr.trna55-GlyTCC (112045-111971) Gly (TCC) 75 bp Sc: 63.54
GCGGGCATCGTATAATGGCTATTACCTTAGCCTTCCAAGCTAATGATGCGGGTTCGATTC
CCGCTGCCCGCTCCA
>Actinobacillus_succinogenes_130Z_chr.trna14-HisGTG (1130231-1130306) His (GTG) 76 bp Sc: 82.05
GTGGCTATAGCTCAGTTGGTAGAGCCCTGGATTGTGATTCCAGTTGTCGTGAGTTCGAGT
CTCATTAGCCACCCCA
>Actinobacillus_succinogenes_130Z_chr.trna16-IleGAT (1132169-1132245) Ile (GAT) 77 bp Sc: 94.77
GGGTCTGTAGCTCAGGTGGTTAGAGCGCACCCCTGATAAGGGTGAGGTCGGTGGTTCAAG
TCCACTCAGACCCACCA
>Actinobacillus_succinogenes_130Z_chr.trna51-IleGAT (322883-322807) Ile (GAT) 77 bp Sc: 94.77
GGGTCTGTAGCTCAGGTGGTTAGAGCGCACCCCTGATAAGGGTGAGGTCGGTGGTTCAAG
TCCACTCAGACCCACCA
>Actinobacillus_succinogenes_130Z_chr.trna57-IleGAT (37587-37511) Ile (GAT) 77 bp Sc: 94.77
GGGTCTGTAGCTCAGGTGGTTAGAGCGCACCCCTGATAAGGGTGAGGTCGGTGGTTCAAG
TCCACTCAGACCCACCA
>Actinobacillus_succinogenes_130Z_chr.trna11-LeuCAA (1024112-1024198) Leu (CAA) 87 bp Sc: 74.45
GCCTGGGTGGCGAAATTGGTAGACGCAGCGGATTCAAAATCCGCCGCCTTTGCGGGTGTG
TCGGTTCGAGTCCGACCCTAGGCACCA
>Actinobacillus_succinogenes_130Z_chr.trna38-LeuCAG (1657325-1657240) Leu (CAG) 86 bp Sc: 63.77
GCTTCGATGGTGGAATTGGTAGACACGCTGTCTTCAGGTGGCAGTGTCCTTAGGATGTGC
GAGTTCGAGTCTCGCTCGAAGCACCA
>Actinobacillus_succinogenes_130Z_chr.trna27-LeuGAG (1876119-1876204) Leu (GAG) 86 bp Sc: 56.69
GCTCTGGTGGTGGAATTGGTAGACACGCTATCTTGAGGGGGTAGTGACCGAAGGTCGTGC
GAGTTCAAGTCTCGCCCAGAGCACCA
>Actinobacillus_succinogenes_130Z_chr.trna29-LeuTAA (1922779-1922865) Leu (TAA) 87 bp Sc: 73.83
GCCCGAGTGGCGGAATCGGTAGACGCAAGGGATTTAAAATCCCTCGCCTTTCGGGGCGTG
CCAGTTCAAGTCTGGCCTCGGGCACCA
>Actinobacillus_succinogenes_130Z_chr.trna31-LeuTAA (2030418-2030504) Leu (TAA) 87 bp Sc: 75.15
GCCCGAGTGGCGGAATCGGTAGACGCAAGGGATTTAAAATCCCTCGCCTTTCGAGGCGTG
CCAGTTCAAGTCTGGCCTCGGGCACCA
>Actinobacillus_succinogenes_130Z_chr.trna20-LeuTAG (1202570-1202652) Leu (TAG) 83 bp Sc: 71.55
GCGGAAGTGGCGAAATTGGTAGACGCACCGGATTTAGGTTCCGGCGCTTGTGCGTGTGGG
TTCAAGTCCCTCCTTCCGCACCA
>Actinobacillus_succinogenes_130Z_chr.trna26-LysCTT (1523540-1523614) Lys (CTT) 75 bp Sc: 82.30
GGGTCGTTAGCTTAGTGGTAGAGCTGCGGACTCTTAATCCGTCGGTCGAGCGTTCGAATC
GCTCACGACCCACCA
>Actinobacillus_succinogenes_130Z_chr.trna40-LysTTT (1365236-1365161) Lys (TTT) 76 bp Sc: 71.48
GGGCGCATAGCTCAACTGGTAGAGCGGCAGACTTTTAATCTGAGGGATAATGGTTCGAGT
CCATCTGCGCGCTCCA
>Actinobacillus_succinogenes_130Z_chr.trna35-LysTTT (1969277-1969202) Lys (TTT) 76 bp Sc: 97.43
GGGTCGTTAGCTCAGTCGGTAGAGCAGCGGACTTTTAATCCGTTGGTCGAAGGTTCGAAT
CCTTCACGACCCACCA
>Actinobacillus_succinogenes_130Z_chr.trna36-LysTTT (1969172-1969097) Lys (TTT) 76 bp Sc: 97.43
GGGTCGTTAGCTCAGTCGGTAGAGCAGCGGACTTTTAATCCGTTGGTCGAAGGTTCGAAT
CCTTCACGACCCACCA
>Actinobacillus_succinogenes_130Z_chr.trna37-LysTTT (1969068-1968993) Lys (TTT) 76 bp Sc: 97.43
GGGTCGTTAGCTCAGTCGGTAGAGCAGCGGACTTTTAATCCGTTGGTCGAAGGTTCGAAT
CCTTCACGACCCACCA
>Actinobacillus_succinogenes_130Z_chr.trna25-MetCAT (1242492-1242568) Met (CAT) 77 bp Sc: 83.87
CGCGGGGTGGAGCAGCTTGGTAGCTCGTCGGGCTCATAACCCGAAGGTCGTTGGTTCAAA
TCCGGCCCCCGCAACCA
>Actinobacillus_succinogenes_130Z_chr.trna42-MetCAT (998864-998788) Met (CAT) 77 bp Sc: 83.87
CGCGGGGTGGAGCAGCTTGGTAGCTCGTCGGGCTCATAACCCGAAGGTCGTTGGTTCAAA
TCCGGCCCCCGCAACCA
>Actinobacillus_succinogenes_130Z_chr.trna34-MetCAT (2211865-2211790) Met (CAT) 76 bp Sc: 88.53
GCCCCCATAGCTCAGCGGTCAGAGCAGACGACTCATAATCGTTTGGTCGCTGGTTCAAAC
CCAGCTGGGGGCACCA
>Actinobacillus_succinogenes_130Z_chr.trna19-MetCAT (1202474-1202550) Met (CAT) 77 bp Sc: 95.27
GGCTACGTAGCTCAGTTGGTTAGAGCACAACACTCATAATGTTGGGGTCACAGGTTCGAA
TCCCGTCGTAGCCACCA
>Actinobacillus_succinogenes_130Z_chr.trna2-PheGAA (596425-596500) Phe (GAA) 76 bp Sc: 85.75
GCCTCGATAGCTCAGTCGGTAGAGCAGGGGATTGAAAATCCCCGTGTCGGTGGTTCGATT
CCGCCTCGAGGCACCA
>Actinobacillus_succinogenes_130Z_chr.trna15-ProTGG (1130328-1130404) Pro (TGG) 77 bp Sc: 90.87
CGGCGAGTAGCGCAGCTTGGTAGCGCAACTGGTTTGGGACCAGTGGGTCGTAGGTTCAAA
TCCTATCTCGCCGACCA

>Actinobacillus_succinogenes_130Z_chr.trna46-ProTGG (821359-821283) Pro (TGG) 77 bp Sc: 90.87
CGGCGAGTAGCGCAGCTTGGTAGCGCAACTGGTTTGGGACCAGTGGGTCGTAGGTTCAAA
TCCTATCTCGCCGACCA
>Actinobacillus_succinogenes_130Z_chr.trna50-SeC(p)TCA (334422-334332) SeC(p) (TCA) 91 bp Sc: 71.65
GGAAGGTCGTCGTCTCCGGTGAGGCGGCAGGATTTCAAATCCTGTTGAGGACGCCAGCGT
TCTTGGGTGGGTTCGACTCCCATGACCTTCC
>Actinobacillus_succinogenes_130Z_chr.trna7-SerGCT (742664-742757) Ser (GCT) 94 bp Sc: 73.33
GGTGAGATGGCCGAGCTGGCTGAAGGCGCTCCCCTGCTAAGGGAGTATGGGGTCAAAAAC
TCCATCGAGGGTTCGAATCCCTCTTTCACCGCCA
>Actinobacillus_succinogenes_130Z_chr.trna33-SerGGA (2261328-2261239) Ser (GGA) 90 bp Sc: 74.47
GGTGAGATGTCCGAGTGGTTGAAGGAGCACGCCTGGAAAGCGTGTATACGGGAAACTGTA
TCGGGGGTTCGAATCCCCCTCTCACCGCCA
>Actinobacillus_succinogenes_130Z_chr.trna12-SerTGA (1083338-1083427) Ser (TGA) 90 bp Sc: 69.05
GGAGGAATGGCAGAGCGGTTGAATGCACCGGTCTTGAAAACCGGCGAGGGTGCGAGCCCT
CCCTGAGTTCGAATCTCAGTTCCTCCGCCA
>Actinobacillus_succinogenes_130Z_chr.trna43-SerTGA (973721-973632) Ser (TGA) 90 bp Sc: 69.05
GGAGGAATGGCAGAGCGGTTGAATGCACCGGTCTTGAAAACCGGCGAGGGTGCGAGCCCT
CCCTGAGTTCGAATCTCAGTTCCTCCGCCA
>Actinobacillus_succinogenes_130Z_chr.trna48-ThrCGT (769262-769187) Thr (CGT) 76 bp Sc: 94.34
GCCGACTTAGCTCAGTCGGTAGAGCAACTGATTCGTAATCAGTAGGTCGTCAGTTCGATT
CTGATAGTCGGCACCA
>Actinobacillus_succinogenes_130Z_chr.trna56-ThrGGT (111966-111891) Thr (GGT) 76 bp Sc: 96.44
GCTGATATAGCTCAGTTGGTAGAGCGCACCCTTGGTAAGGGTGAGGTCGGCGGTTCAAAT
CCGCCTATCAGCACCA
>Actinobacillus_succinogenes_130Z_chr.trna53-ThrTGT (112269-112194) Thr (TGT) 76 bp Sc: 90.20
GCCGACTTAGCTCAGTAGGTAGAGCAACTGACTTGTAATCAGTAGGTCATCAGTTCGATT
CCGATAGTCGGCACCA
>Actinobacillus_succinogenes_130Z_chr.trna60-TrpCCA (33583-33508) Trp (CCA) 76 bp Sc: 79.69
AGGGGCGTAGTTCAATTGGTAGAGCACCGGTCTCCAAAACCGGGTGTTGGGAGTTCGAGC
CTCTCCGCCCCTGCCA
>Actinobacillus_succinogenes_130Z_chr.trna54-TyrGTA (112166-112082) Tyr (GTA) 85 bp Sc: 65.61
GGAGGGGTTCCCGAGCGGCCAAAGGGGGCAGACTGTAAATCTGTTGGCTCAGCCTTCGAA
GGTTCGAATCCTTCTCCCTCCACCA
>Actinobacillus_succinogenes_130Z_chr.trna39-ValGAC (1582794-1582718) Val (GAC) 77 bp Sc: 90.13
GCGACTATAGCTCAGTTGGTTAGAGCACCACCTTGACATGGTGGGGGTCACTGGTTCGAG
TCCAGCTAGTCGCACCA
>Actinobacillus_succinogenes_130Z_chr.trna4-ValTAC (615299-615374) Val (TAC) 76 bp Sc: 90.00
GGGCGATTAGCTCAGTTGGGAGAGCACCTCCCTTACAAGGAGGGGGTCACTGGTTCGAGA
CCGGTATCGCCCACCA
>Actinobacillus_succinogenes_130Z_chr.trna5-ValTAC (615426-615501) Val (TAC) 76 bp Sc: 90.00
GGGCGATTAGCTCAGTTGGGAGAGCACCTCCCTTACAAGGAGGGGGTCACTGGTTCGAGA
CCGGTATCGCCCACCA
>Actinobacillus_succinogenes_130Z_chr.trna6-ValTAC (615616-615691) Val (TAC) 76 bp Sc: 90.00
GGGCGATTAGCTCAGTTGGGAGAGCACCTCCCTTACAAGGAGGGGGTCACTGGTTCGAGA
CCGGTATCGCCCACCA
>Aeromonas_hydrophila_ATCC_7966_chr.trna88-AlaGGC (3876267-3876192) Ala (GGC) 76 bp Sc: 81.36
GGGGTTATAGCTCAGCTGGGAGAGCGCTTGCATGGCATGCAAGAGGTCCGCGGTTCGATC
CCGCGTAGCTCCACCA
>Aeromonas_hydrophila_ATCC_7966_chr.trna89-AlaGGC (3876163-3876088) Ala (GGC) 76 bp Sc: 81.36
GGGGTTATAGCTCAGCTGGGAGAGCGCTTGCATGGCATGCAAGAGGTCCGCGGTTCGATC
CCGCGTAGCTCCACCA
>Aeromonas_hydrophila_ATCC_7966_chr.trna90-AlaGGC (3876024-3875949) Ala (GGC) 76 bp Sc: 81.36
GGGGTTATAGCTCAGCTGGGAGAGCGCTTGCATGGCATGCAAGAGGTCCGCGGTTCGATC
CCGCGTAGCTCCACCA
>Aeromonas_hydrophila_ATCC_7966_chr.trna91-AlaGGC (3875900-3875825) Ala (GGC) 76 bp Sc: 81.36
GGGGTTATAGCTCAGCTGGGAGAGCGCTTGCATGGCATGCAAGAGGTCCGCGGTTCGATC
CCGCGTAGCTCCACCA
>Aeromonas_hydrophila_ATCC_7966_chr.trna92-AlaGGC (3875790-3875715) Ala (GGC) 76 bp Sc: 81.36
GGGGTTATAGCTCAGCTGGGAGAGCGCTTGCATGGCATGCAAGAGGTCCGCGGTTCGATC
CCGCGTAGCTCCACCA
>Aeromonas_hydrophila_ATCC_7966_chr.trna50-AlaTGC (4452741-4452666) Ala (TGC) 76 bp Sc: 88.77
GGGGCTATAGCTCAGCTGGGAGAGCGCCTGCTTTGCACGCAGGAGGTCTGCGGTTCGATC
CCGCATAGCTCCACCA
>Aeromonas_hydrophila_ATCC_7966_chr.trna53-AlaTGC (4369719-4369644) Ala (TGC) 76 bp Sc: 88.77
GGGGCTATAGCTCAGCTGGGAGAGCGCCTGCTTTGCACGCAGGAGGTCTGCGGTTCGATC
CCGCATAGCTCCACCA
>Aeromonas_hydrophila_ATCC_7966_chr.trna9-AlaTGC (216610-216685) Ala (TGC) 76 bp Sc: 88.77

GGGGCTATAGCTCAGCTGGGAGAGCGCCTGCTTTGCACGCAGGAGGTCTGCGGTTCGATC
CCGCATAGCTCCACCA
>Aeromonas_hydrophila_ATCC_7966_chr.trna59-ArgACG (4142172-4142096) Arg (ACG) 77 bp Sc: 91.00
GCGCCCGTAGCTCAGCTGGATAGAGTACCTGGCTACGAACCAGGTGGTCAGAGGTTCGAA
TCCTCTCGGGCGCACCA
>Aeromonas_hydrophila_ATCC_7966_chr.trna60-ArgACG (4141996-4141920) Arg (ACG) 77 bp Sc: 91.00
GCGCCCGTAGCTCAGCTGGATAGAGTACCTGGCTACGAACCAGGTGGTCAGAGGTTCGAA
TCCTCTCGGGCGCACCA
>Aeromonas_hydrophila_ATCC_7966_chr.trna61-ArgACG (4141893-4141817) Arg (ACG) 77 bp Sc: 91.00
GCGCCCGTAGCTCAGCTGGATAGAGTACCTGGCTACGAACCAGGTGGTCAGAGGTTCGAA
TCCTCTCGGGCGCACCA
>Aeromonas_hydrophila_ATCC_7966_chr.trna62-ArgACG (4141788-4141712) Arg (ACG) 77 bp Sc: 91.00
GCGCCCGTAGCTCAGCTGGATAGAGTACCTGGCTACGAACCAGGTGGTCAGAGGTTCGAA
TCCTCTCGGGCGCACCA
>Aeromonas_hydrophila_ATCC_7966_chr.trna63-ArgACG (4141683-4141607) Arg (ACG) 77 bp Sc: 91.00
GCGCCCGTAGCTCAGCTGGATAGAGTACCTGGCTACGAACCAGGTGGTCAGAGGTTCGAA
TCCTCTCGGGCGCACCA
>Aeromonas_hydrophila_ATCC_7966_chr.trna1-ArgCCG (29246-29322) Arg (CCG) 77 bp Sc: 95.23
GCGCCCGTAGCTCAGCTGGATAGAGCGCTGCCCTCCGGAGGCAGAGGTCACAGGTTCGAA
TCCTGTCGGGCGCACCA
>Aeromonas_hydrophila_ATCC_7966_chr.trna125-ArgCCT (1187103-1187027) Arg (CCT) 77 bp Sc: 79.52
GCTCCCGTAGCTCAGTAGGATAGAGCGGTCCCCTCCTAAGGGACAGGCCACTGGTTCGAC
TCCAGTCGGGAGCGCCA
>Aeromonas_hydrophila_ATCC_7966_chr.trna119-ArgTCT (2027039-2026963) Arg (TCT) 77 bp Sc: 97.39
GCGCCCTTAGCTCAGTCGGATAGAGCAACGGCCTTCTAAGCCGTGGGTCGCAGGTTCGAA
TCCTGCAGGGCGCGCCA
>Aeromonas_hydrophila_ATCC_7966_chr.trna22-AsnGTT (2829543-2829618) Asn (GTT) 76 bp Sc: 85.17
TCCTCCTTAGTTCAGTCGGTAGAACGGCGGACTGTTAATCCGTATGTCACTGGTTCAAGT
CCAGTAGGAGGAGCCA
>Aeromonas_hydrophila_ATCC_7966_chr.trna36-AsnGTT (3894450-3894525) Asn (GTT) 76 bp Sc: 85.17
TCCTCCTTAGTTCAGTCGGTAGAACGGCGGACTGTTAATCCGTATGTCACTGGTTCAAGT
CCAGTAGGAGGAGCCA
>Aeromonas_hydrophila_ATCC_7966_chr.trna57-AsnGTT (4268596-4268521) Asn (GTT) 76 bp Sc: 85.17
TCCTCCTTAGTTCAGTCGGTAGAACGGCGGACTGTTAATCCGTATGTCACTGGTTCAAGT
CCAGTAGGAGGAGCCA
>Aeromonas_hydrophila_ATCC_7966_chr.trna23-AsnGTT (2829621-2829696) Asn (GTT) 76 bp Sc: 85.53
TCCCTCGTAGTTCAGTCGGTAGAACGGCGGACTGTTAATCCGTATGTCACAGGTTCAAGT
CCTGTCGAGGGAGCCA
>Aeromonas_hydrophila_ATCC_7966_chr.trna20-AsnGTT (2702867-2702942) Asn (GTT) 76 bp Sc: 87.36
TCCCCCGTAGTTCAGTCGGTAGAACGGCGGACTGTTAATCCGTATGTCACTGGTTCAAGT
CCAGTCGGGGGAGCCA
>Aeromonas_hydrophila_ATCC_7966_chr.trna12-AspGTC (355180-355256) Asp (GTC) 77 bp Sc: 95.34
GGAGCGGTAGTTCAGTCGGTTAGAATACCGGCCTGTCACGCCGGGGGTCGCGGGTTCGAG
TCCCGTCCGCTCCGCCA
>Aeromonas_hydrophila_ATCC_7966_chr.trna14-AspGTC (809122-809198) Asp (GTC) 77 bp Sc: 95.34
GGAGCGGTAGTTCAGTCGGTTAGAATACCGGCCTGTCACGCCGGGGGTCGCGGGTTCGAG
TCCCGTCCGCTCCGCCA
>Aeromonas_hydrophila_ATCC_7966_chr.trna43-AspGTC (4697596-4697520) Asp (GTC) 77 bp Sc: 95.34
GGAGCGGTAGTTCAGTCGGTTAGAATACCGGCCTGTCACGCCGGGGGTCGCGGGTTCGAG
TCCCGTCCGCTCCGCCA
>Aeromonas_hydrophila_ATCC_7966_chr.trna51-AspGTC (4449246-4449170) Asp (GTC) 77 bp Sc: 95.34
GGAGCGGTAGTTCAGTCGGTTAGAATACCGGCCTGTCACGCCGGGGGTCGCGGGTTCGAG
TCCCGTCCGCTCCGCCA
>Aeromonas_hydrophila_ATCC_7966_chr.trna100-CysGCA (3111351-3111278) Cys (GCA) 74 bp Sc: 53.70
GGCGCGTTAGCAAAGCGGTTATGCAGCGGATTGCAAATCCGTTTAGTCCGGTTCGACTCC
GGAACGTGCCTCCA
>Aeromonas_hydrophila_ATCC_7966_chr.trna96-CysGCA (3487523-3487450) Cys (GCA) 74 bp Sc: 53.70
GGCGCGTTAGCAAAGCGGTTATGCAGCGGATTGCAAATCCGTTTAGTCCGGTTCGACTCC
GGAACGTGCCTCCA
>Aeromonas_hydrophila_ATCC_7966_chr.trna29-GlnTTG (3572241-3572315) Gln (TTG) 75 bp Sc: 61.93
TGGGGCATCGCCAAGCGGTAAGGCAGCGGGTTTTGATCTCGCCATACCTAGGTTCGAATC
CTAGTGCCCCAGCCA
>Aeromonas_hydrophila_ATCC_7966_chr.trna32-GlnTTG (3572592-3572666) Gln (TTG) 75 bp Sc: 61.93
TGGGGCATCGCCAAGCGGTAAGGCAGCGGGTTTTGATCTCGCCATACCTAGGTTCGAATC
CTAGTGCCCCAGCCA
>Aeromonas_hydrophila_ATCC_7966_chr.trna28-GlnTTG (3572020-3572094) Gln (TTG) 75 bp Sc: 62.85
TGGGGTATCGCCAAGCGGTAAGGCAGCGGGTTTTGATCTCGCCATCCCTAGGTTCGAATC

CTAGTACCCCAGCCA
>Aeromonas_hydrophila_ATCC_7966_chr.trna30-GlnTTG (3572357-3572431) Gln (TTG) 75 bp Sc: 62.85
TGGGGTATCGCCAAGCGGTAAGGCAGCGGGTTTTGATCTCGCCATCCCTAGGTTCGAATC
CTAGTACCCCAGCCA
>Aeromonas_hydrophila_ATCC_7966_chr.trna26-GlnTTG (3566154-3566228) Gln (TTG) 75 bp Sc: 67.44
TGGGGTATCGCCAAGCGGTAAGGCAGCGGGTTTTGATCTCGCCATTCCTAGGTTCGAATC
CTAGTACCCCAGCCA
>Aeromonas_hydrophila_ATCC_7966_chr.trna11-GluTTC (351690-351765) Glu (TTC) 76 bp Sc: 62.50
GTCCCCTTCGTCTAGAGGCCTAGGACACCGCCCTTTCACGGCGGTAACAGGGGTTCGAAT
CCCCTAGGGGACGCCA
>Aeromonas_hydrophila_ATCC_7966_chr.trna13-GluTTC (805590-805665) Glu (TTC) 76 bp Sc: 62.50
GTCCCCTTCGTCTAGAGGCCTAGGACACCGCCCTTTCACGGCGGTAACAGGGGTTCGAAT
CCCCTAGGGGACGCCA
>Aeromonas_hydrophila_ATCC_7966_chr.trna16-GluTTC (934767-934842) Glu (TTC) 76 bp Sc: 62.50
GTCCCCTTCGTCTAGAGGCCTAGGACACCGCCCTTTCACGGCGGTAACAGGGGTTCGAAT
CCCCTAGGGGACGCCA
>Aeromonas_hydrophila_ATCC_7966_chr.trna42-GluTTC (4701129-4701054) Glu (TTC) 76 bp Sc: 62.50
GTCCCCTTCGTCTAGAGGCCTAGGACACCGCCCTTTCACGGCGGTAACAGGGGTTCGAAT
CCCCTAGGGGACGCCA
>Aeromonas_hydrophila_ATCC_7966_chr.trna5-GluTTC (86944-87019) Glu (TTC) 76 bp Sc: 62.50
GTCCCCTTCGTCTAGAGGCCTAGGACACCGCCCTTTCACGGCGGTAACAGGGGTTCGAAT
CCCCTAGGGGACGCCA
>Aeromonas_hydrophila_ATCC_7966_chr.trna7-GluTTC (165116-165191) Glu (TTC) 76 bp Sc: 62.50
GTCCCCTTCGTCTAGAGGCCTAGGACACCGCCCTTTCACGGCGGTAACAGGGGTTCGAAT
CCCCTAGGGGACGCCA
>Aeromonas_hydrophila_ATCC_7966_chr.trna79-GluTTC (3955008-3954933) Glu (TTC) 76 bp Sc: 62.50
GTCCCCTTCGTCTAGAGGCCTAGGACACCGCCCTTTCACGGCGGTAACAGGGGTTCGAAT
CCCCTAGGGGACGCCA
>Aeromonas_hydrophila_ATCC_7966_chr.trna97-GlyCCC (3414662-3414589) Gly (CCC) 74 bp Sc: 74.92
GCGGGTGTAGTTCAATGGTAGAACGGTAGCTTCCCAAGCTGCATACGTGGGTTCGATTCC
CATCACCCGCTCCA
>Aeromonas_hydrophila_ATCC_7966_chr.trna80-GlyGCC (3882630-3882555) Gly (GCC) 76 bp Sc: 93.74
GCGGGAATAGCTCAGTTGGTAGAGCACGACCTTGCCAAGGTCGGGGTCGCGAGTTCGAGT
CTCGTTTCCCGCTCCA
>Aeromonas_hydrophila_ATCC_7966_chr.trna81-GlyGCC (3882529-3882454) Gly (GCC) 76 bp Sc: 93.74
GCGGGAATAGCTCAGTTGGTAGAGCACGACCTTGCCAAGGTCGGGGTCGCGAGTTCGAGT
CTCGTTTCCCGCTCCA
>Aeromonas_hydrophila_ATCC_7966_chr.trna82-GlyGCC (3882420-3882345) Gly (GCC) 76 bp Sc: 93.74
GCGGGAATAGCTCAGTTGGTAGAGCACGACCTTGCCAAGGTCGGGGTCGCGAGTTCGAGT
CTCGTTTCCCGCTCCA
>Aeromonas_hydrophila_ATCC_7966_chr.trna83-GlyGCC (3882320-3882245) Gly (GCC) 76 bp Sc: 93.74
GCGGGAATAGCTCAGTTGGTAGAGCACGACCTTGCCAAGGTCGGGGTCGCGAGTTCGAGT
CTCGTTTCCCGCTCCA
>Aeromonas_hydrophila_ATCC_7966_chr.trna84-GlyGCC (3882220-3882145) Gly (GCC) 76 bp Sc: 93.74
GCGGGAATAGCTCAGTTGGTAGAGCACGACCTTGCCAAGGTCGGGGTCGCGAGTTCGAGT
CTCGTTTCCCGCTCCA
>Aeromonas_hydrophila_ATCC_7966_chr.trna85-GlyGCC (3882120-3882045) Gly (GCC) 76 bp Sc: 93.74
GCGGGAATAGCTCAGTTGGTAGAGCACGACCTTGCCAAGGTCGGGGTCGCGAGTTCGAGT
CTCGTTTCCCGCTCCA
>Aeromonas_hydrophila_ATCC_7966_chr.trna86-GlyGCC (3882020-3881945) Gly (GCC) 76 bp Sc: 93.74
GCGGGAATAGCTCAGTTGGTAGAGCACGACCTTGCCAAGGTCGGGGTCGCGAGTTCGAGT
CTCGTTTCCCGCTCCA
>Aeromonas_hydrophila_ATCC_7966_chr.trna95-GlyGCC (3487654-3487579) Gly (GCC) 76 bp Sc: 93.74
GCGGGAATAGCTCAGTTGGTAGAGCACGACCTTGCCAAGGTCGGGGTCGCGAGTTCGAGT
CTCGTTTCCCGCTCCA
>Aeromonas_hydrophila_ATCC_7966_chr.trna99-GlyGCC (3111461-3111386) Gly (GCC) 76 bp Sc: 93.74
GCGGGAATAGCTCAGTTGGTAGAGCACGACCTTGCCAAGGTCGGGGTCGCGAGTTCGAGT
CTCGTTTCCCGCTCCA
>Aeromonas_hydrophila_ATCC_7966_chr.trna47-GlyTCC (4476138-4476064) Gly (TCC) 75 bp Sc: 64.85
GCGGGCATCGTATAATGGCTATTACCTCAGCCTTCCAAGCTGATGATGCGGGTTCGATTC
CCGCTGCCCGCTCCA
>Aeromonas_hydrophila_ATCC_7966_chr.trna98-GlyTCC (3414494-3414420) Gly (TCC) 75 bp Sc: 64.85
GCGGGCATCGTATAATGGCTATTACCTCAGCCTTCCAAGCTGATGATGCGGGTTCGATTC
CCGCTGCCCGCTCCA
>Aeromonas_hydrophila_ATCC_7966_chr.trna120-HisGTG (2026913-2026838) His (GTG) 76 bp Sc: 80.26
GTGGCTGTAGCTCAGTTGGTAGAGTCCCGGATTGTGATTCCGGTTGTCGTGGGTTCGAGC
CCCATCAGCCACCCCA

>Aeromonas_hydrophila_ATCC_7966_chr.trna2-HisGTG (29373-29448) His (GTG) 76 bp Sc: 80.26
GTGGCTGTAGCTCAGTTGGTAGAGTCCCGGATTGTGATTCCGGTTGTCGTGGGTTCGAGC
CCCATCAGCCACCCCA
>Aeromonas_hydrophila_ATCC_7966_chr.trna49-IleGAT (4452828-4452752) Ile (GAT) 77 bp Sc: 95.89
GGGTCTGTAGCTCAGGTGGTTAGAGCGCACCCCTGATAAGGGTGAGGTCGGTGGTTCGAG
TCCACTCAGACCCACCA
>Aeromonas_hydrophila_ATCC_7966_chr.trna52-IleGAT (4369806-4369730) Ile (GAT) 77 bp Sc: 95.89
GGGTCTGTAGCTCAGGTGGTTAGAGCGCACCCCTGATAAGGGTGAGGTCGGTGGTTCGAG
TCCACTCAGACCCACCA
>Aeromonas_hydrophila_ATCC_7966_chr.trna8-IleGAT (216523-216599) Ile (GAT) 77 bp Sc: 95.89
GGGTCTGTAGCTCAGGTGGTTAGAGCGCACCCCTGATAAGGGTGAGGTCGGTGGTTCGAG
TCCACTCAGACCCACCA
>Aeromonas_hydrophila_ATCC_7966_chr.trna64-LeuCAA (4129749-4129664) Leu (CAA) 86 bp Sc: 75.64
GCCCGGGTGGCGAAATCGGTAGACGCAGCGGATTCAAAATCCGCCGGTGAATAACCGTGT
CGGTTCGAGTCCGACCCCGGGCACCA
>Aeromonas_hydrophila_ATCC_7966_chr.trna37-LeuCAG (4234485-4234570) Leu (CAG) 86 bp Sc: 63.52
GCGAAGGTGGCGGAATTGGTAGACGCGCTAGCTTCAGGTGTTAGTGTCCCACGGATGTGA
GGGTTCGAGTCCCTCTCTTCGCACCA
>Aeromonas_hydrophila_ATCC_7966_chr.trna39-LeuCAG (4234756-4234841) Leu (CAG) 86 bp Sc: 63.52
GCGAAGGTGGCGGAATTGGTAGACGCGCTAGCTTCAGGTGTTAGTGTCCCACGGATGTGA
GGGTTCGAGTCCCTCTCTTCGCACCA
>Aeromonas_hydrophila_ATCC_7966_chr.trna41-LeuCAG (4235044-4235129) Leu (CAG) 86 bp Sc: 63.52
GCGAAGGTGGCGGAATTGGTAGACGCGCTAGCTTCAGGTGTTAGTGTCCCACGGATGTGA
GGGTTCGAGTCCCTCTCTTCGCACCA
>Aeromonas_hydrophila_ATCC_7966_chr.trna3-LeuCAG (29469-29554) Leu (CAG) 86 bp Sc: 67.52
GCGAAGGTGGCGGAATTGGTAGACGCGCTAGCTTCAGGTGTTAGTGCCCCCCGGGTGTGA
GGGTTCGAGTCCCTCTCTTCGCACCA
>Aeromonas_hydrophila_ATCC_7966_chr.trna38-LeuCAG (4234626-4234711) Leu (CAG) 86 bp Sc: 67.52
GCGAAGGTGGCGGAATTGGTAGACGCGCTAGCTTCAGGTGTTAGTGCCCCCCGGGTGTGA
GGGTTCGAGTCCCTCTCTTCGCACCA
>Aeromonas_hydrophila_ATCC_7966_chr.trna40-LeuCAG (4234887-4234972) Leu (CAG) 86 bp Sc: 67.52
GCGAAGGTGGCGGAATTGGTAGACGCGCTAGCTTCAGGTGTTAGTGCCCCCCGGGTGTGA
GGGTTCGAGTCCCTCTCTTCGCACCA
>Aeromonas_hydrophila_ATCC_7966_chr.trna93-LeuGAG (3727685-3727601) Leu (GAG) 85 bp Sc: 61.40
GCCGTGGTGGTGGAATTGGTAGACACGCTATCTTGAGGGGGTAGTGCTCTAGGGTGTGCG
GGTTCGAGTCCCGCCCACGGCACCA
>Aeromonas_hydrophila_ATCC_7966_chr.trna101-LeuTAA (3111236-3111150) Leu (TAA) 87 bp Sc: 75.24
GCCCGAGTGGTGAAATCGGTAGACACAAGGGATTTAAAATCCCTCGACGTTCGCGTCGTG
CCGGTTCGATTCCGGCCTCGGGCACCA
>Aeromonas_hydrophila_ATCC_7966_chr.trna34-LeuTAG (3572788-3572871) Leu (TAG) 84 bp Sc: 78.55
GCGGAAGTGGCGAAATTGGTAGACGCACCAGATTTAGGTTCTGGCGCCCTAGGCGTGAGA
GTTCGAGTCTCTCCTTCCGCACCA
>Aeromonas_hydrophila_ATCC_7966_chr.trna67-LysCTT (4069641-4069566) Lys (CTT) 76 bp Sc: 91.87
GGGTCGTTAGCTCAGTCGGTAGAGCAGTTGACTCTTAATCAATTGGTCGTAGGTTCGATC
CCTACACGACCCACCA
>Aeromonas_hydrophila_ATCC_7966_chr.trna65-LysTTT (4069872-4069797) Lys (TTT) 76 bp Sc: 96.39
GGGTCGTTAGCTCAGTCGGTAGAGCAGTTGACTTTTAATCAATTGGTCGCTGGTTCGAAT
CCAGCACGACCCACCA
>Aeromonas_hydrophila_ATCC_7966_chr.trna69-LysTTT (4069445-4069370) Lys (TTT) 76 bp Sc: 96.39
GGGTCGTTAGCTCAGTCGGTAGAGCAGTTGACTTTTAATCAATTGGTCGCTGGTTCGAAT
CCAGCACGACCCACCA
>Aeromonas_hydrophila_ATCC_7966_chr.trna71-LysTTT (4069254-4069179) Lys (TTT) 76 bp Sc: 96.39
GGGTCGTTAGCTCAGTCGGTAGAGCAGTTGACTTTTAATCAATTGGTCGCTGGTTCGAAT
CCAGCACGACCCACCA
>Aeromonas_hydrophila_ATCC_7966_chr.trna73-LysTTT (4069062-4068987) Lys (TTT) 76 bp Sc: 96.39
GGGTCGTTAGCTCAGTCGGTAGAGCAGTTGACTTTTAATCAATTGGTCGCTGGTTCGAAT
CCAGCACGACCCACCA
>Aeromonas_hydrophila_ATCC_7966_chr.trna75-LysTTT (4068871-4068796) Lys (TTT) 76 bp Sc: 96.39
GGGTCGTTAGCTCAGTCGGTAGAGCAGTTGACTTTTAATCAATTGGTCGCTGGTTCGAAT
CCAGCACGACCCACCA
>Aeromonas_hydrophila_ATCC_7966_chr.trna77-LysTTT (4068680-4068605) Lys (TTT) 76 bp Sc: 96.39
GGGTCGTTAGCTCAGTCGGTAGAGCAGTTGACTTTTAATCAATTGGTCGCTGGTTCGAAT
CCAGCACGACCCACCA
>Aeromonas_hydrophila_ATCC_7966_chr.trna104-MetCAT (2640467-2640391) Met (CAT) 77 bp Sc: 81.80
CGCGGGGTGGAGCAGCCCGGTAGCTCGTCGGGCTCATAACCCGAAGGTCGTCAGTTCAAA
TCTGGCCCCCGCAACCA
>Aeromonas_hydrophila_ATCC_7966_chr.trna94-MetCAT (3727508-3727432) Met (CAT) 77 bp Sc: 84.66

CGCGGGGTGGAGCAGCATGGTAGCTCGTCGGGCTCATAACCCGAAGGTCGTCGGTTCAAA
TCCGGCCCCCGCAACCA
>Aeromonas_hydrophila_ATCC_7966_chr.trna105-MetCAT (2640298-2640222) Met (CAT) 77 bp Sc: 86.71
CGCGGGGTGGAGCAGCTCGGTAGCTCGTCGGGCTCATAACCCGAAGGTCGTCGGTTCAAA
TCCGGCCCCCGCAACCA
>Aeromonas_hydrophila_ATCC_7966_chr.trna106-MetCAT (2640125-2640049) Met (CAT) 77 bp Sc: 86.71
CGCGGGGTGGAGCAGCTCGGTAGCTCGTCGGGCTCATAACCCGAAGGTCGTCGGTTCAAA
TCCGGCCCCCGCAACCA
>Aeromonas_hydrophila_ATCC_7966_chr.trna107-MetCAT (2639952-2639876) Met (CAT) 77 bp Sc: 86.71
CGCGGGGTGGAGCAGCTCGGTAGCTCGTCGGGCTCATAACCCGAAGGTCGTCGGTTCAAA
TCCGGCCCCCGCAACCA
>Aeromonas_hydrophila_ATCC_7966_chr.trna108-MetCAT (2639779-2639703) Met (CAT) 77 bp Sc: 86.71
CGCGGGGTGGAGCAGCTCGGTAGCTCGTCGGGCTCATAACCCGAAGGTCGTCGGTTCAAA
TCCGGCCCCCGCAACCA
>Aeromonas_hydrophila_ATCC_7966_chr.trna109-MetCAT (2639606-2639530) Met (CAT) 77 bp Sc: 86.71
CGCGGGGTGGAGCAGCTCGGTAGCTCGTCGGGCTCATAACCCGAAGGTCGTCGGTTCAAA
TCCGGCCCCCGCAACCA
>Aeromonas_hydrophila_ATCC_7966_chr.trna110-MetCAT (2639433-2639357) Met (CAT) 77 bp Sc: 86.71
CGCGGGGTGGAGCAGCTCGGTAGCTCGTCGGGCTCATAACCCGAAGGTCGTCGGTTCAAA
TCCGGCCCCCGCAACCA
>Aeromonas_hydrophila_ATCC_7966_chr.trna111-MetCAT (2639259-2639183) Met (CAT) 77 bp Sc: 86.71
CGCGGGGTGGAGCAGCTCGGTAGCTCGTCGGGCTCATAACCCGAAGGTCGTCGGTTCAAA
TCCGGCCCCCGCAACCA
>Aeromonas_hydrophila_ATCC_7966_chr.trna15-MetCAT (897169-897245) Met (CAT) 77 bp Sc: 90.81
GGCCCCTTAGCTCAGTTGGTCAGAGCAGTCGACTCATAATCGATTGGTCGTGGGTTCAAG
TCCCCCAGGGGCCACCA
>Aeromonas_hydrophila_ATCC_7966_chr.trna27-MetCAT (3571935-3572011) Met (CAT) 77 bp Sc: 93.33
GGCTATGTAGCTCAGTTGGTTAGAGCATCGCACTCATAATGCGGGGGTCACAGGTTCGAA
TCCCGTCATAGCCACCA
>Aeromonas_hydrophila_ATCC_7966_chr.trna31-MetCAT (3572470-3572546) Met (CAT) 77 bp Sc: 93.33
GGCTATGTAGCTCAGTTGGTTAGAGCATCGCACTCATAATGCGGGGGTCACAGGTTCGAA
TCCCGTCATAGCCACCA
>Aeromonas_hydrophila_ATCC_7966_chr.trna33-MetCAT (3572703-3572779) Met (CAT) 77 bp Sc: 93.33
GGCTATGTAGCTCAGTTGGTTAGAGCATCGCACTCATAATGCGGGGGTCACAGGTTCGAA
TCCCGTCATAGCCACCA
>Aeromonas_hydrophila_ATCC_7966_chr.trna35-PheGAA (3894370-3894445) Phe (GAA) 76 bp Sc: 89.80
GCCCAGATAGCTCAGTCGGTAGAGCAGGGGATTGAAAATCCCCGTGTCGGCGGTTCGATT
CCGTCTCTGGGCACCA
>Aeromonas_hydrophila_ATCC_7966_chr.trna54-PheGAA (4268924-4268849) Phe (GAA) 76 bp Sc: 89.80
GCCCAGATAGCTCAGTCGGTAGAGCAGGGGATTGAAAATCCCCGTGTCGGCGGTTCGATT
CCGTCTCTGGGCACCA
>Aeromonas_hydrophila_ATCC_7966_chr.trna56-PheGAA (4268717-4268642) Phe (GAA) 76 bp Sc: 89.80
GCCCAGATAGCTCAGTCGGTAGAGCAGGGGATTGAAAATCCCCGTGTCGGCGGTTCGATT
CCGTCTCTGGGCACCA
>Aeromonas_hydrophila_ATCC_7966_chr.trna44-ProCGG (4579934-4579858) Pro (CGG) 77 bp Sc: 89.45
CGGTGTGTAGCGCAGCTTGGTAGCGCACTTCGTTCGGGACGAAGGGGTCGGAGGTTCGAA
TCCTCTCACACCGACCA
>Aeromonas_hydrophila_ATCC_7966_chr.trna24-ProGGG (2900868-2900944) Pro (GGG) 77 bp Sc: 74.34
CGGTGTGTAGCGCAGCCAGGTAGCGCATCTGCATGGGGTGTAGAGGGTCGGAGGTTCGAA
TCCTCTCACACCGACCA
>Aeromonas_hydrophila_ATCC_7966_chr.trna25-ProGGG (3162154-3162230) Pro (GGG) 77 bp Sc: 74.34
CGGTGTGTAGCGCAGCCAGGTAGCGCATCTGCATGGGGTGTAGAGGGTCGGAGGTTCGAA
TCCTCTCACACCGACCA
>Aeromonas_hydrophila_ATCC_7966_chr.trna118-ProTGG (2027152-2027076) Pro (TGG) 77 bp Sc: 86.34
CGGTGATTAGCGCAGCCCGGTAGCGCATCTGGTTTGGGACCAGAGGGTCAAAGGTTCGAA
TCCTTTATCACCGACCA
>Aeromonas_hydrophila_ATCC_7966_chr.trna4-ProTGG (29628-29704) Pro (TGG) 77 bp Sc: 86.34
CGGTGATTAGCGCAGCCCGGTAGCGCATCTGGTTTGGGACCAGAGGGTCAAAGGTTCGAA
TCCTTTATCACCGACCA
>Aeromonas_hydrophila_ATCC_7966_chr.trna115-ValGAC (2322902-2322825) Val (GAC) 78 bp Sc: 31.36
GGCTTATAGCTCAGTTGGTTAGAACAGTACTTTGACATGGCGTAGGCGATGTCGTTGGTT
CGAATCCAATTGAACACA
>Aeromonas_hydrophila_ATCC_7966_chr.trna87-GlyGCC (3881881-3881802) Gly (GCC) 80 bp Sc: 30.09
GCTGCCATATTCAGTTGGTAGAGCATGATCCGCGCTTGCCAAGGCTGGGGTCGCGAGTTC
GAGTCTCGTTTCCCGCTCCA
>Aeromonas_hydrophila_ATCC_7966_chr.trna21-SeC(p)TCA (2791529-2791616) SeC(p) (TCA) 88 bp Sc: 32.53
GGAAGCTGATGGGGTCTGGTGGCCTCCTCGGTCTTCAAAACCGATGAGAGCCGAGAAGGC

TTTGGCAGGTTCGATTCCTGCCTCTTCC
>Aeromonas_hydrophila_ATCC_7966_chr.trna18-SerCGA (1432952-1433041) Ser (CGA) 90 bp Sc: 68.59
GGAGAGATGCCGGAGTGGCCGAACGGGATTGACTCGAAATCAATTGTGGCAGCAATGCCA
CCCAGGGTTCGAATCCCTGTCTCTCCGCCA
>Aeromonas_hydrophila_ATCC_7966_chr.trna58-SerGCT (4142268-4142176) Ser (GCT) 93 bp Sc: 71.74
GGAGAGGTGGCCGAGAGGCTGAAGGCGCTCCCCTGCTAAGGGAGTATGCGGCTTATACCC
GCATCGGGGGTTCGAATCCCCCCTTCTCCGCCA
>Aeromonas_hydrophila_ATCC_7966_chr.trna103-SerGGA (2827066-2826979) Ser (GGA) 88 bp Sc: 68.20
GGTGAGGTGTCCGAGTGGCTGAAGGAGCACGCCTGGAAAGTGTGTATACGGCAACGTATC
GAGGGTTCGAATCCCTCCCTCACCGCCA
>Aeromonas_hydrophila_ATCC_7966_chr.trna121-SerGGA (1955555-1955468) Ser (GGA) 88 bp Sc: 68.20
GGTGAGGTGTCCGAGTGGCTGAAGGAGCACGCCTGGAAAGTGTGTATACGGCAACGTATC
GAGGGTTCGAATCCCTCCCTCACCGCCA
>Aeromonas_hydrophila_ATCC_7966_chr.trna19-SerGGA (1989214-1989301) Ser (GGA) 88 bp Sc: 68.20
GGTGAGGTGTCCGAGTGGCTGAAGGAGCACGCCTGGAAAGTGTGTATACGGCAACGTATC
GAGGGTTCGAATCCCTCCCTCACCGCCA
>Aeromonas_hydrophila_ATCC_7966_chr.trna102-SerTGA (2983787-2983700) Ser (TGA) 88 bp Sc: 68.13
GGAGGGGTGGCAGAGCGGCTGAATGCACCGGTCTTGAAAACCGGCGATGGGCGACCATCC
GTGGGTTCAAATCCCACCTCCTCCGCCA
>Aeromonas_hydrophila_ATCC_7966_chr.trna17-SerTGA (1210105-1210192) Ser (TGA) 88 bp Sc: 68.13
GGAGGGGTGGCAGAGCGGCTGAATGCACCGGTCTTGAAAACCGGCGATGGGCGACCATCC
GTGGGTTCAAATCCCACCTCCTCCGCCA
>Aeromonas_hydrophila_ATCC_7966_chr.trna124-ThrCGT (1458020-1457945) Thr (CGT) 76 bp Sc: 90.80
GCCGGTATAGCTCAGTTGGTAGAGCAGCGCATTCGTAATGCGAAGGTCGGGGGTTCGACT
CCTCTTACCGGCACCA
>Aeromonas_hydrophila_ATCC_7966_chr.trna10-ThrGGT (220152-220227) Thr (GGT) 76 bp Sc: 89.28
GCTGATATGGCTCAGTTGGTAGAGCGCATCCTTGGTAAGGATGAGGTCCCCAGTTCGACT
CTGGGTATCAGCACCA
>Aeromonas_hydrophila_ATCC_7966_chr.trna6-ThrGGT (90487-90562) Thr (GGT) 76 bp Sc: 89.28
GCTGATATGGCTCAGTTGGTAGAGCGCATCCTTGGTAAGGATGAGGTCCCCAGTTCGACT
CTGGGTATCAGCACCA
>Aeromonas_hydrophila_ATCC_7966_chr.trna55-ThrTGT (4268796-4268721) Thr (TGT) 76 bp Sc: 92.56
GCCGGCTTAGCTCAGTAGGTAGAGCAACTGACTTGTAATCAGTAGGTCACCAGTTCGATT
CCGGTAGCCGGCACCA
>Aeromonas_hydrophila_ATCC_7966_chr.trna45-TrpCCA (4526386-4526310) Trp (CCA) 77 bp Sc: 74.57
AGGGGCGTAGTTCCAATTGGTAGAACAGCGGTCTCCAAAACCGATGGTTGCGGGTTCGAG
TCCTGCCGCCCCTGCCA
>Aeromonas_hydrophila_ATCC_7966_chr.trna48-TrpCCA (4474654-4474578) Trp (CCA) 77 bp Sc: 74.57
AGGGGCGTAGTTCGAATTGGTAGAACAGCGGTCTCCAAAACCGATGGTTGCGGGTTCGAG
TCCTGCCGCCCCTGCCA
>Aeromonas_hydrophila_ATCC_7966_chr.trna123-TyrGTA (1702522-1702438) Tyr (GTA) 85 bp Sc: 66.59
GGAGGGGTTCCCGAGCGGCCAAAGGGATCAGACTGTAAATCTGACGGCTCTGCCTTCGAA
GGTTCGAATCCTTCTCCCTCCACCA
>Aeromonas_hydrophila_ATCC_7966_chr.trna126-TyrGTA (1108548-1108464) Tyr (GTA) 85 bp Sc: 66.59
GGAGGGGTTCCCGAGCGGCCAAAGGGATCAGACTGTAAATCTGACGGCTCTGCCTTCGAA
GGTTCGAATCCTTCTCCCTCCACCA
>Aeromonas_hydrophila_ATCC_7966_chr.trna127-TyrGTA (1108432-1108348) Tyr (GTA) 85 bp Sc: 66.59
GGAGGGGTTCCCGAGCGGCCAAAGGGATCAGACTGTAAATCTGACGGCTCTGCCTTCGAA
GGTTCGAATCCTTCTCCCTCCACCA
>Aeromonas_hydrophila_ATCC_7966_chr.trna128-TyrGTA (1108316-1108232) Tyr (GTA) 85 bp Sc: 66.59
GGAGGGGTTCCCGAGCGGCCAAAGGGATCAGACTGTAAATCTGACGGCTCTGCCTTCGAA
GGTTCGAATCCTTCTCCCTCCACCA
>Aeromonas_hydrophila_ATCC_7966_chr.trna46-TyrGTA (4476261-4476177) Tyr (GTA) 85 bp Sc: 66.59
GGAGGGGTTCCCGAGCGGCCAAAGGGATCAGACTGTAAATCTGACGGCTCTGCCTTCGAA
GGTTCGAATCCTTCTCCCTCCACCA
>Aeromonas_hydrophila_ATCC_7966_chr.trna122-TyrGTA (1702658-1702574) Tyr (GTA) 85 bp Sc: 67.45
GGAGGGGTTCCCGAGTGGCCAAAGGGATCAGACTGTAAATCTGACGGCTCTGCCTTCGAA
GGTTCGAATCCTTCTCCCTCCACCA
>Aeromonas_hydrophila_ATCC_7966_chr.trna114-ValGAC (2323017-2322941) Val (GAC) 77 bp Sc: 90.51
GCGTCCTTAGCTCAGCTGGTTAGAGCATCACCTTGACATGGTGGGGGTCGGTGGTTCGAA
TCCACTAGGACGCACCA
>Aeromonas_hydrophila_ATCC_7966_chr.trna117-ValGAC (2322705-2322629) Val (GAC) 77 bp Sc: 90.51
GCGTCCTTAGCTCAGCTGGTTAGAGCATCACCTTGACATGGTGGGGGTCGGTGGTTCGAA
TCCACTAGGACGCACCA
>Aeromonas_hydrophila_ATCC_7966_chr.trna112-ValGAC (2323200-2323124) Val (GAC) 77 bp Sc: 91.82
GGGTTTATAGCTCAGTTGGTTAGAGCACTACCTTGACATGGTAGGGGTCGTTGGTTCGAA
TCCAATTAAACCCACCA

>Aeromonas_hydrophila_ATCC_7966_chr.trna116-ValGAC (2322819-2322743) Val (GAC) 77 bp Sc: 91.82
GGGTTTATAGCTCAGTTGGTTAGAGCACTACCTTGACATGGTAGGGGTCGTTGGTTCGAA
TCCAATTAAACCCACCA
>Aeromonas_hydrophila_ATCC_7966_chr.trna113-ValGAC (2323111-2323035) Val (GAC) 77 bp Sc: 95.18
GCGTCCTTAGCTCAGCTGGTTAGAGCACCACCTTGACATGGTGGGGGTCGGTGGTTCGAA
TCCACTAGGACGCACCA
>Aeromonas_hydrophila_ATCC_7966_chr.trna66-ValTAC (4069765-4069690) Val (TAC) 76 bp Sc: 93.75
GGTCGCTTAGCTCAGCTGGGAGAGCACCTCCCTTACAAGGAGGGGGTCACTGGTTCGATC
CCAGTAGCGACCACCA
>Aeromonas_hydrophila_ATCC_7966_chr.trna70-ValTAC (4069352-4069277) Val (TAC) 76 bp Sc: 93.87
GGTCGTTTAGCTCAGCTGGGAGAGCACCTCCCTTACAAGGAGGGGGTCACTGGTTCGATC
CCAGTAACGACCACCA
>Aeromonas_hydrophila_ATCC_7966_chr.trna72-ValTAC (4069162-4069087) Val (TAC) 76 bp Sc: 93.87
GGTCGTTTAGCTCAGCTGGGAGAGCACCTCCCTTACAAGGAGGGGGTCACTGGTTCGATC
CCAGTAACGACCACCA
>Aeromonas_hydrophila_ATCC_7966_chr.trna74-ValTAC (4068970-4068895) Val (TAC) 76 bp Sc: 93.87
GGTCGTTTAGCTCAGCTGGGAGAGCACCTCCCTTACAAGGAGGGGGTCACTGGTTCGATC
CCAGTAACGACCACCA
>Aeromonas_hydrophila_ATCC_7966_chr.trna76-ValTAC (4068778-4068703) Val (TAC) 76 bp Sc: 93.87
GGTCGTTTAGCTCAGCTGGGAGAGCACCTCCCTTACAAGGAGGGGGTCACTGGTTCGATC
CCAGTAACGACCACCA
>Aeromonas_hydrophila_ATCC_7966_chr.trna78-ValTAC (4068587-4068512) Val (TAC) 76 bp Sc: 93.87
GGTCGTTTAGCTCAGCTGGGAGAGCACCTCCCTTACAAGGAGGGGGTCACTGGTTCGATC
CCAGTAACGACCACCA
>Aeromonas_hydrophila_ATCC_7966_chr.trna68-ValTAC (4069544-4069469) Val (TAC) 76 bp Sc: 94.81
GGTCGTTTAGCTCAGCTGGGAGAGCACCTCCCTTACAAGGAGGGGGTCACTGGTTCGAGC
CCAGTAACGACCACCA
>Aeromonas_salmonicida_A449_chr.trna33-AlaGGC (896653-896728) Ala (GGC) 76 bp Sc: 81.36
GGGGTTATAGCTCAGCTGGGAGAGCGCTTGCATGGCATGCAAGAGGTCCGCGGTTCGATC
CCGCGTAGCTCCACCA
>Aeromonas_salmonicida_A449_chr.trna34-AlaGGC (896757-896832) Ala (GGC) 76 bp Sc: 81.36
GGGGTTATAGCTCAGCTGGGAGAGCGCTTGCATGGCATGCAAGAGGTCCGCGGTTCGATC
CCGCGTAGCTCCACCA
>Aeromonas_salmonicida_A449_chr.trna35-AlaGGC (896881-896956) Ala (GGC) 76 bp Sc: 81.36
GGGGTTATAGCTCAGCTGGGAGAGCGCTTGCATGGCATGCAAGAGGTCCGCGGTTCGATC
CCGCGTAGCTCCACCA
>Aeromonas_salmonicida_A449_chr.trna36-AlaGGC (897005-897080) Ala (GGC) 76 bp Sc: 81.36
GGGGTTATAGCTCAGCTGGGAGAGCGCTTGCATGGCATGCAAGAGGTCCGCGGTTCGATC
CCGCGTAGCTCCACCA
>Aeromonas_salmonicida_A449_chr.trna27-AlaTGC (821126-821201) Ala (TGC) 76 bp Sc: 80.82
GGGGCTAAAGCTCAGCTGGGAGAGCGCCTGCTTTGCACGCAGGAGGTCTGCGGTTCGATC
CCGCATAGCTCCACCA
>Aeromonas_salmonicida_A449_chr.trna64-AlaTGC (4616728-4616653) Ala (TGC) 76 bp Sc: 88.77
GGGGCTATAGCTCAGCTGGGAGAGCGCCTGCTTTGCACGCAGGAGGTCTGCGGTTCGATC
CCGCATAGCTCCACCA
>Aeromonas_salmonicida_A449_chr.trna68-AlaTGC (4486047-4485972) Ala (TGC) 76 bp Sc: 88.77
GGGGCTATAGCTCAGCTGGGAGAGCGCCTGCTTTGCACGCAGGAGGTCTGCGGTTCGATC
CCGCATAGCTCCACCA
>Aeromonas_salmonicida_A449_chr.trna59-ArgACG (4107192-4107268) Arg (ACG) 77 bp Sc: 91.84
GCGCCCGTAGCTCAGCTGGATAGAGTACCTGGCTACGAACCAGGTGGTCGGAGGTTCGAA
TCCTCCCGGGCGCACCA
>Aeromonas_salmonicida_A449_chr.trna60-ArgACG (4107296-4107372) Arg (ACG) 77 bp Sc: 91.84
GCGCCCGTAGCTCAGCTGGATAGAGTACCTGGCTACGAACCAGGTGGTCGGAGGTTCGAA
TCCTCCCGGGCGCACCA
>Aeromonas_salmonicida_A449_chr.trna61-ArgACG (4107399-4107475) Arg (ACG) 77 bp Sc: 91.84
GCGCCCGTAGCTCAGCTGGATAGAGTACCTGGCTACGAACCAGGTGGTCGGAGGTTCGAA
TCCTCCCGGGCGCACCA
>Aeromonas_salmonicida_A449_chr.trna1-ArgCCG (32107-32183) Arg (CCG) 77 bp Sc: 95.23
GCGCCCGTAGCTCAGCTGGATAGAGCGCTGCCCTCCGGAGGCAGAGGTCACAGGTTCGAA
TCCTGTCGGGCGCACCA
>Aeromonas_salmonicida_A449_chr.trna54-ArgCCT (3507115-3507191) Arg (CCT) 77 bp Sc: 81.03
GCTCCCGTAGCTCAGTAGGATAGAGCGGTCCCCTCCTAAGGGACAGGCCACTGGTTCGAC
TCCAGTCGGGAGCACCA
>Aeromonas_salmonicida_A449_chr.trna49-ArgTCT (2613515-2613591) Arg (TCT) 77 bp Sc: 98.90
GCGCCCTTAGCTCAGTCGGATAGAGCAACGGCCTTCTAAGCCGTGGGTCGCAGGTTCGAA
TCCTGCAGGGCGCACCA
>Aeromonas_salmonicida_A449_chr.trna52-AsnGTT (2641321-2641396) Asn (GTT) 76 bp Sc: 79.67

TCCCTCGTAGTTCAGTCGGTAGAACGGCGGACTGTTAATCCGTATGTCACTGGTTCAAGT
CCTGTCGAGGGAGCCA
>Aeromonas_salmonicida_A449_chr.trna51-AsnGTT (2641243-2641318) Asn (GTT) 76 bp Sc: 85.17
TCCTCCTTAGTTCAGTCGGTAGAACGGCGGACTGTTAATCCGTATGTCACTGGTTCAAGT
CCAGTAGGAGGAGCCA
>Aeromonas_salmonicida_A449_chr.trna105-AsnGTT (880436-880361) Asn (GTT) 76 bp Sc: 86.00
TCCTCCTTAGTTCAGTCGGTAGAACGGCGGACTGTTAATCCGTATGTCGCTGGTTCAAGT
CCAGCAGGAGGAGCCA
>Aeromonas_salmonicida_A449_chr.trna16-AsnGTT (456680-456755) Asn (GTT) 76 bp Sc: 86.00
TCCTCCTTAGTTCAGTCGGTAGAACGGCGGACTGTTAATCCGTATGTCGCTGGTTCAAGT
CCAGCAGGAGGAGCCA
>Aeromonas_salmonicida_A449_chr.trna47-AsnGTT (2442944-2443019) Asn (GTT) 76 bp Sc: 87.36
TCCCCCGTAGTTCAGTCGGTAGAACGGCGGACTGTTAATCCGTATGTCACTGGTTCAAGT
CCAGTCGGGGGAGCCA
>Aeromonas_salmonicida_A449_chr.trna6-AspGTC (88626-88702) Asp (GTC) 77 bp Sc: 97.44
GGAGCGGTAGTTCAGTTGGTTAGAATACCGGCCTGTCACGCCGGGGGTCGCGGGTTCGAG
TCCCGTCCGCTCCGCCA
>Aeromonas_salmonicida_A449_chr.trna71-AspGTC (4358383-4358307) Asp (GTC) 77 bp Sc: 97.44
GGAGCGGTAGTTCAGTTGGTTAGAATACCGGCCTGTCACGCCGGGGGTCGCGGGTTCGAG
TCCCGTCCGCTCCGCCA
>Aeromonas_salmonicida_A449_chr.trna73-AspGTC (3888216-3888140) Asp (GTC) 77 bp Sc: 97.44
GGAGCGGTAGTTCAGTTGGTTAGAATACCGGCCTGTCACGCCGGGGGTCGCGGGTTCGAG
TCCCGTCCGCTCCGCCA
>Aeromonas_salmonicida_A449_chr.trna41-CysGCA (1635427-1635500) Cys (GCA) 74 bp Sc: 53.70
GGCGCGTTAGCAAAGCGGTTATGCAGCGGATTGCAAATCCGTTTAGTCCGGTTCGACTCC
GGAACGTGCCTCCA
>Aeromonas_salmonicida_A449_chr.trna78-CysGCA (3340584-3340511) Cys (GCA) 74 bp Sc: 53.70
GGCGCGTTAGCAAAGCGGTTATGCAGCGGATTGCAAATCCGTTTAGTCCGGTTCGACTCC
GGAACGTGCCTCCA
>Aeromonas_salmonicida_A449_chr.trna95-GlnTTG (1210742-1210668) Gln (TTG) 75 bp Sc: 62.60
TGGGATATCGCCAAGCGGTAAGGCAGCGGGTTTTGATCTCGCCATTCCTAGGTTCGAATC
CTAGTACCCCAGCCA
>Aeromonas_salmonicida_A449_chr.trna98-GlnTTG (1201153-1201079) Gln (TTG) 75 bp Sc: 62.85
TGGGGTATCGCCAAGCGGTAAGGCAGCGGGTTTTGATCTCGCCATCCCTAGGTTCGAATC
CTAGTACCCCAGCCA
>Aeromonas_salmonicida_A449_chr.trna101-GlnTTG (1200739-1200665) Gln (TTG) 75 bp Sc: 67.44
TGGGGTATCGCCAAGCGGTAAGGCAGCGGGTTTTGATCTCGCCATTCCTAGGTTCGAATC
CTAGTACCCCAGCCA
>Aeromonas_salmonicida_A449_chr.trna97-GlnTTG (1201268-1201194) Gln (TTG) 75 bp Sc: 67.44
TGGGGTATCGCCAAGCGGTAAGGCAGCGGGTTTTGATCTCGCCATTCCTAGGTTCGAATC
CTAGTACCCCAGCCA
>Aeromonas_salmonicida_A449_chr.trna99-GlnTTG (1201038-1200964) Gln (TTG) 75 bp Sc: 67.44
TGGGGTATCGCCAAGCGGTAAGGCAGCGGGTTTTGATCTCGCCATTCCTAGGTTCGAATC
CTAGTACCCCAGCCA
>Aeromonas_salmonicida_A449_chr.trna12-GluTTC (315033-315108) Glu (TTC) 76 bp Sc: 62.50
GTCCCCTTCGTCTAGAGGCCTAGGACACCGCCCTTTCACGGCGGTAACAGGGGTTCGAAT
CCCCTAGGGGACGCCA
>Aeromonas_salmonicida_A449_chr.trna5-GluTTC (85084-85159) Glu (TTC) 76 bp Sc: 62.50
GTCCCCTTCGTCTAGAGGCCTAGGACACCGCCCTTTCACGGCGGTAACAGGGGTTCGAAT
CCCCTAGGGGACGCCA
>Aeromonas_salmonicida_A449_chr.trna66-GluTTC (4537209-4537134) Glu (TTC) 76 bp Sc: 62.50
GTCCCCTTCGTCTAGAGGCCTAGGACACCGCCCTTTCACGGCGGTAACAGGGGTTCGAAT
CCCCTAGGGGACGCCA
>Aeromonas_salmonicida_A449_chr.trna70-GluTTC (4361925-4361850) Glu (TTC) 76 bp Sc: 62.50
GTCCCCTTCGTCTAGAGGCCTAGGACACCGCCCTTTCACGGCGGTAACAGGGGTTCGAAT
CCCCTAGGGGACGCCA
>Aeromonas_salmonicida_A449_chr.trna72-GluTTC (3891757-3891682) Glu (TTC) 76 bp Sc: 62.50
GTCCCCTTCGTCTAGAGGCCTAGGACACCGCCCTTTCACGGCGGTAACAGGGGTTCGAAT
CCCCTAGGGGACGCCA
>Aeromonas_salmonicida_A449_chr.trna75-GluTTC (3708217-3708142) Glu (TTC) 76 bp Sc: 62.50
GTCCCCTTCGTCTAGAGGCCTAGGACACCGCCCTTTCACGGCGGTAACAGGGGTTCGAAT
CCCCTAGGGGACGCCA
>Aeromonas_salmonicida_A449_chr.trna79-GlyCCC (3283584-3283511) Gly (CCC) 74 bp Sc: 74.92
GCGGGTGTAGTTCAATGGTAGAACGGTAGCTTCCCAAGCTGCATACGTGGGTTCGATTCC
CATCACCCGCTCCA
>Aeromonas_salmonicida_A449_chr.trna28-GlyGCC (890391-890466) Gly (GCC) 76 bp Sc: 93.74
GCGGGAATAGCTCAGTTGGTAGAGCACGACCTTGCCAAGGTCGGGGTCGCGAGTTCGAGT

CTCGTTTCCCGCTCCA
>Aeromonas_salmonicida_A449_chr.trna29-GlyGCC (890503-890578) Gly (GCC) 76 bp Sc: 93.74
GCGGGAATAGCTCAGTTGGTAGAGCACGACCTTGCCAAGGTCGGGGTCGCGAGTTCGAGT
CTCGTTTCCCGCTCCA
>Aeromonas_salmonicida_A449_chr.trna30-GlyGCC (890614-890689) Gly (GCC) 76 bp Sc: 93.74
GCGGGAATAGCTCAGTTGGTAGAGCACGACCTTGCCAAGGTCGGGGTCGCGAGTTCGAGT
CTCGTTTCCCGCTCCA
>Aeromonas_salmonicida_A449_chr.trna31-GlyGCC (890726-890801) Gly (GCC) 76 bp Sc: 93.74
GCGGGAATAGCTCAGTTGGTAGAGCACGACCTTGCCAAGGTCGGGGTCGCGAGTTCGAGT
CTCGTTTCCCGCTCCA
>Aeromonas_salmonicida_A449_chr.trna32-GlyGCC (890828-890903) Gly (GCC) 76 bp Sc: 93.74
GCGGGAATAGCTCAGTTGGTAGAGCACGACCTTGCCAAGGTCGGGGTCGCGAGTTCGAGT
CTCGTTTCCCGCTCCA
>Aeromonas_salmonicida_A449_chr.trna40-GlyGCC (1635316-1635391) Gly (GCC) 76 bp Sc: 93.74
GCGGGAATAGCTCAGTTGGTAGAGCACGACCTTGCCAAGGTCGGGGTCGCGAGTTCGAGT
CTCGTTTCCCGCTCCA
>Aeromonas_salmonicida_A449_chr.trna77-GlyGCC (3340716-3340641) Gly (GCC) 76 bp Sc: 93.74
GCGGGAATAGCTCAGTTGGTAGAGCACGACCTTGCCAAGGTCGGGGTCGCGAGTTCGAGT
CTCGTTTCCCGCTCCA
>Aeromonas_salmonicida_A449_chr.trna10-GlyTCC (291554-291628) Gly (TCC) 75 bp Sc: 61.33
GCGGGCATCGTATAATGGCTATTACCTCAGCCTTCCAAGCTGATGATGCGGGTTCGATTC
CCGCTGCCCGTTCCA
>Aeromonas_salmonicida_A449_chr.trna80-GlyTCC (3283410-3283336) Gly (TCC) 75 bp Sc: 64.85
GCGGGCATCGTATAATGGCTATTACCTCAGCCTTCCAAGCTGATGATGCGGGTTCGATTC
CCGCTGCCCGCTCCA
>Aeromonas_salmonicida_A449_chr.trna2-HisGTG (32234-32309) His (GTG) 76 bp Sc: 80.26
GTGGCTGTAGCTCAGTTGGTAGAGTCCCGGATTGTGATTCCGGTTGTCGTGGGTTCGAGC
CCCATCAGCCACCCCA
>Aeromonas_salmonicida_A449_chr.trna50-HisGTG (2613644-2613719) His (GTG) 76 bp Sc: 80.26
GTGGCTGTAGCTCAGTTGGTAGAGTCCCGGATTGTGATTCCGGTTGTCGTGGGTTCGAGC
CCCATCAGCCACCCCA
>Aeromonas_salmonicida_A449_chr.trna26-IleGAT (821029-821105) Ile (GAT) 77 bp Sc: 92.47
GGGTCTGTAGCTCAGGTGGTTAGAGCTCACCCCTGATAAGGGTGAGGTCGGTGGTTCGAG
TCCACTCAGACCCACCA
>Aeromonas_salmonicida_A449_chr.trna63-IleGAT (4616825-4616749) Ile (GAT) 77 bp Sc: 95.89
GGGTCTGTAGCTCAGGTGGTTAGAGCGCACCCCTGATAAGGGTGAGGTCGGTGGTTCGAG
TCCACTCAGACCCACCA
>Aeromonas_salmonicida_A449_chr.trna67-IleGAT (4486144-4486068) Ile (GAT) 77 bp Sc: 95.89
GGGTCTGTAGCTCAGGTGGTTAGAGCGCACCCCTGATAAGGGTGAGGTCGGTGGTTCGAG
TCCACTCAGACCCACCA
>Aeromonas_salmonicida_A449_chr.trna17-LeuCAA (468166-468233) Leu (CAA) 68 bp Sc: 22.83
GCTGATTTGAACCGGTACATGGACTGTTAATCCGTATGTCGCTGGTTCAAGTCCAGCAGG
AGGAGCCA
>Aeromonas_salmonicida_A449_chr.trna62-LeuCAA (4119115-4119200) Leu (CAA) 86 bp Sc: 73.65
GCCCGGGTGGCGAAATCGGTAGACGCAGCGGATTCAAAATCCGCCGATGAATAATCGTGT
CGGTTCGAGTCCGACCCCGGGCACCA
>Aeromonas_salmonicida_A449_chr.trna106-LeuCAG (502982-502897) Leu (CAG) 86 bp Sc: 63.52
GCGAAGGTGGCGGAATTGGTAGACGCGCTAGCTTCAGGTGTTAGTGTCCCACGGATGTGA
GGGTTCGAGTCCCTCTCTTCGCACCA
>Aeromonas_salmonicida_A449_chr.trna108-LeuCAG (502714-502629) Leu (CAG) 86 bp Sc: 63.52
GCGAAGGTGGCGGAATTGGTAGACGCGCTAGCTTCAGGTGTTAGTGTCCCACGGATGTGA
GGGTTCGAGTCCCTCTCTTCGCACCA
>Aeromonas_salmonicida_A449_chr.trna110-LeuCAG (502427-502342) Leu (CAG) 86 bp Sc: 63.52
GCGAAGGTGGCGGAATTGGTAGACGCGCTAGCTTCAGGTGTTAGTGTCCCACGGATGTGA
GGGTTCGAGTCCCTCTCTTCGCACCA
>Aeromonas_salmonicida_A449_chr.trna107-LeuCAG (502843-502759) Leu (CAG) 85 bp Sc: 66.55
GCGAAGGTGGCGGAATTGGTAGACGCGCTAGCTTCAGGTGTTAGTGCCCCCGGGTGTGAG
GGTTCGAGTCCCTCTCTTCGCACCA
>Aeromonas_salmonicida_A449_chr.trna109-LeuCAG (502584-502499) Leu (CAG) 86 bp Sc: 67.52
GCGAAGGTGGCGGAATTGGTAGACGCGCTAGCTTCAGGTGTTAGTGCCCCCCGGGTGTGA
GGGTTCGAGTCCCTCTCTTCGCACCA
>Aeromonas_salmonicida_A449_chr.trna3-LeuCAG (32330-32415) Leu (CAG) 86 bp Sc: 67.52
GCGAAGGTGGCGGAATTGGTAGACGCGCTAGCTTCAGGTGTTAGTGCCCCCCGGGTGTGA
GGGTTCGAGTCCCTCTCTTCGCACCA
>Aeromonas_salmonicida_A449_chr.trna37-LeuGAG (1035796-1035880) Leu (GAG) 85 bp Sc: 61.40
GCCGTGGTGGTGGAATTGGTAGACACGCTATCTTGAGGGGGTAGTGCTCTAGGGTGTGCG
GGTTCGAGTCCCGCCCACGGCACCA

>Aeromonas_salmonicida_A449_chr.trna42-LeuTAA (1635542-1635628) Leu (TAA) 87 bp Sc: 75.24
GCCCGAGTGGTGAAATCGGTAGACACAAGGGATTTAAAATCCCTCGACGTTCGCGTCGTG
CCGGTTCGATTCCGGCCTCGGGCACCA
>Aeromonas_salmonicida_A449_chr.trna103-LeuTAG (1200421-1200337) Leu (TAG) 85 bp Sc: 77.41
GCGGAAGTGGCGAAATTGGTAGACGCACCAGATTTAGGTTCTGGCGCCGCAAGGTGTGAG
AGTTCGAGTCTCTCCTTCCGCACCA
>Aeromonas_salmonicida_A449_chr.trna20-LysCTT (766579-766654) Lys (CTT) 76 bp Sc: 95.49
GGGTCGTTAGCTCAGTTGGTAGAGCAGTTGACTCTTAATCAATTGGTCGCTGGTTCGAAC
CCAGCACGACCCACCA
>Aeromonas_salmonicida_A449_chr.trna18-LysTTT (766339-766414) Lys (TTT) 76 bp Sc: 96.39
GGGTCGTTAGCTCAGTCGGTAGAGCAGTTGACTTTTAATCAATTGGTCGCTGGTTCGAAT
CCAGCACGACCCACCA
>Aeromonas_salmonicida_A449_chr.trna22-LysTTT (766774-766849) Lys (TTT) 76 bp Sc: 96.39
GGGTCGTTAGCTCAGTCGGTAGAGCAGTTGACTTTTAATCAATTGGTCGCTGGTTCGAAT
CCAGCACGACCCACCA
>Aeromonas_salmonicida_A449_chr.trna24-LysTTT (766967-767042) Lys (TTT) 76 bp Sc: 96.39
GGGTCGTTAGCTCAGTCGGTAGAGCAGTTGACTTTTAATCAATTGGTCGCTGGTTCGAAT
CCAGCACGACCCACCA
>Aeromonas_salmonicida_A449_chr.trna84-MetCAT (2379223-2379147) Met (CAT) 77 bp Sc: 83.89
CGCGGGGTGGAGCAGCTCGGTAGCTCGTCGGGCTCATAACCCGAAGGTCGTCAGTTCAAA
TCTGGCCCCCGCAACCA
>Aeromonas_salmonicida_A449_chr.trna85-MetCAT (2379044-2378968) Met (CAT) 77 bp Sc: 83.89
CGCGGGGTGGAGCAGCTCGGTAGCTCGTCGGGCTCATAACCCGAAGGTCGTCAGTTCAAA
TCTGGCCCCCGCAACCA
>Aeromonas_salmonicida_A449_chr.trna86-MetCAT (2378866-2378790) Met (CAT) 77 bp Sc: 83.89
CGCGGGGTGGAGCAGCTCGGTAGCTCGTCGGGCTCATAACCCGAAGGTCGTCAGTTCAAA
TCTGGCCCCCGCAACCA
>Aeromonas_salmonicida_A449_chr.trna87-MetCAT (2378687-2378611) Met (CAT) 77 bp Sc: 83.89
CGCGGGGTGGAGCAGCTCGGTAGCTCGTCGGGCTCATAACCCGAAGGTCGTCAGTTCAAA
TCTGGCCCCCGCAACCA
>Aeromonas_salmonicida_A449_chr.trna88-MetCAT (2378509-2378433) Met (CAT) 77 bp Sc: 83.89
CGCGGGGTGGAGCAGCTCGGTAGCTCGTCGGGCTCATAACCCGAAGGTCGTCAGTTCAAA
TCTGGCCCCCGCAACCA
>Aeromonas_salmonicida_A449_chr.trna89-MetCAT (2378331-2378255) Met (CAT) 77 bp Sc: 83.89
CGCGGGGTGGAGCAGCTCGGTAGCTCGTCGGGCTCATAACCCGAAGGTCGTCAGTTCAAA
TCTGGCCCCCGCAACCA
>Aeromonas_salmonicida_A449_chr.trna38-MetCAT (1035973-1036049) Met (CAT) 77 bp Sc: 84.66
CGCGGGGTGGAGCAGCATGGTAGCTCGTCGGGCTCATAACCCGAAGGTCGTCGGTTCAAA
TCCGGCCCCCGCAACCA
>Aeromonas_salmonicida_A449_chr.trna83-MetCAT (2379393-2379317) Met (CAT) 77 bp Sc: 86.71
CGCGGGGTGGAGCAGCTCGGTAGCTCGTCGGGCTCATAACCCGAAGGTCGTCGGTTCAAA
TCCGGCCCCCGCAACCA
>Aeromonas_salmonicida_A449_chr.trna74-MetCAT (3750543-3750467) Met (CAT) 77 bp Sc: 92.65
GGCCCCTTAGCTCAGTTGGTTAGAGCAGTCGACTCATAATCGATTGGTCGCCCGTTCAAG
TCGGGCAGGGGCCACCA
>Aeromonas_salmonicida_A449_chr.trna100-MetCAT (1200829-1200753) Met (CAT) 77 bp Sc: 96.50
GGCTATGTAGCTCAGTTGGTTAGAGCATCGCACTCATAATGCGAGGGTCACAGGTTCGAA
TCCCGTCATAGCCACCA
>Aeromonas_salmonicida_A449_chr.trna102-MetCAT (1200506-1200430) Met (CAT) 77 bp Sc: 96.50
GGCTATGTAGCTCAGTTGGTTAGAGCATCGCACTCATAATGCGAGGGTCACAGGTTCGAA
TCCCGTCATAGCCACCA
>Aeromonas_salmonicida_A449_chr.trna96-MetCAT (1201358-1201282) Met (CAT) 77 bp Sc: 96.50
GGCTATGTAGCTCAGTTGGTTAGAGCATCGCACTCATAATGCGAGGGTCACAGGTTCGAA
TCCCGTCATAGCCACCA
>Aeromonas_salmonicida_A449_chr.trna104-PheGAA (880517-880442) Phe (GAA) 76 bp Sc: 89.80
GCCCAGATAGCTCAGTCGGTAGAGCAGGGGATTGAAAATCCCCGTGTCGGCGGTTCGATT
CCGTCTCTGGGCACCA
>Aeromonas_salmonicida_A449_chr.trna13-PheGAA (456383-456458) Phe (GAA) 76 bp Sc: 89.80
GCCCAGATAGCTCAGTCGGTAGAGCAGGGGATTGAAAATCCCCGTGTCGGCGGTTCGATT
CCGTCTCTGGGCACCA
>Aeromonas_salmonicida_A449_chr.trna15-PheGAA (456596-456671) Phe (GAA) 76 bp Sc: 89.80
GCCCAGATAGCTCAGTCGGTAGAGCAGGGGATTGAAAATCCCCGTGTCGGCGGTTCGATT
CCGTCTCTGGGCACCA
>Aeromonas_salmonicida_A449_chr.trna7-ProCGG (190811-190887) Pro (CGG) 77 bp Sc: 89.45
CGGTGTGTAGCGCAGCTTGGTAGCGCACTTCGTTCGGGACGAAGGGGTCGGAGGTTCGAA
TCCTCTCACACCGACCA
>Aeromonas_salmonicida_A449_chr.trna92-ProGGG (1792944-1792868) Pro (GGG) 77 bp Sc: 74.34

CGGTGTGTAGCGCAGCCAGGTAGCGCATCTGCATGGGGTGTAGAGGGTCGGAGGTTCGAA
TCCTCTCACACCGACCA
>Aeromonas_salmonicida_A449_chr.trna93-ProGGG (1587424-1587348) Pro (GGG) 77 bp Sc: 74.34
CGGTGTGTAGCGCAGCCAGGTAGCGCATCTGCATGGGGTGTAGAGGGTCGGAGGTTCGAA
TCCTCTCACACCGACCA
>Aeromonas_salmonicida_A449_chr.trna4-ProTGG (32493-32569) Pro (TGG) 77 bp Sc: 86.34
CGGTGATTAGCGCAGCCCGGTAGCGCATCTGGTTTGGGACCAGAGGGTCAAAGGTTCGAA
TCCTTTATCACCGACCA
>Aeromonas_salmonicida_A449_chr.trna48-ProTGG (2613401-2613477) Pro (TGG) 77 bp Sc: 86.34
CGGTGATTAGCGCAGCCCGGTAGCGCATCTGGTTTGGGACCAGAGGGTCAAAGGTTCGAA
TCCTTTATCACCGACCA
>Aeromonas_salmonicida_A449_chr.trna90-SeC(p)TCA (1890799-1890712) SeC(p) (TCA) 88 bp Sc: 31.31
GGAAGTTGATGGGGTCTGGTGGCCTCCTCGGTCTTCAAAACCGATGTGAGCCGAGAAGGC
TTTGGCAGGTTCGATTCCTGCCTCTTCC
>Aeromonas_salmonicida_A449_chr.trna39-SerCGA (1352749-1352838) Ser (CGA) 90 bp Sc: 69.81
GGAGAGATGCCGGAGTGGCCGAACGGGATTGACTCGAAATCAATTGTGGCAGTAATGCCA
CCCAGGGTTCGAATCCCTGTCTCTCCGCCA
>Aeromonas_salmonicida_A449_chr.trna58-SerGCT (4107096-4107188) Ser (GCT) 93 bp Sc: 71.74
GGAGAGGTGGCCGAGAGGCTGAAGGCGCTCCCCTGCTAAGGGAGTATGCGGCTTATACCC
GCATCGGGGGTTCGAATCCCCCCTTCTCCGCCA
>Aeromonas_salmonicida_A449_chr.trna43-SerGGA (1866847-1866934) Ser (GGA) 88 bp Sc: 68.20
GGTGAGGTGTCCGAGTGGCTGAAGGAGCACGCCTGGAAAGTGTGTATACGGCAACGTATC
GAGGGTTCGAATCCCTCCCTCACCGCCA
>Aeromonas_salmonicida_A449_chr.trna82-SerGGA (2638663-2638576) Ser (GGA) 88 bp Sc: 68.20
GGTGAGGTGTCCGAGTGGCTGAAGGAGCACGCCTGGAAAGTGTGTATACGGCAACGTATC
GAGGGTTCGAATCCCTCCCTCACCGCCA
>Aeromonas_salmonicida_A449_chr.trna91-SerGGA (1827982-1827895) Ser (GGA) 88 bp Sc: 68.20
GGTGAGGTGTCCGAGTGGCTGAAGGAGCACGCCTGGAAAGTGTGTATACGGCAACGTATC
GAGGGTTCGAATCCCTCCCTCACCGCCA
>Aeromonas_salmonicida_A449_chr.trna76-SerTGA (3484129-3484042) Ser (TGA) 88 bp Sc: 68.13
GGAGGGGTGGCAGAGCGGCTGAATGCACCGGTCTTGAAAACCGGCGATGGGCGACCATCC
GTGGGTTCAAATCCCACCTCCTCCGCCA
>Aeromonas_salmonicida_A449_chr.trna81-SerTGA (2693978-2693891) Ser (TGA) 88 bp Sc: 68.13
GGAGGGGTGGCAGAGCGGCTGAATGCACCGGTCTTGAAAACCGGCGATGGGCGACCATCC
GTGGGTTCAAATCCCACCTCCTCCGCCA
>Aeromonas_salmonicida_A449_chr.trna11-SupCTA (293041-293117) Sup (CTA) 77 bp Sc: 74.88
AGGGGCGTAGTTCGAATTGGTAGAACAGCGGTCTCTAAAACCGATGGTTGCGGGTTCGAG
TCCTGCCGCCCCTGCCA
>Aeromonas_salmonicida_A449_chr.trna94-ThrCGT (1368894-1368819) Thr (CGT) 76 bp Sc: 82.74
GCCGGTATAGCTCAGTTGGTAGAGCAGCGCATTCGTAATGCGAAGGTCAGGGGTTCGACT
CCTCTTACCGGCACCG
>Aeromonas_salmonicida_A449_chr.trna65-ThrGGT (4613173-4613098) Thr (GGT) 76 bp Sc: 89.28
GCTGATATGGCTCAGTTGGTAGAGCGCATCCTTGGTAAGGATGAGGTCCCCAGTTCGACT
CTGGGTATCAGCACCA
>Aeromonas_salmonicida_A449_chr.trna69-ThrGGT (4482492-4482417) Thr (GGT) 76 bp Sc: 89.28
GCTGATATGGCTCAGTTGGTAGAGCGCATCCTTGGTAAGGATGAGGTCCCCAGTTCGACT
CTGGGTATCAGCACCA
>Aeromonas_salmonicida_A449_chr.trna14-ThrTGT (456510-456585) Thr (TGT) 76 bp Sc: 90.67
GCCGGCTTAGCTCAGTAGGCAGAGCAACTGACTTGTAATCAGTAGGTCACCAGTTCGATT
CCGGTAGCCGGCACCA
>Aeromonas_salmonicida_A449_chr.trna8-TrpCCA (243581-243657) Trp (CCA) 77 bp Sc: 74.57
AGGGGCGTAGTTCCAATTGGTAGAACAGCGGTCTCCAAAACCGATGGTTGCGGGTTCGAG
TCCTGCCGCCCCTGCCA
>Aeromonas_salmonicida_A449_chr.trna53-TyrGTA (3001139-3001223) Tyr (GTA) 85 bp Sc: 66.59
GGAGGGGTTCCCGAGCGGCCAAAGGGATCAGACTGTAAATCTGACGGCTCTGCCTTCGAA
GGTTCGAATCCTTCTCCCTCCACCA
>Aeromonas_salmonicida_A449_chr.trna55-TyrGTA (3541098-3541182) Tyr (GTA) 85 bp Sc: 66.59
GGAGGGGTTCCCGAGCGGCCAAAGGGATCAGACTGTAAATCTGACGGCTCTGCCTTCGAA
GGTTCGAATCCTTCTCCCTCCACCA
>Aeromonas_salmonicida_A449_chr.trna56-TyrGTA (3541222-3541306) Tyr (GTA) 85 bp Sc: 66.59
GGAGGGGTTCCCGAGCGGCCAAAGGGATCAGACTGTAAATCTGACGGCTCTGCCTTCGAA
GGTTCGAATCCTTCTCCCTCCACCA
>Aeromonas_salmonicida_A449_chr.trna57-TyrGTA (3541351-3541435) Tyr (GTA) 85 bp Sc: 66.59
GGAGGGGTTCCCGAGCGGCCAAAGGGATCAGACTGTAAATCTGACGGCTCTGCCTTCGAA
GGTTCGAATCCTTCTCCCTCCACCA
>Aeromonas_salmonicida_A449_chr.trna9-TyrGTA (291431-291515) Tyr (GTA) 85 bp Sc: 66.59
GGAGGGGTTCCCGAGCGGCCAAAGGGATCAGACTGTAAATCTGACGGCTCTGCCTTCGAA

GGTTCGAATCCTTCTCCCTCCACCA
>Aeromonas_salmonicida_A449_chr.trna46-ValGAC (2319713-2319789) Val (GAC) 77 bp Sc: 90.51
GCGTCCTTAGCTCAGCTGGTTAGAGCATCACCTTGACATGGTGGGGGTCGGTGGTTCGAA
TCCACTAGGACGCACCA
>Aeromonas_salmonicida_A449_chr.trna44-ValGAC (2319528-2319604) Val (GAC) 77 bp Sc: 91.82
GGGTTTATAGCTCAGTTGGTTAGAGCACTACCTTGACATGGTAGGGGTCGTTGGTTCGAA
TCCAATTAAACCCACCA
>Aeromonas_salmonicida_A449_chr.trna45-ValGAC (2319618-2319694) Val (GAC) 77 bp Sc: 95.18
GCGTCCTTAGCTCAGCTGGTTAGAGCACCACCTTGACATGGTGGGGGTCGGTGGTTCGAA
TCCACTAGGACGCACCA
>Aeromonas_salmonicida_A449_chr.trna19-ValTAC (766445-766520) Val (TAC) 76 bp Sc: 93.87
GGTCGTTTAGCTCAGCTGGGAGAGCACCTCCCTTACAAGGAGGGGGTCACTGGTTCGATC
CCAGTAACGACCACCA
>Aeromonas_salmonicida_A449_chr.trna21-ValTAC (766676-766751) Val (TAC) 76 bp Sc: 93.87
GGTCGTTTAGCTCAGCTGGGAGAGCACCTCCCTTACAAGGAGGGGGTCACTGGTTCGATC
CCAGTAACGACCACCA
>Aeromonas_salmonicida_A449_chr.trna23-ValTAC (766868-766943) Val (TAC) 76 bp Sc: 93.87
GGTCGTTTAGCTCAGCTGGGAGAGCACCTCCCTTACAAGGAGGGGGTCACTGGTTCGATC
CCAGTAACGACCACCA
>Aeromonas_salmonicida_A449_chr.trna25-ValTAC (767061-767136) Val (TAC) 76 bp Sc: 93.87
GGTCGTTTAGCTCAGCTGGGAGAGCACCTCCCTTACAAGGAGGGGGTCACTGGTTCGATC
CCAGTAACGACCACCA
>Anopheles_gambiae_chr3R.trna51-AlaAGC (12752402-12752330) Ala (AGC) 73 bp Sc: 59.55
GGGGGTGTAGCTCAGATGGTAGAGCGCTCGCTTAGCATGTGAGAGGCACTGGGATCGATA
CCCAGCATCTCCA
>Anopheles_gambiae_chr3R.trna14-AlaAGC (17865832-17865904) Ala (AGC) 73 bp Sc: 63.54
GGGGATGTAGCTCAGATGGTAGAGCGCTCGCTTAGCATGTGAGAGGTACGGGGATCGATA
CCCCGCATCTCCA
>Anopheles_gambiae_chr3R.trna39-AlaAGC (17866758-17866686) Ala (AGC) 73 bp Sc: 63.54
GGGGATGTAGCTCAGATGGTAGAGCGCTCGCTTAGCATGTGAGAGGTACGGGGATCGATA
CCCCGCATCTCCA
>Anopheles_gambiae_chr3R.trna35-AlaAGC (27632209-27632137) Ala (AGC) 73 bp Sc: 63.64
GGGGGTGTAGCTCAGATGGTAGAGCGCTCGCTTAGCATGTGAGAGGTACTGGGATCGATA
CCCAGCATCTCCA
>Anopheles_gambiae_chr2L.trna126-AlaAGC (22734404-22734332) Ala (AGC) 73 bp Sc: 65.14
GGGGGTGTAGCTCAGATGGTAGAGCGCTCGCTTAGCATGTGAGAGGTACCGGGATCGATA
CCCGGCATCTCCA
>Anopheles_gambiae_chr2L.trna127-AlaAGC (22670884-22670812) Ala (AGC) 73 bp Sc: 65.14
GGGGGTGTAGCTCAGATGGTAGAGCGCTCGCTTAGCATGTGAGAGGTACCGGGATCGATA
CCCGGCATCTCCA
>Anopheles_gambiae_chr2L.trna128-AlaAGC (22668604-22668532) Ala (AGC) 73 bp Sc: 65.14
GGGGGTGTAGCTCAGATGGTAGAGCGCTCGCTTAGCATGTGAGAGGTACCGGGATCGATA
CCCGGCATCTCCA
>Anopheles_gambiae_chr2R.trna105-AlaAGC (22868444-22868372) Ala (AGC) 73 bp Sc: 65.14
GGGGGTGTAGCTCAGATGGTAGAGCGCTCGCTTAGCATGTGAGAGGTACCGGGATCGATA
CCCGGCATCTCCA
>Anopheles_gambiae_chr2R.trna106-AlaAGC (22854590-22854518) Ala (AGC) 73 bp Sc: 65.14
GGGGGTGTAGCTCAGATGGTAGAGCGCTCGCTTAGCATGTGAGAGGTACCGGGATCGATA
CCCGGCATCTCCA
>Anopheles_gambiae_chr2R.trna118-AlaAGC (11395178-11395106) Ala (AGC) 73 bp Sc: 65.14
GGGGGTGTAGCTCAGATGGTAGAGCGCTCGCTTAGCATGTGAGAGGTACCGGGATCGATA
CCCGGCATCTCCA
>Anopheles_gambiae_chr2R.trna12-AlaAGC (11393010-11393082) Ala (AGC) 73 bp Sc: 65.14
GGGGGTGTAGCTCAGATGGTAGAGCGCTCGCTTAGCATGTGAGAGGTACCGGGATCGATA
CCCGGCATCTCCA
>Anopheles_gambiae_chr2R.trna19-AlaAGC (23459367-23459439) Ala (AGC) 73 bp Sc: 65.14
GGGGGTGTAGCTCAGATGGTAGAGCGCTCGCTTAGCATGTGAGAGGTACCGGGATCGATA
CCCGGCATCTCCA
>Anopheles_gambiae_chr2R.trna82-AlaAGC (47828539-47828467) Ala (AGC) 73 bp Sc: 65.14
GGGGGTGTAGCTCAGATGGTAGAGCGCTCGCTTAGCATGTGAGAGGTACCGGGATCGATA
CCCGGCATCTCCA
>Anopheles_gambiae_chr3L.trna16-AlaAGC (12585547-12585619) Ala (AGC) 73 bp Sc: 65.14
GGGGGTGTAGCTCAGATGGTAGAGCGCTCGCTTAGCATGTGAGAGGTACCGGGATCGATA
CCCGGCATCTCCA
>Anopheles_gambiae_chr3R.trna2-AlaAGC (4981962-4982034) Ala (AGC) 73 bp Sc: 65.14
GGGGGTGTAGCTCAGATGGTAGAGCGCTCGCTTAGCATGTGAGAGGTACCGGGATCGATA
CCCGGCATCTCCA

>Anopheles_gambiae_chr3R.trna36-AlaAGC (26006448-26006376) Ala (AGC) 73 bp Sc: 65.14
GGGGGTGTAGCTCAGATGGTAGAGCGCTCGCTTAGCATGTGAGAGGTACCGGGATCGATA
CCCGGCATCTCCA
>Anopheles_gambiae_chr3R.trna37-AlaAGC (17900289-17900217) Ala (AGC) 73 bp Sc: 65.14
GGGGGTGTAGCTCAGATGGTAGAGCGCTCGCTTAGCATGTGAGAGGTACCGGGATCGATA
CCCGGCATCTCCA
>Anopheles_gambiae_chr2L.trna13-AlaCGC (19729816-19729887) Ala (CGC) 72 bp Sc: 75.02
GGGGATGTAGCTCAGTGGTAGAGCGCTCGCTTCGCATGTGAGAAGTCCCGGGTTCAATCC
CCGGCATCTCCA
>Anopheles_gambiae_chr2R.trna34-AlaCGC (37444265-37444336) Ala (CGC) 72 bp Sc: 75.02
GGGGATGTAGCTCAGTGGTAGAGCGCTCGCTTCGCATGTGAGAAGTCCCGGGTTCAATCC
CCGGCATCTCCA
>Anopheles_gambiae_chr2R.trna79-AlaCGC (48604164-48604093) Ala (CGC) 72 bp Sc: 75.02
GGGGATGTAGCTCAGTGGTAGAGCGCTCGCTTCGCATGTGAGAAGTCCCGGGTTCAATCC
CCGGCATCTCCA
>Anopheles_gambiae_chr3L.trna83-AlaCGC (6441843-6441772) Ala (CGC) 72 bp Sc: 75.02
GGGGATGTAGCTCAGTGGTAGAGCGCTCGCTTCGCATGTGAGAAGTCCCGGGTTCAATCC
CCGGCATCTCCA
>Anopheles_gambiae_chr3L.trna84-AlaCGC (6440392-6440321) Ala (CGC) 72 bp Sc: 75.02
GGGGATGTAGCTCAGTGGTAGAGCGCTCGCTTCGCATGTGAGAAGTCCCGGGTTCAATCC
CCGGCATCTCCA
>Anopheles_gambiae_chr2R.trna81-AlaCGC (48460466-48460395) Ala (CGC) 72 bp Sc: 76.28
GGGGACGTAGCTCAGTGGTAGAGCGCTCGCTTCGCATGTGAGAAGTCCCGGGTTCAATCC
CCGGCGTCTCCA
>Anopheles_gambiae_chr2R.trna16-AlaTGC (11446452-11446523) Ala (TGC) 72 bp Sc: 75.14
GGGGATATAGCTCAGTGGTAGAGCGTTCGCTTTGCATGTGAAAGGCCCCGGGTTCGATCC
CCGGTATCTCCA
>Anopheles_gambiae_chr2R.trna7-AlaTGC (11352124-11352195) Ala (TGC) 72 bp Sc: 75.14
GGGGATATAGCTCAGTGGTAGAGCGTTCGCTTTGCATGTGAAAGGCCCCGGGTTCGATCC
CCGGTATCTCCA
>Anopheles_gambiae_chr2R.trna114-AlaTGC (11445770-11445699) Ala (TGC) 72 bp Sc: 75.54
GGGGATGTAGCTCAGTGGTAGAGCGTTCGCTTTGCATGTGAAAGGCCCCGGGTTCGATCC
CCGGCATCTCCA
>Anopheles_gambiae_chr2R.trna116-ArgACG (11444246-11444174) Arg (ACG) 73 bp Sc: 73.12
GGTCCTGTGGCGCAATGGATAACGCGTCTGACTACGGATCAGAAGATTCCAGGTTCGACT
CCTGGCAGGATCG
>Anopheles_gambiae_chr2R.trna124-ArgACG (11289350-11289278) Arg (ACG) 73 bp Sc: 73.12
GGTCCTGTGGCGCAATGGATAACGCGTCTGACTACGGATCAGAAGATTCCAGGTTCGACT
CCTGGCAGGATCG
>Anopheles_gambiae_chr2R.trna6-ArgACG (11337066-11337138) Arg (ACG) 73 bp Sc: 73.12
GGTCCTGTGGCGCAATGGATAACGCGTCTGACTACGGATCAGAAGATTCCAGGTTCGACT
CCTGGCAGGATCG
>Anopheles_gambiae_chr3L.trna30-ArgACG (28681285-28681357) Arg (ACG) 73 bp Sc: 73.12
GGTCCTGTGGCGCAATGGATAACGCGTCTGACTACGGATCAGAAGATTCCAGGTTCGACT
CCTGGCAGGATCG
>Anopheles_gambiae_chr3L.trna38-ArgACG (34356549-34356621) Arg (ACG) 73 bp Sc: 73.12
GGTCCTGTGGCGCAATGGATAACGCGTCTGACTACGGATCAGAAGATTCCAGGTTCGACT
CCTGGCAGGATCG
>Anopheles_gambiae_chr3L.trna49-ArgACG (34313891-34313819) Arg (ACG) 73 bp Sc: 73.12
GGTCCTGTGGCGCAATGGATAACGCGTCTGACTACGGATCAGAAGATTCCAGGTTCGACT
CCTGGCAGGATCG
>Anopheles_gambiae_chr3L.trna50-ArgACG (34306557-34306485) Arg (ACG) 73 bp Sc: 73.12
GGTCCTGTGGCGCAATGGATAACGCGTCTGACTACGGATCAGAAGATTCCAGGTTCGACT
CCTGGCAGGATCG
>Anopheles_gambiae_chr3L.trna57-ArgACG (28429020-28428948) Arg (ACG) 73 bp Sc: 73.12
GGTCCTGTGGCGCAATGGATAACGCGTCTGACTACGGATCAGAAGATTCCAGGTTCGACT
CCTGGCAGGATCG
>Anopheles_gambiae_chr3L.trna59-ArgACG (27208162-27208090) Arg (ACG) 73 bp Sc: 73.12
GGTCCTGTGGCGCAATGGATAACGCGTCTGACTACGGATCAGAAGATTCCAGGTTCGACT
CCTGGCAGGATCG
>Anopheles_gambiae_chr3L.trna85-ArgACG (5719903-5719831) Arg (ACG) 73 bp Sc: 73.12
GGTCCTGTGGCGCAATGGATAACGCGTCTGACTACGGATCAGAAGATTCCAGGTTCGACT
CCTGGCAGGATCG
>Anopheles_gambiae_chr2L.trna58-ArgCCT (47569212-47569284) Arg (CCT) 73 bp Sc: 67.27
GCCCCAGTGGCCTAATGGAGAAGGCACCGGCCTCCTAAGCCGGGGATTGTGGGTTCGAGT
CCCATCTGGGGTA
>Anopheles_gambiae_chr3L.trna52-ArgCCT (34274985-34274913) Arg (CCT) 73 bp Sc: 68.76

GCCCCAGTGGCCTAATGGACAAGGCACCGGCCTCCTAAGCCGGGGATTGTGGGTTCGAGT
CCCATCTGGGGTA
>Anopheles_gambiae_chr3R.trna17-ArgTCG (19628281-19628353) Arg (TCG) 73 bp Sc: 58.44
AGCCGTGTGGCCTAATGGAGAAGGCGTCGGACTTCGGATCCGAAGATTTCAGGTTCGAGT
CCTGTCACGGTCG
>Anopheles_gambiae_chr2R.trna39-ArgTCG (37857999-37858071) Arg (TCG) 73 bp Sc: 68.87
GACCGTGTGGCCTAATGGAGAAGGCGTCGGACTTCGGATCCGAAGATTGCAGGTTCGAGT
CCTGTCACGGTCG
>Anopheles_gambiae_chr3R.trna18-ArgTCG (30826562-30826634) Arg (TCG) 73 bp Sc: 68.87
GACCGTGTGGCCTAATGGAGAAGGCGTCGGACTTCGGATCCGAAGATTGCAGGTTCGAGT
CCTGTCACGGTCG
>Anopheles_gambiae_chr3R.trna53-ArgTCG (10173556-10173484) Arg (TCG) 73 bp Sc: 68.87
GACCGTGTGGCCTAATGGAGAAGGCGTCGGACTTCGGATCCGAAGATTGCAGGTTCGAGT
CCTGTCACGGTCG
>Anopheles_gambiae_chr2L.trna133-ArgTCG (15468290-15468218) Arg (TCG) 73 bp Sc: 69.68
GGCCGTGTGGCCTAATGGAGAAGGCGTCGGACTTCGGATCCGAAGATTGCAGGTTCGAGT
CCTGTCACGGTCG
>Anopheles_gambiae_chr2R.trna56-ArgTCG (50064657-50064729) Arg (TCG) 73 bp Sc: 69.68
GGCCGTGTGGCCTAATGGAGAAGGCGTCGGACTTCGGATCCGAAGATTGCAGGTTCGAGT
CCTGTCACGGTCG
>Anopheles_gambiae_chr2R.trna64-ArgTCG (55850358-55850430) Arg (TCG) 73 bp Sc: 69.68
GGCCGTGTGGCCTAATGGAGAAGGCGTCGGACTTCGGATCCGAAGATTGCAGGTTCGAGT
CCTGTCACGGTCG
>Anopheles_gambiae_chr3L.trna35-ArgTCG (34249079-34249151) Arg (TCG) 73 bp Sc: 71.07
GGCCGTGTGGCCTAATGGAGAAGGCGTCGGACTTCGAATCCGAAGATTGCAGGTTCGAGT
CCTGTCACGGTCG
>Anopheles_gambiae_chr2L.trna52-ArgTCT (46611269-46611341) Arg (TCT) 73 bp Sc: 81.43
GTCCCTCTGGCGCAGCGGATAGCGCGTTGGACTTCTAATCCAAAGGTCGTGGGTTCGATC
CCCACGAGGGATG
>Anopheles_gambiae_chr2R.trna51-ArgTCT (47717201-47717273) Arg (TCT) 73 bp Sc: 83.26
GTCCCTTTGGCGCAGCGGATAGCGCGTTGGACTTCTAATCCAAAGGTCGTGGGTTCGATC
CCCACAAGGGATG
>Anopheles_gambiae_chr2L.trna124-AsnGTT (28157607-28157534) Asn (GTT) 74 bp Sc: 83.46
GCCTTCGTGGCGCAATCGGTTAGCGCGTTCGGCTGTTAACCGAAAGGTTGGTGGTTCGAG
TCCACCCGGGGGCG
>Anopheles_gambiae_chr2L.trna138-AsnGTT (9781204-9781131) Asn (GTT) 74 bp Sc: 83.46
GCCTTCGTGGCGCAATCGGTTAGCGCGTTCGGCTGTTAACCGAAAGGTTGGTGGTTCGAG
TCCACCCGGGGGCG
>Anopheles_gambiae_chr2L.trna2-AsnGTT (9780556-9780629) Asn (GTT) 74 bp Sc: 83.46
GCCTTCGTGGCGCAATCGGTTAGCGCGTTCGGCTGTTAACCGAAAGGTTGGTGGTTCGAG
TCCACCCGGGGGCG
>Anopheles_gambiae_chr2L.trna35-AsnGTT (45501714-45501787) Asn (GTT) 74 bp Sc: 83.46
GCCTTCGTGGCGCAATCGGTTAGCGCGTTCGGCTGTTAACCGAAAGGTTGGTGGTTCGAG
TCCACCCGGGGGCG
>Anopheles_gambiae_chr2L.trna70-AsnGTT (45498846-45498773) Asn (GTT) 74 bp Sc: 83.46
GCCTTCGTGGCGCAATCGGTTAGCGCGTTCGGCTGTTAACCGAAAGGTTGGTGGTTCGAG
TCCACCCGGGGGCG
>Anopheles_gambiae_chr2L.trna82-AsnGTT (33780319-33780246) Asn (GTT) 74 bp Sc: 83.46
GCCTTCGTGGCGCAATCGGTTAGCGCGTTCGGCTGTTAACCGAAAGGTTGGTGGTTCGAG
TCCACCCGGGGGCG
>Anopheles_gambiae_chr2R.trna108-AsnGTT (12921150-12921077) Asn (GTT) 74 bp Sc: 83.46
GCCTTCGTGGCGCAATCGGTTAGCGCGTTCGGCTGTTAACCGAAAGGTTGGTGGTTCGAG
TCCACCCGGGGGCG
>Anopheles_gambiae_chr2R.trna33-AsnGTT (35216147-35216220) Asn (GTT) 74 bp Sc: 83.46
GCCTTCGTGGCGCAATCGGTTAGCGCGTTCGGCTGTTAACCGAAAGGTTGGTGGTTCGAG
TCCACCCGGGGGCG
>Anopheles_gambiae_chr3L.trna17-AsnGTT (13066762-13066835) Asn (GTT) 74 bp Sc: 83.46
GCCTTCGTGGCGCAATCGGTTAGCGCGTTCGGCTGTTAACCGAAAGGTTGGTGGTTCGAG
TCCACCCGGGGGCG
>Anopheles_gambiae_chr3L.trna86-AsnGTT (5669855-5669782) Asn (GTT) 74 bp Sc: 83.46
GCCTTCGTGGCGCAATCGGTTAGCGCGTTCGGCTGTTAACCGAAAGGTTGGTGGTTCGAG
TCCACCCGGGGGCG
>Anopheles_gambiae_chr3R.trna38-AsnGTT (17895633-17895560) Asn (GTT) 74 bp Sc: 83.46
GCCTTCGTGGCGCAATCGGTTAGCGCGTTCGGCTGTTAACCGAAAGGTTGGTGGTTCGAG
TCCACCCGGGGGCG
>Anopheles_gambiae_chr3L.trna2-AspGTC (5646636-5646707) Asp (GTC) 72 bp Sc: 62.35
TCCTCGATAGTATATTGGTCAGTATCCCCGCCTGTCACGCGGGAGACCGGGGTTCGATTC

CCCGTCTGGGAG
>Anopheles_gambiae_chr2R.trna107-AspGTC (14098372-14098301) Asp (GTC) 72 bp Sc: 74.99
TCCTCGATAGTATAGTGGTCAGTATCCCCGCCTGTCACGCGGGAGACCGGGGTTCGATTC
CCCGTCGGGGAG
>Anopheles_gambiae_chr2R.trna111-AspGTC (12101531-12101460) Asp (GTC) 72 bp Sc: 74.99
TCCTCGATAGTATAGTGGTCAGTATCCCCGCCTGTCACGCGGGAGACCGGGGTTCGATTC
CCCGTCGGGGAG
>Anopheles_gambiae_chr2R.trna112-AspGTC (12101075-12101004) Asp (GTC) 72 bp Sc: 74.99
TCCTCGATAGTATAGTGGTCAGTATCCCCGCCTGTCACGCGGGAGACCGGGGTTCGATTC
CCCGTCGGGGAG
>Anopheles_gambiae_chr2R.trna127-AspGTC (6094107-6094036) Asp (GTC) 72 bp Sc: 74.99
TCCTCGATAGTATAGTGGTCAGTATCCCCGCCTGTCACGCGGGAGACCGGGGTTCGATTC
CCCGTCGGGGAG
>Anopheles_gambiae_chr2R.trna17-AspGTC (12101674-12101745) Asp (GTC) 72 bp Sc: 74.99
TCCTCGATAGTATAGTGGTCAGTATCCCCGCCTGTCACGCGGGAGACCGGGGTTCGATTC
CCCGTCGGGGAG
>Anopheles_gambiae_chr2R.trna3-AspGTC (8880032-8880103) Asp (GTC) 72 bp Sc: 74.99
TCCTCGATAGTATAGTGGTCAGTATCCCCGCCTGTCACGCGGGAGACCGGGGTTCGATTC
CCCGTCGGGGAG
>Anopheles_gambiae_chr2R.trna4-AspGTC (11295934-11296005) Asp (GTC) 72 bp Sc: 74.99
TCCTCGATAGTATAGTGGTCAGTATCCCCGCCTGTCACGCGGGAGACCGGGGTTCGATTC
CCCGTCGGGGAG
>Anopheles_gambiae_chr3L.trna1-AspGTC (5645769-5645840) Asp (GTC) 72 bp Sc: 74.99
TCCTCGATAGTATAGTGGTCAGTATCCCCGCCTGTCACGCGGGAGACCGGGGTTCGATTC
CCCGTCGGGGAG
>Anopheles_gambiae_chr3L.trna11-AspGTC (8165478-8165549) Asp (GTC) 72 bp Sc: 74.99
TCCTCGATAGTATAGTGGTCAGTATCCCCGCCTGTCACGCGGGAGACCGGGGTTCGATTC
CCCGTCGGGGAG
>Anopheles_gambiae_chr3L.trna12-AspGTC (8167264-8167335) Asp (GTC) 72 bp Sc: 74.99
TCCTCGATAGTATAGTGGTCAGTATCCCCGCCTGTCACGCGGGAGACCGGGGTTCGATTC
CCCGTCGGGGAG
>Anopheles_gambiae_chr3L.trna13-AspGTC (8168553-8168624) Asp (GTC) 72 bp Sc: 74.99
TCCTCGATAGTATAGTGGTCAGTATCCCCGCCTGTCACGCGGGAGACCGGGGTTCGATTC
CCCGTCGGGGAG
>Anopheles_gambiae_chr3L.trna14-AspGTC (8171430-8171501) Asp (GTC) 72 bp Sc: 74.99
TCCTCGATAGTATAGTGGTCAGTATCCCCGCCTGTCACGCGGGAGACCGGGGTTCGATTC
CCCGTCGGGGAG
>Anopheles_gambiae_chr3L.trna15-AspGTC (8172720-8172791) Asp (GTC) 72 bp Sc: 74.99
TCCTCGATAGTATAGTGGTCAGTATCCCCGCCTGTCACGCGGGAGACCGGGGTTCGATTC
CCCGTCGGGGAG
>Anopheles_gambiae_chr3L.trna3-AspGTC (5648197-5648268) Asp (GTC) 72 bp Sc: 74.99
TCCTCGATAGTATAGTGGTCAGTATCCCCGCCTGTCACGCGGGAGACCGGGGTTCGATTC
CCCGTCGGGGAG
>Anopheles_gambiae_chr3L.trna4-AspGTC (5649132-5649203) Asp (GTC) 72 bp Sc: 74.99
TCCTCGATAGTATAGTGGTCAGTATCCCCGCCTGTCACGCGGGAGACCGGGGTTCGATTC
CCCGTCGGGGAG
>Anopheles_gambiae_chr3L.trna5-AspGTC (5650035-5650106) Asp (GTC) 72 bp Sc: 74.99
TCCTCGATAGTATAGTGGTCAGTATCCCCGCCTGTCACGCGGGAGACCGGGGTTCGATTC
CCCGTCGGGGAG
>Anopheles_gambiae_chr3L.trna67-AspGTC (18088605-18088534) Asp (GTC) 72 bp Sc: 74.99
TCCTCGATAGTATAGTGGTCAGTATCCCCGCCTGTCACGCGGGAGACCGGGGTTCGATTC
CCCGTCGGGGAG
>Anopheles_gambiae_chr3R.trna12-AspGTC (13539371-13539442) Asp (GTC) 72 bp Sc: 74.99
TCCTCGATAGTATAGTGGTCAGTATCCCCGCCTGTCACGCGGGAGACCGGGGTTCGATTC
CCCGTCGGGGAG
>Anopheles_gambiae_chr3R.trna42-AspGTC (13716929-13716858) Asp (GTC) 72 bp Sc: 74.99
TCCTCGATAGTATAGTGGTCAGTATCCCCGCCTGTCACGCGGGAGACCGGGGTTCGATTC
CCCGTCGGGGAG
>Anopheles_gambiae_chr2L.trna29-CysGCA (34057954-34058025) Cys (GCA) 72 bp Sc: 76.52
GGGGGTATAGCTCAGTGGTAGAGCATTCGACTGCAGATCGAGAGGTCCCCGGTTCAAACC
CGGGTGCCCCCT
>Anopheles_gambiae_chr3R.trna20-CysGCA (30913579-30913650) Cys (GCA) 72 bp Sc: 76.52
GGGGGTATAGCTCAGTGGTAGAGCATTCGACTGCAGATCGAGAGGTCCCCGGTTCAAACC
CGGGTGCCCCCT
>Anopheles_gambiae_chr3R.trna33-CysGCA (30915916-30915845) Cys (GCA) 72 bp Sc: 76.52
GGGGGTATAGCTCAGTGGTAGAGCATTCGACTGCAGATCGAGAGGTCCCCGGTTCAAACC
CGGGTGCCCCCT

>Anopheles_gambiae_chr3R.trna44-CysGCA (13548279-13548208) Cys (GCA) 72 bp Sc: 76.52
GGGGGTATAGCTCAGTGGTAGAGCATTCGACTGCAGATCGAGAGGTCCCCGGTTCAAACC
CGGGTGCCCCCT
>Anopheles_gambiae_chr3R.trna47-CysGCA (13317170-13317099) Cys (GCA) 72 bp Sc: 76.52
GGGGGTATAGCTCAGTGGTAGAGCATTCGACTGCAGATCGAGAGGTCCCCGGTTCAAACC
CGGGTGCCCCCT
>Anopheles_gambiae_chr3L.trna27-GlnCTG (28623639-28623710) Gln (CTG) 72 bp Sc: 61.53
GGTTCCATGGTGTAATGGTTAGCACTTAGGACTCTGAATCCAACGATCCGAGTTCAAATC
TCGGTTGAACCT
>Anopheles_gambiae_chr2L.trna60-GlnCTG (49094003-49093932) Gln (CTG) 72 bp Sc: 69.92
GGTTCCATGGTGTAACGGTTAGCACTCAGGACTCTGAATCCTGCGATCCGAGTTCAAATC
TCGGTGGAACCT
>Anopheles_gambiae_chr3R.trna41-GlnCTG (14573148-14573077) Gln (CTG) 72 bp Sc: 71.61
GGTTCCATGGTGTAATGGTTAGCACTTTGGACTCTGAATCCAACGATCCGAGTTCAAGTC
TCGGTGGAACCT
>Anopheles_gambiae_chr3L.trna28-GlnCTG (28664548-28664619) Gln (CTG) 72 bp Sc: 72.00
GGTTCTATGGTGTAATGGTTAGCACTTTGGACTCTGAATCCAACGATCCGAGTTCAAATC
TCGGTAGAACCT
>Anopheles_gambiae_chr3R.trna43-GlnCTG (13549101-13549030) Gln (CTG) 72 bp Sc: 72.37
GGTTCCATGGTGTAATGGTTAGCACTCAGGACTCTGAATCCTGCGATCCGAGTTCAAATC
TCGGTGGAACCT
>Anopheles_gambiae_chr2L.trna76-GlnCTG (38217510-38217439) Gln (CTG) 72 bp Sc: 73.26
GGTTCCATGGTGTAATGGTTAGCACTTTGGACTCTGAATCCAACGATCCGAGTTCAAATC
TCGGTGGAACCT
>Anopheles_gambiae_chr2R.trna58-GlnCTG (54263184-54263255) Gln (CTG) 72 bp Sc: 73.26
GGTTCCATGGTGTAATGGTTAGCACTTTGGACTCTGAATCCAACGATCCGAGTTCAAATC
TCGGTGGAACCT
>Anopheles_gambiae_chr2R.trna67-GlnCTG (57463166-57463095) Gln (CTG) 72 bp Sc: 73.26
GGTTCCATGGTGTAATGGTTAGCACTTTGGACTCTGAATCCAACGATCCGAGTTCAAATC
TCGGTGGAACCT
>Anopheles_gambiae_chr3L.trna31-GlnCTG (29128420-29128491) Gln (CTG) 72 bp Sc: 73.26
GGTTCCATGGTGTAATGGTTAGCACTTTGGACTCTGAATCCAACGATCCGAGTTCAAATC
TCGGTGGAACCT
>Anopheles_gambiae_chr3L.trna32-GlnCTG (29131476-29131547) Gln (CTG) 72 bp Sc: 73.26
GGTTCCATGGTGTAATGGTTAGCACTTTGGACTCTGAATCCAACGATCCGAGTTCAAATC
TCGGTGGAACCT
>Anopheles_gambiae_chr3L.trna78-GlnCTG (13281248-13281177) Gln (CTG) 72 bp Sc: 73.26
GGTTCCATGGTGTAATGGTTAGCACTTTGGACTCTGAATCCAACGATCCGAGTTCAAATC
TCGGTGGAACCT
>Anopheles_gambiae_chr3R.trna21-GlnTTG (30915644-30915715) Gln (TTG) 72 bp Sc: 69.56
GGTTCCATGGTGTAACGGTTAGCACTCAGGACTTTGAATCCTGCGATCCGAGTTCAAATC
TCGGTGGAACCT
>Anopheles_gambiae_chr3R.trna34-GlnTTG (30913845-30913774) Gln (TTG) 72 bp Sc: 69.56
GGTTCCATGGTGTAACGGTTAGCACTCAGGACTTTGAATCCTGCGATCCGAGTTCAAATC
TCGGTGGAACCT
>Anopheles_gambiae_chr2R.trna123-GlnTTG (11377425-11377354) Gln (TTG) 72 bp Sc: 72.01
GGTTCCATGGTGTAATGGTTAGCACTCAGGACTTTGAATCCTGCGATCCGAGTTCAAATC
TCGGTGGAACCT
>Anopheles_gambiae_chr3R.trna19-GlnTTG (30912631-30912702) Gln (TTG) 72 bp Sc: 72.01
GGTTCCATGGTGTAATGGTTAGCACTCAGGACTTTGAATCCTGCGATCCGAGTTCAAATC
TCGGTGGAACCT
>Anopheles_gambiae_chr2L.trna56-GluCTC (47207278-47207351) Glu (CTC) 74 bp Sc: 47.32
TCCCCTATTGTCTAGTGGTTCGCGATCTCCGGCTCTCACCCGGAACGGCCCGGGTTCGAT
TCCCGGTATGGGAA
>Anopheles_gambiae_chr2L.trna12-GluCTC (18784528-18784599) Glu (CTC) 72 bp Sc: 79.25
TCCCATATTGTCTAGTGGTTAGGATATCCGGCTCTCACCCGGAAGGCCCGGGTTCGATTC
CCGGTATGGGAA
>Anopheles_gambiae_chr2L.trna134-GluCTC (12526056-12525985) Glu (CTC) 72 bp Sc: 79.25
TCCCATATTGTCTAGTGGTTAGGATATCCGGCTCTCACCCGGAAGGCCCGGGTTCGATTC
CCGGTATGGGAA
>Anopheles_gambiae_chr2L.trna28-GluCTC (31100715-31100786) Glu (CTC) 72 bp Sc: 79.25
TCCCATATTGTCTAGTGGTTAGGATATCCGGCTCTCACCCGGAAGGCCCGGGTTCGATTC
CCGGTATGGGAA
>Anopheles_gambiae_chr2L.trna30-GluCTC (39363918-39363989) Glu (CTC) 72 bp Sc: 79.25
TCCCATATTGTCTAGTGGTTAGGATATCCGGCTCTCACCCGGAAGGCCCGGGTTCGATTC
CCGGTATGGGAA
>Anopheles_gambiae_chr2L.trna63-GluCTC (47207818-47207747) Glu (CTC) 72 bp Sc: 79.25

TCCCATATTGTCTAGTGGTTAGGATATCCGGCTCTCACCCGGAAGGCCCGGGTTCGATTC
CCGGTATGGGAA
>Anopheles_gambiae_chr2R.trna20-GluCTC (25207247-25207318) Glu (CTC) 72 bp Sc: 79.25
TCCCATATTGTCTAGTGGTTAGGATATCCGGCTCTCACCCGGAAGGCCCGGGTTCGATTC
CCGGTATGGGAA
>Anopheles_gambiae_chr2R.trna30-GluCTC (32444715-32444786) Glu (CTC) 72 bp Sc: 79.25
TCCCATATTGTCTAGTGGTTAGGATATCCGGCTCTCACCCGGAAGGCCCGGGTTCGATTC
CCGGTATGGGAA
>Anopheles_gambiae_chr2R.trna50-GluCTC (47455093-47455164) Glu (CTC) 72 bp Sc: 79.25
TCCCATATTGTCTAGTGGTTAGGATATCCGGCTCTCACCCGGAAGGCCCGGGTTCGATTC
CCGGTATGGGAA
>Anopheles_gambiae_chr3R.trna10-GluCTC (13509144-13509215) Glu (CTC) 72 bp Sc: 79.25
TCCCATATTGTCTAGTGGTTAGGATATCCGGCTCTCACCCGGAAGGCCCGGGTTCGATTC
CCGGTATGGGAA
>Anopheles_gambiae_chr3R.trna7-GluCTC (13440200-13440271) Glu (CTC) 72 bp Sc: 79.25
TCCCATATTGTCTAGTGGTTAGGATATCCGGCTCTCACCCGGAAGGCCCGGGTTCGATTC
CCGGTATGGGAA
>Anopheles_gambiae_chr3R.trna8-GluCTC (13444227-13444298) Glu (CTC) 72 bp Sc: 79.25
TCCCATATTGTCTAGTGGTTAGGATATCCGGCTCTCACCCGGAAGGCCCGGGTTCGATTC
CCGGTATGGGAA
>Anopheles_gambiae_chr2L.trna3-GluCTC (9892709-9892780) Glu (CTC) 72 bp Sc: 79.86
TCCCGTATTGTCTAGTGGTTAGGATATCCGGCTCTCACCCGGAAGGCCCGGGTTCGATTC
CCGGTACGGGAA
>Anopheles_gambiae_chr2L.trna62-GluCTC (47545459-47545388) Glu (CTC) 72 bp Sc: 79.86
TCCCGTATTGTCTAGTGGTTAGGATATCCGGCTCTCACCCGGAAGGCCCGGGTTCGATTC
CCGGTACGGGAA
>Anopheles_gambiae_chrX.trna1-GluCTC (13307851-13307922) Glu (CTC) 72 bp Sc: 79.86
TCCCGTATTGTCTAGTGGTTAGGATATCCGGCTCTCACCCGGAAGGCCCGGGTTCGATTC
CCGGTACGGGAA
>Anopheles_gambiae_chr3L.trna58-GluTTC (28172313-28172242) Glu (TTC) 72 bp Sc: 64.47
TCCCATATGCTCTAGTGGCTAGGATAACTGGCATTCACCCAGTAGGCCCGGGTTCGATTC
CCGGTATGGGAA
>Anopheles_gambiae_chr2R.trna115-GluTTC (11445015-11444944) Glu (TTC) 72 bp Sc: 78.23
TCCCATATGGTCTAGTGGCTAGGATAACTGGCTTTCACCCAGTAGGCCCGGGTTCGATTC
CCGGTATGGGAA
>Anopheles_gambiae_chr2L.trna10-GluTTC (16997688-16997759) Glu (TTC) 72 bp Sc: 80.27
TCCCATATGGTCTAGTGGCTAGGATATCTGGCTTTCACCCAGAAGGCCCGGGTTCGATTC
CCGGTATGGGAA
>Anopheles_gambiae_chr2L.trna125-GluTTC (23437111-23437040) Glu (TTC) 72 bp Sc: 80.27
TCCCATATGGTCTAGTGGCTAGGATATCTGGCTTTCACCCAGAAGGCCCGGGTTCGATTC
CCGGTATGGGAA
>Anopheles_gambiae_chr2L.trna55-GluTTC (46629471-46629542) Glu (TTC) 72 bp Sc: 80.27
TCCCATATGGTCTAGTGGCTAGGATATCTGGCTTTCACCCAGAAGGCCCGGGTTCGATTC
CCGGTATGGGAA
>Anopheles_gambiae_chr2L.trna57-GluTTC (47548690-47548761) Glu (TTC) 72 bp Sc: 80.27
TCCCATATGGTCTAGTGGCTAGGATATCTGGCTTTCACCCAGAAGGCCCGGGTTCGATTC
CCGGTATGGGAA
>Anopheles_gambiae_chr2L.trna64-GluTTC (47206803-47206732) Glu (TTC) 72 bp Sc: 80.27
TCCCATATGGTCTAGTGGCTAGGATATCTGGCTTTCACCCAGAAGGCCCGGGTTCGATTC
CCGGTATGGGAA
>Anopheles_gambiae_chr2R.trna68-GluTTC (55996576-55996505) Glu (TTC) 72 bp Sc: 80.27
TCCCATATGGTCTAGTGGCTAGGATATCTGGCTTTCACCCAGAAGGCCCGGGTTCGATTC
CCGGTATGGGAA
>Anopheles_gambiae_chr3L.trna40-GluTTC (36032748-36032819) Glu (TTC) 72 bp Sc: 80.27
TCCCATATGGTCTAGTGGCTAGGATATCTGGCTTTCACCCAGAAGGCCCGGGTTCGATTC
CCGGTATGGGAA
>Anopheles_gambiae_chr3L.trna80-GluTTC (9769013-9768942) Glu (TTC) 72 bp Sc: 80.27
TCCCATATGGTCTAGTGGCTAGGATATCTGGCTTTCACCCAGAAGGCCCGGGTTCGATTC
CCGGTATGGGAA
>Anopheles_gambiae_chr3L.trna81-GluTTC (9755825-9755754) Glu (TTC) 72 bp Sc: 80.27
TCCCATATGGTCTAGTGGCTAGGATATCTGGCTTTCACCCAGAAGGCCCGGGTTCGATTC
CCGGTATGGGAA
>Anopheles_gambiae_chr2L.trna130-GlyGCC (15482432-15482362) Gly (GCC) 71 bp Sc: 82.25
GCATCGGTGGTTCAGTGGTAGAATGCTCGCCTGCCACGCGGGCGGCCCGGGTTCGATTCC
CGGCCGATGCA
>Anopheles_gambiae_chr2L.trna131-GlyGCC (15480024-15479954) Gly (GCC) 71 bp Sc: 82.25
GCATCGGTGGTTCAGTGGTAGAATGCTCGCCTGCCACGCGGGCGGCCCGGGTTCGATTCC

CGGCCGATGCA
>Anopheles_gambiae_chr2L.trna132-GlyGCC (15473270-15473200) Gly (GCC) 71 bp Sc: 82.25
GCATCGGTGGTTCAGTGGTAGAATGCTCGCCTGCCACGCGGGCGGCCCGGGTTCGATTCC
CGGCCGATGCA
>Anopheles_gambiae_chr2L.trna7-GlyGCC (14668048-14668118) Gly (GCC) 71 bp Sc: 82.25
GCATCGGTGGTTCAGTGGTAGAATGCTCGCCTGCCACGCGGGCGGCCCGGGTTCGATTCC
CGGCCGATGCA
>Anopheles_gambiae_chr2R.trna61-GlyGCC (55542894-55542964) Gly (GCC) 71 bp Sc: 82.25
GCATCGGTGGTTCAGTGGTAGAATGCTCGCCTGCCACGCGGGCGGCCCGGGTTCGATTCC
CGGCCGATGCA
>Anopheles_gambiae_chr2R.trna66-GlyGCC (57612141-57612211) Gly (GCC) 71 bp Sc: 82.25
GCATCGGTGGTTCAGTGGTAGAATGCTCGCCTGCCACGCGGGCGGCCCGGGTTCGATTCC
CGGCCGATGCA
>Anopheles_gambiae_chr2R.trna72-GlyGCC (51448701-51448631) Gly (GCC) 71 bp Sc: 82.25
GCATCGGTGGTTCAGTGGTAGAATGCTCGCCTGCCACGCGGGCGGCCCGGGTTCGATTCC
CGGCCGATGCA
>Anopheles_gambiae_chr2R.trna73-GlyGCC (51401819-51401749) Gly (GCC) 71 bp Sc: 82.25
GCATCGGTGGTTCAGTGGTAGAATGCTCGCCTGCCACGCGGGCGGCCCGGGTTCGATTCC
CGGCCGATGCA
>Anopheles_gambiae_chr2R.trna96-GlyGCC (32435252-32435182) Gly (GCC) 71 bp Sc: 82.25
GCATCGGTGGTTCAGTGGTAGAATGCTCGCCTGCCACGCGGGCGGCCCGGGTTCGATTCC
CGGCCGATGCA
>Anopheles_gambiae_chr2R.trna99-GlyGCC (32429500-32429430) Gly (GCC) 71 bp Sc: 82.25
GCATCGGTGGTTCAGTGGTAGAATGCTCGCCTGCCACGCGGGCGGCCCGGGTTCGATTCC
CGGCCGATGCA
>Anopheles_gambiae_chr3L.trna10-GlyGCC (8129363-8129433) Gly (GCC) 71 bp Sc: 82.25
GCATCGGTGGTTCAGTGGTAGAATGCTCGCCTGCCACGCGGGCGGCCCGGGTTCGATTCC
CGGCCGATGCA
>Anopheles_gambiae_chr3L.trna87-GlyGCC (5637949-5637879) Gly (GCC) 71 bp Sc: 82.25
GCATCGGTGGTTCAGTGGTAGAATGCTCGCCTGCCACGCGGGCGGCCCGGGTTCGATTCC
CGGCCGATGCA
>Anopheles_gambiae_chr3R.trna23-GlyGCC (32850262-32850332) Gly (GCC) 71 bp Sc: 82.25
GCATCGGTGGTTCAGTGGTAGAATGCTCGCCTGCCACGCGGGCGGCCCGGGTTCGATTCC
CGGCCGATGCA
>Anopheles_gambiae_chr3R.trna24-GlyGCC (33021487-33021557) Gly (GCC) 71 bp Sc: 82.25
GCATCGGTGGTTCAGTGGTAGAATGCTCGCCTGCCACGCGGGCGGCCCGGGTTCGATTCC
CGGCCGATGCA
>Anopheles_gambiae_chr3R.trna32-GlyGCC (33706356-33706286) Gly (GCC) 71 bp Sc: 82.25
GCATCGGTGGTTCAGTGGTAGAATGCTCGCCTGCCACGCGGGCGGCCCGGGTTCGATTCC
CGGCCGATGCA
>Anopheles_gambiae_chr2L.trna71-GlyTCC (44792453-44792382) Gly (TCC) 72 bp Sc: 72.21
GCGTCGGTGGTGTAATGGTCAGCATAGTTGCCTTCCAAGCAGTTGATCCGGGTTCGATTC
CCGGCCGACGCA
>Anopheles_gambiae_chr2R.trna27-GlyTCC (27030814-27030885) Gly (TCC) 72 bp Sc: 72.21
GCGTCGGTGGTGTAATGGTCAGCATAGTTGCCTTCCAAGCAGTTGATCCGGGTTCGATTC
CCGGCCGACGCA
>Anopheles_gambiae_chr2R.trna37-GlyTCC (37856478-37856549) Gly (TCC) 72 bp Sc: 72.21
GCGTCGGTGGTGTAATGGTCAGCATAGTTGCCTTCCAAGCAGTTGATCCGGGTTCGATTC
CCGGCCGACGCA
>Anopheles_gambiae_chr2R.trna40-GlyTCC (37884513-37884584) Gly (TCC) 72 bp Sc: 72.21
GCGTCGGTGGTGTAATGGTCAGCATAGTTGCCTTCCAAGCAGTTGATCCGGGTTCGATTC
CCGGCCGACGCA
>Anopheles_gambiae_chr2R.trna47-GlyTCC (46945357-46945428) Gly (TCC) 72 bp Sc: 72.21
GCGTCGGTGGTGTAATGGTCAGCATAGTTGCCTTCCAAGCAGTTGATCCGGGTTCGATTC
CCGGCCGACGCA
>Anopheles_gambiae_chr2R.trna59-GlyTCC (55074547-55074618) Gly (TCC) 72 bp Sc: 72.21
GCGTCGGTGGTGTAATGGTCAGCATAGTTGCCTTCCAAGCAGTTGATCCGGGTTCGATTC
CCGGCCGACGCA
>Anopheles_gambiae_chr2R.trna65-GlyTCC (57467215-57467286) Gly (TCC) 72 bp Sc: 72.21
GCGTCGGTGGTGTAATGGTCAGCATAGTTGCCTTCCAAGCAGTTGATCCGGGTTCGATTC
CCGGCCGACGCA
>Anopheles_gambiae_chr3R.trna54-GlyTCC (9550053-9549982) Gly (TCC) 72 bp Sc: 72.21
GCGTCGGTGGTGTAATGGTCAGCATAGTTGCCTTCCAAGCAGTTGATCCGGGTTCGATTC
CCGGCCGACGCA
>Anopheles_gambiae_chr3L.trna25-HisGTG (20396619-20396687) His (GTG) 69 bp Sc: 42.78
CCCCTGGTCTAGTGGTTAGGACCCTACGTTGTGGCCGTAGTAACCCAGGTTCGAATCCTG
GTCACGGCA

>Anopheles_gambiae_chr2L.trna22-HisGTG (29810350-29810421) His (GTG) 72 bp Sc: 53.59
GCCGTAATCGTCTAGTGATTAGGACCCTACGTTGTGGCCGTAGTAACCCAGGTTCGAATC
CTGGTCACGGCA
>Anopheles_gambiae_chr3L.trna66-HisGTG (20297373-20297302) His (GTG) 72 bp Sc: 59.48
GCCGTGATCGTCTAGTAGTTAGGACCCTACGTTGTGGCCGTAGTAACCCAGGTTCGAATC
CTGGTCACGGCA
>Anopheles_gambiae_chr2L.trna17-HisGTG (29653053-29653124) His (GTG) 72 bp Sc: 60.34
GCCGTGATCGTCTAGTGGTTAGGACCCTACGTTGTGGCCGTAGTAACCCAGGTTCGAATC
CTGGTCACAGCA
>Anopheles_gambiae_chr2L.trna18-HisGTG (29762939-29763010) His (GTG) 72 bp Sc: 67.41
GCCGTGATCGTCTAGTGGTTAGGACCCTACGTTGTGGCCGTAGTAACCCAGGTTCGAATC
CTGGTCACGGCA
>Anopheles_gambiae_chr2L.trna20-HisGTG (29809134-29809205) His (GTG) 72 bp Sc: 67.41
GCCGTGATCGTCTAGTGGTTAGGACCCTACGTTGTGGCCGTAGTAACCCAGGTTCGAATC
CTGGTCACGGCA
>Anopheles_gambiae_chr2L.trna21-HisGTG (29809716-29809787) His (GTG) 72 bp Sc: 67.41
GCCGTGATCGTCTAGTGGTTAGGACCCTACGTTGTGGCCGTAGTAACCCAGGTTCGAATC
CTGGTCACGGCA
>Anopheles_gambiae_chr2L.trna23-HisGTG (29810769-29810840) His (GTG) 72 bp Sc: 67.41
GCCGTGATCGTCTAGTGGTTAGGACCCTACGTTGTGGCCGTAGTAACCCAGGTTCGAATC
CTGGTCACGGCA
>Anopheles_gambiae_chr2L.trna24-HisGTG (29811335-29811406) His (GTG) 72 bp Sc: 67.41
GCCGTGATCGTCTAGTGGTTAGGACCCTACGTTGTGGCCGTAGTAACCCAGGTTCGAATC
CTGGTCACGGCA
>Anopheles_gambiae_chr2L.trna27-HisGTG (30429871-30429942) His (GTG) 72 bp Sc: 67.41
GCCGTGATCGTCTAGTGGTTAGGACCCTACGTTGTGGCCGTAGTAACCCAGGTTCGAATC
CTGGTCACGGCA
>Anopheles_gambiae_chr2L.trna85-HisGTG (29804123-29804052) His (GTG) 72 bp Sc: 67.41
GCCGTGATCGTCTAGTGGTTAGGACCCTACGTTGTGGCCGTAGTAACCCAGGTTCGAATC
CTGGTCACGGCA
>Anopheles_gambiae_chr2R.trna38-HisGTG (37857375-37857446) His (GTG) 72 bp Sc: 67.41
GCCGTGATCGTCTAGTGGTTAGGACCCTACGTTGTGGCCGTAGTAACCCAGGTTCGAATC
CTGGTCACGGCA
>Anopheles_gambiae_chr2R.trna41-HisGTG (37885138-37885209) His (GTG) 72 bp Sc: 67.41
GCCGTGATCGTCTAGTGGTTAGGACCCTACGTTGTGGCCGTAGTAACCCAGGTTCGAATC
CTGGTCACGGCA
>Anopheles_gambiae_chr2R.trna60-HisGTG (55074886-55074957) His (GTG) 72 bp Sc: 67.41
GCCGTGATCGTCTAGTGGTTAGGACCCTACGTTGTGGCCGTAGTAACCCAGGTTCGAATC
CTGGTCACGGCA
>Anopheles_gambiae_chr2R.trna94-HisGTG (37851686-37851615) His (GTG) 72 bp Sc: 67.41
GCCGTGATCGTCTAGTGGTTAGGACCCTACGTTGTGGCCGTAGTAACCCAGGTTCGAATC
CTGGTCACGGCA
>Anopheles_gambiae_chr3L.trna19-HisGTG (19485955-19486026) His (GTG) 72 bp Sc: 67.41
GCCGTGATCGTCTAGTGGTTAGGACCCTACGTTGTGGCCGTAGTAACCCAGGTTCGAATC
CTGGTCACGGCA
>Anopheles_gambiae_chr3L.trna21-HisGTG (20339192-20339263) His (GTG) 72 bp Sc: 67.41
GCCGTGATCGTCTAGTGGTTAGGACCCTACGTTGTGGCCGTAGTAACCCAGGTTCGAATC
CTGGTCACGGCA
>Anopheles_gambiae_chr3L.trna23-HisGTG (20376423-20376494) His (GTG) 72 bp Sc: 67.41
GCCGTGATCGTCTAGTGGTTAGGACCCTACGTTGTGGCCGTAGTAACCCAGGTTCGAATC
CTGGTCACGGCA
>Anopheles_gambiae_chr3L.trna64-HisGTG (20314071-20314000) His (GTG) 72 bp Sc: 67.41
GCCGTGATCGTCTAGTGGTTAGGACCCTACGTTGTGGCCGTAGTAACCCAGGTTCGAATC
CTGGTCACGGCA
>Anopheles_gambiae_chr3R.trna45-HisGTG (13455434-13455363) His (GTG) 72 bp Sc: 67.41
GCCGTGATCGTCTAGTGGTTAGGACCCTACGTTGTGGCCGTAGTAACCCAGGTTCGAATC
CTGGTCACGGCA
>Anopheles_gambiae_chr3R.trna9-HisGTG (13455965-13456036) His (GTG) 72 bp Sc: 67.41
GCCGTGATCGTCTAGTGGTTAGGACCCTACGTTGTGGCCGTAGTAACCCAGGTTCGAATC
CTGGTCACGGCA
>Anopheles_gambiae_chr2R.trna87-IleAAT (46982362-46982289) Ile (AAT) 74 bp Sc: 73.04
GGCCCCATAGCTCAGCTGGTTAGAGCGTCGTGCTAATAACGCGAAGGTCGTGAGTTCGAT
CCTCTCTGGAGCCA
>Anopheles_gambiae_chr2R.trna31-IleAAT (32504775-32504848) Ile (AAT) 74 bp Sc: 77.68
GGCTCCATAGCTCAGCTGGTTAGAGCGTCGTGCTAATAACGCGAAGGTCGTGAGTTCGAT
CCTCTCTGGAGCCA
>Anopheles_gambiae_chr2R.trna45-IleAAT (46941350-46941423) Ile (AAT) 74 bp Sc: 77.68

GGCTCCATAGCTCAGCTGGTTAGAGCGTCGTGCTAATAACGCGAAGGTCGTGAGTTCGAT
CCTCTCTGGAGCCA
>Anopheles_gambiae_chr2R.trna46-IleAAT (46944131-46944204) Ile (AAT) 74 bp Sc: 77.68
GGCTCCATAGCTCAGCTGGTTAGAGCGTCGTGCTAATAACGCGAAGGTCGTGAGTTCGAT
CCTCTCTGGAGCCA
>Anopheles_gambiae_chr2R.trna84-IleAAT (46986810-46986737) Ile (AAT) 74 bp Sc: 77.68
GGCTCCATAGCTCAGCTGGTTAGAGCGTCGTGCTAATAACGCGAAGGTCGTGAGTTCGAT
CCTCTCTGGAGCCA
>Anopheles_gambiae_chr2R.trna85-IleAAT (46983598-46983525) Ile (AAT) 74 bp Sc: 77.68
GGCTCCATAGCTCAGCTGGTTAGAGCGTCGTGCTAATAACGCGAAGGTCGTGAGTTCGAT
CCTCTCTGGAGCCA
>Anopheles_gambiae_chr2R.trna86-IleAAT (46982808-46982735) Ile (AAT) 74 bp Sc: 77.68
GGCTCCATAGCTCAGCTGGTTAGAGCGTCGTGCTAATAACGCGAAGGTCGTGAGTTCGAT
CCTCTCTGGAGCCA
>Anopheles_gambiae_chr2R.trna88-IleAAT (46982076-46982003) Ile (AAT) 74 bp Sc: 77.68
GGCTCCATAGCTCAGCTGGTTAGAGCGTCGTGCTAATAACGCGAAGGTCGTGAGTTCGAT
CCTCTCTGGAGCCA
>Anopheles_gambiae_chr2R.trna89-IleAAT (46981273-46981200) Ile (AAT) 74 bp Sc: 77.68
GGCTCCATAGCTCAGCTGGTTAGAGCGTCGTGCTAATAACGCGAAGGTCGTGAGTTCGAT
CCTCTCTGGAGCCA
>Anopheles_gambiae_chr2R.trna97-IleAAT (32433965-32433892) Ile (AAT) 74 bp Sc: 77.68
GGCTCCATAGCTCAGCTGGTTAGAGCGTCGTGCTAATAACGCGAAGGTCGTGAGTTCGAT
CCTCTCTGGAGCCA
>Anopheles_gambiae_chr2R.trna98-IleAAT (32432028-32431955) Ile (AAT) 74 bp Sc: 77.68
GGCTCCATAGCTCAGCTGGTTAGAGCGTCGTGCTAATAACGCGAAGGTCGTGAGTTCGAT
CCTCTCTGGAGCCA
>Anopheles_gambiae_chr3R.trna52-IleAAT (11753139-11753066) Ile (AAT) 74 bp Sc: 77.68
GGCTCCATAGCTCAGCTGGTTAGAGCGTCGTGCTAATAACGCGAAGGTCGTGAGTTCGAT
CCTCTCTGGAGCCA
>Anopheles_gambiae_chr2R.trna1-IleTAT (170662-170764) Ile (TAT) 103 bp Sc: 65.20
GCTCCAGTGGCGCAATTGGTTAGCGCACGGTACTTATAAGACAGTAATCGTTGCTTGGCT
ATGAGCGATGCCGTGGTTGTGAGTTCGAGCCTCACCTGGAGCA
>Anopheles_gambiae_chr2L.trna81-IleTAT (35416889-35416792) Ile (TAT) 98 bp Sc: 65.28
GCTCCAGTGGCGCAATTGGTTAGCGCACGGTACTTATATGACAGTAATCGTTGCTTCGAG
CGATGCCGTGGTTGTGAGTTCGAGCCTCACCTGGAGCA
>Anopheles_gambiae_chr2L.trna61-LeuAAG (47557460-47557379) Leu (AAG) 82 bp Sc: 65.26
GGTAGCGTGGCCGAGCGGTCTAAGGCGCTGGTTTAAGGCACCAGTCACTTCGGTGGCGTG
GGTTCGAATCCCACCGCTGCCA
>Anopheles_gambiae_chr3L.trna26-LeuAAG (26139049-26139130) Leu (AAG) 82 bp Sc: 65.26
GGTAGCGTGGCCGAGCGGTCTAAGGCGCTGGTTTAAGGCACCAGTCACTTCGGTGGCGTG
GGTTCGAATCCCACCGCTGCCA
>Anopheles_gambiae_chr3L.trna56-LeuAAG (28657217-28657136) Leu (AAG) 82 bp Sc: 65.26
GGTAGCGTGGCCGAGCGGTCTAAGGCGCTGGTTTAAGGCACCAGTCACTTCGGTGGCGTG
GGTTCGAATCCCACCGCTGCCA
>Anopheles_gambiae_chr2R.trna35-LeuAAG (37707745-37707826) Leu (AAG) 82 bp Sc: 71.48
GGTAGCGTGGCCGAGCGGTCTAAGGCGCTGGTTTAAGGCACCAGTCTCCTCGGAGGCGTG
GGTTCGAATCCCACCGCTGCCA
>Anopheles_gambiae_chr2R.trna36-LeuAAG (37708261-37708342) Leu (AAG) 82 bp Sc: 71.48
GGTAGCGTGGCCGAGCGGTCTAAGGCGCTGGTTTAAGGCACCAGTCTCCTCGGAGGCGTG
GGTTCGAATCCCACCGCTGCCA
>Anopheles_gambiae_chr3R.trna13-LeuCAA (17839803-17839968) Leu (CAA) 166 bp Sc: 61.96
GTCAGGATGGCCGAGCGGTCTAAGGCGCCAGACTCAAGAGATAATCTTGCTCACAAAAGC
GGCGTATCGTCGATGGGTGTGAGTTCGCGCCTTCACAGGCGGGAGAGAAAGAGAGAGAGC
GTTCTGGTCCTCTCTGAGGGCGTGGGTTCGAATCCCACTTCTGACA
>Anopheles_gambiae_chr2R.trna75-LeuCAA (49481484-49481363) Leu (CAA) 122 bp Sc: 63.93
GTCAGGATGGCCGAGCGGTCTAAGGCGCCAGACTCAAGAGCAATCTTGCCCGCCTTCATG
CGGGTCTCTCCCGAGCGTTCTGGTCCTCTCTGAGGGCGTGGGTTCGAATCCCACTTCTGA
CA
>Anopheles_gambiae_chr2R.trna74-LeuCAA (49484024-49483908) Leu (CAA) 117 bp Sc: 64.70
GTCAGGATGGCCGAGCGGTCTAAGGCGCCAGACTCAAGAGCAATCTTGCCCGCTACTGCG
CGGGTCCGAGCGTTCTGGTCCTCTCTGAGGGCGTGGGTTCGAATCCCACTTCTGACA
>Anopheles_gambiae_chr2L.trna36-LeuCAG (45502940-45503022) Leu (CAG) 83 bp Sc: 74.76
GTCAGGATGGCCGAGCGGTCTAAGGCGCTACGTTCAGGTCGTAGTCTTCTCTGAAGGCGT
GGGTTCGAATCCCACTTCTGACA
>Anopheles_gambiae_chr2L.trna86-LeuCAG (29580192-29580110) Leu (CAG) 83 bp Sc: 75.17
GTCAGGATGGCCGAGCGGTCTAAGGCGCTACGTTCAGGTCGTAGTCTACTCTGTAGGCGT
GGGTTCGAATCCCACTTCTGACA

>Anopheles_gambiae_chr2R.trna103-LeuCAG (25724064-25723982) Leu (CAG) 83 bp Sc: 75.17
GTCAGGATGGCCGAGCGGTCTAAGGCGCTACGTTCAGGTCGTAGTCTACTCTGTAGGCGT
GGGTTCGAATCCCACTTCTGACA
>Anopheles_gambiae_chr2R.trna22-LeuCAG (25721497-25721579) Leu (CAG) 83 bp Sc: 75.17
GTCAGGATGGCCGAGCGGTCTAAGGCGCTACGTTCAGGTCGTAGTCTACTCTGTAGGCGT
GGGTTCGAATCCCACTTCTGACA
>Anopheles_gambiae_chr2R.trna23-LeuCAG (25721994-25722076) Leu (CAG) 83 bp Sc: 75.17
GTCAGGATGGCCGAGCGGTCTAAGGCGCTACGTTCAGGTCGTAGTCTACTCTGTAGGCGT
GGGTTCGAATCCCACTTCTGACA
>Anopheles_gambiae_chr2R.trna24-LeuCAG (25722329-25722411) Leu (CAG) 83 bp Sc: 75.17
GTCAGGATGGCCGAGCGGTCTAAGGCGCTACGTTCAGGTCGTAGTCTACTCTGTAGGCGT
GGGTTCGAATCCCACTTCTGACA
>Anopheles_gambiae_chr2R.trna25-LeuCAG (25722662-25722744) Leu (CAG) 83 bp Sc: 75.17
GTCAGGATGGCCGAGCGGTCTAAGGCGCTACGTTCAGGTCGTAGTCTACTCTGTAGGCGT
GGGTTCGAATCCCACTTCTGACA
>Anopheles_gambiae_chr2R.trna26-LeuCAG (25723627-25723709) Leu (CAG) 83 bp Sc: 75.17
GTCAGGATGGCCGAGCGGTCTAAGGCGCTACGTTCAGGTCGTAGTCTACTCTGTAGGCGT
GGGTTCGAATCCCACTTCTGACA
>Anopheles_gambiae_chr2R.trna69-LeuCAG (55867434-55867352) Leu (CAG) 83 bp Sc: 75.17
GTCAGGATGGCCGAGCGGTCTAAGGCGCTACGTTCAGGTCGTAGTCTACTCTGTAGGCGT
GGGTTCGAATCCCACTTCTGACA
>Anopheles_gambiae_chr3L.trna6-LeuCAG (5780915-5780997) Leu (CAG) 83 bp Sc: 75.17
GTCAGGATGGCCGAGCGGTCTAAGGCGCTACGTTCAGGTCGTAGTCTACTCTGTAGGCGT
GGGTTCGAATCCCACTTCTGACA
>Anopheles_gambiae_chr3L.trna37-LeuTAA (34355647-34355730) Leu (TAA) 84 bp Sc: 73.09
GTCAGGATGGCCGAGCGGTCTAAGGCGCCAGATTTAAGCTCTGGTTCCCGTAAGGGAGCG
TGGGTTCGAACCCCACTTCTGACA
>Anopheles_gambiae_chr3L.trna48-LeuTAA (34337754-34337671) Leu (TAA) 84 bp Sc: 73.09
GTCAGGATGGCCGAGCGGTCTAAGGCGCCAGATTTAAGCTCTGGTTCCCGTAAGGGAGCG
TGGGTTCGAACCCCACTTCTGACA
>Anopheles_gambiae_chr3R.trna11-LeuTAG (13521462-13521541) Leu (TAG) 80 bp Sc: 65.87
GGCAGCGTGGCCGAGCGGTCTAAGGCGCTGGTTTTAGGCACCAGTCAGAAATGGCGTGGG
TTCGAATCCCACCGCTGTCA
>Anopheles_gambiae_chr3R.trna6-LeuTAG (13341329-13341408) Leu (TAG) 80 bp Sc: 65.87
GGCAGCGTGGCCGAGCGGTCTAAGGCGCTGGTTTTAGGCACCAGTCAGAAATGGCGTGGG
TTCGAATCCCACCGCTGTCA
>Anopheles_gambiae_chr2R.trna113-LeuTAG (11446826-11446747) Leu (TAG) 80 bp Sc: 68.33
GGCAGCGTGGCCGAGTGGTCTAAGGCGCTGGTTTTAGGCACCAGTCAGAAATGGCGTGGG
TTCGAATCCCACCGCTGTCA
>Anopheles_gambiae_chr2R.trna76-LysCTT (49190073-49189998) Lys (CTT) 76 bp Sc: 76.48
GCCCGGCTAGCTCAGTCGGTAGAGCATGAGACTCTTAATCTCAGGGTCGTCGTGGGTTCG
AGCCCCACGTTGGGCG
>Anopheles_gambiae_chr2L.trna142-LysCTT (3876760-3876688) Lys (CTT) 73 bp Sc: 80.47
GCCCGGCTAGCTCAGTCGGTAGAGCATGAGACTCTTAATCTCAGGGTCGTGGGTTCGAGC
CCCACGTTGGGCG
>Anopheles_gambiae_chr2L.trna15-LysCTT (22777748-22777820) Lys (CTT) 73 bp Sc: 80.47
GCCCGGCTAGCTCAGTCGGTAGAGCATGAGACTCTTAATCTCAGGGTCGTGGGTTCGAGC
CCCACGTTGGGCG
>Anopheles_gambiae_chr2L.trna31-LysCTT (41633777-41633849) Lys (CTT) 73 bp Sc: 80.47
GCCCGGCTAGCTCAGTCGGTAGAGCATGAGACTCTTAATCTCAGGGTCGTGGGTTCGAGC
CCCACGTTGGGCG
>Anopheles_gambiae_chr2L.trna59-LysCTT (49093506-49093578) Lys (CTT) 73 bp Sc: 80.47
GCCCGGCTAGCTCAGTCGGTAGAGCATGAGACTCTTAATCTCAGGGTCGTGGGTTCGAGC
CCCACGTTGGGCG
>Anopheles_gambiae_chr2L.trna6-LysCTT (10738148-10738220) Lys (CTT) 73 bp Sc: 80.47
GCCCGGCTAGCTCAGTCGGTAGAGCATGAGACTCTTAATCTCAGGGTCGTGGGTTCGAGC
CCCACGTTGGGCG
>Anopheles_gambiae_chr2L.trna72-LysCTT (44764687-44764615) Lys (CTT) 73 bp Sc: 80.47
GCCCGGCTAGCTCAGTCGGTAGAGCATGAGACTCTTAATCTCAGGGTCGTGGGTTCGAGC
CCCACGTTGGGCG
>Anopheles_gambiae_chr2R.trna28-LysCTT (32434922-32434994) Lys (CTT) 73 bp Sc: 80.47
GCCCGGCTAGCTCAGTCGGTAGAGCATGAGACTCTTAATCTCAGGGTCGTGGGTTCGAGC
CCCACGTTGGGCG
>Anopheles_gambiae_chr2R.trna77-LysCTT (48627118-48627046) Lys (CTT) 73 bp Sc: 80.47
GCCCGGCTAGCTCAGTCGGTAGAGCATGAGACTCTTAATCTCAGGGTCGTGGGTTCGAGC
CCCACGTTGGGCG
>Anopheles_gambiae_chr3R.trna1-LysCTT (4555079-4555151) Lys (CTT) 73 bp Sc: 80.47

GCCCGGCTAGCTCAGTCGGTAGAGCATGAGACTCTTAATCTCAGGGTCGTGGGTTCGAGC
CCCACGTTGGGCG
>Anopheles_gambiae_chr3R.trna15-LysCTT (17867097-17867169) Lys (CTT) 73 bp Sc: 80.47
GCCCGGCTAGCTCAGTCGGTAGAGCATGAGACTCTTAATCTCAGGGTCGTGGGTTCGAGC
CCCACGTTGGGCG
>Anopheles_gambiae_chr3R.trna16-LysCTT (17901902-17901974) Lys (CTT) 73 bp Sc: 80.47
GCCCGGCTAGCTCAGTCGGTAGAGCATGAGACTCTTAATCTCAGGGTCGTGGGTTCGAGC
CCCACGTTGGGCG
>Anopheles_gambiae_chr3R.trna22-LysCTT (31273547-31273619) Lys (CTT) 73 bp Sc: 80.47
GCCCGGCTAGCTCAGTCGGTAGAGCATGAGACTCTTAATCTCAGGGTCGTGGGTTCGAGC
CCCACGTTGGGCG
>Anopheles_gambiae_chr3R.trna4-LysCTT (11156537-11156609) Lys (CTT) 73 bp Sc: 80.47
GCCCGGCTAGCTCAGTCGGTAGAGCATGAGACTCTTAATCTCAGGGTCGTGGGTTCGAGC
CCCACGTTGGGCG
>Anopheles_gambiae_chr3R.trna40-LysCTT (17865495-17865423) Lys (CTT) 73 bp Sc: 80.47
GCCCGGCTAGCTCAGTCGGTAGAGCATGAGACTCTTAATCTCAGGGTCGTGGGTTCGAGC
CCCACGTTGGGCG
>Anopheles_gambiae_chr3R.trna56-LysCTT (6928067-6927995) Lys (CTT) 73 bp Sc: 80.47
GCCCGGCTAGCTCAGTCGGTAGAGCATGAGACTCTTAATCTCAGGGTCGTGGGTTCGAGC
CCCACGTTGGGCG
>Anopheles_gambiae_chr3R.trna58-LysCTT (4551785-4551713) Lys (CTT) 73 bp Sc: 80.47
GCCCGGCTAGCTCAGTCGGTAGAGCATGAGACTCTTAATCTCAGGGTCGTGGGTTCGAGC
CCCACGTTGGGCG
>Anopheles_gambiae_chr2L.trna4-LysTTT (10058609-10058681) Lys (TTT) 73 bp Sc: 56.01
GCCCGGGTAGCTCAGTCGGTAGAGCTTTGGACTTTTTATCCAACGGTCTAGGGTTCAAGT
CCATATTTGGTCG
>Anopheles_gambiae_chr2R.trna55-LysTTT (49483079-49483151) Lys (TTT) 73 bp Sc: 78.68
GCCCGGGTAGCTCAGTCGGTAGAGCGTTGGACTTTTAATCCAACGGTCTAGGGTTCAAGT
CCCTATTCGGGCG
>Anopheles_gambiae_chr2R.trna54-LysTTT (49481924-49481996) Lys (TTT) 73 bp Sc: 81.91
GCCCGGATAGCTCAGTCGGTAGAGCGTTGGACTTTTAATCCAACGGTCTAGGGTTCAAGT
CCCTATTCGGGCG
>Anopheles_gambiae_chr2R.trna93-LysTTT (38990378-38990306) Lys (TTT) 73 bp Sc: 81.91
GCCCGGATAGCTCAGTCGGTAGAGCGTTGGACTTTTAATCCAACGGTCTAGGGTTCAAGT
CCCTATTCGGGCG
>Anopheles_gambiae_chr3R.trna28-LysTTT (39041184-39041112) Lys (TTT) 73 bp Sc: 81.91
GCCCGGATAGCTCAGTCGGTAGAGCGTTGGACTTTTAATCCAACGGTCTAGGGTTCAAGT
CCCTATTCGGGCG
>Anopheles_gambiae_chr3R.trna29-LysTTT (38713479-38713407) Lys (TTT) 73 bp Sc: 81.91
GCCCGGATAGCTCAGTCGGTAGAGCGTTGGACTTTTAATCCAACGGTCTAGGGTTCAAGT
CCCTATTCGGGCG
>Anopheles_gambiae_chr2R.trna32-LysTTT (35213034-35213106) Lys (TTT) 73 bp Sc: 82.41
GCCCGGATAGCTCAGTCGGTAGAGCGTTGGACTTTTAATCCAACGGTCTGGGGTTCAAGT
CCCCATTCGGGCG
>Anopheles_gambiae_chr2R.trna44-LysTTT (46939155-46939227) Lys (TTT) 73 bp Sc: 83.78
GCCCGGATAGCTCAGTCGGTAGAGCGTTGGACTTTTAATCCAACGGTCAAGGGTTCAAGT
CCCTTTTCGGGCG
>Anopheles_gambiae_chr2L.trna32-MetCAT (42159290-42159361) Met (CAT) 72 bp Sc: 63.19
AGCAGAGTGGCGCAGTGGAAGCGTGCTGGGCCCATAACCCAGAGGTCCGTAGATCGAAAC
TATGTTCTGCTA
>Anopheles_gambiae_chr2L.trna136-MetCAT (9784639-9784568) Met (CAT) 72 bp Sc: 65.36
AGCAGAGTGGCGCAGTGGAAGCGTGCTGGGCCCATAACCCAGAGGTCCGTAGATCGAAAC
TACGTTCTGCTA
>Anopheles_gambiae_chr2L.trna137-MetCAT (9782540-9782469) Met (CAT) 72 bp Sc: 65.36
AGCAGAGTGGCGCAGTGGAAGCGTGCTGGGCCCATAACCCAGAGGTCCGTAGATCGAAAC
TACGTTCTGCTA
>Anopheles_gambiae_chr2R.trna29-MetCAT (32435821-32435892) Met (CAT) 72 bp Sc: 65.36
AGCAGAGTGGCGCAGTGGAAGCGTGCTGGGCCCATAACCCAGAGGTCCGTAGATCGAAAC
TACGTTCTGCTA
>Anopheles_gambiae_chr3R.trna30-MetCAT (37767492-37767421) Met (CAT) 72 bp Sc: 65.36
AGCAGAGTGGCGCAGTGGAAGCGTGCTGGGCCCATAACCCAGAGGTCCGTAGATCGAAAC
TACGTTCTGCTA
>Anopheles_gambiae_chr2L.trna77-MetCAT (36768776-36768705) Met (CAT) 72 bp Sc: 68.98
AGCAGAGTGGCGCAGTGGAAGCGTGCTGGGCCCATAACCCAGAGGTCCGTAGATCGAAAC
TACGCTCTGCTA
>Anopheles_gambiae_chr2L.trna79-MetCAT (36763471-36763400) Met (CAT) 72 bp Sc: 68.98
AGCAGAGTGGCGCAGTGGAAGCGTGCTGGGCCCATAACCCAGAGGTCCGTAGATCGAAAC

TACGCTCTGCTA
>Anopheles_gambiae_chr3L.trna33-MetCAT (29453423-29453494) Met (CAT) 72 bp Sc: 68.98
AGCAGAGTGGCGCAGTGGAAGCGTGCTGGGCCCATAACCCAGAGGTCCGTAGATCGAAAC
TACGCTCTGCTA
>Anopheles_gambiae_chr3L.trna34-MetCAT (31025107-31025178) Met (CAT) 72 bp Sc: 68.98
AGCAGAGTGGCGCAGTGGAAGCGTGCTGGGCCCATAACCCAGAGGTCCGTAGATCGAAAC
TACGCTCTGCTA
>Anopheles_gambiae_chr3L.trna54-MetCAT (30229679-30229608) Met (CAT) 72 bp Sc: 68.98
AGCAGAGTGGCGCAGTGGAAGCGTGCTGGGCCCATAACCCAGAGGTCCGTAGATCGAAAC
TACGCTCTGCTA
>Anopheles_gambiae_chr3L.trna7-MetCAT (6504846-6504917) Met (CAT) 72 bp Sc: 68.98
AGCAGAGTGGCGCAGTGGAAGCGTGCTGGGCCCATAACCCAGAGGTCCGTAGATCGAAAC
TACGCTCTGCTA
>Anopheles_gambiae_chr2L.trna135-MetCAT (9892366-9892294) Met (CAT) 73 bp Sc: 79.42
GCCTCGATAGCGCAGTAGGTAGCGCGTAAGTCTCATAATCTTAAGGTCGTGAGTTCGATC
CTCACTCGGGGCA
>Anopheles_gambiae_chr2L.trna78-MetCAT (36764910-36764838) Met (CAT) 73 bp Sc: 79.42
GCCTCGATAGCGCAGTAGGTAGCGCGTAAGTCTCATAATCTTAAGGTCGTGAGTTCGATC
CTCACTCGGGGCA
>Anopheles_gambiae_chrX.trna3-MetCAT (13307540-13307468) Met (CAT) 73 bp Sc: 79.42
GCCTCGATAGCGCAGTAGGTAGCGCGTAAGTCTCATAATCTTAAGGTCGTGAGTTCGATC
CTCACTCGGGGCA
>Anopheles_gambiae_chr2L.trna80-MetCAT (36762792-36762720) Met (CAT) 73 bp Sc: 83.32
GCCTCGATAGCGCAGTCGGTAGCGCGTAAGTCTCATAATCTTAAGGTCGTGAGTTCGATC
CTCACTCGGGGCA
>Anopheles_gambiae_chr2R.trna125-MetCAT (6678571-6678499) Met (CAT) 73 bp Sc: 83.32
GCCTCGATAGCGCAGTCGGTAGCGCGTAAGTCTCATAATCTTAAGGTCGTGAGTTCGATC
CTCACTCGGGGCA
>Anopheles_gambiae_chr2R.trna62-MetCAT (55548615-55548687) Met (CAT) 73 bp Sc: 83.32
GCCTCGATAGCGCAGTCGGTAGCGCGTAAGTCTCATAATCTTAAGGTCGTGAGTTCGATC
CTCACTCGGGGCA
>Anopheles_gambiae_chr2R.trna63-MetCAT (55554655-55554727) Met (CAT) 73 bp Sc: 83.32
GCCTCGATAGCGCAGTCGGTAGCGCGTAAGTCTCATAATCTTAAGGTCGTGAGTTCGATC
CTCACTCGGGGCA
>Anopheles_gambiae_chr2R.trna109-PheGAA (12349341-12349269) Phe (GAA) 73 bp Sc: 82.98
GCCGAAATAGCTCAGTTGGGAGAGCGTTAGACTGAAGATCTAAAGGTCCCCGGTTCAATC
CCGGGTTTCGGCA
>Anopheles_gambiae_chr2R.trna110-PheGAA (12343355-12343283) Phe (GAA) 73 bp Sc: 82.98
GCCGAAATAGCTCAGTTGGGAGAGCGTTAGACTGAAGATCTAAAGGTCCCCGGTTCAATC
CCGGGTTTCGGCA
>Anopheles_gambiae_chr2R.trna129-PheGAA (524561-524489) Phe (GAA) 73 bp Sc: 82.98
GCCGAAATAGCTCAGTTGGGAGAGCGTTAGACTGAAGATCTAAAGGTCCCCGGTTCAATC
CCGGGTTTCGGCA
>Anopheles_gambiae_chr3L.trna18-PheGAA (13532451-13532523) Phe (GAA) 73 bp Sc: 82.98
GCCGAAATAGCTCAGTTGGGAGAGCGTTAGACTGAAGATCTAAAGGTCCCCGGTTCAATC
CCGGGTTTCGGCA
>Anopheles_gambiae_chr3L.trna41-PheGAA (38071954-38072026) Phe (GAA) 73 bp Sc: 82.98
GCCGAAATAGCTCAGTTGGGAGAGCGTTAGACTGAAGATCTAAAGGTCCCCGGTTCAATC
CCGGGTTTCGGCA
>Anopheles_gambiae_chr3L.trna42-PheGAA (38072518-38072590) Phe (GAA) 73 bp Sc: 82.98
GCCGAAATAGCTCAGTTGGGAGAGCGTTAGACTGAAGATCTAAAGGTCCCCGGTTCAATC
CCGGGTTTCGGCA
>Anopheles_gambiae_chr3L.trna43-PheGAA (38073179-38073251) Phe (GAA) 73 bp Sc: 82.98
GCCGAAATAGCTCAGTTGGGAGAGCGTTAGACTGAAGATCTAAAGGTCCCCGGTTCAATC
CCGGGTTTCGGCA
>Anopheles_gambiae_chr3L.trna44-PheGAA (38086228-38086300) Phe (GAA) 73 bp Sc: 82.98
GCCGAAATAGCTCAGTTGGGAGAGCGTTAGACTGAAGATCTAAAGGTCCCCGGTTCAATC
CCGGGTTTCGGCA
>Anopheles_gambiae_chr3L.trna60-PheGAA (24948593-24948521) Phe (GAA) 73 bp Sc: 82.98
GCCGAAATAGCTCAGTTGGGAGAGCGTTAGACTGAAGATCTAAAGGTCCCCGGTTCAATC
CCGGGTTTCGGCA
>Anopheles_gambiae_chr2L.trna143-ProAGG (1929996-1929925) Pro (AGG) 72 bp Sc: 52.43
GGCTCGTTGGTCTAGGAGTATGATTTTCGCTTAGGGTGCGAGAGGTCCTGGGTTAAAATC
CCGGACGAGCCC
>Anopheles_gambiae_chr3L.trna70-ProAGG (13618911-13618840) Pro (AGG) 72 bp Sc: 63.62
GACTTAATGGTCTAGGGGTATGATTTTCGCTTAGGGTGCGAGAGGTCCCGGGTTCAAATC
CCGGTTGAGCCC

>Anopheles_gambiae_chr3R.trna50-ProAGG (12956429-12956358) Pro (AGG) 72 bp Sc: 67.25
GGCTCGTTGGCCTAGGGGTATGATTTTCGCTTAGGGTGCGAGAGGTCCCGGGTTCAAATC
CCGGACGAGCCC
>Anopheles_gambiae_chr2L.trna141-ProAGG (6099779-6099708) Pro (AGG) 72 bp Sc: 72.15
GGCTCAATGGTCTAGGGGTATGATTTTCGCTTAGGGTGCGAGAGGTCCCGGGTTCAAATC
CCGGTTGAGCCC
>Anopheles_gambiae_chr3R.trna26-ProAGG (44892785-44892856) Pro (AGG) 72 bp Sc: 72.57
GGCTCGTTGGTCTAGGGGTATGATTTTCGCTTAGGGTGCGAGAGGTCCCGGGTTCAAATC
CCGGACGAGCCC
>Anopheles_gambiae_chr3R.trna46-ProAGG (13387438-13387367) Pro (AGG) 72 bp Sc: 72.57
GGCTCGTTGGTCTAGGGGTATGATTTTCGCTTAGGGTGCGAGAGGTCCCGGGTTCAAATC
CCGGACGAGCCC
>Anopheles_gambiae_chr3R.trna5-ProAGG (13290398-13290469) Pro (AGG) 72 bp Sc: 72.57
GGCTCGTTGGTCTAGGGGTATGATTTTCGCTTAGGGTGCGAGAGGTCCCGGGTTCAAATC
CCGGACGAGCCC
>Anopheles_gambiae_chr3L.trna73-ProCGG (13618308-13618236) Pro (CGG) 73 bp Sc: 56.89
AGCTCAATGGTCTAGGGGTATGATTTTCGCTTCGGGTGCGAGAGGTCCCCGGGTTCAAAT
CCCGGTTGAGCCC
>Anopheles_gambiae_chr2L.trna139-ProCGG (6101768-6101697) Pro (CGG) 72 bp Sc: 72.75
GGCTCAATGGTCTAGGGGTATGATTTTCGCTTCGGGTGCGAGAGGTCCCGGGTTCAAATC
CCGGTTGAGCCC
>Anopheles_gambiae_chr2L.trna140-ProCGG (6100005-6099934) Pro (CGG) 72 bp Sc: 72.75
GGCTCAATGGTCTAGGGGTATGATTTTCGCTTCGGGTGCGAGAGGTCCCGGGTTCAAATC
CCGGTTGAGCCC
>Anopheles_gambiae_chr3L.trna68-ProCGG (13619346-13619275) Pro (CGG) 72 bp Sc: 72.75
GGCTCAATGGTCTAGGGGTATGATTTTCGCTTCGGGTGCGAGAGGTCCCGGGTTCAAATC
CCGGTTGAGCCC
>Anopheles_gambiae_chr3L.trna71-ProCGG (13618710-13618639) Pro (CGG) 72 bp Sc: 72.75
GGCTCAATGGTCTAGGGGTATGATTTTCGCTTCGGGTGCGAGAGGTCCCGGGTTCAAATC
CCGGTTGAGCCC
>Anopheles_gambiae_chr3L.trna76-ProCGG (13617696-13617625) Pro (CGG) 72 bp Sc: 72.75
GGCTCAATGGTCTAGGGGTATGATTTTCGCTTCGGGTGCGAGAGGTCCCGGGTTCAAATC
CCGGTTGAGCCC
>Anopheles_gambiae_chr3L.trna77-ProCGG (13617495-13617424) Pro (CGG) 72 bp Sc: 72.75
GGCTCAATGGTCTAGGGGTATGATTTTCGCTTCGGGTGCGAGAGGTCCCGGGTTCAAATC
CCGGTTGAGCCC
>Anopheles_gambiae_chr2R.trna42-ProCGG (44033938-44034009) Pro (CGG) 72 bp Sc: 72.94
GGCTCGTTGGTCTAGGGGTATGATTTTCGCTTCGGGTGCGAGAGGTCCCGGGTTCAATTC
CCGGACGAGCCC
>Anopheles_gambiae_chr2R.trna91-ProCGG (44047500-44047429) Pro (CGG) 72 bp Sc: 73.17
GGCTCGTTGGTCTAGGGGTATGATTTTCGCTTCGGGTGCGAGAGGTCCCGGGTTCAAATC
CCGGACGAGCCC
>Anopheles_gambiae_chr2L.trna129-ProCGG (15723520-15723449) Pro (CGG) 72 bp Sc: 76.28
GGCTCGTTGGTCTAGGGGTATGATTCTCGCTTCGGGTGCGAGAGGTCCCGGGTTCAATTC
CCGGACGAGCCC
>Anopheles_gambiae_chr2R.trna80-ProCGG (48533761-48533690) Pro (CGG) 72 bp Sc: 76.28
GGCTCGTTGGTCTAGGGGTATGATTCTCGCTTCGGGTGCGAGAGGTCCCGGGTTCAATTC
CCGGACGAGCCC
>Anopheles_gambiae_chr3L.trna53-ProCGG (34271862-34271791) Pro (CGG) 72 bp Sc: 76.28
GGCTCGTTGGTCTAGGGGTATGATTCTCGCTTCGGGTGCGAGAGGTCCCGGGTTCAATTC
CCGGACGAGCCC
>Anopheles_gambiae_chr3L.trna55-ProCGG (28736472-28736401) Pro (CGG) 72 bp Sc: 76.52
GGCTCGTTGGTCTAGGGGTATGATTCTCGCTTCGGGTGCGAGAGGTCCCGGGTTCAAATC
CCGGACGAGCCC
>Anopheles_gambiae_chr3L.trna74-ProTGG (13618106-13618035) Pro (TGG) 72 bp Sc: 69.20
GGCTCAATGGTCTAGGGGTATGATTCTCGCTTTGGGTGCGAGAGGTCCCGGGTTCAAATC
CCGGTTTAGCCC
>Anopheles_gambiae_chr2L.trna11-ProTGG (18649875-18649946) Pro (TGG) 72 bp Sc: 75.74
GGCTCAATGGTCTAGGGGTATGATTCTCGCTTTGGGTGCGAGAGGTCCCGGGTTCAAATC
CCGGTTGAGCCC
>Anopheles_gambiae_chr2R.trna11-ProTGG (11388289-11388360) Pro (TGG) 72 bp Sc: 75.74
GGCTCAATGGTCTAGGGGTATGATTCTCGCTTTGGGTGCGAGAGGTCCCGGGTTCAAATC
CCGGTTGAGCCC
>Anopheles_gambiae_chr2R.trna119-ProTGG (11394363-11394292) Pro (TGG) 72 bp Sc: 75.74
GGCTCAATGGTCTAGGGGTATGATTCTCGCTTTGGGTGCGAGAGGTCCCGGGTTCAAATC
CCGGTTGAGCCC
>Anopheles_gambiae_chr2R.trna120-ProTGG (11393576-11393505) Pro (TGG) 72 bp Sc: 75.74

GGCTCAATGGTCTAGGGGTATGATTCTCGCTTTGGGTGCGAGAGGTCCCGGGTTCAAATC
CCGGTTGAGCCC
>Anopheles_gambiae_chr3L.trna69-ProTGG (13619112-13619041) Pro (TGG) 72 bp Sc: 75.74
GGCTCAATGGTCTAGGGGTATGATTCTCGCTTTGGGTGCGAGAGGTCCCGGGTTCAAATC
CCGGTTGAGCCC
>Anopheles_gambiae_chr3L.trna72-ProTGG (13618509-13618438) Pro (TGG) 72 bp Sc: 75.74
GGCTCAATGGTCTAGGGGTATGATTCTCGCTTTGGGTGCGAGAGGTCCCGGGTTCAAATC
CCGGTTGAGCCC
>Anopheles_gambiae_chr3L.trna75-ProTGG (13617897-13617826) Pro (TGG) 72 bp Sc: 75.74
GGCTCAATGGTCTAGGGGTATGATTCTCGCTTTGGGTGCGAGAGGTCCCGGGTTCAAATC
CCGGTTGAGCCC
>Anopheles_gambiae_chr2R.trna102-LeuCAA (26626389-26626314) Leu (CAA) 76 bp Sc: 21.30
GGTCTGGTGGTAGAATCGTCAACTCGTACGACTCAAAAACATATGCTCGTCATGGGTTCA
AGCCCCGAATGGAACG
>Anopheles_gambiae_chrX.trna4-ThrCGT (9742899-9742827) Thr (CGT) 73 bp Sc: 24.54
TCCGGAATAGTGCAATGGTAAGCGTTCAGGCTTCGTACGCCACAGTTTTTGTGTTCGAAT
CCCGATTTCAGCA
>Anopheles_gambiae_chrX.trna2-AsnGTT (13578639-13578567) Asn (GTT) 73 bp Sc: 23.09
CCGCTGATAGTGTAGCGGGTAGAGCGCTCGGCTGTTGACCGGCACGTTACAAGTTCGAAC
TGTGCTTAGTGCG
>Anopheles_gambiae_chr3L.trna61-LeuTAA (24497237-24497164) Leu (TAA) 74 bp Sc: 21.96
GGTCTGGTGGTACAGTCATCAATATGTACGTCTTAAAAACATGCCCGTCATGGGTTCAAG
CCCCGAATAGACCG
>Anopheles_gambiae_chrX.trna5-LeuTAA (7949565-7949492) Leu (TAA) 74 bp Sc: 22.18
GGTCTCGTAGTACAGTCGTCAACTCGTACGACTTAACATCATGCTCGTCATGGGTTCGAT
CCCCAAATAGACCG
>Anopheles_gambiae_chr3L.trna62-LeuTAA (22981229-22981156) Leu (TAA) 74 bp Sc: 22.27
GAACCCATGGCACAGTGGTTAGCTCGCATGACTTAAAAACACGCCCGTGGTGGGTTCAAG
CCTCGCATGGTCCG
>Anopheles_gambiae_chr2L.trna5-LeuTAA (10618945-10619018) Leu (TAA) 74 bp Sc: 23.94
GGTCTCGTAGTACAGTCGTCAACTCGTACGACTTAAAAACATAACCGTCATGGGTTCAAT
CCCCAAATAGACCG
>Anopheles_gambiae_chr3R.trna27-SupTTA (45170591-45170517) Sup (TTA) 75 bp Sc: 20.86
GGTCTGGTGGTATAGTTGTCAACTCGTACGACTTTAACAACATGCCCATCATGGGTTCAA
GCCCCGTCTAGACCG
>Anopheles_gambiae_chr2R.trna10-SerAGA (11365429-11365510) Ser (AGA) 82 bp Sc: 83.36
GCAGTCGTGGCCGAGCGGTTAAGGCGTCTGACTAGAAATCAGATTCCCTCTGGGAGCGTA
GGTTCGAATCCTACCGGCTGCG
>Anopheles_gambiae_chr2R.trna9-SerAGA (11364131-11364212) Ser (AGA) 82 bp Sc: 83.36
GCAGTCGTGGCCGAGCGGTTAAGGCGTCTGACTAGAAATCAGATTCCCTCTGGGAGCGTA
GGTTCGAATCCTACCGGCTGCG
>Anopheles_gambiae_chr2L.trna9-SerAGA (16557453-16557534) Ser (AGA) 82 bp Sc: 84.54
GCAGTCGTGGCCGAGCGGTTAAGGCGTCTGACTAGAAATCAGATTCCCTCTGGGAGCGTA
GGTTCGAATCCTACCGACTGCG
>Anopheles_gambiae_chr2R.trna2-SerAGA (1180148-1180229) Ser (AGA) 82 bp Sc: 84.54
GCAGTCGTGGCCGAGCGGTTAAGGCGTCTGACTAGAAATCAGATTCCCTCTGGGAGCGTA
GGTTCGAATCCTACCGACTGCG
>Anopheles_gambiae_chr2R.trna5-SerAGA (11307656-11307737) Ser (AGA) 82 bp Sc: 84.54
GCAGTCGTGGCCGAGCGGTTAAGGCGTCTGACTAGAAATCAGATTCCCTCTGGGAGCGTA
GGTTCGAATCCTACCGACTGCG
>Anopheles_gambiae_chr2R.trna8-SerAGA (11360407-11360488) Ser (AGA) 82 bp Sc: 84.54
GCAGTCGTGGCCGAGCGGTTAAGGCGTCTGACTAGAAATCAGATTCCCTCTGGGAGCGTA
GGTTCGAATCCTACCGACTGCG
>Anopheles_gambiae_chr2L.trna33-SerCGA (44400916-44400997) Ser (CGA) 82 bp Sc: 86.41
GCAGTCGTGGCCGAGTGGTTAAGGCGTCTGACTCGAAATCAGATTCCCTCTGGGAGCGTA
GGTTCGAATCCTACCGGCTGCG
>Anopheles_gambiae_chr3L.trna36-SerCGA (34276995-34277076) Ser (CGA) 82 bp Sc: 86.41
GCAGTCGTGGCCGAGTGGTTAAGGCGTCTGACTCGAAATCAGATTCCCTCTGGGAGCGTA
GGTTCGAATCCTACCGGCTGCG
>Anopheles_gambiae_chr3L.trna39-SerCGA (34540587-34540668) Ser (CGA) 82 bp Sc: 86.41
GCAGTCGTGGCCGAGTGGTTAAGGCGTCTGACTCGAAATCAGATTCCCTCTGGGAGCGTA
GGTTCGAATCCTACCGGCTGCG
>Anopheles_gambiae_chr3L.trna47-SerCGA (35896886-35896805) Ser (CGA) 82 bp Sc: 86.41
GCAGTCGTGGCCGAGTGGTTAAGGCGTCTGACTCGAAATCAGATTCCCTCTGGGAGCGTA
GGTTCGAATCCTACCGGCTGCG
>Anopheles_gambiae_chr3L.trna51-SerCGA (34289527-34289446) Ser (CGA) 82 bp Sc: 86.41
GCAGTCGTGGCCGAGTGGTTAAGGCGTCTGACTCGAAATCAGATTCCCTCTGGGAGCGTA

GGTTCGAATCCTACCGGCTGCG
>Anopheles_gambiae_chr3L.trna8-SerCGA (8001971-8002052) Ser (CGA) 82 bp Sc: 86.41
GCAGTCGTGGCCGAGTGGTTAAGGCGTCTGACTCGAAATCAGATTCCCTCTGGGAGCGTA
GGTTCGAATCCTACCGGCTGCG
>Anopheles_gambiae_chr3L.trna82-SerCGA (8240631-8240550) Ser (CGA) 82 bp Sc: 86.41
GCAGTCGTGGCCGAGTGGTTAAGGCGTCTGACTCGAAATCAGATTCCCTCTGGGAGCGTA
GGTTCGAATCCTACCGGCTGCG
>Anopheles_gambiae_chr3L.trna9-SerCGA (8011139-8011220) Ser (CGA) 82 bp Sc: 86.41
GCAGTCGTGGCCGAGTGGTTAAGGCGTCTGACTCGAAATCAGATTCCCTCTGGGAGCGTA
GGTTCGAATCCTACCGGCTGCG
>Anopheles_gambiae_chr2L.trna74-SerGCT (40037718-40037637) Ser (GCT) 82 bp Sc: 82.84
GACGAGGTGGCCGAGAGGTTAAGGCGTTGGACTGCTAATCCAATGTGCTCTGCACGCGTG
GGTTCGAATCCCATCCTCGTCG
>Anopheles_gambiae_chr2R.trna100-SerGCT (26666161-26666080) Ser (GCT) 82 bp Sc: 82.84
GACGAGGTGGCCGAGAGGTTAAGGCGTTGGACTGCTAATCCAATGTGCTCTGCACGCGTG
GGTTCGAATCCCATCCTCGTCG
>Anopheles_gambiae_chr2R.trna101-SerGCT (26665705-26665624) Ser (GCT) 82 bp Sc: 82.84
GACGAGGTGGCCGAGAGGTTAAGGCGTTGGACTGCTAATCCAATGTGCTCTGCACGCGTG
GGTTCGAATCCCATCCTCGTCG
>Anopheles_gambiae_chr2R.trna18-SerGCT (20698736-20698817) Ser (GCT) 82 bp Sc: 82.84
GACGAGGTGGCCGAGAGGTTAAGGCGTTGGACTGCTAATCCAATGTGCTCTGCACGCGTG
GGTTCGAATCCCATCCTCGTCG
>Anopheles_gambiae_chr2R.trna43-SerGCT (44231696-44231777) Ser (GCT) 82 bp Sc: 87.38
GACGAGGTGGCCGAGTGGTTAAGGCGTTGGACTGCTAATCCAATGTGCTCTGCACGCGTG
GGTTCGAATCCCATCCTCGTCG
>Anopheles_gambiae_chr2R.trna78-SerGCT (48604451-48604370) Ser (GCT) 82 bp Sc: 87.38
GACGAGGTGGCCGAGTGGTTAAGGCGTTGGACTGCTAATCCAATGTGCTCTGCACGCGTG
GGTTCGAATCCCATCCTCGTCG
>Anopheles_gambiae_chr2R.trna122-SerTGA (11382430-11382349) Ser (TGA) 82 bp Sc: 77.94
GCTGTGGTGTCCGAGCGGTTAAGGAGATGGACTTGAAATCCATTGGGTTCTACCCGCACA
GGTTCGAATCCTGTCTACAGCG
>Anopheles_gambiae_chr2R.trna13-SerTGA (11436109-11436190) Ser (TGA) 82 bp Sc: 77.94
GCTGTGGTGTCCGAGCGGTTAAGGAGATGGACTTGAAATCCATTGGGTTCTACCCGCACA
GGTTCGAATCCTGTCTACAGCG
>Anopheles_gambiae_chr2R.trna117-ThrAGT (11437773-11437700) Thr (AGT) 74 bp Sc: 74.61
GGCGCCGTGGCTTAGTTGGTTAAAGCGCCTGTCTAGTAAACAGGAGATCGTGAGTTCGAA
TCTCGCCGGAGCCT
>Anopheles_gambiae_chr2R.trna121-ThrAGT (11383517-11383444) Thr (AGT) 74 bp Sc: 74.61
GGCGCCGTGGCTTAGTTGGTTAAAGCGCCTGTCTAGTAAACAGGAGATCGTGAGTTCGAA
TCTCGCCGGAGCCT
>Anopheles_gambiae_chr2R.trna128-ThrAGT (526107-526034) Thr (AGT) 74 bp Sc: 74.61
GGCGCCGTGGCTTAGTTGGTTAAAGCGCCTGTCTAGTAAACAGGAGATCGTGAGTTCGAA
TCTCGCCGGAGCCT
>Anopheles_gambiae_chr2R.trna14-ThrAGT (11437077-11437150) Thr (AGT) 74 bp Sc: 74.61
GGCGCCGTGGCTTAGTTGGTTAAAGCGCCTGTCTAGTAAACAGGAGATCGTGAGTTCGAA
TCTCGCCGGAGCCT
>Anopheles_gambiae_chr2R.trna15-ThrAGT (11438918-11438991) Thr (AGT) 74 bp Sc: 74.61
GGCGCCGTGGCTTAGTTGGTTAAAGCGCCTGTCTAGTAAACAGGAGATCGTGAGTTCGAA
TCTCGCCGGAGCCT
>Anopheles_gambiae_chr3R.trna25-ThrAGT (33710443-33710516) Thr (AGT) 74 bp Sc: 74.61
GGCGCCGTGGCTTAGTTGGTTAAAGCGCCTGTCTAGTAAACAGGAGATCGTGAGTTCGAA
TCTCGCCGGAGCCT
>Anopheles_gambiae_chr3R.trna3-ThrAGT (5039303-5039376) Thr (AGT) 74 bp Sc: 74.61
GGCGCCGTGGCTTAGTTGGTTAAAGCGCCTGTCTAGTAAACAGGAGATCGTGAGTTCGAA
TCTCGCCGGAGCCT
>Anopheles_gambiae_chr3R.trna31-ThrAGT (33724970-33724897) Thr (AGT) 74 bp Sc: 74.61
GGCGCCGTGGCTTAGTTGGTTAAAGCGCCTGTCTAGTAAACAGGAGATCGTGAGTTCGAA
TCTCGCCGGAGCCT
>Anopheles_gambiae_chr2R.trna104-ThrCGT (25327925-25327854) Thr (CGT) 72 bp Sc: 82.08
GCCTCTTTAGCTCAGTGGTAGAGCGCTGGTCTCGTAAACCAGCGGTCGTGAGTTCAATCC
TCACAGGAGGCA
>Anopheles_gambiae_chr2R.trna126-ThrCGT (6314238-6314167) Thr (CGT) 72 bp Sc: 82.08
GCCTCTTTAGCTCAGTGGTAGAGCGCTGGTCTCGTAAACCAGCGGTCGTGAGTTCAATCC
TCACAGGAGGCA
>Anopheles_gambiae_chr2R.trna52-ThrCGT (48516817-48516888) Thr (CGT) 72 bp Sc: 82.08
GCCTCTTTAGCTCAGTGGTAGAGCGCTGGTCTCGTAAACCAGCGGTCGTGAGTTCAATCC
TCACAGGAGGCA

>Anopheles_gambiae_chr3R.trna57-ThrCGT (5038778-5038707) Thr (CGT) 72 bp Sc: 82.08
GCCTCTTTAGCTCAGTGGTAGAGCGCTGGTCTCGTAAACCAGCGGTCGTGAGTTCAATCC
TCACAGGAGGCA
>Anopheles_gambiae_chr2R.trna53-ThrCGT (48578521-48578592) Thr (CGT) 72 bp Sc: 82.31
GCCTCTTTAGCTCAGTGGTAGAGCGCTGGTCTCGTAAACCAGCGGTCGTGAGTTCAAACC
TCACAGGAGGCA
>Anopheles_gambiae_chr2L.trna73-ThrTGT (44763679-44763607) Thr (TGT) 73 bp Sc: 76.48
GCCTCTTTAGCTCAGTAGGCAGAGCGCTGGTCTTGTAAACCAGCGGTCGTGAGTTCGATT
CTCACAGGAGGCA
>Anopheles_gambiae_chr2L.trna83-ThrTGT (32711177-32711105) Thr (TGT) 73 bp Sc: 76.48
GCCTCTTTAGCTCAGTAGGCAGAGCGCTGGTCTTGTAAACCAGCGGTCGTGAGTTCGATT
CTCACAGGAGGCA
>Anopheles_gambiae_chr2R.trna48-TrpCCA (46957392-46957463) Trp (CCA) 72 bp Sc: 75.41
GACTCCGTGGCGCAACGGTAGCGCGTCTGACTCCAGATCAGAAGGTTGCGTGTTCAAATC
ACGTCGGGGTCA
>Anopheles_gambiae_chr2R.trna49-TrpCCA (46988542-46988613) Trp (CCA) 72 bp Sc: 75.41
GACTCCGTGGCGCAACGGTAGCGCGTCTGACTCCAGATCAGAAGGTTGCGTGTTCAAATC
ACGTCGGGGTCA
>Anopheles_gambiae_chr2R.trna70-TrpCCA (55555262-55555191) Trp (CCA) 72 bp Sc: 75.41
GACTCCGTGGCGCAACGGTAGCGCGTCTGACTCCAGATCAGAAGGTTGCGTGTTCAAATC
ACGTCGGGGTCA
>Anopheles_gambiae_chr2R.trna71-TrpCCA (55550049-55549978) Trp (CCA) 72 bp Sc: 75.41
GACTCCGTGGCGCAACGGTAGCGCGTCTGACTCCAGATCAGAAGGTTGCGTGTTCAAATC
ACGTCGGGGTCA
>Anopheles_gambiae_chr2R.trna90-TrpCCA (46938389-46938318) Trp (CCA) 72 bp Sc: 75.41
GACTCCGTGGCGCAACGGTAGCGCGTCTGACTCCAGATCAGAAGGTTGCGTGTTCAAATC
ACGTCGGGGTCA
>Anopheles_gambiae_chr3R.trna55-TrpCCA (6981083-6981012) Trp (CCA) 72 bp Sc: 75.41
GACTCCGTGGCGCAACGGTAGCGCGTCTGACTCCAGATCAGAAGGTTGCGTGTTCAAATC
ACGTCGGGGTCA
>Anopheles_gambiae_chr2L.trna14-TyrGTA (22061211-22061311) Tyr (GTA) 101 bp Sc: 75.32
CCTTCGATAGCTCAGTTGGTAGAGCGGTGGACTGTAGAGAGAGAGAGATGACGCAGAATG
TAGTAATCCATAGGTCACTGGTTCAAATCCGGTTCGAAGGA
>Anopheles_gambiae_chr2L.trna16-TyrGTA (23993379-23993479) Tyr (GTA) 101 bp Sc: 77.33
CCTTCGATAGCTCAGTTGGTAGAGCGGTGGACTGTAGAGAGTGAACACACACACGATAGA
GAGAAATCCATAGGTCGCTGGTTCAAATCCGGCTCGAAGGA
>Anopheles_gambiae_chr2R.trna21-TyrGTA (25510326-25510421) Tyr (GTA) 96 bp Sc: 75.61
CCTTCGATAGCTCAGTTGGTAGAGCGGTGGACTGTAGAAGCGTGATGTGTTGATAGAGTA
ATCCATAGGTCACTGGTTCAAATCCGGTTCGAAGGA
>Anopheles_gambiae_chr3L.trna46-TyrGTA (36039348-36039241) Tyr (GTA) 108 bp Sc: 71.62
CCTTCGATAGCTCAGTTGGTAGAGCGGTGGACTGTAGACAGCGATAGCGTGCGCGCATCA
TCGGTAGAAGTAATCCATAGGTCACTGGTTCAAATCCGGTTCGAAGGA
>Anopheles_gambiae_chr2L.trna39-TyrGTA (46564059-46564158) Tyr (GTA) 100 bp Sc: 74.42
CCTTCGATAGCTCAGTTGGTAGAGCGGTGGACTGTAGAAGCGCAAGATGCGGTGAAAGAG
AGTAATCCATAGGTCACTGGTTCAAATCCGGTTCGAAGGA
>Anopheles_gambiae_chr2L.trna67-TyrGTA (46574185-46574061) Tyr (GTA) 125 bp Sc: 74.20
CCTTCGATAGCTCAGTTGGTAGAGCGGTGGACTGTAGCGAAAGTAAGGAGAAGTGCGATC
ATGTGATCAAAACTCCGATCATTGTAGAAATCCATAGGTCACTGGTTCAAATCCGGTTCG
AAGGA
>Anopheles_gambiae_chr2L.trna66-TyrGTA (46574587-46574446) Tyr (GTA) 142 bp Sc: 71.06
CCTTCGATAGCTCAGTTGGTAGAGCGGTGGACTGTAGCGAGTTTGGGGGTCGATCATCTG
TTACAATGCATCTCAAAGTGCATCTGATGATCACCTGTGAATAGAAATCCATAGGTCACT
GGTTCAAATCCGGTTCGAAGGA
>Anopheles_gambiae_chr2L.trna65-TyrGTA (46574991-46574835) Tyr (GTA) 157 bp Sc: 77.85
CCTTCGATAGCTCAGTTGGTAGAGCGGTGGACTGTAGAAAATTTGAAGCGAAGTGATTTG
GTAGAGATAATGTAATCATTACAAAGTTGCATTGAGCTCTTATCACTTTCAAACAATAGA
AATCCATAGGTCACTGGTTCAAATCCGGTTCGAAGGA
>Anopheles_gambiae_chr2L.trna40-TyrGTA (46579229-46579379) Tyr (GTA) 151 bp Sc: 63.60
CCTTCGATAGCTAAGTTGGTAGAGCGGTGGACTGTAGCAACGAGCGTGGAACGATTGTGA
GTTGGCAATTGGTACCTTAAATGCCTGCGATCAACCGTCTCCAATGTAGTTAGAAATCCA
TAGGTCACTGGTTCAAATCCGGTTCGAAGGA
>Anopheles_gambiae_chr2L.trna41-TyrGTA (46579460-46579601) Tyr (GTA) 142 bp Sc: 71.06
CCTTCGATAGCTCAGTTGGTAGAGCGGTGGACTGTAGCGAGTTTGGGGGTCGATCATCTG
TTACAATGCATCTCAAAGTGCATCTGATGATCACCTGTGAATAGAAATCCATAGGTCACT
GGTTCAAATCCGGTTCGAAGGA
>Anopheles_gambiae_chr2L.trna42-TyrGTA (46579861-46579986) Tyr (GTA) 126 bp Sc: 74.83
CCTTCGATAGCTCAGTTGGTAGAGCGGTGGACTGTAGAGAAAGTTAGGAGAAGTACGATC

ATGTGATCAAATCTCCCGATCATTGTAGAAATCCATAGGTCACTGGTTCAAATCCGGTTC
GAAGGA
>Anopheles_gambiae_chr2L.trna43-TyrGTA (46580278-46580419) Tyr (GTA) 142 bp Sc: 73.11
CCTTCGATAGCTCAGTTGGTAGAGCGGTGGACTGTAGCGAGTTTAGAGGTCGATCAACTG
TTACAATGCATTTCAAAGTGCATCTGATGATCACCTGTGAATAGAAATCCATAGGTCACT
GGTTCAAATCCGGTTCGAAGGA
>Anopheles_gambiae_chr2L.trna44-TyrGTA (46580678-46580807) Tyr (GTA) 130 bp Sc: 71.96
CCTTCGATAGCTCAGTTGGTAGAGCGGTGGACTGTAGAGAAAGTTTGGAGTGTGAGCGCT
CAAGAAGTGATCAAACCTCCAAATCATTGTAGAAATCCATAGATCACTGGTTCAAATCCG
GTTCGAAGGA
>Anopheles_gambiae_chr2L.trna45-TyrGTA (46584062-46584332) Tyr (GTA) 271 bp Sc: 57.49
CCTTCGATAGCTCAGTTGGTAGAGCGGTGGACTGTAGAAGTTGTTTCAGCGCCGCGCGTT
TGAGTAGTTGTGTTTACGGGATTTGTTGAGGTttgttgtgTTGAGTCAGTAGTCAGTGCG
AGAAAGCTTTGtgttgtAATTGCAAACAATGCACACTTTCACTCCTCCTACTGTCTCAAT
GCAAAGTTTCTCAGCTCTCCACCAACACACGCTCAGCAGCATGTGTGTTTCAGTAATCCA
TAGGTCACTGGTTCAAATCCGGTTCGAAGGA
>Anopheles_gambiae_chr2L.trna46-TyrGTA (46584505-46584639) Tyr (GTA) 135 bp Sc: 75.13
CCTTCGATAGCTCAGTTGGTAGAGCGGTGGACTGTAGAAAGTTTAAGTAGTTGGTCAACT
TAGAAATGTGCATCTGACGATCACATGCATCCAATAGAAATCCATAGGTCACTGGTTCAA
ATCCGGTTCGAAGGA
>Anopheles_gambiae_chr2L.trna47-TyrGTA (46584868-46584992) Tyr (GTA) 125 bp Sc: 74.34
CCTTCGATAGCTCAGTTGGTAGAGCGGTGGACTGTAGAGAAAGTTAGGAGAAGTGCGATC
ATGTGATCAAAGCTCCGATCATTGAAGAAATCCATAGGTCACTGGTTCAAATCCGGTTCG
AAGGA
>Anopheles_gambiae_chr2L.trna48-TyrGTA (46585254-46585399) Tyr (GTA) 146 bp Sc: 70.73
CCTTCGATAGCTCAGTTGGTAGAGCGGTGGACTGTAGCGAGTTTTAGAGGTCGATCAACT
GTTACAATGCATCGCAAAGTGCATCTGACGATCACCTGCAACCAATAGAAATCCATAGGT
CACTGGTTCAAATCCGGTTCGAAGGA
>Anopheles_gambiae_chr2L.trna49-TyrGTA (46585652-46585794) Tyr (GTA) 143 bp Sc: 73.80
CCTTCGATAGCTCAGTTGGTAGAGCGGTGGACTGTAGAGAAAATTCGGAGTGTGTTGCTT
TCAAGTCGAAATGCACTTGAGCCAAACCTTCAAGAGTTGTAAAAGAAATCCATAGGTCAC
TGGTTCAAATCCGGTTCGAAGGA
>Anopheles_gambiae_chr2L.trna50-TyrGTA (46585880-46586009) Tyr (GTA) 130 bp Sc: 72.15
CCTTCGATAGCTCAGTTGGTAGAGCGGTGGACTGTAGCGAGGTTGAGGGTCGATCATCTG
TTACAATGGCATCTCAAAGTGCATCTGAATAGAAATCCATAGGTCACTGGTTCAAATCCG
GTTCGAAGGA
>Anopheles_gambiae_chr2L.trna53-TyrGTA (46622585-46622680) Tyr (GTA) 96 bp Sc: 77.04
CCTTCGATAGCTCAGTTGGTAGAGCGGTGGACTGTAGAAGCAAAAGTTCGATATAGAGTA
ATCCATAGGTCACTGGTTCAAATCCGGTTCGAAGGA
>Anopheles_gambiae_chr2L.trna54-TyrGTA (46623063-46623153) Tyr (GTA) 91 bp Sc: 74.65
CCTTCGATAGCTCAGTTGGTAGAGCGGTGGACTGTAGAAGCGTCTCTCCATAGTAATCCA
TAGGTCACTGGTTCAAATCCGGTTCGAAGGA
>Anopheles_gambiae_chr2R.trna83-TyrGTA (46989195-46989100) Tyr (GTA) 96 bp Sc: 81.14
CCTTCGATAGCTCAGTTGGTAGAGCGGTGGACTGTAGAAGAGGAAAATATGAAAAAAGAA
ATCCATAGGTCGCTGGTTCAAATCCGGCTCGAAGGA
>Anopheles_gambiae_chr2L.trna106-ValAAC (29568261-29568189) Val (AAC) 73 bp Sc: 67.50
GTTTCCGTGGTGTAGCGGTTATCACATCTGCCTAACAGGCAGAAGGCCCCCGGTTCGATC
CCGTGCGGAAACA
>Anopheles_gambiae_chr3L.trna20-ValAAC (20338750-20338822) Val (AAC) 73 bp Sc: 70.43
GTTTCCGTGGTGTAGTGGTTATCACATCAGTCTAACACGCGGAAGGCCCCCGGTTCAATC
CCGGGCGGAAACA
>Anopheles_gambiae_chr2L.trna108-ValAAC (29567793-29567721) Val (AAC) 73 bp Sc: 74.75
GTTTCCGTGGTGTAGCGGTTATCACATCTGCCTAACACGCAGAAGGCCCCCGGTTCGATC
CCAGGCGGAAACA
>Anopheles_gambiae_chr2L.trna8-ValAAC (15481054-15481126) Val (AAC) 73 bp Sc: 77.72
GTTTCCGTGGTGTAGTGGTTATCACATCCGCCTAACACGCGGAAGGCCTCCGGTTCGATC
CCGGGCGGAAACA
>Anopheles_gambiae_chr2L.trna101-ValAAC (29569427-29569355) Val (AAC) 73 bp Sc: 79.44
GTTTCCGTGGTGTAGCGGTTATCACATCTGCCTAACACGCAGAAGGCCCCCGGTTCGATC
CCGGGCGGAAACA
>Anopheles_gambiae_chr2L.trna102-ValAAC (29569197-29569125) Val (AAC) 73 bp Sc: 79.44
GTTTCCGTGGTGTAGCGGTTATCACATCTGCCTAACACGCAGAAGGCCCCCGGTTCGATC
CCGGGCGGAAACA
>Anopheles_gambiae_chr2L.trna104-ValAAC (29568725-29568653) Val (AAC) 73 bp Sc: 79.44
GTTTCCGTGGTGTAGCGGTTATCACATCTGCCTAACACGCAGAAGGCCCCCGGTTCGATC
CCGGGCGGAAACA
>Anopheles_gambiae_chr2L.trna111-ValAAC (29567087-29567015) Val (AAC) 73 bp Sc: 79.44

GTTTCCGTGGTGTAGCGGTTATCACATCTGCCTAACACGCAGAAGGCCCCCGGTTCGATC
CCGGGCGGAAACA
>Anopheles_gambiae_chr2L.trna113-ValAAC (29566619-29566547) Val (AAC) 73 bp Sc: 79.44
GTTTCCGTGGTGTAGCGGTTATCACATCTGCCTAACACGCAGAAGGCCCCCGGTTCGATC
CCGGGCGGAAACA
>Anopheles_gambiae_chr2L.trna118-ValAAC (29565449-29565377) Val (AAC) 73 bp Sc: 79.44
GTTTCCGTGGTGTAGCGGTTATCACATCTGCCTAACACGCAGAAGGCCCCCGGTTCGATC
CCGGGCGGAAACA
>Anopheles_gambiae_chr2L.trna120-ValAAC (29565040-29564968) Val (AAC) 73 bp Sc: 79.44
GTTTCCGTGGTGTAGCGGTTATCACATCTGCCTAACACGCAGAAGGCCCCCGGTTCGATC
CCGGGCGGAAACA
>Anopheles_gambiae_chr2L.trna122-ValAAC (29564635-29564563) Val (AAC) 73 bp Sc: 79.44
GTTTCCGTGGTGTAGCGGTTATCACATCTGCCTAACACGCAGAAGGCCCCCGGTTCGATC
CCGGGCGGAAACA
>Anopheles_gambiae_chr2L.trna87-ValAAC (29572716-29572644) Val (AAC) 73 bp Sc: 79.44
GTTTCCGTGGTGTAGCGGTTATCACATCTGCCTAACACGCAGAAGGCCCCCGGTTCGATC
CCGGGCGGAAACA
>Anopheles_gambiae_chr2L.trna91-ValAAC (29571776-29571704) Val (AAC) 73 bp Sc: 79.44
GTTTCCGTGGTGTAGCGGTTATCACATCTGCCTAACACGCAGAAGGCCCCCGGTTCGATC
CCGGGCGGAAACA
>Anopheles_gambiae_chr2L.trna92-ValAAC (29571542-29571470) Val (AAC) 73 bp Sc: 79.44
GTTTCCGTGGTGTAGCGGTTATCACATCTGCCTAACACGCAGAAGGCCCCCGGTTCGATC
CCGGGCGGAAACA
>Anopheles_gambiae_chr2L.trna95-ValAAC (29570836-29570764) Val (AAC) 73 bp Sc: 79.44
GTTTCCGTGGTGTAGCGGTTATCACATCTGCCTAACACGCAGAAGGCCCCCGGTTCGATC
CCGGGCGGAAACA
>Anopheles_gambiae_chr2L.trna99-ValAAC (29569896-29569824) Val (AAC) 73 bp Sc: 79.44
GTTTCCGTGGTGTAGCGGTTATCACATCTGCCTAACACGCAGAAGGCCCCCGGTTCGATC
CCGGGCGGAAACA
>Anopheles_gambiae_chr3L.trna22-ValAAC (20375982-20376054) Val (AAC) 73 bp Sc: 80.36
GTTTCCGTGGTGTAGTGGTTATCACATCCGCCTAACACGCGGAAGGCCCCCGGTTCAATC
CCGGGCGGAAACA
>Anopheles_gambiae_chr3L.trna24-ValAAC (20395854-20395926) Val (AAC) 73 bp Sc: 80.36
GTTTCCGTGGTGTAGTGGTTATCACATCCGCCTAACACGCGGAAGGCCCCCGGTTCAATC
CCGGGCGGAAACA
>Anopheles_gambiae_chr3L.trna63-ValAAC (20314510-20314438) Val (AAC) 73 bp Sc: 80.36
GTTTCCGTGGTGTAGTGGTTATCACATCCGCCTAACACGCGGAAGGCCCCCGGTTCAATC
CCGGGCGGAAACA
>Anopheles_gambiae_chr3L.trna65-ValAAC (20297804-20297732) Val (AAC) 73 bp Sc: 80.36
GTTTCCGTGGTGTAGTGGTTATCACATCCGCCTAACACGCGGAAGGCCCCCGGTTCAATC
CCGGGCGGAAACA
>Anopheles_gambiae_chr2L.trna19-ValAAC (29808708-29808780) Val (AAC) 73 bp Sc: 82.09
GTTTCCGTGGTGTAGTGGTTATCACATCCGCCTAACACGCGGAAGGCCCCCGGTTCGATC
CCGGGCGGAAACA
>Anopheles_gambiae_chr2L.trna25-ValAAC (29942173-29942245) Val (AAC) 73 bp Sc: 82.09
GTTTCCGTGGTGTAGTGGTTATCACATCCGCCTAACACGCGGAAGGCCCCCGGTTCGATC
CCGGGCGGAAACA
>Anopheles_gambiae_chr2L.trna26-ValAAC (30429346-30429418) Val (AAC) 73 bp Sc: 82.09
GTTTCCGTGGTGTAGTGGTTATCACATCCGCCTAACACGCGGAAGGCCCCCGGTTCGATC
CCGGGCGGAAACA
>Anopheles_gambiae_chr2L.trna51-ValAAC (46602011-46602083) Val (AAC) 73 bp Sc: 82.09
GTTTCCGTGGTGTAGTGGTTATCACATCCGCCTAACACGCGGAAGGCCCCCGGTTCGATC
CCGGGCGGAAACA
>Anopheles_gambiae_chr2L.trna69-ValAAC (45999232-45999160) Val (AAC) 73 bp Sc: 82.09
GTTTCCGTGGTGTAGTGGTTATCACATCCGCCTAACACGCGGAAGGCCCCCGGTTCGATC
CCGGGCGGAAACA
>Anopheles_gambiae_chr2L.trna84-ValAAC (29812041-29811969) Val (AAC) 73 bp Sc: 82.09
GTTTCCGTGGTGTAGTGGTTATCACATCCGCCTAACACGCGGAAGGCCCCCGGTTCGATC
CCGGGCGGAAACA
>Anopheles_gambiae_chr2R.trna57-ValAAC (54258741-54258813) Val (AAC) 73 bp Sc: 82.09
GTTTCCGTGGTGTAGTGGTTATCACATCCGCCTAACACGCGGAAGGCCCCCGGTTCGATC
CCGGGCGGAAACA
>Anopheles_gambiae_chr3L.trna29-ValAAC (28667273-28667345) Val (AAC) 73 bp Sc: 82.09
GTTTCCGTGGTGTAGTGGTTATCACATCCGCCTAACACGCGGAAGGCCCCCGGTTCGATC
CCGGGCGGAAACA
>Anopheles_gambiae_chr3R.trna48-ValAAC (13316495-13316423) Val (AAC) 73 bp Sc: 82.09
GTTTCCGTGGTGTAGTGGTTATCACATCCGCCTAACACGCGGAAGGCCCCCGGTTCGATC

CCGGGCGGAAACA
>Anopheles_gambiae_chr3R.trna49-ValAAC (13280619-13280547) Val (AAC) 73 bp Sc: 82.09
GTTTCCGTGGTGTAGTGGTTATCACATCCGCCTAACACGCGGAAGGCCCCCGGTTCGATC
CCGGGCGGAAACA
>Anopheles_gambiae_chr2L.trna121-ValCAC (29564869-29564797) Val (CAC) 73 bp Sc: 73.69
GTTTCCGTGGTGTAGCGGTTATCACGTATGCTTCACACGCAGAAGGCCCCCGGTTCGATC
CCGGGCGGAAACA
>Anopheles_gambiae_chr2L.trna90-ValCAC (29572014-29571942) Val (CAC) 73 bp Sc: 73.91
GTTTCCGTGGTTTAGCGGTTATCACGTCTGCTTCACACGCAGAAGGCCCCCGGTTCGATC
CCGGGCGGAAACA
>Anopheles_gambiae_chr2L.trna107-ValCAC (29568027-29567955) Val (CAC) 73 bp Sc: 74.46
GATTCCGTGGTGTAGCGGTTATCACGTCTGCTTCACACGCAGAAGGCCCCCGGTTCGATC
CCGGGCGGAAACA
>Anopheles_gambiae_chr2L.trna110-ValCAC (29567321-29567249) Val (CAC) 73 bp Sc: 74.46
GATTCCGTGGTGTAGCGGTTATCACGTCTGCTTCACACGCAGAAGGCCCCCGGTTCGATC
CCGGGCGGAAACA
>Anopheles_gambiae_chr2L.trna123-ValCAC (29564401-29564329) Val (CAC) 73 bp Sc: 75.57
GTTTCCGTGGTGTAGCGGTTATCACGTCTGCTTCACACGCAGAAGGCCTCCGGTTCGATC
CCGGGCGGAAACA
>Anopheles_gambiae_chr2L.trna100-ValCAC (29569661-29569589) Val (CAC) 73 bp Sc: 79.95
GTTTCCGTGGTGTAGCGGTTATCACGTCTGCTTCACACGCAGAAGGCCCCCGGTTCGATC
CCGGGCGGAAACA
>Anopheles_gambiae_chr2L.trna103-ValCAC (29568959-29568887) Val (CAC) 73 bp Sc: 79.95
GTTTCCGTGGTGTAGCGGTTATCACGTCTGCTTCACACGCAGAAGGCCCCCGGTTCGATC
CCGGGCGGAAACA
>Anopheles_gambiae_chr2L.trna105-ValCAC (29568495-29568423) Val (CAC) 73 bp Sc: 79.95
GTTTCCGTGGTGTAGCGGTTATCACGTCTGCTTCACACGCAGAAGGCCCCCGGTTCGATC
CCGGGCGGAAACA
>Anopheles_gambiae_chr2L.trna109-ValCAC (29567555-29567483) Val (CAC) 73 bp Sc: 79.95
GTTTCCGTGGTGTAGCGGTTATCACGTCTGCTTCACACGCAGAAGGCCCCCGGTTCGATC
CCGGGCGGAAACA
>Anopheles_gambiae_chr2L.trna112-ValCAC (29566853-29566781) Val (CAC) 73 bp Sc: 79.95
GTTTCCGTGGTGTAGCGGTTATCACGTCTGCTTCACACGCAGAAGGCCCCCGGTTCGATC
CCGGGCGGAAACA
>Anopheles_gambiae_chr2L.trna114-ValCAC (29566389-29566317) Val (CAC) 73 bp Sc: 79.95
GTTTCCGTGGTGTAGCGGTTATCACGTCTGCTTCACACGCAGAAGGCCCCCGGTTCGATC
CCGGGCGGAAACA
>Anopheles_gambiae_chr2L.trna115-ValCAC (29566155-29566083) Val (CAC) 73 bp Sc: 79.95
GTTTCCGTGGTGTAGCGGTTATCACGTCTGCTTCACACGCAGAAGGCCCCCGGTTCGATC
CCGGGCGGAAACA
>Anopheles_gambiae_chr2L.trna119-ValCAC (29565278-29565206) Val (CAC) 73 bp Sc: 79.95
GTTTCCGTGGTGTAGCGGTTATCACGTCTGCTTCACACGCAGAAGGCCCCCGGTTCGATC
CCGGGCGGAAACA
>Anopheles_gambiae_chr2L.trna88-ValCAC (29572482-29572410) Val (CAC) 73 bp Sc: 79.95
GTTTCCGTGGTGTAGCGGTTATCACGTCTGCTTCACACGCAGAAGGCCCCCGGTTCGATC
CCGGGCGGAAACA
>Anopheles_gambiae_chr2L.trna89-ValCAC (29572248-29572176) Val (CAC) 73 bp Sc: 79.95
GTTTCCGTGGTGTAGCGGTTATCACGTCTGCTTCACACGCAGAAGGCCCCCGGTTCGATC
CCGGGCGGAAACA
>Anopheles_gambiae_chr2L.trna93-ValCAC (29571308-29571236) Val (CAC) 73 bp Sc: 79.95
GTTTCCGTGGTGTAGCGGTTATCACGTCTGCTTCACACGCAGAAGGCCCCCGGTTCGATC
CCGGGCGGAAACA
>Anopheles_gambiae_chr2L.trna94-ValCAC (29571074-29571002) Val (CAC) 73 bp Sc: 79.95
GTTTCCGTGGTGTAGCGGTTATCACGTCTGCTTCACACGCAGAAGGCCCCCGGTTCGATC
CCGGGCGGAAACA
>Anopheles_gambiae_chr2L.trna96-ValCAC (29570602-29570530) Val (CAC) 73 bp Sc: 79.95
GTTTCCGTGGTGTAGCGGTTATCACGTCTGCTTCACACGCAGAAGGCCCCCGGTTCGATC
CCGGGCGGAAACA
>Anopheles_gambiae_chr2L.trna97-ValCAC (29570368-29570296) Val (CAC) 73 bp Sc: 79.95
GTTTCCGTGGTGTAGCGGTTATCACGTCTGCTTCACACGCAGAAGGCCCCCGGTTCGATC
CCGGGCGGAAACA
>Anopheles_gambiae_chr2L.trna98-ValCAC (29570134-29570062) Val (CAC) 73 bp Sc: 79.95
GTTTCCGTGGTGTAGCGGTTATCACGTCTGCTTCACACGCAGAAGGCCCCCGGTTCGATC
CCGGGCGGAAACA
>Anopheles_gambiae_chr2L.trna1-ValCAC (6105713-6105785) Val (CAC) 73 bp Sc: 83.88
GTTTCCGTAGTGTAGCGGTTATCACGTCTGCTTCACACGCAGAAGGTCCCCGGTTCGAAC
CCGGGCGGAAACA

>Anopheles_gambiae_chr2L.trna34-ValCAC (44797285-44797357) Val (CAC) 73 bp Sc: 83.88
GTTTCCGTAGTGTAGCGGTTATCACGTCTGCTTCACACGCAGAAGGTCCCCGGTTCGAAC
CCGGGCGGAAACA
>Anopheles_gambiae_chr2L.trna75-ValCAC (38668172-38668100) Val (CAC) 73 bp Sc: 83.88
GTTTCCGTAGTGTAGCGGTTATCACGTCTGCTTCACACGCAGAAGGTCCCCGGTTCGAAC
CCGGGCGGAAACA
>Anopheles_gambiae_chr2R.trna95-ValCAC (37570399-37570327) Val (CAC) 73 bp Sc: 83.88
GTTTCCGTAGTGTAGCGGTTATCACGTCTGCTTCACACGCAGAAGGTCCCCGGTTCGAAC
CCGGGCGGAAACA
>Anopheles_gambiae_chr3L.trna79-ValCAC (12204478-12204406) Val (CAC) 73 bp Sc: 83.88
GTTTCCGTAGTGTAGCGGTTATCACGTCTGCTTCACACGCAGAAGGTCCCCGGTTCGAAC
CCGGGCGGAAACA
>Anopheles_gambiae_chr2L.trna116-ValCAC (29565921-29565849) Val (CAC) 73 bp Sc: 84.05
GTTTCCGTGGTGTAGCGGTTATCACGTCTGCTTCACACGCAGAAGGTCCCCGGTTCGATC
CCGGGCGGAAACA
>Anopheles_gambiae_chr2L.trna117-ValCAC (29565687-29565615) Val (CAC) 73 bp Sc: 84.05
GTTTCCGTGGTGTAGCGGTTATCACGTCTGCTTCACACGCAGAAGGTCCCCGGTTCGATC
CCGGGCGGAAACA
>Anopheles_gambiae_chr2R.trna92-ValCAC (43444313-43444241) Val (CAC) 73 bp Sc: 86.33
GTTTCCGTAGTGTAGTGGTTATCACGTCTGCTTCACACGCAGAAGGTCCCCGGTTCGAAC
CCGGGCGGAAACA
>Anopheles_gambiae_chr3L.trna45-ValCAC (38069503-38069431) Val (CAC) 73 bp Sc: 86.33
GTTTCCGTAGTGTAGTGGTTATCACGTCTGCTTCACACGCAGAAGGTCCCCGGTTCGAAC
CCGGGCGGAAACA
>Anopheles_gambiae_chr2L.trna37-ValTAC (46001403-46001475) Val (TAC) 73 bp Sc: 76.11
GGTTCCATAGTGTAGCGGTTATCACGTCTGCTTTACACGCAGAAGGTCTCCAGTTCGATC
CTGGATGGAACCA
>Anopheles_gambiae_chr2L.trna38-ValTAC (46052072-46052144) Val (TAC) 73 bp Sc: 76.11
GGTTCCATAGTGTAGCGGTTATCACGTCTGCTTTACACGCAGAAGGTCTCCAGTTCGATC
CTGGATGGAACCA
>Anopheles_gambiae_chr2L.trna68-ValTAC (46003719-46003647) Val (TAC) 73 bp Sc: 76.11
GGTTCCATAGTGTAGCGGTTATCACGTCTGCTTTACACGCAGAAGGTCTCCAGTTCGATC
CTGGATGGAACCA
>Agrobacterium_tumefaciens_C58_UWash_chrlinear.trna9-AlaGGC (1221177-1221102) Ala (GGC) 76 bp Sc: 86.51
GGGGCTATAGCTCAGCTGGGAGAGCGCTTGCATGGCATGCAAGAGGTCAGCGGTTCGATC
CCGCTTAGCTCCACCA
>Agrobacterium_tumefaciens_C58_UWash_chrcircular.trna2-AlaTGC (58688-58763) Ala (TGC) 76 bp Sc: 95.66
GGGGCTGTAGCTCAGCTGGGAGAGCACCTGCTTTGCAAGCAGGGGGTCAGCGGTTCGATC
CCGCTCAGCTCCACCA
>Agrobacterium_tumefaciens_C58_UWash_chrcircular.trna22-AlaTGC (2516592-2516517) Ala (TGC) 76 bp Sc: 95.66
GGGGCTGTAGCTCAGCTGGGAGAGCACCTGCTTTGCAAGCAGGGGGTCAGCGGTTCGATC
CCGCTCAGCTCCACCA
>Agrobacterium_tumefaciens_C58_UWash_chrlinear.trna4-AlaTGC (1043752-1043827) Ala (TGC) 76 bp Sc: 95.66
GGGGCTGTAGCTCAGCTGGGAGAGCACCTGCTTTGCAAGCAGGGGGTCAGCGGTTCGATC
CCGCTCAGCTCCACCA
>Agrobacterium_tumefaciens_C58_UWash_chrlinear.trna7-AlaTGC (1306235-1306310) Ala (TGC) 76 bp Sc: 95.66
GGGGCTGTAGCTCAGCTGGGAGAGCACCTGCTTTGCAAGCAGGGGGTCAGCGGTTCGATC
CCGCTCAGCTCCACCA
>Agrobacterium_tumefaciens_C58_UWash_chrcircular.trna4-ArgACG (382706-382782) Arg (ACG) 77 bp Sc: 89.63
GCACCCGTAGCTCAGCTGGATAGAGCACCAGACTACGAATCTGGGGGTCAGGAGTTCGAA
TCTCTTCGGGTGCGCCA
>Agrobacterium_tumefaciens_C58_UWash_chrcircular.trna19-ArgCCG (2330046-2330122) Arg (CCG) 77 bp Sc: 86.49
GCACCCGTAGCTCAGCTGGATAGAGTGTTGGATTCCGATTCCAAAGGTCACAGGTTCGAA
TCCTGTCGGGTGCGCCA
>Agrobacterium_tumefaciens_C58_UWash_chrcircular.trna18-ArgTCT (1792320-1792396) Arg (TCT) 77 bp Sc: 93.45
GGCCCCTTAGCTCAACTGGATAGAGCAACTGCCTTCTAAGCAGTAGGTCGCAGGTTCGAG
TCCTGCAGGGGTCGCCA
>Agrobacterium_tumefaciens_C58_UWash_chrcircular.trna36-AsnGTT (1217105-1217031) Asn (GTT) 75 bp Sc: 85.96
TCCCTGGTAGCTCAGCGGTAGAGCATTCGACTGTTAATCGACAGGTCGCCGGTTCGAATC
CGGCCCGGGGAGCCA
>Agrobacterium_tumefaciens_C58_UWash_chrcircular.trna13-AspGTC (1255618-1255694) Asp (GTC) 77 bp Sc: 90.74
GCGGGTGTAGCTCAGTCGGTTAGAGTGCCGGCCTGTCACGCCGGAGGTCGCGGGTTCGAG
CCCCGTCACTCGCGCCA
>Agrobacterium_tumefaciens_C58_UWash_chrcircular.trna12-AspGTC (1255500-1255576) Asp (GTC) 77 bp Sc: 92.83
GCGGGTGTAGCTCAGTTGGTTAGAGTGCCGGCCTGTCACGCCGGAGGTCGCGGGTTCGAG
CCCCGTCACTCGCGCCA
>Agrobacterium_tumefaciens_C58_UWash_chrcircular.trna30-CysGCA (1711064-1710991) Cys (GCA) 74 bp Sc: 70.77

GGCCTCGTGGCGGAGTGGTGACGCAGAGGACTGCAAATCCTTGTACCCCGGTTCAATTCC
GGGCGAGGCCTCCA
>Agrobacterium_tumefaciens_C58_UWash_chrlinear.trna10-GlnCTG (1118616-1118543) Gln (CTG) 74 bp Sc: 72.58
TGGGGAATAGTTCAATGGTAGAACGACGGACTCTGACTCCGTTAATCTTGGTTCGAGTCC
AGGTTCCCCAGCCA
>Agrobacterium_tumefaciens_C58_UWash_chrcircular.trna9-GlnTTG (809486-809560) Gln (TTG) 75 bp Sc: 73.94
TGGGGCGTCGCCAAGCGGTAAGGCACCGGTTTTGGTACCGGCATTCCCAGGTTCGAATC
CTGGCGCCCCAGCCA
>Agrobacterium_tumefaciens_C58_UWash_chrcircular.trna14-GluTTC (1567173-1567247) Glu (TTC) 75 bp Sc: 52.80
GCGCCCTTCGTCTATCGGTTAGGACGTCAGATTTTCATTCTGAAAAGAGGGGTTCGACTC
CCCTAGGGCGTGCCA
>Agrobacterium_tumefaciens_C58_UWash_chrcircular.trna15-GluTTC (1567700-1567774) Glu (TTC) 75 bp Sc: 52.80
GCGCCCTTCGTCTATCGGTTAGGACGTCAGATTTTCATTCTGAAAAGAGGGGTTCGACTC
CCCTAGGGCGTGCCA
>Agrobacterium_tumefaciens_C58_UWash_chrcircular.trna5-GlyCCC (430676-430749) Gly (CCC) 74 bp Sc: 80.72
GCGGGTGTAGCTCAATGGTAGAGCAGCAGCTTCCCAAGCTGAATACGAGGGTTCGATTCC
CTTCACCCGCTCCA
>Agrobacterium_tumefaciens_C58_UWash_chrcircular.trna20-GlyGCC (2446476-2446550) Gly (GCC) 75 bp Sc: 87.04
GCGGGTGTAGCTCAGGGGTAGAGCACAACCTTGCCAAGGTTGGGGTCGAGGGTTCAAATC
CCTTCGCCCGCTCCA
>Agrobacterium_tumefaciens_C58_UWash_chrlinear.trna2-GlyGCC (779527-779601) Gly (GCC) 75 bp Sc: 87.04
GCGGGTGTAGCTCAGGGGTAGAGCACAACCTTGCCAAGGTTGGGGTCGAGGGTTCAAATC
CCTTCGCCCGCTCCA
>Agrobacterium_tumefaciens_C58_UWash_chrcircular.trna27-GlyTCC (1956756-1956683) Gly (TCC) 74 bp Sc: 83.28
GCGGGTATAGCTCAATGGTAGAGCAGCAGCCTTCCAAGCTGAATACGCGGGTTCGATTCC
CGCTACCCGCTCCA
>Agrobacterium_tumefaciens_C58_UWash_chrcircular.trna8-HisGTG (606854-606930) His (GTG) 77 bp Sc: 83.76
GCGGTTGTAGCTCAGTTGGTTAGAGCGCAGGTTTGTGGCACCTGAGGTCGGTGGTTCGAC
CCCACTCAACCGTACCA
>Agrobacterium_tumefaciens_C58_UWash_chrcircular.trna1-IleGAT (58553-58629) Ile (GAT) 77 bp Sc: 99.89
GGGCCCGTAGCTCAGTTGGTTAGAGCACACGCTTGATAAGCGTGGGGTCGGTAGTTCGAG
TCTACCCGGGCCCACCA
>Agrobacterium_tumefaciens_C58_UWash_chrcircular.trna21-IleGAT (2516727-2516651) Ile (GAT) 77 bp Sc: 99.89
GGGCCCGTAGCTCAGTTGGTTAGAGCACACGCTTGATAAGCGTGGGGTCGGTAGTTCGAG
TCTACCCGGGCCCACCA
>Agrobacterium_tumefaciens_C58_UWash_chrlinear.trna3-IleGAT (1043617-1043693) Ile (GAT) 77 bp Sc: 99.89
GGGCCCGTAGCTCAGTTGGTTAGAGCACACGCTTGATAAGCGTGGGGTCGGTAGTTCGAG
TCTACCCGGGCCCACCA
>Agrobacterium_tumefaciens_C58_UWash_chrlinear.trna6-IleGAT (1306100-1306176) Ile (GAT) 77 bp Sc: 99.89
GGGCCCGTAGCTCAGTTGGTTAGAGCACACGCTTGATAAGCGTGGGGTCGGTAGTTCGAG
TCTACCCGGGCCCACCA
>Agrobacterium_tumefaciens_C58_UWash_chrlinear.trna1-LeuCAA (576107-576191) Leu (CAA) 85 bp Sc: 72.97
GCGGGTGTGGTGGAATTGGTAGACGCGCCGGACTCAAAATCCGGTTCCGAAAGGAGTGTC
GGTTCGACCCCGACCACCCGCACCA
>Agrobacterium_tumefaciens_C58_UWash_chrcircular.trna39-LeuCAG (230917-230831) Leu (CAG) 87 bp Sc: 77.25
GCCCAGATGGCGGAATTGGTAGACGCGCCAGCTTCAGGTGCTGGTACTCGAAAGGGTGTG
GAGGTTCGAGTCCTCTTCTGGGCACCA
>Agrobacterium_tumefaciens_C58_UWash_chrcircular.trna32-LeuGAG (1543362-1543278) Leu (GAG) 85 bp Sc: 70.40
GCGGTCGTGGCGGAATTGGTAGACGCGCAGCGTTGAGGTCGCTGTGGGGCAACCCGTGGA
AGTTCGAGTCTTCTCGACCGCACCA
>Agrobacterium_tumefaciens_C58_UWash_chrcircular.trna37-LeuTAA (1184739-1184656) Leu (TAA) 84 bp Sc: 72.37
GCCCGCATGGTGAAATTGGTAAACACATCGCACTTAAAATGCGCCGCCTCTGGCTTGCCG
GTTCAAGTCCGGCTGCGGGCACCA
>Agrobacterium_tumefaciens_C58_UWash_chrcircular.trna31-LeuTAG (1652413-1652331) Leu (TAG) 83 bp Sc: 70.05
GCGGGTATGGCGGAATTGGTAGACGCATTGGTTTTAGGTACCAACGCTTCGGCGTGGGAG
TTCGAGTCTCTCTACCCGCACCA
>Agrobacterium_tumefaciens_C58_UWash_chrcircular.trna16-LysCTT (1623522-1623597) Lys (CTT) 76 bp Sc: 97.03
GGGTGATTAGCTCAGTTGGTAGAGCAGCTGACTCTTAATCAGCGGGTCGTAGGTTCGAGC
CCTACATCACCCACCA
>Agrobacterium_tumefaciens_C58_UWash_chrcircular.trna17-LysTTT (1784212-1784287) Lys (TTT) 76 bp Sc: 92.90
GAGAGCGTAGCTCAGCCGGTAGAGCAACTGACTTTTAATCAGTAGGTCCAGGGTTCGAAT
CCCTGCGCTCTCACCA
>Agrobacterium_tumefaciens_C58_UWash_chrcircular.trna23-MetCAT (2512316-2512240) Met (CAT) 77 bp Sc: 81.76
CGCGGGGTGGAGCAGCCCGGTAGCTCGTCAGGCTCATAACCTGAAGGCCGCAGGTTCAAA
TCCTGCCCCCGCAACCA
>Agrobacterium_tumefaciens_C58_UWash_chrcircular.trna3-MetCAT (62964-63040) Met (CAT) 77 bp Sc: 81.76
CGCGGGGTGGAGCAGCCCGGTAGCTCGTCAGGCTCATAACCTGAAGGCCGCAGGTTCAAA

TCCTGCCCCCGCAACCA
>Agrobacterium_tumefaciens_C58_UWash_chrlinear.trna5-MetCAT (1048028-1048104) Met (CAT) 77 bp Sc: 81.76
CGCGGGGTGGAGCAGCCCGGTAGCTCGTCAGGCTCATAACCTGAAGGCCGCAGGTTCAAA
TCCTGCCCCCGCAACCA
>Agrobacterium_tumefaciens_C58_UWash_chrlinear.trna8-MetCAT (1310422-1310498) Met (CAT) 77 bp Sc: 81.76
CGCGGGGTGGAGCAGCCCGGTAGCTCGTCAGGCTCATAACCTGAAGGCCGCAGGTTCAAA
TCCTGCCCCCGCAACCA
>Agrobacterium_tumefaciens_C58_UWash_chrcircular.trna24-MetCAT (2128475-2128400) Met (CAT) 76 bp Sc: 82.10
GGGCCTGTAGCTCAATGGTTAGAGCCGGCGGCTCATAACCGCTTGGTTGGGAGTTCGAGT
CTCTCCGGGCCCACCA
>Agrobacterium_tumefaciens_C58_UWash_chrlinear.trna13-MetCAT (780880-780804) Met (CAT) 77 bp Sc: 83.79
GGCGGGGTAGCTCAGGTGGTTAGAGCAGCGGAATCATAATCCGCGTGTCGGGGGTTCAAG
TCCCTCTCCCGCTACCA
>Agrobacterium_tumefaciens_C58_UWash_chrcircular.trna6-PheGAA (496312-496387) Phe (GAA) 76 bp Sc: 92.15
GCCCGGATAGCTCAGTTGGTAGAGCAGCGGATTGAAAATCCGCGTGTCGGTGGTTCAAAT
CCGCCTCCGGGCACCA
>Agrobacterium_tumefaciens_C58_UWash_chrcircular.trna10-ProCGG (813139-813215) Pro (CGG) 77 bp Sc: 85.78
CGGAGTGTAGCGCAGTCTGGTAGCGCACGTCGTTCGGGACGACGGGGTCGGAGGTTCGAA
TCCTCTCACTCCGACCA
>Agrobacterium_tumefaciens_C58_UWash_chrcircular.trna35-ProGGG (1221105-1221029) Pro (GGG) 77 bp Sc: 81.90
CGGAGCGTAGCGCAGCCCGGTAGCGCACTTGACTGGGGGTCAAGGGGTCGTGGGTTCGAA
TCCCGCCGCTCCGACCA
>Agrobacterium_tumefaciens_C58_UWash_chrcircular.trna29-ProTGG (1791702-1791626) Pro (TGG) 77 bp Sc: 86.97
CGGAGTGTAGCGCAGTCTGGTAGCGCATCTGGTTTGGGACCAGAGGGTCGGGAGTTCGAA
TCTCTCCACTCCGACCA
>Agrobacterium_tumefaciens_C58_UWash_chrlinear.trna12-SerCGA (948260-948171) Ser (CGA) 90 bp Sc: 74.37
GGAGAGGTGGCAGAGTGGTCGAATGCACCGCACTCGAAATGCGGCATACCTGCAAGGGTA
TCGTGGGTTCGAATCCCACCCTCTCCGCCA
>Agrobacterium_tumefaciens_C58_UWash_chrcircular.trna11-SerGCT (1010919-1011009) Ser (GCT) 91 bp Sc: 73.86
GGAGAGGTGGCCGAGTGGTCGAAGGCGCTCCCCTGCTAAGGGAGTATACGTCAAAAGCGT
ATCGTGGGTTCGAATCCCATCCTCTCCGCCA
>Agrobacterium_tumefaciens_C58_UWash_chrcircular.trna40-SerGGA (128502-128413) Ser (GGA) 90 bp Sc: 82.03
GGACAGGTGGCCGAGTGGTTTAAGGCGCACGCCTGGAACGCGTGTGTGCGTGAAAGCGTA
CCGTGGGTTCGAATCCCACCCTGTCCGCCA
>Agrobacterium_tumefaciens_C58_UWash_chrcircular.trna33-SerTGA (1491834-1491745) Ser (TGA) 90 bp Sc: 70.89
GGATGGGTGTCCGAGTGGTTTAAGGAACCGGTCTTGAAAACCGGCGTGCGTGAGAGCGTA
CCGTGGGTTCGAATCCCACCCCATCTGCCA
>Agrobacterium_tumefaciens_C58_UWash_chrcircular.trna38-ThrCGT (411150-411075) Thr (CGT) 76 bp Sc: 91.69
GCCGCTTTAGCTCAGTTGGTAGAGCACGTCATTCGTAATGACGGGGTCACGTGTTCGAGT
CACGTAAGCGGCACCA
>Agrobacterium_tumefaciens_C58_UWash_chrcircular.trna7-ThrGGT (531671-531745) Thr (GGT) 75 bp Sc: 84.06
GCTGCTATAGCTCAGGGGTAGAGCACTCCCTTGGTAAGGGAGAGGCCGAGAGTTCAAATC
TCTCTAGCAGCACCA
>Agrobacterium_tumefaciens_C58_UWash_chrcircular.trna25-ThrTGT (1970102-1970027) Thr (TGT) 76 bp Sc: 90.75
GCCCTTGTAGCTCAGTTGGTAGAGCACCTGATTTGTAATCAGGGGGTCACGAGTTCGAAC
CTTGTCGGGGGCACCA
>Agrobacterium_tumefaciens_C58_UWash_chrcircular.trna28-TrpCCA (1935128-1935053) Trp (CCA) 76 bp Sc: 85.38
AGGGGTATAGCTCAGTTGGTAGAGCGGCGGTCTCCAAAACCGCAGGTCGTAAGTTCGAGC
CTTACTGCCCCTGCCA
>Agrobacterium_tumefaciens_C58_UWash_chrcircular.trna26-TyrGTA (1956866-1956782) Tyr (GTA) 85 bp Sc: 70.96
GGAGGGATGCCCGAGTGGTTAAAGGGGACGGACTGTAAATCCGTTGGCTACGCCTACGTT
GGTTCAAATCCAACTCCCTCCACCA
>Agrobacterium_tumefaciens_C58_UWash_chrlinear.trna11-ValGAC (978026-977952) Val (GAC) 75 bp Sc: 86.72
GGGCGTGTAGCTCAGCGGGAGAGCACTACGTTGACATCGTAGGGGTCACAGGTTCAATCC
CTGTCACGCCCACCA
>Agrobacterium_tumefaciens_C58_UWash_chrcircular.trna34-ValTAC (1255054-1254979) Val (TAC) 76 bp Sc: 94.30
GGGCGATTAGCTCAGTTGGTAGAGCGCCTCGTTTACACCGAGGATGTCGGGAGTTCGAGT
CTCTCATCGCCCACCA
>Alcanivorax_borkumensis_SK2_chr.trna38-AlaGGC (1723436-1723361) Ala (GGC) 76 bp Sc: 84.59
GGGGCTATAGCTCAGCTGGGAGAGCGCAACACTGGCAGTGTTGAGGTCAGCGGTTCGATC
CCGCTTAGCTCCACCA
>Alcanivorax_borkumensis_SK2_chr.trna4-AlaTGC (404901-404976) Ala (TGC) 76 bp Sc: 93.92
GGGGCCTTAGCTCAGCTGGGAGAGCGCCTGCCTTGCACGCAGGAGGTCAGCGGTTCGATC
CCGCTAGGCTCCACCA
>Alcanivorax_borkumensis_SK2_chr.trna14-ArgACG (938279-938355) Arg (ACG) 77 bp Sc: 90.58
GCACCGGTAGCTCAGCTGGATAGAGCAACTGGCTACGAACCAGTAGGTCGGGGGTTCGAC
TCCCTCCCGGTGCACCA

>Alcanivorax_borkumensis_SK2_chr.trna34-ArgCCG (2885700-2885776) Arg (CCG) 77 bp Sc: 89.30
GCGCCCGTAGCTCAGCTGGATAGAGCGTTGCCCTCCGGAGGCAAAGGTCAGAGGTTCGAA
TCCTCTCGGGCGTACCA
>Alcanivorax_borkumensis_SK2_chr.trna29-ArgCCT (2067041-2067117) Arg (CCT) 77 bp Sc: 89.63
GCCCCGGTAGCTCAGTTGGATAGAGCAAGCGCCTCCTAAGCGCTAGGTCGGACGTTCGAA
TCGTCTCCGGGGCACCA
>Alcanivorax_borkumensis_SK2_chr.trna20-ArgTCT (1373594-1373670) Arg (TCT) 77 bp Sc: 93.23
GCGCCTGTAGCTCAACCGGATAGAGCAACGGCCTTCTAAGCCGTCGGTTGCAGGTTCGAG
TCCTGCCAGGCGCGCCA
>Alcanivorax_borkumensis_SK2_chr.trna40-AsnGTT (1057994-1057919) Asn (GTT) 76 bp Sc: 93.75
TCCGCCTTAGCTCAGTTGGTAGAGCAAATGACTGTTAATCATTGGGTCGCTGGTTCGAGC
CCAGCAGGCGGAGCCA
>Alcanivorax_borkumensis_SK2_chr.trna22-AspGTC (1380529-1380605) Asp (GTC) 77 bp Sc: 96.59
GGAGCGGTAGTTCAGCTGGTTAGAATACCGGCCTGTCACGCCGGGGGTCGCGGGTTCGAG
TCCCGTCCGCTCCGCCA
>Alcanivorax_borkumensis_SK2_chr.trna18-AspGTC (1123417-1123493) Asp (GTC) 77 bp Sc: 97.44
GGAGCGGTAGTTCAGTTGGTTAGAATACCGGCCTGTCACGCCGGGGGTCGCGGGTTCGAG
TCCCGTCCGCTCCGCCA
>Alcanivorax_borkumensis_SK2_chr.trna25-CysGCA (1481879-1481952) Cys (GCA) 74 bp Sc: 64.79
GGCGCGGTGGCAGAGTGGTTATGCAGCGGACTGCAACTCCGTGTACGCCGGTTCGATTCC
GACCCGCGCCTCCA
>Alcanivorax_borkumensis_SK2_chr.trna42-GlnTTG (570290-570215) Gln (TTG) 76 bp Sc: 81.16
TGGGGTATAGCCAAGTTGGTAAGGCAGCGGGTTTTGATCCCGTTATTCCCAGGTTCGAGT
CCTGGTACCCCAGCCA
>Alcanivorax_borkumensis_SK2_chr.trna15-GluTTC (972582-972657) Glu (TTC) 76 bp Sc: 60.45
GTCCCCTTCGTCTAGTGGCCCAGGACACCGCCCTTTCACGGCGGTAACAGGGGTTCGACT
CCCCTAGGGGACGCCA
>Alcanivorax_borkumensis_SK2_chr.trna39-GluTTC (1723355-1723280) Glu (TTC) 76 bp Sc: 60.45
GTCCCCTTCGTCTAGTGGCCCAGGACACCGCCCTTTCACGGCGGTAACAGGGGTTCGACT
CCCCTAGGGGACGCCA
>Alcanivorax_borkumensis_SK2_chr.trna37-GlyCCC (1807972-1807899) Gly (CCC) 74 bp Sc: 77.93
GCGGGTGTAGTTCAATGGTAGAACGGCAGCTTCCCAAGCTGCATACGAGAGTTCGATTCT
CTTCACCCGCTCCA
>Alcanivorax_borkumensis_SK2_chr.trna16-GlyGCC (972692-972767) Gly (GCC) 76 bp Sc: 93.74
GCGGGAATAGCTCAGTTGGTAGAGCACGACCTTGCCAAGGTCGGGGTCGCGAGTTCGAAT
CTCGTTTCCCGCTCCA
>Alcanivorax_borkumensis_SK2_chr.trna24-GlyGCC (1481751-1481826) Gly (GCC) 76 bp Sc: 95.06
GCGGGAATAGCTCAGTTGGTAGAGCACAACCTTGCCAAGGTTGGGGTCGCGAGTTCGAAT
CTCGTTTCCCGCTCCA
>Alcanivorax_borkumensis_SK2_chr.trna7-GlyTCC (413664-413737) Gly (TCC) 74 bp Sc: 83.13
GCGGGTATAGTTCAACGGTAGAACCTCAGCCTTCCAAGCTGATGGTGCGGGTTCGATTCC
CGCTACCCGCTCCA
>Alcanivorax_borkumensis_SK2_chr.trna21-HisGTG (1373701-1373776) His (GTG) 76 bp Sc: 80.50
GTGATTGTAGCTCAGTTGGTAGAGCTCCGGATTGTGATTCCGGCGGTCGTGGGTTCGAGC
CCCATCAGTCACCCCA
>Alcanivorax_borkumensis_SK2_chr.trna3-IleGAT (404807-404883) Ile (GAT) 77 bp Sc: 93.51
AGGTCTGTAGCTCAGTTGGTTAGAGCGCACCCCTGATAAGGGTGAGGTCGGCAGTTCAAA
TCTGCCCAGACCTACCA
>Alcanivorax_borkumensis_SK2_chr.trna10-LeuCAA (548346-548432) Leu (CAA) 87 bp Sc: 73.73
GCCAGAGTGGTGGAATTGGTAGACACGACGGATTCAAAATCCGTTGCCTTCACGGGCGTG
CCGGTTCAAGTCCGGCCTCTGGTACCA
>Alcanivorax_borkumensis_SK2_chr.trna32-LeuCAG (2504025-2504111) Leu (CAG) 87 bp Sc: 75.45
GCCGAGGTGGTGAAATTGGTAGACACGCTAGCTTCAGGTGCTAGTGCTCGCAAGGGCGTG
GAGGTTCAAGTCCTCTCCTCGGCACCA
>Alcanivorax_borkumensis_SK2_chr.trna1-LeuGAG (357700-357785) Leu (GAG) 86 bp Sc: 60.29
GCCGAAGTGGTGGAATTGGTAGACACGCTATCTTGAGGGGGTAGTGAGCTTCGCTCGTGC
CGGTTCAAGTCCGGCCTTCGGCACCA
>Alcanivorax_borkumensis_SK2_chr.trna26-LeuTAA (1482100-1482186) Leu (TAA) 87 bp Sc: 80.06
GCGGGTATGGTGAAATTGGTAGACACAACAGACTTAAAATCTGTCGACCGTTAGGTCATG
CCGGTTCAAGTCCGGCTACCCGCACCA
>Alcanivorax_borkumensis_SK2_chr.trna11-LeuTAG (721889-721973) Leu (TAG) 85 bp Sc: 72.50
GCGCCAGTGGTGGAATTGGTAGACACGCCAGATTTAGGTTCTGGTGCCGAGAGGTGTGAA
GGTTCAAGTCCTTCCTGGCGCACCA
>Alcanivorax_borkumensis_SK2_chr.trna12-LysTTT (856687-856762) Lys (TTT) 76 bp Sc: 97.75
GGGTCGTTAGCTCAGTTGGTAGAGCAGTTGGCTTTTAACCAATTGGTCGCTGGTTCGAAT
CCAGCACGACCCACCA
>Alcanivorax_borkumensis_SK2_chr.trna2-MetCAT (357997-358073) Met (CAT) 77 bp Sc: 83.90

TGCGGGGTGGAGCAGTCTGGTAGCTCGTCGGGCTCATAACCCGAAGGTCGTAGGTTCAAA
TCCTGCCCCCGCTACCA
>Alcanivorax_borkumensis_SK2_chr.trna31-MetCAT (2352210-2352286) Met (CAT) 77 bp Sc: 94.15
GGGCCTATAGCTCAGCTGGTTAGAGCAGTCGACTCATAATCGATTGGTCCCAGGTTCAAG
TCCTGGTGGGCCCACCA
>Alcanivorax_borkumensis_SK2_chr.trna30-MetCAT (2149218-2149294) Met (CAT) 77 bp Sc: 94.31
GGCAAGGTAGCTCAGCTGGTTAGAGCACAGCACTCATAATGCTGGGGTCGGCGGTTCAAG
TCCGCCCCTTGCTACCA
>Alcanivorax_borkumensis_SK2_chr.trna33-PheGAA (2607047-2607122) Phe (GAA) 76 bp Sc: 88.50
GCCCGGATAGCTCAGTCGGTAGAGCAGAGGATTGAAAATCCTCGTGTCGGTGGTTCGATT
CCGCCTCCGGGCACCA
>Alcanivorax_borkumensis_SK2_chr.trna27-ProCGG (1651601-1651677) Pro (CGG) 77 bp Sc: 85.28
CGGAGTATAGCTCAGCTTGGTAGAGTACTACGTTCGGGACGTAGGGGTCGCAGGTTCGAA
TCCTGCTACTCCGACCA
>Alcanivorax_borkumensis_SK2_chr.trna35-ProGGG (2104441-2104365) Pro (GGG) 77 bp Sc: 82.41
CGGGGCGTAGCGCAGCCTGGTAGCGCACTTGCATGGGGTGCAAGGGGTCGGAGGTTCGAA
TCCTCCCGTCCCGACCA
>Alcanivorax_borkumensis_SK2_chr.trna19-ProTGG (1373493-1373569) Pro (TGG) 77 bp Sc: 91.84
CGGGGTGTAGCGCAGCCTGGTAGCGCGCCTGCTTTGGGAGCAGGATGTCGGGGGTTCGAA
TCCCTCCACCCCGACCA
>Alcanivorax_borkumensis_SK2_chr.trna36-SerGCT (2044939-2044850) Ser (GCT) 90 bp Sc: 74.98
GGAGAGGTGGGTGAGTGGCTGAAACCAGCTCCCTGCTAAGGAGCCATACGGGAAACTGTA
TCGCGGGTTCGAATCCCGCCCTCTCCGCCA
>Alcanivorax_borkumensis_SK2_chr.trna41-SerGGA (978208-978121) Ser (GGA) 88 bp Sc: 64.75
GGTGAGGTGTCCGAGTGGCCGAAGGAGCACGCCTGGAAAGTGTGTATACCGCAAGGTATC
GAGGGTTCGAATCCCTCCCTCACCGCCA
>Alcanivorax_borkumensis_SK2_chr.trna23-SerTGA (1474964-1475051) Ser (TGA) 88 bp Sc: 71.63
GGAGGGGTGGCAGAGTGGCCGAATGCACCGGTCTTGAAAACCGGCGACGGGAGACCGTCC
GTGGGTTCAAATCCCACCCCCTCCGCCA
>Alcanivorax_borkumensis_SK2_chr.trna28-ThrCGT (1753004-1753079) Thr (CGT) 76 bp Sc: 92.92
GCCGGGATAGCTCAGTTGGTAGAGCAACTGATTCGTAATCAGTAGGTCCGCGGTTCGATT
CCGCGTTCCGGCACCA
>Alcanivorax_borkumensis_SK2_chr.trna8-ThrGGT (413782-413857) Thr (GGT) 76 bp Sc: 91.14
GCTCATGTAGCTCAGTAGGTAGAGCACACCCTTGGTAAGGGTGAGGTCACCGGTTCAAAT
CCGGTCATGAGCTCCA
>Alcanivorax_borkumensis_SK2_chr.trna5-ThrTGT (413351-413426) Thr (TGT) 76 bp Sc: 91.42
GCTGGCGTAGCTCAGTTGGTAGAGCAGCTGATTTGTAATCAGCCGGTCGGGGGTTCGACT
CCTCTCGCCAGCTCCA
>Alcanivorax_borkumensis_SK2_chr.trna9-TrpCCA (415271-415346) Trp (CCA) 76 bp Sc: 83.81
AGGCCAGTAGTTCAATTGGTAGAGCACCGGTCTCCAAAACCGGCTGTTGGGGGTTCGAGT
CCCTCCTGGCCTGCCA
>Alcanivorax_borkumensis_SK2_chr.trna6-TyrGTA (413504-413587) Tyr (GTA) 84 bp Sc: 70.75
GGAGGGGTTCCCGAGTGGCCAAAGGGATCAGACTGTAAATCTGACGCGAAAGCTTCGGAG
GTTCGAATCCTCCCCCCTCCACCA
>Alcanivorax_borkumensis_SK2_chr.trna13-ValGAC (875508-875584) Val (GAC) 77 bp Sc: 93.93
AGGCACGTAGCTCAGTTGGTTAGAGCACCACCTTGACATGGTGGGGGTCGGTGGTTCGAA
TCCACTCGTGCCTACCA
>Alcanivorax_borkumensis_SK2_chr.trna17-ValTAC (1123305-1123380) Val (TAC) 76 bp Sc: 94.49
GGGTGATTAGCTCAGCTGGGAGAGCATCTGCCTTACAAGCAGAGGGTCGGCGGTTCGATC
CCGTCATCACCCACCA
>Alkaliphilus_metalliredigens_QYMF_chr.trna32-AlaGGC (3221892-3221967) Ala (GGC) 76 bp Sc: 88.39
GGGGCATTAGTTCAGTTGGGAGAACGCCACACTGGCAGTGTGGAGGTCAGCGGTTCGAGT
CCGCTATGCTCCACCA
>Alkaliphilus_metalliredigens_QYMF_chr.trna16-AlaTGC (1838777-1838852) Ala (TGC) 76 bp Sc: 91.34
GGGGGCGTAGCTCAGTTGGGAGAGCACCTGCCTTGCAAGCAGGGGGTCAGGAGTTCGACT
CTCCTCGTCTCCACCA
>Alkaliphilus_metalliredigens_QYMF_chr.trna3-AlaTGC (50461-50536) Ala (TGC) 76 bp Sc: 91.34
GGGGGCGTAGCTCAGTTGGGAGAGCACCTGCCTTGCAAGCAGGGGGTCAGGAGTTCGACT
CTCCTCGTCTCCACCA
>Alkaliphilus_metalliredigens_QYMF_chr.trna44-AlaTGC (4450559-4450484) Ala (TGC) 76 bp Sc: 91.34
GGGGGCGTAGCTCAGTTGGGAGAGCACCTGCCTTGCAAGCAGGGGGTCAGGAGTTCGACT
CTCCTCGTCTCCACCA
>Alkaliphilus_metalliredigens_QYMF_chr.trna29-ArgACG (1843948-1844024) Arg (ACG) 77 bp Sc: 87.34
GCGCCCATAGCTCAGCTGGATAGAGTGTCTGACTACGAATCAGAAGGTCGGGAGTTCGAA
TCTCTCTGGGCGCACCA
>Alkaliphilus_metalliredigens_QYMF_chr.trna1-ArgCCT (38852-38928) Arg (CCT) 77 bp Sc: 84.68
GCACCTATAGCTCAGTAGGATAGAGCAACGGTTTCCTAAACCGTGTGCCGGAGGTTCGAA

TCCTCCTAGGTGCACCA
>Alkaliphilus_metalliredigens_QYMF_chr.trna11-ArgTCG (997239-997315) Arg (TCG) 77 bp Sc: 84.18
GCGCCTTTAGCTCAGCTGGATAGAGTTGCAGACTTCGAATCTGAAGGTCGGGGGTTCGAA
TCCCTCAAGGCGCGCCA
>Alkaliphilus_metalliredigens_QYMF_chr.trna10-ArgTCT (997147-997223) Arg (TCT) 77 bp Sc: 83.94
GTGCTCATAGCTCAGCTGGATAGAGCAACGGCCTTCTAAGCCGTAGGCCCGGGGTTCGAA
TCCCTGTGGGCACGCCA
>Alkaliphilus_metalliredigens_QYMF_chr.trna6-ArgTCT (996725-996801) Arg (TCT) 77 bp Sc: 83.94
GTGCTCATAGCTCAGCTGGATAGAGCAACGGCCTTCTAAGCCGTAGGCCCGGGGTTCGAA
TCCCTGTGGGCACGCCA
>Alkaliphilus_metalliredigens_QYMF_chr.trna39-ArgTCT (4452941-4452865) Arg (TCT) 77 bp Sc: 88.53
GTGCTCATAGCTCAGCTGGATAGAGCAACGGCCTTCTAAGCCGTAGGTCCGGGGTTCGAA
TCCCTGTGGGCACGCCA
>Alkaliphilus_metalliredigens_QYMF_chr.trna73-ArgTCT (4437555-4437479) Arg (TCT) 77 bp Sc: 88.53
GTGCTCATAGCTCAGCTGGATAGAGCAACGGCCTTCTAAGCCGTAGGTCCGGGGTTCGAA
TCCCTGTGGGCACGCCA
>Alkaliphilus_metalliredigens_QYMF_chr.trna17-AsnGTT (1842385-1842459) Asn (GTT) 75 bp Sc: 80.01
TCCTGGGTAGCTCAATGGTGGAGCATTCGGCTGTTAACCGACAGGTTGGAGGTTCGAGTC
CTCTCCCAGGAGCCA
>Alkaliphilus_metalliredigens_QYMF_chr.trna45-AsnGTT (4447075-4447001) Asn (GTT) 75 bp Sc: 80.01
TCCTGGGTAGCTCAATGGTGGAGCATTCGGCTGTTAACCGACAGGTTGGAGGTTCGAGTC
CTCTCCCAGGAGCCA
>Alkaliphilus_metalliredigens_QYMF_chr.trna89-AsnGTT (4430624-4430550) Asn (GTT) 75 bp Sc: 80.01
TCCTGGGTAGCTCAATGGTGGAGCATTCGGCTGTTAACCGACAGGTTGGAGGTTCGAGTC
CTCTCCCAGGAGCCA
>Alkaliphilus_metalliredigens_QYMF_chr.trna23-AspGTC (1843020-1843096) Asp (GTC) 77 bp Sc: 88.33
GGCCTGGTAGTTCAGTTGGTTAGAATGCCAGCCTGTCACGCTGGAGGTCGAGGGTTCGAG
TCCCTTCCAGGTCGCCA
>Alkaliphilus_metalliredigens_QYMF_chr.trna50-AspGTC (4446522-4446446) Asp (GTC) 77 bp Sc: 88.33
GGCCTGGTAGTTCAGTTGGTTAGAATGCCAGCCTGTCACGCTGGAGGTCGAGGGTTCGAG
TCCCTTCCAGGTCGCCA
>Alkaliphilus_metalliredigens_QYMF_chr.trna70-AspGTC (4437811-4437735) Asp (GTC) 77 bp Sc: 88.33
GGCCTGGTAGTTCAGTTGGTTAGAATGCCAGCCTGTCACGCTGGAGGTCGAGGGTTCGAG
TCCCTTCCAGGTCGCCA
>Alkaliphilus_metalliredigens_QYMF_chr.trna86-AspGTC (4436263-4436187) Asp (GTC) 77 bp Sc: 88.33
GGCCTGGTAGTTCAGTTGGTTAGAATGCCAGCCTGTCACGCTGGAGGTCGAGGGTTCGAG
TCCCTTCCAGGTCGCCA
>Alkaliphilus_metalliredigens_QYMF_chr.trna94-AspGTC (4430069-4429993) Asp (GTC) 77 bp Sc: 88.33
GGCCTGGTAGTTCAGTTGGTTAGAATGCCAGCCTGTCACGCTGGAGGTCGAGGGTTCGAG
TCCCTTCCAGGTCGCCA
>Alkaliphilus_metalliredigens_QYMF_chr.trna15-CysGCA (1715478-1715552) Cys (GCA) 75 bp Sc: 77.86
GGCGGCATAGCCAAGTGGTAAGGCAGAGGTCTGCAAAACCTCTATTCCCCAGTTCAAATC
TGGGTGCCGCCTCCA
>Alkaliphilus_metalliredigens_QYMF_chr.trna28-CysGCA (1843869-1843943) Cys (GCA) 75 bp Sc: 77.86
GGCGGCATAGCCAAGTGGTAAGGCAGAGGTCTGCAAAACCTCTATTCCCCAGTTCAAATC
TGGGTGCCGCCTCCA
>Alkaliphilus_metalliredigens_QYMF_chr.trna40-GlnTTG (4452858-4452783) Gln (TTG) 76 bp Sc: 80.59
TGGGATATAGCCAAGTCGGTAAGGCAACGGACTTTGACTCCGTCATCCGCAGGTTCGAGT
CCTGCTATCCCAGCCA
>Alkaliphilus_metalliredigens_QYMF_chr.trna74-GlnTTG (4437472-4437397) Gln (TTG) 76 bp Sc: 80.59
TGGGATATAGCCAAGTCGGTAAGGCAACGGACTTTGACTCCGTCATCCGCAGGTTCGAGT
CCTGCTATCCCAGCCA
>Alkaliphilus_metalliredigens_QYMF_chr.trna9-GlnTTG (997036-997111) Gln (TTG) 76 bp Sc: 80.59
TGGGATATAGCCAAGTCGGTAAGGCAACGGACTTTGACTCCGTCATCCGCAGGTTCGAGT
CCTGCTATCCCAGCCA
>Alkaliphilus_metalliredigens_QYMF_chr.trna21-GluTTC (1842736-1842810) Glu (TTC) 75 bp Sc: 73.83
GGCCCCTTGGTCAAGCGGTCAAGACACCGCCCTTTCACGGCGGTAACAGGGGTTCGATTC
CCCTAGGGGTCACCA
>Alkaliphilus_metalliredigens_QYMF_chr.trna34-GluTTC (4894664-4894590) Glu (TTC) 75 bp Sc: 73.83
GGCCCCTTGGTCAAGCGGTCAAGACACCGCCCTTTCACGGCGGTAACAGGGGTTCGATTC
CCCTAGGGGTCACCA
>Alkaliphilus_metalliredigens_QYMF_chr.trna35-GluTTC (4453403-4453329) Glu (TTC) 75 bp Sc: 73.83
GGCCCCTTGGTCAAGCGGTCAAGACACCGCCCTTTCACGGCGGTAACAGGGGTTCGATTC
CCCTAGGGGTCACCA
>Alkaliphilus_metalliredigens_QYMF_chr.trna48-GluTTC (4446810-4446736) Glu (TTC) 75 bp Sc: 73.83
GGCCCCTTGGTCAAGCGGTCAAGACACCGCCCTTTCACGGCGGTAACAGGGGTTCGATTC
CCCTAGGGGTCACCA

>Alkaliphilus_metalliredigens_QYMF_chr.trna68-GluTTC (4438099-4438025) Glu (TTC) 75 bp Sc: 73.83
GGCCCCTTGGTCAAGCGGTCAAGACACCGCCCTTTCACGGCGGTAACAGGGGTTCGATTC
CCCTAGGGGTCACCA
>Alkaliphilus_metalliredigens_QYMF_chr.trna92-GluTTC (4430358-4430284) Glu (TTC) 75 bp Sc: 73.83
GGCCCCTTGGTCAAGCGGTCAAGACACCGCCCTTTCACGGCGGTAACAGGGGTTCGATTC
CCCTAGGGGTCACCA
>Alkaliphilus_metalliredigens_QYMF_chr.trna26-GlyGCC (1843694-1843768) Gly (GCC) 75 bp Sc: 91.02
GCGGAAGTGGCTCAGTGGTAGAGCATCGCCTTGCCAAGGCGAGGGTCGCGAGTTCAAATC
TCGTCTTCCGCTCCA
>Alkaliphilus_metalliredigens_QYMF_chr.trna54-GlyGCC (4446065-4445991) Gly (GCC) 75 bp Sc: 91.02
GCGGAAGTGGCTCAGTGGTAGAGCATCGCCTTGCCAAGGCGAGGGTCGCGAGTTCAAATC
TCGTCTTCCGCTCCA
>Alkaliphilus_metalliredigens_QYMF_chr.trna98-GlyGCC (4429559-4429485) Gly (GCC) 75 bp Sc: 91.02
GCGGAAGTGGCTCAGTGGTAGAGCATCGCCTTGCCAAGGCGAGGGTCGCGAGTTCAAATC
TCGTCTTCCGCTCCA
>Alkaliphilus_metalliredigens_QYMF_chr.trna38-GlyTCC (4453023-4452950) Gly (TCC) 74 bp Sc: 75.29
GCGGGTGTAGTTTAGTGGTAGAACTTCAGCCTTCCAAGCTGATCGCGAGGGTTCGATTCC
CTCTACCCGCTCCA
>Alkaliphilus_metalliredigens_QYMF_chr.trna5-GlyTCC (996643-996716) Gly (TCC) 74 bp Sc: 75.29
GCGGGTGTAGTTTAGTGGTAGAACTTCAGCCTTCCAAGCTGATCGCGAGGGTTCGATTCC
CTCTACCCGCTCCA
>Alkaliphilus_metalliredigens_QYMF_chr.trna66-GlyTCC (4444833-4444760) Gly (TCC) 74 bp Sc: 75.29
GCGGGTGTAGTTTAGTGGTAGAACTTCAGCCTTCCAAGCTGATCGCGAGGGTTCGATTCC
CTCTACCCGCTCCA
>Alkaliphilus_metalliredigens_QYMF_chr.trna72-GlyTCC (4437637-4437564) Gly (TCC) 74 bp Sc: 75.29
GCGGGTGTAGTTTAGTGGTAGAACTTCAGCCTTCCAAGCTGATCGCGAGGGTTCGATTCC
CTCTACCCGCTCCA
>Alkaliphilus_metalliredigens_QYMF_chr.trna88-GlyTCC (4436089-4436016) Gly (TCC) 74 bp Sc: 75.29
GCGGGTGTAGTTTAGTGGTAGAACTTCAGCCTTCCAAGCTGATCGCGAGGGTTCGATTCC
CTCTACCCGCTCCA
>Alkaliphilus_metalliredigens_QYMF_chr.trna27-HisGTG (1843781-1843856) His (GTG) 76 bp Sc: 72.34
GCGGGTGTAGTCAAGTGGTTAAGACACAGGATTGTGGTTCCTGCATGCGTGGGTTCGAAC
CCCATCACCCGCCCCA
>Alkaliphilus_metalliredigens_QYMF_chr.trna7-HisGTG (996844-996919) His (GTG) 76 bp Sc: 72.34
GCGGGTGTAGTCAAGTGGTTAAGACACAGGATTGTGGTTCCTGCATGCGTGGGTTCGAAC
CCCATCACCCGCCCCA
>Alkaliphilus_metalliredigens_QYMF_chr.trna8-HisGTG (996956-997031) His (GTG) 76 bp Sc: 72.34
GCGGGTGTAGTCAAGTGGTTAAGACACAGGATTGTGGTTCCTGCATGCGTGGGTTCGAAC
CCCATCACCCGCCCCA
>Alkaliphilus_metalliredigens_QYMF_chr.trna100-IleGAT (4429399-4429323) Ile (GAT) 77 bp Sc: 98.59
GGGCTCATAGCTCAGCTGGTTAGAGCGCACGCCTGATAAGCGTGAGGTCGGTGGTTCGAG
TCCACTTGAGCCCACCA
>Alkaliphilus_metalliredigens_QYMF_chr.trna4-IleGAT (56089-56165) Ile (GAT) 77 bp Sc: 98.59
GGGCTCATAGCTCAGCTGGTTAGAGCGCACGCCTGATAAGCGTGAGGTCGGTGGTTCGAG
TCCACTTGAGCCCACCA
>Alkaliphilus_metalliredigens_QYMF_chr.trna42-IleGAT (4452698-4452622) Ile (GAT) 77 bp Sc: 98.59
GGGCTCATAGCTCAGCTGGTTAGAGCGCACGCCTGATAAGCGTGAGGTCGGTGGTTCGAG
TCCACTTGAGCCCACCA
>Alkaliphilus_metalliredigens_QYMF_chr.trna62-IleGAT (4445344-4445268) Ile (GAT) 77 bp Sc: 98.59
GGGCTCATAGCTCAGCTGGTTAGAGCGCACGCCTGATAAGCGTGAGGTCGGTGGTTCGAG
TCCACTTGAGCCCACCA
>Alkaliphilus_metalliredigens_QYMF_chr.trna82-IleGAT (4436750-4436674) Ile (GAT) 77 bp Sc: 98.59
GGGCTCATAGCTCAGCTGGTTAGAGCGCACGCCTGATAAGCGTGAGGTCGGTGGTTCGAG
TCCACTTGAGCCCACCA
>Alkaliphilus_metalliredigens_QYMF_chr.trna31-LeuCAA (3174464-3174550) Leu (CAA) 87 bp Sc: 78.62
GCCGCGGTGGCGGAACTGGCAGACGCAGCAGACTCAAAATCTGCCGATGGTAACATCGTG
GGGGTTCGATTCCCTTCCGCGGCACCA
>Alkaliphilus_metalliredigens_QYMF_chr.trna104-LeuGAG (3165365-3165279) Leu (GAG) 87 bp Sc: 63.50
GCGGATATGGCGGAATTGGTATACGCGTAGGCTTGAGGGGTCTATGAGCGAATGCTCGTG
CAGGTTCGAGTCCTGTTATCCGCACCA
>Alkaliphilus_metalliredigens_QYMF_chr.trna18-LeuTAA (1842465-1842553) Leu (TAA) 89 bp Sc: 77.53
GCCGAAGTGGCGGAACTGGCAGACGCACAGGACTTAAAATCCTGCGGTCCTTCAAGATCG
TACCGGTTCGATTCCGGTCTTCGGCACCA
>Alkaliphilus_metalliredigens_QYMF_chr.trna46-LeuTAA (4446995-4446907) Leu (TAA) 89 bp Sc: 77.53
GCCGAAGTGGCGGAACTGGCAGACGCACAGGACTTAAAATCCTGCGGTCCTTCAAGATCG
TACCGGTTCGATTCCGGTCTTCGGCACCA
>Alkaliphilus_metalliredigens_QYMF_chr.trna90-LeuTAA (4430544-4430456) Leu (TAA) 89 bp Sc: 77.53

GCCGAAGTGGCGGAACTGGCAGACGCACAGGACTTAAAATCCTGCGGTCCTTCAAGATCG
TACCGGTTCGATTCCGGTCTTCGGCACCA
>Alkaliphilus_metalliredigens_QYMF_chr.trna25-LeuTAG (1843290-1843372) Leu (TAG) 83 bp Sc: 78.64
GCGGGTGTGGCGGAATTGGCAGACGCACTAGACTTAGGATCTAGCGGGCGACCGTGGGGG
TTCAAGTCCCTCCACCCGCACCA
>Alkaliphilus_metalliredigens_QYMF_chr.trna53-LeuTAG (4446175-4446093) Leu (TAG) 83 bp Sc: 78.64
GCGGGTGTGGCGGAATTGGCAGACGCACTAGACTTAGGATCTAGCGGGCGACCGTGGGGG
TTCAAGTCCCTCCACCCGCACCA
>Alkaliphilus_metalliredigens_QYMF_chr.trna97-LeuTAG (4429669-4429587) Leu (TAG) 83 bp Sc: 78.64
GCGGGTGTGGCGGAATTGGCAGACGCACTAGACTTAGGATCTAGCGGGCGACCGTGGGGG
TTCAAGTCCCTCCACCCGCACCA
>Alkaliphilus_metalliredigens_QYMF_chr.trna33-LysTTT (4894793-4894718) Lys (TTT) 76 bp Sc: 88.29
GACCCATTAGCTCAGTCGGTAGAGCACCTGACTTTTAATCAGGGTGTCCCGCGTTCGAGT
CGCGGATGGGTCACCA
>Alkaliphilus_metalliredigens_QYMF_chr.trna41-LysTTT (4452778-4452703) Lys (TTT) 76 bp Sc: 88.29
GACCCATTAGCTCAGTCGGTAGAGCACCTGACTTTTAATCAGGGTGTCCCGCGTTCGAGT
CGCGGATGGGTCACCA
>Alkaliphilus_metalliredigens_QYMF_chr.trna55-LysTTT (4445986-4445911) Lys (TTT) 76 bp Sc: 88.29
GACCCATTAGCTCAGTCGGTAGAGCACCTGACTTTTAATCAGGGTGTCCCGCGTTCGAGT
CGCGGATGGGTCACCA
>Alkaliphilus_metalliredigens_QYMF_chr.trna75-LysTTT (4437392-4437317) Lys (TTT) 76 bp Sc: 88.29
GACCCATTAGCTCAGTCGGTAGAGCACCTGACTTTTAATCAGGGTGTCCCGCGTTCGAGT
CGCGGATGGGTCACCA
>Alkaliphilus_metalliredigens_QYMF_chr.trna99-LysTTT (4429480-4429405) Lys (TTT) 76 bp Sc: 88.29
GACCCATTAGCTCAGTCGGTAGAGCACCTGACTTTTAATCAGGGTGTCCCGCGTTCGAGT
CGCGGATGGGTCACCA
>Alkaliphilus_metalliredigens_QYMF_chr.trna84-MetCAT (4436511-4436436) Met (CAT) 76 bp Sc: 79.52
CGCGGGGTGGAGCAACTGGTAGCTCGTCGGGCTCATAACCCGAAGGTTGTAGGTTCAAAT
CCTGCCTCCGCAACCA
>Alkaliphilus_metalliredigens_QYMF_chr.trna19-MetCAT (1842569-1842644) Met (CAT) 76 bp Sc: 80.09
CGCGGGGTGGAGCAACTGGTAGCTCGTCGGGCTCATAATCCGAAGGTCGCAGGTTCGAAT
CCTGTCTCCGCAACCA
>Alkaliphilus_metalliredigens_QYMF_chr.trna64-MetCAT (4445105-4445030) Met (CAT) 76 bp Sc: 82.53
CGCGGGGTGGAGCAACTGGTAGCTCGTCGGGCTCATAACCCGAAGGTCGTAGGTTCGAAT
CCTGCCTCCGCAACCA
>Alkaliphilus_metalliredigens_QYMF_chr.trna65-MetCAT (4444946-4444870) Met (CAT) 77 bp Sc: 86.42
GGCGGCATAGCTCAGATGGCTAGAGCGTTCGGTTCATACCCGAAAGGTCACAGGTTCGAC
TCCTGTTGCCGCTACCA
>Alkaliphilus_metalliredigens_QYMF_chr.trna47-MetCAT (4446892-4446817) Met (CAT) 76 bp Sc: 88.48
CGCGGGGTGGAGCAACTGGTAGCTCGTCGGGCTCATAACCCGAAGGTCGTAGGTTCGAAT
CCTGCCCCCGCAACCA
>Alkaliphilus_metalliredigens_QYMF_chr.trna67-MetCAT (4438181-4438106) Met (CAT) 76 bp Sc: 88.48
CGCGGGGTGGAGCAACTGGTAGCTCGTCGGGCTCATAACCCGAAGGTCGTAGGTTCGAAT
CCTGCCCCCGCAACCA
>Alkaliphilus_metalliredigens_QYMF_chr.trna91-MetCAT (4430440-4430365) Met (CAT) 76 bp Sc: 88.48
CGCGGGGTGGAGCAACTGGTAGCTCGTCGGGCTCATAACCCGAAGGTCGTAGGTTCGAAT
CCTGCCCCCGCAACCA
>Alkaliphilus_metalliredigens_QYMF_chr.trna20-MetCAT (1842650-1842726) Met (CAT) 77 bp Sc: 89.47
GGCGGCATAGCTCAGCTGGCTAGAGCGTTCGGTTCATACCCGAAAGGTCACAGGTTCGAC
TCCTGTTGCCGCTACCA
>Alkaliphilus_metalliredigens_QYMF_chr.trna85-MetCAT (4436352-4436276) Met (CAT) 77 bp Sc: 89.47
GGCGGCATAGCTCAGCTGGCTAGAGCGTTCGGTTCATACCCGAAAGGTCACAGGTTCGAC
TCCTGTTGCCGCTACCA
>Alkaliphilus_metalliredigens_QYMF_chr.trna59-MetCAT (4445607-4445531) Met (CAT) 77 bp Sc: 95.67
GGGCCTTTAGCTCAGTTGGTTAGAGCGTCCGGCTCATAACCGGCAGGTCCGGGGTTCGAA
TCCCTGAAGGCCCACCA
>Alkaliphilus_metalliredigens_QYMF_chr.trna79-MetCAT (4437013-4436937) Met (CAT) 77 bp Sc: 95.67
GGGCCTTTAGCTCAGTTGGTTAGAGCGTCCGGCTCATAACCGGCAGGTCCGGGGTTCGAA
TCCCTGAAGGCCCACCA
>Alkaliphilus_metalliredigens_QYMF_chr.trna101-PheGAA (4429313-4429238) Phe (GAA) 76 bp Sc: 87.43
GCCCAGATAGCTCAGTCGGTAGAGCAGGGGACTGAAAATCCCCGTGTCGGTGGTTCGATT
CCGCCTCTGGGCACCA
>Alkaliphilus_metalliredigens_QYMF_chr.trna14-PheGAA (1715384-1715459) Phe (GAA) 76 bp Sc: 87.43
GCCCAGATAGCTCAGTCGGTAGAGCAGGGGACTGAAAATCCCCGTGTCGGTGGTTCGATT
CCGCCTCTGGGCACCA
>Alkaliphilus_metalliredigens_QYMF_chr.trna43-PheGAA (4452612-4452537) Phe (GAA) 76 bp Sc: 87.43
GCCCAGATAGCTCAGTCGGTAGAGCAGGGGACTGAAAATCCCCGTGTCGGTGGTTCGATT

CCGCCTCTGGGCACCA
>Alkaliphilus_metalliredigens_QYMF_chr.trna63-PheGAA (4445258-4445183) Phe (GAA) 76 bp Sc: 87.43
GCCCAGATAGCTCAGTCGGTAGAGCAGGGGACTGAAAATCCCCGTGTCGGTGGTTCGATT
CCGCCTCTGGGCACCA
>Alkaliphilus_metalliredigens_QYMF_chr.trna83-PheGAA (4436664-4436589) Phe (GAA) 76 bp Sc: 87.43
GCCCAGATAGCTCAGTCGGTAGAGCAGGGGACTGAAAATCCCCGTGTCGGTGGTTCGATT
CCGCCTCTGGGCACCA
>Alkaliphilus_metalliredigens_QYMF_chr.trna105-ProGGG (2867859-2867783) Pro (GGG) 77 bp Sc: 84.34
CGGAGCGTGGCGCAGCTTGGTAGCGCGCATGGCTGGGGGCCATGAGGTCGCAGGTTCGAA
TCCTGTCGCTCCGACCA
>Alkaliphilus_metalliredigens_QYMF_chr.trna58-ProTGG (4445698-4445622) Pro (TGG) 77 bp Sc: 90.59
CCGGGTGTAGCGCAGTTTGGTAGCGCACATGGTTTGGGACCATGGGGTCGGGAGTTCGAA
TCTCTTCACCCGGACCA
>Alkaliphilus_metalliredigens_QYMF_chr.trna61-ProTGG (4445426-4445350) Pro (TGG) 77 bp Sc: 90.59
CCGGGTGTAGCGCAGTTTGGTAGCGCACATGGTTTGGGACCATGGGGTCGGGAGTTCGAA
TCTCTTCACCCGGACCA
>Alkaliphilus_metalliredigens_QYMF_chr.trna78-ProTGG (4437104-4437028) Pro (TGG) 77 bp Sc: 90.59
CCGGGTGTAGCGCAGTTTGGTAGCGCACATGGTTTGGGACCATGGGGTCGGGAGTTCGAA
TCTCTTCACCCGGACCA
>Alkaliphilus_metalliredigens_QYMF_chr.trna81-ProTGG (4436832-4436756) Pro (TGG) 77 bp Sc: 90.59
CCGGGTGTAGCGCAGTTTGGTAGCGCACATGGTTTGGGACCATGGGGTCGGGAGTTCGAA
TCTCTTCACCCGGACCA
>Alkaliphilus_metalliredigens_QYMF_chr.trna106-SerCGA (2847546-2847478) Ser (CGA) 69 bp Sc: 33.89
AGGGGTGTCAGGTAGCGCGCATGGCTGGGGGCCATGAGGTCGCAGGTTCGAATCCTGTCG
CTCCGACCA
>Alkaliphilus_metalliredigens_QYMF_chr.trna12-ArgTCG (1009021-1009100) Arg (TCG) 80 bp Sc: 25.68
GCGTTATAAATCTCAATGATACAAAGAACCTCAAACTTCGAATCTGAAGGTCGGGGGTTC
GAATCCCTCAAGGCGCGCCA
>Alkaliphilus_metalliredigens_QYMF_chr.trna30-SeC(p)TCA (2399957-2400049) SeC(p) (TCA) 93 bp Sc: 27.20
GGGGGTAGATGGGTTCTGGTGTGCCCTCTGGTCTTCAAAACCAGCGCGAGGGGTTAGTAG
CCTCTTGGGTGGGTTCGATTCCCACGTACTCCC
>Alkaliphilus_metalliredigens_QYMF_chr.trna57-SerGCT (4445799-4445708) Ser (GCT) 92 bp Sc: 73.42
GGAGAAGTACTCAAGTAGGCTCAAGAGGACGGTTTGCTAAACCGTTAGGTCGTGTATGCG
GCGCGCGGGTTCGAATCCCGCCTTCTCCGCCA
>Alkaliphilus_metalliredigens_QYMF_chr.trna77-SerGCT (4437205-4437114) Ser (GCT) 92 bp Sc: 73.42
GGAGAAGTACTCAAGTAGGCTCAAGAGGACGGTTTGCTAAACCGTTAGGTCGTGTATGCG
GCGCGCGGGTTCGAATCCCGCCTTCTCCGCCA
>Alkaliphilus_metalliredigens_QYMF_chr.trna2-SerGGA (39483-39570) Ser (GGA) 88 bp Sc: 69.03
GGAGAGGTGACCGAGTGGCTAAGGGGCACGCCTGGAAAGCGTGTAAGGCTGAAAGGCCCC
GCGGGTTCGAGTCCCGCTCTCTCCGCCA
>Alkaliphilus_metalliredigens_QYMF_chr.trna56-SerTGA (4445902-4445814) Ser (TGA) 89 bp Sc: 72.53
GGAGAGGTGTCCGAGCGGTTTAAGGAGCTAGTCTTGAAAACTAGTGATTCTGAAAGGGAC
CGTGGGTTCGAATCCCACCCTCTCCGCCA
>Alkaliphilus_metalliredigens_QYMF_chr.trna76-SerTGA (4437308-4437220) Ser (TGA) 89 bp Sc: 72.53
GGAGAGGTGTCCGAGCGGTTTAAGGAGCTAGTCTTGAAAACTAGTGATTCTGAAAGGGAC
CGTGGGTTCGAATCCCACCCTCTCCGCCA
>Alkaliphilus_metalliredigens_QYMF_chr.trna102-ThrGGT (3362174-3362100) Thr (GGT) 75 bp Sc: 86.63
GCTGATGTGGCTCAGGGGTAGAGCACTTCCTTGGTAAGGAAGAGGTCGGCGGTTCAATTC
CGCTCATCAGCTCCA
>Alkaliphilus_metalliredigens_QYMF_chr.trna37-ThrTGT (4453107-4453032) Thr (TGT) 76 bp Sc: 88.94
GCTGGTGTAGCTCAATTGGCAGAGCAGCTGATTTGTAATCAGCAGGTCGCGGGTTCGAAT
CCCATCACCAGCTCCA
>Alkaliphilus_metalliredigens_QYMF_chr.trna71-ThrTGT (4437721-4437646) Thr (TGT) 76 bp Sc: 88.94
GCTGGTGTAGCTCAATTGGCAGAGCAGCTGATTTGTAATCAGCAGGTCGCGGGTTCGAAT
CCCATCACCAGCTCCA
>Alkaliphilus_metalliredigens_QYMF_chr.trna87-ThrTGT (4436173-4436098) Thr (TGT) 76 bp Sc: 88.94
GCTGGTGTAGCTCAATTGGCAGAGCAGCTGATTTGTAATCAGCAGGTCGCGGGTTCGAAT
CCCATCACCAGCTCCA
>Alkaliphilus_metalliredigens_QYMF_chr.trna51-ThrTGT (4446432-4446357) Thr (TGT) 76 bp Sc: 90.83
GCTGGTGTAGCTCAATTGGTAGAGCAGCTGATTTGTAATCAGCAGGTCGCGGGTTCGAAT
CCCATCACCAGCTCCA
>Alkaliphilus_metalliredigens_QYMF_chr.trna95-ThrTGT (4429979-4429904) Thr (TGT) 76 bp Sc: 90.83
GCTGGTGTAGCTCAATTGGTAGAGCAGCTGATTTGTAATCAGCAGGTCGCGGGTTCGAAT
CCCATCACCAGCTCCA
>Alkaliphilus_metalliredigens_QYMF_chr.trna60-TrpCCA (4445508-4445433) Trp (CCA) 76 bp Sc: 83.41
AGGGGTGTAGTTCAGTTGGTAGAACAGTGGTCTCCAAAACCACGTGTCGTGGGTTCAAAT
CCTGTCACCCCTGCCA

>Alkaliphilus_metalliredigens_QYMF_chr.trna80-TrpCCA (4436914-4436839) Trp (CCA) 76 bp Sc: 83.41
AGGGGTGTAGTTCAGTTGGTAGAACAGTGGTCTCCAAAACCACGTGTCGTGGGTTCAAAT
CCTGTCACCCCTGCCA
>Alkaliphilus_metalliredigens_QYMF_chr.trna24-TyrGTA (1843166-1843250) Tyr (GTA) 85 bp Sc: 63.18
GGAGGGGTTCCCGAGCGGCCAAAGGGGGCAGACTGTAAATCtgttgtCAGCGACTTCGAA
GGTTCGAATCCTTCTCCCTCCACCA
>Alkaliphilus_metalliredigens_QYMF_chr.trna52-TyrGTA (4446344-4446260) Tyr (GTA) 85 bp Sc: 63.18
GGAGGGGTTCCCGAGCGGCCAAAGGGGGCAGACTGTAAATCtgttgtCAGCGACTTCGAA
GGTTCGAATCCTTCTCCCTCCACCA
>Alkaliphilus_metalliredigens_QYMF_chr.trna96-TyrGTA (4429891-4429807) Tyr (GTA) 85 bp Sc: 63.18
GGAGGGGTTCCCGAGCGGCCAAAGGGGGCAGACTGTAAATCtgttgtCAGCGACTTCGAA
GGTTCGAATCCTTCTCCCTCCACCA
>Alkaliphilus_metalliredigens_QYMF_chr.trna103-ValGAC (3237075-3236999) Val (GAC) 77 bp Sc: 87.26
GGGGAATTAGCTCAGCTGGCTAGAGTACTTGCTTGACATGCAAGGGGTCGATGGTTCGAG
TCCATTATTTCCCACCA
>Alkaliphilus_metalliredigens_QYMF_chr.trna13-ValTAC (1715308-1715383) Val (TAC) 76 bp Sc: 94.51
GGGCACATAGCTCAGCTGGGAGAGCACCTGCCTTACAAGCAGGGGGTCACAGGTTCGAGC
CCTGTTGCGCCCACCA
>Alkaliphilus_metalliredigens_QYMF_chr.trna22-ValTAC (1842819-1842894) Val (TAC) 76 bp Sc: 99.35
GGGCGCATAGCTCAGCTGGGAGAGCACCTGCCTTACAAGCAGGGGGTCACAGGTTCGAGC
CCTGTTGCGCCCACCA
>Alkaliphilus_metalliredigens_QYMF_chr.trna36-ValTAC (4453316-4453241) Val (TAC) 76 bp Sc: 99.35
GGGCGCATAGCTCAGCTGGGAGAGCACCTGCCTTACAAGCAGGGGGTCACAGGTTCGAGC
CCTGTTGCGCCCACCA
>Alkaliphilus_metalliredigens_QYMF_chr.trna49-ValTAC (4446723-4446648) Val (TAC) 76 bp Sc: 99.35
GGGCGCATAGCTCAGCTGGGAGAGCACCTGCCTTACAAGCAGGGGGTCACAGGTTCGAGC
CCTGTTGCGCCCACCA
>Alkaliphilus_metalliredigens_QYMF_chr.trna69-ValTAC (4438012-4437937) Val (TAC) 76 bp Sc: 99.35
GGGCGCATAGCTCAGCTGGGAGAGCACCTGCCTTACAAGCAGGGGGTCACAGGTTCGAGC
CCTGTTGCGCCCACCA
>Alkaliphilus_metalliredigens_QYMF_chr.trna93-ValTAC (4430271-4430196) Val (TAC) 76 bp Sc: 99.35
GGGCGCATAGCTCAGCTGGGAGAGCACCTGCCTTACAAGCAGGGGGTCACAGGTTCGAGC
CCTGTTGCGCCCACCA
>Alkaliphilus_oremlandii_OhILAs_chr.trna15-AlaTGC (2615869-2615794) Ala (TGC) 76 bp Sc: 91.34
GGGGGCGTAGCTCAGTTGGGAGAGCACCTGCCTTGCAAGCAGGGGGTCAGGAGTTCGACT
CTCCTCGTCTCCACCA
>Alkaliphilus_oremlandii_OhILAs_chr.trna3-AlaTGC (64740-64815) Ala (TGC) 76 bp Sc: 91.34
GGGGGCGTAGCTCAGTTGGGAGAGCACCTGCCTTGCAAGCAGGGGGTCAGGAGTTCGACT
CTCCTCGTCTCCACCA
>Alkaliphilus_oremlandii_OhILAs_chr.trna40-AlaTGC (2608388-2608313) Ala (TGC) 76 bp Sc: 91.34
GGGGGCGTAGCTCAGTTGGGAGAGCACCTGCCTTGCAAGCAGGGGGTCAGGAGTTCGACT
CTCCTCGTCTCCACCA
>Alkaliphilus_oremlandii_OhILAs_chr.trna61-AlaTGC (1927767-1927692) Ala (TGC) 76 bp Sc: 91.34
GGGGGCGTAGCTCAGTTGGGAGAGCACCTGCCTTGCAAGCAGGGGGTCAGGAGTTCGACT
CTCCTCGTCTCCACCA
>Alkaliphilus_oremlandii_OhILAs_chr.trna73-ArgACG (1923452-1923376) Arg (ACG) 77 bp Sc: 83.67
GCGCTCATAGCTCAGCTGGATAGAGTGTCTGACTACGAATCAGAAGGTCGGGAGTTCGAA
TCTCTCTGGGCGCACCA
>Alkaliphilus_oremlandii_OhILAs_chr.trna1-ArgCCT (20517-20593) Arg (CCT) 77 bp Sc: 83.77
GCACCTGTAGCTCAGTAGGATAGAGCAACGGTTTCCTAAACCGCAGGTCGGAGGTTCGAC
TCCTCTCAGGTGCACCA
>Alkaliphilus_oremlandii_OhILAs_chr.trna11-ArgTCG (618086-618162) Arg (TCG) 77 bp Sc: 79.00
GCGCCATTAGCTCAATCGGATAGAGTTGCAGACTTCGAATCTGAAGGTTGGGGGTTCGAG
TCCCTCATGGCGCGCCA
>Alkaliphilus_oremlandii_OhILAs_chr.trna10-ArgTCT (617953-618029) Arg (TCT) 77 bp Sc: 87.34
GTACTCATAGCTCAGCTGGATAGAGCAACGGCCTTCTAAGCCGTAGGTCCGGGGTTCGAA
TCCCTGTGGGTACGCCA
>Alkaliphilus_oremlandii_OhILAs_chr.trna22-ArgTCT (2611993-2611917) Arg (TCT) 77 bp Sc: 87.34
GTACTCATAGCTCAGCTGGATAGAGCAACGGCCTTCTAAGCCGTAGGTCCGGGGTTCGAA
TCCCTGTGGGTACGCCA
>Alkaliphilus_oremlandii_OhILAs_chr.trna5-ArgTCT (617511-617587) Arg (TCT) 77 bp Sc: 87.34
GTACTCATAGCTCAGCTGGATAGAGCAACGGCCTTCTAAGCCGTAGGTCCGGGGTTCGAA
TCCCTGTGGGTACGCCA
>Alkaliphilus_oremlandii_OhILAs_chr.trna16-AsnGTT (2612509-2612435) Asn (GTT) 75 bp Sc: 78.16
TCCAGAGTAGCTCAATGGTGGAGCACCCGGCTGTTAACCGGTAGGTTGGAGGTTCGAGCC
CTCTCTCTGGAGCCA
>Alkaliphilus_oremlandii_OhILAs_chr.trna41-AsnGTT (2605028-2604954) Asn (GTT) 75 bp Sc: 78.16

TCCAGAGTAGCTCAATGGTGGAGCACCCGGCTGTTAACCGGTAGGTTGGAGGTTCGAGCC
CTCTCTCTGGAGCCA
>Alkaliphilus_oremlandii_OhILAs_chr.trna54-AsnGTT (2598388-2598314) Asn (GTT) 75 bp Sc: 81.79
TCCAAAGTAGCTCAATGGTGGAGCAACCGGCTGTTAACCGGTAGGTTGGAGGTTCGAGCC
CTCTCTTTGGAGCCA
>Alkaliphilus_oremlandii_OhILAs_chr.trna62-AsnGTT (1924408-1924334) Asn (GTT) 75 bp Sc: 81.79
TCCAAAGTAGCTCAATGGTGGAGCAACCGGCTGTTAACCGGTAGGTTGGAGGTTCGAGCC
CTCTCTTTGGAGCCA
>Alkaliphilus_oremlandii_OhILAs_chr.trna19-AspGTC (2612265-2612189) Asp (GTC) 77 bp Sc: 87.10
GGTCCGGTAGTTCAGTTGGTTAGAATGCCAGCCTGTCACGCTGGAGGTCGAGGGTTCGAG
CCCCTTCCGGATCGCCA
>Alkaliphilus_oremlandii_OhILAs_chr.trna35-AspGTC (2610794-2610718) Asp (GTC) 77 bp Sc: 87.10
GGTCCGGTAGTTCAGTTGGTTAGAATGCCAGCCTGTCACGCTGGAGGTCGAGGGTTCGAG
CCCCTTCCGGATCGCCA
>Alkaliphilus_oremlandii_OhILAs_chr.trna46-AspGTC (2604471-2604395) Asp (GTC) 77 bp Sc: 87.10
GGTCCGGTAGTTCAGTTGGTTAGAATGCCAGCCTGTCACGCTGGAGGTCGAGGGTTCGAG
CCCCTTCCGGATCGCCA
>Alkaliphilus_oremlandii_OhILAs_chr.trna68-AspGTC (1923894-1923818) Asp (GTC) 77 bp Sc: 87.10
GGTCCGGTAGTTCAGTTGGTTAGAATGCCAGCCTGTCACGCTGGAGGTCGAGGGTTCGAG
CCCCTTCCGGATCGCCA
>Alkaliphilus_oremlandii_OhILAs_chr.trna60-CysGCA (2125280-2125207) Cys (GCA) 74 bp Sc: 71.43
GGCGGCATAGCCAAGTGGTAAGGCAGAGGTCTGCAAAACCTTTATCCCCAGTTCAAATCT
GGGTGTCGCCTCCA
>Alkaliphilus_oremlandii_OhILAs_chr.trna72-CysGCA (1923531-1923457) Cys (GCA) 75 bp Sc: 74.42
GGCGACATAGCCAAGTGGTAAGGCAGAGGTCTGCAAAACCTTTACTCCCCAGTTCAAATC
TGGGTGTCGCCTCCA
>Alkaliphilus_oremlandii_OhILAs_chr.trna23-GlnTTG (2611913-2611837) Gln (TTG) 77 bp Sc: 77.24
TGGGATATAGCCAAGTCGGTAAGGCAACGGACTTTGACTCCGTCATTCCGCAGGTTCGAG
TCCTGCTATCCCAGCCA
>Alkaliphilus_oremlandii_OhILAs_chr.trna7-GlnTTG (617677-617753) Gln (TTG) 77 bp Sc: 77.24
TGGGATATAGCCAAGTCGGTAAGGCAACGGACTTTGACTCCGTCATTCCGCAGGTTCGAG
TCCTGCTATCCCAGCCA
>Alkaliphilus_oremlandii_OhILAs_chr.trna78-GlnTTG (1915849-1915773) Gln (TTG) 77 bp Sc: 77.24
TGGGATATAGCCAAGTCGGTAAGGCAACGGACTTTGACTCCGTCATTCCGCAGGTTCGAG
TCCTGCTATCCCAGCCA
>Alkaliphilus_oremlandii_OhILAs_chr.trna57-GluTTC (2598054-2597980) Glu (TTC) 75 bp Sc: 70.16
GGCCTCTTGGTCAAGCGGTCAAGACACCGCCCTTTCACGGCGGTAACAGGGGTTCGATTC
CCCTAGGGGTCACCA
>Alkaliphilus_oremlandii_OhILAs_chr.trna17-GluTTC (2612427-2612353) Glu (TTC) 75 bp Sc: 73.83
GGCCCCTTGGTCAAGCGGTCAAGACACCGCCCTTTCACGGCGGTAACAGGGGTTCGATTC
CCCTAGGGGTCACCA
>Alkaliphilus_oremlandii_OhILAs_chr.trna44-GluTTC (2604633-2604559) Glu (TTC) 75 bp Sc: 73.83
GGCCCCTTGGTCAAGCGGTCAAGACACCGCCCTTTCACGGCGGTAACAGGGGTTCGATTC
CCCTAGGGGTCACCA
>Alkaliphilus_oremlandii_OhILAs_chr.trna66-GluTTC (1924056-1923982) Glu (TTC) 75 bp Sc: 73.83
GGCCCCTTGGTCAAGCGGTCAAGACACCGCCCTTTCACGGCGGTAACAGGGGTTCGATTC
CCCTAGGGGTCACCA
>Alkaliphilus_oremlandii_OhILAs_chr.trna50-GlyGCC (2604016-2603942) Gly (GCC) 75 bp Sc: 94.96
GCGGAAGTGGCTCAGTGGTAGAGCATCGCCTTGCCAAGGCGAGGGTCGCGGGTTCGAGTC
CCGTCTTCCGCTCCA
>Alkaliphilus_oremlandii_OhILAs_chr.trna76-GlyGCC (1916013-1915939) Gly (GCC) 75 bp Sc: 94.96
GCGGAAGTGGCTCAGTGGTAGAGCATCGCCTTGCCAAGGCGAGGGTCGCGGGTTCGAGTC
CCGTCTTCCGCTCCA
>Alkaliphilus_oremlandii_OhILAs_chr.trna9-GlyGCC (617874-617948) Gly (GCC) 75 bp Sc: 94.96
GCGGAAGTGGCTCAGTGGTAGAGCATCGCCTTGCCAAGGCGAGGGTCGCGGGTTCGAGTC
CCGTCTTCCGCTCCA
>Alkaliphilus_oremlandii_OhILAs_chr.trna21-GlyTCC (2612075-2612002) Gly (TCC) 74 bp Sc: 83.64
GCGGGTGTAGTTCAATGGTAGAACGTCAGCCTTCCAAGCTGATCGCGAGGGTTCGATTCC
CTTCACCCGCTCCA
>Alkaliphilus_oremlandii_OhILAs_chr.trna38-GlyTCC (2610381-2610308) Gly (TCC) 74 bp Sc: 83.64
GCGGGTGTAGTTCAATGGTAGAACGTCAGCCTTCCAAGCTGATCGCGAGGGTTCGATTCC
CTTCACCCGCTCCA
>Alkaliphilus_oremlandii_OhILAs_chr.trna4-GlyTCC (617432-617505) Gly (TCC) 74 bp Sc: 83.64
GCGGGTGTAGTTCAATGGTAGAACGTCAGCCTTCCAAGCTGATCGCGAGGGTTCGATTCC
CTTCACCCGCTCCA
>Alkaliphilus_oremlandii_OhILAs_chr.trna70-GlyTCC (1923704-1923631) Gly (TCC) 74 bp Sc: 83.64
GCGGGTGTAGTTCAATGGTAGAACGTCAGCCTTCCAAGCTGATCGCGAGGGTTCGATTCC

CTTCACCCGCTCCA
>Alkaliphilus_oremlandii_OhILAs_chr.trna77-HisGTG (1915930-1915855) His (GTG) 76 bp Sc: 68.17
GTGGGTGTAGTCAAGTGGTTAAGACACAGGATTGTGGCTCCTGCATGCATGGGTTCGAAT
CCCATCATCCACCCCA
>Alkaliphilus_oremlandii_OhILAs_chr.trna6-HisGTG (617596-617671) His (GTG) 76 bp Sc: 70.97
GTGGGTGTAGTCAAGTGGTTAAGACACAGGATTGTGGCTCCTGCACTCATGGGTTCGAAT
CCCATCATCCACCCCA
>Alkaliphilus_oremlandii_OhILAs_chr.trna31-IleGAT (2611143-2611067) Ile (GAT) 77 bp Sc: 91.14
AGGCTTATAGCTCAGGTGGTTAGAGCGCACGCCTGATAAGCGTGAGGTCGGTGGTTCGAG
TCCACTTAAGCCTACCA
>Alkaliphilus_oremlandii_OhILAs_chr.trna51-IleGAT (2603931-2603855) Ile (GAT) 77 bp Sc: 95.22
GGGCTCATAGCTCAGGTGGTTAGAGCGCACGCCTGATAAGCGTGAGGTCGGTGGTTCGAG
TCCACTTGAGCCCACCA
>Alkaliphilus_oremlandii_OhILAs_chr.trna53-IleGAT (2601744-2601668) Ile (GAT) 77 bp Sc: 95.22
GGGCTCATAGCTCAGGTGGTTAGAGCGCACGCCTGATAAGCGTGAGGTCGGTGGTTCGAG
TCCACTTGAGCCCACCA
>Alkaliphilus_oremlandii_OhILAs_chr.trna86-LeuCAA (1303577-1303495) Leu (CAA) 83 bp Sc: 66.42
GTCGCTGTGGCGGAACTGGCAGACGCATACGACTCAAAATCGTACGGGAAACCATATGGG
TTCGATTCCCATCAGCGGCACCA
>Alkaliphilus_oremlandii_OhILAs_chr.trna14-LeuGAG (1310933-1311018) Leu (GAG) 86 bp Sc: 58.59
GCGGATGTGGTGGAACGGCAGACACGCTATCTTGAGGGGGTAGTACTCGAATGGGTGTGC
GGGTTCAAATCCCGCCATCCGCACCA
>Alkaliphilus_oremlandii_OhILAs_chr.trna42-LeuTAA (2604880-2604792) Leu (TAA) 89 bp Sc: 77.53
GCCGAAGTGGCGGAACTGGCAGACGCACAGGACTTAAAATCCTGCGGTCCTTCAAGATCG
TACCGGTTCGATTCCGGTCTTCGGCACCA
>Alkaliphilus_oremlandii_OhILAs_chr.trna55-LeuTAA (2598240-2598152) Leu (TAA) 89 bp Sc: 77.53
GCCGAAGTGGCGGAACTGGCAGACGCACAGGACTTAAAATCCTGCGGTCCTTCAAGATCG
TACCGGTTCGATTCCGGTCTTCGGCACCA
>Alkaliphilus_oremlandii_OhILAs_chr.trna63-LeuTAA (1924327-1924239) Leu (TAA) 89 bp Sc: 77.53
GCCGAAGTGGCGGAACTGGCAGACGCACAGGACTTAAAATCCTGCGGTCCTTCAAGATCG
TACCGGTTCGATTCCGGTCTTCGGCACCA
>Alkaliphilus_oremlandii_OhILAs_chr.trna75-LeuTAG (1916109-1916027) Leu (TAG) 83 bp Sc: 73.34
GCGGGTGTGGCGGAATTGGCAGACGCACTAGACTTAGGATCTAGCGGGCAACCGTGGGGG
TTCGACTCCCTTCACCCGCACCA
>Alkaliphilus_oremlandii_OhILAs_chr.trna8-LeuTAG (617787-617869) Leu (TAG) 83 bp Sc: 73.34
GCGGGTGTGGCGGAATTGGCAGACGCACTAGACTTAGGATCTAGCGGGCAACCGTGGGGG
TTCGACTCCCTTCACCCGCACCA
>Alkaliphilus_oremlandii_OhILAs_chr.trna49-LeuTAG (2604125-2604043) Leu (TAG) 83 bp Sc: 73.45
GCGGGTGTGGCGGAATTGGCAGACGCACTAGACTTAGGATCTAGCGGGAAACCGTGGGGG
TTCGACTCCCTTCACCCGCACCA
>Alkaliphilus_oremlandii_OhILAs_chr.trna24-LysTTT (2611832-2611757) Lys (TTT) 76 bp Sc: 88.29
GACCCATTAGCTCAGTCGGTAGAGCACCTGACTTTTAATCAGGGTGTCCCGCGTTCGAGT
CGCGGATGGGTCACCA
>Alkaliphilus_oremlandii_OhILAs_chr.trna39-LysTTT (2610298-2610223) Lys (TTT) 76 bp Sc: 88.29
GACCCATTAGCTCAGTCGGTAGAGCACCTGACTTTTAATCAGGGTGTCCCGCGTTCGAGT
CGCGGATGGGTCACCA
>Alkaliphilus_oremlandii_OhILAs_chr.trna71-LysTTT (1923622-1923547) Lys (TTT) 76 bp Sc: 88.29
GACCCATTAGCTCAGTCGGTAGAGCACCTGACTTTTAATCAGGGTGTCCCGCGTTCGAGT
CGCGGATGGGTCACCA
>Alkaliphilus_oremlandii_OhILAs_chr.trna79-LysTTT (1915768-1915693) Lys (TTT) 76 bp Sc: 88.29
GACCCATTAGCTCAGTCGGTAGAGCACCTGACTTTTAATCAGGGTGTCCCGCGTTCGAGT
CGCGGATGGGTCACCA
>Alkaliphilus_oremlandii_OhILAs_chr.trna33-MetCAT (2610968-2610892) Met (CAT) 77 bp Sc: 86.62
GGCGGCATAGCTTAGTTGGCTAGAGCGTTCGGTTCATACCCGAAAGGTCACAGGTTCGAC
TCCTGTTGCCGCTACCA
>Alkaliphilus_oremlandii_OhILAs_chr.trna65-MetCAT (1924141-1924065) Met (CAT) 77 bp Sc: 86.62
GGCGGCATAGCTTAGTTGGCTAGAGCGTTCGGTTCATACCCGAAAGGTCACAGGTTCGAC
TCCTGTTGCCGCTACCA
>Alkaliphilus_oremlandii_OhILAs_chr.trna43-MetCAT (2604715-2604640) Met (CAT) 76 bp Sc: 89.23
CGCGGGGTGGAGCAGTTGGCAGCTCGTCGGGCTCATAACCCGAAGGTCGTAGGTTCAAGT
CCTACCCCCGCAACCA
>Alkaliphilus_oremlandii_OhILAs_chr.trna56-MetCAT (2598136-2598061) Met (CAT) 76 bp Sc: 89.23
CGCGGGGTGGAGCAGTTGGCAGCTCGTCGGGCTCATAACCCGAAGGTCGTAGGTTCAAGT
CCTACCCCCGCAACCA
>Alkaliphilus_oremlandii_OhILAs_chr.trna64-MetCAT (1924223-1924148) Met (CAT) 76 bp Sc: 89.23
CGCGGGGTGGAGCAGTTGGCAGCTCGTCGGGCTCATAACCCGAAGGTCGTAGGTTCAAGT
CCTACCCCCGCAACCA

>Alkaliphilus_oremlandii_OhILAs_chr.trna83-MetCAT (1915389-1915313) Met (CAT) 77 bp Sc: 92.33
GGACCTTTAGCTCAGTTGGTCAGAGCATCCGGCTCATAACCGGCAGGTCCGGGGTTCGAA
TCCCTGAAGGTCCACCA
>Alkaliphilus_oremlandii_OhILAs_chr.trna28-MetCAT (2611406-2611330) Met (CAT) 77 bp Sc: 93.51
GGGCCTTTAGCTCAGTTGGTCAGAGCATCCGGCTCATAACCGGCAGGTCCGGGGTTCGAA
TCCCTGAAGGCCCACCA
>Alkaliphilus_oremlandii_OhILAs_chr.trna32-PheGAA (2611048-2610973) Phe (GAA) 76 bp Sc: 87.43
GCCCAGATAGCTCAGTCGGTAGAGCAGGGGACTGAAAATCCCCGTGTCGGTGGTTCGATT
CCGCCTCTGGGCACCA
>Alkaliphilus_oremlandii_OhILAs_chr.trna52-PheGAA (2603845-2603770) Phe (GAA) 76 bp Sc: 87.43
GCCCAGATAGCTCAGTCGGTAGAGCAGGGGACTGAAAATCCCCGTGTCGGTGGTTCGATT
CCGCCTCTGGGCACCA
>Alkaliphilus_oremlandii_OhILAs_chr.trna59-PheGAA (2125379-2125304) Phe (GAA) 76 bp Sc: 87.43
GCCCAGATAGCTCAGTCGGTAGAGCAGGGGACTGAAAATCCCCGTGTCGGTGGTTCGATT
CCGCCTCTGGGCACCA
>Alkaliphilus_oremlandii_OhILAs_chr.trna84-PheGAA (1915297-1915222) Phe (GAA) 76 bp Sc: 87.43
GCCCAGATAGCTCAGTCGGTAGAGCAGGGGACTGAAAATCCCCGTGTCGGTGGTTCGATT
CCGCCTCTGGGCACCA
>Alkaliphilus_oremlandii_OhILAs_chr.trna27-ProTGG (2611504-2611428) Pro (TGG) 77 bp Sc: 89.27
CCGGGTGTAGCGCAGTTTGGTAGCGCACATGGTTTGGGACCATGGGGCCGGGGGTTCGAA
TCCCTCCACCCGGACCA
>Alkaliphilus_oremlandii_OhILAs_chr.trna30-ProTGG (2611227-2611151) Pro (TGG) 77 bp Sc: 89.27
CCGGGTGTAGCGCAGTTTGGTAGCGCACATGGTTTGGGACCATGGGGCCGGGGGTTCGAA
TCCCTCCACCCGGACCA
>Alkaliphilus_oremlandii_OhILAs_chr.trna82-ProTGG (1915489-1915413) Pro (TGG) 77 bp Sc: 89.27
CCGGGTGTAGCGCAGTTTGGTAGCGCACATGGTTTGGGACCATGGGGCCGGGGGTTCGAA
TCCCTCCACCCGGACCA
>Alkaliphilus_oremlandii_OhILAs_chr.trna85-SeC(p)TCA (1831799-1831707) SeC(p) (TCA) 93 bp Sc: 26.55
GGGAGTAGATGGGTGCTGGTGTGCCCTATGGTCTTCAAAACCATTGTGAGGGGTTAGTAG
CTTCTTAGGTGGGTTCGATTCCCACGTACTCCC
>Alkaliphilus_oremlandii_OhILAs_chr.trna26-SerGCT (2611604-2611513) Ser (GCT) 92 bp Sc: 73.31
GGAGAAGTACTCAAGTAGGCTGAAGAGGACGGTTTGCTAAACCGTTAGGTCGCGTAAGTG
GCGCGCGGGTTCGAATCCCGCCTTCTCCGCCA
>Alkaliphilus_oremlandii_OhILAs_chr.trna81-SerGCT (1915589-1915498) Ser (GCT) 92 bp Sc: 73.31
GGAGAAGTACTCAAGTAGGCTGAAGAGGACGGTTTGCTAAACCGTTAGGTCGCGTAAGTG
GCGCGCGGGTTCGAATCCCGCCTTCTCCGCCA
>Alkaliphilus_oremlandii_OhILAs_chr.trna2-SerGGA (23350-23438) Ser (GGA) 89 bp Sc: 72.81
GGAGAAATGTCCGAGTTGGCTAAGGGGCACGCCTGGAAAGCGTGTAAGGCCGAAAGGCCC
CGTGGGTTCGAGTCCCACTTTCTCCGCCA
>Alkaliphilus_oremlandii_OhILAs_chr.trna25-SerTGA (2611749-2611661) Ser (TGA) 89 bp Sc: 72.90
GGAGAGATGTCCGAGCGGTTTAAGGAGCTGGTCTTGAAAACCAGTGACTCCGAAAGGGGC
CGTGGGTTCGAATCCCACTCTCTCCGCCA
>Alkaliphilus_oremlandii_OhILAs_chr.trna80-SerTGA (1915686-1915598) Ser (TGA) 89 bp Sc: 72.90
GGAGAGATGTCCGAGCGGTTTAAGGAGCTGGTCTTGAAAACCAGTGACTCCGAAAGGGGC
CGTGGGTTCGAATCCCACTCTCTCCGCCA
>Alkaliphilus_oremlandii_OhILAs_chr.trna12-ThrGGT (1146249-1146323) Thr (GGT) 75 bp Sc: 90.86
GCTGATGTGGCTCAGTGGTAGAGCACTTCCTTGGTAAGGAAGAGGTCGGCGGTTCAATTC
CGCTCATCAGCTCCA
>Alkaliphilus_oremlandii_OhILAs_chr.trna20-ThrTGT (2612157-2612081) Thr (TGT) 77 bp Sc: 88.90
GCTGGTGTAGCTCAGTTGGCCAGAGCAGCTGATTTGTAATCAGCAGGTCGCGGGTTCGAA
TCCCATCACCAGCTCCA
>Alkaliphilus_oremlandii_OhILAs_chr.trna36-ThrTGT (2610648-2610572) Thr (TGT) 77 bp Sc: 88.90
GCTGGTGTAGCTCAGTTGGCCAGAGCAGCTGATTTGTAATCAGCAGGTCGCGGGTTCGAA
TCCCATCACCAGCTCCA
>Alkaliphilus_oremlandii_OhILAs_chr.trna47-ThrTGT (2604363-2604287) Thr (TGT) 77 bp Sc: 88.90
GCTGGTGTAGCTCAGTTGGCCAGAGCAGCTGATTTGTAATCAGCAGGTCGCGGGTTCGAA
TCCCATCACCAGCTCCA
>Alkaliphilus_oremlandii_OhILAs_chr.trna69-ThrTGT (1923786-1923710) Thr (TGT) 77 bp Sc: 88.90
GCTGGTGTAGCTCAGTTGGCCAGAGCAGCTGATTTGTAATCAGCAGGTCGCGGGTTCGAA
TCCCATCACCAGCTCCA
>Alkaliphilus_oremlandii_OhILAs_chr.trna29-TrpCCA (2611306-2611231) Trp (CCA) 76 bp Sc: 84.99
AGGGGTGTAGTTCAGTTGGTAGAACAGTGGTCTCCAAAACCACGTGTCGTGGGTTCGAGT
CCTGCCACCCCTGCCA
>Alkaliphilus_oremlandii_OhILAs_chr.trna37-TyrGTA (2610544-2610460) Tyr (GTA) 85 bp Sc: 61.91
GGAGGGGTTCCCGAGCGGCCAAAGGGGGCAGACTGTAAATCtgttgtCAACGACTTCGAA
GGTTCGAATCCTTCTCCCTCCACCA
>Alkaliphilus_oremlandii_OhILAs_chr.trna48-TyrGTA (2604219-2604135) Tyr (GTA) 85 bp Sc: 61.91

GGAGGGGTTCCCGAGCGGCCAAAGGGGGCAGACTGTAAATCtgttgtCAACGACTTCGAA
GGTTCGAATCCTTCTCCCTCCACCA
>Alkaliphilus_oremlandii_OhILAs_chr.trna74-TyrGTA (1916233-1916149) Tyr (GTA) 85 bp Sc: 61.91
GGAGGGGTTCCCGAGCGGCCAAAGGGGGCAGACTGTAAATCtgttgtCAACGACTTCGAA
GGTTCGAATCCTTCTCCCTCCACCA
>Alkaliphilus_oremlandii_OhILAs_chr.trna13-ValGAC (1257317-1257392) Val (GAC) 76 bp Sc: 82.77
GAGGGTATAGCTCAGCTGGCAGAGCGCTACGTTGACATCGTAGAGGTCGTTGGTTCAAGT
CCAATTATCCTCACCA
>Alkaliphilus_oremlandii_OhILAs_chr.trna18-ValTAC (2612345-2612270) Val (TAC) 76 bp Sc: 100.60
GGGCGCATAGCTCAGCTGGGAGAGCACCTGCCTTACAAGCAGGGGGTCACAGGTTCAAGT
CCTGTTGCGCCCACCA
>Alkaliphilus_oremlandii_OhILAs_chr.trna34-ValTAC (2610875-2610800) Val (TAC) 76 bp Sc: 100.60
GGGCGCATAGCTCAGCTGGGAGAGCACCTGCCTTACAAGCAGGGGGTCACAGGTTCAAGT
CCTGTTGCGCCCACCA
>Alkaliphilus_oremlandii_OhILAs_chr.trna45-ValTAC (2604551-2604476) Val (TAC) 76 bp Sc: 100.60
GGGCGCATAGCTCAGCTGGGAGAGCACCTGCCTTACAAGCAGGGGGTCACAGGTTCAAGT
CCTGTTGCGCCCACCA
>Alkaliphilus_oremlandii_OhILAs_chr.trna67-ValTAC (1923974-1923899) Val (TAC) 76 bp Sc: 100.60
GGGCGCATAGCTCAGCTGGGAGAGCACCTGCCTTACAAGCAGGGGGTCACAGGTTCAAGT
CCTGTTGCGCCCACCA
>Alkaliphilus_oremlandii_OhILAs_chr.trna58-ValTAC (2125455-2125380) Val (TAC) 76 bp Sc: 99.35
GGGCGCATAGCTCAGCTGGGAGAGCACCTGCCTTACAAGCAGGGGGTCACAGGTTCGAGC
CCTGTTGCGCCCACCA
>Ailuropoda_melanoleuca_GL194938.1.trna13-AlaAGC (18703-18631) Ala (AGC) 73 bp Sc: 56.27
GGGGAATTAGCTCAATTGGTAGAGCGCTTGCTTAGCACGTGAGAGGTAGTGGGATCGATG
CCCACATTCTCCA
>Ailuropoda_melanoleuca_GL192819.1.trna53-AlaAGC (1311438-1311366) Ala (AGC) 73 bp Sc: 58.48
GGGGAATTAGCTCAAATGGTAGAGCGCTCGCTTAGCATGTGAGAGGTAGCGGGATCGATG
CCCGCATTCTCCA
>Ailuropoda_melanoleuca_GL194901.1.trna5-AlaAGC (71887-71959) Ala (AGC) 73 bp Sc: 63.06
GGGGAATTAGCTCAAATGGTAGAGCGCTCGCTTAGCATGCGAGAGGTAGCGGGATCGATG
CCCGCATTCTCCA
>Ailuropoda_melanoleuca_GL192369.1.trna279-AlaAGC (847198-847126) Ala (AGC) 73 bp Sc: 63.68
GGGGGATTAGCTCAAATGGTAGAGCGCTCGCTTAGCATGCGAGAGGTAGCGGGATCGATG
CCCGCATCCTCCA
>Ailuropoda_melanoleuca_GL192478.1.trna32-AlaAGC (1363633-1363705) Ala (AGC) 73 bp Sc: 63.68
GGGGGATTAGCTCAAATGGTAGAGCGCTCGCTTAGCATGCGAGAGGTAGCGGGATCGATG
CCCGCATCCTCCA
>Ailuropoda_melanoleuca_GL194938.1.trna10-AlaAGC (26002-25930) Ala (AGC) 73 bp Sc: 64.41
GGGGAATTAGCTCAGGTGGTAGAGCGCTCGCTTAGCATGCGAGAGGTGGTGGGATCGATG
CCCACATTCTCCA
>Ailuropoda_melanoleuca_GL193897.1.trna21-AlaAGC (240434-240505) Ala (AGC) 72 bp Sc: 68.18
GGGGGTGTAGCTCAGCGGTAGAGCGCGCGCTTAGCATGCGCGAGGCCCTAGGTTCGATCC
CCAGCACCTCCA
>Ailuropoda_melanoleuca_GL193748.1.trna12-AlaAGC (361038-361109) Ala (AGC) 72 bp Sc: 74.48
GGGGATGTAGCTCAGTGGTAGAGCGCATGCTTAGCATGCATGAGGTCCTGGGTTTGATCC
CCAGCATCTCCA
>Ailuropoda_melanoleuca_GL193897.1.trna47-AlaAGC (238784-238713) Ala (AGC) 72 bp Sc: 75.28
GGGGGTGTAGCTCAGTGGTAGAGCGCGTGCTTAGCATGCACGAGGCCCCGGGTTCAATCC
CCGGCACTTCCA
>Ailuropoda_melanoleuca_GL193897.1.trna26-AlaAGC (272870-272941) Ala (AGC) 72 bp Sc: 75.78
GGGGATGTAGCTCAGTGGTAGAGCGCATGCTTAGCATGCATGAGGTCCCGGGTTCGATCC
CCAGCATCTCCA
>Ailuropoda_melanoleuca_GL193897.1.trna41-AlaAGC (311269-311198) Ala (AGC) 72 bp Sc: 76.69
GGGGGTGTAGCTCAGTGGTAGAGCGCGTGCTTAGCATGTACGAGGTCCCGGGTTCAATCC
CCGGCACCTCCA
>Ailuropoda_melanoleuca_GL193897.1.trna11-AlaAGC (175733-175804) Ala (AGC) 72 bp Sc: 77.18
GGGGGTGTAGCTCAGTGGTAGAGCGCGTGCTTAGCATGCACGAGGCCCCGGGTTCAATCC
CCGGCACCTCCA
>Ailuropoda_melanoleuca_GL193897.1.trna12-AlaAGC (182847-182918) Ala (AGC) 72 bp Sc: 77.18
GGGGGTGTAGCTCAGTGGTAGAGCGCGTGCTTAGCATGCACGAGGCCCCGGGTTCAATCC
CCGGCACCTCCA
>Ailuropoda_melanoleuca_GL193897.1.trna19-AlaAGC (218541-218612) Ala (AGC) 72 bp Sc: 77.18
GGGGGTGTAGCTCAGTGGTAGAGCGCGTGCTTAGCATGCACGAGGCCCCGGGTTCAATCC
CCGGCACCTCCA
>Ailuropoda_melanoleuca_GL193897.1.trna46-AlaAGC (241046-240975) Ala (AGC) 72 bp Sc: 77.42
GGGGGTGTAGCTCAGTGGTAGAGCGCGTGCTTAGCATGCACGAGGCCCTGGGTTCGATCC

CCAGCACCTCCA
>Ailuropoda_melanoleuca_GL193897.1.trna15-AlaAGC (191336-191407) Ala (AGC) 72 bp Sc: 77.92
GGGGGTGTAGCTCAGTGGTAGAGCGCATGCTTAGCATGCATGAGGCCCTGGGTTCGATCC
CCAGCACCTCCA
>Ailuropoda_melanoleuca_GL193666.1.trna55-AlaAGC (155406-155335) Ala (AGC) 72 bp Sc: 81.40
GGGGATGTAGCTCAGTGGTAGAGCGCATGCTTAGCATGCATGAGGTCCTGGGTTCGATCC
CCAGCATCTCCA
>Ailuropoda_melanoleuca_GL193897.1.trna51-AlaCGC (222565-222494) Ala (CGC) 72 bp Sc: 70.09
GGGGGTGTAGCTCAGTGGTAGAGCGCGTGCTTCGCATGTACGAGGCCCCGGGTTCGACCC
CCGGCTCCTCCA
>Ailuropoda_melanoleuca_GL193406.1.trna6-AlaCGC (116391-116462) Ala (CGC) 72 bp Sc: 73.12
GGGGATGTAGCTCAGTGGTAGAGCGCGCGCTTCGCATGTGTGAGGTCCCGGGTTCAATCC
CCGGCATCTCCA
>Ailuropoda_melanoleuca_GL193897.1.trna22-AlaCGC (244752-244823) Ala (CGC) 72 bp Sc: 73.20
GGGGGTGTAGCTCAGTGGTAGAGCGCGTGCTTCGCATGTACGAGGCCCCGGGTTCAATCC
CCGGCACCTCCA
>Ailuropoda_melanoleuca_GL193897.1.trna25-AlaCGC (260938-261009) Ala (CGC) 72 bp Sc: 74.81
GGGGATGTAGCTCAGTGGTAGAGCGCATGCTTCGCATGTATGAGGCCCCGGGTTCGATCC
CCGGCATCTCCA
>Ailuropoda_melanoleuca_GL194901.1.trna11-AlaCGC (88153-88082) Ala (CGC) 72 bp Sc: 74.81
GGGGATGTAGCTCAGTGGTAGAGCGCATGCTTCGCATGTATGAGGCCCCGGGTTCGATCC
CCGGCATCTCCA
>Ailuropoda_melanoleuca_GL193113.1.trna14-AlaTGC (582889-582973) Ala (TGC) 85 bp Sc: 30.66
GCCTGGGTGGCTCAGTCGGTTAAGTGCCTGACTTGCGTTCAGGTCCTGATCTTAGGGCCC
CTAGGATCCAGCCCTGGGTTGGGCT
>Ailuropoda_melanoleuca_GL192816.1.trna52-AlaTGC (629059-628988) Ala (TGC) 72 bp Sc: 59.42
GGGGATGTAGCTCAATGGTAGAGTATATGCTTTGCATGTGTAAGGCCCCAGGTTCAATCC
CTGGCATCTCCA
>Ailuropoda_melanoleuca_GL193897.1.trna43-AlaTGC (298098-298027) Ala (TGC) 72 bp Sc: 63.65
GGGGGTGTAGCTCAGTGGTAGAGCGCATGCTTTGCATGTGTGAGGCCCTGGGTTCGATCC
CCGGCATCTCCA
>Ailuropoda_melanoleuca_GL192354.1.trna187-AlaTGC (4185983-4186054) Ala (TGC) 72 bp Sc: 72.71
GGGGATGTAGCTCAGTGGTAGAGCGCATGCTTTGCATGTATGAGGCCCCGGGTTCAATCC
CCGGCATCTCCA
>Ailuropoda_melanoleuca_GL192354.1.trna215-AlaTGC (4179565-4179494) Ala (TGC) 72 bp Sc: 74.45
GGGGATGTAGCTCAGTGGTAGAGCGCATGCTTTGCATGTATGAGGCCCCGGGTTCGATCC
CCGGCATCTCCA
>Ailuropoda_melanoleuca_GL194954.1.trna12-AlaTGC (86465-86394) Ala (TGC) 72 bp Sc: 74.45
GGGGATGTAGCTCAGTGGTAGAGCGCATGCTTTGCATGTATGAGGCCCCGGGTTCGATCC
CCGGCATCTCCA
>Ailuropoda_melanoleuca_GL193897.1.trna28-AlaTGC (302892-302963) Ala (TGC) 72 bp Sc: 79.03
GGGGATGTAGCTCAGTGGTAGAGCGCATGCTTTGCATGCATGAGGCCCCGGGTTCGATCC
CCGGCATCTCCA
>Ailuropoda_melanoleuca_GL193897.1.trna17-AlaTGC (200837-200908) Ala (TGC) 72 bp Sc: 79.65
GGGGGTGTAGCTCAGTGGTAGAGCGCATGCTTTGCATGCATGAGGCCCCGGGTTCGATCC
CCGGCACCTCCA
>Ailuropoda_melanoleuca_GL193897.1.trna50-AlaTGC (224972-224901) Ala (TGC) 72 bp Sc: 79.65
GGGGGTGTAGCTCAGTGGTAGAGCGCATGCTTTGCATGCATGAGGCCCCGGGTTCGATCC
CCGGCACCTCCA
>Ailuropoda_melanoleuca_GL192478.1.trna95-ArgACG (1362461-1362389) Arg (ACG) 73 bp Sc: 63.19
GGGCCAGTGGCGCAATGGATAGCGCGTCTGATTACGGATCAGAAGATTATAGGTTCGACT
CCTGCCTGGCTCG
>Ailuropoda_melanoleuca_GL192348.1.trna276-ArgACG (3394969-3394897) Arg (ACG) 73 bp Sc: 68.07
GGGCCAGTGGCGCAATGGATAACGCGTCTGACTACGGATCAGAAGATTCTAGGTTCGACT
CCTGGCTGGCTCG
>Ailuropoda_melanoleuca_GL193552.1.trna15-ArgACG (224101-224173) Arg (ACG) 73 bp Sc: 68.07
GGGCCAGTGGCGCAATGGATAACGCGTCTGACTACGGATCAGAAGATTCTAGGTTCGACT
CCTGGCTGGCTCG
>Ailuropoda_melanoleuca_GL193552.1.trna51-ArgACG (547635-547563) Arg (ACG) 73 bp Sc: 68.07
GGGCCAGTGGCGCAATGGATAACGCGTCTGACTACGGATCAGAAGATTCTAGGTTCGACT
CCTGGCTGGCTCG
>Ailuropoda_melanoleuca_GL193372.1.trna22-ArgACG (427073-427145) Arg (ACG) 73 bp Sc: 72.37
GGGCCAGTGGCGCAATGGATAACGCGTCTGACTACGGATCAGAAGATTCCAGGTTCGACT
CCTGGCTGGCTCG
>Ailuropoda_melanoleuca_GL193874.1.trna13-ArgACG (389306-389378) Arg (ACG) 73 bp Sc: 72.37
GGGCCAGTGGCGCAATGGATAACGCGTCTGACTACGGATCAGAAGATTCCAGGTTCGACT
CCTGGCTGGCTCG

>Ailuropoda_melanoleuca_GL194901.1.trna9-ArgACG (105580-105508) Arg (ACG) 73 bp Sc: 72.37
GGGCCAGTGGCGCAATGGATAACGCGTCTGACTACGGATCAGAAGATTCCAGGTTCGACT
CCTGGCTGGCTCG
>Ailuropoda_melanoleuca_GL194938.1.trna8-ArgACG (31574-31502) Arg (ACG) 73 bp Sc: 72.37
GGGCCAGTGGCGCAATGGATAACGCGTCTGACTACGGATCAGAAGATTCCAGGTTCGACT
CCTGGCTGGCTCG
>Ailuropoda_melanoleuca_GL192562.1.trna19-ArgCCG (949917-949999) Arg (CCG) 83 bp Sc: 35.29
GCCTGGGTGGCACAGCGGTTAAGCGTCTGCCTCCGGCTCAGGGTGTGATCCCCGCATTGT
GGGATCGAGGCCCACCTCAGGCT
>Ailuropoda_melanoleuca_GL192810.1.trna46-ArgCCG (1173678-1173750) Arg (CCG) 73 bp Sc: 55.50
ACCTGGCTGGCTCAGTTGGTAGAGCACACGACTCCGGCTCTTGGGGTTGTGAGTTCGAGC
CCCACGTCGGGGG
>Ailuropoda_melanoleuca_GL193167.1.trna61-ArgCCG (877574-877502) Arg (CCG) 73 bp Sc: 65.49
GACCCAGTGGCCTAATGGATAAGGCATCAGCCTCCGGAGCTGGGGATTGTGGGTTCGAGT
CCCATCTGGGTCG
>Ailuropoda_melanoleuca_GL192703.1.trna14-ArgCCG (351983-352055) Arg (CCG) 73 bp Sc: 69.07
GACCGCGTGGCCTAATGGATAAGGCGTCTGATTCCGGATCAGAAGATTGAGGGTTCGAGT
CCCTTCGTGGTCG
>Ailuropoda_melanoleuca_GL193204.1.trna1-ArgCCG (32479-32551) Arg (CCG) 73 bp Sc: 69.88
GGCCGCGTGGCCTAATGGATAAGGCGTCTGATTCCGGATCAGAAGATTGAGGGTTCGAGT
CCCTTCGTGGTCG
>Ailuropoda_melanoleuca_GL193897.1.trna18-ArgCCG (214722-214794) Arg (CCG) 73 bp Sc: 69.88
GGCCGCGTGGCCTAATGGATAAGGCGTCTGATTCCGGATCAGAAGATTGAGGGTTCGAGT
CCCTTCGTGGTCG
>Ailuropoda_melanoleuca_GL193897.1.trna53-ArgCCG (167655-167583) Arg (CCG) 73 bp Sc: 69.88
GGCCGCGTGGCCTAATGGATAAGGCGTCTGATTCCGGATCAGAAGATTGAGGGTTCGAGT
CCCTTCGTGGTCG
>Ailuropoda_melanoleuca_GL192475.1.trna111-ArgCCT (2143159-2143087) Arg (CCT) 73 bp Sc: 22.85
ACCTGGTTGGCTCAGTCAGTAGAGCATGGCACTCCTGATCTCAGCGCTGTAAGTTTGAAC
CCTACGTTGGGTG
>Ailuropoda_melanoleuca_GL194414.1.trna18-ArgCCT (201264-201336) Arg (CCT) 73 bp Sc: 29.02
GCCTGGCTGGCTTAGTCGGTGAGGCAGGTGACTCCTCATCTCCAGGTTGTGAGTTTGAGC
CCCATGTTGGGTG
>Ailuropoda_melanoleuca_GL194599.1.trna7-ArgCCT (57459-57387) Arg (CCT) 73 bp Sc: 29.93
GCCTGGCTGGCCCAGCTGATGAGGCGTATGACTCCTCATCTTGGAGTTGTGAGTTCGAGC
CCCACGCTGGGTG
>Ailuropoda_melanoleuca_GL193605.1.trna29-ArgCCT (154820-154748) Arg (CCT) 73 bp Sc: 37.00
ACCTGGCTGGCTCAGTCAGTAAAGCATGTGACTCCTGATCTCGGGGTTGTGAGTTTGAGT
CCCATGTTGGGTA
>Ailuropoda_melanoleuca_GL192378.1.trna151-ArgCCT (1114354-1114282) Arg (CCT) 73 bp Sc: 37.12
GCCTGGCTGGCTCCGTTGGTGGAGCATGTGACTCCTGATCTTGGGGTTGTGGGTTTGAGC
CCCACGTTGGGTG
>Ailuropoda_melanoleuca_GL192682.1.trna87-ArgCCT (651900-651828) Arg (CCT) 73 bp Sc: 37.49
GCCTGGCTGGCTCAGTCGGTGGAGCACGTGACTCCTGATCTTGGGACTGTGAGTTCAAGC
CCCATGTTGGGTG
>Ailuropoda_melanoleuca_GL192385.1.trna175-ArgCCT (2116808-2116736) Arg (CCT) 73 bp Sc: 37.51
ACCTGGCTGGCTCAGTCAGTAGAGCATGCAATTCCTGATTTCAGGGTTGTGAGTTCAAGC
CCCATGTTGGGTG
>Ailuropoda_melanoleuca_GL194560.1.trna10-ArgCCT (175139-175067) Arg (CCT) 73 bp Sc: 39.84
GCCTAGCTGGCTCAGTGGGAGAAGTGTGTGACTCCTGATCTCAGGGTCATGAGTTTGAGC
CCCATGTTGGGTG
>Ailuropoda_melanoleuca_GL192340.1.trna326-ArgCCT (1868840-1868768) Arg (CCT) 73 bp Sc: 40.26
ACCTGGCTGGCTCAGTCAATAGAGCATGTGACTCCTGATCTTGGGGTTGTGGGTTCAAGC
CCCATGCTGGGTG
>Ailuropoda_melanoleuca_GL192405.1.trna180-ArgCCT (1487388-1487316) Arg (CCT) 73 bp Sc: 43.48
GCCTAACTGGCTCAGTTGGTAGAGCATGTGACTCCTTATCTCAGGGTAGTGAGTTTGAGC
CCCATGTTGGGTA
>Ailuropoda_melanoleuca_GL194216.1.trna12-ArgCCT (16532-16460) Arg (CCT) 73 bp Sc: 44.66
GCCTGGCTGGCTCAGTCGGAAGAGCATGAAACTCCTGATCTCAGGATTGTGAGTTCGAGC
TCCATGTTGGGTA
>Ailuropoda_melanoleuca_GL193139.1.trna32-ArgCCT (848914-848986) Arg (CCT) 73 bp Sc: 44.75
ACCTGGTAGGCTCAGTGGGTAGAGCATGTGATTCCTGATCTCAGAGTCGTGGGTTCAAGC
CCCATGTTGGGTG
>Ailuropoda_melanoleuca_GL193342.1.trna27-ArgCCT (722931-723003) Arg (CCT) 73 bp Sc: 44.92
ACCTGGCTGGTTCAGTTTGAAGAGCGTGAGACTCCTGATCTTGGGGTCGTGAGTTCGAGC
CCCATGCTGGGTG
>Ailuropoda_melanoleuca_GL193680.1.trna18-ArgCCT (449001-449073) Arg (CCT) 73 bp Sc: 44.99

GCCTGGCTGGCTCAGTCGGAAGAGCATGCGACTCCTGATCTCAGGGTTGTGAGTTCCAGC
CCCATGTTGGGTG
>Ailuropoda_melanoleuca_GL193402.1.trna14-ArgCCT (17356-17280) Arg (CCT) 77 bp Sc: 46.07
ACCTGGCTGGCTCAGTTGGTAGAGCAGGCGACTCCTGATCTCTCTCTGGGTTGTGAGTTC
AAGCCCCATGTTGGGTG
>Ailuropoda_melanoleuca_GL194702.1.trna5-ArgCCT (14820-14748) Arg (CCT) 73 bp Sc: 46.22
GCCTGGCTGGCTCAGTCAGAAGAGCGTGGGACTCCTGATCTCAGAGTTGTGAGTTCGAGC
CCCACGTTAGGTG
>Ailuropoda_melanoleuca_GL192483.1.trna118-ArgCCT (1863358-1863286) Arg (CCT) 73 bp Sc: 46.30
GCCTAGCTGGCTCAGTCAGTAGAGCGTGTGACTCCTGATCTCGCGGTTGTGAGTTCAAGC
CCCATGTTGGGTG
>Ailuropoda_melanoleuca_GL192442.1.trna196-ArgCCT (823752-823680) Arg (CCT) 73 bp Sc: 46.61
ACCTGGCTGGCTCAGTCAGTAGAGCGTGTGACTCCTGGTCTCAAGGTCGTGAGTTCAAGC
CCCATGTTGGGTG
>Ailuropoda_melanoleuca_GL193570.1.trna8-ArgCCT (400801-400873) Arg (CCT) 73 bp Sc: 46.79
ACCTGGCTGGCTCAGTCAGTAGAGTATGTGACTCCTGATCTTGGGGTCATGGGTTCAAGC
CCCATGCTGGGCA
>Ailuropoda_melanoleuca_GL192340.1.trna53-ArgCCT (1418685-1418757) Arg (CCT) 73 bp Sc: 47.60
GCCTGGTTGGCTCAGTCAGTGGAGCATGTGACTCCTGATCTCAGGGTTGTGGGTTCGAGC
CCCACGCTGGGTG
>Ailuropoda_melanoleuca_GL192479.1.trna60-ArgCCT (907505-907577) Arg (CCT) 73 bp Sc: 48.50
GCCTGGCTGGCTCAGTGGGTAGACCATGTGACTCCTGATCTCAGGGTCGTGGGCTCAAGC
CCCATGCTGGGTA
>Ailuropoda_melanoleuca_GL192421.1.trna185-ArgCCT (1059586-1059514) Arg (CCT) 73 bp Sc: 49.12
GCCTGGTTGGCTCAGTTGGTAGAGCACGTGACTCCTGATCTCGCGATCGTGAGTTCACGC
CCCATGTTGGGCA
>Ailuropoda_melanoleuca_GL192804.1.trna5-ArgCCT (406896-406968) Arg (CCT) 73 bp Sc: 49.39
GCCTATTTGGCTCAGTTGGTAGAGTGTAAGACTCCTGATCTTGGGGTAGTGAGTTTGAGC
CTCATGTTGGGTG
>Ailuropoda_melanoleuca_GL193724.1.trna11-ArgCCT (99048-99120) Arg (CCT) 73 bp Sc: 50.23
ACCCAGCTGGCTCAGTCGGTGGAGCGTCAGACTCCTGATCTTGGGGTCATGAGTTCAAGC
CTCACGTTGGGTG
>Ailuropoda_melanoleuca_GL193470.1.trna13-ArgCCT (625611-625540) Arg (CCT) 72 bp Sc: 50.76
ACCCAACTGGCTCAGTCGGTAGAGCGTGGGGCTCCTGAACTCAGGTCATGAGTTCAAGCC
CCATGTTGGGTG
>Ailuropoda_melanoleuca_GL192340.1.trna103-ArgCCT (1914722-1914794) Arg (CCT) 73 bp Sc: 51.51
GTCTGGCTGGCTCAGTTGGTAGAGTATGTGACTCCTGATCTCAGGGTCATGAGTTCAAGC
CCCATGTTGGGTG
>Ailuropoda_melanoleuca_GL192466.1.trna31-ArgCCT (1561732-1561804) Arg (CCT) 73 bp Sc: 51.87
GCCTAGCTGGCTCAGTGGGAAGAGTATAAGACTCCTGATCTCAGAGTTGTGAGTTCGATC
CTCATGTTGGGTA
>Ailuropoda_melanoleuca_GL192870.1.trna9-ArgCCT (150149-150221) Arg (CCT) 73 bp Sc: 53.47
ACCTGGCTGGCTCAGTCGGAAGAGCATGTGACTCCTGATCTCGGGGTTGTGAGTTCGAGC
CCCACGTTGGGTG
>Ailuropoda_melanoleuca_GL192749.1.trna48-ArgCCT (1160421-1160349) Arg (CCT) 73 bp Sc: 53.67
ACCTGGCTGGTTCAGTTGGTAGAGCATGTGACTCCTAATCTCGGGGTCATGAGTTCGAGC
CCCATGTTGGGTG
>Ailuropoda_melanoleuca_GL192405.1.trna31-ArgCCT (1676960-1677032) Arg (CCT) 73 bp Sc: 54.84
GCCTGGCTGGCTCAGTTGGTAGAGCACGGGACTCCTGATCTCAGGGTCGTGAGTTCAAGC
CCCACGCTGGGTG
>Ailuropoda_melanoleuca_GL192698.1.trna16-ArgCCT (925354-925425) Arg (CCT) 72 bp Sc: 55.59
ACCTGGCTGGCTCAGTTGGTAGAGCGTGTGACTCCTGATCTCAGGGCGTGAGTTCAAGTC
CCATGTTGGGCA
>Ailuropoda_melanoleuca_GL193570.1.trna23-ArgCCT (490449-490377) Arg (CCT) 73 bp Sc: 56.14
ACCTGGCTGGCTCAGTGGGTAGAGCATGTGACTCCTGATCTCAGGGTCGTGAGTTCAAGC
CCCACGCTGGGCA
>Ailuropoda_melanoleuca_GL192369.1.trna6-ArgCCT (191898-191970) Arg (CCT) 73 bp Sc: 56.72
GCCTAGCTGGCTCAGTCGGTAGAGCACGTGACTCCTGATCTTGGGGTTGTGAGTTCAAGC
CCCACACTGGGCA
>Ailuropoda_melanoleuca_GL194600.1.trna12-ArgCCT (182437-182509) Arg (CCT) 73 bp Sc: 59.54
GCCTGGCTGGCTCAGTTGGTAGAGCGTGTGACTCCTGATCTCAGGGTCATGAGTTTGAGC
CCCATGTTGGGCA
>Ailuropoda_melanoleuca_GL192518.1.trna9-ArgCCT (334904-334976) Arg (CCT) 73 bp Sc: 59.65
GCCTGGCTGGCTCAATTGGAAGAGCATGGGACTCCTAATCTCAGGGTCATGAGTTCGAGC
CCCATGTTGGGCA
>Ailuropoda_melanoleuca_GL192574.1.trna33-ArgCCT (498342-498414) Arg (CCT) 73 bp Sc: 63.78
GCCTGGCTGTCTTAGTTGGTAGAGCGCATGACTCCTGATCATGGGGTTGTGGGTTCAAGC

CCCACATTGGGCA
>Ailuropoda_melanoleuca_GL192383.1.trna189-ArgCCT (1027921-1027849) Arg (CCT) 73 bp Sc: 67.28
GCCCCAGTGGCCTAATGGATAAGGCATTGGCCTCCTAAGCCAGGGATTGTGGGTTCGAGT
CCCATCTGGGGTG
>Ailuropoda_melanoleuca_GL192703.1.trna15-ArgCCT (352303-352375) Arg (CCT) 73 bp Sc: 70.63
GCCCCAGTGGCCTAATGGATAAGGCACTGGCCTCCTAAGCCAGGGATTGTGGGTTCGAGT
CCCATCTGGGGTG
>Ailuropoda_melanoleuca_GL193204.1.trna73-ArgCCT (65031-64959) Arg (CCT) 73 bp Sc: 71.31
GCCCCGGTGGCCTAATGGAGAAGGCACTGGCCTCCTAAGCCAGGGATTGTGGGTTCGAGT
CCCACCCGGGGTA
>Ailuropoda_melanoleuca_GL193204.1.trna2-ArgCCT (34083-34155) Arg (CCT) 73 bp Sc: 71.53
GCCCCGGTGGCCTAATGGATAAGGCATTGGCCTCCTAAGCCAGGGATTGTGGGTTCGAGT
CCCACCCGGGGTA
>Ailuropoda_melanoleuca_GL192703.1.trna158-ArgCCT (351331-351259) Arg (CCT) 73 bp Sc: 73.41
GCCCCAGTGGCCTAATGGATAAGGCACTGGCCTCCTAAGCCAGGGATTGTGGGTTCGAGT
CCCACCTGGGGTG
>Ailuropoda_melanoleuca_GL192703.1.trna13-ArgCCT (350780-350852) Arg (CCT) 73 bp Sc: 73.88
GCCCCAGTGGCCTAATGGATAAGGCACTGGCCTCCTAAGCCAGGGATTGTGGGTTCGAGT
CCCACCTGGGGTA
>Ailuropoda_melanoleuca_GL193204.1.trna14-ArgCCT (78013-78085) Arg (CCT) 73 bp Sc: 73.88
GCCCCAGTGGCCTAATGGATAAGGCACTGGCCTCCTAAGCCAGGGATTGTGGGTTCGAGT
CCCACCTGGGGTA
>Ailuropoda_melanoleuca_GL192664.1.trna67-ArgCCT (40807-40717) Arg (CCT) 91 bp Sc: 37.02
GCCTGGGTGGCTCAGTCGGTTGAGCGCCTGACTCCTGATCTCAGCTCAGATCTTGGTCTC
AGGGTTGTGGGTTCAGGCCCCACATTGGGCT
>Ailuropoda_melanoleuca_GL194222.1.trna10-ArgCCT (8058-7968) Arg (CCT) 91 bp Sc: 37.17
ACCTGGGTGGCTCAGTCGGTTGAGTGTCCGACTCCTGGTTTCATCTCAGGTCATGATCTC
AGGGTCGTGGGATCAAGCCCCATGTCAGGTT
>Ailuropoda_melanoleuca_GL192357.1.trna143-ArgGCG (3881233-3881323) Arg (GCG) 91 bp Sc: 42.98
GCCTGGGTGGCTCAGTTGGTTGAGTGTCTGACTGCGGATTTCGGTTCACGTCCTGATCTC
AGGGTCACGGGATCAAGCCCCGTGTCAGGCT
>Ailuropoda_melanoleuca_GL193601.1.trna16-ArgTCG (587102-587184) Arg (TCG) 83 bp Sc: 23.42
GCCTGGGTGGCTCAGTCGTTTAGTGTCTGCCTTCGGCTCAGGGTGTGATCCCGGCATTAT
GGGATCGAGCCCCATGTCAGGCT
>Ailuropoda_melanoleuca_GL193722.1.trna6-ArgTCG (391799-391881) Arg (TCG) 83 bp Sc: 23.95
GCCTGGGTGGCTCAGTCGTTGAGTGTCTGCCTTCGGCTCAGGGCGTGATGCCAGCGTTGT
GGGATCGAGCCCCATGTCAGGTT
>Ailuropoda_melanoleuca_GL195202.1.trna13-ArgTCG (14503-14421) Arg (TCG) 83 bp Sc: 24.07
GCCTGGGTGGCTCAGTCGCTAAGTGTCTGCCTTCGGCTCAGGGCGTGATCCCTGCGTTAT
GGGATCGAGCCCCATGTCAGGCT
>Ailuropoda_melanoleuca_GL192345.1.trna176-ArgTCG (1659919-1659837) Arg (TCG) 83 bp Sc: 24.54
GCCTGGGTGGCTCAGTCGTTAAGCGTCTGCCTTCGGCTCAGGGCATGATCCCGGCGTTAT
GGGATCGAGCCCCATGTCAGGCT
>Ailuropoda_melanoleuca_GL193379.1.trna15-ArgTCG (718645-718727) Arg (TCG) 83 bp Sc: 24.61
GCCTGGGTGGCTCAGTCGTTAAGTGTCTGCCTTCGGCTCAGGGCGTGATCCCAGCATTAT
GGGATCGAGCCCCATATCAGGCT
>Ailuropoda_melanoleuca_GL192700.1.trna48-ArgTCG (882733-882815) Arg (TCG) 83 bp Sc: 25.64
GCCTGGGTGGCTCAATCGTTAAGCGTCTGCCTTCGGCTCAGGGCGTGATCCTGACATTAT
GGGATCGAGCCCCATGTCAGGCT
>Ailuropoda_melanoleuca_GL192462.1.trna34-ArgTCG (1390281-1390363) Arg (TCG) 83 bp Sc: 26.32
GCCTGGGTGGCTCACTGGTTAAGCGTCTGCCTTCGGCTCAGGGCGTGATCCTGGCATTAT
GGGATCGAGCCCCATATCAGGCT
>Ailuropoda_melanoleuca_GL193042.1.trna76-ArgTCG (105583-105500) Arg (TCG) 84 bp Sc: 26.54
ACCTGGGTGGCTTAGTTGGTTAAGCTTCTGCCTTCGGCTCAGGTCACGATCCCAGAGTCC
TGGGATTGAGCCCCAGGTCGGGTT
>Ailuropoda_melanoleuca_GL192712.1.trna12-ArgTCG (612007-612089) Arg (TCG) 83 bp Sc: 26.66
GCCTGGGTAGCTCAGTCGTTAAGCATCTGCCTTCGGCTCAGGGTGTGATCCCAGCGTTAT
GGGATCGAGCCCCATATCAGGCT
>Ailuropoda_melanoleuca_GL192426.1.trna39-ArgTCG (1402567-1402649) Arg (TCG) 83 bp Sc: 27.70
ACCTGGGTGGCTCAGTGGTTGGGCTTCTGCCTTCGGCTCAGGGCATGGTCCCGGTGTTAT
GGGATCAAGCCCCATATCGGGTT
>Ailuropoda_melanoleuca_GL193342.1.trna21-ArgTCG (564330-564412) Arg (TCG) 83 bp Sc: 27.97
GCCTGGGTGGCTCAGTCGTTAAGCGTCTGCCTTCGGCTCAGGGCGTGATCCTGGCGTTAT
GGGATCGAGCCCCATATCAGGCT
>Ailuropoda_melanoleuca_GL192452.1.trna81-ArgTCG (175744-175673) Arg (TCG) 72 bp Sc: 28.12
GCTTGGTTGGCTCAGTTGGTTGGGCACCTGTCTTCGGCCCAGGTCATGGTCCCAGGATCC
TGGGATTGAGCC

>Ailuropoda_melanoleuca_GL193247.1.trna8-ArgTCG (332589-332672) Arg (TCG) 84 bp Sc: 28.65
GCCTGGGTGGCTCAGTCAGTTAAGCATCTGTCTTCGGCTCAGGTCATGGTCCTGGGTTCC
TGGGATCGAGTCCCGCCTCAGGCT
>Ailuropoda_melanoleuca_GL192420.1.trna54-ArgTCG (2738781-2738863) Arg (TCG) 83 bp Sc: 28.78
GCCTGGGTGGCTCAGTCGTTAAGCGTCTGCCTTCGGCTCAGGGTGTGATCCCGGCGTTAT
GGGATCGAGCCCCATATCAGGCT
>Ailuropoda_melanoleuca_GL193538.1.trna23-ArgTCG (231846-231928) Arg (TCG) 83 bp Sc: 29.03
GCCTGGCTGGCTCAGTCGTTAAGCGTCTGCCTTCGGCTCAGGGCGTGATCCTGGCATTAT
GGGATCGAGTCCCACGTCAGGCG
>Ailuropoda_melanoleuca_GL192922.1.trna24-ArgTCG (630753-630835) Arg (TCG) 83 bp Sc: 30.30
GCCTGGGTGGCTCAGTTGCTAAGTGTCTGCCTTCGGCTCAGGGCGTGGTCCCGGCGTTGT
GGGATCGAGCCCCACATCAGGCT
>Ailuropoda_melanoleuca_GL192974.1.trna37-ArgTCG (1010536-1010454) Arg (TCG) 83 bp Sc: 30.51
GCCTGGGTGGCTCAGTTGTTAAGCGTCTGCCTTCGGCTCAGGGTGTGGTCCCGGCGTTGT
GGGATCGAGCCCCACATCAGGTT
>Ailuropoda_melanoleuca_GL192873.1.trna57-ArgTCG (496449-496367) Arg (TCG) 83 bp Sc: 30.89
GCCTGGGTGGCTCAGTCGTTAAGCGTCTGCCTTCGGCTCAGGGCGTGATCCCAGCTTTCT
GGGATCGAGCCCCAGCTCAGGCT
>Ailuropoda_melanoleuca_GL192423.1.trna114-ArgTCG (1141438-1141356) Arg (TCG) 83 bp Sc: 30.90
GCCTGGGTGGCTCAGTTGTTAAGCGTCTGCCTTCGGCTCAGGGTGTGATCCCAGCGTTGT
GGGATCGAGCCCCACATCAGGCT
>Ailuropoda_melanoleuca_GL192666.1.trna25-ArgTCG (706625-706694) Arg (TCG) 70 bp Sc: 30.91
ACCTGGTTGGTTCAATCGGTAGAGTATATGACTCGATCTCAGGTTGTGAGTTCAAGCCCC
ACATTGGGTG
>Ailuropoda_melanoleuca_GL194160.1.trna13-ArgTCG (153151-153069) Arg (TCG) 83 bp Sc: 31.47
GCCTGGGTGGCTCAGTCGTTAAGCGTCTGCCTTCGGCTCAGGGCATGATCCCCATGTTCT
GGGATCGACCCCCACCTCAGGCT
>Ailuropoda_melanoleuca_GL193921.1.trna10-ArgTCG (100623-100541) Arg (TCG) 83 bp Sc: 31.90
GCCTGGGTGGCTCAGTCGTTAAGCGTCTGCCTTCGGCTCAGGGAGTGATCCCGGCGTTGT
GGGATCGAGCCCCACATCAGGCT
>Ailuropoda_melanoleuca_GL192371.1.trna54-ArgTCG (1370961-1371043) Arg (TCG) 83 bp Sc: 32.02
GCCTGGGTGGCTCAGTCGTTGAGCGTCTGCCTTCGGCTCAGGGCATGGTCCCAGGGTCCT
GGGATCAAGCCCCGGATCAGGCT
>Ailuropoda_melanoleuca_GL192611.1.trna3-ArgTCG (82703-82785) Arg (TCG) 83 bp Sc: 32.23
GCCTGGGTGGCACAGCGGTTATGCGTCTGCCTTCGGCTCAGGGCGTGATCCTGGCATTGT
GGGATCGAGCCCCACATCAGGCT
>Ailuropoda_melanoleuca_GL192392.1.trna161-ArgTCG (2229051-2228969) Arg (TCG) 83 bp Sc: 32.68
GCCTGGGTGGCTCAGTTGTTAAGCGTCTGCCTTCGGCTCAGGGAGTGATCCTGGCATCGT
GGGATCGAGCCCCACATCAGGCT
>Ailuropoda_melanoleuca_GL192369.1.trna136-ArgTCG (3374951-3375021) Arg (TCG) 71 bp Sc: 32.74
ACCTGGCTGGCTCAGGTGGAAGAGCATGTGACTCGATTTCAAGGTCGTGAGTTTGAGACC
CATGTTGGGTG
>Ailuropoda_melanoleuca_GL192857.1.trna23-ArgTCG (909337-909255) Arg (TCG) 83 bp Sc: 32.75
GCCTGGTTGGCACAGCGGTTAAGCATCTGCCTTCGGCTCAGGGTGCGATCCCGGtgttgt
GGGATCGAGCCCCACATCAGGCT
>Ailuropoda_melanoleuca_GL194492.1.trna8-ArgTCG (59291-59209) Arg (TCG) 83 bp Sc: 32.96
GCCTGGGTGGCACAGCCGTTAAGCGTCTGCCTTCGGCTCAGGGTGTGATCCCAGCGTTGT
GGGTTCGAGCCCCACCTCAGGCT
>Ailuropoda_melanoleuca_GL192351.1.trna149-ArgTCG (4297729-4297810) Arg (TCG) 82 bp Sc: 33.31
GCCTGAGTGGCTCAGTCAGTTAAGCGTCTGCCTTCGGCTCAGGTCATGATCAGGGTTGTG
GGATCAAGTCCCACATCAGGCT
>Ailuropoda_melanoleuca_GL192414.1.trna23-ArgTCG (567362-567445) Arg (TCG) 84 bp Sc: 33.34
GCCTGGCTGGCTCAGTCGGTGAAGCGTCTGCCTTCGGCTCAGGCCCTGATCCCAGGGCCC
TGGGATCGAGCCCCATGTCGGGCT
>Ailuropoda_melanoleuca_GL192446.1.trna103-ArgTCG (634657-634575) Arg (TCG) 83 bp Sc: 33.40
GCCTGGGTGGCTCAGTTGTTAAGCGTCTGCCTTCGGCTCAGGGTGTGATCCCAGAGTGGT
GGGATCGAGTCCCACCTCAGGCT
>Ailuropoda_melanoleuca_GL192560.1.trna43-ArgTCG (2020784-2020702) Arg (TCG) 83 bp Sc: 33.54
GCCTGGTTGGCACAGCGGTTAAGCGTCTGCCTTCGGCTCAGGGTGTGATCCTGGCGTTGT
GGGATCAAGACCCACATCAGGCT
>Ailuropoda_melanoleuca_GL192371.1.trna151-ArgTCG (3170691-3170609) Arg (TCG) 83 bp Sc: 33.55
GCCTGGTTGGCACAGCGGTTAAGCGTCTGCCTTCGGCTCAGGGCGTGATCCCGGCGTTAT
GGGATCGAGCCCCATATCAGGCT
>Ailuropoda_melanoleuca_GL193955.1.trna10-ArgTCG (168779-168703) Arg (TCG) 77 bp Sc: 33.78
GCCTGGGGAGCTCAGTCGGTTGGGCCTCTGTCTTCGGCTCAGGTCATGGTCCGTGGGATC
GAGTCCCACCTCGGGCT
>Ailuropoda_melanoleuca_GL192548.1.trna12-ArgTCG (289287-289369) Arg (TCG) 83 bp Sc: 33.80

GCCTGAGTGGCCCAGCGGTTAGGCGTCTGCCTTCGGCTCAGGGCGTGATCCCGGCGTTAT
GGGATCGAGCCCCATGTCAGGCT
>Ailuropoda_melanoleuca_GL193249.1.trna53-ArgTCG (352723-352641) Arg (TCG) 83 bp Sc: 33.87
GCCTGGGTGGCTCAGTCGTTAAGCGTCTGCCTTCGGCTCAGGGAGTGATCACGGCGTTGT
GGGATCGAGTCCCACATCAGGCT
>Ailuropoda_melanoleuca_GL192549.1.trna13-ArgTCG (343636-343718) Arg (TCG) 83 bp Sc: 33.94
GCCTGGTTGGCACAGCGGTTAAGCGTCTGCCTTCGGCTCAGGGCGTGATCCTGGCGTTGT
GGGATCAAGCCCCACATCAGGCT
>Ailuropoda_melanoleuca_GL193489.1.trna9-ArgTCG (269134-269216) Arg (TCG) 83 bp Sc: 34.12
GCCTGGGTGGCACAGCGGTTATGCGTCTGCCTTCGGCTCAGGGCGTGATCCTAGCGTTGT
GGGATCGAGCCCCACATCAGGCT
>Ailuropoda_melanoleuca_GL192442.1.trna23-ArgTCG (374585-374655) Arg (TCG) 71 bp Sc: 34.40
TCATGGCTGGCTCAGCTGGAAGAGCATGTGACTCGATCTCAGGGTCATGAGTTCTATCCT
CGtgttgtGTA
>Ailuropoda_melanoleuca_GL192707.1.trna56-ArgTCG (1168971-1168888) Arg (TCG) 84 bp Sc: 34.48
GCCTGGGTGGCTCAGTCGGGGAAGCGTCTGCCTTCGGCTCAGGTCATGATCCCAGGGTTC
TGGGATCGAGCCCCAACCCAGGCT
>Ailuropoda_melanoleuca_GL194769.1.trna11-ArgTCG (120703-120786) Arg (TCG) 84 bp Sc: 34.50
GCCTGGCTGGCTCAGTTGGTTAAGTGTCTGCCTTCGGCCCAGGTCATGATCCTGGGAGCC
TGGGATCGAGCCCCATGTCAGGCT
>Ailuropoda_melanoleuca_GL192684.1.trna26-ArgTCG (1585907-1585825) Arg (TCG) 83 bp Sc: 34.53
GCCTGGGTGGCGGAGCGGTGACGCGTCTGCCTTCGGCTCAGGGCGTGATCCCGGCGTTGT
GGGATCGAGCCCCACGTCGGGCT
>Ailuropoda_melanoleuca_GL192768.1.trna15-ArgTCG (305050-305132) Arg (TCG) 83 bp Sc: 34.60
GCCTGGTTGGCACAGCGGTTAAGCGTCTGCCTTCGGCTCAGGGTGTGATCCCGGCATTGT
GGGATCGAGCCCCACATCAGGCT
>Ailuropoda_melanoleuca_GL192757.1.trna22-ArgTCG (1448900-1448982) Arg (TCG) 83 bp Sc: 34.62
GCCTGGGTGGCACAGCGGTTAAGCGTCTGCCTTCGGCTCAGGGAGTGATCCCAGCGTTGT
GGGATCGAGCCCCACCTCAGGTT
>Ailuropoda_melanoleuca_GL193020.1.trna9-ArgTCG (555872-555954) Arg (TCG) 83 bp Sc: 34.76
GCCTGGGTGGCACAGCGGTTAGGCGTCTGCCTTCGGCTCAGGGCGTGATCCTGACGTTGT
GGGATTGAGCCCCACCTCGGGCT
>Ailuropoda_melanoleuca_GL192901.1.trna33-ArgTCG (71565-71483) Arg (TCG) 83 bp Sc: 34.85
GCCTGGGTGGCACAGTGGTTATGCATCTGCCTTCGGCTCAGGGCGTGATCCTGGCGTTGT
GGGATCGAGCCCCACATCAGGCT
>Ailuropoda_melanoleuca_GL192460.1.trna288-ArgTCG (343481-343411) Arg (TCG) 71 bp Sc: 35.05
GCCTAGCTGGCTCAGTTGATAGAGCATGTGACTCGATCTCAGGGTCGTGAGTTTGAGCCC
CATGTTGGGTG
>Ailuropoda_melanoleuca_GL193614.1.trna17-ArgTCG (140740-140657) Arg (TCG) 84 bp Sc: 35.36
GCCTGGCTGGCTCAGTTGGTTAAGCGTCTGCCTTCGGCTCAGGGCGTGATCCCGGCGTTA
TGGGATCGAGCCCCACGTCAGGCT
>Ailuropoda_melanoleuca_GL192557.1.trna16-ArgTCG (507617-507700) Arg (TCG) 84 bp Sc: 35.38
GCCTGGGTGGCTCAGTTGGTTAAGCATCTGCTTTCGGCTCAGGTCATGATTCCAGGGTCC
TGGGATCGAGGCCCACCTCGGGCT
>Ailuropoda_melanoleuca_GL192717.1.trna3-ArgTCG (140561-140643) Arg (TCG) 83 bp Sc: 35.59
GCCTGGGTGGCACAGCGGTTAAGCGTCTGCCTTCGGCTCAGGGTGTGATCCCAGCATTGT
GGGATCGAGCCCCACCTCAGGCT
>Ailuropoda_melanoleuca_GL193966.1.trna11-ArgTCG (385810-385889) Arg (TCG) 80 bp Sc: 35.60
GCCTGGGTGGCTCAGTCGTTAAGCGTCTGCCTTCGGCTCAGGGCGTGATCCCGGCGTGGG
ATCGAGCCCCACATCAGGCT
>Ailuropoda_melanoleuca_GL192415.1.trna38-ArgTCG (1701748-1701831) Arg (TCG) 84 bp Sc: 35.63
GCCTGGATGGCTCAGATGGTTGGGTGTCTGCCTTCGGCTCAGGTCATGATCTCCAGGTCC
TGGGGTCGAGCCCCATTTCAGGCT
>Ailuropoda_melanoleuca_GL192877.1.trna39-ArgTCG (347930-347845) Arg (TCG) 86 bp Sc: 35.68
GCCTGGGTGGCACAGCGGTTAAGTGCGTCTGCCTTCGGCTCAGGGCGTGATCCCGGGCGT
TATGGGATCGAGCCCCATATCAGGCT
>Ailuropoda_melanoleuca_GL194295.1.trna9-ArgTCG (72561-72479) Arg (TCG) 83 bp Sc: 36.03
GCCTGGGTGGCACAGCGGTTGTGCATCTGCCTTCGGCTCAGGGCGTGATCCCAGCGTTGT
GGGATCGAGCCCCACGTCAGGCT
>Ailuropoda_melanoleuca_GL193503.1.trna3-ArgTCG (249262-249344) Arg (TCG) 83 bp Sc: 36.57
GCCTGGTTGGCACAGTGGTTGGGCGTCTGCCTTCGGCTCAGAGCGTGATCCCGGCATTGT
GGGATCGAGCCCCACGTCAGGCT
>Ailuropoda_melanoleuca_GL192977.1.trna42-ArgTCG (76539-76457) Arg (TCG) 83 bp Sc: 36.68
GCCTGGGTGGCTCAGTTGTTAAGCGTCTGACTTCGGCTCAGGGCGTGATCCCGGCGTTGT
GGGATCGAGCCCCACATCAGGCT
>Ailuropoda_melanoleuca_GL193122.1.trna63-ArgTCG (360059-359989) Arg (TCG) 71 bp Sc: 37.50
GCCTGGCTGGCTCAGCCAGTAGAGCTTGTGACTCGGTCTCGAGGTCGTGAGTTCAAGCCC

CACGTTGGGTG
>Ailuropoda_melanoleuca_GL194789.1.trna3-ArgTCG (49517-49435) Arg (TCG) 83 bp Sc: 37.74
GCCTGGGTGGCACAGCGGTTGTGCGTCTGCCTTCGGCTCAGGGCGTGATCCCGGCGTTGT
GGGATCGAGCCCCACATCAGGCT
>Ailuropoda_melanoleuca_GL193551.1.trna37-ArgTCG (322258-322176) Arg (TCG) 83 bp Sc: 38.13
GCCTGGTTGGCACAGTGGTTAAGCGTCTGCCTTCGGCTCAGGGCGTGATCCTGGCGTTGT
GGGATCGAGCCCCACATCAGGCT
>Ailuropoda_melanoleuca_GL193603.1.trna6-ArgTCG (291471-291553) Arg (TCG) 83 bp Sc: 38.13
GCCTGGTTGGCACAGTGGTTAAGCGTCTGCCTTCGGCTCAGGGCGTGATCCTGGCGTTGT
GGGATCGAGCCCCACATCAGGCT
>Ailuropoda_melanoleuca_GL192754.1.trna95-ArgTCG (389999-389917) Arg (TCG) 83 bp Sc: 38.21
GCCTGGGTGGCACAGAGGTTAGGCGTCTGCCTTCGGCTCAGGGCGTGATCCCGGCGTTGT
GGGATCGAGCCCCACCTCAGGCT
>Ailuropoda_melanoleuca_GL195007.1.trna3-ArgTCG (48522-48604) Arg (TCG) 83 bp Sc: 38.55
GCCTGGGTGGCACAGCGGTTGTGCGTCTGCCTTCGGCTCAGGGTGTGATCCTGGCGTTGT
GGGATCGAGCCCCACATCAGGCT
>Ailuropoda_melanoleuca_GL194483.1.trna5-ArgTCG (141774-141856) Arg (TCG) 83 bp Sc: 38.68
GCCTGGGTGGCTCAATGGTTGAGCGCCTGCCTTCGGCTCAGGGCGTGATCCCGGtgttgt
GGGATCGAGCCCCACGTCAGGCT
>Ailuropoda_melanoleuca_GL193400.1.trna19-ArgTCG (234193-234111) Arg (TCG) 83 bp Sc: 38.69
GCCTGGTTGGCACAGCGGTTGAGCGTCTGCCTTCGGCTCAGGGCGTGATCCCGGCGTTGT
GGGATCGAGCCCCACATCAGGCT
>Ailuropoda_melanoleuca_GL192350.1.trna142-ArgTCG (4350619-4350701) Arg (TCG) 83 bp Sc: 38.79
GCCTGGGTGGCAGAGCGGTTAAGCGTCTGCCTTCGGCTCAGGGCGTGATCCCGGCGTTGT
GGGATCGAGCCCCACCTCAGGCT
>Ailuropoda_melanoleuca_GL192846.1.trna53-ArgTCG (51435-51352) Arg (TCG) 84 bp Sc: 39.06
GCCTGGGTGGCTCAGCGGTTAAAGTGTCTGCCTTCGGCTCAGGGCGTGATCCTGGCGTTA
TGGGATCGAGCCCCATGTCAGGCT
>Ailuropoda_melanoleuca_GL194891.1.trna7-ArgTCG (19750-19668) Arg (TCG) 83 bp Sc: 39.19
GCCTGGGTGGCTCAGCGGTTGAGCGTCTGCCTTCGGCTCAGGGCGTGATCCCAGCGTTGT
GGGATCGAGCCCCACATCAGGCT
>Ailuropoda_melanoleuca_GL192660.1.trna118-ArgTCG (492709-492627) Arg (TCG) 83 bp Sc: 40.18
GCCTGAGTGGCACAGCGGTTGTGCGTCTGCCTTCGGCTCAGGGCGTGATCCCGACGTTGT
GGGATCGAGCCCCACATCAGGCT
>Ailuropoda_melanoleuca_GL192737.1.trna26-ArgTCG (1466839-1466921) Arg (TCG) 83 bp Sc: 40.20
GCCTGGGTGGCACAGTGGTTGTGCGTCTGCCTTCGGCTCAGGGCGTGATCCCGGCGTTGT
GGGATCGAGCCCCACATCAGGCT
>Ailuropoda_melanoleuca_GL193267.1.trna9-ArgTCG (232081-232150) Arg (TCG) 70 bp Sc: 40.29
GCCTGGCTGGCTCAGTGGTAGAGCACGTGACTCGATCTCGGGGTTGTGAGTTTGAGCCCC
ATGTTGGGTA
>Ailuropoda_melanoleuca_GL192689.1.trna47-ArgTCG (508166-508084) Arg (TCG) 83 bp Sc: 40.29
GCCTGGGTGGCACAGCGGTTAGGCGTCTGCCTTCGGCTCAGGGCGTGATCCCGGCGTTGT
GGGATCGAGCCCCACCTCAGGCT
>Ailuropoda_melanoleuca_GL193917.1.trna15-ArgTCG (19153-19070) Arg (TCG) 84 bp Sc: 40.94
GCCTGGGTGGCTCAGCGGTTAAAGCATCTGCCTTCGGCTCAGGGCGTGATCCCAGCGTTG
TGGGATCGAGCCCCACGTCAGGCT
>Ailuropoda_melanoleuca_GL195318.1.trna1-ArgTCG (42653-42736) Arg (TCG) 84 bp Sc: 42.66
GCCTGGGTGGCTCAGCGGTTAAAGCGTCTGCCTTCGGCTCAGGGCGTGATCCCAGCGTTG
TGGGATCGAGCCCCACGTCAGGCT
>Ailuropoda_melanoleuca_GL192357.1.trna86-ArgTCG (2501222-2501292) Arg (TCG) 71 bp Sc: 45.18
GCCTGGCTGGCTCAGTTGGTAGAGTATGTGACTCGATCTCAGGGTTGTGAGTTTGAGCCC
CACGTTGGGCA
>Ailuropoda_melanoleuca_GL192829.1.trna60-ArgTCG (1045995-1045913) Arg (TCG) 83 bp Sc: 45.98
GCCTGGGTGGCACAGCGGTTGGGCGCCTGACTTCGGCTCAGGGCGTGATCCCGGCGTTGT
GGGATCGAGCCCCACTTCAGGCT
>Ailuropoda_melanoleuca_GL194903.1.trna4-ArgTCG (107940-107857) Arg (TCG) 84 bp Sc: 46.81
GCCTGGGTGGCTCAGTTGGTTAAGCACCTGCCTTCGATTCAGGTCATGATTTCAGGGTCC
TGGGATCGAGTCCCAGGTTGGGCT
>Ailuropoda_melanoleuca_GL193874.1.trna10-ArgTCG (364322-364394) Arg (TCG) 73 bp Sc: 67.10
GACCACGTGGCCTAACGGATAAGGCGTCTGACTTCGGATCAGAAGATTGAGGGTTCGAAT
CCCTTCGTGGTTA
>Ailuropoda_melanoleuca_GL192703.1.trna157-ArgTCG (351763-351691) Arg (TCG) 73 bp Sc: 68.88
GGCCGCGTGGCCTAACGGATAAGGCGTCTGACTTCGGATCAGAAGATTGAGGGTTCGAGT
CCCTTCGTGGTCG
>Ailuropoda_melanoleuca_GL193874.1.trna11-ArgTCG (386134-386206) Arg (TCG) 73 bp Sc: 69.08
GACCACGTGGCCTAATGGATAAGGCGTCTGACTTCGGATCAGAAGATTGAGGGTTCGAAT
CCCTTCGTGGTTG

>Ailuropoda_melanoleuca_GL193897.1.trna33-ArgTCG (362117-362189) Arg (TCG) 73 bp Sc: 69.08
GACCACGTGGCCTAATGGATAAGGCGTCTGACTTCGGATCAGAAGATTGAGGGTTCGAAT
CCCTTCGTGGTTG
>Ailuropoda_melanoleuca_GL192681.1.trna46-ArgTCG (1283728-1283800) Arg (TCG) 73 bp Sc: 76.93
GGCCGCGTGGCCTAATGGATAAGGCGTCTGACTTCGGATCAGAAGATTGCAGGTTCGAGT
CCTGCCGCGGTCG
>Ailuropoda_melanoleuca_GL192757.1.trna40-ArgTCG (1027560-1027477) Arg (TCG) 84 bp Sc: 26.91
ACCTGGGTGGCTCAGTCGGTTAAGCATCTGCCTTCGGCTTGGGTCATGGTCCCAGAGTTG
TGGGATCAAGCCCCATTTCAGGTT
>Ailuropoda_melanoleuca_GL192467.1.trna118-ArgTCG (1935680-1935597) Arg (TCG) 84 bp Sc: 36.37
GCCTGGGTGGCTCAGTAGGTTAAGCTTCTGACTTCGGCTTAGGTCATGATCACAGGGTCT
TGGGATCAAGCCCCAGGTCGGGCT
>Ailuropoda_melanoleuca_GL192346.1.trna94-ArgTCG (4324418-4324506) Arg (TCG) 89 bp Sc: 28.07
ACCCGGGTGGCTCAGTTGGTTGGGCTTCTGATTTCGATTTCCGCTCAGGTCTTGGTCTTG
GGTCGTGGGTTTGAGCCTTACCCTGGGTA
>Ailuropoda_melanoleuca_GL192649.1.trna80-ArgTCT (234832-234762) Arg (TCT) 71 bp Sc: 27.67
GCCTGGTTGGCTCAGTCAATAGAGCATGTGACTCTTTCTCAGGGTCATGAGTTCAAGCCC
CATGTTGGGTG
>Ailuropoda_melanoleuca_GL192769.1.trna86-ArgTCT (10010-9927) Arg (TCT) 84 bp Sc: 29.23
GCCTGGGTGGCTCAGTTGGTTAAGTGCCTGCCTTCTGCTCAGGTCATGATCTCAGGATCT
TGGGATCGGGCCCCAAGTTGGGCT
>Ailuropoda_melanoleuca_GL193685.1.trna24-ArgTCT (6540-6458) Arg (TCT) 83 bp Sc: 33.88
GCCTGGGTGGCTCAGTTGGTTGAGCATCTGCCTTCTGCTCAGGTCATGATCCTGGGTCCT
GGGATTGAGCCCCACCTCGGGTT
>Ailuropoda_melanoleuca_GL192399.1.trna191-ArgTCT (847674-847591) Arg (TCT) 84 bp Sc: 37.18
GCCTGGGTGGCTCAGTTGGTTAAGCATCTGTCTTCTGCTCAGGTCCTGATCCCAGGATCC
TGGGATGGAGCCCCAGGTCAGGCT
>Ailuropoda_melanoleuca_GL193075.1.trna19-ArgTCT (499927-499999) Arg (TCT) 73 bp Sc: 37.68
GCCTGGCTGGCTCAGTGGGTGGGGTGTGCGACTTCTAATCTCAGGGCTGTGGGTTTGAGC
CCCACATTGGGTG
>Ailuropoda_melanoleuca_GL192640.1.trna91-ArgTCT (533009-532936) Arg (TCT) 74 bp Sc: 37.83
GCCTGGCTGGCTTAGTCGGTGGAGCATGAGACTTCTGATCTTGGGGGCTGTGAGTTCGAG
CCCCATGTTGGGCG
>Ailuropoda_melanoleuca_GL193061.1.trna39-ArgTCT (754816-754746) Arg (TCT) 71 bp Sc: 38.11
AACTGACTGGCTCAGTTGGAAGAGCTTGTGACTCTATCTCAGGGTCATGAGTTCTAGCCT
CATGTTGGGTG
>Ailuropoda_melanoleuca_GL192357.1.trna5-ArgTCT (24998-25069) Arg (TCT) 72 bp Sc: 40.91
ACCTGGCTGGCTCAGTAGGTAGAGCATGTGACTTCTGATCTCGGGTCATGAGTTCGCGCC
CCATGTTGGGTG
>Ailuropoda_melanoleuca_GL192364.1.trna239-ArgTCT (303502-303430) Arg (TCT) 73 bp Sc: 44.07
ACCTGGCTGGCTCAGTTGGTGGAGCATGCGACTTCTGATCCCAGGGTTGTGAGTTCGAGG
CCCATGTTGGGTG
>Ailuropoda_melanoleuca_GL192355.1.trna73-ArgTCT (2587691-2587774) Arg (TCT) 84 bp Sc: 46.92
GCCTGGGTGGCTCAGTTGGTTAAGCATCTGACTTCTGCTCAGGTCCTAACCTCAGGGTCC
TGGGATCGAGCCCCATCTCGGGCT
>Ailuropoda_melanoleuca_GL192626.1.trna59-ArgTCT (1156754-1156826) Arg (TCT) 73 bp Sc: 48.37
GCCTGGCAGGCTCAGTCAGTGGAGCGTGAGACTTCTGATCTCAGGGTTGTGGGTTCGAGC
CCCACGTTGGGTG
>Ailuropoda_melanoleuca_GL193579.1.trna11-ArgTCT (413251-413178) Arg (TCT) 74 bp Sc: 76.29
GTCTCTGTGGCGCAATGGACGAGCGCGCTGGACTTCTAATCCAGAGGTTCCGGGTTCGAG
TCCCGGCAGAGATG
>Ailuropoda_melanoleuca_GL192808.1.trna78-ArgTCT (1203830-1203744) Arg (TCT) 87 bp Sc: 71.15
GGCTCTGTGGCGCAATGGATAGCGCATTGGACTTCTAGTGACGAAAAAGCGATTCAAAGG
TTGTGGGTTCGAATCCCACCAGAGTCG
>Ailuropoda_melanoleuca_GL193552.1.trna71-ArgTCT (286176-286088) Arg (TCT) 89 bp Sc: 70.73
GGCTCTGTGGCGCAATGGATAGCGCATTGGACTTCTAGTGCTTGAAGGAAGGAATTCAAA
GGTTGCGGGTTCGAGTCCCGCCAGAGTCG
>Ailuropoda_melanoleuca_GL194275.1.trna2-ArgTCT (30449-30534) Arg (TCT) 86 bp Sc: 68.58
GGCTCTGTGGCGCAATGGATAGCGCATTGGACTTCTAGACAGATGGAGGCATTCAAAGGT
TGTGGGTTCGAGTCCCACCAGAGTCG
>Ailuropoda_melanoleuca_GL192421.1.trna213-ArgTCT (428683-428593) Arg (TCT) 91 bp Sc: 66.03
GGCTCTGTGGCGCAATGGATAGCGCATTGGACTTCTAGCCGATCGTGGTGTGGCGATTCA
AAGGTTGTGGGTTCGAGTCCCACCAGAGTCG
>Ailuropoda_melanoleuca_GL192568.1.trna108-ArgTCT (862272-862188) Arg (TCT) 85 bp Sc: 71.82
GGCTCCGTGGCGCAATGGATAGCGCATTGGACTTCTAGAGGCTGAAGACATTCAAAGGTT
CCGGGTTCGAGTCCCGGCGGAGTCG
>Ailuropoda_melanoleuca_GL192385.1.trna46-AsnATT (1088388-1088459) Asn (ATT) 72 bp Sc: 31.79

GCCTGGCTGGCTCAGTCCGTGGAGCATGGGACTATTGATCTCAGGTGGTGGGTTCAAGCC
TCACCTTGGGTG
>Ailuropoda_melanoleuca_GL193160.1.trna71-AsnATT (165361-165289) Asn (ATT) 73 bp Sc: 39.49
ACTTGGCTGGCTGAGTCGATAGGGCACGTGACTATTGATCACAGGGTCATGAGTTCGAGC
CTCATGTTGGGTG
>Ailuropoda_melanoleuca_GL193448.1.trna5-AsnATT (214971-215043) Asn (ATT) 73 bp Sc: 42.21
ACATGACTGGCTCAGTCGGTGGAGCATGGGACTATTGATCTCGGGGTTGTGAGTTCGAAT
CTCGTGTTGAGTG
>Ailuropoda_melanoleuca_GL192360.1.trna276-AsnATT (984804-984732) Asn (ATT) 73 bp Sc: 47.33
GCCTGGCTGGCTCAGTCGTTAGAGTATGTGACTATTGATTTCGAGGTTGTGAGTTCGAGC
CCCACGTTGGGTG
>Ailuropoda_melanoleuca_GL192524.1.trna90-AsnATT (1021575-1021503) Asn (ATT) 73 bp Sc: 54.73
TCCTGGCTGGCTCAGTCGGTAGAGCATGAGACTATTGATCTTGGGGTCATGAGTTCGAGC
CCCATGTTGGGGG
>Ailuropoda_melanoleuca_GL194239.1.trna2-AsnATT (6153-6226) Asn (ATT) 74 bp Sc: 72.54
GTCTCTGTGGTGCAATTGGTTAGCACATTCGGCTATTAACTGAAAGGTTGGTGGTTCAAT
TCCACCCAGGGATG
>Ailuropoda_melanoleuca_GL192930.1.trna2-AsnGTT (10550-10622) Asn (GTT) 73 bp Sc: 28.02
GCCTGGCTGGCTTAGCTGGCGGAGTGTGTGATTGTTCATCTCAGAGCTGTGGGTTCGAGC
TCCATGTTGGGTG
>Ailuropoda_melanoleuca_GL193394.1.trna44-AsnGTT (15871-15799) Asn (GTT) 73 bp Sc: 38.07
GCCTAGCTGGCTCAGTTAGTAGAGCATGTGACTGTTGATCTTGGGGTTATGAGTTTGAGA
CCCATGTTGGGCT
>Ailuropoda_melanoleuca_GL192886.1.trna13-AsnGTT (562069-562141) Asn (GTT) 73 bp Sc: 38.32
GCCTGGCTGGTTCAGTCAGAAGAGCATGCGACTGTTGATTTCAGGGTCATGAGTTCAAGC
CCCATGCTGGGTG
>Ailuropoda_melanoleuca_GL193652.1.trna14-AsnGTT (363212-363140) Asn (GTT) 73 bp Sc: 38.60
CCCTGGCTGGCTTAGTCTGTACAGCATGAGACTGTTGATCTCGGGGTGGTAGGTTCAAGC
CCTGTGTTGGGTG
>Ailuropoda_melanoleuca_GL192718.1.trna65-AsnGTT (600915-600843) Asn (GTT) 73 bp Sc: 43.55
ACCTGGCTGGCTCAGTCAGTAGAGCATGTGACTGTTAATCTCAGCGTTGTGAGTTCAAGC
CCCATGTTGGGTG
>Ailuropoda_melanoleuca_GL192382.1.trna68-AsnGTT (2519056-2519128) Asn (GTT) 73 bp Sc: 44.24
GCCTGGCTGGCTCAGTCAGTGGAGCATGTGACTGTTGATCTCAGGGTCATGAGTTCAAGC
CCCATGCTGGGTG
>Ailuropoda_melanoleuca_GL192596.1.trna15-AsnGTT (875690-875762) Asn (GTT) 73 bp Sc: 46.89
GCCTGGCTGGCTCAGTCAGCAGAGCGTGAGACTGTTCATCTTGAGGTTGTGGGTTCGAGT
CCCATATTGGGTG
>Ailuropoda_melanoleuca_GL193452.1.trna1-AsnGTT (2381-2453) Asn (GTT) 73 bp Sc: 46.94
ACCTGGCTGGCTTAGTCAGTAGAGCATATGACTGTTGATCTTGGGGTCATGAGTTCAAGC
CCCATGTTGGGTG
>Ailuropoda_melanoleuca_GL192946.1.trna20-AsnGTT (1066543-1066471) Asn (GTT) 73 bp Sc: 47.72
ACCTGGTTGGCTTAGTCAGAAGAGTATGAGACTGTTGATCTCATGGTCGTGGGTTCAGAC
CCCATGTTGGGTA
>Ailuropoda_melanoleuca_GL192439.1.trna166-AsnGTT (949071-949000) Asn (GTT) 72 bp Sc: 49.21
GCCTGGCTGGCTCAGTGGGTGAGCATGTGACTGTTGATCTCAGGGTCGTGAGTTCAAGCC
CCATGTTGGGTA
>Ailuropoda_melanoleuca_GL194364.1.trna9-AsnGTT (171878-171950) Asn (GTT) 73 bp Sc: 50.28
GCCTGGCTGGCTTAGTTGGTAAAGCATGTGACTGTTGATCTCGGGATTATGAGTTCGAGC
CCCATGTTGGGTG
>Ailuropoda_melanoleuca_GL192369.1.trna39-AsnGTT (1009406-1009478) Asn (GTT) 73 bp Sc: 54.56
ACCTGGCTGGCTCAGTCAGTAGAGCGTGAGACTGTTGATCTTGCGGTTGTGAGTTCGAGT
CCCACGTTAGGTA
>Ailuropoda_melanoleuca_GL193650.1.trna5-AsnGTT (108287-108359) Asn (GTT) 73 bp Sc: 56.13
GCCTGGCTGGCTCAGTAGGTAGAGCATGTGACTGTTAATCTCAGGGTTGTGGGTTCGAGC
CCCTTGTTGGGTA
>Ailuropoda_melanoleuca_GL194818.1.trna4-AsnGTT (129366-129295) Asn (GTT) 72 bp Sc: 58.89
ACCTGGCTGGCTCAGTCGGAAGAGTGTGTGACTGTTGATCACAGGTCATGAGTTCAAGCC
CCATGCTGGGTA
>Ailuropoda_melanoleuca_GL194883.1.trna18-AsnGTT (18388-18315) Asn (GTT) 74 bp Sc: 74.75
GTCTCTGTGGCGCAATGGGTTAGCGCGTTCGGCTGTTAACTGAAAGGTTGGTGGTTCGAG
CCCACCCAGGGACG
>Ailuropoda_melanoleuca_GL192355.1.trna220-AsnGTT (2358742-2358669) Asn (GTT) 74 bp Sc: 82.24
GTCTCTGTGGCGCAATCGGTTAGCGCGTTCGGCTGTTAACCGAAAGGTTGGTGGTTCGAG
CCCACCCAGGGACG
>Ailuropoda_melanoleuca_GL192400.1.trna22-AsnGTT (1576363-1576436) Asn (GTT) 74 bp Sc: 82.24
GTCTCTGTGGCGCAATCGGTTAGCGCGTTCGGCTGTTAACCGAAAGGTTGGTGGTTCGAG

CCCACCCAGGGACG
>Ailuropoda_melanoleuca_GL192497.1.trna29-AsnGTT (582682-582755) Asn (GTT) 74 bp Sc: 82.24
GTCTCTGTGGCGCAATCGGTTAGCGCGTTCGGCTGTTAACCGAAAGGTTGGTGGTTCGAG
CCCACCCAGGGACG
>Ailuropoda_melanoleuca_GL192730.1.trna44-AsnGTT (1355757-1355684) Asn (GTT) 74 bp Sc: 82.24
GTCTCTGTGGCGCAATCGGTTAGCGCGTTCGGCTGTTAACCGAAAGGTTGGTGGTTCGAG
CCCACCCAGGGACG
>Ailuropoda_melanoleuca_GL193041.1.trna15-AsnGTT (757159-757232) Asn (GTT) 74 bp Sc: 82.24
GTCTCTGTGGCGCAATCGGTTAGCGCGTTCGGCTGTTAACCGAAAGGTTGGTGGTTCGAG
CCCACCCAGGGACG
>Ailuropoda_melanoleuca_GL193160.1.trna41-AsnGTT (891083-891156) Asn (GTT) 74 bp Sc: 82.24
GTCTCTGTGGCGCAATCGGTTAGCGCGTTCGGCTGTTAACCGAAAGGTTGGTGGTTCGAG
CCCACCCAGGGACG
>Ailuropoda_melanoleuca_GL193160.1.trna48-AsnGTT (907502-907429) Asn (GTT) 74 bp Sc: 82.24
GTCTCTGTGGCGCAATCGGTTAGCGCGTTCGGCTGTTAACCGAAAGGTTGGTGGTTCGAG
CCCACCCAGGGACG
>Ailuropoda_melanoleuca_GL193160.1.trna50-AsnGTT (845093-845020) Asn (GTT) 74 bp Sc: 82.24
GTCTCTGTGGCGCAATCGGTTAGCGCGTTCGGCTGTTAACCGAAAGGTTGGTGGTTCGAG
CCCACCCAGGGACG
>Ailuropoda_melanoleuca_GL194883.1.trna4-AsnGTT (24551-24624) Asn (GTT) 74 bp Sc: 82.24
GTCTCTGTGGCGCAATCGGTTAGCGCGTTCGGCTGTTAACCGAAAGGTTGGTGGTTCGAG
CCCACCCAGGGACG
>Ailuropoda_melanoleuca_GL194883.1.trna8-AsnGTT (66652-66725) Asn (GTT) 74 bp Sc: 82.24
GTCTCTGTGGCGCAATCGGTTAGCGCGTTCGGCTGTTAACCGAAAGGTTGGTGGTTCGAG
CCCACCCAGGGACG
>Ailuropoda_melanoleuca_GL194901.1.trna4-AsnGTT (51645-51718) Asn (GTT) 74 bp Sc: 82.24
GTCTCTGTGGCGCAATCGGTTAGCGCGTTCGGCTGTTAACCGAAAGGTTGGTGGTTCGAG
CCCACCCAGGGACG
>Ailuropoda_melanoleuca_GL193920.1.trna2-AsnGTT (2344-2417) Asn (GTT) 74 bp Sc: 84.31
GTCTCTGTGGCGCAATTGGTTAGCGCGTTCGGCTGTTAACCGAAAGGTTGGTGGTTCGAT
CCCACCCAGGGACG
>Ailuropoda_melanoleuca_GL192358.1.trna114-AsnGTT (2863624-2863526) Asn (GTT) 99 bp Sc: 30.23
GCCTAGGTGGCTCAGTCGGTTAATCGGGTAAGCGTCTGACTGTTGGTTTCGGCTTAGGTC
GTGATCTCACGGTTGTGGGATCAAGCCCCACATTGGGCT
>Ailuropoda_melanoleuca_GL192551.1.trna11-AspATC (673996-674068) Asp (ATC) 73 bp Sc: 62.66
GCCTGGCTGGCTCAGTTGGTAGAGCCTGCGACTATCAATCTCAGGGTCATGAGTTCAAGC
CCCATGCTGGGCA
>Ailuropoda_melanoleuca_GL192425.1.trna6-AspGTC (299957-300029) Asp (GTC) 73 bp Sc: 43.73
GTCTGGCTGGCTTAGTTAGTAGAGCATGTGACTGTCAATCTCAGGGCTGTGAGTTCAAGC
CCCATGCTGGGCA
>Ailuropoda_melanoleuca_GL192592.1.trna46-AspGTC (1261886-1261815) Asp (GTC) 72 bp Sc: 50.96
TCCTCTTTAGTGTAGTGGTGAGTATCCCCGCCTGTCACGCGGGAGACTGGGGTTCGATTC
CTCGACGGGGAG
>Ailuropoda_melanoleuca_GL192817.1.trna6-AspGTC (384569-384641) Asp (GTC) 73 bp Sc: 56.86
GCCTGGCTGGCTCAGTCGGTAGAGCATGAGACTGTCGATCTCGGGGTCCTGTGTTCAAGC
CGCACACTGGGCA
>Ailuropoda_melanoleuca_GL192354.1.trna212-AspGTC (4185733-4185662) Asp (GTC) 72 bp Sc: 71.60
TCCTCGTTAGTATAGTGGTAAGTATCCCCGCCTGTCACGCGGGAGACCGGGGTTCGATTC
CCCGACGGGGAG
>Ailuropoda_melanoleuca_GL192354.1.trna214-AspGTC (4183309-4183238) Asp (GTC) 72 bp Sc: 72.92
TCCTCGTTAGTATAGTGGTGAGTATCCCCGCCTGTCACGCGGGAGACCGGGGTTCGATTC
CCCGACGGGGAG
>Ailuropoda_melanoleuca_GL192660.1.trna120-AspGTC (480819-480748) Asp (GTC) 72 bp Sc: 72.92
TCCTCGTTAGTATAGTGGTGAGTATCCCCGCCTGTCACGCGGGAGACCGGGGTTCGATTC
CCCGACGGGGAG
>Ailuropoda_melanoleuca_GL192808.1.trna54-AspGTC (1126978-1127049) Asp (GTC) 72 bp Sc: 72.92
TCCTCGTTAGTATAGTGGTGAGTATCCCCGCCTGTCACGCGGGAGACCGGGGTTCGATTC
CCCGACGGGGAG
>Ailuropoda_melanoleuca_GL193552.1.trna21-AspGTC (274050-274121) Asp (GTC) 72 bp Sc: 72.92
TCCTCGTTAGTATAGTGGTGAGTATCCCCGCCTGTCACGCGGGAGACCGGGGTTCGATTC
CCCGACGGGGAG
>Ailuropoda_melanoleuca_GL193552.1.trna66-AspGTC (337596-337525) Asp (GTC) 72 bp Sc: 72.92
TCCTCGTTAGTATAGTGGTGAGTATCCCCGCCTGTCACGCGGGAGACCGGGGTTCGATTC
CCCGACGGGGAG
>Ailuropoda_melanoleuca_GL194913.1.trna10-AspGTC (91011-90940) Asp (GTC) 72 bp Sc: 72.92
TCCTCGTTAGTATAGTGGTGAGTATCCCCGCCTGTCACGCGGGAGACCGGGGTTCGATTC
CCCGACGGGGAG

>Ailuropoda_melanoleuca_GL192741.1.trna15-AspGTC (730845-730916) Asp (GTC) 72 bp Sc: 74.76
TCCTCGTTAGTATAGTGGTTAGTATCCCCGCCTGTCACGCGGGAGACCGGGGTTCAATTC
CCCGACGGGGAG
>Ailuropoda_melanoleuca_GL192563.1.trna14-CysGCA (389720-389803) Cys (GCA) 84 bp Sc: 36.71
GCCTGGGTGGCTCAGTAGGTTAAGCATCTGCCTGCAGCTCAGATCGTAATCTCAGTGTCC
TGGGATCAAGCCCCACCTCAGGCT
>Ailuropoda_melanoleuca_GL193067.1.trna90-CysGCA (313321-313250) Cys (GCA) 72 bp Sc: 70.28
GGGGGTATAGCTCAGGGGCAGAGCATTTGACTGCAGATCAAGAGGTCCCTGGTTCAAATC
CAGGTGCCCCCT
>Ailuropoda_melanoleuca_GL193067.1.trna10-CysGCA (304924-304995) Cys (GCA) 72 bp Sc: 70.97
GGGGGTATAGCTCAGGGGCAGAGCATTTGACTGCAGATCAAGAGGTCCCCGGTTCAAATC
CGGGTACCCCCT
>Ailuropoda_melanoleuca_GL193067.1.trna20-CysGCA (431414-431485) Cys (GCA) 72 bp Sc: 71.19
GGGGGTATAGCTCAGGGGTAGAGCATTTGACTGCAGATCAAGAGGTCCCCAGTTCAAATC
TGGGTGCCCCCT
>Ailuropoda_melanoleuca_GL193067.1.trna89-CysGCA (317012-316941) Cys (GCA) 72 bp Sc: 71.19
GGGGGTATAGCTCAGGGGTAGAGCATTTGACTGCAGATCAAGAGGTCCCCAGTTCAAATC
TGGGTGCCCCCT
>Ailuropoda_melanoleuca_GL193067.1.trna79-CysGCA (439199-439128) Cys (GCA) 72 bp Sc: 72.34
GGGGGTATAGCTCAGGGGTAGAGCATTTGACTGCAGATCAAGAGGTCCCTGGTTCAAACC
CGGGTGCCCCCT
>Ailuropoda_melanoleuca_GL193067.1.trna84-CysGCA (330211-330140) Cys (GCA) 72 bp Sc: 73.73
GGGGGTATAGCTCAGGGGTAGAGCATTTGACTGCAGATCAAGAGGTCCCTGGTTCAAATC
CGGGTGCCCCCT
>Ailuropoda_melanoleuca_GL193067.1.trna87-CysGCA (323445-323374) Cys (GCA) 72 bp Sc: 73.73
GGGGGTATAGCTCAGGGGTAGAGCATTTGACTGCAGATCAAGAGGTCCCTGGTTCAAATC
CGGGTGCCCCCT
>Ailuropoda_melanoleuca_GL193067.1.trna91-CysGCA (293835-293764) Cys (GCA) 72 bp Sc: 73.73
GGGGGTATAGCTCAGGGGTAGAGCATTTGACTGCAGATCAAGAGGTCCCTGGTTCAAATC
CGGGTGCCCCCT
>Ailuropoda_melanoleuca_GL193067.1.trna11-CysGCA (309964-310035) Cys (GCA) 72 bp Sc: 74.20
GGGGGTATAGCTCAGGGGTAGAGCATTTGACTGCAGATCAAGAGGTCCCTGGTTCAAATC
CAGGTGCCCCCT
>Ailuropoda_melanoleuca_GL193067.1.trna80-CysGCA (438110-438039) Cys (GCA) 72 bp Sc: 74.20
GGGGGTATAGCTCAGGGGTAGAGCATTTGACTGCAGATCAAGAGGTCCCTGGTTCAAATC
CAGGTGCCCCCT
>Ailuropoda_melanoleuca_GL193067.1.trna85-CysGCA (325915-325844) Cys (GCA) 72 bp Sc: 74.20
GGGGGTATAGCTCAGGGGTAGAGCATTTGACTGCAGATCAAGAGGTCCCTGGTTCAAATC
CAGGTGCCCCCT
>Ailuropoda_melanoleuca_GL193067.1.trna86-CysGCA (324888-324817) Cys (GCA) 72 bp Sc: 74.20
GGGGGTATAGCTCAGGGGTAGAGCATTTGACTGCAGATCAAGAGGTCCCTGGTTCAAATC
CAGGTGCCCCCT
>Ailuropoda_melanoleuca_GL193401.1.trna57-CysGCA (57920-57849) Cys (GCA) 72 bp Sc: 74.20
GGGGGTATAGCTCAGGGGTAGAGCATTTGACTGCAGATCAAGAGGTCCCTGGTTCAAATC
CAGGTGCCCCCT
>Ailuropoda_melanoleuca_GL192497.1.trna22-CysGCA (494006-494077) Cys (GCA) 72 bp Sc: 74.26
GGGGGTATAGCTCAGGGGTAGAGCATTTGACTGCAGATCAAGAGGTCCCCGGTTCAAATC
CGGGTGCCCCCT
>Ailuropoda_melanoleuca_GL192497.1.trna24-CysGCA (511954-512025) Cys (GCA) 72 bp Sc: 74.26
GGGGGTATAGCTCAGGGGTAGAGCATTTGACTGCAGATCAAGAGGTCCCCGGTTCAAATC
CGGGTGCCCCCT
>Ailuropoda_melanoleuca_GL193067.1.trna16-CysGCA (418374-418445) Cys (GCA) 72 bp Sc: 74.26
GGGGGTATAGCTCAGGGGTAGAGCATTTGACTGCAGATCAAGAGGTCCCCGGTTCAAATC
CGGGTGCCCCCT
>Ailuropoda_melanoleuca_GL193067.1.trna8-CysGCA (297144-297215) Cys (GCA) 72 bp Sc: 74.26
GGGGGTATAGCTCAGGGGTAGAGCATTTGACTGCAGATCAAGAGGTCCCCGGTTCAAATC
CGGGTGCCCCCT
>Ailuropoda_melanoleuca_GL193067.1.trna9-CysGCA (301075-301146) Cys (GCA) 72 bp Sc: 74.26
GGGGGTATAGCTCAGGGGTAGAGCATTTGACTGCAGATCAAGAGGTCCCCGGTTCAAATC
CGGGTGCCCCCT
>Ailuropoda_melanoleuca_GL193067.1.trna19-CysGCA (429682-429753) Cys (GCA) 72 bp Sc: 76.00
GGGGGTATAGCTCAGTGGTAGAGCATTTGACTGCAGATCAAGAGGTCCCTGGTTCAAGTC
CAGGTGCCCCCT
>Ailuropoda_melanoleuca_GL192497.1.trna21-CysGCA (488445-488516) Cys (GCA) 72 bp Sc: 77.18
GGGGGTATAGCTCAGTGGTAGAGCATTTGACTGCAGATCAAGAGGTCCCTGGTTCAAATC
CGGGTGCCCCCT
>Ailuropoda_melanoleuca_GL193401.1.trna58-CysGCA (55244-55173) Cys (GCA) 72 bp Sc: 77.65

GGGGGTATAGCTCAGTGGTAGAGCATTTGACTGCAGATCAAGAGGTCCCTGGTTCAAATC
CAGGTGCCCCCT
>Ailuropoda_melanoleuca_GL192497.1.trna10-CysGCA (324251-324322) Cys (GCA) 72 bp Sc: 77.71
GGGGGTATAGCTCAGTGGTAGAGCATTTGACTGCAGATCAAGAGGTCCCCGGTTCAAATC
CGGGTGCCCCCT
>Ailuropoda_melanoleuca_GL192497.1.trna11-CysGCA (325039-325110) Cys (GCA) 72 bp Sc: 77.71
GGGGGTATAGCTCAGTGGTAGAGCATTTGACTGCAGATCAAGAGGTCCCCGGTTCAAATC
CGGGTGCCCCCT
>Ailuropoda_melanoleuca_GL192497.1.trna208-CysGCA (490284-490213) Cys (GCA) 72 bp Sc: 77.71
GGGGGTATAGCTCAGTGGTAGAGCATTTGACTGCAGATCAAGAGGTCCCCGGTTCAAATC
CGGGTGCCCCCT
>Ailuropoda_melanoleuca_GL193054.1.trna27-GlnCTG (748251-748323) Gln (CTG) 73 bp Sc: 26.65
ACCTGACTGGCTCAGTCGGAGGAGCACGGGATTCTGGGTTTCAGGGTCGTGAGTTCGAGC
CCTACGTTGGGTA
>Ailuropoda_melanoleuca_GL193180.1.trna39-GlnCTG (435031-434958) Gln (CTG) 74 bp Sc: 28.91
ACCTGACTGGCTCAGTGGGTGGAGCATGAGATTCTGGGTTTTTGGGCTTGTGGGTTCGAG
TCCCAGGTTGGGTG
>Ailuropoda_melanoleuca_GL193311.1.trna3-GlnCTG (74392-74464) Gln (CTG) 73 bp Sc: 33.31
ACCTGGCTGGCTCAGTCGGGAGGGCATGCAACTCTGGCTTTCAGGGTTGTGAGCTCAAGC
CCCACGTTGGGTG
>Ailuropoda_melanoleuca_GL192474.1.trna106-GlnCTG (361664-361592) Gln (CTG) 73 bp Sc: 36.80
ACCTGGTTGGTTCAATCAGTAGAGTATGAAACTCTGGATCTCAGGGTCATGAGTTCAAGC
CCCATGTTGGGTA
>Ailuropoda_melanoleuca_GL192442.1.trna178-GlnCTG (1055217-1055145) Gln (CTG) 73 bp Sc: 40.33
CCCTGGCTGGCTCAATGGGTACAGCATGCGACTCTGGATCTCAGGGTCATGGGTTGAAGC
CCCGTGTTGGGCG
>Ailuropoda_melanoleuca_GL192797.1.trna46-GlnCTG (808997-808925) Gln (CTG) 73 bp Sc: 40.51
ACCTGGCTGGTTCAGTCTGTGGAGCATGAGACTCTGGATCTCAGGGTTGTGGGTTCAAGC
CCCATGTTGGATG
>Ailuropoda_melanoleuca_GL192808.1.trna36-GlnCTG (981786-981858) Gln (CTG) 73 bp Sc: 41.05
GCCTGGCTGGCTCAGTTGGTGGGGTGTGTGACTCTGGATCTCGGGGTCATGAGTTTGAGC
CCCATGCTGGGTG
>Ailuropoda_melanoleuca_GL192437.1.trna123-GlnCTG (2434749-2434821) Gln (CTG) 73 bp Sc: 43.51
ACCTGGCTGGCTCAGTCTGAAGAGCATGCGGTTCTGGACCTCGGGGTCGTGAGTTCAAGC
CCCACGTTGGGTG
>Ailuropoda_melanoleuca_GL192350.1.trna114-GlnCTG (3136026-3136098) Gln (CTG) 73 bp Sc: 47.22
ACCTGACTGGCTCAGTTGGTAGAGCATGTGACTCTGGATCTCGGGGCTGTGAGTTCAAAC
TCCACGTTGGGTG
>Ailuropoda_melanoleuca_GL192344.1.trna96-GlnCTG (4104003-4104075) Gln (CTG) 73 bp Sc: 51.58
ACCTAGCTGGCTCAGATGGAAGAGCGTGTGACTCTGGATCTCAAGGTCATGAGTTTGAGC
CCCATGTTGGGTG
>Ailuropoda_melanoleuca_GL193552.1.trna9-GlnCTG (180594-180665) Gln (CTG) 72 bp Sc: 66.59
GGCCCCATGGTGTAATGGTCAGCACTCTGGACTCTGAATCCAGCGATCCGAGTTCAAATC
TCGGTGGGACCT
>Ailuropoda_melanoleuca_GL194239.1.trna1-GlnCTG (2087-2158) Gln (CTG) 72 bp Sc: 67.69
GGTTCCATGGTGTAATGGTGAGCACTCTGGACTCTGAATCCAGCGATCCGAGTTCAAGTC
TCGGTGGGACCT
>Ailuropoda_melanoleuca_GL194239.1.trna15-GlnCTG (9715-9644) Gln (CTG) 72 bp Sc: 67.69
GGTTCCATGGTGTAATGGTGAGCACTCTGGACTCTGAATCCAGCGATCCGAGTTCAAGTC
TCGGTGGGACCT
>Ailuropoda_melanoleuca_GL193874.1.trna20-GlnCTG (377087-377016) Gln (CTG) 72 bp Sc: 68.68
GGCCCCATGGTGTAATGGTTAGCACTCTGGACTCTGAATCCAGCGATCCGAGTTCAAATC
TCGGTGGGACCT
>Ailuropoda_melanoleuca_GL193160.1.trna39-GlnCTG (862970-863041) Gln (CTG) 72 bp Sc: 69.34
GGTTCCATGGTGTAATGGTGAGCACTCTGGACTCTGAATCCAGCGATCCGAGTTCAAATC
TCGGTGGGACCT
>Ailuropoda_melanoleuca_GL193431.1.trna47-GlnCTG (85671-85600) Gln (CTG) 72 bp Sc: 70.62
GGTTCCATGGTGTAATGGTCAGCACTCTGGACTCTGAATCCAGTGATCCGAGTTCAAGTC
TCGGTGGGACCT
>Ailuropoda_melanoleuca_GL193552.1.trna24-GlnCTG (299447-299518) Gln (CTG) 72 bp Sc: 72.00
GGTTCCATGGTGTAATGGTTAGCACTCTGGACTCTGAATCCAGCGATCCGAGTTCAAGTC
TCGGTGGAACCT
>Ailuropoda_melanoleuca_GL192385.1.trna166-GlnCTG (2301147-2301076) Gln (CTG) 72 bp Sc: 73.65
GGTTCCATGGTGTAATGGTTAGCACTCTGGACTCTGAATCCAGCGATCCGAGTTCAAATC
TCGGTGGAACCT
>Ailuropoda_melanoleuca_GL192808.1.trna77-GlnCTG (1204911-1204840) Gln (CTG) 72 bp Sc: 73.65
GGTTCCATGGTGTAATGGTTAGCACTCTGGACTCTGAATCCAGCGATCCGAGTTCAAATC

TCGGTGGAACCT
>Ailuropoda_melanoleuca_GL193552.1.trna67-GlnCTG (326954-326883) Gln (CTG) 72 bp Sc: 73.65
GGTTCCATGGTGTAATGGTTAGCACTCTGGACTCTGAATCCAGCGATCCGAGTTCAAATC
TCGGTGGAACCT
>Ailuropoda_melanoleuca_GL193897.1.trna8-GlnCTG (112531-112602) Gln (CTG) 72 bp Sc: 73.65
GGTTCCATGGTGTAATGGTTAGCACTCTGGACTCTGAATCCAGCGATCCGAGTTCAAATC
TCGGTGGAACCT
>Ailuropoda_melanoleuca_GL193552.1.trna59-GlnCTG (489184-489113) Gln (CTG) 72 bp Sc: 74.13
GGTCCCATGGTGTAATGGTTAGCACTCTGGACTCTGAATCCAGCGATCCGAGTTCAAATC
TCGGTGGGACCT
>Ailuropoda_melanoleuca_GL192619.1.trna67-GlnCTG (279344-279253) Gln (CTG) 92 bp Sc: 32.45
GCCTCAGTGGCTCAGTGGGTTGAGCATTGGACTCTGGGTGTCCGCTGAGGTCGTGATCTC
ATGGGTGGTGGGATCCAGTCCCACCTGGGGCT
>Ailuropoda_melanoleuca_GL193410.1.trna13-GlnCTG (366748-366837) Gln (CTG) 90 bp Sc: 42.66
GCCCAGGTGGCTCAGTCAGTTAAGCTTCTGACTCTGATCTCAGCTCAAGTCTTGATCTGA
AGGTCATGAGTTCAAGTCTCATGTTGGGCT
>Ailuropoda_melanoleuca_GL193039.1.trna81-GlnCTG (9901-9811) Gln (CTG) 91 bp Sc: 34.35
GCCTGGGTAGCTTAGCTGGTTAAGCCTCTGGCTCTGGATTTCAGCTCAGGTCATGGTCTC
AGAGTCGTGGGATCGAGCCCCACGCCAGGCT
>Ailuropoda_melanoleuca_GL193564.1.trna21-GlnTTG (37679-37597) Gln (TTG) 83 bp Sc: 21.36
AGCTGGGTGGCTCAGTCGTTAAGCACCTGCCTTTGGCTCAGGGCATGATCCCAGCATTCT
GGGATCGAGCCCCAGTCAGGCTC
>Ailuropoda_melanoleuca_GL192514.1.trna45-GlnTTG (1691302-1691372) Gln (TTG) 71 bp Sc: 22.14
ACCTGGCTGGCTCAGTCAGTGGAGCATGTGACTTGATCTTGGGGTTATGAGTTCAAGCCC
CATGTTGGGTG
>Ailuropoda_melanoleuca_GL192348.1.trna226-GlnTTG (4643461-4643378) Gln (TTG) 84 bp Sc: 22.18
ACCTGGTTGGCTCAGTCGGTTAAGCGTCTGCCTTTGGCTCAGGTCAGGTTGCCAGGCTCC
TGGGATTGAGCCCCACGTCGGGTT
>Ailuropoda_melanoleuca_GL192371.1.trna87-GlnTTG (1920832-1920902) Gln (TTG) 71 bp Sc: 24.14
GCCTGGCTGGCTCAGTCGGTGGAGCATGCGACTTGATCTCGGGGTTGTGAGTTAAAGCCC
CATGTTGGGTG
>Ailuropoda_melanoleuca_GL192431.1.trna36-GlnTTG (1938810-1938894) Gln (TTG) 85 bp Sc: 24.99
GCCTGGGTGGCTCAGTCAGTTAAGCATCTGACTTTGGCTCAGGTCATGATCCCAGGATCT
TGGGATCGAGTCCCCCATCCGGGTC
>Ailuropoda_melanoleuca_GL193020.1.trna1-GlnTTG (11945-12027) Gln (TTG) 83 bp Sc: 25.54
GCCTGGGTGGCTCAGTCGTTAAGCGTCTGCCTTTGGCTCAGGGCATGATCCCGGCGTTAT
GGGATCGAGCCCCATGTCAGGCT
>Ailuropoda_melanoleuca_GL192360.1.trna285-GlnTTG (863210-863127) Gln (TTG) 84 bp Sc: 26.85
GCCTGGGTGGCTCAGTTGGTGAAGTGTCTGTCTTTGGCTCAGGTCATGATCTCATGGACA
TGGGATTGAGTCCCATGTCAGGCT
>Ailuropoda_melanoleuca_GL192699.1.trna24-GlnTTG (429776-429846) Gln (TTG) 71 bp Sc: 27.07
ACCTGGCTGGCTCAGTAGGTGGAGCATGTGACTTGATCTCGGGGGCATGAGTTCAAGCCC
CATGTTGGGTG
>Ailuropoda_melanoleuca_GL192360.1.trna90-GlnTTG (1521173-1521254) Gln (TTG) 82 bp Sc: 27.30
GCCTGGGTGGCTCAGTTGGTGAAGCATCTGCCTTTGGCTCAGGTCATGATCTCAGTCTTG
GGATCAAGGCCCAAGTCAGGCT
>Ailuropoda_melanoleuca_GL192350.1.trna217-GlnTTG (2890038-2889968) Gln (TTG) 71 bp Sc: 28.33
ACCTGGCTGGCTCAGTTGGGGGAGCATGTGACTTGATCTCGGGGTCGTGGGTTTGAGCCT
CATGTTGGGTG
>Ailuropoda_melanoleuca_GL192404.1.trna35-GlnTTG (1470901-1470980) Gln (TTG) 80 bp Sc: 28.52
GCCTGGGTGGCTTAGTCAGTTAAGCGCCTGCCTTTGGCTCAGGTCATGATTCCCAGTGGG
ATCGAGCCCCACCTCAGGCT
>Ailuropoda_melanoleuca_GL192764.1.trna14-GlnTTG (376988-377071) Gln (TTG) 84 bp Sc: 28.90
GCCTGGGTGGCTCAGTTGGTTAAGCATCTGACTTTGTCTCAGGTCATGATCTCAGGGTCG
TGGGATTGAGCCCCATGTCAGGTT
>Ailuropoda_melanoleuca_GL193514.1.trna23-GlnTTG (322333-322251) Gln (TTG) 83 bp Sc: 28.92
GCCTGGGTGGCTCAGTCGTTAAGCGTCTGCCTTTGGCTCAGGGTGTGATTCTGGAGTTCT
GGGATCGAGCCCCAGGTCAGGCT
>Ailuropoda_melanoleuca_GL192399.1.trna184-GlnTTG (1018749-1018666) Gln (TTG) 84 bp Sc: 30.11
GCGTGGGTGGCTCAGTAGGTTAAGCACCTACCTTTGGCTTAGGTCATGATCCCAGGGTCT
TGGGATCGAGCCCCAACTCAGGCT
>Ailuropoda_melanoleuca_GL192967.1.trna37-GlnTTG (115664-115581) Gln (TTG) 84 bp Sc: 30.14
GCCTGGCTGGCTCAGTTGGTTAAGTGTCTGCATTTGGCTCAGGTCATGACTGCGGGGTCC
TGGGATCAAGCCCCGAGTTGGGCT
>Ailuropoda_melanoleuca_GL192610.1.trna18-GlnTTG (837283-837353) Gln (TTG) 71 bp Sc: 30.66
ACCTGGCTGGCTCAGTCAGAAGAGCATGCGACTTGATCTCGGGGTCATGGGTTTGAGCCC
CATATTGGGTA

>Ailuropoda_melanoleuca_GL192415.1.trna40-GlnTTG (1752674-1752744) Gln (TTG) 71 bp Sc: 31.76
GCCTGGCTGGCTCAGTTGGCAGAGCACACAACTTGATCTTGGGATTGTGAGTTTGAGCCC
CATGTTGGGCA
>Ailuropoda_melanoleuca_GL192461.1.trna76-GlnTTG (2062608-2062678) Gln (TTG) 71 bp Sc: 32.10
GTCTGGCTGGCTCAGCCAGTAGAGTGTGTGACTTGGTCTCAGGGTCGTGAGTTCAAGCCC
CACGTTGGGTG
>Ailuropoda_melanoleuca_GL194830.1.trna1-GlnTTG (90001-90084) Gln (TTG) 84 bp Sc: 33.16
ACCTGGGTGGCTCAGATGGTTGGGCCTCTGACTTTGGCTCAGGTCATGATCTCCAAGTCC
TGGGATCGAGCCCCGCTTTGGGTT
>Ailuropoda_melanoleuca_GL192492.1.trna126-GlnTTG (941667-941584) Gln (TTG) 84 bp Sc: 33.78
GCCTGGATGGCTCAGATGGTTGAGCCTCTGCCTTTGGCTCGGGTCATGATCTCCAGGTCC
TGGGATCAAGACCCAGGTCAGGCT
>Ailuropoda_melanoleuca_GL192365.1.trna47-GlnTTG (2092092-2092162) Gln (TTG) 71 bp Sc: 33.83
ACCTGGCTGGCTCAGTCAGTAGAGCATGTGACTTGCTCTTGGGGCTGTGGGTTCGAGCCC
CACATCAGGTG
>Ailuropoda_melanoleuca_GL193494.1.trna21-GlnTTG (151173-151103) Gln (TTG) 71 bp Sc: 35.39
GCCTGGGTGGCTCAGTTGGTTGAGTGTCTGCCTTTGGCTCAGGTCATTGGGATCAAGCCC
CAAGTCAGGCT
>Ailuropoda_melanoleuca_GL193035.1.trna7-GlnTTG (258206-258276) Gln (TTG) 71 bp Sc: 36.08
GCCTGGCTGGCTCAGTCTGTAGAGCATGTGACTTGATCTCAGGGTTGTGGGTTCAGGTCC
CATGTTGGGTG
>Ailuropoda_melanoleuca_GL193465.1.trna3-GlnTTG (83848-83918) Gln (TTG) 71 bp Sc: 36.37
GCCTGGCTGGCTCACTTGGTAGTGCACATGACTTGATCTTGGGGTCATGGGTTGGAGCCC
CATGTTGGGTG
>Ailuropoda_melanoleuca_GL192644.1.trna7-GlnTTG (808264-808346) Gln (TTG) 83 bp Sc: 36.52
GCCTGGGTGGCTCAGTCGTTGAGCGTCTGCCTTTGGCTCAGGGCGTGATCCTGGCGCTGT
GGGATCGAGCCCCACCTCGGGCT
>Ailuropoda_melanoleuca_GL193839.1.trna14-GlnTTG (138702-138620) Gln (TTG) 83 bp Sc: 37.22
ACCTGGGTGGCTCAGTTGGTTAAGCATCTGACTTTGGCTCAAGTCCTGATCCAGGGTCCT
GGGATGAAGCCCCAGCTCGGGCT
>Ailuropoda_melanoleuca_GL192943.1.trna18-GlnTTG (416752-416835) Gln (TTG) 84 bp Sc: 37.33
GCCTGGGTGGCTCAGTGGGCTAAGCGTCTGCCTTTGGCTCAGGTCATGATTTCAGGGTCC
TGGGATCAAGCCCCATCTTGGGCT
>Ailuropoda_melanoleuca_GL193510.1.trna25-GlnTTG (100328-100258) Gln (TTG) 71 bp Sc: 39.64
ACCTGGCTGGCTCAGTCAGTAGAGCATATGACTTGATCTTAGGGTTGTGAGTTCGAGCCC
CATGCTGGGTG
>Ailuropoda_melanoleuca_GL192607.1.trna17-GlnTTG (679460-679543) Gln (TTG) 84 bp Sc: 39.93
GCCTGGTTGGCTCAGTGGGTTAAGCATCTGACTTTGGCTCGGGTCATGATCTCAGGGTCC
TGGGATCGAGCCCCAAATCAGGCT
>Ailuropoda_melanoleuca_GL194797.1.trna1-GlnTTG (83539-83609) Gln (TTG) 71 bp Sc: 40.03
ACCTGGCTGGCTCAGTTGGTGGAGCATGTGACTTGATCTCAGGGTTGTGAGTTCAAGCCC
CACGTTGGGTA
>Ailuropoda_melanoleuca_GL193029.1.trna29-GlnTTG (1046319-1046237) Gln (TTG) 83 bp Sc: 40.07
GTCTGGGTGGCTCAGTTGGTTGAGCGTCTGCCTTTGGCTCAGGTCATGATCCTGGGTCGT
GGGATCCAGCCCCACCTCGGGCT
>Ailuropoda_melanoleuca_GL192904.1.trna71-GlnTTG (612804-612721) Gln (TTG) 84 bp Sc: 42.63
GCCTGGGTGGCTCAGTTGGTTAAGCATCTGACTTTGGCTCAGGTCATGATCTCAGGGTCC
TGGGATTGAGCCCCAACTCAGGCT
>Ailuropoda_melanoleuca_GL193509.1.trna4-GlnTTG (316151-316221) Gln (TTG) 71 bp Sc: 43.22
ACCTGGTTGGCTCAGTTGGCAGAGCGTGTGACTTGATCTTGGGGGTGTGGGTTCGAGCCC
CACATTGGGTG
>Ailuropoda_melanoleuca_GL192513.1.trna68-GlnTTG (484811-484728) Gln (TTG) 84 bp Sc: 46.99
GCCTGGGTGGCTCAGTCGGTTAAGCGTCTGACTTTGGCTCAGGTCATGATCCCAGGGTCC
TGGGATCGAGCCCCAGGTTGGGCT
>Ailuropoda_melanoleuca_GL193167.1.trna22-GlnTTG (481419-481502) Gln (TTG) 84 bp Sc: 48.27
GCCTGGTTGGCTCAGTTGGTTAAGCGCCTGCCTTTGGCTCAGGTCATGATCGCACGGTCC
TGGGATCGAGTCCCATATCGGGCT
>Ailuropoda_melanoleuca_GL192704.1.trna25-GlnTTG (995483-995553) Gln (TTG) 71 bp Sc: 51.72
GCCTGGCTGGCTCAGTTGGTAGAGCATGTGACTTGATCTCAGGGTTGTGGGTTTGAGTCC
CATGTTGGGCA
>Ailuropoda_melanoleuca_GL193431.1.trna48-GlnTTG (85490-85419) Gln (TTG) 72 bp Sc: 64.27
GGTTCTATGGTGTAATGGTCAGCACTCTGGACTTTGAATCCAGCGATCTGAGTTCAAATC
TCGGTGGGACCT
>Ailuropoda_melanoleuca_GL193552.1.trna8-GlnTTG (177206-177277) Gln (TTG) 72 bp Sc: 68.31
GGCCCCATGGTGTAATGGTTAGCACTCTGGACTTTGAATCCAGCGATCCGAGTTCAAATC
TCGGTGGGACCT
>Ailuropoda_melanoleuca_GL193874.1.trna21-GlnTTG (376566-376495) Gln (TTG) 72 bp Sc: 68.31

GGCCCCATGGTGTAATGGTTAGCACTCTGGACTTTGAATCCAGCGATCCGAGTTCAAATC
TCGGTGGGACCT
>Ailuropoda_melanoleuca_GL192497.1.trna115-GlnTTG (2314520-2314591) Gln (TTG) 72 bp Sc: 73.77
GGTCCCATGGTGTAATGGTTAGCACTCTGGACTTTGAATCCAGCGATCCGAGTTCAAATC
TCGGTGGGACCT
>Ailuropoda_melanoleuca_GL192392.1.trna49-GlnTTG (1858976-1859059) Gln (TTG) 84 bp Sc: 37.42
GCCTGGTTGGCTCAGTCGGTTAAGCGTCTGCCTTTGGCTTGGGTCATGATCCCAGAGTGG
TGGGATCGAGCCCCACATCGGGCT
>Ailuropoda_melanoleuca_GL192419.1.trna105-GlnTTG (371652-371569) Gln (TTG) 84 bp Sc: 34.08
GCTTGGGTGGCTCAGTCGGTTAAGCATCTGCCTTTGACTCAGGTCATGATATCAGGGACC
TGGGATCGAGCCCCAACTCAGGCT
>Ailuropoda_melanoleuca_GL192798.1.trna36-GlnTTG (807671-807580) Gln (TTG) 92 bp Sc: 29.06
GCCTGGGTGGCTCAGTCGTTTAAGGGTCTGACCCTTTGATTTTGGCTCAGGTCATGATCT
CAGCCTCATGGGATCGAGTCCCATATCAGGCT
>Ailuropoda_melanoleuca_GL193014.1.trna7-GluCTC (439262-439334) Glu (CTC) 73 bp Sc: 24.22
ACCTGGCTGGCTCAGTGAGTAGAGCATGGGGTTCTCGGTCTCGGGGTTGTGAGTTCAAGC
CGTACGCTGGGTG
>Ailuropoda_melanoleuca_GL192421.1.trna79-GluCTC (1741379-1741451) Glu (CTC) 73 bp Sc: 28.78
AGTTTGCTGGCTTGGTTGGTGGAGCGTGTGACTCTCGATCTCAGAGTTGTGGGTTTGAGC
CCCACGTTGGGTA
>Ailuropoda_melanoleuca_GL192422.1.trna43-GluCTC (2253837-2253908) Glu (CTC) 72 bp Sc: 37.91
ACCTGGCTGGCTCAGGTGGAAGAGTATGTGACTCTCGATCTTGGGTCATGTGTTTGAGCC
CCATGCTGGGTA
>Ailuropoda_melanoleuca_GL192818.1.trna96-GluCTC (107015-106943) Glu (CTC) 73 bp Sc: 38.84
GCCCGGCTGGCTCAGTCAGCAGAGCATGTGACTCTCAGTTTCAGGGTTGTGAGTTCAAGC
CCCATGTTGGGCA
>Ailuropoda_melanoleuca_GL192793.1.trna48-GluCTC (1021470-1021542) Glu (CTC) 73 bp Sc: 41.09
ACCTGGCTGGCTTAACTGGAAGAGTGTGGGACTCTCGATCTCGGGATTTTGAGTTCAAGC
CTCATGTTGGGTG
>Ailuropoda_melanoleuca_GL192420.1.trna111-GluCTC (2290565-2290492) Glu (CTC) 74 bp Sc: 41.33
GCCTGGCTGGCTCAGTCAGTGGAGTGTGCGACTCTCGATCTCAGGGTTTGTGAGTTCAAG
CCCCACGTTGGGCG
>Ailuropoda_melanoleuca_GL193954.1.trna1-GluCTC (10655-10727) Glu (CTC) 73 bp Sc: 41.80
ACCTGGCTGGCTCAGTTGGAGGAGTGTGGGACTCTCGATCTTGGGGTCATGGATTCAAAC
CCCATGTTGGGTG
>Ailuropoda_melanoleuca_GL192338.1.trna124-GluCTC (4413824-4413896) Glu (CTC) 73 bp Sc: 42.98
ACCTGGCTGGCTCAGTTGGTGGAGCATGTGACTCTCGATCTTGGAGTTGTGGGTTCGAGC
CTCATGCTGGGTA
>Ailuropoda_melanoleuca_GL192393.1.trna128-GluCTC (1774427-1774355) Glu (CTC) 73 bp Sc: 44.16
GCCTGGCTAGCTCAGTCAGTAGAGCACGTGACTCTCAATCTTGGGGTCATGAGTTTGAGC
CCCATGTTAGGTG
>Ailuropoda_melanoleuca_GL193335.1.trna13-GluCTC (470664-470592) Glu (CTC) 73 bp Sc: 44.47
GCCTGGCTGGCTCAGTCAGTGGAGCATGTGACTCTCGATCTCAGGGTCATGAGTTCAAGC
CCCATGTCGGGTG
>Ailuropoda_melanoleuca_GL192651.1.trna17-GluCTC (779859-779931) Glu (CTC) 73 bp Sc: 44.61
CCTTGACTGGCTCAGTCAGTAAAGCATGTGACTCTCAATCTCAAAGTCATGAGTTCAAGC
CCCATGTTGAGGT
>Ailuropoda_melanoleuca_GL192929.1.trna48-GluCTC (1048896-1048824) Glu (CTC) 73 bp Sc: 45.54
GCTTGGCTGGTTCAGTTGGTAGAGTGTGAGACTCTCTATCTCCAGATTGTGAGTTCAAGC
CCCATGTTGGGCA
>Ailuropoda_melanoleuca_GL193332.1.trna1-GluCTC (63749-63821) Glu (CTC) 73 bp Sc: 46.07
GCCTGGCTGGCTCAGTCAGTAGAGCATGTGACTCTCGATCTCAGCGTTGTGTGTTCGAGC
CCCATGTCAGGTG
>Ailuropoda_melanoleuca_GL192728.1.trna39-GluCTC (837382-837454) Glu (CTC) 73 bp Sc: 46.44
GCCTGGCTGGCTCAGTTGGAGGAGCGTGTGACTCTCGATCTCGGGGTCATGAGTTCGAGC
CCCGTGTTGGGTG
>Ailuropoda_melanoleuca_GL192942.1.trna26-GluCTC (875917-875989) Glu (CTC) 73 bp Sc: 46.49
ACCTGGCTGGCTCAGTAGGTGGAGCATGTGACTCTCGATTTCAAGGTTGTGGGTTCGAGC
CCCAAGTTGGGTG
>Ailuropoda_melanoleuca_GL193040.1.trna5-GluCTC (172855-172927) Glu (CTC) 73 bp Sc: 48.05
GCCTGGCTGGCTCAGTCAGTAGAGCATGTGACTCTCGATCTCAGGGTCGTGAGTTTGAGT
CCCGTGTTGGGCA
>Ailuropoda_melanoleuca_GL192479.1.trna21-GluCTC (404717-404789) Glu (CTC) 73 bp Sc: 49.09
GCCTGGCTGGCTCAGTTGGTAGAGCATGTGATTCTCAGTCTCAGGGACATGAGTTCAAGC
CCCATGTTGGGCA
>Ailuropoda_melanoleuca_GL192750.1.trna93-GluCTC (1037870-1037798) Glu (CTC) 73 bp Sc: 50.32
GCCTGGCTGGCTCAGTCAGTAGAGCATGTGACTCTCGATCTCAGGGTCGTGAGTTCAAGC

CCCATGTTGGGTG
>Ailuropoda_melanoleuca_GL192386.1.trna127-GluCTC (2378619-2378691) Glu (CTC) 73 bp Sc: 52.25
GCCTGGCTGGCTTAGTCGGTGGAGTGTGTGACTCTCAATCTCAGGGTCGTGAGTTCGAGC
CCCATGTTGGGTG
>Ailuropoda_melanoleuca_GL192475.1.trna138-GluCTC (1227880-1227808) Glu (CTC) 73 bp Sc: 52.57
GCCCAGCTGGCTCAGTCGGTAGAGCGGGTGACTCTCGATCTCTGGGTTGTGAGTTTGAGC
CCCATGTTGGGTG
>Ailuropoda_melanoleuca_GL192736.1.trna6-GluCTC (63940-64012) Glu (CTC) 73 bp Sc: 55.25
GCCTGGCTGGCTCAGTTGGTAGAGCATGTGATTCTCGATCTCAGGGTCATGAGTTCAAGC
CCCATACTGGGTG
>Ailuropoda_melanoleuca_GL193679.1.trna22-GluCTC (448392-448320) Glu (CTC) 73 bp Sc: 55.92
GCCTGGCTGGCTCAAGCGGTAGAGCATGTGACTCTCGATCTCAGGGTCATGAGTTCAAAT
CCCATGTTGGGTG
>Ailuropoda_melanoleuca_GL193028.1.trna35-GluCTC (639830-639902) Glu (CTC) 73 bp Sc: 56.26
GCCTGGCTGGCTCAGTTGGTGGAGCATGTGACTCTCGATCTCAGGGTTGTGAGTTCAAGC
CTCATGTTGGGCA
>Ailuropoda_melanoleuca_GL193384.1.trna19-GluCTC (487490-487562) Glu (CTC) 73 bp Sc: 56.34
GCCTGGCTGGCTCAATCGGTAGAGCATGTGACTCTCGATCTCAGGGTCATGAGTTCAAGC
CCCATGTTGGGTG
>Ailuropoda_melanoleuca_GL193146.1.trna84-GluCTC (57550-57478) Glu (CTC) 73 bp Sc: 56.47
GCCTGGCTGGCTCAGTGGGTGGAGTGTGTGACTCTCGATCTCATGGTCGTGAGTTCAAGC
CCCACGTTGGGCG
>Ailuropoda_melanoleuca_GL192462.1.trna58-GluCTC (1524943-1524871) Glu (CTC) 73 bp Sc: 56.59
ATCTAGCTGGCTCAGTCGGTAGAGCATGTGACTCTCGATCTCAGGGTTGTGAGTTCGAGC
CCCACGCTGGGTG
>Ailuropoda_melanoleuca_GL192573.1.trna155-GluCTC (1349235-1349163) Glu (CTC) 73 bp Sc: 58.24
GTCAGGCTGGCTCAGTTGGTAGAGCATGTGACTCTCAATCTCAGGGTCATGAGTTCAAGC
CTCATGTTGGGCA
>Ailuropoda_melanoleuca_GL192525.1.trna64-GluCTC (1809980-1809908) Glu (CTC) 73 bp Sc: 58.63
GCCTGGCTGGCTCAGTTGGTAGAGCCTCAGACTCTCGATCTCAGGGTTGTGAGTTCAAGC
CCCACACTGGGCA
>Ailuropoda_melanoleuca_GL193485.1.trna5-GluCTC (110431-110503) Glu (CTC) 73 bp Sc: 59.71
TCCCTGGTGGTCTAGTGGTTAGGATTCGGCGCTCTCACCGCTGCGGCCTGGGGTTCGATT
CCCGGTCAGGGAA
>Ailuropoda_melanoleuca_GL192338.1.trna66-GluCTC (2572672-2572744) Glu (CTC) 73 bp Sc: 59.99
TCCTGGCTAGCTTAGCCGGTAGAGCATGTGACTCTCAATCTCAGGGTCGTGAGTTCAAGC
CCCACGTTGGGAA
>Ailuropoda_melanoleuca_GL192817.1.trna17-GluCTC (704546-704618) Glu (CTC) 73 bp Sc: 64.56
GCCCGCCTGGCTCAGTCGGTAGAGCGTGGGACTCTCGATCCCAGGGTTGTGAGTTCGAGC
CCCACGTTGGGTG
>Ailuropoda_melanoleuca_GL193064.1.trna7-GluCTC (916459-916531) Glu (CTC) 73 bp Sc: 66.27
GCCTGGCTGGCTCAATTGGTAGAGCATGAGACTCTCAATCTCAGGGTTGTGAGTTCGAGC
CCCACACTGGGCA
>Ailuropoda_melanoleuca_GL192419.1.trna24-GluCTC (1374287-1374358) Glu (CTC) 72 bp Sc: 76.61
TCCCTGGTGGTCTAGTGGTTAGGATTCGGCGCTCTCACCGCCGCGGCCCGGGTTCGATTC
CCGGTCAGGGAA
>Ailuropoda_melanoleuca_GL193057.1.trna19-GluCTC (707059-707130) Glu (CTC) 72 bp Sc: 76.61
TCCCTGGTGGTCTAGTGGTTAGGATTCGGCGCTCTCACCGCCGCGGCCCGGGTTCGATTC
CCGGTCAGGGAA
>Ailuropoda_melanoleuca_GL193160.1.trna34-GluCTC (831034-831105) Glu (CTC) 72 bp Sc: 76.61
TCCCTGGTGGTCTAGTGGTTAGGATTCGGCGCTCTCACCGCCGCGGCCCGGGTTCGATTC
CCGGTCAGGGAA
>Ailuropoda_melanoleuca_GL193897.1.trna57-GluCTC (94203-94132) Glu (CTC) 72 bp Sc: 76.61
TCCCTGGTGGTCTAGTGGTTAGGATTCGGCGCTCTCACCGCCGCGGCCCGGGTTCGATTC
CCGGTCAGGGAA
>Ailuropoda_melanoleuca_GL194834.1.trna3-GluCTC (64326-64397) Glu (CTC) 72 bp Sc: 80.24
TCCCTGGTGGTCTAGTGGTTAGGATTCGGCGCTCTCACCGCCGCGGCCCGGGTTCGATTC
CCGGCCAGGGAA
>Ailuropoda_melanoleuca_GL192351.1.trna80-GluTTC (2280439-2280511) Glu (TTC) 73 bp Sc: 49.95
GCCTGGCTGGCTCAGTGGGAAGAGCGTGAGACTTTCGATCTCTGGGTCGTGAGTTGGAGC
CCCACGTTGGGTG
>Ailuropoda_melanoleuca_GL192516.1.trna99-GluTTC (139290-139218) Glu (TTC) 73 bp Sc: 61.15
GCCTGGCTGGCTCAGTTGGTAGAGCATGTGACTTTCAGTCTCAGGGTCGTGAGTTCAAGT
CCCACGTTGGGCA
>Ailuropoda_melanoleuca_GL193160.1.trna45-GluTTC (909975-910046) Glu (TTC) 72 bp Sc: 72.33
TCCCTGGTGGTCTAGTGGCTAGGATTCGGCGCTTTCACCGCCGCGGCCCGGGTTCGATTC
CCGGTCAGGGAA

>Ailuropoda_melanoleuca_GL193920.1.trna48-GluTTC (11453-11382) Glu (TTC) 72 bp Sc: 72.33
TCCCTGGTGGTCTAGTGGCTAGGATTCGGCGCTTTCACCGCCGCGGCCCGGGTTCGATTC
CCGGTCAGGGAA
>Ailuropoda_melanoleuca_GL194883.1.trna19-GluTTC (12779-12708) Glu (TTC) 72 bp Sc: 72.33
TCCCTGGTGGTCTAGTGGCTAGGATTCGGCGCTTTCACCGCCGCGGCCCGGGTTCGATTC
CCGGTCAGGGAA
>Ailuropoda_melanoleuca_GL195218.1.trna1-GluTTC (98-169) Glu (TTC) 72 bp Sc: 72.33
TCCCTGGTGGTCTAGTGGCTAGGATTCGGCGCTTTCACCGCCGCGGCCCGGGTTCGATTC
CCGGTCAGGGAA
>Ailuropoda_melanoleuca_GL193610.1.trna8-GluTTC (424690-424619) Glu (TTC) 72 bp Sc: 74.37
TCCCATATGGTCTAGCGGTTAGGATTCCTGGTTTTCACCCAGGCGGCCCGGGTTCGACTC
CCGGTGTGGGAA
>Ailuropoda_melanoleuca_GL192447.1.trna98-GluTTC (1217307-1217236) Glu (TTC) 72 bp Sc: 75.00
TCCCATATGGTCTAGCGGTTAGGATTCCTGGTTTTCACCCAGGCGGCCCGGGTTCGACTC
CCGGTATGGGAA
>Ailuropoda_melanoleuca_GL194986.1.trna1-GluTTC (58726-58797) Glu (TTC) 72 bp Sc: 75.00
TCCCATATGGTCTAGCGGTTAGGATTCCTGGTTTTCACCCAGGCGGCCCGGGTTCGACTC
CCGGTATGGGAA
>Ailuropoda_melanoleuca_GL193920.1.trna3-GluTTC (10473-10544) Glu (TTC) 72 bp Sc: 76.25
TCCCTGGTGGTCTAGTGGTTAGGATTCGGCGCTTTCACCGCCGCGGCCCGGGTTCGATTC
CCGGTCAGGGAA
>Ailuropoda_melanoleuca_GL192408.1.trna162-GluTTC (1017577-1017506) Glu (TTC) 72 bp Sc: 76.26
TCCCACATGGTCTAGCGGTTAGGATTCCTGGTTTTCACCCAGGCGGCCCGGGTTCGACTC
CCGGTGTGGGAA
>Ailuropoda_melanoleuca_GL193488.1.trna37-GlyCCC (585553-585481) Gly (CCC) 73 bp Sc: 44.89
ACCCAGCTGGCTCAGCTGGAAGAGCATGCGACTCCCTATCTCAGGGTTGTGAGTTCAAGC
CCCATGTTGGGTG
>Ailuropoda_melanoleuca_GL194257.1.trna6-GlyCCC (231134-231204) Gly (CCC) 71 bp Sc: 48.80
GCATTGGTGGTTCAGTGGTAGAATTCTTGCCTCCCACGTGGGAGAGCTGTGTTTGATTCC
CAGCCAATGCA
>Ailuropoda_melanoleuca_GL192781.1.trna53-GlyCCC (1229045-1228973) Gly (CCC) 73 bp Sc: 50.08
GCCTGGCTGGCTCAGTCAGAAAAGCGTGTGACTCCCGATCTCGGGGTCATGGGTTCAAGT
CCCACGTTGGGCA
>Ailuropoda_melanoleuca_GL192406.1.trna56-GlyCCC (2450118-2450188) Gly (CCC) 71 bp Sc: 76.98
GCGCCGCTGGTGTAGTGGTATCATGCAAGATTCCCATTCTTGCGACCCGGGTTCGATTCC
CGGGCGGCGCA
>Ailuropoda_melanoleuca_GL192733.1.trna61-GlyCCC (507191-507121) Gly (CCC) 71 bp Sc: 76.98
GCGCCGCTGGTGTAGTGGTATCATGCAAGATTCCCATTCTTGCGACCCGGGTTCGATTCC
CGGGCGGCGCA
>Ailuropoda_melanoleuca_GL193160.1.trna47-GlyCCC (916532-916462) Gly (CCC) 71 bp Sc: 80.05
GCATTGGTGGTTCAGTGGTAGAATTCTCGCCTCCCACGCGGGAGACCCGGGTTCGATTCC
CGGCCAATGCA
>Ailuropoda_melanoleuca_GL195218.1.trna5-GlyCCC (4083-4013) Gly (CCC) 71 bp Sc: 80.05
GCATTGGTGGTTCAGTGGTAGAATTCTCGCCTCCCACGCGGGAGACCCGGGTTCGATTCC
CGGCCAATGCA
>Ailuropoda_melanoleuca_GL193347.1.trna4-GlyGCC (157229-157299) Gly (GCC) 71 bp Sc: 66.28
GCATTGGTGGTTCAGTGGTAGAATTCTCGCCTGCCATGCGGGAGGCCCAGGTTTGATTTC
TGGCCAATGCA
>Ailuropoda_melanoleuca_GL192795.1.trna12-GlyGCC (370472-370542) Gly (GCC) 71 bp Sc: 81.62
GCATTGGTGGTTCAGTGGTAGAATTCTCGCCTGCCACGCGGGAGGCCCGGGTTCGATTCC
CGGCCAATGCA
>Ailuropoda_melanoleuca_GL192795.1.trna13-GlyGCC (371132-371202) Gly (GCC) 71 bp Sc: 81.62
GCATTGGTGGTTCAGTGGTAGAATTCTCGCCTGCCACGCGGGAGGCCCGGGTTCGATTCC
CGGCCAATGCA
>Ailuropoda_melanoleuca_GL192795.1.trna96-GlyGCC (362025-361955) Gly (GCC) 71 bp Sc: 81.62
GCATTGGTGGTTCAGTGGTAGAATTCTCGCCTGCCACGCGGGAGGCCCGGGTTCGATTCC
CGGCCAATGCA
>Ailuropoda_melanoleuca_GL192808.1.trna79-GlyGCC (1199023-1198953) Gly (GCC) 71 bp Sc: 81.62
GCATTGGTGGTTCAGTGGTAGAATTCTCGCCTGCCACGCGGGAGGCCCGGGTTCGATTCC
CGGCCAATGCA
>Ailuropoda_melanoleuca_GL193406.1.trna25-GlyGCC (116810-116740) Gly (GCC) 71 bp Sc: 81.62
GCATTGGTGGTTCAGTGGTAGAATTCTCGCCTGCCACGCGGGAGGCCCGGGTTCGATTCC
CGGCCAATGCA
>Ailuropoda_melanoleuca_GL193552.1.trna5-GlyGCC (104498-104568) Gly (GCC) 71 bp Sc: 81.62
GCATTGGTGGTTCAGTGGTAGAATTCTCGCCTGCCACGCGGGAGGCCCGGGTTCGATTCC
CGGCCAATGCA
>Ailuropoda_melanoleuca_GL193874.1.trna17-GlyGCC (379275-379205) Gly (GCC) 71 bp Sc: 81.62

GCATTGGTGGTTCAGTGGTAGAATTCTCGCCTGCCACGCGGGAGGCCCGGGTTCGATTCC
CGGCCAATGCA
>Ailuropoda_melanoleuca_GL194913.1.trna5-GlyGCC (100377-100447) Gly (GCC) 71 bp Sc: 81.62
GCATTGGTGGTTCAGTGGTAGAATTCTCGCCTGCCACGCGGGAGGCCCGGGTTCGATTCC
CGGCCAATGCA
>Ailuropoda_melanoleuca_GL195041.1.trna9-GlyGCC (40417-40347) Gly (GCC) 71 bp Sc: 81.62
GCATTGGTGGTTCAGTGGTAGAATTCTCGCCTGCCACGCGGGAGGCCCGGGTTCGATTCC
CGGCCAATGCA
>Ailuropoda_melanoleuca_GL192559.1.trna24-GlyTCC (1788749-1788821) Gly (TCC) 73 bp Sc: 32.28
ACCTGGCTGGCTCAGTCAGTAGAGCATGCGACTTCCGATCTTGGGTTTGTGAGTTCAAAG
CCCACGTTGGGTG
>Ailuropoda_melanoleuca_GL193376.1.trna15-GlyTCC (527758-527841) Gly (TCC) 84 bp Sc: 37.22
GCCTGACTGGCTCAGCGGGTTGAGCGTCTGCCTTCCACTCAGGTCATGGTCCCAGGGTCC
TAGGATCGAGTCCTGCGTCAGGCT
>Ailuropoda_melanoleuca_GL192808.1.trna85-GlyTCC (1127539-1127468) Gly (TCC) 72 bp Sc: 73.26
GCGTTGGTGGTATAGTGGTGAGCATAGCTGCCTTCCAAGCAGTTGACCCGGGTTCGATTC
CCGGCCAACGCA
>Ailuropoda_melanoleuca_GL193160.1.trna35-GlyTCC (833047-833118) Gly (TCC) 72 bp Sc: 73.26
GCGTTGGTGGTATAGTGGTGAGCATAGCTGCCTTCCAAGCAGTTGACCCGGGTTCGATTC
CCGGCCAACGCA
>Ailuropoda_melanoleuca_GL194913.1.trna8-GlyTCC (111712-111641) Gly (TCC) 72 bp Sc: 73.26
GCGTTGGTGGTATAGTGGTGAGCATAGCTGCCTTCCAAGCAGTTGACCCGGGTTCGATTC
CCGGCCAACGCA
>Ailuropoda_melanoleuca_GL194913.1.trna9-GlyTCC (91941-91870) Gly (TCC) 72 bp Sc: 73.26
GCGTTGGTGGTATAGTGGTGAGCATAGCTGCCTTCCAAGCAGTTGACCCGGGTTCGATTC
CCGGCCAACGCA
>Ailuropoda_melanoleuca_GL194164.1.trna5-GlyTCC (84198-84269) Gly (TCC) 72 bp Sc: 76.83
GCGTTGGTGGTATAGTGGTTAGCATAGCTGCCTTCCAAGCAGTTGACCCGGGTTCGATTC
CCGGCCAACGCA
>Ailuropoda_melanoleuca_GL193160.1.trna36-HisGTG (833928-833999) His (GTG) 72 bp Sc: 64.63
GCCGTGATCGTATAGTGGTTAGTACTCTGCGTTGTGGCCGCAGCAACCTCGGTTCGAATC
CGAGTCACGGCA
>Ailuropoda_melanoleuca_GL193160.1.trna44-HisGTG (908067-908138) His (GTG) 72 bp Sc: 64.63
GCCGTGATCGTATAGTGGTTAGTACTCTGCGTTGTGGCCGCAGCAACCTCGGTTCGAATC
CGAGTCACGGCA
>Ailuropoda_melanoleuca_GL193552.1.trna48-HisGTG (581521-581450) His (GTG) 72 bp Sc: 64.63
GCCGTGATCGTATAGTGGTTAGTACTCTGCGTTGTGGCCGCAGCAACCTCGGTTCGAATC
CGAGTCACGGCA
>Ailuropoda_melanoleuca_GL193707.1.trna19-HisGTG (494031-494102) His (GTG) 72 bp Sc: 64.63
GCCGTGATCGTATAGTGGTTAGTACTCTGCGTTGTGGCCGCAGCAACCTCGGTTCGAATC
CGAGTCACGGCA
>Ailuropoda_melanoleuca_GL193707.1.trna20-HisGTG (495562-495633) His (GTG) 72 bp Sc: 64.63
GCCGTGATCGTATAGTGGTTAGTACTCTGCGTTGTGGCCGCAGCAACCTCGGTTCGAATC
CGAGTCACGGCA
>Ailuropoda_melanoleuca_GL193707.1.trna21-HisGTG (493366-493295) His (GTG) 72 bp Sc: 64.63
GCCGTGATCGTATAGTGGTTAGTACTCTGCGTTGTGGCCGCAGCAACCTCGGTTCGAATC
CGAGTCACGGCA
>Ailuropoda_melanoleuca_GL194883.1.trna17-HisGTG (23889-23818) His (GTG) 72 bp Sc: 64.63
GCCGTGATCGTATAGTGGTTAGTACTCTGCGTTGTGGCCGCAGCAACCTCGGTTCGAATC
CGAGTCACGGCA
>Ailuropoda_melanoleuca_GL194883.1.trna7-HisGTG (64567-64638) His (GTG) 72 bp Sc: 64.63
GCCGTGATCGTATAGTGGTTAGTACTCTGCGTTGTGGCCGCAGCAACCTCGGTTCGAATC
CGAGTCACGGCA
>Ailuropoda_melanoleuca_GL193552.1.trna58-IleAAT (495545-495472) Ile (AAT) 74 bp Sc: 73.75
GGCCGGTTAGCTCAGTTGGTTAGAGCGTGGTGCTAATAACGCCAAGGTCGCGGGTTTGAT
CCCCGTACGGGCCA
>Ailuropoda_melanoleuca_GL193552.1.trna80-IleAAT (215848-215775) Ile (AAT) 74 bp Sc: 76.56
GGCCGGTTAGCTCAGTTGGTTAGAGCGTGGTGCTAATAACGCCAAGGCCGCGGGTTCGAT
CCCCGTACGGGCCA
>Ailuropoda_melanoleuca_GL193552.1.trna38-IleAAT (500391-500464) Ile (AAT) 74 bp Sc: 78.21
GGCCGGTTAGCTCAGCTGGTTAGAGCGTGGTGCTAATAACGCCAAGGTCGCGGGTTCGAT
CCCCGTACGGGCCA
>Ailuropoda_melanoleuca_GL193552.1.trna54-IleAAT (516370-516297) Ile (AAT) 74 bp Sc: 79.56
GGCCGGTTAGCTCAGCTGGTTAGAGCGTGGTGCTAATAACGCCAAGGTCGCGGGTTCGAG
CCCCGCACGGGCCA
>Ailuropoda_melanoleuca_GL192632.1.trna80-IleAAT (1648098-1648025) Ile (AAT) 74 bp Sc: 80.66
GGCCGGTTAGCTCAGTTGGTTAGAGCGTGGTGCTAATAACGCCAAGGTCGCGGGTTCGAT

CCCCGTACGGGCCA
>Ailuropoda_melanoleuca_GL192808.1.trna50-IleAAT (1122366-1122439) Ile (AAT) 74 bp Sc: 80.66
GGCCGGTTAGCTCAGTTGGTTAGAGCGTGGTGCTAATAACGCCAAGGTCGCGGGTTCGAT
CCCCGTACGGGCCA
>Ailuropoda_melanoleuca_GL192808.1.trna84-IleAAT (1147736-1147663) Ile (AAT) 74 bp Sc: 80.66
GGCCGGTTAGCTCAGTTGGTTAGAGCGTGGTGCTAATAACGCCAAGGTCGCGGGTTCGAT
CCCCGTACGGGCCA
>Ailuropoda_melanoleuca_GL193552.1.trna43-IleAAT (565066-565139) Ile (AAT) 74 bp Sc: 80.66
GGCCGGTTAGCTCAGTTGGTTAGAGCGTGGTGCTAATAACGCCAAGGTCGCGGGTTCGAT
CCCCGTACGGGCCA
>Ailuropoda_melanoleuca_GL193552.1.trna53-IleAAT (516757-516684) Ile (AAT) 74 bp Sc: 80.66
GGCCGGTTAGCTCAGTTGGTTAGAGCGTGGTGCTAATAACGCCAAGGTCGCGGGTTCGAT
CCCCGTACGGGCCA
>Ailuropoda_melanoleuca_GL193552.1.trna79-IleAAT (225692-225619) Ile (AAT) 74 bp Sc: 80.66
GGCCGGTTAGCTCAGTTGGTTAGAGCGTGGTGCTAATAACGCCAAGGTCGCGGGTTCGAT
CCCCGTACGGGCCA
>Ailuropoda_melanoleuca_GL194901.1.trna12-IleAAT (87550-87477) Ile (AAT) 74 bp Sc: 80.66
GGCCGGTTAGCTCAGTTGGTTAGAGCGTGGTGCTAATAACGCCAAGGTCGCGGGTTCGAT
CCCCGTACGGGCCA
>Ailuropoda_melanoleuca_GL194938.1.trna1-IleAAT (15992-16065) Ile (AAT) 74 bp Sc: 80.66
GGCCGGTTAGCTCAGTTGGTTAGAGCGTGGTGCTAATAACGCCAAGGTCGCGGGTTCGAT
CCCCGTACGGGCCA
>Ailuropoda_melanoleuca_GL192786.1.trna49-IleTAT (973398-973326) Ile (TAT) 73 bp Sc: 59.81
GGTTCCATGGTGTAGTGGTTAACACATTTGCTTTATATGCAGAAGATCCTGGGTTTAATT
CCCGGTGTAATCA
>Ailuropoda_melanoleuca_GL193591.1.trna13-IleTAT (156567-156659) Ile (TAT) 93 bp Sc: 67.11
GCTCCAGTGGCGCAATCGGTTAGCGCGCGGTACTTATACGACAGTGCGAGCGGAGCGATG
CCGAGGTTGTGAGTTCGATCCTCACCTGGAGCA
>Ailuropoda_melanoleuca_GL193552.1.trna76-IleTAT (237926-237833) Ile (TAT) 94 bp Sc: 67.58
GCTCCAGTGGCGCAATCGGTTAGCGCGCGGTACTTATACAGCAGTATATGTGCGGGTGAT
GCCGAGGTTGTGAGTTCGAGCCTCACCTGGAGCA
>Ailuropoda_melanoleuca_GL193897.1.trna38-IleTAT (373187-373095) Ile (TAT) 93 bp Sc: 66.82
GCTCCAGTGGCGCAATCGGTTAGCGCGCGGTACTTATAAGACAGTAGCTCGTGGGCGATG
CCGAGGTTGTGAGTTCGAGCCTCACCTGGAGCA
>Ailuropoda_melanoleuca_GL194938.1.trna2-IleTAT (39721-39814) Ile (TAT) 94 bp Sc: 64.63
GCTCCAGTGGCGCAACCGGTTAGCGCGCGGTACTTATAGAGCAGTATGTGTGCGGGTGAT
GCCGAGGTTGTGAGTTCGAGCCTCACCTGGAGCA
>Ailuropoda_melanoleuca_GL192340.1.trna17-IleTAT (472057-472149) Ile (TAT) 93 bp Sc: 68.11
GCTCCAGTGGCGCAATCGGTTAGCGCGCGGTACTTATACAGCAGTACATGCAGAGCAATG
CCGAGGTTGTGAGTTCGAGCCTCACCTGGAGCA
>Ailuropoda_melanoleuca_GL193897.1.trna6-LeuAAG (108816-108897) Leu (AAG) 82 bp Sc: 63.32
GGTAGCGTGGCCGAGCGGTCTAAGGCGCCGGATTAAGGCTCCAGTCTCTTCGGGGGCGTG
GGTTCGAATCCCACCGCTGCCA
>Ailuropoda_melanoleuca_GL193897.1.trna36-LeuAAG (437481-437400) Leu (AAG) 82 bp Sc: 69.34
GGTAGCGTGGCCGAGCGGTCTAAGGCGCTGGATTAAGGCTCCAGTCTCTGCGGGGGCGTG
GGTTCGAATCCCACCGCTGCCA
>Ailuropoda_melanoleuca_GL195202.1.trna9-LeuAAG (28525-28444) Leu (AAG) 82 bp Sc: 69.34
GGTAGCGTGGCCGAGCGGTCTAAGGCGCTGGATTAAGGCTCCAGTCTCTCCGGGGGCGTG
GGTTCGAATCCCACCGCTGCCA
>Ailuropoda_melanoleuca_GL194954.1.trna11-LeuAAG (103714-103633) Leu (AAG) 82 bp Sc: 69.69
GGTAGTGTGGCCGAGCGGTCTAAGGCGCTGGATTAAGGCTCCAGTCCCTTCGGGGGCGTG
GGTTCGAATCCCACCGCTGCCA
>Ailuropoda_melanoleuca_GL193377.1.trna50-LeuAAG (369234-369153) Leu (AAG) 82 bp Sc: 69.84
GGTAGCGTGGCCGAGCGGTCTAAGGCGCTGGATTAAGGCTCCAGTCTCTTCGGGGGCGTG
GGTTCGAATCCCACCGCTGCCA
>Ailuropoda_melanoleuca_GL193897.1.trna58-LeuAAG (90758-90677) Leu (AAG) 82 bp Sc: 69.84
GGTAGCGTGGCCGAGCGGTCTAAGGCGCTGGATTAAGGCTCCAGTCTCTTCGGGGGCGTG
GGTTCGAATCCCACCGCTGCCA
>Ailuropoda_melanoleuca_GL194214.1.trna35-LeuAAG (28491-28410) Leu (AAG) 82 bp Sc: 69.84
GGTAGCGTGGCCGAGCGGTCTAAGGCGCTGGATTAAGGCTCCAGTCTCTTCGGGGGCGTG
GGTTCGAATCCCACCGCTGCCA
>Ailuropoda_melanoleuca_GL193897.1.trna54-LeuCAA (113026-112920) Leu (CAA) 107 bp Sc: 66.60
GTCAGGATGGCCGAGTGGTCTAAGGCGCCAGACTCAAGCTTCGTCTTCCTCGGAAAGAGG
GTTCTGGTCTCCGAATGGAGGCGTGGGTTCGAATCCCACTTCTGACA
>Ailuropoda_melanoleuca_GL193897.1.trna9-LeuCAA (151491-151596) Leu (CAA) 106 bp Sc: 63.51
GTCAGGATGGCCGAGTGGTCTAAGGCGCCAGACTCAAGCTCCGCTTCCCCGCGCTGGGGA
TTCTGGTCTCCGAATGGAGGCGTGGGTTCGAATCCCACTTCTGACA

>Ailuropoda_melanoleuca_GL193552.1.trna18-LeuCAA (256902-257007) Leu (CAA) 106 bp Sc: 66.03
GTCAGGATGGCCGAGTGGTCTAAGGCGCCAGACTCAAGCACCTGCTTCCCGCACTGGGGC
TTCTGGTCTCCGCATGGAGGCGTGGGTTCGAATCCCACTTCTGACA
>Ailuropoda_melanoleuca_GL193552.1.trna74-LeuCAA (260951-260846) Leu (CAA) 106 bp Sc: 66.03
GTCAGGATGGCCGAGTGGTCTAAGGCGCCAGACTCAAGCACCTGCTTCCCGCACTGGGGC
TTCTGGTCTCCGCATGGAGGCGTGGGTTCGAATCCCACTTCTGACA
>Ailuropoda_melanoleuca_GL193552.1.trna22-LeuCAA (279151-279257) Leu (CAA) 107 bp Sc: 63.49
GTCAGGATGGCCGAGTGGTCTAAGGCGCCAGACTCAAGCTTTCGCTTCCCGCGCTCGGGG
CTTCTGGTCTCCAGATGGAGGCGTGGGTTCGAATCCCACTTCTGACA
>Ailuropoda_melanoleuca_GL193474.1.trna20-LeuCAA (378456-378353) Leu (CAA) 104 bp Sc: 23.82
GCCTGGGTGGCTCAGTTGTTGAGCGTCTGCAACGGGCTCAAGGANNNNNNNNNNNGGCTC
AGGGCGTGATCCTGGAGTTGTGGGATCGAGCCCCACCTCGGGCT
>Ailuropoda_melanoleuca_GL194834.1.trna2-LeuCAA (63935-64040) Leu (CAA) 106 bp Sc: 62.89
GTCAGGATGGCCGAGTGGTCTAAGGCGCCAGACTCAAGGTGTTTACCTTCCCTGCGGGCG
TTCTGGTCTCTGAATAGAGGCGTGGGTTCGAATCCCACTTCTGACA
>Ailuropoda_melanoleuca_GL193057.1.trna18-LeuCAA (706668-706773) Leu (CAA) 106 bp Sc: 62.89
GTCAGGATGGCCGAGTGGTCTAAGGCGCCAGACTCAAGGTGTTTACCTTCCCTGCGGGCG
TTCTGGTCTCTGAATAGAGGCGTGGGTTCGAATCCCACTTCTGACA
>Ailuropoda_melanoleuca_GL192736.1.trna41-LeuCAA (859681-859776) Leu (CAA) 96 bp Sc: 29.96
GCCTGGGTGGCTCAGCGGTTAAGCGTCTGACTCAAGTCTCTGCCTTCGGCTCAGGGCGTG
ATCCCGGCATTATGGGATCGAGCCCCATATCAGGCT
>Ailuropoda_melanoleuca_GL192429.1.trna18-LeuCAG (1651497-1651574) Leu (CAG) 78 bp Sc: 31.58
GCCTGGGTGGCTCAGTCGGTTAAGTGTCCAGCTCAGGTCTTGGTCTCATGGTTGTGGGAT
CGAGCCCCACGTTGGGCT
>Ailuropoda_melanoleuca_GL193920.1.trna22-LeuCAG (330725-330797) Leu (CAG) 73 bp Sc: 47.69
GCCCAGCTGGTTCAGTCGGTGGAGCATGTGACTCAGGATCTCAGGGTTGTGGGTTTGAGC
CCCACGTTGGGTG
>Ailuropoda_melanoleuca_GL192338.1.trna89-LeuCAG (3346398-3346480) Leu (CAG) 83 bp Sc: 75.90
GTCAGGATGGCCGAGCGGTCTAAGGCGCTGCGTTCAGGTCGCAGTCTCCCCTGGAGGCGT
GGGTTCGAATCCCACTTCTGACA
>Ailuropoda_melanoleuca_GL194913.1.trna4-LeuCAG (92512-92594) Leu (CAG) 83 bp Sc: 75.90
GTCAGGATGGCCGAGCGGTCTAAGGCGCTGCGTTCAGGTCGCAGTCTCCCCTGGAGGCGT
GGGTTCGAATCCCACTTCTGACA
>Ailuropoda_melanoleuca_GL192338.1.trna225-LeuCAG (3346816-3346734) Leu (CAG) 83 bp Sc: 77.22
GTCAGGATGGCCGAGCGGTCTAAGGCGCTGCGTTCAGGTCGCAGTCTCCCCTGGAGGCGT
GGGTTCGAATCCCACTCCTGACA
>Ailuropoda_melanoleuca_GL194901.1.trna8-LeuCAG (113288-113206) Leu (CAG) 83 bp Sc: 77.22
GTCAGGATGGCCGAGCGGTCTAAGGCGCTGCGTTCAGGTCGCAGTCTCCCCTGGAGGCGT
GGGTTCGAATCCCACTCCTGACA
>Ailuropoda_melanoleuca_GL193915.1.trna2-LeuCAG (109964-110044) Leu (CAG) 81 bp Sc: 30.04
GCCTGGGTGGCACAGCGGTTAAGCGTGTCTTGGGCTCAGGGCATGATCCCGGCGTTGTGG
GATCGAGCCCCATGTCAGGCT
>Ailuropoda_melanoleuca_chrM.trna1-LeuTAA (4040-4114) Leu (TAA) 75 bp Sc: 42.19
GTTAGGGTGGCAGAGCCCGGTAATTGTGCAAAACTTAAACTCTTGTGTCCAGAGGTTCAA
TTCCTCTCCCTAGCA
>Ailuropoda_melanoleuca_GL194275.1.trna3-LeuTAA (30911-30993) Leu (TAA) 83 bp Sc: 74.33
ACCAGAATGGCCGAGTGGTTAAGGCGTTGGACTTAAGATCCAATGGATTTATATCCTCGT
GGGTTCGAACCCCACTTCTGGTA
>Ailuropoda_melanoleuca_GL193552.1.trna11-LeuTAA (198866-198948) Leu (TAA) 83 bp Sc: 81.57
ACCGGGATGGCCGAGTGGTTAAGGCGTTGGACTTAAGATCCAATGGGCGTGTGCCCGCGT
GGGTTCGAACCCCACTCCCGGTA
>Ailuropoda_melanoleuca_GL193552.1.trna41-LeuTAA (539870-539952) Leu (TAA) 83 bp Sc: 81.57
ACCGGGATGGCCGAGTGGTTAAGGCGTTGGACTTAAGATCCAATGGGCGTGTGCCCGCGT
GGGTTCGAACCCCACTCCCGGTA
>Ailuropoda_melanoleuca_GL192345.1.trna205-LeuTAA (288495-288414) Leu (TAA) 82 bp Sc: 81.58
ACCAGGATGGCCGAGTGGTTAAGGCGTTGGACTTAAGATCCAATGGACAATGTCCGCGTG
GGTTCGAACCCCACTCCTGGTA
>Ailuropoda_melanoleuca_GL192733.1.trna64-LeuTAG (360481-360411) Leu (TAG) 71 bp Sc: 32.10
GCCTGGCTGGCTCAGTCAGTGGAGTGTGTGACTAGATCTCAAGGTGGTGAGTTCAAGCCC
CACGCTGGGTG
>Ailuropoda_melanoleuca_GL193377.1.trna27-LeuTAG (453016-453097) Leu (TAG) 82 bp Sc: 68.14
GGTAGCGTGGCCGAGTGGTCTAAGGCGCTGGATTTAGGCTCCAGTCATTTCGATGGCGTG
GGTTCGAATCCCACCGCTGCCA
>Ailuropoda_melanoleuca_GL195202.1.trna8-LeuTAG (34005-33924) Leu (TAG) 82 bp Sc: 70.08
GGTAGCGTGGCCGAGCGGTCTAAGGCGCTGGATTTAGGCTCCAGTCTCTTCGGGGGCGTG
GGTTCGAATCCCACCGCTGCCA
>Ailuropoda_melanoleuca_GL194214.1.trna36-LeuTAG (19681-19600) Leu (TAG) 82 bp Sc: 70.31

GGTAGTGTGGCCGAGCGGTCTAAGGCGCTGGATTTAGGCTCCAGTCTCTTCGGAGGCGTG
GGTTCGAATCCCACCGCTGCCA
>Ailuropoda_melanoleuca_GL192808.1.trna59-LeuTAG (1204317-1204398) Leu (TAG) 82 bp Sc: 72.19
GGTAGCGTGGCCGAGCGGTCTAAGGCGCTGGATTTAGGCTCCAGTCTCTTCGGAGGCGTG
GGTTCGAATCCCACCGCTGCCA
>Ailuropoda_melanoleuca_GL194118.1.trna6-LysCTT (122221-122293) Lys (CTT) 73 bp Sc: 23.39
GCCTGGTTGGCTCAGTCAGTGGAGCTTGTGAGTCTTGATTTCGGAGTTGTGGGTTTGAGC
CCCACGCTGGGTA
>Ailuropoda_melanoleuca_GL192790.1.trna112-LysCTT (517515-517443) Lys (CTT) 73 bp Sc: 24.16
GCCTGGCTGGTTCAGTCGGTGGAACATGCAATTCTTGATTTTGGGGTTGTGAGTTCAGGC
CCCATGCTGGGTG
>Ailuropoda_melanoleuca_GL194483.1.trna8-LysCTT (152939-152867) Lys (CTT) 73 bp Sc: 25.68
ACTTGGCTGGCTCAGTTGTTGGAGCATGCAACTCTTGATTTCAGGGTTGTGGGTTCCAGC
CCCATGTTGGATG
>Ailuropoda_melanoleuca_GL192578.1.trna47-LysCTT (1082581-1082653) Lys (CTT) 73 bp Sc: 26.52
ACCTGGTTGGCTCAGTTGGAGGAGCCTGTGACTCTTGGTCCCAGGGTTGTGAGTTAGAGT
CCCATGTTGGGTG
>Ailuropoda_melanoleuca_GL194771.1.trna2-LysCTT (35796-35868) Lys (CTT) 73 bp Sc: 27.59
ACCTGGCTGGCTCAGTTGGCAGAGCATGTGACTCTTTGTCTTGGGGTTGTGAGTTCAAGT
TCCATGTTGGGTG
>Ailuropoda_melanoleuca_GL192640.1.trna62-LysCTT (1520451-1520379) Lys (CTT) 73 bp Sc: 27.79
ACCTGGCTGGTTCAGTCAGTAGAGCATGCAGTTCTTATTTTCAGGATTGTGAGTTCAAGC
CCCACGTTGGGTG
>Ailuropoda_melanoleuca_GL193450.1.trna48-LysCTT (571761-571689) Lys (CTT) 73 bp Sc: 28.19
ACTTGGCTGGCTCAGTCAGAAGAGCGTGGGACTCTTGATCTTGGCTTTGTGAGTTCGAGC
CTCATGTTGGGTG
>Ailuropoda_melanoleuca_GL192709.1.trna10-LysCTT (379752-379824) Lys (CTT) 73 bp Sc: 28.27
ACCTGGTTGGCTGAGTCAGAGGAGCATGAGACTCTTGACCTCAGGGTCATGAGTTCAAGC
CCCATGTTGGGTG
>Ailuropoda_melanoleuca_GL193480.1.trna12-LysCTT (520921-520993) Lys (CTT) 73 bp Sc: 28.71
GTCTGGCTGGCTCAGTTGGTGGAGCACGCAACTCTTGATTTCGGGGTTGTGAGTTCCATC
CCTATGTTGGATG
>Ailuropoda_melanoleuca_GL193033.1.trna21-LysCTT (439934-439862) Lys (CTT) 73 bp Sc: 29.14
GCCTGGCTGGCTCAGTCAGTACAGCATGTGAGTCTTGATCTTGGGGTGGTGGGTTTGAGC
CCCACGTTGGGTG
>Ailuropoda_melanoleuca_GL192563.1.trna121-LysCTT (1809457-1809385) Lys (CTT) 73 bp Sc: 29.23
ACCTGGCTGGCTCAGTCAGTGGAGCATGTGACTCTTGATCTCGGGGATATGGGTTCGAGC
CCTGTGTTGGGTG
>Ailuropoda_melanoleuca_GL193721.1.trna10-LysCTT (472282-472210) Lys (CTT) 73 bp Sc: 29.46
GCCTGGCTGGCTTAGTTAGTAGAGCATGCAACTCTTGATTTTAGGATTATGAGTTCAAGG
CCCATGTTGGGTG
>Ailuropoda_melanoleuca_GL193888.1.trna37-LysCTT (172743-172671) Lys (CTT) 73 bp Sc: 29.50
ACCTGGCTGGCTCAGTGGGTGGAGTGTGTGGCTCTTGGTCTCGGAGTTGTGGGTTCAAGC
CCCATGTTGGGAG
>Ailuropoda_melanoleuca_GL192356.1.trna345-LysCTT (2025887-2025815) Lys (CTT) 73 bp Sc: 29.51
GCCTGGCTGGCTCAGTCAGAGGAGCATGCGACGCTTGATCTTGGAGTCATGGGTTCGAGC
CCCATGTTGGGTG
>Ailuropoda_melanoleuca_GL192640.1.trna44-LysCTT (1603424-1603495) Lys (CTT) 72 bp Sc: 29.57
ACCTGGCTGGCTCAGCTGGTGGAGCTGTGACCCTTGATCTTGCAGTTGTGGGTTTGAGTC
CCATGTTGGGTA
>Ailuropoda_melanoleuca_GL192392.1.trna231-LysCTT (119542-119470) Lys (CTT) 73 bp Sc: 29.95
ACCTGGCTGGCTCGGTTGGTGGAGCATGTGACTCTTGATTTTGGGGCTGTGGGTTCGAGC
CTCATGTTGGGTG
>Ailuropoda_melanoleuca_GL192685.1.trna7-LysCTT (183045-183117) Lys (CTT) 73 bp Sc: 30.12
ACCTGGCTGGCTCAGTTGGTGCAGCATGTGGCTCTTGGTCTCAAGGTCGTAAGTTTGAGC
CCTATGCTGGGTG
>Ailuropoda_melanoleuca_GL192675.1.trna86-LysCTT (1219093-1219164) Lys (CTT) 72 bp Sc: 30.22
GCCTGGCTGGCTCAGTCAGTGAGCATAAGAATCTTGATCTCGGGGTTATAGGTTCAAGCC
CCATGTTGGGTG
>Ailuropoda_melanoleuca_GL193035.1.trna20-LysCTT (893392-893464) Lys (CTT) 73 bp Sc: 30.27
GCCTGGCTGGCTTAGTCTGTGGAGCATGTGACTCTTGGTCTCAGGGTCGTAGGTTTGAGT
CCCACGTTGGGTT
>Ailuropoda_melanoleuca_GL192667.1.trna153-LysCTT (650570-650498) Lys (CTT) 73 bp Sc: 30.40
ATCTGGCTGGCTCAGTCAGTAGAGCATGTGACTCTTAGTCTCAGGTTTGTGAGTTCAAGC
CCCATGTTGGGTG
>Ailuropoda_melanoleuca_GL192354.1.trna82-LysCTT (1978644-1978715) Lys (CTT) 72 bp Sc: 30.41
GCCTGGCTGGTTCAGTCAGTGGAGCCTGCGACTCTTGATCTTGGGTTGTGAGTTCGAGCC

TCATGTTGGGCA
>Ailuropoda_melanoleuca_GL193126.1.trna9-LysCTT (256202-256274) Lys (CTT) 73 bp Sc: 30.68
ACCTGGCTGGCTCAGCTGGTGAAGTGTGGGACTCTTGATGTCAGGGCTTTGGGTTCAAGC
CCCAGGATGGGTG
>Ailuropoda_melanoleuca_GL192703.1.trna149-LysCTT (445032-444960) Lys (CTT) 73 bp Sc: 30.86
ACCTGGCTGGCTCAGTCAGTGAAGCATGCGACTCTTGATCTCAGCATTGTGAGTTCAAGC
CCCACGTTGGGTG
>Ailuropoda_melanoleuca_GL192906.1.trna30-LysCTT (773707-773778) Lys (CTT) 72 bp Sc: 30.89
GCCTGGCTGGCTCAGTTGGAGAAGCGTGTGAATCTTCATCTCGGGTCGTGAGTTCAAGCC
CCACGTTAGGTA
>Ailuropoda_melanoleuca_GL193391.1.trna14-LysCTT (416561-416633) Lys (CTT) 73 bp Sc: 30.93
GCCTGGCTGGTTCAGTCAGTGGAGCATGTGATTCTTGATCTCAGGATTGTGAGTTCAAGC
CCCATGTTGGGTG
>Ailuropoda_melanoleuca_GL192758.1.trna62-LysCTT (291253-291173) Lys (CTT) 81 bp Sc: 31.02
ACCTGGCTGGTTCAGTTGGTAAAGCATGCAATTCTTGATCTCAAGGTTATGAGTTTATGA
GTTCAAGCCCCATGTTGGGTG
>Ailuropoda_melanoleuca_GL192401.1.trna49-LysCTT (1327908-1327980) Lys (CTT) 73 bp Sc: 31.05
ACCTGGCTGGCTCAGTCAGTGAAGCCTATGACTCTTGATCTTGGGATTGTGAGTTCAAGC
CCCATGCTGGGTG
>Ailuropoda_melanoleuca_GL192346.1.trna229-LysCTT (1149747-1149675) Lys (CTT) 73 bp Sc: 31.10
GCCTAGCTGGCTCAGTCAGTGAAGCACATGGCTCTTGATCTTGGAGTTATGAGTTCGAGC
CCCATGTTGGGTG
>Ailuropoda_melanoleuca_GL193437.1.trna17-LysCTT (411891-411819) Lys (CTT) 73 bp Sc: 31.46
GCCTGGCTGGCTCAGTCAGAGGAGCATGCGATTCTTGATCTCGGGATTGTGAGTTCAAGT
CCCATGTTAGGTG
>Ailuropoda_melanoleuca_GL192808.1.trna88-LysCTT (1035073-1035001) Lys (CTT) 73 bp Sc: 31.52
ACCTCACTGGTTCAGTGGGTGGGACGTGTGACTCTTGGTCTCAGCTTTGTGGGTTCGAGC
CCCACGTTGGGTG
>Ailuropoda_melanoleuca_GL192366.1.trna12-LysCTT (295233-295305) Lys (CTT) 73 bp Sc: 31.53
ACCTGGCTGGCTCAGTTGGAGGAGCATGCGACTCTTGATTTTGGGGTCATGAGTTTGAGC
CCCATGTTGGGTG
>Ailuropoda_melanoleuca_GL192364.1.trna43-LysCTT (1144451-1144523) Lys (CTT) 73 bp Sc: 31.60
GCCCAGTTGGCTCACTTGGTGGAGCACGTGACTCTTGATCTCGGCGTTGTGAGTTAGAGC
CCCATGTTGGGCG
>Ailuropoda_melanoleuca_GL193497.1.trna19-LysCTT (630100-630172) Lys (CTT) 73 bp Sc: 31.74
ACCTGGCTGGCTCAGGGGGTGGAGCCTGTGACTCTTGATTTCAAGGTTGTGGGGTCGAGC
CCCATGGTGGGTT
>Ailuropoda_melanoleuca_GL193093.1.trna12-LysCTT (299234-299306) Lys (CTT) 73 bp Sc: 31.81
ACCTGGTTGGCTCAGTTGGAAGAGTGTGTAATTCTTGATTTCAGGTTTGTGAGTTTGAGC
CCCATGCTGGGTG
>Ailuropoda_melanoleuca_GL193028.1.trna48-LysCTT (709104-709032) Lys (CTT) 73 bp Sc: 31.83
GCCTGGCTGGCTCAGTTGGTGGAGCGTGCAATTCTTGATTTCAGGGCTGTGGGTTTGAGC
CTCACATTGGGTG
>Ailuropoda_melanoleuca_GL193073.1.trna29-LysCTT (804957-804885) Lys (CTT) 73 bp Sc: 31.97
GCCTGGCTGGCTCAGTCAGTAGAGCATGTGACTCTTGGTCTTGGGGTCATGAGTTTGAGC
CCCATGTTGGGTG
>Ailuropoda_melanoleuca_GL192733.1.trna2-LysCTT (46017-46089) Lys (CTT) 73 bp Sc: 32.05
ACCTGGCTGGCTGTGTTGGAAGAGCCTGCGACTCTTGATCTTGGGGCCATGGGTTTGAGC
CCCATGTCGGGTG
>Ailuropoda_melanoleuca_GL194356.1.trna16-LysCTT (251245-251316) Lys (CTT) 72 bp Sc: 32.06
ATCTGGCTGGGTTAGTCGGTAGAGCATGTGACTCTTGATCTTGGGTGGTGGGTTTGAGCC
CCACGTTGGGTG
>Ailuropoda_melanoleuca_GL192548.1.trna16-LysCTT (414496-414568) Lys (CTT) 73 bp Sc: 32.20
ACCTGGCTGGCTCAGTCGGAGAAGCATGTGATTCTTGATCTCAGGATTGTAGGTTTGAGC
CCCATGTTGGGTG
>Ailuropoda_melanoleuca_GL192654.1.trna36-LysCTT (1443930-1444002) Lys (CTT) 73 bp Sc: 32.21
GCCTGGCTGGCTCAGTCAGTGAAGCATGAAACTCTTGATGTTGGGGTTGTGAGTTCAATT
CTCACATTGGGTG
>Ailuropoda_melanoleuca_GL193800.1.trna11-LysCTT (345731-345800) Lys (CTT) 70 bp Sc: 32.23
GCCTGGCTGGCTCAGTTGGTGGAGCATGGAACTCTTGATCTCGTTGTGAGTTCGAGCCTC
ATATTGGGCA
>Ailuropoda_melanoleuca_GL192574.1.trna86-LysCTT (465068-464996) Lys (CTT) 73 bp Sc: 32.32
GCCTGGCTGGCTCAGCCGAAAAAGCATAAGGCTCTTTATCTCAGGGTCATGAGTTCGAGC
CCCATGTTGGGTG
>Ailuropoda_melanoleuca_GL194157.1.trna5-LysCTT (318901-318973) Lys (CTT) 73 bp Sc: 32.54
CCCTGGCTGGTTCAGTTGGGAGAGCATGTGGCTCTTTATCTCAGGGTGGTGAGTTCAAGC
CCCATGTTGGGGG

>Ailuropoda_melanoleuca_GL195565.1.trna1-LysCTT (6283-6355) Lys (CTT) 73 bp Sc: 32.63
GCCTGACTGGCTCAGTGGGTGGAGTATGCGACTCTTGATCTCAGAGTGGTGAGTTCAAGC
CCTACGTTGGGTG
>Ailuropoda_melanoleuca_GL206637.1.trna1-LysCTT (91-163) Lys (CTT) 73 bp Sc: 32.63
GCCTGACTGGCTCAGTGGGTGGAGTATGCGACTCTTGATCTCAGAGTGGTGAGTTCAAGC
CCTACGTTGGGTG
>Ailuropoda_melanoleuca_GL192908.1.trna40-LysCTT (829181-829109) Lys (CTT) 73 bp Sc: 32.65
GCCTGGCTGGCTCAGTCAGCAGAGCATAGGACTCTTGATTTTGGGTTTGTGAGTTCAAGC
CCCACGTTGGGTG
>Ailuropoda_melanoleuca_GL193228.1.trna19-LysCTT (281100-281170) Lys (CTT) 71 bp Sc: 32.66
GCCTGGCTGGCTCAGTCAGTGGAGTGTGTGACTCTTGATCAGGGTTGTGAGTTCAAGCCC
CACGTTGGGTA
>Ailuropoda_melanoleuca_GL193488.1.trna54-LysCTT (16485-16413) Lys (CTT) 73 bp Sc: 32.68
ACCTGGCTGGCTCAGTCGGTGGAGCGTGCGACTCTTGATCTTGGGATTGTGAGTTCGGGA
CCCATGTTGGGTG
>Ailuropoda_melanoleuca_GL194223.1.trna13-LysCTT (162732-162660) Lys (CTT) 73 bp Sc: 32.76
GCCTGGCTGGTTCAGGTGGTAAAGCATGTGACTCTTGATTTCAGGGTTGTGAGTTTGAGC
CCCATGTTAGGTT
>Ailuropoda_melanoleuca_GL192457.1.trna27-LysCTT (323221-323293) Lys (CTT) 73 bp Sc: 32.93
ATCTGGCTGGCTCAGTCGGTGGGGCGTGTGACTCTTGATCTCGGGGTTATGAGTTTGAGC
CCCATGTTGGGTG
>Ailuropoda_melanoleuca_GL192748.1.trna20-LysCTT (561052-561124) Lys (CTT) 73 bp Sc: 33.14
ACCTGGCTGGCTCAAACGGCAGAGCATGTGACTCTTGATTTCGGGGCTGTGAGTTCAAGC
CCCGCGTTGGGTG
>Ailuropoda_melanoleuca_GL192362.1.trna33-LysCTT (1876352-1876424) Lys (CTT) 73 bp Sc: 33.15
GCCTGGCTGGCTCAGTTGGTGGAGCATGTGACTCTTGATTTCGGGGTTGTGAGCTCAAGC
CCCATGTTGGGTG
>Ailuropoda_melanoleuca_GL192701.1.trna14-LysCTT (383802-383874) Lys (CTT) 73 bp Sc: 33.20
GCCTGGCTGGGTCAGTTGGTGAAGCGTGTGACTCTTGATTTTGGGATTGTGAGTTCAAGC
CTCACGTTAGGTG
>Ailuropoda_melanoleuca_GL193633.1.trna54-LysCTT (197926-197854) Lys (CTT) 73 bp Sc: 33.20
ACCTGGCTGGCTCAATCAGTGGAGTGTGTGACTCTTGATCTTGGAGTCGTGAGTTCGATC
CCCATGTTGGGTG
>Ailuropoda_melanoleuca_GL192573.1.trna179-LysCTT (859259-859187) Lys (CTT) 73 bp Sc: 33.23
ACCTGACTGGCTTAGTTGGAGGGGTATGTGACTCTTGATTTCAGGGTTGTGGGTTTGAGC
CCCACGTTGGGCT
>Ailuropoda_melanoleuca_GL194254.1.trna7-LysCTT (187864-187936) Lys (CTT) 73 bp Sc: 33.26
GCCTGGCTGGCTTAGTCAGTGGAGCATGTGACTCTTGATCTTAGGGTTGTAAGTTCAAGC
CCTATGTTGGGTG
>Ailuropoda_melanoleuca_GL192691.1.trna28-LysCTT (1144521-1144593) Lys (CTT) 73 bp Sc: 33.32
ACCTGGCTGGCTCAGTCGGTGGAGCGTGATACTCTTGATCTTGGGGTTGTGAGTTTAAGC
CCCATGTTGGGTG
>Ailuropoda_melanoleuca_GL192450.1.trna35-LysCTT (1354652-1354727) Lys (CTT) 76 bp Sc: 33.38
GCCTGGCTGGCTCAGTTGGCAATGGAGCATGGGACTCTTGATGTCAGGGTTGTGGGTTTG
AGCCCCATGCTGGCCA
>Ailuropoda_melanoleuca_GL193122.1.trna22-LysCTT (434197-434267) Lys (CTT) 71 bp Sc: 33.81
ACCTGGCTGGCTCAGTCCGTAGAGCATGTGACTCTTGATCTCGGTCGTGAGTTCAAGCCC
TACGTTGGGTG
>Ailuropoda_melanoleuca_GL193361.1.trna16-LysCTT (384761-384833) Lys (CTT) 73 bp Sc: 33.85
GCCTGGCTGGCTCAGTCAGTGGAGCGTGAGACTCTTGATATTGGGGTTGTGAGTTCAAAC
CCCATGTTGGGTG
>Ailuropoda_melanoleuca_GL192881.1.trna62-LysCTT (609456-609384) Lys (CTT) 73 bp Sc: 33.96
ACCTGGCTGGCTCAGTCAGAAGAGCATGTGACTCTTCATCTTGAGGTTGTGGGTTCCAGC
CCCATGTGGGGTG
>Ailuropoda_melanoleuca_GL193041.1.trna8-LysCTT (263557-263629) Lys (CTT) 73 bp Sc: 34.09
GCCTAGTTGGCTCGGTCAGTAGAGCATGTGACTCTTGGTCTTGGGGTTGTGAGTTCGAGC
CTCATGTTGGGCA
>Ailuropoda_melanoleuca_GL192360.1.trna69-LysCTT (1184712-1184784) Lys (CTT) 73 bp Sc: 34.12
ACCTGGCTGGCTCAGTTGGAGGAGTATGCAACTCTTGATTTTGGGGTCGTGGGTTCAAGC
CCCATGTTGGATG
>Ailuropoda_melanoleuca_GL193167.1.trna81-LysCTT (585848-585776) Lys (CTT) 73 bp Sc: 34.16
GCCTGGCTGGCTCAGTCCGTAGAGCGTGCGACTCTTGGTCTCAGGGATGTGAGTTCAAGC
CCCATGTTGGGTG
>Ailuropoda_melanoleuca_GL193776.1.trna10-LysCTT (277087-277159) Lys (CTT) 73 bp Sc: 34.33
ACCTGGCTGGCTCAGTCAGAAGAGCATGTGGCTCTTGATCTCGGGATGGTGAGTTCAAGC
CCCACGTTGGGTG
>Ailuropoda_melanoleuca_GL192751.1.trna16-LysCTT (971841-971913) Lys (CTT) 73 bp Sc: 34.45

GCCCAGTTGGCTCAGTTGGTAGAGCACGAAACTCTTGATTTCAGGGCTGTGAGTTTGAGC
CCCGTGTTGGGTG
>Ailuropoda_melanoleuca_GL192359.1.trna156-LysCTT (4173460-4173388) Lys (CTT) 73 bp Sc: 34.49
GCCCAGCTGGCTCAGTGTGTGGAGCATGCGGCTCTTGATCTCGGGGTTGTGAGTTCAAGC
CCCACGTTGGGTG
>Ailuropoda_melanoleuca_GL192471.1.trna97-LysCTT (72230-72158) Lys (CTT) 73 bp Sc: 34.52
ACCTGGCTGGCTCAGTCTATAGAGCATGTGACTCTTGATCTTGGGATTATGAGTTCGAGC
CCCATGTTGGGTG
>Ailuropoda_melanoleuca_GL193536.1.trna11-LysCTT (575829-575901) Lys (CTT) 73 bp Sc: 34.53
ACGTGGCTGGCTTAGTTCGTAGAGCATGTGACTCTTGATCTTGGGGCAGTAGGTTCGAGT
CCCATGTTGCGTG
>Ailuropoda_melanoleuca_GL192391.1.trna241-LysCTT (1714027-1713955) Lys (CTT) 73 bp Sc: 34.64
GCCTGGCTGGCTCAGCTGGTAGAGCATGTGACTCTTGATTTCAGAGTTGTAAGTTAGACC
CCTATGTTGGGTT
>Ailuropoda_melanoleuca_GL193026.1.trna1-LysCTT (17638-17710) Lys (CTT) 73 bp Sc: 34.66
GCCTGGCTGGCTCAGTCAGTGGAGCACGCGAGTCTTGATCTTGAGGTTGTGAGTTCAAGC
CCCACGCTGGGTG
>Ailuropoda_melanoleuca_GL192450.1.trna43-LysCTT (1596590-1596661) Lys (CTT) 72 bp Sc: 34.98
ACCTGGCTGGCTCAGTTGAGGAGTGTGTGACTCTTGATCTCGGGGCTGCAGGTTCGAGCC
CTACGTTGGGTG
>Ailuropoda_melanoleuca_GL192632.1.trna51-LysCTT (1372838-1372910) Lys (CTT) 73 bp Sc: 34.98
GCCTGGCTGGCTCAATCAGTGGAGCATACGACTCTTGATCTTGCAGTTGTGAGTTCGAAC
CCCATGCTGGGTG
>Ailuropoda_melanoleuca_GL192442.1.trna221-LysCTT (199978-199906) Lys (CTT) 73 bp Sc: 35.06
ACCTGGCTGGCTCAGTCTGTGGAGCATGTGACTCTTGATCTTGGGGTTGTGAGTTCGAGC
CCCATGTTGGGTG
>Ailuropoda_melanoleuca_GL193607.1.trna19-LysCTT (12865-12793) Lys (CTT) 73 bp Sc: 35.13
ACTTGGCTGGCTCAGTTGGTAAAGCATGTGAGTCTTGATCTCAGGATTGTGAGTTTGAAC
CCCATGTTGGGTG
>Ailuropoda_melanoleuca_GL192474.1.trna12-LysCTT (414509-414581) Lys (CTT) 73 bp Sc: 35.22
ACCTGGCTGGCTCAGTCAGAGGAGCATGTGACTCTTGATCTCGGGATTGTGGGTTCAAGC
CCCATGTTGGGGG
>Ailuropoda_melanoleuca_GL193010.1.trna84-LysCTT (655169-655097) Lys (CTT) 73 bp Sc: 35.30
GCCTGGCTGGCTCAGTCGGAGGAGTATGTGACTCTTGATCTCAGGTTTGTGAGTTCAAGC
CCCATGTTGGGTT
>Ailuropoda_melanoleuca_GL192486.1.trna16-LysCTT (402786-402858) Lys (CTT) 73 bp Sc: 35.33
ACCTGGCTGGCTCAGTCAGTAGAGCATGTGACTCTTAGTCTTGGGGTTGTGAGTTCAAGC
CCCATGTTGGGTG
>Ailuropoda_melanoleuca_GL192521.1.trna8-LysCTT (623219-623291) Lys (CTT) 73 bp Sc: 35.35
GTCTGGCTGGCTCAGTTGGTAGAGTATGCGACTCTTCATCTCAGGGTTGTAAGTTTGAGA
CCTATGTTGGGTG
>Ailuropoda_melanoleuca_GL193058.1.trna22-LysCTT (853667-853738) Lys (CTT) 72 bp Sc: 35.47
GCCTGACTGGTTCAGTGGGAAAAGCATGTGATTCTTGATCTCGGGTTGTGAGTTCAAGCC
CCACGTTGGGTG
>Ailuropoda_melanoleuca_GL192648.1.trna19-LysCTT (1248389-1248461) Lys (CTT) 73 bp Sc: 35.57
ACCTGGCTGGCTTAGTCAGTGGAGCATGTGACTCTTGATCTCGGGGTTGTGAGTTCAAGC
CCCATGTTGGGTG
>Ailuropoda_melanoleuca_GL193028.1.trna53-LysCTT (496155-496084) Lys (CTT) 72 bp Sc: 35.59
GCCTGGCTGGCTCAGTTGGTGGAGCATGCGACTCTTGATCTCGGGTTGTGAGTTTAAGCC
CCATGCTGGGTA
>Ailuropoda_melanoleuca_GL193619.1.trna16-LysCTT (418662-418734) Lys (CTT) 73 bp Sc: 35.62
GCCCAGCTGGCTTAGTCAGTGGAGCATGTGATTCTTGATCTCGGGGTTGTGAGTTCAAGC
CCCATGTTGGGTG
>Ailuropoda_melanoleuca_GL192421.1.trna15-LysCTT (242504-242576) Lys (CTT) 73 bp Sc: 35.63
ACCTGGTTGGCTCAGTCAGTAGAGCATGTGACTCTTGGTCTCGGGGTTGTGAGTTCGAGC
CCCATGTTGGGTG
>Ailuropoda_melanoleuca_GL193025.1.trna54-LysCTT (94216-94144) Lys (CTT) 73 bp Sc: 35.72
GCCTGGCTGGCTCAGTCAGAAGAGCATGTGACTCTTGATCTCAGAGTCATGGGTTTGAGC
CCTATATTGGGTA
>Ailuropoda_melanoleuca_GL192424.1.trna17-LysCTT (718949-719021) Lys (CTT) 73 bp Sc: 35.82
GCCTGGCTGGCTTAGTCGGTAAAGCATGTGACTCTTGATCTCAGCACTGTGAGTTTGAGC
CCCATGTTGGGTG
>Ailuropoda_melanoleuca_GL193188.1.trna73-LysCTT (600678-600606) Lys (CTT) 73 bp Sc: 35.84
ACTTGGCTGGCTCAGTTGGTGGAGTATGTGACTCTTGATCTCAGGGTTGTAGGTTTGTGC
CCTGCGCTGGGTG
>Ailuropoda_melanoleuca_GL192433.1.trna92-LysCTT (1803249-1803173) Lys (CTT) 77 bp Sc: 35.96
GCCTGGCTGGCTCAGTCAGTAAAGCACGAGACTCTTGTTCTTGGTTGGGGTTGTGAGTTC

AAGACCCACGTTGGGCC
>Ailuropoda_melanoleuca_GL193748.1.trna26-LysCTT (291296-291224) Lys (CTT) 73 bp Sc: 35.97
GCCTGGCTGGCTCAGCTGGAGAAGCATGTGACTCTTGATCTCAGGGTTGTGAGTTTGAAC
CCCATGTTGGGTG
>Ailuropoda_melanoleuca_GL192420.1.trna104-LysCTT (2651127-2651054) Lys (CTT) 74 bp Sc: 35.98
GCCTGGCTCGCTCAGTCAGTAGAGCATGTGACTCTTGATCTCAGGGGTTGTGAGTTCAAG
CCCCATGTTGGGTA
>Ailuropoda_melanoleuca_GL192447.1.trna93-LysCTT (1392550-1392478) Lys (CTT) 73 bp Sc: 36.00
ACCTGGCTGGTTCAGTCAGAAGAGCATGTGACTCTTGATCTCGGCGTCATGAGTTCAAGT
CCCATGTTGGGTG
>Ailuropoda_melanoleuca_GL193457.1.trna15-LysCTT (439402-439474) Lys (CTT) 73 bp Sc: 36.00
GCCTGACTGGCTTAGTTGGTGGAGTGTGAGACTCTTGATCTCAGAGTGGTGAGTTCAAGC
CCTGTGTTGGGTG
>Ailuropoda_melanoleuca_GL192443.1.trna53-LysCTT (2550543-2550615) Lys (CTT) 73 bp Sc: 36.01
ACCTGGCTGGCTCAGTCAGTAGAGTGTGCGAGTCTTGATCTCGGGGTTGTAAGTTCAAGC
CTCACGTTGGGTG
>Ailuropoda_melanoleuca_GL194012.1.trna16-LysCTT (370793-370865) Lys (CTT) 73 bp Sc: 36.01
GCCTGGCTGGCTCAGTGGGTGGAGCGTGTGACTCTTGATCTTGGGGTCATGAGTGCAAGC
CCCATGTTGGGTG
>Ailuropoda_melanoleuca_GL192660.1.trna30-LysCTT (823534-823606) Lys (CTT) 73 bp Sc: 36.03
GCCTGGCTGGCTCAGCCGGTAGAGCATGCAACTCTTGATTTTGGGGTTGTGAGATCAAGC
CCCATGTTGGGTA
>Ailuropoda_melanoleuca_GL192450.1.trna74-LysCTT (1620321-1620249) Lys (CTT) 73 bp Sc: 36.11
GCCAGGCTGGCTCAGTCAGGGGAGCATGTGACTCTTGATCTCGGGGTTGTGGGTTCAAGC
CCCATGTTAGGTG
>Ailuropoda_melanoleuca_GL192567.1.trna143-LysCTT (1066702-1066630) Lys (CTT) 73 bp Sc: 36.11
GTCTGGCTGGCTCAGCTGGTGGAGTGTGTGACTCTTGATCTCACAGTTGTGAGTTTGAGC
CCCATGTTGGGCA
>Ailuropoda_melanoleuca_GL192657.1.trna17-LysCTT (434872-434944) Lys (CTT) 73 bp Sc: 36.43
ACCCGGCTGGCTCAGTCAGTGGAGCATGCGATGCTTGATCTTGGGGTTGTGGGTTCGAGT
CCCACGTTGGGTG
>Ailuropoda_melanoleuca_GL194901.1.trna23-LysCTT (2582-2510) Lys (CTT) 73 bp Sc: 36.44
GCCTGGCTGGCTCAGTTTGTGGAGCATGGGACTCTTGATCTTGGGGTTGTGAGTTCAAGC
CTCATGTTGGGTG
>Ailuropoda_melanoleuca_GL193477.1.trna10-LysCTT (365111-365183) Lys (CTT) 73 bp Sc: 36.45
ACCTGGCTGGCTCAGTTGGCAGAGTATGTGACTCTTGATCTTGGGGTTGTGAGTCCAAGC
CCCATGTTGGGTG
>Ailuropoda_melanoleuca_GL192614.1.trna52-LysCTT (1198516-1198444) Lys (CTT) 73 bp Sc: 36.51
GCCTGGCTGGCTCAGTCGGAAGACCATGGGAGTCTTGATTTCAGGGTCATGGGTTCCAGC
CCCATGTTGGGTG
>Ailuropoda_melanoleuca_GL193092.1.trna40-LysCTT (929910-929838) Lys (CTT) 73 bp Sc: 36.52
ACCTGGCTGGCTTACTGGAAAGAGCCTGTGGCTCTTGACCTCGGAGTCATGAGTTCGAGC
CCCATGTTGGGTG
>Ailuropoda_melanoleuca_GL193629.1.trna36-LysCTT (286518-286446) Lys (CTT) 73 bp Sc: 36.56
ACCTGGCTGGCTCAGTCAGAAAAGCATGTGACTCTTGATCTTGGGGTCGTGAGTTTGAAC
CCCACGTTGGGTG
>Ailuropoda_melanoleuca_GL192489.1.trna112-LysCTT (1934894-1934822) Lys (CTT) 73 bp Sc: 36.59
GCCTGACTGGCTCAGTCAGTAGAGCATATGGCTCTTGACCTTGGGTTCATGAGTTCAAGC
CTTATTTTGGGCC
>Ailuropoda_melanoleuca_GL192497.1.trna36-LysCTT (787140-787212) Lys (CTT) 73 bp Sc: 36.62
GCCCAGCTGGCTCAGTCTGTAGAGCCTGTGACTCTTGATTTCGGGGTTGTGAGCTCGAGC
CCCACGTTGGGTG
>Ailuropoda_melanoleuca_GL193117.1.trna29-LysCTT (698778-698850) Lys (CTT) 73 bp Sc: 36.64
ACCCAGCTGGCTCAGCCGGAAGAGCACGTGACTCTTGGTCTTGGGGTTGTGAGTTTGAGC
CCCATGTTGGGTG
>Ailuropoda_melanoleuca_GL192401.1.trna111-LysCTT (1375350-1375278) Lys (CTT) 73 bp Sc: 36.77
ACCTGGCTGGCTCAGTTGTTGGAGCATGCGACTCTTCATCTCAGGGTTGTGGGTTTGAGC
CCCACGTTGGGAG
>Ailuropoda_melanoleuca_GL192357.1.trna190-LysCTT (4489340-4489412) Lys (CTT) 73 bp Sc: 36.79
GCCCGGCTGGCTCAGTCGGTGGAGCCTGTGATTCTTGATCTCGGGGTTGTGAGTTTGAGC
CCCATGTTGGGTG
>Ailuropoda_melanoleuca_GL193497.1.trna17-LysCTT (623279-623351) Lys (CTT) 73 bp Sc: 36.79
ACCTGGCTGGCTTAGTCAGTAGAGCATGTGACTCTTCATCTTGGAGTCGTGAGTTCGAGT
CCCATGTTGGGTG
>Ailuropoda_melanoleuca_GL193390.1.trna42-LysCTT (529495-529423) Lys (CTT) 73 bp Sc: 36.84
GCCTGGCTGGCTCAGTTAGTGGAGCTTGTGACTCTTGATCTTGGAGTTGTGGGTTCAAGC
CCCATGTTGGGTG

>Ailuropoda_melanoleuca_GL194240.1.trna10-LysCTT (166869-166941) Lys (CTT) 73 bp Sc: 36.87
GCCTGACTAGCTCAGTCAGTAGGGTATGCGATTCTTGATCTCAGGGTTGTGAGTTCGAGC
CATACGTTGGGTA
>Ailuropoda_melanoleuca_GL192507.1.trna111-LysCTT (907503-907431) Lys (CTT) 73 bp Sc: 36.88
GCCCGGCTGGCTCAGTTGGTGGAGCATGCGATTCTTGATCTCGGGATTGTGAGTTTGAGC
CCCATGTTGGGTG
>Ailuropoda_melanoleuca_GL193276.1.trna42-LysCTT (274580-274508) Lys (CTT) 73 bp Sc: 36.89
GCCTGGCTGGCTCAGTCGTTGGAGTATGTGACTCTTGATCTCAGAGTTGTGAGTTCAAGC
CCCATGTTGGGTG
>Ailuropoda_melanoleuca_GL193173.1.trna5-LysCTT (76323-76394) Lys (CTT) 72 bp Sc: 36.90
ACCTGGCTGGCTCAGTTGAAGAGCATGTGACTCTTGATCTTAGGGTTGTGAGTTCAAGTC
CTATGTTAGGTG
>Ailuropoda_melanoleuca_GL192356.1.trna216-LysCTT (4206487-4206559) Lys (CTT) 73 bp Sc: 36.92
ACCTGGCTGGCTCAGTCAGTAGAGCATGGAGCTCTTGACCTTGGGGTTGTGAGTTCAAGC
CCCACGTTGGGTA
>Ailuropoda_melanoleuca_GL192593.1.trna71-LysCTT (1822195-1822123) Lys (CTT) 73 bp Sc: 36.95
GCCTGGCTGGTTCAGTCAGTGGAGTATGTGACTCTTGATCTCAGGGTTGTGAGTTCGAGC
CCCATGCTAGGTG
>Ailuropoda_melanoleuca_GL192703.1.trna51-LysCTT (997639-997711) Lys (CTT) 73 bp Sc: 36.95
ACCTGGCTGGCTTAGTCAGTAGAGCATGTGACTCTTGGTCTTGGGGTTGTGAGTTCGAGC
CCCACGTTGGGTG
>Ailuropoda_melanoleuca_GL192496.1.trna56-LysCTT (2235568-2235496) Lys (CTT) 73 bp Sc: 37.16
GCCTGGCTGGCTCAGTCGATAGAGCATGCGACTCTTGATCTCAGGGTTGTGGGTTTGAGC
CTCATGTTGGGGT
>Ailuropoda_melanoleuca_GL193138.1.trna47-LysCTT (309937-309865) Lys (CTT) 73 bp Sc: 37.17
ACCTGGCTGGCTAAGTAGGAGGAGCATGTGACTCTTGATCTTGGGGTTGTGGGTTCGAGC
CCCATGTTGGGTG
>Ailuropoda_melanoleuca_GL192392.1.trna229-LysCTT (185862-185790) Lys (CTT) 73 bp Sc: 37.28
ACCTGGCTGGCTCAGTCGGAAAAGCATGTGACTCTTCATCTTGGGGTAATGAGTTCAAGC
CCCATGCTGGGTG
>Ailuropoda_melanoleuca_GL193067.1.trna37-LysCTT (610989-611062) Lys (CTT) 74 bp Sc: 37.29
GCCTAGCTGGCTCAGTTGGTGGAGCGTGTGACTCTTGATCTTGTGGATCGTGAGTTTGAG
CCCCACGTTGGGTG
>Ailuropoda_melanoleuca_GL195144.1.trna3-LysCTT (23856-23784) Lys (CTT) 73 bp Sc: 37.30
ACCCGGCTGGCTCAGCTGATAGAGCATGCTACTCTTGATGTCAGCGTTGTGAGTTCAAAC
CCCATGTTGGGTG
>Ailuropoda_melanoleuca_GL192340.1.trna99-LysCTT (1861415-1861487) Lys (CTT) 73 bp Sc: 37.36
ACCTGGCTGGCTCAGTCAGAAGAGCTTGCGACTCTTGATCTTGGGGTTGTGAGTTCAAGA
CCCATGCTGGGTG
>Ailuropoda_melanoleuca_GL192654.1.trna43-LysCTT (1503337-1503409) Lys (CTT) 73 bp Sc: 37.37
GCCTGGCTTGCTCAGTTGGTGGAGCCTGTGACTCTTGATCTCGGGGTTGTTGGTTCAAGC
CCCATGTTGGGTG
>Ailuropoda_melanoleuca_GL192338.1.trna211-LysCTT (3763049-3762977) Lys (CTT) 73 bp Sc: 37.38
GCCTGGCTGGTTCAGTTGCTAGAGCACGTGACTCTTGATCTCGGGGTTGTGAGTTAGAGC
CCCATGTTGGGTG
>Ailuropoda_melanoleuca_GL194023.1.trna5-LysCTT (348329-348257) Lys (CTT) 73 bp Sc: 37.38
GCCTGGCTGGTTCAATTGGTGGAGCATGTGACTCTTGATCTTGAAGTTATGGGTTCAAAG
CCCATGTTGGGTA
>Ailuropoda_melanoleuca_GL192407.1.trna54-LysCTT (2647031-2647103) Lys (CTT) 73 bp Sc: 37.43
ACCTGGCTGGCTCAGTCGGAAGAGCATGCGACTCTTGATCTTGGTGTTATGAGTTTGAGT
CCCATGTTGGGTG
>Ailuropoda_melanoleuca_GL192582.1.trna38-LysCTT (1313848-1313920) Lys (CTT) 73 bp Sc: 37.48
ACTTAGTTAGCTCAGTCAGTACAGCATGTGACTCTTGATCTCGTGGTTGTGAGTTCAAGC
CTCATGCTGGGTG
>Ailuropoda_melanoleuca_GL193343.1.trna14-LysCTT (403011-403085) Lys (CTT) 75 bp Sc: 37.51
GCCTGGCTGGCTCAGTCAGTAGAGCACAAGAAGCTTAATCTTTCGGGGTCGTGAGTTCAA
GCCTCATGTTGGGCA
>Ailuropoda_melanoleuca_GL192539.1.trna137-LysCTT (1337955-1337883) Lys (CTT) 73 bp Sc: 37.64
GCCTGGCTGGCTTAGTCAGTAGAGCATGTGACTCTTCATCTTGGGGTTATGAGTTCGAGC
CCCATGTTAGGTG
>Ailuropoda_melanoleuca_GL193330.1.trna12-LysCTT (689324-689396) Lys (CTT) 73 bp Sc: 37.91
ACCTGGCTGGTTCAGTCAGTAGAGCATGTGACTCTTGATCTCAGGGTTGTGAGTTTGAGC
CCCATGTTGGGTA
>Ailuropoda_melanoleuca_GL192377.1.trna128-LysCTT (1386618-1386546) Lys (CTT) 73 bp Sc: 37.96
ACCTGGCTGGCTCAGTCAGTGGAGCATGTGACTCTTGATCTCGGGATTGTGAGTTCAAGC
CCCACGTTGGGTG
>Ailuropoda_melanoleuca_GL192837.1.trna60-LysCTT (541965-541893) Lys (CTT) 73 bp Sc: 38.08

ACCTGGCTGGCTCAATCGGTGGAGCATGTGATTCTTGATCTTGGGGTTGTAGGTTCGAGC
CCTGTGTTAGGGG
>Ailuropoda_melanoleuca_GL194181.1.trna12-LysCTT (126875-126947) Lys (CTT) 73 bp Sc: 38.20
ACCTGGCTGGCTCAGTGGGTGGAGTGTGCGACTCTTGCTCTCGGGGTTGTGAGTTTGAGC
CCCACGTTGGGTG
>Ailuropoda_melanoleuca_GL192370.1.trna89-LysCTT (2930580-2930652) Lys (CTT) 73 bp Sc: 38.23
ACCTAGTTGGCTGAATTGGTACAGCTTGCGACTCTTGATCTTGGAGTCATGAGTTTGAGC
CCCATATTGGGTG
>Ailuropoda_melanoleuca_GL192844.1.trna47-LysCTT (1155862-1155934) Lys (CTT) 73 bp Sc: 38.28
GCCTGGCTGGCTCAGTCAGTGGAGCATGTGACTCTTGATCTCGGGGTTGTGAGTTCAAGC
CCCATGCTGGGTG
>Ailuropoda_melanoleuca_GL194018.1.trna2-LysCTT (70635-70707) Lys (CTT) 73 bp Sc: 38.31
GCCTGGCTGGCTCAGTTGGTAGAGCATGCGATTCTTGATCTCAGCATTATAAGTTTGAGC
CTCATGTTGGGTG
>Ailuropoda_melanoleuca_GL192886.1.trna25-LysCTT (816403-816475) Lys (CTT) 73 bp Sc: 38.34
ACCTGGCTGGCTCAGCTGGAAAAGCCTGTGACTCTTGATCTCGGGGTTGTGAGTTTGAGC
CCCATGCTGGGTG
>Ailuropoda_melanoleuca_GL192479.1.trna16-LysCTT (277632-277704) Lys (CTT) 73 bp Sc: 38.41
GCCTGGCTGGCTCAGTTGGTGGAGCATGAAACTCTTGATCTTGGGGTTGTGAGTTCATGT
CCCATGTTGGGCA
>Ailuropoda_melanoleuca_GL192728.1.trna129-LysCTT (412913-412841) Lys (CTT) 73 bp Sc: 38.47
GCCTGGCTAGCTCAGTCAGTGGAGCATGTGACTCTTGATCTCAAAGTTGTGAGTTCAAGC
CCCATGTTGGGTG
>Ailuropoda_melanoleuca_GL193067.1.trna98-LysCTT (9079-9007) Lys (CTT) 73 bp Sc: 38.59
ACCTGGCTGGCTCAGTCGGTGGAGCATGTGGCTCTTGATCTCAGGGTTGTGAGTTTGAGT
CCCATGTTGGGTG
>Ailuropoda_melanoleuca_GL192351.1.trna258-LysCTT (2222593-2222521) Lys (CTT) 73 bp Sc: 38.66
GCCTGGCTGGCTCAGTTGGTGGGGTGTGCGACTCTTGGTCTCAGGGTTGTGAGTTCGAGC
CCCATGTTGGGTG
>Ailuropoda_melanoleuca_GL192571.1.trna80-LysCTT (35829-35757) Lys (CTT) 73 bp Sc: 38.67
GCCTGACTGGCTCAGTTGATAGAGCATGTGACTCTTGATCTCAGAGTCATGAGTTCAAGC
CCTGTGTTGGGTG
>Ailuropoda_melanoleuca_GL192504.1.trna61-LysCTT (2000533-2000461) Lys (CTT) 73 bp Sc: 38.73
ACCTGGCTGGCTCAGCTGGCAGAGCATGCGATTCTTGGTTTCAGAGTCATGAGTTCAAGC
CTCATGTTGGGTG
>Ailuropoda_melanoleuca_GL193360.1.trna16-LysCTT (722621-722693) Lys (CTT) 73 bp Sc: 38.77
GCCTGGTTGGCTCAGTTGGTGGAGTGTGTGACTCTTGATCTCGGGGTTATGAGTTTGAGC
CCCATGCTGGGTG
>Ailuropoda_melanoleuca_GL193666.1.trna31-LysCTT (479995-480071) Lys (CTT) 77 bp Sc: 38.81
ACCTGGCTGGCTCAGTCGGAAAAGCATGTGGCTCTTGATCTCAGGGTCGGTCGTGAGTCC
GAGCCCCACGTTGGGTG
>Ailuropoda_melanoleuca_GL192793.1.trna115-LysCTT (1030231-1030159) Lys (CTT) 73 bp Sc: 38.85
ACCTGGCTGGCTCAGTCGGTGGTGCGTGTGACTCTTGATCTTGGGGTTGTGGGTTTGAGC
CCCACGTTGGGTT
>Ailuropoda_melanoleuca_GL194280.1.trna21-LysCTT (34971-34899) Lys (CTT) 73 bp Sc: 38.96
GCCTGGCTGGCTCAGTCTGTAGAGCATGGGACTCTTGATCTTGGGGTCATGAGTTTGAGC
CCCATGTTAGGTG
>Ailuropoda_melanoleuca_GL192615.1.trna35-LysCTT (1172092-1172164) Lys (CTT) 73 bp Sc: 39.15
ACCTGGCTGGCTCAGTTGGAGGAACCTGTGACTCTTGGCCACAGGGTTGTGGGTTCGAGC
CCCACGTTGGGTG
>Ailuropoda_melanoleuca_GL192844.1.trna98-LysCTT (603309-603237) Lys (CTT) 73 bp Sc: 39.28
ACCTGACTGGCTCATTTGGTAGAGCATGTGACTCTTGATCTTGGGGTTGTGAGTTTGAGC
CCCATGTTGGGTA
>Ailuropoda_melanoleuca_GL192515.1.trna34-LysCTT (1301804-1301877) Lys (CTT) 74 bp Sc: 39.29
GCCTGGCTGGCTCAGTCAGTAGAGCATGTGGCTCTTAATCTCAGGAGTCATGAGTTCAAG
CCCCATGTTGGGTG
>Ailuropoda_melanoleuca_GL193005.1.trna24-LysCTT (888093-888165) Lys (CTT) 73 bp Sc: 39.37
GCCTGGCTGGCTCAGTCGGTGAAGTATGCAACTCTTGATTTCAGGGCTGTGGGTTCAAAC
CCCATGTTGGGTG
>Ailuropoda_melanoleuca_GL192795.1.trna45-LysCTT (1165776-1165848) Lys (CTT) 73 bp Sc: 39.37
GCCTGGCTGGCTCAGTCAGTGGAGTGTGCGACTCTTGATCTCAGGGCTGTAGGTTCAAGT
CCCACGTTGGGTA
>Ailuropoda_melanoleuca_GL193160.1.trna58-LysCTT (649302-649230) Lys (CTT) 73 bp Sc: 39.44
GCCTGGCTGGCTCAGTTGGAAGAGCATGTGACTCTTGATCTTGGCGTCATGAGTTTGAGC
CCCATGTTGGGTG
>Ailuropoda_melanoleuca_GL192518.1.trna27-LysCTT (1150807-1150879) Lys (CTT) 73 bp Sc: 39.48
ACCTGACTGGCTTAGTCGGCAGAGCATGTGACTCTTGATCTCACtgttgtGAGTTCAAGC

CTTATGTTGGGCA
>Ailuropoda_melanoleuca_GL192787.1.trna26-LysCTT (765455-765527) Lys (CTT) 73 bp Sc: 39.56
ACCTAGCTGGCTCAGTCAGTGGAGCATGTGACTCTTGATCTCGGGGTTGTGAGTTCGAGC
CCCATGTTGGGTG
>Ailuropoda_melanoleuca_GL192860.1.trna18-LysCTT (755153-755225) Lys (CTT) 73 bp Sc: 39.67
ACCTGGCTGGCTCAGTCGGTAGAGCATGTAACTCTTGATTTCAGGCCTGTGAGTTTGAGT
CCCACGTTGGGTA
>Ailuropoda_melanoleuca_GL192580.1.trna94-LysCTT (609912-609840) Lys (CTT) 73 bp Sc: 39.68
ACCTGGCTGGTTCAGTCCGTGGAGCGTGCGACTCTTGATCTCGGGGTCGTGAGTTCGAGC
CCCACGTTGGGTG
>Ailuropoda_melanoleuca_GL192660.1.trna66-LysCTT (1610381-1610309) Lys (CTT) 73 bp Sc: 39.69
GCCTGACTGGCTCAGTCAGTACAGCATGTGACTCTTGATCTCGGAGTCATGAGTTCAAGC
CCCATGTTGGGTA
>Ailuropoda_melanoleuca_GL192439.1.trna148-LysCTT (1116069-1115997) Lys (CTT) 73 bp Sc: 39.69
GCCTGGCTGGCTCAGTGGGTGGAGTGTGCGACTCTTGATCTCAGGGTTGTGAGTTTGAGC
CCCATGTTGGGTG
>Ailuropoda_melanoleuca_GL193673.1.trna12-LysCTT (182971-182899) Lys (CTT) 73 bp Sc: 39.86
ATCTGGCTGGTTCAGTCAGTAGAGTATGAGACTCTTGATCTCAGGGTGGTGAGTTCAAGC
CTCACATTGGGTG
>Ailuropoda_melanoleuca_GL192723.1.trna113-LysCTT (595512-595440) Lys (CTT) 73 bp Sc: 39.97
ACCTGGCTAGCTCAGTAGGTAGAGCCTGGAACTCTTGATCTTAGGGCTGTGAGTTCGAGC
CTCACACTGGGTG
>Ailuropoda_melanoleuca_GL192844.1.trna69-LysCTT (1194405-1194334) Lys (CTT) 72 bp Sc: 40.20
GCCTGGCTGGCTCAGTAGGTGGAGCTTGTGACTCTTGATCTTGGGTTATGGGTTCGAGCC
CCATGTTGGGTT
>Ailuropoda_melanoleuca_GL193161.1.trna35-LysCTT (407799-407727) Lys (CTT) 73 bp Sc: 40.20
GCCTGGCTGGCTCAGTCAGTAGAGCATGTGACTCTTGATCTCGGtgttgtGAGTTCAAGC
CCCATGTTGGGTG
>Ailuropoda_melanoleuca_GL192398.1.trna237-LysCTT (67029-66957) Lys (CTT) 73 bp Sc: 40.24
GCCTGGCTGGCTCAGTCAGTAGAGCATGTGACTCTTGATCTTGGGGTCATGAGTTCCAAC
CCCATGTTGGGTG
>Ailuropoda_melanoleuca_GL192635.1.trna67-LysCTT (1629812-1629740) Lys (CTT) 73 bp Sc: 40.26
GCCCAGCTGGCTCAGTTGGTGGAGCATGCAACTCTTGATTGTGGAGTTGTGGGTTTGAGC
CCCACACTGGGTG
>Ailuropoda_melanoleuca_GL192439.1.trna132-LysCTT (1654630-1654557) Lys (CTT) 74 bp Sc: 40.28
ACCTGGCTGGCTGAATTGGAAGGGGTGTGAGACTCTTGATCTCAGAGTTGTGAGTTCGAA
CCCCACGTTAGGTG
>Ailuropoda_melanoleuca_GL192421.1.trna222-LysCTT (211211-211139) Lys (CTT) 73 bp Sc: 40.30
ACCTGGCTGGTTCAGTCGGAAGAGTGCGTGACTCTTGGTCTTGGGGTTGTGAGTTCGAGC
CCCATGCTGGGTG
>Ailuropoda_melanoleuca_GL192398.1.trna136-LysCTT (2564854-2564782) Lys (CTT) 73 bp Sc: 40.36
ACCTGGCTGGCTCAGTCAGTGGAGCATGCGACTCTTGATCTCAGGGTTGTGAGTTCAAGC
CCCATGCTGGGTG
>Ailuropoda_melanoleuca_GL192545.1.trna100-LysCTT (641150-641078) Lys (CTT) 73 bp Sc: 40.42
CCCTGGCTGGTTCAGTCAGAAAAGCATGCGACTCTTGATCTCAGGGTCATGAGTTCGAGC
CCCATGTTGGGGG
>Ailuropoda_melanoleuca_GL193093.1.trna45-LysCTT (504978-504906) Lys (CTT) 73 bp Sc: 40.49
ACCTGGCTGGCTTAGTCAGTAGAGCATGTGACTCTTGATCTCGGGGTCATGAGTTCAGAC
CCCATGTTGGGTG
>Ailuropoda_melanoleuca_GL192415.1.trna52-LysCTT (2477447-2477519) Lys (CTT) 73 bp Sc: 40.53
ACCTGGCTGGCTCAGTCGGTGGAGCGTGCGACTCTTGATCTCAGGGTTGTGAGTTCCAGC
CCCGTGTTGGGTA
>Ailuropoda_melanoleuca_GL193001.1.trna35-LysCTT (699817-699889) Lys (CTT) 73 bp Sc: 40.55
GCCTGGCTGGCTCAGTCAGTGAAGCGTGCGACTCTTGATCTCGGGATTGTGGGTTCGAGC
CCCATGTTGGGTG
>Ailuropoda_melanoleuca_GL192580.1.trna16-LysCTT (407496-407568) Lys (CTT) 73 bp Sc: 40.68
GATTGGCTGGCTCAGTCAGTAGAGCATGGGACTCTTGATCTCAGGGTTGTGGGTTTGAGC
CCCATGTTGGACG
>Ailuropoda_melanoleuca_GL192563.1.trna147-LysCTT (1360090-1360018) Lys (CTT) 73 bp Sc: 40.74
ACCCAGCTGGCTCAGTCAGTAGAGCATGTGACTCTTTATCTCGGGATTGTGGGTTTGAGC
CCCACACTGGGTG
>Ailuropoda_melanoleuca_GL194802.1.trna3-LysCTT (82510-82438) Lys (CTT) 73 bp Sc: 40.76
ACCTGGCTGGCTCAGTGGGAGGAGCATGTGACTCTTGATCTCAGGGTTGTGAGTTTGAGC
CCCACGTTGGGTG
>Ailuropoda_melanoleuca_GL193334.1.trna8-LysCTT (363621-363693) Lys (CTT) 73 bp Sc: 40.76
ACCTGGTTGGCTCAATCGGTGGAGCATGTGACTCTTAATCTCAGGATTGTAGGTTCAAGC
CCCATGTTGGGTG

>Ailuropoda_melanoleuca_GL192618.1.trna124-LysCTT (853193-853121) Lys (CTT) 73 bp Sc: 40.77
GCCTGGCTGGTTCAGTCAGTGGAGCATGCGACTCTTGATCTCAGGGTCATGGGTTCAAGC
CCCACGTTGGGCT
>Ailuropoda_melanoleuca_GL193305.1.trna9-LysCTT (348144-348216) Lys (CTT) 73 bp Sc: 40.83
GCTTGGCTGGCTCAGTCAGCAGAGCATGTGACTCTTGATCTCAGGATTGTGAGTTCAAGC
CTCATGTTGGGTG
>Ailuropoda_melanoleuca_GL193225.1.trna21-LysCTT (421945-422017) Lys (CTT) 73 bp Sc: 40.87
GCCTGGCTGGCTTAGTCAGTGAAGCGTGCGACTCTTGATCTCGGGATTGTGGGTTCGAGC
CCCATGTTGGGTG
>Ailuropoda_melanoleuca_GL193012.1.trna43-LysCTT (956467-956395) Lys (CTT) 73 bp Sc: 40.87
GCCTGGCTGGCTCAGTCAGTAGAGTGTATGACTCTTGATTTTGGGGTTATGAGTTCAAGC
CCCGTGTTAGGCC
>Ailuropoda_melanoleuca_GL192385.1.trna206-LysCTT (1500743-1500671) Lys (CTT) 73 bp Sc: 40.88
ACCTGGCTGGCTCAGTTGGCAGAGCACGAGACTCTTGACCTTGGGGGTGTGAGTTTGAGC
CCCACGCTGGGTG
>Ailuropoda_melanoleuca_GL193167.1.trna43-LysCTT (669784-669856) Lys (CTT) 73 bp Sc: 40.88
ACCTGGCTGGCTCAGTCAGTAGAGCATGCGACTCTTGATCTTGGGATTGTGAGTTCAAGC
CCCATGTCAGGTA
>Ailuropoda_melanoleuca_GL192340.1.trna396-LysCTT (41406-41334) Lys (CTT) 73 bp Sc: 41.06
ACCTGGCTGGCTCAGTCAGTGGAGCGTGCGGCTCTTGATCTCAGGGTTGTGGGTTCGATC
CCCACGTTGGCTG
>Ailuropoda_melanoleuca_GL192359.1.trna273-LysCTT (1195320-1195248) Lys (CTT) 73 bp Sc: 41.17
ATCTGGCTGGTTCAGTTGGTAGAGCATGTGACTCTTGATCTCAGACTTGTGGGTTTGAGC
CCCACGTTGGGTG
>Ailuropoda_melanoleuca_GL193453.1.trna23-LysCTT (81846-81774) Lys (CTT) 73 bp Sc: 41.20
ACCTGGCTGGCTTAGTCAGAAGAGTATGGGACTCTTGATCTCGGGGTCGTGAGTTCAAGC
CCCATGTTGGGTA
>Ailuropoda_melanoleuca_GL192383.1.trna44-LysCTT (1102008-1102080) Lys (CTT) 73 bp Sc: 41.24
GCCTAGCTGGCTTAGTTGGAAGAGTATGTGGCTCTTGATCTTGGGGTCATGAGTTCGAGC
TTCATGTTGGGTG
>Ailuropoda_melanoleuca_GL192437.1.trna265-LysCTT (608723-608650) Lys (CTT) 74 bp Sc: 41.25
GCCTGACTGGCTTATTTGGTAGAGCATGTGACTCTTGATCTCAGGGTTGTTAAGTTCAAG
CCTTATGTTGGGTA
>Ailuropoda_melanoleuca_GL192546.1.trna12-LysCTT (320869-320941) Lys (CTT) 73 bp Sc: 41.39
ACCTGGCTGGCTCAGTCAGAAGAGCCTGGAACTCTTGATCTCAGGGTCGTAGGTTCGAAC
CCCATGTTGGGTG
>Ailuropoda_melanoleuca_GL192421.1.trna106-LysCTT (2819553-2819625) Lys (CTT) 73 bp Sc: 41.46
GTCCAGCTGGCTCAATCGGTGGAGCGTGCGACTCTTGATCTCGGGCTTGTGAGTTCAAGC
CCCACGTTGGGCA
>Ailuropoda_melanoleuca_GL192850.1.trna41-LysCTT (1050973-1050901) Lys (CTT) 73 bp Sc: 41.51
GCCTGGCTGGCTCAGTCAGCAGAGTATGCGACTCTTGATCTCGGGGTTGTGAGTTCGAGC
CCCATGCTGGGTG
>Ailuropoda_melanoleuca_GL194842.1.trna6-LysCTT (32130-32059) Lys (CTT) 72 bp Sc: 41.52
ACCTGGCTGGCTCAGTCGGAAGAGCATGCAATTCTTGATTTTGGGTCATGAGTTCGAGTC
CCATGTCAGGTG
>Ailuropoda_melanoleuca_GL193618.1.trna4-LysCTT (40487-40559) Lys (CTT) 73 bp Sc: 41.56
ACCTGGCTGGCTCAGGCGGTGGAGTGTGCGACTCTTGATCTTGGGGTTGTGAGTTCGAGC
CCCACGTTGGGTG
>Ailuropoda_melanoleuca_GL192357.1.trna87-LysCTT (2533482-2533554) Lys (CTT) 73 bp Sc: 41.57
ACCTGGCTGGCTCAGTCAGTAGAGCCTGCGACTCTTGATCTTAGGGTTGTGAGTTCAAGC
CCCATGTTAGGTG
>Ailuropoda_melanoleuca_GL192699.1.trna21-LysCTT (368779-368850) Lys (CTT) 72 bp Sc: 41.64
ACCCGGCTGGCTCAGTTGAAGAGCATGAGATTCTTGATCTTGGGTTTGTGAGTTCGAGCC
CCACGTTGGGTG
>Ailuropoda_melanoleuca_GL192339.1.trna68-LysCTT (1451385-1451457) Lys (CTT) 73 bp Sc: 41.65
GCCTGGCTGGCTCAGTTGGCAGAGCATGTGATTCTTGATCTTGGGGTCATGAGTTCAAGC
TCCATGTTAGGTG
>Ailuropoda_melanoleuca_GL192979.1.trna60-LysCTT (764845-764773) Lys (CTT) 73 bp Sc: 41.73
GCCTAGCTGGCTCATTCGGAAGAGTATGTGATTCTTGTTCTCGGGGTCGTGAGTTCAAGC
CCCATGTTGGGTG
>Ailuropoda_melanoleuca_GL194142.1.trna18-LysCTT (100900-100830) Lys (CTT) 71 bp Sc: 41.80
ACCTGGTTGGCTCAGTTGGTATAGCACACGACTCTTGTTCTTGGGTGTGAGTTTGAGCCC
CACATTGGGTG
>Ailuropoda_melanoleuca_GL192366.1.trna70-LysCTT (1684798-1684870) Lys (CTT) 73 bp Sc: 41.91
ACCTGGCTAGCTCAGTCAGTAGAGCATGTGAGTCTTGATCTCAGGGTTATGAGTTCAAGC
CCCATGCTGGGTG
>Ailuropoda_melanoleuca_GL193082.1.trna73-LysCTT (346111-346039) Lys (CTT) 73 bp Sc: 41.93

ATCTGGCTGGCTCAGTTGGTAGAGCATGTAACTCTTAATTTCAGGGTCGTGAGTTCAAGC
TCCATGTTGGGTG
>Ailuropoda_melanoleuca_GL193362.1.trna15-LysCTT (471944-472016) Lys (CTT) 73 bp Sc: 41.94
ACCTGGCTAGCTCAGTCGGTGAAGCATGTGATTCTTGATCTTGGtgttgtGGGTTCGAGC
CCCACGTTGGGTG
>Ailuropoda_melanoleuca_GL192649.1.trna40-LysCTT (1627249-1627321) Lys (CTT) 73 bp Sc: 41.98
GCCTGGCTGGCTCAGTCGGTGGAGCGTGCAACTCTTGATTTCAGGGTTGTGAGTTCAAGC
CCCATGTTGGGTG
>Ailuropoda_melanoleuca_GL192344.1.trna115-LysCTT (4744983-4745055) Lys (CTT) 73 bp Sc: 42.05
GCCTGGCTGGCTCAGTCGATAGAGCATGTGACTCTTGATCTTGGAGTCGTGAGTTTGAGC
CCCACGTTGGGCA
>Ailuropoda_melanoleuca_GL192520.1.trna55-LysCTT (1481269-1481197) Lys (CTT) 73 bp Sc: 42.14
ACCTGGCTGGCTCAGTAGGTAGAGCATGTGACTCTTCATCTTGGGGCTGTGGGTTTGAGC
CCCACGTCGGGTA
>Ailuropoda_melanoleuca_GL192512.1.trna19-LysCTT (1076676-1076748) Lys (CTT) 73 bp Sc: 42.18
GCCTGGCTGGCTCAGTTGGAAGAGCATGAGACTCTTGATCTTGGGATGATGAGTTTGAGC
CCCATGTTGGGTG
>Ailuropoda_melanoleuca_GL193277.1.trna30-LysCTT (669644-669716) Lys (CTT) 73 bp Sc: 42.25
GCCTGGCTGGCTCAGTCGGAGGAGCATGTGACTCTTGATCTTGGGGTTATGAGTTCGAAC
CCCATGTTGGGTG
>Ailuropoda_melanoleuca_GL192573.1.trna40-LysCTT (710369-710441) Lys (CTT) 73 bp Sc: 42.27
ACCTGGCTGGCTCAGTTGGTGGAGCATGTGACTCTTGGTCTCAGGGTTGTGGGTTTGAGC
CCCACATTGGGTA
>Ailuropoda_melanoleuca_GL194413.1.trna12-LysCTT (35429-35357) Lys (CTT) 73 bp Sc: 42.28
ACCTGGCTGGCTCAGTTCGTAGAGCATGTGACTCTTAATTTCAGAGTTATGAGTTCAAGC
CTCATGTTGGGCG
>Ailuropoda_melanoleuca_GL193447.1.trna23-LysCTT (599880-599952) Lys (CTT) 73 bp Sc: 42.28
ACCTGGCTGGCTTAGTCTGTAGAGCATGAGACTCTTGATCTCGGAGTTGTGAGTTTGAGC
CCCACGTTGGGTG
>Ailuropoda_melanoleuca_GL192351.1.trna292-LysCTT (1260964-1260892) Lys (CTT) 73 bp Sc: 42.35
GCCCGGCTGGCTCAGTCGGGGGAGTGCGTGACTCTTGATCTCGGGGACGTGAGTTCAAGT
CCCATGTTGGGTG
>Ailuropoda_melanoleuca_GL192725.1.trna8-LysCTT (193465-193537) Lys (CTT) 73 bp Sc: 42.37
ACCTGGCTGGCTCAGTCAGAAGAGCATGTGATTCTTGATCTTGGGGTCGTGAGTTCAAGC
CCCACGTTGGGTA
>Ailuropoda_melanoleuca_GL194842.1.trna4-LysCTT (101158-101086) Lys (CTT) 73 bp Sc: 42.37
GCCTGGCTGGCTCAGTCGGTGGAGCATGTGACTCTTGATCTCGGGGTCGTGAGTTTGAGC
CCCATGTTGGGTG
>Ailuropoda_melanoleuca_GL192990.1.trna8-LysCTT (304001-304073) Lys (CTT) 73 bp Sc: 42.38
ACCTAGCTGGCTCATTTGGTGGAGCGCGTGACTCTTGATCTTGAGGTTATGGGTTCCAGC
CCCCTGCTGGGTG
>Ailuropoda_melanoleuca_GL193395.1.trna41-LysCTT (84828-84756) Lys (CTT) 73 bp Sc: 42.40
GCCTGGCTGGCTCAGTAGGAAGGGCATGTGACTCTTGATCTCAGGACTGTGAGTTCGAGC
CCCATGCTGGGTG
>Ailuropoda_melanoleuca_GL193591.1.trna33-LysCTT (571155-571083) Lys (CTT) 73 bp Sc: 42.44
ACCTGGCTGGCTCAGTTTGTAGAGCATGTGACTCTTGATCTTGTGGTCGTGAGTTTGAAC
CCCATGTTGGGTG
>Ailuropoda_melanoleuca_GL192457.1.trna186-LysCTT (1187061-1186989) Lys (CTT) 73 bp Sc: 42.46
GCCTGGCTGGCTCAGTCGGTGGAGTATGTGACTCTTGATCTCAGAGTCATGAGTGCAAGC
CCCATGTTGGGCA
>Ailuropoda_melanoleuca_GL194105.1.trna13-LysCTT (107197-107269) Lys (CTT) 73 bp Sc: 42.46
ACCTGGCTGGCTCAGTTGGAAAAGCACGTGACTCTTGATCTTGGGGCTGTGAGTTCAAAG
CCCACGTTGGGTA
>Ailuropoda_melanoleuca_GL192442.1.trna194-LysCTT (857649-857577) Lys (CTT) 73 bp Sc: 42.48
ACCTGGCTGGCTCAGTTGGTGGAGCGTGTGACTCTTCCTCTCAGAGTTGTGGGTTCAAGC
CCCATGTTAGATG
>Ailuropoda_melanoleuca_GL192385.1.trna136-LysCTT (2778710-2778782) Lys (CTT) 73 bp Sc: 42.48
ACCTGGCTGGCTCAGTCAGTAGAGCATGTGACTCTTGATCTCGAGGTTGTAGGTTTGAGC
CCCATGTTGGGTA
>Ailuropoda_melanoleuca_GL192554.1.trna14-LysCTT (319874-319945) Lys (CTT) 72 bp Sc: 42.51
GCCTGGCTAGCTCAGTCGGTAGAGCATGTGACTCTTGATCTTGAATTGTGGGTTTGAGCC
CCATGTTTGGTA
>Ailuropoda_melanoleuca_GL192825.1.trna26-LysCTT (384018-384090) Lys (CTT) 73 bp Sc: 42.52
GCCTGGTTGGCTCAGCTGGTGGAGCATGTGACTCTTGATCTCAGGTCTGTGGGCTCGAGC
CCCACACTGGGTG
>Ailuropoda_melanoleuca_GL192808.1.trna33-LysCTT (797472-797544) Lys (CTT) 73 bp Sc: 42.57
ACCTGGCTGGCTTAATGGGTAGAGCGTGTGACTCTTGATTTCAGGGCTGTGAGTTCAAGC

CCCATGTTGGGTG
>Ailuropoda_melanoleuca_GL193450.1.trna45-LysCTT (591641-591570) Lys (CTT) 72 bp Sc: 42.58
ACCTGGCTGGCTCAGTCGGTGGAGCATGTGACTCTTGATCTCGGGTTGTAGGTTCGAGCC
CCATGTTGGGTG
>Ailuropoda_melanoleuca_GL192440.1.trna94-LysCTT (2272321-2272392) Lys (CTT) 72 bp Sc: 42.65
ACCTGGCTGGCTCAGTTGAAGGAGCATGTGACTCTTGATCTCAGGGTATGGGTTCAAGCC
CCATGTTGGGTG
>Ailuropoda_melanoleuca_GL192774.1.trna14-LysCTT (326317-326389) Lys (CTT) 73 bp Sc: 42.66
ACCTGGCTGGCTCAGTCAGTGGAGCATGTGGCTCTTGATCTCGGGGTTGTGGGTTCGAGC
CCCACGTTGGGTG
>Ailuropoda_melanoleuca_GL192675.1.trna147-LysCTT (608799-608726) Lys (CTT) 74 bp Sc: 42.76
ATCTGGCTGGCTCAGTCGGTAGAGCAAGGGACTCTTGATCTCGGGGGTCGTGAGTTCAAG
CCTCACCTTGGGTA
>Ailuropoda_melanoleuca_GL192383.1.trna67-LysCTT (1771372-1771444) Lys (CTT) 73 bp Sc: 42.93
ACCTGGCTGGCTCAGTCAGTAGAGCATGTGACTCTTGATCTTGGGGTTGTGAGTTCGAGC
CCCATGTTGGGTG
>Ailuropoda_melanoleuca_GL192339.1.trna92-LysCTT (1894670-1894742) Lys (CTT) 73 bp Sc: 42.96
GCTTGGCTGGCTTAGTTGGTAGAGCATGTGGCTCTTAATCTCAGCGTCATGAGTTTGAGC
CCCATGTTGGGCA
>Ailuropoda_melanoleuca_GL192914.1.trna31-LysCTT (1088263-1088191) Lys (CTT) 73 bp Sc: 42.97
GCCTGGCTGGCTCAGCTGGAGGAGCATGTGACTCTTGATCTCAGGGTCGTGGGTTTGAGC
CCCACGTTGAGTG
>Ailuropoda_melanoleuca_GL192667.1.trna161-LysCTT (440072-440000) Lys (CTT) 73 bp Sc: 42.99
GCCTGGCTGGCTCAGTCGGTGGAGCATGGGACTCTTGATCTCAGGGTTGTGAGTTTGAGC
CCCATGCTGGGTG
>Ailuropoda_melanoleuca_GL192591.1.trna84-LysCTT (134217-134145) Lys (CTT) 73 bp Sc: 43.05
GCCTGGCTGGCTTAGTCGGAGGAGCGTGAGACTCTTGATCTTGGGGTTGTGGGTTTGAGC
CCCAGGTCAGGTG
>Ailuropoda_melanoleuca_GL192641.1.trna55-LysCTT (882434-882362) Lys (CTT) 73 bp Sc: 43.09
ACCTGGCTGGCTCAGTCAGTGGAGCATGCGACTCTTGATCTCAGGGTCGTGAGTTCAAGT
CCCATGTTGGGTG
>Ailuropoda_melanoleuca_GL192382.1.trna87-LysCTT (2804552-2804624) Lys (CTT) 73 bp Sc: 43.13
ACCTGGCTGGCTCAGTTGGTGGAGTGTATGACTCTTGATCTTGGGGTTGTGAGTTCCAGC
CTCACATTGGGTG
>Ailuropoda_melanoleuca_GL192526.1.trna27-LysCTT (1653588-1653660) Lys (CTT) 73 bp Sc: 43.14
GCCTGGCTGGCTCAGTTGGTGGAGCATGTGACTCTTGATCTCGGGCTTGTGAGTTCAAGC
CTCACATTGGGTG
>Ailuropoda_melanoleuca_GL192544.1.trna14-LysCTT (316910-316982) Lys (CTT) 73 bp Sc: 43.17
ACCTGGCTGGCTCAGTTGGTGGAGTGTGTGACTCTTGATTTTGGGGTTGTGGGTTCAAGC
CCCACATTGGGTG
>Ailuropoda_melanoleuca_GL193538.1.trna25-LysCTT (240695-240767) Lys (CTT) 73 bp Sc: 43.22
ACCTGGCTGGCTCAGTTGGTATAGCATGTGACTCTTGATCTCGGGGTTGTGAGCTCGAGT
CCCATGTTGGGTG
>Ailuropoda_melanoleuca_GL192656.1.trna69-LysCTT (429239-429167) Lys (CTT) 73 bp Sc: 43.26
GCCTGGCTGGCTCAGTCGATAGAGCATGTGATTCTTGATCTCAGGGGTGTGAGTTCAAGC
CCCACGTTGGGTG
>Ailuropoda_melanoleuca_GL192543.1.trna65-LysCTT (1529307-1529235) Lys (CTT) 73 bp Sc: 43.32
GCCCGGCTGGCTCAGTCCGAAGAGCATGCGACTCTTGATCTCAGGGCAGTGAGTTCGAGC
CCCACGTTGGGTG
>Ailuropoda_melanoleuca_GL192339.1.trna391-LysCTT (2214108-2214036) Lys (CTT) 73 bp Sc: 43.32
ACCTGGCTGGCTCAGTCTGTGGAGCATGTGACTCTTGATCTCGGGGTCGTGGGTTCAAGC
CCCATCTTAGGTG
>Ailuropoda_melanoleuca_GL192786.1.trna13-LysCTT (254679-254751) Lys (CTT) 73 bp Sc: 43.33
GCCTGGCTGGCTCATTGGGAAGAGCATGTGACTCTTGATCTCAGAATCATGAGTTCGAGC
CCCATGTTGGGTG
>Ailuropoda_melanoleuca_GL192513.1.trna89-LysCTT (165298-165226) Lys (CTT) 73 bp Sc: 43.34
GCCTGGCTGGCTCAATTGGTAGAGCATGTGACTCTTGATCTTGGGATCATGTGTTTGAGC
CCCATGTTGGGTG
>Ailuropoda_melanoleuca_GL192524.1.trna77-LysCTT (1333807-1333735) Lys (CTT) 73 bp Sc: 43.35
GCTTGGCTGGCTCAGTTGGAAGAGCATGTGACTCTTAATTTCAGGGTTATGAGTTCAAGC
CCCGTGTTGGGTG
>Ailuropoda_melanoleuca_GL192850.1.trna42-LysCTT (1042107-1042035) Lys (CTT) 73 bp Sc: 43.36
ACCTGGCTGGCTCAATCAGAAGAGCATGTGACTCTTGATCTTGGGGTCGTGAGTTCGAGC
CCCACGTTGGGTA
>Ailuropoda_melanoleuca_GL193176.1.trna29-LysCTT (786243-786315) Lys (CTT) 73 bp Sc: 43.36
GCCTGGCTGGCTCAGTTAGTAGAGCACGCGACTCTTGATCTTGGGGTCGTGAGTTTGAGC
CCCATGCTGGGTA

>Ailuropoda_melanoleuca_GL193450.1.trna5-LysCTT (74828-74900) Lys (CTT) 73 bp Sc: 43.37
ACCTGGCTGGCTCAGTTGGAAAAGCATGCGACTCTTGATCTCGAGGTTGTGAGTTTGAGC
CCCATGTTGGGTG
>Ailuropoda_melanoleuca_GL193054.1.trna61-LysCTT (562215-562143) Lys (CTT) 73 bp Sc: 43.41
ATCTGGCTGGCTCAGTTGGTAGAGCATGTGACTCTTGATCTCAGGGTAGTGAGTTTGAGC
CCCATGTTGGGTG
>Ailuropoda_melanoleuca_GL192366.1.trna190-LysCTT (1919994-1919922) Lys (CTT) 73 bp Sc: 43.47
ACCTGGCTGGCTCAGTCAGTAGAGCATGTGACTCTTGATCTCAGGGTTGTGAGTTTGAGC
CCCATGTTAGGTG
>Ailuropoda_melanoleuca_GL192395.1.trna7-LysCTT (637804-637876) Lys (CTT) 73 bp Sc: 43.47
ACTTGGCTGGCTCAGTTGGTAAAGCTTGTGACTCTTGATCTCAGGGTTGTGAGTTTGAGC
CCCACGTTGGGTT
>Ailuropoda_melanoleuca_GL194504.1.trna14-LysCTT (176199-176271) Lys (CTT) 73 bp Sc: 43.49
ACCTGGCTGGCTCAGTCAGTAGAGCATGTGACTCTTGATCTCAGAGTGGTGAGTTCGAGC
CCCATGTTGGGTA
>Ailuropoda_melanoleuca_GL192350.1.trna40-LysCTT (1281101-1281177) Lys (CTT) 77 bp Sc: 43.50
TCCTGGCTGGCTCAGTTGGTAGAGCCTGTGACTCTTCCTTCATCTCAGGGTTGTGGGTTT
GAGTCCCACGTTGGGTG
>Ailuropoda_melanoleuca_GL192363.1.trna111-LysCTT (3874617-3874545) Lys (CTT) 73 bp Sc: 43.55
ACCTGGCTGGCTCAGTCAGTAGAGCGTGAGACTCTTGATCTCGGGGTTGTGGGTTCAGGC
CCCATGTTGGCTG
>Ailuropoda_melanoleuca_GL192351.1.trna261-LysCTT (2200071-2199999) Lys (CTT) 73 bp Sc: 43.56
ACCTGGCTGGCTCAGTTGGTATAGCATGTGACTCTTGCTCTCAGGGTTGTGAGTTTGAGT
CTAATGCTGGGTG
>Ailuropoda_melanoleuca_GL192477.1.trna49-LysCTT (2403801-2403873) Lys (CTT) 73 bp Sc: 43.56
GCCTGGCTGGCTCAGTTGGTGGAGCATGCGATTCTTGATCTCAGGATTGTGGGTTCAAGC
TCCACATTGGGTA
>Ailuropoda_melanoleuca_GL192426.1.trna108-LysCTT (412587-412515) Lys (CTT) 73 bp Sc: 43.58
ACCTGGCTGGCTCAGTTGAAAGAGCATGCGGCTCTTGATCTCAGGGTTGTGTGTTCGAGC
CCCACGTTGGGTG
>Ailuropoda_melanoleuca_GL192371.1.trna192-LysCTT (1868001-1867929) Lys (CTT) 73 bp Sc: 43.68
GCCCGGCTGGCTCAGTTGGAAGAGCATGTGACTCTTGATCTTGGGGTTGTGAGCTCGAGC
CCCATGCTGGGTA
>Ailuropoda_melanoleuca_GL192359.1.trna18-LysCTT (886379-886451) Lys (CTT) 73 bp Sc: 43.73
GTCTGGCTGGCTCAGTCAGTATAGCATGTGACTCTTGATCTCAGGGTTGTGAGTTCAAGC
CCCACGTTGGATG
>Ailuropoda_melanoleuca_GL193481.1.trna19-LysCTT (362086-362158) Lys (CTT) 73 bp Sc: 43.74
ACCTGGCTGGCTCAGTCAGTGGAGCGTGTGACCCTTGTTCTCAGGGTTGTGGGTTCGAGC
CCCATGTTGGGTG
>Ailuropoda_melanoleuca_GL194210.1.trna11-LysCTT (286420-286348) Lys (CTT) 73 bp Sc: 43.74
GTCTGGCTGGCTCAGTTGGTAGAGCCTGCGATGCTTGATCTCGGGGTCGTGAGTTCAAGC
CTCACGTTGGGTG
>Ailuropoda_melanoleuca_GL192398.1.trna156-LysCTT (1892636-1892563) Lys (CTT) 74 bp Sc: 43.76
GCCCGGCTGGCTCAGTCAGTGGAGCGTGCGACTCTTGATCTCAGGGGTTGTGGGTTCGAG
CCCCACACTGGGTG
>Ailuropoda_melanoleuca_GL192814.1.trna23-LysCTT (1041361-1041433) Lys (CTT) 73 bp Sc: 43.77
GCCTGGCTGGCTCAGTCTGTGAAGCATGTGACTCTTGATCTCAGGGTTGTGGGTTCAAGT
CCCACATTGGGTA
>Ailuropoda_melanoleuca_GL193960.1.trna10-LysCTT (300066-300138) Lys (CTT) 73 bp Sc: 43.80
ACCCAGCTGGCTGAGTCAGAAAAGCATGAGACTCTTGATCTCAGGGTTGTGAGTTCAAGC
CCTATGTTGGGTA
>Ailuropoda_melanoleuca_GL192383.1.trna157-LysCTT (1430131-1430059) Lys (CTT) 73 bp Sc: 43.80
ACCTGGCTGGCTCAGTCAGAAGAGCATGCGACTCTTGATCTCGGGGTTGTGAGTTCAAGC
CCCACGTTGGGTG
>Ailuropoda_melanoleuca_GL193428.1.trna31-LysCTT (521121-521049) Lys (CTT) 73 bp Sc: 43.81
GCCTGGCTGGCTCAGTCCGTAGAGCATGTGACTCTTGATCCCGGGGTCATGAGCTCGATC
CCCATGTTGGGCC
>Ailuropoda_melanoleuca_GL192428.1.trna52-LysCTT (2449549-2449621) Lys (CTT) 73 bp Sc: 43.87
ACCTGGCTGGCTCAGTTGCTAGAGCATGTGACTCTTGATCTCAGGGTTGTGAGTTGGAGC
CCCATGTTGGGTA
>Ailuropoda_melanoleuca_GL192588.1.trna142-LysCTT (778700-778627) Lys (CTT) 74 bp Sc: 43.87
GCCTGGCTGGCTCAGTTGGTAAGAGCATGTGACTCTTGATCCCGGGGTTGTGAGTTTGAG
CCCCATGTTGGGTG
>Ailuropoda_melanoleuca_GL192398.1.trna13-LysCTT (251562-251634) Lys (CTT) 73 bp Sc: 43.87
GCCTGGCTGGCTCAGTTGGAAGAGCATGTGACTCTTGATCTTGGGGTCATGAGTTAGAGC
CCCATGTTGGGTG
>Ailuropoda_melanoleuca_GL193484.1.trna7-LysCTT (400282-400354) Lys (CTT) 73 bp Sc: 43.95

GCCTGGTTGGTTCATTTGGTACAGCATGTGACTCTTGATCTCAGGGTTGTGAGTTCAAGC
CCCACATTGGGTG
>Ailuropoda_melanoleuca_GL193839.1.trna5-LysCTT (443001-443073) Lys (CTT) 73 bp Sc: 43.98
TCCTGGCTGGCTCAGTCGGCAGAGCGGGAGACTCTTGATCTTGGGGTTGTGAGTTCCAGC
CCCATGTTGGGAG
>Ailuropoda_melanoleuca_GL192603.1.trna12-LysCTT (540131-540203) Lys (CTT) 73 bp Sc: 43.99
GCCCAGCTGGCTCAGTCAGTAGAGCCTGGGACTCTTGATCTTGGGGTTGTGGGTTTGAGC
CCCACGTTGGGTG
>Ailuropoda_melanoleuca_GL192494.1.trna90-LysCTT (1713464-1713536) Lys (CTT) 73 bp Sc: 44.01
ACCTGGCTGGCTTAGTCGGTAGAGCACATGATTCTTGATCTTGGGGTTGTAAGTTTGAGC
CCTGTGTTGGGTG
>Ailuropoda_melanoleuca_GL192969.1.trna81-LysCTT (421580-421508) Lys (CTT) 73 bp Sc: 44.05
ACCAGGCTGGCTCAGTCAGTAGAGTGTGCGACTCTTGATCTCAGGGCTGTGAGTTCAAGC
CCCATGTTTGGTA
>Ailuropoda_melanoleuca_GL192794.1.trna21-LysCTT (944158-944230) Lys (CTT) 73 bp Sc: 44.05
ACCTAACTGGCTCAGTCGGAAGAGCATGTGACTCTTAATCTTGGGGTCATGAGTTCAGGC
CCCATGTTGGGTG
>Ailuropoda_melanoleuca_GL192524.1.trna20-LysCTT (859577-859649) Lys (CTT) 73 bp Sc: 44.12
GCCTGGCTGGCTCAGTTGGTGGAGCGCGTGACTCTTGATTTTGGGGTTGTGGGTTCGAGT
GCCATGTTGGGTG
>Ailuropoda_melanoleuca_GL193724.1.trna3-LysCTT (43109-43181) Lys (CTT) 73 bp Sc: 44.13
GCCTGGCTGGCTCAGCTGGAAGAGCGTGTGACTCTTGATCTTGGGGTCATGAGTTTGAGC
CCCATGTTGGGTG
>Ailuropoda_melanoleuca_GL193098.1.trna16-LysCTT (664232-664304) Lys (CTT) 73 bp Sc: 44.17
ATCTGGCTGGCTCAGTCAGTAGAGCATGTGACTCTTGATCTTGGGGTCATGAGTTCGAGC
CTCACGTTGGATG
>Ailuropoda_melanoleuca_GL192566.1.trna58-LysCTT (1968799-1968727) Lys (CTT) 73 bp Sc: 44.19
GCCTGACTGGCTCAGTTGGTAGAGCATGAAAGTCTTGTTCTTGGGGTCATGAGTTCGAGC
CCCATGTTGGGTG
>Ailuropoda_melanoleuca_GL193473.1.trna2-LysCTT (29267-29339) Lys (CTT) 73 bp Sc: 44.22
GCCCAGCTGGCTCAGTCGGGGGAGCATGGGATTCTTGATCTCAGGGTGGTGAGTTCGAGC
CCCACGTTGGGTG
>Ailuropoda_melanoleuca_GL194620.1.trna6-LysCTT (99169-99241) Lys (CTT) 73 bp Sc: 44.24
GCCTGGCTGGCTCAGTCGTTAGAGCATGAAACTCTTGATCTCAGGGTCATGAGTTCGAGC
CCTATGTTGGGTC
>Ailuropoda_melanoleuca_GL192703.1.trna74-LysCTT (1270953-1271025) Lys (CTT) 73 bp Sc: 44.25
ACCTGGCTGGCTCAGTCGGCAGAGCATGCGACTCTTGATCTCGGGGTTGTGGGCTCAAGC
CTCACGTTGGGTG
>Ailuropoda_melanoleuca_GL192414.1.trna189-LysCTT (1600405-1600333) Lys (CTT) 73 bp Sc: 44.26
ACCTGGCTGGCTCAGTGGGAAGAGCGTGTGACTCTTGATCTCGGGCTTGTGGGTTTGAGC
CCCACATTGGGTG
>Ailuropoda_melanoleuca_GL193304.1.trna29-LysCTT (683701-683629) Lys (CTT) 73 bp Sc: 44.26
GCCTGGCTGGCTCAGTTGGTGGAGCAGGAGGCTCTTGATCTCAGGGTTGTGAGTTCAAGC
CCCACGTTGGGTG
>Ailuropoda_melanoleuca_GL193342.1.trna29-LysCTT (751188-751260) Lys (CTT) 73 bp Sc: 44.28
ACCTGGCTGGCTCAGTTGGTGGAGTATGTGACTCTTGATCTCAGGGTCATGGGTTTAAGC
CCCATGTTGGGCT
>Ailuropoda_melanoleuca_GL192863.1.trna65-LysCTT (399784-399712) Lys (CTT) 73 bp Sc: 44.38
ATCTGGCTAGCTCAGTTGGAAGAGCATGAGACTCTTGATTTCAGGGTTGTGAGTTTGAGA
CTCACTTTGGGTA
>Ailuropoda_melanoleuca_GL192736.1.trna5-LysCTT (47758-47830) Lys (CTT) 73 bp Sc: 44.39
GCCTGGCTGGCTCAGTTGGTGGAGTGTGCGACTCTTGGTCTCGGGATTGTGGGTTCAAGC
CCCATGTCAGGTG
>Ailuropoda_melanoleuca_GL193943.1.trna34-LysCTT (94899-94827) Lys (CTT) 73 bp Sc: 44.39
GCCTGACTGGCTCAGTCAGTAGAGCACGTGACTCTTGATCTTGGTGTCATGAGTTCAAAC
CCCGTGTCAGGTG
>Ailuropoda_melanoleuca_GL193608.1.trna38-LysCTT (463367-463295) Lys (CTT) 73 bp Sc: 44.42
ACCTGGCTGGCTCAGTTGGTGGAGCATGTGGCTCTTGATCTCAGGGTTGTAGGTTCGAGC
CCCATGTTGGGTG
>Ailuropoda_melanoleuca_GL192979.1.trna58-LysCTT (785404-785332) Lys (CTT) 73 bp Sc: 44.45
ACCTGGCTGGCTCAGTCAGTAGAGCGTGTGACTCTTGATCTCAGGGTTGTGAGTTTGAGC
CCCATGTTGGGTG
>Ailuropoda_melanoleuca_GL192924.1.trna116-LysCTT (582309-582237) Lys (CTT) 73 bp Sc: 44.45
GCCTGCCTGGTTCAGTTGGAGGAGTGTGTGACTCTTGATTTCAGGGTCGTGGGTTCAAGC
CCCACGTTGGGTG
>Ailuropoda_melanoleuca_GL192729.1.trna25-LysCTT (852304-852376) Lys (CTT) 73 bp Sc: 44.46
GCCTGGCTAGCTCAGTCAGTAGAGCATGTGACTCTTGGTCTCAGGGTTGTGAGTTCAAGC

CCCATGTTGGGCA
>Ailuropoda_melanoleuca_GL194507.1.trna7-LysCTT (179241-179169) Lys (CTT) 73 bp Sc: 44.46
GCCTGGCTGGCTCAGTTGGTAGAGCATGTGGCTCTTGATCTCAGGGTTGTGAGTTCAAGC
TCCATGTTGGGTG
>Ailuropoda_melanoleuca_GL192414.1.trna32-LysCTT (672795-672867) Lys (CTT) 73 bp Sc: 44.54
GCCTGGCTGGCTCAGTCGGTAGGGCCTGGGACTCTTGACCTTAGGGTCATGAGTTGGAGC
CTCATGTTGGGCA
>Ailuropoda_melanoleuca_GL192924.1.trna137-LysCTT (150089-150017) Lys (CTT) 73 bp Sc: 44.57
ACCCGGCTGGCTCGGTCAGAAGAGTGTGCGACTCTTGATCTCGGGGTTGTGGGTTCGAGC
CCCATGTTGGGTG
>Ailuropoda_melanoleuca_GL192344.1.trna56-LysCTT (3002257-3002329) Lys (CTT) 73 bp Sc: 44.61
GCCCGGCTGGCTCAGCTGGAAGAGCATGGGACTCTTCATCTTGCGGTCATGAGTTTGAGT
CCCATGCTGGGCA
>Ailuropoda_melanoleuca_GL192343.1.trna21-LysCTT (1497069-1497141) Lys (CTT) 73 bp Sc: 44.65
ACCTGGCTGGCTCAGTCGGTGGAGCATGCGACTCTTGATCTTGGGGTCATGAGTTCAAGC
CTCATGTTAGGCA
>Ailuropoda_melanoleuca_GL192395.1.trna17-LysCTT (1316912-1316984) Lys (CTT) 73 bp Sc: 44.66
ACCAGGCTGGTTCAGTTGGAAGAACATGAGACTCTTGATCTTAGAATCGTGGGTTCAGGC
CCCATGTTGGGTG
>Ailuropoda_melanoleuca_GL192660.1.trna50-LysCTT (1374775-1374847) Lys (CTT) 73 bp Sc: 44.67
ACCTGGCTGGCTTAGTGGGTAGGGCATGTGACTCTTGATCTCAGGGTTATGAGTTTGAAC
CTCATGGTGGGTG
>Ailuropoda_melanoleuca_GL192359.1.trna26-LysCTT (1038853-1038925) Lys (CTT) 73 bp Sc: 44.72
ACCCGGCTGGCTCAGTTGGTGGAGTGTGTGACTCTTGATCTCGGGGTTGTGAGTTTGAGC
CCCACGTTGGGTG
>Ailuropoda_melanoleuca_GL193281.1.trna8-LysCTT (664313-664386) Lys (CTT) 74 bp Sc: 44.73
GCCTGGCTGGCTCAGTCCGTAGAGCACGAGACTCTTGATCTCGGGGGTGGTGGGTTTGAG
CCCCATGTTGGGTG
>Ailuropoda_melanoleuca_GL192583.1.trna78-LysCTT (1546162-1546090) Lys (CTT) 73 bp Sc: 44.74
ACCCGGCTGGCTCAGTTGATAGAGCATGCGACTCTTGATCTTGGGGTCGTGAGTTCAAGC
CCCATGTTGGGTG
>Ailuropoda_melanoleuca_GL192342.1.trna202-LysCTT (1229985-1229913) Lys (CTT) 73 bp Sc: 44.75
GCCTGGCTGGCTCAGTCGGTGGAGCGTGTGACTCTTGATCTCAGGGTTGTGAGTTCTAGC
CCCATGTTGGGTG
>Ailuropoda_melanoleuca_GL194201.1.trna3-LysCTT (256907-256979) Lys (CTT) 73 bp Sc: 44.80
ACCTGGCTGGCTCAGTCAGTAGAGTGTGGGACTCTTGATCTCAGGGCTGTGAGTTCGAGC
CCCATGTTGGGTG
>Ailuropoda_melanoleuca_GL192699.1.trna103-LysCTT (1054695-1054623) Lys (CTT) 73 bp Sc: 44.80
GCTTGGCTGGCTCAGTCAGCAGAGCACGTGACTCTTGATCTTGGGGTTATGGGTTCGAGC
CCCATGTTGGGTG
>Ailuropoda_melanoleuca_GL193135.1.trna34-LysCTT (275676-275604) Lys (CTT) 73 bp Sc: 44.84
ACCTGGCTGGCTCAGTCAGTAGAGCATAAGGCTCTTGATCTCGGGGTCATGAGTTCAAGC
CCCATGCTAGGTG
>Ailuropoda_melanoleuca_GL194784.1.trna3-LysCTT (20952-21024) Lys (CTT) 73 bp Sc: 44.84
ACCTGGCTGGCTCAGTCAGAAGAGCATGTGGCTCTTGATCTCAGGGTCGTGAGTTCAAGC
CCCACGTTGGGTG
>Ailuropoda_melanoleuca_GL192392.1.trna109-LysCTT (3423524-3423596) Lys (CTT) 73 bp Sc: 44.87
ACCTGGCTGGCTCAGTCAGTAAAGCATGTGATTCTTGATCATTGGGTTGTGAGTTCAAGC
CTCACGCTGGGTG
>Ailuropoda_melanoleuca_GL194071.1.trna9-LysCTT (253054-252982) Lys (CTT) 73 bp Sc: 44.99
GCCTGGCTGGCTCAGTCAGTAGAGCATGAAACTCTTGATTTCAGGGTGGTGAGTTTGAGC
CCCACGTTGGGCA
>Ailuropoda_melanoleuca_GL193010.1.trna35-LysCTT (469579-469651) Lys (CTT) 73 bp Sc: 45.01
GCCTGGCTGGCTCAATCGGAAGAGCACGTGACTCTTGATCTTGGGGTCATGAGTTTGAGC
CCCATGTTGGGTG
>Ailuropoda_melanoleuca_GL194250.1.trna7-LysCTT (255900-255972) Lys (CTT) 73 bp Sc: 45.06
GCCTGGCTGGCTCAGTCGGTAGAGCATGTGACTCTTCATCTCAGGGTTGTGAGTTGAAGC
CCCATGCTGGGTG
>Ailuropoda_melanoleuca_GL192879.1.trna13-LysCTT (810793-810865) Lys (CTT) 73 bp Sc: 45.11
ACCTGGTTAGTTCAGTCTGTAGGGCATGAGACTCTTGATCTCAGGGTCATGAGTTCGAGC
CTCATGTTGGGCA
>Ailuropoda_melanoleuca_GL192659.1.trna15-LysCTT (287504-287576) Lys (CTT) 73 bp Sc: 45.13
ACCTGGCTGGCTCAGTCAGTAGAGCATGTGACTCTTGATCCCAGGTTTGTGAGTTCAAGT
CCCACGTTGGGTG
>Ailuropoda_melanoleuca_GL192460.1.trna51-LysCTT (942365-942437) Lys (CTT) 73 bp Sc: 45.20
GCCTGGCTGGCTCAGTGGGTATAGCATATGACTCTTGATCACAGCGTCATGAGTTCAAGT
CCTATGTTAGGCA

>Ailuropoda_melanoleuca_GL192357.1.trna385-LysCTT (969291-969219) Lys (CTT) 73 bp Sc: 45.25
ACCTGGCTGGCTCAGTTTGTGGAGCGTGTGACTCTTGGTCTCAGGGTTGTGGGTTCGATC
CCCACACTGGGTG
>Ailuropoda_melanoleuca_GL192858.1.trna71-LysCTT (726093-726021) Lys (CTT) 73 bp Sc: 45.26
ACCTGGCTGGCTCAGTTGTTGGAGCATGTGACTCTTGATCTCAGGGTTGTGAGTTCGAGC
CTCACGTTGGATG
>Ailuropoda_melanoleuca_GL194557.1.trna2-LysCTT (137892-137964) Lys (CTT) 73 bp Sc: 45.26
GCCCGGCTGGCTCAGTTGGTGGAGCGTGCGACTCTTGATCTCAGGGTTGTGAGTTTGAGC
CCCATGTTGGGTG
>Ailuropoda_melanoleuca_GL192830.1.trna13-LysCTT (439786-439858) Lys (CTT) 73 bp Sc: 45.28
ACCTAGCTGGCTCAGTCAGTACAGCACGTGACTCTTGATCTCGGGGTTGTGAGTTCAAGT
CCCATGTTGGGTA
>Ailuropoda_melanoleuca_GL192655.1.trna57-LysCTT (798877-798805) Lys (CTT) 73 bp Sc: 45.30
ACCTGACTGGCTCAGTCGGTACAGTGTGTGACTCTTAATCTTGGGATTGTGAGTTCGAGC
CCCATGTTGGGTG
>Ailuropoda_melanoleuca_GL193614.1.trna11-LysCTT (520412-520484) Lys (CTT) 73 bp Sc: 45.40
ACCTGGCTGGCTCATTTGGTAGAGCATGTGACTCTTGATCTTGGGGTTGTGAGTTCGAGC
CCCATGTTGGGTG
>Ailuropoda_melanoleuca_GL192618.1.trna44-LysCTT (972709-972781) Lys (CTT) 73 bp Sc: 45.42
GCCTGGCTGGCTCAGTCGGGACAGCATGCGACTCTTGATCTCAGAGTTGTGGGTTTGAGC
CCCACGTTGGGTG
>Ailuropoda_melanoleuca_GL193569.1.trna25-LysCTT (521844-521916) Lys (CTT) 73 bp Sc: 45.42
GCCTGGCTGGCTCAGTTGATAGAGCATGTGACTCTTGATCTTAGGGTCATGAGTTTAAGC
CCCATGTTGGGCA
>Ailuropoda_melanoleuca_GL193280.1.trna9-LysCTT (390226-390298) Lys (CTT) 73 bp Sc: 45.42
GCCTGGCTGGCTCAGTTGGAGGAGCATATGACTCTTGATCCTGGGGTCATGAGTTCAAGC
CCCATGTTGGGTG
>Ailuropoda_melanoleuca_GL194667.1.trna9-LysCTT (72988-72916) Lys (CTT) 73 bp Sc: 45.45
ACCCGGCTGGCTCAGTGGGTGAAGCGTGTGACTCTTGATCTCAGGGTTGTGAGTTCGAGG
CTCATGTTGGGTG
>Ailuropoda_melanoleuca_GL193343.1.trna16-LysCTT (443866-443939) Lys (CTT) 74 bp Sc: 45.45
GCCTGGCTGGCTCAGTCAGTAGAGTATGCGACTCTTGATCTCGCACGTTGTGAGTTCGAG
CCTCATGTTGGGTA
>Ailuropoda_melanoleuca_GL192885.1.trna2-LysCTT (353251-353323) Lys (CTT) 73 bp Sc: 45.46
ACCTGGCTGGCTCAGTTGGAAGAGCATGTGACTCTTGATCTTGGGGTCATGAGTTTGAGC
CCCATGTTGGGTA
>Ailuropoda_melanoleuca_GL192486.1.trna70-LysCTT (2275330-2275402) Lys (CTT) 73 bp Sc: 45.50
GCCTGGCTGGCTCAGTCTGTGGAGCATGGGATTCTTGATCTCAGGGTTGTGGGTTCGAGC
CCCATGTTGGGTG
>Ailuropoda_melanoleuca_GL195004.1.trna1-LysCTT (17232-17304) Lys (CTT) 73 bp Sc: 45.52
GCCTGGCTGGCTCAGTCGGTAGAGCAAGGGACTCTTGATCTCAGGTTTGTGAGTTCAAGC
CTCACATTGGGTA
>Ailuropoda_melanoleuca_GL192494.1.trna92-LysCTT (1724406-1724478) Lys (CTT) 73 bp Sc: 45.55
ACCTGGCTGGCTCAGTCGGTGGAGCATGTGGCTCTTGACCTCAGAGTTGTGAGTTCGAGC
CCCATGTTGGGTG
>Ailuropoda_melanoleuca_GL192615.1.trna62-LysCTT (1749995-1749922) Lys (CTT) 74 bp Sc: 45.56
GCCTGACTGGCTCAGTCGGGTAGAGCATGTGACTCTTGATCTTGGGGTTGTGAGTTCAAG
CCACATGTTGGGTG
>Ailuropoda_melanoleuca_GL192643.1.trna92-LysCTT (940687-940615) Lys (CTT) 73 bp Sc: 45.59
GCCTGGCTGGCTCAGTCAGTGGAGTGTGTGACTCTTGATCTCGGGGTTGTGGGTTCGAGC
CCCATGTTGGGTG
>Ailuropoda_melanoleuca_GL193928.1.trna12-LysCTT (157874-157946) Lys (CTT) 73 bp Sc: 45.71
ACCCGGCTGGCTCAGCTGGAAGAGCATATGACTCTTGATTGTGCAGTCATGAGTTCGAGC
CCCATGTTGGGTG
>Ailuropoda_melanoleuca_GL192460.1.trna5-LysCTT (65161-65233) Lys (CTT) 73 bp Sc: 45.77
ACCTGGCTGGTTCAGTGGGTGGAGTACGCGACTCTTGATCTTGGGGTTGTGGGTTCGAGC
CCCACGTTGGGTG
>Ailuropoda_melanoleuca_GL192900.1.trna68-LysCTT (671698-671626) Lys (CTT) 73 bp Sc: 45.84
GCCTGGCTGGCTCAGTCAGAAGAGCATGTGATTCTTGATCTCAGGGTTGTGAGTTCAAGC
CCCACGTTGGGTG
>Ailuropoda_melanoleuca_GL192369.1.trna38-LysCTT (964405-964477) Lys (CTT) 73 bp Sc: 45.84
GCCTGGCTGGCTCAGTCAGTGGAGCGTGTGACTCTTGATCTCGGGGTTGTGGGTTCAAGC
CCCAGGTTGGGCA
>Ailuropoda_melanoleuca_GL192436.1.trna58-LysCTT (1668715-1668643) Lys (CTT) 73 bp Sc: 45.87
GCCTGACTGGCTCAGTGGGTACAGCGTGTGGCTCTTGTTCTCGGGGTTGTGGGTTCAAGC
CCCATGTTGGGTG
>Ailuropoda_melanoleuca_GL192443.1.trna111-LysCTT (1145618-1145546) Lys (CTT) 73 bp Sc: 45.92

GCCTGGCTGGCTCAGTCGGTGGAACGTGTGACTCTTGATCTTGGGGTCATGAGTTCGAGC
CTCATGTTAGGTG
>Ailuropoda_melanoleuca_GL192588.1.trna105-LysCTT (1706755-1706683) Lys (CTT) 73 bp Sc: 45.95
GCCTGGCTGGCTCCATTGGTAAAGCATGTGACTCTTGATCTCGGGGTTGTGGGTTCAAGC
CCCATGTTGGGTG
>Ailuropoda_melanoleuca_GL194150.1.trna10-LysCTT (182538-182610) Lys (CTT) 73 bp Sc: 45.96
GCCTGGCTGGCTCAGTTGGTAGAGCATATCACTCTTGATCATGGCATTGTGAGTTCGAGC
CCCATGTTGGGTA
>Ailuropoda_melanoleuca_GL193246.1.trna62-LysCTT (412331-412259) Lys (CTT) 73 bp Sc: 45.97
ACCTGGCTGGCTCAGTCAGAAGAGCCTGTGACTCTTGATCTCGGGGTCATGAGTTCGAGC
CTCATGCTGGGGG
>Ailuropoda_melanoleuca_GL193109.1.trna39-LysCTT (78640-78568) Lys (CTT) 73 bp Sc: 45.98
GCCTGGCTGGCTTAGTCAGTAGAGCATGAGACTCTTGATCTCAGGGTTGTGAGTTTGAGC
CCCATGTTGGGTG
>Ailuropoda_melanoleuca_GL193800.1.trna8-LysCTT (300885-300957) Lys (CTT) 73 bp Sc: 45.98
ACCTGGCTGGCTCAGTTGGTGAAGCATGTGACTCTTGATCATAGGGTTGTGAGTTCAAGC
CCCATGTTGGGTG
>Ailuropoda_melanoleuca_GL192414.1.trna144-LysCTT (3018641-3018569) Lys (CTT) 73 bp Sc: 46.02
ACCTAGCTGGCTCAGTCTGTGGAGCGTGCGACTCTTGATCTCAGGGTTGTGAGTTCGAGC
CCCACGTTGGGTG
>Ailuropoda_melanoleuca_GL192825.1.trna89-LysCTT (462208-462136) Lys (CTT) 73 bp Sc: 46.05
GCCTGGCTGGCTGAGTTGGAAGAGCCTGCGACTCTTGATCTCAGGGCTGTGGGTTCAAGC
CCCATGTTGGGTG
>Ailuropoda_melanoleuca_GL192347.1.trna75-LysCTT (3612332-3612404) Lys (CTT) 73 bp Sc: 46.08
GCCTGGCTGGCTCAGTCAGTGGAGCGTGTGACTCTTGATCTCAGGGTTGTGAGTTCGAGT
CCCATGCTGGGTG
>Ailuropoda_melanoleuca_GL192460.1.trna116-LysCTT (1739959-1740031) Lys (CTT) 73 bp Sc: 46.10
GCCTGGCTGGCTCAGTTCATAGAGCGTGTGACTCTTGATCTCAGGGTTGTGAGTTCAAGC
CCCATGTTGGGCG
>Ailuropoda_melanoleuca_GL192765.1.trna51-LysCTT (1182926-1182998) Lys (CTT) 73 bp Sc: 46.12
ACCTGGCTGGCTCAGTCGGAAGAGCACGCGACTCTTGATCTCGGGGTTGTGAGTTCGGGC
CCCATGTTGGGTG
>Ailuropoda_melanoleuca_GL192500.1.trna4-LysCTT (47955-48027) Lys (CTT) 73 bp Sc: 46.13
ACCTGGTTGGCTCAGTCGGAAGAGCATGTGACTCTTGATCTTGAAGTCATGAGTTCAAGA
CCCATGCCGGGTG
>Ailuropoda_melanoleuca_GL193153.1.trna42-LysCTT (856588-856660) Lys (CTT) 73 bp Sc: 46.14
GCCTGGCTGGCTCAGTCGGTAGAGCATGAGACTCTTGATCCCAGGGTTGTAAGTTTGAGC
CTCATGTTGGGTG
>Ailuropoda_melanoleuca_GL192679.1.trna82-LysCTT (191384-191312) Lys (CTT) 73 bp Sc: 46.15
ACCCAACTGGTTCAGTGGGTGGAGCATGTGACTCTTGATCTCAGGGTTGTGGGTTCAAGC
CCCATGTTGGGCA
>Ailuropoda_melanoleuca_GL192588.1.trna137-LysCTT (995637-995565) Lys (CTT) 73 bp Sc: 46.18
GCCTTGCTGGCTCAGTTGGTGGAGTGTGTGACTCTTGATCTCGAGGTCGTGAGTTTGAGC
CTCATGTTGGGTG
>Ailuropoda_melanoleuca_GL193400.1.trna1-LysCTT (45813-45885) Lys (CTT) 73 bp Sc: 46.21
ACCTGGCCGGCTCAGTCGGAAGAGCATGCGACTCTTGATCTTGAGGTTGTGAGTTCAAGC
CTCACGTTGGGTG
>Ailuropoda_melanoleuca_GL194199.1.trna7-LysCTT (196108-196180) Lys (CTT) 73 bp Sc: 46.21
ACCTGGCCGGCTCAGTCGGAAGAGCATGCGACTCTTGATCTTGAGGTTGTGAGTTCAAGC
CTCACGTTGGGTG
>Ailuropoda_melanoleuca_GL194559.1.trna4-LysCTT (121116-121188) Lys (CTT) 73 bp Sc: 46.27
ACCTGACTGGTTCAGTTGGTAGAGCATGTGACTCTTGATCTCAGGGTTGTGGGTTCAGAC
CTCACATTAGGTG
>Ailuropoda_melanoleuca_GL192672.1.trna74-LysCTT (722268-722196) Lys (CTT) 73 bp Sc: 46.30
GCCTGGCTGGCTCAGTCAGTAGAGCATGGGACTCTTGATCTCGGGGTCATGAGTTGGAGC
CCCATGTTGGGCA
>Ailuropoda_melanoleuca_GL192494.1.trna8-LysCTT (242810-242881) Lys (CTT) 72 bp Sc: 46.31
GCCTGGCTGGCTCAGTTGGAGAGCATGTGACTCTTGATCTCGGGGTTACGAGTTCAAGCC
TCATGTTGGGCA
>Ailuropoda_melanoleuca_GL192569.1.trna3-LysCTT (104084-104156) Lys (CTT) 73 bp Sc: 46.34
GTCTGACTGGCTCAGTCAGTAGAGCCTGAGACTCTTAATCTCAGGGTTGTGAGTTCAAGC
CCCATGTTGGGTG
>Ailuropoda_melanoleuca_GL192492.1.trna116-LysCTT (1286353-1286281) Lys (CTT) 73 bp Sc: 46.40
GTCTGGCTGGCTCAGTCAGTAGAGAATGCGACTCTTGATCTCAGGGTCATGGGTTCAAGT
CCCATGCTGGGTG
>Ailuropoda_melanoleuca_GL192435.1.trna95-LysCTT (2385749-2385677) Lys (CTT) 73 bp Sc: 46.43
ACCCGGCTGGCTCAGTTGGTGGAGCATGCGACTCTTGATCTCAGGGTTGTGAGTTTGAGC

CCCACGTTGGGTG
>Ailuropoda_melanoleuca_GL192840.1.trna79-LysCTT (630261-630189) Lys (CTT) 73 bp Sc: 46.44
ACCTGGCTGGCTCAGTAGATAGAGCGTGTGACTCTTGATCTCAGGGTTGTGAGTTCAAGC
CCCGTGTTGGGTA
>Ailuropoda_melanoleuca_GL194634.1.trna4-LysCTT (109812-109740) Lys (CTT) 73 bp Sc: 46.51
GCCCAGCTGGCTCAGTGGGTGGAGCATGTGACTCTTGATCTCAGGGTTGTAGGTTCAAGA
CCCATGTTGGGTA
>Ailuropoda_melanoleuca_GL193266.1.trna37-LysCTT (102598-102526) Lys (CTT) 73 bp Sc: 46.62
GCCTGGCTGGCTCAGTCAGTAGAGCATGTGACTCTTGATCTCACGGTTGTGAGTTTAATC
CCCATGTTGGGCA
>Ailuropoda_melanoleuca_GL192676.1.trna52-LysCTT (1249544-1249616) Lys (CTT) 73 bp Sc: 46.62
GCCTGGTTGGCTCAGTTGGAAGAGTATCTGACTCTTGATCTCAAAGTCATGAGTTTGAGC
CCCATATTGGGTA
>Ailuropoda_melanoleuca_GL193867.1.trna17-LysCTT (35987-35915) Lys (CTT) 73 bp Sc: 46.66
ACCTGGCTGGCTCAGCTGGTAGAGCATGTGACTCTTGATCTCAGAATCATGAGTTTGAGC
CCCATGTTGGGTG
>Ailuropoda_melanoleuca_GL192635.1.trna65-LysCTT (1674950-1674878) Lys (CTT) 73 bp Sc: 46.67
GCCTGGCTGGCTCAGTCGGTAGAGCATGGGACACTTAATCTCAGGGTTGTGAGTTTGAGC
CTCATGTTGGGTG
>Ailuropoda_melanoleuca_GL192354.1.trna19-LysCTT (713402-713474) Lys (CTT) 73 bp Sc: 46.69
ACCTGGCTGGCTCAGTCCGTACAGCATGTGACTCTTAATCTCGGGGTTATGAGTTCGAGC
CTCATGTTGGGTG
>Ailuropoda_melanoleuca_GL192420.1.trna40-LysCTT (2409922-2409994) Lys (CTT) 73 bp Sc: 46.70
ACCTGACTGGCTCAGTCAGTAGAGCACGTGACTCTTGATCACAGGGTTGTGAGTTCAAGC
CCCATGTTGGGTG
>Ailuropoda_melanoleuca_GL192840.1.trna21-LysCTT (880142-880214) Lys (CTT) 73 bp Sc: 46.75
ACTTGGCTGGCTCAGTTGGTAGAGCATGTGACTCTTGATCTTGGGGTCATGAGTTTGAGC
CTCATGTTGGGTG
>Ailuropoda_melanoleuca_GL193162.1.trna13-LysCTT (783505-783577) Lys (CTT) 73 bp Sc: 46.80
TCCTGGCTGGCTCAGTCTGTAGAGCGTGTGACTCTTGATCTCGGGGTGGTGAGTTCAAGC
CTCACGTTGGGGA
>Ailuropoda_melanoleuca_GL192443.1.trna56-LysCTT (2577221-2577293) Lys (CTT) 73 bp Sc: 46.83
GCCTGGCTGGCTCAGTCGGTAGAGCACGGAACTCTTGATTTTGGGGTTGTGGGTTCGAGC
TCCATGTTGGGTG
>Ailuropoda_melanoleuca_GL192357.1.trna147-LysCTT (3965204-3965276) Lys (CTT) 73 bp Sc: 46.89
GCCTGGCTGGCTCAGTCAGTGGAGCGTGAGACTCTTTATCTCGGGGTTGTGGGTTCAAGC
CCCACGTTGGGTG
>Ailuropoda_melanoleuca_GL192351.1.trna174-LysCTT (4465023-4464951) Lys (CTT) 73 bp Sc: 46.97
GCCTGGCTGGCTCAGTCGGTAGAGCATGTGACTCTTGATCTCGGAGTTGTGAGGTCAAGC
CCCATGCTGGGCA
>Ailuropoda_melanoleuca_GL192863.1.trna2-LysCTT (74707-74779) Lys (CTT) 73 bp Sc: 47.00
GCCTGGCTAGCTCAGTCACTAGAGCATGAGACTCTTGATCTTGGGGTCATGAGTTCGAGC
CCCATGTTGGGCA
>Ailuropoda_melanoleuca_GL192466.1.trna44-LysCTT (1829587-1829659) Lys (CTT) 73 bp Sc: 47.01
ACCTGGCTGGCTCAGTCAGTAAAGCATGTGACTCTTGATCTCAGGGTCATGAGTTCGAGC
CTCATGTTGGGCT
>Ailuropoda_melanoleuca_GL192355.1.trna119-LysCTT (3470485-3470557) Lys (CTT) 73 bp Sc: 47.06
GCCTGGCTGGCTCAGTTGGGAGAGTGTGCGACTCTTGATCTTGGGGTCATGAGTTTGAGC
CCCATGTTGGGCA
>Ailuropoda_melanoleuca_GL192698.1.trna57-LysCTT (451564-451492) Lys (CTT) 73 bp Sc: 47.11
ACCTGGCTGGCTCAGTTGGAAGAGCATGTGACTCTTGATCTTGGGGTCGTGAGTTGGAGC
CCCACGTTGGGTG
>Ailuropoda_melanoleuca_GL192349.1.trna64-LysCTT (2080823-2080895) Lys (CTT) 73 bp Sc: 47.14
GCCTGGCTGGCTCAGTCAGTAGAGTGTGTGACTCTTGATCTTGGGGTTGTGAGTTCAACC
CCCACGTTGGGCA
>Ailuropoda_melanoleuca_GL192480.1.trna16-LysCTT (1186297-1186369) Lys (CTT) 73 bp Sc: 47.15
GCCTGGCTGGCTCAGTCTGTGGAGTGTGTGACTCTTGATCTCAGGGTCGTGAGTTCAAGC
CCCATGTTGGGCA
>Ailuropoda_melanoleuca_GL192385.1.trna197-LysCTT (1743138-1743066) Lys (CTT) 73 bp Sc: 47.15
ACCTGGCTGGCTCAGTCGGAAGAGCACGTGACTCTTGATCTTGGGGTTGTGAGTTTGAGC
CCCACGTTGGGTG
>Ailuropoda_melanoleuca_GL193800.1.trna14-LysCTT (460508-460580) Lys (CTT) 73 bp Sc: 47.15
GCCTGGCTGGCTCAGTTGGAAGAGCATGAGACTCTTGATCTTGGGATGGTGGGTTTGAGC
CCCACATTGGGTG
>Ailuropoda_melanoleuca_GL193846.1.trna2-LysCTT (110340-110412) Lys (CTT) 73 bp Sc: 47.19
ACCTGGCTAGCTCAGTCAGTAGAGCATGTGACTCTTGATCTCGGGGTTGTAGGTTCAAAC
CCCATGCTGGGTG

>Ailuropoda_melanoleuca_GL193254.1.trna20-LysCTT (475731-475803) Lys (CTT) 73 bp Sc: 47.19
GCCTGGCTGGCTCAGCTGGTGGAGCACGTGACTCTTGATCTTGGGGTTGTGAGTTCGAGC
CCCACGTTGGGTG
>Ailuropoda_melanoleuca_GL192414.1.trna191-LysCTT (1572897-1572825) Lys (CTT) 73 bp Sc: 47.20
GCCTGGCTGGCTCAGTCGATAGAACATGTGACTCTTGATCTCAGAGTCGTGGGTTCAAGC
CCCATGTTGGGTG
>Ailuropoda_melanoleuca_GL194755.1.trna2-LysCTT (79612-79684) Lys (CTT) 73 bp Sc: 47.23
GCCTGGCTGGCTCAGTCAGTGGAGCATGGGACTCTTGATCTCAGGGTCGTGAGTTCAAGC
CCCATGCTGGGCA
>Ailuropoda_melanoleuca_GL192563.1.trna199-LysCTT (674002-673930) Lys (CTT) 73 bp Sc: 47.23
GCCTGGCTGGCTCAGGTGGTGGAGCATGTGACTCTTCATCTCAGGGTTGTGGGTTCAAGC
CCCACATTGGGCT
>Ailuropoda_melanoleuca_GL192740.1.trna25-LysCTT (1198597-1198671) Lys (CTT) 75 bp Sc: 47.27
GCCTGGCTGGCTCAGTTGGTAGAGCGAGTGACTCTTGATCTCTCGGGATTGTGGGTTTGA
GCCCCATGTTGGGTG
>Ailuropoda_melanoleuca_GL192494.1.trna144-LysCTT (1725951-1725879) Lys (CTT) 73 bp Sc: 47.27
GCCTGGTTGGCTCAGTGGGTAGAGCATGTGGCTCTTGACCTCAGAGTTGTGAGTTCGAGC
CCCATGTTGGGTG
>Ailuropoda_melanoleuca_GL192760.1.trna49-LysCTT (878422-878350) Lys (CTT) 73 bp Sc: 47.28
GCCTGGCTGGCTCAGTCGGTAGAGCATGCGACTCTTGATCTCAGGGTTGTGAGTTAAAGC
CCCATGTTGGGTA
>Ailuropoda_melanoleuca_GL193039.1.trna35-LysCTT (978535-978607) Lys (CTT) 73 bp Sc: 47.33
GCCTGGCTGGCTCAGTCAGAAGAGCGTGCGACTCTTGATCTCAGGGTTGTGAGTTCGAGC
CCCATGTTGGGTG
>Ailuropoda_melanoleuca_GL193217.1.trna15-LysCTT (534167-534095) Lys (CTT) 73 bp Sc: 47.34
GCCTGGCTGGCTCAGTAGGCAGAGTATGTGACTCTTGATCTTGGGGTCATGAGTTCAAGC
CCCATGTTGGGCA
>Ailuropoda_melanoleuca_GL194272.1.trna7-LysCTT (286971-287043) Lys (CTT) 73 bp Sc: 47.37
ACCTGGCTGGCTCAGTTGGTAGAGCATGTGACTCTTGATCTTGGGGTTGTGAGTTTGAGC
CCCACGTTGGGTG
>Ailuropoda_melanoleuca_GL192460.1.trna109-LysCTT (1673300-1673372) Lys (CTT) 73 bp Sc: 47.39
GTCTGGCTGGCTTAGTCAGAAGAGCACGCGACCCTTGATCTTGAGGTCATGGGTTCAAGC
CCCATGCTGGATG
>Ailuropoda_melanoleuca_GL192439.1.trna120-LysCTT (1934545-1934473) Lys (CTT) 73 bp Sc: 47.42
GCCTGGCTGGCTCAATGGGTAGAGCCTGGGACTCTTGATCTCCGGGTTGTGAGTTCGAGC
CCCACGTTGGGTG
>Ailuropoda_melanoleuca_GL192338.1.trna242-LysCTT (2479982-2479910) Lys (CTT) 73 bp Sc: 47.47
ACTTGGCTGGCTCAGTTGGTAGAGCGTGTGACTCTTGATCTTGGGGTTGTGAGTTCAAGC
CCCATGTTAGGTG
>Ailuropoda_melanoleuca_GL192610.1.trna7-LysCTT (385612-385684) Lys (CTT) 73 bp Sc: 47.58
GTCTGGCTGGCTCAGTCAGTGGAGCATGAGACTCTTGATCTCAGGGTTGTGGGTTCGAGC
CCCACGTGGGGTG
>Ailuropoda_melanoleuca_GL192376.1.trna67-LysCTT (3524548-3524620) Lys (CTT) 73 bp Sc: 47.59
ACCTGGCTGGCTCAGTTGGAAGAGCACGTGACTCTTGATCTTGAGGTTGTGAGTTTGAGC
CCCATGTTGGGTG
>Ailuropoda_melanoleuca_GL193772.1.trna10-LysCTT (290367-290438) Lys (CTT) 72 bp Sc: 47.68
AGCTGGCTGGCTCAGTCGGAAGAGCATGAGACTCTTGATCTCGGGTCGTGAGTTTGAGCC
CCATGTTGGCTG
>Ailuropoda_melanoleuca_GL193096.1.trna52-LysCTT (852308-852236) Lys (CTT) 73 bp Sc: 47.69
ACCTGGCTGGCTCAGTTGGCAGAGCAGGAAACTCTTGGTCTCTGGGTCATGAGTTCGAGC
CCCATGTTGGGTG
>Ailuropoda_melanoleuca_GL192574.1.trna32-LysCTT (486256-486328) Lys (CTT) 73 bp Sc: 47.75
GCCTGGCTCGCTCAGTTGGTGGAGCGTGTGACTCTTGATCTTGGGGTCGTGAGTTCAAGC
CCCACGTTGGGCA
>Ailuropoda_melanoleuca_GL193612.1.trna50-LysCTT (152363-152291) Lys (CTT) 73 bp Sc: 47.76
ACCTGGCTGGCTCAGTCAGTAGAGCCTGAAACTCTTGATCTCAGGGTCATGCGTTCAAGC
CTCATGTTGGGTG
>Ailuropoda_melanoleuca_GL193622.1.trna59-LysCTT (52160-52087) Lys (CTT) 74 bp Sc: 47.76
GCCTGACTGGCTCAGTTGGTAGAGCAGGTGACTCTTAATCCTCAGGGTTGTGAGTTTGAG
CCCCACGTTGGGCA
>Ailuropoda_melanoleuca_GL192406.1.trna189-LysCTT (20210-20138) Lys (CTT) 73 bp Sc: 47.77
GCCTGGCTGGCTCAGTTGGCAGAGCATGTGACTCTTGATCTTGGGGTTGTGGGTTTGAGC
CCCACGTTGGGTT
>Ailuropoda_melanoleuca_GL193822.1.trna29-LysCTT (465419-465347) Lys (CTT) 73 bp Sc: 47.85
ACCTGGCTGGTTCAGTTGGAAAAGTGTGAGACTCTTGATCTCAGGATTGTGAGTTCAAGC
CCCATGTTGGGTG
>Ailuropoda_melanoleuca_GL193528.1.trna11-LysCTT (625910-625838) Lys (CTT) 73 bp Sc: 47.90

ACCTGGCTGGCTCAGTCAGTAGAGCATGTGACTCTTGATCTCAGGATTGTAGGTTCGAGC
CCCATGTTGGGTA
>Ailuropoda_melanoleuca_GL193438.1.trna64-LysCTT (53854-53782) Lys (CTT) 73 bp Sc: 47.92
GCCTGGCTGGCTCAGTAGGAAAAGCATGAGACTCTTGATCTGAGGGTTGTGAGTTCAAGC
CTCACATTGGGTG
>Ailuropoda_melanoleuca_GL193335.1.trna1-LysCTT (157429-157501) Lys (CTT) 73 bp Sc: 48.01
GCCTGGCTGGCTCAGTTGGAAGAGCATGCGACTCTTGATCTCAGGGTTGTGAGTTTGAGC
CCCATGTTAGGTG
>Ailuropoda_melanoleuca_GL192611.1.trna35-LysCTT (1645786-1645714) Lys (CTT) 73 bp Sc: 48.02
ACCCAGCTGGCTCAGTCGGTGAAGTGTGTGACTCTTGATCTCGGGGTTGTGAGTTCAAAC
CCCACGTTGGGTG
>Ailuropoda_melanoleuca_GL192924.1.trna64-LysCTT (927334-927406) Lys (CTT) 73 bp Sc: 48.05
ACCTGGCTGGCTCAGTCAGAAGAGCATGTGACTCTTGATCTCAGGGTTGTGAGTTCGAGC
CCCATGCTGGGTG
>Ailuropoda_melanoleuca_GL192439.1.trna125-LysCTT (1887110-1887038) Lys (CTT) 73 bp Sc: 48.26
GCCTGGCTGGCTTAGCAGGTAGAGTATGTGACTCTTAATCTCAGGGTTGTGAGTTCAAGC
CCCATGTTGGGTA
>Ailuropoda_melanoleuca_GL192348.1.trna399-LysCTT (570489-570417) Lys (CTT) 73 bp Sc: 48.28
ACCTGGTTGGCTCAGTGGGTGAAGCATGTGACTCTTAATCTCGGGGTCGTGAGTTCAAGC
CCCACATTGGGTA
>Ailuropoda_melanoleuca_GL192927.1.trna17-LysCTT (557177-557249) Lys (CTT) 73 bp Sc: 48.31
GCCTGACTGGCTCAGTTGGTAGAGCATGTGATTCTTGGTCTCAGAGTTGTGAGTTCAAGC
CCCATGTTGGGCA
>Ailuropoda_melanoleuca_GL193109.1.trna3-LysCTT (29484-29556) Lys (CTT) 73 bp Sc: 48.33
GCCCGGCTCGCTCAGTTGGTAGAGCATGTGACTCTTGATCTCAGGGTTGTGAGTTTGATC
CCCATGTTGGGTG
>Ailuropoda_melanoleuca_GL192509.1.trna42-LysCTT (1332864-1332936) Lys (CTT) 73 bp Sc: 48.34
ACCTGGCTGGCTCAGTTGGTAGAGCATGTGACTCTTGGTCTCAGGGTCGTGAGTTCAAGC
TCCACGTTGGGTG
>Ailuropoda_melanoleuca_GL192511.1.trna91-LysCTT (216047-215975) Lys (CTT) 73 bp Sc: 48.34
TCCTGGCTGGCTCAGTTGGTGGAGCGTGCGACTCTTGATCTTGGGGTTGTGGGTTCAAGC
CCCACGTTGGGTG
>Ailuropoda_melanoleuca_GL192338.1.trna103-LysCTT (3916944-3917016) Lys (CTT) 73 bp Sc: 48.37
GCCTGGCTGGCTCAGTCGGTGGAGCCTGAGACTCTTGATCTCGGGATGGTGAGTTCGAGC
CCCACGCTGGGTG
>Ailuropoda_melanoleuca_GL192811.1.trna13-LysCTT (1097257-1097329) Lys (CTT) 73 bp Sc: 48.39
GTCTGGCTGGCTCAGTCTGTAGAGCACATGACTCTTGATCTTGGGGTCATGAGTTCAAGC
CCCATGTCAGGTG
>Ailuropoda_melanoleuca_GL193088.1.trna56-LysCTT (568822-568750) Lys (CTT) 73 bp Sc: 48.48
ACCTGGCTGGCTCAGTTGGAAGAGCACATGACTCTTGATCTTGGGGCCATGAGTTTGAGC
CCCATGTTGGGTG
>Ailuropoda_melanoleuca_GL192626.1.trna126-LysCTT (1193659-1193587) Lys (CTT) 73 bp Sc: 48.50
ACCTGGCTGGCTCAGTCAGTAGAGTGTGTGACTCTTGATCTCGGGGTTGTGAGTTCGAGA
CCCACGTTGGGTG
>Ailuropoda_melanoleuca_GL193318.1.trna30-LysCTT (199198-199126) Lys (CTT) 73 bp Sc: 48.53
GCCTGGCTGGCTCAGTCTGTAGAGCATGCGACTCTTGATCTCAGGGTTGTGAGTTCGAGC
CCCATGTTGGGTG
>Ailuropoda_melanoleuca_GL192391.1.trna34-LysCTT (901521-901593) Lys (CTT) 73 bp Sc: 48.56
ACCTGGCTGGCTCAGTCAGTAGAGCATGTGACTCTTGATCTCAGGGTCATGAGTTCAAGC
CTCATGTTGGGCC
>Ailuropoda_melanoleuca_GL193413.1.trna55-LysCTT (25072-25000) Lys (CTT) 73 bp Sc: 48.59
GCCTGGCTGGCTCAGTTGGTGGAGCTTGTGACTCTTGATCTCGGGATTGTGAGTTCGAGC
CCCACGTTGGGTA
>Ailuropoda_melanoleuca_GL193422.1.trna43-LysCTT (270997-270926) Lys (CTT) 72 bp Sc: 48.64
ACCTGGCTGGCTCAGGTGGTGGAGCGTGTGGCTCTTAATCTCAGGGTGTGGGTTCGAGCC
CCATGTTGGGTG
>Ailuropoda_melanoleuca_GL192643.1.trna14-LysCTT (368194-368266) Lys (CTT) 73 bp Sc: 48.69
ACCTGGTTGGCTCAGTTGGCAGAGTATGAGACTCTTAATCTCGGGATTGTGTGTTCAAGC
CCCATGTTGGGTG
>Ailuropoda_melanoleuca_GL192983.1.trna35-LysCTT (674132-674060) Lys (CTT) 73 bp Sc: 48.72
ACCTGGCTGGCTCAGTCTGTAGAGCATGTGACTCTTGATCTCAGGGTCATGGGTTTGAGC
CCCACGTTGGGTA
>Ailuropoda_melanoleuca_GL193127.1.trna49-LysCTT (417506-417434) Lys (CTT) 73 bp Sc: 48.76
ACCTGGCTGGCTCAGTCAGTAAAGCACAAGACTCTTGATCTCGGGGTCGTGAGTTCAAGC
CCCATGCTGGGTG
>Ailuropoda_melanoleuca_GL192357.1.trna205-LysCTT (4358659-4358587) Lys (CTT) 73 bp Sc: 48.77
ACCTGGCTGGCTCAGTAGGAAGAGTGCGTGGCTCTTAATCTCGGGGTCATGAGTTCAAGC

CCCATGTTGGGTG
>Ailuropoda_melanoleuca_GL194256.1.trna20-LysCTT (149779-149707) Lys (CTT) 73 bp Sc: 48.78
GCCTGACTGGCTTAGTTAGTAGAGCATGTGACTCTTGATCCCAGGATCGTGAGTTCGAGC
CCCATGTTGGGTG
>Ailuropoda_melanoleuca_GL193810.1.trna6-LysCTT (89323-89395) Lys (CTT) 73 bp Sc: 48.83
ACCTGGCTGGCTCAGTTGGAAGAGCGTGTGACTCTTGATTTCAGGGTCATGAGTTTGAGC
CCCATGTTGGGTG
>Ailuropoda_melanoleuca_GL192489.1.trna31-LysCTT (1164673-1164745) Lys (CTT) 73 bp Sc: 48.87
ACCTGGCTGGCTCAGTCGATAGAGCATGTGACTCTTGATCTTAGGGTCATGAGTTCAAGT
CCCATGTTGGGTG
>Ailuropoda_melanoleuca_GL193845.1.trna34-LysCTT (310285-310213) Lys (CTT) 73 bp Sc: 48.89
GCCTGGCTGGCTCAGTAGGAAGAGCATGTGACTCTTGATCTCAGGGTTGTGAGTTCAAGC
CCCATGTTGGGTG
>Ailuropoda_melanoleuca_GL192348.1.trna22-LysCTT (564423-564495) Lys (CTT) 73 bp Sc: 48.89
GCCTGGCTGGCTCAGTCAGTAGAGCATGCGACTCTTGATCTCGGGGTCATGAGTTCGAGC
CCCATGTTGGGTG
>Ailuropoda_melanoleuca_GL193176.1.trna26-LysCTT (731428-731499) Lys (CTT) 72 bp Sc: 48.91
ACCTGGCTGGCTCAGTGGGAGAGCATGAAACTCTTGATCTTAGGGTTGTGAGTTCGAGCC
CCATGTTGGGTG
>Ailuropoda_melanoleuca_GL192543.1.trna36-LysCTT (1427119-1427191) Lys (CTT) 73 bp Sc: 48.98
GCCTGGCTGGCTCAGCTGGAAGAGCATGCGACTCTTTATCTCAGGGTCATGAGTTCGAGC
CCCATGTTGGGTG
>Ailuropoda_melanoleuca_GL192484.1.trna34-LysCTT (2028051-2028123) Lys (CTT) 73 bp Sc: 49.01
ACCCGGCTGGCTCAGTCAGTAGAGCATGAGACTCTTGATCTTGGGGTTGTGAGTTCGAGC
CCCATGTTGGGTG
>Ailuropoda_melanoleuca_GL194591.1.trna7-LysCTT (131271-131343) Lys (CTT) 73 bp Sc: 49.05
ACCTGGCTGGCTCAGTTGGTGGAGCGTGTGACTCTTGATCTTGGGGTCGTGAGTTCAAGC
CCCACGTTGGGTG
>Ailuropoda_melanoleuca_GL192545.1.trna23-LysCTT (804218-804290) Lys (CTT) 73 bp Sc: 49.07
ACCTGGCTGGCTCAGTGGGTAAAGCACGAGACTCTTGATCTTGGGGTTGTGAGTTTGAGC
CCCACGTTGGGTG
>Ailuropoda_melanoleuca_GL192367.1.trna24-LysCTT (744862-744934) Lys (CTT) 73 bp Sc: 49.09
ACCTGGCTGGCTCAGTCGGAGGAGCATGGGACTCTTGATCTCAGGGTCATGAGTTCGAGC
CCCATGTTGGGTG
>Ailuropoda_melanoleuca_GL193203.1.trna7-LysCTT (264527-264599) Lys (CTT) 73 bp Sc: 49.09
GCCTGGCTGGCTCAGTCAGTAGAGCATGCGACTCTTGATCTCGGGGTTGTGAGTTCGAGC
CCCACGTTAGGTG
>Ailuropoda_melanoleuca_GL192421.1.trna119-LysCTT (2843170-2843100) Lys (CTT) 71 bp Sc: 49.10
GCCGGGCTGGCTCAGTCGGTGGAGTGTGTGACTCTTGATCTCAGGTGTGGGTTCGAGCCC
CACGTTGGGTG
>Ailuropoda_melanoleuca_GL192419.1.trna77-LysCTT (2115863-2115792) Lys (CTT) 72 bp Sc: 49.10
ACCTGGCTGGCTCAGTTGGTAGAGCATGTGACTCTTGATCTTGGGTTGTGAGTTCGATCC
CCATGTTGGGTG
>Ailuropoda_melanoleuca_GL192893.1.trna91-LysCTT (208967-208895) Lys (CTT) 73 bp Sc: 49.18
GCCTGGCTGGCTCAGGTGGTAGAGCATGGAACTCTTGATCTTGAGACTGTGAGTTCAAGT
CTCACGCTGGGCA
>Ailuropoda_melanoleuca_GL193072.1.trna1-LysCTT (35175-35247) Lys (CTT) 73 bp Sc: 49.21
GCCTGGCTGGCTCACTTGGTAGAGCATGTGACTCTTGTTCTCAGGGTCGTGAGTTCAAGC
CCCATGTTGGGTG
>Ailuropoda_melanoleuca_GL192723.1.trna136-LysCTT (255653-255581) Lys (CTT) 73 bp Sc: 49.23
ACCTGGCTGGCTTAGTCGGTGGAGCATGTGACTCTTGATCTCAGGGTTGTGAGTTCAAGC
CCCACGTTGGGTG
>Ailuropoda_melanoleuca_GL192408.1.trna154-LysCTT (1140281-1140209) Lys (CTT) 73 bp Sc: 49.23
ACCTGGCTGGTTCAGTTGGAAGAGCATGTGACTCTTGATCTTGGGGTTGTGAGTTCAAGC
CTCACGCTGGGTG
>Ailuropoda_melanoleuca_GL192382.1.trna170-LysCTT (1826932-1826860) Lys (CTT) 73 bp Sc: 49.24
ACCCGGCTGGCTCAGTCTGTAGAGCATGAGACTCTTGATCTGAGGGTTGTGAGTTCAAGC
CCCATGTTGGGTG
>Ailuropoda_melanoleuca_GL192362.1.trna71-LysCTT (2729587-2729659) Lys (CTT) 73 bp Sc: 49.26
GCCTGGCTGGTTCAGTCGGTAAAGCATGGGATTCTTGGTCTCATGGTCGTGAGTTCAAGC
CCCACGCTGGGCA
>Ailuropoda_melanoleuca_GL193205.1.trna31-LysCTT (142251-142179) Lys (CTT) 73 bp Sc: 49.30
GCCTGGCTGGCTCAGTTGGTGGAGCATGCAACTCTTGATTTCAGGATTGTGGGTTCGAGC
CCCACGCTGGGTT
>Ailuropoda_melanoleuca_GL193435.1.trna18-LysCTT (502206-502134) Lys (CTT) 73 bp Sc: 49.30
ATCTGGCTGGCTCAGTTGGAAGAGCATGGGACTCTTGATCCAGGGGTCATGAGTTCAAGC
CCCATGTTAGGTG

>Ailuropoda_melanoleuca_GL192542.1.trna17-LysCTT (637308-637380) Lys (CTT) 73 bp Sc: 49.37
GCCTGGCTGGCTCAGTGAGTAGAGCATGTGACTCTTGATCTCAGGATTGTGAGTTCGAGC
CCCACGTTGGGTG
>Ailuropoda_melanoleuca_GL192750.1.trna89-LysCTT (1087021-1086949) Lys (CTT) 73 bp Sc: 49.39
ACCTGGCTGGCTCAGTCAGAAGAGCATGTGACTCTTGATCTCAGGGTCGTGAGTTCGAGC
CCCATGTTGGGTG
>Ailuropoda_melanoleuca_GL192633.1.trna4-LysCTT (35250-35322) Lys (CTT) 73 bp Sc: 49.43
ACCTGGCTGGCTCAGTCGGAGGAGTGTGCGACTCTTGATCTCAGAGTTGTGGGTTCAAAC
CCCATGTTGGGTA
>Ailuropoda_melanoleuca_GL193080.1.trna11-LysCTT (393652-393724) Lys (CTT) 73 bp Sc: 49.46
GCCTGGCTGGCTCAGTTGGTAGAGCATGCGACTCTTGATCTTGGGGCTGTGAGTTCAAGC
CTCATGCTGGGTG
>Ailuropoda_melanoleuca_GL192794.1.trna39-LysCTT (1307192-1307264) Lys (CTT) 73 bp Sc: 49.48
GTCTGGCTGGCTCAGTTGGAAGAGCATGTGACTCTTGATCTCAGGGTTGTGAGTTCGAGC
CCCATGTTGGGTG
>Ailuropoda_melanoleuca_GL193352.1.trna21-LysCTT (725327-725255) Lys (CTT) 73 bp Sc: 49.53
ACCCGGCTGGCTCAGTCAGAAGAGCATGTGACTCTTGATCTCAGGGTCATGAGTTCAAGC
CCCATGTTGGGTG
>Ailuropoda_melanoleuca_GL192708.1.trna81-LysCTT (1558727-1558655) Lys (CTT) 73 bp Sc: 49.59
ACCTGGCTGGCTCAGTCAGTAGAGCATGTGACTCTTGATCTCGGGGTCATGAGTTCAAGC
CCCATGCTGGGTG
>Ailuropoda_melanoleuca_GL192838.1.trna48-LysCTT (459152-459080) Lys (CTT) 73 bp Sc: 49.63
ACCTGGCTGGCTCAGTTGGTAGAGCTTGTGACTCTTGATCTCGGGGTCGTGAGTTCAAGC
CCTATGTTGGGTA
>Ailuropoda_melanoleuca_GL193258.1.trna13-LysCTT (654232-654304) Lys (CTT) 73 bp Sc: 49.64
GCCTGGCTGGCTCAGCTGGTAGAGCGTGTGACTCTTGATCTCGGGGTTGTGAGTTCCAGC
CCCACGTTGGGTG
>Ailuropoda_melanoleuca_GL193192.1.trna35-LysCTT (665148-665220) Lys (CTT) 73 bp Sc: 49.65
GCCTGGCTGGCTCAGTGGGAGGAGTGTGCGACTCTTGATCTCAGGGTTGTGGGTTCGAGC
CCCACATTGGGTG
>Ailuropoda_melanoleuca_GL193486.1.trna19-LysCTT (572228-572156) Lys (CTT) 73 bp Sc: 49.69
GCCTGGCTGGCTCAGTTGGTAGAGCATGCGACTCTTGATCTCGGGGTCATGAGTGCAATC
CCCATGTTGGGTA
>Ailuropoda_melanoleuca_GL192432.1.trna13-LysCTT (409955-410027) Lys (CTT) 73 bp Sc: 49.76
ACCTGGCTGGCTCAGTCGGAAGAGCACGAAACTCTTGATCTCGGGGTTGTGAGTTTGAGC
CCCACGTTGGGTG
>Ailuropoda_melanoleuca_GL193096.1.trna91-LysCTT (104690-104618) Lys (CTT) 73 bp Sc: 49.76
GCCTGGCTGGCTCAGTTGGTGGGGTGTGTGACTCTTGATCTCAGGGTCGTGAGTTTGAGC
CCCACGTTGGGCA
>Ailuropoda_melanoleuca_GL192557.1.trna6-LysCTT (219686-219758) Lys (CTT) 73 bp Sc: 49.77
GCCTGGCTGGTTCAGTTGGTGAAGCATGTGACTCTTGATCTCAAGATTATGGGTTCGAGC
CCCATGTTGGGTA
>Ailuropoda_melanoleuca_GL192695.1.trna47-LysCTT (1313816-1313888) Lys (CTT) 73 bp Sc: 49.80
GCCTGGCTGGCTCAGTTGGTGGAGCATGTGACTCTTGATCTCAGGGTCATGAGTTCAAGC
TCCATGTTGGGCA
>Ailuropoda_melanoleuca_GL193060.1.trna57-LysCTT (727680-727608) Lys (CTT) 73 bp Sc: 49.92
GCCTGGCTGGCTCAGTCGGTGGAGTGTGCGACTCTTGATCTCAGGGTTGTGGGTTCATGC
CCCACGCTGGGTG
>Ailuropoda_melanoleuca_GL193655.1.trna1-LysCTT (141267-141339) Lys (CTT) 73 bp Sc: 49.93
GCCTGGCTGGCTTAGTCGGTGGAGTGTGTGACTCTTAATCTCAGGGTTGTGGGTTTGAGC
CCCATGTTGGGTA
>Ailuropoda_melanoleuca_GL192354.1.trna251-LysCTT (2933921-2933843) Lys (CTT) 79 bp Sc: 49.98
GCCTGGCTGGCTCAGTCGGAAGAGTGTGCGACTCTTGATCTTGGGATCGGGGTTGTGGGT
TCAAGCCCCACGTTGGGTG
>Ailuropoda_melanoleuca_GL193216.1.trna42-LysCTT (155368-155296) Lys (CTT) 73 bp Sc: 50.02
GCCTGGCTGGCTCAGGTGGTGGAGCATGTGACTCTTGATCTCAGGGTTGTGAGTTCGAGC
CCCACGTTGGGTA
>Ailuropoda_melanoleuca_GL192371.1.trna185-LysCTT (1908628-1908556) Lys (CTT) 73 bp Sc: 50.06
ACCTGGCTGGCTCAGTCATTAGAGCATGTGACTCTTGATCTCAGGGTTGTGGGTTCAAGC
CCCACACTGGGTA
>Ailuropoda_melanoleuca_GL192359.1.trna2-LysCTT (73918-73990) Lys (CTT) 73 bp Sc: 50.08
GCCTAGCTGGCTCAGTTGAAAGAGTGTGTGACTCTTGATCTCAGAGTCATGAGTTCGAGT
CCCATGTTAGGTG
>Ailuropoda_melanoleuca_GL192391.1.trna222-LysCTT (2105871-2105799) Lys (CTT) 73 bp Sc: 50.08
TGCCGGCTGGCTCAGTCGGTGGAGCATGTGACTCTTGATCTCGGGGTCATGAGTTCAAGC
CCCATGTTGGCAG
>Ailuropoda_melanoleuca_GL192420.1.trna36-LysCTT (1848269-1848341) Lys (CTT) 73 bp Sc: 50.10

ACCTGGCTGGCTCAGTTGGAAGAGCGTGTGACTCTTGATCTCAGGGTTGTGAGTTTGAGC
CCCATGTTGGGTG
>Ailuropoda_melanoleuca_GL192533.1.trna54-LysCTT (2159100-2159028) Lys (CTT) 73 bp Sc: 50.23
GCCTGGCTGGCTCAGCTGGAAGAGCATGTGACTCTTGATCTCAGGGTCCTGAGTTCAAGC
TCCAGGTTGGGTG
>Ailuropoda_melanoleuca_GL192686.1.trna15-LysCTT (1123885-1123957) Lys (CTT) 73 bp Sc: 50.23
ACCTGGCTGGCTCAGTTGGTAGAGCATGTGACTCTTGATCTTGGGGTTGTGAGTTCAAGC
CCCATGTTGGGTA
>Ailuropoda_melanoleuca_GL192367.1.trna78-LysCTT (3575595-3575667) Lys (CTT) 73 bp Sc: 50.25
GCCTGGCTGGCTCAATCGGAAGGGCATGAGACTCTTGATCTCGGGGTCGTGAGTTTGATC
CCCACGTTGGGTG
>Ailuropoda_melanoleuca_GL192460.1.trna248-LysCTT (1396017-1395945) Lys (CTT) 73 bp Sc: 50.33
GCCTGGCTGGCTCAGTTGGTAGAGCATGCGACTCTTGATCTCAGGGTTGTAGGTTTGAGC
CCCATGTTGGGTA
>Ailuropoda_melanoleuca_GL193373.1.trna15-LysCTT (324298-324370) Lys (CTT) 73 bp Sc: 50.37
GCCTGGCTGGCTCAGTCTGTAGAGCATGTGACTCTTGATCTCGAGGTTGTGAGTTCAAGC
CCCATGCTGGGCT
>Ailuropoda_melanoleuca_GL194453.1.trna16-LysCTT (115660-115588) Lys (CTT) 73 bp Sc: 50.41
GCCTGGCTGGCTCAGTCGGTGGAGCATGTGACTCTTGATCTCGGGGTCGTGGGTTTGAGT
CCCATGTTGGGTG
>Ailuropoda_melanoleuca_GL193187.1.trna29-LysCTT (646313-646384) Lys (CTT) 72 bp Sc: 50.44
ACCTGGCTGGCTCAGTGGAAGAGCATGGGACTCTTGATCTCAGGGTTGTGGGTTCAAGCT
CCGTGTTGGGTA
>Ailuropoda_melanoleuca_GL192879.1.trna14-LysCTT (821917-821989) Lys (CTT) 73 bp Sc: 50.50
GCCTGGCTGGCTCAGTTGGAAGAGCATGTGACTCTTGATCTCGGGGTAGTGAGTTCGAGC
CCCATGTTGGGTA
>Ailuropoda_melanoleuca_GL192503.1.trna73-LysCTT (767262-767190) Lys (CTT) 73 bp Sc: 50.51
ACCTGGCTGGCTCACTTGGTAGAGCGTGTGACTCTTGATCTCAGGGTTGTGAGTTCAAGC
CCCATGTTGGGTG
>Ailuropoda_melanoleuca_GL193487.1.trna29-LysCTT (196860-196788) Lys (CTT) 73 bp Sc: 50.58
ACCTGGCTGGCTCAATTGGAAGAGCATGTGACTCTTGATCACAGGGTTGTGAGTTTGAGC
CCCATGTTGGGTG
>Ailuropoda_melanoleuca_GL193146.1.trna66-LysCTT (373402-373330) Lys (CTT) 73 bp Sc: 50.64
ACCTGGCTGGCTCAGTGGGTAGAGCATGTGACTCTTGATCTCGGGGTTGTGAGTTCAAGC
CCCATGTTGGGTG
>Ailuropoda_melanoleuca_GL192635.1.trna45-LysCTT (1573055-1573127) Lys (CTT) 73 bp Sc: 50.70
ACCTGGCTGGCTCAGTCAGTAGAGCATGCGACTCTTGATCTCAGGGTCATGAGTTCAAGC
CCCATGTTGGGTA
>Ailuropoda_melanoleuca_GL193192.1.trna16-LysCTT (444930-445002) Lys (CTT) 73 bp Sc: 50.70
GCCTGGCTGGCTCAGTGGGAAGGGCGTGTGACTCTTGATCTCAGGGTCGTGAGTTCAAGC
CCCATGTTGGGTG
>Ailuropoda_melanoleuca_GL193067.1.trna61-LysCTT (808622-808540) Lys (CTT) 83 bp Sc: 50.73
GCCTGGCTGGCTCAGTTGGAAGAGCATGAGGCTCTTGAACTCAGGGTCGTGAGTGGTCAT
GAGTTCGAGCCCCATGCTGGGTG
>Ailuropoda_melanoleuca_GL192640.1.trna73-LysCTT (1275660-1275588) Lys (CTT) 73 bp Sc: 50.73
GCCTGGCTGGCTCAGTTGGAAAAGCATGAGACTCTTGATCTTGGGGTTGTGAGTTCAAGC
CCCACGTTGGGTG
>Ailuropoda_melanoleuca_GL192942.1.trna5-LysCTT (87279-87351) Lys (CTT) 73 bp Sc: 50.74
GCCTGGCTGGCTCAGTCAGTAGAGCATGTGACTCTTGATCTCGGGGTCGTGAGTTGGAGC
CCCACGCTGGGCA
>Ailuropoda_melanoleuca_GL192765.1.trna75-LysCTT (1138700-1138628) Lys (CTT) 73 bp Sc: 50.75
GCCTGGCTAGCTCAGTCAGAAGAGTATGTGATTCTTGATCTCAAGGTCGTGAGTTCAAGC
CCCATGTTGGGCA
>Ailuropoda_melanoleuca_GL193691.1.trna3-LysCTT (368261-368333) Lys (CTT) 73 bp Sc: 50.76
ACCTGGCTGGCTTAGTTGGTAGAGCATGTGACTCTTGATCTTAGGGTTATGAGTTTGAGC
CTCATGTTGGGTA
>Ailuropoda_melanoleuca_GL193160.1.trna28-LysCTT (716959-717031) Lys (CTT) 73 bp Sc: 50.79
GCCTGGCTGGCTCAGCTGGTAGAGCGTGTGACTCTTCATCTCAGGGCTGTGAGTTCAAGC
CCCATGTTGGGCA
>Ailuropoda_melanoleuca_GL192870.1.trna121-LysCTT (259824-259752) Lys (CTT) 73 bp Sc: 50.81
GCCCAGCTGGCTTAGTTGGTGGAGTGTGTGACTCTTGATCTCGGGGCTGTGGGTTCAAAT
CCCACATTGGGTG
>Ailuropoda_melanoleuca_GL192567.1.trna95-LysCTT (1922394-1922466) Lys (CTT) 73 bp Sc: 50.82
GCCCGGCTGGTTCAGTCGGAAGAGCATGTGACTCTTGATCTCGGGGTCATGAGTTCGAGC
CCCATGTTGGGTG
>Ailuropoda_melanoleuca_GL193416.1.trna11-LysCTT (666403-666331) Lys (CTT) 73 bp Sc: 50.83
ACCTGGCTGGCTCAGTTGGAAGAGCATGTGACTCTTGATCTCAGGGTCATGAGTTTGAGC

CTCAGGTTGGGTG
>Ailuropoda_melanoleuca_GL192383.1.trna179-LysCTT (1128890-1128818) Lys (CTT) 73 bp Sc: 50.85
ACCTGGCTTGCTCAGTGGTTAGAGCGTGTGACTCTTGATCTCAGGGTTGTGAGTTCGAGC
TCCATGCTGGGTG
>Ailuropoda_melanoleuca_GL192766.1.trna4-LysCTT (98589-98661) Lys (CTT) 73 bp Sc: 50.86
GCCTGGCTGGCTCAGTCGGCAGAGCATGTGACTCTTGATCTCAGGGTTGTGAGTTTAAAC
CCCATGTTGGGCA
>Ailuropoda_melanoleuca_GL193010.1.trna27-LysCTT (393043-393115) Lys (CTT) 73 bp Sc: 50.91
ACCTGGCTGGCTCAGTCGGCAGAGTATCAGACTCTTGATCTCAGGGTTGTGAGTTTGAGT
CCCACGTCGGGTG
>Ailuropoda_melanoleuca_GL192914.1.trna47-LysCTT (393798-393726) Lys (CTT) 73 bp Sc: 50.93
GCCTGGCTGGCTCAGTCAGTAGAGCATGGGACTCTTGATCTCGGGGTTGTGAGTTCAAGC
CCCACGTTGGGCA
>Ailuropoda_melanoleuca_GL192381.1.trna146-LysCTT (2088110-2088038) Lys (CTT) 73 bp Sc: 50.96
ACCTGGCTGGCTCAGTTGGTAGAGCATGTGACTCTTGATCTCGGGGTTGTGTGTTCCAGC
CCCACGTTGGGTA
>Ailuropoda_melanoleuca_GL192392.1.trna39-LysCTT (1571634-1571706) Lys (CTT) 73 bp Sc: 51.03
ATCTGGCTAGTTCAGTCGGTAGAGCGTGTGACTCTTGATCTCAGAGTCGTGAGTTCGAGC
CCCACGCTGGGTG
>Ailuropoda_melanoleuca_GL192421.1.trna31-LysCTT (503122-503194) Lys (CTT) 73 bp Sc: 51.06
GCCTGGCTGGCTCAGTTGGTAGAGCACATGACTCTTGATCTTGGGGTTGTGAGTCCGAGC
CCCATGTTGGGTG
>Ailuropoda_melanoleuca_GL192457.1.trna33-LysCTT (396400-396472) Lys (CTT) 73 bp Sc: 51.06
GCCTGGCTGGCTCAGTTGGAAGAGCACGTGACTCTTGATCTTGGGGTTGTGGGTTCAAGC
CCTATGCTGGGTG
>Ailuropoda_melanoleuca_GL193214.1.trna34-LysCTT (778000-777928) Lys (CTT) 73 bp Sc: 51.11
GCCTGGCTGGCTCAGTCAGTAGAGCATGTGACTCTTGATCTCAGGGTCATGAGTTCAAGC
CCCATGTTGGGTG
>Ailuropoda_melanoleuca_GL192786.1.trna17-LysCTT (283485-283557) Lys (CTT) 73 bp Sc: 51.13
GCCTGGCTGGCTCAGTAGGTAGAGCATGGGACTCTTGATCTCAGCATCATGAGTTCAAGC
CCCATGTTGGGCA
>Ailuropoda_melanoleuca_GL193218.1.trna19-LysCTT (588098-588170) Lys (CTT) 73 bp Sc: 51.17
ACCTGGCTGGCTCAGTCAGAAGAGCATGGGACTCTTGATCTCAGGGTCGTGAGTTCGAGC
CCCACGTTGGGTG
>Ailuropoda_melanoleuca_GL192962.1.trna10-LysCTT (409233-409307) Lys (CTT) 75 bp Sc: 51.27
GCCCAGCTGGCTCAGGTGGTAGAGAGCATGTGACTCTTGATCTCAGGGTTGTGAGTTCAA
GCCCCATGTTGGGCA
>Ailuropoda_melanoleuca_GL193760.1.trna12-LysCTT (454838-454766) Lys (CTT) 73 bp Sc: 51.28
GCCTGGCTGGCTGAGTTGGTAGAGCATGGGACTCTTGCTTTCAAGGTCATGAGTTCGAGC
CTTATGTTGGGCA
>Ailuropoda_melanoleuca_GL192494.1.trna120-LysCTT (2237493-2237421) Lys (CTT) 73 bp Sc: 51.31
GCCTGGCTGGCTCAGTCAGCAGAGTATGGGACTCTTGATCTCAGGGTCATGAGTTCAATC
CCCATGCTGGGCG
>Ailuropoda_melanoleuca_GL192835.1.trna39-LysCTT (1241937-1241865) Lys (CTT) 73 bp Sc: 51.40
ACCTGGCTGGCTCATTCGGTAGAGTATGCGACTCTTGATCTCAGGGTTGTGAGTTCAAGC
CCCACGTCGGGTG
>Ailuropoda_melanoleuca_GL192414.1.trna193-LysCTT (1559593-1559521) Lys (CTT) 73 bp Sc: 51.40
GCCTGGCTGGCTCAGTAGGTAGAGCATGTGACTCTTGATCTCGGGGTTGTGAGTTCAAGC
CTCACATTGGGTG
>Ailuropoda_melanoleuca_GL193815.1.trna8-LysCTT (311850-311922) Lys (CTT) 73 bp Sc: 51.41
GCCTGGCTGGCTCAGTTGGTAGAGCATGTGACTCTTGATCTCAGGGCTGTGAGTTTGAGC
CCCATGTTGGGCA
>Ailuropoda_melanoleuca_GL192405.1.trna179-LysCTT (1503651-1503579) Lys (CTT) 73 bp Sc: 51.42
GCCTGGCTGGCTCAGTCAGTACAGCATGAGACTCTTGATCTCAGGGTCGTGAGTTCGAGC
CCCATGTTGGGTG
>Ailuropoda_melanoleuca_GL194557.1.trna4-LysCTT (137843-137771) Lys (CTT) 73 bp Sc: 51.45
GCCTGGCTGGCTCAGTTGGTGGAGCGTGCGACTCTTGATCTCAGGGTTGTGGGTTTGAGC
CCCACACTGGGTA
>Ailuropoda_melanoleuca_GL193385.1.trna15-LysCTT (449472-449543) Lys (CTT) 72 bp Sc: 51.46
GCCTGGCTGGCTCAGTGGTGGAGCGTGTGACTCTTGATCTCAGGGTTGCAGGTTCGAGCC
CTATGTTGGGTG
>Ailuropoda_melanoleuca_GL192405.1.trna162-LysCTT (1991269-1991197) Lys (CTT) 73 bp Sc: 51.49
GTGTGGCTGGCTCAGTGGATAGAGCGTGTGGCTCTTGATCTCAGGGTTATGGGTTCAAGC
CCCATGTTGGATA
>Ailuropoda_melanoleuca_GL192659.1.trna57-LysCTT (1251207-1251135) Lys (CTT) 73 bp Sc: 51.54
GCCTGGCTGGCTCAGTCGGGAGAGCGTCAGACTCTTGATCTCGGGGTAGTGAGTTCGAGG
CCCACGTTGGGCG

>Ailuropoda_melanoleuca_GL192747.1.trna37-LysCTT (314150-314078) Lys (CTT) 73 bp Sc: 51.64
GCCTGGCTGGCTCAGTTGGTAGAGTATATGGCTCTTGATCTTAGGGTCGTGAGTTCAGGC
CCCACGTTGGGCA
>Ailuropoda_melanoleuca_GL194181.1.trna5-LysCTT (49023-49095) Lys (CTT) 73 bp Sc: 51.65
ACCTATCTGGCTCAGTTGGTAGAGCGTGCAACTCTTGATCTCAGGGTTGTGGGTTTGAGC
CCCATGTTGGGTG
>Ailuropoda_melanoleuca_GL192426.1.trna18-LysCTT (396274-396346) Lys (CTT) 73 bp Sc: 51.70
ACCTGGCTGGCTCAGTTGGAAGAGCATGTGACTCTTGATCTCAGGGTCATGAGTTTGAGC
CCCATGTTGGGTG
>Ailuropoda_melanoleuca_GL192732.1.trna78-LysCTT (1075278-1075206) Lys (CTT) 73 bp Sc: 51.70
ACCTGGCTGGCTCAGTTGGTAGAGCTTGTGACTCTTAATCTCGGGGTTGTGGGTTCAGGC
CCCATGCTGGGCA
>Ailuropoda_melanoleuca_GL192904.1.trna42-LysCTT (1201335-1201407) Lys (CTT) 73 bp Sc: 51.73
GCCTGGCTGGCTCATTTGGGAGAGCATGAGACTCTTGATCTTGGGGTCATGCGTTCAAGC
CCCATGTTGGGCA
>Ailuropoda_melanoleuca_GL193361.1.trna72-LysCTT (35986-35914) Lys (CTT) 73 bp Sc: 51.82
ACCTGGCTGGCTCAGTCGGTAGAGCATGTGACTCTTGATCTCAGGGTTGTGAGTTAGAGT
CTCGTGTTGGGTG
>Ailuropoda_melanoleuca_GL193823.1.trna40-LysCTT (10136-10064) Lys (CTT) 73 bp Sc: 51.87
GCCTGGCTGGCTCAGTCAGTGGAGCATGAGACTCTTGATCTCAGGGTTGTGAGTTCAAGC
CCCACGTTGGGCA
>Ailuropoda_melanoleuca_GL192559.1.trna16-LysCTT (1237909-1237981) Lys (CTT) 73 bp Sc: 51.87
ACCTGGCTGGCTCAGTTGGTAGAGTATGCGACTCTTGATTTCAGGGTTGTAGGTTCAAGC
CCCACGTTGGGTG
>Ailuropoda_melanoleuca_GL192460.1.trna133-LysCTT (1884306-1884378) Lys (CTT) 73 bp Sc: 51.88
GCCTGGCTGGCTCAGTTGGTGGAGTGTGTGATTCTTGATCTCAGGGTCATGAGTTCAAGC
CCCATGTTGGGCT
>Ailuropoda_melanoleuca_GL193748.1.trna22-LysCTT (385883-385811) Lys (CTT) 73 bp Sc: 51.88
ACCTGGCTGGCTCAGTTGGTAGAGTATGTGACTCTTGATTTCATGGTTGTGAGTTCAAGC
CCCATGTTGGGTG
>Ailuropoda_melanoleuca_GL193862.1.trna5-LysCTT (68795-68866) Lys (CTT) 72 bp Sc: 51.90
ACCTGGCTGGCTCAATGGTAGGGCATGTGACTCTTGCTCTCAAGGTCATGAGTTCAAGCC
CCGTGTTGGGTG
>Ailuropoda_melanoleuca_GL192543.1.trna60-LysCTT (1750542-1750470) Lys (CTT) 73 bp Sc: 51.98
GCCTGGCTGGCTCAGTCGGTAGAGCATGGGACTCTTGATCTCAGGATTGTGAGTTTGAGC
CCCATGTTGGGCA
>Ailuropoda_melanoleuca_GL193204.1.trna6-LysCTT (54595-54667) Lys (CTT) 73 bp Sc: 52.06
GCCCGGCTAGCTCAGTCAGTAGAGCGTGAGACTCTTAACTTCAGGGTTGTGGGTTCAAGC
CCCATGTTGGGTG
>Ailuropoda_melanoleuca_GL194269.1.trna8-LysCTT (235028-234956) Lys (CTT) 73 bp Sc: 52.09
GCCTGGCTGGCTCAGTCAGTAGAGCATGTGACTCTTGATCTCAGGATCGTGAGTTCAAGC
CCCACGTTAGGTG
>Ailuropoda_melanoleuca_GL192388.1.trna109-LysCTT (2515192-2515120) Lys (CTT) 73 bp Sc: 52.09
GCCTGGCTGGCTCAGTGGGGAGAGTGTGTGACTCTTGATCTCAGGGTTGTGAGTTTGAGC
CCCACGTTGGGCA
>Ailuropoda_melanoleuca_GL192573.1.trna156-LysCTT (1341952-1341880) Lys (CTT) 73 bp Sc: 52.10
ACCTGGCTGGCTCAGTCGGTAGAGCATGTGATTCTTGATTTCAGGGTCGTGAGTTCAAGC
CCCATGTTGGGTG
>Ailuropoda_melanoleuca_GL192808.1.trna101-LysCTT (682706-682634) Lys (CTT) 73 bp Sc: 52.13
GCTTGGCTGGCTCAGTTGGTGGAGCATGTGACTCTTGATCTCAGGGTTGTGGGTTCGAGC
CCCATGTTGGGTG
>Ailuropoda_melanoleuca_GL192667.1.trna51-LysCTT (957777-957849) Lys (CTT) 73 bp Sc: 52.19
GCCTGGCTGGTTCAGCGGGTAGAGTATGAGACTCTTGATCTCAGGGTTGTGAGTTCGAGC
CCCATGCTGGGTG
>Ailuropoda_melanoleuca_GL194566.1.trna10-LysCTT (35459-35387) Lys (CTT) 73 bp Sc: 52.20
ACCTGGCTGGCTCAGTTGGTAGAGCATGTGATTCTTGATCTCAGGGTTGTGAGTTCGAGC
CCTACGTTGGGTG
>Ailuropoda_melanoleuca_GL193552.1.trna2-LysCTT (74457-74529) Lys (CTT) 73 bp Sc: 52.23
GCCTGGCTGGCTCAGTCGGAAGAGCGCGAGACTCTTGATCTCGGGGTGATGAGTTTGAGC
CCCATGTTGGGTG
>Ailuropoda_melanoleuca_GL192530.1.trna66-LysCTT (2096655-2096583) Lys (CTT) 73 bp Sc: 52.39
ATCTAGCTGGCTCAGTTGGTAAAGCATGTGACTCTTGATCTCAGGGTTGTGAGTTCAAGC
CCCATGTTGGATG
>Ailuropoda_melanoleuca_GL193085.1.trna23-LysCTT (886675-886747) Lys (CTT) 73 bp Sc: 52.44
ACCCGGCTGGCTCAGTTGGGAGAGTGTGTGACTCTTAATCTCAAGGTTGTGAGTTTGAGC
CCCATGTTGGGTG
>Ailuropoda_melanoleuca_GL193276.1.trna32-LysCTT (701726-701654) Lys (CTT) 73 bp Sc: 52.46

GCCTGGCTGGCTCAGTCGGAAGAGCATGTGACTCTTGATCTCAGGGTTGTGAGTTTGAGC
CCCACGCTAGGTG
>Ailuropoda_melanoleuca_GL192620.1.trna33-LysCTT (1258837-1258909) Lys (CTT) 73 bp Sc: 52.50
GCCTGGCTGGCTCAGTTGGTGGAGCATGTGACTCTTGATCTCAGAGTCATGAGTTCAAGC
CCCATGTTGGGCA
>Ailuropoda_melanoleuca_GL192571.1.trna13-LysCTT (699020-699092) Lys (CTT) 73 bp Sc: 52.55
ACCTGGCTGGCTCAGTCGGTAGAGCATGTGACTCTTGATCTCAGGGTTGTGAGCTCGAGC
CCCACGTTGGGTG
>Ailuropoda_melanoleuca_GL193159.1.trna11-LysCTT (107950-108022) Lys (CTT) 73 bp Sc: 52.61
GCCTGGCTGGCTCAGTCGGTAGAGCCTGTGACTCTTGATCTCAGGGCCATGAGTTCAAGC
CCCATGTTGGGTG
>Ailuropoda_melanoleuca_GL192385.1.trna28-LysCTT (799484-799556) Lys (CTT) 73 bp Sc: 52.61
GCCTGGCTGGCTTAGTTGGTAGAGCATGTGACTCTTGATCTCAGGATCGTGAGTTCCAGC
CCCACGTTGGGTG
>Ailuropoda_melanoleuca_GL192782.1.trna26-LysCTT (773274-773346) Lys (CTT) 73 bp Sc: 52.61
GGCTGGCTGGCTCAGTTGGTAGAGCACATGACTCTTGATCTTGGGGTTGTGGGTTCGAGC
TCCATGTTGGGTG
>Ailuropoda_melanoleuca_GL193622.1.trna54-LysCTT (134188-134116) Lys (CTT) 73 bp Sc: 52.61
GCCTGGCTGGCTCAGTTGGTAGAGCATGTGACTCTTGATCTTAGGGTTGTGAGTTCAAGC
CCCATGTTGGGTG
>Ailuropoda_melanoleuca_GL192362.1.trna87-LysCTT (3124175-3124247) Lys (CTT) 73 bp Sc: 52.70
ACCTGGCTGGCTCAGATGGCAGAGCATGCGACTCTTGATCTCAGGGCTGTGGGTTCGAGT
CCCATGTTGGGTG
>Ailuropoda_melanoleuca_GL192401.1.trna115-LysCTT (1293367-1293295) Lys (CTT) 73 bp Sc: 52.76
GCCTGGCTGGCTCAGTTGGTAGAGCATGTGACTCTTGATCTCAGGGTTGTGAGTTCAGGC
CCCATGTTGGGCA
>Ailuropoda_melanoleuca_GL192387.1.trna113-LysCTT (1698765-1698693) Lys (CTT) 73 bp Sc: 52.78
ACCTGGCTGGCTCAGTGGGAAGAGCATGTGACTCTTGATCTCAGGGTTGTGAGTTCAAGC
CCCACGTTGGGTG
>Ailuropoda_melanoleuca_GL193373.1.trna80-LysCTT (124873-124801) Lys (CTT) 73 bp Sc: 52.79
GCCTGACTGGCTCAGTCAGTAGAGCATGTGACTCTTGATCTCGGGGTCATGAGTTCAAGC
CCCATGTTGGGCA
>Ailuropoda_melanoleuca_GL192793.1.trna98-LysCTT (1177756-1177684) Lys (CTT) 73 bp Sc: 52.90
GCCTGGCTGGCTCAGTCCGGAGAGCGTGCGACTCTTGATCTCAGGGTTGTGGGTTCGAGC
CCCACACTGGGTG
>Ailuropoda_melanoleuca_GL193446.1.trna41-LysCTT (266999-266927) Lys (CTT) 73 bp Sc: 53.01
ACCTGGCTGGCTCAGTTGGTAGAGCATGCGACTCTTGATCTCAGGGTTGTGAGTTCGAGC
CCTACGTTGGGTG
>Ailuropoda_melanoleuca_GL192703.1.trna42-LysCTT (681786-681858) Lys (CTT) 73 bp Sc: 53.10
ACCTGGCTGGCTCAGTGAGTAGAGCATGTGACTCTTGATCTCAAGGTCATGAGTTCAAGC
CTCATGTTGGGCA
>Ailuropoda_melanoleuca_GL192822.1.trna73-LysCTT (1207665-1207593) Lys (CTT) 73 bp Sc: 53.15
GCCTGGCTGGCTCAGTTGGTAGAGCATGTGACTCTTGATCTCAGGTTTGTGAGTTTGAGC
CTCATGTTGGGCA
>Ailuropoda_melanoleuca_GL192343.1.trna165-LysCTT (575532-575460) Lys (CTT) 73 bp Sc: 53.19
GCCTGGCTGGCTTAGTCAGTAGAGCATGTGACTCTTGATCTCAAGGTTGTGAGTTCAAGC
CCCATGTTGGGCA
>Ailuropoda_melanoleuca_GL193697.1.trna21-LysCTT (8549-8477) Lys (CTT) 73 bp Sc: 53.19
GCCTGGCTGGCTTAGTCAGTAGAGCATGTGACTCTTGATCTCAAGGTTGTGAGTTCAAGC
CCCATGTTGGGCA
>Ailuropoda_melanoleuca_GL192348.1.trna297-LysCTT (2974688-2974616) Lys (CTT) 73 bp Sc: 53.29
ACCCGGTTGGCTCAGTCGGTAGAGCGTGTGACTCTTCATCTTGGGGTTGTGGGTTCGAGC
CCCACATTGGCTG
>Ailuropoda_melanoleuca_GL192479.1.trna30-LysCTT (577141-577213) Lys (CTT) 73 bp Sc: 53.30
ACCTGGCTGGCTCAGTTGGTAGAGCATGTGACTCTTGATCTCGGGGTTGTGAGTTCAAGC
CTCAAGTTGGGTG
>Ailuropoda_melanoleuca_GL192425.1.trna20-LysCTT (803540-803612) Lys (CTT) 73 bp Sc: 53.30
GCCTGGCTGGCTCAGTTGGTACAGCATAAGACTCTTGATCTCAGGGTCATGAGTTTGAGC
CCCATGTTGGGCA
>Ailuropoda_melanoleuca_GL192369.1.trna86-LysCTT (2163281-2163353) Lys (CTT) 73 bp Sc: 53.34
ACCTGGCTGGCTCAGTCGGTAGAGCATGTGACTCTTGATCTCAGGGTCATGAGTTCCAGC
CCCATGTTGGGTG
>Ailuropoda_melanoleuca_GL193697.1.trna20-LysCTT (21883-21811) Lys (CTT) 73 bp Sc: 53.35
GCCTGGCTGGCTCAATGAGTAGAGTGTGAGACTCTTGATCTCAGGGTTGTGAGTTCAAGT
CCCATGCCGGGTG
>Ailuropoda_melanoleuca_GL193445.1.trna12-LysCTT (584049-584121) Lys (CTT) 73 bp Sc: 53.42
GCCTGGCTGGCTCAGTGGGTGGAGCGTGTGACTCTTGATCTCAGGGTTGTGGGTTTGAGC

CCCACACTGGGCA
>Ailuropoda_melanoleuca_GL192649.1.trna29-LysCTT (1104603-1104675) Lys (CTT) 73 bp Sc: 53.44
GCCTGGCTGGCTCAGTTGGAAGAGCATGTGACTCTTGATCTCAGGGTTGTGAGTTCAAGC
CCCATGTTGGGTG
>Ailuropoda_melanoleuca_GL192372.1.trna218-LysCTT (2238575-2238502) Lys (CTT) 74 bp Sc: 53.47
GCCTGGCTGGCTCAGTCGGAAGAGCATGTGACTCTTGATCGCATGGGTTGTGAGTTCGAG
TCTCACACTGGGTG
>Ailuropoda_melanoleuca_GL194135.1.trna20-LysCTT (116059-115986) Lys (CTT) 74 bp Sc: 53.57
GCCTGGCTGGCTCAGTTGGTAGGGGTGTGTGACTCTTGATCTCAGGGTTGTGGGTTCAAG
CCCCACGTTGGGTG
>Ailuropoda_melanoleuca_GL192391.1.trna244-LysCTT (1672594-1672522) Lys (CTT) 73 bp Sc: 53.60
ACTTGGCTGGCTCAGTGGGAAGAGCACGTGACTCTTGATCTTGGGGTCATGGGTTCGAGC
CCCATGTTGGGTG
>Ailuropoda_melanoleuca_GL193279.1.trna16-LysCTT (707368-707440) Lys (CTT) 73 bp Sc: 53.62
ACCTGGCTGGCTCAGTTGGAAGAGCATGTGACTCTTGATCTCAAGGTCGTGAGTTTGAGC
CCCATGTTGGGTG
>Ailuropoda_melanoleuca_GL193790.1.trna30-LysCTT (110873-110801) Lys (CTT) 73 bp Sc: 53.69
GCCTGGCTGGCTCAGTTGGTGGGGTGTGCGACTCTTGATCTCAAGGTTGTGGGTTCAAGC
CCCACCCTGGGTG
>Ailuropoda_melanoleuca_GL193228.1.trna54-LysCTT (650694-650622) Lys (CTT) 73 bp Sc: 53.77
ACCTGGCTGGCTCAGTTGGTAGAGCATGCGACTCTTGATCTTAGGGTTATGAGTTCAAGT
CCCATGTTGGGTG
>Ailuropoda_melanoleuca_GL192498.1.trna66-LysCTT (2147659-2147587) Lys (CTT) 73 bp Sc: 53.81
GCCTGGCTGGTTCAGTGGGTAGAGCTTGCGACTCTTGATCTCAGGGTTGTGGGTTCAAGC
CCCATGTTGGGTG
>Ailuropoda_melanoleuca_GL192448.1.trna175-LysCTT (10718-10646) Lys (CTT) 73 bp Sc: 53.84
ACCTGGCTGGCTCAGTTGGTACAGCATGTGACTCTTAATCCCAAGGTCGTGAGTTTGAGC
CCCATGCTAGGTA
>Ailuropoda_melanoleuca_GL192933.1.trna3-LysCTT (135751-135823) Lys (CTT) 73 bp Sc: 53.88
GCCTGGCTGGCTCAGTCGGAAGAGTGTGTGACTCTTGATCTCAGGGTCATGGGTTCAAGC
CTCATATTGGGTG
>Ailuropoda_melanoleuca_GL193544.1.trna48-LysCTT (616855-616927) Lys (CTT) 73 bp Sc: 53.88
GCCTGGCTGGCTCAGTTGGTAGAGCATGGGACTCTTGATCTCAGGGTTGTGAGTTCAAGC
CCCGTGCTGGGTG
>Ailuropoda_melanoleuca_GL192392.1.trna201-LysCTT (1307805-1307733) Lys (CTT) 73 bp Sc: 54.02
ACCCGGCTGGCTCAGTCAGTAGAGCATGTGACTCTTGATCCCAAGGTCATGAGTTCAAGC
CCCATGTTGGGTG
>Ailuropoda_melanoleuca_GL193208.1.trna24-LysCTT (23969-23897) Lys (CTT) 73 bp Sc: 54.04
ACCTGGCTGGCTCAGTTGGCAGAGCATGTGACTCTTGATCTTGGGGTTGTGAGTTCAAGC
CTCACGTCGGGTA
>Ailuropoda_melanoleuca_GL192756.1.trna23-LysCTT (1101240-1101168) Lys (CTT) 73 bp Sc: 54.13
GCCTAGGTGGCTCAGTTGGTGGAGTATGTGACTCTTGATCTCAGGGTTGTGGGTTCAAGC
CCCACATTAGGTG
>Ailuropoda_melanoleuca_GL194076.1.trna45-LysCTT (116538-116466) Lys (CTT) 73 bp Sc: 54.18
GCCTGACTGGCTCAGCTGGTAGAGCACGTGACTCTTGATCTTGGGGTCATGAGTTCAAGC
CCCATGTTGGGTG
>Ailuropoda_melanoleuca_GL192765.1.trna48-LysCTT (1168930-1169002) Lys (CTT) 73 bp Sc: 54.28
GCTTGGCTGGCTCAGTTGGTAGAGCATGTGACTCTTGATCTCAGGGTCGTGAGTTCAAGC
CCCATGTTGGGTG
>Ailuropoda_melanoleuca_GL192700.1.trna92-LysCTT (876740-876668) Lys (CTT) 73 bp Sc: 54.30
GCCTGGCTGGCTCAGTCCGTGGAGCTTGAGACTCTTGATCTCAAGGTCGTGAGTTCAAGC
CCCATGTTGGGCA
>Ailuropoda_melanoleuca_GL192818.1.trna56-LysCTT (1017206-1017134) Lys (CTT) 73 bp Sc: 54.31
ACCTGGCTGGCTCAGTAGGTAGAGCGTGTGACTCTTGATCTTGGGGTTGTGGGTTCAAGC
CCCATGTTAGGTG
>Ailuropoda_melanoleuca_GL194092.1.trna6-LysCTT (98736-98808) Lys (CTT) 73 bp Sc: 54.38
ACCTGGCTGGCTCAGTCGGTAGAGCATGAGACTCTTGGTCTTAGGGTCATGAGTTCAAGT
CCCATGTTGGGTG
>Ailuropoda_melanoleuca_GL192494.1.trna47-LysCTT (737994-738066) Lys (CTT) 73 bp Sc: 54.39
GCCTGGCTGGCTCAGTTGGTAGAGCATGGGACTCTTGATCTTAGGGTTGTGAGTTCAAGC
CCCACGTTGGGTG
>Ailuropoda_melanoleuca_GL192942.1.trna42-LysCTT (1018293-1018222) Lys (CTT) 72 bp Sc: 54.40
ACCTGGCTGGCTCAGTGTTAGAGCATGTGACTCTTGATCTCAAGGTTGTGAGTTCAAGCC
CCATGCTGGGTA
>Ailuropoda_melanoleuca_GL193606.1.trna30-LysCTT (73843-73771) Lys (CTT) 73 bp Sc: 54.41
ACCTGGCTGGCTCAGTTGGTAGAGCATGTGACTCTTGATCTCGGGGTTGTGAGTTTGAAT
CTCACATTGGGTA

>Ailuropoda_melanoleuca_GL194164.1.trna20-LysCTT (214573-214501) Lys (CTT) 73 bp Sc: 54.41
ACCTGGCTGGCTCAGTTGGTAGAGCATGGGACTCTTGATCTCAGGGTTGTGAGTTCGAGC
CCCGTGTTGGGTG
>Ailuropoda_melanoleuca_GL192363.1.trna36-LysCTT (1212237-1212309) Lys (CTT) 73 bp Sc: 54.45
GCCTAGCTGGCTCAGTCGGTAAAGCATGTGACTCTTGGTCCCGGGGTCGTGAGTTCGAGC
CCCACGCTGGGCA
>Ailuropoda_melanoleuca_GL192444.1.trna5-LysCTT (146066-146136) Lys (CTT) 71 bp Sc: 54.56
ACCTGGCTGGCTCAGTCGGTAGAGCATGCGACTCTTGATCTCAGTCGTGGGTTCAAGCCC
CATGTTGGGTG
>Ailuropoda_melanoleuca_GL193226.1.trna32-LysCTT (473796-473724) Lys (CTT) 73 bp Sc: 54.62
GCCTGGCTGGCTCAGTCAGTAGAGCATGTGACTCTTGATCTCAGGGTCATAGGTTCAAGC
CCCATGTTGGGCG
>Ailuropoda_melanoleuca_GL194043.1.trna12-LysCTT (282315-282243) Lys (CTT) 73 bp Sc: 54.63
GCCTGGCTGGCTCAGTCGGTGGAGCATGTGATTCTTGATCACAGGGTTGTGAGTTCAAGC
CCCATGTTGGGCG
>Ailuropoda_melanoleuca_GL193538.1.trna39-LysCTT (302100-302172) Lys (CTT) 73 bp Sc: 54.63
ACCTGGCTGGCTCAGTTGGTAAAGCATGGGACTCTTGATCTCAGGGTTGTGAGTTCAAGC
CCCACGTTGGGTG
>Ailuropoda_melanoleuca_GL192414.1.trna66-LysCTT (1238336-1238408) Lys (CTT) 73 bp Sc: 54.66
ACCTGGCTGGCTCAGTTGGTAGAGCATGTGATTCTTGATCTCAGGGTTGTGAGTTCAAGC
CCCATGTTGGGTG
>Ailuropoda_melanoleuca_GL193656.1.trna25-LysCTT (107249-107177) Lys (CTT) 73 bp Sc: 54.70
GCTTGGCTGGCTCAGTCGGAAGAGCATGCGACTCTTGATCTCAGGGTCGTGAGTTCGAGC
CCCACGTTGGGTA
>Ailuropoda_melanoleuca_GL193583.1.trna16-LysCTT (314407-314479) Lys (CTT) 73 bp Sc: 54.77
ACCTGGCTGGCTCAGTAGGTAGAGCGCAAAACTCTTGATCTTGGGGTTGTGAGTTCAAGT
CCCATGTTGGGTG
>Ailuropoda_melanoleuca_GL192368.1.trna128-LysCTT (3122941-3123013) Lys (CTT) 73 bp Sc: 54.78
GCCTGGCTGGCTCAGTCGGTAGAGCATGTGACTCTTGATCTCAGGGTCGTGAGTTCACGC
CCCATGTTGGGCA
>Ailuropoda_melanoleuca_GL192385.1.trna173-LysCTT (2158225-2158153) Lys (CTT) 73 bp Sc: 55.03
GCCTGGCTGGCTCAGGGGATAGAGCATGTGACTCTTGATCTGAGGGTCATGAGTTCAAGC
CCCATGTTAGGCA
>Ailuropoda_melanoleuca_GL194015.1.trna12-LysCTT (195712-195784) Lys (CTT) 73 bp Sc: 55.08
ACCTGGCTGGCTCAGTCGGTAGAGCATGTGACTCTTGATCTCGGGGTCGTGAGTTCAAGC
CCCACGTTGGGCA
>Ailuropoda_melanoleuca_GL192873.1.trna63-LysCTT (421536-421464) Lys (CTT) 73 bp Sc: 55.09
GCCTGGTTGGCTCAGTTGGTAGAGCGTGTGACTCTTGGTCACACGGTCATGAGTTCAAGC
CCCATGTTGGGTG
>Ailuropoda_melanoleuca_GL192342.1.trna38-LysCTT (1280547-1280619) Lys (CTT) 73 bp Sc: 55.11
GCCTGGCTGGCTCAGTTGGAAGAGCATGTGACTCTTAATCTGGGGGTCGTGAGTTCGAGC
CTCATGTTGGGTG
>Ailuropoda_melanoleuca_GL192443.1.trna49-LysCTT (2533182-2533254) Lys (CTT) 73 bp Sc: 55.12
GCCTGGCTGGCTCAGTTGGAAGAGCATGTGACTCTTGATCTCAGtgttgtGAGTTCGAGC
CCCACGCTGGGTA
>Ailuropoda_melanoleuca_GL193312.1.trna4-LysCTT (127488-127560) Lys (CTT) 73 bp Sc: 55.13
GCCTACCTGGCTCAGTCGGTAGAGCATGTGACTCTTGATCTCAGGGTCATGAGTTCGAAC
CCCACGTTGGGTG
>Ailuropoda_melanoleuca_GL193288.1.trna4-LysCTT (255130-255202) Lys (CTT) 73 bp Sc: 55.14
GTCCGGCTGGCTCAGTCGGTAGAGCACGCGACTCTTGATCTCGGGGTTGTGAGTTCGAGC
CCCACGTTGGGTG
>Ailuropoda_melanoleuca_GL192936.1.trna44-LysCTT (942475-942403) Lys (CTT) 73 bp Sc: 55.17
ACCTGTCTGGCTCAGTCGGTAGAGCATGTGACTCTTGATCTCAGGGTTGTGAGTTCAAGC
CCCACAATGGGTG
>Ailuropoda_melanoleuca_GL192585.1.trna41-LysCTT (1909131-1909203) Lys (CTT) 73 bp Sc: 55.20
GCCTGGCTGGCTCAGTTGGTAGAGCATGCAACTCTTGATCTCAGGGTTGTGAGTTCGAGC
CCCACGCTGGGTG
>Ailuropoda_melanoleuca_GL192826.1.trna18-LysCTT (746977-747049) Lys (CTT) 73 bp Sc: 55.27
GCCCAGCTGGCTCAGTCAGAAGAGTGTGAGACTCTTGATCTCAGGGTCATGAGTTCAAGC
CCCATGTTGGGTG
>Ailuropoda_melanoleuca_GL193168.1.trna21-LysCTT (205807-205735) Lys (CTT) 73 bp Sc: 55.27
ACCTGTCTGGCTCAGTTGGTAGAGCATGAGACTCTTGATCTCAAGTTTGTGAGTTCAAGC
CCCACGTTGGGGG
>Ailuropoda_melanoleuca_GL194011.1.trna1-LysCTT (36196-36268) Lys (CTT) 73 bp Sc: 55.29
GCCTGGCTGGCTCAGTCGGAAGAGCATGAGACTCTTGATCTCGGGGTCATGAGTTCGAGC
CCCACGTTGGGTG
>Ailuropoda_melanoleuca_GL192369.1.trna287-LysCTT (666877-666805) Lys (CTT) 73 bp Sc: 55.37

GCCTGACTGGCTCAGTTGGTAGAGCATGCGACTCTTAATCTCAGGGTCATGAGTTCAAGC
TCCATGTTGGGCC
>Ailuropoda_melanoleuca_GL192401.1.trna96-LysCTT (1747190-1747118) Lys (CTT) 73 bp Sc: 55.42
ACCTGGCTGGCTCAGCTGGTAGAGCATGAGACTCTTGATCTCGGGGTTGTGGGTTCAGGC
CCCACACTGGGTA
>Ailuropoda_melanoleuca_GL192372.1.trna130-LysCTT (3646036-3646108) Lys (CTT) 73 bp Sc: 55.42
GCCTGGCTGGCTCAGTTGGTAGAGCATGTGACTCTTGATCTCAGGGTTGTGAGTTTAAAC
CCCATGTTGGGCA
>Ailuropoda_melanoleuca_GL192343.1.trna129-LysCTT (2873284-2873212) Lys (CTT) 73 bp Sc: 55.45
ACCCTGCTGGCTTAGTTGGTAAAGCGTGTGACTCTTAATCACAGGGTCTTGAGTTCAAGT
CCCACATTGGGTG
>Ailuropoda_melanoleuca_GL192998.1.trna15-LysCTT (835109-835181) Lys (CTT) 73 bp Sc: 55.46
ACCTGGCCGGCTCAGTTGGTAGAGCATGTGACTCTTGATCTCAGGGTCATGAGTTCAAGC
CTCATGTTGGGTG
>Ailuropoda_melanoleuca_GL192460.1.trna237-LysCTT (1486149-1486077) Lys (CTT) 73 bp Sc: 55.46
ACCTGGCTGGCTCAGTCAGTAGAGTGTGTGACTCTTGATCCCAGGGTTGTGGGTTCAAAC
CCCATGTTGGGTG
>Ailuropoda_melanoleuca_GL192818.1.trna9-LysCTT (357585-357657) Lys (CTT) 73 bp Sc: 55.48
ACCTGGCTGGCTCAGTTGGTAGAGCATGCGACTCTTGATCTCAGGGTTGTGAGTTCAAGC
CCCATGTTGGGTG
>Ailuropoda_melanoleuca_GL192604.1.trna58-LysCTT (1504157-1504229) Lys (CTT) 73 bp Sc: 55.57
GCCTGGCTGGTTCAGTCGGTAGAACATGTGACTCTTAATCTCAGGGTCATGAGTTCAAGC
CCCCTGTTAGGTG
>Ailuropoda_melanoleuca_GL192437.1.trna21-LysCTT (342651-342723) Lys (CTT) 73 bp Sc: 55.59
GCCTGACTGGCTCAATTGGTAGAGCATGCGACTCTTGATCTCAGGGTCATGAGTTTGAGC
CCCATGTTGGGCA
>Ailuropoda_melanoleuca_GL193096.1.trna7-LysCTT (151382-151454) Lys (CTT) 73 bp Sc: 55.69
ACCTCGCTGGCTCAGTCGGTAGAGCATGTGACTCTTGATCTCAGGGTTGTGAGTTCAAGC
CCCACGCTGGGTG
>Ailuropoda_melanoleuca_GL192516.1.trna87-LysCTT (614566-614494) Lys (CTT) 73 bp Sc: 55.69
GCCTGGCTGGCTCAGTTGGTAGAGCATGAGACTCTTGATCTCAGGGTCATGAGTTCAAGG
CCCACATTGGGCA
>Ailuropoda_melanoleuca_GL192475.1.trna75-LysCTT (2099032-2099104) Lys (CTT) 73 bp Sc: 55.70
GCCCGGCTGGCTCAGTTGGAAGAATGTGTGACTCTTGATCTCGGGGTCGTGAGTTCGAGC
CCCATGCTGGGCA
>Ailuropoda_melanoleuca_GL193084.1.trna14-LysCTT (191416-191486) Lys (CTT) 71 bp Sc: 55.74
GCCTGGCTGGCTCAGTCGGTAGAGCACGGGACTCTTGATCTCGGGGTCGTGGGTTCAAGC
CACGCTGGGCA
>Ailuropoda_melanoleuca_GL192470.1.trna25-LysCTT (1942908-1942980) Lys (CTT) 73 bp Sc: 55.75
ATCTGGCTGGCTCAGTTGGTAGAGCATGTGACTCTTGATCTCAGGGTTGTGAGTTCAAGT
CTCATGTTGGGTG
>Ailuropoda_melanoleuca_GL193563.1.trna49-LysCTT (369872-369800) Lys (CTT) 73 bp Sc: 55.84
ACCTGGCTGGCTCAGTTGGTAGAGCATGCGACTCTTGATCTCGGGGTCATGAGTTCAAGC
CCCATGTTGGGTG
>Ailuropoda_melanoleuca_GL192640.1.trna4-LysCTT (82469-82541) Lys (CTT) 73 bp Sc: 55.87
GCCTGGCTGGCTCAGCTGGTAGAGCACGTGACTCTTGATCTCGGCGTTGTGAGTTCAAGC
CCCATGCCGGGCA
>Ailuropoda_melanoleuca_GL193386.1.trna19-LysCTT (285511-285439) Lys (CTT) 73 bp Sc: 55.88
GCCTGGCTGGCTCAGTCGGTAAAGCATGTGACTCTTGATCTCAGGGTCGTGAGTTCGAAC
CCCATGCTGGGAG
>Ailuropoda_melanoleuca_GL193852.1.trna29-LysCTT (153152-153080) Lys (CTT) 73 bp Sc: 55.89
GCCTGGCTGGCTCAGTCGGTAGAGCATGTGACTCTTGATCTCAGGGTCATGAGTTCAAGC
CCTATGTTGGGCG
>Ailuropoda_melanoleuca_GL192663.1.trna45-LysCTT (1045473-1045401) Lys (CTT) 73 bp Sc: 55.92
GCCTGGCTGGCTCAGTCGGTAAAGTATGTGACTCTTGATCTCAAGGTCATGAGTTCAAGG
CCCATGTTGGGCA
>Ailuropoda_melanoleuca_GL192612.1.trna23-LysCTT (1281359-1281431) Lys (CTT) 73 bp Sc: 55.98
ACCTGGCTGGCTCAGTCGGTAGAGCATGCGACTCTTGATCTCAAGTTTGTGAGTTCGAGC
CCCACGTTGGGTG
>Ailuropoda_melanoleuca_GL193217.1.trna1-LysCTT (14345-14417) Lys (CTT) 73 bp Sc: 55.98
ACCTGGCTGGCTCAGTTGGTAGAGCGTGCAACTCTTGATCTCAAGGTCGTGAGTTCAAGC
CCCACGTTGGGCA
>Ailuropoda_melanoleuca_GL193037.1.trna41-LysCTT (601394-601322) Lys (CTT) 73 bp Sc: 55.99
GCCTGGCTGGCTCAGTCAGTAGAGCATGTGACTCTTAATCTCAGGGTCGTGAGTTCAAGC
CCCACACTGGGCG
>Ailuropoda_melanoleuca_GL193764.1.trna16-LysCTT (422614-422542) Lys (CTT) 73 bp Sc: 56.00
ACCTGGCTAGCTCAGTCGGAAGAGCATGAGACTCTTGATCTCGAGGTTGTGAGTTTGATC

CCCACGTTGGGTG
>Ailuropoda_melanoleuca_GL192703.1.trna75-LysCTT (1283588-1283660) Lys (CTT) 73 bp Sc: 56.03
GCTTGGCTGGCTCAGTCGGTAGAGCATGTGTCTCTTGATCTCAGGGTTGTGGGTTCAAGA
CCCACACTGGGCA
>Ailuropoda_melanoleuca_GL192431.1.trna69-LysCTT (2216527-2216455) Lys (CTT) 73 bp Sc: 56.12
GCCTGGCTGGCTCAGATGGAAGAGCATGTGACTCTTGATCCCAGGGTTGTGAGTTCAAGT
CTCACGTTGGGTA
>Ailuropoda_melanoleuca_GL192354.1.trna6-LysCTT (82180-82252) Lys (CTT) 73 bp Sc: 56.42
GCCTGGCTGGCTCAGTCGGTGGAGCATGGGACTCTTGATCCCACGGTCATGAGTTCAAGC
CTCCCGCTGGGCA
>Ailuropoda_melanoleuca_GL192573.1.trna15-LysCTT (262111-262183) Lys (CTT) 73 bp Sc: 56.44
ACCTGGCTGGCTCAGTTGGTAAAGCGTGTGACTCTTGATCTCAGGGTTGTAGGTTCGAGC
CCCATGTTGGGTA
>Ailuropoda_melanoleuca_GL192477.1.trna57-LysCTT (2368350-2368278) Lys (CTT) 73 bp Sc: 56.62
ACCTGGCTGGCTCAGTTGGTAGAGCATGTGACTCTTGATCACCGGGTCATGAGTTCAAGC
CCCATGTTGGGCA
>Ailuropoda_melanoleuca_GL192392.1.trna86-LysCTT (3058166-3058239) Lys (CTT) 74 bp Sc: 56.63
GCCTGGCTGGTTCAGTCGGTTAGAGCATGTGACTCTTGATCTCGGGGTCATGAGTTCAAG
CCCCATGTTGGGCA
>Ailuropoda_melanoleuca_GL192651.1.trna33-LysCTT (1242002-1242074) Lys (CTT) 73 bp Sc: 56.65
GCCTGGCTGGCTCAGTCGGTAGAGCATGTGACTCTTGATCTCAGGGTTGTGAATTCGAGC
CCCACGTTGGGCA
>Ailuropoda_melanoleuca_GL192660.1.trna122-LysCTT (444357-444285) Lys (CTT) 73 bp Sc: 56.75
ACCTGGCTGGCTCAGTTGGTAGAGTATGTGACTCTTGATCTCAGGGTTGTGAGTTCGAGC
CCCATGTTGGGTG
>Ailuropoda_melanoleuca_GL193204.1.trna63-LysCTT (353494-353422) Lys (CTT) 73 bp Sc: 56.78
GCCTGGCTGGCTCAGTTGGTAGAGCATGCAACTCTTGTTCTCAGGGTCGTGAGTTCAAGC
CTCACATTGGGCA
>Ailuropoda_melanoleuca_GL193562.1.trna6-LysCTT (196759-196831) Lys (CTT) 73 bp Sc: 56.84
GCCTAGCTGGCTCAGTTGGTAGAGCATGGGACTCTTGATCTCAGGGTCATGAGTTGAAGC
CCCATGTTGGGCA
>Ailuropoda_melanoleuca_GL192479.1.trna211-LysCTT (625470-625398) Lys (CTT) 73 bp Sc: 56.86
ACCTGGTTGGCTCAGTTGGTACAGCATGAGACTCTTAATCTCAGGGTTGTGAGTTCGAGC
CCCACATTGAGTA
>Ailuropoda_melanoleuca_GL193802.1.trna4-LysCTT (425479-425551) Lys (CTT) 73 bp Sc: 56.99
ACCTGGCTGGCTCAGTTGGTGGAGCATGTGACTCTTGATCTCAGGGTTGTGGGTTCGAGC
CCCATGTTGGGTG
>Ailuropoda_melanoleuca_GL193246.1.trna80-LysCTT (106282-106210) Lys (CTT) 73 bp Sc: 57.02
ACCTGGCTGGCTCAGTTGGTAGAGCATGGGACTCTTGATCTCAGGGTTGTGGGTTCAAGC
CCCATGTTGGATG
>Ailuropoda_melanoleuca_GL194686.1.trna15-LysCTT (26298-26226) Lys (CTT) 73 bp Sc: 57.04
GCCTGGCTGGCTCAGTCGGTAGAGCATGTGACTCTTGATCTCAGGGTTGTGAGTTCAAGC
CCCATGCTGGGTG
>Ailuropoda_melanoleuca_GL193203.1.trna52-LysCTT (547265-547193) Lys (CTT) 73 bp Sc: 57.10
GCCTGACTGGCTCAGTCGGTAGAGCGTGTGACTCTTGATCTCGAGGTCATGAATTCAAGC
CCCATGTTGGGCA
>Ailuropoda_melanoleuca_GL193007.1.trna37-LysCTT (190894-190822) Lys (CTT) 73 bp Sc: 57.13
ACCTGGCTGGCTCAGTCGGTAAAGTGTGTGACTCTTGATCTCAAGGTCATGAGTTCAAGA
CCCATGTTGGGTG
>Ailuropoda_melanoleuca_GL192502.1.trna83-LysCTT (1237424-1237352) Lys (CTT) 73 bp Sc: 57.19
GCCTGGCTGGCTCAGTTGGTGGAGCATGCGACTCTTGATCTCAAGGTTGTGGGTTCAAGC
CCCATGTTGGGTA
>Ailuropoda_melanoleuca_GL193617.1.trna23-LysCTT (353366-353294) Lys (CTT) 73 bp Sc: 57.21
ACCTGGCTGGCTCAGCTGGTAGAGCATGCGACTCTTGATCTCAGGGTCTTGAGTTCGAGC
CTCATGTTGGGTG
>Ailuropoda_melanoleuca_GL193563.1.trna68-LysCTT (32003-31931) Lys (CTT) 73 bp Sc: 57.24
GCCTGGCTGGCTCAGTGGGTAGAGCATGCAACTCTTGATCTCAGGGTCATGAGTTCAAGC
CTCATGTTGGGCG
>Ailuropoda_melanoleuca_GL192939.1.trna33-LysCTT (858174-858246) Lys (CTT) 73 bp Sc: 57.26
GCCTGGCTGGCTCAGTCGGTAGAGCATGAGACTCTTGATCTCAGGGTTGTGAGTTTGAGT
CCCATGTTGGGTA
>Ailuropoda_melanoleuca_GL192451.1.trna60-LysCTT (2350999-2351071) Lys (CTT) 73 bp Sc: 57.28
GCCTGGCTGGCTCAGTTGGTAGAGCATGTGACTCTTGATCTTGGGGTCATGAGTTCAAGC
CCCATGTTGGGCA
>Ailuropoda_melanoleuca_GL192351.1.trna257-LysCTT (2224130-2224058) Lys (CTT) 73 bp Sc: 57.30
TCCTGGCTGGCTCAGTTGGTAGAGCATGTGACTCTTGATCTCAGGGTTGTGAGTTCGAGC
CCCACGTTGGGTG

>Ailuropoda_melanoleuca_GL193581.1.trna60-LysCTT (1652-1580) Lys (CTT) 73 bp Sc: 57.31
ACCTGACTGGCTCAGTTGGAAGAGTATGTGATTCTTGATCTCAGGGTCATGAGTTCGAAC
CCCATGTTGGGTG
>Ailuropoda_melanoleuca_GL192339.1.trna371-LysCTT (2622038-2621966) Lys (CTT) 73 bp Sc: 57.31
ACCTGGCTGGCTCAGTCGGTAGAGCATGCGACTCTTGATCTCGGGGTCGTGAGTTCAAGC
CCCACGTTGGGTG
>Ailuropoda_melanoleuca_GL192357.1.trna125-LysCTT (3478007-3478079) Lys (CTT) 73 bp Sc: 57.33
GCCTGGCTGGCTCAGTTGGTAGAGCATGTGATTCTTGATCTCAGGGTTGTGAGTTCAAGC
CCCACGTTGGGTG
>Ailuropoda_melanoleuca_GL192660.1.trna91-LysCTT (1159085-1159013) Lys (CTT) 73 bp Sc: 57.34
GCCTGGCTGGCTCAGTTGGCAGATCACGAGACTCTTGATCTCGGGGTTGTGGGTTTGAGC
CCCACGCCGGGTG
>Ailuropoda_melanoleuca_GL192682.1.trna90-LysCTT (494436-494364) Lys (CTT) 73 bp Sc: 57.52
GCCTGGCTGGCTCAGTTGGTAGAGCATGTGACTCTTGATCTTGGGATCGTGAGTTCAAGC
CCCACGTTGGGCA
>Ailuropoda_melanoleuca_GL193485.1.trna31-LysCTT (514452-514524) Lys (CTT) 73 bp Sc: 57.55
GCCTGGCTGGCTCAGTCGGTAGAACATGTGACTCTTAATCTCAGGGTCATGAGTTCCAGC
CTCATGCTGGGCA
>Ailuropoda_melanoleuca_GL192825.1.trna76-LysCTT (618361-618289) Lys (CTT) 73 bp Sc: 57.55
ACCTGGCTGGCTCAGTTGGTAGAGCCTGTGACTCTTGATCTCAAGGTCATGAGTTCAAGC
CCCATGTTAGGCA
>Ailuropoda_melanoleuca_GL192351.1.trna187-LysCTT (4237125-4237053) Lys (CTT) 73 bp Sc: 57.56
GCCTGGCTGGCTTAGTTGGTAAAGCATGTGACTCTTGATCTTGGGGTCATGAGTTCAAGC
CTCATGTTGGGCA
>Ailuropoda_melanoleuca_GL193141.1.trna5-LysCTT (189864-189936) Lys (CTT) 73 bp Sc: 57.59
ACCTGGATGGCTCACTCGGTAGAGCATGAGACTCTTGATCTCAGAGTCATGGGTTCAAGC
CCCATGCTAGGTG
>Ailuropoda_melanoleuca_GL192542.1.trna82-LysCTT (247843-247771) Lys (CTT) 73 bp Sc: 57.62
ACCTGGCTGGCTCAGTTGGAAGAGCATGTGACTCTTAATCTCAGGGTCGTGAGTTCAAGC
CCCATGTTGGGTG
>Ailuropoda_melanoleuca_GL192791.1.trna44-LysCTT (1120893-1120965) Lys (CTT) 73 bp Sc: 57.64
GCCTGGCTGGCTCACTTGGTAGAGCATGTGACTCTTGATCTTGGGGTCGTGAGTTCAATT
CTCACGTTGGGCG
>Ailuropoda_melanoleuca_GL193619.1.trna5-LysCTT (116193-116265) Lys (CTT) 73 bp Sc: 57.78
GCCTGACTGGCTCAGTTGGTAGAGCACATGACTCTTGATCTTGAGGCTGTGAGTTCGAGC
CCCATGTTGGGTG
>Ailuropoda_melanoleuca_GL192595.1.trna67-LysCTT (917842-917770) Lys (CTT) 73 bp Sc: 57.84
GCCTAGCTGGCTCAGTCGGTAGAGCACGAGACTCTTGATCTTGGGGTTGTGAGTTTGAGC
CCCATGTTAGGCA
>Ailuropoda_melanoleuca_GL193472.1.trna6-LysCTT (321922-321994) Lys (CTT) 73 bp Sc: 57.89
ACCTGGCTGGCTCAGTTGATACAGCATAAGACTCTTGATCTTGGGGTCGTGGGTTCAAGC
CCCACGTTGGGTA
>Ailuropoda_melanoleuca_GL193845.1.trna23-LysCTT (377319-377247) Lys (CTT) 73 bp Sc: 57.91
GCCTGGTTGGTTCAGTCAGTAGAACATGTGACTCTTGATCTCAGGATCCTGAGTTCGAGC
CCCAGATTGGGCG
>Ailuropoda_melanoleuca_GL192723.1.trna112-LysCTT (598556-598484) Lys (CTT) 73 bp Sc: 57.97
GCCTGGCTGGCTCAGTGGGTAGAGCATGTGACTCTTGATCTCAGAGTCATGGGTTTAAGC
CCCATGTTAGGCA
>Ailuropoda_melanoleuca_GL192350.1.trna204-LysCTT (3133351-3133279) Lys (CTT) 73 bp Sc: 58.02
ACCTGGCTGGTTCAGTCGGTAGAGCTTGAGACTCTTGATCTCAGGGTCGTGAGTTCAAGC
CCCATGTTGGGTG
>Ailuropoda_melanoleuca_GL192563.1.trna42-LysCTT (1093336-1093408) Lys (CTT) 73 bp Sc: 58.16
ACCCGGCTGGCTCAGTTGGTAGAGCATGTGACTCTTGATCACAGAGTCATGAGTTCAAGC
CCCACGTTGGGTA
>Ailuropoda_melanoleuca_GL192626.1.trna48-LysCTT (921133-921205) Lys (CTT) 73 bp Sc: 58.20
GCCTGGCTGGCTCAGTTGGTAGAGCCTGTGACTCTTGATTTCGGGGTCATGGGTTCGAGC
CCCATGTTGGGTG
>Ailuropoda_melanoleuca_GL193071.1.trna7-LysCTT (278802-278874) Lys (CTT) 73 bp Sc: 58.31
ACCTGGCTAGCTCAGTTGGTAGAGCATGAAACTCTTGATCTCAGGGTCGTGAGTTCGAGC
CCCACATTGGGTG
>Ailuropoda_melanoleuca_GL192424.1.trna8-LysCTT (431473-431545) Lys (CTT) 73 bp Sc: 58.31
ACCTGGCTGGCTCAGTCGGTAGAGCATGTGACTCTTGATCTCAGGGTTGTGGGTTCGAAA
CTCACATTGGGTG
>Ailuropoda_melanoleuca_GL194160.1.trna19-LysCTT (9005-8933) Lys (CTT) 73 bp Sc: 58.49
GCCTGGCTGGCTCAGTCGGTAGAGCATGTGACTCTTGATCTCAGGGTTGTGAGTTCAAGC
CCCACGTTGGGTG
>Ailuropoda_melanoleuca_GL192531.1.trna41-LysCTT (1848585-1848657) Lys (CTT) 73 bp Sc: 58.54

ACCTGGCTGGCTCAGTTGGTAGAGCATGTGACTCTTGATCCCAGGGTTGTGAGTTCGAGC
CCCATGCTGGGTG
>Ailuropoda_melanoleuca_GL193433.1.trna12-LysCTT (492082-492154) Lys (CTT) 73 bp Sc: 58.54
GCCTGGCTGGCTCAGTTGGTAGAGCATGTGACTCTTGATCCCAGGGTCCTGTGTTCAAGC
CCCATGTTGGGCA
>Ailuropoda_melanoleuca_GL192571.1.trna15-LysCTT (789301-789373) Lys (CTT) 73 bp Sc: 58.70
ACCTGGCTGGCTCAGTCAGAAGAGCATGAGACTCTTGATCTCAGGGTTGTGGGTTCGAGC
CCCATGTTGGGTA
>Ailuropoda_melanoleuca_GL192360.1.trna100-LysCTT (1725846-1725918) Lys (CTT) 73 bp Sc: 58.83
GCCTGGCTGGCTCAGTGGGTAGAGCGTGCGACTCTTGATCTCAGGGTTGTGGTTTCGAGC
CCCACACTGGGCG
>Ailuropoda_melanoleuca_GL193724.1.trna37-LysCTT (1404-1332) Lys (CTT) 73 bp Sc: 58.88
GCCTGGCTGGCTCAGTGGGTAGAGCACATGACTCTTGATCTTGGGGTTGTGAGTTCGAGC
CCCACGTTGGGTG
>Ailuropoda_melanoleuca_GL192423.1.trna23-LysCTT (901061-901133) Lys (CTT) 73 bp Sc: 59.00
GCCTGACTGGCTCAGTTGGTAGAGCATGGGACTCTTGATCTCAGGGTCATGAGTTCAAGC
CCCATGTTGGGTG
>Ailuropoda_melanoleuca_GL193063.1.trna25-LysCTT (505140-505068) Lys (CTT) 73 bp Sc: 59.09
ACCTGGCTGGCTCAGTCGGTAGAGCATGTGACTCTTGATCTCAGGGTTGTAGGTTCGAGT
CCCATGTTGGGTA
>Ailuropoda_melanoleuca_GL193485.1.trna21-LysCTT (472195-472267) Lys (CTT) 73 bp Sc: 59.13
GCCTGGCTGGCTCAGTCGGTAGAGCATGTGACTCTTAATCTCAGGGTCAGGAGTTCAAGC
CCCATGTTGGGCA
>Ailuropoda_melanoleuca_GL192906.1.trna46-LysCTT (999483-999555) Lys (CTT) 73 bp Sc: 59.14
GCCTGGCTGGCTTAGTTGGTAGAGCATGAGACTCTTGATCACAGGGTCTTGAGTTCAAGC
CTCACACTGGGCA
>Ailuropoda_melanoleuca_GL193091.1.trna26-LysCTT (726924-726852) Lys (CTT) 73 bp Sc: 59.21
ACCTGGCTGGCTCAGTCGGTAAAGTATGTGACTCTTGATCTCAAGGTTGTGGGTTCGAGC
CCCAGGCTGGGTG
>Ailuropoda_melanoleuca_GL192356.1.trna395-LysCTT (860449-860377) Lys (CTT) 73 bp Sc: 59.23
GCCTGGCTGGCTCAGTTCGTAGAGCATGAGACTCTTGATCTCAGGGTCATGAGTTCAAGC
CCCATGCTGGGCC
>Ailuropoda_melanoleuca_GL193863.1.trna1-LysCTT (61451-61523) Lys (CTT) 73 bp Sc: 59.30
ACCTGGCTGGCTCGGTCGGTAGAGCATGAGACTCTTAATCTCAGGGTCGTGAGTTCAAGC
CCCACGCTGGGCA
>Ailuropoda_melanoleuca_GL192757.1.trna29-LysCTT (1303406-1303334) Lys (CTT) 73 bp Sc: 59.38
GCCTGGCTGGCTCAGTTGGTAGAGCATGTGACTCTTGATCTCAAGGTTGTGGGTTTGAGC
CCCACATTGGGTG
>Ailuropoda_melanoleuca_GL192447.1.trna31-LysCTT (1309160-1309232) Lys (CTT) 73 bp Sc: 59.42
ACCTGGCTGGCTTAGTTGGTAGAGCATGCGACTCTTGATCTCAGGGTCGTGAGTTCAAGC
CCCACGTTGGGTG
>Ailuropoda_melanoleuca_GL193177.1.trna50-LysCTT (262454-262382) Lys (CTT) 73 bp Sc: 59.50
ACCTGGCTAGCTCAGTTGGTAGAGCTTTAGACTCTTGATCTCGAAGTTGTGGGTTCGAGC
CCCACATTGGGTG
>Ailuropoda_melanoleuca_GL192511.1.trna12-LysCTT (611075-611147) Lys (CTT) 73 bp Sc: 59.54
TCCTGGTTAGTTCAGTCAGTAGAACATGAGACTCTTAATCTCAGGGTCGTGAGTTCAAGT
CCCACACTGGGCA
>Ailuropoda_melanoleuca_GL192356.1.trna160-LysCTT (3095397-3095469) Lys (CTT) 73 bp Sc: 59.57
ACCTGGCTGGCTCAGCTGGTAGAGCACGAGACTCTTGATCTCGGGGTTGTGAGTTCAAGC
CCCACACTGGGTG
>Ailuropoda_melanoleuca_GL193062.1.trna47-LysCTT (110610-110538) Lys (CTT) 73 bp Sc: 59.61
GCCTGGCTGGCTCAGTGGGTAAAGCGTGAGACTCTTGATCTCAGGGTCATGAGTTCAAGC
CCCACGTTGGGCA
>Ailuropoda_melanoleuca_GL194476.1.trna1-LysCTT (14384-14456) Lys (CTT) 73 bp Sc: 59.64
GCCAGGCTGGCTCAGTTGGTAGAGCGTGTGACTCTTGATCTCGGGGTTGTGGGTTCAAGC
CCCACGTTGGGTG
>Ailuropoda_melanoleuca_GL192382.1.trna82-LysCTT (2753370-2753442) Lys (CTT) 73 bp Sc: 59.89
GCCTGGCTGGCTCAGTCGGTAGAGCATGTGACTCTTGATCTGAGGATTATGGGTTCAAAT
CCCATGTTGGGTA
>Ailuropoda_melanoleuca_GL192476.1.trna8-LysCTT (375097-375169) Lys (CTT) 73 bp Sc: 59.97
GCCTGGCTGGCTCAGTTGGAAGAGCATGTGACTCTTGATCTCGTGGTCGTGAGTTCAAGC
CTCATGCTGGGTA
>Ailuropoda_melanoleuca_GL192360.1.trna50-LysCTT (909104-909176) Lys (CTT) 73 bp Sc: 60.07
GCCTGGCTGGCTCAATTGGTAGAGCATGAGACTCTTGATTTCGGGGTCATGAGTTCAAGC
CCCATGCTGGGCA
>Ailuropoda_melanoleuca_GL192356.1.trna297-LysCTT (3239330-3239258) Lys (CTT) 73 bp Sc: 60.12
GCCTAGCTGGCTCAGTTGGTAGAGCATGTGACTCTTGATCTCAGTGTCATGAGTTCAAGC

CCCATGTTGGGCA
>Ailuropoda_melanoleuca_GL193145.1.trna6-LysCTT (352708-352780) Lys (CTT) 73 bp Sc: 60.22
TCCTGGTTGGCTCAGTTGGTAGAGCATGTGACTCTTATTCTCATGGTTGTGAGTTCAAGC
CCCACATCAGGTG
>Ailuropoda_melanoleuca_GL192718.1.trna15-LysCTT (310687-310759) Lys (CTT) 73 bp Sc: 60.39
ACCTGACTGGCTCAGTCGGTAGAGCATGTGACTCTTAATCTCAGGGTTGTGGGTTCAAGC
CCCAGGTTGGGTG
>Ailuropoda_melanoleuca_GL192364.1.trna237-LysCTT (364417-364345) Lys (CTT) 73 bp Sc: 60.60
GCCTGGCTGGCTTAGTTGGTAGAGCACGTGACTCTTGATCTTGAGGTTGTGAGATCGAGC
CTCACACTGGGCA
>Ailuropoda_melanoleuca_GL192830.1.trna19-LysCTT (906171-906243) Lys (CTT) 73 bp Sc: 60.82
GCCTGGCTGGCTCAGTCAGTAGAGCATATGACTCTTGATCTCAGGGTCGTGGGTTCAAGC
CCCACGTCGGGCA
>Ailuropoda_melanoleuca_GL192342.1.trna42-LysCTT (1354975-1355047) Lys (CTT) 73 bp Sc: 60.99
GCCTGGCTGGCTCAGTCGGTAGAGCATGCGACTCTTAATCTCAGGGTTGTGAGTTCAAGC
CCCACACTAGGCG
>Ailuropoda_melanoleuca_GL192432.1.trna51-LysCTT (2488203-2488275) Lys (CTT) 73 bp Sc: 61.10
GCCTGGCTGGCTCAGTCGGTAGAGCGTGTGATTCTTAATCTCAGGATTGTGAGTTCAAAC
CCCATGTTGGGCA
>Ailuropoda_melanoleuca_GL196082.1.trna1-LysCTT (4270-4342) Lys (CTT) 73 bp Sc: 61.12
GCCTGGCTGGCTCAGTCAGTAGAGCACACGACTCTTAATCGTGGGGTTGTGAGTTCAAGC
CACACGTTGGGCA
>Ailuropoda_melanoleuca_GL192440.1.trna200-LysCTT (687287-687215) Lys (CTT) 73 bp Sc: 61.20
GCCTGGCTGGCTCAGTTGGTGGAGCATGTGACTCTTGATCTCAGGGTTGTGGGTTCGAGC
CCCATGTTGGGCA
>Ailuropoda_melanoleuca_GL192365.1.trna128-LysCTT (2395133-2395061) Lys (CTT) 73 bp Sc: 61.28
GCCTGGCTGGCTCAGTTGGTAGAGCTTGTGACTCTTGATCTCAGGGTCATGAGTTCAAGC
CCCATGTTGGGCT
>Ailuropoda_melanoleuca_GL192368.1.trna53-LysCTT (1958455-1958527) Lys (CTT) 73 bp Sc: 61.28
GCCTGGCTGGCTCAGTTGGTAGAGTGTGTGACTCTTGATCTCGGGGTCATGAGTTCAAGC
CCCATGTTGGGCA
>Ailuropoda_melanoleuca_GL192439.1.trna51-LysCTT (1087652-1087724) Lys (CTT) 73 bp Sc: 61.39
GCCTGGCTGGCTCAGTTGGTAGAGCATGTGACTCTTGATCTCAGGGTTGTGAGTTCGAGC
CCCACTTTGGGCA
>Ailuropoda_melanoleuca_GL192536.1.trna97-LysCTT (932359-932287) Lys (CTT) 73 bp Sc: 61.45
GCCTGGCTGGCTCAGTTGGAAGAGCATGTGACTCTTGATCTCGGGGTTGTGGGTTCAAGC
CCCATGTTGGGCA
>Ailuropoda_melanoleuca_GL192497.1.trna13-LysCTT (377255-377327) Lys (CTT) 73 bp Sc: 61.51
ACCTGGTGGGCTCAGTTGGTAGAGCGTGAGATTCTTGATCTCAGGGTTGTGGGTTTGAGC
CCCACATCAGGTG
>Ailuropoda_melanoleuca_GL194632.1.trna2-LysCTT (88934-89006) Lys (CTT) 73 bp Sc: 61.55
GCCTGGCTGGCTCAGTTGGTAGAGCATGTGATTCTTGATCTCGGGGTCATGGGTTCAAGC
CCCATGTTGGGTG
>Ailuropoda_melanoleuca_GL192483.1.trna75-LysCTT (2309632-2309704) Lys (CTT) 73 bp Sc: 61.62
GCCTGGCTGGCTCAGTCGGTAGAGCGTGGGACTCTTGATCTCAGAGTCATGAGTTCAAAC
CCCATGTTGGGCG
>Ailuropoda_melanoleuca_GL192400.1.trna73-LysCTT (2145437-2145365) Lys (CTT) 73 bp Sc: 61.67
GCCTGGCTGGCTCAGTTGGTAGAGCATGCGACTCTTGATCTCAGGGTCATGAGTTCGAGT
CCCACGTTGGGCA
>Ailuropoda_melanoleuca_GL193590.1.trna22-LysCTT (562966-562894) Lys (CTT) 73 bp Sc: 61.95
ACCTGGCTGGCTCAGTTGGTAGAGTATGGGACTCTTAATCTCAGGGTCGTGAGTTCAAGC
CTCACACTGGGTG
>Ailuropoda_melanoleuca_GL192681.1.trna33-LysCTT (1037785-1037857) Lys (CTT) 73 bp Sc: 62.33
GCCCAGCTGGCTCAGTTGGTAGAGCATGCGACTCTTGATCTCAGGGTTGTGAGTTCGAGC
CCCACGTTGGGTA
>Ailuropoda_melanoleuca_GL192618.1.trna79-LysCTT (1651823-1651895) Lys (CTT) 73 bp Sc: 62.56
ACCTGGCTGGCTCAGTTGGAATAGCATGAGACTCTTGATCTCGGGGTCGTGGGTTCGAGT
CCCACACTGGGTG
>Ailuropoda_melanoleuca_GL192405.1.trna135-LysCTT (2672718-2672646) Lys (CTT) 73 bp Sc: 63.15
GCCTGGCTGGCTCAGTTGGTAAAGCGTGAGACTCTTGATCTCAGGGTCATGAGTTCAAGC
CCCATGCTGGACA
>Ailuropoda_melanoleuca_GL194658.1.trna3-LysCTT (115411-115483) Lys (CTT) 73 bp Sc: 63.22
GCCTGGCTGGCTCAGTGGGTAGAGCACGCGACTCTTGATCTCGGGGTCGTGAGTTCAAGC
CTCACGCTGGGTG
>Ailuropoda_melanoleuca_GL192382.1.trna65-LysCTT (2474866-2474938) Lys (CTT) 73 bp Sc: 64.00
GCCTGGCTGGCTCAGTTGGTAGAGCATGTGACTCTTGATCTCAGGGTCATGAGTTCAAGC
CCCATGTTGGGCA

>Ailuropoda_melanoleuca_GL192725.1.trna30-LysCTT (1338799-1338871) Lys (CTT) 73 bp Sc: 64.36
GCCTGGCTGGCTCAGTTGGTAGAACATGTGACTCTTGATCTCAGGGTCATGGGTTCAAGC
CCCATGTTGGGCA
>Ailuropoda_melanoleuca_GL193397.1.trna2-LysCTT (133852-133924) Lys (CTT) 73 bp Sc: 64.66
GCCTGGCTGGCTCAGTTGGTAGAGTATGTGACTCTTGATCTCAGGGTCGTGAGTTCAAGC
CCCACGTTGGGCA
>Ailuropoda_melanoleuca_GL192383.1.trna103-LysCTT (3679712-3679784) Lys (CTT) 73 bp Sc: 67.71
GCCTGGCTGGCTCAGTGGGAAGAGCATGCGACTCTTGATCGCAGGGTCGTGAGTTCGAGC
CCCACGTCGGGCA
>Ailuropoda_melanoleuca_GL193160.1.trna37-LysCTT (834909-834981) Lys (CTT) 73 bp Sc: 80.47
GCCCGGCTAGCTCAGTCGGTAGAGCATGAGACTCTTAATCTCAGGGTCGTGGGTTCGAGC
CCCACGTTGGGCG
>Ailuropoda_melanoleuca_GL193204.1.trna12-LysCTT (74960-75032) Lys (CTT) 73 bp Sc: 80.47
GCCCGGCTAGCTCAGTCGGTAGAGCATGAGACTCTTAATCTCAGGGTCGTGGGTTCGAGC
CCCACGTTGGGCG
>Ailuropoda_melanoleuca_GL193204.1.trna5-LysCTT (50269-50341) Lys (CTT) 73 bp Sc: 80.47
GCCCGGCTAGCTCAGTCGGTAGAGCATGAGACTCTTAATCTCAGGGTCGTGGGTTCGAGC
CCCACGTTGGGCG
>Ailuropoda_melanoleuca_GL193204.1.trna76-LysCTT (47023-46951) Lys (CTT) 73 bp Sc: 80.47
GCCCGGCTAGCTCAGTCGGTAGAGCATGAGACTCTTAATCTCAGGGTCGTGGGTTCGAGC
CCCACGTTGGGCG
>Ailuropoda_melanoleuca_GL193204.1.trna77-LysCTT (42651-42579) Lys (CTT) 73 bp Sc: 80.47
GCCCGGCTAGCTCAGTCGGTAGAGCATGAGACTCTTAATCTCAGGGTCGTGGGTTCGAGC
CCCACGTTGGGCG
>Ailuropoda_melanoleuca_GL193204.1.trna9-LysCTT (65847-65919) Lys (CTT) 73 bp Sc: 80.47
GCCCGGCTAGCTCAGTCGGTAGAGCATGAGACTCTTAATCTCAGGGTCGTGGGTTCGAGC
CCCACGTTGGGCG
>Ailuropoda_melanoleuca_GL194901.1.trna14-LysCTT (85635-85563) Lys (CTT) 73 bp Sc: 80.47
GCCCGGCTAGCTCAGTCGGTAGAGCATGAGACTCTTAATCTCAGGGTCGTGGGTTCGAGC
CCCACGTTGGGCG
>Ailuropoda_melanoleuca_GL194954.1.trna13-LysCTT (85956-85884) Lys (CTT) 73 bp Sc: 80.47
GCCCGGCTAGCTCAGTCGGTAGAGCATGAGACTCTTAATCTCAGGGTCGTGGGTTCGAGC
CCCACGTTGGGCG
>Ailuropoda_melanoleuca_GL194954.1.trna5-LysCTT (76903-76975) Lys (CTT) 73 bp Sc: 80.47
GCCCGGCTAGCTCAGTCGGTAGAGCATGAGACTCTTAATCTCAGGGTCGTGGGTTCGAGC
CCCACGTTGGGCG
>Ailuropoda_melanoleuca_GL192352.1.trna239-LysCTT (1517025-1516953) Lys (CTT) 73 bp Sc: 80.72
GCCCGGCTAGCTCAGTCGGTAGAGCATGGGACTCTTAATCCCAGGGTCGTGGGTTCGAGC
CCCACGTTGGGCG
>Ailuropoda_melanoleuca_GL192987.1.trna35-LysCTT (528919-528847) Lys (CTT) 73 bp Sc: 80.72
GCCCGGCTAGCTCAGTCGGTAGAGCATGGGACTCTTAATCCCAGGGTCGTGGGTTCGAGC
CCCACGTTGGGCG
>Ailuropoda_melanoleuca_GL192944.1.trna15-LysCTT (1106404-1106314) Lys (CTT) 91 bp Sc: 40.34
GCCTGGGTGGCTGAGTCGGTTAAGTGTCTGACTCTTGGTTTCAGCTCAGGTCTCGATCTC
AGGGTCATGAGTTCAAGCCTCATACTGGGCT
>Ailuropoda_melanoleuca_GL192360.1.trna247-LysCTT (1286283-1286192) Lys (CTT) 92 bp Sc: 30.86
GCCTGGGTGGCTCAGTTGGTTAAGCATCCAACTCTTAGTTTGGGCTCAGGTGCTGATCTC
ACAAGTTGTGGGATCAAGCCCCATGTCAGGCT
>Ailuropoda_melanoleuca_GL195009.1.trna1-LysCTT (13621-13711) Lys (CTT) 91 bp Sc: 34.84
GCCTGGGTGGCTCAGCTGGTTAAGCTTCTGACTCTTGGTTTTGGCTCAGGTCATGATTTC
AGGATCATGGGATCAAGCCCCGTGCCAGGCT
>Ailuropoda_melanoleuca_GL193091.1.trna8-LysCTT (160628-160718) Lys (CTT) 91 bp Sc: 42.38
GCCTGGCTGGCTCAGTCGGCGGAGTGTCTGACTCTTGATTTCGGCTCAGGTCATGATCTC
AGGGTCGTGGGATCGAGCCCCACGTTGGGCT
>Ailuropoda_melanoleuca_GL192514.1.trna38-LysCTT (1637253-1637343) Lys (CTT) 91 bp Sc: 30.86
GCCTAGGTGGCTGAGTCGGTTAAGCATCCGACTCTTGATCTCGGCTCAGATAGTGATCTC
AGGGTCATGGGGTCAAGCCCCATGTTGGGTT
>Ailuropoda_melanoleuca_GL192389.1.trna134-LysCTT (16458-16369) Lys (CTT) 90 bp Sc: 28.34
TTCTGGGTAGCTCAGTTGGTTGAGCGTTCAACTCTTGATTTGGCCCAGGTCATGATCTCA
GGGTTGTGGGATTGAGCCCCACATCAGAAT
>Ailuropoda_melanoleuca_GL192868.1.trna50-LysCTT (181644-181553) Lys (CTT) 92 bp Sc: 35.05
GCCTGGGTAGCTCAGTTGGTTGAGCATTGAACTCTTGGTTTTGGCTCAGGTCATGATCTC
CTGAATTGTGGGATCAAACCCCACATTGGGCT
>Ailuropoda_melanoleuca_GL192549.1.trna85-LysCTT (1959510-1959420) Lys (CTT) 91 bp Sc: 27.49
GCCTGGTTGGCTCAGTGGGTTAAGCATCTGAGTCTTGATTTCGGCTCAGGTAATGATCTC
AGGGGCATGGGATCGAGCCCCACGTCGGGCT
>Ailuropoda_melanoleuca_GL192969.1.trna12-LysCTT (201925-202015) Lys (CTT) 91 bp Sc: 35.05

GCCTGGGTGGCTCAGTCAGTTTAGCGTCTGACTCTTGACTTCAGCTCAGGTCTTGACTTC
AGGGTTGTGGGTTCAAGCCCCACGCTGGGCT
>Ailuropoda_melanoleuca_GL192412.1.trna6-LysCTT (202097-202187) Lys (CTT) 91 bp Sc: 38.84
GCCTGGTTGGCTCAGTCGGTTAAGCGGCTGACTCTTGGTTTCAGCTCAGATGATGATCTC
AGTGTCATGGGATCGAGCCCCACACTGGGTT
>Ailuropoda_melanoleuca_GL192430.1.trna66-LysCTT (2254803-2254891) Lys (CTT) 89 bp Sc: 31.07
GTCTGGGTGGCTCAGTTGGTTAAGCACACGACTCTTGTTCAGCTGGGGTCATGATTGTGG
CGTTATGGGATCGAGCCCCATGTTGGGCC
>Ailuropoda_melanoleuca_GL194148.1.trna9-LysCTT (233775-233685) Lys (CTT) 91 bp Sc: 32.61
GCCTGGGTGGCTTAGTTGGTTGGGCATCGGACTCTTGATTTCGGCTCAGGTCCTGGTCTC
AGGGTTGTGGGATCAAGCCCCATGTCAGGCT
>Ailuropoda_melanoleuca_GL192370.1.trna62-LysCTT (2395183-2395275) Lys (CTT) 93 bp Sc: 39.52
GCCTGGGTGGCTCAGTTGGTTGAGTGTCAGACTCTTGATTTCGGCTCAGGTCATGATTTC
TCAGGGTCTTGGGATGGAGCCCCAGGTCAGGCT
>Ailuropoda_melanoleuca_GL192425.1.trna135-LysCTT (2457330-2457240) Lys (CTT) 91 bp Sc: 38.77
ACCTGGCTGGCTCAGTTGGTTGAGTGTCCGACTCTTGGTTTTGGCTCTGGTCATGATCTC
GGGGTTGTGGGATCGAGCCCCACGTCAGGCT
>Ailuropoda_melanoleuca_GL192806.1.trna48-LysCTT (263696-263606) Lys (CTT) 91 bp Sc: 33.86
GCCTGGGTGGCTCAGTGGGCTAAGCATCTGACTCTTGGTTTTGGCTCAGGTTGTGATCTC
GCAGTCATGGGATCGAGCCCCATGTCGGGTT
>Ailuropoda_melanoleuca_GL193064.1.trna24-LysCTT (26418-26328) Lys (CTT) 91 bp Sc: 27.04
ACCTGGGTGGCTCAGTTAGTTAAGTGTCTGACTCTTGATTTCAGCTCAGTTCACGATCTC
AGGGTGGTGGGATCAAACCCCACATCAGGTT
>Ailuropoda_melanoleuca_GL192388.1.trna65-LysCTT (2642183-2642272) Lys (CTT) 90 bp Sc: 38.50
GCCTGGGTGGCTCAGTGGGTTAAGCGGTGACTCTTGGTTTCAGCTCAGGTCATGATCTCA
GGGTCGTGGGATCGAGCCCCGCCTCGGGCT
>Ailuropoda_melanoleuca_GL194126.1.trna2-LysCTT (282289-282379) Lys (CTT) 91 bp Sc: 42.05
GCCTGGGTGGCTCAGTTGGTTGAGCATCTGACTCTTAATTTCACCTCAGGTCTTGATCTC
AGGGTTGTGGGATCAAGCCCCATGTCAGGCT
>Ailuropoda_melanoleuca_GL193873.1.trna3-LysCTT (30786-30866) Lys (CTT) 81 bp Sc: 53.66
ACCTGGCTGGCTCAGTCGGTAGAGCATGCGACTCTTGAGCTCTTGATCGTAGGGTTGTGA
GTTCGAGCCCCACGTTGGGTG
>Ailuropoda_melanoleuca_GL192361.1.trna65-LysCTT (3421868-3421959) Lys (CTT) 92 bp Sc: 44.96
GCCTGGCTGGCTCAGTTGGTTGAGCGCCTGACTCTTGATTTGGGCTCAGGTTGTGATCTC
ATGAGTCATGAGATCGAGCCCCATGTTGGGCT
>Ailuropoda_melanoleuca_GL192992.1.trna8-LysCTT (365391-365481) Lys (CTT) 91 bp Sc: 34.25
GCCTGGGTGGCTCAGCTGGTTGAGCATCTAACTCTTGATTTCAGCTCAGGTCATGATCTC
AGAGTCATGGGATCGAGCCCCGTGTCGGGCT
>Ailuropoda_melanoleuca_GL192373.1.trna75-LysCTT (3805852-3805762) Lys (CTT) 91 bp Sc: 37.64
GCCTGGGTGGCTCAGTTGGTTAAGCATAGGACTCTTGATTTCAGCTCAGGTCATGATCTC
AGGAGCATGGGATCGAACCCCAACTCAGGCT
>Ailuropoda_melanoleuca_GL194281.1.trna35-LysCTT (38103-37981) Lys (CTT) 123 bp Sc: 48.74
GCCTGGGTGGCTCAGTTGGTTAAGCGTCTGACTCTTGATTTCAAAGACTTTCAAGAGTCT
TTCTGACTCTTGATACCTGAGGTCATGATCTCAGAGTTGTGGGATCGAGTCCCACCTTGG
GCT
>Ailuropoda_melanoleuca_GL192340.1.trna147-LysCTT (3848165-3848256) Lys (CTT) 92 bp Sc: 27.69
GCCTAGGTGGCTCAGTAGGTTGAGTGTCTTGACTCTTAATTTCAGCTCAGGCCATGATCT
CAGGATTGTGGGATCAAGCCCCACGTTGGGCT
>Ailuropoda_melanoleuca_GL192355.1.trna136-LysCTT (4086861-4086951) Lys (CTT) 91 bp Sc: 43.24
GCCTGGATGGCTCAGTTGGTTGAGTGTCGGACTCTTGATTTCAGCTCAGGTAGTGATCTC
AGGGTCGTGGGATGGATTCCCACGTCAGGCT
>Ailuropoda_melanoleuca_GL192954.1.trna32-LysCTT (408769-408680) Lys (CTT) 90 bp Sc: 39.76
GCCTGGATGGCTCAGTCGGTTAAGCATCAGACTCTTGATTTGGCTCAAGTTGTGATCTCA
GGGTTGTGGGATCAAGCCCCATGTTGGGCT
>Ailuropoda_melanoleuca_GL192383.1.trna203-LysCTT (414798-414708) Lys (CTT) 91 bp Sc: 33.46
GCCTGGGTGGCTTACTTGGTTGAGCATCTGACTCTTGATTTCAGCTCAGGTCATGATCTG
GGGATCTTAGGATCAAGCCCTACTTCAGGCT
>Ailuropoda_melanoleuca_GL192351.1.trna143-LysCTT (4150297-4150388) Lys (CTT) 92 bp Sc: 33.37
GCCTGGGTGGCTCAGTCGGTTGAGCGTCCAACTCTTGGTTTCGGCTCAGGTCATGATCTC
GGGGGGAGTGGGATCCAGCCCCACACTGGGCT
>Ailuropoda_melanoleuca_GL194786.1.trna1-LysCTT (4659-4747) Lys (CTT) 89 bp Sc: 28.35
GCCTGGGTGGCTCAGTCAGTTAAGCTTCTGACTCTTGGTTCGGGCAGGTCATGGTCTCAG
GGTCGTGGGATCGAGCCCCATGTCAGGCT
>Ailuropoda_melanoleuca_GL193612.1.trna21-LysCTT (474158-474249) Lys (CTT) 92 bp Sc: 37.76
GCCTGGGTGGCTCAGTTGGTTGAGCATCAGACTCTTGGTTTCCGCTTGGGTCGTGATCTT
GGGGGTCGCGGGATCCAGCCCCGCATCAGGCT
>Ailuropoda_melanoleuca_GL192997.1.trna27-LysCTT (489295-489205) Lys (CTT) 91 bp Sc: 39.77

GCCTGGGTGGCTCAGCTGGTTGAGCATCTGACTCTTGATTTTGGCTCAGGTCATGATCTC
AGGGTTGTGAGATCGAGCCTCACATCAGGCT
>Ailuropoda_melanoleuca_GL193444.1.trna10-LysCTT (497269-497359) Lys (CTT) 91 bp Sc: 37.21
GCCTGGGTAGCTCAGTTGGTTGAGCATAAAACTCTTGATTTTGCCTGAGGTCATGATCTC
AAGGTCATGGGATCCAGCCCCATGTTGGGCT
>Ailuropoda_melanoleuca_GL193485.1.trna52-LysCTT (515076-514986) Lys (CTT) 91 bp Sc: 38.74
GCCTGGGTAGCTCAGTTGGTTAAGTGTCTGACTCTTGATTTCAGCTCAGATCGTGATCTC
AGGGTTGTGAGATCAAGTCTCACGTCGGGCT
>Ailuropoda_melanoleuca_GL192547.1.trna73-LysCTT (533356-533252) Lys (CTT) 105 bp Sc: 38.72
GCCTGGGTGGCTCAGTCGGTTAAGCACCCGACTCTTGATCTCAGCTCAGGTCGTGATCTC
GGGGTCATGGTCTCAGGATCGTGAGTTCAAGCCCCACCTTGGGCT
>Ailuropoda_melanoleuca_GL192856.1.trna43-LysCTT (58261-58172) Lys (CTT) 90 bp Sc: 32.15
GCCTAGGTGGCTCAGTCGGTTAAGCATCTGACTCTTGGTTCAGCTCAGGTCATGTTCTTG
CATTTGTGGGATCAAGCCCCACATTGGGCT
>Ailuropoda_melanoleuca_GL194202.1.trna10-LysCTT (61446-61356) Lys (CTT) 91 bp Sc: 35.32
GCCTGGATGGCTCAGTTGGTTAAGCCTCTGATTCTTGGTTTCGGCTCAGGTCATGAACTC
AGGGTGGTGGGATCAAGCCCCACTTCAGGCT
>Ailuropoda_melanoleuca_GL193192.1.trna55-LysCTT (646782-646691) Lys (CTT) 92 bp Sc: 39.22
GCCCAGGTGGCTCAGTCGGTTAAGCATTTGACTCTTGATCTTAGCTCAGGTCTCAAATCT
CTGGGTCGTGGGTTAAAGTCCCATGTTGGGCT
>Ailuropoda_melanoleuca_GL192836.1.trna12-LysCTT (712651-712741) Lys (CTT) 91 bp Sc: 36.77
GCCTGGGTAGCTCAGTTGGTTAAGCATCTGACTCTTGGTTTTGGCTCAGGTCACGAACTC
AGGGTTATGGGATCGAGCCCCATGCCAGGCT
>Ailuropoda_melanoleuca_GL192870.1.trna37-LysCTT (798010-798100) Lys (CTT) 91 bp Sc: 36.04
GCCTGGGTGGCTCAGTTGGTTAAGCCTCCGACCCTTGATTTTAGCTCAGGTCATGATCTC
AGGGACGTGGGATCGAGCCCCACCTTGGGCT
>Ailuropoda_melanoleuca_GL193999.1.trna1-LysCTT (82345-82435) Lys (CTT) 91 bp Sc: 27.93
ACCTGGGTGGCTCAGTTGGTTAAGTATCTGACTCTTGATTTTGGCTCAGGTCATGATCTC
AGGATTGTGGGATCAAGGCCCACATCAGGTT
>Ailuropoda_melanoleuca_GL192682.1.trna75-LysCTT (835453-835363) Lys (CTT) 91 bp Sc: 36.13
GCCTGGCTGGCTCAGTCGGTTGAGCGTCCAACTCTTGATCTTGGCTCAGGTTTTGATCTC
ATGGTGGTGAGTTCGAGCCCCACGTTGGGCT
>Ailuropoda_melanoleuca_GL192353.1.trna16-LysCTT (875386-875475) Lys (CTT) 90 bp Sc: 29.44
GCCTGGATGGCTCAGTCAGTAAGTGTCTGACTCTTGATTTTGGCTCAGATCATGACCTCA
GGGTGCTGGGATCGAGCCCCAGGTCAGGCT
>Ailuropoda_melanoleuca_GL193154.1.trna31-LysCTT (924877-924786) Lys (CTT) 92 bp Sc: 27.51
GCCTGAGTGGCTCAGTCGGCTAAGCATCTGACTCTTGGTTTCGGCTCAGGTCACAATCTC
ATGGGTGGTGGGATCGAGCCCCATGTCAGGCT
>Ailuropoda_melanoleuca_GL192443.1.trna17-LysCTT (950428-950518) Lys (CTT) 91 bp Sc: 38.54
GCCTGGCTGGCTCAGTTGGTTAAGCATCTGACTCTTGGTTTCGGCTCAGGTCATGATCTC
AGGGTCCTGGGATCAAGCCCCATGTCAGGCT
>Ailuropoda_melanoleuca_GL192508.1.trna37-LysCTT (954885-954979) Lys (CTT) 95 bp Sc: 38.99
GCCTGGGTGGCTCAGTTGGTTGAGCTTCTGACTCTTGATTTTGACTCAGGTCATGATCTC
ACTCAGGGTCATGAGATCGAGACTCATGTCAGGCT
>Ailuropoda_melanoleuca_GL192831.1.trna27-LysTTT (545177-545105) Lys (TTT) 73 bp Sc: 28.13
ACCTGGCTGGCTCAGTTGCTAGAGCATGCAGCTTTTGATTTCAGGGTTGTGAGTTTAAGC
CCCATGTTGGGTG
>Ailuropoda_melanoleuca_GL193022.1.trna60-LysTTT (1031276-1031204) Lys (TTT) 73 bp Sc: 30.16
GCCTGACTGGCTCAGTAGGTGGAGCGTGCAACTTTTGATTTTGGGGTTGTGGGTTTGAAC
CCCACGTTGTGTG
>Ailuropoda_melanoleuca_GL193257.1.trna3-LysTTT (76760-76832) Lys (TTT) 73 bp Sc: 30.22
ATTTGGCTGGCTCAGTCAGTACAGCATGGGATTTTTGATCTTGCGGTCATGAGTTCAAGC
CCCATGTTGGATA
>Ailuropoda_melanoleuca_GL193182.1.trna17-LysTTT (414213-414284) Lys (TTT) 72 bp Sc: 30.25
ACCTGGCTGGCTCAGCCAGTACAGCATGTGACTTTTGATCTTGGGTTGTGAGTTCAAGCC
CCATGCTGGGTG
>Ailuropoda_melanoleuca_GL192471.1.trna96-LysTTT (95370-95298) Lys (TTT) 73 bp Sc: 31.73
ACTGGGCTGGCTCAGTTGGTGGAGCATGCGACTTTTGATTTTAGGGTTGTGGGTTTGAGC
CCCATGTTGGGTG
>Ailuropoda_melanoleuca_GL192359.1.trna179-LysTTT (3484397-3484325) Lys (TTT) 73 bp Sc: 32.96
GCCTGGCTAGCTCACTTGGTGGAGCTTATGACTTTTGATCTTGGGGCTGTGAGTTCGAGC
CCCATGTTGGGTT
>Ailuropoda_melanoleuca_GL192521.1.trna48-LysTTT (1540280-1540208) Lys (TTT) 73 bp Sc: 34.20
GCCTGGCTGGCTTGGTTGGTGGAGCTTGTGATTTTTGGTCTTGGGGTTGTGGGTTCGAGC
CCCATGCTGGGTG
>Ailuropoda_melanoleuca_GL192688.1.trna21-LysTTT (876241-876313) Lys (TTT) 73 bp Sc: 34.28
TTCTGGCTGGCTTAGTCAGCGGAGCATGTGACTTTTGATCTCAGGGTTGTGGGTTCAAGC

CCCACGTTGGGTG
>Ailuropoda_melanoleuca_GL192870.1.trna43-LysTTT (857645-857717) Lys (TTT) 73 bp Sc: 35.33
ACCTGGCTGGCTCAGTCAGTGGAGCGTGTGACCTTTGATCTCGGGGTCGTGGGTTCAAGC
CTCGTGTTGGGTG
>Ailuropoda_melanoleuca_GL192924.1.trna74-LysTTT (1179198-1179270) Lys (TTT) 73 bp Sc: 35.72
ACCTGACTGGCTCAGTTGGTGGAGCATACGACTTTTGATCTTGGGGTGGTGAGTTTGAGA
CCCATGTTGGGTG
>Ailuropoda_melanoleuca_GL192556.1.trna81-LysTTT (477497-477425) Lys (TTT) 73 bp Sc: 35.94
ATCTGATTGGCTCAGTTGGTGGAGTGTGTGACTTTTGATCTCGGGATTGTGGGTTTGAGC
CCCATGTTGGGTG
>Ailuropoda_melanoleuca_GL193249.1.trna34-LysTTT (797839-797767) Lys (TTT) 73 bp Sc: 37.76
ACCTGGCTAGCTCAGTCAGAAGAGTGTGTGACTTTTGATCTTGGGGTTGTGAGTTCAAGC
CCCATGTTGGGTG
>Ailuropoda_melanoleuca_GL192489.1.trna125-LysTTT (1658292-1658220) Lys (TTT) 73 bp Sc: 38.99
GCCTGGTTGGCTCAGTTATTAGAGCTTGAGACTTTTGATATCGGGGTCGTGAGTTCAACC
CCCATGTTGGGCA
>Ailuropoda_melanoleuca_GL193447.1.trna24-LysTTT (609067-609139) Lys (TTT) 73 bp Sc: 40.63
ACCTGGCTGGCTCAGTCGGTAGAGCATGTGACTTTTGATTTCAGGGTTGTGAGTGCAAGC
CCCGTGTTGGGTA
>Ailuropoda_melanoleuca_GL192405.1.trna159-LysTTT (1997615-1997543) Lys (TTT) 73 bp Sc: 42.03
GCCTGGCTGGCTCAGTCAGTAGAGCATGTGACTTTTGATCTCAGGGTGGTGAGTTTGAGC
CCCACGTTGGGTG
>Ailuropoda_melanoleuca_GL194909.1.trna6-LysTTT (108176-108248) Lys (TTT) 73 bp Sc: 42.47
ACCTGGTTGGCTTAGTCAGTGGAGCATGAGACTTTTGATCTCAGGGTTGTGAGTTCAAGC
CCCACGTTGGGTA
>Ailuropoda_melanoleuca_GL193649.1.trna4-LysTTT (212994-213066) Lys (TTT) 73 bp Sc: 43.05
ACCTGGCTGGCTCAGTCAGTATAGCATGTGACTTTTGATCTCGGGGTTGTGAGTTCAAGC
CCCACGCTGGGTG
>Ailuropoda_melanoleuca_GL192489.1.trna67-LysTTT (1883107-1883179) Lys (TTT) 73 bp Sc: 43.49
GCCTGACTGGCTCAGTCAGTGGAGCATGTGACTTTTGATCGCAGGGTCATGAGTTCAAGC
CCCATGTTGGGCC
>Ailuropoda_melanoleuca_GL194507.1.trna9-LysTTT (18222-18150) Lys (TTT) 73 bp Sc: 44.23
GCCTGGCTGGCTCAATCAGTGGAGCACATGACTTTTGATCCTGGGGTTGTGGGTTCGAGC
CCCACATTGGGTG
>Ailuropoda_melanoleuca_GL192349.1.trna62-LysTTT (2027453-2027525) Lys (TTT) 73 bp Sc: 44.25
ACCTGACTAGCTCAGTTGGAAGAGCATGTGACTTTTGATCTTGGGGTTGTGAGTTTGAGC
CTCACATTGGGTA
>Ailuropoda_melanoleuca_GL192426.1.trna70-LysTTT (2883775-2883847) Lys (TTT) 73 bp Sc: 44.34
ACCCGGCTGGCTCAGTTGGTGAAGCACGTGACTTTTGATCTCGGAGTTGTGGGTTCAAGG
CCCATGTTGGGTG
>Ailuropoda_melanoleuca_GL192810.1.trna67-LysTTT (1204908-1204836) Lys (TTT) 73 bp Sc: 44.38
ACCTGGCTGGCTCAGTCAGAAGAGCATGGGATTTTTGATCTCGGGGTCATGAGTTCGAGC
CCCATGTTGGGTA
>Ailuropoda_melanoleuca_GL192588.1.trna116-LysTTT (1506922-1506850) Lys (TTT) 73 bp Sc: 45.20
GCCTGGCTGGCTCAGTCGGTAGAGCATGTGACTTTTCATCTTGGGATTGTGTGTTCGAGC
CTCACGTTGGGTG
>Ailuropoda_melanoleuca_GL193741.1.trna8-LysTTT (508787-508859) Lys (TTT) 73 bp Sc: 45.47
GCCTGGCTGGCTCAGTCAGTAAAGCATGTGACTTTTGATCTCAGGGTCGTGAGTTCAAGC
CACATGTTGGGTG
>Ailuropoda_melanoleuca_GL193225.1.trna49-LysTTT (162565-162493) Lys (TTT) 73 bp Sc: 45.57
GCCTGGCTGGCTCAGTTGGTGGAGCATGTGACTTTTGATCTCAGGGTTGTGAGTTCAAGC
CCCATGTTGGGTC
>Ailuropoda_melanoleuca_GL192375.1.trna172-LysTTT (2352673-2352601) Lys (TTT) 73 bp Sc: 46.64
GCCCAGCTGGCTCAGTCTGTGGAGCACGTGACTTTTGATCTTGAGGTTGTGAGTTCGAGT
CCCACGTTGGGTG
>Ailuropoda_melanoleuca_GL192668.1.trna46-LysTTT (1612802-1612874) Lys (TTT) 73 bp Sc: 47.12
ACCTGGCTGGCTCAGTTGGTAAAGCATGTGACCTTTGGTCTCAAGGTTGTGAGTTCAAGC
CTCATGTTGGGTG
>Ailuropoda_melanoleuca_GL192375.1.trna61-LysTTT (2082234-2082306) Lys (TTT) 73 bp Sc: 48.02
GCCTGGCTGGCTCAGTCGGTGGAGTGTGTGGCTTTTGATCTCATGGTTGTGAGTTCAAAC
CCCACGTTGGGTG
>Ailuropoda_melanoleuca_GL193718.1.trna48-LysTTT (225445-225373) Lys (TTT) 73 bp Sc: 49.45
GCCGGGCTGGCTCAGTGGGAGGAGCGTGCGACTTTTGATCTCAGGGTCATGGGTTCAAGC
CCCATGTTGGGTG
>Ailuropoda_melanoleuca_GL193113.1.trna19-LysTTT (819492-819564) Lys (TTT) 73 bp Sc: 49.59
ACTCGGCTGGCTCAGGCGGAAGAGTGTGTGACTTTTGATCTCAGGGTCCTGAGTTCAAGC
CTCACGCTGGGTG

>Ailuropoda_melanoleuca_GL192979.1.trna63-LysTTT (671499-671427) Lys (TTT) 73 bp Sc: 50.67
GCCCAGTTGGCTCAGTCGGTGGAGCGTGCGACTTTTGATTTCAGGGTTGTGAGTTCAAGC
CCCACATTGGGTA
>Ailuropoda_melanoleuca_GL192728.1.trna83-LysTTT (1211187-1211115) Lys (TTT) 73 bp Sc: 50.69
GCCTGGCTGGCTCAGTCAGAAGAGCATGTGACTTTTGATCTCAGGGTCGTAGGTTCAAGC
CCCATGTTGGGCG
>Ailuropoda_melanoleuca_GL192379.1.trna122-LysTTT (2905569-2905497) Lys (TTT) 73 bp Sc: 52.16
GCCTGGCTGGCTCAGTCAGTAGAGCATGAGATTTTTGATCTCAGGTTCATGAGTTCAAGC
CCCATGTTGGGCA
>Ailuropoda_melanoleuca_GL192919.1.trna5-LysTTT (103764-103836) Lys (TTT) 73 bp Sc: 55.11
GCCTGGCTGGCTCAGTCAGAAGAGCGTGAGACTTTTGATCTCAGGGCCATGGGTTCGAGC
CCCATGTTGGGGG
>Ailuropoda_melanoleuca_GL194512.1.trna2-LysTTT (177440-177368) Lys (TTT) 73 bp Sc: 55.11
GCCTGGCTGGCTCAGTCAGAAGAGCGTGAGACTTTTGATCTCAGGGCCATGGGTTCGAGC
CCCATGTTGGGGG
>Ailuropoda_melanoleuca_GL194072.1.trna13-LysTTT (13370-13298) Lys (TTT) 73 bp Sc: 55.91
GCCTGGCTGGCTCAGTGGGTAGAGCATGTGACTTTTGATCTCAGGGTTGTGAGTTCGAGT
CCCACGTTGGGGA
>Ailuropoda_melanoleuca_GL193003.1.trna10-LysTTT (470017-470089) Lys (TTT) 73 bp Sc: 56.10
GCCTGGCTGGCTCAGTTGGTAGAGCATGCAACTTTTGATCTCAGGGTTGTGAGTTCAAGC
CCCACGTTGGGCA
>Ailuropoda_melanoleuca_GL192573.1.trna69-LysTTT (1192860-1192932) Lys (TTT) 73 bp Sc: 57.12
ACCTGGCTGGCTCAGTTGGTAGAGCATGTGACTTTTAATCTCAGGGTCATGAGTTCAAGC
CCCATGTTGGGGG
>Ailuropoda_melanoleuca_GL192502.1.trna22-LysTTT (1215897-1215969) Lys (TTT) 73 bp Sc: 60.59
GCCTAGCTGGCTCAGTTGGTAGAGCGTGTGACTTTTGATCTAAGGGTTGTGAGTTCAAGC
CTCACATTGGGCA
>Ailuropoda_melanoleuca_GL192462.1.trna33-LysTTT (1377651-1377723) Lys (TTT) 73 bp Sc: 60.71
ACCTGGCTGGCTCAGTTGGTAGAGCATGTGACTTTTAATCTCAGGGTCATGAGTTCAAGC
CTCATGTTGGGCG
>Ailuropoda_melanoleuca_GL193552.1.trna33-LysTTT (457733-457805) Lys (TTT) 73 bp Sc: 70.83
GCCTGGGTAGCTCAGGTGGCAGAGCATCAGACTTTTAATCTGAGGGTCCAGGGTTCAAGT
CCCTGTCCAGGTG
>Ailuropoda_melanoleuca_GL193552.1.trna72-LysTTT (277777-277705) Lys (TTT) 73 bp Sc: 76.70
GCCTGGATAGCTCAGTCGGTAGAGCATCAGACTTTTAATCTGAGGGTCCAGGGTTCAAGC
CCCTGCTCAGGCG
>Ailuropoda_melanoleuca_GL193897.1.trna56-LysTTT (106316-106244) Lys (TTT) 73 bp Sc: 77.77
GCCCGGGTAGCTCAGTTGGCAGAGCATCAGACTTTTAATCTGAGGGTCCAGGGTTCAAGT
CCCTGTTCGGGCA
>Ailuropoda_melanoleuca_GL193897.1.trna52-LysTTT (211639-211567) Lys (TTT) 73 bp Sc: 79.39
GCCTGGATAGCTCAGTCGGCAGAGCATCAGACTTTTAATCTGAGGGTCCAGGGTTCAAGT
CCCTGTTCAGGCG
>Ailuropoda_melanoleuca_GL193552.1.trna20-LysTTT (267210-267282) Lys (TTT) 73 bp Sc: 80.08
GCCTGGGTAGCTCAGTCGGTAGAGCATCAGACTTTTAATCTGAGGGTCCAGGGTTCAAGT
CCCTGTTCAGGCG
>Ailuropoda_melanoleuca_GL193897.1.trna23-LysTTT (249131-249203) Lys (TTT) 73 bp Sc: 80.53
GCCCGGATAGCTCAGTTGGCAGAGCATCAGACTTTTAATCTGAGGGTCCAGGGTTCAAGT
CCCTGTTCGGGCG
>Ailuropoda_melanoleuca_GL192583.1.trna27-LysTTT (893900-893972) Lys (TTT) 73 bp Sc: 81.29
GCCCGGATAGCTCAGTCGGTAGAGCATCAGACTTTTAATCTGAGGGTCCAGGGTTCGAGT
CCCCGTTCGGGCG
>Ailuropoda_melanoleuca_GL192583.1.trna97-LysTTT (894315-894243) Lys (TTT) 73 bp Sc: 83.80
GCCCGGATAGCTCAGTCGGTAGAGCATCAGACTTTTAATCTGAGGGTCCAGGGTTCAAGT
CCCTGTTCGGGCG
>Ailuropoda_melanoleuca_GL192808.1.trna76-LysTTT (1205557-1205485) Lys (TTT) 73 bp Sc: 83.80
GCCCGGATAGCTCAGTCGGTAGAGCATCAGACTTTTAATCTGAGGGTCCAGGGTTCAAGT
CCCTGTTCGGGCG
>Ailuropoda_melanoleuca_GL194275.1.trna18-LysTTT (36174-36102) Lys (TTT) 73 bp Sc: 83.80
GCCCGGATAGCTCAGTCGGTAGAGCATCAGACTTTTAATCTGAGGGTCCAGGGTTCAAGT
CCCTGTTCGGGCG
>Ailuropoda_melanoleuca_GL194275.1.trna4-LysTTT (32562-32634) Lys (TTT) 73 bp Sc: 83.80
GCCCGGATAGCTCAGTCGGTAGAGCATCAGACTTTTAATCTGAGGGTCCAGGGTTCAAGT
CCCTGTTCGGGCG
>Ailuropoda_melanoleuca_GL194901.1.trna6-LysTTT (90068-90140) Lys (TTT) 73 bp Sc: 83.80
GCCCGGATAGCTCAGTCGGTAGAGCATCAGACTTTTAATCTGAGGGTCCAGGGTTCAAGT
CCCTGTTCGGGCG
>Ailuropoda_melanoleuca_GL192728.1.trna80-LysTTT (1290013-1289924) Lys (TTT) 90 bp Sc: 25.96

GCCTGGGTGGCTCAGTCGGTCGAGCATTGACTTTTGGTTTCGGCTCAAGTCATAGTCTCA
GGGACATGGGATTGAGCCCCATGTCGGGCT
>Ailuropoda_melanoleuca_GL193882.1.trna14-LysTTT (151932-151843) Lys (TTT) 90 bp Sc: 38.68
ACCTGGGTGGCTCAGTTGGTTAAGCATCTGACTTTTGATCTCAGCTCAGGTCTTGATTTG
GGGTGGTGGGTTCGAACCCCACTCTGGGCT
>Ailuropoda_melanoleuca_GL192868.1.trna48-LysTTT (201482-201392) Lys (TTT) 91 bp Sc: 24.41
GCCTGGGTGGCTCAGTCGGTTAAGCAACTTACTTTTGATTTCAGCTCAGGTCCTGATCTC
AGTCTTGTGAGATCGAGCCCCACTTCAGGCT
>Ailuropoda_melanoleuca_GL192457.1.trna67-MetCAT (718735-718807) Met (CAT) 73 bp Sc: 30.11
GCCTGGCTGGCTCAGTTAGAAGAGCATGCAACTCATTATTTCAGGTTCATGAGTTCAAGC
CCCATGCTGGGTG
>Ailuropoda_melanoleuca_GL192364.1.trna134-MetCAT (3885947-3885874) Met (CAT) 74 bp Sc: 34.67
ACCTGGCTGGTTCAGTCAGTACAGCATGTGACTCATGATCTCAGGGTTTGTGAGTTCGAG
CCCCATGTTAGGTG
>Ailuropoda_melanoleuca_GL194019.1.trna14-MetCAT (223844-223772) Met (CAT) 73 bp Sc: 36.69
GCCTGGCTGGCTCAGTCTGTAGAGCATGGGACTCATGACCTCAGGGTTGTAAGTTTGAGC
CCTACGTTAGGTG
>Ailuropoda_melanoleuca_GL193431.1.trna46-MetCAT (85840-85769) Met (CAT) 72 bp Sc: 58.99
AGCAGAGTGGCGCAGTGGAAGTGTTCTGGGCCCATAACCCAGAGGTCGATGGATCGAAAC
CCTCCTCTGCTA
>Ailuropoda_melanoleuca_GL193874.1.trna18-MetCAT (378788-378717) Met (CAT) 72 bp Sc: 61.41
AGCAGAGTGGCGCAGCGGAAGCGTGCTGGGACCATAACCCAGAGGTCGATGGATCGAAAC
CATCCTCTGCTA
>Ailuropoda_melanoleuca_GL193552.1.trna26-MetCAT (317802-317873) Met (CAT) 72 bp Sc: 67.60
AGCAGAGTGGCGCAGCGGGAGCGTGCTGGGCCCATAACCCAGAGGTCGATGGATCGAAAC
CATCCTCTGCTA
>Ailuropoda_melanoleuca_GL193552.1.trna37-MetCAT (482918-482989) Met (CAT) 72 bp Sc: 67.60
AGCAGAGTGGCGCAGCGGGAGCGTGCTGGGCCCATAACCCAGAGGTCGATGGATCGAAAC
CATCCTCTGCTA
>Ailuropoda_melanoleuca_GL192922.1.trna59-MetCAT (1010783-1010712) Met (CAT) 72 bp Sc: 68.98
AGCAGAGTGGCGCAGCGGAAGCGTGCTGGGCCCATAACCCAGAGGTCGATGGATCGAAAC
CATCCTCTGCTA
>Ailuropoda_melanoleuca_GL193552.1.trna19-MetCAT (265955-266026) Met (CAT) 72 bp Sc: 68.98
AGCAGAGTGGCGCAGCGGAAGCGTGCTGGGCCCATAACCCAGAGGTCGATGGATCGAAAC
CATCCTCTGCTA
>Ailuropoda_melanoleuca_GL193552.1.trna34-MetCAT (462929-463000) Met (CAT) 72 bp Sc: 68.98
AGCAGAGTGGCGCAGCGGAAGCGTGCTGGGCCCATAACCCAGAGGTCGATGGATCGAAAC
CATCCTCTGCTA
>Ailuropoda_melanoleuca_GL193552.1.trna82-MetCAT (186190-186119) Met (CAT) 72 bp Sc: 68.98
AGCAGAGTGGCGCAGCGGAAGCGTGCTGGGCCCATAACCCAGAGGTCGATGGATCGAAAC
CATCCTCTGCTA
>Ailuropoda_melanoleuca_GL193874.1.trna15-MetCAT (390702-390631) Met (CAT) 72 bp Sc: 68.98
AGCAGAGTGGCGCAGCGGAAGCGTGCTGGGCCCATAACCCAGAGGTCGATGGATCGAAAC
CATCCTCTGCTA
>Ailuropoda_melanoleuca_GL193874.1.trna9-MetCAT (360091-360162) Met (CAT) 72 bp Sc: 68.98
AGCAGAGTGGCGCAGCGGAAGCGTGCTGGGCCCATAACCCAGAGGTCGATGGATCGAAAC
CATCCTCTGCTA
>Ailuropoda_melanoleuca_GL192819.1.trna13-MetCAT (286540-286612) Met (CAT) 73 bp Sc: 76.47
GCCCTCTTAGCGCAGTGGGCAGCGCGTCAGTCTCATAATCTGAAGGTCCTGAGTTCAAGC
CTCAGAGAGGGCA
>Ailuropoda_melanoleuca_GL194938.1.trna11-MetCAT (22902-22830) Met (CAT) 73 bp Sc: 76.47
GCCCTCTTAGCGCAGTGGGCAGCGCGTCAGTCTCATAATCTGAAGGTCCTGAGTTCAAGC
CTCAGAGAGGGCA
>Ailuropoda_melanoleuca_GL193897.1.trna5-MetCAT (104009-104081) Met (CAT) 73 bp Sc: 76.58
GCCTCCTTAGCGCAGTAGGCAGCGCGTCAGTCTCATAATCTGAAGGTCCTGAGTTCGAAC
CTCAGAGGGGGCA
>Ailuropoda_melanoleuca_GL193897.1.trna55-MetCAT (108163-108091) Met (CAT) 73 bp Sc: 76.58
GCCTCCTTAGCGCAGTAGGCAGCGCGTCAGTCTCATAATCTGAAGGTCCTGAGTTCGAAC
CTCAGAGGGGGCA
>Ailuropoda_melanoleuca_GL192661.1.trna2-MetCAT (91466-91538) Met (CAT) 73 bp Sc: 76.99
GCCTTCTTAGCGCAGTGGGCAGCGCGTCAGTCTCATAATCTGAAGGTCCTGAGTTCGAGC
CTCAGAGAGGGCA
>Ailuropoda_melanoleuca_GL193771.1.trna18-MetCAT (467303-467375) Met (CAT) 73 bp Sc: 76.99
GCCTTCTTAGCGCAGTGGGCAGCGCGTCAGTCTCATAATCTGAAGGTCCTGAGTTCGAGC
CTCAGAGAGGGCA
>Ailuropoda_melanoleuca_GL194901.1.trna24-MetCAT (327-255) Met (CAT) 73 bp Sc: 76.99
GCCTTCTTAGCGCAGTGGGCAGCGCGTCAGTCTCATAATCTGAAGGTCCTGAGTTCGAGC

CTCAGAGAGGGCA
>Ailuropoda_melanoleuca_GL194931.1.trna1-MetCAT (13591-13663) Met (CAT) 73 bp Sc: 76.99
GCCTTCTTAGCGCAGTGGGCAGCGCGTCAGTCTCATAATCTGAAGGTCCTGAGTTCGAGC
CTCAGAGAGGGCA
>Ailuropoda_melanoleuca_GL193897.1.trna37-MetCAT (434992-434920) Met (CAT) 73 bp Sc: 77.72
GCCTTCTTAGCGCAGTGGGCAGCGCGTCAGTCTCATAATCTGAAGGTCCTGAGTTCGAGC
CTCAGAGAAGGCA
>Ailuropoda_melanoleuca_GL192795.1.trna29-MetCAT (863847-863919) Met (CAT) 73 bp Sc: 78.61
GCCCTCTTGGCGCAGTGGGCAGCGCGTCAGTCTCATAATCTGAAGGTCCTGAGTTCGAGC
CTCAGAGAGGGCA
>Ailuropoda_melanoleuca_GL193135.1.trna45-MetCAT (77347-77275) Met (CAT) 73 bp Sc: 79.85
GCCTCGTTAGCGCAGTAGGTAGCGCGTCAGTCTCATAATCTGAAGGTCGTGAGTTCGATC
CTCACACGGGGCA
>Ailuropoda_melanoleuca_GL192607.1.trna26-PheGAA (1209664-1209736) Phe (GAA) 73 bp Sc: 80.85
GCTGAAATAGCTCAGTTGGGAGAGCGTTAGACTGAAGATCTAAAGGTCCCTGGTTCAATC
CCGGGTTTCAGCA
>Ailuropoda_melanoleuca_GL192354.1.trna213-PheGAA (4183962-4183890) Phe (GAA) 73 bp Sc: 84.19
GCCGAAATAGCTCAGTTGGGAGAGCGTTAGACTGAAGATCTAAAGGTCCCTGGTTCGATC
CCGGGTTTCGGCA
>Ailuropoda_melanoleuca_GL192615.1.trna100-PheGAA (596407-596335) Phe (GAA) 73 bp Sc: 84.19
GCCGAAATAGCTCAGTTGGGAGAGCGTTAGACTGAAGATCTAAAGGTCCCTGGTTCGATC
CCGGGTTTCGGCA
>Ailuropoda_melanoleuca_GL193041.1.trna27-PheGAA (757034-756962) Phe (GAA) 73 bp Sc: 84.19
GCCGAAATAGCTCAGTTGGGAGAGCGTTAGACTGAAGATCTAAAGGTCCCTGGTTCGATC
CCGGGTTTCGGCA
>Ailuropoda_melanoleuca_GL193897.1.trna16-PheGAA (197423-197495) Phe (GAA) 73 bp Sc: 84.19
GCCGAAATAGCTCAGTTGGGAGAGCGTTAGACTGAAGATCTAAAGGTCCCTGGTTCGATC
CCGGGTTTCGGCA
>Ailuropoda_melanoleuca_GL193897.1.trna20-PheGAA (226997-227069) Phe (GAA) 73 bp Sc: 84.19
GCCGAAATAGCTCAGTTGGGAGAGCGTTAGACTGAAGATCTAAAGGTCCCTGGTTCGATC
CCGGGTTTCGGCA
>Ailuropoda_melanoleuca_GL193897.1.trna48-PheGAA (230285-230213) Phe (GAA) 73 bp Sc: 84.19
GCCGAAATAGCTCAGTTGGGAGAGCGTTAGACTGAAGATCTAAAGGTCCCTGGTTCGATC
CCGGGTTTCGGCA
>Ailuropoda_melanoleuca_GL193897.1.trna4-PheGAA (94582-94654) Phe (GAA) 73 bp Sc: 84.19
GCCGAAATAGCTCAGTTGGGAGAGCGTTAGACTGAAGATCTAAAGGTCCCTGGTTCGATC
CCGGGTTTCGGCA
>Ailuropoda_melanoleuca_GL194275.1.trna17-PheGAA (39450-39378) Phe (GAA) 73 bp Sc: 84.19
GCCGAAATAGCTCAGTTGGGAGAGCGTTAGACTGAAGATCTAAAGGTCCCTGGTTCGATC
CCGGGTTTCGGCA
>Ailuropoda_melanoleuca_GL194275.1.trna19-PheGAA (33659-33587) Phe (GAA) 73 bp Sc: 84.19
GCCGAAATAGCTCAGTTGGGAGAGCGTTAGACTGAAGATCTAAAGGTCCCTGGTTCGATC
CCGGGTTTCGGCA
>Ailuropoda_melanoleuca_GL193552.1.trna77-PheGAA (228398-228326) Phe (GAA) 73 bp Sc: 85.19
GCCGAGATAGCTCAGTTGGGAGAGCGTTAGACTGAAGATCTAAAGGTCCCTGGTTCGATC
CCGGGTCTCGGCA
>Ailuropoda_melanoleuca_GL194214.1.trna4-ProAGG (26260-26331) Pro (AGG) 72 bp Sc: 70.27
GGCACGTTGGTCTAGGGGTATGATTCTCGCTTAGGGTGCGAGAGGTCCCGGGTTCAAATC
CCGGACGAGCCC
>Ailuropoda_melanoleuca_GL192359.1.trna50-ProAGG (1908784-1908855) Pro (AGG) 72 bp Sc: 75.92
GGCTCGTTGGTCTAGGGGTATGATTCTCGCTTAGGGTGCGAGAGGTCCCGGGTTCAAATC
CCGGACGAGCCC
>Ailuropoda_melanoleuca_GL192425.1.trna188-ProAGG (522902-522831) Pro (AGG) 72 bp Sc: 75.92
GGCTCGTTGGTCTAGGGGTATGATTCTCGCTTAGGGTGCGAGAGGTCCCGGGTTCAAATC
CCGGACGAGCCC
>Ailuropoda_melanoleuca_GL193204.1.trna11-ProAGG (71330-71401) Pro (AGG) 72 bp Sc: 75.92
GGCTCGTTGGTCTAGGGGTATGATTCTCGCTTAGGGTGCGAGAGGTCCCGGGTTCAAATC
CCGGACGAGCCC
>Ailuropoda_melanoleuca_GL193204.1.trna13-ProAGG (75509-75580) Pro (AGG) 72 bp Sc: 75.92
GGCTCGTTGGTCTAGGGGTATGATTCTCGCTTAGGGTGCGAGAGGTCCCGGGTTCAAATC
CCGGACGAGCCC
>Ailuropoda_melanoleuca_GL193204.1.trna75-ProAGG (50047-49976) Pro (AGG) 72 bp Sc: 75.92
GGCTCGTTGGTCTAGGGGTATGATTCTCGCTTAGGGTGCGAGAGGTCCCGGGTTCAAATC
CCGGACGAGCCC
>Ailuropoda_melanoleuca_GL193775.1.trna44-ProAGG (100603-100532) Pro (AGG) 72 bp Sc: 75.92
GGCTCGTTGGTCTAGGGGTATGATTCTCGCTTAGGGTGCGAGAGGTCCCGGGTTCAAATC
CCGGACGAGCCC

>Ailuropoda_melanoleuca_GL194214.1.trna5-ProAGG (29347-29418) Pro (AGG) 72 bp Sc: 75.92
GGCTCGTTGGTCTAGGGGTATGATTCTCGCTTAGGGTGCGAGAGGTCCCGGGTTCAAATC
CCGGACGAGCCC
>Ailuropoda_melanoleuca_GL194901.1.trna13-ProAGG (86526-86455) Pro (AGG) 72 bp Sc: 75.92
GGCTCGTTGGTCTAGGGGTATGATTCTCGCTTAGGGTGCGAGAGGTCCCGGGTTCAAATC
CCGGACGAGCCC
>Ailuropoda_melanoleuca_GL192808.1.trna53-ProCGG (1126404-1126475) Pro (CGG) 72 bp Sc: 76.52
GGCTCGTTGGTCTAGGGGTATGATTCTCGCTTCGGGTGCGAGAGGTCCCGGGTTCAAATC
CCGGACGAGCCC
>Ailuropoda_melanoleuca_GL193204.1.trna7-ProCGG (58527-58598) Pro (CGG) 72 bp Sc: 76.52
GGCTCGTTGGTCTAGGGGTATGATTCTCGCTTCGGGTGCGAGAGGTCCCGGGTTCAAATC
CCGGACGAGCCC
>Ailuropoda_melanoleuca_GL193775.1.trna3-ProCGG (99823-99894) Pro (CGG) 72 bp Sc: 76.52
GGCTCGTTGGTCTAGGGGTATGATTCTCGCTTCGGGTGCGAGAGGTCCCGGGTTCAAATC
CCGGACGAGCCC
>Ailuropoda_melanoleuca_GL194938.1.trna5-ProCGG (95593-95664) Pro (CGG) 72 bp Sc: 76.52
GGCTCGTTGGTCTAGGGGTATGATTCTCGCTTCGGGTGCGAGAGGTCCCGGGTTCAAATC
CCGGACGAGCCC
>Ailuropoda_melanoleuca_GL193608.1.trna41-ProCGG (214639-214550) Pro (CGG) 90 bp Sc: 30.83
GCCTGGGTGGCTCAGCTGGTTAAGTGTCTGACTCGGTTTGGCTCAGGTCATGATTTCAT
GGGTCGTGGGATCGAGCCCCACGTCAGGCT
>Ailuropoda_melanoleuca_GL192359.1.trna75-ProTGG (2452770-2452840) Pro (TGG) 71 bp Sc: 31.18
ACCTGGCTGGCTCAATCAGTAGAGCATGTGACTGGATCTCAGGGTTGTGAGTTCAAGCCC
CATGTTGGGTT
>Ailuropoda_melanoleuca_GL192946.1.trna25-ProTGG (719213-719131) Pro (TGG) 83 bp Sc: 32.24
GCCTGGGTGGCTGAGTGGTTAAGTGGCTGCCTTGGGCTCAGGTCATGATCTCGGTGTCCT
GGGATCTAGCCCCAGGTCAGGCT
>Ailuropoda_melanoleuca_GL193931.1.trna8-ProTGG (85730-85802) Pro (TGG) 73 bp Sc: 54.80
ACCTGGCTGGCTCAGTCGGTAGAGCATGTGACTTGGGATCTCAGGGTTGTGAGTTCAAGC
CCCATGTTGGGTA
>Ailuropoda_melanoleuca_GL195202.1.trna3-ProTGG (42034-42105) Pro (TGG) 72 bp Sc: 73.09
GGCTCGTTGGTCTAGGGGTATGATTCTCGCTTTGGGTGCGGGAGGTCCCGGGTTCAAATC
CCGGACGAGCCC
>Ailuropoda_melanoleuca_GL193204.1.trna10-ProTGG (69870-69941) Pro (TGG) 72 bp Sc: 76.15
GGCTCGTTGGTCTAGGGGTATGATTCTCGCTTTGGGTGCGAGAGGTCCCGGGTTCAAATC
CCGGACGAGCCC
>Ailuropoda_melanoleuca_GL193204.1.trna3-ProTGG (43745-43816) Pro (TGG) 72 bp Sc: 76.15
GGCTCGTTGGTCTAGGGGTATGATTCTCGCTTTGGGTGCGAGAGGTCCCGGGTTCAAATC
CCGGACGAGCCC
>Ailuropoda_melanoleuca_GL193204.1.trna74-ProTGG (53288-53217) Pro (TGG) 72 bp Sc: 76.15
GGCTCGTTGGTCTAGGGGTATGATTCTCGCTTTGGGTGCGAGAGGTCCCGGGTTCAAATC
CCGGACGAGCCC
>Ailuropoda_melanoleuca_GL194954.1.trna9-ProTGG (102722-102793) Pro (TGG) 72 bp Sc: 76.15
GGCTCGTTGGTCTAGGGGTATGATTCTCGCTTTGGGTGCGAGAGGTCCCGGGTTCAAATC
CCGGACGAGCCC
>Ailuropoda_melanoleuca_GL192425.1.trna14-ProTGG (522566-522637) Pro (TGG) 72 bp Sc: 76.24
GGCTCGTTGGTCTAGGGGTATGATTCTCGGTTTGGGTCCGAGAGGTCCCGGGTTCAAATC
CCGGACGAGCCC
>Ailuropoda_melanoleuca_GL194214.1.trna37-ProTGG (14304-14233) Pro (TGG) 72 bp Sc: 79.61
GGCTCGTTGGTCTAGTGGTATGATTCTCGCTTTGGGTGCGAGAGGTCCCGGGTTCAAATC
CCGGACGAGCCC
>Ailuropoda_melanoleuca_GL193184.1.trna21-ProTGG (328360-328449) Pro (TGG) 90 bp Sc: 29.18
GCCTGGGTAGCTCAGTCAGTTAAGCATCTGACTTGGTTTCAGCTCAGGTCACAATCTCAG
GGGTCATGGGATCGATCCCCATGTCGGGCT
>Ailuropoda_melanoleuca_GL193124.1.trna3-SeC(e)TCA (26915-27000) SeC(e) (TCA) 86 bp Sc: 73.19
GCCCGAATGATCCTCAGTGGTCTGGGGTGCAGGCTTCAAACCTGTAGCTGTCTAGCGACA
GAGTGGTTCAATTCCACCTTTCGGGC
>Ailuropoda_melanoleuca_GL192530.1.trna29-SeCTCA (930778-930848) SeC (TCA) 71 bp Sc: 50.79
ACCTGGCTGGCTCAGTCGGTAGAGCATGTGACTCAATCTCATGGTTGTGAGTTCGAGACC
CACGTTGGGTA
>Ailuropoda_melanoleuca_GL192672.1.trna8-SerACT (206797-206869) Ser (ACT) 73 bp Sc: 38.69
ACCTAGCTGGCTCAGTCAGTGGAGCATATGACTACTGATCTTGGGGTTATGAGTTCAAGC
CCCATGCTGGGTG
>Ailuropoda_melanoleuca_GL192369.1.trna236-SerACT (1803268-1803195) Ser (ACT) 74 bp Sc: 38.94
GCCTGGCTGGCTCAGTTGGTGGGAGCATGAGACTACTGACCTCAGGACTGTGAGTTCAAG
CCCCATGCTGGGTA
>Ailuropoda_melanoleuca_GL193552.1.trna64-SerAGA (342452-342371) Ser (AGA) 82 bp Sc: 71.86

GTAGTGGTGGCCGAGCGGTTAAGGCGACGGGCTAGAAATCCGTTGGGGTCTCCCCGCGCA
GGTTCGAGTCCTGCCGACTACG
>Ailuropoda_melanoleuca_GL192808.1.trna51-SerAGA (1122853-1122934) Ser (AGA) 82 bp Sc: 79.25
GTAGTCGTGGCCGAGTGGTTAAGGAGATGGACTAGAGATCCATTGGGGTCTCCCCGCGCA
GGTTCGAATCCTGCCGACTACG
>Ailuropoda_melanoleuca_GL193552.1.trna68-SerAGA (319641-319560) Ser (AGA) 82 bp Sc: 86.36
GTAGTCGTGGCCGAGTGGTTAAGGCGATGGACTAGAAATCCATTGGGGTCTCCCCGCGCA
GGTTCGAGTCCTGCCGACTACG
>Ailuropoda_melanoleuca_GL193450.1.trna4-SerAGA (71574-71655) Ser (AGA) 82 bp Sc: 88.01
GTAGTCGTGGCCGAGTGGTTAAGGCGATGGACTAGAAATCCATTGGGGTCTCCCCGCGCA
GGTTCGAATCCTGCCGACTACG
>Ailuropoda_melanoleuca_GL193552.1.trna23-SerAGA (292747-292828) Ser (AGA) 82 bp Sc: 88.01
GTAGTCGTGGCCGAGTGGTTAAGGCGATGGACTAGAAATCCATTGGGGTCTCCCCGCGCA
GGTTCGAATCCTGCCGACTACG
>Ailuropoda_melanoleuca_GL193552.1.trna25-SerAGA (312866-312947) Ser (AGA) 82 bp Sc: 88.01
GTAGTCGTGGCCGAGTGGTTAAGGCGATGGACTAGAAATCCATTGGGGTCTCCCCGCGCA
GGTTCGAATCCTGCCGACTACG
>Ailuropoda_melanoleuca_GL193552.1.trna63-SerAGA (355839-355758) Ser (AGA) 82 bp Sc: 88.01
GTAGTCGTGGCCGAGTGGTTAAGGCGATGGACTAGAAATCCATTGGGGTCTCCCCGCGCA
GGTTCGAATCCTGCCGACTACG
>Ailuropoda_melanoleuca_GL193552.1.trna65-SerAGA (337994-337913) Ser (AGA) 82 bp Sc: 88.01
GTAGTCGTGGCCGAGTGGTTAAGGCGATGGACTAGAAATCCATTGGGGTCTCCCCGCGCA
GGTTCGAATCCTGCCGACTACG
>Ailuropoda_melanoleuca_GL193874.1.trna12-SerAGA (388888-388969) Ser (AGA) 82 bp Sc: 88.01
GTAGTCGTGGCCGAGTGGTTAAGGCGATGGACTAGAAATCCATTGGGGTCTCCCCGCGCA
GGTTCGAATCCTGCCGACTACG
>Ailuropoda_melanoleuca_GL193874.1.trna19-SerAGA (378252-378171) Ser (AGA) 82 bp Sc: 88.01
GTAGTCGTGGCCGAGTGGTTAAGGCGATGGACTAGAAATCCATTGGGGTCTCCCCGCGCA
GGTTCGAATCCTGCCGACTACG
>Ailuropoda_melanoleuca_GL192457.1.trna23-SerCGA (298871-298952) Ser (CGA) 82 bp Sc: 89.14
GTCACGGTGGCCGAGTGGTTAAGGCGTTGGACTCGAAATCCAATGGGGTTTCCCCGCACA
GGTTCGAATCCTGTTCGTGACG
>Ailuropoda_melanoleuca_GL193552.1.trna14-SerCGA (222967-223048) Ser (CGA) 82 bp Sc: 90.35
GCTGTGATGGCCGAGTGGTTAAGGCGTTGGACTCGAAATCCAATGGGGTCTCCCCGCGCA
GGTTCAAATCCTGCTCACAGCG
>Ailuropoda_melanoleuca_GL193552.1.trna50-SerCGA (549261-549181) Ser (CGA) 81 bp Sc: 91.15
GCTGTGATGGCCGAGTGGTTAAGGCGTTGGACTCGAAATCCAATGGGGTTTCCCGCGCAG
GTTCAAATCCTGCTCACAGCG
>Ailuropoda_melanoleuca_GL192808.1.trna58-SerCGA (1191793-1191874) Ser (CGA) 82 bp Sc: 92.09
GCTGTGATGGCCGAGTGGTTAAGGCGTTGGACTCGAAATCCAATGGGGTCTCCCCGCGCA
GGTTCGAATCCTGCTCACAGCG
>Ailuropoda_melanoleuca_GL192770.1.trna40-SerGCT (316281-316200) Ser (GCT) 82 bp Sc: 30.88
GCCTGGGTGGCGCAGTTGTTAAGCGTCTCCTTCTGCTCAGGGCGTGATCCCGGAGTCGTG
GGATCGAGCCCCACATCAGGCT
>Ailuropoda_melanoleuca_GL192779.1.trna20-SerGCT (1075400-1075473) Ser (GCT) 74 bp Sc: 44.54
GTCTCTGTGGTGCACCCGGTTAGCACATTCGGTTGCTAGCGGAAAGGTTGGTGGTTCAAA
CTCACCCAGGGATA
>Ailuropoda_melanoleuca_GL192457.1.trna169-SerGCT (1463092-1463020) Ser (GCT) 73 bp Sc: 61.94
GCCTGGTTGGCTCAGTTGGTAGAGCATGTGACTGCTGATCTCAGGGCTGTGAGTTCAAGC
CCCACACTAGGCA
>Ailuropoda_melanoleuca_GL193874.1.trna22-SerGCT (369593-369512) Ser (GCT) 82 bp Sc: 81.10
GACGAGGTGGCCGAGTGGTTAAGGCGATGGACTGCTAATCCATTGTTCTCTGCACGCGTG
GGTTCGAATCCCATCCTCGTCG
>Ailuropoda_melanoleuca_GL193000.1.trna28-SerGCT (1022726-1022645) Ser (GCT) 82 bp Sc: 81.42
GACGAGGTGGCCGAGTGGCTAAGGCGATGGACTGCTAATCCATTGTGCTCTGCACGCGTG
GGTTCGAATCCCATCCTCGTCG
>Ailuropoda_melanoleuca_GL194938.1.trna3-SerGCT (67807-67888) Ser (GCT) 82 bp Sc: 82.98
GACGGAGTGGCCGAGTGGTTAAGGCGATGGACTGCTAATCCATTGTGCTCTGCACGCGTG
GGTTCAAGTCCCACCTTCGTCG
>Ailuropoda_melanoleuca_GL192356.1.trna194-SerGCT (3632227-3632308) Ser (GCT) 82 bp Sc: 85.34
GACGAGGTGGCCGAGTGGTTAAGGCGATGGACTGCTAATCCATTGTGCTCTGCACGCGTG
GGTTCGAATCCCATCCTCGTCG
>Ailuropoda_melanoleuca_GL192808.1.trna82-SerGCT (1148491-1148410) Ser (GCT) 82 bp Sc: 85.34
GACGAGGTGGCCGAGTGGTTAAGGCGATGGACTGCTAATCCATTGTGCTCTGCACGCGTG
GGTTCGAATCCCATCCTCGTCG
>Ailuropoda_melanoleuca_GL194580.1.trna23-SerGCT (36742-36661) Ser (GCT) 82 bp Sc: 85.34
GACGAGGTGGCCGAGTGGTTAAGGCGATGGACTGCTAATCCATTGTGCTCTGCACGCGTG

GGTTCGAATCCCATCCTCGTCG
>Ailuropoda_melanoleuca_GL194938.1.trna6-SerGCT (101378-101459) Ser (GCT) 82 bp Sc: 85.60
GACGAGGTGGCCGAGTGGTTAAGGCGATGGACTGCTAATCCATTGTGTTCTGCACGCGTG
GGTTCGAATCCCACCCTCGTCG
>Ailuropoda_melanoleuca_GL192492.1.trna161-SerGCT (58893-58812) Ser (GCT) 82 bp Sc: 85.83
GACGAGGTGGCCGAGTGGTTAAGGCGATGGACTGCTAATCCATTGTGCTTTGCACGCGTG
GGTTCGAATCCCATCCTCGTCG
>Ailuropoda_melanoleuca_GL193552.1.trna60-SerGCT (485939-485858) Ser (GCT) 82 bp Sc: 88.12
GACGAGGTGGCCGAGTGGTTAAGGCGATGGACTGCTAATCCATTGTGCTCTGCACGCGTG
GGTTCGAATCCCACCCTCGTCG
>Ailuropoda_melanoleuca_GL193897.1.trna29-SerGCT (323226-323307) Ser (GCT) 82 bp Sc: 88.12
GACGAGGTGGCCGAGTGGTTAAGGCGATGGACTGCTAATCCATTGTGCTCTGCACGCGTG
GGTTCGAATCCCACCCTCGTCG
>Ailuropoda_melanoleuca_GL192896.1.trna34-SerTGA (883254-883184) Ser (TGA) 71 bp Sc: 21.16
ACCTGGCTGGCTCAGCAGGTAAGGCATATGACTTGATTTCAGGGTCATGAGTTTGAGCCC
CATGTTGGGTA
>Ailuropoda_melanoleuca_GL193771.1.trna37-SerTGA (307451-307368) Ser (TGA) 84 bp Sc: 49.67
GCCTGAGTGGCTCAGTCGGTTAAGCGTCTGCCTTGAGCTCAGGTCATGATCCCAGGGTCC
TGGGATCGAGCCCCAGCTCAGGCT
>Ailuropoda_melanoleuca_GL193552.1.trna27-SerTGA (333436-333517) Ser (TGA) 82 bp Sc: 87.07
GTAGTCGTGGCCGAGTGGTTAAGGCGATGGACTTGAAATCCATTGGGGTCTCCCCGCGCA
GGTTCGAATCCTGCCGGCTACG
>Ailuropoda_melanoleuca_GL193552.1.trna69-SerTGA (306572-306491) Ser (TGA) 82 bp Sc: 88.73
GTAGTCGTGGCCGAGTGGTTAAGGCGATGGACTTGAAATCCATTGGGGTTTCCCCGCGCA
GGTTCGAATCCTGCCGACTACG
>Ailuropoda_melanoleuca_GL192368.1.trna166-SerTGA (3556459-3556378) Ser (TGA) 82 bp Sc: 90.86
GCAGCGATGGCCGAGTGGTTAAGGCGTTGGACTTGAAATCCAATGGGGTCTCCCCGCGCA
GGTTCGAACCCTGCTCGCTGCG
>Ailuropoda_melanoleuca_GL192512.1.trna46-SerTGA (2013404-2013492) Ser (TGA) 89 bp Sc: 30.71
GCCTGAGTGGCTCAGTAGGTTAAGTGCCCGACTTGATTTTGGCTCAGGTCATGATATCGG
GGTTGTGAGATCGAGCCCCACCTTGGGCT
>Ailuropoda_melanoleuca_GL192757.1.trna18-SupCTA (1221220-1221292) Sup (CTA) 73 bp Sc: 38.38
ACCTGGCTGGCTCAGTAGGTAGAGTGTGCAACTCTAGATTTTGGGGCTGTGAGTTCGAGC
CCCACGTTGGGTG
>Ailuropoda_melanoleuca_GL192790.1.trna61-SupCTA (1289833-1289761) Sup (CTA) 73 bp Sc: 38.85
ATCTGACTGGCTTAGTCAGTAGAGTGTGTGACTCTAGATCTCGGGGTTGTGGGTTTGAGC
CCCACATTGGGTG
>Ailuropoda_melanoleuca_GL194579.1.trna17-SupCTA (153367-153295) Sup (CTA) 73 bp Sc: 42.52
GCCCGGCTGGCTCAGTGGGTAGAGCGTAAGACTCTAAGTCTCAGGGCTGTGAGTTTAAGC
CCCACGTTGGGTG
>Ailuropoda_melanoleuca_GL192766.1.trna7-SupCTA (143169-143241) Sup (CTA) 73 bp Sc: 48.21
GCCTGGCTGGCTCAGTCGGAAGAGCATGGGACTCTAGATCTCAGGGTCATGAGTTCAAGC
CCTATGTTGGGTG
>Ailuropoda_melanoleuca_GL195554.1.trna2-SupCTA (7497-7569) Sup (CTA) 73 bp Sc: 48.47
ACCTGGCTGGCTCAGTTGGAAGAGCATGTGACTCTAGATCTCAGGGTTGTGAGTTTGAGC
CCCATGTTGGGTG
>Ailuropoda_melanoleuca_GL192346.1.trna43-SupCTA (1927391-1927463) Sup (CTA) 73 bp Sc: 50.66
GCCTGGCTGGCTCAGTCAGAAGAGCATGTGACTCTAAATCTCAGGGTTGTGAGTTCGAGC
CCCATGTCAGGTA
>Ailuropoda_melanoleuca_GL194216.1.trna8-SupCTA (82558-82486) Sup (CTA) 73 bp Sc: 52.90
GCCTGGCTGGCTCAGTTAGAAGAGCATGTGATTCTAGATCTCAGGGTCGTGAGTTCGAGT
CGCATGTTGGGCA
>Ailuropoda_melanoleuca_GL192982.1.trna4-SupCTA (323628-323700) Sup (CTA) 73 bp Sc: 53.52
TCCTGGCTGGCTCAGTCAGTAGAGCATGAGATTCTAAATCTCAGGGTTGTGAGTTCAAGC
CTCATGTTAGGCA
>Ailuropoda_melanoleuca_GL192385.1.trna148-SupCTA (3319083-3319011) Sup (CTA) 73 bp Sc: 59.76
GCCTGGCTGGCTCAGTTGGAAGAGCGTGTGACTCTAGATCTCAGGGTTGTGAGTTCGAGC
CCCACGTTGGGTG
>Ailuropoda_melanoleuca_GL194267.1.trna6-SupCTA (165572-165482) Sup (CTA) 91 bp Sc: 36.95
ACCTGGGTGGCTCAGTCGGTTAAGTGTCTTACTCTAGATTTTGTCTCAGATCATGATGTC
AGAGTCGCGGGATCGAGCCCCGCCTCAGGCT
>Ailuropoda_melanoleuca_GL192582.1.trna75-SupTTA (627111-627041) Sup (TTA) 71 bp Sc: 44.18
ACCTGGCTAGCTCAGTTGGTAGAGCATGTGACTTAATCTCGGGGTTGTGAGTTCAACCCC
CACGTTGGGTG
>Ailuropoda_melanoleuca_GL193552.1.trna55-ThrAGT (505414-505341) Thr (AGT) 74 bp Sc: 75.82
GGCTCTGTGGCTCAGCTGGTCAAAGCGCCTGTCTAGTAAACAGGAGATCCTGGGTTCGAC
TCCCAGCGGGGCCT

>Ailuropoda_melanoleuca_GL193552.1.trna49-ThrAGT (571696-571623) Thr (AGT) 74 bp Sc: 79.08
GGCTCTGTGGCTTAGCTGGTCAAAGCGCCTGTCTAGTAAACAGGAGATCCTGGGTTCGAA
TCCCAGCGGGGCCT
>Ailuropoda_melanoleuca_GL193552.1.trna12-ThrAGT (217961-218034) Thr (AGT) 74 bp Sc: 81.31
GGCTCCGTGGCTTAGCTGGTTAAAGCGCCTGTCTAGTAAACAGGAGATCCTGGGTTCAAA
TCCCAGCGGGGCCT
>Ailuropoda_melanoleuca_GL193552.1.trna81-ThrAGT (194487-194414) Thr (AGT) 74 bp Sc: 81.31
GGCTCCGTGGCTTAGCTGGTTAAAGCGCCTGTCTAGTAAACAGGAGATCCTGGGTTCAAA
TCCCAGCGGGGCCT
>Ailuropoda_melanoleuca_GL192808.1.trna57-ThrAGT (1191262-1191335) Thr (AGT) 74 bp Sc: 81.60
GGCGCCGTGGCTTAGCTGGTTAAAGCGCCTGTCTAGTAAACAGGAGATCCTGGGTTCGAA
TCCCAGCGGTGCCT
>Ailuropoda_melanoleuca_GL193897.1.trna49-ThrAGT (228494-228421) Thr (AGT) 74 bp Sc: 83.05
GGCTCCGTGGCTTAGCTGGTTAAAGCGCCTGTCTAGTAAACAGGAGATCCTGGGTTCGAA
TCCCAGCGGGGCCT
>Ailuropoda_melanoleuca_GL194901.1.trna7-ThrAGT (109134-109207) Thr (AGT) 74 bp Sc: 83.05
GGCTCCGTGGCTTAGCTGGTTAAAGCGCCTGTCTAGTAAACAGGAGATCCTGGGTTCGAA
TCCCAGCGGGGCCT
>Ailuropoda_melanoleuca_GL192808.1.trna52-ThrAGT (1123228-1123301) Thr (AGT) 74 bp Sc: 84.06
GGCGCCGTGGCTTAGTTGGTTAAAGCGCCTGTCTAGTAAACAGGAGATCCTGGGTTCGAA
TCCCAGCGGTGCCT
>Ailuropoda_melanoleuca_GL192808.1.trna83-ThrAGT (1148158-1148085) Thr (AGT) 74 bp Sc: 84.06
GGCGCCGTGGCTTAGTTGGTTAAAGCGCCTGTCTAGTAAACAGGAGATCCTGGGTTCGAA
TCCCAGCGGTGCCT
>Ailuropoda_melanoleuca_GL194426.1.trna8-ThrAGT (207449-207376) Thr (AGT) 74 bp Sc: 84.06
GGCGCCGTGGCTTAGTTGGTTAAAGCGCCTGTCTAGTAAACAGGAGATCCTGGGTTCGAA
TCCCAGCGGTGCCT
>Ailuropoda_melanoleuca_GL192596.1.trna81-ThrCGT (1601596-1601524) Thr (CGT) 73 bp Sc: 35.90
GCCTGGCTGGCTCAGTCAGTGGAGCATGGGACTCGTGATCTTGGGGCTGTGGGTTCAAAC
CCCATGTTGGGTG
>Ailuropoda_melanoleuca_GL194240.1.trna9-ThrCGT (157386-157458) Thr (CGT) 73 bp Sc: 39.60
ACCTGGCTGGCTGAATTGGAAGAGCATGCGACTCGTGATTTCAGGGTTGTGAGTTCAAGC
CCCATGCTGGGTG
>Ailuropoda_melanoleuca_GL195194.1.trna2-ThrCGT (39310-39382) Thr (CGT) 73 bp Sc: 46.58
GCCTGGCTGGCTCAGCTGGTAGAGCATATGACTCGTGATCGTGGGGCTGTAAGTTCAAGC
CTCACGTCGGGTG
>Ailuropoda_melanoleuca_GL192464.1.trna72-ThrCGT (1191169-1191241) Thr (CGT) 73 bp Sc: 53.17
ACCTGGCTGGCTCAGTTGGTACAGCTTGAGATTCGTGATTTCAGGGTCGTGAGTTCGAGC
CCCATGTTGGGTG
>Ailuropoda_melanoleuca_GL192439.1.trna57-ThrCGT (1625819-1625891) Thr (CGT) 73 bp Sc: 62.44
GCCTAGTTGGCTCAGTCGGAAGAGCGTGGGACTCGTGATCGCAGGGTCGTGGGTTCGAGC
CCTACATTGGGTG
>Ailuropoda_melanoleuca_GL193552.1.trna75-ThrCGT (248184-248112) Thr (CGT) 73 bp Sc: 76.84
GGCTCCGTAGCTCAGGGGTTAGAGCACTGGTCTCGTAAACCAGGGGTCGTGAGTTCGAAT
CTCACTGGGGCCT
>Ailuropoda_melanoleuca_GL192339.1.trna159-ThrCGT (3515036-3515107) Thr (CGT) 72 bp Sc: 79.77
GGCGCGGTGGCCAAGTGGTAAGGCGTCGGTCTCGTAAACCGAAGATCGCGGGTTCGAACC
CCGTCCGTGCCT
>Ailuropoda_melanoleuca_GL193897.1.trna27-ThrCGT (282097-282170) Thr (CGT) 74 bp Sc: 80.30
GGCTCTGTGGCTTAGTTGGCTAAAGCGCCTGTCTCGTAAACAGGAGATCCTGGGTTCGAA
TCCCAGCGGGGCCT
>Ailuropoda_melanoleuca_GL192574.1.trna84-ThrCGT (585438-585367) Thr (CGT) 72 bp Sc: 80.42
GGCGCGGTGGCCAAGTGGTAAGGCGTCGGTCTCGTAAACCGAAGATCACGGGTTCGAACC
CCGTCCGTGCCT
>Ailuropoda_melanoleuca_GL193897.1.trna35-ThrCGT (423634-423707) Thr (CGT) 74 bp Sc: 81.51
GGCTCTATGGCTTAGTTGGTTAAAGCGCCTGTCTCGTAAACAGGAGATCCTGGGTTCGAC
TCCCAGTAGGGCCT
>Ailuropoda_melanoleuca_GL192444.1.trna68-ThrTGT (2546745-2546673) Thr (TGT) 73 bp Sc: 35.72
GCCTGGCTGGCTCAGTCAGAAGAGCACGGGACTTGTGATCTCCTGGGTGTGAGTTCGAGC
CCCATGCTGGGTG
>Ailuropoda_melanoleuca_GL193897.1.trna32-ThrTGT (353535-353620) Thr (TGT) 86 bp Sc: 47.86
GGCTCCGTGGCTTAGCGAGTCAAGGCGCCTGTCTTGTAAACAGGAGATCCTGGGTTCGAC
CCTGGGTTCGACTCCCAGCGGGGCCT
>Ailuropoda_melanoleuca_GL192539.1.trna167-ThrTGT (233519-233447) Thr (TGT) 73 bp Sc: 51.16
GCCTGGCAGGCTCAGTTGGTACAGCATGAGACTTGTGATCTTGGGGTCGTGAGTTCAAGC
CTCACGCTAGGTG
>Ailuropoda_melanoleuca_GL195202.1.trna1-ThrTGT (39745-39817) Thr (TGT) 73 bp Sc: 78.79

GGCCCTATAGCTCAGGGGTTAGAGCACTGGTCTTGTAAACCAGGGGTCGCGAGTTCAAAT
CTCGCTGGGGCCT
>Ailuropoda_melanoleuca_GL194214.1.trna3-ThrTGT (25871-25943) Thr (TGT) 73 bp Sc: 79.46
GGCTCCATAGCTCAGGGGTTAGAGCACTGGTCTTGTAAACCAGGGGTCGCGAGTTCAAAT
CTCGCTGGGGCCT
>Ailuropoda_melanoleuca_GL194954.1.trna8-ThrTGT (100174-100246) Thr (TGT) 73 bp Sc: 79.46
GGCTCCATAGCTCAGGGGTTAGAGCACTGGTCTTGTAAACCAGGGGTCGCGAGTTCAAAT
CTCGCTGGGGCCT
>Ailuropoda_melanoleuca_GL194214.1.trna1-ThrTGT (15929-16001) Thr (TGT) 73 bp Sc: 80.19
GGCTCCATAGCTCAGGGGTTAGAGCACTGGTCTTGTAAACCAGGGGTCGCGAGTTCAAAT
CTCGCTGGAGCCT
>Ailuropoda_melanoleuca_GL194538.1.trna1-ThrTGT (4819-4892) Thr (TGT) 74 bp Sc: 80.36
GGCTCCGTGGCTTAGCTGGTTAAAGCGCCTGTCTTGTAAACAGGAGATCCTGGGTTCGAC
TCCCAGCGGGGCCT
>Ailuropoda_melanoleuca_GL192382.1.trna128-ThrTGT (2849669-2849597) Thr (TGT) 73 bp Sc: 83.03
GGCTCCATAGCTCAGTGGTTAGAGCACTGGTCTTGTAAACCAGGGGTCGCGAGTTCGATC
CTCGCTGGGGCCT
>Ailuropoda_melanoleuca_GL192501.1.trna45-TrpCCA (1655840-1655912) Trp (CCA) 73 bp Sc: 52.13
ACCTGGCTGGCTCAGTTGGTAGAGCTTGTGACTCCAGATCTTGGGGTCATGAGTTCAAGC
CCCATGTTGGGTG
>Ailuropoda_melanoleuca_GL193165.1.trna20-TrpCCA (841151-841223) Trp (CCA) 73 bp Sc: 61.04
GCCTGGCTGGCTCAGTTGGTAGAGTATGCGACTCCAGATCTCAGGGTCGTGAGTTCAAGC
CCCACATCAGGCA
>Ailuropoda_melanoleuca_GL192741.1.trna16-TrpCCA (731553-731624) Trp (CCA) 72 bp Sc: 71.65
GACCTCGTGGCGCAACGGTAGCGCGTCTGACTCCAGATCAGAAGGCTGCGTGTTCGAATC
ACGTCGGGGTCA
>Ailuropoda_melanoleuca_GL193874.1.trna14-TrpCCA (392060-391989) Trp (CCA) 72 bp Sc: 74.00
GACCTCGTGGCGCAACGGTAGCGCGTCTGACTCCAGATCAGAAGGTTGCGTGTTCAAATC
ACGTCGGGGTCA
>Ailuropoda_melanoleuca_GL192793.1.trna8-TrpCCA (45756-45827) Trp (CCA) 72 bp Sc: 74.80
GACCTCGTGGCGCAATGGTAGCGCGTCTGACTCCAGATCAGAAGGTTGCGTGTTCAAGTC
ACGTCGGGGTCA
>Ailuropoda_melanoleuca_GL192793.1.trna85-TrpCCA (1279081-1279010) Trp (CCA) 72 bp Sc: 74.80
GACCTCGTGGCGCAATGGTAGCGCGTCTGACTCCAGATCAGAAGGTTGCGTGTTCAAGTC
ACGTCGGGGTCA
>Ailuropoda_melanoleuca_GL192808.1.trna55-TrpCCA (1128076-1128147) Trp (CCA) 72 bp Sc: 74.81
GGCCTCGTGGCGCAACGGTAGCGCGTCTGACTCCAGATCAGAAGGTTGCGTGTTCAAATC
ACGTCGGGGTCA
>Ailuropoda_melanoleuca_GL192808.1.trna81-TrpCCA (1148896-1148825) Trp (CCA) 72 bp Sc: 74.81
GGCCTCGTGGCGCAACGGTAGCGCGTCTGACTCCAGATCAGAAGGTTGCGTGTTCAAATC
ACGTCGGGGTCA
>Ailuropoda_melanoleuca_GL193874.1.trna16-TrpCCA (383852-383781) Trp (CCA) 72 bp Sc: 74.81
GGCCTCGTGGCGCAACGGTAGCGCGTCTGACTCCAGATCAGAAGGTTGCGTGTTCAAATC
ACGTCGGGGTCA
>Ailuropoda_melanoleuca_GL193117.1.trna68-TrpCCA (218856-218785) Trp (CCA) 72 bp Sc: 75.74
GACCTCGTGGCGCAACGGTAGCGCGTCTGACTCCAGATCAGAAGGTTGCGTGTTCGAATC
ACGTCGGGGTCA
>Ailuropoda_melanoleuca_GL192478.1.trna30-TyrGTA (1362821-1362913) Tyr (GTA) 93 bp Sc: 73.81
CCTTCGATAGCTCAGCTGGTAGAGCGGAGGACTGTAGCTGACTTCCTGGTAAGAGACATC
CTTAGGTCGCTGGTTCGATTCCGGCTCGAAGGA
>Ailuropoda_melanoleuca_GL192478.1.trna31-TyrGTA (1363421-1363510) Tyr (GTA) 90 bp Sc: 70.45
CCTTCGATAGCTCAGCTGGTAGAGCGGAGGACTGTAGGCGCTGTGCCCGTGGCCATCCTT
AGGTCGCTGGTTCGATTCCGGCTCGAAGGA
>Ailuropoda_melanoleuca_GL195734.1.trna2-TyrGTA (14734-14645) Tyr (GTA) 90 bp Sc: 63.18
CCTTCGATAGCTCAGCTGGTAGAGCGGAGGACTGTAGGCCGGAGAGGCGCGGACATCCTT
AGGTCGCTGGTTCTAGTCCGGCTCGAAGGA
>Ailuropoda_melanoleuca_GL194214.1.trna2-TyrGTA (16647-16737) Tyr (GTA) 91 bp Sc: 74.33
CCTTCGATAGCTCAGCTGGTAGAGCGGAGGACTGTAGAGGGAAACAACTGCTGTCATCCT
TAGGTCGCTGGTTCGATTCCGGCTCGAAGGA
>Ailuropoda_melanoleuca_GL195202.1.trna12-TyrGTA (19895-19806) Tyr (GTA) 90 bp Sc: 71.23
CCTTCGATAGCTCAGCTGGTAGAGCGGAGGACTGTAGACTACACACGTGTGGACATCCTT
AGGTCGCTGGTTCGACTCCGGCTCGAAGGA
>Ailuropoda_melanoleuca_GL195202.1.trna11-TyrGTA (22919-22830) Tyr (GTA) 90 bp Sc: 71.23
CCTTCGATAGCTCAGCTGGTAGAGCGGAGGACTGTAGACTACACACGTGTGGACATCCTT
AGGTCGCTGGTTCGACTCCGGCTCGAAGGA
>Ailuropoda_melanoleuca_GL195202.1.trna2-TyrGTA (40763-40853) Tyr (GTA) 91 bp Sc: 72.49
CCTTCGATAGCTCAGCTGGTAGAGCGGAGGACTGTAGTGCGCTTCCGGTGTAGTCATCCT

TAGGTCGCTGGTTCGATTCCGGCTCGAAGGA
>Ailuropoda_melanoleuca_GL194901.1.trna19-TyrGTA (58511-58423) Tyr (GTA) 89 bp Sc: 76.82
CCTTCGATAGCTCAGTTGGTAGAGCGGAGGACTGTAGATGTTTGGTCGTGGTCATCCTTA
GGTCGCTGGTTCGAATCCGGCTCGAAGGA
>Ailuropoda_melanoleuca_GL194901.1.trna17-TyrGTA (68558-68471) Tyr (GTA) 88 bp Sc: 76.48
CCTTCGATAGCTCAGTTGGTAGAGCGGAGGACTGTAGGTGAGACAGTTGGGCATCCTTAG
GTCGCTGGTTCGAATCCGGCTCGAAGGA
>Ailuropoda_melanoleuca_GL194901.1.trna16-TyrGTA (73733-73645) Tyr (GTA) 89 bp Sc: 75.70
CCTTCGATAGCTCAGTTGGTAGAGCGGAGGACTGTAGTGGAAGCTCTCCAGGCATCCTTA
GGTCGCTGGTTCGAATCCGGCTCGAAGGA
>Ailuropoda_melanoleuca_GL192369.1.trna278-TyrGTA (847626-847537) Tyr (GTA) 90 bp Sc: 76.50
CCTTCGATAGCTCAGTTGGTAGAGCGGAGGACTGTAGTGGCATGAGTTGCGGTAATCCTT
AGGTCGCTGGTTCGATTCCGGCTCGAAGGA
>Ailuropoda_melanoleuca_GL192626.1.trna73-Undet??? (1390198-1390271) Undet (???) 74 bp Sc: 55.15
GCCTGGCTGGCTCAGTTGGTAGAGCCTGAGACTCTTTGATCTCAGGATCGTGAGTTCGAG
CCCCACGTTGGGTA
>Ailuropoda_melanoleuca_GL192350.1.trna13-Undet??? (436508-436579) Undet (???) 72 bp Sc: 56.15
GCCCTGCTGGCTCAGTCGGTAGAGCATGCGACTCTTATCTCAGGGTCGTGAGTTCAATTC
CCACGTTGGGCG
>Ailuropoda_melanoleuca_GL192382.1.trna67-ValAAC (2509179-2509251) Val (AAC) 73 bp Sc: 86.79
GTTTCCGTAGTGTAGTGGTTATCACGTTCGCCTAACACGCGAAAGGTCCCCGGTTCGAAA
CCGGGCGGAAACA
>Ailuropoda_melanoleuca_GL192535.1.trna80-ValAAC (69285-69213) Val (AAC) 73 bp Sc: 86.79
GTTTCCGTAGTGTAGTGGTTATCACGTTCGCCTAACACGCGAAAGGTCCCCGGTTCGAAA
CCGGGCGGAAACA
>Ailuropoda_melanoleuca_GL193552.1.trna13-ValAAC (221357-221429) Val (AAC) 73 bp Sc: 86.79
GTTTCCGTAGTGTAGTGGTTATCACGTTCGCCTAACACGCGAAAGGTCCCCGGTTCGAAA
CCGGGCGGAAACA
>Ailuropoda_melanoleuca_GL193552.1.trna52-ValAAC (536565-536493) Val (AAC) 73 bp Sc: 86.79
GTTTCCGTAGTGTAGTGGTTATCACGTTCGCCTAACACGCGAAAGGTCCCCGGTTCGAAA
CCGGGCGGAAACA
>Ailuropoda_melanoleuca_GL193552.1.trna56-ValAAC (502150-502078) Val (AAC) 73 bp Sc: 86.79
GTTTCCGTAGTGTAGTGGTTATCACGTTCGCCTAACACGCGAAAGGTCCCCGGTTCGAAA
CCGGGCGGAAACA
>Ailuropoda_melanoleuca_GL193552.1.trna57-ValAAC (499024-498952) Val (AAC) 73 bp Sc: 86.79
GTTTCCGTAGTGTAGTGGTTATCACGTTCGCCTAACACGCGAAAGGTCCCCGGTTCGAAA
CCGGGCGGAAACA
>Ailuropoda_melanoleuca_GL194954.1.trna10-ValAAC (103099-103171) Val (AAC) 73 bp Sc: 86.79
GTTTCCGTAGTGTAGTGGTTATCACGTTCGCCTAACACGCGAAAGGTCCCCGGTTCGAAA
CCGGGCGGAAACA
>Ailuropoda_melanoleuca_GL194954.1.trna6-ValAAC (80351-80423) Val (AAC) 73 bp Sc: 86.79
GTTTCCGTAGTGTAGTGGTTATCACGTTCGCCTAACACGCGAAAGGTCCCCGGTTCGAAA
CCGGGCGGAAACA
>Ailuropoda_melanoleuca_GL194938.1.trna12-ValAAC (19212-19140) Val (AAC) 73 bp Sc: 87.18
GTTTCCGTAGTGTAGTGGTTATCACGCTCGCCTAACACGCGAGAGGTCCCCGGTTCGAAA
CCGGGCGGAAACA
>Ailuropoda_melanoleuca_GL194938.1.trna9-ValAAC (26490-26418) Val (AAC) 73 bp Sc: 87.18
GTTTCCGTAGTGTAGTGGTTATCACGCTCGCCTAACACGCGAGAGGTCCCCGGTTCGAAA
CCGGGCGGAAACA
>Ailuropoda_melanoleuca_GL194164.1.trna26-ValCAC (84541-84469) Val (CAC) 73 bp Sc: 85.92
GTTTCCGTAGTGTAGCGGTTATCACGTTCGCCTCACACGCGAAAGGTCCCCGGTTCGATC
CCGGGCGGAAACA
>Ailuropoda_melanoleuca_GL193160.1.trna43-ValCAC (902548-902620) Val (CAC) 73 bp Sc: 87.39
GTTTCCGTAGTGTAGTGGTTATCACGTTCGCCTCACACGCGAAAGGTCCCCGGTTCGAAA
CCGGGCGGAAACA
>Ailuropoda_melanoleuca_GL193552.1.trna10-ValCAC (192962-193034) Val (CAC) 73 bp Sc: 87.39
GTTTCCGTAGTGTAGTGGTTATCACGTTCGCCTCACACGCGAAAGGTCCCCGGTTCGAAA
CCGGGCGGAAACA
>Ailuropoda_melanoleuca_GL193552.1.trna42-ValCAC (555671-555743) Val (CAC) 73 bp Sc: 87.39
GTTTCCGTAGTGTAGTGGTTATCACGTTCGCCTCACACGCGAAAGGTCCCCGGTTCGAAA
CCGGGCGGAAACA
>Ailuropoda_melanoleuca_GL193920.1.trna4-ValCAC (16141-16213) Val (CAC) 73 bp Sc: 87.39
GTTTCCGTAGTGTAGTGGTTATCACGTTCGCCTCACACGCGAAAGGTCCCCGGTTCGAAA
CCGGGCGGAAACA
>Ailuropoda_melanoleuca_GL194883.1.trna16-ValCAC (27420-27348) Val (CAC) 73 bp Sc: 87.39
GTTTCCGTAGTGTAGTGGTTATCACGTTCGCCTCACACGCGAAAGGTCCCCGGTTCGAAA
CCGGGCGGAAACA

>Ailuropoda_melanoleuca_GL194883.1.trna3-ValCAC (16375-16447) Val (CAC) 73 bp Sc: 87.39
GTTTCCGTAGTGTAGTGGTTATCACGTTCGCCTCACACGCGAAAGGTCCCCGGTTCGAAA
CCGGGCGGAAACA
>Ailuropoda_melanoleuca_GL194901.1.trna10-ValCAC (105031-104959) Val (CAC) 73 bp Sc: 87.39
GTTTCCGTAGTGTAGTGGTTATCACGTTCGCCTCACACGCGAAAGGTCCCCGGTTCGAAA
CCGGGCGGAAACA
>Ailuropoda_melanoleuca_GL194954.1.trna4-ValCAC (76400-76472) Val (CAC) 73 bp Sc: 87.39
GTTTCCGTAGTGTAGTGGTTATCACGTTCGCCTCACACGCGAAAGGTCCCCGGTTCGAAA
CCGGGCGGAAACA
>Ailuropoda_melanoleuca_GL192414.1.trna136-ValTAC (3124730-3124802) Val (TAC) 73 bp Sc: 73.86
GGTTCCATAGTGTAGTGGTTATCACGTCTGCTTTACACGCAGAAGGTCCTGTGTTCGAGC
CCCCGTGGAACCA
>Ailuropoda_melanoleuca_GL193552.1.trna44-ValTAC (576187-576259) Val (TAC) 73 bp Sc: 79.89
GGTTCCATAGTGTAGTGGTTATCACGTCTGCTTTACACGCAGAAGGTCCTGTGTTCGATC
CCCAGTGGAACCA
>Ailuropoda_melanoleuca_GL194275.1.trna20-ValTAC (30169-30097) Val (TAC) 73 bp Sc: 81.05
GGTTCCATAGTGTAGCGGTTAGCACGTCTGCTTTACGCGCAGAAGGTCCTGGGTTCGAGC
CCCAGTGGAACCA
>Ailuropoda_melanoleuca_GL193552.1.trna39-ValTAC (511461-511533) Val (TAC) 73 bp Sc: 81.19
GGTTCTGTGGTGTAGTGGTTAGCACATCTGCCTTACACGCAGAAGATCCTGGGTTCGATT
CCCAGTGGAACCA
>Ailuropoda_melanoleuca_GL193455.1.trna13-ValTAC (622446-622518) Val (TAC) 73 bp Sc: 81.83
GGTTCCATGGTGTAGTGGTTAGCACATCTGCCTTACACGCAGAAGCTCCTGGGTTCGAGT
CCCAGTGGAACCA
>Ailuropoda_melanoleuca_GL192372.1.trna103-ValTAC (3393276-3393348) Val (TAC) 73 bp Sc: 83.73
GGTTCCATAGTGTAGTGGTTATCACGTCTGCTTTACGCGCAGAAGGTCCTGGGTTCGAGC
CCCAGTGGAACCA
>Ailuropoda_melanoleuca_GL193235.1.trna4-ValTAC (734849-734921) Val (TAC) 73 bp Sc: 84.42
GGTTCTATGGTGTAGTGGTTAGCACATCTGCCTTACACGCAGAAGATCCTGGGTTCGATT
CCCAGTGGAACCA
>Anabaena_variabilis_ATCC_29413_chr.trna14-AlaCGC (4251344-4251416) Ala (CGC) 73 bp Sc: 75.74
GGGGAATTAGCTCAGTTGGTAGAGCGCTGCGATCGCACCGCAGAGGTCAGGGATTCGAGT
TCCCTATTCTCCA
>Anabaena_variabilis_ATCC_29413_chr.trna29-AlaGGC (4716431-4716359) Ala (GGC) 73 bp Sc: 77.13
GGGGTTATAGCTCAGTTGGTAGAGCGCTGCAATGGCATTGCAGAGGTCAGCGGTTCGAAC
CCGCTTAGCTCCA
>Anabaena_variabilis_ATCC_29413_chr.trna11-AlaTGC (3895832-3895904) Ala (TGC) 73 bp Sc: 91.01
GGGGGTTTAGCTCAGTTGGTAGAGCGCCTGCTTTGCAAGCAGGATGTCAGCGGTTCGAGT
CCGCTAACCTCCA
>Anabaena_variabilis_ATCC_29413_chr.trna26-AlaTGC (5435606-5435534) Ala (TGC) 73 bp Sc: 91.01
GGGGGTTTAGCTCAGTTGGTAGAGCGCCTGCTTTGCAAGCAGGATGTCAGCGGTTCGAGT
CCGCTAACCTCCA
>Anabaena_variabilis_ATCC_29413_chr.trna3-AlaTGC (1004675-1004747) Ala (TGC) 73 bp Sc: 91.01
GGGGGTTTAGCTCAGTTGGTAGAGCGCCTGCTTTGCAAGCAGGATGTCAGCGGTTCGAGT
CCGCTAACCTCCA
>Anabaena_variabilis_ATCC_29413_chr.trna9-AlaTGC (3497796-3497868) Ala (TGC) 73 bp Sc: 91.01
GGGGGTTTAGCTCAGTTGGTAGAGCGCCTGCTTTGCAAGCAGGATGTCAGCGGTTCGAGT
CCGCTAACCTCCA
>Anabaena_variabilis_ATCC_29413_chr.trna30-ArgACG (4227368-4227295) Arg (ACG) 74 bp Sc: 67.68
GGGCGTGTAGCTCAGTGGACTAGAGCACGTGGCTACGGACCACGGTGTCGGGGGTTCGAA
TCCCTCCTCGCCCG
>Anabaena_variabilis_ATCC_29413_chr.trna47-ArgCCG (238386-238314) Arg (CCG) 73 bp Sc: 73.77
GGGCGCGTGGCTCAGTGGATAGAGCAACAGATTCCGGTTCTGTGGGTCGGGGGTTCAAAT
CCCTCCGCGCTCG
>Anabaena_variabilis_ATCC_29413_chr.trna33-ArgCCT (3373777-3373701) Arg (CCT) 77 bp Sc: 76.20
GGGGCTGTAGCTCAGTTGGATAGAGCGAGCGCCTCCTAAGCGCTAGGTCGTGCGTTCAAG
TCGCACCAGTCCCGTCA
>Anabaena_variabilis_ATCC_29413_chr.trna21-ArgTCT (6341598-6341525) Arg (TCT) 74 bp Sc: 77.97
GGGCGCGTAACTCAGTTGGATAGAGTATCCGCCTTCTAAGCGGACTGTCGCAGGTTCAAG
TCCTGCCGCGCCTG
>Anabaena_variabilis_ATCC_29413_chr.trna18-AsnGTT (5802515-5802589) Asn (GTT) 75 bp Sc: 71.61
CGAAAGTTAGCTCACTGGTAGAGCGATCGACTGTTAATCGATttgttgtAGGTTCGATTC
CTATACTTTCTCCCA
>Anabaena_variabilis_ATCC_29413_chr.trna6-AsnGTT (3109711-3109782) Asn (GTT) 72 bp Sc: 78.78
TCCTCAGTAGCTCAGCGGTAGAGCGATCGACTGTTAATCGATTGGTCACTGGTTCGAATC
CAGTCTGGGGAG
>Anabaena_variabilis_ATCC_29413_chr.trna22-AspGTC (6126075-6126002) Asp (GTC) 74 bp Sc: 78.38

GGGACTGTAGTTCAATTGGTTAGAGCACCGCCCTGTCACGGCGGAAGTTGCGGGTTCGAG
CCCCGTCAGTCCCG
>Anabaena_variabilis_ATCC_29413_chr.trna34-CysGCA (3175692-3175621) Cys (GCA) 72 bp Sc: 67.71
GGCGGCATAGCCAAGTGGTAAGGCAGAGGTCTGCAAAACCTCCATCCCCCGGTTCAAATC
CGGGTGCCGCCT
>Anabaena_variabilis_ATCC_29413_chr.trna27-GlnTTG (5292286-5292215) Gln (TTG) 72 bp Sc: 60.00
TGGGGCGTCGCCAAGTGGTAAGGCATCGGGTTTTGGTCCCGACATCCCTAGGTTCGAATC
CTAGCGCCCCAG
>Anabaena_variabilis_ATCC_29413_chr.trna17-GluTTC (5580255-5580330) Glu (TTC) 76 bp Sc: 49.96
GCCCCCATCGTCTAGAGGCCTAGGACACCTCCCTTTCACGGAGGTAACGGGGATTCGAAT
TCCCCTGGGGGTACTA
>Anabaena_variabilis_ATCC_29413_chr.trna28-GlyCCC (5209484-5209413) Gly (CCC) 72 bp Sc: 72.00
GCGGGCGTAATTCAGTGGTAGAATGTCACCTTCCCAAGGTGAACGTCGTGGGTTCGAGTC
CCATCGCCCGCT
>Anabaena_variabilis_ATCC_29413_chr.trna40-GlyGCC (2133971-2133900) Gly (GCC) 72 bp Sc: 77.98
GCGGGTATAGCTCAGTGGTAGAGCGTCACCTTGCCAAGGTGAATGTCGCGCGTTCGAATC
GCGTTACCCGCT
>Anabaena_variabilis_ATCC_29413_chr.trna13-GlyTCC (4023869-4023939) Gly (TCC) 71 bp Sc: 66.79
GCGGGCGTAGTTTAGTGGTAAAACTATAGCCTTCCAAGCTATTAATGCGGGTTCGATTCC
CGCCGCCCGCT
>Anabaena_variabilis_ATCC_29413_chr.trna31-HisGTG (3626113-3626041) His (GTG) 73 bp Sc: 61.85
GCGGGCGTAGCCAAGTGGTTAAGGCAGTGGATTGTGGTTCCACCATTCGGGGGTTCAAGT
CCCCTCGTTCGCC
>Anabaena_variabilis_ATCC_29413_chr.trna10-IleGAT (3895681-3895757) Ile (GAT) 77 bp Sc: 84.18
GGGCTATTAGCTCAGGTGGTTAGAGCGCACCCCTGATAAGGGTGAGGTCCCTGGTTCGAG
TCCAGGATGGCCCACCT
>Anabaena_variabilis_ATCC_29413_chr.trna2-IleGAT (1004524-1004600) Ile (GAT) 77 bp Sc: 84.18
GGGCTATTAGCTCAGGTGGTTAGAGCGCACCCCTGATAAGGGTGAGGTCCCTGGTTCGAG
TCCAGGATGGCCCACCT
>Anabaena_variabilis_ATCC_29413_chr.trna25-IleGAT (5435757-5435681) Ile (GAT) 77 bp Sc: 84.18
GGGCTATTAGCTCAGGTGGTTAGAGCGCACCCCTGATAAGGGTGAGGTCCCTGGTTCGAG
TCCAGGATGGCCCACCT
>Anabaena_variabilis_ATCC_29413_chr.trna41-LeuCAA (1768664-1768581) Leu (CAA) 84 bp Sc: 65.40
GGGCGGGTGGCGAAACTGGTAGACGCACCACACTCAAAATGTGGCGACCTTGCGGTCATA
GGAGTTCGATTCTCCTCCTGCCCA
>Anabaena_variabilis_ATCC_29413_chr.trna4-LeuCAG (1777233-1777314) Leu (CAG) 82 bp Sc: 60.23
GCGGAACTGGCGGAATTGGCAGACGCGCTAGATTCAGGTTCTAGTGCCGCAAGGCTTCCG
GGTTCAAGTCCCGGGTTCCGCA
>Anabaena_variabilis_ATCC_29413_chr.trna46-LeuGAG (649992-649912) Leu (GAG) 81 bp Sc: 60.28
GCGGATGTGGCGGAATTGGTATACGCGCACGCTTGAGGTGCGTGTGGCTTTGCCTTGCGA
GTTCGAGTCTCGCCATCCGCA
>Anabaena_variabilis_ATCC_29413_chr.trna1-LeuTAG (505303-505384) Leu (TAG) 82 bp Sc: 61.46
GCGGATGTGGCGGAATTGGCAGACGCGCTAGATTTAGGTTCTAGTTCCGAGAGGAGTGAA
GGTTCAAGTCCTTTCATCCGCA
>Anabaena_variabilis_ATCC_29413_chr.trna7-LysCTT (3123086-3123157) Lys (CTT) 72 bp Sc: 71.90
GGGTGACTAGCTCAACGGTAGAGCAGTAGACTCTTAATCTATTGGTTGCGGGTTCAAATC
CCTCGTCACCCA
>Anabaena_variabilis_ATCC_29413_chr.trna16-LysTTT (5364031-5364102) Lys (TTT) 72 bp Sc: 66.30
GGGTCGCTAACTCAACGGTAGAGTACTCGGCTTTTAACCGATTAGTTCCGGGTTCGAATC
CCGGGCGACCCA
>Anabaena_variabilis_ATCC_29413_chr.trna35-MetCAT (2938983-2938911) Met (CAT) 73 bp Sc: 48.95
CCAGGGTTGGCCGAGCGGTTGAGGCAGCGAACTCATAATTCGCCCAAGGCAGGTTCAACT
CCTGCACCCTGGA
>Anabaena_variabilis_ATCC_29413_chr.trna37-MetCAT (2508478-2508405) Met (CAT) 74 bp Sc: 79.24
GGCTCAGTAGCTCAGTTGGTTAGAGCACGGGACTCATAAGCCTGGGGTCGTTGGTTCAAA
TCCGACCTGAGCCA
>Anabaena_variabilis_ATCC_29413_chr.trna45-MetCAT (906140-906064) Met (CAT) 77 bp Sc: 83.46
CGCGGGATAGAGCAGCCTGGTAGCTCGTCGGGCTCATAACCCGAAGGTCAGTGGTTCAAA
TCCACTTCCCGCCACCA
>Anabaena_variabilis_ATCC_29413_chr.trna24-PheGAA (5557885-5557813) Phe (GAA) 73 bp Sc: 81.35
GCCGGGATAGCTCAGTTGGTAGAGCAGAGGACTGAAAATCCTCGTGTCACGAGTTCAAGT
CTCGTTCCTGGCA
>Anabaena_variabilis_ATCC_29413_chr.trna42-ProCGG (1424348-1424275) Pro (CGG) 74 bp Sc: 77.90
CGGGATGTAGCGCAGCTTGGTAGCGCACTTCGTTCGGGACGAAGGGGCCGCTGGTTCGAA
TCCAGTCATCCCGA
>Anabaena_variabilis_ATCC_29413_chr.trna38-ProGGG (2494686-2494613) Pro (GGG) 74 bp Sc: 74.78
CGGGGCGTAGCGCAGCTTGGTAGCGCGCCACTTTGGGGTAGTGGAGGTCGTGGGTTCGAA

TCCCGCCGCTCCGA
>Anabaena_variabilis_ATCC_29413_chr.trna5-ProTGG (1828001-1828074) Pro (TGG) 74 bp Sc: 79.94
CGGGATGTAGCGCAGCTTGGTAGCGCGCCTGCTTTGGGAGCAGGATGCCGCAGGTTCAAA
TCCTGTCATCCCGA
>Anabaena_variabilis_ATCC_29413_chr.trna15-SerCGA (4743213-4743297) Ser (CGA) 85 bp Sc: 55.96
GGAGAGGTGTCCGAGTGGTTGATGGTGACGCACTCGAAATGCGTTTTGGGGAAACCCAAC
GGGGGTTCAAATCCCCCCCTCTCCG
>Anabaena_variabilis_ATCC_29413_chr.trna23-SerGCT (6024598-6024507) Ser (GCT) 92 bp Sc: 65.99
GGAGAGGTGGCTGAGTGGTCGAAAGCGGCAGATTGCTAATCtgttgtACGGCAGGCAACT
CCGTACCGAGGGTTCGAATCCCTCCCTCTCCG
>Anabaena_variabilis_ATCC_29413_chr.trna44-SerGGA (1182011-1181925) Ser (GGA) 87 bp Sc: 68.55
GGAGAGATGGCCGAGTGGTTTAAGGCGCAGCACTGGAAATGCTGTAATGCGGAAACGTAT
TCTAGGGTTCAAATCCCTATCTCTCCG
>Anabaena_variabilis_ATCC_29413_chr.trna39-SerTGA (2470973-2470889) Ser (TGA) 85 bp Sc: 58.62
GGAGAGGTGGCAGAGTGGTCGATTGCGTCCGACTTGAAATCGGATGAGGCTAAAACCTCC
GGGAGTTCGAATCTCCCCCTCTCCG
>Anabaena_variabilis_ATCC_29413_chr.trna12-ThrCGT (3948182-3948253) Thr (CGT) 72 bp Sc: 79.94
GCCGATGTGGCTCAGTGGTAGAGCAGCTGATTCGTAATCAGCAGGCCGTGGGTTCAAATC
CCATCATCGGCT
>Anabaena_variabilis_ATCC_29413_chr.trna20-ThrGGT (6278962-6279033) Thr (GGT) 72 bp Sc: 79.24
GCCCGTGTGGCTCAGTGGTAGAGCACACCCTTGGTAAGGGTGAGGTCACGAGTTCAATCC
TCGTCACGGGCT
>Anabaena_variabilis_ATCC_29413_chr.trna36-ThrTGT (2728193-2728122) Thr (TGT) 72 bp Sc: 80.42
GCCAGCATAGCACAGTGGTAGTGCATCCGACTTGTAATCGGAAGGTCGTCGGTTCAAATC
CGACTGCTGGCT
>Anabaena_variabilis_ATCC_29413_chr.trna8-TrpCCA (3152762-3152834) Trp (CCA) 73 bp Sc: 77.93
GCGCTCTTAGTTCAGTTGGTAGAACGCAGGTCTCCAAAACCTGATGTCGGGGGTTCAAGT
CCTCCAGGGCGCG
>Anabaena_variabilis_ATCC_29413_chr.trna19-TyrGTA (6278819-6278904) Tyr (GTA) 86 bp Sc: 57.48
GGGTCGGTGTCCGAGTGGTTAATGGAGACGGACTGTAAATCCGTTGGTTTACACCTACGC
TGGTTCAAATCCAGCCCGGCCCACCT
>Anabaena_variabilis_ATCC_29413_chr.trna43-ValGAC (1219650-1219577) Val (GAC) 74 bp Sc: 86.80
GGACGTATAGCTCAGTTGGTTAGAGCGCTACGTTGACATCGTAGAGGTCACTGGTTCGAA
TCCAGTTACGTCCA
>Anabaena_variabilis_ATCC_29413_chr.trna32-ValTAC (3620894-3620822) Val (TAC) 73 bp Sc: 85.44
GGGCGGTTAGCTCAGTTGGTAGAGCGCCTGCCTTACAAGCAGGATGTCATCAGTTCGAGT
CTGGTACTGCCCA
>Anaplasma_phagocytophilum_HZ_chr.trna3-AlaTGC (48972-49044) Ala (TGC) 73 bp Sc: 82.60
GGGGGCATAGCTCAGTTGGTAGAGCATCTGTTTTGCACACAGAAGGTCAGCGGTTCGACC
CCGCTTGCCTCCA
>Anaplasma_phagocytophilum_HZ_chr.trna33-ArgACG (642405-642332) Arg (ACG) 74 bp Sc: 73.28
GCGCCCTTAGCTCAGTTGGATAGAGCATTTGACTACGGATCAGAAGGTCGGGCGTTCGAG
TCGCTCAGGGCGCA
>Anaplasma_phagocytophilum_HZ_chr.trna9-ArgCCG (361880-361954) Arg (CCG) 75 bp Sc: 72.98
GCACTCGTAGCTCAGTTAGGATAGAGCGCTACCCTCCGGAGGTAGAGGTCGCAGGTTCGA
GTCCTGCTGAGTGCG
>Anaplasma_phagocytophilum_HZ_chr.trna17-ArgCCT (1079546-1079619) Arg (CCT) 74 bp Sc: 70.84
GGGGCCGTAGCTCAGTAGGATAGAGCATTAGATTCCTAATCTGAGGGTCGTGCGTTCGAA
TCGCACCGGTCCCA
>Anaplasma_phagocytophilum_HZ_chr.trna1-ArgTCT (9233-9309) Arg (TCT) 77 bp Sc: 91.04
GTGTCCGTAGCTCAGCTGGATAGAGCAACAGCCTTCTAAGCTGTGGGTCAGGGGTTCGAA
TCCCTTCGGGCACACCA
>Anaplasma_phagocytophilum_HZ_chr.trna30-AsnGTT (821989-821918) Asn (GTT) 72 bp Sc: 77.23
TCCCTGGTAGCTCAGCGGTAGAGCAACTGGCTGTTAACCAGTAGGTCGTTGGTTCGAATC
CGACCCGGGGAG
>Anaplasma_phagocytophilum_HZ_chr.trna10-AspGTC (500646-500719) Asp (GTC) 74 bp Sc: 81.76
GGGGGTGTAGCTCAGTTGGTTAGAGCGCAAGCCTGTCACGTTTGAGGTCGCGAGTTCGAG
TCTCGTCACTCCCG
>Anaplasma_phagocytophilum_HZ_chr.trna18-CysGCA (1330024-1330095) Cys (GCA) 72 bp Sc: 56.39
GGCTGGGTGGCAGAGTGGTTTATGCAGAGGACTGCAAATCCTTTTATACCGGTTCGATTC
CGGTCCCGGCCT
>Anaplasma_phagocytophilum_HZ_chr.trna37-GlnTTG (12233-12162) Gln (TTG) 72 bp Sc: 59.16
TGGGATGTCGCCAAGCGGTAAGGCAACGGTTTTTGGTACCGTTATGCGGAGGTTCGATTC
CTCCCATCCCAG
>Anaplasma_phagocytophilum_HZ_chr.trna8-GluTTC (313821-313893) Glu (TTC) 73 bp Sc: 46.43
GACTCCTTCGTCTAGTGGTCTAGGACCCCACCCTTTCACGGTGGTAACGCGGGTTCGACT
CCCGTGGGAGTCA

>Anaplasma_phagocytophilum_HZ_chr.trna29-GlyGCC (920897-920826) Gly (GCC) 72 bp Sc: 78.14
GCGGGAGTAGCTCAGTGGTAGAGTATAACCTTGCCAAGGTTAGGGTCGAGGGTTCGAACC
CCTTCTCCCGCT
>Anaplasma_phagocytophilum_HZ_chr.trna7-GlyTCC (273587-273657) Gly (TCC) 71 bp Sc: 71.64
GCGGGTATAGCTCAATGGTAGAGTAGCAGCCTTCCAAGCTGCCTGTGTGGGTTCGATTCC
CATTACCCGCT
>Anaplasma_phagocytophilum_HZ_chr.trna4-HisGTG (80704-80779) His (GTG) 76 bp Sc: 73.71
GCGGACGTAGCTCAATCGGTGGAGCGTCAGTTTGTGGCACTGAAGGTCGCCAGTTCGATC
CTGGTCGTTCGCCCCA
>Anaplasma_phagocytophilum_HZ_chr.trna14-IleGAT (720645-720720) Ile (GAT) 76 bp Sc: 87.35
GGGTCTGTAGCTCAGTGGTTAGAGCGCACGCCTGATAAGCGTGAGGTCGGAGGTTCGACT
CTTCCCAGGCCCACCA
>Anaplasma_phagocytophilum_HZ_chr.trna35-LeuCAA (522750-522668) Leu (CAA) 83 bp Sc: 62.69
GCCCTTATGGCGGAATGGTAGACGCGGTAGACTCAAAATCTACTGCCCGTGAGGGTGTGC
TGGTTCGAGTCCGGCTAAGGGCA
>Anaplasma_phagocytophilum_HZ_chr.trna22-LeuCAG (1376553-1376470) Leu (CAG) 84 bp Sc: 60.32
GTCCGAATGGCGGAATTGGTAGACGCGCTAGCTTCAGGTGCTAGTAACCTTACGGTTGTG
GAAGTTCGAGTCTTCTTTCGGGCA
>Anaplasma_phagocytophilum_HZ_chr.trna21-LeuGAG (1382940-1382853) Leu (GAG) 88 bp Sc: 59.07
GCGGTTATGGTGGAAATGGTAGACACGCAGCGTTGAGGTCGCTGTGGCTATATCAGCCTT
GGAAGTTCAAGTCTTCTTAACCGCACCA
>Anaplasma_phagocytophilum_HZ_chr.trna16-LeuTAA (1048915-1049000) Leu (TAA) 86 bp Sc: 66.75
GTCCATGTGGCGGAATGGTAGACGCTGCGGACTTAAAATCCGTTGACCTTTGGGTCGTGG
GAGTTCAAGTCTCCCCATGGGCACCA
>Anaplasma_phagocytophilum_HZ_chr.trna27-LeuTAG (1033518-1033436) Leu (TAG) 83 bp Sc: 55.08
GCGGATATGGCGGAATTGGTAGACGTGCTAGGTTTAGGTCCTAGTGAGTTATGCTCGTGG
GGGTTCAAGTCCCTCTATCCGCA
>Anaplasma_phagocytophilum_HZ_chr.trna26-LysCTT (1033737-1033666) Lys (CTT) 72 bp Sc: 75.59
GGTTCCGTAGCTCAGTGGTAGAGCGCCTGACTCTTAATCAGTGTGTCGCGTGTTCGAATC
ACGCCGGAACCA
>Anaplasma_phagocytophilum_HZ_chr.trna23-LysTTT (1316557-1316486) Lys (TTT) 72 bp Sc: 67.17
GGTCCTGTAGCTCAGTGGTAGAGCAGGTGGCTTTTAATCACCGTGTCGCGCGTTCGATTC
GCGCTGGGACCA
>Anaplasma_phagocytophilum_HZ_chr.trna13-MetCAT (661314-661385) Met (CAT) 72 bp Sc: 58.85
GGCTAGGTAGCTCAGGGGTAGAGCAGGAGCATCATAATCTCCGGGCCGGGGGTTCGAATC
CCTCCTTAGCCA
>Anaplasma_phagocytophilum_HZ_chr.trna15-MetCAT (773874-773945) Met (CAT) 72 bp Sc: 58.85
GGCTAGGTAGCTCAGGGGTAGAGCAGGAGCATCATAATCTCCGGGCCGGGGGTTCGAATC
CCTCCTTAGCCA
>Anaplasma_phagocytophilum_HZ_chr.trna31-MetCAT (783247-783176) Met (CAT) 72 bp Sc: 76.95
CGCGGGGTGGAGCAGTGGTAGCTCATCAGGCTCATAACCTGAAGGTCGATGGTTCGAGTC
CGTCCCCCGCAA
>Anaplasma_phagocytophilum_HZ_chr.trna28-MetCAT (1027436-1027364) Met (CAT) 73 bp Sc: 85.12
GGGGCTGTAGCTCAGTGGTTAGAGCGACTCGCTCATAACGAGTGGGCCGTAGGTTCAAAT
CCTACCAGCCCCA
>Anaplasma_phagocytophilum_HZ_chr.trna32-PheGAA (673023-672953) Phe (GAA) 71 bp Sc: 72.19
GGCCTGGTAGCTCAGTGGTAGAGCAGAGGACTGAAAATCCTTGTGCGTTGGTTCAATTCC
GACCCGGGCCA
>Anaplasma_phagocytophilum_HZ_chr.trna19-ProGGG (1351275-1351352) Pro (GGG) 78 bp Sc: 76.31
CGGAATGTAGCGCAGCCTGGTTAGCGCGTCAGTCTGGGGGACTGGAGGTCATGGGTTCGA
ATCCCGTCATTCCGACCA
>Anaplasma_phagocytophilum_HZ_chr.trna34-ProTGG (526464-526391) Pro (TGG) 74 bp Sc: 80.51
CGGGGTGTGGCGCAGCTTGGTAGCGCACTTGGTTTGGGACCAAGGGGTCGGGAGTTCAAA
TCTCTCCACCCCGA
>Anaplasma_phagocytophilum_HZ_chr.trna11-SerGCT (646353-646444) Ser (GCT) 92 bp Sc: 64.66
GGAGAGGTGGCCGAGAGGCTGAAGGCGGCGGTTTGCTAAACCGTTATACAAGTAACATTG
TATCGAGGGTTCGAATCCCTCTCTCTCCGCCA
>Anaplasma_phagocytophilum_HZ_chr.trna24-SerGGA (1252920-1252836) Ser (GGA) 85 bp Sc: 64.40
GGAGAGATGGCTGAGTGGTCGAAAGCGCACGCCTGGAAAGTGTGTACACAGCAATGTGTC
GGGGGTTCGAATCCCCCTCTTTCCG
>Anaplasma_phagocytophilum_HZ_chr.trna2-SerTGA (31291-31377) Ser (TGA) 87 bp Sc: 65.85
GGACGGATGGCCGAGCGGTTTAAGGCACCAGTCTTGAAAACTGACGTACGCTAACACGTA
CCGTGGGTTCGAATCCCACTCCGTCCG
>Anaplasma_phagocytophilum_HZ_chr.trna36-ThrGGT (426996-426923) Thr (GGT) 74 bp Sc: 72.19
GCCGTTATAGCTTAGTGGTAGAACGCGTCCTTGGTAAGGACGAAGCCCGAGTTCGATTCT
CGGTAACGGCACCA
>Anaplasma_phagocytophilum_HZ_chr.trna12-ThrTGT (650430-650503) Thr (TGT) 74 bp Sc: 68.10

GCCGGCTTAGCTATAATTGGTAGAGCGCCTGACTTGTAATCAGGAtgttgtCGGTTCGAG
TCCGGCAGTCGGCT
>Anaplasma_phagocytophilum_HZ_chr.trna25-TrpCCA (1094172-1094099) Trp (CCA) 74 bp Sc: 69.90
AGGAGTGTAGTTCAATGGTAGAACATCAGTCTCCAAAACTGAGAGTATGGGTTCGACTCC
TGTCACTCCTGCCA
>Anaplasma_phagocytophilum_HZ_chr.trna6-TyrGTA (273497-273579) Tyr (GTA) 83 bp Sc: 60.76
GGAGGGGTGGCCGAGTGGTTAAAGGCAGCAGACTGTAAATCTGCCCACTTCAGTGTACGT
AGGTTCGAATCCTACCTCCTCCA
>Anaplasma_phagocytophilum_HZ_chr.trna20-ValGAC (1456612-1456540) Val (GAC) 73 bp Sc: 76.82
GGGTGATTAGCTCAGGTGGTAGAGCGTTTGCTTGACGTGCAAAAGGCCACTGGTTCGAGT
CCAGTATCACCCA
>Anaplasma_phagocytophilum_HZ_chr.trna5-ValTAC (176879-176951) Val (TAC) 73 bp Sc: 81.11
GGGCGGTTAGCTCAGTTGGTAGAGCGCCTCGTTTACACCGAGGAAGTCAGCAGTTCGAGT
CTGTTACTGCCCA
>Arcobacter_butzleri_RM4018_chr.trna30-AlaTGC (2274575-2274500) Ala (TGC) 76 bp Sc: 92.14
GGGGAATTAGCTCAGCTGGGAGAGCGCCTGCTTTGCACGCAGGAGGTCAGCGGTTCGATC
CCGCTATTCTCCACCA
>Arcobacter_butzleri_RM4018_chr.trna32-AlaTGC (2003372-2003297) Ala (TGC) 76 bp Sc: 92.14
GGGGAATTAGCTCAGCTGGGAGAGCGCCTGCTTTGCACGCAGGAGGTCAGCGGTTCGATC
CCGCTATTCTCCACCA
>Arcobacter_butzleri_RM4018_chr.trna35-AlaTGC (1918431-1918356) Ala (TGC) 76 bp Sc: 92.14
GGGGAATTAGCTCAGCTGGGAGAGCGCCTGCTTTGCACGCAGGAGGTCAGCGGTTCGATC
CCGCTATTCTCCACCA
>Arcobacter_butzleri_RM4018_chr.trna43-AlaTGC (1783589-1783514) Ala (TGC) 76 bp Sc: 92.14
GGGGAATTAGCTCAGCTGGGAGAGCGCCTGCTTTGCACGCAGGAGGTCAGCGGTTCGATC
CCGCTATTCTCCACCA
>Arcobacter_butzleri_RM4018_chr.trna9-AlaTGC (849213-849288) Ala (TGC) 76 bp Sc: 92.14
GGGGAATTAGCTCAGCTGGGAGAGCGCCTGCTTTGCACGCAGGAGGTCAGCGGTTCGATC
CCGCTATTCTCCACCA
>Arcobacter_butzleri_RM4018_chr.trna54-ArgTCG (154500-154424) Arg (TCG) 77 bp Sc: 70.20
GCGTCTGTAGTTCAACTGGATAGAATAACCGGTTTCGACCCGGTGGGTTGTGGGTTCGAC
TCCTACCAGGCGTGCCA
>Arcobacter_butzleri_RM4018_chr.trna3-ArgTCT (169616-169692) Arg (TCT) 77 bp Sc: 76.69
GCTTCCATAGCTCAGCTGGATAGAGCAACGCCCTTCTAAGGCGTAGGCCGTACGTTCGAA
TCGTACTGGGAGTACCA
>Arcobacter_butzleri_RM4018_chr.trna6-ArgTCT (169934-170010) Arg (TCT) 77 bp Sc: 76.69
GCTTCCATAGCTCAGCTGGATAGAGCAACGCCCTTCTAAGGCGTAGGCCGTACGTTCGAA
TCGTACTGGGAGTACCA
>Arcobacter_butzleri_RM4018_chr.trna33-AsnGTT (1920476-1920402) Asn (GTT) 75 bp Sc: 81.23
TCCGGCATAGCTCAGCGGTAGAGTAGATGACTGTTAATCATTTGGTCCCTGGTTCGAACC
CAGGTGCCGGAGCCA
>Arcobacter_butzleri_RM4018_chr.trna41-AsnGTT (1785635-1785561) Asn (GTT) 75 bp Sc: 81.23
TCCGGCATAGCTCAGCGGTAGAGTAGATGACTGTTAATCATTTGGTCCCTGGTTCGAACC
CAGGTGCCGGAGCCA
>Arcobacter_butzleri_RM4018_chr.trna53-AspGTC (519385-519309) Asp (GTC) 77 bp Sc: 91.27
GCGGCCGTAGTTTAGTTGGTTAGAATGCCTGCCTGTCACGCAGGAGGTCGCGAGTTCGAG
TCTCGTCGGCCGCGCCA
>Arcobacter_butzleri_RM4018_chr.trna49-AspGTC (519767-519691) Asp (GTC) 77 bp Sc: 91.38
GCGGCTGTAGTTTAGTTGGTTAGAATGCCTGCCTGTCACGCAGGAGGTCGCGAGTTCGAG
TCTCGTCAGCCGCGCCA
>Arcobacter_butzleri_RM4018_chr.trna28-CysGCA (2103790-2103865) Cys (GCA) 76 bp Sc: 71.82
GGAGGTATAGCAAAGCTGGTAATGCCTCGGATTGCAAATCCGACATGCGTTGGTTCGAGT
CCGACTACCTCCTCCA
>Arcobacter_butzleri_RM4018_chr.trna10-GlnTTG (1234392-1234466) Gln (TTG) 75 bp Sc: 69.73
TGGGGTATCGCCAAGCGGTAAGGCAACGGTTTTTGGTACCGTCACTCGAAGGTTCGAATC
CTTCTACCCCATCCA
>Arcobacter_butzleri_RM4018_chr.trna14-GlnTTG (1541408-1541482) Gln (TTG) 75 bp Sc: 69.73
TGGGGTATCGCCAAGCGGTAAGGCAACGGTTTTTGGTACCGTCACTCGAAGGTTCGAATC
CTTCTACCCCATCCA
>Arcobacter_butzleri_RM4018_chr.trna25-GlnTTG (1882310-1882384) Gln (TTG) 75 bp Sc: 69.73
TGGGGTATCGCCAAGCGGTAAGGCAACGGTTTTTGGTACCGTCACTCGAAGGTTCGAATC
CTTCTACCCCATCCA
>Arcobacter_butzleri_RM4018_chr.trna45-GluTTC (1368730-1368655) Glu (TTC) 76 bp Sc: 38.23
GACCCTGTCGTCTAGTGGCCAAGGACGTTAGGATTTCCTCCTAAACACGCAAGTTCGAAT
CTTGCTGGGGTCGCCA
>Arcobacter_butzleri_RM4018_chr.trna47-GluTTC (520000-519926) Glu (TTC) 75 bp Sc: 54.27
GGCCCCTTCATCTAACGGTTAGGATTCATGGTTTTCATCCATGCCACAGGGGTTCGAATC

CCCTAGGGGTCACCA
>Arcobacter_butzleri_RM4018_chr.trna51-GluTTC (519575-519501) Glu (TTC) 75 bp Sc: 54.27
GGCCCCTTCATCTAACGGTTAGGATTCATGGTTTTCATCCATGCCACAGGGGTTCGAATC
CCCTAGGGGTCACCA
>Arcobacter_butzleri_RM4018_chr.trna21-GlyTCC (1876218-1876294) Gly (TCC) 77 bp Sc: 91.96
GCGGGAGTAGCTCAGTTGGCTAGAGCTTCTGCCTTCCAAGCAGACTGTCGCGAGTTCGAG
TCTCGTCTCCCGCTCCA
>Arcobacter_butzleri_RM4018_chr.trna39-GlyTCC (1898064-1897988) Gly (TCC) 77 bp Sc: 91.96
GCGGGAGTAGCTCAGTTGGCTAGAGCTTCTGCCTTCCAAGCAGACTGTCGCGAGTTCGAG
TCTCGTCTCCCGCTCCA
>Arcobacter_butzleri_RM4018_chr.trna5-GlyTCC (169789-169865) Gly (TCC) 77 bp Sc: 91.96
GCGGGAGTAGCTCAGTTGGCTAGAGCTTCTGCCTTCCAAGCAGACTGTCGCGAGTTCGAG
TCTCGTCTCCCGCTCCA
>Arcobacter_butzleri_RM4018_chr.trna7-GlyTCC (170017-170093) Gly (TCC) 77 bp Sc: 91.96
GCGGGAGTAGCTCAGTTGGCTAGAGCTTCTGCCTTCCAAGCAGACTGTCGCGAGTTCGAG
TCTCGTCTCCCGCTCCA
>Arcobacter_butzleri_RM4018_chr.trna2-HisGTG (169522-169598) His (GTG) 77 bp Sc: 79.00
GTAGGTATAGCTCAGTCGGTTAGAGCATCGGGTTGTGGTTCCGAGGGTCGTGGGTTCGAG
CCCCATTACCTACCCCA
>Arcobacter_butzleri_RM4018_chr.trna29-IleGAT (2274706-2274630) Ile (GAT) 77 bp Sc: 91.94
GGGCCTATAGCTCAGCTGGCTAGAGCGCTCGACTGATAATCGTGAGGTCTCAGGTTCAAG
TCCTGATAGGCCCACCA
>Arcobacter_butzleri_RM4018_chr.trna31-IleGAT (2003503-2003427) Ile (GAT) 77 bp Sc: 91.94
GGGCCTATAGCTCAGCTGGCTAGAGCGCTCGACTGATAATCGTGAGGTCTCAGGTTCAAG
TCCTGATAGGCCCACCA
>Arcobacter_butzleri_RM4018_chr.trna34-IleGAT (1918562-1918486) Ile (GAT) 77 bp Sc: 91.94
GGGCCTATAGCTCAGCTGGCTAGAGCGCTCGACTGATAATCGTGAGGTCTCAGGTTCAAG
TCCTGATAGGCCCACCA
>Arcobacter_butzleri_RM4018_chr.trna42-IleGAT (1783720-1783644) Ile (GAT) 77 bp Sc: 91.94
GGGCCTATAGCTCAGCTGGCTAGAGCGCTCGACTGATAATCGTGAGGTCTCAGGTTCAAG
TCCTGATAGGCCCACCA
>Arcobacter_butzleri_RM4018_chr.trna8-IleGAT (849082-849158) Ile (GAT) 77 bp Sc: 91.94
GGGCCTATAGCTCAGCTGGCTAGAGCGCTCGACTGATAATCGTGAGGTCTCAGGTTCAAG
TCCTGATAGGCCCACCA
>Arcobacter_butzleri_RM4018_chr.trna13-LeuCAA (1324501-1324584) Leu (CAA) 84 bp Sc: 70.33
GCCGCTATGGTGAAATTGGTAGACACAAGGGATTCAAAATCCCTCGCCTTTGGTGTGTCG
GTTCGAGTCCGACTAGCGGTACCA
>Arcobacter_butzleri_RM4018_chr.trna18-LeuGAG (1755476-1755560) Leu (GAG) 85 bp Sc: 64.86
GCGGATGTGGTGGAATGGTAGACACGCCATCTTGAGGTGGTGGTGAGCTAAGCTTGTGCG
GGTTCAAGTCCCGCCATCCGCACCA
>Arcobacter_butzleri_RM4018_chr.trna17-LeuTAA (1541706-1541792) Leu (TAA) 87 bp Sc: 68.34
GCGAGTGTGGCGGAATAGGTAGACGCGCGGGACTTAAAATCCCGTTCCGGTTTCGGAGTG
TGAGTTCGATTCTCACCATTCGCACCA
>Arcobacter_butzleri_RM4018_chr.trna26-LeuTAA (2103540-2103626) Leu (TAA) 87 bp Sc: 68.34
GCGAGTGTGGCGGAATAGGTAGACGCGCGGGACTTAAAATCCCGTTCCGGTTTCGGAGTG
TGAGTTCGATTCTCACCATTCGCACCA
>Arcobacter_butzleri_RM4018_chr.trna4-LeuTAG (169697-169781) Leu (TAG) 85 bp Sc: 76.84
GCGGACGTGGTGAAATTGGTAGACACGCCAGACTTAGGATCTGGTGCCTCACGGTGTGAG
AGTTCGAGTCTCTCCGTCCGCACCA
>Arcobacter_butzleri_RM4018_chr.trna46-LysTTT (520098-520023) Lys (TTT) 76 bp Sc: 84.27
GACCCGTTAGCTCAGCCGGTAGAGCATCTCACTTTTAATGAGGGGGCCAATGGTTCGAAT
CCATTACGGGTCACCA
>Arcobacter_butzleri_RM4018_chr.trna50-LysTTT (519673-519598) Lys (TTT) 76 bp Sc: 84.27
GACCCGTTAGCTCAGCCGGTAGAGCATCTCACTTTTAATGAGGGGGCCAATGGTTCGAAT
CCATTACGGGTCACCA
>Arcobacter_butzleri_RM4018_chr.trna23-MetCAT (1882129-1882205) Met (CAT) 77 bp Sc: 84.09
GTCAAGGTAGCTCAGCTGGTTAGAGCGCTGGTCTCATAAGCCGGAGGTCGAGGGTTCGAG
TCCCTCTCTTGACACCA
>Arcobacter_butzleri_RM4018_chr.trna11-MetCAT (1234491-1234567) Met (CAT) 77 bp Sc: 84.30
CGCGGAATAGAGCAGCTAGGTAGCTCGTCGGGCTCATAACCCGAAGGTCATAGGTTCAAA
TCCTATTTCCGCAACCA
>Arcobacter_butzleri_RM4018_chr.trna22-MetCAT (1876328-1876404) Met (CAT) 77 bp Sc: 84.30
CGCGGAATAGAGCAGCTAGGTAGCTCGTCGGGCTCATAACCCGAAGGTCATAGGTTCAAA
TCCTATTTCCGCAACCA
>Arcobacter_butzleri_RM4018_chr.trna20-MetCAT (1854194-1854270) Met (CAT) 77 bp Sc: 99.94
GGGTCATTAGCTCAGCTGGTTAGAGCACTCGGCTCATAACCGAGTGGTCGAAGGTTCGAG
TCCTTCATGACCCACCA

>Arcobacter_butzleri_RM4018_chr.trna16-PheGAA (1541606-1541681) Phe (GAA) 76 bp Sc: 88.27
GGTTCGATAGCTCAGTCGGTAGAGCAAAGGATTGAAAATCCTTGTGTCGACAGTTCGATT
CTGTCTCGAACCACCA
>Arcobacter_butzleri_RM4018_chr.trna36-PheGAA (1898379-1898304) Phe (GAA) 76 bp Sc: 88.27
GGTTCGATAGCTCAGTCGGTAGAGCAAAGGATTGAAAATCCTTGTGTCGACAGTTCGATT
CTGTCTCGAACCACCA
>Arcobacter_butzleri_RM4018_chr.trna1-ProTGG (169426-169503) Pro (TGG) 78 bp Sc: 90.06
CGGGGCGTAGCGCAGTCTGGTTAGCGCACCTGGTTTGGGACCAGGGGGCCGGAGGTTCGA
ATCCTCTCGCCCCGACCA
>Arcobacter_butzleri_RM4018_chr.trna44-SeCTCA (1529552-1529454) SeC (TCA) 99 bp Sc: 35.69
GGGAGGTTAGTGTACCTGGTGGGCACCACAGGCTTCAACCCTGATTGACGGTTTTGATGA
CAACGCCGTGGGAGGTTCGATTCCTTCACCTTCTCGCCA
>Arcobacter_butzleri_RM4018_chr.trna19-SerGCT (1854099-1854189) Ser (GCT) 91 bp Sc: 71.27
GGACAGTTGGGTGAGTTGGCTGAAACCACCTCCCTGCTAAGGAGACGTACTGGCAACGGT
ACCGAGGGTTCAAATCCCTCACTGTCCGCCA
>Arcobacter_butzleri_RM4018_chr.trna27-SerTGA (2103636-2103724) Ser (TGA) 89 bp Sc: 51.70
AGAGAGTTGACAGAGTTGGTCGATTGTACCGGTTTTGAAAACCGGCGAGGTTCACGCCTC
CGAGGGTTCGAATCCCTTGCTCTCTGCCA
>Arcobacter_butzleri_RM4018_chr.trna12-ThrTGT (1234644-1234720) Thr (TGT) 77 bp Sc: 93.03
GCTGGTGTAGCTCAGTTGGCTAGAGCAGCTGATTTGTAATCAGCAGGTCGGGGGTTCGAC
TCCCTTCACCAGCTCCA
>Arcobacter_butzleri_RM4018_chr.trna24-ThrTGT (1882216-1882292) Thr (TGT) 77 bp Sc: 93.03
GCTGGTGTAGCTCAGTTGGCTAGAGCAGCTGATTTGTAATCAGCAGGTCGGGGGTTCGAC
TCCCTTCACCAGCTCCA
>Arcobacter_butzleri_RM4018_chr.trna37-ThrTGT (1898292-1898216) Thr (TGT) 77 bp Sc: 93.03
GCTGGTGTAGCTCAGTTGGCTAGAGCAGCTGATTTGTAATCAGCAGGTCGGGGGTTCGAC
TCCCTTCACCAGCTCCA
>Arcobacter_butzleri_RM4018_chr.trna40-TrpCCA (1896346-1896271) Trp (CCA) 76 bp Sc: 68.07
AGGTCAGTAGCTCCAATGGTAGAGCGCCGGATTCCAAATCCGATGGTTGTGGGTTCGAAT
CCCCCCTGGCCTGCCA
>Arcobacter_butzleri_RM4018_chr.trna15-TyrGTA (1541504-1541588) Tyr (GTA) 85 bp Sc: 57.82
GGTGAGGTTGGAGAGTGGTCAAATCCTGCGGACTGTAAATCCGCCGCCTACGGCTTCGAA
GGTTCGAATCCTTCTCTCACCACCA
>Arcobacter_butzleri_RM4018_chr.trna38-TyrGTA (1898158-1898074) Tyr (GTA) 85 bp Sc: 57.82
GGTGAGGTTGGAGAGTGGTCAAATCCTGCGGACTGTAAATCCGCCGCCTACGGCTTCGAA
GGTTCGAATCCTTCTCTCACCACCA
>Arcobacter_butzleri_RM4018_chr.trna48-ValTAC (519906-519831) Val (TAC) 76 bp Sc: 92.54
GGTCGATTAGCTCAGTTGGTAGAGCGCTACCCTTACAAGGTAGATGTCATAAGTTCGAGT
CTTATATCGACCACCA
>Arcobacter_butzleri_RM4018_chr.trna52-ValTAC (519481-519406) Val (TAC) 76 bp Sc: 92.54
GGTCGATTAGCTCAGTTGGTAGAGCGCTACCCTTACAAGGTAGATGTCATAAGTTCGAGT
CTTATATCGACCACCA
>Arthrobacter_FB24_chr1.trna31-AlaCGC (3217490-3217415) Ala (CGC) 76 bp Sc: 87.27
GGGGCTATAGCTCAGTTGGTAGAGCGCTTCGTTCGCATCGAAGAGGTCAGGGGTTCGAAT
CCCCTTAGCTCCACAA
>Arthrobacter_FB24_chr1.trna39-AlaGGC (2670906-2670834) Ala (GGC) 73 bp Sc: 84.56
GGGGCTGTGGCGCAGCTGGTAGCGCACCTGCATGGCATGCAGGGGGTCAGGGGTTCGAGT
CCCCTCAGCTCCA
>Arthrobacter_FB24_chr1.trna38-AlaGGC (2671174-2671099) Ala (GGC) 76 bp Sc: 85.18
GGGGCTGTGGCGCAGCTGGTAGCGCACCTGCATGGCATGCAGGGGGTCAGGGGTTCGAGT
CCCCTCAGCTCCACCC
>Arthrobacter_FB24_chr1.trna2-AlaTGC (12163-12238) Ala (TGC) 76 bp Sc: 94.41
GGGGGCATGGCGCAATTGGTAGCGCACCTGCTTTGCAAGCAGGGGGTTCGGGGTTCGAGT
CCCCGTGCCTCCACCA
>Arthrobacter_FB24_chr1.trna50-ArgACG (710744-710671) Arg (ACG) 74 bp Sc: 81.27
GCGCCCATAGCTCAGCTGGATAGAGCGTCTGTCTACGGAACAGAAGGTCAGGGGTTCGAA
TCCCTTTGGGCGCA
>Arthrobacter_FB24_chr1.trna24-ArgCCG (2947636-2947708) Arg (CCG) 73 bp Sc: 77.91
GCCCTCGTAGCTCAGGGGATAGAGCGTCTGCCTCCGGAGCAGAAGGTCGTAGGTTCGAAT
CCTATCGAGGGCA
>Arthrobacter_FB24_chr1.trna46-ArgCCT (877220-877148) Arg (CCT) 73 bp Sc: 75.16
GCCCTCGTAGCTCAGTGGATAGAGCACGGCTCTCCTAAAGCCGGTGTCGTTGGTTCGATT
CCAATCGAGGGCA
>Arthrobacter_FB24_chr1.trna20-ArgTCT (2715875-2715947) Arg (TCT) 73 bp Sc: 75.88
GCCCCAGTAGCTCAGGGGATAGAGCAGCGGCCTTCTAATCCGCCGGTCGGGGGTTCGATT
CCCTCCTGGGGCA
>Arthrobacter_FB24_chr1.trna13-AsnGTT (1502820-1502892) Asn (GTT) 73 bp Sc: 78.80

TCCTCCTTAGCTCAATTGGCAGAGCATTCGACTGTTAATCGAAGGGTTGCTGGTTCAAGT
CCAGCAGGAGGAG
>Arthrobacter_FB24_chr1.trna14-AsnGTT (1503019-1503091) Asn (GTT) 73 bp Sc: 78.80
TCCTCCTTAGCTCAATTGGCAGAGCATTCGACTGTTAATCGAAGGGTTGCTGGTTCAAGT
CCAGCAGGAGGAG
>Arthrobacter_FB24_chr1.trna7-AspGTC (421519-421592) Asp (GTC) 74 bp Sc: 87.50
GGCCCTGTAGCGCAGTTGGTTAGCGCGCCGCCCTGTCACGGCGGAGGTCGCGGGTTCAAG
TCCCGTCAGGGTCG
>Arthrobacter_FB24_chr1.trna42-CysGCA (1863418-1863348) Cys (GCA) 71 bp Sc: 63.57
GGTGGGTTGGCCGAGAGGCGAGGCAGCGGCCTGCAAAGCCGTATACACGGGTTCGAATCC
CGTACCCACCT
>Arthrobacter_FB24_chr1.trna34-GlnCTG (2827326-2827255) Gln (CTG) 72 bp Sc: 50.48
TGGGATATGGTGTAATTGGCAACACTACGGTTTCTGGTACCGTCATTCTAGGTTCGAGTC
CTGGTATCCCAG
>Arthrobacter_FB24_chr1.trna12-GlnTTG (1326164-1326235) Gln (TTG) 72 bp Sc: 48.66
TCCGCTCTGGTGTAATGGCAGCACCCCGGCCTTTGGAGCCGTGGAGTATAGGTTCGAATC
CTATGGGCGGAA
>Arthrobacter_FB24_chr1.trna35-GluCTC (2827127-2827052) Glu (CTC) 76 bp Sc: 59.41
GGCCCCATCGTATAGCGGCCTAGTACGCTGCCCTCTCACGGCGGTAACGCGGGTTCGAAT
CCCGCTGGGGTCACCA
>Arthrobacter_FB24_chr1.trna6-GluTTC (421350-421422) Glu (TTC) 73 bp Sc: 64.51
GCCCCCATCGTCTAGCGGCCTAGGACACCGCCCTTTCACGGCGGCGGCACGGGTTCGAAT
CCCGTTGGGGGTA
>Arthrobacter_FB24_chr1.trna26-GlyCCC (4364506-4364579) Gly (CCC) 74 bp Sc: 81.46
GCGGCTGTAGCTCAATGGTAGAGCGCTAGCTTCCCAAGCTTGATACGCGGGTTCGATTCC
CGTCAGCCGCTCCA
>Arthrobacter_FB24_chr1.trna41-GlyGCC (1863537-1863465) Gly (GCC) 73 bp Sc: 85.80
GCGGACGTAGCTCAGCTGGTAGAGCACCACCTTGCCAAGGTGGATGTCGCGAGTTCGAAT
CTCGTCGTCCGCT
>Arthrobacter_FB24_chr1.trna44-GlyGCC (1863175-1863103) Gly (GCC) 73 bp Sc: 85.80
GCGGACGTAGCTCAGCTGGTAGAGCACCACCTTGCCAAGGTGGATGTCGCGAGTTCGAAT
CTCGTCGTCCGCT
>Arthrobacter_FB24_chr1.trna19-GlyTCC (2701975-2702048) Gly (TCC) 74 bp Sc: 75.34
GGGGACGTAGCTTAATGGTAAAGCCTCAGTCTTCCAAACTGATTACGCGGGTTCGATTCC
CGTCGTCCCCTCCA
>Arthrobacter_FB24_chr1.trna21-HisGTG (2731989-2732061) His (GTG) 73 bp Sc: 69.94
GTGGGCTTAGCTCAGTTGGCAGAGCGCCTGGTTGTGGTCCAGGAGGTCGCGGGTTCAACC
CCCGTAGCTCACC
>Arthrobacter_FB24_chr1.trna1-IleGAT (11842-11915) Ile (GAT) 74 bp Sc: 85.09
GGGCGTATAGCTCAGGCGGTTAGAGCGCTTCGCTGATAACGAAGAGGTCCCAGGTTCAAG
TCCTGGTACGCCCA
>Arthrobacter_FB24_chr1.trna18-LeuCAA (1892239-1892320) Leu (CAA) 82 bp Sc: 70.06
GCCCGAGTGGCGGAATTGGCAGACGCGCCGCACTCAAAATGCGGTATCGAAAGGTGTGTG
GGTTCGAGTCCCACCTCGGGCA
>Arthrobacter_FB24_chr1.trna3-LeuCAG (33132-33214) Leu (CAG) 83 bp Sc: 64.30
GCGCGAGTGGCGGAACGGCAGACGCGCTGGCTTCAGGTGCCAGTGTCCGAAAGGGCGTGG
GGGTTCAAATCCCCCCTCGCGCA
>Arthrobacter_FB24_chr1.trna40-LeuGAG (2436594-2436509) Leu (GAG) 86 bp Sc: 60.42
GCGCGGGTGGCGGAATGGCAGACGCGCTAGCTTGAGGTGCTAGTCCTCGAAAGGGGGTGG
GGGTTCAAGTCCCCCTCCGCGCACAA
>Arthrobacter_FB24_chr1.trna11-LeuTAA (1251644-1251717) Leu (TAA) 74 bp Sc: 69.73
GCCCCAGTAGCCCAATTGGCAGAGGCAGCGGACTTAAAATCCGCGtgttgtGGGTTCGAG
TCCCACCTGGGGTA
>Arthrobacter_FB24_chr1.trna23-LeuTAG (2856320-2856401) Leu (TAG) 82 bp Sc: 61.53
GCGCGAGTGGTGAAATTGGCAGACACGCAGGATTTAGGTTCCTGTGCCTTCGGGCGTGGG
GGTTCAAGTCCCCCCTTGCGCA
>Arthrobacter_FB24_chr1.trna22-LysCTT (2854533-2854608) Lys (CTT) 76 bp Sc: 86.89
GCACCTCTAGCTCAACTGGCAGAGCAATTGACTCTTAATCAATGGGTTCCGGGTTCGAGT
CCCGGGGGGTGCACCA
>Arthrobacter_FB24_chr1.trna4-LysTTT (76568-76640) Lys (TTT) 73 bp Sc: 82.32
GCACCCGTAGCTCAGCTGGGAGAGCAGGGGACTTTTAATCCTCGGGTCGTGGGTTCGAGC
CCCACCGGGTGCA
>Arthrobacter_FB24_chr1.trna5-LysTTT (218923-218995) Lys (TTT) 73 bp Sc: 85.14
GGGCCTTTAGCTCAGTTGGTAGAGCATCGGACTTTTAATCCGTGGGTCGTGGGTTCGATC
CCCACAGGGCCCA
>Arthrobacter_FB24_chr1.trna15-MetCAT (1509118-1509191) Met (CAT) 74 bp Sc: 79.51
GGGGCTATAGCTCAGCTGGTTAGAGCGCGGGACTCATAATCCTAAGGTCCTCGGTTCAAG

TCCGAGTAGCCCTA
>Arthrobacter_FB24_chr1.trna32-MetCAT (3091587-3091514) Met (CAT) 74 bp Sc: 81.23
CGCGGGGTGGAGCAGTTCGGTAGCTCGCTGGGCTCATAACCCAGAGGTCACAGGTTCAAA
TCCTGTCCCCGCAA
>Arthrobacter_FB24_chr1.trna29-MetCAT (3416260-3416184) Met (CAT) 77 bp Sc: 88.40
GGCGGTGTAGCTCAGTTGGTTAGAGCGCACGACTCATAATCGTGAGGTCGGGAGATCGAG
CCTCCCCACCGCTACCA
>Arthrobacter_FB24_chr1.trna33-MetCAT (3091325-3091249) Met (CAT) 77 bp Sc: 89.53
CGCGGGGTGGAGCAGTTCGGTAGCTCGCTGGGCTCATAACCCAGAGGTCACAGGTTCAAA
TCCTGTCCCCGCAACCA
>Arthrobacter_FB24_chr1.trna8-PheGAA (421641-421716) Phe (GAA) 76 bp Sc: 79.10
GGCTCTGTAGCTCAGTTGGTAGAGCGTTCGACTGAAAATCGAAAGGTCACCGGATCGACG
CCGGTCGGAGCCACCA
>Arthrobacter_FB24_chr1.trna25-ProCGG (3828097-3828173) Pro (CGG) 77 bp Sc: 87.48
CGGGATGTGGCGCAGCTTGGTAGCGCGCGTCGTTCGGGACGACGAGGTCGCAGGTTCAAA
TCCTGTCATCCCGACCA
>Arthrobacter_FB24_chr1.trna16-ProGGG (1714644-1714720) Pro (GGG) 77 bp Sc: 88.91
CGGGCTGTAGCGCAGCTTGGTAGCGCACTTGACTGGGGGTCAAGGGGTCGCAGGTTCAAA
TCCTGTCAGCCCGACCA
>Arthrobacter_FB24_chr1.trna37-ProTGG (2701817-2701743) Pro (TGG) 75 bp Sc: 85.57
CGGGGTGTAGCTCAGCTTGGCTAGAGCGCCTGCTTTGGGAGCAGGAAGTCGCAGGTTCAA
ATCCTGTCACCCCGA
>Arthrobacter_FB24_chr1.trna51-SerCGA (687336-687247) Ser (CGA) 90 bp Sc: 50.21
GGTGACGTGTCCGAGCGGCCGAAGGTGCAACACTCGAAAtgttgtTTGGTGTCAAAGCCA
ACGTGGGTTCAAATCCCACCGTCACCGCGA
>Arthrobacter_FB24_chr1.trna49-SerGCT (722414-722327) Ser (GCT) 88 bp Sc: 56.00
GGAGACGTGCCAGAGCGGCCGAATGGGCTTCACTGCTAATGAAGTGTGGGGCACAACTCC
ACCGGGGGTTCAAATCCCCCCGTCTCCG
>Arthrobacter_FB24_chr1.trna9-SerGGA (596911-596995) Ser (GGA) 85 bp Sc: 57.93
GGAGAATTCGCCTAGCGGCCTATGGCGCACGCCTGGAACGCGTGTTGGGTTAACGCCCTC
GGGGGTTCAAATCCCCCATTCTCCG
>Arthrobacter_FB24_chr1.trna48-SerTGA (733196-733106) Ser (TGA) 91 bp Sc: 52.59
GGAAGGTTGTCCGAGCGGCCGAAGGAGCTGGTCTTGAAAACCAGTGTGCGGTAACCCCGT
ACCAAGAGTTCGAATCTCTTACCTTCCGCGA
>Arthrobacter_FB24_chr1.trna47-ThrCGT (760820-760748) Thr (CGT) 73 bp Sc: 77.66
GCTTCCTTAGCTCAGTCGGTAGAGCGTTTCACTCGTAATGAAAAGGTCATCAGTTCGATT
CTGATAGGAAGCT
>Arthrobacter_FB24_chr1.trna28-ThrGGT (3416393-3416322) Thr (GGT) 72 bp Sc: 67.14
GCCCCCCTAGCTCAGTGGTAGAGCGCGTTCTTGGTAAGAACGAGGTCACCGGATCGATTC
CGGTGGGGGGCT
>Arthrobacter_FB24_chr1.trna10-ThrTGT (816148-816224) Thr (TGT) 77 bp Sc: 89.94
GCCTCCTTAGCTCAGTTGGCCAGAGCACCGCTCTTGTAAAGCGGGGGTCGTCGGTTCGAA
TCCGACAGGGGGCTCCA
>Arthrobacter_FB24_chr1.trna30-TrpCCA (3364657-3364585) Trp (CCA) 73 bp Sc: 77.06
AGGGTAGTGGCGCAATTGGTAGCGCAGCGGTCTCCAAAACCGCAGGTTGCAGGTTCGAGT
CCTGTCTGCCCTG
>Arthrobacter_FB24_chr1.trna27-TyrGTA (3444175-3444091) Tyr (GTA) 85 bp Sc: 56.33
GGCAGATTACCCGAGCGGCCAAAGGGGGCTGACTGTAAATCAGCTGGCAACGCCTACGGG
GGTTCGAATCCCTCATCTGCCACCC
>Arthrobacter_FB24_chr1.trna17-ValCAC (1863854-1863928) Val (CAC) 75 bp Sc: 81.53
GGGCGATTGGCGCAGTGGTAGCGCGCTTCGTTCACACCGAAGAGGTCACTGGTTCGAACC
CAGTATCGCCCACCC
>Arthrobacter_FB24_chr1.trna43-ValGAC (1863305-1863231) Val (GAC) 75 bp Sc: 89.88
GGGCGATTGGCGCAGCGGTAGCGCGCTTCCCTGACACGGAAGAGGTCACTGGTTCGATCC
CAGTATCGCCCACCA
>Arthrobacter_FB24_chr1.trna45-ValGAC (1863064-1862990) Val (GAC) 75 bp Sc: 89.88
GGGCGATTGGCGCAGCGGTAGCGCGCTTCCCTGACACGGAAGAGGTCACTGGTTCGATCC
CAGTATCGCCCACCA
>Arthrobacter_FB24_chr1.trna36-ValTAC (2756648-2756573) Val (TAC) 76 bp Sc: 86.12
GGGTCTTTAGCTCAGCTGGTAGAGCGCCACGTTTACACCGTGGATGTCATCGGTTCGATC
CCGGTAGGACCCACCA
>Arthrobacter_aurescens_TC1_chr.trna31-AlaCGC (3123682-3123610) Ala (CGC) 73 bp Sc: 83.84
GGGGCTATAGCTCAGTTGGTAGAGCGCTTCGTTCGCATCGAAGAGGTCAGGAGTTCGAAT
CTCCTTAGCTCCA
>Arthrobacter_aurescens_TC1_chr.trna40-AlaGGC (2593417-2593345) Ala (GGC) 73 bp Sc: 84.56
GGGGCTGTGGCGCAGCTGGTAGCGCACCTGCATGGCATGCAGGGGGTCAGGGGTTCGAGT
CCCCTCAGCTCCA

>Arthrobacter_aurescens_TC1_chr.trna39-AlaGGC (2594538-2594463) Ala (GGC) 76 bp Sc: 92.85
GGGGCTGTGGCGCAGCTGGTAGCGCACCTGCATGGCATGCAGGGGGTCAGGGGTTCGAGT
CCCCTCAGCTCCACCA
>Arthrobacter_aurescens_TC1_chr.trna2-AlaTGC (12161-12236) Ala (TGC) 76 bp Sc: 94.41
GGGGGCATGGCGCAATTGGTAGCGCACCTGCTTTGCAAGCAGGGGGTTCGGGGTTCGAGT
CCCCGTGCCTCCACCA
>Arthrobacter_aurescens_TC1_chr.trna52-ArgACG (869019-868946) Arg (ACG) 74 bp Sc: 81.27
GCGCCCATAGCTCAGCTGGATAGAGCGTCTGTCTACGGAACAGAAGGTCAGGGGTTCGAA
TCCCTTTGGGCGCA
>Arthrobacter_aurescens_TC1_chr.trna23-ArgCCG (2872934-2873009) Arg (CCG) 76 bp Sc: 73.94
GCCCTCGTAGCTCAGGGGATAGAGCGTCTGCCTCCGGAGCAGAAGGCCGTAGGTTCGAAT
CCTATCGAGGGCACAA
>Arthrobacter_aurescens_TC1_chr.trna48-ArgCCT (1100732-1100660) Arg (CCT) 73 bp Sc: 75.16
GCCCTCGTAGCTCAGTGGATAGAGCACGGCTCTCCTAAAGCCGGTGTCGTTGGTTCGATT
CCAATCGAGGGCA
>Arthrobacter_aurescens_TC1_chr.trna37-ArgTCT (2638735-2638663) Arg (TCT) 73 bp Sc: 75.88
GCCCCAGTAGCTCAGGGGATAGAGCAGCGGCCTTCTAATCCGCCGGTCGGGGGTTCGATT
CCCTCCTGGGGCA
>Arthrobacter_aurescens_TC1_chr.trna12-AsnGTT (1634392-1634464) Asn (GTT) 73 bp Sc: 78.69
TCCTCCGTAGCTCAATTGGCAGAGCATTCGACTGTTAATCGAAGGGTTACTGGTTCAAGT
CCAGTCGGAGGAG
>Arthrobacter_aurescens_TC1_chr.trna13-AsnGTT (1634581-1634653) Asn (GTT) 73 bp Sc: 78.69
TCCTCCGTAGCTCAATTGGCAGAGCATTCGACTGTTAATCGAAGGGTTACTGGTTCAAGT
CCAGTCGGAGGAG
>Arthrobacter_aurescens_TC1_chr.trna6-AspGTC (431107-431180) Asp (GTC) 74 bp Sc: 87.50
GGCCCTGTAGCGCAGTTGGTTAGCGCGCCGCCCTGTCACGGCGGAGGTCGCGGGTTCAAG
TCCCGTCAGGGTCG
>Arthrobacter_aurescens_TC1_chr.trna54-CysGCA (417046-416976) Cys (GCA) 71 bp Sc: 52.45
CGTGGTGTACCCGAGAGGCCAGGGAGCTGCCTGCAAAGCAGCGCACGCGGGTTCGAATCC
CGCCACCACGT
>Arthrobacter_aurescens_TC1_chr.trna43-CysGCA (1987753-1987683) Cys (GCA) 71 bp Sc: 63.57
GGTGGGTTGGCCGAGAGGCGAGGCAGCGGCCTGCAAAGCCGTATACACGGGTTCGAATCC
CGTACCCACCT
>Arthrobacter_aurescens_TC1_chr.trna34-GlnCTG (2736077-2736006) Gln (CTG) 72 bp Sc: 50.48
TGGGATATGGTGTAATTGGCAACACTACGGTTTCTGGTACCGTCATTCTAGGTTCGAGTC
CTGGTATCCCAG
>Arthrobacter_aurescens_TC1_chr.trna11-GlnTTG (1435208-1435279) Gln (TTG) 72 bp Sc: 48.66
TCCGCTCTGGTGTAATGGCAGCACCCCGGCCTTTGGAGCCGTGGAGTATAGGTTCGAATC
CTATGGGCGGAA
>Arthrobacter_aurescens_TC1_chr.trna35-GluCTC (2735878-2735803) Glu (CTC) 76 bp Sc: 57.21
GGCCCCATCGTATAGCGGCCTAGTACGCCGCCCTCTCACGGCGGTAACGCGGGTTCGAAT
CCCGCTGGGGTCACAA
>Arthrobacter_aurescens_TC1_chr.trna5-GluTTC (430950-431022) Glu (TTC) 73 bp Sc: 64.51
GCCCCCATCGTCTAGCGGCCTAGGACACCGCCCTTTCACGGCGGCGGCACGGGTTCGAAT
CCCGTTGGGGGTA
>Arthrobacter_aurescens_TC1_chr.trna25-GlyCCC (4045671-4045744) Gly (CCC) 74 bp Sc: 79.70
GCGGGCGTAGCTCAATGGTAGAGCGCTAGCTTCCCAAGCTCGATACGCGGGTTCGATTCC
CGTCGCCCGCTCCA
>Arthrobacter_aurescens_TC1_chr.trna42-GlyGCC (1987872-1987800) Gly (GCC) 73 bp Sc: 85.80
GCGGACGTAGCTCAGCTGGTAGAGCACCACCTTGCCAAGGTGGATGTCGCGAGTTCGAAT
CTCGTCGTCCGCT
>Arthrobacter_aurescens_TC1_chr.trna45-GlyGCC (1987512-1987440) Gly (GCC) 73 bp Sc: 85.80
GCGGACGTAGCTCAGCTGGTAGAGCACCACCTTGCCAAGGTGGATGTCGCGAGTTCGAAT
CTCGTCGTCCGCT
>Arthrobacter_aurescens_TC1_chr.trna47-GlyGCC (1987283-1987208) Gly (GCC) 76 bp Sc: 94.10
GCGGACGTAGCTCAGCTGGTAGAGCACCACCTTGCCAAGGTGGATGTCGCGAGTTCGAAT
CTCGTCGTCCGCTCCA
>Arthrobacter_aurescens_TC1_chr.trna18-GlyTCC (2625965-2626038) Gly (TCC) 74 bp Sc: 75.34
GGGGACGTAGCTTAATGGTAAAGCCTCAGTCTTCCAAACTGATTACGCGGGTTCGATTCC
CGTCGTCCCCTCCA
>Arthrobacter_aurescens_TC1_chr.trna19-HisGTG (2644367-2644442) His (GTG) 76 bp Sc: 78.24
GTGGGCTTAGCTCAGTTGGCAGAGCGCCTGGTTGTGGTCCAGGAGGTCGCGGGTTCAACC
CCCGTAGCTCACCCCA
>Arthrobacter_aurescens_TC1_chr.trna1-IleGAT (11850-11923) Ile (GAT) 74 bp Sc: 85.09
GGGCGTATAGCTCAGGCGGTTAGAGCGCTTCGCTGATAACGAAGAGGTCCCAGGTTCAAG
TCCTGGTACGCCCA
>Arthrobacter_aurescens_TC1_chr.trna17-LeuCAA (2016989-2017070) Leu (CAA) 82 bp Sc: 68.75

GCCCGAGTGGCGGAATTGGCAGACGCGCTGCACTCAAAATGCAGTATCGAAAGGTGTGTG
GGTTCGAGTCCCACCTCGGGCA
>Arthrobacter_aurescens_TC1_chr.trna3-LeuCAG (33474-33556) Leu (CAG) 83 bp Sc: 63.18
GCGCGAGTGGCGGAACGGCAGACGCGCTGGCTTCAGGTGCCAGTATCCGAAAGGGTGTGG
GGGTTCAAATCCCCCCTCGCGCA
>Arthrobacter_aurescens_TC1_chr.trna26-LeuGAG (4546298-4546213) Leu (GAG) 86 bp Sc: 60.42
GCGCGGGTGGCGGAATGGCAGACGCGCTAGCTTGAGGTGCTAGTCCTCGAAAGGGGGTGG
GGGTTCAAGTCCCCCTCCGCGCACAA
>Arthrobacter_aurescens_TC1_chr.trna41-LeuGAG (2359800-2359715) Leu (GAG) 86 bp Sc: 60.42
GCGCGGGTGGCGGAATGGCAGACGCGCTAGCTTGAGGTGCTAGTCCTCGAAAGGGGGTGG
GGGTTCAAGTCCCCCTCCGCGCACAA
>Arthrobacter_aurescens_TC1_chr.trna10-LeuTAA (1355200-1355276) Leu (TAA) 77 bp Sc: 73.87
GCCCCAGTAGCCCAATTGGCAGAGGCAGCGGACTTAAAATCCGCGtgttgtGGGTTCGAG
TCCCACCTGGGGCACAA
>Arthrobacter_aurescens_TC1_chr.trna22-LeuTAG (2766406-2766487) Leu (TAG) 82 bp Sc: 61.53
GCGCGAGTGGTGAAATTGGCAGACACGCAGGATTTAGGTTCCTGTGCCTTCGGGCGTGGG
GGTTCAAGTCCCCCCTTGCGCA
>Arthrobacter_aurescens_TC1_chr.trna20-LysCTT (2764879-2764954) Lys (CTT) 76 bp Sc: 86.89
GCACCTCTAGCTCAACTGGCAGAGCAATTGACTCTTAATCAATGGGTTCCGGGTTCGAGT
CCCGGGGGGTGCACCA
>Arthrobacter_aurescens_TC1_chr.trna21-LysCTT (2765150-2765225) Lys (CTT) 76 bp Sc: 86.89
GCACCTCTAGCTCAACTGGCAGAGCAATTGACTCTTAATCAATGGGTTCCGGGTTCGAGT
CCCGGGGGGTGCACCA
>Arthrobacter_aurescens_TC1_chr.trna4-LysTTT (209827-209902) Lys (TTT) 76 bp Sc: 85.76
GGGCCTTTAGCTCAGTTGGTAGAGCATCGGACTTTTAATCCGTGGGTCGTGGGTTCGATC
CCCACAGGGCCCACCC
>Arthrobacter_aurescens_TC1_chr.trna14-MetCAT (1644503-1644576) Met (CAT) 74 bp Sc: 79.51
GGGGCTATAGCTCAGCTGGTTAGAGCGCGGGACTCATAATCCTAAGGTCCTCGGTTCAAG
TCCGAGTAGCCCTA
>Arthrobacter_aurescens_TC1_chr.trna29-MetCAT (3296611-3296538) Met (CAT) 74 bp Sc: 80.11
GGCGGTGTAGCTCAGTTGGTTAGAGCGCACGACTCATAATCGTGAGGTCGGGAGATCGAG
CCTCCCCACCGCTA
>Arthrobacter_aurescens_TC1_chr.trna32-MetCAT (3004752-3004679) Met (CAT) 74 bp Sc: 81.23
CGCGGGGTGGAGCAGTTCGGTAGCTCGCTGGGCTCATAACCCAGAGGTCACAGGTTCAAA
TCCTGTCCCCGCAA
>Arthrobacter_aurescens_TC1_chr.trna33-MetCAT (3004366-3004293) Met (CAT) 74 bp Sc: 81.23
CGCGGGGTGGAGCAGTTCGGTAGCTCGCTGGGCTCATAACCCAGAGGTCACAGGTTCAAA
TCCTGTCCCCGCAA
>Arthrobacter_aurescens_TC1_chr.trna7-PheGAA (431227-431302) Phe (GAA) 76 bp Sc: 79.10
GGCTCTGTAGCTCAGTTGGTAGAGCGTTCGACTGAAAATCGAAAGGTCACCGGATCGACG
CCGGTCGGAGCCACCA
>Arthrobacter_aurescens_TC1_chr.trna24-ProCGG (3719623-3719699) Pro (CGG) 77 bp Sc: 90.60
CGGGATGTAGCGCAGCTTGGTAGCGCGCTTCGTTCGGGACGAAGAGGTCGCAGGTTCAAA
TCCTGTCATCCCGACCA
>Arthrobacter_aurescens_TC1_chr.trna15-ProGGG (1833188-1833264) Pro (GGG) 77 bp Sc: 88.91
CGGGCTGTAGCGCAGCTTGGTAGCGCACTTGACTGGGGGTCAAGGGGTCGCAGGTTCAAA
TCCTGTCAGCCCGACCA
>Arthrobacter_aurescens_TC1_chr.trna38-ProTGG (2625836-2625762) Pro (TGG) 75 bp Sc: 85.57
CGGGGTGTAGCTCAGCTTGGCTAGAGCGCCTGCTTTGGGAGCAGGAAGTCGCAGGTTCAA
ATCCTGTCACCCCGA
>Arthrobacter_aurescens_TC1_chr.trna53-SerCGA (822934-822845) Ser (CGA) 90 bp Sc: 58.01
GGTGACGTGTCCGAGCGGCCGAAGGTGCAACACTCGAAAtgttgtTTGGTGTAAAAGCCA
ACGTGGGTTCAAATCCCACCGTCACCGCCA
>Arthrobacter_aurescens_TC1_chr.trna51-SerGCT (878944-878854) Ser (GCT) 91 bp Sc: 56.62
GGAGACGTGCCAGAGCGGCCGAATGGGCTTCACTGCTAATGAAGTGTGGGGCACAACTCC
ACCGGGGGTTCAAATCCCCCCGTCTCCGCGA
>Arthrobacter_aurescens_TC1_chr.trna8-SerGGA (688080-688167) Ser (GGA) 88 bp Sc: 58.55
GGAGAATTCGCCTAGCGGCCTATGGCGCACGCCTGGAACGCGTGTTGGGTTAACGCCCTC
GGGGGTTCAAATCCCCCATTCTCCGCCG
>Arthrobacter_aurescens_TC1_chr.trna50-SerTGA (891050-890960) Ser (TGA) 91 bp Sc: 56.50
GGAAGGTTGTCCGAGCGGCCTAAGGAGCTGGTCTTGAAAACCAGTGTGCGGTAACCCCGT
ACCAAGGGTTCAAATCCCTTACCTTCCGCGA
>Arthrobacter_aurescens_TC1_chr.trna49-ThrCGT (930743-930671) Thr (CGT) 73 bp Sc: 77.66
GCTTCCTTAGCTCAGTCGGTAGAGCGTTTCACTCGTAATGAAAAGGTCATCAGTTCGATT
CTGATAGGAAGCT
>Arthrobacter_aurescens_TC1_chr.trna28-ThrGGT (3296744-3296673) Thr (GGT) 72 bp Sc: 67.14
GCCCCCCTAGCTCAGTGGTAGAGCGCGTTCTTGGTAAGAACGAGGTCACCGGATCGATTC

CGGTGGGGGGCT
>Arthrobacter_aurescens_TC1_chr.trna9-ThrTGT (992133-992209) Thr (TGT) 77 bp Sc: 89.94
GCCTCCTTAGCTCAGTTGGCCAGAGCACCGCTCTTGTAAAGCGGGGGTCGTCGGTTCGAA
TCCGACAGGGGGCTCCA
>Arthrobacter_aurescens_TC1_chr.trna30-TrpCCA (3249809-3249737) Trp (CCA) 73 bp Sc: 77.06
AGGGTAGTGGCGCAATTGGTAGCGCAGCGGTCTCCAAAACCGCAGGTTGCAGGTTCGAGT
CCTGTCTGCCCTG
>Arthrobacter_aurescens_TC1_chr.trna27-TyrGTA (3332622-3332538) Tyr (GTA) 85 bp Sc: 56.33
GGCAGATTACCCGAGCGGCCAAAGGGGGCTGACTGTAAATCAGCTGGCAACGCCTACGGG
GGTTCGAATCCCTCATCTGCCACCC
>Arthrobacter_aurescens_TC1_chr.trna16-ValCAC (1988123-1988197) Val (CAC) 75 bp Sc: 89.21
GGGCGATTGGCGCAGTGGTAGCGCGCTTCGTTCACACCGAAGAGGTCACTGGTTCGAACC
CAGTATCGCCCACCA
>Arthrobacter_aurescens_TC1_chr.trna44-ValGAC (1987644-1987570) Val (GAC) 75 bp Sc: 89.88
GGGCGATTGGCGCAGCGGTAGCGCGCTTCCCTGACACGGAAGAGGTCACTGGTTCGATCC
CAGTATCGCCCACCA
>Arthrobacter_aurescens_TC1_chr.trna46-ValGAC (1987416-1987342) Val (GAC) 75 bp Sc: 89.88
GGGCGATTGGCGCAGCGGTAGCGCGCTTCCCTGACACGGAAGAGGTCACTGGTTCGATCC
CAGTATCGCCCACCA
>Arthrobacter_aurescens_TC1_chr.trna36-ValTAC (2666668-2666593) Val (TAC) 76 bp Sc: 86.12
GGGTCTTTAGCTCAGCTGGTAGAGCGCCACGTTTACACCGTGGATGTCATCGGTTCGATC
CCGGTAGGACCCACCA
>Aspergillus_fumigatus_chr2.trna2-AlaAGC (935026-935097) Ala (AGC) 72 bp Sc: 74.84
GGGCTTGTGGTTTAGTGGTATAATACCCCCTTAGCATGGGGGTGGTCCGGGGTTCGATTC
CCCGCGAGTCCA
>Aspergillus_fumigatus_chr2.trna21-AlaAGC (937016-936945) Ala (AGC) 72 bp Sc: 74.84
GGGCTTGTGGTTTAGTGGTATAATACCCCCTTAGCATGGGGGTGGTCCGGGGTTCGATTC
CCCGCGAGTCCA
>Aspergillus_fumigatus_chr2.trna22-AlaAGC (934042-933971) Ala (AGC) 72 bp Sc: 74.84
GGGCTTGTGGTTTAGTGGTATAATACCCCCTTAGCATGGGGGTGGTCCGGGGTTCGATTC
CCCGCGAGTCCA
>Aspergillus_fumigatus_chr2.trna3-AlaAGC (937478-937549) Ala (AGC) 72 bp Sc: 74.84
GGGCTTGTGGTTTAGTGGTATAATACCCCCTTAGCATGGGGGTGGTCCGGGGTTCGATTC
CCCGCGAGTCCA
>Aspergillus_fumigatus_chr3.trna21-AlaAGC (741446-741375) Ala (AGC) 72 bp Sc: 74.84
GGGCTTGTGGTTTAGTGGTATAATACCCCCTTAGCATGGGGGTGGTCCGGGGTTCGATTC
CCCGCGAGTCCA
>Aspergillus_fumigatus_chr3.trna25-AlaAGC (289763-289692) Ala (AGC) 72 bp Sc: 74.84
GGGCTTGTGGTTTAGTGGTATAATACCCCCTTAGCATGGGGGTGGTCCGGGGTTCGATTC
CCCGCGAGTCCA
>Aspergillus_fumigatus_chr3.trna5-AlaAGC (531049-531120) Ala (AGC) 72 bp Sc: 74.84
GGGCTTGTGGTTTAGTGGTATAATACCCCCTTAGCATGGGGGTGGTCCGGGGTTCGATTC
CCCGCGAGTCCA
>Aspergillus_fumigatus_chr7.trna6-AlaAGC (1156256-1156327) Ala (AGC) 72 bp Sc: 74.84
GGGCTTGTGGTTTAGTGGTATAATACCCCCTTAGCATGGGGGTGGTCCGGGGTTCGATTC
CCCGCGAGTCCA
>Aspergillus_fumigatus_chr5.trna27-AlaCGC (2654429-2654358) Ala (CGC) 72 bp Sc: 82.54
GGGCTGTTAGCACAGTGGTAGTGCGCCCGCTTCGCATGCGGGAGGTCGTGGGTTCGATTC
CCACATAGTCCA
>Aspergillus_fumigatus_chr8.trna7-AlaCGC (1422869-1422953) Ala (CGC) 85 bp Sc: 71.56
GGGCTTGTGGTTTAGGGGTATAACGCTCCATTCGCATATGCAATGGGTCTTGGAGAGGTC
CCGGGTTCGATTCCCGGCGAGTCCA
>Aspergillus_fumigatus_chr5.trna4-AlaTGC (1440602-1440673) Ala (TGC) 72 bp Sc: 81.20
GGGCTTGTGGTTTAGTGGTATAATACCTGCCTTGCAAGCAGGTGGTCCGGGGTTCGATTC
CCCGCGAGTCCA
>Aspergillus_fumigatus_chr5.trna3-AlaTGC (1440395-1440499) Ala (TGC) 105 bp Sc: 58.41
GGGCTTGTAGCTCAGTGGTAGTAGCGCTCGCTTTGCATTTAATAAAGGTGCAGGTCCTCG
GACTAGATATGCGAGAGGTCCGGGGTTCAATCCCCCGTGAGTCCA
>Aspergillus_fumigatus_chr2.trna6-AlaTGC (2549759-2549860) Ala (TGC) 102 bp Sc: 60.01
GGGCTTGTAGCTCAGTGGTAGTAGCGCTCGCTTTGCATTTGTAAATGCAGGTTTTTAGAC
CTAGATTGCGAGAGGTCCGGGGTTCAATCCCCCGTGAGTCCA
>Aspergillus_fumigatus_chr2.trna25-ArgACG (87180-87109) Arg (ACG) 72 bp Sc: 75.55
GGCCTGCTGGCCCAATGGTAAGGCGCTTGACTACGGATCAAGAGATTGCAGGTTCGAATC
CTGCGTAGGTCA
>Aspergillus_fumigatus_chr2.trna8-ArgACG (2993163-2993234) Arg (ACG) 72 bp Sc: 75.55
GGCCTGCTGGCCCAATGGTAAGGCGCTTGACTACGGATCAAGAGATTGCAGGTTCGAATC
CTGCGTAGGTCA

>Aspergillus_fumigatus_chr3.trna6-ArgACG (821186-821257) Arg (ACG) 72 bp Sc: 75.55
GGCCTGCTGGCCCAATGGTAAGGCGCTTGACTACGGATCAAGAGATTGCAGGTTCGAATC
CTGCGTAGGTCA
>Aspergillus_fumigatus_chr6.trna10-ArgACG (3587601-3587672) Arg (ACG) 72 bp Sc: 75.55
GGCCTGCTGGCCCAATGGTAAGGCGCTTGACTACGGATCAAGAGATTGCAGGTTCGAATC
CTGCGTAGGTCA
>Aspergillus_fumigatus_chr6.trna11-ArgACG (3438601-3438530) Arg (ACG) 72 bp Sc: 75.55
GGCCTGCTGGCCCAATGGTAAGGCGCTTGACTACGGATCAAGAGATTGCAGGTTCGAATC
CTGCGTAGGTCA
>Aspergillus_fumigatus_chr6.trna14-ArgACG (847831-847760) Arg (ACG) 72 bp Sc: 75.55
GGCCTGCTGGCCCAATGGTAAGGCGCTTGACTACGGATCAAGAGATTGCAGGTTCGAATC
CTGCGTAGGTCA
>Aspergillus_fumigatus_chr6.trna16-ArgACG (713999-713928) Arg (ACG) 72 bp Sc: 75.55
GGCCTGCTGGCCCAATGGTAAGGCGCTTGACTACGGATCAAGAGATTGCAGGTTCGAATC
CTGCGTAGGTCA
>Aspergillus_fumigatus_chr7.trna7-ArgACG (994880-994809) Arg (ACG) 72 bp Sc: 76.08
GGCCGGCTGGCCCAATGGTAAGGCGCTTGACTACGGATCAAGAGATTGCAGGTTCGAATC
CTGCGTCGGTCA
>Aspergillus_fumigatus_chr2.trna19-ArgACG (1651778-1651707) Arg (ACG) 72 bp Sc: 76.87
GGCCTGCTGGCCCAATGGTAAGGCGCTTGACTACGGATCAAGAGATTGCAGGTTCGAATC
CTGCGCAGGTCA
>Aspergillus_fumigatus_chr3.trna9-ArgCCG (2342082-2342153) Arg (CCG) 72 bp Sc: 65.23
GGTCTCGTGGCGCAATGGTAGCGCGTTAGTTTCCGGTACTAAAGGCTGTGCGTTCGAATC
GCACCGGGATCG
>Aspergillus_fumigatus_chr3.trna10-ArgCCG (2345014-2345085) Arg (CCG) 72 bp Sc: 65.96
GGTCTCGTGGCGCAATGGTAGCGCATTAGTTTCCGGTACTAAAGGTTGTGCGTTCGAGTC
GCACCGGGATCG
>Aspergillus_fumigatus_chr6.trna17-ArgCCT (596224-596153) Arg (CCT) 72 bp Sc: 71.52
GGCCGGCTGGCCTAATGGTAAGGCGTTGCTCTCCTAAAGCAAAGATTGCAGGTTCGATCC
CTGCGTCGGTCG
>Aspergillus_fumigatus_chr8.trna17-ArgCCT (648049-647970) Arg (CCT) 80 bp Sc: 60.99
GGTCTATTGGCTCAATGGTAGAGCACCTGTCTCCTATGCTGGAAAACAGGAGGTTGTGGG
TTCGAGTCCCATGTAGATCG
>Aspergillus_fumigatus_chr3.trna23-ArgTCG (493923-493852) Arg (TCG) 72 bp Sc: 69.93
GGCCGGATGGCGCAATGGTAGCGCGTTGGTTTTCGGCACCAAAGGTTGCGCGTTCGAATC
GCGTTTCGGTCG
>Aspergillus_fumigatus_chr3.trna17-ArgTCG (1149522-1149451) Arg (TCG) 72 bp Sc: 75.02
GGCCGGCTGGCCTAATGGTAAGGCGCTTGACTTCGGATCAAGAGATTGCAGGTTCGATCC
CTGCGTCGGTCA
>Aspergillus_fumigatus_chr7.trna2-ArgTCT (114031-114102) Arg (TCT) 72 bp Sc: 77.85
GCCCTGCTGGCGCAATGGTAGCGCGTCAGACTTCTAATCTGAAGGTTGTGGGTTCGACCC
CCACGTAGGGCT
>Aspergillus_fumigatus_chr3.trna26-AsnGTT (179048-178975) Asn (GTT) 74 bp Sc: 79.12
GTCTCCGTGGCGCAATTGGTTAGCGCGTCTGACTGTTAATCAGGAGGTTGGAAGTTCGAG
CCTTCCCGGGGACG
>Aspergillus_fumigatus_chr5.trna15-AsnGTT (3372970-3373059) Asn (GTT) 90 bp Sc: 66.22
GTCTCCATGGCCCAGCTGGTTAAGGCGTCCGACTGTTAAGCAGTGGCTTGTGCAATCGGG
AGATCGGCAGTTCGAGCCTGCCTGGGGACG
>Aspergillus_fumigatus_chr5.trna20-AsnGTT (3373659-3373563) Asn (GTT) 97 bp Sc: 64.72
GTCTCCATGGCCCAGCTGGTTAAGGCGTCCGACTGTTATCGCAGTCCATTTGGACTCGGC
AATCGGGAGATCGGCAGTTCGAGCCTGCCTGGGGACG
>Aspergillus_fumigatus_chr5.trna19-AsnGTT (3374293-3374204) Asn (GTT) 90 bp Sc: 66.22
GTCTCCATGGCCCAGCTGGTTAAGGCGTCCGACTGTTAAGCAGTGGCTTGTGCAATCGGG
AGATCGGCAGTTCGAGCCTGCCTGGGGACG
>Aspergillus_fumigatus_chr5.trna18-AsnGTT (3374656-3374565) Asn (GTT) 92 bp Sc: 67.53
GTCTCCGTGGCCCAGCTGGTTAAGGCGTCCGACTGTTAAGCTAGCTGCTCCTAGCAATCG
GGAGATCGGCAGTTCGATCCTGCCCGGGGACG
>Aspergillus_fumigatus_chr8.trna5-AspGTC (1023658-1023746) Asp (GTC) 89 bp Sc: 57.72
TCCTCGATGGTCTAACGGTCATGATTTCCGCTTGTCACTCTCAATGAGAACGAGCGCGGG
AGACCAGGGTTCGACTCCCTGTCGGGGAG
>Aspergillus_fumigatus_chr2.trna20-AspGTC (1301538-1301452) Asp (GTC) 87 bp Sc: 57.47
TCCTCGATGGTCTAACGGTCATGATTTCCGCTTGTCACTCCAAGAGAACGAGCGCGGGAG
ACCAGGGTTCGACTCCCTGTCGGGGAG
>Aspergillus_fumigatus_chr8.trna6-AspGTC (1409471-1409558) Asp (GTC) 88 bp Sc: 57.73
TCCTCGATGGTCTAACGGTCATGATTTCCGCTTGTCACTCGAATGAGAACGAGCGCGGGA
GACCAGGGTTCGACTCCCTGTCGGGGAG
>Aspergillus_fumigatus_chr1.trna12-AspGTC (2727249-2727336) Asp (GTC) 88 bp Sc: 56.96

TCCTCGATGGTCTAACGGTCATGATTTCCGCTTGTCACTCCAACGAGAACGAGCGCGGGA
GACCAGGGTTCGACTCCCTGTCGGGGAG
>Aspergillus_fumigatus_chr6.trna18-AspGTC (490809-490721) Asp (GTC) 89 bp Sc: 58.49
TCCTCGATGGTCTAACGGTCATGATTTCCGCTTGTCACTCAAATTGAGAACGAGCGCGGG
AGACCAGGGTTCGACTCCCTGTCGGGGAG
>Aspergillus_fumigatus_chr1.trna1-AspGTC (766126-766213) Asp (GTC) 88 bp Sc: 57.73
TCCTCGATGGTCTAACGGTCATGATTTCCGCTTGTCACTCAACAGAGAACGAGCGCGGGA
GACCAGGGTTCGACTCCCTGTCGGGGAG
>Aspergillus_fumigatus_chr1.trna32-AspGTC (767355-767268) Asp (GTC) 88 bp Sc: 57.73
TCCTCGATGGTCTAACGGTCATGATTTCCGCTTGTCACTCAACAGAGAACGAGCGCGGGA
GACCAGGGTTCGACTCCCTGTCGGGGAG
>Aspergillus_fumigatus_chr3.trna20-AspGTC (900660-900573) Asp (GTC) 88 bp Sc: 58.23
TCCTCGATGGTCTAACGGTCATGATTTCCGCTTGTCACTCTAATGAGAACGAGCGCGGGA
GACCAGGGTTCGACTCCCTGTCGGGGAG
>Aspergillus_fumigatus_chr3.trna19-AspGTC (979297-979210) Asp (GTC) 88 bp Sc: 56.95
TCCTCGATGGTCTAACGGTCATGATTTCCGCTTGTCACTCCGATGAGAACGAGCGCGGGA
GACCAGGGTTCGACTCCCTGTCGGGGAG
>Aspergillus_fumigatus_chr3.trna4-CysGCA (395422-395493) Cys (GCA) 72 bp Sc: 71.41
GGACTGGTAGCTCAGTGGTAGAGCGTGAGATTGCAAATCTTAAGGTCGCGTGTTCGATCC
ACGCTTAGTCCT
>Aspergillus_fumigatus_chr3.trna24-CysGCA (401933-401862) Cys (GCA) 72 bp Sc: 71.64
GGGCTGGTAGCTCAGTGGTAGAGCGTGAGATTGCAAATCTTAAGGTCGCGTGTTCGATCC
ACGCTTAGCCCT
>Aspergillus_fumigatus_chr5.trna16-CysGCA (3703632-3703727) Cys (GCA) 96 bp Sc: 58.99
GGACTGGTACCTCAGTGGTAGAGGGTGAGATTGCAATAACATCGCAAGATGTACTGTAAT
ATCTTGAGGTCCCGTGTTCGATCCACGGTCAGTCCT
>Aspergillus_fumigatus_chr8.trna8-GlnCTG (1428297-1428368) Gln (CTG) 72 bp Sc: 63.14
GGTTGTGTAGTGTAATGGTTATCACTCCAGATTCTGATTCTGGCAATCTTGGTTCGATTC
CGAGCACGACCT
>Aspergillus_fumigatus_chr1.trna29-GlnCTG (2279068-2278997) Gln (CTG) 72 bp Sc: 64.64
GGTTGTGTAGTGTAATGGTTATCACTCCAGATTCTGATTCTGGCAATCCTGGTTCGATTC
CGGGCACGACCT
>Aspergillus_fumigatus_chr1.trna9-GlnCTG (2282077-2282148) Gln (CTG) 72 bp Sc: 64.64
GGTTGTGTAGTGTAATGGTTATCACTCCAGATTCTGATTCTGGCAATCCTGGTTCGATTC
CGGGCACGACCT
>Aspergillus_fumigatus_chr1.trna10-GlnCTG (2282224-2282295) Gln (CTG) 72 bp Sc: 65.89
GGTTGCGTAGTGTAATGGTTATCACTCCAGATTCTGATTCTGGCAATCCTGGTTCGATTC
CGGGCGCGACCT
>Aspergillus_fumigatus_chr1.trna28-GlnCTG (2280625-2280554) Gln (CTG) 72 bp Sc: 65.89
GGTTGCGTAGTGTAATGGTTATCACTCCAGATTCTGATTCTGGCAATCCTGGTTCGATTC
CGGGCGCGACCT
>Aspergillus_fumigatus_chr1.trna8-GlnCTG (2281487-2281558) Gln (CTG) 72 bp Sc: 65.89
GGTTGCGTAGTGTAATGGTTATCACTCCAGATTCTGATTCTGGCAATCCTGGTTCGATTC
CGGGCGCGACCT
>Aspergillus_fumigatus_chr8.trna14-GlnTTG (1106893-1106808) Gln (TTG) 86 bp Sc: 63.13
GCTTCTATGGTCTAGTGGTTATGACTCCGGATTTTGAGCATTCGCTGAGTCTTCCGGCAA
CCCCGGTTCGAATCCGGGTAGGAGCT
>Aspergillus_fumigatus_chr6.trna2-GlnTTG (248352-248435) Gln (TTG) 84 bp Sc: 60.03
GGTTCCATGGTCTAGTGGTTATGACTCCGGATTTTGACGCGTGTCAGTTTTCCGGCAACT
CGGGTTCAATTCCCGATGGGACCT
>Aspergillus_fumigatus_chr5.trna12-GluCTC (3206972-3207043) Glu (CTC) 72 bp Sc: 57.70
TCCGATGTGGTGTAATGGCTAACATCGCTGTCTCTCACACAGCAGCTCGGGGTTCGATTC
CCCGCATCGGAA
>Aspergillus_fumigatus_chr5.trna14-GluCTC (3207575-3207646) Glu (CTC) 72 bp Sc: 57.70
TCCGATGTGGTGTAATGGCTAACATCGCTGTCTCTCACACAGCAGCTCGGGGTTCGATTC
CCCGCATCGGAA
>Aspergillus_fumigatus_chr5.trna17-GluCTC (3575251-3575180) Glu (CTC) 72 bp Sc: 57.70
TCCGATGTGGTGTAATGGCTAACATCGCTGTCTCTCACACAGCAGCTCGGGGTTCGATTC
CCCGCATCGGAA
>Aspergillus_fumigatus_chr5.trna21-GluCTC (3208796-3208725) Glu (CTC) 72 bp Sc: 57.70
TCCGATGTGGTGTAATGGCTAACATCGCTGTCTCTCACACAGCAGCTCGGGGTTCGATTC
CCCGCATCGGAA
>Aspergillus_fumigatus_chr5.trna22-GluCTC (3208340-3208269) Glu (CTC) 72 bp Sc: 57.70
TCCGATGTGGTGTAATGGCTAACATCGCTGTCTCTCACACAGCAGCTCGGGGTTCGATTC
CCCGCATCGGAA
>Aspergillus_fumigatus_chr5.trna34-GluCTC (1723131-1723060) Glu (CTC) 72 bp Sc: 57.70
TCCGATGTGGTGTAATGGCTAACATCGCTGTCTCTCACACAGCAGCTCGGGGTTCGATTC

CCCGCATCGGAA
>Aspergillus_fumigatus_chr5.trna5-GluCTC (1723332-1723403) Glu (CTC) 72 bp Sc: 57.70
TCCGATGTGGTGTAATGGCTAACATCGCTGTCTCTCACACAGCAGCTCGGGGTTCGATTC
CCCGCATCGGAA
>Aspergillus_fumigatus_chr1.trna20-GluCTC (4489501-4489430) Glu (CTC) 72 bp Sc: 62.13
TCCGATATGGTGTAGTGGCTAACATCGCCGTCTCTCACACGGCAGCCGGGGGTTCGATTC
CCCCTATCGGAG
>Aspergillus_fumigatus_chr1.trna3-GluTTC (1380646-1380717) Glu (TTC) 72 bp Sc: 51.51
TCCGATATGGTGTAACGGCTAACATCGCCGTCTTTCACACGGCAGCTCGGGGTTCGACTC
CCCGTATCGGAG
>Aspergillus_fumigatus_chr1.trna31-GluTTC (1387319-1387248) Glu (TTC) 72 bp Sc: 51.51
TCCGATATGGTGTAACGGCTAACATCGCCGTCTTTCACACGGCAGCTCGGGGTTCGACTC
CCCGTATCGGAG
>Aspergillus_fumigatus_chr1.trna4-GluTTC (1380814-1380885) Glu (TTC) 72 bp Sc: 56.66
TCCGATATGGTGTAATGGCTAACATCGCCGTCTTTCACACGGCAGCTCGGGGTTCGATTC
CCCGTATCGGAG
>Aspergillus_fumigatus_chr2.trna7-GluTTC (2619790-2619861) Glu (TTC) 72 bp Sc: 65.66
TCCGTTGTAGTGTAACGGCTATCATACCTGTCTTTCACACAGGAGATCGGGGTTCGATTC
CCCGCAACGGAA
>Aspergillus_fumigatus_chr4.trna9-GlyCCC (2307984-2307898) Gly (CCC) 87 bp Sc: 60.39
GCGCCAATGGTCTAGTGGTTAGGATGCGGTCCTCCCATTCCCCTACTGGGCAAGACCGAG
ACACGAGTTCGAATCCCGTTTGGCGCA
>Aspergillus_fumigatus_chr1.trna13-GlyGCC (2907410-2907480) Gly (GCC) 71 bp Sc: 66.83
GCATCATTGGTCTAGTGGTAGAATTCGTCGTTGCCATCGACGAGGCCCGTGTTCGATTCA
CGGATGATGCA
>Aspergillus_fumigatus_chr1.trna7-GlyGCC (1651256-1651326) Gly (GCC) 71 bp Sc: 66.83
GCATCATTGGTCTAGTGGTAGAATTCGTCGTTGCCATCGACGAGGCCCGTGTTCGATTCA
CGGATGATGCA
>Aspergillus_fumigatus_chr2.trna23-GlyGCC (465305-465235) Gly (GCC) 71 bp Sc: 66.83
GCATCATTGGTCTAGTGGTAGAATTCGTCGTTGCCATCGACGAGGCCCGTGTTCGATTCA
CGGATGATGCA
>Aspergillus_fumigatus_chr3.trna13-GlyGCC (3556767-3556837) Gly (GCC) 71 bp Sc: 66.83
GCATCATTGGTCTAGTGGTAGAATTCGTCGTTGCCATCGACGAGGCCCGTGTTCGATTCA
CGGATGATGCA
>Aspergillus_fumigatus_chr4.trna6-GlyGCC (3650153-3650223) Gly (GCC) 71 bp Sc: 66.83
GCATCATTGGTCTAGTGGTAGAATTCGTCGTTGCCATCGACGAGGCCCGTGTTCGATTCA
CGGATGATGCA
>Aspergillus_fumigatus_chr4.trna8-GlyGCC (2409096-2409026) Gly (GCC) 71 bp Sc: 66.83
GCATCATTGGTCTAGTGGTAGAATTCGTCGTTGCCATCGACGAGGCCCGTGTTCGATTCA
CGGATGATGCA
>Aspergillus_fumigatus_chr5.trna33-GlyGCC (1903658-1903588) Gly (GCC) 71 bp Sc: 66.83
GCATCATTGGTCTAGTGGTAGAATTCGTCGTTGCCATCGACGAGGCCCGTGTTCGATTCA
CGGATGATGCA
>Aspergillus_fumigatus_chr5.trna35-GlyGCC (321680-321610) Gly (GCC) 71 bp Sc: 66.83
GCATCATTGGTCTAGTGGTAGAATTCGTCGTTGCCATCGACGAGGCCCGTGTTCGATTCA
CGGATGATGCA
>Aspergillus_fumigatus_chr5.trna9-GlyGCC (3086241-3086311) Gly (GCC) 71 bp Sc: 66.83
GCATCATTGGTCTAGTGGTAGAATTCGTCGTTGCCATCGACGAGGCCCGTGTTCGATTCA
CGGATGATGCA
>Aspergillus_fumigatus_chr6.trna9-GlyGCC (3477652-3477722) Gly (GCC) 71 bp Sc: 66.83
GCATCATTGGTCTAGTGGTAGAATTCGTCGTTGCCATCGACGAGGCCCGTGTTCGATTCA
CGGATGATGCA
>Aspergillus_fumigatus_chr8.trna16-GlyGCC (665850-665780) Gly (GCC) 71 bp Sc: 66.83
GCATCATTGGTCTAGTGGTAGAATTCGTCGTTGCCATCGACGAGGCCCGTGTTCGATTCA
CGGATGATGCA
>Aspergillus_fumigatus_chr1.trna19-GlyTCC (4157779-4157850) Gly (TCC) 72 bp Sc: 71.91
GCGTTAGTGGTGTAGCGGTTAGCATTGTTGCCTTCCAAGCAATAGATCTGGGTTCGACTC
CCAGCTAACGCA
>Aspergillus_fumigatus_chr5.trna30-GlyTCC (2561769-2561698) Gly (TCC) 72 bp Sc: 71.91
GCGTTAGTGGTGTAGCGGTTAGCATTGTTGCCTTCCAAGCAATAGATCTGGGTTCGACTC
CCAGCTAACGCA
>Aspergillus_fumigatus_chr7.trna4-GlyTCC (220338-220409) Gly (TCC) 72 bp Sc: 71.91
GCGTTAGTGGTGTAGCGGTTAGCATTGTTGCCTTCCAAGCAATAGATCTGGGTTCGACTC
CCAGCTAACGCA
>Aspergillus_fumigatus_chr5.trna8-HisGTG (2557308-2557378) His (GTG) 71 bp Sc: 59.31
GGTGCTATGGTCTAGGGGTAGGACGCCACGTTGTGGCCGTGTCAGCCCCGGTTCGATTCC
GGGTAGCGCCA

>Aspergillus_fumigatus_chr5.trna29-HisGTG (2565008-2564938) His (GTG) 71 bp Sc: 67.88
GGTGCTGTGGTCTAGTGGTAGGACGTCACGTTGTGGCCGTGCCAGCCCCGGTTCGATTCC
GGGCAGCACCA
>Aspergillus_fumigatus_chr8.trna11-HisGTG (1618840-1618770) His (GTG) 71 bp Sc: 67.88
GGTGCTGTGGTCTAGTGGTAGGACGTCACGTTGTGGCCGTGCCAGCCCCGGTTCGATTCC
GGGCAGCACCA
>Aspergillus_fumigatus_chr4.trna5-HisGTG (3470061-3470144) His (GTG) 84 bp Sc: 54.03
GGTGCTGTAGTGTAGTGGTTATCATGCGACGTTGTGGCTCCCCACAGCTCCGTCTCGACC
TGGGTTCGATTCCCAGCAGCGCCA
>Aspergillus_fumigatus_chr3.trna1-IleAAT (108846-108919) Ile (AAT) 74 bp Sc: 75.60
GGTCGCTTAGCTCAGTCGGTTAGAGCGTGGTGCTAATAACGCCAAGGTCGCGAGTTCGAC
CCTCGTAGTGACCA
>Aspergillus_fumigatus_chr4.trna10-IleAAT (1817040-1816967) Ile (AAT) 74 bp Sc: 75.60
GGTCGCTTAGCTCAGTCGGTTAGAGCGTGGTGCTAATAACGCCAAGGTCGCGAGTTCGAC
CCTCGTAGTGACCA
>Aspergillus_fumigatus_chr8.trna12-IleAAT (1434776-1434703) Ile (AAT) 74 bp Sc: 75.60
GGTCGCTTAGCTCAGTCGGTTAGAGCGTGGTGCTAATAACGCCAAGGTCGCGAGTTCGAC
CCTCGTAGTGACCA
>Aspergillus_fumigatus_chr3.trna11-IleAAT (2793260-2793333) Ile (AAT) 74 bp Sc: 76.01
GGTCCTTTAGCTCAGTCGGTTAGAGCGTGGTGCTAATAACGCCAAGGTCGCGAGTTCGAC
CCTCGTAGGGACCA
>Aspergillus_fumigatus_chr3.trna12-IleAAT (2793697-2793770) Ile (AAT) 74 bp Sc: 76.01
GGTCCTTTAGCTCAGTCGGTTAGAGCGTGGTGCTAATAACGCCAAGGTCGCGAGTTCGAC
CCTCGTAGGGACCA
>Aspergillus_fumigatus_chr3.trna16-IleAAT (2792050-2791977) Ile (AAT) 74 bp Sc: 76.01
GGTCCTTTAGCTCAGTCGGTTAGAGCGTGGTGCTAATAACGCCAAGGTCGCGAGTTCGAC
CCTCGTAGGGACCA
>Aspergillus_fumigatus_chr8.trna15-IleAAT (970558-970485) Ile (AAT) 74 bp Sc: 76.01
GGTCCTTTAGCTCAGTCGGTTAGAGCGTGGTGCTAATAACGCCAAGGTCGCGAGTTCGAC
CCTCGTAGGGACCA
>Aspergillus_fumigatus_chr4.trna12-IleTAT (913009-912922) Ile (TAT) 88 bp Sc: 65.84
GGACGCGTAGCTCAGTTGGTTAGAGCGACGAGCTTATGAGGTCGTTTGTGATCCTCGTAG
GTCGGGAGTTCGACTCTCCCCGTGTCCA
>Aspergillus_fumigatus_chr3.trna7-LeuAAG (2145343-2145455) Leu (AAG) 113 bp Sc: 57.90
GGCAAGATGGCCGAGTTGGTCCAAGGCGTGAGGTTAAGGTACACCTTAATACCAAACTCT
ATGAGTTTCCTCATCATCGTATGATGGCGTGGGTTCGAATCCCACTCTTGTCA
>Aspergillus_fumigatus_chr2.trna16-LeuAAG (2412140-2412028) Leu (AAG) 113 bp Sc: 57.12
GGCAAGATGGCCGAGTTGGTCCAAGGCGTGAGGTTAAGGTTCACCTTAATACCAAACTCT
TAGAGCTTCCTCATCATCGTATGATGGCGTGGGTTCGAATCCCACTCTTGTCA
>Aspergillus_fumigatus_chr5.trna28-LeuAAG (2570024-2569912) Leu (AAG) 113 bp Sc: 56.98
GGCAAGATGGCCGAGTTGGTCCAAGGCGTGAGGTTAAGGTCTACCTTAATATCAAACTCT
CCGAGTTTCCTCATCATCGTATGATGGCGTGGGTTCGAATCCCACTCTTGTCA
>Aspergillus_fumigatus_chr2.trna9-LeuAAG (3830552-3830664) Leu (AAG) 113 bp Sc: 58.39
GGCAAGATGGCCGAGTTGGTCCAAGGCGTGAGGTTAAGGTATACCTTAATATCAAACTCT
TCGAGTTTCCTCATCATCGTATGATGGCGTGGGTTCGAATCCCACTCTTGTCA
>Aspergillus_fumigatus_chr2.trna13-LeuAAG (4667163-4667051) Leu (AAG) 113 bp Sc: 56.20
GGCAAGATGGCCGAGTTGGTCCAAGGCGTGAGGTTAAGGTCCACCTTAATACCACTCTCT
TAGAGTTTCCTCATCATCGTATGATGGCGTGGGTTCGAATCCCACTCTTGTCA
>Aspergillus_fumigatus_chr7.trna8-LeuAAG (817375-817263) Leu (AAG) 113 bp Sc: 59.66
GGCAAGATGGCCGAGTTGGTCCAAGGCGTGAGGTTAAGGTATACCTTAATAATCAACTTT
TCTAAGTTCCTCATCATCGTATGATGGCGTGGGTTCGAATCCCACTCTTGTCA
>Aspergillus_fumigatus_chr4.trna7-LeuCAA (3456679-3456566) Leu (CAA) 114 bp Sc: 58.46
GGTAAGATGACCGAGTGGTTAAGGTGGTGCACTCAAGCTGGTTAGCTTTCTCTAGAAAAC
TAGATTTGTCATGCACTCTCGCAAGAGGCCTGGGTTCGACTCCCAGTCTTACCA
>Aspergillus_fumigatus_chr4.trna4-LeuCAA (3456951-3457064) Leu (CAA) 114 bp Sc: 58.46
GGTAAGATGACCGAGTGGTTAAGGTGGTGCACTCAAGCTGGTTAGCTTTCTCTAGAAAAC
TAGATTTGTCATGCACTCTCGCAAGAGGCCTGGGTTCGACTCCCAGTCTTACCA
>Aspergillus_fumigatus_chr6.trna12-LeuCAG (3400535-3400425) Leu (CAG) 111 bp Sc: 55.71
GGCAAGGTGTCCGAGTGGTCCAAGGAGCACGGTTCAGGAACTGAACCTACAAACTGATTC
ATCAGTTTCCGTGTCTCGAAAGGGGCGTGGGTTCGAATCCCACCCTTGTCA
>Aspergillus_fumigatus_chr6.trna8-LeuCAG (3401235-3401345) Leu (CAG) 111 bp Sc: 52.62
GGCAAGGTGTCCGAGCGGTCCAAGGAGCACGGTTCAGGAACTGAACCTACAAACTGATTC
GTCAGTTTCCGTGTCTCGAAAGGGGCGTGGGTTCGAATCCCACCCTTGTCA
>Aspergillus_fumigatus_chr2.trna10-LeuCAG (4365818-4365928) Leu (CAG) 111 bp Sc: 57.01
GGCAAGGTGTCCGAGTGGTCTAAGGAGCACGGTTCAGGAACTGAACCTACAAACTGTTTC
GTCAGTTTCCGTGTCTCGCAAGGGGCGTGGGTTCGAATCCCACCCTTGTCA
>Aspergillus_fumigatus_chr7.trna13-LeuCAG (87486-87376) Leu (CAG) 111 bp Sc: 55.20

GGCAAGGTGTCCGAGTGGTCCAAGGAGCACGGTTCAGGAACTGGACCTTAAAACTGAGTC
ATCAGTTTCCGTGTCTCGAAAGGGGCGTGGGTTCGAATCCCACCCTTGTCA
>Aspergillus_fumigatus_chr5.trna25-LeuTAA (2691226-2691096) Leu (TAA) 131 bp Sc: 56.82
GGTAAGATGGCCGAGTGGTTAAGGCGAGTGACTTAAGTGCTGCGTGCTGTACCACAGCTT
TTACATAAGCTGTACAGGCCGCTCAAATTCACTTGACGAAAGTCGCGTGGGTTCGAACCC
CACTCTTACCA
>Aspergillus_fumigatus_chr7.trna10-LysCTT (632409-632337) Lys (CTT) 73 bp Sc: 72.66
GCCGGGCTAGCTCAATCGGTAGAGCGTGAGACTCTTAATCTCAAGGCTGCGGGTTCGACC
CCCGCGTTCGGCT
>Aspergillus_fumigatus_chr2.trna14-LysCTT (4182933-4182861) Lys (CTT) 73 bp Sc: 76.76
GCCGGGCTAGCTCAATCGGTAGAGCGTGAGACTCTTAATCTCAAGGTTGCGGGTTCGACC
CCCGCGTTCGGCT
>Aspergillus_fumigatus_chr3.trna14-LysCTT (3583456-3583528) Lys (CTT) 73 bp Sc: 76.76
GCCGGGCTAGCTCAATCGGTAGAGCGTGAGACTCTTAATCTCAAGGTTGCGGGTTCGACC
CCCGCGTTCGGCT
>Aspergillus_fumigatus_chr3.trna15-LysCTT (3571414-3571342) Lys (CTT) 73 bp Sc: 76.76
GCCGGGCTAGCTCAATCGGTAGAGCGTGAGACTCTTAATCTCAAGGTTGCGGGTTCGACC
CCCGCGTTCGGCT
>Aspergillus_fumigatus_chr3.trna18-LysCTT (1149267-1149195) Lys (CTT) 73 bp Sc: 76.76
GCCGGGCTAGCTCAATCGGTAGAGCGTGAGACTCTTAATCTCAAGGTTGCGGGTTCGACC
CCCGCGTTCGGCT
>Aspergillus_fumigatus_chr5.trna26-LysCTT (2687508-2687436) Lys (CTT) 73 bp Sc: 76.76
GCCGGGCTAGCTCAATCGGTAGAGCGTGAGACTCTTAATCTCAAGGTTGCGGGTTCGACC
CCCGCGTTCGGCT
>Aspergillus_fumigatus_chr7.trna11-LysCTT (631929-631857) Lys (CTT) 73 bp Sc: 76.76
GCCGGGCTAGCTCAATCGGTAGAGCGTGAGACTCTTAATCTCAAGGTTGCGGGTTCGACC
CCCGCGTTCGGCT
>Aspergillus_fumigatus_chr7.trna9-LysCTT (632559-632487) Lys (CTT) 73 bp Sc: 76.76
GCCGGGCTAGCTCAATCGGTAGAGCGTGAGACTCTTAATCTCAAGGTTGCGGGTTCGACC
CCCGCGTTCGGCT
>Aspergillus_fumigatus_chr5.trna1-LysTTT (883190-883272) Lys (TTT) 83 bp Sc: 71.51
GCCCGATTAACTCAGTTGGTTAGAGTGTCAGACTTTTATGGCAAGAGATCTGAAAGTCGC
GAGTTCGACCCTCGCATCGGGCG
>Aspergillus_fumigatus_chr5.trna31-MetCAT (2538536-2538465) Met (CAT) 72 bp Sc: 75.63
AGCATGTTAGCTCAGGGGAAGAGCGCCGGGCTCATAACCCGGAGGTCCCTGGATCGAAAC
CAGGACATGCTA
>Aspergillus_fumigatus_chr5.trna32-MetCAT (2538092-2538021) Met (CAT) 72 bp Sc: 75.63
AGCATGTTAGCTCAGGGGAAGAGCGCCGGGCTCATAACCCGGAGGTCCCTGGATCGAAAC
CAGGACATGCTA
>Aspergillus_fumigatus_chr5.trna7-MetCAT (2535907-2535978) Met (CAT) 72 bp Sc: 75.63
AGCATGTTAGCTCAGGGGAAGAGCGCCGGGCTCATAACCCGGAGGTCCCTGGATCGAAAC
CAGGACATGCTA
>Aspergillus_fumigatus_chr8.trna9-MetCAT (1674926-1674997) Met (CAT) 72 bp Sc: 75.63
AGCATGTTAGCTCAGGGGAAGAGCGCCGGGCTCATAACCCGGAGGTCCCTGGATCGAAAC
CAGGACATGCTA
>Aspergillus_fumigatus_chr1.trna5-MetCAT (1461859-1461966) Met (CAT) 108 bp Sc: 72.25
GCCTCTGTGGTGTAGTTGGTTATCACGTGAGTCTCATATCTTACATATGACTCAGAAGGG
TACTTCTGGGACATCTCAAGGTCGGGAGTTCGATCCTCCCCGGAGGCA
>Aspergillus_fumigatus_chr8.trna10-MetCAT (1756466-1756578) Met (CAT) 113 bp Sc: 68.06
GCCTCTATGGTGTAGTTGGTTATCACGCAGGTCTCATATCACATGACTCTTCTGTCAACA
GAGGGGATGTTCCTGAAATCCTGAGGTCTCCAGTTCGATCCTGGATGGAGGCA
>Aspergillus_fumigatus_chr3.trna8-MetCAT (2210063-2210170) Met (CAT) 108 bp Sc: 72.89
GCCTCTGTGGTGTAGTTGGTTATCACGTGAGTCTCATATTATGTATATGATCAGAAGCTG
CACTTCTGGGACATCTCAAGGTCGGGAGTTCGATCCTCCCCGGAGGCA
>Aspergillus_fumigatus_chr2.trna12-MetCAT (4722232-4722338) Met (CAT) 107 bp Sc: 72.49
GCCTCTGTGGTGTAGTTGGTTATCACGTGAGTCTCATATTGCCTATATGATCAGAAGATC
ACTTCTGAGCCATCTCAAGGTCGGGAGTTCGATCCTCCCCGGAGGCA
>Aspergillus_fumigatus_chr3.trna2-PheGAA (115480-115567) Phe (GAA) 88 bp Sc: 68.72
GGGGTGATAGCTCAGTTGGGAGAGCGTGCGACTGAAGTTACTCAGTCATTTAATCGTAAG
GTCGCCGGTTCAATCCCGGCTCACCCCA
>Aspergillus_fumigatus_chr5.trna24-PheGAA (3169547-3169460) Phe (GAA) 88 bp Sc: 72.33
GGGGTGATAGCTCAGTTGGGAGAGCGTACGACTGAAGTTACTCAGTCACTTAATCGTAAG
GTCGCCGGTTCAATCCCGGCTCACCCCA
>Aspergillus_fumigatus_chr5.trna23-PheGAA (3169866-3169779) Phe (GAA) 88 bp Sc: 72.47
GGGGTGATAGCTCAGTTGGGAGAGCGTACGACTGAAGTTACTCAGTCACATAATCGTAAG
GTCGCCGGTTCAATCCCGGCTCACCCCA
>Aspergillus_fumigatus_chr5.trna10-PheGAA (3171825-3171912) Phe (GAA) 88 bp Sc: 72.33

GGGGTGATAGCTCAGTTGGGAGAGCGTACGACTGAAGTTACTCAGTCACTTAATCGTAAG
GTCGCCGGTTCAATCCCGGCTCACCCCA
>Aspergillus_fumigatus_chr5.trna11-PheGAA (3171999-3172086) Phe (GAA) 88 bp Sc: 71.21
GGGGTGATAGCTCAGTTGGGAGAGCGTACGACTGAAGTTATTCAGTCACATAATCGTAAG
GTCGCCGGTTCAATCCCGGCTCATCCCA
>Aspergillus_fumigatus_chr1.trna14-ProAGG (3107447-3107536) Pro (AGG) 90 bp Sc: 65.92
GCCCGGGTGGTCTAGTGGTATGATTCTCGCTTAGGGAAAAACCCCAAGCATATCTGCGAG
AGGTCCCGCGTTCGATCCGCGGCTCGGGCC
>Aspergillus_fumigatus_chr6.trna7-ProAGG (3140471-3140558) Pro (AGG) 88 bp Sc: 66.16
GCCCGGGTGGTCTAGTGGTATGATTCTCGCTTAGGGAAAACCCAAGCACATTTGCGAGAG
GTCCCGCGTTCGATCCGCGGCTCGGGCC
>Aspergillus_fumigatus_chr1.trna17-ProAGG (3802349-3802437) Pro (AGG) 89 bp Sc: 66.43
GCCCGGGTGGTCTAGTGGTATGATTCTCGCTTAGGGAAAAACCCAAGCATATCTGCGAGA
GGTCCCGCGTTCGATCCGCGGCTCGGGCC
>Aspergillus_fumigatus_chr3.trna22-ProAGG (577205-577117) Pro (AGG) 89 bp Sc: 67.07
GCCCGGGTGGTCTAGTGGTATGATTCTCGCTTAGGGAAAAACCCAAGCATATTTGCGAGA
GGTCCCGCGTTCGATCCGCGGCTCGGGCC
>Aspergillus_fumigatus_chr4.trna13-ProAGG (722485-722398) Pro (AGG) 88 bp Sc: 66.31
GCCCGGGTGGTCTAGTGGTATGATTCTCGCTTAGGGAAAACCCAAGCAAATCTGCGAGAG
GTCCCGCGTTCGATCCGCGGCTCGGGCC
>Aspergillus_fumigatus_chr1.trna21-ProCGG (4333677-4333579) Pro (CGG) 99 bp Sc: 54.28
GCCTGAATAGTCTAGAGGTAGGATTCATCCTTCGGGAACTCCCGGCTCTCAAAAGAGCTA
TGAATGGATGAGGCCCGCGTTCGAATCGCGGTTCAGGCC
>Aspergillus_fumigatus_chr7.trna5-ProCGG (689334-689419) Pro (CGG) 86 bp Sc: 53.62
GCCCGAGTGGTCCAGGGGTAAGATTCATCGTTCGGGTGTGCAAACCTTGTCTCGATGAGA
CCCGCGTTCGAATCGCGGCTCGGGCC
>Aspergillus_fumigatus_chr1.trna25-ProTGG (3698876-3698805) Pro (TGG) 72 bp Sc: 71.85
GCCCGGATGGTCTAGTGGTATGATTCTCGCTTTGGGTGCGAGAGGTCGCGCGTTCGACTC
GCGCTTCGGGCC
>Aspergillus_fumigatus_chr5.trna13-ProTGG (3207244-3207315) Pro (TGG) 72 bp Sc: 71.85
GCCCGGATGGTCTAGTGGTATGATTCTCGCTTTGGGTGCGAGAGGTCGCGCGTTCGACTC
GCGCTTCGGGCC
>Aspergillus_fumigatus_chr1.trna30-SerAGA (1609041-1608961) Ser (AGA) 81 bp Sc: 70.79
GTCAGTGTGCCCGAGTGGTTAAGGGGAGTGATTAGAAATCACTTGGCTTCGGCCGCATAG
GTTCGAGTCCTATCGCTGACG
>Aspergillus_fumigatus_chr1.trna6-SerAGA (1609608-1609688) Ser (AGA) 81 bp Sc: 70.79
GTCAGTGTGCCCGAGTGGTTAAGGGGAGTGATTAGAAATCACTTGGCTTCGGCCGCATAG
GTTCGAGTCCTATCGCTGACG
>Aspergillus_fumigatus_chr2.trna1-SerAGA (463802-463882) Ser (AGA) 81 bp Sc: 70.79
GTCAGTGTGCCCGAGTGGTTAAGGGGAGTGATTAGAAATCACTTGGCTTCGGCCGCATAG
GTTCGAGTCCTATCGCTGACG
>Aspergillus_fumigatus_chr2.trna4-SerAGA (1301238-1301318) Ser (AGA) 81 bp Sc: 70.79
GTCAGTGTGCCCGAGTGGTTAAGGGGAGTGATTAGAAATCACTTGGCTTCGGCCGCATAG
GTTCGAGTCCTATCGCTGACG
>Aspergillus_fumigatus_chr4.trna1-SerAGA (420996-421076) Ser (AGA) 81 bp Sc: 70.79
GTCAGTGTGCCCGAGTGGTTAAGGGGAGTGATTAGAAATCACTTGGCTTCGGCCGCATAG
GTTCGAGTCCTATCGCTGACG
>Aspergillus_fumigatus_chr7.trna12-SerCGA (133143-133017) Ser (CGA) 127 bp Sc: 62.02
GGCAAGGTGGCCGAGTGGTTAAGGCGTTTGATTCGAATTGGGtgttgtAAAGACCGATGC
CGGTCTGGTCATCATCCCCGACTATCAAATTCATTCGTGAGCCGGGGTTCAAATCCCCGC
CTTGTCG
>Aspergillus_fumigatus_chr7.trna3-SerCGA (133533-133629) Ser (CGA) 97 bp Sc: 70.47
GGCAAGGTGGCCGAGTGGTTAAGGCGTCAGTTTCGAATTGAGCAAACTCAATTAGCTGAT
TCCCTAGGGATCGCCGGTTCGAATCCGGTCCTTGTCG
>Aspergillus_fumigatus_chr1.trna11-SerGCT (2426522-2426602) Ser (GCT) 81 bp Sc: 82.08
GACACTTTGGCCGAGTGGTTAAGGCGCAGGCCTGCTAAGCCTGTGGGTTCGCCCGCCTGG
GTTCGAATCCCAGAGGTGTCG
>Aspergillus_fumigatus_chr1.trna26-SerGCT (2425996-2425916) Ser (GCT) 81 bp Sc: 82.08
GACACTTTGGCCGAGTGGTTAAGGCGCAGGCCTGCTAAGCCTGTGGGTTCGCCCGCCTGG
GTTCGAATCCCAGAGGTGTCG
>Aspergillus_fumigatus_chr1.trna27-SerGCT (2425454-2425374) Ser (GCT) 81 bp Sc: 82.08
GACACTTTGGCCGAGTGGTTAAGGCGCAGGCCTGCTAAGCCTGTGGGTTCGCCCGCCTGG
GTTCGAATCCCAGAGGTGTCG
>Aspergillus_fumigatus_chr6.trna4-SerGCT (874333-874413) Ser (GCT) 81 bp Sc: 82.08
GACACTTTGGCCGAGTGGTTAAGGCGCAGGCCTGCTAAGCCTGTGGGTTCGCCCGCCTGG
GTTCGAATCCCAGAGGTGTCG
>Aspergillus_fumigatus_chr1.trna2-SerTGA (917096-917192) Ser (TGA) 97 bp Sc: 68.58

GGCAAGGTGGCCGAGTGGTTAAGGCGTCAGTTTTGAATTTGAGCAAACTCAATAGCTGAT
TCCCTAGGGATCGTCGGTTCGAATCCGGCCCTTGTCG
>Aspergillus_fumigatus_chr2.trna24-ThrAGT (143028-142956) Thr (AGT) 73 bp Sc: 84.01
GCTCGCATAGCTCAGTTGGTAGAGCGCATGACTAGTAATCATGAGGTCAGCGGTTCAAAT
CCGCTTGTGAGCA
>Aspergillus_fumigatus_chr3.trna3-ThrAGT (163221-163293) Thr (AGT) 73 bp Sc: 84.01
GCTCGCATAGCTCAGTTGGTAGAGCGCATGACTAGTAATCATGAGGTCAGCGGTTCAAAT
CCGCTTGTGAGCA
>Aspergillus_fumigatus_chr4.trna2-ThrAGT (2000974-2001046) Thr (AGT) 73 bp Sc: 84.01
GCTCGCATAGCTCAGTTGGTAGAGCGCATGACTAGTAATCATGAGGTCAGCGGTTCAAAT
CCGCTTGTGAGCA
>Aspergillus_fumigatus_chr4.trna3-ThrAGT (2410166-2410238) Thr (AGT) 73 bp Sc: 84.01
GCTCGCATAGCTCAGTTGGTAGAGCGCATGACTAGTAATCATGAGGTCAGCGGTTCAAAT
CCGCTTGTGAGCA
>Aspergillus_fumigatus_chr6.trna13-ThrAGT (2164940-2164868) Thr (AGT) 73 bp Sc: 84.01
GCTCGCATAGCTCAGTTGGTAGAGCGCATGACTAGTAATCATGAGGTCAGCGGTTCAAAT
CCGCTTGTGAGCA
>Aspergillus_fumigatus_chr6.trna6-ThrAGT (2165147-2165219) Thr (AGT) 73 bp Sc: 84.01
GCTCGCATAGCTCAGTTGGTAGAGCGCATGACTAGTAATCATGAGGTCAGCGGTTCAAAT
CCGCTTGTGAGCA
>Aspergillus_fumigatus_chr2.trna11-ThrCGT (4499991-4500062) Thr (CGT) 72 bp Sc: 82.80
GCATCTATGGCTCAGTGGTAGAGCGTTCCACTCGTAATGGAAAGGTCCATGGTTCAATCC
CGTGTAGATGCA
>Aspergillus_fumigatus_chr6.trna5-ThrCGT (1574776-1574860) Thr (CGT) 85 bp Sc: 71.97
GCACCTATAGCTCAGTGGTAGAGCGTTCCACTCGTATGGTGTACGACCTATGGAAAGGTC
CGCGGTTCGATCCCGCGTCGGTGCA
>Aspergillus_fumigatus_chr4.trna11-ThrTGT (1213130-1213043) Thr (TGT) 88 bp Sc: 76.20
GCACCTATGGCTCAGTGGTAGAGCATTCCACTTGTATTGTCAAGAAACAGTGATGGAAAG
GTCCGCGGTTCGATCCCGCGTGGGTGCA
>Aspergillus_fumigatus_chr6.trna3-ThrTGT (617113-617210) Thr (TGT) 98 bp Sc: 63.28
GCACGAGTGGCCAAGTGGCTAAGGCGTCGCACTTGTATCTGCCCGCTTGGGCTAAGATCG
CTATGCGAAGATCGGGGGTTCAATTCCCCTCTTGTGCA
>Aspergillus_fumigatus_chr5.trna2-ThrTGT (883581-883709) Thr (TGT) 129 bp Sc: 56.44
GCCCGATTAACTCAGTTGGCTAGAGTGTCAGACTTGTTCGTACCCCTTCTTAGTCATATC
TTCTTTCGCACATTACTAATAACCACCCAGTTAATCTGAACGTCGGGGGTTCGAGCCCCC
CATCGGGCG
>Aspergillus_fumigatus_chr2.trna17-TrpCCA (1691252-1691180) Trp (CCA) 73 bp Sc: 59.83
GGGGCTGTGGCGTAATTGGTAGCGCGTGAGACTCCAGATCTTAAGGTTATCCGTTCAAGT
CGGGTCAGCCTCA
>Aspergillus_fumigatus_chr2.trna18-TrpCCA (1690720-1690648) Trp (CCA) 73 bp Sc: 59.83
GGGGCTGTGGCGTAATTGGTAGCGCGTGAGACTCCAGATCTTAAGGTTATCCGTTCAAGT
CGGGTCAGCCTCA
>Aspergillus_fumigatus_chr2.trna5-TrpCCA (1691910-1691982) Trp (CCA) 73 bp Sc: 59.83
GGGGCTGTGGCGTAATTGGTAGCGCGTGAGACTCCAGATCTTAAGGTTATCCGTTCAAGT
CGGGTCAGCCTCA
>Aspergillus_fumigatus_chr1.trna15-TyrGTA (3759573-3759657) Tyr (GTA) 85 bp Sc: 69.98
CCCCCGATGGTCTAGTTGGATATGGCGTTCGGCTGTAATGGAAATTGTAACCGAAAGGTC
GCCGGCTCGATTCCGGCTCGGGGGA
>Aspergillus_fumigatus_chr1.trna16-TyrGTA (3760089-3760173) Tyr (GTA) 85 bp Sc: 69.34
CCCCCGATGGTCTAGTTGGATATGGCGTTCGGCTGTAATGGGAATTGTAACCGAAAGGTC
GCCGGCTCGATTCCGGCTCGGGGGA
>Aspergillus_fumigatus_chr1.trna24-TyrGTA (3760797-3760713) Tyr (GTA) 85 bp Sc: 69.98
CCCCCGATGGTCTAGTTGGATATGGCGTTCGGCTGTAATGGAAATTGTAACCGAAAGGTC
GCCGGCTCGATTCCGGCTCGGGGGA
>Aspergillus_fumigatus_chr1.trna23-TyrGTA (3761229-3761145) Tyr (GTA) 85 bp Sc: 69.34
CCCCCGATGGTCTAGTTGGATATGGCGTTCGGCTGTAATGGAAATTGTGACCGAAAGGTC
GCCGGCTCGATTCCGGCTCGGGGGA
>Aspergillus_fumigatus_chr1.trna22-TyrGTA (3761792-3761708) Tyr (GTA) 85 bp Sc: 69.34
CCCCCGATGGTCTAGTTGGATATGGCGTTCGGCTGTAATGGGAATTGTAACCGAAAGGTC
GCCGGCTCGATTCCGGCTCGGGGGA
>Aspergillus_fumigatus_chr6.trna15-Undet??? (809379-809244) Undet (???) 136 bp Sc: 39.26
GGCAAGATGGCCGAGTGGTTAAGGCGGTGAGTGTTTCCATGTGTCCCTTAGTCTTTCGAC
ATTCTATCTAATAAATCAACCAGTTACGTTTAGGCCGTAATGGGAAACCGCGTGGGTTCG
AATCCCACTCTTGTCA
>Aspergillus_fumigatus_chr8.trna3-ValAAC (1011735-1011808) Val (AAC) 74 bp Sc: 73.95
GGCTGGTTGGTGTAGTTGGTTATCACGTATCGTTAACACCGATAAGGTCGCCGGATCGAG
CCCGGCACTGGTCA

>Aspergillus_fumigatus_chr6.trna1-ValAAC (126214-126287) Val (AAC) 74 bp Sc: 75.17
GGCCGGTTGGTGTAGTTGGTTATCACGTATCGTTAACACCGATAAGGTCGCCGGATCGAG
CCCGGCACTGGTCA
>Aspergillus_fumigatus_chr7.trna1-ValAAC (103767-103840) Val (AAC) 74 bp Sc: 75.17
GGCCGGTTGGTGTAGTTGGTTATCACGTATCGTTAACACCGATAAGGTCGCCGGATCGAG
CCCGGCACTGGTCA
>Aspergillus_fumigatus_chr8.trna1-ValAAC (1009806-1009879) Val (AAC) 74 bp Sc: 75.17
GGCCGGTTGGTGTAGTTGGTTATCACGTATCGTTAACACCGATAAGGTCGCCGGATCGAG
CCCGGCACTGGTCA
>Aspergillus_fumigatus_chr8.trna13-ValAAC (1315335-1315262) Val (AAC) 74 bp Sc: 75.17
GGCCGGTTGGTGTAGTTGGTTATCACGTATCGTTAACACCGATAAGGTCGCCGGATCGAG
CCCGGCACTGGTCA
>Aspergillus_fumigatus_chr8.trna4-ValAAC (1016439-1016512) Val (AAC) 74 bp Sc: 75.17
GGCCGGTTGGTGTAGTTGGTTATCACGTATCGTTAACACCGATAAGGTCGCCGGATCGAG
CCCGGCACTGGTCA
>Aspergillus_fumigatus_chr8.trna2-ValAAC (1010165-1010238) Val (AAC) 74 bp Sc: 76.83
GGCCGGTTGGTGTAGTTGGTTATCACGTATCGTTAACACCGATAAGGTCGCCGGATCGAA
CCCGGCACTGGTCA
>Aspergillus_fumigatus_chr2.trna15-ValCAC (3631928-3631830) Val (CAC) 99 bp Sc: 65.23
AGTCACGTGGTCTAATGGTTATGATTTCTCGTTCACAATTAAAAGTGAGCTCCTTAAAGG
ATACCGAGAAGGTTCCCGGTTCGATCCCGGGCGTGACTA
>Aspergillus_fumigatus_chr1.trna18-ValCAC (3909273-3909368) Val (CAC) 96 bp Sc: 62.98
AGTCGCGTGGTCTAATGGTTATGATTTCTCGTTCACAGTCATGTGAGCTTCGCAAGGATT
CCGAGAAGGTTCCCGGTTCGATCCCGGGCGCGACTA
>Aspergillus_fumigatus_chr5.trna6-ValTAC (2114322-2114394) Val (TAC) 73 bp Sc: 82.61
GGCCGGTTGGCGTAGTGGTTATCGCGTTTCGTTTACACCGAAAAGGTCGCCGGTTCGAAC
CCGGCACTGGTCA
>Arabidopsis_thaliana_chr4.trna25-AlaAGC (13454563-13454635) Ala (AGC) 73 bp Sc: 64.02
GGGGATGTAGCTCAGATGGTAGAGCGCTCGCTTAGCATGCGAGAGGCACGGGGATCGATA
CCCCGCATCTCCA
>Arabidopsis_thaliana_chr4.trna71-AlaAGC (6905552-6905480) Ala (AGC) 73 bp Sc: 64.02
GGGGATGTAGCTCAGATGGTAGAGCGCTCGCTTAGCATGCGAGAGGCACGGGGATCGATA
CCCCGCATCTCCA
>Arabidopsis_thaliana_chr5.trna88-AlaAGC (17476475-17476403) Ala (AGC) 73 bp Sc: 64.02
GGGGATGTAGCTCAGATGGTAGAGCGCTCGCTTAGCATGCGAGAGGCACGGGGATCGATA
CCCCGCATCTCCA
>Arabidopsis_thaliana_chr2.trna71-AlaAGC (13144385-13144313) Ala (AGC) 73 bp Sc: 65.56
GGGGATGTAGCTCAAATGGTAGAGCGCTCGCTTAGCATGCGAGAGGTACGGGGATCGATA
CCCCGCATCTCCA
>Arabidopsis_thaliana_chr1.trna11-AlaAGC (2025355-2025427) Ala (AGC) 73 bp Sc: 68.12
GGGGATGTAGCTCAGATGGTAGAGCGCTCGCTTAGCATGCGAGAGGTACGGGGATCGATA
CCCCGCATCTCCA
>Arabidopsis_thaliana_chr1.trna13-AlaAGC (2334819-2334891) Ala (AGC) 73 bp Sc: 68.12
GGGGATGTAGCTCAGATGGTAGAGCGCTCGCTTAGCATGCGAGAGGTACGGGGATCGATA
CCCCGCATCTCCA
>Arabidopsis_thaliana_chr2.trna2-AlaAGC (706795-706867) Ala (AGC) 73 bp Sc: 68.12
GGGGATGTAGCTCAGATGGTAGAGCGCTCGCTTAGCATGCGAGAGGTACGGGGATCGATA
CCCCGCATCTCCA
>Arabidopsis_thaliana_chr2.trna99-AlaAGC (846926-846854) Ala (AGC) 73 bp Sc: 68.12
GGGGATGTAGCTCAGATGGTAGAGCGCTCGCTTAGCATGCGAGAGGTACGGGGATCGATA
CCCCGCATCTCCA
>Arabidopsis_thaliana_chr3.trna29-AlaAGC (10753668-10753740) Ala (AGC) 73 bp Sc: 68.12
GGGGATGTAGCTCAGATGGTAGAGCGCTCGCTTAGCATGCGAGAGGTACGGGGATCGATA
CCCCGCATCTCCA
>Arabidopsis_thaliana_chr3.trna75-AlaAGC (10760702-10760630) Ala (AGC) 73 bp Sc: 68.12
GGGGATGTAGCTCAGATGGTAGAGCGCTCGCTTAGCATGCGAGAGGTACGGGGATCGATA
CCCCGCATCTCCA
>Arabidopsis_thaliana_chr4.trna70-AlaAGC (7232865-7232793) Ala (AGC) 73 bp Sc: 68.12
GGGGATGTAGCTCAGATGGTAGAGCGCTCGCTTAGCATGCGAGAGGTACGGGGATCGATA
CCCCGCATCTCCA
>Arabidopsis_thaliana_chr5.trna111-AlaAGC (2432336-2432264) Ala (AGC) 73 bp Sc: 68.12
GGGGATGTAGCTCAGATGGTAGAGCGCTCGCTTAGCATGCGAGAGGTACGGGGATCGATA
CCCCGCATCTCCA
>Arabidopsis_thaliana_chr5.trna123-AlaAGC (150426-150354) Ala (AGC) 73 bp Sc: 68.12
GGGGATGTAGCTCAGATGGTAGAGCGCTCGCTTAGCATGCGAGAGGTACGGGGATCGATA
CCCCGCATCTCCA
>Arabidopsis_thaliana_chr5.trna30-AlaAGC (15993515-15993587) Ala (AGC) 73 bp Sc: 68.12

GGGGATGTAGCTCAGATGGTAGAGCGCTCGCTTAGCATGCGAGAGGTACGGGGATCGATA
CCCCGCATCTCCA
>Arabidopsis_thaliana_chr5.trna56-AlaAGC (24726892-24726964) Ala (AGC) 73 bp Sc: 68.12
GGGGATGTAGCTCAGATGGTAGAGCGCTCGCTTAGCATGCGAGAGGTACGGGGATCGATA
CCCCGCATCTCCA
>Arabidopsis_thaliana_chr5.trna74-AlaAGC (22502068-22501996) Ala (AGC) 73 bp Sc: 68.12
GGGGATGTAGCTCAGATGGTAGAGCGCTCGCTTAGCATGCGAGAGGTACGGGGATCGATA
CCCCGCATCTCCA
>Arabidopsis_thaliana_chr2.trna27-AlaCGC (9598107-9598179) Ala (CGC) 73 bp Sc: 61.16
GGGGGTGTAGCTCATATGGTAGAGCGCTCGTTTCGCATGCGAGAGGCACGGGGTTCGATT
CCCCGCACCTCCA
>Arabidopsis_thaliana_chr3.trna57-AlaCGC (22871102-22871174) Ala (CGC) 73 bp Sc: 64.52
GGGGATGTAGCTCATATGGTAGAGCGCTCGCTTCGCATGCGAGAGGCACGGGGTTCGATT
CCCCGCATCTCCA
>Arabidopsis_thaliana_chr1.trna164-AlaCGC (23936800-23936872) Ala (CGC) 73 bp Sc: 65.14
GGGGGTGTAGCTCATATGGTAGAGCGCTCGCTTCGCATGCGAGAGGCACGGGGTTCGATT
CCCCGCACCTCCA
>Arabidopsis_thaliana_chr4.trna23-AlaCGC (12429609-12429681) Ala (CGC) 73 bp Sc: 65.14
GGGGGTGTAGCTCATATGGTAGAGCGCTCGCTTCGCATGCGAGAGGCACGGGGTTCGATT
CCCCGCACCTCCA
>Arabidopsis_thaliana_chr4.trna51-AlaCGC (13885269-13885197) Ala (CGC) 73 bp Sc: 65.14
GGGGGTGTAGCTCATATGGTAGAGCGCTCGCTTCGCATGCGAGAGGCACGGGGTTCGATT
CCCCGCACCTCCA
>Arabidopsis_thaliana_chr5.trna110-AlaCGC (2587899-2587827) Ala (CGC) 73 bp Sc: 65.14
GGGGGTGTAGCTCATATGGTAGAGCGCTCGCTTCGCATGCGAGAGGCACGGGGTTCGATT
CCCCGCACCTCCA
>Arabidopsis_thaliana_chr5.trna53-AlaCGC (24547402-24547474) Ala (CGC) 73 bp Sc: 65.14
GGGGGTGTAGCTCATATGGTAGAGCGCTCGCTTCGCATGCGAGAGGCACGGGGTTCGATT
CCCCGCACCTCCA
>Arabidopsis_thaliana_chr3.trna59-AlaTGC (23047855-23047927) Ala (TGC) 73 bp Sc: 63.65
GGGGATGTAGCTCATATGGTAGAGCGCTCGCTTTGCATGCGAGAGGCACAGGGTTCGATT
CCCTGCATCTCCA
>Arabidopsis_thaliana_chr1.trna207-AlaTGC (20416667-20416595) Ala (TGC) 73 bp Sc: 66.31
GGGGATGTAGCTCAAATGGTAGAGCGCTCGCTTTGCATGCGAGAGGCACGGGGTTCGATC
CCCCGCATCTCCA
>Arabidopsis_thaliana_chr1.trna22-AlaTGC (4741996-4742068) Ala (TGC) 73 bp Sc: 66.31
GGGGATGTAGCTCAAATGGTAGAGCGCTCGCTTTGCATGCGAGAGGCACGGGGTTCGATC
CCCCGCATCTCCA
>Arabidopsis_thaliana_chr1.trna23-AlaTGC (4743092-4743164) Ala (TGC) 73 bp Sc: 66.31
GGGGATGTAGCTCAAATGGTAGAGCGCTCGCTTTGCATGCGAGAGGCACGGGGTTCGATC
CCCCGCATCTCCA
>Arabidopsis_thaliana_chr1.trna237-AlaTGC (2846611-2846539) Ala (TGC) 73 bp Sc: 66.31
GGGGATGTAGCTCAAATGGTAGAGCGCTCGCTTTGCATGCGAGAGGCACGGGGTTCGATC
CCCCGCATCTCCA
>Arabidopsis_thaliana_chr1.trna33-AlaTGC (9158170-9158242) Ala (TGC) 73 bp Sc: 66.31
GGGGATGTAGCTCAAATGGTAGAGCGCTCGCTTTGCATGCGAGAGGCACGGGGTTCGATC
CCCCGCATCTCCA
>Arabidopsis_thaliana_chr2.trna59-AlaTGC (19351497-19351569) Ala (TGC) 73 bp Sc: 66.31
GGGGATGTAGCTCAAATGGTAGAGCGCTCGCTTTGCATGCGAGAGGCACGGGGTTCGATC
CCCCGCATCTCCA
>Arabidopsis_thaliana_chr2.trna98-AlaTGC (1054787-1054715) Ala (TGC) 73 bp Sc: 66.31
GGGGATGTAGCTCAAATGGTAGAGCGCTCGCTTTGCATGCGAGAGGCACGGGGTTCGATC
CCCCGCATCTCCA
>Arabidopsis_thaliana_chr3.trna61-AlaTGC (23296801-23296729) Ala (TGC) 73 bp Sc: 66.31
GGGGATGTAGCTCAAATGGTAGAGCGCTCGCTTTGCATGCGAGAGGCACGGGGTTCGATC
CCCCGCATCTCCA
>Arabidopsis_thaliana_chr3.trna62-AlaTGC (23296640-23296568) Ala (TGC) 73 bp Sc: 66.31
GGGGATGTAGCTCAAATGGTAGAGCGCTCGCTTTGCATGCGAGAGGCACGGGGTTCGATC
CCCCGCATCTCCA
>Arabidopsis_thaliana_chr2.trna80-ArgACG (9472733-9472661) Arg (ACG) 73 bp Sc: 70.96
GACTCCATGGCCCAATGGATAAGGCGCTGGTCTACGAAACCAGAGACTCTGGGTTCGATC
CCCAGTGGAGTCG
>Arabidopsis_thaliana_chr1.trna20-ArgACG (4440212-4440284) Arg (ACG) 73 bp Sc: 73.60
GACTTCATGGCCCAATGGATAAGGCGCTGGTCTACGAAACCAGAGATTCTGGGTTCGATC
CCCAGTGAAGTCG
>Arabidopsis_thaliana_chr2.trna55-ArgACG (17880767-17880839) Arg (ACG) 73 bp Sc: 73.60
GACTTCATGGCCCAATGGATAAGGCGCTGGTCTACGAAACCAGAGATTCTGGGTTCGATC

CCCAGTGAAGTCG
>Arabidopsis_thaliana_chr2.trna28-ArgACG (10380656-10380728) Arg (ACG) 73 bp Sc: 74.20
GACTCTGTGGCCCAATGGATAAGGCGCTGGTCTACGAAACCAGAGATTCTGGGTTCGATC
CCCAGCAGAGTCG
>Arabidopsis_thaliana_chr5.trna107-ArgACG (3212424-3212352) Arg (ACG) 73 bp Sc: 74.20
GACTCTGTGGCCCAATGGATAAGGCGCTGGTCTACGAAACCAGAGATTCTGGGTTCGATC
CCCAGCAGAGTCG
>Arabidopsis_thaliana_chr3.trna79-ArgACG (7224296-7224224) Arg (ACG) 73 bp Sc: 74.25
GGTTCCATGGCCCAATGGATAAGGCGCTGGTCTACGAAACCAGAGATTCTGGGTTCGATC
CCCAGTGGAATCG
>Arabidopsis_thaliana_chr4.trna31-ArgACG (15183090-15183162) Arg (ACG) 73 bp Sc: 75.05
GACTCCATGGCCCAATGGATAAGGCGCTGGTCTACGAAACCAGAGATTCTGGGTTCGATC
CCCAGTGGAGTCG
>Arabidopsis_thaliana_chr4.trna44-ArgACG (18501778-18501706) Arg (ACG) 73 bp Sc: 75.05
GACTCCATGGCCCAATGGATAAGGCGCTGGTCTACGAAACCAGAGATTCTGGGTTCGATC
CCCAGTGGAGTCG
>Arabidopsis_thaliana_chr5.trna61-ArgACG (25989904-25989976) Arg (ACG) 73 bp Sc: 75.45
GACTCCGTGGCCCAATGGATAAGGCGCTGGTCTACGAAACCAGAGATTCTGGGTTCGATC
CCCAGCGGAGTCG
>Arabidopsis_thaliana_chr1.trna181-ArgCCG (29809016-29809088) Arg (CCG) 73 bp Sc: 72.20
GACCGCGTGGCCTAATGGATAAGGCGCTCGCCTCCGGAGCGGGAGATTGTGGGTTCGAGT
CCCACCGTGGTCG
>Arabidopsis_thaliana_chr1.trna227-ArgCCG (5619537-5619465) Arg (CCG) 73 bp Sc: 72.20
GACCGCGTGGCCTAATGGATAAGGCGCTCGCCTCCGGAGCGGGAGATTGTGGGTTCGAGT
CCCACCGTGGTCG
>Arabidopsis_thaliana_chr3.trna7-ArgCCG (1220235-1220307) Arg (CCG) 73 bp Sc: 72.92
GTTTGCGTGGCCTAATGGATAAGGCGCTCGCCTCCGAAGCGGGAGATTGTGGGTTCGAAT
CCCATCGCGAACG
>Arabidopsis_thaliana_chr3.trna82-ArgCCG (3995176-3995104) Arg (CCG) 73 bp Sc: 85.01
GATCCCATAGCGGAGTGGATATCGCGTTAGACTCCGAATCTAAAGGTCGTGGGTTCGATT
CCCACTGGGATCA
>Arabidopsis_thaliana_chr5.trna94-ArgCCT (13823284-13823212) Arg (CCT) 73 bp Sc: 57.69
GCGCATGTAGCTCAGTGGATAGAGCGTCTGTTTCCTAAGCAGAAGGTCGTAGGTTTGACC
CCTACCTGGCACG
>Arabidopsis_thaliana_chr4.trna48-ArgCCT (16454773-16454701) Arg (CCT) 73 bp Sc: 71.26
GCGCCTGTAGCTCAGTGGATAGAGCGTCTGTTTCCTAAGCAGAAAGTCGAAGGTTCGACC
CCTTCCTGGCGCG
>Arabidopsis_thaliana_chr5.trna12-ArgCCT (3324703-3324775) Arg (CCT) 73 bp Sc: 75.77
GCGTCTGTAGCTCAGTGGATAGAGCGTCTGTTTCCTAAGCAGAAGGTCGTAGGTTCGACC
CCTACCTGACGCG
>Arabidopsis_thaliana_chr1.trna74-ArgCCT (18391470-18391542) Arg (CCT) 73 bp Sc: 76.26
GCGCCTGTAGCTCAGTGGATAGAGCGTCTGTTTCCTAAGCAGAAGGTCGTAGGTTCGACC
CCTACCTGGCGCG
>Arabidopsis_thaliana_chr3.trna14-ArgCCT (3535152-3535224) Arg (CCT) 73 bp Sc: 76.26
GCGCCTGTAGCTCAGTGGATAGAGCGTCTGTTTCCTAAGCAGAAGGTCGTAGGTTCGACC
CCTACCTGGCGCG
>Arabidopsis_thaliana_chr3.trna23-ArgCCT (6663714-6663786) Arg (CCT) 73 bp Sc: 76.26
GCGCCTGTAGCTCAGTGGATAGAGCGTCTGTTTCCTAAGCAGAAGGTCGTAGGTTCGACC
CCTACCTGGCGCG
>Arabidopsis_thaliana_chr5.trna6-ArgCCT (1742693-1742765) Arg (CCT) 73 bp Sc: 76.26
GCGCCTGTAGCTCAGTGGATAGAGCGTCTGTTTCCTAAGCAGAAGGTCGTAGGTTCGACC
CCTACCTGGCGCG
>Arabidopsis_thaliana_chr5.trna62-ArgCCT (26212685-26212757) Arg (CCT) 73 bp Sc: 76.26
GCGCCTGTAGCTCAGTGGATAGAGCGTCTGTTTCCTAAGCAGAAGGTCGTAGGTTCGACC
CCTACCTGGCGCG
>Arabidopsis_thaliana_chr1.trna200-ArgTCG (22481511-22481438) Arg (TCG) 74 bp Sc: 81.86
GACCGCATAGCGCAGTGGATTAGCGCGTCTGACTTCGGATCAGAAGGTCGTGGGTTCGAC
TCCCACTGTGGTCG
>Arabidopsis_thaliana_chr1.trna210-ArgTCG (19502161-19502088) Arg (TCG) 74 bp Sc: 81.86
GACCGCATAGCGCAGTGGATTAGCGCGTCTGACTTCGGATCAGAAGGTCGTGGGTTCGAC
TCCCACTGTGGTCG
>Arabidopsis_thaliana_chr1.trna235-ArgTCG (3678848-3678775) Arg (TCG) 74 bp Sc: 81.86
GACCGCATAGCGCAGTGGATTAGCGCGTCTGACTTCGGATCAGAAGGTCGTGGGTTCGAC
TCCCACTGTGGTCG
>Arabidopsis_thaliana_chr1.trna186-ArgTCG (30088962-30088889) Arg (TCG) 74 bp Sc: 82.11
GACCGCATAGCGCAGTGGATTAGCGCGTTTGACTTCGGATCAAAAGGTCGTGGGTTCGAC
TCCCACTGTGGTCG

>Arabidopsis_thaliana_chr3.trna86-ArgTCG (2918492-2918419) Arg (TCG) 74 bp Sc: 82.11
GACCGCATAGCGCAGTGGATTAGCGCGTTTGACTTCGGATCAAAAGGTCGTGGGTTCGAC
TCCCACTGTGGTCG
>Arabidopsis_thaliana_chr5.trna119-ArgTCG (588964-588891) Arg (TCG) 74 bp Sc: 82.11
GACCGCATAGCGCAGTGGATTAGCGCGTTTGACTTCGGATCAAAAGGTCGTGGGTTCGAC
TCCCACTGTGGTCG
>Arabidopsis_thaliana_chr5.trna5-ArgTCT (998941-999013) Arg (TCT) 73 bp Sc: 66.87
GCACCCGTGGCCTAATGGATAAGGCGTTTGACTTCTAATCACACGATTGTGGGTTCGAGT
CCCACCGGGTGTG
>Arabidopsis_thaliana_chr2.trna56-ArgTCT (18576986-18577058) Arg (TCT) 73 bp Sc: 70.42
GCACCCGTGGCCTAATGGATAAGGCGTTTGACTTCTAATCAAGCGATTGTGGGTTCGAGT
CCCACCGGGTGTG
>Arabidopsis_thaliana_chr4.trna35-ArgTCT (16304129-16304201) Arg (TCT) 73 bp Sc: 71.62
GCGCTCGTGGCCCAATGGATAAGGCGTCTGACTTCTAATCAGACGATTGTGGGTTCGATC
CCCACCGAGCGTG
>Arabidopsis_thaliana_chr4.trna30-ArgTCT (15120656-15120728) Arg (TCT) 73 bp Sc: 73.37
GCACCCATGGCCTAATGGATAAGGCGCTTGACTTCTAATCAAGCGATTGTGGGTTCGAGT
CCCACTGGGTGTG
>Arabidopsis_thaliana_chr4.trna32-ArgTCT (15576656-15576728) Arg (TCT) 73 bp Sc: 73.37
GCACCCATGGCCTAATGGATAAGGCGCTTGACTTCTAATCAAGCGATTGTGGGTTCGAGT
CCCACTGGGTGTG
>Arabidopsis_thaliana_chr3.trna13-ArgTCT (3096221-3096293) Arg (TCT) 73 bp Sc: 73.39
GCACCCGTGGCCTAATGGATAAGGCGTTTGACTTCTAATCAAACGATTGTGGGTTCGAGT
CCCACCGGGTGTG
>Arabidopsis_thaliana_chr1.trna64-ArgTCT (13687638-13687710) Arg (TCT) 73 bp Sc: 73.62
GCGCCCGTGGCCTAATGGATAAGGCGTTTGACTTCTAATCAAACGATTGTGGGTTCGAGT
CCCACCGGGCGTG
>Arabidopsis_thaliana_chr2.trna51-ArgTCT (15353239-15353311) Arg (TCT) 73 bp Sc: 73.62
GCGCCCGTGGCCTAATGGATAAGGCGTTTGACTTCTAATCAAACGATTGTGGGTTCGAGT
CCCACCGGGCGTG
>Arabidopsis_thaliana_chr2.trna29-ArgTCT (10820918-10820990) Arg (TCT) 73 bp Sc: 75.03
GCACCCATGGCCTAATGGATAAGGCGCTTGACTTCTAATCAAGCGATTGTGGGTTCGAAT
CCCACTGGGTGTG
>Arabidopsis_thaliana_chr2.trna34-AsnGTT (13658351-13658425) Asn (GTT) 75 bp Sc: 72.51
GCTGGAATAGCTCAGTAGGTTTAGAGCGTGTGGCTGTTAACCTCAAGGTCGGAGGTTCGA
CCCCTCCTTTCAGCG
>Arabidopsis_thaliana_chr1.trna230-AsnGTT (5189755-5189682) Asn (GTT) 74 bp Sc: 73.38
GCTGGAATAGCTCAGATGGCTAGAGCGTGTGGCTGTTAACCACAAGGTCGGAGGTTCAAG
CCCTCCTTCTAGCG
>Arabidopsis_thaliana_chr2.trna18-AsnGTT (3496437-3496508) Asn (GTT) 72 bp Sc: 77.17
TCCTCAGTAGCTCAGTGGTAGAGCGGTCGGCTGTTAACTGATTGGTCGTAGGTTCAAATC
CTACTTGGGGAG
>Arabidopsis_thaliana_chr4.trna69-AsnGTT (7341494-7341421) Asn (GTT) 74 bp Sc: 78.40
GCTGGAGTAGCTCAGTTGGTTAGAGCGTGTGGCTGTTAACCACAAGGTCAGAGGTTCGAC
CCCTTTCTCTAGCG
>Arabidopsis_thaliana_chr1.trna77-AsnGTT (19428224-19428297) Asn (GTT) 74 bp Sc: 80.57
GCTGGAGTAGCTCAGTTGGTTAGAGCGTGTGGCTGTTAACCACAAGGTCAGAGGTTCGAC
CCCTCTCTCTAGCG
>Arabidopsis_thaliana_chr2.trna86-AsnGTT (7962832-7962759) Asn (GTT) 74 bp Sc: 80.57
GCTGGAGTAGCTCAGTTGGTTAGAGCGTGTGGCTGTTAACCACAAGGTCAGAGGTTCGAC
CCCTCTCTCTAGCG
>Arabidopsis_thaliana_chr4.trna20-AsnGTT (11910300-11910373) Asn (GTT) 74 bp Sc: 80.57
GCTGGAGTAGCTCAGTTGGTTAGAGCGTGTGGCTGTTAACCACAAGGTCAGAGGTTCGAC
CCCTCTCTCTAGCG
>Arabidopsis_thaliana_chr5.trna67-AsnGTT (26573322-26573249) Asn (GTT) 74 bp Sc: 80.57
GCTGGAGTAGCTCAGTTGGTTAGAGCGTGTGGCTGTTAACCACAAGGTCAGAGGTTCGAC
CCCTCTCTCTAGCG
>Arabidopsis_thaliana_chr1.trna170-AsnGTT (26053141-26053214) Asn (GTT) 74 bp Sc: 82.29
GCTGGAATAGCTCAGTTGGTTAGAGCGTGTGGCTGTTAACCACAAGGTCGGAGGTTCGAC
CCCTCCTTCTAGCG
>Arabidopsis_thaliana_chr2.trna1-AsnGTT (102064-102137) Asn (GTT) 74 bp Sc: 82.29
GCTGGAATAGCTCAGTTGGTTAGAGCGTGTGGCTGTTAACCACAAGGTCGGAGGTTCGAC
CCCTCCTTCTAGCG
>Arabidopsis_thaliana_chr2.trna48-AsnGTT (15175941-15176014) Asn (GTT) 74 bp Sc: 82.29
GCTGGAATAGCTCAGTTGGTTAGAGCGTGTGGCTGTTAACCACAAGGTCGGAGGTTCGAC
CCCTCCTTCTAGCG
>Arabidopsis_thaliana_chr3.trna42-AsnGTT (18928414-18928487) Asn (GTT) 74 bp Sc: 82.29

GCTGGAATAGCTCAGTTGGTTAGAGCGTGTGGCTGTTAACCACAAGGTCGGAGGTTCGAC
CCCTCCTTCTAGCG
>Arabidopsis_thaliana_chr5.trna68-AsnGTT (26238933-26238860) Asn (GTT) 74 bp Sc: 82.29
GCTGGAATAGCTCAGTTGGTTAGAGCGTGTGGCTGTTAACCACAAGGTCGGAGGTTCGAC
CCCTCCTTCTAGCG
>Arabidopsis_thaliana_chr5.trna93-AsnGTT (15049811-15049738) Asn (GTT) 74 bp Sc: 82.29
GCTGGAATAGCTCAGTTGGTTAGAGCGTGTGGCTGTTAACCACAAGGTCGGAGGTTCGAC
CCCTCCTTCTAGCG
>Arabidopsis_thaliana_chr1.trna242-AsnGTT (1583844-1583771) Asn (GTT) 74 bp Sc: 82.78
GCTGGAATAGCTCAGCTGGTTAGAGCGTGTGGCTGTTAACCACAAGGTCGGAGGTTCGAA
CCCTCCTTCTAGCG
>Arabidopsis_thaliana_chr1.trna32-AsnGTT (9143797-9143870) Asn (GTT) 74 bp Sc: 84.99
GCTGGAATAGCTCAGTTGGTTAGAGCGTGTGGCTGTTAACCACAAGGTCGGAGGTTCGAT
CCCTCCTTCTAGCG
>Arabidopsis_thaliana_chr2.trna94-AspGTC (3255902-3255829) Asp (GTC) 74 bp Sc: 60.73
GGGATTGTAGTTCAATCGGTCAGAGCACCGCCCTGTCAAGGCGGAAGCTGCGGGTTCGAG
CCCCGTCAGTCCCG
>Arabidopsis_thaliana_chr5.trna27-AspGTC (11802285-11802356) Asp (GTC) 72 bp Sc: 61.39
GTCGTTATAGTATAGTGGTAAGTATTCCCGCCTGTCATGCGGGTGACCCGGGTTCAATCC
CCGGCAACGGCG
>Arabidopsis_thaliana_chr1.trna213-AspGTC (16588216-16588145) Asp (GTC) 72 bp Sc: 63.92
GTCGTTGTAGTATAGTGGTAAGGATTCCCGCCTGTCACGCGGGTGACCCGGGTTCGATCC
TCGGCAACGACG
>Arabidopsis_thaliana_chr2.trna35-AspGTC (14250207-14250278) Asp (GTC) 72 bp Sc: 64.11
GTCGTTGTAGTATAGTGGTAAGTATTCCCGCCTGTCACGCGGGTGACCCGGGTTCGATCC
CCGGCAAAGGCG
>Arabidopsis_thaliana_chr1.trna174-AspGTC (27792644-27792715) Asp (GTC) 72 bp Sc: 69.46
GTCGTTGTAGTATAGTGGTAAGTATTCCCGCCTGTCACGCGGGTGACCCGGGTTCGATCC
CCGGCAACGGCG
>Arabidopsis_thaliana_chr1.trna176-AspGTC (28188193-28188264) Asp (GTC) 72 bp Sc: 69.46
GTCGTTGTAGTATAGTGGTAAGTATTCCCGCCTGTCACGCGGGTGACCCGGGTTCGATCC
CCGGCAACGGCG
>Arabidopsis_thaliana_chr1.trna179-AspGTC (28531428-28531499) Asp (GTC) 72 bp Sc: 69.46
GTCGTTGTAGTATAGTGGTAAGTATTCCCGCCTGTCACGCGGGTGACCCGGGTTCGATCC
CCGGCAACGGCG
>Arabidopsis_thaliana_chr1.trna212-AspGTC (18139337-18139266) Asp (GTC) 72 bp Sc: 69.46
GTCGTTGTAGTATAGTGGTAAGTATTCCCGCCTGTCACGCGGGTGACCCGGGTTCGATCC
CCGGCAACGGCG
>Arabidopsis_thaliana_chr1.trna26-AspGTC (6075813-6075884) Asp (GTC) 72 bp Sc: 69.46
GTCGTTGTAGTATAGTGGTAAGTATTCCCGCCTGTCACGCGGGTGACCCGGGTTCGATCC
CCGGCAACGGCG
>Arabidopsis_thaliana_chr1.trna27-AspGTC (6345757-6345828) Asp (GTC) 72 bp Sc: 69.46
GTCGTTGTAGTATAGTGGTAAGTATTCCCGCCTGTCACGCGGGTGACCCGGGTTCGATCC
CCGGCAACGGCG
>Arabidopsis_thaliana_chr1.trna5-AspGTC (877649-877720) Asp (GTC) 72 bp Sc: 69.46
GTCGTTGTAGTATAGTGGTAAGTATTCCCGCCTGTCACGCGGGTGACCCGGGTTCGATCC
CCGGCAACGGCG
>Arabidopsis_thaliana_chr2.trna36-AspGTC (14251775-14251846) Asp (GTC) 72 bp Sc: 69.46
GTCGTTGTAGTATAGTGGTAAGTATTCCCGCCTGTCACGCGGGTGACCCGGGTTCGATCC
CCGGCAACGGCG
>Arabidopsis_thaliana_chr2.trna64-AspGTC (16646929-16646858) Asp (GTC) 72 bp Sc: 69.46
GTCGTTGTAGTATAGTGGTAAGTATTCCCGCCTGTCACGCGGGTGACCCGGGTTCGATCC
CCGGCAACGGCG
>Arabidopsis_thaliana_chr3.trna18-AspGTC (4330779-4330850) Asp (GTC) 72 bp Sc: 69.46
GTCGTTGTAGTATAGTGGTAAGTATTCCCGCCTGTCACGCGGGTGACCCGGGTTCGATCC
CCGGCAACGGCG
>Arabidopsis_thaliana_chr3.trna26-AspGTC (10211565-10211636) Asp (GTC) 72 bp Sc: 69.46
GTCGTTGTAGTATAGTGGTAAGTATTCCCGCCTGTCACGCGGGTGACCCGGGTTCGATCC
CCGGCAACGGCG
>Arabidopsis_thaliana_chr3.trna3-AspGTC (476809-476880) Asp (GTC) 72 bp Sc: 69.46
GTCGTTGTAGTATAGTGGTAAGTATTCCCGCCTGTCACGCGGGTGACCCGGGTTCGATCC
CCGGCAACGGCG
>Arabidopsis_thaliana_chr3.trna44-AspGTC (19566014-19566085) Asp (GTC) 72 bp Sc: 69.46
GTCGTTGTAGTATAGTGGTAAGTATTCCCGCCTGTCACGCGGGTGACCCGGGTTCGATCC
CCGGCAACGGCG
>Arabidopsis_thaliana_chr3.trna66-AspGTC (22149895-22149824) Asp (GTC) 72 bp Sc: 69.46
GTCGTTGTAGTATAGTGGTAAGTATTCCCGCCTGTCACGCGGGTGACCCGGGTTCGATCC

CCGGCAACGGCG
>Arabidopsis_thaliana_chr3.trna70-AspGTC (19044443-19044372) Asp (GTC) 72 bp Sc: 69.46
GTCGTTGTAGTATAGTGGTAAGTATTCCCGCCTGTCACGCGGGTGACCCGGGTTCGATCC
CCGGCAACGGCG
>Arabidopsis_thaliana_chr5.trna16-AspGTC (4673903-4673974) Asp (GTC) 72 bp Sc: 69.46
GTCGTTGTAGTATAGTGGTAAGTATTCCCGCCTGTCACGCGGGTGACCCGGGTTCGATCC
CCGGCAACGGCG
>Arabidopsis_thaliana_chr5.trna19-AspGTC (5161515-5161586) Asp (GTC) 72 bp Sc: 69.46
GTCGTTGTAGTATAGTGGTAAGTATTCCCGCCTGTCACGCGGGTGACCCGGGTTCGATCC
CCGGCAACGGCG
>Arabidopsis_thaliana_chr5.trna49-AspGTC (23861432-23861503) Asp (GTC) 72 bp Sc: 69.46
GTCGTTGTAGTATAGTGGTAAGTATTCCCGCCTGTCACGCGGGTGACCCGGGTTCGATCC
CCGGCAACGGCG
>Arabidopsis_thaliana_chr5.trna7-AspGTC (1788652-1788723) Asp (GTC) 72 bp Sc: 69.46
GTCGTTGTAGTATAGTGGTAAGTATTCCCGCCTGTCACGCGGGTGACCCGGGTTCGATCC
CCGGCAACGGCG
>Arabidopsis_thaliana_chr5.trna91-AspGTC (16256937-16256866) Asp (GTC) 72 bp Sc: 69.46
GTCGTTGTAGTATAGTGGTAAGTATTCCCGCCTGTCACGCGGGTGACCCGGGTTCGATCC
CCGGCAACGGCG
>Arabidopsis_thaliana_chr5.trna97-AspGTC (7222131-7222060) Asp (GTC) 72 bp Sc: 69.46
GTCGTTGTAGTATAGTGGTAAGTATTCCCGCCTGTCACGCGGGTGACCCGGGTTCGATCC
CCGGCAACGGCG
>Arabidopsis_thaliana_chr1.trna29-AspGTC (7004505-7004576) Asp (GTC) 72 bp Sc: 71.35
GTCGTTGTAGTATAGTGGTAAGTATTCCCGCCTGTCACGCGGGTGGCCCGGGTTCGATCC
CCGGCAACGGCG
>Arabidopsis_thaliana_chr2.trna17-CysGCA (3495439-3495509) Cys (GCA) 71 bp Sc: 42.92
GGCTAGGTAACATAATGGAAATGTATCGGACTGCAAATCCTGTAATGACGGTTCGACTCC
GTCCTTGGCCT
>Arabidopsis_thaliana_chr5.trna99-CysGCA (7074429-7074358) Cys (GCA) 72 bp Sc: 59.44
AGGTCCATAGCTCAGTGGTAGAGCAATTGACTGCAGATCAATAGGTCACCGGTTTGAACC
TGGTTGGGCCCT
>Arabidopsis_thaliana_chr5.trna98-CysGCA (7074845-7074774) Cys (GCA) 72 bp Sc: 64.57
AGGTCCATAGCTCAGTGGTAGAGCAATTGACTGCAGATCAATAGGTCACCGGTTCGAACC
CGATTGGGCCCT
>Arabidopsis_thaliana_chr4.trna65-CysGCA (9978189-9978118) Cys (GCA) 72 bp Sc: 71.20
GGGTCCATAGCTCAGTGATAGAGCAATTGACTGCAGATCAATAGGTCACCGGTTCGAACC
CGGTTGGGCCCT
>Arabidopsis_thaliana_chr5.trna100-CysGCA (7074016-7073945) Cys (GCA) 72 bp Sc: 71.20
GGGTCCATAGCTCAGTGATAGAGCAATTGACTGCAGATCAATAGGTCACCGGTTCGAACC
CGGTTGGGCCCT
>Arabidopsis_thaliana_chr5.trna90-CysGCA (16524832-16524761) Cys (GCA) 72 bp Sc: 73.07
GGGCCCATAGCTCAGTGGTAGAGCATTCGACTGCAGATCGAGAGGTCACCGGTTCGAACC
CGGTTGAGCCCT
>Arabidopsis_thaliana_chr5.trna101-CysGCA (7073603-7073532) Cys (GCA) 72 bp Sc: 76.48
GGGTTCATAGCTCAGTGGTAGAGCAATTGACTGCAGATCAATAGGTCACCGGTTCGAACC
CGGTTGGGCCCT
>Arabidopsis_thaliana_chr1.trna198-CysGCA (23562921-23562850) Cys (GCA) 72 bp Sc: 76.97
GGGCTCATAGCTCAGTGGTAGAGCATTCGACTGCAGATCGAGAGGTCACCGGTTCGAACC
CGGTTGGGCCCT
>Arabidopsis_thaliana_chr2.trna53-CysGCA (16525847-16525918) Cys (GCA) 72 bp Sc: 77.51
GAGCCTATAGCTCAGTGGTAGAGCAATTGACTGCAGATCAATAGGTCACCGGTTCGAATC
CGGTTGGGCTCT
>Arabidopsis_thaliana_chr2.trna65-CysGCA (16525713-16525642) Cys (GCA) 72 bp Sc: 77.51
GAGCCTATAGCTCAGTGGTAGAGCAATTGACTGCAGATCAATAGGTCACCGGTTCGAATC
CGGTTGGGCTCT
>Arabidopsis_thaliana_chr3.trna19-CysGCA (4998148-4998219) Cys (GCA) 72 bp Sc: 77.60
GGGTCCTTAGCTCAGTGGTAGAGCATTTGACTGCAGATCAAGAGGTCACCGGTTCGAACC
CGGTAGGGCCCT
>Arabidopsis_thaliana_chr3.trna68-CysGCA (19420417-19420346) Cys (GCA) 72 bp Sc: 78.18
GGGTCCATAGCTCAGTGGTAGAGCATTTGACTGCAGATCAAGAGGTCACCGGTTCGAACC
CGGTTGGGCCCT
>Arabidopsis_thaliana_chr5.trna36-CysGCA (18924750-18924821) Cys (GCA) 72 bp Sc: 79.11
GGGTCCATAGCTCAGTGGTAGAGCAATTGACTGCAGATCAATAGGTCACCGGTTCGAACC
CGGTTGGGCCCT
>Arabidopsis_thaliana_chr1.trna208-CysGCA (19929578-19929507) Cys (GCA) 72 bp Sc: 79.91
GGGTCCTTAGCTCAGTGGTAGAGCAATTGACTGCAGATCAATAGGTCACCGGTTCGAATC
CGGTAGGGCCCT

>Arabidopsis_thaliana_chr2.trna30-CysGCA (12336080-12336151) Cys (GCA) 72 bp Sc: 79.91
GGGTCCTTAGCTCAGTGGTAGAGCAATTGACTGCAGATCAATAGGTCACCGGTTCGAATC
CGGTAGGGCCCT
>Arabidopsis_thaliana_chr2.trna21-GlnCTG (7046176-7046247) Gln (CTG) 72 bp Sc: 69.59
GGTTCTATGGTCTAGCGGTTAGGACATTGGACTCTGAATCCAGTAACCCGAGTTCAATTC
TCGGTAGAACCT
>Arabidopsis_thaliana_chr2.trna82-GlnCTG (9277814-9277743) Gln (CTG) 72 bp Sc: 69.59
GGTTCTATGGTCTAGCGGTTAGGACATTGGACTCTGAATCCAGTAACCCGAGTTCAATTC
TCGGTAGAACCT
>Arabidopsis_thaliana_chr4.trna52-GlnCTG (13882994-13882923) Gln (CTG) 72 bp Sc: 69.59
GGTTCTATGGTCTAGCGGTTAGGACATTGGACTCTGAATCCAGTAACCCGAGTTCAATTC
TCGGTAGAACCT
>Arabidopsis_thaliana_chr4.trna79-GlnCTG (424788-424717) Gln (CTG) 72 bp Sc: 69.59
GGTTCTATGGTCTAGCGGTTAGGACATTGGACTCTGAATCCAGTAACCCGAGTTCAATTC
TCGGTAGAACCT
>Arabidopsis_thaliana_chr3.trna58-GlnCTG (22892782-22892853) Gln (CTG) 72 bp Sc: 69.79
GGTTCCATGGTCTAGTGGTCAGGACATTGGACTCTGAATCCAGTAACCCGAGTTCAAGTC
TCGGTGGAACCT
>Arabidopsis_thaliana_chr1.trna6-GlnCTG (892514-892585) Gln (CTG) 72 bp Sc: 70.28
GGTCCCATGGTCTAGTGGTCAGGACATTGGACTCTGAATCCAGTAACCCGAGTTCAAGTC
TCGGTGGGACCT
>Arabidopsis_thaliana_chr5.trna24-GlnCTG (7387891-7387962) Gln (CTG) 72 bp Sc: 70.28
GGTCCCATGGTCTAGTGGTCAGGACATTGGACTCTGAATCCAGTAACCCGAGTTCAAGTC
TCGGTGGGACCT
>Arabidopsis_thaliana_chr4.trna5-GlnCTG (1501606-1501677) Gln (CTG) 72 bp Sc: 70.67
GGTCCTATGGTCTAGTGGTCAGGACATTGGACTCTGAATCCAGTAACCCGAGTTCAAATC
TCGGTAGGACCT
>Arabidopsis_thaliana_chr1.trna245-GlnCTG (111961-111890) Gln (CTG) 72 bp Sc: 72.52
GGTTCCATGGTCTAGCGGTTAGGACACTAGACTCTGAATCTAGTAACCCGAGTTCAAGTC
TCGGTGGAACCT
>Arabidopsis_thaliana_chr2.trna5-GlnTTG (3276437-3276508) Gln (TTG) 72 bp Sc: 54.44
TGGAGTATAGCCAAGTGGTAAGGCACCGGTTTTTGGTACCGGCATGCAAAGGTTCGAATC
CTTTTACTCCAG
>Arabidopsis_thaliana_chr5.trna25-GlnTTG (7981401-7981472) Gln (TTG) 72 bp Sc: 73.55
GGTTCTATGGTGTAGTGGTTAGCACTCAGGACTTTGAATCCTGCGACCTGGGTTCGACTC
CCGGTAGAACCT
>Arabidopsis_thaliana_chr1.trna188-GlnTTG (29148055-29147984) Gln (TTG) 72 bp Sc: 74.09
GGTTCTATGGTGTAGTGGTTAGCACTCTGGACTTTGAATCCAGCGACCTGGGTTCGACTC
CCGGTAGGACCT
>Arabidopsis_thaliana_chr1.trna190-GlnTTG (28952519-28952448) Gln (TTG) 72 bp Sc: 74.09
GGTTCTATGGTGTAGTGGTTAGCACTCTGGACTTTGAATCCAGCGACCTGGGTTCGACTC
CCGGTAGGACCT
>Arabidopsis_thaliana_chr1.trna221-GlnTTG (7656180-7656109) Gln (TTG) 72 bp Sc: 74.09
GGTTCTATGGTGTAGTGGTTAGCACTCTGGACTTTGAATCCAGCGACCTGGGTTCGACTC
CCGGTAGGACCT
>Arabidopsis_thaliana_chr1.trna222-GlnTTG (7499793-7499722) Gln (TTG) 72 bp Sc: 74.09
GGTTCTATGGTGTAGTGGTTAGCACTCTGGACTTTGAATCCAGCGACCTGGGTTCGACTC
CCGGTAGGACCT
>Arabidopsis_thaliana_chr3.trna38-GlnTTG (18080063-18080134) Gln (TTG) 72 bp Sc: 74.09
GGTTCTATGGTGTAGTGGTTAGCACTCTGGACTTTGAATCCAGCGACCTGGGTTCGACTC
CCGGTAGGACCT
>Arabidopsis_thaliana_chr5.trna40-GlnTTG (20692448-20692519) Gln (TTG) 72 bp Sc: 74.09
GGTTCTATGGTGTAGTGGTTAGCACTCTGGACTTTGAATCCAGCGACCTGGGTTCGACTC
CCGGTAGGACCT
>Arabidopsis_thaliana_chr4.trna19-GluCTC (10302012-10302084) Glu (CTC) 73 bp Sc: 70.56
TCCGTTGTAGTCTAGCTGGTCAGGATACTCGGCTCTCACCCAAGAGACCCGGGTTCGAGT
CCCGGCAACGGAG
>Arabidopsis_thaliana_chr1.trna177-GluCTC (28411645-28411717) Glu (CTC) 73 bp Sc: 76.51
TCCGTTGTAGTCTAGCTGGTCAGGATACTCGGCTCTCACCCGAGAGACCCGGGTTCGAGT
CCCGGCAACGGAG
>Arabidopsis_thaliana_chr1.trna192-GluCTC (28377222-28377150) Glu (CTC) 73 bp Sc: 76.51
TCCGTTGTAGTCTAGCTGGTCAGGATACTCGGCTCTCACCCGAGAGACCCGGGTTCGAGT
CCCGGCAACGGAG
>Arabidopsis_thaliana_chr1.trna218-GluCTC (10207364-10207292) Glu (CTC) 73 bp Sc: 76.51
TCCGTTGTAGTCTAGCTGGTCAGGATACTCGGCTCTCACCCGAGAGACCCGGGTTCGAGT
CCCGGCAACGGAG
>Arabidopsis_thaliana_chr1.trna61-GluCTC (10756704-10756776) Glu (CTC) 73 bp Sc: 76.51

TCCGTTGTAGTCTAGCTGGTCAGGATACTCGGCTCTCACCCGAGAGACCCGGGTTCGAGT
CCCGGCAACGGAG
>Arabidopsis_thaliana_chr2.trna58-GluCTC (18963714-18963786) Glu (CTC) 73 bp Sc: 76.51
TCCGTTGTAGTCTAGCTGGTCAGGATACTCGGCTCTCACCCGAGAGACCCGGGTTCGAGT
CCCGGCAACGGAG
>Arabidopsis_thaliana_chr3.trna55-GluCTC (22786897-22786969) Glu (CTC) 73 bp Sc: 76.51
TCCGTTGTAGTCTAGCTGGTCAGGATACTCGGCTCTCACCCGAGAGACCCGGGTTCGAGT
CCCGGCAACGGAG
>Arabidopsis_thaliana_chr4.trna36-GluCTC (16556628-16556700) Glu (CTC) 73 bp Sc: 76.51
TCCGTTGTAGTCTAGCTGGTCAGGATACTCGGCTCTCACCCGAGAGACCCGGGTTCGAGT
CCCGGCAACGGAG
>Arabidopsis_thaliana_chr4.trna38-GluCTC (17130055-17130127) Glu (CTC) 73 bp Sc: 76.51
TCCGTTGTAGTCTAGCTGGTCAGGATACTCGGCTCTCACCCGAGAGACCCGGGTTCGAGT
CCCGGCAACGGAG
>Arabidopsis_thaliana_chr5.trna108-GluCTC (3030890-3030818) Glu (CTC) 73 bp Sc: 76.51
TCCGTTGTAGTCTAGCTGGTCAGGATACTCGGCTCTCACCCGAGAGACCCGGGTTCGAGT
CCCGGCAACGGAG
>Arabidopsis_thaliana_chr2.trna52-GluCTC (15923895-15923967) Glu (CTC) 73 bp Sc: 78.60
TCCGTTGTAGTCTAGCTGGTTAGGATACTCGGCTCTCACCCGAGAGACCCGGGTTCGAGT
CCCGGCAACGGAG
>Arabidopsis_thaliana_chr3.trna71-GluCTC (19008694-19008622) Glu (CTC) 73 bp Sc: 79.86
TCCGTCGTAGTCTAGCTGGTTAGGATACTCGGCTCTCACCCGAGAGACCCGGGTTCGAGT
CCCGGCGACGGAG
>Arabidopsis_thaliana_chr4.trna12-GluCTC (7814198-7814270) Glu (CTC) 73 bp Sc: 79.86
TCCGTCGTAGTCTAGCTGGTTAGGATACTCGGCTCTCACCCGAGAGACCCGGGTTCGAGT
CCCGGCGACGGAG
>Arabidopsis_thaliana_chr4.trna77-GluTTC (611932-611866) Glu (TTC) 67 bp Sc: 24.19
CTTAAACACTCAGCGGTTAGGATATCTGGCTTTCACCCAGGAGACCCGGGTTCCATTCCC
GGTAACG
>Arabidopsis_thaliana_chr2.trna93-GluTTC (3303312-3303241) Glu (TTC) 72 bp Sc: 65.98
GTCCCTTTCGTCCAGTGGTTAGGACATCGTCTTTTCATGTCGAAGACACGGGTTCGATTC
CCGTAAGGGATA
>Arabidopsis_thaliana_chr1.trna191-GluTTC (28523717-28523646) Glu (TTC) 72 bp Sc: 71.73
TCCGTTATCGTCCAGCGGTTAGGATATCTGGCTTTCACCCAGGAGACCCGGGTTCGATTC
CCGGTAACGGAG
>Arabidopsis_thaliana_chr3.trna72-GluTTC (18755860-18755789) Glu (TTC) 72 bp Sc: 71.73
TCCGTTATCGTCCAGCGGTTAGGATATCTGGCTTTCACCCAGGAGACCCGGGTTCGATTC
CCGGTAACGGAG
>Arabidopsis_thaliana_chr1.trna17-GluTTC (2939419-2939490) Glu (TTC) 72 bp Sc: 72.05
TCCGATGTCGTCCAGCGGTTAGGATATCTGGCTTTCACCCAGGAGACCCGGGTTCGATTC
CCGGCATCGGAG
>Arabidopsis_thaliana_chr1.trna224-GluTTC (6995344-6995273) Glu (TTC) 72 bp Sc: 72.05
TCCGATGTCGTCCAGCGGTTAGGATATCTGGCTTTCACCCAGGAGACCCGGGTTCGATTC
CCGGCATCGGAG
>Arabidopsis_thaliana_chr4.trna46-GluTTC (17500872-17500801) Glu (TTC) 72 bp Sc: 72.05
TCCGATGTCGTCCAGCGGTTAGGATATCTGGCTTTCACCCAGGAGACCCGGGTTCGATTC
CCGGCATCGGAG
>Arabidopsis_thaliana_chr5.trna66-GluTTC (26670567-26670496) Glu (TTC) 72 bp Sc: 72.05
TCCGATGTCGTCCAGCGGTTAGGATATCTGGCTTTCACCCAGGAGACCCGGGTTCGATTC
CCGGCATCGGAG
>Arabidopsis_thaliana_chr5.trna84-GluTTC (18560278-18560207) Glu (TTC) 72 bp Sc: 72.05
TCCGATGTCGTCCAGCGGTTAGGATATCTGGCTTTCACCCAGGAGACCCGGGTTCGATTC
CCGGCATCGGAG
>Arabidopsis_thaliana_chr5.trna95-GluTTC (9815477-9815406) Glu (TTC) 72 bp Sc: 72.05
TCCGATGTCGTCCAGCGGTTAGGATATCTGGCTTTCACCCAGGAGACCCGGGTTCGATTC
CCGGCATCGGAG
>Arabidopsis_thaliana_chr3.trna93-GluTTC (1601415-1601344) Glu (TTC) 72 bp Sc: 72.13
TCCGTTGTCGTCCAGCGGTTAGGATATCTGGCTTTCACCCAGGAGACCCGGGTTCGATTC
CCGGCAACGGAG
>Arabidopsis_thaliana_chr3.trna40-GluTTC (18837021-18837092) Glu (TTC) 72 bp Sc: 72.20
TCCATTGTCGTCCAGCGGTTAGGATATCTGGCTTTCACCCAGGAGACCCGGGTTCGATTC
CCGGCAATGGAG
>Arabidopsis_thaliana_chr1.trna215-GlyACC (12097327-12097259) Gly (ACC) 69 bp Sc: 20.99
GCACCAATGGTTCAGTGGTAGATTAATACTCTACCACTATCTAACCCGGGTTCGATCCCA
GACGGTGTA
>Arabidopsis_thaliana_chr2.trna60-GlyCCC (19566995-19566925) Gly (CCC) 71 bp Sc: 68.50
GCGCATCTGGTGTAGTGGTATCATAGTACCCTCCCACGGTACTGACCAGGGTTCGATTCC

CTGGATGCGCA
>Arabidopsis_thaliana_chr3.trna60-GlyCCC (23221683-23221753) Gly (CCC) 71 bp Sc: 68.50
GCGCATCTGGTGTAGTGGTATCATAGTACCCTCCCACGGTACTGACCAGGGTTCGATTCC
CTGGATGCGCA
>Arabidopsis_thaliana_chr4.trna59-GlyCCC (12486282-12486212) Gly (CCC) 71 bp Sc: 68.50
GCGCATCTGGTGTAGTGGTATCATAGTACCCTCCCACGGTACTGACCAGGGTTCGATTCC
CTGGATGCGCA
>Arabidopsis_thaliana_chr4.trna6-GlyCCC (6646426-6646496) Gly (CCC) 71 bp Sc: 68.50
GCGCATCTGGTGTAGTGGTATCATAGTACCCTCCCACGGTACTGACCAGGGTTCGATTCC
CTGGATGCGCA
>Arabidopsis_thaliana_chr5.trna78-GlyCCC (21740410-21740340) Gly (CCC) 71 bp Sc: 68.50
GCGCATCTGGTGTAGTGGTATCATAGTACCCTCCCACGGTACTGACCAGGGTTCGATTCC
CTGGATGCGCA
>Arabidopsis_thaliana_chr1.trna7-GlyGCC (1159022-1159092) Gly (GCC) 71 bp Sc: 57.43
TAACCAGTGGTCTAGTGGTAGAATAGTACCCTGCCACGGTACAGACCCGGGTTCGATTCC
CGGCTGGTGCA
>Arabidopsis_thaliana_chr2.trna88-GlyGCC (3444431-3444360) Gly (GCC) 72 bp Sc: 62.60
GCGGAAATAGCTTAATGGTAGAGCATAGCCTTGCCAAGGCTAAGGTTGAGGGTTCAAGTC
CCTCCTTCCGCT
>Arabidopsis_thaliana_chr3.trna91-GlyGCC (1843400-1843330) Gly (GCC) 71 bp Sc: 65.04
GCACCAGTGGTCTAGTGGTAGAATAGAACCCTGCCACGGTACAGACCCGGGTTCGATTCC
CGGCTGGTGCA
>Arabidopsis_thaliana_chr5.trna122-GlyGCC (389960-389890) Gly (GCC) 71 bp Sc: 66.11
GCACCAGTGGTCTAGTGGCATGATAGTACCCTGCCACGGTACATACCCGGGTTCAATTCC
CGGCTGGTGCA
>Arabidopsis_thaliana_chr1.trna201-GlyGCC (22401327-22401257) Gly (GCC) 71 bp Sc: 67.59
GCACCAGTGGTCTAGTGGTAGAATAGTACTCTGCCACGGTACAGACCCGGGTTCGATTCC
CGGCTGGTGCA
>Arabidopsis_thaliana_chr1.trna161-GlyGCC (22431832-22431902) Gly (GCC) 71 bp Sc: 69.82
GCACCAGTGGTCTAGTGGTAGAATAGTACCCTGCCACGGTACAGACCCGGGTTCAATTCC
CGGCTGGTGCA
>Arabidopsis_thaliana_chr2.trna54-GlyGCC (17667856-17667926) Gly (GCC) 71 bp Sc: 69.82
GCACCAGTGGTCTAGTGGTAGAATAGTACCCTGCCACGGTACAGACCCGGGTTCAATTCC
CGGCTGGTGCA
>Arabidopsis_thaliana_chr1.trna171-GlyGCC (26977051-26977121) Gly (GCC) 71 bp Sc: 70.74
GCACCAGTGGTCTAGTGGCATGATAGTACCCTGCCACGGTACAGACCCGGGTTCAATTCC
CGGCTGGTGCA
>Arabidopsis_thaliana_chr5.trna87-GlyGCC (17512839-17512769) Gly (GCC) 71 bp Sc: 70.74
GCACCAGTGGTCTAGTGGCATGATAGTACCCTGCCACGGTACAGACCCGGGTTCAATTCC
CGGCTGGTGCA
>Arabidopsis_thaliana_chr1.trna202-GlyGCC (22398372-22398302) Gly (GCC) 71 bp Sc: 71.56
GCACCAGTGGTCTAGTGGTAGAATAGTACCCTGCCACGGTACAGACCCGGGTTCGATTCC
CGGCTGGTGCA
>Arabidopsis_thaliana_chr1.trna203-GlyGCC (22317149-22317079) Gly (GCC) 71 bp Sc: 71.56
GCACCAGTGGTCTAGTGGTAGAATAGTACCCTGCCACGGTACAGACCCGGGTTCGATTCC
CGGCTGGTGCA
>Arabidopsis_thaliana_chr1.trna240-GlyGCC (2111217-2111147) Gly (GCC) 71 bp Sc: 71.56
GCACCAGTGGTCTAGTGGTAGAATAGTACCCTGCCACGGTACAGACCCGGGTTCGATTCC
CGGCTGGTGCA
>Arabidopsis_thaliana_chr1.trna241-GlyGCC (2107467-2107397) Gly (GCC) 71 bp Sc: 71.56
GCACCAGTGGTCTAGTGGTAGAATAGTACCCTGCCACGGTACAGACCCGGGTTCGATTCC
CGGCTGGTGCA
>Arabidopsis_thaliana_chr2.trna32-GlyGCC (12888668-12888738) Gly (GCC) 71 bp Sc: 71.56
GCACCAGTGGTCTAGTGGTAGAATAGTACCCTGCCACGGTACAGACCCGGGTTCGATTCC
CGGCTGGTGCA
>Arabidopsis_thaliana_chr2.trna33-GlyGCC (12889811-12889881) Gly (GCC) 71 bp Sc: 71.56
GCACCAGTGGTCTAGTGGTAGAATAGTACCCTGCCACGGTACAGACCCGGGTTCGATTCC
CGGCTGGTGCA
>Arabidopsis_thaliana_chr2.trna72-GlyGCC (12983095-12983025) Gly (GCC) 71 bp Sc: 71.56
GCACCAGTGGTCTAGTGGTAGAATAGTACCCTGCCACGGTACAGACCCGGGTTCGATTCC
CGGCTGGTGCA
>Arabidopsis_thaliana_chr3.trna47-GlyGCC (19995919-19995989) Gly (GCC) 71 bp Sc: 71.56
GCACCAGTGGTCTAGTGGTAGAATAGTACCCTGCCACGGTACAGACCCGGGTTCGATTCC
CGGCTGGTGCA
>Arabidopsis_thaliana_chr3.trna51-GlyGCC (21553259-21553329) Gly (GCC) 71 bp Sc: 71.56
GCACCAGTGGTCTAGTGGTAGAATAGTACCCTGCCACGGTACAGACCCGGGTTCGATTCC
CGGCTGGTGCA

>Arabidopsis_thaliana_chr3.trna56-GlyGCC (22853497-22853567) Gly (GCC) 71 bp Sc: 71.56
GCACCAGTGGTCTAGTGGTAGAATAGTACCCTGCCACGGTACAGACCCGGGTTCGATTCC
CGGCTGGTGCA
>Arabidopsis_thaliana_chr4.trna33-GlyGCC (15671399-15671469) Gly (GCC) 71 bp Sc: 71.56
GCACCAGTGGTCTAGTGGTAGAATAGTACCCTGCCACGGTACAGACCCGGGTTCGATTCC
CGGCTGGTGCA
>Arabidopsis_thaliana_chr5.trna113-GlyGCC (2212749-2212679) Gly (GCC) 71 bp Sc: 71.56
GCACCAGTGGTCTAGTGGTAGAATAGTACCCTGCCACGGTACAGACCCGGGTTCGATTCC
CGGCTGGTGCA
>Arabidopsis_thaliana_chr5.trna23-GlyGCC (6401241-6401311) Gly (GCC) 71 bp Sc: 71.56
GCACCAGTGGTCTAGTGGTAGAATAGTACCCTGCCACGGTACAGACCCGGGTTCGATTCC
CGGCTGGTGCA
>Arabidopsis_thaliana_chr5.trna65-GlyGCC (26700004-26699934) Gly (GCC) 71 bp Sc: 71.56
GCACCAGTGGTCTAGTGGTAGAATAGTACCCTGCCACGGTACAGACCCGGGTTCGATTCC
CGGCTGGTGCA
>Arabidopsis_thaliana_chr1.trna14-GlyTCC (2588522-2588593) Gly (TCC) 72 bp Sc: 75.78
GCGTCTGTAGTCCAACGGTTAGGATAATTGCCTTCCAAGCAATAGACCCGGGTTCGACTC
CCGGCAGACGCA
>Arabidopsis_thaliana_chr1.trna232-GlyTCC (4285956-4285885) Gly (TCC) 72 bp Sc: 75.78
GCGTCTGTAGTCCAACGGTTAGGATAATTGCCTTCCAAGCAATAGACCCGGGTTCGACTC
CCGGCAGACGCA
>Arabidopsis_thaliana_chr2.trna74-GlyTCC (12478921-12478850) Gly (TCC) 72 bp Sc: 75.78
GCGTCTGTAGTCCAACGGTTAGGATAATTGCCTTCCAAGCAATAGACCCGGGTTCGACTC
CCGGCAGACGCA
>Arabidopsis_thaliana_chr2.trna75-GlyTCC (11871302-11871231) Gly (TCC) 72 bp Sc: 75.78
GCGTCTGTAGTCCAACGGTTAGGATAATTGCCTTCCAAGCAATAGACCCGGGTTCGACTC
CCGGCAGACGCA
>Arabidopsis_thaliana_chr3.trna35-GlyTCC (17160737-17160808) Gly (TCC) 72 bp Sc: 75.78
GCGTCTGTAGTCCAACGGTTAGGATAATTGCCTTCCAAGCAATAGACCCGGGTTCGACTC
CCGGCAGACGCA
>Arabidopsis_thaliana_chr3.trna74-GlyTCC (16438097-16438026) Gly (TCC) 72 bp Sc: 75.78
GCGTCTGTAGTCCAACGGTTAGGATAATTGCCTTCCAAGCAATAGACCCGGGTTCGACTC
CCGGCAGACGCA
>Arabidopsis_thaliana_chr4.trna63-GlyTCC (10326047-10325976) Gly (TCC) 72 bp Sc: 75.78
GCGTCTGTAGTCCAACGGTTAGGATAATTGCCTTCCAAGCAATAGACCCGGGTTCGACTC
CCGGCAGACGCA
>Arabidopsis_thaliana_chr5.trna105-GlyTCC (3578367-3578296) Gly (TCC) 72 bp Sc: 75.78
GCGTCTGTAGTCCAACGGTTAGGATAATTGCCTTCCAAGCAATAGACCCGGGTTCGACTC
CCGGCAGACGCA
>Arabidopsis_thaliana_chr5.trna13-GlyTCC (3617059-3617130) Gly (TCC) 72 bp Sc: 75.78
GCGTCTGTAGTCCAACGGTTAGGATAATTGCCTTCCAAGCAATAGACCCGGGTTCGACTC
CCGGCAGACGCA
>Arabidopsis_thaliana_chr5.trna34-GlyTCC (18576072-18576143) Gly (TCC) 72 bp Sc: 75.78
GCGTCTGTAGTCCAACGGTTAGGATAATTGCCTTCCAAGCAATAGACCCGGGTTCGACTC
CCGGCAGACGCA
>Arabidopsis_thaliana_chr5.trna50-GlyTCC (23971168-23971239) Gly (TCC) 72 bp Sc: 75.78
GCGTCTGTAGTCCAACGGTTAGGATAATTGCCTTCCAAGCAATAGACCCGGGTTCGACTC
CCGGCAGACGCA
>Arabidopsis_thaliana_chr5.trna51-GlyTCC (23974656-23974727) Gly (TCC) 72 bp Sc: 75.78
GCGTCTGTAGTCCAACGGTTAGGATAATTGCCTTCCAAGCAATAGACCCGGGTTCGACTC
CCGGCAGACGCA
>Arabidopsis_thaliana_chr4.trna2-HisGTG (901176-901247) His (GTG) 72 bp Sc: 57.37
GTGGTTGTAGTATAGCGGTTAGTATCCCACGTTGTGGCCGTGGGGACCCGGGCTCGAATC
CCGGCAGCCACA
>Arabidopsis_thaliana_chr1.trna239-HisGTG (2406902-2406831) His (GTG) 72 bp Sc: 62.38
GTGGCTGTAGTTTAGTGGTAAGAATTCCACGTTGTGGCCGTGGAGACCTGGGCTCGAATC
CCAGCAGCCACA
>Arabidopsis_thaliana_chr1.trna3-HisGTG (552640-552711) His (GTG) 72 bp Sc: 62.38
GTGGCTGTAGTTTAGTGGTAAGAATTCCACGTTGTGGCCGTGGAGACCTGGGCTCGAATC
CCAGCAGCCACA
>Arabidopsis_thaliana_chr3.trna89-HisGTG (2105033-2104962) His (GTG) 72 bp Sc: 62.38
GTGGCTGTAGTTTAGTGGTAAGAATTCCACGTTGTGGCCGTGGAGACCTGGGCTCGAATC
CCAGCAGCCACA
>Arabidopsis_thaliana_chr4.trna40-HisGTG (18293774-18293845) His (GTG) 72 bp Sc: 62.38
GTGGCTGTAGTTTAGTGGTAAGAATTCCACGTTGTGGCCGTGGAGACCTGGGCTCGAATC
CCAGCAGCCACA
>Arabidopsis_thaliana_chr5.trna120-HisGTG (559399-559328) His (GTG) 72 bp Sc: 62.38

GTGGCTGTAGTTTAGTGGTAAGAATTCCACGTTGTGGCCGTGGAGACCTGGGCTCGAATC
CCAGCAGCCACA
>Arabidopsis_thaliana_chr5.trna39-HisGTG (20064049-20064120) His (GTG) 72 bp Sc: 62.38
GTGGCTGTAGTTTAGTGGTAAGAATTCCACGTTGTGGCCGTGGAGACCTGGGCTCGAATC
CCAGCAGCCACA
>Arabidopsis_thaliana_chr2.trna31-HisGTG (12346828-12346899) His (GTG) 72 bp Sc: 62.41
GTGGCTGTAGTTTAGTGGTTAGAATCCCACGTTGTGGCCGTGGGGACCTGGGCTCGAATC
CCAGCAGCCACA
>Arabidopsis_thaliana_chr4.trna13-HisGTG (8255696-8255767) His (GTG) 72 bp Sc: 62.41
GTGGCTGTAGTTTAGTGGTTAGAATCCCACGTTGTGGCCGTGGGGACCTGGGCTCGAATC
CCAGCAGCCACA
>Arabidopsis_thaliana_chr5.trna71-HisGTG (25892691-25892620) His (GTG) 72 bp Sc: 67.27
GTGGCTGTAGTTTAGTGGTTAGAATTCCACGTTGTGGCCGTGGAGACCTGGGCTCGAATC
CCAGCAGCCACA
>Arabidopsis_thaliana_chr2.trna7-IleAAT (3290963-3291034) Ile (AAT) 72 bp Sc: 42.87
GCATCCATGGCTGAATGGTTAAAGCGCCCAACCTAATAAAGAGGCAAGTAGGTTCGATTC
CTGCTGGATGCA
>Arabidopsis_thaliana_chr2.trna95-IleAAT (3239725-3239654) Ile (AAT) 72 bp Sc: 42.87
GCATCCATGGCTGAATGGTTAAAGCGCCCAACCTAATAAAGAGGCAAGTAGGTTCGATTC
CTGCTGGATGCA
>Arabidopsis_thaliana_chr1.trna169-IleAAT (25993393-25993466) Ile (AAT) 74 bp Sc: 83.92
GGCCTATTAGCTCAGTTGGTTAGAGCGTCGTGCTAATAACGCGAAGGTCACAGGTTCGAT
CCCTGTATAGGCCA
>Arabidopsis_thaliana_chr1.trna62-IleAAT (11104094-11104167) Ile (AAT) 74 bp Sc: 83.92
GGCCTATTAGCTCAGTTGGTTAGAGCGTCGTGCTAATAACGCGAAGGTCACAGGTTCGAT
CCCTGTATAGGCCA
>Arabidopsis_thaliana_chr2.trna26-IleAAT (9509360-9509433) Ile (AAT) 74 bp Sc: 83.92
GGCCTATTAGCTCAGTTGGTTAGAGCGTCGTGCTAATAACGCGAAGGTCACAGGTTCGAT
CCCTGTATAGGCCA
>Arabidopsis_thaliana_chr2.trna81-IleAAT (9456962-9456889) Ile (AAT) 74 bp Sc: 83.92
GGCCTATTAGCTCAGTTGGTTAGAGCGTCGTGCTAATAACGCGAAGGTCACAGGTTCGAT
CCCTGTATAGGCCA
>Arabidopsis_thaliana_chr3.trna76-IleAAT (9373684-9373611) Ile (AAT) 74 bp Sc: 83.92
GGCCTATTAGCTCAGTTGGTTAGAGCGTCGTGCTAATAACGCGAAGGTCACAGGTTCGAT
CCCTGTATAGGCCA
>Arabidopsis_thaliana_chr4.trna53-IleAAT (13341575-13341502) Ile (AAT) 74 bp Sc: 83.92
GGCCTATTAGCTCAGTTGGTTAGAGCGTCGTGCTAATAACGCGAAGGTCACAGGTTCGAT
CCCTGTATAGGCCA
>Arabidopsis_thaliana_chr4.trna54-IleAAT (13336419-13336346) Ile (AAT) 74 bp Sc: 83.92
GGCCTATTAGCTCAGTTGGTTAGAGCGTCGTGCTAATAACGCGAAGGTCACAGGTTCGAT
CCCTGTATAGGCCA
>Arabidopsis_thaliana_chr5.trna18-IleAAT (5157642-5157715) Ile (AAT) 74 bp Sc: 83.92
GGCCTATTAGCTCAGTTGGTTAGAGCGTCGTGCTAATAACGCGAAGGTCACAGGTTCGAT
CCCTGTATAGGCCA
>Arabidopsis_thaliana_chr1.trna10-IleAAT (1978083-1978156) Ile (AAT) 74 bp Sc: 86.52
GGCCCATTAGCTCAGTTGGTTAGAGCGTCGTGCTAATAACGCGAAGGTCGCAGGTTCGAA
ACCTGCATGGGCCA
>Arabidopsis_thaliana_chr1.trna166-IleAAT (25890199-25890272) Ile (AAT) 74 bp Sc: 86.52
GGCCCATTAGCTCAGTTGGTTAGAGCGTCGTGCTAATAACGCGAAGGTCGCAGGTTCGAA
ACCTGCATGGGCCA
>Arabidopsis_thaliana_chr1.trna231-IleAAT (4522779-4522706) Ile (AAT) 74 bp Sc: 86.52
GGCCCATTAGCTCAGTTGGTTAGAGCGTCGTGCTAATAACGCGAAGGTCGCAGGTTCGAA
ACCTGCATGGGCCA
>Arabidopsis_thaliana_chr2.trna22-IleAAT (7749794-7749867) Ile (AAT) 74 bp Sc: 86.52
GGCCCATTAGCTCAGTTGGTTAGAGCGTCGTGCTAATAACGCGAAGGTCGCAGGTTCGAA
ACCTGCATGGGCCA
>Arabidopsis_thaliana_chr3.trna2-IleAAT (469667-469740) Ile (AAT) 74 bp Sc: 86.52
GGCCCATTAGCTCAGTTGGTTAGAGCGTCGTGCTAATAACGCGAAGGTCGCAGGTTCGAA
ACCTGCATGGGCCA
>Arabidopsis_thaliana_chr4.trna43-IleAAT (18542165-18542238) Ile (AAT) 74 bp Sc: 86.52
GGCCCATTAGCTCAGTTGGTTAGAGCGTCGTGCTAATAACGCGAAGGTCGCAGGTTCGAA
ACCTGCATGGGCCA
>Arabidopsis_thaliana_chr5.trna29-IleAAT (15593087-15593160) Ile (AAT) 74 bp Sc: 86.52
GGCCCATTAGCTCAGTTGGTTAGAGCGTCGTGCTAATAACGCGAAGGTCGCAGGTTCGAA
ACCTGCATGGGCCA
>Arabidopsis_thaliana_chr5.trna45-IleAAT (22845357-22845430) Ile (AAT) 74 bp Sc: 86.52
GGCCCATTAGCTCAGTTGGTTAGAGCGTCGTGCTAATAACGCGAAGGTCGCAGGTTCGAA

ACCTGCATGGGCCA
>Arabidopsis_thaliana_chr5.trna63-IleAAT (26585623-26585696) Ile (AAT) 74 bp Sc: 86.52
GGCCCATTAGCTCAGTTGGTTAGAGCGTCGTGCTAATAACGCGAAGGTCGCAGGTTCGAA
ACCTGCATGGGCCA
>Arabidopsis_thaliana_chr3.trna8-IleTAT (1738797-1738870) Ile (TAT) 74 bp Sc: 81.06
GCTCCCGTAGCTCAGTTGGTTAGAGCGTTGGTCTTATGAGCCGAAGGTCGCGGGTTCGAG
CCCCGCCGGAAGCA
>Arabidopsis_thaliana_chr1.trna12-IleTAT (2177884-2177957) Ile (TAT) 74 bp Sc: 85.40
GGTCCCGTAGCTCAGTTGGTTAGAGCGTTGGTCTTATGAGCCGAAGGTCGCGGGTTCGAG
CCCCGCCGGGACCA
>Arabidopsis_thaliana_chr1.trna78-IleTAT (19851641-19851714) Ile (TAT) 74 bp Sc: 85.40
GGTCCCGTAGCTCAGTTGGTTAGAGCGTTGGTCTTATGAGCCGAAGGTCGCGGGTTCGAG
CCCCGCCGGGACCA
>Arabidopsis_thaliana_chr3.trna81-IleTAT (5068374-5068301) Ile (TAT) 74 bp Sc: 85.40
GGTCCCGTAGCTCAGTTGGTTAGAGCGTTGGTCTTATGAGCCGAAGGTCGCGGGTTCGAG
CCCCGCCGGGACCA
>Arabidopsis_thaliana_chr4.trna47-IleTAT (17276779-17276706) Ile (TAT) 74 bp Sc: 85.40
GGTCCCGTAGCTCAGTTGGTTAGAGCGTTGGTCTTATGAGCCGAAGGTCGCGGGTTCGAG
CCCCGCCGGGACCA
>Arabidopsis_thaliana_chr1.trna66-LeuAAG (17159800-17159871) Leu (AAG) 72 bp Sc: 55.79
GGGCATTTGGTCTAGTGGTTTGATTTTCGCTTAAGGTGCGAGAGGTCCCGAGTTCAATTC
TCAGAATGCCCC
>Arabidopsis_thaliana_chr5.trna52-LeuAAG (24279806-24279886) Leu (AAG) 81 bp Sc: 66.01
GTTGATATGGCCGAGTTGGTCCAAGGCGCCAGATTAAGGTTCTGGTCCGAAAGGGCGTGG
GTTCAAATCCCACTGTCAACA
>Arabidopsis_thaliana_chr1.trna193-LeuAAG (28024124-28024044) Leu (AAG) 81 bp Sc: 68.09
GTTGATATGGCCGAGTTGGTCTAAGGCGCCAGATTAAGGTTCTGGTCCGAAAGGGCGTGG
GTTCAAATCCCACTGTCAACA
>Arabidopsis_thaliana_chr2.trna77-LeuAAG (11122837-11122757) Leu (AAG) 81 bp Sc: 68.09
GTTGATATGGCCGAGTTGGTCTAAGGCGCCAGATTAAGGTTCTGGTCCGAAAGGGCGTGG
GTTCAAATCCCACTGTCAACA
>Arabidopsis_thaliana_chr2.trna83-LeuAAG (9273969-9273889) Leu (AAG) 81 bp Sc: 68.09
GTTGATATGGCCGAGTTGGTCTAAGGCGCCAGATTAAGGTTCTGGTCCGAAAGGGCGTGG
GTTCAAATCCCACTGTCAACA
>Arabidopsis_thaliana_chr3.trna17-LeuAAG (4254535-4254615) Leu (AAG) 81 bp Sc: 68.09
GTTGATATGGCCGAGTTGGTCTAAGGCGCCAGATTAAGGTTCTGGTCCGAAAGGGCGTGG
GTTCAAATCCCACTGTCAACA
>Arabidopsis_thaliana_chr3.trna54-LeuAAG (22622385-22622465) Leu (AAG) 81 bp Sc: 68.09
GTTGATATGGCCGAGTTGGTCTAAGGCGCCAGATTAAGGTTCTGGTCCGAAAGGGCGTGG
GTTCAAATCCCACTGTCAACA
>Arabidopsis_thaliana_chr4.trna11-LeuAAG (7730957-7731037) Leu (AAG) 81 bp Sc: 68.09
GTTGATATGGCCGAGTTGGTCTAAGGCGCCAGATTAAGGTTCTGGTCCGAAAGGGCGTGG
GTTCAAATCCCACTGTCAACA
>Arabidopsis_thaliana_chr5.trna11-LeuAAG (3118378-3118458) Leu (AAG) 81 bp Sc: 68.09
GTTGATATGGCCGAGTTGGTCTAAGGCGCCAGATTAAGGTTCTGGTCCGAAAGGGCGTGG
GTTCAAATCCCACTGTCAACA
>Arabidopsis_thaliana_chr5.trna116-LeuAAG (970878-970798) Leu (AAG) 81 bp Sc: 68.09
GTTGATATGGCCGAGTTGGTCTAAGGCGCCAGATTAAGGTTCTGGTCCGAAAGGGCGTGG
GTTCAAATCCCACTGTCAACA
>Arabidopsis_thaliana_chr5.trna31-LeuAAG (16427813-16427893) Leu (AAG) 81 bp Sc: 68.09
GTTGATATGGCCGAGTTGGTCTAAGGCGCCAGATTAAGGTTCTGGTCCGAAAGGGCGTGG
GTTCAAATCCCACTGTCAACA
>Arabidopsis_thaliana_chr5.trna60-LeuAAG (25942292-25942372) Leu (AAG) 81 bp Sc: 68.09
GTTGATATGGCCGAGTTGGTCTAAGGCGCCAGATTAAGGTTCTGGTCCGAAAGGGCGTGG
GTTCAAATCCCACTGTCAACA
>Arabidopsis_thaliana_chr5.trna109-LeuCAA (2898951-2898868) Leu (CAA) 84 bp Sc: 69.94
GTCAGGTTGGCCGAGTGGTCTAAGGCGCCAGACTCAAGTTCTGGTCTTCGAGAGAGGGCG
TGGGTTCAAATCCCACATCTGACA
>Arabidopsis_thaliana_chr5.trna9-LeuCAA (2411149-2411232) Leu (CAA) 84 bp Sc: 69.94
GTCAGGTTGGCCGAGTGGTCTAAGGCGCCAGACTCAAGTTCTGGTCTTCGAGAGAGGGCG
TGGGTTCAAATCCCACATCTGACA
>Arabidopsis_thaliana_chr1.trna163-LeuCAA (23806216-23806299) Leu (CAA) 84 bp Sc: 70.82
GTCAGGATGGCCGAGTGGTCTAAGGCGCCAGACTCAAGTTCTGGTCTTCGAAAGAGGGCG
TGGGTTCAAATCCCACTTCTGACA
>Arabidopsis_thaliana_chr4.trna22-LeuCAA (12320120-12320203) Leu (CAA) 84 bp Sc: 72.13
GTCAGGGTGGCCGAGTGGTCTAAGGCGCCAGACTCAAGTTCTGGTCCTCGTAAGAGGGCG
TGGGTTCAAATCCCACTTCTGACA

>Arabidopsis_thaliana_chr5.trna26-LeuCAA (8906419-8906502) Leu (CAA) 84 bp Sc: 72.13
GTCAGGGTGGCCGAGTGGTCTAAGGCGCCAGACTCAAGTTCTGGTCCTCGTAAGAGGGCG
TGGGTTCAAATCCCACTTCTGACA
>Arabidopsis_thaliana_chr5.trna79-LeuCAA (21318189-21318106) Leu (CAA) 84 bp Sc: 72.13
GTCAGGGTGGCCGAGTGGTCTAAGGCGCCAGACTCAAGTTCTGGTCCTCGTAAGAGGGCG
TGGGTTCAAATCCCACTTCTGACA
>Arabidopsis_thaliana_chr4.trna56-LeuCAA (13076666-13076583) Leu (CAA) 84 bp Sc: 72.24
GTCAGGATGGCCGAGTGGTCTAAGGCGCCAGACTCAAGTTCTGGTCTTCGTAAGAGGGCG
TGGGTTCAAACCCCACTTCTGACA
>Arabidopsis_thaliana_chr1.trna225-LeuCAA (6948934-6948851) Leu (CAA) 84 bp Sc: 73.97
GTCAGGATGGCCGAGTGGTCTAAGGCGCCAGACTCAAGTTCTGGTCCTCGTAAGAGGGCG
TGGGTTCAAACCCCACTTCTGACA
>Arabidopsis_thaliana_chr4.trna72-LeuCAA (6837853-6837770) Leu (CAA) 84 bp Sc: 73.97
GTCAGGATGGCCGAGTGGTCTAAGGCGCCAGACTCAAGTTCTGGTCCTCGTAAGAGGGCG
TGGGTTCAAACCCCACTTCTGACA
>Arabidopsis_thaliana_chr5.trna32-LeuCAA (16655394-16655477) Leu (CAA) 84 bp Sc: 73.97
GTCAGGATGGCCGAGTGGTCTAAGGCGCCAGACTCAAGTTCTGGTCCTCGTAAGAGGGCG
TGGGTTCAAACCCCACTTCTGACA
>Arabidopsis_thaliana_chr1.trna175-LeuCAG (28031621-28031701) Leu (CAG) 81 bp Sc: 68.96
GTCAAGATGGCCGAGTTGGTCTAAGGCGCCAGTTTCAGGTACTGGTCCGAAAGGGCGTGG
GTTCAAATCCCACTCTTGACA
>Arabidopsis_thaliana_chr1.trna28-LeuCAG (6488646-6488726) Leu (CAG) 81 bp Sc: 68.96
GTCAAGATGGCCGAGTTGGTCTAAGGCGCCAGTTTCAGGTACTGGTCCGAAAGGGCGTGG
GTTCAAATCCCACTCTTGACA
>Arabidopsis_thaliana_chr4.trna18-LeuCAG (9289707-9289787) Leu (CAG) 81 bp Sc: 68.96
GTCAAGATGGCCGAGTTGGTCTAAGGCGCCAGTTTCAGGTACTGGTCCGAAAGGGCGTGG
GTTCAAATCCCACTCTTGACA
>Arabidopsis_thaliana_chr2.trna24-LeuGAG (9291114-9291185) Leu (GAG) 72 bp Sc: 49.78
GTGGCTGTAATACAGTGGTTAGTATTCTACGTTGAGGCCGAAGGGACCTGGGCTCGATTC
CCAGCAGACACA
>Arabidopsis_thaliana_chr3.trna12-LeuTAA (3090549-3090631) Leu (TAA) 83 bp Sc: 63.86
GCAGGTTTGCCCGAGAGGTTAAGGGGGAAGACTTAAGTTCTTCTGCACATAAGTGCGCGT
GGGTTCGAACCCCACAGCCTGCA
>Arabidopsis_thaliana_chr3.trna45-LeuTAA (19648106-19648188) Leu (TAA) 83 bp Sc: 63.86
GCAGGTTTGCCCGAGAGGTTAAGGGGGAAGACTTAAGTTCTTCTGCACATAAGTGCGCGT
GGGTTCGAACCCCACAGCCTGCA
>Arabidopsis_thaliana_chr2.trna50-LeuTAA (15327313-15327395) Leu (TAA) 83 bp Sc: 68.40
GCAGGTTTGCCCGAGTGGTTAAGGGGGAAGACTTAAGTTCTTCTGCACATAAGTGCGCGT
GGGTTCGAACCCCACAGCCTGCA
>Arabidopsis_thaliana_chr2.trna67-LeuTAA (15327546-15327464) Leu (TAA) 83 bp Sc: 68.40
GCAGGTTTGCCCGAGTGGTTAAGGGGGAAGACTTAAGTTCTTCTGCACATAAGTGCGCGT
GGGTTCGAACCCCACAGCCTGCA
>Arabidopsis_thaliana_chr5.trna4-LeuTAA (984366-984448) Leu (TAA) 83 bp Sc: 68.40
GCAGGTTTGCCCGAGTGGTTAAGGGGGAAGACTTAAGTTCTTCTGCACATAAGTGCGCGT
GGGTTCGAACCCCACAGCCTGCA
>Arabidopsis_thaliana_chr4.trna73-LeuTAA (6837639-6837556) Leu (TAA) 84 bp Sc: 74.53
GTCAGTATGGCCGAGTGGTCTAAGGCGCCAGACTTAAGTTCTGGTCCTCTTACGAGGGCG
TGGGTTCAAACCCCACTGCTGACA
>Arabidopsis_thaliana_chr4.trna74-LeuTAG (5268210-5268125) Leu (TAG) 86 bp Sc: 41.09
GCCGCTATGGTGAAATTGGTAGACACGCTGCTCTTAGGAAGCAGTGCTAGAGCATCTCGG
TTCGAGTTCGAGTCCGAGTAGCGGCA
>Arabidopsis_thaliana_chr1.trna75-LeuTAG (18556667-18556746) Leu (TAG) 80 bp Sc: 63.97
GATAGTTTGGCCGAGTGGTCTAAGGCGCCAGATTTAGGCTCTGGTCCGAAAGGGCGTGGG
TTCAAATCCCACAGCTGTCA
>Arabidopsis_thaliana_chr1.trna76-LeuTAG (18561568-18561647) Leu (TAG) 80 bp Sc: 63.97
GATAGTTTGGCCGAGTGGTCTAAGGCGCCAGATTTAGGCTCTGGTCCGAAAGGGCGTGGG
TTCAAATCCCACAGCTGTCA
>Arabidopsis_thaliana_chr1.trna162-LeuTAG (22890755-22890834) Leu (TAG) 80 bp Sc: 68.10
GACAATTTGGCCGAGTGGTCTAAGGCGCCAGATTTAGGCTCTGGTCCGAAAGGGCGTGGG
TTCAAATCCCACAGTTGTCA
>Arabidopsis_thaliana_chr2.trna49-LeuTAG (15176657-15176736) Leu (TAG) 80 bp Sc: 68.10
GACAATTTGGCCGAGTGGTCTAAGGCGCCAGATTTAGGCTCTGGTCCGAAAGGGCGTGGG
TTCAAATCCCACAGTTGTCA
>Arabidopsis_thaliana_chr4.trna64-LeuTAG (10093157-10093078) Leu (TAG) 80 bp Sc: 68.10
GACAATTTGGCCGAGTGGTCTAAGGCGCCAGATTTAGGCTCTGGTCCGAAAGGGCGTGGG
TTCAAATCCCACAGTTGTCA
>Arabidopsis_thaliana_chr1.trna19-LeuTAG (3881529-3881608) Leu (TAG) 80 bp Sc: 68.72

GACAGTTTGGCCGAGTGGTCTAAGGCGCCAGATTTAGGCTCTGGTCCGAAAGGGCGTGGG
TTCAAATCCCACAGCTGTCA
>Arabidopsis_thaliana_chr3.trna43-LeuTAG (19511966-19512045) Leu (TAG) 80 bp Sc: 68.72
GACAGTTTGGCCGAGTGGTCTAAGGCGCCAGATTTAGGCTCTGGTCCGAAAGGGCGTGGG
TTCAAATCCCACAGCTGTCA
>Arabidopsis_thaliana_chr5.trna115-LeuTAG (1853382-1853303) Leu (TAG) 80 bp Sc: 68.72
GACAGTTTGGCCGAGTGGTCTAAGGCGCCAGATTTAGGCTCTGGTCCGAAAGGGCGTGGG
TTCAAATCCCACAGCTGTCA
>Arabidopsis_thaliana_chr5.trna35-LeuTAG (18807535-18807614) Leu (TAG) 80 bp Sc: 68.72
GACAGTTTGGCCGAGTGGTCTAAGGCGCCAGATTTAGGCTCTGGTCCGAAAGGGCGTGGG
TTCAAATCCCACAGCTGTCA
>Arabidopsis_thaliana_chr4.trna21-LysCTT (11974952-11975032) Lys (CTT) 81 bp Sc: 33.91
CATCGTCTAGCTCAGTTGGTAGAGCGCAAGGCTCTTAACTTTGTGGACTTGTGGTCATTG
GTTCGAGTTACATGGTGGGCG
>Arabidopsis_thaliana_chr4.trna58-LysCTT (13006316-13006244) Lys (CTT) 73 bp Sc: 77.71
GCCCGTCTAGCTCAGTTGGTAGAGCGCAAGGCTCTTAACCTTCTGGTCGTGGGTTCGAGC
CCCACGGTGGGCG
>Arabidopsis_thaliana_chr1.trna228-LysCTT (5518128-5518056) Lys (CTT) 73 bp Sc: 84.38
GCCCGTCTAGCTCAGTTGGTAGAGCGCAAGGCTCTTAACCTTGTGGTCGTGGGTTCGAGC
CCCACGGTGGGCG
>Arabidopsis_thaliana_chr1.trna244-LysCTT (309347-309275) Lys (CTT) 73 bp Sc: 84.38
GCCCGTCTAGCTCAGTTGGTAGAGCGCAAGGCTCTTAACCTTGTGGTCGTGGGTTCGAGC
CCCACGGTGGGCG
>Arabidopsis_thaliana_chr1.trna30-LysCTT (7016507-7016579) Lys (CTT) 73 bp Sc: 84.38
GCCCGTCTAGCTCAGTTGGTAGAGCGCAAGGCTCTTAACCTTGTGGTCGTGGGTTCGAGC
CCCACGGTGGGCG
>Arabidopsis_thaliana_chr1.trna4-LysCTT (604402-604474) Lys (CTT) 73 bp Sc: 84.38
GCCCGTCTAGCTCAGTTGGTAGAGCGCAAGGCTCTTAACCTTGTGGTCGTGGGTTCGAGC
CCCACGGTGGGCG
>Arabidopsis_thaliana_chr1.trna72-LysCTT (17735510-17735582) Lys (CTT) 73 bp Sc: 84.38
GCCCGTCTAGCTCAGTTGGTAGAGCGCAAGGCTCTTAACCTTGTGGTCGTGGGTTCGAGC
CCCACGGTGGGCG
>Arabidopsis_thaliana_chr2.trna57-LysCTT (18710752-18710824) Lys (CTT) 73 bp Sc: 84.38
GCCCGTCTAGCTCAGTTGGTAGAGCGCAAGGCTCTTAACCTTGTGGTCGTGGGTTCGAGC
CCCACGGTGGGCG
>Arabidopsis_thaliana_chr3.trna31-LysCTT (11961646-11961718) Lys (CTT) 73 bp Sc: 84.38
GCCCGTCTAGCTCAGTTGGTAGAGCGCAAGGCTCTTAACCTTGTGGTCGTGGGTTCGAGC
CCCACGGTGGGCG
>Arabidopsis_thaliana_chr3.trna64-LysCTT (22481288-22481216) Lys (CTT) 73 bp Sc: 84.38
GCCCGTCTAGCTCAGTTGGTAGAGCGCAAGGCTCTTAACCTTGTGGTCGTGGGTTCGAGC
CCCACGGTGGGCG
>Arabidopsis_thaliana_chr3.trna67-LysCTT (20824568-20824496) Lys (CTT) 73 bp Sc: 84.38
GCCCGTCTAGCTCAGTTGGTAGAGCGCAAGGCTCTTAACCTTGTGGTCGTGGGTTCGAGC
CCCACGGTGGGCG
>Arabidopsis_thaliana_chr4.trna1-LysCTT (33800-33872) Lys (CTT) 73 bp Sc: 84.38
GCCCGTCTAGCTCAGTTGGTAGAGCGCAAGGCTCTTAACCTTGTGGTCGTGGGTTCGAGC
CCCACGGTGGGCG
>Arabidopsis_thaliana_chr4.trna10-LysCTT (7256409-7256481) Lys (CTT) 73 bp Sc: 84.38
GCCCGTCTAGCTCAGTTGGTAGAGCGCAAGGCTCTTAACCTTGTGGTCGTGGGTTCGAGC
CCCACGGTGGGCG
>Arabidopsis_thaliana_chr4.trna14-LysCTT (8979657-8979729) Lys (CTT) 73 bp Sc: 84.38
GCCCGTCTAGCTCAGTTGGTAGAGCGCAAGGCTCTTAACCTTGTGGTCGTGGGTTCGAGC
CCCACGGTGGGCG
>Arabidopsis_thaliana_chr4.trna60-LysCTT (11954824-11954752) Lys (CTT) 73 bp Sc: 84.38
GCCCGTCTAGCTCAGTTGGTAGAGCGCAAGGCTCTTAACCTTGTGGTCGTGGGTTCGAGC
CCCACGGTGGGCG
>Arabidopsis_thaliana_chr5.trna121-LysCTT (508500-508428) Lys (CTT) 73 bp Sc: 84.38
GCCCGTCTAGCTCAGTTGGTAGAGCGCAAGGCTCTTAACCTTGTGGTCGTGGGTTCGAGC
CCCACGGTGGGCG
>Arabidopsis_thaliana_chr5.trna15-LysCTT (4530476-4530548) Lys (CTT) 73 bp Sc: 84.38
GCCCGTCTAGCTCAGTTGGTAGAGCGCAAGGCTCTTAACCTTGTGGTCGTGGGTTCGAGC
CCCACGGTGGGCG
>Arabidopsis_thaliana_chr3.trna21-LysCTT (5632858-5632930) Lys (CTT) 73 bp Sc: 86.03
GCCCGTCTAGCTCAGTTGGTAGAGCGCAAGGCTCTTAACCTTGTGGTCGTGGGTTCGAAC
CCCACGGTGGGCG
>Arabidopsis_thaliana_chr3.trna10-LysTTT (2285608-2285679) Lys (TTT) 72 bp Sc: 81.97
GCCGTCTTAGCTCAGCGGTAGAGCGCGTGGCTTTTAACCACGTGGCCGTGGGTTCGATCC

CCACAGACGGCG
>Arabidopsis_thaliana_chr5.trna37-LysTTT (19658829-19658900) Lys (TTT) 72 bp Sc: 83.16
GCCTTCTTAGCTCAGTGGTAGAGCGCGTGGCTTTTAACCACGTGGTCGTGGGTTCGATCC
CCACAGACGGCG
>Arabidopsis_thaliana_chr4.trna27-LysTTT (14032496-14032567) Lys (TTT) 72 bp Sc: 84.12
GCCGTCTTAGCTCAGTGGTAGAGCGCGTGGCTTTTACCCACGAGGCCGTGGGTTCGATCC
CCACAGACGGCG
>Arabidopsis_thaliana_chr1.trna182-LysTTT (29838010-29838081) Lys (TTT) 72 bp Sc: 84.42
GCCGTCTTAGCTCAGTGGTAGAGCGCGTGGCTTTTAACCACGTGGCCGTGGGTTCGATCC
CCACAGACGGCG
>Arabidopsis_thaliana_chr1.trna183-LysTTT (29838611-29838682) Lys (TTT) 72 bp Sc: 84.42
GCCGTCTTAGCTCAGTGGTAGAGCGCGTGGCTTTTAACCACGTGGCCGTGGGTTCGATCC
CCACAGACGGCG
>Arabidopsis_thaliana_chr2.trna76-LysTTT (11782000-11781929) Lys (TTT) 72 bp Sc: 84.42
GCCGTCTTAGCTCAGTGGTAGAGCGCGTGGCTTTTAACCACGTGGCCGTGGGTTCGATCC
CCACAGACGGCG
>Arabidopsis_thaliana_chr3.trna16-LysTTT (3942013-3942084) Lys (TTT) 72 bp Sc: 84.42
GCCGTCTTAGCTCAGTGGTAGAGCGCGTGGCTTTTAACCACGTGGCCGTGGGTTCGATCC
CCACAGACGGCG
>Arabidopsis_thaliana_chr3.trna46-LysTTT (19935355-19935426) Lys (TTT) 72 bp Sc: 84.42
GCCGTCTTAGCTCAGTGGTAGAGCGCGTGGCTTTTAACCACGTGGCCGTGGGTTCGATCC
CCACAGACGGCG
>Arabidopsis_thaliana_chr5.trna14-LysTTT (4471051-4471122) Lys (TTT) 72 bp Sc: 84.42
GCCGTCTTAGCTCAGTGGTAGAGCGCGTGGCTTTTAACCACGTGGCCGTGGGTTCGATCC
CCACAGACGGCG
>Arabidopsis_thaliana_chr5.trna42-LysTTT (21418370-21418441) Lys (TTT) 72 bp Sc: 84.42
GCCGTCTTAGCTCAGTGGTAGAGCGCGTGGCTTTTAACCACGTGGCCGTGGGTTCGATCC
CCACAGACGGCG
>Arabidopsis_thaliana_chr5.trna55-LysTTT (24684508-24684579) Lys (TTT) 72 bp Sc: 84.42
GCCGTCTTAGCTCAGTGGTAGAGCGCGTGGCTTTTAACCACGTGGCCGTGGGTTCGATCC
CCACAGACGGCG
>Arabidopsis_thaliana_chr5.trna82-LysTTT (19761472-19761401) Lys (TTT) 72 bp Sc: 84.42
GCCGTCTTAGCTCAGTGGTAGAGCGCGTGGCTTTTAACCACGTGGCCGTGGGTTCGATCC
CCACAGACGGCG
>Arabidopsis_thaliana_chr5.trna86-LysTTT (17856883-17856812) Lys (TTT) 72 bp Sc: 84.42
GCCGTCTTAGCTCAGTGGTAGAGCGCGTGGCTTTTAACCACGTGGCCGTGGGTTCGATCC
CCACAGACGGCG
>Arabidopsis_thaliana_chr2.trna89-MetCAT (3383474-3383401) Met (CAT) 74 bp Sc: 49.74
GGGCTTATAGTTTAATTGGTTGAAACGTACCGCTCATAACGGTTATATTGTAGGTTCGAG
CCCTACTAAGCCTA
>Arabidopsis_thaliana_chr3.trna49-MetCAT (20720152-20720223) Met (CAT) 72 bp Sc: 57.66
ATCAAAGTGGTGCAGCGGAAGCGTGATGGGCCCATAACCCACAGGTCACAGGATCGAAAC
CTGTCTTTGATA
>Arabidopsis_thaliana_chr2.trna90-MetCAT (3328739-3328667) Met (CAT) 73 bp Sc: 59.47
ACCTACTTGACTCAGCGGTTAGAGTATCGCTTTCATACGGCGAGAGTCATTGGTTCAAAT
CCAATAGTAGGTA
>Arabidopsis_thaliana_chr2.trna12-MetCAT (3462075-3462148) Met (CAT) 74 bp Sc: 61.58
AGCGGGGTAGAGGAATTGGTCAACTCATCAGGCTCATGACCTGAAGATTACAGGTTCGAA
TCCTGTCCCCGCAT
>Arabidopsis_thaliana_chr1.trna214-MetCAT (15733127-15733056) Met (CAT) 72 bp Sc: 65.74
ATCAGAGTGGCGCAGCGGAAGCGTGGTGGGCCCATAACCCACAGGTCCCAAGATCGAAAC
TTGGCTCTGATA
>Arabidopsis_thaliana_chr3.trna48-MetCAT (20704665-20704736) Met (CAT) 72 bp Sc: 65.74
ATCAGAGTGGCGCAGCGGAAGCGTGGTGGGCCCATAACCCACAGGTCCCAAGATCGAAAC
TTGGCTCTGATA
>Arabidopsis_thaliana_chr1.trna168-MetCAT (25944827-25944898) Met (CAT) 72 bp Sc: 68.81
ATCAGAGTGGCGCAGCGGAAGCGTGGTGGGCCCATAACCCACAGGTCCCAGGATCGAAAC
CTGGCTCTGATA
>Arabidopsis_thaliana_chr2.trna78-MetCAT (10066597-10066526) Met (CAT) 72 bp Sc: 68.81
ATCAGAGTGGCGCAGCGGAAGCGTGGTGGGCCCATAACCCACAGGTCCCAGGATCGAAAC
CTGGCTCTGATA
>Arabidopsis_thaliana_chr2.trna79-MetCAT (9810368-9810297) Met (CAT) 72 bp Sc: 68.81
ATCAGAGTGGCGCAGCGGAAGCGTGGTGGGCCCATAACCCACAGGTCCCAGGATCGAAAC
CTGGCTCTGATA
>Arabidopsis_thaliana_chr5.trna47-MetCAT (23463220-23463291) Met (CAT) 72 bp Sc: 68.81
ATCAGAGTGGCGCAGCGGAAGCGTGGTGGGCCCATAACCCACAGGTCCCAGGATCGAAAC
CTGGCTCTGATA

>Arabidopsis_thaliana_chr5.trna64-MetCAT (26938898-26938969) Met (CAT) 72 bp Sc: 68.81
ATCAGAGTGGCGCAGCGGAAGCGTGGTGGGCCCATAACCCACAGGTCCCAGGATCGAAAC
CTGGCTCTGATA
>Arabidopsis_thaliana_chr5.trna77-MetCAT (22304923-22304852) Met (CAT) 72 bp Sc: 68.81
ATCAGAGTGGCGCAGCGGAAGCGTGGTGGGCCCATAACCCACAGGTCCCAGGATCGAAAC
CTGGCTCTGATA
>Arabidopsis_thaliana_chr5.trna89-MetCAT (16684735-16684664) Met (CAT) 72 bp Sc: 69.22
AGCAGAGTGGCGCAGCGGAAGCGTGGTGGGCCCATAACCCACAGGTCCCAGGATCGAAAC
CTGGCTCTGCTA
>Arabidopsis_thaliana_chr1.trna216-MetCAT (11797265-11797181) Met (CAT) 85 bp Sc: 60.80
GGGGTGGTGGCGCAGTTGGCTAGCGCGTAGGTCTCATAGCTACTGAGTGATCCTGAGGTC
GAGAGTTCGAGCCTCTCTCACCCCA
>Arabidopsis_thaliana_chr1.trna8-MetCAT (1324922-1325006) Met (CAT) 85 bp Sc: 60.94
GGGGTGGTGGCGCAGTTGGCTAGCGCGTAGGTCTCATAGCTACAGAGTGATCCTGAGGTC
GAGAGTTCGAGCCTCTCTCACCCCA
>Arabidopsis_thaliana_chr2.trna70-MetCAT (14054503-14054419) Met (CAT) 85 bp Sc: 60.80
GGGGTGGTGGCGCAGTTGGCTAGCGCGTAGGTCTCATAGCTACTGAGTGATCCTGAGGTC
GAGAGTTCGAGCCTCTCTCACCCCA
>Arabidopsis_thaliana_chr2.trna47-MetCAT (14847686-14847770) Met (CAT) 85 bp Sc: 60.80
GGGGTGGTGGCGCAGTTGGCTAGCGCGTAGGTCTCATAGCTACTGAGTGATCCTGAGGTC
GAGAGTTCGAGCCTCTCTCACCCCA
>Arabidopsis_thaliana_chr4.trna50-MetCAT (15345802-15345718) Met (CAT) 85 bp Sc: 61.43
GGGGTGGTGGCGCAGTTGGCTAGCGCGTAGGTCTCATAGCTATTGAGTGATCCTGAGGTC
GAGAGTTCGAGCCTCTCTCACCCCA
>Arabidopsis_thaliana_chr5.trna38-MetCAT (19820361-19820446) Met (CAT) 86 bp Sc: 61.56
GGGGTGGTGGCGCAGTTGGCTAGCGCGTAGGTCTCATAGCTATCTGAGTAATCCTGAGGT
CGAGAGTTCGAGCCTCTCTCACCCCA
>Arabidopsis_thaliana_chr3.trna88-MetCAT (2232556-2232471) Met (CAT) 86 bp Sc: 61.56
GGGGTGGTGGCGCAGTTGGCTAGCGCGTAGGTCTCATAGCTATCTGAGTAATCCTGAGGT
CGAGAGTTCGAGCCTCTCTCACCCCA
>Arabidopsis_thaliana_chr5.trna70-MetCAT (26114793-26114709) Met (CAT) 85 bp Sc: 60.93
GGGGTGGTGGCGCAGTTGGCTAGCGCGTAGGTCTCATAGCTAGTGAGTGATCCTGAGGTC
GAGAGTTCGAGCCTCTCTCACCCCA
>Arabidopsis_thaliana_chr1.trna187-MetCAT (29448903-29448819) Met (CAT) 85 bp Sc: 60.80
GGGGTGGTGGCGCAGTTGGCTAGCGCGTAGGTCTCATAGCTACTGAGTGATCCTGAGGTC
GAGAGTTCGAGCCTCTCTCACCCCA
>Arabidopsis_thaliana_chr1.trna18-MetCAT (3676607-3676691) Met (CAT) 85 bp Sc: 61.43
GGGGTGGTGGCGCAGTTGGCTAGCGCGTAGGTCTCATAGCTATTGAGTGATCCTGAGGTC
GAGAGTTCGAGCCTCTCTCACCCCA
>Arabidopsis_thaliana_chr1.trna220-MetCAT (7884443-7884359) Met (CAT) 85 bp Sc: 60.80
GGGGTGGTGGCGCAGTTGGCTAGCGCGTAGGTCTCATAGCTACTGAGTGATCCTGAGGTC
GAGAGTTCGAGCCTCTCTCACCCCA
>Arabidopsis_thaliana_chr1.trna167-PheGAA (25931859-25931931) Phe (GAA) 73 bp Sc: 63.59
GCGGGGATAGCTCAGTTGGGAGAGCGTGACACTGAAGATCTGAAGGTCGCGTGTTCGATC
CACGCTCACCGCA
>Arabidopsis_thaliana_chr1.trna195-PheGAA (25935812-25935740) Phe (GAA) 73 bp Sc: 77.43
GCGGGGATAGCTCAGTTGGGAGAGCGTCAGACTGAAGATCTGAAGGTCGCGTGTTCGATC
CACGCTCACCGCA
>Arabidopsis_thaliana_chr1.trna2-PheGAA (515494-515566) Phe (GAA) 73 bp Sc: 77.43
GCGGGGATAGCTCAGTTGGGAGAGCGTCAGACTGAAGATCTGAAGGTCGCGTGTTCGATC
CACGCTCACCGCA
>Arabidopsis_thaliana_chr1.trna243-PheGAA (909882-909810) Phe (GAA) 73 bp Sc: 77.43
GCGGGGATAGCTCAGTTGGGAGAGCGTCAGACTGAAGATCTGAAGGTCGCGTGTTCGATC
CACGCTCACCGCA
>Arabidopsis_thaliana_chr3.trna20-PheGAA (5275156-5275228) Phe (GAA) 73 bp Sc: 77.43
GCGGGGATAGCTCAGTTGGGAGAGCGTCAGACTGAAGATCTGAAGGTCGCGTGTTCGATC
CACGCTCACCGCA
>Arabidopsis_thaliana_chr3.trna63-PheGAA (23062517-23062445) Phe (GAA) 73 bp Sc: 77.43
GCGGGGATAGCTCAGTTGGGAGAGCGTCAGACTGAAGATCTGAAGGTCGCGTGTTCGATC
CACGCTCACCGCA
>Arabidopsis_thaliana_chr4.trna26-PheGAA (13704788-13704860) Phe (GAA) 73 bp Sc: 77.43
GCGGGGATAGCTCAGTTGGGAGAGCGTCAGACTGAAGATCTGAAGGTCGCGTGTTCGATC
CACGCTCACCGCA
>Arabidopsis_thaliana_chr4.trna75-PheGAA (1520854-1520782) Phe (GAA) 73 bp Sc: 77.43
GCGGGGATAGCTCAGTTGGGAGAGCGTCAGACTGAAGATCTGAAGGTCGCGTGTTCGATC
CACGCTCACCGCA
>Arabidopsis_thaliana_chr4.trna76-PheGAA (808342-808270) Phe (GAA) 73 bp Sc: 77.43

GCGGGGATAGCTCAGTTGGGAGAGCGTCAGACTGAAGATCTGAAGGTCGCGTGTTCGATC
CACGCTCACCGCA
>Arabidopsis_thaliana_chr5.trna104-PheGAA (3669073-3669001) Phe (GAA) 73 bp Sc: 77.43
GCGGGGATAGCTCAGTTGGGAGAGCGTCAGACTGAAGATCTGAAGGTCGCGTGTTCGATC
CACGCTCACCGCA
>Arabidopsis_thaliana_chr5.trna117-PheGAA (862468-862396) Phe (GAA) 73 bp Sc: 77.43
GCGGGGATAGCTCAGTTGGGAGAGCGTCAGACTGAAGATCTGAAGGTCGCGTGTTCGATC
CACGCTCACCGCA
>Arabidopsis_thaliana_chr5.trna43-PheGAA (22091632-22091704) Phe (GAA) 73 bp Sc: 77.43
GCGGGGATAGCTCAGTTGGGAGAGCGTCAGACTGAAGATCTGAAGGTCGCGTGTTCGATC
CACGCTCACCGCA
>Arabidopsis_thaliana_chr5.trna44-PheGAA (22094088-22094160) Phe (GAA) 73 bp Sc: 77.43
GCGGGGATAGCTCAGTTGGGAGAGCGTCAGACTGAAGATCTGAAGGTCGCGTGTTCGATC
CACGCTCACCGCA
>Arabidopsis_thaliana_chr5.trna75-PheGAA (22357799-22357727) Phe (GAA) 73 bp Sc: 77.43
GCGGGGATAGCTCAGTTGGGAGAGCGTCAGACTGAAGATCTGAAGGTCGCGTGTTCGATC
CACGCTCACCGCA
>Arabidopsis_thaliana_chr5.trna76-PheGAA (22355970-22355898) Phe (GAA) 73 bp Sc: 77.43
GCGGGGATAGCTCAGTTGGGAGAGCGTCAGACTGAAGATCTGAAGGTCGCGTGTTCGATC
CACGCTCACCGCA
>Arabidopsis_thaliana_chr5.trna96-PheGAA (8946899-8946827) Phe (GAA) 73 bp Sc: 77.43
GCGGGGATAGCTCAGTTGGGAGAGCGTCAGACTGAAGATCTGAAGGTCGCGTGTTCGATC
CACGCTCACCGCA
>Arabidopsis_thaliana_chr1.trna36-ProAGG (10089884-10089955) Pro (AGG) 72 bp Sc: 36.67
GGGCATTTGGTCTAGTGGTATGATTCTTGCTTAGGGTGCGAGAGGCCCGGAGCTCAATTC
TCGGAAAACCCC
>Arabidopsis_thaliana_chr1.trna44-ProAGG (10096937-10097008) Pro (AGG) 72 bp Sc: 64.32
TGGCATTTGGTCTAGTGGTATGATTCTCGCTTAGGGTGCGAGAGGTCCCGAGTTCAATTC
TCGGAATGCCCC
>Arabidopsis_thaliana_chr1.trna194-ProAGG (26385888-26385817) Pro (AGG) 72 bp Sc: 69.40
GGGCATTTGGTCTAGTGGTATGATTCTCGCTTAGGGTGCGAGAGGTCCGGAGTTCAATTC
TCCGAATGCCCC
>Arabidopsis_thaliana_chr1.trna165-ProAGG (25833317-25833388) Pro (AGG) 72 bp Sc: 71.75
GGGCATTTGGTCTAGTGGTATGATTCTCGCTTAGGGTGCGAGAGGTCCCGAGTTCAATTC
TCGGAATGCCCC
>Arabidopsis_thaliana_chr1.trna49-ProAGG (10099411-10099482) Pro (AGG) 72 bp Sc: 71.75
GGGCATTTGGTCTAGTGGTATGATTCTCGCTTAGGGTGCGAGAGGTCCCGAGTTCAATTC
TCGGAATGCCCC
>Arabidopsis_thaliana_chr2.trna40-ProAGG (14360222-14360293) Pro (AGG) 72 bp Sc: 71.75
GGGCATTTGGTCTAGTGGTATGATTCTCGCTTAGGGTGCGAGAGGTCCCGAGTTCAATTC
TCGGAATGCCCC
>Arabidopsis_thaliana_chr2.trna44-ProAGG (14363134-14363205) Pro (AGG) 72 bp Sc: 71.75
GGGCATTTGGTCTAGTGGTATGATTCTCGCTTAGGGTGCGAGAGGTCCCGAGTTCAATTC
TCGGAATGCCCC
>Arabidopsis_thaliana_chr3.trna1-ProAGG (259641-259712) Pro (AGG) 72 bp Sc: 71.75
GGGCATTTGGTCTAGTGGTATGATTCTCGCTTAGGGTGCGAGAGGTCCCGAGTTCAATTC
TCGGAATGCCCC
>Arabidopsis_thaliana_chr3.trna30-ProAGG (11076815-11076886) Pro (AGG) 72 bp Sc: 71.75
GGGCATTTGGTCTAGTGGTATGATTCTCGCTTAGGGTGCGAGAGGTCCCGAGTTCAATTC
TCGGAATGCCCC
>Arabidopsis_thaliana_chr3.trna69-ProAGG (19403723-19403652) Pro (AGG) 72 bp Sc: 71.75
GGGCATTTGGTCTAGTGGTATGATTCTCGCTTAGGGTGCGAGAGGTCCCGAGTTCAATTC
TCGGAATGCCCC
>Arabidopsis_thaliana_chr4.trna3-ProAGG (1390895-1390966) Pro (AGG) 72 bp Sc: 71.75
GGGCATTTGGTCTAGTGGTATGATTCTCGCTTAGGGTGCGAGAGGTCCCGAGTTCAATTC
TCGGAATGCCCC
>Arabidopsis_thaliana_chr5.trna92-ProAGG (15844325-15844254) Pro (AGG) 72 bp Sc: 71.75
GGGCATTTGGTCTAGTGGTATGATTCTCGCTTAGGGTGCGAGAGGTCCCGAGTTCAATTC
TCGGAATGCCCC
>Arabidopsis_thaliana_chr1.trna16-ProAGG (2879306-2879377) Pro (AGG) 72 bp Sc: 72.36
GGGCGTTTGGTCTAGTGGTATGATTCTCGCTTAGGGTGCGAGAGGTCCCGAGTTCAATTC
TCGGAACGCCCC
>Arabidopsis_thaliana_chr1.trna204-ProAGG (22044348-22044277) Pro (AGG) 72 bp Sc: 72.36
GGGCGTTTGGTCTAGTGGTATGATTCTCGCTTAGGGTGCGAGAGGTCCCGAGTTCAATTC
TCGGAACGCCCC
>Arabidopsis_thaliana_chr1.trna236-ProAGG (3431257-3431186) Pro (AGG) 72 bp Sc: 72.36
GGGCGTTTGGTCTAGTGGTATGATTCTCGCTTAGGGTGCGAGAGGTCCCGAGTTCAATTC

TCGGAACGCCCC
>Arabidopsis_thaliana_chr3.trna77-ProAGG (7567471-7567400) Pro (AGG) 72 bp Sc: 72.36
GGGCGTTTGGTCTAGTGGTATGATTCTCGCTTAGGGTGCGAGAGGTCCCGAGTTCAATTC
TCGGAACGCCCC
>Arabidopsis_thaliana_chr2.trna43-ProCGG (14362216-14362287) Pro (CGG) 72 bp Sc: 71.52
GGGCATTTGGTCTAGTGGTATGATTCTCGCTTCGGGTGCGAGGGGTCCCGAGTTCGATTC
TCGGAATGCCCC
>Arabidopsis_thaliana_chr3.trna92-ProCGG (1707815-1707744) Pro (CGG) 72 bp Sc: 72.45
GGGTGTTTGGTCTAGTGGTATGATTCGCGCTTCGGGTGCGCGAGGTCCCGAGTTCGATTC
TCGGAACACCCC
>Arabidopsis_thaliana_chr2.trna23-ProCGG (9144436-9144507) Pro (CGG) 72 bp Sc: 74.22
GGGTGTTTGGTCTAGTGGTATGATTCTCGCTTCGGGTGCGAGAGGTCCCGAGTTCGATTC
TCGGAACACCCC
>Arabidopsis_thaliana_chr3.trna83-ProCGG (3622769-3622698) Pro (CGG) 72 bp Sc: 74.22
GGGTGTTTGGTCTAGTGGTATGATTCTCGCTTCGGGTGCGAGAGGTCCCGAGTTCGATTC
TCGGAACACCCC
>Arabidopsis_thaliana_chr4.trna39-ProCGG (17149474-17149545) Pro (CGG) 72 bp Sc: 74.22
GGGTGTTTGGTCTAGTGGTATGATTCTCGCTTCGGGTGCGAGAGGTCCCGAGTTCGATTC
TCGGAACACCCC
>Arabidopsis_thaliana_chr2.trna16-ProTGG (3495009-3495083) Pro (TGG) 75 bp Sc: 54.34
CGAGGTGTAGCGCAGTCTGGTCAGCGCATCTGTTTTGGGTACAGAGGGCCATAGGTTCGA
ATCCTGTCACCTTGA
>Arabidopsis_thaliana_chr1.trna35-ProTGG (10089423-10089494) Pro (TGG) 72 bp Sc: 54.44
CAGCATTTGGTCTAGTGGTATGATTCTCGCTTTGGGTGCAAGAGGTCCCGAGTTCGATTC
TCGGAATGCCCC
>Arabidopsis_thaliana_chr1.trna60-ProTGG (10108696-10108767) Pro (TGG) 72 bp Sc: 67.03
GGGCATTTGGTCTAGTGGTATGATTCTCGCTTTGGGTGCGAGAGGTCCCGAGTTCGATTC
TCGCAATGCCCC
>Arabidopsis_thaliana_chr1.trna37-ProTGG (10090354-10090425) Pro (TGG) 72 bp Sc: 73.73
GGGCATTTGGTCTAGTGGTATGATTCTCGCTTTGGGTGCGAGAGGTCCCGAGTTCGATTC
TCGGAATGCCCC
>Arabidopsis_thaliana_chr1.trna38-ProTGG (10090755-10090826) Pro (TGG) 72 bp Sc: 73.73
GGGCATTTGGTCTAGTGGTATGATTCTCGCTTTGGGTGCGAGAGGTCCCGAGTTCGATTC
TCGGAATGCCCC
>Arabidopsis_thaliana_chr1.trna39-ProTGG (10092311-10092382) Pro (TGG) 72 bp Sc: 73.73
GGGCATTTGGTCTAGTGGTATGATTCTCGCTTTGGGTGCGAGAGGTCCCGAGTTCGATTC
TCGGAATGCCCC
>Arabidopsis_thaliana_chr1.trna40-ProTGG (10092787-10092858) Pro (TGG) 72 bp Sc: 73.73
GGGCATTTGGTCTAGTGGTATGATTCTCGCTTTGGGTGCGAGAGGTCCCGAGTTCGATTC
TCGGAATGCCCC
>Arabidopsis_thaliana_chr1.trna41-ProTGG (10093295-10093366) Pro (TGG) 72 bp Sc: 73.73
GGGCATTTGGTCTAGTGGTATGATTCTCGCTTTGGGTGCGAGAGGTCCCGAGTTCGATTC
TCGGAATGCCCC
>Arabidopsis_thaliana_chr1.trna42-ProTGG (10093732-10093803) Pro (TGG) 72 bp Sc: 73.73
GGGCATTTGGTCTAGTGGTATGATTCTCGCTTTGGGTGCGAGAGGTCCCGAGTTCGATTC
TCGGAATGCCCC
>Arabidopsis_thaliana_chr1.trna43-ProTGG (10094159-10094230) Pro (TGG) 72 bp Sc: 73.73
GGGCATTTGGTCTAGTGGTATGATTCTCGCTTTGGGTGCGAGAGGTCCCGAGTTCGATTC
TCGGAATGCCCC
>Arabidopsis_thaliana_chr1.trna45-ProTGG (10097100-10097171) Pro (TGG) 72 bp Sc: 73.73
GGGCATTTGGTCTAGTGGTATGATTCTCGCTTTGGGTGCGAGAGGTCCCGAGTTCGATTC
TCGGAATGCCCC
>Arabidopsis_thaliana_chr1.trna46-ProTGG (10097704-10097775) Pro (TGG) 72 bp Sc: 73.73
GGGCATTTGGTCTAGTGGTATGATTCTCGCTTTGGGTGCGAGAGGTCCCGAGTTCGATTC
TCGGAATGCCCC
>Arabidopsis_thaliana_chr1.trna47-ProTGG (10098639-10098710) Pro (TGG) 72 bp Sc: 73.73
GGGCATTTGGTCTAGTGGTATGATTCTCGCTTTGGGTGCGAGAGGTCCCGAGTTCGATTC
TCGGAATGCCCC
>Arabidopsis_thaliana_chr1.trna48-ProTGG (10099065-10099136) Pro (TGG) 72 bp Sc: 73.73
GGGCATTTGGTCTAGTGGTATGATTCTCGCTTTGGGTGCGAGAGGTCCCGAGTTCGATTC
TCGGAATGCCCC
>Arabidopsis_thaliana_chr1.trna50-ProTGG (10099813-10099884) Pro (TGG) 72 bp Sc: 73.73
GGGCATTTGGTCTAGTGGTATGATTCTCGCTTTGGGTGCGAGAGGTCCCGAGTTCGATTC
TCGGAATGCCCC
>Arabidopsis_thaliana_chr1.trna51-ProTGG (10100259-10100330) Pro (TGG) 72 bp Sc: 73.73
GGGCATTTGGTCTAGTGGTATGATTCTCGCTTTGGGTGCGAGAGGTCCCGAGTTCGATTC
TCGGAATGCCCC

>Arabidopsis_thaliana_chr1.trna52-ProTGG (10101014-10101085) Pro (TGG) 72 bp Sc: 73.73
GGGCATTTGGTCTAGTGGTATGATTCTCGCTTTGGGTGCGAGAGGTCCCGAGTTCGATTC
TCGGAATGCCCC
>Arabidopsis_thaliana_chr1.trna53-ProTGG (10101586-10101657) Pro (TGG) 72 bp Sc: 73.73
GGGCATTTGGTCTAGTGGTATGATTCTCGCTTTGGGTGCGAGAGGTCCCGAGTTCGATTC
TCGGAATGCCCC
>Arabidopsis_thaliana_chr1.trna54-ProTGG (10101979-10102050) Pro (TGG) 72 bp Sc: 73.73
GGGCATTTGGTCTAGTGGTATGATTCTCGCTTTGGGTGCGAGAGGTCCCGAGTTCGATTC
TCGGAATGCCCC
>Arabidopsis_thaliana_chr1.trna55-ProTGG (10106076-10106147) Pro (TGG) 72 bp Sc: 73.73
GGGCATTTGGTCTAGTGGTATGATTCTCGCTTTGGGTGCGAGAGGTCCCGAGTTCGATTC
TCGGAATGCCCC
>Arabidopsis_thaliana_chr1.trna56-ProTGG (10106514-10106585) Pro (TGG) 72 bp Sc: 73.73
GGGCATTTGGTCTAGTGGTATGATTCTCGCTTTGGGTGCGAGAGGTCCCGAGTTCGATTC
TCGGAATGCCCC
>Arabidopsis_thaliana_chr1.trna57-ProTGG (10106884-10106955) Pro (TGG) 72 bp Sc: 73.73
GGGCATTTGGTCTAGTGGTATGATTCTCGCTTTGGGTGCGAGAGGTCCCGAGTTCGATTC
TCGGAATGCCCC
>Arabidopsis_thaliana_chr1.trna58-ProTGG (10107635-10107706) Pro (TGG) 72 bp Sc: 73.73
GGGCATTTGGTCTAGTGGTATGATTCTCGCTTTGGGTGCGAGAGGTCCCGAGTTCGATTC
TCGGAATGCCCC
>Arabidopsis_thaliana_chr1.trna59-ProTGG (10108375-10108446) Pro (TGG) 72 bp Sc: 73.73
GGGCATTTGGTCTAGTGGTATGATTCTCGCTTTGGGTGCGAGAGGTCCCGAGTTCGATTC
TCGGAATGCCCC
>Arabidopsis_thaliana_chr1.trna65-ProTGG (17159047-17159118) Pro (TGG) 72 bp Sc: 73.73
GGGCATTTGGTCTAGTGGTATGATTCTCGCTTTGGGTGCGAGAGGTCCCGAGTTCGATTC
TCGGAATGCCCC
>Arabidopsis_thaliana_chr1.trna67-ProTGG (17160971-17161042) Pro (TGG) 72 bp Sc: 73.73
GGGCATTTGGTCTAGTGGTATGATTCTCGCTTTGGGTGCGAGAGGTCCCGAGTTCGATTC
TCGGAATGCCCC
>Arabidopsis_thaliana_chr1.trna68-ProTGG (17161419-17161490) Pro (TGG) 72 bp Sc: 73.73
GGGCATTTGGTCTAGTGGTATGATTCTCGCTTTGGGTGCGAGAGGTCCCGAGTTCGATTC
TCGGAATGCCCC
>Arabidopsis_thaliana_chr1.trna69-ProTGG (17162968-17163039) Pro (TGG) 72 bp Sc: 73.73
GGGCATTTGGTCTAGTGGTATGATTCTCGCTTTGGGTGCGAGAGGTCCCGAGTTCGATTC
TCGGAATGCCCC
>Arabidopsis_thaliana_chr1.trna70-ProTGG (17163409-17163480) Pro (TGG) 72 bp Sc: 73.73
GGGCATTTGGTCTAGTGGTATGATTCTCGCTTTGGGTGCGAGAGGTCCCGAGTTCGATTC
TCGGAATGCCCC
>Arabidopsis_thaliana_chr1.trna71-ProTGG (17164462-17164533) Pro (TGG) 72 bp Sc: 73.73
GGGCATTTGGTCTAGTGGTATGATTCTCGCTTTGGGTGCGAGAGGTCCCGAGTTCGATTC
TCGGAATGCCCC
>Arabidopsis_thaliana_chr2.trna37-ProTGG (14357465-14357536) Pro (TGG) 72 bp Sc: 73.73
GGGCATTTGGTCTAGTGGTATGATTCTCGCTTTGGGTGCGAGAGGTCCCGAGTTCGATTC
TCGGAATGCCCC
>Arabidopsis_thaliana_chr2.trna38-ProTGG (14358438-14358509) Pro (TGG) 72 bp Sc: 73.73
GGGCATTTGGTCTAGTGGTATGATTCTCGCTTTGGGTGCGAGAGGTCCCGAGTTCGATTC
TCGGAATGCCCC
>Arabidopsis_thaliana_chr2.trna39-ProTGG (14359270-14359341) Pro (TGG) 72 bp Sc: 73.73
GGGCATTTGGTCTAGTGGTATGATTCTCGCTTTGGGTGCGAGAGGTCCCGAGTTCGATTC
TCGGAATGCCCC
>Arabidopsis_thaliana_chr2.trna41-ProTGG (14360735-14360806) Pro (TGG) 72 bp Sc: 73.73
GGGCATTTGGTCTAGTGGTATGATTCTCGCTTTGGGTGCGAGAGGTCCCGAGTTCGATTC
TCGGAATGCCCC
>Arabidopsis_thaliana_chr2.trna42-ProTGG (14361177-14361248) Pro (TGG) 72 bp Sc: 73.73
GGGCATTTGGTCTAGTGGTATGATTCTCGCTTTGGGTGCGAGAGGTCCCGAGTTCGATTC
TCGGAATGCCCC
>Arabidopsis_thaliana_chr2.trna45-ProTGG (14363600-14363671) Pro (TGG) 72 bp Sc: 73.73
GGGCATTTGGTCTAGTGGTATGATTCTCGCTTTGGGTGCGAGAGGTCCCGAGTTCGATTC
TCGGAATGCCCC
>Arabidopsis_thaliana_chr3.trna90-ProTGG (1920110-1920039) Pro (TGG) 72 bp Sc: 73.73
GGGCATTTGGTCTAGTGGTATGATTCTCGCTTTGGGTGCGAGAGGTCCCGAGTTCGATTC
TCGGAATGCCCC
>Arabidopsis_thaliana_chr1.trna31-ProTGG (7082658-7082729) Pro (TGG) 72 bp Sc: 74.34
GGGCGTTTGGTCTAGTGGTATGATTCTCGCTTTGGGTGCGAGAGGTCCCGAGTTCGATTC
TCGGAACGCCCC
>Arabidopsis_thaliana_chr3.trna27-ProTGG (10319499-10319570) Pro (TGG) 72 bp Sc: 74.34

GGGCGTTTGGTCTAGTGGTATGATTCTCGCTTTGGGTGCGAGAGGTCCCGAGTTCGATTC
TCGGAACGCCCC
>Arabidopsis_thaliana_chr3.trna28-ProTGG (10325876-10325947) Pro (TGG) 72 bp Sc: 74.34
GGGCGTTTGGTCTAGTGGTATGATTCTCGCTTTGGGTGCGAGAGGTCCCGAGTTCGATTC
TCGGAACGCCCC
>Arabidopsis_thaliana_chr4.trna29-ProTGG (14470284-14470355) Pro (TGG) 72 bp Sc: 74.34
GGGCGTTTGGTCTAGTGGTATGATTCTCGCTTTGGGTGCGAGAGGTCCCGAGTTCGATTC
TCGGAACGCCCC
>Arabidopsis_thaliana_chr4.trna55-ProTGG (13285503-13285432) Pro (TGG) 72 bp Sc: 74.34
GGGCGTTTGGTCTAGTGGTATGATTCTCGCTTTGGGTGCGAGAGGTCCCGAGTTCGATTC
TCGGAACGCCCC
>Arabidopsis_thaliana_chr5.trna22-ProTGG (6258147-6258218) Pro (TGG) 72 bp Sc: 74.34
GGGCGTTTGGTCTAGTGGTATGATTCTCGCTTTGGGTGCGAGAGGTCCCGAGTTCGATTC
TCGGAACGCCCC
>Arabidopsis_thaliana_chr5.trna46-ProTGG (22973067-22973138) Pro (TGG) 72 bp Sc: 74.34
GGGCGTTTGGTCTAGTGGTATGATTCTCGCTTTGGGTGCGAGAGGTCCCGAGTTCGATTC
TCGGAACGCCCC
>Arabidopsis_thaliana_chr5.trna83-ProTGG (18715960-18715889) Pro (TGG) 72 bp Sc: 74.34
GGGCGTTTGGTCTAGTGGTATGATTCTCGCTTTGGGTGCGAGAGGTCCCGAGTTCGATTC
TCGGAACGCCCC
>Arabidopsis_thaliana_chr1.trna34-ProAGG (10088850-10088921) Pro (AGG) 72 bp Sc: 38.27
GGGCTTTTGGTCTAGTGGTATGATTCTTGCTTAGGGTGCGGGAGGTCCCGAGTTCAATTA
TAGGAATGACTA
>Arabidopsis_thaliana_chr1.trna15-ArgCCG (2751332-2751404) Arg (CCG) 73 bp Sc: 37.42
GTTCGCGTGGCCCAATGGATAAGGCGCTCGCCTCCGGAGGGGGATATTCTCGGTTCGACC
TCCATCGTGAACG
>Arabidopsis_thaliana_chr2.trna10-PheGAA (3437581-3437649) Phe (GAA) 69 bp Sc: 29.25
AGGTAGTAGCTCTGGTAGAGCGAGGGACTGAAAATCCTTCTGTCTGCGGTTCGAATCCGG
ACTCACTTC
>Arabidopsis_thaliana_chr1.trna63-GlyGCC (12083875-12083945) Gly (GCC) 71 bp Sc: 27.76
GCATTAGTCGTCTAGTGGTTGTATTGTAGCCTGCCACTTGACAGTCCAGGGTTCGATTCC
CGGATGGTGCA
>Arabidopsis_thaliana_chr2.trna13-TyrGTA (3489586-3489654) Tyr (GTA) 69 bp Sc: 22.49
AGGTAGTAGCTCTGGTAGAGTGCAGGACTGTAAATCCTTCTGTGAGCGGTTCGAATCCGC
AATCACTTA
>Arabidopsis_thaliana_chr2.trna85-GluTTC (8002594-8002514) Glu (TTC) 81 bp Sc: 32.64
AGATCTGTAAGCTCAAATTGGTAGAGCGCTCGTTTTCTTCTAGTGGACGAGAGGTTGGTG
GTTCGAATCCACCCAGTTCTG
>Arabidopsis_thaliana_chr1.trna173-GluTTC (27691824-27691895) Glu (TTC) 72 bp Sc: 46.41
TCCGATGTCGTCCAGCGGTTAGGATATTTGGCTTTCACCCAGGAGACCCGGGTTCGATTC
TTGGAATGGGAA
>Arabidopsis_thaliana_chr1.trna238-LysTTT (2464959-2464889) Lys (TTT) 71 bp Sc: 28.25
GGTCGCTTAGCTCAGTTGATAGAGCACCACATTTTTTGTGGTAGAAATCGGTTTGAATCC
GATAGCGGCTT
>Arabidopsis_thaliana_chr1.trna149-SerAGA (21306648-21306729) Ser (AGA) 82 bp Sc: 55.83
GTGGACGTGCCGGAGTGGTTATCGGGAATGACTAGAAATCATGGAGGCTTTGCCCGCGCA
CGTTTGAATCTTGCCGTTTACG
>Arabidopsis_thaliana_chr1.trna155-SerAGA (21309720-21309801) Ser (AGA) 82 bp Sc: 55.83
GTGGACGTGCCGGAGTGGTTATCGGGAATGACTAGAAATCATGGAGGCTTTGCCCGCGCA
CGTTTGAATCTTGCCGTTTACG
>Arabidopsis_thaliana_chr1.trna122-SerAGA (21293777-21293858) Ser (AGA) 82 bp Sc: 57.29
GTGGAAGTGCCGGAGTGGTTATCGGGAATGACTAGAAATCATGGGGGCTTTGCCCGCGCA
GGTTTGAATCTTGCCGTTTACG
>Arabidopsis_thaliana_chr1.trna128-SerAGA (21296657-21296738) Ser (AGA) 82 bp Sc: 57.29
GTGGAAGTGCCGGAGTGGTTATCGGGAATGACTAGAAATCATGGGGGCTTTGCCCGCGCA
GGTTTGAATCTTGCCGTTTACG
>Arabidopsis_thaliana_chr1.trna152-SerAGA (21308185-21308268) Ser (AGA) 84 bp Sc: 57.41
GTGGACATGCCGGAGTGGTGTTATCGGGCATAACTAGAAATCATGTGGGCTTTGCCCGCG
CAGGTTCGAATCATGCCGTTTACG
>Arabidopsis_thaliana_chr1.trna158-SerAGA (21311257-21311340) Ser (AGA) 84 bp Sc: 57.41
GTGGACATGCCGGAGTGGTGTTATCGGGCATAACTAGAAATCATGTGGGCTTTGCCCGCG
CAGGTTCGAATCATGCCGTTTACG
>Arabidopsis_thaliana_chr1.trna134-SerAGA (21299537-21299618) Ser (AGA) 82 bp Sc: 61.91
GTGGACGTGCCGGAGTGGTTATCGGGAATGACTAGAAATCATGGGGGCTTTGCCCGCGCA
GGTTTGAATCTTGCCGTTTACG
>Arabidopsis_thaliana_chr1.trna140-SerAGA (21302418-21302499) Ser (AGA) 82 bp Sc: 61.91
GTGGACGTGCCGGAGTGGTTATCGGGAATGACTAGAAATCATGGGGGCTTTGCCCGCGCA

GGTTTGAATCTTGCCGTTTACG
>Arabidopsis_thaliana_chr1.trna146-SerAGA (21305299-21305380) Ser (AGA) 82 bp Sc: 61.91
GTGGACGTGCCGGAGTGGTTATCGGGAATGACTAGAAATCATGGGGGCTTTGCCCGCGCA
GGTTTGAATCTTGCCGTTTACG
>Arabidopsis_thaliana_chr1.trna80-SerAGA (21272452-21272533) Ser (AGA) 82 bp Sc: 75.14
GTGGAAGTGGCGGAGTGGTTATCGGGCATGACTAGAAATCATGTGGGCTTTGCCCGCGCA
GGTTCGAATCCTGCCGTTCACG
>Arabidopsis_thaliana_chr1.trna86-SerAGA (21275470-21275551) Ser (AGA) 82 bp Sc: 75.14
GTGGAAGTGGCGGAGTGGTTATCGGGCATGACTAGAAATCATGTGGGCTTTGCCCGCGCA
GGTTCGAATCCTGCCGTTCACG
>Arabidopsis_thaliana_chr1.trna101-SerAGA (21283055-21283136) Ser (AGA) 82 bp Sc: 76.22
GTGGACGTGCCGGAGTGGTTATCGGGCATAACTAGAAATCATGTGGGCTTTGCCCGCGCA
GGTTCGAATCCTGCCGTTCACG
>Arabidopsis_thaliana_chr1.trna92-SerAGA (21278488-21278569) Ser (AGA) 82 bp Sc: 79.70
GTGGACGTGCCGGAGTGGTTATCGGGCATGACTAGAAATCATGTGGGTTTTGCCCGCGCA
GGTTCGAATCCTGCCGTTCACG
>Arabidopsis_thaliana_chr1.trna98-SerAGA (21281526-21281607) Ser (AGA) 82 bp Sc: 79.70
GTGGACGTGCCGGAGTGGTTATCGGGCATGACTAGAAATCATGTGGGTTTTGCCCGCGCA
GGTTCGAATCCTGCCGTTCACG
>Arabidopsis_thaliana_chr3.trna4-SerAGA (586093-586174) Ser (AGA) 82 bp Sc: 81.73
GTGGACGTGCCGGAGTGGTTATCGGGCATGACTAGAAATCATGTGGGCTCTGCCCGCGCA
GGTTCGAATCCTGCCGTTCACG
>Arabidopsis_thaliana_chr1.trna104-SerAGA (21284587-21284668) Ser (AGA) 82 bp Sc: 82.22
GTGGACGTGCCGGAGTGGTTATCGGGCATGACTAGAAATCATGTGGGCTTTGCCCGCGCA
GGTTCGAATCCTGCCGTTCACG
>Arabidopsis_thaliana_chr1.trna107-SerAGA (21286119-21286200) Ser (AGA) 82 bp Sc: 82.22
GTGGACGTGCCGGAGTGGTTATCGGGCATGACTAGAAATCATGTGGGCTTTGCCCGCGCA
GGTTCGAATCCTGCCGTTCACG
>Arabidopsis_thaliana_chr1.trna110-SerAGA (21287651-21287732) Ser (AGA) 82 bp Sc: 82.22
GTGGACGTGCCGGAGTGGTTATCGGGCATGACTAGAAATCATGTGGGCTTTGCCCGCGCA
GGTTCGAATCCTGCCGTTCACG
>Arabidopsis_thaliana_chr1.trna113-SerAGA (21289184-21289265) Ser (AGA) 82 bp Sc: 82.22
GTGGACGTGCCGGAGTGGTTATCGGGCATGACTAGAAATCATGTGGGCTTTGCCCGCGCA
GGTTCGAATCCTGCCGTTCACG
>Arabidopsis_thaliana_chr1.trna116-SerAGA (21290715-21290796) Ser (AGA) 82 bp Sc: 82.22
GTGGACGTGCCGGAGTGGTTATCGGGCATGACTAGAAATCATGTGGGCTTTGCCCGCGCA
GGTTCGAATCCTGCCGTTCACG
>Arabidopsis_thaliana_chr1.trna119-SerAGA (21292246-21292327) Ser (AGA) 82 bp Sc: 82.22
GTGGACGTGCCGGAGTGGTTATCGGGCATGACTAGAAATCATGTGGGCTTTGCCCGCGCA
GGTTCGAATCCTGCCGTTCACG
>Arabidopsis_thaliana_chr1.trna125-SerAGA (21295126-21295207) Ser (AGA) 82 bp Sc: 82.22
GTGGACGTGCCGGAGTGGTTATCGGGCATGACTAGAAATCATGTGGGCTTTGCCCGCGCA
GGTTCGAATCCTGCCGTTCACG
>Arabidopsis_thaliana_chr1.trna131-SerAGA (21298006-21298087) Ser (AGA) 82 bp Sc: 82.22
GTGGACGTGCCGGAGTGGTTATCGGGCATGACTAGAAATCATGTGGGCTTTGCCCGCGCA
GGTTCGAATCCTGCCGTTCACG
>Arabidopsis_thaliana_chr1.trna137-SerAGA (21300886-21300967) Ser (AGA) 82 bp Sc: 82.22
GTGGACGTGCCGGAGTGGTTATCGGGCATGACTAGAAATCATGTGGGCTTTGCCCGCGCA
GGTTCGAATCCTGCCGTTCACG
>Arabidopsis_thaliana_chr1.trna143-SerAGA (21303767-21303848) Ser (AGA) 82 bp Sc: 82.22
GTGGACGTGCCGGAGTGGTTATCGGGCATGACTAGAAATCATGTGGGCTTTGCCCGCGCA
GGTTCGAATCCTGCCGTTCACG
>Arabidopsis_thaliana_chr1.trna172-SerAGA (27397047-27397128) Ser (AGA) 82 bp Sc: 82.22
GTGGACGTGCCGGAGTGGTTATCGGGCATGACTAGAAATCATGTGGGCTTTGCCCGCGCA
GGTTCGAATCCTGCCGTTCACG
>Arabidopsis_thaliana_chr1.trna184-SerAGA (30242676-30242757) Ser (AGA) 82 bp Sc: 82.22
GTGGACGTGCCGGAGTGGTTATCGGGCATGACTAGAAATCATGTGGGCTTTGCCCGCGCA
GGTTCGAATCCTGCCGTTCACG
>Arabidopsis_thaliana_chr1.trna196-SerAGA (24490736-24490655) Ser (AGA) 82 bp Sc: 82.22
GTGGACGTGCCGGAGTGGTTATCGGGCATGACTAGAAATCATGTGGGCTTTGCCCGCGCA
GGTTCGAATCCTGCCGTTCACG
>Arabidopsis_thaliana_chr1.trna205-SerAGA (21887589-21887508) Ser (AGA) 82 bp Sc: 82.22
GTGGACGTGCCGGAGTGGTTATCGGGCATGACTAGAAATCATGTGGGCTTTGCCCGCGCA
GGTTCGAATCCTGCCGTTCACG
>Arabidopsis_thaliana_chr1.trna24-SerAGA (5380902-5380983) Ser (AGA) 82 bp Sc: 82.22
GTGGACGTGCCGGAGTGGTTATCGGGCATGACTAGAAATCATGTGGGCTTTGCCCGCGCA
GGTTCGAATCCTGCCGTTCACG

>Arabidopsis_thaliana_chr1.trna83-SerAGA (21273961-21274042) Ser (AGA) 82 bp Sc: 82.22
GTGGACGTGCCGGAGTGGTTATCGGGCATGACTAGAAATCATGTGGGCTTTGCCCGCGCA
GGTTCGAATCCTGCCGTTCACG
>Arabidopsis_thaliana_chr1.trna89-SerAGA (21276979-21277060) Ser (AGA) 82 bp Sc: 82.22
GTGGACGTGCCGGAGTGGTTATCGGGCATGACTAGAAATCATGTGGGCTTTGCCCGCGCA
GGTTCGAATCCTGCCGTTCACG
>Arabidopsis_thaliana_chr1.trna95-SerAGA (21280017-21280098) Ser (AGA) 82 bp Sc: 82.22
GTGGACGTGCCGGAGTGGTTATCGGGCATGACTAGAAATCATGTGGGCTTTGCCCGCGCA
GGTTCGAATCCTGCCGTTCACG
>Arabidopsis_thaliana_chr5.trna20-SerAGA (5358919-5359000) Ser (AGA) 82 bp Sc: 82.22
GTGGACGTGCCGGAGTGGTTATCGGGCATGACTAGAAATCATGTGGGCTTTGCCCGCGCA
GGTTCGAATCCTGCCGTTCACG
>Arabidopsis_thaliana_chr5.trna41-SerAGA (20773556-20773637) Ser (AGA) 82 bp Sc: 82.22
GTGGACGTGCCGGAGTGGTTATCGGGCATGACTAGAAATCATGTGGGCTTTGCCCGCGCA
GGTTCGAATCCTGCCGTTCACG
>Arabidopsis_thaliana_chr3.trna36-SerAGA (17275565-17275646) Ser (AGA) 82 bp Sc: 82.83
GTGGGCGTGCCGGAGTGGTTATCGGGCATGACTAGAAATCATGTGGGCTTTGCCCGCGCA
GGTTCGAATCCTGCCGCTCACG
>Arabidopsis_thaliana_chr1.trna25-SerAGA (6038750-6038831) Ser (AGA) 82 bp Sc: 84.50
GTAGGCGTGCCGGAGTGGTTATCGGGCATGACTAGAAATCATGTGGGCTTTGCCCGCGCA
GGTTCGAATCCTGCCGCCTACG
>Arabidopsis_thaliana_chr5.trna48-SerCGA (23661937-23662018) Ser (CGA) 82 bp Sc: 81.35
GTCAATATGTCCGAGTGGTTAAGGAGACAGACTCGAAATCTGTTGGGCTTTGCCTGCGCA
GGTTCGAATCCTGCTGTTGACG
>Arabidopsis_thaliana_chr5.trna2-SerCGA (614642-614723) Ser (CGA) 82 bp Sc: 85.19
GTCGATATGTCCGAGTGGTTAAGGAGATTGACTCGAAATCAATTGGGCTTTGCCCGCGCA
GGTTCGAATCCTGCTGTCGACG
>Arabidopsis_thaliana_chr3.trna11-SerCGA (2944617-2944698) Ser (CGA) 82 bp Sc: 85.25
GTCAATATGTCCGAGTGGTTAAGGAGATTGACTCGAAATCAATTGGGCTTTGCCCGCGCA
GGTTCGAATCCTGCTGTTGACG
>Arabidopsis_thaliana_chr4.trna34-SerCGA (15804554-15804635) Ser (CGA) 82 bp Sc: 85.25
GTCAATATGTCCGAGTGGTTAAGGAGATTGACTCGAAATCAATTGGGCTTTGCCCGCGCA
GGTTCGAATCCTGCTGTTGACG
>Arabidopsis_thaliana_chr3.trna65-SerGCT (22197892-22197811) Ser (GCT) 82 bp Sc: 69.54
GTCGCTTTGGCCGAGTGGTTAAGGCGTGTTCCTGCTAAGTACATGGGGTTTCCCCGCGAG
AGTTCGAATCTGTCAGGCGACG
>Arabidopsis_thaliana_chr3.trna78-SerGCT (7319664-7319583) Ser (GCT) 82 bp Sc: 76.07
GTCGCTTTGGCCGAGTGGTTAAGGCGTGTGCCTGCTAAGTACATGGGATTTTCCCGCGAG
AGTTCGAATCTCTCAGGCGACG
>Arabidopsis_thaliana_chr5.trna69-SerGCT (26174496-26174415) Ser (GCT) 82 bp Sc: 78.42
GTCGCTTTGGCCGAGTGGTTAAGGCGCTCGCCTGCTAAGTGAGTGGGATTTTCCCGCGAG
AGTTCGAATCTCTCAGGCGACG
>Arabidopsis_thaliana_chr3.trna50-SerGCT (21498294-21498375) Ser (GCT) 82 bp Sc: 79.46
GACACTTTGGCCGAGTGGTTAAGGCGTTTGCCTGCTAAGTAAATGGGGTTTCCCCGCGAG
AGTTCGAATCTCTCAGGTGTCG
>Arabidopsis_thaliana_chr2.trna61-SerGCT (18317134-18317053) Ser (GCT) 82 bp Sc: 79.53
GTCGCTTTGGCCGAGTGGTTAAGGCGTGTGCCTGCTAAGTACATGGGGTTTCCCCGCGAG
AGTTCGAATCTCTCAGGCGACG
>Arabidopsis_thaliana_chr4.trna28-SerGCT (14267287-14267368) Ser (GCT) 82 bp Sc: 79.53
GTCGCTTTGGCCGAGTGGTTAAGGCGTGTGCCTGCTAAGTACATGGGGTTTCCCCGCGAG
AGTTCGAATCTCTCAGGCGACG
>Arabidopsis_thaliana_chr5.trna106-SerGCT (3287635-3287554) Ser (GCT) 82 bp Sc: 79.53
GTCGCTTTGGCCGAGTGGTTAAGGCGTGTGCCTGCTAAGTACATGGGGTTTCCCCGCGAG
AGTTCGAATCTCTCAGGCGACG
>Arabidopsis_thaliana_chr1.trna189-SerGCT (29007180-29007099) Ser (GCT) 82 bp Sc: 80.11
GTCGCTTTGGCCGAGTGGTTAAGGCGTGTGCCTGCTAAGTACATGGGCTCTGCCCGCGAG
AGTTCGAATCTCTCAGGCGACG
>Arabidopsis_thaliana_chr4.trna24-SerGCT (12686149-12686230) Ser (GCT) 82 bp Sc: 80.11
GTCGCTTTGGCCGAGTGGTTAAGGCGTGTGCCTGCTAAGTACATGGGCTCTGCCCGCGAG
AGTTCGAATCTCTCAGGCGACG
>Arabidopsis_thaliana_chr2.trna63-SerGCT (17545862-17545781) Ser (GCT) 82 bp Sc: 81.30
GTCGCTTTGGCCGAGTGGTTAAGGCGTTTGCCTGCTAAGTAAATGGGGTTTCCCCGCGAG
AGTTCGAATCTCTCAGGCGACG
>Arabidopsis_thaliana_chr5.trna114-SerGCT (2060235-2060154) Ser (GCT) 82 bp Sc: 81.30
GTCGCTTTGGCCGAGTGGTTAAGGCGTTTGCCTGCTAAGTAAATGGGGTTTCCCCGCGAG
AGTTCGAATCTCTCAGGCGACG
>Arabidopsis_thaliana_chr2.trna91-SerGCT (3303819-3303732) Ser (GCT) 88 bp Sc: 40.19

GGAGGGATGGCTGAGTGGCTTAAGGCATTGGTTTGCTAAATCGACATACAAGAAGATTGT
ATCATGGGTTCGAATCCCATTTCCTCCG
>Arabidopsis_thaliana_chr2.trna14-SerGCT (3494379-3494466) Ser (GCT) 88 bp Sc: 40.19
GGAGGGATGGCTGAGTGGCTTAAGGCATTGGTTTGCTAAATCGACATACAAGAAGATTGT
ATCATGGGTTCGAATCCCATTTCCTCCG
>Arabidopsis_thaliana_chr2.trna11-SerGGA (3452974-3453060) Ser (GGA) 87 bp Sc: 58.88
GGAGAGATGGCCGAGTGGTTTAAGGCGTAGCATTGGAACTGCTATGTAGGCTTTTGTTTA
CCGAGGGTTCGAATCCCTCTCTTTCCG
>Arabidopsis_thaliana_chr2.trna20-SerTGA (3503519-3503605) Ser (TGA) 87 bp Sc: 56.81
GGATGGATGTCTGAGCGGTTGAAAGAGTCGGTCTTGAAAACCGAAGTATTTCTAGGAATA
CCGGGGGTTCGAATCCCTCTCCATCCG
>Arabidopsis_thaliana_chr2.trna9-SerTGA (3369351-3369437) Ser (TGA) 87 bp Sc: 56.81
GGATGGATGTCTGAGCGGTTGAAAGAGTCGGTCTTGAAAACCGAAGTATTTCTAGGAATA
CCGGGGGTTCGAATCCCTCTCCATCCG
>Arabidopsis_thaliana_chr1.trna79-SerTGA (20794977-20795058) Ser (TGA) 82 bp Sc: 80.82
GTCGATATGTCCGAGTGGTTAAGGAGACAGACTTGAAATCTGTTGGGCTTCGCCCGCGCA
GGTTCGAACCCTGCTGTCGACG
>Arabidopsis_thaliana_chr4.trna57-SerTGA (13058922-13058841) Ser (TGA) 82 bp Sc: 80.82
GTCGATATGTCCGAGTGGTTAAGGAGACAGACTTGAAATCTGTTGGGCTTCGCCCGCGCA
GGTTCGAACCCTGCTGTCGACG
>Arabidopsis_thaliana_chr5.trna80-SerTGA (21275141-21275060) Ser (TGA) 82 bp Sc: 80.82
GTCGATATGTCCGAGTGGTTAAGGAGACAGACTTGAAATCTGTTGGGCTTCGCCCGCGCA
GGTTCGAACCCTGCTGTCGACG
>Arabidopsis_thaliana_chr3.trna87-SerTGA (2253762-2253681) Ser (TGA) 82 bp Sc: 80.89
GTCAATATGTCCGAGTGGTTAAGGAGACAGACTTGAAATCTGTTGGGCTTCGCCCGCGCA
GGTTCGAACCCTGCTGTTGACG
>Arabidopsis_thaliana_chr3.trna85-SerTGA (2945782-2945701) Ser (TGA) 82 bp Sc: 81.08
GTCGATATGTCCGAGTGGTTAAGGAGATAGACTTGAAATCTATTGGGCTTCGCCCGCGCA
GGTTCGAACCCTGCTGTCGACG
>Arabidopsis_thaliana_chr5.trna118-SerTGA (642479-642398) Ser (TGA) 82 bp Sc: 81.08
GTCGATATGTCCGAGTGGTTAAGGAGATAGACTTGAAATCTATTGGGCTTCGCCCGCGCA
GGTTCGAACCCTGCTGTCGACG
>Arabidopsis_thaliana_chr1.trna180-SerTGA (28639586-28639667) Ser (TGA) 82 bp Sc: 81.15
GTCAATATGTCCGAGTGGTTAAGGAGATAGACTTGAAATCTATTGGGCTTCGCCCGCGCA
GGTTCGAACCCTGCTGTTGACG
>Arabidopsis_thaliana_chr1.trna211-ThrAGT (18234220-18234147) Thr (AGT) 74 bp Sc: 70.04
GCTCTCGTAGCTCAGTTGGTTAGAGCACCCGTTTAGTAAGCGGGAGGTCTTGAGTTCAAC
TCTCAACGAAAGCA
>Arabidopsis_thaliana_chr4.trna37-ThrAGT (16655291-16655364) Thr (AGT) 74 bp Sc: 70.04
GCTCTCGTAGCTCAGTTGGTTAGAGCACCCGTTTAGTAAGCGGGAGGTCTTGAGTTCAAC
TCTCAACGAAAGCA
>Arabidopsis_thaliana_chr2.trna69-ThrAGT (14750627-14750554) Thr (AGT) 74 bp Sc: 74.28
GCTTTCATAGCTCAGTTGGTTAGAGCACCCGTTTAGTAAGCGGGAGGTCTTGAGTTCAAC
TCTCAATGAAAGCA
>Arabidopsis_thaliana_chr4.trna17-ThrAGT (9287231-9287304) Thr (AGT) 74 bp Sc: 74.28
GCTTTCATAGCTCAGTTGGTTAGAGCACCCGTTTAGTAAGCGGGAGGTCTTGAGTTCAAC
TCTCAATGAAAGCA
>Arabidopsis_thaliana_chr4.trna49-ThrAGT (15430303-15430230) Thr (AGT) 74 bp Sc: 74.28
GCTTTCATAGCTCAGTTGGTTAGAGCACCCGTTTAGTAAGCGGGAGGTCTTGAGTTCAAC
TCTCAATGAAAGCA
>Arabidopsis_thaliana_chr4.trna62-ThrAGT (10878807-10878734) Thr (AGT) 74 bp Sc: 74.28
GCTTTCATAGCTCAGTTGGTTAGAGCACCCGTTTAGTAAGCGGGAGGTCTTGAGTTCAAC
TCTCAATGAAAGCA
>Arabidopsis_thaliana_chr5.trna33-ThrAGT (18058015-18058088) Thr (AGT) 74 bp Sc: 74.28
GCTTTCATAGCTCAGTTGGTTAGAGCACCCGTTTAGTAAGCGGGAGGTCTTGAGTTCAAC
TCTCAATGAAAGCA
>Arabidopsis_thaliana_chr3.trna80-ThrAGT (6484019-6483946) Thr (AGT) 74 bp Sc: 75.17
GCTCTCGTAGCTCAGTTGGTTAGAGCACCCGTTTAGTAAGCGGGAGGTCTTGAGTTCAAC
TCTCAACGAGAGCA
>Arabidopsis_thaliana_chr4.trna15-ThrAGT (9108318-9108391) Thr (AGT) 74 bp Sc: 75.17
GCTCTCGTAGCTCAGTTGGTTAGAGCACCCGTTTAGTAAGCGGGAGGTCTTGAGTTCAAC
TCTCAACGAGAGCA
>Arabidopsis_thaliana_chr4.trna68-ThrAGT (8301794-8301721) Thr (AGT) 74 bp Sc: 75.17
GCTCTCGTAGCTCAGTTGGTTAGAGCACCCGTTTAGTAAGCGGGAGGTCTTGAGTTCAAC
TCTCAACGAGAGCA
>Arabidopsis_thaliana_chr2.trna46-ThrCGT (14757143-14757213) Thr (CGT) 71 bp Sc: 22.63
CTTAGGATGGCCGAGTGGACACAAGTTCTGGTCCTCGTAAGAGGGTGTGGGTTCAAACCC

CACTTCTGACA
>Arabidopsis_thaliana_chr2.trna97-ThrCGT (1114052-1113981) Thr (CGT) 72 bp Sc: 77.36
GCCTCCGTAGCATAGTGGTATTGCGTTCGCTTCGTAAGCGAAAGGTCGTGAGTTCGATCC
TCACCGTAGGCT
>Arabidopsis_thaliana_chr2.trna96-ThrCGT (1123458-1123387) Thr (CGT) 72 bp Sc: 79.41
GCCTCTGTAGCATAGTGGTATTGCGTTCGCTTCGTAAGCGAAAGGCCGCGAGTTCGATCC
TCGCCGGAGGCT
>Arabidopsis_thaliana_chr1.trna178-ThrCGT (28444550-28444621) Thr (CGT) 72 bp Sc: 80.56
GCCTCCGTAGCATAGTGGTATTGCGTTCGCTTCGTAAGCGAAAGGCCGCGAGTTCGATCC
TCGCCGGGGGCT
>Arabidopsis_thaliana_chr1.trna21-ThrCGT (4709632-4709703) Thr (CGT) 72 bp Sc: 80.56
GCCTCCGTAGCATAGTGGTATTGCGTTCGCTTCGTAAGCGAAAGGCCGCGAGTTCGATCC
TCGCCGGGGGCT
>Arabidopsis_thaliana_chr5.trna54-ThrCGT (24548893-24548964) Thr (CGT) 72 bp Sc: 80.56
GCCTCCGTAGCATAGTGGTATTGCGTTCGCTTCGTAAGCGAAAGGCCGCGAGTTCGATCC
TCGCCGGGGGCT
>Arabidopsis_thaliana_chr3.trna52-ThrTGT (21970487-21970557) Thr (TGT) 71 bp Sc: 67.75
GCCCGTATAGCTCAGTGGTAGAGCGTCAGTCTTGTAAACTGAAGGTCCGTAGTTCGATCC
TGCGTGTGGCA
>Arabidopsis_thaliana_chr2.trna62-ThrTGT (18002721-18002650) Thr (TGT) 72 bp Sc: 73.80
GCCCGTATAGCTCAGTGGTAGAGCGTCAGTCTTGTAAACTGAAGGTCCGTAGTTCTATCC
TGCGTGTGGGCA
>Arabidopsis_thaliana_chr1.trna9-ThrTGT (1817399-1817470) Thr (TGT) 72 bp Sc: 80.35
GCCCGTATAGCTCAGTGGTAGAGCGTCAGTCTTGTAAACTGAAGGTCCGTAGTTCGATCC
TGCGTGTGGGCA
>Arabidopsis_thaliana_chr4.trna4-ThrTGT (1442005-1442076) Thr (TGT) 72 bp Sc: 80.35
GCCCGTATAGCTCAGTGGTAGAGCGTCAGTCTTGTAAACTGAAGGTCCGTAGTTCGATCC
TGCGTGTGGGCA
>Arabidopsis_thaliana_chr4.trna8-ThrTGT (6944722-6944793) Thr (TGT) 72 bp Sc: 80.35
GCCCGTATAGCTCAGTGGTAGAGCGTCAGTCTTGTAAACTGAAGGTCCGTAGTTCGATCC
TGCGTGTGGGCA
>Arabidopsis_thaliana_chr5.trna58-ThrTGT (25347262-25347333) Thr (TGT) 72 bp Sc: 80.35
GCCCGTATAGCTCAGTGGTAGAGCGTCAGTCTTGTAAACTGAAGGTCCGTAGTTCGATCC
TGCGTGTGGGCA
>Arabidopsis_thaliana_chr1.trna197-ThrTGT (23671219-23671148) Thr (TGT) 72 bp Sc: 81.19
GCCCTTATAGCTCAGTGGTAGAGCGTCAGTCTTGTAAACTGAAGGTCCGTAGTTCGATCC
TGCGTGAGGGCA
>Arabidopsis_thaliana_chr3.trna37-ThrTGT (17905396-17905467) Thr (TGT) 72 bp Sc: 81.19
GCCCTTATAGCTCAGTGGTAGAGCGTCAGTCTTGTAAACTGAAGGTCCGTAGTTCGATCC
TGCGTGAGGGCA
>Arabidopsis_thaliana_chr2.trna6-TrpCCA (3280860-3280933) Trp (CCA) 74 bp Sc: 67.55
GCGCTCTTAGTTCAGTTCGGTAGAACGTGGGTCTCCAAAACCCAATGTCGTAGGTTCAAA
TCCTACAGAGCGTG
>Arabidopsis_thaliana_chr5.trna21-TrpCCA (5984379-5984450) Trp (CCA) 72 bp Sc: 70.65
GGATCCGTGGCGCAATGGTAGCGCGTCTGACTCCAGATCAGAAGGTTGCTTGTTCGATTC
ACGTCGGGTTCA
>Arabidopsis_thaliana_chr1.trna223-TrpCCA (7242821-7242750) Trp (CCA) 72 bp Sc: 75.00
GGATTCGTGGCGCAATGGTAGCGCGTCTGACTCCAGATCAGAAGGTTGCGTGTTCGATTC
ACGTCGGGTTCA
>Arabidopsis_thaliana_chr1.trna185-TrpCCA (30178977-30178906) Trp (CCA) 72 bp Sc: 77.63
GGATCCGTGGCGCAATGGTAGCGCGTCTGACTCCAGATCAGAAGGTTGCGTGTTCGATTC
ACGTCGGGTTCA
>Arabidopsis_thaliana_chr1.trna199-TrpCCA (22870494-22870423) Trp (CCA) 72 bp Sc: 77.63
GGATCCGTGGCGCAATGGTAGCGCGTCTGACTCCAGATCAGAAGGTTGCGTGTTCGATTC
ACGTCGGGTTCA
>Arabidopsis_thaliana_chr1.trna229-TrpCCA (5309713-5309642) Trp (CCA) 72 bp Sc: 77.63
GGATCCGTGGCGCAATGGTAGCGCGTCTGACTCCAGATCAGAAGGTTGCGTGTTCGATTC
ACGTCGGGTTCA
>Arabidopsis_thaliana_chr1.trna234-TrpCCA (3914700-3914629) Trp (CCA) 72 bp Sc: 77.63
GGATCCGTGGCGCAATGGTAGCGCGTCTGACTCCAGATCAGAAGGTTGCGTGTTCGATTC
ACGTCGGGTTCA
>Arabidopsis_thaliana_chr3.trna5-TrpCCA (981976-982047) Trp (CCA) 72 bp Sc: 77.63
GGATCCGTGGCGCAATGGTAGCGCGTCTGACTCCAGATCAGAAGGTTGCGTGTTCGATTC
ACGTCGGGTTCA
>Arabidopsis_thaliana_chr3.trna6-TrpCCA (984106-984177) Trp (CCA) 72 bp Sc: 77.63
GGATCCGTGGCGCAATGGTAGCGCGTCTGACTCCAGATCAGAAGGTTGCGTGTTCGATTC
ACGTCGGGTTCA

>Arabidopsis_thaliana_chr4.trna41-TrpCCA (18395022-18395093) Trp (CCA) 72 bp Sc: 77.63
GGATCCGTGGCGCAATGGTAGCGCGTCTGACTCCAGATCAGAAGGTTGCGTGTTCGATTC
ACGTCGGGTTCA
>Arabidopsis_thaliana_chr4.trna66-TrpCCA (9873596-9873525) Trp (CCA) 72 bp Sc: 77.63
GGATCCGTGGCGCAATGGTAGCGCGTCTGACTCCAGATCAGAAGGTTGCGTGTTCGATTC
ACGTCGGGTTCA
>Arabidopsis_thaliana_chr4.trna67-TrpCCA (9815287-9815216) Trp (CCA) 72 bp Sc: 77.63
GGATCCGTGGCGCAATGGTAGCGCGTCTGACTCCAGATCAGAAGGTTGCGTGTTCGATTC
ACGTCGGGTTCA
>Arabidopsis_thaliana_chr5.trna102-TrpCCA (5966044-5965973) Trp (CCA) 72 bp Sc: 77.63
GGATCCGTGGCGCAATGGTAGCGCGTCTGACTCCAGATCAGAAGGTTGCGTGTTCGATTC
ACGTCGGGTTCA
>Arabidopsis_thaliana_chr5.trna103-TrpCCA (5962771-5962700) Trp (CCA) 72 bp Sc: 77.63
GGATCCGTGGCGCAATGGTAGCGCGTCTGACTCCAGATCAGAAGGTTGCGTGTTCGATTC
ACGTCGGGTTCA
>Arabidopsis_thaliana_chr2.trna8-TyrGTA (3332675-3332756) Tyr (GTA) 82 bp Sc: 32.67
GGGAAAGGGGAGAGTGGTCAAAAGCGGCAGACTGTAAATCTGTTGAAGTTTTTCTACGTA
GGTTCGAATCCTGCCTCTCCCA
>Arabidopsis_thaliana_chr2.trna4-TyrGTA (3268804-3268883) Tyr (GTA) 80 bp Sc: 39.02
TCACAGGTAGCTCAGATGGTTAGAGCAAAGGACTGTAAATCCTTGTGTTAGTGGTTCGAT
TCCACAACCACTTCTATGCG
>Arabidopsis_thaliana_chr2.trna15-TyrGTA (3494773-3494847) Tyr (GTA) 75 bp Sc: 52.74
AAGTGGTTCAGCTCAGCTGGTTAGAGCAAAGGACTGTAAATCCTTGTGTCAGTGGTTCGA
ATCCACAACCACTTC
>Arabidopsis_thaliana_chr2.trna92-TyrGTA (3303425-3303351) Tyr (GTA) 75 bp Sc: 52.74
AAGTGGTTCAGCTCAGCTGGTTAGAGCAAAGGACTGTAAATCCTTGTGTCAGTGGTTCGA
ATCCACAACCACTTC
>Arabidopsis_thaliana_chr3.trna24-TyrGTA (7104315-7104398) Tyr (GTA) 84 bp Sc: 53.74
GGGTCGATGCCCGAGCGGTTAATGGGGACGGACTGTAAATTCGTTGGCAATATGTCTACG
CTGGTTCAAATCCAGCTCGGCCCA
>Arabidopsis_thaliana_chr2.trna19-TyrGTA (3497355-3497437) Tyr (GTA) 83 bp Sc: 56.98
GGGAGAGTGGCCGAGTGGTCAAAAGCGGCAGACTGTAAATCTGTTGAAGTTTTTCTACGT
AGGTTCGAATCCTGCCTCTCCCA
>Arabidopsis_thaliana_chr4.trna61-TyrGTA (11774558-11774473) Tyr (GTA) 86 bp Sc: 77.17
CCGACCTTAGCTCAGTTGGTAGAGCGGAAGACTGTAGTCTTTGACAGATAATCTTTAGGT
CGCTGGTTCGATTCCGGCAGGTCGGA
>Arabidopsis_thaliana_chr2.trna68-TyrGTA (15215517-15215434) Tyr (GTA) 84 bp Sc: 76.77
CCGACCTTAGCTCAGTTGGTAGAGCGGAAGACTGTAGTAGTTGCTGTAATCTTTAGGTCG
CTGGTTCGATTCCGGCAGGTCGGA
>Arabidopsis_thaliana_chr2.trna66-TyrGTA (15477324-15477241) Tyr (GTA) 84 bp Sc: 76.91
CCGACCTTAGCTCAGTTGGTAGAGCGGAAGACTGTAGTAGTAGCTGTAATCTTTAGGTCG
CTGGTTCGATTCCGGCAGGTCGGA
>Arabidopsis_thaliana_chr3.trna73-TyrGTA (17985661-17985576) Tyr (GTA) 86 bp Sc: 76.04
CCGACCTTAGCTCAGTTGGTAGAGCGGAAGACTGTAGTCTGTGACAGCAAATCTTTAGGT
CGCTGGTTCGATTCCGGCAGGTCGGA
>Arabidopsis_thaliana_chr4.trna45-TyrGTA (18255066-18254983) Tyr (GTA) 84 bp Sc: 76.77
CCGACCTTAGCTCAGTTGGTAGAGCGGAAGACTGTAGTAGTTGCTGTAATCTTTAGGTCG
CTGGTTCGATTCCGGCAGGTCGGA
>Arabidopsis_thaliana_chr1.trna73-TyrGTA (18312571-18312655) Tyr (GTA) 85 bp Sc: 76.40
CCGACCTTAGCTCAGTTGGTAGAGCGGAAGACTGTAGTGTTTGCAGACAATCTTTAGGTC
GCTGGTTCGATTCCGGCAGGTCGGA
>Arabidopsis_thaliana_chr1.trna209-TyrGTA (19688887-19688802) Tyr (GTA) 86 bp Sc: 77.17
CCGACCTTAGCTCAGTTGGTAGAGCGGAAGACTGTAGTCTTTGACAGATAATCTTTAGGT
CGCTGGTTCGATTCCGGCAGGTCGGA
>Arabidopsis_thaliana_chr5.trna81-TyrGTA (20758940-20758852) Tyr (GTA) 89 bp Sc: 77.34
CCGACCTTAGCTCAGTTGGTAGAGCGGAAGACTGTAGTTGTTTGCAACAGAAAATCTTTA
GGTCGCTGGTTCGATTCCGGCAGGTCGGA
>Arabidopsis_thaliana_chr1.trna81-TyrGTA (21272787-21272871) Tyr (GTA) 85 bp Sc: 68.66
CCGACCTTAGCTCAGTTGGTAGAGCGGAGGACTGTAGTTGACGCAGATTATCCTTAGGTC
ATTGGTTCGAATCCGCTAGGTCGGA
>Arabidopsis_thaliana_chr1.trna82-TyrGTA (21273217-21273301) Tyr (GTA) 85 bp Sc: 74.91
CCGACCTTAGCTCAGTTGGTAGAGCGGAGGACTGTAGTAGACGCAGATTATCCTTAGGTC
ACTGGTTCGAATCCGGTAGGTCGGA
>Arabidopsis_thaliana_chr1.trna84-TyrGTA (21274296-21274380) Tyr (GTA) 85 bp Sc: 68.66
CCGACCTTAGCTCAGTTGGTAGAGCGGAGGACTGTAGTTGACGCAGATTATCCTTAGGTC
ATTGGTTCGAATCCGCTAGGTCGGA
>Arabidopsis_thaliana_chr1.trna85-TyrGTA (21274726-21274810) Tyr (GTA) 85 bp Sc: 74.91

CCGACCTTAGCTCAGTTGGTAGAGCGGAGGACTGTAGTAGACGCAGATTATCCTTAGGTC
ACTGGTTCGAATCCGGTAGGTCGGA
>Arabidopsis_thaliana_chr1.trna87-TyrGTA (21275805-21275889) Tyr (GTA) 85 bp Sc: 68.66
CCGACCTTAGCTCAGTTGGTAGAGCGGAGGACTGTAGTTGACGCAGATTATCCTTAGGTC
ATTGGTTCGAATCCGCTAGGTCGGA
>Arabidopsis_thaliana_chr1.trna88-TyrGTA (21276235-21276319) Tyr (GTA) 85 bp Sc: 74.91
CCGACCTTAGCTCAGTTGGTAGAGCGGAGGACTGTAGTAGACGCAGATTATCCTTAGGTC
ACTGGTTCGAATCCGGTAGGTCGGA
>Arabidopsis_thaliana_chr1.trna90-TyrGTA (21277314-21277398) Tyr (GTA) 85 bp Sc: 68.66
CCGACCTTAGCTCAGTTGGTAGAGCGGAGGACTGTAGTTGACGCAGATTATCCTTAGGTC
ATTGGTTCGAATCCGCTAGGTCGGA
>Arabidopsis_thaliana_chr1.trna91-TyrGTA (21277744-21277828) Tyr (GTA) 85 bp Sc: 74.91
CCGACCTTAGCTCAGTTGGTAGAGCGGAGGACTGTAGTAGACGCAGATTATCCTTAGGTC
ACTGGTTCGAATCCGGTAGGTCGGA
>Arabidopsis_thaliana_chr1.trna93-TyrGTA (21278823-21278907) Tyr (GTA) 85 bp Sc: 74.76
CCGACCTTAGCTCAGTTGGTAGAGCGGAGGACTGTAGTTGACGCAGATTATCCTTAGGTC
ACTGGTTCGAATCCGGTAGGTCGGA
>Arabidopsis_thaliana_chr1.trna94-TyrGTA (21279274-21279358) Tyr (GTA) 85 bp Sc: 75.54
CCGACCTTAGCTCAGTTGGTAGAGCGGAGGACTGTAGTAGACGTAGATTATCCTTAGGTC
ACTGGTTCGAATCCGGTAGGTCGGA
>Arabidopsis_thaliana_chr1.trna96-TyrGTA (21280352-21280436) Tyr (GTA) 85 bp Sc: 68.66
CCGACCTTAGCTCAGTTGGTAGAGCGGAGGACTGTAGTTGACGCAGATTATCCTTAGGTC
ATTGGTTCGAATCCGCTAGGTCGGA
>Arabidopsis_thaliana_chr1.trna97-TyrGTA (21280782-21280866) Tyr (GTA) 85 bp Sc: 74.91
CCGACCTTAGCTCAGTTGGTAGAGCGGAGGACTGTAGTAGACGCAGATTATCCTTAGGTC
ACTGGTTCGAATCCGGTAGGTCGGA
>Arabidopsis_thaliana_chr1.trna99-TyrGTA (21281861-21281945) Tyr (GTA) 85 bp Sc: 74.76
CCGACCTTAGCTCAGTTGGTAGAGCGGAGGACTGTAGTTGACGCAGATTATCCTTAGGTC
ACTGGTTCGAATCCGGTAGGTCGGA
>Arabidopsis_thaliana_chr1.trna100-TyrGTA (21282312-21282396) Tyr (GTA) 85 bp Sc: 75.54
CCGACCTTAGCTCAGTTGGTAGAGCGGAGGACTGTAGTAGACGTAGATTATCCTTAGGTC
ACTGGTTCGAATCCGGTAGGTCGGA
>Arabidopsis_thaliana_chr1.trna102-TyrGTA (21283385-21283469) Tyr (GTA) 85 bp Sc: 74.91
CCGACCTTAGCTCAGTTGGTAGAGCGGAGGACTGTAGTTGACGCAGATAATCCTTAGGTC
ACTGGTTCGAATCCGGTAGGTCGGA
>Arabidopsis_thaliana_chr1.trna103-TyrGTA (21283843-21283927) Tyr (GTA) 85 bp Sc: 74.91
CCGACCTTAGCTCAGTTGGTAGAGCGGAGGACTGTAGTAGACGCAGATTATCCTTAGGTC
ACTGGTTCGAATCCGGTAGGTCGGA
>Arabidopsis_thaliana_chr1.trna105-TyrGTA (21284917-21285001) Tyr (GTA) 85 bp Sc: 74.91
CCGACCTTAGCTCAGTTGGTAGAGCGGAGGACTGTAGTTGACGCAGATAATCCTTAGGTC
ACTGGTTCGAATCCGGTAGGTCGGA
>Arabidopsis_thaliana_chr1.trna106-TyrGTA (21285375-21285459) Tyr (GTA) 85 bp Sc: 63.52
CCGACCTTAGCTCAGTTGGTAGAGCGGAGGACTGTAGTAGACGCAGATTATCCTTAGGTC
ACTGGTTCGAATCTGGTAGGTTTGA
>Arabidopsis_thaliana_chr1.trna108-TyrGTA (21286449-21286533) Tyr (GTA) 85 bp Sc: 74.91
CCGACCTTAGCTCAGTTGGTAGAGCGGAGGACTGTAGTTGACGCAGATAATCCTTAGGTC
ACTGGTTCGAATCCGGTAGGTCGGA
>Arabidopsis_thaliana_chr1.trna109-TyrGTA (21286907-21286991) Tyr (GTA) 85 bp Sc: 74.91
CCGACCTTAGCTCAGTTGGTAGAGCGGAGGACTGTAGTAGACGCAGATTATCCTTAGGTC
ACTGGTTCGAATCCGGTAGGTCGGA
>Arabidopsis_thaliana_chr1.trna111-TyrGTA (21287981-21288065) Tyr (GTA) 85 bp Sc: 74.91
CCGACCTTAGCTCAGTTGGTAGAGCGGAGGACTGTAGTTGACGCAGATAATCCTTAGGTC
ACTGGTTCGAATCCGGTAGGTCGGA
>Arabidopsis_thaliana_chr1.trna112-TyrGTA (21288439-21288523) Tyr (GTA) 85 bp Sc: 74.91
CCGACCTTAGCTCAGTTGGTAGAGCGGAGGACTGTAGTAGACGCAGATTATCCTTAGGTC
ACTGGTTCGAATCCGGTAGGTCGGA
>Arabidopsis_thaliana_chr1.trna114-TyrGTA (21289514-21289598) Tyr (GTA) 85 bp Sc: 74.91
CCGACCTTAGCTCAGTTGGTAGAGCGGAGGACTGTAGTTGACGCAGATAATCCTTAGGTC
ACTGGTTCGAATCCGGTAGGTCGGA
>Arabidopsis_thaliana_chr1.trna115-TyrGTA (21289971-21290055) Tyr (GTA) 85 bp Sc: 74.91
CCGACCTTAGCTCAGTTGGTAGAGCGGAGGACTGTAGTAGACGCAGATTATCCTTAGGTC
ACTGGTTCGAATCCGGTAGGTCGGA
>Arabidopsis_thaliana_chr1.trna117-TyrGTA (21291045-21291129) Tyr (GTA) 85 bp Sc: 74.91
CCGACCTTAGCTCAGTTGGTAGAGCGGAGGACTGTAGTTGACGCAGATAATCCTTAGGTC
ACTGGTTCGAATCCGGTAGGTCGGA
>Arabidopsis_thaliana_chr1.trna118-TyrGTA (21291502-21291586) Tyr (GTA) 85 bp Sc: 74.91
CCGACCTTAGCTCAGTTGGTAGAGCGGAGGACTGTAGTAGACGCAGATTATCCTTAGGTC

ACTGGTTCGAATCCGGTAGGTCGGA
>Arabidopsis_thaliana_chr1.trna120-TyrGTA (21292575-21292659) Tyr (GTA) 85 bp Sc: 74.91
CCGACCTTAGCTCAGTTGGTAGAGCGGAGGACTGTAGTTGACGCAGATAATCCTTAGGTC
ACTGGTTCGAATCCGGTAGGTCGGA
>Arabidopsis_thaliana_chr1.trna121-TyrGTA (21293033-21293117) Tyr (GTA) 85 bp Sc: 74.91
CCGACCTTAGCTCAGTTGGTAGAGCGGAGGACTGTAGTAGACGCAGATTATCCTTAGGTC
ACTGGTTCGAATCCGGTAGGTCGGA
>Arabidopsis_thaliana_chr1.trna123-TyrGTA (21294110-21294194) Tyr (GTA) 85 bp Sc: 70.61
CCGACCTTAGCTCAGTTGGTAGAGCGGAGGACTGTAGTTGACGCAGATAATCCTTAGGTC
ATTGGTTCGAATCCGGTAGGTCGGA
>Arabidopsis_thaliana_chr1.trna124-TyrGTA (21294568-21294652) Tyr (GTA) 85 bp Sc: 65.30
CCGACTTTAGCTCAGTTGGTAGAGCGGAGGACAGTAGAAGACGCAGATTATCCTTAGGTC
ACTGGTTCGAATCCGGTAGGTCGGA
>Arabidopsis_thaliana_chr1.trna126-TyrGTA (21295456-21295540) Tyr (GTA) 85 bp Sc: 68.58
CCGACCTTAGCTCAGTTGGTAGAGCGGAGGACTGTAGTTGACGCAGATAATCCTTAGGTC
ACTGGTTCGAATTCGGTAGGTCGGA
>Arabidopsis_thaliana_chr1.trna127-TyrGTA (21295913-21295997) Tyr (GTA) 85 bp Sc: 74.91
CCGACCTTAGCTCAGTTGGTAGAGCGGAGGACTGTAGTAGACGCAGATTATCCTTAGGTC
ACTGGTTCGAATCCGGTAGGTCGGA
>Arabidopsis_thaliana_chr1.trna129-TyrGTA (21296990-21297074) Tyr (GTA) 85 bp Sc: 70.61
CCGACCTTAGCTCAGTTGGTAGAGCGGAGGACTGTAGTTGACGCAGATAATCCTTAGGTC
ATTGGTTCGAATCCGGTAGGTCGGA
>Arabidopsis_thaliana_chr1.trna130-TyrGTA (21297448-21297532) Tyr (GTA) 85 bp Sc: 65.30
CCGACTTTAGCTCAGTTGGTAGAGCGGAGGACAGTAGAAGACGCAGATTATCCTTAGGTC
ACTGGTTCGAATCCGGTAGGTCGGA
>Arabidopsis_thaliana_chr1.trna132-TyrGTA (21298336-21298420) Tyr (GTA) 85 bp Sc: 68.58
CCGACCTTAGCTCAGTTGGTAGAGCGGAGGACTGTAGTTGACGCAGATAATCCTTAGGTC
ACTGGTTCGAATTCGGTAGGTCGGA
>Arabidopsis_thaliana_chr1.trna133-TyrGTA (21298793-21298877) Tyr (GTA) 85 bp Sc: 74.91
CCGACCTTAGCTCAGTTGGTAGAGCGGAGGACTGTAGTAGACGCAGATTATCCTTAGGTC
ACTGGTTCGAATCCGGTAGGTCGGA
>Arabidopsis_thaliana_chr1.trna135-TyrGTA (21299870-21299954) Tyr (GTA) 85 bp Sc: 70.61
CCGACCTTAGCTCAGTTGGTAGAGCGGAGGACTGTAGTTGACGCAGATAATCCTTAGGTC
ATTGGTTCGAATCCGGTAGGTCGGA
>Arabidopsis_thaliana_chr1.trna136-TyrGTA (21300328-21300412) Tyr (GTA) 85 bp Sc: 63.02
CCGACCTTAGCTCAGTTGGTAGAGCGGAGGACAGTAGAAGACGCAGATTATCCTTAGGTC
ACAGGTTCGAATCCGGTAGGTCGGA
>Arabidopsis_thaliana_chr1.trna138-TyrGTA (21301216-21301300) Tyr (GTA) 85 bp Sc: 58.50
CCAACCTTAGCTCAGTTGGTAGAGCGGAGGACTGTAGTTGACGCAGATAATCCTTAGGTC
ACTGGTTCGAATTTGGTAGGTCGGA
>Arabidopsis_thaliana_chr1.trna139-TyrGTA (21301674-21301758) Tyr (GTA) 85 bp Sc: 74.91
CCGACCTTAGCTCAGTTGGTAGAGCGGAGGACTGTAGTAGACGCAGATTATCCTTAGGTC
ACTGGTTCGAATCCGGTAGGTCGGA
>Arabidopsis_thaliana_chr1.trna141-TyrGTA (21302751-21302835) Tyr (GTA) 85 bp Sc: 70.61
CCGACCTTAGCTCAGTTGGTAGAGCGGAGGACTGTAGTTGACGCAGATAATCCTTAGGTC
ATTGGTTCGAATCCGGTAGGTCGGA
>Arabidopsis_thaliana_chr1.trna142-TyrGTA (21303209-21303293) Tyr (GTA) 85 bp Sc: 63.02
CCGACCTTAGCTCAGTTGGTAGAGCGGAGGACAGTAGAAGACGCAGATTATCCTTAGGTC
ACAGGTTCGAATCCGGTAGGTCGGA
>Arabidopsis_thaliana_chr1.trna144-TyrGTA (21304097-21304181) Tyr (GTA) 85 bp Sc: 58.50
CCAACCTTAGCTCAGTTGGTAGAGCGGAGGACTGTAGTTGACGCAGATAATCCTTAGGTC
ACTGGTTCGAATTTGGTAGGTCGGA
>Arabidopsis_thaliana_chr1.trna145-TyrGTA (21304555-21304639) Tyr (GTA) 85 bp Sc: 74.91
CCGACCTTAGCTCAGTTGGTAGAGCGGAGGACTGTAGTAGACGCAGATTATCCTTAGGTC
ACTGGTTCGAATCCGGTAGGTCGGA
>Arabidopsis_thaliana_chr1.trna147-TyrGTA (21305632-21305716) Tyr (GTA) 85 bp Sc: 70.61
CCGACCTTAGCTCAGTTGGTAGAGCGGAGGACTGTAGTTGACGCAGATAATCCTTAGGTC
ATTGGTTCGAATCCGGTAGGTCGGA
>Arabidopsis_thaliana_chr1.trna148-TyrGTA (21306090-21306174) Tyr (GTA) 85 bp Sc: 58.73
CCGACCTTAGCTCAGTTGGTAGAGCGGAGGACAGTAGAAGACGCAGATTATCCTTAGGTC
ATAGGTTCGAATCCGGTAGGTCGGA
>Arabidopsis_thaliana_chr1.trna150-TyrGTA (21306982-21307066) Tyr (GTA) 85 bp Sc: 75.68
CCGACCTTAGCTCAGTTGGTAGAGCGGAGGACTGTAGTTGAAGCAGATAATCCTTAGGTC
ACTGGTTCGAATCCGGTAGGTCGGA
>Arabidopsis_thaliana_chr1.trna151-TyrGTA (21307442-21307526) Tyr (GTA) 85 bp Sc: 74.91
CCGACCTTAGCTCAGTTGGTAGAGCGGAGGACTGTAGTAGACGCAGATTATCCTTAGGTC
ACTGGTTCGAATCCGGTAGGTCGGA

>Arabidopsis_thaliana_chr1.trna153-TyrGTA (21308521-21308600) Tyr (GTA) 80 bp Sc: 55.49
CCGACCTTAGCTCTGTTGGTATAGCGGAGGACTGTAGTTGACGCATCCTTAGGTCACTGG
TTCGAATTCGGTAGGTCGGA
>Arabidopsis_thaliana_chr1.trna154-TyrGTA (21308976-21309060) Tyr (GTA) 85 bp Sc: 74.91
CCGACCTTAGCTCAGTTGGTAGAGCGGAGGACTGTAGTAGACGCAGATTATCCTTAGGTC
ACTGGTTCGAATCCGGTAGGTCGGA
>Arabidopsis_thaliana_chr1.trna156-TyrGTA (21310054-21310138) Tyr (GTA) 85 bp Sc: 75.68
CCGACCTTAGCTCAGTTGGTAGAGCGGAGGACTGTAGTTGAAGCAGATAATCCTTAGGTC
ACTGGTTCGAATCCGGTAGGTCGGA
>Arabidopsis_thaliana_chr1.trna157-TyrGTA (21310514-21310598) Tyr (GTA) 85 bp Sc: 74.91
CCGACCTTAGCTCAGTTGGTAGAGCGGAGGACTGTAGTAGACGCAGATTATCCTTAGGTC
ACTGGTTCGAATCCGGTAGGTCGGA
>Arabidopsis_thaliana_chr1.trna159-TyrGTA (21311593-21311677) Tyr (GTA) 85 bp Sc: 56.05
CCGACCTTAGCTCTGTTGGTATAGCGGAGGACTGTAGTTGACGCAGATAATCCTTAGGTC
ACTGGTTCGAATTCGGTAGGTCGGA
>Arabidopsis_thaliana_chr1.trna160-TyrGTA (21312052-21312136) Tyr (GTA) 85 bp Sc: 74.91
CCGACCTTAGCTCAGTTGGTAGAGCGGAGGACTGTAGTAGACGCAGATTATCCTTAGGTC
ACTGGTTCGAATCCGGTAGGTCGGA
>Arabidopsis_thaliana_chr5.trna112-TyrGTA (2309549-2309464) Tyr (GTA) 86 bp Sc: 76.04
CCGACCTTAGCTCAGTTGGTAGAGCGGAAGACTGTAGTCTGTGACAGCAAATCTTTAGGT
CGCTGGTTCGATTCCGGCAGGTCGGA
>Arabidopsis_thaliana_chr5.trna57-TyrGTA (24856206-24856290) Tyr (GTA) 85 bp Sc: 78.43
CCGACCTTAGCTCAGTTGGTAGAGCGGAAGACTGTAATGTTTGCAGATAATCTTTAGGTC
GCTGGTTCGATTCCGGCAGGTCGGA
>Arabidopsis_thaliana_chr3.trna84-TyrGTA (3238461-3238372) Tyr (GTA) 90 bp Sc: 73.56
CCGACCTTAGCTCAGTTGGTAGAGCGGAAGACTGTAATGGGTAATTCCAAAGAAATCTTT
AGGTCGCTGGTTCGATTCCGGCAGGTTGAA
>Arabidopsis_thaliana_chr3.trna22-TyrGTA (6540637-6540721) Tyr (GTA) 85 bp Sc: 75.91
CCGACCTTAGCTCAGTTGGTAGAGCGGAAGACTGTAGGCGTTGCAGATAATCTTTAGGTC
GCTGGTTCGATTCCGGCAGGTCGGA
>Arabidopsis_thaliana_chr4.trna7-TyrGTA (6819288-6819371) Tyr (GTA) 84 bp Sc: 76.91
CCGACCTTAGCTCAGTTGGTAGAGCGGAAGACTGTAGTAGTTGCTGAAATCTTTAGGTCG
CTGGTTCGATTCCGGCAGGTCGGA
>Arabidopsis_thaliana_chr2.trna87-TyrGTA (6953961-6953873) Tyr (GTA) 89 bp Sc: 78.61
CCGACCTTAGCTCAGTTGGTAGAGCGGAAGACTGTAGTTGTTTAAAACAGATAATCTTTA
GGTCGCTGGTTCGATTCCGGCAGGTCGGA
>Arabidopsis_thaliana_chr5.trna3-TyrGTA (858535-858618) Tyr (GTA) 84 bp Sc: 76.91
CCGACCTTAGCTCAGTTGGTAGAGCGGAAGACTGTAGTAGTAGCTGTAATCTTTAGGTCG
CTGGTTCGATTCCGGCAGGTCGGA
>Arabidopsis_thaliana_chr2.trna84-TyrGTA (9241356-9241273) Tyr (GTA) 84 bp Sc: 76.13
CCGACCTTAGCTCAGTTGGTAGAGCGGAAGACTGTAGTAGTTGCTGTGATCTTTAGGTCG
CTGGTTCGATTCCGGCAGGTCGGA
>Arabidopsis_thaliana_chr1.trna206-Undet??? (21378054-21377984) Undet (???) 71 bp Sc: 27.18
TCCGCTGTAGCACAATGGTTAAAACAGCTGTCTAAACATCTGATTTCCGAGTTCAAAGCT
CGGCAGTGGAA
>Arabidopsis_thaliana_chr4.trna78-ValAAC (562634-562561) Val (AAC) 74 bp Sc: 43.60
GGTTTCGTGGTGTAGTTGTTTATCACGTCATTCTAACACAACTAAGGTTTCTGGTTCGAA
CCCGGTCAAAACCA
>Arabidopsis_thaliana_chr3.trna33-ValAAC (16902624-16902697) Val (AAC) 74 bp Sc: 70.95
GGTTTCGTGGTGTAGTTGGTTATCACGTCAGTCTAACACACTAAAGGTCTCCGGTTCGAA
CCCGGGCGAAGCCA
>Arabidopsis_thaliana_chr1.trna217-ValAAC (10963627-10963554) Val (AAC) 74 bp Sc: 77.22
GGTTTCGTGGTGTAGTTGGTTATCACGTCAGTCTAACACACTGAAGGTCTCCGGTTCGAA
CCCGGGCGAAGCCA
>Arabidopsis_thaliana_chr1.trna219-ValAAC (8520352-8520279) Val (AAC) 74 bp Sc: 77.22
GGTTTCGTGGTGTAGTTGGTTATCACGTCAGTCTAACACACTGAAGGTCTCCGGTTCGAA
CCCGGGCGAAGCCA
>Arabidopsis_thaliana_chr1.trna226-ValAAC (6076011-6075938) Val (AAC) 74 bp Sc: 77.22
GGTTTCGTGGTGTAGTTGGTTATCACGTCAGTCTAACACACTGAAGGTCTCCGGTTCGAA
CCCGGGCGAAGCCA
>Arabidopsis_thaliana_chr1.trna233-ValAAC (4267059-4266986) Val (AAC) 74 bp Sc: 77.22
GGTTTCGTGGTGTAGTTGGTTATCACGTCAGTCTAACACACTGAAGGTCTCCGGTTCGAA
CCCGGGCGAAGCCA
>Arabidopsis_thaliana_chr3.trna32-ValAAC (16896876-16896949) Val (AAC) 74 bp Sc: 77.22
GGTTTCGTGGTGTAGTTGGTTATCACGTCAGTCTAACACACTGAAGGTCTCCGGTTCGAA
CCCGGGCGAAGCCA
>Arabidopsis_thaliana_chr3.trna34-ValAAC (16905148-16905221) Val (AAC) 74 bp Sc: 77.22

GGTTTCGTGGTGTAGTTGGTTATCACGTCAGTCTAACACACTGAAGGTCTCCGGTTCGAA
CCCGGGCGAAGCCA
>Arabidopsis_thaliana_chr3.trna39-ValAAC (18518797-18518870) Val (AAC) 74 bp Sc: 77.22
GGTTTCGTGGTGTAGTTGGTTATCACGTCAGTCTAACACACTGAAGGTCTCCGGTTCGAA
CCCGGGCGAAGCCA
>Arabidopsis_thaliana_chr3.trna53-ValAAC (22149700-22149773) Val (AAC) 74 bp Sc: 77.22
GGTTTCGTGGTGTAGTTGGTTATCACGTCAGTCTAACACACTGAAGGTCTCCGGTTCGAA
CCCGGGCGAAGCCA
>Arabidopsis_thaliana_chr4.trna9-ValAAC (7185698-7185771) Val (AAC) 74 bp Sc: 77.22
GGTTTCGTGGTGTAGTTGGTTATCACGTCAGTCTAACACACTGAAGGTCTCCGGTTCGAA
CCCGGGCGAAGCCA
>Arabidopsis_thaliana_chr5.trna1-ValAAC (530820-530893) Val (AAC) 74 bp Sc: 77.22
GGTTTCGTGGTGTAGTTGGTTATCACGTCAGTCTAACACACTGAAGGTCTCCGGTTCGAA
CCCGGGCGAAGCCA
>Arabidopsis_thaliana_chr5.trna10-ValAAC (2993328-2993401) Val (AAC) 74 bp Sc: 77.22
GGTTTCGTGGTGTAGTTGGTTATCACGTCAGTCTAACACACTGAAGGTCTCCGGTTCGAA
CCCGGGCGAAGCCA
>Arabidopsis_thaliana_chr5.trna59-ValAAC (25801341-25801414) Val (AAC) 74 bp Sc: 77.22
GGTTTCGTGGTGTAGTTGGTTATCACGTCAGTCTAACACACTGAAGGTCTCCGGTTCGAA
CCCGGGCGAAGCCA
>Arabidopsis_thaliana_chr5.trna72-ValAAC (24157245-24157172) Val (AAC) 74 bp Sc: 77.22
GGTTTCGTGGTGTAGTTGGTTATCACGTCAGTCTAACACACTGAAGGTCTCCGGTTCGAA
CCCGGGCGAAGCCA
>Arabidopsis_thaliana_chr3.trna15-ValCAC (3575850-3575923) Val (CAC) 74 bp Sc: 85.02
GTCTGGGTAGTGTAGTCGGTTATCACGCTAGTCTCACACACTAGAGGTCCCCGGTTCGAA
CCCGGGCTCAGACA
>Arabidopsis_thaliana_chr2.trna25-ValCAC (9400750-9400823) Val (CAC) 74 bp Sc: 85.42
GTCTGGGTGGTGTAGTCGGTTATCACGCTAGTCTCACACACTAGAGGTCCCCGGTTCGAA
CCCGGGCTCAGACA
>Arabidopsis_thaliana_chr3.trna25-ValCAC (9840421-9840494) Val (CAC) 74 bp Sc: 85.42
GTCTGGGTGGTGTAGTCGGTTATCACGCTAGTCTCACACACTAGAGGTCCCCGGTTCGAA
CCCGGGCTCAGACA
>Arabidopsis_thaliana_chr4.trna42-ValCAC (18411259-18411332) Val (CAC) 74 bp Sc: 85.42
GTCTGGGTGGTGTAGTCGGTTATCACGCTAGTCTCACACACTAGAGGTCCCCGGTTCGAA
CCCGGGCTCAGACA
>Arabidopsis_thaliana_chr5.trna17-ValCAC (4929563-4929636) Val (CAC) 74 bp Sc: 85.42
GTCTGGGTGGTGTAGTCGGTTATCACGCTAGTCTCACACACTAGAGGTCCCCGGTTCGAA
CCCGGGCTCAGACA
>Arabidopsis_thaliana_chr5.trna28-ValCAC (15242548-15242621) Val (CAC) 74 bp Sc: 85.42
GTCTGGGTGGTGTAGTCGGTTATCACGCTAGTCTCACACACTAGAGGTCCCCGGTTCGAA
CCCGGGCTCAGACA
>Arabidopsis_thaliana_chr5.trna73-ValCAC (23072070-23071997) Val (CAC) 74 bp Sc: 85.42
GTCTGGGTGGTGTAGTCGGTTATCACGCTAGTCTCACACACTAGAGGTCCCCGGTTCGAA
CCCGGGCTCAGACA
>Arabidopsis_thaliana_chr5.trna8-ValCAC (1804617-1804690) Val (CAC) 74 bp Sc: 85.42
GTCTGGGTGGTGTAGTCGGTTATCACGCTAGTCTCACACACTAGAGGTCCCCGGTTCGAA
CCCGGGCTCAGACA
>Arabidopsis_thaliana_chr3.trna41-ValTAC (18907852-18907924) Val (TAC) 73 bp Sc: 76.93
GGTGCTGTGGTGTAGTGGTTATCACGTTTGCCTTACACGCAAAAGGTCTCCAGTTCGACC
CTGGGCAGCACCA
>Arabidopsis_thaliana_chr4.trna16-ValTAC (9191591-9191663) Val (TAC) 73 bp Sc: 77.60
GATTCTGTGGTGTAGTGGTTATCACGTTTGCCTTACACGCAAAAGGTCTCCAGTTCGATC
CTGGGCAGAATCA
>Arabidopsis_thaliana_chr1.trna1-ValTAC (306384-306456) Val (TAC) 73 bp Sc: 79.63
GGTGCTGTGGTGTAGTGGTTATCACGTTTGCCTTACACGCAAAAGGTCTCCAGTTCGATC
CTGGGCAGCACCA
>Arabidopsis_thaliana_chr2.trna3-ValTAC (1154382-1154454) Val (TAC) 73 bp Sc: 79.63
GGTGCTGTGGTGTAGTGGTTATCACGTTTGCCTTACACGCAAAAGGTCTCCAGTTCGATC
CTGGGCAGCACCA
>Arabidopsis_thaliana_chr2.trna73-ValTAC (12645305-12645233) Val (TAC) 73 bp Sc: 79.63
GGTGCTGTGGTGTAGTGGTTATCACGTTTGCCTTACACGCAAAAGGTCTCCAGTTCGATC
CTGGGCAGCACCA
>Arabidopsis_thaliana_chr3.trna9-ValTAC (2222252-2222324) Val (TAC) 73 bp Sc: 79.63
GGTGCTGTGGTGTAGTGGTTATCACGTTTGCCTTACACGCAAAAGGTCTCCAGTTCGATC
CTGGGCAGCACCA
>Arabidopsis_thaliana_chr5.trna85-ValTAC (17894979-17894907) Val (TAC) 73 bp Sc: 79.63
GGTGCTGTGGTGTAGTGGTTATCACGTTTGCCTTACACGCAAAAGGTCTCCAGTTCGATC

CTGGGCAGCACCA
>Azoarcus_BH72_chr.trna21-AlaCGC (1508492-1508567) Ala (CGC) 76 bp Sc: 82.95
GGGCCGATAGCTCAGCTGGGAGAGCGCTGCGTTCGCAATGCAGAGGTCGGGAGTTCGATC
CTCCTTCGGTCCACCA
>Azoarcus_BH72_chr.trna12-AlaGGC (1105287-1105362) Ala (GGC) 76 bp Sc: 86.46
GGGGGTATAGCTCAGCTGGGAGAGCGCTTGCATGGCATGCAAGAGGTCAGCGGTTCGATC
CCGCTTACCTCCACCA
>Azoarcus_BH72_chr.trna9-AlaGGC (1104890-1104965) Ala (GGC) 76 bp Sc: 86.46
GGGGGTATAGCTCAGCTGGGAGAGCGCTTGCATGGCATGCAAGAGGTCAGCGGTTCGATC
CCGCTTACCTCCACCA
>Azoarcus_BH72_chr.trna20-AlaTGC (1393621-1393696) Ala (TGC) 76 bp Sc: 92.07
GGGGCTGTAGCTCAGCTGGGAGAGCGGCGGCTTTGCAAGCCGTAGGTCGTCGGTTCGATC
CCGACCAGCTCCACCA
>Azoarcus_BH72_chr.trna3-AlaTGC (206517-206592) Ala (TGC) 76 bp Sc: 92.07
GGGGCTGTAGCTCAGCTGGGAGAGCGGCGGCTTTGCAAGCCGTAGGTCGTCGGTTCGATC
CCGACCAGCTCCACCA
>Azoarcus_BH72_chr.trna34-AlaTGC (3515226-3515151) Ala (TGC) 76 bp Sc: 92.07
GGGGCTGTAGCTCAGCTGGGAGAGCGGCGGCTTTGCAAGCCGTAGGTCGTCGGTTCGATC
CCGACCAGCTCCACCA
>Azoarcus_BH72_chr.trna5-AlaTGC (772439-772514) Ala (TGC) 76 bp Sc: 92.07
GGGGCTGTAGCTCAGCTGGGAGAGCGGCGGCTTTGCAAGCCGTAGGTCGTCGGTTCGATC
CCGACCAGCTCCACCA
>Azoarcus_BH72_chr.trna48-ArgACG (1466348-1466272) Arg (ACG) 77 bp Sc: 76.44
GCGCCCGTAGCTCATCTGGATAGAGTACCTGGCTACGAACCAGGGGGTAGGAGGTTCGAA
TCCTTCCGGGCGCGCCA
>Azoarcus_BH72_chr.trna1-ArgCCG (204448-204524) Arg (CCG) 77 bp Sc: 84.33
GCGCCCGTAGCTCAGCTGGATAGAGTACTGCCCTCCGAAGGCAGGGGTCGGACGTTCGAA
TCGTCTCGGGCGCGCCA
>Azoarcus_BH72_chr.trna27-ArgCCT (3499971-3500045) Arg (CCT) 75 bp Sc: 68.43
CTCGCCATAGTTCAATGGATAGAACACGGACCTCCTAAGTCTGAGATGCAGGTTCGATTC
CTGCTGGCGAGGCCA
>Azoarcus_BH72_chr.trna40-ArgTCT (2308196-2308120) Arg (TCT) 77 bp Sc: 88.11
CTGCCCGTAGCTCAATTGGATAGAGCACCGGCCTTCTAAGCCGGGGGTTGCAGGTTCGAT
CCCTGCCGGGCAGGCCA
>Azoarcus_BH72_chr.trna46-AsnGTT (1772190-1772115) Asn (GTT) 76 bp Sc: 84.40
TCCTCGATAGCTCAGTCGGTAGAGCGCCGGACTGTTAATCCGTAGGTCCCTGGTTCGAGC
CCAGGTCGAGGAGCCA
>Azoarcus_BH72_chr.trna47-AsnGTT (1772049-1771974) Asn (GTT) 76 bp Sc: 85.87
TCCCCGATAGCTCAGTCGGTAGAGCGCCGGACTGTTAATCCGTAGGTCCCTGGTTCGAGC
CCAGGTCGGGGAGCCA
>Azoarcus_BH72_chr.trna11-AspGTC (1105124-1105200) Asp (GTC) 77 bp Sc: 92.46
GGAGTGGTAGTTCAGTCGGTTAGAATACCGGCCTGTCACGCCGGGGGTCGCGGGTTCGAG
TCCCGTCCACTCCGCCA
>Azoarcus_BH72_chr.trna14-AspGTC (1105508-1105584) Asp (GTC) 77 bp Sc: 92.46
GGAGTGGTAGTTCAGTCGGTTAGAATACCGGCCTGTCACGCCGGGGGTCGCGGGTTCGAG
TCCCGTCCACTCCGCCA
>Azoarcus_BH72_chr.trna24-CysGCA (1810049-1810122) Cys (GCA) 74 bp Sc: 58.52
GGCGCGATAGCAAAGCGGTTATGCACCGGATTGCAAATCCGTGTAGGTCGGTTCGACTCC
GGCTCGCGCCTCCA
>Azoarcus_BH72_chr.trna53-GlnTTG (807679-807603) Gln (TTG) 77 bp Sc: 79.11
TGGGGAGTCGCCAAGTCGGTTAAGGCACCGGATTTTGATTCCGGCATTCGAGGGTTCGAA
TCCTTCCTCCCCAGCCA
>Azoarcus_BH72_chr.trna10-GluTTC (1104986-1105061) Glu (TTC) 76 bp Sc: 55.53
GTCCCCATCGTCTAGAGGCCTAGGACACCGCCCTTTCACGGCGATAACCGGGGTTCGAAT
CCCCGTGGGGACGCCA
>Azoarcus_BH72_chr.trna13-GluTTC (1105372-1105447) Glu (TTC) 76 bp Sc: 55.53
GTCCCCATCGTCTAGAGGCCTAGGACACCGCCCTTTCACGGCGATAACCGGGGTTCGAAT
CCCCGTGGGGACGCCA
>Azoarcus_BH72_chr.trna28-GlyCCC (4144876-4144950) Gly (CCC) 75 bp Sc: 60.99
GCGGGCGTCGTATAACGGCTATTACCTCAGCTTCCCAAGCTGATGACGAGGGTTCGACTC
CCTTCGCCCGCTCCA
>Azoarcus_BH72_chr.trna23-GlyGCC (1809899-1809974) Gly (GCC) 76 bp Sc: 88.26
GCGGGAATAGCTCAGTTGGTAGAGCGCAACCTTGCCAAGGTTGAGGTCGCGAGTTCGAGA
CTCGTTTCCCGCTCCA
>Azoarcus_BH72_chr.trna25-GlyGCC (1810132-1810207) Gly (GCC) 76 bp Sc: 88.26
GCGGGAATAGCTCAGTTGGTAGAGCGCAACCTTGCCAAGGTTGAGGTCGCGAGTTCGAGA
CTCGTTTCCCGCTCCA

>Azoarcus_BH72_chr.trna30-GlyTCC (3760855-3760782) Gly (TCC) 74 bp Sc: 82.17
GCGGGTGTAGCTCAATGGTAGAGCAGAAGCCTTCCAAGCTTACGACGAGGGTTCGATTCC
CTTCACCCGCTCCA
>Azoarcus_BH72_chr.trna41-HisGTG (2308074-2307999) His (GTG) 76 bp Sc: 86.36
GCGGCATTAGCTCAGTTGGTAGAGCACTGGATTGTGGCTCCAGGTGTCACCGGTTCGATT
CCGGTATGTCGCCCCA
>Azoarcus_BH72_chr.trna19-IleGAT (1393517-1393593) Ile (GAT) 77 bp Sc: 92.16
GGGTCTGTAGCTCAGCTGGTTAGAGCACCGTCTTGATAAGGCGGGGGTCGTTGGTTCGAA
CCCAACCAGACCCACCA
>Azoarcus_BH72_chr.trna2-IleGAT (206413-206489) Ile (GAT) 77 bp Sc: 92.16
GGGTCTGTAGCTCAGCTGGTTAGAGCACCGTCTTGATAAGGCGGGGGTCGTTGGTTCGAA
CCCAACCAGACCCACCA
>Azoarcus_BH72_chr.trna33-IleGAT (3515330-3515254) Ile (GAT) 77 bp Sc: 92.16
GGGTCTGTAGCTCAGCTGGTTAGAGCACCGTCTTGATAAGGCGGGGGTCGTTGGTTCGAA
CCCAACCAGACCCACCA
>Azoarcus_BH72_chr.trna4-IleGAT (772335-772411) Ile (GAT) 77 bp Sc: 92.16
GGGTCTGTAGCTCAGCTGGTTAGAGCACCGTCTTGATAAGGCGGGGGTCGTTGGTTCGAA
CCCAACCAGACCCACCA
>Azoarcus_BH72_chr.trna6-LeuCAA (973414-973498) Leu (CAA) 85 bp Sc: 79.74
GCCGGGATGGCGGAATCGGTAGACGCAGCGGATTCAAAATCCGCCGCCGCAAGGTGTGTG
GGTTCGAGTCCCACTCCCGGCACCA
>Azoarcus_BH72_chr.trna50-LeuCAG (1005437-1005353) Leu (CAG) 85 bp Sc: 69.74
GCCCAGATGGCGGAATTGGTAGACGCACTAGTTTCAGGTACTAGCGCTGCGAGGCGTGGA
GGTTCGAGTCCTCTTCTGGGCACCA
>Azoarcus_BH72_chr.trna51-LeuCAG (1005179-1005095) Leu (CAG) 85 bp Sc: 72.23
GCCCAGATGGCGGAATTGGTAGACGCACTAGTTTCAGGTACTAGCGCCGCGAGGCGTGGA
GGTTCGAGTCCTCTTCTGGGCACCA
>Azoarcus_BH72_chr.trna22-LeuGAG (1524791-1524875) Leu (GAG) 85 bp Sc: 63.82
GCCGACGTGGTGAAATTGGTAGACACGCTATCTTGAGGGGGTAGTGGCGAAAGCCGTGTG
AGTTCGAGTCTCACCGTCGGCACCA
>Azoarcus_BH72_chr.trna45-LeuTAA (1812305-1812221) Leu (TAA) 85 bp Sc: 70.17
GCCCGGGTGATGAAATCGGTAACCATAGCGGACTTAAAATCCGCCGGCGCAAGCCTTACG
GGTTCGAGTCCCGTCCCGGGCACCA
>Azoarcus_BH72_chr.trna26-LeuTAG (2045941-2046025) Leu (TAG) 85 bp Sc: 76.14
GCGAGGGTGGCGGAATTGGTAGACGCACTGGATTTAGGTTCCAGCGCCGCAAGGCGTGAG
AGTTCGAGTCTCTTCCCTCGCACCA
>Azoarcus_BH72_chr.trna54-LysCTT (601766-601691) Lys (CTT) 76 bp Sc: 97.03
GGGGGTATAGCTCAGTTGGTAGAGCAGCTGACTCTTAATCAGTAGGTCCAAGGTTCGAAT
CCTTGTGCCCCCACCA
>Azoarcus_BH72_chr.trna55-LysTTT (438288-438216) Lys (TTT) 73 bp Sc: 90.50
GGGTCGGTAGCTCAGTCGGTAGAGCAGCGGACTTTTAATCCGTTGGTCGAGGGTTCGAGT
CCCTCCCGACCCA
>Azoarcus_BH72_chr.trna37-MetCAT (2869448-2869372) Met (CAT) 77 bp Sc: 87.64
CGCGGGGTGGAGCAGTCTGGCAGCTCGTCGGGCTCATAACCCGAAGGTCGCAGGTTCAAA
TCCTGCCCCCGCAACCA
>Azoarcus_BH72_chr.trna52-MetCAT (832527-832451) Met (CAT) 77 bp Sc: 89.34
GGTGATGTAGCTCAGACGGTTAGAGCGACGGATTCATAACCCGTAGGTCGGCGGTTCGAT
TCCGCCCATCACCACCA
>Azoarcus_BH72_chr.trna7-PheGAA (1052967-1053042) Phe (GAA) 76 bp Sc: 90.03
GGCCAGTTAGCTCAGTTGGTAGAGCAGCGGATTGAAAATCCGCGTGTCCGTGGTTCGATT
CCGCGACTGGCCACCA
>Azoarcus_BH72_chr.trna8-ProCGG (1091451-1091527) Pro (CGG) 77 bp Sc: 85.19
CGGGGAATAGCTCAGCCTGGTAGAGCACTGCGTTCGGGACGCAGGGGCCGGAGGTTCGAA
TCCTCTTTCCCCGACCA
>Azoarcus_BH72_chr.trna16-ProGGG (1170058-1170134) Pro (GGG) 77 bp Sc: 81.03
CGGGGCGTAGCGCAGCCTGGTAGCGCACTTGCATGGGGTGCAAGGGGTCGCGAGTTCGAA
TCCCGCCGCCCCGACCA
>Azoarcus_BH72_chr.trna39-ProTGG (2308300-2308224) Pro (TGG) 77 bp Sc: 89.92
CGGGGTGTAGCGCAGTCCGGTAGCGCGCTTGCTTTGGGAGCAAGATGTCGGGGGTTCGAA
TCCCTCCACCCCGACCA
>Azoarcus_BH72_chr.trna49-SerCGA (1445408-1445319) Ser (CGA) 90 bp Sc: 74.76
GGAGAGGTGGCAGAGTGGTCGAATGTACCTGACTCGAAATCAGGCGTAGGTGCAAGCCTA
CCGTGGGTTCGAATCCCACCCTCTCCGCCA
>Azoarcus_BH72_chr.trna35-SerGCT (3423134-3423041) Ser (GCT) 94 bp Sc: 75.50
GGAGACGTGGCCGAGAGGTCGAAGGCACTCCCCTGCTAAGGGAGCATGCGGGCTAAAACT
CGCATCGAGGGTTCGAATCCCTCCGTCTCCGCCA
>Azoarcus_BH72_chr.trna38-SerGGA (2508863-2508773) Ser (GGA) 91 bp Sc: 71.71

GGAGAGGTGGATGAGCGGTTTAAGTCGCACGCCTGGAAAGCGTGTTCAGGGTAATACCCT
GACGCGGGTTCGAATCCCGCCCTCTCCGCCA
>Azoarcus_BH72_chr.trna17-SerTGA (1184957-1185046) Ser (TGA) 90 bp Sc: 76.28
GGATGGGTGGCAGAGCGGTTTAATGCACTGGTCTTGAAAACCAGCGTGCATGTGAGTGCA
CCGTGGGTTCGAATCCCACCCCATCCGCCA
>Azoarcus_BH72_chr.trna18-ThrCGT (1283196-1283271) Thr (CGT) 76 bp Sc: 91.51
GCTGCTGTAGCTCAGTCGGTAGAGCAACTGATTCGTAATCAGTAGGTCACCAGTTCGATT
CCGGTCAGCAGCACCA
>Azoarcus_BH72_chr.trna31-ThrGGT (3760760-3760686) Thr (GGT) 75 bp Sc: 81.50
GCCCATGTAGCTCAGTGGTAGAGCACTCCCTTGGTAAGGGAGAGGCCACGTGTTCAATCC
ACGTCATGGGCACCA
>Azoarcus_BH72_chr.trna36-ThrTGT (2962675-2962600) Thr (TGT) 76 bp Sc: 88.19
GCCCTTGTAGCTCAGTTGGTAGAGCAACGGTTTTGTAAACCGAAGGTCGCGGGTTCGAGT
CCTGCCGGGGGCACCA
>Azoarcus_BH72_chr.trna32-TrpCCA (3759359-3759284) Trp (CCA) 76 bp Sc: 87.97
AGGGGCATAGCTCAATTGGCAGAGCGTCGGTCTCCAAAACCGAAGGTTGGGGGTTCGATT
CCCTCTGCCCCTGCCA
>Azoarcus_BH72_chr.trna29-TyrGTA (3761025-3760941) Tyr (GTA) 85 bp Sc: 72.08
GGAGGGGTTCCCGAGCGGTCAAAGGGATCAGACTGTAAATCTGACGGCTCTGCCTTCGAA
GGTTCGAATCCTTCCCCCTCCACCA
>Azoarcus_BH72_chr.trna43-ValCAC (2269337-2269263) Val (CAC) 75 bp Sc: 91.15
GGGCGGTTAGCTCAGCGGTAGAGCACTGCCTTCACACGGCAGGGGTCACTGGTTCGATCC
CAGTACCGCCCACCA
>Azoarcus_BH72_chr.trna44-ValCAC (2269224-2269150) Val (CAC) 75 bp Sc: 91.15
GGGCGGTTAGCTCAGCGGTAGAGCACTGCCTTCACACGGCAGGGGTCACTGGTTCGATCC
CAGTACCGCCCACCA
>Azoarcus_BH72_chr.trna15-ValGAC (1162391-1162467) Val (GAC) 77 bp Sc: 93.92
AGGCGCGTAGCTCAGTTGGTTAGAGCACCACCTTGACATGGTGGGGGTCGTTGGTTCGAG
TCCAATCGCGCCTACCA
>Azoarcus_BH72_chr.trna42-ValTAC (2269493-2269418) Val (TAC) 76 bp Sc: 91.62
GGGTGCTTAGCTCAGTTGGTAGAGCGTCGCCCTTACAAGGCGAATGTCGGCGGTTCGACC
CCGTCAGCACCCACCA
>Azoarcus_sp_EbN1_chr.trna58-AlaCGC (55689-55614) Ala (CGC) 76 bp Sc: 85.36
GGGCCGATAGCTCAGCTGGGAGAGCGCTGCGTTCGCAATGCAGAGGTCGAGGGTTCGATC
CCCTTTCGGTCCACCA
>Azoarcus_sp_EbN1_chr.trna24-AlaGGC (2803127-2803202) Ala (GGC) 76 bp Sc: 86.46
GGGGGTATAGCTCAGCTGGGAGAGCGCTTGCATGGCATGCAAGAGGTCAGCGGTTCGATC
CCGCTTACCTCCACCA
>Azoarcus_sp_EbN1_chr.trna27-AlaGGC (2803552-2803627) Ala (GGC) 76 bp Sc: 86.46
GGGGGTATAGCTCAGCTGGGAGAGCGCTTGCATGGCATGCAAGAGGTCAGCGGTTCGATC
CCGCTTACCTCCACCA
>Azoarcus_sp_EbN1_chr.trna14-AlaTGC (1847570-1847645) Ala (TGC) 76 bp Sc: 95.18
GGGGGATTAGCTCAGCTGGGAGAGCACCTGCTTTGCAAGCAGGGGGTCGACGGTTCGATC
CCGTCATCCTCCACCA
>Azoarcus_sp_EbN1_chr.trna20-AlaTGC (2413420-2413495) Ala (TGC) 76 bp Sc: 95.18
GGGGGATTAGCTCAGCTGGGAGAGCACCTGCTTTGCAAGCAGGGGGTCGACGGTTCGATC
CCGTCATCCTCCACCA
>Azoarcus_sp_EbN1_chr.trna22-AlaTGC (2622691-2622766) Ala (TGC) 76 bp Sc: 95.18
GGGGGATTAGCTCAGCTGGGAGAGCACCTGCTTTGCAAGCAGGGGGTCGACGGTTCGATC
CCGTCATCCTCCACCA
>Azoarcus_sp_EbN1_chr.trna51-AlaTGC (548735-548660) Ala (TGC) 76 bp Sc: 95.18
GGGGGATTAGCTCAGCTGGGAGAGCACCTGCTTTGCAAGCAGGGGGTCGACGGTTCGATC
CCGTCATCCTCCACCA
>Azoarcus_sp_EbN1_chr.trna57-ArgACG (340005-339929) Arg (ACG) 77 bp Sc: 72.96
GCGCCCGTAGCTCATCTGGATAGAGTACTTGGCTACGAACCAAGGGGTAGGGAGTTCGAA
TCTCTCCGGGCGCGCCA
>Azoarcus_sp_EbN1_chr.trna12-ArgCCG (1833251-1833327) Arg (CCG) 77 bp Sc: 84.33
GCGCCCGTAGCTCAGCTGGATAGAGTACTGCCCTCCGAAGGCAGGGGTCGGACGTTCGAA
TCGTCTCGGGCGCGCCA
>Azoarcus_sp_EbN1_chr.trna1-ArgCCT (13221-13295) Arg (CCT) 75 bp Sc: 66.53
CTCCTCGTAGTTCAATGGATAGAACGAGCGCCTCCTAAGCGCTAGATGTGAGTTCGATTC
TCGCCGAGGAGACCA
>Azoarcus_sp_EbN1_chr.trna37-ArgTCT (3482360-3482436) Arg (TCT) 77 bp Sc: 87.27
CTGCCCGTAGCTCAATTGGATAGAGCACCGGCCTTCTAAGCCGGGGGTTACAGGTTCGAT
CCCTGTCGGGCAGGCCA
>Azoarcus_sp_EbN1_chr.trna44-AsnGTT (3240506-3240431) Asn (GTT) 76 bp Sc: 87.97
TCCCCGATAGCTCAGTCGGTAGAGCAACGGACTGTTAATCCGTGTGTCCCTGGTTCGAGC

CCAGGTCGGGGAGCCA
>Azoarcus_sp_EbN1_chr.trna45-AsnGTT (3240391-3240316) Asn (GTT) 76 bp Sc: 87.97
TCCCCGATAGCTCAGTCGGTAGAGCAACGGACTGTTAATCCGTGTGTCCCTGGTTCGAGC
CCAGGTCGGGGAGCCA
>Azoarcus_sp_EbN1_chr.trna26-AspGTC (2803363-2803439) Asp (GTC) 77 bp Sc: 94.55
GGAGTGGTAGTTCAGTTGGTTAGAATACCGGCCTGTCACGCCGGGGGTCGCGGGTTCGAG
TCCCGTCCACTCCGCCA
>Azoarcus_sp_EbN1_chr.trna29-AspGTC (2803788-2803864) Asp (GTC) 77 bp Sc: 94.55
GGAGTGGTAGTTCAGTTGGTTAGAATACCGGCCTGTCACGCCGGGGGTCGCGGGTTCGAG
TCCCGTCCACTCCGCCA
>Azoarcus_sp_EbN1_chr.trna34-CysGCA (3380747-3380816) Cys (GCA) 70 bp Sc: 21.54
CGCGCCAATGCTGGAAATCCCACGGCCTGCAAAGCCGTGTACATCGGTTCGATTCCGGTT
CGCGCCTCCA
>Azoarcus_sp_EbN1_chr.trna33-CysGCA (3305084-3305157) Cys (GCA) 74 bp Sc: 69.09
GGCGCGATGGCAGAGTGGTTATGCAGCGGCCTGCAAAGCCGTGTACATCGGTTCGATTCC
GGTTCGCGCCTCCA
>Azoarcus_sp_EbN1_chr.trna7-GlnTTG (826265-826341) Gln (TTG) 77 bp Sc: 77.07
TGGGGAGTCGCCAAGTTGGTCAAGGCACCGGATTTTGATTCCGGCATTCGAAGGTTCGAA
TCCTTCTTCCCCAGCCA
>Azoarcus_sp_EbN1_chr.trna25-GluTTC (2803221-2803296) Glu (TTC) 76 bp Sc: 56.27
GTCCCCATCGTCTAGAGGCCTAGGACATCGCCCTTTCACGGCGGTAACCGGGGTTCGAAT
CCCCGTGGGGACGCCA
>Azoarcus_sp_EbN1_chr.trna28-GluTTC (2803646-2803721) Glu (TTC) 76 bp Sc: 56.27
GTCCCCATCGTCTAGAGGCCTAGGACATCGCCCTTTCACGGCGGTAACCGGGGTTCGAAT
CCCCGTGGGGACGCCA
>Azoarcus_sp_EbN1_chr.trna8-GlyCCC (1383723-1383796) Gly (CCC) 74 bp Sc: 65.01
GCGGGCGTCGTATAATGGTAATACCCTAGCTTCCCAAGCTAGAGCCGTGGGTTCGATTCC
CATCGCCCGCTCCA
>Azoarcus_sp_EbN1_chr.trna32-GlyGCC (3304938-3305013) Gly (GCC) 76 bp Sc: 88.26
GCGGGAATAGCTCAGTTGGTAGAGCGCAACCTTGCCAAGGTTGAGGTCGCGAGTTCGAGA
CTCGTTTCCCGCTCCA
>Azoarcus_sp_EbN1_chr.trna35-GlyGCC (3400810-3400885) Gly (GCC) 76 bp Sc: 88.26
GCGGGAATAGCTCAGTTGGTAGAGCGCAACCTTGCCAAGGTTGAGGTCGCGAGTTCGAGA
CTCGTTTCCCGCTCCA
>Azoarcus_sp_EbN1_chr.trna16-GlyTCC (2242786-2242859) Gly (TCC) 74 bp Sc: 82.26
GCGGGTGTAGCTCAATGGTAGAGCAGAAGCCTTCCAAGCTTATGACGAGGGTTCGATTCC
CTTCACCCGCTCCA
>Azoarcus_sp_EbN1_chr.trna38-HisGTG (3482477-3482552) His (GTG) 76 bp Sc: 83.72
GCGGCATTAGCTCAGTTGGTAGAGCACTGGATTGTGGCTCCAGTTGTCACCGGTTCGATC
CCGGTATGTCGCCCCA
>Azoarcus_sp_EbN1_chr.trna13-IleGAT (1847482-1847558) Ile (GAT) 77 bp Sc: 92.16
GGGTCTGTAGCTCAGCTGGTTAGAGCACCGTCTTGATAAGGCGGGGGTCGTTGGTTCGAA
CCCAACCAGACCCACCA
>Azoarcus_sp_EbN1_chr.trna19-IleGAT (2413332-2413408) Ile (GAT) 77 bp Sc: 92.16
GGGTCTGTAGCTCAGCTGGTTAGAGCACCGTCTTGATAAGGCGGGGGTCGTTGGTTCGAA
CCCAACCAGACCCACCA
>Azoarcus_sp_EbN1_chr.trna21-IleGAT (2622603-2622679) Ile (GAT) 77 bp Sc: 92.16
GGGTCTGTAGCTCAGCTGGTTAGAGCACCGTCTTGATAAGGCGGGGGTCGTTGGTTCGAA
CCCAACCAGACCCACCA
>Azoarcus_sp_EbN1_chr.trna50-IleGAT (548823-548747) Ile (GAT) 77 bp Sc: 92.16
GGGTCTGTAGCTCAGCTGGTTAGAGCACCGTCTTGATAAGGCGGGGGTCGTTGGTTCGAA
CCCAACCAGACCCACCA
>Azoarcus_sp_EbN1_chr.trna48-LeuCAA (828913-828827) Leu (CAA) 87 bp Sc: 80.90
GCCGGGATGGCGGAATCGGTAGACGCAGCGGATTCAAAATCCGCCGGTGGTAACACTGTG
AGGGTTCGAGTCCCTCTCCCGGCACCA
>Azoarcus_sp_EbN1_chr.trna4-LeuCAG (735160-735244) Leu (CAG) 85 bp Sc: 69.74
GCCCAGATGGCGGAATTGGTAGACGCACTAGTTTCAGGTACTAGCGCTGCGAGGCGTGGA
GGTTCGAGTCCTCTTCTGGGCACCA
>Azoarcus_sp_EbN1_chr.trna5-LeuCAG (735370-735454) Leu (CAG) 85 bp Sc: 72.23
GCCCAGATGGCGGAATTGGTAGACGCACTAGTTTCAGGTACTAGCGCCGCGAGGCGTGGA
GGTTCGAGTCCTCTTCTGGGCACCA
>Azoarcus_sp_EbN1_chr.trna30-LeuGAG (2866309-2866393) Leu (GAG) 85 bp Sc: 65.14
GCCGACGTGGTGAAATTGGTAGACACGCCATCTTGAGGGGGTGGTGGCGAAAGCCGTGTG
AGTTCGAGTCTCACCGTCGGCACCA
>Azoarcus_sp_EbN1_chr.trna43-LeuTAA (3402745-3402661) Leu (TAA) 85 bp Sc: 72.36
GCCGGGGTGGTGAAATCGGTATACACAGCGGATTTAAAATCCGCCGCCTTCGGGCTTACG
GGTTCAAGTCCCGTCCCCGGCACCA

>Azoarcus_sp_EbN1_chr.trna42-LeuTAG (4035847-4035763) Leu (TAG) 85 bp Sc: 76.14
GCGAGGGTGGCGGAATTGGTAGACGCACTGGATTTAGGTTCCAGCGCCGCAAGGCGTGAG
AGTTCGAGTCTCTTCCCTCGCACCA
>Azoarcus_sp_EbN1_chr.trna11-LysCTT (1599533-1599600) Lys (CTT) 68 bp Sc: 33.58
GGCGGAATTGATGGAAATTGTGACTCTTAATCAGTAGGTCGTAGGTTCGAATCCTACTGC
CCCCACCA
>Azoarcus_sp_EbN1_chr.trna10-LysCTT (1583319-1583394) Lys (CTT) 76 bp Sc: 99.01
GGGGGTATAGCTCAGTTGGTAGAGCAGCTGACTCTTAATCAGTAGGTCGTAGGTTCGAAT
CCTACTGCCCCCACCA
>Azoarcus_sp_EbN1_chr.trna9-LysTTT (1583229-1583304) Lys (TTT) 76 bp Sc: 90.00
GGGTCGTTAGCTCAGCTGGTAGAGCAGCGGACTTTTAATCCGTTGGTCGCAGGTTCGAAT
CCCGCACGGCCTACCA
>Azoarcus_sp_EbN1_chr.trna3-MetCAT (139763-139839) Met (CAT) 77 bp Sc: 87.64
CGCGGGGTGGAGCAGTCTGGCAGCTCGTCGGGCTCATAACCCGAAGGTCGCAGGTTCAAA
TCCTGCCCCCGCAACCA
>Azoarcus_sp_EbN1_chr.trna6-MetCAT (792356-792432) Met (CAT) 77 bp Sc: 88.03
GGTGATGTAGCTCAGACGGTTAGAGCGATGGATTCATAACCCATAGGTCGGCGGTTCGAT
TCCGCCCATCACCACCA
>Azoarcus_sp_EbN1_chr.trna47-MetCAT (2581896-2581821) Met (CAT) 76 bp Sc: 94.30
GGGCCCATAGCTCAATGGTTAGAGCAGAGGACTCATAATCCTTTGGTTGCAGGTTCGAGT
CCTGCTGGGCCCACCA
>Azoarcus_sp_EbN1_chr.trna49-PheGAA (689528-689453) Phe (GAA) 76 bp Sc: 96.42
GGCCAGGTAGCTCAGTTGGTAGAGCAGCGGATTGAAAATCCGCGTGTCGGCGGTTCGATT
CCGTCCCTGGCCACCA
>Azoarcus_sp_EbN1_chr.trna23-ProCGG (2677689-2677765) Pro (CGG) 77 bp Sc: 85.19
CGGGGAATAGCTCAGCCTGGTAGAGCACTGCGTTCGGGACGCAGGGGCCGGAGGTTCGAA
TCCTCTTTCCCCGACCA
>Azoarcus_sp_EbN1_chr.trna53-ProGGG (504198-504122) Pro (GGG) 77 bp Sc: 81.03
CGGGGCGTAGCGCAGCCTGGTAGCGCACTTGCATGGGGTGCAAGGGGTCGCGAGTTCGAA
TCCCGCCGCCCCGACCA
>Azoarcus_sp_EbN1_chr.trna36-ProTGG (3482256-3482332) Pro (TGG) 77 bp Sc: 89.92
CGGGGTGTAGCGCAGTCCGGTAGCGCGCTTGCTTTGGGAGCAAGATGTCGGGGGTTCGAA
TCCCTCCACCCCGACCA
>Azoarcus_sp_EbN1_chr.trna39-SerCGA (4155777-4155688) Ser (CGA) 90 bp Sc: 71.26
GGAGAGGTGGCAGAGTGGTCGAATGTACCTGACTCGAAATCAGGCGTAGGTGCGAGCCTA
CCGTGGGTTCGAATCCCACCCTCTCCGCCA
>Azoarcus_sp_EbN1_chr.trna56-SerGCT (372265-372172) Ser (GCT) 94 bp Sc: 74.87
GGAGAGTTGGCCGAGAGGTCGAAGGCACTCCCCTGCTAAGGGAGCATGCGGGCTAAAACT
CGCATCGAGGGTTCGAATCCCTCACTCTCCGCCA
>Azoarcus_sp_EbN1_chr.trna46-SerGGA (2695011-2694921) Ser (GGA) 91 bp Sc: 72.00
GGAGAGGTGGATGAGTGGTTTAAGTCGCACGCCTGGAAAGCGTGTTTGGGATAATATCCC
AACGCGGGTTCGAATCCCGCCCTCTCCGCCA
>Azoarcus_sp_EbN1_chr.trna55-SerTGA (440758-440671) Ser (TGA) 88 bp Sc: 66.83
GGAAGCGTGGCAGAGTGGTCGATTGCACCGGTCTTGAAAACCGGCAACGGGCAACCGTTC
GTGAGTTCGAATCTCACCGCTTCCGCCA
>Azoarcus_sp_EbN1_chr.trna54-ThrCGT (469570-469495) Thr (CGT) 76 bp Sc: 91.51
GCTGCTGTAGCTCAGTCGGTAGAGCAACTGATTCGTAATCAGTAGGTCACCAGTTCGATT
CCGGTCAGCAGCACCA
>Azoarcus_sp_EbN1_chr.trna17-ThrGGT (2242886-2242960) Thr (GGT) 75 bp Sc: 86.08
GCCCATGTAGCTCAGTGGTAGAGCACTCCCTTGGTAAGGGAGAGGTCACGTGTTCAATCC
ACGTCATGGGCACCA
>Azoarcus_sp_EbN1_chr.trna2-ThrTGT (119914-119989) Thr (TGT) 76 bp Sc: 90.15
GCCGGCATAGCTCAGTTGGTAGAGCAACCGCCTTGTAAGCGGTAGGTCATCTGTTCGAGT
CAGATTGTCGGCACCA
>Azoarcus_sp_EbN1_chr.trna18-TrpCCA (2244297-2244372) Trp (CCA) 76 bp Sc: 87.97
AGGGGCATAGCTCAATTGGCAGAGCGTCGGTCTCCAAAACCGAAGGTTGGGGGTTCGATT
CCCTCTGCCCCTGCCA
>Azoarcus_sp_EbN1_chr.trna15-TyrGTA (2242628-2242712) Tyr (GTA) 85 bp Sc: 70.88
GGAGGGGTTCCCGAGCGGTCAAAGGGATCAGACTGTAAATCTGACGGCACTGCCTTCGAA
GGTTCGAATCCTTCCCCCTCCACCA
>Azoarcus_sp_EbN1_chr.trna40-ValCAC (4068953-4068879) Val (CAC) 75 bp Sc: 91.15
GGGCGGTTAGCTCAGCGGTAGAGCACTGCCTTCACACGGCAGGGGTCACTGGTTCGATCC
CAGTACCGCCCACCA
>Azoarcus_sp_EbN1_chr.trna41-ValCAC (4068850-4068776) Val (CAC) 75 bp Sc: 91.15
GGGCGGTTAGCTCAGCGGTAGAGCACTGCCTTCACACGGCAGGGGTCACTGGTTCGATCC
CAGTACCGCCCACCA
>Azoarcus_sp_EbN1_chr.trna52-ValGAC (511820-511744) Val (GAC) 77 bp Sc: 93.06

AGGCGCGTAGCTCAGCTGGTTAGAGCACCACCTTGACATGGTGGGGGTCGTTGGTTCGAG
TCCAATCGCGCCTACCA
>Azoarcus_sp_EbN1_chr.trna31-ValTAC (2959537-2959612) Val (TAC) 76 bp Sc: 92.87
GGGTGCTTAGCTCAGTTGGTAGAGCGCCGCCCTTACAAGGCGGATGTCGGCGGTTCGAGC
CCGTCAGCACCTACCA
>Azorhizobium_caulinodans_ORS_571_chr.trna38-AlaCGC (4254192-4254117) Ala (CGC) 76 bp Sc: 85.66
GGGGCCGTAGCTCAGATGGGAGAGCGCTGCAATCGCACTGCAGAGGTCAGGGGTTCGATT
CCCCTCGGCTCCACCA
>Azorhizobium_caulinodans_ORS_571_chr.trna37-AlaGGC (4492652-4492577) Ala (GGC) 76 bp Sc: 86.53
GGGGTCATAGCTCAGTTGGGAGAGCGCTTGAATGGCATTCAAGAGGTCGGCGGTTCGATT
CCGCCTGGCTCCACCA
>Azorhizobium_caulinodans_ORS_571_chr.trna36-AlaGGC (4492943-4492868) Ala (GGC) 76 bp Sc: 90.20
GGGGCCATAGCTCAGTTGGGAGAGCGCTTGAATGGCATTCAAGAGGTCGGCGGTTCGATT
CCGCCTGGCTCCACCA
>Azorhizobium_caulinodans_ORS_571_chr.trna3-AlaTGC (682344-682419) Ala (TGC) 76 bp Sc: 87.21
GGGGCCATAGCTCAGTTGGGAGAGCGCGTGCTTTGCAAGCATGAGGTCGTCGGTTCGATC
CCGTCTGGCTCCACCA
>Azorhizobium_caulinodans_ORS_571_chr.trna33-AlaTGC (5045414-5045339) Ala (TGC) 76 bp Sc: 87.21
GGGGCCATAGCTCAGTTGGGAGAGCGCGTGCTTTGCAAGCATGAGGTCGTCGGTTCGATC
CCGTCTGGCTCCACCA
>Azorhizobium_caulinodans_ORS_571_chr.trna35-AlaTGC (4778952-4778877) Ala (TGC) 76 bp Sc: 87.21
GGGGCCATAGCTCAGTTGGGAGAGCGCGTGCTTTGCAAGCATGAGGTCGTCGGTTCGATC
CCGTCTGGCTCCACCA
>Azorhizobium_caulinodans_ORS_571_chr.trna27-ArgACG (4641635-4641711) Arg (ACG) 77 bp Sc: 92.98
GCGCCCGTAGCTCAGCTGGATAGAGCACCAGACTACGAATCTGGGGGTCAGAGGTTCGAA
TCCTTTCGGGCGCGCCA
>Azorhizobium_caulinodans_ORS_571_chr.trna40-ArgCCG (3766370-3766294) Arg (CCG) 77 bp Sc: 87.21
GCACCCGTAGCTCAGCTGGATAGAGCGTTGCCCTCCGAAGGCAAAGGTCGCACGTTCGAA
TCGTGCCGGGTGCGCCA
>Azorhizobium_caulinodans_ORS_571_chr.trna25-ArgCCT (4270441-4270517) Arg (CCT) 77 bp Sc: 82.13
CTCCCGGTAGCTCAGCAGGATAGAGCACAGGTTTCCTAAACCTGGGGTCAGGGGTTCGAT
TCCCTTCCGGGAGGCCA
>Azorhizobium_caulinodans_ORS_571_chr.trna10-ArgTCT (1777284-1777360) Arg (TCT) 77 bp Sc: 90.66
GGCCCCGTAGCTCAGCTGGATAGAGCAAGTGCCTTCTAAGCACTAGGTCGCAGGTTCGAG
CCCTGCCGGGGTCGCCA
>Azorhizobium_caulinodans_ORS_571_chr.trna20-AsnGTT (3210345-3210419) Asn (GTT) 75 bp Sc: 90.41
TCCCCGGTAGCTCAGTGGTAGAGCAACCGGCTGTTAACCGGTTGGTCGCTGGTTCGAATC
CGGCCCGGGGAGCCA
>Azorhizobium_caulinodans_ORS_571_chr.trna47-AspGTC (1846897-1846821) Asp (GTC) 77 bp Sc: 97.83
GGGGGTGTAGCTCAGTTGGTTAGAGCGCCGGCCTGTCACGCCGGAGGTCGCGGGTTCGAG
CCCCGTCACTCCCGCCA
>Azorhizobium_caulinodans_ORS_571_chr.trna19-CysGCA (3208033-3208106) Cys (GCA) 74 bp Sc: 68.35
GGCCACGTGGCGGAGTGGTTACGCAGAGGACTGCAAATCCTTGCACCCCGGTTCGATTCC
GGGCGTGGCCTCCA
>Azorhizobium_caulinodans_ORS_571_chr.trna4-GlnCTG (740234-740307) Gln (CTG) 74 bp Sc: 69.00
TGGGGGATAGTTCAACGGTAGAACCGCGGACTCTGACTCCGTTAATCCTGGTTCGAATCC
AGGTCCCCCAGCCA
>Azorhizobium_caulinodans_ORS_571_chr.trna48-GlnTTG (1744594-1744520) Gln (TTG) 75 bp Sc: 77.41
TGGGGCGTAGCCAAGTGGTAAGGCAGGGGATTTTGATTCCCCCATTCCCAGGTTCGAATC
CTGGCGCCCCAGCCA
>Azorhizobium_caulinodans_ORS_571_chr.trna52-GluCTC (322656-322581) Glu (CTC) 76 bp Sc: 66.94
GCTCCCATCGTCTAGCGGTCTAGGACGTCGCCCTCTCACGGCGAAAACAGGGGTTCGAGT
CCCCTTGGGAGCGCCA
>Azorhizobium_caulinodans_ORS_571_chr.trna46-GluTTC (2235680-2235606) Glu (TTC) 75 bp Sc: 60.47
GCGCCCTTCGTCTATCGGTTAGGACGCCACCCTTTCACGGTGGAGAGAGGGGTTCGACTC
CCCTAGGGCGCGCCA
>Azorhizobium_caulinodans_ORS_571_chr.trna53-GlyCCC (260304-260230) Gly (CCC) 75 bp Sc: 90.16
GCGGGCGTAGTTCAGTGGTAGAACGACAGCTTCCCAAGCTGTATGTCGTGGGTTCGATTC
CCATCGCCCGCTCCA
>Azorhizobium_caulinodans_ORS_571_chr.trna26-GlyGCC (4346061-4346135) Gly (GCC) 75 bp Sc: 88.17
GCGGGTGTAGCTCAGGGGTAGAGCACAACCTTGCCAAGGTTGGGGTCGAGGGTTCGAATC
CCTTCGCCCGCTCCA
>Azorhizobium_caulinodans_ORS_571_chr.trna7-GlyTCC (981811-981884) Gly (TCC) 74 bp Sc: 82.42
GCGGGTGTAGCTCAATGGTAGAGCAACAGCCTTCCAAGCTGATGACGAGGGTTCGATTCC
CTTCACCCGCTCCA
>Azorhizobium_caulinodans_ORS_571_chr.trna18-HisGTG (2944201-2944277) His (GTG) 77 bp Sc: 93.68
GCAGCTGTAGCTCAGTTGGTTAGAGCGCCGGTCTGTGGAACCGGAGGTCGGTGGTTCGAG

CCCACCCAGCTGTACCA
>Azorhizobium_caulinodans_ORS_571_chr.trna2-IleGAT (682210-682286) Ile (GAT) 77 bp Sc: 92.10
GGGCCTGTAGCTCAGGTGGTTAGAGCGCACCCCTGATAAGGGTGAGGTCGGACGTTCGAG
TCGTCCCAGGCCCACCA
>Azorhizobium_caulinodans_ORS_571_chr.trna32-IleGAT (5045548-5045472) Ile (GAT) 77 bp Sc: 92.10
GGGCCTGTAGCTCAGGTGGTTAGAGCGCACCCCTGATAAGGGTGAGGTCGGACGTTCGAG
TCGTCCCAGGCCCACCA
>Azorhizobium_caulinodans_ORS_571_chr.trna34-IleGAT (4779086-4779010) Ile (GAT) 77 bp Sc: 92.10
GGGCCTGTAGCTCAGGTGGTTAGAGCGCACCCCTGATAAGGGTGAGGTCGGACGTTCGAG
TCGTCCCAGGCCCACCA
>Azorhizobium_caulinodans_ORS_571_chr.trna29-LeuCAA (4773690-4773776) Leu (CAA) 87 bp Sc: 79.42
GCGGGTGTGGCGGAACTGGTAGACGCGACGGACTCAAAATCCGTTTCTGGTGACAGAGTG
TCGGTTCGAGTCCGACCACCCGCACCA
>Azorhizobium_caulinodans_ORS_571_chr.trna5-LeuCAG (906185-906269) Leu (CAG) 85 bp Sc: 73.31
GCCCAGGTGGCGGAATTGGTAGACGCGCTGGTTTCAGGTACCAGTAGTGAAAGCTGTGGA
GGTTCGAGTCCTCTCCTGGGCACCA
>Azorhizobium_caulinodans_ORS_571_chr.trna42-LeuGAG (3233045-3232961) Leu (GAG) 85 bp Sc: 68.46
GCGGTCGTGGCGGAATTGGTAGACGCGCTAGCTTGAGGTGCTAGTGGGGAGACCCGTGGA
GGTTCGAGTCCTCTCGTCCGCACCA
>Azorhizobium_caulinodans_ORS_571_chr.trna39-LeuTAA (4097057-4096969) Leu (TAA) 89 bp Sc: 77.16
GCGGGCGTGGCGAAACTGGTAAACGCAGCGGACTTAAAATTCGCCGCCCTTTCAATGGCT
TGCGGGTTCGAGCCCCGCCGCCCGCACCA
>Azorhizobium_caulinodans_ORS_571_chr.trna11-LeuTAG (1833437-1833521) Leu (TAG) 85 bp Sc: 70.01
GCGGGCGTGGCGGAACTGGTAGACGCGCGGGATTTAGGTTCCCGTGACGAAAGTCGTGGG
GGTTCGAAACCCTCCGCCCGCACCA
>Azorhizobium_caulinodans_ORS_571_chr.trna23-LysCTT (4079261-4079336) Lys (CTT) 76 bp Sc: 96.14
GGGCGCATAGCTCAGTTGGTAGAGCAGCTGACTCTTAATCAGCGGGTCCTAGGTTCGAGC
CCTAGTGCGCCCACCA
>Azorhizobium_caulinodans_ORS_571_chr.trna24-LysCTT (4079417-4079492) Lys (CTT) 76 bp Sc: 96.14
GGGCGCATAGCTCAGTTGGTAGAGCAGCTGACTCTTAATCAGCGGGTCCTAGGTTCGAGC
CCTAGTGCGCCCACCA
>Azorhizobium_caulinodans_ORS_571_chr.trna16-LysTTT (2509899-2509974) Lys (TTT) 76 bp Sc: 90.38
GAGCCCGTAGCTCAGCCGGTAGAGCACGTGACTTTTAATCATGGGGTCTCGGGTTCGAAT
CCCGACGGGCTCACCA
>Azorhizobium_caulinodans_ORS_571_chr.trna49-MetCAT (1660418-1660343) Met (CAT) 76 bp Sc: 85.45
GGGCCTGTAGCTCAATGGTTAGAGCTGACCGCTCATAACGGTCTGGTTGGGGGTTCGAGT
CCCTCCGGGCCCACCA
>Azorhizobium_caulinodans_ORS_571_chr.trna15-MetCAT (2138705-2138781) Met (CAT) 77 bp Sc: 85.51
CGCGGGGTGGAGCAGCCCGGTAGCTCGTCAGGCTCATAACCTGAAGGTCACAGGTTCAAA
TCCTGTCCCCGCAACCA
>Azorhizobium_caulinodans_ORS_571_chr.trna31-MetCAT (5086224-5086148) Met (CAT) 77 bp Sc: 90.53
GGCAGCGTAGCTCAGTTGGTGAGAGCGCCGGATTCATAACCCGGAGGTCGGCGGTTCAAG
TCCGCCCGCTGCTACCA
>Azorhizobium_caulinodans_ORS_571_chr.trna9-PheGAA (1710750-1710825) Phe (GAA) 76 bp Sc: 91.10
GCCCAGGTAGCTCAGTTGGTAGAGCATGCGACTGAAAATCGCAGTGTCGGTGGTTCGATT
CCGCCCCTGGGCACCA
>Azorhizobium_caulinodans_ORS_571_chr.trna13-ProCGG (2005036-2005112) Pro (CGG) 77 bp Sc: 88.17
CGGAGTGTAGCGCAGTCTGGTAGCGCACCACGTTCGGGACGTGGGGGTCGCAGGTTCAAA
TCCTGCCACTCCGACCA
>Azorhizobium_caulinodans_ORS_571_chr.trna14-ProCGG (2005292-2005368) Pro (CGG) 77 bp Sc: 88.17
CGGAGTGTAGCGCAGTCTGGTAGCGCACCACGTTCGGGACGTGGGGGTCGCAGGTTCAAA
TCCTGCCACTCCGACCA
>Azorhizobium_caulinodans_ORS_571_chr.trna44-ProGGG (2614230-2614153) Pro (GGG) 78 bp Sc: 81.55
CGGGCAGTAGCGCAGCCCGGTTAGCGCACTAGTCTGGGGGACTAGGGGTCGTGGGTTCAA
ATCCCGCCTGCCCGACCA
>Azorhizobium_caulinodans_ORS_571_chr.trna43-ProTGG (3132381-3132305) Pro (TGG) 77 bp Sc: 89.09
CGGAGCATAGCGCAGCCTGGTAGCGCATCTGCTTTGGGAGCAGAGGGTCGCAGGTTCAAA
TCCTGCTGCTCCGACCA
>Azorhizobium_caulinodans_ORS_571_chr.trna50-SeC(p)TCA (1285115-1285024) SeC(p) (TCA) 92 bp Sc: 54.71
GGAGGAGAAGCGTCCCTGGTGGGGCGTCCGGACTTCAAATCCGGGAGGGGCCGCGAGCCG
GTCCTTGGTGGGTTCGACTCCCACTCTCTTCC
>Azorhizobium_caulinodans_ORS_571_chr.trna28-SerCGA (4663988-4664077) Ser (CGA) 90 bp Sc: 81.19
GGAGAGGTGGCAGAGTGGTTGAATGCACCGCACTCGAAATGCGGCATACGGGCAACCGTA
TCGGGGGTTCGAATCCCCCCCTCTCCGCCA
>Azorhizobium_caulinodans_ORS_571_chr.trna41-SerGCT (3345883-3345792) Ser (GCT) 92 bp Sc: 68.39
GGAGACGTGGCCGAGAGGCTGAAGGCACTCGTTTGCTAAATGAGCAGACCCCTAAAAGGG
TCTCGAGGGTTCGAATCCCTCCGTCTCCGCCA

>Azorhizobium_caulinodans_ORS_571_chr.trna1-SerGGA (258233-258322) Ser (GGA) 90 bp Sc: 77.71
GGAGAGGTGGCCGAGTGGTTGAAGGCGCACGCCTGGAACGCGTGTATACGGGAAACCGTA
TCGAGGGTTCGAATCCCTCTCTCTCCGCCA
>Azorhizobium_caulinodans_ORS_571_chr.trna45-SerTGA (2487298-2487209) Ser (TGA) 90 bp Sc: 80.63
GGAAGGGTGGCCGAGTGGTTTAAGGCAGCGGTCTTGAAAACCGCCGTGGGTGCAAGCCCA
CCGTGGGTTCGAATCCCACCCCTTCCGCCA
>Azorhizobium_caulinodans_ORS_571_chr.trna21-ThrCGT (3606742-3606817) Thr (CGT) 76 bp Sc: 100.66
GCCGCTTTAGCTCAGCTGGTAGAGCACCTCATTCGTAATGAGGGGGTCGGGGGTTCGAAT
CCCTCAAGCGGCACCA
>Azorhizobium_caulinodans_ORS_571_chr.trna22-ThrGGT (4041028-4041102) Thr (GGT) 75 bp Sc: 86.73
GCTGCGGTAGCTCAGTGGTAGAGCACTCCCTTGGTAAGGGAGAGGTCGAGAGTTCAATCC
TCTCTCGCAGCACCA
>Azorhizobium_caulinodans_ORS_571_chr.trna51-ThrTGT (934686-934612) Thr (TGT) 75 bp Sc: 85.16
GCCGGCTTAGCACAGCGGTAGTGCAGCGGTTTTGTAAACCGAAGGTCGGGGGTTCGATCC
CCTCAGCCGGCACCA
>Azorhizobium_caulinodans_ORS_571_chr.trna8-TrpCCA (983455-983531) Trp (CCA) 77 bp Sc: 90.34
AGGAGTGTAGCTCAACTGGTTAGAGCACCGGTCTCCAAAACCGGGGGTTGGGGGTTCGAG
TCCCTCCACTCCTGCCA
>Azorhizobium_caulinodans_ORS_571_chr.trna6-TyrGTA (981689-981773) Tyr (GTA) 85 bp Sc: 72.88
GGAGGGGTGCCCGAGTGGTTAAAGGGGACGGACTGTAAATCCGTTGGCTCTGCCTACGTT
GGTTCGAATCCAACCCCCTCCACCA
>Azorhizobium_caulinodans_ORS_571_chr.trna17-ValCAC (2688478-2688552) Val (CAC) 75 bp Sc: 88.51
GGGCGATTAGCTCAGCGGGAGAGCACTCCCTTCACACGGGAGGGGTCGCAGGTTCAATCC
CTGCATCGCCCACCA
>Azorhizobium_caulinodans_ORS_571_chr.trna30-ValGAC (4865454-4865528) Val (GAC) 75 bp Sc: 87.73
GGGCGCGTAGCTCAGCGGGAGAGCACTACGTTGACATCGTAGGGGTCACAGGTTCGATCC
CTGTCGCGCCCACCA
>Azorhizobium_caulinodans_ORS_571_chr.trna12-ValTAC (1845381-1845456) Val (TAC) 76 bp Sc: 95.21
GGGCGGTTAGCTCAGTTGGTAGAGCATCTCGTTTACACCGAGAGGGTCGGCGGTTCGAGC
CCGTCACCGCCCACCA
>Bacillus_amyloliquefaciens_FZB42_chr.trna63-AlaGGC (2940796-2940724) Ala (GGC) 73 bp Sc: 77.43
GGGGCCATAGCTCAGCTGGGAGAGCGCTACGCTGGCAGCGTAGAGGTCAGGGGTTCGAGC
CCCCTTGGCTCCA
>Bacillus_amyloliquefaciens_FZB42_chr.trna18-AlaTGC (167353-167425) Ala (TGC) 73 bp Sc: 84.64
GGGGCCTTAGCTCAGCTGGGAGAGCGCCTGCTTTGCACGCAGGAGGTCAGCGGTTCGATC
CCGCTAGGCTCCA
>Bacillus_amyloliquefaciens_FZB42_chr.trna74-AlaTGC (2921864-2921792) Ala (TGC) 73 bp Sc: 84.64
GGGGCCTTAGCTCAGCTGGGAGAGCGCCTGCTTTGCACGCAGGAGGTCAGCGGTTCGATC
CCGCTAGGCTCCA
>Bacillus_amyloliquefaciens_FZB42_chr.trna12-AlaTGC (96824-96899) Ala (TGC) 76 bp Sc: 92.94
GGGGCCTTAGCTCAGCTGGGAGAGCGCCTGCTTTGCACGCAGGAGGTCAGCGGTTCGATC
CCGCTAGGCTCCACCA
>Bacillus_amyloliquefaciens_FZB42_chr.trna22-AlaTGC (168370-168445) Ala (TGC) 76 bp Sc: 92.94
GGGGCCTTAGCTCAGCTGGGAGAGCGCCTGCTTTGCACGCAGGAGGTCAGCGGTTCGATC
CCGCTAGGCTCCACCA
>Bacillus_amyloliquefaciens_FZB42_chr.trna65-AlaTGC (2925902-2925827) Ala (TGC) 76 bp Sc: 92.94
GGGGCCTTAGCTCAGCTGGGAGAGCGCCTGCTTTGCACGCAGGAGGTCAGCGGTTCGATC
CCGCTAGGCTCCACCA
>Bacillus_amyloliquefaciens_FZB42_chr.trna10-ArgACG (96650-96726) Arg (ACG) 77 bp Sc: 81.43
GCGCCCGTAGCTCAATTGGATAGAGCGTTTGACTACGGATCAAAAGGTTAGGGGTTCGAC
TCCTCTCGGGCGCGCCA
>Bacillus_amyloliquefaciens_FZB42_chr.trna16-ArgACG (167179-167255) Arg (ACG) 77 bp Sc: 81.43
GCGCCCGTAGCTCAATTGGATAGAGCGTTTGACTACGGATCAAAAGGTTAGGGGTTCGAC
TCCTCTCGGGCGCGCCA
>Bacillus_amyloliquefaciens_FZB42_chr.trna35-ArgACG (604799-604875) Arg (ACG) 77 bp Sc: 81.43
GCGCCCGTAGCTCAATTGGATAGAGCGTTTGACTACGGATCAAAAGGTTAGGGGTTCGAC
TCCTCTCGGGCGCGCCA
>Bacillus_amyloliquefaciens_FZB42_chr.trna72-ArgACG (2922039-2921963) Arg (ACG) 77 bp Sc: 81.43
GCGCCCGTAGCTCAATTGGATAGAGCGTTTGACTACGGATCAAAAGGTTAGGGGTTCGAC
TCCTCTCGGGCGCGCCA
>Bacillus_amyloliquefaciens_FZB42_chr.trna62-ArgCCG (3296286-3296211) Arg (CCG) 76 bp Sc: 66.66
GCGCTCGTAGCTCAGTTGGATAGAGCGGTGGTTTCCGGTACCACGTCTGTCGGGGGTTCG
AATCCCTCCGAGCGCG
>Bacillus_amyloliquefaciens_FZB42_chr.trna89-ArgCCT (1974432-1974360) Arg (CCT) 73 bp Sc: 69.33
GCTCTAGTAGCACAGCGGATAGTGCAGCAGTTTCCTAAACTGCAGGTCGGGAGTTCGAAT
CTCTCCTAGAGCG
>Bacillus_amyloliquefaciens_FZB42_chr.trna88-ArgTCT (2663208-2663135) Arg (TCT) 74 bp Sc: 81.02

GTCCCAGTAGCTCAGCCGGATAGAGCAACGGCCTTCTAAGCCGTCGGTCGGGAGTTCGAA
TCTCTCCTGGGACG
>Bacillus_amyloliquefaciens_FZB42_chr.trna87-ArgTCT (2663406-2663333) Arg (TCT) 74 bp Sc: 83.12
GTCCCAGTAGCTCAGCTGGATAGAGCAACGGCCTTCTAAGCCGTCGGTCGGGAGTTCGAA
TCTCTCCTGGGACG
>Bacillus_amyloliquefaciens_FZB42_chr.trna28-AsnGTT (501647-501721) Asn (GTT) 75 bp Sc: 87.08
TCCACAGTAGCTCAGTGGTAGAGCTATCGGCTGTTAACCGATCGGTCGCAGGTTCGAATC
CTGCCTGTGGAGCCA
>Bacillus_amyloliquefaciens_FZB42_chr.trna84-AsnGTT (2920970-2920896) Asn (GTT) 75 bp Sc: 87.08
TCCACAGTAGCTCAGTGGTAGAGCTATCGGCTGTTAACCGATCGGTCGCAGGTTCGAATC
CTGCCTGTGGAGCCA
>Bacillus_amyloliquefaciens_FZB42_chr.trna13-AsnGTT (166580-166654) Asn (GTT) 75 bp Sc: 88.32
TCCGCAGTAGCTCAGTGGTAGAGCTATCGGCTGTTAACCGATCGGTCGTAGGTTCGAGTC
CTACCTGCGGAGCCA
>Bacillus_amyloliquefaciens_FZB42_chr.trna39-AsnGTT (881058-881132) Asn (GTT) 75 bp Sc: 88.32
TCCGCAGTAGCTCAGTGGTAGAGCTATCGGCTGTTAACCGATCGGTCGTAGGTTCGAATC
CTACCTGCGGAGCCA
>Bacillus_amyloliquefaciens_FZB42_chr.trna38-AspGTC (609983-610059) Asp (GTC) 77 bp Sc: 95.80
GGTCCGGTAGTTCAGTTGGTTAGAATGCCTGCCTGTCACGCAGGAGGTCGCGGGTTCGAG
TCCCGTCCGGACCGCCA
>Bacillus_amyloliquefaciens_FZB42_chr.trna44-AspGTC (881514-881590) Asp (GTC) 77 bp Sc: 95.80
GGTCCGGTAGTTCAGTTGGTTAGAATGCCTGCCTGTCACGCAGGAGGTCGCGGGTTCGAG
TCCCGTCCGGACCGCCA
>Bacillus_amyloliquefaciens_FZB42_chr.trna60-AspGTC (3852563-3852487) Asp (GTC) 77 bp Sc: 95.80
GGTCCGGTAGTTCAGTTGGTTAGAATGCCTGCCTGTCACGCAGGAGGTCGCGGGTTCGAG
TCCCGTCCGGACCGCCA
>Bacillus_amyloliquefaciens_FZB42_chr.trna79-AspGTC (2921413-2921337) Asp (GTC) 77 bp Sc: 95.80
GGTCCGGTAGTTCAGTTGGTTAGAATGCCTGCCTGTCACGCAGGAGGTCGCGGGTTCGAG
TCCCGTCCGGACCGCCA
>Bacillus_amyloliquefaciens_FZB42_chr.trna52-CysGCA (882253-882323) Cys (GCA) 71 bp Sc: 67.07
GGCGGCATAGCCAAGTGGTAAGGCAGAGGTCTGCAAAACCTTTATCCCCGGTTCGAATCC
GGGTGTCGCCT
>Bacillus_amyloliquefaciens_FZB42_chr.trna57-GlnTTG (2436822-2436892) Gln (TTG) 71 bp Sc: 65.47
TGGGCTATAGCCAAGCGGTAAGGCAATGGACTTTGACTCCGTGATCGTTGGTTCGAATCC
AGCTAGCCCAG
>Bacillus_amyloliquefaciens_FZB42_chr.trna27-GlnTTG (198400-198471) Gln (TTG) 72 bp Sc: 67.67
TGGGCTATAGCCAAGCGGTAAGGCAACGGACTTTGACTCCGTCATGCGTTGGTTCGAATC
CAGCTAGCCCAG
>Bacillus_amyloliquefaciens_FZB42_chr.trna50-GlnTTG (882047-882118) Gln (TTG) 72 bp Sc: 67.67
TGGGCTATAGCCAAGCGGTAAGGCAACGGACTTTGACTCCGTCATGCGTTGGTTCGAATC
CAGCTAGCCCAG
>Bacillus_amyloliquefaciens_FZB42_chr.trna31-GlnTTG (501921-501995) Gln (TTG) 75 bp Sc: 75.97
TGGGCTATAGCCAAGCGGTAAGGCAACGGACTTTGACTCCGTCATGCGTTGGTTCGAATC
CAGCTAGCCCAGCCA
>Bacillus_amyloliquefaciens_FZB42_chr.trna23-GluTTC (198063-198134) Glu (TTC) 72 bp Sc: 70.15
GGCCCGTTGGTCAAGCGGTTAAGACACCGCCCTTTCACGGCGGTAACACGGGTTCGAATC
CCGTACGGGTCA
>Bacillus_amyloliquefaciens_FZB42_chr.trna41-GluTTC (881265-881336) Glu (TTC) 72 bp Sc: 70.15
GGCCCGTTGGTCAAGCGGTTAAGACACCGCCCTTTCACGGCGGTAACACGGGTTCGAATC
CCGTACGGGTCA
>Bacillus_amyloliquefaciens_FZB42_chr.trna59-GluTTC (3852719-3852648) Glu (TTC) 72 bp Sc: 70.15
GGCCCGTTGGTCAAGCGGTTAAGACACCGCCCTTTCACGGCGGTAACACGGGTTCGAATC
CCGTACGGGTCA
>Bacillus_amyloliquefaciens_FZB42_chr.trna86-GluTTC (2920798-2920727) Glu (TTC) 72 bp Sc: 70.15
GGCCCGTTGGTCAAGCGGTTAAGACACCGCCCTTTCACGGCGGTAACACGGGTTCGAATC
CCGTACGGGTCA
>Bacillus_amyloliquefaciens_FZB42_chr.trna3-GluTTC (70956-71030) Glu (TTC) 75 bp Sc: 70.77
GGCCCGTTGGTCAAGCGGTTAAGACACCGCCCTTTCACGGCGGTAACACGGGTTCGAATC
CCGTACGGGTCATCA
>Bacillus_amyloliquefaciens_FZB42_chr.trna30-GluTTC (501835-501909) Glu (TTC) 75 bp Sc: 78.45
GGCCCGTTGGTCAAGCGGTTAAGACACCGCCCTTTCACGGCGGTAACACGGGTTCGAATC
CCGTACGGGTCACCA
>Bacillus_amyloliquefaciens_FZB42_chr.trna15-GlyGCC (167072-167146) Gly (GCC) 75 bp Sc: 90.25
GCGGAAGTAGTTCAGTGGTAGAACATCACCTTGCCAAGGTGGGGGTCGCGGGTTCGAATC
CCGTCTTCCGCTCCA
>Bacillus_amyloliquefaciens_FZB42_chr.trna51-GlyGCC (882172-882246) Gly (GCC) 75 bp Sc: 90.25
GCGGAAGTAGTTCAGTGGTAGAACATCACCTTGCCAAGGTGGGGGTCGCGGGTTCGAATC

CCGTCTTCCGCTCCA
>Bacillus_amyloliquefaciens_FZB42_chr.trna70-GlyGCC (2922228-2922154) Gly (GCC) 75 bp Sc: 90.25
GCGGAAGTAGTTCAGTGGTAGAACATCACCTTGCCAAGGTGGGGGTCGCGGGTTCGAATC
CCGTCTTCCGCTCCA
>Bacillus_amyloliquefaciens_FZB42_chr.trna8-GlyGCC (96464-96538) Gly (GCC) 75 bp Sc: 94.93
GCGGAAGTAGTTCAGTGGTAGAACACCACCTTGCCAAGGTGGGGGTCGCGGGTTCGAATC
CCGTCTTCCGCTCCA
>Bacillus_amyloliquefaciens_FZB42_chr.trna36-GlyTCC (604884-604957) Gly (TCC) 74 bp Sc: 78.55
GCGGGTGTAGTTTAGTGGTAAAACCTCAGCCTTCCAAGCTGATGTCGTGAGTTCGATTCT
CATCACCCGCTCCA
>Bacillus_amyloliquefaciens_FZB42_chr.trna55-GlyTCC (897337-897410) Gly (TCC) 74 bp Sc: 78.55
GCGGGTGTAGTTTAGTGGTAAAACCTCAGCCTTCCAAGCTGATGTCGTGAGTTCGATTCT
CATCACCCGCTCCA
>Bacillus_amyloliquefaciens_FZB42_chr.trna82-GlyTCC (2921146-2921073) Gly (TCC) 74 bp Sc: 81.36
GCGGGTGTAGTTTAGTGGTAAAACCTCAGCCTTCCAAGCTGATGTCGTGGGTTCGATTCC
CATCACCCGCTCCA
>Bacillus_amyloliquefaciens_FZB42_chr.trna49-HisGTG (881962-882037) His (GTG) 76 bp Sc: 72.51
GCGGTTGTGGCGAAGTGGTTAACGCACCAGATTGTGGCTCTGGCACTCGTGGGTTCGATT
CCCATCAATCGCCCCA
>Bacillus_amyloliquefaciens_FZB42_chr.trna81-HisGTG (2921230-2921155) His (GTG) 76 bp Sc: 72.51
GCGGTTGTGGCGAAGTGGTTAACGCACCAGATTGTGGCTCTGGCACTCGTGGGTTCGATT
CCCATCAATCGCCCCA
>Bacillus_amyloliquefaciens_FZB42_chr.trna21-IleGAT (168279-168355) Ile (GAT) 77 bp Sc: 102.39
GGGCCTGTAGCTCAGCTGGTTAGAGCGCACGCCTGATAAGCGTGAGGTCGGTGGTTCGAG
TCCACTCAGGCCCACCA
>Bacillus_amyloliquefaciens_FZB42_chr.trna64-IleGAT (2925993-2925917) Ile (GAT) 77 bp Sc: 102.39
GGGCCTGTAGCTCAGCTGGTTAGAGCGCACGCCTGATAAGCGTGAGGTCGGTGGTTCGAG
TCCACTCAGGCCCACCA
>Bacillus_amyloliquefaciens_FZB42_chr.trna83-IleGAT (2921057-2920981) Ile (GAT) 77 bp Sc: 102.39
GGGCCTGTAGCTCAGCTGGTTAGAGCGCACGCCTGATAAGCGTGAGGTCGGTGGTTCGAG
TCCACTCAGGCCCACCA
>Bacillus_amyloliquefaciens_FZB42_chr.trna54-LeuCAA (882665-882746) Leu (CAA) 82 bp Sc: 61.23
GCCGGTGTGGCGGAATTGGCAGACGCGCACGACTCAAAATCGTGTTCCTTCTGGAGTGCC
GGTTCGACCCCGGCCACCGGTA
>Bacillus_amyloliquefaciens_FZB42_chr.trna69-LeuCAG (2922321-2922238) Leu (CAG) 84 bp Sc: 64.19
GCGGATGTGGCGGAATTGGCAGACGCGCTAGAATCAGGCTCTAGTGTCTTTACAGACGTG
GGGGTTCAAGTCCCTTCATCCGCA
>Bacillus_amyloliquefaciens_FZB42_chr.trna34-LeuGAG (502263-502346) Leu (GAG) 84 bp Sc: 53.11
GCGGTCGTGGCGGAATGGCAGACGCGCTAGGTTGAGGGCCTAGTGGGTGAATAACCCGTG
GAGGTTCAAGTCCTCTCGGCCGCA
>Bacillus_amyloliquefaciens_FZB42_chr.trna71-LeuTAA (2922134-2922049) Leu (TAA) 86 bp Sc: 68.21
GCCGGGGTGGTGGAATTGGCAGACACACAGGACTTAAAATCCTGCGGTAGGTGACTACCG
TGCCGGTTCAAGTCCGGCCCTCGGCA
>Bacillus_amyloliquefaciens_FZB42_chr.trna9-LeuTAA (96553-96638) Leu (TAA) 86 bp Sc: 68.21
GCCGGGGTGGTGGAATTGGCAGACACACAGGACTTAAAATCCTGCGGTAGGTGACTACCG
TGCCGGTTCAAGTCCGGCCCTCGGCA
>Bacillus_amyloliquefaciens_FZB42_chr.trna53-LeuTAA (882331-882419) Leu (TAA) 89 bp Sc: 76.51
GCCGGGGTGGTGGAATTGGCAGACACACAGGACTTAAAATCCTGCGGTAGGTGACTACCG
TGCCGGTTCAAGTCCGGCCCTCGGCACCA
>Bacillus_amyloliquefaciens_FZB42_chr.trna7-LeuTAG (96358-96439) Leu (TAG) 82 bp Sc: 71.53
GCGGGTGTGGCGGAATTGGCAGACGCGCTAGACTTAGGATCTAGTGTCTTATGACGTGGG
GGTTCAAGTCCCTTCACCCGCA
>Bacillus_amyloliquefaciens_FZB42_chr.trna33-LeuTAG (502113-502194) Leu (TAG) 82 bp Sc: 73.07
GCGGGTGTGGCGGAATTGGCAGACGCGCTAGACTTAGGATCTAGTGTCTTACGACGTGGG
GGTTCGAGTCCCTTCACCCGCA
>Bacillus_amyloliquefaciens_FZB42_chr.trna32-LysTTT (502029-502104) Lys (TTT) 76 bp Sc: 98.67
GAGCCATTAGCTCAGTTGGTAGAGCATCTGACTTTTAATCAGAGGGTCGAAGGTTCGAGT
CCTTCATGGCTCACCA
>Bacillus_amyloliquefaciens_FZB42_chr.trna58-LysTTT (3852803-3852728) Lys (TTT) 76 bp Sc: 98.67
GAGCCATTAGCTCAGTTGGTAGAGCATCTGACTTTTAATCAGAGGGTCGAAGGTTCGAGT
CCTTCATGGCTCACCA
>Bacillus_amyloliquefaciens_FZB42_chr.trna6-LysTTT (96276-96351) Lys (TTT) 76 bp Sc: 98.67
GAGCCATTAGCTCAGTTGGTAGAGCATCTGACTTTTAATCAGAGGGTCGAAGGTTCGAGT
CCTTCATGGCTCACCA
>Bacillus_amyloliquefaciens_FZB42_chr.trna68-LysTTT (2922407-2922332) Lys (TTT) 76 bp Sc: 98.67
GAGCCATTAGCTCAGTTGGTAGAGCATCTGACTTTTAATCAGAGGGTCGAAGGTTCGAGT
CCTTCATGGCTCACCA

>Bacillus_amyloliquefaciens_FZB42_chr.trna20-MetCAT (167525-167594) Met (CAT) 70 bp Sc: 36.46
GGACCTTTAGCTCAGTTGGTTAGAGCAGACGGCTCATAACCGTCCGGTCGTAGGTTGAGC
ATGTGGTTTA
>Bacillus_amyloliquefaciens_FZB42_chr.trna19-MetCAT (167446-167522) Met (CAT) 77 bp Sc: 83.99
GGCGGTGTAGCTCAGCTGGCTAGAGCGTACGGTTCATACCCGTGAGGTCGGGGGTTCGAT
CCCCTCCGCCGCTACCA
>Bacillus_amyloliquefaciens_FZB42_chr.trna2-MetCAT (70870-70946) Met (CAT) 77 bp Sc: 83.99
GGCGGTGTAGCTCAGCTGGCTAGAGCGTACGGTTCATACCCGTGAGGTCGGGGGTTCGAT
CCCCTCCGCCGCTACCA
>Bacillus_amyloliquefaciens_FZB42_chr.trna75-MetCAT (2921771-2921695) Met (CAT) 77 bp Sc: 83.99
GGCGGTGTAGCTCAGCTGGCTAGAGCGTACGGTTCATACCCGTGAGGTCGGGGGTTCGAT
CCCCTCCGCCGCTACCA
>Bacillus_amyloliquefaciens_FZB42_chr.trna37-MetCAT (609896-609972) Met (CAT) 77 bp Sc: 90.17
CGCGGGGTGGAGCAGTTCGGTAGCTCGTCGGGCTCATAACCCGAAGGTCGCAGGTTCAAA
TCCTGCCCCCGCAACCA
>Bacillus_amyloliquefaciens_FZB42_chr.trna43-MetCAT (881430-881506) Met (CAT) 77 bp Sc: 90.17
CGCGGGGTGGAGCAGTTCGGTAGCTCGTCGGGCTCATAACCCGAAGGTCGCAGGTTCAAA
TCCTGCCCCCGCAACCA
>Bacillus_amyloliquefaciens_FZB42_chr.trna78-MetCAT (2921496-2921420) Met (CAT) 77 bp Sc: 90.17
CGCGGGGTGGAGCAGTTCGGTAGCTCGTCGGGCTCATAACCCGAAGGTCGCAGGTTCAAA
TCCTGCCCCCGCAACCA
>Bacillus_amyloliquefaciens_FZB42_chr.trna76-MetCAT (2921692-2921616) Met (CAT) 77 bp Sc: 97.54
GGACCTTTAGCTCAGTTGGTTAGAGCAGACGGCTCATAACCGTCCGGTCGTAGGTTCGAG
TCCTACAAGGTCCACCA
>Bacillus_amyloliquefaciens_FZB42_chr.trna61-PheGAA (3852450-3852378) Phe (GAA) 73 bp Sc: 89.60
GGCTCGGTAGCTCAGTTGGTAGAGCAACGGACTGAAAATCCGTGTGTCGGCGGTTCGATT
CCGTCCCGAGCCA
>Bacillus_amyloliquefaciens_FZB42_chr.trna45-PheGAA (881601-881676) Phe (GAA) 76 bp Sc: 97.89
GGCTCGGTAGCTCAGTTGGTAGAGCAACGGACTGAAAATCCGTGTGTCGGCGGTTCGATT
CCGTCCCGAGCCACCA
>Bacillus_amyloliquefaciens_FZB42_chr.trna80-PheGAA (2921326-2921251) Phe (GAA) 76 bp Sc: 97.89
GGCTCGGTAGCTCAGTTGGTAGAGCAACGGACTGAAAATCCGTGTGTCGGCGGTTCGATT
CCGTCCCGAGCCACCA
>Bacillus_amyloliquefaciens_FZB42_chr.trna11-ProTGG (96743-96819) Pro (TGG) 77 bp Sc: 94.72
CGGGAAGTAGCTCAGCTTGGTAGAGCACATGGTTTGGGACCATGGGGTCGCAGGTTCGAA
TCCTGTCTTCCCGACCA
>Bacillus_amyloliquefaciens_FZB42_chr.trna17-ProTGG (167272-167348) Pro (TGG) 77 bp Sc: 94.72
CGGGAAGTAGCTCAGCTTGGTAGAGCACATGGTTTGGGACCATGGGGTCGCAGGTTCGAA
TCCTGTCTTCCCGACCA
>Bacillus_amyloliquefaciens_FZB42_chr.trna73-ProTGG (2921945-2921869) Pro (TGG) 77 bp Sc: 94.72
CGGGAAGTAGCTCAGCTTGGTAGAGCACATGGTTTGGGACCATGGGGTCGCAGGTTCGAA
TCCTGTCTTCCCGACCA
>Bacillus_amyloliquefaciens_FZB42_chr.trna29-SerGCT (501725-501815) Ser (GCT) 91 bp Sc: 71.43
GGAGAAGTACTCAAGTGGCTGAAGAGGCGCCCCTGCTAAGGGTGTAGGTCGCGTAAGCGG
CGCGAGGGTTCAAATCCCTCCTTCTCCGCCA
>Bacillus_amyloliquefaciens_FZB42_chr.trna85-SerGCT (2920892-2920802) Ser (GCT) 91 bp Sc: 72.43
GGAGAAGTACTCAAGTGGCTGAAGAGGCGCCCCTGCTAAGGGTGTAGGTCGTGTAAGCGG
CGCGAGGGTTCAAATCCCTCCTTCTCCGCCA
>Bacillus_amyloliquefaciens_FZB42_chr.trna40-SerGGA (881138-881228) Ser (GGA) 91 bp Sc: 61.49
GGAGAGCTGTCCGAGTGGCCGAAGGAGCACGATTGGAAATCGTGTAGGCGGTAACCCCGT
CTCAAGGGTTCGAATCCCTTGCTCTCCGCCA
>Bacillus_amyloliquefaciens_FZB42_chr.trna1-SerTGA (23312-23404) Ser (TGA) 93 bp Sc: 72.49
GGAGGAATACCCAAGTCTGGCTGAAGGGATCGGTCTTGAAAACCGACAGGGGTGTCAAAG
CCCGCGGGGGTTCGAATCCCTCTTCCTCCGCCA
>Bacillus_amyloliquefaciens_FZB42_chr.trna77-SerTGA (2921608-2921516) Ser (TGA) 93 bp Sc: 72.49
GGAGGAATACCCAAGTCTGGCTGAAGGGATCGGTCTTGAAAACCGACAGGGGTGTCAAAG
CCCGCGGGGGTTCGAATCCCTCTTCCTCCGCCA
>Bacillus_amyloliquefaciens_FZB42_chr.trna14-ThrGGT (166657-166729) Thr (GGT) 73 bp Sc: 75.20
GCTTCCATAGCTCAGCAGGTAGAGCACTTCCATGGTAAGGAAGAGGTCAGCGGTTCGAGC
CCGCTTGGAAGCT
>Bacillus_amyloliquefaciens_FZB42_chr.trna46-ThrTGT (881680-881752) Thr (TGT) 73 bp Sc: 82.36
GCCGGTCTAGCTCAATTGGTAGAGCAACTGACTTGTAATCAGTAGGTTGGGGGTTCAAGT
CCTCTGGCCGGCA
>Bacillus_amyloliquefaciens_FZB42_chr.trna25-ThrTGT (198219-198291) Thr (TGT) 73 bp Sc: 84.64
GCCGGCCTAGCTCAATTGGTAGAGCAACTGACTTGTAATCAGTAGGTTGGGGGTTCAAGT
CCTCTGGCCGGCA
>Bacillus_amyloliquefaciens_FZB42_chr.trna67-ThrTGT (2922520-2922445) Thr (TGT) 76 bp Sc: 91.03

GCCGGTGTAGCTCAATTGGTAGAGCAACTGACTTGTAATCAGTAGGTTGGGGGTTCAAGT
CCTCTTGCCGGCACCA
>Bacillus_amyloliquefaciens_FZB42_chr.trna5-ThrTGT (96164-96239) Thr (TGT) 76 bp Sc: 92.93
GCCGGCCTAGCTCAATTGGTAGAGCAACTGACTTGTAATCAGTAGGTTGGGGGTTCAAGT
CCTCTGGCCGGCACCA
>Bacillus_amyloliquefaciens_FZB42_chr.trna48-TrpCCA (881859-881932) Trp (CCA) 74 bp Sc: 71.01
AGGGGCATAGTTTAACGGTAGAACAGAGGTCTCCAAAACCTCCGGTGTGGGTTCGATTCC
TACTGCCCCTGCCA
>Bacillus_amyloliquefaciens_FZB42_chr.trna26-TyrGTA (198311-198395) Tyr (GTA) 85 bp Sc: 78.86
GGAGGGGTAGCGAAGTGGCTAAACGCGGCGGACTGTAAATCCGCTCCCTCAGGGTTCGGC
AGTTCGAATCTGCCCCCCTCCACCA
>Bacillus_amyloliquefaciens_FZB42_chr.trna47-TyrGTA (881764-881848) Tyr (GTA) 85 bp Sc: 78.86
GGAGGGGTAGCGAAGTGGCTAAACGCGGCGGACTGTAAATCCGCTCCCTCAGGGTTCGGC
AGTTCGAATCTGCCCCCCTCCACCA
>Bacillus_amyloliquefaciens_FZB42_chr.trna56-ValGAC (1164134-1164206) Val (GAC) 73 bp Sc: 71.64
GATTCCGTAGCTCAGCTGGGAGAGCGCTACCTTGACAGGGTAGAGGTCGCTGGTTCGAGC
CCAGTCGGAATCA
>Bacillus_amyloliquefaciens_FZB42_chr.trna24-ValTAC (198138-198213) Val (TAC) 76 bp Sc: 96.46
GGAGGATTAGCTCAGCTGGGAGAGCATCTGCCTTACAAGCAGAGGGTCGGCGGTTCGAGC
CCGTCATCCTCCACCA
>Bacillus_amyloliquefaciens_FZB42_chr.trna4-ValTAC (96083-96158) Val (TAC) 76 bp Sc: 96.46
GGAGGATTAGCTCAGCTGGGAGAGCATCTGCCTTACAAGCAGAGGGTCGGCGGTTCGAGC
CCGTCATCCTCCACCA
>Bacillus_amyloliquefaciens_FZB42_chr.trna42-ValTAC (881347-881422) Val (TAC) 76 bp Sc: 96.46
GGAGGATTAGCTCAGCTGGGAGAGCATCTGCCTTACAAGCAGAGGGTCGGCGGTTCGAGC
CCGTCATCCTCCACCA
>Bacillus_amyloliquefaciens_FZB42_chr.trna66-ValTAC (2922625-2922550) Val (TAC) 76 bp Sc: 96.46
GGAGGATTAGCTCAGCTGGGAGAGCATCTGCCTTACAAGCAGAGGGTCGGCGGTTCGAGC
CCGTCATCCTCCACCA
>Bacillus_anthracis_A2012_,_unfinished_sequence.trna34-ArgCCG (209104-209026) Arg (CCG) 79 bp Sc: 62.69
GCGCCCATAGCTCAGTCGGATAGAGCGGTGGTTTCCGGTACCACGTCTGCCGGGGGTTCG
AATCCCTCTGGGCGCGTCA
>Bacillus_anthracis_A2012_,_unfinished_sequence.trna4-AsnGTT (784437-784508) Asn (GTT) 72 bp Sc: 45.62
TCCGCAGTAGCTCAGTGGTAGAGCTATCGGATGTTAACCGATCGGTCGTAGGTTCGANNN
NNNNNNNNNNNN
>Bacillus_anthracis_A2012_,_unfinished_sequence.trna19-AsnGTT (1250303-1250376) Asn (GTT) 74 bp Sc: 77.34
TCCGCAGTAGCTCAGTGGTAGAGCTATCGGCTGTTAACCGATCGGTCGTAGGTTCAGTCC
TACCTGCGGAGCCA
>Bacillus_anthracis_A2012_,_unfinished_sequence.trna25-AsnGTT (5092950-5092876) Asn (GTT) 75 bp Sc: 82.05
TCCGCAGTAGCTCAGTGGTAGAGCTATCGGCTGTTAACCGATCGGTCGTAGGTTCGAGTC
CTACCTGCKGAGCCA
>Bacillus_anthracis_A2012_,_unfinished_sequence.trna32-AspGTC (547398-547323) Asp (GTC) 76 bp Sc: 76.06
GGTCCCGTGGTGTAGTGGTTAACATGCCTGCCTGTCACGCAGGAGATCGCCGGTTCGACC
CCGGTCGGGACCGCCA
>Bacillus_anthracis_A2012_,_unfinished_sequence.trna6-AspGTC (784682-784757) Asp (GTC) 76 bp Sc: 76.06
GGTCCCGTGGTGTAGTGGTTAACATGCCTGCCTGTCACGCAGGAGATCGCCGGTTCGACC
CCGGTCGGGACCGCCA
>Bacillus_anthracis_A2012_,_unfinished_sequence.trna8-AspGTC (1048640-1048715) Asp (GTC) 76 bp Sc: 76.06
GGTCCCGTGGTGTAGTGGTTAACATGCCTGCCTGTCACGCAGGAGATCGCCGGTTCGACC
CCGGTCGGGACCGCCA
>Bacillus_anthracis_A2012_,_unfinished_sequence.trna16-CysGCA (1049396-1049466) Cys (GCA) 71 bp Sc: 71.27
GGCGGCATAGCCAAGTGGTAAGGCAGAGGTCTGCAAAACCTTTATCACCGGTTCAAATCC
GGTTGCCGCCT
>Bacillus_anthracis_A2012_,_unfinished_sequence.trna14-GlnTTG (1049227-1049301) Gln (TTG) 75 bp Sc: 78.60
TGGGCTATAGCCAAGCGGTAAGGCAACGGACTTTGACTCCGTCATGCGCTGGTTCGAATC
CAGCTAGCCCAGCCA
>Bacillus_anthracis_A2012_,_unfinished_sequence.trna20-GluTTC (1250378-1250449) Glu (TTC) 72 bp Sc: 71.01
GGCCCGTTGGTCAAGTGGTTAAGACACCGCCCTTTCACGGCGGTAACACGGGTTCGAATC
CCGTACGGGTCA
>Bacillus_anthracis_A2012_,_unfinished_sequence.trna27-GluTTC (5092771-5092700) Glu (TTC) 72 bp Sc: 71.01
GGCCCGTTGGTCAAGTGGTTAAGACACCGCCCTTTCACGGCGGTAACACGGGTTCGAATC
CCGTACGGGTCA
>Bacillus_anthracis_A2012_,_unfinished_sequence.trna3-GluTTC (621633-621704) Glu (TTC) 72 bp Sc: 71.01
GGCCCGTTGGTCAAGTGGTTAAGACACCGCCCTTTCACGGCGGTAACACGGGTTCGAATC
CCGTACGGGTCA
>Bacillus_anthracis_A2012_,_unfinished_sequence.trna31-GluTTC (547490-547419) Glu (TTC) 72 bp Sc: 71.01
GGCCCGTTGGTCAAGTGGTTAAGACACCGCCCTTTCACGGCGGTAACACGGGTTCGAATC

CCGTACGGGTCA
>Bacillus_anthracis_A2012_,_unfinished_sequence.trna15-GlyGCC (1049307-1049381) Gly (GCC) 75 bp Sc: 88.86
GCGGAAGTAGTTCAGTGGTAGAATACAACCTTGCCAAGGTTGGGGTCGCGGGTTCGAATC
CCGTCTTCCGCTCCA
>Bacillus_anthracis_A2012_,_unfinished_sequence.trna18-GlyTCC (1053988-1054061) Gly (TCC) 74 bp Sc: 60.03
TCCGGAGTAGTTTAGTGGTAAAACAAGAGCCTTCCAAGCTCTGGTCGAGAGTTCGATTCT
CTTCACCCGCTCCA
>Bacillus_anthracis_A2012_,_unfinished_sequence.trna13-HisGTG (1049088-1049163) His (GTG) 76 bp Sc: 74.61
GCGGTTGTGGCGAAGTGGTTAACGCACCGGATTGTGGCTCCGGCATTCGTGGGTTCGATT
CCCATCAGTCGCCCCA
>Bacillus_anthracis_A2012_,_unfinished_sequence.trna17-LeuCAA (1049477-1049561) Leu (CAA) 85 bp Sc: 60.08
GCCGATGTGGCGGAATTGGCAGACGCGCACGACTCAAAATCGTGTTCCTTCGGGAGTGTC
GGTTCGACCCCGACCATCGGTATCA
>Bacillus_anthracis_A2012_,_unfinished_sequence.trna30-LysTTT (547579-547504) Lys (TTT) 76 bp Sc: 98.67
GAGCCATTAGCTCAGTTGGTAGAGCATCTGACTTTTAATCAGAGGGTCGAAGGTTCGAGT
CCTTCATGGCTCACCA
>Bacillus_anthracis_A2012_,_unfinished_sequence.trna7-MetCAT (1048561-1048636) Met (CAT) 76 bp Sc: 69.29
CGCGGGGTGGAGCAGCACGTTAGCTCACGGGCTCATAACCCGAAGGTCGCAGGTTCAAAT
CCTGTCCCCGCAACCA
>Bacillus_anthracis_A2012_,_unfinished_sequence.trna2-MetCAT (621546-621619) Met (CAT) 74 bp Sc: 75.69
GGCGGTGTAGCTCAGCTGGCTAGAGCGTACGGTTCATACCCGTGAGGTCGGGGGTTCGAT
CCCCTCCGCCGCTA
>Bacillus_anthracis_A2012_,_unfinished_sequence.trna5-MetCAT (784602-784678) Met (CAT) 77 bp Sc: 85.35
CGCGGGGTGGAGCAGCACGGTAGCTCGTCGGGCTCATAACCCGAAGGTCGCAGGTTCAAA
TCCTGTCCCCGCAACCA
>Bacillus_anthracis_A2012_,_unfinished_sequence.trna28-MetCAT (4540320-4540247) Met (CAT) 74 bp Sc: 88.27
GGACCCTTAGCTCAGCTGGTTAGAGCAGACGGCTCATAACCGTCCGGTCGTAGGTTCGAG
TCCTACAGGGTCCA
>Bacillus_anthracis_A2012_,_unfinished_sequence.trna33-PheGAA (547281-547209) Phe (GAA) 73 bp Sc: 84.57
GGCTCGGTAGCTCAGTCGGTAGAGCAGAGGACTGAAAATCCTCGTGTCGGCGGTTCGATT
CCGTCCCGAGCCA
>Bacillus_anthracis_A2012_,_unfinished_sequence.trna22-PheGAA (5093322-5093247) Phe (GAA) 76 bp Sc: 86.60
GGCTCGGTAGCTCAGTCGGTAGAGCAGAGGACTGAAAATCCTCGTGTCGGCGGTTCGATT
CCGTCCCGATCCACCA
>Bacillus_anthracis_A2012_,_unfinished_sequence.trna9-PheGAA (1048725-1048800) Phe (GAA) 76 bp Sc: 92.86
GGCTCGGTAGCTCAGTCGGTAGAGCAGAGGACTGAAAATCCTCGTGTCGGCGGTTCGATT
CCGTCCCGAGCCACCA
>Bacillus_anthracis_A2012_,_unfinished_sequence.trna24-LysTTT (5093146-5093088) Lys (TTT) 59 bp Sc: 27.20
GAGCCATTAGCTCAGTTGGTAGAGCATCTGACTTTTAATCAGAGGGGTCGAAGNNNNNN
>Bacillus_anthracis_A2012_,_unfinished_sequence.trna26-SerGCT (5092868-5092778) Ser (GCT) 91 bp Sc: 67.93
AGAGAAGTACCCAAGTGGCTCAAGGGGCTCCCCTGCTAAGGGAGTAGATCGCGAACGCGG
TGCGAGGGTTCGAATCCCTTCTTCTCTGCCA
>Bacillus_anthracis_A2012_,_unfinished_sequence.trna1-SerTGA (584432-584524) Ser (TGA) 93 bp Sc: 71.57
GGAGGTATACCCAAGTCTGGCTGAAGGGATCGGTCTTGAAAACCGACAGGCGGCGAGAGT
CGCGCGGGGGTTCGAATCCCTCTACCTCCTCCA
>Bacillus_anthracis_A2012_,_unfinished_sequence.trna21-SerTGA (5093554-5093462) Ser (TGA) 93 bp Sc: 71.57
GGAGGTATACCCAAGTCTGGCTGAAGGGATCGGTCTTGAAAACCGACAGGCGGCGAGAGT
CGCGCGGGGGTTCGAATCCCTCTACCTCCTCCA
>Bacillus_anthracis_A2012_,_unfinished_sequence.trna10-ThrTGT (1048819-1048894) Thr (TGT) 76 bp Sc: 96.17
GCCGGCTTAGCTCAATTGGTAGAGCAACTGACTTGTAATCAGTAGGTTGGGGGTTCAAGT
CCTCTAGCCGGCACCA
>Bacillus_anthracis_A2012_,_unfinished_sequence.trna23-ThrTGT (5093232-5093157) Thr (TGT) 76 bp Sc: 96.17
GCCGGCTTAGCTCAATTGGTAGAGCAACTGACTTGTAATCAGTAGGTTGGGGGTTCAAGT
CCTCTAGCCGGCACCA
>Bacillus_anthracis_A2012_,_unfinished_sequence.trna11-TyrGTA (1048905-1048988) Tyr (GTA) 84 bp Sc: 77.59
GGAGGGGTAGCGAAGTGGCTAAACGCGGCGGACTGTAAATCCGCTCCTTCGGGTTCGGCA
GTTCGAATCTGCCCCCCTCCACCA
>Bacillus_anthracis_A2012_,_unfinished_sequence.trna12-Undet??? (1048996-1049068) Undet (???) 73 bp Sc: 53.73
AGGGGCATAGTTTAAAGGTAGAACTGAGGTCTGAAAACCTCCAGTGTGGGTTCGATTCCT
ACTGCCCCTGCCA
>Bacillus_anthracis_A2012_,_unfinished_sequence.trna29-ValGAC (1725129-1725057) Val (GAC) 73 bp Sc: 64.72
GATCCCGTAGCTCAGCAGGGAGAGCGCCACCTTGACAGGGTGGAGGTCGTGAGTTCGAGC
CTCTCCGGGATCA
>Bacillus_anthracis_Ames_chr.trna16-AlaTGC (151060-151132) Ala (TGC) 73 bp Sc: 85.62
GGGGCCTTAGCTCAGCTGGGAGAGCGCCTGCCTTGCACGCAGGAGGTCAGCGGTTCGATC
CCGCTAGGCTCCA
>Bacillus_anthracis_Ames_chr.trna54-AlaTGC (747102-747174) Ala (TGC) 73 bp Sc: 85.62

GGGGCCTTAGCTCAGCTGGGAGAGCGCCTGCCTTGCACGCAGGAGGTCAGCGGTTCGATC
CCGCTAGGCTCCA
>Bacillus_anthracis_Ames_chr.trna78-AlaTGC (4651174-4651102) Ala (TGC) 73 bp Sc: 85.62
GGGGCCTTAGCTCAGCTGGGAGAGCGCCTGCCTTGCACGCAGGAGGTCAGCGGTTCGATC
CCGCTAGGCTCCA
>Bacillus_anthracis_Ames_chr.trna2-AlaTGC (11077-11152) Ala (TGC) 76 bp Sc: 93.92
GGGGCCTTAGCTCAGCTGGGAGAGCGCCTGCCTTGCACGCAGGAGGTCAGCGGTTCGATC
CCGCTAGGCTCCACCA
>Bacillus_anthracis_Ames_chr.trna5-AlaTGC (30871-30946) Ala (TGC) 76 bp Sc: 93.92
GGGGCCTTAGCTCAGCTGGGAGAGCGCCTGCCTTGCACGCAGGAGGTCAGCGGTTCGATC
CCGCTAGGCTCCACCA
>Bacillus_anthracis_Ames_chr.trna52-ArgACG (746928-747001) Arg (ACG) 74 bp Sc: 69.97
GCGCCCGTAGCTCAATTGGATAGAGCGTTTGACTACGGATCAAGAGGTTAGGGGTTCGAC
TCCTCTCGGGCGCG
>Bacillus_anthracis_Ames_chr.trna76-ArgACG (4651347-4651274) Arg (ACG) 74 bp Sc: 69.97
GCGCCCGTAGCTCAATTGGATAGAGCGTTTGACTACGGATCAAGAGGTTAGGGGTTCGAC
TCCTCTCGGGCGCG
>Bacillus_anthracis_Ames_chr.trna25-ArgACG (245902-245978) Arg (ACG) 77 bp Sc: 78.27
GCGCCCGTAGCTCAATTGGATAGAGCGTTTGACTACGGATCAAGAGGTTAGGGGTTCGAC
TCCTCTCGGGCGCGCCA
>Bacillus_anthracis_Ames_chr.trna69-ArgCCG (4867440-4867362) Arg (CCG) 79 bp Sc: 62.69
GCGCCCATAGCTCAGTCGGATAGAGCGGTGGTTTCCGGTACCACGTCTGCCGGGGGTTCG
AATCCCTCTGGGCGCGTCA
>Bacillus_anthracis_Ames_chr.trna93-ArgTCT (4287577-4287501) Arg (TCT) 77 bp Sc: 79.49
GTCCCAGTAGCTCAGCCGGATAGAGCATACGCCTTCTAAGCGTACGGTCGGGAGTTCGAA
TCTCTCCTGGGACGCTA
>Bacillus_anthracis_Ames_chr.trna30-AsnGTT (537272-537346) Asn (GTT) 75 bp Sc: 86.53
TCCGCAGTAGCTCAGCGGTAGAGCTATCGGCTGTTAACCGATCGGTCGTAGGTTCGATTC
CTACCTGCGGAGCCA
>Bacillus_anthracis_Ames_chr.trna17-AsnGTT (245009-245083) Asn (GTT) 75 bp Sc: 88.32
TCCGCAGTAGCTCAGTGGTAGAGCTATCGGCTGTTAACCGATCGGTCGTAGGTTCGAGTC
CTACCTGCGGAGCCA
>Bacillus_anthracis_Ames_chr.trna63-AsnGTT (747950-748024) Asn (GTT) 75 bp Sc: 88.32
TCCGCAGTAGCTCAGTGGTAGAGCTATCGGCTGTTAACCGATCGGTCGTAGGTTCGAGTC
CTACCTGCGGAGCCA
>Bacillus_anthracis_Ames_chr.trna8-AsnGTT (150319-150393) Asn (GTT) 75 bp Sc: 88.32
TCCGCAGTAGCTCAGTGGTAGAGCTATCGGCTGTTAACCGATCGGTCGTAGGTTCGAGTC
CTACCTGCGGAGCCA
>Bacillus_anthracis_Ames_chr.trna89-AsnGTT (4650189-4650115) Asn (GTT) 75 bp Sc: 88.32
TCCGCAGTAGCTCAGTGGTAGAGCTATCGGCTGTTAACCGATCGGTCGTAGGTTCGAGTC
CTACCTGCGGAGCCA
>Bacillus_anthracis_Ames_chr.trna21-AspGTC (245387-245462) Asp (GTC) 76 bp Sc: 76.06
GGTCCCGTGGTGTAGTGGTTAACATGCCTGCCTGTCACGCAGGAGATCGCCGGTTCGACC
CCGGTCGGGACCGCCA
>Bacillus_anthracis_Ames_chr.trna29-AspGTC (251085-251160) Asp (GTC) 76 bp Sc: 76.06
GGTCCCGTGGTGTAGTGGTTAACATGCCTGCCTGTCACGCAGGAGATCGCCGGTTCGACC
CCGGTCGGGACCGCCA
>Bacillus_anthracis_Ames_chr.trna35-AspGTC (537718-537793) Asp (GTC) 76 bp Sc: 76.06
GGTCCCGTGGTGTAGTGGTTAACATGCCTGCCTGTCACGCAGGAGATCGCCGGTTCGACC
CCGGTCGGGACCGCCA
>Bacillus_anthracis_Ames_chr.trna58-AspGTC (747536-747611) Asp (GTC) 76 bp Sc: 76.06
GGTCCCGTGGTGTAGTGGTTAACATGCCTGCCTGTCACGCAGGAGATCGCCGGTTCGACC
CCGGTCGGGACCGCCA
>Bacillus_anthracis_Ames_chr.trna67-AspGTC (5206864-5206789) Asp (GTC) 76 bp Sc: 76.06
GGTCCCGTGGTGTAGTGGTTAACATGCCTGCCTGTCACGCAGGAGATCGCCGGTTCGACC
CCGGTCGGGACCGCCA
>Bacillus_anthracis_Ames_chr.trna83-AspGTC (4650707-4650632) Asp (GTC) 76 bp Sc: 76.06
GGTCCCGTGGTGTAGTGGTTAACATGCCTGCCTGTCACGCAGGAGATCGCCGGTTCGACC
CCGGTCGGGACCGCCA
>Bacillus_anthracis_Ames_chr.trna43-CysGCA (538475-538545) Cys (GCA) 71 bp Sc: 71.27
GGCGGCATAGCCAAGTGGTAAGGCAGAGGTCTGCAAAACCTTTATCACCGGTTCAAATCC
GGTTGCCGCCT
>Bacillus_anthracis_Ames_chr.trna13-GlnTTG (150817-150891) Gln (TTG) 75 bp Sc: 78.60
TGGGCTATAGCCAAGCGGTAAGGCAACGGACTTTGACTCCGTCATGCGCTGGTTCGAATC
CAGCTAGCCCAGCCA
>Bacillus_anthracis_Ames_chr.trna22-GlnTTG (245551-245625) Gln (TTG) 75 bp Sc: 78.60
TGGGCTATAGCCAAGCGGTAAGGCAACGGACTTTGACTCCGTCATGCGCTGGTTCGAATC

CAGCTAGCCCAGCCA
>Bacillus_anthracis_Ames_chr.trna41-GlnTTG (538306-538380) Gln (TTG) 75 bp Sc: 78.60
TGGGCTATAGCCAAGCGGTAAGGCAACGGACTTTGACTCCGTCATGCGCTGGTTCGAATC
CAGCTAGCCCAGCCA
>Bacillus_anthracis_Ames_chr.trna47-GlnTTG (746466-746540) Gln (TTG) 75 bp Sc: 78.60
TGGGCTATAGCCAAGCGGTAAGGCAACGGACTTTGACTCCGTCATGCGCTGGTTCGAATC
CAGCTAGCCCAGCCA
>Bacillus_anthracis_Ames_chr.trna64-GluTTC (748026-748097) Glu (TTC) 72 bp Sc: 71.01
GGCCCGTTGGTCAAGTGGTTAAGACACCGCCCTTTCACGGCGGTAACACGGGTTCGAATC
CCGTACGGGTCA
>Bacillus_anthracis_Ames_chr.trna66-GluTTC (5206956-5206885) Glu (TTC) 72 bp Sc: 71.01
GGCCCGTTGGTCAAGTGGTTAAGACACCGCCCTTTCACGGCGGTAACACGGGTTCGAATC
CCGTACGGGTCA
>Bacillus_anthracis_Ames_chr.trna7-GluTTC (64512-64583) Glu (TTC) 72 bp Sc: 71.01
GGCCCGTTGGTCAAGTGGTTAAGACACCGCCCTTTCACGGCGGTAACACGGGTTCGAATC
CCGTACGGGTCA
>Bacillus_anthracis_Ames_chr.trna91-GluTTC (4650010-4649939) Glu (TTC) 72 bp Sc: 71.01
GGCCCGTTGGTCAAGTGGTTAAGACACCGCCCTTTCACGGCGGTAACACGGGTTCGAATC
CCGTACGGGTCA
>Bacillus_anthracis_Ames_chr.trna10-GluTTC (150495-150569) Glu (TTC) 75 bp Sc: 72.70
GGCCCGTTGGTCAAGTGGTTAAGACACCGCCCTTTCACGGCGGTAAAACGGGTTCGAATC
CCGTACGGGTCACCA
>Bacillus_anthracis_Ames_chr.trna19-GluTTC (245186-245260) Glu (TTC) 75 bp Sc: 79.30
GGCCCGTTGGTCAAGTGGTTAAGACACCGCCCTTTCACGGCGGTAACACGGGTTCGAATC
CCGTACGGGTCACCA
>Bacillus_anthracis_Ames_chr.trna32-GluTTC (537458-537532) Glu (TTC) 75 bp Sc: 79.30
GGCCCGTTGGTCAAGTGGTTAAGACACCGCCCTTTCACGGCGGTAACACGGGTTCGAATC
CCGTACGGGTCACCA
>Bacillus_anthracis_Ames_chr.trna15-GlyGCC (150978-151049) Gly (GCC) 72 bp Sc: 80.56
GCGGAAGTAGTTCAGTGGTAGAATACAACCTTGCCAAGGTTGGGGTCGCGGGTTCGAATC
CCGTCTTCCGCT
>Bacillus_anthracis_Ames_chr.trna42-GlyGCC (538386-538460) Gly (GCC) 75 bp Sc: 88.86
GCGGAAGTAGTTCAGTGGTAGAATACAACCTTGCCAAGGTTGGGGTCGCGGGTTCGAATC
CCGTCTTCCGCTCCA
>Bacillus_anthracis_Ames_chr.trna50-GlyGCC (746745-746819) Gly (GCC) 75 bp Sc: 88.86
GCGGAAGTAGTTCAGTGGTAGAATACAACCTTGCCAAGGTTGGGGTCGCGGGTTCGAATC
CCGTCTTCCGCTCCA
>Bacillus_anthracis_Ames_chr.trna74-GlyGCC (4651530-4651456) Gly (GCC) 75 bp Sc: 88.86
GCGGAAGTAGTTCAGTGGTAGAATACAACCTTGCCAAGGTTGGGGTCGCGGGTTCGAATC
CCGTCTTCCGCTCCA
>Bacillus_anthracis_Ames_chr.trna27-GlyTCC (246061-246131) Gly (TCC) 71 bp Sc: 71.78
GCGGGTGTAGTTTAGTGGTAAAACAAGAGCCTTCCAAGCTCTGGTCGAGAGTTCGATTCT
CTTCACCCGCT
>Bacillus_anthracis_Ames_chr.trna87-GlyTCC (4650354-4650284) Gly (TCC) 71 bp Sc: 71.78
GCGGGTGTAGTTTAGTGGTAAAACAAGAGCCTTCCAAGCTCTGGTCGAGAGTTCGATTCT
CTTCACCCGCT
>Bacillus_anthracis_Ames_chr.trna45-GlyTCC (543071-543144) Gly (TCC) 74 bp Sc: 80.08
GCGGGTGTAGTTTAGTGGTAAAACAAGAGCCTTCCAAGCTCTGGTCGAGAGTTCGATTCT
CTTCACCCGCTCCA
>Bacillus_anthracis_Ames_chr.trna92-GlyTCC (4287652-4287579) Gly (TCC) 74 bp Sc: 80.08
GCGGGTGTAGTTTAGTGGTAAAACAAGAGCCTTCCAAGCTCTGGTCGAGAGTTCGATTCT
CTTCACCCGCTCCA
>Bacillus_anthracis_Ames_chr.trna40-HisGTG (538167-538242) His (GTG) 76 bp Sc: 74.61
GCGGTTGTGGCGAAGTGGTTAACGCACCGGATTGTGGCTCCGGCATTCGTGGGTTCGATT
CCCATCAGTCGCCCCA
>Bacillus_anthracis_Ames_chr.trna72-HisGTG (4651738-4651663) His (GTG) 76 bp Sc: 74.61
GCGGTTGTGGCGAAGTGGTTAACGCACCGGATTGTGGCTCCGGCATTCGTGGGTTCGATT
CCCATCAGTCGCCCCA
>Bacillus_anthracis_Ames_chr.trna1-IleGAT (10992-11068) Ile (GAT) 77 bp Sc: 101.21
GGGCCTATAGCTCAGCTGGTTAGAGCGCACGCCTGATAAGCGTGAGGTCGATGGTTCGAG
TCCATTTAGGCCCACCA
>Bacillus_anthracis_Ames_chr.trna4-IleGAT (30786-30862) Ile (GAT) 77 bp Sc: 101.21
GGGCCTATAGCTCAGCTGGTTAGAGCGCACGCCTGATAAGCGTGAGGTCGATGGTTCGAG
TCCATTTAGGCCCACCA
>Bacillus_anthracis_Ames_chr.trna88-IleGAT (4650273-4650197) Ile (GAT) 77 bp Sc: 101.21
GGGCCTATAGCTCAGCTGGTTAGAGCGCACGCCTGATAAGCGTGAGGTCGATGGTTCGAG
TCCATTTAGGCCCACCA

>Bacillus_anthracis_Ames_chr.trna62-IleGAT (747865-747941) Ile (GAT) 77 bp Sc: 94.98
GGGCCTATAGCTCAGCTGGTTAGAGCGCACGCCTGATAAGCGTGAGGTCGATGGTTCGAG
TCCATTTAGACCCACCA
>Bacillus_anthracis_Ames_chr.trna44-LeuCAA (538556-538640) Leu (CAA) 85 bp Sc: 60.08
GCCGATGTGGCGGAATTGGCAGACGCGCACGACTCAAAATCGTGTTCCTTCGGGAGTGTC
GGTTCGACCCCGACCATCGGTATCA
>Bacillus_anthracis_Ames_chr.trna24-LeuGAG (245721-245806) Leu (GAG) 86 bp Sc: 57.19
GCGGTCGTGGCGGAACGGCAGACGCGCTAGGTTGAGGGCCTAGTGGGGGAAACCCCGTGG
AGGTTCAAGTCCTCTCGGCCGCATCA
>Bacillus_anthracis_Ames_chr.trna51-LeuTAA (746836-746924) Leu (TAA) 89 bp Sc: 71.28
GCCGGGGTGGCGGAACAGGCAGACGCACAGGACTTAAAATCCTGCGGTGGGTGACCACCG
TGCGGGTTCGACCCCCGCCCTCGGCACCA
>Bacillus_anthracis_Ames_chr.trna75-LeuTAA (4651439-4651351) Leu (TAA) 89 bp Sc: 71.28
GCCGGGGTGGCGGAACAGGCAGACGCACAGGACTTAAAATCCTGCGGTGGGTGACCACCG
TGCGGGTTCGACCCCCGCCCTCGGCACCA
>Bacillus_anthracis_Ames_chr.trna49-LeuTAG (746635-746715) Leu (TAG) 81 bp Sc: 66.65
GCGGGTGTGGCGGAATTGGCAGACGCACTAGACTTAGGATCTAGCGCCTTTGGCGTGGGG
GTTCGACTCCCTTCACCCGCA
>Bacillus_anthracis_Ames_chr.trna73-LeuTAG (4651640-4651560) Leu (TAG) 81 bp Sc: 67.96
GCGGGTGTGGCGGAATTGGCAGACGCACCAGACTTAGGATCTGGCGCCTTTGGCGTGGGG
GTTCGACTCCCTTCACCCGCA
>Bacillus_anthracis_Ames_chr.trna23-LysTTT (245631-245703) Lys (TTT) 73 bp Sc: 90.38
GAGCCATTAGCTCAGTTGGTAGAGCATCTGACTTTTAATCAGAGGGTCGAAGGTTCGAGT
CCTTCATGGCTCA
>Bacillus_anthracis_Ames_chr.trna14-LysTTT (150897-150972) Lys (TTT) 76 bp Sc: 98.67
GAGCCATTAGCTCAGTTGGTAGAGCATCTGACTTTTAATCAGAGGGTCGAAGGTTCGAGT
CCTTCATGGCTCACCA
>Bacillus_anthracis_Ames_chr.trna48-LysTTT (746545-746620) Lys (TTT) 76 bp Sc: 98.67
GAGCCATTAGCTCAGTTGGTAGAGCATCTGACTTTTAATCAGAGGGTCGAAGGTTCGAGT
CCTTCATGGCTCACCA
>Bacillus_anthracis_Ames_chr.trna65-LysTTT (5207045-5206970) Lys (TTT) 76 bp Sc: 98.67
GAGCCATTAGCTCAGTTGGTAGAGCATCTGACTTTTAATCAGAGGGTCGAAGGTTCGAGT
CCTTCATGGCTCACCA
>Bacillus_anthracis_Ames_chr.trna86-LysTTT (4650443-4650368) Lys (TTT) 76 bp Sc: 98.67
GAGCCATTAGCTCAGTTGGTAGAGCATCTGACTTTTAATCAGAGGGTCGAAGGTTCGAGT
CCTTCATGGCTCACCA
>Bacillus_anthracis_Ames_chr.trna79-MetCAT (4651081-4651004) Met (CAT) 78 bp Sc: 72.67
GGCGGTGTAGCTCAGCTAGGCTAGAGCGTATGGTTCATACCCGTGAGGTCGGGGGTTCGA
TCCCCTCCGCCGCTACCA
>Bacillus_anthracis_Ames_chr.trna6-MetCAT (64425-64498) Met (CAT) 74 bp Sc: 75.69
GGCGGTGTAGCTCAGCTGGCTAGAGCGTACGGTTCATACCCGTGAGGTCGGGGGTTCGAT
CCCCTCCGCCGCTA
>Bacillus_anthracis_Ames_chr.trna34-MetCAT (537638-537714) Met (CAT) 77 bp Sc: 80.72
CGCGGGGTGGAGCAGCACGTTAGCTCGTCGGGCTCATAACCCGAAGGTCGCAGGTTCAAA
TCCTGTCCCCGCAACCA
>Bacillus_anthracis_Ames_chr.trna28-MetCAT (251005-251081) Met (CAT) 77 bp Sc: 85.35
CGCGGGGTGGAGCAGCACGGTAGCTCGTCGGGCTCATAACCCGAAGGTCGCAGGTTCAAA
TCCTGTCCCCGCAACCA
>Bacillus_anthracis_Ames_chr.trna57-MetCAT (747455-747531) Met (CAT) 77 bp Sc: 85.35
CGCGGGGTGGAGCAGCACGGTAGCTCGTCGGGCTCATAACCCGAAGGTCGCAGGTTCAAA
TCCTGTCCCCGCAACCA
>Bacillus_anthracis_Ames_chr.trna94-MetCAT (4094657-4094584) Met (CAT) 74 bp Sc: 88.27
GGACCCTTAGCTCAGCTGGTTAGAGCAGACGGCTCATAACCGTCCGGTCGTAGGTTCGAG
TCCTACAGGGTCCA
>Bacillus_anthracis_Ames_chr.trna82-MetCAT (4650785-4650709) Met (CAT) 77 bp Sc: 89.08
CGCGGGGTGGAGCAGTCTGGTAGCTCGTCGGGCTCATAACCCGAAGGTCGCAGGTTCAAA
TCCTGTCCCCGCAACCA
>Bacillus_anthracis_Ames_chr.trna80-MetCAT (4650999-4650923) Met (CAT) 77 bp Sc: 96.69
GGACCTTTAGCTCAGCTGGTTAGAGCAGACGGCTCATAACCGTCCGGTCGTAGGTTCGAG
TCCTACAAGGTCCACCA
>Bacillus_anthracis_Ames_chr.trna68-PheGAA (5206747-5206675) Phe (GAA) 73 bp Sc: 84.57
GGCTCGGTAGCTCAGTCGGTAGAGCAGAGGACTGAAAATCCTCGTGTCGGCGGTTCGATT
CCGTCCCGAGCCA
>Bacillus_anthracis_Ames_chr.trna84-PheGAA (4650619-4650544) Phe (GAA) 76 bp Sc: 86.60
GGCTCGGTAGCTCAGTCGGTAGAGCAGAGGACTGAAAATCCTCGTGTCGGCGGTTCGATT
CCGTCCCGATCCACCA
>Bacillus_anthracis_Ames_chr.trna36-PheGAA (537803-537878) Phe (GAA) 76 bp Sc: 92.86

GGCTCGGTAGCTCAGTCGGTAGAGCAGAGGACTGAAAATCCTCGTGTCGGCGGTTCGATT
CCGTCCCGAGCCACCA
>Bacillus_anthracis_Ames_chr.trna59-PheGAA (747620-747695) Phe (GAA) 76 bp Sc: 92.86
GGCTCGGTAGCTCAGTCGGTAGAGCAGAGGACTGAAAATCCTCGTGTCGGCGGTTCGATT
CCGTCCCGAGCCACCA
>Bacillus_anthracis_Ames_chr.trna53-ProTGG (747012-747085) Pro (TGG) 74 bp Sc: 85.68
CGGGAAGTGGCTCAGCTTGGTAGAGCACCTGGTTTGGGACCAGGGGGTCGCAGGTTCAAA
TCCTGTCTTCCCGA
>Bacillus_anthracis_Ames_chr.trna77-ProTGG (4651263-4651190) Pro (TGG) 74 bp Sc: 85.68
CGGGAAGTGGCTCAGCTTGGTAGAGCACCTGGTTTGGGACCAGGGGGTCGCAGGTTCAAA
TCCTGTCTTCCCGA
>Bacillus_anthracis_Ames_chr.trna26-ProTGG (245983-246059) Pro (TGG) 77 bp Sc: 93.98
CGGGAAGTGGCTCAGCTTGGTAGAGCACCTGGTTTGGGACCAGGGGGTCGCAGGTTCAAA
TCCTGTCTTCCCGACCA
>Bacillus_anthracis_Ames_chr.trna18-SerGCT (245087-245177) Ser (GCT) 91 bp Sc: 67.93
AGAGAAGTACCCAAGTGGCTCAAGGGGCTCCCCTGCTAAGGGAGTAGATCGCGAACGCGG
TGCGAGGGTTCGAATCCCTTCTTCTCTGCCA
>Bacillus_anthracis_Ames_chr.trna90-SerGCT (4650107-4650017) Ser (GCT) 91 bp Sc: 67.93
AGAGAAGTACCCAAGTGGCTCAAGGGGCTCCCCTGCTAAGGGAGTAGATCGCGAACGCGG
TGCGAGGGTTCGAATCCCTTCTTCTCTGCCA
>Bacillus_anthracis_Ames_chr.trna31-SerGGA (537349-537440) Ser (GGA) 92 bp Sc: 63.66
GGAGAGCTGTCCGAGTTGGCCGAAGGAGCACGATTGGAAATCGTGTATACGTCACAAGCG
TATCAAGGGTTCGAATCCCTTGCTCTCCGCCA
>Bacillus_anthracis_Ames_chr.trna55-SerTGA (747195-747287) Ser (TGA) 93 bp Sc: 71.21
GGAGGTATACCCAAGTTCGGCTGAAGGGATCGGTCTTGAAAACCGACAGGCGGTGAGAAT
CGCGCGGGGGTTCGAATCCCTCTACCTCCTCCA
>Bacillus_anthracis_Ames_chr.trna3-SerTGA (21769-21861) Ser (TGA) 93 bp Sc: 71.57
GGAGGTATACCCAAGTCTGGCTGAAGGGATCGGTCTTGAAAACCGACAGGCGGCGAGAGT
CGCGCGGGGGTTCGAATCCCTCTACCTCCTCCA
>Bacillus_anthracis_Ames_chr.trna56-SerTGA (747342-747434) Ser (TGA) 93 bp Sc: 71.91
GGAGGTATACCCAAGCTCGGCTGAAGGGATCGGTCTTGAAAACCGACAGGCGGCGAGAGT
CGCGCGAGGGTTCGAATCCCTCTACCTCCTCCA
>Bacillus_anthracis_Ames_chr.trna81-SerTGA (4650905-4650813) Ser (TGA) 93 bp Sc: 72.21
GGAGGTATACCCAAGTTCGGCTGAAGGGATCGGTCTTGAAAACCGACAGGCGGCGAGAGT
CGCGCGGGGGTTCGAATCCCTCTACCTCCTCCA
>Bacillus_anthracis_Ames_chr.trna9-ThrGGT (150398-150470) Thr (GGT) 73 bp Sc: 78.48
GCTTCCATAGCTCAGCTGGTAGAGCACTTCCATGGTAAGGAAGAGGTCACCGGTTCAAGC
CCGGTTGGAAGCT
>Bacillus_anthracis_Ames_chr.trna60-ThrTGT (747705-747777) Thr (TGT) 73 bp Sc: 87.87
GCCGGCTTAGCTCAATTGGTAGAGCAACTGACTTGTAATCAGTAGGTTGGGGGTTCAAGT
CCTCTAGCCGGCA
>Bacillus_anthracis_Ames_chr.trna71-ThrTGT (4651823-4651748) Thr (TGT) 76 bp Sc: 92.70
GCCGACTTAGCTCAATTGGGAGAGCAACTGACTTGTAATCAGTAGGTTGGGGGTTCAAGT
CCTCTAGTCGGCACCA
>Bacillus_anthracis_Ames_chr.trna37-ThrTGT (537897-537972) Thr (TGT) 76 bp Sc: 96.17
GCCGGCTTAGCTCAATTGGTAGAGCAACTGACTTGTAATCAGTAGGTTGGGGGTTCAAGT
CCTCTAGCCGGCACCA
>Bacillus_anthracis_Ames_chr.trna85-ThrTGT (4650529-4650454) Thr (TGT) 76 bp Sc: 96.17
GCCGGCTTAGCTCAATTGGTAGAGCAACTGACTTGTAATCAGTAGGTTGGGGGTTCAAGT
CCTCTAGCCGGCACCA
>Bacillus_anthracis_Ames_chr.trna61-TrpCCA (747784-747854) Trp (CCA) 71 bp Sc: 53.62
AGGGGCATAGTTTAAAGGTAGAACTGAGGTCTCCAAAACCTCCAGTGTGGGTTCGATTCC
TACTGCCCCTG
>Bacillus_anthracis_Ames_chr.trna39-TrpCCA (538074-538147) Trp (CCA) 74 bp Sc: 61.92
AGGGGCATAGTTTAAAGGTAGAACTGAGGTCTCCAAAACCTCCAGTGTGGGTTCGATTCC
TACTGCCCCTGCCA
>Bacillus_anthracis_Ames_chr.trna12-TyrGTA (150668-150751) Tyr (GTA) 84 bp Sc: 77.59
GGAGGGGTAGCGAAGTGGCTAAACGCGGCGGACTGTAAATCCGCTCCTTCGGGTTCGGCA
GTTCGAATCTGCCCCCCTCCACCA
>Bacillus_anthracis_Ames_chr.trna38-TyrGTA (537983-538066) Tyr (GTA) 84 bp Sc: 77.59
GGAGGGGTAGCGAAGTGGCTAAACGCGGCGGACTGTAAATCCGCTCCTTCGGGTTCGGCA
GTTCGAATCTGCCCCCCTCCACCA
>Bacillus_anthracis_Ames_chr.trna95-ValGAC (1232550-1232478) Val (GAC) 73 bp Sc: 64.72
GATCCCGTAGCTCAGCAGGGAGAGCGCCACCTTGACAGGGTGGAGGTCGTGAGTTCGAGC
CTCTCCGGGATCA
>Bacillus_anthracis_Ames_chr.trna11-ValTAC (150575-150650) Val (TAC) 76 bp Sc: 97.03
GGAGGATTAGCTCAGCTGGGAGAGCACCTGCCTTACAAGCAGGGGGTCGGCGGTTCGATC

CCGTCATCCTCCACCA
>Bacillus_anthracis_Ames_chr.trna20-ValTAC (245265-245340) Val (TAC) 76 bp Sc: 97.03
GGAGGATTAGCTCAGCTGGGAGAGCACCTGCCTTACAAGCAGGGGGTCGGCGGTTCGATC
CCGTCATCCTCCACCA
>Bacillus_anthracis_Ames_chr.trna33-ValTAC (537538-537613) Val (TAC) 76 bp Sc: 97.03
GGAGGATTAGCTCAGCTGGGAGAGCACCTGCCTTACAAGCAGGGGGTCGGCGGTTCGATC
CCGTCATCCTCCACCA
>Bacillus_anthracis_Ames_chr.trna46-ValTAC (746379-746454) Val (TAC) 76 bp Sc: 97.03
GGAGGATTAGCTCAGCTGGGAGAGCACCTGCCTTACAAGCAGGGGGTCGGCGGTTCGATC
CCGTCATCCTCCACCA
>Bacillus_anthracis_Ames_chr.trna70-ValTAC (4651903-4651828) Val (TAC) 76 bp Sc: 97.03
GGAGGATTAGCTCAGCTGGGAGAGCACCTGCCTTACAAGCAGGGGGTCGGCGGTTCGATC
CCGTCATCCTCCACCA
>Bacillus_cereus_ATCC_10987_chr.trna16-AlaTGC (151066-151138) Ala (TGC) 73 bp Sc: 85.62
GGGGCCTTAGCTCAGCTGGGAGAGCGCCTGCCTTGCACGCAGGAGGTCAGCGGTTCGATC
CCGCTAGGCTCCA
>Bacillus_cereus_ATCC_10987_chr.trna57-AlaTGC (823794-823866) Ala (TGC) 73 bp Sc: 85.62
GGGGCCTTAGCTCAGCTGGGAGAGCGCCTGCCTTGCACGCAGGAGGTCAGCGGTTCGATC
CCGCTAGGCTCCA
>Bacillus_cereus_ATCC_10987_chr.trna81-AlaTGC (4650152-4650080) Ala (TGC) 73 bp Sc: 85.62
GGGGCCTTAGCTCAGCTGGGAGAGCGCCTGCCTTGCACGCAGGAGGTCAGCGGTTCGATC
CCGCTAGGCTCCA
>Bacillus_cereus_ATCC_10987_chr.trna2-AlaTGC (11076-11151) Ala (TGC) 76 bp Sc: 93.92
GGGGCCTTAGCTCAGCTGGGAGAGCGCCTGCCTTGCACGCAGGAGGTCAGCGGTTCGATC
CCGCTAGGCTCCACCA
>Bacillus_cereus_ATCC_10987_chr.trna5-AlaTGC (30850-30925) Ala (TGC) 76 bp Sc: 93.92
GGGGCCTTAGCTCAGCTGGGAGAGCGCCTGCCTTGCACGCAGGAGGTCAGCGGTTCGATC
CCGCTAGGCTCCACCA
>Bacillus_cereus_ATCC_10987_chr.trna55-ArgACG (823621-823694) Arg (ACG) 74 bp Sc: 69.97
GCGCCCGTAGCTCAATTGGATAGAGCGTTTGACTACGGATCAAGAGGTTAGGGGTTCGAC
TCCTCTCGGGCGCG
>Bacillus_cereus_ATCC_10987_chr.trna79-ArgACG (4650325-4650252) Arg (ACG) 74 bp Sc: 69.97
GCGCCCGTAGCTCAATTGGATAGAGCGTTTGACTACGGATCAAGAGGTTAGGGGTTCGAC
TCCTCTCGGGCGCG
>Bacillus_cereus_ATCC_10987_chr.trna25-ArgACG (279293-279369) Arg (ACG) 77 bp Sc: 78.27
GCGCCCGTAGCTCAATTGGATAGAGCGTTTGACTACGGATCAAGAGGTTAGGGGTTCGAC
TCCTCTCGGGCGCGCCA
>Bacillus_cereus_ATCC_10987_chr.trna72-ArgCCG (4843854-4843776) Arg (CCG) 79 bp Sc: 62.69
GCGCCCATAGCTCAGTCGGATAGAGCGGTGGTTTCCGGTACCACGTCTGCCGGGGGTTCG
AATCCCTCTGGGCGCGTCA
>Bacillus_cereus_ATCC_10987_chr.trna32-AsnGTT (609775-609849) Asn (GTT) 75 bp Sc: 86.53
TCCGCAGTAGCTCAGCGGTAGAGCTATCGGCTGTTAACCGATCGGTCGTAGGTTCGATTC
CTACCTGCGGAGCCA
>Bacillus_cereus_ATCC_10987_chr.trna17-AsnGTT (278404-278478) Asn (GTT) 75 bp Sc: 88.32
TCCGCAGTAGCTCAGTGGTAGAGCTATCGGCTGTTAACCGATCGGTCGTAGGTTCGAGTC
CTACCTGCGGAGCCA
>Bacillus_cereus_ATCC_10987_chr.trna66-AsnGTT (824646-824720) Asn (GTT) 75 bp Sc: 88.32
TCCGCAGTAGCTCAGTGGTAGAGCTATCGGCTGTTAACCGATCGGTCGTAGGTTCGAGTC
CTACCTGCGGAGCCA
>Bacillus_cereus_ATCC_10987_chr.trna8-AsnGTT (150325-150399) Asn (GTT) 75 bp Sc: 88.32
TCCGCAGTAGCTCAGTGGTAGAGCTATCGGCTGTTAACCGATCGGTCGTAGGTTCGAGTC
CTACCTGCGGAGCCA
>Bacillus_cereus_ATCC_10987_chr.trna92-AsnGTT (4649168-4649094) Asn (GTT) 75 bp Sc: 88.32
TCCGCAGTAGCTCAGTGGTAGAGCTATCGGCTGTTAACCGATCGGTCGTAGGTTCGAGTC
CTACCTGCGGAGCCA
>Bacillus_cereus_ATCC_10987_chr.trna21-AspGTC (278782-278857) Asp (GTC) 76 bp Sc: 76.06
GGTCCCGTGGTGTAGTGGTTAACATGCCTGCCTGTCACGCAGGAGATCGCCGGTTCGACC
CCGGTCGGGACCGCCA
>Bacillus_cereus_ATCC_10987_chr.trna29-AspGTC (284475-284550) Asp (GTC) 76 bp Sc: 76.06
GGTCCCGTGGTGTAGTGGTTAACATGCCTGCCTGTCACGCAGGAGATCGCCGGTTCGACC
CCGGTCGGGACCGCCA
>Bacillus_cereus_ATCC_10987_chr.trna31-AspGTC (285007-285082) Asp (GTC) 76 bp Sc: 76.06
GGTCCCGTGGTGTAGTGGTTAACATGCCTGCCTGTCACGCAGGAGATCGCCGGTTCGACC
CCGGTCGGGACCGCCA
>Bacillus_cereus_ATCC_10987_chr.trna37-AspGTC (610220-610295) Asp (GTC) 76 bp Sc: 76.06
GGTCCCGTGGTGTAGTGGTTAACATGCCTGCCTGTCACGCAGGAGATCGCCGGTTCGACC
CCGGTCGGGACCGCCA

>Bacillus_cereus_ATCC_10987_chr.trna61-AspGTC (824231-824306) Asp (GTC) 76 bp Sc: 76.06
GGTCCCGTGGTGTAGTGGTTAACATGCCTGCCTGTCACGCAGGAGATCGCCGGTTCGACC
CCGGTCGGGACCGCCA
>Bacillus_cereus_ATCC_10987_chr.trna70-AspGTC (5203863-5203788) Asp (GTC) 76 bp Sc: 76.06
GGTCCCGTGGTGTAGTGGTTAACATGCCTGCCTGTCACGCAGGAGATCGCCGGTTCGACC
CCGGTCGGGACCGCCA
>Bacillus_cereus_ATCC_10987_chr.trna86-AspGTC (4649686-4649611) Asp (GTC) 76 bp Sc: 76.06
GGTCCCGTGGTGTAGTGGTTAACATGCCTGCCTGTCACGCAGGAGATCGCCGGTTCGACC
CCGGTCGGGACCGCCA
>Bacillus_cereus_ATCC_10987_chr.trna45-CysGCA (610977-611047) Cys (GCA) 71 bp Sc: 71.27
GGCGGCATAGCCAAGTGGTAAGGCAGAGGTCTGCAAAACCTTTATCACCGGTTCAAATCC
GGTTGCCGCCT
>Bacillus_cereus_ATCC_10987_chr.trna13-GlnTTG (150823-150897) Gln (TTG) 75 bp Sc: 78.60
TGGGCTATAGCCAAGCGGTAAGGCAACGGACTTTGACTCCGTCATGCGCTGGTTCGAATC
CAGCTAGCCCAGCCA
>Bacillus_cereus_ATCC_10987_chr.trna22-GlnTTG (278945-279019) Gln (TTG) 75 bp Sc: 78.60
TGGGCTATAGCCAAGCGGTAAGGCAACGGACTTTGACTCCGTCATGCGCTGGTTCGAATC
CAGCTAGCCCAGCCA
>Bacillus_cereus_ATCC_10987_chr.trna43-GlnTTG (610808-610882) Gln (TTG) 75 bp Sc: 78.60
TGGGCTATAGCCAAGCGGTAAGGCAACGGACTTTGACTCCGTCATGCGCTGGTTCGAATC
CAGCTAGCCCAGCCA
>Bacillus_cereus_ATCC_10987_chr.trna50-GlnTTG (823159-823233) Gln (TTG) 75 bp Sc: 78.60
TGGGCTATAGCCAAGCGGTAAGGCAACGGACTTTGACTCCGTCATGCGCTGGTTCGAATC
CAGCTAGCCCAGCCA
>Bacillus_cereus_ATCC_10987_chr.trna67-GluTTC (824722-824793) Glu (TTC) 72 bp Sc: 71.01
GGCCCGTTGGTCAAGTGGTTAAGACACCGCCCTTTCACGGCGGTAACACGGGTTCGAATC
CCGTACGGGTCA
>Bacillus_cereus_ATCC_10987_chr.trna69-GluTTC (5203956-5203885) Glu (TTC) 72 bp Sc: 71.01
GGCCCGTTGGTCAAGTGGTTAAGACACCGCCCTTTCACGGCGGTAACACGGGTTCGAATC
CCGTACGGGTCA
>Bacillus_cereus_ATCC_10987_chr.trna7-GluTTC (64526-64597) Glu (TTC) 72 bp Sc: 71.01
GGCCCGTTGGTCAAGTGGTTAAGACACCGCCCTTTCACGGCGGTAACACGGGTTCGAATC
CCGTACGGGTCA
>Bacillus_cereus_ATCC_10987_chr.trna94-GluTTC (4648989-4648918) Glu (TTC) 72 bp Sc: 71.01
GGCCCGTTGGTCAAGTGGTTAAGACACCGCCCTTTCACGGCGGTAACACGGGTTCGAATC
CCGTACGGGTCA
>Bacillus_cereus_ATCC_10987_chr.trna10-GluTTC (150501-150575) Glu (TTC) 75 bp Sc: 79.30
GGCCCGTTGGTCAAGTGGTTAAGACACCGCCCTTTCACGGCGGTAACACGGGTTCGAATC
CCGTACGGGTCACCA
>Bacillus_cereus_ATCC_10987_chr.trna19-GluTTC (278581-278655) Glu (TTC) 75 bp Sc: 79.30
GGCCCGTTGGTCAAGTGGTTAAGACACCGCCCTTTCACGGCGGTAACACGGGTTCGAATC
CCGTACGGGTCACCA
>Bacillus_cereus_ATCC_10987_chr.trna34-GluTTC (609961-610035) Glu (TTC) 75 bp Sc: 79.30
GGCCCGTTGGTCAAGTGGTTAAGACACCGCCCTTTCACGGCGGTAACACGGGTTCGAATC
CCGTACGGGTCACCA
>Bacillus_cereus_ATCC_10987_chr.trna15-GlyGCC (150984-151055) Gly (GCC) 72 bp Sc: 80.56
GCGGAAGTAGTTCAGTGGTAGAATACAACCTTGCCAAGGTTGGGGTCGCGGGTTCGAATC
CCGTCTTCCGCT
>Bacillus_cereus_ATCC_10987_chr.trna44-GlyGCC (610888-610962) Gly (GCC) 75 bp Sc: 88.86
GCGGAAGTAGTTCAGTGGTAGAATACAACCTTGCCAAGGTTGGGGTCGCGGGTTCGAATC
CCGTCTTCCGCTCCA
>Bacillus_cereus_ATCC_10987_chr.trna53-GlyGCC (823438-823512) Gly (GCC) 75 bp Sc: 88.86
GCGGAAGTAGTTCAGTGGTAGAATACAACCTTGCCAAGGTTGGGGTCGCGGGTTCGAATC
CCGTCTTCCGCTCCA
>Bacillus_cereus_ATCC_10987_chr.trna77-GlyGCC (4650508-4650434) Gly (GCC) 75 bp Sc: 88.86
GCGGAAGTAGTTCAGTGGTAGAATACAACCTTGCCAAGGTTGGGGTCGCGGGTTCGAATC
CCGTCTTCCGCTCCA
>Bacillus_cereus_ATCC_10987_chr.trna27-GlyTCC (279452-279522) Gly (TCC) 71 bp Sc: 71.78
GCGGGTGTAGTTTAGTGGTAAAACAAGAGCCTTCCAAGCTCTGGTCGAGAGTTCGATTCT
CTTCACCCGCT
>Bacillus_cereus_ATCC_10987_chr.trna90-GlyTCC (4649333-4649263) Gly (TCC) 71 bp Sc: 71.78
GCGGGTGTAGTTTAGTGGTAAAACAAGAGCCTTCCAAGCTCTGGTCGAGAGTTCGATTCT
CTTCACCCGCT
>Bacillus_cereus_ATCC_10987_chr.trna47-GlyTCC (615179-615252) Gly (TCC) 74 bp Sc: 80.08
GCGGGTGTAGTTTAGTGGTAAAACAAGAGCCTTCCAAGCTCTGGTCGAGAGTTCGATTCT
CTTCACCCGCTCCA
>Bacillus_cereus_ATCC_10987_chr.trna95-GlyTCC (4248374-4248301) Gly (TCC) 74 bp Sc: 80.08

GCGGGTGTAGTTTAGTGGTAAAACAAGAGCCTTCCAAGCTCTGGTCGAGAGTTCGATTCT
CTTCACCCGCTCCA
>Bacillus_cereus_ATCC_10987_chr.trna42-HisGTG (610669-610744) His (GTG) 76 bp Sc: 74.61
GCGGTTGTGGCGAAGTGGTTAACGCACCGGATTGTGGCTCCGGCATTCGTGGGTTCGATT
CCCATCAGTCGCCCCA
>Bacillus_cereus_ATCC_10987_chr.trna75-HisGTG (4650716-4650641) His (GTG) 76 bp Sc: 74.61
GCGGTTGTGGCGAAGTGGTTAACGCACCGGATTGTGGCTCCGGCATTCGTGGGTTCGATT
CCCATCAGTCGCCCCA
>Bacillus_cereus_ATCC_10987_chr.trna1-IleGAT (10991-11067) Ile (GAT) 77 bp Sc: 101.21
GGGCCTATAGCTCAGCTGGTTAGAGCGCACGCCTGATAAGCGTGAGGTCGATGGTTCGAG
TCCATTTAGGCCCACCA
>Bacillus_cereus_ATCC_10987_chr.trna4-IleGAT (30765-30841) Ile (GAT) 77 bp Sc: 101.21
GGGCCTATAGCTCAGCTGGTTAGAGCGCACGCCTGATAAGCGTGAGGTCGATGGTTCGAG
TCCATTTAGGCCCACCA
>Bacillus_cereus_ATCC_10987_chr.trna65-IleGAT (824561-824637) Ile (GAT) 77 bp Sc: 101.21
GGGCCTATAGCTCAGCTGGTTAGAGCGCACGCCTGATAAGCGTGAGGTCGATGGTTCGAG
TCCATTTAGGCCCACCA
>Bacillus_cereus_ATCC_10987_chr.trna91-IleGAT (4649252-4649176) Ile (GAT) 77 bp Sc: 101.21
GGGCCTATAGCTCAGCTGGTTAGAGCGCACGCCTGATAAGCGTGAGGTCGATGGTTCGAG
TCCATTTAGGCCCACCA
>Bacillus_cereus_ATCC_10987_chr.trna46-LeuCAA (611058-611142) Leu (CAA) 85 bp Sc: 60.08
GCCGATGTGGCGGAATTGGCAGACGCGCACGACTCAAAATCGTGTTCCTTCGGGAGTGTC
GGTTCGACCCCGACCATCGGTATCA
>Bacillus_cereus_ATCC_10987_chr.trna24-LeuGAG (279114-279199) Leu (GAG) 86 bp Sc: 57.19
GCGGTCGTGGCGGAACGGCAGACGCGCTAGGTTGAGGGCCTAGTGGGGGAAACCCCGTGG
AGGTTCAAGTCCTCTCGGCCGCATCA
>Bacillus_cereus_ATCC_10987_chr.trna54-LeuTAA (823529-823617) Leu (TAA) 89 bp Sc: 71.28
GCCGGGGTGGCGGAACAGGCAGACGCACAGGACTTAAAATCCTGCGGTGGGTGACCACCG
TGCGGGTTCGACCCCCGCCCTCGGCACCA
>Bacillus_cereus_ATCC_10987_chr.trna78-LeuTAA (4650417-4650329) Leu (TAA) 89 bp Sc: 71.28
GCCGGGGTGGCGGAACAGGCAGACGCACAGGACTTAAAATCCTGCGGTGGGTGACCACCG
TGCGGGTTCGACCCCCGCCCTCGGCACCA
>Bacillus_cereus_ATCC_10987_chr.trna52-LeuTAG (823328-823408) Leu (TAG) 81 bp Sc: 66.65
GCGGGTGTGGCGGAATTGGCAGACGCACTAGACTTAGGATCTAGCGCCTTTGGCGTGGGG
GTTCGACTCCCTTCACCCGCA
>Bacillus_cereus_ATCC_10987_chr.trna76-LeuTAG (4650618-4650538) Leu (TAG) 81 bp Sc: 67.96
GCGGGTGTGGCGGAATTGGCAGACGCACCAGACTTAGGATCTGGCGCCTTTGGCGTGGGG
GTTCGACTCCCTTCACCCGCA
>Bacillus_cereus_ATCC_10987_chr.trna23-LysTTT (279025-279097) Lys (TTT) 73 bp Sc: 90.38
GAGCCATTAGCTCAGTTGGTAGAGCATCTGACTTTTAATCAGAGGGTCGAAGGTTCGAGT
CCTTCATGGCTCA
>Bacillus_cereus_ATCC_10987_chr.trna14-LysTTT (150903-150978) Lys (TTT) 76 bp Sc: 98.67
GAGCCATTAGCTCAGTTGGTAGAGCATCTGACTTTTAATCAGAGGGTCGAAGGTTCGAGT
CCTTCATGGCTCACCA
>Bacillus_cereus_ATCC_10987_chr.trna51-LysTTT (823238-823313) Lys (TTT) 76 bp Sc: 98.67
GAGCCATTAGCTCAGTTGGTAGAGCATCTGACTTTTAATCAGAGGGTCGAAGGTTCGAGT
CCTTCATGGCTCACCA
>Bacillus_cereus_ATCC_10987_chr.trna68-LysTTT (5204045-5203970) Lys (TTT) 76 bp Sc: 98.67
GAGCCATTAGCTCAGTTGGTAGAGCATCTGACTTTTAATCAGAGGGTCGAAGGTTCGAGT
CCTTCATGGCTCACCA
>Bacillus_cereus_ATCC_10987_chr.trna89-LysTTT (4649422-4649347) Lys (TTT) 76 bp Sc: 98.67
GAGCCATTAGCTCAGTTGGTAGAGCATCTGACTTTTAATCAGAGGGTCGAAGGTTCGAGT
CCTTCATGGCTCACCA
>Bacillus_cereus_ATCC_10987_chr.trna30-MetCAT (284925-285003) Met (CAT) 79 bp Sc: 66.09
CGCGGGGTGGAGCAGCACGGTAGCTCGTCGGGCTCATAACCCCGAAGGTCGCAGGTTCAA
ATCCTNGTCCCCGCAACCA
>Bacillus_cereus_ATCC_10987_chr.trna6-MetCAT (64439-64512) Met (CAT) 74 bp Sc: 75.69
GGCGGTGTAGCTCAGCTGGCTAGAGCGTACGGTTCATACCCGTGAGGTCGGGGGTTCGAT
CCCCTCCGCCGCTA
>Bacillus_cereus_ATCC_10987_chr.trna82-MetCAT (4650059-4649983) Met (CAT) 77 bp Sc: 83.99
GGCGGTGTAGCTCAGCTGGCTAGAGCGTACGGTTCATACCCGTGAGGTCGGGGGTTCGAT
CCCCTCCGCCGCTACCA
>Bacillus_cereus_ATCC_10987_chr.trna28-MetCAT (284395-284471) Met (CAT) 77 bp Sc: 85.35
CGCGGGGTGGAGCAGCACGGTAGCTCGTCGGGCTCATAACCCGAAGGTCGCAGGTTCAAA
TCCTGTCCCCGCAACCA
>Bacillus_cereus_ATCC_10987_chr.trna36-MetCAT (610140-610216) Met (CAT) 77 bp Sc: 85.35
CGCGGGGTGGAGCAGCACGGTAGCTCGTCGGGCTCATAACCCGAAGGTCGCAGGTTCAAA

TCCTGTCCCCGCAACCA
>Bacillus_cereus_ATCC_10987_chr.trna60-MetCAT (824151-824227) Met (CAT) 77 bp Sc: 85.35
CGCGGGGTGGAGCAGCACGGTAGCTCGTCGGGCTCATAACCCGAAGGTCGCAGGTTCAAA
TCCTGTCCCCGCAACCA
>Bacillus_cereus_ATCC_10987_chr.trna97-MetCAT (4043277-4043204) Met (CAT) 74 bp Sc: 88.27
GGACCCTTAGCTCAGCTGGTTAGAGCAGACGGCTCATAACCGTCCGGTCGTAGGTTCGAG
TCCTACAGGGTCCA
>Bacillus_cereus_ATCC_10987_chr.trna85-MetCAT (4649764-4649688) Met (CAT) 77 bp Sc: 89.08
CGCGGGGTGGAGCAGTCTGGTAGCTCGTCGGGCTCATAACCCGAAGGTCGCAGGTTCAAA
TCCTGTCCCCGCAACCA
>Bacillus_cereus_ATCC_10987_chr.trna83-MetCAT (4649978-4649902) Met (CAT) 77 bp Sc: 96.69
GGACCTTTAGCTCAGCTGGTTAGAGCAGACGGCTCATAACCGTCCGGTCGTAGGTTCGAG
TCCTACAAGGTCCACCA
>Bacillus_cereus_ATCC_10987_chr.trna71-PheGAA (5203746-5203674) Phe (GAA) 73 bp Sc: 84.57
GGCTCGGTAGCTCAGTCGGTAGAGCAGAGGACTGAAAATCCTCGTGTCGGCGGTTCGATT
CCGTCCCGAGCCA
>Bacillus_cereus_ATCC_10987_chr.trna38-PheGAA (610305-610380) Phe (GAA) 76 bp Sc: 92.86
GGCTCGGTAGCTCAGTCGGTAGAGCAGAGGACTGAAAATCCTCGTGTCGGCGGTTCGATT
CCGTCCCGAGCCACCA
>Bacillus_cereus_ATCC_10987_chr.trna62-PheGAA (824316-824391) Phe (GAA) 76 bp Sc: 92.86
GGCTCGGTAGCTCAGTCGGTAGAGCAGAGGACTGAAAATCCTCGTGTCGGCGGTTCGATT
CCGTCCCGAGCCACCA
>Bacillus_cereus_ATCC_10987_chr.trna87-PheGAA (4649598-4649523) Phe (GAA) 76 bp Sc: 92.86
GGCTCGGTAGCTCAGTCGGTAGAGCAGAGGACTGAAAATCCTCGTGTCGGCGGTTCGATT
CCGTCCCGAGCCACCA
>Bacillus_cereus_ATCC_10987_chr.trna56-ProTGG (823705-823778) Pro (TGG) 74 bp Sc: 85.68
CGGGAAGTGGCTCAGCTTGGTAGAGCACCTGGTTTGGGACCAGGGGGTCGCAGGTTCAAA
TCCTGTCTTCCCGA
>Bacillus_cereus_ATCC_10987_chr.trna80-ProTGG (4650241-4650168) Pro (TGG) 74 bp Sc: 85.68
CGGGAAGTGGCTCAGCTTGGTAGAGCACCTGGTTTGGGACCAGGGGGTCGCAGGTTCAAA
TCCTGTCTTCCCGA
>Bacillus_cereus_ATCC_10987_chr.trna26-ProTGG (279374-279450) Pro (TGG) 77 bp Sc: 93.98
CGGGAAGTGGCTCAGCTTGGTAGAGCACCTGGTTTGGGACCAGGGGGTCGCAGGTTCAAA
TCCTGTCTTCCCGACCA
>Bacillus_cereus_ATCC_10987_chr.trna96-GlnTTG (4246559-4246487) Gln (TTG) 73 bp Sc: 28.25
GAGGTCGTGGAGTAACGGTTTAACTCGCCTATACTTTGTATAGGAGATGGGGGTTCGATT
CCCTTCGACCAAA
>Bacillus_cereus_ATCC_10987_chr.trna18-SerGCT (278482-278572) Ser (GCT) 91 bp Sc: 67.93
AGAGAAGTACCCAAGTGGCTCAAGGGGCTCCCCTGCTAAGGGAGTAGATCGCGAACGCGG
TGCGAGGGTTCGAATCCCTTCTTCTCTGCCA
>Bacillus_cereus_ATCC_10987_chr.trna93-SerGCT (4649086-4648996) Ser (GCT) 91 bp Sc: 67.93
AGAGAAGTACCCAAGTGGCTCAAGGGGCTCCCCTGCTAAGGGAGTAGATCGCGAACGCGG
TGCGAGGGTTCGAATCCCTTCTTCTCTGCCA
>Bacillus_cereus_ATCC_10987_chr.trna33-SerGGA (609852-609943) Ser (GGA) 92 bp Sc: 63.66
GGAGAGCTGTCCGAGTTGGCCGAAGGAGCACGATTGGAAATCGTGTATACGTCACAAGCG
TATCAAGGGTTCGAATCCCTTGCTCTCCGCCA
>Bacillus_cereus_ATCC_10987_chr.trna58-SerTGA (823887-823979) Ser (TGA) 93 bp Sc: 71.21
GGAGGTATACCCAAGTTCGGCTGAAGGGATCGGTCTTGAAAACCGACAGGCGGTGAGAAT
CGCGCGGGGGTTCGAATCCCTCTACCTCCTCCA
>Bacillus_cereus_ATCC_10987_chr.trna3-SerTGA (21771-21863) Ser (TGA) 93 bp Sc: 71.57
GGAGGTATACCCAAGTCTGGCTGAAGGGATCGGTCTTGAAAACCGACAGGCGGCGAGAGT
CGCGCGGGGGTTCGAATCCCTCTACCTCCTCCA
>Bacillus_cereus_ATCC_10987_chr.trna84-SerTGA (4649884-4649792) Ser (TGA) 93 bp Sc: 72.21
GGAGGTATACCCAAGTTCGGCTGAAGGGATCGGTCTTGAAAACCGACAGGCGGCGAGAGT
CGCGCGGGGGTTCGAATCCCTCTACCTCCTCCA
>Bacillus_cereus_ATCC_10987_chr.trna59-SerTGA (824031-824123) Ser (TGA) 93 bp Sc: 72.21
GGAGGTATACCCAAGTTCGGCTGAAGGGATCGGTCTTGAAAACCGACAGGCGGCGAGAGT
CGCGCGGGGGTTCGAATCCCTCTACCTCCTCCA
>Bacillus_cereus_ATCC_10987_chr.trna9-ThrGGT (150404-150476) Thr (GGT) 73 bp Sc: 78.48
GCTTCCATAGCTCAGCTGGTAGAGCACTTCCATGGTAAGGAAGAGGTCACCGGTTCAAGC
CCGGTTGGAAGCT
>Bacillus_cereus_ATCC_10987_chr.trna63-ThrTGT (824401-824473) Thr (TGT) 73 bp Sc: 87.15
GCCGACTTAGCTCAATTGGTAGAGCAACTGACTTGTAATCAGTAGGTTGGGGGTTCAAGT
CCTCTAGTCGGCA
>Bacillus_cereus_ATCC_10987_chr.trna74-ThrTGT (4650801-4650726) Thr (TGT) 76 bp Sc: 95.44
GCCGACTTAGCTCAATTGGTAGAGCAACTGACTTGTAATCAGTAGGTTGGGGGTTCAAGT
CCTCTAGTCGGCACCA

>Bacillus_cereus_ATCC_10987_chr.trna39-ThrTGT (610399-610474) Thr (TGT) 76 bp Sc: 96.17
GCCGGCTTAGCTCAATTGGTAGAGCAACTGACTTGTAATCAGTAGGTTGGGGGTTCAAGT
CCTCTAGCCGGCACCA
>Bacillus_cereus_ATCC_10987_chr.trna88-ThrTGT (4649508-4649433) Thr (TGT) 76 bp Sc: 96.17
GCCGGCTTAGCTCAATTGGTAGAGCAACTGACTTGTAATCAGTAGGTTGGGGGTTCAAGT
CCTCTAGCCGGCACCA
>Bacillus_cereus_ATCC_10987_chr.trna64-TrpCCA (824480-824550) Trp (CCA) 71 bp Sc: 53.62
AGGGGCATAGTTTAAAGGTAGAACTGAGGTCTCCAAAACCTCCAGTGTGGGTTCGATTCC
TACTGCCCCTG
>Bacillus_cereus_ATCC_10987_chr.trna41-TrpCCA (610576-610649) Trp (CCA) 74 bp Sc: 61.92
AGGGGCATAGTTTAAAGGTAGAACTGAGGTCTCCAAAACCTCCAGTGTGGGTTCGATTCC
TACTGCCCCTGCCA
>Bacillus_cereus_ATCC_10987_chr.trna12-TyrGTA (150674-150757) Tyr (GTA) 84 bp Sc: 77.59
GGAGGGGTAGCGAAGTGGCTAAACGCGGCGGACTGTAAATCCGCTCCTTCGGGTTCGGCA
GTTCGAATCTGCCCCCCTCCACCA
>Bacillus_cereus_ATCC_10987_chr.trna40-TyrGTA (610485-610568) Tyr (GTA) 84 bp Sc: 77.59
GGAGGGGTAGCGAAGTGGCTAAACGCGGCGGACTGTAAATCCGCTCCTTCGGGTTCGGCA
GTTCGAATCTGCCCCCCTCCACCA
>Bacillus_cereus_ATCC_10987_chr.trna49-TyrGTA (823064-823147) Tyr (GTA) 84 bp Sc: 77.59
GGAGGGGTAGCGAAGTGGCTAAACGCGGCGGACTGTAAATCCGCTCCTTCGGGTTCGGCA
GTTCGAATCTGCCCCCCTCCACCA
>Bacillus_cereus_ATCC_10987_chr.trna98-ValGAC (1389582-1389510) Val (GAC) 73 bp Sc: 61.20
GATCCCGTAGCTCAGCAGGGAGAGCGCCACCTTGACAGGGTGGAGGTCGTGAGTTCGAGC
CTCTCCGGGGTCA
>Bacillus_cereus_ATCC_10987_chr.trna11-ValTAC (150581-150656) Val (TAC) 76 bp Sc: 97.03
GGAGGATTAGCTCAGCTGGGAGAGCACCTGCCTTACAAGCAGGGGGTCGGCGGTTCGATC
CCGTCATCCTCCACCA
>Bacillus_cereus_ATCC_10987_chr.trna20-ValTAC (278660-278735) Val (TAC) 76 bp Sc: 97.03
GGAGGATTAGCTCAGCTGGGAGAGCACCTGCCTTACAAGCAGGGGGTCGGCGGTTCGATC
CCGTCATCCTCCACCA
>Bacillus_cereus_ATCC_10987_chr.trna35-ValTAC (610040-610115) Val (TAC) 76 bp Sc: 97.03
GGAGGATTAGCTCAGCTGGGAGAGCACCTGCCTTACAAGCAGGGGGTCGGCGGTTCGATC
CCGTCATCCTCCACCA
>Bacillus_cereus_ATCC_10987_chr.trna48-ValTAC (822980-823055) Val (TAC) 76 bp Sc: 97.03
GGAGGATTAGCTCAGCTGGGAGAGCACCTGCCTTACAAGCAGGGGGTCGGCGGTTCGATC
CCGTCATCCTCCACCA
>Bacillus_cereus_ATCC_10987_chr.trna73-ValTAC (4650881-4650806) Val (TAC) 76 bp Sc: 97.03
GGAGGATTAGCTCAGCTGGGAGAGCACCTGCCTTACAAGCAGGGGGTCGGCGGTTCGATC
CCGTCATCCTCCACCA
>Bacillus_licheniformis_ATCC_14580_chr.trna50-AlaGGC (3143484-3143412) Ala (GGC) 73 bp Sc: 77.59
GGGGCATTAGCTCAGCTGGGAGAGCGCTACACTGGCAGTGTAGAGGTCAGCGGTTCGAGC
CCGCTATGCTCCA
>Bacillus_licheniformis_ATCC_14580_chr.trna58-AlaTGC (3116676-3116604) Ala (TGC) 73 bp Sc: 84.64
GGGGCCTTAGCTCAGCTGGGAGAGCGCCTGCTTTGCACGCAGGAGGTCAGCGGTTCGATC
CCGCTAGGCTCCA
>Bacillus_licheniformis_ATCC_14580_chr.trna2-AlaTGC (11641-11716) Ala (TGC) 76 bp Sc: 85.87
GGGGCCTTAGCTCAGCTGGGAGAGCGCCTGCTTTGCACGCATGAGGTCAGCGGTTCGATC
CCGCTAGGCTCCACCA
>Bacillus_licheniformis_ATCC_14580_chr.trna5-AlaTGC (36344-36419) Ala (TGC) 76 bp Sc: 92.94
GGGGCCTTAGCTCAGCTGGGAGAGCGCCTGCTTTGCACGCAGGAGGTCAGCGGTTCGATC
CCGCTAGGCTCCACCA
>Bacillus_licheniformis_ATCC_14580_chr.trna22-ArgACG (611686-611762) Arg (ACG) 77 bp Sc: 81.43
GCGCCCGTAGCTCAATTGGATAGAGCGTTTGACTACGGATCAAAAGGTTAGGGGTTCGAC
TCCTCTCGGGCGCGCCA
>Bacillus_licheniformis_ATCC_14580_chr.trna56-ArgACG (3116844-3116768) Arg (ACG) 77 bp Sc: 81.43
GCGCCCGTAGCTCAATTGGATAGAGCGTTTGACTACGGATCAAAAGGTTAGGGGTTCGAC
TCCTCTCGGGCGCGCCA
>Bacillus_licheniformis_ATCC_14580_chr.trna49-ArgCCG (3538188-3538113) Arg (CCG) 76 bp Sc: 61.22
GCGCTCGTAGCTCAGCTGGATAGAGCGGTGGTTTCCGGTACCACGTCTGCCGGGGGTTCG
AATCCCTCCGAGCGCG
>Bacillus_licheniformis_ATCC_14580_chr.trna43-ArgCCT (2175934-2176007) Arg (CCT) 74 bp Sc: 76.22
GTCCCAGTAGCTCAGGTGGATAGAGCAACAGCCTCCTAAGCTGTGTGTCGGGAGTTCGAA
TCTCTCCTGGGACG
>Bacillus_licheniformis_ATCC_14580_chr.trna71-ArgTCT (2865376-2865300) Arg (TCT) 77 bp Sc: 83.73
GTCCCAGTAGCTCAGCTGGATAGAGCAACGGCCTTCTAAGCCGTCGGTCGGGAGTTCGAA
TCTCTCCTGGGACGTCA
>Bacillus_licheniformis_ATCC_14580_chr.trna15-AsnGTT (572258-572332) Asn (GTT) 75 bp Sc: 87.08

TCCACAGTAGCTCAGTGGTAGAGCTATCGGCTGTTAACCGATCGGTCGCAGGTTCGAATC
CTGCCTGTGGAGCCA
>Bacillus_licheniformis_ATCC_14580_chr.trna26-AsnGTT (925436-925510) Asn (GTT) 75 bp Sc: 89.95
TCCGCAGTAGCTCAGTGGTAGAGCTATCGGCTGTTAACCGATCGGTCGCAGGTTCGAATC
CTGCCTGCGGAGCCA
>Bacillus_licheniformis_ATCC_14580_chr.trna68-AsnGTT (3115738-3115664) Asn (GTT) 75 bp Sc: 89.95
TCCGCAGTAGCTCAGTGGTAGAGCTATCGGCTGTTAACCGATCGGTCGCAGGTTCGAATC
CTGCCTGCGGAGCCA
>Bacillus_licheniformis_ATCC_14580_chr.trna25-AspGTC (617041-617117) Asp (GTC) 77 bp Sc: 95.80
GGTCCGGTAGTTCAGTTGGTTAGAATGCCTGCCTGTCACGCAGGAGGTCGCGGGTTCGAG
TCCCGTCCGGACCGCCA
>Bacillus_licheniformis_ATCC_14580_chr.trna31-AspGTC (926003-926079) Asp (GTC) 77 bp Sc: 95.80
GGTCCGGTAGTTCAGTTGGTTAGAATGCCTGCCTGTCACGCAGGAGGTCGCGGGTTCGAG
TCCCGTCCGGACCGCCA
>Bacillus_licheniformis_ATCC_14580_chr.trna47-AspGTC (4187251-4187175) Asp (GTC) 77 bp Sc: 95.80
GGTCCGGTAGTTCAGTTGGTTAGAATGCCTGCCTGTCACGCAGGAGGTCGCGGGTTCGAG
TCCCGTCCGGACCGCCA
>Bacillus_licheniformis_ATCC_14580_chr.trna63-AspGTC (3116176-3116100) Asp (GTC) 77 bp Sc: 95.80
GGTCCGGTAGTTCAGTTGGTTAGAATGCCTGCCTGTCACGCAGGAGGTCGCGGGTTCGAG
TCCCGTCCGGACCGCCA
>Bacillus_licheniformis_ATCC_14580_chr.trna39-CysGCA (926729-926799) Cys (GCA) 71 bp Sc: 67.07
GGCGGCATAGCCAAGTGGTAAGGCAGAGGTCTGCAAAACCTTTATCCCCGGTTCGAATCC
GGGTGTCGCCT
>Bacillus_licheniformis_ATCC_14580_chr.trna44-GlnTTG (2558249-2558323) Gln (TTG) 75 bp Sc: 57.71
TGGGCTATAGCCAAGCGGTAAGGCAACGGACTTTGACTCCGTGACGCGCTGGTTCGAATC
CACCAGCTAGCCCAG
>Bacillus_licheniformis_ATCC_14580_chr.trna14-GlnTTG (176973-177044) Gln (TTG) 72 bp Sc: 70.30
TGGGCTATAGCCAAGCGGTAAGGCAACGGACTTTGACTCCGTCATGCGCTGGTTCGAATC
CAGCTAGCCCAG
>Bacillus_licheniformis_ATCC_14580_chr.trna37-GlnTTG (926516-926587) Gln (TTG) 72 bp Sc: 70.30
TGGGCTATAGCCAAGCGGTAAGGCAACGGACTTTGACTCCGTCATGCGCTGGTTCGAATC
CAGCTAGCCCAG
>Bacillus_licheniformis_ATCC_14580_chr.trna18-GlnTTG (572538-572612) Gln (TTG) 75 bp Sc: 75.97
TGGGCTATAGCCAAGCGGTAAGGCAACGGACTTTGACTCCGTCATGCGTTGGTTCGAATC
CAGCTAGCCCAGCCA
>Bacillus_licheniformis_ATCC_14580_chr.trna10-GluTTC (176445-176518) Glu (TTC) 74 bp Sc: 66.32
GGCCCGTTGGTCAAGTGGTTAAGACACCGCCCTTTCACGGCGGTAACACGGTTCGAATCC
CGTACGGGTCACCA
>Bacillus_licheniformis_ATCC_14580_chr.trna17-GluTTC (572454-572525) Glu (TTC) 72 bp Sc: 71.01
GGCCCGTTGGTCAAGTGGTTAAGACACCGCCCTTTCACGGCGGTAACACGGGTTCGAATC
CCGTACGGGTCA
>Bacillus_licheniformis_ATCC_14580_chr.trna46-GluTTC (4187411-4187340) Glu (TTC) 72 bp Sc: 71.01
GGCCCGTTGGTCAAGTGGTTAAGACACCGCCCTTTCACGGCGGTAACACGGGTTCGAATC
CCGTACGGGTCA
>Bacillus_licheniformis_ATCC_14580_chr.trna7-GluTTC (75033-75104) Glu (TTC) 72 bp Sc: 71.01
GGCCCGTTGGTCAAGTGGTTAAGACACCGCCCTTTCACGGCGGTAACACGGGTTCGAATC
CCGTACGGGTCA
>Bacillus_licheniformis_ATCC_14580_chr.trna70-GluTTC (3115559-3115485) Glu (TTC) 75 bp Sc: 71.62
GGCCCGTTGGTCAAGTGGTTAAGACACCGCCCTTTCACGGCGGTAACACGGGTTCGAATC
CCGTACGGGTCATCA
>Bacillus_licheniformis_ATCC_14580_chr.trna28-GluTTC (925623-925697) Glu (TTC) 75 bp Sc: 79.30
GGCCCGTTGGTCAAGTGGTTAAGACACCGCCCTTTCACGGCGGTAACACGGGTTCGAATC
CCGTACGGGTCACCA
>Bacillus_licheniformis_ATCC_14580_chr.trna55-GlyGCC (3116926-3116855) Gly (GCC) 72 bp Sc: 86.63
GCGGAAGTAGTTCAGTGGTAGAACACCACCTTGCCAAGGTGGGGGTCGCGGGTTCGAATC
CCGTCTTCCGCT
>Bacillus_licheniformis_ATCC_14580_chr.trna38-GlyGCC (926641-926715) Gly (GCC) 75 bp Sc: 94.93
GCGGAAGTAGTTCAGTGGTAGAACACCACCTTGCCAAGGTGGGGGTCGCGGGTTCGAATC
CCGTCTTCCGCTCCA
>Bacillus_licheniformis_ATCC_14580_chr.trna23-GlyTCC (611775-611848) Gly (TCC) 74 bp Sc: 78.55
GCGGGTGTAGTTTAGTGGTAAAACCTCAGCCTTCCAAGCTGATGTCGTGAGTTCGATTCT
CATCACCCGCTCCA
>Bacillus_licheniformis_ATCC_14580_chr.trna42-GlyTCC (953171-953244) Gly (TCC) 74 bp Sc: 81.36
GCGGGTGTAGTTTAGTGGTAAAACCTCAGCCTTCCAAGCTGATGTCGTGGGTTCGATTCC
CATCACCCGCTCCA
>Bacillus_licheniformis_ATCC_14580_chr.trna66-GlyTCC (3115907-3115834) Gly (TCC) 74 bp Sc: 81.36
GCGGGTGTAGTTTAGTGGTAAAACCTCAGCCTTCCAAGCTGATGTCGTGGGTTCGATTCC

CATCACCCGCTCCA
>Bacillus_licheniformis_ATCC_14580_chr.trna36-HisGTG (926435-926507) His (GTG) 73 bp Sc: 64.21
GCGGTTGTGGCGAAGTGGTTAACGCACCAGATTGTGGCTCTGGCACTCGTGGGTTCGATT
CCCATCAATCGCC
>Bacillus_licheniformis_ATCC_14580_chr.trna65-HisGTG (3115991-3115916) His (GTG) 76 bp Sc: 79.15
GCGGTTGTGGCGAAGTGGTTAACGCACCAGATTGTGGCTCTGGCATTCGTGGGTTCGATT
CCCATCAACCGCCCCA
>Bacillus_licheniformis_ATCC_14580_chr.trna67-IleGAT (3115821-3115745) Ile (GAT) 77 bp Sc: 102.00
GGGCCTGTAGCTCAGCTGGTTAGAGCGCACGCCTGATAAGCGTGAGGTCGATGGTTCGAG
TCCATTCAGGCCCACCA
>Bacillus_licheniformis_ATCC_14580_chr.trna1-IleGAT (11553-11629) Ile (GAT) 77 bp Sc: 102.39
GGGCCTGTAGCTCAGCTGGTTAGAGCGCACGCCTGATAAGCGTGAGGTCGGTGGTTCGAG
TCCACTCAGGCCCACCA
>Bacillus_licheniformis_ATCC_14580_chr.trna4-IleGAT (36256-36332) Ile (GAT) 77 bp Sc: 102.39
GGGCCTGTAGCTCAGCTGGTTAGAGCGCACGCCTGATAAGCGTGAGGTCGGTGGTTCGAG
TCCACTCAGGCCCACCA
>Bacillus_licheniformis_ATCC_14580_chr.trna41-LeuCAA (926913-926997) Leu (CAA) 85 bp Sc: 60.81
GCCGGTGTGGCGGAATTGGCAGACGCGCACGACTCAAAATCGTGTTCCTTCGGGAGTGTC
GGTTCGACCCCGACCACCGGTATCA
>Bacillus_licheniformis_ATCC_14580_chr.trna54-LeuCAG (3117026-3116942) Leu (CAG) 85 bp Sc: 62.54
GCGGATGTGGCGGAATTGGCAGACGCGCTAGAATCAGGCTCTAGTGTCTTTTAAAGACGT
GGGGGTTCAAGTCCCTTCATCCGCA
>Bacillus_licheniformis_ATCC_14580_chr.trna21-LeuGAG (572812-572895) Leu (GAG) 84 bp Sc: 53.11
GCGGTCGTGGCGGAATGGCAGACGCGCTAGGTTGAGGGCCTAGTGGGTGAATAACCCGTG
GAGGTTCAAGTCCTCTCGGCCGCA
>Bacillus_licheniformis_ATCC_14580_chr.trna40-LeuTAA (926810-926898) Leu (TAA) 89 bp Sc: 76.51
GCCGGGGTGGTGGAATTGGCAGACACACAGGACTTAAAATCCTGCGGTAGGTGACTACCG
TGCCGGTTCAAGTCCGGCCCTCGGCACCA
>Bacillus_licheniformis_ATCC_14580_chr.trna20-LeuTAG (572709-572791) Leu (TAG) 83 bp Sc: 69.61
GCGGGTGTGGCGGAATTGGCAGACGCGCTAGACTTAGGATCTAGTGTCTTTACGGCGTGG
GGGTTCGAGTCCCTTCACCCGCA
>Bacillus_licheniformis_ATCC_14580_chr.trna19-LysTTT (572621-572696) Lys (TTT) 76 bp Sc: 98.67
GAGCCATTAGCTCAGTTGGTAGAGCATCTGACTTTTAATCAGAGGGTCGAAGGTTCGAGT
CCTTCATGGCTCACCA
>Bacillus_licheniformis_ATCC_14580_chr.trna45-LysTTT (4187494-4187419) Lys (TTT) 76 bp Sc: 98.67
GAGCCATTAGCTCAGTTGGTAGAGCATCTGACTTTTAATCAGAGGGTCGAAGGTTCGAGT
CCTTCATGGCTCACCA
>Bacillus_licheniformis_ATCC_14580_chr.trna53-LysTTT (3117108-3117033) Lys (TTT) 76 bp Sc: 98.67
GAGCCATTAGCTCAGTTGGTAGAGCATCTGACTTTTAATCAGAGGGTCGAAGGTTCGAGT
CCTTCATGGCTCACCA
>Bacillus_licheniformis_ATCC_14580_chr.trna6-MetCAT (74949-75022) Met (CAT) 74 bp Sc: 75.69
GGCGGTGTAGCTCAGCTGGCTAGAGCGTACGGTTCATACCCGTGAGGTCGGGGGTTCGAT
CCCCTCCGCCGCTA
>Bacillus_licheniformis_ATCC_14580_chr.trna59-MetCAT (3116578-3116505) Met (CAT) 74 bp Sc: 78.06
GGCGGTGTAGCTCAGCTGGCTAGAGCGTACGGTTCATACCCGTGAGGTCGGGGGTTCGAT
TCCCTCCGCCGCTA
>Bacillus_licheniformis_ATCC_14580_chr.trna24-MetCAT (616923-616999) Met (CAT) 77 bp Sc: 90.17
CGCGGGGTGGAGCAGTTCGGTAGCTCGTCGGGCTCATAACCCGAAGGTCGCAGGTTCAAA
TCCTGCCCCCGCAACCA
>Bacillus_licheniformis_ATCC_14580_chr.trna30-MetCAT (925912-925988) Met (CAT) 77 bp Sc: 90.17
CGCGGGGTGGAGCAGTTCGGTAGCTCGTCGGGCTCATAACCCGAAGGTCGCAGGTTCAAA
TCCTGCCCCCGCAACCA
>Bacillus_licheniformis_ATCC_14580_chr.trna62-MetCAT (3116298-3116222) Met (CAT) 77 bp Sc: 90.17
CGCGGGGTGGAGCAGTTCGGTAGCTCGTCGGGCTCATAACCCGAAGGTCGCAGGTTCAAA
TCCTGCCCCCGCAACCA
>Bacillus_licheniformis_ATCC_14580_chr.trna60-MetCAT (3116499-3116423) Met (CAT) 77 bp Sc: 97.54
GGACCTTTAGCTCAGTTGGTTAGAGCAGACGGCTCATAACCGTCCGGTCGTAGGTTCGAG
TCCTACAAGGTCCACCA
>Bacillus_licheniformis_ATCC_14580_chr.trna48-PheGAA (4187148-4187076) Phe (GAA) 73 bp Sc: 89.60
GGCTCGGTAGCTCAGTTGGTAGAGCAACGGACTGAAAATCCGTGTGTCGGCGGTTCGATT
CCGTCCCGAGCCA
>Bacillus_licheniformis_ATCC_14580_chr.trna32-PheGAA (926090-926165) Phe (GAA) 76 bp Sc: 97.89
GGCTCGGTAGCTCAGTTGGTAGAGCAACGGACTGAAAATCCGTGTGTCGGCGGTTCGATT
CCGTCCCGAGCCACCA
>Bacillus_licheniformis_ATCC_14580_chr.trna64-PheGAA (3116089-3116014) Phe (GAA) 76 bp Sc: 97.89
GGCTCGGTAGCTCAGTTGGTAGAGCAACGGACTGAAAATCCGTGTGTCGGCGGTTCGATT
CCGTCCCGAGCCACCA

>Bacillus_licheniformis_ATCC_14580_chr.trna57-ProTGG (3116759-3116683) Pro (TGG) 77 bp Sc: 94.72
CGGGAAGTAGCTCAGCTTGGTAGAGCACATGGTTTGGGACCATGGGGTCGCAGGTTCGAA
TCCTGTCTTCCCGACCA
>Bacillus_licheniformis_ATCC_14580_chr.trna16-SerGCT (572335-572425) Ser (GCT) 91 bp Sc: 71.43
GGAGAAGTACTCAAGTGGCTGAAGAGGCGCCCCTGCTAAGGGTGTAGGTCGCGTAAGCGG
CGCGAGGGTTCAAATCCCTCCTTCTCCGCCA
>Bacillus_licheniformis_ATCC_14580_chr.trna69-SerGCT (3115662-3115572) Ser (GCT) 91 bp Sc: 71.43
GGAGAAGTACTCAAGTGGCTGAAGAGGCGCCCCTGCTAAGGGTGTAGGTCGCGTAAGCGG
CGCGAGGGTTCAAATCCCTCCTTCTCCGCCA
>Bacillus_licheniformis_ATCC_14580_chr.trna27-SerGGA (925517-925608) Ser (GGA) 92 bp Sc: 63.95
GGAGAGCTGTCCGAGTGGTCGAAGGAGCACGATTGGAAATCGTGTAGGCGGCCAACGCCG
TCTCAAGGGTTCGAATCCCTTGCTCTCCGCCA
>Bacillus_licheniformis_ATCC_14580_chr.trna3-SerTGA (26549-26641) Ser (TGA) 93 bp Sc: 72.49
GGAGGAATACCCAAGTCTGGCTGAAGGGATCGGTCTTGAAAACCGACAGGGGTGTCAAAG
CCCGCGGGGGTTCGAATCCCTCTTCCTCCGCCA
>Bacillus_licheniformis_ATCC_14580_chr.trna61-SerTGA (3116415-3116323) Ser (TGA) 93 bp Sc: 72.49
GGAGGAATACCCAAGTCTGGCTGAAGGGATCGGTCTTGAAAACCGACAGGGGTGTCAAAG
CCCGCGGGGGTTCGAATCCCTCTTCCTCCGCCA
>Bacillus_licheniformis_ATCC_14580_chr.trna52-ThrCGT (3117265-3117190) Thr (CGT) 76 bp Sc: 85.36
GCCGGTGTAGCTCAATTGGTAGAGCAACTGAATCGTAATCAGTAGGTTGGGGGTTCAAGT
CCTCTTGCCGGCACCA
>Bacillus_licheniformis_ATCC_14580_chr.trna9-ThrGGT (163318-163390) Thr (GGT) 73 bp Sc: 75.20
GCTTCCATAGCTCAGCAGGTAGAGCACTTCCATGGTAAGGAAGAGGTCAGCGGTTCGAGC
CCGCTTGGAAGCT
>Bacillus_licheniformis_ATCC_14580_chr.trna12-ThrTGT (176803-176875) Thr (TGT) 73 bp Sc: 82.36
GCCGGTCTAGCTCAATTGGTAGAGCAACTGACTTGTAATCAGTAGGTTGGGGGTTCAAGT
CCTCTGGCCGGCA
>Bacillus_licheniformis_ATCC_14580_chr.trna33-ThrTGT (926169-926241) Thr (TGT) 73 bp Sc: 82.36
GCCGGTCTAGCTCAATTGGTAGAGCAACTGACTTGTAATCAGTAGGTTGGGGGTTCAAGT
CCTCTGGCCGGCA
>Bacillus_licheniformis_ATCC_14580_chr.trna35-TrpCCA (926341-926414) Trp (CCA) 74 bp Sc: 71.01
AGGGGCATAGTTTAACGGTAGAACAGAGGTCTCCAAAACCTCCGGTGTGGGTTCGATTCC
TACTGCCCCTGCCA
>Bacillus_licheniformis_ATCC_14580_chr.trna13-TyrGTA (176884-176968) Tyr (GTA) 85 bp Sc: 80.73
GGAGGGGTAGCGAAGTGGCTAAACGCGGCGGACTGTAAATCCGCTCCCTCAGGGTTCGGC
GGTTCGAATCCGTCCCCCTCCACCA
>Bacillus_licheniformis_ATCC_14580_chr.trna34-TyrGTA (926249-926333) Tyr (GTA) 85 bp Sc: 80.73
GGAGGGGTAGCGAAGTGGCTAAACGCGGCGGACTGTAAATCCGCTCCCTCAGGGTTCGGC
GGTTCGAATCCGTCCCCCTCCACCA
>Bacillus_licheniformis_ATCC_14580_chr.trna8-Undet??? (163242-163315) Undet (???) 74 bp Sc: 80.44
TCCGCAGTAGCTCAGTGGTAGAGCTATCGGCTGTAACCGATCGGTCGCAGGTTCGAATCC
TGCCTGCGGAGCCA
>Bacillus_licheniformis_ATCC_14580_chr.trna72-ValGAC (1299799-1299727) Val (GAC) 73 bp Sc: 71.64
GATTCCGTAGCTCAGCTGGGAGAGCGCTACCTTGACAGGGTAGAGGTCGCTGGTTCGAGC
CCAGTCGGAATCA
>Bacillus_licheniformis_ATCC_14580_chr.trna11-ValTAC (176669-176744) Val (TAC) 76 bp Sc: 95.52
GGAGGATTAGCTCAGCTGGGAGAGCATCTGCCTTACAAGCAGAGGGTCGGCGGTTCGATC
CCGTCATCCTCCACCA
>Bacillus_licheniformis_ATCC_14580_chr.trna29-ValTAC (925815-925890) Val (TAC) 76 bp Sc: 95.52
GGAGGATTAGCTCAGCTGGGAGAGCATCTGCCTTACAAGCAGAGGGTCGGCGGTTCGATC
CCGTCATCCTCCACCA
>Bacillus_licheniformis_ATCC_14580_chr.trna51-ValTAC (3117356-3117281) Val (TAC) 76 bp Sc: 95.52
GGAGGATTAGCTCAGCTGGGAGAGCATCTGCCTTACAAGCAGAGGGTCGGCGGTTCGATC
CCGTCATCCTCCACCA
>Bacillus_licheniformis_DSM_13_chr.trna50-AlaGGC (3143658-3143586) Ala (GGC) 73 bp Sc: 77.59
GGGGCATTAGCTCAGCTGGGAGAGCGCTACACTGGCAGTGTAGAGGTCAGCGGTTCGAGC
CCGCTATGCTCCA
>Bacillus_licheniformis_DSM_13_chr.trna58-AlaTGC (3116853-3116781) Ala (TGC) 73 bp Sc: 84.64
GGGGCCTTAGCTCAGCTGGGAGAGCGCCTGCTTTGCACGCAGGAGGTCAGCGGTTCGATC
CCGCTAGGCTCCA
>Bacillus_licheniformis_DSM_13_chr.trna2-AlaTGC (11446-11521) Ala (TGC) 76 bp Sc: 92.94
GGGGCCTTAGCTCAGCTGGGAGAGCGCCTGCTTTGCACGCAGGAGGTCAGCGGTTCGATC
CCGCTAGGCTCCACCA
>Bacillus_licheniformis_DSM_13_chr.trna5-AlaTGC (36144-36219) Ala (TGC) 76 bp Sc: 92.94
GGGGCCTTAGCTCAGCTGGGAGAGCGCCTGCTTTGCACGCAGGAGGTCAGCGGTTCGATC
CCGCTAGGCTCCACCA
>Bacillus_licheniformis_DSM_13_chr.trna22-ArgACG (611478-611554) Arg (ACG) 77 bp Sc: 81.43

GCGCCCGTAGCTCAATTGGATAGAGCGTTTGACTACGGATCAAAAGGTTAGGGGTTCGAC
TCCTCTCGGGCGCGCCA
>Bacillus_licheniformis_DSM_13_chr.trna56-ArgACG (3117021-3116945) Arg (ACG) 77 bp Sc: 81.43
GCGCCCGTAGCTCAATTGGATAGAGCGTTTGACTACGGATCAAAAGGTTAGGGGTTCGAC
TCCTCTCGGGCGCGCCA
>Bacillus_licheniformis_DSM_13_chr.trna49-ArgCCG (3538357-3538282) Arg (CCG) 76 bp Sc: 61.22
GCGCTCGTAGCTCAGCTGGATAGAGCGGTGGTTTCCGGTACCACGTCTGCCGGGGGTTCG
AATCCCTCCGAGCGCG
>Bacillus_licheniformis_DSM_13_chr.trna43-ArgCCT (2176780-2176853) Arg (CCT) 74 bp Sc: 76.22
GTCCCAGTAGCTCAGGTGGATAGAGCAACAGCCTCCTAAGCTGTGTGTCGGGAGTTCGAA
TCTCTCCTGGGACG
>Bacillus_licheniformis_DSM_13_chr.trna71-ArgTCT (2866237-2866161) Arg (TCT) 77 bp Sc: 83.73
GTCCCAGTAGCTCAGCTGGATAGAGCAACGGCCTTCTAAGCCGTCGGTCGGGAGTTCGAA
TCTCTCCTGGGACGTCA
>Bacillus_licheniformis_DSM_13_chr.trna15-AsnGTT (572074-572148) Asn (GTT) 75 bp Sc: 87.08
TCCACAGTAGCTCAGTGGTAGAGCTATCGGCTGTTAACCGATCGGTCGCAGGTTCGAATC
CTGCCTGTGGAGCCA
>Bacillus_licheniformis_DSM_13_chr.trna26-AsnGTT (925729-925803) Asn (GTT) 75 bp Sc: 89.95
TCCGCAGTAGCTCAGTGGTAGAGCTATCGGCTGTTAACCGATCGGTCGCAGGTTCGAATC
CTGCCTGCGGAGCCA
>Bacillus_licheniformis_DSM_13_chr.trna68-AsnGTT (3115915-3115841) Asn (GTT) 75 bp Sc: 89.95
TCCGCAGTAGCTCAGTGGTAGAGCTATCGGCTGTTAACCGATCGGTCGCAGGTTCGAATC
CTGCCTGCGGAGCCA
>Bacillus_licheniformis_DSM_13_chr.trna8-AsnGTT (163048-163122) Asn (GTT) 75 bp Sc: 89.95
TCCGCAGTAGCTCAGTGGTAGAGCTATCGGCTGTTAACCGATCGGTCGCAGGTTCGAATC
CTGCCTGCGGAGCCA
>Bacillus_licheniformis_DSM_13_chr.trna25-AspGTC (616831-616907) Asp (GTC) 77 bp Sc: 95.80
GGTCCGGTAGTTCAGTTGGTTAGAATGCCTGCCTGTCACGCAGGAGGTCGCGGGTTCGAG
TCCCGTCCGGACCGCCA
>Bacillus_licheniformis_DSM_13_chr.trna31-AspGTC (926296-926372) Asp (GTC) 77 bp Sc: 95.80
GGTCCGGTAGTTCAGTTGGTTAGAATGCCTGCCTGTCACGCAGGAGGTCGCGGGTTCGAG
TCCCGTCCGGACCGCCA
>Bacillus_licheniformis_DSM_13_chr.trna47-AspGTC (4187366-4187290) Asp (GTC) 77 bp Sc: 95.80
GGTCCGGTAGTTCAGTTGGTTAGAATGCCTGCCTGTCACGCAGGAGGTCGCGGGTTCGAG
TCCCGTCCGGACCGCCA
>Bacillus_licheniformis_DSM_13_chr.trna63-AspGTC (3116353-3116277) Asp (GTC) 77 bp Sc: 95.80
GGTCCGGTAGTTCAGTTGGTTAGAATGCCTGCCTGTCACGCAGGAGGTCGCGGGTTCGAG
TCCCGTCCGGACCGCCA
>Bacillus_licheniformis_DSM_13_chr.trna39-CysGCA (927022-927092) Cys (GCA) 71 bp Sc: 67.07
GGCGGCATAGCCAAGTGGTAAGGCAGAGGTCTGCAAAACCTTTATCCCCGGTTCGAATCC
GGGTGTCGCCT
>Bacillus_licheniformis_DSM_13_chr.trna44-GlnTTG (2559110-2559181) Gln (TTG) 72 bp Sc: 68.66
TGGGCTATAGCCAAGCGGTAAGGCAACGGACTTTGACTCCGTGACGCGCTGGTTCGAATC
CAGCTAGCCCAG
>Bacillus_licheniformis_DSM_13_chr.trna14-GlnTTG (176781-176852) Gln (TTG) 72 bp Sc: 70.30
TGGGCTATAGCCAAGCGGTAAGGCAACGGACTTTGACTCCGTCATGCGCTGGTTCGAATC
CAGCTAGCCCAG
>Bacillus_licheniformis_DSM_13_chr.trna37-GlnTTG (926809-926880) Gln (TTG) 72 bp Sc: 70.30
TGGGCTATAGCCAAGCGGTAAGGCAACGGACTTTGACTCCGTCATGCGCTGGTTCGAATC
CAGCTAGCCCAG
>Bacillus_licheniformis_DSM_13_chr.trna18-GlnTTG (572354-572428) Gln (TTG) 75 bp Sc: 75.97
TGGGCTATAGCCAAGCGGTAAGGCAACGGACTTTGACTCCGTCATGCGTTGGTTCGAATC
CAGCTAGCCCAGCCA
>Bacillus_licheniformis_DSM_13_chr.trna17-GluTTC (572270-572341) Glu (TTC) 72 bp Sc: 71.01
GGCCCGTTGGTCAAGTGGTTAAGACACCGCCCTTTCACGGCGGTAACACGGGTTCGAATC
CCGTACGGGTCA
>Bacillus_licheniformis_DSM_13_chr.trna46-GluTTC (4187526-4187455) Glu (TTC) 72 bp Sc: 71.01
GGCCCGTTGGTCAAGTGGTTAAGACACCGCCCTTTCACGGCGGTAACACGGGTTCGAATC
CCGTACGGGTCA
>Bacillus_licheniformis_DSM_13_chr.trna7-GluTTC (74834-74905) Glu (TTC) 72 bp Sc: 71.01
GGCCCGTTGGTCAAGTGGTTAAGACACCGCCCTTTCACGGCGGTAACACGGGTTCGAATC
CCGTACGGGTCA
>Bacillus_licheniformis_DSM_13_chr.trna70-GluTTC (3115736-3115662) Glu (TTC) 75 bp Sc: 71.62
GGCCCGTTGGTCAAGTGGTTAAGACACCGCCCTTTCACGGCGGTAACACGGGTTCGAATC
CCGTACGGGTCATCA
>Bacillus_licheniformis_DSM_13_chr.trna10-GluTTC (176252-176326) Glu (TTC) 75 bp Sc: 79.30
GGCCCGTTGGTCAAGTGGTTAAGACACCGCCCTTTCACGGCGGTAACACGGGTTCGAATC

CCGTACGGGTCACCA
>Bacillus_licheniformis_DSM_13_chr.trna28-GluTTC (925916-925990) Glu (TTC) 75 bp Sc: 79.30
GGCCCGTTGGTCAAGTGGTTAAGACACCGCCCTTTCACGGCGGTAACACGGGTTCGAATC
CCGTACGGGTCACCA
>Bacillus_licheniformis_DSM_13_chr.trna55-GlyGCC (3117103-3117032) Gly (GCC) 72 bp Sc: 86.63
GCGGAAGTAGTTCAGTGGTAGAACACCACCTTGCCAAGGTGGGGGTCGCGGGTTCGAATC
CCGTCTTCCGCT
>Bacillus_licheniformis_DSM_13_chr.trna38-GlyGCC (926934-927008) Gly (GCC) 75 bp Sc: 94.93
GCGGAAGTAGTTCAGTGGTAGAACACCACCTTGCCAAGGTGGGGGTCGCGGGTTCGAATC
CCGTCTTCCGCTCCA
>Bacillus_licheniformis_DSM_13_chr.trna23-GlyTCC (611567-611640) Gly (TCC) 74 bp Sc: 78.55
GCGGGTGTAGTTTAGTGGTAAAACCTCAGCCTTCCAAGCTGATGTCGTGAGTTCGATTCT
CATCACCCGCTCCA
>Bacillus_licheniformis_DSM_13_chr.trna42-GlyTCC (953465-953538) Gly (TCC) 74 bp Sc: 81.36
GCGGGTGTAGTTTAGTGGTAAAACCTCAGCCTTCCAAGCTGATGTCGTGGGTTCGATTCC
CATCACCCGCTCCA
>Bacillus_licheniformis_DSM_13_chr.trna66-GlyTCC (3116084-3116011) Gly (TCC) 74 bp Sc: 81.36
GCGGGTGTAGTTTAGTGGTAAAACCTCAGCCTTCCAAGCTGATGTCGTGGGTTCGATTCC
CATCACCCGCTCCA
>Bacillus_licheniformis_DSM_13_chr.trna36-HisGTG (926728-926800) His (GTG) 73 bp Sc: 64.21
GCGGTTGTGGCGAAGTGGTTAACGCACCAGATTGTGGCTCTGGCACTCGTGGGTTCGATT
CCCATCAATCGCC
>Bacillus_licheniformis_DSM_13_chr.trna65-HisGTG (3116168-3116093) His (GTG) 76 bp Sc: 79.15
GCGGTTGTGGCGAAGTGGTTAACGCACCAGATTGTGGCTCTGGCATTCGTGGGTTCGATT
CCCATCAACCGCCCCA
>Bacillus_licheniformis_DSM_13_chr.trna67-IleGAT (3115998-3115922) Ile (GAT) 77 bp Sc: 102.00
GGGCCTGTAGCTCAGCTGGTTAGAGCGCACGCCTGATAAGCGTGAGGTCGATGGTTCGAG
TCCATTCAGGCCCACCA
>Bacillus_licheniformis_DSM_13_chr.trna1-IleGAT (11358-11434) Ile (GAT) 77 bp Sc: 102.39
GGGCCTGTAGCTCAGCTGGTTAGAGCGCACGCCTGATAAGCGTGAGGTCGGTGGTTCGAG
TCCACTCAGGCCCACCA
>Bacillus_licheniformis_DSM_13_chr.trna4-IleGAT (36056-36132) Ile (GAT) 77 bp Sc: 102.39
GGGCCTGTAGCTCAGCTGGTTAGAGCGCACGCCTGATAAGCGTGAGGTCGGTGGTTCGAG
TCCACTCAGGCCCACCA
>Bacillus_licheniformis_DSM_13_chr.trna41-LeuCAA (927206-927290) Leu (CAA) 85 bp Sc: 60.81
GCCGGTGTGGCGGAATTGGCAGACGCGCACGACTCAAAATCGTGTTCCTTCGGGAGTGTC
GGTTCGACCCCGACCACCGGTATCA
>Bacillus_licheniformis_DSM_13_chr.trna54-LeuCAG (3117203-3117119) Leu (CAG) 85 bp Sc: 62.54
GCGGATGTGGCGGAATTGGCAGACGCGCTAGAATCAGGCTCTAGTGTCTTTTAAAGACGT
GGGGGTTCAAGTCCCTTCATCCGCA
>Bacillus_licheniformis_DSM_13_chr.trna21-LeuGAG (572628-572711) Leu (GAG) 84 bp Sc: 53.11
GCGGTCGTGGCGGAATGGCAGACGCGCTAGGTTGAGGGCCTAGTGGGTGAATAACCCGTG
GAGGTTCAAGTCCTCTCGGCCGCA
>Bacillus_licheniformis_DSM_13_chr.trna40-LeuTAA (927103-927191) Leu (TAA) 89 bp Sc: 76.51
GCCGGGGTGGTGGAATTGGCAGACACACAGGACTTAAAATCCTGCGGTAGGTGACTACCG
TGCCGGTTCAAGTCCGGCCCTCGGCACCA
>Bacillus_licheniformis_DSM_13_chr.trna20-LeuTAG (572525-572607) Leu (TAG) 83 bp Sc: 69.61
GCGGGTGTGGCGGAATTGGCAGACGCGCTAGACTTAGGATCTAGTGTCTTTACGGCGTGG
GGGTTCGAGTCCCTTCACCCGCA
>Bacillus_licheniformis_DSM_13_chr.trna19-LysTTT (572437-572512) Lys (TTT) 76 bp Sc: 98.67
GAGCCATTAGCTCAGTTGGTAGAGCATCTGACTTTTAATCAGAGGGTCGAAGGTTCGAGT
CCTTCATGGCTCACCA
>Bacillus_licheniformis_DSM_13_chr.trna45-LysTTT (4187609-4187534) Lys (TTT) 76 bp Sc: 98.67
GAGCCATTAGCTCAGTTGGTAGAGCATCTGACTTTTAATCAGAGGGTCGAAGGTTCGAGT
CCTTCATGGCTCACCA
>Bacillus_licheniformis_DSM_13_chr.trna53-LysTTT (3117285-3117210) Lys (TTT) 76 bp Sc: 98.67
GAGCCATTAGCTCAGTTGGTAGAGCATCTGACTTTTAATCAGAGGGTCGAAGGTTCGAGT
CCTTCATGGCTCACCA
>Bacillus_licheniformis_DSM_13_chr.trna6-MetCAT (74750-74823) Met (CAT) 74 bp Sc: 75.69
GGCGGTGTAGCTCAGCTGGCTAGAGCGTACGGTTCATACCCGTGAGGTCGGGGGTTCGAT
CCCCTCCGCCGCTA
>Bacillus_licheniformis_DSM_13_chr.trna59-MetCAT (3116755-3116682) Met (CAT) 74 bp Sc: 78.06
GGCGGTGTAGCTCAGCTGGCTAGAGCGTACGGTTCATACCCGTGAGGTCGGGGGTTCGAT
TCCCTCCGCCGCTA
>Bacillus_licheniformis_DSM_13_chr.trna24-MetCAT (616713-616789) Met (CAT) 77 bp Sc: 90.17
CGCGGGGTGGAGCAGTTCGGTAGCTCGTCGGGCTCATAACCCGAAGGTCGCAGGTTCAAA
TCCTGCCCCCGCAACCA

>Bacillus_licheniformis_DSM_13_chr.trna30-MetCAT (926205-926281) Met (CAT) 77 bp Sc: 90.17
CGCGGGGTGGAGCAGTTCGGTAGCTCGTCGGGCTCATAACCCGAAGGTCGCAGGTTCAAA
TCCTGCCCCCGCAACCA
>Bacillus_licheniformis_DSM_13_chr.trna62-MetCAT (3116475-3116399) Met (CAT) 77 bp Sc: 90.17
CGCGGGGTGGAGCAGTTCGGTAGCTCGTCGGGCTCATAACCCGAAGGTCGCAGGTTCAAA
TCCTGCCCCCGCAACCA
>Bacillus_licheniformis_DSM_13_chr.trna60-MetCAT (3116676-3116600) Met (CAT) 77 bp Sc: 97.54
GGACCTTTAGCTCAGTTGGTTAGAGCAGACGGCTCATAACCGTCCGGTCGTAGGTTCGAG
TCCTACAAGGTCCACCA
>Bacillus_licheniformis_DSM_13_chr.trna48-PheGAA (4187263-4187191) Phe (GAA) 73 bp Sc: 89.60
GGCTCGGTAGCTCAGTTGGTAGAGCAACGGACTGAAAATCCGTGTGTCGGCGGTTCGATT
CCGTCCCGAGCCA
>Bacillus_licheniformis_DSM_13_chr.trna32-PheGAA (926383-926458) Phe (GAA) 76 bp Sc: 97.89
GGCTCGGTAGCTCAGTTGGTAGAGCAACGGACTGAAAATCCGTGTGTCGGCGGTTCGATT
CCGTCCCGAGCCACCA
>Bacillus_licheniformis_DSM_13_chr.trna64-PheGAA (3116266-3116191) Phe (GAA) 76 bp Sc: 97.89
GGCTCGGTAGCTCAGTTGGTAGAGCAACGGACTGAAAATCCGTGTGTCGGCGGTTCGATT
CCGTCCCGAGCCACCA
>Bacillus_licheniformis_DSM_13_chr.trna57-ProTGG (3116936-3116860) Pro (TGG) 77 bp Sc: 94.72
CGGGAAGTAGCTCAGCTTGGTAGAGCACATGGTTTGGGACCATGGGGTCGCAGGTTCGAA
TCCTGTCTTCCCGACCA
>Bacillus_licheniformis_DSM_13_chr.trna16-SerGCT (572151-572241) Ser (GCT) 91 bp Sc: 71.43
GGAGAAGTACTCAAGTGGCTGAAGAGGCGCCCCTGCTAAGGGTGTAGGTCGCGTAAGCGG
CGCGAGGGTTCAAATCCCTCCTTCTCCGCCA
>Bacillus_licheniformis_DSM_13_chr.trna69-SerGCT (3115839-3115749) Ser (GCT) 91 bp Sc: 71.43
GGAGAAGTACTCAAGTGGCTGAAGAGGCGCCCCTGCTAAGGGTGTAGGTCGCGTAAGCGG
CGCGAGGGTTCAAATCCCTCCTTCTCCGCCA
>Bacillus_licheniformis_DSM_13_chr.trna27-SerGGA (925810-925901) Ser (GGA) 92 bp Sc: 63.95
GGAGAGCTGTCCGAGTGGTCGAAGGAGCACGATTGGAAATCGTGTAGGCGGCCAACGCCG
TCTCAAGGGTTCGAATCCCTTGCTCTCCGCCA
>Bacillus_licheniformis_DSM_13_chr.trna3-SerTGA (26352-26444) Ser (TGA) 93 bp Sc: 72.49
GGAGGAATACCCAAGTCTGGCTGAAGGGATCGGTCTTGAAAACCGACAGGGGTGTCAAAG
CCCGCGGGGGTTCGAATCCCTCTTCCTCCGCCA
>Bacillus_licheniformis_DSM_13_chr.trna61-SerTGA (3116592-3116500) Ser (TGA) 93 bp Sc: 72.49
GGAGGAATACCCAAGTCTGGCTGAAGGGATCGGTCTTGAAAACCGACAGGGGTGTCAAAG
CCCGCGGGGGTTCGAATCCCTCTTCCTCCGCCA
>Bacillus_licheniformis_DSM_13_chr.trna52-ThrCGT (3117442-3117367) Thr (CGT) 76 bp Sc: 85.36
GCCGGTGTAGCTCAATTGGTAGAGCAACTGAATCGTAATCAGTAGGTTGGGGGTTCAAGT
CCTCTTGCCGGCACCA
>Bacillus_licheniformis_DSM_13_chr.trna9-ThrGGT (163125-163197) Thr (GGT) 73 bp Sc: 75.20
GCTTCCATAGCTCAGCAGGTAGAGCACTTCCATGGTAAGGAAGAGGTCAGCGGTTCGAGC
CCGCTTGGAAGCT
>Bacillus_licheniformis_DSM_13_chr.trna12-ThrTGT (176611-176683) Thr (TGT) 73 bp Sc: 82.36
GCCGGTCTAGCTCAATTGGTAGAGCAACTGACTTGTAATCAGTAGGTTGGGGGTTCAAGT
CCTCTGGCCGGCA
>Bacillus_licheniformis_DSM_13_chr.trna33-ThrTGT (926462-926534) Thr (TGT) 73 bp Sc: 82.36
GCCGGTCTAGCTCAATTGGTAGAGCAACTGACTTGTAATCAGTAGGTTGGGGGTTCAAGT
CCTCTGGCCGGCA
>Bacillus_licheniformis_DSM_13_chr.trna35-TrpCCA (926634-926707) Trp (CCA) 74 bp Sc: 71.01
AGGGGCATAGTTTAACGGTAGAACAGAGGTCTCCAAAACCTCCGGTGTGGGTTCGATTCC
TACTGCCCCTGCCA
>Bacillus_licheniformis_DSM_13_chr.trna13-TyrGTA (176692-176776) Tyr (GTA) 85 bp Sc: 80.73
GGAGGGGTAGCGAAGTGGCTAAACGCGGCGGACTGTAAATCCGCTCCCTCAGGGTTCGGC
GGTTCGAATCCGTCCCCCTCCACCA
>Bacillus_licheniformis_DSM_13_chr.trna34-TyrGTA (926542-926626) Tyr (GTA) 85 bp Sc: 80.73
GGAGGGGTAGCGAAGTGGCTAAACGCGGCGGACTGTAAATCCGCTCCCTCAGGGTTCGGC
GGTTCGAATCCGTCCCCCTCCACCA
>Bacillus_licheniformis_DSM_13_chr.trna72-ValGAC (1300661-1300589) Val (GAC) 73 bp Sc: 71.64
GATTCCGTAGCTCAGCTGGGAGAGCGCTACCTTGACAGGGTAGAGGTCGCTGGTTCGAGC
CCAGTCGGAATCA
>Bacillus_licheniformis_DSM_13_chr.trna11-ValTAC (176477-176552) Val (TAC) 76 bp Sc: 95.52
GGAGGATTAGCTCAGCTGGGAGAGCATCTGCCTTACAAGCAGAGGGTCGGCGGTTCGATC
CCGTCATCCTCCACCA
>Bacillus_licheniformis_DSM_13_chr.trna29-ValTAC (926108-926183) Val (TAC) 76 bp Sc: 95.52
GGAGGATTAGCTCAGCTGGGAGAGCATCTGCCTTACAAGCAGAGGGTCGGCGGTTCGATC
CCGTCATCCTCCACCA
>Bacillus_licheniformis_DSM_13_chr.trna51-ValTAC (3117533-3117458) Val (TAC) 76 bp Sc: 95.52

GGAGGATTAGCTCAGCTGGGAGAGCATCTGCCTTACAAGCAGAGGGTCGGCGGTTCGATC
CCGTCATCCTCCACCA
>Bacillus_thuringiensis_Al_Hakam_chr.trna88-AlaTGC (4667458-4667385) Ala (TGC) 74 bp Sc: 76.07
GGGGCCTTAGCTCAGCTGGGAGAGCGCCTGCCTTGCACGCAGGAGGTCAGCGGTTCGCAT
CCCGCTAGGCTCCA
>Bacillus_thuringiensis_Al_Hakam_chr.trna25-AlaTGC (156787-156859) Ala (TGC) 73 bp Sc: 85.62
GGGGCCTTAGCTCAGCTGGGAGAGCGCCTGCCTTGCACGCAGGAGGTCAGCGGTTCGATC
CCGCTAGGCTCCA
>Bacillus_thuringiensis_Al_Hakam_chr.trna64-AlaTGC (767640-767712) Ala (TGC) 73 bp Sc: 85.62
GGGGCCTTAGCTCAGCTGGGAGAGCGCCTGCCTTGCACGCAGGAGGTCAGCGGTTCGATC
CCGCTAGGCTCCA
>Bacillus_thuringiensis_Al_Hakam_chr.trna16-AlaTGC (87995-88070) Ala (TGC) 76 bp Sc: 93.92
GGGGCCTTAGCTCAGCTGGGAGAGCGCCTGCCTTGCACGCAGGAGGTCAGCGGTTCGATC
CCGCTAGGCTCCACCA
>Bacillus_thuringiensis_Al_Hakam_chr.trna2-AlaTGC (11078-11153) Ala (TGC) 76 bp Sc: 93.92
GGGGCCTTAGCTCAGCTGGGAGAGCGCCTGCCTTGCACGCAGGAGGTCAGCGGTTCGATC
CCGCTAGGCTCCACCA
>Bacillus_thuringiensis_Al_Hakam_chr.trna5-AlaTGC (30878-30953) Ala (TGC) 76 bp Sc: 93.92
GGGGCCTTAGCTCAGCTGGGAGAGCGCCTGCCTTGCACGCAGGAGGTCAGCGGTTCGATC
CCGCTAGGCTCCACCA
>Bacillus_thuringiensis_Al_Hakam_chr.trna14-ArgACG (87830-87903) Arg (ACG) 74 bp Sc: 69.97
GCGCCCGTAGCTCAATTGGATAGAGCGTTTGACTACGGATCAAGAGGTTAGGGGTTCGAC
TCCTCTCGGGCGCG
>Bacillus_thuringiensis_Al_Hakam_chr.trna62-ArgACG (767466-767539) Arg (ACG) 74 bp Sc: 69.97
GCGCCCGTAGCTCAATTGGATAGAGCGTTTGACTACGGATCAAGAGGTTAGGGGTTCGAC
TCCTCTCGGGCGCG
>Bacillus_thuringiensis_Al_Hakam_chr.trna86-ArgACG (4667632-4667559) Arg (ACG) 74 bp Sc: 69.97
GCGCCCGTAGCTCAATTGGATAGAGCGTTTGACTACGGATCAAGAGGTTAGGGGTTCGAC
TCCTCTCGGGCGCG
>Bacillus_thuringiensis_Al_Hakam_chr.trna34-ArgACG (257898-257974) Arg (ACG) 77 bp Sc: 78.27
GCGCCCGTAGCTCAATTGGATAGAGCGTTTGACTACGGATCAAGAGGTTAGGGGTTCGAC
TCCTCTCGGGCGCGCCA
>Bacillus_thuringiensis_Al_Hakam_chr.trna79-ArgCCG (4880634-4880559) Arg (CCG) 76 bp Sc: 62.08
GCGCCCATAGCTCAGTCGGATAGAGCGGTGGTTTCCGGTACCACGTCTGCCGGGGGTTCG
AATCCCTCTGGGCGCG
>Bacillus_thuringiensis_Al_Hakam_chr.trna39-AsnGTT (539300-539374) Asn (GTT) 75 bp Sc: 86.53
TCCGCAGTAGCTCAGCGGTAGAGCTATCGGCTGTTAACCGATCGGTCGTAGGTTCGATTC
CTACCTGCGGAGCCA
>Bacillus_thuringiensis_Al_Hakam_chr.trna17-AsnGTT (156046-156120) Asn (GTT) 75 bp Sc: 88.32
TCCGCAGTAGCTCAGTGGTAGAGCTATCGGCTGTTAACCGATCGGTCGTAGGTTCGAGTC
CTACCTGCGGAGCCA
>Bacillus_thuringiensis_Al_Hakam_chr.trna26-AsnGTT (257007-257081) Asn (GTT) 75 bp Sc: 88.32
TCCGCAGTAGCTCAGTGGTAGAGCTATCGGCTGTTAACCGATCGGTCGTAGGTTCGAGTC
CTACCTGCGGAGCCA
>Bacillus_thuringiensis_Al_Hakam_chr.trna73-AsnGTT (768497-768571) Asn (GTT) 75 bp Sc: 88.32
TCCGCAGTAGCTCAGTGGTAGAGCTATCGGCTGTTAACCGATCGGTCGTAGGTTCGAGTC
CTACCTGCGGAGCCA
>Bacillus_thuringiensis_Al_Hakam_chr.trna99-AsnGTT (4666473-4666399) Asn (GTT) 75 bp Sc: 88.32
TCCGCAGTAGCTCAGTGGTAGAGCTATCGGCTGTTAACCGATCGGTCGTAGGTTCGAGTC
CTACCTGCGGAGCCA
>Bacillus_thuringiensis_Al_Hakam_chr.trna30-AspGTC (257385-257460) Asp (GTC) 76 bp Sc: 76.06
GGTCCCGTGGTGTAGTGGTTAACATGCCTGCCTGTCACGCAGGAGATCGCCGGTTCGACC
CCGGTCGGGACCGCCA
>Bacillus_thuringiensis_Al_Hakam_chr.trna38-AspGTC (263080-263155) Asp (GTC) 76 bp Sc: 76.06
GGTCCCGTGGTGTAGTGGTTAACATGCCTGCCTGTCACGCAGGAGATCGCCGGTTCGACC
CCGGTCGGGACCGCCA
>Bacillus_thuringiensis_Al_Hakam_chr.trna44-AspGTC (539746-539821) Asp (GTC) 76 bp Sc: 76.06
GGTCCCGTGGTGTAGTGGTTAACATGCCTGCCTGTCACGCAGGAGATCGCCGGTTCGACC
CCGGTCGGGACCGCCA
>Bacillus_thuringiensis_Al_Hakam_chr.trna68-AspGTC (768081-768156) Asp (GTC) 76 bp Sc: 76.06
GGTCCCGTGGTGTAGTGGTTAACATGCCTGCCTGTCACGCAGGAGATCGCCGGTTCGACC
CCGGTCGGGACCGCCA
>Bacillus_thuringiensis_Al_Hakam_chr.trna77-AspGTC (5236660-5236585) Asp (GTC) 76 bp Sc: 76.06
GGTCCCGTGGTGTAGTGGTTAACATGCCTGCCTGTCACGCAGGAGATCGCCGGTTCGACC
CCGGTCGGGACCGCCA
>Bacillus_thuringiensis_Al_Hakam_chr.trna93-AspGTC (4666991-4666916) Asp (GTC) 76 bp Sc: 76.06
GGTCCCGTGGTGTAGTGGTTAACATGCCTGCCTGTCACGCAGGAGATCGCCGGTTCGACC

CCGGTCGGGACCGCCA
>Bacillus_thuringiensis_Al_Hakam_chr.trna52-CysGCA (540503-540573) Cys (GCA) 71 bp Sc: 71.27
GGCGGCATAGCCAAGTGGTAAGGCAGAGGTCTGCAAAACCTTTATCACCGGTTCAAATCC
GGTTGCCGCCT
>Bacillus_thuringiensis_Al_Hakam_chr.trna22-GlnTTG (156544-156618) Gln (TTG) 75 bp Sc: 78.60
TGGGCTATAGCCAAGCGGTAAGGCAACGGACTTTGACTCCGTCATGCGCTGGTTCGAATC
CAGCTAGCCCAGCCA
>Bacillus_thuringiensis_Al_Hakam_chr.trna31-GlnTTG (257548-257622) Gln (TTG) 75 bp Sc: 78.60
TGGGCTATAGCCAAGCGGTAAGGCAACGGACTTTGACTCCGTCATGCGCTGGTTCGAATC
CAGCTAGCCCAGCCA
>Bacillus_thuringiensis_Al_Hakam_chr.trna50-GlnTTG (540334-540408) Gln (TTG) 75 bp Sc: 78.60
TGGGCTATAGCCAAGCGGTAAGGCAACGGACTTTGACTCCGTCATGCGCTGGTTCGAATC
CAGCTAGCCCAGCCA
>Bacillus_thuringiensis_Al_Hakam_chr.trna57-GlnTTG (767004-767078) Gln (TTG) 75 bp Sc: 78.60
TGGGCTATAGCCAAGCGGTAAGGCAACGGACTTTGACTCCGTCATGCGCTGGTTCGAATC
CAGCTAGCCCAGCCA
>Bacillus_thuringiensis_Al_Hakam_chr.trna101-GluTTC (4666294-4666223) Glu (TTC) 72 bp Sc: 71.01
GGCCCGTTGGTCAAGTGGTTAAGACACCGCCCTTTCACGGCGGTAACACGGGTTCGAATC
CCGTACGGGTCA
>Bacillus_thuringiensis_Al_Hakam_chr.trna7-GluTTC (64523-64594) Glu (TTC) 72 bp Sc: 71.01
GGCCCGTTGGTCAAGTGGTTAAGACACCGCCCTTTCACGGCGGTAACACGGGTTCGAATC
CCGTACGGGTCA
>Bacillus_thuringiensis_Al_Hakam_chr.trna74-GluTTC (768573-768644) Glu (TTC) 72 bp Sc: 71.01
GGCCCGTTGGTCAAGTGGTTAAGACACCGCCCTTTCACGGCGGTAACACGGGTTCGAATC
CCGTACGGGTCA
>Bacillus_thuringiensis_Al_Hakam_chr.trna76-GluTTC (5236753-5236682) Glu (TTC) 72 bp Sc: 71.01
GGCCCGTTGGTCAAGTGGTTAAGACACCGCCCTTTCACGGCGGTAACACGGGTTCGAATC
CCGTACGGGTCA
>Bacillus_thuringiensis_Al_Hakam_chr.trna19-GluTTC (156222-156296) Glu (TTC) 75 bp Sc: 79.30
GGCCCGTTGGTCAAGTGGTTAAGACACCGCCCTTTCACGGCGGTAACACGGGTTCGAATC
CCGTACGGGTCACCA
>Bacillus_thuringiensis_Al_Hakam_chr.trna28-GluTTC (257184-257258) Glu (TTC) 75 bp Sc: 79.30
GGCCCGTTGGTCAAGTGGTTAAGACACCGCCCTTTCACGGCGGTAACACGGGTTCGAATC
CCGTACGGGTCACCA
>Bacillus_thuringiensis_Al_Hakam_chr.trna41-GluTTC (539486-539560) Glu (TTC) 75 bp Sc: 79.30
GGCCCGTTGGTCAAGTGGTTAAGACACCGCCCTTTCACGGCGGTAACACGGGTTCGAATC
CCGTACGGGTCACCA
>Bacillus_thuringiensis_Al_Hakam_chr.trna24-GlyGCC (156705-156776) Gly (GCC) 72 bp Sc: 80.56
GCGGAAGTAGTTCAGTGGTAGAATACAACCTTGCCAAGGTTGGGGTCGCGGGTTCGAATC
CCGTCTTCCGCT
>Bacillus_thuringiensis_Al_Hakam_chr.trna12-GlyGCC (87647-87721) Gly (GCC) 75 bp Sc: 88.86
GCGGAAGTAGTTCAGTGGTAGAATACAACCTTGCCAAGGTTGGGGTCGCGGGTTCGAATC
CCGTCTTCCGCTCCA
>Bacillus_thuringiensis_Al_Hakam_chr.trna51-GlyGCC (540414-540488) Gly (GCC) 75 bp Sc: 88.86
GCGGAAGTAGTTCAGTGGTAGAATACAACCTTGCCAAGGTTGGGGTCGCGGGTTCGAATC
CCGTCTTCCGCTCCA
>Bacillus_thuringiensis_Al_Hakam_chr.trna60-GlyGCC (767283-767357) Gly (GCC) 75 bp Sc: 88.86
GCGGAAGTAGTTCAGTGGTAGAATACAACCTTGCCAAGGTTGGGGTCGCGGGTTCGAATC
CCGTCTTCCGCTCCA
>Bacillus_thuringiensis_Al_Hakam_chr.trna84-GlyGCC (4667815-4667741) Gly (GCC) 75 bp Sc: 88.86
GCGGAAGTAGTTCAGTGGTAGAATACAACCTTGCCAAGGTTGGGGTCGCGGGTTCGAATC
CCGTCTTCCGCTCCA
>Bacillus_thuringiensis_Al_Hakam_chr.trna36-GlyTCC (258057-258127) Gly (TCC) 71 bp Sc: 71.78
GCGGGTGTAGTTTAGTGGTAAAACAAGAGCCTTCCAAGCTCTGGTCGAGAGTTCGATTCT
CTTCACCCGCT
>Bacillus_thuringiensis_Al_Hakam_chr.trna97-GlyTCC (4666638-4666568) Gly (TCC) 71 bp Sc: 71.78
GCGGGTGTAGTTTAGTGGTAAAACAAGAGCCTTCCAAGCTCTGGTCGAGAGTTCGATTCT
CTTCACCCGCT
>Bacillus_thuringiensis_Al_Hakam_chr.trna102-GlyTCC (4294741-4294668) Gly (TCC) 74 bp Sc: 80.08
GCGGGTGTAGTTTAGTGGTAAAACAAGAGCCTTCCAAGCTCTGGTCGAGAGTTCGATTCT
CTTCACCCGCTCCA
>Bacillus_thuringiensis_Al_Hakam_chr.trna54-GlyTCC (552778-552851) Gly (TCC) 74 bp Sc: 80.08
GCGGGTGTAGTTTAGTGGTAAAACAAGAGCCTTCCAAGCTCTGGTCGAGAGTTCGATTCT
CTTCACCCGCTCCA
>Bacillus_thuringiensis_Al_Hakam_chr.trna49-HisGTG (540195-540270) His (GTG) 76 bp Sc: 74.61
GCGGTTGTGGCGAAGTGGTTAACGCACCGGATTGTGGCTCCGGCATTCGTGGGTTCGATT
CCCATCAGTCGCCCCA

>Bacillus_thuringiensis_Al_Hakam_chr.trna82-HisGTG (4668023-4667948) His (GTG) 76 bp Sc: 74.61
GCGGTTGTGGCGAAGTGGTTAACGCACCGGATTGTGGCTCCGGCATTCGTGGGTTCGATT
CCCATCAGTCGCCCCA
>Bacillus_thuringiensis_Al_Hakam_chr.trna1-IleGAT (10993-11069) Ile (GAT) 77 bp Sc: 101.21
GGGCCTATAGCTCAGCTGGTTAGAGCGCACGCCTGATAAGCGTGAGGTCGATGGTTCGAG
TCCATTTAGGCCCACCA
>Bacillus_thuringiensis_Al_Hakam_chr.trna4-IleGAT (30793-30869) Ile (GAT) 77 bp Sc: 101.21
GGGCCTATAGCTCAGCTGGTTAGAGCGCACGCCTGATAAGCGTGAGGTCGATGGTTCGAG
TCCATTTAGGCCCACCA
>Bacillus_thuringiensis_Al_Hakam_chr.trna72-IleGAT (768410-768486) Ile (GAT) 77 bp Sc: 101.21
GGGCCTATAGCTCAGCTGGTTAGAGCGCACGCCTGATAAGCGTGAGGTCGATGGTTCGAG
TCCATTTAGGCCCACCA
>Bacillus_thuringiensis_Al_Hakam_chr.trna98-IleGAT (4666557-4666481) Ile (GAT) 77 bp Sc: 101.21
GGGCCTATAGCTCAGCTGGTTAGAGCGCACGCCTGATAAGCGTGAGGTCGATGGTTCGAG
TCCATTTAGGCCCACCA
>Bacillus_thuringiensis_Al_Hakam_chr.trna53-LeuCAA (540584-540668) Leu (CAA) 85 bp Sc: 60.08
GCCGATGTGGCGGAATTGGCAGACGCGCACGACTCAAAATCGTGTTCCTTCGGGAGTGTC
GGTTCGACCCCGACCATCGGTATCA
>Bacillus_thuringiensis_Al_Hakam_chr.trna33-LeuGAG (257717-257802) Leu (GAG) 86 bp Sc: 57.19
GCGGTCGTGGCGGAACGGCAGACGCGCTAGGTTGAGGGCCTAGTGGGGGAAACCCCGTGG
AGGTTCAAGTCCTCTCGGCCGCATCA
>Bacillus_thuringiensis_Al_Hakam_chr.trna13-LeuTAA (87738-87826) Leu (TAA) 89 bp Sc: 71.28
GCCGGGGTGGCGGAACAGGCAGACGCACAGGACTTAAAATCCTGCGGTGGGTGACCACCG
TGCGGGTTCGACCCCCGCCCTCGGCACCA
>Bacillus_thuringiensis_Al_Hakam_chr.trna61-LeuTAA (767374-767462) Leu (TAA) 89 bp Sc: 71.28
GCCGGGGTGGCGGAACAGGCAGACGCACAGGACTTAAAATCCTGCGGTGGGTGACCACCG
TGCGGGTTCGACCCCCGCCCTCGGCACCA
>Bacillus_thuringiensis_Al_Hakam_chr.trna85-LeuTAA (4667724-4667636) Leu (TAA) 89 bp Sc: 71.28
GCCGGGGTGGCGGAACAGGCAGACGCACAGGACTTAAAATCCTGCGGTGGGTGACCACCG
TGCGGGTTCGACCCCCGCCCTCGGCACCA
>Bacillus_thuringiensis_Al_Hakam_chr.trna11-LeuTAG (87537-87617) Leu (TAG) 81 bp Sc: 66.65
GCGGGTGTGGCGGAATTGGCAGACGCACTAGACTTAGGATCTAGCGCCTTTGGCGTGGGG
GTTCGACTCCCTTCACCCGCA
>Bacillus_thuringiensis_Al_Hakam_chr.trna59-LeuTAG (767173-767253) Leu (TAG) 81 bp Sc: 66.65
GCGGGTGTGGCGGAATTGGCAGACGCACTAGACTTAGGATCTAGCGCCTTTGGCGTGGGG
GTTCGACTCCCTTCACCCGCA
>Bacillus_thuringiensis_Al_Hakam_chr.trna83-LeuTAG (4667925-4667845) Leu (TAG) 81 bp Sc: 67.96
GCGGGTGTGGCGGAATTGGCAGACGCACCAGACTTAGGATCTGGCGCCTTTGGCGTGGGG
GTTCGACTCCCTTCACCCGCA
>Bacillus_thuringiensis_Al_Hakam_chr.trna32-LysTTT (257628-257700) Lys (TTT) 73 bp Sc: 90.38
GAGCCATTAGCTCAGTTGGTAGAGCATCTGACTTTTAATCAGAGGGTCGAAGGTTCGAGT
CCTTCATGGCTCA
>Bacillus_thuringiensis_Al_Hakam_chr.trna10-LysTTT (87446-87521) Lys (TTT) 76 bp Sc: 98.67
GAGCCATTAGCTCAGTTGGTAGAGCATCTGACTTTTAATCAGAGGGTCGAAGGTTCGAGT
CCTTCATGGCTCACCA
>Bacillus_thuringiensis_Al_Hakam_chr.trna23-LysTTT (156624-156699) Lys (TTT) 76 bp Sc: 98.67
GAGCCATTAGCTCAGTTGGTAGAGCATCTGACTTTTAATCAGAGGGTCGAAGGTTCGAGT
CCTTCATGGCTCACCA
>Bacillus_thuringiensis_Al_Hakam_chr.trna58-LysTTT (767083-767158) Lys (TTT) 76 bp Sc: 98.67
GAGCCATTAGCTCAGTTGGTAGAGCATCTGACTTTTAATCAGAGGGTCGAAGGTTCGAGT
CCTTCATGGCTCACCA
>Bacillus_thuringiensis_Al_Hakam_chr.trna75-LysTTT (5236842-5236767) Lys (TTT) 76 bp Sc: 98.67
GAGCCATTAGCTCAGTTGGTAGAGCATCTGACTTTTAATCAGAGGGTCGAAGGTTCGAGT
CCTTCATGGCTCACCA
>Bacillus_thuringiensis_Al_Hakam_chr.trna96-LysTTT (4666727-4666652) Lys (TTT) 76 bp Sc: 98.67
GAGCCATTAGCTCAGTTGGTAGAGCATCTGACTTTTAATCAGAGGGTCGAAGGTTCGAGT
CCTTCATGGCTCACCA
>Bacillus_thuringiensis_Al_Hakam_chr.trna6-MetCAT (64436-64509) Met (CAT) 74 bp Sc: 75.69
GGCGGTGTAGCTCAGCTGGCTAGAGCGTACGGTTCATACCCGTGAGGTCGGGGGTTCGAT
CCCCTCCGCCGCTA
>Bacillus_thuringiensis_Al_Hakam_chr.trna89-MetCAT (4667364-4667288) Met (CAT) 77 bp Sc: 83.99
GGCGGTGTAGCTCAGCTGGCTAGAGCGTACGGTTCATACCCGTGAGGTCGGGGGTTCGAT
CCCCTCCGCCGCTACCA
>Bacillus_thuringiensis_Al_Hakam_chr.trna37-MetCAT (263000-263076) Met (CAT) 77 bp Sc: 85.35
CGCGGGGTGGAGCAGCACGGTAGCTCGTCGGGCTCATAACCCGAAGGTCGCAGGTTCAAA
TCCTGTCCCCGCAACCA
>Bacillus_thuringiensis_Al_Hakam_chr.trna43-MetCAT (539666-539742) Met (CAT) 77 bp Sc: 85.35

CGCGGGGTGGAGCAGCACGGTAGCTCGTCGGGCTCATAACCCGAAGGTCGCAGGTTCAAA
TCCTGTCCCCGCAACCA
>Bacillus_thuringiensis_Al_Hakam_chr.trna67-MetCAT (768001-768077) Met (CAT) 77 bp Sc: 85.35
CGCGGGGTGGAGCAGCACGGTAGCTCGTCGGGCTCATAACCCGAAGGTCGCAGGTTCAAA
TCCTGTCCCCGCAACCA
>Bacillus_thuringiensis_Al_Hakam_chr.trna103-MetCAT (4081704-4081631) Met (CAT) 74 bp Sc: 88.27
GGACCCTTAGCTCAGCTGGTTAGAGCAGACGGCTCATAACCGTCCGGTCGTAGGTTCGAG
TCCTACAGGGTCCA
>Bacillus_thuringiensis_Al_Hakam_chr.trna92-MetCAT (4667069-4666993) Met (CAT) 77 bp Sc: 89.08
CGCGGGGTGGAGCAGTCTGGTAGCTCGTCGGGCTCATAACCCGAAGGTCGCAGGTTCAAA
TCCTGTCCCCGCAACCA
>Bacillus_thuringiensis_Al_Hakam_chr.trna90-MetCAT (4667283-4667207) Met (CAT) 77 bp Sc: 96.69
GGACCTTTAGCTCAGCTGGTTAGAGCAGACGGCTCATAACCGTCCGGTCGTAGGTTCGAG
TCCTACAAGGTCCACCA
>Bacillus_thuringiensis_Al_Hakam_chr.trna78-PheGAA (5236543-5236471) Phe (GAA) 73 bp Sc: 84.57
GGCTCGGTAGCTCAGTCGGTAGAGCAGAGGACTGAAAATCCTCGTGTCGGCGGTTCGATT
CCGTCCCGAGCCA
>Bacillus_thuringiensis_Al_Hakam_chr.trna45-PheGAA (539831-539906) Phe (GAA) 76 bp Sc: 92.86
GGCTCGGTAGCTCAGTCGGTAGAGCAGAGGACTGAAAATCCTCGTGTCGGCGGTTCGATT
CCGTCCCGAGCCACCA
>Bacillus_thuringiensis_Al_Hakam_chr.trna69-PheGAA (768165-768240) Phe (GAA) 76 bp Sc: 92.86
GGCTCGGTAGCTCAGTCGGTAGAGCAGAGGACTGAAAATCCTCGTGTCGGCGGTTCGATT
CCGTCCCGAGCCACCA
>Bacillus_thuringiensis_Al_Hakam_chr.trna94-PheGAA (4666903-4666828) Phe (GAA) 76 bp Sc: 92.86
GGCTCGGTAGCTCAGTCGGTAGAGCAGAGGACTGAAAATCCTCGTGTCGGCGGTTCGATT
CCGTCCCGAGCCACCA
>Bacillus_thuringiensis_Al_Hakam_chr.trna15-ProTGG (87912-87985) Pro (TGG) 74 bp Sc: 85.68
CGGGAAGTGGCTCAGCTTGGTAGAGCACCTGGTTTGGGACCAGGGGGTCGCAGGTTCAAA
TCCTGTCTTCCCGA
>Bacillus_thuringiensis_Al_Hakam_chr.trna63-ProTGG (767550-767623) Pro (TGG) 74 bp Sc: 85.68
CGGGAAGTGGCTCAGCTTGGTAGAGCACCTGGTTTGGGACCAGGGGGTCGCAGGTTCAAA
TCCTGTCTTCCCGA
>Bacillus_thuringiensis_Al_Hakam_chr.trna87-ProTGG (4667548-4667475) Pro (TGG) 74 bp Sc: 85.68
CGGGAAGTGGCTCAGCTTGGTAGAGCACCTGGTTTGGGACCAGGGGGTCGCAGGTTCAAA
TCCTGTCTTCCCGA
>Bacillus_thuringiensis_Al_Hakam_chr.trna35-ProTGG (257979-258055) Pro (TGG) 77 bp Sc: 93.98
CGGGAAGTGGCTCAGCTTGGTAGAGCACCTGGTTTGGGACCAGGGGGTCGCAGGTTCAAA
TCCTGTCTTCCCGACCA
>Bacillus_thuringiensis_Al_Hakam_chr.trna100-SerGCT (4666391-4666301) Ser (GCT) 91 bp Sc: 67.93
AGAGAAGTACCCAAGTGGCTCAAGGGGCTCCCCTGCTAAGGGAGTAGATCGCGAACGCGG
TGCGAGGGTTCGAATCCCTTCTTCTCTGCCA
>Bacillus_thuringiensis_Al_Hakam_chr.trna27-SerGCT (257085-257175) Ser (GCT) 91 bp Sc: 67.93
AGAGAAGTACCCAAGTGGCTCAAGGGGCTCCCCTGCTAAGGGAGTAGATCGCGAACGCGG
TGCGAGGGTTCGAATCCCTTCTTCTCTGCCA
>Bacillus_thuringiensis_Al_Hakam_chr.trna40-SerGGA (539377-539468) Ser (GGA) 92 bp Sc: 63.66
GGAGAGCTGTCCGAGTTGGCCGAAGGAGCACGATTGGAAATCGTGTATACGTCACAAGCG
TATCAAGGGTTCGAATCCCTTGCTCTCCGCCA
>Bacillus_thuringiensis_Al_Hakam_chr.trna65-SerTGA (767733-767825) Ser (TGA) 93 bp Sc: 71.21
GGAGGTATACCCAAGTTCGGCTGAAGGGATCGGTCTTGAAAACCGACAGGCGGTGAGAAT
CGCGCGGGGGTTCGAATCCCTCTACCTCCTCCA
>Bacillus_thuringiensis_Al_Hakam_chr.trna91-SerTGA (4667189-4667097) Ser (TGA) 93 bp Sc: 72.21
GGAGGTATACCCAAGTTCGGCTGAAGGGATCGGTCTTGAAAACCGACAGGCGGCGAGAGT
CGCGCGGGGGTTCGAATCCCTCTACCTCCTCCA
>Bacillus_thuringiensis_Al_Hakam_chr.trna66-SerTGA (767881-767973) Ser (TGA) 93 bp Sc: 72.21
GGAGGTATACCCAAGTTCGGCTGAAGGGATCGGTCTTGAAAACCGACAGGCGGCGAGAGT
CGCGCGGGGGTTCGAATCCCTCTACCTCCTCCA
>Bacillus_thuringiensis_Al_Hakam_chr.trna3-SerTGA (21776-21868) Ser (TGA) 93 bp Sc: 72.57
GGAGGTATACCCAAGTCTGGCTGAAGGGATCGGTCTTGAAAACCGACAGGCGGTGAGAGT
CGCGCGGGGGTTCGAATCCCTCTACCTCCTCCA
>Bacillus_thuringiensis_Al_Hakam_chr.trna18-ThrGGT (156125-156197) Thr (GGT) 73 bp Sc: 78.48
GCTTCCATAGCTCAGCTGGTAGAGCACTTCCATGGTAAGGAAGAGGTCACCGGTTCAAGC
CCGGTTGGAAGCT
>Bacillus_thuringiensis_Al_Hakam_chr.trna70-ThrTGT (768250-768322) Thr (TGT) 73 bp Sc: 87.15
GCCGACTTAGCTCAATTGGTAGAGCAACTGACTTGTAATCAGTAGGTTGGGGGTTCAAGT
CCTCTAGTCGGCA
>Bacillus_thuringiensis_Al_Hakam_chr.trna81-ThrTGT (4668108-4668033) Thr (TGT) 76 bp Sc: 95.44
GCCGACTTAGCTCAATTGGTAGAGCAACTGACTTGTAATCAGTAGGTTGGGGGTTCAAGT

CCTCTAGTCGGCACCA
>Bacillus_thuringiensis_Al_Hakam_chr.trna9-ThrTGT (87357-87432) Thr (TGT) 76 bp Sc: 95.44
GCCGACTTAGCTCAATTGGTAGAGCAACTGACTTGTAATCAGTAGGTTGGGGGTTCAAGT
CCTCTAGTCGGCACCA
>Bacillus_thuringiensis_Al_Hakam_chr.trna46-ThrTGT (539925-540000) Thr (TGT) 76 bp Sc: 96.17
GCCGGCTTAGCTCAATTGGTAGAGCAACTGACTTGTAATCAGTAGGTTGGGGGTTCAAGT
CCTCTAGCCGGCACCA
>Bacillus_thuringiensis_Al_Hakam_chr.trna95-ThrTGT (4666813-4666738) Thr (TGT) 76 bp Sc: 96.17
GCCGGCTTAGCTCAATTGGTAGAGCAACTGACTTGTAATCAGTAGGTTGGGGGTTCAAGT
CCTCTAGCCGGCACCA
>Bacillus_thuringiensis_Al_Hakam_chr.trna71-TrpCCA (768329-768399) Trp (CCA) 71 bp Sc: 53.62
AGGGGCATAGTTTAAAGGTAGAACTGAGGTCTCCAAAACCTCCAGTGTGGGTTCGATTCC
TACTGCCCCTG
>Bacillus_thuringiensis_Al_Hakam_chr.trna48-TrpCCA (540102-540175) Trp (CCA) 74 bp Sc: 61.92
AGGGGCATAGTTTAAAGGTAGAACTGAGGTCTCCAAAACCTCCAGTGTGGGTTCGATTCC
TACTGCCCCTGCCA
>Bacillus_thuringiensis_Al_Hakam_chr.trna21-TyrGTA (156395-156478) Tyr (GTA) 84 bp Sc: 77.59
GGAGGGGTAGCGAAGTGGCTAAACGCGGCGGACTGTAAATCCGCTCCTTCGGGTTCGGCA
GTTCGAATCTGCCCCCCTCCACCA
>Bacillus_thuringiensis_Al_Hakam_chr.trna47-TyrGTA (540011-540094) Tyr (GTA) 84 bp Sc: 77.59
GGAGGGGTAGCGAAGTGGCTAAACGCGGCGGACTGTAAATCCGCTCCTTCGGGTTCGGCA
GTTCGAATCTGCCCCCCTCCACCA
>Bacillus_thuringiensis_Al_Hakam_chr.trna56-TyrGTA (766909-766992) Tyr (GTA) 84 bp Sc: 77.59
GGAGGGGTAGCGAAGTGGCTAAACGCGGCGGACTGTAAATCCGCTCCTTCGGGTTCGGCA
GTTCGAATCTGCCCCCCTCCACCA
>Bacillus_thuringiensis_Al_Hakam_chr.trna104-ValGAC (1292474-1292402) Val (GAC) 73 bp Sc: 61.20
GATCCCGTAGCTCAGCAGGGAGAGCGCCACCTTGACAGGGTGGAGGTCGTGAGTTCGAGC
CTCTCCGGGGTCA
>Bacillus_thuringiensis_Al_Hakam_chr.trna20-ValTAC (156302-156377) Val (TAC) 76 bp Sc: 97.03
GGAGGATTAGCTCAGCTGGGAGAGCACCTGCCTTACAAGCAGGGGGTCGGCGGTTCGATC
CCGTCATCCTCCACCA
>Bacillus_thuringiensis_Al_Hakam_chr.trna29-ValTAC (257263-257338) Val (TAC) 76 bp Sc: 97.03
GGAGGATTAGCTCAGCTGGGAGAGCACCTGCCTTACAAGCAGGGGGTCGGCGGTTCGATC
CCGTCATCCTCCACCA
>Bacillus_thuringiensis_Al_Hakam_chr.trna42-ValTAC (539566-539641) Val (TAC) 76 bp Sc: 97.03
GGAGGATTAGCTCAGCTGGGAGAGCACCTGCCTTACAAGCAGGGGGTCGGCGGTTCGATC
CCGTCATCCTCCACCA
>Bacillus_thuringiensis_Al_Hakam_chr.trna55-ValTAC (766825-766900) Val (TAC) 76 bp Sc: 97.03
GGAGGATTAGCTCAGCTGGGAGAGCACCTGCCTTACAAGCAGGGGGTCGGCGGTTCGATC
CCGTCATCCTCCACCA
>Bacillus_thuringiensis_Al_Hakam_chr.trna8-ValTAC (87277-87352) Val (TAC) 76 bp Sc: 97.03
GGAGGATTAGCTCAGCTGGGAGAGCACCTGCCTTACAAGCAGGGGGTCGGCGGTTCGATC
CCGTCATCCTCCACCA
>Bacillus_thuringiensis_Al_Hakam_chr.trna80-ValTAC (4668188-4668113) Val (TAC) 76 bp Sc: 97.03
GGAGGATTAGCTCAGCTGGGAGAGCACCTGCCTTACAAGCAGGGGGTCGGCGGTTCGATC
CCGTCATCCTCCACCA
>Bacillus_weihenstephanensis_KBAB4_chr.trna25-AlaTGC (156674-156746) Ala (TGC) 73 bp Sc: 85.62
GGGGCCTTAGCTCAGCTGGGAGAGCGCCTGCCTTGCACGCAGGAGGTCAGCGGTTCGATC
CCGCTAGGCTCCA
>Bacillus_weihenstephanensis_KBAB4_chr.trna66-AlaTGC (733984-734056) Ala (TGC) 73 bp Sc: 85.62
GGGGCCTTAGCTCAGCTGGGAGAGCGCCTGCCTTGCACGCAGGAGGTCAGCGGTTCGATC
CCGCTAGGCTCCA
>Bacillus_weihenstephanensis_KBAB4_chr.trna90-AlaTGC (4694623-4694551) Ala (TGC) 73 bp Sc: 85.62
GGGGCCTTAGCTCAGCTGGGAGAGCGCCTGCCTTGCACGCAGGAGGTCAGCGGTTCGATC
CCGCTAGGCTCCA
>Bacillus_weihenstephanensis_KBAB4_chr.trna16-AlaTGC (87840-87915) Ala (TGC) 76 bp Sc: 93.92
GGGGCCTTAGCTCAGCTGGGAGAGCGCCTGCCTTGCACGCAGGAGGTCAGCGGTTCGATC
CCGCTAGGCTCCACCA
>Bacillus_weihenstephanensis_KBAB4_chr.trna2-AlaTGC (10790-10865) Ala (TGC) 76 bp Sc: 93.92
GGGGCCTTAGCTCAGCTGGGAGAGCGCCTGCCTTGCACGCAGGAGGTCAGCGGTTCGATC
CCGCTAGGCTCCACCA
>Bacillus_weihenstephanensis_KBAB4_chr.trna5-AlaTGC (30624-30699) Ala (TGC) 76 bp Sc: 93.92
GGGGCCTTAGCTCAGCTGGGAGAGCGCCTGCCTTGCACGCAGGAGGTCAGCGGTTCGATC
CCGCTAGGCTCCACCA
>Bacillus_weihenstephanensis_KBAB4_chr.trna14-ArgACG (87675-87748) Arg (ACG) 74 bp Sc: 69.97
GCGCCCGTAGCTCAATTGGATAGAGCGTTTGACTACGGATCAAGAGGTTAGGGGTTCGAC
TCCTCTCGGGCGCG

>Bacillus_weihenstephanensis_KBAB4_chr.trna64-ArgACG (733811-733884) Arg (ACG) 74 bp Sc: 69.97
GCGCCCGTAGCTCAATTGGATAGAGCGTTTGACTACGGATCAAGAGGTTAGGGGTTCGAC
TCCTCTCGGGCGCG
>Bacillus_weihenstephanensis_KBAB4_chr.trna88-ArgACG (4694796-4694723) Arg (ACG) 74 bp Sc: 69.97
GCGCCCGTAGCTCAATTGGATAGAGCGTTTGACTACGGATCAAGAGGTTAGGGGTTCGAC
TCCTCTCGGGCGCG
>Bacillus_weihenstephanensis_KBAB4_chr.trna35-ArgACG (247496-247572) Arg (ACG) 77 bp Sc: 78.27
GCGCCCGTAGCTCAATTGGATAGAGCGTTTGACTACGGATCAAGAGGTTAGGGGTTCGAC
TCCTCTCGGGCGCGCCA
>Bacillus_weihenstephanensis_KBAB4_chr.trna81-ArgCCG (4900468-4900393) Arg (CCG) 76 bp Sc: 62.08
GCGCCCATAGCTCAGTCGGATAGAGCGGTGGTTTCCGGTACCACGTCTGCCGGGGGTTCG
AATCCCTCTGGGCGCG
>Bacillus_weihenstephanensis_KBAB4_chr.trna105-ArgTCT (4322774-4322698) Arg (TCT) 77 bp Sc: 79.49
GTCCCAGTAGCTCAGCCGGATAGAGCATACGCCTTCTAAGCGTACGGTCGGGAGTTCGAA
TCTCTCCTGGGACGCTA
>Bacillus_weihenstephanensis_KBAB4_chr.trna17-AsnGTT (155935-156009) Asn (GTT) 75 bp Sc: 86.53
TCCGCAGTAGCTCAGCGGTAGAGCTATCGGCTGTTAACCGATCGGTCGTAGGTTCGATTC
CTACCTGCGGAGCCA
>Bacillus_weihenstephanensis_KBAB4_chr.trna40-AsnGTT (520791-520865) Asn (GTT) 75 bp Sc: 86.53
TCCGCAGTAGCTCAGCGGTAGAGCTATCGGCTGTTAACCGATCGGTCGTAGGTTCGATTC
CTACCTGCGGAGCCA
>Bacillus_weihenstephanensis_KBAB4_chr.trna101-AsnGTT (4693639-4693565) Asn (GTT) 75 bp Sc: 88.32
TCCGCAGTAGCTCAGTGGTAGAGCTATCGGCTGTTAACCGATCGGTCGTAGGTTCGAGTC
CTACCTGCGGAGCCA
>Bacillus_weihenstephanensis_KBAB4_chr.trna26-AsnGTT (246549-246623) Asn (GTT) 75 bp Sc: 88.32
TCCGCAGTAGCTCAGTGGTAGAGCTATCGGCTGTTAACCGATCGGTCGTAGGTTCGAGTC
CTACCTGCGGAGCCA
>Bacillus_weihenstephanensis_KBAB4_chr.trna75-AsnGTT (734849-734923) Asn (GTT) 75 bp Sc: 88.32
TCCGCAGTAGCTCAGTGGTAGAGCTATCGGCTGTTAACCGATCGGTCGTAGGTTCGAGTC
CTACCTGCGGAGCCA
>Bacillus_weihenstephanensis_KBAB4_chr.trna31-AspGTC (246985-247060) Asp (GTC) 76 bp Sc: 76.06
GGTCCCGTGGTGTAGTGGTTAACATGCCTGCCTGTCACGCAGGAGATCGCCGGTTCGACC
CCGGTCGGGACCGCCA
>Bacillus_weihenstephanensis_KBAB4_chr.trna39-AspGTC (252731-252806) Asp (GTC) 76 bp Sc: 76.06
GGTCCCGTGGTGTAGTGGTTAACATGCCTGCCTGTCACGCAGGAGATCGCCGGTTCGACC
CCGGTCGGGACCGCCA
>Bacillus_weihenstephanensis_KBAB4_chr.trna46-AspGTC (521373-521448) Asp (GTC) 76 bp Sc: 76.06
GGTCCCGTGGTGTAGTGGTTAACATGCCTGCCTGTCACGCAGGAGATCGCCGGTTCGACC
CCGGTCGGGACCGCCA
>Bacillus_weihenstephanensis_KBAB4_chr.trna70-AspGTC (734425-734500) Asp (GTC) 76 bp Sc: 76.06
GGTCCCGTGGTGTAGTGGTTAACATGCCTGCCTGTCACGCAGGAGATCGCCGGTTCGACC
CCGGTCGGGACCGCCA
>Bacillus_weihenstephanensis_KBAB4_chr.trna79-AspGTC (5242040-5241965) Asp (GTC) 76 bp Sc: 76.06
GGTCCCGTGGTGTAGTGGTTAACATGCCTGCCTGTCACGCAGGAGATCGCCGGTTCGACC
CCGGTCGGGACCGCCA
>Bacillus_weihenstephanensis_KBAB4_chr.trna95-AspGTC (4694155-4694080) Asp (GTC) 76 bp Sc: 76.06
GGTCCCGTGGTGTAGTGGTTAACATGCCTGCCTGTCACGCAGGAGATCGCCGGTTCGACC
CCGGTCGGGACCGCCA
>Bacillus_weihenstephanensis_KBAB4_chr.trna54-CysGCA (522129-522199) Cys (GCA) 71 bp Sc: 67.53
GGCGGCATAGCCAAGTGGTAAGGCAGAGGTCTGCAAAACCTTTACCACCGGTTCAAATCC
GGTTGCCGCCT
>Bacillus_weihenstephanensis_KBAB4_chr.trna22-GlnTTG (156431-156505) Gln (TTG) 75 bp Sc: 78.60
TGGGCTATAGCCAAGCGGTAAGGCAACGGACTTTGACTCCGTCATGCGCTGGTTCGAATC
CAGCTAGCCCAGCCA
>Bacillus_weihenstephanensis_KBAB4_chr.trna32-GlnTTG (247148-247222) Gln (TTG) 75 bp Sc: 78.60
TGGGCTATAGCCAAGCGGTAAGGCAACGGACTTTGACTCCGTCATGCGCTGGTTCGAATC
CAGCTAGCCCAGCCA
>Bacillus_weihenstephanensis_KBAB4_chr.trna52-GlnTTG (521960-522034) Gln (TTG) 75 bp Sc: 78.60
TGGGCTATAGCCAAGCGGTAAGGCAACGGACTTTGACTCCGTCATGCGCTGGTTCGAATC
CAGCTAGCCCAGCCA
>Bacillus_weihenstephanensis_KBAB4_chr.trna59-GlnTTG (733347-733421) Gln (TTG) 75 bp Sc: 78.60
TGGGCTATAGCCAAGCGGTAAGGCAACGGACTTTGACTCCGTCATGCGCTGGTTCGAATC
CAGCTAGCCCAGCCA
>Bacillus_weihenstephanensis_KBAB4_chr.trna103-GluTTC (4693461-4693390) Glu (TTC) 72 bp Sc: 71.01
GGCCCGTTGGTCAAGTGGTTAAGACACCGCCCTTTCACGGCGGTAACACGGGTTCGAATC
CCGTACGGGTCA
>Bacillus_weihenstephanensis_KBAB4_chr.trna7-GluTTC (64377-64448) Glu (TTC) 72 bp Sc: 71.01

GGCCCGTTGGTCAAGTGGTTAAGACACCGCCCTTTCACGGCGGTAACACGGGTTCGAATC
CCGTACGGGTCA
>Bacillus_weihenstephanensis_KBAB4_chr.trna76-GluTTC (734925-734996) Glu (TTC) 72 bp Sc: 71.01
GGCCCGTTGGTCAAGTGGTTAAGACACCGCCCTTTCACGGCGGTAACACGGGTTCGAATC
CCGTACGGGTCA
>Bacillus_weihenstephanensis_KBAB4_chr.trna78-GluTTC (5242134-5242063) Glu (TTC) 72 bp Sc: 71.01
GGCCCGTTGGTCAAGTGGTTAAGACACCGCCCTTTCACGGCGGTAACACGGGTTCGAATC
CCGTACGGGTCA
>Bacillus_weihenstephanensis_KBAB4_chr.trna19-GluTTC (156110-156184) Glu (TTC) 75 bp Sc: 79.30
GGCCCGTTGGTCAAGTGGTTAAGACACCGCCCTTTCACGGCGGTAACACGGGTTCGAATC
CCGTACGGGTCACCA
>Bacillus_weihenstephanensis_KBAB4_chr.trna28-GluTTC (246726-246800) Glu (TTC) 75 bp Sc: 79.30
GGCCCGTTGGTCAAGTGGTTAAGACACCGCCCTTTCACGGCGGTAACACGGGTTCGAATC
CCGTACGGGTCACCA
>Bacillus_weihenstephanensis_KBAB4_chr.trna42-GluTTC (520979-521053) Glu (TTC) 75 bp Sc: 79.30
GGCCCGTTGGTCAAGTGGTTAAGACACCGCCCTTTCACGGCGGTAACACGGGTTCGAATC
CCGTACGGGTCACCA
>Bacillus_weihenstephanensis_KBAB4_chr.trna24-GlyGCC (156592-156663) Gly (GCC) 72 bp Sc: 80.56
GCGGAAGTAGTTCAGTGGTAGAATACAACCTTGCCAAGGTTGGGGTCGCGGGTTCGAATC
CCGTCTTCCGCT
>Bacillus_weihenstephanensis_KBAB4_chr.trna12-GlyGCC (87492-87566) Gly (GCC) 75 bp Sc: 88.86
GCGGAAGTAGTTCAGTGGTAGAATACAACCTTGCCAAGGTTGGGGTCGCGGGTTCGAATC
CCGTCTTCCGCTCCA
>Bacillus_weihenstephanensis_KBAB4_chr.trna53-GlyGCC (522040-522114) Gly (GCC) 75 bp Sc: 88.86
GCGGAAGTAGTTCAGTGGTAGAATACAACCTTGCCAAGGTTGGGGTCGCGGGTTCGAATC
CCGTCTTCCGCTCCA
>Bacillus_weihenstephanensis_KBAB4_chr.trna62-GlyGCC (733628-733702) Gly (GCC) 75 bp Sc: 88.86
GCGGAAGTAGTTCAGTGGTAGAATACAACCTTGCCAAGGTTGGGGTCGCGGGTTCGAATC
CCGTCTTCCGCTCCA
>Bacillus_weihenstephanensis_KBAB4_chr.trna86-GlyGCC (4694979-4694905) Gly (GCC) 75 bp Sc: 88.86
GCGGAAGTAGTTCAGTGGTAGAATACAACCTTGCCAAGGTTGGGGTCGCGGGTTCGAATC
CCGTCTTCCGCTCCA
>Bacillus_weihenstephanensis_KBAB4_chr.trna37-GlyTCC (247654-247724) Gly (TCC) 71 bp Sc: 71.78
GCGGGTGTAGTTTAGTGGTAAAACAAGAGCCTTCCAAGCTCTGGTCGAGAGTTCGATTCT
CTTCACCCGCT
>Bacillus_weihenstephanensis_KBAB4_chr.trna99-GlyTCC (4693800-4693730) Gly (TCC) 71 bp Sc: 71.78
GCGGGTGTAGTTTAGTGGTAAAACAAGAGCCTTCCAAGCTCTGGTCGAGAGTTCGATTCT
CTTCACCCGCT
>Bacillus_weihenstephanensis_KBAB4_chr.trna104-GlyTCC (4322849-4322776) Gly (TCC) 74 bp Sc: 80.08
GCGGGTGTAGTTTAGTGGTAAAACAAGAGCCTTCCAAGCTCTGGTCGAGAGTTCGATTCT
CTTCACCCGCTCCA
>Bacillus_weihenstephanensis_KBAB4_chr.trna56-GlyTCC (527074-527147) Gly (TCC) 74 bp Sc: 80.08
GCGGGTGTAGTTTAGTGGTAAAACAAGAGCCTTCCAAGCTCTGGTCGAGAGTTCGATTCT
CTTCACCCGCTCCA
>Bacillus_weihenstephanensis_KBAB4_chr.trna84-HisGTG (4695189-4695114) His (GTG) 76 bp Sc: 71.48
GCGGTTGTGGCGAAGTGGTTAACGCACCGGATTGTGGCTCCGGCACTCGTGGGTTCAATT
CCCATCAGTCGCCCCA
>Bacillus_weihenstephanensis_KBAB4_chr.trna51-HisGTG (521819-521894) His (GTG) 76 bp Sc: 73.48
GCGGTTGTGGCGAAGTGGTTAACGCACCGGATTGTGGCTCCGGCATTCGTGGGTTCAATT
CCCATCAGTCGCCCCA
>Bacillus_weihenstephanensis_KBAB4_chr.trna1-IleGAT (10703-10779) Ile (GAT) 77 bp Sc: 101.21
GGGCCTATAGCTCAGCTGGTTAGAGCGCACGCCTGATAAGCGTGAGGTCGATGGTTCGAG
TCCATTTAGGCCCACCA
>Bacillus_weihenstephanensis_KBAB4_chr.trna100-IleGAT (4693723-4693647) Ile (GAT) 77 bp Sc: 101.21
GGGCCTATAGCTCAGCTGGTTAGAGCGCACGCCTGATAAGCGTGAGGTCGATGGTTCGAG
TCCATTTAGGCCCACCA
>Bacillus_weihenstephanensis_KBAB4_chr.trna4-IleGAT (30537-30613) Ile (GAT) 77 bp Sc: 101.21
GGGCCTATAGCTCAGCTGGTTAGAGCGCACGCCTGATAAGCGTGAGGTCGATGGTTCGAG
TCCATTTAGGCCCACCA
>Bacillus_weihenstephanensis_KBAB4_chr.trna74-IleGAT (734765-734841) Ile (GAT) 77 bp Sc: 101.21
GGGCCTATAGCTCAGCTGGTTAGAGCGCACGCCTGATAAGCGTGAGGTCGATGGTTCGAG
TCCATTTAGGCCCACCA
>Bacillus_weihenstephanensis_KBAB4_chr.trna55-LeuCAA (522209-522293) Leu (CAA) 85 bp Sc: 60.08
GCCGATGTGGCGGAATTGGCAGACGCGCACGACTCAAAATCGTGTTCCTTCGGGAGTGTC
GGTTCGACCCCGACCATCGGTATCA
>Bacillus_weihenstephanensis_KBAB4_chr.trna34-LeuGAG (247317-247402) Leu (GAG) 86 bp Sc: 57.19
GCGGTCGTGGCGGAACGGCAGACGCGCTAGGTTGAGGGCCTAGTGGGGGAAACCCCGTGG

AGGTTCAAGTCCTCTCGGCCGCATCA
>Bacillus_weihenstephanensis_KBAB4_chr.trna13-LeuTAA (87583-87671) Leu (TAA) 89 bp Sc: 71.28
GCCGGGGTGGCGGAACAGGCAGACGCACAGGACTTAAAATCCTGCGGTGGGTGACCACCG
TGCGGGTTCGACCCCCGCCCTCGGCACCA
>Bacillus_weihenstephanensis_KBAB4_chr.trna63-LeuTAA (733719-733807) Leu (TAA) 89 bp Sc: 71.28
GCCGGGGTGGCGGAACAGGCAGACGCACAGGACTTAAAATCCTGCGGTGGGTGACCACCG
TGCGGGTTCGACCCCCGCCCTCGGCACCA
>Bacillus_weihenstephanensis_KBAB4_chr.trna87-LeuTAA (4694888-4694800) Leu (TAA) 89 bp Sc: 71.28
GCCGGGGTGGCGGAACAGGCAGACGCACAGGACTTAAAATCCTGCGGTGGGTGACCACCG
TGCGGGTTCGACCCCCGCCCTCGGCACCA
>Bacillus_weihenstephanensis_KBAB4_chr.trna11-LeuTAG (87382-87462) Leu (TAG) 81 bp Sc: 66.65
GCGGGTGTGGCGGAATTGGCAGACGCACTAGACTTAGGATCTAGCGCCTTTGGCGTGGGG
GTTCGACTCCCTTCACCCGCA
>Bacillus_weihenstephanensis_KBAB4_chr.trna61-LeuTAG (733518-733598) Leu (TAG) 81 bp Sc: 66.65
GCGGGTGTGGCGGAATTGGCAGACGCACTAGACTTAGGATCTAGCGCCTTTGGCGTGGGG
GTTCGACTCCCTTCACCCGCA
>Bacillus_weihenstephanensis_KBAB4_chr.trna85-LeuTAG (4695089-4695009) Leu (TAG) 81 bp Sc: 66.65
GCGGGTGTGGCGGAATTGGCAGACGCACTAGACTTAGGATCTAGCGCCTTTGGCGTGGGG
GTTCGACTCCCTTCACCCGCA
>Bacillus_weihenstephanensis_KBAB4_chr.trna33-LysTTT (247228-247300) Lys (TTT) 73 bp Sc: 90.38
GAGCCATTAGCTCAGTTGGTAGAGCATCTGACTTTTAATCAGAGGGTCGAAGGTTCGAAT
CCTTCATGGCTCA
>Bacillus_weihenstephanensis_KBAB4_chr.trna10-LysTTT (87292-87367) Lys (TTT) 76 bp Sc: 98.67
GAGCCATTAGCTCAGTTGGTAGAGCATCTGACTTTTAATCAGAGGGTCGAAGGTTCGAAT
CCTTCATGGCTCACCA
>Bacillus_weihenstephanensis_KBAB4_chr.trna23-LysTTT (156511-156586) Lys (TTT) 76 bp Sc: 98.67
GAGCCATTAGCTCAGTTGGTAGAGCATCTGACTTTTAATCAGAGGGTCGAAGGTTCGAAT
CCTTCATGGCTCACCA
>Bacillus_weihenstephanensis_KBAB4_chr.trna60-LysTTT (733426-733501) Lys (TTT) 76 bp Sc: 98.67
GAGCCATTAGCTCAGTTGGTAGAGCATCTGACTTTTAATCAGAGGGTCGAAGGTTCGAAT
CCTTCATGGCTCACCA
>Bacillus_weihenstephanensis_KBAB4_chr.trna77-LysTTT (5242223-5242148) Lys (TTT) 76 bp Sc: 98.67
GAGCCATTAGCTCAGTTGGTAGAGCATCTGACTTTTAATCAGAGGGTCGAAGGTTCGAAT
CCTTCATGGCTCACCA
>Bacillus_weihenstephanensis_KBAB4_chr.trna98-LysTTT (4693889-4693814) Lys (TTT) 76 bp Sc: 98.67
GAGCCATTAGCTCAGTTGGTAGAGCATCTGACTTTTAATCAGAGGGTCGAAGGTTCGAAT
CCTTCATGGCTCACCA
>Bacillus_weihenstephanensis_KBAB4_chr.trna6-MetCAT (64290-64363) Met (CAT) 74 bp Sc: 75.69
GGCGGTGTAGCTCAGCTGGCTAGAGCGTACGGTTCATACCCGTGAGGTCGGGGGTTCGAT
CCCCTCCGCCGCTA
>Bacillus_weihenstephanensis_KBAB4_chr.trna69-MetCAT (734346-734422) Met (CAT) 77 bp Sc: 81.59
CGCGGGGTGGAGCAGTACGGTAGCTCGTCGGGCTCATAATCCGAAGGTCGCAGGTTCAAA
TCCTGTCCCCGCAACCA
>Bacillus_weihenstephanensis_KBAB4_chr.trna91-MetCAT (4694530-4694454) Met (CAT) 77 bp Sc: 83.99
GGCGGTGTAGCTCAGCTGGCTAGAGCGTACGGTTCATACCCGTGAGGTCGGGGGTTCGAT
CCCCTCCGCCGCTACCA
>Bacillus_weihenstephanensis_KBAB4_chr.trna30-MetCAT (246905-246981) Met (CAT) 77 bp Sc: 86.20
CGCGGGGTGGAGCAGTACGGTAGCTCGTCGGGCTCATAACCCGAAGGTCGCAGGTTCAAA
TCCTGTCCCCGCAACCA
>Bacillus_weihenstephanensis_KBAB4_chr.trna38-MetCAT (252651-252727) Met (CAT) 77 bp Sc: 86.20
CGCGGGGTGGAGCAGTACGGTAGCTCGTCGGGCTCATAACCCGAAGGTCGCAGGTTCAAA
TCCTGTCCCCGCAACCA
>Bacillus_weihenstephanensis_KBAB4_chr.trna44-MetCAT (521159-521235) Met (CAT) 77 bp Sc: 86.20
CGCGGGGTGGAGCAGTACGGTAGCTCGTCGGGCTCATAACCCGAAGGTCGCAGGTTCAAA
TCCTGTCCCCGCAACCA
>Bacillus_weihenstephanensis_KBAB4_chr.trna45-MetCAT (521293-521369) Met (CAT) 77 bp Sc: 86.20
CGCGGGGTGGAGCAGTACGGTAGCTCGTCGGGCTCATAACCCGAAGGTCGCAGGTTCAAA
TCCTGTCCCCGCAACCA
>Bacillus_weihenstephanensis_KBAB4_chr.trna94-MetCAT (4694235-4694159) Met (CAT) 77 bp Sc: 86.20
CGCGGGGTGGAGCAGTACGGTAGCTCGTCGGGCTCATAACCCGAAGGTCGCAGGTTCAAA
TCCTGTCCCCGCAACCA
>Bacillus_weihenstephanensis_KBAB4_chr.trna106-MetCAT (4133101-4133028) Met (CAT) 74 bp Sc: 89.13
GGACCCTTAGCTCAGTTGGTTAGAGCAGACGGCTCATAACCGTCCGGTCGTAGGTTCGAG
TCCTACAGGGTCCA
>Bacillus_weihenstephanensis_KBAB4_chr.trna92-MetCAT (4694449-4694373) Met (CAT) 77 bp Sc: 96.69
GGACCTTTAGCTCAGCTGGTTAGAGCAGACGGCTCATAACCGTCCGGTCGTAGGTTCGAG
TCCTACAAGGTCCACCA

>Bacillus_weihenstephanensis_KBAB4_chr.trna80-PheGAA (5241923-5241851) Phe (GAA) 73 bp Sc: 84.57
GGCTCGGTAGCTCAGTCGGTAGAGCAGAGGACTGAAAATCCTCGTGTCGGCGGTTCGATT
CCGTCCCGAGCCA
>Bacillus_weihenstephanensis_KBAB4_chr.trna47-PheGAA (521458-521533) Phe (GAA) 76 bp Sc: 92.86
GGCTCGGTAGCTCAGTCGGTAGAGCAGAGGACTGAAAATCCTCGTGTCGGCGGTTCGATT
CCGTCCCGAGCCACCA
>Bacillus_weihenstephanensis_KBAB4_chr.trna71-PheGAA (734510-734585) Phe (GAA) 76 bp Sc: 92.86
GGCTCGGTAGCTCAGTCGGTAGAGCAGAGGACTGAAAATCCTCGTGTCGGCGGTTCGATT
CCGTCCCGAGCCACCA
>Bacillus_weihenstephanensis_KBAB4_chr.trna96-PheGAA (4694065-4693990) Phe (GAA) 76 bp Sc: 92.86
GGCTCGGTAGCTCAGTCGGTAGAGCAGAGGACTGAAAATCCTCGTGTCGGCGGTTCGATT
CCGTCCCGAGCCACCA
>Bacillus_weihenstephanensis_KBAB4_chr.trna15-ProTGG (87757-87830) Pro (TGG) 74 bp Sc: 85.68
CGGGAAGTGGCTCAGCTTGGTAGAGCACCTGGTTTGGGACCAGGGGGTCGCAGGTTCAAA
TCCTGTCTTCCCGA
>Bacillus_weihenstephanensis_KBAB4_chr.trna65-ProTGG (733895-733968) Pro (TGG) 74 bp Sc: 85.68
CGGGAAGTGGCTCAGCTTGGTAGAGCACCTGGTTTGGGACCAGGGGGTCGCAGGTTCAAA
TCCTGTCTTCCCGA
>Bacillus_weihenstephanensis_KBAB4_chr.trna89-ProTGG (4694711-4694638) Pro (TGG) 74 bp Sc: 85.68
CGGGAAGTGGCTCAGCTTGGTAGAGCACCTGGTTTGGGACCAGGGGGTCGCAGGTTCAAA
TCCTGTCTTCCCGA
>Bacillus_weihenstephanensis_KBAB4_chr.trna36-ProTGG (247576-247652) Pro (TGG) 77 bp Sc: 93.98
CGGGAAGTGGCTCAGCTTGGTAGAGCACCTGGTTTGGGACCAGGGGGTCGCAGGTTCAAA
TCCTGTCTTCCCGACCA
>Bacillus_weihenstephanensis_KBAB4_chr.trna27-SerGCT (246627-246717) Ser (GCT) 91 bp Sc: 67.68
AGAGAAGTACCCAAGTGGCTCAAGGGGCTCCCCTGCTAAGGGAGTAGATCGCTAACGCGG
TGCGAGGGTTCGAATCCCTTCTTCTCTGCCA
>Bacillus_weihenstephanensis_KBAB4_chr.trna102-SerGCT (4693558-4693468) Ser (GCT) 91 bp Sc: 67.93
AGAGAAGTACCCAAGTGGCTCAAGGGGCTCCCCTGCTAAGGGAGTAGATCGCGAACGCGG
TGCGAGGGTTCGAATCCCTTCTTCTCTGCCA
>Bacillus_weihenstephanensis_KBAB4_chr.trna41-SerGGA (520868-520959) Ser (GGA) 92 bp Sc: 63.66
GGAGAGCTGTCCGAGTTGGCCGAAGGAGCACGATTGGAAATCGTGTATACGTCACAAGCG
TATCAAGGGTTCGAATCCCTTGCTCTCCGCCA
>Bacillus_weihenstephanensis_KBAB4_chr.trna3-SerTGA (21481-21573) Ser (TGA) 93 bp Sc: 71.57
GGAGGTATACCCAAGTCTGGCTGAAGGGATCGGTCTTGAAAACCGACAGGCGGCGAGAGT
CGCGCGGGGGTTCGAATCCCTCTACCTCCTCCA
>Bacillus_weihenstephanensis_KBAB4_chr.trna67-SerTGA (734075-734167) Ser (TGA) 93 bp Sc: 72.21
GGAGGTATACCCAAGTTCGGCTGAAGGGATCGGTCTTGAAAACCGACAGGCGGCGAGAGT
CGCGCGGGGGTTCGAATCCCTCTACCTCCTCCA
>Bacillus_weihenstephanensis_KBAB4_chr.trna68-SerTGA (734229-734321) Ser (TGA) 93 bp Sc: 72.21
GGAGGTATACCCAAGTTCGGCTGAAGGGATCGGTCTTGAAAACCGACAGGCGGCGAGAGT
CGCGCGGGGGTTCGAATCCCTCTACCTCCTCCA
>Bacillus_weihenstephanensis_KBAB4_chr.trna93-SerTGA (4694355-4694263) Ser (TGA) 93 bp Sc: 72.21
GGAGGTATACCCAAGTTCGGCTGAAGGGATCGGTCTTGAAAACCGACAGGCGGCGAGAGT
CGCGCGGGGGTTCGAATCCCTCTACCTCCTCCA
>Bacillus_weihenstephanensis_KBAB4_chr.trna18-ThrGGT (156014-156086) Thr (GGT) 73 bp Sc: 74.15
GCTTCCATAGCTCAGCTGGTAGAGTACTTCCATGGTAAGGAAGAGGTCACCGGTTCAAGC
CCGGTTGGAAGCT
>Bacillus_weihenstephanensis_KBAB4_chr.trna83-ThrTGT (4695275-4695200) Thr (TGT) 76 bp Sc: 95.44
GCCGACTTAGCTCAATTGGTAGAGCAACTGACTTGTAATCAGTAGGTTGGGGGTTCAAGT
CCTCTAGTCGGCACCA
>Bacillus_weihenstephanensis_KBAB4_chr.trna9-ThrTGT (87203-87278) Thr (TGT) 76 bp Sc: 95.44
GCCGACTTAGCTCAATTGGTAGAGCAACTGACTTGTAATCAGTAGGTTGGGGGTTCAAGT
CCTCTAGTCGGCACCA
>Bacillus_weihenstephanensis_KBAB4_chr.trna48-ThrTGT (521548-521623) Thr (TGT) 76 bp Sc: 96.17
GCCGGCTTAGCTCAATTGGTAGAGCAACTGACTTGTAATCAGTAGGTTGGGGGTTCAAGT
CCTCTAGCCGGCACCA
>Bacillus_weihenstephanensis_KBAB4_chr.trna72-ThrTGT (734605-734680) Thr (TGT) 76 bp Sc: 96.17
GCCGGCTTAGCTCAATTGGTAGAGCAACTGACTTGTAATCAGTAGGTTGGGGGTTCAAGT
CCTCTAGCCGGCACCA
>Bacillus_weihenstephanensis_KBAB4_chr.trna97-ThrTGT (4693975-4693900) Thr (TGT) 76 bp Sc: 96.17
GCCGGCTTAGCTCAATTGGTAGAGCAACTGACTTGTAATCAGTAGGTTGGGGGTTCAAGT
CCTCTAGCCGGCACCA
>Bacillus_weihenstephanensis_KBAB4_chr.trna73-TrpCCA (734684-734754) Trp (CCA) 71 bp Sc: 52.50
AGGGGCATAGTTTAAAGGTAGAACTGAGGTCTCCAAAACCTCCAGTGTGGGTTCAATTCC
TACTGCCCCTG
>Bacillus_weihenstephanensis_KBAB4_chr.trna50-TrpCCA (521726-521799) Trp (CCA) 74 bp Sc: 60.79

AGGGGCATAGTTTAAAGGTAGAACTGAGGTCTCCAAAACCTCCAGTGTGGGTTCAATTCC
TACTGCCCCTGCCA
>Bacillus_weihenstephanensis_KBAB4_chr.trna21-TyrGTA (156282-156365) Tyr (GTA) 84 bp Sc: 77.59
GGAGGGGTAGCGAAGTGGCTAAACGCGGCGGACTGTAAATCCGCTCCTTTGGGTTCGGCA
GTTCGAATCTGCCCCCCTCCACCA
>Bacillus_weihenstephanensis_KBAB4_chr.trna49-TyrGTA (521634-521717) Tyr (GTA) 84 bp Sc: 77.59
GGAGGGGTAGCGAAGTGGCTAAACGCGGCGGACTGTAAATCCGCTCCTTCGGGTTCGGCA
GTTCGAATCTGCCCCCCTCCACCA
>Bacillus_weihenstephanensis_KBAB4_chr.trna58-TyrGTA (733251-733334) Tyr (GTA) 84 bp Sc: 77.59
GGAGGGGTAGCGAAGTGGCTAAACGCGGCGGACTGTAAATCCGCTCCTTCGGGTTCGGCA
GTTCGAATCTGCCCCCCTCCACCA
>Bacillus_weihenstephanensis_KBAB4_chr.trna108-ValGAC (1243656-1243584) Val (GAC) 73 bp Sc: 53.67
CATCCCGTAGCTCAGCAGGGAGAGCGCCACCTTGACAGGGTGGAGGTCGTGAGTTCGAGC
CTCTCCGGGGTCA
>Bacillus_weihenstephanensis_KBAB4_chr.trna107-ValGAC (1260461-1260389) Val (GAC) 73 bp Sc: 61.20
GATCCCGTAGCTCAGCAGGGAGAGCGCCACCTTGACAGGGTGGAGGTCGTGAGTTCGAGC
CTCTCCGGGGTCA
>Bacillus_weihenstephanensis_KBAB4_chr.trna20-ValTAC (156190-156265) Val (TAC) 76 bp Sc: 97.03
GGAGGATTAGCTCAGCTGGGAGAGCACCTGCCTTACAAGCAGGGGGTCGGCGGTTCGATC
CCGTCATCCTCCACCA
>Bacillus_weihenstephanensis_KBAB4_chr.trna29-ValTAC (246805-246880) Val (TAC) 76 bp Sc: 97.03
GGAGGATTAGCTCAGCTGGGAGAGCACCTGCCTTACAAGCAGGGGGTCGGCGGTTCGATC
CCGTCATCCTCCACCA
>Bacillus_weihenstephanensis_KBAB4_chr.trna43-ValTAC (521059-521134) Val (TAC) 76 bp Sc: 97.03
GGAGGATTAGCTCAGCTGGGAGAGCACCTGCCTTACAAGCAGGGGGTCGGCGGTTCGATC
CCGTCATCCTCCACCA
>Bacillus_weihenstephanensis_KBAB4_chr.trna57-ValTAC (733167-733242) Val (TAC) 76 bp Sc: 97.03
GGAGGATTAGCTCAGCTGGGAGAGCACCTGCCTTACAAGCAGGGGGTCGGCGGTTCGATC
CCGTCATCCTCCACCA
>Bacillus_weihenstephanensis_KBAB4_chr.trna8-ValTAC (87123-87198) Val (TAC) 76 bp Sc: 97.03
GGAGGATTAGCTCAGCTGGGAGAGCACCTGCCTTACAAGCAGGGGGTCGGCGGTTCGATC
CCGTCATCCTCCACCA
>Bacillus_weihenstephanensis_KBAB4_chr.trna82-ValTAC (4695355-4695280) Val (TAC) 76 bp Sc: 97.03
GGAGGATTAGCTCAGCTGGGAGAGCACCTGCCTTACAAGCAGGGGGTCGGCGGTTCGATC
CCGTCATCCTCCACCA
>Bacteroides_fragilis_YCH46_chr.trna16-AlaGGC (3713658-3713731) Ala (GGC) 74 bp Sc: 62.47
GGGGGGTTAGCTCATCTGGCTAGAGCGTAACACTGGCAGTGTTAAGGTGATCGGTTCGAG
TCCGATACTCTCCA
>Bacteroides_fragilis_YCH46_chr.trna26-AlaTGC (5195246-5195173) Ala (TGC) 74 bp Sc: 84.82
GGGGGATTAGCTCAGCTGGCTAGAGCATCTGCCTTGCACGCAGAGGGTCAACGGTTCGAA
TCCGTTATTCTCCA
>Bacteroides_fragilis_YCH46_chr.trna35-AlaTGC (4735486-4735413) Ala (TGC) 74 bp Sc: 84.82
GGGGGATTAGCTCAGCTGGCTAGAGCATCTGCCTTGCACGCAGAGGGTCAACGGTTCGAA
TCCGTTATTCTCCA
>Bacteroides_fragilis_YCH46_chr.trna37-AlaTGC (4676129-4676056) Ala (TGC) 74 bp Sc: 84.82
GGGGGATTAGCTCAGCTGGCTAGAGCATCTGCCTTGCACGCAGAGGGTCAACGGTTCGAA
TCCGTTATTCTCCA
>Bacteroides_fragilis_YCH46_chr.trna42-AlaTGC (4526710-4526637) Ala (TGC) 74 bp Sc: 84.82
GGGGGATTAGCTCAGCTGGCTAGAGCATCTGCCTTGCACGCAGAGGGTCAACGGTTCGAA
TCCGTTATTCTCCA
>Bacteroides_fragilis_YCH46_chr.trna46-AlaTGC (3947586-3947513) Ala (TGC) 74 bp Sc: 84.82
GGGGGATTAGCTCAGCTGGCTAGAGCATCTGCCTTGCACGCAGAGGGTCAACGGTTCGAA
TCCGTTATTCTCCA
>Bacteroides_fragilis_YCH46_chr.trna48-AlaTGC (3143990-3143917) Ala (TGC) 74 bp Sc: 84.82
GGGGGATTAGCTCAGCTGGCTAGAGCATCTGCCTTGCACGCAGAGGGTCAACGGTTCGAA
TCCGTTATTCTCCA
>Bacteroides_fragilis_YCH46_chr.trna56-ArgACG (2780447-2780374) Arg (ACG) 74 bp Sc: 54.07
GGTCGCGTAGCTCAACTGAATAGAGTAGCTGACTACGGATCAGCCGGTTACAGGTTTGAA
TCCTGTCGCGATCA
>Bacteroides_fragilis_YCH46_chr.trna58-ArgACG (2780259-2780183) Arg (ACG) 77 bp Sc: 54.69
GGTCGCGTAGCTCAACTGAATAGAGTAGCTGACTACGGATCAGCCGGTTACAGGTTTGAA
TCCTGTCGCGATCACAA
>Bacteroides_fragilis_YCH46_chr.trna57-ArgACG (2780355-2780282) Arg (ACG) 74 bp Sc: 55.81
GGCCGCGTAGCTCAACTGAATAGAGTAGCTGACTACGGATCAGCCGGTTACAGGTTTGAA
TCCTGTCGCGGTCA
>Bacteroides_fragilis_YCH46_chr.trna43-ArgCCG (4350434-4350360) Arg (CCG) 75 bp Sc: 36.35
GCTCCTGTAGTTCAACGAATAGAATGTAGGTTTCCGGTACCTAAGATAAGGGTTTGATTC

CCTTCGGGAGTACAA
>Bacteroides_fragilis_YCH46_chr.trna27-ArgCCT (5081077-5081003) Arg (CCT) 75 bp Sc: 51.34
GGCCCTGTAGTTCAACGGATAGAATAGATGTTTCCTAAACATTAGATAGGGGTTCGATTC
TCCTCGGGGCTACAA
>Bacteroides_fragilis_YCH46_chr.trna12-ArgTCT (2609966-2610042) Arg (TCT) 77 bp Sc: 76.34
GGTTCCGTAGCTCAGCTGGATAGAGCAACGCCCTTCTAAGGCGTGGGTCAAGCGTTCGAA
TCGCTTCGGAATCACAA
>Bacteroides_fragilis_YCH46_chr.trna22-AsnGTT (4989982-4990055) Asn (GTT) 74 bp Sc: 79.33
TCTTCCTTAGCTCAGTCGGTTAGAGCATCTGACTGTTAATCAGAGGGTCCTTGGTTCAAG
TCCAAGAGGAAGAG
>Bacteroides_fragilis_YCH46_chr.trna23-AsnGTT (4990085-4990161) Asn (GTT) 77 bp Sc: 79.94
TCTTCCTTAGCTCAGTCGGTTAGAGCATCTGACTGTTAATCAGAGGGTCCTTGGTTCAAG
TCCAAGAGGAAGAGCAA
>Bacteroides_fragilis_YCH46_chr.trna13-AspGTC (3453440-3453513) Asp (GTC) 74 bp Sc: 85.50
GGTGCGTTAGTTCAGTTGGTTAGAATACATGCCTGTCACGCATGGGGTCACGGGTTCGAG
TCCCGTACGCACCG
>Bacteroides_fragilis_YCH46_chr.trna11-AspGTC (2410290-2410366) Asp (GTC) 77 bp Sc: 86.11
GGTGCGTTAGTTCAGTTGGTTAGAATACATGCCTGTCACGCATGGGGTCACGGGTTCGAG
TCCCGTACGCACCGCGA
>Bacteroides_fragilis_YCH46_chr.trna14-AspGTC (3453544-3453620) Asp (GTC) 77 bp Sc: 86.11
GGTGCGTTAGTTCAGTTGGTTAGAATACATGCCTGTCACGCATGGGGTCACGGGTTCGAG
TCCCGTACGCACCGCAA
>Bacteroides_fragilis_YCH46_chr.trna8-CysGCA (1662518-1662589) Cys (GCA) 72 bp Sc: 51.74
GGTTCCTTGGATGAGTGGCTTAGTCAGCGGTCTGCAAAACCGTGTACGGCGGTTCGAATC
CGCCAGGAACCT
>Bacteroides_fragilis_YCH46_chr.trna4-GlnCTG (923978-924051) Gln (CTG) 74 bp Sc: 47.13
TGGGCTATGGTGTAATGGTAACACTACAGATTCTGGTCCTGTCATTTCTGGTTCGAGTCC
AGATAGCCCAACAA
>Bacteroides_fragilis_YCH46_chr.trna1-GlnTTG (74043-74113) Gln (TTG) 71 bp Sc: 47.95
TGTCCTATGGTGTAATGGTAGCACAACAGTTTTTGGTTCTGTTTGTCTAAGTTCGAATCT
TGGTAGGACAA
>Bacteroides_fragilis_YCH46_chr.trna10-GluCTC (2136722-2136796) Glu (CTC) 75 bp Sc: 57.41
GGTGGGTTCGTCTAACGGTTAGGACACATGCCTCTCACGCATGTAATACGAGTTCGATTC
TCGTACCCACTACTA
>Bacteroides_fragilis_YCH46_chr.trna74-GluTTC (331696-331622) Glu (TTC) 75 bp Sc: 50.05
GGCCCGTTCGTCTATCGGTTAGGACGCAAGATTTTCATTCTTGAAAGGGGGGTTCGATTC
CCCCACGGGCTACAA
>Bacteroides_fragilis_YCH46_chr.trna55-GlyCCC (2858492-2858417) Gly (CCC) 76 bp Sc: 72.47
GCGGTAGTAGCTCAGTTGGCAGAGCGGCGGCTTCCCAAGCCGCAGGTCACGAGTTCGACC
CTCGCCTACCGCTCTA
>Bacteroides_fragilis_YCH46_chr.trna2-GlyGCC (589029-589101) Gly (GCC) 73 bp Sc: 84.53
GCGGAAATAGCTCAGTTGGTAGAGCATAACCTTGCCAAGGTTAGGGTCGCGAGTTCGAGT
CTCGTTTTCCGCT
>Bacteroides_fragilis_YCH46_chr.trna49-GlyGCC (2904824-2904752) Gly (GCC) 73 bp Sc: 84.53
GCGGAAATAGCTCAGTTGGTAGAGCATAACCTTGCCAAGGTTAGGGTCGCGAGTTCGAGT
CTCGTTTTCCGCT
>Bacteroides_fragilis_YCH46_chr.trna54-GlyGCC (2904274-2904202) Gly (GCC) 73 bp Sc: 84.53
GCGGAAATAGCTCAGTTGGTAGAGCATAACCTTGCCAAGGTTAGGGTCGCGAGTTCGAGT
CTCGTTTTCCGCT
>Bacteroides_fragilis_YCH46_chr.trna52-GlyGCC (2904501-2904426) Gly (GCC) 76 bp Sc: 85.15
GCGGAAATAGCTCAGTTGGTAGAGCATAACCTTGCCAAGGTTAGGGTCGCGAGTTCGAGT
CTCGTTTTCCGCTCAA
>Bacteroides_fragilis_YCH46_chr.trna31-GlyTCC (4795253-4795181) Gly (TCC) 73 bp Sc: 68.88
GCGGAAGTAGCTCAGTTGATAGAGCATTAGCCTTCCAAGCTGAGGGTCGCGGGTTTGAGC
CCCGTCTTCCGCT
>Bacteroides_fragilis_YCH46_chr.trna67-GlyTCC (1157452-1157377) Gly (TCC) 76 bp Sc: 69.50
GCGGAAGTAGCTCAGTTGATAGAGCATTAGCCTTCCAAGCTGAGGGTCGCGGGTTTGAGC
CCCGTCTTCCGCTCTA
>Bacteroides_fragilis_YCH46_chr.trna15-HisGTG (3655080-3655155) His (GTG) 76 bp Sc: 69.34
GTGGCTGTAGTTCAGTTGGTTAGAGCGTCAGATTGTGGTTCTGAATGTCGTGGGTTCGAG
TCCCATCTGCCACCCA
>Bacteroides_fragilis_YCH46_chr.trna25-IleGAT (5195351-5195275) Ile (GAT) 77 bp Sc: 86.36
AGTCCTATAGCTCAGTTGGTTAGAGCGCTACACTGATAATGTAGAGGTCGGCAGTTCAAC
TCTGCCTGGGACTACCA
>Bacteroides_fragilis_YCH46_chr.trna34-IleGAT (4735591-4735515) Ile (GAT) 77 bp Sc: 86.36
AGTCCTATAGCTCAGTTGGTTAGAGCGCTACACTGATAATGTAGAGGTCGGCAGTTCAAC
TCTGCCTGGGACTACCA

>Bacteroides_fragilis_YCH46_chr.trna36-IleGAT (4676234-4676158) Ile (GAT) 77 bp Sc: 86.36
AGTCCTATAGCTCAGTTGGTTAGAGCGCTACACTGATAATGTAGAGGTCGGCAGTTCAAC
TCTGCCTGGGACTACCA
>Bacteroides_fragilis_YCH46_chr.trna41-IleGAT (4526815-4526739) Ile (GAT) 77 bp Sc: 86.36
AGTCCTATAGCTCAGTTGGTTAGAGCGCTACACTGATAATGTAGAGGTCGGCAGTTCAAC
TCTGCCTGGGACTACCA
>Bacteroides_fragilis_YCH46_chr.trna45-IleGAT (3947691-3947615) Ile (GAT) 77 bp Sc: 86.36
AGTCCTATAGCTCAGTTGGTTAGAGCGCTACACTGATAATGTAGAGGTCGGCAGTTCAAC
TCTGCCTGGGACTACCA
>Bacteroides_fragilis_YCH46_chr.trna47-IleGAT (3144095-3144019) Ile (GAT) 77 bp Sc: 86.36
AGTCCTATAGCTCAGTTGGTTAGAGCGCTACACTGATAATGTAGAGGTCGGCAGTTCAAC
TCTGCCTGGGACTACCA
>Bacteroides_fragilis_YCH46_chr.trna28-LeuCAA (4919485-4919402) Leu (CAA) 84 bp Sc: 50.22
GCCCAGATGGCGGAATCGGTAGACGCGCTGGTCTCAAACACCAGTGGATTCACTTCCATC
CCGGTTCGACCCCGGGTCTGGGTA
>Bacteroides_fragilis_YCH46_chr.trna50-LeuCAG (2904726-2904642) Leu (CAG) 85 bp Sc: 51.53
GCCCAGGTGGCGGAATTGGTAGACGCGCACGTTTCAGGTGCGTGTGTCGAGAGGCATGCA
GGTTCGAGTCCTGTTCTGGGCACAA
>Bacteroides_fragilis_YCH46_chr.trna53-LeuCAG (2904363-2904279) Leu (CAG) 85 bp Sc: 51.53
GCCCAGGTGGCGGAATTGGTAGACGCGCACGTTTCAGGTGCGTGTGTCGAGAGGCATGCA
GGTTCGAGTCCTGTTCTGGGCACTA
>Bacteroides_fragilis_YCH46_chr.trna51-LeuGAG (2904607-2904524) Leu (GAG) 84 bp Sc: 47.76
GGAGAGGTGGCGGAATTGGTAGACGCGCTACTTTGAGGGGGTAGTGACATTACTGTCGTG
GGAGTTCGAGTCTCCTTCTCTTCA
>Bacteroides_fragilis_YCH46_chr.trna3-LeuTAA (589116-589199) Leu (TAA) 84 bp Sc: 60.93
GCTCGAATGGTGGAATGGTAGACACGAAGGACTTAAAATCCTTTGGCCATTGCGGCTGTG
CGGGTTCAAGTCCCGCTTCGAGTA
>Bacteroides_fragilis_YCH46_chr.trna73-LeuTAG (473498-473414) Leu (TAG) 85 bp Sc: 67.89
GCGGATGTGGCGTAATTGGTAGCCGCGCCAGACTTAGGATCTGGTGTCTCGCGACGTGTA
GGTTCGAGTCCTATCATCCGCACAA
>Bacteroides_fragilis_YCH46_chr.trna61-LysCTT (2663522-2663447) Lys (CTT) 76 bp Sc: 81.92
GCACTCTTAGCTCAGCTGGTAGAGCAATTGACTCTTAATCAATGGGTCCAGGGTTCGAGT
CCCTGAGGGTGTACAA
>Bacteroides_fragilis_YCH46_chr.trna62-LysCTT (2660162-2660087) Lys (CTT) 76 bp Sc: 81.92
GCACTCTTAGCTCAGCTGGTAGAGCAATTGACTCTTAATCAATGGGTCCAGGGTTCGAGT
CCCTGAGGGTGTACAA
>Bacteroides_fragilis_YCH46_chr.trna63-LysTTT (2330116-2330044) Lys (TTT) 73 bp Sc: 70.05
GATTCGCTAGCTCAGCAGGTAGAGCACAACACTTTTAATGTTGGGGTCCTGGGTTCGAGC
CCCAGGCGGATCA
>Bacteroides_fragilis_YCH46_chr.trna69-LysTTT (770262-770190) Lys (TTT) 73 bp Sc: 70.05
GATTCGCTAGCTCAGCAGGTAGAGCACAACACTTTTAATGTTGGGGTCCTGGGTTCGAGC
CCCAGGCGGATCA
>Bacteroides_fragilis_YCH46_chr.trna24-MetCAT (5184390-5184463) Met (CAT) 74 bp Sc: 73.67
GGGCTTATAGCTCAGTTGGTTAGAGCAACAGACTCATAATCTGGAGGTCCTAGGTTCAAG
CCCTAGTTGGCCCA
>Bacteroides_fragilis_YCH46_chr.trna20-MetCAT (4717266-4717341) Met (CAT) 76 bp Sc: 74.39
CGCGGAGTGGAGCAGTTGGTAGCTCGTTGGGCTCATAACCCAAAGGTCGTCTGTTCGAGT
CAGGCCTCCGCAACTA
>Bacteroides_fragilis_YCH46_chr.trna70-MetCAT (695614-695538) Met (CAT) 77 bp Sc: 92.99
GGCGGGATAGCTCAGCTGGTTAGAGCGCATGATTCATAATCATGAGGTCCCCGGTTCAAT
CCCGGGTCCCGCTACCA
>Bacteroides_fragilis_YCH46_chr.trna17-PheGAA (3956374-3956446) Phe (GAA) 73 bp Sc: 82.07
GGTGCCATAGCTCAGTTGGTAGAGCAAAGGACTGAAAATCCTTGTGTCCCCGGTTCGATT
CCTGGTGGCACCA
>Bacteroides_fragilis_YCH46_chr.trna71-PheGAA (614218-614146) Phe (GAA) 73 bp Sc: 82.07
GGTGCCATAGCTCAGTTGGTAGAGCAAAGGACTGAAAATCCTTGTGTCCCCGGTTCGATT
CCTGGTGGCACCA
>Bacteroides_fragilis_YCH46_chr.trna72-ProCGG (614133-614061) Pro (CGG) 73 bp Sc: 74.71
CGGAATGTAGCGCAGTTGGTAGCGCACTACGTTCGGGACGTAGGGGTCGGGCGTTCGAGT
CGCCTCATTCCGA
>Bacteroides_fragilis_YCH46_chr.trna68-ProGGG (879366-879289) Pro (GGG) 78 bp Sc: 65.93
CGGGGTGTAGCTCAGCCCGGTTAGAGTACGCGTCTGGGGGGCGTGTGGTCGCTGGTTCGA
ATCCAGTCACCCCGACTA
>Bacteroides_fragilis_YCH46_chr.trna60-ProTGG (2726331-2726254) Pro (TGG) 78 bp Sc: 81.48
CGGGGTGTAGCGCAGTCCGGTTAGCGCACCAGCTTTGGGAGCTGGGGGTCGTGGGTTCGA
ATCCCGCTACCCCGACAA
>Bacteroides_fragilis_YCH46_chr.trna59-ProTGG (2726442-2726365) Pro (TGG) 78 bp Sc: 82.39

CGGGGTGTAGCGCAGTCCGGTTAGCGCACCTGCTTTGGGAGCAGGGGGTCGTGGGTTCGA
ATCCCGCTACCCCGACAA
>Bacteroides_fragilis_YCH46_chr.trna21-SerCGA (4810635-4810722) Ser (CGA) 88 bp Sc: 37.65
GGAAAGATGCCAGAGTGATCGAATGGGCCGGACTCGAAATCCGGTGAACGGCTTGTCCGT
TCCGTGGGTTTGAATCCCACTCTCTCCG
>Bacteroides_fragilis_YCH46_chr.trna18-PheGAA (3972876-3972949) Phe (GAA) 74 bp Sc: 24.48
GCGAGGCTTTTAAAGGTCTTGAGAGCAAAGGACTGAAAATCCTTGTGTCCCCGGTTCGAT
TCCTGGTGGCACCA
>Bacteroides_fragilis_YCH46_chr.trna9-SerGCT (2136557-2136643) Ser (GCT) 87 bp Sc: 59.32
GGAAGTTTGGGTGAGTGGCTGAAACCACCAGTTTGCTAAACTGACGTACGGGTAACCGTA
CCGGGGGTTCGAATCCCCCAGCTTCCG
>Bacteroides_fragilis_YCH46_chr.trna44-SerGGA (4249228-4249141) Ser (GGA) 88 bp Sc: 62.11
GGAGAGATGCTCGAGTGGTTGAAGAGGCACGCCTGGAAAGCGTGTATACGCCAAAAGTGT
ATCGCGGGTTCGAATCCCGCTCTCTCCG
>Bacteroides_fragilis_YCH46_chr.trna7-SerTGA (1584834-1584918) Ser (TGA) 85 bp Sc: 48.26
GGAGAGGTGGCAGAGTGGTCGATTGCGGCGGTCTTGAAAACCGTTGTACTGCGAGGTACC
CGGGGTTCGAATCCCTGTCTCTCCG
>Bacteroides_fragilis_YCH46_chr.trna6-SerTGA (1506263-1506350) Ser (TGA) 88 bp Sc: 48.87
GGAGAGGTGGCAGAGTGGTCGATTGCGGCGGTCTTGAAAACCGTTGTACTGCGAGGTACC
CGGGGTTCGAATCCCTGTCTCTCCGCAA
>Bacteroides_fragilis_YCH46_chr.trna64-ThrCGT (1970190-1970118) Thr (CGT) 73 bp Sc: 79.29
GCCGCTATAGCTCAGTCGGTAGAGCAACGCATTCGTAACGCGTAGGTCGCCAGTTCAAGT
CTGGCTAGCGGCT
>Bacteroides_fragilis_YCH46_chr.trna32-ThrGGT (4795170-4795099) Thr (GGT) 72 bp Sc: 81.61
GCTGTTATAGCTCAGTGGTAGAGCACTTCCTTGGTAAGGAAGAGGTCCCGGGTTCAAATC
CCGGTAACAGCT
>Bacteroides_fragilis_YCH46_chr.trna29-ThrTGT (4795515-4795439) Thr (TGT) 77 bp Sc: 73.56
GCCTCTTTAGCTCAGTTGGCCAGAGCACGTGATTTGTAATCTCGGGGTCGTTGGTTCGAA
TCCGACAAGAGGCTCAA
>Bacteroides_fragilis_YCH46_chr.trna5-ThrTGT (1422325-1422401) Thr (TGT) 77 bp Sc: 73.56
GCCTCTTTAGCTCAGTTGGCCAGAGCACGTGATTTGTAATCTCGGGGTCGTTGGTTCGAA
TCCGACAAGAGGCTCAA
>Bacteroides_fragilis_YCH46_chr.trna65-ThrTGT (1691024-1690948) Thr (TGT) 77 bp Sc: 73.56
GCCTCTTTAGCTCAGTTGGCCAGAGCACGTGATTTGTAATCTCGGGGTCGTTGGTTCGAA
TCCGACAAGAGGCTCAA
>Bacteroides_fragilis_YCH46_chr.trna19-TrpCCA (4095270-4095342) Trp (CCA) 73 bp Sc: 80.46
ACGGGAGTAGCTCAGTTGGTAGAGCACCGGTCTCCAAAACCGGGTGTCGGGAGTTCGAGC
CTCTCCTCCCGTG
>Bacteroides_fragilis_YCH46_chr.trna33-TrpCCA (4793810-4793735) Trp (CCA) 76 bp Sc: 81.08
ACGGGAGTAGCTCAGTTGGTAGAGCACCGGTCTCCAAAACCGGGTGTCGGGAGTTCGAGC
CTCTCCTCCCGTGCTA
>Bacteroides_fragilis_YCH46_chr.trna66-TyrGTA (1157543-1157458) Tyr (GTA) 86 bp Sc: 63.89
GGGCAAATACCAGAGTGGCCAAATGGGGCAGACTGTAAATCTGCTGTCTTTCGACTTCGG
TGGTTCGAATCCATCTTTGCCCACAA
>Bacteroides_fragilis_YCH46_chr.trna30-TyrGTA (4795367-4795282) Tyr (GTA) 86 bp Sc: 65.64
GGGCAAATACCAGAGTGGCCAAATGGGGCAGACTGTAAATCTGCTGGCTTACGCCTTCGG
TGGTTCGAATCCATCTTTGCCCACAA
>Bacteroides_fragilis_YCH46_chr.trna39-ValTAC (4590711-4590637) Val (TAC) 75 bp Sc: 90.62
GGGCGTTTAGCTCAGCTGGTTCAGAGCATCTGCCTTACAAGCAGAGGGTCGGCGGTTCGA
ATCCGTCAACGCCCA
>Bacteroides_fragilis_YCH46_chr.trna40-ValTAC (4590585-4590511) Val (TAC) 75 bp Sc: 90.62
GGGCGTTTAGCTCAGCTGGTTCAGAGCATCTGCCTTACAAGCAGAGGGTCGGCGGTTCGA
ATCCGTCAACGCCCA
>Bacteroides_fragilis_YCH46_chr.trna38-ValTAC (4590815-4590738) Val (TAC) 78 bp Sc: 91.24
GGGCGTTTAGCTCAGCTGGTTCAGAGCATCTGCCTTACAAGCAGAGGGTCGGCGGTTCGA
ATCCGTCAACGCCCACAA
>Bacteroides_vulgatus_ATCC_8482_chr.trna28-AlaGGC (3838191-3838264) Ala (GGC) 74 bp Sc: 64.94
GGGGTATTAGCTCATCTGGCTAGAGCGTTAGACTGGCAGTCTAAAGGTGGCGAGTTCGAG
TCTCGCATGCTCCA
>Bacteroides_vulgatus_ATCC_8482_chr.trna13-AlaTGC (2097198-2097271) Ala (TGC) 74 bp Sc: 84.82
GGGGGATTAGCTCAGCTGGCTAGAGCATCTGCCTTGCACGCAGAGGGTCAACGGTTCGAA
TCCGTTATTCTCCA
>Bacteroides_vulgatus_ATCC_8482_chr.trna15-AlaTGC (2185290-2185363) Ala (TGC) 74 bp Sc: 84.82
GGGGGATTAGCTCAGCTGGCTAGAGCATCTGCCTTGCACGCAGAGGGTCAACGGTTCGAA
TCCGTTATTCTCCA
>Bacteroides_vulgatus_ATCC_8482_chr.trna19-AlaTGC (2589478-2589551) Ala (TGC) 74 bp Sc: 84.82
GGGGGATTAGCTCAGCTGGCTAGAGCATCTGCCTTGCACGCAGAGGGTCAACGGTTCGAA

TCCGTTATTCTCCA
>Bacteroides_vulgatus_ATCC_8482_chr.trna42-AlaTGC (4742891-4742818) Ala (TGC) 74 bp Sc: 84.82
GGGGGATTAGCTCAGCTGGCTAGAGCATCTGCCTTGCACGCAGAGGGTCAACGGTTCGAA
TCCGTTATTCTCCA
>Bacteroides_vulgatus_ATCC_8482_chr.trna44-AlaTGC (4430229-4430156) Ala (TGC) 74 bp Sc: 84.82
GGGGGATTAGCTCAGCTGGCTAGAGCATCTGCCTTGCACGCAGAGGGTCAACGGTTCGAA
TCCGTTATTCTCCA
>Bacteroides_vulgatus_ATCC_8482_chr.trna78-AlaTGC (647594-647521) Ala (TGC) 74 bp Sc: 84.82
GGGGGATTAGCTCAGCTGGCTAGAGCATCTGCCTTGCACGCAGAGGGTCAACGGTTCGAA
TCCGTTATTCTCCA
>Bacteroides_vulgatus_ATCC_8482_chr.trna83-AlaTGC (346495-346422) Ala (TGC) 74 bp Sc: 84.82
GGGGGATTAGCTCAGCTGGCTAGAGCATCTGCCTTGCACGCAGAGGGTCAACGGTTCGAA
TCCGTTATTCTCCA
>Bacteroides_vulgatus_ATCC_8482_chr.trna35-ArgACG (4140540-4140616) Arg (ACG) 77 bp Sc: 54.69
GGTCGCGTAGCTCAACTGAATAGAGTAGCTGACTACGGATCAGCCGGTTACAGGTTTGAA
TCCTGTCGCGATCACAA
>Bacteroides_vulgatus_ATCC_8482_chr.trna33-ArgACG (4140343-4140419) Arg (ACG) 77 bp Sc: 56.43
GGCCGCGTAGCTCAACTGAATAGAGTAGCTGACTACGGATCAGCCGGTTACAGGTTTGAA
TCCTGTCGCGGTCACTA
>Bacteroides_vulgatus_ATCC_8482_chr.trna34-ArgACG (4140446-4140522) Arg (ACG) 77 bp Sc: 56.43
GGCCGCGTAGCTCAACTGAATAGAGTAGCTGACTACGGATCAGCCGGTTACAGGTTTGAA
TCCTGTCGCGGTCACTA
>Bacteroides_vulgatus_ATCC_8482_chr.trna36-ArgCCG (5109298-5109224) Arg (CCG) 75 bp Sc: 52.64
GCACCCGTAGTTCAATGGATAGAATACCGGATTCCGGTTCCGACGATATGAGTTCGATTC
TCATCGGGTGTACAA
>Bacteroides_vulgatus_ATCC_8482_chr.trna7-ArgCCT (1295598-1295672) Arg (CCT) 75 bp Sc: 54.54
GGCCTTGTAGTTCAACGGATAGAATAGAAGTTTCCTAAACTTTAGATAGGGGTTCGATTC
CCCTCGGGGCTACAA
>Bacteroides_vulgatus_ATCC_8482_chr.trna8-ArgCCT (1304273-1304347) Arg (CCT) 75 bp Sc: 54.54
GGCCTTGTAGTTCAACGGATAGAATAGAAGTTTCCTAAACTTTAGATAGGGGTTCGATTC
CCCTCGGGGCTACAA
>Bacteroides_vulgatus_ATCC_8482_chr.trna26-ArgTCT (3460103-3460176) Arg (TCT) 74 bp Sc: 71.16
GGTTCCGTAGCTCAGCTGGATAGAGCAACGCCCTTCTAAGGCGTGGGTCTTGCGTTCGAA
TCGCAACGGAATCA
>Bacteroides_vulgatus_ATCC_8482_chr.trna5-AsnGTT (1133552-1133625) Asn (GTT) 74 bp Sc: 79.42
TCTTCGTTAGCTCAGTCGGTTAGAGCATCTGACTGTTAATCAGAGGGTCCTTGGTTCAAG
TCCAAGACGAAGAG
>Bacteroides_vulgatus_ATCC_8482_chr.trna6-AsnGTT (1133651-1133724) Asn (GTT) 74 bp Sc: 79.42
TCTTCGTTAGCTCAGTCGGTTAGAGCATCTGACTGTTAATCAGAGGGTCCTTGGTTCAAG
TCCAAGACGAAGAG
>Bacteroides_vulgatus_ATCC_8482_chr.trna27-AspGTC (3770740-3770813) Asp (GTC) 74 bp Sc: 85.50
GGTGCGTTAGTTCAGTTGGTTAGAATACATGCCTGTCACGCATGGGGTCACGGGTTCGAG
TCCCGTACGCACCG
>Bacteroides_vulgatus_ATCC_8482_chr.trna40-AspGTC (4751005-4750932) Asp (GTC) 74 bp Sc: 85.50
GGTGCGTTAGTTCAGTTGGTTAGAATACATGCCTGTCACGCATGGGGTCACGGGTTCGAG
TCCCGTACGCACCG
>Bacteroides_vulgatus_ATCC_8482_chr.trna38-AspGTC (4751212-4751136) Asp (GTC) 77 bp Sc: 86.11
GGTGCGTTAGTTCAGTTGGTTAGAATACATGCCTGTCACGCATGGGGTCACGGGTTCGAG
TCCCGTACGCACCGCAA
>Bacteroides_vulgatus_ATCC_8482_chr.trna39-AspGTC (4751108-4751032) Asp (GTC) 77 bp Sc: 86.11
GGTGCGTTAGTTCAGTTGGTTAGAATACATGCCTGTCACGCATGGGGTCACGGGTTCGAG
TCCCGTACGCACCGCAA
>Bacteroides_vulgatus_ATCC_8482_chr.trna54-CysGCA (3649745-3649671) Cys (GCA) 75 bp Sc: 60.03
GGTTCCTTGGATGAGTGGCTTAGTCAGCGGTCTGCAAAACCGTGTACGGCGGTTCGAATC
CGCCAGGAACCTCCA
>Bacteroides_vulgatus_ATCC_8482_chr.trna16-GlnCTG (2499345-2499415) Gln (CTG) 71 bp Sc: 53.75
TGGGATATGGTGTAATGGTAACACAACAGATTCTGGTCCTGTTTTTCCAGGTTCGAATCC
TGGTATCCCAA
>Bacteroides_vulgatus_ATCC_8482_chr.trna9-GlnTTG (1609248-1609318) Gln (TTG) 71 bp Sc: 51.46
TGGACTATGGTGTAATGGTAGCACAACAGGTTTTGGTTCTGTTTGTCCAAGTTCGAATCT
TGGTAGTCCAA
>Bacteroides_vulgatus_ATCC_8482_chr.trna56-GluCTC (3648550-3648475) Glu (CTC) 76 bp Sc: 44.64
GGCGGTATCGTCTAGTGGCCTAGGACGCGGCCCTCTCACGGCTGAAACTCGGGTTCGATT
CCCGGTTCCGCTACCT
>Bacteroides_vulgatus_ATCC_8482_chr.trna68-GluTTC (1862716-1862642) Glu (TTC) 75 bp Sc: 50.05
GGCCCGTTCGTCTATCGGTTAGGACGCAAGATTTTCATTCTTGAAAGGGGGGTTCGATTC
CCCCACGGGCTACAA

>Bacteroides_vulgatus_ATCC_8482_chr.trna63-GluTTC (3129481-3129407) Glu (TTC) 75 bp Sc: 57.73
GGCCCGTTCGTCTATCGGTTAGGACGCAAGATTTTCATTCTTGAAAGGGGGGTTCGATTC
CCCCACGGGCTACCA
>Bacteroides_vulgatus_ATCC_8482_chr.trna30-GlyCCC (3958542-3958639) Gly (CCC) 98 bp Sc: 53.14
GCGGCAGTAGCTCAGTTGGTCACTTTTAATCCGCAGGATTAAAAGGCATCAGCTTCCCAA
GCTGAGGGTCGCGGGTTCGAATCCCGTTTGCCGCTCCA
>Bacteroides_vulgatus_ATCC_8482_chr.trna29-GlyCCC (3943164-3943236) Gly (CCC) 73 bp Sc: 76.85
GCGGCAGTAGCTCAGTTGGTAGAGCATCAGCTTCCCAAGCTGAGGGTCACGAGTTCGAAC
CTCGCTTGCCGCT
>Bacteroides_vulgatus_ATCC_8482_chr.trna59-GlyCCC (3633774-3633700) Gly (CCC) 75 bp Sc: 83.20
GGACGATTAGCTCAGAGGCAGAGCATCAGCTTCCCAAGCTGAGGGTCGCGGGTTCAAGTC
CCGTATCGTCCACGA
>Bacteroides_vulgatus_ATCC_8482_chr.trna45-GlyGCC (4390009-4389937) Gly (GCC) 73 bp Sc: 84.53
GCGGAAATAGCTCAGTTGGTAGAGCATAACCTTGCCAAGGTTAGGGTCGCGAGTTCGAGT
CTCGTTTTCCGCT
>Bacteroides_vulgatus_ATCC_8482_chr.trna48-GlyGCC (4389685-4389613) Gly (GCC) 73 bp Sc: 84.53
GCGGAAATAGCTCAGTTGGTAGAGCATAACCTTGCCAAGGTTAGGGTCGCGAGTTCGAGT
CTCGTTTTCCGCT
>Bacteroides_vulgatus_ATCC_8482_chr.trna69-GlyGCC (1160616-1160544) Gly (GCC) 73 bp Sc: 84.53
GCGGAAATAGCTCAGTTGGTAGAGCATAACCTTGCCAAGGTTAGGGTCGCGAGTTCGAGT
CTCGTTTTCCGCT
>Bacteroides_vulgatus_ATCC_8482_chr.trna50-GlyGCC (4389469-4389394) Gly (GCC) 76 bp Sc: 85.15
GCGGAAATAGCTCAGTTGGTAGAGCATAACCTTGCCAAGGTTAGGGTCGCGAGTTCGAGT
CTCGTTTTCCGCTCAA
>Bacteroides_vulgatus_ATCC_8482_chr.trna73-GlyTCC (1120213-1120141) Gly (TCC) 73 bp Sc: 68.88
GCGGAAGTAGCTCAGTTGATAGAGCATTAGCCTTCCAAGCTGAGGGTCGCGGGTTTGAGC
CCCGTCTTCCGCT
>Bacteroides_vulgatus_ATCC_8482_chr.trna62-GlyTCC (3328976-3328901) Gly (TCC) 76 bp Sc: 69.50
GCGGAAGTAGCTCAGTTGATAGAGCATTAGCCTTCCAAGCTGAGGGTCGCGGGTTTGAGC
CCCGTCTTCCGCTCTA
>Bacteroides_vulgatus_ATCC_8482_chr.trna21-HisGTG (2864348-2864424) His (GTG) 77 bp Sc: 68.54
GTGACTATAGTTCAGTCGGTTAGAGCGTCAGATTGTGGTTCTGAATGTCGTGGGTTCGAA
TCCCACTAGTCACCCCT
>Bacteroides_vulgatus_ATCC_8482_chr.trna12-IleGAT (2097107-2097183) Ile (GAT) 77 bp Sc: 78.68
AGTCCTATAGCTCAGTTGGTTAGAGCGCTACACTGATAATGTAGAGGTCGGCAGTTCAAC
TCTGCCTGGGACTACGA
>Bacteroides_vulgatus_ATCC_8482_chr.trna14-IleGAT (2185199-2185275) Ile (GAT) 77 bp Sc: 78.68
AGTCCTATAGCTCAGTTGGTTAGAGCGCTACACTGATAATGTAGAGGTCGGCAGTTCAAC
TCTGCCTGGGACTACGA
>Bacteroides_vulgatus_ATCC_8482_chr.trna18-IleGAT (2589387-2589463) Ile (GAT) 77 bp Sc: 78.68
AGTCCTATAGCTCAGTTGGTTAGAGCGCTACACTGATAATGTAGAGGTCGGCAGTTCAAC
TCTGCCTGGGACTACGA
>Bacteroides_vulgatus_ATCC_8482_chr.trna41-IleGAT (4742982-4742906) Ile (GAT) 77 bp Sc: 78.68
AGTCCTATAGCTCAGTTGGTTAGAGCGCTACACTGATAATGTAGAGGTCGGCAGTTCAAC
TCTGCCTGGGACTACGA
>Bacteroides_vulgatus_ATCC_8482_chr.trna43-IleGAT (4430320-4430244) Ile (GAT) 77 bp Sc: 78.68
AGTCCTATAGCTCAGTTGGTTAGAGCGCTACACTGATAATGTAGAGGTCGGCAGTTCAAC
TCTGCCTGGGACTACGA
>Bacteroides_vulgatus_ATCC_8482_chr.trna77-IleGAT (647685-647609) Ile (GAT) 77 bp Sc: 78.68
AGTCCTATAGCTCAGTTGGTTAGAGCGCTACACTGATAATGTAGAGGTCGGCAGTTCAAC
TCTGCCTGGGACTACGA
>Bacteroides_vulgatus_ATCC_8482_chr.trna82-IleGAT (346586-346510) Ile (GAT) 77 bp Sc: 78.68
AGTCCTATAGCTCAGTTGGTTAGAGCGCTACACTGATAATGTAGAGGTCGGCAGTTCAAC
TCTGCCTGGGACTACGA
>Bacteroides_vulgatus_ATCC_8482_chr.trna11-LeuCAA (1916377-1916463) Leu (CAA) 87 bp Sc: 50.84
GCCCAGATGGCGGAATCGGTAGACGCGCTGGTCTCAAACACCAGTGGATTCACTTCCATC
CCGGTTCGACCCCGGGTCTGGGTACAA
>Bacteroides_vulgatus_ATCC_8482_chr.trna46-LeuCAG (4389889-4389806) Leu (CAG) 84 bp Sc: 52.96
GCCCAGGTGGCGGAATGGTAGACGCGCACGTTTCAGGTGCGTGTGTCGAGAGACATGCAG
GTTCGAGTCCTGTTCTGGGCACTA
>Bacteroides_vulgatus_ATCC_8482_chr.trna49-LeuCAG (4389565-4389482) Leu (CAG) 84 bp Sc: 56.47
GCCCAGGTGGCGGAATGGTAGACGCGCACGTTTCAGGTGCGTGTGTCGAGAGGCATGCAG
GTTCGAGTCCTGTTCTGGGCACCA
>Bacteroides_vulgatus_ATCC_8482_chr.trna47-LeuGAG (4389785-4389699) Leu (GAG) 87 bp Sc: 49.96
GGAGGAATGGCGGAATTGGTAGACGCGCCACTTTGAGGGGGTGGTGACAATTATGTCGTG
TGAGTTCGAGTCTCATTTCCTTCACGA
>Bacteroides_vulgatus_ATCC_8482_chr.trna70-LeuTAA (1160527-1160440) Leu (TAA) 88 bp Sc: 60.87

GCTCGAATGGTGGAATCGGTAGACACGAGGGACTTAAAATCCCTTGGCCATTGCGGCTGT
GCGGGTTCAAGTCCCGCTTCGAGTACAA
>Bacteroides_vulgatus_ATCC_8482_chr.trna1-LeuTAG (325238-325322) Leu (TAG) 85 bp Sc: 68.96
GCGCGTGTGGCGTAATTGGTAGCCGCGCCAGACTTAGGATCTGGTGTCGTGAGACGTGTA
GGTTCGAGTCCTATCACGCGCACAA
>Bacteroides_vulgatus_ATCC_8482_chr.trna52-LysCTT (3881640-3881565) Lys (CTT) 76 bp Sc: 81.92
GCACTCTTAGCTCAGCTGGTAGAGCAATTGACTCTTAATCAATGGGTCCAGGGTTCGAGT
CCCTGAGGGTGTACAA
>Bacteroides_vulgatus_ATCC_8482_chr.trna51-LysCTT (3881737-3881662) Lys (CTT) 76 bp Sc: 84.08
GCACTCTTAGCTCAGTTGGTAGAGCAACTGACTCTTAATCAGTGGGTCCAGGGTTCGAAT
CCCTGAGGGTGTACGA
>Bacteroides_vulgatus_ATCC_8482_chr.trna53-LysCTT (3881547-3881472) Lys (CTT) 76 bp Sc: 84.08
GCACTCTTAGCTCAGTTGGTAGAGCAACTGACTCTTAATCAGTGGGTCCAGGGTTCGAAT
CCCTGAGGGTGTACAA
>Bacteroides_vulgatus_ATCC_8482_chr.trna25-LysTTT (3442271-3442343) Lys (TTT) 73 bp Sc: 67.31
GATTCGCTAGCTCAGCAGGTAGAGCACAACACTTTTAATGTTGGGGTCTTGGGTTCGAGC
CCCAAGCGGATCA
>Bacteroides_vulgatus_ATCC_8482_chr.trna66-LysTTT (2380170-2380095) Lys (TTT) 76 bp Sc: 75.61
GATTCGCTAGCTCAGCAGGTAGAGCACAACACTTTTAATGTTGGGGTCTTGGGTTCGAGC
CCCAAGCGGATCACCA
>Bacteroides_vulgatus_ATCC_8482_chr.trna3-MetCAT (559150-559225) Met (CAT) 76 bp Sc: 74.39
CGCGGAGTGGAGCAGTTGGTAGCTCGTTGGGCTCATAACCCAAAGGTCGTCTGTTCGAGT
CAGGCCTCCGCAACTA
>Bacteroides_vulgatus_ATCC_8482_chr.trna10-MetCAT (1753704-1753780) Met (CAT) 77 bp Sc: 74.47
GGACTTGTAGCTCAGTTGGTTAGAGCAACAGACTCATAATCTGGAGGTCCCTGGTTCAAG
CCCAGGCTGGTCCACAA
>Bacteroides_vulgatus_ATCC_8482_chr.trna84-MetCAT (212472-212396) Met (CAT) 77 bp Sc: 82.50
GGCGGGATAGCTCAGCTGGTTAGAGCGCATGATTCATAATCATGAGGTCCCCAGTTCAAT
CCTGGGTCCCGCTACAA
>Bacteroides_vulgatus_ATCC_8482_chr.trna85-MetCAT (212336-212263) Met (CAT) 74 bp Sc: 84.69
GGCGGGATAGCTCAGCTGGTTAGAGCGCATGATTCATAATCATGAGGTCCCCGGTTCAAT
CCCGGGTCCCGCTA
>Bacteroides_vulgatus_ATCC_8482_chr.trna4-PheGAA (920578-920653) Phe (GAA) 76 bp Sc: 90.36
GGTGCCATAGCTCAGTTGGTAGAGCAAAGGACTGAAAATCCTTGTGTCCCCGGTTCGATT
CCTGGTGGCACCACCA
>Bacteroides_vulgatus_ATCC_8482_chr.trna67-PheGAA (2084844-2084769) Phe (GAA) 76 bp Sc: 90.36
GGTGCCATAGCTCAGTTGGTAGAGCAAAGGACTGAAAATCCTTGTGTCCCCGGTTCGATT
CCTGGTGGCACCACCA
>Bacteroides_vulgatus_ATCC_8482_chr.trna76-ProCGG (920397-920325) Pro (CGG) 73 bp Sc: 74.71
CGGAATGTAGCGCAGTTGGTAGCGCACTACGTTCGGGACGTAGGGGTCGGGCGTTCGAGT
CGCCTCATTCCGA
>Bacteroides_vulgatus_ATCC_8482_chr.trna65-ProGGG (2471235-2471158) Pro (GGG) 78 bp Sc: 65.93
CGGGGTGTAGCTCAGCCCGGTTAGAGTACGCGTCTGGGGGGCGTGTGGTCGCTGGTTCGA
ATCCAGTCACCCCGACAA
>Bacteroides_vulgatus_ATCC_8482_chr.trna31-ProTGG (4005155-4005229) Pro (TGG) 75 bp Sc: 81.77
CGGGGTGTAGCGCAGTCCGGTTAGCGCACCTGCTTTGGGAGCAGGGGGTCGTGGGTTCGA
ATCCCGCTACCCCGA
>Bacteroides_vulgatus_ATCC_8482_chr.trna32-ProTGG (4018554-4018627) Pro (TGG) 74 bp Sc: 22.00
GATGATATTAATAAGGTTAGCGCACCTGCTTTGGGAGCAGGGGGTCGTGGGTTCGAATCC
CGCTACCCCGACAA
>Bacteroides_vulgatus_ATCC_8482_chr.trna60-ProTGG (3633243-3633137) Pro (TGG) 107 bp Sc: 23.77
GGAGAAATGGCGGAATAGGCAGACGCACCATTAGATGACAGGAAGGCCAACCTTGGATGT
GGCGGACCTGGCAACTCATCCCGGTTCGAATCCGGGTTTCTCCACCA
>Bacteroides_vulgatus_ATCC_8482_chr.trna55-SerGCT (3648992-3648903) Ser (GCT) 90 bp Sc: 59.94
GGAAGTTTGGGTGAGTGGCTGAAACCACCAGTTTGCTAAACTGACGTACGGGTAACCGTA
CCGGGGGTTCGAATCCCCCAGCTTCCGCAA
>Bacteroides_vulgatus_ATCC_8482_chr.trna22-SerGGA (2907991-2908081) Ser (GGA) 91 bp Sc: 58.00
GGAGAGGTGCTCGAGTGGTTGAAGAGGCACGCCTGGAAAGCGTGTATACCCCAAAAGGGT
ATCCGGGGTTCGAATCCCCGTCTCTCCGCAA
>Bacteroides_vulgatus_ATCC_8482_chr.trna17-SerGGA (2522607-2522694) Ser (GGA) 88 bp Sc: 60.38
GGAGAGATGCTCGAGTGGTTGAAGAGGCACGCCTGGAAAGCGTGTAAACGCCAAAAGTGT
TTCGCGGGTTCGAATCCCGCTCTCTCCG
>Bacteroides_vulgatus_ATCC_8482_chr.trna23-SerTGA (3152477-3152564) Ser (TGA) 88 bp Sc: 48.87
GGAGAGGTGGCAGAGTGGTCGATTGCGGCGGTCTTGAAAACCGTTGTACTGCGAGGTACC
CGGGGTTCGAATCCCTGTCTCTCCGCAA
>Bacteroides_vulgatus_ATCC_8482_chr.trna20-SerTGA (2710261-2710348) Ser (TGA) 88 bp Sc: 50.28
GGAGAGGTGGCAGAGTGGTCGATTGCGGCGGTCTTGAAAACCGTTGTACCGCGAGGTACC

CGGGGTTCGAATCCCTGTCTCTCCGCAA
>Bacteroides_vulgatus_ATCC_8482_chr.trna37-ThrCGT (4945142-4945067) Thr (CGT) 76 bp Sc: 86.05
GCCGAAATGGCTCAGTTGGTAGAGCAATTCATTCGTAATGAATAGGTCCCGGGTTCGAGT
CCCGGTTTCGGCTCAA
>Bacteroides_vulgatus_ATCC_8482_chr.trna74-ThrGGT (1120128-1120057) Thr (GGT) 72 bp Sc: 80.28
GCTGTTATAGCTCAGTGGTAGAGCACTTCCTTGGTAAGGAAGAGGTCACGAGTTCAAGTC
TCGTTAACAGCT
>Bacteroides_vulgatus_ATCC_8482_chr.trna2-ThrTGT (501218-501291) Thr (TGT) 74 bp Sc: 72.94
GCCTCTTTAGCTCAGTTGGCCAGAGCACGTGATTTGTAATCTCGGGGTCGTTGGTTCGAA
TCCGACAAGAGGCT
>Bacteroides_vulgatus_ATCC_8482_chr.trna24-ThrTGT (3345414-3345487) Thr (TGT) 74 bp Sc: 72.94
GCCTCTTTAGCTCAGTTGGCCAGAGCACGTGATTTGTAATCTCGGGGTCGTTGGTTCGAA
TCCGACAAGAGGCT
>Bacteroides_vulgatus_ATCC_8482_chr.trna71-ThrTGT (1120446-1120370) Thr (TGT) 77 bp Sc: 73.56
GCCTCTTTAGCTCAGTTGGCCAGAGCACGTGATTTGTAATCTCGGGGTCGTTGGTTCGAA
TCCGACAAGAGGCTCAA
>Bacteroides_vulgatus_ATCC_8482_chr.trna64-TrpCCA (2696640-2696565) Trp (CCA) 76 bp Sc: 81.08
ACGGGAGTAGCTCAGTTGGTAGAGCACCGGTCTCCAAAACCGGGTGTCGGGAGTTCGAGC
CTCTCCTCCCGTGCAA
>Bacteroides_vulgatus_ATCC_8482_chr.trna75-TrpCCA (1118763-1118688) Trp (CCA) 76 bp Sc: 81.08
ACGGGAGTAGCTCAGTTGGTAGAGCACCGGTCTCCAAAACCGGGTGTCGGGAGTTCGAGC
CTCTCCTCCCGTGCTA
>Bacteroides_vulgatus_ATCC_8482_chr.trna72-TyrGTA (1120329-1120247) Tyr (GTA) 83 bp Sc: 63.27
GGGCAAATACCAGAGTGGCCAAATGGGGCAGACTGTAAATCTGCTGTCTTTCGACTTCGG
TGGTTCGAATCCATCTTTGCCCA
>Bacteroides_vulgatus_ATCC_8482_chr.trna61-TyrGTA (3329068-3328983) Tyr (GTA) 86 bp Sc: 63.89
GGGCAAATACCAGAGTGGCCAAATGGGGCAGACTGTAAATCTGCTGTCTTTCGACTTCGG
TGGTTCGAATCCATCTTTGCCCACAA
>Bacteroides_vulgatus_ATCC_8482_chr.trna57-Undet??? (3635321-3635249) Undet (???) 73 bp Sc: 50.49
GGTGATGTAGTTCAGTCAGGCAGAGCACATGGTTCCCATGAGGCCGGCGGTTCGAGTCCG
CCCGTCACCTCTA
>Bacteroides_vulgatus_ATCC_8482_chr.trna58-Undet??? (3633924-3633851) Undet (???) 74 bp Sc: 67.83
GCGGATGTAGCTCAGTCAGGCAGAGCGCATGGTTTTCCGTGAGGTCGGCGGTTCGAGTCC
GCCCGTCCGCACCA
>Bacteroides_vulgatus_ATCC_8482_chr.trna79-ValTAC (587016-586942) Val (TAC) 75 bp Sc: 90.62
GGGCGTTTAGCTCAGCTGGTTCAGAGCATCTGCCTTACAAGCAGAGGGTCGGCGGTTCGA
ATCCGTCAACGCCCA
>Bacteroides_vulgatus_ATCC_8482_chr.trna80-ValTAC (586903-586826) Val (TAC) 78 bp Sc: 91.24
GGGCGTTTAGCTCAGCTGGTTCAGAGCATCTGCCTTACAAGCAGAGGGTCGGCGGTTCGA
ATCCGTCAACGCCCACCG
>Bacteroides_vulgatus_ATCC_8482_chr.trna81-ValTAC (586788-586711) Val (TAC) 78 bp Sc: 91.24
GGGCGTTTAGCTCAGCTGGTTCAGAGCATCTGCCTTACAAGCAGAGGGTCGGCGGTTCGA
ATCCGTCAACGCCCACCG
>Bartonella_bacilliformis_KC583_chr.trna25-AlaGGC (1345411-1345336) Ala (GGC) 76 bp Sc: 86.51
GGGGCTATAGCTCAGCTGGGAGAGCGCTTGCATGGCATGCAAGAGGTCAGCGGTTCGATC
CCGCTTAGCTCCACCA
>Bartonella_bacilliformis_KC583_chr.trna27-AlaTGC (1111896-1111821) Ala (TGC) 76 bp Sc: 91.73
GGGGCCGTAGCTCAGCTGGGAGAGCACCTGCTTTGCAAGCAGGGGGTCGTCGGTTCGATC
CCGTCCGGCTCCACCA
>Bartonella_bacilliformis_KC583_chr.trna3-AlaTGC (253896-253971) Ala (TGC) 76 bp Sc: 91.73
GGGGCCGTAGCTCAGCTGGGAGAGCACCTGCTTTGCAAGCAGGGGGTCGTCGGTTCGATC
CCGTCCGGCTCCACCA
>Bartonella_bacilliformis_KC583_chr.trna41-ArgACG (292219-292143) Arg (ACG) 77 bp Sc: 89.63
GCACCCGTAGCTCAGCTGGATAGAGCACCAGACTACGAATCTGGGGGTCAGGAGTTCGAA
TCTCTTCGGGTGCGCCA
>Bartonella_bacilliformis_KC583_chr.trna12-ArgCCG (628819-628895) Arg (CCG) 77 bp Sc: 89.22
GCACCCGTAGCTCAGCTGGATAGAGCGCTGCCCTCCGAAGGCAGAGGTCACAGATTCGAA
TTCTGTCGGGTGCACCA
>Bartonella_bacilliformis_KC583_chr.trna17-ArgTCT (784570-784646) Arg (TCT) 77 bp Sc: 94.56
GGTCCCGTAGCTCAGCTGGATAGAGCAACGGCCTTCTAAGCCGTGGGTCACAGGTTCGAA
TCCTGTCGGGATCACCA
>Bartonella_bacilliformis_KC583_chr.trna36-AsnGTT (695670-695596) Asn (GTT) 75 bp Sc: 85.91
TCCTCGGTAGCTCAGCGGTAGAGCAACCGGCTGTTAACCGGTTGGTCGCTGGTTCGAATC
CGGCCCGGGGAGCCA
>Bartonella_bacilliformis_KC583_chr.trna37-AsnGTT (667917-667843) Asn (GTT) 75 bp Sc: 85.91
TCCTCGGTAGCTCAGCGGTAGAGCAACCGGCTGTTAACCGGTTGGTCGCTGGTTCGAATC
CGGCCCGGGGAGCCA

>Bartonella_bacilliformis_KC583_chr.trna18-AspGTC (789082-789158) Asp (GTC) 77 bp Sc: 87.35
GCGGGTGTAGCTCAGTTGGTTAGAGTGCTGGCCTGTCACGCCGGAGGTCGCGGGTTCGAG
CCCCGTCACTCGCGCCA
>Bartonella_bacilliformis_KC583_chr.trna14-CysGCA (668105-668178) Cys (GCA) 74 bp Sc: 62.66
GGCCTTGTGGCGGAATGGTTACGCAGAGGACTGCAAATCCTTGTATCCCGGTTCGATTCC
GGGCGAGGCCTCCA
>Bartonella_bacilliformis_KC583_chr.trna15-CysGCA (695858-695931) Cys (GCA) 74 bp Sc: 62.66
GGCCTTGTGGCGGAATGGTTACGCAGAGGACTGCAAATCCTTGTATCCCGGTTCGATTCC
GGGCGAGGCCTCCA
>Bartonella_bacilliformis_KC583_chr.trna23-GlnCTG (1153813-1153886) Gln (CTG) 74 bp Sc: 67.51
TGGGGAATAGTTTAATGGTAGAACAGCGGACTCTGACTCCGTTAATCTTGGTTCGAATCC
AGGTTCCCCAGCCA
>Bartonella_bacilliformis_KC583_chr.trna6-GlnTTG (423255-423329) Gln (TTG) 75 bp Sc: 76.64
TGGGGTATAGCCAAGCGGTAAGGCACCGGTTTTTGGTACCGGCATTCCCTGGTTCGAATC
CAGGTACCCCAGCCA
>Bartonella_bacilliformis_KC583_chr.trna35-GluTTC (754788-754714) Glu (TTC) 75 bp Sc: 67.99
GCGCCCATCGTCTAGCGGTTAGGACATCGCCCTTTCACGGCGGAAACAGGGGTTCGATTC
CCCTTGGGCGTACCA
>Bartonella_bacilliformis_KC583_chr.trna44-GlyGCC (50872-50798) Gly (GCC) 75 bp Sc: 87.83
GCGGGTGTAGCTCAGGGGTAGAGCACAACCTTGCCAAGGTTGGGGTCGTGGGTTCGAATC
CCATCGCCCGCTCCA
>Bartonella_bacilliformis_KC583_chr.trna10-GlyTCC (572451-572524) Gly (TCC) 74 bp Sc: 81.40
GCGGGTATAGCTCAATGGTAGAGCAGCAGCCTTCCAAGCTGAATATGCGGGTTCGATTCC
CGCTACCCGCTCCA
>Bartonella_bacilliformis_KC583_chr.trna5-HisGTG (282066-282142) His (GTG) 77 bp Sc: 84.34
GCGGTTGTAGCTCAGCTGGTTAGAGCGCTGGTTTGTGGCACCAGAGGTCGTAGGTTCAAA
TCCCACCAACCGTACCA
>Bartonella_bacilliformis_KC583_chr.trna2-IleGAT (253697-253773) Ile (GAT) 77 bp Sc: 96.25
GGGCTTGTAGCTCAGTTGGTTAGAGCGCGCGCTTGATAAGCGTGAGGTCGGAGGTTCAAG
TCCTCCCAGGCCCACCA
>Bartonella_bacilliformis_KC583_chr.trna26-IleGAT (1112095-1112019) Ile (GAT) 77 bp Sc: 96.25
GGGCTTGTAGCTCAGTTGGTTAGAGCGCGCGCTTGATAAGCGTGAGGTCGGAGGTTCAAG
TCCTCCCAGGCCCACCA
>Bartonella_bacilliformis_KC583_chr.trna1-LeuCAA (191477-191561) Leu (CAA) 85 bp Sc: 73.37
GCGGGTGTGGTGGAACTGGTAGACGCGCCAGACTCAAAATCTGGTTCCGAGAGGAGTGTC
GGTTCGAGTCCGACCACCCGCACCA
>Bartonella_bacilliformis_KC583_chr.trna22-LeuCAG (1088890-1088974) Leu (CAG) 85 bp Sc: 65.29
GCCCAGATGGCGGAATTGGTAGACGCGCAGGTTTCAGGTATCTGTGCTGCGAAGCGTGGA
GGTTCGAGTCCTCTTCTGGGCACCA
>Bartonella_bacilliformis_KC583_chr.trna13-LeuGAG (662845-662929) Leu (GAG) 85 bp Sc: 66.41
GCGGTCGTGGCGGAATTGGTAGACGCGCAGCGTTGAGGTCGCTGTGGGGCAACTCGTGGA
AGTTCGAGTCTTCTCGACCGCACCA
>Bartonella_bacilliformis_KC583_chr.trna19-LeuTAA (903483-903568) Leu (TAA) 86 bp Sc: 72.65
GCGGACGTGATGAAATTGGTAAACATAGCAGACTTAAAATCTGCCGGTCAAAGACCTTGC
GGGTTCAAGTCCCGCCGCCCGCACCA
>Bartonella_bacilliformis_KC583_chr.trna8-LeuTAG (565396-565478) Leu (TAG) 83 bp Sc: 70.69
GCGGGTGTGGCGAAATTGGTAGACGCATCAGATTTAGGTTCTGGCGGGAGACCGTGGGGG
TTCGAGTCCCTCCACCCGCACCA
>Bartonella_bacilliformis_KC583_chr.trna20-LysCTT (911355-911430) Lys (CTT) 76 bp Sc: 92.83
GGGTGATTAGCTCAGTTGGTAGAGCAGCTGACTCTTAATCAGCGGGTCGTGGGTTCGATC
CCCTCATCACCCACCA
>Bartonella_bacilliformis_KC583_chr.trna31-LysTTT (844319-844244) Lys (TTT) 76 bp Sc: 97.14
GAGCGCGTAGCTCAGCTGGTAGAGCAACTGACTTTTAATCAGTAGGTCCAGGGTTCGAAT
CCCTGCGCGCTCACCA
>Bartonella_bacilliformis_KC583_chr.trna28-MetCAT (1108355-1108279) Met (CAT) 77 bp Sc: 81.76
CGCGGGGTGGAGCAGCCCGGTAGCTCGTCAGGCTCATAACCTGAAGGCCGCAGGTTCAAA
TCCTGCCCCCGCAACCA
>Bartonella_bacilliformis_KC583_chr.trna4-MetCAT (257437-257513) Met (CAT) 77 bp Sc: 81.76
CGCGGGGTGGAGCAGCCCGGTAGCTCGTCAGGCTCATAACCTGAAGGCCGCAGGTTCAAA
TCCTGCCCCCGCAACCA
>Bartonella_bacilliformis_KC583_chr.trna21-MetCAT (1061073-1061149) Met (CAT) 77 bp Sc: 86.65
GGCGGAGTAGCTCAGTAGGTTAGAGCAGAGGAATCATAATCCTTGTGTCGGGGGTTCGAA
TCCCTCCTCCGCTACCA
>Bartonella_bacilliformis_KC583_chr.trna29-MetCAT (996091-996015) Met (CAT) 77 bp Sc: 89.54
GGGCCTGTAGCTCAATTGGTTAGAGCCAGCGGCTCATAACCGCTTGGTTGGGGGTTCGAG
TCCCTCCGGGCCCACCA
>Bartonella_bacilliformis_KC583_chr.trna42-PheGAA (260898-260823) Phe (GAA) 76 bp Sc: 91.21

GCCCAGATAGCTCAGTTGGTAGAGCAGCGGACTGAAAATCCGCGTGTCCGTGGTTCGAAT
CCGCGTCTGGGCACCA
>Bartonella_bacilliformis_KC583_chr.trna38-ProGGG (631293-631217) Pro (GGG) 77 bp Sc: 79.52
CGGAGCGTAGCGCAGCCTGGTAGCGCACTTGACTGGGGGTCAAGGGGTCGTGGGTTCGAA
TCCCGCCGTTCCGACCA
>Bartonella_bacilliformis_KC583_chr.trna16-ProTGG (784065-784141) Pro (TGG) 77 bp Sc: 91.02
CGGAGCGTAGCGCAGTCTGGTAGCGCACCTGATTTGGGATCAGGGGGTCGTAGGTTCGAA
TCCTATCGCTCCGACCA
>Bartonella_bacilliformis_KC583_chr.trna40-SerCGA (298834-298745) Ser (CGA) 90 bp Sc: 80.66
GGAGAGATGGCTGAGTGGTTGAAAGCACCGCACTCGAAATGCGGCATAGGGGCAACTCTA
TCGGGGGTTCAAATCCCTCTCTCTCCGCCA
>Bartonella_bacilliformis_KC583_chr.trna7-SerGCT (454701-454791) Ser (GCT) 91 bp Sc: 70.31
GGAGAGGTGGCCGAGTGGTCGAAGGCGCTCCCCTGCTAAGGGAGTAGGGCTCAAAAGGCT
CTCGAGAGTTCGAATCTCTTCCTCTCCGCCA
>Bartonella_bacilliformis_KC583_chr.trna30-SerGGA (935519-935430) Ser (GGA) 90 bp Sc: 77.71
GGAGAGGTGGCCGAGTGGTTGAAGGCGCACGCCTGGAACGCGTGTATACGGGAAACCGTA
TCGAGGGTTCGAATCCCTCTCTCTCCGCCA
>Bartonella_bacilliformis_KC583_chr.trna32-SerTGA (835359-835270) Ser (TGA) 90 bp Sc: 80.78
GGAGGGGTGGCCGAGCGGTTTAAGGCACCGGTCTTGAAAACCGGCGTGCAGGAGACTGTA
CCGTGGGTTCGAATCCCACCCCCTCCGCCA
>Bartonella_bacilliformis_KC583_chr.trna24-ThrCGT (1240034-1240109) Thr (CGT) 76 bp Sc: 94.45
GCCGCATTAGCTCAGTCGGTAGAGCACATCATTCGTAATGATGGGGTCGCTGGTTCGAAT
CCGGCATGCGGCACCA
>Bartonella_bacilliformis_KC583_chr.trna43-ThrGGT (201897-201823) Thr (GGT) 75 bp Sc: 84.84
GCTGCGGTAGCTCAGTGGCAGAGCACTCCCTTGGTAAGGGAGAGGTCGAGAGTTCAATCC
TCTCTCGCAGCACCA
>Bartonella_bacilliformis_KC583_chr.trna34-ThrTGT (776412-776337) Thr (TGT) 76 bp Sc: 94.13
GCCCTTATAGCTCAGTTGGTAGAGCACCTGATTTGTAATCAGGGGGTCGGGAGTTCGAGT
CTCTCTGGGGGCACCA
>Bartonella_bacilliformis_KC583_chr.trna11-TrpCCA (573914-573989) Trp (CCA) 76 bp Sc: 80.53
AGGGGTATAGCTCAGTTGGTAGAGCGGCGGTCTCCAAAACCGTAGGTCGCGGGTTCAAAT
CCTGCTGCCCCTGCCG
>Bartonella_bacilliformis_KC583_chr.trna9-TyrGTA (572344-572427) Tyr (GTA) 84 bp Sc: 74.29
GGAGGGGTGCCCGAGTGGTTAAAGGGGGCGGACTGTAAATCCGTTGCGTATGCTACGTTG
GTTCGAATCCAACCCCCTCCACCA
>Bartonella_bacilliformis_KC583_chr.trna39-ValGAC (629346-629272) Val (GAC) 75 bp Sc: 87.15
GGGCGTGTAGCTCAGCGGGAGAGCACTACGTTGACATCGTAGAGGTCACAGGTTCAATCC
CTGTCACGCCCACCA
>Bartonella_bacilliformis_KC583_chr.trna33-ValTAC (788859-788784) Val (TAC) 76 bp Sc: 93.45
GGGCGATTAGCTCAGCTGGTAGAGCGCCTCGTTTACACCGAGGATGTCGGGAGTTCGAGT
CTCTCATCGCCCACCA
>Bartonella_quintana_Toulouse_chr.trna1-AlaGGC (121934-122009) Ala (GGC) 76 bp Sc: 87.36
GGGGCTATAGCTCAGTTGGGAGAGCGCTTGCATGGCATGCAAGAGGTCAGCGGTTCGATC
CCGCTTAGCTCCACCA
>Bartonella_quintana_Toulouse_chr.trna20-AlaTGC (1295304-1295229) Ala (TGC) 76 bp Sc: 91.73
GGGGCCGTAGCTCAGCTGGGAGAGCACCTGCTTTGCAAGCAGGGGGTCGTCGGTTCGATC
CCGTCCGGCTCCACCA
>Bartonella_quintana_Toulouse_chr.trna23-AlaTGC (1176590-1176515) Ala (TGC) 76 bp Sc: 91.73
GGGGCCGTAGCTCAGCTGGGAGAGCACCTGCTTTGCAAGCAGGGGGTCGTCGGTTCGATC
CCGTCCGGCTCCACCA
>Bartonella_quintana_Toulouse_chr.trna42-ArgACG (164300-164224) Arg (ACG) 77 bp Sc: 89.63
GCACCCGTAGCTCAGCTGGATAGAGCACCAGACTACGAATCTGGGGGTCAGGAGTTCGAA
TCTCTTCGGGTGCGCCA
>Bartonella_quintana_Toulouse_chr.trna33-ArgCCG (796830-796754) Arg (CCG) 77 bp Sc: 89.22
GCACCCGTAGCTCAGCTGGATAGAGCGCTGCCCTCCGAAGGCAGAGGTCACAGATTCGAA
TTCTGTCGGGTGCACCA
>Bartonella_quintana_Toulouse_chr.trna29-ArgTCT (865164-865088) Arg (TCT) 77 bp Sc: 93.05
GGTCCCGTAGCTCAGCTGGATAGAGCAACGGCCTTCTAAGCCGTGGGTCACAGGTTCGAA
TCCTGTCGGGATCGCCA
>Bartonella_quintana_Toulouse_chr.trna10-AsnGTT (983247-983321) Asn (GTT) 75 bp Sc: 90.41
TCCCCGGTAGCTCAGTGGTAGAGCAACCGGCTGTTAACCGGTTGGTCGCTGGTTCGAATC
CGGCCCGGGGAGCCA
>Bartonella_quintana_Toulouse_chr.trna7-AspGTC (669195-669271) Asp (GTC) 77 bp Sc: 87.35
GCGGGTGTAGCTCAGTTGGTTAGAGTGCTGGCCTGTCACGCCGGAGGTCGCGGGTTCGAG
CCCCGTCACTCGCGCCA
>Bartonella_quintana_Toulouse_chr.trna27-CysGCA (983061-982988) Cys (GCA) 74 bp Sc: 64.93
GGCCTCGTGGCGGAATGGTTACGCAGAGGACTGCAAATCCTTGTATCCCGGTTCGATTCC

GGGCGAGGCCTCCA
>Bartonella_quintana_Toulouse_chr.trna15-GlnCTG (1223802-1223875) Gln (CTG) 74 bp Sc: 65.45
TGGGGAATAGTTTAAGGGTAGAACAACGGACTCTGACTCCGTGAATCTTGGTTCGAATCC
AGGTTCCCCAGCCA
>Bartonella_quintana_Toulouse_chr.trna4-GlnTTG (473911-473985) Gln (TTG) 75 bp Sc: 76.64
TGGGGTATAGCCAAGCGGTAAGGCACCGGTTTTTGGTACCGGCATTCCCTGGTTCGAATC
CAGGTACCCCAGCCA
>Bartonella_quintana_Toulouse_chr.trna9-GluTTC (921688-921762) Glu (TTC) 75 bp Sc: 72.14
GCGCCCATCGTCTAGCGGTTAGGACGCCGCCCTTTCACGGCGGAAACAGGGGTTCGATTC
CCCTTGGGCGTACCA
>Bartonella_quintana_Toulouse_chr.trna16-GlyGCC (1535763-1535837) Gly (GCC) 75 bp Sc: 87.83
GCGGGTGTAGCTCAGGGGTAGAGCACAACCTTGCCAAGGTTGGGGTCGTGGGTTCGAATC
CCATCGCCCGCTCCA
>Bartonella_quintana_Toulouse_chr.trna31-GlyTCC (855787-855714) Gly (TCC) 74 bp Sc: 81.40
GCGGGTATAGCTCAATGGTAGAGCAGCAGCCTTCCAAGCTGAATATGCGGGTTCGATTCC
CGCTACCCGCTCCA
>Bartonella_quintana_Toulouse_chr.trna3-HisGTG (360936-361012) His (GTG) 77 bp Sc: 86.32
GCGGTTGTAGCTCAGTTGGTTAGAGCGCTGGTTTGTGGCACCAGAGGTCGTAGGTTCGAA
TCCCACCAACCGTACCA
>Bartonella_quintana_Toulouse_chr.trna19-IleGAT (1295645-1295569) Ile (GAT) 77 bp Sc: 95.39
GGGCTTGTAGCTCAGCTGGTTAGAGCGCGCGCTTGATAAGCGTGAGGTCGGAGGTTCAAG
TCCTCCCAGGCCCACCA
>Bartonella_quintana_Toulouse_chr.trna22-IleGAT (1176931-1176855) Ile (GAT) 77 bp Sc: 95.39
GGGCTTGTAGCTCAGCTGGTTAGAGCGCGCGCTTGATAAGCGTGAGGTCGGAGGTTCAAG
TCCTCCCAGGCCCACCA
>Bartonella_quintana_Toulouse_chr.trna17-LeuCAA (1367920-1367836) Leu (CAA) 85 bp Sc: 73.37
GCGGGTGTGGTGGAACTGGTAGACGCGCCAGACTCAAAATCTGGTTCCGAGAGGAGTGTC
GGTTCGAGTCCGACCACCCGCACCA
>Bartonella_quintana_Toulouse_chr.trna14-LeuCAG (1160971-1161055) Leu (CAG) 85 bp Sc: 70.84
GCCCAGATGGCGGAATTGGTAGACGCGCAGGTTTCAGGTACCTGTGCCGTAAGGCGTGGA
GGTTCGAGTCCTCTTTGGGCACCA
>Bartonella_quintana_Toulouse_chr.trna6-LeuGAG (665057-665141) Leu (GAG) 85 bp Sc: 61.85
GCGGTCGTGGCGGAAGTGGTAGACGTGCAGCGTTGAGGTCGCTGTGGGGCAACCCGTGGA
AGTTCGAGTCTTCTCGACCGCACCA
>Bartonella_quintana_Toulouse_chr.trna11-LeuTAA (985365-985450) Leu (TAA) 86 bp Sc: 74.47
GCGGATGTGATGAAATCGGTAAACATAGCAGACTTAAAATCTGCCGGTCTAAGACCTTGC
GGGTTCAAGTCCCGCCGTCCGCACCA
>Bartonella_quintana_Toulouse_chr.trna5-LeuTAG (620443-620525) Leu (TAG) 83 bp Sc: 72.86
GCGGATGTGGCGAAATTGGTAGACGCACCAGATTTAGGTTCTGGCGGGAGACTGTGGGGG
TTCGAGTCCCTCCATCCGCACCA
>Bartonella_quintana_Toulouse_chr.trna12-LysCTT (999088-999163) Lys (CTT) 76 bp Sc: 93.11
GGGTGATTAGCTCAGTCGGTAGAGCAGCTGACTCTTAATCAGCGGGTCGTGGGTTCGATT
CCCTCATCACCCACCA
>Bartonella_quintana_Toulouse_chr.trna34-LysTTT (729960-729885) Lys (TTT) 76 bp Sc: 96.01
GAGCGCGTAGCTCAGCTGGTAGAGCAACTGACTTTTAATCAGTAGGTCCAGGGTTCAAAT
CCCTGCGCGCTCACCA
>Bartonella_quintana_Toulouse_chr.trna21-MetCAT (1291713-1291637) Met (CAT) 77 bp Sc: 81.76
CGCGGGGTGGAGCAGCCCGGTAGCTCGTCAGGCTCATAACCTGAAGGCCGCAGGTTCAAA
TCCTGCCCCCGCAACCA
>Bartonella_quintana_Toulouse_chr.trna24-MetCAT (1172999-1172923) Met (CAT) 77 bp Sc: 81.76
CGCGGGGTGGAGCAGCCCGGTAGCTCGTCAGGCTCATAACCTGAAGGCCGCAGGTTCAAA
TCCTGCCCCCGCAACCA
>Bartonella_quintana_Toulouse_chr.trna13-MetCAT (1129906-1129982) Met (CAT) 77 bp Sc: 85.53
GGCGGAGTAGCTCAGTAGGTTAGAGCAGAGGAATCATAATCCTTGTGTCGGGGGTTCAAA
TCCCTCCTCCGCTACCA
>Bartonella_quintana_Toulouse_chr.trna26-MetCAT (1072651-1072575) Met (CAT) 77 bp Sc: 89.54
GGGCCTGTAGCTCAATTGGTTAGAGCCAGCGGCTCATAACCGCTTGGTTGGGGGTTCGAG
TCCCTCCGGGCCCACCA
>Bartonella_quintana_Toulouse_chr.trna39-PheGAA (344708-344633) Phe (GAA) 76 bp Sc: 91.21
GCCCAGATAGCTCAGTTGGTAGAGCAGCGGACTGAAAATCCGCGTGTCCGTGGTTCGAAT
CCGCGTCTGGGCACCA
>Bartonella_quintana_Toulouse_chr.trna36-ProGGG (630460-630384) Pro (GGG) 77 bp Sc: 82.24
CGGAGCGTAGCGCAGCCTGGTAGCGCACTTGACTGGGGGTCAAGGGGTCGTGGGTTCAAA
TCCCGCCGCTCCGACCA
>Bartonella_quintana_Toulouse_chr.trna28-ProTGG (865673-865597) Pro (TGG) 77 bp Sc: 90.17
CGGAGCGTAGCGCAGCCTGGTAGCGCACCTGATTTGGGATCAGGGGGTCGTAGGTTCGAA
TCCTATCGCTCCGACCA

>Bartonella_quintana_Toulouse_chr.trna41-SerCGA (170943-170854) Ser (CGA) 90 bp Sc: 80.66
GGAGAGATGGCTGAGTGGTTGAAAGCACCGCACTCGAAATGCGGCATAGGGGCAACTCTA
TCGGGGGTTCAAATCCCTCTCTCTCCGCCA
>Bartonella_quintana_Toulouse_chr.trna25-SerGCT (1164015-1163925) Ser (GCT) 91 bp Sc: 70.31
GGAGAGGTGGCCGAGTGGTCGAAGGCGCTCCCCTGCTAAGGGAGTAGGGCTCAAAGGCT
CTCGAGAGTTCGAATCTCTTCCTCTCCGCCA
>Bartonella_quintana_Toulouse_chr.trna2-SerGGA (176397-176486) Ser (GGA) 90 bp Sc: 74.44
GGAGAGGTGGCCGAGTGGTTGAAGGCGCACGCCTGGAACGCGTGTATATAGGAAACTATA
TCGAGGGTTCGAATCCCTCTCTCTCCGCCA
>Bartonella_quintana_Toulouse_chr.trna38-SerTGA (622670-622581) Ser (TGA) 90 bp Sc: 73.81
GGATGGGTGGCCGAGCGGTTTAAGGCACCGGTCTTGAAAACCGGCGTGCAGGAGACTGTA
CCGTGGGTTCGAATCCCACCCCCTCCGCCA
>Bartonella_quintana_Toulouse_chr.trna40-ThrCGT (336370-336295) Thr (CGT) 76 bp Sc: 86.50
GCCGCATTAGCTCAGTTGGTAGAGCACATCATTCGTAATGATGGGGTCGCTGGTTCGAAC
CCGGCATGCGGCACCG
>Bartonella_quintana_Toulouse_chr.trna18-ThrGGT (1342282-1342208) Thr (GGT) 75 bp Sc: 84.84
GCTGCGGTAGCTCAGTGGCAGAGCACTCCCTTGGTAAGGGAGAGGTCGAGAGTTCAATCC
TCTCTCGCAGCACCA
>Bartonella_quintana_Toulouse_chr.trna8-ThrTGT (770509-770584) Thr (TGT) 76 bp Sc: 94.13
GCCCTTATAGCTCAGTTGGTAGAGCACCTGATTTGTAATCAGGGGGTCGGGAGTTCGAGT
CTCTCTGGGGGCACCA
>Bartonella_quintana_Toulouse_chr.trna32-TrpCCA (854293-854218) Trp (CCA) 76 bp Sc: 86.54
AGGGGTATAGCTCAGTTGGTAGAGCGGCGGTCTCCAAAACCGCAGGTCGCGGGTTCAAGC
CCTGCTGCCCCTGCCA
>Bartonella_quintana_Toulouse_chr.trna30-TyrGTA (855900-855817) Tyr (GTA) 84 bp Sc: 74.29
GGAGGGGTGCCCGAGTGGTTAAAGGGGGCGGACTGTAAATCCGTTGCGTATGCTACGTTG
GTTCGAATCCAACCCCCTCCACCA
>Bartonella_quintana_Toulouse_chr.trna37-ValGAC (627358-627284) Val (GAC) 75 bp Sc: 87.84
GGGCGTGTAGCTCAGCGGGAGAGCACTACGTTGACATCGTAGGGGTCACAGGTTCGATCC
CTGTCACGCCCACCA
>Bartonella_quintana_Toulouse_chr.trna35-ValTAC (669021-668946) Val (TAC) 76 bp Sc: 92.14
GGGCGATTAGCTCAGCTGGTAGAGCGCTTCGTTTACACCGAAGATGTCGGGAGTTCGAGT
CTCTCATCGCCCACCA
>Bartonella_tribocorum_CIP_105476_chr.trna42-AlaGGC (102580-102505) Ala (GGC) 76 bp Sc: 86.51
GGGGCTATAGCTCAGCTGGGAGAGCGCTTGCATGGCATGCAAGAGGTCAGCGGTTCGATC
CCGCTTAGCTCCACCA
>Bartonella_tribocorum_CIP_105476_chr.trna24-AlaTGC (2060928-2060853) Ala (TGC) 76 bp Sc: 91.73
GGGGCCGTAGCTCAGCTGGGAGAGCACCTGCTTTGCAAGCAGGGGGTCGTCGGTTCGATC
CCGTCCGGCTCCACCA
>Bartonella_tribocorum_CIP_105476_chr.trna28-AlaTGC (1854531-1854456) Ala (TGC) 76 bp Sc: 91.73
GGGGCCGTAGCTCAGCTGGGAGAGCACCTGCTTTGCAAGCAGGGGGTCGTCGGTTCGATC
CCGTCCGGCTCCACCA
>Bartonella_tribocorum_CIP_105476_chr.trna41-ArgACG (178038-177962) Arg (ACG) 77 bp Sc: 89.63
GCACCCGTAGCTCAGCTGGATAGAGCACCAGACTACGAATCTGGGGGTCAGGAGTTCGAA
TCTCTTCGGGTGCGCCA
>Bartonella_tribocorum_CIP_105476_chr.trna8-ArgCCG (1009656-1009732) Arg (CCG) 77 bp Sc: 89.22
GCACCCGTAGCTCAGCTGGATAGAGCGCTGCCCTCCGAAGGCAGAGGTCACAGATTCGAA
TTCTGTCGGGTGCACCA
>Bartonella_tribocorum_CIP_105476_chr.trna34-ArgTCT (1426041-1425965) Arg (TCT) 77 bp Sc: 94.56
GGTCCCGTAGCTCAGCTGGATAGAGCAACGGCCTTCTAAGCCGTGGGTCACAGGTTCGAA
TCCTGTCGGGATCACCA
>Bartonella_tribocorum_CIP_105476_chr.trna15-AsnGTT (1625105-1625179) Asn (GTT) 75 bp Sc: 89.56
TCCCCGGTAGCTCAGCGGTAGAGCAACCGGCTGTTAACCGGTTGGTCGCTGGTTCGAATC
CGGCCCGGGGAGCCA
>Bartonella_tribocorum_CIP_105476_chr.trna36-AspGTC (1305738-1305662) Asp (GTC) 77 bp Sc: 89.12
GCGAGTGTAGCTCAGTTGGTTAGAGTGCTGGCCTGTCACGCCGGAGGTCGCGGGTTCGAG
CCCCGTCACTCGCGCCA
>Bartonella_tribocorum_CIP_105476_chr.trna32-CysGCA (1624918-1624845) Cys (GCA) 74 bp Sc: 62.66
GGCCTTGTGGCGGAATGGTTACGCAGAGGACTGCAAATCCTTGTATCCCGGTTCGATTCC
GGGCGAGGCCTCCA
>Bartonella_tribocorum_CIP_105476_chr.trna18-GlnCTG (1932867-1932940) Gln (CTG) 74 bp Sc: 68.42
TGGGGAATAGTTTAATGGTAGAACAGCGGACTCTGACTCCGTCAATCTTGGTTCGAATCC
AAGTTCCCCAGCCA
>Bartonella_tribocorum_CIP_105476_chr.trna3-GlnTTG (723322-723396) Gln (TTG) 75 bp Sc: 76.64
TGGGGTATAGCCAAGCGGTAAGGCACCGGTTTTTGGTACCGGCATTCCCTGGTTCGAATC
CAGGTACCCCAGCCA
>Bartonella_tribocorum_CIP_105476_chr.trna14-GluTTC (1481765-1481839) Glu (TTC) 75 bp Sc: 67.99

GCGCCCATCGTCTAGCGGTTAGGACATCGCCCTTTCACGGCGGAAACAGGGGTTCGATTC
CCCTTGGGCGTACCA
>Bartonella_tribocorum_CIP_105476_chr.trna21-GlyGCC (2530958-2531032) Gly (GCC) 75 bp Sc: 87.83
GCGGGTGTAGCTCAGGGGTAGAGCACAACCTTGCCAAGGTTGGGGTCGTGGGTTCGAATC
CCATCGCCCGCTCCA
>Bartonella_tribocorum_CIP_105476_chr.trna6-GlyTCC (949166-949239) Gly (TCC) 74 bp Sc: 81.40
GCGGGTATAGCTCAATGGTAGAGCAGCAGCCTTCCAAGCTGAATATGCGGGTTCGATTCC
CGCTACCCGCTCCA
>Bartonella_tribocorum_CIP_105476_chr.trna2-HisGTG (604758-604834) His (GTG) 77 bp Sc: 85.20
GCGGTTGTAGCTCAGTTGGTTAGAGCGCTGGTTTGTGGCACCAGAGGTCGTAGGTTCAAA
TCCCACCAACCGTACCA
>Bartonella_tribocorum_CIP_105476_chr.trna23-IleGAT (2061326-2061250) Ile (GAT) 77 bp Sc: 96.25
GGGCTTGTAGCTCAGTTGGTTAGAGCGCGCGCTTGATAAGCGTGAGGTCGGAGGTTCAAG
TCCTCCCAGGCCCACCA
>Bartonella_tribocorum_CIP_105476_chr.trna27-IleGAT (1854929-1854853) Ile (GAT) 77 bp Sc: 96.25
GGGCTTGTAGCTCAGTTGGTTAGAGCGCGCGCTTGATAAGCGTGAGGTCGGAGGTTCAAG
TCCTCCCAGGCCCACCA
>Bartonella_tribocorum_CIP_105476_chr.trna22-LeuCAA (2277078-2276994) Leu (CAA) 85 bp Sc: 76.87
GCGGGTGTGGTGGAACTGGTAGACGCGCCAGACTCAAAATCTGGTTCCGAAAGGAGTGTC
GGTTCGAGTCCGACCACCCGCACCA
>Bartonella_tribocorum_CIP_105476_chr.trna19-LeuCAG (2018302-2018386) Leu (CAG) 85 bp Sc: 71.46
GCCCAGATGGCGGAATTGGTAGACGCGCAGGTTTCAGGTACCTGTGCCGCAAGGCGTGGA
GGTTCGAGTCCTCTTCCGGGCACCA
>Bartonella_tribocorum_CIP_105476_chr.trna35-LeuGAG (1385101-1385017) Leu (GAG) 85 bp Sc: 70.40
GCGGTCGTGGCGGAATTGGTAGACGCGCAGCGTTGAGGTCGCTGTGGGGCAACCCGTGGA
AGTTCGAGTCTTCTCGACCGCACCA
>Bartonella_tribocorum_CIP_105476_chr.trna16-LeuTAA (1630267-1630352) Leu (TAA) 86 bp Sc: 73.08
GCGGACGTGATGAAAACGGTAAACATAGCAGACTTAAAATCTGCCGGTCGAAGACCTTGC
GGGTTCAAGTCCCGCCGTCCGCACCA
>Bartonella_tribocorum_CIP_105476_chr.trna4-LeuTAG (941712-941794) Leu (TAG) 83 bp Sc: 74.64
GCGGATGTGGCGAAATTGGTAGACGCACCAGATTTAGGTTCTGGCGGGAGACCGTGGGGG
TTCGAGTCCCTCCATCCGCACCA
>Bartonella_tribocorum_CIP_105476_chr.trna31-LysCTT (1641520-1641445) Lys (CTT) 76 bp Sc: 90.73
GGGTGATTAGCTCAGTCGGTAGAGCAGCTGACTCTTAATCAGCGGGTCGTGGGTTCGATC
CCCTCATCACCCACCA
>Bartonella_tribocorum_CIP_105476_chr.trna9-LysTTT (1235938-1236013) Lys (TTT) 76 bp Sc: 97.14
GAGCGCGTAGCTCAGCTGGTAGAGCAACTGACTTTTAATCAGTAGGTCCAGGGTTCGAAT
CCCTGCGCGCTCACCA
>Bartonella_tribocorum_CIP_105476_chr.trna17-MetCAT (1758516-1758592) Met (CAT) 77 bp Sc: 81.69
GGCGGAGTAGCTCAGTAGGTTAGAGCAGAGGAATCATAATCCTTGTGTCGGGGGTTCAAA
TCCCTCCTTCGCTACCA
>Bartonella_tribocorum_CIP_105476_chr.trna25-MetCAT (2057411-2057335) Met (CAT) 77 bp Sc: 81.76
CGCGGGGTGGAGCAGCCCGGTAGCTCGTCAGGCTCATAACCTGAAGGCCGCAGGTTCAAA
TCCTGCCCCCGCAACCA
>Bartonella_tribocorum_CIP_105476_chr.trna29-MetCAT (1851014-1850938) Met (CAT) 77 bp Sc: 81.76
CGCGGGGTGGAGCAGCCCGGTAGCTCGTCAGGCTCATAACCTGAAGGCCGCAGGTTCAAA
TCCTGCCCCCGCAACCA
>Bartonella_tribocorum_CIP_105476_chr.trna30-MetCAT (1725700-1725624) Met (CAT) 77 bp Sc: 89.54
GGGCCTGTAGCTCAATTGGTTAGAGCCAGCGGCTCATAACCGCTTGGTTGGGGGTTCGAG
TCCCTCCGGGCCCACCA
>Bartonella_tribocorum_CIP_105476_chr.trna38-PheGAA (586990-586915) Phe (GAA) 76 bp Sc: 87.70
GCCCAGATAGCTCAGTAGGTAGAGCAGCGGACTGAAAATCCGCGTGTCCGTGGTTCGAAT
CCGCGTCTGGGCACCA
>Bartonella_tribocorum_CIP_105476_chr.trna12-ProGGG (1420725-1420801) Pro (GGG) 77 bp Sc: 84.33
CGGAGCGTAGCGCAGCTTGGTAGCGCACTTGACTGGGGGTCAAGGGGTCGTGGGTTCAAA
TCCCGCCGCTCCGACCA
>Bartonella_tribocorum_CIP_105476_chr.trna33-ProTGG (1426559-1426483) Pro (TGG) 77 bp Sc: 89.64
CGGAGCGTAGCGCAGCCTGGTAGCGCGCCTGATTTGGGATCAGGGGGTCGTAGGTTCGAA
TCCTATCGCTCCGACCA
>Bartonella_tribocorum_CIP_105476_chr.trna40-SerCGA (184929-184840) Ser (CGA) 90 bp Sc: 76.12
GGAGAGGTGGCTGAGTGGTTGAAAGCACCGCACTCGAAATGCGGCATAGGGGTGACTCTA
TCGAGGGTTCAAATCCCTCTCTCTCCGCCA
>Bartonella_tribocorum_CIP_105476_chr.trna26-SerGCT (2025419-2025329) Ser (GCT) 91 bp Sc: 70.31
GGAGAGGTGGCCGAGTGGTCGAAGGCGCTCCCCTGCTAAGGGAGTAGGGCTCAAAAGGCT
CTCGAGAGTTCGAATCTCTTCCTCTCCGCCA
>Bartonella_tribocorum_CIP_105476_chr.trna1-SerGGA (190497-190586) Ser (GGA) 90 bp Sc: 76.03
GGAGAGGTGGCCGAGTGGTTGAAGGCGCACGCCTGGAACGCGTGTATATGGGAAACCATA

TCGAGGGTTCGAATCCCTCTCTCTCCGCCA
>Bartonella_tribocorum_CIP_105476_chr.trna11-SerTGA (1307069-1307158) Ser (TGA) 90 bp Sc: 80.78
GGAGGGGTGGCCGAGCGGTTTAAGGCACCGGTCTTGAAAACCGGCGTGCAGGAGACTGTA
CCGTGGGTTCGAATCCCACCCCCTCCGCCA
>Bartonella_tribocorum_CIP_105476_chr.trna39-ThrCGT (423186-423111) Thr (CGT) 76 bp Sc: 96.55
GCCGCATTAGCTCAGTTGGTAGAGCACATCATTCGTAATGATGGGGTCGCTGGTTCGAAT
CCGGCATGCGGCACCA
>Bartonella_tribocorum_CIP_105476_chr.trna20-ThrGGT (2158311-2158385) Thr (GGT) 75 bp Sc: 86.73
GCTGCGGTAGCTCAGTGGTAGAGCACTCCCTTGGTAAGGGAGAGGTCGAGAGTTCAATCC
TCTCTCGCAGCACCA
>Bartonella_tribocorum_CIP_105476_chr.trna37-ThrTGT (1191859-1191784) Thr (TGT) 76 bp Sc: 94.13
GCCCTTATAGCTCAGTTGGTAGAGCACCTGATTTGTAATCAGGGGGTCGGGAGTTCGAGT
CTCTCTGGGGGCACCA
>Bartonella_tribocorum_CIP_105476_chr.trna7-TrpCCA (950644-950719) Trp (CCA) 76 bp Sc: 88.91
AGGGGTATAGCTCAGTTGGTAGAGCGGCGGTCTCCAAAACCGCAGGTCGCGGGTTCAAGT
CCTGCTGCCCCTGCCA
>Bartonella_tribocorum_CIP_105476_chr.trna5-TyrGTA (949051-949134) Tyr (GTA) 84 bp Sc: 74.29
GGAGGGGTGCCCGAGTGGTTAAAGGGGGCGGACTGTAAATCCGTTGCGTATGCTACGTTG
GTTCGAATCCAACCCCCTCCACCA
>Bartonella_tribocorum_CIP_105476_chr.trna13-ValGAC (1423970-1424044) Val (GAC) 75 bp Sc: 86.72
GGGCGTGTAGCTCAGCGGGAGAGCACTACGTTGACATCGTAGGGGTCACAGGTTCAATCC
CTGTCACGCCCACCA
>Bartonella_tribocorum_CIP_105476_chr.trna10-ValTAC (1305918-1305993) Val (TAC) 76 bp Sc: 93.45
GGGCGATTAGCTCAGCTGGTAGAGCGCCTCGTTTACACCGAGGATGTCGGGAGTTCGAGT
CTCTCATCGCCCACCA
>Brachypodium_distachyon_chr4.trna90-AlaAGC (15207225-15207153) Ala (AGC) 73 bp Sc: 57.37
GGGGATGTAGCTCAGATGGTAGAGCGCTCGCTTAGCATGCGAGAGGCACGGTGATCGATA
CCCCGCATCTCCA
>Brachypodium_distachyon_chr1.trna120-AlaAGC (61989288-61989216) Ala (AGC) 73 bp Sc: 64.02
GGGGATGTAGCTCAGATGGTAGAGCGCTCGCTTAGCATGCGAGAGGCACGGGGATCGATA
CCCCGCATCTCCA
>Brachypodium_distachyon_chr1.trna159-AlaAGC (15937200-15937128) Ala (AGC) 73 bp Sc: 64.02
GGGGATGTAGCTCAGATGGTAGAGCGCTCGCTTAGCATGCGAGAGGCACGGGGATCGATA
CCCCGCATCTCCA
>Brachypodium_distachyon_chr1.trna56-AlaAGC (46913635-46913707) Ala (AGC) 73 bp Sc: 64.02
GGGGATGTAGCTCAGATGGTAGAGCGCTCGCTTAGCATGCGAGAGGCACGGGGATCGATA
CCCCGCATCTCCA
>Brachypodium_distachyon_chr1.trna57-AlaAGC (46920993-46921065) Ala (AGC) 73 bp Sc: 64.02
GGGGATGTAGCTCAGATGGTAGAGCGCTCGCTTAGCATGCGAGAGGCACGGGGATCGATA
CCCCGCATCTCCA
>Brachypodium_distachyon_chr1.trna83-AlaAGC (63855207-63855279) Ala (AGC) 73 bp Sc: 64.02
GGGGATGTAGCTCAGATGGTAGAGCGCTCGCTTAGCATGCGAGAGGCACGGGGATCGATA
CCCCGCATCTCCA
>Brachypodium_distachyon_chr3.trna131-AlaAGC (1139589-1139517) Ala (AGC) 73 bp Sc: 64.02
GGGGATGTAGCTCAGATGGTAGAGCGCTCGCTTAGCATGCGAGAGGCACGGGGATCGATA
CCCCGCATCTCCA
>Brachypodium_distachyon_chr3.trna42-AlaAGC (43981410-43981482) Ala (AGC) 73 bp Sc: 64.02
GGGGATGTAGCTCAGATGGTAGAGCGCTCGCTTAGCATGCGAGAGGCACGGGGATCGATA
CCCCGCATCTCCA
>Brachypodium_distachyon_chr4.trna20-AlaAGC (15151867-15151939) Ala (AGC) 73 bp Sc: 64.02
GGGGATGTAGCTCAGATGGTAGAGCGCTCGCTTAGCATGCGAGAGGCACGGGGATCGATA
CCCCGCATCTCCA
>Brachypodium_distachyon_chr4.trna48-AlaAGC (46553572-46553644) Ala (AGC) 73 bp Sc: 64.02
GGGGATGTAGCTCAGATGGTAGAGCGCTCGCTTAGCATGCGAGAGGCACGGGGATCGATA
CCCCGCATCTCCA
>Brachypodium_distachyon_chr1.trna18-AlaAGC (4590279-4590351) Ala (AGC) 73 bp Sc: 68.12
GGGGATGTAGCTCAGATGGTAGAGCGCTCGCTTAGCATGCGAGAGGTACGGGGATCGATA
CCCCGCATCTCCA
>Brachypodium_distachyon_chr1.trna55-AlaAGC (46901642-46901714) Ala (AGC) 73 bp Sc: 68.12
GGGGATGTAGCTCAGATGGTAGAGCGCTCGCTTAGCATGCGAGAGGTACGGGGATCGATA
CCCCGCATCTCCA
>Brachypodium_distachyon_chr1.trna77-AlaAGC (60974559-60974631) Ala (AGC) 73 bp Sc: 68.12
GGGGATGTAGCTCAGATGGTAGAGCGCTCGCTTAGCATGCGAGAGGTACGGGGATCGATA
CCCCGCATCTCCA
>Brachypodium_distachyon_chr2.trna80-AlaAGC (56635004-56634932) Ala (AGC) 73 bp Sc: 68.12
GGGGATGTAGCTCAGATGGTAGAGCGCTCGCTTAGCATGCGAGAGGTACGGGGATCGATA
CCCCGCATCTCCA

>Brachypodium_distachyon_chr3.trna32-AlaAGC (37543514-37543586) Ala (AGC) 73 bp Sc: 68.12
GGGGATGTAGCTCAGATGGTAGAGCGCTCGCTTAGCATGCGAGAGGTACGGGGATCGATA
CCCCGCATCTCCA
>Brachypodium_distachyon_chr3.trna91-AlaAGC (31494715-31494643) Ala (AGC) 73 bp Sc: 68.12
GGGGATGTAGCTCAGATGGTAGAGCGCTCGCTTAGCATGCGAGAGGTACGGGGATCGATA
CCCCGCATCTCCA
>Brachypodium_distachyon_chr4.trna65-AlaAGC (41853058-41852986) Ala (AGC) 73 bp Sc: 68.12
GGGGATGTAGCTCAGATGGTAGAGCGCTCGCTTAGCATGCGAGAGGTACGGGGATCGATA
CCCCGCATCTCCA
>Brachypodium_distachyon_chr4.trna91-AlaAGC (14430576-14430504) Ala (AGC) 73 bp Sc: 68.12
GGGGATGTAGCTCAGATGGTAGAGCGCTCGCTTAGCATGCGAGAGGTACGGGGATCGATA
CCCCGCATCTCCA
>Brachypodium_distachyon_chr5.trna8-AlaAGC (9795827-9795899) Ala (AGC) 73 bp Sc: 68.12
GGGGATGTAGCTCAGATGGTAGAGCGCTCGCTTAGCATGCGAGAGGTACGGGGATCGATA
CCCCGCATCTCCA
>Brachypodium_distachyon_chr1.trna119-AlaCGC (63261119-63261047) Ala (CGC) 73 bp Sc: 65.14
GGGGGTGTAGCTCATATGGTAGAGCGCTCGCTTCGCATGCGAGAGGCACGGGGTTCGATT
CCCCGCACCTCCA
>Brachypodium_distachyon_chr1.trna27-AlaCGC (13804012-13804084) Ala (CGC) 73 bp Sc: 65.14
GGGGGTGTAGCTCATATGGTAGAGCGCTCGCTTCGCATGCGAGAGGCACGGGGTTCGATT
CCCCGCACCTCCA
>Brachypodium_distachyon_chr3.trna120-AlaCGC (11514917-11514845) Ala (CGC) 73 bp Sc: 65.14
GGGGGTGTAGCTCATATGGTAGAGCGCTCGCTTCGCATGCGAGAGGCACGGGGTTCGATT
CCCCGCACCTCCA
>Brachypodium_distachyon_chr3.trna132-AlaCGC (967505-967433) Ala (CGC) 73 bp Sc: 65.14
GGGGGTGTAGCTCATATGGTAGAGCGCTCGCTTCGCATGCGAGAGGCACGGGGTTCGATT
CCCCGCACCTCCA
>Brachypodium_distachyon_chr5.trna33-AlaCGC (26907234-26907162) Ala (CGC) 73 bp Sc: 65.14
GGGGGTGTAGCTCATATGGTAGAGCGCTCGCTTCGCATGCGAGAGGCACGGGGTTCGATT
CCCCGCACCTCCA
>Brachypodium_distachyon_chr5.trna41-AlaCGC (23991988-23991916) Ala (CGC) 73 bp Sc: 65.14
GGGGGTGTAGCTCATATGGTAGAGCGCTCGCTTCGCATGCGAGAGGCACGGGGTTCGATT
CCCCGCACCTCCA
>Brachypodium_distachyon_chr3.trna37-AlaCGC (40013896-40013968) Ala (CGC) 73 bp Sc: 65.78
GGGGACGTAGCTCATATGGTAGAGCGCTCGCTTCGCATGCGAGAGGCACGGGGTTCGATT
CCCCGCGTCTCCA
>Brachypodium_distachyon_chr3.trna49-AlaCGC (48122984-48123056) Ala (CGC) 73 bp Sc: 65.78
GGGGACGTAGCTCATATGGTAGAGCGCTCGCTTCGCATGCGAGAGGCACGGGGTTCGATT
CCCCGCGTCTCCA
>Brachypodium_distachyon_chr3.trna63-AlaCGC (59117222-59117150) Ala (CGC) 73 bp Sc: 65.78
GGGGACGTAGCTCATATGGTAGAGCGCTCGCTTCGCATGCGAGAGGCACGGGGTTCGATT
CCCCGCGTCTCCA
>Brachypodium_distachyon_chr4.trna39-AlaCGC (36876269-36876341) Ala (CGC) 73 bp Sc: 65.78
GGGGACGTAGCTCATATGGTAGAGCGCTCGCTTCGCATGCGAGAGGCACGGGGTTCGATT
CCCCGCGTCTCCA
>Brachypodium_distachyon_chr5.trna53-AlaCGC (14649994-14649922) Ala (CGC) 73 bp Sc: 65.78
GGGGACGTAGCTCATATGGTAGAGCGCTCGCTTCGCATGCGAGAGGCACGGGGTTCGATT
CCCCGCGTCTCCA
>Brachypodium_distachyon_chr4.trna17-AlaTGC (14646954-14647026) Ala (TGC) 73 bp Sc: 30.80
GGGGATGTAGCTCAATTGGTAGAGTGAATGTCCTGCTTTCATTAGGTATAGGGTTCAATT
CTTCGCATCTCCT
>Brachypodium_distachyon_chr4.trna25-AlaTGC (26001278-26001350) Ala (TGC) 73 bp Sc: 31.06
GGGGATGTAGCTCAATTGGTAGAGTACAAAGTATGCTTCTTTGAGGTATGGGGTTTGATT
CCTCGCGTCTCCA
>Brachypodium_distachyon_chr4.trna114-AlaTGC (401874-401803) Ala (TGC) 72 bp Sc: 43.53
GCGGATGTAGCTCAAATGGTAGAGCGCTCACTTTGCATGCGAGAGGCACTGGGTTCGATC
CCCGCATCTCCA
>Brachypodium_distachyon_chr4.trna57-AlaTGC (45002307-45002235) Ala (TGC) 73 bp Sc: 66.06
GGGGATGTAGCTCAAATGGTAGAGCGCCCGCTTTGCATGCGGGAGGCACGGGGTTCGATC
CCCCGCATCTCCA
>Brachypodium_distachyon_chr4.trna78-AlaTGC (31514755-31514683) Ala (TGC) 73 bp Sc: 66.06
GGGGATGTAGCTCAAATGGTAGAGCGCCCGCTTTGCATGCGGGAGGCACGGGGTTCGATC
CCCCGCATCTCCA
>Brachypodium_distachyon_chr2.trna98-AlaTGC (39835351-39835279) Ala (TGC) 73 bp Sc: 66.31
GGGGATGTAGCTCAAATGGTAGAGCGCTCGCTTTGCATGCGAGAGGCACGGGGTTCGATC
CCCCGCATCTCCA
>Brachypodium_distachyon_chr3.trna30-AlaTGC (36427064-36427136) Ala (TGC) 73 bp Sc: 66.31

GGGGATGTAGCTCAAATGGTAGAGCGCTCGCTTTGCATGCGAGAGGCACGGGGTTCGATC
CCCCGCATCTCCA
>Brachypodium_distachyon_chr3.trna55-AlaTGC (50982203-50982275) Ala (TGC) 73 bp Sc: 66.31
GGGGATGTAGCTCAAATGGTAGAGCGCTCGCTTTGCATGCGAGAGGCACGGGGTTCGATC
CCCCGCATCTCCA
>Brachypodium_distachyon_chr3.trna78-AlaTGC (47790217-47790145) Ala (TGC) 73 bp Sc: 66.31
GGGGATGTAGCTCAAATGGTAGAGCGCTCGCTTTGCATGCGAGAGGCACGGGGTTCGATC
CCCCGCATCTCCA
>Brachypodium_distachyon_chr4.trna49-AlaTGC (47450861-47450933) Ala (TGC) 73 bp Sc: 66.31
GGGGATGTAGCTCAAATGGTAGAGCGCTCGCTTTGCATGCGAGAGGCACGGGGTTCGATC
CCCCGCATCTCCA
>Brachypodium_distachyon_chr5.trna21-AlaTGC (19709538-19709610) Ala (TGC) 73 bp Sc: 66.31
GGGGATGTAGCTCAAATGGTAGAGCGCTCGCTTTGCATGCGAGAGGCACGGGGTTCGATC
CCCCGCATCTCCA
>Brachypodium_distachyon_chr5.trna55-AlaTGC (14079477-14079405) Ala (TGC) 73 bp Sc: 66.31
GGGGATGTAGCTCAAATGGTAGAGCGCTCGCTTTGCATGCGAGAGGCACGGGGTTCGATC
CCCCGCATCTCCA
>Brachypodium_distachyon_chr4.trna19-ArgACG (14650211-14650282) Arg (ACG) 72 bp Sc: 23.11
GGGGATGTAGCTCAATTGGTAGAGCAATCGCAACGCTTGTGTTAGGTTCGGGGTTTGATT
CCTCCATCTCCA
>Brachypodium_distachyon_scaffold_6.trna25-ArgACG (124545-124474) Arg (ACG) 72 bp Sc: 52.32
GGCCTGTAGCTCAGAGGATTAGAGCACGTGGCTACGAACCACGGTGTCGGGGGTTCGAAT
CCCTCCTCGCCA
>Brachypodium_distachyon_chr2.trna97-ArgACG (42002717-42002644) Arg (ACG) 74 bp Sc: 61.16
GGGCCTGTAGCTCAGAGGATTAGAGCACGTGGCTACGAACCACGGTGTCGGGGGTTCGAA
TCCCTCCTCGCCCA
>Brachypodium_distachyon_chr4.trna9-ArgACG (7037102-7037175) Arg (ACG) 74 bp Sc: 61.16
GGGCCTGTAGCTCAGAGGATTAGAGCACGTGGCTACGAACCACGGTGTCGGGGGTTCGAA
TCCCTCCTCGCCCA
>Brachypodium_distachyon_scaffold_6.trna5-ArgACG (106887-106960) Arg (ACG) 74 bp Sc: 61.16
GGGCCTGTAGCTCAGAGGATTAGAGCACGTGGCTACGAACCACGGTGTCGGGGGTTCGAA
TCCCTCCTCGCCCA
>Brachypodium_distachyon_chr1.trna105-ArgACG (69699692-69699620) Arg (ACG) 73 bp Sc: 74.06
GACTCCGTGGCCCAATGGATAAGGCGCTGGTCTACGGAACCAGAGATTCTGGGTTCGATC
CCCAGCGGAGTCG
>Brachypodium_distachyon_chr1.trna167-ArgACG (5798492-5798420) Arg (ACG) 73 bp Sc: 74.06
GACTCCGTGGCCCAATGGATAAGGCGCTGGTCTACGGAACCAGAGATTCTGGGTTCGATC
CCCAGCGGAGTCG
>Brachypodium_distachyon_chr1.trna17-ArgACG (4579162-4579234) Arg (ACG) 73 bp Sc: 74.06
GACTCCGTGGCCCAATGGATAAGGCGCTGGTCTACGGAACCAGAGATTCTGGGTTCGATC
CCCAGCGGAGTCG
>Brachypodium_distachyon_chr2.trna127-ArgACG (5125576-5125504) Arg (ACG) 73 bp Sc: 74.06
GACTCCGTGGCCCAATGGATAAGGCGCTGGTCTACGGAACCAGAGATTCTGGGTTCGATC
CCCAGCGGAGTCG
>Brachypodium_distachyon_chr2.trna17-ArgACG (14465396-14465468) Arg (ACG) 73 bp Sc: 74.06
GACTCCGTGGCCCAATGGATAAGGCGCTGGTCTACGGAACCAGAGATTCTGGGTTCGATC
CCCAGCGGAGTCG
>Brachypodium_distachyon_chr2.trna24-ArgACG (17439705-17439777) Arg (ACG) 73 bp Sc: 74.06
GACTCCGTGGCCCAATGGATAAGGCGCTGGTCTACGGAACCAGAGATTCTGGGTTCGATC
CCCAGCGGAGTCG
>Brachypodium_distachyon_chr2.trna48-ArgACG (46368365-46368437) Arg (ACG) 73 bp Sc: 74.06
GACTCCGTGGCCCAATGGATAAGGCGCTGGTCTACGGAACCAGAGATTCTGGGTTCGATC
CCCAGCGGAGTCG
>Brachypodium_distachyon_chr2.trna54-ArgACG (49964187-49964259) Arg (ACG) 73 bp Sc: 74.06
GACTCCGTGGCCCAATGGATAAGGCGCTGGTCTACGGAACCAGAGATTCTGGGTTCGATC
CCCAGCGGAGTCG
>Brachypodium_distachyon_chr4.trna108-ArgACG (3066089-3066017) Arg (ACG) 73 bp Sc: 74.06
GACTCCGTGGCCCAATGGATAAGGCGCTGGTCTACGGAACCAGAGATTCTGGGTTCGATC
CCCAGCGGAGTCG
>Brachypodium_distachyon_chr5.trna1-ArgACG (385423-385495) Arg (ACG) 73 bp Sc: 74.06
GACTCCGTGGCCCAATGGATAAGGCGCTGGTCTACGGAACCAGAGATTCTGGGTTCGATC
CCCAGCGGAGTCG
>Brachypodium_distachyon_chr5.trna17-ArgACG (15581039-15581111) Arg (ACG) 73 bp Sc: 74.06
GACTCCGTGGCCCAATGGATAAGGCGCTGGTCTACGGAACCAGAGATTCTGGGTTCGATC
CCCAGCGGAGTCG
>Brachypodium_distachyon_chr3.trna82-ArgCCG (44162787-44162715) Arg (CCG) 73 bp Sc: 67.58
GATCGCGTGGCCTAATGGATAAGGCGCTCGCCTCCGGAGCGGGAGATTCTGGGTTCGAGT

CCCAGCGTGGTCG
>Brachypodium_distachyon_chr1.trna147-ArgCCG (28072547-28072475) Arg (CCG) 73 bp Sc: 71.52
GGTCGCGTGGCCTAATGGATAAGGCGCTCGCCTCCGGAGCGGGAGATTCTGGGTTCGAGT
CCCAGCGTGATCG
>Brachypodium_distachyon_chr2.trna89-ArgCCG (50732740-50732668) Arg (CCG) 73 bp Sc: 72.32
GACCGCGTGGCCTAATGGATAAGGCGCTCGCCTCCGGAGCGGGAGATTCTGGGTTCGAGT
CCCAGCGTGGTCG
>Brachypodium_distachyon_chr4.trna44-ArgCCG (43297813-43297885) Arg (CCG) 73 bp Sc: 72.32
GACCGCGTGGCCTAATGGATAAGGCGCTCGCCTCCGGAGCGGGAGATTCTGGGTTCGAGT
CCCAGCGTGGTCG
>Brachypodium_distachyon_chr4.trna61-ArgCCG (43199399-43199327) Arg (CCG) 73 bp Sc: 72.32
GACCGCGTGGCCTAATGGATAAGGCGCTCGCCTCCGGAGCGGGAGATTCTGGGTTCGAGT
CCCAGCGTGGTCG
>Brachypodium_distachyon_chr2.trna61-ArgCCG (53419344-53419416) Arg (CCG) 73 bp Sc: 72.55
GATCATATAGCGAAGTGGATATCGCGTTAGATTCCGAATCTATAGGTCGTGGGTTCGAAT
CCCACTATGATCA
>Brachypodium_distachyon_chr1.trna21-ArgCCT (7415979-7416051) Arg (CCT) 73 bp Sc: 70.66
GCGCTTGTAGCTCAGCGGATAGAGCATTTGTTTCCTAAACAAAAAGTCGAAGGTTCGATC
CCTTCCTAGCGCA
>Brachypodium_distachyon_chr1.trna166-ArgCCT (6010907-6010835) Arg (CCT) 73 bp Sc: 72.16
GCGCCTGTAGCTCAGTGGATAGAGCGTCTGTTTCCTAAGCAGAAGGCCGTAGGTTCGACC
CCTACCTGGCGCG
>Brachypodium_distachyon_chr3.trna107-ArgCCT (22890191-22890119) Arg (CCT) 73 bp Sc: 72.16
GCGCCTGTAGCTCAGTGGATAGAGCGTCTGTTTCCTAAGCAGAAGGCCGTAGGTTCGACC
CCTACCTGGCGCG
>Brachypodium_distachyon_chr4.trna2-ArgCCT (510874-510946) Arg (CCT) 73 bp Sc: 72.16
GCGCCTGTAGCTCAGTGGATAGAGCGTCTGTTTCCTAAGCAGAAGGCCGTAGGTTCGACC
CCTACCTGGCGCG
>Brachypodium_distachyon_chr1.trna23-ArgCCT (9682917-9682989) Arg (CCT) 73 bp Sc: 72.36
GCGCCTGTAGCTCAGTGGATAGAGCGTCCGTTTCCTAAGCGGAAGGCCGTAGGTTCGACC
CCTACCTGGCGCG
>Brachypodium_distachyon_chr1.trna111-ArgCCT (67536386-67536314) Arg (CCT) 73 bp Sc: 72.63
GCGCCTGTAGCTCAGTGGATAGAGCGTCTGTTTCCTAAGCAGAAAGTCGTAGGTTCGACC
CCTACCTGGCGCG
>Brachypodium_distachyon_chr2.trna16-ArgCCT (13630974-13631046) Arg (CCT) 73 bp Sc: 72.63
GCGCCTGTAGCTCAGTGGATAGAGCGTCTGTTTCCTAAGCAGAAAGTCGTAGGTTCGACC
CCTACCTGGCGCG
>Brachypodium_distachyon_chr2.trna37-ArgCCT (25503480-25503552) Arg (CCT) 73 bp Sc: 72.63
GCGCCTGTAGCTCAGTGGATAGAGCGTCTGTTTCCTAAGCAGAAAGTCGTAGGTTCGACC
CCTACCTGGCGCG
>Brachypodium_distachyon_chr3.trna108-ArgCCT (20546990-20546918) Arg (CCT) 73 bp Sc: 76.45
GCGCCTGTAGCTCAGTGGATAGAGCGTCCGTTTCCTAAGCGGAAGGTCGTAGGTTCGACC
CCTACCTGGCGCG
>Brachypodium_distachyon_chr2.trna114-ArgGCG (16637404-16637333) Arg (GCG) 72 bp Sc: 27.18
GGGGATGTAGCTAATTGGTAGAGCAAACGCAGCGCTTGTGTCAGGTACGGGGTTCAATTC
CTCGCATCTCCA
>Brachypodium_distachyon_chr1.trna115-ArgTCG (65079478-65079405) Arg (TCG) 74 bp Sc: 81.86
GACCGCATAGCGCAGTGGATTAGCGCGTCTGACTTCGGATCAGAAGGTCGTGGGTTCGAC
TCCCACTGTGGTCG
>Brachypodium_distachyon_chr1.trna25-ArgTCG (13339445-13339518) Arg (TCG) 74 bp Sc: 81.86
GACCGCATAGCGCAGTGGATTAGCGCGTCTGACTTCGGATCAGAAGGTCGTGGGTTCGAC
TCCCACTGTGGTCG
>Brachypodium_distachyon_chr2.trna43-ArgTCG (34812136-34812209) Arg (TCG) 74 bp Sc: 81.86
GACCGCATAGCGCAGTGGATTAGCGCGTCTGACTTCGGATCAGAAGGTCGTGGGTTCGAC
TCCCACTGTGGTCG
>Brachypodium_distachyon_chr2.trna46-ArgTCG (37819368-37819441) Arg (TCG) 74 bp Sc: 81.86
GACCGCATAGCGCAGTGGATTAGCGCGTCTGACTTCGGATCAGAAGGTCGTGGGTTCGAC
TCCCACTGTGGTCG
>Brachypodium_distachyon_chr2.trna73-ArgTCG (59188324-59188397) Arg (TCG) 74 bp Sc: 81.86
GACCGCATAGCGCAGTGGATTAGCGCGTCTGACTTCGGATCAGAAGGTCGTGGGTTCGAC
TCCCACTGTGGTCG
>Brachypodium_distachyon_chr4.trna100-ArgTCT (7034934-7034864) Arg (TCT) 71 bp Sc: 39.92
GCGTCCATTGTCTAATGGATAGGACAGAGGTCTTCTAAACCTTTGGTATAGGTTCAAATC
CTATTGATTTA
>Brachypodium_distachyon_chr3.trna15-ArgTCT (16309909-16309980) Arg (TCT) 72 bp Sc: 59.70
GCGTCCATTGTCTAATGGATAGGACAGAGGTCTTCTAAACCTTTGGTATAGGTTCAAATC
CTGTTGGACGCA

>Brachypodium_distachyon_chr3.trna111-ArgTCT (17523513-17523441) Arg (TCT) 73 bp Sc: 70.00
GCGCCCATGGCCTAATGGATAAGGCGTCTGACTTCTAATCAGGCGATTGTGGGTTCGAGT
CCCACTGGGCGTG
>Brachypodium_distachyon_chr3.trna14-ArgTCT (15122101-15122173) Arg (TCT) 73 bp Sc: 70.00
GCGCCCATGGCCTAATGGATAAGGCGTCTGACTTCTAATCAGGCGATTGTGGGTTCGAGT
CCCACTGGGCGTG
>Brachypodium_distachyon_chr4.trna43-ArgTCT (42821868-42821940) Arg (TCT) 73 bp Sc: 70.00
GCGCCCATGGCCTAATGGATAAGGCGTCTGACTTCTAATCAGGCGATTGTGGGTTCGAGT
CCCACTGGGCGTG
>Brachypodium_distachyon_chr2.trna78-ArgTCT (58536954-58536882) Arg (TCT) 73 bp Sc: 70.40
GCGCCCGTGGCCTAATGGATAAGGCGTCTGACTTCTAATCAGGCGATTGTGGGTTCGAGT
CCCACCGGGCGTG
>Brachypodium_distachyon_chr3.trna10-ArgTCT (11745707-11745779) Arg (TCT) 73 bp Sc: 70.45
GCGCTCGTGGCCTAATGGATAAGGCATTTGACTTCTAATCAAAGGATTGTGGGTTCGAGT
CCCACCGAGCGTG
>Brachypodium_distachyon_chr4.trna82-ArgTCT (28744617-28744545) Arg (TCT) 73 bp Sc: 70.66
GCGCCCGTGGCCTAATGGATAAGGCGTTTGACTTCTAATCAAGCGATTGTGGGTTCGAGT
CCCACCGGGCGTG
>Brachypodium_distachyon_chr4.trna56-ArgTCT (45400635-45400563) Arg (TCT) 73 bp Sc: 72.36
GCGCCTGTGGCCTAATGGATAAGGCGTTTGACTTCTAATCAAACGATTGTGGGTTCGAGT
CCCACCAGGCGTG
>Brachypodium_distachyon_chr2.trna39-AsnGTT (27777640-27777711) Asn (GTT) 72 bp Sc: 51.20
TCCTCGGTAGCTCACTGGTAGAGCGGTCTATTGTTAACTGACTGGTCGTAGGTTCGAATC
CTACTTGGAGAG
>Brachypodium_distachyon_scaffold_6.trna6-AsnGTT (124147-124217) Asn (GTT) 71 bp Sc: 63.35
TCCTCAGTAGCTCAGTGGTAGAGCGGTCGGCTGTTAACTGACTGGTCGTAGTTCGAATCC
TACTTGGGGAG
>Brachypodium_distachyon_chr1.trna89-AsnGTT (73156645-73156718) Asn (GTT) 74 bp Sc: 75.83
GCTGGAATAGCTCAGTTGGCCAGAGCGTGTGGCTGTTAACCACAAGGTCGGAGGTTCAAG
CCCTCCTTCTAGCG
>Brachypodium_distachyon_chr1.trna91-AsnGTT (74089196-74089269) Asn (GTT) 74 bp Sc: 75.83
GCTGGAATAGCTCAGTTGGCCAGAGCGTGTGGCTGTTAACCACAAGGTCGGAGGTTCAAG
CCCTCCTTCTAGCG
>Brachypodium_distachyon_chr2.trna23-AsnGTT (17212759-17212832) Asn (GTT) 74 bp Sc: 75.83
GCTGGAATAGCTCAGTTGGCCAGAGCGTGTGGCTGTTAACCACAAGGTCGGAGGTTCAAG
CCCTCCTTCTAGCG
>Brachypodium_distachyon_chr2.trna45-AsnGTT (36712861-36712934) Asn (GTT) 74 bp Sc: 75.83
GCTGGAATAGCTCAGTTGGCCAGAGCGTGTGGCTGTTAACCACAAGGTCGGAGGTTCAAG
CCCTCCTTCTAGCG
>Brachypodium_distachyon_chr3.trna93-AsnGTT (30227304-30227231) Asn (GTT) 74 bp Sc: 75.83
GCTGGAATAGCTCAGTTGGCCAGAGCGTGTGGCTGTTAACCACAAGGTCGGAGGTTCAAG
CCCTCCTTCTAGCG
>Brachypodium_distachyon_chr3.trna21-AsnGTT (28276041-28276112) Asn (GTT) 72 bp Sc: 77.16
TCCTCAGTAGCTCAGTGGTAGAGCGGTCGGCTGTTAACTGACTGGTCGTAGGTTCGAATC
CTACTTGGGGAG
>Brachypodium_distachyon_scaffold_6.trna2-AsnGTT (40188-40259) Asn (GTT) 72 bp Sc: 77.16
TCCTCAGTAGCTCAGTGGTAGAGCGGTCGGCTGTTAACTGACTGGTCGTAGGTTCGAATC
CTACTTGGGGAG
>Brachypodium_distachyon_scaffold_6.trna27-AsnGTT (107291-107220) Asn (GTT) 72 bp Sc: 77.16
TCCTCAGTAGCTCAGTGGTAGAGCGGTCGGCTGTTAACTGACTGGTCGTAGGTTCGAATC
CTACTTGGGGAG
>Brachypodium_distachyon_chr1.trna33-AsnGTT (17870882-17870955) Asn (GTT) 74 bp Sc: 77.57
GCTGGAATAGCTCAGTTGGCCAGAGCGTGTGGCTGTTAACCACAAGGTCGGAGGTTCGAG
CCCTCCTTCTAGCG
>Brachypodium_distachyon_chr2.trna100-AsnGTT (34996655-34996582) Asn (GTT) 74 bp Sc: 77.57
GCTGGAATAGCTCAGTTGGCCAGAGCGTGTGGCTGTTAACCACAAGGTCGGAGGTTCGAG
CCCTCCTTCTAGCG
>Brachypodium_distachyon_chr2.trna38-AsnGTT (26257764-26257837) Asn (GTT) 74 bp Sc: 77.57
GCTGGAATAGCTCAGTTGGCCAGAGCGTGTGGCTGTTAACCACAAGGTCGGAGGTTCGAG
CCCTCCTTCTAGCG
>Brachypodium_distachyon_chr4.trna69-AsnGTT (40357031-40356958) Asn (GTT) 74 bp Sc: 77.57
GCTGGAATAGCTCAGTTGGCCAGAGCGTGTGGCTGTTAACCACAAGGTCGGAGGTTCGAG
CCCTCCTTCTAGCG
>Brachypodium_distachyon_chr1.trna122-AsnGTT (61760521-61760448) Asn (GTT) 74 bp Sc: 81.83
GCTGGAATAGCTCAGTTGGTTAGAGCGTGTGGCTGTTAACCACAAGGTCGGAGGTTCAAG
CCCTCCTTCTAGCG
>Brachypodium_distachyon_chr5.trna12-AsnGTT (12050936-12051009) Asn (GTT) 74 bp Sc: 81.83

GCTGGAATAGCTCAGTTGGTTAGAGCGTGTGGCTGTTAACCACAAGGTCGGAGGTTCAAG
CCCTCCTTCTAGCG
>Brachypodium_distachyon_chr2.trna125-AspGTC (6913871-6913798) Asp (GTC) 74 bp Sc: 51.70
GTGATTGTAGCTCAATTGGTCAGAGCACCGCCCTGTCAAGGCGGAAGCTGCGGGTTCGAG
CCTGGCCAGTCCCA
>Brachypodium_distachyon_chr1.trna51-AspGTC (35763915-35763988) Asp (GTC) 74 bp Sc: 61.37
GGGATTGTAGTTCAATTGGTCAGAGCACCGCCCTGTCAAGGCGGAAGCTGCGGGTTCGAG
CCCCGTCAGTCCCG
>Brachypodium_distachyon_chr1.trna68-AspGTC (55191976-55192049) Asp (GTC) 74 bp Sc: 61.37
GGGATTGTAGTTCAATTGGTCAGAGCACCGCCCTGTCAAGGCGGAAGCTGCGGGTTCGAG
CCCCGTCAGTCCCG
>Brachypodium_distachyon_chr3.trna102-AspGTC (28292859-28292786) Asp (GTC) 74 bp Sc: 61.37
GGGATTGTAGTTCAATTGGTCAGAGCACCGCCCTGTCAAGGCGGAAGCTGCGGGTTCGAG
CCCCGTCAGTCCCG
>Brachypodium_distachyon_chr1.trna139-AspGTC (47379825-47379754) Asp (GTC) 72 bp Sc: 69.46
GTCGTTGTAGTATAGTGGTAAGTATTCCCGCCTGTCACGCGGGTGACCCGGGTTCGATCC
CCGGCAACGGCG
>Brachypodium_distachyon_chr1.trna162-AspGTC (12063211-12063140) Asp (GTC) 72 bp Sc: 69.46
GTCGTTGTAGTATAGTGGTAAGTATTCCCGCCTGTCACGCGGGTGACCCGGGTTCGATCC
CCGGCAACGGCG
>Brachypodium_distachyon_chr1.trna32-AspGTC (17689894-17689965) Asp (GTC) 72 bp Sc: 69.46
GTCGTTGTAGTATAGTGGTAAGTATTCCCGCCTGTCACGCGGGTGACCCGGGTTCGATCC
CCGGCAACGGCG
>Brachypodium_distachyon_chr1.trna58-AspGTC (48912751-48912822) Asp (GTC) 72 bp Sc: 69.46
GTCGTTGTAGTATAGTGGTAAGTATTCCCGCCTGTCACGCGGGTGACCCGGGTTCGATCC
CCGGCAACGGCG
>Brachypodium_distachyon_chr1.trna60-AspGTC (50222028-50222099) Asp (GTC) 72 bp Sc: 69.46
GTCGTTGTAGTATAGTGGTAAGTATTCCCGCCTGTCACGCGGGTGACCCGGGTTCGATCC
CCGGCAACGGCG
>Brachypodium_distachyon_chr1.trna73-AspGTC (58370791-58370862) Asp (GTC) 72 bp Sc: 69.46
GTCGTTGTAGTATAGTGGTAAGTATTCCCGCCTGTCACGCGGGTGACCCGGGTTCGATCC
CCGGCAACGGCG
>Brachypodium_distachyon_chr1.trna87-AspGTC (69705565-69705636) Asp (GTC) 72 bp Sc: 69.46
GTCGTTGTAGTATAGTGGTAAGTATTCCCGCCTGTCACGCGGGTGACCCGGGTTCGATCC
CCGGCAACGGCG
>Brachypodium_distachyon_chr2.trna111-AspGTC (18137381-18137310) Asp (GTC) 72 bp Sc: 69.46
GTCGTTGTAGTATAGTGGTAAGTATTCCCGCCTGTCACGCGGGTGACCCGGGTTCGATCC
CCGGCAACGGCG
>Brachypodium_distachyon_chr2.trna117-AspGTC (15346531-15346460) Asp (GTC) 72 bp Sc: 69.46
GTCGTTGTAGTATAGTGGTAAGTATTCCCGCCTGTCACGCGGGTGACCCGGGTTCGATCC
CCGGCAACGGCG
>Brachypodium_distachyon_chr2.trna118-AspGTC (15345496-15345425) Asp (GTC) 72 bp Sc: 69.46
GTCGTTGTAGTATAGTGGTAAGTATTCCCGCCTGTCACGCGGGTGACCCGGGTTCGATCC
CCGGCAACGGCG
>Brachypodium_distachyon_chr2.trna129-AspGTC (3214977-3214906) Asp (GTC) 72 bp Sc: 69.46
GTCGTTGTAGTATAGTGGTAAGTATTCCCGCCTGTCACGCGGGTGACCCGGGTTCGATCC
CCGGCAACGGCG
>Brachypodium_distachyon_chr2.trna25-AspGTC (17694575-17694646) Asp (GTC) 72 bp Sc: 69.46
GTCGTTGTAGTATAGTGGTAAGTATTCCCGCCTGTCACGCGGGTGACCCGGGTTCGATCC
CCGGCAACGGCG
>Brachypodium_distachyon_chr2.trna26-AspGTC (17721420-17721491) Asp (GTC) 72 bp Sc: 69.46
GTCGTTGTAGTATAGTGGTAAGTATTCCCGCCTGTCACGCGGGTGACCCGGGTTCGATCC
CCGGCAACGGCG
>Brachypodium_distachyon_chr2.trna3-AspGTC (1977258-1977329) Asp (GTC) 72 bp Sc: 69.46
GTCGTTGTAGTATAGTGGTAAGTATTCCCGCCTGTCACGCGGGTGACCCGGGTTCGATCC
CCGGCAACGGCG
>Brachypodium_distachyon_chr2.trna57-AspGTC (50722739-50722810) Asp (GTC) 72 bp Sc: 69.46
GTCGTTGTAGTATAGTGGTAAGTATTCCCGCCTGTCACGCGGGTGACCCGGGTTCGATCC
CCGGCAACGGCG
>Brachypodium_distachyon_chr2.trna88-AspGTC (51403877-51403806) Asp (GTC) 72 bp Sc: 69.46
GTCGTTGTAGTATAGTGGTAAGTATTCCCGCCTGTCACGCGGGTGACCCGGGTTCGATCC
CCGGCAACGGCG
>Brachypodium_distachyon_chr2.trna92-AspGTC (49271200-49271129) Asp (GTC) 72 bp Sc: 69.46
GTCGTTGTAGTATAGTGGTAAGTATTCCCGCCTGTCACGCGGGTGACCCGGGTTCGATCC
CCGGCAACGGCG
>Brachypodium_distachyon_chr3.trna88-AspGTC (37178844-37178773) Asp (GTC) 72 bp Sc: 69.46
GTCGTTGTAGTATAGTGGTAAGTATTCCCGCCTGTCACGCGGGTGACCCGGGTTCGATCC

CCGGCAACGGCG
>Brachypodium_distachyon_chr4.trna33-AspGTC (33713673-33713744) Asp (GTC) 72 bp Sc: 69.46
GTCGTTGTAGTATAGTGGTAAGTATTCCCGCCTGTCACGCGGGTGACCCGGGTTCGATCC
CCGGCAACGGCG
>Brachypodium_distachyon_scaffold_6.trna18-AspGTC (456938-457011) Asp (GTC) 74 bp Sc: 77.79
GGGGAAATAGCTCAGTTGGTTAGAGTGCTGGTCTGTCACGCCAGAAGTCGCGGGTTCGAA
CCCCGTTTTCCCCG
>Brachypodium_distachyon_scaffold_6.trna28-AspGTC (40972-40899) Asp (GTC) 74 bp Sc: 77.79
GGGGAAATAGCTCAGTTGGTTAGAGTGCTGGTCTGTCACGCCAGAAGTCGCGGGTTCGAA
CCCCGTTTTCCCCG
>Brachypodium_distachyon_chr1.trna137-CysGCA (48299567-48299484) Cys (GCA) 84 bp Sc: 32.10
CGGTCGATGCCCGAGCGGTTAATGGGGACGGACTGCAAATTTGTTGACAATATGTCTACG
CTGGTTCAAATCCAACTCGGCCCA
>Brachypodium_distachyon_chr3.trna68-CysGCA (56006742-56006672) Cys (GCA) 71 bp Sc: 59.84
GGGTCCATAGCTCAGTGGTAGAGCATTTGACTGCAGATCAAGAGGTCTCTGGTTTGAACC
CGGTGGGCCCT
>Brachypodium_distachyon_scaffold_6.trna8-CysGCA (169528-169598) Cys (GCA) 71 bp Sc: 62.84
GGCGGCATGGCCAAGCGGTAAGGCAGGGGACTGCAAATCCTTTATCCCCAGTTCAAATCT
GGGTGTCGCCT
>Brachypodium_distachyon_chr1.trna143-CysGCA (35766774-35766704) Cys (GCA) 71 bp Sc: 66.65
GGCGGCATGGCCAAGTGGTAAGGCAGGGGACTGCAAATCCTTTATCCCCAGTTCAAATCT
GGGTGCCGCCT
>Brachypodium_distachyon_chr3.trna26-CysGCA (33554811-33554881) Cys (GCA) 71 bp Sc: 66.65
GGCGGCATGGCCAAGTGGTAAGGCAGGGGACTGCAAATCCTTTATCCCCAGTTCAAATCT
GGGTGCCGCCT
>Brachypodium_distachyon_chr1.trna15-CysGCA (4216669-4216740) Cys (GCA) 72 bp Sc: 73.78
GGGTCCATAGCTCAGTGGTAGAGCAATTGACTGCAGATCAATAGGTCACCGGTTCGAACC
TGGTTGGGCCCT
>Brachypodium_distachyon_chr3.trna80-CysGCA (46874580-46874509) Cys (GCA) 72 bp Sc: 77.23
GGGTCCATAGCTCAGTGGTAGAGCAATTGACTGCAGATCAATAGGTCTCCGGTTCGAACC
CGGATGGGCCCT
>Brachypodium_distachyon_chr5.trna23-CysGCA (21137820-21137891) Cys (GCA) 72 bp Sc: 78.18
GGGTCCATAGCTCAGTGGTAGAGCATTTGACTGCAGATCAAGAGGTCACCGGTTCGAACC
CGGTTGGGCCCT
>Brachypodium_distachyon_chr1.trna126-CysGCA (55676885-55676814) Cys (GCA) 72 bp Sc: 79.11
GGGTCCATAGCTCAGTGGTAGAGCAATTGACTGCAGATCAATAGGTCACCGGTTCGAACC
CGGTTGGGCCCT
>Brachypodium_distachyon_chr1.trna127-CysGCA (55675756-55675685) Cys (GCA) 72 bp Sc: 79.11
GGGTCCATAGCTCAGTGGTAGAGCAATTGACTGCAGATCAATAGGTCACCGGTTCGAACC
CGGTTGGGCCCT
>Brachypodium_distachyon_chr1.trna174-CysGCA (1309065-1308994) Cys (GCA) 72 bp Sc: 79.11
GGGTCCATAGCTCAGTGGTAGAGCAATTGACTGCAGATCAATAGGTCACCGGTTCGAACC
CGGTTGGGCCCT
>Brachypodium_distachyon_chr1.trna65-CysGCA (53173251-53173322) Cys (GCA) 72 bp Sc: 79.11
GGGTCCATAGCTCAGTGGTAGAGCAATTGACTGCAGATCAATAGGTCACCGGTTCGAACC
CGGTTGGGCCCT
>Brachypodium_distachyon_chr2.trna130-CysGCA (2951235-2951164) Cys (GCA) 72 bp Sc: 79.11
GGGTCCATAGCTCAGTGGTAGAGCAATTGACTGCAGATCAATAGGTCACCGGTTCGAACC
CGGTTGGGCCCT
>Brachypodium_distachyon_chr2.trna77-CysGCA (58758356-58758285) Cys (GCA) 72 bp Sc: 79.11
GGGTCCATAGCTCAGTGGTAGAGCAATTGACTGCAGATCAATAGGTCACCGGTTCGAACC
CGGTTGGGCCCT
>Brachypodium_distachyon_chr2.trna10-GlnCTG (6431756-6431827) Gln (CTG) 72 bp Sc: 69.43
GGTTCCATGGTCTAGCGGTTAGGACATTGGACTCTGAATCCAGTAACCCGAGTTCAAGTC
TCGGTGGAACCT
>Brachypodium_distachyon_chr2.trna11-GlnCTG (6432429-6432500) Gln (CTG) 72 bp Sc: 71.88
GGTTCCATGGTCTAGTGGTTAGGACATTGGACTCTGAATCCAGTAACCCGAGTTCAAGTC
TCGGTGGAACCT
>Brachypodium_distachyon_chr2.trna40-GlnCTG (31590021-31590092) Gln (CTG) 72 bp Sc: 71.88
GGTTCCATGGTCTAGTGGTTAGGACATTGGACTCTGAATCCAGTAACCCGAGTTCAAGTC
TCGGTGGAACCT
>Brachypodium_distachyon_chr1.trna168-GlnCTG (5044583-5044512) Gln (CTG) 72 bp Sc: 72.36
GGTCCCATGGTCTAGTGGTTAGGACATTGGACTCTGAATCCAGTAACCCGAGTTCAAGTC
TCGGTGGGACCT
>Brachypodium_distachyon_chr1.trna42-GlnCTG (30719971-30720042) Gln (CTG) 72 bp Sc: 72.36
GGTCCCATGGTCTAGTGGTTAGGACATTGGACTCTGAATCCAGTAACCCGAGTTCAAGTC
TCGGTGGGACCT

>Brachypodium_distachyon_chr1.trna48-GlnCTG (34356637-34356708) Gln (CTG) 72 bp Sc: 72.36
GGTCCCATGGTCTAGTGGTTAGGACATTGGACTCTGAATCCAGTAACCCGAGTTCAAGTC
TCGGTGGGACCT
>Brachypodium_distachyon_chr2.trna41-GlnCTG (31986634-31986705) Gln (CTG) 72 bp Sc: 72.36
GGTCCCATGGTCTAGTGGTTAGGACATTGGACTCTGAATCCAGTAACCCGAGTTCAAGTC
TCGGTGGGACCT
>Brachypodium_distachyon_chr2.trna56-GlnCTG (50679753-50679824) Gln (CTG) 72 bp Sc: 72.36
GGTCCCATGGTCTAGTGGTTAGGACATTGGACTCTGAATCCAGTAACCCGAGTTCAAGTC
TCGGTGGGACCT
>Brachypodium_distachyon_chr3.trna57-GlnCTG (54012205-54012276) Gln (CTG) 72 bp Sc: 72.36
GGTCCCATGGTCTAGTGGTTAGGACATTGGACTCTGAATCCAGTAACCCGAGTTCAAGTC
TCGGTGGGACCT
>Brachypodium_distachyon_chr3.trna9-GlnCTG (7108537-7108608) Gln (CTG) 72 bp Sc: 72.36
GGTCCCATGGTCTAGTGGTTAGGACATTGGACTCTGAATCCAGTAACCCGAGTTCAAGTC
TCGGTGGGACCT
>Brachypodium_distachyon_chr4.trna112-GlnCTG (500681-500610) Gln (CTG) 72 bp Sc: 72.36
GGTCCCATGGTCTAGTGGTTAGGACATTGGACTCTGAATCCAGTAACCCGAGTTCAAGTC
TCGGTGGGACCT
>Brachypodium_distachyon_chr5.trna7-GlnTTG (9572094-9572165) Gln (TTG) 72 bp Sc: 49.58
TGGAGTATAGCCAAGTGGTAAGGCATCGGTTTTTGGTACCGGCATGCAAAGGTTCGAATC
CTTTTACTCTAG
>Brachypodium_distachyon_scaffold_6.trna9-GlnTTG (244600-244671) Gln (TTG) 72 bp Sc: 51.09
TGGAGTATAGCCAAGTGGTAAGGCATCGGTTTTTGGTACCGGCATGCAAAGGTTCGAATC
CTTTTACTCCAG
>Brachypodium_distachyon_chr3.trna19-GlnTTG (26072257-26072328) Gln (TTG) 72 bp Sc: 61.03
TGGGGCGTGGCCAAGTGGTAAGGCAGCGGGTTTTGGTCCCGTTACTCGGAGGTTCGAATC
CTTCCGTCCCAG
>Brachypodium_distachyon_scaffold_108.trna2-GlnTTG (3401-3472) Gln (TTG) 72 bp Sc: 61.03
TGGGGCGTGGCCAAGTGGTAAGGCAGCGGGTTTTGGTCCCGTTACTCGGAGGTTCGAATC
CTTCCGTCCCAG
>Brachypodium_distachyon_scaffold_116.trna1-GlnTTG (90-161) Gln (TTG) 72 bp Sc: 61.03
TGGGGCGTGGCCAAGTGGTAAGGCAGCGGGTTTTGGTCCCGTTACTCGGAGGTTCGAATC
CTTCCGTCCCAG
>Brachypodium_distachyon_scaffold_120.trna2-GlnTTG (2921-2850) Gln (TTG) 72 bp Sc: 61.03
TGGGGCGTGGCCAAGTGGTAAGGCAGCGGGTTTTGGTCCCGTTACTCGGAGGTTCGAATC
CTTCCGTCCCAG
>Brachypodium_distachyon_scaffold_127.trna2-GlnTTG (1147-1076) Gln (TTG) 72 bp Sc: 61.03
TGGGGCGTGGCCAAGTGGTAAGGCAGCGGGTTTTGGTCCCGTTACTCGGAGGTTCGAATC
CTTCCGTCCCAG
>Brachypodium_distachyon_scaffold_149.trna2-GlnTTG (2670-2599) Gln (TTG) 72 bp Sc: 61.03
TGGGGCGTGGCCAAGTGGTAAGGCAGCGGGTTTTGGTCCCGTTACTCGGAGGTTCGAATC
CTTCCGTCCCAG
>Brachypodium_distachyon_scaffold_55.trna2-GlnTTG (2352-2423) Gln (TTG) 72 bp Sc: 61.03
TGGGGCGTGGCCAAGTGGTAAGGCAGCGGGTTTTGGTCCCGTTACTCGGAGGTTCGAATC
CTTCCGTCCCAG
>Brachypodium_distachyon_chr3.trna121-GlnTTG (7597830-7597759) Gln (TTG) 72 bp Sc: 68.99
GGTTCCATAGTGTAGTGGCTAGCACCCCAGACTTTGAATCTGGTAACCTGGGTTCGAATC
CCGGTGGGACCT
>Brachypodium_distachyon_chr3.trna79-GlnTTG (47474389-47474318) Gln (TTG) 72 bp Sc: 76.11
GGTTCTATAGTGTAGTGGTTAGCACTCCAGACTTTGAATCTGGCGACCTGGGTTCGAATC
CCGGTAGGACCT
>Brachypodium_distachyon_chr2.trna84-GlnTTG (54637690-54637619) Gln (TTG) 72 bp Sc: 76.32
GGTTTCGTAGTGTAGTGGTTAGCACTCCAGACTTTGAATCTGGCGACCTGGGTTCGAATC
CCGGCGAGACCT
>Brachypodium_distachyon_chr3.trna119-GlnTTG (11703219-11703148) Gln (TTG) 72 bp Sc: 76.52
GGTTCTATGGTGTAGTGGTTAGCACTCCAGACTTTGAATCTGGCGACCTGGGTTCGAATC
CCGGTAGGACCT
>Brachypodium_distachyon_chr5.trna31-GlnTTG (28168258-28168187) Gln (TTG) 72 bp Sc: 76.52
GGTTCTATGGTGTAGTGGTTAGCACTCCAGACTTTGAATCTGGCGACCTGGGTTCGAATC
CCGGTAGGACCT
>Brachypodium_distachyon_chr1.trna5-GlnTTG (1656154-1656225) Gln (TTG) 72 bp Sc: 77.37
GGTTCCATAGTGTAGTGGTTAGCACTCCAGACTTTGAATCTGGCGACCTGGGTTCGAATC
CCGGTGGGACCT
>Brachypodium_distachyon_chr1.trna40-GluCTC (26560508-26560581) Glu (CTC) 74 bp Sc: 57.24
TCCGTCGTAGTCTAGGTGGTTAGGATACTCGGCTCTCACCCGAGAGAACCCGGGTTCAAG
TCCCAGCAATGGAG
>Brachypodium_distachyon_chr4.trna62-GluCTC (42918624-42918552) Glu (CTC) 73 bp Sc: 71.68

TCCGTCGTACTCTAGGTGGTTAGGATACTCGGCTCTCACCCGAGAGACCCGGGTTCAAGT
CCCGGCGACGGAA
>Brachypodium_distachyon_chr1.trna146-GluCTC (29582441-29582369) Glu (CTC) 73 bp Sc: 77.59
TCCGTCGTAGTCTAGGTGGTTAGGATACTCGGCTCTCACCCGAGAGACCCGGGTTCAAGT
CCCGGCGACGGAA
>Brachypodium_distachyon_chr1.trna171-GluCTC (2918684-2918612) Glu (CTC) 73 bp Sc: 77.59
TCCGTCGTAGTCTAGGTGGTTAGGATACTCGGCTCTCACCCGAGAGACCCGGGTTCAAGT
CCCGGCGACGGAA
>Brachypodium_distachyon_chr1.trna41-GluCTC (26573445-26573517) Glu (CTC) 73 bp Sc: 77.59
TCCGTCGTAGTCTAGGTGGTTAGGATACTCGGCTCTCACCCGAGAGACCCGGGTTCAAGT
CCCGGCGACGGAA
>Brachypodium_distachyon_chr1.trna6-GluCTC (2586359-2586431) Glu (CTC) 73 bp Sc: 77.59
TCCGTCGTAGTCTAGGTGGTTAGGATACTCGGCTCTCACCCGAGAGACCCGGGTTCAAGT
CCCGGCGACGGAA
>Brachypodium_distachyon_chr1.trna7-GluCTC (2593290-2593362) Glu (CTC) 73 bp Sc: 77.59
TCCGTCGTAGTCTAGGTGGTTAGGATACTCGGCTCTCACCCGAGAGACCCGGGTTCAAGT
CCCGGCGACGGAA
>Brachypodium_distachyon_chr2.trna1-GluCTC (410517-410589) Glu (CTC) 73 bp Sc: 77.59
TCCGTCGTAGTCTAGGTGGTTAGGATACTCGGCTCTCACCCGAGAGACCCGGGTTCAAGT
CCCGGCGACGGAA
>Brachypodium_distachyon_chr2.trna18-GluCTC (15335622-15335694) Glu (CTC) 73 bp Sc: 77.59
TCCGTCGTAGTCTAGGTGGTTAGGATACTCGGCTCTCACCCGAGAGACCCGGGTTCAAGT
CCCGGCGACGGAA
>Brachypodium_distachyon_chr2.trna32-GluCTC (20333016-20333088) Glu (CTC) 73 bp Sc: 77.59
TCCGTCGTAGTCTAGGTGGTTAGGATACTCGGCTCTCACCCGAGAGACCCGGGTTCAAGT
CCCGGCGACGGAA
>Brachypodium_distachyon_chr2.trna36-GluCTC (24233023-24233095) Glu (CTC) 73 bp Sc: 77.59
TCCGTCGTAGTCTAGGTGGTTAGGATACTCGGCTCTCACCCGAGAGACCCGGGTTCAAGT
CCCGGCGACGGAA
>Brachypodium_distachyon_chr2.trna47-GluCTC (38126383-38126455) Glu (CTC) 73 bp Sc: 77.59
TCCGTCGTAGTCTAGGTGGTTAGGATACTCGGCTCTCACCCGAGAGACCCGGGTTCAAGT
CCCGGCGACGGAA
>Brachypodium_distachyon_chr2.trna49-GluCTC (47121681-47121753) Glu (CTC) 73 bp Sc: 77.59
TCCGTCGTAGTCTAGGTGGTTAGGATACTCGGCTCTCACCCGAGAGACCCGGGTTCAAGT
CCCGGCGACGGAA
>Brachypodium_distachyon_chr2.trna7-GluCTC (4945030-4945102) Glu (CTC) 73 bp Sc: 77.59
TCCGTCGTAGTCTAGGTGGTTAGGATACTCGGCTCTCACCCGAGAGACCCGGGTTCAAGT
CCCGGCGACGGAA
>Brachypodium_distachyon_chr2.trna87-GluCTC (53193837-53193765) Glu (CTC) 73 bp Sc: 77.59
TCCGTCGTAGTCTAGGTGGTTAGGATACTCGGCTCTCACCCGAGAGACCCGGGTTCAAGT
CCCGGCGACGGAA
>Brachypodium_distachyon_chr2.trna9-GluCTC (6419624-6419696) Glu (CTC) 73 bp Sc: 77.59
TCCGTCGTAGTCTAGGTGGTTAGGATACTCGGCTCTCACCCGAGAGACCCGGGTTCAAGT
CCCGGCGACGGAA
>Brachypodium_distachyon_chr3.trna127-GluCTC (4186781-4186709) Glu (CTC) 73 bp Sc: 77.59
TCCGTCGTAGTCTAGGTGGTTAGGATACTCGGCTCTCACCCGAGAGACCCGGGTTCAAGT
CCCGGCGACGGAA
>Brachypodium_distachyon_chr3.trna73-GluCTC (51896138-51896066) Glu (CTC) 73 bp Sc: 77.59
TCCGTCGTAGTCTAGGTGGTTAGGATACTCGGCTCTCACCCGAGAGACCCGGGTTCAAGT
CCCGGCGACGGAA
>Brachypodium_distachyon_chr3.trna83-GluCTC (42511049-42510977) Glu (CTC) 73 bp Sc: 77.59
TCCGTCGTAGTCTAGGTGGTTAGGATACTCGGCTCTCACCCGAGAGACCCGGGTTCAAGT
CCCGGCGACGGAA
>Brachypodium_distachyon_chr1.trna50-GluTTC (35763355-35763427) Glu (TTC) 73 bp Sc: 50.26
GCCCCTATCGTCTAGTGGTTCAGGACATCTCTCTTTCAAGGAGGCAGCGGGGATTCGACT
TCCCCTGGGGGTA
>Brachypodium_distachyon_chr1.trna132-GluTTC (54725024-54724952) Glu (TTC) 73 bp Sc: 50.26
GCCCCTATCGTCTAGTGGTTCAGGACATCTCTCTTTCAAGGAGGCAGCGGGGATTCGACT
TCCCCTGGGGGTA
>Brachypodium_distachyon_chr3.trna100-GluTTC (28293429-28293357) Glu (TTC) 73 bp Sc: 50.26
GCCCCTATCGTCTAGTGGTTCAGGACATCTCTCTTTCAAGGAGGCAGCGGGGATTCGACT
TCCCCTGGGGGTA
>Brachypodium_distachyon_chr5.trna59-GluTTC (9568491-9568420) Glu (TTC) 72 bp Sc: 54.67
GTCCCTTTCATCCAGAGGTTAGGATATCGGTTTTTCATGTCGAAGACACGGGTTCGATTC
CCGTAAGGGATA
>Brachypodium_distachyon_scaffold_6.trna16-GluTTC (329847-329918) Glu (TTC) 72 bp Sc: 58.65
GTCCCTTTCATCCAGAGGTTAGGATATCGGCTTTTCATGTCGAAGACACGGGTTCGATTC

CCGTAAGGGATA
>Brachypodium_distachyon_chr4.trna47-GluTTC (46274830-46274903) Glu (TTC) 74 bp Sc: 63.93
TCCATTGTCGTCCAGCCCGGTTAGGATATCTGGCTTTCACCCAGACGACCCGGGTTCAAA
TCCCGGCAATGGAG
>Brachypodium_distachyon_chr1.trna125-GluTTC (56247642-56247569) Glu (TTC) 74 bp Sc: 64.32
TCCATTGTCGTCCAGCCCGGTTAGGATACCTGGCTTTCACCCAGGCGACCCGGGTTCAAA
TCCCGGCAATGGAG
>Brachypodium_distachyon_chr3.trna115-GluTTC (14148888-14148815) Glu (TTC) 74 bp Sc: 64.32
TCCATTGTCGTCCAGCCCGGTTAGGATACCTGGCTTTCACCCAGGCGACCCGGGTTCAAA
TCCCGGCAATGGAG
>Brachypodium_distachyon_chr3.trna116-GluTTC (14144590-14144517) Glu (TTC) 74 bp Sc: 64.32
TCCATTGTCGTCCAGCCCGGTTAGGATACCTGGCTTTCACCCAGGCGACCCGGGTTCAAA
TCCCGGCAATGGAG
>Brachypodium_distachyon_chr3.trna117-GluTTC (14141900-14141827) Glu (TTC) 74 bp Sc: 64.32
TCCATTGTCGTCCAGCCCGGTTAGGATACCTGGCTTTCACCCAGGCGACCCGGGTTCAAA
TCCCGGCAATGGAG
>Brachypodium_distachyon_chr1.trna20-GluTTC (5186760-5186833) Glu (TTC) 74 bp Sc: 65.35
TCCATTGTCGTCTAGTCCGGTTAGGATATCTGGCTTTCACCCAGACGACCCGGGTTCAAA
TCCCGGCAATGGAA
>Brachypodium_distachyon_chr4.trna3-GluTTC (2077666-2077739) Glu (TTC) 74 bp Sc: 65.35
TCCATTGTCGTCTAGTCCGGTTAGGATATCTGGCTTTCACCCAGACGACCCGGGTTCAAA
TCCCGGCAATGGAA
>Brachypodium_distachyon_chr4.trna88-GluTTC (17066933-17066860) Glu (TTC) 74 bp Sc: 65.35
TCCATTGTCGTCTAGTCCGGTTAGGATATCTGGCTTTCACCCAGACGACCCGGGTTCAAA
TCCCGGCAATGGAA
>Brachypodium_distachyon_chr5.trna37-GluTTC (25542104-25542031) Glu (TTC) 74 bp Sc: 65.35
TCCATTGTCGTCTAGTCCGGTTAGGATATCTGGCTTTCACCCAGACGACCCGGGTTCAAA
TCCCGGCAATGGAA
>Brachypodium_distachyon_chr1.trna112-GluTTC (67455025-67454952) Glu (TTC) 74 bp Sc: 66.61
TCCATCGTCGTCTAGTCCGGTTAGGATATCTGGCTTTCACCCAGACGACCCGGGTTCAAA
TCCCGGCGATGGAA
>Brachypodium_distachyon_chr1.trna109-GlyCCC (67836802-67836732) Gly (CCC) 71 bp Sc: 68.50
GCGCATCTGGTGTAGTGGTATCATAGTACCCTCCCACGGTACTGACCAGGGTTCGATTCC
CTGGATGCGCA
>Brachypodium_distachyon_chr1.trna161-GlyCCC (14510054-14509984) Gly (CCC) 71 bp Sc: 68.50
GCGCATCTGGTGTAGTGGTATCATAGTACCCTCCCACGGTACTGACCAGGGTTCGATTCC
CTGGATGCGCA
>Brachypodium_distachyon_chr1.trna29-GlyCCC (14682083-14682153) Gly (CCC) 71 bp Sc: 68.50
GCGCATCTGGTGTAGTGGTATCATAGTACCCTCCCACGGTACTGACCAGGGTTCGATTCC
CTGGATGCGCA
>Brachypodium_distachyon_chr1.trna80-GlyCCC (62214183-62214253) Gly (CCC) 71 bp Sc: 68.50
GCGCATCTGGTGTAGTGGTATCATAGTACCCTCCCACGGTACTGACCAGGGTTCGATTCC
CTGGATGCGCA
>Brachypodium_distachyon_chr2.trna93-GlyCCC (48559532-48559462) Gly (CCC) 71 bp Sc: 68.50
GCGCATCTGGTGTAGTGGTATCATAGTACCCTCCCACGGTACTGACCAGGGTTCGATTCC
CTGGATGCGCA
>Brachypodium_distachyon_chr3.trna7-GlyCCC (5483812-5483882) Gly (CCC) 71 bp Sc: 68.50
GCGCATCTGGTGTAGTGGTATCATAGTACCCTCCCACGGTACTGACCAGGGTTCGATTCC
CTGGATGCGCA
>Brachypodium_distachyon_chr3.trna85-GlyCCC (38548253-38548183) Gly (CCC) 71 bp Sc: 68.50
GCGCATCTGGTGTAGTGGTATCATAGTACCCTCCCACGGTACTGACCAGGGTTCGATTCC
CTGGATGCGCA
>Brachypodium_distachyon_chr4.trna104-GlyCCC (4667360-4667290) Gly (CCC) 71 bp Sc: 68.50
GCGCATCTGGTGTAGTGGTATCATAGTACCCTCCCACGGTACTGACCAGGGTTCGATTCC
CTGGATGCGCA
>Brachypodium_distachyon_chr4.trna72-GlyCCC (35285672-35285602) Gly (CCC) 71 bp Sc: 68.50
GCGCATCTGGTGTAGTGGTATCATAGTACCCTCCCACGGTACTGACCAGGGTTCGATTCC
CTGGATGCGCA
>Brachypodium_distachyon_chr4.trna113-GlyGCC (407298-407228) Gly (GCC) 71 bp Sc: 54.35
GCGAGTGTAGTTCAATGGTAAACCATCTCCTTGCCAAGGAGAAGATACGGGTTCGATTCC
CGCCGCTCGCC
>Brachypodium_distachyon_chr5.trna58-GlyGCC (9608389-9608319) Gly (GCC) 71 bp Sc: 55.65
GCGAGCGTAGTTCAATGGTAAAACATCTCCTTGCCAAGGAGAAGATACGGGTTTGATTCC
CGCCGCTCGCC
>Brachypodium_distachyon_chr1.trna175-GlyGCC (778570-778500) Gly (GCC) 71 bp Sc: 55.92
GCAAGTGTAGTTCAATGGTAAAACATCTCCTTGCCAAGGAGAAGATACGGGTTCGATTCC
CGCCGCTCGCC

>Brachypodium_distachyon_chr3.trna98-GlyGCC (28296252-28296182) Gly (GCC) 71 bp Sc: 61.61
GCGAGCGTAGTTCAATGGTAAAACATCTCCTTGCCAAGGAGAAGATACGGGTTCGATTCC
CGCCGCTTGCC
>Brachypodium_distachyon_chr1.trna130-GlyGCC (54727850-54727780) Gly (GCC) 71 bp Sc: 62.56
GCGAGCGTAGTTCAATGGTAAAACATCTCCTTGCCAAGGAGAAGATACGGGTTCGATTCC
CGCCGCTCGCC
>Brachypodium_distachyon_chr1.trna92-GlyGCC (74163058-74163128) Gly (GCC) 71 bp Sc: 62.56
GCGAGCGTAGTTCAATGGTAAAACATCTCCTTGCCAAGGAGAAGATACGGGTTCGATTCC
CGCCGCTCGCC
>Brachypodium_distachyon_chr2.trna116-GlyGCC (16388847-16388777) Gly (GCC) 71 bp Sc: 62.56
GCGAGCGTAGTTCAATGGTAAAACATCTCCTTGCCAAGGAGAAGATACGGGTTCGATTCC
CGCCGCTCGCC
>Brachypodium_distachyon_chr3.trna27-GlyGCC (35358440-35358510) Gly (GCC) 71 bp Sc: 65.59
GCACCAGTGGTCTAGTGGTAGAATAGTACCCTGCCACTGTACAGACCCGGGTTCGATTCC
CGGCTGGTGCA
>Brachypodium_distachyon_chr5.trna45-GlyGCC (18805152-18805082) Gly (GCC) 71 bp Sc: 67.64
GCACCAGTGGTCTAGTGGCAGAATAGTACCCTGCCACGGTACAGACCCGGGTTCGATTCC
CGGCTGGTGCA
>Brachypodium_distachyon_chr1.trna101-GlyGCC (72602209-72602139) Gly (GCC) 71 bp Sc: 71.56
GCACCAGTGGTCTAGTGGTAGAATAGTACCCTGCCACGGTACAGACCCGGGTTCGATTCC
CGGCTGGTGCA
>Brachypodium_distachyon_chr1.trna102-GlyGCC (72597467-72597397) Gly (GCC) 71 bp Sc: 71.56
GCACCAGTGGTCTAGTGGTAGAATAGTACCCTGCCACGGTACAGACCCGGGTTCGATTCC
CGGCTGGTGCA
>Brachypodium_distachyon_chr1.trna117-GlyGCC (64237306-64237236) Gly (GCC) 71 bp Sc: 71.56
GCACCAGTGGTCTAGTGGTAGAATAGTACCCTGCCACGGTACAGACCCGGGTTCGATTCC
CGGCTGGTGCA
>Brachypodium_distachyon_chr1.trna129-GlyGCC (54918233-54918163) Gly (GCC) 71 bp Sc: 71.56
GCACCAGTGGTCTAGTGGTAGAATAGTACCCTGCCACGGTACAGACCCGGGTTCGATTCC
CGGCTGGTGCA
>Brachypodium_distachyon_chr1.trna163-GlyGCC (10042289-10042219) Gly (GCC) 71 bp Sc: 71.56
GCACCAGTGGTCTAGTGGTAGAATAGTACCCTGCCACGGTACAGACCCGGGTTCGATTCC
CGGCTGGTGCA
>Brachypodium_distachyon_chr1.trna169-GlyGCC (4075009-4074939) Gly (GCC) 71 bp Sc: 71.56
GCACCAGTGGTCTAGTGGTAGAATAGTACCCTGCCACGGTACAGACCCGGGTTCGATTCC
CGGCTGGTGCA
>Brachypodium_distachyon_chr1.trna47-GlyGCC (32957780-32957850) Gly (GCC) 71 bp Sc: 71.56
GCACCAGTGGTCTAGTGGTAGAATAGTACCCTGCCACGGTACAGACCCGGGTTCGATTCC
CGGCTGGTGCA
>Brachypodium_distachyon_chr1.trna52-GlyGCC (42995888-42995958) Gly (GCC) 71 bp Sc: 71.56
GCACCAGTGGTCTAGTGGTAGAATAGTACCCTGCCACGGTACAGACCCGGGTTCGATTCC
CGGCTGGTGCA
>Brachypodium_distachyon_chr1.trna59-GlyGCC (49670122-49670192) Gly (GCC) 71 bp Sc: 71.56
GCACCAGTGGTCTAGTGGTAGAATAGTACCCTGCCACGGTACAGACCCGGGTTCGATTCC
CGGCTGGTGCA
>Brachypodium_distachyon_chr1.trna88-GlyGCC (72190585-72190655) Gly (GCC) 71 bp Sc: 71.56
GCACCAGTGGTCTAGTGGTAGAATAGTACCCTGCCACGGTACAGACCCGGGTTCGATTCC
CGGCTGGTGCA
>Brachypodium_distachyon_chr2.trna128-GlyGCC (3857235-3857165) Gly (GCC) 71 bp Sc: 71.56
GCACCAGTGGTCTAGTGGTAGAATAGTACCCTGCCACGGTACAGACCCGGGTTCGATTCC
CGGCTGGTGCA
>Brachypodium_distachyon_chr3.trna124-GlyGCC (6185863-6185793) Gly (GCC) 71 bp Sc: 71.56
GCACCAGTGGTCTAGTGGTAGAATAGTACCCTGCCACGGTACAGACCCGGGTTCGATTCC
CGGCTGGTGCA
>Brachypodium_distachyon_chr3.trna31-GlyGCC (36642001-36642071) Gly (GCC) 71 bp Sc: 71.56
GCACCAGTGGTCTAGTGGTAGAATAGTACCCTGCCACGGTACAGACCCGGGTTCGATTCC
CGGCTGGTGCA
>Brachypodium_distachyon_chr3.trna33-GlyGCC (37560538-37560608) Gly (GCC) 71 bp Sc: 71.56
GCACCAGTGGTCTAGTGGTAGAATAGTACCCTGCCACGGTACAGACCCGGGTTCGATTCC
CGGCTGGTGCA
>Brachypodium_distachyon_chr3.trna94-GlyGCC (29683449-29683379) Gly (GCC) 71 bp Sc: 71.56
GCACCAGTGGTCTAGTGGTAGAATAGTACCCTGCCACGGTACAGACCCGGGTTCGATTCC
CGGCTGGTGCA
>Brachypodium_distachyon_chr4.trna105-GlyGCC (3885550-3885480) Gly (GCC) 71 bp Sc: 71.56
GCACCAGTGGTCTAGTGGTAGAATAGTACCCTGCCACGGTACAGACCCGGGTTCGATTCC
CGGCTGGTGCA
>Brachypodium_distachyon_chr4.trna111-GlyGCC (752951-752881) Gly (GCC) 71 bp Sc: 71.56

GCACCAGTGGTCTAGTGGTAGAATAGTACCCTGCCACGGTACAGACCCGGGTTCGATTCC
CGGCTGGTGCA
>Brachypodium_distachyon_chr4.trna59-GlyGCC (44583073-44583003) Gly (GCC) 71 bp Sc: 71.56
GCACCAGTGGTCTAGTGGTAGAATAGTACCCTGCCACGGTACAGACCCGGGTTCGATTCC
CGGCTGGTGCA
>Brachypodium_distachyon_chr1.trna82-GlyTCC (63387217-63387287) Gly (TCC) 71 bp Sc: 49.97
GTGTCTGTAGTCCAACGGTTAGGATAATTGCCTTCCAAGCAATAGACCCTGTTCGACTCC
CGGCAGACGCA
>Brachypodium_distachyon_chr1.trna34-GlyTCC (20063451-20063522) Gly (TCC) 72 bp Sc: 73.14
GCGTTTGTAGTCCAACGGTTAGGATAATTGCCTTCCAAGCAATAGACCCGGGTTCGACTC
CCGGCAGACGCA
>Brachypodium_distachyon_chr2.trna31-GlyTCC (20084762-20084833) Gly (TCC) 72 bp Sc: 73.14
GCGTTTGTAGTCCAACGGTTAGGATAATTGCCTTCCAAGCAATAGACCCGGGTTCGACTC
CCGGCAGACGCA
>Brachypodium_distachyon_chr2.trna60-GlyTCC (52940374-52940445) Gly (TCC) 72 bp Sc: 73.14
GCGTTTGTAGTCCAACGGTTAGGATAATTGCCTTCCAAGCAATAGACCCGGGTTCGACTC
CCGGCAGACGCA
>Brachypodium_distachyon_chr2.trna96-GlyTCC (42096168-42096097) Gly (TCC) 72 bp Sc: 73.14
GCGTTTGTAGTCCAACGGTTAGGATAATTGCCTTCCAAGCAATAGACCCGGGTTCGACTC
CCGGCAGACGCA
>Brachypodium_distachyon_chr1.trna69-GlyTCC (55523598-55523669) Gly (TCC) 72 bp Sc: 75.78
GCGTCTGTAGTCCAACGGTTAGGATAATTGCCTTCCAAGCAATAGACCCGGGTTCGACTC
CCGGCAGACGCA
>Brachypodium_distachyon_chr3.trna11-GlyTCC (12376076-12376147) Gly (TCC) 72 bp Sc: 75.78
GCGTCTGTAGTCCAACGGTTAGGATAATTGCCTTCCAAGCAATAGACCCGGGTTCGACTC
CCGGCAGACGCA
>Brachypodium_distachyon_chr4.trna107-GlyTCC (3767461-3767390) Gly (TCC) 72 bp Sc: 75.78
GCGTCTGTAGTCCAACGGTTAGGATAATTGCCTTCCAAGCAATAGACCCGGGTTCGACTC
CCGGCAGACGCA
>Brachypodium_distachyon_chr5.trna10-GlyTCC (10925504-10925575) Gly (TCC) 72 bp Sc: 75.78
GCGTCTGTAGTCCAACGGTTAGGATAATTGCCTTCCAAGCAATAGACCCGGGTTCGACTC
CCGGCAGACGCA
>Brachypodium_distachyon_chr3.trna5-HisATG (3083067-3083138) His (ATG) 72 bp Sc: 20.85
GGGGATGTAGCTCAATTGGTAGAGCACAAATGATGCATGTTCGAGACACAGGTTCAACGC
CTCGCGTCTCCA
>Brachypodium_distachyon_chr1.trna95-HisATG (73289380-73289308) His (ATG) 73 bp Sc: 21.24
GCGGATGTAGCTCAATTTGTAGAGCAAATGTCATGCTTTCATTAGGTACGGGGTTCGAAT
CCTCAAATCTCCA
>Brachypodium_distachyon_chr3.trna3-HisATG (3080249-3080321) His (ATG) 73 bp Sc: 32.44
GGGGATGTAGCTCAATTGGTAGAGCGCAAATCATGCTTGTTTGAGGCATGGGGTTCAATT
CCTTGCATCTCCA
>Brachypodium_distachyon_chr1.trna106-HisGTG (69467106-69467033) His (GTG) 74 bp Sc: 45.59
GCGGATGTAGCCAAGTGGATCAAGGCAGTGGATTGTGAATCCACCATGCGCTGGTTCAAT
TCCCGTCGTTCGCC
>Brachypodium_distachyon_chr5.trna54-HisGTG (14474031-14473958) His (GTG) 74 bp Sc: 45.59
GCGGATGTAGCCAAGTGGATCAAGGCAGTGGATTGTGAATCCACCATGCGCAGGTTCAAT
TCCCGTCGTTCGCC
>Brachypodium_distachyon_chr4.trna24-HisGTG (22611111-22611184) His (GTG) 74 bp Sc: 48.58
GCGGATGTAGCCAAGTGGATCAAGGCAGTGGATTGTGAATCCACCATGCGCGGGTTCAAT
TCCCGTCGCTCGCC
>Brachypodium_distachyon_chr1.trna170-HisGTG (3929213-3929140) His (GTG) 74 bp Sc: 52.58
GCGGATGTAGCCAAGTGGATCAAGGCAGTGGATTGTGAATCCACCATGCGCGGGTTCAAT
TCCCGTCGTTCGCC
>Brachypodium_distachyon_chr3.trna16-HisGTG (16803262-16803335) His (GTG) 74 bp Sc: 52.58
GCGGATGTAGCCAAGTGGATCAAGGCAGTGGATTGTGAATCCACCATGCGCGGGTTCAAT
TCCCGTCGTTCGCC
>Brachypodium_distachyon_chr4.trna89-HisGTG (16071083-16071010) His (GTG) 74 bp Sc: 52.58
GCGGATGTAGCCAAGTGGATCAAGGCAGTGGATTGTGAATCCACCATGCGCGGGTTCAAT
TCCCGTCGTTCGCC
>Brachypodium_distachyon_scaffold_55.trna3-HisGTG (9149-9222) His (GTG) 74 bp Sc: 52.58
GCGGATGTAGCCAAGTGGATCAAGGCAGTGGATTGTGAATCCACCATGCGCGGGTTCAAT
TCCCGTCGTTCGCC
>Brachypodium_distachyon_scaffold_6.trna4-HisGTG (64035-64108) His (GTG) 74 bp Sc: 52.58
GCGGATGTAGCCAAGTGGATCAAGGCAGTGGATTGTGAATCCACCATGCGCGGGTTCAAT
TCCCGTCGTTCGCC
>Brachypodium_distachyon_scaffold_84.trna3-HisGTG (5531-5458) His (GTG) 74 bp Sc: 52.58
GCGGATGTAGCCAAGTGGATCAAGGCAGTGGATTGTGAATCCACCATGCGCGGGTTCAAT

TCCCGTCGTTCGCC
>Brachypodium_distachyon_chr3.trna67-HisGTG (56995534-56995461) His (GTG) 74 bp Sc: 55.60
GCGGATGTAGCCAAGTGGATCAAGGCAGTGGATTGTGAATCCACCATGCGCGGGTTCAAT
TCCCGTCGTTCGCA
>Brachypodium_distachyon_scaffold_186.trna1-HisGTG (49-121) His (GTG) 73 bp Sc: 56.45
GCGGATGTAGCCAAGTGGATTAAGGCAGTGGATTGTGAATTCACCATCGCGGGTTCAATT
CCCGTCGTTCGCC
>Brachypodium_distachyon_chr1.trna116-HisGTG (64449771-64449700) His (GTG) 72 bp Sc: 63.70
GTGGCTGTAGTTTAGTGGTGAGAATTCCACGTTGTGGCCGTGGAGACCTGGGCTCGAATC
CCAGCAGCCACA
>Brachypodium_distachyon_chr1.trna145-HisGTG (30017782-30017711) His (GTG) 72 bp Sc: 63.70
GTGGCTGTAGTTTAGTGGTGAGAATTCCACGTTGTGGCCGTGGAGACCTGGGCTCGAATC
CCAGCAGCCACA
>Brachypodium_distachyon_chr1.trna151-HisGTG (21653466-21653395) His (GTG) 72 bp Sc: 63.70
GTGGCTGTAGTTTAGTGGTGAGAATTCCACGTTGTGGCCGTGGAGACCTGGGCTCGAATC
CCAGCAGCCACA
>Brachypodium_distachyon_chr1.trna158-HisGTG (16228656-16228585) His (GTG) 72 bp Sc: 63.70
GTGGCTGTAGTTTAGTGGTGAGAATTCCACGTTGTGGCCGTGGAGACCTGGGCTCGAATC
CCAGCAGCCACA
>Brachypodium_distachyon_chr1.trna90-HisGTG (73311513-73311584) His (GTG) 72 bp Sc: 63.70
GTGGCTGTAGTTTAGTGGTGAGAATTCCACGTTGTGGCCGTGGAGACCTGGGCTCGAATC
CCAGCAGCCACA
>Brachypodium_distachyon_chr2.trna101-HisGTG (34898647-34898576) His (GTG) 72 bp Sc: 63.70
GTGGCTGTAGTTTAGTGGTGAGAATTCCACGTTGTGGCCGTGGAGACCTGGGCTCGAATC
CCAGCAGCCACA
>Brachypodium_distachyon_chr2.trna12-HisGTG (10380106-10380177) His (GTG) 72 bp Sc: 63.70
GTGGCTGTAGTTTAGTGGTGAGAATTCCACGTTGTGGCCGTGGAGACCTGGGCTCGAATC
CCAGCAGCCACA
>Brachypodium_distachyon_chr3.trna129-HisGTG (2239588-2239517) His (GTG) 72 bp Sc: 63.70
GTGGCTGTAGTTTAGTGGTGAGAATTCCACGTTGTGGCCGTGGAGACCTGGGCTCGAATC
CCAGCAGCCACA
>Brachypodium_distachyon_chr4.trna35-HisGTG (34309271-34309342) His (GTG) 72 bp Sc: 63.70
GTGGCTGTAGTTTAGTGGTGAGAATTCCACGTTGTGGCCGTGGAGACCTGGGCTCGAATC
CCAGCAGCCACA
>Brachypodium_distachyon_chr4.trna81-HisGTG (29872294-29872223) His (GTG) 72 bp Sc: 63.70
GTGGCTGTAGTTTAGTGGTGAGAATTCCACGTTGTGGCCGTGGAGACCTGGGCTCGAATC
CCAGCAGCCACA
>Brachypodium_distachyon_chr4.trna26-IleAAT (26437653-26437726) Ile (AAT) 74 bp Sc: 20.05
GGGGATGTAGCTCATTTGGTAGAGCGCCGCTAATACGCACGACAGAGGTACGGGGTTTGA
GTCTCGCATCTTCA
>Brachypodium_distachyon_chr3.trna25-IleAAT (30183820-30183893) Ile (AAT) 74 bp Sc: 72.36
TGCCTATTAGCTCAGCTGGTTAGAGCGTCGTGCTAATAACGCGAAGGTCGCAGGTTCGAG
ACCTGCATGGGCCA
>Brachypodium_distachyon_chr2.trna134-IleAAT (31892-31819) Ile (AAT) 74 bp Sc: 72.97
GGCCTATTAGCTTAGCTGGTAAGAGCGTGGTCCTAATAAGGCCAAGGTCGCAGGTTCGAG
ACCTGCATAGGCCA
>Brachypodium_distachyon_chr1.trna157-IleAAT (16369721-16369648) Ile (AAT) 74 bp Sc: 77.66
GGCCTATTAGCTCAGCTGGTTAGAGCGTCGTGCTAATAACGCGAAGGTCACAGGTTCGAG
ACCTGTATGGGCCA
>Brachypodium_distachyon_chr2.trna30-IleAAT (19712848-19712921) Ile (AAT) 74 bp Sc: 77.66
GGCCTATTAGCTCAGCTGGTTAGAGCGTCGTGCTAATAACGCGAAGGTCACAGGTTCGAG
ACCTGTATGGGCCA
>Brachypodium_distachyon_chr2.trna64-IleAAT (55276388-55276461) Ile (AAT) 74 bp Sc: 77.66
GGCCTATTAGCTCAGCTGGTTAGAGCGTCGTGCTAATAACGCGAAGGTCACAGGTTCGAG
ACCTGTATGGGCCA
>Brachypodium_distachyon_chr3.trna38-IleAAT (41572273-41572346) Ile (AAT) 74 bp Sc: 77.66
GGCCTATTAGCTCAGCTGGTTAGAGCGTCGTGCTAATAACGCGAAGGTCACAGGTTCGAG
ACCTGTATGGGCCA
>Brachypodium_distachyon_chr4.trna71-IleAAT (35761796-35761723) Ile (AAT) 74 bp Sc: 77.66
GGCCTATTAGCTCAGCTGGTTAGAGCGTCGTGCTAATAACGCGAAGGTCACAGGTTCGAG
ACCTGTATGGGCCA
>Brachypodium_distachyon_chr5.trna47-IleAAT (18242240-18242167) Ile (AAT) 74 bp Sc: 77.66
GGCCTATTAGCTCAGCTGGTTAGAGCGTCGTGCTAATAACGCGAAGGTCACAGGTTCGAG
ACCTGTATGGGCCA
>Brachypodium_distachyon_chr2.trna59-IleAAT (52561829-52561902) Ile (AAT) 74 bp Sc: 79.79
GGCCTATTAGCTCAGCTGGTTAGAGCGTCGTGCTAATAACGCGAAGGTCGCAGGTTCGAG
ACCTGCATGGGCCA

>Brachypodium_distachyon_chr2.trna95-IleAAT (45477975-45477902) Ile (AAT) 74 bp Sc: 79.79
GGCCTATTAGCTCAGCTGGTTAGAGCGTCGTGCTAATAACGCGAAGGTCGCAGGTTCGAG
ACCTGCATGGGCCA
>Brachypodium_distachyon_chr3.trna52-IleAAT (48904610-48904683) Ile (AAT) 74 bp Sc: 79.79
GGCCTATTAGCTCAGCTGGTTAGAGCGTCGTGCTAATAACGCGAAGGTCGCAGGTTCGAG
ACCTGCATGGGCCA
>Brachypodium_distachyon_chr4.trna40-IleAAT (38988368-38988441) Ile (AAT) 74 bp Sc: 79.79
GGCCTATTAGCTCAGCTGGTTAGAGCGTCGTGCTAATAACGCGAAGGTCGCAGGTTCGAG
ACCTGCATGGGCCA
>Brachypodium_distachyon_chr4.trna51-IleAAT (48176308-48176235) Ile (AAT) 74 bp Sc: 79.79
GGCCTATTAGCTCAGCTGGTTAGAGCGTCGTGCTAATAACGCGAAGGTCGCAGGTTCGAG
ACCTGCATGGGCCA
>Brachypodium_distachyon_chr4.trna76-IleAAT (31865255-31865182) Ile (AAT) 74 bp Sc: 79.79
GGCCTATTAGCTCAGCTGGTTAGAGCGTCGTGCTAATAACGCGAAGGTCGCAGGTTCGAG
ACCTGCATGGGCCA
>Brachypodium_distachyon_chr5.trna18-IleAAT (16544101-16544174) Ile (AAT) 74 bp Sc: 79.79
GGCCTATTAGCTCAGCTGGTTAGAGCGTCGTGCTAATAACGCGAAGGTCGCAGGTTCGAG
ACCTGCATGGGCCA
>Brachypodium_distachyon_chr5.trna46-IleAAT (18789094-18789021) Ile (AAT) 74 bp Sc: 79.79
GGCCTATTAGCTCAGCTGGTTAGAGCGTCGTGCTAATAACGCGAAGGTCGCAGGTTCGAG
ACCTGCATGGGCCA
>Brachypodium_distachyon_chr2.trna65-IleAAT (55554250-55554323) Ile (AAT) 74 bp Sc: 81.06
GGCCTATTAGCTCAGTTGGTTAGAGCGTCGCTCTAATAAGGCGAAGGTCGCAGGTTCGAG
ACCTGCATGGGCCA
>Brachypodium_distachyon_chr2.trna67-IleAAT (55906781-55906854) Ile (AAT) 74 bp Sc: 82.42
GGCCCATTAGCTCAGCTGGTTAGAGCGTCGTGCTAATAACGCGAAGGTCGCAGGTTCGAG
ACCTGCATGGGCCA
>Brachypodium_distachyon_chr2.trna75-IleAAT (59007551-59007478) Ile (AAT) 74 bp Sc: 82.42
GGCCCATTAGCTCAGCTGGTTAGAGCGTCGTGCTAATAACGCGAAGGTCGCAGGTTCGAG
ACCTGCATGGGCCA
>Brachypodium_distachyon_chr4.trna95-IleGAT (13905444-13905371) Ile (GAT) 74 bp Sc: 64.57
GGTCCTGTAGCTCAGTTGGTTACAGTATTGGTCTGATATGCCAAAGGTCTTGGGTTCAAG
CCCCAACAGGAGCA
>Brachypodium_distachyon_chr5.trna42-IleGAT (22879886-22879813) Ile (GAT) 74 bp Sc: 71.73
GGTCCCGTAGCTCAGTTGGTTAGAGTGCTGGTCTGATATGCCATAGGCCTTGGGTTCAAA
TCCCAACGGGACCA
>Brachypodium_distachyon_chr2.trna106-IleTAT (22886234-22886161) Ile (TAT) 74 bp Sc: 74.63
GCCCCCGTAGCTCAGTTGGTTAGAGCGTTGGTCTTATGAGCCGAAGGTCGTGGGTTCGAG
CCCCCCCGGGAGCA
>Brachypodium_distachyon_chr2.trna81-IleTAT (55415741-55415668) Ile (TAT) 74 bp Sc: 83.43
GGTCCCGTAGCTCAGTTGGTTAGAGCGTTGGTCTTATGAGCCGAAAGTCGCGGGTTCGAA
CCCCGCCGGGACCA
>Brachypodium_distachyon_chr2.trna107-IleTAT (22880080-22880007) Ile (TAT) 74 bp Sc: 86.19
GCTCCCGTAGCTCAGTTGGTTAGAGCGTTGGTCTTATGAGCCGAAGGTCGCGGGTTCGAG
CCCCGCCGGGAGCA
>Brachypodium_distachyon_chr2.trna126-IleTAT (6793972-6793899) Ile (TAT) 74 bp Sc: 86.19
GCTCCCGTAGCTCAGTTGGTTAGAGCGTTGGTCTTATGAGCCGAAGGTCGCGGGTTCGAG
CCCCGCCGGGAGCA
>Brachypodium_distachyon_chr5.trna11-LeuAAG (11114395-11114475) Leu (AAG) 81 bp Sc: 65.09
GTTGAGATGGTCGAGTTGGTCTAAGGCGCCAGATTAAGGCTCTGGTCCGAAAGGGCGTGG
GTTCAAATCCCACTCTCAACA
>Brachypodium_distachyon_chr3.trna28-LeuAAG (35476740-35476820) Leu (AAG) 81 bp Sc: 65.41
GATCAGATGGCCGAGTTGGTCTAAGGCGCCAGATTAAGGTTCTGGTCCGAAAGGGCGTGG
GTTCAAATCCCACTCTGGTCA
>Brachypodium_distachyon_chr2.trna42-LeuAAG (33028250-33028330) Leu (AAG) 81 bp Sc: 66.11
GATCAGATGGCCGAGTTGGTCTAAGGCGCCAGATTAAGGCTCTGGTCCGAAAGGGCGTGG
GTTCAAATCCCACTCTGGTCA
>Brachypodium_distachyon_chr3.trna29-LeuAAG (35478689-35478769) Leu (AAG) 81 bp Sc: 66.11
GATCAGATGGCCGAGTTGGTCTAAGGCGCCAGATTAAGGCTCTGGTCCGAAAGGGCGTGG
GTTCAAATCCCACTCTGGTCA
>Brachypodium_distachyon_chr1.trna153-LeuAAG (19626935-19626855) Leu (AAG) 81 bp Sc: 68.09
GTTGATATGGCCGAGTTGGTCTAAGGCGCCAGATTAAGGTTCTGGTCCGAAAGGGCGTGG
GTTCAAATCCCACTGTCAACA
>Brachypodium_distachyon_chr1.trna43-LeuAAG (32016414-32016494) Leu (AAG) 81 bp Sc: 68.09
GTTGATATGGCCGAGTTGGTCTAAGGCGCCAGATTAAGGTTCTGGTCCGAAAGGGCGTGG
GTTCAAATCCCACTGTCAACA
>Brachypodium_distachyon_chr1.trna44-LeuAAG (32024389-32024469) Leu (AAG) 81 bp Sc: 68.09

GTTGATATGGCCGAGTTGGTCTAAGGCGCCAGATTAAGGTTCTGGTCCGAAAGGGCGTGG
GTTCAAATCCCACTGTCAACA
>Brachypodium_distachyon_chr2.trna69-LeuAAG (57245777-57245857) Leu (AAG) 81 bp Sc: 68.09
GTTGATATGGCCGAGTTGGTCTAAGGCGCCAGATTAAGGTTCTGGTCCGAAAGGGCGTGG
GTTCAAATCCCACTGTCAACA
>Brachypodium_distachyon_chr2.trna79-LeuAAG (57240403-57240323) Leu (AAG) 81 bp Sc: 68.09
GTTGATATGGCCGAGTTGGTCTAAGGCGCCAGATTAAGGTTCTGGTCCGAAAGGGCGTGG
GTTCAAATCCCACTGTCAACA
>Brachypodium_distachyon_chr3.trna6-LeuAAG (5447167-5447247) Leu (AAG) 81 bp Sc: 68.09
GTTGATATGGCCGAGTTGGTCTAAGGCGCCAGATTAAGGTTCTGGTCCGAAAGGGCGTGG
GTTCAAATCCCACTGTCAACA
>Brachypodium_distachyon_chr5.trna60-LeuAAG (477422-477342) Leu (AAG) 81 bp Sc: 68.09
GTTGATATGGCCGAGTTGGTCTAAGGCGCCAGATTAAGGTTCTGGTCCGAAAGGGCGTGG
GTTCAAATCCCACTGTCAACA
>Brachypodium_distachyon_chr3.trna45-LeuAAG (46256811-46256891) Leu (AAG) 81 bp Sc: 68.80
GTTGATATGGCCGAGTTGGTCTAAGGCGCCAGATTAAGGCTCTGGTCCGAAAGGGCGTGG
GTTCAAATCCCACTGTCAACA
>Brachypodium_distachyon_chr2.trna13-LeuAAG (11204167-11204247) Leu (AAG) 81 bp Sc: 69.68
GTTGAGATGGCCGAGTTGGTCTAAGGCGCCAGATTAAGGTTCTGGTCCGAAAGGGCGTGG
GTTCAAATCCCACTCTCAACA
>Brachypodium_distachyon_chr1.trna84-LeuAAG (64181816-64181896) Leu (AAG) 81 bp Sc: 70.38
GTTGAGATGGCCGAGTTGGTCTAAGGCGCCAGATTAAGGCTCTGGTCCGAAAGGGCGTGG
GTTCAAATCCCACTCTCAACA
>Brachypodium_distachyon_chr5.trna14-LeuCAA (14469394-14469476) Leu (CAA) 83 bp Sc: 45.38
GCCTTGATGGTGAAATGGTAGACACGCGAGACTCAAAATCTCATGCTGAAGAGCGTGTGG
AGGTTCGAGTCCTCTTCAAGGCA
>Brachypodium_distachyon_chr1.trna152-LeuCAA (21460239-21460159) Leu (CAA) 81 bp Sc: 54.16
GCCTTGATGGTGAAATGGTAGACACGCGAGACTCAAAATCTCGTGCTGAAGAACGTGGAG
GTTCGAGTCCTCTTCAAGGCA
>Brachypodium_distachyon_chr1.trna10-LeuCAA (3886087-3886167) Leu (CAA) 81 bp Sc: 55.48
GCCTTGATGGTGAAATGGTAGACACGCGAGACTCAAAATCTCGTGCTGAAGAGCGTGGAG
GTTCGAGTCCTCTTCAAGGCA
>Brachypodium_distachyon_chr1.trna13-LeuCAA (3924480-3924560) Leu (CAA) 81 bp Sc: 55.48
GCCTTGATGGTGAAATGGTAGACACGCGAGACTCAAAATCTCGTGCTGAAGAGCGTGGAG
GTTCGAGTCCTCTTCAAGGCA
>Brachypodium_distachyon_chr3.trna17-LeuCAA (17460817-17460897) Leu (CAA) 81 bp Sc: 55.48
GCCTTGATGGTGAAATGGTAGACACGCGAGACTCAAAATCTCGTGCTGAAGAGCGTGGAG
GTTCGAGTCCTCTTCAAGGCA
>Brachypodium_distachyon_chr3.trna96-LeuCAA (28316420-28316340) Leu (CAA) 81 bp Sc: 55.48
GCCTTGATGGTGAAATGGTAGACACGCGAGACTCAAAATCTCGTGCTGAAGAGCGTGGAG
GTTCGAGTCCTCTTCAAGGCA
>Brachypodium_distachyon_chr4.trna85-LeuCAA (22615598-22615518) Leu (CAA) 81 bp Sc: 55.48
GCCTTGATGGTGAAATGGTAGACACGCGAGACTCAAAATCTCGTGCTGAAGAGCGTGGAG
GTTCGAGTCCTCTTCAAGGCA
>Brachypodium_distachyon_scaffold_84.trna1-LeuCAA (794-874) Leu (CAA) 81 bp Sc: 55.48
GCCTTGATGGTGAAATGGTAGACACGCGAGACTCAAAATCTCGTGCTGAAGAGCGTGGAG
GTTCGAGTCCTCTTCAAGGCA
>Brachypodium_distachyon_chr3.trna59-LeuCAA (56642538-56642618) Leu (CAA) 81 bp Sc: 58.95
GCCTTGATGGTGAAATGGTAGACACGCGAGACTCAAAATCTCGTGCTGAAGAGCTTGGAG
GTTCGAGTCCTCTTCAAGGCA
>Brachypodium_distachyon_chr1.trna103-LeuCAA (71768049-71767966) Leu (CAA) 84 bp Sc: 75.15
GTCAGGATGGCCGAGTGGTCTAAGGCGCCAGACTCAAGTTCTGGTCCTCTTACGAGGGCG
TGGGTTCAAACCCCACTTCTGACA
>Brachypodium_distachyon_chr4.trna54-LeuCAA (47741968-47741885) Leu (CAA) 84 bp Sc: 75.15
GTCAGGATGGCCGAGTGGTCTAAGGCGCCAGACTCAAGTTCTGGTCCTCTTACGAGGGCG
TGGGTTCAAACCCCACTTCTGACA
>Brachypodium_distachyon_chr1.trna172-LeuCAA (2800687-2800604) Leu (CAA) 84 bp Sc: 76.89
GTCAGGATGGCCGAGTGGTCTAAGGCGCCAGACTCAAGTTCTGGTCCTCTTACGAGGGCG
TGGGTTCGAACCCCACTTCTGACA
>Brachypodium_distachyon_chr1.trna45-LeuCAA (32226901-32226984) Leu (CAA) 84 bp Sc: 76.89
GTCAGGATGGCCGAGTGGTCTAAGGCGCCAGACTCAAGTTCTGGTCCTCTTACGAGGGCG
TGGGTTCGAACCCCACTTCTGACA
>Brachypodium_distachyon_chr3.trna69-LeuCAA (55916199-55916116) Leu (CAA) 84 bp Sc: 76.89
GTCAGGATGGCCGAGTGGTCTAAGGCGCCAGACTCAAGTTCTGGTCCTCTTACGAGGGCG
TGGGTTCGAACCCCACTTCTGACA
>Brachypodium_distachyon_chr4.trna30-LeuCAA (31280958-31281041) Leu (CAA) 84 bp Sc: 76.89
GTCAGGATGGCCGAGTGGTCTAAGGCGCCAGACTCAAGTTCTGGTCCTCTTACGAGGGCG

TGGGTTCGAACCCCACTTCTGACA
>Brachypodium_distachyon_chr1.trna123-LeuCAG (60506877-60506797) Leu (CAG) 81 bp Sc: 68.96
GTCAAGATGGCCGAGTTGGTCTAAGGCGCCAGTTTCAGGTACTGGTCCGAAAGGGCGTGG
GTTCAAATCCCACTCTTGACA
>Brachypodium_distachyon_chr1.trna144-LeuCAG (31347273-31347193) Leu (CAG) 81 bp Sc: 68.96
GTCAAGATGGCCGAGTTGGTCTAAGGCGCCAGTTTCAGGTACTGGTCCGAAAGGGCGTGG
GTTCAAATCCCACTCTTGACA
>Brachypodium_distachyon_chr1.trna30-LeuCAG (16409166-16409246) Leu (CAG) 81 bp Sc: 68.96
GTCAAGATGGCCGAGTTGGTCTAAGGCGCCAGTTTCAGGTACTGGTCCGAAAGGGCGTGG
GTTCAAATCCCACTCTTGACA
>Brachypodium_distachyon_chr1.trna35-LeuCAG (20701832-20701912) Leu (CAG) 81 bp Sc: 68.96
GTCAAGATGGCCGAGTTGGTCTAAGGCGCCAGTTTCAGGTACTGGTCCGAAAGGGCGTGG
GTTCAAATCCCACTCTTGACA
>Brachypodium_distachyon_chr2.trna132-LeuCAG (2143876-2143796) Leu (CAG) 81 bp Sc: 68.96
GTCAAGATGGCCGAGTTGGTCTAAGGCGCCAGTTTCAGGTACTGGTCCGAAAGGGCGTGG
GTTCAAATCCCACTCTTGACA
>Brachypodium_distachyon_chr2.trna33-LeuCAG (20968458-20968538) Leu (CAG) 81 bp Sc: 68.96
GTCAAGATGGCCGAGTTGGTCTAAGGCGCCAGTTTCAGGTACTGGTCCGAAAGGGCGTGG
GTTCAAATCCCACTCTTGACA
>Brachypodium_distachyon_chr3.trna76-LeuCAG (48574638-48574558) Leu (CAG) 81 bp Sc: 68.96
GTCAAGATGGCCGAGTTGGTCTAAGGCGCCAGTTTCAGGTACTGGTCCGAAAGGGCGTGG
GTTCAAATCCCACTCTTGACA
>Brachypodium_distachyon_chr4.trna38-LeuCAG (35860057-35860137) Leu (CAG) 81 bp Sc: 68.96
GTCAAGATGGCCGAGTTGGTCTAAGGCGCCAGTTTCAGGTACTGGTCCGAAAGGGCGTGG
GTTCAAATCCCACTCTTGACA
>Brachypodium_distachyon_chr4.trna10-LeuTAA (11787188-11787270) Leu (TAA) 83 bp Sc: 68.63
GCTGGTTTGCCCGAGTGGTTAAGGGGGAAGACTTAAGATCTTCTGCACAGAAGTGCGCGT
GGGTTCGAACCCCACAGCCAGCA
>Brachypodium_distachyon_chr4.trna7-LeuTAA (5063490-5063572) Leu (TAA) 83 bp Sc: 68.63
GCTGGTTTGCCCGAGTGGTTAAGGGGGAAGACTTAAGATCTTCTGCACAGAAGTGCGCGT
GGGTTCGAACCCCACAGCCAGCA
>Brachypodium_distachyon_chr5.trna49-LeuTAA (17555926-17555844) Leu (TAA) 83 bp Sc: 68.63
GCTGGTTTGCCCGAGTGGTTAAGGGGGAAGACTTAAGATCTTCTGCACAGAAGTGCGCGT
GGGTTCGAACCCCACAGCCAGCA
>Brachypodium_distachyon_chr2.trna71-LeuTAA (58807352-58807434) Leu (TAA) 83 bp Sc: 71.47
GCTGGTTTGCCCGAGTGGTTAAGGGGGAAGACTTAAGATCTTCTGCACATAAGTGCGCGT
GGGTTCGAACCCCACAGCCAGCA
>Brachypodium_distachyon_chr4.trna97-LeuTAG (9310472-9310393) Leu (TAG) 80 bp Sc: 52.01
GCCGCCATGGTGAAATTGGTAGACACGCTGCTCTTAGGAAGCAGTGCTCAAGCATCTCGG
TTTGAATCCGAGTGGCGGCA
>Brachypodium_distachyon_chr1.trna128-LeuTAG (55191815-55191736) Leu (TAG) 80 bp Sc: 58.92
GCCGCCATGGTGAAATTGGTAGACACGCTGCTCTTAGGAAGCAGTGCTCAAGCATCTCGG
TTCGAATCCGAGTGGCGGCA
>Brachypodium_distachyon_chr2.trna14-LeuTAG (11487374-11487453) Leu (TAG) 80 bp Sc: 58.92
GCCGCCATGGTGAAATTGGTAGACACGCTGCTCTTAGGAAGCAGTGCTCAAGCATCTCGG
TTCGAATCCGAGTGGCGGCA
>Brachypodium_distachyon_chr4.trna27-LeuTAG (28202134-28202213) Leu (TAG) 80 bp Sc: 58.92
GCCGCCATGGTGAAATTGGTAGACACGCTGCTCTTAGGAAGCAGTGCTCAAGCATCTCGG
TTCGAATCCGAGTGGCGGCA
>Brachypodium_distachyon_scaffold_6.trna26-LeuTAG (118010-117931) Leu (TAG) 80 bp Sc: 58.92
GCCGCCATGGTGAAATTGGTAGACACGCTGCTCTTAGGAAGCAGTGCTCAAGCATCTCGG
TTCGAATCCGAGTGGCGGCA
>Brachypodium_distachyon_chr2.trna109-LeuTAG (21701689-21701610) Leu (TAG) 80 bp Sc: 62.05
GACAGTTTGGCCGAGTGGTCTAAGGCGCCAGATTTAGGCTCTGTTCCGAAAGGGCGTGGG
TTCAAATCCCACAGCTGTCA
>Brachypodium_distachyon_chr2.trna70-LeuTAG (58754033-58754112) Leu (TAG) 80 bp Sc: 68.72
GACAGTTTGGCCGAGTGGTCTAAGGCGCCAGATTTAGGCTCTGGTCCGAAAGGGCGTGGG
TTCAAATCCCACAGCTGTCA
>Brachypodium_distachyon_chr2.trna85-LeuTAG (54261834-54261755) Leu (TAG) 80 bp Sc: 68.72
GACAGTTTGGCCGAGTGGTCTAAGGCGCCAGATTTAGGCTCTGGTCCGAAAGGGCGTGGG
TTCAAATCCCACAGCTGTCA
>Brachypodium_distachyon_chr2.trna99-LeuTAG (39520224-39520145) Leu (TAG) 80 bp Sc: 68.72
GACAGTTTGGCCGAGTGGTCTAAGGCGCCAGATTTAGGCTCTGGTCCGAAAGGGCGTGGG
TTCAAATCCCACAGCTGTCA
>Brachypodium_distachyon_chr3.trna46-LeuTAG (46954060-46954139) Leu (TAG) 80 bp Sc: 68.72
GACAGTTTGGCCGAGTGGTCTAAGGCGCCAGATTTAGGCTCTGGTCCGAAAGGGCGTGGG
TTCAAATCCCACAGCTGTCA

>Brachypodium_distachyon_chr2.trna105-LysCTT (27485808-27485736) Lys (CTT) 73 bp Sc: 84.38
GCCCGTCTAGCTCAGTTGGTAGAGCGCAAGGCTCTTAACCTTGTGGTCGTGGGTTCGAGC
CCCACGGTGGGCG
>Brachypodium_distachyon_chr2.trna21-LysCTT (16690355-16690427) Lys (CTT) 73 bp Sc: 84.38
GCCCGTCTAGCTCAGTTGGTAGAGCGCAAGGCTCTTAACCTTGTGGTCGTGGGTTCGAGC
CCCACGGTGGGCG
>Brachypodium_distachyon_chr2.trna22-LysCTT (17137037-17137109) Lys (CTT) 73 bp Sc: 84.38
GCCCGTCTAGCTCAGTTGGTAGAGCGCAAGGCTCTTAACCTTGTGGTCGTGGGTTCGAGC
CCCACGGTGGGCG
>Brachypodium_distachyon_chr2.trna52-LysCTT (48891053-48891125) Lys (CTT) 73 bp Sc: 84.38
GCCCGTCTAGCTCAGTTGGTAGAGCGCAAGGCTCTTAACCTTGTGGTCGTGGGTTCGAGC
CCCACGGTGGGCG
>Brachypodium_distachyon_chr2.trna53-LysCTT (49650721-49650793) Lys (CTT) 73 bp Sc: 84.38
GCCCGTCTAGCTCAGTTGGTAGAGCGCAAGGCTCTTAACCTTGTGGTCGTGGGTTCGAGC
CCCACGGTGGGCG
>Brachypodium_distachyon_chr2.trna62-LysCTT (53494490-53494562) Lys (CTT) 73 bp Sc: 84.38
GCCCGTCTAGCTCAGTTGGTAGAGCGCAAGGCTCTTAACCTTGTGGTCGTGGGTTCGAGC
CCCACGGTGGGCG
>Brachypodium_distachyon_chr3.trna12-LysCTT (14055378-14055450) Lys (CTT) 73 bp Sc: 84.38
GCCCGTCTAGCTCAGTTGGTAGAGCGCAAGGCTCTTAACCTTGTGGTCGTGGGTTCGAGC
CCCACGGTGGGCG
>Brachypodium_distachyon_chr3.trna13-LysCTT (14057649-14057721) Lys (CTT) 73 bp Sc: 84.38
GCCCGTCTAGCTCAGTTGGTAGAGCGCAAGGCTCTTAACCTTGTGGTCGTGGGTTCGAGC
CCCACGGTGGGCG
>Brachypodium_distachyon_chr3.trna35-LysCTT (37905937-37906009) Lys (CTT) 73 bp Sc: 84.38
GCCCGTCTAGCTCAGTTGGTAGAGCGCAAGGCTCTTAACCTTGTGGTCGTGGGTTCGAGC
CCCACGGTGGGCG
>Brachypodium_distachyon_chr3.trna36-LysCTT (38104418-38104490) Lys (CTT) 73 bp Sc: 84.38
GCCCGTCTAGCTCAGTTGGTAGAGCGCAAGGCTCTTAACCTTGTGGTCGTGGGTTCGAGC
CCCACGGTGGGCG
>Brachypodium_distachyon_chr3.trna95-LysCTT (28920681-28920609) Lys (CTT) 73 bp Sc: 84.38
GCCCGTCTAGCTCAGTTGGTAGAGCGCAAGGCTCTTAACCTTGTGGTCGTGGGTTCGAGC
CCCACGGTGGGCG
>Brachypodium_distachyon_chr4.trna36-LysCTT (34530662-34530734) Lys (CTT) 73 bp Sc: 84.38
GCCCGTCTAGCTCAGTTGGTAGAGCGCAAGGCTCTTAACCTTGTGGTCGTGGGTTCGAGC
CCCACGGTGGGCG
>Brachypodium_distachyon_chr4.trna37-LysCTT (34832025-34832097) Lys (CTT) 73 bp Sc: 84.38
GCCCGTCTAGCTCAGTTGGTAGAGCGCAAGGCTCTTAACCTTGTGGTCGTGGGTTCGAGC
CCCACGGTGGGCG
>Brachypodium_distachyon_chr4.trna4-LysCTT (3028993-3029065) Lys (CTT) 73 bp Sc: 84.38
GCCCGTCTAGCTCAGTTGGTAGAGCGCAAGGCTCTTAACCTTGTGGTCGTGGGTTCGAGC
CCCACGGTGGGCG
>Brachypodium_distachyon_chr4.trna5-LysCTT (3516416-3516488) Lys (CTT) 73 bp Sc: 84.38
GCCCGTCTAGCTCAGTTGGTAGAGCGCAAGGCTCTTAACCTTGTGGTCGTGGGTTCGAGC
CCCACGGTGGGCG
>Brachypodium_distachyon_chr4.trna74-LysCTT (33858056-33857984) Lys (CTT) 73 bp Sc: 84.38
GCCCGTCTAGCTCAGTTGGTAGAGCGCAAGGCTCTTAACCTTGTGGTCGTGGGTTCGAGC
CCCACGGTGGGCG
>Brachypodium_distachyon_chr4.trna98-LysCTT (8580123-8580051) Lys (CTT) 73 bp Sc: 84.38
GCCCGTCTAGCTCAGTTGGTAGAGCGCAAGGCTCTTAACCTTGTGGTCGTGGGTTCGAGC
CCCACGGTGGGCG
>Brachypodium_distachyon_chr2.trna74-LysCTT (59246779-59246851) Lys (CTT) 73 bp Sc: 84.85
GCCCGTCTAGCTCAGTTGGTAGAGCGCAAGGCTCTTAACCTTGTGGTCGTGGGTTCGAGC
CCCACGGTGGGCA
>Brachypodium_distachyon_chr3.trna106-LysTTT (26124667-26124595) Lys (TTT) 73 bp Sc: 54.70
ATTGGCTTAGCTCAGAGGTTAGAGCATCGCATTTTTAATGCGAGGGTCATCGGTTCAAAT
CCGATAGTCGGCT
>Brachypodium_distachyon_chr4.trna84-LysTTT (26776601-26776530) Lys (TTT) 72 bp Sc: 58.49
GCTGACCTAGCTCAGTGGTAGAGCGCGTGGCTTTTAACCACGTGGCCGTGGGTTTGATCG
CCACGGTTGGCC
>Brachypodium_distachyon_scaffold_6.trna10-LysTTT (248492-248564) Lys (TTT) 73 bp Sc: 77.00
GGGTGTATAGCTCAGTTGGTAGAGCATTGGGCTTTTAACCTAATGGTCGCAGGTTCAAGT
CCTGCTATACCCA
>Brachypodium_distachyon_chr2.trna8-LysTTT (6124706-6124777) Lys (TTT) 72 bp Sc: 82.49
GCCGACCTAGCTCAGTGGTAGAGCGCGTGGCTTTTAACCACGTGGCCGTGGGTTCGATCC
CCACGGTCGGCG
>Brachypodium_distachyon_chr4.trna45-LysTTT (45004335-45004406) Lys (TTT) 72 bp Sc: 82.49

GCCGACCTAGCTCAGTGGTAGAGCGCGTGGCTTTTAACCACGTGGCCGTGGGTTCGATCC
CCACGGTCGGCG
>Brachypodium_distachyon_chr4.trna83-LysTTT (26890086-26890015) Lys (TTT) 72 bp Sc: 82.49
GCCGACCTAGCTCAGTGGTAGAGCGCGTGGCTTTTAACCACGTGGCCGTGGGTTCGATCC
CCACGGTCGGCG
>Brachypodium_distachyon_chr4.trna67-LysTTT (41084085-41084014) Lys (TTT) 72 bp Sc: 82.58
GCCGTCCTAGCTCAGTGGTAGAGCGCGTGGCTTTTAACCACGTGGCCGTGGGTTCGATCC
CCACGGACGGCG
>Brachypodium_distachyon_chr2.trna68-LysTTT (57130574-57130645) Lys (TTT) 72 bp Sc: 86.68
GCCGTCCTAGCTCAGTGGTAGAGCGCGTGGCTTTTAACCACGTGGTCGTGGGTTCGATCC
CCACGGACGGCG
>Brachypodium_distachyon_chr3.trna128-LysTTT (3430445-3430373) Lys (TTT) 73 bp Sc: 87.57
GCCGTCTTAGCTCAGCTGGTAGAGCGCGTGGCTTTTAACCACGTGGTCGTGGGTTCGATT
CCCACAGACGGCG
>Brachypodium_distachyon_chr3.trna48-LysTTT (47920220-47920292) Lys (TTT) 73 bp Sc: 89.09
GCCGTCTTAGCTCAGTTGGTAGAGCGCATGGCTTTTAACCATGTGGTCGTGGGTTCGAGT
CCCACAGACGGCG
>Brachypodium_distachyon_chr5.trna52-LysTTT (14885233-14885161) Lys (TTT) 73 bp Sc: 89.09
GCCGTCTTAGCTCAGTTGGTAGAGCGCATGGCTTTTAACCATGTGGTCGTGGGTTCGAGT
CCCACAGACGGCG
>Brachypodium_distachyon_chr1.trna54-LysTTT (45444968-45445040) Lys (TTT) 73 bp Sc: 89.11
GCCGTCCTAGCTCAGTTGGTAGAGCGCACGGCTTTTAACCGTGTGGTCGTGGGTTCGAAT
CCCACGGACGGCG
>Brachypodium_distachyon_chr1.trna118-MetCAT (63716068-63715990) Met (CAT) 79 bp Sc: 28.46
ATCAGAGTGGCGAAGCGGAAGTGTGGTGGGCCCATAAACCACAGGCCACAGGTCCCAAGA
TCCAAACTTGGCTCTGATA
>Brachypodium_distachyon_chr5.trna35-MetCAT (25630023-25629950) Met (CAT) 74 bp Sc: 31.50
AGAGGATAGAGGAGTCTGGTAGCTCACAAGTCTCATAACCTTGGGGTTGTGGGTTCGATT
CCCACTAGCATTTG
>Brachypodium_distachyon_chr2.trna29-MetCAT (18375155-18375227) Met (CAT) 73 bp Sc: 48.94
GACTACTTAACTCAGTGGTTAGAGTATTGCTTTCATACGGCGGGAGTCATTGGTTCAAAT
CCAATAGTAGGTA
>Brachypodium_distachyon_scaffold_6.trna13-MetCAT (277865-277938) Met (CAT) 74 bp Sc: 48.95
AGCGGGGTAGAGGAATTGGTCGACTCATCAGGCTCATGACCTGAAGACTGCAGGTTCGAA
TCCTGTCCCCGCCT
>Brachypodium_distachyon_scaffold_6.trna23-MetCAT (235820-235747) Met (CAT) 74 bp Sc: 48.95
AGCGGGGTAGAGGAATTGGTCGACTCATCAGGCTCATGACCTGAAGACTGCAGGTTCGAA
TCCTGTCCCCGCCT
>Brachypodium_distachyon_scaffold_6.trna17-MetCAT (354304-354377) Met (CAT) 74 bp Sc: 48.95
CGCGGGGTAGAGGAATTGGTCGACTCATCAGGCTCATGACCTGAAGACTGCAGGTTCGAA
TCCTGTCCCCGCCT
>Brachypodium_distachyon_chr4.trna58-MetCAT (44948704-44948631) Met (CAT) 74 bp Sc: 51.21
GCATCCATGGCTGAATGGTTAAAGCGCCCAACTCATAATTGGTAAATTTGTGGGTTCAAT
TCCTGTTGGATGCA
>Brachypodium_distachyon_chr2.trna124-MetCAT (11497772-11497700) Met (CAT) 73 bp Sc: 55.64
GCCTACTTAACTCAGTGGTTAGAGTATTGCTTTCATACGGCGGGAGTCATTGGTTCAAAT
CCAATAGTAGGTA
>Brachypodium_distachyon_chr3.trna103-MetCAT (28280520-28280448) Met (CAT) 73 bp Sc: 55.64
GCCTACTTAACTCAGTGGTTAGAGTATTGCTTTCATACGGCGGGAGTCATTGGTTCAAAT
CCAATAGTAGGTA
>Brachypodium_distachyon_chr4.trna80-MetCAT (29903552-29903480) Met (CAT) 73 bp Sc: 55.64
GCCTACTTAACTCAGTGGTTAGAGTATTGCTTTCATACGGCGGGAGTCATTGGTTCAAAT
CCAATAGTAGGTA
>Brachypodium_distachyon_scaffold_6.trna3-MetCAT (41670-41743) Met (CAT) 74 bp Sc: 56.91
GGGCTTATAGTTTAATTGGTTGAAACGTACCGCTCATAACGGTGATATTGTAGGTTCGAG
CCCTACTAAGCCCA
>Brachypodium_distachyon_chr1.trna14-MetCAT (3927053-3927126) Met (CAT) 74 bp Sc: 58.29
GCATCCATGGCTGAATGGTTAAAGCGCCCAACTCATAATTGGTAAATTTGCGGGTTCAAT
TCCTGCTGGATGCA
>Brachypodium_distachyon_chr1.trna148-MetCAT (27802125-27802052) Met (CAT) 74 bp Sc: 58.29
GCATCCATGGCTGAATGGTTAAAGCGCCCAACTCATAATTGGTAAATTTGCGGGTTCAAT
TCCTGCTGGATGCA
>Brachypodium_distachyon_chr1.trna8-MetCAT (2616618-2616691) Met (CAT) 74 bp Sc: 58.29
GCATCCATGGCTGAATGGTTAAAGCGCCCAACTCATAATTGGTAAATTTGCGGGTTCAAT
TCCTGCTGGATGCA
>Brachypodium_distachyon_chr2.trna104-MetCAT (30709157-30709084) Met (CAT) 74 bp Sc: 58.29
GCATCCATGGCTGAATGGTTAAAGCGCCCAACTCATAATTGGTAAATTTGCGGGTTCAAT

TCCTGCTGGATGCA
>Brachypodium_distachyon_chr3.trna113-MetCAT (16805422-16805349) Met (CAT) 74 bp Sc: 58.29
GCATCCATGGCTGAATGGTTAAAGCGCCCAACTCATAATTGGTAAATTTGCGGGTTCAAT
TCCTGCTGGATGCA
>Brachypodium_distachyon_chr3.trna18-MetCAT (17463393-17463466) Met (CAT) 74 bp Sc: 58.29
GCATCCATGGCTGAATGGTTAAAGCGCCCAACTCATAATTGGTAAATTTGCGGGTTCAAT
TCCTGCTGGATGCA
>Brachypodium_distachyon_chr3.trna97-MetCAT (28313849-28313776) Met (CAT) 74 bp Sc: 58.29
GCATCCATGGCTGAATGGTTAAAGCGCCCAACTCATAATTGGTAAATTTGCGGGTTCAAT
TCCTGCTGGATGCA
>Brachypodium_distachyon_chr4.trna86-MetCAT (22613039-22612966) Met (CAT) 74 bp Sc: 58.29
GCATCCATGGCTGAATGGTTAAAGCGCCCAACTCATAATTGGTAAATTTGCGGGTTCAAT
TCCTGCTGGATGCA
>Brachypodium_distachyon_chr5.trna15-MetCAT (14471922-14471995) Met (CAT) 74 bp Sc: 58.29
GCATCCATGGCTGAATGGTTAAAGCGCCCAACTCATAATTGGTAAATTTGCGGGTTCAAT
TCCTGCTGGATGCA
>Brachypodium_distachyon_chr5.trna2-MetCAT (4856156-4856229) Met (CAT) 74 bp Sc: 58.29
GCATCCATGGCTGAATGGTTAAAGCGCCCAACTCATAATTGGTAAATTTGCGGGTTCAAT
TCCTGCTGGATGCA
>Brachypodium_distachyon_chr5.trna5-MetCAT (7217193-7217266) Met (CAT) 74 bp Sc: 58.29
GCATCCATGGCTGAATGGTTAAAGCGCCCAACTCATAATTGGTAAATTTGCGGGTTCAAT
TCCTGCTGGATGCA
>Brachypodium_distachyon_scaffold_84.trna2-MetCAT (3371-3444) Met (CAT) 74 bp Sc: 58.29
GCATCCATGGCTGAATGGTTAAAGCGCCCAACTCATAATTGGTAAATTTGCGGGTTCAAT
TCCTGCTGGATGCA
>Brachypodium_distachyon_chr4.trna70-MetCAT (40042659-40042587) Met (CAT) 73 bp Sc: 59.47
ACCTACTTGACTCAGCGGTTAGAGTATCGCTTTCATACGGCGAGAGTCATTGGTTCAAAT
CCAATAGTAGGTA
>Brachypodium_distachyon_scaffold_6.trna29-MetCAT (24697-24625) Met (CAT) 73 bp Sc: 59.47
ACCTACTTGACTCAGCGGTTAGAGTATCGCTTTCATACGGCGAGAGTCATTGGTTCAAAT
CCAATAGTAGGTA
>Brachypodium_distachyon_chr1.trna66-MetCAT (54727271-54727344) Met (CAT) 74 bp Sc: 61.45
AGCGGAGTAGAGCAGTTTGGTAGCTCACGAGGCTCATAACCTTGAGGTCACGGGTTCGAT
TCCCGTCTCCGCAC
>Brachypodium_distachyon_chr2.trna19-MetCAT (16388267-16388340) Met (CAT) 74 bp Sc: 61.45
AGCGGAGTAGAGCAGTTTGGTAGCTCACGAGGCTCATAACCTTGAGGTCACGGGTTCGAT
TCCCGTCTCCGCAC
>Brachypodium_distachyon_chr3.trna23-MetCAT (28295672-28295745) Met (CAT) 74 bp Sc: 61.45
AGCGGAGTAGAGCAGTTTGGTAGCTCACGAGGCTCATAACCTTGAGGTCACGGGTTCGAT
TCCCGTCTCCGCAC
>Brachypodium_distachyon_scaffold_111.trna1-MetCAT (3398-3471) Met (CAT) 74 bp Sc: 62.47
GCATCCATGGCTGAATGGTTAAAGCGCCCAACTCATAATTGGCGAATTCGTAGGTTCAAT
TCCTACTGGATGCA
>Brachypodium_distachyon_chr1.trna108-MetCAT (69079862-69079791) Met (CAT) 72 bp Sc: 68.81
ATCAGAGTGGCGCAGCGGAAGCGTGGTGGGCCCATAACCCACAGGTCCCAGGATCGAAAC
CTGGCTCTGATA
>Brachypodium_distachyon_chr1.trna121-MetCAT (61779977-61779906) Met (CAT) 72 bp Sc: 68.81
ATCAGAGTGGCGCAGCGGAAGCGTGGTGGGCCCATAACCCACAGGTCCCAGGATCGAAAC
CTGGCTCTGATA
>Brachypodium_distachyon_chr1.trna142-MetCAT (41470220-41470149) Met (CAT) 72 bp Sc: 68.81
ATCAGAGTGGCGCAGCGGAAGCGTGGTGGGCCCATAACCCACAGGTCCCAGGATCGAAAC
CTGGCTCTGATA
>Brachypodium_distachyon_chr1.trna64-MetCAT (52934017-52934088) Met (CAT) 72 bp Sc: 68.81
ATCAGAGTGGCGCAGCGGAAGCGTGGTGGGCCCATAACCCACAGGTCCCAGGATCGAAAC
CTGGCTCTGATA
>Brachypodium_distachyon_chr1.trna85-MetCAT (64283476-64283547) Met (CAT) 72 bp Sc: 68.81
ATCAGAGTGGCGCAGCGGAAGCGTGGTGGGCCCATAACCCACAGGTCCCAGGATCGAAAC
CTGGCTCTGATA
>Brachypodium_distachyon_chr1.trna86-MetCAT (69014429-69014500) Met (CAT) 72 bp Sc: 68.81
ATCAGAGTGGCGCAGCGGAAGCGTGGTGGGCCCATAACCCACAGGTCCCAGGATCGAAAC
CTGGCTCTGATA
>Brachypodium_distachyon_chr3.trna58-MetCAT (56240012-56240083) Met (CAT) 72 bp Sc: 68.81
ATCAGAGTGGCGCAGCGGAAGCGTGGTGGGCCCATAACCCACAGGTCCCAGGATCGAAAC
CTGGCTCTGATA
>Brachypodium_distachyon_chr3.trna64-MetCAT (58553480-58553409) Met (CAT) 72 bp Sc: 68.81
ATCAGAGTGGCGCAGCGGAAGCGTGGTGGGCCCATAACCCACAGGTCCCAGGATCGAAAC
CTGGCTCTGATA

>Brachypodium_distachyon_chr3.trna71-MetCAT (53032877-53032806) Met (CAT) 72 bp Sc: 68.81
ATCAGAGTGGCGCAGCGGAAGCGTGGTGGGCCCATAACCCACAGGTCCCAGGATCGAAAC
CTGGCTCTGATA
>Brachypodium_distachyon_chr4.trna110-MetCAT (1340669-1340580) Met (CAT) 90 bp Sc: 60.72
GGGGTGGTGGCGCAGTTGGCTAGCGCGTAGGTCTCATAGCTAAATACGCGAGTGATCCTG
AGGTCGAGAGTTCGAGCCTCTCTCACCCCA
>Brachypodium_distachyon_chr2.trna115-MetCAT (16500517-16500431) Met (CAT) 87 bp Sc: 47.77
GGGGTAGTGGCGCAGTTGGCTAGCGAGTAGGTCTCATAGCTTTGTTGAGTGATCCTGAGG
TCGAGAGTTCGAGCCTCTCTCACCCCA
>Brachypodium_distachyon_chr1.trna31-MetCAT (17165947-17166034) Met (CAT) 88 bp Sc: 61.74
GGGGTGGTGGCGCAGTTGGCTAGCGCGTAGGTCTCATAGCTAAAAGTGAGTGATCCTGAG
GTCGAGAGTTCGAGCCTCTCTCACCCCA
>Brachypodium_distachyon_chr2.trna27-MetCAT (17774050-17774139) Met (CAT) 90 bp Sc: 61.36
GGGGTGGTGGCGCAGTTGGCTAGCGCGTAGGTCTCATAGCTATAAACGTGAGTGATCCTG
AGGTCGAGAGTTCGAGCCTCTCTCACCCCA
>Brachypodium_distachyon_chr3.trna110-MetCAT (18248851-18248766) Met (CAT) 86 bp Sc: 60.92
GGGGTGGTGGCGCAGTTGGCTAGCGCGTAGGTCTCATAGCTTGTTGAGTGATCCTGAGGT
CGAGAGTTCGAGCCTCTCTCACCCCA
>Brachypodium_distachyon_chr3.trna109-MetCAT (18251716-18251626) Met (CAT) 91 bp Sc: 42.91
GGGTGGATGGCGCAGTTGGCTAGCGCGTATGTCGCATAGGTTATCATCAGGAGTGATCGT
GAGGTCGAGAGTTCGAACCTCTCTCACCCCA
>Brachypodium_distachyon_chr5.trna25-MetCAT (22921886-22921972) Met (CAT) 87 bp Sc: 60.84
GGGGTGGTGGCGCAGTTGGCTAGCGCGTAGGTCTCATAGCTAAAGCGAGTGATCCTGAGG
TCGAGAGTTCGAGCCTCTCTCACCCCA
>Brachypodium_distachyon_chr1.trna9-MetCAT (3076312-3076399) Met (CAT) 88 bp Sc: 60.96
GGGGTGGTGGCGCAGTTGGCTAGCGCGTAGGTCTCATAGCTTAAAGCGAGTGATCCTGAG
GTCGAGAGTTCGAGCCTCTCTCACCCCA
>Brachypodium_distachyon_chr2.trna6-MetCAT (3616171-3616258) Met (CAT) 88 bp Sc: 60.96
GGGGTGGTGGCGCAGTTGGCTAGCGCGTAGGTCTCATAGCTAAACGTGAGTGATCCTGAG
GTCGAGAGTTCGAGCCTCTCTCACCCCA
>Brachypodium_distachyon_chr3.trna90-MetCAT (36514123-36514037) Met (CAT) 87 bp Sc: 61.47
GGGGTGGTGGCGCAGTTGGCTAGCGCGTAGGTCTCATAGCTAAAGTGAGTGATCCTGAGG
TCGAGAGTTCGAGCCTCTCTCACCCCA
>Brachypodium_distachyon_chr3.trna50-MetCAT (48136444-48136531) Met (CAT) 88 bp Sc: 41.17
GGGATGGTGGCGCAGTTGGCTGGCGCGGTGGTCTCATAGCTGGATGTGAGTGATCCTGAG
GTCGAGAGTTCGAACCTCTCTCACCCCA
>Brachypodium_distachyon_chr2.trna58-MetCAT (50781671-50781756) Met (CAT) 86 bp Sc: 64.14
GGGGTGATGGCGCAGTTGGCTAGCGCGTAGGTCTCATAGCTTGTTGAGTGATCCTGAGGT
CGAGAGTTCGAGCCTCTCTCACCCCA
>Brachypodium_distachyon_chr1.trna136-MetCAT (52386688-52386601) Met (CAT) 88 bp Sc: 60.82
GGGGTGGTGGCGCAGTTGGCTAGCGCGTAGGTCTCATAGCTAATTGCGAGTGATCCTGAG
GTCGAGAGTTCGAGCCTCTCTCACCCCA
>Brachypodium_distachyon_chr3.trna70-MetCAT (55399908-55399822) Met (CAT) 87 bp Sc: 61.83
GGGGTGGTGGCGCAGTTGGCTAGCGCGTAGGTCTCATAGCTAACTTGAGTAATCCTGAGG
TCGAGAGTTCGAGCCTCTCTCACCCCA
>Brachypodium_distachyon_chr1.trna71-MetCAT (57071369-57071458) Met (CAT) 90 bp Sc: 60.72
GGGGTGGTGGCGCAGTTGGCTAGCGCGTAGGTCTCATAGCTAAGTACGTGAGTGATCCTG
AGGTCGAGAGTTCGAGCCTCTCTCACCCCA
>Brachypodium_distachyon_chr2.trna76-MetCAT (59006317-59006231) Met (CAT) 87 bp Sc: 61.97
GGGGTGGTGGCGCAGTTGGCTAGCGCGTAGGTCTCATAGCTAAATTGAGTGATCCTGAGG
TCGAGAGTTCGAGCCTCTCTCACCCCA
>Brachypodium_distachyon_chr1.trna75-MetCAT (60156000-60156086) Met (CAT) 87 bp Sc: 60.69
GGGGTGGTGGCGCAGTTGGCTAGCGCGTAGGTCTCATAGCTAATGCGAGTGATCCTGAGG
TCGAGAGTTCGAGCCTCTCTCACCCCA
>Brachypodium_distachyon_chr1.trna36-PheGAA (21634890-21634962) Phe (GAA) 73 bp Sc: 63.65
GTCAGGATAGCTCAGTTGGTAGAGCAGAGGACTGAAAATCCTCGTGCCACCAGTTCAAAT
CTGGTTCCTGGCA
>Brachypodium_distachyon_chr1.trna78-PheGAA (61408109-61408181) Phe (GAA) 73 bp Sc: 65.48
GTCAGGATAGCTCAGTTGGTAGAGCAGAGGACTGAAAATCCTCGTGTCACCGGTTCAAAT
CTGGTTCCTGGCA
>Brachypodium_distachyon_chr2.trna123-PheGAA (11501759-11501687) Phe (GAA) 73 bp Sc: 67.75
GTCAGGATAGCTCAGTTGGTAGAGCAGAGGACTGAAAATCCTCGTGTCACCAGTTCAAAT
CTGGTTCCTGGCA
>Brachypodium_distachyon_chr5.trna28-PheGAA (25641472-25641544) Phe (GAA) 73 bp Sc: 67.75
GTCAGGATAGCTCAGTTGGTAGAGCAGAGGACTGAAAATCCTCGTGTCACCAGTTCAAAT
CTGGTTCCTGGCA
>Brachypodium_distachyon_scaffold_6.trna21-PheGAA (459699-459627) Phe (GAA) 73 bp Sc: 67.75

GTCAGGATAGCTCAGTTGGTAGAGCAGAGGACTGAAAATCCTCGTGTCACCAGTTCAAAT
CTGGTTCCTGGCA
>Brachypodium_distachyon_chr5.trna13-PheGAA (13925632-13925704) Phe (GAA) 73 bp Sc: 70.52
GCGGGGATAGCTCAGTTGGGAGAGCGTCAGACTGAAGATCTGAAGGTCGCGTGTTTGATC
CACGCTCACCGCA
>Brachypodium_distachyon_chr1.trna107-PheGAA (69342262-69342190) Phe (GAA) 73 bp Sc: 77.43
GCGGGGATAGCTCAGTTGGGAGAGCGTCAGACTGAAGATCTGAAGGTCGCGTGTTCGATC
CACGCTCACCGCA
>Brachypodium_distachyon_chr1.trna135-PheGAA (54274244-54274172) Phe (GAA) 73 bp Sc: 77.43
GCGGGGATAGCTCAGTTGGGAGAGCGTCAGACTGAAGATCTGAAGGTCGCGTGTTCGATC
CACGCTCACCGCA
>Brachypodium_distachyon_chr2.trna112-PheGAA (17136249-17136177) Phe (GAA) 73 bp Sc: 77.43
GCGGGGATAGCTCAGTTGGGAGAGCGTCAGACTGAAGATCTGAAGGTCGCGTGTTCGATC
CACGCTCACCGCA
>Brachypodium_distachyon_chr2.trna133-PheGAA (1545873-1545801) Phe (GAA) 73 bp Sc: 77.43
GCGGGGATAGCTCAGTTGGGAGAGCGTCAGACTGAAGATCTGAAGGTCGCGTGTTCGATC
CACGCTCACCGCA
>Brachypodium_distachyon_chr2.trna91-PheGAA (49630424-49630352) Phe (GAA) 73 bp Sc: 77.43
GCGGGGATAGCTCAGTTGGGAGAGCGTCAGACTGAAGATCTGAAGGTCGCGTGTTCGATC
CACGCTCACCGCA
>Brachypodium_distachyon_chr3.trna39-PheGAA (42291484-42291556) Phe (GAA) 73 bp Sc: 77.43
GCGGGGATAGCTCAGTTGGGAGAGCGTCAGACTGAAGATCTGAAGGTCGCGTGTTCGATC
CACGCTCACCGCA
>Brachypodium_distachyon_chr3.trna47-PheGAA (47767951-47768023) Phe (GAA) 73 bp Sc: 77.43
GCGGGGATAGCTCAGTTGGGAGAGCGTCAGACTGAAGATCTGAAGGTCGCGTGTTCGATC
CACGCTCACCGCA
>Brachypodium_distachyon_chr3.trna54-PheGAA (50957010-50957082) Phe (GAA) 73 bp Sc: 77.43
GCGGGGATAGCTCAGTTGGGAGAGCGTCAGACTGAAGATCTGAAGGTCGCGTGTTCGATC
CACGCTCACCGCA
>Brachypodium_distachyon_chr3.trna86-PheGAA (37769642-37769570) Phe (GAA) 73 bp Sc: 77.43
GCGGGGATAGCTCAGTTGGGAGAGCGTCAGACTGAAGATCTGAAGGTCGCGTGTTCGATC
CACGCTCACCGCA
>Brachypodium_distachyon_chr4.trna34-PheGAA (33867732-33867804) Phe (GAA) 73 bp Sc: 77.43
GCGGGGATAGCTCAGTTGGGAGAGCGTCAGACTGAAGATCTGAAGGTCGCGTGTTCGATC
CACGCTCACCGCA
>Brachypodium_distachyon_chr4.trna41-PheGAA (39510482-39510554) Phe (GAA) 73 bp Sc: 77.43
GCGGGGATAGCTCAGTTGGGAGAGCGTCAGACTGAAGATCTGAAGGTCGCGTGTTCGATC
CACGCTCACCGCA
>Brachypodium_distachyon_chr5.trna20-PheGAA (19663123-19663195) Phe (GAA) 73 bp Sc: 77.43
GCGGGGATAGCTCAGTTGGGAGAGCGTCAGACTGAAGATCTGAAGGTCGCGTGTTCGATC
CACGCTCACCGCA
>Brachypodium_distachyon_chr4.trna11-ProAGG (14247912-14247984) Pro (AGG) 73 bp Sc: 29.66
TAGAATTTAGTCTAGTGGTAAGATGCTCACTTAGGGAGTGGATGGTCCTGAGTTCAATCC
TTAGAATCTCCAA
>Brachypodium_distachyon_chr3.trna105-ProAGG (27495972-27495899) Pro (AGG) 74 bp Sc: 54.36
GGGCCTGTAGCTCAGAGGATTAGACCACGTGGCTAGGAACCACGGTGTCGGGGGTTCGAA
TCCCTCCTCGCCCA
>Brachypodium_distachyon_chr1.trna173-ProAGG (2613567-2613496) Pro (AGG) 72 bp Sc: 71.75
GGGCATTTGGTCTAGTGGTATGATTCTCGCTTAGGGTGCGAGAGGTCCCGAGTTCAATTC
TCGGAATGCCCC
>Brachypodium_distachyon_chr1.trna46-ProAGG (32296995-32297066) Pro (AGG) 72 bp Sc: 71.75
GGGCATTTGGTCTAGTGGTATGATTCTCGCTTAGGGTGCGAGAGGTCCCGAGTTCAATTC
TCGGAATGCCCC
>Brachypodium_distachyon_chr2.trna103-ProAGG (34329161-34329090) Pro (AGG) 72 bp Sc: 71.75
GGGCATTTGGTCTAGTGGTATGATTCTCGCTTAGGGTGCGAGAGGTCCCGAGTTCAATTC
TCGGAATGCCCC
>Brachypodium_distachyon_chr2.trna5-ProAGG (3156244-3156315) Pro (AGG) 72 bp Sc: 71.75
GGGCATTTGGTCTAGTGGTATGATTCTCGCTTAGGGTGCGAGAGGTCCCGAGTTCAATTC
TCGGAATGCCCC
>Brachypodium_distachyon_chr3.trna60-ProAGG (59416528-59416599) Pro (AGG) 72 bp Sc: 71.75
GGGCATTTGGTCTAGTGGTATGATTCTCGCTTAGGGTGCGAGAGGTCCCGAGTTCAATTC
TCGGAATGCCCC
>Brachypodium_distachyon_chr4.trna102-ProAGG (5860302-5860231) Pro (AGG) 72 bp Sc: 71.75
GGGCATTTGGTCTAGTGGTATGATTCTCGCTTAGGGTGCGAGAGGTCCCGAGTTCAATTC
TCGGAATGCCCC
>Brachypodium_distachyon_chr4.trna103-ProAGG (4898563-4898492) Pro (AGG) 72 bp Sc: 71.75
GGGCATTTGGTCTAGTGGTATGATTCTCGCTTAGGGTGCGAGAGGTCCCGAGTTCAATTC

TCGGAATGCCCC
>Brachypodium_distachyon_chr4.trna106-ProAGG (3872146-3872075) Pro (AGG) 72 bp Sc: 71.75
GGGCATTTGGTCTAGTGGTATGATTCTCGCTTAGGGTGCGAGAGGTCCCGAGTTCAATTC
TCGGAATGCCCC
>Brachypodium_distachyon_chr4.trna13-ProAGG (14249738-14249809) Pro (AGG) 72 bp Sc: 71.75
GGGCATTTGGTCTAGTGGTATGATTCTCGCTTAGGGTGCGAGAGGTCCCGAGTTCAATTC
TCGGAATGCCCC
>Brachypodium_distachyon_chr4.trna79-ProAGG (30278984-30278913) Pro (AGG) 72 bp Sc: 71.75
GGGCATTTGGTCTAGTGGTATGATTCTCGCTTAGGGTGCGAGAGGTCCCGAGTTCAATTC
TCGGAATGCCCC
>Brachypodium_distachyon_chr4.trna93-ProAGG (13927247-13927176) Pro (AGG) 72 bp Sc: 71.75
GGGCATTTGGTCTAGTGGTATGATTCTCGCTTAGGGTGCGAGAGGTCCCGAGTTCAATTC
TCGGAATGCCCC
>Brachypodium_distachyon_chr4.trna12-ProAGG (14249364-14249435) Pro (AGG) 72 bp Sc: 73.34
GGGGATTTAGTCTAGTGGTAAGATATTCGCTTAGGGAGCGAAAGGTCCCGAGTTCAATTC
TCGGAATCCCCA
>Brachypodium_distachyon_chr4.trna92-ProAGG (13927644-13927573) Pro (AGG) 72 bp Sc: 73.34
GGGGATTTAGTCTAGTGGTAAGATATTCGCTTAGGGAGCGAAAGGTCCCGAGTTCAATTC
TCGGAATCCCCA
>Brachypodium_distachyon_chr2.trna86-ProCGG (54249393-54249322) Pro (CGG) 72 bp Sc: 63.45
GGGCGTTTGGTCTAGTGGTATGATTCCCGCTTCGGGTGCGGGAGGTCATGAGTTCGATTC
TCGCAACGCCCC
>Brachypodium_distachyon_chr4.trna50-ProCGG (48009796-48009867) Pro (CGG) 72 bp Sc: 69.81
GGGTGTTTGGTCTAGTGGTATGATTCTCGCTTCGGGTGCGAGAGGTCGTGAGTTCGATTC
TCGCAACACCCC
>Brachypodium_distachyon_chr1.trna37-ProCGG (23951885-23951956) Pro (CGG) 72 bp Sc: 74.11
GGGTGTTTGGTCTAGTGGTATGATTCTCGCTTCGGGTGCGAGAGGTCGCGAGTTCGATTC
TCGCAACACCCC
>Brachypodium_distachyon_chr4.trna29-ProCGG (30951986-30952057) Pro (CGG) 72 bp Sc: 74.11
GGGTGTTTGGTCTAGTGGTATGATTCTCGCTTCGGGTGCGAGAGGTCGCGAGTTCGATTC
TCGCAACACCCC
>Brachypodium_distachyon_chr4.trna52-ProCGG (48146440-48146369) Pro (CGG) 72 bp Sc: 74.11
GGGTGTTTGGTCTAGTGGTATGATTCTCGCTTCGGGTGCGAGAGGTCGCGAGTTCGATTC
TCGCAACACCCC
>Brachypodium_distachyon_chr4.trna60-ProCGG (43423498-43423427) Pro (CGG) 72 bp Sc: 74.11
GGGTGTTTGGTCTAGTGGTATGATTCTCGCTTCGGGTGCGAGAGGTCGCGAGTTCGATTC
TCGCAACACCCC
>Brachypodium_distachyon_chr4.trna77-ProCGG (31856994-31856923) Pro (CGG) 72 bp Sc: 74.11
GGGTGTTTGGTCTAGTGGTATGATTCTCGCTTCGGGTGCGAGAGGTCGCGAGTTCGATTC
TCGCAACACCCC
>Brachypodium_distachyon_chr5.trna22-ProCGG (20300770-20300841) Pro (CGG) 72 bp Sc: 74.11
GGGTGTTTGGTCTAGTGGTATGATTCTCGCTTCGGGTGCGAGAGGTCGCGAGTTCGATTC
TCGCAACACCCC
>Brachypodium_distachyon_chr5.trna3-ProTGG (4857576-4857649) Pro (TGG) 74 bp Sc: 43.92
AGGGACGTAGCACAGCTTGGTAGCGCGTTTGTTTTGGGTAAAAAATGTCACAGGTTCAAA
TCCTGTCATCCCTA
>Brachypodium_distachyon_chr4.trna109-ProTGG (1728830-1728759) Pro (TGG) 72 bp Sc: 51.95
GGGCGTTTGGTATAGTGGTGTGATTCTTGCTTTGGGTGCGAGAGGTCGCGGGTTCGATTC
TCGCAACGCCCC
>Brachypodium_distachyon_scaffold_6.trna1-ProTGG (5512-5586) Pro (TGG) 75 bp Sc: 54.34
CGAGGTGTAGCGCAGTCTGGTCAGCGCATCTGTTTTGGGTACAGAGGGCCATAGGTTCGA
ATCCTGTCACCTTGA
>Brachypodium_distachyon_scaffold_6.trna15-ProTGG (309692-309766) Pro (TGG) 75 bp Sc: 54.34
CGAGGTGTAGCGCAGTCTGGTCAGCGCATCTGTTTTGGGTACAGAGGGCCATAGGTTCGA
ATCCTGTCACCTTGA
>Brachypodium_distachyon_chr1.trna164-ProTGG (9836753-9836680) Pro (TGG) 74 bp Sc: 54.68
AGGGATGTAGCGCAGCTTGGTAGCGCATTTGTTTTGGGTACAAAATGCCACAGGTTCAAA
TCCTGTCATCCCTA
>Brachypodium_distachyon_scaffold_6.trna11-ProTGG (251226-251299) Pro (TGG) 74 bp Sc: 54.91
AGGGATGTAGCGCAGCTTGGTAGCGCGTTTGTTTTGGGTACAAAATGTCACGGGTTCAAA
TCCTGTCATCCCTA
>Brachypodium_distachyon_chr4.trna22-ProTGG (19571402-19571475) Pro (TGG) 74 bp Sc: 58.78
AGGGATGTAGCGCAGCTTGGTAGCGCATTTGTTTTGGGTACAAAATGTCACAGGTTCAAA
TCCTGTCATCCCTA
>Brachypodium_distachyon_chr1.trna113-ProTGG (66524063-66523990) Pro (TGG) 74 bp Sc: 59.74
AGGGATGTAGCGCAGCTTGTTAGCGCGTTTGTTTTGGGTACAAAATGTCACAGGTTCAAA
TCCTGTCATCCCTA

>Brachypodium_distachyon_chr1.trna11-ProTGG (3914450-3914523) Pro (TGG) 74 bp Sc: 60.50
AGGGATGTAGCGCAGCTTGGTAGCGCGTTTGTTTTGGGTACAAAATGTCACAGGTTCAAA
TCCTGTCATCCCTA
>Brachypodium_distachyon_chr5.trna29-ProTGG (25652808-25652881) Pro (TGG) 74 bp Sc: 60.50
AGGGATGTAGCGCAGCTTGGTAGCGCGTTTGTTTTGGGTACAAAATGTCACAGGTTCAAA
TCCTGTCATCCCTA
>Brachypodium_distachyon_chr1.trna160-ProTGG (14944840-14944769) Pro (TGG) 72 bp Sc: 73.73
GGGCATTTGGTCTAGTGGTATGATTCTCGCTTTGGGTGCGAGAGGTCCCGAGTTCGATTC
TCGGAATGCCCC
>Brachypodium_distachyon_chr1.trna79-ProTGG (61630288-61630359) Pro (TGG) 72 bp Sc: 73.73
GGGCATTTGGTCTAGTGGTATGATTCTCGCTTTGGGTGCGAGAGGTCCCGAGTTCGATTC
TCGGAATGCCCC
>Brachypodium_distachyon_chr3.trna56-ProTGG (51218308-51218379) Pro (TGG) 72 bp Sc: 73.73
GGGCATTTGGTCTAGTGGTATGATTCTCGCTTTGGGTGCGAGAGGTCCCGAGTTCGATTC
TCGGAATGCCCC
>Brachypodium_distachyon_chr4.trna21-ProTGG (15158072-15158143) Pro (TGG) 72 bp Sc: 73.73
GGGCATTTGGTCTAGTGGTATGATTCTCGCTTTGGGTGCGAGAGGTCCCGAGTTCGATTC
TCGGAATGCCCC
>Brachypodium_distachyon_chr1.trna140-ProTGG (47146919-47146848) Pro (TGG) 72 bp Sc: 74.23
GGGCGTTTGGTCTAGTGGTATGATTCTCGCTTTGGGTGCGAGAGGTCGCGAGTTCGATTC
TCGCAACGCCCC
>Brachypodium_distachyon_chr1.trna150-ProTGG (24807816-24807745) Pro (TGG) 72 bp Sc: 74.23
GGGCGTTTGGTCTAGTGGTATGATTCTCGCTTTGGGTGCGAGAGGTCGCGAGTTCGATTC
TCGCAACGCCCC
>Brachypodium_distachyon_chr3.trna43-ProTGG (43999429-43999500) Pro (TGG) 72 bp Sc: 74.23
GGGCGTTTGGTCTAGTGGTATGATTCTCGCTTTGGGTGCGAGAGGTCGCGAGTTCGATTC
TCGCAACGCCCC
>Brachypodium_distachyon_chr4.trna66-ProTGG (41732553-41732482) Pro (TGG) 72 bp Sc: 74.23
GGGCGTTTGGTCTAGTGGTATGATTCTCGCTTTGGGTGCGAGAGGTCGCGAGTTCGATTC
TCGCAACGCCCC
>Brachypodium_distachyon_chr1.trna138-Undet??? (47381918-47381848) Undet (???) 71 bp Sc: 20.61
GACTCTCTAGCCTAGTGGTTAGAGGGTCTAGTAGCACCTCTTTGGTCTTGGGTTCGATTC
CCGCGGGAGCG
>Brachypodium_distachyon_chr1.trna99-Undet??? (73288822-73288749) Undet (???) 74 bp Sc: 25.74
GGGGACGTAGCTCAATTGGTAGAGCAAATGCAAATGCTTGTGTTAGGTACGGGGTTCGAT
TCATTGCATCTCCA
>Brachypodium_distachyon_chr1.trna63-Undet??? (52781493-52781572) Undet (???) 80 bp Sc: 28.69
GGGGGtgttgtCAAATGGAATGACAGACGGATTAATAACCGTTGCTCTTTGAGCTGAGAG
TTCAATTCTCTCCATTTCCC
>Brachypodium_distachyon_chr2.trna2-Undet??? (1893901-1893980) Undet (???) 80 bp Sc: 28.69
GGGGGtgttgtCAAATGGAATGACAGACGGATTAATAACCGTTGCTCTTTGAGCTGAGAG
TTCAATTCTCTCCATTTCCC
>Brachypodium_distachyon_chr1.trna100-Undet??? (73288682-73288610) Undet (???) 73 bp Sc: 29.10
GGGGATGTAGCTCAATTGGTAGAGCAATTGCAGCGCTTGTGTTAGGTACGGGGTTCAATT
CCTTGCATCTCCA
>Brachypodium_distachyon_chr4.trna18-Undet??? (14647472-14647544) Undet (???) 73 bp Sc: 30.84
GGGGATGTAGCTCAATTGGTAGAGCAATTGCAGCGCTTGTGTTAGGTACGGGGTTCGATT
CCTTGCATCTCCA
>Brachypodium_distachyon_chr5.trna26-ValAAC (23012386-23012457) Val (AAC) 72 bp Sc: 37.37
AGGGATATAACTCATCGGTAGAGTGTCACCTTAACGTGATGGAAGTCATCAGATCGAACC
TGATTATCTCTA
>Brachypodium_distachyon_chr3.trna104-LeuCAA (28134878-28134794) Leu (CAA) 85 bp Sc: 44.47
GCTAGGATGGTTGAGTGGTCTAAGGCGCTAGACTCAAGTTTTGGTCCTCTTACGAGGGGT
GTGGGTTCAAACCCCACTTCTGACA
>Brachypodium_distachyon_chr4.trna94-MetCAT (13912905-13912829) Met (CAT) 77 bp Sc: 28.74
GGTGCCTTATCTCAGTTGGTTCAGAGCATTGGTCTCATATGCCAAAAGGTCAATGGGTTC
AAACCCCAACAGGACTA
>Brachypodium_distachyon_chr3.trna81-ArgCCT (46482776-46482701) Arg (CCT) 76 bp Sc: 20.07
TGCCGGTTGGTGCAGTGGTGCTTGCTTGCCTCCTAGTTGCGAGCTAGAGGTAGTGAGTTC
GAACCCACGTCTGTCT
>Brachypodium_distachyon_chr4.trna101-ValGAC (7032116-7032045) Val (GAC) 72 bp Sc: 45.14
AGGGATATAACTCAGCAGTAGAGTGTCACCTTGACATGGTGGAAGTCATCAGTTCGAGCC
TGATTATCCCTA
>Brachypodium_distachyon_chr4.trna8-CysGCA (6031624-6031697) Cys (GCA) 74 bp Sc: 21.02
GAGCCTCTAGCCTAGTGGTCAAGGGCTCTAGTAGCACCCTTTTGGTCCCGGGTTCGACTC
CTCCGGGAGGTGAA
>Brachypodium_distachyon_chr1.trna26-GlyGCC (13487392-13487459) Gly (GCC) 68 bp Sc: 28.93

GCACCAGTGGTCTTATGGTAGAATAGTACCCTGCCACGGTACAGACCCGGGTTCGATTCC
GGGGTACA
>Brachypodium_distachyon_chr1.trna134-AspGTC (54724454-54724382) Asp (GTC) 73 bp Sc: 33.60
GGGATTGTAGTTCAATTGGTCAGAGCACCGCCCTGTCAAGGCGGAAGCTGCGGGTTCGAG
CCCGGAATTACCT
>Brachypodium_distachyon_chr3.trna4-AlaTGC (3082872-3082944) Ala (TGC) 73 bp Sc: 22.54
GGGGATGTAGCTCGTTTGGTAGAGCACAAATTATGCATGTTTGAGGGACGGGGTTTGATT
CCTCGCATCTCCC
>Brachypodium_distachyon_chr5.trna16-SerAGA (14863899-14863979) Ser (AGA) 81 bp Sc: 49.52
GTGGACGTGTCAGAGTGGTTATTGGGTATGACTAGAAATCATATGGGTTTTGCCCGCGTA
GGTTCGAATCCTTCTTACACG
>Brachypodium_distachyon_chr3.trna51-SerAGA (48192407-48192488) Ser (AGA) 82 bp Sc: 83.72
GTGGTCGTGCCGGAGTGGTTATCGGGCATGACTAGAAATCATGTGGGCTCTGCCCGCGCA
GGTTCGAATCCTGCCGACCACG
>Brachypodium_distachyon_chr1.trna2-SerAGA (1436131-1436212) Ser (AGA) 82 bp Sc: 84.20
GTGGTCGTGCCGGAGTGGTTATCGGGCATGACTAGAAATCATGTGGGCTTTGCCCGCGCA
GGTTCGAATCCTGCCGACCACG
>Brachypodium_distachyon_chr1.trna39-SerAGA (26251071-26251152) Ser (AGA) 82 bp Sc: 84.20
GTGGTCGTGCCGGAGTGGTTATCGGGCATGACTAGAAATCATGTGGGCTTTGCCCGCGCA
GGTTCGAATCCTGCCGACCACG
>Brachypodium_distachyon_chr1.trna61-SerAGA (51423901-51423982) Ser (AGA) 82 bp Sc: 84.20
GTGGTCGTGCCGGAGTGGTTATCGGGCATGACTAGAAATCATGTGGGCTTTGCCCGCGCA
GGTTCGAATCCTGCCGACCACG
>Brachypodium_distachyon_chr2.trna108-SerAGA (22824386-22824305) Ser (AGA) 82 bp Sc: 84.20
GTGGTCGTGCCGGAGTGGTTATCGGGCATGACTAGAAATCATGTGGGCTTTGCCCGCGCA
GGTTCGAATCCTGCCGACCACG
>Brachypodium_distachyon_chr2.trna131-SerAGA (2579385-2579304) Ser (AGA) 82 bp Sc: 84.20
GTGGTCGTGCCGGAGTGGTTATCGGGCATGACTAGAAATCATGTGGGCTTTGCCCGCGCA
GGTTCGAATCCTGCCGACCACG
>Brachypodium_distachyon_chr2.trna82-SerAGA (55364119-55364038) Ser (AGA) 82 bp Sc: 84.20
GTGGTCGTGCCGGAGTGGTTATCGGGCATGACTAGAAATCATGTGGGCTTTGCCCGCGCA
GGTTCGAATCCTGCCGACCACG
>Brachypodium_distachyon_chr3.trna126-SerAGA (4359787-4359706) Ser (AGA) 82 bp Sc: 84.20
GTGGTCGTGCCGGAGTGGTTATCGGGCATGACTAGAAATCATGTGGGCTTTGCCCGCGCA
GGTTCGAATCCTGCCGACCACG
>Brachypodium_distachyon_chr3.trna53-SerAGA (49728683-49728764) Ser (AGA) 82 bp Sc: 84.20
GTGGTCGTGCCGGAGTGGTTATCGGGCATGACTAGAAATCATGTGGGCTTTGCCCGCGCA
GGTTCGAATCCTGCCGACCACG
>Brachypodium_distachyon_chr4.trna63-SerAGA (42772790-42772709) Ser (AGA) 82 bp Sc: 84.20
GTGGTCGTGCCGGAGTGGTTATCGGGCATGACTAGAAATCATGTGGGCTTTGCCCGCGCA
GGTTCGAATCCTGCCGACCACG
>Brachypodium_distachyon_chr1.trna3-SerCGA (1506575-1506656) Ser (CGA) 82 bp Sc: 81.28
GTCGATATGTCCGAGTGGTTAAGGAGACAGACTCGAAATCTGTTGGGCTTTGCCTGCGCA
GGTTCGAATCCTGCTGTCGACG
>Brachypodium_distachyon_chr2.trna119-SerCGA (14766543-14766462) Ser (CGA) 82 bp Sc: 81.28
GTCGATATGTCCGAGTGGTTAAGGAGACAGACTCGAAATCTGTTGGGCTTTGCCTGCGCA
GGTTCGAATCCTGCTGTCGACG
>Brachypodium_distachyon_chr2.trna34-SerCGA (22535393-22535474) Ser (CGA) 82 bp Sc: 81.28
GTCGATATGTCCGAGTGGTTAAGGAGACAGACTCGAAATCTGTTGGGCTTTGCCTGCGCA
GGTTCGAATCCTGCTGTCGACG
>Brachypodium_distachyon_chr2.trna63-SerCGA (55047140-55047221) Ser (CGA) 82 bp Sc: 81.28
GTCGATATGTCCGAGTGGTTAAGGAGACAGACTCGAAATCTGTTGGGCTTTGCCTGCGCA
GGTTCGAATCCTGCTGTCGACG
>Brachypodium_distachyon_chr2.trna94-SerCGA (46837951-46837870) Ser (CGA) 82 bp Sc: 81.28
GTCGATATGTCCGAGTGGTTAAGGAGACAGACTCGAAATCTGTTGGGCTTTGCCTGCGCA
GGTTCGAATCCTGCTGTCGACG
>Brachypodium_distachyon_scaffold_127.trna1-SerGCT (2407-2321) Ser (GCT) 87 bp Sc: 47.37
GGAGAGATGGCTGAGTGGACTAAAGCGGCGGATTGCTAATCCGTTGTACAATTTTTTTTG
TACGAGGGTTCGATCCCTCTCTTTCCG
>Brachypodium_distachyon_scaffold_149.trna1-SerGCT (3930-3843) Ser (GCT) 88 bp Sc: 54.57
GGAGAGATGGCTGAGTGGACCAAGGCGGCGGATTGCTAATCCGTTGTTCAATTTTTTTGT
ACCGAGGGTTCGAATCCCTCTCTTTCCG
>Brachypodium_distachyon_chr5.trna4-SerGCT (6651810-6651897) Ser (GCT) 88 bp Sc: 59.95
GGAGAGATGGCTGAGTGGACTAAAGCGGCGGATTGCTAATCCGTTGTACAATTTTTTTGT
ACCGAGGGTTCGAATCCCTCTCTTTCCG
>Brachypodium_distachyon_scaffold_108.trna1-SerGCT (2140-2227) Ser (GCT) 88 bp Sc: 59.95
GGAGAGATGGCTGAGTGGACTAAAGCGGCGGATTGCTAATCCGTTGTACAATTTTTTTGT

ACCGAGGGTTCGAATCCCTCTCTTTCCG
>Brachypodium_distachyon_scaffold_120.trna1-SerGCT (4182-4095) Ser (GCT) 88 bp Sc: 59.95
GGAGAGATGGCTGAGTGGACTAAAGCGGCGGATTGCTAATCCGTTGTACAATTTTTTTGT
ACCGAGGGTTCGAATCCCTCTCTTTCCG
>Brachypodium_distachyon_scaffold_55.trna1-SerGCT (1091-1178) Ser (GCT) 88 bp Sc: 59.95
GGAGAGATGGCTGAGTGGACTAAAGCGGCGGATTGCTAATCCGTTGTACAATTTTTTTGT
ACCGAGGGTTCGAATCCCTCTCTTTCCG
>Brachypodium_distachyon_chr1.trna154-SerGCT (17425440-17425359) Ser (GCT) 82 bp Sc: 77.62
GACGCTTTGGCCGAGTGGTTAAGGCGTGTGCCTGCTAAGTACATGGGGTTTCCCCGCGAG
AGTTCGAATCTCTCAGGCGTCG
>Brachypodium_distachyon_chr1.trna155-SerGCT (17424289-17424208) Ser (GCT) 82 bp Sc: 77.62
GACGCTTTGGCCGAGTGGTTAAGGCGTGTGCCTGCTAAGTACATGGGGTTTCCCCGCGAG
AGTTCGAATCTCTCAGGCGTCG
>Brachypodium_distachyon_chr1.trna53-SerGCT (44829766-44829847) Ser (GCT) 82 bp Sc: 77.62
GACGCTTTGGCCGAGTGGTTAAGGCGTGTGCCTGCTAAGTACATGGGGTTTCCCCGCGAG
AGTTCGAATCTCTCAGGCGTCG
>Brachypodium_distachyon_chr1.trna74-SerGCT (59380988-59381069) Ser (GCT) 82 bp Sc: 77.62
GACGCTTTGGCCGAGTGGTTAAGGCGTGTGCCTGCTAAGTACATGGGGTTTCCCCGCGAG
AGTTCGAATCTCTCAGGCGTCG
>Brachypodium_distachyon_chr3.trna112-SerGCT (17500655-17500574) Ser (GCT) 82 bp Sc: 77.62
GACGCTTTGGCCGAGTGGTTAAGGCGTGTGCCTGCTAAGTACATGGGGTTTCCCCGCGAG
AGTTCGAATCTCTCAGGCGTCG
>Brachypodium_distachyon_chr3.trna130-SerGCT (1696260-1696179) Ser (GCT) 82 bp Sc: 77.62
GACGCTTTGGCCGAGTGGTTAAGGCGTGTGCCTGCTAAGTACATGGGGTTTCCCCGCGAG
AGTTCGAATCTCTCAGGCGTCG
>Brachypodium_distachyon_chr3.trna2-SerGCT (1162248-1162329) Ser (GCT) 82 bp Sc: 77.62
GACGCTTTGGCCGAGTGGTTAAGGCGTGTGCCTGCTAAGTACATGGGGTTTCCCCGCGAG
AGTTCGAATCTCTCAGGCGTCG
>Brachypodium_distachyon_chr3.trna84-SerGCT (40332077-40331996) Ser (GCT) 82 bp Sc: 77.62
GACGCTTTGGCCGAGTGGTTAAGGCGTGTGCCTGCTAAGTACATGGGGTTTCCCCGCGAG
AGTTCGAATCTCTCAGGCGTCG
>Brachypodium_distachyon_chr4.trna96-SerGCT (13572113-13572032) Ser (GCT) 82 bp Sc: 77.62
GACGCTTTGGCCGAGTGGTTAAGGCGTGTGCCTGCTAAGTACATGGGGTTTCCCCGCGAG
AGTTCGAATCTCTCAGGCGTCG
>Brachypodium_distachyon_scaffold_6.trna24-SerGCT (143272-143185) Ser (GCT) 88 bp Sc: 38.42
GGAGGTATGGCTGAGTGGCTTAAGGCATTGGTTTGCTAAATCGACATACAAGAAGATTGT
ATCATGGGTTCGAATCCCATTTCCTCCG
>Brachypodium_distachyon_chr3.trna24-SerGCT (28830394-28830481) Ser (GCT) 88 bp Sc: 40.56
GGAGGTATGGCTGAGTGGCTTAAGGCATTGGTTTGCTAAATCGACATACAAGAAGATTGC
ATCATGGGTTCGAATCCCATTTCCTCCG
>Brachypodium_distachyon_scaffold_6.trna19-SerGCT (489019-489106) Ser (GCT) 88 bp Sc: 38.42
GGAGGTATGGCTGAGTGGCTTAAGGCATTGGTTTGCTAAATCGACATACAAGAAGATTGT
ATCATGGGTTCGAATCCCATTTCCTCCG
>Brachypodium_distachyon_chr2.trna28-SerGGA (18188711-18188797) Ser (GGA) 87 bp Sc: 49.82
GGAGAGATGGCCGAGCGGTTCAAGGCATAGCATTGGAACTGCTATGTAGACTTTTGTTCA
CCGAGGGTTCGAATCCCTCTCTTTCTG
>Brachypodium_distachyon_chr2.trna122-SerGGA (11504917-11504831) Ser (GGA) 87 bp Sc: 55.11
GGAGAGATGGCCGAGCGGTTCAAGGCGTAGCATTGGAACTGCTATGTAGACTTTTGTTTA
CCGAGGGTTCGAATCCCTCTCTTTCCG
>Brachypodium_distachyon_chr5.trna27-SerGGA (25638321-25638407) Ser (GGA) 87 bp Sc: 55.11
GGAGAGATGGCCGAGCGGTTCAAGGCGTAGCATTGGAACTGCTATGTAGACTTTTGTTTA
CCGAGGGTTCGAATCCCTCTCTTTCCG
>Brachypodium_distachyon_scaffold_6.trna20-SerGGA (461351-461265) Ser (GGA) 87 bp Sc: 56.80
GGAGAGATGGCCGAGTGGTTCAAGGCGTAGCATTGGAACTGCTATGTAGGCTTTTGTTTA
CCGAGGGTTCGAATCCCTCTCTTTCCG
>Brachypodium_distachyon_scaffold_6.trna14-SerTGA (290336-290422) Ser (TGA) 87 bp Sc: 53.41
GGATGGATGTCTGAGCGGTTGAAAGAGTCGGTCTTGAAAACCGAAGTATTGATAGGAATA
CCGGGGGTTCGAATCCCTCTCCATCCG
>Brachypodium_distachyon_chr5.trna9-SerTGA (10657657-10657744) Ser (TGA) 88 bp Sc: 53.43
GGAGAGATGGCTGAGTGGTTGATAGCTCCGGTCTTGAAAACCGGTATAGTTCTAGTAACT
ATCGAGGGTTCGAATCCCTCTCTCTCCT
>Brachypodium_distachyon_chr1.trna67-SerTGA (54728665-54728752) Ser (TGA) 88 bp Sc: 54.93
GGAGAGATGGCTGAGTGGTTGATAGCTCCGGTCTTGAAAACCGGTATAGTTCTAGGAACT
ATCGAGGGTTCGAATCCCTCTCTCTCCT
>Brachypodium_distachyon_chr1.trna93-SerTGA (74162243-74162156) Ser (TGA) 88 bp Sc: 54.93
GGAGAGATGGCTGAGTGGTTGATAGCTCCGGTCTTGAAAACCGGTATAGTTCTAGGAACT
ATCGAGGGTTCGAATCCCTCTCTCTCCT

>Brachypodium_distachyon_chr2.trna121-SerTGA (12981974-12981887) Ser (TGA) 88 bp Sc: 54.93
GGAGAGATGGCTGAGTGGTTGATAGCTCCGGTCTTGAAAACCGGTATAGTTCTAGGAACT
ATCGAGGGTTCGAATCCCTCTCTCTCCT
>Brachypodium_distachyon_chr1.trna28-SerTGA (14428888-14428969) Ser (TGA) 82 bp Sc: 77.78
GTCGATATGTCCGAGTGGTTAAGGAGACAGACTTGAAATCTGTTGGGCTACGCCCGCGCA
GGTTCGATCCCTGCTGTCGACG
>Brachypodium_distachyon_chr2.trna102-SerTGA (34567465-34567384) Ser (TGA) 82 bp Sc: 77.78
GTCGATATGTCCGAGTGGTTAAGGAGACAGACTTGAAATCTGTTGGGCTACGCCCGCGCA
GGTTCGATCCCTGCTGTCGACG
>Brachypodium_distachyon_chr4.trna53-SerTGA (47878315-47878234) Ser (TGA) 82 bp Sc: 77.78
GTCGATATGTCCGAGTGGTTAAGGAGACAGACTTGAAATCTGTTGGGCTACGCCCGCGCA
GGTTCGATCCCTGCTGTCGACG
>Brachypodium_distachyon_chr1.trna81-SerTGA (62282106-62282187) Ser (TGA) 82 bp Sc: 78.02
GTCGATATGTCCGAGTGGTTAAGGAGACAGACTTGAAATCTGTTGGGCTACGCCCGCGCA
GGTTCGAACCCTGCTGTCGACG
>Brachypodium_distachyon_chr2.trna4-SerTGA (2881620-2881701) Ser (TGA) 82 bp Sc: 78.02
GTCGATATGTCCGAGTGGTTAAGGAGACAGACTTGAAATCTGTTGGGCTACGCCCGCGCA
GGTTCGAACCCTGCTGTCGACG
>Brachypodium_distachyon_chr5.trna19-ThrAGT (18263979-18264055) Thr (AGT) 77 bp Sc: 51.14
GCTTTCATAGCTCAGTTGGGTTAGGAGCACCCCGTTTAGTAAGCGGGAGGTCTTGAGTTC
AACTCTCAATGAAAGCA
>Brachypodium_distachyon_chr1.trna70-ThrAGT (56682862-56682933) Thr (AGT) 72 bp Sc: 54.10
GCTTTCGTAGCTCAGTTGGTTAGAGCACCCGTTTAGTAAGCGGGAGGTCTTGAGTTCAAC
TCTCACGAAGCA
>Brachypodium_distachyon_chr2.trna44-ThrAGT (35184008-35184081) Thr (AGT) 74 bp Sc: 61.37
GCTCTCGTAGCTCAGTTGGTTAGAGCACCCGTTAAGTAAACGGGAGTTCCTGAGTTCAAC
TCTCAACGAGAGCA
>Brachypodium_distachyon_chr1.trna22-ThrAGT (9629726-9629798) Thr (AGT) 73 bp Sc: 61.86
GCTTTCATAGCTCAGTTGGTTAGAGCACCGTTTAGTAAGCGGGAGGTCTTGAGTTCAACT
CTCAATGAAAGCA
>Brachypodium_distachyon_chr1.trna110-ThrAGT (67616328-67616255) Thr (AGT) 74 bp Sc: 74.28
GCTTTCATAGCTCAGTTGGTTAGAGCACCCGTTTAGTAAGCGGGAGGTCTTGAGTTCAAC
TCTCAATGAAAGCA
>Brachypodium_distachyon_chr4.trna1-ThrAGT (441647-441720) Thr (AGT) 74 bp Sc: 74.28
GCTTTCATAGCTCAGTTGGTTAGAGCACCCGTTTAGTAAGCGGGAGGTCTTGAGTTCAAC
TCTCAATGAAAGCA
>Brachypodium_distachyon_chr4.trna46-ThrAGT (45715081-45715154) Thr (AGT) 74 bp Sc: 74.28
GCTTTCATAGCTCAGTTGGTTAGAGCACCCGTTTAGTAAGCGGGAGGTCTTGAGTTCAAC
TCTCAATGAAAGCA
>Brachypodium_distachyon_chr2.trna55-ThrAGT (50679605-50679678) Thr (AGT) 74 bp Sc: 74.68
GCTTTCGTAGCTCAGTTGGTTAGAGCACCCGTTTAGTAAGCGGGAGGTCTTGAGTTCAAC
TCTCAACGAAAGCA
>Brachypodium_distachyon_chr1.trna19-ThrAGT (4997295-4997498) Thr (AGT) 204 bp Sc: 37.05
GCTTTCATAGCTCAGTGTTAGAGCACCGTTTAGTAGCGGGGAGGTCTTGAGTCACTCTCA
TGAATGCGTTttgttgtTTTTTTCAATCGTAGTCAGGAACGAATTTGGTCTCTCTTTTGT
GTTTGTGTGTGTTttgttgtCTCTAACCATTTCGGCAATCTTTGACTAAGCGGGAGGTCT
TGAGTTCAACTCTCAATGAAAGCA
>Brachypodium_distachyon_chr4.trna64-ThrCGT (42431030-42430959) Thr (CGT) 72 bp Sc: 78.75
GCTTCCGTAGCATAGTGGTATTGCGTTCGCTTCGTAAGCGAAAGGTCGTGAGTTCGATCC
TCGCCGGGAGCT
>Brachypodium_distachyon_chr1.trna149-ThrCGT (25266830-25266759) Thr (CGT) 72 bp Sc: 80.56
GCCTCCGTAGCATAGTGGTAGTGCGTTCCCTTCGTAAGGGAAAGGTCGTGAGTTCGATCC
TCACCGGGGGCT
>Brachypodium_distachyon_chr1.trna72-ThrCGT (57107413-57107484) Thr (CGT) 72 bp Sc: 80.56
GCCTCCGTAGCATAGTGGTAGTGCGTTCCCTTCGTAAGGGAAAGGTCGTGAGTTCGATCC
TCACCGGGGGCT
>Brachypodium_distachyon_chr3.trna62-ThrCGT (59638413-59638342) Thr (CGT) 72 bp Sc: 80.56
GCCTCCGTAGCATAGTGGTAGTGCGTTCCCTTCGTAAGGGAAAGGTCGTGAGTTCGATCC
TCACCGGGGGCT
>Brachypodium_distachyon_chr3.trna72-ThrCGT (52249200-52249129) Thr (CGT) 72 bp Sc: 81.33
GCTTCCGTAGCATAGTGGTAGTGCGTTCGCTTCGTAAGCGAAAGGTCGTGAGTTCGATCC
TCACCGGGAGCT
>Brachypodium_distachyon_chr5.trna24-ThrCGT (21477049-21477120) Thr (CGT) 72 bp Sc: 82.82
GCTTCCGTAGCATAGTGGTAGTGCGTTCGCTTCGTAAGCGAAAGGTCGCGAGTTCGATCC
TCGCCGGGAGCT
>Brachypodium_distachyon_chr1.trna131-ThrGGT (54725620-54725549) Thr (GGT) 72 bp Sc: 57.42
GCCCTTTTAACTCAGTGGTAGAGTAATGCCATGGTAAGGCGTAAGTCATCGGTTCAAATC

CGATAAAGGGCT
>Brachypodium_distachyon_chr1.trna49-ThrGGT (35762760-35762831) Thr (GGT) 72 bp Sc: 57.42
GCCCTTTTAACTCAGTGGTAGAGTAATGCCATGGTAAGGCGTAAGTCATCGGTTCAAATC
CGATAAAGGGCT
>Brachypodium_distachyon_chr3.trna99-ThrGGT (28294024-28293953) Thr (GGT) 72 bp Sc: 57.42
GCCCTTTTAACTCAGTGGTAGAGTAATGCCATGGTAAGGCGTAAGTCATCGGTTCAAATC
CGATAAAGGGCT
>Brachypodium_distachyon_chr5.trna57-ThrTGT (10539747-10539675) Thr (TGT) 73 bp Sc: 54.66
GACCACTTAGCTCAGAGGTTAGAGCATCGCATTTGTAATGTGAGGGTCATCGGTTCAAAT
CCGATAGTCGGCT
>Brachypodium_distachyon_chr3.trna125-ThrTGT (5635079-5635008) Thr (TGT) 72 bp Sc: 55.08
GCCCACTTAGCTTAGAGGTTAGAGCATCGCATTTGTAATGCGAGGTCATCGGTTCAAATC
CGATAGTCTGCT
>Brachypodium_distachyon_chr5.trna34-ThrTGT (25639665-25639593) Thr (TGT) 73 bp Sc: 64.66
GCCCACTTAGCTCAGAGGTTAGAGCATCGCATTTGTAATGCGAGGGTCATCGGTTCAAAT
CCGGTAGTCGGCT
>Brachypodium_distachyon_chr2.trna15-ThrTGT (11503571-11503643) Thr (TGT) 73 bp Sc: 67.46
GCCCACTTAGCTCAGAGGTTAGAGCATCGCATTTGTAATGCGAGGGTCATCGGTTCAAAT
CCGATAGTCGGCT
>Brachypodium_distachyon_chr1.trna38-ThrTGT (24018510-24018581) Thr (TGT) 72 bp Sc: 78.52
GCCCCTATAGCTCAGTGGTAGAGCGTCAGTCTTGTAAACTGAAGGTCTGTAGTTCGATCC
TGCATGGGGGCA
>Brachypodium_distachyon_chr3.trna74-ThrTGT (50960940-50960869) Thr (TGT) 72 bp Sc: 78.52
GCCCCTATAGCTCAGTGGTAGAGCGTCAGTCTTGTAAACTGAAGGTCTGTAGTTCGATCC
TGCATGGGGGCA
>Brachypodium_distachyon_chr3.trna92-ThrTGT (31450973-31450902) Thr (TGT) 72 bp Sc: 78.52
GCCCCTATAGCTCAGTGGTAGAGCGTCAGTCTTGTAAACTGAAGGTCTGTAGTTCGATCC
TGCATGGGGGCA
>Brachypodium_distachyon_chr4.trna68-ThrTGT (40617450-40617379) Thr (TGT) 72 bp Sc: 80.01
GCCCTTATAGCTCAGTGGTAGAGCGTCAGTCTTGTAAACTGAAGGTCCGTAGTTCGATCC
TGCGTGGGGGCA
>Brachypodium_distachyon_chr1.trna94-ThrTGT (74061976-74061905) Thr (TGT) 72 bp Sc: 81.19
GCCCTTATAGCTCAGTGGTAGAGCGTCAGTCTTGTAAACTGAAGGTCCGTAGTTCGATCC
TGCGTGAGGGCA
>Brachypodium_distachyon_chr3.trna41-ThrTGT (43623107-43623178) Thr (TGT) 72 bp Sc: 82.64
GCCCCTATAGCTCAGTGGTAGAGCGTCAGTCTTGTAAACTGAAGGTCCGTAGTTCGATCC
TGCGTGGGGGCA
>Brachypodium_distachyon_chr4.trna23-TrpCCA (19571616-19571689) Trp (CCA) 74 bp Sc: 56.77
ATGCTCTTAGTTCAGTTCGGTAGAACGCGGGTCTCCAAAACCCGATGTCGTGGGTTCAAA
TCCTACAGAGCGTG
>Brachypodium_distachyon_chr5.trna43-TrpCCA (22215750-22215677) Trp (CCA) 74 bp Sc: 61.75
ACGCTCTTAGTTCAGTTCGGTAGAACGCGGGTCTCCAAAACCTGATGTCGTAGGTTCAAA
TCCTACAGAGCGTC
>Brachypodium_distachyon_chr1.trna114-TrpCCA (66523859-66523786) Trp (CCA) 74 bp Sc: 62.41
ACGCTCTTAGTTCAGTTCGGTAGAACGCGGGTCTCCAAAACCCGATGTCGTAGGTTCAAA
TCCTACGGAGCGTG
>Brachypodium_distachyon_scaffold_6.trna12-TrpCCA (251442-251515) Trp (CCA) 74 bp Sc: 67.55
GCGCTCTTAGTTCAGTTCGGTAGAACGTGGGTCTCCAAAACCCAATGTCGTAGGTTCAAA
TCCTACAGAGCGTG
>Brachypodium_distachyon_chr1.trna12-TrpCCA (3914664-3914737) Trp (CCA) 74 bp Sc: 67.94
GCGCTCTTAGTTCAGTTCGGTAGAACGCGGGTCTCCAAAACCCGATGTCGTAGGTTCAAA
TCCTACAGAGCGTG
>Brachypodium_distachyon_chr1.trna165-TrpCCA (9836539-9836466) Trp (CCA) 74 bp Sc: 67.94
GCGCTCTTAGTTCAGTTCGGTAGAACGCGGGTCTCCAAAACCCGATGTCGTAGGTTCAAA
TCCTACAGAGCGTG
>Brachypodium_distachyon_chr5.trna30-TrpCCA (25653022-25653095) Trp (CCA) 74 bp Sc: 67.94
GCGCTCTTAGTTCAGTTCGGTAGAACGCGGGTCTCCAAAACCCGATGTCGTAGGTTCAAA
TCCTACAGAGCGTG
>Brachypodium_distachyon_chr1.trna141-TrpCCA (43595658-43595587) Trp (CCA) 72 bp Sc: 77.63
GGATCCGTGGCGCAATGGTAGCGCGTCTGACTCCAGATCAGAAGGTTGCGTGTTCGATTC
ACGTCGGGTTCA
>Brachypodium_distachyon_chr1.trna156-TrpCCA (16496552-16496481) Trp (CCA) 72 bp Sc: 77.63
GGATCCGTGGCGCAATGGTAGCGCGTCTGACTCCAGATCAGAAGGTTGCGTGTTCGATTC
ACGTCGGGTTCA
>Brachypodium_distachyon_chr1.trna76-TrpCCA (60384326-60384397) Trp (CCA) 72 bp Sc: 77.63
GGATCCGTGGCGCAATGGTAGCGCGTCTGACTCCAGATCAGAAGGTTGCGTGTTCGATTC
ACGTCGGGTTCA

>Brachypodium_distachyon_chr3.trna118-TrpCCA (13896382-13896311) Trp (CCA) 72 bp Sc: 77.63
GGATCCGTGGCGCAATGGTAGCGCGTCTGACTCCAGATCAGAAGGTTGCGTGTTCGATTC
ACGTCGGGTTCA
>Brachypodium_distachyon_chr3.trna65-TrpCCA (58368565-58368494) Trp (CCA) 72 bp Sc: 77.63
GGATCCGTGGCGCAATGGTAGCGCGTCTGACTCCAGATCAGAAGGTTGCGTGTTCGATTC
ACGTCGGGTTCA
>Brachypodium_distachyon_chr3.trna75-TrpCCA (48636944-48636873) Trp (CCA) 72 bp Sc: 77.63
GGATCCGTGGCGCAATGGTAGCGCGTCTGACTCCAGATCAGAAGGTTGCGTGTTCGATTC
ACGTCGGGTTCA
>Brachypodium_distachyon_chr3.trna77-TrpCCA (48510642-48510571) Trp (CCA) 72 bp Sc: 77.63
GGATCCGTGGCGCAATGGTAGCGCGTCTGACTCCAGATCAGAAGGTTGCGTGTTCGATTC
ACGTCGGGTTCA
>Brachypodium_distachyon_chr4.trna99-TrpCCA (7424590-7424519) Trp (CCA) 72 bp Sc: 77.63
GGATCCGTGGCGCAATGGTAGCGCGTCTGACTCCAGATCAGAAGGTTGCGTGTTCGATTC
ACGTCGGGTTCA
>Brachypodium_distachyon_chr5.trna38-TrpCCA (25320530-25320459) Trp (CCA) 72 bp Sc: 77.63
GGATCCGTGGCGCAATGGTAGCGCGTCTGACTCCAGATCAGAAGGTTGCGTGTTCGATTC
ACGTCGGGTTCA
>Brachypodium_distachyon_chr5.trna50-TrpCCA (15882606-15882535) Trp (CCA) 72 bp Sc: 77.63
GGATCCGTGGCGCAATGGTAGCGCGTCTGACTCCAGATCAGAAGGTTGCGTGTTCGATTC
ACGTCGGGTTCA
>Brachypodium_distachyon_chr5.trna51-TrpCCA (15680893-15680822) Trp (CCA) 72 bp Sc: 77.63
GGATCCGTGGCGCAATGGTAGCGCGTCTGACTCCAGATCAGAAGGTTGCGTGTTCGATTC
ACGTCGGGTTCA
>Brachypodium_distachyon_chr2.trna51-TyrATA (48880360-48880432) Tyr (ATA) 73 bp Sc: 45.86
GCGGTGATAGCTCAGATGGGAGAGCGTCGGAAATAACATCTGAAGGACCCGTGTTCGATC
CACGGTCACTGCA
>Brachypodium_distachyon_chr1.trna133-TyrGTA (54724890-54724807) Tyr (GTA) 84 bp Sc: 50.98
GGGTCGATGCCCGAGCGGTTAATGGGGACGGACTGTAAATTCGTTGACAAAATGTCTACG
CTGGTTCAAATCCAGCTCGGCCCA
>Brachypodium_distachyon_chr3.trna101-TyrGTA (28293295-28293212) Tyr (GTA) 84 bp Sc: 50.98
GGGTCGATGCCCGAGCGGTTAATGGGGACGGACTGTAAATTCGTTGACAAAATGTCTACG
CTGGTTCAAATCCAGCTCGGCCCA
>Brachypodium_distachyon_chr3.trna122-TyrGTA (7501619-7501536) Tyr (GTA) 84 bp Sc: 50.98
GGGTCGATGCCCGAGCGGTTAATGGGGACGGACTGTAAATTCGTTGACAAAATGTCTACG
CTGGTTCAAATCCAGCTCGGCCCA
>Brachypodium_distachyon_chr5.trna6-TyrGTA (9243386-9243469) Tyr (GTA) 84 bp Sc: 50.98
GGGTCGATGCCCGAGCGGTTAATGGGGACGGACTGTAAATTCGTTGACAAAATGTCTACG
CTGGTTCAAATCCAGCTCGGCCCA
>Brachypodium_distachyon_scaffold_6.trna22-TyrGTA (340549-340467) Tyr (GTA) 83 bp Sc: 53.60
GGGAGAGTGGCCGAGCGGTCAAAAGCGACAGACTGTAAATCTGTTGAAGGTTTTCTACGT
AGGTTCGAATCCTGCCTCTCCCA
>Brachypodium_distachyon_chr4.trna14-TyrGTA (14431578-14431665) Tyr (GTA) 88 bp Sc: 74.93
CCGACCTTAGCTCAGTTGGTAGAGCGGAGGACTGTAGTGCGTTGCAGATAAAATCCTTAG
GTCACTGGTTCGAATCCGGTAGGTCGGA
>Brachypodium_distachyon_chr3.trna114-TyrGTA (15427109-15427025) Tyr (GTA) 85 bp Sc: 77.66
CCGACCTTAGCTCAGTTGGTAGAGCGGAGGACTGTAGTGTTGCAGATAAATCCTTAGGTC
GCTGGTTCGAATCCGGCAGGTCGGA
>Brachypodium_distachyon_chr5.trna40-TyrGTA (24434148-24434063) Tyr (GTA) 86 bp Sc: 77.16
CCGACCTTAGCTCAGTTGGTAGAGCGGAGGACTGTAGTGTTCGCAGATAAATCCTTAGGT
CGCTGGTTCGAATCCGGCAGGTCGGA
>Brachypodium_distachyon_chr5.trna32-TyrGTA (27764422-27764336) Tyr (GTA) 87 bp Sc: 77.92
CCGACCTTAGCTCAGTTGGTAGAGCGGAGGACTGTAGTGTTTGCAGATTAAATCCTTAGG
TCGCTGGTTCGAATCCGGCAGGTCGGA
>Brachypodium_distachyon_chr4.trna32-TyrGTA (33318988-33319074) Tyr (GTA) 87 bp Sc: 74.52
CCGACCTTAGCTCAGTTGGTAGAGCGGAGGACTGTAGTGCGTTGCAGATTAATCCTTAGG
TCACTGGTTCGAATCCGGTAGGTCGGA
>Brachypodium_distachyon_chr4.trna73-TyrGTA (34372500-34372414) Tyr (GTA) 87 bp Sc: 78.06
CCGACCTTAGCTCAGTTGGTAGAGCGGAGGACTGTAGTTTGAAGCAGATTAATCCTTAGG
TCGCTGGTTCGAATCCGGCAGGTCGGA
>Brachypodium_distachyon_chr3.trna89-TyrGTA (37033788-37033704) Tyr (GTA) 85 bp Sc: 76.89
CCGACCTTAGCTCAGTTGGTAGAGCGGAGGACTGTAGTCGTTGCAGATAATCCTTAGGTC
GCTGGTTCGAATCCGGCAGGTCGGA
>Brachypodium_distachyon_chr3.trna87-TyrGTA (37542375-37542289) Tyr (GTA) 87 bp Sc: 75.44
CCGACCTTAGCTCAGTTGGTAGAGCGGAGGACTGTAGTACGTTGCAGAAAAATCCTTAGG
TCACTGGTTCGAATCCGGTAGGTCGGA
>Brachypodium_distachyon_chr3.trna34-TyrGTA (37822417-37822502) Tyr (GTA) 86 bp Sc: 75.03

CCGACCTTAGCTCAGTTGGTAGAGCGGAGGACTGTAGTCGTTGCAGATAAATCCTTAGGT
CACTGGTTCGAATCCGGTAGGTCGGA
>Brachypodium_distachyon_chr2.trna90-TyrGTA (50360167-50360082) Tyr (GTA) 86 bp Sc: 75.40
CCGACCTTAGCTCAGTTGGTAGAGCGGAGGACTGTAGTATTTCCAGATCAATCCTTAGGT
CACTGGTTCGAATCCGGTAGGTCGGA
>Brachypodium_distachyon_chr3.trna66-TyrGTA (58083907-58083822) Tyr (GTA) 86 bp Sc: 76.38
CCGACCTTAGCTCAGTTGGTAGAGCGGAGGACTGTAGTCGTTGCAGATCAATCCTTAGGT
CGCTGGTTCGAATCCGGCAGGTCGGA
>Brachypodium_distachyon_chr1.trna104-TyrGTA (69768723-69768638) Tyr (GTA) 86 bp Sc: 77.16
CCGACCTTAGCTCAGTTGGTAGAGCGGAGGACTGTAGTCGTTGCAGATAAATCCTTAGGT
CGCTGGTTCGAATCCGGCAGGTCGGA
>Brachypodium_distachyon_chr1.trna96-Undet??? (73289240-73289160) Undet (???) 81 bp Sc: 23.64
GGTGGTGTAGCTCAATTGGTAGAGCAAACGCAAACGCTTGTGTTAGGTATGGGGTATGGG
GTTCGATTCCTCGCATCTCCA
>Brachypodium_distachyon_chr2.trna113-Undet??? (16637543-16637470) Undet (???) 74 bp Sc: 26.05
GGGGATGTAGCTCAATTGGTAGAGCAAATGCAAATGCTTGTGTTAGGTATGGGGTTTGAT
TCCTTGCATCTCCA
>Brachypodium_distachyon_chr1.trna97-Undet??? (73289091-73289018) Undet (???) 74 bp Sc: 31.13
GGGGATGTAGCTCAATTGGTAGAGCAAATGCAAACGCTTGTGTTAGGTACGGGGTTTGAT
TCCTTGCATCTCCA
>Brachypodium_distachyon_chr4.trna42-Undet??? (41011373-41011446) Undet (???) 74 bp Sc: 33.04
GGGGATGTAGCTCAATTGGTAGAGAAAAAACAAACGCTTGTGTTAAGTTCGGGGTTCGAT
TCCTCGCATCTCCA
>Brachypodium_distachyon_chr2.trna35-Undet??? (23881057-23881130) Undet (???) 74 bp Sc: 33.37
GGGGATGTAGCTCAGTTGGTAGAGCGATTGAAATTGCTTTCAGTAGGCTTGGGGTTTGAC
TCCTCACATCTCCA
>Brachypodium_distachyon_chr1.trna98-Undet??? (73288951-73288878) Undet (???) 74 bp Sc: 40.21
GGGGATGTAGCTCAATTGGTAGAGCAAATGCAAACGCTTGTGTTAGGTACGGGGTTCGAT
TCCTCGCATCTCCA
>Brachypodium_distachyon_chr5.trna36-Undet??? (25629842-25629772) Undet (???) 71 bp Sc: 53.03
GCGTCCATTGTCTAATGGATAGGACAGAGGTCTTCTAACCTTTGGTATAGGTTCAAATCC
TATTGGACGCA
>Brachypodium_distachyon_chr1.trna124-ValAAC (57070584-57070511) Val (AAC) 74 bp Sc: 75.48
GGTTTCGTGGTGTAGTTGGTTATCACGTCAGTCTAACACACTGAAGGTCTCCGGTTCAAA
CCCGGGCGAAGCCA
>Brachypodium_distachyon_chr2.trna72-ValAAC (59007354-59007427) Val (AAC) 74 bp Sc: 75.48
GGTTTCGTGGTGTAGTTGGTTATCACGTCAGTCTAACACACTGAAGGTCTCCGGTTCAAA
CCCGGGCGAAGCCA
>Brachypodium_distachyon_chr1.trna16-ValAAC (4573767-4573840) Val (AAC) 74 bp Sc: 77.22
GGTTTCGTGGTGTAGTTGGTTATCACGTCAGTCTAACACACTGAAGGTCTCCGGTTCGAA
CCCGGGCGAAGCCA
>Brachypodium_distachyon_chr1.trna4-ValAAC (1530755-1530828) Val (AAC) 74 bp Sc: 77.22
GGTTTCGTGGTGTAGTTGGTTATCACGTCAGTCTAACACACTGAAGGTCTCCGGTTCGAA
CCCGGGCGAAGCCA
>Brachypodium_distachyon_chr1.trna62-ValAAC (51468509-51468582) Val (AAC) 74 bp Sc: 77.22
GGTTTCGTGGTGTAGTTGGTTATCACGTCAGTCTAACACACTGAAGGTCTCCGGTTCGAA
CCCGGGCGAAGCCA
>Brachypodium_distachyon_chr2.trna110-ValAAC (20779116-20779043) Val (AAC) 74 bp Sc: 77.22
GGTTTCGTGGTGTAGTTGGTTATCACGTCAGTCTAACACACTGAAGGTCTCCGGTTCGAA
CCCGGGCGAAGCCA
>Brachypodium_distachyon_chr2.trna83-ValAAC (55025738-55025665) Val (AAC) 74 bp Sc: 77.22
GGTTTCGTGGTGTAGTTGGTTATCACGTCAGTCTAACACACTGAAGGTCTCCGGTTCGAA
CCCGGGCGAAGCCA
>Brachypodium_distachyon_chr3.trna1-ValAAC (956323-956396) Val (AAC) 74 bp Sc: 77.22
GGTTTCGTGGTGTAGTTGGTTATCACGTCAGTCTAACACACTGAAGGTCTCCGGTTCGAA
CCCGGGCGAAGCCA
>Brachypodium_distachyon_chr3.trna123-ValAAC (7331140-7331067) Val (AAC) 74 bp Sc: 77.22
GGTTTCGTGGTGTAGTTGGTTATCACGTCAGTCTAACACACTGAAGGTCTCCGGTTCGAA
CCCGGGCGAAGCCA
>Brachypodium_distachyon_chr3.trna40-ValAAC (43445447-43445520) Val (AAC) 74 bp Sc: 77.22
GGTTTCGTGGTGTAGTTGGTTATCACGTCAGTCTAACACACTGAAGGTCTCCGGTTCGAA
CCCGGGCGAAGCCA
>Brachypodium_distachyon_chr3.trna8-ValAAC (6959115-6959188) Val (AAC) 74 bp Sc: 77.22
GGTTTCGTGGTGTAGTTGGTTATCACGTCAGTCTAACACACTGAAGGTCTCCGGTTCGAA
CCCGGGCGAAGCCA
>Brachypodium_distachyon_chr4.trna28-ValAAC (29159127-29159200) Val (AAC) 74 bp Sc: 77.22
GGTTTCGTGGTGTAGTTGGTTATCACGTCAGTCTAACACACTGAAGGTCTCCGGTTCGAA

CCCGGGCGAAGCCA
>Brachypodium_distachyon_chr5.trna39-ValAAC (24474183-24474110) Val (AAC) 74 bp Sc: 77.22
GGTTTCGTGGTGTAGTTGGTTATCACGTCAGTCTAACACACTGAAGGTCTCCGGTTCGAA
CCCGGGCGAAGCCA
>Brachypodium_distachyon_scaffold_6.trna7-ValAAC (139575-139659) Val (AAC) 85 bp Sc: 28.24
GCATCCATGGCTGAATGGTGAAAGCGCCCACCAACCTAGAAAGGAAAAATGGGTAAAAAA
GTAGGTTCGATTCCTGCCGGATGCA
>Brachypodium_distachyon_chr3.trna61-ValAAC (59854599-59854515) Val (AAC) 85 bp Sc: 24.77
GCATCCATGGCTGAATGGTGAAAGCGCCCACCAACCTAGAAAGGCAAAATGGGTCAAAAA
GTAGGTTCGACTCCTGCCGGATGCA
>Brachypodium_distachyon_chr5.trna56-ValCAC (10672193-10672120) Val (CAC) 74 bp Sc: 68.51
GTCAGGGTGGTGTAGTTGGTTATCACGTTAGTCTCACACACTAAAGGTCCCCAGTTCGAA
CCTGAGCTTAGACA
>Brachypodium_distachyon_chr4.trna16-ValCAC (14518582-14518655) Val (CAC) 74 bp Sc: 81.25
GTCTGGGTGGTGTAGTTGGTTATCACGTTAGTCTCACACACTAAAGGTCCCCAGTTCGAA
CCTGGGCTTAGACA
>Brachypodium_distachyon_chr4.trna75-ValCAC (33454049-33453976) Val (CAC) 74 bp Sc: 81.25
GTCTGGGTGGTGTAGTTGGTTATCACGTTAGTCTCACACACTAAAGGTCCCCAGTTCGAA
CCTGGGCTTAGACA
>Brachypodium_distachyon_chr1.trna1-ValCAC (1247156-1247229) Val (CAC) 74 bp Sc: 82.62
GTCTGGGTGGTGTAGTTGGTTATCACGTTAGTCTCACACACTAAAGGTCCCCAGTTCGAA
CCTGGGCTCAGACA
>Brachypodium_distachyon_chr1.trna24-ValCAC (10735326-10735399) Val (CAC) 74 bp Sc: 82.62
GTCTGGGTGGTGTAGTTGGTTATCACGTTAGTCTCACACACTAAAGGTCCCCAGTTCGAA
CCTGGGCTCAGACA
>Brachypodium_distachyon_chr2.trna66-ValCAC (55583945-55584018) Val (CAC) 74 bp Sc: 82.62
GTCTGGGTGGTGTAGTTGGTTATCACGTTAGTCTCACACACTAAAGGTCCCCAGTTCGAA
CCTGGGCTCAGACA
>Brachypodium_distachyon_chr3.trna44-ValCAC (44312619-44312692) Val (CAC) 74 bp Sc: 82.62
GTCTGGGTGGTGTAGTTGGTTATCACGTTAGTCTCACACACTAAAGGTCCCCAGTTCGAA
CCTGGGCTCAGACA
>Brachypodium_distachyon_chr4.trna15-ValCAC (14505423-14505496) Val (CAC) 74 bp Sc: 82.62
GTCTGGGTGGTGTAGTTGGTTATCACGTTAGTCTCACACACTAAAGGTCCCCAGTTCGAA
CCTGGGCTCAGACA
>Brachypodium_distachyon_chr4.trna31-ValCAC (31538163-31538236) Val (CAC) 74 bp Sc: 82.62
GTCTGGGTGGTGTAGTTGGTTATCACGTTAGTCTCACACACTAAAGGTCCCCAGTTCGAA
CCTGGGCTCAGACA
>Brachypodium_distachyon_chr4.trna55-ValCAC (47333237-47333164) Val (CAC) 74 bp Sc: 82.62
GTCTGGGTGGTGTAGTTGGTTATCACGTTAGTCTCACACACTAAAGGTCCCCAGTTCGAA
CCTGGGCTCAGACA
>Brachypodium_distachyon_chr5.trna48-ValCAC (18013959-18013886) Val (CAC) 74 bp Sc: 82.62
GTCTGGGTGGTGTAGTTGGTTATCACGTTAGTCTCACACACTAAAGGTCCCCAGTTCGAA
CCTGGGCTCAGACA
>Brachypodium_distachyon_chr3.trna20-ValGAC (26471540-26471611) Val (GAC) 72 bp Sc: 51.67
AGGGATATAACTCAGCGGTAGAGTGTCACCTTGACGTGGTGGAAGTCATCAGTTCGAGCC
TGATTATCCCTA
>Brachypodium_distachyon_chr3.trna22-ValGAC (28288141-28288212) Val (GAC) 72 bp Sc: 51.67
AGGGATATAACTCAGCGGTAGAGTGTCACCTTGACGTGGTGGAAGTCATCAGTTCGAGCC
TGATTATCCCTA
>Brachypodium_distachyon_chr4.trna87-ValGAC (19566463-19566392) Val (GAC) 72 bp Sc: 51.67
AGGGATATAACTCAGCGGTAGAGTGTCACCTTGACGTGGTGGAAGTCATCAGTTCGAGCC
TGATTATCCCTA
>Brachypodium_distachyon_chr5.trna44-ValTAC (21887935-21887863) Val (TAC) 73 bp Sc: 74.81
GTTTCTGTGGTGTAGTGGTCATCACGTCAGTTTTACACACTGAAGATCCCCAGTTCGATC
CTGGGCAGGAACA
>Brachypodium_distachyon_chr2.trna120-ValTAC (13139877-13139805) Val (TAC) 73 bp Sc: 76.67
GTTGCTGTGGTGTAGTGGTTATCACGTCAGTCTTACACACTGAAGGTCTCCAGTTCGATC
CTGGGCAGCAACA
>Brachypodium_distachyon_chr2.trna20-ValTAC (16676349-16676421) Val (TAC) 73 bp Sc: 76.67
GTTGCTGTGGTGTAGTGGTTATCACGTCAGTCTTACACACTGAAGGTCTCCAGTTCGATC
CTGGGCAGCAACA
>Brachypodium_distachyon_chr2.trna50-ValTAC (48786753-48786825) Val (TAC) 73 bp Sc: 76.67
GTTGCTGTGGTGTAGTGGTTATCACGTCAGTCTTACACACTGAAGGTCTCCAGTTCGATC
CTGGGCAGCAACA
>Brachypodium_distachyon_chr4.trna6-ValTAC (3855861-3855934) Val (TAC) 74 bp Sc: 79.77
GGTGCTGTGGTGTAGTCGGTTATCACGTTAGTCTTACACACTAAAGGTCCCCAGTTCGAG
CCTGGGCAGCACCA

>Beijerinckia_indica_ATCC_9039_chr.trna20-AlaCGC (3289472-3289547) Ala (CGC) 76 bp Sc: 85.78
GGGGCTGTAGCTCAGATGGGAGAGCGCTGCAATCGCACTGCAGAGGTCAGGGGTTCGATT
CCCCTCAGCTCCACCA
>Beijerinckia_indica_ATCC_9039_chr.trna21-AlaGGC (3352815-3352890) Ala (GGC) 76 bp Sc: 84.87
GGGGTCATAGCTCAGTTGGGAGAGCGCTTCAATGGCATTGAAGAGGTCGGCGGTTCGATT
CCGCCTGGCTCCACCA
>Beijerinckia_indica_ATCC_9039_chr.trna26-AlaTGC (3931734-3931659) Ala (TGC) 76 bp Sc: 87.21
GGGGCCATAGCTCAGTTGGGAGAGCGCGTGCTTTGCAAGCATGAGGTCGTCGGTTCGATC
CCGTCTGGCTCCACCA
>Beijerinckia_indica_ATCC_9039_chr.trna32-AlaTGC (3418064-3417989) Ala (TGC) 76 bp Sc: 87.21
GGGGCCATAGCTCAGTTGGGAGAGCGCGTGCTTTGCAAGCATGAGGTCGTCGGTTCGATC
CCGTCTGGCTCCACCA
>Beijerinckia_indica_ATCC_9039_chr.trna4-AlaTGC (682425-682500) Ala (TGC) 76 bp Sc: 87.21
GGGGCCATAGCTCAGTTGGGAGAGCGCGTGCTTTGCAAGCATGAGGTCGTCGGTTCGATC
CCGTCTGGCTCCACCA
>Beijerinckia_indica_ATCC_9039_chr.trna23-ArgACG (4009812-4009736) Arg (ACG) 77 bp Sc: 89.50
GCGCCCGTAGCTCAGCTGGATAGAGCACTAGACTACGAATCTAGGGGTCAGGAGTTCGAA
TCTCTTCGGGCGCGCCA
>Beijerinckia_indica_ATCC_9039_chr.trna30-ArgCCG (3529719-3529643) Arg (CCG) 77 bp Sc: 89.17
GCACCCGTAGCTCAGCTGGATAGAGCGCTGCCCTCCGAAGGCAGAGGCCAGAGGTTCGAA
TCCTCTCGGGTGCGCCA
>Beijerinckia_indica_ATCC_9039_chr.trna22-ArgCCT (3610522-3610598) Arg (CCT) 77 bp Sc: 83.59
CTCCCGGTAGCTCAGCAGGATAGAGCAACGGTTTCCTAAACCGTAGGTCAGGGGTTCGAA
TCCCTTCCGGGAGGCCA
>Beijerinckia_indica_ATCC_9039_chr.trna49-ArgTCT (1751788-1751712) Arg (TCT) 77 bp Sc: 91.17
GGCCCCATAGCTCAGCTGGATAGAGCAGCCGCCTTCTAAGCGGCAGGTCGCAGGTTCGAA
CCCTGCTGGGGTCGCCA
>Beijerinckia_indica_ATCC_9039_chr.trna17-AsnGTT (2438863-2438938) Asn (GTT) 76 bp Sc: 90.13
TCCCTGATAGCTCAGCCGGTAGAGCAATCGACTGTTAATCGATCGGTCGCAGGTTCGAGT
CCTGCTCAGGGAGCCA
>Beijerinckia_indica_ATCC_9039_chr.trna37-AspGTC (2740885-2740809) Asp (GTC) 77 bp Sc: 92.70
GGGGGAGTAGCTCAGCTGGTTAGAGCGCCGGCCTGTCACGCCGGAGGTCGCGGGTTCGAG
CCCCGTCTCTCTCGCCA
>Beijerinckia_indica_ATCC_9039_chr.trna41-CysGCA (2438654-2438581) Cys (GCA) 74 bp Sc: 66.24
GGCCTCGTGGCGGAGTGGTTACGCAGAGGACTGCAAATCCTTGCACCCCGGTTCGATTCC
GGGCGAGGCCTCCA
>Beijerinckia_indica_ATCC_9039_chr.trna43-GlnCTG (2160025-2159952) Gln (CTG) 74 bp Sc: 71.34
TGGGGGATAGTTCAATGGTAGAACAGCGGACTCTGACTCCGTTAATCTTGGTTCGAATCC
AGGTCCCCCAGCCA
>Beijerinckia_indica_ATCC_9039_chr.trna16-GlnTTG (2422185-2422259) Gln (TTG) 75 bp Sc: 81.90
TGGGGCGTAGCCAAGTGGTAAGGCAGCGGATTTTGATTCCGCCATTCGGAGGTTCGAGTC
CTCCCGCCCCAGCCA
>Beijerinckia_indica_ATCC_9039_chr.trna6-GluCTC (932762-932837) Glu (CTC) 76 bp Sc: 67.01
GCTCCCTTCGTCTAGCGGTCTAGGACGTCGCCCTCTCACGGCGAAAACAGGGGTTCGAGT
CCCCTAGGGAGCGCCA
>Beijerinckia_indica_ATCC_9039_chr.trna47-GluTTC (1764532-1764458) Glu (TTC) 75 bp Sc: 57.07
GCGCTCTTCGTCTATCGGTTAGGACGGCACCCTTTCACGGTGCAGAGAGGGGTTCGATTC
CCCTAGAGCGCGCCA
>Beijerinckia_indica_ATCC_9039_chr.trna1-GlyCCC (431977-432050) Gly (CCC) 74 bp Sc: 80.62
GCGGGCGTAGTTCAATGGTAGAACGGCAGCTTCCCAAGCTGCATACGAGGGTTCGATTCC
CTTCGCCCGCTCCA
>Beijerinckia_indica_ATCC_9039_chr.trna34-GlyGCC (3178840-3178766) Gly (GCC) 75 bp Sc: 88.17
GCGGGTGTAGCTCAGGGGTAGAGCACAACCTTGCCAAGGTTGGGGTCGAGGGTTCGAATC
CCTTCGCCCGCTCCA
>Beijerinckia_indica_ATCC_9039_chr.trna13-GlyTCC (1547422-1547495) Gly (TCC) 74 bp Sc: 82.33
GCGGGTGTAGCTCAATGGTAGAGCAGCAGCCTTCCAAGCTGAATACGAGGGTTCGATTCC
CTTCACCCGCTCCA
>Beijerinckia_indica_ATCC_9039_chr.trna51-HisGTG (759445-759369) His (GTG) 77 bp Sc: 85.36
GCCGCCATAGCTCAGTTGGTTAGAGCGCTAGATTGTGGATCTAGAGGTCCCCCGTTCGAG
CCGGGGTGGCGGTACCA
>Beijerinckia_indica_ATCC_9039_chr.trna25-IleGAT (3931940-3931864) Ile (GAT) 77 bp Sc: 93.43
GGGCTTGTAGCTCAGTTGGTTAGAGCGCGCGCTTGATAAGCGTGAGGTCGGAAGTTCAAG
TCTTCCCAGGCCCACCA
>Beijerinckia_indica_ATCC_9039_chr.trna3-IleGAT (682219-682295) Ile (GAT) 77 bp Sc: 93.43
GGGCTTGTAGCTCAGTTGGTTAGAGCGCGCGCTTGATAAGCGTGAGGTCGGAAGTTCAAG
TCTTCCCAGGCCCACCA
>Beijerinckia_indica_ATCC_9039_chr.trna31-IleGAT (3418291-3418215) Ile (GAT) 77 bp Sc: 93.43

GGGCTTGTAGCTCAGTTGGTTAGAGCGCGCTTGATAAGCGTGAGGTCGGAAGTTCAAG
TCTTCCCAGGCCCACCA
>Beijerinckia_indica_ATCC_9039_chr.trna42-LeuCAA (2391696-2391610) Leu (CAA) 87 bp Sc: 79.42
GCGGGCGTGGCGGAACAGGTAGACGCGCCGGACTCAAAATCCGGTTCTGGTAACAGAGTG
TCGGTTCGATTCCGACCGCCCGCACCA
>Beijerinckia_indica_ATCC_9039_chr.trna10-LeuCAG (1515738-1515822) Leu (CAG) 85 bp Sc: 69.76
GCCCTGGTGGCGGAATTGGTAGACGCGCTGGTTTCAGGTACCAGTGGGTAACACCGTGGA
GGTTCGAGTCCTCTCCAGGGCACCA
>Beijerinckia_indica_ATCC_9039_chr.trna46-LeuGAG (1832213-1832129) Leu (GAG) 85 bp Sc: 71.14
GCGGCCATGGCGGAATTGGTAGACGCGCTAGCTTGAGGTGCTAGTTGGGAGACCAGTGGA
GGTTCGAGTCCTCTTGGCCGCACCA
>Beijerinckia_indica_ATCC_9039_chr.trna39-LeuTAG (2499512-2499428) Leu (TAG) 85 bp Sc: 77.94
GCGGGCGTGGCGGAATTGGTAGACGCGCCGGATTTAGGTTCCGGTGATGAAAATCGTGGG
GGTTCGAGTCCCTTCGCCCGCACCA
>Beijerinckia_indica_ATCC_9039_chr.trna2-LysCTT (667706-667781) Lys (CTT) 76 bp Sc: 91.42
GGGCGTATAGCTCAGTTGGTAGAGCAGCTGACTCTTAATCAGCGGGTCCACAGTTCGAGC
CTGTGTGCGCCCACCA
>Beijerinckia_indica_ATCC_9039_chr.trna45-LysTTT (2021140-2021065) Lys (TTT) 76 bp Sc: 96.75
GAGCGCGTAGCTCAGTCGGTAGAGCATCTGACTTTTAATCAGAGGGTCATGGGTTCGAAT
CCCATCGCGCTCACCA
>Beijerinckia_indica_ATCC_9039_chr.trna35-MetCAT (3051495-3051419) Met (CAT) 77 bp Sc: 85.32
GGCGGAGTAGCTCAGCCGGCTAGAGCAGAGGAATCATAATCCTTGTGTCGGGGGTTCGAG
TCCCTCCTCCGCTACCA
>Beijerinckia_indica_ATCC_9039_chr.trna27-MetCAT (3928066-3927990) Met (CAT) 77 bp Sc: 85.51
CGCGGGGTGGAGCAGCCCGGTAGCTCGTCAGGCTCATAACCTGAAGGTCACAGGTTCAAA
TCCTGTCCCCGCAACCA
>Beijerinckia_indica_ATCC_9039_chr.trna33-MetCAT (3414406-3414330) Met (CAT) 77 bp Sc: 85.51
CGCGGGGTGGAGCAGCCCGGTAGCTCGTCAGGCTCATAACCTGAAGGTCACAGGTTCAAA
TCCTGTCCCCGCAACCA
>Beijerinckia_indica_ATCC_9039_chr.trna5-MetCAT (686093-686169) Met (CAT) 77 bp Sc: 85.51
CGCGGGGTGGAGCAGCCCGGTAGCTCGTCAGGCTCATAACCTGAAGGTCACAGGTTCAAA
TCCTGTCCCCGCAACCA
>Beijerinckia_indica_ATCC_9039_chr.trna24-MetCAT (3968812-3968737) Met (CAT) 76 bp Sc: 86.11
GGGCCGGTAGCTCAATGGTTAGAGCCGGCCGCTCATAACGGTCTGGTTGCAGGTTCGAGT
CCTGCCCGGCCCACCA
>Beijerinckia_indica_ATCC_9039_chr.trna7-PheGAA (1164649-1164724) Phe (GAA) 76 bp Sc: 91.10
GCCCAGGTAGCTCAGTTGGTAGAGCATGCGACTGAAAATCGCAGTGTCGGTGGTTCGATT
CCGCCCCTGGGCACCA
>Beijerinckia_indica_ATCC_9039_chr.trna36-ProCGG (2763339-2763263) Pro (CGG) 77 bp Sc: 88.00
CGGAGTGTAGCGCAGCCTGGTAGCGCACCTCGTTCGGGACGAGGGGGTCGGAGGTTCGAA
TCCTCTCACTCCGACCA
>Beijerinckia_indica_ATCC_9039_chr.trna28-ProGGG (3830915-3830838) Pro (GGG) 78 bp Sc: 79.89
CGGAGTGTAGCGCAGCCCGGTTAGCGCACTAGTCTGGGGGACTAGGGGTCGTGGGTTCGA
ATCCCGCCACTCCGACCA
>Beijerinckia_indica_ATCC_9039_chr.trna48-ProTGG (1752260-1752184) Pro (TGG) 77 bp Sc: 85.37
CGGGGTATAGCGCAGTCTGGTAGCGCGGAAGTTTTGGGAACTTCAGGTCGCAGGTTCGAA
TCCTGCTGCCCCGACCA
>Beijerinckia_indica_ATCC_9039_chr.trna18-ThrCGT (2869646-2869721) Thr (CGT) 76 bp Sc: 53.13
GGCTGAGTAGCTCAGCCGGCTAGAGCACGTCATTCGTAATGAGAGGGTCGGGGTTCGAGT
CCCTCCCCTGCAGCCA
>Beijerinckia_indica_ATCC_9039_chr.trna52-SerCGA (45536-45447) Ser (CGA) 90 bp Sc: 84.29
GGAGAGGTGGCTGAGTGGTTGAAAGCACCGCACTCGAAATGCGGCATAGGTGCAAGCCTA
TCGGGGGTTCGAATCCCCCCCTCTCCGCCA
>Beijerinckia_indica_ATCC_9039_chr.trna15-SerGCT (2137115-2137208) Ser (GCT) 94 bp Sc: 72.33
GGAGACGTGGCCGAGAGGCTGAAGGCGGCGGTTTGCTAAACCGTTATAGGGTTGTAAAGC
CCTATCGAGGGTTCGAATCCCTCCGTCTCCGCCA
>Beijerinckia_indica_ATCC_9039_chr.trna50-SerGGA (1198394-1198305) Ser (GGA) 90 bp Sc: 76.79
GGAGGGGTGGCCGAGTGGTTGAAGGCGCACGCCTGGAAAGTGTGTATACGGGAAACCGTA
TCGCGGGTTCGAATCCCGCTCCCTCCGCCA
>Beijerinckia_indica_ATCC_9039_chr.trna38-SerTGA (2623984-2623895) Ser (TGA) 90 bp Sc: 73.46
GGAAGGGTGGCCGAGTGGTTTAAGGCAGCGGTCTTGAAAACCGCCGTGGGTGCAAGCCTA
CCGTGGGTTCGAATCCCACCTCTTCCGCCA
>Beijerinckia_indica_ATCC_9039_chr.trna9-ThrCGT (1382498-1382573) Thr (CGT) 76 bp Sc: 100.45
GCCGCAATAGCTCAGCTGGTAGAGCACCTCATTCGTAATGAGGGGGTCGGGGGTTCGAAT
CCCTCTTGCGGCACCA
>Beijerinckia_indica_ATCC_9039_chr.trna19-ThrGGT (3100608-3100682) Thr (GGT) 75 bp Sc: 87.76
GCTGCCGTAGCTCAGTGGTAGAGCACTCCCTTGGTAAGGGAGAGGTCGAGAGTTCGATCC

TCTCTGGCAGCACCA
>Beijerinckia_indica_ATCC_9039_chr.trna44-ThrTGT (2070017-2069942) Thr (TGT) 76 bp Sc: 98.31
GCCGGTTTAGCTCAGTTGGTAGAGCAACTGATTTGTAATCAGTAGGTCGCGGGTTCGACT
CCTGCAACCGGCACCA
>Beijerinckia_indica_ATCC_9039_chr.trna8-TrpCCA (1307425-1307502) Trp (CCA) 78 bp Sc: 84.09
AGGAGTGTAGCTCAATTTGGTTAGAGCACCGGTCTCCAAAACCGGGGGTTGGGGGTTCGA
GTCCCTCCTCTCCTGCCA
>Beijerinckia_indica_ATCC_9039_chr.trna12-TyrGTA (1547235-1547317) Tyr (GTA) 83 bp Sc: 59.64
GGGAGTGTCCCGAGCGGCAAAGGGGGCGGACTGTAAATCCGCTGGCTATGCCTTCGTAGG
TTCGAGTCCTACCTCCCCCACCA
>Beijerinckia_indica_ATCC_9039_chr.trna11-ValCAC (1516464-1516535) Val (CAC) 72 bp Sc: 67.08
GGGTGCGTAGCTCAGCGGGAGAGCACTCCCTTCACACGGGAGGGGTCATAGGTTCAATCC
CTGTGGCGCCCA
>Beijerinckia_indica_ATCC_9039_chr.trna14-ValCAC (1899325-1899399) Val (CAC) 75 bp Sc: 88.42
GGGCGCGTAGCTCAGCGGGAGAGCACTCCCTTCACACGGGAGGGGTCACAGGTTCAATCC
CTGTCGCGCCCACCA
>Beijerinckia_indica_ATCC_9039_chr.trna29-ValGAC (3660181-3660107) Val (GAC) 75 bp Sc: 88.73
GGGCGCGTAGCTCAGCGGGAGAGCACTACCTTGACATGGTAGGGGTCACAGGTTCGATCC
CTGTCGCGCCCACCA
>Beijerinckia_indica_ATCC_9039_chr.trna40-ValTAC (2491813-2491738) Val (TAC) 76 bp Sc: 94.06
GGGCGGTTAGCTCAGTTGGTAGAGCATCTCGTTTACACCGAGAGGGTCGGCGGTTCGAGT
CCGTCACCGCCTACCA
>Bifidobacterium_adolescentis_ATCC_15703_chr.trna28-AlaCGC (1951626-1951701) Ala (CGC) 76 bp Sc: 78.13
GGGGCTGTAGCTCAGTTGGTAGAGCGCTTCGTTCGCATCGAAGAGGTCGCGGTTTCGACT
ACCGTCAGCTCCACGA
>Bifidobacterium_adolescentis_ATCC_15703_chr.trna54-AlaGGC (13132-13060) Ala (GGC) 73 bp Sc: 80.60
GGGGCTATGGCGCAGCTGGTAGCGCATCTCCATGGCATGGAGAGGGTCAGGGGTTCGAAT
CCCCTTAGCTCCA
>Bifidobacterium_adolescentis_ATCC_15703_chr.trna33-AlaGGC (1084245-1084170) Ala (GGC) 76 bp Sc: 81.22
GGGGCTATGGCGCAGCTGGTAGCGCATCTCCATGGCATGGAGAGGGTCAGGGGTTCGAAT
CCCCTTAGCTCCACGA
>Bifidobacterium_adolescentis_ATCC_15703_chr.trna53-AlaTGC (13206-13134) Ala (TGC) 73 bp Sc: 89.29
GGGGCTATAGCGCAGCTGGTAGCGCATCTGCTTTGCAAGCAGAGGGTCGCCGGTTCGAAC
CCGGCTAGCTCCA
>Bifidobacterium_adolescentis_ATCC_15703_chr.trna18-ArgACG (738577-738650) Arg (ACG) 74 bp Sc: 73.52
GCGCGAGTAGCCCAGCGGATTAGAGCAGCTGACTACGGATCAGCAGGTCGCAGGTTCGAA
TCCTGTCTCGCGCA
>Bifidobacterium_adolescentis_ATCC_15703_chr.trna17-ArgACG (738459-738535) Arg (ACG) 77 bp Sc: 81.82
GCGCGAGTAGCCCAGCGGATTAGAGCAGCTGACTACGGATCAGCAGGTCGCAGGTTCGAA
TCCTGTCTCGCGCACCA
>Bifidobacterium_adolescentis_ATCC_15703_chr.trna32-ArgCCG (1796439-1796364) Arg (CCG) 76 bp Sc: 74.44
GGGTTTGTAGCTCAGCGGATAGAGCGTCTGTCTCCGGAACAGAAGGTCGTGGGTTCGATC
CCCATCAAGCCCACCG
>Bifidobacterium_adolescentis_ATCC_15703_chr.trna26-ArgCCT (1522409-1522481) Arg (CCT) 73 bp Sc: 77.22
GCCTCAGTAGCTCAGTGGATAGAGCACTTCTCTCCTAAAGAAGGTGTCGTAGGTTCGATT
CCTATCTGGGGCA
>Bifidobacterium_adolescentis_ATCC_15703_chr.trna16-ArgTCT (626466-626542) Arg (TCT) 77 bp Sc: 83.49
GGCCCTGTAGCTCAGCTGGATAGAGCATGTGACTTCTAATCTCAAGGTCGCCGGTTCGAG
CCCGGCCGGGGTCACCA
>Bifidobacterium_adolescentis_ATCC_15703_chr.trna23-AsnGTT (972247-972319) Asn (GTT) 73 bp Sc: 79.71
TCCTGCGTAGCTCAGTTGGCAGAGCATTCGACTGTTAATCGAACGGTCACTGGTTCAAGC
CCAGTCGCAGGAG
>Bifidobacterium_adolescentis_ATCC_15703_chr.trna22-AsnGTT (972147-972219) Asn (GTT) 73 bp Sc: 81.02
TCCTGCGTAGCTCAGTTGGCAGAGCATCCGACTGTTAATCGGACGGTCACTGGTTCAAGC
CCAGTCGCAGGAG
>Bifidobacterium_adolescentis_ATCC_15703_chr.trna34-AsnGTT (1071366-1071291) Asn (GTT) 76 bp Sc: 89.32
TCCTGCGTAGCTCAGTTGGCAGAGCATCCGACTGTTAATCGGACGGTCACTGGTTCAAGC
CCAGTCGCAGGAGCCA
>Bifidobacterium_adolescentis_ATCC_15703_chr.trna13-AspGTC (493171-493244) Asp (GTC) 74 bp Sc: 77.86
GGCCGCGTAGCTCAGTTGGTTAGAGCGCCGCCCTGTCACGGCGGAGGTCACCGGTTCAAG
TCCGGCCGTGGTCG
>Bifidobacterium_adolescentis_ATCC_15703_chr.trna46-AspGTC (490738-490662) Asp (GTC) 77 bp Sc: 86.15
GGCCGCGTAGCTCAGTTGGTTAGAGCGCCGCCCTGTCACGGCGGAGGTCACCGGTTCAAG
TCCGGCCGTGGTCGCCA
>Bifidobacterium_adolescentis_ATCC_15703_chr.trna37-CysGCA (1029044-1028974) Cys (GCA) 71 bp Sc: 54.73
GGTGGGTTAGCCAAGCGGTTAGGCAGCGGCCTGCAAAGCCGTATAGACGAGTTCGACTCT
CGTACCCACCT

>Bifidobacterium_adolescentis_ATCC_15703_chr.trna3-GlnCTG (240596-240670) Gln (CTG) 75 bp Sc: 63.28
TGGGATGTGGTGTAATTGGCAACACAGCTGATTCTGGTTCAGCCATTCTTGGTTCGAGTC
CAGGCATCCCAGCCA
>Bifidobacterium_adolescentis_ATCC_15703_chr.trna24-GlnTTG (1107262-1107335) Gln (TTG) 74 bp Sc: 49.45
TCCCCCATGGTGTAATGGCAGCACACGGGTCTTTGGAACCCTTTGTCTTGGTTCGAGTCC
AGGTGGGGGAGCAA
>Bifidobacterium_adolescentis_ATCC_15703_chr.trna2-GluCTC (240485-240560) Glu (CTC) 76 bp Sc: 49.25
GCCCCCGTCGTCTAGCGGTCTAGGACTACGCCCTCTCACGGCGCCAACACCGGTTCAAAT
CCGGTCGGGGGTACGA
>Bifidobacterium_adolescentis_ATCC_15703_chr.trna12-GluTTC (493074-493145) Glu (TTC) 72 bp Sc: 53.66
GCCCCCTTCGTCTAACGGTTAGGACACCAGACTTTCAATCTGACAACGAGAGTTCGACTC
TCTCAGGGGGTA
>Bifidobacterium_adolescentis_ATCC_15703_chr.trna1-GlyCCC (81167-81240) Gly (CCC) 74 bp Sc: 67.14
GCGGATGTAGTTCATCGGTAGAACGAAAGCTTCCCAAGCTTTAAAGGCGGGTTCGACTCC
CGTCATCCGCTCCA
>Bifidobacterium_adolescentis_ATCC_15703_chr.trna35-GlyGCC (1060616-1060541) Gly (GCC) 76 bp Sc: 76.41
GCGGACATAGCTTAGTTGGTAAAGCGCAACCTTGCCAAGGTTGAGACCGCGGGTCCGAGT
CCCGTTGTCCGCTCCA
>Bifidobacterium_adolescentis_ATCC_15703_chr.trna45-GlyGCC (628805-628730) Gly (GCC) 76 bp Sc: 76.41
GCGGACATAGCTTAGTTGGTAAAGCGCAACCTTGCCAAGGTTGAGACCGCGGGTCCGAGT
CCCGTTGTCCGCTCCA
>Bifidobacterium_adolescentis_ATCC_15703_chr.trna36-GlyGCC (1029147-1029072) Gly (GCC) 76 bp Sc: 76.69
GCGGACATAGCTTAGTTGGTAAAGCGCAACCTTGCCAAGGTTGAGACCGCGGGTTCGAGT
CCCGTTGTCCGCTCTA
>Bifidobacterium_adolescentis_ATCC_15703_chr.trna7-GlyTCC (342497-342570) Gly (TCC) 74 bp Sc: 79.76
GCGGATGTAGCTCAATGGTAGAGCCTCAGTCTTCCAAACTGATTACGCGGGTTCGATTCC
CGTCATCCGCTCCA
>Bifidobacterium_adolescentis_ATCC_15703_chr.trna42-HisGTG (662696-662621) His (GTG) 76 bp Sc: 74.58
GTGGCTATAGCTCAGTTGGTAGAGCATCTGATTGTGGTTCAGAAGGTCGCGCGTTCGAGC
CGCGTTAGCCACCCCG
>Bifidobacterium_adolescentis_ATCC_15703_chr.trna52-IleGAT (13322-13249) Ile (GAT) 74 bp Sc: 89.90
GGGCCCGTAGCTCAGGTGGTTAGAGCGCATCCCTGATAAGGATGAGGTCGGAGGTTCAAG
TCCTCCCGGGCCCA
>Bifidobacterium_adolescentis_ATCC_15703_chr.trna15-IleTAT (553096-553169) Ile (TAT) 74 bp Sc: 82.79
GCCCTCGTAGCTCAGTTGGTTAGAGCAGCACCCTTATAAGGTGTTTGTCCTGGGTTCAAG
TCCCAGCGAGGGCA
>Bifidobacterium_adolescentis_ATCC_15703_chr.trna20-LeuCAA (847728-847801) Leu (CAA) 74 bp Sc: 48.19
GCCCTCGTATCCCAATTGGTAGAGGAAGCAGCCTCAAAATCTGCGCAGTGTGGGTTCGAG
TCCCACCGAGGGCA
>Bifidobacterium_adolescentis_ATCC_15703_chr.trna51-LeuCAG (64761-64679) Leu (CAG) 83 bp Sc: 50.26
GCGAGCGTGGCGGAATGGTAGACGCGCTGTCTTCAGGTGGCAGTGAGTGTATGCTCGTGG
GGGTTCAACTCCCCCCGCTCGCA
>Bifidobacterium_adolescentis_ATCC_15703_chr.trna41-LeuGAG (904886-904796) Leu (GAG) 91 bp Sc: 58.25
GTCCGGGTGGCGGAATGGTAGACGCGCTAGCTTGAGGTGCTAGTGCCTATTTTATACAGG
CGTGCGGGTTCAAGTCCCGCTCCGGACACCG
>Bifidobacterium_adolescentis_ATCC_15703_chr.trna19-LeuTAA (812748-812827) Leu (TAA) 80 bp Sc: 59.60
GCCTCCATGGCGAAATCGGTATACGCAGTCGACTTAAAATCGACCTCTTCGGATTGTGGG
TTCGAGTCCCGCTGGAGGCA
>Bifidobacterium_adolescentis_ATCC_15703_chr.trna5-LeuTAG (302204-302284) Leu (TAG) 81 bp Sc: 66.45
GCGCGAGTGGCGGAATTGGTAGACGCGCAGGATTTAGGTTCCTGTGTCTTTGACGTGTGG
GTTCAAGTCCCATCTCGCGCA
>Bifidobacterium_adolescentis_ATCC_15703_chr.trna31-LysCTT (1861680-1861607) Lys (CTT) 74 bp Sc: 86.48
GCCCCTTTAGCTCAGTTGGTTAGAGCAGCTGACTCTTAATCAGCGTGTCCAGGGTTCGAA
TCCCTGAGGGGGCA
>Bifidobacterium_adolescentis_ATCC_15703_chr.trna50-LysTTT (152339-152267) Lys (TTT) 73 bp Sc: 73.84
GCCCCTCTAGCTCAACGGTTAGAGCAGCGTCCTTTTAAGTCGTGGGTTGTGGGTTCGAAT
CCCACGGGGGGCA
>Bifidobacterium_adolescentis_ATCC_15703_chr.trna10-MetCAT (388109-388185) Met (CAT) 77 bp Sc: 80.92
GGCGGGATAGCTCAGCTGGCTAGAGCGTACGACTCATAATCGTAAGGTCAAGAGTTCGAG
TCTCTTTCTCGCTACAA
>Bifidobacterium_adolescentis_ATCC_15703_chr.trna27-MetCAT (1810564-1810640) Met (CAT) 77 bp Sc: 83.43
CGCGGGGTGGAGCAGCTCGGTAGCTCGCTGGGCTCATAACCCAGAGGTCCATGGTTCAAA
TCCATGCCCCGCTACCA
>Bifidobacterium_adolescentis_ATCC_15703_chr.trna14-PheGAA (493268-493343) Phe (GAA) 76 bp Sc: 79.96
GGCTCTGTAGCTCAGTTGGTAGAGCGAACGACTGAAAATCGTTAGGTCAGCGGATCGATG
CCGCTCGGAGCCACCA
>Bifidobacterium_adolescentis_ATCC_15703_chr.trna47-ProCGG (474265-474189) Pro (CGG) 77 bp Sc: 80.22

CGGGCTGTAGCGCAGCTTGGTAGCGCGCTTCGTTCGGGACGAAGAGGCCGCGGGTTCAAA
TCCCGCCAGCCCGACAA
>Bifidobacterium_adolescentis_ATCC_15703_chr.trna40-ProGGG (1018241-1018165) Pro (GGG) 77 bp Sc: 87.08
CGGACCATAGCGCAGTTTGGTAGCGCACTTGACTGGGGGTCAAGGGGTCGCGGGTTCAAA
TCCCGCTGGTCCGACCA
>Bifidobacterium_adolescentis_ATCC_15703_chr.trna21-ProTGG (856660-856736) Pro (TGG) 77 bp Sc: 85.27
CGGGCTGTAGCGCAGTTTGGTAGCGCGTCTGCTTTGGGAGCAGAATGTCGCAGGTTCAAA
TCCTGTCAGCCCGACAA
>Bifidobacterium_adolescentis_ATCC_15703_chr.trna48-SerCGA (442196-442111) Ser (CGA) 86 bp Sc: 58.08
GGATAAGTGTCCGAGCGGTCTAAGGAGCACGCCTCGAAAGCGTGTGAGGGCGAACCCCTC
CGCGAGTTCGAATCTCGCCTTATCCG
>Bifidobacterium_adolescentis_ATCC_15703_chr.trna49-SerGCT (400828-400738) Ser (GCT) 91 bp Sc: 52.97
GGAGTCATGCCTGAGTGGCCGATAGGGGCACCCTGCTAAGGTGTTAGTCGTGTTTAGCGG
CTCGAGGGTTCGAATCCCTCTGACTCCGCCT
>Bifidobacterium_adolescentis_ATCC_15703_chr.trna30-SerGGA (1905059-1904972) Ser (GGA) 88 bp Sc: 59.29
GGAGAATTCGCCTAGTGGTCTATGGCGCACGCTTGGAAAGCGTGTTGGTGTAACAGCCTC
ACGAGTTCGAATCTCGTATTCTCCGCCC
>Bifidobacterium_adolescentis_ATCC_15703_chr.trna11-SerTGA (465693-465777) Ser (TGA) 85 bp Sc: 59.36
GGGTAGATGTCTGAGCGGTCTAAAGAGACGGTCTTGAAAACCGTTGAGGTGCAAGCCTCC
GCGAGTTCGAATCTCGCTCTGCCCG
>Bifidobacterium_adolescentis_ATCC_15703_chr.trna4-ThrCGT (302036-302111) Thr (CGT) 76 bp Sc: 88.32
GCCACTTTAGCTCAGTCGGTAGAGCGGCTCACTCGTAATGAGCAGGTCGACAGTTCGATT
CTGTCAAGTGGCTCCA
>Bifidobacterium_adolescentis_ATCC_15703_chr.trna9-ThrGGT (388031-388103) Thr (GGT) 73 bp Sc: 65.14
GCCTCGATAGCTCAGTGGTAGAGCACTTCCTTGGTAAAGGAAGAGGTCGTGAGTCCGATT
CTCACTCGAGGCT
>Bifidobacterium_adolescentis_ATCC_15703_chr.trna29-ThrTGT (2069586-2069662) Thr (TGT) 77 bp Sc: 77.62
GCCTCCTTAGCTCAGATGGCCAGAGCGGCCGCCTTGTAAGCGGCAGGTCGTCGGTTCGAT
CCCGACAGGAGGCTCGA
>Bifidobacterium_adolescentis_ATCC_15703_chr.trna6-TrpCCA (320827-320902) Trp (CCA) 76 bp Sc: 87.91
AGGGAAGTGGCGCAATTGGTAGCGCAACGGTCTCCAAAACCGTAGGTTGTGGGTTCGAGT
CCCGCCTTCCCTGCCA
>Bifidobacterium_adolescentis_ATCC_15703_chr.trna8-TyrGTA (387948-388029) Tyr (GTA) 82 bp Sc: 64.35
GGCGGATTTCCCAAGCGGTCAAAGGGAACTGACTGTAAATCAGTCGCCTCGTGCTTCAGT
GGTTCGAATCCACTATCCGCCA
>Bifidobacterium_adolescentis_ATCC_15703_chr.trna44-ValCAC (628911-628839) Val (CAC) 73 bp Sc: 79.39
GGGTGATTGGCGCAGCGGCTAGCGCACTTCCTTCACACGGAAGGGGTCATAGGTTCGATT
CCTATATCACCCA
>Bifidobacterium_adolescentis_ATCC_15703_chr.trna39-ValCAC (1028831-1028756) Val (CAC) 76 bp Sc: 80.01
GGGTGATTGGCGCAGCGGCTAGCGCACTTCCTTCACACGGAAGGGGTCATAGGTTCGATT
CCTATATCACCCACCG
>Bifidobacterium_adolescentis_ATCC_15703_chr.trna38-ValGAC (1028933-1028862) Val (GAC) 72 bp Sc: 74.35
GGGCGGTTGGCGCAGAGGTAGCGCACTTCCCTGACACGGAAGGGGTCACAAGTTCGAATC
TTGTATCGCCCA
>Bifidobacterium_adolescentis_ATCC_15703_chr.trna43-ValGAC (629028-628957) Val (GAC) 72 bp Sc: 74.35
GGGCGGTTGGCGCAGAGGTAGCGCACTTCCCTGACACGGAAGGGGTCACAAGTTCGAATC
TTGTATCGCCCA
>Bifidobacterium_adolescentis_ATCC_15703_chr.trna25-ValTAC (1435985-1436058) Val (TAC) 74 bp Sc: 89.66
GGGCGATTAGCTCAGTTGGTTAGAGCGCCACCTTTACACGGTGGATGTCGGGGGTTCGAA
TCCCTCATCGCCCA
>Blochmannia_floridanus_chr.trna20-AlaTGC (664314-664242) Ala (TGC) 73 bp Sc: 80.93
GGGGCTATAGCTCAATTGGGAGAGCATCTGTTTTGCACACAGAAGGTTAGCGGTTCGATT
CCGCTTAGCTCCA
>Blochmannia_floridanus_chr.trna6-ArgACG (204800-204873) Arg (ACG) 74 bp Sc: 79.10
GCACCCGTAGCTCAGTTGGATAGAGCACTCGGCTACGAACCGAGAGGTCGGGGGTTCAAA
TCCTCTCGGGTGTA
>Blochmannia_floridanus_chr.trna23-ArgCCG (653133-653060) Arg (CCG) 74 bp Sc: 66.11
GCGTCCGTAGCTTAATTGGATAGAGCATTGCCCTCCGAAGGCAAAGGTGTCAGGTTCGAT
CCCTGTCGGGCGCA
>Blochmannia_floridanus_chr.trna16-ArgCCT (556861-556932) Arg (CCT) 72 bp Sc: 52.37
GTCTTCGTAGTTAAACGGATATAACAAGCCTCTCCTAAAGGCTAATTACAGGTTCGATCC
CTGTCGAAGACA
>Blochmannia_floridanus_chr.trna12-ArgTCT (338731-338804) Arg (TCT) 74 bp Sc: 71.59
GCGCTCTTAACTCAATAGGATAGAGTAACGGCCTTCTAAGCCGTAAGTTATAGGTTCGAA
TCCTATAGAGCGCA
>Blochmannia_floridanus_chr.trna15-AsnGTT (505897-505972) Asn (GTT) 76 bp Sc: 85.94
TCCTCTGTAGTTCAGTTGGTAGAACGGCGGACTGTTAATCCGTATGTCACTGGTTCAAAT

CCAGTCAGGGGAGCCA
>Blochmannia_floridanus_chr.trna21-AspGTC (664177-664101) Asp (GTC) 77 bp Sc: 87.47
GGTGCGGTAGTTCAGATGGTTAGAATATCGGCTTGTCACGCCGGAGGTCGCGGGTTCGAA
TCCCGTCCGCACCGCCA
>Blochmannia_floridanus_chr.trna30-CysGCA (455468-455398) Cys (GCA) 71 bp Sc: 56.37
GGCGCGTTAACAAAGTGGTAATGTAGCGGACTGCAAATCCGTATAGCTCGGTTCGAATCC
GAGACGCGCCT
>Blochmannia_floridanus_chr.trna35-GlnTTG (353907-353836) Gln (TTG) 72 bp Sc: 67.90
TGGGGTATAGCCAAGCGGTAAGGCAGCGGGTTTTGATCCCGCCACTCCCAGGTTCGAATC
CTGGTACCCCAG
>Blochmannia_floridanus_chr.trna27-GluTTC (616408-616337) Glu (TTC) 72 bp Sc: 49.34
GTCCCCTTCGTCTAGAGGTTAGGACACTGCCCTTTCACGGCGGCAACAGGGGTTCGAAAC
CCCTAGGGGACA
>Blochmannia_floridanus_chr.trna2-GlyGCC (82596-82669) Gly (GCC) 74 bp Sc: 79.17
GCGGGAATAGCTCAGATGGTTAGAGTGCAACCTTGCCAAGGTTGAGGTCGCGAGTTCGAA
TCTCGTTTCCCGCT
>Blochmannia_floridanus_chr.trna9-GlyTCC (220138-220209) Gly (TCC) 72 bp Sc: 42.59
GTGGGCATCGTATAATGGTTATTACCTCAACCTTCCAAGTTGATGATGTGGGTTCGATTC
CCACTGTCCGCT
>Blochmannia_floridanus_chr.trna24-HisGTG (652988-652914) His (GTG) 75 bp Sc: 73.32
GTGATTGTAGCTCAGTTGGTAGAGCTCTGGATTGTGGTTCCAGTGGTCGTGGGTTCGAAT
CCCATCAGTCACCCA
>Blochmannia_floridanus_chr.trna19-IleGAT (705085-705012) Ile (GAT) 74 bp Sc: 81.35
AGGCTTGTAGCTCAGCCGGTTAGAGCGCACCCCTGATAAGGGTGAGGTCGGTGGTTCAAG
TCCACTCAGGCCTA
>Blochmannia_floridanus_chr.trna1-LeuCAA (46904-46985) Leu (CAA) 82 bp Sc: 57.72
GCCGAAGTGGCGGAAAAGGTAGACGCAATTGATTCAAAATCAATCACTGTGAGGTGTGTC
GGTTCGAGTCCGACCTTCGGTA
>Blochmannia_floridanus_chr.trna25-LeuCAG (652885-652799) Leu (CAG) 87 bp Sc: 45.40
GCGAAGGTGGCGAAATAGGTAGACGCACTAGCTTCAGGGGTTAGTGTCTTGTATGTAGAC
GTAAGGGTTCAAATCCCTTCCTTCGTA
>Blochmannia_floridanus_chr.trna3-LeuGAG (110685-110768) Leu (GAG) 84 bp Sc: 44.99
GCCGAGATGGTGAAATTTGGTATACACGCTACTTTGAGGGGGTAGTCCGGTTAATGGTTG
CGGGTTCAAATCCCGTTCTCGGCA
>Blochmannia_floridanus_chr.trna31-LeuTAA (455378-455296) Leu (TAA) 83 bp Sc: 59.80
GCCCGGGTGGTGGAATTGGTAGACACAAGGGACTTAAAATCCCTCAGTTTTATACTGTAC
GAGTTCAAGTCTCGTCCCGGGTA
>Blochmannia_floridanus_chr.trna34-LeuTAG (354041-353957) Leu (TAG) 85 bp Sc: 47.33
ACGGGAGTGGCGAAATAGGTAGACGCGTCAGAGTTAGAGTCTGATATCCTTAGATATGCG
AGTTCAAATCTCGCCTCCCGTATCA
>Blochmannia_floridanus_chr.trna13-LysCTT (456786-456857) Lys (CTT) 72 bp Sc: 79.31
GGGTCGTTAGCTCAATGGTAGAGCAGTTGACTCTTAATCAATTGATTGTAGGTTCGAGTC
CTACACGACCCA
>Blochmannia_floridanus_chr.trna18-LysTTT (561984-562056) Lys (TTT) 73 bp Sc: 90.12
GGGTCGTTAGCTCAGTTGGTAGAGCAGTTGACTTTTAATCAATTGGTCGCAGGTTCAAGT
CCTGCACGACCCA
>Blochmannia_floridanus_chr.trna33-MetCAT (354170-354097) Met (CAT) 74 bp Sc: 78.66
GGCTACGTAGCTCAGATGGTTAGAGCGCAGCACTCATAATGCTGAGGGCACAGGTTCAAA
TCCTGTCGTAGCTA
>Blochmannia_floridanus_chr.trna4-MetCAT (110836-110912) Met (CAT) 77 bp Sc: 82.21
CGCGGGGTGGAGCAGTATGGTAGCTCGTCGGGCTCATAACCCGAAGGTCGTTGGTTCAAA
TCCGACCCCCGCAACCA
>Blochmannia_floridanus_chr.trna37-MetCAT (59570-59497) Met (CAT) 74 bp Sc: 87.58
GGCCCTTTAGCTCAGTTGGTTAGAGCAGGCGACTCATAATCGCTTGGTCACTGGTTCAAA
TCCAGTAAGGGCCA
>Blochmannia_floridanus_chr.trna36-PheGAA (73366-73291) Phe (GAA) 76 bp Sc: 85.67
GCCCAGATAGCTCAGTTGGTAGAGCAGAGGACTGAAGATCCTCGTGTCCTTGGTTCGATT
CCGAGTCTGGGCACCA
>Blochmannia_floridanus_chr.trna26-ProTGG (652786-652713) Pro (TGG) 74 bp Sc: 77.60
CGGCGAGTGGCGCAGTTTGGTAGCGTGGCTGGTTTGGGACCAGTAGGTCGGAGGTTCGAA
TCCTCTCTCGCCGA
>Blochmannia_floridanus_chr.trna28-SerCGA (505721-505634) Ser (CGA) 88 bp Sc: 52.03
GGAGAGATGCCGGAGTGGATGAACGGGACGGTTTCGAAAATCGTTAAAGAATGTAATTTT
TTCAAGGGTTCGAATCCCTTTCTCTCCT
>Blochmannia_floridanus_chr.trna5-SerGCT (204652-204742) Ser (GCT) 91 bp Sc: 57.55
AGTGAGGTGGCCGAGAGGCTAAAGGCACTCCCCTGCTAAGGGAGTATATAGTCTAAATAC
TGTATCGAGGGTTCGAATCCCTCCCTCACTG

>Blochmannia_floridanus_chr.trna14-SerGGA (505174-505261) Ser (GGA) 88 bp Sc: 53.70
GGTGAGGTGTCCGAGAGGCTCAAGGAGCATGCCTGGAAAGCATGTATACATATAATATGT
ATCAAGGGTTCGAATCCCTTCCTCACCG
>Blochmannia_floridanus_chr.trna29-SerTGA (457082-456999) Ser (TGA) 84 bp Sc: 50.49
GGAGGAGTGGCCGAGAGGATGAAGGCGCCGGTCTTGAAAATCGGTAATGTAAAAAATTCT
AGAGTTCGAATCTCTACTCCTCCG
>Blochmannia_floridanus_chr.trna11-ThrCGT (241865-241937) Thr (CGT) 73 bp Sc: 73.67
GCCGATATAGCTTAATTGGTAGAGCAATGCATTCGTAATGCAAAGGTTGTAGGTTCGAAT
CCTATTGTCGGCA
>Blochmannia_floridanus_chr.trna10-ThrGGT (220246-220317) Thr (GGT) 72 bp Sc: 73.01
GCTGATATAGCTCAGGGGCAGAGCACTCCCTTGGTAAGGGTGAGGTCGGCAGTTCGATTC
TGCTTATCAGCA
>Blochmannia_floridanus_chr.trna7-ThrTGT (219918-219989) Thr (TGT) 72 bp Sc: 77.81
GCCGGTGTAGCTCAATGGTAGAGCAACTGATTTGTAATCAGTGGGTTAAGGGTTCAAATC
CTTTTGCCGGCA
>Blochmannia_floridanus_chr.trna22-TrpCCA (664046-663975) Trp (CCA) 72 bp Sc: 70.05
AGGGGTGTAGTTCAATGGTAGAATATCGGTCTCCAAAACCGAGGGTTGGGAGTTCAAGTC
TCTTCACCCCTG
>Blochmannia_floridanus_chr.trna8-TyrGTA (220004-220085) Tyr (GTA) 82 bp Sc: 66.39
GATGGGGTGCCCGAGTGGTTAAAGGGAGCAGACTGTAAATCTGCCGTCGTAGACTTCGAA
GGTTCAAATCCTTCCCCCATCA
>Blochmannia_floridanus_chr.trna32-ValGAC (402258-402185) Val (GAC) 74 bp Sc: 72.33
GCGTTCTTAGCTCAGTTGGTTAGAGTGCTACCGTGACATGGTAGAGGTCGATGGTTCGAG
TCCATTAGAACGCA
>Blochmannia_floridanus_chr.trna17-ValTAC (561868-561940) Val (TAC) 73 bp Sc: 72.12
AGGTGATTAGCTCAATAGGTAGAGTATCTCTCTTACAAGGAGAAGGTCGGCGGTTCAAAT
CCGCCATCACCTA
>Bordetella_avium_197N_chr.trna51-AlaCGC (1703594-1703519) Ala (CGC) 76 bp Sc: 84.28
GGGGCGGTAGCTCAGCTGGGAGAGCGTCGCGTTCGCAATGCGAAGGTCGGGAGTTCGATC
CTCCTCCGCTCCACCA
>Bordetella_avium_197N_chr.trna53-AlaGGC (877634-877559) Ala (GGC) 76 bp Sc: 86.46
GGGGGTATAGCTCAGCTGGGAGAGCGCTTGCATGGCATGCAAGAGGTCAGCGGTTCGATC
CCGCTTACCTCCACCA
>Bordetella_avium_197N_chr.trna56-AlaGGC (877059-876984) Ala (GGC) 76 bp Sc: 86.46
GGGGGTATAGCTCAGCTGGGAGAGCGCTTGCATGGCATGCAAGAGGTCAGCGGTTCGATC
CCGCTTACCTCCACCA
>Bordetella_avium_197N_chr.trna8-AlaGGC (885428-885503) Ala (GGC) 76 bp Sc: 86.46
GGGGGTATAGCTCAGCTGGGAGAGCGCTTGCATGGCATGCAAGAGGTCAGCGGTTCGATC
CCGCTTACCTCCACCA
>Bordetella_avium_197N_chr.trna30-AlaTGC (3150360-3150285) Ala (TGC) 76 bp Sc: 88.49
GGGGGTGTAGCTCAGCTGGGAGAGCGCCTGCTTTGCAAGCAGGATGTCATCGGTTCGATC
CCGTTCACCTCCACCA
>Bordetella_avium_197N_chr.trna41-AlaTGC (2699255-2699180) Ala (TGC) 76 bp Sc: 88.49
GGGGGTGTAGCTCAGCTGGGAGAGCGCCTGCTTTGCAAGCAGGATGTCATCGGTTCGATC
CCGTTCACCTCCACCA
>Bordetella_avium_197N_chr.trna48-AlaTGC (2398053-2397978) Ala (TGC) 76 bp Sc: 88.49
GGGGGTGTAGCTCAGCTGGGAGAGCGCCTGCTTTGCAAGCAGGATGTCATCGGTTCGATC
CCGTTCACCTCCACCA
>Bordetella_avium_197N_chr.trna37-ArgACG (2768511-2768435) Arg (ACG) 77 bp Sc: 86.25
GCGCCCGTAGCTCAGTTGGATAGAGTACTTGGCTACGAACCAAGGGGTCGTGGGTTCGAA
TCCTGCCGGGCGCGCCA
>Bordetella_avium_197N_chr.trna38-ArgACG (2768300-2768224) Arg (ACG) 77 bp Sc: 86.25
GCGCCCGTAGCTCAGTTGGATAGAGTACTTGGCTACGAACCAAGGGGTCGTGGGTTCGAA
TCCTGCCGGGCGCGCCA
>Bordetella_avium_197N_chr.trna39-ArgACG (2766465-2766389) Arg (ACG) 77 bp Sc: 86.25
GCGCCCGTAGCTCAGTTGGATAGAGTACTTGGCTACGAACCAAGGGGTCGTGGGTTCGAA
TCCTGCCGGGCGCGCCA
>Bordetella_avium_197N_chr.trna26-ArgCCG (3468618-3468544) Arg (CCG) 75 bp Sc: 61.31
GTGCCCGTAGTTCAATGGATAGAATGAGAGTTTCCGAAGCTCTTGATACAGGTTCGATTC
CTGTCGGGCGCGCCA
>Bordetella_avium_197N_chr.trna11-ArgCCT (962825-962899) Arg (CCT) 75 bp Sc: 58.85
GTCCCTGTAGTTCAATGGATAGAACAGGTTCCTCCTAAGAATCAGATCCAGGTTCGATTC
CTGGTGGGGACGCCA
>Bordetella_avium_197N_chr.trna52-ArgTCT (1380288-1380212) Arg (TCT) 77 bp Sc: 78.38
GCGCCCGTAGCTCAAATGGATAGAGCAGAGGATTTCTACCCCTCAGGTTGCAGGTTCGAC
TCCTGCCGGGCGCGCCA
>Bordetella_avium_197N_chr.trna34-ArgTCT (2815281-2815205) Arg (TCT) 77 bp Sc: 85.87

CCGCCCTTAGCTCAGTCGGATAGAGCACTCGCCTTCTAAGCGAGCGGTCACACGTTCGAA
TCGTGTAGGGCGGGCCA
>Bordetella_avium_197N_chr.trna25-AsnGTT (3619019-3619094) Asn (GTT) 76 bp Sc: 87.19
TCCCCGATAGCTCAGTCGGTAGAGCGACGGACTGTTAATCCGCAGGTCGCTGGTTCGAGC
CCAGCTCGGGGAGCCA
>Bordetella_avium_197N_chr.trna43-AsnGTT (2594270-2594195) Asn (GTT) 76 bp Sc: 87.19
TCCCCGATAGCTCAGTCGGTAGAGCGACGGACTGTTAATCCGCAGGTCGCTGGTTCGAGC
CCAGCTCGGGGAGCCA
>Bordetella_avium_197N_chr.trna10-AspGTC (885641-885717) Asp (GTC) 77 bp Sc: 95.07
GGAGCGGTAGTTCAGTTGGTTAGAATACCGGCCTGTCACGCCGGGGGTCGCGGGTTCGAG
CCCCGTCCGCTCCGCCA
>Bordetella_avium_197N_chr.trna55-AspGTC (877420-877344) Asp (GTC) 77 bp Sc: 95.07
GGAGCGGTAGTTCAGTTGGTTAGAATACCGGCCTGTCACGCCGGGGGTCGCGGGTTCGAG
CCCCGTCCGCTCCGCCA
>Bordetella_avium_197N_chr.trna58-AspGTC (876845-876769) Asp (GTC) 77 bp Sc: 95.07
GGAGCGGTAGTTCAGTTGGTTAGAATACCGGCCTGTCACGCCGGGGGTCGCGGGTTCGAG
CCCCGTCCGCTCCGCCA
>Bordetella_avium_197N_chr.trna23-CysGCA (2974965-2975038) Cys (GCA) 74 bp Sc: 68.57
GGCGCAATGGCAGAGTGGTTATGCAGCGGATTGCAAATCCGTGTACGTCGGTTCGATTCC
GGCTTGCGCCTCCA
>Bordetella_avium_197N_chr.trna7-GlnTTG (558750-558826) Gln (TTG) 77 bp Sc: 77.70
TGGGGAGTCGCCAAGCTGGTTAAGGCACCGGATTTGATTCCGGCATGCGAAGGTTCGAA
TCCTTCCTCCCCAGCCA
>Bordetella_avium_197N_chr.trna54-GluTTC (877534-877459) Glu (TTC) 76 bp Sc: 60.10
GTCCCCTTCGTCTAGAGGCCTAGGACATCACCCTTTCACGGTGAGTACAGGGGTTCGAAT
CCCCTAGGGGACGCCA
>Bordetella_avium_197N_chr.trna57-GluTTC (876959-876884) Glu (TTC) 76 bp Sc: 60.10
GTCCCCTTCGTCTAGAGGCCTAGGACATCACCCTTTCACGGTGAGTACAGGGGTTCGAAT
CCCCTAGGGGACGCCA
>Bordetella_avium_197N_chr.trna9-GluTTC (885527-885602) Glu (TTC) 76 bp Sc: 60.10
GTCCCCTTCGTCTAGAGGCCTAGGACATCACCCTTTCACGGTGAGTACAGGGGTTCGAAT
CCCCTAGGGGACGCCA
>Bordetella_avium_197N_chr.trna6-GlyCCC (455847-455920) Gly (CCC) 74 bp Sc: 77.64
GCGGGTGTAGTTCAATGGTAGAACGGCGGCTTCCCAAGCCTCAAGCGTGGGTTCGATTCC
CATCACCCGCTCCA
>Bordetella_avium_197N_chr.trna21-GlyGCC (2953742-2953817) Gly (GCC) 76 bp Sc: 89.05
GCGGGAGTAGCTCAGTTGGTAGAGCGCAACCTTGCCAAGGTTGAGGTCGCGAGTTCGAGA
CTCGTCTCCCGCTCCA
>Bordetella_avium_197N_chr.trna22-GlyGCC (2962130-2962205) Gly (GCC) 76 bp Sc: 89.05
GCGGGAGTAGCTCAGTTGGTAGAGCGCAACCTTGCCAAGGTTGAGGTCGCGAGTTCGAGA
CTCGTCTCCCGCTCCA
>Bordetella_avium_197N_chr.trna2-GlyTCC (5642-5715) Gly (TCC) 74 bp Sc: 82.17
GCGGGTGTAGCTCAATGGTAGAGCAGAAGCCTTCCAAGCTTACGACGAGGGTTCGATTCC
CTTCACCCGCTCCA
>Bordetella_avium_197N_chr.trna35-HisGTG (2811770-2811695) His (GTG) 76 bp Sc: 80.26
GTGGCTGTAGCTCAGTTGGTAGAGTCCCGGATTGTGATTCCGGTTGTCGTGGGTTCGAGC
CCCATCAGCCACCCCA
>Bordetella_avium_197N_chr.trna29-IleGAT (3150444-3150368) Ile (GAT) 77 bp Sc: 93.29
GGGTCTGTAGCTCAGTCGGTTAGAGCACCGTCTTGATAAGGCGGGGGTCGTTGGTTCGAA
TCCAACCAGACCCACCA
>Bordetella_avium_197N_chr.trna40-IleGAT (2699339-2699263) Ile (GAT) 77 bp Sc: 93.29
GGGTCTGTAGCTCAGTCGGTTAGAGCACCGTCTTGATAAGGCGGGGGTCGTTGGTTCGAA
TCCAACCAGACCCACCA
>Bordetella_avium_197N_chr.trna47-IleGAT (2398137-2398061) Ile (GAT) 77 bp Sc: 93.29
GGGTCTGTAGCTCAGTCGGTTAGAGCACCGTCTTGATAAGGCGGGGGTCGTTGGTTCGAA
TCCAACCAGACCCACCA
>Bordetella_avium_197N_chr.trna59-LeuCAA (443768-443684) Leu (CAA) 85 bp Sc: 75.82
GCCCGGGTGGTGAAATTGGTAGACGCAGGGGACTCAAAATCCCCCGCCGCAAGGCGTGCC
GGTTCGATTCCGGCCCCGGGCACCA
>Bordetella_avium_197N_chr.trna14-LeuCAG (2273831-2273915) Leu (CAG) 85 bp Sc: 75.85
GCCCAGGTGGCGGAATTGGTAGACGCGCATGGTTCAGGTCCATGTGCCGCAAGGTGTGGA
GGTTCGAGTCCTCTCCTGGGCACCA
>Bordetella_avium_197N_chr.trna15-LeuCAG (2274782-2274866) Leu (CAG) 85 bp Sc: 75.85
GCCCAGGTGGCGGAATTGGTAGACGCGCATGGTTCAGGTCCATGTGCCGCAAGGTGTGGA
GGTTCGAGTCCTCTCCTGGGCACCA
>Bordetella_avium_197N_chr.trna16-LeuCAG (2275016-2275100) Leu (CAG) 85 bp Sc: 75.85
GCCCAGGTGGCGGAATTGGTAGACGCGCATGGTTCAGGTCCATGTGCCGCAAGGTGTGGA

GGTTCGAGTCCTCTCCTGGGCACCA
>Bordetella_avium_197N_chr.trna33-LeuGAG (2871644-2871560) Leu (GAG) 85 bp Sc: 63.68
GCCGACGTGGTGAAATTGGTAGACACGCTATCTTGAGGGGGTAGTGGCGAAAGCTGTGCG
AGTTCGAGTCTCGCCGTCGGCACCA
>Bordetella_avium_197N_chr.trna13-LeuTAG (1496530-1496616) Leu (TAG) 87 bp Sc: 68.96
GCGAAGGTGGCGAAATTGGTAGACGCACCAGGTTTAGGTCCTGACGCCGTAACAGGTGTG
CGGGTTCGAGTCCCGCCCTTCGCACCA
>Bordetella_avium_197N_chr.trna32-LysCTT (3093135-3093060) Lys (CTT) 76 bp Sc: 88.69
GGGCTGTTAGCTCAGTTGGTAGAGCAGCGGACTCTTAATCCGTAGGTCGAGTGTTCGAGC
CACTCACAGCCCACCA
>Bordetella_avium_197N_chr.trna31-LysTTT (3098700-3098625) Lys (TTT) 76 bp Sc: 92.60
GGGTCGTTAGCTCAGTCGGTAGAGCAGCGGACTTTTAATCCGTTGGTCGCGCGTTCGAGT
CGCGCACGACCCACCA
>Bordetella_avium_197N_chr.trna44-MetCAT (2507391-2507313) Met (CAT) 79 bp Sc: 66.21
GGGCCTCTAGCTCATGCCTGGTTAGAGCAGCGGACTCATAATCCGTTGGTGCCGAGTTCG
ACTCTCGGGGGGCCTACCA
>Bordetella_avium_197N_chr.trna17-MetCAT (2281839-2281915) Met (CAT) 77 bp Sc: 81.73
GGCGCATTAGCTCAGCCGGTTAGAGCGACGGAATCATAATCCGCAGGTCCCCTGTTCGAA
TCAGGGATGCGCCACCA
>Bordetella_avium_197N_chr.trna18-MetCAT (2592127-2592203) Met (CAT) 77 bp Sc: 85.01
CGCGGGGTGGAGCAGTCTGGCAGCTCGTCGGGCTCATAACCCGAAGGTCGTAGGTTCAAA
TCCTGCCCCCGCAACCA
>Bordetella_avium_197N_chr.trna45-MetCAT (2500626-2500550) Met (CAT) 77 bp Sc: 85.01
CGCGGGGTGGAGCAGTCTGGCAGCTCGTCGGGCTCATAACCCGAAGGTCGTAGGTTCAAA
TCCTGCCCCCGCAACCA
>Bordetella_avium_197N_chr.trna61-PheGAA (309323-309248) Phe (GAA) 76 bp Sc: 86.41
GGCCTGGTAGCTCAGTCGGTAGAGCAGAGGATTGAAAATCCTTGTGTCGGTGGTTCGATT
CCGCCCCAGGCCACCA
>Bordetella_avium_197N_chr.trna5-ProCGG (267998-268074) Pro (CGG) 77 bp Sc: 85.02
CGGGGCGTAGCTCAGTCTGGTAGAGTACTGCGTTCGGGACGCAGGAGTCGGAGGTTCGAA
TCCTCTCGCCCCGACCA
>Bordetella_avium_197N_chr.trna50-ProGGG (2204244-2204168) Pro (GGG) 77 bp Sc: 77.19
CGGGGCGTAGCGCAGCCTGGTAGCGCACTTGCATGGGGTGCAAGGGGTCGCGAGTTCGAA
TCCCGCCGTCCCGACCA
>Bordetella_avium_197N_chr.trna12-ProTGG (1013977-1014053) Pro (TGG) 77 bp Sc: 87.76
CGGGGCGTAGCGCAGCCTGGTAGCGCATCTGCTTTGGGAGCAGAGGGTCGCGAGTTCGAA
TCCCGCCGCCCCGACCA
>Bordetella_avium_197N_chr.trna60-Undet??? (402562-402479) Undet (???) 84 bp Sc: 25.82
ACCCGCGTATTTACTGGTTTCTTTGGTGGGACTCAAAATCCCCCGCCGCAAGGCGTGCCG
GTTCGATTCCGGCCCCGGGCACCA
>Bordetella_avium_197N_chr.trna20-SerCGA (2896943-2897032) Ser (CGA) 90 bp Sc: 77.54
GGAGAGGTGGCAGAGTGGTCGAATGCGCCGGACTCGAAATCCGGTATACGTTTAGGCGTA
TCGTGGGTTCGAATCCCACCCTCTCCGCCA
>Bordetella_avium_197N_chr.trna36-SerGCT (2768859-2768767) Ser (GCT) 93 bp Sc: 69.39
GGAGACGTGCCCGAGAGGCTGAAGGGGCTCCCCTGCTAAGGGAGTATGTGGCTAAAAACT
GCATCGTGGGTTCGAATCCCACCGTCTCCGCCA
>Bordetella_avium_197N_chr.trna27-SerGGA (3208335-3208245) Ser (GGA) 91 bp Sc: 75.09
GGACAGGTGGCCGAGTGGTTGAAGGCGCACGCCTGGAAAGCGTGTATACGTGATAAGCGT
ATCGGGGGTTCGAATCCCCCTCTGTCCGCCA
>Bordetella_avium_197N_chr.trna19-SerTGA (2878073-2878162) Ser (TGA) 90 bp Sc: 75.83
GGAAGCGTGGCCGAGCGGTTGAAGGCACCGGTCTTGAAAACCGGCAAGGGTTTATACCCT
TCGTGGGTTCGAATCCCACCGCTTCCGCCA
>Bordetella_avium_197N_chr.trna42-ThrCGT (2594536-2594461) Thr (CGT) 76 bp Sc: 90.68
GCCGGCATAGCTCAGTTGGTAGAGCAGCGCATTCGTAATGCGAAGGTCGGGGGTTCGACT
CCTCTTGCCGGCACCA
>Bordetella_avium_197N_chr.trna3-ThrGGT (5719-5793) Thr (GGT) 75 bp Sc: 86.85
GCCCATGTGGCTCAGTGGTAGAGCACTCCCTTGGTAAGGGAGAGGTCACGCGTTCGATCC
GCGTCATGGGCACCA
>Bordetella_avium_197N_chr.trna24-ThrTGT (3618347-3618422) Thr (TGT) 76 bp Sc: 95.16
GCCGGTGTAGCTCAGTTGGTAGAGCAGCTCACTTGTAATGAGAAGGTCGAGGGTTCGATT
CCTTTCACCGGCACCA
>Bordetella_avium_197N_chr.trna4-TrpCCA (7199-7274) Trp (CCA) 76 bp Sc: 85.26
AGGGGTATAGCTCAATTGGCAGAGCGTCGGTCTCCAAAACCGAAGGTTGTAGGTTCGATT
CCTACTGCCCCTGCCA
>Bordetella_avium_197N_chr.trna1-TyrGTA (5439-5525) Tyr (GTA) 87 bp Sc: 66.36
GGAGGGGTGCCCGAGTGGTTAAAGGGGGCAGACTGTAAATCTGTTGGCCTTGCGCCTACG
TTGGTTCGAATCCAACCTCCTCCACCA

>Bordetella_avium_197N_chr.trna28-ValCAC (3187668-3187594) Val (CAC) 75 bp Sc: 85.38
GGGTGGTTAGCTCAGTGGTAGAGCACTGCCTTCACACGGCAGGGGTCACAAGTTCGAAAC
TTGTACCACCCACCA
>Bordetella_avium_197N_chr.trna46-ValGAC (2442088-2442012) Val (GAC) 77 bp Sc: 86.03
AGGCGGTTAGCTCAGATGGTTAGAGCGCTACGTTGACATCGTAGAGGTCGTTGGTTCGAT
TCCAATACCGCCTACCA
>Bordetella_avium_197N_chr.trna49-ValTAC (2268394-2268319) Val (TAC) 76 bp Sc: 92.94
GGGTGCTTAGCTCAGTTGGTAGAGCGGCGCCCTTACAAGGCGTAGGTCACAGGTTCGACC
CCTGTAGCACCCACCA
>Borrelia_afzelii_PKo_chr.trna31-AlaTGC (448255-448182) Ala (TGC) 74 bp Sc: 91.03
GGGGGTTTAGCTCAGTTGGCTAGAGCATCGGCTTTGCAAGCCGAGGGTCAAGGGTTCGAG
TCCCTTAACCTCCA
>Borrelia_afzelii_PKo_chr.trna10-ArgGCG (486081-486154) Arg (GCG) 74 bp Sc: 72.81
GTGTCCATAGCTCAGTTGGATAGAGCGTTAGATTGCGATTCTTAAGGTCGGAGGTTCAAG
TCCTCTTGGACACG
>Borrelia_afzelii_PKo_chr.trna13-ArgTCG (640003-640076) Arg (TCG) 74 bp Sc: 69.91
GCATTCATAGCTCAATTGGATAGAGCAGTGGACTTCGAATCCGAAGGTTGCAGGTTCGAA
TCCTGCTGAGTGCG
>Borrelia_afzelii_PKo_chr.trna22-ArgTCT (792833-792760) Arg (TCT) 74 bp Sc: 78.73
GCACCAATAGCTCAATTGGATAGAGCAACAGACTTCTAATCTGTAGGTTTTAGGTTCGAG
TCCTAATTGGTGCG
>Borrelia_afzelii_PKo_chr.trna17-AsnGTT (660719-660790) Asn (GTT) 72 bp Sc: 76.04
TCCCCTATAGCTCAGTGGTAGAGCGGGTGGCTGTTAACCACTAGGTCGGAGGTTCAAGTC
CTTCTGGGGGAG
>Borrelia_afzelii_PKo_chr.trna25-AspGTC (666899-666826) Asp (GTC) 74 bp Sc: 79.20
GGGGGCGTAGTTCAGATGGTTAGAACGCCTGCCTGTCACGCAGGAGGTCGCGGGTTCGAG
ACCCGTCGCTCCCG
>Borrelia_afzelii_PKo_chr.trna2-CysGCA (202115-202187) Cys (GCA) 73 bp Sc: 69.14
GGCGCTGTACCCAAGTGGCTAAGGGAGAAGTCTGCAAAACTTTGATTCGCCGGTTCGATT
CCGGTCAGCGCCT
>Borrelia_afzelii_PKo_chr.trna21-GlnTTG (826673-826744) Gln (TTG) 72 bp Sc: 59.77
TGGGACGTCGACAAGCGGTAAGTCAACTGGTTTTGGTCCAGTCATTCGAGGGTTCGAATC
CTTCCGTCCCAG
>Borrelia_afzelii_PKo_chr.trna15-GlyGCC (640242-640313) Gly (GCC) 72 bp Sc: 66.76
GCGAAAGTAACTCAGGGGTAGAGTGTCACCTTGCCAAGGTGGAAGTCGCGGGTTCAAATC
CCGTCTTTCGCT
>Borrelia_afzelii_PKo_chr.trna1-GlyTCC (46313-46384) Gly (TCC) 72 bp Sc: 50.44
GCGTCCTTCGTATAATGGCTATTACCTTAGCCTTCCAAGCTAATGATGTCGGTTCGATTC
CGATAGGACGCT
>Borrelia_afzelii_PKo_chr.trna12-HisGTG (639907-639979) His (GTG) 73 bp Sc: 64.57
GTGGTTGTAGCTCAGTTGGTAGAGCGTCGGGTTGTGGTTCCGAATGTCGCGGGTTCAAGC
CCCGTCAATCACC
>Borrelia_afzelii_PKo_chr.trna7-IleGAT (446076-446149) Ile (GAT) 74 bp Sc: 71.67
GGGATCATAGCTCAGGTGGTTAGAGCGCAGGTCTGATAAACCTGAGGTCGGATGTTCAAC
TCATCCTGGTCCCA
>Borrelia_afzelii_PKo_chr.trna27-LeuCAA (587950-587867) Leu (CAA) 84 bp Sc: 63.10
GCCGGTATGGCGGAATTGGTAGACGCGTCAGACTCAAAATCTGATGGGGGCAACTTCGTG
TCGGTTCGACTCCGACTACCGGTA
>Borrelia_afzelii_PKo_chr.trna6-LeuGAG (255201-255285) Leu (GAG) 85 bp Sc: 46.32
GCTGTGGTGGTGGAAGTGGTAGACACGCTAGCTTGAGGGGCTAGTGGGCGATAAGCCCAT
GCTGGTTCAAGTCCAGTTCACAGCA
>Borrelia_afzelii_PKo_chr.trna20-LeuTAA (805331-805414) Leu (TAA) 84 bp Sc: 65.47
GCCGAAGTGGTGGAAGTGGTAGACACACAGGACTTAAAATCCTGAGGAGGAAGCTCCGTA
CCGGTTCAAGTCCGGTCTTCGGTA
>Borrelia_afzelii_PKo_chr.trna14-LeuTAG (640156-640236) Leu (TAG) 81 bp Sc: 53.67
GCAGGAGTGGTGGAATTGGCAGACACGCTAGACTTAGGATCTAGTGCTTTTGGCGTGTGG
GTTCGACTCCCACCTTCTGTA
>Borrelia_afzelii_PKo_chr.trna28-LysCTT (551697-551625) Lys (CTT) 73 bp Sc: 70.67
GGGCTGCTAGCTCAAGTGGTAGAGCATCGGACTCTTAATCCGTTGGTTACAGGTTCAAGT
CCTGTGCAGCTCA
>Borrelia_afzelii_PKo_chr.trna29-LysTTT (551517-551445) Lys (TTT) 73 bp Sc: 77.18
GGGCTCATAGCTCAGGTGGTAGAGCAGCGCCCTTTTAAGGCGTTTGTCGTAGGTTCGAGT
CCTACTGAGCTCA
>Borrelia_afzelii_PKo_chr.trna26-MetCAT (646988-646916) Met (CAT) 73 bp Sc: 80.18
CGCAGGGTAGAGCAGTTGGTAGCTCGTCGGGCTCATAACCCGAAGGTCATAGGTTCGAGT
CCTATCCCTGCTA
>Borrelia_afzelii_PKo_chr.trna16-MetCAT (652244-652317) Met (CAT) 74 bp Sc: 82.28

GCTGATGTAGCTCAGTTGGTTAGAGCACTCGGCTCATATCCGAGTTGTCGTGGGTTCAAG
TCCCTCCATCAGCA
>Borrelia_afzelii_PKo_chr.trna5-MetCAT (217430-217503) Met (CAT) 74 bp Sc: 88.53
GGGCCCATAGCTCAGTTGGTTAGAGCACCCGACTCATAATCGGTAGGTCCCAGGTTCAAG
TCCTGGTGGGCCCA
>Borrelia_afzelii_PKo_chr.trna4-PheGAA (205829-205901) Phe (GAA) 73 bp Sc: 77.30
GTCGCTGTAGCTCAGTTGGTAGAGCATAGGACTGAAAATCCTTGTGTCAGGAGTTCGATT
CTCCTCGGCGACA
>Borrelia_afzelii_PKo_chr.trna3-PheGAA (205737-205809) Phe (GAA) 73 bp Sc: 78.84
GCTGCTGTAGCTCAGTTGGTAGAGCATAGGACTGAAAATCCTTGTGTCAGGAGTTCGATT
CTCCTCGGCAGCA
>Borrelia_afzelii_PKo_chr.trna23-ProTGG (792752-792680) Pro (TGG) 73 bp Sc: 68.55
CGGGATGTGGCCTAGTGGCTAAGGCACCTGCTTTGGGAGCAGGGGATCGTGAGTTCGAAT
CCCACCATCCCGA
>Borrelia_afzelii_PKo_chr.trna30-GluTTC (551439-551368) Glu (TTC) 72 bp Sc: 34.25
GTCCTCTTCGTCTATCGGTCAGGACTCCAGGTTTTCATCCTGGCAAGAGGGGTTCGATTC
CCCTAGAGGATG
>Borrelia_afzelii_PKo_chr.trna8-SerGCT (485038-485127) Ser (GCT) 90 bp Sc: 56.66
GGAGAGATGCCAGAGTGGCCGAATGGGGCTTCCTGCTAAGAAGTTGTCCTTTTAAAAAGG
GGACCATGGGTTCGAATCCCATTCTCTCCG
>Borrelia_afzelii_PKo_chr.trna11-SerGGA (486170-486256) Ser (GGA) 87 bp Sc: 68.51
GGAGAGATGGCCGAGTGGCTTAAGGCGCACGCTTGGAAAGCGTGTATACGGTAAAATGTA
TCATGGGTTCGAATCCCATTCTCTCCG
>Borrelia_afzelii_PKo_chr.trna9-SerTGA (485184-485270) Ser (TGA) 87 bp Sc: 67.23
GGAGAGGTGGCAGAGTGGTTTAATGCTACGGTCTTGAAAACCGTTGTAGGTGTAAGCCTA
CCGTGAGTTCGAATCTCACCCTCTCCG
>Borrelia_afzelii_PKo_chr.trna32-ThrGGT (410685-410613) Thr (GGT) 73 bp Sc: 78.49
GCCCTTATAGCTCAGTTGGTAGAGCACCACCATGGTAAGGTGGGGGTCGTCGGTTCAAGT
CCGATTGAGGGCT
>Borrelia_afzelii_PKo_chr.trna18-ThrTGT (748734-748807) Thr (TGT) 74 bp Sc: 80.38
GCCGACTTAGCTCAGCTGGCCAGAGCAGCGGTCTTGTAAACCGCAGGTCGTCGGTTCGAC
TCCGACAGTCGGCT
>Borrelia_afzelii_PKo_chr.trna33-TrpCCA (410397-410325) Trp (CCA) 73 bp Sc: 58.17
AGGTCAGTAGTTCCAACGGTAGAACGACAGTCTCCAAAACTGTATGCTGGGGGTTCGAAT
CCCTCCTGACCTG
>Borrelia_afzelii_PKo_chr.trna19-TyrGTA (748812-748893) Tyr (GTA) 82 bp Sc: 57.25
GGGGCGGTACCGAAGTGGTTAACCGGGGCAGACTGTAAATCTGTTGGCTTTGCCTACGTG
GGTTCGAATCCCACCCTCCCCA
>Borrelia_afzelii_PKo_chr.trna24-ValTAC (734390-734317) Val (TAC) 74 bp Sc: 85.81
GGGCTCATAGCTCAGTTGGTCAGAGCGCCTGCCTTACAAGCAGGATGTCGGGAGTTCGAA
TCTCTCTGGGCCCA
>Borrelia_garinii_PBi_chrlinear.trna31-AlaTGC (446598-446525) Ala (TGC) 74 bp Sc: 91.03
GGGGGTTTAGCTCAGTTGGCTAGAGCATCGGCTTTGCAAGCCGAGGGTCAAGGGTTCGAG
TCCCTTAACCTCCA
>Borrelia_garinii_PBi_chrlinear.trna10-ArgGCG (484443-484516) Arg (GCG) 74 bp Sc: 72.81
GTGTCCATAGCTCAGTTGGATAGAGCGTTAGATTGCGATTCTTAAGGTCGGAGGTTCAAG
TCCTCTTGGACACG
>Borrelia_garinii_PBi_chrlinear.trna13-ArgTCG (638176-638249) Arg (TCG) 74 bp Sc: 69.91
GCATTCATAGCTCAATTGGATAGAGCAGTGGACTTCGAATCCGAAGGTTGCAGGTTCGAG
TCCTGCTGAGTGCG
>Borrelia_garinii_PBi_chrlinear.trna22-ArgTCT (791200-791127) Arg (TCT) 74 bp Sc: 75.09
GCATCAATAGCTCAATTGGATAGAGCAACAGACTTCTAATCTGTAGGTTTTAGGTTCGAG
TCCTAATTGGTGCG
>Borrelia_garinii_PBi_chrlinear.trna17-AsnGTT (658879-658950) Asn (GTT) 72 bp Sc: 76.04
TCCCCTATAGCTCAGTGGTAGAGCGGGTGGCTGTTAACCACTAGGTCGGAGGTTCAAGTC
CTTCTGGGGGAG
>Borrelia_garinii_PBi_chrlinear.trna25-AspGTC (665077-665004) Asp (GTC) 74 bp Sc: 79.20
GGGGGCGTAGTTCAGATGGTTAGAACGCCTGCCTGTCACGCAGGAGGTCGCGGGTTCGAG
ACCCGTCGCTCCCG
>Borrelia_garinii_PBi_chrlinear.trna2-CysGCA (202268-202340) Cys (GCA) 73 bp Sc: 69.14
GGCGCTGTACCCAAGTGGCTAAGGGAGAAGTCTGCAAAACTTTGATTCGCCGGTTCGATT
CCGGTCAGCGCCT
>Borrelia_garinii_PBi_chrlinear.trna21-GlnTTG (825030-825101) Gln (TTG) 72 bp Sc: 59.77
TGGGACGTCGACAAGCGGTAAGTCAACTGGTTTTGGTCCAGTCATTCGAGGGTTCGAATC
CTTCCGTCCCAG
>Borrelia_garinii_PBi_chrlinear.trna15-GlyGCC (638415-638486) Gly (GCC) 72 bp Sc: 66.76
GCGAAAGTAACTCAGGGGTAGAGTGTCACCTTGCCAAGGTGGAAGTCGCGGGTTCAAATC

CCGTCTTTCGCT
>Borrelia_garinii_PBi_chrlinear.trna1-GlyTCC (46379-46450) Gly (TCC) 72 bp Sc: 50.44
GCGTCCTTCGTATAATGGCTATTACCTTAGCCTTCCAAGCTAATGATGTCGGTTCGATTC
CGATAGGACGCT
>Borrelia_garinii_PBi_chrlinear.trna12-HisGTG (638080-638152) His (GTG) 73 bp Sc: 64.57
GTGGTTGTAGCTCAGTTGGTAGAGCGTCGGGTTGTGGTTCCGAATGTCGCGGGTTCAAGC
CCCGTCAATCACC
>Borrelia_garinii_PBi_chrlinear.trna7-IleGAT (444915-444988) Ile (GAT) 74 bp Sc: 71.67
GGGATCATAGCTCAGGTGGTTAGAGCGCAGGTCTGATAAACCTGAGGTCGGATGTTCAAC
TCATCCTGGTCCCA
>Borrelia_garinii_PBi_chrlinear.trna27-LeuCAA (586110-586027) Leu (CAA) 84 bp Sc: 65.10
GCCGGTATGGCGGAATTGGTAGACGCGTCAGACTCAAAATCTGATGAGGGCAACTTCGTG
TCGGTTCGACTCCGACTACCGGTA
>Borrelia_garinii_PBi_chrlinear.trna6-LeuGAG (255077-255163) Leu (GAG) 87 bp Sc: 51.29
GCTGTGGTGGTGGAAGTGGTAGACACGCTAGCTTGAGGGGCTAGTGGGCGTAAGCCCATG
CTGGTTCAAGTCCAGTTCACAGCATCA
>Borrelia_garinii_PBi_chrlinear.trna20-LeuTAA (803728-803811) Leu (TAA) 84 bp Sc: 65.47
GCCGAAGTGGTGGAAGTGGTAGACACACAGGACTTAAAATCCTGAGGAGGAAGCTCCGTA
CCGGTTCAAGTCCGGTCTTCGGTA
>Borrelia_garinii_PBi_chrlinear.trna14-LeuTAG (638329-638409) Leu (TAG) 81 bp Sc: 53.67
GCAGGAGTGGTGGAATTGGCAGACACGCTAGACTTAGGATCTAGTGCTTTTGGCGTGTGG
GTTCGACTCCCACCTTCTGTA
>Borrelia_garinii_PBi_chrlinear.trna28-LysCTT (549950-549878) Lys (CTT) 73 bp Sc: 70.67
GGGCTGCTAGCTCAAGTGGTAGAGCATCGGACTCTTAATCCGTTGGTTACAGGTTCAAGT
CCTGTGCAGCTCA
>Borrelia_garinii_PBi_chrlinear.trna29-LysTTT (549767-549695) Lys (TTT) 73 bp Sc: 77.18
GGGCTCATAGCTCAGGTGGTAGAGCAGCGCCCTTTTAAGGCGTTTGTCGTAGGTTCGAGT
CCTACTGAGCTCA
>Borrelia_garinii_PBi_chrlinear.trna26-MetCAT (645152-645080) Met (CAT) 73 bp Sc: 80.18
CGCAGGGTAGAGCAGTTGGTAGCTCGTCGGGCTCATAACCCGAAGGTCATAGGTTCGAGT
CCTATCCCTGCTA
>Borrelia_garinii_PBi_chrlinear.trna16-MetCAT (650409-650482) Met (CAT) 74 bp Sc: 82.28
GCTGATGTAGCTCAGTTGGTTAGAGCACTCGGCTCATATCCGAGTTGTCGTGGGTTCAAG
TCCCTCCATCAGCA
>Borrelia_garinii_PBi_chrlinear.trna5-MetCAT (217290-217363) Met (CAT) 74 bp Sc: 88.53
GGGCCCATAGCTCAGTTGGTTAGAGCACCCGACTCATAATCGGTAGGTCCCAGGTTCAAG
TCCTGGTGGGCCCA
>Borrelia_garinii_PBi_chrlinear.trna4-PheGAA (206023-206095) Phe (GAA) 73 bp Sc: 77.30
GTCGCTGTAGCTCAGTTGGTAGAGCATAGGACTGAAAATCCTTGTGTCAGGAGTTCGATT
CTCCTCGGCGACA
>Borrelia_garinii_PBi_chrlinear.trna3-PheGAA (205930-206002) Phe (GAA) 73 bp Sc: 78.84
GCTGCTGTAGCTCAGTTGGTAGAGCATAGGACTGAAAATCCTTGTGTCAGGAGTTCGATT
CTCCTCGGCAGCA
>Borrelia_garinii_PBi_chrlinear.trna23-ProTGG (791119-791047) Pro (TGG) 73 bp Sc: 68.55
CGGGATGTGGCCTAGTGGCTAAGGCACCTGCTTTGGGAGCAGGGGATCGTGAGTTCGAAT
CCCACCATCCCGA
>Borrelia_garinii_PBi_chrlinear.trna30-GluTTC (549689-549618) Glu (TTC) 72 bp Sc: 36.94
GTCCTCTTCGTCTATCGGTTAGGACTCCAGGTTTTCATCCTGGCAAGAGGGGTTCGATTC
CCCTAGAGGATG
>Borrelia_garinii_PBi_chrlinear.trna8-SerGCT (483406-483494) Ser (GCT) 89 bp Sc: 51.84
GGAGAGATGCCAGAGTGGCCGAATGGGGCTTCCTGCTAAGAAGTTGTCCTTTTGAAAGGG
GACCATGGGTTCGAATCCCATTCTCTCCG
>Borrelia_garinii_PBi_chrlinear.trna11-SerGGA (484533-484619) Ser (GGA) 87 bp Sc: 68.51
GGAGAGATGGCCGAGTGGCTTAAGGCGCACGCTTGGAAAGCGTGTATACGGTAAAATGTA
TCATGGGTTCGAATCCCATTCTCTCCG
>Borrelia_garinii_PBi_chrlinear.trna9-SerTGA (483551-483637) Ser (TGA) 87 bp Sc: 67.23
GGAGAGGTGGCAGAGTGGTTTAATGCTACGGTCTTGAAAACCGTTGTAGGTGTAAGCCTA
CCGTGAGTTCGAATCTCACCCTCTCCG
>Borrelia_garinii_PBi_chrlinear.trna32-ThrGGT (409127-409055) Thr (GGT) 73 bp Sc: 78.49
GCCCTTATAGCTCAGTTGGTAGAGCACCACCATGGTAAGGTGGGGGTCGTCGGTTCAAGT
CCGATTGAGGGCT
>Borrelia_garinii_PBi_chrlinear.trna18-ThrTGT (746829-746902) Thr (TGT) 74 bp Sc: 80.38
GCCGACTTAGCTCAGCTGGCCAGAGCAGCGGTCTTGTAAACCGCAGGTCGTCGGTTCGAC
TCCGACAGTCGGCT
>Borrelia_garinii_PBi_chrlinear.trna33-TrpCCA (408839-408767) Trp (CCA) 73 bp Sc: 58.17
AGGTCAGTAGTTCCAACGGTAGAACGACAGTCTCCAAAACTGTATGCTGGGGGTTCGAAT
CCCTCCTGACCTG

>Borrelia_garinii_PBi_chrlinear.trna19-TyrGTA (746907-746988) Tyr (GTA) 82 bp Sc: 57.25
GGGGCGGTACCGAAGTGGTTAACCGGGGCAGACTGTAAATCTGTTGGCTTTGCCTACGTG
GGTTCGAATCCCACCCTCCCCA
>Borrelia_garinii_PBi_chrlinear.trna24-ValTAC (732507-732434) Val (TAC) 74 bp Sc: 85.81
GGGCTCATAGCTCAGTTGGTCAGAGCGCCTGCCTTACAAGCAGGATGTCGGGAGTTCGAA
TCTCTCTGGGCCCA
>Bradyrhizobium_BTAi1_chr.trna3-AlaCGC (276743-276818) Ala (CGC) 76 bp Sc: 85.83
GGGGCCATAGCTCAGCTGGGAGAGCGCATCGTTCGCAATGATGAGGTCGGCGGTTCGATC
CCGCCTGGCTCCACCA
>Bradyrhizobium_BTAi1_chr.trna1-AlaGGC (112686-112761) Ala (GGC) 76 bp Sc: 87.23
GGGGCCATAGCTCAGCTGGGAGAGCGCTTGCATGGCATGCAAGAGGTCGGCGGTTCGATC
CCGCCTGGCTCCACCA
>Bradyrhizobium_BTAi1_chr.trna10-AlaTGC (1275627-1275702) Ala (TGC) 76 bp Sc: 90.33
GGGGCCTTAGCTCAGCTGGGAGAGCGCGTGCTTTGCAAGCATGAGGTCGTCGGTTCGATC
CCGACAGGCTCCACCA
>Bradyrhizobium_BTAi1_chr.trna18-AlaTGC (3362428-3362503) Ala (TGC) 76 bp Sc: 90.33
GGGGCCTTAGCTCAGCTGGGAGAGCGCGTGCTTTGCAAGCATGAGGTCGTCGGTTCGATC
CCGACAGGCTCCACCA
>Bradyrhizobium_BTAi1_chr.trna32-ArgACG (7688295-7688219) Arg (ACG) 77 bp Sc: 85.54
GCGCCCGTAGCTCAGCTGGATAGAGCATCAGACTACGAATCTGAGGGTCGGACGTTCGAA
TCGTTCCGGGCGCGCCA
>Bradyrhizobium_BTAi1_chr.trna30-ArgCCG (8089157-8089233) Arg (CCG) 77 bp Sc: 94.29
GCACCCATAGCTCAGCTGGATAGAGCGCCACCCTCCGAAGGTGGAGGTCACAGGTTCGAA
TCCTGTTGGGTGCGCCA
>Bradyrhizobium_BTAi1_chr.trna31-ArgCCT (7905048-7904972) Arg (CCT) 77 bp Sc: 80.28
GGTCCCGTAGCTCAGCTGGATAGAGCGGCGGTTTCCTAAACCGTAGGTCGCATGTTCGAG
TCATGCCGGGATCGCCA
>Bradyrhizobium_BTAi1_chr.trna24-ArgTCG (5191948-5192024) Arg (TCG) 77 bp Sc: 87.99
GCGCTCGTAGCTCAGCTGGATAGAGCATCGGATTTCGATTCCGAGGGTCGGGAGTTCGAA
TCTCTCCGAGCGCGCCA
>Bradyrhizobium_BTAi1_chr.trna46-ArgTCT (4096206-4096130) Arg (TCT) 77 bp Sc: 80.78
GGTCCCATAGCTCAACTGGATAGAGTAGCGGATTTCTACTCCGCGGGTTGCAGGTTCGAG
TCCTGCTGGGATCGCCA
>Bradyrhizobium_BTAi1_chr.trna48-AsnGTT (3926576-3926502) Asn (GTT) 75 bp Sc: 33.52
GCGCCGTGGTGAGGAGTGCTGATACTTCGACTGTTAATCGAGAGGTCGTGGGTTCGAGTC
CCACCCGGCGTTCCA
>Bradyrhizobium_BTAi1_chr.trna44-AsnGTT (4137815-4137741) Asn (GTT) 75 bp Sc: 88.70
TCCTCGGTAGCTCAGCGGTAGAGCATTCGACTGTTAATCGAATGGTCGCTGGTTCGAATC
CAGCCCGGGGAGCCA
>Bradyrhizobium_BTAi1_chr.trna43-AspGTC (4783835-4783759) Asp (GTC) 77 bp Sc: 97.02
GCGGGAGTAGCTCAGTTGGTTAGAGCGCCGGCCTGTCACGCCGGAGGTCGCGGGTTCGAG
CCCCGTCTCTCGCGCCA
>Bradyrhizobium_BTAi1_chr.trna45-CysGCA (4131244-4131171) Cys (GCA) 74 bp Sc: 67.89
GGCCACGTGGCGGAGTGGTTACGCAGCGGTCTGCAAAACCGTTTACACCAGTTCAATTCT
GGTCGTGGCCTCCA
>Bradyrhizobium_BTAi1_chr.trna49-GlnCTG (3688300-3688227) Gln (CTG) 74 bp Sc: 68.00
TGGGGAATGGTGTAACGGTAGCACAACAGACTCTGACTCTGTTTGTCTTGGTTCGAATCC
AGGTTCCCCAGCCA
>Bradyrhizobium_BTAi1_chr.trna16-GlnTTG (2583194-2583268) Gln (TTG) 75 bp Sc: 77.73
TGGGGCGTAGCCAAGCGGTAAGGCAGCGGATTTTGATTCCGCCATTCGGAGGTTCGATCC
CTCCCGCCCCAGCCA
>Bradyrhizobium_BTAi1_chr.trna34-GluCTC (7041998-7041924) Glu (CTC) 75 bp Sc: 68.86
GCTCCCTTCGTCTAGCGGTTAGGACGCGGCCCTCTCAAGGCTGAAACAGGGGTTCGATTC
CCCTAGGGAGCGCCA
>Bradyrhizobium_BTAi1_chr.trna19-GluTTC (4231469-4231543) Glu (TTC) 75 bp Sc: 60.15
GCTCCCTTCGTCTATCGGTTAGGACGCCACCCTTTCACGGTGGAGAGAGCGGTTCGATTC
CGCTAGGGAGCGCCA
>Bradyrhizobium_BTAi1_chr.trna7-GlyCCC (799661-799734) Gly (CCC) 74 bp Sc: 80.62
GCGGGCGTAGTTCAATGGTAGAACGGCAGCTTCCCAAGCTGCATACGAGGGTTCGATTCC
CTTCGCCCGCTCCA
>Bradyrhizobium_BTAi1_chr.trna4-GlyGCC (310428-310502) Gly (GCC) 75 bp Sc: 88.70
GCGGGTGTAGCTCAGTGGTAGAGCACGACCTTGCCAAGGTCGGGGTCGAGGGTTCGAACC
CCTTCGCCCGCTCCA
>Bradyrhizobium_BTAi1_chr.trna37-GlyTCC (5356535-5356462) Gly (TCC) 74 bp Sc: 82.33
GCGGGTGTAGCTCAATGGTAGAGCAGCAGCCTTCCAAGCTGAATACGAGGGTTCGATTCC
CTTCACCCGCTCCA
>Bradyrhizobium_BTAi1_chr.trna33-HisGTG (7140394-7140318) His (GTG) 77 bp Sc: 90.83

GCAGTTGTAGCTCAGTTGGTTAGAGCGCCTGTCTGTGGAACAGGAGGTCGGTGGTTCGAG
CCCACCCAACTGTACCA
>Bradyrhizobium_BTAi1_chr.trna17-IleGAT (3362208-3362284) Ile (GAT) 77 bp Sc: 93.43
GGGCTTGTAGCTCAGTTGGTTAGAGCGCGCGCTTGATAAGCGTGAGGTCGGAAGTTCAAG
TCTTCCCAGGCCCACCA
>Bradyrhizobium_BTAi1_chr.trna9-IleGAT (1275407-1275483) Ile (GAT) 77 bp Sc: 93.43
GGGCTTGTAGCTCAGTTGGTTAGAGCGCGCGCTTGATAAGCGTGAGGTCGGAAGTTCAAG
TCTTCCCAGGCCCACCA
>Bradyrhizobium_BTAi1_chr.trna50-LeuCAA (1169298-1169214) Leu (CAA) 85 bp Sc: 77.17
GCCCCTGTGGCGGAACTGGTAGACGCGCTCGACTCAAAATCGAGTTCCGCAAGGAGTGCT
GGTTCGATTCCGGCCAGGGGCACCA
>Bradyrhizobium_BTAi1_chr.trna2-LeuCAG (230495-230581) Leu (CAG) 87 bp Sc: 75.99
GCCCAGGTGGCGGAATTGGTAGACGCGCTGGCTTCAGGTGCCAGTGGCTTAACGGCCGTG
AAGGTTCGAGTCCTTTCCTGGGCACCA
>Bradyrhizobium_BTAi1_chr.trna39-LeuGAG (5131078-5130994) Leu (GAG) 85 bp Sc: 71.15
GCGCTCGTGGCGGAATTGGTAGACGCGCTGCCTTGAGGTGGCAGTGGGTAACACCGTGGG
GGTTCGAGTCCCTCCGAGCGCACCA
>Bradyrhizobium_BTAi1_chr.trna26-LeuTAA (5463122-5463208) Leu (TAA) 87 bp Sc: 76.40
GCGGACGTGGCGGAATTGGTAGACGCAAGGGACTTAAAATCCCTCGATGGCAACGTCGTG
TGGGTTCGAGTCCCACCGCCCGCACCA
>Bradyrhizobium_BTAi1_chr.trna41-LeuTAG (4796235-4796151) Leu (TAG) 85 bp Sc: 76.68
GCGGGCGTGGCGGAACTGGTAGACGCGCTGGATTTAGGTTCCAGTGACGAAAGTTGTGGG
GGTTCGAGTCCCTCCGCCCGCACCA
>Bradyrhizobium_BTAi1_chr.trna35-LysCTT (6260780-6260705) Lys (CTT) 76 bp Sc: 96.14
GGGCGCATAGCTCAGTTGGTAGAGCAGCTGACTCTTAATCAGCGGGTCCTAGGTTCGAGC
CCTAGTGCGCCCACCA
>Bradyrhizobium_BTAi1_chr.trna22-LysTTT (4388572-4388647) Lys (TTT) 76 bp Sc: 90.48
GAGCGCGTAGCTCAGCCGGTAGAGCACGTGACTTTTAATCACGGGGTCCTGGGTTCGAGC
CCCAGCGCGCTCACCA
>Bradyrhizobium_BTAi1_chr.trna27-MetCAT (6615111-6615186) Met (CAT) 76 bp Sc: 83.74
GGGCCTGTAGCTCAATGGTTAGAGCCGGCCGCTCATAACGGTCTGGTTGCAGGTTCGAGT
CCTGCCGGGCCCACCA
>Bradyrhizobium_BTAi1_chr.trna11-MetCAT (1294885-1294961) Met (CAT) 77 bp Sc: 83.88
CGCGGGGTGGAGCAGCCCGGTAGCTCGTCAGGCTCATAACCTGAAGGTCATAGGTTCAAA
TCCTATCCCCGCAACCA
>Bradyrhizobium_BTAi1_chr.trna14-MetCAT (1951990-1952066) Met (CAT) 77 bp Sc: 92.61
GGCGGGGTAGCTCAGCTGGTTAGAGCACGGGAATCATAATCCTGGGGTCGGGGGTTCGAG
TCCCTCCCCCGCTACCA
>Bradyrhizobium_BTAi1_chr.trna8-PheGAA (935782-935857) Phe (GAA) 76 bp Sc: 91.10
GCCCAGGTAGCTCAGTTGGTAGAGCATGCGACTGAAAATCGCAGTGTCGGTGGTTCGATT
CCGCCCCTGGGCACCA
>Bradyrhizobium_BTAi1_chr.trna15-ProCGG (2477299-2477375) Pro (CGG) 77 bp Sc: 86.24
CGGAGCGTGGCTCAGCCCGGTAGAGCACTGCGTTCGGGACGCAGGGGTCGCAGGTTCGAA
TCCTGCCGCTCCGACCA
>Bradyrhizobium_BTAi1_chr.trna40-ProGGG (4855481-4855404) Pro (GGG) 78 bp Sc: 78.41
CGGAGCGTGGCGCAGCCCGGTTAGCGCACTAGTCTGGGGGACTAGGGGTCGTGGGTTCGA
ATCCCGCCGCTCCGACCA
>Bradyrhizobium_BTAi1_chr.trna20-ProTGG (4237965-4238041) Pro (TGG) 77 bp Sc: 79.83
CGGGGTATAGCGCAGCCTGGTAGCGCGGCAGTTTTGGGTACTGCAGGTCGTTGGTTCGAA
TCCAGCTGCCCCGACCA
>Bradyrhizobium_BTAi1_chr.trna29-Undet??? (7078602-7078678) Undet (???) 77 bp Sc: 22.69
GGCCCGGTGGCGGAAGCTACGACGCGAAGAGCGGTGCAACGCTCTTGAACCTGGTTCAAG
TCCAGGCCGGGCCTCCA
>Bradyrhizobium_BTAi1_chr.trna12-MetCAT (1326564-1326631) Met (CAT) 68 bp Sc: 22.61
GGCGGGAATGAGCAATCATCGTACTCATAACCTGAAGGTCATAGGTTCAAATCCTATCCC
CGCAACCA
>Bradyrhizobium_BTAi1_chr.trna47-GluTTC (3926805-3926738) Glu (TTC) 68 bp Sc: 27.12
GCCGTGGTGAAGATGACTGTTACTTCGCCTTTCACGCGAGAGGTCGCGGGTTCGAATCCC
GCCCGGCG
>Bradyrhizobium_BTAi1_chr.trna5-SerCGA (432947-433036) Ser (CGA) 90 bp Sc: 78.76
GGAGAGGTGGCAGAGTGGTTGAATGCACCGCACTCGAAATGCGGCATAGGTGCAAGCCTA
TCGGGGGTTCGAATCCCTCCCTCTCCGCCA
>Bradyrhizobium_BTAi1_chr.trna23-SerGCT (4931548-4931641) Ser (GCT) 94 bp Sc: 76.24
GGAGACGTGGCCGAGTGGCTGAAGGCGGCGGTTTGCTAAACCGTTATAGGGTTGTAAAGC
CCTATCGAGGGTTCGAATCCCTCCGTCTCCGCCA
>Bradyrhizobium_BTAi1_chr.trna6-SerGGA (587810-587899) Ser (GGA) 90 bp Sc: 81.68
GGAGAGATGGCCGAGTGGCTTAAGGCGCACGCTTGGAAAGCGTGTGTGCGGGAAACCGTA

CCGTGGGTTCGAATCCCACTCTCTCCGCCA
>Bradyrhizobium_BTAi1_chr.trna21-SerTGA (4309469-4309558) Ser (TGA) 90 bp Sc: 79.34
GGAAGGGTGGCCGAGTGGTTTAAGGCACCGGTCTTGAAAACCGGCGTGCCCGCAAGGGTA
CCGTGGGTTCGAATCCCACCCCTTCCGCCA
>Bradyrhizobium_BTAi1_chr.trna52-ThrCGT (289723-289648) Thr (CGT) 76 bp Sc: 97.39
GCCGCAATAGCTCAGCTGGTAGAGCACGTCATTCGTAATGACGGGGTCGGGGGTTCGAGT
CCCTCTTGCGGCACCA
>Bradyrhizobium_BTAi1_chr.trna51-ThrGGT (927963-927889) Thr (GGT) 75 bp Sc: 89.61
GCTGCCGTAGCTCAGTGGTAGAGCACTCCCTTGGTAAGGGAGAGGTCGACAGTTCAATCC
TGTCCGGCAGCACCA
>Bradyrhizobium_BTAi1_chr.trna25-ThrTGT (5392519-5392593) Thr (TGT) 75 bp Sc: 88.63
GCCGGCTTAGCTCAGCGGTAGAGCAGCGGTTTTGTAAACCGAAGGTCGGGGGTTCAATCC
CCTCAGCCGGCACCA
>Bradyrhizobium_BTAi1_chr.trna38-TrpCCA (5321801-5321726) Trp (CCA) 76 bp Sc: 85.27
AGGAGTGTAGCTCAATTGGTAGAGCACCGGTCTCCAAAACCGGGGGTTGGGAGTTCGAGC
CTCTCCACTCCTGCCA
>Bradyrhizobium_BTAi1_chr.trna36-TyrGTA (5389349-5389264) Tyr (GTA) 86 bp Sc: 74.36
GGGGGAGTGTCCCGAGTGGCAAAGGGAGCTGACTGTAAATCAGCCGCCTCATGGCTTCGC
AGGTTCGAGTCCTGCCTCCCCCACCA
>Bradyrhizobium_BTAi1_chr.trna28-ValCAC (6851026-6851100) Val (CAC) 75 bp Sc: 81.20
GGGCGCATAGCTCAGCGGGAGAGCGTTCCCTTCACACGGGAGAGGTCCAAGGTTCGATCC
CTTGTGCGCCCACCA
>Bradyrhizobium_BTAi1_chr.trna13-ValGAC (1490439-1490513) Val (GAC) 75 bp Sc: 88.00
GGGCACGTAGCTCAGCGGGAGAGCACTACCTTGACATGGTAGGGGTCACAGGTTCGATCC
CTGTCGTGCCCACCA
>Bradyrhizobium_BTAi1_chr.trna42-ValTAC (4788972-4788896) Val (TAC) 77 bp Sc: 91.77
GGGCGGTTAGCTCAGCTGGTTAGAGCATCTCGTTTACACCGAGAGGGTCGGGAGTTCGAA
TCTCTCACCGCCTACCA
>Brucella_canis_ATCC_23365_chrI.trna21-AlaCGC (1917379-1917304) Ala (CGC) 76 bp Sc: 85.21
GGGGCTGTAGCTCAGCTGGGAGAGCGCGTCGTTCGCAATGACGAGGTCAGGGGTTCGATC
CCCCTCAGCTCCACCA
>Brucella_canis_ATCC_23365_chrI.trna41-AlaGGC (28391-28316) Ala (GGC) 76 bp Sc: 87.70
GGGGCCATAGCTCAGCTGGGAGAGCGCCTGCATGGCATGCAGGAGGTCAGCGGTTCGATC
CCGCTTGGCTCCACCA
>Brucella_canis_ATCC_23365_chrI.trna23-AlaTGC (1791467-1791392) Ala (TGC) 76 bp Sc: 91.73
GGGGCCGTAGCTCAGCTGGGAGAGCACCTGCTTTGCAAGCAGGGGGTCGTCGGTTCGATC
CCGTCCGGCTCCACCA
>Brucella_canis_ATCC_23365_chrI.trna26-AlaTGC (1587062-1586987) Ala (TGC) 76 bp Sc: 91.73
GGGGCCGTAGCTCAGCTGGGAGAGCACCTGCTTTGCAAGCAGGGGGTCGTCGGTTCGATC
CCGTCCGGCTCCACCA
>Brucella_canis_ATCC_23365_chrII.trna8-AlaTGC (1107201-1107126) Ala (TGC) 76 bp Sc: 91.73
GGGGCCGTAGCTCAGCTGGGAGAGCACCTGCTTTGCAAGCAGGGGGTCGTCGGTTCGATC
CCGTCCGGCTCCACCA
>Brucella_canis_ATCC_23365_chrI.trna39-ArgACG (279486-279410) Arg (ACG) 77 bp Sc: 91.80
GCACCCGTAGCTCAGCTGGATAGAGCACCAGACTACGAATCTGGGGGTCAGAGGTTCGAA
TCCTTTCGGGTGCGCCA
>Brucella_canis_ATCC_23365_chrI.trna8-ArgCCG (935320-935396) Arg (CCG) 77 bp Sc: 90.06
GCACCCGTAGCTCAGCTGGATAGAGCGCTGCCCTCCGAAGGCAGAGGCCACAGGTTCGAA
TCCTGTCGGGTGCGCCA
>Brucella_canis_ATCC_23365_chrI.trna20-ArgCCT (1961622-1961698) Arg (CCT) 77 bp Sc: 88.11
GGTCCCGTAGCTCAGTAGGATAGAGCGACAGATTCCTAATCTGTAGGTCACAGGTTCGAT
TCCTGTCGGGATCACCA
>Brucella_canis_ATCC_23365_chrI.trna5-ArgTCT (704407-704483) Arg (TCT) 77 bp Sc: 90.69
GGTCCCGTAGCTCAGTTGGATAGAGCACCGGCCTTCTAAGCCGATGGTCGCAGGTTCGAA
TCCTGCCGGGATCGCCA
>Brucella_canis_ATCC_23365_chrII.trna10-AsnGTT (608777-608703) Asn (GTT) 75 bp Sc: 89.56
TCCCCGGTAGCTCAGCGGTAGAGCAACCGGCTGTTAACCGGTTGGTCGCTGGTTCGAATC
CGGCCCGGGGAGCCA
>Brucella_canis_ATCC_23365_chrI.trna6-AspGTC (776844-776920) Asp (GTC) 77 bp Sc: 92.83
GCGGGTGTAGCTCAGTTGGTTAGAGTGCCGGCCTGTCACGCCGGAGGTCGCGGGTTCGAG
CCCCGTCACTCGCGCCA
>Brucella_canis_ATCC_23365_chrII.trna13-AspGTC (305172-305096) Asp (GTC) 77 bp Sc: 92.83
GCGGGTGTAGCTCAGTTGGTTAGAGTGCCGGCCTGTCACGCCGGAGGTCGCGGGTTCGAG
CCCCGTCACTCGCGCCA
>Brucella_canis_ATCC_23365_chrII.trna1-CysGCA (604341-604414) Cys (GCA) 74 bp Sc: 66.24
GGCCTCGTGGCGGAGTGGTTACGCAGAGGACTGCAAATCCTTGCACCCCGGTTCGATTCC
GGGCGAGGCCTCCA

>Brucella_canis_ATCC_23365_chrI.trna16-GlnCTG (1562849-1562922) Gln (CTG) 74 bp Sc: 66.55
TGGGGGATAGTTTAAGGGTAGAACAGCGGACTCTGACTCCGTTAGTCTTGGTTCGAATCC
AGGTCCCCCAGCCA
>Brucella_canis_ATCC_23365_chrI.trna3-GlnTTG (511737-511811) Gln (TTG) 75 bp Sc: 74.48
TGGGGCGTAGCCAAGCGGTAAGGCAGCGGTTTTTGGTACCGCCATTCCCTGGTTCGAATC
CAGGCGCCCCAGCCA
>Brucella_canis_ATCC_23365_chrI.trna17-GluCTC (1602102-1602176) Glu (CTC) 75 bp Sc: 65.98
GCGCCCATCGTCTAGCGGTCAGGACGGCGCCCTCTCACGGCGCAAACAGGGGTTCGATTC
CCCTTGGGCGTACCA
>Brucella_canis_ATCC_23365_chrI.trna12-GluTTC (1239950-1240024) Glu (TTC) 75 bp Sc: 72.14
GCGCCCATCGTCTAGCGGTTAGGACGCCGCCCTTTCACGGCGGAAACAGGGGTTCGATTC
CCCTTGGGCGTACCA
>Brucella_canis_ATCC_23365_chrI.trna13-GluTTC (1240172-1240246) Glu (TTC) 75 bp Sc: 72.14
GCGCCCATCGTCTAGCGGTTAGGACGCCGCCCTTTCACGGCGGAAACAGGGGTTCGATTC
CCCTTGGGCGTACCA
>Brucella_canis_ATCC_23365_chrI.trna33-GlyCCC (936171-936098) Gly (CCC) 74 bp Sc: 65.12
GCGGGTATGATGTAATGGTAGCCTGTCAGCTTCCCAAGCTGAACGCGCGGGTTCGATTCC
CGCTACCCGCTCCA
>Brucella_canis_ATCC_23365_chrII.trna3-GlyGCC (877831-877905) Gly (GCC) 75 bp Sc: 88.17
GCGGGTGTAGCTCAGGGGTAGAGCACAACCTTGCCAAGGTTGGGGTCGAGGGTTCGAATC
CCTTCGCCCGCTCCA
>Brucella_canis_ATCC_23365_chrII.trna4-GlyGCC (1006981-1007055) Gly (GCC) 75 bp Sc: 88.17
GCGGGTGTAGCTCAGGGGTAGAGCACAACCTTGCCAAGGTTGGGGTCGAGGGTTCGAATC
CCTTCGCCCGCTCCA
>Brucella_canis_ATCC_23365_chrI.trna31-GlyTCC (1219037-1218964) Gly (TCC) 74 bp Sc: 83.28
GCGGGTATAGCTCAATGGTAGAGCAGCAGCCTTCCAAGCTGAATACGCGGGTTCGATTCC
CGCTACCCGCTCCA
>Brucella_canis_ATCC_23365_chrI.trna28-HisGTG (1548547-1548471) His (GTG) 77 bp Sc: 86.57
GCGGTCGTAGCTCAGTTGGTTAGAGCGCAGGATTGTGGCTCCTGAGGTCGTTGGTTCAAC
TCCAACCGACCGTACCA
>Brucella_canis_ATCC_23365_chrI.trna22-IleGAT (1791557-1791481) Ile (GAT) 77 bp Sc: 99.09
GGGCTTGTAGCTCAGTTGGTTAGAGCACACGCTTGATAAGCGTGGGGTCGGAGGTTCAAG
TCCTCCCAGGCCCACCA
>Brucella_canis_ATCC_23365_chrI.trna25-IleGAT (1587152-1587076) Ile (GAT) 77 bp Sc: 99.09
GGGCTTGTAGCTCAGTTGGTTAGAGCACACGCTTGATAAGCGTGGGGTCGGAGGTTCAAG
TCCTCCCAGGCCCACCA
>Brucella_canis_ATCC_23365_chrII.trna7-IleGAT (1107291-1107215) Ile (GAT) 77 bp Sc: 99.09
GGGCTTGTAGCTCAGTTGGTTAGAGCACACGCTTGATAAGCGTGGGGTCGGAGGTTCAAG
TCCTCCCAGGCCCACCA
>Brucella_canis_ATCC_23365_chrI.trna19-LeuCAA (1879854-1879938) Leu (CAA) 85 bp Sc: 76.80
GCGGGTGTGGTGGAACTGGTAGACGCGCCGGACTCAAAATCCGGTTCCGAAAGGAGTGTC
GGTTCGATTCCGACCACCCGCACCA
>Brucella_canis_ATCC_23365_chrII.trna5-LeuCAG (1037011-1037097) Leu (CAG) 87 bp Sc: 78.71
GCCCAGATGGCGGAATTGGTAGACGCACCAGCTTCAGGTGCTGGCGCTCGCAAGGGCGTG
GAGGTTCGAGTCCTCTTCTGGGCACCA
>Brucella_canis_ATCC_23365_chrI.trna10-LeuGAG (1046638-1046722) Leu (GAG) 85 bp Sc: 70.40
GCGGTCGTGGCGGAATTGGTAGACGCGCAGCGTTGAGGTCGCTGTGGGGCAACCCGTGGA
AGTTCGAGTCTTCTCGACCGCACCA
>Brucella_canis_ATCC_23365_chrI.trna11-LeuGAG (1054822-1054906) Leu (GAG) 85 bp Sc: 70.40
GCGGTCGTGGCGGAATTGGTAGACGCGCAGCGTTGAGGTCGCTGTGGGGCAACCCGTGGA
AGTTCGAGTCTTCTCGACCGCACCA
>Brucella_canis_ATCC_23365_chrI.trna9-LeuTAA (1017887-1017972) Leu (TAA) 86 bp Sc: 77.49
GCGGTCGTGGCGAAATTGGTATACGCAACGGACTTAAAATCCGTCGTCTTTAAGACTTGC
GGGTTCAAGTCCCGCCGACCGCACCA
>Brucella_canis_ATCC_23365_chrI.trna7-LeuTAG (869755-869837) Leu (TAG) 83 bp Sc: 74.64
GCGGATGTGGCGAAATTGGTAGACGCACCAGATTTAGGTTCTGGCGGGAGACCGTGGGGG
TTCGAGTCCCTCCATCCGCACCA
>Brucella_canis_ATCC_23365_chrI.trna14-LysCTT (1296312-1296387) Lys (CTT) 76 bp Sc: 96.09
GGGTGATTAGCTCAGTTGGTAGAGCAGCTGACTCTTAATCAGCGGGTCGTAGGTTCGATC
CCTACATCACCCACCA
>Brucella_canis_ATCC_23365_chrII.trna11-LysTTT (572477-572402) Lys (TTT) 76 bp Sc: 95.04
GAGCGCGTAGCTCAGCCGGTAGAGCAACTGACTTTTAATCAGTAGGTCCAGGGTTCGAAT
CCCTGCGCGCTCACCA
>Brucella_canis_ATCC_23365_chrI.trna24-MetCAT (1787726-1787650) Met (CAT) 77 bp Sc: 81.76
CGCGGGGTGGAGCAGCCCGGTAGCTCGTCAGGCTCATAACCTGAAGGCCGCAGGTTCAAA
TCCTGCCCCCGCAACCA
>Brucella_canis_ATCC_23365_chrI.trna27-MetCAT (1583321-1583245) Met (CAT) 77 bp Sc: 81.76

CGCGGGGTGGAGCAGCCCGGTAGCTCGTCAGGCTCATAACCTGAAGGCCGCAGGTTCAAA
TCCTGCCCCCGCAACCA
>Brucella_canis_ATCC_23365_chrII.trna9-MetCAT (1103460-1103384) Met (CAT) 77 bp Sc: 81.76
CGCGGGGTGGAGCAGCCCGGTAGCTCGTCAGGCTCATAACCTGAAGGCCGCAGGTTCAAA
TCCTGCCCCCGCAACCA
>Brucella_canis_ATCC_23365_chrI.trna15-MetCAT (1546715-1546791) Met (CAT) 77 bp Sc: 86.65
GGCGGAGTAGCTCAGTAGGTTAGAGCAGAGGAATCATAATCCTTGTGTCGGGGGTTCGAA
TCCCTCCTCCGCTACCA
>Brucella_canis_ATCC_23365_chrI.trna29-MetCAT (1428336-1428260) Met (CAT) 77 bp Sc: 87.64
GGGCCTGTAGCTCAATTGGTTAGAGCCGGCGGCTCATAACCGCTTGGTTGGGGGTTCGAG
TCCCTCCGGGCCCACCA
>Brucella_canis_ATCC_23365_chrI.trna40-PheGAA (258978-258903) Phe (GAA) 76 bp Sc: 93.28
GCCCGGATAGCTCAGTTGGTAGAGCAGCGGATTGAAAATCCGCGTGTCGGTGGTTCGAAT
CCGCCTCCGGGCACCA
>Brucella_canis_ATCC_23365_chrI.trna1-ProCGG (207682-207758) Pro (CGG) 77 bp Sc: 88.00
CGGAGTGTAGCGCAGCCTGGTAGCGCACCTCGTTCGGGACGAGGGGGTCGGAGGTTCGAA
TCCTCTCACTCCGACCA
>Brucella_canis_ATCC_23365_chrI.trna37-ProGGG (762786-762710) Pro (GGG) 77 bp Sc: 81.90
CGGAGCGTAGCGCAGCCCGGTAGCGCACTTGACTGGGGGTCAAGGGGTCGTGGGTTCGAA
TCCCGCCGCTCCGACCA
>Brucella_canis_ATCC_23365_chrI.trna4-ProTGG (703769-703845) Pro (TGG) 77 bp Sc: 93.11
CGGAGCGTAGCGCAGTCTGGTAGCGCACCTGATTTGGGATCAGGGGGTCGCAGGTTCGAA
TCCTGCCGCTCCGACCA
>Brucella_canis_ATCC_23365_chrI.trna18-SerCGA (1779599-1779688) Ser (CGA) 90 bp Sc: 83.06
GGAGAGGTGGCTGAGTGGTTGAAAGCACCGCACTCGAAATGCGGCATGGGGGCAACTCCA
TCGGGGGTTCGAATCCCTCCCTCTCCGCCA
>Brucella_canis_ATCC_23365_chrI.trna38-SerGCT (630792-630702) Ser (GCT) 91 bp Sc: 74.16
GGAGAGGTGGCCGAGTGGTCGAAGGCGCTCCCCTGCTAAGGGAGTAGACCTCAAAAGGGT
CTCGTGGGTTCGAATCCCATCCTCTCCGCCA
>Brucella_canis_ATCC_23365_chrII.trna6-SerGGA (1054438-1054527) Ser (GGA) 90 bp Sc: 77.71
GGAGAGGTGGCCGAGTGGTTGAAGGCGCACGCCTGGAACGCGTGTATACGGGAAACCGTA
TCGAGGGTTCGAATCCCTCTCTCTCCGCCA
>Brucella_canis_ATCC_23365_chrII.trna12-SerTGA (540026-539937) Ser (TGA) 90 bp Sc: 74.18
GGAAGGGTGGCCGAGCGGTTTAAGGCACCGGTCTTGAAAACCGGCGTGCGAGAGATCGTA
CCGTGGGTTCGAATCCCACCCCCTCCGCCA
>Brucella_canis_ATCC_23365_chrII.trna2-ThrCGT (817541-817616) Thr (CGT) 76 bp Sc: 93.84
GCCGCTTTAGCTCAGTTGGTAGAGCACATCATTCGTAATGATGGGGTCGCGTGTTCGAGT
CACGCAAGCGGCACCA
>Brucella_canis_ATCC_23365_chrI.trna2-ThrGGT (265265-265339) Thr (GGT) 75 bp Sc: 86.73
GCTGCGGTAGCTCAGTGGTAGAGCACTCCCTTGGTAAGGGAGAGGTCGAGAGTTCAATCC
TCTCTCGCAGCACCA
>Brucella_canis_ATCC_23365_chrI.trna35-ThrTGT (909013-908938) Thr (TGT) 76 bp Sc: 94.13
GCCCTTATAGCTCAGTTGGTAGAGCACCTGATTTGTAATCAGGGGGTCGGGAGTTCGAGT
CTCTCTGGGGGCACCA
>Brucella_canis_ATCC_23365_chrI.trna32-TrpCCA (1217541-1217466) Trp (CCA) 76 bp Sc: 88.85
AGGGGTATAGCTCAGTTGGTAGAGCGACGGTCTCCAAAACCGTAGGTCGCGGGTTCGAAC
CCTGCTGCCCCTGCCA
>Brucella_canis_ATCC_23365_chrI.trna30-TyrGTA (1219146-1219062) Tyr (GTA) 85 bp Sc: 73.28
GGAGGGATGCCCGAGTGGTTAAAGGGGACGGACTGTAAATCCGTTGGCTATGCCTACGTT
GGTTCAAATCCAACTCCCTCCACCA
>Brucella_canis_ATCC_23365_chrI.trna34-ValCAC (934903-934829) Val (CAC) 75 bp Sc: 88.42
GGGCGCGTAGCTCAGCGGGAGAGCACTCCCTTCACACGGGAGGGGTCACAGGTTCAATCC
CTGTCGCGCCCACCA
>Brucella_canis_ATCC_23365_chrII.trna14-ValGAC (39500-39426) Val (GAC) 75 bp Sc: 86.72
GGGCGTGTAGCTCAGCGGGAGAGCACTACGTTGACATCGTAGGGGTCACAGGTTCAATCC
CTGTCACGCCCACCA
>Brucella_canis_ATCC_23365_chrI.trna36-ValTAC (776434-776359) Val (TAC) 76 bp Sc: 92.99
GGGCGATTAGCTCAGTTGGTAGAGCGCTTCGTTTACACCGAAGATGTCGGGAGTTCGAGT
CTCTCATCGCCCACCA
>Brucella_melitensis_chrI.trna1-AlaCGC (74618-74693) Ala (CGC) 76 bp Sc: 85.21
GGGGCTGTAGCTCAGCTGGGAGAGCGCGTCGTTCGCAATGACGAGGTCAGGGGTTCGATC
CCCCTCAGCTCCACCA
>Brucella_melitensis_chrI.trna21-AlaGGC (1974970-1975045) Ala (GGC) 76 bp Sc: 87.70
GGGGCCATAGCTCAGCTGGGAGAGCGCCTGCATGGCATGCAGGAGGTCAGCGGTTCGATC
CCGCTTGGCTCCACCA
>Brucella_melitensis_chrI.trna3-AlaTGC (200503-200578) Ala (TGC) 76 bp Sc: 91.73
GGGGCCGTAGCTCAGCTGGGAGAGCACCTGCTTTGCAAGCAGGGGGTCGTCGGTTCGATC

CCGTCCGGCTCCACCA
>Brucella_melitensis_chrI.trna6-AlaTGC (400131-400206) Ala (TGC) 76 bp Sc: 91.73
GGGGCCGTAGCTCAGCTGGGAGAGCACCTGCTTTGCAAGCAGGGGGTCGTCGGTTCGATC
CCGTCCGGCTCCACCA
>Brucella_melitensis_chrII.trna3-AlaTGC (191618-191693) Ala (TGC) 76 bp Sc: 91.73
GGGGCCGTAGCTCAGCTGGGAGAGCACCTGCTTTGCAAGCAGGGGGTCGTCGGTTCGATC
CCGTCCGGCTCCACCA
>Brucella_melitensis_chrI.trna19-ArgACG (1702809-1702885) Arg (ACG) 77 bp Sc: 91.80
GCACCCGTAGCTCAGCTGGATAGAGCACCAGACTACGAATCTGGGGGTCAGAGGTTCGAA
TCCTTTCGGGTGCGCCA
>Brucella_melitensis_chrI.trna29-ArgCCG (1051438-1051362) Arg (CCG) 77 bp Sc: 90.06
GCACCCGTAGCTCAGCTGGATAGAGCGCTGCCCTCCGAAGGCAGAGGCCACAGGTTCGAA
TCCTGTCGGGTGCGCCA
>Brucella_melitensis_chrI.trna40-ArgCCT (30404-30328) Arg (CCT) 77 bp Sc: 88.11
GGTCCCGTAGCTCAGTAGGATAGAGCGACAGATTCCTAATCTGTAGGTCACAGGTTCGAT
TCCTGTCGGGATCACCA
>Brucella_melitensis_chrI.trna26-ArgTCT (1280694-1280618) Arg (TCT) 77 bp Sc: 90.69
GGTCCCGTAGCTCAGTTGGATAGAGCACCGGCCTTCTAAGCCGATGGTCGCAGGTTCGAA
TCCTGCCGGGATCGCCA
>Brucella_melitensis_chrII.trna5-AsnGTT (681164-681238) Asn (GTT) 75 bp Sc: 89.56
TCCCCGGTAGCTCAGCGGTAGAGCAACCGGCTGTTAACCGGTTGGTCGCTGGTTCGAATC
CGGCCCGGGGAGCCA
>Brucella_melitensis_chrI.trna27-AspGTC (1207180-1207104) Asp (GTC) 77 bp Sc: 92.83
GCGGGTGTAGCTCAGTTGGTTAGAGTGCCGGCCTGTCACGCCGGAGGTCGCGGGTTCGAG
CCCCGTCACTCGCGCCA
>Brucella_melitensis_chrII.trna8-AspGTC (965580-965656) Asp (GTC) 77 bp Sc: 92.83
GCGGGTGTAGCTCAGTTGGTTAGAGTGCCGGCCTGTCACGCCGGAGGTCGCGGGTTCGAG
CCCCGTCACTCGCGCCA
>Brucella_melitensis_chrII.trna9-CysGCA (685600-685527) Cys (GCA) 74 bp Sc: 66.24
GGCCTCGTGGCGGAGTGGTTACGCAGAGGACTGCAAATCCTTGCACCCCGGTTCGATTCC
GGGCGAGGCCTCCA
>Brucella_melitensis_chrI.trna36-GlnCTG (424109-424036) Gln (CTG) 74 bp Sc: 66.55
TGGGGGATAGTTTAAGGGTAGAACAGCGGACTCTGACTCCGTTAGTCTTGGTTCGAATCC
AGGTCCCCCAGCCA
>Brucella_melitensis_chrI.trna24-GlnTTG (1469946-1469872) Gln (TTG) 75 bp Sc: 74.48
TGGGGCGTAGCCAAGCGGTAAGGCAGCGGTTTTTGGTACCGCCATTCCCTGGTTCGAATC
CAGGCGCCCCAGCCA
>Brucella_melitensis_chrI.trna37-GluCTC (385091-385017) Glu (CTC) 75 bp Sc: 65.98
GCGCCCATCGTCTAGCGGTCAGGACGGCGCCCTCTCACGGCGCAAACAGGGGTTCGATTC
CCCTTGGGCGTACCA
>Brucella_melitensis_chrI.trna33-GluTTC (746963-746889) Glu (TTC) 75 bp Sc: 72.14
GCGCCCATCGTCTAGCGGTTAGGACGCCGCCCTTTCACGGCGGAAACAGGGGTTCGATTC
CCCTTGGGCGTACCA
>Brucella_melitensis_chrI.trna13-GlyCCC (1050587-1050660) Gly (CCC) 74 bp Sc: 65.12
GCGGGTATGATGTAATGGTAGCCTGTCAGCTTCCCAAGCTGAACGCGCGGGTTCGATTCC
CGCTACCCGCTCCA
>Brucella_melitensis_chrII.trna11-GlyGCC (419834-419760) Gly (GCC) 75 bp Sc: 88.17
GCGGGTGTAGCTCAGGGGTAGAGCACAACCTTGCCAAGGTTGGGGTCGAGGGTTCGAATC
CCTTCGCCCGCTCCA
>Brucella_melitensis_chrII.trna12-GlyGCC (291445-291371) Gly (GCC) 75 bp Sc: 88.17
GCGGGTGTAGCTCAGGGGTAGAGCACAACCTTGCCAAGGTTGGGGTCGAGGGTTCGAATC
CCTTCGCCCGCTCCA
>Brucella_melitensis_chrI.trna11-GlyTCC (767877-767950) Gly (TCC) 74 bp Sc: 83.28
GCGGGTATAGCTCAATGGTAGAGCAGCAGCCTTCCAAGCTGAATACGCGGGTTCGATTCC
CGCTACCCGCTCCA
>Brucella_melitensis_chrI.trna8-HisGTG (438537-438613) His (GTG) 77 bp Sc: 86.57
GCGGTCGTAGCTCAGTTGGTTAGAGCGCAGGATTGTGGCTCCTGAGGTCGTTGGTTCAAC
TCCAACCGACCGTACCA
>Brucella_melitensis_chrI.trna2-IleGAT (200413-200489) Ile (GAT) 77 bp Sc: 99.09
GGGCTTGTAGCTCAGTTGGTTAGAGCACACGCTTGATAAGCGTGGGGTCGGAGGTTCAAG
TCCTCCCAGGCCCACCA
>Brucella_melitensis_chrI.trna5-IleGAT (400041-400117) Ile (GAT) 77 bp Sc: 99.09
GGGCTTGTAGCTCAGTTGGTTAGAGCACACGCTTGATAAGCGTGGGGTCGGAGGTTCAAG
TCCTCCCAGGCCCACCA
>Brucella_melitensis_chrII.trna2-IleGAT (191528-191604) Ile (GAT) 77 bp Sc: 99.09
GGGCTTGTAGCTCAGTTGGTTAGAGCACACGCTTGATAAGCGTGGGGTCGGAGGTTCAAG
TCCTCCCAGGCCCACCA

>Brucella_melitensis_chrI.trna39-LeuCAA (112135-112051) Leu (CAA) 85 bp Sc: 76.80
GCGGGTGTGGTGGAACTGGTAGACGCGCCGGACTCAAAATCCGGTTCCGAAAGGAGTGTC
GGTTCGATTCCGACCACCCGCACCA
>Brucella_melitensis_chrII.trna13-LeuCAG (261394-261308) Leu (CAG) 87 bp Sc: 78.71
GCCCAGATGGCGGAATTGGTAGACGCACCAGCTTCAGGTGCTGGCGCTCGCAAGGGCGTG
GAGGTTCGAGTCCTCTTCTGGGCACCA
>Brucella_melitensis_chrI.trna32-LeuGAG (932703-932619) Leu (GAG) 85 bp Sc: 63.63
GCGGTCATGGCGGAATTGGTAGACGCGCAGCGTTGAGGTCGCTGTGGGGCAACCCGTGGA
AGTTCGAGTCTTCTCGACCGCACCA
>Brucella_melitensis_chrI.trna31-LeuGAG (940948-940864) Leu (GAG) 85 bp Sc: 70.40
GCGGTCGTGGCGGAATTGGTAGACGCGCAGCGTTGAGGTCGCTGTGGGGCAACCCGTGGA
AGTTCGAGTCTTCTCGACCGCACCA
>Brucella_melitensis_chrI.trna30-LeuTAA (968916-968831) Leu (TAA) 86 bp Sc: 77.49
GCGGTCGTGGCGAAATTGGTATACGCAACGGACTTAAAATCCGTCGTCTTTAAGACTTGC
GGGTTCAAGTCCCGCCGACCGCACCA
>Brucella_melitensis_chrI.trna28-LeuTAG (1114259-1114177) Leu (TAG) 83 bp Sc: 74.64
GCGGATGTGGCGAAATTGGTAGACGCACCAGATTTAGGTTCTGGCGGGAGACCGTGGGGG
TTCGAGTCCCTCCATCCGCACCA
>Brucella_melitensis_chrI.trna34-LysCTT (690819-690744) Lys (CTT) 76 bp Sc: 96.09
GGGTGATTAGCTCAGTTGGTAGAGCAGCTGACTCTTAATCAGCGGGTCGTAGGTTCGATC
CCTACATCACCCACCA
>Brucella_melitensis_chrII.trna6-LysTTT (717481-717556) Lys (TTT) 76 bp Sc: 95.04
GAGCGCGTAGCTCAGCCGGTAGAGCAACTGACTTTTAATCAGTAGGTCCAGGGTTCGAAT
CCCTGCGCGCTCACCA
>Brucella_melitensis_chrI.trna4-MetCAT (204245-204321) Met (CAT) 77 bp Sc: 81.76
CGCGGGGTGGAGCAGCCCGGTAGCTCGTCAGGCTCATAACCTGAAGGCCGCAGGTTCAAA
TCCTGCCCCCGCAACCA
>Brucella_melitensis_chrI.trna7-MetCAT (403871-403947) Met (CAT) 77 bp Sc: 81.76
CGCGGGGTGGAGCAGCCCGGTAGCTCGTCAGGCTCATAACCTGAAGGCCGCAGGTTCAAA
TCCTGCCCCCGCAACCA
>Brucella_melitensis_chrII.trna4-MetCAT (195360-195436) Met (CAT) 77 bp Sc: 81.76
CGCGGGGTGGAGCAGCCCGGTAGCTCGTCAGGCTCATAACCTGAAGGCCGCAGGTTCAAA
TCCTGCCCCCGCAACCA
>Brucella_melitensis_chrI.trna35-MetCAT (440369-440293) Met (CAT) 77 bp Sc: 86.65
GGCGGAGTAGCTCAGTAGGTTAGAGCAGAGGAATCATAATCCTTGTGTCGGGGGTTCGAA
TCCCTCCTCCGCTACCA
>Brucella_melitensis_chrI.trna9-MetCAT (558758-558834) Met (CAT) 77 bp Sc: 87.64
GGGCCTGTAGCTCAATTGGTTAGAGCCGGCGGCTCATAACCGCTTGGTTGGGGGTTCGAG
TCCCTCCGGGCCCACCA
>Brucella_melitensis_chrI.trna20-PheGAA (1723207-1723282) Phe (GAA) 76 bp Sc: 93.28
GCCCGGATAGCTCAGTTGGTAGAGCAGCGGATTGAAAATCCGCGTGTCGGTGGTTCGAAT
CCGCCTCCGGGCACCA
>Brucella_melitensis_chrI.trna22-ProCGG (1795375-1795299) Pro (CGG) 77 bp Sc: 88.00
CGGAGTGTAGCGCAGCCTGGTAGCGCACCTCGTTCGGGACGAGGGGGTCGGAGGTTCGAA
TCCTCTCACTCCGACCA
>Brucella_melitensis_chrI.trna17-ProGGG (1222081-1222157) Pro (GGG) 77 bp Sc: 81.90
CGGAGCGTAGCGCAGCCCGGTAGCGCACTTGACTGGGGGTCAAGGGGTCGTGGGTTCGAA
TCCCGCCGCTCCGACCA
>Brucella_melitensis_chrI.trna25-ProTGG (1281332-1281256) Pro (TGG) 77 bp Sc: 93.11
CGGAGCGTAGCGCAGTCTGGTAGCGCACCTGATTTGGGATCAGGGGGTCGCAGGTTCGAA
TCCTGCCGCTCCGACCA
>Brucella_melitensis_chrI.trna38-SerCGA (208409-208320) Ser (CGA) 90 bp Sc: 83.06
GGAGAGGTGGCTGAGTGGTTGAAAGCACCGCACTCGAAATGCGGCATGGGGGCAACTCCA
TCGGGGGTTCGAATCCCTCCCTCTCCGCCA
>Brucella_melitensis_chrI.trna18-SerGCT (1354525-1354615) Ser (GCT) 91 bp Sc: 74.16
GGAGAGGTGGCCGAGTGGTCGAAGGCGCTCCCCTGCTAAGGGAGTAGACCTCAAAAGGGT
CTCGTGGGTTCGAATCCCATCCTCTCCGCCA
>Brucella_melitensis_chrII.trna14-SerGGA (243985-243896) Ser (GGA) 90 bp Sc: 77.71
GGAGAGGTGGCCGAGTGGTTGAAGGCGCACGCCTGGAACGCGTGTATACGGGAAACCGTA
TCGAGGGTTCGAATCCCTCTCTCTCCGCCA
>Brucella_melitensis_chrII.trna7-SerTGA (749928-750017) Ser (TGA) 90 bp Sc: 74.18
GGAAGGGTGGCCGAGCGGTTTAAGGCACCGGTCTTGAAAACCGGCGTGCGAGAGATCGTA
CCGTGGGTTCGAATCCCACCCCCTCCGCCA
>Brucella_melitensis_chrII.trna10-ThrCGT (480131-480056) Thr (CGT) 76 bp Sc: 93.84
GCCGCTTTAGCTCAGTTGGTAGAGCACATCATTCGTAATGATGGGGTCGCGTGTTCGAGT
CACGCAAGCGGCACCA
>Brucella_melitensis_chrI.trna23-ThrGGT (1716920-1716846) Thr (GGT) 75 bp Sc: 86.73

GCTGCGGTAGCTCAGTGGTAGAGCACTCCCTTGGTAAGGGAGAGGTCGAGAGTTCAATCC
TCTCTCGCAGCACCA
>Brucella_melitensis_chrI.trna15-ThrTGT (1075111-1075186) Thr (TGT) 76 bp Sc: 94.13
GCCCTTATAGCTCAGTTGGTAGAGCACCTGATTTGTAATCAGGGGGTCGGGAGTTCGAGT
CTCTCTGGGGGCACCA
>Brucella_melitensis_chrI.trna12-TrpCCA (769373-769448) Trp (CCA) 76 bp Sc: 88.85
AGGGGTATAGCTCAGTTGGTAGAGCGACGGTCTCCAAAACCGTAGGTCGCGGGTTCGAAC
CCTGCTGCCCCTGCCA
>Brucella_melitensis_chrI.trna10-TyrGTA (767768-767852) Tyr (GTA) 85 bp Sc: 73.28
GGAGGGATGCCCGAGTGGTTAAAGGGGACGGACTGTAAATCCGTTGGCTATGCCTACGTT
GGTTCAAATCCAACTCCCTCCACCA
>Brucella_melitensis_chrI.trna14-ValCAC (1051855-1051929) Val (CAC) 75 bp Sc: 88.42
GGGCGCGTAGCTCAGCGGGAGAGCACTCCCTTCACACGGGAGGGGTCACAGGTTCAATCC
CTGTCGCGCCCACCA
>Brucella_melitensis_chrII.trna1-ValGAC (52564-52638) Val (GAC) 75 bp Sc: 86.72
GGGCGTGTAGCTCAGCGGGAGAGCACTACGTTGACATCGTAGGGGTCACAGGTTCAATCC
CTGTCACGCCCACCA
>Brucella_melitensis_chrI.trna16-ValTAC (1207591-1207666) Val (TAC) 76 bp Sc: 92.99
GGGCGATTAGCTCAGTTGGTAGAGCGCTTCGTTTACACCGAAGATGTCGGGAGTTCGAGT
CTCTCATCGCCCACCA
>Brucella_suis_1330_chrI.trna21-AlaCGC (1919204-1919129) Ala (CGC) 76 bp Sc: 85.21
GGGGCTGTAGCTCAGCTGGGAGAGCGCGTCGTTCGCAATGACGAGGTCAGGGGTTCGATC
CCCCTCAGCTCCACCA
>Brucella_suis_1330_chrI.trna41-AlaGGC (28391-28316) Ala (GGC) 76 bp Sc: 87.70
GGGGCCATAGCTCAGCTGGGAGAGCGCCTGCATGGCATGCAGGAGGTCAGCGGTTCGATC
CCGCTTGGCTCCACCA
>Brucella_suis_1330_chrI.trna23-AlaTGC (1793292-1793217) Ala (TGC) 76 bp Sc: 91.73
GGGGCCGTAGCTCAGCTGGGAGAGCACCTGCTTTGCAAGCAGGGGGTCGTCGGTTCGATC
CCGTCCGGCTCCACCA
>Brucella_suis_1330_chrI.trna26-AlaTGC (1588230-1588155) Ala (TGC) 76 bp Sc: 91.73
GGGGCCGTAGCTCAGCTGGGAGAGCACCTGCTTTGCAAGCAGGGGGTCGTCGGTTCGATC
CCGTCCGGCTCCACCA
>Brucella_suis_1330_chrII.trna8-AlaTGC (1107769-1107694) Ala (TGC) 76 bp Sc: 91.73
GGGGCCGTAGCTCAGCTGGGAGAGCACCTGCTTTGCAAGCAGGGGGTCGTCGGTTCGATC
CCGTCCGGCTCCACCA
>Brucella_suis_1330_chrI.trna39-ArgACG (279497-279421) Arg (ACG) 77 bp Sc: 91.80
GCACCCGTAGCTCAGCTGGATAGAGCACCAGACTACGAATCTGGGGGTCAGAGGTTCGAA
TCCTTTCGGGTGCGCCA
>Brucella_suis_1330_chrI.trna8-ArgCCG (936870-936946) Arg (CCG) 77 bp Sc: 90.06
GCACCCGTAGCTCAGCTGGATAGAGCGCTGCCCTCCGAAGGCAGAGGCCACAGGTTCGAA
TCCTGTCGGGTGCGCCA
>Brucella_suis_1330_chrI.trna20-ArgCCT (1963450-1963526) Arg (CCT) 77 bp Sc: 88.11
GGTCCCGTAGCTCAGTAGGATAGAGCGACAGATTCCTAATCTGTAGGTCACAGGTTCGAT
TCCTGTCGGGATCACCA
>Brucella_suis_1330_chrI.trna5-ArgTCT (705770-705846) Arg (TCT) 77 bp Sc: 90.69
GGTCCCGTAGCTCAGTTGGATAGAGCACCGGCCTTCTAAGCCGATGGTCGCAGGTTCGAA
TCCTGCCGGGATCGCCA
>Brucella_suis_1330_chrII.trna10-AsnGTT (608824-608750) Asn (GTT) 75 bp Sc: 89.56
TCCCCGGTAGCTCAGCGGTAGAGCAACCGGCTGTTAACCGGTTGGTCGCTGGTTCGAATC
CGGCCCGGGGAGCCA
>Brucella_suis_1330_chrI.trna6-AspGTC (778480-778556) Asp (GTC) 77 bp Sc: 92.83
GCGGGTGTAGCTCAGTTGGTTAGAGTGCCGGCCTGTCACGCCGGAGGTCGCGGGTTCGAG
CCCCGTCACTCGCGCCA
>Brucella_suis_1330_chrII.trna13-AspGTC (305058-304982) Asp (GTC) 77 bp Sc: 92.83
GCGGGTGTAGCTCAGTTGGTTAGAGTGCCGGCCTGTCACGCCGGAGGTCGCGGGTTCGAG
CCCCGTCACTCGCGCCA
>Brucella_suis_1330_chrII.trna1-CysGCA (604389-604462) Cys (GCA) 74 bp Sc: 66.24
GGCCTCGTGGCGGAGTGGTTACGCAGAGGACTGCAAATCCTTGCACCCCGGTTCGATTCC
GGGCGAGGCCTCCA
>Brucella_suis_1330_chrI.trna16-GlnCTG (1564017-1564090) Gln (CTG) 74 bp Sc: 66.55
TGGGGGATAGTTTAAGGGTAGAACAGCGGACTCTGACTCCGTTAGTCTTGGTTCGAATCC
AGGTCCCCCAGCCA
>Brucella_suis_1330_chrI.trna3-GlnTTG (513096-513170) Gln (TTG) 75 bp Sc: 74.48
TGGGGCGTAGCCAAGCGGTAAGGCAGCGGTTTTTGGTACCGCCATTCCCTGGTTCGAATC
CAGGCGCCCCAGCCA
>Brucella_suis_1330_chrI.trna17-GluCTC (1603269-1603343) Glu (CTC) 75 bp Sc: 65.98
GCGCCCATCGTCTAGCGGTCAGGACGGCGCCCTCTCACGGCGCAAACAGGGGTTCGATTC

CCCTTGGGCGTACCA
>Brucella_suis_1330_chrI.trna12-GluTTC (1241104-1241178) Glu (TTC) 75 bp Sc: 72.14
GCGCCCATCGTCTAGCGGTTAGGACGCCGCCCTTTCACGGCGGAAACAGGGGTTCGATTC
CCCTTGGGCGTACCA
>Brucella_suis_1330_chrI.trna13-GluTTC (1241326-1241400) Glu (TTC) 75 bp Sc: 72.14
GCGCCCATCGTCTAGCGGTTAGGACGCCGCCCTTTCACGGCGGAAACAGGGGTTCGATTC
CCCTTGGGCGTACCA
>Brucella_suis_1330_chrI.trna33-GlyCCC (937721-937648) Gly (CCC) 74 bp Sc: 65.12
GCGGGTATGATGTAATGGTAGCCTGTCAGCTTCCCAAGCTGAACGCGCGGGTTCGATTCC
CGCTACCCGCTCCA
>Brucella_suis_1330_chrII.trna3-GlyGCC (878530-878604) Gly (GCC) 75 bp Sc: 88.17
GCGGGTGTAGCTCAGGGGTAGAGCACAACCTTGCCAAGGTTGGGGTCGAGGGTTCGAATC
CCTTCGCCCGCTCCA
>Brucella_suis_1330_chrII.trna4-GlyGCC (1007558-1007632) Gly (GCC) 75 bp Sc: 88.17
GCGGGTGTAGCTCAGGGGTAGAGCACAACCTTGCCAAGGTTGGGGTCGAGGGTTCGAATC
CCTTCGCCCGCTCCA
>Brucella_suis_1330_chrI.trna31-GlyTCC (1220146-1220073) Gly (TCC) 74 bp Sc: 83.28
GCGGGTATAGCTCAATGGTAGAGCAGCAGCCTTCCAAGCTGAATACGCGGGTTCGATTCC
CGCTACCCGCTCCA
>Brucella_suis_1330_chrI.trna28-HisGTG (1549590-1549514) His (GTG) 77 bp Sc: 86.57
GCGGTCGTAGCTCAGTTGGTTAGAGCGCAGGATTGTGGCTCCTGAGGTCGTTGGTTCAAC
TCCAACCGACCGTACCA
>Brucella_suis_1330_chrI.trna22-IleGAT (1793382-1793306) Ile (GAT) 77 bp Sc: 99.09
GGGCTTGTAGCTCAGTTGGTTAGAGCACACGCTTGATAAGCGTGGGGTCGGAGGTTCAAG
TCCTCCCAGGCCCACCA
>Brucella_suis_1330_chrI.trna25-IleGAT (1588320-1588244) Ile (GAT) 77 bp Sc: 99.09
GGGCTTGTAGCTCAGTTGGTTAGAGCACACGCTTGATAAGCGTGGGGTCGGAGGTTCAAG
TCCTCCCAGGCCCACCA
>Brucella_suis_1330_chrII.trna7-IleGAT (1107859-1107783) Ile (GAT) 77 bp Sc: 99.09
GGGCTTGTAGCTCAGTTGGTTAGAGCACACGCTTGATAAGCGTGGGGTCGGAGGTTCAAG
TCCTCCCAGGCCCACCA
>Brucella_suis_1330_chrI.trna19-LeuCAA (1881678-1881762) Leu (CAA) 85 bp Sc: 76.80
GCGGGTGTGGTGGAACTGGTAGACGCGCCGGACTCAAAATCCGGTTCCGAAAGGAGTGTC
GGTTCGATTCCGACCACCCGCACCA
>Brucella_suis_1330_chrII.trna5-LeuCAG (1037602-1037688) Leu (CAG) 87 bp Sc: 78.71
GCCCAGATGGCGGAATTGGTAGACGCACCAGCTTCAGGTGCTGGCGCTCGCAAGGGCGTG
GAGGTTCGAGTCCTCTTCTGGGCACCA
>Brucella_suis_1330_chrI.trna10-LeuGAG (1048185-1048269) Leu (GAG) 85 bp Sc: 70.40
GCGGTCGTGGCGGAATTGGTAGACGCGCAGCGTTGAGGTCGCTGTGGGGCAACCCGTGGA
AGTTCGAGTCTTCTCGACCGCACCA
>Brucella_suis_1330_chrI.trna11-LeuGAG (1055930-1056014) Leu (GAG) 85 bp Sc: 70.40
GCGGTCGTGGCGGAATTGGTAGACGCGCAGCGTTGAGGTCGCTGTGGGGCAACCCGTGGA
AGTTCGAGTCTTCTCGACCGCACCA
>Brucella_suis_1330_chrI.trna9-LeuTAA (1019437-1019522) Leu (TAA) 86 bp Sc: 77.49
GCGGTCGTGGCGAAATTGGTATACGCAACGGACTTAAAATCCGTCGTCTTTAAGACTTGC
GGGTTCAAGTCCCGCCGACCGCACCA
>Brucella_suis_1330_chrI.trna7-LeuTAG (871385-871467) Leu (TAG) 83 bp Sc: 74.64
GCGGATGTGGCGAAATTGGTAGACGCACCAGATTTAGGTTCTGGCGGGAGACCGTGGGGG
TTCGAGTCCCTCCATCCGCACCA
>Brucella_suis_1330_chrI.trna14-LysCTT (1297468-1297543) Lys (CTT) 76 bp Sc: 96.09
GGGTGATTAGCTCAGTTGGTAGAGCAGCTGACTCTTAATCAGCGGGTCGTAGGTTCGATC
CCTACATCACCCACCA
>Brucella_suis_1330_chrII.trna11-LysTTT (572492-572417) Lys (TTT) 76 bp Sc: 95.04
GAGCGCGTAGCTCAGCCGGTAGAGCAACTGACTTTTAATCAGTAGGTCCAGGGTTCGAAT
CCCTGCGCGCTCACCA
>Brucella_suis_1330_chrI.trna24-MetCAT (1789551-1789475) Met (CAT) 77 bp Sc: 81.76
CGCGGGGTGGAGCAGCCCGGTAGCTCGTCAGGCTCATAACCTGAAGGCCGCAGGTTCAAA
TCCTGCCCCCGCAACCA
>Brucella_suis_1330_chrI.trna27-MetCAT (1584489-1584413) Met (CAT) 77 bp Sc: 81.76
CGCGGGGTGGAGCAGCCCGGTAGCTCGTCAGGCTCATAACCTGAAGGCCGCAGGTTCAAA
TCCTGCCCCCGCAACCA
>Brucella_suis_1330_chrII.trna9-MetCAT (1104028-1103952) Met (CAT) 77 bp Sc: 81.76
CGCGGGGTGGAGCAGCCCGGTAGCTCGTCAGGCTCATAACCTGAAGGCCGCAGGTTCAAA
TCCTGCCCCCGCAACCA
>Brucella_suis_1330_chrI.trna15-MetCAT (1547758-1547834) Met (CAT) 77 bp Sc: 86.65
GGCGGAGTAGCTCAGTAGGTTAGAGCAGAGGAATCATAATCCTTGTGTCGGGGGTTCGAA
TCCCTCCTCCGCTACCA

>Brucella_suis_1330_chrI.trna29-MetCAT (1429378-1429302) Met (CAT) 77 bp Sc: 87.64
GGGCCTGTAGCTCAATTGGTTAGAGCCGGCGGCTCATAACCGCTTGGTTGGGGGTTCGAG
TCCCTCCGGGCCCACCA
>Brucella_suis_1330_chrI.trna40-PheGAA (258990-258915) Phe (GAA) 76 bp Sc: 93.28
GCCCGGATAGCTCAGTTGGTAGAGCAGCGGATTGAAAATCCGCGTGTCGGTGGTTCGAAT
CCGCCTCCGGGCACCA
>Brucella_suis_1330_chrI.trna1-ProCGG (207695-207771) Pro (CGG) 77 bp Sc: 88.00
CGGAGTGTAGCGCAGCCTGGTAGCGCACCTCGTTCGGGACGAGGGGGTCGGAGGTTCGAA
TCCTCTCACTCCGACCA
>Brucella_suis_1330_chrI.trna37-ProGGG (764424-764348) Pro (GGG) 77 bp Sc: 81.90
CGGAGCGTAGCGCAGCCCGGTAGCGCACTTGACTGGGGGTCAAGGGGTCGTGGGTTCGAA
TCCCGCCGCTCCGACCA
>Brucella_suis_1330_chrI.trna4-ProTGG (705132-705208) Pro (TGG) 77 bp Sc: 93.11
CGGAGCGTAGCGCAGTCTGGTAGCGCACCTGATTTGGGATCAGGGGGTCGCAGGTTCGAA
TCCTGCCGCTCCGACCA
>Brucella_suis_1330_chrI.trna18-SerCGA (1781424-1781513) Ser (CGA) 90 bp Sc: 83.06
GGAGAGGTGGCTGAGTGGTTGAAAGCACCGCACTCGAAATGCGGCATGGGGGCAACTCCA
TCGGGGGTTCGAATCCCTCCCTCTCCGCCA
>Brucella_suis_1330_chrI.trna38-SerGCT (632055-631965) Ser (GCT) 91 bp Sc: 74.16
GGAGAGGTGGCCGAGTGGTCGAAGGCGCTCCCCTGCTAAGGGAGTAGACCTCAAAAGGGT
CTCGTGGGTTCGAATCCCATCCTCTCCGCCA
>Brucella_suis_1330_chrII.trna6-SerGGA (1055010-1055099) Ser (GGA) 90 bp Sc: 77.71
GGAGAGGTGGCCGAGTGGTTGAAGGCGCACGCCTGGAACGCGTGTATACGGGAAACCGTA
TCGAGGGTTCGAATCCCTCTCTCTCCGCCA
>Brucella_suis_1330_chrII.trna12-SerTGA (540041-539952) Ser (TGA) 90 bp Sc: 74.18
GGAAGGGTGGCCGAGCGGTTTAAGGCACCGGTCTTGAAAACCGGCGTGCGAGAGATCGTA
CCGTGGGTTCGAATCCCACCCCCTCCGCCA
>Brucella_suis_1330_chrII.trna2-ThrCGT (818236-818311) Thr (CGT) 76 bp Sc: 93.84
GCCGCTTTAGCTCAGTTGGTAGAGCACATCATTCGTAATGATGGGGTCGCGTGTTCGAGT
CACGCAAGCGGCACCA
>Brucella_suis_1330_chrI.trna2-ThrGGT (265276-265350) Thr (GGT) 75 bp Sc: 86.73
GCTGCGGTAGCTCAGTGGTAGAGCACTCCCTTGGTAAGGGAGAGGTCGAGAGTTCAATCC
TCTCTCGCAGCACCA
>Brucella_suis_1330_chrI.trna35-ThrTGT (910625-910550) Thr (TGT) 76 bp Sc: 94.13
GCCCTTATAGCTCAGTTGGTAGAGCACCTGATTTGTAATCAGGGGGTCGGGAGTTCGAGT
CTCTCTGGGGGCACCA
>Brucella_suis_1330_chrI.trna32-TrpCCA (1218650-1218575) Trp (CCA) 76 bp Sc: 88.85
AGGGGTATAGCTCAGTTGGTAGAGCGACGGTCTCCAAAACCGTAGGTCGCGGGTTCGAAC
CCTGCTGCCCCTGCCA
>Brucella_suis_1330_chrI.trna30-TyrGTA (1220255-1220171) Tyr (GTA) 85 bp Sc: 73.28
GGAGGGATGCCCGAGTGGTTAAAGGGGACGGACTGTAAATCCGTTGGCTATGCCTACGTT
GGTTCAAATCCAACTCCCTCCACCA
>Brucella_suis_1330_chrI.trna34-ValCAC (936453-936379) Val (CAC) 75 bp Sc: 88.42
GGGCGCGTAGCTCAGCGGGAGAGCACTCCCTTCACACGGGAGGGGTCACAGGTTCAATCC
CTGTCGCGCCCACCA
>Brucella_suis_1330_chrII.trna14-ValGAC (39495-39421) Val (GAC) 75 bp Sc: 86.72
GGGCGTGTAGCTCAGCGGGAGAGCACTACGTTGACATCGTAGGGGTCACAGGTTCAATCC
CTGTCACGCCCACCA
>Brucella_suis_1330_chrI.trna36-ValTAC (778070-777995) Val (TAC) 76 bp Sc: 92.99
GGGCGATTAGCTCAGTTGGTAGAGCGCTTCGTTTACACCGAAGATGTCGGGAGTTCGAGT
CTCTCATCGCCCACCA
>Brucella_suis_ATCC_23445_chrI.trna18-AlaCGC (1735202-1735127) Ala (CGC) 76 bp Sc: 85.21
GGGGCTGTAGCTCAGCTGGGAGAGCGCGTCGTTCGCAATGACGAGGTCAGGGGTTCGATC
CCCCTCAGCTCCACCA
>Brucella_suis_ATCC_23445_chrI.trna35-AlaGGC (28385-28310) Ala (GGC) 76 bp Sc: 87.70
GGGGCCATAGCTCAGCTGGGAGAGCGCCTGCATGGCATGCAGGAGGTCAGCGGTTCGATC
CCGCTTGGCTCCACCA
>Brucella_suis_ATCC_23445_chrI.trna20-AlaTGC (1609250-1609175) Ala (TGC) 76 bp Sc: 91.73
GGGGCCGTAGCTCAGCTGGGAGAGCACCTGCTTTGCAAGCAGGGGGTCGTCGGTTCGATC
CCGTCCGGCTCCACCA
>Brucella_suis_ATCC_23445_chrII.trna10-AlaTGC (1301399-1301324) Ala (TGC) 76 bp Sc: 91.73
GGGGCCGTAGCTCAGCTGGGAGAGCACCTGCTTTGCAAGCAGGGGGTCGTCGGTTCGATC
CCGTCCGGCTCCACCA
>Brucella_suis_ATCC_23445_chrII.trna13-AlaTGC (1096226-1096151) Ala (TGC) 76 bp Sc: 91.73
GGGGCCGTAGCTCAGCTGGGAGAGCACCTGCTTTGCAAGCAGGGGGTCGTCGGTTCGATC
CCGTCCGGCTCCACCA
>Brucella_suis_ATCC_23445_chrI.trna33-ArgACG (297017-296941) Arg (ACG) 77 bp Sc: 91.80

GCACCCGTAGCTCAGCTGGATAGAGCACCAGACTACGAATCTGGGGGTCAGAGGTTCGAA
TCCTTTCGGGTGCGCCA
>Brucella_suis_ATCC_23445_chrI.trna8-ArgCCG (956623-956699) Arg (CCG) 77 bp Sc: 90.06
GCACCCGTAGCTCAGCTGGATAGAGCGCTGCCCTCCGAAGGCAGAGGCCACAGGTTCGAA
TCCTGTCGGGTGCGCCA
>Brucella_suis_ATCC_23445_chrI.trna17-ArgCCT (1779432-1779508) Arg (CCT) 77 bp Sc: 88.11
GGTCCCGTAGCTCAGTAGGATAGAGCGACAGATTCCTAATCTGTAGGTCACAGGTTCGAT
TCCTGTCGGGATCACCA
>Brucella_suis_ATCC_23445_chrI.trna5-ArgTCT (724684-724760) Arg (TCT) 77 bp Sc: 90.69
GGTCCCGTAGCTCAGTTGGATAGAGCACCGGCCTTCTAAGCCGATGGTCGCAGGTTCGAA
TCCTGCCGGGATCGCCA
>Brucella_suis_ATCC_23445_chrII.trna15-AsnGTT (609409-609335) Asn (GTT) 75 bp Sc: 89.56
TCCCCGGTAGCTCAGCGGTAGAGCAACCGGCTGTTAACCGGTTGGTCGCTGGTTCGAATC
CGGCCCGGGGAGCCA
>Brucella_suis_ATCC_23445_chrI.trna6-AspGTC (798325-798401) Asp (GTC) 77 bp Sc: 92.83
GCGGGTGTAGCTCAGTTGGTTAGAGTGCCGGCCTGTCACGCCGGAGGTCGCGGGTTCGAG
CCCCGTCACTCGCGCCA
>Brucella_suis_ATCC_23445_chrII.trna18-AspGTC (306405-306329) Asp (GTC) 77 bp Sc: 92.83
GCGGGTGTAGCTCAGTTGGTTAGAGTGCCGGCCTGTCACGCCGGAGGTCGCGGGTTCGAG
CCCCGTCACTCGCGCCA
>Brucella_suis_ATCC_23445_chrII.trna1-CysGCA (604974-605047) Cys (GCA) 74 bp Sc: 66.24
GGCCTCGTGGCGGAGTGGTTACGCAGAGGACTGCAAATCCTTGCACCCCGGTTCGATTCC
GGGCGAGGCCTCCA
>Brucella_suis_ATCC_23445_chrI.trna15-GlnCTG (1585034-1585107) Gln (CTG) 74 bp Sc: 66.55
TGGGGGATAGTTTAAGGGTAGAACAGCGGACTCTGACTCCGTTAGTCTTGGTTCGAATCC
AGGTCCCCCAGCCA
>Brucella_suis_ATCC_23445_chrI.trna3-GlnTTG (531086-531160) Gln (TTG) 75 bp Sc: 74.48
TGGGGCGTAGCCAAGCGGTAAGGCAGCGGTTTTTGGTACCGCCATTCCCTGGTTCGAATC
CAGGCGCCCCAGCCA
>Brucella_suis_ATCC_23445_chrII.trna7-GluCTC (1111264-1111338) Glu (CTC) 75 bp Sc: 65.98
GCGCCCATCGTCTAGCGGTCAGGACGGCGCCCTCTCACGGCGCAAACAGGGGTTCGATTC
CCCTTGGGCGTACCA
>Brucella_suis_ATCC_23445_chrI.trna12-GluTTC (1261800-1261874) Glu (TTC) 75 bp Sc: 72.14
GCGCCCATCGTCTAGCGGTTAGGACGCCGCCCTTTCACGGCGGAAACAGGGGTTCGATTC
CCCTTGGGCGTACCA
>Brucella_suis_ATCC_23445_chrI.trna27-GlyCCC (957474-957401) Gly (CCC) 74 bp Sc: 65.12
GCGGGTATGATGTAATGGTAGCCTGTCAGCTTCCCAAGCTGAACGCGCGGGTTCGATTCC
CGCTACCCGCTCCA
>Brucella_suis_ATCC_23445_chrII.trna3-GlyGCC (866152-866226) Gly (GCC) 75 bp Sc: 88.17
GCGGGTGTAGCTCAGGGGTAGAGCACAACCTTGCCAAGGTTGGGGTCGAGGGTTCGAATC
CCTTCGCCCGCTCCA
>Brucella_suis_ATCC_23445_chrII.trna4-GlyGCC (996011-996085) Gly (GCC) 75 bp Sc: 88.17
GCGGGTGTAGCTCAGGGGTAGAGCACAACCTTGCCAAGGTTGGGGTCGAGGGTTCGAATC
CCTTCGCCCGCTCCA
>Brucella_suis_ATCC_23445_chrI.trna25-GlyTCC (1240896-1240823) Gly (TCC) 74 bp Sc: 83.28
GCGGGTATAGCTCAATGGTAGAGCAGCAGCCTTCCAAGCTGAATACGCGGGTTCGATTCC
CGCTACCCGCTCCA
>Brucella_suis_ATCC_23445_chrI.trna22-HisGTG (1570351-1570275) His (GTG) 77 bp Sc: 86.57
GCGGTCGTAGCTCAGTTGGTTAGAGCGCAGGATTGTGGCTCCTGAGGTCGTTGGTTCAAC
TCCAACCGACCGTACCA
>Brucella_suis_ATCC_23445_chrI.trna19-IleGAT (1609340-1609264) Ile (GAT) 77 bp Sc: 99.09
GGGCTTGTAGCTCAGTTGGTTAGAGCACACGCTTGATAAGCGTGGGGTCGGAGGTTCAAG
TCCTCCCAGGCCCACCA
>Brucella_suis_ATCC_23445_chrII.trna12-IleGAT (1096316-1096240) Ile (GAT) 77 bp Sc: 99.09
GGGCTTGTAGCTCAGTTGGTTAGAGCACACGCTTGATAAGCGTGGGGTCGGAGGTTCAAG
TCCTCCCAGGCCCACCA
>Brucella_suis_ATCC_23445_chrII.trna9-IleGAT (1301489-1301413) Ile (GAT) 77 bp Sc: 99.09
GGGCTTGTAGCTCAGTTGGTTAGAGCACACGCTTGATAAGCGTGGGGTCGGAGGTTCAAG
TCCTCCCAGGCCCACCA
>Brucella_suis_ATCC_23445_chrI.trna16-LeuCAA (1697674-1697758) Leu (CAA) 85 bp Sc: 76.80
GCGGGTGTGGTGGAACTGGTAGACGCGCCGGACTCAAAATCCGGTTCCGAAAGGAGTGTC
GGTTCGATTCCGACCACCCGCACCA
>Brucella_suis_ATCC_23445_chrII.trna5-LeuCAG (1026050-1026136) Leu (CAG) 87 bp Sc: 78.71
GCCCAGATGGCGGAATTGGTAGACGCACCAGCTTCAGGTGCTGGCGCTCGCAAGGGCGTG
GAGGTTCGAGTCCTCTTCTGGGCACCA
>Brucella_suis_ATCC_23445_chrI.trna10-LeuGAG (1067774-1067858) Leu (GAG) 85 bp Sc: 70.40
GCGGTCGTGGCGGAATTGGTAGACGCGCAGCGTTGAGGTCGCTGTGGGGCAACCCGTGGA

AGTTCGAGTCTTCTCGACCGCACCA
>Brucella_suis_ATCC_23445_chrI.trna11-LeuGAG (1076030-1076114) Leu (GAG) 85 bp Sc: 70.40
GCGGTCGTGGCGGAATTGGTAGACGCGCAGCGTTGAGGTCGCTGTGGGGCAACCCGTGGA
AGTTCGAGTCTTCTCGACCGCACCA
>Brucella_suis_ATCC_23445_chrI.trna9-LeuTAA (1039197-1039282) Leu (TAA) 86 bp Sc: 77.49
GCGGTCGTGGCGAAATTGGTATACGCAACGGACTTAAAATCCGTCGTCTTTAAGACTTGC
GGGTTCAAGTCCCGCCGACCGCACCA
>Brucella_suis_ATCC_23445_chrI.trna7-LeuTAG (891251-891333) Leu (TAG) 83 bp Sc: 74.64
GCGGATGTGGCGAAATTGGTAGACGCACCAGATTTAGGTTCTGGCGGGAGACCGTGGGGG
TTCGAGTCCCTCCATCCGCACCA
>Brucella_suis_ATCC_23445_chrI.trna13-LysCTT (1317939-1318014) Lys (CTT) 76 bp Sc: 96.09
GGGTGATTAGCTCAGTTGGTAGAGCAGCTGACTCTTAATCAGCGGGTCGTAGGTTCGATC
CCTACATCACCCACCA
>Brucella_suis_ATCC_23445_chrII.trna16-LysTTT (573078-573003) Lys (TTT) 76 bp Sc: 95.04
GAGCGCGTAGCTCAGCCGGTAGAGCAACTGACTTTTAATCAGTAGGTCCAGGGTTCGAAT
CCCTGCGCGCTCACCA
>Brucella_suis_ATCC_23445_chrI.trna21-MetCAT (1605509-1605433) Met (CAT) 77 bp Sc: 81.76
CGCGGGGTGGAGCAGCCCGGTAGCTCGTCAGGCTCATAACCTGAAGGCCGCAGGTTCAAA
TCCTGCCCCCGCAACCA
>Brucella_suis_ATCC_23445_chrII.trna11-MetCAT (1297658-1297582) Met (CAT) 77 bp Sc: 81.76
CGCGGGGTGGAGCAGCCCGGTAGCTCGTCAGGCTCATAACCTGAAGGCCGCAGGTTCAAA
TCCTGCCCCCGCAACCA
>Brucella_suis_ATCC_23445_chrII.trna14-MetCAT (1092485-1092409) Met (CAT) 77 bp Sc: 81.76
CGCGGGGTGGAGCAGCCCGGTAGCTCGTCAGGCTCATAACCTGAAGGCCGCAGGTTCAAA
TCCTGCCCCCGCAACCA
>Brucella_suis_ATCC_23445_chrI.trna14-MetCAT (1568520-1568596) Met (CAT) 77 bp Sc: 86.65
GGCGGAGTAGCTCAGTAGGTTAGAGCAGAGGAATCATAATCCTTGTGTCGGGGGTTCGAA
TCCCTCCTCCGCTACCA
>Brucella_suis_ATCC_23445_chrI.trna23-MetCAT (1450179-1450103) Met (CAT) 77 bp Sc: 87.64
GGGCCTGTAGCTCAATTGGTTAGAGCCGGCGGCTCATAACCGCTTGGTTGGGGGTTCGAG
TCCCTCCGGGCCCACCA
>Brucella_suis_ATCC_23445_chrI.trna34-PheGAA (276651-276576) Phe (GAA) 76 bp Sc: 93.28
GCCCGGATAGCTCAGTTGGTAGAGCAGCGGATTGAAAATCCGCGTGTCGGTGGTTCGAAT
CCGCCTCCGGGCACCA
>Brucella_suis_ATCC_23445_chrI.trna1-ProCGG (204388-204464) Pro (CGG) 77 bp Sc: 88.00
CGGAGTGTAGCGCAGCCTGGTAGCGCACCTCGTTCGGGACGAGGGGGTCGGAGGTTCGAA
TCCTCTCACTCCGACCA
>Brucella_suis_ATCC_23445_chrI.trna31-ProGGG (783457-783381) Pro (GGG) 77 bp Sc: 81.90
CGGAGCGTAGCGCAGCCCGGTAGCGCACTTGACTGGGGGTCAAGGGGTCGTGGGTTCGAA
TCCCGCCGCTCCGACCA
>Brucella_suis_ATCC_23445_chrI.trna4-ProTGG (724046-724122) Pro (TGG) 77 bp Sc: 93.11
CGGAGCGTAGCGCAGTCTGGTAGCGCACCTGATTTGGGATCAGGGGGTCGCAGGTTCGAA
TCCTGCCGCTCCGACCA
>Brucella_suis_ATCC_23445_chrII.trna8-SerCGA (1289543-1289632) Ser (CGA) 90 bp Sc: 83.06
GGAGAGGTGGCTGAGTGGTTGAAAGCACCGCACTCGAAATGCGGCATGGGGGCAACTCCA
TCGGGGGTTCGAATCCCTCCCTCTCCGCCA
>Brucella_suis_ATCC_23445_chrI.trna32-SerGCT (650111-650021) Ser (GCT) 91 bp Sc: 74.16
GGAGAGGTGGCCGAGTGGTCGAAGGCGCTCCCCTGCTAAGGGAGTAGACCTCAAAAGGGT
CTCGTGGGTTCGAATCCCATCCTCTCCGCCA
>Brucella_suis_ATCC_23445_chrII.trna6-SerGGA (1043478-1043567) Ser (GGA) 90 bp Sc: 77.71
GGAGAGGTGGCCGAGTGGTTGAAGGCGCACGCCTGGAACGCGTGTATACGGGAAACCGTA
TCGAGGGTTCGAATCCCTCTCTCTCCGCCA
>Brucella_suis_ATCC_23445_chrII.trna17-SerTGA (540638-540549) Ser (TGA) 90 bp Sc: 74.18
GGAAGGGTGGCCGAGCGGTTTAAGGCACCGGTCTTGAAAACCGGCGTGCGAGAGATCGTA
CCGTGGGTTCGAATCCCACCCCCTCCGCCA
>Brucella_suis_ATCC_23445_chrII.trna2-ThrCGT (806043-806118) Thr (CGT) 76 bp Sc: 93.84
GCCGCTTTAGCTCAGTTGGTAGAGCACATCATTCGTAATGATGGGGTCGCGTGTTCGAGT
CACGCAAGCGGCACCA
>Brucella_suis_ATCC_23445_chrI.trna2-ThrGGT (282938-283012) Thr (GGT) 75 bp Sc: 86.73
GCTGCGGTAGCTCAGTGGTAGAGCACTCCCTTGGTAAGGGAGAGGTCGAGAGTTCAATCC
TCTCTCGCAGCACCA
>Brucella_suis_ATCC_23445_chrI.trna29-ThrTGT (930302-930227) Thr (TGT) 76 bp Sc: 94.13
GCCCTTATAGCTCAGTTGGTAGAGCACCTGATTTGTAATCAGGGGGTCGGGAGTTCGAGT
CTCTCTGGGGGCACCA
>Brucella_suis_ATCC_23445_chrI.trna26-TrpCCA (1239400-1239325) Trp (CCA) 76 bp Sc: 88.85
AGGGGTATAGCTCAGTTGGTAGAGCGACGGTCTCCAAAACCGTAGGTCGCGGGTTCGAAC
CCTGCTGCCCCTGCCA

>Brucella_suis_ATCC_23445_chrI.trna24-TyrGTA (1241005-1240921) Tyr (GTA) 85 bp Sc: 73.28
GGAGGGATGCCCGAGTGGTTAAAGGGGACGGACTGTAAATCCGTTGGCTATGCCTACGTT
GGTTCAAATCCAACTCCCTCCACCA
>Brucella_suis_ATCC_23445_chrI.trna28-ValCAC (956206-956132) Val (CAC) 75 bp Sc: 88.42
GGGCGCGTAGCTCAGCGGGAGAGCACTCCCTTCACACGGGAGGGGTCACAGGTTCAATCC
CTGTCGCGCCCACCA
>Brucella_suis_ATCC_23445_chrII.trna19-ValGAC (39527-39453) Val (GAC) 75 bp Sc: 86.72
GGGCGTGTAGCTCAGCGGGAGAGCACTACGTTGACATCGTAGGGGTCACAGGTTCAATCC
CTGTCACGCCCACCA
>Brucella_suis_ATCC_23445_chrI.trna30-ValTAC (797915-797840) Val (TAC) 76 bp Sc: 88.04
GGGCGATTAGCTCAGTTGGTAGAGCGCTTCGTTTACACCGAAGATGTCGGGGGTTCGAGT
CTCTCATCGCCCACCA
>Bos_taurus_chrX.trna1652-AlaAGC (45858011-45858082) Ala (AGC) 72 bp Sc: 37.04
GGGGGTATAGCTTAGTGGCAGATTACATGCTTAGCATGTATGAAGCCCTGGGTTCTGTCC
CCAGTACTTCCA
>Bos_taurus_chr25.trna198-AlaAGC (4622205-4622276) Ala (AGC) 72 bp Sc: 47.44
GGGGGTGTAGTTCAATGGTAGAGCGTGTGCTTAGCATGCTCGAGACCCCGGGTTCAATGC
CTGGCACCTCCA
>Bos_taurus_chr18.trna929-AlaAGC (23066520-23066592) Ala (AGC) 73 bp Sc: 55.26
GGGGAATTAGCTCAAGTGGTAGAGCGCTCGCTTAGCATGTGAGAGGTAGTGGGATCGATG
CCCGCATTCTCCA
>Bos_taurus_chr29.trna2134-AlaAGC (47749412-47749340) Ala (AGC) 73 bp Sc: 55.26
GGGGAATTAGCTCAAGTGGTAGAGCGCTCGCTTAGCATGTGAGAGGTAGTGGGATCGATG
CCCGCATTCTCCA
>Bos_taurus_chr21.trna2790-AlaAGC (67007914-67007985) Ala (AGC) 72 bp Sc: 55.39
TCCCTGGCAGTCCAGTGGTTAGGACTTGGCACCAGCACTGCCAGGGCCCAGGTTCGATCC
TTGGTTGGGGAA
>Bos_taurus_chr18.trna1164-AlaAGC (29068166-29068236) Ala (AGC) 71 bp Sc: 56.59
GGGGGTGTGGCTCAGTGGTAGAGTGCATGCTTAGCATGTATGAGACCCTGGGTTCAATCC
CAGTACTCCCA
>Bos_taurus_chr23.trna1407-AlaAGC (31430264-31430336) Ala (AGC) 73 bp Sc: 58.07
GGGGAATTAGCTCAAGTGGTAGAGCGCTCGCTTAGCATGTGAGAGGTAGTGGGATCGATG
CCCACATTCTCCA
>Bos_taurus_chr23.trna1411-AlaAGC (31443135-31443207) Ala (AGC) 73 bp Sc: 58.07
GGGGAATTAGCTCAAGTGGTAGAGCGCTCGCTTAGCATGTGAGAGGTAGTGGGATCGATG
CCCACATTCTCCA
>Bos_taurus_chr23.trna3386-AlaAGC (31454510-31454438) Ala (AGC) 73 bp Sc: 58.07
GGGGAATTAGCTCAAGTGGTAGAGCGCTCGCTTAGCATGTGAGAGGTAGTGGGATCGATG
CCCACATTCTCCA
>Bos_taurus_chr23.trna1415-AlaAGC (31552098-31552170) Ala (AGC) 73 bp Sc: 58.48
GGGGAATTAGCTCAAATGGTAGAGCGCTCGCTTAGCATGTGAGAGGTAGCGGGATCGATG
CCCGCATTCTCCA
>Bos_taurus_chr23.trna1414-AlaAGC (31447554-31447626) Ala (AGC) 73 bp Sc: 59.41
GGGGGATTAGCTCAAATGGTAGAGCGCTCGCTTAGCATGCGAGAGGTAGTGGGATCGATG
CCCATATCCTCCA
>Bos_taurus_chrUn.004.7.trna78-AlaAGC (481340-481269) Ala (AGC) 72 bp Sc: 61.64
GGGGGTATAGCTCAGTGGCAGAGCACATGCTTAGCATGCACGAGACCCTGGGTTCAATCC
CCAGTATCTCCA
>Bos_taurus_chr26.trna3034-AlaAGC (26435796-26435725) Ala (AGC) 72 bp Sc: 62.92
GGGGGTATAGCTCAGTGGTAGAGTGCGTGCTTAGCATGTATGAGGTCCTGAGTTCAATCC
CCAGTACCTCCA
>Bos_taurus_chr11.trna6186-AlaAGC (74675776-74675704) Ala (AGC) 73 bp Sc: 63.68
GGGGGATTAGCTCAAATGGTAGAGCGCTCGCTTAGCATGCGAGAGGTAGCGGGATCGATG
CCCGCATCCTCCA
>Bos_taurus_chr14.trna1329-AlaAGC (30404774-30404846) Ala (AGC) 73 bp Sc: 63.68
GGGGGATTAGCTCAAATGGTAGAGCGCTCGCTTAGCATGCGAGAGGTAGCGGGATCGATG
CCCGCATCCTCCA
>Bos_taurus_chrUn.004.3373.trna1-AlaAGC (2471-2543) Ala (AGC) 73 bp Sc: 66.70
GGGGGTGTAGCTCAGTGGTTAGAGTGTATGCTTAGCATGCACGAGGTACCAGGTTCAAAT
CCTGGCACTTCCA
>Bos_taurus_chr7.trna2098-AlaAGC (46262593-46262664) Ala (AGC) 72 bp Sc: 73.53
GGCGGTATAGCTCAGTGGTAGAGCACATGCTTAGCATGCATGAGACCCTGGGTTCAATCC
CCAGTACTGCCA
>Bos_taurus_chr18.trna3623-AlaAGC (53513763-53513692) Ala (AGC) 72 bp Sc: 75.78
GGGGGTATAGCTCAGTGGTAGAGCGCATGCTTAGCATGCATGAGGCCCTGGGTTCAATCC
CCAGTACCTCCA
>Bos_taurus_chr19.trna3780-AlaAGC (57572329-57572258) Ala (AGC) 72 bp Sc: 75.78

GGGGGTATAGCTCAGTGGTAGAGCGCATGCTTAGCATGCATGAGGCCCTGGGTTCAATCC
CCAGTACCTCCA
>Bos_taurus_chr23.trna1332-AlaAGC (30232216-30232287) Ala (AGC) 72 bp Sc: 75.78
GGGGATGTAGCTCAGTGGTAGAGCGCATGCTTAGCATGCATGAGGTCCCGGGTTCGATCC
CCAGCATCTCCA
>Bos_taurus_chr23.trna3454-AlaAGC (30247870-30247799) Ala (AGC) 72 bp Sc: 76.69
GGGGGTGTAGCTCAGTGGTAGAGCGCGTGCTTAGCATGTACGAGGTCCCGGGTTCAATCC
CCGGCACCTCCA
>Bos_taurus_chr23.trna1321-AlaAGC (30132286-30132357) Ala (AGC) 72 bp Sc: 77.18
GGGGGTGTAGCTCAGTGGTAGAGCGCGTGCTTAGCATGCACGAGGCCCCGGGTTCAATCC
CCGGCACCTCCA
>Bos_taurus_chr23.trna1322-AlaAGC (30140470-30140541) Ala (AGC) 72 bp Sc: 77.18
GGGGGTGTAGCTCAGTGGTAGAGCGCGTGCTTAGCATGCACGAGGCCCCGGGTTCAATCC
CCGGCACCTCCA
>Bos_taurus_chr23.trna1326-AlaAGC (30211049-30211120) Ala (AGC) 72 bp Sc: 77.18
GGGGGTGTAGCTCAGTGGTAGAGCGCGTGCTTAGCATGCACGAGGCCCCGGGTTCAATCC
CCGGCACCTCCA
>Bos_taurus_chr23.trna1328-AlaAGC (30216453-30216524) Ala (AGC) 72 bp Sc: 77.18
GGGGGTGTAGCTCAGTGGTAGAGCGCGTGCTTAGCATGCACGAGGCCCCGGGTTCAATCC
CCGGCACCTCCA
>Bos_taurus_chr23.trna1339-AlaAGC (30346292-30346363) Ala (AGC) 72 bp Sc: 77.18
GGGGGTGTAGCTCAGTGGTAGAGCGCGTGCTTAGCATGCACGAGGCCCCGGGTTCAATCC
CCGGCACCTCCA
>Bos_taurus_chr23.trna1364-AlaAGC (31087529-31087600) Ala (AGC) 72 bp Sc: 77.18
GGGGGTGTAGCTCAGTGGTAGAGCGCGTGCTTAGCATGCACGAGGCCCCGGGTTCAATCC
CCGGCACCTCCA
>Bos_taurus_chr23.trna3457-AlaAGC (30210219-30210148) Ala (AGC) 72 bp Sc: 77.18
GGGGGTGTAGCTCAGTGGTAGAGCGCGTGCTTAGCATGCACGAGGCCCCGGGTTCAATCC
CCGGCACCTCCA
>Bos_taurus_chr23.trna3460-AlaAGC (30202745-30202674) Ala (AGC) 72 bp Sc: 77.18
GGGGGTGTAGCTCAGTGGTAGAGCGCGTGCTTAGCATGCACGAGGCCCCGGGTTCAATCC
CCGGCACCTCCA
>Bos_taurus_chr11.trna4634-AlaAGC (108404673-108404594) Ala (AGC) 80 bp Sc: 35.84
TCCCGGGTGGCTCAGAGGCGAAGAGTCCACCTGCCAAGCAGGAGATGCAGGAGACCCAGG
TTCGATCCCTGGCTTGGGAA
>Bos_taurus_chr1.trna804-AlaCGC (19451371-19451443) Ala (CGC) 73 bp Sc: 33.89
TCCCTTGTGGTCCAGTAGTTAAGACTTGGTACTCGCAATGCAAGGGGTCTGGGTTCAGTC
CCTGGCTAGGGAA
>Bos_taurus_chrUn.004.62.trna1-AlaCGC (82445-82517) Ala (CGC) 73 bp Sc: 39.74
TCCCTGGTGGTCCAGTGGCTAAGACTCCGTATTCGCAATGCAGGGGGCTGAGGTTTGATC
CCTGGTCAGGGAA
>Bos_taurus_chr1.trna7008-AlaCGC (128921300-128921228) Ala (CGC) 73 bp Sc: 40.65
CTCCTGGTGGTCCAGTGGCTAAGACTCTGTGCTCGCAATGCAGGGGGCCTGGGTTTGATC
CCTAGTCAGGAAA
>Bos_taurus_chr18.trna3122-AlaCGC (62789961-62789889) Ala (CGC) 73 bp Sc: 40.94
TCTGTGATGGTCCAGTGACTAAGACTCTGGGTTCGCAATGCAGGGGGTCCAGGTTCAATC
CCTGGTCAGAGAA
>Bos_taurus_chrUn.004.663.trna3-AlaCGC (21401-21329) Ala (CGC) 73 bp Sc: 40.94
TCTGTGATGGTCCAGTGACTAAGACTCTGGGTTCGCAATGCAGGGGGTCCAGGTTCAATC
CCTGGTCAGAGAA
>Bos_taurus_chr11.trna8323-AlaCGC (20163446-20163374) Ala (CGC) 73 bp Sc: 46.16
TCCCTGGTAGTCCAGTGGCTAAGACTCCACAATCGCAATGTGGAGGGATCAGGTTCAATC
TCTGGTCAGGGAA
>Bos_taurus_chr9.trna1445-AlaCGC (44687629-44687700) Ala (CGC) 72 bp Sc: 50.24
TTCCTGTTGGTCCAGTGACTAAGACTCTGCACTCGCAATGCAGGGGCCCAGGTTCAATCC
TTGGTCAGGAAC
>Bos_taurus_chr22.trna3245-AlaCGC (32918115-32918043) Ala (CGC) 73 bp Sc: 52.35
TTCTGGGTGGTCTAGTGGCTAAGACTCTGCACTCGCAATGCAGGGGGCCCAGGTTTGATC
CCTGGTCAGGGAA
>Bos_taurus_chr3.trna8591-AlaCGC (22569693-22569623) Ala (CGC) 71 bp Sc: 55.09
GCATTGGTGGTTCAGTGGTAGAATTCTCACCTCGCACGCGGGAAACCCCGGTTCAATTCC
CAGCCAATGCA
>Bos_taurus_chrUn.004.1856.trna7-AlaCGC (5865-5795) Ala (CGC) 71 bp Sc: 55.09
GCATTGGTGGTTCAGTGGTAGAATTCTCACCTCGCACGCGGGAAACCCCGGTTCAATTCC
CAGCCAATGCA
>Bos_taurus_chr24.trna129-AlaCGC (4585197-4585268) Ala (CGC) 72 bp Sc: 71.19
GGGGATGTAGCTCAGTGGTAGAGCATATGCTTCGCATGCATGAGGCCCCAGGTTCAATCC

CTGGCATCTCCA
>Bos_taurus_chr23.trna1329-AlaCGC (30218379-30218450) Ala (CGC) 72 bp Sc: 72.31
GGGGGTGTAGCTCAGTGGTAGAGCGCGTGCTTCGCATGTACGAGGTCCCTGGTTCAATCC
CTGGCACCTCCA
>Bos_taurus_chr2.trna9125-AlaCGC (41255525-41255454) Ala (CGC) 72 bp Sc: 73.12
GGGGATGTAGCTCAGTGGTAGAGCGCGCGCTTCGCATGTGTGAGGTCCCGGGTTCAATCC
CCGGCATCTCCA
>Bos_taurus_chr23.trna1323-AlaCGC (30145095-30145166) Ala (CGC) 72 bp Sc: 73.20
GGGGGTGTAGCTCAGTGGTAGAGCGCGTGCTTCGCATGTACGAGGCCCCGGGTTCAATCC
CCGGCACCTCCA
>Bos_taurus_chr23.trna1331-AlaCGC (30226305-30226376) Ala (CGC) 72 bp Sc: 74.81
GGGGATGTAGCTCAGTGGTAGAGCGCATGCTTCGCATGTATGAGGCCCCGGGTTCGATCC
CCGGCATCTCCA
>Bos_taurus_chr23.trna3378-AlaCGC (31569141-31569070) Ala (CGC) 72 bp Sc: 74.81
GGGGATGTAGCTCAGTGGTAGAGCGCATGCTTCGCATGTATGAGGCCCCGGGTTCGATCC
CCGGCATCTCCA
>Bos_taurus_chr6.trna7482-AlaCGC (37938164-37938093) Ala (CGC) 72 bp Sc: 74.81
GGGGATGTAGCTCAGTGGTAGAGCGCATGCTTCGCATGTATGAGGCCCCGGGTTCGATCC
CCGGCATCTCCA
>Bos_taurus_chr6.trna4455-AlaGGC (116270063-116269991) Ala (GGC) 73 bp Sc: 53.39
TCCCTGGTGGTCCAGTGGTTAAGACGCTGCACTGGCAGTTCAGAGGGCTCAGGTTCGATC
CCTGGTCAGAGAA
>Bos_taurus_chr29.trna191-AlaGGC (5889912-5889983) Ala (GGC) 72 bp Sc: 55.27
TCCCTAATAGCTCAGCTGGTAAAGAATCTGCCTGGCATGCAGGAGACCCAGGTTCGAATC
CTGGATTGGGAA
>Bos_taurus_chr5.trna8220-AlaTGC (54793966-54793894) Ala (TGC) 73 bp Sc: 27.94
CCCCTGGTGGTTCAGGGGTTAAGACTCAGTGCTTGCACTACAGGGAGACTAGGTTTGATC
CCTAGTCAGGGAA
>Bos_taurus_chr15.trna4347-AlaTGC (52719060-52718988) Ala (TGC) 73 bp Sc: 28.91
TTCCTGATGGTCCAGTGATTAAGACTCTGCACTTGCAGTGCAGTGGACATGGGTTCAGTC
CCTGGTCAGGGAA
>Bos_taurus_chr19.trna5078-AlaTGC (32945048-32944957) Ala (TGC) 92 bp Sc: 31.18
TCCCTGGTGGTCCAGTGGCTAAGACTCCACACTTGCTATGAGGGGTTCGGGGGTGGGGAG
GGTGGGCCTGGGTTAGATCCCCAGTCAGGGAA
>Bos_taurus_chr13.trna3195-AlaTGC (74191447-74191518) Ala (TGC) 72 bp Sc: 33.47
TCCCTAGTGGTCCACTGGCTAAGACTCTGTACTTGCAGTGCAGGAGGCCGGGTTCGATCC
CTGGTCAGGGAA
>Bos_taurus_chr2.trna4722-AlaTGC (132583219-132583299) Ala (TGC) 81 bp Sc: 34.76
TCCCTGGTGGTCCAGTGTTTAGGACTCTGTGCTTGCACTGCAGGACTGTGGGTGGCACAG
GTTTGATACCTGGTCAGGGAA
>Bos_taurus_chr26.trna352-AlaTGC (12742415-12742487) Ala (TGC) 73 bp Sc: 34.95
TCCCTGGTGGTCCAGTGGTTAAGATTCTGCACTTGCATTGCAAGGGGGGCAGGCTCCATC
CCTGGTCAGGGAA
>Bos_taurus_chr23.trna4511-AlaTGC (8917672-8917602) Ala (TGC) 71 bp Sc: 37.79
TCCATGGTGGTCCAGTGGGCAGGACTCTGTACTTGCATTGCAGGGCCTGAGTTCCATCCT
TGGTCAGGGAA
>Bos_taurus_chr18.trna90-AlaTGC (2106221-2106292) Ala (TGC) 72 bp Sc: 39.58
TCCCTGGCAGTTCAGGGGTTAGGACTCAGTACTTGCACTGCTGTAGCCCGGGTTTAATCC
CTGGTCAGGGAT
>Bos_taurus_chrUn.004.140.trna7-AlaTGC (229779-229707) Ala (TGC) 73 bp Sc: 40.57
TCTGTGATGGTCCAGTGACTAAGACTCTGGGTTTGCAATGCAGGGGGTCCAGGTTCAATC
CCTGGTCAGAGAA
>Bos_taurus_chrUn.004.4132.trna1-AlaTGC (2072-2144) Ala (TGC) 73 bp Sc: 40.57
TCTGTGATGGTCCAGTGACTAAGACTCTGGGTTTGCAATGCAGGGGGTCCAGGTTCAATC
CCTGGTCAGAGAA
>Bos_taurus_chr15.trna5331-AlaTGC (27592415-27592343) Ala (TGC) 73 bp Sc: 40.92
TCCCCGGTGGTCCAGAGGCTAAGACTCTGCACTTGCAAGGCAGGGGGCCCAGGTTCCATC
CTTGGTCAGGGAA
>Bos_taurus_chr19.trna6300-AlaTGC (12726590-12726518) Ala (TGC) 73 bp Sc: 40.98
TCCCTGGTAGTCTAGTGGCTAAGACTTTGTGGTTGCAATGCAAGGGGTCGAGGTTCAATC
CCTGGTCAGGGAA
>Bos_taurus_chr24.trna1653-AlaTGC (40243015-40243086) Ala (TGC) 72 bp Sc: 41.80
GGGGATGTAGCTCAGTGGTAGAACACATGCTTTGCAGGTATGAGGTCTCGGGTTTGATCC
TTGGCATCTCCA
>Bos_taurus_chr7.trna714-AlaTGC (13361753-13361826) Ala (TGC) 74 bp Sc: 42.53
TCCCTGGTGGTCTAGTGGCTAAGACTCTGTGTTTGCAATGCAGGGGGCCCTGGACTCGAT
CCCTGGTCAGGGAA

>Bos_taurus_chr19.trna3755-AlaTGC (57999131-57999059) Ala (TGC) 73 bp Sc: 43.44
TGCCTGGTGGTCCAGTGCTTAAGACTCTGCACTTGCACTGCAGTGGGACCAGGTTCCATC
CCTGGTTGGGGAA
>Bos_taurus_chr7.trna6442-AlaTGC (50005889-50005817) Ala (TGC) 73 bp Sc: 43.45
TCTCTGGTGGTCTAGTGGCTAAGACTCGGTGCTTGCAATGCAGGGAATCCAGGTTCGATC
CCTGGTCAGGGAA
>Bos_taurus_chr11.trna1786-AlaTGC (45106152-45106222) Ala (TGC) 71 bp Sc: 43.90
TCCCTGGTGGTCCAGTGGTTAAGGATTTGCCTTGCAAGCAGGGAACTCAGGTTCAATCCC
TGGTCAGGGAA
>Bos_taurus_chr14.trna2420-AlaTGC (59103638-59103710) Ala (TGC) 73 bp Sc: 45.76
TCTCTGGTGGTCCAGTGTTAGGATGTAGCATTTGCACTGCTAGGGGGTCCAGGTTCAACC
CCTGGTCGGGGAA
>Bos_taurus_chr11.trna2916-AlaTGC (73248168-73248240) Ala (TGC) 73 bp Sc: 49.74
TCCCTGGTGGTCCAGTAGTTAAGACTCTGTGCTTGCAATGCAGAGGGTGCAGGTTCGATC
CCTGATCAGGGAA
>Bos_taurus_chr2.trna714-AlaTGC (23726946-23727017) Ala (TGC) 72 bp Sc: 57.69
TCCCTGGTGGTCCAGTGGTTAGGACTCAGCACTTGCACTGCAGTGGCCTAGGTTGAATCC
CTGGTTGGGGAA
>Bos_taurus_chr6.trna1311-AlaTGC (46755846-46755918) Ala (TGC) 73 bp Sc: 58.06
GCCCTGATGGTCCAGTGGCTAAGACTCTGCGCTTGCAATGCAGGGGGCCCAAGTTCGATC
CTTGGTCAGGGAG
>Bos_taurus_chr23.trna3058-AlaTGC (39690950-39690879) Ala (TGC) 72 bp Sc: 66.75
GGGGATGTAGCTCAGTGCTAGAGCGCATGCTTTGCATGCATGAGGTCCCAGGTTCAATCC
TTGGCATCTCCA
>Bos_taurus_chr9.trna449-AlaTGC (17132348-17132419) Ala (TGC) 72 bp Sc: 68.22
GGGGGTGTAGCTCAGTGGTAGAGCATGTGCTTTGCATGCACAAGGCCCTGGGTTCAATTC
CTAGCACCTCCA
>Bos_taurus_chr23.trna799-AlaTGC (18288142-18288214) Ala (TGC) 73 bp Sc: 69.55
TCCCTGGTGGTCCAGTGGTTAGGACTCTGCACTTGCACTGCAGGGGGCACAGGTTCGATC
CCTGGCTGGGGAA
>Bos_taurus_chr17.trna4791-AlaTGC (53704357-53704286) Ala (TGC) 72 bp Sc: 72.71
GGGGATGTAGCTCAGTGGTAGAGCGCATGCTTTGCATGTATGAGGCCCCGGGTTCAATCC
CCGGCATCTCCA
>Bos_taurus_chr3.trna5327-AlaTGC (114092325-114092254) Ala (TGC) 72 bp Sc: 72.71
GGGGATGTAGCTCAGTGGTAGAGCGCATGCTTTGCATGTATGAGGCCCCGGGTTCAATCC
CCGGCATCTCCA
>Bos_taurus_chrUn.004.8164.trna2-AlaTGC (2292-2221) Ala (TGC) 72 bp Sc: 72.71
GGGGATGTAGCTCAGTGGTAGAGCGCATGCTTTGCATGTATGAGGCCCCGGGTTCAATCC
CCGGCATCTCCA
>Bos_taurus_chr6.trna4758-AlaTGC (110544890-110544819) Ala (TGC) 72 bp Sc: 73.60
GGGGATGTAGCTCAGTGGTAGAGCACATGCTTTGCATGTATGAGGTCCTGGGTTCAATCC
CCAGCATCTCCA
>Bos_taurus_chr23.trna3455-AlaTGC (30241368-30241297) Ala (TGC) 72 bp Sc: 73.79
GGGGATGTAGCTCAGTGGTAGAGCGCCTGCTTTGCATGCATGAGGCCCCGGGTTCGATCC
CCGGCATCTCCA
>Bos_taurus_chr17.trna2068-AlaTGC (53711095-53711166) Ala (TGC) 72 bp Sc: 74.45
GGGGATGTAGCTCAGTGGTAGAGCGCATGCTTTGCATGTATGAGGCCCCGGGTTCGATCC
CCGGCATCTCCA
>Bos_taurus_chr7.trna1884-AlaTGC (39691465-39691536) Ala (TGC) 72 bp Sc: 74.45
GGGGATGTAGCTCAGTGGTAGAGCGCATGCTTTGCATGTATGAGGCCCCGGGTTCGATCC
CCGGCATCTCCA
>Bos_taurus_chr5.trna7500-AlaTGC (72915262-72915191) Ala (TGC) 72 bp Sc: 74.98
GGGGATGTAGCTCAGTGGTAGAGCACATGCTTTGCATGTATGAGGTCCTGGGTTCAATTC
CCAGCATCTCCA
>Bos_taurus_chr6.trna2908-AlaTGC (94952293-94952364) Ala (TGC) 72 bp Sc: 75.09
GGGGATGTAGCTCAGTGGTAGAGCACATGCTTTGCATGTATGAGGTCCCGGGTTCAATCC
CCGGCATCTCCA
>Bos_taurus_chr23.trna1334-AlaTGC (30245378-30245449) Ala (TGC) 72 bp Sc: 78.55
GGGGATGTAGCTCAGTGGTAGAGCGCATGCTTTGCATGTATGAGGTCCCGGGTTCGATCC
CCGGCATCTCCA
>Bos_taurus_chr14.trna4272-ArgACG (57965972-57965901) Arg (ACG) 72 bp Sc: 48.19
TCTCAGGTGGCTCAGATGGTAAAGCGTCTGTCTACGATGCAGGAGACCCAGGTTCTATCC
CTGGGTTGGGAA
>Bos_taurus_chr28.trna424-ArgACG (10886847-10886917) Arg (ACG) 71 bp Sc: 55.03
TCCCTGGTGGCTCAGTTGGTAAAGCGTCTGCCTACGATGCAGGAGACCCCGGTTCGAACC
TGGGTGGGGAA
>Bos_taurus_chr4.trna5777-ArgACG (93312796-93312725) Arg (ACG) 72 bp Sc: 57.31

TCCCTGGTAGCTCAGATGGTAAAGCTTCTGCCTACGATGCAGGAGACCCAGGTTCGATCC
CTGGGTTGGGAA
>Bos_taurus_chr3.trna5710-ArgACG (105049413-105049342) Arg (ACG) 72 bp Sc: 57.74
TCCCTGGTGGCTCAGATGGTAAAGCATCTGCCTACGATGCAGGAGACCCAGGTTCGATCC
CTGGGTTGGGAA
>Bos_taurus_chr22.trna1979-ArgACG (54988296-54988368) Arg (ACG) 73 bp Sc: 68.07
GGGCCAGTGGCGCAATGGATAACGCGTCTGACTACGGATCAGAAGATTCTAGGTTCGACT
CCTGGCTGGCTCG
>Bos_taurus_chr23.trna1369-ArgACG (31115504-31115576) Arg (ACG) 73 bp Sc: 68.07
GGGCCAGTGGCGCAATGGATAACGCGTCTGACTACGGATCAGAAGATTCTAGGTTCGACT
CCTGGCTGGCTCG
>Bos_taurus_chr23.trna1392-ArgACG (31319544-31319616) Arg (ACG) 73 bp Sc: 68.07
GGGCCAGTGGCGCAATGGATAACGCGTCTGACTACGGATCAGAAGATTCTAGGTTCGACT
CCTGGCTGGCTCG
>Bos_taurus_chr23.trna3398-ArgACG (31340615-31340543) Arg (ACG) 73 bp Sc: 68.07
GGGCCAGTGGCGCAATGGATAACGCGTCTGACTACGGATCAGAAGATTCTAGGTTCGACT
CCTGGCTGGCTCG
>Bos_taurus_chrUn.004.1183.trna6-ArgACG (14814-14742) Arg (ACG) 73 bp Sc: 68.07
GGGCCAGTGGCGCAATGGATAACGCGTCTGACTACGGATCAGAAGATTCTAGGTTCGACT
CCTGGCTGGCTCG
>Bos_taurus_chr10.trna7307-ArgACG (22206885-22206813) Arg (ACG) 73 bp Sc: 72.37
GGGCCAGTGGCGCAATGGATAACGCGTCTGACTACGGATCAGAAGATTCCAGGTTCGACT
CCTGGCTGGCTCG
>Bos_taurus_chr23.trna1404-ArgACG (31423586-31423658) Arg (ACG) 73 bp Sc: 72.37
GGGCCAGTGGCGCAATGGATAACGCGTCTGACTACGGATCAGAAGATTCCAGGTTCGACT
CCTGGCTGGCTCG
>Bos_taurus_chr23.trna3371-ArgACG (31694862-31694790) Arg (ACG) 73 bp Sc: 72.37
GGGCCAGTGGCGCAATGGATAACGCGTCTGACTACGGATCAGAAGATTCCAGGTTCGACT
CCTGGCTGGCTCG
>Bos_taurus_chr23.trna3376-ArgACG (31587323-31587251) Arg (ACG) 73 bp Sc: 72.37
GGGCCAGTGGCGCAATGGATAACGCGTCTGACTACGGATCAGAAGATTCCAGGTTCGACT
CCTGGCTGGCTCG
>Bos_taurus_chrUn.004.1183.trna12-ArgACG (665-593) Arg (ACG) 73 bp Sc: 72.37
GGGCCAGTGGCGCAATGGATAACGCGTCTGACTACGGATCAGAAGATTCCAGGTTCGACT
CCTGGCTGGCTCG
>Bos_taurus_chrUn.004.298.trna3-ArgCCG (113069-113141) Arg (CCG) 73 bp Sc: 32.46
GCCCTGGTGGTCCAGTGCCTAAGACTCTGCAATCCGAATGCAGAGGGATTAGGTTCAACC
CCTGGTCAGGGAA
>Bos_taurus_chr13.trna4754-ArgCCG (65903376-65903304) Arg (CCG) 73 bp Sc: 35.26
TCCCTGATGGTCCAGTGGCTAAGACTCTGTGTTCCGCATGCAGGGGGCCCGGGTTTGATC
CCTAGTCAGGGAA
>Bos_taurus_chr1.trna8475-ArgCCG (82793917-82793845) Arg (CCG) 73 bp Sc: 37.84
TCCCTGGTGGTCCAGTAGCTAAGACTCTGTGCTCCGAATGCAGGGGGCCTGGGTTCAATC
CTTAGTCAGGGAA
>Bos_taurus_chr2.trna4346-ArgCCG (126148037-126148109) Arg (CCG) 73 bp Sc: 39.76
TCCCTGGTAGTCCAGTGGCTAAGACTCTGCGCTCCGAATGCAGGGGGCCTGGATTCTATC
CCTGGTCAGGGAA
>Bos_taurus_chr19.trna1665-ArgCCG (32861616-32861688) Arg (CCG) 73 bp Sc: 48.87
TCCTTGGTGGTCCACTGGCTAAGACTCCGCACTCCGAATGCAGAGGGCCCAGGTTCAATC
CCTGGTCATGGAA
>Bos_taurus_chrX.trna238-ArgCCG (5903250-5903322) Arg (CCG) 73 bp Sc: 49.42
TCCCTGGTGGTCCAGTGGCTAAGACTCTGCACTCCGAATGCTGGGGACCAGGGTTCAATC
CCTGGTCAGGGAA
>Bos_taurus_chr10.trna7600-ArgCCG (14953276-14953204) Arg (CCG) 73 bp Sc: 50.75
TCCCTGGTGGTCCAGTGGCTAAGACTCTGCATTCCGAATACAGGGGGCCCAGATTCGACC
CCTGGTCAGGGAA
>Bos_taurus_chr10.trna7934-ArgCCG (7868730-7868658) Arg (CCG) 73 bp Sc: 51.27
TCCCTGGTAGTCCAACGGCTAAGACTCTGCGCTCCGAATGTAGGAGGCCCAGGTTCGATC
CCTGGCCAGGGAA
>Bos_taurus_chr8.trna3573-ArgCCG (103748990-103749062) Arg (CCG) 73 bp Sc: 54.46
TCCCTGGTGGTCCAGTGGCTAAGACTCCGCGTTCCGAATGCAGGGGGCCCAGGTTCGATC
CCTGGTCAGGGAA
>Bos_taurus_chr7.trna7545-ArgCCG (18377876-18377804) Arg (CCG) 73 bp Sc: 57.48
TCCCTGGTGGTCCAGTGGTTGAGACTCTGAACTCCGAATGCAGGGGGCCCAGGTTCAATC
CCTGGTCAGGGAA
>Bos_taurus_chr16.trna4776-ArgCCG (40105966-40105894) Arg (CCG) 73 bp Sc: 61.75
TCCCTGGTGGTCCAGTGGCTAAGACTCTGCACTCCGAATGCAGGGAGCCCAGGTTCAATC

CCTGGGCAGGGAA
>Bos_taurus_chr19.trna2573-ArgCCG (50472850-50472922) Arg (CCG) 73 bp Sc: 65.49
GACCCAGTGGCCTAATGGATAAGGCATCAGCCTCCGGAGCTGGGGATTGTGGGTTCGAGT
CCCATCTGGGTCG
>Bos_taurus_chr23.trna3462-ArgCCG (30127706-30127634) Arg (CCG) 73 bp Sc: 69.88
GGCCGCGTGGCCTAATGGATAAGGCGTCTGATTCCGGATCAGAAGATTGAGGGTTCGAGT
CCCTTCGTGGTCG
>Bos_taurus_chr25.trna105-ArgCCG (3147401-3147473) Arg (CCG) 73 bp Sc: 69.88
GGCCGCGTGGCCTAATGGATAAGGCGTCTGATTCCGGATCAGAAGATTGAGGGTTCGAGT
CCCTTCGTGGTCG
>Bos_taurus_chr17.trna2203-ArgCCT (54871603-54871675) Arg (CCT) 73 bp Sc: 24.33
TCCCTGGTGGCCCAGAGACTGGGACTCCATGTTCCCTGTGCAGGGGTCCCAGGTTCGAGC
CCTGGTCAGGGAA
>Bos_taurus_chr24.trna3355-ArgCCT (50970904-50970832) Arg (CCT) 73 bp Sc: 25.30
TCTCTGGTGGTCCAGTGACTAAGATTCCGTGCTCCTAACGCAGGGGAGTCAGGTTCGACC
CTTGGTCAGGGAA
>Bos_taurus_chr4.trna5652-ArgCCT (96122958-96122886) Arg (CCT) 73 bp Sc: 26.83
TCCCTGGGGGTCCAGTGGCTAAGACACTGTGCTCCCTGTGCAGGGGTCCTGGGTTTGATC
CCTGGTCAGGGAA
>Bos_taurus_chr9.trna936-ArgCCT (29785021-29785093) Arg (CCT) 73 bp Sc: 26.99
TTCCTGGTGATCCAATGGTTAAGATTCTGAGCTCCCTGTGCAGGGGACTCAGGTTCTATC
CCTGGCCAGGGAA
>Bos_taurus_chr3.trna9101-ArgCCT (9113915-9113842) Arg (CCT) 74 bp Sc: 27.21
TCCCTGGTGGTCCAGTGGCTAAGACTTTGTGCCCCTAATGCAGGGGAAGCCAGGTTCCAT
CCTTGGTCAGGGAA
>Bos_taurus_chr22.trna1544-ArgCCT (43751162-43751233) Arg (CCT) 72 bp Sc: 27.92
TCCCTGGTGGTCCAGAGGCGAGACTCTGCGCTCCTAGTGCAGGGGGCCCAGGTTCGGTCC
CTGGTGAGGGAG
>Bos_taurus_chr18.trna4055-ArgCCT (47718834-47718762) Arg (CCT) 73 bp Sc: 29.47
TCCCTGGTAGTCTAGTGGCTAAGATTCTGTGCTCCTGGTACTGAGGACCCGGGCTCAATC
CCTGGTCAGGGAA
>Bos_taurus_chr10.trna1839-ArgCCT (47703208-47703280) Arg (CCT) 73 bp Sc: 29.95
TCCCTGATGGTCCAGAGGCTAAGATTTTGCACTCCTCATGCAGGGGGCCTGTGTTTGATC
CCTGGTCAGGGAC
>Bos_taurus_chr8.trna4239-ArgCCT (112873355-112873283) Arg (CCT) 73 bp Sc: 30.35
TCCCTGGTGGTCCGGTGGTTAAGACTCTGTTCTCCTGATGCAGGTGGGCTGGGTTTGACC
CCTAGTCAGGGAA
>Bos_taurus_chr7.trna723-ArgCCT (13467401-13467474) Arg (CCT) 74 bp Sc: 31.00
TCCCTGGTGGTCCAGTGTCTAAGACTCCATGCTCCTAATGCGGGGGACCCCAGGTTGGAT
CCCTGGTCAGGGAT
>Bos_taurus_chr11.trna6148-ArgCCT (75488268-75488196) Arg (CCT) 73 bp Sc: 31.10
CTTCTGGTGGTCCAGTGGCTGAGACTCCGCACTCCTACTGCAGGAGGCCCAGGTTCAGTT
CCTGGTCAGGGAA
>Bos_taurus_chrUn.004.405.trna13-ArgCCT (78460-78388) Arg (CCT) 73 bp Sc: 32.21
TCCCTGGTGGTCCAGTCTCTAAGACTCTGCACTCCTAATGCAACAGACTTGGGTTCAATT
CCTAGTCAGGGAA
>Bos_taurus_chr23.trna831-ArgCCT (18857875-18857947) Arg (CCT) 73 bp Sc: 33.93
TCCCTGTTGGTTCAGCGGCAAAGACTCCGCACTCCTAATGCAGGTGGTCCGGGTTTGATC
CCTGGTCAGGGAA
>Bos_taurus_chr9.trna3459-ArgCCT (97753440-97753512) Arg (CCT) 73 bp Sc: 34.18
TCCTTGATAGTCCAGCGGCTAAGACTCTGTGCTCCTAGTACATGGAGCTCAGGTTCAGTT
CCTGATTGGGGAA
>Bos_taurus_chr18.trna5059-ArgCCT (24284461-24284389) Arg (CCT) 73 bp Sc: 35.54
TTACTGGTGGTCCAGAGACTAAGACTGTGCACTCCTAATGCAGGGGACCCAGGTTCGATC
CCTGGTCAGGGAA
>Bos_taurus_chr1.trna6285-ArgCCT (149313253-149313181) Arg (CCT) 73 bp Sc: 35.57
TTCCTGGTGGTTATGCGGCTAAGACTCTGCGTTCCTAATGCAGGGGGCCCAGGTTCCATC
CCTGGTCAGGGAA
>Bos_taurus_chr17.trna521-ArgCCT (14300428-14300500) Arg (CCT) 73 bp Sc: 36.23
TCCCTGGTAGCCCAGTGGCTGAGGTTCTGTCCTCCTGCTGCAGAGGGCCCATGTTCGATC
TCTGGTTAGGGAA
>Bos_taurus_chr6.trna1682-ArgCCT (60140814-60140886) Arg (CCT) 73 bp Sc: 36.55
TCCCTGGTGGTCTAGTGGCTAAGACTTGGAGCTCCTGATTCAGGGGACCCAGGTTCAACC
CCTGATCAGGGAA
>Bos_taurus_chr13.trna4013-ArgCCT (78583606-78583534) Arg (CCT) 73 bp Sc: 36.59
TCTCTGGTGGTCCAGTGGCTAAGACTCCGTGCTCCTAATACGTGTGGCTCAGGTTCCATC
CCTGGTCAGGGAA

>Bos_taurus_chr1.trna5122-ArgCCT (147349916-147349987) Arg (CCT) 72 bp Sc: 37.32
TCCCTGGTGGTTCAGTGGCTGGGACTCTGTGTTCCTGATGCAGGGGCCCCGGTTCAGTCC
CTGGTCAGGGAA
>Bos_taurus_chr8.trna7878-ArgCCT (9997161-9997089) Arg (CCT) 73 bp Sc: 37.54
TCCCTGGTGGTCCCACGGCTAAGACTCCGTGTTCCTAATGCAGGGGGCCCAGGTTCAATC
CCTGGTCAGGGAA
>Bos_taurus_chr19.trna6035-ArgCCT (17341976-17341904) Arg (CCT) 73 bp Sc: 37.55
TCCCTGGTCGTCCAGTGGCTAAGACTCTCTGCTCCTAACGCAGAGGGCCTGGGTTCAGTC
CCTGGTCAGGGAG
>Bos_taurus_chr24.trna1410-ArgCCT (35691482-35691554) Arg (CCT) 73 bp Sc: 37.68
TCCTTGATGGTCCATTGGTTAAGACTCTGTTTTCCTATTGCAGGGAGCCTGGGTTCCATC
CCTGGTCAGGGAA
>Bos_taurus_chr26.trna1089-ArgCCT (32271737-32271808) Arg (CCT) 72 bp Sc: 38.77
TCCCTGGTGGTCCAGGGGCTGAGACTCTGCACTCCTAATGCAAGATCCAGGGTTCAATCC
CTAGTCAGGGAA
>Bos_taurus_chr11.trna118-ArgCCT (1987422-1987494) Arg (CCT) 73 bp Sc: 39.18
TCTCTGGTGGTCCAGTAGTTGGGACTCTGAGCTCCTGATGCAGGGGCCACAGGTTCGATC
CCTGCTTGGAGAA
>Bos_taurus_chr11.trna9072-ArgCCT (2184814-2184742) Arg (CCT) 73 bp Sc: 39.18
TCTCTGGTGGTCCAGTAGTTGGGACTCTGAGCTCCTGATGCAGGGGCCACAGGTTCGATC
CCTGCTTGGAGAA
>Bos_taurus_chr15.trna2767-ArgCCT (77383818-77383890) Arg (CCT) 73 bp Sc: 39.55
TCCCTGGTGGTCCAGTGGCTACGACTCCGCACTCCTGATGCAGGGGGCCTGGGTCCAATC
CCTGGTCAGGGAA
>Bos_taurus_chr3.trna8631-ArgCCT (21583852-21583780) Arg (CCT) 73 bp Sc: 39.65
TCCTTGGTGGTTCAGTGGCTAAGACACTATGCTCCTGATATAGTGAATGCAGGTTCAATC
CCTGGTCAGGGAA
>Bos_taurus_chr8.trna4932-ArgCCT (94895802-94895730) Arg (CCT) 73 bp Sc: 40.02
TTCCTGGTGGTCTAGTAGTTAAGATTTTGCACTCCTAATGCAGGGGGCACAGGTTTGATC
CCTGGTCGGGGAA
>Bos_taurus_chr16.trna3686-ArgCCT (65436562-65436490) Arg (CCT) 73 bp Sc: 40.11
TCTCTGGTGGTCCAGTGGCTAAGACTTTGCAATCCTAATGCAGGGGGCACAGGTTCGTTT
CCTGGTCAGGGAA
>Bos_taurus_chr19.trna1344-ArgCCT (27226677-27226749) Arg (CCT) 73 bp Sc: 40.90
TACCTGGTGGTCCAGTGGCTAAGACTCTGCACTCCTAATACAGGGGGCCTGGGTTCGATC
TCTGGTCAGGGAA
>Bos_taurus_chr13.trna1264-ArgCCT (31155663-31155735) Arg (CCT) 73 bp Sc: 41.85
TCCCTGGTGGTCCAGTGGTTAAGACCCTGAGCTCCTAACATAGGGGGCCCAGATTCTATC
CCTGGTTGGGGAA
>Bos_taurus_chr22.trna305-ArgCCT (7070990-7071062) Arg (CCT) 73 bp Sc: 42.03
TCCCTGGTGGTCCAGTGGCTAAGACCTTGTGTTCCTAATGCAGGAGGTCCAAGTTCAATC
CCTGGCCAGGGAA
>Bos_taurus_chrX.trna1172-ArgCCT (30662561-30662633) Arg (CCT) 73 bp Sc: 42.31
TCCCTGGTGGTCCAGTGGCTGAAACTCTGCATTCCTAATGCAGGGAGCCCAGGTTCCATC
CCTGGTTGGGGAG
>Bos_taurus_chr2.trna2800-ArgCCT (89280615-89280687) Arg (CCT) 73 bp Sc: 42.39
TCCCTGGTGGTCCAGTGGCTAAGATTCTGCAGTCCTAATGCAGGGGTGCTGGGTTCCATC
CCTGGTCAGGGAA
>Bos_taurus_chr19.trna5438-ArgCCT (26945866-26945794) Arg (CCT) 73 bp Sc: 42.56
TTCTTGGTGGTCTAGTGGTTAAGACTCTGTGCTCCTAACACAGGGGGCCCAGGTTTGATC
CCTGGTGGGGGGA
>Bos_taurus_chr3.trna8112-ArgCCT (32923193-32923121) Arg (CCT) 73 bp Sc: 42.73
TCCATGGTGGTCCAGTGGCTGAGACTCTGTGCTCCTAACACAGGGGGCCCAGGTTCCATC
CCTGGTCAGGGAA
>Bos_taurus_chr7.trna2507-ArgCCT (57934861-57934933) Arg (CCT) 73 bp Sc: 42.76
TCCCTGGTGGTCCACTGGCTAAGACCCTGCACTCCTGATACAGGGGGCCCAGGTGCAATC
CCTGGTCAGGGAA
>Bos_taurus_chr23.trna3564-ArgCCT (27428177-27428105) Arg (CCT) 73 bp Sc: 43.06
TCCCTGGTGGTCCAGTGGCCAAGACTCTGAGATCCTAATGCAGGGGCCCCAGGTTTGATC
CCTGGTCAGGGAA
>Bos_taurus_chr4.trna1704-ArgCCT (53144647-53144719) Arg (CCT) 73 bp Sc: 43.55
TGCCTGGTGGTCCAGTGGCTAAGACTCTGTGCTCCTAACGCAGGGGGCCTGGGTTCAGTT
CCTAGTCAGGGAA
>Bos_taurus_chr25.trna1654-ArgCCT (28543944-28544016) Arg (CCT) 73 bp Sc: 43.61
TCCCTGGTGGTCCAGTGGTTAAGACTCTGCGCTTCCTATGCAAGGGGCACAGGTTTGATC
CCTGCTCAGGGAA
>Bos_taurus_chr27.trna1340-ArgCCT (35655127-35655199) Arg (CCT) 73 bp Sc: 43.68

TCCCTGGTGGTCCAGTGGATAAGGCTCTGTGCCCCTACCGCAGGGGGCCTGGGTTCGATC
CCTGGTCAGGGAC
>Bos_taurus_chr6.trna3219-ArgCCT (101100410-101100482) Arg (CCT) 73 bp Sc: 43.90
TCCCTGGAGGTCCAGTGATTAAGACTCTGTGCTCCTAATGTAGGGGGCCCAGGTTCGATT
CCTGGTCAGGGAA
>Bos_taurus_chr11.trna330-ArgCCT (5711380-5711452) Arg (CCT) 73 bp Sc: 44.09
TCCCTGGTGGGCCAGTGGCTAAGACCCTGTGCTCCTGATGCAGGGGAACCAGGTTCGATC
CCTGGTCAGGGAA
>Bos_taurus_chr5.trna3795-ArgCCT (100259832-100259904) Arg (CCT) 73 bp Sc: 44.38
TCCCTGGTGGTCCAGTAGCAAGGATTCTGCACTCCTAATGCAGGGGGCCCGAGTTCAATC
CCTGGTCAGGGAA
>Bos_taurus_chr14.trna3841-ArgCCT (67371728-67371656) Arg (CCT) 73 bp Sc: 44.53
TCCCTGGTAGTCCTGTGGTTAAGACTCTGAGCTCCTAATTCAGGGGGCTCAGGTTTGATC
CCTGGTCAGGGAA
>Bos_taurus_chr1.trna6733-ArgCCT (135562688-135562616) Arg (CCT) 73 bp Sc: 44.81
TCCCTGGTGGTCCAGTGGCTGAGACTCCGTACTCCTAATGCAGGGGGCCCAGGTTTGATC
CCTGGTCAGGGAA
>Bos_taurus_chr22.trna3971-ArgCCT (12954484-12954412) Arg (CCT) 73 bp Sc: 45.63
TCCCTGGTGGTCCAGTGGCTAAGATTCTGTGTTCCTAATGCAGGGGGCCTGGGTTCAATC
CCAGGTCAGGGAA
>Bos_taurus_chr20.trna915-ArgCCT (26040853-26040925) Arg (CCT) 73 bp Sc: 46.03
TCCCTGGTGGTCCAATGGCTAAGACTCTGTACTCCTGATGCAGCAGGCCAGGGTTCGATC
CCTGGTCAGGGAA
>Bos_taurus_chr18.trna1091-ArgCCT (26897857-26897929) Arg (CCT) 73 bp Sc: 46.15
TTCCTGGTGGTCCAGTGGCTAAGACTCTGTGCTCCTAATGCAGGGGTCCCAAGTTCCATC
CCTGGTCAGGGAA
>Bos_taurus_chr15.trna5215-ArgCCT (29794012-29793940) Arg (CCT) 73 bp Sc: 46.55
TCCCTGGTGGTCCAGTGGCTAAGACTCTGCACTCCTAATGCAGGAGGGCTGGGTTTGATC
CCTGGCCAGGGAA
>Bos_taurus_chr22.trna4051-ArgCCT (11339814-11339742) Arg (CCT) 73 bp Sc: 46.63
TCCCTGGTGGTCCAGTGGCTAAGACTCTGCGCTCCTAATGCAGGGGGCCTGGGTTTGATC
CCTGGTCAGGGAA
>Bos_taurus_chr13.trna3076-ArgCCT (72713757-72713829) Arg (CCT) 73 bp Sc: 46.81
TCCCTGGTGGTCCAGTGGCTAAGACTCTGTGCTCCTCATGCTGCGGGCCCAGGTTCAATC
CCTGGTCAGGGAA
>Bos_taurus_chr7.trna3036-ArgCCT (72457931-72458003) Arg (CCT) 73 bp Sc: 47.02
TCCCTGATGGTCCAGTGGCCAAGACTTTGTGTTCCTAAGGCAGGGGGCCCAGGTTCCAAC
CCTGGTCGGGGAA
>Bos_taurus_chr5.trna2680-ArgCCT (72558254-72558325) Arg (CCT) 72 bp Sc: 47.22
TCCCTGGTGGTCCAGTGGCTAAGTCTCTGTGCTCCTAATGCAGAAGCCCAGGTTCGACCT
CTGGTCAGGGAA
>Bos_taurus_chr7.trna7783-ArgCCT (15448415-15448343) Arg (CCT) 73 bp Sc: 47.61
TCCCTGGAAGTCCAGTGATTAAGACTTTGCACTCCTGATGCAGAGGGCCCAGGTTTGATC
CCTGGTCAGGGAA
>Bos_taurus_chr16.trna1846-ArgCCT (47907799-47907870) Arg (CCT) 72 bp Sc: 47.68
TTCCTTGTGGTCCAGTGGGTAGGACTCAGTGCTCCTCCTGCTGGGGCCCAGGTTCAATCC
CTGGTCGGGAAA
>Bos_taurus_chrUn.004.1214.trna3-ArgCCT (6320-6249) Arg (CCT) 72 bp Sc: 47.87
TCCCTGATGGTCCAGTGGTGAGACTCTGCATTCCTGATGTAGGGGGCGCAGGTTCAATTC
CTGGTTGGGGAA
>Bos_taurus_chr19.trna2797-ArgCCT (56077484-56077556) Arg (CCT) 73 bp Sc: 48.02
TCCCTGGTGGTCCTGTGACTAGGACTCTGTGCTCCTAATACAGGGGGCCCAGGTTCGATC
CCTGGTCAGGGAA
>Bos_taurus_chrUn.004.815.trna2-ArgCCT (31479-31551) Arg (CCT) 73 bp Sc: 48.18
TCCCTGGTGGTTCAGTGGGTAAGACTCTACACTCCTAATGCAGGGAGCCCAGGTTCAATC
CCTAGTCAGGGAA
>Bos_taurus_chr11.trna4577-ArgCCT (109465558-109465629) Arg (CCT) 72 bp Sc: 48.21
TCCCTGGGGGTCGAGTGGCTAAGACTCCGTGTTCCTAATGCAGGGGTCCAGGTTCGATTC
CTGGTCAGGGAA
>Bos_taurus_chr21.trna476-ArgCCT (14312554-14312626) Arg (CCT) 73 bp Sc: 48.27
TCCCTGGGGGTCCAGTGGCTAAGACTCTGCTCTCCTGGTGCAGAGGGCCCAGGTTCAATC
CCTGGTCAGGGAA
>Bos_taurus_chr3.trna8974-ArgCCT (13578903-13578831) Arg (CCT) 73 bp Sc: 48.27
TCCTTGGTGGTTCAGTGGCTAAGACTCTGCACTCCTAAAGCAGGGGTCCCAGGTTTGGTC
CCTGGTCAGGGAA
>Bos_taurus_chr7.trna8133-ArgCCT (10899528-10899456) Arg (CCT) 73 bp Sc: 48.28
TCCCTGGTGGTCCAGTAGTTAGGACTCCACATGCCTAATGTAGGAGGCCCGGGTTCGATC

CCTGGTCAGGGAA
>Bos_taurus_chr19.trna2492-ArgCCT (49417528-49417600) Arg (CCT) 73 bp Sc: 48.30
TCCCTGGTGGTCCAGTGGTTAAGACTCTGCCCTCCTAATGCAGGCGGCCCAGGTTTGATC
CCTGATCAGGGAA
>Bos_taurus_chr19.trna2301-ArgCCT (46282745-46282817) Arg (CCT) 73 bp Sc: 48.89
TCTTTGGTGGTCCAGTGGCTAAGACTCTGTGCTCCTAATGCTGGGGTGCCAGGTTCGATC
CCTGGTCAGGGAA
>Bos_taurus_chrX.trna5208-ArgCCT (47812560-47812489) Arg (CCT) 72 bp Sc: 49.06
TCCCTGGTGGTTCAGTGGCTGGGATTCTGCACTCCTAGTGAAGGGGCCCAGGTTCAATTC
CTGGTCAGGGAA
>Bos_taurus_chr13.trna3374-ArgCCT (77732132-77732204) Arg (CCT) 73 bp Sc: 49.67
TCCCTGGTAGTCCAGCGGCTAAGACTCTGCACTCCTAATGCAGGGGGTCCGGGTTTGATC
CCTGGTTAGGGAA
>Bos_taurus_chr3.trna5643-ArgCCT (106777628-106777556) Arg (CCT) 73 bp Sc: 50.24
TCCCTGGTGGTTCAGTGGCTAAGACTCTGCACTCCTAATGCAGAGGACTAGGGTTCAATC
CCTGGTCAGGGAA
>Bos_taurus_chr4.trna5910-ArgCCT (89866077-89866005) Arg (CCT) 73 bp Sc: 50.42
ACTCTGGTGGTCTAATGGCTAAGACTCTGCACTCCTAATGCAGAGGACCCAGGTTCCATC
CCTGGTCAGGGAA
>Bos_taurus_chr12.trna6609-ArgCCT (11139509-11139437) Arg (CCT) 73 bp Sc: 50.42
TCCCTGGTGGTTCAGTGGCTAAGACTCTGCACTCCTAATACAGGGGGTCCAGGTTCGATC
CCTAGTCAGGGAA
>Bos_taurus_chr7.trna7791-ArgCCT (15317957-15317885) Arg (CCT) 73 bp Sc: 50.48
TTCCTGGTGGTCCAGTGGCTCAGACTCTGAACTCCTAATTCAGGGGGCCCAGGTTCAATC
CCTGGTCAGGGAA
>Bos_taurus_chr11.trna4770-ArgCCT (105000739-105000666) Arg (CCT) 74 bp Sc: 51.17
TCCCTGGTGGTTCAGTGGCTAAGACTCTGTACTCCTAATACAGGGGCTCCCAGGTTCGAT
CCCTAGTCAGGGAA
>Bos_taurus_chr17.trna2362-ArgCCT (56621253-56621325) Arg (CCT) 73 bp Sc: 51.45
TCCCTGGTGGTCTAGTGGGTAAGACTCTGCTCTCCTAATGCAGGGGGCCCGGGTTTGATC
CCTGGTCAGGGAA
>Bos_taurus_chr2.trna8497-ArgCCT (62842538-62842466) Arg (CCT) 73 bp Sc: 51.56
TCCCTGATGGTCCAATGGCTAAGACTTTGCTCTCCTAATGCAGGGGGCCTGGGTTCAATC
CCCGGTCAAGGAA
>Bos_taurus_chr13.trna5718-ArgCCT (41372787-41372715) Arg (CCT) 73 bp Sc: 51.84
TCCCTGGTGGTCCAGTGGCTAAGACTCCACACTCCTAGTGTAGGGGTCCCGGGTTCGATC
CCTGTCCAGGGAA
>Bos_taurus_chr8.trna146-ArgCCT (5174633-5174705) Arg (CCT) 73 bp Sc: 52.14
GCCCCAGAAGCCTAATGGATAAGGCACTGTCCTCCTAAGCCAGGGACTGTGGGTTCAAGT
CCCACCTGGGGTA
>Bos_taurus_chr7.trna3787-ArgCCT (96236910-96236982) Arg (CCT) 73 bp Sc: 52.23
TCCCTGGTGGTCCAGTGGCTAGCACTCTGCACTCCTAATGCAAGGGGCCCAGGTTCCATC
CCTGGTCAGGGAA
>Bos_taurus_chr15.trna4875-ArgCCT (37267535-37267463) Arg (CCT) 73 bp Sc: 52.32
TCCCTGGTGGCCCAGCGGCTAAGACTCTGTACTCCTGATGCAGGGAGCCCAGGTTCGAGC
CCTGGTTAGGGAA
>Bos_taurus_chr4.trna4453-ArgCCT (121343964-121343892) Arg (CCT) 73 bp Sc: 52.32
TCCCTGGTGGTCCAGTGGTTAAGACTCTGCACTCCTAATGGAGGGGGCCCTGGTTTGATC
CCGGATCAGGGAG
>Bos_taurus_chr26.trna785-ArgCCT (23065287-23065359) Arg (CCT) 73 bp Sc: 52.39
TCCCTGGTGGTCCAATGGCTAAGACTCTGTACTCCTAATGCAGGGGGTCTGGGTTCGATT
CCTGGTCAGGGAA
>Bos_taurus_chr11.trna177-ArgCCT (2685120-2685192) Arg (CCT) 73 bp Sc: 52.75
TCCCTGGTGGTCCAATGGCTAAGACTCTGCCCTCCTGATGCAGTGGCCCCGGGTTCGATC
CCTGGACAGGGAA
>Bos_taurus_chr13.trna4949-ArgCCT (62967946-62967874) Arg (CCT) 73 bp Sc: 53.09
TCCTTGGTGGTCCAGTGGCTAAGACTCTGAGCTCCTGATGCAGGGGGCCCAGGTTCGATC
CCTGGTTGGGGAA
>Bos_taurus_chr15.trna5200-ArgCCT (30092951-30092879) Arg (CCT) 73 bp Sc: 53.18
TCCTTGGTGGTCCAGTGGTTAAGACTCTGTGCTCCTAATGTAGGGGGCCCAGGTTCGATT
CTTGGTCAGGGAA
>Bos_taurus_chr3.trna5371-ArgCCT (113139451-113139379) Arg (CCT) 73 bp Sc: 53.25
TCCTTGGTGGTCCAGTGGCTAAGACTCTGCACTCCTAATGCAGGGGACCTGGGTTCAATC
CCTGGTTAGGGAA
>Bos_taurus_chr12.trna6335-ArgCCT (15265415-15265343) Arg (CCT) 73 bp Sc: 53.59
TCCCTGGTGGTCCAGTGGCTAAGACTCTGCACTCCTAATACAGGGGCCCTGGGTTCAATC
CCTGGTCAGGGAA

>Bos_taurus_chr10.trna3035-ArgCCT (80367271-80367343) Arg (CCT) 73 bp Sc: 55.33
TCTCTGGTGGTCCAGTGGCTAAGACTGCACGCTCCTAATGTGCAGGGCCCAGGTTCAATC
CCTGGTCAGGGAA
>Bos_taurus_chrUn.004.2159.trna2-ArgCCT (915-843) Arg (CCT) 73 bp Sc: 55.33
TCTCTGGTGGTCCAGTGGCTAAGACTGCACGCTCCTAATGTGCAGGGCCCAGGTTCAATC
CCTGGTCAGGGAA
>Bos_taurus_chr17.trna711-ArgCCT (17950319-17950391) Arg (CCT) 73 bp Sc: 55.48
TCCCTGGTAGTCCAGTGGCTAAGACTCTGCACTCCTAATGCAGGGGGCCCCGGTTCCATC
CCTGGTCAGGGAA
>Bos_taurus_chr1.trna4070-ArgCCT (119027655-119027727) Arg (CCT) 73 bp Sc: 56.51
TCCCTGGTGGTCCAGTGGCTAAGACTCTGCGCTCCTAATGCAGGGGGCCCAGGTTTGATC
CCTGGTCAGGGAA
>Bos_taurus_chr13.trna3284-ArgCCT (76035135-76035207) Arg (CCT) 73 bp Sc: 56.75
ACCCAGCTAGTTCAGCTGGTAGAGCGTGGGACTCCTGATCCCAGCATTGTGGGTTCGAGC
CCCACGTTGGGCA
>Bos_taurus_chr7.trna6845-ArgCCT (37833334-37833262) Arg (CCT) 73 bp Sc: 56.97
TCCCTGGTGGTTCAGTGGTTAAGACTTTGCACCCCTAATGCAGGGGGCTCAGGTTCAATT
CCTGGTCAGGGAA
>Bos_taurus_chr28.trna2925-ArgCCT (6893889-6893817) Arg (CCT) 73 bp Sc: 57.20
TCCCTGGTGGTCTAGTGGCTAAGACTCTGCACTCCTAATGCAGGAGGCCTGGGTTCGATT
CCTGGTCAGGGAA
>Bos_taurus_chr12.trna4735-ArgCCT (53248885-53248813) Arg (CCT) 73 bp Sc: 57.29
TCCCTGGTGGTCCAGTGGTTAAGATTCTACACTCCTAATGCAGGGGGCCCAGGTTCAATC
TCTGGTCAGGGAA
>Bos_taurus_chr19.trna5916-ArgCCT (19053988-19053916) Arg (CCT) 73 bp Sc: 57.41
TTCCTGGTGGTCCAGTGGCTAAGACTCTGTGCTCCTAATGCAGAGGGCCCAGGTTCGATC
CCTGGTCAGGGAA
>Bos_taurus_chr2.trna4200-ArgCCT (123653497-123653567) Arg (CCT) 71 bp Sc: 57.48
TCCCTGGTGGTCCAGTGGCTAAGACTCTGTGCTCCTAAGACAGGGCCCAGGTTCGATCCC
TGGTCAGGGAA
>Bos_taurus_chr12.trna6687-ArgCCT (9811499-9811428) Arg (CCT) 72 bp Sc: 58.04
TCCCTGGTGGTCCAGTGGCTAAGACTCTGTACTCCTAATGCAGGGGTCTGGGTTCGATTC
CTGGTCAGGGAA
>Bos_taurus_chr29.trna1848-ArgCCT (48673144-48673216) Arg (CCT) 73 bp Sc: 58.25
TCCCTGGTGGTCCAGTGGCTAGGACTCCGGGCTCCTAATGCAGGGGACCCAGGTTCGATC
CCTGGCCAGGGAA
>Bos_taurus_chr9.trna506-ArgCCT (18640518-18640590) Arg (CCT) 73 bp Sc: 59.05
TCCTTGGTGGTTCAGTGGTTAAGACGCTGAGCTCCTAATGCAGGGGGCCCAGGTTCGATC
CCTGGTCAGGGAA
>Bos_taurus_chr7.trna1231-ArgCCT (19970280-19970352) Arg (CCT) 73 bp Sc: 59.09
TCCCTGGTGGTCCAGTGGCTAAGACTCCACACTCCTGATGCAGGAGGCCCTGGTTCGATT
CCTGGCCAGGGAA
>Bos_taurus_chr5.trna7241-ArgCCT (78686042-78685970) Arg (CCT) 73 bp Sc: 59.30
GTCCCAGTGGCCTAATGGACAAGGCACTGGCCTCCTAAGTCAGGGATTGTGGGTTCAAGT
CCCACCTGGGGTG
>Bos_taurus_chr15.trna446-ArgCCT (18287132-18287204) Arg (CCT) 73 bp Sc: 60.10
TCCCTGGTGGTCCAGTGGCTAGGACTCTGCACTCCTAATGCAGGTGGCCCAGTTTCGATC
CCTGGTCAGGGAA
>Bos_taurus_chr7.trna8382-ArgCCT (6987007-6986935) Arg (CCT) 73 bp Sc: 60.76
TCCCTGGTGGTCCAGTGGCTAGGACTCCGCACTCCTAATGCGGGGGGCCTGGGTTCAATC
CCTGGTCAGGGAA
>Bos_taurus_chr25.trna1135-ArgCCT (19922975-19923047) Arg (CCT) 73 bp Sc: 61.70
TCCTTGGTGGTCCAGTGGTTAAGACTTTGCACTCCTAATGCAGGGGGCCCAAGTTCGATC
CCTGGTCAGGGAA
>Bos_taurus_chr4.trna3598-ArgCCT (106528023-106528095) Arg (CCT) 73 bp Sc: 67.28
GCCCCAGTGGCCTAATGGATAAGGCATTGGCCTCCTAAGCCAGGGATTGTGGGTTCGAGT
CCCATCTGGGGTG
>Bos_taurus_chr19.trna3754-ArgCCT (58027365-58027293) Arg (CCT) 73 bp Sc: 70.63
GCCCCAGTGGCCTAATGGATAAGGCACTGGCCTCCTAAGCCAGGGATTGTGGGTTCGAGT
CCCATCTGGGGTG
>Bos_taurus_chr25.trna4870-ArgCCT (3183109-3183037) Arg (CCT) 73 bp Sc: 71.31
GCCCCGGTGGCCTAATGGAGAAGGCACTGGCCTCCTAAGCCAGGGATTGTGGGTTCGAGT
CCCACCCGGGGTA
>Bos_taurus_chr25.trna106-ArgCCT (3149703-3149775) Arg (CCT) 73 bp Sc: 71.53
GCCCCGGTGGCCTAATGGATAAGGCATTGGCCTCCTAAGCCAGGGATTGTGGGTTCGAGT
CCCACCCGGGGTA
>Bos_taurus_chr19.trna2966-ArgCCT (58028239-58028311) Arg (CCT) 73 bp Sc: 73.41

GCCCCAGTGGCCTAATGGATAAGGCACTGGCCTCCTAAGCCAGGGATTGTGGGTTCGAGT
CCCACCTGGGGTG
>Bos_taurus_chr19.trna3753-ArgCCT (58028797-58028725) Arg (CCT) 73 bp Sc: 73.88
GCCCCAGTGGCCTAATGGATAAGGCACTGGCCTCCTAAGCCAGGGATTGTGGGTTCGAGT
CCCACCTGGGGTA
>Bos_taurus_chr25.trna127-ArgCCT (3201694-3201766) Arg (CCT) 73 bp Sc: 73.88
GCCCCAGTGGCCTAATGGATAAGGCACTGGCCTCCTAAGCCAGGGATTGTGGGTTCGAGT
CCCACCTGGGGTA
>Bos_taurus_chr2.trna9934-ArgGCG (16538034-16537963) Arg (GCG) 72 bp Sc: 39.43
TCCCTGGTGGCTCAGAAGGTAAAGTGTCTGCCTGCGATGTGGGAGACCCAGGTTTGATCC
CTGGCTCGGGAT
>Bos_taurus_chr5.trna1905-ArgGCG (53057886-53057957) Arg (GCG) 72 bp Sc: 40.19
TCCCGGGTGGCTCAGATGGTCAAGAATCTGCCTGCGATGCAGGAGACCTAGGTTTGATCC
CTGGTTCGGGAA
>Bos_taurus_chr8.trna2847-ArgGCG (82918334-82918405) Arg (GCG) 72 bp Sc: 45.78
TCCCTGGTGGCTCAGTCAGTAAAGTTTCTGCCTGCGATTCAGGAGACCTAAGTTCGATCC
TTGGCTTGGGAA
>Bos_taurus_chr10.trna2338-ArgGCG (61688442-61688513) Arg (GCG) 72 bp Sc: 52.87
TCCCTGGTGGCTCAGAGGGTAAAGCGTCTGCCTGCGATGCAGGAGACCCAGGTTTGATCC
CTGGGTGGGGAA
>Bos_taurus_chr4.trna5277-ArgGCG (103625780-103625709) Arg (GCG) 72 bp Sc: 55.17
TCCCTGGTGGCTCAGTTGGTAAAGAGTCTGCCTGCGATGCAGGAGACCCAGGTTCAATCC
CTGGGTTGGGAA
>Bos_taurus_chrUn.004.3951.trna1-ArgGCG (2941-2870) Arg (GCG) 72 bp Sc: 55.17
TCCCTGGTGGCTCAGTTGGTAAAGAGTCTGCCTGCGATGCAGGAGACCCAGGTTCAATCC
CTGGGTTGGGAA
>Bos_taurus_chr1.trna2186-ArgGCG (63315648-63315719) Arg (GCG) 72 bp Sc: 55.32
TCCTTGGTGGCTCAGATGGTAAAGTGTCTGCCTGCGATGCAGGAGACCCAGGTTCAATCC
CTGGGTTGGGAA
>Bos_taurus_chr20.trna635-ArgGCG (18794365-18794436) Arg (GCG) 72 bp Sc: 56.03
TCCCTGGTAGCTCAGCTGGTAAAGTGTCTGCCTGCGATGCAGGAGACCCTGGTTCAATTC
CTGGGTTGGGAA
>Bos_taurus_chr16.trna2871-ArgGCG (70282726-70282797) Arg (GCG) 72 bp Sc: 58.43
TCCCTGGTGGCTCAGAGGTTAAAGCGTCTGCCTGCGATGCAGGAGACCCAGGTTCAATCC
CTGGGTCGGGAA
>Bos_taurus_chr11.trna2121-ArgTCG (50795868-50795940) Arg (TCG) 73 bp Sc: 42.55
TCCCTGGTGGTCCAGTGGCTGAGACTCTGCACTTCGAATCCGGGGGTCCCAGGTTTGCTC
CCTGGTCAGGGAA
>Bos_taurus_chrUn.004.34.trna39-ArgTCG (221166-221095) Arg (TCG) 72 bp Sc: 45.49
TTCCTGGTGGTCCAGTGGTTAGGACTCTGTGCTTCGAATACAAGGGGCCTGGTTCAATCC
CTGGTCAGGGAA
>Bos_taurus_chr8.trna4147-ArgTCG (115616111-115616040) Arg (TCG) 72 bp Sc: 45.77
TCCCTGGTGGTCCAGTGGCTAAGACTCTGCACTTCGAATGCAGGGGCCTGGTTTCCATCC
CTGGTCAGGGAA
>Bos_taurus_chr25.trna1641-ArgTCG (28441111-28441183) Arg (TCG) 73 bp Sc: 63.83
GGCTGTGTGGCCTAATGGATAAGGCGTATGACTTCGGATCAGAAGTTTGCAGGTTCGAGT
CCTGCCACAGCCC
>Bos_taurus_chr23.trna3368-ArgTCG (31710995-31710923) Arg (TCG) 73 bp Sc: 67.10
GACCACGTGGCCTAACGGATAAGGCGTCTGACTTCGGATCAGAAGATTGAGGGTTCGAAT
CCCTTCGTGGTTA
>Bos_taurus_chr20.trna5459-ArgTCG (7763983-7763911) Arg (TCG) 73 bp Sc: 67.54
AACCACGTGGCCTAATGGATAAGGCGTCTGACTTCGGATCAGAAGATTGAGGGTTCGAGT
CCCTTCGTGGTTG
>Bos_taurus_chr19.trna2965-ArgTCG (58027840-58027912) Arg (TCG) 73 bp Sc: 68.07
GACCGCGTGGCCTAACGGATAAGGCGTCTGACTTCGGATCAGAAGATTGAGGGTTCGAGT
CCCTTCGTGGTCG
>Bos_taurus_chr23.trna1337-ArgTCG (30290193-30290265) Arg (TCG) 73 bp Sc: 69.08
GACCACGTGGCCTAATGGATAAGGCGTCTGACTTCGGATCAGAAGATTGAGGGTTCGAAT
CCCTTCGTGGTTG
>Bos_taurus_chr23.trna3369-ArgTCG (31697964-31697892) Arg (TCG) 73 bp Sc: 69.08
GACCACGTGGCCTAATGGATAAGGCGTCTGACTTCGGATCAGAAGATTGAGGGTTCGAAT
CCCTTCGTGGTTG
>Bos_taurus_chr25.trna1642-ArgTCG (28441811-28441883) Arg (TCG) 73 bp Sc: 72.33
GGCTGTGTGGCCTAATGGATAAGGCGTCTGACTTCGGATCAGAAGATTACAGGTTCGAGT
CCTGTCACGGTCG
>Bos_taurus_chr25.trna1644-ArgTCG (28447037-28447109) Arg (TCG) 73 bp Sc: 72.94
GGCCATGTGGCCTAATGGATAAGGCGTCTGACTTCGGATCAGAAGATTACAGGTTCGAGT

CCTGTCATGGTCG
>Bos_taurus_chr21.trna787-ArgTCG (20568002-20568074) Arg (TCG) 73 bp Sc: 76.93
GGCCGCGTGGCCTAATGGATAAGGCGTCTGACTTCGGATCAGAAGATTGCAGGTTCGAGT
CCTGCCGCGGTCG
>Bos_taurus_chr20.trna155-ArgTCT (4240898-4240975) Arg (TCT) 78 bp Sc: 31.80
TCCCTGGTGGTCCAGTGGTTAAGATTCTGTGCTTCTACTGCAGGGGGCTGGGCACGGGTC
CAACCCCTGATCAGGGAA
>Bos_taurus_chr25.trna2391-ArgTCT (40280459-40280531) Arg (TCT) 73 bp Sc: 31.97
TCCCTGGCAGTCTAGTGGTTGAGACTCTGCATTTCTAATGCAGGGGACTCGGGTTCCACT
CCTGGTTGGGGAA
>Bos_taurus_chr23.trna4110-ArgTCT (16304233-16304162) Arg (TCT) 72 bp Sc: 34.41
TCCCTGGTGGTCCAGTGATTAAGATTCTGTGCTTCTGCTGCAGGGGTGCAGGTTTGGTCC
CTGGCCAGGGAA
>Bos_taurus_chr29.trna1471-ArgTCT (41598921-41598993) Arg (TCT) 73 bp Sc: 37.09
TCCCTGGTGGTCCAGTGGCGAAGACTCTATGCTTCTACTTTAGGGGGCATGGGTTCAATC
CCTAGTCAGGGAA
>Bos_taurus_chr18.trna1508-ArgTCT (37998794-37998866) Arg (TCT) 73 bp Sc: 37.25
TCCCTGGCAGTCCAATGGTAAAGACTCCGTGCTTCTACTGCAGGGGCCCCAGGTTCAAAC
CCTGGTCAGGGAC
>Bos_taurus_chr17.trna4419-ArgTCT (57557678-57557606) Arg (TCT) 73 bp Sc: 37.98
TCCTTGGTGGTCCAGTGATTAAGACTCTGCACTTCTACTGCAGGGGGCTTAGGCTCCATC
CCTGGTCAGGGAA
>Bos_taurus_chr23.trna3702-ArgTCT (23801769-23801697) Arg (TCT) 73 bp Sc: 38.27
TCCCTGGTGGCCCAGGGGTTAGGGCTCTGTGCTTCTCCTGCAGGGGGCATAGGTTTGATC
CCTGGTCGGGGAA
>Bos_taurus_chr13.trna5480-ArgTCT (48199277-48199206) Arg (TCT) 72 bp Sc: 38.90
TCCCTGGCAGTCCAGAGGTGAGGACTCTGTGGTTCTACTGCAGGGGCACAGGTTCAATCC
CTGTTTGGGGAA
>Bos_taurus_chr9.trna762-ArgTCT (25684269-25684341) Arg (TCT) 73 bp Sc: 38.91
TCCCTGGTAGTCAAGTGGTTAAGACTTGGCAGTTCTGCTGCAGAGGGCCCAGGTTTGATA
CCTGGTTGGGAAA
>Bos_taurus_chr5.trna9806-ArgTCT (10406338-10406266) Arg (TCT) 73 bp Sc: 39.28
TCCCTGGGGGTCCAGTGGCTAAGACTCTGTGCTTCTAATGCAGGGGGCCCGGGTTTGATC
CCTGGTCAGGGAG
>Bos_taurus_chr19.trna4393-ArgTCT (46581860-46581788) Arg (TCT) 73 bp Sc: 39.51
TCCCTGGTGGTCCAGTGGTTAAGACTCTGTGCTTCTAATGCTGGGGGCACAGGTTCAGCC
CCTGGTCAGGGAA
>Bos_taurus_chr28.trna404-ArgTCT (10054508-10054579) Arg (TCT) 72 bp Sc: 39.65
TCCTTGATGGTCCAGTGACTAAGACTCTGCACTTCTAAGGCAGGGGCTTGGGTTCGATCT
CTGGTCAGGGAA
>Bos_taurus_chr25.trna3300-ArgTCT (29623866-29623794) Arg (TCT) 73 bp Sc: 39.69
TCCTTGGTGGTCCAGTGGTTAAGACTCTGCACTTCTACTGCAGGGAGTTCGGGTACCATT
CCTGACTGGGGAC
>Bos_taurus_chr13.trna4856-ArgTCT (64851816-64851744) Arg (TCT) 73 bp Sc: 40.12
TCCCTGGTGGTCCAGTGGCTAAGGGTCTGTGTTTCTAATGCAGGGGGCCCAGGTTCCATC
CCTGGTCAGGGAA
>Bos_taurus_chr5.trna9183-ArgTCT (29563362-29563290) Arg (TCT) 73 bp Sc: 40.80
TTCCTGGTGGTCTAGTGGTTAAGACTCTGTATTTCTGCTGCAGGGGGCTTGGGTTTGATC
CCTGGCTAGGGAA
>Bos_taurus_chrX.trna736-ArgTCT (16796876-16796948) Arg (TCT) 73 bp Sc: 40.95
TCCCTGGTGGTGCAGTGGTTAGCATTCTACATTTCTATTGCAGGGGGTGCAGGCTCCATC
CCTGGCTGGGGAA
>Bos_taurus_chr5.trna2780-ArgTCT (74479964-74480037) Arg (TCT) 74 bp Sc: 41.16
TCCTTGATGGTCCAGTGGTTAGGACTCTGTGCTTCTGATGCAGTGAGGCCTGGGTTTTAT
CCCTGGTCAGGGAA
>Bos_taurus_chr21.trna5172-ArgTCT (15604281-15604209) Arg (TCT) 73 bp Sc: 42.56
TCCCTGGTGGTCCAGTGACTAAGACTCCGTGCTCTCTATGCAGGGGGCCCGGGTTCAATC
CCTGGCCAGGGAA
>Bos_taurus_chr2.trna6227-ArgTCT (123377315-123377243) Arg (TCT) 73 bp Sc: 43.29
TCCCTGGTGGTCCAGTGGTTAAGACTCTGAGCTTCTACTGCAGGGGGCATGGGTTTGATC
CCTAGCCAGGGAA
>Bos_taurus_chr15.trna1476-ArgTCT (42129062-42129134) Arg (TCT) 73 bp Sc: 44.68
TCCCTGAAGGTCCAGTGGTTAAGACTCTGCCCTTCTACTGCAGCGGGCATGGGTTCAATC
CCTGTTTAGGGAA
>Bos_taurus_chr29.trna1430-ArgTCT (39005501-39005573) Arg (TCT) 73 bp Sc: 44.80
TCCCTGGTGGTCCAGTGGTTAAGACTCTGCCCTTCTAATGCAGGAGGCACAGGTTTGATT
CTTGGTCAGGGAA

>Bos_taurus_chr15.trna3335-ArgTCT (77849984-77849911) Arg (TCT) 74 bp Sc: 46.52
TCCCTGGTGGTCCAGTGGTTAGGACTCTGCACTTCTGCTGCAGGGGACCACAGGTTTGAT
CCTTGTTCAGGAAA
>Bos_taurus_chr27.trna2989-ArgTCT (26399550-26399478) Arg (TCT) 73 bp Sc: 47.83
TCCCTGGTGGTCCAGTGGTTAGGATTCTGTGCTTCTACAGCAGGGGGCTTGGGTTCAATC
CCTGGTCAGGGAA
>Bos_taurus_chr27.trna3003-ArgTCT (26200573-26200501) Arg (TCT) 73 bp Sc: 47.83
TCCCTGGTGGTCCAGTGGTTAGGATTCTGTGCTTCTACAGCAGGGGGCTTGGGTTCAATC
CCTGGTCAGGGAA
>Bos_taurus_chr1.trna5889-ArgTCT (157131210-157131138) Arg (TCT) 73 bp Sc: 47.97
TCCCTGATGGTCCAGTGGTTAAGACTCTGCACTTCTGGTGCAGGGGGCCTGGGTTTGATC
CCTAGTTGGGGAT
>Bos_taurus_chr5.trna2873-ArgTCT (77176813-77176885) Arg (TCT) 73 bp Sc: 48.59
TCCCTGGTGGTCCAGTGGTTAAGACTCTGTGTTTCTACTACAGGGGGCACAGGTTCAATC
CCTGGTTGGGGAA
>Bos_taurus_chr1.trna9540-ArgTCT (47599453-47599381) Arg (TCT) 73 bp Sc: 49.65
TCCCTGGTGGTCCAGTGGTTAAGACTCTGTGCTTCTAATTCAGAGGCCACAGGTTTGATC
CCTGATTGGGGAA
>Bos_taurus_chrX.trna4207-ArgTCT (74360420-74360348) Arg (TCT) 73 bp Sc: 51.02
TCCCTGGTGGTCCAGTGGTTAAGACTCTGAGCTTCTACTGCAGGAGGCTCAGGTTCGATC
CCTGGTTAGGGAA
>Bos_taurus_chr13.trna4227-ArgTCT (75196396-75196324) Arg (TCT) 73 bp Sc: 51.11
TCCCTGGTGGTCCAGTGGTTAAGACTTTGCACTTCTGATGCAGGGGGCACAGGTTTGATC
CCTGTTAGGGGAG
>Bos_taurus_chr9.trna4994-ArgTCT (83981312-83981240) Arg (TCT) 73 bp Sc: 51.53
TCCTTGGTGGTCTAGTGGTTAAGACTCTGCACTTCTAATATAGGGGCCACAGGTTCAATA
CCTGGTTAAGGAA
>Bos_taurus_chr13.trna2972-ArgTCT (70467807-70467879) Arg (TCT) 73 bp Sc: 52.37
TCCTTGGTGGTCCAGTGGTTAGGACTCTGAGCTTCTACTGCAGGGTGCCTGGGTTCGATC
CCTAGTCAGGGAA
>Bos_taurus_chr6.trna7216-ArgTCT (46450097-46450025) Arg (TCT) 73 bp Sc: 54.10
TTTCTGGTGGTCCAGTGGCTAGGACTCTGCACTTCTAATGCAGGGGGCCCAGGTTCGATC
CCTGGTCAGGGCT
>Bos_taurus_chr9.trna277-ArgTCT (12590577-12590649) Arg (TCT) 73 bp Sc: 55.39
TCCCTGATGGTCCAGTGGTTAGAACTCTGCACTTCTACTGTAGGAGACCCAGGTTGGAAC
CCTGGTCAGGGAA
>Bos_taurus_chr13.trna4522-ArgTCT (69464607-69464535) Arg (TCT) 73 bp Sc: 56.48
TCCCTGGTGGTCCAGTGGTTAAAACTCTGCACTTCTACTGCAGAGGGTGCAGGTTCAACC
CCTGCTTTGGGAA
>Bos_taurus_chr17.trna4792-ArgTCT (53685409-53685337) Arg (TCT) 73 bp Sc: 58.87
TCCCTGGTGGTCCAGTGGTTAGGACTCAGCACTTCTACTGCTGAGGACCGGGGTTCAATC
CCTGGTCGGGGAA
>Bos_taurus_chr1.trna2984-ArgTCT (87187848-87187920) Arg (TCT) 73 bp Sc: 59.00
TCCCTGGTGGTCCAGTGGCTAAGACTCTGCACTTCTAATGCAGGGGGCCCAGGTTCAATC
ACTGGTCAGGGAA
>Bos_taurus_chr2.trna9558-ArgTCT (27575881-27575810) Arg (TCT) 72 bp Sc: 59.06
TCCCTGGTGGTCTAGTGGTTAGGACTTGGCATTTCTACTGCCATGGCCCAGATTCAATCC
CTGGTCATGGAA
>Bos_taurus_chrUn.004.113.trna13-ArgTCT (172512-172440) Arg (TCT) 73 bp Sc: 66.22
GCCCGGATAGCTCAGTCAGTAGAGCTTCAGACTTCTAGTCTGAGGGTCCAGGGTTCAAGT
CCCTGTTTGGGCG
>Bos_taurus_chr3.trna381-ArgTCT (11562645-11562718) Arg (TCT) 74 bp Sc: 76.29
GTCTCTGTGGCGCAATGGACGAGCGCGCTGGACTTCTAATCCAGAGGTTCCGGGTTCGAG
TCCCGGCAGAGATG
>Bos_taurus_chr23.trna1024-ArgTCT (22846699-22846789) Arg (TCT) 91 bp Sc: 36.52
TCCCTGATAGTTCAGTGGTGAGGACTCCATGCTTCTACTGCAGGGGGAATCCCTGGTTGG
GTTTCTCCAGGTTCAATCCCTGGTTGGGGAA
>Bos_taurus_chr19.trna1408-ArgTCT (28306599-28306685) Arg (TCT) 87 bp Sc: 70.50
GGCTCTGTGGCGCAATGGATAGCGCATTGGACTTCTAGTGACGAAAGAGCGATTCAAAGG
TTGTGGGTTCGAATCCCACCAGAGTCG
>Bos_taurus_chr23.trna3409-ArgTCT (31168655-31168568) Arg (TCT) 88 bp Sc: 69.06
GGCTCTGTGGCGCAATGGATAGCGCATTGGACTTCTAGCACAGCAGTGAGGCATTCAAAG
GTTGCGGGTTCGAGTCCCGCCAGAGTCG
>Bos_taurus_chr3.trna7471-ArgTCT (52947209-52947125) Arg (TCT) 85 bp Sc: 71.81
GGCTCCGTGGCGCAATGGATAGCGCATTGGACTTCTAGAGGTTGAAGGCATTCAAAGGTT
CCGGGTTCGAGTCCCGGCGGAGTCG
>Bos_taurus_chr15.trna2991-ArgTCT (83646112-83646197) Arg (TCT) 86 bp Sc: 68.58

GGCTCTGTGGCGCAATGGATAGCGCATTGGACTTCTAGATAGACGGAGGCATTCAAAGGT
TGTGGGTTCGAGTCCCACCAGAGTCG
>Bos_taurus_chr4.trna849-AsnGTT (25116559-25116632) Asn (GTT) 74 bp Sc: 56.05
GTCTCTGTGGTGCAATCGGTTATCATGTTCAGCTGTTAACTGAAAAATTGGTGGTTCGAG
CCCATCCAGGGATG
>Bos_taurus_chr3.trna854-AsnGTT (22900179-22900252) Asn (GTT) 74 bp Sc: 60.19
GTCTCTGTGGTGCAGTCGTTTAGCGCATGCGGCTGTTAACCGAAAGGTTGCTGGTTCGAG
CCCACCCAGGGACG
>Bos_taurus_chr3.trna8565-AsnGTT (22865776-22865703) Asn (GTT) 74 bp Sc: 60.19
GTCTCTGTGGTGCAGTCGTTTAGCGCATGCGGCTGTTAACCGAAAGGTTGCTGGTTCGAG
CCCACCCAGGGACG
>Bos_taurus_chr23.trna3385-AsnGTT (31530492-31530419) Asn (GTT) 74 bp Sc: 62.46
GGCTCTGTGGCGCAATCAGTTAGCACCTTTGGCTGTTAACCAAAAGGTTGGTGGTTCAAG
CCCACCCAGGGCCG
>Bos_taurus_chr3.trna8572-AsnGTT (22687949-22687876) Asn (GTT) 74 bp Sc: 63.85
GTCTCTGTGGCACAATCGGTTAGCGCTCTCGGCTGTTAACCGAAAGGCTGGTGGTTCGAG
CCCACCCAGGGACT
>Bos_taurus_chr3.trna8580-AsnGTT (22637176-22637103) Asn (GTT) 74 bp Sc: 63.85
GTCTCTGTGGCACAATCGGTTAGCGCTCTCGGCTGTTAACCGAAAGGCTGGTGGTTCGAG
CCCACCCAGGGACT
>Bos_taurus_chr3.trna861-AsnGTT (22941622-22941695) Asn (GTT) 74 bp Sc: 63.85
GTCTCTGTGGCACAATCGGTTAGCGCTCTCGGCTGTTAACCGAAAGGCTGGTGGTTCGAG
CCCACCCAGGGACT
>Bos_taurus_chrUn.004.185.trna24-AsnGTT (175322-175249) Asn (GTT) 74 bp Sc: 63.85
GTCTCTGTGGCACAATCGGTTAGCGCTCTCGGCTGTTAACCGAAAGGCTGGTGGTTCGAG
CCCACCCAGGGACT
>Bos_taurus_chr3.trna863-AsnGTT (22958009-22958082) Asn (GTT) 74 bp Sc: 73.80
GTCTCTGTGGCGCAATCGGTTAGCGCGTTCGGCTGTTAACTGAAAGGTTGGTGGTTCGAG
CCCACCCAGGGATG
>Bos_taurus_chr3.trna866-AsnGTT (22966480-22966553) Asn (GTT) 74 bp Sc: 74.46
GTCTCTGTGGCGCAATCGGTTAGTGCGTTCGGCTGTTAACCGAAAGGTTGGTGGTTCAAG
CCCACCCAGGGACG
>Bos_taurus_chr3.trna938-AsnGTT (24580280-24580353) Asn (GTT) 74 bp Sc: 75.33
GTCTCTGTGGTGCAATTGGTTAGCACGTTCAGCTGTTAACTGAAAGGTTGGTGGTTCGAG
CCCACCCAGGGACG
>Bos_taurus_chr3.trna8597-AsnGTT (22497936-22497863) Asn (GTT) 74 bp Sc: 78.61
GTCTCTGTGGCGCAATCGGTTAGCGCGTTCGGCTGTTAACCGAAAAGTTGGTGGTTCGAG
CCCACCCAGGGACG
>Bos_taurus_chrUn.004.3551.trna1-AsnGTT (914-987) Asn (GTT) 74 bp Sc: 78.61
GTCTCTGTGGCGCAATCGGTTAGCGCGTTCGGCTGTTAACCGAAAAGTTGGTGGTTCGAG
CCCACCCAGGGACG
>Bos_taurus_chr3.trna8544-AsnGTT (23042135-23042062) Asn (GTT) 74 bp Sc: 78.67
GTCTCTGTGGCGCAATCGGTGAGCGCGTTCGGCTGTTAACCGAAAGGTTGGTGGTTCGAG
CCCACCCAGGGACG
>Bos_taurus_chr3.trna8540-AsnGTT (23078294-23078221) Asn (GTT) 74 bp Sc: 79.17
GTCTCTGTGGCGCAATCGGTTAGCGCGTTCGGCTGTTAACCGGAAGGTTGGTGGTTCGAG
CCCACCCAGGGACG
>Bos_taurus_chr13.trna6721-AsnGTT (22801183-22801110) Asn (GTT) 74 bp Sc: 79.28
GTCTCTGTGGCGCAATCGGTTAGCGCGTTCGGCTGTTAACCGAGAGGTTGGTGGTTCGAG
CCCACCCAGGGACG
>Bos_taurus_chr3.trna8481-AsnGTT (24597900-24597827) Asn (GTT) 74 bp Sc: 80.50
GTCTCTGTGGCGCAATCGGTTAGCGCGTTCGGCTGTTAACCGAAAGGTTGGTGGTTCAAG
CCCACCCAGGGACG
>Bos_taurus_chrUn.004.185.trna29-AsnGTT (18070-17997) Asn (GTT) 74 bp Sc: 80.50
GTCTCTGTGGCGCAATCGGTTAGCGCGTTCGGCTGTTAACCGAAAGGTTGGTGGTTCAAG
CCCACCCAGGGACG
>Bos_taurus_chr12.trna1315-AsnGTT (29986018-29986091) Asn (GTT) 74 bp Sc: 82.24
GTCTCTGTGGCGCAATCGGTTAGCGCGTTCGGCTGTTAACCGAAAGGTTGGTGGTTCGAG
CCCACCCAGGGACG
>Bos_taurus_chr19.trna4772-AsnGTT (40586074-40586001) Asn (GTT) 74 bp Sc: 82.24
GTCTCTGTGGCGCAATCGGTTAGCGCGTTCGGCTGTTAACCGAAAGGTTGGTGGTTCGAG
CCCACCCAGGGACG
>Bos_taurus_chr3.trna815-AsnGTT (22504764-22504837) Asn (GTT) 74 bp Sc: 82.24
GTCTCTGTGGCGCAATCGGTTAGCGCGTTCGGCTGTTAACCGAAAGGTTGGTGGTTCGAG
CCCACCCAGGGACG
>Bos_taurus_chr3.trna821-AsnGTT (22556580-22556653) Asn (GTT) 74 bp Sc: 82.24
GTCTCTGTGGCGCAATCGGTTAGCGCGTTCGGCTGTTAACCGAAAGGTTGGTGGTTCGAG

CCCACCCAGGGACG
>Bos_taurus_chr3.trna824-AsnGTT (22566601-22566674) Asn (GTT) 74 bp Sc: 82.24
GTCTCTGTGGCGCAATCGGTTAGCGCGTTCGGCTGTTAACCGAAAGGTTGGTGGTTCGAG
CCCACCCAGGGACG
>Bos_taurus_chr3.trna829-AsnGTT (22591608-22591681) Asn (GTT) 74 bp Sc: 82.24
GTCTCTGTGGCGCAATCGGTTAGCGCGTTCGGCTGTTAACCGAAAGGTTGGTGGTTCGAG
CCCACCCAGGGACG
>Bos_taurus_chr3.trna833-AsnGTT (22616545-22616618) Asn (GTT) 74 bp Sc: 82.24
GTCTCTGTGGCGCAATCGGTTAGCGCGTTCGGCTGTTAACCGAAAGGTTGGTGGTTCGAG
CCCACCCAGGGACG
>Bos_taurus_chr3.trna839-AsnGTT (22655088-22655161) Asn (GTT) 74 bp Sc: 82.24
GTCTCTGTGGCGCAATCGGTTAGCGCGTTCGGCTGTTAACCGAAAGGTTGGTGGTTCGAG
CCCACCCAGGGACG
>Bos_taurus_chr3.trna846-AsnGTT (22701141-22701214) Asn (GTT) 74 bp Sc: 82.24
GTCTCTGTGGCGCAATCGGTTAGCGCGTTCGGCTGTTAACCGAAAGGTTGGTGGTTCGAG
CCCACCCAGGGACG
>Bos_taurus_chr3.trna8537-AsnGTT (23081019-23080946) Asn (GTT) 74 bp Sc: 82.24
GTCTCTGTGGCGCAATCGGTTAGCGCGTTCGGCTGTTAACCGAAAGGTTGGTGGTTCGAG
CCCACCCAGGGACG
>Bos_taurus_chr3.trna8541-AsnGTT (23056985-23056912) Asn (GTT) 74 bp Sc: 82.24
GTCTCTGTGGCGCAATCGGTTAGCGCGTTCGGCTGTTAACCGAAAGGTTGGTGGTTCGAG
CCCACCCAGGGACG
>Bos_taurus_chr3.trna8545-AsnGTT (23011460-23011387) Asn (GTT) 74 bp Sc: 82.24
GTCTCTGTGGCGCAATCGGTTAGCGCGTTCGGCTGTTAACCGAAAGGTTGGTGGTTCGAG
CCCACCCAGGGACG
>Bos_taurus_chr3.trna8554-AsnGTT (22928722-22928649) Asn (GTT) 74 bp Sc: 82.24
GTCTCTGTGGCGCAATCGGTTAGCGCGTTCGGCTGTTAACCGAAAGGTTGGTGGTTCGAG
CCCACCCAGGGACG
>Bos_taurus_chr3.trna8601-AsnGTT (22472090-22472017) Asn (GTT) 74 bp Sc: 82.24
GTCTCTGTGGCGCAATCGGTTAGCGCGTTCGGCTGTTAACCGAAAGGTTGGTGGTTCGAG
CCCACCCAGGGACG
>Bos_taurus_chr7.trna1970-AsnGTT (42831094-42831167) Asn (GTT) 74 bp Sc: 82.24
GTCTCTGTGGCGCAATCGGTTAGCGCGTTCGGCTGTTAACCGAAAGGTTGGTGGTTCGAG
CCCACCCAGGGACG
>Bos_taurus_chrUn.004.185.trna16-AsnGTT (223917-223844) Asn (GTT) 74 bp Sc: 82.24
GTCTCTGTGGCGCAATCGGTTAGCGCGTTCGGCTGTTAACCGAAAGGTTGGTGGTTCGAG
CCCACCCAGGGACG
>Bos_taurus_chrUn.004.185.trna28-AsnGTT (27331-27258) Asn (GTT) 74 bp Sc: 82.24
GTCTCTGTGGCGCAATCGGTTAGCGCGTTCGGCTGTTAACCGAAAGGTTGGTGGTTCGAG
CCCACCCAGGGACG
>Bos_taurus_chrUn.004.185.trna8-AsnGTT (190635-190708) Asn (GTT) 74 bp Sc: 82.24
GTCTCTGTGGCGCAATCGGTTAGCGCGTTCGGCTGTTAACCGAAAGGTTGGTGGTTCGAG
CCCACCCAGGGACG
>Bos_taurus_chrUn.004.1856.trna1-AsnGTT (2776-2849) Asn (GTT) 74 bp Sc: 82.24
GTCTCTGTGGCGCAATCGGTTAGCGCGTTCGGCTGTTAACCGAAAGGTTGGTGGTTCGAG
CCCACCCAGGGACG
>Bos_taurus_chrUn.004.2930.trna2-AsnGTT (7835-7908) Asn (GTT) 74 bp Sc: 82.24
GTCTCTGTGGCGCAATCGGTTAGCGCGTTCGGCTGTTAACCGAAAGGTTGGTGGTTCGAG
CCCACCCAGGGACG
>Bos_taurus_chrUn.004.2930.trna5-AsnGTT (12727-12800) Asn (GTT) 74 bp Sc: 82.24
GTCTCTGTGGCGCAATCGGTTAGCGCGTTCGGCTGTTAACCGAAAGGTTGGTGGTTCGAG
CCCACCCAGGGACG
>Bos_taurus_chr3.trna9120-AsnGTT (8728537-8728464) Asn (GTT) 74 bp Sc: 84.88
GTCTCTGTAGCGCAATCGGTTAGCGCGTTCGGCTGTTAACCGAAAGGTTGGTGGTTCGAA
TCCACCCAGGGACG
>Bos_taurus_chrUn.004.248.trna9-AspATC (160254-160182) Asp (ATC) 73 bp Sc: 43.64
TCCCTGGTGGTCCAGTGGTTTTGACTCAGTGCTATCACTGCTGAGGGCTCAGATTCGATT
CCTGGTTGGGGAA
>Bos_taurus_chr14.trna1538-AspATC (34520787-34520859) Asp (ATC) 73 bp Sc: 46.41
TCCCTGGTGGTTCAGTAGCTAAGACTCTGCACTATCAATGCAGAGAGCCCAGGTTCGATC
TCTGATCAGGGAA
>Bos_taurus_chr12.trna3043-AspATC (76905381-76905451) Asp (ATC) 71 bp Sc: 50.62
TCCCTGGTGGTCCGGTGTTAAGACTCCGCACTATCAATGCAGGGCCCCAGGTTCGATCCC
TGGTCAGGGAA
>Bos_taurus_chr17.trna4986-AspATC (50327196-50327124) Asp (ATC) 73 bp Sc: 51.74
TCCCTGGGGGTCCAGTGGCTCGGACTCCACACTATCAATGCGGGGGGCCCAGGTTCGATC
CCTGGTCAGGGAA

>Bos_taurus_chr13.trna6411-AspATC (27913443-27913371) Asp (ATC) 73 bp Sc: 55.43
TCCCTGATGGTCCAGGGGATAAGCCTCTGCACTATCAATGCAGGGGACCCAGGTTTGATT
CCTGGTCGGGGAA
>Bos_taurus_chr3.trna4266-AspATC (117067925-117067997) Asp (ATC) 73 bp Sc: 56.78
TCCCTGGTGGTCTGGTGGTTAGGACTCTACACTATCACTGCTGAGGGCCCAGGTTCAATC
CCTGGTCAGGGAA
>Bos_taurus_chr23.trna148-AspGTC (3786757-3786828) Asp (GTC) 72 bp Sc: 33.11
CTTTTGTTAGTACAGTGTTGAGTATCCTTGCCTGTCACTCAGGAGACTGGGGTTTGATTC
CCCAACGGAGAG
>Bos_taurus_chr17.trna429-AspGTC (12850434-12850506) Asp (GTC) 73 bp Sc: 34.92
TCCCTGGTAGTCCAGTGGCTAAGATTCTGTGCTGTCAGCACAGGGAGTCTGGGTTCAATC
CCTGGCCGGAGAA
>Bos_taurus_chr19.trna1575-AspGTC (30941423-30941495) Asp (GTC) 73 bp Sc: 35.42
TCCCCGGTGGTCAAGTGGTTGGGACTCTGTGCTGTCAATGAAGGGGGCTCAGGTTTGACC
CCTGATTGGGGAA
>Bos_taurus_chr19.trna1866-AspGTC (37454153-37454225) Asp (GTC) 73 bp Sc: 36.34
TCCCTGGTAGTCCAGTGGCTAAGATTCCGTGCTGTCAGCACAGGGAATCTGGGTTCGATC
CCTGGTCAGGGAA
>Bos_taurus_chr18.trna2493-AspGTC (55342716-55342787) Asp (GTC) 72 bp Sc: 38.07
TCTTTGGCTGTCCAGTGGTCAGGACTCGGCACTGTCACTTCTGGGGCCTGGGTTCAATCC
CTGGTCAGAGAA
>Bos_taurus_chr18.trna2511-AspGTC (55529606-55529677) Asp (GTC) 72 bp Sc: 38.07
TCTTTGGCTGTCCAGTGGTCAGGACTCGGCACTGTCACTTCTGGGGCCTGGGTTCAATCC
CTGGTCAGAGAA
>Bos_taurus_chrUn.004.1143.trna3-AspGTC (38611-38682) Asp (GTC) 72 bp Sc: 39.59
TCCCTGGTGGCTCAGATGGGAAAGAATCTGCCTGTCATGCAGGAAACCCAGGTTCCATCC
CTGGATGGGGAA
>Bos_taurus_chr11.trna789-AspGTC (18000274-18000345) Asp (GTC) 72 bp Sc: 40.19
TTCCCAGTGGTCTAATGGTTAAGATTTAGTACTGTCACTGCTATGACCTGGGTTTGATTC
CTGGTTGGAAAC
>Bos_taurus_chr10.trna6187-AspGTC (52710793-52710722) Asp (GTC) 72 bp Sc: 41.50
TCCCTGATGGCCTTGTGGTTAGGGTTTGGGGCTGTCACTGCCGTGGCCCAGGTTTCATCC
CTGGTCAGGGAT
>Bos_taurus_chr27.trna3064-AspGTC (25164314-25164243) Asp (GTC) 72 bp Sc: 42.31
TCCTCATTAGTATAGTGGTGAGTATCCCCACCTGTCACGTGGGAGACTGGGGTTTGATTC
CTGGATGGGGAG
>Bos_taurus_chr21.trna1272-AspGTC (27322693-27322763) Asp (GTC) 71 bp Sc: 43.88
TCCCTGGTTGTCCAGTGGTTAGGACTCAGTGCTGTCACCGTGGGACCTGGGTTCAATTCC
TGGTCAGGGAA
>Bos_taurus_chr14.trna692-AspGTC (15485831-15485902) Asp (GTC) 72 bp Sc: 44.81
TTCCCAGTGGTTCAGTGGTTAAGACTCAGCACTGTCACTACTGGGGCCCAGGTCCAATCC
TTGGTTGGGGAA
>Bos_taurus_chr21.trna2286-AspGTC (56680807-56680879) Asp (GTC) 73 bp Sc: 45.11
TCCCTGGTGGTCCGGTGACTAAGACTCTGTGCTGTCAATGCAGGGGGCCCAGGTTCAATC
CCTGGTCAGGGAA
>Bos_taurus_chr8.trna4086-AspGTC (116742429-116742500) Asp (GTC) 72 bp Sc: 45.26
TCCTCGTTAGTATAGTGGTAAGTATCCCTACCTGTCACGCAGGAGATGGGGGTTTGATTC
CCCAATGGGGAC
>Bos_taurus_chr1.trna1122-AspGTC (28682554-28682625) Asp (GTC) 72 bp Sc: 45.74
TCCTCATTAGTATAGTGGTTAGTATCCTTACCAGTCATGTGGGAGACTGGGGTTCAATTC
CCCAATGGGGAG
>Bos_taurus_chr18.trna3262-AspGTC (58367075-58367004) Asp (GTC) 72 bp Sc: 48.22
TCCCTGGTGGTCTAGTGTTTAGGATTTGGCACTGTCACTGCTATGGCCTGGGTTTGATCC
CTGGTCAGGGAA
>Bos_taurus_chr15.trna133-AspGTC (5145680-5145751) Asp (GTC) 72 bp Sc: 49.80
TCCTCCTTAGTATAGTGGTGAATATCCCTGCTTGTCACACAGGAGACCAGGGTTTGATTC
CCTGACGGGGAG
>Bos_taurus_chr25.trna3813-AspGTC (21418078-21418005) Asp (GTC) 74 bp Sc: 51.62
TCCCTGGTGGTCCAGTGGCTAGGACTCGGCACTGTCACTGCCGTGGGCCTGGGGTTCAAT
CCCTGGTCAGGGAA
>Bos_taurus_chr12.trna371-AspGTC (11831868-11831939) Asp (GTC) 72 bp Sc: 52.27
TCCTTGTTAGTACAGTGGTGACTATCCCTGCCTGTCACGCAGGAGTCAGGGGTTTGATTC
CCCAATGAGGAG
>Bos_taurus_chr11.trna8063-AspGTC (26676873-26676801) Asp (GTC) 73 bp Sc: 52.72
TCCCTGGCAGTCCAGTGGTTAGGATTCCGTACTGTCGTTGCTGAGGGCCCAGGTTCAATC
CCTGGTTGGGGAA
>Bos_taurus_chr1.trna10412-AspGTC (19043698-19043627) Asp (GTC) 72 bp Sc: 52.78

TCCCTGTTAGTCTAGTGTTGAGTATCCCCGCCTGTCACTCGGGAGACCGGGGTTCAATTC
CCCGACGGGGAG
>Bos_taurus_chr3.trna2132-AspGTC (59486256-59486327) Asp (GTC) 72 bp Sc: 55.16
TCTTCGTTAGTATAGTGGTGAATATCCCCGCCTGTCACGCCAGAGACCGGGGTTCGATTC
CCCGACGGGGAG
>Bos_taurus_chrUn.004.22.trna32-AspGTC (638255-638184) Asp (GTC) 72 bp Sc: 56.44
TCCTCGTTAGTACAGTGGTGAGTATCCCTGCCTGTCACGTGGGAGACCTGGGTTCGACTC
CCTGACGGGGAG
>Bos_taurus_chr3.trna9267-AspGTC (3984175-3984104) Asp (GTC) 72 bp Sc: 56.75
TCCTCGTTAGTATAGTGGTGAATATCCCTGCCTGTCACGCGGGAGACCAGGATTCGATTC
CCTGACGGGGAG
>Bos_taurus_chr19.trna4331-AspGTC (47408827-47408755) Asp (GTC) 73 bp Sc: 57.52
TCCCTGGTGGTTCAGTGGTTAGGACTCTGTGCTGTCACTGCTGAGGTCCCAGGTTGAATC
CCTGGTCAGGGAA
>Bos_taurus_chrUn.004.1939.trna4-AspGTC (17769-17841) Asp (GTC) 73 bp Sc: 57.52
TCCCTGGTGGTTCAGTGGTTAGGACTCTGTGCTGTCACTGCTGAGGTCCCAGGTTGAATC
CCTGGTCAGGGAA
>Bos_taurus_chr10.trna770-AspGTC (18333532-18333603) Asp (GTC) 72 bp Sc: 57.66
TCCTCGTTAATGTAGTGGTGAGTATCCCCGCCTGTCACGCGGGAGACCGAGGTTCGATTC
CCCGACGAGGAG
>Bos_taurus_chrX.trna3155-AspGTC (82627279-82627350) Asp (GTC) 72 bp Sc: 57.78
TCCCTGGTGGTCCAGTGGTTAGGACTTGGCACTGTCACTGCCATGGCCCAGGTTCAATCT
CTCGTCAGGAAA
>Bos_taurus_chr18.trna4609-AspGTC (35640774-35640703) Asp (GTC) 72 bp Sc: 57.79
TCCTTGTTAGTATAGTGGTGATTATCCCTGCCTGTCATGCAGGAGACCGGGGTTTGAGTC
CCTGATGGGGAG
>Bos_taurus_chr7.trna8002-AspGTC (12985494-12985422) Asp (GTC) 73 bp Sc: 58.06
TCCCTGACAGTCCAGTGGTTAAGACTCTGTACTGTCACTACTGAGGGCCCAGGTTCAATC
CCTGGTCAGGGAA
>Bos_taurus_chr3.trna6514-AspGTC (82774394-82774323) Asp (GTC) 72 bp Sc: 62.73
TCCTCTTTAGTATAGTGGTGAGTATCCCCACCTGTCACGTGGGAGACCAGGGTTCGATTC
CCTGATGGGGAG
>Bos_taurus_chr3.trna281-AspGTC (8961140-8961211) Asp (GTC) 72 bp Sc: 65.38
TCCTCGTTAGTATAGTGGTGAGTATCCCCGCCTGTCACGCGGGAGACCGGGGTTCCATTC
CCCGACGGGGAG
>Bos_taurus_chr3.trna9108-AspGTC (8825514-8825443) Asp (GTC) 72 bp Sc: 67.28
TCCTCGATAGTATAGTGGTGAGTATCCCCGCCTGTCACGCGGGAGACCGGGGTTCGATTC
CCCGACGGGGAG
>Bos_taurus_chr3.trna9111-AspGTC (8816058-8815987) Asp (GTC) 72 bp Sc: 71.60
TCCTCGTTAGTATAGTGGTAAGTATCCCCGCCTGTCACGCGGGAGACCGGGGTTCGATTC
CCCGACGGGGAG
>Bos_taurus_chr3.trna9123-AspGTC (8629859-8629788) Asp (GTC) 72 bp Sc: 71.60
TCCTCGTTAGTATAGTGGTAAGTATCCCCGCCTGTCACGCGGGAGACCGGGGTTCGATTC
CCCGACGGGGAG
>Bos_taurus_chr17.trna2065-AspGTC (53704617-53704688) Asp (GTC) 72 bp Sc: 72.92
TCCTCGTTAGTATAGTGGTGAGTATCCCCGCCTGTCACGCGGGAGACCGGGGTTCGATTC
CCCGACGGGGAG
>Bos_taurus_chr17.trna2067-AspGTC (53707588-53707659) Asp (GTC) 72 bp Sc: 72.92
TCCTCGTTAGTATAGTGGTGAGTATCCCCGCCTGTCACGCGGGAGACCGGGGTTCGATTC
CCCGACGGGGAG
>Bos_taurus_chr19.trna5354-AspGTC (28390837-28390766) Asp (GTC) 72 bp Sc: 72.92
TCCTCGTTAGTATAGTGGTGAGTATCCCCGCCTGTCACGCGGGAGACCGGGGTTCGATTC
CCCGACGGGGAG
>Bos_taurus_chr22.trna1575-AspGTC (44267620-44267691) Asp (GTC) 72 bp Sc: 72.92
TCCTCGTTAGTATAGTGGTGAGTATCCCCGCCTGTCACGCGGGAGACCGGGGTTCGATTC
CCCGACGGGGAG
>Bos_taurus_chr23.trna1375-AspGTC (31152996-31153067) Asp (GTC) 72 bp Sc: 72.92
TCCTCGTTAGTATAGTGGTGAGTATCCCCGCCTGTCACGCGGGAGACCGGGGTTCGATTC
CCCGACGGGGAG
>Bos_taurus_chr23.trna3406-AspGTC (31194217-31194146) Asp (GTC) 72 bp Sc: 72.92
TCCTCGTTAGTATAGTGGTGAGTATCCCCGCCTGTCACGCGGGAGACCGGGGTTCGATTC
CCCGACGGGGAG
>Bos_taurus_chr3.trna9105-AspGTC (8948842-8948771) Asp (GTC) 72 bp Sc: 72.92
TCCTCGTTAGTATAGTGGTGAGTATCCCCGCCTGTCACGCGGGAGACCGGGGTTCGATTC
CCCGACGGGGAG
>Bos_taurus_chr3.trna9119-AspGTC (8740977-8740906) Asp (GTC) 72 bp Sc: 72.92
TCCTCGTTAGTATAGTGGTGAGTATCCCCGCCTGTCACGCGGGAGACCGGGGTTCGATTC

CCCGACGGGGAG
>Bos_taurus_chr5.trna2359-AspGTC (64870209-64870280) Asp (GTC) 72 bp Sc: 72.92
TCCTCGTTAGTATAGTGGTGAGTATCCCCGCCTGTCACGCGGGAGACCGGGGTTCGATTC
CCCGACGGGGAG
>Bos_taurus_chr5.trna7823-AspGTC (65017985-65017914) Asp (GTC) 72 bp Sc: 72.92
TCCTCGTTAGTATAGTGGTGAGTATCCCCGCCTGTCACGCGGGAGACCGGGGTTCGATTC
CCCGACGGGGAG
>Bos_taurus_chrUn.004.4089.trna2-AspGTC (1109-1180) Asp (GTC) 72 bp Sc: 72.92
TCCTCGTTAGTATAGTGGTGAGTATCCCCGCCTGTCACGCGGGAGACCGGGGTTCGATTC
CCCGACGGGGAG
>Bos_taurus_chrUn.004.8164.trna1-AspGTC (2552-2623) Asp (GTC) 72 bp Sc: 72.92
TCCTCGTTAGTATAGTGGTGAGTATCCCCGCCTGTCACGCGGGAGACCGGGGTTCGATTC
CCCGACGGGGAG
>Bos_taurus_chr5.trna2468-AspGTC (67443721-67443792) Asp (GTC) 72 bp Sc: 74.76
TCCTCGTTAGTATAGTGGTTAGTATCCCCGCCTGTCACGCGGGAGACCGGGGTTCAATTC
CCCGACGGGGAG
>Bos_taurus_chr20.trna4651-CysACA (30003367-30003291) Cys (ACA) 77 bp Sc: 31.68
CTTTTTAGGTTGGCTCAGATGGTAAAGCATCTGCCTACAGTGTGGGAGACCCAGGTTCAA
TCCCTGGGTTGGGAAGA
>Bos_taurus_chrUn.004.3983.trna1-CysACA (415-486) Cys (ACA) 72 bp Sc: 34.09
TCCCTGGTGGCTCATAGGGTAAAGTGTCTGCCTACAATGTGGGAGACTCAGGTTCCATCC
CTGGCTTGGGAA
>Bos_taurus_chr29.trna342-CysACA (9384909-9384981) Cys (ACA) 73 bp Sc: 34.47
TCCCTGGTGGCTCAGACGGTAAAATATCTGCCCACAATGCAGGAGACCCAGGTTTGATCC
CTGGGTCAGGAAG
>Bos_taurus_chr9.trna3675-CysACA (101384644-101384715) Cys (ACA) 72 bp Sc: 36.74
TCCCTATTGGCTCAGACAGTAAAGCATCTGCCTACAGTGTGGGAGACCCAGGTTCAATCC
CTGGGTTGGGAA
>Bos_taurus_chr8.trna3292-CysACA (95262760-95262830) Cys (ACA) 71 bp Sc: 37.03
TCCCTGGTGGCTCAGATGGCAAAGCATCTGCCTACACTGCAGAAGACTGGGTTCGAGCCC
TGGGTAGGGAA
>Bos_taurus_chr11.trna1367-CysACA (31838487-31838558) Cys (ACA) 72 bp Sc: 37.12
TCCCTGGTGGCTCAGACAGTAAGGCATCTGCCTACAATGCAGGAAACCCAGGTTCAGTCC
CTGGGTAGGGAA
>Bos_taurus_chrUn.004.44.trna87-CysACA (253308-253238) Cys (ACA) 71 bp Sc: 37.59
TCCCTGGTGGCTCAGAGGGTAAAGCATCTGCCTACAATGCGGAAGACCGGGTTTGATCCC
TGGGTAGGGAA
>Bos_taurus_chr21.trna2155-CysACA (53097388-53097459) Cys (ACA) 72 bp Sc: 37.78
TCCCTGCTGGCTCAGACAGTAAAGCGTCTGCCTACAATGTGGGAAATCCAGGTTCGATCC
CTGGGTCGGGAA
>Bos_taurus_chr3.trna201-CysACA (7036542-7036613) Cys (ACA) 72 bp Sc: 38.57
TCCCTGGTGGCTCAGACGTTAAAGCATCTGTCTACAATGTGGGAGACCCAGGTTCAAGCC
CTGGGTAGGGAA
>Bos_taurus_chr16.trna3850-CysACA (61884661-61884590) Cys (ACA) 72 bp Sc: 39.02
TCCCTGGTGGCTCAGACAGTAAAGCATCTGCCTACACTGGAGGTGACCCAGGTTCGATTC
CTGGGTGGGGAA
>Bos_taurus_chr3.trna2162-CysACA (60930328-60930399) Cys (ACA) 72 bp Sc: 40.63
TCCCTGGTGGCTCAGATGGTAAAGCATCTACCTACAGTGTGGGAGACCCAGGTTTGATCC
CTGGTCAGGAAG
>Bos_taurus_chr5.trna438-CysACA (13420074-13420145) Cys (ACA) 72 bp Sc: 40.83
TCCCTGGTGGCTCAGACCTTAAAGTGTCCACCTACAATGTGGGAGACCCAGGTTCAATCC
CTGGGTAGGGAA
>Bos_taurus_chr19.trna1859-CysACA (37188775-37188847) Cys (ACA) 73 bp Sc: 41.26
TCCCTGGTGGCTCAGATGGTAAAAGCATGTGCCTACAAAGCAGGAGACCCGGGTTTGGTC
CCTGGCTAGGGAA
>Bos_taurus_chr12.trna1987-CysACA (50921217-50921288) Cys (ACA) 72 bp Sc: 41.63
TCCCTGGTGGCTCATAGGGTAAAGTGTCTGCCTACAATGTGGGAGACTCAGGTTCGATCC
CTGGCTTGGGAA
>Bos_taurus_chr23.trna1869-CysACA (42083337-42083409) Cys (ACA) 73 bp Sc: 42.66
TCCCTGGTGGCTCAGACAGTAAAAGCATCTGCCTACAAGGCAGGAGACCCAGGTTCAATC
CCTGGTTTGGGAA
>Bos_taurus_chr28.trna1766-CysACA (38985009-38984938) Cys (ACA) 72 bp Sc: 42.70
TTCCTGGTAGCTCAGACAGTAAAGCGCCTGCCTACAATGTGGGAGACCCAGGTTCGATCC
CTGGTCGGAAAG
>Bos_taurus_chr13.trna7147-CysACA (12697263-12697193) Cys (ACA) 71 bp Sc: 42.87
TCCCTGGTGGCTCACATGGTAAAGCATCTGCCTACAATGCGGAAGACCAGGTTCGATCCC
TGGTCGGGAAG

>Bos_taurus_chr3.trna2245-CysACA (63314789-63314859) Cys (ACA) 71 bp Sc: 42.87
TCCCTGGTGGCTCACATGGTAAAGCATCTGCCTACAATGCGGAAGACCAGGTTCGATCCC
TGGTCGGGAAG
>Bos_taurus_chr5.trna6483-CysACA (97934260-97934188) Cys (ACA) 73 bp Sc: 43.26
TCCCTGGTGGCTCAGATGGTAAAAGCATCTGCCTACAATGTGGGAGACCCAGGTTTGATC
CCTGGGCAGGGAA
>Bos_taurus_chr25.trna3389-CysACA (28433477-28433406) Cys (ACA) 72 bp Sc: 43.39
TCCCTGGTGGCTCAGACAGTAAAGTGTCTGCCTACAATGCAGGAAACTCAGGTTCAATCC
CTGGTTTGGGAA
>Bos_taurus_chr1.trna4518-CysACA (130381839-130381910) Cys (ACA) 72 bp Sc: 43.97
TCCCTGCTGGCTCAGACGGTAAAGTGTCTGTCTACAATGCAGGAGACCCGGGTTTGAGCC
CTGGGTTGGGAA
>Bos_taurus_chr3.trna6204-CysACA (91486450-91486379) Cys (ACA) 72 bp Sc: 44.16
TCCCTGGTAGCTCAGATGGTAAAGCGTCTGCCTACAATGAGGGATACCCAGGTTCAATCC
CTGGGTAGGGAA
>Bos_taurus_chr5.trna3893-CysACA (101695559-101695630) Cys (ACA) 72 bp Sc: 44.73
TCCCTGGTGGCTCAGACAGTAAAGTGTCTGCCTACAGTGCAGGAGACCCAGGTTTGATCC
CTGGGTGGGGAA
>Bos_taurus_chr29.trna264-CysACA (7569896-7569967) Cys (ACA) 72 bp Sc: 44.74
TCCCTGGTGGCTCAGACGGTAAAGTGTCTGTCTACAATGCAGGAGACCCAGGTTTGATCC
TTGGGTGGGGAA
>Bos_taurus_chr9.trna3282-CysACA (93795788-93795859) Cys (ACA) 72 bp Sc: 44.96
TCCCTGGTGGCTCAGACAGTAAAGTGTATGCTTACAATGCAGGAGACCCAGGTTCGATCC
CTGGGTGGGGAA
>Bos_taurus_chr16.trna3906-CysACA (60678714-60678643) Cys (ACA) 72 bp Sc: 46.43
TCCCTGGTGGCTCAGACGGTAAAGCATCTGCCTACAATGTGGAAGACCCAGGTTTGATCC
CTGGGTGGGGAA
>Bos_taurus_chrUn.004.51.trna6-CysACA (275782-275853) Cys (ACA) 72 bp Sc: 46.52
TCCCTGGTGGCTCAGATGGTAAAGTGTCTGTCTACAATGCGGGAGACCCAGGTTCTATCC
CTGGGTAGGGAA
>Bos_taurus_chr13.trna1902-CysACA (46061919-46061990) Cys (ACA) 72 bp Sc: 47.14
TCCCTAGTGGCTCAGACGGTAAAGCATCTGCCTACAATGAGGGAGACCCAGGTTCAATCC
CTGGGTAGGGAA
>Bos_taurus_chr22.trna1652-CysACA (46450458-46450529) Cys (ACA) 72 bp Sc: 47.24
TCCCTGCTGGCTCAGACAGTAAAGCGTTTGCCTACAATGCAGAAGACCCGGGTTCAATCC
CTGGGTCGGGAA
>Bos_taurus_chr24.trna2110-CysACA (50851182-50851252) Cys (ACA) 71 bp Sc: 47.28
TCCCTGGTAGCTCAGAGGGTAAAGCGTCTGCCTACAATGCAGGAGACCAGGTTTGATCCC
TGGGTAGGGAA
>Bos_taurus_chr16.trna6061-CysACA (5777013-5776942) Cys (ACA) 72 bp Sc: 47.38
TCCCTGGTGGCTTAGACAGTAAAGCATCTGCCTACAATGCAGGAGACCCAGGTTTGATCC
CTGGGTAGGGAA
>Bos_taurus_chr1.trna3547-CysACA (103886927-103886998) Cys (ACA) 72 bp Sc: 48.40
TCCCTGGTGGCTCAGATGGTAAAGCATCTGTCTACAATGCAGAAGACCCGGGTTCCATCC
CTGGGTAGGGAA
>Bos_taurus_chr16.trna702-CysACA (22428054-22428126) Cys (ACA) 73 bp Sc: 49.35
TCCCGGGTGGCTCAGATGGGAAAAGTGTCTGCTTACAATGCGGGAGACCCAGGTTCAATC
CCTGGTTCGGGAA
>Bos_taurus_chr20.trna3950-CysACA (47728606-47728535) Cys (ACA) 72 bp Sc: 49.36
TCCCTGTTGGCTTAGACAGTAAAGCATCTGCCTACAATGCAGGAGGCCCAGGTTCAATCC
CTGGGTCGGGAA
>Bos_taurus_chr4.trna7593-CysACA (40586891-40586820) Cys (ACA) 72 bp Sc: 49.40
TCCCTGTTGGCTCAGATGGTAAAGCATCTGCCTACAATGCTGGAGACCCAAGTTCAATCC
CTGGGTAGGGAA
>Bos_taurus_chr4.trna7596-CysACA (40526829-40526758) Cys (ACA) 72 bp Sc: 49.40
TCCCTGTTGGCTCAGATGGTAAAGCATCTGCCTACAATGCTGGAGACCCAAGTTCAATCC
CTGGGTAGGGAA
>Bos_taurus_chr28.trna1802-CysACA (37988242-37988171) Cys (ACA) 72 bp Sc: 49.68
TCCCAGGTGGCTCAGATGGTAAAGCGTCTGCGTACAATGCAGAAGACCTGGGTTCGATCC
CTGGGTTGGGAA
>Bos_taurus_chr6.trna4640-CysACA (113087134-113087063) Cys (ACA) 72 bp Sc: 49.83
TCCCAGATGGCTCACATGGTAAAGCATCTGACTACAATGCAGGAGACCCAGGTTCGATCC
CTGGGTTGGGAA
>Bos_taurus_chr9.trna678-CysACA (23450405-23450476) Cys (ACA) 72 bp Sc: 49.90
TCCCTGGTGGCTCAGAGGATAAAGTGTCTGTCTACAATGTAGGAGACCCAGGTTCAATCC
CTGGTTTGGGAA
>Bos_taurus_chr22.trna1651-CysACA (46444644-46444715) Cys (ACA) 72 bp Sc: 50.05

TCCCTGCTGGCTCAGACAGTAAAGCGTCTGCCTACAATGCAGAAGACCCGGGTTCAATCC
CTGGGTCGGGAA
>Bos_taurus_chr29.trna1337-CysACA (36468998-36469069) Cys (ACA) 72 bp Sc: 50.05
TCCCTGCTGGCTCAGACAGTAAAGCGTCTGCCTACAATGCAGAAGACCCGGGTTCAATCC
CTGGGTCGGGAA
>Bos_taurus_chr16.trna2657-CysACA (66375386-66375457) Cys (ACA) 72 bp Sc: 50.16
TCCTTGGTGGCTCAAACAGTAAAGCGTCTGCCTACAATGCAGGAGACCCAGGTTCAATCC
CTGGGTAGGGAA
>Bos_taurus_chr17.trna3873-CysACA (66450461-66450390) Cys (ACA) 72 bp Sc: 50.38
TCCCGAATAGCTCAGACAGTAAAGCGTCTGCCTACAATGCGGGAGACCCAGGTTCGATCC
CTGGGTCGGGAA
>Bos_taurus_chr6.trna6035-CysACA (83842074-83842003) Cys (ACA) 72 bp Sc: 50.51
TCCCTACTGGCTCAGATGGTAAAGCATCTGCCTACAATGTGGGAGACCCAGGTTCAATCC
CTGGGTTGGGAA
>Bos_taurus_chr20.trna2671-CysACA (69538927-69538998) Cys (ACA) 72 bp Sc: 50.66
TCCCTGGTGGCTCAGATGGTAAAGTGTCTGCCTACAATGCAGGAGACCCGGGTTCTATCC
CTGGGTAGGGAA
>Bos_taurus_chr21.trna620-CysACA (17240564-17240636) Cys (ACA) 73 bp Sc: 51.05
TTCCTGGTGGCTCAGATGGTAAAGTGTCTGCCTACAATGCAGGAGACCCGGGTTCAATCC
CTGGTCCGGGAAG
>Bos_taurus_chr3.trna6712-CysACA (76529865-76529794) Cys (ACA) 72 bp Sc: 51.56
TCCCTGGTGGCTCAGACGGTAAAGCATCTGCCTACAATGTGGGAGACCCAGGTTCGATCC
CTGGGTAGGGAA
>Bos_taurus_chr6.trna6317-CysACA (73807910-73807839) Cys (ACA) 72 bp Sc: 51.84
TTCCTGCTAGCTCAGACGGTAAAGCGTCTGCCTACAATGCAGGAGACCTGGGTTCGATCC
CTGGGTTGGAAA
>Bos_taurus_chrUn.004.1227.trna5-CysACA (31069-30998) Cys (ACA) 72 bp Sc: 51.86
TCCCTGGTGGCTCAGACAGTAAAGCGTCTGCCTACAATGCAGGAGACCCAGGTTCAATCC
CTGGTTCGGGAA
>Bos_taurus_chr1.trna1919-CysACA (54828299-54828370) Cys (ACA) 72 bp Sc: 52.51
TCCCTGGTGGCTCAGATGGTAAAGCGTCTACCTACAATGCGGGAGACCCAGGTTCGATCC
CTGGGTAGGGAA
>Bos_taurus_chr11.trna5039-CysACA (100432991-100432920) Cys (ACA) 72 bp Sc: 52.56
TCCCTGGTGGCTCAGACGGTAAAGCGTCTGCCTACAATGTAAGAGACCCAGGTTCAATCC
CTGGCTCGGGAA
>Bos_taurus_chr20.trna4015-CysACA (45056284-45056213) Cys (ACA) 72 bp Sc: 52.67
TCCCTAGTGGCTCAGATGGTAAAGCATCTGTCTACAACACAGGAGACCCGGGTTCAATCC
CTGGGTGGGGAA
>Bos_taurus_chr20.trna4017-CysACA (45022266-45022195) Cys (ACA) 72 bp Sc: 52.67
TCCCTAGTGGCTCAGATGGTAAAGCATCTGTCTACAACACAGGAGACCCGGGTTCAATCC
CTGGGTGGGGAA
>Bos_taurus_chr28.trna1150-CysACA (32642087-32642158) Cys (ACA) 72 bp Sc: 52.94
TCCCTGGTGGCTCAGACGGTAAAGCATCTGCCTACAATGTAGGAGACCCAGGTTTGATCC
CTGGCTTGGGAA
>Bos_taurus_chr6.trna7920-CysACA (24235717-24235646) Cys (ACA) 72 bp Sc: 53.09
TCCCTGGTGGCTCAGACGGTAAAGCATCTGCCTACAATGCGGGAGACCCAGGTTCAACTC
CTGGGTAGGGAA
>Bos_taurus_chr7.trna3959-CysACA (100718197-100718268) Cys (ACA) 72 bp Sc: 53.62
TCCCTGGTGGCTCAGAGGGTAAAGCATCTGCCTACAATGCAGGAGACCTAGGTTCAATCC
CTGGGTAGGGAA
>Bos_taurus_chr7.trna6103-CysACA (60438286-60438214) Cys (ACA) 73 bp Sc: 53.78
TCCTTGGTGGCTCAGACGGTAAAAGCGTCTGCCTACAAAGTGGAAGACCCAGGTTCGATC
CCTGGGTAGGGAA
>Bos_taurus_chr5.trna9404-CysACA (23664052-23663981) Cys (ACA) 72 bp Sc: 54.01
TCCCTGGTGGCTCAGATGGTATAGCGTCTGCCTACAATGCAGGAGACCCAGGTTCAATCC
TTGGGTGGGGAA
>Bos_taurus_chr7.trna3837-CysACA (97751830-97751901) Cys (ACA) 72 bp Sc: 54.02
TCCCCGCTGGCTCAGATGGTAAAGTGTCTGCCTACAATGCGGGAGACCCAGGTTCAATCC
CTGGGTTGGGAA
>Bos_taurus_chr28.trna258-CysACA (6534562-6534633) Cys (ACA) 72 bp Sc: 54.36
TCCCTGGTGGCTCAGATGGTAAAGTGTCTGTCTACAATGTAGAAGACCCAGGTTCAATCC
CTGGTTTGGGAA
>Bos_taurus_chr2.trna4536-CysACA (129776591-129776662) Cys (ACA) 72 bp Sc: 54.72
TCCCTGGTGGCTCAGACGGTAAAGCATCTACCTACAATGCAGGAGACCCAGGTTCGATCC
CTGGGTGGGGAA
>Bos_taurus_chr15.trna4945-CysACA (35037882-35037811) Cys (ACA) 72 bp Sc: 54.89
TCCCTGGTAGCTCAGACAGTAAAGCATCTGCCTACAATGCAGGAGACCCAGGTTCGATCC

CTGGGCAGGGAA
>Bos_taurus_chr11.trna5800-CysACA (83557868-83557797) Cys (ACA) 72 bp Sc: 54.98
TCCCTGATGGCTTAGACAGTAAAGCGTCTGCCTACAACGCAGGAGACCCAGGTTCAATCC
CTGGGTAGGGAA
>Bos_taurus_chr21.trna4729-CysACA (23469845-23469774) Cys (ACA) 72 bp Sc: 54.99
TCCCTGGTGGCTCAGGTGGTAAAGCGTCTGTCTACAACACGGGAGACCCAGGTTCAATCC
CTGGGTGGGGAA
>Bos_taurus_chr27.trna3492-CysACA (15171191-15171119) Cys (ACA) 73 bp Sc: 55.05
TTCCTGGTGGCTCAGATGGTAAAGCATCTGCCTACAATGCAGGAGACCCAGGTTCAATCC
CTGGGTCGGGAAG
>Bos_taurus_chr23.trna1952-CysACA (43698653-43698724) Cys (ACA) 72 bp Sc: 55.05
TCCCTGGTGGCTCAGATGGTAAACCATCTGCCTACAATGCAGGAGACCCAGGTTCAATCC
CTGGCTCGGGAA
>Bos_taurus_chr13.trna3394-CysACA (78029367-78029438) Cys (ACA) 72 bp Sc: 55.07
TCCCTGGTGGCTCAGACGGTAAAGCATCTGCCTACAATGCAGGAGACCCAGGTTCAATCC
CTGGATCGGGAA
>Bos_taurus_chr17.trna342-CysACA (10873414-10873485) Cys (ACA) 72 bp Sc: 55.09
TCCTGGGTGGCTCAGATGGTAAAGCATCTGCCTACAATGCGGGAGACCCAGGTTCAATCC
CTGGGTCAGGAA
>Bos_taurus_chr3.trna9022-CysACA (11281344-11281272) Cys (ACA) 73 bp Sc: 55.10
TCCCTGGTGGCTCAGATGGTAAAAGCATCTGCCTACAATGCAGGAGACCCAGGTTCGATC
CCTGGGTTGGGAA
>Bos_taurus_chr18.trna4824-CysACA (29843658-29843587) Cys (ACA) 72 bp Sc: 55.12
TCCCTGGTGGCTCAGACGGTAAAGCGTCTGCCTACAATGCAGGAGATCCAGGTTCAATCC
CTGGGTTGGGAA
>Bos_taurus_chr10.trna1631-CysACA (43007612-43007683) Cys (ACA) 72 bp Sc: 55.12
TCCCTGGTGGCTCAGATGGTAAAGTATCTGCCTACAATGCGGGAGACCCAGGTTCAATCC
CTGGCTCGGGAA
>Bos_taurus_chr24.trna4137-CysACA (34242517-34242446) Cys (ACA) 72 bp Sc: 55.15
TCCCTGGTGGCTCAGATGGTAAAGTGTCTGCCTACACTGCAGGAGACCCAGGTTCGATCC
CTGGGTGGGGAA
>Bos_taurus_chr8.trna4493-CysACA (106369413-106369342) Cys (ACA) 72 bp Sc: 55.18
TCCCTGGTGGCTCAGAAGGTAAAGCGTCTGCCTACAATGCGGAAGACCCAGGTTCAATCC
CTGGGTAGGGAA
>Bos_taurus_chr3.trna5523-CysACA (109225919-109225849) Cys (ACA) 71 bp Sc: 55.20
CCCTGGTGGCTTAGAGGTTAAAGCGTCTGCCTACAATGCAGGAGACCCAGGTTCGATCCC
TGGATCAGGAA
>Bos_taurus_chr12.trna5008-CysACA (46968066-46967995) Cys (ACA) 72 bp Sc: 55.22
TCCCTGGTGGCTCAGACGGTAAAGCGTCTGCCTACAATGCAGGAGACCTAGGTTCGATCC
CTGGGTTGGGAA
>Bos_taurus_chr1.trna4130-CysACA (120212104-120212175) Cys (ACA) 72 bp Sc: 55.25
TCCCTGATGGCTCAGATGGTAAAGTATCTGCCTACAATGCAGGAGACCCAGGTTCAATCC
CTGGGTTGGGAA
>Bos_taurus_chr20.trna2515-CysACA (66621214-66621285) Cys (ACA) 72 bp Sc: 55.34
TCCCTGGTGGCTCAGACGGTAAAGTGTCTGTCTACAATGCAGGAGACCCAGGTTCAATCC
CTGGTTTGGGAA
>Bos_taurus_chr11.trna3473-CysACA (86064449-86064521) Cys (ACA) 73 bp Sc: 55.42
TCCCTGGTGGCTCAGATGGTAAAGCGTCTGCCTACAATGCAGGAGACCCAGGTTCAATTC
CTGTGTCAGGAAG
>Bos_taurus_chr27.trna3118-CysACA (23626669-23626597) Cys (ACA) 73 bp Sc: 55.42
TCCCTGGTGGCTCAGATGGTAAAGCGTCTGCCTACAATGCAGGAGACCCAGGTTCAATTC
CTGGGTCAGGAAG
>Bos_taurus_chr11.trna3035-CysACA (75492184-75492256) Cys (ACA) 73 bp Sc: 55.44
TCCCTGGTGGCTCAGATGGTAAAGCGTCTGCCTACAATGCAGGAGACCCGGGTTCAATCC
CCGGGTCAGGAAG
>Bos_taurus_chr3.trna2375-CysACA (67810792-67810863) Cys (ACA) 72 bp Sc: 55.48
ACCCTGCTGGCTCAGATGGTAAAGCATCTGCCTACAATGCAGGAGACCCAGGTTCAATCC
CTGGGTTGGGAA
>Bos_taurus_chr24.trna4879-CysACA (14728416-14728344) Cys (ACA) 73 bp Sc: 55.51
TCCCGGGTGGCCCAGACGGTAAAAGTGTCTGCCTACAAAGCAGGAGACCCAGGTTCGATC
CCTGGGTTGGGAA
>Bos_taurus_chr16.trna4914-CysACA (36933290-36933218) Cys (ACA) 73 bp Sc: 55.53
TCCCTGGTGGCTCAGATGGTAAAGTGTCTGCCTACAATGCAGAAGACCCAGGTTCAATCC
CTGGGTCAGGAAG
>Bos_taurus_chr15.trna730-CysACA (26171528-26171599) Cys (ACA) 72 bp Sc: 55.55
TCCCTGGTGGCTCAGACGGTAAAGCGTCTGTCTACAATGCAGGAGACCCAGGTTCGATCC
CTGGGTTGGGAA

>Bos_taurus_chr20.trna2678-CysACA (69672474-69672545) Cys (ACA) 72 bp Sc: 55.55
TCCCTGGTGGCTCAGACGGTAAAGCGTCTGTCTACAATGCAGGAGACCCAGGTTCGATCC
CTGGGTTGGGAA
>Bos_taurus_chr9.trna3449-CysACA (97456745-97456816) Cys (ACA) 72 bp Sc: 55.55
TCCCTGGTGGCTCAGACGGTAAAGCGTCTGTCTACAATGCAGGAGACCCAGGTTCGATCC
CTGGGTTGGGAA
>Bos_taurus_chrUn.004.1.trna223-CysACA (3358394-3358323) Cys (ACA) 72 bp Sc: 55.55
TCCCTGGTGGCTCAGACGGTAAAGCGTCTGTCTACAATGCAGGAGACCCAGGTTCGATCC
CTGGGTTGGGAA
>Bos_taurus_chr7.trna5308-CysACA (84091086-84091014) Cys (ACA) 73 bp Sc: 55.65
TTCCTGGTGGCTCAGATGGTAAAGCGTCTGCCTACAATGCAGGAGACCCAGGTTCTATTC
CTGGGCTGGGAAG
>Bos_taurus_chr20.trna5390-CysACA (9737053-9736982) Cys (ACA) 72 bp Sc: 55.65
TCCCTGGTGGCTCAGATGGTAAAGCATTTGCCTACAATGCAGGAGACCCAGGTTCGATCC
CTGGGTTGGGAA
>Bos_taurus_chr11.trna5384-CysACA (92113778-92113707) Cys (ACA) 72 bp Sc: 55.72
TCCCTGGTGGCTCAGATGGTAAAGCATCTGCCTACAATGCAGGAGACCCAGGTTCAATCC
CTGGGTCGGGAA
>Bos_taurus_chrX.trna259-CysACA (6336755-6336826) Cys (ACA) 72 bp Sc: 55.86
TCCCTGGTGGCTCAGGTGGTAAAGCGTCTGCCTACAATGCAGGAGATCCAGGTTCAATCC
CTGGGTCGGGAA
>Bos_taurus_chr20.trna3546-CysACA (60511129-60511058) Cys (ACA) 72 bp Sc: 55.87
TCCCTGGTGGCTCAGATGGTAAAGCCTCTGCCTACAATGCGGGAGACCCGGGTTCGATCC
CCGGGTAGGGAA
>Bos_taurus_chr15.trna3148-CysACA (82271794-82271722) Cys (ACA) 73 bp Sc: 55.97
TCCCTGATGGCTCAGATGGTAAAGCGTCTGCCTACAATGCAGGAGACCCAGGTTCGATCC
CTTGGTCAGGAAG
>Bos_taurus_chrUn.004.1072.trna2-CysACA (13175-13103) Cys (ACA) 73 bp Sc: 55.97
TCCCTGATGGCTCAGATGGTAAAGCGTCTGCCTACAATGCAGGAGACCCAGGTTCGATCC
CTTGGTCAGGAAG
>Bos_taurus_chr16.trna3946-CysACA (59571826-59571755) Cys (ACA) 72 bp Sc: 56.04
TCCCTGGTGGCTTAGATGGTAAAGCATCTGCCTACAATGCAGGAGACCCAGGTTCAATCC
CTGGGTCGGGAA
>Bos_taurus_chr1.trna4862-CysACA (139800422-139800493) Cys (ACA) 72 bp Sc: 56.07
TCCTTGGTGGCTCAGAGGGTAAAGCGTCTGCCTACAATGCAGGAGACCCAGGTTCGATCC
CTGGGTTGGGAA
>Bos_taurus_chr8.trna7731-CysACA (12407324-12407253) Cys (ACA) 72 bp Sc: 56.11
TCCCTGGTAGCTCAGATGGTAAAGCGTCTGCCTACAATGCAGGAGATCCAGGTTCGATCC
CTGGGTCGGGAA
>Bos_taurus_chr4.trna21-CysACA (696802-696873) Cys (ACA) 72 bp Sc: 56.26
TCCCTGGTGGCTCAGACGGTAAAGCGCCTGCCTACAATGCAGGAGACCCAGGTTCCATCC
CTGACTTGGGAA
>Bos_taurus_chr10.trna3893-CysACA (100696405-100696476) Cys (ACA) 72 bp Sc: 56.28
TCCCTGGTAGCTCAGATGGTAAAGCTTCTGCCTACAATGCAGGAGACCCAGGTTCAATCC
CTGGATTGGGAA
>Bos_taurus_chr10.trna5594-CysACA (70441808-70441737) Cys (ACA) 72 bp Sc: 56.29
TCCCTGGTGGCTCAGACGGTAAAGCGTCTGCCTACAATGCAGGAGACCCGGGTTCAATCC
CTGGGTAGGGAA
>Bos_taurus_chr26.trna1705-CysACA (44678169-44678241) Cys (ACA) 73 bp Sc: 56.34
TCCCTGGTGGCTCAGTTGGTAAAGCGTCTGTCTACAATGCAGGAGACCCAGGTTCGATCC
CTGGGTCAGGAAG
>Bos_taurus_chr8.trna499-CysACA (11652484-11652555) Cys (ACA) 72 bp Sc: 56.35
TCCCTGGTGGCTCAGATGGTAGAGCGTCTGCTTACAATGCAGGAGACCTGGGTTCAATCC
CCGGGTCGGGAA
>Bos_taurus_chr20.trna1838-CysACA (50378239-50378311) Cys (ACA) 73 bp Sc: 56.48
TCCCTGGTGGCTTAGATGGTAAAGCGTCTGCCTACAATGCAGGAGGCCCAGGTTCAAACC
CTGGGTCAGGAAG
>Bos_taurus_chr27.trna849-CysACA (26217827-26217898) Cys (ACA) 72 bp Sc: 56.61
TCCCTGATAGCTCAGTTGGTAAAGAATCTGCCTACAATGCAGGAGACCCAGGTTCGATTC
CTGGGTTGGGAA
>Bos_taurus_chr21.trna1185-CysACA (25935989-25936060) Cys (ACA) 72 bp Sc: 56.64
TCCCTGGTGGCTCAGATGGTAAAGCATCTGCTTACAATGCAGGAGACCCAGGTTCGATCC
CTGGGTTGGGAA
>Bos_taurus_chr9.trna4535-CysACA (93603111-93603040) Cys (ACA) 72 bp Sc: 56.68
TCCCTGATGGCTCAGATGGTAAAGCTTCTGCCTACAATGCAGGAGACCCAGGTTCAATCC
CTGGATTGGGAA
>Bos_taurus_chr18.trna5229-CysACA (21163365-21163294) Cys (ACA) 72 bp Sc: 56.72

TCCCTGGTGGCTCAGATGGTAAAGCATCTGCCTACAATGCAGGAGACCCAGGTTCAATCC
CTGGATTGGGAA
>Bos_taurus_chr4.trna2844-CysACA (89031809-89031880) Cys (ACA) 72 bp Sc: 56.77
TCCCTGGTGGCTCAGATGGTATAGCGTCTGCCTACAATGCAGGCGACCCAGGTTCAATCC
CTGGGTGGGGAA
>Bos_taurus_chrX.trna1779-CysACA (49251376-49251448) Cys (ACA) 73 bp Sc: 56.81
TCCCTGGTGGCTCAGATGGTAAAAGCGTCTGCCTACAATGCAGGAGACCCAGGTTCGATC
CCTGGGTTGGGAA
>Bos_taurus_chr2.trna5261-CysACA (139101590-139101519) Cys (ACA) 72 bp Sc: 56.85
TCCCTGGTGGCTCAGACGGTAAAGTGTCTGCCTACAATGCAGGAGACCCAGGTTCGATAC
CTGGGTTGGGAA
>Bos_taurus_chr2.trna3602-CysACA (110271651-110271722) Cys (ACA) 72 bp Sc: 56.96
TCCCTGGTGGCTCAGATGGTAAAGTGTCTGCCTACAATGCAGGAGACCCAGGTTCAATCC
CTGGGTTGGGAA
>Bos_taurus_chr11.trna1979-CysACA (49141615-49141686) Cys (ACA) 72 bp Sc: 56.98
TCCCTGGTGGCTCAGACGGTAAAGTGTCTGTCTACAATGCAGGAGACCCAGGTTCGATCC
CTGGGTGGGGAA
>Bos_taurus_chr14.trna218-CysACA (6231978-6232049) Cys (ACA) 72 bp Sc: 56.98
TCCCTGGTGGCTCAGACGGTAAAGCGTCCGCCTACAATGCGGGAGACCCAGGTTCAATCC
CTGGGTCGGGAA
>Bos_taurus_chr5.trna728-CysACA (22913433-22913504) Cys (ACA) 72 bp Sc: 57.04
TCCCTAGTGGCTCAGATGGTAAAGCATCTGCCTACAATGCAGGAGACCCAGGTTCAATCC
CTGGGTTGGGAA
>Bos_taurus_chr14.trna3913-CysACA (65217044-65216973) Cys (ACA) 72 bp Sc: 57.04
TCCCTGGTGGCTTAGATGGTAAAGCATCTGCCTACAATGCAGGAGACCCAGGTTCAATCC
CTGGGTTGGGAA
>Bos_taurus_chr5.trna1335-CysACA (37553135-37553206) Cys (ACA) 72 bp Sc: 57.08
TCCCTGGTGGCTCAGACGGTAAAGTGTCTGTCTACAATGCAGGAGACCCAGGTTCGATCC
CTGGTTTGGGAA
>Bos_taurus_chr3.trna6284-CysACA (89509523-89509452) Cys (ACA) 72 bp Sc: 57.22
TCCTTGGTGGCTCAGATGGTAAAGTGTCTGCCTACAATGCAGGAGACCCAGGTTCGATCC
CTGGGTCAGGAA
>Bos_taurus_chr2.trna6012-CysACA (126630426-126630355) Cys (ACA) 72 bp Sc: 57.22
TCCCTGGTGGCTCAGATGGTAAAGCATCTGCCTACAATGCAGGAGACCCAGGTTCAATCC
CTGGCTCAGGAA
>Bos_taurus_chrUn.004.255.trna6-CysACA (148545-148474) Cys (ACA) 72 bp Sc: 57.28
TCCCTGGTGGCTCAGATGGTAAAGTGTCTGTCTACAATGCAGGAGACCCAGGTTCAATTC
CTGGGTGGGGAA
>Bos_taurus_chrUn.004.10938.trna1-CysACA (541-613) Cys (ACA) 73 bp Sc: 57.30
TCCCTGGTGGCTCAGACGGTAAAAGCGTCTGCCTACAAAGCAGGAGACCCAGGTTCGATC
CCTGGGTCGGGAA
>Bos_taurus_chr5.trna6476-CysACA (98081855-98081784) Cys (ACA) 72 bp Sc: 57.74
TCCCTGGTGGCTCAGATGGTAAAGCGTCTGTCTACAATGCAGAAGACCCAGGTTCGAGCC
CTGGGTTGGGAA
>Bos_taurus_chr10.trna2717-CysACA (71777075-71777146) Cys (ACA) 72 bp Sc: 57.75
TCCCTTGTGGCTCAGACGGTAAAGCGTCTGCCTACAATGCAGGAGACCCAGGTTCGATCC
CTGGGTTGGGAA
>Bos_taurus_chr13.trna1834-CysACA (43676797-43676868) Cys (ACA) 72 bp Sc: 57.78
TCCCTGATGGCTCAGACGGTAAAGCGTCTGCCTACAATGCAGGAGACCCAGGTTCAATCC
CTGGGTTGGGAA
>Bos_taurus_chr13.trna7237-CysACA (10159138-10159067) Cys (ACA) 72 bp Sc: 57.84
TCCCTGGTGGCTCAGACGGTAAAGCGTCTGTCTACAACACAGGAGACCCAGGTTCGATCC
CTGGGTTGGGAA
>Bos_taurus_chr25.trna3630-CysACA (24737705-24737634) Cys (ACA) 72 bp Sc: 58.03
TCCCTGGTAGCTCAGATGGTAAAGCGTCTGCCTACAATGCAGGAGACCCAGGTTCAATCC
CTGGGTTGGGAA
>Bos_taurus_chr3.trna5560-CysACA (108166378-108166307) Cys (ACA) 72 bp Sc: 58.03
TCCCTGGTGGCTCAGACGGTAAAGCGTCTGCCTACAATGCAGGAGACCCGGGTTCGATCC
CTGGGTAGGGAA
>Bos_taurus_chr3.trna7877-CysACA (39238088-39238017) Cys (ACA) 72 bp Sc: 58.16
TCCCTGGTGGCTCAGATGGTAAAGTATCTGCCTACAATGCAGGAGACCCAGGTTCAATCC
CTGGATGGGGAA
>Bos_taurus_chr27.trna719-CysACA (22849776-22849848) Cys (ACA) 73 bp Sc: 58.36
TCCCTGGTGGCTCAGGTGGTAAAGTGTCTGCCTACAATGCAGAAGACCCAGGTTCGATCC
CTGGGTCAGGAAG
>Bos_taurus_chr1.trna8292-CysACA (88193512-88193441) Cys (ACA) 72 bp Sc: 58.43
TCCCTGGTGGCTCAGATGGTAAAGCGTCTGCCTACAATGCAGGAGACCCAGGTTCAATCC

CTGGGTTGGGAA
>Bos_taurus_chr18.trna2610-CysACA (57020482-57020553) Cys (ACA) 72 bp Sc: 58.43
TCCCTGGTGGCTCAGATGGTAAAGCGTCTGCCTACAATGCAGGAGACCCAGGTTCAATCC
CTGGGTTGGGAA
>Bos_taurus_chr2.trna1324-CysACA (40711640-40711711) Cys (ACA) 72 bp Sc: 58.43
TCCCTGGTGGCTCAGATGGTAAAGCGTCTGCCTACAATGCAGGAGACCCAGGTTCAATCC
CTGGGTTGGGAA
>Bos_taurus_chr16.trna5036-CysACA (33845276-33845205) Cys (ACA) 72 bp Sc: 58.45
TCCCTGATGGCTCAGATGGTAAAGCATCTGCCTACAATGCAGGAGACCCAGGTTCGATCC
CTGGGTTGGGAA
>Bos_taurus_chr2.trna9903-CysACA (17664027-17663956) Cys (ACA) 72 bp Sc: 58.45
TCCCTGGTGGCTCAGATGGTAAAGCATCTGCCTACAATGCAGGAGACCCAGGTTCGATCC
CTGGGTTGGGAA
>Bos_taurus_chr4.trna3378-CysACA (101675487-101675558) Cys (ACA) 72 bp Sc: 58.45
TCCCTGGTGGCTCAGATGGTAAAGCATCTGCCTACAATGCAGGAGACCCAGGTTCGATCC
CTGGGTTGGGAA
>Bos_taurus_chr4.trna4637-CysACA (117548974-117548903) Cys (ACA) 72 bp Sc: 58.45
TCCCTGGTGGCTCAGATGGTAAAGCATCTGCCTACAATGCAGGAGACCCAGGTTCGATCC
CTGGGTTGGGAA
>Bos_taurus_chr6.trna1885-CysACA (64857606-64857677) Cys (ACA) 72 bp Sc: 58.45
TCCCTGGTGGCTCAGATGGTAAAGCATCTGCCTACAATGCAGGAGACCCAGGTTCGATCC
CTGGATTGGGAA
>Bos_taurus_chr5.trna3783-CysACA (100034458-100034529) Cys (ACA) 72 bp Sc: 58.62
TCCCTGGTGGCTCAGATGGTAAAGTGTCTGCCTACAAAGCAGGAGACCCAGGTTCAATCC
CTGGGTTGGGAG
>Bos_taurus_chr4.trna7208-CysACA (52772366-52772294) Cys (ACA) 73 bp Sc: 58.67
TTCCTGATGGCTCAGATGGTAAAGCGTCTGCCTACAATGCAGGAGACCCAGGTTCAATCC
CTGGGTTGGGAAG
>Bos_taurus_chr7.trna4200-CysACA (108113772-108113843) Cys (ACA) 72 bp Sc: 58.72
TCCCTGGTGGCTTAGATGGTAAAGCACCTGCCTACAATGCGGGAGACCCAGGTTCAATCC
CTGGGTAGGGAA
>Bos_taurus_chr23.trna4663-CysACA (4044336-4044265) Cys (ACA) 72 bp Sc: 58.93
TCCCTGGTGGCTCAGATGGTAAAGTGTCTGCCTACAATGCAGAAGACCCAGGTTCAATCC
CTGGGTCGGGAA
>Bos_taurus_chr3.trna1965-CysACA (54265750-54265821) Cys (ACA) 72 bp Sc: 59.14
TCCCTGGTGGCTCAAATGGTAAAGTGTCTGCCTACAATGCAGGAGACCCAGGTTCGATCC
CTGGTTTGGGAA
>Bos_taurus_chrUn.004.856.trna5-CysACA (35532-35603) Cys (ACA) 72 bp Sc: 59.55
TCCCTGCTGGCTCAGATGGTAAAGCATCTGCTTACAATGCAGGAGACCCAGGTTCGATCC
CTGGATGGGGAA
>Bos_taurus_chr22.trna2124-CysACA (58232940-58233011) Cys (ACA) 72 bp Sc: 59.82
TCCCTGGTGGCTCAGATGGTAAAGCGTCTGCCTACAATGCAGGAGACCCAGGTTCAATTC
CTGGGTTGGGAA
>Bos_taurus_chr1.trna3063-CysACA (89404997-89405068) Cys (ACA) 72 bp Sc: 59.88
TCCCTGTTGGCTCAGAGGTTAAAGCATCTGCCTACAATGCAGGAGACCCAGGTTCGATCC
CTGGGTTGGGAA
>Bos_taurus_chr27.trna2386-CysACA (36964778-36964707) Cys (ACA) 72 bp Sc: 59.91
TCCCTGGTGGCTCAGACGGTAAAGCGTCTGCCTACAATGCAGGAGACCCAGGTTCGATTC
CTGGGTCGGGAA
>Bos_taurus_chr27.trna2387-CysACA (36959730-36959659) Cys (ACA) 72 bp Sc: 59.91
TCCCTGGTGGCTCAGACGGTAAAGCGTCTGCCTACAATGCAGGAGACCCAGGTTCGATTC
CTGGGTCGGGAA
>Bos_taurus_chr15.trna3917-CysACA (63712041-63711970) Cys (ACA) 72 bp Sc: 60.17
TCCCTGGTGGCTCAGATGGTAAAGCGTCTGCCTACAATGCAGGAGACCCAGGTTCGATCC
CTGGGTTGGGAA
>Bos_taurus_chr7.trna5484-CysACA (78460105-78460034) Cys (ACA) 72 bp Sc: 60.35
TCCCTGGTGGCTTAGATGGTAAAGCATCTGCCTACAGTGCAGAAGACCCAGGTTCGATCC
CTGGGTTGGGAA
>Bos_taurus_chrUn.004.618.trna10-CysACA (233-162) Cys (ACA) 72 bp Sc: 60.35
TCCCTGGTGGCTTAGATGGTAAAGCATCTGCCTACAGTGCAGAAGACCCAGGTTCGATCC
CTGGGTTGGGAA
>Bos_taurus_chr29.trna3393-CysACA (18604248-18604177) Cys (ACA) 72 bp Sc: 60.71
TCCCTTGTGGCTCAGACGGTAAAGCGTCTGCCTACAATGCAGAAGACCCAGGTTCGATCC
CTGGGTTGGGAA
>Bos_taurus_chrUn.004.80.trna13-CysACA (33763-33691) Cys (ACA) 73 bp Sc: 60.77
TCCCGGGTGGCTCAGATGGTAAAAGCGTCTGCCTACAAAGCGGAAGACCCAGGTTCGATC
CCTGGGTCGGGAA

>Bos_taurus_chr7.trna5757-CysACA (70951296-70951225) Cys (ACA) 72 bp Sc: 61.18
TCCCTTGTAACTCAGTTGGTAAAGAACCTGCCTACAATGCAGGAGACCCAGGTTCGATTC
CTGGTTTGGGAA
>Bos_taurus_chr11.trna838-CysACA (19843145-19843216) Cys (ACA) 72 bp Sc: 61.45
TCCCTGGTGGCTCAGATGGTAAAGCATCTGCCTACAATGCAGGAGACCCAGGTTCGATCC
CTGGTTTGGGAA
>Bos_taurus_chr14.trna4497-CysACA (51204740-51204669) Cys (ACA) 72 bp Sc: 62.35
TCCCTGGTGGCTCAGACGGTAAAGTGTCTGCCTACAATGCAGGAGACCCAGGTTCGATTC
CTGGGTGGGGAA
>Bos_taurus_chr16.trna5725-CysACA (18306130-18306059) Cys (ACA) 72 bp Sc: 63.00
TCCCTAGTGGCTCAGATGGTAAAGCATCTGCCTACAATGCAGAAGACCCAGGTTCAATCC
CTGGTTTGGGAA
>Bos_taurus_chr4.trna310-CysACA (9722292-9722363) Cys (ACA) 72 bp Sc: 63.17
TCCCTGGTGGCTCAGATGGTAAAGCGTCTGCCTACAATGCAGGAGACCCAGGTTCGATCC
CTGGTTTGGGAA
>Bos_taurus_chrUn.004.5340.trna1-CysACA (1219-1290) Cys (ACA) 72 bp Sc: 63.17
TCCCTGGTGGCTCAGATGGTAAAGCGTCTGCCTACAATGCAGGAGACCCAGGTTCGATCC
CTGGTTTGGGAA
>Bos_taurus_chr18.trna5402-CysGCA (17118450-17118378) Cys (GCA) 73 bp Sc: 23.90
TCCCTGGTGGCTCAGACAGTGAAGAATCCACCTGCAATGCAGGAGACCCAGGTTCGATCC
CTGGGTCAGGGAG
>Bos_taurus_chr23.trna3636-CysGCA (25114656-25114587) Cys (GCA) 70 bp Sc: 24.28
TTCATAGCTCAGGCAGTAAAGAACCTGCCTGCAATGCAGGAGACCCAGGTTCAATTCTTG
GGTTGGGAAG
>Bos_taurus_chr8.trna571-CysGCA (14044377-14044447) Cys (GCA) 71 bp Sc: 24.53
TCCCTGTTGGCTCAGATGGTAAAGAATCCACCTGCAATGTGGGAACCTGGGTTCAGTCCC
TGGGTGGGGAA
>Bos_taurus_chr15.trna4000-CysGCA (61518311-61518240) Cys (GCA) 72 bp Sc: 27.82
TCCCAGGTAACTCAGACAGTAGAGAATCTGTTTGCAATGTTGGAGACCTGGGTTCGATCC
CTAGGTTGGGAA
>Bos_taurus_chr7.trna8606-CysGCA (2422020-2421948) Cys (GCA) 73 bp Sc: 28.95
TTCCTGGTGGCTCAGATGGTAAAGAATCTGCCTGCAATGCAGGCAACCCAGGTTTGATCT
CTGGGTTGGGAAG
>Bos_taurus_chr12.trna2952-CysGCA (75250327-75250398) Cys (GCA) 72 bp Sc: 30.03
TCCCTGGTGGCTCAGATGATAAAGAATCTTCCTGCAATGTAGGAGACCTGGGTTTGAACC
CTGGCTAGGGAA
>Bos_taurus_chr12.trna1861-CysGCA (47396956-47397028) Cys (GCA) 73 bp Sc: 30.66
TCCCTGGTGGCTCATATGGTAAAGAATCTGCCTGCAATGCAGGAGATCCAGGTTCCATCC
CTGGGTCAGGAAG
>Bos_taurus_chr2.trna6562-CysGCA (116619052-116618982) Cys (GCA) 71 bp Sc: 32.23
CTCTGGTGTTTCAGATGGTAAAGAATCTGCCTGCAAAGAAGGAGACCCAGGTTCAATCCC
TGGGTCGGAAG
>Bos_taurus_chr3.trna7445-CysGCA (53671954-53671883) Cys (GCA) 72 bp Sc: 32.68
TCCCTGGTGGCTTAGAGGGTAAAGCCTCTGCCTGCAGTGTGGAAGTCCTGGGTTCAGTCC
CTGGCTTGGGAA
>Bos_taurus_chr19.trna3301-CysGCA (63445473-63445544) Cys (GCA) 72 bp Sc: 34.85
TCCCAGGTGGCTCAGACGATAAAGCATCCACCTGCAACGTGGGAAACCCGGGTTCAACCC
CTGGGTTGGGAA
>Bos_taurus_chrUn.004.295.trna6-CysGCA (122679-122751) Cys (GCA) 73 bp Sc: 34.94
TTTCTGGTGGCTCAGAGATTAAAGTGTCTGCCTGCAATGTGGAAGACCCAGGTTTGATCC
CTGGGTCGGAAAG
>Bos_taurus_chr4.trna4363-CysGCA (124124521-124124592) Cys (GCA) 72 bp Sc: 35.26
TCATGGCTGGCTCAGACGGTAAAGCATCTGCCTGCAGTGCAGAAAACCTGGGTTCAGTCC
CTGGGTTGGGAA
>Bos_taurus_chr9.trna5619-CysGCA (68221416-68221345) Cys (GCA) 72 bp Sc: 35.45
TCCCAGGTGGCTCAGACCGTAAAGCATCTTCCTGCAAGGCAGGAGAGCCAGGTTCAATTC
CTGGGTTGGGAA
>Bos_taurus_chr17.trna6159-CysGCA (15821594-15821523) Cys (GCA) 72 bp Sc: 35.61
TTCCTGGTAGCTCAAAGGGTAAAGCATCTGCCTGCAATGAAGGAGACCCAGGTTTGATCC
CTGGGTGGGGAA
>Bos_taurus_chr17.trna1344-CysGCA (36023165-36023236) Cys (GCA) 72 bp Sc: 36.23
TTCCGGCTGGCTCACATGGCAAAGTATCTGCCTGCAATGCAGAAGACCCGGGTTCAACCC
CTGGGTCAGGAA
>Bos_taurus_chr2.trna341-CysGCA (12200671-12200742) Cys (GCA) 72 bp Sc: 37.11
TCCCTGGTGGTCCAGTGGTTAAGATTCTGACTTGCAATGTAAGAGACGCCGGTTTGATCC
TGGTCCAGGAGG
>Bos_taurus_chr6.trna3705-CysGCA (110873349-110873420) Cys (GCA) 72 bp Sc: 37.85

TCCTGGCTGGCTCAGACGGTAAAGCATCTGTCTGCAATGTGGGAGACCCAGGTTCCATCT
CTGGGTCAGGAA
>Bos_taurus_chr21.trna501-CysGCA (14646073-14646144) Cys (GCA) 72 bp Sc: 37.94
TCCCTGGTGGCTCAGACGGTAAAGCATCTGCCTGCAATGCAGGAGACTCGGGTTTTATCC
CTGATTTGGGAA
>Bos_taurus_chr6.trna7056-CysGCA (51496964-51496893) Cys (GCA) 72 bp Sc: 38.23
TCCCTGGTGGCTCAAATGGTAAAGTATCTACCTGCAATGCAGGAGACCCAGGTTCAATCT
TTGGGTGGGGAA
>Bos_taurus_chrX.trna2556-CysGCA (69534781-69534852) Cys (GCA) 72 bp Sc: 38.35
TCCCAGGTAGCTCAGCTGGTATAGAATCTGCCTGCAATGCAGGAGGCCCTGGTTTGATTT
TGGGGTTGGGAA
>Bos_taurus_chr9.trna80-CysGCA (5522597-5522668) Cys (GCA) 72 bp Sc: 38.41
TCCCTGGTGGCTCAGAGGATAAAGTGTCTTCCTGCAATGAAGGAGACCCGGGTTTGATCC
CTGGGTTGGGAA
>Bos_taurus_chr13.trna1213-CysGCA (30479726-30479794) Cys (GCA) 69 bp Sc: 38.78
GACACTTTTAGCTCAGTCGGTAAAGAATCTGCCTGCAATGCAGGAGACCTAGGTTCGATT
CCTAGGTCA
>Bos_taurus_chr13.trna6760-CysGCA (21896852-21896781) Cys (GCA) 72 bp Sc: 39.12
TCCCTGGTGGCTGAGATGGTAAAGAATCCTCCTGCAATGCAGAAGACCCAGGTTTGATCC
CTGGGTGGGGAA
>Bos_taurus_chr15.trna1660-CysGCA (48510367-48510438) Cys (GCA) 72 bp Sc: 39.45
TCCCTGGTAGCTCAGAGGGTAAAGCATCTGCCTGCAATGCAGGAGACCTAGGCTTGATCC
CTGGTTTGGGAA
>Bos_taurus_chr2.trna7825-CysGCA (82936923-82936852) Cys (GCA) 72 bp Sc: 39.50
TCCCTGCTGGCTCAGACTGTAAAGCATCTGCTTGCAATGCAGGAGATCTGGGTTCAAATC
CTGGGTTGGGAA
>Bos_taurus_chr11.trna5676-CysGCA (85931140-85931069) Cys (GCA) 72 bp Sc: 40.91
TCCCTGGTGGCTCAAAGGGTGAAGCATCTGCCTGCAGTGCAGGAGACTCAGGTTCAATCC
CTGGTTTGGGAA
>Bos_taurus_chr12.trna4535-CysGCA (57709374-57709303) Cys (GCA) 72 bp Sc: 40.94
TCCCAAGTGGCTTAGATGGTCAAGAATCTGTCTGCAATGCAGGGGACCCAGGTCCAATTC
CTGGCTTGGGAA
>Bos_taurus_chrUn.004.21.trna63-CysGCA (522058-521989) Cys (GCA) 70 bp Sc: 40.94
TCCCTGGTGGCTTAGATGGTAAGGAATCTGCCTGCAATGTAGGACCCAGGTTCAATCCCT
GGTCAGGGAA
>Bos_taurus_chr6.trna6076-CysGCA (82351732-82351661) Cys (GCA) 72 bp Sc: 41.26
TCCCTGGTGGCTCAAACAGTAAAGCGTCTGCCTGCAATGCGGGAGACCCAGGTTCTATCC
CTGGGCGGGGAA
>Bos_taurus_chr14.trna532-CysGCA (12183451-12183522) Cys (GCA) 72 bp Sc: 41.42
TCCCTGGTGGTCCAGTGGTTAAGAATCCACCTTGCAGTGGAGGGGACTCAGGTTTGATCC
CTGGTTGGGGAA
>Bos_taurus_chrUn.004.1288.trna8-CysGCA (3690-3619) Cys (GCA) 72 bp Sc: 41.50
TCCCTGCTGGCTCAGACAGTAAAGCGTCTGTCTGCAATGCAGGAGACCCAGGTTCCATCC
CCGGGTGGGGAA
>Bos_taurus_chrUn.004.9.trna28-CysGCA (197850-197921) Cys (GCA) 72 bp Sc: 41.50
TCCCTGCTGGCTCAGACAGTAAAGCGTCTGTCTGCAATGCAGGAGACCCAGGTTCCATCC
CCGGGTGGGGAA
>Bos_taurus_chr9.trna1721-CysGCA (53205039-53205110) Cys (GCA) 72 bp Sc: 41.75
TCCTAGCTGGCTCAGATGGTAAAGAATCTGCCTGCAATGCAGGAGACCTGGGTTCGATCT
CTGGGTTGGGAA
>Bos_taurus_chr25.trna2582-CysGCA (41035211-41035139) Cys (GCA) 73 bp Sc: 42.02
TCCCTGGTGGCTCAGATGGTAAAGCGTCTGCCTGCAATGCAGGAGACTCAGGTTCAGTCC
CTGGGTCAGGAAG
>Bos_taurus_chr19.trna1911-CysGCA (38777695-38777766) Cys (GCA) 72 bp Sc: 42.27
TCTCAGGTGGCTCAGACAGTAAAGCGTCTGCTTGCAATGCAGAAGACCCGGGTTCAACCC
CTGGGTTGGGAA
>Bos_taurus_chr20.trna3825-CysGCA (51893345-51893274) Cys (GCA) 72 bp Sc: 42.55
TCCCTGGTGGCTGAGAGGGTAAAGCGTCTGCCTGCATTGCAGAAAACCCGGGTTCCATCC
CTGGGTAGGGAA
>Bos_taurus_chr5.trna595-CysGCA (18595453-18595525) Cys (GCA) 73 bp Sc: 42.58
TTCCTGGTGGCTCAGAGGATAAAGCATCTGCCTGCAAGGCAGGAGACCCAGGTTCTATCC
CTGGGTTGGGAAG
>Bos_taurus_chr9.trna5793-CysGCA (63559635-63559565) Cys (GCA) 71 bp Sc: 42.59
TCCCTGGTGGTCCAGTGGTTAAGACTCATGCTTGCAAAGCAGGGGCTCTGGGTTGAACCC
TGGTCAGGGAA
>Bos_taurus_chr26.trna1964-CysGCA (50180584-50180655) Cys (GCA) 72 bp Sc: 42.67
TCCCTGGTGGCTCAAATGGTAGAGCGTCTGCCTGCAGTGTGGGAGACCTAGGTTTGATCC

CTGGGTAGGGAA
>Bos_taurus_chr20.trna3926-CysGCA (48823704-48823632) Cys (GCA) 73 bp Sc: 43.37
TCCCTCCTGGCTCAGATGGTAAAAGCATCTGCTTGCAATGAGGGAGACCCAGGTTCGATC
CCTGGGTGGGGAA
>Bos_taurus_chr11.trna343-CysGCA (6062415-6062486) Cys (GCA) 72 bp Sc: 43.39
TCCCTGCTAGCTCAGTTGGTAAAGAATCTGTCTGCAATGAAGAAGACCTGGGTTCAATTC
CTGGGTGGGGAA
>Bos_taurus_chrUn.004.1.trna352-CysGCA (1183576-1183505) Cys (GCA) 72 bp Sc: 43.45
TCCCTGGTAGCTCAGATGATAAAGAATCTGCCTGCAAAGCAGGAGACCCAGGTTCAATCC
CTGGGTTGGGAA
>Bos_taurus_chrUn.004.9.trna177-CysGCA (335653-335582) Cys (GCA) 72 bp Sc: 43.78
TCCCCAGTGGCTCAGACAGTAAAGCATCTGCCTGCAATGCAGGACACCCAGGTTTGATCC
CTGGCTTGGGAA
>Bos_taurus_chr12.trna6929-CysGCA (916773-916702) Cys (GCA) 72 bp Sc: 43.90
TCCCTGGTGGCTCAGACAGTAAAGCGTCTGCCTGCAATGTGGGAGACCCAGGTTCAATCC
CTGGGTAGGGAA
>Bos_taurus_chr6.trna1774-CysGCA (61916219-61916290) Cys (GCA) 72 bp Sc: 44.21
TCCCTAGTAGCTCAGATGGTAAAGCATCTGCCTGCAGTGCACGAGGCCCAGGTTCAGTCC
CTTGTTAGGGAA
>Bos_taurus_chr13.trna5206-CysGCA (57287426-57287355) Cys (GCA) 72 bp Sc: 44.25
TCCCTGCTGGCTCAGATGGTAAAGAATCTGCCTGCAATGCAGAAGACCTGGGTTTGATCC
CTGGGTGGGGAA
>Bos_taurus_chrUn.004.141.trna1-CysGCA (272441-272370) Cys (GCA) 72 bp Sc: 44.33
TCCCTGTTGGCTCAGAGGTTAAAGTGTCTGCCTGCAATGTGGCAGACCCAGGTTTGATCC
CTGGGTGGGGAA
>Bos_taurus_chr22.trna451-CysGCA (10110638-10110709) Cys (GCA) 72 bp Sc: 44.33
TCCCTGGTGGCTCAGAGGGCAAAGCATCTGCCTGCAATGCAGAAAACCTGGGTTTGATTC
CTGGCTTGGGAA
>Bos_taurus_chr21.trna5381-CysGCA (10931343-10931272) Cys (GCA) 72 bp Sc: 44.86
TCCCTGGTGGCTCAGATGGTAAAGAACCTACCTGCAACGCAGGAGACCCAGGTTCGATCT
TTGGTTAGGGAA
>Bos_taurus_chr12.trna1991-CysGCA (51026037-51026108) Cys (GCA) 72 bp Sc: 44.87
TCTCTGGTGGTCCAGTGGTTAAGAATCCACCTTGCAATGGAGGAGACACGGGTTCAATCC
CTGTTCAGGGAA
>Bos_taurus_chr11.trna6422-CysGCA (68450985-68450914) Cys (GCA) 72 bp Sc: 44.90
TCCCTGGTGGCTCAGACGGTAAAGCTTCTGCCTGCAATGTGGGAGACCCAGGTTTGATCC
CTGGGTAGGGAA
>Bos_taurus_chr25.trna4923-CysGCA (2290373-2290302) Cys (GCA) 72 bp Sc: 45.24
TCCCTGGTGGCTCAGACAGTACAGCATCTGTCTGCAATGCAGGAGACCCGGGTTCGATCC
CTGGCTTGGGAA
>Bos_taurus_chr10.trna4059-CysGCA (104386481-104386552) Cys (GCA) 72 bp Sc: 45.39
TCCCTGCTGGCTCAGACAGTAAGGAATCTGCCTGCAATGCAGGAGACCCAGGTTCAATCC
CTGGGTAGGGAA
>Bos_taurus_chr6.trna7896-CysGCA (24874097-24874026) Cys (GCA) 72 bp Sc: 45.42
TCCCTGGTGGCTCAAACGGTAAAGAATCTGCCTGCAATGCAGGAGACCCAGGTTCAATCC
CTGGGTCGGGAA
>Bos_taurus_chr5.trna6383-CysGCA (100487464-100487393) Cys (GCA) 72 bp Sc: 45.61
TCCCTGCTGGCTCAGTTGGTAAAGAATCTGCCTGCAGTGCAGGAGACCTGGGTTTGATCC
CTGGGTAGGGAA
>Bos_taurus_chr27.trna1679-CysGCA (43264578-43264649) Cys (GCA) 72 bp Sc: 45.66
TCCCTGTTTGCTCAGAGGGTAGAGCATTTGCCTGCAATGCAGGAGACCCAGGTTTGATTC
CTGGGTTGGGAA
>Bos_taurus_chr9.trna4400-CysGCA (96692637-96692566) Cys (GCA) 72 bp Sc: 45.97
TCCCTGGTGGCTCAGATGGTAAAGCATCTGCTTGCAATGCAGGAGACCCAGGTTCCATCT
CTGGGTGGGGAA
>Bos_taurus_chrUn.004.7145.trna2-CysGCA (986-1057) Cys (GCA) 72 bp Sc: 45.97
TCCCTGGTGGCTCAGATGGTAAAGCATCTGCTTGCAATGCAGGAGACCCAGGTTCCATCT
CTGGGTGGGGAA
>Bos_taurus_chr6.trna2312-CysGCA (76325823-76325894) Cys (GCA) 72 bp Sc: 46.31
TTCCTGGTGGCTCAGACAGTAAAGTGACTGCCCGCAATGCAGGAGACCCAGGTTCAATCC
CTGGTTGGGAAG
>Bos_taurus_chr15.trna2447-CysGCA (68749146-68749217) Cys (GCA) 72 bp Sc: 46.65
TCCCAAGTGGCTCAGACAGTAAAGCGTCTGTCTGCAATGCAGGAGACCCGGGTTCGATCC
CTGGGTTGGGAA
>Bos_taurus_chr2.trna2159-CysGCA (68692755-68692826) Cys (GCA) 72 bp Sc: 46.73
TCCCTGGTGGCTCAGACAGTAAAGCGTCTGCCTGCAATGCAGGCAACCCGGGTTCGATCC
CTGGCTTGGGAA

>Bos_taurus_chr5.trna3591-CysGCA (96029533-96029615) Cys (GCA) 83 bp Sc: 46.75
TCCCTGGTGGCTCAGATGGTAAAGCGTCTGCCTGCAGGGCAGATCTAACCAGGGAGACCC
AGGTTCAATCCCTGGGTGGGGAA
>Bos_taurus_chr6.trna2755-CysGCA (92053973-92054044) Cys (GCA) 72 bp Sc: 46.82
TCCTGAGTGGCTCAGACAGGAAAGTGTCTGTCTGCAATGCAGGAGACCTAGGTTCGATCC
CTGGCTTGGGAA
>Bos_taurus_chr18.trna5579-CysGCA (11189985-11189915) Cys (GCA) 71 bp Sc: 47.20
TCCCCGCTGGTTCAGCGGTAAAGAATCCGCCTGCAATGCAGGAGACCCAGGTTCAACCCC
TGGGTGGGGAA
>Bos_taurus_chr15.trna1652-CysGCA (48295526-48295597) Cys (GCA) 72 bp Sc: 47.23
TTCCTGATAGCTCAGCTAGTAAAGAATCTGCCTGCAATGCAGGAGACCCTGGTTCGATTC
TGGGTTGGGAAG
>Bos_taurus_chr15.trna1673-CysGCA (49237284-49237355) Cys (GCA) 72 bp Sc: 47.23
TTCCTGATAGCTCAGCTAGTAAAGAATCTGCCTGCAATGCAGGAGACCCTGGTTCGATTC
TGGGTTGGGAAG
>Bos_taurus_chrUn.004.123.trna2-CysGCA (59776-59847) Cys (GCA) 72 bp Sc: 47.23
TTCCTGATAGCTCAGCTAGTAAAGAATCTGCCTGCAATGCAGGAGACCCTGGTTCGATTC
TGGGTTGGGAAG
>Bos_taurus_chrUn.004.26.trna18-CysGCA (624197-624126) Cys (GCA) 72 bp Sc: 47.23
TTCCTGATAGCTCAGCTAGTAAAGAATCTGCCTGCAATGCAGGAGACCCTGGTTCGATTC
TGGGTTGGGAAG
>Bos_taurus_chrUn.004.743.trna3-CysGCA (36290-36219) Cys (GCA) 72 bp Sc: 47.23
TTCCTGATAGCTCAGCTAGTAAAGAATCTGCCTGCAATGCAGGAGACCCTGGTTCGATTC
TGGGTTGGGAAG
>Bos_taurus_chr11.trna5232-CysGCA (95748843-95748773) Cys (GCA) 71 bp Sc: 47.58
TCCCAGCTGGCTCAGGGGTAAAGAATCAGCCTGCAATGCAGGAGGTGCAGGTTGGATCCC
TGCGTTGGGAA
>Bos_taurus_chr14.trna6228-CysGCA (10340680-10340609) Cys (GCA) 72 bp Sc: 47.84
TCCCTGGTAGCTCAGATGGTAAAGAATCTGCCTGCAGTGCAGGAGACCCAGGTTCAATCC
CTGGGTTGGGAA
>Bos_taurus_chr21.trna2339-CysGCA (57872394-57872465) Cys (GCA) 72 bp Sc: 48.39
TCCCTGCTGGCTTAGATGGTAGAGTGTCTGCCTGCAGTGTGGGAGTCCTAGGTTCGATCC
CTGGGTCGGGAA
>Bos_taurus_chrUn.004.1.trna25-CysGCA (517292-517363) Cys (GCA) 72 bp Sc: 48.39
TCCCTGCTGGCTCAGCTGGTAAAGAATCCGCCTGCAATGTGGAAGACCTGGGTTCGATCC
CTGGGTGGGGAG
>Bos_taurus_chr9.trna2965-CysGCA (87613626-87613697) Cys (GCA) 72 bp Sc: 48.47
TCCCTGCTGGCTCAGACAGTAAAGTGTCTGCCTGCAATGCGGGAGACCCAGGTTCGATCC
CTGGGTTGGGAA
>Bos_taurus_chr27.trna1472-CysGCA (38651309-38651380) Cys (GCA) 72 bp Sc: 48.49
TCCCTGGTGGCTCAGAAGGTAAAGCGTCTGCCTGCAATACAGGAGACCCAGGTTCAACCC
CTGGTTTGGGAA
>Bos_taurus_chr9.trna5015-CysGCA (83718951-83718880) Cys (GCA) 72 bp Sc: 48.62
TCCCTGGTGGCTCAGACGGTAAAGCGTCTGCCTGCAATGCAGGGGACCCGGGTTCAGTCC
CTGGCTTGGGAA
>Bos_taurus_chr1.trna4396-CysGCA (127960327-127960398) Cys (GCA) 72 bp Sc: 48.88
TCTTTGTTGGCTCAGATGGTAAAGAACCTGCCTGCAATGTGGGAGACCCAGGTTCGATCC
CTGGATAAGGAA
>Bos_taurus_chr8.trna2061-CysGCA (65683625-65683696) Cys (GCA) 72 bp Sc: 48.89
TCCCTAGTAGCTCAGATGGTAAAGTATCTGCCTGCAGTGCAGGAGACCCAGGTTTGATCC
CTGGGTTGGGAA
>Bos_taurus_chr13.trna6888-CysGCA (18998027-18997956) Cys (GCA) 72 bp Sc: 50.05
TCCCTAGTGGCTCAAAGGGTAAAGCATCTGCATGCAATGCAGGAGACCTGGGTTCGATCC
CCAGGTAGGGAA
>Bos_taurus_chr12.trna5736-CysGCA (27063651-27063580) Cys (GCA) 72 bp Sc: 50.20
TCCCTGGTGGCTCAGACGGTAAAGCGTCTGCCTGCAGTGCAGGAGACCTAGGTTTGATCC
CTGGTTTGGGAA
>Bos_taurus_chr4.trna1584-CysGCA (49924983-49925054) Cys (GCA) 72 bp Sc: 50.64
TCCCTGGTGGCTCAAATGGTAAAGAATCCGCCTGCAGTGCAGGAGACCCAGGTTCAATCC
CTGGCTAGGGAA
>Bos_taurus_chr17.trna6238-CysGCA (14344481-14344410) Cys (GCA) 72 bp Sc: 51.33
TCCCTGGTGGCTCAGATGGTAAAGAATCTGCCTGCAATGCAGGAGATCCAGGTTTGATCC
CTGGCTGGGGAA
>Bos_taurus_chr10.trna5381-CysGCA (76065348-76065277) Cys (GCA) 72 bp Sc: 51.42
TCCCTGGTGGCTCAGACGGTAAAGAGTCTGCCTGCAGTGTGGGAGACCCAGGTTCGATCC
CTGGCTAGGGAA
>Bos_taurus_chr10.trna1002-CysGCA (22844952-22845023) Cys (GCA) 72 bp Sc: 51.54

TCTCAGGTGGCTCAGATGGTAAAGAATCTGTCTGCAATGCAGGAGACCCAGGTTCAATCC
CTGGCTTGGGAA
>Bos_taurus_chrUn.004.769.trna4-CysGCA (34429-34358) Cys (GCA) 72 bp Sc: 51.54
TCTCAGGTGGCTCAGATGGTAAAGAATCTGTCTGCAATGCAGGAGACCCAGGTTCAATCC
CTGGCTTGGGAA
>Bos_taurus_chr29.trna3642-CysGCA (11937377-11937306) Cys (GCA) 72 bp Sc: 51.56
TCCCTACTGGCTCAGTTGGTAAAGAATCTGCCTGCAATGCAGGAGACCTGGGTTCGATCC
CTGGGTAGGGAT
>Bos_taurus_chr13.trna46-CysGCA (1807663-1807734) Cys (GCA) 72 bp Sc: 51.84
TCCCTGGTGGCTCAAATGGTCAAGAATCTGCCTGCAATGCAGGAGACCCAGGTTCAATCC
CTGGCTAGGGAA
>Bos_taurus_chr19.trna3995-CysGCA (54187397-54187326) Cys (GCA) 72 bp Sc: 51.86
TCCCTGGTGGCTCAGAGGGTAAAGCATCTGCCTGCAATGCAGGAGACCCAGGTTTGATCC
CTGGGTGGGGAA
>Bos_taurus_chr28.trna555-CysGCA (13834705-13834776) Cys (GCA) 72 bp Sc: 51.98
TCCCTGGTGGCTCAGAGGTTAAAGCGTCTGCCTGCAATGCAGGAGACCTGGGTTTGATCC
CTAGGTAGGGAA
>Bos_taurus_chr9.trna5215-CysGCA (78013492-78013421) Cys (GCA) 72 bp Sc: 52.08
TCCCAGGTGGCTCAGATGGTAAAGTATCTGCCTGCATTGCAGGAGACCCAGGTTCAATCC
CTGGATTGGGAA
>Bos_taurus_chr10.trna5777-CysGCA (65681389-65681318) Cys (GCA) 72 bp Sc: 52.11
TTCCTGATAGCTCAGTTGGTAAAGAATCTGCCTGCAATGCAGGAGACTCTGGTTTGATTC
CGGGTTGGGAAA
>Bos_taurus_chr1.trna2592-CysGCA (75383010-75383081) Cys (GCA) 72 bp Sc: 52.48
TCCCAGGTGGCTCAGAGGTTAAAGCGTCTGCCTGCAATGCAGGAGACCTGGGTTCGATCT
CTAGGTTGGGAA
>Bos_taurus_chr7.trna3526-CysGCA (88915281-88915352) Cys (GCA) 72 bp Sc: 52.79
TCCCAGGTGGCTCAGAGGGTAAAGCATCTGCCTGCAATGCAGGAGACCTGGGTTCGATCC
CTAGGTTGGGAA
>Bos_taurus_chr9.trna3058-CysGCA (89442895-89442966) Cys (GCA) 72 bp Sc: 52.86
TCCCTGGTGGCTCAGACGGTCAAGCGTCTGCCTGCAATGCAGGTGACCCGGGTTCGATCC
CTGGCTTGGGAA
>Bos_taurus_chr19.trna319-CysGCA (10447852-10447923) Cys (GCA) 72 bp Sc: 52.98
TCCCTGGTGGCTCAGACGGTAAAGCGTCTGCCTGCAATGCGGGAGACCCAGGTTCTATTC
CTGGGTAGGGAA
>Bos_taurus_chr15.trna1576-CysGCA (45120872-45120943) Cys (GCA) 72 bp Sc: 53.02
TCCCTGGTGGCTCAGATGGTAAAGCATCTGCCTGCAATGCAGGGGACCTGGGTTCGACCC
CTAGCTTGGGAA
>Bos_taurus_chr17.trna990-CysGCA (24488675-24488746) Cys (GCA) 72 bp Sc: 53.05
TCCCTGATGGCTCAGAGGGTAAAGCATCTGTCTGCAATGCAGGAGACCCAGGTTCAATCC
CTGGGTGGGGAA
>Bos_taurus_chr15.trna2362-CysGCA (66472625-66472696) Cys (GCA) 72 bp Sc: 53.08
TCCCTGGTGGCTCAGACAGTAAAGCATCTGCCTGCAATGCAGGAGACCCAGGTTCGATCC
CTGGGTGGGGAA
>Bos_taurus_chr15.trna3810-CysGCA (65455683-65455612) Cys (GCA) 72 bp Sc: 53.26
TCCCTGGTGGCTTAGACGGTAAAGCGTCTGCCTGCAATGCAGGAGACCCGGGTACGATCC
CTGGCTTGGGAA
>Bos_taurus_chr23.trna2170-CysGCA (48220275-48220346) Cys (GCA) 72 bp Sc: 53.67
TCCCTGGTGGCTCAGAGGGTAAAGCGTCTGCCTGCAATGCAGGAGACCCAGGTTTGATCC
CTGGTTTGGGAA
>Bos_taurus_chr14.trna846-CysGCA (19575731-19575802) Cys (GCA) 72 bp Sc: 53.68
TCCCTGGTGGCTCAGATGGTAAAGAATCTGCCTGCAATGCAGGAGACCTAGGTTCGATCC
CTGGTTAGGGAG
>Bos_taurus_chr8.trna3965-CysGCA (112880979-112881050) Cys (GCA) 72 bp Sc: 54.33
TCCCAGGTGGCTCAGATGGTAAAGTGTCTGCCTGCAATGCAGAAGACCTGGGTTCAATCC
CTGGGTTGGGAA
>Bos_taurus_chr12.trna5122-CysGCA (41756012-41755941) Cys (GCA) 72 bp Sc: 54.33
TCCCTGCTGGCTCAGAGGTTAAAGTGTCTGCCTGCAATGCAGGAGACCTGGGTTCGATCC
CTGGGTTGGGAA
>Bos_taurus_chr21.trna1704-CysGCA (39375761-39375832) Cys (GCA) 72 bp Sc: 54.50
TCCCTGGTAGCTCAGTTGGTAAAGAATCTGCCTGCAATGCAGGAGACCCAGGTTTGATCC
CTGGTTGGGGAA
>Bos_taurus_chr1.trna10124-CysGCA (27445431-27445360) Cys (GCA) 72 bp Sc: 55.00
TCCCTGGTGGCTCAGATGGTACAGCATCTGCCTGCAATGCAGGAGACCCAGGTTCAATCC
CTGGGTTGGGAA
>Bos_taurus_chr14.trna3778-CysGCA (68854023-68853952) Cys (GCA) 72 bp Sc: 55.00
TCCCTGGTGGCTCAGATGGTAGAGAATCTGCCTGCAATGCAGGAGACCCAGGTTCGATCC

CTGGGTTGGGAA
>Bos_taurus_chr5.trna1709-CysGCA (48132571-48132641) Cys (GCA) 71 bp Sc: 55.00
TCCCTGGTGGCTCAGTGGTAAAGAAACTGCCTGCAATGCAGGAGACCCAGGTTCGATCCC
TGGTTCGGGAG
>Bos_taurus_chr12.trna4348-CysGCA (65210953-65210882) Cys (GCA) 72 bp Sc: 55.01
TCCCTGGTGGCTCAGAGGTTAAAGCGTCTGCCTGCAAGGCAGGAGACCCAGGTTCAATCC
CTGGGTTGGGAA
>Bos_taurus_chr22.trna1141-CysGCA (30570757-30570828) Cys (GCA) 72 bp Sc: 55.01
TCCCTGGTGGCTCAGATGGTAAAGTGTCTGTCTGCAATGCAGGAGACCCAGGTTCGATCC
CTGGGTTGGGAA
>Bos_taurus_chr8.trna6333-CysGCA (59702652-59702581) Cys (GCA) 72 bp Sc: 55.01
TCCCTGGTGGCTCAGATGGTAAAGTGTCTGTCTGCAATGCAGGAGACCCAGGTTCGATCC
CTGGGTTGGGAA
>Bos_taurus_chr26.trna1214-CysGCA (35565650-35565721) Cys (GCA) 72 bp Sc: 55.03
TCCCTGGTGGCTCAGATGGTAAAGCATCCGCCTGCAATGCGGGAGACCCAGGTTTGATCC
CTGGTTTGGGAA
>Bos_taurus_chr19.trna2125-CysGCA (43493821-43493891) Cys (GCA) 71 bp Sc: 55.05
TCCCAGCTGGCTCAGTGGTAAAGAATCTGCCTGCAGTGCAGAAGACTCAGGTTCAATCCC
TGGGTCGGGAA
>Bos_taurus_chr3.trna5128-CysGCA (118637573-118637501) Cys (GCA) 73 bp Sc: 55.06
TCCCTGGTGGCTCAGACGGTAAAGCGTCTGCCTGCAATGCAGGAGACCCAGGTTCAATTC
CTGGGTCAGGAAG
>Bos_taurus_chr24.trna522-CysGCA (15515548-15515619) Cys (GCA) 72 bp Sc: 55.12
TCCCTGATACCTCAGTTGGTAGAGAATCTGCCTGCAGTGCAGGAGACCCAGGTTTGATTC
CTGGGTGGGGAA
>Bos_taurus_chr9.trna5241-CysGCA (77589977-77589905) Cys (GCA) 73 bp Sc: 55.14
TCCCTGATAGCTCAGTTGGTAAAGCATCTGCCTGCAATGCAGGAGACTCTGGTTCGATTC
CTGGGTCAGGAAG
>Bos_taurus_chrX.trna329-CysGCA (7757885-7757956) Cys (GCA) 72 bp Sc: 55.15
TCCCTGATAGCTCAGTTGGTAAAGAATCTGCCTGCAATGCAGGAGACCCAGGTTCAATTC
CTGGGTTGGGAA
>Bos_taurus_chr29.trna3666-CysGCA (11259435-11259364) Cys (GCA) 72 bp Sc: 55.15
TCCCTGATAGCTCAGTTGGTAAAGAACCTGCCTGCAATGCAGGAGACCCTGGTTCAAATC
CTGGGTTGGGAA
>Bos_taurus_chr9.trna5629-CysGCA (67789528-67789457) Cys (GCA) 72 bp Sc: 55.16
TCCCTGGTGGCTTAGAGGGTAAAGCGTCTGCCTGCAATGCAGGAGACCCAGGTTCAATCC
CTGGGTCGGGAA
>Bos_taurus_chr11.trna5972-CysGCA (80122639-80122568) Cys (GCA) 72 bp Sc: 55.16
TCCCTGGTGGCTCAGATGGTAAAGCATCTGCCTGCAATGCTGGAGACCCAGGTTCAATCC
CTGGGTGGGGAA
>Bos_taurus_chr24.trna4965-CysGCA (12445458-12445387) Cys (GCA) 72 bp Sc: 55.17
TCCCTAGTAGTTCAGTTGGTAAAGCATCTGCCTGCAATGCAGGAGACCCAGGTTCAATCC
CTGGGTCGGGAA
>Bos_taurus_chrX.trna2224-CysGCA (62240764-62240835) Cys (GCA) 72 bp Sc: 55.19
TCCCTGCTGGCTCAGATGGTAAAGCTTCTGCCTGCAATGCAGGAGACCCAGGTTCAATCC
CTGGGTTGGAAG
>Bos_taurus_chr28.trna1712-CysGCA (40643923-40643852) Cys (GCA) 72 bp Sc: 55.20
TCCCTGGTGGCTCAGGCGGTAAAGCATCTGTCTGCAATGCAGGAGACCCAGGTTCGATCC
CTGGGTTGGGAA
>Bos_taurus_chr16.trna1191-CysGCA (33896376-33896447) Cys (GCA) 72 bp Sc: 55.21
TCCCTGGTAGCTCAGCTGGTAAAGAATCTGCCTGCAATGCAGAAGACCCTGGTTCGACTC
CTGGATAGGGAA
>Bos_taurus_chr20.trna1940-CysGCA (53643330-53643401) Cys (GCA) 72 bp Sc: 55.24
TCCCCACTGGCTCAGACGGTAAAGGATCTGCCTGCAAAGCAGGAGACCCAGGTTCAATCC
CTGGGTTGGGAA
>Bos_taurus_chr20.trna3794-CysGCA (53612579-53612508) Cys (GCA) 72 bp Sc: 55.24
TCCCCACTGGCTCAGACGGTAAAGGATCTGCCTGCAAAGCAGGAGACCCAGGTTCAATCC
CTGGGTTGGGAA
>Bos_taurus_chr20.trna3171-CysGCA (68047916-68047845) Cys (GCA) 72 bp Sc: 55.30
TCCCTCCTAGCTCAGTTGGTAAAGAATCTGCTTGCAATGCAGGAGACCCAGGTTCGATTC
CTGGGTTGGGAA
>Bos_taurus_chr10.trna2890-CysGCA (76749172-76749243) Cys (GCA) 72 bp Sc: 55.31
TCCCAGATAGCTCAGTTGGTAAAGAATCTGCCTGCAATGCAGGAGACCCCGGTTCGATTC
CTGGGTTGGGAA
>Bos_taurus_chr11.trna9152-CysGCA (795730-795659) Cys (GCA) 72 bp Sc: 55.32
TCCCTGATGGCTCAGTTGGTAAAGAACCTGCCTGCAATGCAGGAGACCCTGGTTCAATTC
CTGGGTTGGGAA

>Bos_taurus_chr12.trna3749-CysGCA (76604038-76603966) Cys (GCA) 73 bp Sc: 55.33
TCCCTGGTGGCTCAGATGGTAGAGCGTCTGCCTGCAATGCAGAAGACCCGGGTTCAATCC
CTGGGTCAGGAAG
>Bos_taurus_chr22.trna607-CysGCA (13821306-13821377) Cys (GCA) 72 bp Sc: 55.34
TCCCTGGTAGCTCAGTTGGTAAAGAATCTGCCTGCAGTGCAGAAGACCCAGGTTCAATCC
CTGGATTGGGAA
>Bos_taurus_chrX.trna5070-CysGCA (50625014-50624943) Cys (GCA) 72 bp Sc: 55.35
TCCCCAGTAGCTCAGTTGGTAAAGAATCTGCCTGCAATGCAGGAGGCCCAGATTCGATCC
CTGGGTGGGGAA
>Bos_taurus_chr14.trna6480-CysGCA (6071188-6071117) Cys (GCA) 72 bp Sc: 55.36
TCCCCGGTGGCTCAGACGGTAAAGCATCTGCCTGCAATGCAGGAGACCCAGGTTCAATCC
CTGGGTTGGGAA
>Bos_taurus_chr19.trna4667-CysGCA (42365979-42365909) Cys (GCA) 71 bp Sc: 55.37
TCCCTGATGACTCAGTGGTAAAGAATCTGCCTGCAATGCAGGAGACCCAGGTTCAATCCC
TGGGCTGGGAA
>Bos_taurus_chr11.trna6746-CysGCA (58906152-58906081) Cys (GCA) 72 bp Sc: 55.39
TCCCTGATGGCTCAGACGGTAAAGCGTCCTCCTGCAATGTGGAAGACCCAGGTTCGATCC
CTGGGTTGGGAA
>Bos_taurus_chrUn.004.206.trna14-CysGCA (114027-113955) Cys (GCA) 73 bp Sc: 55.40
TCCCTGATAACTAAGTTGGTAAAGAATCTGCCTGCAATGCAGGAGACCCCAGGTTCGATT
CCTGGGTTGGGAA
>Bos_taurus_chr23.trna1380-CysGCA (31215725-31215796) Cys (GCA) 72 bp Sc: 55.40
TCCCTGGTGGCTCAGATGGTAAAGCGGCTGCCTGCAATGCAGGAGACCCAGGTTCAATCC
CTGGGTTGGGAA
>Bos_taurus_chr15.trna2045-CysGCA (58572845-58572916) Cys (GCA) 72 bp Sc: 55.43
TCCCTGGTGGCTCAGAGGATAAAGCATCTGCTTGCAATGCAGGAGACCCAGGTTCGATCC
CTGGGTTGGGAA
>Bos_taurus_chr11.trna3801-CysGCA (92804804-92804875) Cys (GCA) 72 bp Sc: 55.43
TTCTGGGTGGCTCAGATGGTAAAGCATCTGCCTGCAATGCAGGAGACCCAGGTTCAATCC
CTGGGTCAGGAA
>Bos_taurus_chr29.trna169-CysGCA (5285675-5285745) Cys (GCA) 71 bp Sc: 55.49
TCCTTGGTGGCTCAGAGGTTAAAGCGTCTGCCTGCAATGCAGAGACCCAGGTTCAATCCC
TGGGTTGGGAA
>Bos_taurus_chrUn.004.11.trna42-CysGCA (319591-319520) Cys (GCA) 72 bp Sc: 55.50
TCCCTGGTGGCTCAGAGGATAAAGCATCTGCCTGCAATGCAGGAGACCCAGGTTCAATCC
CTGGGTTGGGAA
>Bos_taurus_chr25.trna1215-CysGCA (21858427-21858499) Cys (GCA) 73 bp Sc: 55.52
TCCCTGATAGCTCAGTTGGTAAAGAATCTGCCTGCAATGCAGGAGACCCTGGTTCAATTC
CTGGGTCAGGGAG
>Bos_taurus_chr11.trna3068-CysGCA (75982676-75982747) Cys (GCA) 72 bp Sc: 55.52
TCCCTGGTGGCTCAGATGGCAAAGCGTCTGTCTGCAATGCAGAAGACCCAGGTTCGATCC
CTGGGTTGGGAA
>Bos_taurus_chr15.trna1728-CysGCA (50563846-50563917) Cys (GCA) 72 bp Sc: 55.54
TCCCTTGTAGCTCAGTTGGTAAAGTATCTGCCTGCAATGCAGGAGACCCACGTTCAATTC
TTGGGTGGGGAA
>Bos_taurus_chr3.trna1510-CysGCA (40070137-40070208) Cys (GCA) 72 bp Sc: 55.54
TCCCTAGTGGCTCAGTTGGTAAAGCATTTGCCTGCAATGCGGGAGTCCCAGGTTCGATCC
CTGGGTTGGGAA
>Bos_taurus_chr10.trna4062-CysGCA (104406834-104406905) Cys (GCA) 72 bp Sc: 55.65
TCCCACATAGCTCAGTTGGTAAATCATCTGCCTGCAATGCAGGAGACCCAGGTTCAATTC
CTGGGTTGGGAA
>Bos_taurus_chrUn.004.707.trna9-CysGCA (12785-12713) Cys (GCA) 73 bp Sc: 55.65
TCCCTGGTGGCTCAGTCGGTAAAGCGTCTGCCTGCAATGCAGGAGACCTAGGTTCGATCC
CTGGGTCAGGAAG
>Bos_taurus_chr4.trna7855-CysGCA (31147729-31147658) Cys (GCA) 72 bp Sc: 55.66
TCCCTGATGGCTCAGTTGGTAAAGAATCTGTCTGCAATGCAGGAGACCCAGGTTCAATTC
CTGGGTAGGGAA
>Bos_taurus_chr4.trna7857-CysGCA (31144191-31144120) Cys (GCA) 72 bp Sc: 55.66
TCCCTGATGGCTCAGTTGGTAAAGAATCTGTCTGCAATGCAGGAGACCCAGGTTCAATTC
CTGGGTAGGGAA
>Bos_taurus_chr17.trna4895-CysGCA (52027285-52027214) Cys (GCA) 72 bp Sc: 55.67
TCCCTGGTGGCTCAGATGGTAAAGCGTCTGTCTGCAGTACAGGAGACCCAGGTTCGATCC
CTGGGTCGGGAA
>Bos_taurus_chr21.trna2928-CysGCA (68172500-68172429) Cys (GCA) 72 bp Sc: 55.68
TCCTGGGTAGCTCAGCTGGTAGAGAATCTGCCTGCAAAGCAGGAGACCCCAGTTCGATTC
TTGGGTCAGGAA
>Bos_taurus_chr21.trna1953-CysGCA (46810299-46810370) Cys (GCA) 72 bp Sc: 55.70

TCCCTAATGACTCAGTTGGTAAAGAACCTGCCTGCAATGCAGGAGACATGGGTTCGATCC
CCAGTGTGGGAA
>Bos_taurus_chr6.trna3414-CysGCA (105314489-105314560) Cys (GCA) 72 bp Sc: 55.71
TCCCTGGTGGCTCAGATGGTAAAGCGTCTGCCCGCAATGCAGGAGACCCAGGTTCGATCC
CTAGCTTGGGAA
>Bos_taurus_chr4.trna2411-CysGCA (75667379-75667450) Cys (GCA) 72 bp Sc: 55.73
TCCCTGCTGGCTAAGATGGTAAAGAATCTGCCTGCAAAGCAGCAGACCCAGGTTCGATCC
CTGGGTTGGGAA
>Bos_taurus_chr8.trna4611-CysGCA (103612377-103612305) Cys (GCA) 73 bp Sc: 55.73
TCCCTGGTGGCTCAGATGGTAAAGCATCTGCCTGCAATGCAGGAGACCCAGGTTCGATTC
CTGGGTCAGGAAG
>Bos_taurus_chr12.trna2760-CysGCA (72082039-72082110) Cys (GCA) 72 bp Sc: 55.74
TCCCTGGTGGCTCAGAGGATAAAGTTTCTGCCTGCAATGCAGGAGACCCAGGTTCGATCC
CTGGGTTGGGAA
>Bos_taurus_chr4.trna8235-CysGCA (17387706-17387634) Cys (GCA) 73 bp Sc: 55.77
TCCCTGGTGGCTCAGAGGTTAAAGCATCTGCCTGCAATGCAGGAGACCCAGGTTCGATCC
CTGGGTCAGGAAG
>Bos_taurus_chr21.trna2963-CysGCA (67196910-67196839) Cys (GCA) 72 bp Sc: 55.81
TCCCTCGTAGCTCAGTTGGTAAAGAATCTGCCTGCAATGCAGGAGACCCAGGTTCAATCC
CTGGCTCGGGAA
>Bos_taurus_chr6.trna8501-CysGCA (6927944-6927873) Cys (GCA) 72 bp Sc: 55.83
TCCCTAGTGGCTCAGAGGATAAAGCATCTGCCTGCAATGCAGGAGACCCAGGTTCAATCC
CTGGGTTGGGAA
>Bos_taurus_chr26.trna2034-CysGCA (50179639-50179568) Cys (GCA) 72 bp Sc: 55.83
TCCCTAGTAGCTCAGTTGGTAAAGAATCTGCCTGCAATGCAGGAGACCCAGGTTCGATCC
CTGGGTTGGGAA
>Bos_taurus_chr17.trna5794-CysGCA (26663255-26663184) Cys (GCA) 72 bp Sc: 55.83
TCCCTGGTGGCTCAGACGGTAAAGCGTCTGTCTGCAATGCAGGAGACCCAGGTTCGATCC
CTGGGTTGGGAA
>Bos_taurus_chr9.trna7218-CysGCA (20109039-20108968) Cys (GCA) 72 bp Sc: 55.83
TCCCTGGTGGCTCAGACGGTAAAGCGTCTGTCTGCAATGCAGGAGACCCAGGTTCGATCC
CTGGGTTGGGAA
>Bos_taurus_chr13.trna6201-CysGCA (31897213-31897142) Cys (GCA) 72 bp Sc: 55.84
TCCCTGGTGGCTCAGAGGGTAAAGCGTCTGCCTGCAATGCAGGAGACCCAGGTTCAATCC
CTGGGTTGGGAA
>Bos_taurus_chr14.trna6183-CysGCA (11246326-11246255) Cys (GCA) 72 bp Sc: 55.86
TCCCTGGTGGCTCAGAGGTTAAAGCATCTGCCTGCAATGCAGGAGACCTAGGTTCGATCC
CTGGGTTGGGAA
>Bos_taurus_chr20.trna2848-CysGCA (74261368-74261439) Cys (GCA) 72 bp Sc: 55.87
TCCCTGGTGTCTGAGATGGTAAAGCGTCTGCCTGCAGTGTAGGAGACCCAGGTTCGATCC
CTGGCTTGGGAA
>Bos_taurus_chr4.trna7691-CysGCA (36271202-36271131) Cys (GCA) 72 bp Sc: 55.87
TTCCTGGTGGCTCAGATGGTAAAGTGTCTGCCTGCAATGCAGGAGACCCAGGTTCAAACC
CTGGGTAGGGAA
>Bos_taurus_chr2.trna7007-CysGCA (105597284-105597213) Cys (GCA) 72 bp Sc: 55.93
TCCCTAGTAGCTCAGATGGTAAAGCATCTGCCTGCAATGCAGGAGACCCAGGTTCAATCC
CTGGGTCGGGAA
>Bos_taurus_chr8.trna2802-CysGCA (81506377-81506448) Cys (GCA) 72 bp Sc: 55.93
TCCCTGGTGGCTCAGACGGTAAAGCGTCTGCCCGCAATGCAGGAGACCCAGGTTCGATTC
CTGGGTCGGGAA
>Bos_taurus_chr14.trna2588-CysGCA (61894189-61894260) Cys (GCA) 72 bp Sc: 55.95
TCCCAGGTGGCTCAGATGGTAAAGTGTCTGCCTGCAATGCAGAAGACCCGGGTTCAATCC
CTGGGCCGGGAA
>Bos_taurus_chrUn.004.7.trna48-CysGCA (1156834-1156764) Cys (GCA) 71 bp Sc: 55.97
TCCCTGGTGGCTCAGCGGTAGAGAATCTGCCTGCAATGCAGGAGACCCAGGTTCGATCCC
TGGGTCGGGAA
>Bos_taurus_chr11.trna4170-CysGCA (101990666-101990737) Cys (GCA) 72 bp Sc: 56.05
TCCCTGATGGCTCAGATGGTAAAGAGTCTGCCTGCAATGCAGAAGACCCAGGTTCGATCC
CTGGATTGGGAA
>Bos_taurus_chr22.trna2285-CysGCA (60102891-60102819) Cys (GCA) 73 bp Sc: 56.06
TCCCTGGTGGCTCAGATGGTAAAGCGTCTGCCTGCAATGCAGGAGACCCAGGTTCGATCC
CTGGGTCAGGAAG
>Bos_taurus_chr1.trna9267-CysGCA (57324057-57323986) Cys (GCA) 72 bp Sc: 56.10
TCCCTGGTGGCTCAGATGGTAAAGAACCTGCCTGCAATGCAGGAGACCCAGGTTCGATTC
CTGGGTTGGGAA
>Bos_taurus_chr3.trna113-CysGCA (3708531-3708602) Cys (GCA) 72 bp Sc: 56.11
TCCCTGGTGGCTCAGAGGGTAAAGTGTCTGCCTGCAATGCAGGAGACCCAGGTTCGATCC

CTGGGTTGGGAA
>Bos_taurus_chr13.trna5579-CysGCA (44585068-44584996) Cys (GCA) 73 bp Sc: 56.12
TTCCTGATAGCTCAGTTGGTAAAGAATCTGCCTGCAATGCAGAAGACCCTGGTTCGATTC
CTAGGTTGGGAAG
>Bos_taurus_chr9.trna3840-CysGCA (105164851-105164922) Cys (GCA) 72 bp Sc: 56.15
TCCCTGGTGGTCCAGTGGTTAAGAATCCTCCTTGCAATGCAGGGGACCCAGGTTCGATCC
CTGGTCAGGGAA
>Bos_taurus_chrUn.004.670.trna3-CysGCA (7133-7204) Cys (GCA) 72 bp Sc: 56.16
TCCCTAGTGGCTCAGACGGTAAAGCGTCTGTCTGCAATGCAGGAGACCCAGGTTCGATCC
CTGGGTTGGGAA
>Bos_taurus_chr9.trna6139-CysGCA (51558554-51558483) Cys (GCA) 72 bp Sc: 56.16
TCCCTGGTGGCTTAGACGGTAAAGCGTCTGTCTGCAATGCAGGAGACCCAGGTTCGATCC
CTGGGTTGGGAA
>Bos_taurus_chr8.trna911-CysGCA (26352328-26352399) Cys (GCA) 72 bp Sc: 56.19
TCCCTGGTGGCTTAGAGGGTAAAGCATCTGCCTGCAATGCAGGAGACCCAGGTTCGATCC
CTGGGTTGGGAA
>Bos_taurus_chr29.trna371-CysGCA (9878089-9878160) Cys (GCA) 72 bp Sc: 56.20
TCCCTGGTGGCTCAGAGGATAAAGCATCTGCCTGCAATGCAGAAGACCCAGGTTTGATCC
CTGGGTGGGGAA
>Bos_taurus_chr8.trna7554-CysGCA (17730050-17729979) Cys (GCA) 72 bp Sc: 56.27
TCCCTGGTAGCTCAGTTGGTAAAGAATCTGCCTGCAATGCAGAAGACCCTGGTTCGATTC
CTGGGTTGGGAA
>Bos_taurus_chr27.trna149-CysGCA (5741858-5741929) Cys (GCA) 72 bp Sc: 56.29
TCCATGGTGGCTCAGTTGGTAGAGAATCTGCCTGCAATGCAGAAGACCCAGGTTCGATTC
CTGGGTCGGGAG
>Bos_taurus_chr9.trna2744-CysGCA (82796427-82796498) Cys (GCA) 72 bp Sc: 56.32
TCCCAGGTGGCTCAGACGGTAAAGCGTCTGCCTGCAATGCGGGAGACCCAGGTTCAATCC
CTGGGTTGGGAA
>Bos_taurus_chr13.trna584-CysGCA (16707179-16707251) Cys (GCA) 73 bp Sc: 56.36
TCCCTGATGGCTCAGTTGGTAAAGCATCCGCCTGCAGTGCAGGAGACCCCGGTTCGATTC
CTGAGTCAGGGAA
>Bos_taurus_chr4.trna3693-CysGCA (108137353-108137424) Cys (GCA) 72 bp Sc: 56.37
TCCTTGGTGGCTCAGATGGTAAAGTGTCTGCCTGCAGTGCAGGAGACCCAGGTTCGATCC
CTGGGTTGGGAA
>Bos_taurus_chr13.trna3733-CysGCA (83901520-83901449) Cys (GCA) 72 bp Sc: 56.38
TCCCTGCTGGCTCAGAGGTTAAAGCGTCTGCCTGCAATGCGGCAGACCCAGGTTCGATTC
CTTGGTGGGGAA
>Bos_taurus_chr2.trna459-CysGCA (15883013-15883084) Cys (GCA) 72 bp Sc: 56.38
TCCCTGGTGGCTCAGAGGATAAAGCGTCTGCCTGCAATGCAGGGGACCCAGGTTCGATCC
CTGGGTTGGGAA
>Bos_taurus_chr1.trna1163-CysGCA (29920978-29921050) Cys (GCA) 73 bp Sc: 56.41
TTCCTGATAGCTCAGTTGGTAAAGAATCTGCCTGCAATGCAGAAGACCCTAGTTCGATTC
CTGGGTCAGGAAG
>Bos_taurus_chr11.trna5818-CysGCA (83302183-83302112) Cys (GCA) 72 bp Sc: 56.41
TCCCTAGTGGCTCAGAGGTTAAAGCGTCTGCCTGCAATGCAGGAGACCTGGGTTCGATCC
CTGGGTAGGGAA
>Bos_taurus_chr1.trna4648-CysGCA (133262726-133262797) Cys (GCA) 72 bp Sc: 56.43
TCCTTGGTGACTCAGATGGTAGAGAATCTGCCTGCAATGCAGGAGACCCAGGTTCAATCT
CTGGCTAGGGAA
>Bos_taurus_chr5.trna9015-CysGCA (32995725-32995654) Cys (GCA) 72 bp Sc: 56.45
TCCCTAGTGGCTCAGTTGGTAAAGAATCTGCCTGCAATGCAGAAGACCCAGGTTCAATCC
CTGGGTCGGGAA
>Bos_taurus_chr16.trna2828-CysGCA (69668101-69668172) Cys (GCA) 72 bp Sc: 56.48
TCCCTGGTGGCTCAGATGGTAAAGCGTCTGTCTGCAATGCAGGAGACCCAGGTTCGATCC
CTGGGTTGGGAA
>Bos_taurus_chr4.trna6479-CysGCA (74467229-74467158) Cys (GCA) 72 bp Sc: 56.56
TCCCTGGTGGCTCAGACGGTAAAGCGTCTGCCTGCAATGTGGGAGACCCAGGTTCGATCC
CTGGTTAGGGAA
>Bos_taurus_chr6.trna8123-CysGCA (18731937-18731866) Cys (GCA) 72 bp Sc: 56.59
TCCCAGGTGGCTCAGATGGTAAAGAATCTGCCTGCAATGCAGAAGACCCAGGTTCAATCC
CTGGGTTGGGAA
>Bos_taurus_chr6.trna1640-CysGCA (59129146-59129217) Cys (GCA) 72 bp Sc: 56.60
TCCCTGGTGGCTCAGAGGATAAAGCGTCTGCCTGCAATGCAGAAGACCCTGGTTCAATCC
CTGGGTTGGGAA
>Bos_taurus_chr19.trna2460-CysGCA (48877435-48877506) Cys (GCA) 72 bp Sc: 56.60
TCCCTGGTGGCTCAGACGGTAAAGTGTCTGCCTGCAATGCAGGAGACCCAGGTTCAATCC
CTGGGTTGGGAA

>Bos_taurus_chr24.trna3211-CysGCA (54885903-54885832) Cys (GCA) 72 bp Sc: 56.61
TCCCTGGTGGCTCAGAGGTTAAAGCGTCTGCCCGCAATGCAGGAGACCCAGGTTCGATCC
CTGGGTCGGGAA
>Bos_taurus_chr21.trna1159-CysGCA (25564391-25564462) Cys (GCA) 72 bp Sc: 56.65
TCCCTGGTAGCTCAGCGGTTAAAGCGTCTGCCTGCAATGCAGGAGACCCAGGTTTGATCC
CTGGGTTGGGAA
>Bos_taurus_chr7.trna2385-CysGCA (54858933-54859004) Cys (GCA) 72 bp Sc: 56.66
TCCCTGATAGCTCAGTTGGTAAAGAATCTGCCTGCAATGCAGGAGACCCTGGTTCGACTC
CTGGTCAGGAAG
>Bos_taurus_chrX.trna1366-CysGCA (35331654-35331725) Cys (GCA) 72 bp Sc: 56.74
TCCCTGGTGGCTTAGAGGATAAAGCATCTGCCTGCAATGCAGGAGACCCAGGTTCAATTC
CTGGGTTGGGAG
>Bos_taurus_chrUn.004.2876.trna1-CysGCA (5340-5412) Cys (GCA) 73 bp Sc: 56.80
TCCCTGGTGGCTCAGACGGTAAAGCGTCTGCCTGCAATGCAGGAGACCCAGGTTCGATTC
CTGGGTCAGGAAG
>Bos_taurus_chrX.trna4189-CysGCA (74915117-74915045) Cys (GCA) 73 bp Sc: 56.80
TCCCTGGTGGCTCAGACGGTAAAGCGTCTGCCTGCAATGCAGGAGACCCAGGTTCGATTC
CTGGGTCAGGAAG
>Bos_taurus_chr17.trna1582-CysGCA (43907827-43907898) Cys (GCA) 72 bp Sc: 56.80
TCCCTAGTGGCTCAGATGGTAAAGCGTCTGTCTGCAATGCAGGAGACCCAGGTTCGATCC
CTGGGTTGGGAA
>Bos_taurus_chr16.trna513-CysGCA (17770605-17770676) Cys (GCA) 72 bp Sc: 56.85
TCCCTGATGGCTCAGATGGTAAAGTGTCTGCCTGCAACGCGGGAGACCCAGGTTCGATCC
CTGGGTGGGGAA
>Bos_taurus_chr27.trna1325-CysGCA (35428696-35428767) Cys (GCA) 72 bp Sc: 56.90
TCCCTAGTGGCTCAGAGGGTAAAGCGTCTGCCTGCAATGCAGGAGACCCAGGTTCGATCC
CTGGGTCGGGAA
>Bos_taurus_chr5.trna17-CysGCA (780535-780607) Cys (GCA) 73 bp Sc: 56.94
TTCCTGATAGCTCAGTTGGTAAAGAATCTGCCTGCAATGCAGAAGACCCCGGTTCAATTC
CTGGGTCAGGAAG
>Bos_taurus_chr7.trna6345-CysGCA (52388678-52388607) Cys (GCA) 72 bp Sc: 56.99
TCCCTGTTGGCTCAGTTGGTAAAGTGTCTGCCTGCAATACTGATGACCCAGGTTCAATCC
CTGGGTAGGGAA
>Bos_taurus_chr12.trna2324-CysGCA (60610295-60610366) Cys (GCA) 72 bp Sc: 56.99
TCCCTGATGGCTCAGATGGTAAGGTGTCTGCCTGCAGTGCAGAAGACCCAGGTTCGATCC
CTGGGTTGGGAA
>Bos_taurus_chr3.trna8333-CysGCA (28404534-28404463) Cys (GCA) 72 bp Sc: 57.00
TCCCTGGTGGCTCAGATGGTAAAGCATCTGCCTGCAATGCAGGAGACCCAGGTTCAATCC
CTGGGTTGGGAA
>Bos_taurus_chr1.trna10479-CysGCA (16510620-16510548) Cys (GCA) 73 bp Sc: 57.02
TCCCTGGTGGCTCAGATGGTAAAGCATCTGCCTGCAATGCAGGAGACCCAGGTTCAATCC
CTGGGTCGGGGAA
>Bos_taurus_chr24.trna4635-CysGCA (23640822-23640751) Cys (GCA) 72 bp Sc: 57.09
TCCCTGGTGGCTCAGAGGTTAAAGCGTCTGCTTGCAATGCAGGAGACCTAGGTTCGATCC
CTGGGCTGGGAA
>Bos_taurus_chr13.trna1996-CysGCA (48648105-48648176) Cys (GCA) 72 bp Sc: 57.10
TCCCAGGTGACTCAGCTGGTAAAGAATCTGCCTGCAAAGCAGGAGACCCTGGTTTGATTC
CTGGCTTGGGAA
>Bos_taurus_chr15.trna1846-CysGCA (53375204-53375275) Cys (GCA) 72 bp Sc: 57.14
TCCCTGGTGGCTCAGAGGATAAAGCGTCTGCTTGCAATGCAGGAGACCCAGGTTCGATCC
CTGGGTTGGGAA
>Bos_taurus_chr2.trna10042-CysGCA (13727780-13727709) Cys (GCA) 72 bp Sc: 57.22
TCCCTGGTGGCTCAGAGGATAAAGCGTCTGCCTGCAATGCAGGAGACCCAGGTTCAATCC
CTGGGTTGGGAA
>Bos_taurus_chr10.trna7603-CysGCA (14914861-14914790) Cys (GCA) 72 bp Sc: 57.24
TCCCTGGTGGCTCAGAGGATAAAGCATCTGCCTGCAATGCAGGAGACCCAGGTTCGATCC
CTGGGTTGGGAA
>Bos_taurus_chr11.trna1366-CysGCA (31824202-31824273) Cys (GCA) 72 bp Sc: 57.24
TCCCTGGTGGCTCAGAGGATAAAGCATCTGCCTGCAATGCAGGAGACCCAGGTTCGATCC
CTGGGTTGGGAA
>Bos_taurus_chr23.trna2983-CysGCA (40652672-40652601) Cys (GCA) 72 bp Sc: 57.24
TCCCTGGTGGCTCAGAGGATAAAGCATCTGCCTGCAATGCAGGAGACCCAGGTTCGATCC
CTGGGTTGGGAA
>Bos_taurus_chr24.trna4231-CysGCA (31968227-31968156) Cys (GCA) 72 bp Sc: 57.24
TCCCTGGTGGCTCAGAGGATAAAGCATCTGCCTGCAATGCAGGAGACCCAGGTTCGATCC
CTGGGTTGGGAA
>Bos_taurus_chrUn.004.90.trna5-CysGCA (56691-56762) Cys (GCA) 72 bp Sc: 57.24

TCCCTGGTGGCTCAGAGGATAAAGCATCTGCCTGCAATGCAGGAGACCCAGGTTCGATCC
CTGGGTTGGGAA
>Bos_taurus_chr10.trna5961-CysGCA (59989929-59989858) Cys (GCA) 72 bp Sc: 57.25
TCCCTGGTGGCTCAGATGGTAAAGTGTCTGCCTGCAATGCAGGAGACCCAGGTTCAATCC
CTGGGTTGGGAA
>Bos_taurus_chr28.trna391-CysGCA (9476056-9476127) Cys (GCA) 72 bp Sc: 57.25
TCCCTGGTGTCTCAGGTGGTAAAGCATCTGCCTGCAATGCAGGAGGCCCAGGTTCGATCC
CTGGATGGGGAA
>Bos_taurus_chr7.trna3532-CysGCA (89138129-89138200) Cys (GCA) 72 bp Sc: 57.28
TCCCTGGTGGCTTAGAGGTTAAAGTATCTGCCTGCAATGCAGGAGACCCAGGTTCAATCC
CTGGGTTGGGAA
>Bos_taurus_chr23.trna4060-CysGCA (17023104-17023033) Cys (GCA) 72 bp Sc: 57.30
TCCCGGATGGCTCAGACGGTAAAGCGTCTGCCTGCAATGCGGGAGACCCAGGTTCGATCC
CTGGGTTGGGAA
>Bos_taurus_chr13.trna4762-CysGCA (65864195-65864125) Cys (GCA) 71 bp Sc: 57.36
TCCCCAATGGCTCAGTGGTAGAGAATTTGCCTGCAATGCAGGAGACCCAGGTTCGATCCC
TGGGTGGGAAA
>Bos_taurus_chr20.trna3979-CysGCA (46445281-46445210) Cys (GCA) 72 bp Sc: 57.40
TCCCTAATAGCTCAGTAGGTAAAGAATCTGCCTGCAAAGCAGGAGACCTGGGTTCGATCC
CCAGGTAGGGAA
>Bos_taurus_chr8.trna5888-CysGCA (72166086-72166015) Cys (GCA) 72 bp Sc: 57.43
TCCCTGGTGGCTCAGACGGTAGAGTATCTGCCTGCAATGCAGGAGACCCAGGTTTGATCC
CTGGGTAGGGAA
>Bos_taurus_chr2.trna8099-CysGCA (74917431-74917360) Cys (GCA) 72 bp Sc: 57.43
TCCCTGGTGGCTCAGATGGTAAAGCATCTGCCTGCAATGCAGGCAACCCAGGTTCAATCC
CTGGCTTGGGAA
>Bos_taurus_chr6.trna6258-CysGCA (75253443-75253372) Cys (GCA) 72 bp Sc: 57.43
TCCCTGGTGGCTCAGAGGTTAAAGCATCTGCCTGCAATGCAGGAGACCCAGGTTCAATCC
CTGGGTCGGGAA
>Bos_taurus_chr13.trna1262-CysGCA (31143301-31143372) Cys (GCA) 72 bp Sc: 57.44
TCCTGGATAGCTCAGTTGGTAAAGAATCTGCCTGCAATGCAGGAGACCCTGGTTCGATTC
CTGGGTCAGGAA
>Bos_taurus_chr12.trna191-CysGCA (8138454-8138526) Cys (GCA) 73 bp Sc: 57.47
TCCCTGGTGGCTCAGATGGTAAAGCGTCTGCCTGCAATGCAGGAGACCCGGGTTCGATCC
CCGAGTCAGGAAG
>Bos_taurus_chr12.trna4198-CysGCA (69447153-69447082) Cys (GCA) 72 bp Sc: 57.56
TCCCTGGTGGCTCAGAGGTTAAAGCGTCTGCCTGCAATGCAGGAGACCTAAGTTCGATCC
CTGGCTTGGGAA
>Bos_taurus_chr10.trna7013-CysGCA (31363777-31363705) Cys (GCA) 73 bp Sc: 57.64
TTCCTGATAGCTCAGTTGGTAAAGAATCTGCCTGCAATGCAGGCGACCCCTGGTTCGATC
CCTGGTCAGGAAG
>Bos_taurus_chrUn.004.124.trna48-CysGCA (180370-180299) Cys (GCA) 72 bp Sc: 57.64
TCCCTGGTGGCTCAGATGGTAAAGTGTCTGCCTGCAATGCAGGAGACCCAGGTTCAATTC
CTGGGTCGGGAA
>Bos_taurus_chrX.trna644-CysGCA (13826126-13826198) Cys (GCA) 73 bp Sc: 57.68
TCCCTGGTGGCTCAGATGGTAAAGTGTCTGCCTGCAATGCAGGAGACCCAGGTTCGATCC
CTGGGTTGGGGAA
>Bos_taurus_chr15.trna1260-CysGCA (36850987-36851058) Cys (GCA) 72 bp Sc: 57.68
TCCCTAATAGCTCAGTTGGTAAAGGATCTGCCTGCAACGCAGGAGACCCTGGTTCAATTC
CTGGGTAGGGAA
>Bos_taurus_chrUn.004.124.trna17-CysGCA (175554-175625) Cys (GCA) 72 bp Sc: 57.71
TCCCTGGTGGCTTAGACGGTAAAGCGTCTGCCTGCAATGCAGGAGACCCAGGTTCGATCC
TTGGGTGGGGAA
>Bos_taurus_chr9.trna6012-CysGCA (55524569-55524498) Cys (GCA) 72 bp Sc: 57.74
TCCCTGGTGGCTCAGACGGTAAAGCATCTGCCTGCAATGCAGGAGACCCAGGTTCAATTC
CTGGGTTGGGAA
>Bos_taurus_chr2.trna7812-CysGCA (83634865-83634794) Cys (GCA) 72 bp Sc: 57.75
TCCCTGGTGGCTCAGAGGATAAAGCGTCTGCCTGCAATGCAGGAGACCCAGGTTCGATAC
CTGGGTTGGGAA
>Bos_taurus_chr8.trna8010-CysGCA (7328554-7328483) Cys (GCA) 72 bp Sc: 57.85
TCCCTGATAGCTCAGTTGGTAAAGAATCTGCCTGCAGTGCAGGAGACCCAGGTTCAATTC
CTGGGTAGGGAA
>Bos_taurus_chrUn.004.2220.trna1-CysGCA (19938-20009) Cys (GCA) 72 bp Sc: 57.85
TCCCTGATAGCTCAGTTGGTAAAGAATCTGCCTGCAGTGCAGGAGACCCAGGTTCAATTC
CTGGGTAGGGAA
>Bos_taurus_chr12.trna4727-CysGCA (53492723-53492651) Cys (GCA) 73 bp Sc: 57.94
TTCCTGATAGCTCAGTTGGTAGAGAATCTGCCTGCAATGCAGGAGACCCTGGTTCAATTC

CTGGGTCAGGAAG
>Bos_taurus_chr9.trna6947-CysGCA (26945382-26945311) Cys (GCA) 72 bp Sc: 58.08
TCCGTGGTGGCTCAGACGGTAAAGCGTCTGCCTGCAACGCGGGAGACCCAGGTTCGATCC
CTGGGTACGGAA
>Bos_taurus_chr23.trna1890-CysGCA (42412538-42412609) Cys (GCA) 72 bp Sc: 58.09
TCCCTGGTGGCTCAGACGGTAAAGCATCTGCCTGCAATGCAGGAGACCCAGGTTCGATCC
CTGGGTTGGGAA
>Bos_taurus_chr14.trna6647-CysGCA (1652621-1652551) Cys (GCA) 71 bp Sc: 58.13
TCCCTGGTGGCTCAGTGGTAAAGAACCTGCCTGCAGTGCAGGAGACCCAGGTTCGATTCC
TGGGTCGGGAA
>Bos_taurus_chr3.trna6786-CysGCA (73694583-73694512) Cys (GCA) 72 bp Sc: 58.18
TCCCTGATAGCTCAGTTGGTAAAGAATCTGCCTGCAATGCAGGAGACCCTGGTTCGATTC
CTGGTCGGGAAG
>Bos_taurus_chr2.trna1974-CysGCA (63647629-63647700) Cys (GCA) 72 bp Sc: 58.20
TCCCTGGTGGCTCAGAGGTTAAAGTGTCTGCCTGCAATGCAGCAGACCCAGGTTCGATCC
CTGGGTTGGGAG
>Bos_taurus_chr10.trna3103-CysGCA (82119455-82119526) Cys (GCA) 72 bp Sc: 58.22
TCCCTGGTGGCTCAGTTGGTAAAGCCTCTGCCTGCAATGCAGGAGACCCAGGTTCAATCC
CTGGATCGGGAA
>Bos_taurus_chr11.trna7912-CysGCA (30527870-30527799) Cys (GCA) 72 bp Sc: 58.28
TCCCTGGTGGCTCAGAAGGTAAAGCATCTGCCTGCAATGCAGGAGACCCAGGTTCGATCC
CTGGGTAGGGAA
>Bos_taurus_chr24.trna4551-CysGCA (25520349-25520278) Cys (GCA) 72 bp Sc: 58.31
TCCCCAGTGGCTCAGATGGTAAAGTGTCTGCCTGCAATGCAGGAGACCCAGGTTCGATCC
CTGGGTCGGGAA
>Bos_taurus_chr1.trna7436-CysGCA (115520253-115520182) Cys (GCA) 72 bp Sc: 58.39
TCCCTGGTGGCTCAGATGGTAAAGCATCTGCCTGCAATGCAGGAGACCCAGGTTCAATTC
CTGGGTTGGGAA
>Bos_taurus_chr10.trna3020-CysGCA (79936129-79936200) Cys (GCA) 72 bp Sc: 58.42
TCCCTGGTGGCTCAGACGGTAAAGCGTCTGCCTGCAGTGCAGGAGACCCAGGTTCGATCC
CTGGGTTGGGAA
>Bos_taurus_chr9.trna5138-CysGCA (79832294-79832222) Cys (GCA) 73 bp Sc: 58.44
TCCCTGGTGGCTCAGACGGTAAAGCACCTGCCTGCAATGCAGGAGACCCAGGTTCGATTC
CTGGGTCAGGAAG
>Bos_taurus_chr12.trna215-CysGCA (8683957-8684028) Cys (GCA) 72 bp Sc: 58.44
TCCCAGGTAGCTCAGCTGGTAAAGAATCTGCCTGCAATGCAGGAGACCCAGGTTCGATTC
CTGGGTTGGGAA
>Bos_taurus_chr11.trna7978-CysGCA (28657744-28657673) Cys (GCA) 72 bp Sc: 58.56
TCCCTGGTGGCTCAGAGGATAAAGCATCTGCCTGCAATGCAGGAGACCCAGGTTCGATCC
CTGGGCTGGGAA
>Bos_taurus_chr4.trna1059-CysGCA (31805162-31805234) Cys (GCA) 73 bp Sc: 58.57
TCCCTGGTGGCTCAGTCGGTAAAGTGTCTGCCTGCAATGCAGGAGACCCAGGTTCGATCC
CTGCGTTGGGAAG
>Bos_taurus_chr12.trna1796-CysGCA (44734189-44734260) Cys (GCA) 72 bp Sc: 58.64
TCCCTGGTGGCTAAAACGGTAAAGCGTCTGCCTGCAATGCAGAAGACCCAGGTTCAATCC
CTGGGTTGGGAA
>Bos_taurus_chr11.trna4638-CysGCA (108319213-108319142) Cys (GCA) 72 bp Sc: 58.64
TCCCTGGTGGCTCAGATGGTAAAGTGTCTGCCTGCAATGCAGGAGACCCAGGTTCAATTC
CTGGGTTGGGAA
>Bos_taurus_chr5.trna5240-CysGCA (121461940-121461869) Cys (GCA) 72 bp Sc: 58.71
TCCCTGGTAGCTCAGCTGGTAAAGAATCTGCCTGCAATGCAGAAGACCCTGGTTCAATTC
CTGGCTTGGGAA
>Bos_taurus_chr12.trna5471-CysGCA (32149796-32149725) Cys (GCA) 72 bp Sc: 58.72
TCCCTGGTGGCTCAGATGGTAAAGCGTCTGCCTGCAATGCAGGAGACCCAGGTTCAATCC
CTGGGTTGGGAA
>Bos_taurus_chr3.trna2931-CysGCA (84924980-84925051) Cys (GCA) 72 bp Sc: 58.72
TCCCTGGTGGCTCAGATGGTAAAGCGTCTGCCTGCAATGCAGGAGACCCAGGTTCAATCC
CTGGGTTGGGAA
>Bos_taurus_chr7.trna5356-CysGCA (82352663-82352592) Cys (GCA) 72 bp Sc: 58.72
TCCCTGGTGGCTCAGATGGTAAAGCGTCTGCCTGCAATGCAGGAGACCCAGGTTCAATCC
CTGGGTTGGGAA
>Bos_taurus_chr20.trna459-CysGCA (12487884-12487955) Cys (GCA) 72 bp Sc: 58.77
TCCCAGATAGCTCAGTTGGTAAAGTATCTGCCTGCAATGCAGGAGACCCTGGTTCAACTC
CTGGGTTGGGAA
>Bos_taurus_chr13.trna4909-CysGCA (63875605-63875534) Cys (GCA) 72 bp Sc: 58.96
TCCCTGGTGGCTCAGAGGATAAAGCGTCTGCCTGCAATGCAGGAGACCCAGGTTCGATCC
CTGGGTTGGGAA

>Bos_taurus_chr27.trna791-CysGCA (24772592-24772663) Cys (GCA) 72 bp Sc: 58.96
TCCCTGGTGGCTCAGAGGATAAAGCGTCTGCCTGCAATGCAGGAGACCCAGGTTCGATCC
CTGGGTTGGGAA
>Bos_taurus_chr29.trna3054-CysGCA (27719440-27719369) Cys (GCA) 72 bp Sc: 58.96
TCCCTGGTGGCTCAGAGGATAAAGCGTCTGCCTGCAATGCAGGAGACCCAGGTTCGATCC
CTGGGTTGGGAA
>Bos_taurus_chr22.trna4585-CysGCA (1480305-1480234) Cys (GCA) 72 bp Sc: 58.97
TCCCTGGTGGCTCAGAGGATAAAGCGTCTGCCTGCAGTGCAGGAGACCCGGGTTCGATCC
CCGGGTTGGGAA
>Bos_taurus_chr28.trna901-CysGCA (24814529-24814600) Cys (GCA) 72 bp Sc: 58.98
TCCCTGGTGGCTAAGACGGTAAAGCGTCTGCCTGCAATGCAGGAGACCCAGGTTCGATCC
CTGGGTCGGGAA
>Bos_taurus_chr8.trna6961-CysGCA (39806603-39806532) Cys (GCA) 72 bp Sc: 59.11
TCCCTGGTGGCTCAGATGGTAAAGCGTCTGCCTGCAATGCAGGAGACCCAGGTTCAATTC
CTGGGTCGGGAA
>Bos_taurus_chr19.trna1006-CysGCA (21512356-21512427) Cys (GCA) 72 bp Sc: 59.14
TCCCTGGTGGCTTAGTTGGTAAAGAATCTGCCTGCAATGCAGGAGACCCAGGTTCGATCC
CTGGGTGGGGAA
>Bos_taurus_chr24.trna2666-CysGCA (63469140-63469210) Cys (GCA) 71 bp Sc: 59.15
TCCCTGATGGCTCAGTGGTAAAGAATCTGCCTGCAGTGCAGGAGACGCAGGTTCGATCCC
TGCTCGGGAAG
>Bos_taurus_chr1.trna2685-CysGCA (78769882-78769953) Cys (GCA) 72 bp Sc: 59.15
TCCCTGGTGGCTCAGTCGGTAAAGCGTCTGCCTGCAATGCAGGAGACCCAGGTTTGATTC
CTGGGCCGGGAA
>Bos_taurus_chr1.trna2687-CysGCA (78850692-78850763) Cys (GCA) 72 bp Sc: 59.15
TCCCTGGTGGCTCAGTCGGTAAAGCGTCTGCCTGCAATGCAGGAGACCCAGGTTTGATTC
CTGGGCCGGGAA
>Bos_taurus_chr12.trna6754-CysGCA (8481361-8481290) Cys (GCA) 72 bp Sc: 59.19
TCCCTGATGGCTCAGATGGTAAAGCGTCTGCCTGCAATGCGGGAGACCCAGGTTCAATCC
CTGGTTTGGGAA
>Bos_taurus_chr10.trna6911-CysGCA (35329471-35329400) Cys (GCA) 72 bp Sc: 59.21
TCCCTGGTAGCTCAGTTGGTAAAGTATCTGCCTGCAATGCAGGAGACCCTGGTTCGATTC
CTGGGTTGGGAA
>Bos_taurus_chrUn.004.1529.trna1-CysGCA (23096-23167) Cys (GCA) 72 bp Sc: 59.33
TCCCTGGTAGCTTAGAGGTTAAAGCGTCTGCCTGCAATGCAGAAGGCCCGGGTTCAATCC
CTGGGTTGGGAA
>Bos_taurus_chr10.trna3504-CysGCA (90622571-90622642) Cys (GCA) 72 bp Sc: 59.46
TCCCTGGTGGCTCAGATGGTAAAGCGTCTGCCTGCAATGCAGGAGACCCAGGTTCGATCC
CTGGGTCGGGAA
>Bos_taurus_chr28.trna127-CysGCA (4140046-4140117) Cys (GCA) 72 bp Sc: 59.51
GGGAGTATAGCTCAGTGGTAGAGCATTTGATTGCAGATCAAGAGGTCTCCAGTTCAAATC
CGGGTGCCCCCT
>Bos_taurus_chr10.trna404-CysGCA (9940763-9940834) Cys (GCA) 72 bp Sc: 59.55
TCCCTGGTGGCTCAGATGGTAAAGCGCCTGTCTGCAATGCAGGAGACCCAGGTTTGATCC
CTGGCTTGGGAA
>Bos_taurus_chrUn.004.1283.trna3-CysGCA (27536-27607) Cys (GCA) 72 bp Sc: 59.55
TCCCTGGTGGCTCAGATGGTAAAGCGCCTGTCTGCAATGCAGGAGACCCAGGTTTGATCC
CTGGCTTGGGAA
>Bos_taurus_chr5.trna1818-CysGCA (51038922-51038993) Cys (GCA) 72 bp Sc: 59.69
TCCCTGGTAGCTCAGCGGTTAAAGCATCTGCCTGCAATGCAGATGACCCGGGTTCGAGCC
CTGGTTTGGGAA
>Bos_taurus_chr6.trna4294-CysGCA (120999207-120999137) Cys (GCA) 71 bp Sc: 59.71
TCCCTGGTGGCTCAGTGGTAGAGAATCTGCCTGCAATGCAGAAGACCCGGGTTCGATCCC
TGGGTGGGGAA
>Bos_taurus_chr27.trna3544-CysGCA (13209647-13209576) Cys (GCA) 72 bp Sc: 59.76
TCCCTGGTAGCTCAGAGGTTAAAGCATCTGCCTGCAATGCAGGAGACCCAGGTTCGATCC
CTGGGTTGGGAA
>Bos_taurus_chrUn.004.1535.trna2-CysGCA (794-723) Cys (GCA) 72 bp Sc: 59.81
TCCCTGGTAGCTCAGATGGTAAAGTGTCTGCCTGCAATGCAGAAGACCCAGGTTCAATCC
CTGGGTTGGGAA
>Bos_taurus_chr2.trna8644-CysGCA (58261454-58261383) Cys (GCA) 72 bp Sc: 59.81
TCCCTGGTGGCTCAGACGGTAAAGCGTCTGCCTGCAATGCAGGAGACCCAGGTTCGATCC
CTGGGTTGGGAA
>Bos_taurus_chr14.trna5560-CysGCA (25032460-25032389) Cys (GCA) 72 bp Sc: 59.81
TCCCTGATAGCTCAGTTGGTAGAGAATCTGCCTGCAATGCAGGAGACCCCGGTTCGATTC
CTGGTCGGGAAG
>Bos_taurus_chr21.trna1354-CysGCA (28928036-28928107) Cys (GCA) 72 bp Sc: 60.03

TCCCTGGTGGCTCAGAGGTTAAAGCGTCTGCCTGCAATGCTGGAGACCCAGGTTCGATCC
CTGGGTGGGGAA
>Bos_taurus_chr12.trna1145-CysGCA (26307490-26307561) Cys (GCA) 72 bp Sc: 60.06
TCCCTGGTAGCTCAGATGGTAAAGCGTCTGCCTGCAATGCAGGAGACCCAGGTTCGATCC
CTGGGTTGGGAA
>Bos_taurus_chr15.trna2373-CysGCA (66607885-66607956) Cys (GCA) 72 bp Sc: 60.14
TCCCTGGTGGCTCAGAGGTTAAAGCGTCTGCCTGCAATGCAGGAGACCCAGGTTCAATCC
CTGGGTTGGGAA
>Bos_taurus_chr6.trna8069-CysGCA (19986045-19985974) Cys (GCA) 72 bp Sc: 60.14
TCCCTGGTGGCTCAGAGGTTAAAGCGTCTGCCTGCAATGCAGGAGACCCAGGTTCAATCC
CTGGGTTGGGAA
>Bos_taurus_chr1.trna57-CysGCA (761584-761655) Cys (GCA) 72 bp Sc: 60.17
TCCCTGATGGCTCAGATGGTAAAGCATCTGCCTGCAGTGCAGGAGACCTGGGTTCGATCC
CCAGGTGGGGAA
>Bos_taurus_chr26.trna1896-CysGCA (47976031-47976102) Cys (GCA) 72 bp Sc: 60.20
TCCCTGGTGGCTCAGACGGTAAAGCGTCTGCCTGCAATGCAGGAGACCCAGGTTCGATTC
CTGGGTCGGGAA
>Bos_taurus_chrUn.004.79.trna5-CysGCA (80279-80350) Cys (GCA) 72 bp Sc: 60.20
TCCCTGGTGGCTCAGACGGTAAAGCGTCTGCCTGCAATGCAGGAGACCCAGGTTCGATTC
CTGGGTCGGGAA
>Bos_taurus_chr19.trna4687-CysGCA (41920302-41920230) Cys (GCA) 73 bp Sc: 60.23
TCCCTGGTGGCTCAGACGGTAAAAGCGTCTGTCTGCAACGCAGAAGACCCAGGTTCGATC
CCTGGGTAGGGAA
>Bos_taurus_chr18.trna4667-CysGCA (34613670-34613599) Cys (GCA) 72 bp Sc: 60.30
TCCCTGGTGGCTCAGAGGTTAAAGTACCTGCCTGCAATGCAGGAGACCCAGGTTCAATCC
CTGGGTTGGGAA
>Bos_taurus_chr2.trna7448-CysGCA (94793770-94793699) Cys (GCA) 72 bp Sc: 60.32
TCCCTAGTGGCTCAGTTGGTAAAGAATCTGCCTGCAATGCAGGAGACCCAGGTTCGATCC
CTGGGTAGGGAA
>Bos_taurus_chr14.trna1369-CysGCA (31396180-31396250) Cys (GCA) 71 bp Sc: 60.41
TCCCTGGTGGCTAAACGGTAAAGCATCTGCCTGCAACGCAGGAGACCCAGGTTCGATTCC
TGGGTTGGGAA
>Bos_taurus_chr5.trna8584-CysGCA (46499144-46499073) Cys (GCA) 72 bp Sc: 60.41
TCCCTGGTGGCTCAGAGGTTAAAGTGTCTGCCTGCAATGCAGGAGACCCAGGTTCGATCC
CTGGGTTGGGAA
>Bos_taurus_chrUn.004.1902.trna2-CysGCA (15462-15391) Cys (GCA) 72 bp Sc: 60.46
TCCCTGGTGGCTCAGATGGTAAAGCGTCTGCCTGCAATGCAGGAGACCCAGGTTCGATCC
CTGGGTTGGGAA
>Bos_taurus_chr5.trna3856-CysGCA (101151794-101151865) Cys (GCA) 72 bp Sc: 60.49
TCCCTGATGGCTCAGAGGTTAAAGCGTCTGCCTGCAGTGCAGGAGACCCAGGTTCGATCC
CTGGGTTGGGAA
>Bos_taurus_chrUn.004.5914.trna2-CysGCA (2352-2281) Cys (GCA) 72 bp Sc: 60.49
TCCCTGATGGCTCAGAGGTTAAAGCGTCTGCCTGCAGTGCAGGAGACCCAGGTTCGATCC
CTGGGTTGGGAA
>Bos_taurus_chr8.trna4530-CysGCA (105391124-105391053) Cys (GCA) 72 bp Sc: 60.49
TCCCTGGTGGCTCAGAGGATAAAGTGTCTGCCTGCAATGCAGGAGACCCAGGTTCGATCC
CTGGTTTGGGAA
>Bos_taurus_chr17.trna6423-CysGCA (9429941-9429870) Cys (GCA) 72 bp Sc: 60.53
TCCCTGGTGGCTTAGATGGCAAAGCGTCTGCCTGCAACGCAGAAGACCCAGGTTCGATCC
CTGGGTTGGGAA
>Bos_taurus_chr19.trna4250-CysGCA (49006452-49006381) Cys (GCA) 72 bp Sc: 60.68
TCCCGGGTGGCTCAGACGGTAAAGCGTCTGCCTGCAAGGCAGGAGACCCAGGTTCGATTC
CTGGGTCGGGAA
>Bos_taurus_chr6.trna5792-CysGCA (90643387-90643316) Cys (GCA) 72 bp Sc: 60.73
TCCCTACTAGCTCAGATGGTAAAGCATCTGCCTGCAATGCAGGAGACCCAGGTTCAATCC
CTGGGTTGGGAA
>Bos_taurus_chr14.trna937-CysGCA (22001258-22001328) Cys (GCA) 71 bp Sc: 60.78
TCCTGGGTGGCTCAGTGGTAAAGAATCTGCCTGCAATGCAGAAGACCCAGGTTCGATCCC
TGGATTGGGAA
>Bos_taurus_chrUn.004.46.trna60-CysGCA (5916-5845) Cys (GCA) 72 bp Sc: 60.93
CCCCAGGTGGCTCAGATGGTAGAGCGTCTGCCTGCAATGCAGGAGGCCCAGGTTCGATCC
CTGGGTCGGGAA
>Bos_taurus_chr27.trna463-CysGCA (16291588-16291659) Cys (GCA) 72 bp Sc: 61.05
TCCCAGGTGGCTCAGATGGTAAAGGGTCTGCCTGCAATGCAGAAGACCCAGGTTCGATCC
CTGGATTGGGAA
>Bos_taurus_chrUn.004.9050.trna1-CysGCA (1765-1836) Cys (GCA) 72 bp Sc: 61.05
TCCCAGGTGGCTCAGATGGTAAAGGGTCTGCCTGCAATGCAGAAGACCCAGGTTCGATCC

CTGGATTGGGAA
>Bos_taurus_chr11.trna1687-CysGCA (41777821-41777892) Cys (GCA) 72 bp Sc: 61.17
TCCCTAGTGGCTCAGATGGTAAAGCGTCTGCCTGCAATGCAGGAGACCCAGGTTCGATTC
CTGGGTCGGGAA
>Bos_taurus_chr12.trna2267-CysGCA (58877439-58877510) Cys (GCA) 72 bp Sc: 61.19
TCCCTGGTGGCTCAGAGGATAGAGCGTCTGCCTGCAGTGCAGGAGACCCAGGTTCGATCC
CTGGGTTGGGAA
>Bos_taurus_chr20.trna738-CysGCA (21530430-21530501) Cys (GCA) 72 bp Sc: 61.20
TCCCTGATAGCTCAGTTGGTAGAGAACCTGCCTGCAATGCAGGAGACCCCGGTTCGATTC
CTGGGTGGGGAA
>Bos_taurus_chr10.trna3684-CysGCA (94326174-94326245) Cys (GCA) 72 bp Sc: 61.20
TCCCTGGTGGCTCAGACGGTAAAGCGTCTGCCTGCAATGCAGGAGACCCAGGTTCGATTC
CTGGGTTGGGAA
>Bos_taurus_chrUn.004.1553.trna1-CysGCA (18325-18254) Cys (GCA) 72 bp Sc: 61.20
TCCCTGGTGGCTCAGACGGTAAAGCGTCTGCCTGCAATGCAGGAGACCCAGGTTCGATTC
CTGGGTTGGGAA
>Bos_taurus_chr10.trna6004-CysGCA (58797434-58797363) Cys (GCA) 72 bp Sc: 61.24
TCCCTGGTGGCTCAGACGGTAAAGCCTCTGCCTGCAATGCAGGAGACCCAGGTTCGATTC
CTGGGTAGGGAA
>Bos_taurus_chr20.trna3739-CysGCA (55822856-55822785) Cys (GCA) 72 bp Sc: 61.31
TCCCTGGTGACTCAGTTGGTAAAGAATCTGTCTGCAATACAGGAGACCCAGGTTCAATCC
CTGGCTGGGGAA
>Bos_taurus_chr9.trna3778-CysGCA (103658028-103658099) Cys (GCA) 72 bp Sc: 61.70
TCCCTGGTGGCTCAGACGGTAAAGCGTCTGCCTGCAATGCAGGAGGCCCAGGTTCGATCC
CTGGGTTGGGAA
>Bos_taurus_chr8.trna5934-CysGCA (71111307-71111236) Cys (GCA) 72 bp Sc: 61.84
TCCCTGGTGGCTCAGACGGTAAAGCATCTGCCCGCAATGCAGGAGACCCAGGTTCGATTC
CTGGCTTGGGAA
>Bos_taurus_chr1.trna9088-CysGCA (62355371-62355300) Cys (GCA) 72 bp Sc: 61.84
TCCCTGGTGGCTCAGATGGTAAAGCGTCTGCCTGCAATGCAGGAGACCCAGGTTCGATTC
CTGGGTTGGGAA
>Bos_taurus_chr2.trna3175-CysGCA (99498723-99498794) Cys (GCA) 72 bp Sc: 62.16
TCCCCGGTGGCTCAGACGGTAAAGCGCCTGCCTGCAATGCAGGAGACCCAGGTTCGATCC
CTGGGTTGGGAA
>Bos_taurus_chr3.trna7191-CysGCA (60880132-60880061) Cys (GCA) 72 bp Sc: 62.51
TCCCTGGTGGCTCAGAGGTTAAAGCACCTGCCTGCAATGCAGGAGACCCAGGTTCGATCC
CTGGGTCGGGAA
>Bos_taurus_chr10.trna5676-CysGCA (68306881-68306811) Cys (GCA) 71 bp Sc: 62.76
TCCCCGCTGGCTCAGTGGTAAAGTATCTGCCTGCAATGCAGGAGACCCAGGTTCAATCCC
TGGGTCGGGAA
>Bos_taurus_chr9.trna5533-CysGCA (70389668-70389597) Cys (GCA) 72 bp Sc: 62.77
TCCCTGGTGGCTCAGACGGTAAAGCGTCTGCCTGCAGTGCAGAAGACCCAGGTTCGATTC
CTGGGTTGGGAA
>Bos_taurus_chr4.trna8059-CysGCA (23794171-23794100) Cys (GCA) 72 bp Sc: 63.00
TCCCTGGTGGCTCAGTCGGTAAAGCGTCTGCCTGCAATGCAGGAGACCCAGGTTCAATTC
CTGGGTCGGGAA
>Bos_taurus_chr1.trna6352-CysGCA (146544438-146544367) Cys (GCA) 72 bp Sc: 63.07
TCCCTGGTGGCTCAGAGGTTAAAGCATCTGCCTGCAATGCAGGAGACCCAGGTTCGATCC
CTGGGTGGGGAA
>Bos_taurus_chr12.trna4511-CysGCA (58383188-58383117) Cys (GCA) 72 bp Sc: 63.68
TCCCTGGTGGCTCAGAGGTTAAAGCATCTGCCTGCAAAGCAGGAGACCCAGGTTCGATTC
CTGGGTTGGGAA
>Bos_taurus_chrUn.004.81.trna26-CysGCA (266046-266117) Cys (GCA) 72 bp Sc: 65.63
TCCCTGGTGGCTCAGGTGGTAAAGCGTCTGCCTGCAATGCAGGAGACCCAGGTTCGATCC
CTGGGTAGGGAA
>Bos_taurus_chr18.trna3914-CysGCA (49726228-49726157) Cys (GCA) 72 bp Sc: 66.00
TCCCCGGTGGCTCAGATGGTAAAGCGTCTGCCTGCAATGCAGGAGACCCAGGTTCGATCC
CTGGGTGGGGAA
>Bos_taurus_chr3.trna8483-CysGCA (24578471-24578400) Cys (GCA) 72 bp Sc: 69.26
GGGGGTACAGCTCAGTGGTAGAGCATTTGACTGCAGATCAAGAGGTCCCTGGTTCAAATC
CGGGTGCCCCCT
>Bos_taurus_chr4.trna4048-CysGCA (116826499-116826570) Cys (GCA) 72 bp Sc: 69.63
GGGGGTATAGCTCAGGGGTAGAGCATTTGACTGCAGATCAAGATGTCCCCGGTTCAAATC
CGGGTGCCCCCT
>Bos_taurus_chr4.trna4664-CysGCA (116855339-116855268) Cys (GCA) 72 bp Sc: 70.63
GGGGGTATAGCTCAGGGGTAAAGCATTTGACTGCAGATCAAGAGGTCCCCGGTTCAAATC
CGGGTGCCCCCT

>Bos_taurus_chr4.trna4050-CysGCA (116836769-116836840) Cys (GCA) 72 bp Sc: 71.19
GGGGGTATAGCTCAGGGGTAGAGCATTTGACTGCAGATCAAGAGGTCCCCAGTTCAAATC
TGGGTGCCCCCT
>Bos_taurus_chr4.trna4684-CysGCA (116697263-116697192) Cys (GCA) 72 bp Sc: 71.19
GGGGGTATAGCTCAGGGGTAGAGCATTTGACTGCAGATCAAGAGGTCCCCAGTTCAAATC
TGGGTGCCCCCT
>Bos_taurus_chr4.trna4683-CysGCA (116698937-116698866) Cys (GCA) 72 bp Sc: 71.36
GGGGGTATAGCTCAGTGGTAGAGCATTTGACTGCAGATCAAGAGGTCCCTGGTTCAAGTC
CCGGTGCCCCCT
>Bos_taurus_chr4.trna4680-CysGCA (116718501-116718430) Cys (GCA) 72 bp Sc: 73.73
GGGGGTATAGCTCAGGGGTAGAGCATTTGACTGCAGATCAAGAGGTCCCTGGTTCAAATC
CGGGTGCCCCCT
>Bos_taurus_chr4.trna4038-CysGCA (116692200-116692271) Cys (GCA) 72 bp Sc: 74.20
GGGGGTATAGCTCAGGGGTAGAGCATTTGACTGCAGATCAAGAGGTCCCTGGTTCAAATC
CAGGTGCCCCCT
>Bos_taurus_chr4.trna4047-CysGCA (116824235-116824306) Cys (GCA) 72 bp Sc: 74.20
GGGGGTATAGCTCAGGGGTAGAGCATTTGACTGCAGATCAAGAGGTCCCTGGTTCAAATC
CAGGTGCCCCCT
>Bos_taurus_chr4.trna4049-CysGCA (116827886-116827957) Cys (GCA) 72 bp Sc: 74.20
GGGGGTATAGCTCAGGGGTAGAGCATTTGACTGCAGATCAAGAGGTCCCTGGTTCAAATC
CAGGTGCCCCCT
>Bos_taurus_chr4.trna4051-CysGCA (116840891-116840962) Cys (GCA) 72 bp Sc: 74.20
GGGGGTATAGCTCAGGGGTAGAGCATTTGACTGCAGATCAAGAGGTCCCTGGTTCAAATC
CAGGTGCCCCCT
>Bos_taurus_chr4.trna4052-CysGCA (116863947-116864018) Cys (GCA) 72 bp Sc: 74.20
GGGGGTATAGCTCAGGGGTAGAGCATTTGACTGCAGATCAAGAGGTCCCTGGTTCAAATC
CAGGTGCCCCCT
>Bos_taurus_chr4.trna4656-CysGCA (117035318-117035247) Cys (GCA) 72 bp Sc: 74.20
GGGGGTATAGCTCAGGGGTAGAGCATTTGACTGCAGATCAAGAGGTCCCTGGTTCAAATC
CAGGTGCCCCCT
>Bos_taurus_chr4.trna4682-CysGCA (116712416-116712345) Cys (GCA) 72 bp Sc: 74.20
GGGGGTATAGCTCAGGGGTAGAGCATTTGACTGCAGATCAAGAGGTCCCTGGTTCAAATC
CAGGTGCCCCCT
>Bos_taurus_chr12.trna1326-CysGCA (30099283-30099354) Cys (GCA) 72 bp Sc: 74.26
GGGGGTATAGCTCAGGGGTAGAGCATTTGACTGCAGATCAAGAGGTCCCCGGTTCAAATC
CGGGTGCCCCCT
>Bos_taurus_chr19.trna4764-CysGCA (40691438-40691367) Cys (GCA) 72 bp Sc: 74.26
GGGGGTATAGCTCAGGGGTAGAGCATTTGACTGCAGATCAAGAGGTCCCCGGTTCAAATC
CGGGTGCCCCCT
>Bos_taurus_chr19.trna4767-CysGCA (40674236-40674165) Cys (GCA) 72 bp Sc: 74.26
GGGGGTATAGCTCAGGGGTAGAGCATTTGACTGCAGATCAAGAGGTCCCCGGTTCAAATC
CGGGTGCCCCCT
>Bos_taurus_chr4.trna4063-CysGCA (117038765-117038836) Cys (GCA) 72 bp Sc: 74.26
GGGGGTATAGCTCAGGGGTAGAGCATTTGACTGCAGATCAAGAGGTCCCCGGTTCAAATC
CGGGTGCCCCCT
>Bos_taurus_chr4.trna4663-CysGCA (116860226-116860155) Cys (GCA) 72 bp Sc: 74.26
GGGGGTATAGCTCAGGGGTAGAGCATTTGACTGCAGATCAAGAGGTCCCCGGTTCAAATC
CGGGTGCCCCCT
>Bos_taurus_chr4.trna4678-CysGCA (116724648-116724577) Cys (GCA) 72 bp Sc: 74.26
GGGGGTATAGCTCAGGGGTAGAGCATTTGACTGCAGATCAAGAGGTCCCCGGTTCAAATC
CGGGTGCCCCCT
>Bos_taurus_chr4.trna4035-CysGCA (116679513-116679584) Cys (GCA) 72 bp Sc: 75.53
GGGGGTATAGCTCAGTGGTAGAGCATTTGACTGCAGATCAAGAGGTCCCTGGTTCAAGTC
CGGGTGCCCCCT
>Bos_taurus_chr4.trna4037-CysGCA (116690900-116690971) Cys (GCA) 72 bp Sc: 77.65
GGGGGTATAGCTCAGTGGTAGAGCATTTGACTGCAGATCAAGAGGTCCCTGGTTCAAATC
CAGGTGCCCCCT
>Bos_taurus_chr19.trna1986-CysGCA (40694740-40694811) Cys (GCA) 72 bp Sc: 77.71
GGGGGTATAGCTCAGTGGTAGAGCATTTGACTGCAGATCAAGAGGTCCCCGGTTCAAATC
CGGGTGCCCCCT
>Bos_taurus_chr19.trna4743-CysGCA (40920705-40920634) Cys (GCA) 72 bp Sc: 77.71
GGGGGTATAGCTCAGTGGTAGAGCATTTGACTGCAGATCAAGAGGTCCCCGGTTCAAATC
CGGGTGCCCCCT
>Bos_taurus_chr19.trna4744-CysGCA (40919971-40919900) Cys (GCA) 72 bp Sc: 77.71
GGGGGTATAGCTCAGTGGTAGAGCATTTGACTGCAGATCAAGAGGTCCCCGGTTCAAATC
CGGGTGCCCCCT
>Bos_taurus_chr26.trna2497-CysGCA (40060393-40060304) Cys (GCA) 90 bp Sc: 57.00

CCTTCAATAGCTCAGCTAGTAGAGCGGAGGACTGCAGACTTGATAAATGTGGACATCCTT
AGGTTGCCGGTTTGATTCCGGCTTGAAGGA
>Bos_taurus_chr3.trna2268-CysGCA (63863184-63863264) Cys (GCA) 81 bp Sc: 31.29
TCCCTGATGGCTCAATGAGTAAAGCATCTTCCTGCAATGCAAGAGCTGCAGAAGACTCAG
GTTTGATCCCTGGGTAGGGAA
>Bos_taurus_chr21.trna1999-GlnCTG (48045905-48045976) Gln (CTG) 72 bp Sc: 21.20
TCGCTAGTGGTCCAATGGCAAAGATTCTGAGCTCTGGATGCAGGGGGCCAGGATTCAATC
CTGGTTGGGGAA
>Bos_taurus_chr3.trna7394-GlnCTG (54632342-54632271) Gln (CTG) 72 bp Sc: 32.11
GGTTCCATGGTGTAATGATGAGTGCTCTGGAATCTGAATCCAGCAATCCGAGTTCAAGTC
TCTGTGGGACCT
>Bos_taurus_chr16.trna1891-GlnCTG (49240603-49240675) Gln (CTG) 73 bp Sc: 32.42
TCCTTGGTGATCTAGTGGCTGAGATTCTGCGTTCTGAAGGCAGGGGCCCTGGGTTTGATT
CCTGGTCAGGGAA
>Bos_taurus_chr2.trna8397-GlnCTG (65582914-65582841) Gln (CTG) 74 bp Sc: 32.87
TCCCTGATGGTCCAGTGGCTAAGACTCTGTGCTCTGGATGCAGTGGGGGCCAGGTTTGAT
CCTTGGTCAGGAAC
>Bos_taurus_chr5.trna7524-GlnCTG (72185431-72185359) Gln (CTG) 73 bp Sc: 34.96
TCCCTGATGGTCCAGTGGCCAAGACTCTGTGTTCCTGATGCAAGGGGCCCAGGTTTGATC
CTTGGTCAGGAAA
>Bos_taurus_chr19.trna4029-GlnCTG (53532297-53532225) Gln (CTG) 73 bp Sc: 40.48
ACCCTGGTGGTTCAGTGGCTGTGACTCTGCACTCTGAATGCAGCAGGACCAGGTTTGATT
CCTGGCCAGGGAA
>Bos_taurus_chr13.trna721-GlnCTG (20063453-20063526) Gln (CTG) 74 bp Sc: 43.65
TCCCTGGTGGTCCAGTGGCTATGACCATGTGCTCTGAACGCAGGGGGGCCTGGGTTCGAT
CCCTAGTCAGGGAA
>Bos_taurus_chr3.trna4301-GlnCTG (117678950-117679022) Gln (CTG) 73 bp Sc: 45.64
TCCCTGGTGGTCCAGTGGCTAAGACTCTGCGCTCTGAATGCAGGAGGCCCAGGTTTGATC
CCTGGTTGGGTAA
>Bos_taurus_chr5.trna9766-GlnCTG (11001628-11001556) Gln (CTG) 73 bp Sc: 48.74
TCCCTGGTGGCCCAGTGTCTAGGACTCTGCGCTCTGAAGGCAGGGGGCCTAGGTTCAATC
CCTGGTCAGGGAA
>Bos_taurus_chrUn.004.2095.trna2-GlnCTG (16679-16751) Gln (CTG) 73 bp Sc: 48.74
TCCCTGGTGGCCCAGTGTCTAGGACTCTGCGCTCTGAAGGCAGGGGGCCTAGGTTCAATC
CCTGGTCAGGGAA
>Bos_taurus_chr25.trna412-GlnCTG (8307560-8307632) Gln (CTG) 73 bp Sc: 49.02
GAGCCAGTGGCCTAATGGATAAGGCATCAGTATCTGGAGCTGGGGGTTGTGGGTTCAAGT
CCCATCTGGGTCG
>Bos_taurus_chr3.trna8785-GlnCTG (17820262-17820190) Gln (CTG) 73 bp Sc: 49.88
TCACTGGTGGTCCAGTGGCTACGACTCTGTACTCTGAATGCAGGGGGCCCAGGTTCCATC
CCTGGTCAGGGAA
>Bos_taurus_chr3.trna8852-GlnCTG (16480169-16480097) Gln (CTG) 73 bp Sc: 50.44
TCCCTGGTAGTCCAGTGGATAAGACCCTGTGCTCTGAATGTAGGGGGTCCAGGTTCGATC
CCTGGTCAGGGAA
>Bos_taurus_chr14.trna3945-GlnCTG (64610680-64610608) Gln (CTG) 73 bp Sc: 52.91
TCCCTGGTGGTCCAGTGGTTAAGACTCTGAACTCTGAATTCATGGGACCCAGGTTCGTTC
CCTGGTCAGGGAA
>Bos_taurus_chr7.trna107-GlnCTG (3191636-3191708) Gln (CTG) 73 bp Sc: 53.87
TCCCTGGTGGTCCAGTGGCTAAGATTCTGCACTCTGAATGCAGGGGGCTCAGGTTCAGTC
CCTGGTCAGGGAA
>Bos_taurus_chr26.trna3745-GlnCTG (7494965-7494894) Gln (CTG) 72 bp Sc: 55.01
GGTTCCATGGTGTAGTGATTAGCACTCTAGACTCTGAATCCAGTGATCCAAGTTCAAGTC
TCGGTGGAACCT
>Bos_taurus_chr12.trna6013-GlnCTG (21315199-21315127) Gln (CTG) 73 bp Sc: 56.72
TTCCTGGTGGTTCAGTGGCTAAGACTCTGCACTCTGAATGCAGAGAGCCCAGGTACAATC
CCTGGCTGGGAAA
>Bos_taurus_chr4.trna3463-GlnCTG (103442359-103442430) Gln (CTG) 72 bp Sc: 57.08
GGTTCCATGGTGTAATGGTTAGCACTCTGGACTCTGAATCCAGCAATCTGAGTTCAAGTC
TTGGTGGAACCT
>Bos_taurus_chrUn.004.2.trna8-GlnCTG (329218-329289) Gln (CTG) 72 bp Sc: 59.02
TGTTCCATGGTGTAATGGTTAGCACTCTGGACTCTGAATCCAGCGATCTGAGTTCAAATC
TCAATGGAACCT
>Bos_taurus_chr18.trna2433-GlnCTG (54332200-54332272) Gln (CTG) 73 bp Sc: 59.38
TCCCTAGTGGTCCAGTGGTTAGGACTCTGCACTCTGATTGCCAAGGTTCCAGGTTCAATC
CCTGGTCAGGGAA
>Bos_taurus_chr1.trna7504-GlnCTG (113224740-113224669) Gln (CTG) 72 bp Sc: 59.44
AGTTCCATGGTGTAATGGTTAGCACTCTGGACTCTGAATCCAGCAATCTGAGTTCAAGTC

TCAGTGGAACCT
>Bos_taurus_chr5.trna8694-GlnCTG (42777509-42777437) Gln (CTG) 73 bp Sc: 60.90
GGCCGTGTGGCCTAATGGATAAGGCGTCTGATTCTGGATCAGAAGATTGAAGGTTCAAGT
CCATTCATGGTCG
>Bos_taurus_chr23.trna2156-GlnCTG (48113950-48114021) Gln (CTG) 72 bp Sc: 61.66
GGTTCCATGGTGTAATGGTTAGCACTCTGGACTCTGAATCCAGCCATCCGAGTTCAAGTC
TCCGTGGAACCG
>Bos_taurus_chr23.trna1363-GlnCTG (31074628-31074699) Gln (CTG) 72 bp Sc: 68.68
GGCCCCATGGTGTAATGGTTAGCACTCTGGACTCTGAATCCAGCGATCCGAGTTCAAATC
TCGGTGGGACCT
>Bos_taurus_chr3.trna8569-GlnCTG (22699955-22699884) Gln (CTG) 72 bp Sc: 69.34
GGTTCCATGGTGTAATGGTGAGCACTCTGGACTCTGAATCCAGCGATCCGAGTTCAAATC
TCGGTGGGACCT
>Bos_taurus_chr3.trna8576-GlnCTG (22653901-22653830) Gln (CTG) 72 bp Sc: 69.34
GGTTCCATGGTGTAATGGTGAGCACTCTGGACTCTGAATCCAGCGATCCGAGTTCAAATC
TCGGTGGGACCT
>Bos_taurus_chr3.trna8584-GlnCTG (22615359-22615288) Gln (CTG) 72 bp Sc: 69.34
GGTTCCATGGTGTAATGGTGAGCACTCTGGACTCTGAATCCAGCGATCCGAGTTCAAATC
TCGGTGGGACCT
>Bos_taurus_chr3.trna8587-GlnCTG (22590416-22590345) Gln (CTG) 72 bp Sc: 69.34
GGTTCCATGGTGTAATGGTGAGCACTCTGGACTCTGAATCCAGCGATCCGAGTTCAAATC
TCGGTGGGACCT
>Bos_taurus_chr3.trna873-GlnCTG (23084477-23084548) Gln (CTG) 72 bp Sc: 69.34
GGTTCCATGGTGTAATGGTGAGCACTCTGGACTCTGAATCCAGCGATCCGAGTTCAAATC
TCGGTGGGACCT
>Bos_taurus_chrUn.004.185.trna15-GlnCTG (228823-228894) Gln (CTG) 72 bp Sc: 69.34
GGTTCCATGGTGTAATGGTGAGCACTCTGGACTCTGAATCCAGCGATCCGAGTTCAAATC
TCGGTGGGACCT
>Bos_taurus_chrUn.004.185.trna22-GlnCTG (189441-189370) Gln (CTG) 72 bp Sc: 69.34
GGTTCCATGGTGTAATGGTGAGCACTCTGGACTCTGAATCCAGCGATCCGAGTTCAAATC
TCGGTGGGACCT
>Bos_taurus_chrUn.004.2930.trna11-GlnCTG (6648-6577) Gln (CTG) 72 bp Sc: 69.34
GGTTCCATGGTGTAATGGTGAGCACTCTGGACTCTGAATCCAGCGATCCGAGTTCAAATC
TCGGTGGGACCT
>Bos_taurus_chrUn.004.2930.trna9-GlnCTG (11535-11464) Gln (CTG) 72 bp Sc: 69.34
GGTTCCATGGTGTAATGGTGAGCACTCTGGACTCTGAATCCAGCGATCCGAGTTCAAATC
TCGGTGGGACCT
>Bos_taurus_chr3.trna8593-GlnCTG (22552211-22552140) Gln (CTG) 72 bp Sc: 70.79
GGTTCCATGGTGTAATGGTGAGCACTCTGGACTCTGAATCCAGTGATCCGAGTTCAAATC
TCGGTGGGACCT
>Bos_taurus_chr3.trna940-GlnCTG (24595650-24595721) Gln (CTG) 72 bp Sc: 71.08
GGTTCCATGGTGTAATGGTGAGCACTCTGGACTCTGAATCCAGCGATCCGAGTTCGAATC
TCGGTGGGACCT
>Bos_taurus_chr23.trna1378-GlnCTG (31177801-31177872) Gln (CTG) 72 bp Sc: 71.26
GGTTCCATGGTGTAATGGTTAGCACTCTGGACTCTGAATCCAGCGATCCGAGTTCAAGTC
TCGGTGGGACCT
>Bos_taurus_chrUn.004.2552.trna1-GlnCTG (2200-2271) Gln (CTG) 72 bp Sc: 71.26
GGTTCCATGGTGTAATGGTTAGCACTCTGGACTCTGAATCCAGCGATCCGAGTTCAAGTC
TCGGTGGGACCT
>Bos_taurus_chr10.trna7732-GlnCTG (12568418-12568347) Gln (CTG) 72 bp Sc: 73.65
GGTTCCATGGTGTAATGGTTAGCACTCTGGACTCTGAATCCAGCGATCCGAGTTCAAATC
TCGGTGGAACCT
>Bos_taurus_chr19.trna1407-GlnCTG (28305497-28305568) Gln (CTG) 72 bp Sc: 73.65
GGTTCCATGGTGTAATGGTTAGCACTCTGGACTCTGAATCCAGCGATCCGAGTTCAAATC
TCGGTGGAACCT
>Bos_taurus_chr23.trna1319-GlnCTG (30091346-30091417) Gln (CTG) 72 bp Sc: 73.65
GGTTCCATGGTGTAATGGTTAGCACTCTGGACTCTGAATCCAGCGATCCGAGTTCAAATC
TCGGTGGAACCT
>Bos_taurus_chr23.trna3407-GlnCTG (31184470-31184399) Gln (CTG) 72 bp Sc: 73.65
GGTTCCATGGTGTAATGGTTAGCACTCTGGACTCTGAATCCAGCGATCCGAGTTCAAATC
TCGGTGGAACCT
>Bos_taurus_chr6.trna639-GlnCTG (23604882-23605004) Gln (CTG) 123 bp Sc: 20.94
TCTCTGGTAGTCCAGTGGTACAGACTTCAGTGCTGTGCAGGATCAATGATAACAGCACTT
ATCAATGCTGATCCTGGTACAGGATCAATGCTGGGGACCCAGGTTTGATCCCTGGTCAGG
GAA
>Bos_taurus_chr3.trna746-GlnTTG (21243831-21243903) Gln (TTG) 73 bp Sc: 39.71
TCCCTGGTGGTCCATTGGTTAAGACTCTGCACTTTGAATGCAGGGAGCATGGGTTTAATC

CCTGGTCAGGGAA
>Bos_taurus_chr23.trna1249-GlnTTG (28312758-28312829) Gln (TTG) 72 bp Sc: 40.09
TCCCTGGTGGTCCAGTGGTTAGGATTCAGTAGTTTGACTGCTGAGACCTGGGTTTGATCT
CTGGTTGGGGAA
>Bos_taurus_chr23.trna4404-GlnTTG (10602971-10602899) Gln (TTG) 73 bp Sc: 40.32
TCTCTGACAGTCCAGTGGTTGGGACTCAGTGCTTTGACTGCTGTGGGCCTGGGTTCAATC
CCTAGTCAGGGAA
>Bos_taurus_chr19.trna4923-GlnTTG (36475346-36475274) Gln (TTG) 73 bp Sc: 40.40
TCCCTGGCTGTTCAGTGGTTAGGACTTGGTACTTTGACTGCCGTGGATCCAGGTTTGATC
CCTGGTCAGGGAA
>Bos_taurus_chrM.trna3-GlnTTG (4194-4123) Gln (TTG) 72 bp Sc: 43.85
TAGAATTTGGTGTAATTGGGAGCACGAAGAGTTTTGGATTCTTAGGAGTAGGTTCGATTC
CTATAGTTCTAG
>Bos_taurus_chr9.trna4482-GlnTTG (94902874-94902802) Gln (TTG) 73 bp Sc: 47.16
TCCCTGGTGGTCCAGTGGTTAGGACTCAGTACTTTGACTGCCGAGGGCTGAGGTTCAGTC
CCTGGTCAGGGAA
>Bos_taurus_chr5.trna4073-GlnTTG (105019822-105019894) Gln (TTG) 73 bp Sc: 54.29
TCCTTGATGGTCCAGTGATTAGGACTTGGCACTTTGACTGCCAAGGGCCCAGGTTCAGTC
CCTGATCAGGGAA
>Bos_taurus_chr10.trna5443-GlnTTG (74387554-74387483) Gln (TTG) 72 bp Sc: 55.87
TCCCTGGTGGTCTAGTGGTTAGGACTCAGAGCTTTGACTACTGTGGCCTGGGTTCAATTC
CTGGTCAGGGAA
>Bos_taurus_chr1.trna10643-GlnTTG (10098320-10098249) Gln (TTG) 72 bp Sc: 56.11
TTCCTGGTGGTCTAGTGGTTAGGATTCGGCACTTTGACTGCTGTGGCCTGGGTTCAATCC
CCAGTTGGGGGA
>Bos_taurus_chr19.trna6013-GlnTTG (17609832-17609761) Gln (TTG) 72 bp Sc: 59.07
TCCCTGGTAGTCTAGTGGTTAGGATTCAACACTTTGACTGCTGTGGCCCGGGTTCAATAC
CTGGTTAGGGAA
>Bos_taurus_chr7.trna1307-GlnTTG (23037775-23037846) Gln (TTG) 72 bp Sc: 59.35
TCCCTGGTGGTCCAGTGGTTAGGACTCCATGCTTTGACTGTTGGGACCCAGGTTCAATCC
CTGGTCAGGGAA
>Bos_taurus_chr23.trna1362-GlnTTG (31070717-31070788) Gln (TTG) 72 bp Sc: 68.31
GGCCCCATGGTGTAATGGTTAGCACTCTGGACTTTGAATCCAGCGATCCGAGTTCAAATC
TCGGTGGGACCT
>Bos_taurus_chr23.trna1426-GlnTTG (31705398-31705469) Gln (TTG) 72 bp Sc: 68.31
GGCCCCATGGTGTAATGGTTAGCACTCTGGACTTTGAATCCAGCGATCCGAGTTCAAATC
TCGGTGGGACCT
>Bos_taurus_chr23.trna1427-GlnTTG (31705961-31706032) Gln (TTG) 72 bp Sc: 68.31
GGCCCCATGGTGTAATGGTTAGCACTCTGGACTTTGAATCCAGCGATCCGAGTTCAAATC
TCGGTGGGACCT
>Bos_taurus_chr23.trna3453-GlnTTG (30256962-30256891) Gln (TTG) 72 bp Sc: 69.80
GGTCCCATGGTGTAATGGTCAGCACTCTGGACTTTGAATCCAGCAATCCGAGTTCGAATC
TCGGTGGGACCT
>Bos_taurus_chr19.trna2628-GlnTTG (51584964-51585035) Gln (TTG) 72 bp Sc: 72.03
GGTTCTATGGTGTAATGGTTAGCACTCTGGACTTTGAATCCAGCGATCCGAGTTCAAATC
TCGGTAGAACCT
>Bos_taurus_chr19.trna4845-GlnTTG (38583266-38583195) Gln (TTG) 72 bp Sc: 73.77
GGTCCCATGGTGTAATGGTTAGCACTCTGGACTTTGAATCCAGCGATCCGAGTTCAAATC
TCGGTGGGACCT
>Bos_taurus_chr25.trna2907-GlnTTG (35596830-35596751) Gln (TTG) 80 bp Sc: 56.77
TCCCTGGTGGTCCAGTGGTTAGGACTTGGCACTTTGGCGCTTTCACTGCCATGGCTTGGG
TTCAATCCCCGTTCAGGGAA
>Bos_taurus_chrUn.004.725.trna2-GluCTC (19880-19952) Glu (CTC) 73 bp Sc: 26.89
TCTCTGGCAGTTCAATGGCTAAGACTCTGTACTCTCACTACAGGGGACCTGGGTTCAATG
CCTGGTCAGGGAA
>Bos_taurus_chr7.trna6689-GluCTC (42398321-42398249) Glu (CTC) 73 bp Sc: 28.69
TCCCTGGCAGTTCAGTAGTTAGAACTAGGTGGTCTCACTGCTCTGGGCCCAGGTTCAATC
CCTGGTTGGGGAA
>Bos_taurus_chr6.trna5651-GluCTC (93982250-93982179) Glu (CTC) 72 bp Sc: 29.10
TCTCTGGTGGTCCAGTGGCTAAGACTTTGCGCTCTCAAAGCAGGGAGACTGGATTCGATC
CTGTTCAGGGAA
>Bos_taurus_chr24.trna1306-GluCTC (34219316-34219389) Glu (CTC) 74 bp Sc: 29.34
TCCCTGGTGGTCCAGTGACTAAGAACTCCGTGTTCTCGATGCAGGGGGCCCAGGTTCCAT
CCCTGGTCAGGGAA
>Bos_taurus_chr6.trna6448-GluCTC (70233922-70233851) Glu (CTC) 72 bp Sc: 30.00
TCCCTGATGGTCCAGCGTTAGGACATGGTTCTCTCACTGCTATAGGTCTGGGTTTGATCT
CTAGTCAGGGAA

>Bos_taurus_chr6.trna8142-GluCTC (18202643-18202571) Glu (CTC) 73 bp Sc: 30.80
TCCCTGGTTGTCCAGTGGTTAAGACTCTGCACCCTCAATACAGGGTGTCTGGGTTCAGTC
CCTGGTTGGGGAA
>Bos_taurus_chr10.trna1370-GluCTC (34963449-34963521) Glu (CTC) 73 bp Sc: 31.81
TCCCTGGTGGTCCAGGGGCTAAGACTCTGAGCTCTCAATGCAGGGAGTCTAGGTTCCACC
CCTAGTCAGAGAG
>Bos_taurus_chr16.trna1195-GluCTC (34039094-34039173) Glu (CTC) 80 bp Sc: 31.86
TCCCTGATGGTCCATTGGTTAGGACTTGGTGCTCTCACTGCAGGGGTGTAGGGGCCTAGG
CTCAATCCCTGGTCAGGGTA
>Bos_taurus_chr28.trna1237-GluCTC (35091974-35092044) Glu (CTC) 71 bp Sc: 31.98
TCCTGGAGGTCCAGGGGTTAGGACTCGGTGTTCTCACTGCCAGGCCTCAGGTTCAATCCC
TGATCGGGGAC
>Bos_taurus_chr26.trna857-GluCTC (24928716-24928787) Glu (CTC) 72 bp Sc: 32.01
TCCCTGGAGGAGCAGTGGTTAAGACTCCCCACTCTCCCTGGGAGGGCTCAGGTTCCATCC
CTGGCCGGGGAA
>Bos_taurus_chr11.trna892-GluCTC (20909741-20909812) Glu (CTC) 72 bp Sc: 32.23
TCCCTGGTGGTCCAGTGGCTAAGATGCTGTGCTCTCAATGCAGGGTACCTGGGTTTGATC
CTGGTCAGGGAA
>Bos_taurus_chr23.trna4336-GluCTC (11628584-11628512) Glu (CTC) 73 bp Sc: 32.48
TCCCTGGCCGTCCAATTGTTAGGACTCTGCACTCTCACTGCTGAAGACCTGGGTTCAATC
CCTGGTCAGGGAA
>Bos_taurus_chr25.trna4157-GluCTC (13969582-13969510) Glu (CTC) 73 bp Sc: 33.44
TCCCTGATGGTCCAGTAGTTAAGACTCTGTGTGCTCAATTCAGGGGGCCCGGGTTTGACC
CCTGGTCAGGGAA
>Bos_taurus_chr7.trna698-GluCTC (13238000-13238072) Glu (CTC) 73 bp Sc: 33.62
TCCCTGGTGGTCCAGTAGCTAAGACTCTGCGCTCTCAGTGCAGGGGGCCCGGGTTGGATC
CCTGGGCAGGGAA
>Bos_taurus_chrX.trna2652-GluCTC (71714643-71714715) Glu (CTC) 73 bp Sc: 33.97
TCCCTGGTAGTCCAGTGGCTAAGACTCCGCACTCTCAATGCAGGGGGGCTGGGTTCCTTC
CCTGCTCAGGGAA
>Bos_taurus_chr7.trna7541-GluCTC (18412057-18411984) Glu (CTC) 74 bp Sc: 33.99
TCCCTGGTGATCCAGTCGCTAAGACTCTGAGCTCTCAATGCAGGGGGCCCAGGGTTCAAT
CCCTGGTCAGGGAA
>Bos_taurus_chr18.trna1224-GluCTC (30610748-30610820) Glu (CTC) 73 bp Sc: 34.10
TCTCTGGTGGTCCAGTGGCTAAGACTCTGTTCTCTCAGTGCAGGGGGTTCAGGTTCAATC
CCTAGTCAGGGAA
>Bos_taurus_chr12.trna755-GluCTC (18616739-18616811) Glu (CTC) 73 bp Sc: 34.17
TCCCTGGTGATCCAGTGGCTGTGATTCTGCACTCTCCATGCAAGGGGCCCAGGTTTGATC
CCTGGTCAGGGAA
>Bos_taurus_chr15.trna3896-GluCTC (64092817-64092745) Glu (CTC) 73 bp Sc: 34.33
TCCCTGGTGGTCCAGTGATTAAGACTCTGTGCTCTCAATGCACTGGATACAGGTTTGATC
CCTGGTCAGGGAA
>Bos_taurus_chr6.trna1592-GluCTC (57267578-57267648) Glu (CTC) 71 bp Sc: 34.47
TCCCTGATGGTCCAATGGGAGGACTTGGCAATCTCACTGCTAGAACCTGGGTTCAATCTC
TGGTTGGGGAA
>Bos_taurus_chr8.trna4885-GluCTC (96130743-96130671) Glu (CTC) 73 bp Sc: 34.94
TCCCTGGTGGCCCAGTGATTAGGGCTCTGTGTTCTCTCTGCTGATGGCCCAGGTTCAATC
CCTGGTCAGAGAA
>Bos_taurus_chr8.trna8149-GluCTC (3683348-3683276) Glu (CTC) 73 bp Sc: 34.97
TTCCTGGTGGTCCAGTGGCTAAGATTCTGCCCTCTCAATGCAGGGGGCTCAGCTTCCATC
CCTGGTTAGGGAA
>Bos_taurus_chr22.trna4114-GluCTC (10010044-10009973) Glu (CTC) 72 bp Sc: 35.12
TCCCTTGTGGTCTACTGGTTAGGATTCAGTGTTCTCTCCACTGCAGCCTGGGTTCGATTC
CTGGTCAGGGAA
>Bos_taurus_chr13.trna4866-GluCTC (64615421-64615349) Glu (CTC) 73 bp Sc: 35.28
TCCCTGGTCGTCCAGTGGTTAAGACTCTATGTTCTCAATGCAGGGGGCTCAGGTTTGATC
CTTGGTCAGGGAA
>Bos_taurus_chr1.trna7898-GluCTC (99933323-99933250) Glu (CTC) 74 bp Sc: 35.29
TCCCTGGTGGTCCAGCAATTAGGACTCTGCACACTCACTGCTGAGGGGTTCAGGTTCAAT
CCCTGGCCGGGGAA
>Bos_taurus_chrUn.004.1.trna143-GluCTC (2251538-2251610) Glu (CTC) 73 bp Sc: 36.03
TCCCTGGTGGTCCAGTGTCTAAGACTCTGAGCTCTCAATGCAGGGGACCTGGGTTCAATC
TCTAGTCAGGGAA
>Bos_taurus_chr17.trna2269-GluCTC (55643896-55643968) Glu (CTC) 73 bp Sc: 36.05
TCCCTGGCAGTCCAGTGGCTAAGACACTGCAATCTCAATGCAAGGGGCTCAGGTTTGATC
CCTGGTCAGGGAA
>Bos_taurus_chr14.trna5401-GluCTC (28350770-28350698) Glu (CTC) 73 bp Sc: 36.29

TCCCTGGTGGTCCAGTGGCCTAGATTCTGCAGTCTCAATGCAGGGGAGCCAGGTTCAGTC
CCTGGTCAGGGAA
>Bos_taurus_chr7.trna7878-GluCTC (14180782-14180710) Glu (CTC) 73 bp Sc: 36.83
TCCCTGGTGGTCCAGTGGCTAAGACGCTGTTCTCTCAATGCAGGAGGGTCAGGTTTGATC
TCTGGTCAGGGAA
>Bos_taurus_chr22.trna4185-GluCTC (8473439-8473367) Glu (CTC) 73 bp Sc: 36.83
TTCCTGGTGGCCCAGGGGCTCAGGCTTCGCATCCTCAATGCAGGGGTCCCAGGTTCCATC
CCTGGTCAGGGAA
>Bos_taurus_chr8.trna2729-GluCTC (79627635-79627707) Glu (CTC) 73 bp Sc: 37.00
TCTCTGGTGGTCCAGTGGCTAAGACTCTGTGCTCTCAATGCAGGGCGCCCAGGCTTGATC
CCTGGTCAGGGAA
>Bos_taurus_chr16.trna2045-GluCTC (52400585-52400656) Glu (CTC) 72 bp Sc: 37.77
TCCCTGGCAGTCCAATGGTTAGGACTTGGTGCTCTCACTGCAGGGGCCCAGGTTCAACCT
CTGGTTAGGGAA
>Bos_taurus_chr20.trna4910-GluCTC (22101783-22101711) Glu (CTC) 73 bp Sc: 37.84
TCCCTGGTGGTGCAGTGGCTAGGACTCTACACTCTCAGTGCGGGGGCCCCAGGTTCTGTC
CCTGGTCAGGGAA
>Bos_taurus_chrUn.004.465.trna1-GluCTC (14315-14387) Glu (CTC) 73 bp Sc: 37.86
TCCCTGGTGGTCCAGTGGCTAAGACTCTGCACTCTCAGTACAGAGGGACTGGGTTTGATC
CCTGGTCAGGGAG
>Bos_taurus_chr17.trna1176-GluCTC (31551005-31551076) Glu (CTC) 72 bp Sc: 37.98
TCCCTGGTGGTCCAGTGACTGGGACTCTGCACTCTCAATGCAGGAGCCCGGGTTGGATCC
CTGTTCAGGGAA
>Bos_taurus_chr4.trna4721-GluCTC (116096332-116096260) Glu (CTC) 73 bp Sc: 38.59
TCCCTGGTGGTCCAGTGGCTGAGACTCTGTGTTCTCAATGCAGGGGGCCCGGGTTCCATC
CCTGGTCAGGGAA
>Bos_taurus_chr24.trna668-GluCTC (20959413-20959485) Glu (CTC) 73 bp Sc: 38.85
TCCCTGATGGTCCAGTGGTTAAGACTTGGTGCTCTCATTGCCATGGGCCTGGGTTAAATA
CCTGGTCACGGAA
>Bos_taurus_chr5.trna1984-GluCTC (54724004-54724076) Glu (CTC) 73 bp Sc: 38.92
TCCCTGGTGGTCCAGTGATTAGGACTTGGTGCTCTCACTGCCAGGGACCTGGGTTCAATC
CTGGGTCAGGGAA
>Bos_taurus_chr5.trna4487-GluCTC (113491728-113491799) Glu (CTC) 72 bp Sc: 39.06
TCCCAGGTGGTCCAGAGCTAAGACTCTGTACTCTCAACACAGGGGACCCAGGTTTGATCC
CTGGCCAGGGAA
>Bos_taurus_chr3.trna3772-GluCTC (106880769-106880841) Glu (CTC) 73 bp Sc: 39.08
TTCCTGGCGGTCCAGTGTTTAGGACTCTGCACTCTCACTGCAGAGGGCCTGGGTTCAGTC
CCTGGTCAGGGAA
>Bos_taurus_chr25.trna4280-GluCTC (11952071-11951999) Glu (CTC) 73 bp Sc: 39.38
TCCCTGGTGGTCCAGTGGCTAAGACTCCATGCTCTCAACGTCGGGGGCTCAGGTTCCCTC
CCTGGTCAGGGAG
>Bos_taurus_chr22.trna487-GluCTC (10789231-10789303) Glu (CTC) 73 bp Sc: 39.69
TCCCTAGTGGTCCAGTGACTAAGACTCTGCACTCTCAATGTAGGGGACTTAGGTTCGATC
CCTGGTCAGGGAA
>Bos_taurus_chr12.trna5506-GluCTC (31530919-31530847) Glu (CTC) 73 bp Sc: 39.77
CCCCTGGTGGTCCAGTGGCTAAGACTCTGTACTCTCAATACAGGCAGGCCAGGTTCTATC
CCTGGTTAGGGAA
>Bos_taurus_chr23.trna1481-GluCTC (33074973-33075045) Glu (CTC) 73 bp Sc: 40.72
TCTCTGGTGGTCCAGTGGCTGGGACTCTGCGCTCTCAGCGCCGAGGGCCCAGGTTCAATC
CCTGCTCAGGGAA
>Bos_taurus_chr8.trna1430-GluCTC (43986665-43986737) Glu (CTC) 73 bp Sc: 40.73
TCTCTGGTAGTCCAGTGGTTAAGACTCTGTTCTCTCAGTGTAGGGGGCCCAGGTTCAATT
TCTGGTCAGGGAA
>Bos_taurus_chr25.trna816-GluCTC (13750321-13750393) Glu (CTC) 73 bp Sc: 41.27
TCTCTGGTGGTCCAGTGGCTAAGACTCTGCACTCTCAGGGCAGGGAACCCAGGTTCCATC
CCTGGTCAGGGAA
>Bos_taurus_chr7.trna3094-GluCTC (74918155-74918226) Glu (CTC) 72 bp Sc: 41.28
TCCCTGGTGGTCTAGTGGTTAGGATTCGGTGCTCTCACTGCCACAGCCAGGGTTGAATTC
CCAGTTAGGGAA
>Bos_taurus_chr1.trna6856-GluCTC (132298853-132298781) Glu (CTC) 73 bp Sc: 41.72
TCCCTGGTGGTCCAGTGGCTAGGACTCTGTGCTCTCAGTGCAGGGGGTCCAGGTTCATTT
CCTGGTCAGGAAA
>Bos_taurus_chr9.trna4959-GluCTC (84535491-84535419) Glu (CTC) 73 bp Sc: 41.94
TATCTGGTGGTCTAGAGGCTAAGACTCTGAGCTCTCAACGCAGGGGGCCCAGGTTCTATC
CCTGGTCAGGAAA
>Bos_taurus_chr20.trna1552-GluCTC (42136910-42136981) Glu (CTC) 72 bp Sc: 42.16
CCCTTGCTGGTCTGGTGGCTAGGATTCCTGGTTCTCATCCAGGCTTTCCAGGTTCAGCTC

CTGGGCAGGGGA
>Bos_taurus_chr28.trna1212-GluCTC (34521151-34521223) Glu (CTC) 73 bp Sc: 42.31
TCCTTGGCAGTCCAGTGGTTAGGACTTCATGCTCTCCCTGTCAAAGGCCCAGGTTCAATC
CCTGGTCAAGGAA
>Bos_taurus_chr2.trna3176-GluCTC (99515947-99516019) Glu (CTC) 73 bp Sc: 42.38
TCCCTGGTGGTCCAGTGGGTAAGACTCTATGCTCTCAATGCAGGGGACCCAGGATCCATC
CCTGGTCAGGGAA
>Bos_taurus_chr21.trna4453-GluCTC (26994339-26994267) Glu (CTC) 73 bp Sc: 42.47
TCCCTGGTGGTCCAGTGGCTAAGACTCTGAGTTCTCAGTGCAGGGGGCCCAGGTTCCATC
CCTGGTCAGGGAA
>Bos_taurus_chr10.trna2247-GluCTC (58962949-58963021) Glu (CTC) 73 bp Sc: 42.55
TTCCTGATGGTCCAGTGGCTAAGACTCCGTGCTCTCAATGCAGGGGGCCCAGGTTTAATA
CCTGGTCAGGGAA
>Bos_taurus_chr19.trna6066-GluCTC (16892643-16892571) Glu (CTC) 73 bp Sc: 42.90
TCCCTGATGGTCCAGTGGCTGAGACTCTGAGCTCTCAATGCAGGGAGCCCAGATTCAATC
CCTGGTCAGGGAA
>Bos_taurus_chrX.trna6605-GluCTC (5978651-5978579) Glu (CTC) 73 bp Sc: 43.27
TCCCTGGTGGTCCAGTGGCTAAGACACTGTGCTCTCAATGCAGGGGGCCTGGGTTCAGTT
CCTGGTCAGGGAA
>Bos_taurus_chrUn.004.475.trna13-GluCTC (18810-18738) Glu (CTC) 73 bp Sc: 43.61
TCCCTGGTGGTCCAGTGGCTAAGACTCTGTACTCTCCATGCAGGGGGCTTAGGTTTGATC
CCTGGTTAGGGAA
>Bos_taurus_chr3.trna5156-GluCTC (118057029-118056957) Glu (CTC) 73 bp Sc: 43.67
TCCCTGATGGTCCTGTGGGTAGGATGTGGTGCTCTCGCTGCCTGGAGCCCAGGTTCAACC
CCTGGTCAGGGAA
>Bos_taurus_chr15.trna1936-GluCTC (55715031-55715103) Glu (CTC) 73 bp Sc: 43.75
TCCCCGGTGGTTCAGTAGTTAGGACTCCGCACTCTCGCTGCTGAGGGCTCTGGTTCCATC
CCTGGTTGGGGAA
>Bos_taurus_chr10.trna3374-GluCTC (88280785-88280857) Glu (CTC) 73 bp Sc: 44.14
TCCCTGGTGATCCAGTGGTTAGGATTTGGTCCTCTCACTGCCAGCGGCCTAGGTTCAATC
CCTGGTTGGGGAG
>Bos_taurus_chr25.trna4326-GluCTC (11556248-11556176) Glu (CTC) 73 bp Sc: 44.18
TCCCTTGTGGTCCAGTGGCTAAGACTCTGAGCTCTCAGTGCAGGGGGCCCAGGTTCAATC
CCTGGTCAGGGAA
>Bos_taurus_chr1.trna5721-GluCTC (160713082-160713153) Glu (CTC) 72 bp Sc: 44.21
TCCTTGGTGGTCCAGTGGTTAGGACTTGGTGCTCTCACTGCCAAGGGCCGGGTTTGATTT
CTGGTTGGGGAA
>Bos_taurus_chr12.trna1108-GluCTC (25144977-25145049) Glu (CTC) 73 bp Sc: 44.43
TCCCTGGCAGTCCAGTGGCTAAGACTCTGTACTCTCAATTCAGGGGTCCTGGGTTCCATC
CCTGGTCAGGGAA
>Bos_taurus_chr11.trna6536-GluCTC (65434744-65434672) Glu (CTC) 73 bp Sc: 44.44
TCCCTGGTGGTCCAGTGGCTAAGACTCTGAGCTCTCAGTGCAGGGGGCCCAGGTTTGATC
CCTGGTCAGGGAG
>Bos_taurus_chr17.trna2948-GluCTC (66923262-66923334) Glu (CTC) 73 bp Sc: 44.63
TCCCTGGTGGTCCAGTGGCTAAGACTCTGCACTCTCAATGCAGGGGGCCTGGGTCCAATC
CCTGATCAGGGAA
>Bos_taurus_chr24.trna3297-GluCTC (52287460-52287389) Glu (CTC) 72 bp Sc: 44.80
TCCCTGATGGTCCACTGGTTGGGACTAGGTGCTCTCACTGCCATAGCCCAGGTTCAATTC
CTGGTTGGGGAA
>Bos_taurus_chr14.trna3058-GluCTC (74320235-74320306) Glu (CTC) 72 bp Sc: 45.07
TCCCTGGTAGTCCAGTGGCTAAGATCCTGAGCTCTCAGTGCAGGGGCCCAGGTTCAATTC
TTGGTCAGGGAA
>Bos_taurus_chr5.trna5819-GluCTC (111445376-111445304) Glu (CTC) 73 bp Sc: 45.09
TCCCTGGTGGTCCAGTGGCTAAGACTCTGCATTCTCAGTTCAGGGGCCTCAGGTTCCATC
CCTGGTCAGGGAA
>Bos_taurus_chr17.trna4272-GluCTC (60868267-60868195) Glu (CTC) 73 bp Sc: 45.10
TCCCTGGTCGTCCAGTGGCTGAGACTCTGCCCTCTCAATGCAGAGGGCCCAGGTTTGATC
CCTGGTCAGGGAG
>Bos_taurus_chr10.trna3372-GluCTC (88241020-88241092) Glu (CTC) 73 bp Sc: 45.23
TCCCTGGTGGTCCAGTGACTAAGACTCCGTGCTCTCAATGCAGGGGGCCCAGGTTCAATC
CCTGGTCAGGGAA
>Bos_taurus_chr9.trna6679-GluCTC (35106489-35106417) Glu (CTC) 73 bp Sc: 45.29
TCCCTGGTGGTCCAGTGGTTAGGATTTGGTGTTCTCACTGCCAGGGGCCTGGGTTTGATC
CCTGGTCAGGGAA
>Bos_taurus_chr3.trna3738-GluCTC (106057206-106057278) Glu (CTC) 73 bp Sc: 45.53
TCCCTGGTGGTCCAATGGCTAAGACTCTGCACTCTCAAGGCAGGGCCCCCAGGTTCCATC
CCTGGTCAGGAAA

>Bos_taurus_chr29.trna3756-GluCTC (9175232-9175161) Glu (CTC) 72 bp Sc: 45.57
TCCCTGGCGGTCCAGTGGCTAAGACTCTGCACTCTCAATGCAGGGGTCTGGGTTGGATCC
CTGGTCAGGGAA
>Bos_taurus_chr1.trna7317-GluCTC (119621992-119621920) Glu (CTC) 73 bp Sc: 45.95
TCCCTGGTGGTCCAGTGGCTAAGATTCTGCCCTCTCAATGCAGGGAGCCCAGGTTTAATC
CTTGGTCAGGGAA
>Bos_taurus_chrUn.004.7.trna10-GluCTC (302998-303069) Glu (CTC) 72 bp Sc: 46.03
TCCCTGGTGGTCCAACAGTTAGGACTTGGTGCTCTCACTGCCATGGCCCAGGTTCAATCC
CTGCTTGGGGAA
>Bos_taurus_chr1.trna8338-GluCTC (86652884-86652813) Glu (CTC) 72 bp Sc: 46.05
TCCCTGGTTGTCCAGTGGCTAAAACTCTATGCTCTCAATGCAGGGGCTCAGGTTCAATCC
CTGACCAGGGAA
>Bos_taurus_chr2.trna4175-GluCTC (123364835-123364906) Glu (CTC) 72 bp Sc: 46.16
TCCCTGATGGCCTAATGGTTAAGATGTGGTCCTCTCATCACTATGGCCTGGGTTCAATCC
CTAGTCAGGGAG
>Bos_taurus_chr21.trna4490-GluCTC (26728157-26728085) Glu (CTC) 73 bp Sc: 46.35
TCCCTGATGGTCCAGTGGCTAAGACTCTGCATTCTCAGTGCAGGGGGACTGGGTTTGATA
CCCCGTCAGGGAA
>Bos_taurus_chr5.trna9802-GluCTC (10451389-10451318) Glu (CTC) 72 bp Sc: 46.37
TCCCTGGAGGTCCAGTGGTTAGGACTTGGTGCTCTCAATGTTGAGACCCAAGTTCAAACC
TTGGCTGGGGAA
>Bos_taurus_chr2.trna8095-GluCTC (75035727-75035655) Glu (CTC) 73 bp Sc: 46.76
TCCCTGGTGGTCCAGTGGCTAAGACTCTGTGCTCTCAGTGCAGGGGGCCCAGGTTTGATC
CCTGGTCAGGGAG
>Bos_taurus_chr11.trna4883-GluCTC (103435269-103435197) Glu (CTC) 73 bp Sc: 46.81
TCCCTGGTGGTCCAGTGGCTAAGACTCTGCGTTCTCAGTGCAAGGGGCCCAGGTTCAATC
CCTGGTCAGGGAA
>Bos_taurus_chr9.trna3781-GluCTC (103895823-103895895) Glu (CTC) 73 bp Sc: 46.89
TCCCTGGCAGTCCAGTGGCTAAGACTCTGTATTCTCAATGCAAGGATCCCAGGTTTGATC
CCTGGTCAGGGAA
>Bos_taurus_chr3.trna8805-GluCTC (17558626-17558555) Glu (CTC) 72 bp Sc: 47.07
TCCCTGGTGGTCCAGTGGCTAAGACTCTGCATTCTCAATGCAGGGGGCCTGGGTTCATCC
CTGGTCAGGGAA
>Bos_taurus_chr1.trna7903-GluCTC (99896417-99896346) Glu (CTC) 72 bp Sc: 47.10
TCCCTGGTAGTTTAGTGGCTAAGATCCTGTGCTCTCAATGCAGGGGCCCAGGTTCAATCC
CTGATTGGGGAA
>Bos_taurus_chr29.trna2158-GluCTC (47554827-47554755) Glu (CTC) 73 bp Sc: 47.12
TCCCTGGTGGTCCAGTGGCTAAGACCCTACATTCTCCATGCAGAGGGCCCAGGTTTAATC
CCTGGTCAGGGAA
>Bos_taurus_chr19.trna1310-GluCTC (26551238-26551310) Glu (CTC) 73 bp Sc: 47.26
TCCCTGGCAGTCCAGTGGTTAGGACTCTGTGCTCTCACTGCCGAGGGCCTGGGTTCAATC
CCTGGCCAGGGAA
>Bos_taurus_chr20.trna1504-GluCTC (41037256-41037328) Glu (CTC) 73 bp Sc: 47.37
TCCCTGGTGGTCCAGTGGCTAAGACTCTACACTCTCAATGCAGGGGACCCAGGTTAGATC
CCAGGTTAGGGAA
>Bos_taurus_chr3.trna6425-GluCTC (85445220-85445149) Glu (CTC) 72 bp Sc: 47.70
TCTTTGCTGGTCTGGTGGTTAGGATTTCTGGTTCTCATCCAGACTACCTAGGTTTAATTC
CTAGGCAGGGAA
>Bos_taurus_chrUn.004.3458.trna3-GluCTC (1800-1729) Glu (CTC) 72 bp Sc: 47.70
TCTTTGCTGGTCTGGTGGTTAGGATTTCTGGTTCTCATCCAGACTACCTAGGTTTAATTC
CTAGGCAGGGAA
>Bos_taurus_chrUn.004.7373.trna3-GluCTC (1506-1435) Glu (CTC) 72 bp Sc: 47.70
TCTTTGCTGGTCTGGTGGTTAGGATTTCTGGTTCTCATCCAGACTACCTAGGTTTAATTC
CTAGGCAGGGAA
>Bos_taurus_chr14.trna6364-GluCTC (8206014-8205943) Glu (CTC) 72 bp Sc: 47.70
TCCCTGGTGGTCCAGTGGCTAAGACTCTGCACTCTCAGTGCAGGGGCCTGGGTTTGATAC
CTGGTCAGGGAA
>Bos_taurus_chr18.trna3228-GluCTC (60048075-60048003) Glu (CTC) 73 bp Sc: 47.71
TCCCTGGTGGTCCAGTGGCTAAGACACTACATTCTCAATGCAGCTGGCCCAGATTCAATC
CCTGGTCAGGGAA
>Bos_taurus_chr7.trna8386-GluCTC (6844857-6844785) Glu (CTC) 73 bp Sc: 48.26
TTCCTGACAGTCCAGTGGTTAGGACTCTGCACTCTCACTGCCGAGGGCCCAGGTTCTATT
CTTGGTTGGGGAA
>Bos_taurus_chrX.trna3265-GluCTC (85160805-85160877) Glu (CTC) 73 bp Sc: 48.47
TCCTTGATGGCCCAGTCGCTAAGACTCTGTGTTCTCAATGCAGGGGGCCCAGGTTCAATC
CCTGGTCAGGGAA
>Bos_taurus_chr5.trna5281-GluCTC (120776571-120776499) Glu (CTC) 73 bp Sc: 48.69

TCCCTGGTGGTCCAGTGGCTAAGATTCTGCACTCTCAGTGCAGAGGGCCCAGGTTTGAGC
CCTGCTCAGGGAA
>Bos_taurus_chr7.trna3035-GluCTC (72447356-72447428) Glu (CTC) 73 bp Sc: 48.73
TCCCTGGTGGTCCAGTTGGAAAGACTCTGTGCTCTCAATGCAGGGGGCCCAGGTTCTATC
CCTGATCAGGGAA
>Bos_taurus_chr1.trna4809-GluCTC (138264526-138264598) Glu (CTC) 73 bp Sc: 49.00
TTTCTGGTGGTCCAGTGGTTAGGAATTGGCATTCTCACTACCAGGGGCCCAGGTTCGATC
CCTGGTCAGGGAA
>Bos_taurus_chr1.trna4159-GluCTC (120638404-120638476) Glu (CTC) 73 bp Sc: 49.10
TCCCTGGTAGTCCAGTGGACAAGACTTTGCACTCTCGATGCAGAAGGCCCGGGTTCGATC
ACTGGTCAGGGAA
>Bos_taurus_chr16.trna6174-GluCTC (1569574-1569502) Glu (CTC) 73 bp Sc: 49.11
TCCCTGGTGGTCCAGTGGCTAAGACTCTGCGCTCTCAGTGCAGGGGGCCCAGGTTCGTTC
CCTGGTCAGGGAA
>Bos_taurus_chr8.trna5671-GluCTC (76191467-76191395) Glu (CTC) 73 bp Sc: 49.11
TCTCTGGTGGTCCAGTGGCTAAGATTCTACACTCTCAATGCAGGGGACCCAGGTTCAATC
CCTGGTCAGGAAA
>Bos_taurus_chr21.trna2505-GluCTC (60798758-60798830) Glu (CTC) 73 bp Sc: 49.23
TCCCTGATGGTCCAGTGCCTAAGACTCTGCACTCTCAATGCAGGTGGCATGGGTTCAATC
CCCGGTCAGGGAA
>Bos_taurus_chr22.trna311-GluCTC (7088838-7088910) Glu (CTC) 73 bp Sc: 49.40
TCTCTGGTGGTCCAGTGGCTAAGACGCTGCACTCTCAATGCAGGGGACCCAGGTTCAGTG
CCTGGTTAGGGAA
>Bos_taurus_chr20.trna890-GluCTC (25448092-25448164) Glu (CTC) 73 bp Sc: 49.62
TCCCTGGTGGTCCAGTGGCTAAGACTCTGAGCTCTCAGTGCAGGGGGCCCAGGTTCAATC
CCTGGTCAGGGAG
>Bos_taurus_chr19.trna1119-GluCTC (23696914-23696984) Glu (CTC) 71 bp Sc: 49.64
TCCCTGGTGGTCTAGTGTTTAGGATTCGGCGCTCTCACCGCCGTGGCCTGGGTTCGTTTC
CGGTCAGGGAA
>Bos_taurus_chr5.trna3501-GluCTC (93927523-93927595) Glu (CTC) 73 bp Sc: 49.74
TCCCTGGTAGTCCAGTGACTAGGACTAGGCACTCTCACTGCTAAGGGTCCAGGTTCAATC
CCTGGTCAGGGAA
>Bos_taurus_chr5.trna4773-GluCTC (119175227-119175299) Glu (CTC) 73 bp Sc: 49.89
TCCCTGGTGGTCCAGTGGCTAAGATTCTGAGCTCTCAGTGCAGGGGGCCCAGGTTCGATC
CCTGGTCAGGGAG
>Bos_taurus_chr22.trna1322-GluCTC (36420122-36420194) Glu (CTC) 73 bp Sc: 50.35
TCCCTGGTGGTCCAGTGGTTAGGACTCTGCAGTCTCATTGCTGAGGGCCGGGGTTCAATC
CCTGGTCAGGGAA
>Bos_taurus_chr13.trna1182-GluCTC (29688661-29688732) Glu (CTC) 72 bp Sc: 50.46
TCCTTGGTGGTCCAGTGGTAGGACTCTATGCTCTCAATGCAGGGGGCACAGGTTTGATAC
CTGTTCAAGGAA
>Bos_taurus_chr21.trna3849-GluCTC (43645737-43645665) Glu (CTC) 73 bp Sc: 50.87
TTTCCGGTGGTCTAGTGGCTAAGACTCTGCACTCTCAATGCAGGGGGCCTGGGTTCGATA
CCTAGTCAGGAAA
>Bos_taurus_chr4.trna4556-GluCTC (119338292-119338220) Glu (CTC) 73 bp Sc: 51.02
TCCCTGGTGGTCCAGTGGTTAGGACTCTGCCCTCTCAATGCAGGGGAACCAGGTTCAATT
TCTGCTCAGGGAA
>Bos_taurus_chr13.trna1122-GluCTC (28534532-28534604) Glu (CTC) 73 bp Sc: 51.05
TCCCTGGTGGTCCAGTGGATAAGACTCTGTGCTCTCAATACAGGGAGCCCAGGTTCGATC
CCTGGTCAGGAAC
>Bos_taurus_chrUn.004.783.trna7-GluCTC (1083-1011) Glu (CTC) 73 bp Sc: 51.40
CCCCTGATGGTCCAGTGGCTAAGACTCTGTGCTCTCAATGCCGAGGGCCCAGGTTCAATC
CCTGGTTGGGGAA
>Bos_taurus_chr10.trna195-GluCTC (5993005-5993077) Glu (CTC) 73 bp Sc: 51.41
TCCCTGGTAGTCCAGTGGTTAAGACTCTGCCCACTCAATGCAGCGGGCCCAGGTTCCATC
CCTGGTCAGGGAA
>Bos_taurus_chr9.trna6692-GluCTC (34947196-34947125) Glu (CTC) 72 bp Sc: 51.54
TCCCTGGTGGTCTAGTGGGTAAGACTCCTCAGTCTCAATGAAGGGGCCCAGGTTGGATCC
CTGGTCAGGGAA
>Bos_taurus_chrX.trna590-GluCTC (12575414-12575486) Glu (CTC) 73 bp Sc: 51.64
TCCTTGGCTGTCCAGTGGTTAAGACTCTGCATTCTCAATGCCGAGGGCCCAGGTTCAATC
CCTGGTCAGGGAA
>Bos_taurus_chr16.trna5588-GluCTC (21223932-21223861) Glu (CTC) 72 bp Sc: 51.68
TCCTTGGTGGTCTAGTGGTTAGGATTCAGTGCTCTCACTGCAGCAGTCTGGGTTCAATTC
CCAGCCAGGAAA
>Bos_taurus_chr21.trna4854-GluCTC (21253904-21253832) Glu (CTC) 73 bp Sc: 51.84
TCCCTGGTGGTCCAGTGGTTAAGACTCTGAACTCTCAATGCAGAGGGCCTGGGTTTGATC

CTGAGTCAGGGAA
>Bos_taurus_chr11.trna915-GluCTC (21395254-21395326) Glu (CTC) 73 bp Sc: 51.91
TCCCTAATAGTCTAGTGGTTAGGACTTGGTGCTCTCACTGCCAGGGCCACGGGTTCAATC
CCTGATTGGGGAA
>Bos_taurus_chr13.trna2288-GluCTC (59189316-59189388) Glu (CTC) 73 bp Sc: 53.12
TCCTTGCTGGTCCAGTGGCTAAGACTCTGAGCTCTCAATGCAGGGGGCCCAGGTTCAATC
CCTGGTCAGGGAA
>Bos_taurus_chr9.trna5331-GluCTC (75486408-75486336) Glu (CTC) 73 bp Sc: 53.22
TCTCTGGTGGTCCAGTAGTTAGGACTCAGCACTCTCACTGCTGAGGACCCGGGTTCAATC
CCTGGTTGGGGAA
>Bos_taurus_chr7.trna791-GluCTC (14141290-14141361) Glu (CTC) 72 bp Sc: 53.23
TCCCTGGTGGTCCAGTGCTTACAACTTTGCACTCTCAATGCAAGGGCCTAGGTTCAACCC
CTGGTCAGGGAA
>Bos_taurus_chrUn.004.3787.trna2-GluCTC (8580-8651) Glu (CTC) 72 bp Sc: 53.23
TCCCTGGTGGTCCAGTGCTTACAACTTTGCACTCTCAATGCAAGGGCCTAGGTTCAACCC
CTGGTCAGGGAA
>Bos_taurus_chr11.trna4492-GluCTC (106733462-106733534) Glu (CTC) 73 bp Sc: 53.57
TCCCTGGTGGTCCAGTGGTTAGGACTCGGTGTTCTCACTGCTGGGGCCCCAGGTTCAATC
CCTGGTCGGGAAA
>Bos_taurus_chr19.trna2791-GluCTC (55969969-55970041) Glu (CTC) 73 bp Sc: 53.58
TCCCTGGTGGTCCAGTGGTTAGGACTTGGCATTCTCCCTGCCAGGGACCCAGATTCGATC
CCTGGTTGGGGAA
>Bos_taurus_chr13.trna3035-GluCTC (71769370-71769442) Glu (CTC) 73 bp Sc: 53.58
CCCTTGATGGTCCAGTGGCTAAGACTCTGTTCTCTCAATGCAGGAGGCCCAGGTTCAATC
CCTGGTCAGGGAA
>Bos_taurus_chr16.trna4757-GluCTC (40419560-40419488) Glu (CTC) 73 bp Sc: 53.75
TCCCTGATAGTCCAATGGTTAGGACTCTGTGCTCTCACTGCTGAGGGCCTGGGTTCGATA
CCTGGTCAGGGAA
>Bos_taurus_chr16.trna4639-GluCTC (41980531-41980459) Glu (CTC) 73 bp Sc: 53.86
TTCCTGGTGGTCCAGTGGCTAAGACTCTGCATTCTCAATGCAGGCGGCCCAGGTTCAATC
CCTGGTTAGGGAA
>Bos_taurus_chr25.trna2577-GluCTC (41180052-41179980) Glu (CTC) 73 bp Sc: 54.23
TCCCTGGTGGTCCAACGGTTAGGACTCTGTGCTCTCATTGCCGAAGGCCCAGGTTCGATC
CCTGGTCAGGGAA
>Bos_taurus_chr19.trna4944-GluCTC (35708394-35708322) Glu (CTC) 73 bp Sc: 54.49
TTCCTGGTGGTCCAGTGGCTAAGACTCTGCACTCTCCATGCAGAGGGCCCTGGTTCAATC
CCTGGTCAGGGAG
>Bos_taurus_chr11.trna250-GluCTC (4229738-4229810) Glu (CTC) 73 bp Sc: 54.51
TCCTTGGTGGTCCAGTGGCCAAGACTCTGCATTCTCAATGCAGGGGGCCTGGGTTCAATT
CCTGGTCAGGGAA
>Bos_taurus_chr19.trna3800-GluCTC (57237344-57237273) Glu (CTC) 72 bp Sc: 54.67
TCCCTCATGGTCCAGTAGTTAGGACTCAGCGCTCTCACTTCTGGGGCCCAGGTTCAATCC
CTGGTCAGGGAA
>Bos_taurus_chr7.trna7655-GluCTC (17213576-17213504) Glu (CTC) 73 bp Sc: 54.81
TCCCTGGTGGTCCAGTGGCTAAGACTCCACACTCTCAATGTAGGGGACCCAGGTTCGATC
CCTGTTCAGGGAA
>Bos_taurus_chr17.trna4399-GluCTC (58212405-58212333) Glu (CTC) 73 bp Sc: 54.84
TCCCTGGTGGTCCAGTGGCCAAGACTCTGCACTCTCAATGCGGGGGGCCTGGGTTCGATC
CCTGGTCAGGGAA
>Bos_taurus_chr25.trna2006-GluCTC (34286579-34286651) Glu (CTC) 73 bp Sc: 54.84
TCCCTGGTGGTCCAGTGGTTAAGACTCTGCACTCTCAATGCAGGGAACCTGGGTTCGATC
CCTGGTCAAGGAG
>Bos_taurus_chr25.trna1724-GluCTC (29520890-29520962) Glu (CTC) 73 bp Sc: 55.37
TCCCTGGTAGTCCAGTGGCTAAGACTCCTCACTCTCAATGCAGGGGACCCAGGTTCAACC
CCTGGTCAGGGAA
>Bos_taurus_chr29.trna2978-GluCTC (30038191-30038119) Glu (CTC) 73 bp Sc: 55.66
TTCCTGGTGGTCCAGTGGCTAAGACTCTGCACTCTCAATGCAGAGGCCCCGGGTTTGATC
CCTGGTCAGGGAA
>Bos_taurus_chr16.trna3825-GluCTC (62467929-62467857) Glu (CTC) 73 bp Sc: 55.74
TCCCTGGTGGTCCAGTGGTTAGGATTCTGCACTCTCACTGCAGAGGGCCCAGGTTCAACC
CCTGCTTGGGGTT
>Bos_taurus_chr17.trna5985-GluCTC (19728510-19728438) Glu (CTC) 73 bp Sc: 55.84
TCCCTCATGGTCCAGTGGTTAGGACTTCATGTTCTCATCATGGAAGGCCCAGGATCAATC
CCTGGTTGGGGAA
>Bos_taurus_chr17.trna3216-GluCTC (71420711-71420782) Glu (CTC) 72 bp Sc: 55.88
TTCCTAGCAGTCCAGTGGTTAGGACTCAGTGCTCTCACTGCTGGGGCCCAGGTTCAATCC
CTGGTTGGGAAA

>Bos_taurus_chr10.trna6492-GluCTC (46532454-46532382) Glu (CTC) 73 bp Sc: 55.98
CCCCTGGTGGTCCAGTGGTTAGGACTCAGCACTCTCACTGCTAGGGCCCCAGGTTCAATC
CCTGCTCAGGGAA
>Bos_taurus_chr11.trna1593-GluCTC (39456729-39456801) Glu (CTC) 73 bp Sc: 56.29
TCCCTGGTGGTCCAGTGGTTAGGTCTTAGTGCTCTCACTGCTGTGGGCCCAGGTTCAATC
CCTGGTCAGGGAA
>Bos_taurus_chr19.trna2569-GluCTC (50429676-50429747) Glu (CTC) 72 bp Sc: 56.39
TCCCCGGTGGTCCAGTGATTAGGATGCGGTACTCTCACTGCTGGGGCCCAGGTTCAATCC
CTGGTTGGGGTA
>Bos_taurus_chr13.trna1114-GluCTC (28301611-28301683) Glu (CTC) 73 bp Sc: 56.54
TTCCTGGTGGTCCAGTAGTTAAGACTCTGCACTCTCAATGCAGGAGGCCCAGGTTCGATC
CCTGGTCAGGGAA
>Bos_taurus_chr20.trna410-GluCTC (11019407-11019479) Glu (CTC) 73 bp Sc: 56.55
TCCCTGGTGGTTCAGTGGTTAGAACTCTGCACTCTCACTGCCAGCAGCTCAGGTTCAATC
CCTGGTTGGGGAA
>Bos_taurus_chr22.trna3230-GluCTC (33138753-33138682) Glu (CTC) 72 bp Sc: 56.90
TCCCTGGTGGTCTAGTGGTTACGATTTGGTGCTCTCACTGCCATGGCCTAGGTTCAATCC
CTGGCTGGGGAA
>Bos_taurus_chr10.trna6424-GluCTC (47739109-47739038) Glu (CTC) 72 bp Sc: 57.05
TCCCTGGTGGTCTAGTGCTTAGGACGTGGCACTCTCACTACTGTGGCCCAGGTTCAATCC
CTGGTCAGGGAA
>Bos_taurus_chrUn.004.8.trna92-GluCTC (42853-42782) Glu (CTC) 72 bp Sc: 57.07
TCCCTGGTGGTCCAGTGGTTAGAACTTGGTGCTCTCACTGCCAGGGCCCAGGTTCAATCC
CTGGTCAGGGCA
>Bos_taurus_chr15.trna5655-GluCTC (20053899-20053829) Glu (CTC) 71 bp Sc: 57.31
TCCTTGGTGGTCTAGTGGTAGGATTCAGTGCTCTCACCACTGCAGCCCAGGCTCGATTCC
TGGTCAGGGAA
>Bos_taurus_chr24.trna2467-GluCTC (59544510-59544582) Glu (CTC) 73 bp Sc: 57.77
TCCCTGGTGGTCCAGTGGCTAGGATTCTGCACTCTCAATGTAGGGGGCCCAAGTTCAAAC
CCTGGTCAGGGAA
>Bos_taurus_chr18.trna863-GluCTC (22066163-22066234) Glu (CTC) 72 bp Sc: 57.78
TCCCTGGTGGTCCAGTGGTTAGGACTCTGAGCTCTCACTGCTGAAGTCTGGGTTCGATCC
CCAGTCGGGGAA
>Bos_taurus_chr25.trna3748-GluCTC (22626753-22626681) Glu (CTC) 73 bp Sc: 60.28
TCCCTGGCAGTCCAGTGGTTAGGATTCTGTACTCTCACTGCAGAGGGTCCAGGTTCAATA
CCTGGTCAGGGAA
>Bos_taurus_chr5.trna5622-GluCTC (114389042-114388970) Glu (CTC) 73 bp Sc: 60.75
TCCCTGGTGGTCCAGTGGTTAGGACTCTGCCCTCTCACTGCCGAGGGCCCAGGTTCAATC
CCTGGTTGGGGAG
>Bos_taurus_chr2.trna738-GluCTC (24144135-24144207) Glu (CTC) 73 bp Sc: 61.01
TCCCTGGTGGTCCAGTGGTTAGGACTCTGCAGTCTCAATGCAGGGGGCCCAGGTTCAATC
CCTAGTCAGGGAA
>Bos_taurus_chr29.trna1734-GluCTC (46388482-46388554) Glu (CTC) 73 bp Sc: 61.04
TCCCTGATGGTTTGGTGGCTAAAACTCTGTGCTCTCAATGCAGGGGTCCCAGGTTCAATC
CCTGGTTAGGGAA
>Bos_taurus_chr27.trna2963-GluCTC (26747788-26747716) Glu (CTC) 73 bp Sc: 61.30
TTCCTGGTGGTTCAGTGGTTAGGACTCTGCACTCTCATTGCTGAGGGCCCAGGTTCAATC
CCTGGTCAGGGAA
>Bos_taurus_chr4.trna5023-GluCTC (108411535-108411463) Glu (CTC) 73 bp Sc: 61.39
TCCCTGGTGGTCCAGTGGTTAAAACTCTGCACTCTCAATGCAGGGGGCCCAGGCTCGATC
CCTGGTCAGGGAA
>Bos_taurus_chr18.trna4031-GluCTC (48006039-48005968) Glu (CTC) 72 bp Sc: 62.06
TCCCTGGTGGTCCAGTGGTGAGGACTCAGCACTCTCATAGCCGAGGCCCAGGTTCAATCC
CTGGTCAGGGAG
>Bos_taurus_chr20.trna4418-GluCTC (36109223-36109151) Glu (CTC) 73 bp Sc: 63.58
TCCCTGATGGTCCAGTGGTTAGGACTCAGCACTCTCACTGCTGAGGGCCTGGGTTCGATC
CCTGGTCAGAGAA
>Bos_taurus_chr2.trna11-GluCTC (859260-859332) Glu (CTC) 73 bp Sc: 64.37
TCCCTGGCAGTCCAGTGGTTAGGACTCTGCACTCTCACTGCTGAGGGCCCAGGTTCAATT
CCTGGTCAGGGAA
>Bos_taurus_chr22.trna4198-GluCTC (8324689-8324617) Glu (CTC) 73 bp Sc: 64.49
TCCCTGATGGTCCAGTGGTTAAGACTCCACACTCTCAATGCAGGGGGCCCAGGTTCGATC
CCTGGTCAGGGAA
>Bos_taurus_chr7.trna2266-GluCTC (50929599-50929671) Glu (CTC) 73 bp Sc: 64.51
TCCCTGGTGGTCCAGTGGTTAGGACTCTGCACTCTCACTGCTGAGGGCCCAGGTTCATTC
CCTGGCTGGGGAA
>Bos_taurus_chrUn.004.1392.trna1-GluCTC (9711-9783) Glu (CTC) 73 bp Sc: 72.43

TCCCTGGTGGTCCAGTGGTTAGGACTCTGCACTCTCACTGCTGAGGGCCCAGGTTCAATC
CCTGGCTGGGGAA
>Bos_taurus_chr3.trna9113-GluCTC (8814852-8814781) Glu (CTC) 72 bp Sc: 75.29
TCCCTGGTGGTCTAGTGGTTAGGATTCGGCGCTCTCACCGCCGCGGCCCGGGTTCGATTC
CCGGTTAGGGAA
>Bos_taurus_chr23.trna3467-GluCTC (30068557-30068486) Glu (CTC) 72 bp Sc: 76.61
TCCCTGGTGGTCTAGTGGTTAGGATTCGGCGCTCTCACCGCCGCGGCCCGGGTTCGATTC
CCGGTCAGGGAA
>Bos_taurus_chr3.trna8534-GluCTC (23224570-23224499) Glu (CTC) 72 bp Sc: 76.61
TCCCTGGTGGTCTAGTGGTTAGGATTCGGCGCTCTCACCGCCGCGGCCCGGGTTCGATTC
CCGGTCAGGGAA
>Bos_taurus_chr3.trna9107-GluCTC (8947627-8947556) Glu (CTC) 72 bp Sc: 76.61
TCCCTGGTGGTCTAGTGGTTAGGATTCGGCGCTCTCACCGCCGCGGCCCGGGTTCGATTC
CCGGTCAGGGAA
>Bos_taurus_chr3.trna9110-GluCTC (8824316-8824245) Glu (CTC) 72 bp Sc: 76.61
TCCCTGGTGGTCTAGTGGTTAGGATTCGGCGCTCTCACCGCCGCGGCCCGGGTTCGATTC
CCGGTCAGGGAA
>Bos_taurus_chr3.trna9125-GluCTC (8628653-8628582) Glu (CTC) 72 bp Sc: 76.61
TCCCTGGTGGTCTAGTGGTTAGGATTCGGCGCTCTCACCGCCGCGGCCCGGGTTCGATTC
CCGGTCAGGGAA
>Bos_taurus_chr7.trna1949-GluCTC (42028609-42028680) Glu (CTC) 72 bp Sc: 76.61
TCCCTGGTGGTCTAGTGGTTAGGATTCGGCGCTCTCACCGCCGCGGCCCGGGTTCGATTC
CCGGTCAGGGAA
>Bos_taurus_chr18.trna3207-GluCTC (60525358-60525235) Glu (CTC) 124 bp Sc: 22.46
TCCTTGGTGGTCCAGTGATTAAGACACCTGGCTCTCAATGCTAGCTTCGGCGAGGGCGGG
GGTGGGGGGTGGGAGGGGATCAGGACTTGGGGGAAGTGCCTAGGTTCGATCCCTGATCAG
GGAA
>Bos_taurus_chr18.trna2747-GluCTC (60700327-60700450) Glu (CTC) 124 bp Sc: 22.46
TCCTTGGTGGTCCAGTGATTAAGACACCTGGCTCTCAATGCTAGCTTCGGCGAGGGCGGG
GGTGGGGGGTGGGAGGGGATCAGGACTTGGGGGAAGTGCCTAGGTTCGATCCCTGATCAG
GGAA
>Bos_taurus_chr18.trna4125-GluTTC (46926347-46926276) Glu (TTC) 72 bp Sc: 25.42
TCTCTGGCTGTCCAGGGATTAGGACTCCATGCTTTCACTGTGGAGGGTCGGGTTCAATCC
CTGGTCAGGGAA
>Bos_taurus_chr10.trna5965-GluTTC (59784280-59784209) Glu (TTC) 72 bp Sc: 25.91
TCCCTCGTGGTCCAGTAGTTAGGACTAGGTGCTTTCTCTACCAGAGCCCAGGTTCAGTCC
CTGGTCAGGGAA
>Bos_taurus_chrUn.004.551.trna7-GluTTC (42365-42293) Glu (TTC) 73 bp Sc: 27.01
TCCTTGGCAGTCTAGTGGGTAGGACTCGGTGCTTTCATTGCCCCAGGCCCAGGCTCAATC
CCTGGTTGGGGAA
>Bos_taurus_chrUn.004.897.trna3-GluTTC (2713-2641) Glu (TTC) 73 bp Sc: 27.01
TCCTTGGCAGTCTAGTGGGTAGGACTCGGTGCTTTCATTGCCCCAGGCCCAGGCTCAATC
CCTGGTTGGGGAA
>Bos_taurus_chr17.trna4973-GluTTC (50728208-50728137) Glu (TTC) 72 bp Sc: 27.93
TCCCCAGTGGTCCAGTCATTAAGACTCTGTGCTTTCACTGCAGGGAATGCGGGTTCGATC
CCTACTGGGGAA
>Bos_taurus_chr18.trna4276-GluTTC (44528996-44528925) Glu (TTC) 72 bp Sc: 27.98
TCTCTGGCTGTCCAGTGGTTAGGACTTGGTGTTTTCATTGCCAGGGCCTGGGCTCAATCC
CTGGTCAGGGAA
>Bos_taurus_chr28.trna2889-GluTTC (7582726-7582654) Glu (TTC) 73 bp Sc: 28.02
TCCCTGGTCGTTCAGTGGTTAAGATTCTGCACTTTCACTACAGGGGGAACAGGTTCCTTC
CCTGGTCGGGGAA
>Bos_taurus_chr5.trna2227-GluTTC (61242053-61242124) Glu (TTC) 72 bp Sc: 28.37
TCCCTGGTTATCCAGTGGTTAGGATTCAGTGCTTTCACTGCTGTAACTTGGGTTCAGTCC
CTGGTTAGGGAA
>Bos_taurus_chr5.trna2902-GluTTC (78087303-78087373) Glu (TTC) 71 bp Sc: 28.40
CCCTAGCTGTCCAATGGTTAAGATTCAGTGCTTTCACAGCTGTGGCCCTGGGTTCAATCT
CTGGTTGGGGA
>Bos_taurus_chr17.trna1801-GluTTC (49875387-49875457) Glu (TTC) 71 bp Sc: 28.82
TCCCTGGAGGTCCAGTGGTTAGGACTTGGAGCTTTCACTGCCAGGGCTGGGTTCAGTCCC
TGGTCAGGGAA
>Bos_taurus_chr5.trna2361-GluTTC (64947860-64947932) Glu (TTC) 73 bp Sc: 28.95
TCCCTGGCGGTCCAGTGGTTAGGACCTTGCGATTTCACTGCGGACAGCCCAGGTTCAGTC
CCTGGTTGGGGAA
>Bos_taurus_chr1.trna11014-GluTTC (2428680-2428610) Glu (TTC) 71 bp Sc: 29.07
TCCCTGGTGGTTCAGTGGTAAGGACTTGGTGCTTTCACTACTGGGTCCAGCTTCTATCCC
TGGCTGGGGAT

>Bos_taurus_chr22.trna3874-GluTTC (14768844-14768772) Glu (TTC) 73 bp Sc: 29.15
TCCCTGGCAGTTCAGTGGTTAGGACTTTGTGATTTCACTGCAGGGGATGCAGGTTTGATT
CCTGTTTGGGGAA
>Bos_taurus_chr11.trna7219-GluTTC (48782125-48782053) Glu (TTC) 73 bp Sc: 29.50
TCCTTGGCAGTCCAGTGGTTACGATTTGGTGCTTTCATTGCCAGGGGCCTGGGTTCAGTT
CCTGGTCAGGGAA
>Bos_taurus_chr17.trna4676-GluTTC (54778628-54778556) Glu (TTC) 73 bp Sc: 29.61
TTCCTGGTTGTCCAGCGGTTAGGACTAGGTATTTTCATTGTTCTGGGCCCAGATTCAATC
CCTGGTCAGGGAG
>Bos_taurus_chr3.trna5352-GluTTC (113439378-113439306) Glu (TTC) 73 bp Sc: 29.66
TCTCTGGCAGTCCAGTGGTGAGGACTCTGTGCTTTCACTACAGGGGGCCTGGGTTCAGTC
CCTGGTCAGGGAA
>Bos_taurus_chr8.trna423-GluTTC (10307391-10307462) Glu (TTC) 72 bp Sc: 29.85
TCCCTGGCCGTCCAGTGGTTAGGACTCTGTAGTTTCATTGCTGAAGGCCAGGGTCAATCC
CTGGTTGGGGAA
>Bos_taurus_chr2.trna4210-GluTTC (123818784-123818856) Glu (TTC) 73 bp Sc: 30.56
TCCCTGGTGGTTCAGTGATTGGGACTTGGTGGTTTCGCTTCCATAGCTCCAGGTTCAATT
CCTGGTCAGGGAA
>Bos_taurus_chr11.trna7094-GluTTC (50590779-50590707) Glu (TTC) 73 bp Sc: 30.60
TCCCTGGCGGTCCAGTGCTTAGGACTAGGCACTTTCATTGCCGAGGACCTGGGTTCAGTC
CCTGGTTGGGGAA
>Bos_taurus_chr24.trna3352-GluTTC (50983526-50983455) Glu (TTC) 72 bp Sc: 30.75
TCCTGGAAGTCCAGTGGTTAAGACTCAGCGCTTTCACTACTGAGGGCCTGGGTTCAGTCC
CTGGTCAGGGAA
>Bos_taurus_chr29.trna1919-GluTTC (50169469-50169539) Glu (TTC) 71 bp Sc: 30.80
CCCTGGCCGTCCAGTGGTTAGGACTCTGCACTTTCACTGCTGAGGGCTTGGGTTCAGTCC
CTGATTAGGGG
>Bos_taurus_chr12.trna727-GluTTC (18049191-18049263) Glu (TTC) 73 bp Sc: 31.33
TCCCTGGTGGTCCAGTAGTTAGGACTCAGTGTTTTCACTGCTGACAGTGTGGGTTCAAAC
CCTCTTTAGGGAA
>Bos_taurus_chr5.trna3048-GluTTC (81354204-81354276) Glu (TTC) 73 bp Sc: 32.08
TCTCTGGTGGTCCAGCGGTTAGGACCTGGTGCTTTCACTGCCAAGGGCTTGGATTCTATT
CCTGGTCAGGGAG
>Bos_taurus_chr9.trna5442-GluTTC (72503102-72503031) Glu (TTC) 72 bp Sc: 32.10
TCCCTGGTGGTTCAGTCATTAGGACTCTGTGCTTTCACTGCAGGGGCACAGGTTCCATCC
CTGGTCAGGGGA
>Bos_taurus_chr2.trna10414-GluTTC (2228739-2228669) Glu (TTC) 71 bp Sc: 32.43
TTCTTGATGGTCCAGTGGTTAGGACTTGGTGCTTTCACTGCCAGAGCCAGAATCAATCCC
TGGTTGGGGGA
>Bos_taurus_chr18.trna4105-GluTTC (47231004-47230933) Glu (TTC) 72 bp Sc: 32.44
TCCTTGGCAGTCCAGTGCTTAAGACTTGGAGTTTTCACTGCTGAAGTCCAGGTTCGATCC
CTGGTCAGGGAA
>Bos_taurus_chr20.trna2227-GluTTC (60886644-60886714) Glu (TTC) 71 bp Sc: 32.50
TCCTCGTGGTCCAGTGATTAGGACTTGGCACTTTCATTACTGTGGCCCAGGTTCAGCTCT
TGGTCGGGGAA
>Bos_taurus_chr12.trna1782-GluTTC (43659741-43659813) Glu (TTC) 73 bp Sc: 32.69
TCCCTTGTAGTCCAGAGGTGAGGATTCTGCGCTTTCACTGTAGAAGGGCCAGGTTTAATC
CCTGGTCAGGGAA
>Bos_taurus_chr14.trna4010-GluTTC (63583660-63583588) Glu (TTC) 73 bp Sc: 33.13
TCTCTGGCTGTCCAGTGGTTGGGACTTGGCACTTTCACTGCTGTGGGCCTAGGTTCCATC
CCTAGTCAGGGAA
>Bos_taurus_chrX.trna21-GluTTC (343756-343826) Glu (TTC) 71 bp Sc: 34.05
TCCCTGGTGGTCCAGTGGTTAGGACTCCGCGCTTTCACTGCTGGGCCTGGGTTGTATCCC
TAATTGGGGAA
>Bos_taurus_chr19.trna5807-GluTTC (20905354-20905282) Glu (TTC) 73 bp Sc: 34.06
TCCCTGGTGGTCTAGCGGTAAGGATTCTGCGTTTTCACTGCTGAAGGTCTGGGTTCCATC
CCTAGTCAGGGAA
>Bos_taurus_chrUn.004.761.trna8-GluTTC (22752-22680) Glu (TTC) 73 bp Sc: 34.14
TTCTTGGTGGTCCAGTGGTTAAGACTTGGTGATTTCACTGCCATGGACCCAGGTTTGATC
CCTGATCAGGGAA
>Bos_taurus_chr5.trna3039-GluTTC (81134991-81135063) Glu (TTC) 73 bp Sc: 34.37
TCTCTGGTGGTCCAGCGGTTAGGACTTGGTGCTTTCACTGCCAAGGGCTTGGATTCTATT
CCTGGTCAGGGAG
>Bos_taurus_chr21.trna3730-GluTTC (46961052-46960980) Glu (TTC) 73 bp Sc: 34.90
TCCCTGGTGGTTCAGTGATGAGGACCCGGTGCTTTCACTGCCGTGGGCCCGGGTTTGATC
CCTGACCAGGGAA
>Bos_taurus_chr5.trna9756-GluTTC (11205798-11205726) Glu (TTC) 73 bp Sc: 34.90

TTCCTGGTGGTTCACTGGTTAAGAATCTGTGCTTTCACTGCAGGGGACCCAGGTTTGATT
CCTGGTCGGGAAG
>Bos_taurus_chr7.trna260-GluTTC (5876867-5876938) Glu (TTC) 72 bp Sc: 35.65
TCCCTGGCAGTCCACTGGTTAGGACTCTGTGATTTCACTGCTGTGGCCCAGGTTCAACCC
CTGGTTGGGGAA
>Bos_taurus_chr10.trna6253-GluTTC (51344828-51344757) Glu (TTC) 72 bp Sc: 35.66
TCCCTGATGGTCCAGTGGCTAAGACTCCACGTTTTCAATGTAGGCGGTCCTGGTTCGATC
CTAGGTCAGGAG
>Bos_taurus_chr16.trna4811-GluTTC (39251849-39251778) Glu (TTC) 72 bp Sc: 36.02
TCCTGGGTTGTCTAGAGGTTAGGACTCAGTGCTTTCACTGCTGTGCCTTGGGTTCAATCC
CTAGTCAGGGAA
>Bos_taurus_chr16.trna5339-GluTTC (26945073-26945001) Glu (TTC) 73 bp Sc: 36.32
TCCTTGGCAGTCCAGTGACTAGGACTCGGCACTTTCACTGCCAGGGGCCTGGGTTCAATT
CCTGGTCAAGGAA
>Bos_taurus_chr24.trna2511-GluTTC (60272011-60272082) Glu (TTC) 72 bp Sc: 36.56
TCCCTGGCGGTCCAGTGGTTGGGACTTGGCGCTTTCCCTGCCAGGGCCCAGGTTCAGTCC
CTGGTTGGGGAA
>Bos_taurus_chr11.trna2510-GluTTC (62088051-62088123) Glu (TTC) 73 bp Sc: 36.78
TTCCTGGTGGTCCAGTAGTTAGGACTCCATGCTTTCACTGTAGTGAGCCTTGGTTCCATT
CCTGGTCAGGAAA
>Bos_taurus_chr12.trna5328-GluTTC (34578638-34578566) Glu (TTC) 73 bp Sc: 36.96
TCCCTGGCGGTCCAGTGGTTAGGACTTGGCACTTTCACTGCTGAGGGCCTGGGTTCAGTG
CCTGGTCAGGGAA
>Bos_taurus_chr22.trna3311-GluTTC (30585668-30585597) Glu (TTC) 72 bp Sc: 37.25
TCCCTGGCAGTCTAGTGGTTAAGATTTGGTGATTTCACTGCCATGGCCTGGGTTCAATCC
CTGGTCAGGGAA
>Bos_taurus_chr5.trna1021-GluTTC (29923278-29923350) Glu (TTC) 73 bp Sc: 37.27
TCCCTGGTGGTCCAGTGGTTGAGACTCCATGCTTTCACTGTGTAGGGCCCAGGTTTAATC
CCTGGTCAAGGAA
>Bos_taurus_chr3.trna7797-GluTTC (42129742-42129670) Glu (TTC) 73 bp Sc: 37.37
TCCCTGGCTGTCCAGTGGTTAGGACTCAGTGCTTTCACTGCTGTGGGCCTAGGTTTGATC
CTTGGTCAGGGAA
>Bos_taurus_chr2.trna3439-GluTTC (106055529-106055601) Glu (TTC) 73 bp Sc: 37.50
TCCCTGGTGGTTCAGTTGTTAGGATTCAGAGCTTTCACTGCTGAGGGCCCAGGTTCAGTT
CCTGGTTGGGGGA
>Bos_taurus_chr9.trna7226-GluTTC (19865739-19865667) Glu (TTC) 73 bp Sc: 37.52
TCCCTGGTGGTCCAGTGGTCAAGACTCTGTGCTTTCATTGCAGGGGGTACAGGTTCCATC
TCTGATCAGGGAA
>Bos_taurus_chr10.trna6275-GluTTC (50950324-50950252) Glu (TTC) 73 bp Sc: 37.76
TTCCTGGTGGTCCAGTGGTTGGGATTTGGCACTTTCATTTCTGGGGCCCCAGGTTCAATC
CCTGGTCAGGGTT
>Bos_taurus_chr19.trna1580-GluTTC (31236351-31236424) Glu (TTC) 74 bp Sc: 37.97
TCCCTGATGGTCCAGTGGTTAAGACTCAGTGCTTTCACtgttgtGGGCCCAGGGTCCAAC
TCCTGGTTGGGGAA
>Bos_taurus_chr3.trna1013-GluTTC (26604798-26604870) Glu (TTC) 73 bp Sc: 38.03
TCCCTGAATGTCCAGTGGTTAGGACTCTGTGCTTTCACTGCTGAGGACACAGGTCCAATC
CCTGTTTGGGGAA
>Bos_taurus_chr15.trna3535-GluTTC (73377760-73377688) Glu (TTC) 73 bp Sc: 38.03
TCTCTGGTGGTCTGGTGGTTAGGACCCTGTGCTTTCACTGCTGATGGCCCAGGTTTGATT
CCTGGTCGGGGAA
>Bos_taurus_chr25.trna671-GluTTC (11722401-11722473) Glu (TTC) 73 bp Sc: 38.13
TTCCTGGTGGTCCAGTAGCTAGGATTTGGCTCTTTCATTGCCAAGGACCTGGGTTCAATC
CCTGGTCAGGGAA
>Bos_taurus_chr7.trna2824-GluTTC (66232707-66232778) Glu (TTC) 72 bp Sc: 38.14
TCCCTGCTGGTCCAATGGTTAAGACCTGGTACTTTCACTGCTGGAGCCTAGGTTTGATCC
CTGGTTGGGGAA
>Bos_taurus_chrX.trna4536-GluTTC (65535252-65535181) Glu (TTC) 72 bp Sc: 38.22
TCCTTGGTGGTGCAGTGGTTAGGACTTGGTGCTTTCTCTGCAATGGCTCAGGTTCAATCT
CTGGTCAAGGAA
>Bos_taurus_chr1.trna7433-GluTTC (115704330-115704258) Glu (TTC) 73 bp Sc: 38.24
TCTCTGGTGGTCCACTGATTAGGACTCAGCCCTTTCATTGCTGAGGGCCTAGGTTCAACC
CCTGGTCAGTGAA
>Bos_taurus_chrX.trna4723-GluTTC (62504905-62504834) Glu (TTC) 72 bp Sc: 38.39
TCCCTGATGGTTCAGTGGTTAGGACTCAGTGCTTTCCCTGCTGTGGCCTAGGTGCAATCC
TTGGTGAGGGAA
>Bos_taurus_chr9.trna5294-GluTTC (76358111-76358040) Glu (TTC) 72 bp Sc: 38.40
TCTCTGATGGTCCAGTGGTTGGGATTTGGCATTTTCACTGGCAAGGTCTGGGTTTGATCC

CTGGTTGGGGAA
>Bos_taurus_chr7.trna872-GluTTC (15065579-15065651) Glu (TTC) 73 bp Sc: 38.58
TCTCTGGTGGTCCGGTGGTTAAGACTCCGTGCTTTCAGTGCTGAGGGCTCAGGTTCGATC
CCTGATCAGGGAA
>Bos_taurus_chr4.trna2552-GluTTC (80445634-80445707) Glu (TTC) 74 bp Sc: 38.71
TCCCTGGTTGTCCAGTGGAAAGGACTTGGTACTTTCATTTCTATGGGCCCCAGGTTCAAT
CCCTGGTCACGGAA
>Bos_taurus_chr9.trna1561-GluTTC (48484238-48484309) Glu (TTC) 72 bp Sc: 38.78
TCTCATATGGTCTAGCTGTTAGGATTCCTGGTTTTCACCCAAGCAGCCAGCGTTCAACAC
CCTGTATGGGAA
>Bos_taurus_chr27.trna1719-GluTTC (44974412-44974484) Glu (TTC) 73 bp Sc: 38.81
TCCCTGGTTGTCCAGTGGTTAGGACTCGGCTCTTTCACTGCTGGAGGCCCAGGGTCAATC
CCTGGTTGAGGAA
>Bos_taurus_chr18.trna3196-GluTTC (61027818-61027746) Glu (TTC) 73 bp Sc: 38.81
TCCCTGGTGGTCCAATGGTTAGGACACTGTGATTTCACTGCTGAAGGTTTGGGTTCAATC
CCCGGTCAGGGAA
>Bos_taurus_chr1.trna8921-GluTTC (67707734-67707655) Glu (TTC) 80 bp Sc: 38.94
TTCCTGGTGGTTCAGTGGTTAGAACTTGGCACTTTCACTTCCAGGGCCCAGAGACCGGGG
TTCAGTCCCTGGTCAGGGAA
>Bos_taurus_chr25.trna2548-GluTTC (42067126-42067054) Glu (TTC) 73 bp Sc: 39.01
TCCTCAGTGGTCCAGTGGTTAAGACTCCATGCTTTCATTGTCGGGGGTGCAGGTTTGATC
CCTGATTGGGGAA
>Bos_taurus_chrUn.004.46.trna17-GluTTC (314783-314853) Glu (TTC) 71 bp Sc: 39.05
TCCCTGGTGGTCCAGGGTTAAGATGTGCTGCTTTCAATGCCAGGGCCCACGTTCCATCCC
TGGCTGGGGAA
>Bos_taurus_chr25.trna2297-GluTTC (38077035-38077107) Glu (TTC) 73 bp Sc: 39.06
TCCCTAGTGGTCCAGTGGTCAGGACCCGGCATTTTCACTGCCTTGGCCCCACGTTCATTC
TCTGGTTGGGGAA
>Bos_taurus_chr18.trna4051-GluTTC (47735447-47735376) Glu (TTC) 72 bp Sc: 39.10
TCCCTGGTGGTCCAGTGGTTAGGACTTCGTGCTTTCACTACCAGGGCCCGGGTGTGATCC
CTGGTTGGGGAA
>Bos_taurus_chr3.trna8446-GluTTC (25584428-25584357) Glu (TTC) 72 bp Sc: 39.13
TCCCTGGTGGTCCAGTAGTTAGGACTCAGTGCTTTCACTACTGTGGCCTGGGTTCAGTCC
CTGGTTGGGGAA
>Bos_taurus_chr11.trna5587-GluTTC (88802955-88802883) Glu (TTC) 73 bp Sc: 39.15
TCTCTTGCTGGTCTAGTGGCTGGGATTCCTGGTTTTCATCCAGACCACCCAGGTTCGATT
CCTGGGCAGGGAA
>Bos_taurus_chr15.trna712-GluTTC (25833011-25833082) Glu (TTC) 72 bp Sc: 39.16
TCCCTGATGGTCCAGAGGTGGGGACGTGGCGCTTTCACTGCCATGGCCTGGGTTCAATCC
CTGGTTGGGAAC
>Bos_taurus_chr13.trna1172-GluTTC (29489543-29489616) Glu (TTC) 74 bp Sc: 39.21
TTCTCTAGTGGTCCAGTGGTTAGGACTTGGCATTTTCACTGCTGAAGATCCAGGTTCAGT
CCCTGGTTGGGAAA
>Bos_taurus_chr12.trna348-GluTTC (11297923-11297994) Glu (TTC) 72 bp Sc: 39.21
TTCTTGATGGTCCGGTGGCTAAGACTTTGTGCTTTCAATGCAGGGACCCAGGTTCATTCC
CTGGTCAGGGAA
>Bos_taurus_chr4.trna7234-GluTTC (51958328-51958256) Glu (TTC) 73 bp Sc: 39.26
TCCTTGGCAGTCCAGTGATTAGGACTTGGCTCTTTCACTGTCAAGGACCCAGGTTCAATG
CCTGGTCAGGGAA
>Bos_taurus_chr17.trna2523-GluTTC (59707140-59707212) Glu (TTC) 73 bp Sc: 39.31
TCCCTGGTGGTCCAGTGGTTGGGACTTGGAGCTTTCACTGCTGTAGCCCCAGGTTTGATT
CCTGGTTGGGGAA
>Bos_taurus_chr17.trna2525-GluTTC (59735018-59735090) Glu (TTC) 73 bp Sc: 39.31
TCCCTGGTGGTCCAGTGGTTGGGACTTGGAGCTTTCACTGCTGTAGCCCCAGGTTTGATT
CCTGGTTGGGGAA
>Bos_taurus_chr14.trna239-GluTTC (6533610-6533681) Glu (TTC) 72 bp Sc: 39.32
TCCCTGGTGGTCTAGTGGTTGGGATTCAGCACTTTCACTGCCATGGGCCAGGTTTAATCC
CTGGTTGGGGAA
>Bos_taurus_chr24.trna4718-GluTTC (22193020-22192948) Glu (TTC) 73 bp Sc: 39.51
TCCCTGGAAGTCCAGTGGCTAGGACTCAGTGCTTTCATTGCTGAGGGCTTGGGTTCAATC
CCTGGTCAGGGAA
>Bos_taurus_chr18.trna4677-GluTTC (34409151-34409079) Glu (TTC) 73 bp Sc: 39.51
TCCCTGGTGGTCCAGTGGTAAAGACTCTGTGTTTTCACTGCAGGGGGCACAGGTTTGATA
CCTGGTTGGGGAA
>Bos_taurus_chr5.trna7976-GluTTC (61548833-61548761) Glu (TTC) 73 bp Sc: 39.61
TTCTTGGTAGTCCAATGGTTAAGACTGGGTACTTTCACTGCCATGGGCTCAGGTTCAATT
GCTGGTCAGGGAA

>Bos_taurus_chr8.trna216-GluTTC (6485187-6485259) Glu (TTC) 73 bp Sc: 39.71
TCCCTGGTGGTTCAGAGGTTAAGACTCTGCTGTTTCAATGCAGGGGGCACAGGTTCTAAC
CCTGGTCAGGGAA
>Bos_taurus_chr21.trna979-GluTTC (22933201-22933273) Glu (TTC) 73 bp Sc: 39.72
TCCCTGGTAGTCCACTGGTTTAGACTCTGTGCTTTCAATGCAGGGGACACAGGTTCAACC
CCTGGCTGGGGAA
>Bos_taurus_chrUn.004.1353.trna2-GluTTC (35761-35833) Glu (TTC) 73 bp Sc: 39.73
TCCCTGGTAGTCCAATGACTAGGACTTGATGCTTTCACTGCCATGGCCCCAGGTTCAATC
CCTGGATGGGGAA
>Bos_taurus_chr11.trna8421-GluTTC (16850196-16850125) Glu (TTC) 72 bp Sc: 39.75
TCCCTGGTGGTCCTGTGGCTAAGACTTCATGTTTTCAATGTTGGGGCCCAGGTTGGATTC
CTGGTCAGGGAA
>Bos_taurus_chr16.trna4813-GluTTC (39237765-39237694) Glu (TTC) 72 bp Sc: 39.77
TCCCTGGTGGTCCAGTGATTAGGACTTGGTACTTTCACTGCTAGGGCCTGGGTTCAGCCC
CTGGTCAGGGAA
>Bos_taurus_chr1.trna6321-GluTTC (147722412-147722340) Glu (TTC) 73 bp Sc: 39.87
TCCCTGGTGGTCCACTGGTTAGGATTTGGTGCTTTCACCGCCAGGTGTCCAGGTTCAGTC
CCTGGTTTGGGAA
>Bos_taurus_chr21.trna4374-GluTTC (28641304-28641232) Glu (TTC) 73 bp Sc: 39.96
TCCCTGGTGGTCCAATGGTTAGGACTCTGTACTTTCACTGCAGGGAGCATAGGTTTGTTC
CCTGGTTGGGGAA
>Bos_taurus_chrUn.004.8.trna25-GluTTC (783983-784056) Glu (TTC) 74 bp Sc: 40.08
TTCCTGGTAGTCCAGTAGTTAAGACTCCGTACTTTCACTGCAGGGGGACACAGGTTCGAT
CCCTGGTCGGGAAA
>Bos_taurus_chr14.trna4881-GluTTC (40719635-40719565) Glu (TTC) 71 bp Sc: 40.11
TCCCTGGTTGTCCAATGGTTAGGACTCAGTGTTTTCACTGCTAGGCCTGGGTTCAGTTCC
CAGTTGGGGAA
>Bos_taurus_chr13.trna5383-GluTTC (51708856-51708785) Glu (TTC) 72 bp Sc: 40.17
TCCCTGGCGGTCCAGTGGTTAGGACTTGGCATTTTCACTGTAATAGCCTGGGTTCTATCC
CTAGTCAGGGAA
>Bos_taurus_chr22.trna1110-GluTTC (29445677-29445750) Glu (TTC) 74 bp Sc: 40.23
TTCCTTGTAGTCCAGTGGTTAGGACTCTGAACTTTCACTGCAGAAGGCCTGGGTTCAGTC
CCTGGTCGGGGAAG
>Bos_taurus_chr25.trna1025-GluTTC (17447582-17447654) Glu (TTC) 73 bp Sc: 40.43
TCTCTGGTGGTCCAATCATAAGGACTCTGCATTTTCACTGCTGTGGCCCCGGGTTCAATC
CCTGGTCAGAGAA
>Bos_taurus_chr10.trna7986-GluTTC (6677934-6677863) Glu (TTC) 72 bp Sc: 40.46
TCCCTGGAGGTCCAGTGGTTAGGATTCAGTGTTTTCACTGGTGGGGTCCAGGTTCAATCT
CTGGTCAGGGAG
>Bos_taurus_chr21.trna609-GluTTC (16935383-16935455) Glu (TTC) 73 bp Sc: 40.50
TCCCTGGCAGTCCAGTGGTTAGGACTCTGTGCTTTCTCCATAAAGGTCCCAGGTTTGATC
CCTGGTCAGGGAC
>Bos_taurus_chrUn.004.66.trna31-GluTTC (330835-330763) Glu (TTC) 73 bp Sc: 40.67
TGCCTGGTGGTCCAGTGGTTAGGACTCTGTGCTTTCATGGCAGGGGATGCAGGTGCGATC
CCTGTTCAGGAAC
>Bos_taurus_chr2.trna6056-GluTTC (126104170-126104098) Glu (TTC) 73 bp Sc: 40.71
TCGCTGGTGGCCAAGTGGTTAGGGCTCAGTGCTTTCACTGCTGTGGACCCAGGTTTGATG
CCTGGTTGGGGAA
>Bos_taurus_chr15.trna1808-GluTTC (52663529-52663600) Glu (TTC) 72 bp Sc: 40.77
TCCCTGGTGGTCCAGTGGTAAGGACTCAGCGCTTTCACTGCTGGGGCCTGAGTTCAGTCC
CTGGTCAGGGAA
>Bos_taurus_chr2.trna1938-GluTTC (62247754-62247826) Glu (TTC) 73 bp Sc: 40.93
TCCCTGGTGGTCCAGTGGTTAGGACATGCTGCTTTCATTACCAAGGGCCTGGGTTCAGTC
CCTGGTCAGGGAA
>Bos_taurus_chr10.trna4381-GluTTC (100264162-100264090) Glu (TTC) 73 bp Sc: 41.01
TCCCTGGTGGTCCCGTGGTTAAGACTCCACGTTTTCACTGTAGAGCGCCCAGGTTCTATC
CCTGGTCAGGGAA
>Bos_taurus_chr3.trna9276-GluTTC (3881396-3881325) Glu (TTC) 72 bp Sc: 41.03
TCCCTGCTGGTCTAGTAGTTAGGATTTGGTGCTTTCACCATGGAGGCCCAGGTTCAAGCC
CTGGTTGGGGAA
>Bos_taurus_chr11.trna6558-GluTTC (65182634-65182562) Glu (TTC) 73 bp Sc: 41.05
TCCCTGATGGTCTACTGATTAGGATTCAGTGCTTTCACTGCTGTAGGCCCCGGTTCAATC
CCTGGTCAGGGAA
>Bos_taurus_chr25.trna4318-GluTTC (11604478-11604407) Glu (TTC) 72 bp Sc: 41.06
TCCCTGGAGGTCCAGTGGTTGGACTCGGCGCTTTCACTGCCATGGTCCCGGGTTTGATCC
CTGGTCAGGGAA
>Bos_taurus_chr29.trna1215-GluTTC (33293529-33293600) Glu (TTC) 72 bp Sc: 41.11

TCCCTGGCTGTCCAGTGATTAGGACTTGGCACTTTCACTGGCATGACCTGGGTTCGATCC
CTGGCTAGGGAA
>Bos_taurus_chr7.trna64-GluTTC (1950054-1950125) Glu (TTC) 72 bp Sc: 41.15
TCCCTGGTGGTCCAGTTGTGAGGACTTGGTACTTTCGTTGGTGAGTCTCAGGTTCAATCC
CTGGTCGGGGAA
>Bos_taurus_chr8.trna5241-GluTTC (85113353-85113281) Glu (TTC) 73 bp Sc: 41.17
TCCCTGGTGGTCCAGTGGTTAGGACTCAGTGCTTTCACCACTGAGGGCCTGGGATTGATC
CTTGGGTGGGGAA
>Bos_taurus_chr19.trna4471-GluTTC (45516425-45516353) Glu (TTC) 73 bp Sc: 41.20
TCCCTGGTGGGCCAATGGTTAGGACTTGGCACTTTCACTGCTGTGGCCCTGGGTTCAGTC
CCTGGTCAGGGAA
>Bos_taurus_chr19.trna1768-GluTTC (34755882-34755954) Glu (TTC) 73 bp Sc: 41.26
TCCCTGGTGGTTTAGTGGTTAGGACTTGGCACTTTCAGTGCCATGGACTTGGGTTCAATC
CCTGATTTGGGAA
>Bos_taurus_chr18.trna2106-GluTTC (49688357-49688429) Glu (TTC) 73 bp Sc: 41.29
TCCCTGGTGGCCCAGTGGTTAGGATTGGCACTTTCACTGTCGAGGTCCCCAGGTTCCATC
CCTGGTCAGGGAA
>Bos_taurus_chr29.trna267-GluTTC (7603476-7603548) Glu (TTC) 73 bp Sc: 41.33
CCCTTAGTGGTCTAGTTGTTAAGACTTGGTACTTTCACTGCCATGGGCCTAGGTTTGATC
CCTGGTTGGGGAA
>Bos_taurus_chr3.trna5210-GluTTC (116826342-116826271) Glu (TTC) 72 bp Sc: 41.37
TCCTTGGAGGTCCAGTGGTTAGGATTCGGTGCTTTCATTACTGTGGCCCAGGTTCAGTCC
CTGGTTGGGGAA
>Bos_taurus_chr28.trna13-GluTTC (540730-540801) Glu (TTC) 72 bp Sc: 41.38
TTCCTGGTGGTCTACTGGTTAGGATTCAGGGCTTTCATTGCTGTGGCCCAGGTTTAATCC
CTGGATGGGAAA
>Bos_taurus_chr19.trna4476-GluTTC (45469960-45469889) Glu (TTC) 72 bp Sc: 41.44
TCCCTGGTGGTCTTGTAGTTAGGATTCAGTGCTTTCACTTCTGTTGCCCAGGTTCAATCC
CTGGTTGGGGAA
>Bos_taurus_chr19.trna2981-GluTTC (58369732-58369804) Glu (TTC) 73 bp Sc: 41.48
TCCCTGATGGTTCAGTGGTTAGGATTCCATGATTTCACTGTGGAGGGAGCAGGTTCAATC
CCTGGTCAGGGGA
>Bos_taurus_chr19.trna4391-GluTTC (46596050-46595979) Glu (TTC) 72 bp Sc: 41.51
TCCCTGGCAGTCCAGTGGTTAGGACCTGGTGCTTTCCCTGCTGTGGCCCAGGTTCAATCC
CTGGTTGGGGAA
>Bos_taurus_chr7.trna3708-GluTTC (94223857-94223928) Glu (TTC) 72 bp Sc: 41.51
TCTCTGGTGGTTCAGTCACTAGGACTCAACACTTTCGCTGTAGTGGCCCAGGTTCAATCC
CTGGTTGGGGAA
>Bos_taurus_chr18.trna2298-GluTTC (52441202-52441272) Glu (TTC) 71 bp Sc: 41.53
TCTCTGGTGGTCCAGTGGTTAGGACTTGGCGCTTTCACTGCCGGGCCTGGGTTCAGTCCC
TAGTCGGGGAA
>Bos_taurus_chr1.trna8293-GluTTC (88146020-88145948) Glu (TTC) 73 bp Sc: 41.60
TCCTTGGTGGTCCAGTGGTTAAGACTCTGCACTTTCACTGCTGAGGACCTGAGTTTGATT
CTTGATCAGGGAG
>Bos_taurus_chr26.trna853-GluTTC (24861588-24861659) Glu (TTC) 72 bp Sc: 41.71
TCCCTGGCAGTCCAGTGGTTAAGACTTGGCACTTTCACTGTCAGGGCCTGGGTTTGATCC
CTGGTCAGGGAA
>Bos_taurus_chr25.trna2945-GluTTC (35120387-35120315) Glu (TTC) 73 bp Sc: 41.87
TCTCTGGTGGTCCAGTGGTTAAGACTCTGTGCTTTCACTGCTGAGGGCCCAGGCTCGATC
CCTGATCAGGGAG
>Bos_taurus_chr14.trna4892-GluTTC (40578744-40578672) Glu (TTC) 73 bp Sc: 42.02
TCCCTGATGGTCCAGTGGTTCAGACTCGGCATTTTCACTGCTGGGGACTGGGGTTCAATT
CCTGGTCAGGGAA
>Bos_taurus_chr1.trna11033-GluTTC (2162452-2162381) Glu (TTC) 72 bp Sc: 42.21
TTCCTGGCAGTCCAGTGGTTAGGACTTGGCACTTTCGCTGCCGTGGCCTGGGTTCCATCC
CTGGTCAGGGAA
>Bos_taurus_chr13.trna1355-GluTTC (32998605-32998677) Glu (TTC) 73 bp Sc: 42.25
TCTCTGGTGGTCGAATGGCTAAGACTCTGCACTTTCAGTGCAGGGGGCCAAGGTTTGATT
CTTGGTCAGAGAA
>Bos_taurus_chr5.trna2211-GluTTC (60832415-60832486) Glu (TTC) 72 bp Sc: 42.50
TGCCTGGTGGTCCAGTGCTTAGGACTTGGCACTTTCACAGCTGAGGGCCAGGTTCAATCC
CTGGTTAGGGAA
>Bos_taurus_chr12.trna245-GluTTC (9611941-9612012) Glu (TTC) 72 bp Sc: 42.56
TCCCTGACAGTCCAATGGTTAGGACTCAGTGCTTTCACTGCCGGGACCTGGGTTCAATCC
CTGGTCAGGGAA
>Bos_taurus_chr13.trna980-GluTTC (25328844-25328915) Glu (TTC) 72 bp Sc: 42.79
TCCTTGGCTGTCCAGTGGTTAGGACTCTGCACTTTCACTGCTGTGGCCCAGGTTCAGTCC

CTGGTTGGGGGA
>Bos_taurus_chr8.trna1232-GluTTC (36269465-36269539) Glu (TTC) 75 bp Sc: 42.80
TTCCTGGCAGTCCAGCGGTGAAGACTCTGCTCTTTCAATGCAGTGGAGGCCCAGGTTCAA
TCCCTGGTCAGGGAA
>Bos_taurus_chr10.trna6217-GluTTC (51975579-51975508) Glu (TTC) 72 bp Sc: 43.05
TCCCTGGTGGTTCAGTGGTTGGGACTTGGCACTTTCACTGCCAGGGCCTGGATTCAATCC
CTGGTTGGGGAA
>Bos_taurus_chr16.trna127-GluTTC (3538534-3538604) Glu (TTC) 71 bp Sc: 43.08
TCCCTGGTGGTCCAGTGGTGAGGATTCAGCACTTTCACTGTGGGGCCCGAGTTTGATCCT
TGGTCGGGGAA
>Bos_taurus_chr23.trna3630-GluTTC (25255218-25255146) Glu (TTC) 73 bp Sc: 43.13
TCCCTGGCAGTCCAGTGGTTAGGACTCAGCACTTTCACTGCAGCAGTCCTGGGTCCAATC
CCTGGTCAGGGAA
>Bos_taurus_chr12.trna2638-GluTTC (68972897-68972969) Glu (TTC) 73 bp Sc: 43.31
TCCCTGGTAGTTCAGTGGATAGAACTCAGTGCTTTCATTGCTGAGGATGTGGGTTCAATC
CCTAGTTAGGGAA
>Bos_taurus_chr14.trna5134-GluTTC (34410034-34409963) Glu (TTC) 72 bp Sc: 43.42
TCCCTGGCAGTCCAGTGGTTAGGATTTGGTGCTTTCACTGCTGGGGTTCAGGTTCGATTC
CTGGTTGGGGAA
>Bos_taurus_chr27.trna3868-GluTTC (2398579-2398507) Glu (TTC) 73 bp Sc: 43.49
TCCCTGGCAGTCCAGTGGTAAAGACTCTGCACTTTCACTGCTGACGGCCCAGGTTTGATC
CCTGGTCAGGGAA
>Bos_taurus_chrUn.004.5935.trna1-GluTTC (2039-2111) Glu (TTC) 73 bp Sc: 43.57
TCCCTGGCAGTCCAGTGGTTGGGACTCTGCGCTTTCATTGCAGGGGGCCTGGGTTCGATC
CCTAGTTGGGGAA
>Bos_taurus_chr6.trna2761-GluTTC (92193253-92193325) Glu (TTC) 73 bp Sc: 43.58
TCCCTGGTGATTCAGTGGTTAAGATTCAGTGCTTTCACTACTGTGGGCCCAGGTTTGATC
CCTGGTTGGGGAA
>Bos_taurus_chr13.trna3436-GluTTC (78570083-78570156) Glu (TTC) 74 bp Sc: 43.63
TCCCTGGCAGTCCAGTTGTTAGGACTCTGCACTTTCACTACAGACAGTTCCAGGTTCAAT
CCCTGGTTGGGGAG
>Bos_taurus_chr13.trna6119-GluTTC (33211335-33211263) Glu (TTC) 73 bp Sc: 43.64
TCCCTGGAGGTCCAGGGGTTAAGACTCTGAACTTTCAATGCAGGGGACCTGGGTTCAATC
CCTGGTCAGGGAA
>Bos_taurus_chr26.trna419-GluTTC (14527751-14527824) Glu (TTC) 74 bp Sc: 43.72
TCCCTGTTGGTCCAGTGGCTAAGACTCTGCACTTTCACCGCAGGGGGCCCCAGGTTTGAT
CCCTGGTTGGGGAA
>Bos_taurus_chr19.trna5752-GluTTC (21774446-21774375) Glu (TTC) 72 bp Sc: 43.82
TTCCTGGTGGTCCAGTGGTTAGGACTCGGCGCTTTCAGTACCGTGGTGCAGGTTCGATCT
GTGCTCAGGAAA
>Bos_taurus_chr10.trna4579-GluTTC (94464519-94464449) Glu (TTC) 71 bp Sc: 43.83
TCCCTGGAGGTCCAGGGTTGGGACTTGGAACTTTCACTGCCATGGCCCAGGTTCAATCCC
TGGTTGGGGAA
>Bos_taurus_chr19.trna6537-GluTTC (8718684-8718613) Glu (TTC) 72 bp Sc: 43.88
TCCCTGGTGGTCCAGTGATTAGGATTTGGTGCTTTCAATGCCACTGCCTGGGTTCAATCC
CTGGTCAGGGAA
>Bos_taurus_chr13.trna4782-GluTTC (65719852-65719782) Glu (TTC) 71 bp Sc: 43.96
TCCCTGGTGGTCCAGTGGTTAGGATTTGGCACTTTCACTGCCGGGGCCACGGTCAATCCC
TGGTTGGGGAA
>Bos_taurus_chr25.trna1715-GluTTC (29318990-29319061) Glu (TTC) 72 bp Sc: 44.01
TCCCTGGTGGTCTAATGGTTAGGACTTGGTGCTTTCACTGCTGGGATCCAGGTTCCATAC
CTGGTCAGGGAA
>Bos_taurus_chr26.trna589-GluTTC (18613510-18613581) Glu (TTC) 72 bp Sc: 44.04
TCCATGGTGGTCCAGTGGTTAGGACTCGGAGCTTTCACTTCAGGGGCCCAGGCTCAACCC
CTGGTCAGGGAA
>Bos_taurus_chrUn.004.206.trna1-GluTTC (4-76) Glu (TTC) 73 bp Sc: 44.22
TCCCTGGCAGTCCAGTGGTTAGGACTTGGTGCTTTCATTGCCATGGACCTGGGTTCAATC
CCTGGTCAGGGAA
>Bos_taurus_chr2.trna9975-GluTTC (15524819-15524748) Glu (TTC) 72 bp Sc: 44.26
TCCCTGGTGGTCTAGTGTCTAGGATTCAGTGCTTTCACTCCTGCGGCTGGGGTTCAATTC
CCTGTTAGGGAA
>Bos_taurus_chr8.trna6272-GluTTC (62038399-62038328) Glu (TTC) 72 bp Sc: 44.27
TCCCTGGCAGTCCAGTAGTTAGGACTCTGTGCTTTCACTGCAAAGTCCCAGGTTCAATCC
CTGGTTGGGGAA
>Bos_taurus_chr16.trna4004-GluTTC (58030387-58030315) Glu (TTC) 73 bp Sc: 44.43
TCCCTGGTAGTCCAGTGGTTAAGACTCTGTACTTTCAATGCAGGGAGCATAGGATGGATC
CCTGGTTGGGGAA

>Bos_taurus_chr2.trna4470-GluTTC (128657827-128657898) Glu (TTC) 72 bp Sc: 44.54
TCCCTGGATGTTCAGTGGTTAGGACTCGGCACTTTCACTGCTGGGGCCTGGGTTCAATCC
CTGGTCAGGGAA
>Bos_taurus_chr13.trna1931-GluTTC (47093099-47093171) Glu (TTC) 73 bp Sc: 44.55
TCCCTGATGGTCCAGAGGTTAGGACTCGGCGTTTTCACTGCTGAGGGCCTGGGCTCAATC
CCTGGTCAGGGAA
>Bos_taurus_chr1.trna10305-GluTTC (21698808-21698737) Glu (TTC) 72 bp Sc: 44.59
TCCCTGATGGTCCAGTGATTAGGACTCGGCACTTTCACTGTGGTGGCCTGCGTTCAATCC
TTGGTCAGGGAA
>Bos_taurus_chrX.trna2206-GluTTC (61909587-61909657) Glu (TTC) 71 bp Sc: 44.63
TCCCTGGCAGTCCAGTGGTTAGGACTTGTCGCTTTCACTGCCAGGCCCAGGTTTGATCCC
TGGTCGGGGAA
>Bos_taurus_chrUn.004.79.trna14-GluTTC (200857-200928) Glu (TTC) 72 bp Sc: 44.65
TCCCTGGAGGTCCAGTGGTTAGGACTTGGTGCTTTCACTGCAGTGGCTGAGGTTCAATTC
CTTGTCAGGGAA
>Bos_taurus_chr26.trna1862-GluTTC (47104419-47104490) Glu (TTC) 72 bp Sc: 44.70
TCCTTGGTGGTTCAGTGGTTAGGATTTGGTGCTTTCACTGCTAGGCCCCAAGTTCAATCC
CTGGTCAGGGAA
>Bos_taurus_chr10.trna5657-GluTTC (68939073-68939001) Glu (TTC) 73 bp Sc: 44.72
TCCCTGGTGGTCCAGTGGTTAACACTCAGTGCTTTCACTGCTGGGGCACCAGGTTAGATC
CCTGGTCAGGGAA
>Bos_taurus_chrUn.004.3466.trna2-GluTTC (7603-7675) Glu (TTC) 73 bp Sc: 44.72
TCCCTGGTGGTCCAGTGGTTAACACTCAGTGCTTTCACTGCTGGGGCACCAGGTTAGATC
CCTGGTCAGGGAA
>Bos_taurus_chr11.trna3785-GluTTC (92396981-92397053) Glu (TTC) 73 bp Sc: 44.77
TCCCTGGCAGTCCAGAGGTTTAGACCTGGAACTTTCACTGCCAAGGGCCCAGGTTCAATC
CCTGGTCAGGGAA
>Bos_taurus_chr19.trna2293-GluTTC (46180963-46181033) Glu (TTC) 71 bp Sc: 44.78
TCCCTGGTGGTCCAGAGGTTAGGACTTGGTGCTTTCACTGCCAGGGTTAGGTTCAATCCC
TGGCTGGGGAA
>Bos_taurus_chr8.trna2538-GluTTC (76469257-76469328) Glu (TTC) 72 bp Sc: 44.79
TCCCTGATGGTCCAGTGGTTAGGACTCTGCGCTTTCATTGCTGTAGTCCGAGTTCAGTTC
CTGGTCAGGGAA
>Bos_taurus_chr25.trna1740-GluTTC (29688740-29688812) Glu (TTC) 73 bp Sc: 44.98
TCCCTGGTGGTCCAGTGGTTAGGACTTGGTGCTTTCACTGCTGTGGCCCTGGGTTCAACC
TTCAGTCAGGGAA
>Bos_taurus_chrX.trna724-GluTTC (16291148-16291220) Glu (TTC) 73 bp Sc: 45.00
TCCCTAGTGGTCCAATGGTTAGGACATAGCACTTTCACTGCCAGGGCCCTGGGTCTGATA
CCTGGTTGGGGAA
>Bos_taurus_chr3.trna569-GluTTC (17429790-17429862) Glu (TTC) 73 bp Sc: 45.31
TCCCTGGAAGTCCAGGGGTTAGGACTTGGCACTTTCAGTGCCAGGGGCCCAGGTTCAATC
TCTGGTTGGGGAA
>Bos_taurus_chr6.trna1067-GluTTC (37973401-37973472) Glu (TTC) 72 bp Sc: 45.37
TCTCTGGTGGTCCAGTGGTTAGAACTCAGTGCTTTCACTGCTGGGACCCAGGTTCAGCCC
CTGGTCAGGGAA
>Bos_taurus_chr5.trna5748-GluTTC (112420901-112420829) Glu (TTC) 73 bp Sc: 45.37
TCCTTGGTGGTCCAGTGGCTAAGACTCTGAGCTTTCAATACAGGAGACCCAGGTTTGATC
CCTGGTCAGGGAA
>Bos_taurus_chr13.trna5998-GluTTC (35539884-35539812) Glu (TTC) 73 bp Sc: 45.42
TCCCTGGTGGTTCGGTGGTTAAGACTCAGCATTTTCACTGCTGAGGGCCCGGGTTCAATC
TCTGGTTGGGGAA
>Bos_taurus_chr7.trna3263-GluTTC (81335640-81335711) Glu (TTC) 72 bp Sc: 45.45
TCCCTGGTGGACCAATGGTTAGGACTTGGCACTTTCCCTGCTGAGGCCTGGGTTCAATCC
CTGGTCAGGGAA
>Bos_taurus_chr8.trna5376-GluTTC (81359615-81359544) Glu (TTC) 72 bp Sc: 45.46
TTCCTGGTGGTCTAGTGCTTATGATTTGGTGTTTTCACCACCATGGCCCAGGTTCAGTCC
CTGGTCAGGGAA
>Bos_taurus_chr23.trna4056-GluTTC (17093695-17093624) Glu (TTC) 72 bp Sc: 45.59
TCCTTGGTGGTCCAGCAGTTAGGACCTGGTATTTTCACTGCCAGGACCTGGGTTCAATCC
CTGGCTGGGGAA
>Bos_taurus_chr5.trna4313-GluTTC (110997273-110997345) Glu (TTC) 73 bp Sc: 45.60
TCTCTGGTGGTCCAGTGGTTAGGAGTCTGCACTTTCACTGAGGAAAGCCCCAGTTCAATT
CTGGGTTGGGGAA
>Bos_taurus_chr25.trna4539-GluTTC (8736916-8736845) Glu (TTC) 72 bp Sc: 45.65
TCCCTGATGGTCCAGTGGTTAGGATTCTGTGCTTTCACTGCTGAGGGCCGGGTTCAGTCC
CTGGTTGGGGAA
>Bos_taurus_chr3.trna1446-GluTTC (37957277-37957349) Glu (TTC) 73 bp Sc: 45.72

TCCCTGGTGGTCCAGTGGTTAAGACTCTGTGCTTTCATTGCAGGGGGCACAGGTTAGATC
CCTGGTTGGGGAA
>Bos_taurus_chr5.trna9197-GluTTC (29347473-29347401) Glu (TTC) 73 bp Sc: 45.78
TCCCTGGAAGTCCAGTGGTTAGGACTCAGTGCTTTCACTGCCGTGGGCCTGGGTTCAATC
CCTGGCCAGGGAA
>Bos_taurus_chr26.trna650-GluTTC (19977785-19977857) Glu (TTC) 73 bp Sc: 46.00
TCCCTGGTGGTCCAGTGGATAAGGACTCAGCACTTTCACTGCTGTCACCTGGGTTCAATC
CCTGGTCAAGGAA
>Bos_taurus_chr3.trna4276-GluTTC (117254423-117254495) Glu (TTC) 73 bp Sc: 46.02
TCCCTTGTGGTCCAGTGGTTCGGACTCCATGCTTTCACTGTTGACGGCCCAGGTTCAATT
CCTGGTTGGGGAA
>Bos_taurus_chr21.trna5497-GluTTC (7129594-7129524) Glu (TTC) 71 bp Sc: 46.07
CCCTGGCAGTCCAGTGGTTAGGACTCAGCACTTTCACAGCTGTGGGCCCAGGTTCCATCC
CTGGTCAGGGG
>Bos_taurus_chr5.trna7985-GluTTC (61375432-61375361) Glu (TTC) 72 bp Sc: 46.18
TCCCTGCTTGTCTAGTGGTTAGGACTCGGTACTTTCACTACTGGGGCCTGGGTTCAATCC
CTGGTCGGGGAA
>Bos_taurus_chr4.trna2944-GluTTC (91231310-91231382) Glu (TTC) 73 bp Sc: 46.21
TCCTTGGTGGTCCAGTGATTGGGATTTGGTGCTTTCACTGCCACGGCCCCAGGTTCAATC
CCTGGTCAGGGAA
>Bos_taurus_chrUn.004.1.trna333-GluTTC (1519434-1519363) Glu (TTC) 72 bp Sc: 46.28
TCCCTGGTAGTCCAGTGGTTAGGATTTGGCGCTTTCACTGCTGGGGTCCAGGTTCCATCC
CTGGTAAGGGAA
>Bos_taurus_chrUn.004.2681.trna1-GluTTC (2639-2710) Glu (TTC) 72 bp Sc: 46.28
TCCCTGGTAGTCCAGTGGTTAGGATTTGGCGCTTTCACTGCTGGGGTCCAGGTTCCATCC
CTGGTAAGGGAA
>Bos_taurus_chr2.trna5878-GluTTC (129193792-129193721) Glu (TTC) 72 bp Sc: 46.28
TCCCTGGTGGCTCAGTGGTTAGGGTTTAGCACTTTCGGTGCTGTGGCCTGGGTTCAATCT
CTGGTCAGGGAA
>Bos_taurus_chr20.trna5443-GluTTC (8266653-8266581) Glu (TTC) 73 bp Sc: 46.41
TCTCTGATGGTCCAGTGGTTAAGACCCTGTTCTTTCAATGCAGAAGATGCAGGTTCAATT
CCTGGTTGGGGAG
>Bos_taurus_chr5.trna4677-GluTTC (117600125-117600195) Glu (TTC) 71 bp Sc: 46.49
TCCCTGGTGGCCCAGTGGTTAGGATTTGTCACTTTCACTGCCATGGTCAGGTTCAATCCC
TGGTCAGGGAC
>Bos_taurus_chr8.trna2698-GluTTC (78971824-78971895) Glu (TTC) 72 bp Sc: 46.51
TCTCTGGCAGTCCAGTGGTTAAGACTTGGCACTTTCACTGCCTGGGCCCAGGTTCAATCC
TTGGTCAGGGAA
>Bos_taurus_chr2.trna9181-GluTTC (39257970-39257898) Glu (TTC) 73 bp Sc: 46.68
TCTCTGGTGGTCCAGTGGTTAAGAGTCTGTGCTTTCACCGCAAGGAGCACAGGTTCGATC
CCTGTTCAGGGAA
>Bos_taurus_chr3.trna8908-GluTTC (15524755-15524683) Glu (TTC) 73 bp Sc: 46.69
TCCCTGGTGGTCCAGTGGTTAGGACTTGGCGTTTTCACTGCAGAGAGCCCAGGTTCAGCC
CCTGGTCAGGGAA
>Bos_taurus_chr7.trna1236-GluTTC (20148023-20148095) Glu (TTC) 73 bp Sc: 46.78
TCCCTGATGGTCCAGTGGTTAGGACTCTGTGCTTTCACTGTTGAGGGCCCAGGTTTGATC
TCTGGTTAGGGAA
>Bos_taurus_chrUn.004.416.trna3-GluTTC (23238-23309) Glu (TTC) 72 bp Sc: 46.81
TCTCTGGTGGTCCAGTGGTTACAACTTTGTAATTTCACTGCAATGGCCCAGGTTCCATCC
CTGGTCAGGGAA
>Bos_taurus_chr1.trna5091-GluTTC (146233437-146233509) Glu (TTC) 73 bp Sc: 46.87
TTCCTGGTGGTCCACGGGTTAGGACTCAGCGCTTTCACTGCTGAGGGCCCAGGTTCGATC
CCTGGTTGAGGAA
>Bos_taurus_chrUn.004.614.trna11-GluTTC (75499-75427) Glu (TTC) 73 bp Sc: 46.97
TCCCTGGTGGCCCAGTGGTTAGGACTCTGTACTTTCACTGCAGCAGACCCGGGTTCAGTC
CCTGGTCAGGGAA
>Bos_taurus_chrUn.004.563.trna5-GluTTC (35366-35438) Glu (TTC) 73 bp Sc: 47.00
TCCCTGGTGGTCCAGTGGTTAGGACTAGGTGCTTTCACTGCTGTGGCCCTCGGTTCAATC
CCTAGTCAGGGAC
>Bos_taurus_chrX.trna1445-GluTTC (37155106-37155178) Glu (TTC) 73 bp Sc: 47.00
TCCCTGGTGGTCCAGTGGTTAGGACTAGGTGCTTTCACTGCTGTGGCCCTCGGTTCAATC
CCTAGTCAGGGAC
>Bos_taurus_chr13.trna7086-GluTTC (14741272-14741201) Glu (TTC) 72 bp Sc: 47.01
TCCCTGATGGCCTAGTGATTAGGACTCGGCACTTTCGTTGCCAGGGCCCAGGTTCAGTCC
CTGGTCAGGGAA
>Bos_taurus_chr6.trna1794-GluTTC (62263073-62263145) Glu (TTC) 73 bp Sc: 47.03
TCCCTGGTGGTACAGAGGTTAGGACTTGGTGCTTTCACTGCCAAGGGCCTAGGTTCGATC

CCTGGTTGGGGAC
>Bos_taurus_chr4.trna5583-GluTTC (97198088-97198016) Glu (TTC) 73 bp Sc: 47.06
TCCCTGGTGGTCCAGTGGTTAGGACTCACTGCTTTCACTGCTGTAAGCCCAGGTTCAGTC
CCTGGTCAGGGAA
>Bos_taurus_chr20.trna659-GluTTC (19260961-19261033) Glu (TTC) 73 bp Sc: 47.17
TCCCTGGTGGTCCAGTGGTTAAGATTCCACACTTTCAATGTAGGGGGTGCAGGTTTAATC
CCTGGTCAGGGAA
>Bos_taurus_chr2.trna5905-GluTTC (128860030-128859958) Glu (TTC) 73 bp Sc: 47.20
TTCCTGGTGGTCCAGTGGTTAGGACTTGTCATTTTCACTGCCAAGGGCCTGGGTTCAATC
CCTACTCGGGAAC
>Bos_taurus_chr25.trna3951-GluTTC (18030775-18030703) Glu (TTC) 73 bp Sc: 47.25
TCCCTGGCAGTTCAGTGGTTAGGACTCAGCGCTTTCATTGCTGAGGGCCCAGGTTCCAGC
CCTGGTTGGGGAA
>Bos_taurus_chr10.trna2291-GluTTC (60521779-60521850) Glu (TTC) 72 bp Sc: 47.32
TCCCTGGTGGTCCAGTGGTTAGGACTTGGCACTTTCGTTGCCATCACTCAGGTTTAATCC
CTGGTCGGAGAA
>Bos_taurus_chr24.trna2529-GluTTC (60516722-60516800) Glu (TTC) 79 bp Sc: 47.48
TCCCTGGTGGCCTAGGGGTTAAGATTCTGGGCTTTCACTGCAGTGACCTGGGGCCCAGGT
TCAATCCCTGGTCAGGGAA
>Bos_taurus_chrX.trna2408-GluTTC (65773286-65773357) Glu (TTC) 72 bp Sc: 47.55
TCCCTGGTGGTCTAGTGGTGAGGATTTGCTACTTTCAATGCCACGGCCTGGCTTCAATCC
CTGGCCAGGGAA
>Bos_taurus_chr11.trna1598-GluTTC (39554324-39554395) Glu (TTC) 72 bp Sc: 47.62
TCCCTGGTGGTCCAGTGGTTAGGACTCGGTGCTTTCACTGCTGGGTCCCAGGTTTAATCC
CTGGTCAGTGAA
>Bos_taurus_chrX.trna2462-GluTTC (67021773-67021845) Glu (TTC) 73 bp Sc: 47.62
TCCCTGGTGGTTCAGTGGTTAGGACTTGGCATTTTCACTGCCAAAAGCTCGGGTTCCATC
CCTGGTCAGGGAA
>Bos_taurus_chrUn.004.108.trna4-GluTTC (336540-336468) Glu (TTC) 73 bp Sc: 47.66
TCCCTGGTGGTCCAGAGGTTAAGACTTGGTGCTTTCACTGCTAAGGCCCCAGGTTTGATC
CCTGGTTGGGGAA
>Bos_taurus_chr20.trna2201-GluTTC (60407621-60407693) Glu (TTC) 73 bp Sc: 47.66
TGCCTGGTGGTCCAGCGGTTAGGACTTTGCACTTTCACTGCTGGGGCCCTGGGTTGGATC
CCTGGTCAGGCAG
>Bos_taurus_chr9.trna1459-GluTTC (44964995-44965066) Glu (TTC) 72 bp Sc: 47.79
TCCCTGGTGGCCCACTGGTTAGGGCTTGGCATTTTCACTTCCATGACCTGGGTTCAATCC
CTGGTTGGGGAG
>Bos_taurus_chr9.trna6332-GluTTC (44829409-44829338) Glu (TTC) 72 bp Sc: 47.79
TCCCTGGTGGCCCACTGGTTAGGGCTTGGCATTTTCACTTCCATGACCTGGGTTCAATCC
CTGGTTGGGGAG
>Bos_taurus_chrUn.004.9016.trna1-GluTTC (944-1015) Glu (TTC) 72 bp Sc: 47.87
TCCTTGGCTGTCCAGTGGTTAGGACTTGGCGCTTTCACTGCTGGGGCCCAGGTTCAATCC
CTGGTCAGGGAA
>Bos_taurus_chr2.trna4186-GluTTC (123428795-123428866) Glu (TTC) 72 bp Sc: 47.93
TCCCTGGCAGTTCAGTGGTTAGGATTCCATGCTTTCACTGTGAGGGCCCAGGTTCAATCC
CTGGTCAGGGAA
>Bos_taurus_chr8.trna5774-GluTTC (74000027-73999955) Glu (TTC) 73 bp Sc: 47.95
TCCCTAGTGGTCCAGTGGTTAAGACTTTGTGTTTTCACTGCAGGGGGCCCAGGTTCAATC
CCTAGCTGGGGAC
>Bos_taurus_chr11.trna2856-GluTTC (71316082-71316154) Glu (TTC) 73 bp Sc: 48.04
TCCCTGGTGGTCCAGTGATTAGTACTTGGTCCTTTCACTGCCATGGACCCAGGTTCAATC
CCTGGTCAGGGAA
>Bos_taurus_chr25.trna1676-GluTTC (28770854-28770926) Glu (TTC) 73 bp Sc: 48.10
TCCCTGGTTGTCCAGTGGTTAGGACTCTGCGCTTTCACTGCCGAGGGCCCGGGTTTGATC
CCTGGTCAGGGAA
>Bos_taurus_chr14.trna4121-GluTTC (60873858-60873787) Glu (TTC) 72 bp Sc: 48.28
TCCCTGATAGTCCAGAGGTTAGGACTTGGCAGTTTCACTGCAGGGGCTCAGGTTTGATCC
CTGGTCAGGGAA
>Bos_taurus_chr22.trna4498-GluTTC (3359255-3359182) Glu (TTC) 74 bp Sc: 48.29
TCCCTGGTGGTCCAGTGGTTAAGATTCTGCACTTTCCCTGCAGAGGGCACAGGGTTCAAT
CCCTGGTCAGGGAA
>Bos_taurus_chr5.trna1095-GluTTC (31653996-31654067) Glu (TTC) 72 bp Sc: 48.33
TCCCTGGTGGTCCAGTGGTTAGGACTTGACACTTTCACTGCTGTGGCCTAGGTTCAGTCC
CTAATCAGGGAA
>Bos_taurus_chr22.trna4067-GluTTC (10780394-10780322) Glu (TTC) 73 bp Sc: 48.37
TCCTTGGTGGTTCACTGGTTAGGACTTGGCGCTTTCACTGCTCTGGGCCCAGGTTCGATC
CCTGGTCAGGGAA

>Bos_taurus_chr24.trna2901-GluTTC (60886680-60886608) Glu (TTC) 73 bp Sc: 48.47
TCTCTGACAGTCTAGCAGTTAGGACTCTGCACTTTCACTGCCGAGGGCCCAGGTTCAAAC
CCTGGTCAGGGAA
>Bos_taurus_chr28.trna2054-GluTTC (29542645-29542574) Glu (TTC) 72 bp Sc: 48.48
TCCCTGGCAGTCCAGTGGTTAGGACTCAGCACTTTCACTGCTGGTACCTGGGTTCAATCC
CTGGTTGGGGAA
>Bos_taurus_chr28.trna213-GluTTC (5720068-5720139) Glu (TTC) 72 bp Sc: 48.52
TCCCTGGCTGTCCAGTGGTTAGGACTTGGCGCTTTCACTGCTGGGGCCCAGGTTCAATCC
CTGGCCGGGGAC
>Bos_taurus_chr19.trna5504-GluTTC (25437983-25437911) Glu (TTC) 73 bp Sc: 48.56
TCCCTGGCGGTCCAGTGGTTAGGACTTGGCGCTTTCACTGCCAAGGGCCCAGGTTTAATC
CTTGGTCAGGGAA
>Bos_taurus_chr25.trna4600-GluTTC (8033813-8033741) Glu (TTC) 73 bp Sc: 48.56
TCCCTGGTGGTCCAGCGGTTAGGACTCTGCACGTTCACTGCAGGGGGCTTGGGTTCGATC
CCTGGTTGGGGAA
>Bos_taurus_chr29.trna2162-GluTTC (47522212-47522140) Glu (TTC) 73 bp Sc: 48.58
TCCCTGGTGGTCTAGTGGTTAGGATTTGACACTTTCACTGTCATGGGCCTGGGTTCAATC
TCTGGTTGGGGAT
>Bos_taurus_chr15.trna273-GluTTC (11885725-11885796) Glu (TTC) 72 bp Sc: 48.62
TCCCTGATGGTCCAGTAGTTAGGACTCTACTTTTTCACTGTGGTGGCCCAGGTTCAGTCC
CTGGTCAGAGAA
>Bos_taurus_chr10.trna7319-GluTTC (22069034-22068963) Glu (TTC) 72 bp Sc: 48.74
TCCCTGGTGGTCCAGTGGTTAGGACTCAGCATTTTCATCGCTGGGGCCGGGGTTTGATCC
CTGGTCAGGGAA
>Bos_taurus_chr7.trna8021-GluTTC (12839042-12838971) Glu (TTC) 72 bp Sc: 48.77
TCTCTGGTTGTTCAGTGGTTAGGACTCAGCGCTTTCACTGCTGGGGCCCAGGTTCAATCT
CTGGTCGGGGAA
>Bos_taurus_chrUn.004.3373.trna2-GluTTC (10531-10603) Glu (TTC) 73 bp Sc: 48.82
TCCCTGGTGGTCCAGTGGTTAGGACTCGTTGCTTTCACTGACAGGGACCCAGGTTCAATC
CCTGGTCGGGGGA
>Bos_taurus_chr21.trna2837-GluTTC (68046271-68046343) Glu (TTC) 73 bp Sc: 48.87
TCCCTGGTGGTCCAGTGGTTAGGACTTGGCGCTTTCACTGCTGAGGGCCCAAGTTTGATC
CCTGGTTAGGGAA
>Bos_taurus_chr4.trna4570-GluTTC (119145290-119145218) Glu (TTC) 73 bp Sc: 48.88
TCCCTGGCAGTCCAGTGGTTAGGACTCTGTGCTTTCACTGCTGGGGGCCCAGGTTCAACC
CCTGGTCAGGGAA
>Bos_taurus_chr11.trna577-GluTTC (10782726-10782798) Glu (TTC) 73 bp Sc: 49.00
TCCCTGGTGGTCCAATGGTTAGGACTAAGCACTTTCACTGCTGAGAGCACAGGTTGGATC
CCTGGTCGGGGAC
>Bos_taurus_chrUn.004.1.trna292-GluTTC (2080679-2080607) Glu (TTC) 73 bp Sc: 49.02
TCCCTGGTGGTCCAGTGGTTAGGACTTTGCACTTTCAGTATGGAGGGCCTATGTTCGATC
CTTGGCTGGGGAA
>Bos_taurus_chr5.trna203-GluTTC (6538988-6539059) Glu (TTC) 72 bp Sc: 49.07
CCCCTGGTGGTCCAGTGGTTAGGATTTGGTGCTTTCAACACTGGGTCCCAGGTTCAATCC
TTGGTTGGGGAA
>Bos_taurus_chr9.trna1470-GluTTC (45285095-45285166) Glu (TTC) 72 bp Sc: 49.07
TCCCTGGTGGTCCAGTGGTTAGAACTCAGTGTTTTCACTTCTGGGGCTCAGGTTCAATCC
CTGGTCAGGGGA
>Bos_taurus_chr10.trna4751-GluTTC (90686035-90685964) Glu (TTC) 72 bp Sc: 49.09
TCCCTGGCAGTCTAGCGGTTAGGACTTGGCGCTTTCACTGCCCGGGCCTAGGTTCAATCC
CTGGCTGGGGAA
>Bos_taurus_chr3.trna8943-GluTTC (13962951-13962880) Glu (TTC) 72 bp Sc: 49.09
TTTCTGGTGGTCTAGCGGTTAGGATTTGGCACTTTCACTGCCAAGGCCTGGGTTCAATCC
CTGATCAGAGAA
>Bos_taurus_chr21.trna3033-GluTTC (65190133-65190062) Glu (TTC) 72 bp Sc: 49.10
TCTCTGGTGGTTCGGTGGTTAGGACTTGGTGTTTTCACTGCCAGGGCCCAGGTTCGATCC
CTGGTTGGGGAA
>Bos_taurus_chrUn.004.1293.trna2-GluTTC (28120-28049) Glu (TTC) 72 bp Sc: 49.13
TCCCTGATGGTCCAGTGCTTATGACTCAGCACTTTCACTGCTGTGGCCTGAGTTCAATCC
CTGGTCGGGGAA
>Bos_taurus_chr1.trna9685-GluTTC (44168902-44168830) Glu (TTC) 73 bp Sc: 49.17
TCCCTGATGGTCCAGTGGTTAGGACATGGTGCTTTCATTGCCAAGGGCCTGGGTTCAGGC
CCTGGTCAGGGAA
>Bos_taurus_chr17.trna6475-GluTTC (8416742-8416670) Glu (TTC) 73 bp Sc: 49.19
TCCCTGGTGGTCCAGTGGTTAGGACTCTGCACTTTCACTGCTGAGGGCGTGGGTTTGATC
CCTGATCAGGGAA
>Bos_taurus_chrUn.004.1520.trna3-GluTTC (17303-17374) Glu (TTC) 72 bp Sc: 49.20

TCCCTGGTGGTCCAGTGGTTAGGACTTGGCACTTTCACTGCCTTGGCCTGGGTTAGATCC
CTAGTTGGGGGA
>Bos_taurus_chr5.trna4835-GluTTC (120157749-120157820) Glu (TTC) 72 bp Sc: 49.24
TTCTTGGCAGTCCAGTGGTTAGGACTTGGCATTTTCATTGCTGAGGGCCAGGTTCAATCC
CTGGTTGGGAAA
>Bos_taurus_chr3.trna3138-GluTTC (90278286-90278357) Glu (TTC) 72 bp Sc: 49.28
TTCTTGACAGTCCAGTGGTTAGGACTCAGCACTTTCAGTGCTGTGGCCTGGGTTCAATTT
CTAGTTGGGAAA
>Bos_taurus_chr2.trna1476-GluTTC (46161631-46161703) Glu (TTC) 73 bp Sc: 49.36
TCCCTGGTGGTCCAGTGCTTAGGACACTGTGCTTTCACTGCTGGGGGTCCAGGTTCAATC
CCTGGTCAGGGAA
>Bos_taurus_chr28.trna2713-GluTTC (12391548-12391476) Glu (TTC) 73 bp Sc: 49.40
TTCCTGGTGGTCCAGTGGTTAGGATTCTGTGCTTTCACTCCAGGGGGCTCAAGTTCAATC
CCTGGTCAGGAAA
>Bos_taurus_chr3.trna5495-GluTTC (110227417-110227344) Glu (TTC) 74 bp Sc: 49.55
TCTCTGGTGGTCCAGTGGTTAGGACTCAGCACTTTCACTGCTGTGCACCTGGGGTTCAAT
CCCTGGCCGGGGAA
>Bos_taurus_chr6.trna3348-GluTTC (103801108-103801179) Glu (TTC) 72 bp Sc: 49.65
TCCCTGGTTGTCCAGTGGTTAAGACTTAGCACTTTCACTGCTGGGGGCCAGATTCAATCC
CTGGTCAGGGAA
>Bos_taurus_chr18.trna2407-GluTTC (54103873-54103945) Glu (TTC) 73 bp Sc: 49.70
TCCCTGGCAGTCTAGTGGTTAAGACTCGGCACTTTCACTGCTGTGGGCCTGGGTTCAATT
CCTGGTCAGGGAA
>Bos_taurus_chrUn.004.2306.trna1-GluTTC (12395-12467) Glu (TTC) 73 bp Sc: 49.70
TCCCTGGCAGTCTAGTGGTTAAGACTCGGCACTTTCACTGCTGTGGGCCTGGGTTCAATT
CCTGGTCAGGGAA
>Bos_taurus_chr29.trna1785-GluTTC (47069387-47069458) Glu (TTC) 72 bp Sc: 49.85
TCCCTGGTGGTCCAGTGGTTAGGACTCTGTGCTTTCACTGCCGTGATCCAGGTTCAGTTC
CTGGTTGGGGAA
>Bos_taurus_chr5.trna10148-GluTTC (913547-913475) Glu (TTC) 73 bp Sc: 49.95
TTCCTGGTGGTCCAGTGGTTAAGACTCTACACTTTCAATGCAGAGGGCACAGGTTCCATC
CCTGATCAGGGAA
>Bos_taurus_chr7.trna2827-GluTTC (66326975-66327047) Glu (TTC) 73 bp Sc: 49.97
TCCTTGGTGGTCCAGTGGTTTGGACTGAGCGCTTTCACTGCCTAGGGCCCAGGTTCAATC
CCTGATCAGGGAA
>Bos_taurus_chr10.trna6857-GluTTC (36640116-36640045) Glu (TTC) 72 bp Sc: 50.00
TCCTTGGTGGTCCAGTGGTTAAGACTTGGCCCTTTCACTGCCAGGGCCCAGGTCCAAATC
CTGGTAGGGGAA
>Bos_taurus_chr22.trna189-GluTTC (5657987-5658058) Glu (TTC) 72 bp Sc: 50.03
TCCCCAGTGGTCCAGTGGTTAGGACTCGGCCCTTTCACTGTCGGAGCTCAGGTTCAATTT
CTGGTTGGGGAA
>Bos_taurus_chr4.trna1140-GluTTC (33864268-33864339) Glu (TTC) 72 bp Sc: 50.31
TCCCTGATGGTCCAGTGCTTATGACTCAGCACTTTCACTGCTGTGGCCTGAGTTCAATCC
CTGGTCAGGGAA
>Bos_taurus_chrUn.004.2195.trna2-GluTTC (9067-8996) Glu (TTC) 72 bp Sc: 50.31
TCCCTGATGGTCCAGTGCTTATGACTCAGCACTTTCACTGCTGTGGCCTGAGTTCAATCC
CTGGTCAGGGAA
>Bos_taurus_chr22.trna1927-GluTTC (53601692-53601763) Glu (TTC) 72 bp Sc: 50.34
TCCCCGGTGGTCTAGTAGTTAGGATTTGGGGCTTTCACTGCCATGGCTCAGGTTCAATCC
CTGGTTGGGGAA
>Bos_taurus_chr22.trna2545-GluTTC (53482295-53482224) Glu (TTC) 72 bp Sc: 50.34
TCCCCGGTGGTCTAGTAGTTAGGATTTGGGGCTTTCACTGCCATGGCTCAGGTTCAATCC
CTGGTTGGGGAA
>Bos_taurus_chr7.trna1011-GluTTC (17093780-17093852) Glu (TTC) 73 bp Sc: 50.44
TCCCTGGCAGTCCAGTGGTTAGGACTTTGCACTTTCACTGCTGGGGCCCCAGGTTTGATC
CCTGGTTGGGGAA
>Bos_taurus_chr12.trna4576-GluTTC (56551452-56551381) Glu (TTC) 72 bp Sc: 50.52
TCCCTGGTGGTCCAGTGGCTAGGACTTGGCACTTTCACTGCTGCAGCCCAGGTTCAATGC
CTGGTCAAGGAA
>Bos_taurus_chr22.trna1801-GluTTC (50699698-50699770) Glu (TTC) 73 bp Sc: 50.85
TTCCCTGTGGTCCAGTGGTTAGGACTTGGTGCTTTCACTGCCATGGTCCCGGGTTCAGTC
CCTGGTTGGGAAC
>Bos_taurus_chr15.trna1539-GluTTC (43559997-43560069) Glu (TTC) 73 bp Sc: 50.88
TCCCTGATGGTCCAGTGGTTAGGACACAGTGCTTTCATTGCTATGGGCCCAGATTCAACC
CCTGGTCAGGGAA
>Bos_taurus_chr25.trna4400-GluTTC (10540505-10540433) Glu (TTC) 73 bp Sc: 50.89
TCCCTGGTGGTCCAGTGGTTAGGACTCTGTACTTTCACTGCCGAGGGTCCGGGTTCATTC

CCAGGTCAGGGAA
>Bos_taurus_chr15.trna1058-GluTTC (32300013-32300085) Glu (TTC) 73 bp Sc: 50.92
TCCCTGGCAGTCCAGTGGTTAGGACTCAGCGCTTTCACCGCTGGGGGCCTGGGTTCAATC
CCTGGTTGGGGAG
>Bos_taurus_chr21.trna1529-GluTTC (33850935-33851006) Glu (TTC) 72 bp Sc: 50.98
TTCCTGGTGGTCTAGTGGTTGAGATTCAGCACTTTCACTGCTGTGACCCAGGTTCAGTCC
CTGGTTGGGAAA
>Bos_taurus_chr29.trna4011-GluTTC (1189134-1189063) Glu (TTC) 72 bp Sc: 50.99
TCCCTGACAGTCCAGTGGTTGGGACTCGGTGCTTTCACTGCTGTGGCCCAGGTTCAATCC
CTGGTCAGGGAG
>Bos_taurus_chr29.trna4027-GluTTC (891307-891236) Glu (TTC) 72 bp Sc: 50.99
TCCCTGACAGTCCAGTGGTTGGGACTCGGTGCTTTCACTGCTGTGGCCCAGGTTCAATCC
CTGGTCAGGGAG
>Bos_taurus_chr4.trna2121-GluTTC (66577073-66577144) Glu (TTC) 72 bp Sc: 50.99
TCCCTGGTAGTCCAGTGATTAGGACTCGGTGCTTTCACTGCTGTGACTCAGGTTCAATCC
CTGGCTGGGGAA
>Bos_taurus_chr1.trna9390-GluTTC (52693061-52692990) Glu (TTC) 72 bp Sc: 51.18
TCTCTGGTGGTCTAGTGGTTAGGATTTGGCACTTTCATTGCCATGGCCTGGGTTCAGTCC
CTGGCTGGGGAA
>Bos_taurus_chr29.trna1339-GluTTC (36521815-36521887) Glu (TTC) 73 bp Sc: 51.18
TCCCTGGTTGTCCAGTGGTTAGGACTTGGCGCTTTCACTGCTGAGGGCCCAGGTTCAATC
CTTGGTCAGGGAA
>Bos_taurus_chr13.trna5022-GluTTC (61608381-61608309) Glu (TTC) 73 bp Sc: 51.24
TCCCCAGGGGTCCAGTGGTTAGGACTCAGAACTTTCGCTGCTGAGGGCCTGGGTTCAATC
CCTGGTTGGGGAA
>Bos_taurus_chr3.trna4946-GluTTC (122392949-122392878) Glu (TTC) 72 bp Sc: 51.45
TCCCTAGTGGCCTAGTGGTTAGGGTTTGGCACTTTCACTGACATGGCCTGGGTTCAGTCC
CCGGTCAGGGAA
>Bos_taurus_chr6.trna1806-GluTTC (62506693-62506765) Glu (TTC) 73 bp Sc: 51.54
TCCCTGGTGGTCCGATGGTTAGGACTCAGTGCTTTCACTGCTGTGGGCCCAGGTTTGATC
CCTGGTCAGGGAA
>Bos_taurus_chr7.trna489-GluTTC (10157152-10157223) Glu (TTC) 72 bp Sc: 51.62
TCCCTGGCAGTCCAGTGTTTAGGACTTGGCGCTTTCACTGCCGCGGCCCAGGTTCAATCC
CTGGTCAGGGAA
>Bos_taurus_chr17.trna4577-GluTTC (55629812-55629740) Glu (TTC) 73 bp Sc: 51.63
TCTCTGGTGGTCCAGTGGCTAAGACGCTGCATTTTCAATGCAGGGGACCCAGGTTCCATT
CTTGGTCAGGGAA
>Bos_taurus_chr29.trna2901-GluTTC (31126432-31126361) Glu (TTC) 72 bp Sc: 51.69
TCCCTGGTTGTTCAGTGGTTAGGATTTGGTGCTTTCACTGCGGCGGCCCAGGTTCAATCC
CTGGCCAGGGAA
>Bos_taurus_chr13.trna5276-GluTTC (54245926-54245854) Glu (TTC) 73 bp Sc: 51.73
TCCCTGGTGGTCCAGTGGTTAAGACACTGCACTTTCACTGCCGAGGGCACAGGTTCAGTC
CCTGGTCAGGGAA
>Bos_taurus_chr13.trna583-GluTTC (16698269-16698341) Glu (TTC) 73 bp Sc: 51.76
TCCCTGGTGGTCCAATGTTTAAGACACTGCACTTTCACTGCTGAGGGCACAGGTTCAATC
CCTGGTCAGGGAA
>Bos_taurus_chrUn.004.6872.trna1-GluTTC (1151-1079) Glu (TTC) 73 bp Sc: 51.76
TCCCTGGTGGTCCAATGTTTAAGACACTGCACTTTCACTGCTGAGGGCACAGGTTCAATC
CCTGGTCAGGGAA
>Bos_taurus_chrUn.004.8.trna88-GluTTC (115095-115023) Glu (TTC) 73 bp Sc: 51.76
TCCCTGGTGGTCCAATGTTTAAGACACTGCACTTTCACTGCTGAGGGCACAGGTTCAATC
CCTGGTCAGGGAA
>Bos_taurus_chr16.trna3758-GluTTC (63854487-63854416) Glu (TTC) 72 bp Sc: 51.81
TTCCATATGGTCTAATGGTTAGGATTCCTGGTTTTCACCCAGGTGGCCTGGGTTTGACTC
CTGGTGTGGGAA
>Bos_taurus_chrUn.004.15.trna32-GluTTC (769219-769291) Glu (TTC) 73 bp Sc: 51.97
TCCCTGATGGTCCAGTGGTTAGGACTCTGTGCTTTCACTGCTGAGGGCCCAGGTTTGATC
CCAGGTTGGGGAG
>Bos_taurus_chr7.trna8384-GluTTC (6963320-6963249) Glu (TTC) 72 bp Sc: 52.13
TCCCTGGTGGTCCAGTGGTTAAGACTCTGCACTTTCACTTCAGGGTCACAGGTTCAGTCC
CTGGTCAGGGAA
>Bos_taurus_chr6.trna6446-GluTTC (70244846-70244774) Glu (TTC) 73 bp Sc: 52.18
TCCCTGGTGGTCCAATGGTTAAGACTCTGCACTTTCACTGCAGGGGGCACAGGTTCAATC
TCTGGTTGGGGAA
>Bos_taurus_chr12.trna559-GluTTC (15153078-15153150) Glu (TTC) 73 bp Sc: 52.18
TCCCTGGTGGTGCAGTGGTTAGGACCCAGCGTTTTCACTGCTAGGGACCCAGGTTCAATT
CCTGGCTAGGGAA

>Bos_taurus_chr11.trna4826-GluTTC (104146024-104145953) Glu (TTC) 72 bp Sc: 52.18
TTCCTGGTGGTCCAGTGGTTAGGACTTAGTGCTTTCACTGCCATGGTCCAGGTTCAATCC
CTGGTTGGGGAA
>Bos_taurus_chr20.trna5337-GluTTC (10656823-10656752) Glu (TTC) 72 bp Sc: 52.24
TCCCTGGTGGTCCAGTGGTTGGAACTCTGTGCTTTCACTGCTGGGGCCCAGGTTCGATCC
CTGGTCAGGGAA
>Bos_taurus_chr5.trna8818-GluTTC (38613459-38613388) Glu (TTC) 72 bp Sc: 52.26
CTCTTGCTGGTCTAGTGGCCAGGATTCCTGGCTTTCATCCAGGCTACCCAGGTTCAATTC
CTGGGCAGGGAA
>Bos_taurus_chr11.trna4176-GluTTC (102052822-102052893) Glu (TTC) 72 bp Sc: 52.37
TCCATGGTAGTCCAGTGGTTAGGACTTGGTGCTTTCACTGCCAGGCCCCAGGTTCAATCC
CTGGTTGGGGAA
>Bos_taurus_chr13.trna1238-GluTTC (30911001-30911072) Glu (TTC) 72 bp Sc: 52.45
TTTTTGATGGTCCAGTGGTTAGGACTCAGTGCTTTCACAGCTGGGGCCCAGGTTCGATCC
CTGGTGGGGGAA
>Bos_taurus_chr13.trna6937-GluTTC (18031573-18031501) Glu (TTC) 73 bp Sc: 52.69
TCCCTGGCAGTCCAGGGGTTAGGACTCAGCACTTTCACTGTTGAGGGTCCGGGTTCAATC
CCTGGCCAGGGAA
>Bos_taurus_chrUn.004.9640.trna1-GluTTC (1947-1875) Glu (TTC) 73 bp Sc: 52.69
TCCCTGGCAGTCCAGGGGTTAGGACTCAGCACTTTCACTGTTGAGGGTCCGGGTTCAATC
CCTGGCCAGGGAA
>Bos_taurus_chr19.trna6005-GluTTC (17796157-17796086) Glu (TTC) 72 bp Sc: 52.72
TCCCTGGTGGTCTAGTGGTTAGGATTCAGCGCTTTCAGTGCCGTGGCCCAGGTTCTATCC
CTGGTCAGGGAA
>Bos_taurus_chr15.trna3950-GluTTC (62987260-62987189) Glu (TTC) 72 bp Sc: 52.72
TCCCTGGTGGTCCAGTGGTTAGGACTTGGTGCTTTCACTGCTGGGGCCCAGGTTGAATCC
CTGGTCAGGGAA
>Bos_taurus_chr18.trna3432-GluTTC (55878506-55878435) Glu (TTC) 72 bp Sc: 52.78
TCCTTGGTGGTCTAGTGGTTAGGATTTGGTGCTTTCACTGCTGGGGTCCAGGTTCAATCC
CTGGTCAGGGAA
>Bos_taurus_chr3.trna8622-GluTTC (21731558-21731486) Glu (TTC) 73 bp Sc: 53.06
TTCCTAATGGTCCAGTGGTTAGGACTCGGCACTTTCACAGCTGAGGGCCCAAGTTCAATC
CCTGGTTGGGGAA
>Bos_taurus_chr11.trna4413-GluTTC (105389339-105389410) Glu (TTC) 72 bp Sc: 53.13
TCCCTGGCGGTCCAGTGGTTAGGACTCAGCATTTTCACAGCTGGGGCCTGGGTTCAATCC
CTGGCCAGGGAA
>Bos_taurus_chr26.trna451-GluTTC (15198195-15198267) Glu (TTC) 73 bp Sc: 53.16
TCCCTGGTAGTCCAGTGGTTAAGACTCTGCACTTTCACTTCAGGGGACCCAGGTTTAATC
CCTGGGCAGGGAA
>Bos_taurus_chr21.trna4953-GluTTC (19890639-19890568) Glu (TTC) 72 bp Sc: 53.30
TCCCTGGTGGTCTAGTGGCTAGGATTCGGTGCTTTCGCCACCGCAGCCAGGGTTCGACTC
CCAGTCAGGGAA
>Bos_taurus_chr21.trna740-GluTTC (19936973-19937044) Glu (TTC) 72 bp Sc: 53.30
TCCCTGGTGGTCTAGTGGCTAGGATTCGGTGCTTTCGCCACCGCAGCCAGGGTTCGACTC
CCAGTCAGGGAA
>Bos_taurus_chr8.trna1890-GluTTC (60548348-60548419) Glu (TTC) 72 bp Sc: 53.37
TCCCTGGTGGTCCAGTGGTTAGAACTCAGTGCTTTCACTGCTGAGACCTAGGTTTGATCC
CTGGTCAGGGAG
>Bos_taurus_chr19.trna2863-GluTTC (56674054-56674125) Glu (TTC) 72 bp Sc: 53.40
TCCCTGGTGGTTCAGTGATTAGGACTCAGGGCTTTCACTACTGGGGCCCAGGTTCAATCC
CTGGTCAGGGAA
>Bos_taurus_chr19.trna4534-GluTTC (44805687-44805615) Glu (TTC) 73 bp Sc: 53.53
TTCCTGGCAGTTCAGTGGTTAGAACTTGGCACTTTCACTGCTGAGGGCCCAGGTTCAATC
CCTGGTCAGGGAA
>Bos_taurus_chr10.trna3242-GluTTC (85645621-85645692) Glu (TTC) 72 bp Sc: 53.55
TCCCTGGTGGTCCAGTAGTTAGGACTTGGCACTTTCACTGCTGAGGCCCAGGTTCAATCC
CTGCTTGGGGAA
>Bos_taurus_chr9.trna5819-GluTTC (63197449-63197378) Glu (TTC) 72 bp Sc: 53.59
TCCCTGGCAGTCCAGTGGTTAGGACTTGGCGCTTTCACTGCAGCTGCCCAGGTTCAATTC
CTGGCCAGGGAA
>Bos_taurus_chr22.trna2852-GluTTC (44712127-44712055) Glu (TTC) 73 bp Sc: 53.68
TCCCTGGTGGTCCAGTGGTTAAGACTCTGTACTTTCAATGCAGGGAGCGCAGGTTCAGTT
CCTGGTCAGGGAA
>Bos_taurus_chr12.trna1155-GluTTC (26677548-26677619) Glu (TTC) 72 bp Sc: 53.75
TCCCTGGTAGTCCAGTGGTTAGGACTTGGAGCTTTCACTGCCAGGGCCTGGGTTCAATCC
CTGGTCAGGGAA
>Bos_taurus_chrUn.004.11234.trna1-GluTTC (844-772) Glu (TTC) 73 bp Sc: 53.77

TCCCTGGTGGTCCAGTGGTTAGGACTCAGTGCTTTCACTGCTGAGAGCCTGGGTTCAATC
CCTGGTCGGGGAA
>Bos_taurus_chrUn.004.1591.trna7-GluTTC (32665-32593) Glu (TTC) 73 bp Sc: 53.77
TCCCTGGTGGTCCAGTGGTTAGGACTCAGTGCTTTCACTGCTGAGAGCCTGGGTTCAATC
CCTGGTCGGGGAA
>Bos_taurus_chr22.trna775-GluTTC (17579367-17579439) Glu (TTC) 73 bp Sc: 53.93
TCCCTGGTGGTCCAGTGGTTAGGACTTTGTGCTTTCAACACCGAGGCCCTGGGTTCAATT
CCTAGTCCGGGAA
>Bos_taurus_chr14.trna5672-GluTTC (23011373-23011302) Glu (TTC) 72 bp Sc: 54.03
TCCCTGGTGGTCCAGTGGTTAGGACTTGGCACTTTCACTGCTCCGGCCCAGGTTCCAGCC
CTGGTCAGGAAA
>Bos_taurus_chrUn.004.2.trna86-GluTTC (1825479-1825551) Glu (TTC) 73 bp Sc: 54.10
TCCCTGGTGGTCCAGTGGTTAGGGGCTCTGTGCTTTCACTGCAGGGGCCCAGGTTCAATC
CCTGGTCGGGGAA
>Bos_taurus_chr11.trna5502-GluTTC (90064781-90064709) Glu (TTC) 73 bp Sc: 54.11
TCCCTGGTGGTCTAGGGGTTAGGACTTGGCACTTTCACTGCCTAGGGCCTGGGTTCAATT
CCTGGTCAGGGAG
>Bos_taurus_chr2.trna6174-GluTTC (124155645-124155574) Glu (TTC) 72 bp Sc: 54.14
TCCCTAGTGGTCCAGTGGTTAGGACTTGGCCCTTTCACTGCTAGGGCTCAGGTTTGATTC
CTGGTTGGGGAA
>Bos_taurus_chr18.trna4136-GluTTC (46782242-46782171) Glu (TTC) 72 bp Sc: 54.20
TCTCTGGCGGTCCAGTGGTTAGGACTTTGCACTTTCATTGCCAGGGCCCAGGTTCAATCC
CTGGTTGGGGAA
>Bos_taurus_chr15.trna3140-GluTTC (82499502-82499430) Glu (TTC) 73 bp Sc: 54.23
TCCCTAGTGGTCCATTGGCTAAGATTCTGCACTTTCAATGCAGGGGGCCCAGGTTTGATT
CCTGGTTGGGGAA
>Bos_taurus_chr9.trna76-GluTTC (5362565-5362637) Glu (TTC) 73 bp Sc: 54.34
TCCCTGGTGGTCCAGTGGTTAGGACTCAGTGCTTTCAGCGCAGAGGGCCCAGGTTCAATC
CCTGGTTAGGGGA
>Bos_taurus_chr22.trna4036-GluTTC (11686085-11686013) Glu (TTC) 73 bp Sc: 54.37
TCCTTGGTGGTCCAGTGGTTAGGACTCTGTGCTTTCACTGCTGAGGGCTCAGGTTCAATC
CCTGGTTGGGGAA
>Bos_taurus_chr25.trna167-GluTTC (4161051-4161123) Glu (TTC) 73 bp Sc: 54.52
TCACTGGTGGTCCAGTGGTTAGGACCTGGTGTTTTCATTGCCATGGTTCCAGGTTCAATC
CCTGGTCGGGGAA
>Bos_taurus_chr23.trna141-GluTTC (3629395-3629467) Glu (TTC) 73 bp Sc: 54.58
TCCCTGGTTGTCCAGTGGCTAAGACTCTGCGCTTTCAATGCAGAGGGCCCAGGTTCCATC
CCTGGCTGGGGAA
>Bos_taurus_chr23.trna4009-GluTTC (17745415-17745343) Glu (TTC) 73 bp Sc: 54.59
TCCCTGGTGGTCCAGTGGTTAAGACTCAGTGCTTTCACTGCTGAGGGCCTGGGTTCAATT
CCTGGTCAGGGAA
>Bos_taurus_chr7.trna570-GluTTC (11306272-11306343) Glu (TTC) 72 bp Sc: 54.63
TCCCCAGTGGTCCAGTGGTTAAGATTCAGCACTTTCACtgttgtGACCTGGGTTCAATCC
CTGGTTGGGGAA
>Bos_taurus_chr15.trna2926-GluTTC (81700538-81700609) Glu (TTC) 72 bp Sc: 54.67
TCCCTGGTGGTCCAGTGGGTAGGATGGAGCACTTTCACTGCTGGGGTCCAGGTTTGATCC
CTGGTTGGGGAA
>Bos_taurus_chr29.trna2146-GluTTC (47695403-47695333) Glu (TTC) 71 bp Sc: 54.74
TCCTTGACGGTCCAGTGGTTAGGACTCGGAACTTTCACTGCTGGGCCCAGGTTCAATCCC
TGGTTGAGGAA
>Bos_taurus_chr5.trna2578-GluTTC (70571318-70571390) Glu (TTC) 73 bp Sc: 54.77
TCCCTGGTGGTCCAGTGGTTAAGACTCTGCACTTTCACTGCAGGAGGCACAGGTTTGATC
CCTGGTCAGGGAA
>Bos_taurus_chr4.trna6491-GluTTC (73979597-73979526) Glu (TTC) 72 bp Sc: 54.84
TCTTTAGTAGTCTAGTGGTTAAGACTCAGTGCTTTCACTGCTGTGGCCCAGGTTCGATCC
CTGGTCAGGGAA
>Bos_taurus_chrUn.004.53.trna31-GluTTC (478124-478052) Glu (TTC) 73 bp Sc: 54.95
TCCCTGGTGGTCCAGTGGTTAAGACTCAGCACTTTCACTGCTGTGGGCCTGGGTTCGATC
CCTAGTCAGGAGA
>Bos_taurus_chr16.trna4520-GluTTC (44193182-44193110) Glu (TTC) 73 bp Sc: 55.02
TCTCTGGCAGTTCAATGGTTAGGATTTGGCACTTTCACTGCCAAGGCCCCAGGTTCAATC
CCTGGTCAGGGAA
>Bos_taurus_chr13.trna296-GluTTC (10260522-10260594) Glu (TTC) 73 bp Sc: 55.13
TCCTTGGTGGTTCAGTGGTTAGGACTCAGCGCTTTCACTGCTGAGAGCCTGGGTTCAATT
CCTGGTCAAGGAA
>Bos_taurus_chrUn.004.815.trna4-GluTTC (62131-62060) Glu (TTC) 72 bp Sc: 55.14
TCCTTGGCAGTCCAGTGGTTAGGACTTGGCACTTTCAATGCCGGGGCCCAGGTCCAATCC

CTGGCCAAGGGA
>Bos_taurus_chrUn.004.9.trna202-GluTTC (128977-128905) Glu (TTC) 73 bp Sc: 55.31
TTCCTGGTGGTCCAGTGGTTAGGACTTGGCCCTTTCACTGCTGAGGGCCCAGGTTCAATC
CCTGGTCAGGGAA
>Bos_taurus_chr13.trna2820-GluTTC (67834264-67834336) Glu (TTC) 73 bp Sc: 55.39
TCCCTAGTGGTCCAGTGGTTAGGACTCAGCACTTTCACTGCTGAGGGCCCACGTTCAATC
CCTAGTTAGGGTA
>Bos_taurus_chr16.trna3524-GluTTC (68672364-68672293) Glu (TTC) 72 bp Sc: 55.39
TCCCTGGTGGTCCAGTGGTTAGGACTTGGTGCTTTCACTGCCAAGGCCCAGGTTCAATTC
CTGATCTAGGAA
>Bos_taurus_chr1.trna9302-GluTTC (56008587-56008516) Glu (TTC) 72 bp Sc: 55.66
TCCCTGGTGGTCCAGTGGTTAGGACTCAGTGCTTTCACTGCTGTGGTCTGGGTTCAATCC
CTGGTCAGGGAG
>Bos_taurus_chr7.trna2183-GluTTC (49004341-49004412) Glu (TTC) 72 bp Sc: 55.67
TCCCTGGTAGTTCAGTGGTTAGGACTCAGCGTTTTCATTGCTGGGGCCCAGGTCCAATCC
CTGGTCAGGGAA
>Bos_taurus_chr26.trna2803-GluTTC (33362196-33362124) Glu (TTC) 73 bp Sc: 55.67
TCCCTGGTGGTCTAGTGGTTAAGACTCTGCACTTTCAATGCAGAGGTCTTGGGTTCAGTC
CCTGATTGGGGAA
>Bos_taurus_chrX.trna2740-GluTTC (73442161-73442233) Glu (TTC) 73 bp Sc: 55.70
TCCCTGGTGGTTCAGTGATTAGAACTTGGCACTTTCACTGCTGAGGGCCCAGGTTCAATT
CTTGGTCAGGGAA
>Bos_taurus_chr8.trna6953-GluTTC (40285746-40285675) Glu (TTC) 72 bp Sc: 55.73
CCCCTGGTGGTCCAGTGGTTAGGACTCTGCACTTTCACTGCTGAGGGCCCAGTTCAATCC
CTGGCCAGGGAA
>Bos_taurus_chr19.trna4513-GluTTC (44984111-44984040) Glu (TTC) 72 bp Sc: 55.81
TCCCTGGTGGCCCAGCGGTTAGGACTCAGAGCTTTCACTGCTGGGGCCCAGGTTCAATCC
CTGGTTAGGGAA
>Bos_taurus_chr1.trna2854-GluTTC (83220962-83221034) Glu (TTC) 73 bp Sc: 55.82
TTTCTGGTGGTCCAGTGGTTAGGACTTGGCACTTTCACTGCCATGGCCCTAGGTTCAAAC
CCTAGTAGGGGAA
>Bos_taurus_chr18.trna4619-GluTTC (35462454-35462383) Glu (TTC) 72 bp Sc: 55.87
TCCCTGGCAGTCCAATGGGTAGGATTCAGCACTTTCACTGCTGTGGCCTGGGTTCAAATC
CTGGCCAGGGAA
>Bos_taurus_chr8.trna1108-GluTTC (31695308-31695380) Glu (TTC) 73 bp Sc: 55.93
TCCCTGGTGGTTCAGTGGTTAGGACTCTGCACTTTCCCTGCTGAAGGCCCAGGTTCAATC
CCTGGTTAGGGAA
>Bos_taurus_chr12.trna6229-GluTTC (17478673-17478601) Glu (TTC) 73 bp Sc: 56.07
TCCCTGGTGGTCCAATGGTTAGCACTCAGCTCTTTCACTGCTGGGGTCCCAGGTTCCATC
CCTGGTTGGGGAA
>Bos_taurus_chr3.trna2971-GluTTC (86184039-86184111) Glu (TTC) 73 bp Sc: 56.11
TCCCTAGTGGTCCAGTGGTTAGAACTCGGCACTTTCACTGCCAAGGGCCCAGGTTCAATC
CCTGGTTGAGGAA
>Bos_taurus_chr10.trna5438-GluTTC (74489000-74488929) Glu (TTC) 72 bp Sc: 56.12
TCCCTGGTGGTCCAGTGGTTAGGACTTGTCTTTTTCACTGCCAGGGCCCAGGTTTAATCC
CTGGCTGGGGAA
>Bos_taurus_chr16.trna4332-GluTTC (49251002-49250931) Glu (TTC) 72 bp Sc: 56.13
ACCCTGGCAGTTCAGTGGTTAGGACTTGGCACTTTCACTGCCAAGGCCCAGGTTCAGTCC
CTGGCCAGGGAA
>Bos_taurus_chr29.trna2341-GluTTC (44450044-44449973) Glu (TTC) 72 bp Sc: 56.13
TCCTTGGCAGTCCAGTGGTTAGGACTTGGCACTTTCACTGCCAGGGCCTGGGTTCAATCC
CTGGTCAAGGAA
>Bos_taurus_chr27.trna3185-GluTTC (21644241-21644169) Glu (TTC) 73 bp Sc: 56.16
TCCCTGGTGGTCTAGTGGTTAAGACTCTGCACTTTCAATGCAGGGGACTTGGGTTCAATC
CCTGGTCAGGGAA
>Bos_taurus_chr5.trna8031-GluTTC (60405254-60405183) Glu (TTC) 72 bp Sc: 56.42
TCCCTGGTGGTCCAGTGGTTAGAACTCTGCACTTTCACTGCTGGGGCCCAGGTTCAGTCC
CTGGTTGGGGAA
>Bos_taurus_chr23.trna2061-GluTTC (46116978-46117050) Glu (TTC) 73 bp Sc: 56.43
TCCCTGGTGGTTCAGTGGTTAGGACTTGGCACTTTCACTGCCAAGGCCCTGGGTTCCATC
CCTGGTCAGGGAA
>Bos_taurus_chr25.trna2192-GluTTC (36638425-36638496) Glu (TTC) 72 bp Sc: 56.47
TCCCTGGTGGTCCAGTGGTTAGGACTTGGTGCTTTCACTGCCAGGGCCTGGGTTCAATCC
CTGGTCAGGGAA
>Bos_taurus_chr11.trna358-GluTTC (6279191-6279263) Glu (TTC) 73 bp Sc: 56.55
TCCCCAGTGGTCCAGTGGTTAAGACTCTGAGCTTTCAACACAGGGAGCACAAGTTCGATC
CCTGGCTGGGGAA

>Bos_taurus_chr2.trna7802-GluTTC (84028390-84028318) Glu (TTC) 73 bp Sc: 56.55
TCCCTGGTGGTCCAGTGGTTAGGACTCTGCACTTTCACTTCTGAGGGTCCAGTTTCAATT
CCTGGTCAGGGAA
>Bos_taurus_chrUn.004.6.trna79-GluTTC (1415336-1415408) Glu (TTC) 73 bp Sc: 56.67
TCCCTGGTAGTCCAGTGGTTAGGACTCTGCACTTTCACTGCTGAGGACTCAGATTCAATC
CCTGGTCAGGGAA
>Bos_taurus_chrUn.004.3.trna64-GluTTC (1718766-1718838) Glu (TTC) 73 bp Sc: 56.81
TCCCTGGTGGTCCAGTGGTTAAGACTCTGCACTTTCACTGCAGGGGACACAGGTTTGATC
CCTGGTCAGGGAA
>Bos_taurus_chr3.trna7392-GluTTC (54660634-54660563) Glu (TTC) 72 bp Sc: 56.95
TCCCTGGTGGTCCAGTGGTTAGGACTCCGTGCTTTCAATGCTGTGGCCCAGATTCAATCC
CTGGTCAGGGAA
>Bos_taurus_chrUn.004.4148.trna1-GluTTC (4545-4616) Glu (TTC) 72 bp Sc: 56.95
TCCCTGGTGGTCCAGTGGTTAGGACTCCGTGCTTTCAATGCTGTGGCCCAGATTCAATCC
CTGGTCAGGGAA
>Bos_taurus_chr29.trna2408-GluTTC (42946326-42946254) Glu (TTC) 73 bp Sc: 57.06
TCCCTAATGGTCCAGTGGTTAGGACTCAACACTTTCACTGCCAGGGCCCCAGGTTCAATC
CCTGGTTGGGGAA
>Bos_taurus_chr3.trna3463-GluTTC (98997525-98997596) Glu (TTC) 72 bp Sc: 57.11
TCCCTGGCAGTCCAGTGGATAGGACTCAGTGCTTTCACTGCTGGGGCCCAGGTTCAATCC
CTGGCTGGGGAA
>Bos_taurus_chr5.trna4227-GluTTC (108627759-108627831) Glu (TTC) 73 bp Sc: 57.11
TCCCTGGCAGTCCAATGGTTAGGACTTGGCACTTTCACTGCCAAGGTCCCAGGTTCAATC
CCTGTTCAAGGAA
>Bos_taurus_chr23.trna429-GluTTC (11176913-11176985) Glu (TTC) 73 bp Sc: 57.12
TCCCTGGTGGTCCAGTGGTGAGAACTCGGCACTTTCACTGCCGAGGGCACAGGTTCAACC
CCTGGTCAGGGAA
>Bos_taurus_chr2.trna8006-GluTTC (77062399-77062328) Glu (TTC) 72 bp Sc: 57.16
TCCCTGGTGGTCCAGTGGCTAGGACTCAGCACTTTCACTGCCAGGGCCCAGGTTCAACCC
CTGGTCAGGGAA
>Bos_taurus_chr29.trna3343-GluTTC (19413485-19413413) Glu (TTC) 73 bp Sc: 57.40
TCCCTGGTAGTCTAGTGGCTGAGACTCTGCACTTTCAATGCAGGGGTCCCAGGTTCACTC
CCTGGTCAGGGAA
>Bos_taurus_chr7.trna4628-GluTTC (102989929-102989858) Glu (TTC) 72 bp Sc: 57.42
TCCCTGGTGGTCTAGTGGCTAGGATTTGGCACTTTCACCACCGTGGCCCGGGTTCAATTC
CCGGTCAGGGAA
>Bos_taurus_chrUn.004.1127.trna9-GluTTC (9976-9904) Glu (TTC) 73 bp Sc: 57.44
TCCTTGGTGGCCCAGTGGTTAAGACTTGGCACTTTCACTGCCAAGGGCACAGGTTCAATC
CCTGGTCAGGGAA
>Bos_taurus_chr11.trna6167-GluTTC (75021445-75021373) Glu (TTC) 73 bp Sc: 57.55
TCCCTGGTGGTCCAGTGGTTAGGACTCTGCACTTTCACTGCAGGGGGTACAGGTTCCATC
CCTGATCGGGGAA
>Bos_taurus_chr1.trna8357-GluTTC (86195569-86195498) Glu (TTC) 72 bp Sc: 57.61
TCCCTGGTGGTCTAGTGGTTAGGATTCTGCGCTTTCACTGCTGTGAACCAGGTTCAATCC
CTGGTCAGGGAA
>Bos_taurus_chr6.trna163-GluTTC (7298644-7298715) Glu (TTC) 72 bp Sc: 57.71
TCCCTGATGGCCAGTGGTTAGGACTTGGTGCTTTCACTGCCGTGGTCCCAGGTTCAATCC
CTGGTCAGGGAA
>Bos_taurus_chr27.trna921-GluTTC (27762350-27762420) Glu (TTC) 71 bp Sc: 57.72
TCCCTGTGGTCCAGTGGTTAGGACTTGGTGCTTTCACTGCCATGGCCCAGGTTCAATCCC
TGGTCGGGGAA
>Bos_taurus_chr4.trna5526-GluTTC (98352135-98352064) Glu (TTC) 72 bp Sc: 57.74
TCCCTGGTGGTCCAGTGGTTAGGACTTGGTGCTTTCACTGCTGGGGCCCAGGTTTAATTC
CTGGCCAGGGAA
>Bos_taurus_chr10.trna2630-GluTTC (69685359-69685431) Glu (TTC) 73 bp Sc: 57.77
TCCCTAGTGGTCCAGTGGTTAGGACTCTGCACTTTCACTACTGAGGGCACAGGTTCAATT
CCTGGTTGGGGAG
>Bos_taurus_chr6.trna758-GluTTC (27393993-27394064) Glu (TTC) 72 bp Sc: 57.88
TCCCTGGTGGTCCAGTGGTTAGGACTCAGCGCTTTCACCGCCGTGGGTCAGGTTCCATTC
CTGGCCAGGGAT
>Bos_taurus_chr23.trna1799-GluTTC (40572317-40572389) Glu (TTC) 73 bp Sc: 57.90
TCCCTGGCAGTCAAATGGTTAAGACTCTGCACTTTCAATGCAGGGGGCGCAGGTTCAATC
CCTGACCAGGGAA
>Bos_taurus_chrUn.004.1260.trna3-GluTTC (19409-19481) Glu (TTC) 73 bp Sc: 57.93
TCCCTGGTAGTTCAGTGGTCAGGACTTGACACTTTCACTGCCATGAGCCCAGGTTCAATC
CCTGGTCGGGGAA
>Bos_taurus_chr2.trna136-GluTTC (5117585-5117656) Glu (TTC) 72 bp Sc: 58.08

TCCCTGGTGGTCCAGTGGTTAGGACTCGGTGCTTTCACCGCAGTGGCCCAGGTTCAGTCC
CTGGTTGGGGAA
>Bos_taurus_chrUn.004.19.trna20-GluTTC (200042-199970) Glu (TTC) 73 bp Sc: 58.16
TCCTTGATGGTCTAGTAGTTAGGACTCTACACTTTCACTGCTGAGGGCCCAGGTTCAATC
CCTGGTCAAGGAA
>Bos_taurus_chr11.trna2119-GluTTC (50778719-50778791) Glu (TTC) 73 bp Sc: 58.19
TTCCTGGTGGTCCAGTGGTTAGGACTCTGTGCTTTCACTGCTGAGGCCCCAGGTTCAATC
CCTGGTCAGGGAA
>Bos_taurus_chr10.trna6198-GluTTC (52441007-52440936) Glu (TTC) 72 bp Sc: 58.28
TCCCTGGTGGTCTAGTGGTTAGGATTCAAGGCTTTCACTGATGTGGCCCAGGTTCAATCC
CTGGTCAGGGAA
>Bos_taurus_chr9.trna2303-GluTTC (71290650-71290721) Glu (TTC) 72 bp Sc: 58.44
TCCCTGATGGTCCAGTGGTTAGGACTTGGCAGTTTCACTGCTGGGGCCCAGGTTCAATCC
TTGGTCAGGGAA
>Bos_taurus_chrX.trna6705-GluTTC (3562434-3562362) Glu (TTC) 73 bp Sc: 58.55
TCCCTGGTGGTCCAGTGTTTAGGACTCAGCACTTTCACTGCTGTGGCCCCAGGTTCAGTC
CCTGGTCAGGGAT
>Bos_taurus_chr18.trna1388-GluTTC (34683053-34683125) Glu (TTC) 73 bp Sc: 58.55
TCTCTGGTGGTCCAGTGGTTAGGACTCTGTGCTTTCACAGCTGAGGGCCCAGGTTCAATC
CCTGGTTAGAGAA
>Bos_taurus_chrX.trna1143-GluTTC (29952604-29952676) Glu (TTC) 73 bp Sc: 58.72
CCCCTGATGGTCCAGTGGTTAGGACTCAGCACTTTCACTGCTGAAGGCCCAGGTTCAATC
CTTGATCAGGGAA
>Bos_taurus_chr19.trna2581-GluTTC (50619663-50619735) Glu (TTC) 73 bp Sc: 58.78
TCCCTGGTGGTCCAGGGGTTAAGACTTGGCACTTTCACTGCCACGGGCCCAGGTTCGATC
CCTGGTTGGGAAA
>Bos_taurus_chr23.trna3907-GluTTC (19412767-19412695) Glu (TTC) 73 bp Sc: 58.84
TCCCTGGTGGTCCAGTGGTTAGGACTTGGCGCTTTCACTGCCAAGGGCCTGGGTTCAATC
CCTGGTCAGGGAA
>Bos_taurus_chrUn.004.234.trna5-GluTTC (194469-194541) Glu (TTC) 73 bp Sc: 58.91
CCCCTGATGGTCCAGTGGTTAGGACTTGGTGCTTTCACTGCCAAGGGCCCAGGTTCAATC
CCTGGTCAGGAAA
>Bos_taurus_chr17.trna250-GluTTC (8507940-8508011) Glu (TTC) 72 bp Sc: 58.99
TCCTGGAAGTCCAGTGGTTAGGACTCGGCACTTTCACTGCTGAGGGCCCAGGTTCAATTC
CTGGTCAGGGAA
>Bos_taurus_chr18.trna3030-GluTTC (65098506-65098435) Glu (TTC) 72 bp Sc: 59.00
TTCCTGATAGTTCAGTGGTTAGGATTCTGCATTTTCCCTGCAGTGACCCAGGTTCAATCC
CTGGTTAGGGAA
>Bos_taurus_chr2.trna8101-GluTTC (74890617-74890545) Glu (TTC) 73 bp Sc: 59.05
TCCCTGGTGGTCCAGTGGTTAGGTCCTGGCACTTTCACTGCCAAGGGCCCAAGTTCAATC
CCTGGTCAGGGAA
>Bos_taurus_chr11.trna1221-GluTTC (28411969-28412041) Glu (TTC) 73 bp Sc: 59.28
TCCTTAGTATTCCAGTTGTTAGGACTTGGCACTTTCAATGCCTTGGTCCCAGGTTCAATC
CCTGGCTGAGGAA
>Bos_taurus_chrUn.004.95.trna21-GluTTC (180796-180724) Glu (TTC) 73 bp Sc: 59.40
TCCTTGATGGTCCAGTGGCTAGGACTCAGCATTTTCACTGCTGAGGTCCCAGGTTCAATG
CCTGGGTAGGTAA
>Bos_taurus_chr1.trna7499-GluTTC (113324631-113324559) Glu (TTC) 73 bp Sc: 59.42
TCCCTGGTGGTCCAGTGGTTAGGATCCTGGGCTTTCACTGCAGAGGGCCCAGGTTCAATC
CCTGGTTGGGGAA
>Bos_taurus_chr2.trna5751-GluTTC (131373168-131373097) Glu (TTC) 72 bp Sc: 59.55
TCCCTGACAGTCCAGTGGTTAGGACTCAGCACTTTCACTGCAGGGGCCTGGGTTCAACCC
CCAGTCAGGGAC
>Bos_taurus_chr15.trna1837-GluTTC (53167526-53167597) Glu (TTC) 72 bp Sc: 59.57
TTTCTGGTGGTCCAGTGGTTAGGACTTGGCACTTTCACTGCTATGGCCCAGGTTCAATCC
CTGGTTGGGGAA
>Bos_taurus_chr29.trna2619-GluTTC (38077192-38077121) Glu (TTC) 72 bp Sc: 59.62
TCCCTGGTGGTCCAGCGGTTAGGACTTGGCATTTTCACTGCCATGGCCTGGGTTCAATCC
CTGGTTGGGGAA
>Bos_taurus_chr5.trna2694-GluTTC (72803927-72803998) Glu (TTC) 72 bp Sc: 59.70
CCCTTGGTGGTCTAGTGGTTAGGATTTGGCACTTTCACTGCTATGGCCCTGGTTCAATCC
CTGGTCAGGGAA
>Bos_taurus_chrUn.004.4854.trna2-GluTTC (6559-6630) Glu (TTC) 72 bp Sc: 59.70
CCCTTGGTGGTCTAGTGGTTAGGATTTGGCACTTTCACTGCTATGGCCCTGGTTCAATCC
CTGGTCAGGGAA
>Bos_taurus_chr15.trna3903-GluTTC (63917571-63917500) Glu (TTC) 72 bp Sc: 59.71
TCCCTGATGGTCCAGTGGTTAAGATTCAGTGCTTTCACTGCTGTGGCCCAGGTTCAATCC

CTAGTCAGGGAA
>Bos_taurus_chr17.trna4512-GluTTC (56352960-56352889) Glu (TTC) 72 bp Sc: 59.78
TCCCTGGTGGTCTAGTGGTTAGGACTCAGCACTTTCACTGCCAGGGCCCAGGTTCAATCC
CTGGTTGGGGAA
>Bos_taurus_chr21.trna2431-GluTTC (59729032-59729103) Glu (TTC) 72 bp Sc: 59.88
TCCCTGGTGGTCCAGTGGTTAGGACTCAGAGCTTTCATTTCTGGGGCCCAGGTTCAATCC
TTGGTCAGGGAA
>Bos_taurus_chr12.trna2543-GluTTC (67180845-67180917) Glu (TTC) 73 bp Sc: 59.99
TCCCTGATGGTCCAGTGGTTAGGACTTGGTACTTTCACTGCCAGGAACCTAGGTTCAATT
CCTGATCAGGGAA
>Bos_taurus_chr3.trna8336-GluTTC (28295302-28295230) Glu (TTC) 73 bp Sc: 60.32
TCCCTGGTGGTCCAGTGGTTAAGACACTGCACTTTCAATGCAGGGGGCCCAGGTTCGACC
CTTGGTCAGGAAA
>Bos_taurus_chr6.trna938-GluTTC (32749488-32749559) Glu (TTC) 72 bp Sc: 60.39
TCCCTGGTGGTCTAGTGGTTAGGACTTGGCACTTTCACTGCTATGGCCCGGGTTCAATCC
CTGGTCAGGGGA
>Bos_taurus_chr13.trna2734-GluTTC (66503138-66503210) Glu (TTC) 73 bp Sc: 60.42
TCCCTGGTGGTCCAGTGGTTAGGACTCCACGCTTTCTCTGTGGAGGGCCCAGGTTCAATC
CCTGGTTGGGGAA
>Bos_taurus_chr3.trna4898-GluTTC (123297823-123297751) Glu (TTC) 73 bp Sc: 60.51
TCCCTGGTGGTCCAGTGGTTAGGACTTAGTGCTTTCACTGCTCTGGGCCCAGGTTCAATC
CCTGGTCAGGGAA
>Bos_taurus_chr19.trna6481-GluTTC (9577244-9577173) Glu (TTC) 72 bp Sc: 60.51
TCCCTGGTGGTCCAGTGGTTACGACGTGGCACTTTCACTGCTGGTGCCCAGGTTCAATTC
CTGGTCAGGGAG
>Bos_taurus_chrUn.004.623.trna6-GluTTC (1032-960) Glu (TTC) 73 bp Sc: 60.53
CCCCTGATGGTCCAGTGGTTAAGATTCTGCACTTTCAATGCAGGGGGCCCAGGTTTGACC
CCTGGTCAGGGAA
>Bos_taurus_chrUn.004.1535.trna1-GluTTC (27084-27155) Glu (TTC) 72 bp Sc: 60.95
TTCCCAGTGGTCTAGTGGTTAGGACTCGGCACTTTCACTCCTGTGGCCCAGGTTCAATCC
CTGGTTGGGGAA
>Bos_taurus_chr28.trna344-GluTTC (8040419-8040491) Glu (TTC) 73 bp Sc: 61.23
TCCCTGGTGGTCCAGTGGTTAGGACTTGGCACTTTCAGTGCAAAGGGCCCAGGTTCAATC
CCTGGTTGGGGAA
>Bos_taurus_chr11.trna7907-GluTTC (30681641-30681569) Glu (TTC) 73 bp Sc: 61.34
TCCCTGGCAGTCCAGCGGTTAGGACTTGGCACTTTCAATGCCATGGGCCCAGGTTCAATC
CCTGGTTGGGGAA
>Bos_taurus_chr7.trna1959-GluTTC (42379038-42379110) Glu (TTC) 73 bp Sc: 61.60
TCCCTGGTGGTCCAGTGGTTAGGACTTGGCTCTTTCACTGCAGAGGGCCCAGGTTCAATC
CCTGGTCAGGGAA
>Bos_taurus_chr18.trna4134-GluTTC (46787607-46787535) Glu (TTC) 73 bp Sc: 61.72
TCCCTGGTGGTCCAGTGGCTAAGACTCTGTGCTTTCAACGCAGGGGGCTCAGGTTCGATC
CCTGGTCAGGGAA
>Bos_taurus_chrUn.004.1.trna65-GluTTC (1253596-1253668) Glu (TTC) 73 bp Sc: 61.77
TCCCTGGTGGTCCAGTGGTTAGGACTCCACACTTTCACTGCCGAGGGCCCAGGTTCAATC
CCTGACCAGGGAA
>Bos_taurus_chr9.trna6372-GluTTC (43774659-43774588) Glu (TTC) 72 bp Sc: 61.78
TCCCTGATGGTCTAGTGGTTAGGATTCTGGGTTTTCACTACAGTGGCCTAGGTTCAATCC
CTGGTCAGGGAA
>Bos_taurus_chr19.trna4562-GluTTC (44220821-44220749) Glu (TTC) 73 bp Sc: 61.83
TCCCTGGTGGTCTAGGGGTTAGGACTCGGCACTTTCACTGCCAGAGGCCCAGGTTCGATC
CCTGGTTGGGGAA
>Bos_taurus_chr3.trna813-GluTTC (22489650-22489721) Glu (TTC) 72 bp Sc: 61.92
TCCCTGGTGGTCTAGTGGCTAGGATTTGGCGCTTTCACCGCCGCGGCCCGGGTTCGATTC
CCGGTCAGAGAA
>Bos_taurus_chr5.trna5544-GluTTC (116107804-116107733) Glu (TTC) 72 bp Sc: 62.02
TCCCTGGTGGTCCAGTGGTTAGGATTTGGCGCTTTCACTGCCCTGGCCCAGGTTCAATCC
CTGGTTGGGGAA
>Bos_taurus_chr10.trna3272-GluTTC (86395940-86396011) Glu (TTC) 72 bp Sc: 62.18
TCCCTGGTGGTCCAGTGGTTAGGACTCAGCACTTTCACTGCTGGGATGCAGGTTCAAACC
CTGGTTGGGGAA
>Bos_taurus_chr14.trna4501-GluTTC (51111036-51110965) Glu (TTC) 72 bp Sc: 62.87
TTCCTGGTGGTCCAGTGGTTAGGACTCAGCACTTTCACTGCTGTGACACAGGTTCAATCC
CTGGTCAGGGAA
>Bos_taurus_chr7.trna5588-GluTTC (75712038-75711966) Glu (TTC) 73 bp Sc: 63.28
TCCCTGGTAGTCCAGTGGTTAGGACTTGGAACTTTCACTACCATGGGCCTAGGTTCAATC
CCTGGTCAGGGAA

>Bos_taurus_chr29.trna2271-GluTTC (45674281-45674209) Glu (TTC) 73 bp Sc: 63.51
TCCCTGGTGGTCTAGTGGTTAGGACTCTGCACTTTCACTGCAGAGGTCCTGGGTTCGATC
TCTGGTTGGGGAA
>Bos_taurus_chrUn.004.315.trna8-GluTTC (72134-72205) Glu (TTC) 72 bp Sc: 63.79
TCCCGGATGGTCCAGTGGTTAGGACTCAGCACTTTCACTGCTGTGACCTGGGTTCAATCC
CTGGTTGGGGAA
>Bos_taurus_chr3.trna8602-GluTTC (22461777-22461706) Glu (TTC) 72 bp Sc: 63.83
TCTCTGGTGGTCTAGTGGCTAGGATTCGGCGCTTTCACCGCTGCGGCCCGGGTTCGATTC
CCGGTCAGGGAA
>Bos_taurus_chr9.trna2040-GluTTC (63961107-63961179) Glu (TTC) 73 bp Sc: 63.84
TCCCTGGTGGTCCAGTGGTTAGGATTCTGCACTTTCAATGCTGAGGGCCCAGGTTCAATC
CCTGGTCAGAGAA
>Bos_taurus_chr8.trna3556-GluTTC (103162661-103162731) Glu (TTC) 71 bp Sc: 64.06
TCCCTGGTGGCCCAGTGGTTAGGACTCAGCACTTTCACTGCTGGGCCCAGGTTCAATCCC
TGGTTGGGGAA
>Bos_taurus_chrUn.004.6913.trna1-GluTTC (353-423) Glu (TTC) 71 bp Sc: 64.06
TCCCTGGTGGCCCAGTGGTTAGGACTCAGCACTTTCACTGCTGGGCCCAGGTTCAATCCC
TGGTTGGGGAA
>Bos_taurus_chr17.trna4956-GluTTC (50977884-50977812) Glu (TTC) 73 bp Sc: 64.08
TCCCTGGTGGTCCAGTGGTCAGGGCTCAGCACTTTCACTGCTGAGGGCCCTGGTTCAATT
CCTGGTTGGGGAA
>Bos_taurus_chr18.trna2908-GluTTC (64533640-64533711) Glu (TTC) 72 bp Sc: 64.09
TCCCTGGTGGCCTAGTGGTTAAGACTCAGCACTTTCACTGCTGAGGCTCAGGTTCAATCC
CTGGTCGGGGAA
>Bos_taurus_chr2.trna4572-GluTTC (130316274-130316345) Glu (TTC) 72 bp Sc: 64.31
TCCCTGATGGTCTAGTGGTTAGGATTCGGCACTTTCACTGTCGCAGCCCAGGTTCAAGCC
CTGGTCAGGGAA
>Bos_taurus_chr14.trna4179-GluTTC (59870820-59870749) Glu (TTC) 72 bp Sc: 65.48
TCCCTGGTGGTCCAGTGGTTAGGACTTGGGGCTTTCACTGCCATGGCCCAGGTTCAATCC
CTGGTCAGGGAA
>Bos_taurus_chr15.trna3154-GluTTC (81988357-81988285) Glu (TTC) 73 bp Sc: 66.69
TCCCTGAGGGTCCAGTGGTTAGGACTCTGCACTTTCACTGCTGGGGTCCCAGGTTCAATC
CCTGGTTGGGGAA
>Bos_taurus_chr13.trna3429-GluTTC (78512913-78512985) Glu (TTC) 73 bp Sc: 66.87
TCCCTGGTGGTCCAGTGGCTAAGACTCTGTACTTTCAATTCAGAGGGCCCAGGTTCGATC
CCTGGTCAGGGAA
>Bos_taurus_chr19.trna1066-GluTTC (22668253-22668325) Glu (TTC) 73 bp Sc: 67.00
TCCCTGATGGTCCAGTGGTTAGGACTCGGCATTTTCACTGCCATGGGCCCAGGTTCAATC
CCTGGTTAGGGAA
>Bos_taurus_chr22.trna1809-GluTTC (51031882-51031953) Glu (TTC) 72 bp Sc: 67.10
TCCCTGGTTGTCCAGTGGTTAGGACGTAGTACTTTCACTGCTGGGGCCCAGGTTCAATTC
CTGGTCAGGGAA
>Bos_taurus_chr12.trna6684-GluTTC (9913661-9913590) Glu (TTC) 72 bp Sc: 67.86
TCCCATATGGTCTAGCGGTTAGGATTCCTGGTTTTCACCCAGACGGCCCGGGTTCGACTC
CCGGTATGGGAN
>Bos_taurus_chr5.trna7191-GluTTC (80571049-80570978) Glu (TTC) 72 bp Sc: 68.68
TCCCTGGTGGTCCAGTGGTTAAGACTCAGCACTTTCACTGCTGGGGCCCAGGTTCGATCC
CTGGTCAAGGAA
>Bos_taurus_chr29.trna1069-GluTTC (30647393-30647465) Glu (TTC) 73 bp Sc: 69.41
TCCCTGGTGGTCCAGTGGTTAGGACTCTGTGCTTTCACTGCAGAAGTCCCAGGTTCAATC
CCTGGTCAGGGAG
>Bos_taurus_chr3.trna856-GluTTC (22901448-22901519) Glu (TTC) 72 bp Sc: 70.59
TCCCTGGTGGTCTAGTGGCTAGGATTCGGCGCTTTCACCGCCGCGGCCCGGGTTCAATTC
CCGGTCAGGGAA
>Bos_taurus_chr3.trna8567-GluTTC (22864507-22864436) Glu (TTC) 72 bp Sc: 70.59
TCCCTGGTGGTCTAGTGGCTAGGATTCGGCGCTTTCACCGCCGCGGCCCGGGTTCAATTC
CCGGTCAGGGAA
>Bos_taurus_chr3.trna841-GluTTC (22656357-22656428) Glu (TTC) 72 bp Sc: 72.33
TCCCTGGTGGTCTAGTGGCTAGGATTCGGCGCTTTCACCGCCGCGGCCCGGGTTCGATTC
CCGGTCAGGGAA
>Bos_taurus_chr3.trna8539-GluTTC (23079352-23079281) Glu (TTC) 72 bp Sc: 72.33
TCCCTGGTGGTCTAGTGGCTAGGATTCGGCGCTTTCACCGCCGCGGCCCGGGTTCGATTC
CCGGTCAGGGAA
>Bos_taurus_chr3.trna8556-GluTTC (22927455-22927384) Glu (TTC) 72 bp Sc: 72.33
TCCCTGGTGGTCTAGTGGCTAGGATTCGGCGCTTTCACCGCCGCGGCCCGGGTTCGATTC
CCGGTCAGGGAA
>Bos_taurus_chrUn.004.185.trna18-GluTTC (222647-222576) Glu (TTC) 72 bp Sc: 72.33

TCCCTGGTGGTCTAGTGGCTAGGATTCGGCGCTTTCACCGCCGCGGCCCGGGTTCGATTC
CCGGTCAGGGAA
>Bos_taurus_chrUn.004.185.trna3-GluTTC (34287-34358) Glu (TTC) 72 bp Sc: 72.33
TCCCTGGTGGTCTAGTGGCTAGGATTCGGCGCTTTCACCGCCGCGGCCCGGGTTCGATTC
CCGGTCAGGGAA
>Bos_taurus_chrUn.004.2930.trna4-GluTTC (9104-9175) Glu (TTC) 72 bp Sc: 72.33
TCCCTGGTGGTCTAGTGGCTAGGATTCGGCGCTTTCACCGCCGCGGCCCGGGTTCGATTC
CCGGTCAGGGAA
>Bos_taurus_chr2.trna10440-GluTTC (1279366-1279295) Glu (TTC) 72 bp Sc: 75.00
TCCCATATGGTCTAGCGGTTAGGATTCCTGGTTTTCACCCAGGCGGCCCGGGTTCGACTC
CCGGTATGGGAA
>Bos_taurus_chr23.trna2215-GluTTC (48749201-48749273) Glu (TTC) 73 bp Sc: 75.48
TCCCTGGTGGTCCAGTGGTTAGGACTCAGCACTTTCACTGCTGTGGCCCCAGGTTCAATC
CCTGGTCAGGGAA
>Bos_taurus_chr3.trna8598-GluTTC (22497376-22497305) Glu (TTC) 72 bp Sc: 76.25
TCCCTGGTGGTCTAGTGGTTAGGATTCGGCGCTTTCACCGCCGCGGCCCGGGTTCGATTC
CCGGTCAGGGAA
>Bos_taurus_chr9.trna799-GluTTC (26564227-26564298) Glu (TTC) 72 bp Sc: 76.25
TCCCTGGTGGTCTAGTGGTTAGGATTCGGCGCTTTCACCGCCGCGGCCCGGGTTCGATTC
CCGGTCAGGGAA
>Bos_taurus_chrUn.004.3551.trna2-GluTTC (1474-1545) Glu (TTC) 72 bp Sc: 76.25
TCCCTGGTGGTCTAGTGGTTAGGATTCGGCGCTTTCACCGCCGCGGCCCGGGTTCGATTC
CCGGTCAGGGAA
>Bos_taurus_chr12.trna6427-GluTTC (14082847-14082776) Glu (TTC) 72 bp Sc: 76.26
TCCCACATGGTCTAGCGGTTAGGATTCCTGGTTTTCACCCAGGCGGCCCGGGTTCGACTC
CCGGTGTGGGAA
>Bos_taurus_chr21.trna5667-GluTTC (1206237-1206166) Glu (TTC) 72 bp Sc: 76.26
TCCCACATGGTCTAGCGGTTAGGATTCCTGGTTTTCACCCAGGCGGCCCGGGTTCGACTC
CCGGTGTGGGAA
>Bos_taurus_chrUn.004.4221.trna2-GluTTC (9619-9690) Glu (TTC) 72 bp Sc: 76.26
TCCCACATGGTCTAGCGGTTAGGATTCCTGGTTTTCACCCAGGCGGCCCGGGTTCGACTC
CCGGTGTGGGAA
>Bos_taurus_chrUn.004.4872.trna1-GluTTC (5933-6004) Glu (TTC) 72 bp Sc: 76.26
TCCCACATGGTCTAGCGGTTAGGATTCCTGGTTTTCACCCAGGCGGCCCGGGTTCGACTC
CCGGTGTGGGAA
>Bos_taurus_chr2.trna5690-GluTTC (132707894-132707823) Glu (TTC) 72 bp Sc: 77.71
TCCCACATGGTCTAGCGGTTAGGATTCCTGGTTTTCACCCAGGTGGCCCGGGTTCGACTC
CCGGTGTGGGAA
>Bos_taurus_chr3.trna5633-GluTTC (106902805-106902724) Glu (TTC) 82 bp Sc: 38.58
TCCCTGGAGGTCCAGTGGTTAGGACTCAGTGCTTTCAGGCTTTCACTGCTCTGTGGCCTG
GGTTCGATCCCTGGTCAGGGAT
>Bos_taurus_chr10.trna865-GlyACC (20039325-20039397) Gly (ACC) 73 bp Sc: 30.58
TCTCTGATGGTCCAGGGCTTAAGACGATGTATTACCAGTACAGGAGGCCCAGGTTAGATC
CCTGGTCAGGGAA
>Bos_taurus_chr9.trna3118-GlyACC (90597498-90597570) Gly (ACC) 73 bp Sc: 33.61
TCCCTGGTGGTCCAGTGGCTGAGATCCTGAGCTACCAATCCAGGGACCTCAGGTTAGATC
CCTGATTAGGGAA
>Bos_taurus_chr25.trna630-GlyACC (11264063-11264135) Gly (ACC) 73 bp Sc: 33.84
TCCCTGGTGGTCCAGTGGTTAAGATTCTGTGCTACCAGTACAGGGGGTCTAGGTTCCATC
CTTGGTCAGGGAA
>Bos_taurus_chr22.trna1672-GlyACC (47319352-47319424) Gly (ACC) 73 bp Sc: 36.83
TCCCTGGTGGTCCAGCAGCTAAGACTCTGCACCACCAATGCAGGGGGCCTGGGTTTGATC
CCTGGTCAGGGAA
>Bos_taurus_chr19.trna2490-GlyACC (49326425-49326496) Gly (ACC) 72 bp Sc: 37.80
TCCCTGGTGGTCCAGTGGCTGAGACTCTGCGCTACCAATGCAGGGGTCTGGGTTCCATCC
CTGGTCAGGGAA
>Bos_taurus_chr5.trna5618-GlyACC (114430852-114430780) Gly (ACC) 73 bp Sc: 39.12
TCCCTGGTGGTCCAGTGGCTAAGTCTCTGTGCTACCAATGCAGGGTGCTCAGGTTTGAAC
CCTGGTCAGGGAA
>Bos_taurus_chr22.trna113-GlyACC (3385873-3385945) Gly (ACC) 73 bp Sc: 39.64
TCCCTCATGGTCCAGTGGCTAAGATTCTGTGCTACCAATGTAGGGGGCTCAGGTTTGATT
CCTGGTCAGGGAA
>Bos_taurus_chr15.trna5149-GlyACC (30970782-30970710) Gly (ACC) 73 bp Sc: 42.71
TCCTTAGTGGTTCAATGGCTAAGACTCTGTGCTACCAATGCAGATGGCCCAGGTTCAATC
CCTGGTCAGGGAA
>Bos_taurus_chr20.trna1184-GlyACC (33508899-33508971) Gly (ACC) 73 bp Sc: 43.40
TCCTTGATGGTCCAGTGGCTAAGATTCTGTGCCACCACTGCAGGGGGCCCGGGTTCAAGC

CCTGGTTGGGGAA
>Bos_taurus_chr14.trna289-GlyACC (7423277-7423349) Gly (ACC) 73 bp Sc: 44.88
TCCCTGGTGGTGCAATGGCTAAGACTCTGCACTACCAGTGCAGAGGGCTCGGGTTCGATC
CCTGGTCAGGGAA
>Bos_taurus_chrX.trna2724-GlyACC (73152721-73152793) Gly (ACC) 73 bp Sc: 45.65
TCCCTGGTGGTCCAGTGGCTAAAACTCTGAGCTACCAATGCAGAAGTCCTAGGTTTGATC
CCTGGTCAGGGAG
>Bos_taurus_chr11.trna4048-GlyACC (99239103-99239175) Gly (ACC) 73 bp Sc: 47.06
TCTCTGGTGGTCCAGTGGCTAAGACTCTGTGCTACCAATGCAGGGGGCCTGGGTTCAATC
CCTGGCCAGGGAA
>Bos_taurus_chr13.trna6530-GlyACC (25688027-25687955) Gly (ACC) 73 bp Sc: 47.54
TCCCTGGTGGTCCAGTGGCCAAGACTCCATGTTACCAATGTAGGGGACCCAGGTTCGATC
CCTGGTCAGGGAA
>Bos_taurus_chr4.trna4061-GlyACC (117003365-117003437) Gly (ACC) 73 bp Sc: 50.18
TCCCTGGTGGTCTAGTGGTTAAGACTCTGCCCTACCACTGCAGGGGACATAGGTTCAATC
CCTGGTCAGGGAA
>Bos_taurus_chr22.trna4309-GlyACC (6815948-6815876) Gly (ACC) 73 bp Sc: 52.52
TCCTAGGTGGTCTAGAGGCTAAGACTCTGCACTACCAAGGCAGGGGGCCTGGGTTCAATC
CCCGGTCTGGGAA
>Bos_taurus_chrX.trna1341-GlyACC (34808900-34808972) Gly (ACC) 73 bp Sc: 53.22
TCCCTGATTGTCCAGTGGCTAAGACTCTGTGCTACCAATGCAGGGGGCCTAGGTTCGATC
CCTGGTCAGGGAA
>Bos_taurus_chr12.trna3870-GlyACC (74898435-74898363) Gly (ACC) 73 bp Sc: 55.45
GGGCCAGTGGCACAATGGGTAATGCATCTGACTACCCATCAGAAGATTCCAAGTTCAACT
CCTGGCTGGCTCA
>Bos_taurus_chrUn.004.891.trna9-GlyACC (51647-51719) Gly (ACC) 73 bp Sc: 55.74
TCCCTGGTGGTCCAGTGGCTACGACTCTGTGCTACCAATGCAGGGGGCCTGGGTTCGATT
CCTAGCCAGGGAA
>Bos_taurus_chr4.trna4265-GlyACC (120631359-120631439) Gly (ACC) 81 bp Sc: 49.30
TCCCAGGTGGCTTACTGGTAAAGCATCTGCCTACCAATGTAGAAGATGCAGGAGACGCAG
GTTCGATCCCTGGTTTGGGAA
>Bos_taurus_chr15.trna1863-GlyACC (53760639-53760727) Gly (ACC) 89 bp Sc: 36.15
TTCCAGTTGGCTCAGTGGTAAAGAATCTGTCTACCAGTGGTTAAAGAATTGAACCAAAGG
AGATGCAGGTTCGATCCCTGTGTTGGAAG
>Bos_taurus_chr28.trna1008-GlyCCC (27712875-27712948) Gly (CCC) 74 bp Sc: 21.36
TCCCTGATGGTCCAAGTGGCTAAGGCTCTGCTGTCCCATAGAGGGGGGTACAGGTTAGAT
CCCTGTTCAGGCAA
>Bos_taurus_chr11.trna7020-GlyCCC (51357939-51357868) Gly (CCC) 72 bp Sc: 21.83
TCCCTGGTGGTCCAGTGGCTGAGGCTCTGTGCTCCCTATACAGGGAGCCCGGTTCCATCC
CTGGTCAGGGAA
>Bos_taurus_chr14.trna1278-GlyCCC (29536419-29536491) Gly (CCC) 73 bp Sc: 22.39
TCTCTGGTGGTCCAGTGCCTAAGAGTCTGTGCTCCCGATGCAGGGGGGTCAGGTTCCATC
CCTGGTCAGGGAA
>Bos_taurus_chr7.trna8147-GlyCCC (10678868-10678798) Gly (CCC) 71 bp Sc: 23.21
TCCTTGGTGGTTTAGTGGCTGAGACTCTGTGCTCCCAGTGCAAGGGCCTGGTTTGATCCC
TGGCCAGGGAA
>Bos_taurus_chr5.trna4914-GlyCCC (121374003-121374074) Gly (CCC) 72 bp Sc: 24.04
TCCCTGGTGGTTCAGTGGAGAAGACTCTGCACTCCCAATGCACGGGGCTGGGTCTAGTCC
CTGGTCAGGGAA
>Bos_taurus_chr5.trna5246-GlyCCC (121258068-121257997) Gly (CCC) 72 bp Sc: 24.04
TCCCTGGTGGTTCAGTGGAGAAGACTCTGCACTCCCAATGCACGGGGCTGGGTCTAGTCC
CTGGTCAGGGAA
>Bos_taurus_chr1.trna781-GlyCCC (19050095-19050167) Gly (CCC) 73 bp Sc: 24.07
TCCCTGGTTGTCCAGTGGCTGAGACTCTGCATTCCCAGTGTAAGGGGTCCAGGTTTGATC
CCTGGTCAGGGAA
>Bos_taurus_chr1.trna2695-GlyCCC (79185717-79185790) Gly (CCC) 74 bp Sc: 24.18
TCCCTGGTGGTCCAATGGCTAAGACTCTGTGCTCCCAATGCAGGGGACTTTAAGTTTAAA
CCCTGGTCAGGGAA
>Bos_taurus_chr19.trna5941-GlyCCC (18790877-18790805) Gly (CCC) 73 bp Sc: 24.94
TCTCTGGTGGTCCAGTGACCAAGACTCTGAACTCCCCATGCAGGCAGTCCAGGTTCCATC
CCTGGTCAGGGAA
>Bos_taurus_chr12.trna6294-GlyCCC (16048422-16048350) Gly (CCC) 73 bp Sc: 25.37
TCCCTGGTGGTCCAGTGGCTGAGACTCTGCGCTCCCAACGCAGGGGGGCTGGGTTCCATC
TCTGATCAGGGAA
>Bos_taurus_chr7.trna8290-GlyCCC (9341356-9341284) Gly (CCC) 73 bp Sc: 25.43
TCTCTGGTGGTCCAGTAGCTAAGACTCTGTGCTCCCAATACAGGGTGCCCAGGTTTGATT
CCTAGTCAGGGGA

>Bos_taurus_chr2.trna4315-GlyCCC (125661715-125661787) Gly (CCC) 73 bp Sc: 25.46
TCCCTGGTGGTCCACTGACTATGTCTCTGTGCTCCCAATGCAGATAGCCTGGGTTCGATC
CCTAGTCAGGGAA
>Bos_taurus_chr29.trna2770-GlyCCC (33718462-33718390) Gly (CCC) 73 bp Sc: 25.50
TCTCTGGTGGTCCAGTGGCTAAGATTCCATGGTCCCAGCGCGGGGGCCCTTGGTTTGATC
CCCAGTCAGGGAA
>Bos_taurus_chr3.trna8832-GlyCCC (16840182-16840109) Gly (CCC) 74 bp Sc: 25.57
TCCCTGGTAGTCCAGTGGCTAAGACTCTGTGCTCCCAGGGCAGGGGGCCTCAGGATAGAT
CCCTGGTCAGGAAA
>Bos_taurus_chr3.trna1894-GlyCCC (51849870-51849942) Gly (CCC) 73 bp Sc: 25.61
TCCCTTGTGGTCCAGGGGCTGAGACTCTGAGTTCCCAATTCAGGGGGCCTAGGTTCCACC
CCTGGTTAGGGAT
>Bos_taurus_chr12.trna6683-GlyCCC (9933513-9933441) Gly (CCC) 73 bp Sc: 25.65
TCCCTGGCAGTCCAGTGGCTAAGACTCTGTGTTCCCAATGCAGGGGAGCCAGGCTCAATC
CTTGGTTGGGGAA
>Bos_taurus_chr7.trna3254-GlyCCC (81131023-81131095) Gly (CCC) 73 bp Sc: 25.80
TTCCTGGTGGTCCAGCGGCTGAGACTCTGCATTCCCAGTACAGGGGGCTCAAGTTCAATC
CCTGGTCAGGGAA
>Bos_taurus_chr6.trna5669-GlyCCC (93704615-93704542) Gly (CCC) 74 bp Sc: 25.86
TCTCCTGATGGTCCAGTGACTAAGACCCCGTACTCCCAATGCTGCAGGCCCGGGTTTGAT
CCCTGGTCAGGGAA
>Bos_taurus_chr7.trna7971-GlyCCC (13335281-13335208) Gly (CCC) 74 bp Sc: 25.95
TCCCTGGTGGTTCAGTGACTAAGACTCTGTGCTCCCAATGCAGGGGGCCTCACGTTCTAT
CCTTGGTCAGGGAA
>Bos_taurus_chr29.trna3340-GlyCCC (19448852-19448780) Gly (CCC) 73 bp Sc: 26.05
TCCCTGATGGTCCAGTGGCTCGGACTATGTACTCCCAATGCAAGTAATCTGGGTTTGATT
CCTGGTCAGGGGC
>Bos_taurus_chr29.trna3345-GlyCCC (19389163-19389091) Gly (CCC) 73 bp Sc: 26.05
TCCCTGATGGTCCAGTGGCTCGGACTATGTACTCCCAATGCAAGTAATCTGGGTTTGATT
CCTGGTCAGGGGC
>Bos_taurus_chr1.trna3337-GlyCCC (97482414-97482486) Gly (CCC) 73 bp Sc: 26.15
TCCCTGGTGGTCCGGCAGGCGGGACTCTGAGCTCCCGATGCAGGGTGTCCGGGTTCGATC
CCTGGTCAGGGAA
>Bos_taurus_chr5.trna9084-GlyCCC (31663926-31663854) Gly (CCC) 73 bp Sc: 26.39
TCCCTGGTGGTCCAGTGGCTGGGCCTCTATGCTCCCAATGCAGGGAACCCAGGTTCCATC
TCTGGTCAGGGAA
>Bos_taurus_chr11.trna3030-GlyCCC (75469807-75469880) Gly (CCC) 74 bp Sc: 26.92
TCCCTGATGGTCCAGTGACTGAGATTCTGAGCTCCCAATGCAGGGAGCCCAGGGTTCGAT
CCTTGGTCAGGGAA
>Bos_taurus_chr7.trna8139-GlyCCC (10855599-10855527) Gly (CCC) 73 bp Sc: 27.00
TCCCTGGTGGTCCAGTGGCTGAGTCTCTGTGCTCCCAGTGCAGGGGGCCCAGGTTTGATC
CCTGGTCAGGGGA
>Bos_taurus_chr9.trna1675-GlyCCC (52124825-52124899) Gly (CCC) 75 bp Sc: 27.03
TCCCTGGTGGTCCAAGTGGCTGAGACTCCGTGCTCCCAACACAGGGGGTGCAGGGTTCGA
TCCCTAGTCAGGGAA
>Bos_taurus_chr19.trna1623-GlyCCC (31984601-31984673) Gly (CCC) 73 bp Sc: 27.15
TCCCTGGTGGTCCACAGGCTAAGACTCTGTGCTCCCACTGCAGGGGGCCAGGGTTTGATC
CCTCGTCAGGGAA
>Bos_taurus_chrX.trna4259-GlyCCC (72988999-72988928) Gly (CCC) 72 bp Sc: 27.16
TCCCTGGTTGTCCAGTGACTAAGACTCCGCATTCCCAGTGCAGGGGTCCGGGTTCCATCC
CTGGTCAGGGAA
>Bos_taurus_chr12.trna4167-GlyCCC (70667324-70667252) Gly (CCC) 73 bp Sc: 27.22
TCCCTGGTGGTCCAGTGGCTAAGACCCTGTGCTCCCAGTGCGGGGGGCCCAGGTTCTGTC
CCTTGTCAGGGAA
>Bos_taurus_chr6.trna4156-GlyCCC (120880809-120880881) Gly (CCC) 73 bp Sc: 27.26
TTCTTGCTGGTCCAGAGGCTCAGACTCTGTCCTCCCCACGCAGGGGGCCCAGGTTCGACC
CCTGGTCGGGGAG
>Bos_taurus_chr7.trna4247-GlyCCC (109437776-109437847) Gly (CCC) 72 bp Sc: 27.28
TCCCTGGTGGTCCAGCGGCCAAGACTCGGCGCTCCCAGTGCAGGCGCCCAGGCTCCATCC
CTGGTCAGGGAA
>Bos_taurus_chr2.trna4749-GlyCCC (133077987-133078059) Gly (CCC) 73 bp Sc: 27.33
TCCCTGGTGGTTCAGTGGCTAAGACTCTGCACTCCCAGTGCAGGGGAGCAGGGTTTGATG
CCTGGTCAGGGAG
>Bos_taurus_chr7.trna5157-GlyCCC (88740306-88740233) Gly (CCC) 74 bp Sc: 27.49
TCCCTGGTGGTCCAGAGGCTAAGATTCCACACTCCCAAGGTGGATTGCCCTGGGGTTGAT
CCCTCGTCAGGGAA
>Bos_taurus_chr6.trna7873-GlyCCC (25611148-25611077) Gly (CCC) 72 bp Sc: 27.55

TCCCTGGTGGTTCAAGGCTGAGATTCTGTGTTCCCAATGCAGAGGGTCCAGGTTCAATCC
CTGGTCAAGGAA
>Bos_taurus_chrUn.004.966.trna5-GlyCCC (55333-55403) Gly (CCC) 71 bp Sc: 27.72
TCCCTGGTGGTGCACTGGCTAAGACTGCACTCCCAACGCAGGGGGCCCAGGTTTGATCCC
TGGTCAGGGAA
>Bos_taurus_chr25.trna2153-GlyCCC (36114556-36114629) Gly (CCC) 74 bp Sc: 27.98
TCCCTGGTGGTCCAGTGTCTAAGACTCTGCACTCCCCATGCAGTGGGGACTGGGCTCAAT
CCCTGGTCAGGGAA
>Bos_taurus_chr18.trna5841-GlyCCC (4165534-4165462) Gly (CCC) 73 bp Sc: 27.99
TCCCTGATGGTCCAGTGCCCAAGACGCTGCGCTCCCCACACAGGGGGCCTGAGTTCAATT
TCTGGTCAGGGAG
>Bos_taurus_chr19.trna5264-GlyCCC (29614215-29614143) Gly (CCC) 73 bp Sc: 28.06
TCCCTGGTGGTCCAGTGGCTAAGACTCCACACTCCCAGTGTAGGTGGCCTGGGGTCAGTC
CCTGGTCAGGGAA
>Bos_taurus_chr21.trna171-GlyCCC (6033388-6033459) Gly (CCC) 72 bp Sc: 28.17
TCCCTGGTGGTCCAGTGGCTAAGTCTCTGTACTCCCAACGCAGGGGGCCAGGGTTGGATC
CTGGTTAGGGAA
>Bos_taurus_chr21.trna5535-GlyCCC (5928533-5928462) Gly (CCC) 72 bp Sc: 28.17
TCCCTGGTGGTCCAGTGGCTAAGTCTCTGTACTCCCAACGCAGGGGGCCAGGGTTGGATC
CTGGTTAGGGAA
>Bos_taurus_chr20.trna4765-GlyCCC (26192143-26192071) Gly (CCC) 73 bp Sc: 28.25
TCTCTGGTGGTCCAGTAGCTGGCACCCTGAGCTCCCAATGCAGGGGGCCCAGGTTCAACC
CCTGGTCAGGGAA
>Bos_taurus_chr23.trna2034-GlyCCC (45582264-45582336) Gly (CCC) 73 bp Sc: 28.31
TCCCTGGTGGTCCAGTAGCTGAGACTCTGAGTTCCCCATGCAGGGGGCCCAGGTTCGATC
CCTGATCAGGGAA
>Bos_taurus_chr17.trna1534-GlyCCC (42298463-42298535) Gly (CCC) 73 bp Sc: 28.32
TCCCTGGTGGTCCACTGGCTAAGACTCCGTGCTCCCCTTGCAGGGGGTCCAGATTCAATT
CCTGGTCAGGGAA
>Bos_taurus_chr4.trna4912-GlyCCC (110866347-110866275) Gly (CCC) 73 bp Sc: 28.32
TCCCTGGTGGTCCAGTGGCTAAGACTCTGTGCTCCCAGCGCAGGGGGACCGGGCTTGATT
CCTGGTCACGGAA
>Bos_taurus_chr18.trna4944-GlyCCC (26674096-26674024) Gly (CCC) 73 bp Sc: 28.50
TCCTTGGTGGCCCATTGGTTAAGACTCTGTGCTCCCACTGCAGGGGGTTCAGGCTTGATC
CCTGGTCAGGGAA
>Bos_taurus_chr29.trna1644-GlyCCC (45167673-45167745) Gly (CCC) 73 bp Sc: 28.96
CCCTTGGTGGTTCAGTGGCTAAGACTTTGAGCTCCCAACACAGGGGCCCTGGGTTTGCTC
CCTAGTCAGGGAG
>Bos_taurus_chrX.trna790-GlyCCC (18701825-18701897) Gly (CCC) 73 bp Sc: 29.11
TCCCTGGTAGTCCAGTGGGTGAGATTCTGTGCTCCCAATGCATGAGGCCCGGGTCTGATT
CCTGGCCGGGGAA
>Bos_taurus_chr6.trna915-GlyCCC (32041000-32041073) Gly (CCC) 74 bp Sc: 29.12
TCCCTGGTGGTCCATTTGGCTGGGACTCTGTGCTCCCAGGGCAGGGGACCTAGGTTTGAT
CCCTGGTCAGGGAA
>Bos_taurus_chr12.trna5931-GlyCCC (22550067-22549995) Gly (CCC) 73 bp Sc: 29.26
TCCCTGATGGTCCAGTGGCTACGACTCTACACTCCCAGTGCAGGGGGCCCGGGTTTGGTC
CCTGGTCAGTGAA
>Bos_taurus_chrUn.004.6729.trna2-GlyCCC (3065-2993) Gly (CCC) 73 bp Sc: 29.26
TCCCTGATGGTCCAGTGGCTACGACTCTACACTCCCAGTGCAGGGGGCCCGGGTTTGGTC
CCTGGTCAGTGAA
>Bos_taurus_chr29.trna2725-GlyCCC (34985790-34985718) Gly (CCC) 73 bp Sc: 29.39
TACCTGGTGGTCCAGTGGCTAAGATTCTGTAATCCCAATACAGGGGGTTCGGGTTTGATC
CCTGGTCAGGGAA
>Bos_taurus_chr10.trna5630-GlyCCC (69438652-69438580) Gly (CCC) 73 bp Sc: 29.43
TCCCTGGTGGTCCAGTGGCTGGGACTCTGTGTTCCCAATGCAGGGGGCCCAGGTTTGGTC
CCTCGTTGGGGAG
>Bos_taurus_chr13.trna3519-GlyCCC (80088900-80088973) Gly (CCC) 74 bp Sc: 29.55
TCCCTGGCAGTCCAGTGGCTGAGACTCTGCTCTCCCAGTGCAGGGGGCCCCGGGTTTGAT
CCCCGGTCAGGGAA
>Bos_taurus_chr16.trna4017-GlyCCC (57793991-57793919) Gly (CCC) 73 bp Sc: 29.59
TCCCTGGTGGTCCAGCGGTTACGACTCTGTGTTCCCAGGGCAGGGGGCCTGGATTCCATC
CCTAGCTAGGGAA
>Bos_taurus_chr5.trna2203-GlyCCC (60763572-60763644) Gly (CCC) 73 bp Sc: 29.67
TCTCTGACAGTCCAGTGGCTAAGACTCTGTGTTCCCCAGGCAGGGGGCCTGGGTTCAATC
CCTGGTCAGGGAA
>Bos_taurus_chr10.trna5216-GlyCCC (80286134-80286062) Gly (CCC) 73 bp Sc: 29.68
TCCCTGGTGGTCCGGTAGCTAAGACTCTGTGCTCCCAATGCAGGGGGACTGGGTTCAATC

CCTGGTCAGGGAA
>Bos_taurus_chr3.trna727-GlyCCC (20968220-20968293) Gly (CCC) 74 bp Sc: 29.76
TTCCTGGTGGTCCAGTGGCTGAGACTCTGCACTCCCCATGCAGGGGGCCCAGGTTCAATT
TTTGGTTGAGGAAA
>Bos_taurus_chr23.trna592-GlyCCC (14776874-14776945) Gly (CCC) 72 bp Sc: 29.93
TCCCTGGTGGCCTGGTGGCTGAGGTGCTGTGCTCCCCATGCAGGGGGCTGGGTTCAATCC
CTAGTCAGGGAA
>Bos_taurus_chr19.trna3765-GlyCCC (57831138-57831066) Gly (CCC) 73 bp Sc: 30.00
TCCCTGGTGGCCCAGTGACTGGGGCTCTGTACTCCCAGTCCAGAGGGCCTGGGTTCGTTC
CCTGGTCAGGGAA
>Bos_taurus_chr5.trna9126-GlyCCC (30650903-30650832) Gly (CCC) 72 bp Sc: 30.13
TCCCTGGTGGTCCAGTGGCTGAGACTCTTCACTCCCCATGCAGAGGGCCTGGGTTGATCC
CTGGTCAGGGAA
>Bos_taurus_chr25.trna4255-GlyCCC (12214270-12214198) Gly (CCC) 73 bp Sc: 30.35
TCCCTGGTGGTCCAGTGACTAAGACTCTGAGCTCCCTATGCAGGGGGCCCAGGTTCAGCC
CCTGGTCAGGGAA
>Bos_taurus_chr24.trna4097-GlyCCC (34595734-34595646) Gly (CCC) 89 bp Sc: 30.40
TCCCTGATGGTCCTATGGGTAAGACTCTGCACTCCCAATGAAGAGGGTTCATCCTTTATC
CAGCCTGGGTTCCATCCCTGGTCAGGGAA
>Bos_taurus_chr6.trna5092-GlyCCC (103690765-103690693) Gly (CCC) 73 bp Sc: 30.48
TCCCTGGTGGTCCAATGGCTAAGACTCTGAACTCCCAATGTAGGGCACCCAGGTTCGGTT
TCTGGTCAAGGAA
>Bos_taurus_chr9.trna2371-GlyCCC (73147144-73147216) Gly (CCC) 73 bp Sc: 30.48
TCCCTGGTGGTCCAATGGCTAAGACTCTGAACTCCCAATGTAGGGCACCCAGGTTCGGTT
TCTGGTCAAGGAA
>Bos_taurus_chr22.trna2574-GlyCCC (52967197-52967125) Gly (CCC) 73 bp Sc: 30.60
TCCCTGGTAGTCCAGTAGCTAAGACTCCGTACTCCCAATGCAGGGTACACAGGTTTAATC
CCTGGTCAGGGAA
>Bos_taurus_chr27.trna47-GlyCCC (2460984-2461056) Gly (CCC) 73 bp Sc: 30.71
TCCCTGGTGGTCCAGTGGCTGAGACATTGTGCTCCCAAAGCAGGGCGCCCAAGCTCAATC
CCTGGCCAGGGAA
>Bos_taurus_chr15.trna2261-GlyCCC (63718146-63718218) Gly (CCC) 73 bp Sc: 30.76
TCCCTGGTGGTCCAGTGGCTAAGACTATGTGCTCCCAATGCAGGGTACCCAAGTTTAATC
CTTGGTCAGAGAA
>Bos_taurus_chr22.trna1152-GlyCCC (31246142-31246214) Gly (CCC) 73 bp Sc: 30.78
TCCCTGGTGGTCCAGTGGCTAAGACTCTGTGCTCCCAATACAGGGGAGCTGGGTTCGATC
TCTGATCAGGGAA
>Bos_taurus_chr19.trna4494-GlyCCC (45160924-45160852) Gly (CCC) 73 bp Sc: 30.82
TCCCTGGCAGTCCAGTGGCTAAGACTCTGAGCTCCCAATGCAGGAAGCCCAGGTTTGATC
CTTGGTCGGGGAA
>Bos_taurus_chr13.trna5693-GlyCCC (41963872-41963800) Gly (CCC) 73 bp Sc: 30.90
TCTCTGGTGGTCTTGAGGCTAAGACTCCATGCTCCCAATGTGGGGGGCCCGGGTTCCATC
CCTGGTCAGGGAA
>Bos_taurus_chr3.trna2479-GlyCCC (71271996-71272072) Gly (CCC) 77 bp Sc: 30.96
TCCCTGGTGGTTCAGTGGCTGGGACTCCATGCTCCCAATGTGGGGGGCCTACCTAGGTTT
GATTCGTGGTCAGGGAA
>Bos_taurus_chr17.trna2285-GlyCCC (55881744-55881816) Gly (CCC) 73 bp Sc: 31.12
TCCCTGGTGGTCCAGTGGCTAAGACTCTGTGCTCCCGATGCAGTTGGCCTGGGTGTGATC
CCTGGTCAGGGAA
>Bos_taurus_chr11.trna8846-GlyCCC (6423962-6423890) Gly (CCC) 73 bp Sc: 31.25
TCCCTGGTAGTCCACAGGCTAAGACTCTGAATTCCCAATGCAGGGGGCCTGGGTTTGATC
CCTGGTCGGGGAA
>Bos_taurus_chr8.trna5867-GlyCCC (72411735-72411663) Gly (CCC) 73 bp Sc: 31.27
TCCCTGGTGGTCCAGTGGCCAAGACTTTGTGTTCCCAATTCAGGGCACTCGAGTTCGATC
CTTGGTCAGGGAA
>Bos_taurus_chr11.trna7661-GlyCCC (38089439-38089365) Gly (CCC) 75 bp Sc: 31.39
TCCCTAGTGGTCTAGAGGCTAAGACTCTGTGCTCCCCAAACAGGGTGGGCCCAGGTTCGA
TCTCTGGTCAGGGAA
>Bos_taurus_chr18.trna3935-GlyCCC (49470431-49470359) Gly (CCC) 73 bp Sc: 31.49
TCCCTGGTAGTCCAGTGACTAAGACTCTGTGCTCCCAATGCAGGGACCCTGGGTTCCACC
CCTGGTCAGGGAA
>Bos_taurus_chr14.trna2698-GlyCCC (64603987-64604059) Gly (CCC) 73 bp Sc: 31.52
TCCCTGGCAGTCCAGTGGCTAAGACTCCGTACTCCCAATGCAGGGGGCTCGGGTCCAATC
CCTGGTCAGGGAA
>Bos_taurus_chr14.trna1028-GlyCCC (24003461-24003533) Gly (CCC) 73 bp Sc: 31.53
TCCCTGGTGGTCTAGCCACTAAGATTCCATGCTCCCGATGTAGGAGACCTAGGTTCGATT
CCTGGTCAGGGAA

>Bos_taurus_chr5.trna9758-GlyCCC (11180743-11180671) Gly (CCC) 73 bp Sc: 31.59
TCCCTGGTGGTCCGCTGGCTAAGACTCTGCGCTCCCAGTGCAAAGGACTCAGGTTCAAAT
CCTGGTCAGGGAG
>Bos_taurus_chr21.trna4133-GlyCCC (34379171-34379099) Gly (CCC) 73 bp Sc: 31.59
TCCCTGGTGGCCCAGTGACTAAGACTCTGTATTCCCAATGGAGGAGGCCCAGGTTTGACC
CCTGGTCAGGGAA
>Bos_taurus_chr5.trna3970-GlyCCC (103372230-103372302) Gly (CCC) 73 bp Sc: 31.63
TCGCTGGTGGTCCAGTGGCTGAGACTCTGTGCTCCCAATGCAGCGGGCCCAGGTTTGGTC
CCTGGTCAGGGAA
>Bos_taurus_chrUn.004.3715.trna4-GlyCCC (8110-8038) Gly (CCC) 73 bp Sc: 31.63
TCGCTGGTGGTCCAGTGGCTGAGACTCTGTGCTCCCAATGCAGCGGGCCCAGGTTTGGTC
CCTGGTCAGGGAA
>Bos_taurus_chr24.trna4300-GlyCCC (30530844-30530772) Gly (CCC) 73 bp Sc: 31.75
TCCCTGGTGGTCCAGTGACTAGGATTTAGTGCTCCCAATGCAGGGGGCTCAGGTTTAATC
CCTGGTCAGGGAA
>Bos_taurus_chr16.trna5397-GlyCCC (25849093-25849021) Gly (CCC) 73 bp Sc: 31.75
TCCCTGGTGGTCCAGTGACTAAGACCTTGTGCTCCCAGTGTAGGGGGCCCAGGTTCAATC
CCTGGTCAGGGAA
>Bos_taurus_chr12.trna1613-GlyCCC (36466900-36466972) Gly (CCC) 73 bp Sc: 31.79
TCTCTGGAAGTCCAGTGGCTGTGACTCCACATTCCCAATGTCGGGGGCCCAGGTTCAACT
CCTGGTTGGGGAA
>Bos_taurus_chr5.trna2467-GlyCCC (67441127-67441198) Gly (CCC) 72 bp Sc: 31.85
TCCCTGGTGGTCCAGTGGCTAAGATTCTGTGCTCCCAGTGCAGGGAGCCAGGTTCAGTCC
CTGATCAGGGAA
>Bos_taurus_chr17.trna2965-GlyCCC (67056360-67056432) Gly (CCC) 73 bp Sc: 31.86
TCCCTGGCGGTCTAGTGGCTGAGACTCTGAGCTCCCAATGCAGGGAGCCCGGGTTGGATC
CCTGGCCAGGGAA
>Bos_taurus_chrUn.004.4114.trna2-GlyCCC (1119-1047) Gly (CCC) 73 bp Sc: 31.86
TCCCTGGCGGTCTAGTGGCTGAGACTCTGAGCTCCCAATGCAGGGAGCCCGGGTTGGATC
CCTGGCCAGGGAA
>Bos_taurus_chr2.trna1871-GlyCCC (60178997-60179069) Gly (CCC) 73 bp Sc: 31.96
TCCCTGGTGGTTCAGTGGCTAAGACTCTGCATTCCCAATGCAGGGCCCTTAAGTTCAGTC
CCTGGTTGGGGAG
>Bos_taurus_chr12.trna1337-GlyCCC (30172549-30172621) Gly (CCC) 73 bp Sc: 31.99
TCCCTGGTGGTCCTGGGGCTGAGACTCTAAGCTCCCAGTGTAGGGGGCCCAGGTTCAATT
CCTGGTCAGGGAA
>Bos_taurus_chr14.trna6043-GlyCCC (14797860-14797788) Gly (CCC) 73 bp Sc: 32.12
TCCCTGGTGGTCCGGAGGCTAAGACTCTGTGCTCCCAATGCAGGGGGCCTGGGTTCCATC
CCTGGTCAGGGAA
>Bos_taurus_chr21.trna542-GlyCCC (15486308-15486380) Gly (CCC) 73 bp Sc: 32.18
TCCCTGGTGGTTCAGCAGCTAAGACTCTGTGCTCCCAATGCAGGGGGCCCAGGTTCCATC
CCTGGTCAGGCAA
>Bos_taurus_chr25.trna1702-GlyCCC (29089329-29089401) Gly (CCC) 73 bp Sc: 32.25
TCCCTGATGGTCCAGCGGCTGAGACTCTGTACTCCCAAGGCAGGAGGCCTGGGTTCCATC
CCTGGTCAGGGAA
>Bos_taurus_chr16.trna4160-GlyCCC (53701137-53701066) Gly (CCC) 72 bp Sc: 32.37
TCTCTGGTGGTTTAGTGACTGGGACTCTGCACTCCCAATTCAGGGTGCCTGGTTCAATCC
CTGGTCAGGGAA
>Bos_taurus_chr6.trna1078-GlyCCC (38358269-38358341) Gly (CCC) 73 bp Sc: 32.40
TCCCTGGTGGTCCAGTGGCCAAGACTCTGTGGTCCCAATGTAGGGGGACCAGGTTTGATC
CCTGGTCAGGGAG
>Bos_taurus_chr7.trna1170-GlyCCC (19179281-19179352) Gly (CCC) 72 bp Sc: 32.47
TCTCTGGTGGTCCAGAGGCTAGACTCTGAGGTCCCAATGCAGGGAACCTGGGTTCAATCC
CCAGCCAGGGAA
>Bos_taurus_chr20.trna5346-GlyCCC (10510415-10510343) Gly (CCC) 73 bp Sc: 32.52
TCCCTGGTGGTCCAGTGGATAAGACTTTGTAATCCCAATGCAGGTGACCTAGGTGCGATC
CCTGGTCAGGAAA
>Bos_taurus_chrX.trna1765-GlyCCC (49080543-49080615) Gly (CCC) 73 bp Sc: 32.53
TCCCTGGTGGTCCGGTGGCAAAGACTCTGTGCTCCCAATTTAGGGGGCCCAGGTTTGATC
CCTGGTTAGGGAA
>Bos_taurus_chr13.trna4131-GlyCCC (76679872-76679800) Gly (CCC) 73 bp Sc: 32.56
TCCCAGGTGGTCCACTGGCTGGGACTCTGAGCTCCCAATGCAGGGGACCCAGGTTTGATC
CCTGGTCAGGGAA
>Bos_taurus_chr10.trna3501-GlyCCC (90565764-90565836) Gly (CCC) 73 bp Sc: 32.56
TCCTTGGTAGGCCACTGGCTAAGGCTCTGTGCTCCCAAGGCAGGGGGCCTAGGTTTGATC
CCTGGTCAGGGAA
>Bos_taurus_chr6.trna5663-GlyCCC (93807792-93807719) Gly (CCC) 74 bp Sc: 32.60

TTCCTGGTGGTCCAATGGTTAAGACTCTGCGATCCCAATGCAGGTGGCCCAGGTCCGATC
CCTGGTCAGAGAAG
>Bos_taurus_chr28.trna2982-GlyCCC (5757001-5756928) Gly (CCC) 74 bp Sc: 32.64
TTCCTGATGGTCCAGTGGCTAAGACTCTGCTCTCCCAATGCAGGGAGCCTGGGGTTCAAC
CCCTGGTTAGGGAC
>Bos_taurus_chr18.trna1920-GlyCCC (46635831-46635903) Gly (CCC) 73 bp Sc: 32.67
TCCCTGGTAGCCCAATGGTTGAGACTCTGAGCTCCCAGTTCAGGGGGCCCAGGTTTGATC
CCTGGTCAGGGAA
>Bos_taurus_chr4.trna8275-GlyCCC (16236613-16236541) Gly (CCC) 73 bp Sc: 32.70
TCCCTGGTGGCCTAGGGGCTAAGACTCTGCATTCCCAGTGCAGGGCACCCGGGTTCTATC
CCTGGTCAGGGAA
>Bos_taurus_chr5.trna256-GlyCCC (8093764-8093836) Gly (CCC) 73 bp Sc: 32.82
TCCCTGGTGGTCCAGTGGCTGAGACTCTGAGCTCCCAGTGCAGGGGGCCCAGGTTCAGTC
CCTGGTCAGGGAG
>Bos_taurus_chr3.trna5861-GlyCCC (100315597-100315526) Gly (CCC) 72 bp Sc: 32.88
TCCCTCATGGTCCAGTGACTAAGACTCTGTGCTCCCAGAGCAGGGGACCAGGTTTGATCC
CTGGTCAGGGAA
>Bos_taurus_chrUn.004.5.trna3-GlyCCC (134629-134701) Gly (CCC) 73 bp Sc: 32.91
TTCCTGGTGGTCCAATAGCTAAGACTCTGAACTCCCAGTGCAGGGGGCCCAGGTTCGATC
TCTGGTCAGGCAA
>Bos_taurus_chr10.trna4547-GlyCCC (95422937-95422865) Gly (CCC) 73 bp Sc: 32.93
TCCCTGGCAGTCCAGTGGCTGAGACTCTGTGCTCCCGATGCAGGGGGCCTGGGTTCGATC
CCTGGTCAGGGAA
>Bos_taurus_chr15.trna5293-GlyCCC (28472622-28472551) Gly (CCC) 72 bp Sc: 33.01
TCCCTGGTGGCCCAGAGGCTAAGATTCTGAGCTCCCAGTGCAGGGGTCCAGGTTGGATCC
CTGGTCAGGGAA
>Bos_taurus_chr24.trna2762-GlyCCC (63962520-63962448) Gly (CCC) 73 bp Sc: 33.20
TCCCTGGTGGCCCCGTGGCTAAGGCTCTGTGCTCCCAGTGGAGGGGGCCCAGGTTTGATC
CCTGGTCAGGGAA
>Bos_taurus_chr25.trna2307-GlyCCC (38249566-38249638) Gly (CCC) 73 bp Sc: 33.24
TCCTTGGTGGTCCAATGGCTAGGACTCTGTGCTCCCCGTGCAGGGGGCCCAAGTTCCAGC
CCTGGTCGGGGAA
>Bos_taurus_chr24.trna2930-GlyCCC (60401794-60401722) Gly (CCC) 73 bp Sc: 33.30
TCCCTGAGGGTCCAGTGGCTAAGACTCCACACTCCCAGTGTGGGAGGCCTGGGTTTGACC
CCTGGTCAGGGAA
>Bos_taurus_chr23.trna329-GlyCCC (9438756-9438828) Gly (CCC) 73 bp Sc: 33.34
TCCCTGGTGGTCCTATGGCTACGACCCCACACTCCCAATGTAGGGGGCCCGGGTTTGATC
CCTGGTTGGGGAA
>Bos_taurus_chr29.trna1195-GlyCCC (32905197-32905270) Gly (CCC) 74 bp Sc: 33.47
TCCCTGGTGGTTCAGTGGCTAAAGACTCTGTAGTCCCAATTCAGGGGGCCCGGGTTCCAT
CCCTGGTCAGGGAA
>Bos_taurus_chr25.trna1875-GlyCCC (31735516-31735587) Gly (CCC) 72 bp Sc: 33.52
TTCCTGGTGGTCCTGTGGCTAAGACTCTGAACTCCCCGTGCAGGGGCCCAGGTTTGATCC
CTGGTCAGGGAA
>Bos_taurus_chr23.trna3438-GlyCCC (30525414-30525343) Gly (CCC) 72 bp Sc: 33.52
TCCTTGATTGTCCAGTGGCTAAGATTCTGTGCTCCCAATGCAGGGACCTGGGTTTGATCT
CTGGTCAGGGAA
>Bos_taurus_chr14.trna3704-GlyCCC (71018916-71018845) Gly (CCC) 72 bp Sc: 33.59
TCCCTGGCGGTCCACTGGCTAAGACTATGTGCTCCCAATGCAGGGGGCCAGGTTCAATAC
CTGGTCAGGGAA
>Bos_taurus_chr14.trna710-GlyCCC (15860302-15860373) Gly (CCC) 72 bp Sc: 33.72
TCCCTGGTGGTCCAATGGCTGAGACTCTGAGTTCCCAATGCAGGGGCCCAGGTTCCATCC
GTGGTCAGGGAA
>Bos_taurus_chr28.trna1515-GlyCCC (44861795-44861867) Gly (CCC) 73 bp Sc: 33.83
TCCCTGGTGGTTAAGTGGCTAAGACTCTATGCTCCCAATGCAGCGGACCCAGATTCCATC
CCTGGTCAGGGAA
>Bos_taurus_chr19.trna6190-GlyCCC (14707932-14707860) Gly (CCC) 73 bp Sc: 34.02
TCTCTGGCAGTCCAGTGGTTAAGACTTTACACTCCCAGTATAGGGGATGCAGGTTCGATC
CCTGTTTGGGGAA
>Bos_taurus_chr7.trna8514-GlyCCC (4616637-4616563) Gly (CCC) 75 bp Sc: 34.03
TCCCTGGTGGTCCAGTGGTTAACACTCTGAGCTCCCAATGCAGGGGAGCCCCAGGTTTGG
TCCCTGGTCAGGGAA
>Bos_taurus_chr26.trna246-GlyCCC (9742258-9742330) Gly (CCC) 73 bp Sc: 34.04
TCCCTGGGGGTCCAGTGGCTAAGACTTGGTGCTCCCAATGCAAGGGGCCTAGGTTCCACT
CCTAGTCGGGGAA
>Bos_taurus_chr13.trna1586-GlyCCC (37977500-37977572) Gly (CCC) 73 bp Sc: 34.07
TCCCTGGTGGTCCAGTGGCTGAGATTCTGTGCTCCCAGTGCAGGGGTCCCGGGTTCAGTC

CCTGGTCAGGGAA
>Bos_taurus_chr13.trna5877-GlyCCC (37999288-37999216) Gly (CCC) 73 bp Sc: 34.07
TCCCTGGTGGTCCAGTGGCTGAGATTCTGTGCTCCCAGTGCAGGGGTCCCGGGTTCAGTC
CCTGGTCAGGGAA
>Bos_taurus_chr24.trna3996-GlyCCC (36358577-36358505) Gly (CCC) 73 bp Sc: 34.11
TCTCTGGTGGCCCAGTGGCCGGGACTCTGAGCTCCCAGTGCAGGGGCCCCAGGTTCAATC
CCTGGTCAGGGAA
>Bos_taurus_chr2.trna6083-GlyCCC (125717208-125717136) Gly (CCC) 73 bp Sc: 34.13
TCCCTGATGGTCCAATGGCTGAGACTTTGTACTCCCATTGCAGGCAGCCTAGGTTCCATC
CCTGGTCAGGGAA
>Bos_taurus_chr11.trna651-GlyCCC (13402559-13402631) Gly (CCC) 73 bp Sc: 34.13
TCTCTGGTAGTCCAGTAGCTAAGACTCTGTGCTCCCAAAGCAGAGGGCCTGGGTTCAGTC
CCTAGTCGGAGAA
>Bos_taurus_chrUn.004.3620.trna1-GlyCCC (6980-7052) Gly (CCC) 73 bp Sc: 34.13
TCTCTGGTAGTCCAGTAGCTAAGACTCTGTGCTCCCAAAGCAGAGGGCCTGGGTTCAGTC
CCTAGTCGGAGAA
>Bos_taurus_chrX.trna4679-GlyCCC (63234096-63234024) Gly (CCC) 73 bp Sc: 34.20
TTCTTGGTGGCCCAATGGTTAAGATTCCGTGCTTCCCTTGCAGGGGGTACAGGTTCAATC
CCTGTTTGGGAAA
>Bos_taurus_chr13.trna4758-GlyCCC (65882194-65882122) Gly (CCC) 73 bp Sc: 34.27
TCCCTGGTGGTCCAGTGACTGAGGCTCTGCACTCCCAACGCAGGGGGACCAGGTTCAATC
CCTGGTTAGGGAA
>Bos_taurus_chr4.trna5109-GlyCCC (107118586-107118514) Gly (CCC) 73 bp Sc: 34.28
TCCCTGGTGGTCTGGCGGCTAAGATACTGTGCTCCCATTGCAGGGGGCCCAGGTTCAACC
CTTGGTTAGGGAC
>Bos_taurus_chr3.trna8793-GlyCCC (17727380-17727303) Gly (CCC) 78 bp Sc: 34.29
TCCCTGTTGGTCTAGTGGCTAAGACTCTGAGCTCCCAATGCAGGGAAGGGAGCCCAGGTT
TGCTTCCTGGTCAGGGAA
>Bos_taurus_chrUn.004.2675.trna1-GlyCCC (1148-1078) Gly (CCC) 71 bp Sc: 34.30
TCCCTGGTGGTGCACTGGCTAAGACTGCACTCCCAATGCAGGGGGCCCAGGTTTGATCCC
TGGTCAGGGAA
>Bos_taurus_chrUn.004.4719.trna1-GlyCCC (1191-1121) Gly (CCC) 71 bp Sc: 34.30
TCCCTGGTGGTGCACTGGCTAAGACTGCACTCCCAATGCAGGGGGCCCAGGTTTGATCCC
TGGTCAGGGAA
>Bos_taurus_chr16.trna4833-GlyCCC (38820201-38820129) Gly (CCC) 73 bp Sc: 34.33
TCCCTGGTGGTCCAGTGGCTGGGACTCTGCACTCCCAGTACAGAGGACCCGGGTTCCATT
CCTGGTAGGGGAA
>Bos_taurus_chrUn.004.6483.trna1-GlyCCC (2812-2881) Gly (CCC) 70 bp Sc: 34.36
TCCTGGTGGTCCAGTGGCCAAGACTTTGTGCTCCCAATTCAGGGGACCCAGGTTCGATCC
CTGGTCGGAG
>Bos_taurus_chr11.trna66-GlyCCC (1217264-1217336) Gly (CCC) 73 bp Sc: 34.40
TCCTTGGTGGTCCAAAGGCTAAGACTCTGTGCTCCCAATGCAGGGGGACCAAGTTTGATC
CCTGGTCAAGGAA
>Bos_taurus_chrX.trna4623-GlyCCC (64308305-64308233) Gly (CCC) 73 bp Sc: 34.45
TCCCTGGTGGTCCAGTGGCTAAGTCTCTGTACTCCCAATGCAGGAGGACTGGGTACAATC
CCTAGTCAGGGAA
>Bos_taurus_chr18.trna1692-GlyCCC (42935327-42935399) Gly (CCC) 73 bp Sc: 34.46
TCCCCAGTGGTCCAGTGATTAAGACTCTGTGCTCCCAATACAGAACACCCAGGTTTGATC
CCTGGTTGAGGAT
>Bos_taurus_chr23.trna971-GlyCCC (21494123-21494194) Gly (CCC) 72 bp Sc: 34.48
TGCCTGGTGGTCCAGTGGCTAAGACTCTGCACTCCCAGTGCAGAGAGCTAGGTTCGTTCT
CTGGTCAGGGAA
>Bos_taurus_chr22.trna4027-GlyCCC (11874295-11874224) Gly (CCC) 72 bp Sc: 34.48
TCCTTGGTGGTCCTGTGGCTAAGACTCTGTACTCCCAATGCAGGGGGCTGGGTTTGACTC
CTGGTCAGGGAT
>Bos_taurus_chr5.trna906-GlyCCC (27358943-27359015) Gly (CCC) 73 bp Sc: 34.57
TCCCTGGTCGTCCAGTGGCTAAGACTCTCTGCTCCCCATGCAGGGGGCCTGGGTTCAACC
CCTGGTCAGGGAA
>Bos_taurus_chr13.trna877-GlyCCC (23442796-23442868) Gly (CCC) 73 bp Sc: 34.66
TTCCTGGTGGTCCAGTTGCTAAGACTCTGTGCTCCCAATGCAGGGGACCCGGGTTTGATC
CCTGGTCAGGAGA
>Bos_taurus_chr5.trna7923-GlyCCC (62197655-62197583) Gly (CCC) 73 bp Sc: 34.67
TCTCTGGTGGTCCAGTGACTAAGACTCTGTGCTCCCAATGCAGGGCACCCAGGTTCTATC
CCTGGTCAGGGAA
>Bos_taurus_chr13.trna4814-GlyCCC (65315361-65315289) Gly (CCC) 73 bp Sc: 34.69
TCCCTGGTAGTCCAGTGTCTTAGACTCTGTGTTCCCAATGCAGGGGGCCTGGGTTCAATC
CCTGGTCAGGGAA

>Bos_taurus_chr7.trna6372-GlyCCC (51792176-51792104) Gly (CCC) 73 bp Sc: 34.82
TCCCTGGTGGTCCTGTGGCTGAGACTCTGCACTCCCAATGCAAGGGGTCCAGGTTGGATC
CCTGGTCAGGGAC
>Bos_taurus_chr27.trna2819-GlyCCC (29076408-29076336) Gly (CCC) 73 bp Sc: 34.83
TCCCTGGTGGTCCTGTGACTAAGACTCTGAGCTCCCAATACAGTGGGCCTGGGTTCGATC
CCTGGTCAGGGAA
>Bos_taurus_chr13.trna7071-GlyCCC (15036510-15036438) Gly (CCC) 73 bp Sc: 34.88
TCCCTGGTGGTCCAGTGGCTACGACTCTGTGCTCCCAATGCAGGGGGCTCGAGTTTGATC
CCTGGTCAGGGAA
>Bos_taurus_chr24.trna4631-GlyCCC (23727722-23727650) Gly (CCC) 73 bp Sc: 34.90
TCCCTGGTGGTTCGGTGGCTACGATTCTGTGCTCCCAATGCAGGAAGCCCAGGTTCCATC
CCTGGCCAGGGAT
>Bos_taurus_chr11.trna9059-GlyCCC (2493042-2492970) Gly (CCC) 73 bp Sc: 34.91
TCCCTGATGGTCCAGCGGCTGGGACTCTGCATTCCCAGTGCAGGGTGCCTGGGTTTGATC
CCTGGTTAGGGAA
>Bos_taurus_chr17.trna6275-GlyCCC (13596913-13596841) Gly (CCC) 73 bp Sc: 34.93
TCTTTGGTGGTTCAGTGGCTGAGACTCTGCACTCCCAATACAGGGGGCTCAGGTTTGATC
CCTGGTCAGGGAA
>Bos_taurus_chr26.trna288-GlyCCC (10978516-10978588) Gly (CCC) 73 bp Sc: 34.93
TCCCTGGTGGTCCAGCAGTTAAGACTCTGTGCTCCCAATGCAGGGCACACAGGTTTGATC
CCTGGCTGGGGAA
>Bos_taurus_chr26.trna3643-GlyCCC (10899078-10899006) Gly (CCC) 73 bp Sc: 34.93
TCCCTGGTGGTCCAGCAGTTAAGACTCTGTGCTCCCAATGCAGGGCACACAGGTTTGATC
CCTGGCTGGGGAA
>Bos_taurus_chrUn.004.7002.trna1-GlyCCC (3019-3091) Gly (CCC) 73 bp Sc: 34.93
TCCCTGGTGGTCCAGCAGTTAAGACTCTGTGCTCCCAATGCAGGGCACACAGGTTTGATC
CCTGGCTGGGGAA
>Bos_taurus_chr13.trna7234-GlyCCC (10189276-10189204) Gly (CCC) 73 bp Sc: 34.95
TTCCTGGTGGTCCAATGGCTAAGACTCTACAATCCCCATGCAGGGGGCCCAGGTTTGATT
CCTGGTCAGGGAA
>Bos_taurus_chr9.trna6648-GlyCCC (36096990-36096918) Gly (CCC) 73 bp Sc: 34.95
TCCCTGATGGTCCTGTGGCTATGACTCTATGCTCCCAATGCAGGGGGACTGGGTTCAATC
CCTAGTCAGGGAA
>Bos_taurus_chr19.trna5690-GlyCCC (22435334-22435262) Gly (CCC) 73 bp Sc: 35.22
TTCCTGGTGGTCCCGTGGTTAAGACTCCCCATTCCCAGTGGAGGGGGTTCAGGTTCGATC
CCTGGTCGGGGAA
>Bos_taurus_chr27.trna535-GlyCCC (18200450-18200521) Gly (CCC) 72 bp Sc: 35.23
TCCCTGGTCGTCCAATGGCTGAGACTCTGTGCTCCCAATGCAGGGAGCCTGGTTCGATCC
CTGGTCGGGGAA
>Bos_taurus_chr18.trna2102-GlyCCC (49563849-49563921) Gly (CCC) 73 bp Sc: 35.31
TCCCTGGTGGTCCAGTGGGTTAGATTCTGCAGTCCCAATGCAGGAGGCCCGGGTTCAATC
CGTGGTCAGGGAA
>Bos_taurus_chr10.trna5398-GlyCCC (75509031-75508959) Gly (CCC) 73 bp Sc: 35.34
TCCCTGGTGGTCCAGTGTCTAAGACTCTTTGCTCCCAATGCAGGGGGCTCAGGTTCTATC
CCTGACCAAGGAG
>Bos_taurus_chrUn.004.8128.trna1-GlyCCC (906-834) Gly (CCC) 73 bp Sc: 35.34
TCCCTGGTGGTCCAGTGTCTAAGACTCTTTGCTCCCAATGCAGGGGGCTCAGGTTCTATC
CCTGACCAAGGAG
>Bos_taurus_chr7.trna596-GlyCCC (11622476-11622548) Gly (CCC) 73 bp Sc: 35.41
TCTCTGGTAGTCCAGTGGCTAAGACTCTGAGCTCCCAATGCAGGGGAACCACGTTCAACC
CCTGGTCAGGGAA
>Bos_taurus_chr7.trna1173-GlyCCC (19188789-19188861) Gly (CCC) 73 bp Sc: 35.49
TCCCTGGGGGTCCAGTGGTTAAGACTCTGAGTTCCCACTGCAGGGTACTCAGGTTTGATC
CCTGGCTGGGGAA
>Bos_taurus_chr11.trna1303-GlyCCC (30683214-30683286) Gly (CCC) 73 bp Sc: 35.51
TCCCTGGTGGTCTAGTGACTAAGATTCTGAGCTCCCACTGCAGAGGGCCCTGGTTCAGGC
CCTGGTCAGGGAA
>Bos_taurus_chr11.trna1664-GlyCCC (41326438-41326510) Gly (CCC) 73 bp Sc: 35.58
TCCCTGGTGGGCCAGTGACTAAGACTTTACACTCCCAATGCAGGGGGCCCAGGTTTGACC
CCTGGTCAGGGAA
>Bos_taurus_chr16.trna5599-GlyCCC (20873413-20873341) Gly (CCC) 73 bp Sc: 35.59
TCCCTGGTGGCCCAGTGGCTAAGACTCTGTGCTCCCAGTGCAGGGGGTCCAGGTTCTATC
CCTGGTCAGGGAC
>Bos_taurus_chr6.trna2254-GlyCCC (74707463-74707535) Gly (CCC) 73 bp Sc: 35.62
TCCCTGGTGGTCCAGTGGCTAAGACCCTGCAATCCCAAAGCAGGGGACCCGGTTTCAATC
CCTGGTCAGGGAA
>Bos_taurus_chr19.trna3164-GlyCCC (60980506-60980578) Gly (CCC) 73 bp Sc: 35.64

TCCCTGGTGGTCCAGTGGCTGGGACTCTGCGCTCCCAATGCAGGGGCTCTGTGTTCCATC
CCTGGTCAGGGAG
>Bos_taurus_chr19.trna3877-GlyCCC (56488122-56488050) Gly (CCC) 73 bp Sc: 35.65
TCCCTGGTGGTCCAGTGGCTGAGACTCTGTCCTCCCCATGCAGGGGGCTCAGGTTCGATC
CCTGGTCAGGGGA
>Bos_taurus_chr22.trna155-GlyCCC (4638281-4638353) Gly (CCC) 73 bp Sc: 35.66
TCCCTGGTGGTCCAGTGGCTCAGACTCTGTGCTCCCAGTGCAGGGGGCCCAGGTTAGACC
CCTGGTCAGGGAA
>Bos_taurus_chr1.trna9631-GlyCCC (45528076-45528004) Gly (CCC) 73 bp Sc: 35.67
TCCCTGGTGGTCCAGTGGCTAAGACTCTGTGCTCCCAATTCAGGGGGCCTGGGTTTGCTC
CCTGGCTAGGGAA
>Bos_taurus_chr3.trna8812-GlyCCC (17236909-17236837) Gly (CCC) 73 bp Sc: 35.68
TCCCTTATGGTCCAGTTGCCAAGATGCTGTGTTCCCAATGCAGGAGGCCAGGGTTCGATC
CCTGGTAAGGGAA
>Bos_taurus_chr19.trna3775-GlyCCC (57638240-57638168) Gly (CCC) 73 bp Sc: 35.77
TCCCTGGTGGTCCAGTGGCTAAGAGTCTGTGCTCCCGATACAGGGGGGCTGGGTTCGATC
CCTGGTCAGGGAA
>Bos_taurus_chr17.trna4279-GlyCCC (60720154-60720082) Gly (CCC) 73 bp Sc: 35.79
TTCCTGGTGGTCCAGTGGCTAAGACTCTGCACTCCCAATGTAGGTGGTCCAGGTTCCACT
CCTGGTCAAGGAA
>Bos_taurus_chr3.trna737-GlyCCC (21106015-21106087) Gly (CCC) 73 bp Sc: 35.79
TCCCTGGTGGTTCAGTGGCTAAGATTCTGTGCTCCCAATGCAGGGGGCCCAGGTTCCATT
TCTGGTCAGAGAA
>Bos_taurus_chr9.trna4985-GlyCCC (84155176-84155104) Gly (CCC) 73 bp Sc: 35.81
TCTCTGGTGGTCCAGTGGCCAAGAGTCTGTGCTCCCAATGCAGTGCTCCCGGGCTCAAGC
CCTGGTCAGAGAA
>Bos_taurus_chr14.trna3252-GlyCCC (79987400-79987472) Gly (CCC) 73 bp Sc: 35.87
TCCCTGGTGGTCCAGTGGCTGAGACTCTGCGCTCCCAGAGCAGGGGGCTCAGGTTTGATC
CCTGGTCAGGGAA
>Bos_taurus_chrX.trna1510-GlyCCC (39055191-39055262) Gly (CCC) 72 bp Sc: 35.95
TCCCTGGTGGTCCATTGGCTAAGACTCTGTGCTCCCAATGCAGGGCACTGGGTTCAATCC
CTGGTCAGGGAA
>Bos_taurus_chr8.trna7141-GlyCCC (31719512-31719439) Gly (CCC) 74 bp Sc: 35.98
TCCCTGGTGGTCCAGTAGCTAAGATTCCGTACTCCCAATGCAGGAGGGCCAGGGTTCGAT
CCCTAGTCAGGGAA
>Bos_taurus_chr3.trna378-GlyCCC (11504051-11504123) Gly (CCC) 73 bp Sc: 36.13
TCCCTGGGTGTTCAGTGGTTAAGATTCTGTGTTCCCAACACAGGGGGCCTGGGTTCAATC
CCTGGTCAGGGAA
>Bos_taurus_chr19.trna2873-GlyCCC (56790093-56790165) Gly (CCC) 73 bp Sc: 36.19
TCCCTGGTGGTCCAGTGGTTGAGATTTTGCCTTCCCATGTTGGGGGGTGCAGGTTCAATC
CCTGCTCAGGGAG
>Bos_taurus_chrX.trna2855-GlyCCC (76263339-76263411) Gly (CCC) 73 bp Sc: 36.26
TCCCTGGTGGTCCAGTAGCTAAGACTCTGTGCTCCCAATGCAGGGGGCTTAGGTTCCATC
CCTGGTCAGGGAA
>Bos_taurus_chrX.trna2877-GlyCCC (76861565-76861637) Gly (CCC) 73 bp Sc: 36.26
TCCCTGGTGGTCCAGTAGCTAAGACTCTGTGCTCCCAATGCAGGGGGCTTAGGTTCCATC
CCTGGTCAGGGAA
>Bos_taurus_chr13.trna4356-GlyCCC (72989447-72989375) Gly (CCC) 73 bp Sc: 36.26
TCCCTGGTGGTCCAGTGTCTAAGATTCTGAGCTCCCAATGCAGGGGGCCCAGGTTCGGTC
CCTTGTCAGGGAA
>Bos_taurus_chrUn.004.864.trna2-GlyCCC (9616-9544) Gly (CCC) 73 bp Sc: 36.31
TCCCTAGTGGTCCATTGGCTGCAACTCTGTGCTCCCAATACAGGGGGCCCAGGTTCGATC
CCTGGTTGGGGAA
>Bos_taurus_chrX.trna2618-GlyCCC (71166177-71166249) Gly (CCC) 73 bp Sc: 36.31
TCCCTAGTGGTCCATTGGCTGCAACTCTGTGCTCCCAATACAGGGGGCCCAGGTTCGATC
CCTGGTTGGGGAA
>Bos_taurus_chr11.trna8066-GlyCCC (26659452-26659380) Gly (CCC) 73 bp Sc: 36.33
TCCCTGGTGGTTCAGTGGCTGGGACTCTGAGCTCCCAATGCAGGGGGCCCACGTTCAATC
CCTGGTCAGGGGG
>Bos_taurus_chr17.trna4605-GlyCCC (55381659-55381587) Gly (CCC) 73 bp Sc: 36.42
TCCCTGGCAGTCCAGTGGTTAAGACTCTGTGCTCCCAATGCAAAGGACCTAGGTTTGATC
CCTGGTCAGGGAT
>Bos_taurus_chr25.trna2234-GlyCCC (37398327-37398403) Gly (CCC) 77 bp Sc: 36.44
TCCCTGGTGGTTCAGTGGCTGAGACTCCGCACTCCCAATGCAGGGGTAGGGGCGAGGTTC
AATCCCTGGCCAGGGAA
>Bos_taurus_chr11.trna3276-GlyCCC (81254746-81254818) Gly (CCC) 73 bp Sc: 36.45
TTCCTGGTGGTCCAAAGGTTTAGACTCTGTCCTCCCAATGCAGGGGCCCCAGGTTTGATC

CCTGGTCAGGGAC
>Bos_taurus_chr10.trna1453-GlyCCC (36753467-36753539) Gly (CCC) 73 bp Sc: 36.48
TCCCTGGTGGTCCAGTGGCTGGGACTCCGTGCTCCCAGTACAGGGAATCCAGGTTCAATC
CCTGGTCAGGAAG
>Bos_taurus_chr14.trna1170-GlyCCC (27100664-27100736) Gly (CCC) 73 bp Sc: 36.49
TCCCTGGTGGTCCAGTGGCTAAGACTCGGTGCTCCCAATGCAGTGAGCCCAGATTCTATC
CCTGGTTGGGGAA
>Bos_taurus_chr18.trna1806-GlyCCC (44808684-44808755) Gly (CCC) 72 bp Sc: 36.50
TCCCTGGTGGTCCAGTGGCTGGGACCCTGAGCTCCCAATGCAGGGGCTGAGGTTGGATCC
CTGATCAGGGAA
>Bos_taurus_chrUn.004.953.trna1-GlyCCC (2753-2823) Gly (CCC) 71 bp Sc: 36.54
TCCTTGGTGGTCCAGTGGCAAGACTCTGTGTTCCCAATGCAGGGTCCCAGGTTCCTTCCC
TGGTCAGGGTG
>Bos_taurus_chr5.trna8020-GlyCCC (60566555-60566484) Gly (CCC) 72 bp Sc: 36.57
TCTCTGGTAGTCCAGTGGCTAAGACTCTGCACTCCCAATGCAGGGAATCTGGGTTCAGTC
CTGGTCAGGGAA
>Bos_taurus_chr17.trna5145-GlyCCC (47541421-47541349) Gly (CCC) 73 bp Sc: 36.69
TCCCTGGTGGTCCAGTGGCTAAGACTCTGCACTCCCAATGGAGGGCGGCCAGGTTCAATG
TCTGGTTGGGGAA
>Bos_taurus_chr10.trna1756-GlyCCC (46210482-46210554) Gly (CCC) 73 bp Sc: 36.75
TCCCTGGTGGTCCAGTGGCTAAGACTCTGTGCTCCCAGTACAGGGGACCCGGATTCGATC
CCTGGTCAGGGAA
>Bos_taurus_chr25.trna515-GlyCCC (9371146-9371218) Gly (CCC) 73 bp Sc: 36.77
TCTCTGGTGGTCCATTGGCTAAGACTCTGTGATCCCAATGCAGGGAACCCAGGTTCAATC
CCTGGTCAGGGAA
>Bos_taurus_chr11.trna3931-GlyCCC (96100684-96100756) Gly (CCC) 73 bp Sc: 36.77
TCCCTGGTGGGCCAGTGTATAAGACTCTGAGCTCCCAATGCAGGGGGCCCAGGTCCAATC
CCTGGTCAGGGAA
>Bos_taurus_chr7.trna1896-GlyCCC (39947494-39947565) Gly (CCC) 72 bp Sc: 36.77
TCCCTGGTGGTCCAGCGTTTAAGACTCCATGCTCCCAATGTAGGGGCTCAGATTCAATCC
CTGATTAGGGAA
>Bos_taurus_chr19.trna165-GlyCCC (7254568-7254640) Gly (CCC) 73 bp Sc: 36.77
TCCCTGATGGTCCAGTGGCTAAGACTTTGCGCTCCCAAAGCAGGGGACCTGAGCTCAATC
CCTGGTCAGGGAA
>Bos_taurus_chr29.trna3752-GlyCCC (9215373-9215301) Gly (CCC) 73 bp Sc: 36.79
TCCCTGGTGGTCCAGTGGCTGAGACTCTGCACTCCCAGTGCAGGGGGCCTGGGTTCAATC
CTTGGTCAGGGAA
>Bos_taurus_chrX.trna399-GlyCCC (8918370-8918442) Gly (CCC) 73 bp Sc: 36.85
TCCCTGGTGGTCCAGTGGCTAAGATTCTGTGCTCCCCATGCAGGGGACTCAGGTTCAGTC
CCTGGTCAGGGAA
>Bos_taurus_chr23.trna1896-GlyCCC (42492445-42492514) Gly (CCC) 70 bp Sc: 36.85
TTCTTGGTGGTCCAGCGGTTAAGACTCAGTGCTCCCAACGCGGGCCCAGGTTCAGTCCCT
GGTCAGGGAG
>Bos_taurus_chrX.trna4835-GlyCCC (59392720-59392648) Gly (CCC) 73 bp Sc: 36.85
TCCCTGATGGTCCAGTGTCTAAGACTCTGTTTTCCCAATGCAGGGGGCACGGGTTCAACC
CCTGGTTGGGGAA
>Bos_taurus_chr19.trna4121-GlyCCC (51161989-51161917) Gly (CCC) 73 bp Sc: 36.87
TCCCTGGTGGTCCAGTGAGTAAGATTCTGAGCTCCCAATGCAGGGGGCCCAGGTTTGATC
CCTGATCAGGGAA
>Bos_taurus_chrUn.004.1096.trna13-GlyCCC (5691-5619) Gly (CCC) 73 bp Sc: 36.89
TTCCTGGTGGTCTGGTGGCTAAGACTCTGCACTCCCAATGCAGGGGCCCTGGGTTGGATG
CCTGGTCAGGGAA
>Bos_taurus_chrUn.004.1030.trna5-GlyCCC (49046-48974) Gly (CCC) 73 bp Sc: 36.89
TCCCTGGTGGTCCAGTGGCTAAGATTCTGCCCTCCCAATGCAGGAGGCCTGGGTTTGATC
TCTAGTCAGGGAA
>Bos_taurus_chrUn.004.2470.trna3-GlyCCC (12273-12345) Gly (CCC) 73 bp Sc: 36.89
TTCTTGGTGGTCCAGTGGCTTAGACTCTGCACTCCCAATGCAGAGGGCCTAGGTTCCATC
CTTGGTCACGGAA
>Bos_taurus_chr21.trna4290-GlyCCC (31049276-31049204) Gly (CCC) 73 bp Sc: 36.90
TCCCTGGTGGTCCAGTGGCTGAGACTCTGCAGTCCCAATGCAGGGGGCCTGAGTTCGATC
CCTGGTCAGGGAA
>Bos_taurus_chr22.trna4208-GlyCCC (8252936-8252864) Gly (CCC) 73 bp Sc: 36.91
TCCCTGGTGGTCCTGTGGCTAGGACTCTGTGTTCCCAACGCAAGGGGCCTGGGTTTGATC
CCTGGTCAGGGAA
>Bos_taurus_chr19.trna5536-GlyCCC (25025075-25025002) Gly (CCC) 74 bp Sc: 36.92
TCCCCGGTAGTCCAGTGAGTAGGTCTCCACACTCCCAATGGAGGGGGCCTCAGGTTTGAT
CCCTGGCTGGGGAA

>Bos_taurus_chr11.trna41-GlyCCC (896975-897046) Gly (CCC) 72 bp Sc: 36.93
TCCCTGGTGGTCCAGTGGTTGGGACTCTGTGCTCCCAATGCAGGGGGCCTGGGTCCATTC
CTGGTCAGGGAC
>Bos_taurus_chr14.trna1426-GlyCCC (32678831-32678902) Gly (CCC) 72 bp Sc: 36.93
TCCCTGGTGGTCCAGTGGCTGAGACTCTGTGCTCCCAATGCAGGGACCTGGGTTTGATCC
CTGGTCAGGGAA
>Bos_taurus_chr2.trna3537-GlyCCC (108668029-108668101) Gly (CCC) 73 bp Sc: 36.96
TCCCTGGTGGTCCAGTGGCTAAGACCCTATGCTCCCAATGCAGGGGGCCCAGGTTTGATC
CCTGGTCAGGAAC
>Bos_taurus_chr17.trna4437-GlyCCC (57364912-57364829) Gly (CCC) 84 bp Sc: 36.98
TCCCTGGTGGTCCAGTGGCTGGGACGCCACGCTCCCAAAGTGGAATGGGGTGGAGAGCCC
CAGGCTCGATCCCTGGTCGGGGAA
>Bos_taurus_chr14.trna2679-GlyCCC (64323388-64323459) Gly (CCC) 72 bp Sc: 37.00
TCCCTGGTGGTCTGGTGGTAAGACTCAGTGCTCCCACTGCAGGGGGCCTAGGTTCCATCC
CTGGTCAGGGAA
>Bos_taurus_chr4.trna6212-GlyCCC (82053861-82053789) Gly (CCC) 73 bp Sc: 37.03
TCCCTGGTGGTCCAATGACTAAGACTCTGCACTCCCAGTGCAGAGGGTCCAGGTTTGATC
CCTAGTCAGGGAA
>Bos_taurus_chr21.trna4139-GlyCCC (34243037-34242965) Gly (CCC) 73 bp Sc: 37.03
TCTCTGGTGGTCCAGTGGCTAAGACTCGGCACTCCCAGTGCAGGGGGCCTGGGTTCAATC
CGTAGTCAGAGAA
>Bos_taurus_chr7.trna8273-GlyCCC (9549043-9548971) Gly (CCC) 73 bp Sc: 37.05
TCCCTGATGGTCCAGTGGCTAAGATTCTGTGTTCCCAATGCAGGGGCCCTGGGTTTGTTC
CCTGGTCAGGGAA
>Bos_taurus_chr16.trna4816-GlyCCC (39145437-39145365) Gly (CCC) 73 bp Sc: 37.08
CCCCTAGTGGTCTACTGTTTAAGACTCTGTGCTCCCAATGCAGGAGGCGCAGGTTCGATC
CCTGGTTGGGGGA
>Bos_taurus_chr23.trna3958-GlyCCC (18591476-18591404) Gly (CCC) 73 bp Sc: 37.08
TCCCTGGCAGTCCAGTGGCTAAGACTCTGCGCTCCCAACTCAGGGGGCCTGGGTTTGAAC
CCTGGTCGGGGAA
>Bos_taurus_chr3.trna2457-GlyCCC (70925298-70925370) Gly (CCC) 73 bp Sc: 37.09
TCCCTGGTAGTCCAATAGGTAAGACTCTGCATTCCCAATGCAGAGAGCTTGGGATCAATG
CCTGGTTAGGGAA
>Bos_taurus_chr25.trna2912-GlyCCC (35521296-35521223) Gly (CCC) 74 bp Sc: 37.10
TACCTGGTGGTCCAGTGGCGAAGACTCTGTGCTCCCAAGACAGGGGTCCCCGGGTTCAAT
CCCTGGTCAGGGAG
>Bos_taurus_chr17.trna4522-GlyCCC (56192424-56192352) Gly (CCC) 73 bp Sc: 37.10
TCCCTGGTGGTCCAGTAGCTAAGACTCTGTGCTCCCAATGCAGGGGCCCTGGGTTTGATC
CCTGGTCAGGGAA
>Bos_taurus_chr15.trna1860-GlyCCC (53694854-53694926) Gly (CCC) 73 bp Sc: 37.13
TCCCTGGTGGTCCAGTGACTGAGACTCCGCATTCCCAATGCTGGGGACCTAGGTTCAATC
CCTGGTCAGGGAA
>Bos_taurus_chr10.trna3813-GlyCCC (97744140-97744211) Gly (CCC) 72 bp Sc: 37.14
TCCCTGGTGGTTCAGTGGCTAAGACTCCATGCTCCCATTGTGGGGGCTTGGGTTTAATCC
CAAGTCAGGGAA
>Bos_taurus_chr17.trna6344-GlyCCC (11726350-11726277) Gly (CCC) 74 bp Sc: 37.17
TCTCTGGTGGCCCAGTGGCTAAGACTCCGTGCTCCCCATGCAGGGAGGGCCAGGTTCAAT
CCCTGGTCAGGGAA
>Bos_taurus_chr13.trna3070-GlyCCC (72607861-72607933) Gly (CCC) 73 bp Sc: 37.18
TCCCTGATGGTCCAGTGGGTCAGACTCTGCTTTCCCAATGCAGGGGGCCTGGATTCAATC
CCTGGTCAGGGAC
>Bos_taurus_chr25.trna907-GlyCCC (15278634-15278706) Gly (CCC) 73 bp Sc: 37.22
TCCCTGGTGGTCCAGTGGCTGAGACTCTGAGCTCCCAATGCAGGGGGGCCAGGTTCAATC
CCAGGTCAGGGAA
>Bos_taurus_chr19.trna3934-GlyCCC (55560283-55560211) Gly (CCC) 73 bp Sc: 37.24
TTCCTGGTGGTCCAGTGGCCAAGAGTCTGTGCTCCCAATGAAGGGGGCCCAGGTTTGATC
CCTGGTCAGGAAG
>Bos_taurus_chr11.trna4524-GlyCCC (107561178-107561250) Gly (CCC) 73 bp Sc: 37.24
TCCCTGGTGGTCCAGTGGGTGAGACTCTTCACTCCCAGTGCAGGGGGCCCGGGTTCAACC
CCTGGTCAGGGAA
>Bos_taurus_chr10.trna6934-GlyCCC (34223146-34223074) Gly (CCC) 73 bp Sc: 37.29
TCCCTGGTGGTCCAGTGGCTAAGACTCTGCGCTCCCCATGCAGGGGGCCTGGGTACAATC
CCTGGTCAGGGAA
>Bos_taurus_chr5.trna9801-GlyCCC (10461926-10461854) Gly (CCC) 73 bp Sc: 37.39
TCCCTGGTGGTCCAGTGGCTCAGACTCTGTGCTCCCAATTCAGGGGGCCCGGGTTTGATC
CCTGGTTAGGGAA
>Bos_taurus_chr9.trna1178-GlyCCC (36950579-36950651) Gly (CCC) 73 bp Sc: 37.40

TCTCTGGTGGTCTAGTGGCTGAGACTCTGCACTCCCAATGCAGGGCACCCCGGTTCAATC
CTTGGTCAGGGAA
>Bos_taurus_chr13.trna4950-GlyCCC (62951606-62951534) Gly (CCC) 73 bp Sc: 37.45
TTCCTGGTGGTCCAGTGGCTAAGACTCTGCACTCCCAATGCAGGGGGCCTGGGTCCGCTC
CCTGGTCAGGGAA
>Bos_taurus_chr2.trna7147-GlyCCC (101660663-101660591) Gly (CCC) 73 bp Sc: 37.50
TCCCTGGTGGTCCAGTGGCTAAGACTCTGAGCTCCCAGTGCAGGGGCCCCAGGTTTGATC
CTTGGTTGGGGAA
>Bos_taurus_chr25.trna1190-GlyCCC (21314396-21314468) Gly (CCC) 73 bp Sc: 37.50
TCCCTGGTGGTCCATTGGCTAAGACTCTGTGCTCCCAATGCAGGGGGCCTGGGTTTGATC
CCTGGTCAGGGAA
>Bos_taurus_chr2.trna7421-GlyCCC (95391307-95391236) Gly (CCC) 72 bp Sc: 37.51
TCCTTGGTGGTCCAATGGCTAGGACTCTGTGCTCCCAGTGCAGGGGCACAGGTTCGTTTC
TTGGTCAGGGAA
>Bos_taurus_chr29.trna2437-GlyCCC (42679436-42679364) Gly (CCC) 73 bp Sc: 37.60
TCCCTGGTGATCCAGTGGCTAAGATTCTGAGCTCCCAATGCAGGGGGCCTGAGTTCAATC
CTTGGTCAGGGAA
>Bos_taurus_chr27.trna2309-GlyCCC (38672371-38672299) Gly (CCC) 73 bp Sc: 37.62
TCCCTGGTGGTCCAGGGGCTGAGACTTTGAGCTCCCAATGCAGGGGGCTCTGGTTCGATC
CCAGGTCAGGGAG
>Bos_taurus_chr11.trna7077-GlyCCC (50759049-50758977) Gly (CCC) 73 bp Sc: 37.66
TCCATGGTGGTCCAGGGGCTAAGACTCTGTGCTCCCAATGCAGGAGGCCCAGGGTCAATC
CCTGGTCAGGGAA
>Bos_taurus_chr28.trna304-GlyCCC (7149380-7149451) Gly (CCC) 72 bp Sc: 37.67
TCCCTGGTTGTCCAGTGGCTAAGACTCTGTGCTCCCAATGCATACGGCCAGGTTCGATCC
CTGGTCAGGAAA
>Bos_taurus_chr21.trna4879-GlyCCC (20915733-20915661) Gly (CCC) 73 bp Sc: 37.70
TCCCTGGAGGTCCAGTAGCTAAGACTCTATGCTCCCAATGCAGAGGACCCAGGTTCAATC
CCTGGTCAGGGAA
>Bos_taurus_chr25.trna862-GlyCCC (14569352-14569426) Gly (CCC) 75 bp Sc: 37.70
TCCCTGGTAGTCCAGTGTCTAAGACTCTGCACTCCCAGTGCAGGGGGGCCCTGGGTTCAA
TTCCTGGTCAGGGAA
>Bos_taurus_chr18.trna4238-GlyCCC (45422792-45422720) Gly (CCC) 73 bp Sc: 37.79
ACCCTGGTGGTCCAGTGGCTAAGACTCTTTGCTCCCGATACAGGGGGCCCAGGTTCACTT
CCTGGTCAGGGAG
>Bos_taurus_chr12.trna3546-GlyCCC (80717644-80717572) Gly (CCC) 73 bp Sc: 37.81
TCCCTGGTGGTCTGGTGGCTAAGATTCTGCACTCCCAGTGCAAGAGGTCTGGGTTCGATC
TCCAGTCAGGGAT
>Bos_taurus_chr28.trna1151-GlyCCC (32651715-32651788) Gly (CCC) 74 bp Sc: 37.84
TCCCTGGTGGTCAGGTGGCTAAGACTCTGAGTTCCCAATGCAGGGGGCCCCAGGTTTGAT
CCCTGGTCAGGGAA
>Bos_taurus_chr25.trna3100-GlyCCC (33195984-33195912) Gly (CCC) 73 bp Sc: 37.87
TCCCTGGTGGTCTTGTGACTAAGACTCAGCACTCCCAATGCAGGGGGTCCGGGTTCAATC
CCTGGTCAGGGAA
>Bos_taurus_chr23.trna3102-GlyCCC (38738678-38738606) Gly (CCC) 73 bp Sc: 37.88
TCCCTGGTGGTTAAGTGACTAAGACTTTACATTCCCAATGCAGTGGGCCCAGGTTTAATC
CCTGGTCGGGGAA
>Bos_taurus_chr17.trna536-GlyCCC (14616526-14616598) Gly (CCC) 73 bp Sc: 37.89
TCCCTGATGGTCCAGTGGCTGGGACCCTGTGCTCCCAGTGCAGGGGGCCCAGGTTTGATC
CCTGGTCAGGGGA
>Bos_taurus_chr25.trna987-GlyCCC (16747021-16747092) Gly (CCC) 72 bp Sc: 37.91
TCCCTGGTGGTCCAATGACTAAGACTCTGAGCTCCCAGTGCAGGGGCCCAGGTTTGATTC
CTGGTCAGGGAA
>Bos_taurus_chr9.trna5689-GlyCCC (65942917-65942845) Gly (CCC) 73 bp Sc: 37.97
TCCTTGGTGGTCCAGGGGTCAAGACTCTGAACTCCCAATACAGGGGGCCTGAGTTCGATC
TCTGGTCAGGGAA
>Bos_taurus_chr9.trna5692-GlyCCC (65893767-65893695) Gly (CCC) 73 bp Sc: 37.97
TCCTTGGTGGTCCAGGGGTCAAGACTCTGAACTCCCAATACAGGGGGCCTGAGTTCGATC
TCTGGTCAGGGAA
>Bos_taurus_chrUn.004.3863.trna1-GlyCCC (3779-3707) Gly (CCC) 73 bp Sc: 37.97
TCCTTGGTGGTCCAGGGGTCAAGACTCTGAACTCCCAATACAGGGGGCCTGAGTTCGATC
TCTGGTCAGGGAA
>Bos_taurus_chr27.trna333-GlyCCC (13042916-13042988) Gly (CCC) 73 bp Sc: 38.08
TCCCTGGTGGTCCAGTGGTCACGACTCTGTGCTCCCAGTGTAGGGAGCCCAGGTTTGATC
CCTGGTCAGGGAA
>Bos_taurus_chr19.trna5695-GlyCCC (22418383-22418311) Gly (CCC) 73 bp Sc: 38.09
TCCCTGATGGTCCAGTGGCTAAGACACTGTGCTCCCGAGGCAGGTGGCCCGAGTTCGATC

TCTGGTCAGGGAA
>Bos_taurus_chr13.trna4157-GlyCCC (76326016-76325944) Gly (CCC) 73 bp Sc: 38.11
TCCCTGGTGGTCCAGTGGCTAAGACTCTGCATTCCCAATGAAGGGGGCCCGGGTTTGACC
CGTGGTCAGGGAA
>Bos_taurus_chr9.trna3835-GlyCCC (105129960-105130034) Gly (CCC) 75 bp Sc: 38.12
TCCATGGTGGTCCAGTGGCTAAGACTCTGCACTCCCAATGCAGGGAGCCCCCAGGGTTGA
TCCCTGGTCAGGGAA
>Bos_taurus_chr10.trna1815-GlyCCC (47203318-47203390) Gly (CCC) 73 bp Sc: 38.13
TCCCTGGTGGTCCAGTGGCTAAGATTCTGTGGTCCCAATGCAGAGGGCTCAGGTTTGATT
CCTGGTGAGGGAA
>Bos_taurus_chr21.trna1525-GlyCCC (33789299-33789371) Gly (CCC) 73 bp Sc: 38.18
TCCCTGGTGGTCCAGTGGTTAAGACTCTGTTCTCCCAATGCAGGGGGCCCGGGCTTGATC
CCTGGTTAGGTAA
>Bos_taurus_chr4.trna5508-GlyCCC (98684374-98684304) Gly (CCC) 71 bp Sc: 38.19
TCCCTGGTGGTCCAGTGGCTAAGACTCTGAGCTCCCAGTGCAGGGTCCAGGTTCAATCTC
TGGTCGGGGAA
>Bos_taurus_chr18.trna3407-GlyCCC (56252354-56252282) Gly (CCC) 73 bp Sc: 38.20
TCCCTGGTTGTCCAGCGGCAAAGACTCTACGCTCCCAATGCAGGGGCCCCAGGTTCCATC
CCTGGTCAGGGAA
>Bos_taurus_chr25.trna2098-GlyCCC (35458262-35458335) Gly (CCC) 74 bp Sc: 38.26
TCCCTGGTGGTCTAGCGGCCAAGATTCTGAGCTCCCAGTGCAGGGGGGCCCAAGGTTCGAT
CCCTGGCCAGGGAA
>Bos_taurus_chr27.trna2413-GlyCCC (36317371-36317299) Gly (CCC) 73 bp Sc: 38.27
TCCCTGGTCGTCTAGTGGCTAAGACTCTGCACTCCCATTGCATGGGGCTCAGGTTTGATC
CTTGGTCAGGGAA
>Bos_taurus_chr12.trna3661-GlyCCC (78437324-78437252) Gly (CCC) 73 bp Sc: 38.27
TCCCTGGTAGTCCAGTTACTAAGATTTTGCATTCCCAATGCAGGGGGCCTGGGTTCAATT
CCTGGTCAGGGAA
>Bos_taurus_chr17.trna1924-GlyCCC (51809202-51809274) Gly (CCC) 73 bp Sc: 38.34
TCCCTGGTGGTCCAGTGGCTAAGACTCTGCATTCCCAATGCAGGGGACTTGGGTTCCATC
CCTAGTCAGGAAC
>Bos_taurus_chrX.trna830-GlyCCC (19611789-19611861) Gly (CCC) 73 bp Sc: 38.35
TCCCTAGTGGTCCAGCAGCTAAGACTCTGCACTCCCAAAGCAGGGGGCTCAGGTTTGATC
CCTGGCCAGGGAA
>Bos_taurus_chr10.trna3535-GlyCCC (91250286-91250358) Gly (CCC) 73 bp Sc: 38.35
TCCCTGGTAGTCCAGAGGCTAAGACTCTGTGCTCCCAATGCAGGGGGCCCAGGTTCCATC
CCTGGTTGGAGAA
>Bos_taurus_chr1.trna7266-GlyCCC (120764378-120764306) Gly (CCC) 73 bp Sc: 38.36
TCCCTGGTGGTCCAGTGGCTAAGACTCTGTGCTCCCAATGCAGGTGACCCAGGTTAGCTC
CCTGGTCAGGGAA
>Bos_taurus_chrUn.004.6187.trna1-GlyCCC (3957-3885) Gly (CCC) 73 bp Sc: 38.36
TCCCTGGTGGTCCAGTGGCTAAGACTCTGTGCTCCCAATGCAGGTGACCCAGGTTAGCTC
CCTGGTCAGGGAA
>Bos_taurus_chr13.trna2976-GlyCCC (70654643-70654715) Gly (CCC) 73 bp Sc: 38.36
TCCCTGGTGGTCTAGTGGCTAAGACTCTGCACTCCCAGTGCAGGGGTCTTGGGTCTGATC
CCTGGTCAGGGAA
>Bos_taurus_chr7.trna7527-GlyCCC (18535047-18534975) Gly (CCC) 73 bp Sc: 38.43
TCCCTAGTGGTCCAGTGGCTAAGACTCTGAGCTCCCAATGCAGGCCAGCCAGGTTCGATC
CCTGGTCAGGGAA
>Bos_taurus_chr1.trna4771-GlyCCC (137110519-137110590) Gly (CCC) 72 bp Sc: 38.44
TCTCTGGTGGTCCCGTGGCTAAGACTCTGAGCTCCCAATGCAGGGATCCAGGTTTGATTC
CTGGTCAGGGAA
>Bos_taurus_chr9.trna618-GlyCCC (21517574-21517646) Gly (CCC) 73 bp Sc: 38.46
TCACTGGTGGTCCAGTGGCTAAGACTCTGAGCTCCCATCTCAAGGAGCCTAGGTTCAATC
CCTGGCTGGGGAC
>Bos_taurus_chr25.trna4714-GlyCCC (6088600-6088528) Gly (CCC) 73 bp Sc: 38.51
TTCCTGGTGGTCCAGTGGTGAAGACTCTGAGCTCCCAATGCAGAGGGGCCTGGTTCCATC
CCTGGTCAGGGAA
>Bos_taurus_chr21.trna4870-GlyCCC (21088953-21088881) Gly (CCC) 73 bp Sc: 38.53
TCCCTGGCAGTCCAGTGGGTAAGACTCTTTACTCCCAATACAGAGGGGCCAGGTTTGATC
CCTGGTTAGGGAA
>Bos_taurus_chr22.trna550-GlyCCC (12507787-12507859) Gly (CCC) 73 bp Sc: 38.56
TCCCTGTTGGTCCAGTGGCTAAGACTCTGTGCTCCCAATGCAGGGGGACTGGGTTTGATC
CCTAGTCAGGGAA
>Bos_taurus_chr3.trna8965-GlyCCC (13648188-13648116) Gly (CCC) 73 bp Sc: 38.57
TCCCTGGTGGTCCAGTGGCTAAGACTCTCTGCTCCCAATGCAGGGAGCCCAGGTGTGATC
CCTGGTCAGGGAA

>Bos_taurus_chr23.trna1505-GlyCCC (33427942-33428015) Gly (CCC) 74 bp Sc: 38.59
TTCCTGGTGGTCCAGTGGCTAAGATTCTGTGCTCCCAATGCAGGGGGCCCCCAGTTCAAT
CCCGGGTCAGGGAA
>Bos_taurus_chr23.trna4672-GlyCCC (3783265-3783193) Gly (CCC) 73 bp Sc: 38.64
TCCCTGGTGGTCCAGTGGCTAAGACTCTGTGCTCCCAATACAGGGGGCTGGTGTTCAATC
CCTCATCAGGGAA
>Bos_taurus_chrUn.004.2789.trna1-GlyCCC (11335-11407) Gly (CCC) 73 bp Sc: 38.65
TCCCTGGTAGTCCACTGGCTAAGATTCTGCATTCCCAATGCATGGGGCCCACGTTTGATT
CCTGGTCAGGGAA
>Bos_taurus_chr15.trna762-GlyCCC (26683442-26683512) Gly (CCC) 71 bp Sc: 38.66
TCCCTGGTGGTCCAGTGGCTAAGACTCCATGCCCCCTGCAGGGGCCCCAGGTTCAATCCC
TGGTCAGGGAA
>Bos_taurus_chr29.trna1641-GlyCCC (45118969-45119041) Gly (CCC) 73 bp Sc: 38.66
TCCCTGGTGGTCCAGTGGCTAAGACTCCGTATTCCCAATGCAGGTGGCCTGGGTTCAGTC
CCTGGTCAGGGAA
>Bos_taurus_chr11.trna4951-GlyCCC (102296396-102296324) Gly (CCC) 73 bp Sc: 38.71
TCCCTGATGGTCCAGGGGCTAAGACTTCATATTCCCAATGTAGGAGGCCTGGGTTTGATC
CCTGGTCAGGGAA
>Bos_taurus_chrX.trna2683-GlyCCC (72278356-72278428) Gly (CCC) 73 bp Sc: 38.72
TTCCTGGTGGTCTAATGGCTAAGACTCTGTGCTCCCAATGCAGGGGGCCCAGGTTTGAAC
CCTGGTCAGAGAA
>Bos_taurus_chr15.trna721-GlyCCC (26010839-26010911) Gly (CCC) 73 bp Sc: 38.73
TCCCTGGTGGTTCTGTGGCTAAGACTCTGTGCTCCCAATGCAGGCAGTCCAGGTTCGATC
CCTGGTCGGGGAA
>Bos_taurus_chr14.trna1159-GlyCCC (26887844-26887916) Gly (CCC) 73 bp Sc: 38.73
TCCCTGGTGGTCCAGTGGCAAGACTTTGAGCTCCCAGTACAGGAGGCCCCAGGTTCAACC
CCTGGTCAGGGAA
>Bos_taurus_chr3.trna5924-GlyCCC (98878858-98878784) Gly (CCC) 75 bp Sc: 38.74
TCCCTGACAGTCCAGTGGTTAAGACTCTGCACTCCCAATGCAGGGGCGCATTGGGTTCAG
TCCCTGATCAGGGAA
>Bos_taurus_chrUn.004.1405.trna5-GlyCCC (33527-33601) Gly (CCC) 75 bp Sc: 38.74
TCCCTGACAGTCCAGTGGTTAAGACTCTGCACTCCCAATGCAGGGGCGCATTGGGTTCAG
TCCCTGATCAGGGAA
>Bos_taurus_chr8.trna3425-GlyCCC (99771786-99771857) Gly (CCC) 72 bp Sc: 38.74
TCCCTGGTAGTCTAGTGGTTAGGACTCCGCACTCCCAGTGCAGGGGGCTGGGCTCAATCC
CTGGTCAGGGAA
>Bos_taurus_chr7.trna6106-GlyCCC (60404733-60404661) Gly (CCC) 73 bp Sc: 38.77
TCCCTGGTGGTCCAGCGGCTAAGACTCTGCACTCCCAGTGCAGGGGACCCGGGTTTGATC
TCTGGTCAGGGAA
>Bos_taurus_chr7.trna7801-GlyCCC (15149938-15149866) Gly (CCC) 73 bp Sc: 38.77
TCCCTGGTGGTCCAGTGGCAAAGACTCGGTGCTCCCAATGCTGGAAGCCCAGGTTCGATC
CCTGATCAGGGAA
>Bos_taurus_chr10.trna2984-GlyCCC (79061209-79061281) Gly (CCC) 73 bp Sc: 38.78
TCCCTGGTGGTCCAGGGGCTAAGACTCCGTGCTCCCCATGCAGGTAGTCCGGGTTCAATC
CCTGGTCAGGGAA
>Bos_taurus_chr19.trna6657-GlyCCC (6129927-6129854) Gly (CCC) 74 bp Sc: 38.78
TCTCTGGTGGTCCAGTGGCCAGGACTCTGCATTCCCAATGAAGGGAGGCCCAGGTTTAAT
CCCTGGTCAGGGGA
>Bos_taurus_chr2.trna1298-GlyCCC (39861470-39861543) Gly (CCC) 74 bp Sc: 38.81
TCCCTGGTGGTCCAGTGGCTACGACTCTGTGCTCCCAAAGCAGGGGGACCTAAGTTCGAT
CCCTGGTCAGGGAG
>Bos_taurus_chr1.trna5173-GlyCCC (148999422-148999494) Gly (CCC) 73 bp Sc: 38.87
TACCTGGTTGTTCAGTGGCTAAGACCCTGCGCTCCCAATGCAGGGGGCCCAGGTTCGATC
TCTGGTCAGGGAA
>Bos_taurus_chr11.trna4269-GlyCCC (103204953-103205025) Gly (CCC) 73 bp Sc: 38.87
TCCCTGGTGGGCCAGTGGCTAAGACTCTGCACTCCCAGTGCAGGGGGCCTGGGTTCTATC
CCTGGTCAGGGAA
>Bos_taurus_chr8.trna2752-GlyCCC (80040964-80041036) Gly (CCC) 73 bp Sc: 38.90
TCCCTGGTGGTCCAGTGGCTGAGACTCTGTGCTCCCAATGCAGGGATCCCAGGTTCTACC
CTTGGTCAGGGAT
>Bos_taurus_chr1.trna7355-GlyCCC (118687193-118687121) Gly (CCC) 73 bp Sc: 38.90
TCCCTGGGGGTCCAGTGGGTAGGATTCCGCGCTCCCAACGCAGGGGGCCTGGGTTTGATC
CCTGGTCAGGGAA
>Bos_taurus_chr5.trna3890-GlyCCC (101643772-101643844) Gly (CCC) 73 bp Sc: 38.94
TCCCTGGTGGTCCAGTGGCTAAGACGCTGCACTCCCAATGCATGGGGTTCAGGTTCCGTC
CCTGGTTGGGGAA
>Bos_taurus_chr5.trna3892-GlyCCC (101679530-101679602) Gly (CCC) 73 bp Sc: 38.94

TCCCTGGTGGTCCAGTGGCTAAGACGCTGCACTCCCAATGCATGGGGTTCAGGTTCCGTC
CCTGGTTGGGGAA
>Bos_taurus_chr18.trna2630-GlyCCC (57296928-57296999) Gly (CCC) 72 bp Sc: 38.99
TCCCTGGTAGACCAGTGGTTAAGACTTTTCCTTCCCATGCAGGGGACTCAGGTTTGATCC
CTGGTCAGGGAG
>Bos_taurus_chr10.trna715-GlyCCC (16979762-16979834) Gly (CCC) 73 bp Sc: 38.99
TCCCTGATGGTTTAGTGGTTAAGACTCTGTTCTCCCAATGCAGGGAGCTGGGGTTTGATC
CCTGGTCAGGGAA
>Bos_taurus_chr5.trna5259-GlyCCC (121045181-121045109) Gly (CCC) 73 bp Sc: 39.02
TCCTTGGTGGTCCAATGACTAAGATTCTGAGTTCCCAATGCAGGGGGTCCAGGTTCGAGC
CCTGGTCAGGGAA
>Bos_taurus_chr10.trna2102-GlyCCC (53513828-53513899) Gly (CCC) 72 bp Sc: 39.02
TCCCTGGTGGTCTAATGTATAAGACTCTGCACTCCCAATGCAGGGCCCTGGGTTCCATCC
CTGGGCAGGGAA
>Bos_taurus_chr13.trna4210-GlyCCC (75442650-75442578) Gly (CCC) 73 bp Sc: 39.06
TCCCTGGAGGTCCAGTGGCTAGGACTCTGTCCTCCCAGTGCAGGGAGCCCAGGTTTGATC
CCTGGTCAGGGAA
>Bos_taurus_chr15.trna4767-GlyCCC (39962513-39962441) Gly (CCC) 73 bp Sc: 39.07
TCCCTGGTGGTCCAGTGACTAGGACTCTGCCTTCCCAATGCAGGGGGCCTGGGTTCAACC
CCTTGTCAGGGAA
>Bos_taurus_chr7.trna2799-GlyCCC (65783582-65783654) Gly (CCC) 73 bp Sc: 39.08
TCCCTGGTGGTCCAGTGACTAAGACTCTGCACTCCCATTTTAGGGGACCCAGGTTCAATC
CCTGGTCAGGAAA
>Bos_taurus_chr2.trna6772-GlyCCC (111485413-111485341) Gly (CCC) 73 bp Sc: 39.09
TCCCTGGTGGTCCAGTGGCTAAGACTCTGTGTTCCCAATGCAGGGGCCTTGGGTTTGATC
CCTGATCAGGGAA
>Bos_taurus_chr19.trna3373-GlyCCC (65036586-65036657) Gly (CCC) 72 bp Sc: 39.11
TCCCTGGTGGTCCATGGCTAAGACTCTGAGCTCCCAGTGCAGGGGGCCCAGGTTCGATCC
CTGGTCAGGGAG
>Bos_taurus_chr2.trna3309-GlyCCC (102372877-102372949) Gly (CCC) 73 bp Sc: 39.13
TCCCTGGTGGTCCAGTAGCTAAGATTCAGCGCTCCCAATGCAGGGGCCCCAGGTTCATTC
CCTGGTCAGGGAA
>Bos_taurus_chr28.trna2513-GlyCCC (16271730-16271658) Gly (CCC) 73 bp Sc: 39.17
TCCCCGATGGTCCAGTGACTAAGATTCTGTCCTCCCAGTGCAGGGGGCCCAGGTTCAATC
CCTGGTCAGGGAA
>Bos_taurus_chr7.trna6879-GlyCCC (36818345-36818274) Gly (CCC) 72 bp Sc: 39.18
GCCCTGGTGGTCCAGTGGCTAAGATTCTGTGCTCCCAGTGCAGGAGCCCAGGTTTGATCC
CTGGTCAGGGAA
>Bos_taurus_chr29.trna2251-GlyCCC (46101771-46101700) Gly (CCC) 72 bp Sc: 39.19
TCCCTGGTGGTCCAGTGGCTAGACTCTGTGCTCCCAAAGCAGGGGGCCCGGGTTCCATCC
CTGGTCAGGGAA
>Bos_taurus_chr10.trna1627-GlyCCC (42860787-42860859) Gly (CCC) 73 bp Sc: 39.20
TCCCTGGTGGTCCAGTGGCTAAGACTCTGTACTCCCAATGCAGGGGGACTGGGTTTGATC
CCTGGTCAAGGAA
>Bos_taurus_chr18.trna3269-GlyCCC (58263415-58263343) Gly (CCC) 73 bp Sc: 39.21
TCCCTGGTGGTCCAGTGGCTAAGACTCTGCACTCCCAGTGCAGGGAGCCTGGGTTTGATT
CCTGGTCAGGAAA
>Bos_taurus_chr10.trna708-GlyCCC (16627381-16627453) Gly (CCC) 73 bp Sc: 39.22
TCCCTGGTGGTCCAGTGGCTGAGACTCTGCGCTCCCAATGCTGGGGACCCAGGTTAGATC
CCTGGTCGGGGAA
>Bos_taurus_chr5.trna2875-GlyCCC (77214741-77214813) Gly (CCC) 73 bp Sc: 39.23
TCCCTGGTGGTCCAGCAGCTAAGACTCCACACTCCCAATGTAGGGGGCCCGGGTTTGATC
CCTGGTCAGGGAA
>Bos_taurus_chr19.trna2553-GlyCCC (50231119-50231190) Gly (CCC) 72 bp Sc: 39.24
TCCTTGGTAGTTAAGTGGCTAAGACTCTGTACTCCCAATACAGGGGCCTGAGTTTGATCC
CTGGTCAGGGAA
>Bos_taurus_chr19.trna679-GlyCCC (16493638-16493710) Gly (CCC) 73 bp Sc: 39.24
TCCGTGGTGGTCCAGTGGCTAAGACTCTGTGCTCCCAGTGCAGGGGGTTCAGGTTCAATC
CCTGGTCAGGGAA
>Bos_taurus_chr4.trna4011-GlyCCC (116369804-116369875) Gly (CCC) 72 bp Sc: 39.26
TCTCTGGTGGTCCAGGGTCAAAGACTCTGCACTCCCAATGCACGGAACCAGGTTCAATTC
CTGGTCGGGGAA
>Bos_taurus_chr15.trna1552-GlyCCC (44338680-44338752) Gly (CCC) 73 bp Sc: 39.26
TCCCTGGTGGTTCAGTGGCTGAAACTCTGGGCTCCCAATGCAGGGGACCCAGGTTCGATC
TCTGGGCAGGGAA
>Bos_taurus_chr11.trna1300-GlyCCC (30526576-30526648) Gly (CCC) 73 bp Sc: 39.30
TTCCTGATGGTCCAGAGTCTAAGACTCTGCACTCCCAATGCAGGAGGCTCAGGTTTGATC

CCTGGTTAGGGAA
>Bos_taurus_chr25.trna3087-GlyCCC (33362299-33362226) Gly (CCC) 74 bp Sc: 39.31
TCCTTGGTGGTCAAAGTGGCTAAGACTCTGTGCTCCCTAAGCAGAGGGCCTGGGTTCGAT
CCCTAGTCGGGGAA
>Bos_taurus_chr16.trna861-GlyCCC (25918206-25918278) Gly (CCC) 73 bp Sc: 39.33
TCCCTGGTGGTCCAGTGGCTAAGACTCCATGCTCCCGATGTGCGGGGCCCGGGTTTGATC
CCTGGTCAGGGAA
>Bos_taurus_chr19.trna4575-GlyCCC (44031938-44031866) Gly (CCC) 73 bp Sc: 39.37
TCCCTGGTGGTCCAGTGTTTAAGACTCTGCACTCCCAATTCGGGGAGCACAGGTTCCACC
CCTGGTCAGGGAA
>Bos_taurus_chr8.trna5349-GlyCCC (81865699-81865627) Gly (CCC) 73 bp Sc: 39.40
TCCCTGGTGGTCCGGTGGCTAAGACTCCACACTCCCAATGTAGGAGTCCCGGGTTCAGTC
CCTGGTCAGGGAC
>Bos_taurus_chr7.trna7708-GlyCCC (16621688-16621616) Gly (CCC) 73 bp Sc: 39.41
TCCCTGGTGGTCCAGTAGCTAAGACTCTGCACTCCCAGTGCAGGGGGTCTGGGTTCAATC
CCTGGTCAGGGAA
>Bos_taurus_chr8.trna2087-GlyCCC (66234745-66234817) Gly (CCC) 73 bp Sc: 39.42
TCCCTGGTGGTCCAGTGGCCAAGGCCCTGCATTCCCAATACAGGGGACCCAGGTTCAATC
GCTGGTTGGGGAA
>Bos_taurus_chr14.trna5504-GlyCCC (26186151-26186080) Gly (CCC) 72 bp Sc: 39.44
TCCCTGGTGGTCCAGTGGCTAAGACTCTGTGCTCCCAGTGCAGGGGCCTGGGTTTGATCC
CTGGTCAGGGAA
>Bos_taurus_chr7.trna980-GlyCCC (16601574-16601646) Gly (CCC) 73 bp Sc: 39.45
TCCCTGGTGGTCCAGTGGCTAAGCCTCAGCACTCCCTATGCAGGGGACCCAGGTTCCATC
CCTGGTCAGGGAA
>Bos_taurus_chr15.trna1305-GlyCCC (37956570-37956642) Gly (CCC) 73 bp Sc: 39.45
TCTCTGGTGGTCCAGTGGCTAAGACTCTGCCCACCCAATGCAGGCGGCCCAAGTTCAATC
CTTGGTCAGGGAA
>Bos_taurus_chr17.trna2288-GlyCCC (55923097-55923169) Gly (CCC) 73 bp Sc: 39.46
TCTCTGGTAGTCCAGTGGCTAAGACTTTGTTCTCCCAATACAGGGGCCCCGGGTTCCATC
CCTGGTCAGGGAA
>Bos_taurus_chr18.trna2406-GlyCCC (54096279-54096351) Gly (CCC) 73 bp Sc: 39.48
TCCCTGGGGGTCCAGTGGCTAAGACTGTGCACTCCCAGTGCAGGGGGCCTGGGTTCGATC
CCTAGTCAGGGAG
>Bos_taurus_chr3.trna5211-GlyCCC (116818022-116817950) Gly (CCC) 73 bp Sc: 39.56
TCCCTGGTGGTCCAGTGGCTGAGACTCTGAGCTCCCAGTGCAGGGGGCCCAGGTTCAATC
CCTGGTCGGGGAG
>Bos_taurus_chr24.trna436-GlyCCC (12549181-12549253) Gly (CCC) 73 bp Sc: 39.60
TCCCTGGTAGTTCAGTGGCTGAGACTCTGTGCTCCCAATGCAGGGGACCCAGGTTCAATC
CCTGGTCAAGGAG
>Bos_taurus_chr18.trna2431-GlyCCC (54322718-54322790) Gly (CCC) 73 bp Sc: 39.64
TCCCTGGTGGTCCAGTGGCTAAGACTCCGTGTTCCCAATGCAGGCAGCCCAGGTTCGATC
GCTGGTCAGGGAA
>Bos_taurus_chrUn.004.5804.trna5-GlyCCC (2511-2439) Gly (CCC) 73 bp Sc: 39.64
TCCCTGGTGGTCCAGTGGCTAAGACTCCGTGTTCCCAATGCAGGCAGCCCAGGTTCGATC
GCTGGTCAGGGAA
>Bos_taurus_chr16.trna78-GlyCCC (2442578-2442650) Gly (CCC) 73 bp Sc: 39.66
TCCCTCGTGGCCCAGCGGCTAAGGCTCTGCACTCCCAGTGCAGGAGGCCCAGGTTCGGTC
CCTGGTCAGGGAC
>Bos_taurus_chr9.trna5247-GlyCCC (77424050-77423978) Gly (CCC) 73 bp Sc: 39.73
TCCCTGGTGGTGTAGGGGCTAAGACCCTGCACTCCCAATGCAGGCGGCCCGGGTTAGATT
CCTGGTCAGGGAA
>Bos_taurus_chr11.trna3929-GlyCCC (96088850-96088922) Gly (CCC) 73 bp Sc: 39.85
TCCCTGATGGTCCAATGGCTAAGATTCTGAGCTCCCAATGCAGGGGGCCTGGGTTCCATC
CCTGGTCAGGGAA
>Bos_taurus_chr8.trna2570-GlyCCC (76928327-76928399) Gly (CCC) 73 bp Sc: 39.87
TCCCTGGTGGTCCAGTGGCTGAGATTCTGAGCTCCCAATGCAGGGGGCCCAGGTTCCATC
CCTGGTTAGGGAA
>Bos_taurus_chr11.trna307-GlyCCC (5263737-5263809) Gly (CCC) 73 bp Sc: 39.90
TCCCTAGTGGTCCAGTGGCTAAGACTCCGCACTCCCAGTGCAGGGAGGCCAGGTTCAATC
CCTGGTCAGGGAA
>Bos_taurus_chr11.trna8477-GlyCCC (15393232-15393158) Gly (CCC) 75 bp Sc: 39.95
CCCCTGGTGGTCCACAGTGGTTAAGACTCTGAGCTCCCAATTCAGGGGGCCCAGGTTTGA
TCCCTGGTCAGGGAA
>Bos_taurus_chr2.trna5775-GlyCCC (131049705-131049633) Gly (CCC) 73 bp Sc: 40.02
TCTCTGGTCATCCAGTGGCTAAGATGCTGAGCTCCCAATACAGGGGGCCCAGGTTTGATC
CCTGGTCAGAGAA

>Bos_taurus_chr13.trna878-GlyCCC (23469716-23469788) Gly (CCC) 73 bp Sc: 40.08
TTGCTGATGGTCCAGTGGCTAAGATTCTGAGCTCCCAATACAGTAGACCCAGGTTCAATC
CCTGGTTGGGGAA
>Bos_taurus_chr3.trna4153-GlyCCC (114514328-114514400) Gly (CCC) 73 bp Sc: 40.09
TCCCTGGTGGTCCAAGGGGTGAGACTCTGCACTCCCACTGCGGGTGGGCCAGGTTCGATC
CCTGGTCAGGGAA
>Bos_taurus_chr2.trna4264-GlyCCC (124878460-124878532) Gly (CCC) 73 bp Sc: 40.10
TCCCTGGTAGTCCAGTGGCTAAGAAGCTGCATTCCCGATGCAGGGGGCCTGGGTCCAATC
CCTGGTCAGGGAA
>Bos_taurus_chr19.trna6491-GlyCCC (9485905-9485833) Gly (CCC) 73 bp Sc: 40.10
GCCCTGGTGGTCCAGTGGCTAAGACTCTGTGTTCCCAATGCAGGGCACCCAGGTTTGATC
CCTGGTCAGGGAA
>Bos_taurus_chr24.trna4009-GlyCCC (35979696-35979624) Gly (CCC) 73 bp Sc: 40.12
TCCCTGGTGGTCCAGTGGCTAAGACTCTGTAGTCCCAATACAGAGGGCCTGGGTACAATC
CCTGGTCAGGGAA
>Bos_taurus_chr19.trna2753-GlyCCC (55175642-55175714) Gly (CCC) 73 bp Sc: 40.12
TCCCTGGTGGTCCAGGGGCCGAGACTCTGCACTCCCAATGCAGTGGGCCTGGGTTTGATC
CCTGGTCAGGGAA
>Bos_taurus_chr9.trna6446-GlyCCC (41991558-41991486) Gly (CCC) 73 bp Sc: 40.12
TCCCTGGTGGTCCAGTGGTTTAGACTCTGCACTCCCAATGCAGCAGGCATGGGTTTGATC
CCTGGTCAGGGAA
>Bos_taurus_chr19.trna4506-GlyCCC (45043084-45043012) Gly (CCC) 73 bp Sc: 40.13
TCCCTGGTGGTCCAGTGGCTAAGACTCTGTGGTCCCAATGTAGGGGGACCAGGTTCAATC
CCTGGTCAGGGAA
>Bos_taurus_chr25.trna2126-GlyCCC (35881958-35882029) Gly (CCC) 72 bp Sc: 40.16
TTCCTGGTGGTCCAGTGGCTAAGGCTCTGAATTCCCAATACAGGGGGCCAGGTTAGATCC
CTGGTCGGGGAA
>Bos_taurus_chr5.trna5984-GlyCCC (108304550-108304478) Gly (CCC) 73 bp Sc: 40.16
TTCCTGGTGGTCCACTGCCTAAGACTCTGCGCTCCCAATGCAGAGGGCCCAGGTTTGATT
CCTGGCTGGGGAA
>Bos_taurus_chr5.trna2900-GlyCCC (78072613-78072685) Gly (CCC) 73 bp Sc: 40.19
TCCCTGGTGGTCCAGTGGCTAAGACTCTGTGGTCCCAATTCAGGGAGCACAGGTTCAATC
CCTGGTCAGGGAA
>Bos_taurus_chr5.trna7271-GlyCCC (78101166-78101094) Gly (CCC) 73 bp Sc: 40.19
TCCCTGGTGGTCCAGTGGCTAAGACTCTGTGGTCCCAATTCAGGGAGCACAGGTTCAATC
CCTGGTCAGGGAA
>Bos_taurus_chr9.trna7392-GlyCCC (14803269-14803197) Gly (CCC) 73 bp Sc: 40.20
TCCCTGGTGGTCCAGTGGCTAAGATTTTGAGCTCCCCATGCAGGGGGCCTGGGTTCGATT
ACCAGTCAGGGAA
>Bos_taurus_chr2.trna3758-GlyCCC (115078803-115078875) Gly (CCC) 73 bp Sc: 40.21
TCCCTGGTGGTCCAGTGGCTAAGACTCTGAATTCCCAATGCAGAGGATCCAGGTTTGTTC
CCTGGTCAAGGAA
>Bos_taurus_chr6.trna5628-GlyCCC (94345971-94345899) Gly (CCC) 73 bp Sc: 40.30
TCCCTGGTGGTCTAATGGCTAAGACTCTGAGCTCCCAATTCAGGGGGCCTTGGTTTAATC
CCTGGTCAGGGAA
>Bos_taurus_chr16.trna813-GlyCCC (24705874-24705946) Gly (CCC) 73 bp Sc: 40.34
TTCCTGGTGGTCCAGCAGCTAAGACTCTGCACCCCCAATGCAGGGGGTCCAGGTTTGATT
CCTGGTCAGGGAA
>Bos_taurus_chr6.trna1317-GlyCCC (46863999-46864071) Gly (CCC) 73 bp Sc: 40.34
TCCTTGATGGTCCAGTGGCTAAGACTCCAAGCTCCCAACTCGGGGGGTCTAGGTTCAATC
CCTGATCAGGGGA
>Bos_taurus_chr25.trna2203-GlyCCC (36902193-36902264) Gly (CCC) 72 bp Sc: 40.37
CCCCTGGTGGTCCAATGGTTAAGACTCTGTGCTCCCAGTGCAGGGGGCCAGGTTCGATCC
TTGGTCGGGGAA
>Bos_taurus_chr9.trna7011-GlyCCC (25192943-25192871) Gly (CCC) 73 bp Sc: 40.39
TCCCTGGTGGTCCAGGGACTAAGGCTTTGCACTCCCAATGCAGGGGCCCCTGGTTCGACG
CCTGGTCAGGGAA
>Bos_taurus_chr25.trna743-GlyCCC (12770093-12770165) Gly (CCC) 73 bp Sc: 40.39
TCCCTGGTGGTCCAGTGGCTAAGACTCCGCACTCCCAATGCAGGGCACCTGGATTCAATC
CCTGGTCAGGGAA
>Bos_taurus_chrUn.004.175.trna3-GlyCCC (126299-126371) Gly (CCC) 73 bp Sc: 40.40
TCCCTGGTGGTCCAGTGGCTAAGACTCTGTGCTCCCGATACAGGGGGGCTGGGTTCAATC
CCTGGTCAGGGAA
>Bos_taurus_chr21.trna3744-GlyCCC (46473294-46473222) Gly (CCC) 73 bp Sc: 40.41
TCCCTGGTGGTCCAGTAGCTAAGACTCCACACTCCCAATGTAGGGGGCCCAGGTTTAATC
CCTGGTCAGGAAA
>Bos_taurus_chr20.trna332-GlyCCC (8730908-8730980) Gly (CCC) 73 bp Sc: 40.41

TCCCTGGTGGTCCAGTGGCTAAGATTCTGCACTCCCAGTGCAGGGGACCTGGGTTCACTT
CCTGGTCAGGGAA
>Bos_taurus_chr18.trna305-GlyCCC (7632775-7632847) Gly (CCC) 73 bp Sc: 40.41
TCCCTGGTGGTTCAGTGGTTCAGACTCCGTGCTCCCACTGCGGATGGCTCGGGTTCGATC
CCTGATCAGGGAA
>Bos_taurus_chr13.trna2335-GlyCCC (60010463-60010535) Gly (CCC) 73 bp Sc: 40.44
TCCCTCGTGGTCCAGTGGCTAAGACTCAGCTTTCCCAAGGCAGGGGGCCCAGGTTTGACC
CCTGGTCAGGGAA
>Bos_taurus_chr16.trna1640-GlyCCC (42514563-42514634) Gly (CCC) 72 bp Sc: 40.45
TCCCTGGTGGTCCAGAGTCTAAGATTCAGCGCTCCCAATGCAGGGGCCCAGGTTCAATCC
TTGGTCAGGGAA
>Bos_taurus_chr5.trna6519-GlyCCC (97246373-97246300) Gly (CCC) 74 bp Sc: 40.52
TCCCTGGTGGTTCGGTGGCTAAAGACTCTGTGCTCCCAAGACAGGGGGCCCAGGTTCAAT
CCCTGGTCAGGGAA
>Bos_taurus_chr21.trna4269-GlyCCC (31455218-31455146) Gly (CCC) 73 bp Sc: 40.53
TCCCTTGTGGTCCAGCGGTTACGACTCTGCATTCCCAATGCAGAGGGACTGGGTTCGGTC
CCTGGTCAGGGAA
>Bos_taurus_chr8.trna2076-GlyCCC (65956539-65956610) Gly (CCC) 72 bp Sc: 40.54
TACCTGGTGGTCCAGCGGCTAAGACTCTGCACTCCCAATGAGGGGACCTAGGTTGGATCC
CTGGTCAGGGAA
>Bos_taurus_chr19.trna4446-GlyCCC (45886521-45886449) Gly (CCC) 73 bp Sc: 40.59
TCCTTGGTGGTCCAGTGGCCAAGACTCTGAGCTCCCAGTGCAGGGAGCCCAGGTTCGATC
CCTGGTCAGGGGA
>Bos_taurus_chr19.trna2892-GlyCCC (57030277-57030349) Gly (CCC) 73 bp Sc: 40.61
TCCCTGGTGGTCTAGTGGCTAGGACTCTGCTCTCCCAATGCAGGGAGCCTGGGTTCGTTC
CCTGATCAGGGAA
>Bos_taurus_chr17.trna4391-GlyCCC (58473034-58472962) Gly (CCC) 73 bp Sc: 40.61
TCCCTGGTGGTCCAGTGGCTGAGACGCTGCACTCCCAGTGCGGGGGCCCTGGGTTCAATT
CCTGGACAGGGAA
>Bos_taurus_chr10.trna7667-GlyCCC (13737265-13737193) Gly (CCC) 73 bp Sc: 40.66
TTCCTGGTAGTCCAGTAGCTAAGACTCTGTGCTCCCAATGCAGGGGGTCCAGGTTCCATC
CCTGGTCAGGAAA
>Bos_taurus_chr7.trna7490-GlyCCC (19119134-19119062) Gly (CCC) 73 bp Sc: 40.66
TCCCTGGTGGTCCAGTGGCTAAGACGCCATGCTCCCAACATAGGGGACTCAGGTTTGATC
CCGGGTCAGGGAA
>Bos_taurus_chr5.trna5094-GlyCCC (125735566-125735494) Gly (CCC) 73 bp Sc: 40.66
TCCCTGGTGGTCCAGTGGCTAAGAtgttgtGCTCCCAATGCAGGGGGCCCAGGTTTGACC
CCTGGTCAGTGAA
>Bos_taurus_chr19.trna4192-GlyCCC (50054166-50054094) Gly (CCC) 73 bp Sc: 40.66
TCCCTGGTAGTCCAGTGGCTAAGACTCTATGCTCCCAATGCAGGGGGCCCAGGTTTGATA
CCTGGTCAGGTAA
>Bos_taurus_chr3.trna3591-GlyCCC (102097378-102097449) Gly (CCC) 72 bp Sc: 40.70
TCCCTGGTGGTCCAGTGATTAAGATTCTGTGTTCCCAATTCAGGGGCCCAGGTTCAATCT
CTGGTCATGGAA
>Bos_taurus_chr10.trna1889-GlyCCC (48404481-48404553) Gly (CCC) 73 bp Sc: 40.71
TCCCTGGTGGTCCAGTGACTAAGATTCTGTGCTCCCAGTACAGGGGGCCCAGGTTCAATC
CCTGGTCAGGGAA
>Bos_taurus_chr1.trna5300-GlyCCC (151862853-151862924) Gly (CCC) 72 bp Sc: 40.75
TCCCTGGTGGTCCTGTGGCTAGGACTCTGCGATCCCAATGCAGAGGCCTGGGTTTCATCC
CCGGTCAGGGAA
>Bos_taurus_chr25.trna497-GlyCCC (9185157-9185230) Gly (CCC) 74 bp Sc: 40.77
TCCCTGGTGGTCCAGTGGCTAAGACTCTGAGCTCCCAACGCAGGGGGCCCTGGGTTCCAT
CCCTGGTTAGGGAA
>Bos_taurus_chr22.trna1585-GlyCCC (44523871-44523943) Gly (CCC) 73 bp Sc: 40.80
TCCCTGGTGGTCCAGTTGCTAAGACTCTCTATTCCCAATGCAGGGGGCCCAGGTTCGATC
CCTGGTCAAGGGT
>Bos_taurus_chr17.trna2687-GlyCCC (63180760-63180833) Gly (CCC) 74 bp Sc: 40.86
TCCCTGATAGTCCAGTGGCTAAGACTCTGTGTTCCCAAAGCAGGGATCCCTGGGTTCAAT
CCCTCGTTAGGGAA
>Bos_taurus_chr12.trna4165-GlyCCC (70687290-70687218) Gly (CCC) 73 bp Sc: 40.89
TCCCTGGTGGTCTAATGGCTAAGACTCTGAGCTCCCAATGCAGGGGGCCCAGGTTTAATC
CCTGGTCGGGAAA
>Bos_taurus_chr16.trna2374-GlyCCC (60876903-60876975) Gly (CCC) 73 bp Sc: 40.91
TCCCTGTTGGTCCAGTGGCTAAGACTCCGTGCTCCCCATGCAGGGAGCCCAGGTTTGATC
CCTGGTCAGGGAA
>Bos_taurus_chr12.trna3794-GlyCCC (75824740-75824668) Gly (CCC) 73 bp Sc: 40.92
TCCCTGCTGGTCCAGTGGTAAAGACTCTGAGTTCCCAGTGCAGGGGGCTCAGGTTCGATC

CCTGATCAGGGAA
>Bos_taurus_chr10.trna693-GlyCCC (16145820-16145892) Gly (CCC) 73 bp Sc: 40.94
TCCCTGGTGGCCCAGTGGCTAAGACTCTGTACTCCCAGTGCCGGGGGTCCAGGTTTGATC
CCTGGCCAGGGAA
>Bos_taurus_chr17.trna4400-GlyCCC (58209195-58209123) Gly (CCC) 73 bp Sc: 40.95
TCCCTGGTTGTCCAGTGGCTAAGACTCTGTGCTCCCAATGCAGGGGGTGCACGTTCAATC
CCTGTTCAGGGAA
>Bos_taurus_chr13.trna6150-GlyCCC (32836285-32836213) Gly (CCC) 73 bp Sc: 40.95
TCCTTGGTGGTCTAGTGGCTGAGACTCCATACTCCCAATGCGGAGGACCTGGGTTCAATC
CCTGGTCAAGGAA
>Bos_taurus_chr5.trna5262-GlyCCC (121025927-121025855) Gly (CCC) 73 bp Sc: 40.96
TCCCTGGCGGTCCAGTGGCTAAGGCTCTGCACTCCCAGTGCAAGGGGCCCAGGTTCAATC
CCTGGTCAGGGAA
>Bos_taurus_chrX.trna6794-GlyCCC (988341-988268) Gly (CCC) 74 bp Sc: 40.97
TCCCTGGTAGCTCAGGTGGCTAAGACTCTGCGCTCCCAATGCAGGGGGCCCAGGTTCACT
CCCTGGTCAGGGAA
>Bos_taurus_chr7.trna6002-GlyCCC (62613839-62613767) Gly (CCC) 73 bp Sc: 41.00
TTCCTGGTAGTCCAGTGGCTAAGACTCTGTGCTCCCAAGGCAGGGGGCCTGGGTTGGATC
CCTGGTCAGGAAA
>Bos_taurus_chr2.trna5779-GlyCCC (131014205-131014133) Gly (CCC) 73 bp Sc: 41.01
TCCCTCGTGGTCCAGTGGCTAAGACTCTGTGCTCCCAAAGCAGAGGGACCAGGTTCTATT
CCTGGTCAGGGGA
>Bos_taurus_chrX.trna3458-GlyCCC (87708329-87708401) Gly (CCC) 73 bp Sc: 41.02
TTCCTGGTGGTCCAGTGGCTAAGACTCTGTGCTCCCAACACAGGGGGCCTGGGTTCCATC
CCTGGTCAGGGAA
>Bos_taurus_chr29.trna2214-GlyCCC (46709835-46709763) Gly (CCC) 73 bp Sc: 41.02
TCTCTGATGGTCCAGTAGCTAAGACTCTGCACTCCCAATGCAGGGAGCCTGTGTTCGATC
CCTGGTCAGGGAA
>Bos_taurus_chr4.trna7444-GlyCCC (45440714-45440642) Gly (CCC) 73 bp Sc: 41.05
TCCCTGGTGGTCCAGTGGCTATGACTCCGCACTCCCACTGTAGGGGGACCAGGTTTGATC
CCTGGTCAGGGAA
>Bos_taurus_chr7.trna2877-GlyCCC (68718956-68719028) Gly (CCC) 73 bp Sc: 41.06
TCCCTGGTGGTCCAGTGGCTAAGACTGTGTGCTCCCGATACAGGGTGCCCATGTTCGATC
CCTGGTCAGGGAA
>Bos_taurus_chr16.trna3189-GlyCCC (77280914-77280843) Gly (CCC) 72 bp Sc: 41.06
TCCCTGGTGGTCCAGTGGCTAAGACTCTGTGCTCCCAATGCAGGGTCCTGGGTTCAGTCC
CTGGCCAGGTAA
>Bos_taurus_chr16.trna1390-GlyCCC (38729719-38729791) Gly (CCC) 73 bp Sc: 41.07
TCCCTGGTGGTCCAGTGGCTAAGACTCTGCACTCCCAATGGAGGGGGTTCAGGTTCCATC
CCTCGTCAGGGAA
>Bos_taurus_chr13.trna4677-GlyCCC (67152516-67152444) Gly (CCC) 73 bp Sc: 41.08
TCCCTGGTGGTCCAGGGGCTACGATTCCACACTCCCAATGTAGGGGGGCCAGGTTCAATC
CCTGTTCAGGGAA
>Bos_taurus_chr8.trna451-GlyCCC (10820819-10820891) Gly (CCC) 73 bp Sc: 41.11
TCCCTGGTGGTCCAGTGGTTAAGATTCTGAGCTCCCAGTGCAGAGGGCCCAGGTTTGATC
CTTGGTTGGGGAA
>Bos_taurus_chr23.trna2167-GlyCCC (48201964-48202036) Gly (CCC) 73 bp Sc: 41.12
TTCCTGGTGGTCCAGGGGCTAAGACTCTGTACTCCCAGTTCAGAGGACCTGGGTTCAATC
CCTGGTCAGGAAA
>Bos_taurus_chrUn.004.161.trna22-GlyCCC (107198-107126) Gly (CCC) 73 bp Sc: 41.12
TCCTTGGCGGTCCAGTGGCTAAGACTCTGCACTCCCAATGCAGTAGGCCTGGGTTCTATC
CCTGGTCAGGGAA
>Bos_taurus_chr22.trna3915-GlyCCC (13862827-13862755) Gly (CCC) 73 bp Sc: 41.13
TCCCTGAAGGTCCAGTGGCTAAGACTCTGTGCTCCCAATGCAGGAGGCCTAGGTTCAATG
CCTGGTCAGGGAA
>Bos_taurus_chr25.trna4346-GlyCCC (11301239-11301167) Gly (CCC) 73 bp Sc: 41.14
TCGCTGGTGGTCCAGTGGTTAAGACACTGTACTCCCAGTACAGGGGATTCAGGTTTGATT
CCTGGTCAGGGAA
>Bos_taurus_chr22.trna1185-GlyCCC (32653645-32653718) Gly (CCC) 74 bp Sc: 41.17
TCCCTGGTGGTCCAGTGGCTAAGACACTGCACTCCCAGTGCAGGGGGGGCCAGGCTCAAT
CCCTGGTCAGGGAA
>Bos_taurus_chr15.trna1417-GlyCCC (40882387-40882459) Gly (CCC) 73 bp Sc: 41.17
TCCCTGGTGGTCCAATGGCTAAGACTCTGCACTCCCATGGCAGGGGGCCTGGGTTCCATC
CCTGGTCAGGGAA
>Bos_taurus_chr12.trna893-GlyCCC (20944322-20944395) Gly (CCC) 74 bp Sc: 41.19
TCTCTGGTGGTCCAGAGGCTTACGACTCTGTGCTCCCAATGAAGGGGGCCCAGGTTCAAT
TCCTGGTCAGGGAG

>Bos_taurus_chr29.trna2404-GlyCCC (42988281-42988209) Gly (CCC) 73 bp Sc: 41.21
TCCCTGGAGGTCCAGTGGCTAAGGCTCTGTGCTCCCAATGCAGGAGACCCAGGTTCAATC
CCTGGTCAGGGAA
>Bos_taurus_chr9.trna6677-GlyCCC (35166737-35166665) Gly (CCC) 73 bp Sc: 41.24
TCCCTGGTGGTCCAGTGGCTAAGACCCTGTGCTCCCAGTGCAGGGGGCCCAGGTTTGACC
CCTGGTCAGGGAA
>Bos_taurus_chrUn.004.7821.trna1-GlyCCC (3917-3989) Gly (CCC) 73 bp Sc: 41.24
TCCCTGGTGGTCCAGTGGCTAAGACCCTGTGCTCCCAGTGCAGGGGGCCCAGGTTTGACC
CCTGGTCAGGGAA
>Bos_taurus_chr7.trna4329-GlyCCC (111952774-111952846) Gly (CCC) 73 bp Sc: 41.35
TCCCTGGTGGTCCAGTGGCTAAGACTCTGTGCTCCCAACACAGGGAGCTTGAGTTCGATC
CCTGGTCAGGGAA
>Bos_taurus_chr14.trna483-GlyCCC (10952151-10952223) Gly (CCC) 73 bp Sc: 41.39
TCCATGTTGGTCCAGTGGCTAAGACTCTGTACTCCCAATGCAGGGGGCCCAGGTTCAATG
TCTGGGTGGGGAA
>Bos_taurus_chr13.trna1218-GlyCCC (30575866-30575938) Gly (CCC) 73 bp Sc: 41.42
TCCTTGATGGTCTAGTGACTGAGACTCTGCACTCCCAATGCAGGGGCCCTGGGTTTGATT
CCTGGTCAGGGAA
>Bos_taurus_chr8.trna4608-GlyCCC (103676941-103676869) Gly (CCC) 73 bp Sc: 41.50
TCCCTGGTGGTCCAGTTGCTAAGACTTTGTGCTCCCAATGCAGGGGGCCCAGGTTCAACC
CCTGATCAGGGAA
>Bos_taurus_chr27.trna1130-GlyCCC (31475005-31475077) Gly (CCC) 73 bp Sc: 41.51
TCTCTGATGGTCCAGTGGTTAAGATTCTGAGCTCCCACTGCAGGGAACCCAGGTTTGATC
CCTGATCAGGGAA
>Bos_taurus_chrUn.004.2207.trna1-GlyCCC (17422-17494) Gly (CCC) 73 bp Sc: 41.53
TCCCTGGTGGTGGAGTGGCTAAGACTCTGCGCTCCCAATGCAGGGGATCCAGGTTTGCTT
CCTGGTCAGGGAA
>Bos_taurus_chr5.trna5147-GlyCCC (123788974-123788902) Gly (CCC) 73 bp Sc: 41.55
TCCCTCGTGGTCCAGTGGCTAAGACTCCACACTCCCAGTGCGGGGGGCCCAGGCTCGATC
CCTGGTCAGGGAA
>Bos_taurus_chr13.trna2666-GlyCCC (65563030-65563102) Gly (CCC) 73 bp Sc: 41.56
TTCCTGGTGGTCCAGTGGTTAAGATTCTGAGCTCCCAATGCAGAGGGCCCAGGTGCGTTC
CCTGGTCAGGGAA
>Bos_taurus_chr11.trna3103-GlyCCC (76629489-76629561) Gly (CCC) 73 bp Sc: 41.56
TCCCTGGTTGTCCAGTGGCTAAGACTCCATGCTCCCAATGTGGGGAGCCCAGGTTCAATC
CCTGTTTGGGGAA
>Bos_taurus_chr6.trna4179-GlyCCC (121335711-121335783) Gly (CCC) 73 bp Sc: 41.58
TCCCTGGTGGTCCAGCAGCTAAGACTCTGTGCTCCCAACGCAGGAGGCCTGGGTTCGATC
CCTGGTCAGGGAA
>Bos_taurus_chr17.trna2925-GlyCCC (66707074-66707146) Gly (CCC) 73 bp Sc: 41.59
TCCGTGGTGGTCCAGTGGCTGAGACTCCGCACTCCCAGTGCAGGGAGCCCAGGTTCGATT
CCTGGTCAGGGAA
>Bos_taurus_chr3.trna1878-GlyCCC (51350142-51350214) Gly (CCC) 73 bp Sc: 41.59
TCCCTGGTGGTCCAGTGGCTAAGACTCTACACTCCCAGTGCAGGGGACTCAGGTTCGAGA
CTTGGTCAGGGAA
>Bos_taurus_chr13.trna5884-GlyCCC (37813979-37813906) Gly (CCC) 74 bp Sc: 41.62
TCCCTGGCAGTCCAGTGGTTAAGACTCTGCATTCCCAATGCAGGGGGCTTCGGGTTCAAC
CCCTGACTGGGGAA
>Bos_taurus_chr11.trna4415-GlyCCC (105502462-105502534) Gly (CCC) 73 bp Sc: 41.64
TCCCTGGTGGTCTAGTGGCTAAGACTCTGTGCTCCCAATGCAGGGGACCAGGGTCCGATC
CCTGGTCAGGGAA
>Bos_taurus_chr5.trna3843-GlyCCC (100945330-100945402) Gly (CCC) 73 bp Sc: 41.64
TCCCTGGTGGTCCAGTGGCTAGTACTCTACACTCCCAGTGCAGGGGACTCGGGTTCGAGT
CCTGATCAGGGAA
>Bos_taurus_chrX.trna103-GlyCCC (2371321-2371393) Gly (CCC) 73 bp Sc: 41.64
TCCCTGGTAGTCCAGTGGCTAGGACTCCGTGCTCCCAATACAGGGGGCCTGGGTTCAATC
CTTAGTCAGGGAA
>Bos_taurus_chr16.trna2489-GlyCCC (63184259-63184331) Gly (CCC) 73 bp Sc: 41.65
TCCCTGGTGGTTCAGTGGCTCAGACTCTGCATTCCCAATGCAGGGGGCCCAGGTTTGATC
CCTGCTTAGGGAA
>Bos_taurus_chr10.trna5923-GlyCCC (61195132-61195060) Gly (CCC) 73 bp Sc: 41.67
TTCCTGGTGGTCTAGTGGTTAAGACACTGTGCTCCCAATACAGGGAGGGCAGGTTCAATC
CCTGGTCAGGGAA
>Bos_taurus_chr27.trna2636-GlyCCC (32410530-32410458) Gly (CCC) 73 bp Sc: 41.71
TCCCTGGTGGTCCAGTAGTTAAGACTCTGTGCTCCCAAAGCAGGGGGTTCAGGTTTGATC
CCTGATCAGGGAA
>Bos_taurus_chr10.trna5275-GlyCCC (79051474-79051402) Gly (CCC) 73 bp Sc: 41.73

TCCCTGGTGGTCCAGTGGCTAAGACTCTGTGCTCCCAATACAGGGGGCCTGGTTTCTATC
CCCGGTCAGGGAA
>Bos_taurus_chr3.trna6836-GlyCCC (72350675-72350603) Gly (CCC) 73 bp Sc: 41.73
TCCCTGGTGGTCCAGTGGCTAAGACTCTGCACTCCCAATGCACAAGGCCAGGGTTTGATC
CCTGGTCAGGGAA
>Bos_taurus_chr12.trna2969-GlyCCC (75470570-75470641) Gly (CCC) 72 bp Sc: 41.76
TCCCTGGTGGTCCAGTGGCTAAGATCCTGTTCTCCCAGTGCGGGGGCCCAGGTTTGATCC
CTGGTTAGGGAA
>Bos_taurus_chr5.trna3936-GlyCCC (102448367-102448439) Gly (CCC) 73 bp Sc: 41.76
TCTCTGGTGGTCCAGTGGCCCAGATCCTGCACTCCCAATCCAGAGAGCCCAGGTTCGATC
CCTGGTCATGGAA
>Bos_taurus_chr19.trna4919-GlyCCC (36580263-36580191) Gly (CCC) 73 bp Sc: 41.79
TCCCTGGTGGTCCAGTGGCTAAGACTCTGTGCTCCCAATACAGGGGGGCTGGGTTCAATC
CCTGGTCAGGGAA
>Bos_taurus_chr2.trna4952-GlyCCC (135931794-135931866) Gly (CCC) 73 bp Sc: 41.79
TCCCTGGTGGTCCAGTGGCTAAGACTCTGTGCTCCCAATACAGGGGGGCTGGGTTCAATC
CCTGGTCAGGGAA
>Bos_taurus_chr25.trna2294-GlyCCC (38073168-38073237) Gly (CCC) 70 bp Sc: 41.81
TCCCTGGTGGTCCAGTGGCTAAGACTCTGCATTCCCAATGCAGGGGCGGGTTGGATCCCT
GGTCAGGGAA
>Bos_taurus_chr2.trna7452-GlyCCC (94780341-94780269) Gly (CCC) 73 bp Sc: 41.82
TCCCTGGTGGTCCAGTGGCTAAGACTCTGCCCTCCCAAAGCAGGGGACCAGGGTTCGATC
TCTCGTCAGGGAA
>Bos_taurus_chr17.trna2358-GlyCCC (56590694-56590766) Gly (CCC) 73 bp Sc: 41.89
TCCCTGGTGGTCCAGAGGCTAAGACTTTGTTCTCCCAATGCAGGGGGCCTAGGTTTGATC
CCTGGTCAGGGAA
>Bos_taurus_chr10.trna5399-GlyCCC (75496289-75496217) Gly (CCC) 73 bp Sc: 41.90
TCCCTGGTGGTCTAGTGGCTAACACTCTGAGTTCCCAATGCAGGGGACCCAGGTTGGATC
CCTGGTCAGGGAA
>Bos_taurus_chr11.trna7098-GlyCCC (50546045-50545973) Gly (CCC) 73 bp Sc: 41.93
TCCCTGGTGGTCCAGTGGCTAAGACTCTGTGCTCCCAACGCAGGGTGCCAGGGTTCCATC
CCTAGTCAGGGAA
>Bos_taurus_chr16.trna1614-GlyCCC (42128275-42128347) Gly (CCC) 73 bp Sc: 41.96
TCCCTGCTGGTCCAGTGGCTAAGACTCTGAGCTCCCAATGCAGGGGGCCCGGGTTCCATC
CCTGGTCAGGGAA
>Bos_taurus_chrUn.004.248.trna13-GlyCCC (37879-37807) Gly (CCC) 73 bp Sc: 41.98
TCCCTGGTGGTCCAGTGGCTAAGACTCTGTGCTCCCAAGGCAGGCAGTCCAGGTTCAATC
CCTGATCAGGGAA
>Bos_taurus_chrX.trna3988-GlyCCC (79931327-79931255) Gly (CCC) 73 bp Sc: 41.98
TCCCTGGTGGTCCAGTGGCTAAGACTCTGTGCTCCCAAGGCAGGCAGTCCAGGTTCAATC
CCTGATCAGGGAA
>Bos_taurus_chr6.trna4936-GlyCCC (106507965-106507894) Gly (CCC) 72 bp Sc: 42.00
TCCCTGGTGGTTCAGTGGTTAAGACCCTGCAGTCCCAGTGCAGGGGCCCAGGTTCGACCC
TTGGTCAAGGAA
>Bos_taurus_chr6.trna6361-GlyCCC (72836747-72836675) Gly (CCC) 73 bp Sc: 42.00
TCCCTGGTGGTCTAGTGGCTAAGACTCTATGCTCCCAATGCAGGGGGCCCAGGTTTGATC
CCTGATCAGGGAA
>Bos_taurus_chr18.trna3025-GlyCCC (65198984-65198911) Gly (CCC) 74 bp Sc: 42.12
TCCCTGATGGTCCAGTAGTTAAGATCCTGCATTCCCAATGCAAGAGGGCATAGGTTCAAT
CCCTGGTCAGGGAA
>Bos_taurus_chr18.trna4469-GlyCCC (39657414-39657342) Gly (CCC) 73 bp Sc: 42.14
TCCCTAGTGGTCCAGTGGTTAGGACTCTGTGCTCCCACAGCAGGGGGATTAGGTTCCACT
CCTGGTTAGGGAA
>Bos_taurus_chr18.trna5374-GlyCCC (17597883-17597811) Gly (CCC) 73 bp Sc: 42.15
TCCCTGGTGGTCCAGGGGTTAAGACTCCGTGATCCCAATGCAGGGGTCCCAGGTTCAACC
CCTGATCAGGGAA
>Bos_taurus_chr17.trna2262-GlyCCC (55551916-55551988) Gly (CCC) 73 bp Sc: 42.22
TCTCTGGTGGTCCAGAGGCTCAGCCTCTGCACTCCCAATGCAGAGGGCCTGGGTTCGATC
CCTGGTCAGAGAA
>Bos_taurus_chr7.trna2431-GlyCCC (56461148-56461220) Gly (CCC) 73 bp Sc: 42.22
TCCCTGGTGGTCTAGTGGCTAATACTCTATGCTCCCAATGCAGAACGCCCAGGTTCAATC
CCTGGTCAGGGAA
>Bos_taurus_chr2.trna5837-GlyCCC (130027392-130027322) Gly (CCC) 71 bp Sc: 42.23
TCCCTGGTAGTCCAGTGGCTAAGACTTTGAGCTCCCAACACAGGGCCTGGGTTCTATCCC
TGGTCAGGGAA
>Bos_taurus_chr5.trna1206-GlyCCC (33684012-33684084) Gly (CCC) 73 bp Sc: 42.24
TCCCTGGTGGTCCAGAGGCTAAGACTCTGTGCTCCCAAAGCAGAGGACCCAGGTTGGATC

CCTGCTCAGGGAA
>Bos_taurus_chr24.trna4050-GlyCCC (35342014-35341942) Gly (CCC) 73 bp Sc: 42.24
TCCCTGGCGGTCCAGTGGTTAAGACTTCACATTCCCAATGCGGTGGGCCTGGGTTCAGTC
CCCGGTCAGGGAA
>Bos_taurus_chr1.trna6300-GlyCCC (148748076-148748004) Gly (CCC) 73 bp Sc: 42.25
TCCCTGGTGGTCCAGTGATTAAGACTCTGTGCTCCCAATTCAGTGGGAACAGGTTCAATC
CCTGGTCAGGGAA
>Bos_taurus_chr21.trna835-GlyCCC (21109879-21109951) Gly (CCC) 73 bp Sc: 42.27
TCCCTGGTGGTCTAGTGGCTAAGATTCTGTGTTCCCAATGCAGGAAGCCCAGGTTCATTC
CCTGGTCAGGGAA
>Bos_taurus_chr29.trna1035-GlyCCC (29890643-29890715) Gly (CCC) 73 bp Sc: 42.27
TCCCTGGTGGTCCAGTGGCTAAGACTCTGCACTCCCTATGCAGGGGGCCCGTGTTTGATC
CCTGGTCAGGGAA
>Bos_taurus_chr25.trna210-GlyCCC (4726938-4727010) Gly (CCC) 73 bp Sc: 42.29
TCGCTGGTGGTCCAGTGGCTAAGACTCTGCGCTCCCAATGTAGGGGCCCCAGGTTCCACC
CCTGGTCAGGGAA
>Bos_taurus_chr25.trna2243-GlyCCC (37473005-37473077) Gly (CCC) 73 bp Sc: 42.30
TCCCTGGTGGTCCAATGGGTAAGACTCTGTGCTCCCAATGCAGGGGGTCCGGGTTTGATC
CCTGGTCAGGGAA
>Bos_taurus_chr15.trna2147-GlyCCC (61197389-61197460) Gly (CCC) 72 bp Sc: 42.38
TCCCTGGGGGTCCAGTGGTTAAGACTCTGTGTTCCCGATGCAGGGGGCCAGGTTTGATCC
CTGGTTAGGGAA
>Bos_taurus_chr19.trna6060-GlyCCC (17029148-17029076) Gly (CCC) 73 bp Sc: 42.39
TCCCTGGTGGTCCAGTGGCTAAGACTCCGTACTCCCTATGCAGGGGACCCAGGTTTGATT
CCTGGTTGGGGAA
>Bos_taurus_chr12.trna2425-GlyCCC (64038394-64038466) Gly (CCC) 73 bp Sc: 42.40
TCCCTGGTAGTCCAGTGACTAAGACTCTGCACTCCCAGTGCAGGGAACCCAGGTTCAATT
CCTGGTGGGGGAA
>Bos_taurus_chr28.trna3162-GlyCCC (1396274-1396202) Gly (CCC) 73 bp Sc: 42.40
TTCCTGGTGGTCCAGTGGCTAAGATTCTGTGCTCCCACTGCAGGGGGCCCAGGTTTGATC
CCTGGTTGGGGAA
>Bos_taurus_chr3.trna1278-GlyCCC (33073445-33073517) Gly (CCC) 73 bp Sc: 42.42
GCCCTGGTGGTCCACTGGCTAAGATTCTGCATTCCCAATGCAGCGGGACCGGGTTCGATC
CCTGGTCAGGGAA
>Bos_taurus_chr1.trna8396-GlyCCC (85050924-85050852) Gly (CCC) 73 bp Sc: 42.43
TCCCTGGTGGTCCAGTGGCTAAGACTCTGAGCTCCCAATGCAGGGAGCACAGGTTCAATC
CTTGGTCAGGGAA
>Bos_taurus_chrUn.004.6.trna27-GlyCCC (589506-589578) Gly (CCC) 73 bp Sc: 42.44
TCCCTCATAGTTTAGTAGCTAAGATTCTGTGCTCCCAATGCAGGGACCCCAGGTTCAATC
CCTGGTCAGGGAA
>Bos_taurus_chr13.trna4576-GlyCCC (68583947-68583874) Gly (CCC) 74 bp Sc: 42.45
TCCCTGGAGGTCCAGTGGCTAAGACTCTGCCCTCCCAACGCAGGGGGCCCCAGGTTCTAT
CCCTGGTCAGGGAA
>Bos_taurus_chr17.trna4815-GlyCCC (53345545-53345473) Gly (CCC) 73 bp Sc: 42.46
TCCCTGGTGGTCTGGTGGTTAAGACTCCGCGCTCCCAGTGCAGAGGGCCCAGGTTCCATC
CCTGGTCAGGGAA
>Bos_taurus_chr11.trna2959-GlyCCC (74076563-74076634) Gly (CCC) 72 bp Sc: 42.47
TCCCTGGTGGTCTAGTGGCTAAGACCCCATGCTCCCAACAAGGGGGCCTTGGCTCAATCC
CTGGTCAGGGAA
>Bos_taurus_chr5.trna7719-GlyCCC (67615036-67614964) Gly (CCC) 73 bp Sc: 42.47
TTCCTGGTGGGCCAGTGGCTAAGACTCCACACTCCCAATGTAGGGGGCTTAGGTTCAATC
CCTAGTCAGGGAA
>Bos_taurus_chr24.trna5297-GlyCCC (3952091-3952019) Gly (CCC) 73 bp Sc: 42.49
TCCCTGGTGGTCCAGCGGCTAAGACACTGCGCTCCCAATGCAGGGGACCTGGGTTTGATC
CCTGGTCAGGGAA
>Bos_taurus_chr13.trna381-GlyCCC (12241587-12241658) Gly (CCC) 72 bp Sc: 42.50
TCCCTGGTAGTTCAGGGCTAAGACTCTACGCTCCCAATGCAGGGGGCCCAGGTTCAATCC
CTGGTCAGGGAA
>Bos_taurus_chr24.trna3280-GlyCCC (52619752-52619680) Gly (CCC) 73 bp Sc: 42.58
TCCCTGGTGGTCCAATGGCTAAGACTCTGAACTCCCAATACAGGGGACCAGGGTTCAACA
CCTGGTCGGGGAA
>Bos_taurus_chr8.trna6190-GlyCCC (64296104-64296031) Gly (CCC) 74 bp Sc: 42.59
TCCCTGGTGGTCCAGTGGCTAGGACCCTGAGCTCCCAATGCAGGGGGCCCAGGGTTTGAT
CCCTGGTCAGGGAA
>Bos_taurus_chrUn.004.2154.trna3-GlyCCC (4051-3979) Gly (CCC) 73 bp Sc: 42.64
TCCCTGGTGGTCCAGTGGCTGAGACTCTGTGCTCCCAATGCAGGGGGCCTGGGTTCGATC
CCTGGTCAGGGAA

>Bos_taurus_chr11.trna187-GlyCCC (2918199-2918271) Gly (CCC) 73 bp Sc: 42.65
TCCCTGGTGGTCTAGCGGCTGAGACTCTGCACTCCCTGTGCAGGGGCCCCAGGTTCAATC
CCTGGTCGGGGAA
>Bos_taurus_chr19.trna6304-GlyCCC (12700242-12700170) Gly (CCC) 73 bp Sc: 42.67
TCCCTGGTTGTCCTGTGGCTCAGACCCTGTACTCCCAATGCAGGGGGCCCAGGTTCAATC
CCTGGTCAGGGAA
>Bos_taurus_chr19.trna6308-GlyCCC (12651747-12651675) Gly (CCC) 73 bp Sc: 42.67
TCCCTGGTTGTCCTGTGGCTCAGACCCTGTACTCCCAATGCAGGGGGCCCAGGTTCAATC
CCTGGTCAGGGAA
>Bos_taurus_chr10.trna2999-GlyCCC (79433984-79434056) Gly (CCC) 73 bp Sc: 42.68
TCCCTGGTGGTCCAGTGGCTAAGATTCTGTGCTCCCAATGCGTGGGGCCCAGCTTCGATT
CCTGGTCAGGGAA
>Bos_taurus_chr17.trna3882-GlyCCC (66301452-66301381) Gly (CCC) 72 bp Sc: 42.71
TCTCTGGTGGTCCAGCGGCTAGGACCATGTGCTCCCAATGCAGGGGGCCTGGTTCGATCC
CTGGTCAGGGAA
>Bos_taurus_chr17.trna2226-GlyCCC (55241468-55241539) Gly (CCC) 72 bp Sc: 42.72
TCCCTGGCGGTCCAGTGGCTAAGACTCAGCGCTCCCAGTGCAGGGGTCCAGGTTCAATCC
CTGGCCGGGGAA
>Bos_taurus_chr18.trna3492-GlyCCC (55109991-55109919) Gly (CCC) 73 bp Sc: 42.76
TCCCTGGTGGTCCAGTGGCTAAGACTCTGAGTTCCCAATGCAGGTGGCCTGGGTTTGACC
CCCGGCCAGGGAA
>Bos_taurus_chr11.trna360-GlyCCC (6286634-6286706) Gly (CCC) 73 bp Sc: 42.76
TCCTTGGTGGTTCAGTGGCTTAGACTCTGTGCTCCCAATGCTGGGGGCCCAGGTTCAATC
CCTGGTCAGGGAA
>Bos_taurus_chr11.trna4533-GlyCCC (107941567-107941639) Gly (CCC) 73 bp Sc: 42.78
TCCCTGGCGGTCCAACGGCTAAGACTCTGCAATCCCAATGCAGGGGGTCCAGGTTCAATC
CCTGGTCGGGGAA
>Bos_taurus_chr16.trna3593-GlyCCC (67170110-67170038) Gly (CCC) 73 bp Sc: 42.79
TTCCTGGTAGTTCAGTGGCTAAAACTCTGTGCTCCCAATGCAGAGGGCCTGGGTTTAATC
CCTGGTCAGGAAA
>Bos_taurus_chr11.trna6523-GlyCCC (65560708-65560636) Gly (CCC) 73 bp Sc: 42.81
TCCCTGGTGGTCCAGTGGCTAAGACTCCATACTCCCGGTGTAGGGGGCCCAGGTTCTATA
CCTGGTCAGGGAA
>Bos_taurus_chr19.trna5419-GlyCCC (27137400-27137328) Gly (CCC) 73 bp Sc: 42.81
TCCCTGGTGGTTCAGTGGCTAAGACTTTGTCCTCCCAATGCAGGGGGCCCAGATTCAATC
CCTGGTCAGGGAA
>Bos_taurus_chr27.trna609-GlyCCC (20190707-20190779) Gly (CCC) 73 bp Sc: 42.82
TCCCTGGTGGTCCAGTGGCTAAGACTCTGTGCTCCCAGTGCAGGGGACCTAGGTTCAACC
CCTGGTCAGGGAA
>Bos_taurus_chr7.trna881-GlyCCC (15264275-15264347) Gly (CCC) 73 bp Sc: 42.83
TCCCTGGTGGTCCAGTCATTAAGACTCTGTGCTCCCACTGCAGGGATCACAGGTTCAATC
CCTGGTCAGGGAA
>Bos_taurus_chr21.trna4192-GlyCCC (33419320-33419248) Gly (CCC) 73 bp Sc: 42.84
TCCCTGATGGTCCAGTGGCTAAGACTCTGTGCTCCCAATGAAGGAAGACCAGGTTCGATT
CCTGATCAGGGAA
>Bos_taurus_chrUn.004.4536.trna2-GlyCCC (9933-9861) Gly (CCC) 73 bp Sc: 42.84
TCCCTGATGGTCCAGTGGCTAAGACTCTGTGCTCCCAATGAAGGAAGACCAGGTTCGATT
CCTGATCAGGGAA
>Bos_taurus_chr22.trna883-GlyCCC (21269379-21269451) Gly (CCC) 73 bp Sc: 42.88
TTCCTGGTGGTCCAGAGGCTAAGACTCTGCACTCCCAGTGCAGGGGACCCAGGTTTGATC
CCTGGTCAGGGAA
>Bos_taurus_chr17.trna3228-GlyCCC (71558763-71558835) Gly (CCC) 73 bp Sc: 42.89
TTCCTGGTGGTCCAGTGGCTAAGACTCTGCGCTCCCAAGGCAGGAGGCCCGGGATCAAGG
CCCGGTCAGGGAA
>Bos_taurus_chrUn.004.428.trna14-GlyCCC (81692-81620) Gly (CCC) 73 bp Sc: 42.89
TTCCTGGTGGTCCAGTGGCTAAGACTCTGCGCTCCCAAGGCAGGAGGCCCGGGATCAAGG
CCCGGTCAGGGAA
>Bos_taurus_chr3.trna8024-GlyCCC (35522698-35522626) Gly (CCC) 73 bp Sc: 42.91
TCCCTGGTGGTCCAGTGGCTAAGACTCTGTGCTCCCAATGCAGGGGATACAGGTTCTATC
CCTGGTCAGGGAA
>Bos_taurus_chr26.trna2493-GlyCCC (40117544-40117472) Gly (CCC) 73 bp Sc: 42.91
TTCTTGGTGGTCCAGTGGCTAAGACTCTGCTCTCCCAGTGCAGGGGGCCCAGGTTCAATC
CCTGGTCAGGGGA
>Bos_taurus_chr19.trna300-GlyCCC (10078124-10078196) Gly (CCC) 73 bp Sc: 42.92
TCCCTGGTGGTCTAGTGGCTAAGACTCTGTGCTCCCAATGCAGGGAGCCTGGGTTCAGTC
CCTGGCCAGGGAA
>Bos_taurus_chrX.trna3653-GlyCCC (86908558-86908486) Gly (CCC) 73 bp Sc: 42.95

TCCCTCGTCGTCCAGTGGCTAAGACTCTGTGCTCCCAAAGCAGCGGACCCAGGTTTGATC
CCTGGCTGGGGAA
>Bos_taurus_chrUn.004.1748.trna3-GlyCCC (18984-18912) Gly (CCC) 73 bp Sc: 42.95
TCCCTGGTGGTCCAGTGGCTGGGACTCTGCATTCCCAATGCAGGGAGCCTGGGTTTGATC
CCTGGTCGGGGAA
>Bos_taurus_chr8.trna2075-GlyCCC (65952249-65952321) Gly (CCC) 73 bp Sc: 42.95
TTCCTGGTGGTCCAGTGGCTGAGACTCTGTGCTCCCAATGCAGAGGGCACAGGTTCAATC
CCTGGTTGGGAAA
>Bos_taurus_chr17.trna3426-GlyCCC (75640071-75639999) Gly (CCC) 73 bp Sc: 42.96
TCCCTGGCGGTCCAGTGGCTAAGACTCTTTGCTCCCAATGCAGGGGGCCCAGGATCGATC
CCTGGTCAGGGAA
>Bos_taurus_chr19.trna6364-GlyCCC (11861099-11861027) Gly (CCC) 73 bp Sc: 43.00
TCCCTGGTGGTCCAATGGCTAGGACTCTGCCTTCCCAATGCAGGGGGCTGGGGTTCAATC
CCTGATCAGGGAA
>Bos_taurus_chr19.trna5375-GlyCCC (27955761-27955689) Gly (CCC) 73 bp Sc: 43.01
TCTTTGGTGGTCCAGTGGCTAAGACTCTGCATTCCCCATGCAGGGGGCCTGGGTTCAATC
CCTGGTCAGGGAA
>Bos_taurus_chr19.trna435-GlyCCC (12561699-12561771) Gly (CCC) 73 bp Sc: 43.02
TCCCTGGTGGTCCAGTGGCTAAGACTCCGTGCTCCCAATGTGGGCAGCCCGGGTTCAATC
CCTGGCCAGGGAA
>Bos_taurus_chr25.trna2021-GlyCCC (34435790-34435862) Gly (CCC) 73 bp Sc: 43.05
TCTCTGGTGGTCCAGCGGCTAAGCCTCTGCTCTCCCAATGCAGGGGGCCCAGGTTCCAAC
CCTGGTCAGGGAA
>Bos_taurus_chr5.trna6842-GlyCCC (90492478-90492406) Gly (CCC) 73 bp Sc: 43.12
TCCCTGGTGGTCCAGTGGCTAAGACTCAGCACTCCCAATGCAGGGGGCCTAGGTTTGATC
CCTGTTTAGGGAA
>Bos_taurus_chr17.trna2976-GlyCCC (67174810-67174882) Gly (CCC) 73 bp Sc: 43.13
TCCCTGGTGGTCCAGTGGCTAAGACTCTGTGCTCCCAATGCAGGGGGCCTGGGTTTGATC
CCTGGTCAGGGAG
>Bos_taurus_chr1.trna3409-GlyCCC (99411019-99411091) Gly (CCC) 73 bp Sc: 43.14
TCCCTGGTGGTCCAGTGGCTGAGACTCTGCATTCCCAATGCAGGGGTCCCAGGTTCTCTC
CCTGGTGGGGGAA
>Bos_taurus_chr4.trna6219-GlyCCC (81858380-81858308) Gly (CCC) 73 bp Sc: 43.16
TCCCTGGTGGTCCAGCGGCTAAGACTCTGAACTCCCACTGCAGGAGGCACAGGTTCAATT
ACTGTTTGGGGAA
>Bos_taurus_chr1.trna5309-GlyCCC (151926844-151926916) Gly (CCC) 73 bp Sc: 43.16
TCCCTGGTGGTCCAGTAGTTAAGACTCTGTGCTCCCACTGCAGGGGACCTGGGTTCAATT
CCTGGTCAGGGAA
>Bos_taurus_chr15.trna1468-GlyCCC (42047893-42047965) Gly (CCC) 73 bp Sc: 43.21
TCTCTGGTGGTCCAGTGGCTAAGACTCTGTACTCCCAATCCAGGGAACCCAGGTTTGATC
CTTGGTCAGGGAA
>Bos_taurus_chr19.trna4038-GlyCCC (53323118-53323046) Gly (CCC) 73 bp Sc: 43.22
TCCCTGGTGGTCCAGTGGGCAAGACTCTGTGCTCCCAGCACAGGGGGCCTGGGTTCGATC
CCTGGTTAGGGAA
>Bos_taurus_chr10.trna3143-GlyCCC (83036746-83036818) Gly (CCC) 73 bp Sc: 43.25
TTCCTGGCGGTCCAGCGGCTAAGATTCCACACTCCCGATGCGGGGGCCCCAGGTTCAATC
CCTGGTCAGGGAA
>Bos_taurus_chr22.trna797-GlyCCC (18242717-18242789) Gly (CCC) 73 bp Sc: 43.31
TCCCTGGTAGTCCAGTGGCTAAGATTCTGTGCTCCCAAAGCAGGGGCCCAGGGTTTGATC
CCTGGTCAGGGAG
>Bos_taurus_chr25.trna4199-GlyCCC (13127349-13127277) Gly (CCC) 73 bp Sc: 43.34
TCCCTGGTGGTCCAATGGCTAAGACTCTGTGTTCCCAATGCCGCGGGCCCAGGTTCCATT
CCTGGTCAGGGAA
>Bos_taurus_chrUn.004.1497.trna3-GlyCCC (31180-31108) Gly (CCC) 73 bp Sc: 43.37
TCCCTGGTGGTCCATTGGCTAAGACTCTGTGCTCCCAATGCAGGGGGTCCAGATTCAATT
CCTGGTCAGGGAA
>Bos_taurus_chr5.trna5412-GlyCCC (118619180-118619108) Gly (CCC) 73 bp Sc: 43.38
TCCCTGGTGGTCCAGTGGCTAAGACTCCGTGCTCCCAACACAGGGGGCCAGGGTTCAATC
CCTGGTCAGGGAA
>Bos_taurus_chr11.trna6221-GlyCCC (74046513-74046441) Gly (CCC) 73 bp Sc: 43.41
TCCCTGGTTGTCCAGTGGTTGGGACTCTGTCCTCCCAATGCAGAGGGCATAGGTTCAATC
CCTGGTCAGGGAA
>Bos_taurus_chr17.trna827-GlyCCC (19989945-19990017) Gly (CCC) 73 bp Sc: 43.44
TCCCTGGTGGTCCAGTGGCTAAGACTCTGAGCTCCCAGTGCAGGGGGCCCAGGTTTGATC
CCTGGTCAGGGAG
>Bos_taurus_chr2.trna1500-GlyCCC (46712045-46712117) Gly (CCC) 73 bp Sc: 43.44
TCCCTGGTGGTCCAGTGGCTAAGACTCTGAGCTCCCAGTGCAGGGGGCCCAGGTTTGATC

CCTGGTCAGGGAG
>Bos_taurus_chr26.trna1345-GlyCCC (38035015-38035087) Gly (CCC) 73 bp Sc: 43.44
TCCCTGGTGGTCCAGTGGCTAAGACTCTGAGCTCCCAGTGCAGGGGGCCCAGGTTTGATC
CCTGGTCAGGGAG
>Bos_taurus_chr25.trna2797-GlyCCC (37290218-37290146) Gly (CCC) 73 bp Sc: 43.44
TCCCTGGTGGTCCAGTGGCTAAGACTCTGCACTCCCAATGCAGGGAATCTGGGTTTGATC
CCTGGTTGGGGAA
>Bos_taurus_chr22.trna411-GlyCCC (9078583-9078656) Gly (CCC) 74 bp Sc: 43.46
TCCCTGGTGGTCCAGTGGCTAAGACCCTGCACTCCCAGTGCAGGGGGCCCCAGGTTCGAT
CTCTGGGCGGGGAA
>Bos_taurus_chr25.trna185-GlyCCC (4437486-4437558) Gly (CCC) 73 bp Sc: 43.48
TCTCTGGTGGTCCAGTGGCTAAGACTCTGTCCCCCCAATGCAGGGGGCCCAGGTTTGATC
CCTGGTCAGGGAA
>Bos_taurus_chr18.trna707-GlyCCC (18581384-18581456) Gly (CCC) 73 bp Sc: 43.51
TCCCTGGTGGTCCAGTGTCTAAGACTTTGTATTCCCAATGCAGAGGGCCCAAGTTTGATC
CCTGGTCAGGGAA
>Bos_taurus_chr2.trna2414-GlyCCC (76437628-76437700) Gly (CCC) 73 bp Sc: 43.51
TCCCTGGTGGTCCAGTGGCTAAGATTCTATGCTCCCAGTGAAGGGGGCCCAGGTTCAATC
CCTGGTTAGGGAA
>Bos_taurus_chr25.trna2882-GlyCCC (36014264-36014192) Gly (CCC) 73 bp Sc: 43.51
TCCCGGGTGGTTCAGTGGCTAAGACTCTGAGCTCCCAACGCAGGGGGCCTAGGTTTGATC
CCTGGTTAGGGAA
>Bos_taurus_chr1.trna5793-GlyCCC (160117551-160117479) Gly (CCC) 73 bp Sc: 43.52
TCCCTGGTGGTCCGGTGGCTAAGACTCTGCACTCCCAATTCAGGGGACCCAGGTTGGATC
CTTGGTCAGGGAA
>Bos_taurus_chr19.trna1441-GlyCCC (28777849-28777921) Gly (CCC) 73 bp Sc: 43.52
TCCCTGGCAGTCCAGTGGCTAAGACTTCACACTCCCAATGTAGGGGGCCCAGGTTTGATC
CCTGGTCAGGGAA
>Bos_taurus_chr16.trna1716-GlyCCC (44083715-44083787) Gly (CCC) 73 bp Sc: 43.55
TTCCTGGTGGTCCAGTGGCTAAGATTCTGCGCTCCCAATGCAGGGATCCCGAGTTCCATC
CTTGGTCAGGGAA
>Bos_taurus_chr23.trna748-GlyCCC (17343006-17343078) Gly (CCC) 73 bp Sc: 43.57
CCCCTGGTGGTCCAATGGCTAAGACTCTGCACTCCCAGTGCGGGGGGCCCAGGTTTGATC
CCTGGTCAGGGAG
>Bos_taurus_chr15.trna3037-GlyCCC (84623589-84623517) Gly (CCC) 73 bp Sc: 43.59
TTCCTGGTGGTCCAGTGGCTAAGACTCTGTGCTCCCGAGGCAGGGGGCCCAGGTTCCATC
CCTGGTCGGGGAA
>Bos_taurus_chr29.trna1807-GlyCCC (47648240-47648312) Gly (CCC) 73 bp Sc: 43.59
TTCCTGGTGGTCCAGTGGCTAAGACTCTGTGCTCCCGAGGCAGGGGGCCCAGGTTCCATC
CCTGGTCGGGGAA
>Bos_taurus_chr16.trna3737-GlyCCC (64205623-64205551) Gly (CCC) 73 bp Sc: 43.61
TCCCTGGTGGTCCAGTGGCTAAGACTCTGTGCTCCCAATGCAGGGGGCCTGGGTTTGATC
CCTGGTCAGGGAA
>Bos_taurus_chr5.trna4616-GlyCCC (116571187-116571259) Gly (CCC) 73 bp Sc: 43.61
TCCCTGGTGGTCCAGTGGCTAAGACTCTGTGCTCCCAATGCAGGGGGCCTGGGTTTGATC
CCTGGTCAGGGAA
>Bos_taurus_chrUn.004.216.trna1-GlyCCC (6834-6906) Gly (CCC) 73 bp Sc: 43.69
TCCCTGGTTGTCCAGTGGCTAAGACTCTGTGCTCCCAATGCAGGGGGCCTGGGTTCGATC
CTCCGTCGGGGAA
>Bos_taurus_chr18.trna476-GlyCCC (12328024-12328096) Gly (CCC) 73 bp Sc: 43.70
TCCCTGGTGGTCCAGTGGCTAAGACTCTGAGCTCCCAATACAGGGAGCCCGGGTTTGATC
CCTGGTCAGGGAA
>Bos_taurus_chr3.trna4046-GlyCCC (112849308-112849380) Gly (CCC) 73 bp Sc: 43.70
TCCCTAGCGGTCCAGTGGCTAAGATTCTGCACTCCCAATGCAGGGGGCCTGGGTTCGATC
CCTGGTCAGGGAA
>Bos_taurus_chrX.trna516-GlyCCC (10976152-10976224) Gly (CCC) 73 bp Sc: 43.71
TCCCTGGTGGTCCAATGACTAAGACACTGCAATCCCAATGCAGAGGGTCCAGGTTCAATC
CTTGGTCAGGGAA
>Bos_taurus_chr5.trna4131-GlyCCC (106279282-106279354) Gly (CCC) 73 bp Sc: 43.71
TCCCTGGTGGTCCAGTGGCTAAGACTCAGCATTCCCAGTGCAGGGGGTCCAGGTTTGATC
CCTGGTCAGGGAA
>Bos_taurus_chrUn.004.6639.trna2-GlyCCC (1409-1337) Gly (CCC) 73 bp Sc: 43.71
TCCCTGGTGGTCCAGTGGCTAAGACTCAGCATTCCCAGTGCAGGGGGTCCAGGTTTGATC
CCTGGTCAGGGAA
>Bos_taurus_chr14.trna5292-GlyCCC (30587338-30587266) Gly (CCC) 73 bp Sc: 43.75
TCCCTGGTGGTCCAGTGTCTAAGACTCTGCTCTCCCAATGCAGTAGGCCTGGGTTCAATC
CCTGGTCAGGGAA

>Bos_taurus_chr21.trna4546-GlyCCC (26146677-26146605) Gly (CCC) 73 bp Sc: 43.79
TCCCTGGTGGTCCAGTGGCTAAGACTCTGTGCTCCCAATGCAGGGGGCCTGGGGTCAATC
CCTAGTCAGGGAA
>Bos_taurus_chr22.trna1424-GlyCCC (40321756-40321828) Gly (CCC) 73 bp Sc: 43.81
TCCTTGGTGGTCCAGTGGCTAAGACTCTGTGCTCCCAATGCAGGCGGCCCAGGTTTGATC
CCTGGTCAGGGAC
>Bos_taurus_chr24.trna1814-GlyCCC (43059990-43060062) Gly (CCC) 73 bp Sc: 43.81
TCCCTGGTGGTCCAGTGGCTAAGACTCTGAGCTCCCAGTGCAGGGGGCCCAGGTTCTATC
CCTGGTCAGGGAG
>Bos_taurus_chr18.trna4676-GlyCCC (34418043-34417971) Gly (CCC) 73 bp Sc: 43.83
TCCTTGGTGGTTCAATGGTTGGGACTCTGCACTCCCACTGCAGGGGGCTCAGGTTCAACC
CCTGGTGGGGGAA
>Bos_taurus_chr14.trna4271-GlyCCC (57970025-57969953) Gly (CCC) 73 bp Sc: 43.84
TGCCTGGTGGTCCAGCCGCTAAGACTCTGCGCTCCCAACGCAGGGGGCCCAGGTTTGATA
CCTGGTCGGGAAA
>Bos_taurus_chr13.trna2351-GlyCCC (60206015-60206087) Gly (CCC) 73 bp Sc: 43.87
TCCCTTATCGTCCAGTGGCTAAGACTCTGAACTCCCAATGCAGGGGGCCTGGGTTCAATC
CCTGGTCAGGGAA
>Bos_taurus_chr11.trna7135-GlyCCC (49855769-49855697) Gly (CCC) 73 bp Sc: 43.89
TCCTTAATAGTCCAGTGGCTAAGACTCTGTGCTCCCAATGCAGGGAGCCCAGGATTGATC
CCTGGTCAGGGAA
>Bos_taurus_chr18.trna1011-GlyCCC (25489346-25489418) Gly (CCC) 73 bp Sc: 43.91
GCCCTGGTGGTCTAGCAGCTAAGACTCTGTGCTCCCAATGCAGGGGTCCCAGGTTTGATC
CCTGGTCAGGGAA
>Bos_taurus_chr5.trna2241-GlyCCC (61459553-61459625) Gly (CCC) 73 bp Sc: 43.91
CCCCTGGTGGTCCAGTGGCTAAGACTTTGTGCTCCCAACGCAGGGGGCCTGGGTTTGATC
CCTGGCCAGGGAA
>Bos_taurus_chr10.trna7624-GlyCCC (14471041-14470969) Gly (CCC) 73 bp Sc: 43.93
TCCCTGGTAGTCCAGTGGCCAAGATCCTGCATTCCCAATGCAAGGGGTCCAGGTTCCATC
CCTGGTCAGGGAA
>Bos_taurus_chr23.trna2220-GlyCCC (48804766-48804837) Gly (CCC) 72 bp Sc: 43.93
TCCTTGGCGGTCCAGTGGCTAAGACTCTGCACTCCCAGTGCAGGGGCCTGGGTTCAATCC
CTGGTCAGGGAA
>Bos_taurus_chr11.trna8880-GlyCCC (5992338-5992267) Gly (CCC) 72 bp Sc: 43.94
TTCCTGCTGGTCCAGTGGCTAAGACTCTGTGCTCCCAATGCAGGGGCCCAGGTTCAGTCC
CTGGTCGGGGAA
>Bos_taurus_chr26.trna2455-GlyCCC (40621851-40621779) Gly (CCC) 73 bp Sc: 43.95
TCCCTGGTGGTCCAGGGACTAAGACTTTGCACTCCCAATGCAGGAGGCCCAGGTTTGATC
CCTGGTCAGGGAA
>Bos_taurus_chr19.trna5951-GlyCCC (18633687-18633615) Gly (CCC) 73 bp Sc: 43.96
TCCCTGGTGGTCCAGGGGCTAAGACTCTGTGCTCCCAATGCAGAGGACCCCGGTCCGATC
CCAGGTCAGGGAG
>Bos_taurus_chr2.trna4833-GlyCCC (134095774-134095846) Gly (CCC) 73 bp Sc: 43.98
TCCCAGATTGTCCAGTGGCTAAGACCCTGCGCTCCCAATGCAGGGGGCCCAGGTTCAGTC
CCTGGTCAGGGAA
>Bos_taurus_chr23.trna2059-GlyCCC (46097322-46097394) Gly (CCC) 73 bp Sc: 43.98
TCCCTGGTGGCCCAGTGGCTAAGACTCTGAGCTCCCAATGCAGAGGGCCCAGGTTTAACC
CCTGGTCAGGGAA
>Bos_taurus_chr17.trna2727-GlyCCC (63777428-63777500) Gly (CCC) 73 bp Sc: 44.00
TCCCTGGTGGACCAGTGGCTAAGACTCTGTACTCCCAGTGCAAGGGGCCCAGGTTCAATC
CCTGGTCAGGGAA
>Bos_taurus_chr25.trna4770-GlyCCC (5131564-5131492) Gly (CCC) 73 bp Sc: 44.01
TCCCTGGTGGTCTAGTGGCTAAGACTCTGTGCTCCCAGCACAGGGGACCTGGGTTCAATC
CCTGGTCAGGGAA
>Bos_taurus_chr13.trna6490-GlyCCC (26268990-26268918) Gly (CCC) 73 bp Sc: 44.01
TCCCTGGTGGTCCAGTTCCTAAGACTTTGCGCTCCCAATGCAGGGGTCCCAGGTTCAATC
CCTGGTTAGGGGA
>Bos_taurus_chr14.trna927-GlyCCC (21782019-21782091) Gly (CCC) 73 bp Sc: 44.02
TCCCTGGTGGTCCAGTGTCTAAGACTCTGTGCTCCCAATGCAGGGTGCCTTGGTTCAATT
CCTAGTTAGGGAA
>Bos_taurus_chr4.trna477-GlyCCC (13979958-13980030) Gly (CCC) 73 bp Sc: 44.05
TCCTTGGTGGTCCAGTGGCTAAGACTCTGTTCTCCCTGTGCAGGGGGCCCAGGTTCAATC
CCTGGTCAAGGAA
>Bos_taurus_chr5.trna4891-GlyCCC (120902343-120902415) Gly (CCC) 73 bp Sc: 44.07
TCCCTGGTGGTCCAGAGGTTAAGACTCTGCACTCCCGATGCAAGGTGCCCAGGTTTGATC
CCTGGTAAGGGAA
>Bos_taurus_chr5.trna5980-GlyCCC (108336954-108336882) Gly (CCC) 73 bp Sc: 44.08

TTCCTGGTGGTCCACTGCTTAAGACTCTGCGCTCCCAATGCAGAGGGCCCAGGTTTGATT
CCTGGCTGGGGAA
>Bos_taurus_chr11.trna4058-GlyCCC (99495624-99495695) Gly (CCC) 72 bp Sc: 44.12
TCCCTGGTGGTCCAGTGGAAAAGACTCTGTGCTCCCAATGCAGGGGCCCAGGTTCCGTCC
CTGGTCAGGGAA
>Bos_taurus_chr22.trna2355-GlyCCC (58329249-58329177) Gly (CCC) 73 bp Sc: 44.12
TCCCTGGTGGTCCAGTGGCTAAGACTGTGCACTCCCCATGCAGGGGGCCTGGGTTCAATC
CCTGGTCAGGGAA
>Bos_taurus_chr17.trna3857-GlyCCC (66678433-66678361) Gly (CCC) 73 bp Sc: 44.15
TCCCTGGTGGTCCAATGGCTAAGACTCTGTGCTCCCAGTACAGGGGGCCCAGGTTCGATC
CCTGGTCAGGAAA
>Bos_taurus_chr27.trna2748-GlyCCC (30336024-30335952) Gly (CCC) 73 bp Sc: 44.17
TCCCTGATGGTCTAGTGGCTAAGACTCTGAGTTCCCCTTGCAGGGGGCCCAGGTTCGATC
CCTGGTCAGAGAA
>Bos_taurus_chr25.trna1789-GlyCCC (30312305-30312376) Gly (CCC) 72 bp Sc: 44.18
TCCCTGGTGGTTCAGTGGCTAAGACTCCGTACTCCCAGTGCGGGCTCCCAGGTTCAATCC
CTGATCAGGGAA
>Bos_taurus_chr1.trna2929-GlyCCC (85209613-85209685) Gly (CCC) 73 bp Sc: 44.20
TCCCTGGTGGTCCAGTGGCTTAGACTCTATACTCCCAATGCAGGGGGTCAAGGTTCAATT
CCTGGTCAGGGAA
>Bos_taurus_chr19.trna950-GlyCCC (20602666-20602738) Gly (CCC) 73 bp Sc: 44.21
TTCCTGGTGGTCCAGTGGTTAAGACTCTGTGCTCCCAATGCAGGGAACACAGGTTCAATC
CCTGATTGGGGAA
>Bos_taurus_chr18.trna2806-GlyCCC (61981237-61981309) Gly (CCC) 73 bp Sc: 44.22
TCTCTGGTGGTCCAGTGGCTAAGACTCTGTGCTCCCAGTGCAGGGGGCCCGGGTTCAATC
CCTGGTCAGAGAA
>Bos_taurus_chr28.trna49-GlyCCC (1724633-1724705) Gly (CCC) 73 bp Sc: 44.28
TCCCTAGTGGTCCAGTGGTTAAGACTATGCGCTCCCAATGCAGGGGGCCCAGGTTTAACC
CCTGGTCAGGGAA
>Bos_taurus_chr17.trna4557-GlyCCC (55821263-55821191) Gly (CCC) 73 bp Sc: 44.30
TCCCTGGTGGTCCAGTGGCTAAGACTCAGCAATCCCAATGCAGGGGGCCTGGGATCGATC
CCTAGTCAGGGAA
>Bos_taurus_chr1.trna5178-GlyCCC (149308922-149308993) Gly (CCC) 72 bp Sc: 44.30
TCCCTGACAGTCCAGTGGTTAAGGCACTGTGCTCCCCATGCTGGGGCCCAGGTTCAATCC
CTGGTCAGGGAA
>Bos_taurus_chr11.trna8986-GlyCCC (3675944-3675872) Gly (CCC) 73 bp Sc: 44.31
TCCCTGGTGGTCCAGGGGCTAAAACTCTGTGATCCCAATGCAGGGGGCCCAGGTTCGATC
CCTGGTCAGGGAG
>Bos_taurus_chr21.trna698-GlyCCC (18849780-18849852) Gly (CCC) 73 bp Sc: 44.31
TCCCTGGTGGTCCAGTGGCTAAGACTCTGTGCTCCCAAAGCGGGGGGCCCAGGTTCGATC
CTTGTTCAGGGAA
>Bos_taurus_chr23.trna1830-GlyCCC (41242149-41242224) Gly (CCC) 76 bp Sc: 44.33
TCTCTGGTGGTCCAGTGGTCAGCGGGATTCTGAGCTCCCAATCCAGAGGGCCCAGGTTCG
ATACCTGGTCAGGGGA
>Bos_taurus_chr7.trna660-GlyCCC (12950963-12951035) Gly (CCC) 73 bp Sc: 44.36
TCCCTGGTGGTCCAGTGGCTAAGACTCTGTGCTCCCAATGCAGGGGGCCTGGGTTCCATT
CCTGGTCAGGGAA
>Bos_taurus_chr4.trna8577-GlyCCC (9381891-9381819) Gly (CCC) 73 bp Sc: 44.37
TCTCTGGTGGTCCAGTGGCTAAGATTCTGTGCTCCCAATGCAGGGGACCCAGGTTTGATC
CCTGGTTAGGGAA
>Bos_taurus_chrUn.004.249.trna22-GlyCCC (123021-122949) Gly (CCC) 73 bp Sc: 44.38
TCCCTTGTGGTCCAGTGGCTAAGACTCTGTGCTCCCAATACAGGGAGCCCAGGTTTGATC
CCTGGTCAGGGAA
>Bos_taurus_chr29.trna2019-GlyCCC (50701366-50701294) Gly (CCC) 73 bp Sc: 44.41
TCCCTGGCGGTCCAGTGGCTAAGACTCTGTGTTCCCAATGCAGGGGGCCTGGGTTCGATC
CCTGGCCAGGGAA
>Bos_taurus_chr25.trna1569-GlyCCC (27638439-27638511) Gly (CCC) 73 bp Sc: 44.42
TTCTTAATGGTCCAGTGGCTAGGACTCTGCACTCCCAATGCAGGGGGTCTGGGTTCAGTC
CCTGGTTGGGGAA
>Bos_taurus_chr29.trna3430-GlyCCC (17788647-17788575) Gly (CCC) 73 bp Sc: 44.44
TCCTTGGTGGTCCAGTGGCTAAGACTCTGAGCTCCCAGTGCAGGGGGTCCAGGTTCGATC
CCTGGTTGGGGAA
>Bos_taurus_chr8.trna6267-GlyCCC (62076412-62076342) Gly (CCC) 71 bp Sc: 44.45
TCCTTGTGGTCCAGTGGCTAGGACTCTGTGCTCCCAATGCAGGGGTCTGGGTTCGATCCC
TGGTCAGGGAA
>Bos_taurus_chr1.trna319-GlyCCC (5089915-5089987) Gly (CCC) 73 bp Sc: 44.48
TTTCTGGTGGTCCAGTAGCTAAGACTCTGCATTCCCAATGCAGGGGTCTCAGGTTCGATC

CCTGGTCAGTGAC
>Bos_taurus_chr27.trna1324-GlyCCC (35425646-35425718) Gly (CCC) 73 bp Sc: 44.49
TCCCTGGTAGTCCAGTGGCTAAGACTCTGAACTCCCAATGCAGGATTCCCAGGCTTGATC
CCTGGTCAGGGAA
>Bos_taurus_chr14.trna6029-GlyCCC (15113699-15113626) Gly (CCC) 74 bp Sc: 44.50
TCCCTGGTGGTCCAGTGGCTAAGACTCTGCACTCCCAATGCAGGGGGGCTCAGGCTTGAT
CCCTGGTCAGGGAA
>Bos_taurus_chr22.trna3822-GlyCCC (16188081-16188009) Gly (CCC) 73 bp Sc: 44.51
CCCTTGGTGGTCCAGTGGCTAAGACACTGTGCTCCCAATGCAGGGGGCCCAGGTTCAGTC
CCTGGTCAGGGAA
>Bos_taurus_chr29.trna314-GlyCCC (8882881-8882953) Gly (CCC) 73 bp Sc: 44.52
CACTTGGTGGTCCAGTGGCTAAGACTCTGTGCTCCCAATGCAGGGGGCCCAGGTTTGATC
CCTGGTCAGGGGA
>Bos_taurus_chr25.trna3000-GlyCCC (34673072-34672999) Gly (CCC) 74 bp Sc: 44.56
TCCCTGGTGGTCCAGTGGCTAAGACTCTGTGCTCCCAATGCAGAGGGGCCCAGGTTCCAT
CCCAGGTCAGGGAA
>Bos_taurus_chr21.trna1050-GlyCCC (24188041-24188112) Gly (CCC) 72 bp Sc: 44.57
TCCCTGGTGGTCCAGTAGCTAAGACTCTGTGCTCCCAATGCAGGGGGCCTGGTTCAATCC
CTGGTCAGGGAA
>Bos_taurus_chr10.trna7661-GlyCCC (13803301-13803229) Gly (CCC) 73 bp Sc: 44.57
TCCCTGGTAGTCCAGGGGCTAAGACTCTGCACTCCCAATGCACGGGGCATGGGTTCGATC
CCTAGTCAGGGAA
>Bos_taurus_chr14.trna4871-GlyCCC (40862663-40862591) Gly (CCC) 73 bp Sc: 44.59
TCCCTGGTAGTTCTGTGGCTAAGACTCTGCACTCCCAGTGCAGGGGGCTCAGGTTCAATC
CCTGGTCAGGGAA
>Bos_taurus_chr14.trna2563-GlyCCC (61522513-61522585) Gly (CCC) 73 bp Sc: 44.60
TCCCTGATGGACTAGTGGCTAAGACTTTGTGCTCCCACTGCAGCAGGCCTGGGTTCAATC
CCCGGTCAGGGAA
>Bos_taurus_chr16.trna4354-GlyCCC (49059163-49059091) Gly (CCC) 73 bp Sc: 44.60
TCTCTGGTGGTCCAGTGGCTAAGACTCTGCATTCCCAATGCAGGGAGCTCAGGTTCGATC
TCTGGTCAAGGAA
>Bos_taurus_chr22.trna2992-GlyCCC (41046916-41046844) Gly (CCC) 73 bp Sc: 44.62
TCCCTGGTGGTCCAGTGGCTAAGACTCTGCATTCCCAATGCAGGCGGCCCAGGTTCCACC
CCAGGTCGGGGAA
>Bos_taurus_chr2.trna4721-GlyCCC (132581200-132581272) Gly (CCC) 73 bp Sc: 44.62
TCCTTGGTGGTCCAATGGCTAAGACTCTGCATTCCCAATGAAGGCGGCCAAGGTTCGATC
CCTGGTCAGGGAA
>Bos_taurus_chr19.trna3711-GlyCCC (58692447-58692375) Gly (CCC) 73 bp Sc: 44.66
TCCTTGGTAGTCTGGTGGCTAAGACTCTGTGCTCCCAACGCCGGAGGCCCAGGTTTGATT
CCTGGTCAGGGAA
>Bos_taurus_chr16.trna1719-GlyCCC (44093628-44093700) Gly (CCC) 73 bp Sc: 44.69
TCCCTGGTGGTCCAGTGGCCAAGACTCTGCTCTCCCAATGCAGGGGACATGGGTTCAATC
CCTAGTCAGGGAA
>Bos_taurus_chr2.trna5455-GlyCCC (135784407-135784335) Gly (CCC) 73 bp Sc: 44.73
TCCCTGATGGTTCCGTGGTTAAGACTCTGAGCTCCCAGTACAGGGGGCCCAGGTTCAATC
CCTGGTCAGGGAA
>Bos_taurus_chr23.trna1650-GlyCCC (37860952-37861023) Gly (CCC) 72 bp Sc: 44.79
TTCCTGGTGGTCTAGTGGCTAAGTCTCTGTACTCCCAATGCAGGGGGCCAGGTTGGATCC
CTGGTCAGGGAA
>Bos_taurus_chr13.trna365-GlyCCC (11928424-11928496) Gly (CCC) 73 bp Sc: 44.79
TCCCTGGTGGTTCAGTGGCTAAGACTTTGTACTCCCAATGCAGGGGGCCCAGGTTCCATC
CCTGTTCAGGGAA
>Bos_taurus_chr2.trna4353-GlyCCC (126204331-126204403) Gly (CCC) 73 bp Sc: 44.79
TCCCTGGTGGTCCAGTGGCTAAGACTCTGTACTCCCAGTGCAGGGGGCCTGGGTTCAATC
CCTAGTCAAGGAA
>Bos_taurus_chr11.trna175-GlyCCC (2668630-2668702) Gly (CCC) 73 bp Sc: 44.82
TCCCTGGTGGTCCAGTGGCTAAGACCCTGAGCTCCCAATGCAGGGGGATCAGGTTCAATC
CCTGGTCAGGGAA
>Bos_taurus_chrX.trna4333-GlyCCC (71335876-71335804) Gly (CCC) 73 bp Sc: 44.82
TCCCTGGTGGTCCAGTGGCTAAGACGCCATGCTCCCAATGTAGGAGGCCCGGGTTCAATG
CCTGGTCAGGGAA
>Bos_taurus_chr19.trna2626-GlyCCC (51569579-51569651) Gly (CCC) 73 bp Sc: 44.84
TCCCTGGTGGTCCAGTGGTTAAGACTCTGTGCTCCCAATGCTGGGGGCCTGGGTTCCATC
TCCAGTCAGGGAA
>Bos_taurus_chr8.trna4951-GlyCCC (94619060-94618988) Gly (CCC) 73 bp Sc: 44.85
TCCCTGGTGGTCCAGTGGCTAAGACTCTGAGCTCCCAATGCAGGGGGCCCAGGTTTGATC
TCTGGTCAGGGAA

>Bos_taurus_chrX.trna4182-GlyCCC (75016030-75015959) Gly (CCC) 72 bp Sc: 44.88
TCCCTGGTGGTCCAGTGGCTAAGACTCTGTGTTCCCAATGCAGGGGCCTGGGTTGGATCC
CTGGTCAGGGAA
>Bos_taurus_chr5.trna4772-GlyCCC (119161956-119162028) Gly (CCC) 73 bp Sc: 44.89
TCCCTGGTGGTTGAGTGGCTAAGACTCTGCGCTCCCAGTGCAGAGGGCCCAGGTTTGATC
CTTGGTCAGGGAA
>Bos_taurus_chr6.trna483-GlyCCC (18536969-18537041) Gly (CCC) 73 bp Sc: 44.89
TCCCTGGTGGTCCAGTCGCTAAGACTCTGCACTCCCAATGCAGTGGACCTGGGTTAGATC
CCTGGTCAGGGAA
>Bos_taurus_chr8.trna3516-GlyCCC (102385681-102385753) Gly (CCC) 73 bp Sc: 44.94
TCCCGGGTGGTCCAGCAGCTAAGACTCTGTGCTCCCAATGCAGGGGACCCAGGTTCGATC
CCTGGTCAGGGAA
>Bos_taurus_chr2.trna5445-GlyCCC (135906026-135905955) Gly (CCC) 72 bp Sc: 44.98
TTCCCAGTGGTCCAGTGGCTAAGACTCTGTACTCCCAAAGCAGGGGCCCCGGTTTGAACC
CTGGTTGGGGAA
>Bos_taurus_chr11.trna5730-GlyCCC (84960710-84960638) Gly (CCC) 73 bp Sc: 44.99
TCCCTGGTGGTCCAGTGGTTAAGACACTGTACTCCCAACGCAGAGGGCTGGGGTTGAATC
CCTGGCCAGGGAA
>Bos_taurus_chr2.trna1634-GlyCCC (50364448-50364520) Gly (CCC) 73 bp Sc: 45.01
TCCCTGGTGGTCCAGTGGCTAAGACTCTACACTCCCAGTGCAGGGGGCCCAAGTTCAATC
CTTGATCAGGGAA
>Bos_taurus_chr5.trna4865-GlyCCC (120543966-120544038) Gly (CCC) 73 bp Sc: 45.01
TCCCTGATGGTCCAGTGGCTAAGACTCTGAGCTCCCAGTGCAGAGGGCCTGGGTTCAATC
CCTGGTCAGGGAA
>Bos_taurus_chr13.trna666-GlyCCC (18706954-18707026) Gly (CCC) 73 bp Sc: 45.02
TCCCTGGTGGTCCAGAGGCTACGACTCTGTGCTCCCAGTGCAGGGGACCCAGGTTCGATC
CCTGGTCAGGGAA
>Bos_taurus_chr18.trna4000-GlyCCC (48420875-48420803) Gly (CCC) 73 bp Sc: 45.02
TCCCTGCTAGTCCAGTGGCTAAGACTCTGGGCTCCCAATGCAGGGGGCCCAGGTGCAATC
CCTGGTCAGGGAA
>Bos_taurus_chr11.trna4171-GlyCCC (101997023-101997095) Gly (CCC) 73 bp Sc: 45.05
TCCCTGGTGGGCCAGTGGCTTAAGACTCAGCGTTCCCAATGCAGGGGGCCAGGTTCGATC
CCTGGTCAGGGAA
>Bos_taurus_chr2.trna5011-GlyCCC (137566812-137566883) Gly (CCC) 72 bp Sc: 45.06
TCCCTGGTGGTCCAGGGCTAAGACTCTGCGCTCCCAATGCAGGGGGCTCAGGTTCAACCC
CTGGTCAGGGAG
>Bos_taurus_chr3.trna1361-GlyCCC (35253199-35253271) Gly (CCC) 73 bp Sc: 45.07
TCCCTGATAGTCCAATGGCTAAGACTCTGCGCTCCCAGTGCAGGGGACCCAGTTTCAATC
CCTGGTCAGGGAA
>Bos_taurus_chr17.trna4079-GlyCCC (63455582-63455511) Gly (CCC) 72 bp Sc: 45.08
TCCCTGGTGGTCCAGTGGTTAAGACTCTGAACTCCCAGTTAGGGGGCCCAGGTTCAATCC
CTAGTTAGGGAA
>Bos_taurus_chr23.trna4011-GlyCCC (17738315-17738243) Gly (CCC) 73 bp Sc: 45.09
TCCCAGGTAGTCCAGTGGCTAAGACTCTGAGCTCCCAATTCAGGGGACCCAGGTTTGATC
CCTGGTCAGGGAA
>Bos_taurus_chr18.trna4924-GlyCCC (27070009-27069937) Gly (CCC) 73 bp Sc: 45.09
TCCTTGGTGGTCTAGTGGTTAAGACTCTGCACTCCCAATGAAGGGAGCCTGGGTTCAGTC
CCTGGTCAAGGAA
>Bos_taurus_chr19.trna834-GlyCCC (18735431-18735502) Gly (CCC) 72 bp Sc: 45.10
TCCCTGCTGGTCCAGTAGCTAAGACTCTGCATTCCCAAAGCAGGGGACCTGGTTCAATCC
CAGGTCAGGGAA
>Bos_taurus_chrUn.004.60.trna2-GlyCCC (4874-4945) Gly (CCC) 72 bp Sc: 45.12
TCCTTGGTGGTCCACTGGCTAAGACTCTGCACTCCCAATGTAGGGGCCCAGGTTTGATCC
CTGGTCACGGAA
>Bos_taurus_chrX.trna3810-GlyCCC (84362731-84362660) Gly (CCC) 72 bp Sc: 45.12
TCCTTGGTGGTCCACTGGCTAAGACTCTGCACTCCCAATGTAGGGGCCCAGGTTTGATCC
CTGGTCACGGAA
>Bos_taurus_chr23.trna473-GlyCCC (11873033-11873105) Gly (CCC) 73 bp Sc: 45.13
TCCCTGGTGGTCCAGTGGCTAAGGCTGTGCACTCCCAGTGCAGGGGCCCCAGGTTTGATC
CCTGGTCAGGGAA
>Bos_taurus_chr3.trna5592-GlyCCC (107511529-107511457) Gly (CCC) 73 bp Sc: 45.14
TCCCTGGTGGTCTAATGGTTAAGACTCTGAGCTCCCAATGCAGGGGGCACAGGTTCGCTC
CCTGGTCAGGGAA
>Bos_taurus_chr2.trna4544-GlyCCC (129874700-129874772) Gly (CCC) 73 bp Sc: 45.15
TCCCTAGTGGTCCAGTGGCGAAGACTCTGTGCTCCCAATACAGGGGTGCTAGGTTCGATC
CCTGGTCAGGGAA
>Bos_taurus_chr9.trna5312-GlyCCC (75864220-75864148) Gly (CCC) 73 bp Sc: 45.16

TCCCTGGTAGTCAAGTGGCTAAGATTCTGCACTCCCAATGCAAGGGGCCCAGGCTCCATC
CCTGGTCAGGGAA
>Bos_taurus_chrX.trna4062-GlyCCC (78221208-78221136) Gly (CCC) 73 bp Sc: 45.17
TCCCTGGTGGTCCAGTGGCTCAGGCTCTGCACTCCCAATACAGGGTTCCCAGGTTCAATC
CCTGGTCAAGGAA
>Bos_taurus_chr10.trna6374-GlyCCC (48693756-48693684) Gly (CCC) 73 bp Sc: 45.19
TCCTTGGTGGTCCAGTGGCTAAGACTCTGTGCTCCCAATGCAGGGGGCCTGGGTTTGATC
CCTAGTCAGGGAA
>Bos_taurus_chr7.trna1222-GlyCCC (19815991-19816063) Gly (CCC) 73 bp Sc: 45.20
TCCCTGCTGGTCCAGTGGTTAAGACTCTGAGCTCCCAGTTCAGGGGGCCCAGGTTCCATC
CCTGGTCAGGGAA
>Bos_taurus_chrUn.004.478.trna6-GlyCCC (26167-26095) Gly (CCC) 73 bp Sc: 45.23
TCCCTGGTGGTCCTGTGGCTGGGACTCTGCACTCCCAATGCAGGGGGCCCGGGTTTGATC
CCTGGTCAGGGAA
>Bos_taurus_chr3.trna5482-GlyCCC (110430876-110430804) Gly (CCC) 73 bp Sc: 45.28
TCCCTGGTGGTCCAGTGGCTAAGACTCTGTGCTCCCAATGCAGGGAGCCCAGGTTTGATC
TCTGGTCAGGGAA
>Bos_taurus_chr2.trna1506-GlyCCC (46902128-46902200) Gly (CCC) 73 bp Sc: 45.29
TCCCTGGTGGTTCAGTGCCTAAGACTCTGCACTCCCAATGCAGGGAGCCAGGGTTCAATC
CCTTGTCAGGGAA
>Bos_taurus_chr21.trna3022-GlyCCC (65365343-65365271) Gly (CCC) 73 bp Sc: 45.31
TCCCTGGTGGTCCAGTGGCTAAGACTCTGAGCTCCCAGTGCAGGGGGCCCAGGTTCGATC
CCTGGTCAGGGGG
>Bos_taurus_chr19.trna4274-GlyCCC (48603562-48603489) Gly (CCC) 74 bp Sc: 45.32
TCCCTGGTGGTCCAGTGGCTAAGACTCTGAGCTCCCAATGCAGGGGGGCCCAGGTTCCAT
TCCTGGTTAGGGAA
>Bos_taurus_chrUn.004.3631.trna2-GlyCCC (11187-11259) Gly (CCC) 73 bp Sc: 45.34
TCCCTGGTGGTCCAGTGGCTGAGACTCCGCATTCCCAATGCAGGGGGCCCGGGTTCAATC
CCTGGTCAGGGAA
>Bos_taurus_chr2.trna4057-GlyCCC (121407648-121407720) Gly (CCC) 73 bp Sc: 45.42
TCCCTGGTGGCCCACTGGCTAAGACTTTGTTTTCCCAATGCAAGGGGCCCAGGTTCAATT
CCTGGTCAGGGAA
>Bos_taurus_chr18.trna1773-GlyCCC (44111976-44112048) Gly (CCC) 73 bp Sc: 45.43
TCCCTGGTGGTCCAGTGGCTAAGACTCTGTGCTCCCAATGCAGGCAGCCTAGGTTCAATT
CCTGGGCAGGGAA
>Bos_taurus_chr2.trna2529-GlyCCC (79816937-79817009) Gly (CCC) 73 bp Sc: 45.43
TTCCTGGTAGACCAGTGGCTAAGACTCTGTGCTCCCAATGCAGGGGTCCCAGGTTCAATC
TCTGGTCAGGGAA
>Bos_taurus_chr1.trna6306-GlyCCC (148352755-148352684) Gly (CCC) 72 bp Sc: 45.43
TCCCTGGTGGTCCAGTGGCTAAGACTCAGTGCTCCCAATGCAGGTGTCCAGGTTCAATCC
CTGGTTGGGGAA
>Bos_taurus_chrUn.004.2042.trna3-GlyCCC (20436-20364) Gly (CCC) 73 bp Sc: 45.44
TCCCTGGTGGTCCAGTGGCTGGGATTCTGCTTTCCCAGTGCAGGGGGCCCAGGTTCGATT
CCTGGTCAGGGGA
>Bos_taurus_chr29.trna2429-GlyCCC (42772359-42772287) Gly (CCC) 73 bp Sc: 45.45
TCACTGGTGGTCCACTGGCTAAGACTCTGCACTCCCTATGCAGTGGGTCCAGGTTCGATC
CCTGGTCAGGGAA
>Bos_taurus_chr13.trna407-GlyCCC (12866105-12866177) Gly (CCC) 73 bp Sc: 45.47
TCCCTGATGGTCCAGTGGCTAAGACTCTGTGCTCCCAACACAGGAGGCCTGAGTTCAATC
CCTGGTCAGGGAA
>Bos_taurus_chr22.trna755-GlyCCC (17231167-17231240) Gly (CCC) 74 bp Sc: 45.48
TCCCTGGTGGTCCAGTGGCTAAGACTCTGTGCTCCCAATGCAGGGGGGCCCAGTTTCAAT
CCCTGGTCAGGGAA
>Bos_taurus_chr25.trna2903-GlyCCC (35662775-35662703) Gly (CCC) 73 bp Sc: 45.49
TCCCTGGTGGTCCAGTGGCTAAGACTCTGCACTCCCAATGCAGGGGGTTTAGGCTCAATC
CCTGGTTGGGGAA
>Bos_taurus_chr2.trna8347-GlyCCC (66682307-66682236) Gly (CCC) 72 bp Sc: 45.52
TCCCTGGTGGTCCAGTGGCTAAGACTCTGAGTTCCCAATGCAGGAGTCCAGGTTCCATCC
CTGGTCAGGGAA
>Bos_taurus_chrUn.004.324.trna9-GlyCCC (134175-134104) Gly (CCC) 72 bp Sc: 45.54
TCCCTGGTGGCCCAGTGGCTAGGACTCTGCGCTCCCAGCACAGGGGCCCAGGTTCCATCC
CTGGTCGGGGAC
>Bos_taurus_chr11.trna4112-GlyCCC (100479816-100479888) Gly (CCC) 73 bp Sc: 45.54
TCCCTGGTGGTCCAGTGGCTAAGACTCTGAGCTCCCAACACAGGGGGCCCGGGTTTGATC
CCTGGTCAGGAAA
>Bos_taurus_chr5.trna828-GlyCCC (25684460-25684532) Gly (CCC) 73 bp Sc: 45.55
TCCTTGGTGGTCCAATGGCTAAGACTCTGCACTCCCAATGCAGGGGACCTGGGTTCAATC

CCTGGTCAAAGAA
>Bos_taurus_chrX.trna2764-GlyCCC (74121314-74121386) Gly (CCC) 73 bp Sc: 45.58
TCCCTGGTGGTCCAGTGACTAAGACTCTGCACTCCCAATGCAGGGGCCCTGGGTTTGATC
CCTGGTCAGGGAA
>Bos_taurus_chr3.trna4577-GlyCCC (122936112-122936184) Gly (CCC) 73 bp Sc: 45.62
TTCCTGGTGGTCCAGTGGCTAGGACTCTGCAGTCCCAATGCAGGGGGCACAGGTTTGATC
CCTGGTCAGGGAA
>Bos_taurus_chr19.trna5750-GlyCCC (21778001-21777929) Gly (CCC) 73 bp Sc: 45.64
TTCCTGGTGGGCCAGTGGCTAAGACTCTGTGCTCCCAAAGCAGGGGGCCCAGGTTCAATC
CCTGGTCAGGGAA
>Bos_taurus_chr11.trna7648-GlyCCC (38307908-38307836) Gly (CCC) 73 bp Sc: 45.67
TCCCTGGTGGTCCGGTGGCTAAGACTGTGCACTCCCAATGCAGGGGGTCCAGGTTCCATC
CCTGGTCAGGGAA
>Bos_taurus_chr4.trna3118-GlyCCC (95640886-95640958) Gly (CCC) 73 bp Sc: 45.71
TCCCTAGTGGTCTAGTGGCTAAGACTCTGTGCTCCCAATGCAGGGGGCCCAGGCTCGACT
CCTGGTCAGGGAA
>Bos_taurus_chr19.trna1912-GlyCCC (38779025-38779097) Gly (CCC) 73 bp Sc: 45.71
TCCCTGGTGGTCCAATGGCTAAGACTCTGCACTCCCAAAGCAGGGGGCCCAGGTTTGATC
CCTAGTCAGGGAA
>Bos_taurus_chr5.trna7415-GlyCCC (74773644-74773572) Gly (CCC) 73 bp Sc: 45.72
TCCCTGGTGGTCCAGTGGCTAAGATTCTCTACTCCCAGTGCAGGGGACTCAGGTTCGATC
CCTGGTCAGGGAG
>Bos_taurus_chr16.trna1270-GlyCCC (35763935-35764007) Gly (CCC) 73 bp Sc: 45.76
TCCCTGGTGGTCCAGTGGCTAAGACTCTGTGCTCCCAATGCAGGGGACCCAGGTTCGTTC
CCTGGTCAAGGAA
>Bos_taurus_chr4.trna5651-GlyCCC (96129538-96129466) Gly (CCC) 73 bp Sc: 45.77
TCCCTGATGGTACAGTGGCTAAGACTCTGTGCTCCCAATGCAGGGGGCCCAGGTTTAATC
CCTGGTCAGGGAC
>Bos_taurus_chrX.trna4180-GlyCCC (75036664-75036592) Gly (CCC) 73 bp Sc: 45.77
TCCCTGGTGGTCCAGTGGCTAAGACTCTGAGCTCCCAATGCAGGGGGTCCAGATTCAATC
CCTGGTCAGGGAA
>Bos_taurus_chr17.trna3828-GlyCCC (66993407-66993335) Gly (CCC) 73 bp Sc: 45.78
TCCCTGGTGGCCCAGTGGCTGAGACTTTGTGTTCCCAACACAGGGGGCCCAGGTTCGATC
CCTGGTCAGGGAA
>Bos_taurus_chr28.trna239-GlyCCC (6320695-6320767) Gly (CCC) 73 bp Sc: 45.79
TCCCTGGTGGTCCAGTGGCTAAGACTCTGCAGTCCCACTGCAGGGGTCTCGGCTTCAATC
CCTGGTCAGGGAA
>Bos_taurus_chr10.trna1670-GlyCCC (44047213-44047285) Gly (CCC) 73 bp Sc: 45.80
TTCCTGGTGGTCCAGTGGCTAAGGCTCTGTGCTCCCAACGCAGGGGGCCCAGATTCAATC
CCTGGTCAGGGAA
>Bos_taurus_chr3.trna7924-GlyCCC (37988773-37988702) Gly (CCC) 72 bp Sc: 45.81
TCCCTGACAGTCCAGTGGCTAAGACTCTGTGCTCCCAATGCAGGGGGCCAGGTTTGATCC
CTGGTCAGGGAA
>Bos_taurus_chr18.trna4893-GlyCCC (27702413-27702341) Gly (CCC) 73 bp Sc: 45.82
TCCCTGGTGGTCCAGTGGCTGGGACTCTGCGCTCCCGACGCAGGGGGCCCACGTTCCACC
CCTGGTCAGGGAA
>Bos_taurus_chr3.trna8451-GlyCCC (25514094-25514023) Gly (CCC) 72 bp Sc: 45.87
TCCCTTGTGGTCTAGTGGCTAAGACTCTGTGCTCCCAATGCAGGTGGCCAGGTTCAATCC
CTGGTCAGGGAA
>Bos_taurus_chr19.trna1818-GlyCCC (36089940-36090012) Gly (CCC) 73 bp Sc: 45.87
TTCCTGATGGTCCAGTGGCTAAGACACTGTGCTCCCAATGCAGGGGGCACAGGTTCAATT
CCTGGTCAGGGGA
>Bos_taurus_chr1.trna4657-GlyCCC (133635008-133635080) Gly (CCC) 73 bp Sc: 45.88
TCCCTGGTGGTCCAGTGGTGAAGATTCTGCACTCCCAATGTAGGGAGTCTAGGTTTGATC
CCTGGTCAGGGAA
>Bos_taurus_chr5.trna6116-GlyCCC (105107372-105107300) Gly (CCC) 73 bp Sc: 45.88
TCCCTAGTGGTCTAGAGGTAAAGGCTCTGCACTCCCAGTGCAGGGGCCCCAGGTTCAATC
CCTGGTCAGGGAA
>Bos_taurus_chr19.trna2671-GlyCCC (53195660-53195732) Gly (CCC) 73 bp Sc: 45.89
TCTCTGGTGGTCCAATGGCTAAGACTCCATGCTCCCAATGTGGGGGGCCCAGGTTCGATC
CCTGTTCAGGGAA
>Bos_taurus_chr10.trna6847-GlyCCC (36808300-36808228) Gly (CCC) 73 bp Sc: 45.91
TCCCTGGTGGTTCAGTGGCGAAGACTCTGCACTCCCAATGCAGGTGGCCTAGGTTCAATC
CCTGGTCAGGAAA
>Bos_taurus_chr19.trna2915-GlyCCC (57265687-57265760) Gly (CCC) 74 bp Sc: 45.93
TCCCTGGTGGTCCAGTGGCTACGACTCTGTGCTCCCAAAGCAGGGGGACCTAGGTTCGAT
CCCTGGTCAGGGAA

>Bos_taurus_chr23.trna4415-GlyCCC (10474963-10474891) Gly (CCC) 73 bp Sc: 45.97
TCCCTGGTGGTCCAGTGACTAAGACTCTGAGCTCCCAATGCAGGGGGCCCGGGTTCGATT
CCTGGTCAGGGAA
>Bos_taurus_chr4.trna1117-GlyCCC (33346202-33346274) Gly (CCC) 73 bp Sc: 45.98
TCCCTGGTGGTCCAGTGGCTAAGACCCTGCGTTCCCAATGCAGGGGACCTGGGTTCAATC
CCTGGTCAGGGAA
>Bos_taurus_chrUn.004.6.trna113-GlyCCC (1008087-1008015) Gly (CCC) 73 bp Sc: 45.99
TCCTTGGTGGTCCAGCGGCTAAGACTCTGCACTCCCAATGCAGGGGGTCTAGGTTTGATC
CTTGGTCAGGGAA
>Bos_taurus_chr1.trna2474-GlyCCC (71923100-71923172) Gly (CCC) 73 bp Sc: 46.01
TCTCCGGTGGTCCAGTGGCTAAGACTCTGCGCTCCCAATGCAGGGGGCCTGGGTTTGATC
CCTAGTCGGGGAA
>Bos_taurus_chr10.trna6506-GlyCCC (46370864-46370792) Gly (CCC) 73 bp Sc: 46.09
TCCCTGACAGTCCAGTGGCTAAGACTCCACGCTCCCAATGTAGGGGGCCCAGGTTCGATC
CTTGGTCAGGGAA
>Bos_taurus_chr27.trna2811-GlyCCC (29148413-29148341) Gly (CCC) 73 bp Sc: 46.10
TCCCTGGTGGTCCAGAGGCTAAGACTGTATGCTCCCAATGCATGGGGCCCAGGTTCAATC
CCTGGTCAGGGAA
>Bos_taurus_chr11.trna8798-GlyCCC (7417223-7417151) Gly (CCC) 73 bp Sc: 46.11
TCCCTGGTGGTCCAGTGGCTAAGACTCTGTGCTCCCAATGCAGAAGGCCTGGGTTCCATT
CCTAGTCAGGGAA
>Bos_taurus_chr10.trna6589-GlyCCC (44838243-44838171) Gly (CCC) 73 bp Sc: 46.12
TCTCTGATGGTCCAGTGGTTAAGACTCTGTGCTCCCAATGCAGGGGGTCCAGGTTTGATC
CCTAGTCAGGGAA
>Bos_taurus_chr10.trna6594-GlyCCC (44695985-44695913) Gly (CCC) 73 bp Sc: 46.12
TCTCTGATGGTCCAGTGGTTAAGACTCTGTGCTCCCAATGCAGGGGGTCCAGGTTTGATC
CCTAGTCAGGGAA
>Bos_taurus_chrUn.004.425.trna5-GlyCCC (54290-54362) Gly (CCC) 73 bp Sc: 46.12
TCCCTGATGGTCCAGTGGCTAAGACTTTGTGCTCCCAATACAGAGGGCCTGGGTTTGATT
CCTGGTCAGGGAA
>Bos_taurus_chrUn.004.5865.trna1-GlyCCC (1879-1951) Gly (CCC) 73 bp Sc: 46.12
TCCCTGATGGTCCAGTGGCTAAGACTTTGTGCTCCCAATACAGAGGGCCTGGGTTTGATT
CCTGGTCAGGGAA
>Bos_taurus_chr28.trna2976-GlyCCC (5902520-5902448) Gly (CCC) 73 bp Sc: 46.13
TCTCTGGTGGTCCGGTGGCTAAGATTCTGTGCTCCCAATGCAGGGGGCCCAGGTTCAATC
CCTGGTCAGGGAA
>Bos_taurus_chr4.trna3267-GlyCCC (98482044-98482116) Gly (CCC) 73 bp Sc: 46.14
TCCCTGGTGGTCCAGTGGTTAAGACTCTGTGCTCCCAATGCAGGGAGCACTGGTTTAATC
CCAGGTCAGGGAA
>Bos_taurus_chr2.trna6218-GlyCCC (123478639-123478567) Gly (CCC) 73 bp Sc: 46.15
TCCCTGGTGGTCTAGGGGTTAGGACTCTGTGTTCCCATTGCAGGGGGCCTGGGTTTGATC
CCCAGTAAGGGAA
>Bos_taurus_chrUn.004.145.trna21-GlyCCC (245781-245853) Gly (CCC) 73 bp Sc: 46.15
TCCCGGGTGGTCCAATGGCTAGGACTCTGAGCTCCCAATGCAGGGGACCCAGGTTAGATC
CCTGGTCGGGGAA
>Bos_taurus_chr19.trna3089-GlyCCC (59941265-59941337) Gly (CCC) 73 bp Sc: 46.15
TCCCTGGTGGTCTAGTGGTTAAGACTCTGTGCTCCCAATGTGGGGAGCCCAGGTTCAATC
CCTGATCAGGGAA
>Bos_taurus_chr19.trna3141-GlyCCC (60602802-60602874) Gly (CCC) 73 bp Sc: 46.15
TCCCTGGTGGTCTAGTGGTTAAGACTCTGTGCTCCCAATGTGGGGAGCCCAGGTTCAATC
CCTGATCAGGGAA
>Bos_taurus_chr2.trna8027-GlyCCC (76560896-76560824) Gly (CCC) 73 bp Sc: 46.24
TCCCTGATGGTCCAGTGGCTTAGACTCTGCTCTCCCAATGCAGGGGGCCTGGGTTTGATC
CCTAGTTAGGGAA
>Bos_taurus_chr2.trna8030-GlyCCC (76493334-76493262) Gly (CCC) 73 bp Sc: 46.24
TCCCTGATGGTCCAGTGGCTTAGACTCTGCTCTCCCAATGCAGGGGGCCTGGGTTTGATC
CCTAGTTAGGGAA
>Bos_taurus_chr8.trna4131-GlyCCC (116074784-116074712) Gly (CCC) 73 bp Sc: 46.24
TCCCTGGTGGTTCAGTGGTTAGAACTCTGTGCTCCCAATGTAGGGGGCACAGGTTCCATC
CCTGGTTAGGGAA
>Bos_taurus_chr13.trna6105-GlyCCC (33384887-33384814) Gly (CCC) 74 bp Sc: 46.30
TCCCTGGTAGTTCAAGTGGCTAAGACTCTGCACTCCCAATGCAGGGGGTCTGGGTTCAAA
TCCTGGTCAGGGAA
>Bos_taurus_chr24.trna743-GlyCCC (22503685-22503757) Gly (CCC) 73 bp Sc: 46.32
TCCCTGGTGGTCCAGTGGCTGAGACTTTGGACTCCCAATGCAGGGGGCTCAGGTTCAATC
CCTGGTCAGGGAA
>Bos_taurus_chr3.trna8894-GlyCCC (15746746-15746674) Gly (CCC) 73 bp Sc: 46.32

TCCCTGATGGTGCAGTGGCTAAGACTTTGTGCTCCCAATGCAGGGGGACCAGGTTCAATC
CCTGGTCGGGGAA
>Bos_taurus_chr6.trna4967-GlyCCC (106185771-106185699) Gly (CCC) 73 bp Sc: 46.36
TCCCTGGTAGTCCAATGGCTAGGACTCTGCGCTCCCAAGGCAGGGAGCCCAGGGTTGATC
CCTGGTCAGGGAA
>Bos_taurus_chr26.trna2458-GlyCCC (40616342-40616270) Gly (CCC) 73 bp Sc: 46.36
TCCCTTATGGTTCAGGGGCTAAGACTCTGCACTCCCCATGCAAGAGGCCCAGGTTCAATC
CCTGGTCAGGGAA
>Bos_taurus_chr23.trna2049-GlyCCC (45844161-45844233) Gly (CCC) 73 bp Sc: 46.37
TCCCTGGTGGTCCAGTGGCTAAGACCCTGTGCTCCCAATACAGGGGGCCCAGGTTCGCTC
CCTGGTTGGGGAA
>Bos_taurus_chr22.trna2602-GlyCCC (52328405-52328333) Gly (CCC) 73 bp Sc: 46.41
TCTCTGGTGGTCCAGCGGCTAAGACTCCGTGCTCCCAATGCGGGGTGCCTAGGTTCAATC
CCTAGTCAGGGAA
>Bos_taurus_chrUn.004.1412.trna1-GlyCCC (4120-4192) Gly (CCC) 73 bp Sc: 46.41
TCTCTGGTGGTCCAGCGGCTAAGACTCCGTGCTCCCAATGCGGGGTGCCTAGGTTCAATC
CCTAGTCAGGGAA
>Bos_taurus_chr6.trna8639-GlyCCC (144013-143941) Gly (CCC) 73 bp Sc: 46.44
TCCCTGGTGGTCCAGTGGCTAAGACTCTGTGCTCCCAATGAAGGGGCCCCAGGTTCAATA
TCTGGTCAGGGAA
>Bos_taurus_chr2.trna712-GlyCCC (23710566-23710638) Gly (CCC) 73 bp Sc: 46.44
TCCCTGGTGGTCCAGTGGCTAAGACTCTGTGCTCCCAATGCAGTGGGCCTGGGTTTGATT
CCTGGTCAGGGAA
>Bos_taurus_chr2.trna9726-GlyCCC (23465275-23465203) Gly (CCC) 73 bp Sc: 46.44
TCCCTGGTGGTCCAGTGGCTAAGACTCTGTGCTCCCAATGCAGTGGGCCTGGGTTTGATT
CCTGGTCAGGGAA
>Bos_taurus_chr8.trna2253-GlyCCC (70724745-70724817) Gly (CCC) 73 bp Sc: 46.46
TCCCTGGTGGTCCAGTGGCCAAGACTCTGCACTCCCAAAGTAGGGGACCCAGGTTCAATT
CCTGATCAGGGAA
>Bos_taurus_chr23.trna2041-GlyCCC (45705744-45705815) Gly (CCC) 72 bp Sc: 46.47
TCCCTGGTAGTCCAGTGGCTAAGACTCTGCGCTCCCAGTGCAGGGGCCCGGGTTCTATCC
CCAGTCAGGGAA
>Bos_taurus_chr26.trna3104-GlyCCC (23988564-23988492) Gly (CCC) 73 bp Sc: 46.55
TCCCTGGTGGTCCAGTGGTTAGAACTCTGTACTCCCACTATAGGGGACACAGGTTAGATC
CCTGTTTAGGGAG
>Bos_taurus_chr21.trna678-GlyCCC (18525857-18525929) Gly (CCC) 73 bp Sc: 46.57
TCCCTGGTGGTTCAGTGGCTAAGACTCTGCACTCCCAATGCAGGGGGCCTGGGGTTCGAT
CCCGGTCAGGGAA
>Bos_taurus_chr8.trna5662-GlyCCC (76276397-76276325) Gly (CCC) 73 bp Sc: 46.58
TCCTTGGTGGTCTGGTGGTTAAGACTCTGCTCTCCCAATGCAGGGGGCCTGGGTTGGATC
CCTAGTCAGGGAA
>Bos_taurus_chr4.trna2521-GlyCCC (79511547-79511619) Gly (CCC) 73 bp Sc: 46.66
TCCCTGGTGGTTCAGTGGCTAAGACTCTGCATTCCCAATGCAGGGGGCCTGGGTTGGATC
CCTGGTCAGGGAA
>Bos_taurus_chr19.trna5679-GlyCCC (22780328-22780256) Gly (CCC) 73 bp Sc: 46.67
TCCCTGGTGGTCCATTGGCTAAGACTCTGTACTCCCAATGCAGGGGACCTGGGTTCAATC
CCTGGTCAGGGAA
>Bos_taurus_chr22.trna2006-GlyCCC (55593543-55593615) Gly (CCC) 73 bp Sc: 46.69
TCCCTTGTGGTCCAGTGGTTAAGACTCTGTGCTCCCAACGCAGGGGGCCTGGGTTGGATC
CCTGGTCAGGGAA
>Bos_taurus_chr17.trna4230-GlyCCC (61707459-61707387) Gly (CCC) 73 bp Sc: 46.73
TTCCTGGTGGTCCAGTGGCTAAGACTCTGAGCTCCCAACACAGGGGGCCCGGGTTCGATC
CCTGGTCAAGGAA
>Bos_taurus_chrX.trna933-GlyCCC (22223589-22223661) Gly (CCC) 73 bp Sc: 46.76
TCCCTGGTAGTCCAGTGGCTAAGACTCTGTGCTCCCAATGCAGGGGGCCCAGGTTAGATC
CTTGGTCAGGGAA
>Bos_taurus_chr5.trna2174-GlyCCC (60021446-60021518) Gly (CCC) 73 bp Sc: 46.77
TCCCTGGTGGTCCAGTGGCTAAGACTCTGTGCTCCCCATGCAGGGGACCCGGGTTCGATC
CCTGGTCAGGGAA
>Bos_taurus_chr10.trna3359-GlyCCC (88076579-88076651) Gly (CCC) 73 bp Sc: 46.78
TCCCTGATGGTCCAGTGGCTAAGACTCTGTTCTCCCAAAGCAGGAGGCCCAGGTGCAATT
CCTGGTCAGAGAG
>Bos_taurus_chr15.trna693-GlyCCC (25473447-25473519) Gly (CCC) 73 bp Sc: 46.81
TCCCTGATGGTCCAGTGACTAAGATTTTGCACTCCCAATGCAGTGGGCCCAGGTTTGATC
CCTAGTCAGGGAA
>Bos_taurus_chr15.trna4114-GlyCCC (58684350-58684278) Gly (CCC) 73 bp Sc: 46.82
TCCCTGGTGGTCCAGTGGCTATGACTCTGCCTTCCCAATGCAGGGGACCCAGGTTCCATA

CCTGGTTAGGGAA
>Bos_taurus_chr13.trna4752-GlyCCC (65935639-65935567) Gly (CCC) 73 bp Sc: 46.84
TCCCTGGTGGTCCAGTGGCTAAGACTCTGCACTCCCAATGCAGGGGGCCTGGGTTCATTC
CCTGCTCAGGGAA
>Bos_taurus_chrX.trna4777-GlyCCC (61452620-61452548) Gly (CCC) 73 bp Sc: 46.86
TCTCTGGTGGTCCAGTGGTTAAGGCTCTGCACTCCCAATGCAGGGGACCCTGGTTTGTTC
CCTGGTCAGAGAA
>Bos_taurus_chr3.trna3744-GlyCCC (106362530-106362603) Gly (CCC) 74 bp Sc: 46.86
TCCCTGGTGGTCCAATGGCTTAAGACTCTGTGCTCCCAATGCAGAGGGTCTGGGTTCGAT
CCCTGGTCAGGGAA
>Bos_taurus_chr10.trna5520-GlyCCC (72442436-72442364) Gly (CCC) 73 bp Sc: 46.87
TCTCTGGTGGTTCAGTGGCTAAGACTCTGCACTCCCAATGCAGGGGGCCTGGGTTTGATC
CCTGGTCAGAGAA
>Bos_taurus_chr10.trna5292-GlyCCC (78806218-78806146) Gly (CCC) 73 bp Sc: 46.89
TCCCTGGTGGTCCAGTGCCTAGGACTCTGAGCTCCCAATGCAGGGGGCCCAGGTTTGATC
CCTGGTCAGGGAA
>Bos_taurus_chr24.trna1401-GlyCCC (35634754-35634826) Gly (CCC) 73 bp Sc: 46.89
TCCCTGGTGGTCCAGTGGCTAAGACTCTGTGCTCCCAATGCAGGGAGCCTGGGTTCAATC
CCTGGTCAGGGAA
>Bos_taurus_chr22.trna3891-GlyCCC (14364467-14364395) Gly (CCC) 73 bp Sc: 46.93
TTTCTGGTGGTCCAGTGGCTAAGGCTCTGCACTCCCAATGCAGGCGGCCCAGGTTCAATC
CCTGGTCAGGGAA
>Bos_taurus_chr19.trna4381-GlyCCC (46704193-46704121) Gly (CCC) 73 bp Sc: 46.95
TCCCTGGTGGTCCAGTGGCTAAGATGCCGCACTCCCAATGTAGGTGGCCCAGGTTCAGTC
CCTGGTCAGGGAG
>Bos_taurus_chr10.trna2739-GlyCCC (72395361-72395433) Gly (CCC) 73 bp Sc: 46.95
TCCCTGATGGTCCAGTGACTAAGATTCCGCACTCCCAATGCAGGGGCCCCAGGTTCAACC
CCTGGACAGGGAA
>Bos_taurus_chr22.trna4150-GlyCCC (9269361-9269289) Gly (CCC) 73 bp Sc: 46.97
TCCCTAGTAGTCCAGTGGCTAAGATGCTGCACTCCCGAAGCAGAGGACCCAGGTTTGATC
CCTGATTGGGGAA
>Bos_taurus_chr5.trna9289-GlyCCC (27325034-27324962) Gly (CCC) 73 bp Sc: 47.01
TCCCTGGCAGTCCAGTGGTTAAGATGCTGTGCTCCCAATGCAGAGGGTCCAGGTTTGAAC
CCTGGTTGGGGAA
>Bos_taurus_chrUn.004.95.trna27-GlyCCC (61339-61267) Gly (CCC) 73 bp Sc: 47.01
TCCTTGGTGGTCCAGTGGCTAAGACCGTGTGCTCCCAATGCAGAGGGCCCAGGTTTGATC
CCTGGCTGGGGAA
>Bos_taurus_chr19.trna818-GlyCCC (18405998-18406070) Gly (CCC) 73 bp Sc: 47.01
TCCCTGGTGGTCCAGTGGCTAAGACTCCACACTCCCAATGTAGGGGACCCAGGTCCGATC
CCCGGTCAGGGAA
>Bos_taurus_chr22.trna1798-GlyCCC (50672334-50672406) Gly (CCC) 73 bp Sc: 47.03
TCCCTGATGGTCCAGTGGCTAAGACTCCACACTCCCAATGTAGGGAGCCAGGGTTCTAGC
CCTTGTCAGGGAA
>Bos_taurus_chr19.trna5367-GlyCCC (28202733-28202661) Gly (CCC) 73 bp Sc: 47.04
TCCCTGGTGGTCCAGTGGCTAAGACTCTGCACTCCCAATGCATGGGGTCCAGGTTTGATC
CCCGGTCAGGGAA
>Bos_taurus_chr13.trna2704-GlyCCC (66149849-66149921) Gly (CCC) 73 bp Sc: 47.04
TCCCTGGTGGCCCAGTGGCTAAGACTCTGCGCTCCCTATGCAGAGGGCCTAGGTTCAATC
CCTGGTCAGGGAA
>Bos_taurus_chr1.trna4511-GlyCCC (130184259-130184331) Gly (CCC) 73 bp Sc: 47.07
TCCCTGGTGGTTTAGTGGCTAAGACTCTGAGCTCCCAGTGCAGGGGGCCCAGGTTCGATC
CCTGGTCAGGGAG
>Bos_taurus_chr17.trna4036-GlyCCC (63991970-63991898) Gly (CCC) 73 bp Sc: 47.09
TCCCTGGTGGTCCAGCGGCTAAGATCCTGTGCTCCCAGTGCAGGAGTCCCAGGTTCAATC
CCTGGTCAGGGAA
>Bos_taurus_chr19.trna5656-GlyCCC (23099407-23099336) Gly (CCC) 72 bp Sc: 47.11
TCCTTGGTGGTCCAGTGGCTGGGACTCTGTACTCCCAAGGTGGGGGCCCAGGTTCAATCC
CTGGTCAGGGAA
>Bos_taurus_chr29.trna3872-GlyCCC (6067163-6067091) Gly (CCC) 73 bp Sc: 47.13
TCCCTGGTGGTCTAGTGGCTAAGATTCTGTGCTCCCAATGCAGGAACCCCTGGTTCGAGC
CCTGGTCAGGGAC
>Bos_taurus_chr3.trna8548-GlyCCC (22986169-22986099) Gly (CCC) 71 bp Sc: 47.14
GCACTGGTGGTTTAATGGTAGAATTCTCACCTCCCATGTGGGAGACCTGGGTTTGATTCC
TGGCCAGTGTA
>Bos_taurus_chr19.trna5207-GlyCCC (30725426-30725353) Gly (CCC) 74 bp Sc: 47.14
TCCCTGATGGTTCAGTGGCTAAAACTCTGCACTCCCAATGCAGAGGGGTCTGGGTTCAGT
CCCTGGTCAGGGAA

>Bos_taurus_chr13.trna2493-GlyCCC (62899961-62900033) Gly (CCC) 73 bp Sc: 47.14
TCCCTGGTGGTTCAGTGGCTAAGACTCTGCTCTCCCAATGCAGGGGGCCCGGGTTCCATC
CCTGGTCAGGGAA
>Bos_taurus_chr1.trna2435-GlyCCC (71048518-71048590) Gly (CCC) 73 bp Sc: 47.30
TCTCTGGTGGTCCAGCGGCTAAGACACTGCTCTCCCAGTGCAGAGGGCCCAGGTTCAATC
CCTGGTCACAGAA
>Bos_taurus_chr25.trna1396-GlyCCC (25063431-25063502) Gly (CCC) 72 bp Sc: 47.32
TCCCCAGTGGTCCAGTGGCTAGGACTCTGCGTTCCCAGTGCCGGGGCCCAGGTTCCATCC
CTGGTTGGGGAA
>Bos_taurus_chr7.trna7902-GlyCCC (13961725-13961653) Gly (CCC) 73 bp Sc: 47.37
TCCTTGATGGTCCAGTGGCTGAGACTCTGCTCTCCCAATGCAGAGGGGCCAGGTTCCATC
CCTGGTCAGGGAA
>Bos_taurus_chr7.trna229-GlyCCC (5471932-5472004) Gly (CCC) 73 bp Sc: 47.37
TCCCTTATGGTCCAGTGGCTAAGACTCTGAGTTCCCAATGCAGAGAGCCCAGGTTTGATC
CCTGGTCAGGGAA
>Bos_taurus_chr15.trna2153-GlyCCC (61368836-61368908) Gly (CCC) 73 bp Sc: 47.38
TCCCTGGTGGTCCAGTGGCTAAGACTCTGTGCTCCCAATGCAGGGGGCCCAGGTTTGATC
CCTTGTCAGGGAA
>Bos_taurus_chr11.trna10-GlyCCC (222960-223032) Gly (CCC) 73 bp Sc: 47.40
CCCCTGGTGGTCCAGTAGCTAAGACTCTGAACTCCCAATACAGGGTGCCCAGGTTCGATC
CCTGGTCAGGGAA
>Bos_taurus_chr19.trna5010-GlyCCC (34161709-34161637) Gly (CCC) 73 bp Sc: 47.42
TTCCTGGTGGTCCAGTGGTTAAGACTCTGTACTCCCAATGCAAGGGGCACAGGTTAGATC
CCTGGTCAGGAAA
>Bos_taurus_chr26.trna2864-GlyCCC (31781496-31781425) Gly (CCC) 72 bp Sc: 47.44
TCCCTGGTGGTCCAGTGGCTAAGACTCTGCAATCCCAGTGCAGGGACCCAGGTTCGATCC
CTGGTCAAGGAT
>Bos_taurus_chr23.trna115-GlyCCC (3007805-3007877) Gly (CCC) 73 bp Sc: 47.44
TCCCTGGTGGTCCAGTGGCTACGACTCTGTGCTCCCAATGCAGGGGGCCTGGGTTCAATC
TCCGGTCAGGGAA
>Bos_taurus_chr11.trna3400-GlyCCC (84483945-84484017) Gly (CCC) 73 bp Sc: 47.45
TCACTGGTGGTCCAGTGGCTAAGACTCTGTGCTCCCAATGCAGGGGACCTAGGTTCGATC
CCTGGTCAGAGAA
>Bos_taurus_chr10.trna860-GlyCCC (19964000-19964071) Gly (CCC) 72 bp Sc: 47.45
TTTCTGATGGTCCAGTGGCTAAGACTCTGCACTCCCAATGCAGGGGGCTGGGTTCAATCC
CTGGTCAGGGAA
>Bos_taurus_chr11.trna9197-GlyCCC (228439-228367) Gly (CCC) 73 bp Sc: 47.52
TCCCTGGTGGTCCAGTGGCCGAGACTCAGCACTCCCGGTGCAGGGGTCCCAGGTTCAATC
CCTGGTCAGGGAA
>Bos_taurus_chrUn.004.998.trna9-GlyCCC (7739-7668) Gly (CCC) 72 bp Sc: 47.57
TCCTTGGTGGTCCAGTGGCTAAGACTCAGCACTCCCAATGCAGGGGCCCAGGGTCAATCC
CTAGTCAGGGAA
>Bos_taurus_chr1.trna7968-GlyCCC (98463656-98463583) Gly (CCC) 74 bp Sc: 47.57
TCCCTGGTGGTCCAGTGGCTAAGACTCTGTGCTCCCAATGCAGGGGACCCCGGGTTCGAT
CCCTGGTCAGGGAA
>Bos_taurus_chr25.trna4919-GlyCCC (2335958-2335886) Gly (CCC) 73 bp Sc: 47.59
TCCTTGGTGGTCCAGTGGCTAAGACTCTGCGTTCCCAATGCAGGGGGCCCAGGTTCCATC
CCTGGTCAGGAAA
>Bos_taurus_chr2.trna1342-GlyCCC (41265404-41265476) Gly (CCC) 73 bp Sc: 47.60
TCCCCAATGGTCCAGTGGCTAAGACTCTGCACTCCCAATGCAGGGGGCCAGGGTTCTGTC
CCTGGTTGGGGAA
>Bos_taurus_chr17.trna5467-GlyCCC (36945300-36945228) Gly (CCC) 73 bp Sc: 47.62
TCCCTGGTGGTCCAGTGGCTAAGACTCTGTGCTCCCAGTGCAGGGGGCCCAGGTTTGATT
CCTGGTCAGGGAA
>Bos_taurus_chr21.trna3438-GlyCCC (56770275-56770203) Gly (CCC) 73 bp Sc: 47.62
TCCCTGGTGGTGCAGTGGCTAAGACTCTGCATTCCCAATACAGGAGGCCCAGGTTCAATC
CCTGGTCAGGGAT
>Bos_taurus_chr10.trna527-GlyCCC (12640119-12640191) Gly (CCC) 73 bp Sc: 47.63
TCCCTGGTGGTCCAGCAGTTAAGACTCTGTGCTCCCAATGCAGGGGGCCCAGGTTCGATC
TCTGGTCAGGGAA
>Bos_taurus_chr16.trna6201-GlyCCC (683162-683090) Gly (CCC) 73 bp Sc: 47.64
TCCCTGGTGGTCCAGTGGCCAGGACTCTGTGCTCCCAGTGCAGGGGACCCAGGTTCAATT
CCTGGTCAGGTAA
>Bos_taurus_chr3.trna4456-GlyCCC (120518070-120518142) Gly (CCC) 73 bp Sc: 47.67
TCCCTGGTGGTTCAGTGGCAAAGACTCTGCACTCCCAATGCAGAGGGTCCAGGTTTGATC
CCTGATCAGGGAA
>Bos_taurus_chr6.trna125-GlyCCC (5775391-5775463) Gly (CCC) 73 bp Sc: 47.72

TCCCTGGTGGTCGAGTGGCTAAGACTCTGCGCTCCCAATGCAGGGGATCCAGGTTTGCTT
CCTGGTCAGGGAA
>Bos_taurus_chrUn.004.337.trna13-GlyCCC (113653-113582) Gly (CCC) 72 bp Sc: 47.74
TCCCTGGTGGTCCAGTGCTTAAGACTCTGCACTCCCAATGCAGGGGCCTGGGTTTGATCC
CTGGCCAGAGAA
>Bos_taurus_chrUn.004.494.trna1-GlyCCC (47511-47582) Gly (CCC) 72 bp Sc: 47.74
TCCCTGGTGGTCCAGTGCTTAAGACTCTGCACTCCCAATGCAGGGGCCTGGGTTTGATCC
CTGGCCAGAGAA
>Bos_taurus_chr28.trna3197-GlyCCC (475235-475163) Gly (CCC) 73 bp Sc: 47.77
TTCCTGGTGGTTCAGTGGCTAAGACTCTGTGCTCCCAATGCAGGGGACCCAGGTTCAATC
CCTGGTCAGGGAA
>Bos_taurus_chrX.trna2108-GlyCCC (59367170-59367242) Gly (CCC) 73 bp Sc: 47.78
TCCATGGTGGTCCAGTGGTTAAGACTATGTGCTCCCAATGCAGGGGACCCAGGTTCAAGC
CCTGGTCAGGGAA
>Bos_taurus_chr13.trna6584-GlyCCC (24871216-24871144) Gly (CCC) 73 bp Sc: 47.79
TCCCTGGTGGTCCAGTAGCTAAGACACTGTGCTCCCAATGCAGGGGTCCTGGGTTCAATT
CCTGGTCAGGGAA
>Bos_taurus_chr13.trna3172-GlyCCC (73947299-73947371) Gly (CCC) 73 bp Sc: 47.81
CCCCTGGTGGTCCAGTGGCTAAGACTCTGTGCTCCCAATGCAGGGGGCCCAGGTTCCATC
CCTGGTCAGGGAA
>Bos_taurus_chr29.trna490-GlyCCC (13012140-13012212) Gly (CCC) 73 bp Sc: 47.84
TCTCTGGTGGTCCAGTGGCTAAGACTCTGAGCTCCCAATGCAGAAGGCCCAGGTTCAACC
CCTGGTCAGGGAA
>Bos_taurus_chrUn.004.429.trna12-GlyCCC (9973-9901) Gly (CCC) 73 bp Sc: 47.84
TCTCTGGTGGTCCAGTGGCTAAGACTCTGAGCTCCCAATGCAGAAGGCCCAGGTTCAACC
CCTGGTCAGGGAA
>Bos_taurus_chr2.trna5145-GlyCCC (140127916-140127987) Gly (CCC) 72 bp Sc: 47.84
TCCCTGGTGGTCCAGTGGCTAAGACTCCGTACTCCCAATGCAGGGGCCCAGGTACGACCC
CTGGTCTGGGAA
>Bos_taurus_chr7.trna2246-GlyCCC (50554101-50554173) Gly (CCC) 73 bp Sc: 47.87
TCCCTGGTGGTCCAGTGGCTAAGACTCTGAGCTCCCAATGCAGGGGGTCCAGGTTCCATC
CCTGGTCAGGGAA
>Bos_taurus_chr7.trna2174-GlyCCC (48833799-48833871) Gly (CCC) 73 bp Sc: 47.87
TCTCTGATGGTCCAGTGGTTAAGACTCTGCACTCCCAAGGCAGGGGGCCTGGATTCAATC
CCTGGTCAGGGAA
>Bos_taurus_chr20.trna128-GlyCCC (3509510-3509582) Gly (CCC) 73 bp Sc: 47.88
TCCTTGGTGGCCCAGTGGTTAAGACTCTGCACTCCCCATACAGGGGCCCCAGGTTTGATA
CCTGGTCAGGGAA
>Bos_taurus_chr15.trna1005-GlyCCC (31416464-31416536) Gly (CCC) 73 bp Sc: 47.88
TCCCTGGTGGTCCAGTGGCTAAGACTGTGCACTCCCAATGCAGGGGGCCTGGGTTCAACC
CCTGGTCAGGGAA
>Bos_taurus_chr22.trna4170-GlyCCC (8758114-8758042) Gly (CCC) 73 bp Sc: 47.91
TCCCTGGTGGTCCAGTGGCTAAGACTCTGCACTCCCAATGCAGGGGGTCTGGGCTCAATC
CCTGGCTGGGGAA
>Bos_taurus_chr23.trna4093-GlyCCC (16492894-16492821) Gly (CCC) 74 bp Sc: 47.91
TCCCTGGTGGTTCAGTGGCTAAGAGCTCTGAGCTCCCAATGCAGAGGGCTCAGGTTCAAT
CCCTGGTCAGGGAA
>Bos_taurus_chrX.trna4908-GlyCCC (56430125-56430053) Gly (CCC) 73 bp Sc: 47.95
TCCCTGGTGGTCCAGTGTTTAAGATTCCGTGATCCCAATGCAGGGGTCCCAGGTTCGATC
CCTGGTCAGGGAA
>Bos_taurus_chr1.trna4054-GlyCCC (118703306-118703378) Gly (CCC) 73 bp Sc: 48.01
TCCCTCATGGTCCAATGGCTAAGACTCTGAGCTCCCAATGCAGAGGGCCAGGGTTAGATC
CCCAGTGAGGGAA
>Bos_taurus_chr19.trna4616-GlyCCC (43457325-43457253) Gly (CCC) 73 bp Sc: 48.02
TCCCTGGTAGTCCAATGGCTAAGACTCTGCACTCCCAATGCAGGGAGCTTGGGTTCAATC
CCTGGTCAGGGAA
>Bos_taurus_chr16.trna239-GlyCCC (8654108-8654180) Gly (CCC) 73 bp Sc: 48.03
TCCCTGATAGTCCAGTGGTTAAGACTCTGTGCTCCCAATTCAGGGGGCCTGGGTTTGATC
CCTGGTCAGGGAA
>Bos_taurus_chr14.trna713-GlyCCC (15928767-15928839) Gly (CCC) 73 bp Sc: 48.05
TCCCTGGTGGTCCAGTGGCTAAGACTCTGTGCTCCCAATGCAGGCGGCCCAGGTTTGATC
CCTGGTCAGGGAA
>Bos_taurus_chr1.trna2539-GlyCCC (73794786-73794858) Gly (CCC) 73 bp Sc: 48.05
TTCCTGGTGGTCCAGTGGCTAAGACTCTTCACTCCCAATGCAGGGAGCCTGGGTTCAATC
CCTGGTCAGGAAA
>Bos_taurus_chr25.trna2963-GlyCCC (35054045-35053973) Gly (CCC) 73 bp Sc: 48.07
TCCCTAGTGGTCCAGTGGATAGGACTCTATGCTCCCAATGCAGGGCGCCCAGGTTCGATC

CCTGGTCAGGGAA
>Bos_taurus_chr23.trna3971-GlyCCC (18307267-18307195) Gly (CCC) 73 bp Sc: 48.08
TCCCTGGTGGTCCAGTGGCTAAGACTCTGCGCTCCCAACGCGTGGAGCCCAGGTTTAATC
CCTGGTCAGGGAA
>Bos_taurus_chr5.trna6590-GlyCCC (95566402-95566330) Gly (CCC) 73 bp Sc: 48.10
TCCCTGGTGGTCCAGTGGCTAAGATTCTGAACCCCCAATGCAGGGGACCCAGGTTTGATC
CCTGGTTAGGGAA
>Bos_taurus_chr7.trna6194-GlyCCC (57520855-57520783) Gly (CCC) 73 bp Sc: 48.11
TCCCTGGTGGTCTAATGGCTAAGACTCTGAGCTCCCAGTGCAGGGGGCCCAGGTTCGATC
CCTGGTCAGGGAG
>Bos_taurus_chrX.trna6808-GlyCCC (721327-721255) Gly (CCC) 73 bp Sc: 48.11
TCCCTGGTGGTCCAGTGGCTAAGACTCTGCACTCCCAAGGCAGAGGGCCCAGGTTTGACC
CCTGGTCAGGAAC
>Bos_taurus_chr11.trna311-GlyCCC (5368226-5368298) Gly (CCC) 73 bp Sc: 48.14
TCCCTGGTGGTCTAATGGCTAGAACTCCGTACTCCCAATGCAGGGAGTCCAGGTTCAATC
CCTGGTCAGGGAA
>Bos_taurus_chr11.trna7649-GlyCCC (38303002-38302931) Gly (CCC) 72 bp Sc: 48.16
TCCCTGGTGGTCCAGTGGCTAAGACTCTATGTTCCCAATGCAGGGGGCCAGGTTCAATCC
CTGGTCAGGGAA
>Bos_taurus_chr23.trna1464-GlyCCC (32744289-32744361) Gly (CCC) 73 bp Sc: 48.17
TCCCTGGTGGTCCAGGGGCTAAGAATCTGCACTCCCAATGCAGGGGGCCTGGGTTCGATC
CCTGGTCAGGGAA
>Bos_taurus_chr12.trna6599-GlyCCC (11351028-11350956) Gly (CCC) 73 bp Sc: 48.20
TCCCTGGTGGTCCAGTGGCTAAGACTCTGTGCTCCCAATGCAGGGGCCACAGGTTTGATC
CCTGCTCAGGGAA
>Bos_taurus_chr16.trna1916-GlyCCC (49576685-49576757) Gly (CCC) 73 bp Sc: 48.23
GCCCTGGTGGTCCAGTGGTTAAGACTCTGAGTTCCCAATGCAGGGGGCCCAGGTTTGATC
CCTGGTCAGGGAA
>Bos_taurus_chr3.trna790-GlyCCC (21949487-21949559) Gly (CCC) 73 bp Sc: 48.25
TCCCTGGTGGTCCAGTGGGCAAGACTCTGTACTCCCAATGCAAGAGACCCAGGTTCAATC
CCTGGTCAGAGAA
>Bos_taurus_chr26.trna1168-GlyCCC (34715753-34715825) Gly (CCC) 73 bp Sc: 48.25
TCCCTGGTGGTCCAGTGGCTAAGACTCTGTGTTCCCAATGCAGGGGGCCTGGGTTCAATC
CCCGATCAGGGAA
>Bos_taurus_chr1.trna4485-GlyCCC (129819879-129819951) Gly (CCC) 73 bp Sc: 48.31
TCCCTGATGGTCCAGGGACTATGACCCTGTGCTCCCAATGCAGGGGGCCCAGGTTCGATC
CCTGGTCAGGGAA
>Bos_taurus_chrUn.004.213.trna5-GlyCCC (25907-25978) Gly (CCC) 72 bp Sc: 48.34
TCTTTGGTGGTCCAGTGGCTAAGACTCTGAACTCCCAATTCAGGAGCCTGGGTTTGATCC
CCGGTCAGGGAA
>Bos_taurus_chrUn.004.6.trna36-GlyCCC (678217-678288) Gly (CCC) 72 bp Sc: 48.34
TCTTTGGTGGTCCAGTGGCTAAGACTCTGAACTCCCAATTCAGGAGCCTGGGTTTGATCC
CCGGTCAGGGAA
>Bos_taurus_chr24.trna3046-GlyCCC (58610910-58610837) Gly (CCC) 74 bp Sc: 48.36
TCCCTGGCGGTCCAGTTGGCTAAGACTCTGTGCTCCCAATGCAGAGGGTTCAGGTTCGAA
CCCTGGTCAGGGAA
>Bos_taurus_chr25.trna1688-GlyCCC (28978163-28978235) Gly (CCC) 73 bp Sc: 48.36
TCCCTGGTGGTCCAGTGGCCAACACTCTGCACTCCCAATGCAGGGGGCCCAGGTTTGATC
CTTGGTCAGGGAA
>Bos_taurus_chr9.trna1409-GlyCCC (43673355-43673427) Gly (CCC) 73 bp Sc: 48.38
TCCCTGGTGGTCCAGTGGCTAAGATTCTCCACTCCCAATAGAGGGAGCCCAGGTTCAATG
CCTGGTCATGGAA
>Bos_taurus_chr2.trna6187-GlyCCC (123909512-123909441) Gly (CCC) 72 bp Sc: 48.38
TCCCTGGTGGTCCAGTGGCTAAGACTTTGTGCTCCCAATGCAAAGGGCTGGGTTCAATCC
CTGGTCAGGGAA
>Bos_taurus_chr17.trna590-GlyCCC (15559505-15559577) Gly (CCC) 73 bp Sc: 48.41
TACCTGGTGGTTCAGTGGCTAAGACTCTGCGCTCCCAACGCAGGGGACCCAGGTTCAATC
TCTGGTCAGGAAA
>Bos_taurus_chr21.trna4830-GlyCCC (21601917-21601845) Gly (CCC) 73 bp Sc: 48.41
TCCCTGGTGGTCCAGTGGCTAAGACTCTGTGCTCCCAGTGCAGGGAATCCGGGTTCGATC
CCCGGTCAGGGAA
>Bos_taurus_chr19.trna317-GlyCCC (10434237-10434309) Gly (CCC) 73 bp Sc: 48.45
CCCCTGGTGGTCCAGTGGCTAAGACTCTGTGCTCCCAATGCAGGGGGCCCAGGTTTGATC
CCTGGTCAGGGAA
>Bos_taurus_chr17.trna2732-GlyCCC (63807910-63807982) Gly (CCC) 73 bp Sc: 48.50
TCCCTGGTGGTCCAGTGGTTAAGGCTCTTCACTCCCAATGCAGGGGGCCCAGGTTCAGTA
CCTGGACAGGGAA

>Bos_taurus_chr9.trna1998-GlyCCC (63275689-63275761) Gly (CCC) 73 bp Sc: 48.51
TCCCTGGTGGTCCAGTGGCTTGGACTCTGGGCTCCCAATGCAGAGTGCCCAGGTTTGATC
CCTGGTCAGGGAA
>Bos_taurus_chr12.trna6496-GlyCCC (13260567-13260495) Gly (CCC) 73 bp Sc: 48.52
TCCCTGGTGGTCCAGTGGCTAAGACTCTGTACCCCCACTGCAGGGAGCCTGGGTTCGATC
CCTGGTCAGGGAA
>Bos_taurus_chr13.trna555-GlyCCC (15986706-15986778) Gly (CCC) 73 bp Sc: 48.54
TCCCTGGTGGTCCAGTGGCTAAGACTCTCCACTCCCAATGCAGGGGGCCCGGATTCAATC
CCTGGTCAGGGAA
>Bos_taurus_chrX.trna3364-GlyCCC (86584453-86584533) Gly (CCC) 81 bp Sc: 48.57
TCCCTGGTAGTCCAGTGGCTAAGACTCTGCACTCCCAATGCAGGGGGGAGGGGGGTCCAG
GTTAGATTCCTGGTCAGGGAA
>Bos_taurus_chr29.trna1841-GlyCCC (48326400-48326472) Gly (CCC) 73 bp Sc: 48.57
TCCCTGGTGGTCCAGAGGCTAACACTCTGCACTCCCAATGCAGGGGACCTGGGTTCAATC
CCTGGCCAGGGAA
>Bos_taurus_chr4.trna4326-GlyCCC (122589726-122589797) Gly (CCC) 72 bp Sc: 48.61
TCCCTGGTGGTCCAGTGGCTAAGAGTGTGCACTCCCAATGCAGGGGGCTGGGTTCAATCC
CCGGTCAGGGAA
>Bos_taurus_chr2.trna421-GlyCCC (14734046-14734118) Gly (CCC) 73 bp Sc: 48.62
TCCCTGGTGGTCCAGTGGCTAAGACTCTGAGCTCCCAGTGCAGGGGGCCCAGGTTCAATC
CCTGGTCAGGGAG
>Bos_taurus_chr1.trna3425-GlyCCC (99701480-99701552) Gly (CCC) 73 bp Sc: 48.63
TCCCTGATGGTCCAGTGGCTAAGACTCTGCGCTCCCAATGCAGGGAGCTTGGGTTCAATC
CCTGGTCAGGGAA
>Bos_taurus_chr9.trna745-GlyCCC (25147292-25147364) Gly (CCC) 73 bp Sc: 48.65
TCCCTGGTGGTCCAGTGGTTGAGACTCTGTGCTCCCAATGCAGGGGGCCCAGGCTCGATC
CCTGGTCAGGGAA
>Bos_taurus_chr19.trna2985-GlyCCC (58424620-58424692) Gly (CCC) 73 bp Sc: 48.65
TCCCTGATGGTCCAGCGGGTAAGGCTCTGCACTCCCGGTGCGGGGGGCCCAGGATCGATC
CCTGGTCGGGGAA
>Bos_taurus_chr19.trna1807-GlyCCC (35607273-35607345) Gly (CCC) 73 bp Sc: 48.66
TTCCTGGTGGTCCAGTGGCCAAGACTCTGCACTCCCACTGCAGGGGACCCAGGTTTGATC
CCTGGTTGGGGAA
>Bos_taurus_chr6.trna4928-GlyCCC (106576023-106575951) Gly (CCC) 73 bp Sc: 48.67
TCCCTGGTGGTCCAGTGGCTAAGACTCAGTGCTCCCAATGCAGGGAACCCAGGTTCAATC
CCTGGTCAGGGAA
>Bos_taurus_chr2.trna5126-GlyCCC (139789553-139789624) Gly (CCC) 72 bp Sc: 48.68
TCCCTGGCAGTCCAGTGGTTAAGACTCTGCACTCCCAGTGCAGGGGCCTGGGTTCAATCC
CTGGTCAGGGAA
>Bos_taurus_chr8.trna329-GlyCCC (8751276-8751348) Gly (CCC) 73 bp Sc: 48.69
TCCCTGGTAGTCCAGCGGCTAAGACTCTGTACTCCCAATGCAGGGAGCCTGGGTTCAGTC
CCCGGTCAGGGAA
>Bos_taurus_chr8.trna7940-GlyCCC (8622712-8622640) Gly (CCC) 73 bp Sc: 48.69
TCCCTGGTAGTCCAGCGGCTAAGACTCTGTACTCCCAATGCAGGGAGCCTGGGTTCAGTC
CCCGGTCAGGGAA
>Bos_taurus_chr18.trna4608-GlyCCC (35645495-35645423) Gly (CCC) 73 bp Sc: 48.73
TCCCTGGTAGTCCAGTGGCTAAGACTCTGCACTCCCAATGCACGGGGCCCAGATTCCATT
TCTGGTCAGGGAA
>Bos_taurus_chr19.trna1744-GlyCCC (34153255-34153327) Gly (CCC) 73 bp Sc: 48.74
TCCCTGGTAGTCCAGTGGCTAGGACTCTGCACTCCCAGTGTGGTGGGCCCAGGTTCAATC
CTTGGTTAGGGAA
>Bos_taurus_chr19.trna4576-GlyCCC (44025972-44025900) Gly (CCC) 73 bp Sc: 48.74
TCCCTGGTGGTCCAGTGGCTAAGACTATGCACTCCCAATGCAGGGGCCCTGGGTTTGATT
CCTGGTCAGGGAG
>Bos_taurus_chr16.trna1339-GlyCCC (37885052-37885124) Gly (CCC) 73 bp Sc: 48.76
TCCCTGGTGGTCCAGTGGCTAAGACTCTGTACTCCCAATGTAGGGGACCCAGGCTCAATC
CCTGGTCAGGGAA
>Bos_taurus_chr17.trna4824-GlyCCC (53129944-53129872) Gly (CCC) 73 bp Sc: 48.79
TTCCTGATGATCCAGTGGCTAAGATTTTGTGCTCCCAATGCAGGGGGCCCAGGTTCGATC
CCTGGTCAGGGAA
>Bos_taurus_chr25.trna4553-GlyCCC (8576447-8576374) Gly (CCC) 74 bp Sc: 48.79
TCCCTGGTGGTCCAGTGGCTAAGACCCCGCTCTCCCAAAGCAGGGGGCCCGGGGTTCGAT
CCCCGGTCAGGGAA
>Bos_taurus_chr15.trna4174-GlyCCC (57151423-57151351) Gly (CCC) 73 bp Sc: 48.81
TCCCTGGAGGTTCAGGGGCTAAGACTTTGCACTCCCAATGCAGGGGGCCCAGGTTCAATC
CCTGGTCAGGGAA
>Bos_taurus_chr8.trna1043-GlyCCC (30035980-30036052) Gly (CCC) 73 bp Sc: 48.83

TCCCTGGTAGTCTAGTGGCTAAGACCCTGTACTCCCAATGCAGGGGGCCCAGGTACAATC
CCTGGTCATGGAA
>Bos_taurus_chrUn.004.5916.trna1-GlyCCC (2375-2303) Gly (CCC) 73 bp Sc: 48.83
TCCCTGGTAGTCTAGTGGCTAAGACCCTGTACTCCCAATGCAGGGGGCCCAGGTACAATC
CCTGGTCATGGAA
>Bos_taurus_chr10.trna7109-GlyCCC (28297900-28297828) Gly (CCC) 73 bp Sc: 48.88
TCTCTGGTGGTCCAGTGGCTAAGACTCTGCACTCCCAATGCAGGAGGTCTGGGTTCAATC
CCTGGTCAGAGAG
>Bos_taurus_chr13.trna1499-GlyCCC (35676048-35676120) Gly (CCC) 73 bp Sc: 48.91
TCCTTGGTGGTCCAGTGGCTAAGACTCTGTGCTCCCAATGCAGGAGGCCCAGGTTCAATC
CCTGGTCATGGAA
>Bos_taurus_chr19.trna5570-GlyCCC (24423327-24423255) Gly (CCC) 73 bp Sc: 48.92
TCCTTGGTGGTCCAGTGTCTGAGACTCTGCACTCCCAATGCAGGGGACCCAGGTTCAATA
CCTGGTCAGGGAA
>Bos_taurus_chr11.trna3254-GlyCCC (80802946-80803018) Gly (CCC) 73 bp Sc: 48.92
TCCCTGATGGTCCAGTGGCTAAGATACCATGCTCCCAATGCGGGGGGCCCAGGTTTGATC
CCTGGTCAGGGAA
>Bos_taurus_chr8.trna496-GlyCCC (11606049-11606121) Gly (CCC) 73 bp Sc: 48.95
TCCGTGGTGGTCCAGTGGTTAAGACTCTGAGCTCCCGATTCAGAGGGCCTAGGTTCGATC
CTTGGTCAGGGAG
>Bos_taurus_chr10.trna6895-GlyCCC (35718235-35718163) Gly (CCC) 73 bp Sc: 48.98
TCCCTGGTGGTCCAGTGGCTAAGACTCTGAACTCCCAATGCAGGGGGCCTGAGTTCAATC
CTTGGTCAGGGAA
>Bos_taurus_chr10.trna1820-GlyCCC (47286676-47286748) Gly (CCC) 73 bp Sc: 49.00
TCCCTGGTGGTCCAGTGGCTAAGACTCTGCGCTCCCAATGCAGGGGGTCTGGGTTCAATC
CCTGGTCAGGGAA
>Bos_taurus_chr5.trna4483-GlyCCC (113384896-113384968) Gly (CCC) 73 bp Sc: 49.02
TCCCTGGTGGTCCAGTGGCTTAGACTCTGCACTCCCATTGTGGGGGGCCCAGGTTCGATC
CCTGGGCAGGGAA
>Bos_taurus_chr21.trna1284-GlyCCC (27439823-27439895) Gly (CCC) 73 bp Sc: 49.02
TCCCTGGTGGTCCAGTGGTTAAGACTCTACATTCCCGATGCAGAGAGCCCAGGTTCAATC
TCTGGTAGGGGAA
>Bos_taurus_chr13.trna2418-GlyCCC (61451691-61451763) Gly (CCC) 73 bp Sc: 49.08
TCCCTGGTGGTCCAGTGGCTAAGGCTCTGAGCTCCCAGTGCAGAGGGCCCAGGTTCGATC
CCTGGTCAGGGAA
>Bos_taurus_chr11.trna1831-GlyCCC (45840722-45840793) Gly (CCC) 72 bp Sc: 49.09
TCCCTGGTGGTCCAGTGGCTAAGACTCTGTGCTCCCAATGCAGGGGGCCTGGATCAATCC
CTGGTCAGGGAA
>Bos_taurus_chr3.trna676-GlyCCC (20216530-20216602) Gly (CCC) 73 bp Sc: 49.10
TCCCTGGTGGTCCAGGGGCTAAGACTCTGCATTCCCAATGCAGGGATCCTGGGTTCAATC
CTTGGTCGGGGAA
>Bos_taurus_chr16.trna3620-GlyCCC (66699182-66699110) Gly (CCC) 73 bp Sc: 49.12
TCCCTGGTGGTCCAGTGGTTAAGATTCTGCGCTCCCAAGGCAGAGGGCCCGGGTTTGATC
CCTGATCAGGGAA
>Bos_taurus_chr15.trna2583-GlyCCC (73153598-73153670) Gly (CCC) 73 bp Sc: 49.13
TCCCTGGTGGTCCAGTGGCTAGGGCTCTGTGCTCCCAATGCAGGGACTCTGGGTTCAATC
CCCTGTCAGGGAA
>Bos_taurus_chr3.trna3995-GlyCCC (111878977-111879049) Gly (CCC) 73 bp Sc: 49.14
TCCCTGATGGTCCCGTGGCTAAGACTCTGAGTTCCCATTACAGGGGCCCCAGGTTCAATC
CCTGGTCAGGGAA
>Bos_taurus_chr18.trna1310-GlyCCC (33074248-33074320) Gly (CCC) 73 bp Sc: 49.15
ACTTTGGTAGTCCATTGGCTAAGACTCTGCACTCCCAATGCAGGGGGCCCAGGTTCAATC
CCTGGTCAGGGAG
>Bos_taurus_chr18.trna4618-GlyCCC (35527590-35527518) Gly (CCC) 73 bp Sc: 49.16
TCCCTGGTGGTCCAGTGGCTGGGACTCTGCGCTCCCAATGCAGGGGGCCTGGGTTCGATC
CCTGGTCAGGGAA
>Bos_taurus_chr19.trna1018-GlyCCC (21681407-21681478) Gly (CCC) 72 bp Sc: 49.17
TCCCTGGTGGTTCAGCGGCTAAGACTCAGCACTCCCAATGCAGGGTCCTGGGTTCAATCC
CTAGTCAGGGAA
>Bos_taurus_chr15.trna3337-GlyCCC (77763365-77763293) Gly (CCC) 73 bp Sc: 49.17
TCCCTGGTGGTCCAGTGGCTAAGACTCTGAGCTCCCAGTGCAGGGGGCCCAGGTTCGATC
CCTGGTCGGGGAG
>Bos_taurus_chr20.trna412-GlyCCC (11079771-11079843) Gly (CCC) 73 bp Sc: 49.17
TCCCTGGTGGTCCAGTGGCTAAGACTCTGAGCTCCCAGTGCAGGGGGCCCAGGTTCGATC
CCTGGTCGGGGAG
>Bos_taurus_chr8.trna4401-GlyCCC (108218040-108217968) Gly (CCC) 73 bp Sc: 49.22
TCCCTGGTAGCCTAGTGGCTAAGGCTCTGCACTCCCCATGCAGGGGGCCCAGGTTCTATC

CCTGGTTGGGGTA
>Bos_taurus_chr3.trna4481-GlyCCC (120967723-120967795) Gly (CCC) 73 bp Sc: 49.25
TCCCTGGTGGTCCAGTGGCTAAGACTCTGTACTCCCAATGCAGGGGGCCTGGGTTCAATC
CCTGGTCAGGTAA
>Bos_taurus_chr11.trna2913-GlyCCC (73139071-73139143) Gly (CCC) 73 bp Sc: 49.26
TCCCTGGTGGTCCAGCGGCTAAGATTCTGTGCTCCCAACGCAGGGGACCCGGGTTCGATC
CCTGGTCAGGAAA
>Bos_taurus_chr26.trna3469-GlyCCC (14816904-14816832) Gly (CCC) 73 bp Sc: 49.31
TCCCTGGTGGTCCAGTGGCTAAGGCACTGTGTTCCCAATTCAGGGGGTCCAGGTTCGATC
CCTGGTCAGGGAA
>Bos_taurus_chr25.trna2740-GlyCCC (38130073-38130001) Gly (CCC) 73 bp Sc: 49.32
TCCCTGGTGGTCCAGTGGCTAAGACTCTCTACTCCCAATACAGGGGGCCTGGGTTCAATT
CCTGGTCAGGGAA
>Bos_taurus_chr4.trna2168-GlyCCC (68181306-68181378) Gly (CCC) 73 bp Sc: 49.32
TGCCTGGTGGTCCAGTGGCTAAGACTCTGCGCTCCCAATGCAGGGGACCCAGGTTCAATT
CTTGGTCAGGGAA
>Bos_taurus_chr18.trna4483-GlyCCC (39010142-39010070) Gly (CCC) 73 bp Sc: 49.40
TCCCTGGTGGTCCAATGGCTAAGACTCCACACTCCCAATGTAGGGGGCCAGGGTTCAACC
CCCGGTCAGGGAA
>Bos_taurus_chr22.trna3012-GlyCCC (40349659-40349587) Gly (CCC) 73 bp Sc: 49.41
TCCCTGGTGGTCCAGTGGCTGAAACCCGGCACTCCCAATGCAGAGGGCTCAGGTTCGATC
CCTGGCCAGGGAA
>Bos_taurus_chr15.trna5047-GlyCCC (32919197-32919125) Gly (CCC) 73 bp Sc: 49.44
TCCCTGGTTGTCCAGTGGCTAAGACTCTGCACTCCCCATGCAGGGAGCCCGGGTTCGATC
CCTGGTCAGGGAA
>Bos_taurus_chr5.trna5831-GlyCCC (111298769-111298697) Gly (CCC) 73 bp Sc: 49.44
TCCCTGGTGGTCCAGTGGCTAAGACTCTGCACTCCCAGTGCAGGGGGCCCAGGTTCAATC
CTTGGTCAGGAAA
>Bos_taurus_chr9.trna3200-GlyCCC (92473950-92474022) Gly (CCC) 73 bp Sc: 49.46
TCCCTGGTGGTCCAGTGGCTAAGACCCTGCACTCCCAATGCAGAGGGCCCAGGTTTGACC
CCTGGTCAGAGAC
>Bos_taurus_chr17.trna2035-GlyCCC (53430606-53430677) Gly (CCC) 72 bp Sc: 49.46
TCCCTGGTGGTCCAGTGTTTATGACTCTGTGCTCCCAATGCAGGGGCACAGGTTCAATCC
CTGGTCGGGGAA
>Bos_taurus_chr16.trna3824-GlyCCC (62475314-62475242) Gly (CCC) 73 bp Sc: 49.47
TTCCTCGTAGTCCAGTGGCTAAGACTCTGCGTTCCCAATGCAGGGGGCCCAGGTTCGATC
CCTGGTCAGGGAA
>Bos_taurus_chr1.trna3086-GlyCCC (90099810-90099882) Gly (CCC) 73 bp Sc: 49.48
TTCCTGGTGGTCCAGTGGCTAAGACTCTGCACTCCCAATGCAGAGGGCCCAGGTTCTACC
CCTGATTAGGGAA
>Bos_taurus_chr1.trna4428-GlyCCC (128565841-128565913) Gly (CCC) 73 bp Sc: 49.48
TCCCTGGTGGTCCAGTGGCTAAGACTCTGCACTCCCAATGCAAGGGGCCCAAGTTCAACT
CCTGGTCAGGGAC
>Bos_taurus_chr23.trna3616-GlyCCC (25610746-25610674) Gly (CCC) 73 bp Sc: 49.52
TCCCTGGTGGTCCAGTGGCTAAGACTCTGCATTCCCAATGCTGGGGACTCCGGTTCGATC
CCTGGTCAGGGAG
>Bos_taurus_chr7.trna780-GlyCCC (14050716-14050789) Gly (CCC) 74 bp Sc: 49.56
TCCCTGATGGTCCAGTGGTTAAGACTCTGAGCTCCCAATGCAGGGGGCCCAGGGTTCAAT
CCCTGGTTAGGGAT
>Bos_taurus_chr7.trna977-GlyCCC (16597518-16597590) Gly (CCC) 73 bp Sc: 49.57
TCCCTAGTGGTCCAGTGGCTAAGACTCTGCACACCCAATGCAGGGGACCCGGGTTTGATC
CCTGGCTAGGGAA
>Bos_taurus_chr9.trna5378-GlyCCC (74204697-74204625) Gly (CCC) 73 bp Sc: 49.58
TCCCTGGTGGTCCAGTGGCTAAGACTCTGAGCTCCCAATGCAGGAGGTCCAGGTTCAATC
CCTGGTCAGGGAG
>Bos_taurus_chr1.trna3410-GlyCCC (99422914-99422986) Gly (CCC) 73 bp Sc: 49.59
TCCCTGGTGGTCCAGTGCCTAAGATTCTGCACTCCCAATGTAGGGGACCCAGGTTCAATC
CCTGGTCAGGGAG
>Bos_taurus_chr21.trna4915-GlyCCC (20379576-20379504) Gly (CCC) 73 bp Sc: 49.60
TCCCTGGTGGTCCAGTGGCTAAGACTCCGTGCTCCCAATACAGAGGGCCCAGGTTTGATT
CCTGGTCAGGGAA
>Bos_taurus_chr1.trna4786-GlyCCC (137513416-137513487) Gly (CCC) 72 bp Sc: 49.61
TCCCTGGTTGTCCAGTGGCTAAGACTCTGTGCTCCCAATGCAGGGGCCCAGTTTCGATCC
CTGGTCAGGGAG
>Bos_taurus_chr17.trna4735-GlyCCC (54123496-54123423) Gly (CCC) 74 bp Sc: 49.62
TCCCTGGTAGTCCAGTGGCTTAAGACTCTGCGCTCCCAATGCAGGGGCCCCGGGTTTGAT
CCCTGGTCGGGGAA

>Bos_taurus_chr2.trna9534-GlyCCC (27958808-27958736) Gly (CCC) 73 bp Sc: 49.62
TCCCTGGTGGTTCAGTGGCTAAGACTCTGCACTCCCAATGCAGGGGGCCTGGGTTCAACC
CCTGGTTAGGGAA
>Bos_taurus_chr9.trna2005-GlyCCC (63329651-63329723) Gly (CCC) 73 bp Sc: 49.65
TCCCTGGTGGTCCAGTGGTTACGACTCTGTGCTCCCAATGCAGGGGGCCCAGGTTCCATC
CCTGGTCAAGGAA
>Bos_taurus_chrUn.004.791.trna3-GlyCCC (50382-50454) Gly (CCC) 73 bp Sc: 49.65
TCCCTGGTGGTCAAGTGGTTAAGACTCTGAGCTCCCAATACAGGGGACACAGGTTCAATC
CCTGGTTGGGGAA
>Bos_taurus_chr8.trna2725-GlyCCC (79361809-79361881) Gly (CCC) 73 bp Sc: 49.65
TCCCTGATGGTCCAGTGGCTAAGATTCTGCACTCCCAATGCAGGGGGTGTGGGTTCAATC
CCTGGTCAGGGAA
>Bos_taurus_chr14.trna5521-GlyCCC (25943374-25943302) Gly (CCC) 73 bp Sc: 49.66
TCCCTGCTGGTCCAGTGGTTAAGACTCTGTGCTCCCAATGCAGAGGGCCCGGGTTTAATC
CCTGGTCAGGGAA
>Bos_taurus_chr17.trna4162-GlyCCC (62586992-62586920) Gly (CCC) 73 bp Sc: 49.67
TCCCTGGTGGTCCAGTGGTTAGGACTCTGCGCTCCCACTGCAGGAGGCACAGGTTGGATC
CCTGGTTGGGGAA
>Bos_taurus_chr17.trna5048-GlyCCC (49494583-49494511) Gly (CCC) 73 bp Sc: 49.75
TCCCTGGTGGTCCAGTGGTTAGGACTCTGTTTTCCCAATGCAGAGGGCCTGGGTTCATTC
CCTGGTCAGGGAA
>Bos_taurus_chr1.trna7947-GlyCCC (98939445-98939372) Gly (CCC) 74 bp Sc: 49.77
TCCCTGGTGGTCCAGTGGCTAAGACTCTGTGCTCCCAATGCAGACAAGCCCAGGTTCAAT
CCCTGGTCAGGAAA
>Bos_taurus_chr11.trna2979-GlyCCC (74435635-74435705) Gly (CCC) 71 bp Sc: 49.80
TCCCTGGTGGTCCAGTGGCTAAGACTCTGAGCTCCCAATGCAGGGCCCAGGTTCCATCCC
TGGTCAGGGAA
>Bos_taurus_chr27.trna2716-GlyCCC (30987921-30987849) Gly (CCC) 73 bp Sc: 49.80
TCCCTGGTGGTCCAGTGGCTAAGACTCTATGCTCCCAATGCAGTTGGCCCAGGTTCGATC
CCTGGTTAGGGAA
>Bos_taurus_chr1.trna10927-GlyCCC (3605709-3605637) Gly (CCC) 73 bp Sc: 49.81
TTCCTGGTGGTCTAGTGGCCAAGATTTCGCACTCCCAATGCAAGGGCCCTGGGTTCAATC
CCCGGTCAGGGAA
>Bos_taurus_chr13.trna5113-GlyCCC (59905042-59904970) Gly (CCC) 73 bp Sc: 49.83
TCACTGATGGTCCAGTGGCTAAGACACTGCACTCCCAATGCAGGGAAACCAGGTTTGATC
CCTGGTCAGGGAA
>Bos_taurus_chr8.trna5627-GlyCCC (76768799-76768727) Gly (CCC) 73 bp Sc: 49.84
TCCCTGGTGGTCCAGTGGCTAAGACTTTGTTCTCCCAATGCAGGGGGCCCAGGCTCGATC
CCTGGTCAGGGAA
>Bos_taurus_chr25.trna1501-GlyCCC (26783757-26783829) Gly (CCC) 73 bp Sc: 49.84
TCCCTGGTGGTCCAGTAGTTAAGACTCAGTGCTCCCAATGCAGGGGGCCCAGGTTCGAGT
CCTGGTCAGGGAA
>Bos_taurus_chrUn.004.1132.trna3-GlyCCC (47203-47275) Gly (CCC) 73 bp Sc: 49.86
TCCCTGGTGGTCCAGTGGCTAAGACTCTGCACTCCCAATGCAGGGTGCCGGGGTTCGATC
CCTGGTCAGGGAC
>Bos_taurus_chr6.trna6283-GlyCCC (74556514-74556442) Gly (CCC) 73 bp Sc: 49.89
TCCCTGGTGGTTCAGTGGGTAGGACTCTGCACTCCCAAAGCAGAGGGCCTGGGTTCCATC
CCTAGTCAGGGAA
>Bos_taurus_chr26.trna2122-GlyCCC (47461983-47461911) Gly (CCC) 73 bp Sc: 49.90
TCCTTGGTGGTCCAGTGGTTAAGACTCTGCACTCCCAGTGCAGAGGGCCTGGGTTTGATC
CCTAGGCAGGGAA
>Bos_taurus_chr1.trna9601-GlyCCC (46207653-46207581) Gly (CCC) 73 bp Sc: 49.90
TCCCAGGTGGTCCAGTAGTTAAGACTCTGCACTCCCAATGCAGGGGGCCTGGGTTCGATC
CCTGGTCAGGGAA
>Bos_taurus_chr22.trna1833-GlyCCC (51606712-51606784) Gly (CCC) 73 bp Sc: 49.91
TCCCTGGTGGTCCAGTGGGTAAGAGTCTGCACTCCCAATGCAGGGGGCTCAGGTTCAATG
CCTGGTCGGGGAA
>Bos_taurus_chr27.trna2179-GlyCCC (40809319-40809247) Gly (CCC) 73 bp Sc: 49.92
TCCCTGGTAGTCCAGTGGCTAAGACTCTGCACTCCCAGTGCAGGGAGCCCAGGTTTGATC
CCTGGTTGGGGAA
>Bos_taurus_chrUn.004.1754.trna1-GlyCCC (24902-24974) Gly (CCC) 73 bp Sc: 49.93
TCCCTGGTGGTCCAGTGGCTAAGATTCTGCACTCCCACTGCAGAGAGCCTGGGTTCAATC
CCTGGTCAGGAAA
>Bos_taurus_chrX.trna4712-GlyCCC (62810370-62810298) Gly (CCC) 73 bp Sc: 49.95
TCCCTGATGGTCCAGTGACTAAGACTCTGCACTCCCAATGCAGGGGGCCAGGGTTCAATC
CCTGGTCAGGGAA
>Bos_taurus_chr16.trna947-GlyCCC (27613579-27613651) Gly (CCC) 73 bp Sc: 49.96

TCCCTGATGGTCCAGTGGCTAAGATTCTGAGCTCCCAATGCAGGGGGCCTGGGTTCGATC
CCTGGTCAGGGAA
>Bos_taurus_chr15.trna5297-GlyCCC (28297346-28297274) Gly (CCC) 73 bp Sc: 49.98
TCCCTGATTGTCCAGTGGCTAAGATTCTGCACTCCCAATGCAGGGCCCCTGGGTTCAATC
CCTGGTCAGGGAA
>Bos_taurus_chr13.trna2646-GlyCCC (65192341-65192413) Gly (CCC) 73 bp Sc: 50.00
TCCCTGGTGGTCCAGCGGCTAAGACTCTGCACTCCCAATACAGGGGTCTCAGTTTCAACC
CCTGGTCAGGGAA
>Bos_taurus_chrUn.004.2869.trna3-GlyCCC (13122-13194) Gly (CCC) 73 bp Sc: 50.00
TCCCTGGTGGTCCAGTGGCTAAGACTCCATGCTCCCAATGTAGGGAGCCCAGGTTCAATC
CCTGGTCAGGGAA
>Bos_taurus_chr16.trna765-GlyCCC (23897631-23897703) Gly (CCC) 73 bp Sc: 50.01
TCCCTGATGGTCCAGTGGCTAAGACTCTGCGTTCCCAATGTAGGGGACCTAGGTTCGACC
CCTGGTCAGGGAA
>Bos_taurus_chrUn.004.161.trna27-GlyCCC (83657-83585) Gly (CCC) 73 bp Sc: 50.02
TCCCTGGTGGTCCAGTGGCTAAGATTCTTCATTCCCAATGAAGGGAGCCCAGGCTTGATT
CCTGGTCAGGGAA
>Bos_taurus_chr10.trna7456-GlyCCC (18605141-18605069) Gly (CCC) 73 bp Sc: 50.03
TCCTTGGTGGTCCAGTAGCTAAGACTCTGCACTCCCAATGCAGGGGACCCAGGTTCAATC
CCTAGTCAAGGAA
>Bos_taurus_chr10.trna8014-GlyCCC (5720524-5720452) Gly (CCC) 73 bp Sc: 50.03
TCCCTGGTGGTCCAATGGCTAACACTCTGCACTCCCAATGCAGTTGGCCCAGGTTTGATC
CCTGGTCAGGGAA
>Bos_taurus_chr17.trna2628-GlyCCC (62571472-62571544) Gly (CCC) 73 bp Sc: 50.06
TCCCTGGTGGTTCAGTGGTTTGGACTCAGTGCTCCCAATGCAGGGGGCCCAGGTTCAATC
CCTGGTCAGGGAG
>Bos_taurus_chrUn.004.1497.trna6-GlyCCC (18247-18175) Gly (CCC) 73 bp Sc: 50.07
TCCCTGGTGGTCCAGTGGCTAAGACCCTGAGCTCCCAATGCAGGGGTCTCAGGTTCAATC
CCTGGTCAGGAAA
>Bos_taurus_chr29.trna1118-GlyCCC (31457227-31457299) Gly (CCC) 73 bp Sc: 50.07
TTCCTGGTGGTCCAGTGGCTAAAACTCTGCACTCCCAATGCAAAGGGTCCAGGTTCAATC
CCTGGTCAGGGAA
>Bos_taurus_chr12.trna701-GlyCCC (17770784-17770856) Gly (CCC) 73 bp Sc: 50.11
TCCCTGGTGGTCCAGTGGCTAAGACTCTGAGTTCCCACTACAGGGGGCCCAGGTTCAATC
CCTGGACAGGGAA
>Bos_taurus_chr8.trna857-GlyCCC (24987301-24987373) Gly (CCC) 73 bp Sc: 50.12
TCCCTGGTGGTCCAGTGGCTAAGACCCTACATTCCCAATGCAGGGGGCCTAGGTTCAATC
CCTGGTTGGGGAA
>Bos_taurus_chr21.trna2743-GlyCCC (66172831-66172903) Gly (CCC) 73 bp Sc: 50.16
TCTCTGGTGGTCCAGTGGCTAAGACTCTGTGCTCCCAATGCAGGGGCCCCAGGTTTGATC
CCTGGTCAGGGAA
>Bos_taurus_chr12.trna2132-GlyCCC (54972099-54972171) Gly (CCC) 73 bp Sc: 50.17
TCCCTGATTGTCCAGTGGCTAAGATTCCGCACTCCCAATGCAGGGGGCACAGGTTCAATC
CCTGGTCAGGGAA
>Bos_taurus_chr2.trna6766-GlyCCC (111544919-111544847) Gly (CCC) 73 bp Sc: 50.21
TCCTTGGTGGTTCAGTGGCTAAGACTCTGAGCTCCCAACACAGGGGCCCTGGGTTCCATT
CCCGGTCAGGGAG
>Bos_taurus_chr1.trna4067-GlyCCC (118948848-118948921) Gly (CCC) 74 bp Sc: 50.22
TCCCTGGTAGTCCATTGGATAAGACTCTGCCCTCCCAATGCAGGAGGTTCCAGGTTCAAT
CCCTGGTCAGGGAA
>Bos_taurus_chr2.trna28-GlyCCC (1266509-1266581) Gly (CCC) 73 bp Sc: 50.27
TTCTTGGTGGTCCAGTGGCTAAGACTCTGCACTCCCAATGCAGGGGACACAGGTTCAATC
CCTTTTCAGGGAA
>Bos_taurus_chr16.trna2205-GlyCCC (57159588-57159660) Gly (CCC) 73 bp Sc: 50.28
TCCCTGGTGGTTCAGGGGCTAAGACTCTGTGCTCCCAATACAGGGGGCCCAGGTTCAATC
CCTGGTCAGGGAA
>Bos_taurus_chr3.trna9227-GlyCCC (5195824-5195752) Gly (CCC) 73 bp Sc: 50.29
TCCCTGGTGGTTCAGTGGCTAAGACTCTGCACTCCCAGTGCAGGGGGCCCAGGTTCTATC
CCTGGTCGGGGAA
>Bos_taurus_chr13.trna96-GlyCCC (3968388-3968460) Gly (CCC) 73 bp Sc: 50.29
TCCCTGGTGGTCCAGTGGCTGAGACTCTGCACTCCCAATGCAGGGGGCCCGGGTTCGATC
CCTGGTCATGGAA
>Bos_taurus_chr16.trna1008-GlyCCC (29338722-29338794) Gly (CCC) 73 bp Sc: 50.31
TCACTGGTGGTCCAGTGGCTAAGACACTGAGCTCCCAATTCAGGGGGCCCAGGTTCAATC
CCTGGTCAGGGAA
>Bos_taurus_chr7.trna5406-GlyCCC (81105501-81105430) Gly (CCC) 72 bp Sc: 50.33
TCCCTGGTTGTCTAGTGGCTAAGATGCTGAGCTCCCAATGCAGGGGCCCGGGTTCAATTC

CTGGTCAGGGAA
>Bos_taurus_chr15.trna2743-GlyCCC (76918492-76918564) Gly (CCC) 73 bp Sc: 50.34
TCCCTGGTGGTCCAGTGACCAAGACTCTGTACTCCCAATGCAGGGGCCCCAGGTTCAATC
CTTGGTCAGGGAA
>Bos_taurus_chr10.trna889-GlyCCC (20688414-20688486) Gly (CCC) 73 bp Sc: 50.35
TCCCTGGTGGTCCAGTGGCTAAGACTCTGAGCTCCCAGTGCAGGGGGCCCAGGTTCGATC
CCTGGTCAGGGAG
>Bos_taurus_chr14.trna5505-GlyCCC (26168054-26167982) Gly (CCC) 73 bp Sc: 50.35
TCCCTGGTGGTCCAGTGGCTAAGACTCTGAGCTCCCAGTGCAGGGGGCCCAGGTTCGATC
CCTGGTCAGGGAG
>Bos_taurus_chr17.trna6518-GlyCCC (7262534-7262462) Gly (CCC) 73 bp Sc: 50.35
TCCCTGGTGGTCCAGTGGCTAAGACTCTGAGCTCCCAGTGCAGGGGGCCCAGGTTCGATC
CCTGGTCAGGGAG
>Bos_taurus_chr18.trna4520-GlyCCC (38356643-38356571) Gly (CCC) 73 bp Sc: 50.35
TCCCTGGTGGTCCAGTGGCTAAGACTCTGAGCTCCCAGTGCAGGGGGCCCAGGTTCGATC
CCTGGTCAGGGAG
>Bos_taurus_chr18.trna5969-GlyCCC (1340823-1340751) Gly (CCC) 73 bp Sc: 50.35
TCCCTGGTGGTCCAGTGGCTAAGACTCTGAGCTCCCAGTGCAGGGGGCCCAGGTTCGATC
CCTGGTCAGGGAG
>Bos_taurus_chr2.trna6794-GlyCCC (110939166-110939094) Gly (CCC) 73 bp Sc: 50.35
TCCCTGGTGGTCCAGTGGCTAAGACTCTGAGCTCCCAGTGCAGGGGGCCCAGGTTCGATC
CCTGGTCAGGGAG
>Bos_taurus_chr20.trna1634-GlyCCC (43794023-43794095) Gly (CCC) 73 bp Sc: 50.35
TCCCTGGTGGTCCAGTGGCTAAGACTCTGAGCTCCCAGTGCAGGGGGCCCAGGTTCGATC
CCTGGTCAGGGAG
>Bos_taurus_chr25.trna2894-GlyCCC (35760231-35760159) Gly (CCC) 73 bp Sc: 50.35
TCCCTGGTGGTCCAGTGGCTAAGACTCTGAGCTCCCAGTGCAGGGGGCCCAGGTTCGATC
CCTGGTCAGGGAG
>Bos_taurus_chr6.trna4942-GlyCCC (106463102-106463030) Gly (CCC) 73 bp Sc: 50.35
TCCCTGGTGGTCCAGTGGCTAAGACTCTGAGCTCCCAGTGCAGGGGGCCCAGGTTCGATC
CCTGGTCAGGGAG
>Bos_taurus_chr9.trna1506-GlyCCC (46412773-46412845) Gly (CCC) 73 bp Sc: 50.35
TCCCTGGTGGTCCAGTGGCTAAGACTCTGAGCTCCCAGTGCAGGGGGCCCAGGTTCGATC
CCTGGTCAGGGAG
>Bos_taurus_chr21.trna4869-GlyCCC (21093452-21093380) Gly (CCC) 73 bp Sc: 50.37
TCCCTAGTGGTCCAGTGGCTAAGACTCTGCACTCCCAATTCAGGGAACCCAGGTTCCATC
CCTGGCCAGGGAA
>Bos_taurus_chr15.trna3746-GlyCCC (67383460-67383388) Gly (CCC) 73 bp Sc: 50.38
TCCCTGGTAGTCCAGTGGCTAGAACTCTGTGCTCCCAATGCAGAGGGCCTAGGTTTGATC
CCTGGTCAGGGAA
>Bos_taurus_chr10.trna1828-GlyCCC (47448015-47448087) Gly (CCC) 73 bp Sc: 50.39
TCCCTGGTGGTCCAGTGTCTAAGGCTCTGTGCTCCCAATGCAGAGGACCCAGGTTCAATT
CCTGGTCAGGGAA
>Bos_taurus_chr29.trna1041-GlyCCC (29941532-29941604) Gly (CCC) 73 bp Sc: 50.44
TCCTTGGTGGTCCAGTGGCTAAGACTCTGCACGCCCGATGCAGAAGGCCCAGGTTTGATC
CCTGGTCAGGGAA
>Bos_taurus_chr9.trna4962-GlyCCC (84508227-84508156) Gly (CCC) 72 bp Sc: 50.45
TCCCTGGTGGTCCAACCGCTAAGACTCCAGACTCCCAATCAGGGGGCCCAGGTTCAATCC
CTGATCAGGGAA
>Bos_taurus_chr13.trna5110-GlyCCC (59917456-59917384) Gly (CCC) 73 bp Sc: 50.47
TCCCTGATGGTCCAGTGGCTAAGACTCTGCACTCCCAATGCAGGGGGCCAGGGTCCAATC
CCTGGTCAGGGAA
>Bos_taurus_chr5.trna2880-GlyCCC (77403221-77403293) Gly (CCC) 73 bp Sc: 50.47
TCCCTGGTGGTCCAGTGGTTAAGACTCTGTGTTCCCAATGCAGGGGGCCCAGGTTGGATC
CCTGGTCAAGGAA
>Bos_taurus_chr18.trna4172-GlyCCC (46172770-46172698) Gly (CCC) 73 bp Sc: 50.50
TCCCTGGTGGTCCAGTGGTTAAGACTCTGTGCTCCCAATACAAGGGGCCCAGGTTCAATC
CCTGGTCTGGGAA
>Bos_taurus_chr1.trna4042-GlyCCC (118355851-118355923) Gly (CCC) 73 bp Sc: 50.56
TCCCTGGTGGTCCAGTGGTTAAGACACTGCGCTCCCAATGCAGGAGCCACGGGTTCAATG
CCTGTTCAGGGAA
>Bos_taurus_chr13.trna948-GlyCCC (24753634-24753706) Gly (CCC) 73 bp Sc: 50.56
TCCTTGGTGGCCCAGGGGTTAAGACTCTGCATTCCCAAAGCAGCGGGTCCAGGTTCGATC
CCTGGTCGGGGAA
>Bos_taurus_chr13.trna5523-GlyCCC (46573183-46573111) Gly (CCC) 73 bp Sc: 50.57
TCCTTGGTGGTCCAGTGGCTAAGACTCTGAGCTCCCAATGCAGGGAGTCCAGGTTCAATC
CCTGGTCAGGGAA

>Bos_taurus_chrUn.004.49.trna32-GlyCCC (167440-167369) Gly (CCC) 72 bp Sc: 50.60
TCCCTGGTGGTCCAATGGCTAAGGCTCTGCACTCCCAATGCAGGGGGCCAGGTTCAATCC
CTGGTCAGAGAA
>Bos_taurus_chr7.trna2512-GlyCCC (58111650-58111721) Gly (CCC) 72 bp Sc: 50.64
TCCCTGGTGGTCCAGTGGCTAACACTCTGAGCTCCCATTGCAGGGGCCCAGGTTCAATCC
CTGGTCAGGGAG
>Bos_taurus_chr25.trna720-GlyCCC (12563893-12563965) Gly (CCC) 73 bp Sc: 50.71
TCCCTGGTGGTCCAGTGGCTAAGACTCTGCACTCCCAATGCAGGGGGGCCGGGTTTGATC
CCTGGTCAGGGAA
>Bos_taurus_chr2.trna10232-GlyCCC (7201058-7200986) Gly (CCC) 73 bp Sc: 50.73
TCCCTGGCAGTCCAGTGGCTAAGACTCTGTGCTCCCAAAGCAGGGGGCCCAGGTTCAATT
CCTGGTCAGGGAA
>Bos_taurus_chrUn.004.81.trna13-GlyCCC (102912-102984) Gly (CCC) 73 bp Sc: 50.77
TCCCTGGTGGTCCAGTGGCTAAGACTTTGCATTCCCAATGCAGGGGGCCTGGGTTCAATC
CCTGGTTAGGGAA
>Bos_taurus_chr7.trna423-GlyCCC (9385000-9385072) Gly (CCC) 73 bp Sc: 50.79
TTCCTGGTGGTCCAGTGGCTAAGATTTTGCACTCCCAATGCACGGGGCCCAGGTTCAATC
CCTGGTCTGGGAA
>Bos_taurus_chr28.trna2496-GlyCCC (16730212-16730140) Gly (CCC) 73 bp Sc: 50.82
TCCCTGGTGGTCCAGTGGCTAAGACTGTGTGCTCCCAATACAGAGCTCTCAGGTTCAATT
CCTGGTCAGGGAA
>Bos_taurus_chr19.trna265-GlyCCC (9494597-9494669) Gly (CCC) 73 bp Sc: 50.96
TCCCTGGTGGTCCAGTGGCTACGACTCTGCACTCCCAAAGCAGGGGACCCAGGTTCGACC
CCTGGTCAGGGGA
>Bos_taurus_chr12.trna5382-GlyCCC (33342066-33341995) Gly (CCC) 72 bp Sc: 50.96
TCCCTGGTGGTCCAGTGGCTAAGACTTTGTGCTCCCAATGCACGGGGCCAGGTTCAAGTC
CTGGTCAGGGAA
>Bos_taurus_chr22.trna3942-GlyCCC (13605271-13605199) Gly (CCC) 73 bp Sc: 50.97
TCCCTGGTGGTCCAGTGGCTAAGACTCTGTGCTCCCAACGCAGAAGGCCCAGGTTCAATC
CTTGGTAAGGGAG
>Bos_taurus_chr24.trna4118-GlyCCC (34372852-34372780) Gly (CCC) 73 bp Sc: 50.97
TCCCTGGTGGTCCAGTGGCTAAGACTCTGAGCTCCCAATGCAGGGGGCCTAGGTTCGATC
CCTTGTCAGGGAA
>Bos_taurus_chr25.trna744-GlyCCC (12775389-12775461) Gly (CCC) 73 bp Sc: 50.97
TCCCTGGTGGTCCAGTGGCTAAGACTCTGTGCTCCCAATGTAGGGGACCCAGGTTCAATC
CCTGGTCAGGGAA
>Bos_taurus_chr16.trna5465-GlyCCC (24295235-24295163) Gly (CCC) 73 bp Sc: 51.00
TCCGTGGTGGTCCAGTGGCTAAGATTTTGCACTCCCAATGCAGGGAGCCTAGGTTCAATC
CCTGGTCAGGGAA
>Bos_taurus_chrX.trna2262-GlyCCC (63012476-63012548) Gly (CCC) 73 bp Sc: 51.01
TCTCTGGTGGTCCAGTGGCTAAGACTCTGCTCTCCCAATGCAGGGGGCCCAGGTTTGATA
CCTGGTCAGGGAA
>Bos_taurus_chr8.trna5967-GlyCCC (70641495-70641423) Gly (CCC) 73 bp Sc: 51.02
TCCCTGGTGGTCCAATGGCTAAGACCCTGAGCTCCCAATGCAGGGGGCCCAGGTTCAATC
CCTGGTCAGGGAG
>Bos_taurus_chr3.trna7098-GlyCCC (63675901-63675829) Gly (CCC) 73 bp Sc: 51.02
TCCCTGGTGGTCCAGTGGTTAAGACTCTGCTCTCCCAATGCAGGGGGCCCAGGTTCATTC
CCTCGTCAGGGAA
>Bos_taurus_chr21.trna1094-GlyCCC (24557917-24557989) Gly (CCC) 73 bp Sc: 51.02
TCCCTGGTGGCCCAGTGGCTAAGACTCTGAGCTCCCAATGCAGGGGGCCCAGGTTCAATC
CCTGGTCAGGGAA
>Bos_taurus_chr25.trna4514-GlyCCC (8959477-8959405) Gly (CCC) 73 bp Sc: 51.03
TCCCTGGTGGTCCAGTGGCTAAGACGCTGCACTCCCAATGCAGTGGATCTGGGTTTGATC
CCTGGTCAGGGAA
>Bos_taurus_chr10.trna1811-GlyCCC (47160832-47160904) Gly (CCC) 73 bp Sc: 51.03
TCCCTGATGGTCCAGTGGCTAGGACCCTGAGCTCCCAATGCAGGGGGCCTGGGTTCAATC
CCTGGTCAGGGAA
>Bos_taurus_chr20.trna1772-GlyCCC (47739197-47739269) Gly (CCC) 73 bp Sc: 51.04
TCCCTGGTAGTCCACTGGCTAAGACTCTGCACTCCCAATGCAGGAGGCTCAGGTTCAATC
CCTGGTCAGGGAA
>Bos_taurus_chr6.trna3590-GlyCCC (108601067-108601139) Gly (CCC) 73 bp Sc: 51.04
TCCCTGGTGGTCCAGTGGCTAAGACTCTGCACTCCCAATGCAGGGGGCCTGGATTCAATC
CCTGGTCAGGGAA
>Bos_taurus_chr19.trna2696-GlyCCC (53969506-53969578) Gly (CCC) 73 bp Sc: 51.05
TCCCTGGCGGTTCAGTGGTTAAGACTCTGTGCTCCCAATGCAGGGGGCCCAGGTTCAATC
CCTGGTCAGGGAA
>Bos_taurus_chr11.trna2783-GlyCCC (70017368-70017440) Gly (CCC) 73 bp Sc: 51.08

TCCCTGGTGGTCCAGTGGTTAAGACTCTGCCCTCCCAATGCAGGGAGACCAAGTTCAATC
CCTGGTCAGGGAA
>Bos_taurus_chr17.trna3512-GlyCCC (73133761-73133689) Gly (CCC) 73 bp Sc: 51.09
TCCCTGGTTGTCCAGTGGTTAGGATGCTGTGCTCCCAGTGCAGGGAGCCCAGGTTCGATC
CCTGGTTGGGGAA
>Bos_taurus_chr7.trna2372-GlyCCC (54455526-54455598) Gly (CCC) 73 bp Sc: 51.16
TCCCTGGTGGTCCAATGGTTAAGACTCTGCACTCCCAATGCAGGGAGCTTGGGTTCAATC
CCTGGTCGGGGAA
>Bos_taurus_chr4.trna459-GlyCCC (13716676-13716748) Gly (CCC) 73 bp Sc: 51.20
TCCCTGGTGGTCCAATGGCTAAGGCTCTGAGCTCCCAATGCAGGGGGCCCAGGTTCGATC
CCTGGTCAGGGAA
>Bos_taurus_chr27.trna3150-GlyCCC (22950562-22950490) Gly (CCC) 73 bp Sc: 51.21
TCCCTGGTAGTCCAGTGGCTAAGACTCTGCACTCCCAATGCAGGGGTCCTGGGTCTGATC
CCCGGTCAGGGGA
>Bos_taurus_chr2.trna4336-GlyCCC (125857475-125857547) Gly (CCC) 73 bp Sc: 51.23
TCCCTGGTGGTCCAGCGGCTAAGATTCTGCTCTCCCAATGCAGGGGCCCCAGGTTCGAAC
CTTGGTCAGGGCA
>Bos_taurus_chrX.trna270-GlyCCC (6487533-6487605) Gly (CCC) 73 bp Sc: 51.27
TCCCTGGTGGTCCAGCAGTTAAGACTCTGAGCTCCCAATGCAGGGGGCCCAGGTTCAATT
CCTGGTCAGGGAA
>Bos_taurus_chr16.trna1960-GlyCCC (50331065-50331137) Gly (CCC) 73 bp Sc: 51.28
TCCCTGGTGGTCCAGTGGCTAAGAATCTGTACTCCCAATGCAGGGAGCCCAGGTTCAATA
CCTGGTTGGGGAA
>Bos_taurus_chr27.trna3072-GlyCCC (25043156-25043084) Gly (CCC) 73 bp Sc: 51.35
TCCCTGGTGGTCCAGTGGGTACGACTCTGTGTTCCCAATGCAGGGGGACCAGGTTCGATC
CCTGGTCAGGGAA
>Bos_taurus_chr14.trna3296-GlyCCC (81190709-81190781) Gly (CCC) 73 bp Sc: 51.35
TCCCTGGTGGTGCAGTGGCTAAGGACTCTGAGCTCCCAACCCAGGAGCTCAGGTTCGATC
CCTGGTCAGGGAA
>Bos_taurus_chr17.trna4747-GlyCCC (54034151-54034080) Gly (CCC) 72 bp Sc: 51.37
TCCCTGATGGTCCAGTGGCTAAGACTCTGCACTCCCAACGCAGGAGCCCAGGTTCTATCC
CTGGTCAGAGAA
>Bos_taurus_chr5.trna4404-GlyCCC (112220799-112220871) Gly (CCC) 73 bp Sc: 51.38
TGCCTGGTGGTCCAGTGGCTAAGACTCTGGGCTCCCAATACAGGGGGCCCAGGTTCGATC
CCTGGTCAGGGAA
>Bos_taurus_chr8.trna3641-GlyCCC (105317123-105317195) Gly (CCC) 73 bp Sc: 51.41
TCCCTGATGGTCCAGTGGCTAAGACTCTGAGCTCCCAATGCAGAGGTCCCAGGTTCCATC
CTTGGTCAGGAAA
>Bos_taurus_chr1.trna4410-GlyCCC (128219503-128219575) Gly (CCC) 73 bp Sc: 51.42
TCTCTGGTGGTCCAGTGGCTAAGACTCTGCACTCCCAGTGCAGGGGATCCAGGTTCGATA
CCTGGTCAGGGAA
>Bos_taurus_chr1.trna4414-GlyCCC (128268445-128268517) Gly (CCC) 73 bp Sc: 51.42
TCTCTGGTGGTCCAGTGGCTAAGACTCTGCACTCCCAGTGCAGGGGATCCAGGTTCGATA
CCTGGTCAGGGAA
>Bos_taurus_chr29.trna1230-GlyCCC (33732952-33733024) Gly (CCC) 73 bp Sc: 51.42
TCCCTGGTGGTCCAGTGGCCAAGACTCAGCACTCCCAGTGCAGGGGACCCAGGTTCGATC
CCTGGTCAGGGAA
>Bos_taurus_chr22.trna4190-GlyCCC (8363607-8363535) Gly (CCC) 73 bp Sc: 51.47
TCCCTGGTGGTCTAGTGGCTAAGACTCTGTGCTCCCAACGCAGGGGGCCTGGGTTCAACC
CCTGGTCAGGGAA
>Bos_taurus_chr3.trna911-GlyCCC (23953462-23953534) Gly (CCC) 73 bp Sc: 51.48
TCCCTGGTGGTCCAGTGGTTAAGACTCAGTGCTCCCAATGCAGGGGGCCCAGGTTCGATC
TCTGGTCAGGGAA
>Bos_taurus_chrX.trna6827-GlyCCC (492511-492439) Gly (CCC) 73 bp Sc: 51.52
TCTCTGGTGGTCCAGTGGCTAAGACCCTGTGCTCCCAACGCAGCGGGCCCAGGTTTGATC
CCTGGTCAGGGAA
>Bos_taurus_chrUn.004.1664.trna4-GlyCCC (22172-22100) Gly (CCC) 73 bp Sc: 51.56
TCCCTGGTAGTCCAGTGGCAAAGACTCGGCGCTCCCAACGCAGAGGGCCCAGGTTCCATC
CCTGGTCAGGGAA
>Bos_taurus_chrUn.004.5967.trna1-GlyCCC (6965-7037) Gly (CCC) 73 bp Sc: 51.56
TCCCTGGTAGTCCAGTGGCAAAGACTCGGCGCTCCCAACGCAGAGGGCCCAGGTTCCATC
CCTGGTCAGGGAA
>Bos_taurus_chr13.trna1794-GlyCCC (42400544-42400616) Gly (CCC) 73 bp Sc: 51.56
TCCCTGATGGTCCAGTGGCTAAGGCTCTGTGCTCCCAATGCAGAGGGCCCAGGATTGATC
CCTGGTCAGGGAA
>Bos_taurus_chr22.trna3812-GlyCCC (16400536-16400464) Gly (CCC) 73 bp Sc: 51.58
TCCCTGGTGGTCCAGTGGCTAAGACTCTGAATTCCCAATGCAGGGAGCCCACGTTCAATC

CCTGGTCAGGGAA
>Bos_taurus_chrX.trna440-GlyCCC (9886939-9887011) Gly (CCC) 73 bp Sc: 51.58
TCCCTGGTGGTCCAGTGGCTAAGACTCTGTGCTCCCAATGCAGGGGGCTCAGGTTCAACC
CCTGGTCAGGGAA
>Bos_taurus_chr17.trna5013-GlyCCC (49973095-49973023) Gly (CCC) 73 bp Sc: 51.59
TCCCTGTTGGTCCAGTGGCTAAGACTCTGTGCTCCCAAAGCAGGGGACCTAGGTTCAATC
CCTAGTCAGGGAA
>Bos_taurus_chr1.trna4134-GlyCCC (120394007-120394079) Gly (CCC) 73 bp Sc: 51.67
TCCCTGGTGGTCTAATGGTTAAGACTCTGTGCTCCCAATGCAGAGGTTCCAGGTTCCATC
CCTAGTCGGGGAA
>Bos_taurus_chrUn.004.229.trna8-GlyCCC (91250-91322) Gly (CCC) 73 bp Sc: 51.71
TCCCTGGTGGTCCAGTGGCTAAGACTCTACACTCCCAATGCAGGGGGCTCAGGTTCAATT
TCTGGTCAGGGAA
>Bos_taurus_chr15.trna4706-GlyCCC (41532267-41532196) Gly (CCC) 72 bp Sc: 51.71
TCCCTGGTGGTCCAGTGGCTAAGACTCTGAGTTCCCAATGCAGGGGCCCAGGTTCAATCC
CTGGTGAGGGAA
>Bos_taurus_chrUn.004.7980.trna1-GlyCCC (2119-2190) Gly (CCC) 72 bp Sc: 51.71
TCCCTGGTGGTCCAGTGGCTAAGACTCTGAGTTCCCAATGCAGGGGCCCAGGTTCAATCC
CTGGTGAGGGAA
>Bos_taurus_chr25.trna1547-GlyCCC (27338427-27338499) Gly (CCC) 73 bp Sc: 51.78
TCCCTGGTGGTCCAGTGGCTAGGACTCTGTGCTCCCAATGCATGGGGCCCGGGTTCGATC
CCTGGTCAGGGAA
>Bos_taurus_chr9.trna5686-GlyCCC (66029548-66029476) Gly (CCC) 73 bp Sc: 51.80
TCTCTGGTGGTCCAGTGGTTAAGACTCTGCGCTCCCAATGCAGGGGGCCTGGGTTCCATC
CCTGGCCAGAGAA
>Bos_taurus_chr17.trna2862-GlyCCC (65687796-65687868) Gly (CCC) 73 bp Sc: 51.84
TCCCTGGTGGTCCAGTGGCTAAGACTCTGCACTCCCAATGCAGAGGGCCCGGGTTTGATC
CCTCGTCAGGGAA
>Bos_taurus_chr17.trna572-GlyCCC (15394041-15394113) Gly (CCC) 73 bp Sc: 51.88
TCCCTGTTGGTCCAGTGGCTAAGACTCTGTGCTCCCAATGCAGGGGGCCCAGGTTTGATT
CCTGGTCAGGGAA
>Bos_taurus_chr21.trna1767-GlyCCC (42197225-42197297) Gly (CCC) 73 bp Sc: 51.89
TCCCTGGTGGTCCAGTGGTTAAGACTCTGTGCTCCCACTGCAGGGGTCTCAGATTCAATC
CCTGGTTGGGGAA
>Bos_taurus_chr5.trna8829-GlyCCC (38035396-38035324) Gly (CCC) 73 bp Sc: 51.90
TCTCTGATGGTCCAGTGGCTAAAACTCTGCACTCCCAATGCAGGGGGCCTGGGTTGGATC
CCTAGTCAGAGAA
>Bos_taurus_chr13.trna4443-GlyCCC (71059335-71059263) Gly (CCC) 73 bp Sc: 51.94
TCCCTGGTGGTCCAGTGGCTAAGACTCTGCATTCCCAATGCAGGAGGCCCTGGTTAGATC
CCTGGTCAGGGAA
>Bos_taurus_chr7.trna4274-GlyCCC (110110551-110110623) Gly (CCC) 73 bp Sc: 51.95
TCCCTGGTGGTCCAGTGGTTACGACTCTGTGCTCCCAACGCAGAGGACCTGGGTTCTATC
CCTGGTCAGGGAA
>Bos_taurus_chrUn.004.53.trna23-GlyCCC (397629-397701) Gly (CCC) 73 bp Sc: 51.97
TCCGTGGTGGTCCAGTGGCTAAGACTCTGCACTCCCAATGCAGGGAGCCTGGGTTCGATC
CCTGGTCAGGGAA
>Bos_taurus_chr4.trna2194-GlyCCC (68857508-68857580) Gly (CCC) 73 bp Sc: 52.01
TCCCTGGTTGTCCAGTGGCTAAGACTCTGCGCTCCCGATGCAGGGGGCTCAGGTTCGATC
CCTGGTCAGGGAA
>Bos_taurus_chr18.trna284-GlyCCC (6802390-6802462) Gly (CCC) 73 bp Sc: 52.01
TCCCTGGTGGTCCAGTGGCTAAGACTCTGTACTCCCAATGCAGGGAGCCTGGGTTCAATC
CCTGGTCAGGGAG
>Bos_taurus_chr25.trna3507-GlyCCC (27034962-27034890) Gly (CCC) 73 bp Sc: 52.02
TCTCTGGTGGTCCAGTGTCTAGGACTCTGCACTCCCAATGCAGGGGGCCCAGGTTTGATC
CCTGGTTGGGGAA
>Bos_taurus_chr21.trna661-GlyCCC (18243974-18244046) Gly (CCC) 73 bp Sc: 52.02
TCCCTGATGGTCCAATGGCTAAGACTCTGTGCTCCCAATGCAGAGGGCCTGGGTTCAATC
CCTGGTCAGGGAA
>Bos_taurus_chr2.trna4263-GlyCCC (124867110-124867182) Gly (CCC) 73 bp Sc: 52.05
TCCCTGGTGGTCCAGTGGCTAAGACACTGCACTCCCAGTGCAGGGGACCCAGGTTCCATC
CCTGGTCAGGGAG
>Bos_taurus_chr21.trna3345-GlyCCC (58803621-58803549) Gly (CCC) 73 bp Sc: 52.06
TCCCTGGTGGTCCAGTGTCTAAGACACTGCACTCCCAATGCAGGGGACCCAGGATGGATT
CCTGGTCAGGGAA
>Bos_taurus_chr18.trna1058-GlyCCC (26260940-26261012) Gly (CCC) 73 bp Sc: 52.06
TCCCTGGTGGTCCAGTGGGTAAGACTCTGCACTCCCAATGCAGGGGGCCTGGGTTCTATC
CCTGGTCAGGGAA

>Bos_taurus_chr5.trna7910-GlyCCC (62420357-62420285) Gly (CCC) 73 bp Sc: 52.06
TTCCTGGTGGTCCAATGGCTAAGACTCTGCTTTCCCAATGCAGAGGTCTCGGGTTCAAAT
CCTGATCAGGGAA
>Bos_taurus_chrUn.004.1325.trna4-GlyCCC (28578-28650) Gly (CCC) 73 bp Sc: 52.08
TCCCTGGTGGTCCAGTGGCTAAGACTCTGTGCTCCCAATGCAGGGGGCCTGGGTTTGATC
CCCAGTTAGGGAA
>Bos_taurus_chr2.trna10385-GlyCCC (3740779-3740707) Gly (CCC) 73 bp Sc: 52.09
TCCCTGGTGGTCCAATGTTTAAGACTCTGAGCTCCCAATGCAGGGGGCCTAGGTTCAATT
CCTGGCCAGGGAA
>Bos_taurus_chr24.trna1385-GlyCCC (35430768-35430840) Gly (CCC) 73 bp Sc: 52.14
TCCCTGATGGTCCAGTGTCTAAGACTCTGCACTCCCAATGCAAGGGCCCCAGGTTAGATC
CCTGGTCAGGGAG
>Bos_taurus_chr13.trna6158-GlyCCC (32665117-32665045) Gly (CCC) 73 bp Sc: 52.18
TCTCTGGTGGTCCAGTGGCTAAGACTCTGTGCTCCCAATGCAGAGGGCCCAGGTTCAATT
CCTGATCAGGGAA
>Bos_taurus_chr10.trna663-GlyCCC (15325917-15325990) Gly (CCC) 74 bp Sc: 52.22
TCCCTGGTGGTCCAGTGGCTAGGACGCTGCACTCCCAATGCAGGGGGCTCCAGGTTTGAT
CCCTGGTAGGGGAA
>Bos_taurus_chr19.trna470-GlyCCC (13119568-13119640) Gly (CCC) 73 bp Sc: 52.25
TCTCTGGTAGTCCAGTGGCTAAGACTCTGCATGCCCAATGCAGGGGTCCCGGGTTCAATC
CCTGGTCAGGGAA
>Bos_taurus_chr1.trna8602-GlyCCC (78595697-78595625) Gly (CCC) 73 bp Sc: 52.27
TCCCTGGTGGTCCAGTGGCCAAGACTCTGCACTCCCGGTGCAGGGGAGCCAGGTTCGATT
CCTGGTCAGGGAA
>Bos_taurus_chrUn.004.658.trna12-GlyCCC (56599-56671) Gly (CCC) 73 bp Sc: 52.27
TCCTTGGTGGTCCAGGGCCTAAGACTCTGCACTCCCAAGGCAGAGGGCCCAGGTTCAATC
CCTGGTCAAGGAA
>Bos_taurus_chr1.trna11058-GlyCCC (1985207-1985135) Gly (CCC) 73 bp Sc: 52.27
TCCCTGCTGGTCCAGTGGCTAAGACTCCGTGCTCCCAATGCAGCGGGCCCAGGTTCGATT
CCTGGTCAGGGAA
>Bos_taurus_chr22.trna3815-GlyCCC (16372951-16372879) Gly (CCC) 73 bp Sc: 52.31
TCCCTGGTGGTCCAGTGGCTAGCACTCTGTACTCCCAATGCTGGGGTCCCGGGTTTGATT
CCTGGTCAGGGAA
>Bos_taurus_chr22.trna365-GlyCCC (7859909-7859981) Gly (CCC) 73 bp Sc: 52.34
TCCCTGGTGGTCCAGTGGCTAAGTCTCTGTGCTCCCAATGCAGGGGGCCCAGGTTCAATC
CCTGGTCAGGGAA
>Bos_taurus_chr21.trna1199-GlyCCC (26078034-26078106) Gly (CCC) 73 bp Sc: 52.36
TCCCTGGTGGTCCAGTGGCTAAGACTTTGAGCTCCCAATGCAGGGATCCCAGGTTTGATT
CCTGGTCAGGGAG
>Bos_taurus_chr19.trna835-GlyCCC (18746013-18746084) Gly (CCC) 72 bp Sc: 52.40
TCCCTGGTGGTCTAGCGGCTAAGACTCTGCACTCCCAATATAGGGGCTCAGGTTCAATCC
CTGGTCAGGGAA
>Bos_taurus_chr17.trna2718-GlyCCC (63639006-63639078) Gly (CCC) 73 bp Sc: 52.40
TCCCTGGTGGCCCAGTGGCTAAGACTCTGCACTCCCAATGCAGAGGGCTCAGGTTCGATC
TCTGGTCAGGGAC
>Bos_taurus_chr11.trna2218-GlyCCC (51850210-51850282) Gly (CCC) 73 bp Sc: 52.40
TCTCTGGTGGTCCAGTGGTTAAGACTCTGTGTTCCCAATGCAGGAGGCCCAGGTTCAATC
CCTGGTCAGGGAA
>Bos_taurus_chr6.trna3631-GlyCCC (109468299-109468372) Gly (CCC) 74 bp Sc: 52.43
TCCCTGATGGTCCAGTGGCCAAGAACTCTGTTCTCCCACTGCAGGGGTCCCAGGTTTGAT
CCCTGGTCAGGGAA
>Bos_taurus_chr21.trna1241-GlyCCC (26712537-26712609) Gly (CCC) 73 bp Sc: 52.47
TCCCTGATAGTCCAGTGGCTAAGACTCTGTAGTCCCAATGCAGAGGACCCAGGTTCAACC
CTTGGTCAGGGAA
>Bos_taurus_chr17.trna6370-GlyCCC (11185891-11185819) Gly (CCC) 73 bp Sc: 52.47
TCCTTGGTGGTCCAGTGGCTAAGACATTGCACTCCCAATGCAGGGGACTCAGGTTCGATC
CCTGGTGAGGGAA
>Bos_taurus_chr14.trna1746-GlyCCC (40313237-40313309) Gly (CCC) 73 bp Sc: 52.52
TCCTTGGTGGTCCAGTGGTTAAGATGCTACACTCCCAATGCAGGAGGCCCACGTTCAATA
CCTGGTCAGGGAA
>Bos_taurus_chr19.trna3594-GlyCCC (60588979-60588907) Gly (CCC) 73 bp Sc: 52.53
TCCCTGCTGGTCCAGTGGCTAAGACTCTGCACTCCCAATGCAGGCAACCCAGGTTCAACC
CCTGGTCAGGGAA
>Bos_taurus_chr19.trna3641-GlyCCC (59927438-59927366) Gly (CCC) 73 bp Sc: 52.53
TCCCTGCTGGTCCAGTGGCTAAGACTCTGCACTCCCAATGCAGGCAACCCAGGTTCAACC
CCTGGTCAGGGAA
>Bos_taurus_chr13.trna2600-GlyCCC (64575239-64575311) Gly (CCC) 73 bp Sc: 52.53

TCCCTGGTGGTCCAGTGGCTAAGATTCTGAGCTCCCAATGCAGAGGGCCCAAGTTCGATC
CCTGGTCAGGGAA
>Bos_taurus_chrUn.004.8.trna31-GlyCCC (959874-959946) Gly (CCC) 73 bp Sc: 52.55
TCCCTGGTGGTCCAGTGGCTAAGACTCTGTGCTCCCAATGTAGGGGGCCCAGGTTCAATC
CCTGGTCAGGGAA
>Bos_taurus_chr5.trna7915-GlyCCC (62287647-62287575) Gly (CCC) 73 bp Sc: 52.60
TTCCTGGTGGTCCAATGGCTAAGACTCTGCTTTCCCAATGCAGAGGTCTCAGGTTCAAAT
CCTGATCAGGGGA
>Bos_taurus_chr7.trna2298-GlyCCC (51804212-51804284) Gly (CCC) 73 bp Sc: 52.61
TCCCTGGTGGTCCAGTGGCTAAGACTCTGAGCTCCCAATGCAGGGAGCCCTGGTTCGATC
CCTGGTCAGGGAA
>Bos_taurus_chrUn.004.818.trna8-GlyCCC (34463-34391) Gly (CCC) 73 bp Sc: 52.63
TCTTTGGTGGTTCAGTGGCTAAGACTCTGCACTCCCAATGCAAGGGGCCCAGGTTCGATC
CCTGGTCAGGGAA
>Bos_taurus_chr16.trna2845-GlyCCC (69916778-69916850) Gly (CCC) 73 bp Sc: 52.74
TCCCTGGTGGTCCAGTGGCTAAGACTCCGTGCTCCCAATGCAGAAGGCCTGGGTTCGATC
CCCAGTCAGGGAA
>Bos_taurus_chr5.trna8889-GlyCCC (36407784-36407712) Gly (CCC) 73 bp Sc: 52.78
TCCCTGCTGGTCCAGTGGCTAAGACTCTGCTCTCCCAATGCAGGGGGCCCAGGTTTGATC
CCTGGTCGGGGAA
>Bos_taurus_chr18.trna1522-GlyCCC (38225817-38225889) Gly (CCC) 73 bp Sc: 52.80
TCCCTGGTGGTCTAGTGGGTAAGACTCTGTGCTCCCAATGCAGGGGGCCCAGGTTCCATC
CCTGGTCAGGGAA
>Bos_taurus_chr17.trna2459-GlyCCC (58254716-58254788) Gly (CCC) 73 bp Sc: 52.82
TCCCTGGTGGTCTGGTGGCTAAGACTCTGTGCTCCCAATGCAGGGGACCCAGGTTCGATC
CCTGGTCAGGGAA
>Bos_taurus_chr29.trna1665-GlyCCC (45339873-45339945) Gly (CCC) 73 bp Sc: 52.82
TCCCTGGTGGTCCAGTGGCTAAGACTCTGTGCTCCCAATACAGGGATCCTGGGTTCGATC
CCTGGTCAGGGAA
>Bos_taurus_chr10.trna2696-GlyCCC (71236614-71236686) Gly (CCC) 73 bp Sc: 52.89
TTCCTGGTGGTCCAGTGGCTAAGACTCTGTGTTCCCAATGCAGGGGGCCCAGGTTCGATC
CCTGGTCAGGGAA
>Bos_taurus_chr27.trna1782-GlyCCC (46208303-46208375) Gly (CCC) 73 bp Sc: 52.93
TCCCTGGTGGTCTAGTGGCTAAGACTCTGTGCTCCCAATGCAGGAGCCCCGGGTTCGATC
CCTGGTCAGGGAA
>Bos_taurus_chr11.trna2179-GlyCCC (51377207-51377279) Gly (CCC) 73 bp Sc: 52.99
TCCCTGGTGGCCCAGTGGGTAAGACTCTGCACTCCCAATGCAGGGGACCCAAGTTTGATT
CTTGGTCAGGGAA
>Bos_taurus_chr18.trna376-GlyCCC (9687487-9687559) Gly (CCC) 73 bp Sc: 53.02
TCCCTGCTGGTCTAGGGGCTAAGACTCTGAGCTCCCAATGCAGAGGGCCCAGGTTCAAAC
CCTGGTCAGGGAA
>Bos_taurus_chr22.trna740-GlyCCC (16891213-16891285) Gly (CCC) 73 bp Sc: 53.07
TCTCTGGTGGTCCAGTGGTTAAGACTCTGTGCTCCCAACCCAGGGGCCAAAGGTTCAATC
CCTTGTTGGAGAA
>Bos_taurus_chr19.trna520-GlyCCC (13934199-13934270) Gly (CCC) 72 bp Sc: 53.08
TCCCTGGTGGTCCAGTGGCTAGGACTCTGTGCTCCCAGTGCAGGGGCCCAGGTTCAATCC
CTGGTCAGGGGA
>Bos_taurus_chr8.trna2068-GlyCCC (65774922-65774994) Gly (CCC) 73 bp Sc: 53.08
TCCCTGGTGGTCCAGTGGCTAAGACTCTGTGCTCCCAATGCAGGGGGCCCGGGTTCAATC
CCTGGTCAGGGAA
>Bos_taurus_chr17.trna4053-GlyCCC (63665313-63665241) Gly (CCC) 73 bp Sc: 53.09
TCCCAGGTAGTCCAGCGGCTAAGACTCTACACTCCCAATGCAGCGGGCCCAGGTTCAATC
CCTGGTCAGGGAA
>Bos_taurus_chrUn.004.18.trna4-GlyCCC (20956-21028) Gly (CCC) 73 bp Sc: 53.10
TCTCTGGTAGTCCAATGGCTAAGACTCTGCACTCCCAATGCAGGGGGCCCAGGTTCAATA
CCTGGTCAGGAAA
>Bos_taurus_chr8.trna4673-GlyCCC (102147767-102147697) Gly (CCC) 71 bp Sc: 53.10
GCATTGGTGGTTCAGTGGTAGAATTCTTGCCTCCCACACGGGAGACCCGGGTTCGTTTCC
CAGCCAATGCA
>Bos_taurus_chr2.trna6098-GlyCCC (125499575-125499503) Gly (CCC) 73 bp Sc: 53.15
TCCCTGGTGGTCCAGTGGCTAAGACTCTGTGCTCCCAGTGCAGGGGGCCCAGGTTCGATC
CCTGGTCAGGGAA
>Bos_taurus_chr27.trna3488-GlyCCC (15188327-15188255) Gly (CCC) 73 bp Sc: 53.17
TCCCTGGTGGTCCAGTGGCTAAGACTCTGCACTCCCAATGCAGGGGACCCGGGATCGATC
CCTGGTCAAGGAA
>Bos_taurus_chr5.trna4498-GlyCCC (113609278-113609350) Gly (CCC) 73 bp Sc: 53.27
TCCCTGGTGGTCCAGTGGCTAAGACTCTGCGCTCCCAATGCAGGGGACGCAGGTTCAATC

CCTGGTCAGGGAA
>Bos_taurus_chr22.trna2537-GlyCCC (53668404-53668333) Gly (CCC) 72 bp Sc: 53.34
TCCCTGGTGGTCCAGTGGTTAGGACTCTGCACTCCCAATGCAGAGGCCCAGGGTTTGATC
CTAGTCAGGGAA
>Bos_taurus_chr10.trna6423-GlyCCC (47764839-47764767) Gly (CCC) 73 bp Sc: 53.42
TCCCTGGTAGTCCAGTGGTTAAGACTCTGTGCTCCCAACGCAGGGGGCCAGGGTTCAATC
CCTGGTTAGGGAA
>Bos_taurus_chr3.trna7409-GlyCCC (54337557-54337486) Gly (CCC) 72 bp Sc: 53.44
TCCCTGGTGGTCCAGTGGCTAAGACTCTGAGCTCCCAGTGCAGGGGCCCAGGTTCGATCC
CTGGTCAGGGAG
>Bos_taurus_chrUn.004.7990.trna1-GlyCCC (484-413) Gly (CCC) 72 bp Sc: 53.44
TCCCTGGTGGTCCAGTGGCTAAGACTCTGAGCTCCCAGTGCAGGGGCCCAGGTTCGATCC
CTGGTCAGGGAG
>Bos_taurus_chr16.trna3919-GlyCCC (60442580-60442508) Gly (CCC) 73 bp Sc: 53.45
TCCCTGGTGGTCCAGTGGCTAAGATTCTGAGCTCCCAATGCAGGGGGCCCAGGTTCAAGC
CCTGGTCAGGGAA
>Bos_taurus_chr15.trna2818-GlyCCC (78237398-78237470) Gly (CCC) 73 bp Sc: 53.47
TCCCTGGTGGTCCAGTGGCTAGGACTCTGCGCTCCCGGTGCGGGGGGCCCAGGTTCAATC
CCTGGTCAGGGAA
>Bos_taurus_chr16.trna4663-GlyCCC (41588026-41587954) Gly (CCC) 73 bp Sc: 53.50
TCCCTGGTGGTCCAGTGGCTAAGACTCTGCACTCCCAATGCAGAGGGCCTGGGTTCAATC
TCTGGTCAGGGAA
>Bos_taurus_chr5.trna2506-GlyCCC (68687745-68687817) Gly (CCC) 73 bp Sc: 53.54
TCCCTGGTAGTCCAGTGGCTAAGACTCTGTACTCCCAATGCAGGGGGCCCAGGTTTGATC
CCTGGTCAAGGAA
>Bos_taurus_chr5.trna5441-GlyCCC (118068989-118068917) Gly (CCC) 73 bp Sc: 53.54
TCCCTGGTGGTCCAGTGGCTAAGACTCTGCACTCCCAATGCAGGGAGCCCGGGTTCAATC
CCTGGTCACGGAA
>Bos_taurus_chrUn.004.237.trna4-GlyCCC (78853-78924) Gly (CCC) 72 bp Sc: 53.55
TCCCTGGTGGTCCAGTGGCTAAGACTCTGCCCTCCCAAGGCAGGGGCCTGGGTTAGATCC
CTGGTCAGGGAA
>Bos_taurus_chr14.trna3643-GlyCCC (72550502-72550430) Gly (CCC) 73 bp Sc: 53.58
TCCCTGGTAGTCCAGTGGCTAGGACTCTGAGCTCCCAGTGCAGGGGGCCCAGGTTCGATC
CCTGGTCAGGGAG
>Bos_taurus_chr13.trna4934-GlyCCC (63250688-63250616) Gly (CCC) 73 bp Sc: 53.58
TCTCTGGTGGTCCAGTGGCCAAGACTCTGCACTCCCAATGCAGGGGCCCTGGGTTCGATC
CCTGGTCAGGGAA
>Bos_taurus_chr3.trna427-GlyCCC (13587316-13587388) Gly (CCC) 73 bp Sc: 53.62
TCCCTGGTGGTCCAGTGGCTAAGACTCAGCATTCCCAATGCAGGGGGCCCAGGTTTGATC
CCTGGTCAGGGAA
>Bos_taurus_chr8.trna2358-GlyCCC (73180692-73180764) Gly (CCC) 73 bp Sc: 53.62
TCTCTGGTGGTCCAGTGGCTAAGACTCTACACTCCCAATGCAGGGGGTCCAGGTTCAATC
CCTGGTCAGGGAA
>Bos_taurus_chr5.trna2208-GlyCCC (60825893-60825965) Gly (CCC) 73 bp Sc: 53.62
TCCCTGGTGGTCCAGTGGTTAAGACTCTGCACTCCCAATACAGGGGACCTGGGTTCGATC
CCTGGTTGGGGAA
>Bos_taurus_chr4.trna5295-GlyCCC (103198205-103198133) Gly (CCC) 73 bp Sc: 53.63
TCCCTGGTGGTCCAGTGGCTAAGATTCTGTGCTCCCAATGCAGAGGGCACAGGTTCGATC
CCTGGTCGGGGAA
>Bos_taurus_chr16.trna2180-GlyCCC (56384124-56384196) Gly (CCC) 73 bp Sc: 53.65
TCCTTGGTGGTTCAGTGGCTAGGACTCTGCACTCCCAAAGCAGGGGGCCCAGGTTCAATC
CCTGATCAGGGAA
>Bos_taurus_chr13.trna3159-GlyCCC (73799937-73800009) Gly (CCC) 73 bp Sc: 53.66
TCCCTGTTGGTCCAGTGACTAAGATTCTGTGCTCCCAATGCAGGGGGCCCAGGTTCGATC
CCTGGACAGGGAA
>Bos_taurus_chr21.trna5080-GlyCCC (16942408-16942336) Gly (CCC) 73 bp Sc: 53.82
TCCCTGGTGGTCCAGTGGCTAAGGCTCCATGCTCCCAAGGTGGGGGGCTCAGGTTCGATC
CCTGGCCAGGGAA
>Bos_taurus_chr8.trna6288-GlyCCC (61207126-61207054) Gly (CCC) 73 bp Sc: 53.83
TCCCTGGTGGTCCAGTGGCTAAGACTTTGCATTCCCAATGCAGGGGGCCTGGGTTCGATC
CCTGGTCAGGGAA
>Bos_taurus_chr3.trna5842-GlyCCC (100682958-100682886) Gly (CCC) 73 bp Sc: 53.87
TCCCTGGTAGTCCAGTGGCTAAGACTCTGTACTCCCAATGCAGAGGGTCTGGGTTCAATC
CCTGGTCAGGGAA
>Bos_taurus_chr17.trna4547-GlyCCC (55997281-55997209) Gly (CCC) 73 bp Sc: 53.92
TCTCTGGTGGTCCAGTGGCTAAGACTCTGCACTCCCAAAGCAGGAGGCCCAGGTTCGATC
CCTGGTCAGGGAA

>Bos_taurus_chrX.trna4323-GlyCCC (71659038-71658966) Gly (CCC) 73 bp Sc: 53.95
TCCCTGATGGTCTAATGGATAAGACTCTGTGTTCCCAAAACAGGGGGCCTAGGTTCGATC
CCTGGTCAGGGAA
>Bos_taurus_chr12.trna3564-GlyCCC (80433260-80433188) Gly (CCC) 73 bp Sc: 53.95
TCCCTGATGGTTTAGTGGTTAAGATTCTGCTCTCCCAATGCAGGGAGCTGGGGTTCAATT
CCCGGTCAGGGAA
>Bos_taurus_chr19.trna5714-GlyCCC (22204427-22204356) Gly (CCC) 72 bp Sc: 54.06
TCCCTGGTGGTCCAGTGGCTAAGACTCTGAGCTCCCAATGCAGGGGCCCAGGTTCGATCC
CTAGTCAGGGAA
>Bos_taurus_chr4.trna887-GlyCCC (26270741-26270813) Gly (CCC) 73 bp Sc: 54.08
TCCCTGGTGGTCCAGTGGTTAAGACTCTGTGCTCCCAATGCAGGGGACCCAGGTTTAATC
CCTGGTCAGGGAA
>Bos_taurus_chr13.trna1071-GlyCCC (27660162-27660234) Gly (CCC) 73 bp Sc: 54.13
TCCCTGGTAGTCCAGTGGCTAAGACTCTGAGTTCCCAATGCAGGGGGCCCAGGTTCAATC
CCTGGTCAGGGAA
>Bos_taurus_chr3.trna6250-GlyCCC (90434224-90434152) Gly (CCC) 73 bp Sc: 54.13
TCCCTGGTGGTTCAGTGGCTAAGACTCTGTGCTCCCAATGCAGGGGGTCCAGGTTCGATC
CCTGGTCAGGGAA
>Bos_taurus_chr29.trna2955-GlyCCC (30310737-30310666) Gly (CCC) 72 bp Sc: 54.25
TCCCTGGTGGTCCATGGATAAGACTCTGTACTCCCAATGCAGGGGGCCCAGGTTCGATCC
CTGGCCAGGAAA
>Bos_taurus_chr4.trna5002-GlyCCC (108746847-108746775) Gly (CCC) 73 bp Sc: 54.28
TCCCTGGTGGTCCAGTGGCTAAGACGCTGAGCTCCCAATGCAGAGGGCCCAGGTTTGATA
CCTGGTCAGGGAA
>Bos_taurus_chrUn.004.2.trna77-GlyCCC (1663368-1663440) Gly (CCC) 73 bp Sc: 54.34
TCCCTGTTGGTCCAGTGGCTAAGACTCTGTGCTCCCAATGCAGGGGGCCCAGGTTCAATC
CCTGGTTAGGGAA
>Bos_taurus_chr23.trna3989-GlyCCC (18088029-18087957) Gly (CCC) 73 bp Sc: 54.41
TCCCTGGTGGTCCAGTGGCTAACACTCTGCACTCCCAATGCAGGGGGCCCAGGTTCAGTT
CCTGGTCAGGGAA
>Bos_taurus_chr13.trna4438-GlyCCC (71086114-71086042) Gly (CCC) 73 bp Sc: 54.42
TCCCTGGTGGTCCAGTGGTTAAGACTCTACACTCCCAATGCAGAGGGCCCAGGTATGATC
CCTGGTCAGGGAA
>Bos_taurus_chrUn.004.276.trna10-GlyCCC (171318-171246) Gly (CCC) 73 bp Sc: 54.52
TCCCCGGTAGTCCAGTGGCTAGGACTCTGCGCTCCCAATGCAGGGGGCCCAGGTTGGATC
CCTGGTCAGGGAA
>Bos_taurus_chr25.trna1314-GlyCCC (23190351-23190423) Gly (CCC) 73 bp Sc: 54.55
TCCCTGGTGGTCCAGTGGCTAAGACTTTGTGCTCCCAATGCAGGGGGCCCAGGTTCGATC
CCTGGTTGGGGAA
>Bos_taurus_chr10.trna6392-GlyCCC (48334966-48334894) Gly (CCC) 73 bp Sc: 54.56
TCCCTGGTGGTCCAGTGGCTAAGACTCTGCGTTCCCAATGCAGGGGGCCCAGGTTTGATC
CCTGGTCAGGGAA
>Bos_taurus_chr17.trna2775-GlyCCC (64389511-64389583) Gly (CCC) 73 bp Sc: 54.56
TCCCTGGTGGTCCGGTGGCTAAGACTCTGCACTCCCAATGCAGGGGGCCTGGGTTCAATC
CCTGGCCAGGGAA
>Bos_taurus_chrUn.004.6624.trna1-GlyCCC (469-541) Gly (CCC) 73 bp Sc: 54.56
TCCCTGGTGGTCCGGTGGCTAAGACTCTGCACTCCCAATGCAGGGGGCCTGGGTTCAATC
CCTGGCCAGGGAA
>Bos_taurus_chr11.trna2202-GlyCCC (51688900-51688972) Gly (CCC) 73 bp Sc: 54.58
TCCCTGGTGGTCCAGTGGCTAGGACTCTGTGCTCCCAATGCAGAGGGCCCAGGTTTGATC
CCTGGTCAGGAAA
>Bos_taurus_chr24.trna2869-GlyCCC (61504594-61504523) Gly (CCC) 72 bp Sc: 54.67
TCCCGGGTGGTCCAGTGGCTAGGACTCTGTGCTCCCGATGCAGGGGCCTGGGTTCGATCC
CTAGTCAGGGAG
>Bos_taurus_chr26.trna2817-GlyCCC (33069664-33069592) Gly (CCC) 73 bp Sc: 54.69
TCCCTGATGGTCCAGTGGCTAAGACTCTGTGCTCCCAATGCAGGGGCCCTGGGTTCGATC
CCTGGTCAGGGAG
>Bos_taurus_chr7.trna3250-GlyCCC (81007716-81007788) Gly (CCC) 73 bp Sc: 54.72
TCCCTGGTAGTCCAGTGGATAAGACTCTGCACTCCCGATGCAGGGGACCCAGGTTCAATG
CCTGGTCATGGAA
>Bos_taurus_chr14.trna5725-GlyCCC (21981858-21981786) Gly (CCC) 73 bp Sc: 54.72
TCCCTGGTGGTCCAGTGGTTAAGGCTCTGCACTCCCAATGCAGAGGGCCTAGGTTTGATC
CCTGGTCAGGAAA
>Bos_taurus_chr11.trna7119-GlyCCC (50272072-50272000) Gly (CCC) 73 bp Sc: 54.76
TCCCTGGTGGTCCAGTGGCTAAGACTCTGTGCTCCCAATCCAGGGGACCCAGGTTCAATC
CCTGGTCAGGGAA
>Bos_taurus_chr5.trna2829-GlyCCC (75949884-75949956) Gly (CCC) 73 bp Sc: 54.79

TCCCTTATGGTCCAGTGGCTAAGACTCTGCATTCCCAATGCAGGAGACTCAGGTTCGATC
CCTGGTCAGGGAA
>Bos_taurus_chrUn.004.18.trna35-GlyCCC (635472-635400) Gly (CCC) 73 bp Sc: 54.80
TCCCTCGTGGTCTAGGGGCTAAGATTCTACACTCCCAATGTAGGGGACCCAGGTTCAATC
CCTGGTCAGGGAA
>Bos_taurus_chr3.trna710-GlyCCC (20722548-20722619) Gly (CCC) 72 bp Sc: 54.83
TCCCTGGTGGTCCAGTGGCTAAGACTCTGTGCTCCCAATGCAGGGGCCCAGGTTTAATCC
CTGGTCAGGGAA
>Bos_taurus_chr22.trna977-GlyCCC (24126779-24126851) Gly (CCC) 73 bp Sc: 54.83
TCCCTGGTGGTGCAGTGGTTAAGACTCTGCATTCCCAATGCAGGGGGCTCGGGTTCGATC
CCTGGTCAGGGAA
>Bos_taurus_chrX.trna4724-GlyCCC (62495601-62495529) Gly (CCC) 73 bp Sc: 54.87
TCCCTGGTGGTCCAGTGGCCGAGACTCTGCACTCCCAATGCAGTGGGTCCAGGTTCAAAC
CCTGGTTAGGGAA
>Bos_taurus_chr1.trna4610-GlyCCC (132647916-132647988) Gly (CCC) 73 bp Sc: 54.88
CCCCTGGTGGTCCAGTGGCTAAGACTCTGCACTCCCAATGCAGGGAGCCCAGGTTCAATC
CTTGGTCAGGGAA
>Bos_taurus_chr2.trna4629-GlyCCC (130989954-130990026) Gly (CCC) 73 bp Sc: 54.93
TCTCTGGTGGTCCAGTGGTTAAGACTCTGCACTCCCAGTGCAGAGGGCACAGGTTCAATC
CCTGGTCAGGGAA
>Bos_taurus_chr7.trna7909-GlyCCC (13869523-13869451) Gly (CCC) 73 bp Sc: 54.94
TCCCTGGTGGTCCAGTGGTTAGGACTCTGAGCTCCCAATGCAGGAGGCCCAGGTTCGATC
CCTGGTCAGAGAA
>Bos_taurus_chr18.trna3957-GlyCCC (49043658-49043586) Gly (CCC) 73 bp Sc: 54.98
TCTCTGGTGGTCCAGTGGTTAAGACTCTGTGCTCCCAACGCAGGGGGCCCAGGTTCAGTC
CCTGGTCAGGGAA
>Bos_taurus_chr19.trna2843-GlyCCC (56578085-56578157) Gly (CCC) 73 bp Sc: 54.98
TCCCTGGTGGTCCAGTGGCTAAGACTCTGAGCTCCCAATGCAGGGTGCCCAGGTTCGATT
CCTGGTCGGGGAA
>Bos_taurus_chr5.trna4702-GlyCCC (117964330-117964402) Gly (CCC) 73 bp Sc: 55.01
TCCCTGGTGGTCCAGTGGCTAAGACTGTGCACTCCCAATGCAGGGGGTCTGGGTTCAACC
CCCAGTCAGGGAA
>Bos_taurus_chr21.trna1190-GlyCCC (25966525-25966597) Gly (CCC) 73 bp Sc: 55.08
TCTCTGGTGGTCCAGTGGCTAAGACTCTGCACTCCCAATGCAGGGGACCCAGGTTCAATT
CCTAGTCAGGGAA
>Bos_taurus_chr6.trna3636-GlyCCC (109522474-109522546) Gly (CCC) 73 bp Sc: 55.13
TCCCTGGTGGGCCAGTGGTTAAGACTCTGCACTCCCAACGCAGGGGGCCCAGGTTCAATC
CCTGGTCAGGGAC
>Bos_taurus_chr19.trna4020-GlyCCC (53793238-53793167) Gly (CCC) 72 bp Sc: 55.13
TCCCTGGTGGTCCAGTGGCTAAGACTCTGCACTCCCAATACAGGGGCCTGGGTTCAATCC
CTGGTCAGGGAA
>Bos_taurus_chr8.trna5592-GlyCCC (77420431-77420360) Gly (CCC) 72 bp Sc: 55.16
TCCCTTGTGGTCCAGTGGTTAAAACTCTGTGCTCCCAATGCAGGGGCCCAGGTTCAATCC
CTGGTCAGGGAA
>Bos_taurus_chr14.trna214-GlyCCC (6195117-6195189) Gly (CCC) 73 bp Sc: 55.19
TCCTTGGTGGTCCGGTGGATAAGACTTTGAACTCCCAATGCAGGGGCCCCAGGTTCGATC
CCTGGTCAGGGAA
>Bos_taurus_chr3.trna4258-GlyCCC (116897763-116897835) Gly (CCC) 73 bp Sc: 55.20
TCCCTGGTGGTCCAGTGGTTAAGATTCTGAGCTCCCAATGCAGAGGGCCCAGGTTAGATC
CCTGGTCAGGGAA
>Bos_taurus_chr19.trna1189-GlyCCC (24589574-24589646) Gly (CCC) 73 bp Sc: 55.23
TTCCTGATGGTCCAGTGGTTAAGACTCTGCACTCCCAATACAGGGGGCCTGGGTTCGATC
CCTGGTCAGGGAA
>Bos_taurus_chr6.trna134-GlyCCC (6128581-6128653) Gly (CCC) 73 bp Sc: 55.30
TCCCTGATGGTCCAATGGCTAAGACTCTGCACCCCCAATGCAGGGGGCCTGGGTTCAATC
CTCGGTCAGGGAA
>Bos_taurus_chr27.trna2415-GlyCCC (36301296-36301224) Gly (CCC) 73 bp Sc: 55.31
TCTCTGGTGGTCCAATGGCTAGGACTCTGCACTCCCAATGCAGGGGGCCTGGGTTCGATC
CCTGGTCAGGGAA
>Bos_taurus_chr23.trna400-GlyCCC (10822202-10822274) Gly (CCC) 73 bp Sc: 55.32
TCCCCGGTGGTCCAGTGGCTAAGACTCTGCACTCCCACGGCAGGGGTCCCAGGTTCAATC
CTTGGTTGGGAAA
>Bos_taurus_chr23.trna1113-GlyCCC (25122589-25122661) Gly (CCC) 73 bp Sc: 55.34
TCCCTGGTGGTCCAGTGGCTAAGACTCTGAGCTCCCAAGGCAGGGGGCCCAGGTTCAATC
CCTGGTCAGGGAA
>Bos_taurus_chr27.trna1288-GlyCCC (34800854-34800927) Gly (CCC) 74 bp Sc: 55.36
TCCCTGGTGGTCCAGTGGTCTAAGACTCCACATTCCCAATGCAGGCGGCCCAGGTTCGAT

CCCTGGTCAGGGAA
>Bos_taurus_chr13.trna1803-GlyCCC (42635163-42635235) Gly (CCC) 73 bp Sc: 55.36
TCCCTGATGGTCCAGTGGCTAAGACTCTGCACTCCCAATGCAGGGGGCCTGGGTTCAATC
CCTGGTCAGGAAA
>Bos_taurus_chr12.trna4120-GlyCCC (71389478-71389407) Gly (CCC) 72 bp Sc: 55.37
TCCCTGGTGGTCCAGTGGCTAAGACTCTGCACTCCCAATGCAGGGGCACAAGTTCAATCC
CTGGCCATGGAA
>Bos_taurus_chr19.trna943-GlyCCC (20540434-20540506) Gly (CCC) 73 bp Sc: 55.39
TCTCTGATGGTCCAGTGGCTAAGACTCTTCACTCCCAATGTAGGGGGCCCAGGTTCAATC
CCTGGTCAGGAAA
>Bos_taurus_chr12.trna2896-GlyCCC (74275018-74275090) Gly (CCC) 73 bp Sc: 55.44
TCCCTGGTGGTCCAGTGGCTAAGACTCAGCACTCCCAATGCAGGGGGCCCAGGTTTGATC
CCTGGTCAGGGAA
>Bos_taurus_chr4.trna323-GlyCCC (10081149-10081221) Gly (CCC) 73 bp Sc: 55.45
TCCCTGATGGTCCAGTGGCTAAGACTCTGCATTCCCAATGCAGGGGGCCCAGGTCTGATC
CCTGGTCAGGGAA
>Bos_taurus_chr15.trna4660-GlyCCC (42545818-42545746) Gly (CCC) 73 bp Sc: 55.45
TCCCTAGTGGTCCAGTGGCTAAGACTCTGCACTCCCAATGCAGGGGACCCAGGTTCAGTC
CCTGGTTGGGGAA
>Bos_taurus_chr5.trna2229-GlyCCC (61258402-61258475) Gly (CCC) 74 bp Sc: 55.46
TCCCTGGTGGTCCAGTGGTTAAGATTCTGGCATTCCCAATGCAGAGGGCTCAGGTTCAAT
CCCTGGTCAGGGAA
>Bos_taurus_chr20.trna637-GlyCCC (18847154-18847226) Gly (CCC) 73 bp Sc: 55.47
TCCCTGGTAGTTCAGTGGCTAAGACACTACACTCCCAATGCAGGGGACCCAGGTTCAATC
CCTGGTCAGGGAA
>Bos_taurus_chr22.trna256-GlyCCC (6528791-6528863) Gly (CCC) 73 bp Sc: 55.47
TCCCTGGTGGTCCAGAGGCTAAGACTCTGCACTCCCAATCAAGGGGTCCCAAGTTCGATC
CTTGGTCAGGGAA
>Bos_taurus_chrX.trna3221-GlyCCC (83982906-83982977) Gly (CCC) 72 bp Sc: 55.50
TCCCTGGTGGTCCAGTGGCTAAGACTCTGTACTCCCAGTGCAGGGGCCCAGGTTCGATCC
CTGGTCGAGGAA
>Bos_taurus_chr3.trna4895-GlyCCC (123465552-123465480) Gly (CCC) 73 bp Sc: 55.50
TCCCTGGTGGTCCAGTGGTTAAGACTTTGCACTCCCAATGCAGGGGACACAGGTTCAAGC
CCTGATTGGGGAA
>Bos_taurus_chr7.trna2998-GlyCCC (71794818-71794890) Gly (CCC) 73 bp Sc: 55.53
TCCCTGGTGGTCTAGTGGCTAAGATACTGTTCTCCCAATGCAGAGGGCCCAGGTTGGATC
CCTGGTCAGGGAA
>Bos_taurus_chr1.trna6679-GlyCCC (137710134-137710063) Gly (CCC) 72 bp Sc: 55.55
TCCTTGGTGGTCCAATGGCTAAGATTCTTCACTCCCAATGCAGGGGCCCAGGTTCGATCC
CTGGTCAGGGTA
>Bos_taurus_chrUn.004.302.trna1-GlyCCC (27646-27717) Gly (CCC) 72 bp Sc: 55.55
TCCTTGGTGGTCCAATGGCTAAGATTCTTCACTCCCAATGCAGGGGCCCAGGTTCGATCC
CTGGTCAGGGTA
>Bos_taurus_chr2.trna8086-GlyCCC (75251261-75251189) Gly (CCC) 73 bp Sc: 55.58
TCCCTGGTGGTCCAGTGGCTAAGACGCTGTGCTCCCAATGCAGGAGGCCCAGGTTCAATA
CCTGGTCAGGGAA
>Bos_taurus_chr5.trna4870-GlyCCC (120589351-120589423) Gly (CCC) 73 bp Sc: 55.68
TCCCTTGTGGTCCAGTGGCTAAGACTCTGCGCCCCCAATGCAGAGGGCCCAGGTTCGATC
CCTGGTCAGGGAA
>Bos_taurus_chr7.trna7570-GlyCCC (17995223-17995151) Gly (CCC) 73 bp Sc: 55.74
TCCCTGGTGGTCTAGTGGCTAAGACTCTGTGCTCCCAACACAGGGACCCAGGGTTCGATC
CCTGGTCAGGGAA
>Bos_taurus_chr22.trna2698-GlyCCC (49776817-49776745) Gly (CCC) 73 bp Sc: 55.75
TCCCTGGTGGTCCAATGGTTAAGACTCTGTGCCCCCAATGCAGGGGGCCCAGGTTCAATC
CCTGGTCAGGGAA
>Bos_taurus_chr27.trna134-GlyCCC (5392363-5392435) Gly (CCC) 73 bp Sc: 55.85
TCCCTGGTGGTCCAGTGGCTAAGACTCTACACTCCCAATGCAGGGGGCCCAGGTTTGATC
CCTGGTCAGGGAA
>Bos_taurus_chr13.trna4778-GlyCCC (65749520-65749448) Gly (CCC) 73 bp Sc: 55.87
TCCCTGGTGGTCCAGTCGCTAAGACTCTGAACTCCCAATGCAGGGGGCCCAGGTTCAATC
CCTGGTCAGGGAA
>Bos_taurus_chr28.trna269-GlyCCC (6746994-6747066) Gly (CCC) 73 bp Sc: 55.87
TCCCTGGTAGTCCAGTGGCTAAGACTCTGGGTTCCCAATACAGGGGGCCCAGGTTCGATC
CCTGGTCAGGGAA
>Bos_taurus_chr6.trna5650-GlyCCC (93990309-93990237) Gly (CCC) 73 bp Sc: 55.87
TCCCTGGTGGTCCAGTGGTTAAGACTTTGCGCTCCCATTGCAGAGGGCACAGGTTCAATC
CCTGGTCAGGGAA

>Bos_taurus_chr2.trna6237-GlyCCC (123294986-123294914) Gly (CCC) 73 bp Sc: 55.91
TCCCTGGTGGTCCAGTGGCTAAGACTCTGTGCTCCCAATGCAGGGGGTCTGGGTTCAATC
CCCAGTCAGGGAA
>Bos_taurus_chr17.trna5262-GlyCCC (43554459-43554387) Gly (CCC) 73 bp Sc: 55.94
TCCCTGGTTGTCTAGTGGCTAAGACTCAGCACTCCCAATGCAGGGGTCCTGAGTTCGATC
CCCAGTCAGGGAA
>Bos_taurus_chr6.trna5333-GlyCCC (99738607-99738535) Gly (CCC) 73 bp Sc: 56.03
TCCCTGGTGGTCCAGTGGCTAAGACTCTGTGCTCCCAATGCAGGGGTCCTGGGTTCGATC
CCTGGTCAGGGAA
>Bos_taurus_chr17.trna3919-GlyCCC (65646440-65646368) Gly (CCC) 73 bp Sc: 56.05
TCCCTGATGGTCCAGTGGCTAAGACTCCACGTTCCCAACATAGGGGGCCCAGGTTCAATC
CCTGGTCAGGGAA
>Bos_taurus_chr16.trna1498-GlyCCC (40624683-40624755) Gly (CCC) 73 bp Sc: 56.05
TCCCTGGTGGTCCAGTGGCTAAGACTCCACACTCCCAATGCAGGGGACCCAGGTTCGATT
CCTGGTCAGGGAA
>Bos_taurus_chr13.trna4839-GlyCCC (65082798-65082726) Gly (CCC) 73 bp Sc: 56.10
TCCCTGGTGGTCCAATGGCTAAGACTCTGTGCTCCCAATGCAGGGGGCCCAGGTTCAATC
CCTGGTCAGGGAA
>Bos_taurus_chr14.trna1892-GlyCCC (43178766-43178838) Gly (CCC) 73 bp Sc: 56.10
TCCCTGGTGGTCCAGTGGCTAAGACTCTGTGCTCCCAATGCAGAGGGCCCAGGTTCAATC
CCTGGTCAAGGAA
>Bos_taurus_chr20.trna1627-GlyCCC (43647705-43647777) Gly (CCC) 73 bp Sc: 56.10
TCCCTGATGGTCCAGTGGCTAAGACTCTGTGCTCCCACTGCAGGGGTCCTGGGTTCAATC
CCTGGTCAGGGAA
>Bos_taurus_chrUn.004.1819.trna8-GlyCCC (4921-4849) Gly (CCC) 73 bp Sc: 56.11
TTCCTGGTGGTCCAGTGGCTAGGACTCTGCACTCCCAATGCAGGGAGCCCAGGTTCAATC
CCTGGTCAGAGAA
>Bos_taurus_chr25.trna940-GlyCCC (15743397-15743468) Gly (CCC) 72 bp Sc: 56.12
TGCCTGGTAGTCTAGTGGCTAAGACTCTACGCTCCCAAAGCAGGGGCCCAGGTTCGATCC
CTGGCCAGGGAA
>Bos_taurus_chr16.trna1219-GlyCCC (34551016-34551088) Gly (CCC) 73 bp Sc: 56.16
TCTCTGGTGGTCCAGTGGCTAAGACTCCACGCTCCCAACATGGGGGGCCCAGGTTCAATC
CCTGGTCAGGGAA
>Bos_taurus_chr14.trna5719-GlyCCC (22117526-22117454) Gly (CCC) 73 bp Sc: 56.18
TCATTGGTGGTCCAGTGGCTAAGACTCTGCACTCCCAATGCAGGGGGTCCAGGTTCAATC
CCTGGTCAGGGAA
>Bos_taurus_chr18.trna3943-GlyCCC (49388350-49388279) Gly (CCC) 72 bp Sc: 56.19
TCCCTGGTGGTCCCGTGGTTAAGACTCTGCACTCCCAATGCAGGGGCCCAAGTTCGATCC
CTGGTCAAGGAA
>Bos_taurus_chr17.trna5054-GlyCCC (49349534-49349462) Gly (CCC) 73 bp Sc: 56.19
TCCCTGGTGGTCTAGTGGCTAAGATGCTGCATTCCCAATGCAGGGGACCTGGGTTCGATC
CCTGGTCAGGGAA
>Bos_taurus_chr23.trna1229-GlyCCC (27990502-27990574) Gly (CCC) 73 bp Sc: 56.21
TCCTTGGTGGTCCAGTGGCTAAGACTCTGCTCTCCCAAAGCAGGGGGCTCAGGTTCAATC
CCTGCTCAGGGAA
>Bos_taurus_chr1.trna7290-GlyCCC (120391192-120391120) Gly (CCC) 73 bp Sc: 56.22
TCCCTGGTGGTCCAGTGGCTAAGACTCTACACTCCCAATGCAGGGTGCCCAGGTTCAATC
CCTGGCCAAGGAA
>Bos_taurus_chr18.trna3327-GlyCCC (57367493-57367421) Gly (CCC) 73 bp Sc: 56.22
TCCCTTGTGGTCCAGTGGCTAGGACTCTGAACTCCCAATGCAGGGGACCCTGGTTCGATC
CCTGGTCAGGGAA
>Bos_taurus_chr6.trna492-GlyCCC (18734252-18734324) Gly (CCC) 73 bp Sc: 56.23
TCCCTGGTGGTCGAGTGTCTAAGACTCTGCGCTCCCAATGCAGGGGATCCAGGTTCGATT
CCTGGTCAGGGAA
>Bos_taurus_chrUn.004.1429.trna3-GlyCCC (29044-29116) Gly (CCC) 73 bp Sc: 56.27
TCCCTGGTGGTCCAGTGGCTAAGACTCTACACTCCCAATGCAGATGGCCCAGGTTCAACC
CCTGGTCAGGGAA
>Bos_taurus_chr16.trna1345-GlyCCC (38148304-38148376) Gly (CCC) 73 bp Sc: 56.27
TCCCTAGTGGTCCAGTGGCTAAGACTCTGCACTCCCAATGCAGGGGGGCCCAGGTTCGATC
CCTGCTCAGGGAA
>Bos_taurus_chr21.trna4539-GlyCCC (26226972-26226900) Gly (CCC) 73 bp Sc: 56.28
TCCCTGGTGGTCCAGTGGCTAAGAGTCTGCATTCCCAATGCAGGGGGTCCAGGTTCAATC
CCTGGTCAGGGAA
>Bos_taurus_chr3.trna6898-GlyCCC (71105469-71105397) Gly (CCC) 73 bp Sc: 56.32
TCCCTGGTGGTCCAGTAGCTAAGACTCTGTACTCCCAATGCAGGGGGCCCAGGTTCAATC
CCTGGTCAGGGAA
>Bos_taurus_chr12.trna5546-GlyCCC (30801034-30800962) Gly (CCC) 73 bp Sc: 56.34

TCCCTGGTGGTCCAGTGGCTAAGACTCTGAGCTCCCAATGCAGGGGGCCCAGGTTCAATC
CCTGGTCAGGGAA
>Bos_taurus_chr3.trna4143-GlyCCC (114394378-114394450) Gly (CCC) 73 bp Sc: 56.38
TCGCTGGTGGTCCAGTGGCTAGGACTCTGTGCTCCCAATGCAGGGGACCCAGGTTCGATC
CCTGGTCAGGGAA
>Bos_taurus_chr22.trna1555-GlyCCC (43979100-43979172) Gly (CCC) 73 bp Sc: 56.43
TCCCTGGTGGTCCAGTGGCTAAGACTCTGTGCTCCCAATGCAGAGGGCCCAGGTTCTATC
CCTGGTCAGGGAA
>Bos_taurus_chr1.trna25-GlyCCC (363540-363612) Gly (CCC) 73 bp Sc: 56.43
TCCTTGGTGGTCCAGTGGCTAAGACTCCGTGCTCCCAATGCAGGGGTCCCAGGTTCAATC
CCTGGTCAGGGAA
>Bos_taurus_chr22.trna237-GlyCCC (6420050-6420126) Gly (CCC) 77 bp Sc: 56.46
TCCCTCATGGTCCAGTGGCTAAGACTCTGCACTCCCAATCAATGCAGGGGACCCAGGTTC
AATTCCTGGTCGGGGAA
>Bos_taurus_chr28.trna661-GlyCCC (16784063-16784135) Gly (CCC) 73 bp Sc: 56.52
TCCCTGGTGGTCCAGTGGTTAAGACTCTGCACTCCCAATACAGGGGGCCTGGGTTCGATC
CCTGGTCGGGGAA
>Bos_taurus_chr21.trna584-GlyCCC (16422114-16422186) Gly (CCC) 73 bp Sc: 56.55
TCCTTGGTTGTCTAGTGGCTAAGACTCTGTGCTCCCAACACAGGGGTCACAGGTTCGATC
CCTGGTCAGGGAA
>Bos_taurus_chr17.trna3595-GlyCCC (71587740-71587669) Gly (CCC) 72 bp Sc: 56.57
TCCCTGGTGGTCCAGTGGCTAAGACTCTGTGCTCCCAATGCAGGGGCCCAGGTTTGATCC
CTGGTCAGGGAA
>Bos_taurus_chrUn.004.428.trna3-GlyCCC (52681-52752) Gly (CCC) 72 bp Sc: 56.57
TCCCTGGTGGTCCAGTGGCTAAGACTCTGTGCTCCCAATGCAGGGGCCCAGGTTTGATCC
CTGGTCAGGGAA
>Bos_taurus_chr9.trna3833-GlyCCC (105017037-105017109) Gly (CCC) 73 bp Sc: 56.58
TCCCTGGTGGTCCAGTGGCTAACACTCTGCACTCCCAATGGAGGGGGCCCAGGTTCGATC
CCTGGTCAGGGAA
>Bos_taurus_chr28.trna522-GlyCCC (12903216-12903288) Gly (CCC) 73 bp Sc: 56.59
TTCCTGGTGGTCCAGTGGTTAAGATTCTGCACTCCCAATGCAGGGGGCCTAGGTTCAATC
CCTGGGCAGGGAA
>Bos_taurus_chr19.trna1816-GlyCCC (36062597-36062669) Gly (CCC) 73 bp Sc: 56.61
TCCCTGGTGGTCCAGTGGCTAAGATTCTGAGCTCCCAATGCAGGGGGCCCAGGTTCGATC
CCTGGTCAGGGAA
>Bos_taurus_chr5.trna9100-GlyCCC (31286104-31286032) Gly (CCC) 73 bp Sc: 56.66
TCCTTGGTGGTCCAATGGCTAAGACTCTGCACTCCCAATGCAGGGAGCCCAGGTTCGGTT
CCTGGTCAGGGAA
>Bos_taurus_chrX.trna3697-GlyCCC (86545686-86545615) Gly (CCC) 72 bp Sc: 56.67
TCCCTGGTGGTTCAGTGGCTAAGACTCTGCACTCCCATTGCAGCGGCCTAGGTTCAATCT
CTAGTCAGGGAA
>Bos_taurus_chr15.trna3066-GlyCCC (83919333-83919261) Gly (CCC) 73 bp Sc: 56.67
TCCCTGGTGGTTGAGTGTTTAAGACTCTGCGCTCCCAAGGCAGGGGGCCCAGGTTCAATC
CCTGGTCAGGGAA
>Bos_taurus_chr27.trna1381-GlyCCC (36251647-36251719) Gly (CCC) 73 bp Sc: 56.69
TCCCTGGTGGTCCAGTGGCGAAGACTCTGCACTCCCAGTGCAGGGGTCCCAGGTTCAATC
CCTGGTCAAGGAA
>Bos_taurus_chr5.trna9094-GlyCCC (31514363-31514291) Gly (CCC) 73 bp Sc: 56.70
TCCCTGGTGATCCAGTGGCTAATACTCTGCACTCCCAATGCAGGGGGCCCAGGTTCGATT
CCTGGTCAGGGAA
>Bos_taurus_chr12.trna1537-GlyCCC (33833165-33833237) Gly (CCC) 73 bp Sc: 56.71
TCCCTGGTGGTCCAGTGGCTAGGACTCTGTGCTCCCAATGCAGGGGGCCCGGGTTCAATC
CCTGGTCAGGGAA
>Bos_taurus_chr23.trna57-GlyCCC (1213026-1213098) Gly (CCC) 73 bp Sc: 56.72
TCCTTGGTGGTTCAGTGGCTAAGACTCTGCACTCCCAATGCAGGGGGCCCGGGTTCAATC
CCTGGTCAGGGAA
>Bos_taurus_chr18.trna644-GlyCCC (17191654-17191726) Gly (CCC) 73 bp Sc: 56.76
TCCCTGGTGGTCCAGTGGTTAAGACTCCGCACTCCCAATGCAGGGGACCCAGGTTCGCTC
CCTGGTCAGGGAA
>Bos_taurus_chr25.trna4059-GlyCCC (15596515-15596444) Gly (CCC) 72 bp Sc: 56.77
TCCCTGGCGGTCCAGTGGCTAGGACTCTGTACTCCCAATCCAAGGTCCCAGGTTCGATCC
CTGGTCAGGGAA
>Bos_taurus_chr2.trna4674-GlyCCC (131748691-131748763) Gly (CCC) 73 bp Sc: 56.77
TCCCTGGTGGTCCAGTGGCTAAGACTCTGTGCTCCCAATGCAGGGAGCCCAGGTTCAATC
CCTGGTCAGGGAA
>Bos_taurus_chr4.trna4527-GlyCCC (119771657-119771585) Gly (CCC) 73 bp Sc: 56.77
TCCCTGGTGGTCCAGTGGCTAAGACTCTGTGCTCCCAATGCAGGAGGCCCAGGTTCGATC

CCTGGTCAGGGAA
>Bos_taurus_chr28.trna695-GlyCCC (17916873-17916945) Gly (CCC) 73 bp Sc: 56.88
TCCCTGGGGGTCCAGTGGTTAGGACTCTGCACTCCCAATGCAGGGGGCCTGGGTTCAATC
CCTGGTCAGGGAA
>Bos_taurus_chr17.trna2755-GlyCCC (64204848-64204920) Gly (CCC) 73 bp Sc: 56.89
TCCTTGGTGGCCCAGTGGCTAAGACTCTGCACTCCCAATGCAGGCGGCCCAGGTTCGATC
CCTGGTCAGGGAA
>Bos_taurus_chr12.trna1470-GlyCCC (32517892-32517964) Gly (CCC) 73 bp Sc: 56.92
TCCATGGTGGTCCGGTGGCTAAGACTCTGCGCTCCCAACGCAGGGGTTCTGGGTTCAATC
CCCAGTCAGGGAA
>Bos_taurus_chr15.trna2014-GlyCCC (57843045-57843117) Gly (CCC) 73 bp Sc: 56.99
TCCCTGGTGGTCCAGTGGTTAAGACTCTGCACTCCCAATGTAGGGGGTGCAGGTTTGATC
CCTGCTCAGGGAA
>Bos_taurus_chr16.trna2189-GlyCCC (56722761-56722833) Gly (CCC) 73 bp Sc: 57.00
TCCCTGGTGGTCCAGTGGCTAAGACTCCACATTCCCAATGCAGAGGGCCCAGGTTCGATC
CCTGGTCAGGGAA
>Bos_taurus_chrX.trna4739-GlyCCC (62258905-62258833) Gly (CCC) 73 bp Sc: 57.18
TCCCTGGTGGTCCAGTGGCCAAGACACCACATTCCCAATGTAGTGGGCCCAGGTTCGATC
CCTGGTTGGGGAA
>Bos_taurus_chr15.trna601-GlyCCC (22845380-22845452) Gly (CCC) 73 bp Sc: 57.25
CCCCTGGTGGTCCAATGGCTAAGACTCTGCACTCCCAATGCAGGCGCCCCAGGTTCGATC
CCTGGTCAGGGAA
>Bos_taurus_chr7.trna8291-GlyCCC (9340662-9340588) Gly (CCC) 75 bp Sc: 57.25
TCCCTAGTGGTCCAGTGGCTAAGACTCTGCACTCCCAATGCAGAGAGGGCCCAGGTTCAA
TCCCTGGTCAGGGAA
>Bos_taurus_chr25.trna2687-GlyCCC (39147417-39147345) Gly (CCC) 73 bp Sc: 57.25
TCCCTGGTGGTCCAGTGGCTAAGACTCTGCACTCCCAATGCAGGGGGCCTGGGTTCAATC
CCTGGTCAGGGAA
>Bos_taurus_chr25.trna4475-GlyCCC (9278026-9277954) Gly (CCC) 73 bp Sc: 57.29
TCCCTGGTGGTCCAGTGGTTAAGACTCTGCACTCCCAATGCAGGGGGCACAGGTTCAATC
CCTGGTCAGAGAA
>Bos_taurus_chr11.trna4382-GlyCCC (104891982-104892054) Gly (CCC) 73 bp Sc: 57.34
TCCCTGGTGGTCCAGTGGCTAAGACTCGGCATTCCCAATGCTGCGGGCCCGGGTTCGATT
CCTGGTCAGGGAA
>Bos_taurus_chr2.trna4795-GlyCCC (133684976-133685048) Gly (CCC) 73 bp Sc: 57.41
TCCCTGGTGGTCCAGTGGCTAAGACTCTGAACTCCCAATGCAGGGGGCCTGGGTTCGATC
CCTGGCCAGGGAA
>Bos_taurus_chr1.trna7254-GlyCCC (120903350-120903279) Gly (CCC) 72 bp Sc: 57.45
TCCCTAGTGGTCCAGTGGTTAAGACTCTGTGATCCCAATGCAGGGTCCCAGGTTCGATCC
CTGGTTAGGGAA
>Bos_taurus_chr14.trna5584-GlyCCC (24487475-24487403) Gly (CCC) 73 bp Sc: 57.46
TCCCTGGTGGTCCAGTGGCTAAGACTCTGCACTCCCAATGCAGAGGGCCTGGGTTTGATC
CCTAGTCAGGGAA
>Bos_taurus_chr28.trna297-GlyCCC (7092798-7092869) Gly (CCC) 72 bp Sc: 57.49
TCCCTGTGATCCAGAGGTTAAGATTCTGCACTCCCAATGCAGGGGTCCCAGGTTCAATCC
CTGGCCAGGGAA
>Bos_taurus_chr21.trna4753-GlyCCC (22960429-22960357) Gly (CCC) 73 bp Sc: 57.52
TCCCTGGTGGTCCAGTGGCTAAGATTCTGCACTCCCAATGCAGGGGGCCTGGGTTCGATC
CCTGGTCAGGGAA
>Bos_taurus_chr5.trna6654-GlyCCC (94060370-94060298) Gly (CCC) 73 bp Sc: 57.54
TCCCTGGTAGTCCAGTGGCTAAGACTCTGAACTCCCAATGCAGGGGGCCCAGGTTCGACC
CCTGGTCAGGGAC
>Bos_taurus_chr16.trna3736-GlyCCC (64239744-64239672) Gly (CCC) 73 bp Sc: 57.55
TGCCTGGTGGTCCAGTGGCTAAGACTCTGTACTCCCAATGCAGGGGGCCCAGGTTCAATC
CCTGGTCAGGGAA
>Bos_taurus_chr26.trna3506-GlyCCC (14120924-14120852) Gly (CCC) 73 bp Sc: 57.67
TCCCTGGTGGTCCAGTGGCTAAGACTCTGCACTCCCAATGCAGGGGGCCTGGGTTCGATC
CCTGGTTAGGGAA
>Bos_taurus_chrUn.004.288.trna24-GlyCCC (83172-83100) Gly (CCC) 73 bp Sc: 57.68
TTCCTACTGGTCTAGTGGCTAAGACTCTGCACTCCCAATGCAGGGGTCCCAGGTTTGATC
CCTGGTAAGGAAA
>Bos_taurus_chrX.trna354-GlyCCC (8196163-8196235) Gly (CCC) 73 bp Sc: 57.68
TCCCTGATGGTCCAGTGGCTAAGACTCCTCACTCCCAATGCAGGGAGTCCAGGTTCAATT
CCTGGTCAGGGAA
>Bos_taurus_chr8.trna152-GlyCCC (5224152-5224224) Gly (CCC) 73 bp Sc: 57.70
TCCCTGATGGTCCAGTGGCTAAGACTCTGTGCTCCCAATGCAGAGGGCCTGGGTTCGATT
CCTGGTCAGGGAA

>Bos_taurus_chrUn.004.4789.trna4-GlyCCC (9319-9391) Gly (CCC) 73 bp Sc: 57.70
TCCCTGATGGTCCAGTGGCTAAGACTCTGTGCTCCCAATGCAGAGGGCCTGGGTTCGATT
CCTGGTCAGGGAA
>Bos_taurus_chrUn.004.281.trna6-GlyCCC (65062-65134) Gly (CCC) 73 bp Sc: 57.80
TCCCTGGTGGTCCAGTGGATAAGACTCTGCACTCCCAATACAGGGGGCCCAGGTTCAATC
CCTGGTCAAGGAA
>Bos_taurus_chr7.trna7421-GlyCCC (20018430-20018359) Gly (CCC) 72 bp Sc: 57.86
TCCCTGGTGGTCCAGTGGCTTAGACTCCGTGCTCCCAACGCAGGGGCCCAGGTTCGATTC
CTGGTCAGGGAA
>Bos_taurus_chr7.trna7828-GlyCCC (14812194-14812122) Gly (CCC) 73 bp Sc: 57.88
TCCCTGGTGGTCCAGTGGCTAAGACTCTGCATTCCCAATGCAGGGGGCCCAAGTTCGATC
CTTTGTCAGGGAA
>Bos_taurus_chr15.trna4732-GlyCCC (40979371-40979299) Gly (CCC) 73 bp Sc: 57.99
TCCCTGGTGGTCCAATGGCTAAGACTCTGCACTCCCAATGCAGAGGGACTAGGTTCAATC
CCTGGTCAGGGAA
>Bos_taurus_chr2.trna7463-GlyCCC (94454951-94454879) Gly (CCC) 73 bp Sc: 58.01
TCCCTGGTGGTCCAGTGGTTGAGACTCTGCACTCCCAATGCAGGGGGTCCAGGTTCAATC
CCTGGTTGGGGAA
>Bos_taurus_chr5.trna644-GlyCCC (20984712-20984784) Gly (CCC) 73 bp Sc: 58.02
TCCTTGGTTGTCCAGTGGCTAAGACTCTGCACTCCCAATGCAGAGGGCCCAGGTTCGATC
CCTGTTCAGGGAA
>Bos_taurus_chr13.trna4917-GlyCCC (63706634-63706562) Gly (CCC) 73 bp Sc: 58.08
TCCCTGGTGGTCCAGTGGCTAAGACTCTGAGCTCCCAATGCAGGGGGCCCAGGTTCGATC
CCTGGTCAGGGAA
>Bos_taurus_chr17.trna4147-GlyCCC (62704002-62703930) Gly (CCC) 73 bp Sc: 58.20
TCCCTGTTGGTCCAGTGGTTAAGACTCTGTGCTCCCAACACAGGGGGCCCAGGTTCAATC
CCTGGTCAAGGAA
>Bos_taurus_chr12.trna364-GlyCCC (11773912-11773984) Gly (CCC) 73 bp Sc: 58.25
TCCCTGGTGGTCCAGTGGCTAAGACTCTGCACTCCCAATGCATGAGGCCTAGGTTCAATC
CCTGGTCAGGGAA
>Bos_taurus_chr6.trna3563-GlyCCC (107776343-107776415) Gly (CCC) 73 bp Sc: 58.26
TCCCTGGTGGTCCAGTGGCTAAGACTCTGCATTCCCAATGCAGGGAACCCTGGTTCAATC
CCTGGTCAGGGAA
>Bos_taurus_chr12.trna4856-GlyCCC (50723546-50723475) Gly (CCC) 72 bp Sc: 58.27
TCCCTGGTGGTCCAGTGGATAAGACTCCATACTCCCAATGCAGAGGCCCAGGTTCAATCC
CTGGTCAGGGAA
>Bos_taurus_chr3.trna8642-GlyCCC (21360158-21360086) Gly (CCC) 73 bp Sc: 58.31
TCCCTGGTGGTCCAATGGCTAAGACTCTGCACTCCCAATGCAGGGATCCTGGGTTCAATC
CCTGGTCAGGGAA
>Bos_taurus_chr9.trna5280-GlyCCC (76792974-76792902) Gly (CCC) 73 bp Sc: 58.53
TCCCTGGTGATCCAGTGGTTAAGACTCTGCTCTCCCAATGCAGGGAGCCCAGGTTCGATC
CCTGGTCAGGGAA
>Bos_taurus_chr22.trna4319-GlyCCC (6704183-6704111) Gly (CCC) 73 bp Sc: 58.55
TCCCTGGTGGTCCAATGGTTAAGATTCTGTGCTCCCAATGCAGGGGGCCCAGGTTCAATC
CCTGGTCAGGGAA
>Bos_taurus_chr22.trna4326-GlyCCC (6665018-6664946) Gly (CCC) 73 bp Sc: 58.55
TCCCTGGTGGTCCAATGGTTAAGATTCTGTGCTCCCAATGCAGGGGGCCCAGGTTCAATC
CCTGGTCAGGGAA
>Bos_taurus_chr27.trna2583-GlyCCC (33601031-33600959) Gly (CCC) 73 bp Sc: 58.57
TCACTGGTGGTCCAGTGGTTAGGACTCTGAGCTCCCAATGCAGGGGGCCCAGGTTCGATC
CCTGGTCAGAGAA
>Bos_taurus_chrUn.004.53.trna65-GlyCCC (5206-5134) Gly (CCC) 73 bp Sc: 58.85
TCCTTGGTGGTCCAGTGGCTAAGACTCTGCACTCCCAATGCAGGGGGCCCAGGTTCAATC
CCTGGTCAGAGAA
>Bos_taurus_chr6.trna3061-GlyCCC (98507652-98507724) Gly (CCC) 73 bp Sc: 58.88
TCCCTGGTGGTCCAGTGGCTAAGACTCCGCATTCCCAATGCCGGTGGCCCAGGTTCAATC
CCTGGTCAGGGAG
>Bos_taurus_chr24.trna4000-GlyCCC (36217142-36217070) Gly (CCC) 73 bp Sc: 58.90
TCCCTGGTGGTCCAGTGGTTAAGACTCTGAGCTCCCAATGCAGAGGGCCCAGGTTCGATT
CCTGGTCAGAGAA
>Bos_taurus_chr23.trna243-GlyCCC (7724623-7724695) Gly (CCC) 73 bp Sc: 58.94
TCCCTGGTGGTCCAGTGGTTAAGACTCCACACTCCCAATGCAGGGGGCCCAGGTTCGATC
CCTGGTTAGGGAA
>Bos_taurus_chr14.trna1721-GlyCCC (39978029-39978101) Gly (CCC) 73 bp Sc: 58.96
TCCCTGATGGTCCAGAGGTTAAGGTTCTGTGCTCCCAACGCAGGGGACCCAGGTTCAATC
CCTGGTCAGGGAA
>Bos_taurus_chr13.trna5836-GlyCCC (38609290-38609218) Gly (CCC) 73 bp Sc: 58.96

TCCCGGGTGGTCCAGTGGCTAAGACTCTGCACTCCCAATGCAGGGGGCCCAGGCTCGATT
CCTGGTCAGGGAA
>Bos_taurus_chr14.trna520-GlyCCC (12020207-12020279) Gly (CCC) 73 bp Sc: 59.08
TCCCTGGTGGTCCAGTGGATAAGACTCTGCACTCCCAATCCAGGGGGCCCAGGTTCGATC
CCCGGTCAGGGAA
>Bos_taurus_chr25.trna2178-GlyCCC (36534947-36535019) Gly (CCC) 73 bp Sc: 59.31
TCCCTGGTGGTCCAGTGCTTAAGACTCTGCATTCCCAATGCAGGGGGCCCAGGTTTGATC
CCTGGCCAGGGAG
>Bos_taurus_chr12.trna2887-GlyCCC (74174023-74174095) Gly (CCC) 73 bp Sc: 59.42
TCCCAGGTGGTCTAGTGGCTAAGACTCTGCACTCCCAACGCAGGGGGCCCAGGTTCAATC
CCTGGCCAGGGAA
>Bos_taurus_chr6.trna6766-GlyCCC (61314596-61314525) Gly (CCC) 72 bp Sc: 59.52
TCCCTAGTGGTCCAGTGGTTAAGACTCTACACTCCCAATGCAGAGGCCCAGGTTTGATCC
CTGGTCAGGGAA
>Bos_taurus_chr22.trna674-GlyCCC (15380938-15381010) Gly (CCC) 73 bp Sc: 59.55
TCCCTGATGGTCCAGTGGCTAAGACTCTGCGCTCCCAATGCAGGGGGCCCAGGTTCAATC
CCTGGCCAGGGAA
>Bos_taurus_chr7.trna8138-GlyCCC (10857832-10857760) Gly (CCC) 73 bp Sc: 59.68
TCCTTGGTGGTCCAGTGGCTAGGACTCTGCACTCCCAGTGCAGAGGGCCCAGGTTTGATC
CCTGGTCAGGGAA
>Bos_taurus_chr20.trna2273-GlyCCC (61654229-61654301) Gly (CCC) 73 bp Sc: 59.72
TCCCTGGTGGTCCAGTGGCTAAGACTCTGCACTCCCAGTGCAGGGGGCCCAGGTTTGATT
CCTGGCCAGGGAA
>Bos_taurus_chrUn.004.206.trna3-GlyCCC (39698-39770) Gly (CCC) 73 bp Sc: 59.84
TCCCTGATGGTCCAGTGGCTAAGACTCTGCACTCCCAATGCAGAGGGCCCAGGTCTGATC
CCTGGTCAGGGAA
>Bos_taurus_chr14.trna5157-GlyCCC (33968212-33968140) Gly (CCC) 73 bp Sc: 59.88
TCCCTGGTGGTCCAGTGGCTAAGACTCTGCACTCCCAGTGCAGGGGGCCCAGGTTCAATC
CCTGGTCAGGGAA
>Bos_taurus_chr3.trna3277-GlyCCC (93533118-93533190) Gly (CCC) 73 bp Sc: 59.95
TCCCTGGTGGTCCAGTGGCTAAGACTCTGCACTCCCAATGCAGGGGACCCAGGTTCAATC
CCTGGTCAAGGAG
>Bos_taurus_chr6.trna6688-GlyCCC (62944216-62944145) Gly (CCC) 72 bp Sc: 60.14
TCCCTGGTGATCCAGTGGCTAAGACTCTGCACTCCCAATGCAGGGGCCCAGGTTCAACCC
CTGGTCAGGGAA
>Bos_taurus_chrX.trna3954-GlyCCC (80509444-80509372) Gly (CCC) 73 bp Sc: 60.21
TCCCTGGTGCTCCAGTGGCTAAGATGCTGCACTCCCAATGCAGGGGGCCTGGGTTCAATA
CCCAGTCAGGGAA
>Bos_taurus_chrUn.004.107.trna6-GlyCCC (259943-260015) Gly (CCC) 73 bp Sc: 60.32
TCTCTGGTGGTCCAGTGGCTAAGACTCTACATTCCCAATGTAGGGGGCCCAGGTTCAATC
CCTGGTCAGGGAA
>Bos_taurus_chrUn.004.1484.trna1-GlyCCC (8833-8905) Gly (CCC) 73 bp Sc: 60.32
TCTCTGGTGGTCCAGTGGCTAAGACTCTACATTCCCAATGTAGGGGGCCCAGGTTCAATC
CCTGGTCAGGGAA
>Bos_taurus_chr8.trna161-GlyCCC (5330653-5330725) Gly (CCC) 73 bp Sc: 60.38
TCCCTGGTGGTCCAATGGCTAAGACTCTGCACTCCCAATGCAGAGGGCCTAAGTTCAATT
CCTAGTCAGGGAA
>Bos_taurus_chr7.trna7593-GlyCCC (17887621-17887549) Gly (CCC) 73 bp Sc: 60.53
TCCTTGGTGGTCCAGTGGTTAGGACTATGCACTCCCAATGCAGGGAGCCCAGGTTCGATC
CCTAGTCAGGGAA
>Bos_taurus_chr28.trna248-GlyCCC (6379816-6379888) Gly (CCC) 73 bp Sc: 60.66
TCCCTGATGGTCCAGTGGCTAAGACTCTGCACTCCCAATGCAGGGAGCCCAGGTTCAACC
CCTGGTCAGGAAA
>Bos_taurus_chr27.trna2710-GlyCCC (31049591-31049519) Gly (CCC) 73 bp Sc: 60.71
TCCCTGATGGTCCAATGGCTAAGACTCTGTGCTCCCAATGCAGGGGGCCCAGGTTCAATT
CCTGGTCAGGGAA
>Bos_taurus_chrX.trna1600-GlyCCC (43602633-43602704) Gly (CCC) 72 bp Sc: 60.72
TCCCTGGTGGTCCAGTGGTTAAGACTCTGCACTCCCAATGCAGGGGTCCGGGTTTGATCC
CTGGTCAGGGAA
>Bos_taurus_chr15.trna1757-GlyCCC (51306112-51306184) Gly (CCC) 73 bp Sc: 60.79
TCCCTGGTGGTCCAGTGGTTAAGACTCTATGCTCCCAATGCAGAGGGCCCAGGTTCGATC
CCTGGTCAGGGAA
>Bos_taurus_chr26.trna1419-GlyCCC (39485574-39485646) Gly (CCC) 73 bp Sc: 61.22
TCCCTGATAGTCCAGTGGCTAAGACTCTGGACTCCCAATGTAGAGGGCCCAGGTTCAATC
CCTGGTCAGGGAA
>Bos_taurus_chrUn.004.2256.trna6-GlyCCC (4907-4835) Gly (CCC) 73 bp Sc: 61.23
TCCCTGGTGGTCCAGTGGTTAAGACTCTGCACTCCCAATGCAGGGGACACAGGTTTGATC

CCTGGCCAGGGAA
>Bos_taurus_chr12.trna562-GlyCCC (15199663-15199735) Gly (CCC) 73 bp Sc: 61.41
TCCCTGATAGTCCAGTGGTTAAGACTCTGCATTCCCAATGCAGGGGGCACAGGTTCGATT
CCTGGTAGGGGAA
>Bos_taurus_chrX.trna4174-GlyCCC (75423908-75423836) Gly (CCC) 73 bp Sc: 61.43
TCCCTGGTGGTCCAGTGGCTAAGACTCTGCACTCCCAATGCAGGGGGCCCAGGTTCAATC
CCTGATCAGGGAA
>Bos_taurus_chr29.trna1272-GlyCCC (34536382-34536454) Gly (CCC) 73 bp Sc: 61.48
TCCCTGATGGTCCAGTGGCTAGGACTCCGCACTCCCAATGCAGGGGTCCCAGGTTCCATC
CCTGATCAGGGAA
>Bos_taurus_chr5.trna1123-GlyCCC (32221407-32221479) Gly (CCC) 73 bp Sc: 61.55
TCCCTGGTGGTCCAGTGGCTAAGACTCTGCGCTCCCAATGCAGGGGGCCCAGGTTCAATC
CCTGGTCAGGGAA
>Bos_taurus_chrUn.004.26.trna26-GlyCCC (442212-442140) Gly (CCC) 73 bp Sc: 61.80
TCCCTGGTGGTCCAGTGGCTAAGACTCTACATTCCCAGTGTAGAGGCCCCAGGTTCAATC
CCTGGTCAGGGAA
>Bos_taurus_chr25.trna4529-GlyCCC (8844230-8844158) Gly (CCC) 73 bp Sc: 61.90
TCCCTGGTGGTCCAGTGGCTAAGACTCTGCACTCCCGATGCAGGGGGCCCAGGTTCGATC
CCAGGTCAGGGAA
>Bos_taurus_chr5.trna6673-GlyCCC (93560958-93560887) Gly (CCC) 72 bp Sc: 62.12
TCCCTGGTGGTCCAGTGGTTAGGACTCTGCACTCCCAGTGCAGTGGGCAAGGTTCAATCC
CTTGTTAGGGAA
>Bos_taurus_chr1.trna6789-GlyCCC (133737468-133737396) Gly (CCC) 73 bp Sc: 62.28
TCCCTGGTGGTCCAGTGGCTAAGATTCTGTACTCCCAATGCAGGGAGCCCAGGTTCAATT
CCTGGTCAGGGAA
>Bos_taurus_chr28.trna2109-GlyCCC (27807779-27807707) Gly (CCC) 73 bp Sc: 62.39
TCTCTGGTGGTCCAGTGGCTAAGACTCTGCACTCCCAATGCAGGGGGCCCAGGTTCAATC
CCTGGTCAGGGAA
>Bos_taurus_chr10.trna2997-GlyCCC (79364467-79364539) Gly (CCC) 73 bp Sc: 62.46
TCCCTGGTGGTCCAGTTGCTAAGACTCTGTACTCCCAATGCAGGGATCCCAGGTTCAATC
CCTGGTCAGGGAA
>Bos_taurus_chr25.trna256-GlyCCC (5897891-5897963) Gly (CCC) 73 bp Sc: 62.47
TCCCTGGTGGTCCAGTGGCTACGACTCTGCACTCCCAATGCAGGGGGTCCAGGTTCAATC
CCTGGTCAGGGAA
>Bos_taurus_chr10.trna486-GlyCCC (12023653-12023725) Gly (CCC) 73 bp Sc: 62.55
TCCTTGATAGTCCAGTGGCTAAGACTCTGCATTCCCAATGCAGGGGTCCTGGGTTCAATC
CCTGGTCAGGGAA
>Bos_taurus_chr3.trna8652-GlyCCC (21257145-21257076) Gly (CCC) 70 bp Sc: 62.56
TCCCTGGTGGTCCAGTGGAAGACTCTGCACTCCCAATGCAGGGGCCCAGGTTCAATCCCT
GGTCAGGGAA
>Bos_taurus_chr22.trna3720-GlyCCC (18515242-18515170) Gly (CCC) 73 bp Sc: 62.79
TCCCTGGTGGTCCAGTGGCTAAAACTCTCCACTCCCAATGCAGGGGGCCCAGGTTCGATC
CCTGGCCAGGGAA
>Bos_taurus_chrUn.004.4513.trna1-GlyCCC (9416-9344) Gly (CCC) 73 bp Sc: 62.79
TCCCTGGTGGTCCAGTGGCTAAAACTCTCCACTCCCAATGCAGGGGGCCCAGGTTCGATC
CCTGGCCAGGGAA
>Bos_taurus_chr22.trna3717-GlyCCC (18636998-18636926) Gly (CCC) 73 bp Sc: 63.20
TCCCTGGTGGTCTAGAGGCTAAGATGCTGCACTCCCAATGCAGGGGGCCCAGGTTCAATC
CCTGGTCAGGGAA
>Bos_taurus_chr3.trna426-GlyCCC (13579708-13579780) Gly (CCC) 73 bp Sc: 63.66
TCCCTGATGGTCCAGTGGCTAAGATTCTGCACTCCCAATGCAGGGGGCCCAGGTTCAATC
CCTGGCCAGGGAA
>Bos_taurus_chr6.trna2895-GlyCCC (94817156-94817228) Gly (CCC) 73 bp Sc: 63.78
TCCCTGGTGGTCCAGTGGCTAAGACTTTGCACTCCCAATGCAGGGGGCCCAGGTTCAATC
CCTGGTCAGGGAA
>Bos_taurus_chr21.trna4836-GlyCCC (21433369-21433297) Gly (CCC) 73 bp Sc: 63.88
TCCTTGATGGTCTAGTGGCTAAGACTCTGCGCTCCCAATGCAGGGGGCCCAGGTTCAATC
CCTGGTCAGGGAA
>Bos_taurus_chrX.trna5742-GlyCCC (30498158-30498087) Gly (CCC) 72 bp Sc: 64.03
TCCCTGGTGGTCCAGTGGCTAAGACTCTGCACTCCCAATGCAGGGGCCCAGGTTCGTTCC
CTGGTCAGGGAA
>Bos_taurus_chr25.trna1291-GlyCCC (22869498-22869570) Gly (CCC) 73 bp Sc: 64.17
TCCCTGATGGTCCAATGGCTAGGACTCTGCACTCCCAGTGCAGGGGGCCCAGGTTCAATC
CCTGGTCAGGGAA
>Bos_taurus_chr25.trna2164-GlyCCC (36178238-36178309) Gly (CCC) 72 bp Sc: 65.43
TCCCTGGTGGTCCAGTGGTTAAGACTCTGCACTCCCAAGGCAGGGGACCAGGTTCGATCC
CTGGTCAGGGAA

>Bos_taurus_chr3.trna8583-GlyCCC (22618697-22618627) Gly (CCC) 71 bp Sc: 65.48
GCATTGGTGGTTCAGTGGTAGAATTCTCTCCTCCCACGCGGGAGACCCGGGTTCGATTCC
TGGCCAATGCA
>Bos_taurus_chr3.trna8586-GlyCCC (22594168-22594098) Gly (CCC) 71 bp Sc: 65.48
GCATTGGTGGTTCAGTGGTAGAATTCTCTCCTCCCACGCGGGAGACCCGGGTTCGATTCC
TGGCCAATGCA
>Bos_taurus_chr3.trna8592-GlyCCC (22558723-22558653) Gly (CCC) 71 bp Sc: 65.48
GCATTGGTGGTTCAGTGGTAGAATTCTCTCCTCCCACGCGGGAGACCCGGGTTCGATTCC
TGGCCAATGCA
>Bos_taurus_chrUn.004.2930.trna8-GlyCCC (14871-14801) Gly (CCC) 71 bp Sc: 65.48
GCATTGGTGGTTCAGTGGTAGAATTCTCTCCTCCCACGCGGGAGACCCGGGTTCGATTCC
TGGCCAATGCA
>Bos_taurus_chr19.trna3749-GlyCCC (58065195-58065123) Gly (CCC) 73 bp Sc: 65.52
TCCCTGATGGTCCAGTGGCTAAGACTCTGTGCTCCCAATGCAGGGATCCCAGGTTCAATC
CCTGGTCAGGGAA
>Bos_taurus_chr15.trna614-GlyCCC (23112723-23112795) Gly (CCC) 73 bp Sc: 65.72
TCCCTGGTGGTCCAGTGGCTAAGACTCTGCACTCCCAATGCAGAGGGTCCAGGTTCAATC
CCTGGTTAGGGAA
>Bos_taurus_chr23.trna118-GlyCCC (3130951-3131023) Gly (CCC) 73 bp Sc: 65.98
TCCCTGGTGGTCCAGTGGCTAAGACTCTGTACTCCCAATGCAGGGGGCCCAGGTTCGATC
CCTGGTCAGGGAA
>Bos_taurus_chr18.trna5094-GlyCCC (23479068-23478996) Gly (CCC) 73 bp Sc: 66.92
TCCCTGGTGGTCCAGTGGCTAAGACTCTGCACTCCCAATGCAGGGGGCCCGGGTTCGATC
CCTGGCCAGGGAA
>Bos_taurus_chr16.trna2752-GlyCCC (68288618-68288690) Gly (CCC) 73 bp Sc: 67.14
TCCCTGGTGGTCCAGTGGCTAAGACTCTGCACTCCCAATGCAGAGGGCCTAGGTTCGATC
CCTGGTCAGGGAA
>Bos_taurus_chr11.trna2503-GlyCCC (61992485-61992557) Gly (CCC) 73 bp Sc: 67.77
TCCCTGATGGTCCAGTGGCTAAAACTTTGCACTCCCAATGCAGGGATCCCAGGTTCGATC
CCTGGTCAGGGAA
>Bos_taurus_chr26.trna2487-GlyCCC (40258270-40258199) Gly (CCC) 72 bp Sc: 68.63
TCCCTGATGGTCCAGTGGCTAAGATTTTGCACTCCCAATGCAGGGGCCCAGGTTCAATCC
CTGGTCAGGGAA
>Bos_taurus_chr28.trna1382-GlyCCC (39799700-39799770) Gly (CCC) 71 bp Sc: 68.72
GCATTGGTGGTTCAGTGGTAGAATTCTCGCCTCCCATGTGGGAGACCTGGGTTCAATTCC
CGGCCAATGCA
>Bos_taurus_chr25.trna2651-GlyCCC (40069911-40069839) Gly (CCC) 73 bp Sc: 68.83
TCCCTGGTGGTCCAGTGGCTAAGACACTGCACTCCCAATGCAGGGGTCCCCGGTTCGATC
CCTGGTCAGGGAA
>Bos_taurus_chr6.trna4529-GlyCCC (115254779-115254709) Gly (CCC) 71 bp Sc: 69.49
GCATTGGTGGTTCAGAGGTAGAATTCTCGCCTCCCATGTGGGAGACCCGGGTTCGATTCC
CGGCCAGTGCA
>Bos_taurus_chr18.trna1628-GlyCCC (41783170-41783242) Gly (CCC) 73 bp Sc: 73.68
TCCCTGGTGGTCCAGTGGTTAAGACTCTGTACTCCCAATGCAGGGGTCCCAGGTTCAATC
CCTGGTCAGGGAA
>Bos_taurus_chr3.trna848-GlyCCC (22863655-22863725) Gly (CCC) 71 bp Sc: 74.10
GCATTGGTGGTTCAGTGGTAGAATTCTCGCCTCCCACGCAGGAGACCCGGGTTCGATTCC
CGGCCAATGCA
>Bos_taurus_chr3.trna8559-GlyCCC (22902300-22902230) Gly (CCC) 71 bp Sc: 74.10
GCATTGGTGGTTCAGTGGTAGAATTCTCGCCTCCCACGCAGGAGACCCGGGTTCGATTCC
CGGCCAATGCA
>Bos_taurus_chr3.trna8575-GlyCCC (22657198-22657128) Gly (CCC) 71 bp Sc: 74.10
GCATTGGTGGTTCAGTGGTAGAATTCTCGCCTCCCACGCAGGAGACCCGGGTTCGATTCC
CGGCCAATGCA
>Bos_taurus_chrUn.004.185.trna21-GlyCCC (192783-192713) Gly (CCC) 71 bp Sc: 74.10
GCATTGGTGGTTCAGTGGTAGAATTCTCGCCTCCCACGCAGGAGACCCGGGTTCGATTCC
CGGCCAATGCA
>Bos_taurus_chrUn.004.2930.trna10-GlyCCC (9945-9875) Gly (CCC) 71 bp Sc: 74.10
GCATTGGTGGTTCAGTGGTAGAATTCTCGCCTCCCACGCAGGAGACCCGGGTTCGATTCC
CGGCCAATGCA
>Bos_taurus_chr3.trna812-GlyCCC (22477091-22477161) Gly (CCC) 71 bp Sc: 75.47
GCATTGGTGGTTCAGTGGTAGAATTCTCGCCTCCCACGTGGGAGACCCGGGTTCGATTCC
CGGCCAATGCA
>Bos_taurus_chr11.trna6329-GlyCCC (70496893-70496823) Gly (CCC) 71 bp Sc: 76.98
GCGCCGCTGGTGTAGTGGTATCATGCAAGATTCCCATTCTTGCGACCCGGGTTCGATTCC
CGGGCGGCGCA
>Bos_taurus_chr3.trna859-GlyCCC (22926610-22926680) Gly (CCC) 71 bp Sc: 79.31

GCATTGGTGGTTCAGTGGTAGAATTCTCGCCTCCCACGCGGGAGACCCGGGTTCGATTCC
CGGCCAGTGCA
>Bos_taurus_chrUn.004.185.trna13-GlyCCC (221802-221872) Gly (CCC) 71 bp Sc: 79.31
GCATTGGTGGTTCAGTGGTAGAATTCTCGCCTCCCACGCGGGAGACCCGGGTTCGATTCC
CGGCCAGTGCA
>Bos_taurus_chrUn.004.185.trna27-GlyCCC (36684-36614) Gly (CCC) 71 bp Sc: 80.05
GCATTGGTGGTTCAGTGGTAGAATTCTCGCCTCCCACGCGGGAGACCCGGGTTCGATTCC
CGGCCAATGCA
>Bos_taurus_chrUn.004.185.trna4-GlyCCC (38387-38457) Gly (CCC) 71 bp Sc: 80.05
GCATTGGTGGTTCAGTGGTAGAATTCTCGCCTCCCACGCGGGAGACCCGGGTTCGATTCC
CGGCCAATGCA
>Bos_taurus_chr1.trna3749-GlyCCC (110495878-110495957) Gly (CCC) 80 bp Sc: 54.72
TCCCTGGTGGTCCAGTGGCTAGGACTCCCCACTCCCAGAGAAAGGGTTAGGGGGCCCAGG
TTCAATCCCTGGTCAGGGAA
>Bos_taurus_chr4.trna8423-GlyCCC (13190255-13190165) Gly (CCC) 91 bp Sc: 28.49
TCCTTGGTAGTCCAGAGGCTAAGACTCTGGGCTCCCAGTTCAGGCAGCACAGGTTTGACC
TCTGACCCAGGTTTGACCCCTGGTCAGGGAA
>Bos_taurus_chr27.trna3275-GlyCCC (19303945-19303861) Gly (CCC) 85 bp Sc: 28.06
TCCCAGGTGGCTTGGTGGTAAAGCATTCATCTCCCAATGCAGGAGATGAAGGTTTCATCC
CTGGGTTCAATTTCTGGCTTGGGAA
>Bos_taurus_chr13.trna6455-GlyCCC (27119952-27119870) Gly (CCC) 83 bp Sc: 33.28
TCCTTGATGGTCTAGCAGCTAAGATTCTGCACTCCCAATGCAGCTGGCCTGCATTGAACC
AGGTTCAATCCCTGGTCAGGGGA
>Bos_taurus_chr13.trna5472-GlyCCC (48383443-48383363) Gly (CCC) 81 bp Sc: 28.73
TCCCTGGTGGTCCAGTGGCTAAGACTTCATCCTCCCAATGCAGGGCAGGGGGGGCCCCAA
GTTAAATTCTTGGTCAAGGAA
>Bos_taurus_chr22.trna143-GlyGCC (4414226-4414298) Gly (GCC) 73 bp Sc: 31.09
TCCCTGGTGGTCCAGTGATTAAGACTCTGTGCTGCCAATACAGGGAGTATGGGTTCGACC
CCTCTTTGGGGAA
>Bos_taurus_chr27.trna2488-GlyGCC (35287916-35287844) Gly (GCC) 73 bp Sc: 31.29
TCCCTGGTAGTCCAGTGGTTAAGACTCTGAGTTGCCCATGCAGGGGTCTTGGGTTTGATC
TCTGATCAGGGAA
>Bos_taurus_chr11.trna3416-GlyGCC (84991155-84991227) Gly (GCC) 73 bp Sc: 31.51
TCCCTGGTGGTCCAGTGGCTAAGACTTCATACTGCCAATATAGGGGGCCTAGGTTTAACC
CCTGGTCAGCGAA
>Bos_taurus_chr13.trna926-GlyGCC (24437844-24437914) Gly (GCC) 71 bp Sc: 32.30
TCCCTGGTGGTCCAGTGGCCAAGACTCTGTGCTGCCAGCGCAGGGGCCAGGTTCAGTCCT
CGGTCAGGGAA
>Bos_taurus_chr13.trna842-GlyGCC (22867577-22867649) Gly (GCC) 73 bp Sc: 33.51
TTCCTGGTGGTCCAATGGTTAGGACTCTGTGCTGCCACTACAGGGGGCCTGGGTTTGATC
TCTGGTCAGGGAA
>Bos_taurus_chr7.trna415-GlyGCC (9193403-9193475) Gly (GCC) 73 bp Sc: 34.86
TTCCTGGTGGTCCAGTGGCTTGGACTCTGCACTGCCAATGCAGGGAGCTCAGGTTCGTTC
CCTAATCAGGGGA
>Bos_taurus_chr22.trna1441-GlyGCC (40919835-40919907) Gly (GCC) 73 bp Sc: 35.45
TCCCTGGTGGTCCAGTATCTAAGACTCTGAGTTGCCAATGCAGGGGGCCTGGGTTCGATC
CCTGGCCAGGGAA
>Bos_taurus_chr16.trna894-GlyGCC (26376932-26377004) Gly (GCC) 73 bp Sc: 35.73
TCCCTGGTGGTCCAGTGGTTAAGACTCTGTGCTGCCAATGCAGGGTATCCGGGTCTGATC
CCTGGTCAGGGAA
>Bos_taurus_chr28.trna2500-GlyGCC (16682481-16682409) Gly (GCC) 73 bp Sc: 36.64
TCCTTAGTGGTCCCGTGGCTAAGACTATGCATTGCCAATGCAGGTGACCCAGGTTTGATC
CCTGGTTGGGGAA
>Bos_taurus_chr11.trna5460-GlyGCC (90787823-90787751) Gly (GCC) 73 bp Sc: 37.06
TCCCTGGTGGTCCAGGGGTTAAGACTCTGAGCTGCCACTGCAGGGAATGCAGGTTTGATC
CCTGGTCAGGGAA
>Bos_taurus_chr5.trna5024-GlyGCC (123381315-123381387) Gly (GCC) 73 bp Sc: 38.82
TCCCTGGTGGCCCAGTGGTTAGGATTTGGTTCTGCCACTGCCAAGGACCTGGGTTCAGTC
CCTGGTCAGGGAG
>Bos_taurus_chr11.trna8811-GlyGCC (7196046-7195974) Gly (GCC) 73 bp Sc: 38.90
TCCCTGGTGGCTCAGTCGGTAAAGATCCCGCCTGCCAATGCAGGAGACTCAGGTTTGATC
CCTGGGTAGGGAA
>Bos_taurus_chr17.trna4142-GlyGCC (62738694-62738621) Gly (GCC) 74 bp Sc: 39.37
TCCCTGGTGGTCCACTGGCTAAAGACTCTGAGCTGCCAATGCGGGGGGCCCAGGTTCAAT
CCCTGGTCAGGGAA
>Bos_taurus_chr13.trna4340-GlyGCC (73383939-73383865) Gly (GCC) 75 bp Sc: 39.63
TCCCTGGTGGTCCAGCGGCTAAGGCTCTGTGCTGCCAATGCAGATTGGTATCAGGTTCAA

CACCTGGTCAGGGAA
>Bos_taurus_chr2.trna8333-GlyGCC (67079038-67078964) Gly (GCC) 75 bp Sc: 40.37
TCCCTGGTGGTCCAGCGGCTAAAAGACTCAGCAATGCCAATGCAGGGGGCCCAGGTTCAA
TCCCTGGTCAGGGAA
>Bos_taurus_chr13.trna3017-GlyGCC (71262409-71262480) Gly (GCC) 72 bp Sc: 40.38
TCCCTGGTGGTCCAGTGGTTAAGACTCTGTGCTGCCAGTGCAGGGGTCTGGGTTTGATCC
CTGGTCAGGGAA
>Bos_taurus_chr8.trna310-GlyGCC (8342043-8342114) Gly (GCC) 72 bp Sc: 45.36
TTTCTACTGGCTCAGACGGTAAAGAATCTGCCTGCCATGCAGGAGACCCAGGTTCAATCC
CTGGGTGGGGAA
>Bos_taurus_chrUn.004.75.trna10-GlyGCC (162402-162474) Gly (GCC) 73 bp Sc: 45.37
TCCCttgttgtCCAGTGGTTAAGATTCTGTGCTGCCAATGCAAAGGGCCCAGGTTTGATC
CCTGGCTGGGGAA
>Bos_taurus_chr18.trna2862-GlyGCC (62991276-62991349) Gly (GCC) 74 bp Sc: 45.40
TCCCTGGTGGTCCAGGGGCTAAGACTCCGTGCTGCCACTGCAGGGGGGCCCAGGTTCAAT
TCCTGGTCAGGGAA
>Bos_taurus_chr15.trna1211-GlyGCC (35991918-35991996) Gly (GCC) 79 bp Sc: 45.64
TCCCCAGTGGTCCAGTTGTTAAGACTTTGCACTGCCACTGCAGGGGGGCACGGGTTAGGT
TCAATCCCTAATTGGGGAA
>Bos_taurus_chrX.trna719-GlyGCC (16213473-16213545) Gly (GCC) 73 bp Sc: 46.12
TCCCTGGTGGTCAAGTGGTTAAGACTCTGCACTGCCAATGCATGGGGCACAGGTTGGATA
CCTGGTCAGGGGA
>Bos_taurus_chr26.trna1853-GlyGCC (47012730-47012802) Gly (GCC) 73 bp Sc: 46.21
TTCCTGGTGGCCCAGTGGTTAAGGCTCTGCTCTGCCAAAGCAGGGAGCACAGGTTCAGGC
CCTGGCTGGGGAA
>Bos_taurus_chr12.trna479-GlyGCC (14072328-14072400) Gly (GCC) 73 bp Sc: 46.62
TCCCTGGTGGTCGAGTGGTTAAGACTCTGTGCTGCCAACACAGGGGGCATGGGTTTGAAC
CCTGGTCAGGGAA
>Bos_taurus_chr10.trna227-GlyGCC (6490906-6490978) Gly (GCC) 73 bp Sc: 47.55
TTCCTGGTTGTCCAGTGGCTAAGACTCTGCACTGCCAATGCAGGGGGCCTGGGTTTGATC
CCCGGTCAGGGAA
>Bos_taurus_chr17.trna3577-GlyGCC (71988247-71988176) Gly (GCC) 72 bp Sc: 47.59
TCCCTGGTGGTCCAGTGGCTAAGAGTCTGTACTGCCAATGCAGGGGGCCAGGTTTAATCC
CTGGTCAGGGAG
>Bos_taurus_chrUn.004.428.trna9-GlyGCC (89168-89239) Gly (GCC) 72 bp Sc: 47.59
TCCCTGGTGGTCCAGTGGCTAAGAGTCTGTACTGCCAATGCAGGGGGCCAGGTTTAATCC
CTGGTCAGGGAG
>Bos_taurus_chr22.trna3485-GlyGCC (25395275-25395203) Gly (GCC) 73 bp Sc: 47.90
TCTCTGATGGTCTGCTGGCTAAGACTCTGCACTGCCAATGCAGGGGGGCCAGGTTCGATC
CCTGGTCAGGGAA
>Bos_taurus_chr1.trna8456-GlyGCC (83350963-83350891) Gly (GCC) 73 bp Sc: 48.09
TCCCTGGTGGTCCAGTGGCTAAGACTCTGTGCTGCCAAAGCAGGGGGCCTAGGTTCGATC
CCTGCTCAGGGAA
>Bos_taurus_chr2.trna181-GlyGCC (6289102-6289172) Gly (GCC) 71 bp Sc: 48.23
GCATAGGTGGTTCAGTGGTAGAATTCTTGCTTGCCACATGGGGGGCCTGGGTTTGATTCC
CAGCCCATGCA
>Bos_taurus_chr19.trna4069-GlyGCC (52507144-52507072) Gly (GCC) 73 bp Sc: 48.31
TCCCTGGTGGTCCAGTGGTTAAGATTCTGAGCTGCCAATGCAGGGGGAACAGGTTCAATT
CCTGGTCAGGGAA
>Bos_taurus_chr16.trna3234-GlyGCC (76151092-76151020) Gly (GCC) 73 bp Sc: 49.72
TCCCTGATGGTCCAGTGGTTAGGACTTGGCACTGCCACTGCAGGGGGCATGGGTTCAATC
CCTGATCAGGGAA
>Bos_taurus_chr24.trna2043-GlyGCC (49624304-49624374) Gly (GCC) 71 bp Sc: 49.99
TCCCAGCTGGCTCAGTGGTTAAGAATCTGCCTGCCAAGCAGGAGACTCGGGTTCGATCCC
TGGGTTGGGAA
>Bos_taurus_chr23.trna4570-GlyGCC (7869087-7869015) Gly (GCC) 73 bp Sc: 50.21
TCCCTGGTGGTCCAGTGGTTAAGATTCCGTGCTGCCACTGCAGGGGGCTCAGGTTCGATC
CCTGGTCAGGGAA
>Bos_taurus_chr18.trna2120-GlyGCC (49962265-49962337) Gly (GCC) 73 bp Sc: 50.38
ACCCTGGTGGTCCAGTGGCTAAGACTCTGCGCTGCCAATGCAGGGGGCCCAGGTTCCATC
CCTGGTCAGGGAA
>Bos_taurus_chr21.trna974-GlyGCC (22893829-22893901) Gly (GCC) 73 bp Sc: 50.48
TCCCTGGTGGTCCAGTGGCTAAGACTCTGTGCTGCCAATGCAGGGGACCCAGGTTCCATC
CCTGGTCAGGGAG
>Bos_taurus_chr18.trna3165-GlyGCC (61943024-61942953) Gly (GCC) 72 bp Sc: 50.97
TTCCTGCTGGCTCAGTTGGTAAAGAATCTGCCTGCCGTGCAGGAGACCCAGGTTTGATTC
CTGGGTAGGGAA

>Bos_taurus_chr23.trna3986-GlyGCC (18113158-18113086) Gly (GCC) 73 bp Sc: 52.48
TCCCTGGTGGTCGAGTGGGTAAGACTCTGCGCTGCCAATGCAGGGGGCCCAGGTTTGATC
CTTGGTCAGGGAA
>Bos_taurus_chr29.trna28-GlyGCC (1022300-1022372) Gly (GCC) 73 bp Sc: 54.37
TCCCTGGTGGTCCAGTGGTTAAGACTCTGCGCTGCCAATGCAGGGGACACAGGTTCAATC
CCTGGTTGGGGAA
>Bos_taurus_chr29.trna39-GlyGCC (1429324-1429396) Gly (GCC) 73 bp Sc: 54.37
TCCCTGGTGGTCCAGTGGTTAAGACTCTGCGCTGCCAATGCAGGGGACACAGGTTCAATC
CCTGGTTGGGGAA
>Bos_taurus_chr1.trna7467-GlyGCC (114410195-114410123) Gly (GCC) 73 bp Sc: 55.34
TCCCTGGTGGTCCAGTGGCTAAGACTCTGGACTGCCAATGCAGAAGGCCTGGGTTCAATC
CCCAGTCAAGGAA
>Bos_taurus_chr4.trna2020-GlyGCC (63774130-63774202) Gly (GCC) 73 bp Sc: 55.74
TCTCTGGTGGTCCAGTGGTTAAGATTCTGCACTGCCAATGCATGGGTTACAGGTTCGATC
CCTGGTCAGGGAA
>Bos_taurus_chr5.trna2122-GlyGCC (58785546-58785617) Gly (GCC) 72 bp Sc: 56.86
TCCCTGGTGGCTCAGACGGTAAAGCGTCTGCCTGCCATGCAGGAGACCCAGGTTCAATCC
CTGGGTCGGGAA
>Bos_taurus_chr17.trna4080-GlyGCC (63453252-63453180) Gly (GCC) 73 bp Sc: 57.78
TTCCTGGTGGTCCAGTGGTCAAGACTCTCCACTGCCAATGCAGAGGGCTCAGGTTCGATC
CCTGGTCAGGGAA
>Bos_taurus_chr27.trna1901-GlyGCC (48285783-48285712) Gly (GCC) 72 bp Sc: 57.82
TCCCTGCTGGCTCAGACGGTAAAGCGTCTGCCTGCCATGCAGGAGACCCGGGTTCGATCC
CTGGGTTGGGAA
>Bos_taurus_chr28.trna3102-GlyGCC (3150077-3150006) Gly (GCC) 72 bp Sc: 59.49
TCCCTGGTGGTCCAGTGGCTAGGACTCTGCACTGCCAATGCAGTGACTCTGGTTTGATCC
CTGGTCAGGGAG
>Bos_taurus_chr27.trna2093-GlyGCC (43147196-43147126) Gly (GCC) 71 bp Sc: 59.70
GCATGGGTGCTTCAGTGGTAGGATTCCCGCCTGCCACGCGGGAGGCCCGGGTTCAATTTC
TGGCCCGTGCA
>Bos_taurus_chr3.trna2984-GlyGCC (86322865-86322938) Gly (GCC) 74 bp Sc: 60.75
TCCCTGGTGGTCTAGTGGTTTAAGACTCTGCACTGCCAATACAGCAGGCCCAGGTTCAAT
CCCTGGTCAGGGAG
>Bos_taurus_chr1.trna8310-GlyGCC (87549283-87549213) Gly (GCC) 71 bp Sc: 66.42
GCATTGGTGGTTCAGTGGTAGAATTCTCACCTGCCACTCGGGAGGCCTGGGTTCAATTCC
CAGCCAATGCA
>Bos_taurus_chr6.trna6754-GlyGCC (61649573-61649503) Gly (GCC) 71 bp Sc: 67.09
GCATTGGTGGTTCAGTGGTAGAATTCTTGCCTGCCACGTGGGAGGCCTGGGTTCGATTCC
CAGCTGATGCA
>Bos_taurus_chr18.trna27-GlyGCC (774792-774862) Gly (GCC) 71 bp Sc: 81.62
GCATTGGTGGTTCAGTGGTAGAATTCTCGCCTGCCACGCGGGAGGCCCGGGTTCGATTCC
CGGCCAATGCA
>Bos_taurus_chr18.trna37-GlyGCC (1170238-1170308) Gly (GCC) 71 bp Sc: 81.62
GCATTGGTGGTTCAGTGGTAGAATTCTCGCCTGCCACGCGGGAGGCCCGGGTTCGATTCC
CGGCCAATGCA
>Bos_taurus_chr18.trna5998-GlyGCC (767760-767690) Gly (GCC) 71 bp Sc: 81.62
GCATTGGTGGTTCAGTGGTAGAATTCTCGCCTGCCACGCGGGAGGCCCGGGTTCGATTCC
CGGCCAATGCA
>Bos_taurus_chr18.trna5999-GlyGCC (767120-767050) Gly (GCC) 71 bp Sc: 81.62
GCATTGGTGGTTCAGTGGTAGAATTCTCGCCTGCCACGCGGGAGGCCCGGGTTCGATTCC
CGGCCAATGCA
>Bos_taurus_chr19.trna1409-GlyGCC (28311388-28311458) Gly (GCC) 71 bp Sc: 81.62
GCATTGGTGGTTCAGTGGTAGAATTCTCGCCTGCCACGCGGGAGGCCCGGGTTCGATTCC
CGGCCAATGCA
>Bos_taurus_chr2.trna1341-GlyGCC (41255098-41255168) Gly (GCC) 71 bp Sc: 81.62
GCATTGGTGGTTCAGTGGTAGAATTCTCGCCTGCCACGCGGGAGGCCCGGGTTCGATTCC
CGGCCAATGCA
>Bos_taurus_chr23.trna1359-GlyGCC (31005308-31005378) Gly (GCC) 71 bp Sc: 81.62
GCATTGGTGGTTCAGTGGTAGAATTCTCGCCTGCCACGCGGGAGGCCCGGGTTCGATTCC
CGGCCAATGCA
>Bos_taurus_chr23.trna1423-GlyGCC (31703264-31703334) Gly (GCC) 71 bp Sc: 81.62
GCATTGGTGGTTCAGTGGTAGAATTCTCGCCTGCCACGCGGGAGGCCCGGGTTCGATTCC
CGGCCAATGCA
>Bos_taurus_chr3.trna274-GlyGCC (8751409-8751479) Gly (GCC) 71 bp Sc: 81.62
GCATTGGTGGTTCAGTGGTAGAATTCTCGCCTGCCACGCGGGAGGCCCGGGTTCGATTCC
CGGCCAATGCA
>Bos_taurus_chr3.trna272-GlyGCC (8631775-8631845) Gly (GCC) 71 bp Sc: 82.15

GCATGGGTGGTTCAGTGGTAGAATTCTCGCCTGCCACGCGGGAGGCCCGGGTTCGATTCC
CGGCCCATGCA
>Bos_taurus_chr3.trna276-GlyGCC (8817913-8817983) Gly (GCC) 71 bp Sc: 82.15
GCATGGGTGGTTCAGTGGTAGAATTCTCGCCTGCCACGCGGGAGGCCCGGGTTCGATTCC
CGGCCCATGCA
>Bos_taurus_chr3.trna278-GlyGCC (8941221-8941291) Gly (GCC) 71 bp Sc: 82.15
GCATGGGTGGTTCAGTGGTAGAATTCTCGCCTGCCACGCGGGAGGCCCGGGTTCGATTCC
CGGCCCATGCA
>Bos_taurus_chr21.trna3271-GlyGCC (60062795-60062673) Gly (GCC) 123 bp Sc: 54.61
TCCCTGGTGGTCCAGTGGCTAAGACTCTGCACTGCCAAGACCAGGGGACATTCCTGGTGG
ACTAGTTGCTAAGACTCCACACTGCCAATGCAGGGGGCCCAGGTTCGATCCCTGGTCAGG
GAA
>Bos_taurus_chr14.trna192-GlyTCC (5693404-5693474) Gly (TCC) 71 bp Sc: 23.47
TCCGTGGTAGTCCAGTGGCTAAGACTCTGTGCTTCCAGTGCAGAAGCTGGGTTTGATGCC
TGGCCAGGGAA
>Bos_taurus_chr7.trna5164-GlyTCC (88599620-88599549) Gly (TCC) 72 bp Sc: 23.51
TCCTGGAAGTCCAGTGGTTGAGACCCTGTACTTCCAGTGCAGGGGGTTCAGGTTTGATCC
CTGGTTGGGGAA
>Bos_taurus_chr9.trna376-GlyTCC (14924258-14924330) Gly (TCC) 73 bp Sc: 23.75
TCCCTGGTGGTCCAGTGGCTCCGATTCTGCATTTCCAGTGCAGGGGGCTTGAGTTCAACC
CCTGGTCAGGGAA
>Bos_taurus_chr29.trna1097-GlyTCC (31051143-31051214) Gly (TCC) 72 bp Sc: 25.20
TCCCTGATGGTCCAGTGGTTAAGACTCTGTGTTTCCAGGGCAGGGGGCACGGGTTCTATC
CATGTCAGGGAA
>Bos_taurus_chr24.trna1342-GlyTCC (34823868-34823940) Gly (TCC) 73 bp Sc: 26.07
TCTCTGGATGTCCAGTGGCTAAGACTTTGTGCTTCCATTGCACAGGGCCCAGGTTTAATC
CCTGGTTGGGGAA
>Bos_taurus_chr23.trna1418-GlyTCC (31615575-31615646) Gly (TCC) 72 bp Sc: 26.15
TTCCTGGTGGTCTAGTGATTAGGACTCTGAGCTTCCATTGCAGGCGCCCAGGTTTCATCC
CTGGTTGGCAAA
>Bos_taurus_chr10.trna3137-GlyTCC (82857477-82857549) Gly (TCC) 73 bp Sc: 26.19
TCCCTGGCAGTCCAGTGGTTAAGACTGAGTTCTTCCACTGTTCTAGACTCAGGTTTGATC
CCTGGTTGGGGAA
>Bos_taurus_chr4.trna5462-GlyTCC (99418748-99418676) Gly (TCC) 73 bp Sc: 27.13
TCCCTGGTGGTCCAGTTTTTAAGACTCTGAGCTTCCACTGCAGGGGGTGCAGGTTTGATC
CCTGTCTGGGAAC
>Bos_taurus_chr18.trna4990-GlyTCC (26042264-26042191) Gly (TCC) 74 bp Sc: 27.46
TCCCTGGTAATTCAGTGGTTAGGATGCTGAGCTTCCATTGTGGCAAGGCCCAGGGTTGAT
CCCTGGTCAGGGAA
>Bos_taurus_chr7.trna7976-GlyTCC (13299129-13299057) Gly (TCC) 73 bp Sc: 27.72
TTCCTGGTGGTCCAGTGATTAAGACTCTGCGCTTCCAGTGCAGGGGGCCTGAGTTTGATC
CTTAATCAGGAAA
>Bos_taurus_chr26.trna3142-GlyTCC (23120775-23120703) Gly (TCC) 73 bp Sc: 27.90
TCTCTGGTGGTCCATTTGTTAAGATTCTGTACTTCCACTGCAGGAGGCATAGGTTTGATT
CCTGGTCAGGGAA
>Bos_taurus_chr26.trna1576-GlyTCC (42417040-42417112) Gly (TCC) 73 bp Sc: 28.02
TCCCTAGTGGTCCAGTGCTTAAGACCCTGCGCTTCCAACACAGGGCCTTCAGGTTCAGCT
CCTAGTTGGGGAA
>Bos_taurus_chr20.trna5278-GlyTCC (11965431-11965359) Gly (TCC) 73 bp Sc: 29.46
TCCCTGGTGGTCCACTGATTAAGACTCTGTGCTTCCACTGCAGGGGGCACAGGTTCAGTT
CCTGGTTGGGGAA
>Bos_taurus_chr13.trna5129-GlyTCC (59643643-59643571) Gly (TCC) 73 bp Sc: 29.52
TCCCTGGCGGTCCAGTGGTTAAGACTCCGTGCTTCCACTACAGGGGGCTCAGGTTCTATC
TCTGGTCAGGGAA
>Bos_taurus_chr11.trna8040-GlyTCC (27176562-27176492) Gly (TCC) 71 bp Sc: 29.54
CCCTGGCTGTCCAGTGGTTAGGACTCTGTGCTTCCATTGCAGCAGGCCTGGGTTTGATCC
CTGGTTGGGGA
>Bos_taurus_chr3.trna2591-GlyTCC (74287597-74287669) Gly (TCC) 73 bp Sc: 29.75
TCTCTGGTGGTCCAGTGGCTAAGATTCTTCACTTCCAGTGCAGGGGATGCAGGTTTGATC
CCTGATTAGGGAA
>Bos_taurus_chrUn.004.12.trna13-GlyTCC (260028-260100) Gly (TCC) 73 bp Sc: 29.75
TCTCTGGTGGTCCAGTGGCTAAGATTCTTCACTTCCAGTGCAGGGGATGCAGGTTTGATC
CCTGATTAGGGAA
>Bos_taurus_chr2.trna4559-GlyTCC (130223108-130223180) Gly (TCC) 73 bp Sc: 30.08
TCCCTGGAGGTCCAGTGGTTAAGATTCTGTACTTCCAACACAGGGGGTATGGGTTTGATC
CCTGTTCAGGGAG
>Bos_taurus_chr25.trna4472-GlyTCC (9283517-9283445) Gly (TCC) 73 bp Sc: 30.24

TCCCTGGCAGTCCAGTGGTTAAGACTCTGCGCTTCCACTGCAGGGGGTTCGGGTTCTGAC
CCTGGTCAGGGAA
>Bos_taurus_chr16.trna3676-GlyTCC (65600609-65600537) Gly (TCC) 73 bp Sc: 30.41
TCCCTAGTGGTTCAGTGGCTAAGACTCCACGCTTCCAGCGTAGGGGGCTCAGGTCCAATC
CCTGGTCAGGGAA
>Bos_taurus_chr17.trna5017-GlyTCC (49932891-49932819) Gly (TCC) 73 bp Sc: 30.42
TCCCTGGTGGTCCAGTGGCTAAGACTCTGTGCTTCCAATGCAGGGGACCTGGGTACCATC
CCTGGTCAGGGAT
>Bos_taurus_chr5.trna1992-GlyTCC (54888095-54888167) Gly (TCC) 73 bp Sc: 30.52
TCCCTGGCAGTCCAGTGGTTCAGACTGTGCACTTCCACTGCAGGGGGCTCGGGTTTAATC
CCTGGTCAGGGAA
>Bos_taurus_chr5.trna981-GlyTCC (29204495-29204567) Gly (TCC) 73 bp Sc: 30.75
TCTCTGGAGGTCCAGTGGTTGGGACTTGGTACTTCCACTGCCGTGGGCCTGGGTTTGATC
CCTGGTCAGGGAA
>Bos_taurus_chr15.trna832-GlyTCC (27852991-27853062) Gly (TCC) 72 bp Sc: 31.32
TCCCTGGTGGTCTAGTTGTTAGGACTCCTTGCTTCCACTTAGGGGTCATAGGTTTGATTC
TTGGTTGGGGAA
>Bos_taurus_chr2.trna2804-GlyTCC (89377631-89377703) Gly (TCC) 73 bp Sc: 31.40
TCCCTGGCAGTCCATTGGTTAAGACTCTGAGCTTCCTCTGTAGGGGCCACAGGTTCGATC
CCTGTTCAGGGAA
>Bos_taurus_chrUn.004.44.trna3-GlyTCC (31910-31982) Gly (TCC) 73 bp Sc: 31.47
TTTTTGGTCAGTCAGTGGTTAAGACTCTGTGCTTCCGAGGCAGGGGGCTCAGGTTCAATC
CCTGGTCAAAGAA
>Bos_taurus_chrUn.004.245.trna14-GlyTCC (149601-149673) Gly (TCC) 73 bp Sc: 32.13
TCCCTGGTGGTCCACTGGTTAAGATGCTGAGCTTCCACTGCAGAGGCAACAAGTTCCATC
CTTGGTTGGGGAA
>Bos_taurus_chr5.trna7925-GlyTCC (62178321-62178252) Gly (TCC) 70 bp Sc: 32.22
TTCCTGGTGGTCCAATGGCTAAGACTCTGCATTTCCAATGCAGACCCGGGTTCAATCTCT
GGTCAGAGAA
>Bos_taurus_chr1.trna797-GlyTCC (19291698-19291770) Gly (TCC) 73 bp Sc: 32.38
TCCCTGGTGGTCCAGTGGTTAAGACTCAGTCCTTCCACGGCAGGGGGCATGGGTTTGTTC
CCTAATCAGGGAA
>Bos_taurus_chr15.trna1924-GlyTCC (55415040-55415111) Gly (TCC) 72 bp Sc: 32.83
TCCCTGGTGGTCCAGTGGTTAGATTCAGTGCTTCCATTGCAGGGACCCCAGGTTTGGTTC
CTGGTCAGGGAA
>Bos_taurus_chr9.trna5545-GlyTCC (70086725-70086653) Gly (TCC) 73 bp Sc: 33.05
TCCCTGGTGGTCAAGATGTTAAGACTCTGCACTTCCAAAGCAGGGGACATAGGTTTGATC
CCTGGTCGGGGAA
>Bos_taurus_chrX.trna451-GlyTCC (10037030-10037111) Gly (TCC) 82 bp Sc: 33.06
TCCCTGGTGGTCCAGTGTCCAAAACTCTGCATTTCCAATGCAGGAGTGAGATGGGGCCCA
GGTTTGATCCCTGGTCAGGGAA
>Bos_taurus_chr21.trna3018-GlyTCC (65457361-65457289) Gly (TCC) 73 bp Sc: 33.14
TTCCTGGTGGTCCAGTGGCTGAGATTCTGCGTTTCCACTGCAGAGGGCACAGGTTGGATC
CCTGCTCAGGGAG
>Bos_taurus_chr22.trna3786-GlyTCC (16882340-16882262) Gly (TCC) 79 bp Sc: 33.20
TCCCTGGTGGTCCAGTGGTTAAGACTCTGCATTTCCACTGCATTTCCATGGAGGATGGGT
TTGATCCCTGGTCAGGGAG
>Bos_taurus_chr25.trna2258-GlyTCC (37602367-37602441) Gly (TCC) 75 bp Sc: 33.41
TCCCTGGTGGTCCAATGGTTGAGGTTCCGTGCTTCCACCACAGAGGGTCGCCAGGTTCGA
TCCCTGGTCGGGGAA
>Bos_taurus_chr1.trna2849-GlyTCC (83104005-83104076) Gly (TCC) 72 bp Sc: 33.61
TCCCTGGTGGTCCAGTGGTCAAGACTCTATGCTTCCAGTAAAGGGGGTTTGGTTCGATCC
CTGATCAGGGAA
>Bos_taurus_chr7.trna1845-GlyTCC (38583758-38583830) Gly (TCC) 73 bp Sc: 33.79
TCTCTGGTGGTCCAGTGCTTAGAACTCTGTAATTCCATTGCAGGGGACCTAGGTTCTATC
CCTGGTCAGGGAA
>Bos_taurus_chr27.trna982-GlyTCC (28876858-28876936) Gly (TCC) 79 bp Sc: 34.03
TCCCTGGTGGTTCAGTGGTTAGGACCCTGCATTTCCATTGCAGGGGGCATGGGCCATGGG
TTCAATCCATGGCAGGGAA
>Bos_taurus_chr25.trna4928-GlyTCC (1983041-1982969) Gly (TCC) 73 bp Sc: 34.31
TCCCTGGTGGTCCAGTGGCTGAAACCTTGCCCTTCCAATGCAGGGGACCCAGGTTAGATC
CCTGGTCAGGGAA
>Bos_taurus_chr13.trna919-GlyTCC (24330307-24330379) Gly (TCC) 73 bp Sc: 34.62
TCCCTGGTGGTCCAGTGGCTAAGACTCTGCACTTCCAGTGCAGCGGGCCTGGGTTCCACC
CTTGGTCGGGGAA
>Bos_taurus_chr18.trna1844-GlyTCC (45549546-45549618) Gly (TCC) 73 bp Sc: 34.67
TTCCTGGTTGTCCAAGGGTGAAGATTCTGTGCTTCCACTGCAGGGGTCACAGGTTCAATC

CCTGGTCAGGGAT
>Bos_taurus_chrUn.004.738.trna2-GlyTCC (18601-18673) Gly (TCC) 73 bp Sc: 34.70
TCCCTGGTGGTCTAGTGGCTAAGATTCCATGATTCCACTATGCGGGGCACAGGTTCGATC
CCTGGTCAGGGAA
>Bos_taurus_chrUn.004.7730.trna1-GlyTCC (3327-3255) Gly (TCC) 73 bp Sc: 34.70
TCCCTGGTGGTCTAGTGGCTAAGATTCCATGATTCCACTATGCGGGGCACAGGTTCGATC
CCTGGTCAGGGAA
>Bos_taurus_chr28.trna2929-GlyTCC (6799468-6799393) Gly (TCC) 76 bp Sc: 34.82
TTCCTGGTGGTCCAGTGCTTAGGACCCTGCACTTCCACTGCAGGAGGCCCTGCAGGTTTG
ATCCCTGGTCGGGGAA
>Bos_taurus_chr3.trna9344-GlyTCC (1429399-1429327) Gly (TCC) 73 bp Sc: 34.99
TCCCTGGTGGTGTAGTGGTTTAGACTCTGAGCTTCCACTGCAGGGAGCTCAGGTTCCATC
CCTGGTCAGGGAA
>Bos_taurus_chr18.trna4362-GlyTCC (42955064-42954992) Gly (TCC) 73 bp Sc: 35.10
CCCCTGGTGGTCCAGTAGTTAAGACTCTGTGCTTCCAACACAGGGAGTATGGGTTCGATC
CCTGGTTGGGGGA
>Bos_taurus_chr5.trna7898-GlyTCC (62728372-62728300) Gly (TCC) 73 bp Sc: 35.17
TCCCTGGAGGTCCAATGATTAGGACTCCATGCTTCCACTGTAGAGGGCACAGGTTCCATT
CCTGTTCAGGGAA
>Bos_taurus_chrX.trna358-GlyTCC (8243957-8244029) Gly (TCC) 73 bp Sc: 35.25
TCCTTGGCAGTCCAGTGGTTAGGACTCTGTGCTTCCTCTGCAGGGGGCCTGGGTTCAATC
CCTGGTCAAGGAC
>Bos_taurus_chr11.trna6821-GlyTCC (56893232-56893160) Gly (TCC) 73 bp Sc: 35.32
TCCTTGGTGGTCCAGTAGTTAAGACTCTGTACTTCCATTGCATGGGGTGCAGGTTTGATC
CCTGGTCAGGGAA
>Bos_taurus_chr11.trna855-GlyTCC (20187242-20187314) Gly (TCC) 73 bp Sc: 35.44
TCCCTGGTGGTCCACTGGCTAAGACTCTGTACTTCCAATGCAGGGGGCCTGGGTTTGATC
CCTGATCAGGGAA
>Bos_taurus_chr1.trna2602-GlyTCC (75569637-75569709) Gly (TCC) 73 bp Sc: 35.83
TTCTTGATAGTCCAGTGGTTAGGACTCTGTTCTTCCATTACAGGAGCCACGGGCTCGATC
CCTGGTTGGGGAA
>Bos_taurus_chrUn.004.1144.trna5-GlyTCC (9809-9737) Gly (TCC) 73 bp Sc: 35.83
TTCTTGATAGTCCAGTGGTTAGGACTCTGTTCTTCCATTACAGGAGCCACGGGCTCGATC
CCTGGTTGGGGAA
>Bos_taurus_chr12.trna4996-GlyTCC (47243477-47243405) Gly (TCC) 73 bp Sc: 35.86
TCCCTGGTGGTCCAGTGGCTAAGGCTCTGTACTTCCAGTGCAGAGGGTCTAGGTTTGATC
CCTGATCGGGGAA
>Bos_taurus_chr6.trna2069-GlyTCC (70295380-70295452) Gly (TCC) 73 bp Sc: 35.88
TCCCTAGTGGTCCAGTGGCTAAGACTCTGCTCTTCCAGTGCAGGGGGCCCAGGTCCTATC
CCTGGTCAGGGAA
>Bos_taurus_chr13.trna3432-GlyTCC (78539661-78539733) Gly (TCC) 73 bp Sc: 35.90
TCCCTGGTGGTCTAGTGTTTAAGACCCTGCCCTTCCACTGCGGGGAACGTGGGTTTGATC
CCCAGTCAGGGAA
>Bos_taurus_chr7.trna8271-GlyTCC (9550903-9550831) Gly (TCC) 73 bp Sc: 35.97
TCCCTGATGGTCCAGTAGTTAAGACTCTGTGCTTCCACTGCATGGGACACAGGTTTGATC
CCTGGTCAGGGAA
>Bos_taurus_chr25.trna4471-GlyTCC (9291711-9291639) Gly (TCC) 73 bp Sc: 36.04
TCCCTGGAGGTCCAGCGATTAGGACTCAGCACTTCCACTGCAGGGGGCTCAGGTTTGATC
CCTGGTTGGGGAA
>Bos_taurus_chr26.trna2760-GlyTCC (34309381-34309309) Gly (TCC) 73 bp Sc: 36.23
TCCCTGGTGGTTCAGTGGTTAAGACTCTGTTCTTCCCCTGCAGGGGGCTCTGGTTCCATC
CCTGGTTAGGGAA
>Bos_taurus_chr3.trna6798-GlyTCC (73465972-73465900) Gly (TCC) 73 bp Sc: 36.40
TCCCTAGTGGTCCAGTGGCTAAGACTCAGTGCTTCCAATACAGGGGGCTCAGGTTTGATC
CCTGCTTGGGGAA
>Bos_taurus_chr25.trna3439-GlyTCC (27901640-27901568) Gly (TCC) 73 bp Sc: 36.47
TCCCTGGCAGTCCAGTGGTTGGGACTCTGCGCTTCCAATGCAGGAGGCTCGGGTTCAAAC
CCTGATTGGGGAG
>Bos_taurus_chr16.trna848-GlyTCC (25694869-25694941) Gly (TCC) 73 bp Sc: 36.63
TCCTTGGTGGTCCAGTGGTTCGGACTCTGTGCTTCCACTGCAGAGGACATGGGTTTAATC
CCTAGTCAGGGAA
>Bos_taurus_chr16.trna6184-GlyTCC (1288058-1287986) Gly (TCC) 73 bp Sc: 36.72
TCCCTGGTGGTCCCGAGGCTGGGACTCAGCACTTCCAGTGCAGGGGGCCCAGGTTCGATC
CCTGGTTAGGGAA
>Bos_taurus_chr2.trna2148-GlyTCC (68533876-68533947) Gly (TCC) 72 bp Sc: 36.74
TCTAGGGTGGTCTAGTGGCTATGACTCTGTGCTTCCAATGCAGGGGGCCAGGTTTAATCC
CTGGTCTTGAAC

>Bos_taurus_chr5.trna7135-GlyTCC (82065803-82065731) Gly (TCC) 73 bp Sc: 36.85
TCCCTGGTGGTCCAGTGGCTGAGACTCTGCACTTCCAAAGCAGGGAGCCCAGGTTTGACC
CCTGATCAGGGAA
>Bos_taurus_chrX.trna2805-GlyTCC (74673904-74673976) Gly (TCC) 73 bp Sc: 36.90
TCCTTGGTGATCCAGTGGTTAAGATTCCGTGCTTCCGTTGTGGGCGGCTCAGGTTCAATC
CCTGACTGGGGAA
>Bos_taurus_chr6.trna5187-GlyTCC (101869409-101869336) Gly (TCC) 74 bp Sc: 36.92
TCCTTGGTGGTCCAGTGGTTAAGATTCTGTGCTTCCATTTCAGGGCGGCCCGGGTTAGGT
CCCTGGCCAGGGAA
>Bos_taurus_chr5.trna3031-GlyTCC (81055299-81055375) Gly (TCC) 77 bp Sc: 37.18
TCCCTGGTGGTCCAGTGGCTAAGACTCTACGCTTCCAACTTGGGCAGGGGGCCCAAGTTG
GATCCCTGGTTAGGGAA
>Bos_taurus_chr13.trna3953-GlyTCC (79381500-79381428) Gly (TCC) 73 bp Sc: 37.22
TCCTTGGTGGTCCAGTGGTTGAGACTCCGTACTTCCACTGCAGAGGGCACAAGTTTGATC
CTTGGTCGGGGAA
>Bos_taurus_chr17.trna6588-GlyTCC (5714022-5713947) Gly (TCC) 76 bp Sc: 37.25
TCCCTGGTGGTCCAGTGGTTAAGACTCTGGAATTCCATTGCAGGGCACAACTCAGGTTTG
ATCCCTGGTTGGGGAG
>Bos_taurus_chrUn.004.3303.trna4-GlyTCC (3192-3120) Gly (TCC) 73 bp Sc: 37.42
TCCCTGGCAGTCCAGTGGTTCAGACTCTACACTTCCATTGTAGGGGGCCCGGGTTCGGGC
CCTGGTTGGGGAA
>Bos_taurus_chr23.trna1348-GlyTCC (30507032-30507105) Gly (TCC) 74 bp Sc: 37.48
TCCTTGATGGTCCAGTGGTTGAGACTCTACACTTCCACTGCAGGGGAGTGCAGGTTTGAT
CCCTGATCAGGGAA
>Bos_taurus_chr8.trna7765-GlyTCC (11441086-11441014) Gly (TCC) 73 bp Sc: 37.60
TCCCTGGTGGTCCAGTGGTTAGCACTCTGTGCTTCCACTGCTGTGAACCCAGGTTGGGTC
CCTGGTCAGGGAA
>Bos_taurus_chr14.trna6267-GlyTCC (9838618-9838546) Gly (TCC) 73 bp Sc: 37.61
TTCCTAGTGGGCCAGTGGCTAAGACTCTGCGCTTCCACTGCAGGGGGTGCAGGTTTGATC
CCTGCTTGGGGAA
>Bos_taurus_chrX.trna1315-GlyTCC (34451813-34451885) Gly (TCC) 73 bp Sc: 37.67
TCCCTGGTGGTCCAATGGTTAAGACTCCACAGTTCCAATGTAGGGGATGCAGGTTGGATC
CCTGGTCAGGGAA
>Bos_taurus_chr27.trna2648-GlyTCC (32132329-32132257) Gly (TCC) 73 bp Sc: 37.84
TCCCTGGTGGTCCAGTGGTTAAGTCTCCATGCTTCCACTGCGGGGGGCACAGGTTGAATT
CCTGTTTGGGGAA
>Bos_taurus_chrX.trna3861-GlyTCC (82944955-82944883) Gly (TCC) 73 bp Sc: 37.86
TCTCTGGCGGTCCAAAGGTTATGACTCAGCACTTCCACTGCAGGGGGACCAGGTTCGATC
CCTGGTCAGGGAA
>Bos_taurus_chr6.trna7716-GlyTCC (30741749-30741679) Gly (TCC) 71 bp Sc: 37.92
TTACTGGTGGTCCAGTGCTAAGACTCTGTGCTTCCAATGCAGGGGCCCAGGTTTGATCCC
TGGTCAGGGAA
>Bos_taurus_chr18.trna816-GlyTCC (21093072-21093144) Gly (TCC) 73 bp Sc: 38.09
TCCCTGGTGGTCCAGGGGGTAAGACTCTGTGCTTCCACTGCAGGGGGCACAGGTTTGATC
CCTGTTTGGGGTA
>Bos_taurus_chrUn.004.74.trna1-GlyTCC (5820-5891) Gly (TCC) 72 bp Sc: 38.10
TCCCTGATGGTCCAGGGTTAAGACTCTGCACTTCCACTGCAGGCGATTCGGGTTCCATCC
CTGGTCAGGGAA
>Bos_taurus_chr16.trna2301-GlyTCC (58903532-58903603) Gly (TCC) 72 bp Sc: 38.37
TCCCTGGTAGTCCAGTGGTTAGGACTTGGCACTTCCACTGCCAGGGATTGGGTTCAGCCC
CTTGTTGGGGAA
>Bos_taurus_chr27.trna591-GlyTCC (19565578-19565650) Gly (TCC) 73 bp Sc: 38.43
TCCCTGGTGGTCCAGTGACCAAGACGCTGGGCTTCCAATGCAGGAAGCCCAGGTTTGATT
CCTGGTCAGGGAA
>Bos_taurus_chr1.trna9179-GlyTCC (59446501-59446429) Gly (TCC) 73 bp Sc: 38.52
TCCCTGGCAGTCCAGTGGCTAAGACTCTGCGCTTCCAATGCAAGGGGCCCAGGTTTGATC
CCTGGTTGGGGAA
>Bos_taurus_chr13.trna2165-GlyTCC (55195749-55195819) Gly (TCC) 71 bp Sc: 38.53
TCCCTGGTGGTCCAGTGGTTAAGACTCCTCGCTTCCTCAGAGGAGTTTGGGTTCAATCCC
CGATCGGTGAA
>Bos_taurus_chr14.trna5433-GlyTCC (27762521-27762449) Gly (TCC) 73 bp Sc: 38.53
TCCCTGGGGGTTCAGTGGTTATGACTCTGAGCTTCCAATGCAGGGGGTCCAGGTTTGATC
CCTGGTCAGGGAA
>Bos_taurus_chr15.trna1557-GlyTCC (44445149-44445224) Gly (TCC) 76 bp Sc: 38.58
TCCCTGGTGGTCTAGTGGCTAAGACTCTACACTTCCAATGTGAGATTAAATCCAGGTTCG
ACCCTTAGTCAGGGAA
>Bos_taurus_chr3.trna5161-GlyTCC (117921628-117921556) Gly (TCC) 73 bp Sc: 38.74

TCCCTGGTAGTCCAGTGAGTAAGACTCTGTGCTTCCAATGCAGGGGGCACAGGTTCAATC
CTTGGTCAGGGAA
>Bos_taurus_chr27.trna1060-GlyTCC (30311833-30311905) Gly (TCC) 73 bp Sc: 39.26
TCCCTGGTGGTCCAAGTGTTAAGACTCTTTACTTCCATTGCAGGGGACACAGGTTCGATC
CCTGGTCAGGGAA
>Bos_taurus_chrUn.004.41.trna23-GlyTCC (266271-266343) Gly (TCC) 73 bp Sc: 39.28
TCCCTGGCAGTCCAGTGGTTAAGACTCTATGCTTCCACTGCAGAGAGCCCAGGTTTGAGC
CCTGGTCGGGGAA
>Bos_taurus_chr14.trna6475-GlyTCC (6141482-6141410) Gly (TCC) 73 bp Sc: 39.34
TCCCTGGTGGTCCAGTGGCTAGGATTCTGTGTTTCCACTGCAGGGTACCAGGGTTCAATC
CCTAGTCAGGGAA
>Bos_taurus_chr5.trna4418-GlyTCC (112311776-112311848) Gly (TCC) 73 bp Sc: 39.34
TCTCTGATGGTCCAGTGGCTGAGACTCTGCACTTCCAGTTCAGGGGGCCCGGGTTCAATC
CCTGGTCAGGGAA
>Bos_taurus_chr17.trna4687-GlyTCC (54606519-54606447) Gly (TCC) 73 bp Sc: 39.43
TCCCTGATGGTCCAGGTGTTAAGACTCTGTGCTTCCAATACAGGGATCTCAGGTTTGATC
CTTGATTGGGGAA
>Bos_taurus_chr20.trna1698-GlyTCC (44974552-44974623) Gly (TCC) 72 bp Sc: 39.43
TCCCTGGTAGTCTAGTGGTTAAGACTCTGTGCTTCCACTGCAGGGGCATAAGGTCAATCC
TTGGTTGGGGAA
>Bos_taurus_chr13.trna5381-GlyTCC (51762459-51762387) Gly (TCC) 73 bp Sc: 39.49
TCCCTGATGGTCCAGTGGTAAGGACTTTGTTCTTCCATTGCAGGAGGCATAGGTTTGATC
CCTGGTCAGGGAA
>Bos_taurus_chr10.trna327-GlyTCC (8464872-8464944) Gly (TCC) 73 bp Sc: 39.50
TCCCTGGTGGTCCAGTGGTTACGACTCTGGGCTTCCAATGCAGGGCACACAGGTTTGATC
CCTGATCAGGGAA
>Bos_taurus_chr12.trna1580-GlyTCC (35396660-35396732) Gly (TCC) 73 bp Sc: 39.51
TCCCTGGTGGTCCAGTGGTTGGGACTCTGCTCTTCCACTGCAGGGGGCCTGGATTCCATT
CCTGGTCAGGGAA
>Bos_taurus_chr10.trna6462-GlyTCC (47089376-47089304) Gly (TCC) 73 bp Sc: 39.54
TCCCTGGTGGTCCAGTGGTTAGGACTCTGAGCTTCCACTGCAGGTTTCCTGGGTTCAGTC
CCTAGTTGGGGAA
>Bos_taurus_chr2.trna10046-GlyTCC (13545826-13545754) Gly (TCC) 73 bp Sc: 39.55
TCCTTGGTGGTCCAGTGGTTAGGGCTCTGTGCTTCCTCTGCAGGGGGCACAGGTTTGATC
CCTGCTCAGGGAA
>Bos_taurus_chr3.trna6472-GlyTCC (83985831-83985759) Gly (TCC) 73 bp Sc: 39.56
TCCCTGGTGGTCCAGCTGTTAGGACTCTGTGCTTCCACTTCAGGGGACCCAGGTTTGATC
CCTGGTCAGGAAG
>Bos_taurus_chr19.trna2599-GlyTCC (50938660-50938731) Gly (TCC) 72 bp Sc: 39.60
TCCCTGGCAGTCCAATGGACAGGACTCTGTGCTTCCACTGTAGGGACCCAGGTTCAACCC
CTGGTTAGGGAA
>Bos_taurus_chr18.trna2280-GlyTCC (52193169-52193241) Gly (TCC) 73 bp Sc: 39.68
TCTCTGGTGGTCCAGTGGCTAGGACCCTGTGCTTCCAACGCAGGGGACGTGGGTTCAGTC
CCCGGTCGGGGAA
>Bos_taurus_chr7.trna3347-GlyTCC (83449769-83449841) Gly (TCC) 73 bp Sc: 39.71
TCTCTGGCAGTCCAGTGGTTAGGATTCCATGCTTCCACTGTTGGGGGCCCAGGATCAATC
CCTGGTCAGGGAA
>Bos_taurus_chr11.trna4969-GlyTCC (102016469-102016397) Gly (TCC) 73 bp Sc: 40.04
TCTCTGGTGGTCCAGTGGTTTAGACTCTGTGCTTCCACTGCAGGGGCCACAGGTTTGATC
CCTGGTCAGGGAA
>Bos_taurus_chr29.trna503-GlyTCC (13361512-13361582) Gly (TCC) 71 bp Sc: 40.05
TTTCTGGTGGTTCAGTGGTAAGACTTGGTATTTCCATTGCTGTGGCCCAGCTTCAATCCC
TGGTCAGGAAG
>Bos_taurus_chr19.trna771-GlyTCC (17783436-17783507) Gly (TCC) 72 bp Sc: 40.25
TCCCTGGTGGACCAGTGGCTTGGACTCCGTGCTTCCAACACAGGGGCCTAGGTTCCATCC
CTGGTCAGGGAA
>Bos_taurus_chr14.trna258-GlyTCC (6872702-6872772) Gly (TCC) 71 bp Sc: 40.34
TCCCTGGTGGTCTAGTGGTTAAGACTCTGTGCTTCCACTGCAGGGGGCAGGTTTGATCCC
TGGTTGGGGAA
>Bos_taurus_chr19.trna3054-GlyTCC (59357059-59357131) Gly (TCC) 73 bp Sc: 40.38
TCCCTGGCGGTCCAGTGGTTAGGACTCAGCATTTCCTCTGCAGGAGGCCCAGGTTTGATC
CCTGGTCAGGGAA
>Bos_taurus_chr5.trna1211-GlyTCC (33732724-33732796) Gly (TCC) 73 bp Sc: 40.44
TCCCTGGTGGTCCAGTGGTTGGGACTCTGTGCTTCCACTGCAGGGGGCACAGGTTTAATC
CCTGGTTGGGGAA
>Bos_taurus_chr5.trna5644-GlyTCC (114232498-114232426) Gly (TCC) 73 bp Sc: 40.55
TCCCTGGCGGTCCAGTGGTCAGGACTCTGTGCTTCCACTGCAGGGAGCCTAGGTTTGATC

CCTGGTTGGGGAA
>Bos_taurus_chr28.trna3097-GlyTCC (3214654-3214582) Gly (TCC) 73 bp Sc: 40.59
TCCCTGGTGGTCCAGTGGTTAAGACTCTGCACTTCCAGTGCAGGGGCCATGGGTTTGATC
CCTGATTGGGGAA
>Bos_taurus_chrUn.004.8.trna94-GlyTCC (4374-4302) Gly (TCC) 73 bp Sc: 40.61
TCCCAGGTGGTCTAGTGGCTAAGACTCTGAGCTTCCAATACAGGCGGCCCAGGTTTGATC
CCTGGTCAGGGAA
>Bos_taurus_chr19.trna2207-GlyTCC (44819799-44819871) Gly (TCC) 73 bp Sc: 40.82
TCCCTGGTGGTTAAGTGGTTAGGACTCTGAGCTTCCACTACAGGGAGCACAGGTTCGACC
CCTGCTTGGGGAA
>Bos_taurus_chr24.trna4472-GlyTCC (26661752-26661681) Gly (TCC) 72 bp Sc: 41.03
TCCCTGGTGGTCCAGTGGTTAGGATTCAGTGCTTCCACTGCAGGGAACGTGGTTCCATCC
CTCGTCAGGGAA
>Bos_taurus_chr22.trna2550-GlyTCC (53445969-53445896) Gly (TCC) 74 bp Sc: 41.10
TCCCTGGTGGTCCAGTGGTCAGAAGACTCTGCACTTCCAATGCAGGGTCATGGGTTCTAT
CCCTGGCTGGGGAA
>Bos_taurus_chr17.trna2245-GlyTCC (55407448-55407520) Gly (TCC) 73 bp Sc: 41.11
TTCCTGGTGGTCCATTGGTTAAGACTCTGTGCTTCCAATGCAGAGGGCACAGGTTTGATC
CCTGGTCAGGGAA
>Bos_taurus_chrX.trna4388-GlyTCC (69674050-69673980) Gly (TCC) 71 bp Sc: 41.14
TCCCTGGTGGTCCAGTGGTTAGGACTTGGCACTTCCACTGTTAGGACCAGGATCAATCCC
TGATCAGGGGA
>Bos_taurus_chr1.trna3756-GlyTCC (110555157-110555228) Gly (TCC) 72 bp Sc: 41.46
TCCCTGGCAGTCCACTGGGAGGACTCCACACTTCCACTGTTGAGGCCCTAGGTTCAATCC
CTGGTTGGGGAA
>Bos_taurus_chr29.trna1285-GlyTCC (35056952-35057024) Gly (TCC) 73 bp Sc: 41.50
TCCCTGACAGTCCAGTGGTTAAGACTCTGACCTTCCAAGGCAGGGACCCTGGGTTCAGTC
CCTGGTCAGGGAA
>Bos_taurus_chr13.trna6214-GlyTCC (31620771-31620699) Gly (TCC) 73 bp Sc: 41.65
TCCCTGGTGGTCCAGTGGTTAAGACACTATGCTTCCATTGTACGGGGAGCAGGTTCAATC
CCTGGTCAGGGAA
>Bos_taurus_chr11.trna3079-GlyTCC (76107243-76107315) Gly (TCC) 73 bp Sc: 41.76
TCCCTGGTGGTCCAGTAGTTAAGACTCTGCACTTCCAGTGCAGGGAGCACAGGTTTGATC
CCTGGTCAGGGAA
>Bos_taurus_chr20.trna4781-GlyTCC (25852538-25852468) Gly (TCC) 71 bp Sc: 41.83
TCCCTGGTGGTTCAGTGGTTGACTCTGCACTTCCATTGCAGGGGGCATGGGTTCAATCCC
TGTTCAGGGAA
>Bos_taurus_chr16.trna6197-GlyTCC (759630-759558) Gly (TCC) 73 bp Sc: 41.87
TCCCTGGTGGTCCAGTGGCTAAGACTCTGCACTTCCAATTCAGGGAGCCTGGGTTCAGTC
CCTGGTCAGGGAA
>Bos_taurus_chr18.trna2160-GlyTCC (50670063-50670135) Gly (TCC) 73 bp Sc: 41.90
TCCCTGGTGGTCCAGTGGCCAAGACTCTGCACGTCCAGTGCAGGGGGCCCAGGTTTGATC
CCTGGTTGGGGAA
>Bos_taurus_chrUn.004.2437.trna2-GlyTCC (19626-19698) Gly (TCC) 73 bp Sc: 41.90
TCCCTGGTGGTCCAGTGGCCAAGACTCTGCACGTCCAGTGCAGGGGGCCCAGGTTTGATC
CCTGGTTGGGGAA
>Bos_taurus_chr2.trna9769-GlyTCC (22378832-22378760) Gly (TCC) 73 bp Sc: 41.93
TCTTTGGTGGTCCAATGGTTAAGGCTCTGTACTTCCAATGCAGGGGGCACAGGTTCAATC
TCTGGTCAGGGAA
>Bos_taurus_chr11.trna8431-GlyTCC (16545562-16545490) Gly (TCC) 73 bp Sc: 41.95
TCCCTGGTAGTCCAGTGGCTCAGACTCTGCTCTTCCAATGCAGGGAGCCTAGGTTTGATC
CCTGGTCGGGGAA
>Bos_taurus_chr2.trna6073-GlyTCC (125815389-125815317) Gly (TCC) 73 bp Sc: 41.99
TCCCTGGCAGTCCAGTGGTTAGGACGCTGTGCTTCCACTGCTGGGGGTCCAGGTTTGATC
CCTGGTTGGGGAA
>Bos_taurus_chr7.trna7455-GlyTCC (19711152-19711079) Gly (TCC) 74 bp Sc: 42.08
TCCCTGGTGGTCCAGTGGCTAAGACTCTGTGCTTCCAATGCAGGGAGCCCCGGGTTCCAT
TCCTGGTCAGGGAA
>Bos_taurus_chr5.trna913-GlyTCC (27479921-27479994) Gly (TCC) 74 bp Sc: 42.13
TCCCTGGCAGTCCAGTGGCTAGGGCTCCACACTTCCACTGTAGGGGGCACCAGGTTCAAT
CCCTGGTCAGGGAA
>Bos_taurus_chr20.trna2620-GlyTCC (68898382-68898454) Gly (TCC) 73 bp Sc: 42.25
TCCCTAATGGTCCAGTGGTTAAGACTCAGTATTTCCACTGCAGAGGGCACAGGTTTCATC
CCTGGTTGGGGAA
>Bos_taurus_chr12.trna2863-GlyTCC (73696974-73697046) Gly (TCC) 73 bp Sc: 42.25
TCCCTGGTGGTCCAGTGGTTGAGACTCTGAGCTTCCGACTCAGGGGGCCTGGGTTCGGAT
CCTGGTCAGGGAA

>Bos_taurus_chr29.trna2138-GlyTCC (47738199-47738126) Gly (TCC) 74 bp Sc: 42.29
TCCCTGGTGGTCCAGTGGTTAGGACCCTGCACTTCCAATGCAGGAGGTGTTGGGTTCAGT
CCCTGATCAGGGAA
>Bos_taurus_chr10.trna7377-GlyTCC (20741860-20741788) Gly (TCC) 73 bp Sc: 42.34
TTCCTGGCAGTCCAATGGCTAGGACTCTGAGCTTCCAATGCAGGGGGCCCAGGTTTGATC
CCTGGTCAGGAAA
>Bos_taurus_chr2.trna631-GlyTCC (21608425-21608497) Gly (TCC) 73 bp Sc: 42.38
TCCCTGGTGGTCCAGTGGTTAGGACTCTGTACTTCCACTGCAGGGGATATGGGTTCAGTC
CCTGGCTGGGGAA
>Bos_taurus_chr19.trna3832-GlyTCC (56840328-56840256) Gly (TCC) 73 bp Sc: 42.41
TCCCTGGTGGTCCAGTGGCTAAGACCCTGAGCTTCCAATGTAGGGGGCCCAGGTTTGATC
CCTGGTCAGGGAA
>Bos_taurus_chr16.trna2970-GlyTCC (72374960-72375032) Gly (TCC) 73 bp Sc: 42.44
TCCCTGGCGGTCTAGTGGTTAAGATTCTGTGCTTCCAATGCAAGGGTCTCAGGTTTGATC
CCTGGTCAGGGAA
>Bos_taurus_chrX.trna469-GlyTCC (10229781-10229853) Gly (TCC) 73 bp Sc: 42.50
TCCCTGGTGGTCCAGTGGTTAGGATTCCTCACTTCCACTGGTGGGGGCCCAGGTTTGATC
CCTGATTGGGGAC
>Bos_taurus_chr5.trna5655-GlyTCC (113830391-113830311) Gly (TCC) 81 bp Sc: 42.61
TCCCTGGTGGTCCAGTGGTTAAGACACCACACTTCCAATGTAGGGGACCCAGGTACCTGG
GTTTGATCCCTGGTTGGGGAA
>Bos_taurus_chr8.trna7862-GlyTCC (10147526-10147454) Gly (TCC) 73 bp Sc: 42.63
TCCTTGGTAGTCCAGTGGCTGAGACTCTGTGCTTCCAAAGCAGGGGACCCAGGTTCGATC
CTTGGTCAGGGAA
>Bos_taurus_chr21.trna1327-GlyTCC (28207234-28207306) Gly (TCC) 73 bp Sc: 42.63
TCTCTGGTGGTCCAGTGGTTAAGATTCTGAGCTTCCAATTCAGGGGGCTCAGGTTTGATC
CCTGGTTGGGGAA
>Bos_taurus_chr16.trna5003-GlyTCC (34620848-34620776) Gly (TCC) 73 bp Sc: 42.71
TCCCTGGTAGTCCAGTAGCTACGACTCTGCACTTCCAGTGCAGAGAACCCGGGTTCAATC
CCTGGTCAGGGAA
>Bos_taurus_chr17.trna3401-GlyTCC (76019204-76019275) Gly (TCC) 72 bp Sc: 42.78
TTCCTGGCGGTCCAGTGGTGGGGACTCTGCACTTCCAATGCAGGGGCTCAGGTTTGATCC
CTGGTCAGGGAA
>Bos_taurus_chr23.trna1665-GlyTCC (38105143-38105215) Gly (TCC) 73 bp Sc: 42.78
TCCCTGGTGGTCCAATGGCTAAGACACTGCATTTCCAATGCAAGGGACCCCGGTTCCATC
CCTGGTCAGGGAA
>Bos_taurus_chr18.trna5309-GlyTCC (19264975-19264904) Gly (TCC) 72 bp Sc: 42.81
TCTCCAGTAGTCTAATGGTTAAGACTCTGCACTTCCAATGCAGGGGTGTGGGTTCATTCC
CTGGTTGGAGAA
>Bos_taurus_chr28.trna595-GlyTCC (14660307-14660379) Gly (TCC) 73 bp Sc: 42.87
TCCCTGGCAGTCCAGTGGTTAGGACTCTGTGCTTCCACTGCAGGGGGTGCAGGTTGGATC
CCTGTTTGGGGAA
>Bos_taurus_chr11.trna8482-GlyTCC (15261193-15261121) Gly (TCC) 73 bp Sc: 42.88
TCCTTGGTGGTTCAGTGGCTAAGATTCTGTGCTTCCAATGCAGGGGCTCCAGGTTTGACC
CCTGGTCAGGGAA
>Bos_taurus_chr19.trna4644-GlyTCC (43035451-43035379) Gly (TCC) 73 bp Sc: 42.92
TCCCTGGTGGTCCAGTGGCTAAGACTCTGTGCTTCCAATGCAGGGAGTCTGGGTTCGATC
CCTGGTCAGGGAT
>Bos_taurus_chr7.trna762-GlyTCC (13855673-13855745) Gly (TCC) 73 bp Sc: 42.99
TCCCCAGTGGTCCAGTGGGTAAGGCTCTGCACTTCCACTGCAGGGGGCTTGGGTTCACTC
CCCGGCTTGGGAA
>Bos_taurus_chr17.trna3248-GlyTCC (72199042-72199114) Gly (TCC) 73 bp Sc: 43.08
TCCCTGGTGGTCCAGTGGTTAAGACTCTGAACTTCCACTTCAGGGGGCTAGGGTTTGATC
CCTGGTCAGGGAA
>Bos_taurus_chr17.trna3564-GlyTCC (72256813-72256741) Gly (TCC) 73 bp Sc: 43.08
TCCCTGGTGGTCCAGTGGTTAAGACTCTGAACTTCCACTTCAGGGGGCTAGGGTTTGATC
CCTGGTCAGGGAA
>Bos_taurus_chr1.trna5084-GlyTCC (145977558-145977630) Gly (TCC) 73 bp Sc: 43.18
TCTCTGGTGGTCCAATGGCTAAGACTCTATACTTCCAATGCAGGGGACCCAGGTTCAATC
CCTGATCAGGGAA
>Bos_taurus_chr23.trna3624-GlyTCC (25292043-25291971) Gly (TCC) 73 bp Sc: 43.34
TCCCTGGTGGTCCAGTTGCTAAGACTCTGTGCTTCCAATGCAGGGGGCTCAGGTTTGATC
CCTGGTCAGGGAA
>Bos_taurus_chr10.trna4440-GlyTCC (98542679-98542607) Gly (TCC) 73 bp Sc: 43.39
TCCCTGGTGGTCCAGTGGTCAAGACTTTGTGCTTCCAATACAGGGGACACAGGTTCGATC
CCTGGTTGGGGAC
>Bos_taurus_chr24.trna1839-GlyTCC (43973022-43973094) Gly (TCC) 73 bp Sc: 43.41

TCCCTGGTGGTCCAGTGATTGGGACTCTGCACTTCCAATGCAGGGGAGGCAGGTTCAATC
CCTGGTCGGGGAA
>Bos_taurus_chr2.trna973-GlyTCC (30171817-30171888) Gly (TCC) 72 bp Sc: 43.47
TCTTTGATGGTCCAGTGGTTAAGACTCTGCACTTCCAAAGCAGGGGTACAGGTTTGATTC
TTGATTGGGGAA
>Bos_taurus_chr13.trna6987-GlyTCC (17003409-17003337) Gly (TCC) 73 bp Sc: 43.50
TCCCTGGTAGTCCAGCGGTTAAGATTCTGCCCTTCCGCTGCAGGGGACCTGGGTTCTATC
CCTAGTCAGGGAA
>Bos_taurus_chr16.trna5511-GlyTCC (23311561-23311489) Gly (TCC) 73 bp Sc: 43.65
TCTCTGGTGGTCCAATGGCTAAGACTCTGCACTTCCAATTCAGGGGGTCCAGGTTGGATC
CCTGGTAAGAGAA
>Bos_taurus_chr11.trna3633-GlyTCC (89660999-89661071) Gly (TCC) 73 bp Sc: 43.69
TCCCTGGTGGTCCAGTGACGAGGACTCCCTGCTTCCAATGTGGTGGGCCCTGGTTCGATC
CCTGGTCAGGGAA
>Bos_taurus_chr21.trna4179-GlyTCC (33691758-33691686) Gly (TCC) 73 bp Sc: 43.72
TCCCTGGAAGTCCAATGGTTAAGACTCTATACTTCCACTGCAGGGAGTCCAGGTTCAATT
CCTGGTTGGGGAA
>Bos_taurus_chr20.trna5520-GlyTCC (6168442-6168371) Gly (TCC) 72 bp Sc: 43.77
TCCTTGGTGGTCTAGTGGTTAAGACTCTGTGCTTCCAATGCAGGAGCACAGGCTCAATCC
CTGGTTGGGGAA
>Bos_taurus_chr17.trna5152-GlyTCC (47385623-47385551) Gly (TCC) 73 bp Sc: 43.79
TCCCTGGTGGTCCAGTGGCTAAGACTCTGCACTTCCAATGCAGGGGGCCTGGGTTTGGTC
CCTGGTCAGGGAA
>Bos_taurus_chr5.trna4591-GlyTCC (115947496-115947569) Gly (TCC) 74 bp Sc: 43.81
TTCCTTGTGGTCCAGTGGTTAGGACTCTGTGCTTCCACTGCTGAGAGCACAGGTTCGATC
CTTGTTCAGGGAAC
>Bos_taurus_chr20.trna2598-GlyTCC (68252569-68252641) Gly (TCC) 73 bp Sc: 43.86
TCCCTGGTGGTTCAGTGGTTAAGACTCTGCACTTCCACTACAGGGGACACAGGTTCCATC
CCTGGTTGGGGAA
>Bos_taurus_chr1.trna5274-GlyTCC (151622382-151622454) Gly (TCC) 73 bp Sc: 43.86
TCCCTGGTGGTCCAGTGGTTAAGACTCTGTGCTTCCACTGCAGGGGGCATGGGTTCTATC
CCTGTTCAGGGAA
>Bos_taurus_chr5.trna2751-GlyTCC (73789445-73789517) Gly (TCC) 73 bp Sc: 43.86
TCCCTGGTGGTCCAGTGGTTAAGACTCTGTGCTTCCACTGCAGGGGGCATGGGTTCTATC
CCTGTTCAGGGAA
>Bos_taurus_chr3.trna4085-GlyTCC (113503749-113503820) Gly (TCC) 72 bp Sc: 43.94
TCCCTGGTGGTCCAGCGTTAAGACTCTGTGCTTCCACTGCAGGGGACACAGGTTCGATCC
CTGGTCAGGGAA
>Bos_taurus_chr18.trna5381-GlyTCC (17524558-17524486) Gly (TCC) 73 bp Sc: 44.11
TCCCCGGTGGTCCAGTGCTTAGGACTTTGCGCTTCCACTGCTGGGGGCCCAGGTTTGACC
CCTGGTCGGGGAA
>Bos_taurus_chr27.trna2419-GlyTCC (36247778-36247706) Gly (TCC) 73 bp Sc: 44.15
CCCCTGGTGGTCTACTGGTTAAGACTCTGCACTTCCACTGCAGGGGGCCCGGGTTCCATC
CCTGGTTAGGGAA
>Bos_taurus_chr12.trna6193-GlyTCC (18061283-18061211) Gly (TCC) 73 bp Sc: 44.22
TCTCTGGTAGTCCAGTGGTTTAGACTCTGAGCTTCCACTGCAGGGGGCACAGGTTCAATC
CCTGGTCAGAGAA
>Bos_taurus_chr14.trna5989-GlyTCC (15724308-15724236) Gly (TCC) 73 bp Sc: 44.34
TCCCTGGTGGTCCAGTGGTTGGGACTCTGTGCTTCCAATGCAGGGAGCTTGGATTCGATT
CCCAGTTGGGGAA
>Bos_taurus_chr19.trna3698-GlyTCC (58837660-58837588) Gly (TCC) 73 bp Sc: 44.40
TCCCTGGTGGTCCAGTGGTTAAGACTCTGCATTTCCAGGGCAGGGGGCACAGGTTCAATT
TCTGGTCAGGGAA
>Bos_taurus_chr15.trna927-GlyTCC (29955050-29955122) Gly (TCC) 73 bp Sc: 44.40
TCCCTGGTAGCCCAGTGGCTAAGACTCCACACTTCCAATGCAGGGGGCTCAGGTTCAATC
CCTGATCAGGGAA
>Bos_taurus_chr1.trna8394-GlyTCC (85150641-85150569) Gly (TCC) 73 bp Sc: 44.52
TCCTTGGTGGTCCAATGGTTAGGACTCTGCGCTTCCACTGCAGGGGGCAAGGGTTCTATC
CCTGTTCAAGGAA
>Bos_taurus_chr28.trna2746-GlyTCC (11501289-11501217) Gly (TCC) 73 bp Sc: 44.54
TCCTTGGTAGTCCTGTGGTTAGGACTCTGTGCTTCCATGGCAGGGGGCCTGGGTTCAAAC
CCTGGTCAGGGAA
>Bos_taurus_chr11.trna4759-GlyTCC (105122868-105122796) Gly (TCC) 73 bp Sc: 44.57
TCCCTGGTGGTCCAGCAGCCAAGACTCTGCACGTCCAATGCAGGGAGCCCAGGTTCAATC
CCTGGTCAGGGAA
>Bos_taurus_chr10.trna1783-GlyTCC (46841390-46841462) Gly (TCC) 73 bp Sc: 44.62
TCCCTGGTGGTCCAGTGGTTAAGACTCTACACTTCCAATGCAGGAAATGCAGGTTCCATC

CCTGGCTAGGGAA
>Bos_taurus_chr25.trna1625-GlyTCC (28274496-28274568) Gly (TCC) 73 bp Sc: 44.63
TCCCTGGTGGTCCAGTGGTTAGGACTCCGCACTTCCACTGCAGGGAGAACAGGTTCCATC
CCTGGTTGGGGAA
>Bos_taurus_chr12.trna6212-GlyTCC (17777366-17777294) Gly (TCC) 73 bp Sc: 44.72
TCCCTGGTGGTCCAGTGGTCATGACTCTGCACCTCCACTGCAGGGAGCCTGGGTTCCATT
CCTGGTCAGGGAA
>Bos_taurus_chr1.trna5101-GlyTCC (146549642-146549713) Gly (TCC) 72 bp Sc: 44.78
TCCCTGGCAGTCCAGTGGTTAGGACTCGGCACTTCCACTGCCAGGATCCAGGTTCAAATT
CTGGTTGGGGAT
>Bos_taurus_chr15.trna3502-GlyTCC (73937646-73937574) Gly (TCC) 73 bp Sc: 44.79
TCCTTGGTGGTCCAGTGGTTAAGACTCTGTACTTCCACTGCAGGGGGTGCAGGTTCCACC
CCTGGTTGGGGAA
>Bos_taurus_chr1.trna878-GlyTCC (21364086-21364158) Gly (TCC) 73 bp Sc: 44.97
TCCTTGGCAGTCCAGTGGTTAAGACTCTGAGCTTCCACTGCAGAGGGCCCAGGTTCAGTT
CCTGGTCAGGGAA
>Bos_taurus_chr11.trna3262-GlyTCC (80976787-80976859) Gly (TCC) 73 bp Sc: 44.97
TCCCTGGTGGTCCAGTGGTTAAGACTCAGCACTTCCACTGCAGGGGGTATAGGTTCCATT
CCTAGTTGGGGAA
>Bos_taurus_chr1.trna5248-GlyTCC (151191388-151191459) Gly (TCC) 72 bp Sc: 44.98
TCCCTGGTGGTCCAGTGTTTAGGACTCTGCACTTCCACTGCCGGGGCCTGGGTTTGATCC
CTAGTGAGGGAA
>Bos_taurus_chr19.trna4469-GlyTCC (45529974-45529902) Gly (TCC) 73 bp Sc: 45.01
TCCCTGATAGTCTAGTGGTTAGGACTCTGAACTTCCACTTCAGGGAGCTTGGGTTCTATC
CCTCATTGGGGAA
>Bos_taurus_chr7.trna409-GlyTCC (9046096-9046168) Gly (TCC) 73 bp Sc: 45.02
TTCCTGGTGGTTCAGTGGCTAGGACTCTGCCCTTCCAGTGCAGAGGGCCCAGGTTCTATC
CCTGGTCAGGGAA
>Bos_taurus_chr7.trna413-GlyTCC (9174406-9174478) Gly (TCC) 73 bp Sc: 45.02
TTCCTGGTGGTTCAGTGGCTAGGACTCTGCCCTTCCAGTGCAGAGGGCCCAGGTTCTATC
CCTGGTCAGGGAA
>Bos_taurus_chr13.trna6602-GlyTCC (24692773-24692701) Gly (TCC) 73 bp Sc: 45.09
TCCCTGATGGTCCAATGGTTAAGACTCTGTGCTTCCAATGCAGTGGGTGTGGGTTCAATC
CCTGATCAGGGAA
>Bos_taurus_chrX.trna6685-GlyTCC (3862467-3862395) Gly (TCC) 73 bp Sc: 45.10
TCCCTGGAAGTCCAGTGGTTAGGACTCTGTGCTTCCACTACAGGGGGTCTGGGTTCGATC
CCTGGCTGGGGAA
>Bos_taurus_chr4.trna2306-GlyTCC (72326941-72327013) Gly (TCC) 73 bp Sc: 45.30
TCCCTGGTGGTCCAGTGGTTGGGACTCTGTGCTTCCACTGCAGGGAGTGCAGGTTCAATC
CCTGGTCAGGGAA
>Bos_taurus_chr16.trna2138-GlyTCC (54926605-54926677) Gly (TCC) 73 bp Sc: 45.35
TCCCTGGTGGTCCAGTGGTTAAGACTCTGAGCTTCCACTGCAGAGACCTCAGGTTGGATT
CCTGGTCAGAGAA
>Bos_taurus_chr15.trna4440-GlyTCC (50393774-50393702) Gly (TCC) 73 bp Sc: 45.40
TCCCTGGTGGTCCAGTGGCTAAGACTCTGAGCTTCCAATGCAGGGGGCCCAGGTTTGACT
CCTGGTCTGGGAA
>Bos_taurus_chr14.trna6588-GlyTCC (3844636-3844564) Gly (TCC) 73 bp Sc: 45.43
TCCCTGGTGGTCCAGTGGCTAAGATGCTGCACTTCCTATGCAGGGAGCCCAGGTTTGATC
CCTAGTCAGGGAA
>Bos_taurus_chr6.trna5225-GlyTCC (101384748-101384676) Gly (TCC) 73 bp Sc: 45.47
TCCCTGGTGGTCCAGCAGTTAAGACTCTGCACTTCCAGTGCGGAAGCCACAGGTTCGATT
CCTGGTTAGGGAA
>Bos_taurus_chr12.trna449-GlyTCC (13814048-13814120) Gly (TCC) 73 bp Sc: 45.51
TCCCTGGTGGTCCAGTGGCTAAGACTCTGTGCTTCCAATGCAGGGGGCCTGGGTGCAATT
CCTGGCCAGGGAA
>Bos_taurus_chrX.trna2211-GlyTCC (61993047-61993119) Gly (TCC) 73 bp Sc: 45.55
TCCCTGGTGGTCCAGTGATTAAGACTCTGCACTTCCACTGCAGGGTGCACAGGTTCCATC
CCTGGTCAGGGAA
>Bos_taurus_chrUn.004.36.trna8-GlyTCC (546664-546592) Gly (TCC) 73 bp Sc: 45.74
TCCCTGGTGGTCCAGTGGCTAAGACTCTGTGCTTCCAATACAGAGGGGCTGGGTTCGATC
CCTGGTCAGGGAA
>Bos_taurus_chr15.trna4439-GlyTCC (50497624-50497553) Gly (TCC) 72 bp Sc: 45.75
TCCCTGGTGGTCCAGTGGTTAGGACTCTGTGCTTCCACTGCAGGGTCCTGGGTTTGATCC
CTTGTTGGGGAA
>Bos_taurus_chr12.trna1446-GlyTCC (31704040-31704112) Gly (TCC) 73 bp Sc: 45.79
TCCCTGGTGGTCCAGTGGTTAAGACTCTGTGCTTCCAACGCAGGGGGCATGGGTTCAATG
CCTGGTCAGGGAA

>Bos_taurus_chr1.trna2838-GlyTCC (82934266-82934338) Gly (TCC) 73 bp Sc: 45.81
TCCCTGGTGGTCCAGTGGCTAAGATTCTGTGTTTCCAATGCAGGGGTTTCAGGTTTGATC
CCTGGTTGGGGAA
>Bos_taurus_chr17.trna2045-GlyTCC (53552239-53552311) Gly (TCC) 73 bp Sc: 45.93
TCCTTGGCAGTCCAGCGGCTAAGACTCTGCACTTCCAGTGCAGGGGACCCAGGTTCAATC
CCTGGTCAGGGAA
>Bos_taurus_chr10.trna7778-GlyTCC (11756994-11756922) Gly (TCC) 73 bp Sc: 46.13
TCCCTGGCAGTCCAGTGGTTAGGGTTCTGTGCTTCCACCACAGGGGGCCCAGGTTCAGTC
CCTGGTCAGGGAA
>Bos_taurus_chr17.trna3119-GlyTCC (69312903-69312975) Gly (TCC) 73 bp Sc: 46.21
TCCCTGGTAGTCTAGAGGTTAGGATTCTGCGCTTCCACTTCAGGGGCCACAGGTTCAACC
CCTGGTCAGGGAA
>Bos_taurus_chr23.trna927-GlyTCC (20927497-20927569) Gly (TCC) 73 bp Sc: 46.22
TCCCTGGTGGCCCAGTGGTTAAGATTCTGTGCTTCCAATGCAGGGGCCCCAGGTTCAGCC
CCTGGTCAGGGAA
>Bos_taurus_chr25.trna573-GlyTCC (10240393-10240465) Gly (TCC) 73 bp Sc: 46.22
TCCCTGGTGGTGCAGTGGTTAGGACTCTGCACTTCCACTGCAGGCGGCCTGGGTTTGATC
CCTGGTCAGGGAA
>Bos_taurus_chr17.trna2697-GlyTCC (63360995-63361067) Gly (TCC) 73 bp Sc: 46.24
TCCATGGTGGTCCAGTGGCTAAGACTGTGCACTTCCACTGCAGGGAGCCCAGGTTCCATT
CCTGGTCAGGGAA
>Bos_taurus_chr21.trna829-GlyTCC (21060467-21060539) Gly (TCC) 73 bp Sc: 46.30
TCCCTGATGGTACAGTGGTTAGGACTCCGTACTTCCACTGCAGGGAACCCGGGTTTGATC
CCTGGTCAGGGAA
>Bos_taurus_chr22.trna2263-GlyTCC (60944540-60944468) Gly (TCC) 73 bp Sc: 46.30
TCCCAGGTGGTCGAGTGGCTGGGACTCTGCGTTTCCACTGCAGAGGGCCCAGGTTCGATC
CCTGGTCAGGGAA
>Bos_taurus_chr6.trna4839-GlyTCC (108571677-108571605) Gly (TCC) 73 bp Sc: 46.59
TCCCTGGTGGTCCAGTAGCTAAGACTCTGTGCTTCCAATGCAGGGGGCCCAGGTTTGATT
CCTGGTCAGGGAA
>Bos_taurus_chr26.trna433-GlyTCC (14817716-14817788) Gly (TCC) 73 bp Sc: 46.61
TCCCTGGTGGTCAAGGTGTTAAGACTCTGCACTTCCAATGCAGGGGGCACAGGTTTGATC
CCTGGTCGGGGAA
>Bos_taurus_chr26.trna795-GlyTCC (23119925-23119997) Gly (TCC) 73 bp Sc: 46.62
TCCCTGGTGATCCAGTGATTAGGATTCTGTGCTTCCAATGCTGGGGGCCCAGGTTCAATC
CCTGGTCAGGGAA
>Bos_taurus_chr18.trna1864-GlyTCC (45842514-45842586) Gly (TCC) 73 bp Sc: 46.67
TCCCTGGTGGTCCAGTGGTTAGGACTCTGCACTTCCACCGCAGGGGGCTCAGGTTCTATC
CTTGGTCATGGAA
>Bos_taurus_chrX.trna2799-GlyTCC (74582178-74582250) Gly (TCC) 73 bp Sc: 46.89
TCCCTGATGGTCTAGTGGTTGGGACTCTGTGCTTCCACTGCAGGGGGCCCGGGTTCCATC
CCTGGTCAGGGAA
>Bos_taurus_chr1.trna8970-GlyTCC (66021682-66021610) Gly (TCC) 73 bp Sc: 46.89
TCCCTGGTGGTCCAGTGGTTAAGATTATGCACTTCCATTGCAGGGGGTGCAGGTTTGATC
CCTGGTCAGGGAA
>Bos_taurus_chr17.trna6240-GlyTCC (14304510-14304437) Gly (TCC) 74 bp Sc: 47.04
TCCCTGGTGGTCCAGTGGTTAGGACTCTGTGCTTCCACTGCAGGGGGCACAGGGTTCGAT
CCCTGGTTGGGGAA
>Bos_taurus_chr17.trna4812-GlyTCC (53467895-53467823) Gly (TCC) 73 bp Sc: 47.09
TCCTTGATGGTCCAGTGGTTAAGACTTTGTTCTTCCAATGCAGGGGGCACAGGTTTGATC
CCTGGTTGGGGAA
>Bos_taurus_chr18.trna2536-GlyTCC (55908816-55908888) Gly (TCC) 73 bp Sc: 47.19
TCCCTGGTGGTCCAGTGGCTAAGACTCTGCGATTCCAACGCAGGAGGCCCAGGTTTGATC
TCTGGCCAGGGAA
>Bos_taurus_chr2.trna3012-GlyTCC (94967172-94967244) Gly (TCC) 73 bp Sc: 47.32
TCCCTGGTGGTCCAGTGGCTAAGACCCTGTGCTTCCAACGCAGGGAGCCCAGGTTTGATC
CCTGTTCAGGGAA
>Bos_taurus_chr9.trna122-GlyTCC (7313852-7313924) Gly (TCC) 73 bp Sc: 47.36
TCCCTGGTGGTCCAGTGGGTCAGACTCTGCACTTCCACTACAGGGGGCCCAAGTTCAATC
CCTGGCTGGGGAA
>Bos_taurus_chr22.trna4274-GlyTCC (7282471-7282399) Gly (TCC) 73 bp Sc: 47.38
TCCCTGGTGGTCCAGTGGTTACGACTCTGCACTTCCATCGCAGAGGGTGCAGGTTCCATC
CCTGGTCAGGGAA
>Bos_taurus_chr19.trna4759-GlyTCC (40766410-40766339) Gly (TCC) 72 bp Sc: 47.47
TCCCTGGTGGTCCAATGGTTAGGATTCTGTGCTTCCACTGCTGGGGCCCAGGTTCGATCT
CTGGTAAGGGAA
>Bos_taurus_chr25.trna656-GlyTCC (11542431-11542503) Gly (TCC) 73 bp Sc: 47.53

TCCCTGGTGGTCCAGTGGTTAAGACTCTGCACTTCCAATGAAGGGAGTACAGGTTCCATC
CCTGGTCAGGGAA
>Bos_taurus_chr5.trna6875-GlyTCC (89840132-89840060) Gly (TCC) 73 bp Sc: 47.59
TCCCTGGTGGTCCAATGGTTAAGACTCTGCACTTCCAATGCAGGGGACGTGGGTTTGATC
CCTAGTCAGGGAA
>Bos_taurus_chr9.trna4969-GlyTCC (84456389-84456316) Gly (TCC) 74 bp Sc: 47.63
TCCCTGGTGGTCCAGTGGCTAAGACTCTGCACTTCCAAGGCAGGAGGCCCAGGGTTCAAT
CCCTGGTCAGGGAA
>Bos_taurus_chr3.trna2384-GlyTCC (68084172-68084244) Gly (TCC) 73 bp Sc: 47.75
TCCCTGGTAGTCTAGTGGTTAAGACTCTGTGCTTCCACTGCAGGGGGCTCAGGTTCAAAC
TCTGGTTGGGGAA
>Bos_taurus_chr10.trna3988-GlyTCC (103228335-103228407) Gly (TCC) 73 bp Sc: 47.77
TCCCTGGTGGTCCAGTGGTTAGGATTCTGCTCTTCCAGTGCAGGGGACTCAGGTTTGATC
CCAGACCAGGGAA
>Bos_taurus_chr10.trna3995-GlyTCC (103311664-103311736) Gly (TCC) 73 bp Sc: 47.77
TCCCTGGTGGTCCAGTGGTTAGGATTCTGCTCTTCCAGTGCAGGGGACTCAGGTTTGATC
CCAGACCAGGGAA
>Bos_taurus_chr18.trna4284-GlyTCC (44467032-44466960) Gly (TCC) 73 bp Sc: 47.81
TCCCTGGTGGTTTGGTGGCTAAAACTTTGTGTTTCCAATGCAAAGGTCCCAGGTTTTATT
CCTGGTCAGGGAA
>Bos_taurus_chr11.trna2791-GlyTCC (70242206-70242278) Gly (TCC) 73 bp Sc: 47.82
TCCCTGGTGGTCCAGTAGTTAAGACTCTGCGCTTCCAATGCAGGGGGTGCAGGTTCAATC
CCTGGTCAGGGAA
>Bos_taurus_chr2.trna4176-GlyTCC (123377674-123377746) Gly (TCC) 73 bp Sc: 47.86
TCCCTGGTGGTCCAGTGGTTAAGACTCTGTGCTTCCATCGCAGGGGGCATGGGTTGGATC
CCTGTTCAGGGAA
>Bos_taurus_chr22.trna4476-GlyTCC (3914163-3914091) Gly (TCC) 73 bp Sc: 47.98
TCCCCGGTGGTCCAGTGACTAAGACTCTGCACCTCCAATGCAGGGGTCCTGGGTTTGATC
CCTAGTTGGGGAA
>Bos_taurus_chr27.trna3782-GlyTCC (5092807-5092736) Gly (TCC) 72 bp Sc: 48.03
TCTCTGGTGGTCCAGTGGTTAAGACTCTGCACTTCCAATGCAGGGGGCATGGGTTCAATC
CCTGTCGGAGAA
>Bos_taurus_chr7.trna2284-GlyTCC (51480482-51480554) Gly (TCC) 73 bp Sc: 48.13
TCCCTGGTCGTCCAGTGGTTAGGATGCTGTGCTTCCATTTCAGAGGGCCCAGGTTCATCC
CCTGGTCAGGGAA
>Bos_taurus_chr8.trna932-GlyTCC (26612068-26612140) Gly (TCC) 73 bp Sc: 48.66
TTCCTGGTGGTCCAGTGATTAGGACTCTGTGCTTCCACTGCTGGGGGCCCAGGTTCAATT
CCTGGTTGGGAAA
>Bos_taurus_chr26.trna2586-GlyTCC (38281852-38281781) Gly (TCC) 72 bp Sc: 48.66
TCCCTGGTGGTTCAGGGGTTAAGATGCTGCACTTCCAGTGCAGGAGCACAGGTTCGATCC
CTGGTTGGGGAA
>Bos_taurus_chr7.trna3327-GlyTCC (82985078-82985150) Gly (TCC) 73 bp Sc: 48.77
TCCTTGGTGGTCCAGTGGTTAAGACTCTGCGTTTCCACTGCAGGGGGCACAGGTTGGATC
CCTGGTCAGGGAA
>Bos_taurus_chr6.trna5176-GlyTCC (101965807-101965735) Gly (TCC) 73 bp Sc: 48.87
TCCCTGGTGGTCCAGTGGTTAAGACTCTGTGCTTCCAATGCAGGGGGTGTGGGTTTGATC
CCCGCTTGGGGAA
>Bos_taurus_chr21.trna929-GlyTCC (22307748-22307820) Gly (TCC) 73 bp Sc: 48.90
TCCCTGGTGGTCCAGTGGTTAAGACTCTGCACTTCCAGTGCAGGGGGTGCAGGTTTGATC
CCTGGTCAGGGAA
>Bos_taurus_chr18.trna3992-GlyTCC (48470361-48470288) Gly (TCC) 74 bp Sc: 48.91
TCCCTGGTGGTCCAATGGTGAAGATGCCATGCTTCCAACGTGGGGGTGTGCGGGTTCAAT
CCCTGCTCGGGGAA
>Bos_taurus_chr11.trna1937-GlyTCC (48300437-48300509) Gly (TCC) 73 bp Sc: 48.91
TCCCAGGTGGTCCAGTGGTTAGGACTCTGCACTTCCACTGCAGGGAGCCTGGGTTCCATC
CCTGGTTGGGGAA
>Bos_taurus_chr20.trna3067-GlyTCC (69945000-69944928) Gly (TCC) 73 bp Sc: 48.93
TCCCTGGTGGTCCAGTGGTTAGGATTCTGTGCTTCCACTGGAGGGGGCCCAGGTTCAGTC
CCTGGTCAGGGAA
>Bos_taurus_chr17.trna6367-GlyTCC (11242440-11242369) Gly (TCC) 72 bp Sc: 48.97
TCCCTGGTGGTCCAGTGATTTGGACTCTGCCCTTCCACTGCAGGGTCACAGGTTCAATCC
CTGACTGGGGAA
>Bos_taurus_chr6.trna4516-GlyTCC (115339366-115339294) Gly (TCC) 73 bp Sc: 49.07
TCCCTGGTGGTTCAGTGGGTATGATTCTGCACTTCCAATACAGGGGGCCCAGGTTCAATC
CCTGGTCAGGGGA
>Bos_taurus_chr11.trna7896-GlyTCC (30955110-30955038) Gly (TCC) 73 bp Sc: 49.12
TCCCCAGTAGTCCAGTGGTTAAGACTCTGAGCTTCCACCACAGAGGGCTTGGGTTCAATC

CCTAGCTAGGGAT
>Bos_taurus_chr9.trna571-GlyTCC (20120981-20121053) Gly (TCC) 73 bp Sc: 49.34
TCCCTGATGGCCCAGTGGTTAGGGTGCTGAGCTTCCACTGCAGGAGACATGGGTTCGATC
CCTAGTCAGGGAA
>Bos_taurus_chr2.trna767-GlyTCC (24804843-24804913) Gly (TCC) 71 bp Sc: 49.40
TCCTTGGTGGTCCAGTGGCTAGGACTCTGTGCTTCCAATGCAGGGACCCAGGTTCAATAC
CTGGTCAGGAG
>Bos_taurus_chr18.trna5003-GlyTCC (25826099-25826028) Gly (TCC) 72 bp Sc: 49.41
TCCCTGGTGGTCCAGTGGTTAGCATGTGGTATTTCCACTGCTGTGACCCAGGTTCAATCC
TTGGTTAGGGAA
>Bos_taurus_chr1.trna6453-GlyTCC (144251698-144251626) Gly (TCC) 73 bp Sc: 49.56
TCCCTGGTGGTTCAGTGGTTAAGACTTTGCACTTCCATTGCAGGGGGAGTGGGTTCAATC
CCTACTCAGGGAA
>Bos_taurus_chr21.trna1320-GlyTCC (28127754-28127825) Gly (TCC) 72 bp Sc: 49.59
TCCCTGGTGGTCCAGTGGTTAAGACCCTGTGCTTCCACAGCAGGGGCATAGGTTCAATCC
CTGGTCAGGGAA
>Bos_taurus_chrUn.004.2.trna168-GlyTCC (1030665-1030593) Gly (TCC) 73 bp Sc: 49.60
TCCCTGGTGGTCCAGTGGTTAAGACTCTGCATTTCCACTACAGTTGGCCTAGGTTTGATC
CCTGGTCAGGGAA
>Bos_taurus_chr18.trna3012-GlyTCC (65597076-65597005) Gly (TCC) 72 bp Sc: 49.66
TTCCTGATGGTCCAGTGGTTAAGACTCTGCTCTTCCAATGCAGGGGCTCAGGTTTGATCC
CTGGTGGGGGAA
>Bos_taurus_chr8.trna1367-GlyTCC (41852184-41852256) Gly (TCC) 73 bp Sc: 49.76
TCCCTGGTAGTCCAGTGGTTAAGGCTCTGCACTTCCACTGCAGGGGGCATAGGTTTGATT
CCTGGTCAGGGAA
>Bos_taurus_chr11.trna1285-GlyTCC (30091494-30091566) Gly (TCC) 73 bp Sc: 49.85
TTCTTGGTGGTCCAGTGGCTAAGACTCTGGGCCTCCAACGCAGGGGGCCCAGGTTCAATC
CCTGGTCAGGGAA
>Bos_taurus_chr17.trna446-GlyTCC (13261272-13261344) Gly (TCC) 73 bp Sc: 49.92
TCCCTGGTGGTCCAGTGGTTAGGATGCTGTGCTTCCACTGCAGGGGGCACAGGTTTTATC
CCTGGCCAGGGAA
>Bos_taurus_chr18.trna669-GlyTCC (17594232-17594304) Gly (TCC) 73 bp Sc: 50.08
TTCCTGGTGGTCCAGTGGCTAAGACTCTGTGCTTCCAACGCAGGGATCCCAGCTTCGATC
CCTGGTCAGGGGA
>Bos_taurus_chr1.trna11015-GlyTCC (2375663-2375591) Gly (TCC) 73 bp Sc: 50.10
TCCCTGGTGGTTCAGTGGCTAAGACTCTGTTCTTCCAATGCAGGGGGCCCAGGTTGGATC
CCTGGTCAGGGAA
>Bos_taurus_chr11.trna3678-GlyTCC (90429261-90429332) Gly (TCC) 72 bp Sc: 50.11
TCCCTGCTGGTCCAATGGTTAAGACTCTGAGCTTCCAATTCAGGGGCCCAGGTTCCATCC
CTGGCTGGGGAA
>Bos_taurus_chr3.trna9288-GlyTCC (3596604-3596532) Gly (TCC) 73 bp Sc: 50.16
TCCCTGGTGGTCCAGTAGTTAAGACTCTGCACTTCCGATGCAGGGGGCATGGGTTCGATC
CCTGGCCAGGGAA
>Bos_taurus_chr19.trna3744-GlyTCC (58181285-58181213) Gly (TCC) 73 bp Sc: 50.22
TCCCTGGTAGTCCAGGGGTGAAGACCCTGCGCTTCCAATGCAGGGGGCCCAGGTTTGATC
CCTGGTCAGGGAA
>Bos_taurus_chr4.trna1541-GlyTCC (48707072-48707144) Gly (TCC) 73 bp Sc: 50.29
TCCCTGGTGGTCCAGTGGCTAAGACTCTGCACTTCCACTGCAGGGGGCCTGGGTTTGATC
CCTGGTCAGGGAA
>Bos_taurus_chr2.trna8973-GlyTCC (47020760-47020688) Gly (TCC) 73 bp Sc: 50.38
TCCCTGGCAGTCCAGTGGTTAGGACTCGGCACTTCCACTGCAGGGGGCCCAGGTTTGATC
CCTGGTTGGGGAA
>Bos_taurus_chr22.trna653-GlyTCC (14831746-14831818) Gly (TCC) 73 bp Sc: 50.40
TCCCTGGTGGTTCAGTGGTTAAGACTCTGCGCTTCCAATGCAGGGGTCTCAGGATCAATC
TCTGGTTGGGGAA
>Bos_taurus_chr6.trna640-GlyTCC (23651667-23651739) Gly (TCC) 73 bp Sc: 50.54
TCCCTGGTGGTCCAGTGGTTAGGACTCTACACTTCCACTGTAGAGGATACAGGTTCCGTC
CCTGGTCAGGGAA
>Bos_taurus_chrUn.004.2284.trna1-GlyTCC (17168-17096) Gly (TCC) 73 bp Sc: 50.54
TCCCTGGTGGTCCAGTGGTTAGGACTCTACACTTCCACTGTAGAGGATACAGGTTCCGTC
CCTGGTCAGGGAA
>Bos_taurus_chrUn.004.5644.trna1-GlyTCC (7435-7363) Gly (TCC) 73 bp Sc: 50.54
TCCCTGGTGGTCCAGTGGTTAGGACTCTACACTTCCACTGTAGAGGATACAGGTTCCGTC
CCTGGTCAGGGAA
>Bos_taurus_chrUn.004.6077.trna1-GlyTCC (6524-6452) Gly (TCC) 73 bp Sc: 50.54
TCCCTGGTGGTCCAGTGGTTAGGACTCTACACTTCCACTGTAGAGGATACAGGTTCCGTC
CCTGGTCAGGGAA

>Bos_taurus_chr26.trna3485-GlyTCC (14647620-14647548) Gly (TCC) 73 bp Sc: 50.63
TCCCTGGCAGTCCAGTGGTTAAGACTCTGCATTTCCACTGCAGGGGCCACAGGTTTGATC
CCTGTTTGGGGAA
>Bos_taurus_chrUn.004.125.trna1-GlyTCC (61294-61366) Gly (TCC) 73 bp Sc: 50.77
TCCCTGGTGGTCCAGTGGTTTAGACTCCACACTTCCAATGTAGGCAGCACAGGTTCAATC
CCTGGCCAGGGAA
>Bos_taurus_chr22.trna1848-GlyTCC (51956972-51957044) Gly (TCC) 73 bp Sc: 50.77
TCCCTGGTGGTCCAGTGGTTAGGGCTCTGTGCTTCCACTGCAGGGGCCACAGGTTTGATC
CCTGGCTGGGGAA
>Bos_taurus_chr11.trna1286-GlyTCC (30102233-30102305) Gly (TCC) 73 bp Sc: 50.89
TCCCTGGTGGTTCAGTGGTTTGGATTCTGCACTTCCAATGCAGGGGGCCCAGGTTTGATC
CCTGGTTGAGGAA
>Bos_taurus_chr7.trna6176-GlyTCC (57889845-57889773) Gly (TCC) 73 bp Sc: 51.02
TCCCTGGTGGTCCAGTGGTGAAGACGCTGCACTTCCACTGCAGGGAGCATGGGTTCGATC
CCTAGTTGGGGAA
>Bos_taurus_chrUn.004.6.trna22-GlyTCC (513296-513368) Gly (TCC) 73 bp Sc: 51.06
TCCCTGGTGGTCCAGTGGTTAAGACGCTGCGCTTCCACTGCAGGGGGCACAGGTTTGATC
CCTGGTTGGGGAA
>Bos_taurus_chr9.trna6653-GlyTCC (35959930-35959858) Gly (TCC) 73 bp Sc: 51.08
TCCCTGGTGGTCCAGTGGTTAGGATTCTGTGCTTCCACTGCAGGGGGCACAGGTTTGATC
CCTGGTCAGGGAA
>Bos_taurus_chr14.trna5777-GlyTCC (20910450-20910378) Gly (TCC) 73 bp Sc: 51.25
TCCCCAATGGTCTAGTGGTTAAGACTCTGCACTTCCAATGCAGAAGGCATGGGTTCCATC
CCTGGTTGGGGAA
>Bos_taurus_chr21.trna2608-GlyTCC (62628622-62628694) Gly (TCC) 73 bp Sc: 51.26
TCCCTGGTGGTCCAGTGGCTAAGACTCTGCACTTCCAATGTAGGGGGCACAGGTTCAACC
CCTGGTCAGGGAA
>Bos_taurus_chr11.trna4880-GlyTCC (103448059-103447987) Gly (TCC) 73 bp Sc: 51.31
TCCCTGGTGGTCCAGTGGTTAAGACTCTGTGCTTCCAATGCAGGGGGTGCAGGTTTGATC
CCTGGCCAGGGAA
>Bos_taurus_chr11.trna3980-GlyTCC (97295437-97295509) Gly (TCC) 73 bp Sc: 51.34
TCCTTGGTGGTCCAGCTGTTAGGATGCTGTTCTTCCAATGCAGGGGGCCTGGGTTCGATT
CCTAGTCAGGGAA
>Bos_taurus_chr17.trna802-GlyTCC (19391859-19391931) Gly (TCC) 73 bp Sc: 51.39
TCCCTGGCAGTCCAGTGGTTAAGGCTTTGCACTTCCACTGCAGGGAGCCCAGGTTCAATC
CCTGGTCAGGGAA
>Bos_taurus_chr8.trna5106-GlyTCC (89913094-89913022) Gly (TCC) 73 bp Sc: 51.45
TCCCTGGTGGTCCAGTGGCTAGGACTCCACACTTCCAATGTAGGCTGCCCAAGTTCAATC
CCTGGTCAGGGAA
>Bos_taurus_chr8.trna1392-GlyTCC (42791127-42791199) Gly (TCC) 73 bp Sc: 51.58
TCCCTGGTGGTCCAGTGGTTAAGGCTCTGCACTTCCACTGCATGGGGCACAGGTTCAATC
CCTGGTCAGGGAA
>Bos_taurus_chr13.trna6646-GlyTCC (24146783-24146712) Gly (TCC) 72 bp Sc: 51.80
TCCCTGCTGGTCCAGTGGCTAAGACTCTGCACTTCCAATGCAGGGGCCTGGGTTGGATCC
CTGGTCAGGGAA
>Bos_taurus_chrUn.004.2057.trna1-GlyTCC (15073-15001) Gly (TCC) 73 bp Sc: 51.90
TCTCTGATGGTCCAGTGGTGAAGACTCTGCGCTTCCACTGCAGGGGGCACAGGTTCAATC
CCTGGTCAGGGAA
>Bos_taurus_chr17.trna5087-GlyTCC (48808049-48807977) Gly (TCC) 73 bp Sc: 51.95
TCCCTGGTGGTGCAGTGGTTAATATTCTGCACTTCCACTGCAGGGGTTGCAGGTTCCATC
CCTGGTCAGGGAA
>Bos_taurus_chr10.trna4968-GlyTCC (86620416-86620344) Gly (TCC) 73 bp Sc: 52.01
TCTCTGGTGGTCCAGTGGTTAAGACTCTGTGCTTCCACTGCAGGGGTCTCAGGTTTGATC
CCTGGTCAGGGAA
>Bos_taurus_chr13.trna808-GlyTCC (22071497-22071568) Gly (TCC) 72 bp Sc: 52.02
TCCCTGGTGGTCCAATGGTTAAGACTTGGCACTTCCACTGCAGTGGCCCATGTTCAACCC
CTGGTCAGGGAA
>Bos_taurus_chr23.trna3898-GlyTCC (19482018-19481946) Gly (TCC) 73 bp Sc: 52.13
TCCCTGGTGGTCTAGTGGTTAAGACTCTGTGCTTCCAATGCAGGGGGCCCAGGTTCAATC
ACTGGTTGGGGAA
>Bos_taurus_chr2.trna2108-GlyTCC (67270832-67270903) Gly (TCC) 72 bp Sc: 52.14
TCCCTGATGGTCTAGTCGTTAAGACAGTGCACTTCCACTGCAGGGGCACAGGTTCAATCC
CTGGTTGGGGAA
>Bos_taurus_chr22.trna3237-GlyTCC (33010416-33010345) Gly (TCC) 72 bp Sc: 52.20
TCCCTGGTGGTCCAGTGCTTAGGACTCAGCACTTCCACTGCTGTGGTCCAGGTTCAATCT
CTGGTCAGAGAA
>Bos_taurus_chr4.trna3669-GlyTCC (107629935-107630007) Gly (TCC) 73 bp Sc: 52.32

TCCCCGGTGGTCCAATGGTTAGGACTCCACACTTCCACTGTGGGGACATGGGTTCGATA
CCTGGTTGGGGAA
>Bos_taurus_chr11.trna2834-GlyTCC (70869324-70869396) Gly (TCC) 73 bp Sc: 52.34
TCCCTGGTGGTCCAGTGGTTAAGACTCTGCACTTCCACTGCAGGGTCCGTGGGTTCGATC
CCTGGTCAGGGAA
>Bos_taurus_chr18.trna4654-GlyTCC (34985649-34985577) Gly (TCC) 73 bp Sc: 52.35
TCCCTGGTGGTCCAGTGGCTAAGACGCTGCACTTCCAATTCAGGGGGCCTGGGTTTGATC
CCCAATCAGGGAA
>Bos_taurus_chr11.trna8837-GlyTCC (6654582-6654510) Gly (TCC) 73 bp Sc: 52.42
TCCCTGGTGGTCCAGTGGCTAAGACTTTGCGCTTCCAACGCAGAGGGCCTGGGTTCGATG
CCTGGTCAGGGAA
>Bos_taurus_chr13.trna6650-GlyTCC (24050821-24050749) Gly (TCC) 73 bp Sc: 52.43
TCCTTGGTGGTCCAGTGGTTAAGACTCTGCACTTCCAATGCAGGGGGTGCAGGTTTGATC
CCTGGTTGGGGAA
>Bos_taurus_chrUn.004.21.trna79-GlyTCC (322528-322457) Gly (TCC) 72 bp Sc: 52.53
TCCCTGGTGGTCCAGTAGCTAAGACTCTGTGCTTCCAATGCAGGGGTCCAGGTTCGATCC
CTGGTCAGGGAA
>Bos_taurus_chr13.trna1846-GlyTCC (44051177-44051249) Gly (TCC) 73 bp Sc: 52.56
TCCCTGGTAGTCCAGTGGTTAAGACTCTGCACTTCCATTGCAGAGGGCGTAGGTTCAAAG
CCTGGTTGGGGAA
>Bos_taurus_chr11.trna4989-GlyTCC (101514014-101513942) Gly (TCC) 73 bp Sc: 52.57
TCCCTGGTGGTCTAGTGGTTAGGACTCTGTGCTTCCAATGCAGGGGCCACAGGTTTGATC
CCTGGTTAGGGAA
>Bos_taurus_chr24.trna3418-GlyTCC (49945762-49945690) Gly (TCC) 73 bp Sc: 52.62
TCCCTGGTGGTCTAGTCGCTAGGACTCTGTGCTTCCAATGCAGGGGGCCCGGGTTCAATT
CCTGGCCGGGGAA
>Bos_taurus_chr12.trna6139-GlyTCC (19075796-19075725) Gly (TCC) 72 bp Sc: 52.67
TCCCTGGTGGTCCAGGGTTAGGACTCTGCACTTCCAGTGCAGGGGCCCAGGGTTCAATTC
CTGGTCAGGGAA
>Bos_taurus_chr13.trna4196-GlyTCC (75659455-75659383) Gly (TCC) 73 bp Sc: 53.07
TCCCTGCTGGTCCAGTGGTTAAGACTCTGCGCTTCCACTGCAGGAGGCACGGGTTCAATC
CCCGAGTGGGGAA
>Bos_taurus_chr22.trna720-GlyTCC (16573962-16574034) Gly (TCC) 73 bp Sc: 53.10
TCCCTGGTGGTCAAGTGGTTGGGACTCTGCACTTCCACTGCAGGGGGCCCAGGTTTGATT
CCTGGTTGGGGAA
>Bos_taurus_chr23.trna820-GlyTCC (18685036-18685108) Gly (TCC) 73 bp Sc: 53.10
CCCTTGGTGGTCCAGTGGTTAAGACTCTGCTCTTCCAAAGCAGGGGGCCTGGGTTCGATC
CCTGGGCGGGGGA
>Bos_taurus_chr3.trna6121-GlyTCC (93693139-93693067) Gly (TCC) 73 bp Sc: 53.29
ACCCTGGTGGTCCAGTGGTTAGGACTCTGCTCTTCCACTGCAGAGGGTCTAGGTTTGATC
CCTGGTCAGGGAA
>Bos_taurus_chr20.trna1413-GlyTCC (39125012-39125083) Gly (TCC) 72 bp Sc: 53.33
TCCCTGGTGGTCCAGTGGTTAAGACTCTGCACTTCCTCTGCAGAGGCCTGGGTTCAATCC
CTAGTCAGAGAA
>Bos_taurus_chr1.trna8836-GlyTCC (70538849-70538777) Gly (TCC) 73 bp Sc: 53.34
TCCCTGGTGGTCCAGTGGCTAAGACTCTGTGCTTCCAATGCAGGGGGCGCAGGTTCGATC
CCTGGTCAGGGAA
>Bos_taurus_chr13.trna1699-GlyTCC (40257852-40257924) Gly (TCC) 73 bp Sc: 53.41
TCCCTGGTGGTCCAGTGGTGAAGACTCTGCACTTCCAATGCAGGGGGCCCAGATTCCATC
CCTGGTCAGGGAA
>Bos_taurus_chr18.trna5070-GlyTCC (24099824-24099752) Gly (TCC) 73 bp Sc: 53.41
TCCCTGGTGGTCCAGTGGTTAGGACTCTGTGCTTCCAATGCTGGGGGCCCAGGTTTAATC
CCTGGTCAGGGAA
>Bos_taurus_chr24.trna4587-GlyTCC (24859388-24859317) Gly (TCC) 72 bp Sc: 53.44
GCGTTGGTGGTGTAGTGGTGAGTATAGCTGCCTTCCAAGCACTTGATCCAGGTTCTATTC
CTGGCCAATGCA
>Bos_taurus_chr11.trna6395-GlyTCC (69182616-69182544) Gly (TCC) 73 bp Sc: 53.52
TCCCTGGTGGTTCAGTGGCTAAGGCTCTGTACTTCCAATGCAGGGGGTCCAGGTTCAAAC
CCTGGTCAGGGAA
>Bos_taurus_chr24.trna5142-GlyTCC (7201194-7201122) Gly (TCC) 73 bp Sc: 53.55
TCCCTGGTGGTCCAGTGGTTAAGACCCTGTGCTTCCACTGCAGGGGGCGCAGGTTCGATC
CCTGGTCAGGGAA
>Bos_taurus_chr12.trna1587-GlyTCC (35792927-35792999) Gly (TCC) 73 bp Sc: 53.63
TCCCTGGTGGTCCAGTGGCTAAGACTCTGCACTTCCAATGCAGGGGGCCCAGGTTCTATC
CTTGGACAGGGAA
>Bos_taurus_chr18.trna3346-GlyTCC (57016681-57016609) Gly (TCC) 73 bp Sc: 53.83
TCCCTGGTGGTCCAGTGGTTAAGACTCTGTGCTTCCAACGCAGGGGACGCAGGTTTGATC

CCTGGTCAGGGAA
>Bos_taurus_chr5.trna1675-GlyTCC (47362529-47362601) Gly (TCC) 73 bp Sc: 53.92
TCCCTGGCAGTCCAGTGGTTAAGACTCTGCATTTCCACTGCAGGAGGCTCGGGTTCAATC
CCCGACTGGGGAA
>Bos_taurus_chr5.trna1117-GlyTCC (32140816-32140888) Gly (TCC) 73 bp Sc: 53.92
TCCCTGGTAGTCCAGTGGTTAAGACTCTGTGCTTCCACTGCAGAGGACACAGGTTCGATC
CCTGGTTGGGGAA
>Bos_taurus_chr5.trna5736-GlyTCC (112552075-112552003) Gly (TCC) 73 bp Sc: 53.93
TCCCTGATGGTCCAGTGGTTAGGACTCTGCACTTCCACTGCTGAGGGTGTGGGTTCAATC
CCTGGTCAGGGAA
>Bos_taurus_chr18.trna947-GlyTCC (23285845-23285918) Gly (TCC) 74 bp Sc: 54.19
TCCTTGGTGGGTCCAGTGGCTAAGACTCTGTGCTTCCAATGCAGGGGCCCCAGGTTCAAT
CCCTGGTCAGGGAA
>Bos_taurus_chr23.trna299-GlyTCC (8777674-8777746) Gly (TCC) 73 bp Sc: 54.25
TCCCTGGTGGTCCAGCGGTTAAGACTCTACACTTCCAATGCAGGGGGCACAGGTTCAATC
CCTGGTCGGGGAA
>Bos_taurus_chr11.trna7180-GlyTCC (49426379-49426307) Gly (TCC) 73 bp Sc: 54.34
TCCCTGGTGGTCCAGTGGCTAAGACTCTGCACTTCCAATGTAGGGGGCCCAGGTTCAATC
TCTGGTCAGGGAA
>Bos_taurus_chr17.trna3802-GlyTCC (67340975-67340903) Gly (TCC) 73 bp Sc: 54.39
TCCCTGGTGGTCCAGTGGTTAAGACTCTGTGCTTCCAATGCAGGGGGCCCAGGTTCGATC
CCTGGTTGGAGAA
>Bos_taurus_chr28.trna508-GlyTCC (12687269-12687341) Gly (TCC) 73 bp Sc: 54.72
TCCCTGGTGGTCCAGTGGTTAAGACTCTGCACTTCCACTGCAGGGAGTGTGGGTTCAATC
CCTGCTCAGGGAA
>Bos_taurus_chr1.trna4557-GlyTCC (131490292-131490364) Gly (TCC) 73 bp Sc: 54.87
TCCCTGGTGGTCCAGTGGCTAAGACTCTGCACTTCCAATGTAGGGGGCCCAGGTTCGATA
TCTGGTCAGGGAA
>Bos_taurus_chr27.trna2425-GlyTCC (36065727-36065655) Gly (TCC) 73 bp Sc: 54.94
TCCCTGGTGGTCCAGTGGCTAAGACCCTGCGCTTCCAATGCAGGGGGCCTGGGTTCGATT
CCTAGTCAGGGAA
>Bos_taurus_chr27.trna832-GlyTCC (25851512-25851584) Gly (TCC) 73 bp Sc: 54.99
TCTCTGGTTGTCCAGTGGTTAAGACTCTGCGCTTCCAATGCAGGGGGCACAGGTTCAATT
CCTGGCTAGAGAA
>Bos_taurus_chr14.trna6666-GlyTCC (628117-628045) Gly (TCC) 73 bp Sc: 55.04
TCCTTGGTGGTCCAATGGTTAAGACTTTGCATTTCCAATGCAGGGGGCACAGGTTCAATC
CCTGGTCAGGGAA
>Bos_taurus_chrUn.004.2377.trna1-GlyTCC (20202-20130) Gly (TCC) 73 bp Sc: 55.04
TCCTTGGTGGTCCAATGGTTAAGACTTTGCATTTCCAATGCAGGGGGCACAGGTTCAATC
CCTGGTCAGGGAA
>Bos_taurus_chr17.trna6215-GlyTCC (14993290-14993218) Gly (TCC) 73 bp Sc: 55.08
TCCCTAGTGGTCCAGTGGTTAAGACTCTGCACTTCCACTGCAGGGGACGCAGGTTCAATC
CCTGGTCAGGGAA
>Bos_taurus_chr13.trna4038-GlyTCC (78412038-78411966) Gly (TCC) 73 bp Sc: 55.14
TCCTTGGCAGTCCAGTGGTTAAGTCTCTGCACTTCCACTGCAGGGGGCCCAGGTTCGATC
CCTGGTCAGGGAA
>Bos_taurus_chrX.trna1886-GlyTCC (54061538-54061610) Gly (TCC) 73 bp Sc: 55.40
TCCCTGATGGTCCAGTGGTTAGGACTCTGTGCTTCCACTGCAGGGGGCCTGGGTTTGATC
CCTAGTCAGGGAA
>Bos_taurus_chr18.trna1531-GlyTCC (38344919-38344991) Gly (TCC) 73 bp Sc: 55.42
TCCCTGGTGGTCCAATGGTTAGGACTCTGCACTTCCACTACAGGGGGCTCAGGTTCAATC
CTTGGTCAGGGAA
>Bos_taurus_chrUn.004.437.trna6-GlyTCC (57269-57341) Gly (TCC) 73 bp Sc: 55.46
TCCGTGGTGGTCCAGTGGCTAAGACTCTGCACTTCCAATGCAGGGGGCCCAGGTTCGCTC
CCTGGTCAGGGAA
>Bos_taurus_chr11.trna7884-GlyTCC (31122496-31122424) Gly (TCC) 73 bp Sc: 55.48
TCCCTGGTGGTCCAGTGGTTAAGACTCTGAGCTTCCACTGCAGGGGGCCCAGGTTCAATC
CCTGGACAGGGAA
>Bos_taurus_chr27.trna3306-GlyTCC (18985647-18985575) Gly (TCC) 73 bp Sc: 55.52
TCCCTGGTGGTCCAGTGGTTAGGACTCTGTGTTTCCACTGCAGGGGGCCCAGGTTCAATC
CCTGGTGGGGGAA
>Bos_taurus_chr2.trna4688-GlyTCC (132056903-132056974) Gly (TCC) 72 bp Sc: 55.71
TCCCTGGTGGCCCAGTGGTTAGGACTCTGCACTTCCACTTCAGCGTCGCAGGTTCAATCC
CTGGTCGGGGAG
>Bos_taurus_chr15.trna4729-GlyTCC (41072021-41071949) Gly (TCC) 73 bp Sc: 55.71
TCCCTGGTGGTCCAGTGGCTAAGACTCTGGGTTTCCAATGCAGGGGTCCCAGGTTCGATC
CCTGATCAGGGAA

>Bos_taurus_chr7.trna8144-GlyTCC (10706427-10706355) Gly (TCC) 73 bp Sc: 55.84
TCCCTGGTGGTCCAGTGGTTAAGACTCTGCACTTCCACTGCAGGGGGCCTGGGTTTGATC
CCTAGTCGGGGAA
>Bos_taurus_chr1.trna4425-GlyTCC (128541245-128541317) Gly (TCC) 73 bp Sc: 56.09
TCCCTGGTGGTCCAGTGGTTAAGACTCTACGCTTCCAACACAGGGGACCCAGGTTCAATC
CCTGGTCGGGGAA
>Bos_taurus_chr9.trna1800-GlyTCC (55747909-55747981) Gly (TCC) 73 bp Sc: 56.10
TCCCTGGTGGTCCAGTGGTTAGGACTCTGCATTTCCATTGCTGATGGCACAGGTTCAATC
CCTGATCAGGGAA
>Bos_taurus_chrUn.004.1.trna321-GlyTCC (1750083-1750011) Gly (TCC) 73 bp Sc: 56.12
TCCCTGGTGGCCCAGTGGTTAAGGTGCCACACTTCCACTGTAGGGGGTGCAGGTTCGATC
CCTGGTCAGGGAA
>Bos_taurus_chr19.trna5696-GlyTCC (22408349-22408277) Gly (TCC) 73 bp Sc: 56.14
TCCTCGGTGGTCCAGTGGTTAAGACTCTGCACTTCCACTGCAGGGGGCATGGGTTCGATC
CCTAGTTGGGGAA
>Bos_taurus_chr15.trna3226-GlyTCC (80849237-80849166) Gly (TCC) 72 bp Sc: 56.19
TCCCTGGTGGTCCAGTGGTTAAGACTCCACACTTCCAATGCAGGGGCACAGGTTCGATCC
CTGATCAGGGAA
>Bos_taurus_chrUn.004.4759.trna3-GlyTCC (5826-5755) Gly (TCC) 72 bp Sc: 56.19
TCCCTGGTGGTCCAGTGGTTAAGACTCCACACTTCCAATGCAGGGGCACAGGTTCGATCC
CTGATCAGGGAA
>Bos_taurus_chr15.trna4768-GlyTCC (39961784-39961712) Gly (TCC) 73 bp Sc: 56.21
TCCCTGGTGGTTCGGTGGTTAGGACTCTGCACTTCCACTGCAAGGGGCCTGGGTTCAATC
CCCAGTCAGGGAA
>Bos_taurus_chr1.trna1031-GlyTCC (25808955-25809026) Gly (TCC) 72 bp Sc: 56.43
TCCCTGGTGGTCCAGTGGTTAAGACTCTGAGTTTCCACTTCAGGGGCTCAGGTTCAATTC
CTGGTTAGGGAA
>Bos_taurus_chr12.trna4612-GlyTCC (55880584-55880513) Gly (TCC) 72 bp Sc: 56.44
TCCCTGGTGGTCCAGTGGTTAAGACTCTGAACTTCCAATGCAGGGGGCCAGGTTCAAACC
CTGTTCAGGGAA
>Bos_taurus_chr15.trna4031-GlyTCC (60868740-60868667) Gly (TCC) 74 bp Sc: 56.45
TCCCTGATGGTCTAGTGGCTAGGATTCTGGCACTTCCAATGCAGGGGCCCCAGGTTTGAT
TCCTGGTTAGGGAA
>Bos_taurus_chr29.trna3333-GlyTCC (19555058-19554986) Gly (TCC) 73 bp Sc: 56.60
TCCCTGGTGGTCCAGTGGTTAGGACTCTGTGCTTCCACTGCAGGGAGCCCAGGTTAGAAC
CCTGGTCAGGGAA
>Bos_taurus_chr19.trna5804-GlyTCC (20924919-20924847) Gly (TCC) 73 bp Sc: 56.70
TCCCTAGTGGTCCAGTGGCTAAGACTCTGTGCTTCCAATGCAGAGGGCCCAGGTTCGATC
CCTGGTCAGGGAA
>Bos_taurus_chr11.trna895-GlyTCC (20983490-20983562) Gly (TCC) 73 bp Sc: 56.82
TCCCTGGTGGTCCAATGGCTAAGACTCTGCACTTCCACTGCAGGGGTCACAGGTTCGATT
GCTGGTCAGGGAA
>Bos_taurus_chr15.trna2875-GlyTCC (80804829-80804901) Gly (TCC) 73 bp Sc: 56.92
TCCCTGGTGGCCCAGTGGTTAGGACTCTGTGCTTCCACAGCAGGGGTCACAGGTTCAATC
CCTGGTCAGGGAA
>Bos_taurus_chr11.trna51-GlyTCC (1022099-1022171) Gly (TCC) 73 bp Sc: 56.96
TCCCTGGTGGTCCAGTGGTTAGGACTCTGTGCTTCCACTGCAGGGGGCACAGGTTCGATC
CCTGGTTGGGGAA
>Bos_taurus_chr19.trna2521-GlyTCC (49866781-49866853) Gly (TCC) 73 bp Sc: 56.97
TCCCTGGTGGTCCAGTGGTTAGGACTCTGTGCTTCCACTGAAGGGGACCCAGGTTCAAAC
CCTGGTCAGGGAA
>Bos_taurus_chrUn.004.1196.trna1-GlyTCC (26447-26519) Gly (TCC) 73 bp Sc: 56.98
TCCCTGATGGTCTAATGGATAAGACTCTGTGCTTCCAATGCAGGGTGCCCAGGTTCAATC
CCTGGTCAGGGAA
>Bos_taurus_chr27.trna871-GlyTCC (26880606-26880678) Gly (TCC) 73 bp Sc: 57.20
TCCCTGGTGGTCTAGTGGTTAAGATTCTGCACTTCCACTGCACGGGGCACAGGTTCAATC
CCTGGCCGGGGAA
>Bos_taurus_chr22.trna2827-GlyTCC (45670205-45670133) Gly (TCC) 73 bp Sc: 57.25
TCCTTGATGGTCCAGTGGTTAGGACTTTGCACTTCCACTACAGGGGTCACAGGTTCAATC
ACTGGTCAGGGAA
>Bos_taurus_chr5.trna5347-GlyTCC (119598805-119598733) Gly (TCC) 73 bp Sc: 57.30
TCCCTGGTGGTCCAGTGGTTAAGACTCTGTACTTCCAATGCAGGGGGTGCAGGTTCGATC
CTTGGCCGGGGAA
>Bos_taurus_chr22.trna287-GlyTCC (6825085-6825157) Gly (TCC) 73 bp Sc: 57.33
TCCCTGGTGGTCCAGTGGTTAAGACTCTGTGCTTCCAATGCAGAGGGCACAGGTTCGATC
CCTGGTTGGGGAA
>Bos_taurus_chr25.trna3519-GlyTCC (26949218-26949146) Gly (TCC) 73 bp Sc: 57.42

TCCCTGGTGATCCAGTGGTTAGGATTCTGTGCTTCCAATGCAGGGGGCTCAGGTTCGATC
CCTGGTCAGGGAA
>Bos_taurus_chr13.trna631-GlyTCC (17717452-17717524) Gly (TCC) 73 bp Sc: 57.45
TTCCTGGTGGTCCAGTGGTTAGGACTCTGAACTTCCAATGCAGGGGACTCAGGTTCAATC
CCTGGTCAGGGAA
>Bos_taurus_chr7.trna2170-GlyTCC (48750970-48751042) Gly (TCC) 73 bp Sc: 57.77
TCCCCAGTGGTCCAGTGGTTAGGACTCTGCATTTCCACTGCAGGGGGCACAGGTTCAATC
CCTGGTTGGAGAA
>Bos_taurus_chr1.trna3103-GlyTCC (90425742-90425814) Gly (TCC) 73 bp Sc: 57.86
TCCCTGGTGGTCCAGTGGGTAAGATTCTGCACTTCCAATGCAGGGAGCCCAGGTTTGATC
CCTGGTCAGGGAA
>Bos_taurus_chr7.trna521-GlyTCC (10560503-10560575) Gly (TCC) 73 bp Sc: 58.06
CCCCTGGTGGTACAGTGGTTAAGACTCTGCATTTCCAATGCAGAGGCCACAGGTTCGATT
CCTGGTCAGGGAA
>Bos_taurus_chr29.trna1541-GlyTCC (42619822-42619894) Gly (TCC) 73 bp Sc: 58.11
TCCCTGGTGGTCCAGTGGTTAGGACTCTGCACTTCCACTGCAGGGGGCATAGGTTTGATT
CCTGGTCAGGGAA
>Bos_taurus_chr12.trna229-GlyTCC (9083078-9083150) Gly (TCC) 73 bp Sc: 58.55
TCCCAGGTGGTCCAGTGGTTAAGACTCTGCACTTCCAATGCAGGGGGTGCAGGTTCAATC
CCTGGCTGGGGAA
>Bos_taurus_chr19.trna924-GlyTCC (20121272-20121344) Gly (TCC) 73 bp Sc: 58.59
TCCTTGGTGGTTCAGTGGCTAAGATTCTGCACTTCCAATGCAGGAGGCCCAGGTTCGATC
CCTGGTCAGGGAA
>Bos_taurus_chr18.trna4022-GlyTCC (48229702-48229630) Gly (TCC) 73 bp Sc: 58.78
TCCCTGATGGTTCAGTGGTTAAGACGTTGCACTTCCAATGCAGAGGGCCAGGGTTCAATC
CCTGGTCAGGGAA
>Bos_taurus_chr28.trna105-GlyTCC (3513103-3513175) Gly (TCC) 73 bp Sc: 58.92
TCCCTGGTGGTCCAGTGGTTAGGACTCTGAACGTCCAATGCAGAGGGCCCAAGTTCGATC
CCTGGTCAGGGAA
>Bos_taurus_chr28.trna94-GlyTCC (3401682-3401754) Gly (TCC) 73 bp Sc: 58.92
TCCCTGGTGGTCCAGTGGTTAGGACTCTGAACGTCCAATGCAGAGGGCCCAAGTTCGATC
CCTGGTCAGGGAA
>Bos_taurus_chr7.trna498-GlyTCC (10300164-10300236) Gly (TCC) 73 bp Sc: 59.07
TCCCTGATGGTCCAATGGTTAAGACTCTGCACTTCCAATGCAGGGGGCACGGGTTCAATC
CCTGGTCAGGGAA
>Bos_taurus_chr18.trna1415-GlyTCC (35174892-35174964) Gly (TCC) 73 bp Sc: 59.30
TCCCTGGTGGTCCAGTGGTTAGGACTCTGTACTTCCAATGTAGGGGGCTCAGGTTCAATC
CCTGGTCGGGGAG
>Bos_taurus_chr7.trna5819-GlyTCC (68170077-68170005) Gly (TCC) 73 bp Sc: 59.35
TCCCTGATGATCCAGTGGTTAAGACTCTGCACTTCCAATGCAGAGTGCACAGGTTCAAAC
CCTGGTCAGGGAA
>Bos_taurus_chr14.trna4007-GlyTCC (63644842-63644770) Gly (TCC) 73 bp Sc: 59.72
CCTCTGGTGGTCCAGTGGTTAAAACTCTGCACTTCCACTGCAGGGGTCCTAGGTTCAATC
CCTGGCCAGGGAA
>Bos_taurus_chr19.trna3166-GlyTCC (61016359-61016431) Gly (TCC) 73 bp Sc: 59.73
TCCCTAGTGGTCCAGTGGTTAAGACTCTGCACTTCCAATGCAGAGGGTGCAGGTTCGATC
CCTGGTCAGGGAA
>Bos_taurus_chr8.trna2311-GlyTCC (72216033-72216105) Gly (TCC) 73 bp Sc: 59.76
TCCCTGATGGTCCAGTGGTTACGACTCTGCACTTCCACTGCAGGAGGCCCAGGTTCAATC
CCTAGTCAGGGAA
>Bos_taurus_chrUn.004.74.trna50-GlyTCC (42667-42595) Gly (TCC) 73 bp Sc: 59.97
TCCCTGGTGGTCCAGTGGTTAAGACTCTGCACTTCCACTGCAGAGGGCACAGGTTTGATC
CCTGGTCAGGGAA
>Bos_taurus_chr14.trna5722-GlyTCC (21992232-21992160) Gly (TCC) 73 bp Sc: 60.09
TCCCCAGTGGTCCAGTGGTTAAGACTCTGCATTTCCAATGCAGGGGTTACATGTTCGATC
CCTGGTTGGGGAG
>Bos_taurus_chr12.trna1361-GlyTCC (30458042-30458114) Gly (TCC) 73 bp Sc: 60.29
TCCCTGGTGGTCTAGTGGTTATGAGTCTGCACTTCCACTGCAGGGGGCACAGGTTCGATC
CCTGGTCAGGGAA
>Bos_taurus_chr22.trna1727-GlyTCC (48837345-48837417) Gly (TCC) 73 bp Sc: 60.39
TCCCTGGTGGTCCAGTGGCTAAGAGTCTGCACTTCCAATGCAGGGGGCCCAGGTTCAATC
CCTGGTCAGGGAA
>Bos_taurus_chr25.trna3509-GlyTCC (27013244-27013172) Gly (TCC) 73 bp Sc: 60.50
TCCGTAGTGGTCCAGTGGTTAGGACTCTGCACTTCCACTGCAGGGGTCCCGGGTTCAATT
CCTGGTCAGGGAT
>Bos_taurus_chr28.trna2582-GlyTCC (15045122-15045052) Gly (TCC) 71 bp Sc: 60.80
TCCCAGGTGGTCCAGTGGTTAGGACTCAGCACTTCCACTGCTGGGGCCTGGGTTCAGTCC

CAGGTTGGGAA
>Bos_taurus_chr20.trna2287-GlyTCC (61951815-61951887) Gly (TCC) 73 bp Sc: 61.54
TCCCTGATGGTCCAGTGGTTAGGACTCTACCCTTCCATTGCAGGGGTCACAGGTTCAATC
CCTGATCAGGGAA
>Bos_taurus_chr5.trna7781-GlyTCC (66118341-66118270) Gly (TCC) 72 bp Sc: 61.60
TCCCTGATGGTCCAGTGGTTAAGACTCTGCACTTCCACTGCAGAAGCACAGGTTCAATCC
CTGGTCGGGGGA
>Bos_taurus_chr5.trna7787-GlyTCC (66008243-66008172) Gly (TCC) 72 bp Sc: 61.60
TCCCTGATGGTCCAGTGGTTAAGACTCTGCACTTCCACTGCAGAAGCACAGGTTCAATCC
CTGGTCGGGGGA
>Bos_taurus_chr2.trna10132-GlyTCC (10394475-10394403) Gly (TCC) 73 bp Sc: 62.52
TCCCTGGTGGTCCAGTGGTTAAGACTCTGCACTTCCATTGGAGAGGCCCCAGGTTCAAGC
CCTGGTTGGGGAA
>Bos_taurus_chr25.trna2401-GlyTCC (40597711-40597783) Gly (TCC) 73 bp Sc: 63.74
TCCCTGATGGTCCAGTGGTCAGGACTCAGCGATTCCACCGCCGAGGGCCCAGGTTCGATC
CCTGGTCAGGGAA
>Bos_taurus_chr9.trna4189-GlyTCC (100778605-100778534) Gly (TCC) 72 bp Sc: 64.88
TTCCTGGTAGTCCAGTGGCTAAGACTCTGCACTTCCAATGCAGGGGCCCAGGTTCAATCC
CTGGCTGGGGAA
>Bos_taurus_chr27.trna1366-GlyTCC (36069172-36069244) Gly (TCC) 73 bp Sc: 65.41
TCCCTGGTGGTCCAGTGGTTAAGACTCTGCACTTCCACTGCAGGGGTCACAGGTTCAATC
CCTGACCGGGAAA
>Bos_taurus_chr6.trna3436-GlyTCC (105499867-105499939) Gly (TCC) 73 bp Sc: 68.51
TCCCTGGTGGTCCAGTGGCTAAGACTCTGCACTTCCAATGCAGGGGGCCCAGGTTCGATC
CCTGGTCAGGGAA
>Bos_taurus_chr6.trna2313-GlyTCC (76394728-76394799) Gly (TCC) 72 bp Sc: 71.29
GCATTGGTGGTATAGTGGTGAGCATAGCTGCCTTCCAAGCAGTTGACCCGGGTTCAATTC
CCGGCCAATGCA
>Bos_taurus_chr19.trna1416-GlyTCC (28390273-28390344) Gly (TCC) 72 bp Sc: 73.26
GCGTTGGTGGTATAGTGGTGAGCATAGCTGCCTTCCAAGCAGTTGACCCGGGTTCGATTC
CCGGCCAACGCA
>Bos_taurus_chr3.trna8535-GlyTCC (23222187-23222116) Gly (TCC) 72 bp Sc: 73.26
GCGTTGGTGGTATAGTGGTGAGCATAGCTGCCTTCCAAGCAGTTGACCCGGGTTCGATTC
CCGGCCAACGCA
>Bos_taurus_chr3.trna9106-GlyTCC (8948000-8947929) Gly (TCC) 72 bp Sc: 73.26
GCGTTGGTGGTATAGTGGTGAGCATAGCTGCCTTCCAAGCAGTTGACCCGGGTTCGATTC
CCGGCCAACGCA
>Bos_taurus_chr3.trna9109-GlyTCC (8824689-8824618) Gly (TCC) 72 bp Sc: 73.26
GCGTTGGTGGTATAGTGGTGAGCATAGCTGCCTTCCAAGCAGTTGACCCGGGTTCGATTC
CCGGCCAACGCA
>Bos_taurus_chr3.trna9112-GlyTCC (8815222-8815151) Gly (TCC) 72 bp Sc: 73.26
GCGTTGGTGGTATAGTGGTGAGCATAGCTGCCTTCCAAGCAGTTGACCCGGGTTCGATTC
CCGGCCAACGCA
>Bos_taurus_chr3.trna9118-GlyTCC (8741938-8741867) Gly (TCC) 72 bp Sc: 73.26
GCGTTGGTGGTATAGTGGTGAGCATAGCTGCCTTCCAAGCAGTTGACCCGGGTTCGATTC
CCGGCCAACGCA
>Bos_taurus_chr3.trna9124-GlyTCC (8629023-8628952) Gly (TCC) 72 bp Sc: 73.26
GCGTTGGTGGTATAGTGGTGAGCATAGCTGCCTTCCAAGCAGTTGACCCGGGTTCGATTC
CCGGCCAACGCA
>Bos_taurus_chrUn.004.4089.trna1-GlyTCC (148-219) Gly (TCC) 72 bp Sc: 73.26
GCGTTGGTGGTATAGTGGTGAGCATAGCTGCCTTCCAAGCAGTTGACCCGGGTTCGATTC
CCGGCCAACGCA
>Bos_taurus_chr15.trna1363-GlyTCC (39230675-39230747) Gly (TCC) 73 bp Sc: 74.45
TCGCTGGTGGTCCAGTGGTTAAGACTCTGCACTTCCAATGCAGAGGTCCCAGGTTCGATC
CCTGGTCAGGGAA
>Bos_taurus_chr7.trna7571-GlyTCC (17992863-17992792) Gly (TCC) 72 bp Sc: 76.83
GCGTTGGTGGTATAGTGGTTAGCATAGCTGCCTTCCAAGCAGTTGACCCGGGTTCGATTC
CCGGCCAACGCA
>Bos_taurus_chr11.trna7542-GlyTCC (42055486-42055405) Gly (TCC) 82 bp Sc: 37.33
TTCCTGGTGGTCCAGTGGTTAAGACTTCATGCTTCCAGTGCAGGTGGTGGTGATGGGATG
GGTTCGATCCCCAGTCAGGGAA
>Bos_taurus_chr22.trna4443-GlyTCC (4540645-4540565) Gly (TCC) 81 bp Sc: 38.10
TCCCGGGTGGTTCAGTCATTAAGACTCTGCACTTCCAATGCAAGGATGCAGAGGGTGCAG
GTTCAATCCCTGGTCCGGGAA
>Bos_taurus_chr5.trna3095-GlyTCC (82986884-82986970) Gly (TCC) 87 bp Sc: 28.08
TCCTTGGCTGTCCAGTGGTTAAGACCCCATGCTTCCACTGAAGGGGAATCATGGTTCAAT
CACCAGGTTCAATCCCTGGTCAGGGAA

>Bos_taurus_chr10.trna2433-HisATG (64353999-64354070) His (ATG) 72 bp Sc: 55.70
TCCCTGGTGGCTCAGATGGTAAAGTGTCTCCCTATGATGCAGGAGACCCAGGTTCGATTC
CTGGGTGGGGAA
>Bos_taurus_chr7.trna8658-HisATG (830951-830880) His (ATG) 72 bp Sc: 60.40
TCCCTGATGGCTCAGTTGGTAAAGAATCTGCCTATGATGCAGGAGACCCAGGTTCAATCC
CTGGTTTGGGAA
>Bos_taurus_chrX.trna4500-HisATG (66217655-66217584) His (ATG) 72 bp Sc: 61.30
TCCCTGGTGGCTCAGATGGTAAAGCATCTGCCTATGATGCAGGAGACCCAGGTTCAATTC
CTGGGTGGGGAA
>Bos_taurus_chr18.trna1562-HisGTG (39141111-39141182) His (GTG) 72 bp Sc: 36.09
GCCGTGATTGTACAGTGGTTAGTACTCGGtgttgtGGCCACAGCAACCTCGGTTTGAATC
TGAGTCACAGCA
>Bos_taurus_chr23.trna3394-HisGTG (31360552-31360481) His (GTG) 72 bp Sc: 51.96
TCTGTGATAGTATAGTGGTTAGTACTCTGCGTTGTGGCTGCAGCAACCTTGGTTCGAATC
CGAGTCACGGCA
>Bos_taurus_chr25.trna3249-HisGTG (30269550-30269479) His (GTG) 72 bp Sc: 55.16
TCCCTGGTGGCTCAGAGGGTAAAGCATCTGCCTGTGATGCAGGAGACCCAGGTTCGATCC
CTGGGTCGGGAA
>Bos_taurus_chr11.trna7744-HisGTG (35104433-35104362) His (GTG) 72 bp Sc: 57.38
TCCCTGGTGGCTCAGAGGGTAAAGCATCTGCCTGTGATGCAGAAGACCCAGGTTCAATCC
CTGGGTTGGGAA
>Bos_taurus_chr10.trna2504-HisGTG (66856406-66856477) His (GTG) 72 bp Sc: 64.63
GCCGTGATCGTATAGTGGTTAGTACTCTGCGTTGTGGCCGCAGCAACCTCGGTTCGAATC
CGAGTCACGGCA
>Bos_taurus_chr10.trna2505-HisGTG (66857938-66858009) His (GTG) 72 bp Sc: 64.63
GCCGTGATCGTATAGTGGTTAGTACTCTGCGTTGTGGCCGCAGCAACCTCGGTTCGAATC
CGAGTCACGGCA
>Bos_taurus_chr10.trna5734-HisGTG (66855742-66855671) His (GTG) 72 bp Sc: 64.63
GCCGTGATCGTATAGTGGTTAGTACTCTGCGTTGTGGCCGCAGCAACCTCGGTTCGAATC
CGAGTCACGGCA
>Bos_taurus_chr3.trna811-HisGTG (22472769-22472840) His (GTG) 72 bp Sc: 64.63
GCCGTGATCGTATAGTGGTTAGTACTCTGCGTTGTGGCCGCAGCAACCTCGGTTCGAATC
CGAGTCACGGCA
>Bos_taurus_chr3.trna814-HisGTG (22498590-22498661) His (GTG) 72 bp Sc: 64.63
GCCGTGATCGTATAGTGGTTAGTACTCTGCGTTGTGGCCGCAGCAACCTCGGTTCGAATC
CGAGTCACGGCA
>Bos_taurus_chr3.trna8543-HisGTG (23046465-23046394) His (GTG) 72 bp Sc: 64.63
GCCGTGATCGTATAGTGGTTAGTACTCTGCGTTGTGGCCGCAGCAACCTCGGTTCGAATC
CGAGTCACGGCA
>Bos_taurus_chr3.trna8557-HisGTG (22919173-22919102) His (GTG) 72 bp Sc: 64.63
GCCGTGATCGTATAGTGGTTAGTACTCTGCGTTGTGGCCGCAGCAACCTCGGTTCGAATC
CGAGTCACGGCA
>Bos_taurus_chr3.trna8573-HisGTG (22678779-22678708) His (GTG) 72 bp Sc: 64.63
GCCGTGATCGTATAGTGGTTAGTACTCTGCGTTGTGGCCGCAGCAACCTCGGTTCGAATC
CGAGTCACGGCA
>Bos_taurus_chr3.trna8582-HisGTG (22627598-22627527) His (GTG) 72 bp Sc: 64.63
GCCGTGATCGTATAGTGGTTAGTACTCTGCGTTGTGGCCGCAGCAACCTCGGTTCGAATC
CGAGTCACGGCA
>Bos_taurus_chr3.trna865-HisGTG (22962665-22962736) His (GTG) 72 bp Sc: 64.63
GCCGTGATCGTATAGTGGTTAGTACTCTGCGTTGTGGCCGCAGCAACCTCGGTTCGAATC
CGAGTCACGGCA
>Bos_taurus_chr3.trna868-HisGTG (23012150-23012221) His (GTG) 72 bp Sc: 64.63
GCCGTGATCGTATAGTGGTTAGTACTCTGCGTTGTGGCCGCAGCAACCTCGGTTCGAATC
CGAGTCACGGCA
>Bos_taurus_chr8.trna1103-HisGTG (31506575-31506646) His (GTG) 72 bp Sc: 64.63
GCCGTGATCGTATAGTGGTTAGTACTCTGCGTTGTGGCCGCAGCAACCTCGGTTCGAATC
CGAGTCACGGCA
>Bos_taurus_chrUn.004.185.trna1-HisGTG (18880-18951) His (GTG) 72 bp Sc: 64.63
GCCGTGATCGTATAGTGGTTAGTACTCTGCGTTGTGGCCGCAGCAACCTCGGTTCGAATC
CGAGTCACGGCA
>Bos_taurus_chrUn.004.185.trna19-HisGTG (214351-214280) His (GTG) 72 bp Sc: 64.63
GCCGTGATCGTATAGTGGTTAGTACTCTGCGTTGTGGCCGCAGCAACCTCGGTTCGAATC
CGAGTCACGGCA
>Bos_taurus_chrUn.004.185.trna2-HisGTG (28021-28092) His (GTG) 72 bp Sc: 64.63
GCCGTGATCGTATAGTGGTTAGTACTCTGCGTTGTGGCCGCAGCAACCTCGGTTCGAATC
CGAGTCACGGCA
>Bos_taurus_chrUn.004.185.trna25-HisGTG (166089-166018) His (GTG) 72 bp Sc: 64.63

GCCGTGATCGTATAGTGGTTAGTACTCTGCGTTGTGGCCGCAGCAACCTCGGTTCGAATC
CGAGTCACGGCA
>Bos_taurus_chrUn.004.1856.trna4-HisGTG (13087-13158) His (GTG) 72 bp Sc: 64.63
GCCGTGATCGTATAGTGGTTAGTACTCTGCGTTGTGGCCGCAGCAACCTCGGTTCGAATC
CGAGTCACGGCA
>Bos_taurus_chrUn.004.3551.trna3-HisGTG (260-189) His (GTG) 72 bp Sc: 64.63
GCCGTGATCGTATAGTGGTTAGTACTCTGCGTTGTGGCCGCAGCAACCTCGGTTCGAATC
CGAGTCACGGCA
>Bos_taurus_chr4.trna6366-IleAAT (77437422-77437349) Ile (AAT) 74 bp Sc: 56.73
GGCCGGTTAGCTCAGTTGATTAGAGTGTGGTGCTAATAACGCCAGGGTCACGGGTTCGAT
CCCTGTGCAGGCCA
>Bos_taurus_chr23.trna3103-IleAAT (38722821-38722748) Ile (AAT) 74 bp Sc: 70.00
GGCCAGTTAGCTCAGTTGGTTAGAGCGTGGTGCTAATAATGCCAAGGTCACAGGTTCAAT
CCCTGTACGGGTCA
>Bos_taurus_chr19.trna5350-IleAAT (28395968-28395895) Ile (AAT) 74 bp Sc: 75.36
GGCCGGTTAGTTCAGTTGGTTAGAGCGTGGTGCTAATAACGCCAAGGTCGCGGGTTCGAT
CCCCGTACGGGCCA
>Bos_taurus_chr19.trna1414-IleAAT (28374885-28374958) Ile (AAT) 74 bp Sc: 79.24
GGCCGGTTAGCTCAGTTGGTTAGAGCGTGGTGCTAATAACGCCAAGGTCGCGGGTTCGAG
CCCCGTACGGGCCA
>Bos_taurus_chr21.trna2799-IleAAT (67186117-67186190) Ile (AAT) 74 bp Sc: 80.66
GGCCGGTTAGCTCAGTTGGTTAGAGCGTGGTGCTAATAACGCCAAGGTCGCGGGTTCGAT
CCCCGTACGGGCCA
>Bos_taurus_chr23.trna1389-IleAAT (31304603-31304676) Ile (AAT) 74 bp Sc: 80.66
GGCCGGTTAGCTCAGTTGGTTAGAGCGTGGTGCTAATAACGCCAAGGTCGCGGGTTCGAT
CCCCGTACGGGCCA
>Bos_taurus_chr23.trna1394-IleAAT (31322007-31322080) Ile (AAT) 74 bp Sc: 80.66
GGCCGGTTAGCTCAGTTGGTTAGAGCGTGGTGCTAATAACGCCAAGGTCGCGGGTTCGAT
CCCCGTACGGGCCA
>Bos_taurus_chr23.trna1401-IleAAT (31352446-31352519) Ile (AAT) 74 bp Sc: 80.66
GGCCGGTTAGCTCAGTTGGTTAGAGCGTGGTGCTAATAACGCCAAGGTCGCGGGTTCGAT
CCCCGTACGGGCCA
>Bos_taurus_chr23.trna1429-IleAAT (31740296-31740369) Ile (AAT) 74 bp Sc: 80.66
GGCCGGTTAGCTCAGTTGGTTAGAGCGTGGTGCTAATAACGCCAAGGTCGCGGGTTCGAT
CCCCGTACGGGCCA
>Bos_taurus_chr23.trna3379-IleAAT (31568596-31568523) Ile (AAT) 74 bp Sc: 80.66
GGCCGGTTAGCTCAGTTGGTTAGAGCGTGGTGCTAATAACGCCAAGGTCGCGGGTTCGAT
CCCCGTACGGGCCA
>Bos_taurus_chr23.trna3399-IleAAT (31320338-31320265) Ile (AAT) 74 bp Sc: 80.66
GGCCGGTTAGCTCAGTTGGTTAGAGCGTGGTGCTAATAACGCCAAGGTCGCGGGTTCGAT
CCCCGTACGGGCCA
>Bos_taurus_chr23.trna3415-IleAAT (31117160-31117087) Ile (AAT) 74 bp Sc: 80.66
GGCCGGTTAGCTCAGTTGGTTAGAGCGTGGTGCTAATAACGCCAAGGTCGCGGGTTCGAT
CCCCGTACGGGCCA
>Bos_taurus_chr23.trna3416-IleAAT (31103282-31103209) Ile (AAT) 74 bp Sc: 80.66
GGCCGGTTAGCTCAGTTGGTTAGAGCGTGGTGCTAATAACGCCAAGGTCGCGGGTTCGAT
CCCCGTACGGGCCA
>Bos_taurus_chr23.trna3417-IleAAT (31101539-31101466) Ile (AAT) 74 bp Sc: 80.66
GGCCGGTTAGCTCAGTTGGTTAGAGCGTGGTGCTAATAACGCCAAGGTCGCGGGTTCGAT
CCCCGTACGGGCCA
>Bos_taurus_chrUn.004.1183.trna1-IleAAT (14020-14093) Ile (AAT) 74 bp Sc: 80.66
GGCCGGTTAGCTCAGTTGGTTAGAGCGTGGTGCTAATAACGCCAAGGTCGCGGGTTCGAT
CCCCGTACGGGCCA
>Bos_taurus_chrUn.004.1183.trna3-IleAAT (30398-30325) Ile (AAT) 74 bp Sc: 80.66
GGCCGGTTAGCTCAGTTGGTTAGAGCGTGGTGCTAATAACGCCAAGGTCGCGGGTTCGAT
CCCCGTACGGGCCA
>Bos_taurus_chrUn.004.1183.trna8-IleAAT (12351-12278) Ile (AAT) 74 bp Sc: 80.66
GGCCGGTTAGCTCAGTTGGTTAGAGCGTGGTGCTAATAACGCCAAGGTCGCGGGTTCGAT
CCCCGTACGGGCCA
>Bos_taurus_chr7.trna1180-IleTAT (19288271-19288341) Ile (TAT) 71 bp Sc: 33.97
TCCCGGGTGGTCCAGTGGATAAGACTCCATGCTTATCTCAGGGAGCCCAGGCTCAATCCT
TGGTCAGGGAA
>Bos_taurus_chr10.trna5602-IleTAT (70128531-70128459) Ile (TAT) 73 bp Sc: 63.95
GGCTCCATAGCTTGGGGGTTAGAGTGCTGGTCTTATAAACCAGGGGTCACAAGTTCAAAT
CTTGCTGGGGCCT
>Bos_taurus_chr23.trna4168-IleTAT (15174070-15173998) Ile (TAT) 73 bp Sc: 68.82
GCCCTCTTAGTGCAGTAGGCAGCACGTTAGTCTTATAATCTGAAGGTCCTGAGTTCAAGC

CTCAGAGAGGGCA
>Bos_taurus_chr11.trna1143-IleTAT (26502587-26502679) Ile (TAT) 93 bp Sc: 68.11
GCTCCAGTGGCGCAATCGGTTAGCGCGCGGTACTTATACAGCAGTACATGCAGAGCAATG
CCGAGGTTGTGAGTTCGAGCCTCACCTGGAGCA
>Bos_taurus_chr23.trna3450-IleTAT (30294018-30293925) Ile (TAT) 94 bp Sc: 68.87
GCTCCAGTGGCGCAATCGGTTAGCGCGCGGTACTTATAAGGCAGTACATTTGTGAGCAAT
GCCGAGGTTGTGAGTTCGAGCCTCACCTGGAGCA
>Bos_taurus_chr23.trna3413-IleTAT (31130443-31130350) Ile (TAT) 94 bp Sc: 67.58
GCTCCAGTGGCGCAATCGGTTAGCGCGCGGTACTTATACAGCAGTATATGTGCGGGTGAT
GCCGAGGTTGTGAGTTCGAGCCTCACCTGGAGCA
>Bos_taurus_chr23.trna3390-IleTAT (31407996-31407903) Ile (TAT) 94 bp Sc: 69.00
GCTCCAGTGGCGCAATCGGTTAGCGCGCGGTACTTATACAGCAGTATGTGTGCGAGTGAT
GCCGAGGTTGTGAGTTCGATCCTCACCTGGAGCA
>Bos_taurus_chr18.trna3980-IleTAT (48608932-48608840) Ile (TAT) 93 bp Sc: 66.61
GCTCCAGTGGCGCAATCGGTTAGCGCGCGGTACTTATAGGGCAGTGCGAGCGGAGCGATG
CCGAGGTTGTGAGTTCGATCCTCACCTGGAGCA
>Bos_taurus_chr23.trna3358-LeuAAG (31897886-31897805) Leu (AAG) 82 bp Sc: 55.18
GGTAGTGTGGCCGAGCGGTCTAAGGCGCTGGATTAAGGCTCCAGTCTCTTTGGGGATGTG
GGTTTGAATCCCACTGCTGCCA
>Bos_taurus_chr10.trna7198-LeuAAG (25895952-25895871) Leu (AAG) 82 bp Sc: 55.18
GGTAGCATGGCCGAGTGGTCTAAGGCGCTGGATTAAGGCTCCAGTCACTTTGGGGGCGTA
GGTTCGAATCGCGCTGCTGCCA
>Bos_taurus_chr7.trna1882-LeuAAG (39665899-39665980) Leu (AAG) 82 bp Sc: 69.34
GGTAGCGTGGCCGAGCGGTCTAAGGCGCTGGATTAAGGCTCCAGTCTCTCCGGGGGCGTG
GGTTCGAATCCCACCGCTGCCA
>Bos_taurus_chr10.trna1094-LeuAAG (25841215-25841296) Leu (AAG) 82 bp Sc: 69.84
GGTAGCGTGGCCGAGCGGTCTAAGGCGCTGGATTAAGGCTCCAGTCTCTTCGGGGGCGTG
GGTTCGAATCCCACCGCTGCCA
>Bos_taurus_chr10.trna7199-LeuAAG (25886390-25886309) Leu (AAG) 82 bp Sc: 69.84
GGTAGCGTGGCCGAGCGGTCTAAGGCGCTGGATTAAGGCTCCAGTCTCTTCGGGGGCGTG
GGTTCGAATCCCACCGCTGCCA
>Bos_taurus_chr23.trna1318-LeuAAG (30086744-30086825) Leu (AAG) 82 bp Sc: 69.84
GGTAGCGTGGCCGAGCGGTCTAAGGCGCTGGATTAAGGCTCCAGTCTCTTCGGGGGCGTG
GGTTCGAATCCCACCGCTGCCA
>Bos_taurus_chr23.trna1325-LeuAAG (30197607-30197688) Leu (AAG) 82 bp Sc: 69.84
GGTAGCGTGGCCGAGCGGTCTAAGGCGCTGGATTAAGGCTCCAGTCTCTTCGGGGGCGTG
GGTTCGAATCCCACCGCTGCCA
>Bos_taurus_chr23.trna1341-LeuAAG (30354799-30354880) Leu (AAG) 82 bp Sc: 69.84
GGTAGCGTGGCCGAGCGGTCTAAGGCGCTGGATTAAGGCTCCAGTCTCTTCGGGGGCGTG
GGTTCGAATCCCACCGCTGCCA
>Bos_taurus_chr23.trna3468-LeuAAG (30065291-30065210) Leu (AAG) 82 bp Sc: 69.84
GGTAGCGTGGCCGAGCGGTCTAAGGCGCTGGATTAAGGCTCCAGTCTCTTCGGGGGCGTG
GGTTCGAATCCCACCGCTGCCA
>Bos_taurus_chr25.trna1184-LeuAAG (21154623-21154704) Leu (AAG) 82 bp Sc: 69.84
GGTAGCGTGGCCGAGCGGTCTAAGGCGCTGGATTAAGGCTCCAGTCTCTTCGGGGGCGTG
GGTTCGAATCCCACCGCTGCCA
>Bos_taurus_chr13.trna6136-LeuAAG (33050295-33050208) Leu (AAG) 88 bp Sc: 21.82
TCCCCAGTGGGGAAGTGGTGATCCCGTGCGTGGTTAAGACTCTGTGCTTCCACTGCAGGG
GGCATGGGTTCAATCCCTATTTGGGGAA
>Bos_taurus_chr18.trna4149-LeuCAA (46484487-46484417) Leu (CAA) 71 bp Sc: 36.37
TCTCTGATGGTCTAGTGGCTAAGACTATCTGCCCAATGCAGGGGGCCTAGGTTTGATCCC
TGGTCAGAGAA
>Bos_taurus_chr14.trna5735-LeuCAA (21791508-21791438) Leu (CAA) 71 bp Sc: 37.09
TCTCTGATGGTCTAATGGCTAAGACTATCTGCCCAATGCAGGGGGCCCAGGTTCGTTCCC
TGGTCAGAGAA
>Bos_taurus_chr19.trna1317-LeuCAA (26689952-26690024) Leu (CAA) 73 bp Sc: 45.12
TCCCTGGTGGTTCAGTGGCTAAGACTCTGTACTCAAAATACACGGGGCCCGGGTTCAACC
CCTGGTCAGGGAA
>Bos_taurus_chr10.trna3222-LeuCAA (84665468-84665540) Leu (CAA) 73 bp Sc: 53.83
TCCCTGGTGGTCCAGTGGCTAAGACTCCGTGCTCCAAATGCAGGCGACCCAGGTTCAATC
CCTGGCTAGGGAA
>Bos_taurus_chr23.trna485-LeuCAA (12232540-12232621) Leu (CAA) 82 bp Sc: 56.50
GCTGTGATGGTCAAGTGGTTAAGGCCTTGGACTCAAGATCCTACGGGGTCTCCCCAAGCA
GGTTCAAACCCTGATCACAGCA
>Bos_taurus_chr2.trna2416-LeuCAA (76456315-76456387) Leu (CAA) 73 bp Sc: 58.85
GCCTTCTTAGCTCAGTAGGCAGTGCATCAGTCTCAAAATCTGAAGGTCCTGAGTTCAAGC
CTCAGATAGGGCA

>Bos_taurus_chr2.trna2422-LeuCAA (76523780-76523852) Leu (CAA) 73 bp Sc: 58.85
GCCTTCTTAGCTCAGTAGGCAGTGCATCAGTCTCAAAATCTGAAGGTCCTGAGTTCAAGC
CTCAGATAGGGCA
>Bos_taurus_chr2.trna9287-LeuCAA (36238515-36238434) Leu (CAA) 82 bp Sc: 58.98
GCTGTGATGGCTGGGTGGTCAAGGCATTGGATTCAAAATCCAATGGGGTCTGCCTGCTCA
GGTTAGAATCCTGCTCACAGCA
>Bos_taurus_chr9.trna1416-LeuCAA (43883890-43883971) Leu (CAA) 82 bp Sc: 62.21
GCTGTGATGACTGAGTGGTTAAGATGTTAGACTCAAAATCCAATGGGGTATCCCCATGCA
GGTTCAAATCCTGCTCACAGCA
>Bos_taurus_chr23.trna3464-LeuCAA (30091947-30091837) Leu (CAA) 111 bp Sc: 67.66
GTCAGGATGGCCGAGTGGTCTAAGGCGCCAGACTCAAGCTCAAACAAGCTTCCTCTGATA
GAGGTTTCTGGTCCCCGAATGGGGGCGTGGGTTCGAATCCCACTTCTGACA
>Bos_taurus_chr23.trna1320-LeuCAA (30121336-30121441) Leu (CAA) 106 bp Sc: 68.07
GTCAGGATGGCCGAGTGGTCTAAGGCGCCAGACTCAAGCGAAGCTTCCCCACCTTGGGGA
TTCTGGTCTCCGTATGGAGGCGTGGGTTCGAATCCCACTTCTGACA
>Bos_taurus_chr23.trna1372-LeuCAA (31143743-31143849) Leu (CAA) 107 bp Sc: 65.27
GTCAGGATGGCCGAGTGGTCTAAGGCGCCAGACTCAAGCTTCTGCTTCCCACCTTTGGGG
CTTCTGGTCTCCGAATGGAGGCGTGGGTTCGAATCCCACTTCTGACA
>Bos_taurus_chr23.trna3411-LeuCAA (31146407-31146300) Leu (CAA) 108 bp Sc: 66.67
GTCAGGATGGCCGAGTGGTCTAAGGCGCCAGACTCAAGATTCTATCTTCCCTCTCTTGGG
GCTTCTGGTCTCCGAATGGAGGCGTGGGTTCGAATCCCACTTCTGACA
>Bos_taurus_chr23.trna1376-LeuCAA (31161357-31161463) Leu (CAA) 107 bp Sc: 70.03
GTCAGGATGGCCGAGTGGTCTAAGGCGCCAGACTCAAGTTCCTACTTCCCCTACTTGGGG
TTTCTGGTCTCCACTTGGAGGCGTGGGTTCGAATCCCACTTCTGACA
>Bos_taurus_chr7.trna1948-LeuCAA (42028223-42028328) Leu (CAA) 106 bp Sc: 66.06
GTCAGGATGGCCGAGTGGTCTAAGGCGCCAGACTCAAGGTGTCTACCTTCCCCTAGGGCA
TTCTGGTCTCCGAATGGAGGCGTGGGTTCGAATCCCACTTCTGACA
>Bos_taurus_chr6.trna2513-LeuCAG (85623486-85623556) Leu (CAG) 71 bp Sc: 42.60
GGCTCATTGGTCTAGGGTTATGATTCTAGCTTCAGATGCGAGGGTTCCGGGTTCAAATCC
TGGACGAGCTT
>Bos_taurus_chr18.trna5030-LeuCAG (24936401-24936319) Leu (CAG) 83 bp Sc: 60.18
GTCAGGATGGCCGAGAGGAATAAGGCGCTGCGTTCAGGTCGCAGTCTCCCCTGGAGGCGT
GGGTTCGAATCCCACTTCTGANN
>Bos_taurus_chr3.trna271-LeuCAG (8630409-8630491) Leu (CAG) 83 bp Sc: 63.92
GTCAGGATGGCCGAGCGGTCTAAGGCGCTGCGTTCAGGTCGCAGTCTCCCCTGGAGGCGT
GGGTTCGAATTCTACTCCTGACA
>Bos_taurus_chr18.trna996-LeuCAG (24935795-24935877) Leu (CAG) 83 bp Sc: 75.90
GTCAGGATGGCCGAGCGGTCTAAGGCGCTGCGTTCAGGTCGCAGTCTCCCCTGGAGGCGT
GGGTTCGAATCCCACTTCTGACA
>Bos_taurus_chr3.trna273-LeuCAG (8742588-8742670) Leu (CAG) 83 bp Sc: 75.90
GTCAGGATGGCCGAGCGGTCTAAGGCGCTGCGTTCAGGTCGCAGTCTCCCCTGGAGGCGT
GGGTTCGAATCCCACTTCTGACA
>Bos_taurus_chr23.trna3375-LeuCAG (31595661-31595579) Leu (CAG) 83 bp Sc: 77.22
GTCAGGATGGCCGAGCGGTCTAAGGCGCTGCGTTCAGGTCGCAGTCTCCCCTGGAGGCGT
GGGTTCGAATCCCACTCCTGACA
>Bos_taurus_chr3.trna275-LeuCAG (8816549-8816631) Leu (CAG) 83 bp Sc: 77.22
GTCAGGATGGCCGAGCGGTCTAAGGCGCTGCGTTCAGGTCGCAGTCTCCCCTGGAGGCGT
GGGTTCGAATCCCACTCCTGACA
>Bos_taurus_chr3.trna277-LeuCAG (8939849-8939931) Leu (CAG) 83 bp Sc: 77.22
GTCAGGATGGCCGAGCGGTCTAAGGCGCTGCGTTCAGGTCGCAGTCTCCCCTGGAGGCGT
GGGTTCGAATCCCACTCCTGACA
>Bos_taurus_chr3.trna279-LeuCAG (8949436-8949518) Leu (CAG) 83 bp Sc: 77.22
GTCAGGATGGCCGAGCGGTCTAAGGCGCTGCGTTCAGGTCGCAGTCTCCCCTGGAGGCGT
GGGTTCGAATCCCACTCCTGACA
>Bos_taurus_chr15.trna2912-LeuTAA (81443919-81443991) Leu (TAA) 73 bp Sc: 32.53
TCCCTGGTAGTCCAGGGGTTAAGACTCTGTACTTAACTTGCAGGGGGCCTGGGTTCCATC
GCTGGTCAGGGAA
>Bos_taurus_chr4.trna8096-LeuTAA (22488592-22488510) Leu (TAA) 83 bp Sc: 46.41
ATTATAATGGCCAAGTGGTTAAGGCATTGGACTTAATATCCAATGGATTTATATCCTCAT
GGGTTCGAACCCCATTTCTGGTA
>Bos_taurus_chr6.trna4226-LeuTAA (122482634-122482715) Leu (TAA) 82 bp Sc: 48.66
GCTGAGATTGCTGAGTGGTTAAGCACTGGACTTAAGATCCGATGGACATGTGTCTGCGTG
GGTTCGAACCCCACTCCCAGCA
>Bos_taurus_chr18.trna4543-LeuTAA (37934709-37934629) Leu (TAA) 81 bp Sc: 55.80
GCAGGATGGCCGAGTGGTTAAGGTGTTGGACTTAAGATCCAATGGGCTAGTGCCTTGTGG
GTTCGAATCCCACTCTCAGTA
>Bos_taurus_chr23.trna1399-LeuTAA (31334263-31334345) Leu (TAA) 83 bp Sc: 70.66

ATCGGGATGGCCGAGTGGTTAAGGCGTTGGACTTAAGATCCAATAGACATCTGTCTGCGT
GGGTTCGAACCCCACTCCCGGTA
>Bos_taurus_chr15.trna2992-LeuTAA (83646565-83646647) Leu (TAA) 83 bp Sc: 74.33
ACCAGAATGGCCGAGTGGTTAAGGCGTTGGACTTAAGATCCAATGGATTTATATCCTCGT
GGGTTCGAACCCCACTTCTGGTA
>Bos_taurus_chr23.trna3401-LeuTAA (31300209-31300127) Leu (TAA) 83 bp Sc: 74.84
ACCGGGATGGCCGAGTGGTTAAGGCGTTGGACTTAAGATCCAATAGACATGTGTCTGCGT
GGGTTCGAACCCCACTCCCGGTA
>Bos_taurus_chr23.trna1365-LeuTAA (31088163-31088245) Leu (TAA) 83 bp Sc: 79.48
ACCGGGATGGCCGAGTGGTTAAGGCGTTGGACTTAAGATCCAATGGGCTAGTGCCCGCGT
GGGTTCGAACCCCACTCTCGGTA
>Bos_taurus_chr9.trna2830-LeuTAA (84404212-84404294) Leu (TAA) 83 bp Sc: 81.03
ACCAGGATGGCCGAGTGGTTAAGGCGTTGGACTTAAGATCCAATGGACGTATGTCCGCGT
GGGTTCGAACCCCACTCCTGGTA
>Bos_taurus_chr25.trna3830-LeuTAG (21056217-21056136) Leu (TAG) 82 bp Sc: 68.14
GGTAGCGTGGCCGAGTGGTCTAAGGCGCTGGATTTAGGCTCCAGTCATTTCGATGGCGTG
GGTTCGAATCCCACCGCTGCCA
>Bos_taurus_chrUn.004.2658.trna1-LeuTAG (13077-13158) Leu (TAG) 82 bp Sc: 68.14
GGTAGCGTGGCCGAGTGGTCTAAGGCGCTGGATTTAGGCTCCAGTCATTTCGATGGCGTG
GGTTCGAATCCCACCGCTGCCA
>Bos_taurus_chr10.trna7200-LeuTAG (25874307-25874226) Leu (TAG) 82 bp Sc: 68.82
GGTAGTGTGGCCGAGCGGTCTAAGGCGCTGGATTTAGGCTCCAGTCTCTTCGGGGGCGTG
GGTTCGAATCCCACCACTGCCA
>Bos_taurus_chr19.trna5362-LeuTAG (28306082-28306001) Leu (TAG) 82 bp Sc: 72.19
GGTAGCGTGGCCGAGCGGTCTAAGGCGCTGGATTTAGGCTCCAGTCTCTTCGGAGGCGTG
GGTTCGAATCCCACCGCTGCCA
>Bos_taurus_chr10.trna1093-LeuTAG (25839548-25839629) Leu (TAG) 82 bp Sc: 74.02
GGTAGCGTGGCCGAGCGGTCTAAGGCGCTGGATTTAGGCTCCAGTCTCTCTGGAGGCGTG
GGTTCGAATCCCACCGCTGCCA
>Bos_taurus_chr19.trna5243-LysCTT (29884488-29884417) Lys (CTT) 72 bp Sc: 31.98
TCCCTGGTGGTCCAGTGGTTAGGATTTGGTGTTCTTCCTGCCAGGACCTGAGTTGGATCC
TTGGTCAGGAAA
>Bos_taurus_chr6.trna1779-LysCTT (61987169-61987242) Lys (CTT) 74 bp Sc: 39.62
TCCCTGATGGTCCAGTGTCTAAGACACTGTGCTCTTAATGCAGGGGGGCCCAAGTTTGAT
CCCTGGTCAGGGAA
>Bos_taurus_chr3.trna514-LysCTT (16279313-16279385) Lys (CTT) 73 bp Sc: 40.70
TCACTGGTAGTCCAGTGGCTAAGACTCTGTGCTCTTCATGCAGGGGGCCCAGGTTTGATC
CCTGGTCAGGGAA
>Bos_taurus_chr10.trna23-LysCTT (536758-536829) Lys (CTT) 72 bp Sc: 42.76
TCCCTGGTGGTCTAGTGCTAAGACTCTGCAGTCTTAATGCAGGGGTTTCAGGTTCAAACT
CTGGTCAGGAAA
>Bos_taurus_chr22.trna3949-LysCTT (13460370-13460298) Lys (CTT) 73 bp Sc: 45.22
TCTCTGGCAGTCCAGTGGTTAGGACTCAGCATTCTTACTGCTGGGACCCTGGGTTGGAGC
CCTAGCTGGGGAA
>Bos_taurus_chr3.trna4556-LysCTT (122411510-122411582) Lys (CTT) 73 bp Sc: 45.35
CCCTTGGTAGTCCAGTGTCTAAGACTCTACACTCTTAATGCAGAGGACCCAGGTTCTATC
CCTGGTCAGGGAA
>Bos_taurus_chr23.trna2490-LysCTT (50028341-50028270) Lys (CTT) 72 bp Sc: 45.55
TCCCTGGTAGTCCAGTGGGTAAGACCCTGAGCTCTTGATGCAGGGGTCTTGGTTCAATCC
CTGGTCAGGGAA
>Bos_taurus_chr4.trna7227-LysCTT (52261065-52260993) Lys (CTT) 73 bp Sc: 45.85
TCCCTGGTGGCCCAATGGCTAAGATTCTGCGTTCTTGATGCAGAGGGGCCAGGTTCAATC
CCTGGTCGGGGAA
>Bos_taurus_chr10.trna3100-LysCTT (82035832-82035904) Lys (CTT) 73 bp Sc: 48.01
TCTCTGATGGTCCAGTGGTTATGACTCAGCGTTCTTACTGCTGGGGCCCTAGGTTTGATC
CCTGGTTGGGGAA
>Bos_taurus_chr16.trna293-LysCTT (10695146-10695218) Lys (CTT) 73 bp Sc: 53.01
TCCCTGGTGGTCCAGTGGTTAGGACTCTGTGCTCTTACTGCTGAGGGGCTAGGTTCAATC
CCTGGTCAGGGAA
>Bos_taurus_chr15.trna3974-LysCTT (62448980-62448894) Lys (CTT) 87 bp Sc: 53.24
GCCTGGCTAGCTCAGTTGGTAGACCATGAGACTCTTAATCTCAGGGTTCAAGCCCTGAGC
TCCTGGGTTCAAGCCCCAGGTTGGGCA
>Bos_taurus_chr21.trna821-LysCTT (20922472-20922544) Lys (CTT) 73 bp Sc: 53.78
TCCCTGGTGGTCCAGTGGTTAGGACTCAGCGCTCTTACTGCAGGGGCCCCAGGTTCTATC
CCTGGTCAGGAAA
>Bos_taurus_chr11.trna3210-LysCTT (79759679-79759751) Lys (CTT) 73 bp Sc: 54.00
TTCCTGATGGTCCAGTGGTTAGGACTCTGCACTCTTAGTGCTGAGGGCCCAGGTTCAATG

CCTGATCAGGAAC
>Bos_taurus_chr19.trna717-LysCTT (17032717-17032789) Lys (CTT) 73 bp Sc: 57.10
CCCCTGGTGGTCCAGTGGTTAAGACCCTGCACTCTTAGTGCAGGGAGCCCAGGTTCAATT
CCTGGTCAGGGAA
>Bos_taurus_chr9.trna1429-LysCTT (44455627-44455699) Lys (CTT) 73 bp Sc: 61.22
TCTCTGGTGGTCCAGTGGTTAAGACTCCACACTCTTTATGTGGGGGGCCCAGGTTCGATC
CCTGGTCAGGGAA
>Bos_taurus_chr7.trna6796-LysCTT (39001391-39001319) Lys (CTT) 73 bp Sc: 61.51
TCCCTGGTGATCCAGTGGTTAGGACTCAGCACTCTTACTGCTGAGGGCCTAGGTTCAATC
CCTGGTTGGGGAA
>Bos_taurus_chr5.trna8375-LysCTT (51500668-51500596) Lys (CTT) 73 bp Sc: 62.60
GCCTGGCTGACTCAGTTGGTAGAGCATGAGACTCTTAATCTCAGGGTTGTGGGTTCGAGC
CCCACATTGGGTG
>Bos_taurus_chr10.trna4514-LysCTT (95902678-95902606) Lys (CTT) 73 bp Sc: 62.92
GCCCAGCTAGCTCAGTCGGTAGAGCATGGGACTCTTAATCCCAGGGCTGGGGGTTCAAGG
CCCACGTTGGGCA
>Bos_taurus_chr6.trna7711-LysCTT (30955503-30955431) Lys (CTT) 73 bp Sc: 63.21
TCCCTGGTGGTCCAGTGGCTAAGATTCTGCACTCTTAATGCAGGGGGCCCTGGTTCAATC
CCTGGTCAGGGAA
>Bos_taurus_chr1.trna3417-LysCTT (99516551-99516623) Lys (CTT) 73 bp Sc: 63.72
TCCCTAGTGGTCCAGTGGTTAGGAATCCACACTCTTATTGTGGACGACCCAGGTTCAATT
CCTGGCTGGGGAG
>Bos_taurus_chr25.trna122-LysCTT (3193600-3193672) Lys (CTT) 73 bp Sc: 74.14
GCCCGGCTAGCTCAGTCGGTAGAGCATGAGACTCTTAATCTCAGGGTCGTGGGTTCGAGC
TCCACGTTGGGCG
>Bos_taurus_chr4.trna6355-LysCTT (78120658-78120586) Lys (CTT) 73 bp Sc: 78.78
GCCCAGCTAGCTCAGTTGGTAGAGCATGGGACTCTTAATCCCAGGGTCGTGGGTTCGAGC
CCCACATTGGGCA
>Bos_taurus_chr23.trna3381-LysCTT (31566115-31566043) Lys (CTT) 73 bp Sc: 80.47
GCCCGGCTAGCTCAGTCGGTAGAGCATGAGACTCTTAATCTCAGGGTCGTGGGTTCGAGC
CCCACGTTGGGCG
>Bos_taurus_chr25.trna109-LysCTT (3166064-3166136) Lys (CTT) 73 bp Sc: 80.47
GCCCGGCTAGCTCAGTCGGTAGAGCATGAGACTCTTAATCTCAGGGTCGTGGGTTCGAGC
CCCACGTTGGGCG
>Bos_taurus_chr25.trna111-LysCTT (3179690-3179762) Lys (CTT) 73 bp Sc: 80.47
GCCCGGCTAGCTCAGTCGGTAGAGCATGAGACTCTTAATCTCAGGGTCGTGGGTTCGAGC
CCCACGTTGGGCG
>Bos_taurus_chr25.trna113-LysCTT (3184276-3184348) Lys (CTT) 73 bp Sc: 80.47
GCCCGGCTAGCTCAGTCGGTAGAGCATGAGACTCTTAATCTCAGGGTCGTGGGTTCGAGC
CCCACGTTGGGCG
>Bos_taurus_chr25.trna117-LysCTT (3189240-3189312) Lys (CTT) 73 bp Sc: 80.47
GCCCGGCTAGCTCAGTCGGTAGAGCATGAGACTCTTAATCTCAGGGTCGTGGGTTCGAGC
CCCACGTTGGGCG
>Bos_taurus_chr25.trna120-LysCTT (3191861-3191933) Lys (CTT) 73 bp Sc: 80.47
GCCCGGCTAGCTCAGTCGGTAGAGCATGAGACTCTTAATCTCAGGGTCGTGGGTTCGAGC
CCCACGTTGGGCG
>Bos_taurus_chr25.trna125-LysCTT (3198209-3198281) Lys (CTT) 73 bp Sc: 80.47
GCCCGGCTAGCTCAGTCGGTAGAGCATGAGACTCTTAATCTCAGGGTCGTGGGTTCGAGC
CCCACGTTGGGCG
>Bos_taurus_chr25.trna128-LysCTT (3203168-3203240) Lys (CTT) 73 bp Sc: 80.47
GCCCGGCTAGCTCAGTCGGTAGAGCATGAGACTCTTAATCTCAGGGTCGTGGGTTCGAGC
CCCACGTTGGGCG
>Bos_taurus_chr25.trna4871-LysCTT (3172987-3172915) Lys (CTT) 73 bp Sc: 80.47
GCCCGGCTAGCTCAGTCGGTAGAGCATGAGACTCTTAATCTCAGGGTCGTGGGTTCGAGC
CCCACGTTGGGCG
>Bos_taurus_chr25.trna4873-LysCTT (3167580-3167508) Lys (CTT) 73 bp Sc: 80.47
GCCCGGCTAGCTCAGTCGGTAGAGCATGAGACTCTTAATCTCAGGGTCGTGGGTTCGAGC
CCCACGTTGGGCG
>Bos_taurus_chr25.trna4875-LysCTT (3160539-3160467) Lys (CTT) 73 bp Sc: 80.47
GCCCGGCTAGCTCAGTCGGTAGAGCATGAGACTCTTAATCTCAGGGTCGTGGGTTCGAGC
CCCACGTTGGGCG
>Bos_taurus_chr25.trna4876-LysCTT (3155949-3155877) Lys (CTT) 73 bp Sc: 80.47
GCCCGGCTAGCTCAGTCGGTAGAGCATGAGACTCTTAATCTCAGGGTCGTGGGTTCGAGC
CCCACGTTGGGCG
>Bos_taurus_chr3.trna872-LysCTT (23049295-23049367) Lys (CTT) 73 bp Sc: 80.47
GCCCGGCTAGCTCAGTCGGTAGAGCATGAGACTCTTAATCTCAGGGTCGTGGGTTCGAGC
CCCACGTTGGGCG

>Bos_taurus_chr7.trna1885-LysCTT (39692005-39692077) Lys (CTT) 73 bp Sc: 80.47
GCCCGGCTAGCTCAGTCGGTAGAGCATGAGACTCTTAATCTCAGGGTCGTGGGTTCGAGC
CCCACGTTGGGCG
>Bos_taurus_chr7.trna6778-LysCTT (39704157-39704085) Lys (CTT) 73 bp Sc: 80.47
GCCCGGCTAGCTCAGTCGGTAGAGCATGAGACTCTTAATCTCAGGGTCGTGGGTTCGAGC
CCCACGTTGGGCG
>Bos_taurus_chr10.trna5511-LysCTT (72540246-72540174) Lys (CTT) 73 bp Sc: 80.72
GCCCGGCTAGCTCAGTCGGTAGAGCATGGGACTCTTAATCCCAGGGTCGTGGGTTCGAGC
CCCACGTTGGGCG
>Bos_taurus_chr21.trna4312-LysCTT (30332259-30332187) Lys (CTT) 73 bp Sc: 80.72
GCCCGGCTAGCTCAGTCGGTAGAGCATGGGACTCTTAATCCCAGGGTCGTGGGTTCGAGC
CCCACGTTGGGCG
>Bos_taurus_chr8.trna2505-LysCTT (75836991-75837072) Lys (CTT) 82 bp Sc: 43.82
TCCCTGGTGGTCTAGTGGGTAGGATTCAGTGCTCTTGGGTTGGAGAACTGCCGTGGCCTG
GGTTTGATTCCCAGTCAGGGAA
>Bos_taurus_chr28.trna1816-LysTTT (37299340-37299259) Lys (TTT) 82 bp Sc: 27.48
TTCTTGGTAGTCCAGTGTTTATGACTCTGTATTTTAGGGCTTCCAGGGTGTGGAGTGCA
GGTTTGATTCCTGACCAGGAAC
>Bos_taurus_chr24.trna3361-LysTTT (50847136-50847067) Lys (TTT) 70 bp Sc: 27.49
TCCCTGGTGGTCCAGTGGTTACGACTCGGTGCTTTTACTGCCAGATCAGGTTTGATGCCT
GGTTGGGGAA
>Bos_taurus_chr25.trna4970-LysTTT (629978-629907) Lys (TTT) 72 bp Sc: 33.60
TCCCTGATGGTCTAGTGGTAAGGACTTGGGGCTTTTTCACCGGGGCCCAGGTTCAGTCC
TCGGTTGGGGAA
>Bos_taurus_chr3.trna603-LysTTT (18039319-18039390) Lys (TTT) 72 bp Sc: 35.00
TCTCTGGTGGTCCAGGGGTTAGGACTTGGCGCTTTTACTACCAGGGCCTGGGTTTTATCC
CTGGTTGGAGAA
>Bos_taurus_chr23.trna3452-LysTTT (30264440-30264366) Lys (TTT) 75 bp Sc: 36.32
TTTTGGATAGCTCAGTCAGTAGAGCATCAGACTTTTAATCTGAGACTCCACAGGGTTCAA
ACCCCTGTTCAGGCT
>Bos_taurus_chr10.trna12-LysTTT (170386-170458) Lys (TTT) 73 bp Sc: 36.36
TCCATGGTGGTTCTCTGGTTAGAACTTGGCACTTTTTCTGCCGTGGGCCTGGGTTCAATC
CCTGGTTATGGAA
>Bos_taurus_chrUn.004.360.trna7-LysTTT (76484-76412) Lys (TTT) 73 bp Sc: 36.36
TCCATGGTGGTTCTCTGGTTAGAACTTGGCACTTTTTCTGCCGTGGGCCTGGGTTCAATC
CCTGGTTATGGAA
>Bos_taurus_chr16.trna4364-LysTTT (48969165-48969094) Lys (TTT) 72 bp Sc: 37.38
TCCCTGGTGGTCCAGTGGTTGGATTTGGTGCTTTTACTGCCAGGGCTCCAGCTTCCATCC
CTGGTTGGGGAA
>Bos_taurus_chrX.trna4253-LysTTT (73179421-73179349) Lys (TTT) 73 bp Sc: 39.16
TCCCTGGTGGTCCAGTTGTGAGGACTTGGAGCTTTTGCTGCCAAGGGCCTAGGATCAATC
CCTATTCAGGGAA
>Bos_taurus_chr3.trna8914-LysTTT (15234326-15234256) Lys (TTT) 71 bp Sc: 40.80
TCCCTGGTGGTCCAGTGGTTAGGACTTGGCGCTTTTACTGTCAGGACTGGGTTCAACACC
TGGTCAGGGAA
>Bos_taurus_chr19.trna386-LysTTT (11862409-11862479) Lys (TTT) 71 bp Sc: 41.55
TCCCTGGTGGTCCAGTAGTTATGACTGGGCACTTTTACTGCTGGGCCCAGGTTCAATCCC
TAGTCAGGGAA
>Bos_taurus_chr24.trna933-LysTTT (26352112-26352183) Lys (TTT) 72 bp Sc: 41.88
TCCCTGGTGGTCCAGTGGTTAGGACTCTGTGCATTTACTATCGGGACCCAAGTTCAATCC
TTGGTCAGGGAA
>Bos_taurus_chr21.trna883-LysTTT (21856849-21856921) Lys (TTT) 73 bp Sc: 43.21
TCCCTGGTGGTCCAGCAGTTAGGACTTGGTACTTTATTGCCCGGGGCCTAGGTTCAATT
CCTGGTGGGGGAA
>Bos_taurus_chr9.trna5798-LysTTT (63465467-63465397) Lys (TTT) 71 bp Sc: 43.93
TCCCTGGTGGTCCAGTGGTTAGACTTAGTGCTTTTATTAATGTGGCCCAGGTTCAATCCC
TGGTCAGGGAT
>Bos_taurus_chr16.trna5395-LysTTT (25870551-25870479) Lys (TTT) 73 bp Sc: 44.15
TCCCTGGTGGTCCAGTGACTAAGACTCTGCATTTTTAATGTAGGGGGCCCAGGTTCATTC
CCTGGTCAGGGAA
>Bos_taurus_chr25.trna2223-LysTTT (37246362-37246434) Lys (TTT) 73 bp Sc: 45.19
TCCCTGGTGGTCCAGTGGTTAGGACTTGCTGCTTTTACTGCTAAGAGCCCAGGTTCAATA
TCTGGTTGGGGAA
>Bos_taurus_chr17.trna2842-LysTTT (65231377-65231448) Lys (TTT) 72 bp Sc: 46.10
TCCCTGGCAGTCCAGTGGTTAGGACTCAGTGCTTTTGCTGCTGGAGCCCAGGTTCAGTCC
CTGGTCAGGGAA
>Bos_taurus_chr15.trna2265-LysTTT (63844496-63844567) Lys (TTT) 72 bp Sc: 46.16

TTCCTGATGGTCTAGTGGTTAGGATTTGGCGCTTTTACTTTCATGGCCCAGTTTCAATCC
CTGGTCAGGGAA
>Bos_taurus_chrUn.004.1778.trna2-LysTTT (29242-29313) Lys (TTT) 72 bp Sc: 46.16
TTCCTGATGGTCTAGTGGTTAGGATTTGGCGCTTTTACTTTCATGGCCCAGTTTCAATCC
CTGGTCAGGGAA
>Bos_taurus_chr3.trna5163-LysTTT (117850461-117850389) Lys (TTT) 73 bp Sc: 46.25
TCCCTGGTGGTTCAGTAGTTAGCACTCGGCACTTTTGCTGCTGCGGATCCAGGTTCAATC
CCTGGTTGGGGAA
>Bos_taurus_chr16.trna2300-LysTTT (58902772-58902843) Lys (TTT) 72 bp Sc: 46.47
TCCTTGGTGGTCCAGTGGTTAGGATTTGGTGCTTTTATTGCTGTGGTCCAGGTTCGGTCC
CTGGTTGGGGAA
>Bos_taurus_chr9.trna5554-LysTTT (69927898-69927827) Lys (TTT) 72 bp Sc: 47.08
TCCCAGGCGGTCCAGTGGTTAGGACTCCGTGCTTTTACTTTGGTGGCCCAGGTTCAATCT
CTGGTCTGGGAA
>Bos_taurus_chr16.trna902-LysTTT (26655742-26655813) Lys (TTT) 72 bp Sc: 47.93
TCCCTGGTGGTCCAATGGTTAGGGCTTGGTGCTTTTACTACCAGGATTCAGGTTCAAGCC
CTGGTCAGGGAA
>Bos_taurus_chr1.trna2803-LysTTT (82211317-82211389) Lys (TTT) 73 bp Sc: 48.37
GCCTGGGTAGCTCAGCTGGTGGAGCATCAGACTTTTAATCTGGGGATCTAGGGTTTGAGT
CCCTGTCCAGTTG
>Bos_taurus_chr19.trna2511-LysTTT (49786807-49786879) Lys (TTT) 73 bp Sc: 48.69
TTCCTGATGGTCCAGGGGTTAAGACTCTGCACTTTTACTGCAGGGGCCACAGGTTTGATC
CTTGGTCAGGGAA
>Bos_taurus_chr8.trna3009-LysTTT (87142220-87142292) Lys (TTT) 73 bp Sc: 48.88
GCTCTGGTGGTCCAGTTGTGAGGACTTAGCACTTTTACTGCCATGATCCCAGGTTTGATC
CCTGGTCAGAGAA
>Bos_taurus_chr21.trna1405-LysTTT (30553494-30553566) Lys (TTT) 73 bp Sc: 49.09
TCCCTGCTAGTCCAATAGGTAGGACTCAGTGCTTTTAGTGCTGAGGGCCCAGGTTCAATC
CCTGGTCAGGGAA
>Bos_taurus_chr17.trna3036-LysTTT (67966409-67966480) Lys (TTT) 72 bp Sc: 49.44
TCCCTGGTGGTCCAGTGCTTACGACGCGGCACTTTTACTGCTGAGGCCCGGATTCGATCC
CTGGTCGGGGAA
>Bos_taurus_chr15.trna259-LysTTT (11520586-11520658) Lys (TTT) 73 bp Sc: 49.70
TTCCTGGTGGTCCAGTGGTTAAGACTCAGTGCTTTTACTGCTGTGGGCGCAGGTTCAATC
CCTGGTCAGGGAA
>Bos_taurus_chr4.trna4980-LysTTT (109236757-109236685) Lys (TTT) 73 bp Sc: 50.50
TCCCTGGCTGTCCAGTGGTTAAGATGCTGCACTTTTACTGCAGGGGACCCAGGTTTGATC
CCTGGTCAGGGAA
>Bos_taurus_chr1.trna784-LysTTT (19119028-19119100) Lys (TTT) 73 bp Sc: 50.59
TCCCTGGTAGCCCAGTGGTTAGGACTCTGTGCTTTTACTGCTGTGGGCCCAGGTTTGATC
CCTGGTCAGGGAA
>Bos_taurus_chr20.trna5005-LysTTT (19267916-19267844) Lys (TTT) 73 bp Sc: 50.72
TCCTTGATGGTCCAGTGGTTAAGACTCTGCACTTTTAACGCAGAGGACTCAAGTTTGACT
CCTGGTCAGGGAA
>Bos_taurus_chr3.trna299-LysTTT (9308409-9308480) Lys (TTT) 72 bp Sc: 51.38
TCTCTGATGGTTCAGTGGTTAGGACTTGGTGCTTTTCCTGCTGTGGCCCAGGTTCGATCC
CTGGTCAGGGAA
>Bos_taurus_chr24.trna3524-LysTTT (47553625-47553553) Lys (TTT) 73 bp Sc: 52.09
TCCCTGGTGGTCCAGTGGTCAGGATTTGGCACTTTTACTGCCAGGGGCTCAGGCTGGATC
CCTGGCTGGGGAA
>Bos_taurus_chr17.trna4302-LysTTT (60038577-60038506) Lys (TTT) 72 bp Sc: 53.26
TCCCTGGCAGTCCAGTGGTTAGGACATAGCACTTTTACTGCTGTGGCCTGGGTTCAATCC
CTGGTTGGGGAA
>Bos_taurus_chr19.trna5648-LysTTT (23285674-23285602) Lys (TTT) 73 bp Sc: 53.37
TCCCTGATGGTCTAGTGGTCAAGACTCAGTACTTTTACTACTGAGGGCCTGGGTTCAATC
CCTGATCAGGGAA
>Bos_taurus_chr20.trna398-LysTTT (10818656-10818728) Lys (TTT) 73 bp Sc: 53.55
TCCTTAGTCGTCTAGAGGTTAGGACTCTGCACTTTTACTGCCGAGGACCCAGGTTCAATC
CCTGGTTGGGGAA
>Bos_taurus_chr6.trna6259-LysTTT (75246057-75245985) Lys (TTT) 73 bp Sc: 55.39
TCTCTGATAGTTCAGTGGTTAGAACCTTGTGCTTTTACTGCCAAGGACCCAGGTTCAATC
CCTGGTCAGGGAA
>Bos_taurus_chr24.trna588-LysTTT (18149387-18149457) Lys (TTT) 71 bp Sc: 55.48
TCCCTGGTGGTCCAGTGATTAGGACTTGCCACTTTTACTTCCAAGCCCAGGTTCAATCCC
TGGTCAGGGAA
>Bos_taurus_chr2.trna4199-LysTTT (123638279-123638351) Lys (TTT) 73 bp Sc: 55.59
TCCCTTGTGGTCCAGTGGTCAGGACTCGGCACTTTTACTGCTGTGGCCCTAGGTTCAATC

CCTGGTTGGGAAA
>Bos_taurus_chr22.trna2019-LysTTT (55807503-55807575) Lys (TTT) 73 bp Sc: 55.80
CCCTGGATAGCTCAGTCAGTAGAGCATCAGACTTTTAATCTGAGGGTCCAGGGTTCAAGT
CCTTGTTCTGGCA
>Bos_taurus_chr1.trna8431-LysTTT (84004369-84004297) Lys (TTT) 73 bp Sc: 56.12
GCCCAGGTAGCTCAGCTGGTAGAGCATCAGACTTTTACTCAGAGGGCCCAGGGTTCAAGT
CCCTGTCCAGGTG
>Bos_taurus_chr4.trna5201-LysTTT (105655989-105655917) Lys (TTT) 73 bp Sc: 56.22
TCCCTGGTGGTCCAGTGGTTAGGACTTCACACTTTTACAGTGAAGGCTCCAGGTTCAATC
TTTGGTCAGGGAA
>Bos_taurus_chr4.trna5726-LysTTT (94763110-94763039) Lys (TTT) 72 bp Sc: 56.53
TCCCTGGTGGTCTAGTGGTTAGGATCTGGTACTTTTACTGCTATGGCCCAAGTTCAATCC
CTGGTTAGGGAA
>Bos_taurus_chr9.trna6760-LysTTT (33216141-33216068) Lys (TTT) 74 bp Sc: 58.66
TCCCTGATAGTCCAGTGGTTAGGATTCGGTGCTTTTCACTGCCGAGGTCCTAGGTTCGAT
CCCTGGTTGGGGAA
>Bos_taurus_chr20.trna121-LysTTT (3195007-3195077) Lys (TTT) 71 bp Sc: 58.69
TCCCTGGGGGTCCAGTGGCAGGACTCAGCACTTTTACTGCTGACTCCCAGGTTCAATCCC
TGGTCAGGGAA
>Bos_taurus_chrUn.004.1838.trna3-LysTTT (6110-6040) Lys (TTT) 71 bp Sc: 58.69
TCCCTGGGGGTCCAGTGGCAGGACTCAGCACTTTTACTGCTGACTCCCAGGTTCAATCCC
TGGTCAGGGAA
>Bos_taurus_chr1.trna9095-LysTTT (62006367-62006295) Lys (TTT) 73 bp Sc: 59.09
GCCTGGATAGCTCATTTGGTAGAGCATCAGATTTTTAATCTGAGTGCCTAGGGTTCAAGT
CCCTGTTCAGGTG
>Bos_taurus_chr24.trna47-LysTTT (1560281-1560353) Lys (TTT) 73 bp Sc: 59.26
TCCCTGGGGGTCTAGTGGTTAGGACTCAGAGCTTTTACCACTGTGGCCCCAGGTTCAATA
CCTGGTCAGGGAA
>Bos_taurus_chr22.trna2221-LysTTT (61055572-61055644) Lys (TTT) 73 bp Sc: 59.78
GCCTGGGTAGCTCAGTCGGCAGAGCATCAGAGCTTTACTCTGAGGGTCCAGGGTTCGAGT
CACTGTCCAGGCA
>Bos_taurus_chr24.trna4933-LysTTT (13017037-13016965) Lys (TTT) 73 bp Sc: 60.10
GCCTGGATAGCTCAGTTGGTAGAGCATCAGACGTTTAATCTGAGGGGCCAGGGTTCATAA
CCCTGTTTGGGCA
>Bos_taurus_chr1.trna7078-LysTTT (127401386-127401314) Lys (TTT) 73 bp Sc: 60.45
GCCTAGGTAGCTCAGCTGGTAGAGCATCAGACTTTTAATCTGAGGGTCCAGGGTTCATGC
CCCTATCCAGGCA
>Bos_taurus_chr22.trna4154-LysTTT (9179873-9179801) Lys (TTT) 73 bp Sc: 61.63
GCCCAGATGGCTCAGTCAGTAGAGCATCAGACTTTTAATCTGAAGGTCCAAGGTTCAAGT
CCCTGTTTGAGCC
>Bos_taurus_chr21.trna3365-LysTTT (58224930-58224858) Lys (TTT) 73 bp Sc: 62.10
TCCTTCATAGTCCAGTGGTTAGGACTCGGCACTTTTACTGCAGGGAATCCAGGTTCAATC
CCTGGTGGGGGAA
>Bos_taurus_chr5.trna674-LysTTT (21757226-21757298) Lys (TTT) 73 bp Sc: 63.60
GCCCAGATAGCTCAGTCAGTAGAGCATCAGACTTTTAATCTGAGGGTCCAGGGTTCAAGT
CCCTGTTCGGACC
>Bos_taurus_chr19.trna6072-LysTTT (16833841-16833770) Lys (TTT) 72 bp Sc: 65.38
TCCCTGATGGTCCAGTGGTTAGGACTTGGCACTTTTACTGCCAGGGCCCAGGTTTGATCC
CTGGTTGAGGAA
>Bos_taurus_chr13.trna4090-LysTTT (77396588-77396516) Lys (TTT) 73 bp Sc: 67.22
GTCTGGGTAGCTCAGCTGGTATAGCATCAGACTTTTAATCTGAAGGCCCAGGGTTCAAGT
CCCTGTCCAGGCA
>Bos_taurus_chr10.trna4528-LysTTT (95699340-95699268) Lys (TTT) 73 bp Sc: 67.23
GCCTGGGTAGCTCTGTTGGTAGAGCATCAGACTTTTAATCTGAGGGTCCAGGGTTCATGT
CCCTGTCCAGGCG
>Bos_taurus_chr16.trna6004-LysTTT (8169122-8169050) Lys (TTT) 73 bp Sc: 68.73
ACCTAGATAGCTCAGTCAGTAGAGCATCAGACTTTTAATCTGAGGGTCCAGGGTTCAAAT
CCCTGTTTGGGCA
>Bos_taurus_chr19.trna1258-LysTTT (25496372-25496444) Lys (TTT) 73 bp Sc: 70.21
GGCTAAGTAGCTCAGTTGGTAGAGCATCAGACTTTTAATCTGAGGGTCCAGGGTTCAAGT
CCCTGTTCAGGCA
>Bos_taurus_chr5.trna6666-LysTTT (93765542-93765470) Lys (TTT) 73 bp Sc: 71.27
GCCTGAATAGCTCAACTGGTAGAGCATCAGACTTTTAACCTGAGGATCCAGGGTTCAAGT
CCCTGTTCAGGCA
>Bos_taurus_chr12.trna5294-LysTTT (35193122-35193050) Lys (TTT) 73 bp Sc: 75.98
GCCTGGGTAGCTCAGCTGGTAGAGCATCAGACTTTTAATCTGAGGGCCCAGGGTTCAAGT
CCCTGTCCAGGCA

>Bos_taurus_chrX.trna4586-LysTTT (64739035-64738963) Lys (TTT) 73 bp Sc: 75.98
GCCTGGGTAGCTCAGCTGGTAGAGCATCAGACTTTTAATCTGAGGGCCCAGGGTTCAAGT
CCCTGTCCAGGCA
>Bos_taurus_chr23.trna3451-LysTTT (30264526-30264454) Lys (TTT) 73 bp Sc: 76.04
GCCTGGATAGCTCAGTTAGTAGAGCATCAGACTTTTAATCTGAGGGTCCAGGGTTCAAGT
CCCTGTTCAGGCG
>Bos_taurus_chr23.trna1416-LysTTT (31569959-31570031) Lys (TTT) 73 bp Sc: 76.37
CCCCGGATAGCTCAGTCGGTAGAGCATCAGACTTTTAATCTGAGGGTCCAGGGTTCAAGT
CCCTGTTCGGGCG
>Bos_taurus_chr7.trna7650-LysTTT (17245640-17245568) Lys (TTT) 73 bp Sc: 78.41
GCCTGGATAGCTCAGTAGGTAGAGTATCAGACTTTTAATCTGAGGGTCCAGGGTTCAAGT
CCCTGTTCAGGCA
>Bos_taurus_chrUn.004.1148.trna3-LysTTT (24597-24525) Lys (TTT) 73 bp Sc: 78.41
GCCTGGATAGCTCAGTAGGTAGAGTATCAGACTTTTAATCTGAGGGTCCAGGGTTCAAGT
CCCTGTTCAGGCA
>Bos_taurus_chr3.trna1368-LysTTT (35349247-35349319) Lys (TTT) 73 bp Sc: 78.43
GCCTGGGTAGCTCAGTTGGTAGAGCATCAGACTTTTAATCTGAGGGCCCAGGGTTCAAGT
CCCTGTCCAGGCA
>Bos_taurus_chr1.trna3921-LysTTT (115209171-115209243) Lys (TTT) 73 bp Sc: 79.55
GCCTGGGTAGCTCAGTTGGTAGAGCATCAGACTTTTAATCTGAGTGTCCAGGGTTCAAAT
CCCTGTCCAGGCA
>Bos_taurus_chr17.trna588-LysTTT (15542329-15542401) Lys (TTT) 73 bp Sc: 80.16
GCCTGGATAGCTTAGTTGGTAGAGCATCAGACTTTTAATCTGAGGGCCCAGGGTTCAAGT
CCCTGTCCAGGCA
>Bos_taurus_chr23.trna1384-LysTTT (31281285-31281357) Lys (TTT) 73 bp Sc: 81.41
GCCTGGGTAGCTCAGTCGGTAGAGCATCAGACTTTTAATCTGAGGGTCCAGGGTTCAAGT
CCCTGTCCAGGCG
>Bos_taurus_chr23.trna1374-LysTTT (31151470-31151542) Lys (TTT) 73 bp Sc: 83.31
GCCTGGATAGCTCAGTCGGTAGAGCATCAGACTTTTAATCTGAGGGTCCAGGGTTCAAGT
CCCTGTTCAGGCG
>Bos_taurus_chr23.trna3410-LysTTT (31154821-31154749) Lys (TTT) 73 bp Sc: 83.31
GCCTGGATAGCTCAGTCGGTAGAGCATCAGACTTTTAATCTGAGGGTCCAGGGTTCAAGT
CCCTGTTCAGGCG
>Bos_taurus_chr15.trna2993-LysTTT (83651194-83651266) Lys (TTT) 73 bp Sc: 83.80
GCCCGGATAGCTCAGTCGGTAGAGCATCAGACTTTTAATCTGAGGGTCCAGGGTTCAAGT
CCCTGTTCGGGCG
>Bos_taurus_chr15.trna3084-LysTTT (83653008-83652936) Lys (TTT) 73 bp Sc: 83.80
GCCCGGATAGCTCAGTCGGTAGAGCATCAGACTTTTAATCTGAGGGTCCAGGGTTCAAGT
CCCTGTTCGGGCG
>Bos_taurus_chr16.trna41-LysTTT (1247867-1247939) Lys (TTT) 73 bp Sc: 83.80
GCCCGGATAGCTCAGTCGGTAGAGCATCAGACTTTTAATCTGAGGGTCCAGGGTTCAAGT
CCCTGTTCGGGCG
>Bos_taurus_chr16.trna6187-LysTTT (1248273-1248201) Lys (TTT) 73 bp Sc: 83.80
GCCCGGATAGCTCAGTCGGTAGAGCATCAGACTTTTAATCTGAGGGTCCAGGGTTCAAGT
CCCTGTTCGGGCG
>Bos_taurus_chr19.trna1406-LysTTT (28304842-28304914) Lys (TTT) 73 bp Sc: 83.80
GCCCGGATAGCTCAGTCGGTAGAGCATCAGACTTTTAATCTGAGGGTCCAGGGTTCAAGT
CCCTGTTCGGGCG
>Bos_taurus_chr23.trna1330-LysTTT (30220122-30220194) Lys (TTT) 73 bp Sc: 83.80
GCCCGGATAGCTCAGTCGGTAGAGCATCAGACTTTTAATCTGAGGGTCCAGGGTTCAAGT
CCCTGTTCGGGCG
>Bos_taurus_chr23.trna1370-LysTTT (31116328-31116400) Lys (TTT) 73 bp Sc: 83.80
GCCCGGATAGCTCAGTCGGTAGAGCATCAGACTTTTAATCTGAGGGTCCAGGGTTCAAGT
CCCTGTTCGGGCG
>Bos_taurus_chr23.trna3466-LysTTT (30083683-30083611) Lys (TTT) 73 bp Sc: 83.80
GCCCGGATAGCTCAGTCGGTAGAGCATCAGACTTTTAATCTGAGGGTCCAGGGTTCAAGT
CCCTGTTCGGGCG
>Bos_taurus_chr4.trna7336-MetCAT (48964256-48964185) Met (CAT) 72 bp Sc: 51.57
GGCAGAGTAGTGCAACAGGAGCATGCTGGGCTCATAACCCAGAGGTCAATGGATCAAAAC
CATCCTCTGCTA
>Bos_taurus_chr11.trna829-MetCAT (19588491-19588563) Met (CAT) 73 bp Sc: 55.96
TCCCTGGTGGTCCAGTGGCTAAGACTCTGCACTCATAACGCAGGGGGCCCCGGTTCGAGC
CCTGGTCAGGGAA
>Bos_taurus_chr25.trna1779-MetCAT (30215167-30215239) Met (CAT) 73 bp Sc: 56.05
GCTCTCTTAGTGCAGTAGGCAGCATGTCAGTCTCATAATCTGAAGGTCCTGAGTTTGAAC
CTCAGACAGGGCA
>Bos_taurus_chr13.trna2652-MetCAT (65277880-65277952) Met (CAT) 73 bp Sc: 57.37

TCCCTGGTGGTCCAGTGGCTAAGATTCTGCATTCATAATGCAGGGAGCCCCGGTTCGAGC
CCTGGTCAGGGAA
>Bos_taurus_chr9.trna6228-MetCAT (48410609-48410537) Met (CAT) 73 bp Sc: 57.87
GCCCTCTTAGTGCAGTAGGCAGCACGTCAGTCTCATAATCTGAAGGTGCTGAGTTCGAAC
CTCAGGAAGGGCA
>Bos_taurus_chr7.trna1184-MetCAT (19308136-19308208) Met (CAT) 73 bp Sc: 58.69
TCTCTGGTGGTCCAGTGGCTAAGACTCTGCACTCATAATGCAGGGGTCCTGGGTTCAATC
CCTGGTCAGGGAA
>Bos_taurus_chr5.trna4826-MetCAT (119785917-119785989) Met (CAT) 73 bp Sc: 63.43
TCCTTGGTGGTTCAGTGGTTAAGACTCTGAACTCATAATGCAGGGGGCCCAGGTTCGATC
CCTGGTCAGGGAA
>Bos_taurus_chr23.trna1408-MetCAT (31431819-31431891) Met (CAT) 73 bp Sc: 65.90
GCCCTCTTAGCGCAGTAGGCAGCGCGTCAGTCTCATAATCTGAAGGTCCTGAGTTCAAGC
CTCAGAGAGAGCA
>Bos_taurus_chr18.trna3550-MetCAT (54519406-54519335) Met (CAT) 72 bp Sc: 66.85
AGCAGAGTGGCGCAGCGGAAGCGTGCTGGGCCCATAACCCAGAGGTCAATGGATCGAAAC
CATTCTCTGCTA
>Bos_taurus_chr19.trna2627-MetCAT (51584661-51584732) Met (CAT) 72 bp Sc: 67.60
AGCAGAGTGGCGCAGCGGGAGCGTGCTGGGCCCATAACCCAGAGGTCGATGGATCGAAAC
CATCCTCTGCTA
>Bos_taurus_chr23.trna1373-MetCAT (31149585-31149656) Met (CAT) 72 bp Sc: 67.60
AGCAGAGTGGCGCAGCGGGAGCGTGCTGGGCCCATAACCCAGAGGTCGATGGATCGAAAC
CATCCTCTGCTA
>Bos_taurus_chr23.trna1395-MetCAT (31325452-31325523) Met (CAT) 72 bp Sc: 67.60
AGCAGAGTGGCGCAGCGGGAGCGTGCTGGGCCCATAACCCAGAGGTCGATGGATCGAAAC
CATCCTCTGCTA
>Bos_taurus_chr23.trna1421-MetCAT (31692755-31692826) Met (CAT) 72 bp Sc: 67.60
AGCAGAGTGGCGCAGCGGGAGCGTGCTGGGCCCATAACCCAGAGGTCGATGGATCGAAAC
CATCCTCTGCTA
>Bos_taurus_chrUn.004.1183.trna9-MetCAT (8892-8821) Met (CAT) 72 bp Sc: 67.60
AGCAGAGTGGCGCAGCGGGAGCGTGCTGGGCCCATAACCCAGAGGTCGATGGATCGAAAC
CATCCTCTGCTA
>Bos_taurus_chr23.trna1360-MetCAT (31005735-31005806) Met (CAT) 72 bp Sc: 68.98
AGCAGAGTGGCGCAGCGGAAGCGTGCTGGGCCCATAACCCAGAGGTCGATGGATCGAAAC
CATCCTCTGCTA
>Bos_taurus_chr23.trna1385-MetCAT (31285490-31285561) Met (CAT) 72 bp Sc: 68.98
AGCAGAGTGGCGCAGCGGAAGCGTGCTGGGCCCATAACCCAGAGGTCGATGGATCGAAAC
CATCCTCTGCTA
>Bos_taurus_chr23.trna1424-MetCAT (31703698-31703769) Met (CAT) 72 bp Sc: 68.98
AGCAGAGTGGCGCAGCGGAAGCGTGCTGGGCCCATAACCCAGAGGTCGATGGATCGAAAC
CATCCTCTGCTA
>Bos_taurus_chr23.trna3367-MetCAT (31712364-31712293) Met (CAT) 72 bp Sc: 68.98
AGCAGAGTGGCGCAGCGGAAGCGTGCTGGGCCCATAACCCAGAGGTCGATGGATCGAAAC
CATCCTCTGCTA
>Bos_taurus_chr23.trna3420-MetCAT (31081217-31081146) Met (CAT) 72 bp Sc: 68.98
AGCAGAGTGGCGCAGCGGAAGCGTGCTGGGCCCATAACCCAGAGGTCGATGGATCGAAAC
CATCCTCTGCTA
>Bos_taurus_chr3.trna8775-MetCAT (18109806-18109735) Met (CAT) 72 bp Sc: 68.98
AGCAGAGTGGCGCAGCGGAAGCGTGCTGGGCCCATAACCCAGAGGTCGATGGATCGAAAC
CATCCTCTGCTA
>Bos_taurus_chr7.trna8317-MetCAT (8712558-8712487) Met (CAT) 72 bp Sc: 68.98
AGCAGAGTGGCGCAGCGGAAGCGTGCTGGGCCCATAACCCAGAGGTCGATGGATCGAAAC
CATCCTCTGCTA
>Bos_taurus_chrUn.004.2552.trna6-MetCAT (9791-9720) Met (CAT) 72 bp Sc: 68.98
AGCAGAGTGGCGCAGCGGAAGCGTGCTGGGCCCATAACCCAGAGGTCGATGGATCGAAAC
CATCCTCTGCTA
>Bos_taurus_chr14.trna5863-MetCAT (18045367-18045295) Met (CAT) 73 bp Sc: 73.13
GCCTCCTTAGCGCAGTAGGCAGCGCATCAGTCTCATAATCTGAAGGTCCTGAGTTCAAAC
CTCAGAGGGGGCA
>Bos_taurus_chr23.trna1412-MetCAT (31444889-31444961) Met (CAT) 73 bp Sc: 73.48
GCCTTCTTAGCGCAGTAGGCAGCGCGTCAGTCTCATAATCTGAAGGTCCTGAGTTCGAGC
CTCAGAGAGGGCA
>Bos_taurus_chr23.trna1410-MetCAT (31440173-31440245) Met (CAT) 73 bp Sc: 74.70
GCCCTCTTAGCGCAGTAGGCAGCGCGTCAGTCTCATAATCTGAAGGTCCTGAGTTCGAGC
CTCAGAGAGGGCA
>Bos_taurus_chr23.trna3448-MetCAT (30349810-30349738) Met (CAT) 73 bp Sc: 75.87
GCCTTCTTAGCGCAGTAGGCAGCGCGTCAGTCTCATAATCTGAAGGTCCTGAGTTCGAAC

CTCAGAGAAGGCA
>Bos_taurus_chr23.trna1317-MetCAT (30080601-30080673) Met (CAT) 73 bp Sc: 76.58
GCCTCCTTAGCGCAGTAGGCAGCGCGTCAGTCTCATAATCTGAAGGTCCTGAGTTCGAAC
CTCAGAGGGGGCA
>Bos_taurus_chr23.trna3421-MetCAT (31067831-31067759) Met (CAT) 73 bp Sc: 76.58
GCCTCCTTAGCGCAGTAGGCAGCGCGTCAGTCTCATAATCTGAAGGTCCTGAGTTCGAAC
CTCAGAGGGGGCA
>Bos_taurus_chr23.trna3465-MetCAT (30085934-30085862) Met (CAT) 73 bp Sc: 76.58
GCCTCCTTAGCGCAGTAGGCAGCGCGTCAGTCTCATAATCTGAAGGTCCTGAGTTCGAAC
CTCAGAGGGGGCA
>Bos_taurus_chr23.trna1405-MetCAT (31427810-31427882) Met (CAT) 73 bp Sc: 76.72
GCCCTCTTAGCGCAGCTGGCAGCGCGTCAGTCTCATAATCTGAAGGTCCTGAGTTCAAAC
CTCAGAGAGGGCA
>Bos_taurus_chr18.trna4500-MetCAT (38743183-38743111) Met (CAT) 73 bp Sc: 76.99
GCCTTCTTAGCGCAGTGGGCAGCGCGTCAGTCTCATAATCTGAAGGTCCTGAGTTCGAGC
CTCAGAGAGGGCA
>Bos_taurus_chr23.trna3387-MetCAT (31448924-31448852) Met (CAT) 73 bp Sc: 76.99
GCCTTCTTAGCGCAGTGGGCAGCGCGTCAGTCTCATAATCTGAAGGTCCTGAGTTCGAGC
CTCAGAGAGGGCA
>Bos_taurus_chr14.trna721-MetCAT (16175577-16175649) Met (CAT) 73 bp Sc: 79.85
GCCTCGTTAGCGCAGTAGGTAGCGCGTCAGTCTCATAATCTGAAGGTCGTGAGTTCGATC
CTCACACGGGGCA
>Bos_taurus_chr15.trna1867-PheAAA (53803603-53803674) Phe (AAA) 72 bp Sc: 26.52
TTCCCTAGAGCTCAGTTGGTAAGAATCTGCCTAAAAAGCAGGAGTCCTGGGTTCAATCCT
TAGGTTAGGAAG
>Bos_taurus_chr8.trna6024-PheAAA (69381715-69381644) Phe (AAA) 72 bp Sc: 26.52
TTCCCTAGAGCTCAGTTGGTAAGAATCTGCCTAAAAAGCAGGAGTCCTGGGTTCAATCCT
TAGGTTAGGAAG
>Bos_taurus_chr27.trna3300-PheAAA (19061132-19061060) Phe (AAA) 73 bp Sc: 55.51
TTCCTGGTGGCTCAGATGGTAAAGCATCTGCTTAAAATGCAGGAGACCCAGGTTCGATCC
CTGGGTCGGGAAG
>Bos_taurus_chr5.trna8264-PheAAA (53577304-53577233) Phe (AAA) 72 bp Sc: 56.04
TCCCTGGTAGCTCAGACGGTAAAGCGTCTGCCTAAAACGCGGGAGATCCAGGTTCGATCC
CTGGGTAGGGAA
>Bos_taurus_chr20.trna1872-PheAAA (51431398-51431470) Phe (AAA) 73 bp Sc: 57.09
GCTGAAATAACTCAACTGGGAGAGTGTTAGACTAAAGATCTAAAGGCCCCTGGTTCAATT
CCATGTTTCAGTG
>Bos_taurus_chr1.trna1707-PheAAA (48771802-48771873) Phe (AAA) 72 bp Sc: 58.43
TCCCTGGTGGCTCAGACGGTAAAGCGTCTGTCTAAAATGCAGGAGACCCAGGTTCAATCC
CTGGGTAGGGAA
>Bos_taurus_chr17.trna952-PheAAA (22985585-22985656) Phe (AAA) 72 bp Sc: 58.97
TCCCTGGTGGCTCAGATGGTAAAGCGTCTGCCTAAAATGCAGGAGACCCAGGTTCAATCC
CTGGGTTGGGAA
>Bos_taurus_chrX.trna5155-PheGAA (48892168-48892097) Phe (GAA) 72 bp Sc: 45.24
TCCCTGGTGGCTCAGATGGTAAAGCATCTGCCTGAAATGTGGAAGATCCAGGTTTGATCC
CTGGGTGGGGAA
>Bos_taurus_chr13.trna6212-PheGAA (31647846-31647774) Phe (GAA) 73 bp Sc: 50.38
GCTGAAATAGCTAAGTTGGGAGAGTGTTAGAGTGAAGATCTGAAGGTCCCCAGTTTGATC
CTGGGTTTTGGCA
>Bos_taurus_chr8.trna7653-PheGAA (14971033-14970962) Phe (GAA) 72 bp Sc: 52.10
TCCCTGGTGGCTCAGACAGTAAAGCGTCCACCTGAAATGCGGGAGACCCAGGTTCGATTC
CTGGGTAGGGAA
>Bos_taurus_chrX.trna2914-PheGAA (77452896-77452967) Phe (GAA) 72 bp Sc: 55.69
GCTGAATAGCTCAGTTGGGAGAGCGTTAGATTGAAGATCTAAAGGTCCCTGGTTTGATCC
CAGGTTCCAGAA
>Bos_taurus_chr12.trna3695-PheGAA (77594170-77594099) Phe (GAA) 72 bp Sc: 56.86
TCCCTAGTGGCTCAGATGGTAAAGTGTCTGCCTGAAATGCAGGAGACTCAGGTTCGATTC
CTGGGTTGGGAA
>Bos_taurus_chr6.trna8237-PheGAA (15774337-15774266) Phe (GAA) 72 bp Sc: 57.28
TCCCTGGTGGCTCAGACGGTAAAGCATCTGCCTGAAATGCAGGAGACCCAGGTTCAATTC
CTGGGTCGGGAA
>Bos_taurus_chrX.trna2917-PheGAA (77455069-77455143) Phe (GAA) 75 bp Sc: 58.36
TCCAGAATAGCTCAGTTGGCAGAGCGTTTAGACTGAAAAATCTAAAGGTCCCTGGTTCGA
TCCCGGGTTCCGGCA
>Bos_taurus_chr13.trna548-PheGAA (15861874-15861946) Phe (GAA) 73 bp Sc: 61.89
GCTGAAATAGCTCTGTTGGGAGAGTGTTAGACTGAAGATCTAAAGGTCCCTGGTTCAGTC
CCAGGTTTCGGCA

>Bos_taurus_chrX.trna2908-PheGAA (77444725-77444797) Phe (GAA) 73 bp Sc: 61.95
GCTGGAAAAGCTCAGTTGGGAGAGCATTAGCCTGAAGATCTAAAGGTCCCTGGTTCGATC
CCGGGTTCTGGCA
>Bos_taurus_chrX.trna2913-PheGAA (77447835-77447907) Phe (GAA) 73 bp Sc: 62.94
TCCGGAATAGCTCAGTTAGCAGAGCGTTAGACTGAAAATCTAAAGGTTCCCGGTTCAATT
CTGGGTTCCGGTG
>Bos_taurus_chr15.trna3123-PheGAA (82740404-82740332) Phe (GAA) 73 bp Sc: 64.56
GTTGAAATAGCTCAGTTGGGAGAGCGTTAGACTGAAGATCTAAAGGTCCCTGGTTCAGTC
CCAGGTTTCGGCA
>Bos_taurus_chr23.trna1343-PheGAA (30367210-30367282) Phe (GAA) 73 bp Sc: 66.66
GCTGAAATAGCTCAGTTGAGAGAGCATTAGACTGAAGATCTAAAGGTCCCTGGTTCAATC
CCAGGTTTTGGCA
>Bos_taurus_chr18.trna953-PheGAA (23381564-23381636) Phe (GAA) 73 bp Sc: 67.01
GCTGAAATAGCTCAATTGGGAGAGCATTAGTCTGAAGATCTAAAGGTCCCTGGTTCAATC
CCAGGTTTTGGCA
>Bos_taurus_chr23.trna2454-PheGAA (50943395-50943323) Phe (GAA) 73 bp Sc: 68.97
GCCGGAATAGCTCAGCTGGGAGAGCGTTACACTGAAGATCTAAAGGTCCCTGGTTCAACC
CCGGGTTTCGGCA
>Bos_taurus_chr15.trna3083-PheGAA (83657602-83657530) Phe (GAA) 73 bp Sc: 82.45
GCCGAAATAGCTCAGTTGGGAGAGCGTTAGACTGAAGATCTAAAGGTCCCTGGTTCAATC
CCGGGTTTCGGCA
>Bos_taurus_chr15.trna3085-PheGAA (83652181-83652109) Phe (GAA) 73 bp Sc: 82.45
GCCGAAATAGCTCAGTTGGGAGAGCGTTAGACTGAAGATCTAAAGGTCCCTGGTTCAATC
CCGGGTTTCGGCA
>Bos_taurus_chr23.trna1327-PheGAA (30215227-30215299) Phe (GAA) 73 bp Sc: 82.45
GCCGAAATAGCTCAGTTGGGAGAGCGTTAGACTGAAGATCTAAAGGTCCCTGGTTCAATC
CCGGGTTTCGGCA
>Bos_taurus_chr23.trna3414-PheGAA (31118865-31118793) Phe (GAA) 73 bp Sc: 82.45
GCCGAAATAGCTCAGTTGGGAGAGCGTTAGACTGAAGATCTAAAGGTCCCTGGTTCAATC
CCGGGTTTCGGCA
>Bos_taurus_chr23.trna3459-PheGAA (30205211-30205139) Phe (GAA) 73 bp Sc: 82.45
GCCGAAATAGCTCAGTTGGGAGAGCGTTAGACTGAAGATCTAAAGGTCCCTGGTTCAATC
CCGGGTTTCGGCA
>Bos_taurus_chr29.trna1429-PheGAA (38941234-38941306) Phe (GAA) 73 bp Sc: 82.45
GCCGAAATAGCTCAGTTGGGAGAGCGTTAGACTGAAGATCTAAAGGTCCCTGGTTCAATC
CCGGGTTTCGGCA
>Bos_taurus_chrX.trna2911-PheGAA (77446113-77446185) Phe (GAA) 73 bp Sc: 82.68
GCCGGAATAGCTCAGTTGGGAGAGCGTTAGACTGAAGTTCTAAAGGTCCCTGGTTCGATC
CCGGGTTCCGGCA
>Bos_taurus_chrX.trna2912-PheGAA (77447356-77447428) Phe (GAA) 73 bp Sc: 82.68
GCCGGAATAGCTCAGTTGGGAGAGCGTTAGACTGAAGTTCTAAAGGTCCCTGGTTCGATC
CCGGGTTCCGGCA
>Bos_taurus_chrX.trna2915-PheGAA (77454098-77454170) Phe (GAA) 73 bp Sc: 82.68
GCCGGAATAGCTCAGTTGGGAGAGCGTTAGACTGAAGTTCTAAAGGTCCCTGGTTCGATC
CCGGGTTCCGGCA
>Bos_taurus_chr12.trna4251-PheGAA (68043790-68043718) Phe (GAA) 73 bp Sc: 84.19
GCCGAAATAGCTCAGTTGGGAGAGCGTTAGACTGAAGATCTAAAGGTCCCTGGTTCGATC
CCGGGTTTCGGCA
>Bos_taurus_chr12.trna4260-PheGAA (67940872-67940800) Phe (GAA) 73 bp Sc: 84.19
GCCGAAATAGCTCAGTTGGGAGAGCGTTAGACTGAAGATCTAAAGGTCCCTGGTTCGATC
CCGGGTTTCGGCA
>Bos_taurus_chr17.trna2066-PheGAA (53707009-53707081) Phe (GAA) 73 bp Sc: 84.19
GCCGAAATAGCTCAGTTGGGAGAGCGTTAGACTGAAGATCTAAAGGTCCCTGGTTCGATC
CCGGGTTTCGGCA
>Bos_taurus_chr23.trna1316-PheGAA (30069308-30069380) Phe (GAA) 73 bp Sc: 84.19
GCCGAAATAGCTCAGTTGGGAGAGCGTTAGACTGAAGATCTAAAGGTCCCTGGTTCGATC
CCGGGTTTCGGCA
>Bos_taurus_chr7.trna6672-PheGAA (42830961-42830889) Phe (GAA) 73 bp Sc: 84.19
GCCGAAATAGCTCAGTTGGGAGAGCGTTAGACTGAAGATCTAAAGGTCCCTGGTTCGATC
CCGGGTTTCGGCA
>Bos_taurus_chr3.trna5950-ProAGG (98222467-98222396) Pro (AGG) 72 bp Sc: 56.30
GGCTTGTTGGTCTAGGGGTATGATTCTCGCTTAGGGTGAGAGAGGTCCTGGCTTCAAATC
CCAGACGAGCCC
>Bos_taurus_chr10.trna1102-ProAGG (25884544-25884615) Pro (AGG) 72 bp Sc: 75.92
GGCTCGTTGGTCTAGGGGTATGATTCTCGCTTAGGGTGCGAGAGGTCCCGGGTTCAAATC
CCGGACGAGCCC
>Bos_taurus_chr10.trna1103-ProAGG (25887284-25887355) Pro (AGG) 72 bp Sc: 75.92

GGCTCGTTGGTCTAGGGGTATGATTCTCGCTTAGGGTGCGAGAGGTCCCGGGTTCAAATC
CCGGACGAGCCC
>Bos_taurus_chr15.trna1917-ProAGG (55208951-55209022) Pro (AGG) 72 bp Sc: 75.92
GGCTCGTTGGTCTAGGGGTATGATTCTCGCTTAGGGTGCGAGAGGTCCCGGGTTCAAATC
CCGGACGAGCCC
>Bos_taurus_chr23.trna3380-ProAGG (31567434-31567363) Pro (AGG) 72 bp Sc: 75.92
GGCTCGTTGGTCTAGGGGTATGATTCTCGCTTAGGGTGCGAGAGGTCCCGGGTTCAAATC
CCGGACGAGCCC
>Bos_taurus_chr25.trna108-ProAGG (3159859-3159930) Pro (AGG) 72 bp Sc: 75.92
GGCTCGTTGGTCTAGGGGTATGATTCTCGCTTAGGGTGCGAGAGGTCCCGGGTTCAAATC
CCGGACGAGCCC
>Bos_taurus_chr25.trna116-ProAGG (3188741-3188812) Pro (AGG) 72 bp Sc: 75.92
GGCTCGTTGGTCTAGGGGTATGATTCTCGCTTAGGGTGCGAGAGGTCCCGGGTTCAAATC
CCGGACGAGCCC
>Bos_taurus_chr25.trna119-ProAGG (3191058-3191129) Pro (AGG) 72 bp Sc: 75.92
GGCTCGTTGGTCTAGGGGTATGATTCTCGCTTAGGGTGCGAGAGGTCCCGGGTTCAAATC
CCGGACGAGCCC
>Bos_taurus_chr25.trna124-ProAGG (3195832-3195903) Pro (AGG) 72 bp Sc: 75.92
GGCTCGTTGGTCTAGGGGTATGATTCTCGCTTAGGGTGCGAGAGGTCCCGGGTTCAAATC
CCGGACGAGCCC
>Bos_taurus_chr25.trna126-ProAGG (3198825-3198896) Pro (AGG) 72 bp Sc: 75.92
GGCTCGTTGGTCTAGGGGTATGATTCTCGCTTAGGGTGCGAGAGGTCCCGGGTTCAAATC
CCGGACGAGCCC
>Bos_taurus_chr25.trna4874-ProAGG (3163847-3163776) Pro (AGG) 72 bp Sc: 75.92
GGCTCGTTGGTCTAGGGGTATGATTCTCGCTTAGGGTGCGAGAGGTCCCGGGTTCAAATC
CCGGACGAGCCC
>Bos_taurus_chr4.trna3145-ProAGG (95994627-95994698) Pro (AGG) 72 bp Sc: 75.92
GGCTCGTTGGTCTAGGGGTATGATTCTCGCTTAGGGTGCGAGAGGTCCCGGGTTCAAATC
CCGGACGAGCCC
>Bos_taurus_chr19.trna5353-ProCGG (28391517-28391446) Pro (CGG) 72 bp Sc: 76.52
GGCTCGTTGGTCTAGGGGTATGATTCTCGCTTCGGGTGCGAGAGGTCCCGGGTTCAAATC
CCGGACGAGCCC
>Bos_taurus_chr23.trna3392-ProCGG (31393477-31393406) Pro (CGG) 72 bp Sc: 76.52
GGCTCGTTGGTCTAGGGGTATGATTCTCGCTTCGGGTGCGAGAGGTCCCGGGTTCAAATC
CCGGACGAGCCC
>Bos_taurus_chr25.trna112-ProCGG (3180959-3181030) Pro (CGG) 72 bp Sc: 76.52
GGCTCGTTGGTCTAGGGGTATGATTCTCGCTTCGGGTGCGAGAGGTCCCGGGTTCAAATC
CCGGACGAGCCC
>Bos_taurus_chr25.trna4872-ProCGG (3171718-3171647) Pro (CGG) 72 bp Sc: 76.52
GGCTCGTTGGTCTAGGGGTATGATTCTCGCTTCGGGTGCGAGAGGTCCCGGGTTCAAATC
CCGGACGAGCCC
>Bos_taurus_chr3.trna9334-ProCGG (1566698-1566627) Pro (CGG) 72 bp Sc: 76.52
GGCTCGTTGGTCTAGGGGTATGATTCTCGCTTCGGGTGCGAGAGGTCCCGGGTTCAAATC
CCGGACGAGCCC
>Bos_taurus_chr13.trna2947-ProTGG (70135424-70135496) Pro (TGG) 73 bp Sc: 47.25
TCCCTGGTAGTCCAGTGGTTAAGACTTTGCACTTGGGATACAGTGGGTGCAGGTTTGATC
CCTGGTCAGGGAA
>Bos_taurus_chr10.trna7204-ProTGG (25815869-25815798) Pro (TGG) 72 bp Sc: 76.15
GGCTCGTTGGTCTAGGGGTATGATTCTCGCTTTGGGTGCGAGAGGTCCCGGGTTCAAATC
CCGGACGAGCCC
>Bos_taurus_chr25.trna107-ProTGG (3156934-3157005) Pro (TGG) 72 bp Sc: 76.15
GGCTCGTTGGTCTAGGGGTATGATTCTCGCTTTGGGTGCGAGAGGTCCCGGGTTCAAATC
CCGGACGAGCCC
>Bos_taurus_chr25.trna115-ProTGG (3187886-3187957) Pro (TGG) 72 bp Sc: 76.15
GGCTCGTTGGTCTAGGGGTATGATTCTCGCTTTGGGTGCGAGAGGTCCCGGGTTCAAATC
CCGGACGAGCCC
>Bos_taurus_chr25.trna118-ProTGG (3190202-3190273) Pro (TGG) 72 bp Sc: 76.15
GGCTCGTTGGTCTAGGGGTATGATTCTCGCTTTGGGTGCGAGAGGTCCCGGGTTCAAATC
CCGGACGAGCCC
>Bos_taurus_chr25.trna121-ProTGG (3193095-3193166) Pro (TGG) 72 bp Sc: 76.15
GGCTCGTTGGTCTAGGGGTATGATTCTCGCTTTGGGTGCGAGAGGTCCCGGGTTCAAATC
CCGGACGAGCCC
>Bos_taurus_chr25.trna123-ProTGG (3194490-3194561) Pro (TGG) 72 bp Sc: 76.15
GGCTCGTTGGTCTAGGGGTATGATTCTCGCTTTGGGTGCGAGAGGTCCCGGGTTCAAATC
CCGGACGAGCCC
>Bos_taurus_chr15.trna4237-ProTGG (55209235-55209164) Pro (TGG) 72 bp Sc: 76.24
GGCTCGTTGGTCTAGGGGTATGATTCTCGGTTTGGGTCCGAGAGGTCCCGGGTTCAAATC

CCGGACGAGCCC
>Bos_taurus_chr10.trna7201-ProTGG (25869025-25868954) Pro (TGG) 72 bp Sc: 79.61
GGCTCGTTGGTCTAGTGGTATGATTCTCGCTTTGGGTGCGAGAGGTCCCGGGTTCAAATC
CCGGACGAGCCC
>Bos_taurus_chr4.trna418-SeCTCA (12402013-12402084) SeC (TCA) 72 bp Sc: 50.54
TCCCTGGTGGTCCAGTGGTTAAGACGCCACGCTTCACTACGAGGAGCACAGGTTCGATCC
CTGGTCAGGGAA
>Bos_taurus_chr1.trna1373-SeCTCA (37419820-37419891) SeC (TCA) 72 bp Sc: 50.55
TCCCTGGTGGCTCAGATGGTAAAGAATCTGCCTTCAATGCAGGAGACCCTGATTCGATCC
CTGGCTAGGGAA
>Bos_taurus_chr15.trna4403-SeCTCA (51197506-51197435) SeC (TCA) 72 bp Sc: 50.56
TCCCTGGTGGCTCAGAGGGTAAAGCGTCTGCCTTCAATGCAGGAGACCTGGGTTCGATCC
CTGGGTGGGGAA
>Bos_taurus_chr28.trna240-SeCTCA (6322983-6323055) SeC (TCA) 73 bp Sc: 50.87
TCCCTGGTGGTCCAGTGGTTAAGATTCTGTGCTTCAACGGCAGGAGGCACAGGTTCGATT
CCTGGTCAGGGAG
>Bos_taurus_chr11.trna4556-SeCTCA (108646933-108647004) SeC (TCA) 72 bp Sc: 51.07
TCCCTGATGGTCCAGTGGCTAAGACTCTGCTCTCAAAGCAGGGGGCCCCGGGTTCGATCC
CTGGTCAGGGAA
>Bos_taurus_chr2.trna2642-SeCTCA (84066260-84066331) SeC (TCA) 72 bp Sc: 51.28
TCCTTGGTGGTCCAGTGGTTAAGACTTGTGCTTTCAATGCAGAAGGCACAGGTTCAGTCC
CTGGTCAGGGAA
>Bos_taurus_chr18.trna2079-SeCTCA (49382195-49382266) SeC (TCA) 72 bp Sc: 51.36
TCCCTGGTGGTCCAGTGGTTAGGACTCCGAGCTTTCAGTGTCGCGGCCCAGGTTCAATCC
CTGGCTAGGGAA
>Bos_taurus_chr2.trna5142-SeCTCA (140109040-140109112) SeC (TCA) 73 bp Sc: 51.39
TCCCTGGTGGTCCAGTGGTTAGGACGCCACGCTTTCAGTGCTAAGGGCCCAGGTTCAATC
CCTGGCCTGGGAA
>Bos_taurus_chrUn.004.257.trna10-SeCTCA (119878-119950) SeC (TCA) 73 bp Sc: 51.48
TCCCTGGTGGTCCAGTGGTTAGGACTTTGGCTTTCAACGCAGGGGGTAACAGGTTCAATC
CCTGCTCAGGGAC
>Bos_taurus_chr18.trna5499-SeCTCA (14005818-14005747) SeC (TCA) 72 bp Sc: 51.71
TCCCTTATAGCTCAGTTGGTAAAGAATCTGCCTTCAATGCAGGAGACCCTGGTTCGATTC
CTGGGTGGGGAT
>Bos_taurus_chr2.trna7777-SeCTCA (84868867-84868795) SeC (TCA) 73 bp Sc: 52.34
TCCCTGGTGGCGCAGATGGTAAAGCATCTGCCTTCAATGCGGAAGACCCAGGTTCGATTC
CTGGGTCAGGGAA
>Bos_taurus_chr10.trna8050-SeCTCA (4807207-4807136) SeC (TCA) 72 bp Sc: 52.53
TCCCTGGTGGCTCAGATGGTAAAGCGTCTGCCTTCAATGCAGGAGACCTGGGTTCGATCC
CTGGTTCGGGAA
>Bos_taurus_chr12.trna3974-SeCTCA (73355479-73355407) SeC (TCA) 73 bp Sc: 52.62
TCCCTGATATCTGAGCTGGTAAAGAATCTGCCTTCAATGCAGGAGACCCTGGTTCAATTC
CTGGGTCAGGAAG
>Bos_taurus_chr22.trna3502-SeCTCA (24920792-24920721) SeC (TCA) 72 bp Sc: 52.65
TCCCTGGTGGCTCAGAAGGTAAAGTGTCTGCCTTCAATGCAGGAGACCCAGGTTCAATCC
CTGGGTTGGGAA
>Bos_taurus_chr4.trna7713-SeCTCA (35308850-35308778) SeC (TCA) 73 bp Sc: 53.86
TTCCTGATAGCTCAGTTGGTAAAGAATCTGCTTTCAATGCAGGAGACCCCGGTTCGATTC
CTGGGTCAGGAAG
>Bos_taurus_chr24.trna1521-SeCTCA (37560676-37560747) SeC (TCA) 72 bp Sc: 54.38
TCCCTGGTGGCTCAGAGGTTAAAGTATCTGCCCTCAATGCAGGAGACCCAGGTTCGATCC
CTGGGTTGGGAA
>Bos_taurus_chr4.trna909-SeCTCA (27065180-27065251) SeC (TCA) 72 bp Sc: 55.25
TCCCTGGTGGCTCAGAGGTTAAAGCGTCTGCCTTCAATGCAGGAGACCCGGGTTCGATCC
CTGGGTCGGGAA
>Bos_taurus_chr13.trna843-SeCTCA (22939864-22939936) SeC (TCA) 73 bp Sc: 55.59
TTCCTGGTGGCTCAGAGGTTAAAGCGTCTGCCTTCAATGCGGGAGACCCAGGTTCGATCC
CTGGGTCAGGAAG
>Bos_taurus_chr15.trna3166-SeCTCA (81716097-81716026) SeC (TCA) 72 bp Sc: 56.22
TCCCTGGTAGCTCAGCGGTTAAAGCATCTGCCTTCAATGCAGGAGACCCGGGTTCGATCC
CTGGGTTGGGAA
>Bos_taurus_chr2.trna469-SeCTCA (16284952-16285023) SeC (TCA) 72 bp Sc: 56.22
TCCCTGGTAGCTCAGCGGTTAAAGCATCTGCCTTCAATGCAGGAGACCCGGGTTCGATCC
CTGGGTTGGGAA
>Bos_taurus_chr18.trna5089-SeCTCA (23589036-23588964) SeC (TCA) 73 bp Sc: 58.04
TCCCTGGTGTTCCAGTGGTTAGTACTCTGCACTTCAGCTGCAGGGGGCGCAGGTTCAATC
CCTGCTCAGGGAA

>Bos_taurus_chr10.trna6109-SeCTCA (55199481-55199410) SeC (TCA) 72 bp Sc: 60.12
TCCCTGGTGGCTCAGAGGTTAAAGCATCTGCCTTCAATGCAGGAGACCCAGGTTCGATCC
CTGGGTTGGGAA
>Bos_taurus_chr21.trna3464-SeCTCA (55960063-55959994) SeC (TCA) 70 bp Sc: 60.45
TCCCTGGTGGTCCAGTGGTTAAGACACTGCTCTCAATGCAGGGGTCCAGGTTCAATCCCT
GGCCAGGGAA
>Bos_taurus_chr6.trna2027-SeCTCA (69501263-69501335) SeC (TCA) 73 bp Sc: 61.39
TCCCTGGTGGTCCAGTGGTTAGGACTCCGTGCTTTCAGTGCAGGGGGCCCAGGTTTGATC
CCTGGCCAGGGAA
>Bos_taurus_chr18.trna3675-SeC(e)TCA (52865801-52865716) SeC(e) (TCA) 86 bp Sc: 75.99
GCCCGGATGATCCTCAGTGGTCTGGGGTGCAGGCTTCAAACCTGTAGCTGTCTAGCGACA
GAGTGGTTCAATTCCACCTTTCGGGC
>Bos_taurus_chr8.trna2181-SerACT (69454460-69454531) Ser (ACT) 72 bp Sc: 52.02
TCCCTGGTGGCTCAGATGGTAAAGTGTCTGCCTACTATGTGGGAGACCCAGGTTCAATTC
CTGGGTAGGGAA
>Bos_taurus_chr12.trna6227-SerACT (17498205-17498133) Ser (ACT) 73 bp Sc: 53.16
TTCCTGATGGTCCAGGGGCTACGACTCTGCACTACTAATGCAGGGGGCCCCGGTTCAATC
CCTGGTCAGGGAA
>Bos_taurus_chr11.trna3858-SerACT (94484516-94484587) Ser (ACT) 72 bp Sc: 56.20
TCCCTCATGGCTCAGATGGTAAAGTGTCTGCCTACTATGCAGGAGACCCAGGTTCAATCC
CTGGGTGGGGAA
>Bos_taurus_chr27.trna3570-SerACT (12533842-12533771) Ser (ACT) 72 bp Sc: 56.23
TCCCTGGTGGCTCAGACGGTAAAGTGTCTGCCTACTATGCAGGAGACCCAGGTTCAATCC
CTGGGTTGGGAA
>Bos_taurus_chr10.trna1266-SerAGA (31001393-31001464) Ser (AGA) 72 bp Sc: 62.08
TCCCTGGTGGCTCAGAGGTTAAAGCGTCTGCCTAGAATGCAGGAGACCCAGGTTCGATCC
CTGGATTGGGAA
>Bos_taurus_chr18.trna1372-SerAGA (34409373-34409454) Ser (AGA) 82 bp Sc: 81.17
GTAGTCGTGGCCGAGTGGTTAAGGCGATGGGCTAGAAATCCATTGGGGTTTCCCCGCGCA
GGTTCAAATCCTGCCGACTACG
>Bos_taurus_chr23.trna3404-SerAGA (31198490-31198409) Ser (AGA) 82 bp Sc: 86.36
GTAGTCGTGGCCGAGTGGTTAAGGCGATGGACTAGAAATCCATTGGGGTCTCCCCGCGCA
GGTTCGAGTCCTGCCGACTACG
>Bos_taurus_chr14.trna2817-SerAGA (67353675-67353756) Ser (AGA) 82 bp Sc: 88.01
GTAGTCGTGGCCGAGTGGTTAAGGCGATGGACTAGAAATCCATTGGGGTCTCCCCGCGCA
GGTTCGAATCCTGCCGACTACG
>Bos_taurus_chr19.trna5351-SerAGA (28395395-28395314) Ser (AGA) 82 bp Sc: 88.01
GTAGTCGTGGCCGAGTGGTTAAGGCGATGGACTAGAAATCCATTGGGGTCTCCCCGCGCA
GGTTCGAATCCTGCCGACTACG
>Bos_taurus_chr23.trna1377-SerAGA (31174334-31174415) Ser (AGA) 82 bp Sc: 88.01
GTAGTCGTGGCCGAGTGGTTAAGGCGATGGACTAGAAATCCATTGGGGTCTCCCCGCGCA
GGTTCGAATCCTGCCGACTACG
>Bos_taurus_chr23.trna3370-SerAGA (31695265-31695184) Ser (AGA) 82 bp Sc: 88.01
GTAGTCGTGGCCGAGTGGTTAAGGCGATGGACTAGAAATCCATTGGGGTCTCCCCGCGCA
GGTTCGAATCCTGCCGACTACG
>Bos_taurus_chr23.trna3403-SerAGA (31206838-31206757) Ser (AGA) 82 bp Sc: 88.01
GTAGTCGTGGCCGAGTGGTTAAGGCGATGGACTAGAAATCCATTGGGGTCTCCCCGCGCA
GGTTCGAATCCTGCCGACTACG
>Bos_taurus_chr23.trna3405-SerAGA (31195000-31194919) Ser (AGA) 82 bp Sc: 88.01
GTAGTCGTGGCCGAGTGGTTAAGGCGATGGACTAGAAATCCATTGGGGTCTCCCCGCGCA
GGTTCGAATCCTGCCGACTACG
>Bos_taurus_chr23.trna3408-SerAGA (31171801-31171720) Ser (AGA) 82 bp Sc: 88.01
GTAGTCGTGGCCGAGTGGTTAAGGCGATGGACTAGAAATCCATTGGGGTCTCCCCGCGCA
GGTTCGAATCCTGCCGACTACG
>Bos_taurus_chrUn.004.2552.trna4-SerAGA (17604-17523) Ser (AGA) 82 bp Sc: 88.01
GTAGTCGTGGCCGAGTGGTTAAGGCGATGGACTAGAAATCCATTGGGGTCTCCCCGCGCA
GGTTCGAATCCTGCCGACTACG
>Bos_taurus_chrUn.004.2552.trna5-SerAGA (13104-13023) Ser (AGA) 82 bp Sc: 88.01
GTAGTCGTGGCCGAGTGGTTAAGGCGATGGACTAGAAATCCATTGGGGTCTCCCCGCGCA
GGTTCGAATCCTGCCGACTACG
>Bos_taurus_chr19.trna4150-SerAGA (50699469-50699388) Ser (AGA) 82 bp Sc: 88.50
GTAGTCGTGGCCGAGTGGTTAAGGCGATGGACTAGAAATCCATTGGGGTTTCCCCGCGCA
GGTTCGAATCCTGCCGACTACG
>Bos_taurus_chrUn.004.2552.trna2-SerAGA (7529-7610) Ser (AGA) 82 bp Sc: 88.50
GTAGTCGTGGCCGAGTGGTTAAGGCGATGGACTAGAAATCCATTGGGGTTTCCCCGCGCA
GGTTCGAATCCTGCCGACTACG
>Bos_taurus_chr3.trna7970-SerCGA (36907946-36907874) Ser (CGA) 73 bp Sc: 42.28

TCTCTGGTGGTCGAGTGTCTAAGACTCTGTGCTCGAAATGCAGGGGGCCTGGGTTCGATC
CCTGGTCAGGGAA
>Bos_taurus_chr25.trna4948-SerCGA (1366932-1366860) Ser (CGA) 73 bp Sc: 45.79
TCCCTGGTGGTCCAGTGGCTAGGACTCCGCGTCCCGAGTGCAGGGGACCCAGGTTCAATC
CCTGGTCAGGGAA
>Bos_taurus_chr23.trna1276-SerCGA (28832552-28832633) Ser (CGA) 82 bp Sc: 64.25
CCTGTGATGGTCGAGTGGTTAAGGCATTGGACTCGAAATCCAATGGGGTTTGCCCGGGCA
GGTTCAAATCCTACTCACAGTA
>Bos_taurus_chr5.trna7967-SerCGA (61705470-61705389) Ser (CGA) 82 bp Sc: 89.14
GTCACGGTGGCCGAGTGGTTAAGGCGTTGGACTCGAAATCCAATGGGGTTTCCCCGCACA
GGTTCGAATCCTGTTCGTGACG
>Bos_taurus_chr23.trna1368-SerCGA (31114259-31114340) Ser (CGA) 82 bp Sc: 89.19
GCTGTGATGGCCGAGTGGTTAAGGCGTTGGACTCGAAATCCAATGGGGTTTCCCCGCGCA
GGTTCAAGTCCTGCTCACAGCG
>Bos_taurus_chr23.trna3397-SerCGA (31341920-31341839) Ser (CGA) 82 bp Sc: 90.35
GCTGTGATGGCCGAGTGGTTAAGGCGTTGGACTCGAAATCCAATGGGGTCTCCCCGCGCA
GGTTCAAATCCTGCTCACAGCG
>Bos_taurus_chr19.trna5361-SerCGA (28321349-28321268) Ser (CGA) 82 bp Sc: 92.09
GCTGTGATGGCCGAGTGGTTAAGGCGTTGGACTCGAAATCCAATGGGGTCTCCCCGCGCA
GGTTCGAATCCTGCTCACAGCG
>Bos_taurus_chr2.trna3084-SerGCT (97020406-97020480) Ser (GCT) 75 bp Sc: 34.19
TCTCTGGTAGTCCAGTGGCTAAGAAGCCATGCTGCTAATATAGGAGGTACCCAGGTTCAA
TCCCTGGTCAGGGAA
>Bos_taurus_chr20.trna2107-SerGCT (58734826-58734908) Ser (GCT) 83 bp Sc: 46.52
AATGAGGTGGCTGAGTGGTTAAGGTGATGGACTGCTAATCCATTGTGCTCCTGCACGTGT
GGGCTTGAATCCCATCCTCGTCG
>Bos_taurus_chr6.trna2789-SerGCT (92920241-92920313) Ser (GCT) 73 bp Sc: 50.46
TCCCTGGTGGTCCAGTGGTTAAGACTCTGCACTGCTAATACAGGAGGTCTGGGTTCAATA
CCCAGTTGGGAAA
>Bos_taurus_chr1.trna6589-SerGCT (139886532-139886461) Ser (GCT) 72 bp Sc: 55.24
TCCCTGGTGACTCAGTGGTAAAGAACCTGCCTGCTGATGCAGGAGACCCAGGTTCGATCC
CTGGGTAGGGAA
>Bos_taurus_chr10.trna2631-SerGCT (69698192-69698273) Ser (GCT) 82 bp Sc: 81.72
GACGAGGTGGCCGAGTGGTTAAGGCGATGGACTGCTAATCCATTGTGCTCTGCACACGTG
GGTTCAAATCCCATCCTCGTCG
>Bos_taurus_chr13.trna3977-SerGCT (79043270-79043189) Ser (GCT) 82 bp Sc: 81.74
GACGAGGTGGCTGAGTGGTTAAGGCGATGGACTGCTAATCCATTGTGCTCTGCACGCGTG
GGTTCGAATCCCATCCTCGTCG
>Bos_taurus_chr10.trna6878-SerGCT (36090364-36090283) Ser (GCT) 82 bp Sc: 85.34
GACGAGGTGGCCGAGTGGTTAAGGCGATGGACTGCTAATCCATTGTGCTCTGCACGCGTG
GGTTCGAATCCCATCCTCGTCG
>Bos_taurus_chr19.trna1412-SerGCT (28374117-28374198) Ser (GCT) 82 bp Sc: 85.34
GACGAGGTGGCCGAGTGGTTAAGGCGATGGACTGCTAATCCATTGTGCTCTGCACGCGTG
GGTTCGAATCCCATCCTCGTCG
>Bos_taurus_chr23.trna1396-SerGCT (31326047-31326128) Ser (GCT) 82 bp Sc: 85.34
GACGAGGTGGCCGAGTGGTTAAGGCGATGGACTGCTAATCCATTGTGCTCTGCACGCGTG
GGTTCGAATCCCATCCTCGTCG
>Bos_taurus_chr23.trna1397-SerGCT (31326676-31326757) Ser (GCT) 82 bp Sc: 85.34
GACGAGGTGGCCGAGTGGTTAAGGCGATGGACTGCTAATCCATTGTGCTCTGCACGCGTG
GGTTCGAATCCCATCCTCGTCG
>Bos_taurus_chr23.trna1428-SerGCT (31707653-31707734) Ser (GCT) 82 bp Sc: 85.34
GACGAGGTGGCCGAGTGGTTAAGGCGATGGACTGCTAATCCATTGTGCTCTGCACGCGTG
GGTTCGAATCCCATCCTCGTCG
>Bos_taurus_chr23.trna3437-SerGCT (30547531-30547450) Ser (GCT) 82 bp Sc: 85.34
GACGAGGTGGCCGAGTGGTTAAGGCGATGGACTGCTAATCCATTGTGCTCTGCACGCGTG
GGTTCGAATCCCATCCTCGTCG
>Bos_taurus_chrUn.004.1183.trna10-SerGCT (8297-8216) Ser (GCT) 82 bp Sc: 85.34
GACGAGGTGGCCGAGTGGTTAAGGCGATGGACTGCTAATCCATTGTGCTCTGCACGCGTG
GGTTCGAATCCCATCCTCGTCG
>Bos_taurus_chrUn.004.1183.trna11-SerGCT (7668-7587) Ser (GCT) 82 bp Sc: 85.34
GACGAGGTGGCCGAGTGGTTAAGGCGATGGACTGCTAATCCATTGTGCTCTGCACGCGTG
GGTTCGAATCCCATCCTCGTCG
>Bos_taurus_chr29.trna1724-SerGCT (46283764-46283845) Ser (GCT) 82 bp Sc: 85.83
GACGAGGTGGCCGAGTGGTTAAGGCGATGGACTGCTAATCCATTGTGCTTTGCACGCGTG
GGTTCGAATCCCATCCTCGTCG
>Bos_taurus_chr23.trna3393-SerGCT (31386025-31385944) Ser (GCT) 82 bp Sc: 86.80
GACGAGGTGGCCGAGTGGTTAAGGCGATGGACTGCTAATCCATTGTGCTCTGCACGCGTG

GGTTCGAATCCCACCTTCGTCG
>Bos_taurus_chr23.trna3391-SerGCT (31402091-31402010) Ser (GCT) 82 bp Sc: 87.28
GACGAGGTGGCCGAGTGGTTAAGGCGATGGACTGCTAATCCATTGTGCTTTGCACGCGTG
GGTTCGAATCCCACCTTCGTCG
>Bos_taurus_chr23.trna1335-SerGCT (30250990-30251071) Ser (GCT) 82 bp Sc: 88.12
GACGAGGTGGCCGAGTGGTTAAGGCGATGGACTGCTAATCCATTGTGCTCTGCACGCGTG
GGTTCGAATCCCACCCTCGTCG
>Bos_taurus_chr25.trna3691-SerGGA (23309525-23309455) Ser (GGA) 71 bp Sc: 41.85
TCCCTGCTGGCTCAGAGATTAAAGCATCTGCCTGGAATGCAGGAGACCAGGTTTGATCCC
TGGGTCGGGAA
>Bos_taurus_chr6.trna1241-SerGGA (44807614-44807685) Ser (GGA) 72 bp Sc: 51.41
TCCCTGGTGGCTCAGATGTTAAAGCATCTGCCTGGAATGCAGGAGACCCGGGTTCGATCC
CTGGGTAGGGAA
>Bos_taurus_chr26.trna617-SerGGA (19242354-19242425) Ser (GGA) 72 bp Sc: 52.01
TCCCTGTTGGCTCAGAGGTTAAAGCATCTGCCTGGAATGCAGGAGACCCGAGTTCGATCC
TTGGGTTGGGAA
>Bos_taurus_chr1.trna4398-SerGGA (128038028-128038099) Ser (GGA) 72 bp Sc: 54.38
TCCCTGGTGGCTCAGAGGTTAAAGCGTCTGCCTGGAATGCGGGAGACCCAGGTTCGATCT
CTGGGTAGGGAA
>Bos_taurus_chr8.trna7575-SerGGA (17392017-17391945) Ser (GGA) 73 bp Sc: 54.43
TCCCTGGTGGCTCAGAGGTTAAAGCATCTGCCTGGAATGCAGGAGACCTGGGTTCAATCC
CCAGGTCAGGAAG
>Bos_taurus_chrX.trna3066-SerGGA (80643619-80643690) Ser (GGA) 72 bp Sc: 55.18
TCCCTGGTAGCTCAGAAGTTAAAGCGTCTGCCTGGAAAGCAGGAGACCCAGGTTCGATCC
CTGGGTCGGGAA
>Bos_taurus_chr4.trna1912-SerGGA (60750359-60750430) Ser (GGA) 72 bp Sc: 55.68
TCCCTGGTGGCTCAGAGGTTAAAGCATCTGTCTGGAATGCAGGAGACCCAGGTTCGATCC
CTGGGTCGGGAA
>Bos_taurus_chr14.trna3102-SerGGA (75646634-75646705) Ser (GGA) 72 bp Sc: 56.05
TCCCTGGTGGCTCAGAGGTTAAAGCGTCTGCCTGGAATGTAGGAGACCCAGGTTCAATCC
CTGGGTTGGGAA
>Bos_taurus_chr11.trna8025-SerGGA (27568596-27568524) Ser (GGA) 73 bp Sc: 56.24
TCCCTGGTGGCTCAGAGGTTAAAGCGTCTGCCTGGAATGCAGGAGACCCAGGTTCAATCC
CTGGGTCAGGAAG
>Bos_taurus_chr1.trna2107-SerGGA (60908536-60908607) Ser (GGA) 72 bp Sc: 56.84
TCCCTGGTGGCTCAGAGGTTAAAGCGTCTGCCTGGAATGCAGGAGACCCAGGTTTGATTC
CTGGGTTGGGAA
>Bos_taurus_chr17.trna3295-SerGGA (73034018-73034089) Ser (GGA) 72 bp Sc: 57.11
TCCCTGGTGGCTCAGAGGTTAAAGCGTCTGCCTGGAATGCCGGAGTCCCAGGTTCGATCC
CTGGGTTGGGAA
>Bos_taurus_chrUn.004.3426.trna2-SerGGA (3515-3444) Ser (GGA) 72 bp Sc: 57.11
TCCCTGGTGGCTCAGAGGTTAAAGCGTCTGCCTGGAATGCCGGAGTCCCAGGTTCGATCC
CTGGGTTGGGAA
>Bos_taurus_chr4.trna6950-SerGGA (61115898-61115827) Ser (GGA) 72 bp Sc: 57.40
TCCCTGGTGGCTCAGAGGTTAAAGCGTCTGTCTGGAATGCAGGAGACCCAGGTTCGATCC
CTGGGTCGGGAA
>Bos_taurus_chr3.trna464-SerGGA (15082363-15082434) Ser (GGA) 72 bp Sc: 57.44
TCCCTGGTGGCTCAGAGGTTAAAGCGTCTGCCTGGAATGCAGGACACCCAGGTTCGATCC
CTGGCTCAGGAA
>Bos_taurus_chr11.trna2894-SerGGA (72758403-72758475) Ser (GGA) 73 bp Sc: 57.98
TCCCTGGTGGCTCAGAGGTTAAAGCGTCTGCCTGGAATGCAGGAGACCCAGGTTCGATCC
CTGGGTCAGGAAG
>Bos_taurus_chr9.trna1145-SerGGA (36215479-36215551) Ser (GGA) 73 bp Sc: 57.98
TCCCTGGTGGCTCAGAGGTTAAAGCGTCTGCCTGGAATGCAGGAGACCCAGGTTCGATCC
CTGGGTCAGGAAG
>Bos_taurus_chrUn.004.11.trna18-SerGGA (848155-848227) Ser (GGA) 73 bp Sc: 57.99
TCCCTGATGGCTCAGAGGTTAAAGCGTCTGCCTGGAATGCAGGAGACCCAGGTTCAATCC
CTGGGTCAGAGAA
>Bos_taurus_chr24.trna4391-SerGGA (28314163-28314093) Ser (GGA) 71 bp Sc: 58.87
ACCTGGTGGCTCAGAGGTTAAAGCGCCTGCCTGGAACGCAGGAGACCCAGGTTCGATCCC
TGGGTCGGGAA
>Bos_taurus_chr6.trna438-SerGGA (17442315-17442386) Ser (GGA) 72 bp Sc: 59.16
TCCCTGGTGGCTCAGAGGTTAAAGTGTCTGCCTGGAATGCAGGAGACCCAGGTTCAATCC
CTGGGTTGGGAA
>Bos_taurus_chrUn.004.6296.trna3-SerGGA (4592-4521) Ser (GGA) 72 bp Sc: 59.16
TCCCTGGTGGCTCAGAGGTTAAAGTGTCTGCCTGGAATGCAGGAGACCCAGGTTCAATCC
CTGGGTTGGGAA

>Bos_taurus_chr1.trna2967-SerGGA (86301877-86301948) Ser (GGA) 72 bp Sc: 59.56
TCCCTGGTGGCTCAGAGGTTAAAGCGTCTGCTTGGAATGCAGGAGACCCAGGTTCGATCC
CTGGGTCGGGAA
>Bos_taurus_chr10.trna580-SerGGA (13442983-13443054) Ser (GGA) 72 bp Sc: 59.66
TCCCTGGTGGCTCAGAGGTTAAAGCATCTGCCTGGAATGCAGGAGACCCAGGTTCGATCC
CTGGGTCGGGAA
>Bos_taurus_chr9.trna6059-SerGGA (54029189-54029118) Ser (GGA) 72 bp Sc: 60.26
TCCCTGGTGGCTTAGAGGTTAAAGCATCTGCCTGGAATGCAGGAAACCCAGGTTCGATCC
CTGGGTGGGGAA
>Bos_taurus_chrUn.004.3234.trna1-SerGGA (7513-7584) Ser (GGA) 72 bp Sc: 60.26
TCCCTGGTGGCTTAGAGGTTAAAGCATCTGCCTGGAATGCAGGAAACCCAGGTTCGATCC
CTGGGTGGGGAA
>Bos_taurus_chr26.trna510-SerGGA (16581774-16581845) Ser (GGA) 72 bp Sc: 61.37
TCCCTGGTGGCTCAGAGGTTAAAGCGTCTGCCTGGAATGCAGGAGACCCAGGTTCGATCC
CTGGGTCGGGAA
>Bos_taurus_chr11.trna7211-SerGGA (49036444-49036373) Ser (GGA) 72 bp Sc: 62.37
TCCCTGATGGCTCAGAGGTTAAAGCGTCTGCCTGGAATGCAGGAGACCCAGGTTCGATCC
CTGGGTTGGGAA
>Bos_taurus_chr4.trna5415-SerGGA (100460552-100460481) Ser (GGA) 72 bp Sc: 62.37
TCCCTGGTGGCTCAGAGGTTAAAGCGTCTGCCTGGAATGCAGGAGACCCAGGTTCGATCC
CTGGGTTGGGAA
>Bos_taurus_chrX.trna5017-SerGGA (53299159-53299088) Ser (GGA) 72 bp Sc: 65.28
TCCCTGGTGGCTCAGAGGTTAAAGCGTCTGCCTGGAATGCAGGAGACCCAGGTTCGATCC
CTGGGTGGGGAA
>Bos_taurus_chrUn.004.225.trna8-SerGGA (209685-209604) Ser (GGA) 82 bp Sc: 84.60
GTAGTCGTGGCCGAGCGGTTAAGGCGGTGGACTGGAAATCCACTGGGGTCTCCCCGCGCA
GGTTCGAATCCTGCCGACTACG
>Bos_taurus_chrUn.004.90.trna15-SerGGA (362522-362603) Ser (GGA) 82 bp Sc: 84.60
GTAGTCGTGGCCGAGCGGTTAAGGCGGTGGACTGGAAATCCACTGGGGTCTCCCCGCGCA
GGTTCGAATCCTGCCGACTACG
>Bos_taurus_chr21.trna701-SerTGA (18888797-18888869) Ser (TGA) 73 bp Sc: 34.70
TCCCTGGTGGTCCAGTAGCTGAGACTCCGTGCTCTGAATGCAGGGGGCCCAGGTTCAATC
CCTGGTTGGGGAA
>Bos_taurus_chr4.trna2772-SerTGA (87198858-87198929) Ser (TGA) 72 bp Sc: 51.24
TCCTGGGTGGCTCAGATGGTAAAGCATCTGCCTTGAATGTGGGAGACCCAGGTTCAATCC
CTGGGTCAGGAA
>Bos_taurus_chrUn.004.2552.trna7-SerTGA (4470-4389) Ser (TGA) 82 bp Sc: 84.76
GTAGTCGTGGCCGAGTGGTTAAGGCGATGGACTTGAAATCCATTGGGGGCTCCCCGCGCA
GGTTCGAATCCTGTCGACTACG
>Bos_taurus_chr23.trna1425-SerTGA (31704214-31704295) Ser (TGA) 82 bp Sc: 87.07
GTAGTCGTGGCCGAGTGGTTAAGGCGATGGACTTGAAATCCATTGGGGTCTCCCCGCGCA
GGTTCGAATCCTGCCGGCTACG
>Bos_taurus_chr23.trna1379-SerTGA (31190382-31190463) Ser (TGA) 82 bp Sc: 88.73
GTAGTCGTGGCCGAGTGGTTAAGGCGATGGACTTGAAATCCATTGGGGTTTCCCCGCGCA
GGTTCGAATCCTGCCGACTACG
>Bos_taurus_chr28.trna843-SerTGA (23420768-23420849) Ser (TGA) 82 bp Sc: 90.86
GCAGCGATGGCCGAGTGGTTAAGGCGTTGGACTTGAAATCCAATGGGGTCTCCCCGCGCA
GGTTCGAACCCTGCTCGCTGCG
>Bos_taurus_chr11.trna6414-SupCTA (68611775-68611702) Sup (CTA) 74 bp Sc: 29.11
TCCCTGACTGTCCAGTGGTTAAGACTCTGTGCTTCTAATGCAGGAGGGCTTGGGTTTGAT
CCCTGGTCAGGGAA
>Bos_taurus_chrUn.004.15.trna36-SupCTA (811129-811057) Sup (CTA) 73 bp Sc: 40.06
TCCCTGGAGGTCCAGTGGCTAGGACTCTGTGCTCCTAGTGCCGGGGGGCCAGGTTCAATC
CCTGGTCAGGGAA
>Bos_taurus_chr9.trna7253-SupCTA (19286229-19286158) Sup (CTA) 72 bp Sc: 42.09
TTCCTGGTGGTCCAGTGGCTAAGATTCCTATTCCTAACGCAGGGGGCCTAGGTTTGATCC
CTGGCCAGGGAA
>Bos_taurus_chr9.trna5041-SupCTA (83202805-83202733) Sup (CTA) 73 bp Sc: 43.01
TCCCTGGTGGTCCAGTGGTTAAGACCTTTTGCTCCTAATGCAGGTGGTCCAGGTTTGATC
CCTGGTTGGGGAA
>Bos_taurus_chr1.trna10984-SupCTA (2967084-2967012) Sup (CTA) 73 bp Sc: 43.35
TCTCTGGTTGTCCAGTGGTTAAGACCCCGTGCTCCTAATGCAGGAGACCTGGGTTCAATC
CCCAGTTGGGGAA
>Bos_taurus_chr11.trna281-SupCTA (4746685-4746757) Sup (CTA) 73 bp Sc: 44.77
TCCCTGACGGTCCAGTGGCAAGGATTCAGCACTCTAACTGCCGAGGGCCCGGGTTCAACC
CCTGGTTGGGGAA
>Bos_taurus_chr3.trna8169-SupCTA (31598647-31598575) Sup (CTA) 73 bp Sc: 49.83

TCCCTGGTGGTCCAGTGGTTAAGACTTTTTGCTCCTAATGCAAGTGGCCCAGCTTCAATC
CCTGGTCAGGGAA
>Bos_taurus_chr8.trna6219-SupCTA (63585071-63585001) Sup (CTA) 71 bp Sc: 54.30
TCCCTGGTGGTCCAGTGGCTAGGACTCCTGCTCCTAATGCAGGGGCCTGGGTTCAATCCC
TGGTCAGGGAA
>Bos_taurus_chrUn.004.384.trna4-SupCTA (77653-77581) Sup (CTA) 73 bp Sc: 57.05
TCCCTAGTGGTCTAGTGGTTAAGATTCCATGCTTCTAATGCAGGGGGCACAGGTTCAATT
CCTGGCTGGGGAA
>Bos_taurus_chr8.trna5607-SupCTA (77117027-77116957) Sup (CTA) 71 bp Sc: 59.38
TCCCTGGTGGCTCAGTGGTAAAGAATCTGCCTCTAATGCAGGAGACTCAGGTTCAATCCC
TGCCTAGGGAA
>Bos_taurus_chr10.trna7646-SupCTA (14030664-14030592) Sup (CTA) 73 bp Sc: 60.78
TCCCTGCTGGTCCGATGGTTAGGACTCTGCACTCTAAATGCAGAGTGCCCAGGTTCAATC
CCTGGTTGGGGAA
>Bos_taurus_chr6.trna8111-SupCTA (18835197-18835126) Sup (CTA) 72 bp Sc: 61.73
TCCCTGGTGGCTCAGAGGTTAAAGTGTCTGCCTCTAATGCAGGAGACCCAGGTTCGATCC
CTGGGTTGGGAA
>Bos_taurus_chrX.trna4744-SupTTA (62161685-62161613) Sup (TTA) 73 bp Sc: 45.65
TCCCTGGTGGTCCAGTGATTAGGACTCTGTGCTTTAACTGCTGAGGGTGCAGGTTCAAAC
CCTGGTCAGGGAA
>Bos_taurus_chr10.trna2271-SupTTA (60006846-60006917) Sup (TTA) 72 bp Sc: 46.41
TCCCTGGTGGGCCAGTGGTTAGGACTTGATTCTTTAACTGTCAGGGCCTGGGTTCAGTCC
CTGGCCAGGGAA
>Bos_taurus_chr27.trna2805-SupTTA (29209948-29209877) Sup (TTA) 72 bp Sc: 49.47
TCCCTGGTAGTCCAGTGATTAGGACTCATCACTTTAACTGCTGTGGCCCGGATTCAATCC
CTGGTCAGGGAA
>Bos_taurus_chr8.trna2077-SupTTA (65960959-65961030) Sup (TTA) 72 bp Sc: 57.29
TCCCTGGTGGTCCAGTGGTTAGGACTCGTCACTTTAACTATGGTGGCCCAGGTTCAATTC
CTGGTGAGGGAA
>Bos_taurus_chrUn.004.7473.trna1-ThrAGT (3568-3641) Thr (AGT) 74 bp Sc: 46.56
GGTGCTGTGGCTTAGCTAGTTAAAGCACCTGTCTAGTAAACAGGAGATCCTGGGTTTGAA
TCTCAGCGATGCCT
>Bos_taurus_chrX.trna3714-ThrAGT (86372220-86372147) Thr (AGT) 74 bp Sc: 60.32
CTCGCCGTGGCTTAGCTGGTTAAAGCGCCTGTCTAGTAAACAGGAGATCCTGGGTTCAAA
TCCCAGCAGTGCCT
>Bos_taurus_chr3.trna5718-ThrAGT (104873225-104873152) Thr (AGT) 74 bp Sc: 76.30
GGCTCCGTGGCTTAGCTGGTTAAAGCGCCTGTCTAGTAAACAGGAGATCCTGGGTTCAAA
CCCCAGTGGGGCCT
>Bos_taurus_chr23.trna1390-ThrAGT (31307982-31308055) Thr (AGT) 74 bp Sc: 80.19
GGCTCCGTGGCTTAGTTGGTGAAAGCGCCTGTCTAGTAAACAGGAGATCCTGGGTTCAAA
TCCCAGCGGGGCCT
>Bos_taurus_chrUn.004.1183.trna4-ThrAGT (27027-26954) Thr (AGT) 74 bp Sc: 80.19
GGCTCCGTGGCTTAGTTGGTGAAAGCGCCTGTCTAGTAAACAGGAGATCCTGGGTTCAAA
TCCCAGCGGGGCCT
>Bos_taurus_chr23.trna3395-ThrAGT (31355143-31355070) Thr (AGT) 74 bp Sc: 80.30
GGCCCTGTGGCTTAGCTGGTCAAAGCGCCTGTCTAGTAAACAGGAGATCCTGGGTTCGAA
TCCCAGCGGGGCCT
>Bos_taurus_chr19.trna5360-ThrAGT (28321855-28321782) Thr (AGT) 74 bp Sc: 81.60
GGCGCCGTGGCTTAGCTGGTTAAAGCGCCTGTCTAGTAAACAGGAGATCCTGGGTTCGAA
TCCCAGCGGTGCCT
>Bos_taurus_chr23.trna1366-ThrAGT (31108072-31108145) Thr (AGT) 74 bp Sc: 83.05
GGCTCCGTGGCTTAGCTGGTTAAAGCGCCTGTCTAGTAAACAGGAGATCCTGGGTTCGAA
TCCCAGCGGGGCCT
>Bos_taurus_chr23.trna1417-ThrAGT (31590939-31591012) Thr (AGT) 74 bp Sc: 83.05
GGCTCCGTGGCTTAGCTGGTTAAAGCGCCTGTCTAGTAAACAGGAGATCCTGGGTTCGAA
TCCCAGCGGGGCCT
>Bos_taurus_chr18.trna1701-ThrAGT (43080199-43080272) Thr (AGT) 74 bp Sc: 84.06
GGCGCCGTGGCTTAGTTGGTTAAAGCGCCTGTCTAGTAAACAGGAGATCCTGGGTTCGAA
TCCCAGCGGTGCCT
>Bos_taurus_chr19.trna1413-ThrAGT (28374420-28374493) Thr (AGT) 74 bp Sc: 84.06
GGCGCCGTGGCTTAGTTGGTTAAAGCGCCTGTCTAGTAAACAGGAGATCCTGGGTTCGAA
TCCCAGCGGTGCCT
>Bos_taurus_chr19.trna5352-ThrAGT (28395029-28394956) Thr (AGT) 74 bp Sc: 84.06
GGCGCCGTGGCTTAGTTGGTTAAAGCGCCTGTCTAGTAAACAGGAGATCCTGGGTTCGAA
TCCCAGCGGTGCCT
>Bos_taurus_chr19.trna5357-ThrAGT (28373042-28372969) Thr (AGT) 74 bp Sc: 84.06
GGCGCCGTGGCTTAGTTGGTTAAAGCGCCTGTCTAGTAAACAGGAGATCCTGGGTTCGAA

TCCCAGCGGTGCCT
>Bos_taurus_chr23.trna3418-ThrAGT (31084776-31084703) Thr (AGT) 74 bp Sc: 85.27
GGCCTCGTGGCTTAGTTGGTTAAAGCGCCTGTCTAGTAAACAGGAGATCCTGGGTTCGAA
TCCCAGCGAGGCCT
>Bos_taurus_chr13.trna4971-ThrCGT (62595118-62595046) Thr (CGT) 73 bp Sc: 44.92
TCCCTGGTTGTCCAGTGGCTTAGACTCTGTGCTCGTAATGCAGGGTGCCCAGGTTCAATC
CCTGGTCAGGGAA
>Bos_taurus_chr23.trna3412-ThrCGT (31137357-31137285) Thr (CGT) 73 bp Sc: 77.58
GGCTCCGTAGCTCAGGGGTTAGAGCACTGGTCTCGTAAACCAGGGGTCGTGAGTTCGAAT
CTCACTGGAGCCT
>Bos_taurus_chr19.trna5965-ThrCGT (18320702-18320631) Thr (CGT) 72 bp Sc: 79.77
GGCGCGGTGGCCAAGTGGTAAGGCGTCGGTCTCGTAAACCGAAGATCGCGGGTTCGAACC
CCGTCCGTGCCT
>Bos_taurus_chr23.trna1333-ThrCGT (30238296-30238369) Thr (CGT) 74 bp Sc: 80.30
GGCTCTGTGGCTTAGTTGGCTAAAGCGCCTGTCTCGTAAACAGGAGATCCTGGGTTCGAA
TCCCAGCGGGGCCT
>Bos_taurus_chr25.trna843-ThrCGT (14301086-14301157) Thr (CGT) 72 bp Sc: 80.42
GGCGCGGTGGCCAAGTGGTAAGGCGTCGGTCTCGTAAACCGAAGATCACGGGTTCGAACC
CCGTCCGTGCCT
>Bos_taurus_chr23.trna1340-ThrCGT (30346865-30346938) Thr (CGT) 74 bp Sc: 81.51
GGCTCTATGGCTTAGTTGGTTAAAGCGCCTGTCTCGTAAACAGGAGATCCTGGGTTCGAC
TCCCAGTAGGGCCT
>Bos_taurus_chr8.trna7981-ThrCGT (7964835-7964755) Thr (CGT) 81 bp Sc: 28.96
TCTCTGGTGGTCCAGTGGCTAAGACTCTGTGCTCGTAATACATGGAGCACGGGGGTATAG
GTTTGATCCCTAGTTGGAGAA
>Bos_taurus_chrUn.004.1648.trna2-ThrGGT (16575-16647) Thr (GGT) 73 bp Sc: 39.56
TTCCTGGTGGTCCAGTGGCTAAGACTTTGCAGTGGTGATGCAGGCGGCCCAGGTTCGATC
CCTGATCAGGGAG
>Bos_taurus_chr18.trna4204-ThrTGT (45811508-45811437) Thr (TGT) 72 bp Sc: 55.17
TCCCTGATGGTCCAGGGTTAGGACTTGGTACTTGTATTGCCAAGGGTCTAGGTTCAATCC
CTGGTTGGGGAA
>Bos_taurus_chr20.trna4138-ThrTGT (42681612-42681541) Thr (TGT) 72 bp Sc: 55.69
GGCCCTATAGCTCAGGGGTTAGAGCACTGGTCTTGTAAACCAGGGTCGTGAGTTCATATC
TCGCTGGGCCCT
>Bos_taurus_chr4.trna123-ThrTGT (5359855-5359927) Thr (TGT) 73 bp Sc: 69.24
GTCTCCATAGCTCAGTGGTTAGAGCACTGGTCTTGTAAACCAGGGGTCGTGAGTTCGATC
CCCGCTGGGGCCT
>Bos_taurus_chr16.trna726-ThrTGT (22980748-22980820) Thr (TGT) 73 bp Sc: 73.06
GGGGCCATAGCTCAGTGGTTAGAGCACTGGTCTTGTAAACCAGGGGTCGCGAGTTCGATC
CTCGCTGGGGCCT
>Bos_taurus_chr10.trna7202-ThrTGT (25818739-25818667) Thr (TGT) 73 bp Sc: 78.79
GGCCCTATAGCTCAGGGGTTAGAGCACTGGTCTTGTAAACCAGGGGTCGCGAGTTCAAAT
CTCGCTGGGGCCT
>Bos_taurus_chr10.trna1099-ThrTGT (25870573-25870645) Thr (TGT) 73 bp Sc: 79.46
GGCTCCATAGCTCAGGGGTTAGAGCACTGGTCTTGTAAACCAGGGGTCGCGAGTTCAAAT
CTCGCTGGGGCCT
>Bos_taurus_chr10.trna1101-ThrTGT (25884134-25884206) Thr (TGT) 73 bp Sc: 79.46
GGCTCCATAGCTCAGGGGTTAGAGCACTGGTCTTGTAAACCAGGGGTCGCGAGTTCAAAT
CTCGCTGGGGCCT
>Bos_taurus_chr7.trna6780-ThrTGT (39670836-39670764) Thr (TGT) 73 bp Sc: 79.46
GGCTCCATAGCTCAGGGGTTAGAGCACTGGTCTTGTAAACCAGGGGTCGCGAGTTCAAAT
CTCGCTGGGGCCT
>Bos_taurus_chr23.trna1336-ThrTGT (30284589-30284662) Thr (TGT) 74 bp Sc: 84.08
GGCTCTATGGCTTAGTTGGTTAAAGCGCCTGTCTTGTAAACAGGAGATCCTGGGTTCGAA
TCCCAGTAGGGCCT
>Bos_taurus_chr23.trna1342-ThrTGT (30358819-30358892) Thr (TGT) 74 bp Sc: 85.34
GGCTCCATGGCTTAGTTGGTTAAAGCGCCTGTCTTGTAAACAGGAGATCCTGGGTTCGAA
TCCCAGTGGGGCCT
>Bos_taurus_chr11.trna121-TrpCCA (2025931-2026003) Trp (CCA) 73 bp Sc: 22.27
TCCCTGGTTGTCCAGGGGCTAAGACTCTGAGCCCCCAGTGCAGGCGGCTCGGGTTCTATC
CCTGATCAGGGAA
>Bos_taurus_chr7.trna8550-TrpCCA (4160729-4160658) Trp (CCA) 72 bp Sc: 24.35
CCCCTGGCGGTCCACTGGTTAAGATCCATGCTTCCACTGCAGGGAGTGCAGGTTTGATCC
CTGCTTGGGGGA
>Bos_taurus_chrUn.004.5569.trna2-TrpCCA (4850-4779) Trp (CCA) 72 bp Sc: 24.35
CCCCTGGCGGTCCACTGGTTAAGATCCATGCTTCCACTGCAGGGAGTGCAGGTTTGATCC
CTGCTTGGGGGA

>Bos_taurus_chr11.trna1893-TrpCCA (47757960-47758029) Trp (CCA) 70 bp Sc: 26.66
CCCTGGCAGTTCAATGGTTAAGACTTTACCTTCCAATGCTAGCAATTCAGGTTCAATCCC
TGGTCAGGGG
>Bos_taurus_chr15.trna3068-TrpCCA (83874700-83874628) Trp (CCA) 73 bp Sc: 26.72
TCCCTGGAGGTCCAGCGGCTACGACTCCCTGCTCCCAGTGCGGGGGGCCTGGGTTCAACC
CCTGGTCAGGGAA
>Bos_taurus_chr26.trna3362-TrpCCA (17238406-17238334) Trp (CCA) 73 bp Sc: 26.74
TCCCTGGTGGTTCAGTGGCTAAAACTTTATGTTCCCAATGCAGGAGACCCAGCGTCGATC
CCTGGTCAGGGAT
>Bos_taurus_chr19.trna1889-TrpCCA (38247269-38247341) Trp (CCA) 73 bp Sc: 27.11
TCCCTGGTGGTCTAGTGCCTAAGATTCTCTGGTCCCAATGCAGGAGGCCCAGGTTTGATC
CCTGGTCAGGAAC
>Bos_taurus_chr19.trna1890-TrpCCA (38256309-38256381) Trp (CCA) 73 bp Sc: 27.11
TCCCTGGTGGTCTAGTGCCTAAGATTCTCTGGTCCCAATGCAGGAGGCCCAGGTTTGATC
CCTGGTCAGGAAC
>Bos_taurus_chr6.trna1823-TrpCCA (62845461-62845533) Trp (CCA) 73 bp Sc: 28.55
TCCCTGGTGGTCCAGTGACTAAGATTCCGTGCTCCCAATGCAGGGGACCCAGGTTCAGTC
CCTGGTCAAGGAA
>Bos_taurus_chr3.trna5157-TrpCCA (118042668-118042596) Trp (CCA) 73 bp Sc: 28.89
TCCCTGGTGGTCCAGTGACTAAGATTCCTCACTTCCAGTGCGGGTGGCCCAGGGTCAATC
CTTGGTCAGGGAA
>Bos_taurus_chr9.trna7559-TrpCCA (9924878-9924806) Trp (CCA) 73 bp Sc: 30.36
GGCTGTGTGGCCTAATGGATAAGGTGCCTGATTCCAGGTCAGATGATTGAAGGTTTTAGT
CCCTTCACGGTCC
>Bos_taurus_chr23.trna3563-TrpCCA (27506322-27506250) Trp (CCA) 73 bp Sc: 31.06
TCCCTGATGGTCCAGTGGCTGAGATTCTGTGCTCCCAGTGCAGACGGCACAGGTTTGATC
CCTGGTCAGGGAA
>Bos_taurus_chr21.trna4090-TrpCCA (35615161-35615089) Trp (CCA) 73 bp Sc: 31.98
TCCCTGGTGGCCCAGGGGCTCAGATTCTGTGTTCCAAATGCAGGGGGCTCAGGTTCGATC
CTTGGTCAGGGAA
>Bos_taurus_chr10.trna1303-TrpCCA (32506233-32506304) Trp (CCA) 72 bp Sc: 32.20
TCCCTGATGGTCCAGTGGTTGAGACCCATGCTTCCACTGCAGGTGGCATGGGTTCACTTC
CTGTTTGGGGAA
>Bos_taurus_chr23.trna3322-TrpCCA (32798824-32798752) Trp (CCA) 73 bp Sc: 32.63
TCCCTGGTGGTTCAGTAGCTAAGACTCCGTGCTCCCAGTGCAGGAAGCCCAGGTTCTATC
CCTGGCCCGGGAA
>Bos_taurus_chr3.trna3323-TrpCCA (94863561-94863632) Trp (CCA) 72 bp Sc: 33.13
TCTCTGGTGGCTCAGAGGGTAAAGCATCTACCTCCAATGTGGGAGACTTGGGTTTGATCC
CCAGGCAGGGAA
>Bos_taurus_chr3.trna3333-TrpCCA (95037866-95037937) Trp (CCA) 72 bp Sc: 33.13
TCTCTGGTGGCTCAGAGGGTAAAGCATCTACCTCCAATGTGGGAGACTTGGGTTTGATCC
CCAGGCAGGGAA
>Bos_taurus_chr28.trna15-TrpCCA (611102-611174) Trp (CCA) 73 bp Sc: 33.27
TCCCTGGTGGTCCAGAGACTAAGACCTGTGCTCCCAATGCAGGGGGCTCCAGGTTCAAGC
CCTGGTCAGGGAA
>Bos_taurus_chr25.trna2101-TrpCCA (35508056-35508126) Trp (CCA) 71 bp Sc: 33.30
TCCCTGGTGGCTCAGTGGCTGAGACTCTGCATCCAGTGTAGGGGGCCTGGGTTCAATCCC
TAGTCAGGGAA
>Bos_taurus_chr13.trna1106-TrpCCA (28220892-28220965) Trp (CCA) 74 bp Sc: 33.84
TCCCTGGTGGTCCAGTAGCTAAGACTTGGGTGCTTCCAATGTAGGAGGCCCAGGTTCAAT
CCCTGGTCGGGGAA
>Bos_taurus_chr18.trna4020-TrpCCA (48231975-48231903) Trp (CCA) 73 bp Sc: 34.08
TCCCTGGTGGTCCAGCGGCTAAGACTCCGTGCTCCCAATGCAGGGGGCTCGGGTTCGATC
GCTGATCAGGGAG
>Bos_taurus_chr2.trna8424-TrpCCA (65242795-65242724) Trp (CCA) 72 bp Sc: 34.60
TCCCTGGTGGTCCAGCAGTTAAGACTTTGCCTTCCAGTGGAGAGGGTGCAGGTTTGATCC
CTGACTGGGGAG
>Bos_taurus_chr29.trna1258-TrpCCA (34215279-34215351) Trp (CCA) 73 bp Sc: 34.79
TTCCTGGTGGTCCAGTGGCTAAGACCCCGTGTTCCCAGTGCAGGGGGCCCAGGTACGATC
CCTGGTCAGGAAC
>Bos_taurus_chr8.trna2343-TrpCCA (72890307-72890379) Trp (CCA) 73 bp Sc: 35.46
TCTCTGGTGGTCCATTGGTTAAGATTCTATGCTCCCAGTGCAGGGGGCCCAGGTTCTATC
CCTGGTCAGGGAA
>Bos_taurus_chrX.trna5195-TrpCCA (47918836-47918764) Trp (CCA) 73 bp Sc: 35.56
TCTCTGGTGGTCCAGTGGCTAAGACTCTGTGCTTCCAATGCAGGAGATTCAGTTTCAAAC
CCTGGTCAGGGAA
>Bos_taurus_chr4.trna3731-TrpCCA (108937685-108937757) Trp (CCA) 73 bp Sc: 35.62

TTCCTAATGGTCCAGTGGCTAAGACCCTGTGCTCCCAGTGCACAGGGCCCAGGTTTGATC
CCTGGTCAGGGAG
>Bos_taurus_chr14.trna5952-TrpCCA (16338446-16338376) Trp (CCA) 71 bp Sc: 35.68
TCCCTGGTGGTCCAGTGTTAAGATTTCACTGTCCAATGCTGGGGACTCAGGTTCAATCCC
TGATCAGGGAA
>Bos_taurus_chr18.trna5521-TrpCCA (13318449-13318378) Trp (CCA) 72 bp Sc: 35.80
TACCTGGTGGTCCAGTGGCTAAGACTGCTATTTCCAACACAGTGGGGCTGGGTTCAATCC
CTGGTCAGGGAA
>Bos_taurus_chr12.trna5314-TrpCCA (34809655-34809583) Trp (CCA) 73 bp Sc: 35.90
TCCCTGGTGGTCCAGCGGTGAAGACTCTGTGCTTCCAATGCAGGAGGCCTGGGTTTGATC
CATAGTCAGGGAA
>Bos_taurus_chr3.trna5215-TrpCCA (116694907-116694832) Trp (CCA) 76 bp Sc: 35.92
TCCCTGGTGGTCCAGTGGCCAATAAGACTCCGTGCTTCCAGTGCAGGGGGCCTGGGTTCG
ATCCCTGGTCAGGGAA
>Bos_taurus_chr21.trna830-TrpCCA (21065018-21065090) Trp (CCA) 73 bp Sc: 36.00
CTTCTGGTGGTCCAGTGGTTAGGACCCTGTGCTTCCAATGCAAGGGACTCAGGTTTGATC
CCTGATCAGGGAA
>Bos_taurus_chr13.trna1686-TrpCCA (40003860-40003933) Trp (CCA) 74 bp Sc: 36.13
TCCCTGGTGGTCCAGTGGCCAAGACCCCGTGCTCCCAGTGCAGGTGGCCCCAGGTTTGAT
CCCAGGTCAGGGAA
>Bos_taurus_chr22.trna1995-TrpCCA (55408412-55408486) Trp (CCA) 75 bp Sc: 36.88
TCCCTGGTGGCCCAGTGGCTGCGGGGCTCTGTGCTCCAAACGCAGGGTGCCTGGGTTTGA
TCCCTGGTCAGGGAA
>Bos_taurus_chr3.trna487-TrpCCA (15753082-15753154) Trp (CCA) 73 bp Sc: 37.38
TCTCTGGTGGTCCAGTGGCTAAGACTCTGTGCTCCCAGTGCAGGAGGCCTGGGTTTGATC
CCTAGTCAGAGAA
>Bos_taurus_chr14.trna950-TrpCCA (22252534-22252606) Trp (CCA) 73 bp Sc: 37.56
TCCCTGGCGGTCCAGTGGCTACGACTCTGTGCTCCCAATGCAAGGGGCCCAGGTTTGATC
CCTGGTCAGGGAG
>Bos_taurus_chr18.trna3544-TrpCCA (54573465-54573394) Trp (CCA) 72 bp Sc: 37.57
TCCCTGGTGGTCTGGTGCTAAGACTCTGTGCTTCCAGTGCAGGGGGCCCGGGTTCGATCC
CTGGTCAGGGAA
>Bos_taurus_chr22.trna1248-TrpCCA (34245562-34245634) Trp (CCA) 73 bp Sc: 37.76
TCCCTTGTGGTCCAGTGGTTAAGACTCCGTGCTTCCACTGCAGGCAGCATGGGTTCAGTC
CCCAGCAGGGGAA
>Bos_taurus_chrX.trna594-TrpCCA (12648739-12648811) Trp (CCA) 73 bp Sc: 37.81
TCCCTGGTTGTCCAGTGGCTAAGACTTTATGGTCCCAATGCAGGAGGCCCAGGTTCAATC
CCTGGTCAGGGAA
>Bos_taurus_chr6.trna8092-TrpCCA (19189771-19189698) Trp (CCA) 74 bp Sc: 38.18
TTCCTGATGGTCCAGGTGGCCAAGACTCTGTGTTCCAAATGCAGGGAGCCCAGGATCAAT
CCCTGATCAGGGAA
>Bos_taurus_chr2.trna8178-TrpCCA (72789856-72789776) Trp (CCA) 81 bp Sc: 38.53
TCCCTGATGGTCCAGTGGAAGTGATGGAAACACTCTGTGCTTCCAATGCTCAGAGCCCAG
GTTCGATCCCTGGTCAGGGAA
>Bos_taurus_chr11.trna4182-TrpCCA (102105895-102105967) Trp (CCA) 73 bp Sc: 38.54
TCCCTGGTGGTCCAGCGGCTAAGACTCTGAGCCCCCAGTGCAGGGGGCCCAGGTTTGATC
CCTGGTCAGGGAA
>Bos_taurus_chr21.trna679-TrpCCA (18583485-18583557) Trp (CCA) 73 bp Sc: 38.65
TCCCTGGTGGTCCAGCAGCTAAGACTCTGTGCTCCCAGTGCCGGGGGCCCAGGTTCAATC
CCTGGTCAGGGAA
>Bos_taurus_chr21.trna5076-TrpCCA (17044885-17044814) Trp (CCA) 72 bp Sc: 38.89
TCCCTGGTGGTTCAGTGACTAAGACATATGCTTCCAAAGCAGGGGACCCAGTTTCAATCC
CTGGTCAGGGAA
>Bos_taurus_chr7.trna7562-TrpCCA (18143624-18143554) Trp (CCA) 71 bp Sc: 39.24
TCCCTGGTGGTCCAGTGGCTATGACTCCAGCTTCCAGTGCAGGGGTGTGGGTTCAATCCC
TAATCAGGGAA
>Bos_taurus_chrUn.004.2720.trna2-TrpCCA (8000-8070) Trp (CCA) 71 bp Sc: 39.24
TCCCTGGTGGTCCAGTGGCTATGACTCCAGCTTCCAGTGCAGGGGTGTGGGTTCAATCCC
TAATCAGGGAA
>Bos_taurus_chr11.trna2411-TrpCCA (57713510-57713582) Trp (CCA) 73 bp Sc: 39.42
TTCCTGGTGGTCCAGTGGCTAAGACTCTGTGCTTCCAATGCAAGGGGCCAAGGTTTGATC
CCTGGTCAGGGAA
>Bos_taurus_chr19.trna6112-TrpCCA (16221935-16221864) Trp (CCA) 72 bp Sc: 39.84
TCCCTGGTGGTCCAGTGGCTAAGACCCCGTGCTCCCAATGCAGGGGACCGGGGTTCGATC
CCTGTCAGGGAA
>Bos_taurus_chr19.trna4313-TrpCCA (47838834-47838762) Trp (CCA) 73 bp Sc: 40.07
TCCCTGGCAGTCCAGTGGTTAAGACTCTGTGCTTCCAATGCAGGAGACACAGGTTCAATC

CTTGTTTGGGGAG
>Bos_taurus_chr7.trna2760-TrpCCA (64831111-64831183) Trp (CCA) 73 bp Sc: 40.44
TCCCTGATGGTCCAGTGGCTGGGACTCCGTGCTCCCAGTGCAGGGGGCCCAGGTTTGATT
CCTGGACAGGGAA
>Bos_taurus_chr15.trna1065-TrpCCA (32383919-32383991) Trp (CCA) 73 bp Sc: 40.52
TCCCTGGTGGTCCAGTGGCTAAGAGTCCATGCTCCCAGTGTAGGGGGCCCAGGTTCAATC
CCTGGTCAGGGAA
>Bos_taurus_chr7.trna7919-TrpCCA (13800828-13800756) Trp (CCA) 73 bp Sc: 40.73
TCCCTGGTGGTCCAGTGGCCACGACTCCGTGCCCCCAGTGCAGGGTGCCCGGGTTCGATC
CCTGGTCAGGGAA
>Bos_taurus_chr22.trna3437-TrpCCA (26689303-26689232) Trp (CCA) 72 bp Sc: 41.14
TCCCTGGTGGTCCAGTGGTTGAGATCTGTGCTCCCAATGCAGGGGGCCCAGGTTCAATGC
CTGGTCAGGGAA
>Bos_taurus_chr19.trna1891-TrpCCA (38267092-38267165) Trp (CCA) 74 bp Sc: 41.20
TCCCTGGTGGTCCAGTGGTTAAGATTCTCTGCTTCCAATGCAGGGGGCAACAGGTTTGAT
CCCTGGTCAGGGAA
>Bos_taurus_chr21.trna5223-TrpCCA (14751300-14751230) Trp (CCA) 71 bp Sc: 41.54
TCCCTGGTGGTCCAGTGGTTAAGAATCTGCCTTCCAATGCCAGGGACCAGGTTCAGTTCC
TGGTCAGGGAG
>Bos_taurus_chr21.trna777-TrpCCA (20423004-20423076) Trp (CCA) 73 bp Sc: 41.70
TCCTTGGTGGGTCACTGGTTCAGACTCCATGCTTCCAATGCAGGGGGCCAAGGTTCAATC
CCTGGTCAGGGAA
>Bos_taurus_chr7.trna2974-TrpCCA (71496372-71496443) Trp (CCA) 72 bp Sc: 41.76
TCCCTGATGGTCCAGTGGTTAAGATTTCGCCTTCCAATGCCGGGCAAGGAGGTTCGATCC
CTCCTTGGGAAG
>Bos_taurus_chrUn.004.359.trna2-TrpCCA (72057-72129) Trp (CCA) 73 bp Sc: 41.84
TCCCTGTTGGTCCAGTGGCTAAGACTCCATGCTTCCAGTGTAGGGGGCCCAGGTTCAATC
TCTGGTCAGGGAA
>Bos_taurus_chr7.trna8209-TrpCCA (10157682-10157610) Trp (CCA) 73 bp Sc: 42.03
TCCCTGGTGGTCCCATGGCTAAGATATTCCGCTTCCAATGCAGGAGGCCCAGGTTCGATC
CCTGGTCAGGGAA
>Bos_taurus_chr19.trna5014-TrpCCA (34058110-34058038) Trp (CCA) 73 bp Sc: 42.09
TCCCTGGTAGTCAAATGGCTAAGACTCTGTGCTCCCAATGCAAGGGGCCCAAGTTTGATC
CTTGGTCAGGGAA
>Bos_taurus_chr27.trna2262-TrpCCA (39440279-39440207) Trp (CCA) 73 bp Sc: 42.09
TCCCTGGTGGTCCAGTGGCTAAGATTCTGTGCTCCCAGTGCAAGGGGCCCAGGTTTGATC
CCTGGGCAGGGAA
>Bos_taurus_chr15.trna2738-TrpCCA (76647475-76647547) Trp (CCA) 73 bp Sc: 42.15
TCCTTGGTGGTCTAGTGGTTACGACTCTGTGCTCCCAGTGCAAGGGGCACAGGTTCAAAT
CCTGGTCGGGGAC
>Bos_taurus_chr19.trna5427-TrpCCA (27076072-27076000) Trp (CCA) 73 bp Sc: 42.16
TCCCTAGTGGTCCAGTGGCTAAGACTCCGTGCTCCCAATGCAGGGGGCCCAGGTTCTATC
CCTGGTCAGGGAA
>Bos_taurus_chr18.trna4326-TrpCCA (43468398-43468328) Trp (CCA) 71 bp Sc: 42.80
TTCCTGGTGGTCTAGTGGTTAGGATTCCAGCTTCCAGTGCAGGGACACAGGTTTGATCCC
TGGTCAGGGAA
>Bos_taurus_chr19.trna4130-TrpCCA (50969344-50969271) Trp (CCA) 74 bp Sc: 42.98
TCCCTAGTGGTCCAGTGGTTAAGACCCTGTGCTCCCAATGCAAGGGGCCCCAGGTTCAGT
CCCTGGTTGGGGAA
>Bos_taurus_chr13.trna4328-TrpCCA (73521064-73520985) Trp (CCA) 80 bp Sc: 43.09
TCCCTGAAGGTTCAGTGGTTACGACTTCATGCTTCCAGTGTAGAGGGTGCAGGTTCCAGG
TTCGATCCCTGGTCAGGGAA
>Bos_taurus_chr17.trna466-TrpCCA (13568409-13568481) Trp (CCA) 73 bp Sc: 43.33
TCCCTGGTGGTCCGGTGGCGAAGACTCTCTGCTCCCAGTGCAGGGGGCCCAGGTTCGATC
CCTGGTCAGGGAA
>Bos_taurus_chr3.trna5316-TrpCCA (114331139-114331067) Trp (CCA) 73 bp Sc: 43.48
TCCTTGGTGGTCCAGTGGCTAGGACTCTGTGCTTCCAGTGCAGGGGACCTAGGTTCAATT
CCTGGTCAGAGAA
>Bos_taurus_chr11.trna274-TrpCCA (4559316-4559387) Trp (CCA) 72 bp Sc: 43.54
TCCCTGGTGGTCCAGTGGTTGGGATTTGGTGACTCCAATTCTGGGGCCCAGGTTCAATCC
CTGGTTAGGGAA
>Bos_taurus_chr21.trna1104-TrpCCA (24792295-24792366) Trp (CCA) 72 bp Sc: 43.69
TCCCTGGTGGTCCAGTGGTTAAGACTTCAGCTTCCACTGCAGGAGGTTCAGGCTCGATCC
CTGTTCAGGGAG
>Bos_taurus_chr16.trna1709-TrpCCA (43943285-43943356) Trp (CCA) 72 bp Sc: 43.75
TCCCTGGTGATCCAGTGGTTAGGATTTTTACTTCCAATGCAGGGGGCCCGGGTTCAGTCC
CTGGTCAGGGAA

>Bos_taurus_chr7.trna7645-TrpCCA (17351593-17351522) Trp (CCA) 72 bp Sc: 43.87
TCCCTGGTGGTCCAGTGGTTAAGACTCCTACTTCCAATGTAGGAGACGTGGGTTCAACCT
CTGTTCAGGGAA
>Bos_taurus_chrUn.004.1.trna53-TrpCCA (1117638-1117710) Trp (CCA) 73 bp Sc: 43.97
TCCCTGGTGGTCCAGTGGCTAAGACTCTACACTCCAAGTGTTGGGGGCCTGGGTTCAATC
CCTGGTCAGGGAG
>Bos_taurus_chr4.trna6664-TrpCCA (69055048-69054976) Trp (CCA) 73 bp Sc: 44.20
TGCCTGGTGGTCCAGTGGCTAAGACTCTGAGCTCCAAATGCAGGGGGCTCAGGTTCGATC
CCTGGTCAGGAAC
>Bos_taurus_chr19.trna5343-TrpCCA (28511221-28511149) Trp (CCA) 73 bp Sc: 44.51
TCCCTGATGGTCCAGTGGTTAAGACTCTGTGCTTCCAGTACAAGGGGTACAGGTTCGATC
CCTGGTCAGGGGA
>Bos_taurus_chr10.trna7681-TrpCCA (13557864-13557793) Trp (CCA) 72 bp Sc: 44.62
TCCCTGATGGTTCAGGGGCTAGGACTCCTGTTCCCAATTCAGGGGGCCCGGGATGGATCC
CTGGTCAGGGAA
>Bos_taurus_chr19.trna449-TrpCCA (12832887-12832959) Trp (CCA) 73 bp Sc: 44.63
TCCCTGGTGGTTCAGTGGCTGAGACCTCGTGCTCCCAATGCAGGGGGCCCAGGTTTGATC
CCTGGTCAGGGAA
>Bos_taurus_chr19.trna452-TrpCCA (12883837-12883909) Trp (CCA) 73 bp Sc: 44.63
TCCCTGGTGGTTCAGTGGCTGAGACCTCGTGCTCCCAATGCAGGGGGCCCAGGTTTGATC
CCTGGTCAGGGAA
>Bos_taurus_chr5.trna4809-TrpCCA (119428304-119428376) Trp (CCA) 73 bp Sc: 45.00
TCCCTGGTGGTCCAGTGGCTAAGACTCTGTGCTCCAAAAGCAGGGGGCCTGGGTTCAATC
CCTGGGCAGGGAA
>Bos_taurus_chrX.trna2049-TrpCCA (57570046-57570118) Trp (CCA) 73 bp Sc: 45.09
TCTCTGGTGGTCCAGTGGTTAAGACTTTATGCTTCCAGTGCAAATGGTGCAGGTTCAATC
CCTGGTTAGGGAG
>Bos_taurus_chr12.trna3191-TrpCCA (80188668-80188740) Trp (CCA) 73 bp Sc: 45.11
TCCTTGGTGGTCCAGTGGCTAAGACTCCGCGCCACACGCAGGGGTCCCAGGTTTGATC
CCTGGTCAGGGAA
>Bos_taurus_chr16.trna1697-TrpCCA (43847171-43847243) Trp (CCA) 73 bp Sc: 45.22
TTCCTGGTGGTCCAGTGGCTAAGACCCCGTGCTCCCAGTGCAGGGGGCCCAGGTTCGATC
CCTGGTCAGGGAA
>Bos_taurus_chr23.trna2917-TrpCCA (41931989-41931917) Trp (CCA) 73 bp Sc: 45.55
TCCCTGGTGGTCTGGTGGCTAAGACTCCGTGCTCCCAATGCAGGGGGCCCAGGTTCAATC
CCTGGTTAGGGAA
>Bos_taurus_chr22.trna194-TrpCCA (5739657-5739729) Trp (CCA) 73 bp Sc: 45.99
TCCCTGGTGGTCCAGTGGTTAAGACTCTGTGCTTCCAATGCAGGAGGTGCAGGTCCAATC
CCTGGCCGGGGAA
>Bos_taurus_chr24.trna4113-TrpCCA (34423924-34423852) Trp (CCA) 73 bp Sc: 46.08
TCCCTGGTGGTTCAGTGGTTAAGACTCCGTGCTTCCAATGCAGGGGACATGGGTTCAACC
CCCAGTCAGGGAA
>Bos_taurus_chr14.trna6225-TrpCCA (10367292-10367220) Trp (CCA) 73 bp Sc: 46.20
TCCCTGGTGGTCCAGTGGCTAAGACTCTGTGCTCCCAGTGCAAGGGGCCCAGGTTTAATT
CCTGGTCAGGGAA
>Bos_taurus_chr28.trna798-TrpCCA (21840788-21840860) Trp (CCA) 73 bp Sc: 46.59
TCCCTGGTGGACCAGTGGCTAAGACTCAGCACTCCAAATGCAGGGGGCCCAGGTTCACTC
CCTGGTCAGGGAA
>Bos_taurus_chr8.trna5271-TrpCCA (84011943-84011872) Trp (CCA) 72 bp Sc: 46.82
TCCCTGATGGTTCAGTGGTTAAGACTTCCCCTTCCAATGGAGGGGATACAGGTTCAATCC
CTGGTCGGGGAG
>Bos_taurus_chr22.trna3585-TrpCCA (22541039-22540967) Trp (CCA) 73 bp Sc: 47.85
TCCCTGGCAGTCCAGTGGTTAAGACTCTGTGCTTCCAATGCAAGGGGCTCAGGTTCGATC
CCTGGTTGGGGAA
>Bos_taurus_chr7.trna6004-TrpCCA (62589711-62589640) Trp (CCA) 72 bp Sc: 48.19
TTCCTGGTGGTCCAGTGGCTAAGACTCTTGTTCCCAATGCAGGGGGCCCAGGTTCAACCC
CTGGTCAGGGAA
>Bos_taurus_chr5.trna4038-TrpCCA (104126170-104126242) Trp (CCA) 73 bp Sc: 48.55
TCCCTGGTGGTCCAGTGGCCAAGACTCCGTGCTCCCAATGCAGGAGGCCCAGGTTCTATC
CCTGGTCAGGGAA
>Bos_taurus_chr4.trna295-TrpCCA (8882030-8882102) Trp (CCA) 73 bp Sc: 48.56
TCCCTGATGGTCCAGTGTCTAAGATTCTGTGCTCCCAATGCAAGGGGCCCAGGTTCAATC
CCTGGTCAGGGAA
>Bos_taurus_chr7.trna193-TrpCCA (4777378-4777450) Trp (CCA) 73 bp Sc: 48.60
TCCCTGGTGGTCCAGTGGTTAAGATGCTGTGCTCCAAATGCAGGAGGCCCAGGTTCCATC
CCTGGTCAGGGCA
>Bos_taurus_chr13.trna4364-TrpCCA (72864745-72864673) Trp (CCA) 73 bp Sc: 48.70

TCCCTGGTGGCCCAGTGGCTAAGACTCCGTGCTCCCAATGCAGGAGGCCCAGGTTCAAGC
CCTGGTCAGGGAA
>Bos_taurus_chr7.trna6057-TrpCCA (61303151-61303079) Trp (CCA) 73 bp Sc: 48.70
TCCCTAGTGGTCCAGTGGCTAAGACTCCGTGCTCCCAATGCAGGGGGCCCAGGTTCGATC
CCTGGTCAGGGAA
>Bos_taurus_chr19.trna1974-TrpCCA (40512069-40512141) Trp (CCA) 73 bp Sc: 48.80
TCCCTGGTAGTCCAGTGGCTAAGACACTCTGCTGCCAGTGCAGGGGGCCCAGGTTCGATC
CCTGGTCAGGGAG
>Bos_taurus_chr5.trna8100-TrpCCA (58271042-58270970) Trp (CCA) 73 bp Sc: 48.93
TCCCTGGTGGTTCAGTGGCTAATACTCCATGCTTCCAGTGCAGGGGGCCCAGGTTTGATT
CCTGGCCAGGGAG
>Bos_taurus_chr2.trna7504-TrpCCA (93735226-93735154) Trp (CCA) 73 bp Sc: 48.97
TCCCTGATGGGTCAGTGGCTAAGACTCCATGCTCCCAATGCAGGAGACCCAGGATCGATC
CCTGGTCAGGGAA
>Bos_taurus_chr24.trna3991-TrpCCA (36382257-36382185) Trp (CCA) 73 bp Sc: 49.14
TCCCTGGTGGTCCAGTGGCTACCACACTGTACTCCAAATGCAGGGGACCCGGGTTCAATC
CCTGGTCAGGGAA
>Bos_taurus_chr12.trna750-TrpCCA (18560557-18560629) Trp (CCA) 73 bp Sc: 49.44
TCCCTGGTGGTCCAGTGGTTAAGACTCTGTGCTTCCAATGCAAGGGGCGCAGGTTTGATC
CCTGGTCAGGGAA
>Bos_taurus_chrUn.004.5.trna50-TrpCCA (704292-704220) Trp (CCA) 73 bp Sc: 49.51
TCCCTGCTGGTCCAGTGGTTAAGACTCTGTGCTTCCAGTGTAGGGGGCCCAGGTTCAAAC
CCTGGTCAGGGAA
>Bos_taurus_chr10.trna3444-TrpCCA (89757044-89757116) Trp (CCA) 73 bp Sc: 49.55
TCCCCGGTGGTCCAGTGGCTAAGACTCTGTGCTCCCAGTGCAAGCAGCCCAGGTTCAATC
CCTGGTCGGGGAA
>Bos_taurus_chr27.trna3481-TrpCCA (15240761-15240690) Trp (CCA) 72 bp Sc: 49.63
TCCTTGGTTGTCCAGTGGCTAAGACTCCTGCTCCCAATGCAGGGGGCCTGGGTTCGATCC
CTGGCTGGGGAA
>Bos_taurus_chr23.trna709-TrpCCA (16822255-16822327) Trp (CCA) 73 bp Sc: 49.89
TCTCTGGTGGTCCAGTGGCTGGGACTCTGTGCTCCAAATGCAGGGGGCCCAGGTTCAATC
CCTGGTCAGGGAA
>Bos_taurus_chr11.trna4213-TrpCCA (102503775-102503847) Trp (CCA) 73 bp Sc: 50.24
TCCCTGGTGGTCCATTGGTTAAGACTCTGTGCTCCAAATGCAGGGGACCTGGATTCGATT
CCCAGTCAGGGAA
>Bos_taurus_chr11.trna4923-TrpCCA (102664633-102664561) Trp (CCA) 73 bp Sc: 50.24
TCCCTGGTGGTCCATTGGTTAAGACTCTGTGCTCCAAATGCAGGGGACCTGGATTCGATT
CCCAGTCAGGGAA
>Bos_taurus_chr11.trna687-TrpCCA (14330120-14330192) Trp (CCA) 73 bp Sc: 50.28
TCCCTGGTGGTCCAGTGGCTAAGACTCTGTGCTTCCAATGCAGGAAGCCCAGGTTCAATC
CCTGGACAGGGAA
>Bos_taurus_chr10.trna882-TrpCCA (20415195-20415267) Trp (CCA) 73 bp Sc: 50.49
TCCCTGGTGGTCCAGTGGTTAGGACTCTGTGCTTCCAATGCAAGGGGTTCAAGTTCGATC
CCTGGTTGGGGAA
>Bos_taurus_chr29.trna1799-TrpCCA (47477691-47477763) Trp (CCA) 73 bp Sc: 50.72
TCCCTGATAGTCCAGTGGCTAAGACTCTGTGCTCCCAATGCAAGGGGCCCAGGTTCAATC
CCTGGCCAGGGAA
>Bos_taurus_chr19.trna2140-TrpCCA (43723176-43723248) Trp (CCA) 73 bp Sc: 50.77
TCCCTGATGGTCCAGTGGCTAAGACTCTGTGCTCCCAATGCAAGGGGCCCGGGTTCAATC
CCTGGTCAGGGAA
>Bos_taurus_chr11.trna2260-TrpCCA (53119033-53119104) Trp (CCA) 72 bp Sc: 50.77
TCCCTGGTGGTCCAGTGGTTAAGACTTCAGCTTCCAATGCAGAGGGTACAGGTTTGATTC
CTGGTTGGGGAA
>Bos_taurus_chr3.trna4992-TrpCCA (121626580-121626508) Trp (CCA) 73 bp Sc: 50.98
TCCCTGGTGGTCCAGTGGCTAAGACTCCGTGCTCCCAATGCAGGGGACCCAGGTTCAATC
CCTGGTCAGGGAA
>Bos_taurus_chr13.trna1510-TrpCCA (35936201-35936273) Trp (CCA) 73 bp Sc: 51.15
TCCCTGGTGGTCCAGTGGCTAGGATTTTGTGCTTCCAGTGCAGGAAGCCCAGGTTCAATC
CCTGGTCAGGGAA
>Bos_taurus_chr7.trna6743-TrpCCA (40313694-40313623) Trp (CCA) 72 bp Sc: 51.21
TCCCTGCTGGCTCAGAGGTTAAAGCATCTGCCTCCAATGTGGGAGACCCAGGTTCAATCC
CTGGGTCGGGAA
>Bos_taurus_chrUn.004.2618.trna1-TrpCCA (10518-10447) Trp (CCA) 72 bp Sc: 51.21
TCCCTGCTGGCTCAGAGGTTAAAGCATCTGCCTCCAATGTGGGAGACCCAGGTTCAATCC
CTGGGTCGGGAA
>Bos_taurus_chr1.trna4687-TrpCCA (134360457-134360529) Trp (CCA) 73 bp Sc: 51.35
TCCCTGGTGGTCCAGTGGCTAAGACTCTGTGCCCCCAATGCAGGAGACCCAGGTTCAATA

CCTGGTCAGGGAA
>Bos_taurus_chr19.trna3019-TrpCCA (58916114-58916186) Trp (CCA) 73 bp Sc: 51.49
TCCCTGGTGGTCCAGTAGCTAGGACTCCGTGCTCCCAGTGCAGGAGGCCCAGGTTCGATC
CCTGGTCAGGGAA
>Bos_taurus_chr15.trna2659-TrpCCA (74710626-74710697) Trp (CCA) 72 bp Sc: 51.62
TCCCGGGTAGCTCAGACGGTGAAGCATCTGCCTCCAGTGCAGGAGACCCAGGTTCAATCC
CTGGATCGGGAA
>Bos_taurus_chr17.trna3656-TrpCCA (70235801-70235729) Trp (CCA) 73 bp Sc: 51.71
TCCCTGGTGGTCCAGTGGCTAAGACTCTGTGCTCCCAGTGCACGGGGCCCAGGTTCGATC
CCTGGTCAGGGAA
>Bos_taurus_chr18.trna1327-TrpCCA (33405185-33405257) Trp (CCA) 73 bp Sc: 52.87
TCCCTGGTGGTCCAGTGGCTAAGACTCCGTGCTCCCAATGCAGGGGGCCCAGGTTCAATC
CCTGGTCAGGGAA
>Bos_taurus_chr27.trna2258-TrpCCA (39468861-39468788) Trp (CCA) 74 bp Sc: 53.25
TCCCTGGTGGTCCAGTGGTTTAAGACCCTGTGCTCCAAATGCAGGGGGCCTGGGTTCAAT
CCCTGGCCAGGGAA
>Bos_taurus_chr22.trna1657-TrpCCA (46703863-46703934) Trp (CCA) 72 bp Sc: 53.51
TCCCTGGTGGTCCAGTGGCTAAGACTCTTGCTCCCAATGCAGGGGACCCAGGTTTGATCC
CTGGTCAGGGAA
>Bos_taurus_chr16.trna3964-TrpCCA (58937752-58937681) Trp (CCA) 72 bp Sc: 53.64
TCCCTGGTGGTCTAGTGGCTACGACTTGTGCTTCCAGTGCAGGGGGCCCAGGTTCAATCC
CTGGTCAGGGAA
>Bos_taurus_chr11.trna7168-TrpCCA (49513374-49513303) Trp (CCA) 72 bp Sc: 53.76
TCCTTGGTGGTCCAGTGGTTAAGACTCCGGCTTCCACTGCAGGGGGCTCAGGTTCAATCC
CTGGTCAGGGAA
>Bos_taurus_chr6.trna6929-TrpCCA (56583028-56582957) Trp (CCA) 72 bp Sc: 54.85
TCCCTGGTGGCTCAGAGGTTAAAGTGTCTGCCTCCAATGCAGGAGACTCGGGTTCGATCC
CTGGGTAGGGAA
>Bos_taurus_chr6.trna6931-TrpCCA (56485458-56485387) Trp (CCA) 72 bp Sc: 54.85
TCCCTGGTGGCTCAGAGGTTAAAGTGTCTGCCTCCAATGCAGGAGACTCGGGTTCGATCC
CTGGGTAGGGAA
>Bos_taurus_chr14.trna6250-TrpCCA (10066839-10066767) Trp (CCA) 73 bp Sc: 54.95
TCCCTGGTGGTCCAGTGGCTAAGACTCTGCACTCCAAATGCAGAGGGCTCAGGTTCGAGC
CTCGGTCAGGGAA
>Bos_taurus_chr18.trna3647-TrpCCA (53323710-53323638) Trp (CCA) 73 bp Sc: 55.10
TCCCTGATGGCTCAGAGGTTAAAGCGTCTACTTCCAATGCAGGAGGCCCAGGTTCGATCC
CTGGGTCAGGAAG
>Bos_taurus_chr27.trna1720-TrpCCA (44995431-44995503) Trp (CCA) 73 bp Sc: 55.10
TCCCTGATGGCTCAGAGGTTAAAGCGTCTACTTCCAATGCAGGAGGCCCAGGTTCGATCC
CTGGGTCAGGAAG
>Bos_taurus_chr1.trna3827-TrpCCA (112872145-112872217) Trp (CCA) 73 bp Sc: 55.18
TCCCTGGTGGCTCAGAGGTTAAAGCGTCTGCCTCCAATGCAGAAGACCCGGGTTCGATCC
CTGGGTCAGGAAG
>Bos_taurus_chr25.trna2820-TrpCCA (36887919-36887848) Trp (CCA) 72 bp Sc: 55.21
TCCCTGGTGGTCCAGTGGTTAAGACTCTGCCTCCACTGCAGGGGGCCCCAGGTTCGATCC
CTGGTCAGGGAA
>Bos_taurus_chr6.trna8579-TrpCCA (3405847-3405776) Trp (CCA) 72 bp Sc: 55.21
TCCCTGGTGGCTCAGAGGTTAAAGCATCTGCCCCCAATGCAGGAGACCCAGGTTCGATCC
CTGGGTCGGGAA
>Bos_taurus_chr21.trna1091-TrpCCA (24469263-24469334) Trp (CCA) 72 bp Sc: 55.28
TCCCTGGTGGCTCAGAGGTTAAAGCGTCTGCCTCCAATGCAGGAGACCCAGGTTTGATCC
CTGGGTTGGGAA
>Bos_taurus_chrX.trna3504-TrpCCA (88296773-88296844) Trp (CCA) 72 bp Sc: 55.58
TCCCTGATAGCTGAGTTGGTAAAGCATCTGCCTCCAATGCAGGAGACCCTGGTTCCATTC
CTGGGTTGGGAA
>Bos_taurus_chr17.trna1442-TrpCCA (39010358-39010430) Trp (CCA) 73 bp Sc: 55.77
TTCCTGGTGGCTCAGAGGTTAAAGCGTCTGCCTCCAATGCTGGAGACCCAGGTTCGATCC
CTGGGTCGGGAAG
>Bos_taurus_chr11.trna4224-TrpCCA (102607992-102608064) Trp (CCA) 73 bp Sc: 55.95
TCCCTGATGGTCCAGTGGCTAAGACTCCATGCTCCCAATGCAGGGGACCCAGGTTCGATC
CCTGGTCAGGGAA
>Bos_taurus_chr11.trna4933-TrpCCA (102560871-102560799) Trp (CCA) 73 bp Sc: 55.95
TCCCTGATGGTCCAGTGGCTAAGACTCCATGCTCCCAATGCAGGGGACCCAGGTTCGATC
CCTGGTCAGGGAA
>Bos_taurus_chr24.trna607-TrpCCA (18664241-18664312) Trp (CCA) 72 bp Sc: 56.07
TCCCTGGTGGCTCAGAGGTTAAAGCGTCTGCCTCCAAGGCAGGAGACCCAGGTTCGATCC
CTGGGTCGGGAA

>Bos_taurus_chr13.trna1477-TrpCCA (35267910-35267982) Trp (CCA) 73 bp Sc: 56.09
TCCCTGATGGTCCAAGGGCTAAGACTCTGCACTCCAAATGCAGGGGGTCCAGGTTCCATC
CCTGGTCAGGGAA
>Bos_taurus_chr23.trna684-TrpCCA (16491624-16491696) Trp (CCA) 73 bp Sc: 56.18
TCCCTGGTGGTCCAGTGGCTAAGACTTCCTGCTCCCAGTGCAGGGGGCCCAGGTTCGATT
CCTGGTCAGGGAA
>Bos_taurus_chr6.trna7902-TrpCCA (24717036-24716964) Trp (CCA) 73 bp Sc: 56.33
TCCCTGGTGGCTCAGAGGTTAAAGTGTCTGCCTCCAATGCAGGAGACCCAGGTTCGATCC
CTGGGTCAGGAAG
>Bos_taurus_chrX.trna2976-TrpCCA (78519971-78520042) Trp (CCA) 72 bp Sc: 56.69
TCCTTGGTGGTCCAGTGGTAGGATACCATGCTCCCAATGCATGGGGCCTGGGTTCAATCC
CCAGTCAGGGAA
>Bos_taurus_chr28.trna2308-TrpCCA (22601007-22600935) Trp (CCA) 73 bp Sc: 57.31
TCCCTGGTGGCTCAGAGGTTAAAGCATCTGCCTCCAATGCAGAAGACCCAGGTTCAATCC
CTGAGTCAGGAAG
>Bos_taurus_chrUn.004.3263.trna2-TrpCCA (11935-11863) Trp (CCA) 73 bp Sc: 57.31
TCCCTGGTGGCTCAGAGGTTAAAGCATCTGCCTCCAATGCAGAAGACCCAGGTTCAATCC
CTGAGTCAGGAAG
>Bos_taurus_chr3.trna2897-TrpCCA (83551929-83552000) Trp (CCA) 72 bp Sc: 57.45
TCTCTGGTGGCTCAGAGGTTAAAGCGTCTGCCTCCAATGCAGGAGACCCAGGTTCGATCC
CTGGGTTGGGAA
>Bos_taurus_chr14.trna2048-TrpCCA (47749089-47749160) Trp (CCA) 72 bp Sc: 57.58
TCCCTGGTGGCTCAGAGGTTAAAGCATCTGCCTCCAGCGCAGGAGACCCAGGTTCGATCC
CTGGGTGGGAAA
>Bos_taurus_chr12.trna731-TrpCCA (18124319-18124390) Trp (CCA) 72 bp Sc: 57.64
TCCCTGGTGGCTCAGAGGTTAAAGCGTCTGCCTCCAATGCAGGAGACCGAGGTTCAATTC
CTGGGTGGGGAA
>Bos_taurus_chr12.trna4631-TrpCCA (55602812-55602740) Trp (CCA) 73 bp Sc: 57.80
TCCCTGGTGGCTCAGAGGTTAAAGCGTCTGCCTCCAATGCAGGAGACCCAGGTTCGATCC
CTGGGTCAGGAAG
>Bos_taurus_chr12.trna4636-TrpCCA (55546115-55546043) Trp (CCA) 73 bp Sc: 57.80
TCCCTGGTGGCTCAGAGGTTAAAGCGTCTGCCTCCAATGCAGGAGACCCAGGTTCGATCC
CTGGGTCAGGAAG
>Bos_taurus_chr1.trna7719-TrpCCA (107636551-107636480) Trp (CCA) 72 bp Sc: 57.99
GACCTTGTGGTGTAATGGTAGCATGTCTGACTCCAAGTCAGAAGGTTGTGTGTTCAAGTC
ACATTGGGGTCA
>Bos_taurus_chr19.trna2010-TrpCCA (41086345-41086416) Trp (CCA) 72 bp Sc: 58.13
TCCCTGGTGGCTCAGAGGTTAAAGCGTCTGCCTCCAATGCAGGGGACCCGGGTTCGATCC
CTGGGTAGGGAA
>Bos_taurus_chr13.trna5253-TrpCCA (55891248-55891176) Trp (CCA) 73 bp Sc: 58.25
TCCCTGGTGGTCCAGTGGCTAAGACTCCTCACTCCAAATGCAGGGGGCCCAGGTTCAATC
CCTGGTTAGGGAA
>Bos_taurus_chrX.trna1434-TrpCCA (37048356-37048428) Trp (CCA) 73 bp Sc: 58.29
TCCCTGGTGGTCCAGTGGCTAAGACTCTTGGCTTCCAATGCAGGAGGCCCAGGTTCAATT
CCTGGTCAGGGAA
>Bos_taurus_chr17.trna1739-TrpCCA (48680392-48680464) Trp (CCA) 73 bp Sc: 58.31
TCCCTGGTGGTCCAGTGGCTAAGATTCCACACTCCAAATGCGGAGGGTCCAGGTTCAATT
CCTGGTTGGGGAA
>Bos_taurus_chrUn.004.211.trna5-TrpCCA (118736-118665) Trp (CCA) 72 bp Sc: 58.74
TCCCTGGTGGCTCAGAGGTTAAAGCATCTGCCTCCAATGCAGGAGACCCAGGTTCAATCC
CTGGGTTGGGAA
>Bos_taurus_chr24.trna4347-TrpCCA (29607965-29607893) Trp (CCA) 73 bp Sc: 58.83
TCCCTGGTGGTTCAGTGGCTAAGACTCTGCACTCCAAATGCAGGGGGCCCAAGTTCGATC
CCTGGTCAGGGAA
>Bos_taurus_chr18.trna896-TrpCCA (22529402-22529473) Trp (CCA) 72 bp Sc: 59.06
TCCCTGGTGGCTCAGAGGTTAAAGCGTCTGCCTCCAGTGCAGGAGACCCAGGTTCAATCC
CTGGGTTGGGAA
>Bos_taurus_chr26.trna932-TrpCCA (27605926-27605997) Trp (CCA) 72 bp Sc: 59.20
TCCCTGGTGGCTCAGAGGTTACAGCGTCTGCCTCCAATGCAGGAGACCCAGGTTCGATCC
CTGGGTCGGGAA
>Bos_taurus_chr21.trna4823-TrpCCA (21741661-21741590) Trp (CCA) 72 bp Sc: 59.46
TCCCTGGTGGCTCAGAGGTTAAAGCGTCTGCCTCCAATGCAGGAGACCCAGGTTCAATCC
CTGGGTCGGGAA
>Bos_taurus_chr12.trna5593-TrpCCA (30170013-30169942) Trp (CCA) 72 bp Sc: 59.48
TCCCTCATGGTCCAGTGGCTAAGACTCTGGCTTCCAATGCAAGGGGCCCAGGTTCAATCC
CTGGTCAGGGAA
>Bos_taurus_chr5.trna6607-TrpCCA (95121376-95121305) Trp (CCA) 72 bp Sc: 59.59

TCCCTGGTGGCTCAGAGGTTAAAGCGTCTGCCTCCAATGCAGGAGACCCGGGTTTGATCC
CCGGGTGGGGAA
>Bos_taurus_chr15.trna255-TrpCCA (11322307-11322378) Trp (CCA) 72 bp Sc: 60.37
TCCCTGGTGGCTCAGAGGTTAAAGTGTCTGCCTCCAATGCAGGAGACCCAGGTTCAATTC
CTGGGTTGGGAA
>Bos_taurus_chrUn.004.136.trna30-TrpCCA (1021-949) Trp (CCA) 73 bp Sc: 60.53
TTCCTGGTGGCTCAGAGGTTAAAGCGTCTGCCTCCAATGCAGGAGACCCAGGTTCGATCC
CTGGGTCGGGAAG
>Bos_taurus_chr19.trna5355-TrpCCA (28389661-28389590) Trp (CCA) 72 bp Sc: 60.75
GGCCTCGTGGCGCAACGGTAGCGCGTCTGACTCCAGATCAGAAGTTGCGTGTTCAAATCA
CGTCGGGGGTCA
>Bos_taurus_chr27.trna3688-TrpCCA (8669135-8669064) Trp (CCA) 72 bp Sc: 60.78
TCCCTAGTGGCTCAGAGGTTAAAGCGTCTGCCTCCAATGCAGGAGACCCAGGTTCAATCC
CTGGGTTGGGAA
>Bos_taurus_chr16.trna3351-TrpCCA (72622490-72622419) Trp (CCA) 72 bp Sc: 60.80
TCCCTGGTGGCTTAGAGGTTAAAGCATCTGCCTCCAATGCAGGAGACCCAGGTTCGATCC
CTGGGTTGGGAA
>Bos_taurus_chr18.trna1096-TrpCCA (26959534-26959605) Trp (CCA) 72 bp Sc: 61.52
TCCCTAGTGGCTCAGAGGTTAAAGCGTCTGCCTCCAATGCAGGAGACCCAGGTTCGATCC
CTGGGTCGGGAA
>Bos_taurus_chr8.trna2605-TrpCCA (77374493-77374564) Trp (CCA) 72 bp Sc: 61.86
TCCTTGGTGGCTCAGAGGTTAAAGCGTCTGCCTCCAATGCAGGAGGCCCAGGTTCGATCC
CTGGGTCGGGAA
>Bos_taurus_chr25.trna3911-TrpCCA (19064405-19064334) Trp (CCA) 72 bp Sc: 62.20
TCCCTGGTGGCTCAGAGGTTAAAGCGTCTGCCTCCAATGCAGGAGACCCAGGTTCGATCC
CTGGATTGGGAA
>Bos_taurus_chr3.trna8612-TrpCCA (22148523-22148452) Trp (CCA) 72 bp Sc: 63.52
TCCCTGGTGGCTCAGTGGGTAAAGCGTCCGCCTCCAATGCGGGAGGCCCAGGTTCGATCC
CTGGGTCGGGAA
>Bos_taurus_chr15.trna4934-TrpCCA (35687145-35687074) Trp (CCA) 72 bp Sc: 63.76
TCCCTGGTGGCTCAGAGGTTAAAGCGTCTGCCTCCAGTGCAGAAGACCCAGGTTCGATCC
CTGGGTTGGGAA
>Bos_taurus_chr3.trna3377-TrpCCA (96177012-96177084) Trp (CCA) 73 bp Sc: 65.70
TCCTTGGTGGTCCAGTGGTTAAGACTCTGCACCCCAAATGCAGAGAGCCCAGGTTCGATC
CCTGGTTGGGGAA
>Bos_taurus_chr3.trna2227-TrpCCA (62707958-62708029) Trp (CCA) 72 bp Sc: 65.82
TCCCTGTTGGCTCAGAGGTTAAAGCATCCGCCTCCAATGTGGGAGACCCAGGTTCGATCC
CTGGATAGGGAA
>Bos_taurus_chr3.trna7141-TrpCCA (62381816-62381745) Trp (CCA) 72 bp Sc: 65.82
TCCCTGTTGGCTCAGAGGTTAAAGCATCCGCCTCCAATGTGGGAGACCCAGGTTCGATCC
CTGGATAGGGAA
>Bos_taurus_chr20.trna4066-TrpCCA (43940108-43940037) Trp (CCA) 72 bp Sc: 69.48
GGCCTCGTGGCGCAGTGGTAGAGCGTCTGACTCCAGATCAGAAGGTTGTGTGTTCAAATC
ACGTCGGGGTCA
>Bos_taurus_chr19.trna1411-TrpCCA (28373611-28373682) Trp (CCA) 72 bp Sc: 74.00
GACCTCGTGGCGCAACGGTAGCGCGTCTGACTCCAGATCAGAAGGTTGCGTGTTCAAATC
ACGTCGGGGTCA
>Bos_taurus_chr23.trna1420-TrpCCA (31691612-31691683) Trp (CCA) 72 bp Sc: 74.00
GACCTCGTGGCGCAACGGTAGCGCGTCTGACTCCAGATCAGAAGGTTGCGTGTTCAAATC
ACGTCGGGGTCA
>Bos_taurus_chr23.trna1422-TrpCCA (31700375-31700446) Trp (CCA) 72 bp Sc: 74.00
GACCTCGTGGCGCAACGGTAGCGCGTCTGACTCCAGATCAGAAGGTTGCGTGTTCAAATC
ACGTCGGGGTCA
>Bos_taurus_chr25.trna2713-TrpCCA (38760610-38760539) Trp (CCA) 72 bp Sc: 74.00
GACCTCGTGGCGCAACGGTAGCGCGTCTGACTCCAGATCAGAAGGTTGCGTGTTCAAATC
ACGTCGGGGTCA
>Bos_taurus_chr25.trna2716-TrpCCA (38669845-38669774) Trp (CCA) 72 bp Sc: 74.00
GACCTCGTGGCGCAACGGTAGCGCGTCTGACTCCAGATCAGAAGGTTGCGTGTTCAAATC
ACGTCGGGGTCA
>Bos_taurus_chr19.trna4961-TrpCCA (35068433-35068362) Trp (CCA) 72 bp Sc: 74.80
GACCTCGTGGCGCAATGGTAGCGCGTCTGACTCCAGATCAGAAGGTTGCGTGTTCAAGTC
ACGTCGGGGTCA
>Bos_taurus_chr19.trna1732-TrpCCA (34003678-34003749) Trp (CCA) 72 bp Sc: 75.98
GACCTCGTGGCGCAATGGTAGCGCGTCTGACTCCAGATCAGAAGGTTGCGTGTTCAAGTC
ACGTCGAGGTCA
>Bos_taurus_chr11.trna12-TrpCCA (244474-244566) Trp (CCA) 93 bp Sc: 29.77
TTCCCAATGGTCTACTGTTTAAGACTCTGCTTTCCAGTGTTTAAGACTCTGCTTCCACTG

CAGGGGATGCAGGTTCGATTCCTGGTTGGGGAA
>Bos_taurus_chr9.trna1681-TyrATA (52260909-52260980) Tyr (ATA) 72 bp Sc: 44.35
TCCTTGCTGGCTCAGACAGTAAAGCATCTGCCTATAGTGCAGGAGCTCCAGGTTCAATCC
CTGGGTCAGGAA
>Bos_taurus_chr21.trna4552-TyrATA (26075925-26075854) Tyr (ATA) 72 bp Sc: 49.91
TCCCTGGTGGCTCAGACGGTAAAGCATCTGCCTATAATGCAGGAGAGCCGGGTTCAATCC
CTGGGTAGGGAA
>Bos_taurus_chr15.trna95-TyrATA (4204993-4205065) Tyr (ATA) 73 bp Sc: 55.06
TCCCTGGTGGCTCAGATGGTAAAGCATCTGCCTATAATGCAGGAGACCCAGGTTCGATCC
CTGGGTCAGGAAG
>Bos_taurus_chr12.trna1139-TyrATA (25969653-25969724) Tyr (ATA) 72 bp Sc: 55.78
TCCCTGGTGGCTCAGATGGTAGGGCATCTGCCTATAATGCAGGAGACCCATGTTCGATCC
CTGGGTGGGGAA
>Bos_taurus_chr12.trna3312-TyrATA (82290770-82290841) Tyr (ATA) 72 bp Sc: 57.40
TCCCTAGTAGCTCAGATGGTAAAGCATCTGTCTATAATGCAGGAGACCCAGGTTCGATCC
CTGGTTCGGGAA
>Bos_taurus_chr23.trna1072-TyrATA (23991188-23991259) Tyr (ATA) 72 bp Sc: 59.60
TCCCTGGTGGCTCAGAAGGTAAAGCATCTGCCTATAATGCAGGAGGCCTGGGTTCGATCC
CCAGGTGGGGAA
>Bos_taurus_chr11.trna1661-TyrATA (41268333-41268405) Tyr (ATA) 73 bp Sc: 59.73
TTCCTGGTGGCTCAGATGGTAAAGCGTCTGCCTATAATGCAGAAGACCCCGGTTCGATCC
CTGGGTCAGGAAG
>Bos_taurus_chr9.trna2219-TyrATA (68857649-68857720) Tyr (ATA) 72 bp Sc: 59.95
TCCTTGGTGGCTCAGATGGTAAAGCGTCTGCCTATAATGCAGGAGACCCAGGTTCGATCC
CTGGGTTGGGAA
>Bos_taurus_chr4.trna7-TyrATA (240980-241051) Tyr (ATA) 72 bp Sc: 60.02
TCCCTAGTGGCTCAGATGGTAAAGTGTCTGCCTATAATGCAGGAGACCCAGGTTCGATCC
CTGGGTTGGGAA
>Bos_taurus_chr16.trna5700-TyrATA (19192687-19192616) Tyr (ATA) 72 bp Sc: 64.25
TCCCTGGTGGCTTAGATGGTAAAGTGTCTGCCTATAATGCAGAAGACCCAGGTTCAATCC
CTGGTTTGGGAA
>Bos_taurus_chr26.trna2716-TyrGTA (35344352-35344279) Tyr (GTA) 74 bp Sc: 21.93
TTCCTGGTGGCTCAGACAGTAAAGGATCTGCCTGTAATGTGAGAAAACTCAGGTTCGATC
CCTGGGTTGGGAAG
>Bos_taurus_chr5.trna6192-TyrGTA (103873816-103873745) Tyr (GTA) 72 bp Sc: 28.30
TCCTTGTTGGCTCAGAAGGTAAAGACTCCACCTGTAATATGGAGGACCCAGGTTTGATCC
CTGGGTGGGGAA
>Bos_taurus_chr1.trna3297-TyrGTA (96876149-96876220) Tyr (GTA) 72 bp Sc: 35.24
TCCTGGGTGGCTCAGACACTAAAGTGTCTGTCTGTAATGCGGGAGATCCAGGTTCGATCC
CTGGGTCAGGAA
>Bos_taurus_chr24.trna4138-TyrGTA (34234332-34234260) Tyr (GTA) 73 bp Sc: 39.46
TCCCTGGTGGTCCAGTGGCTGAGACTCTCTGCTCGTAATGCAGGAAGCCCAGGTTTGATC
CCTGGTCAGGGAA
>Bos_taurus_chr14.trna4741-TyrGTA (43888913-43888842) Tyr (GTA) 72 bp Sc: 40.94
TCCCTAGTGGCTCAGATGGTAAAGTATCTGCCTGTAATGAGGGAACCCCAGGTTCAATCC
CTGGGTGGGGAA
>Bos_taurus_chr7.trna2768-TyrGTA (65123030-65123101) Tyr (GTA) 72 bp Sc: 42.11
TCCTTGGTGGCTCAGACAGTAAAGTGTCTGCCTGTAATGCAGGAGCCCTGGGTTCGACCT
CCAGGTAGGGAA
>Bos_taurus_chr8.trna1476-TyrGTA (45997658-45997729) Tyr (GTA) 72 bp Sc: 49.17
TCCCTGGTAGCTCAGTCAGTAAAGAACCTGCCTGTAGTGCAGGAAACCCAGGTTCGATCC
CTGGGTGGGGAA
>Bos_taurus_chr23.trna3573-TyrGTA (27119474-27119403) Tyr (GTA) 72 bp Sc: 50.57
TCCCTAGTGGCTCAGATGGTAAAGCATCTGCCTGTAATGCAGGAGACCTAGGTTGGACCC
CTGGCTGGGAAA
>Bos_taurus_chr10.trna8126-TyrGTA (2488616-2488545) Tyr (GTA) 72 bp Sc: 55.38
TCCCTGCTAGCTCAGTTGGTAAAGAATCTGCCTGTAATGCAGGAGACCCTGGTTCAATTC
CTGGGTCGGGAA
>Bos_taurus_chr2.trna1450-TyrGTA (45556863-45556934) Tyr (GTA) 72 bp Sc: 55.44
TCCCTAGTGGCTCAGACGGTAAAGCATCTGTCTGTAATGCAGGAGACCCAGGTTCGATCC
CTGGGTTGGGAA
>Bos_taurus_chr15.trna1231-TyrGTA (36414383-36414453) Tyr (GTA) 71 bp Sc: 55.79
TCCCTGGTGGCTCATTGGTAAAGAGTCTGCCTGTAATGCAGGAGGTCCAGGTTCAATCCC
TGGTTGGGGAA
>Bos_taurus_chr5.trna7402-TyrGTA (75121036-75120966) Tyr (GTA) 71 bp Sc: 56.10
TCCTGGCTGGCTCAGCGGTAAAGAATCTGCCTGTAATGCAGGAGGCCCAGGTTCAATCTC
TGGGTCAGGAA

>Bos_taurus_chr3.trna3750-TyrGTA (106523838-106523909) Tyr (GTA) 72 bp Sc: 56.29
TCCCTGATAGCTCAGTTGGTAAAGTATCTGCCTGTAGTGCAGGAGACCTGGGTTCAATCC
CTAGGTAGGGAA
>Bos_taurus_chr3.trna7519-TyrGTA (51687011-51686940) Tyr (GTA) 72 bp Sc: 56.51
TCCCTTGTAGCTGAGTTGGTAAAGAATCTGCCTGTAATGCAGGAGACCCAGGTTCAATTC
CTGGGTTGGGAA
>Bos_taurus_chr1.trna8178-TyrGTA (91805218-91805147) Tyr (GTA) 72 bp Sc: 56.66
CCCCTGCTAGCTCAGTTGGTAAAGAATCTGCCTGTAATGCAGGAGACCCAGGTTCGATTC
CTGGGTTGGGAA
>Bos_taurus_chr22.trna2510-TyrGTA (54473765-54473694) Tyr (GTA) 72 bp Sc: 56.95
TCCCTGGTGGCTCAGATGGTAAAGCATCTGCCTGTAATGCGGAAGACCCAGGTTCGATCC
CTGGGTTGGGAA
>Bos_taurus_chr5.trna4580-TyrGTA (115536488-115536559) Tyr (GTA) 72 bp Sc: 57.05
TCCCTGGTGGCTCAGATGGTAAAGCGTCTGCCTGTAATGCAGGAGATCCAGGTTCAATCC
CTGGGTTGGGAA
>Bos_taurus_chr17.trna150-TyrGTA (5514888-5514959) Tyr (GTA) 72 bp Sc: 57.24
TCCCCGGTGGCTCAGAGGATAAAGCATCTGCCTGTAATGCAGGAGACCCAGGTTCGATCC
CTGGGTTGGGAA
>Bos_taurus_chrUn.004.1176.trna4-TyrGTA (26242-26171) Tyr (GTA) 72 bp Sc: 57.89
TCCCTGGTAGCTCAGATGGTAAAGAATCTGCTTGTAATGCAGAAGACCCAGGTTCAATCC
CTGGCTGGGGAC
>Bos_taurus_chr5.trna134-TyrGTA (4759686-4759757) Tyr (GTA) 72 bp Sc: 58.66
TCCCTAGTGGTTCAGATGGTAAAGCATCTGCCTGTAATGCAGGAGACCCAGGTTCAATCC
CTGGCTCGGGAA
>Bos_taurus_chrX.trna4077-TyrGTA (77839964-77839893) Tyr (GTA) 72 bp Sc: 59.46
TCCCTGGTGGCTCAGACGGTAAAGCGTCTGCCTGTAATGCAGGAGACCCAGGTTCAATTC
CTGGGTCGGGAA
>Bos_taurus_chr10.trna7203-TyrGTA (25817095-25817006) Tyr (GTA) 90 bp Sc: 75.19
CCTTCGATAGCTCAGCTGGTAGAGCGGAGGACTGTAGTAATTCCAAGTGTGGACATCCTT
AGGTCGCTGGTTCGATTCCGGCTCGAAGGA
>Bos_taurus_chr10.trna1095-TyrGTA (25844468-25844557) Tyr (GTA) 90 bp Sc: 71.72
CCTTCGATAGCTCAGCTGGTAGAGCGGAGGACTGTAGGTCTACTACATGTGGACATCCTT
AGGTCGCTGGTTCGACTCCGGCTCGAAGGA
>Bos_taurus_chr10.trna1096-TyrGTA (25845137-25845226) Tyr (GTA) 90 bp Sc: 72.64
CCTTTGATAGCTCAGCTGGTAGAGCAGAGGACTGTAGACCTGATAAATGTGGACATCCTT
AGGTCGCTGGTTCGATTCCAGCTCAAAGGA
>Bos_taurus_chr10.trna1097-TyrGTA (25847905-25847995) Tyr (GTA) 91 bp Sc: 77.87
CCTTCGATAGCTCAGCTGGTAGAGCGGAGGACTGTAGTATTTCATGAATATAGATATCCT
TAGGTCGCTGGTTCGATTCCGGCTCGAAGGA
>Bos_taurus_chr10.trna1098-TyrGTA (25849753-25849842) Tyr (GTA) 90 bp Sc: 75.48
CCTTCGATAGCTCAGCTGGTAGAGCGGAGGACTGTAGATACGAAGCATGTAGACATCCTT
AGGTCGCTGGTTCGATTCCGGCTCGAAGGA
>Bos_taurus_chr14.trna1327-TyrGTA (30403968-30404061) Tyr (GTA) 94 bp Sc: 73.83
CCTTCGATAGCTCAGTTGGTAGAGCGGAGGACTGTAGTTGATACTCTAGCCGAGAGACAT
CCTTAGGTCGCTGGTTCGACTCCGGCTCGAAGGA
>Bos_taurus_chr14.trna1328-TyrGTA (30404552-30404640) Tyr (GTA) 89 bp Sc: 72.24
CCTTCGATAGCTCAGCTGGTAGAGCGGAGGACTGTAGACGTATGTCGCCGGCCATCCTTA
GGTCGCTGGTTCGATTCCGGCTCGAAGGA
>Bos_taurus_chr23.trna3384-TyrGTA (31548315-31548226) Tyr (GTA) 90 bp Sc: 77.86
CCTTCGATAGCTCAGTTGGTAGAGCGGAGGACTGTAGGAACTTGGTTGATGGTTATCCTT
AGGTCGCTGGTTCGAATCCGGCTCGAAGGA
>Bos_taurus_chr23.trna3383-TyrGTA (31548723-31548634) Tyr (GTA) 90 bp Sc: 77.74
CCTTCGATAGCTCAGTTGGTAGAGCGGAGGACTGTAGGTATTTGACTAATGGCCATCCTT
AGGTCGCTGGTTCGAATCCGGCTCGAAGGA
>Bos_taurus_chr23.trna3382-TyrGTA (31556019-31555928) Tyr (GTA) 92 bp Sc: 77.63
CCTTCGATAGCTCAGTTGGTAGAGCGGAGGACTGTAGTGTGTTAAACGTCATAGGCATCC
TTAGGTCGCTGGTTCGAATCCGGCTCGAAGGA
>Bos_taurus_chr20.trna1231-TyrGTA (35115043-35115131) Tyr (GTA) 89 bp Sc: 69.50
CCTTCGGTAGCTCAGCTGGTAGAGCGGAGGACTGTAGATGCCTGTCTGTGGCCATCCTTA
GGTCGCTGGTTCGATTCCGGCTCGAAGGA
>Bos_taurus_chr8.trna8123-TyrGTA (4372578-4372489) Tyr (GTA) 90 bp Sc: 48.39
CCTTTGATGGCTCAGCTGGTAGAGTGGAGGACTGTAGTATATCCCAGTATAGTCATCCTT
AGGTCACTGGTTTGATACTGGGTTGGAGGA
>Bos_taurus_chr21.trna3782-TyrGTA (45762023-45761934) Tyr (GTA) 90 bp Sc: 73.14
CCTTCGATAGCTCAGCTGGTAGAGCGGAGGACTGTAGATCCGACACGTGTGGTCATCCTT
AGGTCGCTGGTTCGATTCCGGCTCGAAGGA
>Bos_taurus_chr21.trna1904-TyrGTA (45781070-45781159) Tyr (GTA) 90 bp Sc: 73.78

CCTTCGATAGCTCAGCTGGTAGAGCGGAGGACTGTAGATCTGACACGTGTGGTCATCCTT
AGGTCGCTGGTTCGATTCCGGCTCGAAGGA
>Bos_taurus_chr21.trna1905-TyrGTA (45787448-45787537) Tyr (GTA) 90 bp Sc: 73.14
CCTTCGATAGCTCAGCTGGTAGAGCGGAGGACTGTAGATCCGACACGTGTGGTCATCCTT
AGGTCGCTGGTTCGATTCCGGCTCGAAGGA
>Bos_taurus_chr11.trna6185-TyrGTA (74676215-74676127) Tyr (GTA) 89 bp Sc: 76.37
CCTTCGATAGCTCAGTTGGTAGAGCGGAGGACTGTAGTGAGTGAGCTGTGGCAATCCTTA
GGTCGCTGGTTCGATTCCGGCTCGAAGGA
>Bos_taurus_chr1.trna7902-TyrGTA (99901875-99901786) Tyr (GTA) 90 bp Sc: 77.09
CCTTCGATAGCTCAGTTGGTAGAGCGGAGGACTGTAGTGTGAAGTTTCTCAGGCATCCTT
AGGTCGCTGGTTCGAATCCGGCTCGAAGGA
>Bos_taurus_chr3.trna4520-Undet??? (121671791-121671863) Undet (???) 73 bp Sc: 31.40
TCCCTGGTGGTCCAGTGGCTAAGACTCAGCATGCCCCGTGCAGGGGGCCCAGGTTCCATC
CCTGGTTGGGGAA
>Bos_taurus_chr19.trna4552-Undet??? (44408029-44407960) Undet (???) 70 bp Sc: 31.99
TCCCTGGTGGCCTACTGGTGAGGGTTCCAGGTTTACGGCTGGGACCCAGGTTCAGTTCCT
GGTTGGGGAA
>Bos_taurus_chr2.trna10428-Undet??? (1742656-1742586) Undet (???) 71 bp Sc: 32.21
TCCCTAGAAGTTCAATGGTTTAGGACTTGGTACTGCTGCTGTGGGCCCAGGTTCGATACC
TGGTTGGGGAA
>Bos_taurus_chr4.trna4287-Undet??? (121361806-121361877) Undet (???) 72 bp Sc: 44.33
TCCCTGGCGGTCTAGTGGTTAGGACTCAGTGCTTTCACTGTGGTTGTCCAGGTTCAATAC
CTGGTCAGGGAA
>Bos_taurus_chr11.trna65-Undet??? (1204765-1204837) Undet (???) 73 bp Sc: 44.44
TCCCTGGTGGGCCAGTGGCTAAGGTTCCTTGCTCCCAATACAAGGGTCCCAGGTTCAATC
CCTGGTCAGGGAA
>Bos_taurus_chrUn.004.1097.trna1-Undet??? (15986-16067) Undet (???) 82 bp Sc: 53.85
TCCCTGATGGTCCAGTGGCTAAGACTCTGTGCTCCCACACAGAGTCTTGCTGGGGGCCCA
GGTTCGATTCCTGGTCAAGGAA
>Bos_taurus_chr3.trna628-Undet??? (18682362-18682432) Undet (???) 71 bp Sc: 57.11
GCATTGGTGATATAGTGGTGAGCATAGCTGCCTTCAAGCAGCTGACCCAGGTTCGATTCC
TGGCCAATGCA
>Bos_taurus_chr3.trna280-Undet??? (8950824-8950895) Undet (???) 72 bp Sc: 73.13
GCATGGGTGGTTCAGTGGTAGAATTCTCGCCTGGCCACGCGGGAGGCCCGGGTTCGATTC
CCGGCCCATGCA
>Bos_taurus_chr18.trna2560-ValAAC (56292738-56292810) Val (AAC) 73 bp Sc: 39.31
TCCCTGGTGGTTGAGTGGTTAGGACATGGCACTAACACTGCCCTGGTCCCAGGGTTCAAT
CCAGGTTGGGGAA
>Bos_taurus_chr1.trna372-ValAAC (6187072-6187144) Val (AAC) 73 bp Sc: 47.34
GTTTCCATTGTGTAGTGGTCATCATGCTTGCCTAACTTGTGAGAAGTCCTTGGTTTGAAA
CCAAGTGGAAACA
>Bos_taurus_chr3.trna842-ValAAC (22665109-22665179) Val (AAC) 71 bp Sc: 61.56
GTTTCCGGTGTAGTGGTTATCACGTTCACCTAACACGTGAAAGGTCCCCGGTTCGAAACC
AGGCGGGAACG
>Bos_taurus_chrUn.004.185.trna11-ValAAC (200693-200763) Val (AAC) 71 bp Sc: 61.56
GTTTCCGGTGTAGTGGTTATCACGTTCACCTAACACGTGAAAGGTCCCCGGTTCGAAACC
AGGCGGGAACG
>Bos_taurus_chr23.trna1406-ValAAC (31429749-31429821) Val (AAC) 73 bp Sc: 63.72
GTTTCTGTAGTGTAGTGGTTATCATGCTTGCCTAACACGCTGGAGGCCCTTGGTTCAAAA
CTAAGCAGAAACA
>Bos_taurus_chr3.trna837-ValAAC (22636210-22636282) Val (AAC) 73 bp Sc: 71.31
GTTTCCGTAGTGTAGGGGTTATCACGTTCGCCTAACATGCGAAAGGTCCCCGGTTCGAAA
CCAGGAGGAAACA
>Bos_taurus_chr3.trna8550-ValAAC (22975574-22975502) Val (AAC) 73 bp Sc: 77.59
GTTTCTGTAGTGTAGTGGTTATCACATTTGCCTAACACACAAAAGGTCCCTGGTTCGAAA
CCAGGCAGAAACA
>Bos_taurus_chr3.trna8568-ValAAC (22855464-22855392) Val (AAC) 73 bp Sc: 79.36
ATTTCCGTAGTGTAGTGGTTATCACGTTCGCCTAACACGCGAAAGGTCCCCGGTTCGAAA
CCGGGCGGAAACA
>Bos_taurus_chr3.trna857-ValAAC (22910491-22910563) Val (AAC) 73 bp Sc: 79.36
ATTTCCGTAGTGTAGTGGTTATCACGTTCGCCTAACACGCGAAAGGTCCCCGGTTCGAAA
CCGGGCGGAAACA
>Bos_taurus_chr23.trna3419-ValAAC (31082207-31082135) Val (AAC) 73 bp Sc: 83.16
GTTTCCGTAGTGTAGTGGTTATCACGTTCGCCTAACACGCGAAAGGTCCCCGGTTCGAAA
CCGGGTGGAAACA
>Bos_taurus_chr23.trna1413-ValAAC (31447034-31447106) Val (AAC) 73 bp Sc: 83.60
GTTTCCGTAGTGTAGTGGTCATCACGCTCGCCTAACACGCGAGAGGTCCTCGGTTCGAAA

CCGAGCGGAAACA
>Bos_taurus_chr3.trna827-ValAAC (22577619-22577691) Val (AAC) 73 bp Sc: 84.90
GTTTCTGTAGTGTAGTGGTTATCACGTTCGCCTAACACGCGAAAGGTCCCCGGTTCGAAA
CCGGGCGGAAACA
>Bos_taurus_chr3.trna832-ValAAC (22602416-22602488) Val (AAC) 73 bp Sc: 84.90
GTTTCTGTAGTGTAGTGGTTATCACGTTCGCCTAACACGCGAAAGGTCCCCGGTTCGAAA
CCGGGCGGAAACA
>Bos_taurus_chr1.trna7897-ValAAC (99989332-99989260) Val (AAC) 73 bp Sc: 86.79
GTTTCCGTAGTGTAGTGGTTATCACGTTCGCCTAACACGCGAAAGGTCCCCGGTTCGAAA
CCGGGCGGAAACA
>Bos_taurus_chr23.trna1367-ValAAC (31112537-31112609) Val (AAC) 73 bp Sc: 86.79
GTTTCCGTAGTGTAGTGGTTATCACGTTCGCCTAACACGCGAAAGGTCCCCGGTTCGAAA
CCGGGCGGAAACA
>Bos_taurus_chr3.trna843-ValAAC (22671993-22672065) Val (AAC) 73 bp Sc: 86.79
GTTTCCGTAGTGTAGTGGTTATCACGTTCGCCTAACACGCGAAAGGTCCCCGGTTCGAAA
CCGGGCGGAAACA
>Bos_taurus_chr3.trna844-ValAAC (22687151-22687223) Val (AAC) 73 bp Sc: 86.79
GTTTCCGTAGTGTAGTGGTTATCACGTTCGCCTAACACGCGAAAGGTCCCCGGTTCGAAA
CCGGGCGGAAACA
>Bos_taurus_chr3.trna8552-ValAAC (22942420-22942348) Val (AAC) 73 bp Sc: 86.79
GTTTCCGTAGTGTAGTGGTTATCACGTTCGCCTAACACGCGAAAGGTCCCCGGTTCGAAA
CCGGGCGGAAACA
>Bos_taurus_chr3.trna870-ValAAC (23035542-23035614) Val (AAC) 73 bp Sc: 86.79
GTTTCCGTAGTGTAGTGGTTATCACGTTCGCCTAACACGCGAAAGGTCCCCGGTTCGAAA
CCGGGCGGAAACA
>Bos_taurus_chr7.trna6779-ValAAC (39699947-39699875) Val (AAC) 73 bp Sc: 86.79
GTTTCCGTAGTGTAGTGGTTATCACGTTCGCCTAACACGCGAAAGGTCCCCGGTTCGAAA
CCGGGCGGAAACA
>Bos_taurus_chrUn.004.185.trna12-ValAAC (207583-207655) Val (AAC) 73 bp Sc: 86.79
GTTTCCGTAGTGTAGTGGTTATCACGTTCGCCTAACACGCGAAAGGTCCCCGGTTCGAAA
CCGGGCGGAAACA
>Bos_taurus_chrUn.004.185.trna6-ValAAC (174524-174596) Val (AAC) 73 bp Sc: 86.79
GTTTCCGTAGTGTAGTGGTTATCACGTTCGCCTAACACGCGAAAGGTCCCCGGTTCGAAA
CCGGGCGGAAACA
>Bos_taurus_chr4.trna4902-ValAAC (111362165-111362093) Val (AAC) 73 bp Sc: 87.13
GTTTCCGTGGTGTAGTGGTTATCACGTTCGCCTAACACGCGAAAGGTCCCTGGTTCGAAA
CCAGGCGGAAACA
>Bos_taurus_chr16.trna208-ValCAC (7002274-7002345) Val (CAC) 72 bp Sc: 28.03
TCCTGGAGGTCCAGTGATTAGGATCCTGTGCTCACAAGGCAGAGGGCCTGGGTTCCATCC
CTGGTCAGGGAA
>Bos_taurus_chr2.trna5017-ValCAC (137652705-137652777) Val (CAC) 73 bp Sc: 30.53
TCCCTGGTGGTCCTGTGGCTAAGACCCTGTGATCACAATGTAGGGGGCCCAGGTTCCATC
CCTGGTCAGGGAA
>Bos_taurus_chr18.trna1176-ValCAC (29288781-29288853) Val (CAC) 73 bp Sc: 35.23
TTCCTGATAGTCCAATGACTAAGACTCTGCACTCACAATGCTGGGGGCCCAGGTTTGATT
CCTAGTCAGGGAT
>Bos_taurus_chr28.trna2060-ValCAC (29417566-29417494) Val (CAC) 73 bp Sc: 35.78
TCCCTCGTGGTTCAGTGGCTAAGACTCTGTGTTCACAATGCAGGGGGCTCAGGCTCAATC
CCTGATTGGGGAA
>Bos_taurus_chr12.trna5785-ValCAC (25887604-25887532) Val (CAC) 73 bp Sc: 39.52
TCCCTGATGGTCCAGTGGTTAAGACTCCATGCTCACATTGTAGGAGGCCTGGGTTTAATC
CCTTGTCAGGGAA
>Bos_taurus_chr20.trna1194-ValCAC (33718951-33719022) Val (CAC) 72 bp Sc: 41.11
TCCCTGATGGTCAAGTGACTAAGACTCTGAGCTCACAATGCAGGGGCCCAGGGTCGATCC
CTAGTCGGGGAA
>Bos_taurus_chr8.trna5929-ValCAC (71265850-71265778) Val (CAC) 73 bp Sc: 41.98
TCCCTGGTGGTCCAGTGACTAAGACTCTGCACCCACAGTGCAGGGGGCCTGGGTTCAATC
CCTGGCCAGGGAA
>Bos_taurus_chr28.trna450-ValCAC (11820160-11820232) Val (CAC) 73 bp Sc: 43.36
TCCCTGATGGTCCAATGGATAAGACTCCATGCTCACAATGCGGGGAGGCCAGGTTCGATC
CTTGGTCAGGGAA
>Bos_taurus_chr9.trna3559-ValCAC (99604047-99604119) Val (CAC) 73 bp Sc: 45.77
TCTCTGGTGGTCCAATGGCTAAGACTCTGTGCTCACAACGCAGGGAGCCTGGGATCAATC
CCCGGACAGGGAA
>Bos_taurus_chr16.trna1848-ValCAC (48002909-48002981) Val (CAC) 73 bp Sc: 48.33
TCCCTGGTGGTCCAGTGGCTAAGACTCTGCACTCACAATGCAAGGGGCCCTGGTTTCATC
CCTGGCCAGGGAA

>Bos_taurus_chr25.trna2723-ValCAC (38573360-38573288) Val (CAC) 73 bp Sc: 53.62
TCCCTGGTGGTCCAGTGGCTAAGACTCTGTGCTCACAATGCAGGGGGCCCGGGTTCAATC
CCTGGTCAGGGAA
>Bos_taurus_chr7.trna7710-ValCAC (16536641-16536569) Val (CAC) 73 bp Sc: 53.66
TCCCTGGTGGTCCAGTGGCTAAGACTCTGCACTCACAGTGCAGGGGGCCCGGGTTCAATC
CCTGGTCGGGGAA
>Bos_taurus_chr15.trna1370-ValCAC (39525817-39525889) Val (CAC) 73 bp Sc: 55.11
TTCTTGGTGGTCCAATGGCTAAGACTCTGCAATCACAATGCAGGGGGCCCAGGTTCAATC
CCTGGTCAGGAAA
>Bos_taurus_chr3.trna864-ValCAC (22958665-22958737) Val (CAC) 73 bp Sc: 56.42
CTTTCCGTGGTGTAGTGGTTATCACGTTTGCCTCACATGTGAAAAGTCTCCGGTTCGAAA
CCAGGCGGAAATA
>Bos_taurus_chr3.trna8549-ValCAC (22979121-22979049) Val (CAC) 73 bp Sc: 56.73
ATTTCTGTAGTGTAGTGGTTATCACGTTCGCCTCACACGCGAAAGGTCCTCGGTTTGAAA
CCACGTGGAAACA
>Bos_taurus_chr29.trna3028-ValCAC (28702023-28701951) Val (CAC) 73 bp Sc: 56.85
GTTTCTATAGTGTAGTAGTTATCACTTTCGCCTCACATGCCAAAGGTCCCTGGTTCAAAA
CCAGGCATAAACA
>Bos_taurus_chr18.trna4235-ValCAC (45439782-45439711) Val (CAC) 72 bp Sc: 61.01
TCCCTGGTGGTCCAGTGGCTAAGACCCTGCACTCACAATGCAGGGGGCCAGGTTCAATCC
CTGGTTAGGGAG
>Bos_taurus_chr2.trna5577-ValCAC (134053534-134053462) Val (CAC) 73 bp Sc: 62.16
TCCCTGGTGGTCCAGTGGCTAAGACTCTGCACTCACAGTGCAGGGGGCCCAGGTTCGATC
CCTGGTCAGGGAA
>Bos_taurus_chr9.trna1086-ValCAC (34406821-34406893) Val (CAC) 73 bp Sc: 65.66
GTTTCTGTAGTGTAGTGGTTATCACGTTCGCCTCACATGCCAGAGGTCCCTGGTTCAAAA
CTGGGTGGAAACA
>Bos_taurus_chr3.trna852-ValCAC (22894967-22895039) Val (CAC) 73 bp Sc: 70.03
GTTTCCGTAGTGTAGTGGTTATCACGTTCGCCTCACATGAGAAAGGTCCCCAGTTCGAAC
CCGGGTGGAAACA
>Bos_taurus_chr3.trna8563-ValCAC (22870988-22870916) Val (CAC) 73 bp Sc: 70.03
GTTTCCGTAGTGTAGTGGTTATCACGTTCGCCTCACATGAGAAAGGTCCCCAGTTCGAAC
CCGGGTGGAAACA
>Bos_taurus_chr3.trna858-ValCAC (22915543-22915615) Val (CAC) 73 bp Sc: 77.39
GTTTCTGTAGTGTAGTGGTTATCACGTTCGCCTCACACGAGAAAGGTCCCTGGTTCGAAA
CCGGGCGGAAACA
>Bos_taurus_chrUn.004.185.trna5-ValCAC (162481-162553) Val (CAC) 73 bp Sc: 77.39
GTTTCTGTAGTGTAGTGGTTATCACGTTCGCCTCACACGAGAAAGGTCCCTGGTTCGAAA
CCGGGCGGAAACA
>Bos_taurus_chrUn.004.1856.trna6-ValCAC (16698-16626) Val (CAC) 73 bp Sc: 77.39
GTTTCTGTAGTGTAGTGGTTATCACGTTCGCCTCACACGAGAAAGGTCCCTGGTTCGAAA
CCGGGCGGAAACA
>Bos_taurus_chr3.trna8555-ValCAC (22928046-22927974) Val (CAC) 73 bp Sc: 80.44
GTTTCTGTAGTGTAGTGGTTATCACGTTCGCCTCACACGCGAAAGGTCCCCGGTTCGAAA
CCGGACAGAAACA
>Bos_taurus_chr3.trna869-ValCAC (23032838-23032910) Val (CAC) 73 bp Sc: 85.40
GTTTCTGTAGTGTAGTGGTTATCACGTTCGCCTCACACGCGAAAGGTCCCCGGTTCGAAA
CCGGGCAGGAACA
>Bos_taurus_chr23.trna1393-ValCAC (31321404-31321476) Val (CAC) 73 bp Sc: 86.66
GTTTCCGTAGTGTAGTGGTTATCACGTTCGCCTCACACGCGAAAGGTCCCCGGTTCGAAA
CCGGGCGGGAACA
>Bos_taurus_chrUn.004.1183.trna7-ValCAC (12954-12882) Val (CAC) 73 bp Sc: 86.66
GTTTCCGTAGTGTAGTGGTTATCACGTTCGCCTCACACGCGAAAGGTCCCCGGTTCGAAA
CCGGGCGGGAACA
>Bos_taurus_chrUn.004.185.trna17-ValCAC (223238-223166) Val (CAC) 73 bp Sc: 86.66
GTTTCCGTAGTGTAGTGGTTATCACGTTCGCCTCACACGCGAAAGGTCCCCGGTTCGAAA
CCGGGCGGGAACA
>Bos_taurus_chr3.trna822-ValCAC (22557256-22557328) Val (CAC) 73 bp Sc: 87.16
GTTTCCGTAGTGTAGTGGTTATCACGTTCGCCTCACACGCGAAAGGTCCCCGGTTCGATA
CCGGGCGGAAACA
>Bos_taurus_chr3.trna830-ValCAC (22592284-22592356) Val (CAC) 73 bp Sc: 87.16
GTTTCCGTAGTGTAGTGGTTATCACGTTCGCCTCACACGCGAAAGGTCCCCGGTTCGATA
CCGGGCGGAAACA
>Bos_taurus_chr3.trna834-ValCAC (22617228-22617300) Val (CAC) 73 bp Sc: 87.16
GTTTCCGTAGTGTAGTGGTTATCACGTTCGCCTCACACGCGAAAGGTCCCCGGTTCGATA
CCGGGCGGAAACA
>Bos_taurus_chr3.trna840-ValCAC (22655765-22655837) Val (CAC) 73 bp Sc: 87.16

GTTTCCGTAGTGTAGTGGTTATCACGTTCGCCTCACACGCGAAAGGTCCCCGGTTCGATA
CCGGGCGGAAACA
>Bos_taurus_chrUn.004.185.trna9-ValCAC (191312-191384) Val (CAC) 73 bp Sc: 87.16
GTTTCCGTAGTGTAGTGGTTATCACGTTCGCCTCACACGCGAAAGGTCCCCGGTTCGATA
CCGGGCGGAAACA
>Bos_taurus_chrUn.004.1856.trna2-ValCAC (3450-3522) Val (CAC) 73 bp Sc: 87.16
GTTTCCGTAGTGTAGTGGTTATCACGTTCGCCTCACACGCGAAAGGTCCCCGGTTCGATA
CCGGGCGGAAACA
>Bos_taurus_chrUn.004.2930.trna3-ValCAC (8512-8584) Val (CAC) 73 bp Sc: 87.16
GTTTCCGTAGTGTAGTGGTTATCACGTTCGCCTCACACGCGAAAGGTCCCCGGTTCGATA
CCGGGCGGAAACA
>Bos_taurus_chrUn.004.2930.trna6-ValCAC (13403-13475) Val (CAC) 73 bp Sc: 87.16
GTTTCCGTAGTGTAGTGGTTATCACGTTCGCCTCACACGCGAAAGGTCCCCGGTTCGATA
CCGGGCGGAAACA
>Bos_taurus_chr23.trna3377-ValCAC (31586445-31586373) Val (CAC) 73 bp Sc: 87.39
GTTTCCGTAGTGTAGTGGTTATCACGTTCGCCTCACACGCGAAAGGTCCCCGGTTCGAAA
CCGGGCGGAAACA
>Bos_taurus_chr23.trna3396-ValCAC (31347431-31347359) Val (CAC) 73 bp Sc: 87.39
GTTTCCGTAGTGTAGTGGTTATCACGTTCGCCTCACACGCGAAAGGTCCCCGGTTCGAAA
CCGGGCGGAAACA
>Bos_taurus_chr3.trna284-ValCAC (8993689-8993761) Val (CAC) 73 bp Sc: 87.39
GTTTCCGTAGTGTAGTGGTTATCACGTTCGCCTCACACGCGAAAGGTCCCCGGTTCGAAA
CCGGGCGGAAACA
>Bos_taurus_chr3.trna825-ValCAC (22567276-22567348) Val (CAC) 73 bp Sc: 87.39
GTTTCCGTAGTGTAGTGGTTATCACGTTCGCCTCACACGCGAAAGGTCCCCGGTTCGAAA
CCGGGCGGAAACA
>Bos_taurus_chr3.trna8538-ValCAC (23080314-23080242) Val (CAC) 73 bp Sc: 87.39
GTTTCCGTAGTGTAGTGGTTATCACGTTCGCCTCACACGCGAAAGGTCCCCGGTTCGAAA
CCGGGCGGAAACA
>Bos_taurus_chr3.trna855-ValCAC (22900856-22900928) Val (CAC) 73 bp Sc: 87.39
GTTTCCGTAGTGTAGTGGTTATCACGTTCGCCTCACACGCGAAAGGTCCCCGGTTCGAAA
CCGGGCGGAAACA
>Bos_taurus_chr3.trna8566-ValCAC (22865099-22865027) Val (CAC) 73 bp Sc: 87.39
GTTTCCGTAGTGTAGTGGTTATCACGTTCGCCTCACACGCGAAAGGTCCCCGGTTCGAAA
CCGGGCGGAAACA
>Bos_taurus_chr7.trna6777-ValCAC (39704889-39704817) Val (CAC) 73 bp Sc: 87.39
GTTTCCGTAGTGTAGTGGTTATCACGTTCGCCTCACACGCGAAAGGTCCCCGGTTCGAAA
CCGGGCGGAAACA
>Bos_taurus_chr3.trna5974-ValTAC (97555728-97555656) Val (TAC) 73 bp Sc: 37.57
TCCCTCGTGGTCCAGTGTCTAAGACACTGCACTTACAATGCAGGGGGCCTAGGTTAAATC
CTTGGTCAGGGAA
>Bos_taurus_chr4.trna8532-ValTAC (10603336-10603264) Val (TAC) 73 bp Sc: 42.68
TCCTTGGTCGTCCAATGGTTAGGACTTGGTATTTACACTGCCATGGGCCCAGGTTCAGTC
CCTGGTCAGGAAC
>Bos_taurus_chr19.trna2921-ValTAC (57376987-57377059) Val (TAC) 73 bp Sc: 43.81
TCCTTGGTGGTCCAACTGTTAGGACTCTGCACTTACACTGCTGAGGGCCTGGGTTCAATC
CCTTGTCAGGGAA
>Bos_taurus_chr21.trna1139-ValTAC (25311573-25311645) Val (TAC) 73 bp Sc: 46.46
TCCCTGGTGGTCCAGTGGTTAAGACTTTGCGCTTACAACGCAGGGGGCCTGGATTCTATC
CCTGGTCAGGGAA
>Bos_taurus_chr20.trna5325-ValTAC (10818611-10818539) Val (TAC) 73 bp Sc: 46.64
TCCCTGGTGGTCCAGTGGCCAAGTTTCTGAGCTTACAATACAGGGGGCCCAGGTTCAATC
CCTGGTCAGGGAA
>Bos_taurus_chr5.trna1001-ValTAC (29665180-29665252) Val (TAC) 73 bp Sc: 47.06
TCCCTGGTGGTCCAATGACTAAGATTCTGTACTTACAATACAAAGGGCCCAGGTTCAATC
CCTGGTCAGGGAA
>Bos_taurus_chr9.trna674-ValTAC (23279774-23279846) Val (TAC) 73 bp Sc: 52.41
TCCCTGGTGGTCCAGTGGTTAAGACTCTGCACTTACGATGCAGGGTATACAGGTTTGATC
CCTGACTAGGGAA
>Bos_taurus_chr17.trna4222-ValTAC (61900613-61900542) Val (TAC) 72 bp Sc: 53.26
TCCCTGGTGGTCCAGTGGTAGGACTCAGCACTTACACTGCTGGGGGCCTGGGTTTGATCC
CTAGTCAGGAAA
>Bos_taurus_chr19.trna2509-ValTAC (49757079-49757151) Val (TAC) 73 bp Sc: 54.14
TCCCTGGTGGTCCAGTGGTTAAGACTCTGCACTTACACTCCAGGGGCCACAGGTTTGATC
CCTGGTCAGGGAA
>Bos_taurus_chr3.trna8553-ValTAC (22931542-22931470) Val (TAC) 73 bp Sc: 62.31
GATTCCGTAGTGTAGTGGTTATCACGTTCGCCTTACACGCGAAAGATGCCGGGTTCGAGA

CTGGGCGGAAACA
>Bos_taurus_chr11.trna6529-ValTAC (65482046-65481974) Val (TAC) 73 bp Sc: 64.63
TCCCTGGTGATCCAGTGGTTAGGATTCTGCGCTTACACTGCAGAGGGCCCAGGTTCAATC
CCTGGTCAGGGAA
>Bos_taurus_chr3.trna838-ValTAC (22652251-22652323) Val (TAC) 73 bp Sc: 67.07
GTTTCCGTAGTGTAGTGGTTATCACGTTCGCCTTACACGCGAAAGCTCCCCAGTTCAAAA
CCGGGAGGAAACA
>Bos_taurus_chrUn.004.185.trna7-ValTAC (187791-187863) Val (TAC) 73 bp Sc: 67.07
GTTTCCGTAGTGTAGTGGTTATCACGTTCGCCTTACACGCGAAAGCTCCCCAGTTCAAAA
CCGGGAGGAAACA
>Bos_taurus_chrUn.004.2930.trna1-ValTAC (4998-5070) Val (TAC) 73 bp Sc: 67.07
GTTTCCGTAGTGTAGTGGTTATCACGTTCGCCTTACACGCGAAAGCTCCCCAGTTCAAAA
CCGGGAGGAAACA
>Bos_taurus_chr3.trna828-ValTAC (22588570-22588642) Val (TAC) 73 bp Sc: 80.49
GTTTCCGTAGTGTAGTGGTTATCACGTTCGCCTTACACGCGAAAGGTCCCCGGTTCGAAA
CCGGGCGAAAACA
>Bos_taurus_chr3.trna845-ValTAC (22698109-22698181) Val (TAC) 73 bp Sc: 80.49
GTTTCCGTAGTGTAGTGGTTATCACGTTCGCCTTACACGCGAAAGGTCCCCGGTTCGAAA
CCGGGCGAAAACA
>Bos_taurus_chrX.trna2907-ValTAC (77440766-77440838) Val (TAC) 73 bp Sc: 81.89
GGTTCCGTAGTGTAGTGGTTATCACGTCTGCTTTACACGCAGAAGGTCCTGGGTTCGAGC
CCCAGTGGAACCA
>Bos_taurus_chr15.trna3086-ValTAC (83645832-83645760) Val (TAC) 73 bp Sc: 82.67
GGTTCCATAGTGTAGCGGTTATCACGTCTGCTTTACACGCAGAAGGTCCTGGGTTCGAGC
CCCAGTGGAACCA
>Bos_taurus_chr23.trna1391-ValTAC (31310368-31310440) Val (TAC) 73 bp Sc: 83.03
GTTTCCGTGGTGTAGCGGTTAGCACATTCGCCTTACACGCGAAAGGTCCCCGGTTCGAAA
CCGGGCGGAAACA
>Bos_taurus_chrUn.004.1183.trna5-ValTAC (24660-24588) Val (TAC) 73 bp Sc: 83.03
GTTTCCGTGGTGTAGCGGTTAGCACATTCGCCTTACACGCGAAAGGTCCCCGGTTCGAAA
CCGGGCGGAAACA
>Bos_taurus_chr23.trna1402-ValTAC (31359861-31359933) Val (TAC) 73 bp Sc: 84.60
GGTTCCATAGTGTAGTGGTTAGCACATCTGCTTTACACGCAGAAGGTCCTGGGTTCGATC
CCCAGTGGAACCA
>Bos_taurus_chr3.trna819-ValTAC (22547660-22547732) Val (TAC) 73 bp Sc: 87.03
GTTTCCGTAGTGTAGTGGTTATCACGTTCGCCTTACACGCGAAAGGTCCCCGGTTCGAAA
CCGGGCGGAAACA
>Bos_taurus_chrX.trna2840-AlaAGC (80920741-80920812) Ala (AGC) 72 bp Sc: 37.04
GGGGGTATAGCTTAGTGGCAGATTACATGCTTAGCATGTATGAAGCCCTGGGTTCTGTCC
CCAGTACTTCCA
>Bos_taurus_chr25.trna181-AlaAGC (4015702-4015773) Ala (AGC) 72 bp Sc: 47.44
GGGGGTGTAGTTCAATGGTAGAGCGTGTGCTTAGCATGCTCGAGACCCCGGGTTCAATGC
CTGGCACCTCCA
>Bos_taurus_chr18.trna968-AlaAGC (23631560-23631632) Ala (AGC) 73 bp Sc: 55.26
GGGGAATTAGCTCAAGTGGTAGAGCGCTCGCTTAGCATGTGAGAGGTAGTGGGATCGATG
CCCGCATTCTCCA
>Bos_taurus_chr29.trna2234-AlaAGC (46581925-46581853) Ala (AGC) 73 bp Sc: 55.26
GGGGAATTAGCTCAAGTGGTAGAGCGCTCGCTTAGCATGTGAGAGGTAGTGGGATCGATG
CCCGCATTCTCCA
>Bos_taurus_chr21.trna2972-AlaAGC (68579158-68579229) Ala (AGC) 72 bp Sc: 55.39
TCCCTGGCAGTCCAGTGGTTAGGACTTGGCACCAGCACTGCCAGGGCCCAGGTTCGATCC
TTGGTTGGGGAA
>Bos_taurus_chr18.trna1227-AlaAGC (29339449-29339519) Ala (AGC) 71 bp Sc: 56.59
GGGGGTGTGGCTCAGTGGTAGAGTGCATGCTTAGCATGTATGAGACCCTGGGTTCAATCC
CAGTACTCCCA
>Bos_taurus_chr23.trna1474-AlaAGC (31175615-31175687) Ala (AGC) 73 bp Sc: 58.07
GGGGAATTAGCTCAAGTGGTAGAGCGCTCGCTTAGCATGTGAGAGGTAGTGGGATCGATG
CCCACATTCTCCA
>Bos_taurus_chr23.trna1478-AlaAGC (31187064-31187136) Ala (AGC) 73 bp Sc: 58.07
GGGGAATTAGCTCAAGTGGTAGAGCGCTCGCTTAGCATGTGAGAGGTAGTGGGATCGATG
CCCACATTCTCCA
>Bos_taurus_chr23.trna3465-AlaAGC (31198853-31198781) Ala (AGC) 73 bp Sc: 58.07
GGGGAATTAGCTCAAGTGGTAGAGCGCTCGCTTAGCATGTGAGAGGTAGTGGGATCGATG
CCCACATTCTCCA
>Bos_taurus_chr23.trna1482-AlaAGC (31296524-31296596) Ala (AGC) 73 bp Sc: 58.48
GGGGAATTAGCTCAAATGGTAGAGCGCTCGCTTAGCATGTGAGAGGTAGCGGGATCGATG
CCCGCATTCTCCA

>Bos_taurus_chr23.trna1481-AlaAGC (31191483-31191555) Ala (AGC) 73 bp Sc: 59.41
GGGGGATTAGCTCAAATGGTAGAGCGCTCGCTTAGCATGCGAGAGGTAGTGGGATCGATG
CCCATATCCTCCA
>Bos_taurus_chr7.trna2708-AlaAGC (60871678-60871749) Ala (AGC) 72 bp Sc: 61.64
GGGGGTATAGCTCAGTGGCAGAGCACATGCTTAGCATGCACGAGACCCTGGGTTCAATCC
CCAGTATCTCCA
>Bos_taurus_chr26.trna3132-AlaAGC (26720523-26720452) Ala (AGC) 72 bp Sc: 62.92
GGGGGTATAGCTCAGTGGTAGAGTGCGTGCTTAGCATGTATGAGGTCCTGAGTTCAATCC
CCAGTACCTCCA
>Bos_taurus_chr11.trna6285-AlaAGC (72573837-72573765) Ala (AGC) 73 bp Sc: 63.68
GGGGGATTAGCTCAAATGGTAGAGCGCTCGCTTAGCATGCGAGAGGTAGCGGGATCGATG
CCCGCATCCTCCA
>Bos_taurus_chr14.trna1445-AlaAGC (32154328-32154400) Ala (AGC) 73 bp Sc: 63.68
GGGGGATTAGCTCAAATGGTAGAGCGCTCGCTTAGCATGCGAGAGGTAGCGGGATCGATG
CCCGCATCCTCCA
>Bos_taurus_chr18.trna3987-AlaAGC (51973843-51973771) Ala (AGC) 73 bp Sc: 66.70
GGGGGTGTAGCTCAGTGGTTAGAGTGTATGCTTAGCATGCACGAGGTACCAGGTTCAAAT
CCTGGCACTTCCA
>Bos_taurus_chr7.trna2263-AlaAGC (48654604-48654675) Ala (AGC) 72 bp Sc: 73.53
GGCGGTATAGCTCAGTGGTAGAGCACATGCTTAGCATGCATGAGACCCTGGGTTCAATCC
CCAGTACTGCCA
>Bos_taurus_chr23.trna1386-AlaAGC (29976892-29976963) Ala (AGC) 72 bp Sc: 75.68
GGGGGTGTAGCTCAGTGGTAGAGCGCGTGCTTAGCATGCACGAGGCCCTGGGTTCAATCC
CCAGCACCTCCA
>Bos_taurus_chr18.trna3801-AlaAGC (54151907-54151836) Ala (AGC) 72 bp Sc: 75.78
GGGGGTATAGCTCAGTGGTAGAGCGCATGCTTAGCATGCATGAGGCCCTGGGTTCAATCC
CCAGTACCTCCA
>Bos_taurus_chr19.trna3847-AlaAGC (56483738-56483667) Ala (AGC) 72 bp Sc: 75.78
GGGGGTATAGCTCAGTGGTAGAGCGCATGCTTAGCATGCATGAGGCCCTGGGTTCAATCC
CCAGTACCTCCA
>Bos_taurus_chr23.trna1396-AlaAGC (30033617-30033688) Ala (AGC) 72 bp Sc: 75.78
GGGGATGTAGCTCAGTGGTAGAGCGCATGCTTAGCATGCATGAGGTCCCGGGTTCGATCC
CCAGCATCTCCA
>Bos_taurus_chr23.trna3539-AlaAGC (30049271-30049200) Ala (AGC) 72 bp Sc: 76.69
GGGGGTGTAGCTCAGTGGTAGAGCGCGTGCTTAGCATGTACGAGGTCCCGGGTTCAATCC
CCGGCACCTCCA
>Bos_taurus_chr1.trna3385-AlaAGC (95174246-95174317) Ala (AGC) 72 bp Sc: 77.18
GGGGGTGTAGCTCAGTGGTAGAGCGCGTGCTTAGCATGCACGAGGCCCCGGGTTCAATCC
CCGGCACCTCCA
>Bos_taurus_chr23.trna1379-AlaAGC (29937386-29937457) Ala (AGC) 72 bp Sc: 77.18
GGGGGTGTAGCTCAGTGGTAGAGCGCGTGCTTAGCATGCACGAGGCCCCGGGTTCAATCC
CCGGCACCTCCA
>Bos_taurus_chr23.trna1380-AlaAGC (29945279-29945350) Ala (AGC) 72 bp Sc: 77.18
GGGGGTGTAGCTCAGTGGTAGAGCGCGTGCTTAGCATGCACGAGGCCCCGGGTTCAATCC
CCGGCACCTCCA
>Bos_taurus_chr23.trna1390-AlaAGC (30012420-30012491) Ala (AGC) 72 bp Sc: 77.18
GGGGGTGTAGCTCAGTGGTAGAGCGCGTGCTTAGCATGCACGAGGCCCCGGGTTCAATCC
CCGGCACCTCCA
>Bos_taurus_chr23.trna1392-AlaAGC (30017824-30017895) Ala (AGC) 72 bp Sc: 77.18
GGGGGTGTAGCTCAGTGGTAGAGCGCGTGCTTAGCATGCACGAGGCCCCGGGTTCAATCC
CCGGCACCTCCA
>Bos_taurus_chr23.trna1420-AlaAGC (30791064-30791135) Ala (AGC) 72 bp Sc: 77.18
GGGGGTGTAGCTCAGTGGTAGAGCGCGTGCTTAGCATGCACGAGGCCCCGGGTTCAATCC
CCGGCACCTCCA
>Bos_taurus_chr23.trna3542-AlaAGC (30011590-30011519) Ala (AGC) 72 bp Sc: 77.18
GGGGGTGTAGCTCAGTGGTAGAGCGCGTGCTTAGCATGCACGAGGCCCCGGGTTCAATCC
CCGGCACCTCCA
>Bos_taurus_chr23.trna3545-AlaAGC (30004116-30004045) Ala (AGC) 72 bp Sc: 77.18
GGGGGTGTAGCTCAGTGGTAGAGCGCGTGCTTAGCATGCACGAGGCCCCGGGTTCAATCC
CCGGCACCTCCA
>Bos_taurus_chr11.trna4763-AlaAGC (104532629-104532550) Ala (AGC) 80 bp Sc: 35.84
TCCCGGGTGGCTCAGAGGCGAAGAGTCCACCTGCCAAGCAGGAGATGCAGGAGACCCAGG
TTCGATCCCTGGCTTGGGAA
>Bos_taurus_chr1.trna810-AlaCGC (19247775-19247847) Ala (CGC) 73 bp Sc: 33.89
TCCCTTGTGGTCCAGTAGTTAAGACTTGGTACTCGCAATGCAAGGGGTCTGGGTTCAGTC
CCTGGCTAGGGAA
>Bos_taurus_chrX.trna9863-AlaCGC (59330782-59330710) Ala (CGC) 73 bp Sc: 39.74

TCCCTGGTGGTCCAGTGGCTAAGACTCCGTATTCGCAATGCAGGGGGCTGAGGTTTGATC
CCTGGTCAGGGAA
>Bos_taurus_chr1.trna7153-AlaCGC (127787557-127787485) Ala (CGC) 73 bp Sc: 40.65
CTCCTGGTGGTCCAGTGGCTAAGACTCTGTGCTCGCAATGCAGGGGGCCTGGGTTTGATC
CCTAGTCAGGAAA
>Bos_taurus_chrX.trna10475-AlaCGC (39324151-39324079) Ala (CGC) 73 bp Sc: 40.94
TCTGTGATGGTCCAGTGACTAAGACTCTGGGTTCGCAATGCAGGGGGTCCAGGTTCAATC
CCTGGTCAGAGAA
>Bos_taurus_chr11.trna8435-AlaCGC (19526455-19526383) Ala (CGC) 73 bp Sc: 46.16
TCCCTGGTAGTCCAGTGGCTAAGACTCCACAATCGCAATGTGGAGGGATCAGGTTCAATC
TCTGGTCAGGGAA
>Bos_taurus_chr9.trna1473-AlaCGC (43306935-43307006) Ala (CGC) 72 bp Sc: 50.24
TTCCTGTTGGTCCAGTGACTAAGACTCTGCACTCGCAATGCAGGGGCCCAGGTTCAATCC
TTGGTCAGGAAC
>Bos_taurus_chr22.trna3341-AlaCGC (32293873-32293801) Ala (CGC) 73 bp Sc: 52.35
TTCTGGGTGGTCTAGTGGCTAAGACTCTGCACTCGCAATGCAGGGGGCCCAGGTTTGATC
CCTGGTCAGGGAA
>Bos_taurus_chr3.trna8663-AlaCGC (21006944-21006874) Ala (CGC) 71 bp Sc: 55.09
GCATTGGTGGTTCAGTGGTAGAATTCTCACCTCGCACGCGGGAAACCCCGGTTCAATTCC
CAGCCAATGCA
>Bos_taurus_chr23.trna3549-AlaCGC (29983448-29983377) Ala (CGC) 72 bp Sc: 68.35
GGGGGTGTAGCTCAGTGGTAGAGCGCGTGCTTCGCATGTACGAGGCCCCGGGTTCAACCC
CCGGCTCCTCCA
>Bos_taurus_chr24.trna140-AlaCGC (4954492-4954563) Ala (CGC) 72 bp Sc: 71.19
GGGGATGTAGCTCAGTGGTAGAGCATATGCTTCGCATGCATGAGGCCCCAGGTTCAATCC
CTGGCATCTCCA
>Bos_taurus_chr23.trna1393-AlaCGC (30019750-30019821) Ala (CGC) 72 bp Sc: 72.31
GGGGGTGTAGCTCAGTGGTAGAGCGCGTGCTTCGCATGTACGAGGTCCCTGGTTCAATCC
CTGGCACCTCCA
>Bos_taurus_chr2.trna9286-AlaCGC (39950662-39950591) Ala (CGC) 72 bp Sc: 73.12
GGGGATGTAGCTCAGTGGTAGAGCGCGCGCTTCGCATGTGTGAGGTCCCGGGTTCAATCC
CCGGCATCTCCA
>Bos_taurus_chr23.trna1381-AlaCGC (29949904-29949975) Ala (CGC) 72 bp Sc: 73.20
GGGGGTGTAGCTCAGTGGTAGAGCGCGTGCTTCGCATGTACGAGGCCCCGGGTTCAATCC
CCGGCACCTCCA
>Bos_taurus_chr23.trna1395-AlaCGC (30027676-30027747) Ala (CGC) 72 bp Sc: 74.81
GGGGATGTAGCTCAGTGGTAGAGCGCATGCTTCGCATGTATGAGGCCCCGGGTTCGATCC
CCGGCATCTCCA
>Bos_taurus_chr23.trna3457-AlaCGC (31313506-31313435) Ala (CGC) 72 bp Sc: 74.81
GGGGATGTAGCTCAGTGGTAGAGCGCATGCTTCGCATGTATGAGGCCCCGGGTTCGATCC
CCGGCATCTCCA
>Bos_taurus_chr6.trna7717-AlaCGC (38552214-38552143) Ala (CGC) 72 bp Sc: 74.81
GGGGATGTAGCTCAGTGGTAGAGCGCATGCTTCGCATGTATGAGGCCCCGGGTTCGATCC
CCGGCATCTCCA
>Bos_taurus_chr6.trna4563-AlaGGC (116486926-116486854) Ala (GGC) 73 bp Sc: 53.39
TCCCTGGTGGTCCAGTGGTTAAGACGCTGCACTGGCAGTTCAGAGGGCTCAGGTTCGATC
CCTGGTCAGAGAA
>Bos_taurus_chr29.trna189-AlaGGC (5882762-5882833) Ala (GGC) 72 bp Sc: 55.27
TCCCTAATAGCTCAGCTGGTAAAGAATCTGCCTGGCATGCAGGAGACCCAGGTTCGAATC
CTGGATTGGGAA
>Bos_taurus_chr5.trna8459-AlaTGC (50873222-50873150) Ala (TGC) 73 bp Sc: 27.94
CCCCTGGTGGTTCAGGGGTTAAGACTCAGTGCTTGCACTACAGGGAGACTAGGTTTGATC
CCTAGTCAGGGAA
>Bos_taurus_chr15.trna4532-AlaTGC (53951300-53951228) Ala (TGC) 73 bp Sc: 28.91
TTCCTGATGGTCCAGTGATTAAGACTCTGCACTTGCAGTGCAGTGGACATGGGTTCAGTC
CCTGGTCAGGGAA
>Bos_taurus_chr19.trna1705-AlaTGC (32691165-32691256) Ala (TGC) 92 bp Sc: 31.18
TCCCTGGTGGTCCAGTGGCTAAGACTCCACACTTGCTATGAGGGGTTCGGGGGTGGGGAG
GGTGGGCCTGGGTTAGATCCCCAGTCAGGGAA
>Bos_taurus_chr13.trna3369-AlaTGC (74145295-74145366) Ala (TGC) 72 bp Sc: 33.47
TCCCTAGTGGTCCACTGGCTAAGACTCTGTACTTGCAGTGCAGGAGGCCGGGTTCGATCC
CTGGTCAGGGAA
>Bos_taurus_chr2.trna4815-AlaTGC (128756355-128756435) Ala (TGC) 81 bp Sc: 34.76
TCCCTGGTGGTCCAGTGTTTAGGACTCTGTGCTTGCACTGCAGGACTGTGGGTGGCACAG
GTTTGATACCTGGTCAGGGAA
>Bos_taurus_chr26.trna372-AlaTGC (12361993-12362065) Ala (TGC) 73 bp Sc: 34.95
TCCCTGGTGGTCCAGTGGTTAAGATTCTGCACTTGCATTGCAAGGGGGGCAGGCTCCATC

CCTGGTCAGGGAA
>Bos_taurus_chr23.trna4605-AlaTGC (8592845-8592775) Ala (TGC) 71 bp Sc: 37.79
TCCATGGTGGTCCAGTGGGCAGGACTCTGTACTTGCATTGCAGGGCCTGAGTTCCATCCT
TGGTCAGGGAA
>Bos_taurus_chr18.trna102-AlaTGC (2729646-2729717) Ala (TGC) 72 bp Sc: 39.58
TCCCTGGCAGTTCAGGGGTTAGGACTCAGTACTTGCACTGCTGTAGCCCGGGTTTAATCC
CTGGTCAGGGAT
>Bos_taurus_chrX.trna4828-AlaTGC (128748230-128748303) Ala (TGC) 74 bp Sc: 39.85
TTCCTGGTGGTCCAATGGCTAAGATTCAGCACTTGCAATACTGAGGGCCCGGGGTTCGAT
CCCTGGTCAGGGAA
>Bos_taurus_chr15.trna5517-AlaTGC (29684631-29684559) Ala (TGC) 73 bp Sc: 40.92
TCCCCGGTGGTCCAGAGGCTAAGACTCTGCACTTGCAAGGCAGGGGGCCCAGGTTCCATC
CTTGGTCAGGGAA
>Bos_taurus_chr19.trna6403-AlaTGC (13656029-13655957) Ala (TGC) 73 bp Sc: 40.98
TCCCTGGTAGTCTAGTGGCTAAGACTTTGTGGTTGCAATGCAAGGGGTCGAGGTTCAATC
CCTGGTCAGGGAA
>Bos_taurus_chr24.trna1687-AlaTGC (39185510-39185581) Ala (TGC) 72 bp Sc: 41.80
GGGGATGTAGCTCAGTGGTAGAACACATGCTTTGCAGGTATGAGGTCTCGGGTTTGATCC
TTGGCATCTCCA
>Bos_taurus_chr7.trna821-AlaTGC (16125878-16125951) Ala (TGC) 74 bp Sc: 42.53
TCCCTGGTGGTCTAGTGGCTAAGACTCTGTGTTTGCAATGCAGGGGGCCCTGGACTCGAT
CCCTGGTCAGGGAA
>Bos_taurus_chr19.trna3808-AlaTGC (57004556-57004484) Ala (TGC) 73 bp Sc: 43.44
TGCCTGGTGGTCCAGTGCTTAAGACTCTGCACTTGCACTGCAGTGGGACCAGGTTCCATC
CCTGGTTGGGGAA
>Bos_taurus_chr7.trna6714-AlaTGC (52319749-52319677) Ala (TGC) 73 bp Sc: 43.45
TCTCTGGTGGTCTAGTGGCTAAGACTCGGTGCTTGCAATGCAGGGAATCCAGGTTCGATC
CCTGGTCAGGGAA
>Bos_taurus_chr11.trna1830-AlaTGC (43317738-43317808) Ala (TGC) 71 bp Sc: 43.90
TCCCTGGTGGTCCAGTGGTTAAGGATTTGCCTTGCAAGCAGGGAACTCAGGTTCAATCCC
TGGTCAGGGAA
>Bos_taurus_chr14.trna4530-AlaTGC (63234143-63234071) Ala (TGC) 73 bp Sc: 45.76
TCTCTGGTGGTCCAGTGTTAGGATGTAGCATTTGCACTGCTAGGGGGTCCAGGTTCAACC
CCTGGTCGGGGAA
>Bos_taurus_chr1.trna10625-AlaTGC (22075040-22074968) Ala (TGC) 73 bp Sc: 49.74
TCCCTGGTGGTCCAGTAGTTAAGACTCTGTGCTTGCAATGCAGAGGGTGCAGGTTCGATC
CCTGATCAGGGAA
>Bos_taurus_chr11.trna2997-AlaTGC (71016568-71016640) Ala (TGC) 73 bp Sc: 49.74
TCCCTGGTGGTCCAGTAGTTAAGACTCTGTGCTTGCAATGCAGAGGGTGCAGGTTCGATC
CCTGATCAGGGAA
>Bos_taurus_chr2.trna722-AlaTGC (23006991-23007062) Ala (TGC) 72 bp Sc: 57.69
TCCCTGGTGGTCCAGTGGTTAGGACTCAGCACTTGCACTGCAGTGGCCTAGGTTGAATCC
CTGGTTGGGGAA
>Bos_taurus_chr6.trna1394-AlaTGC (47008906-47008978) Ala (TGC) 73 bp Sc: 58.06
GCCCTGATGGTCCAGTGGCTAAGACTCTGCGCTTGCAATGCAGGGGGCCCAAGTTCGATC
CTTGGTCAGGGAG
>Bos_taurus_chr23.trna3136-AlaTGC (39054638-39054567) Ala (TGC) 72 bp Sc: 66.75
GGGGATGTAGCTCAGTGCTAGAGCGCATGCTTTGCATGCATGAGGTCCCAGGTTCAATCC
TTGGCATCTCCA
>Bos_taurus_chr9.trna483-AlaTGC (17473021-17473092) Ala (TGC) 72 bp Sc: 68.22
GGGGGTGTAGCTCAGTGGTAGAGCATGTGCTTTGCATGCACAAGGCCCTGGGTTCAATTC
CTAGCACCTCCA
>Bos_taurus_chr23.trna815-AlaTGC (17653318-17653390) Ala (TGC) 73 bp Sc: 69.55
TCCCTGGTGGTCCAGTGGTTAGGACTCTGCACTTGCACTGCAGGGGGCACAGGTTCGATC
CCTGGCTGGGGAA
>Bos_taurus_chr17.trna4876-AlaTGC (53127960-53127889) Ala (TGC) 72 bp Sc: 72.71
GGGGATGTAGCTCAGTGGTAGAGCGCATGCTTTGCATGTATGAGGCCCCGGGTTCAATCC
CCGGCATCTCCA
>Bos_taurus_chr3.trna5428-AlaTGC (107627974-107627903) Ala (TGC) 72 bp Sc: 72.71
GGGGATGTAGCTCAGTGGTAGAGCGCATGCTTTGCATGTATGAGGCCCCGGGTTCAATCC
CCGGCATCTCCA
>Bos_taurus_chr23.trna1383-AlaTGC (29966807-29966878) Ala (TGC) 72 bp Sc: 73.33
GGGGGTGTAGCTCAGTGGTAGAGCGCATGCTTTGCATGTATGAGGCCCCGGGTTCAATCC
CCGGCACCTCCA
>Bos_taurus_chr6.trna4862-AlaTGC (110935447-110935376) Ala (TGC) 72 bp Sc: 73.60
GGGGATGTAGCTCAGTGGTAGAGCACATGCTTTGCATGTATGAGGTCCTGGGTTCAATCC
CCAGCATCTCCA

>Bos_taurus_chr23.trna3540-AlaTGC (30042769-30042698) Ala (TGC) 72 bp Sc: 73.79
GGGGATGTAGCTCAGTGGTAGAGCGCCTGCTTTGCATGCATGAGGCCCCGGGTTCGATCC
CCGGCATCTCCA
>Bos_taurus_chr17.trna2136-AlaTGC (53134698-53134769) Ala (TGC) 72 bp Sc: 74.45
GGGGATGTAGCTCAGTGGTAGAGCGCATGCTTTGCATGTATGAGGCCCCGGGTTCGATCC
CCGGCATCTCCA
>Bos_taurus_chr23.trna3548-AlaTGC (29987688-29987617) Ala (TGC) 72 bp Sc: 74.45
GGGGATGTAGCTCAGTGGTAGAGCGCATGCTTTGCATGTATGAGGCCCCGGGTTCGATCC
CCGGCATCTCCA
>Bos_taurus_chr7.trna2033-AlaTGC (41735751-41735822) Ala (TGC) 72 bp Sc: 74.45
GGGGATGTAGCTCAGTGGTAGAGCGCATGCTTTGCATGTATGAGGCCCCGGGTTCGATCC
CCGGCATCTCCA
>Bos_taurus_chr5.trna7746-AlaTGC (68229746-68229675) Ala (TGC) 72 bp Sc: 74.98
GGGGATGTAGCTCAGTGGTAGAGCACATGCTTTGCATGTATGAGGTCCTGGGTTCAATTC
CCAGCATCTCCA
>Bos_taurus_chr6.trna3040-AlaTGC (93461519-93461590) Ala (TGC) 72 bp Sc: 75.09
GGGGATGTAGCTCAGTGGTAGAGCACATGCTTTGCATGTATGAGGTCCCGGGTTCAATCC
CCGGCATCTCCA
>Bos_taurus_chr23.trna1398-AlaTGC (30046779-30046850) Ala (TGC) 72 bp Sc: 78.55
GGGGATGTAGCTCAGTGGTAGAGCGCATGCTTTGCATGTATGAGGTCCCGGGTTCGATCC
CCGGCATCTCCA
>Bos_taurus_chr14.trna4583-ArgACG (62086917-62086846) Arg (ACG) 72 bp Sc: 48.19
TCTCAGGTGGCTCAGATGGTAAAGCGTCTGTCTACGATGCAGGAGACCCAGGTTCTATCC
CTGGGTTGGGAA
>Bos_taurus_chr28.trna520-ArgACG (12353762-12353832) Arg (ACG) 71 bp Sc: 55.35
TCCCTGGTGGCTTAGTTGGTAAAGCGTCTGCCTACGATGCAGGAGACCCCGGTTCGAACC
TGGGTGGGGAA
>Bos_taurus_chr4.trna5845-ArgACG (90963589-90963518) Arg (ACG) 72 bp Sc: 57.31
TCCCTGGTAGCTCAGATGGTAAAGCTTCTGCCTACGATGCAGGAGACCCAGGTTCGATCC
CTGGGTTGGGAA
>Bos_taurus_chr3.trna3724-ArgACG (98670614-98670685) Arg (ACG) 72 bp Sc: 57.74
TCCCTGGTGGCTCAGATGGTAAAGCATCTGCCTACGATGCAGGAGACCCAGGTTCGATCC
CTGGGTTGGGAA
>Bos_taurus_chr22.trna1997-ArgACG (54252476-54252548) Arg (ACG) 73 bp Sc: 68.07
GGGCCAGTGGCGCAATGGATAACGCGTCTGACTACGGATCAGAAGATTCTAGGTTCGACT
CCTGGCTGGCTCG
>Bos_taurus_chr23.trna1425-ArgACG (30819036-30819108) Arg (ACG) 73 bp Sc: 68.07
GGGCCAGTGGCGCAATGGATAACGCGTCTGACTACGGATCAGAAGATTCTAGGTTCGACT
CCTGGCTGGCTCG
>Bos_taurus_chr23.trna1437-ArgACG (30906150-30906222) Arg (ACG) 73 bp Sc: 68.07
GGGCCAGTGGCGCAATGGATAACGCGTCTGACTACGGATCAGAAGATTCTAGGTTCGACT
CCTGGCTGGCTCG
>Bos_taurus_chr23.trna1438-ArgACG (30907150-30907222) Arg (ACG) 73 bp Sc: 68.07
GGGCCAGTGGCGCAATGGATAACGCGTCTGACTACGGATCAGAAGATTCTAGGTTCGACT
CCTGGCTGGCTCG
>Bos_taurus_chr23.trna3477-ArgACG (31086280-31086208) Arg (ACG) 73 bp Sc: 68.07
GGGCCAGTGGCGCAATGGATAACGCGTCTGACTACGGATCAGAAGATTCTAGGTTCGACT
CCTGGCTGGCTCG
>Bos_taurus_chr10.trna7485-ArgACG (21752430-21752358) Arg (ACG) 73 bp Sc: 72.37
GGGCCAGTGGCGCAATGGATAACGCGTCTGACTACGGATCAGAAGATTCCAGGTTCGACT
CCTGGCTGGCTCG
>Bos_taurus_chr23.trna1471-ArgACG (31168994-31169066) Arg (ACG) 73 bp Sc: 72.37
GGGCCAGTGGCGCAATGGATAACGCGTCTGACTACGGATCAGAAGATTCCAGGTTCGACT
CCTGGCTGGCTCG
>Bos_taurus_chr23.trna3450-ArgACG (31438450-31438378) Arg (ACG) 73 bp Sc: 72.37
GGGCCAGTGGCGCAATGGATAACGCGTCTGACTACGGATCAGAAGATTCCAGGTTCGACT
CCTGGCTGGCTCG
>Bos_taurus_chr23.trna3455-ArgACG (31331688-31331616) Arg (ACG) 73 bp Sc: 72.37
GGGCCAGTGGCGCAATGGATAACGCGTCTGACTACGGATCAGAAGATTCCAGGTTCGACT
CCTGGCTGGCTCG
>Bos_taurus_chrX.trna2546-ArgCCG (67411607-67411679) Arg (CCG) 73 bp Sc: 32.46
GCCCTGGTGGTCCAGTGCCTAAGACTCTGCAATCCGAATGCAGAGGGATTAGGTTCAACC
CCTGGTCAGGGAA
>Bos_taurus_chr13.trna4969-ArgCCG (65975780-65975708) Arg (CCG) 73 bp Sc: 35.26
TCCCTGATGGTCCAGTGGCTAAGACTCTGTGTTCCGCATGCAGGGGGCCCGGGTTTGATC
CCTAGTCAGGGAA
>Bos_taurus_chr1.trna8724-ArgCCG (81512080-81512008) Arg (CCG) 73 bp Sc: 37.84

TCCCTGGTGGTCCAGTAGCTAAGACTCTGTGCTCCGAATGCAGGGGGCCTGGGTTCAATC
CTTAGTCAGGGAA
>Bos_taurus_chr2.trna4455-ArgCCG (122729034-122729106) Arg (CCG) 73 bp Sc: 39.76
TCCCTGGTAGTCCAGTGGCTAAGACTCTGCGCTCCGAATGCAGGGGGCCTGGATTCTATC
CCTGGTCAGGGAA
>Bos_taurus_chr19.trna1694-ArgCCG (32531318-32531390) Arg (CCG) 73 bp Sc: 48.87
TCCTTGGTGGTCCACTGGCTAAGACTCCGCACTCCGAATGCAGAGGGCCCAGGTTCAATC
CCTGGTCATGGAA
>Bos_taurus_chrX.trna563-ArgCCG (13180602-13180674) Arg (CCG) 73 bp Sc: 49.42
TCCCTGGTGGTCCAGTGGCTAAGACTCTGCACTCCGAATGCTGGGGACCAGGGTTCAATC
CCTGGTCAGGGAA
>Bos_taurus_chr10.trna7767-ArgCCG (15110720-15110648) Arg (CCG) 73 bp Sc: 50.75
TCCCTGGTGGTCCAGTGGCTAAGACTCTGCATTCCGAATACAGGGGGCCCAGATTCGACC
CCTGGTCAGGGAA
>Bos_taurus_chr10.trna8097-ArgCCG (8214787-8214715) Arg (CCG) 73 bp Sc: 51.27
TCCCTGGTAGTCCAACGGCTAAGACTCTGCGCTCCGAATGTAGGAGGCCCAGGTTCGATC
CCTGGCCAGGGAA
>Bos_taurus_chr8.trna3636-ArgCCG (100369865-100369937) Arg (CCG) 73 bp Sc: 54.46
TCCCTGGTGGTCCAGTGGCTAAGACTCCGCGTTCCGAATGCAGGGGGCCCAGGTTCGATC
CCTGGTCAGGGAA
>Bos_taurus_chr7.trna7868-ArgCCG (21000461-21000389) Arg (CCG) 73 bp Sc: 57.48
TCCCTGGTGGTCCAGTGGTTGAGACTCTGAACTCCGAATGCAGGGGGCCCAGGTTCAATC
CCTGGTCAGGGAA
>Bos_taurus_chr16.trna5143-ArgCCG (44063177-44063105) Arg (CCG) 73 bp Sc: 61.75
TCCCTGGTGGTCCAGTGGCTAAGACTCTGCACTCCGAATGCAGGGAGCCCAGGTTCAATC
CCTGGGCAGGGAA
>Bos_taurus_chr19.trna2612-ArgCCG (49585336-49585408) Arg (CCG) 73 bp Sc: 65.49
GACCCAGTGGCCTAATGGATAAGGCATCAGCCTCCGGAGCTGGGGATTGTGGGTTCGAGT
CCCATCTGGGTCG
>Bos_taurus_chr23.trna1384-ArgCCG (29971502-29971574) Arg (CCG) 73 bp Sc: 69.88
GGCCGCGTGGCCTAATGGATAAGGCGTCTGATTCCGGATCAGAAGATTGAGGGTTCGAGT
CCCTTCGTGGTCG
>Bos_taurus_chr23.trna1385-ArgCCG (29971886-29971958) Arg (CCG) 73 bp Sc: 69.88
GGCCGCGTGGCCTAATGGATAAGGCGTCTGATTCCGGATCAGAAGATTGAGGGTTCGAGT
CCCTTCGTGGTCG
>Bos_taurus_chr23.trna3552-ArgCCG (29932898-29932826) Arg (CCG) 73 bp Sc: 69.88
GGCCGCGTGGCCTAATGGATAAGGCGTCTGATTCCGGATCAGAAGATTGAGGGTTCGAGT
CCCTTCGTGGTCG
>Bos_taurus_chr25.trna88-ArgCCG (2584569-2584641) Arg (CCG) 73 bp Sc: 69.88
GGCCGCGTGGCCTAATGGATAAGGCGTCTGATTCCGGATCAGAAGATTGAGGGTTCGAGT
CCCTTCGTGGTCG
>Bos_taurus_chr17.trna2287-ArgCCT (54455295-54455367) Arg (CCT) 73 bp Sc: 24.33
TCCCTGGTGGCCCAGAGACTGGGACTCCATGTTCCCTGTGCAGGGGTCCCAGGTTCGAGC
CCTGGTCAGGGAA
>Bos_taurus_chr24.trna3401-ArgCCT (49706446-49706374) Arg (CCT) 73 bp Sc: 25.30
TCTCTGGTGGTCCAGTGACTAAGATTCCGTGCTCCTAACGCAGGGGAGTCAGGTTCGACC
CTTGGTCAGGGAA
>Bos_taurus_chr4.trna5719-ArgCCT (93697661-93697589) Arg (CCT) 73 bp Sc: 26.83
TCCCTGGGGGTCCAGTGGCTAAGACACTGTGCTCCCTGTGCAGGGGTCCTGGGTTTGATC
CCTGGTCAGGGAA
>Bos_taurus_chr9.trna940-ArgCCT (28953546-28953618) Arg (CCT) 73 bp Sc: 26.99
TTCCTGGTGATCCAATGGTTAAGATTCTGAGCTCCCTGTGCAGGGGACTCAGGTTCTATC
CCTGGCCAGGGAA
>Bos_taurus_chr3.trna9173-ArgCCT (8454710-8454637) Arg (CCT) 74 bp Sc: 27.21
TCCCTGGTGGTCCAGTGGCTAAGACTTTGTGCCCCTAATGCAGGGGAAGCCAGGTTCCAT
CCTTGGTCAGGGAA
>Bos_taurus_chr22.trna1569-ArgCCT (43443356-43443427) Arg (CCT) 72 bp Sc: 27.92
TCCCTGGTGGTCCAGAGGCGAGACTCTGCGCTCCTAGTGCAGGGGGCCCAGGTTCGGTCC
CTGGTGAGGGAG
>Bos_taurus_chr8.trna4328-ArgCCT (109276710-109276638) Arg (CCT) 73 bp Sc: 28.76
TCCCTGGTGGTCCCGTGGTTAAGACTCTGTTCTCCTGATGCAGGTGGGCTGGGTTTGACC
CCTAGTCAGGGAA
>Bos_taurus_chr18.trna4246-ArgCCT (48519402-48519330) Arg (CCT) 73 bp Sc: 29.47
TCCCTGGTAGTCTAGTGGCTAAGATTCTGTGCTCCTGGTACTGAGGACCCGGGCTCAATC
CCTGGTCAGGGAA
>Bos_taurus_chr10.trna1878-ArgCCT (47374945-47375017) Arg (CCT) 73 bp Sc: 29.95
TCCCTGATGGTCCAGAGGCTAAGATTTTGCACTCCTCATGCAGGGGGCCTGTGTTTGATC

CCTGGTCAGGGAC
>Bos_taurus_chr7.trna833-ArgCCT (16234340-16234413) Arg (CCT) 74 bp Sc: 31.00
TCCCTGGTGGTCCAGTGTCTAAGACTCCATGCTCCTAATGCGGGGGACCCCAGGTTGGAT
CCCTGGTCAGGGAT
>Bos_taurus_chr11.trna6249-ArgCCT (73385018-73384946) Arg (CCT) 73 bp Sc: 31.10
CTTCTGGTGGTCCAGTGGCTGAGACTCCGCACTCCTACTGCAGGAGGCCCAGGTTCAGTT
CCTGGTCAGGGAA
>Bos_taurus_chrX.trna11842-ArgCCT (4165126-4165054) Arg (CCT) 73 bp Sc: 32.21
TCCCTGGTGGTCCAGTCTCTAAGACTCTGCACTCCTAATGCAACAGACTTGGGTTCAATT
CCTAGTCAGGGAA
>Bos_taurus_chr23.trna850-ArgCCT (18192851-18192923) Arg (CCT) 73 bp Sc: 33.93
TCCCTGTTGGTTCAGCGGCAAAGACTCCGCACTCCTAATGCAGGTGGTCCGGGTTTGATC
CCTGGTCAGGGAA
>Bos_taurus_chr9.trna3504-ArgCCT (95424990-95425062) Arg (CCT) 73 bp Sc: 34.18
TCCTTGATAGTCCAGCGGCTAAGACTCTGTGCTCCTAGTACATGGAGCTCAGGTTCAGTT
CCTGATTGGGGAA
>Bos_taurus_chr18.trna5314-ArgCCT (24790986-24790914) Arg (CCT) 73 bp Sc: 35.54
TTACTGGTGGTCCAGAGACTAAGACTGTGCACTCCTAATGCAGGGGACCCAGGTTCGATC
CCTGGTCAGGGAA
>Bos_taurus_chr1.trna6379-ArgCCT (148075271-148075199) Arg (CCT) 73 bp Sc: 35.57
TTCCTGGTGGTTATGCGGCTAAGACTCTGCGTTCCTAATGCAGGGGGCCCAGGTTCCATC
CCTGGTCAGGGAA
>Bos_taurus_chr17.trna531-ArgCCT (13526121-13526193) Arg (CCT) 73 bp Sc: 36.23
TCCCTGGTAGCCCAGTGGCTGAGGTTCTGTCCTCCTGCTGCAGAGGGCCCATGTTCGATC
TCTGGTTAGGGAA
>Bos_taurus_chr6.trna7056-ArgCCT (59471031-59470959) Arg (CCT) 73 bp Sc: 36.55
TCCCTGGTGGTCTAGTGGCTAAGACTTGGAGCTCCTGATTCAGGGGACCCAGGTTCAACC
CCTGATCAGGGAA
>Bos_taurus_chr13.trna4209-ArgCCT (78298278-78298206) Arg (CCT) 73 bp Sc: 36.59
TCTCTGGTGGTCCAGTGGCTAAGACTCCGTGCTCCTAATACGTGTGGCTCAGGTTCCATC
CCTGGTCAGGGAA
>Bos_taurus_chr1.trna6434-ArgCCT (145651610-145651539) Arg (CCT) 72 bp Sc: 37.32
TCCCTGGTGGTTCAGTGGCTGGGACTCTGTGTTCCTGATGCAGGGGCCCCGGTTCAGTCC
CTGGTCAGGGAA
>Bos_taurus_chr8.trna8064-ArgCCT (9788151-9788079) Arg (CCT) 73 bp Sc: 37.54
TCCCTGGTGGTCCCACGGCTAAGACTCCGTGTTCCTAATGCAGGGGGCCCAGGTTCAATC
CCTGGTCAGGGAA
>Bos_taurus_chr19.trna6149-ArgCCT (17891817-17891745) Arg (CCT) 73 bp Sc: 37.55
TCCCTGGTCGTCCAGTGGCTAAGACTCTCTGCTCCTAACGCAGAGGGCCTGGGTTCAGTC
CCTGGTCAGGGAG
>Bos_taurus_chr24.trna1453-ArgCCT (34953093-34953165) Arg (CCT) 73 bp Sc: 37.68
TCCTTGATGGTCCATTGGTTAAGACTCTGTTTTCCTATTGCAGGGAGCCTGGGTTCCATC
CCTGGTCAGGGAA
>Bos_taurus_chrX.trna5052-ArgCCT (133702249-133702320) Arg (CCT) 72 bp Sc: 38.15
TCCCTGGAGGTCCAGTGGCTAGGACTCTGTGCTCCTAGTGCCGGGGGCCAGGTTCAATCC
CTGGTCAGGGAA
>Bos_taurus_chr26.trna1128-ArgCCT (32271633-32271704) Arg (CCT) 72 bp Sc: 38.77
TCCCTGGTGGTCCAGGGGCTGAGACTCTGCACTCCTAATGCAAGATCCAGGGTTCAATCC
CTAGTCAGGGAA
>Bos_taurus_chr15.trna2489-ArgCCT (66896317-66896389) Arg (CCT) 73 bp Sc: 39.14
TCCCTGGTGGTCCAGTGGCTAAGACTCTGTGCTCCTAATGCAGGGAACCCGGATTCAACC
CCTGGTCAGGGAA
>Bos_taurus_chr11.trna127-ArgCCT (1836829-1836901) Arg (CCT) 73 bp Sc: 39.18
TCTCTGGTGGTCCAGTAGTTGGGACTCTGAGCTCCTGATGCAGGGGCCACAGGTTCGATC
CCTGCTTGGAGAA
>Bos_taurus_chr15.trna2932-ArgCCT (78224706-78224778) Arg (CCT) 73 bp Sc: 39.55
TCCCTGGTGGTCCAGTGGCTACGACTCCGCACTCCTGATGCAGGGGGCCTGGGTCCAATC
CCTGGTCAGGGAA
>Bos_taurus_chr3.trna8701-ArgCCT (20089857-20089785) Arg (CCT) 73 bp Sc: 39.65
TCCTTGGTGGTTCAGTGGCTAAGACACTATGCTCCTGATATAGTGAATGCAGGTTCAATC
CCTGGTCAGGGAA
>Bos_taurus_chr8.trna5052-ArgCCT (91657692-91657620) Arg (CCT) 73 bp Sc: 40.02
TTCCTGGTGGTCTAGTAGTTAAGATTTTGCACTCCTAATGCAGGGGGCACAGGTTTGATC
CCTGGTCGGGGAA
>Bos_taurus_chr16.trna3964-ArgCCT (69188009-69187937) Arg (CCT) 73 bp Sc: 40.11
TCTCTGGTGGTCCAGTGGCTAAGACTTTGCAATCCTAATGCAGGGGGCACAGGTTCGTTT
CCTGGTCAGGGAA

>Bos_taurus_chr19.trna1387-ArgCCT (27481947-27482019) Arg (CCT) 73 bp Sc: 40.90
TACCTGGTGGTCCAGTGGCTAAGACTCTGCACTCCTAATACAGGGGGCCTGGGTTCGATC
TCTGGTCAGGGAA
>Bos_taurus_chr13.trna1365-ArgCCT (31625994-31626066) Arg (CCT) 73 bp Sc: 41.85
TCCCTGGTGGTCCAGTGGTTAAGACCCTGAGCTCCTAACATAGGGGGCCCAGATTCTATC
CCTGGTTGGGGAA
>Bos_taurus_chr22.trna318-ArgCCT (7129259-7129331) Arg (CCT) 73 bp Sc: 42.03
TCCCTGGTGGTCCAGTGGCTAAGACCTTGTGTTCCTAATGCAGGAGGTCCAAGTTCAATC
CCTGGCCAGGGAA
>Bos_taurus_chrX.trna2066-ArgCCT (55163369-55163441) Arg (CCT) 73 bp Sc: 42.31
TCCCTGGTGGTCCAGTGGCTGAAACTCTGCATTCCTAATGCAGGGAGCCCAGGTTCCATC
CCTGGTTGGGGAG
>Bos_taurus_chr2.trna2823-ArgCCT (85704123-85704195) Arg (CCT) 73 bp Sc: 42.39
TCCCTGGTGGTCCAGTGGCTAAGATTCTGCAGTCCTAATGCAGGGGTGCTGGGTTCCATC
CCTGGTCAGGGAA
>Bos_taurus_chr19.trna5524-ArgCCT (27212025-27211953) Arg (CCT) 73 bp Sc: 42.56
TTCTTGGTGGTCTAGTGGTTAAGACTCTGTGCTCCTAACACAGGGGGCCCAGGTTTGATC
CCTGGTGGGGGGA
>Bos_taurus_chr3.trna8187-ArgCCT (30617445-30617373) Arg (CCT) 73 bp Sc: 42.73
TCCATGGTGGTCCAGTGGCTGAGACTCTGTGCTCCTAACACAGGGGGCCCAGGTTCCATC
CCTGGTCAGGGAA
>Bos_taurus_chr7.trna2668-ArgCCT (59831879-59831951) Arg (CCT) 73 bp Sc: 42.76
TCCCTGGTGGTCCACTGGCTAAGACCCTGCACTCCTGATACAGGGGGCCCAGGTGCAATC
CCTGGTCAGGGAA
>Bos_taurus_chr23.trna3650-ArgCCT (27442421-27442349) Arg (CCT) 73 bp Sc: 43.06
TCCCTGGTGGTCCAGTGGCCAAGACTCTGAGATCCTAATGCAGGGGCCCCAGGTTTGATC
CCTGGTCAGGGAA
>Bos_taurus_chr4.trna1705-ArgCCT (51379119-51379191) Arg (CCT) 73 bp Sc: 43.55
TGCCTGGTGGTCCAGTGGCTAAGACTCTGTGCTCCTAACGCAGGGGGCCTGGGTTCAGTT
CCTAGTCAGGGAA
>Bos_taurus_chr25.trna1659-ArgCCT (26975929-26976001) Arg (CCT) 73 bp Sc: 43.61
TCCCTGGTGGTCCAGTGGTTAAGACTCTGCGCTTCCTATGCAAGGGGCACAGGTTTGATC
CCTGCTCAGGGAA
>Bos_taurus_chr27.trna1319-ArgCCT (33079628-33079700) Arg (CCT) 73 bp Sc: 43.68
TCCCTGGTGGTCCAGTGGATAAGGCTCTGTGCCCCTACCGCAGGGGGCCTGGGTTCGATC
CCTGGTCAGGGAC
>Bos_taurus_chr6.trna3346-ArgCCT (99192020-99192092) Arg (CCT) 73 bp Sc: 43.90
TCCCTGGAGGTCCAGTGATTAAGACTCTGTGCTCCTAATGTAGGGGGCCCAGGTTCGATT
CCTGGTCAGGGAA
>Bos_taurus_chr11.trna341-ArgCCT (5537208-5537280) Arg (CCT) 73 bp Sc: 44.09
TCCCTGGTGGGCCAGTGGCTAAGACCCTGTGCTCCTGATGCAGGGGAACCAGGTTCGATC
CCTGGTCAGGGAA
>Bos_taurus_chr25.trna781-ArgCCT (12322174-12322246) Arg (CCT) 73 bp Sc: 44.18
TCCCTGGTTGTCCAGTGGCTAAGACTCTGCACTCCTGATGCAGGGGTCCTGAGTTTGATC
CCTGGTCAGGGAA
>Bos_taurus_chr25.trna819-ArgCCT (12850779-12850851) Arg (CCT) 73 bp Sc: 44.18
TCCCTGGTTGTCCAGTGGCTAAGACTCTGCACTCCTGATGCAGGGGTCCTGAGTTTGATC
CCTGGTCAGGGAA
>Bos_taurus_chr5.trna3726-ArgCCT (93829241-93829313) Arg (CCT) 73 bp Sc: 44.38
TCCCTGGTGGTCCAGTAGCAAGGATTCTGCACTCCTAATGCAGGGGGCCCGAGTTCAATC
CCTGGTCAGGGAA
>Bos_taurus_chr14.trna4137-ArgCCT (71414406-71414334) Arg (CCT) 73 bp Sc: 44.53
TCCCTGGTAGTCCTGTGGTTAAGACTCTGAGCTCCTAATTCAGGGGGCTCAGGTTTGATC
CCTGGTCAGGGAA
>Bos_taurus_chr1.trna6853-ArgCCT (134408700-134408628) Arg (CCT) 73 bp Sc: 44.81
TCCCTGGTGGTCCAGTGGCTGAGACTCCGTACTCCTAATGCAGGGGGCCCAGGTTTGATC
CCTGGTCAGGGAA
>Bos_taurus_chr22.trna4074-ArgCCT (12797672-12797600) Arg (CCT) 73 bp Sc: 45.63
TCCCTGGTGGTCCAGTGGCTAAGATTCTGTGTTCCTAATGCAGGGGGCCTGGGTTCAATC
CCAGGTCAGGGAA
>Bos_taurus_chr6.trna5460-ArgCCT (99198498-99198426) Arg (CCT) 73 bp Sc: 45.92
TCCCTGGTGGTCCAGTGGCTATGACCCTGCACTCCTAATGTAGGGGGTCCAAGTTCAATT
CCTGGTCAGGGAC
>Bos_taurus_chr20.trna930-ArgCCT (24405952-24406024) Arg (CCT) 73 bp Sc: 46.03
TCCCTGGTGGTCCAATGGCTAAGACTCTGTACTCCTGATGCAGCAGGCCAGGGTTCGATC
CCTGGTCAGGGAA
>Bos_taurus_chr18.trna1130-ArgCCT (26969400-26969472) Arg (CCT) 73 bp Sc: 46.15

TTCCTGGTGGTCCAGTGGCTAAGACTCTGTGCTCCTAATGCAGGGGTCCCAAGTTCCATC
CCTGGTCAGGGAA
>Bos_taurus_chr15.trna5399-ArgCCT (31774403-31774331) Arg (CCT) 73 bp Sc: 46.55
TCCCTGGTGGTCCAGTGGCTAAGACTCTGCACTCCTAATGCAGGAGGGCTGGGTTTGATC
CCTGGCCAGGGAA
>Bos_taurus_chr22.trna4154-ArgCCT (11333010-11332938) Arg (CCT) 73 bp Sc: 46.63
TCCCTGGTGGTCCAGTGGCTAAGACTCTGCGCTCCTAATGCAGGGGGCCTGGGTTTGATC
CCTGGTCAGGGAA
>Bos_taurus_chr13.trna3246-ArgCCT (72667773-72667845) Arg (CCT) 73 bp Sc: 46.81
TCCCTGGTGGTCCAGTGGCTAAGACTCTGTGCTCCTCATGCTGCGGGCCCAGGTTCAATC
CCTGGTCAGGGAA
>Bos_taurus_chr7.trna3254-ArgCCT (74712010-74712082) Arg (CCT) 73 bp Sc: 47.02
TCCCTGATGGTCCAGTGGCCAAGACTTTGTGTTCCTAAGGCAGGGGGCCCAGGTTCCAAC
CCTGGTCGGGGAA
>Bos_taurus_chr5.trna2601-ArgCCT (67872764-67872835) Arg (CCT) 72 bp Sc: 47.22
TCCCTGGTGGTCCAGTGGCTAAGTCTCTGTGCTCCTAATGCAGAAGCCCAGGTTCGACCT
CTGGTCAGGGAA
>Bos_taurus_chr7.trna8103-ArgCCT (18219343-18219271) Arg (CCT) 73 bp Sc: 47.61
TCCCTGGAAGTCCAGTGATTAAGACTTTGCACTCCTGATGCAGAGGGCCCAGGTTTGATC
CCTGGTCAGGGAA
>Bos_taurus_chr16.trna2041-ArgCCT (51884730-51884801) Arg (CCT) 72 bp Sc: 47.68
TTCCTTGTGGTCCAGTGGGTAGGACTCAGTGCTCCTCCTGCTGGGGCCCAGGTTCAATCC
CTGGTCGGGAAA
>Bos_taurus_chr11.trna9067-ArgCCT (4539273-4539202) Arg (CCT) 72 bp Sc: 47.87
TCCCTGATGGTCCAGTGGTGAGACTCTGCATTCCTGATGTAGGGGGCGCAGGTTCAATTC
CTGGTTGGGGAA
>Bos_taurus_chr19.trna2845-ArgCCT (55057939-55058011) Arg (CCT) 73 bp Sc: 48.02
TCCCTGGTGGTCCTGTGACTAGGACTCTGTGCTCCTAATACAGGGGGCCCAGGTTCGATC
CCTGGTCAGGGAA
>Bos_taurus_chrX.trna2465-ArgCCT (64809331-64809403) Arg (CCT) 73 bp Sc: 48.18
TCCCTGGTGGTTCAGTGGGTAAGACTCTACACTCCTAATGCAGGGAGCCCAGGTTCAATC
CCTAGTCAGGGAA
>Bos_taurus_chr11.trna4687-ArgCCT (106400942-106401013) Arg (CCT) 72 bp Sc: 48.21
TCCCTGGGGGTCGAGTGGCTAAGACTCCGTGTTCCTAATGCAGGGGTCCAGGTTCGATTC
CTGGTCAGGGAA
>Bos_taurus_chr21.trna510-ArgCCT (15354374-15354446) Arg (CCT) 73 bp Sc: 48.27
TCCCTGGGGGTCCAGTGGCTAAGACTCTGCTCTCCTGGTGCAGAGGGCCCAGGTTCAATC
CCTGGTCAGGGAA
>Bos_taurus_chr3.trna9052-ArgCCT (12377149-12377077) Arg (CCT) 73 bp Sc: 48.27
TCCTTGGTGGTTCAGTGGCTAAGACTCTGCACTCCTAAAGCAGGGGTCCCAGGTTTGGTC
CCTGGTCAGGGAA
>Bos_taurus_chr7.trna8461-ArgCCT (13747885-13747813) Arg (CCT) 73 bp Sc: 48.28
TCCCTGGTGGTCCAGTAGTTAGGACTCCACATGCCTAATGTAGGAGGCCCGGGTTCGATC
CCTGGTCAGGGAA
>Bos_taurus_chr19.trna2525-ArgCCT (48524015-48524087) Arg (CCT) 73 bp Sc: 48.30
TCCCTGGTGGTCCAGTGGTTAAGACTCTGCCCTCCTAATGCAGGCGGCCCAGGTTTGATC
CCTGATCAGGGAA
>Bos_taurus_chr3.trna4876-ArgCCT (119209755-119209683) Arg (CCT) 73 bp Sc: 48.49
TCCCTGGTGGCCCAGTGGCTGAGACTCTGCGCTCCTAATGCAGGGGGCCCAGGTTCAATC
CCTGGTCAGGGAA
>Bos_taurus_chr19.trna2335-ArgCCT (45475332-45475404) Arg (CCT) 73 bp Sc: 48.89
TCTTTGGTGGTCCAGTGGCTAAGACTCTGTGCTCCTAATGCTGGGGTGCCAGGTTCGATC
CCTGGTCAGGGAA
>Bos_taurus_chrX.trna2908-ArgCCT (82693191-82693262) Arg (CCT) 72 bp Sc: 49.06
TCCCTGGTGGTTCAGTGGCTGGGATTCTGCACTCCTAGTGAAGGGGCCCAGGTTCAATTC
CTGGTCAGGGAA
>Bos_taurus_chr13.trna3550-ArgCCT (77451428-77451500) Arg (CCT) 73 bp Sc: 49.67
TCCCTGGTAGTCCAGCGGCTAAGACTCTGCACTCCTAATGCAGGGGGTCCGGGTTTGATC
CCTGGTTAGGGAA
>Bos_taurus_chr3.trna5741-ArgCCT (100476872-100476800) Arg (CCT) 73 bp Sc: 50.24
TCCCTGGTGGTTCAGTGGCTAAGACTCTGCACTCCTAATGCAGAGGACTAGGGTTCAATC
CCTGGTCAGGGAA
>Bos_taurus_chr4.trna5979-ArgCCT (87601908-87601836) Arg (CCT) 73 bp Sc: 50.42
ACTCTGGTGGTCTAATGGCTAAGACTCTGCACTCCTAATGCAGAGGACCCAGGTTCCATC
CCTGGTCAGGGAA
>Bos_taurus_chr12.trna7093-ArgCCT (12432591-12432519) Arg (CCT) 73 bp Sc: 50.42
TCCCTGGTGGTTCAGTGGCTAAGACTCTGCACTCCTAATACAGGGGGTCCAGGTTCGATC

CCTAGTCAGGGAA
>Bos_taurus_chr7.trna8113-ArgCCT (18082621-18082549) Arg (CCT) 73 bp Sc: 50.48
TTCCTGGTGGTCCAGTGGCTCAGACTCTGAACTCCTAATTCAGGGGGCCCAGGTTCAATC
CCTGGTCAGGGAA
>Bos_taurus_chr11.trna4893-ArgCCT (101287140-101287067) Arg (CCT) 74 bp Sc: 51.17
TCCCTGGTGGTTCAGTGGCTAAGACTCTGTACTCCTAATACAGGGGCTCCCAGGTTCGAT
CCCTAGTCAGGGAA
>Bos_taurus_chr11.trna175-ArgCCT (2370695-2370767) Arg (CCT) 73 bp Sc: 51.34
TCCCTGGTGGTCCAATGGCTAAGACTCTGCCCTCCTGATGCAGTGGGCCCGGGTTCGATC
CCTGGACAGGGAA
>Bos_taurus_chr17.trna2444-ArgCCT (56134270-56134342) Arg (CCT) 73 bp Sc: 51.45
TCCCTGGTGGTCTAGTGGGTAAGACTCTGCTCTCCTAATGCAGGGGGCCCGGGTTTGATC
CCTGGTCAGGGAA
>Bos_taurus_chr2.trna8638-ArgCCT (60255968-60255896) Arg (CCT) 73 bp Sc: 51.56
TCCCTGATGGTCCAATGGCTAAGACTTTGCTCTCCTAATGCAGGGGGCCTGGGTTCAATC
CCCGGTCAAGGAA
>Bos_taurus_chr13.trna5944-ArgCCT (41665961-41665889) Arg (CCT) 73 bp Sc: 51.84
TCCCTGGTGGTCCAGTGGCTAAGACTCCACACTCCTAGTGTAGGGGTCCCGGGTTCGATC
CCTGTCCAGGGAA
>Bos_taurus_chr8.trna156-ArgCCT (5155243-5155315) Arg (CCT) 73 bp Sc: 52.14
GCCCCAGAAGCCTAATGGATAAGGCACTGTCCTCCTAAGCCAGGGACTGTGGGTTCAAGT
CCCACCTGGGGTA
>Bos_taurus_chr7.trna4014-ArgCCT (97296777-97296849) Arg (CCT) 73 bp Sc: 52.23
TCCCTGGTGGTCCAGTGGCTAGCACTCTGCACTCCTAATGCAAGGGGCCCAGGTTCCATC
CCTGGTCAGGGAA
>Bos_taurus_chr15.trna5058-ArgCCT (39017478-39017406) Arg (CCT) 73 bp Sc: 52.32
TCCCTGGTGGCCCAGCGGCTAAGACTCTGTACTCCTGATGCAGGGAGCCCAGGTTCGAGC
CCTGGTTAGGGAA
>Bos_taurus_chr4.trna4497-ArgCCT (117891579-117891507) Arg (CCT) 73 bp Sc: 52.32
TCCCTGGTGGTCCAGTGGTTAAGACTCTGCACTCCTAATGGAGGGGGCCCTGGTTTGATC
CCGGATCAGGGAG
>Bos_taurus_chr26.trna810-ArgCCT (22733763-22733835) Arg (CCT) 73 bp Sc: 52.39
TCCCTGGTGGTCCAATGGCTAAGACTCTGTACTCCTAATGCAGGGGGTCTGGGTTCGATT
CCTGGTCAGGGAA
>Bos_taurus_chr13.trna2626-ArgCCT (62643271-62643343) Arg (CCT) 73 bp Sc: 53.09
TCCTTGGTGGTCCAGTGGCTAAGACTCTGAGCTCCTGATGCAGGGGGCCCAGGTTCGATC
CCTGGTTGGGGAA
>Bos_taurus_chr15.trna5387-ArgCCT (32014015-32013943) Arg (CCT) 73 bp Sc: 53.18
TCCTTGGTGGTCCAGTGGTTAAGACTCTGTGCTCCTAATGTAGGGGGCCCAGGTTCGATT
CTTGGTCAGGGAA
>Bos_taurus_chr3.trna5472-ArgCCT (106643930-106643858) Arg (CCT) 73 bp Sc: 53.25
TCCTTGGTGGTCCAGTGGCTAAGACTCTGCACTCCTAATGCAGGGGACCTGGGTTCAATC
CCTGGTTAGGGAA
>Bos_taurus_chr15.trna2345-ArgCCT (63633463-63633535) Arg (CCT) 73 bp Sc: 53.49
TCCCTGGTGGTCCAGTGGCTAAGATTCTGTACTCCTAATGCAGGGGGTCTGGGTTCGATT
CCTGGTCAGGGAA
>Bos_taurus_chr12.trna6816-ArgCCT (16293611-16293539) Arg (CCT) 73 bp Sc: 53.59
TCCCTGGTGGTCCAGTGGCTAAGACTCTGCACTCCTAATACAGGGGCCCTGGGTTCAATC
CCTGGTCAGGGAA
>Bos_taurus_chr10.trna3101-ArgCCT (78471977-78472049) Arg (CCT) 73 bp Sc: 55.33
TCTCTGGTGGTCCAGTGGCTAAGACTGCACGCTCCTAATGTGCAGGGCCCAGGTTCAATC
CCTGGTCAGGGAA
>Bos_taurus_chr17.trna726-ArgCCT (17169899-17169971) Arg (CCT) 73 bp Sc: 55.48
TCCCTGGTAGTCCAGTGGCTAAGACTCTGCACTCCTAATGCAGGGGGCCCCGGTTCCATC
CCTGGTCAGGGAA
>Bos_taurus_chr1.trna4211-ArgCCT (118331059-118331131) Arg (CCT) 73 bp Sc: 56.51
TCCCTGGTGGTCCAGTGGCTAAGACTCTGCGCTCCTAATGCAGGGGGCCCAGGTTTGATC
CCTGGTCAGGGAA
>Bos_taurus_chr13.trna3464-ArgCCT (75985782-75985854) Arg (CCT) 73 bp Sc: 56.75
ACCCAGCTAGTTCAGCTGGTAGAGCGTGGGACTCCTGATCCCAGCATTGTGGGTTCGAGC
CCCACGTTGGGCA
>Bos_taurus_chr7.trna7152-ArgCCT (39893718-39893646) Arg (CCT) 73 bp Sc: 56.97
TCCCTGGTGGTTCAGTGGTTAAGACTTTGCACCCCTAATGCAGGGGGCTCAGGTTCAATT
CCTGGTCAGGGAA
>Bos_taurus_chr28.trna3039-ArgCCT (8457120-8457048) Arg (CCT) 73 bp Sc: 57.20
TCCCTGGTGGTCTAGTGGCTAAGACTCTGCACTCCTAATGCAGGAGGCCTGGGTTCGATT
CCTGGTCAGGGAA

>Bos_taurus_chr19.trna6032-ArgCCT (19570925-19570853) Arg (CCT) 73 bp Sc: 57.41
TTCCTGGTGGTCCAGTGGCTAAGACTCTGTGCTCCTAATGCAGAGGGCCCAGGTTCGATC
CCTGGTCAGGGAA
>Bos_taurus_chr2.trna4303-ArgCCT (120297753-120297823) Arg (CCT) 71 bp Sc: 57.48
TCCCTGGTGGTCCAGTGGCTAAGACTCTGTGCTCCTAAGACAGGGCCCAGGTTCGATCCC
TGGTCAGGGAA
>Bos_taurus_chr12.trna7166-ArgCCT (11203641-11203570) Arg (CCT) 72 bp Sc: 58.04
TCCCTGGTGGTCCAGTGGCTAAGACTCTGTACTCCTAATGCAGGGGTCTGGGTTCGATTC
CTGGTCAGGGAA
>Bos_taurus_chr29.trna1918-ArgCCT (47476503-47476575) Arg (CCT) 73 bp Sc: 58.25
TCCCTGGTGGTCCAGTGGCTAGGACTCCGGGCTCCTAATGCAGGGGACCCAGGTTCGATC
CCTGGCCAGGGAA
>Bos_taurus_chr9.trna538-ArgCCT (18951903-18951975) Arg (CCT) 73 bp Sc: 59.05
TCCTTGGTGGTTCAGTGGTTAAGACGCTGAGCTCCTAATGCAGGGGGCCCAGGTTCGATC
CCTGGTCAGGGAA
>Bos_taurus_chr7.trna1352-ArgCCT (22546287-22546359) Arg (CCT) 73 bp Sc: 59.09
TCCCTGGTGGTCCAGTGGCTAAGACTCCACACTCCTGATGCAGGAGGCCCTGGTTCGATT
CCTGGCCAGGGAA
>Bos_taurus_chr5.trna7502-ArgCCT (73646029-73645957) Arg (CCT) 73 bp Sc: 59.30
GTCCCAGTGGCCTAATGGACAAGGCACTGGCCTCCTAAGTCAGGGATTGTGGGTTCAAGT
CCCACCTGGGGTG
>Bos_taurus_chr15.trna562-ArgCCT (20399874-20399946) Arg (CCT) 73 bp Sc: 60.10
TCCCTGGTGGTCCAGTGGCTAGGACTCTGCACTCCTAATGCAGGTGGCCCAGTTTCGATC
CCTGGTCAGGGAA
>Bos_taurus_chr7.trna8782-ArgCCT (6463120-6463048) Arg (CCT) 73 bp Sc: 60.76
TCCCTGGTGGTCCAGTGGCTAGGACTCCGCACTCCTAATGCGGGGGGCCTGGGTTCAATC
CCTGGTCAGGGAA
>Bos_taurus_chr25.trna1145-ArgCCT (18985181-18985253) Arg (CCT) 73 bp Sc: 61.70
TCCTTGGTGGTCCAGTGGTTAAGACTTTGCACTCCTAATGCAGGGGGCCCAAGTTCGATC
CCTGGTCAGGGAA
>Bos_taurus_chr12.trna5226-ArgCCT (53100221-53100149) Arg (CCT) 73 bp Sc: 63.61
TCCCTGGTGGTCCAGTGGTTAAGATTCTACACTCCTAATGCAGGGGGCCCAGGTTCAATC
CCTGGTCAGGGAA
>Bos_taurus_chr19.trna3010-ArgCCT (57033478-57033551) Arg (CCT) 74 bp Sc: 66.90
GCCCCAGTGGCCTAATGGATAAGGCACTGGCCTCCTAAAGCCAGGGATTGTGGGTTCGAG
TCCCACCTGGGGTG
>Bos_taurus_chr4.trna3603-ArgCCT (103714740-103714812) Arg (CCT) 73 bp Sc: 67.28
GCCCCAGTGGCCTAATGGATAAGGCATTGGCCTCCTAAGCCAGGGATTGTGGGTTCGAGT
CCCATCTGGGGTG
>Bos_taurus_chr19.trna3807-ArgCCT (57032604-57032532) Arg (CCT) 73 bp Sc: 70.63
GCCCCAGTGGCCTAATGGATAAGGCACTGGCCTCCTAAGCCAGGGATTGTGGGTTCGAGT
CCCATCTGGGGTG
>Bos_taurus_chr25.trna5070-ArgCCT (2612241-2612169) Arg (CCT) 73 bp Sc: 71.31
GCCCCGGTGGCCTAATGGAGAAGGCACTGGCCTCCTAAGCCAGGGATTGTGGGTTCGAGT
CCCACCCGGGGTA
>Bos_taurus_chr25.trna89-ArgCCT (2586871-2586943) Arg (CCT) 73 bp Sc: 71.53
GCCCCGGTGGCCTAATGGATAAGGCATTGGCCTCCTAAGCCAGGGATTGTGGGTTCGAGT
CCCACCCGGGGTA
>Bos_taurus_chr19.trna3806-ArgCCT (57034037-57033965) Arg (CCT) 73 bp Sc: 73.88
GCCCCAGTGGCCTAATGGATAAGGCACTGGCCTCCTAAGCCAGGGATTGTGGGTTCGAGT
CCCACCTGGGGTA
>Bos_taurus_chr25.trna113-ArgCCT (2633690-2633762) Arg (CCT) 73 bp Sc: 73.88
GCCCCAGTGGCCTAATGGATAAGGCACTGGCCTCCTAAGCCAGGGATTGTGGGTTCGAGT
CCCACCTGGGGTA
>Bos_taurus_chr2.trna10090-ArgGCG (16039028-16038957) Arg (GCG) 72 bp Sc: 39.43
TCCCTGGTGGCTCAGAAGGTAAAGTGTCTGCCTGCGATGTGGGAGACCCAGGTTTGATCC
CTGGCTCGGGAT
>Bos_taurus_chr5.trna1833-ArgGCG (49356408-49356479) Arg (GCG) 72 bp Sc: 40.19
TCCCGGGTGGCTCAGATGGTCAAGAATCTGCCTGCGATGCAGGAGACCTAGGTTTGATCC
CTGGTTCGGGAA
>Bos_taurus_chr8.trna2876-ArgGCG (80171696-80171767) Arg (GCG) 72 bp Sc: 45.78
TCCCTGGTGGCTCAGTCAGTAAAGTTTCTGCCTGCGATTCAGGAGACCTAAGTTCGATCC
TTGGCTTGGGAA
>Bos_taurus_chr10.trna2397-ArgGCG (60631092-60631163) Arg (GCG) 72 bp Sc: 52.87
TCCCTGGTGGCTCAGAGGGTAAAGCGTCTGCCTGCGATGCAGGAGACCCAGGTTTGATCC
CTGGGTGGGGAA
>Bos_taurus_chr4.trna5359-ArgGCG (100836666-100836595) Arg (GCG) 72 bp Sc: 55.17

TCCCTGGTGGCTCAGTTGGTAAAGAGTCTGCCTGCGATGCAGGAGACCCAGGTTCAATCC
CTGGGTTGGGAA
>Bos_taurus_chr1.trna2239-ArgGCG (63023621-63023692) Arg (GCG) 72 bp Sc: 55.32
TCCTTGGTGGCTCAGATGGTAAAGTGTCTGCCTGCGATGCAGGAGACCCAGGTTCAATCC
CTGGGTTGGGAA
>Bos_taurus_chr20.trna653-ArgGCG (17460221-17460292) Arg (GCG) 72 bp Sc: 56.03
TCCCTGGTAGCTCAGCTGGTAAAGTGTCTGCCTGCGATGCAGGAGACCCTGGTTCAATTC
CTGGGTTGGGAA
>Bos_taurus_chr11.trna2167-ArgTCG (48896385-48896457) Arg (TCG) 73 bp Sc: 42.55
TCCCTGGTGGTCCAGTGGCTGAGACTCTGCACTTCGAATCCGGGGGTCCCAGGTTTGCTC
CCTGGTCAGGGAA
>Bos_taurus_chrX.trna10571-ArgTCG (37344387-37344316) Arg (TCG) 72 bp Sc: 45.49
TTCCTGGTGGTCCAGTGGTTAGGACTCTGTGCTTCGAATACAAGGGGCCTGGTTCAATCC
CTGGTCAGGGAA
>Bos_taurus_chr8.trna4226-ArgTCG (112134749-112134678) Arg (TCG) 72 bp Sc: 45.77
TCCCTGGTGGTCCAGTGGCTAAGACTCTGCACTTCGAATGCAGGGGCCTGGTTTCCATCC
CTGGTCAGGGAA
>Bos_taurus_chr25.trna1646-ArgTCG (26871493-26871565) Arg (TCG) 73 bp Sc: 64.87
GGCTGTGTGGCCTAATGGATAAGGCGTATGACTTCGGATCAGAAGTTTGCAGGTTCGAGT
CCTGCCACAGTCG
>Bos_taurus_chr23.trna3447-ArgTCG (31454583-31454511) Arg (TCG) 73 bp Sc: 67.10
GACCACGTGGCCTAACGGATAAGGCGTCTGACTTCGGATCAGAAGATTGAGGGTTCGAAT
CCCTTCGTGGTTA
>Bos_taurus_chr20.trna5538-ArgTCG (7388151-7388079) Arg (TCG) 73 bp Sc: 67.54
AACCACGTGGCCTAATGGATAAGGCGTCTGACTTCGGATCAGAAGATTGAGGGTTCGAGT
CCCTTCGTGGTTG
>Bos_taurus_chr19.trna3009-ArgTCG (57033079-57033151) Arg (TCG) 73 bp Sc: 68.07
GACCGCGTGGCCTAACGGATAAGGCGTCTGACTTCGGATCAGAAGATTGAGGGTTCGAGT
CCCTTCGTGGTCG
>Bos_taurus_chr1.trna3383-ArgTCG (95118151-95118223) Arg (TCG) 73 bp Sc: 69.08
GACCACGTGGCCTAATGGATAAGGCGTCTGACTTCGGATCAGAAGATTGAGGGTTCGAAT
CCCTTCGTGGTTG
>Bos_taurus_chr23.trna3448-ArgTCG (31441552-31441480) Arg (TCG) 73 bp Sc: 69.08
GACCACGTGGCCTAATGGATAAGGCGTCTGACTTCGGATCAGAAGATTGAGGGTTCGAAT
CCCTTCGTGGTTG
>Bos_taurus_chr25.trna1647-ArgTCG (26872202-26872274) Arg (TCG) 73 bp Sc: 72.33
GGCTGTGTGGCCTAATGGATAAGGCGTCTGACTTCGGATCAGAAGATTACAGGTTCGAGT
CCTGTCACGGTCG
>Bos_taurus_chr25.trna1649-ArgTCG (26877428-26877500) Arg (TCG) 73 bp Sc: 72.94
GGCCATGTGGCCTAATGGATAAGGCGTCTGACTTCGGATCAGAAGATTACAGGTTCGAGT
CCTGTCATGGTCG
>Bos_taurus_chr21.trna829-ArgTCG (21215853-21215925) Arg (TCG) 73 bp Sc: 76.93
GGCCGCGTGGCCTAATGGATAAGGCGTCTGACTTCGGATCAGAAGATTGCAGGTTCGAGT
CCTGCCGCGGTCG
>Bos_taurus_chr20.trna153-ArgTCT (4112596-4112673) Arg (TCT) 78 bp Sc: 31.80
TCCCTGGTGGTCCAGTGGTTAAGATTCTGTGCTTCTACTGCAGGGGGCTGGGCACGGGTC
CAACCCCTGATCAGGGAA
>Bos_taurus_chr25.trna2460-ArgTCT (38986930-38987002) Arg (TCT) 73 bp Sc: 31.97
TCCCTGGCAGTCTAGTGGTTGAGACTCTGCATTTCTAATGCAGGGGACTCGGGTTCCACT
CCTGGTTGGGGAA
>Bos_taurus_chr23.trna4216-ArgTCT (15690122-15690051) Arg (TCT) 72 bp Sc: 34.41
TCCCTGGTGGTCCAGTGATTAAGATTCTGTGCTTCTGCTGCAGGGGTGCAGGTTTGGTCC
CTGGCCAGGGAA
>Bos_taurus_chr29.trna1518-ArgTCT (40634665-40634737) Arg (TCT) 73 bp Sc: 37.09
TCCCTGGTGGTCCAGTGGCGAAGACTCTATGCTTCTACTTTAGGGGGCATGGGTTCAATC
CCTAGTCAGGGAA
>Bos_taurus_chr18.trna1595-ArgTCT (39039297-39039369) Arg (TCT) 73 bp Sc: 37.25
TCCCTGGCAGTCCAATGGTAAAGACTCCGTGCTTCTACTGCAGGGGCCCCAGGTTCAAAC
CCTGGTCAGGGAC
>Bos_taurus_chr17.trna4509-ArgTCT (56994843-56994771) Arg (TCT) 73 bp Sc: 37.98
TCCTTGGTGGTCCAGTGATTAAGACTCTGCACTTCTACTGCAGGGGGCTTAGGCTCCATC
CCTGGTCAGGGAA
>Bos_taurus_chr23.trna3807-ArgTCT (23045933-23045861) Arg (TCT) 73 bp Sc: 38.27
TCCCTGGTGGCCCAGGGGTTAGGGCTCTGTGCTTCTCCTGCAGGGGGCATAGGTTTGATC
CCTGGTCGGGGAA
>Bos_taurus_chr13.trna5714-ArgTCT (48329221-48329150) Arg (TCT) 72 bp Sc: 38.90
TCCCTGGCAGTCCAGAGGTGAGGACTCTGTGGTTCTACTGCAGGGGCACAGGTTCAATCC

CTGTTTGGGGAA
>Bos_taurus_chr9.trna771-ArgTCT (24983969-24984041) Arg (TCT) 73 bp Sc: 38.91
TCCCTGGTAGTCAAGTGGTTAAGACTTGGCAGTTCTGCTGCAGAGGGCCCAGGTTTGATA
CCTGGTTGGGAAA
>Bos_taurus_chr5.trna6105-ArgTCT (103789033-103788961) Arg (TCT) 73 bp Sc: 39.28
TCCCTGGGGGTCCAGTGGCTAAGACTCTGTGCTTCTAATGCAGGGGGCCCGGGTTTGATC
CCTGGTCAGGGAG
>Bos_taurus_chr19.trna4465-ArgTCT (45750528-45750456) Arg (TCT) 73 bp Sc: 39.51
TCCCTGGTGGTCCAGTGGTTAAGACTCTGTGCTTCTAATGCTGGGGGCACAGGTTCAGCC
CCTGGTCAGGGAA
>Bos_taurus_chr28.trna500-ArgTCT (11524545-11524616) Arg (TCT) 72 bp Sc: 39.65
TCCTTGATGGTCCAGTGACTAAGACTCTGCACTTCTAAGGCAGGGGCTTGGGTTCGATCT
CTGGTCAGGGAA
>Bos_taurus_chr25.trna3431-ArgTCT (28055950-28055878) Arg (TCT) 73 bp Sc: 39.69
TCCTTGGTGGTCCAGTGGTTAAGACTCTGCACTTCTACTGCAGGGAGTTCGGGTACCATT
CCTGACTGGGGAC
>Bos_taurus_chr13.trna5071-ArgTCT (64877458-64877386) Arg (TCT) 73 bp Sc: 40.12
TCCCTGGTGGTCCAGTGGCTAAGGGTCTGTGTTTCTAATGCAGGGGGCCCAGGTTCCATC
CCTGGTCAGGGAA
>Bos_taurus_chr5.trna9426-ArgTCT (26776285-26776213) Arg (TCT) 73 bp Sc: 40.80
TTCCTGGTGGTCTAGTGGTTAAGACTCTGTATTTCTGCTGCAGGGGGCTTGGGTTTGATC
CCTGGCTAGGGAA
>Bos_taurus_chrX.trna10883-ArgTCT (27485818-27485746) Arg (TCT) 73 bp Sc: 40.95
TCCCTGGTGGTGCAGTGGTTAGCATTCTACATTTCTATTGCAGGGGGTGCAGGCTCCATC
CCTGGCTGGGGAA
>Bos_taurus_chr5.trna2702-ArgTCT (69676209-69676282) Arg (TCT) 74 bp Sc: 41.16
TCCTTGATGGTCCAGTGGTTAGGACTCTGTGCTTCTGATGCAGTGAGGCCTGGGTTTTAT
CCCTGGTCAGGGAA
>Bos_taurus_chr21.trna5468-ArgTCT (16573594-16573522) Arg (TCT) 73 bp Sc: 42.56
TCCCTGGTGGTCCAGTGACTAAGACTCCGTGCTCTCTATGCAGGGGGCCCGGGTTCAATC
CCTGGCCAGGGAA
>Bos_taurus_chr2.trna6319-ArgTCT (120014195-120014123) Arg (TCT) 73 bp Sc: 43.29
TCCCTGGTGGTCCAGTGGTTAAGACTCTGAGCTTCTACTGCAGGGGGCATGGGTTTGATC
CCTAGCCAGGGAA
>Bos_taurus_chr15.trna1597-ArgTCT (43702522-43702594) Arg (TCT) 73 bp Sc: 44.68
TCCCTGAAGGTCCAGTGGTTAAGACTCTGCCCTTCTACTGCAGCGGGCATGGGTTCAATC
CCTGTTTAGGGAA
>Bos_taurus_chr29.trna1475-ArgTCT (37846559-37846631) Arg (TCT) 73 bp Sc: 44.80
TCCCTGGTGGTCCAGTGGTTAAGACTCTGCCCTTCTAATGCAGGAGGCACAGGTTTGATT
CTTGGTCAGGGAA
>Bos_taurus_chr15.trna3506-ArgTCT (78707458-78707385) Arg (TCT) 74 bp Sc: 46.52
TCCCTGGTGGTCCAGTGGTTAGGACTCTGCACTTCTGCTGCAGGGGACCACAGGTTTGAT
CCTTGTTCAGGAAA
>Bos_taurus_chr27.trna3007-ArgTCT (23281755-23281683) Arg (TCT) 73 bp Sc: 47.83
TCCCTGGTGGTCCAGTGGTTAGGATTCTGTGCTTCTACAGCAGGGGGCTTGGGTTCAATC
CCTGGTCAGGGAA
>Bos_taurus_chr1.trna5977-ArgTCT (155371610-155371538) Arg (TCT) 73 bp Sc: 47.97
TCCCTGATGGTCCAGTGGTTAAGACTCTGCACTTCTGGTGCAGGGGGCCTGGGTTTGATC
CCTAGTTGGGGAT
>Bos_taurus_chr5.trna2796-ArgTCT (72274813-72274885) Arg (TCT) 73 bp Sc: 48.59
TCCCTGGTGGTCCAGTGGTTAAGACTCTGTGTTTCTACTACAGGGGGCACAGGTTCAATC
CCTGGTTGGGGAA
>Bos_taurus_chr1.trna9831-ArgTCT (46841692-46841620) Arg (TCT) 73 bp Sc: 49.65
TCCCTGGTGGTCCAGTGGTTAAGACTCTGTGCTTCTAATTCAGAGGCCACAGGTTTGATC
CCTGATTGGGGAA
>Bos_taurus_chrX.trna7312-ArgTCT (128373010-128372938) Arg (TCT) 73 bp Sc: 51.02
TCCCTGGTGGTCCAGTGGTTAAGACTCTGAGCTTCTACTGCAGGAGGCTCAGGTTCGATC
CCTGGTTAGGGAA
>Bos_taurus_chr13.trna4423-ArgTCT (75143929-75143857) Arg (TCT) 73 bp Sc: 51.11
TCCCTGGTGGTCCAGTGGTTAAGACTTTGCACTTCTGATGCAGGGGGCACAGGTTTGATC
CCTGTTAGGGGAG
>Bos_taurus_chr9.trna5087-ArgTCT (82234315-82234243) Arg (TCT) 73 bp Sc: 51.53
TCCTTGGTGGTCTAGTGGTTAAGACTCTGCACTTCTAATATAGGGGCCACAGGTTCAATA
CCTGGTTAAGGAA
>Bos_taurus_chr13.trna3137-ArgTCT (70459997-70460069) Arg (TCT) 73 bp Sc: 52.37
TCCTTGGTGGTCCAGTGGTTAGGACTCTGAGCTTCTACTGCAGGGTGCCTGGGTTCGATC
CCTAGTCAGGGAA

>Bos_taurus_chr6.trna7454-ArgTCT (46678517-46678445) Arg (TCT) 73 bp Sc: 54.10
TTTCTGGTGGTCCAGTGGCTAGGACTCTGCACTTCTAATGCAGGGGGCCCAGGTTCGATC
CCTGGTCAGGGCT
>Bos_taurus_chr9.trna305-ArgTCT (13209876-13209948) Arg (TCT) 73 bp Sc: 55.39
TCCCTGATGGTCCAGTGGTTAGAACTCTGCACTTCTACTGTAGGAGACCCAGGTTGGAAC
CCTGGTCAGGGAA
>Bos_taurus_chr13.trna4725-ArgTCT (69462732-69462660) Arg (TCT) 73 bp Sc: 56.48
TCCCTGGTGGTCCAGTGGTTAAAACTCTGCACTTCTACTGCAGAGGGTGCAGGTTCAACC
CCTGCTTTGGGAA
>Bos_taurus_chr17.trna4877-ArgTCT (53109012-53108940) Arg (TCT) 73 bp Sc: 58.87
TCCCTGGTGGTCCAGTGGTTAGGACTCAGCACTTCTACTGCTGAGGACCGGGGTTCAATC
CCTGGTCGGGGAA
>Bos_taurus_chr1.trna3053-ArgTCT (85737930-85738002) Arg (TCT) 73 bp Sc: 59.00
TCCCTGGTGGTCCAGTGGCTAAGACTCTGCACTTCTAATGCAGGGGGCCCAGGTTCAATC
ACTGGTCAGGGAA
>Bos_taurus_chr2.trna9721-ArgTCT (26829055-26828984) Arg (TCT) 72 bp Sc: 59.06
TCCCTGGTGGTCTAGTGGTTAGGACTTGGCATTTCTACTGCCATGGCCCAGATTCAATCC
CTGGTCATGGAA
>Bos_taurus_chr16.trna5-ArgTCT (442114-442186) Arg (TCT) 73 bp Sc: 66.22
GCCCGGATAGCTCAGTCAGTAGAGCTTCAGACTTCTAGTCTGAGGGTCCAGGGTTCAAGT
CCCTGTTTGGGCG
>Bos_taurus_chr3.trna376-ArgTCT (10711897-10711970) Arg (TCT) 74 bp Sc: 76.29
GTCTCTGTGGCGCAATGGACGAGCGCGCTGGACTTCTAATCCAGAGGTTCCGGGTTCGAG
TCCCGGCAGAGATG
>Bos_taurus_chr23.trna1046-ArgTCT (22111809-22111899) Arg (TCT) 91 bp Sc: 36.52
TCCCTGATAGTTCAGTGGTGAGGACTCCATGCTTCTACTGCAGGGGGAATCCCTGGTTGG
GTTTCTCCAGGTTCAATCCCTGGTTGGGGAA
>Bos_taurus_chr19.trna1438-ArgTCT (28374366-28374452) Arg (TCT) 87 bp Sc: 70.50
GGCTCTGTGGCGCAATGGATAGCGCATTGGACTTCTAGTGACGAAAGAGCGATTCAAAGG
TTGTGGGTTCGAATCCCACCAGAGTCG
>Bos_taurus_chr23.trna3496-ArgTCT (30875385-30875298) Arg (TCT) 88 bp Sc: 69.06
GGCTCTGTGGCGCAATGGATAGCGCATTGGACTTCTAGCACAGCAGTGAGGCATTCAAAG
GTTGCGGGTTCGAGTCCCGCCAGAGTCG
>Bos_taurus_chr3.trna7544-ArgTCT (49796487-49796403) Arg (TCT) 85 bp Sc: 71.81
GGCTCCGTGGCGCAATGGATAGCGCATTGGACTTCTAGAGGTTGAAGGCATTCAAAGGTT
CCGGGTTCGAGTCCCGGCGGAGTCG
>Bos_taurus_chr15.trna3179-ArgTCT (84095760-84095845) Arg (TCT) 86 bp Sc: 68.58
GGCTCTGTGGCGCAATGGATAGCGCATTGGACTTCTAGATAGACGGAGGCATTCAAAGGT
TGTGGGTTCGAGTCCCACCAGAGTCG
>Bos_taurus_chr11.trna5050-ArgTCT (98957779-98957689) Arg (TCT) 91 bp Sc: 66.52
GGCTCTGTGGCGCAATGGATAGCGCATTGGACTTCTAGCTGGCCTGGGTGTGGTTATTCA
AAGGTTGTGGGTTCGAGTCCCACCAGAGTCG
>Bos_taurus_chr4.trna830-AsnGTT (24000361-24000434) Asn (GTT) 74 bp Sc: 56.05
GTCTCTGTGGTGCAATCGGTTATCATGTTCAGCTGTTAACTGAAAAATTGGTGGTTCGAG
CCCATCCAGGGATG
>Bos_taurus_chr3.trna8652-AsnGTT (21067603-21067530) Asn (GTT) 74 bp Sc: 60.19
GTCTCTGTGGTGCAGTCGTTTAGCGCATGCGGCTGTTAACCGAAAGGTTGCTGGTTCGAG
CCCACCCAGGGACG
>Bos_taurus_chr23.trna3464-AsnGTT (31274918-31274845) Asn (GTT) 74 bp Sc: 62.46
GGCTCTGTGGCGCAATCAGTTAGCACCTTTGGCTGTTAACCAAAAGGTTGGTGGTTCAAG
CCCACCCAGGGCCG
>Bos_taurus_chr3.trna852-AsnGTT (21242779-21242852) Asn (GTT) 74 bp Sc: 63.85
GTCTCTGTGGCACAATCGGTTAGCGCTCTCGGCTGTTAACCGAAAGGCTGGTGGTTCGAG
CCCACCCAGGGACT
>Bos_taurus_chr3.trna854-AsnGTT (21260760-21260833) Asn (GTT) 74 bp Sc: 63.85
GTCTCTGTGGCACAATCGGTTAGCGCTCTCGGCTGTTAACCGAAAGGCTGGTGGTTCGAG
CCCACCCAGGGACT
>Bos_taurus_chr3.trna8643-AsnGTT (21147520-21147447) Asn (GTT) 74 bp Sc: 73.80
GTCTCTGTGGCGCAATCGGTTAGCGCGTTCGGCTGTTAACTGAAAGGTTGGTGGTTCGAG
CCCACCCAGGGATG
>Bos_taurus_chr3.trna8646-AsnGTT (21139130-21139057) Asn (GTT) 74 bp Sc: 74.46
GTCTCTGTGGCGCAATCGGTTAGTGCGTTCGGCTGTTAACCGAAAGGTTGGTGGTTCAAG
CCCACCCAGGGACG
>Bos_taurus_chr3.trna947-AsnGTT (22836908-22836981) Asn (GTT) 74 bp Sc: 75.33
GTCTCTGTGGTGCAATTGGTTAGCACGTTCAGCTGTTAACTGAAAGGTTGGTGGTTCGAG
CCCACCCAGGGACG
>Bos_taurus_chr3.trna8668-AsnGTT (20944200-20944127) Asn (GTT) 74 bp Sc: 78.61

GTCTCTGTGGCGCAATCGGTTAGCGCGTTCGGCTGTTAACCGAAAAGTTGGTGGTTCGAG
CCCACCCAGGGACG
>Bos_taurus_chr3.trna8617-AsnGTT (21378605-21378532) Asn (GTT) 74 bp Sc: 78.67
GTCTCTGTGGCGCAATCGGTGAGCGCGTTCGGCTGTTAACCGAAAGGTTGGTGGTTCGAG
CCCACCCAGGGACG
>Bos_taurus_chr3.trna8610-AsnGTT (21442962-21442889) Asn (GTT) 74 bp Sc: 79.17
GTCTCTGTGGCGCAATCGGTTAGCGCGTTCGGCTGTTAACCGGAAGGTTGGTGGTTCGAG
CCCACCCAGGGACG
>Bos_taurus_chr13.trna6960-AsnGTT (23624370-23624297) Asn (GTT) 74 bp Sc: 79.28
GTCTCTGTGGCGCAATCGGTTAGCGCGTTCGGCTGTTAACCGAGAGGTTGGTGGTTCGAG
CCCACCCAGGGACG
>Bos_taurus_chr3.trna8553-AsnGTT (22854529-22854456) Asn (GTT) 74 bp Sc: 80.50
GTCTCTGTGGCGCAATCGGTTAGCGCGTTCGGCTGTTAACCGAAAGGTTGGTGGTTCAAG
CCCACCCAGGGACG
>Bos_taurus_chr3.trna8618-AsnGTT (21350629-21350556) Asn (GTT) 74 bp Sc: 80.50
GTCTCTGTGGCGCAATCGGTTAGCGCGTTCGGCTGTTAACCGAAAGGTTGGTGGTTCAAG
CCCACCCAGGGACG
>Bos_taurus_chr12.trna1373-AsnGTT (30199504-30199577) Asn (GTT) 74 bp Sc: 82.24
GTCTCTGTGGCGCAATCGGTTAGCGCGTTCGGCTGTTAACCGAAAGGTTGGTGGTTCGAG
CCCACCCAGGGACG
>Bos_taurus_chr19.trna4859-AsnGTT (39979601-39979528) Asn (GTT) 74 bp Sc: 82.24
GTCTCTGTGGCGCAATCGGTTAGCGCGTTCGGCTGTTAACCGAAAGGTTGGTGGTTCGAG
CCCACCCAGGGACG
>Bos_taurus_chr3.trna811-AsnGTT (20951393-20951466) Asn (GTT) 74 bp Sc: 82.24
GTCTCTGTGGCGCAATCGGTTAGCGCGTTCGGCTGTTAACCGAAAGGTTGGTGGTTCGAG
CCCACCCAGGGACG
>Bos_taurus_chr3.trna818-AsnGTT (21003852-21003925) Asn (GTT) 74 bp Sc: 82.24
GTCTCTGTGGCGCAATCGGTTAGCGCGTTCGGCTGTTAACCGAAAGGTTGGTGGTTCGAG
CCCACCCAGGGACG
>Bos_taurus_chr3.trna823-AsnGTT (21028854-21028927) Asn (GTT) 74 bp Sc: 82.24
GTCTCTGTGGCGCAATCGGTTAGCGCGTTCGGCTGTTAACCGAAAGGTTGGTGGTTCGAG
CCCACCCAGGGACG
>Bos_taurus_chr3.trna833-AsnGTT (21094078-21094151) Asn (GTT) 74 bp Sc: 82.24
GTCTCTGTGGCGCAATCGGTTAGCGCGTTCGGCTGTTAACCGAAAGGTTGGTGGTTCGAG
CCCACCCAGGGACG
>Bos_taurus_chr3.trna841-AsnGTT (21176822-21176895) Asn (GTT) 74 bp Sc: 82.24
GTCTCTGTGGCGCAATCGGTTAGCGCGTTCGGCTGTTAACCGAAAGGTTGGTGGTTCGAG
CCCACCCAGGGACG
>Bos_taurus_chr3.trna844-AsnGTT (21208570-21208643) Asn (GTT) 74 bp Sc: 82.24
GTCTCTGTGGCGCAATCGGTTAGCGCGTTCGGCTGTTAACCGAAAGGTTGGTGGTTCGAG
CCCACCCAGGGACG
>Bos_taurus_chr3.trna8607-AsnGTT (21445687-21445614) Asn (GTT) 74 bp Sc: 82.24
GTCTCTGTGGCGCAATCGGTTAGCGCGTTCGGCTGTTAACCGAAAGGTTGGTGGTTCGAG
CCCACCCAGGGACG
>Bos_taurus_chr3.trna8614-AsnGTT (21403071-21402998) Asn (GTT) 74 bp Sc: 82.24
GTCTCTGTGGCGCAATCGGTTAGCGCGTTCGGCTGTTAACCGAAAGGTTGGTGGTTCGAG
CCCACCCAGGGACG
>Bos_taurus_chr3.trna8620-AsnGTT (21318588-21318515) Asn (GTT) 74 bp Sc: 82.24
GTCTCTGTGGCGCAATCGGTTAGCGCGTTCGGCTGTTAACCGAAAGGTTGGTGGTTCGAG
CCCACCCAGGGACG
>Bos_taurus_chr3.trna8631-AsnGTT (21227331-21227258) Asn (GTT) 74 bp Sc: 82.24
GTCTCTGTGGCGCAATCGGTTAGCGCGTTCGGCTGTTAACCGAAAGGTTGGTGGTTCGAG
CCCACCCAGGGACG
>Bos_taurus_chr3.trna863-AsnGTT (21326888-21326961) Asn (GTT) 74 bp Sc: 82.24
GTCTCTGTGGCGCAATCGGTTAGCGCGTTCGGCTGTTAACCGAAAGGTTGGTGGTTCGAG
CCCACCCAGGGACG
>Bos_taurus_chr3.trna8672-AsnGTT (20918354-20918281) Asn (GTT) 74 bp Sc: 82.24
GTCTCTGTGGCGCAATCGGTTAGCGCGTTCGGCTGTTAACCGAAAGGTTGGTGGTTCGAG
CCCACCCAGGGACG
>Bos_taurus_chr7.trna2138-AsnGTT (45426984-45427057) Asn (GTT) 74 bp Sc: 82.24
GTCTCTGTGGCGCAATCGGTTAGCGCGTTCGGCTGTTAACCGAAAGGTTGGTGGTTCGAG
CCCACCCAGGGACG
>Bos_taurus_chr3.trna9204-AsnGTT (8007586-8007513) Asn (GTT) 74 bp Sc: 84.88
GTCTCTGTAGCGCAATCGGTTAGCGCGTTCGGCTGTTAACCGAAAGGTTGGTGGTTCGAA
TCCACCCAGGGACG
>Bos_taurus_chrX.trna7005-AspATC (135252109-135252037) Asp (ATC) 73 bp Sc: 43.64
TCCCTGGTGGTCCAGTGGTTTTGACTCAGTGCTATCACTGCTGAGGGCTCAGATTCGATT

CCTGGTTGGGGAA
>Bos_taurus_chr14.trna1639-AspATC (36354085-36354157) Asp (ATC) 73 bp Sc: 46.41
TCCCTGGTGGTTCAGTAGCTAAGACTCTGCACTATCAATGCAGAGAGCCCAGGTTCGATC
TCTGATCAGGGAA
>Bos_taurus_chr12.trna3360-AspATC (83101378-83101448) Asp (ATC) 71 bp Sc: 50.62
TCCCTGGTGGTCCGGTGTTAAGACTCCGCACTATCAATGCAGGGCCCCAGGTTCGATCCC
TGGTCAGGGAA
>Bos_taurus_chr17.trna5115-AspATC (48732630-48732558) Asp (ATC) 73 bp Sc: 51.74
TCCCTGGGGGTCCAGTGGCTCGGACTCCACACTATCAATGCGGGGGGCCCAGGTTCGATC
CCTGGTCAGGGAA
>Bos_taurus_chr13.trna6642-AspATC (28560456-28560384) Asp (ATC) 73 bp Sc: 55.43
TCCCTGATGGTCCAGGGGATAAGCCTCTGCACTATCAATGCAGGGGACCCAGGTTTGATT
CCTGGTCGGGGAA
>Bos_taurus_chr3.trna4332-AspATC (110433757-110433829) Asp (ATC) 73 bp Sc: 56.78
TCCCTGGTGGTCTGGTGGTTAGGACTCTACACTATCACTGCTGAGGGCCCAGGTTCAATC
CCTGGTCAGGGAA
>Bos_taurus_chr23.trna4868-AspGTC (2746589-2746518) Asp (GTC) 72 bp Sc: 33.11
CTTTTGTTAGTACAGTGTTGAGTATCCTTGCCTGTCACTCAGGAGACTGGGGTTTGATTC
CCCAACGGAGAG
>Bos_taurus_chr17.trna437-AspGTC (12065269-12065341) Asp (GTC) 73 bp Sc: 34.92
TCCCTGGTAGTCCAGTGGCTAAGATTCTGTGCTGTCAGCACAGGGAGTCTGGGTTCAATC
CCTGGCCGGAGAA
>Bos_taurus_chr19.trna1612-AspGTC (31055786-31055858) Asp (GTC) 73 bp Sc: 35.42
TCCCCGGTGGTCAAGTGGTTGGGACTCTGTGCTGTCAATGAAGGGGGCTCAGGTTTGACC
CCTGATTGGGGAA
>Bos_taurus_chr19.trna1897-AspGTC (36906718-36906790) Asp (GTC) 73 bp Sc: 36.34
TCCCTGGTAGTCCAGTGGCTAAGATTCCGTGCTGTCAGCACAGGGAATCTGGGTTCGATC
CCTGGTCAGGGAA
>Bos_taurus_chr28.trna790-AspGTC (19046357-19046428) Asp (GTC) 72 bp Sc: 37.94
TTCTTGGTTGTCCAGTCGTTAGGACTCAGTGCTGTCACTGCTGTAAGCCAGGTTCAATCC
CTGGTCAAGGAA
>Bos_taurus_chr18.trna2622-AspGTC (55975580-55975651) Asp (GTC) 72 bp Sc: 38.07
TCTTTGGCTGTCCAGTGGTCAGGACTCGGCACTGTCACTTCTGGGGCCTGGGTTCAATCC
CTGGTCAGAGAA
>Bos_taurus_chr7.trna2755-AspGTC (61773958-61774029) Asp (GTC) 72 bp Sc: 39.59
TCCCTGGTGGCTCAGATGGGAAAGAATCTGCCTGTCATGCAGGAAACCCAGGTTCCATCC
CTGGATGGGGAA
>Bos_taurus_chr11.trna817-AspGTC (17120094-17120165) Asp (GTC) 72 bp Sc: 40.19
TTCCCAGTGGTCTAATGGTTAAGATTTAGTACTGTCACTGCTATGACCTGGGTTTGATTC
CTGGTTGGAAAC
>Bos_taurus_chr10.trna6300-AspGTC (52217002-52216931) Asp (GTC) 72 bp Sc: 41.50
TCCCTGATGGCCTTGTGGTTAGGGTTTGGGGCTGTCACTGCCGTGGCCCAGGTTTCATCC
CTGGTCAGGGAT
>Bos_taurus_chr27.trna3063-AspGTC (22265689-22265618) Asp (GTC) 72 bp Sc: 42.31
TCCTCATTAGTATAGTGGTGAGTATCCCCACCTGTCACGTGGGAGACTGGGGTTTGATTC
CTGGATGGGGAG
>Bos_taurus_chr21.trna1338-AspGTC (28056477-28056547) Asp (GTC) 71 bp Sc: 43.88
TCCCTGGTTGTCCAGTGGTTAGGACTCAGTGCTGTCACCGTGGGACCTGGGTTCAATTCC
TGGTCAGGGAA
>Bos_taurus_chr14.trna787-AspGTC (17583779-17583850) Asp (GTC) 72 bp Sc: 44.81
TTCCCAGTGGTTCAGTGGTTAAGACTCAGCACTGTCACTACTGGGGCCCAGGTCCAATCC
TTGGTTGGGGAA
>Bos_taurus_chr21.trna2407-AspGTC (56784355-56784427) Asp (GTC) 73 bp Sc: 45.11
TCCCTGGTGGTCCGGTGACTAAGACTCTGTGCTGTCAATGCAGGGGGCCCAGGTTCAATC
CCTGGTCAGGGAA
>Bos_taurus_chr8.trna4163-AspGTC (113196383-113196454) Asp (GTC) 72 bp Sc: 45.60
TCCTCGTTAGTATAGTGGTAAGTATCCCTACCTGTCACGCAGGAGATGGGGGTTTGATTC
CCCAATGGGGAT
>Bos_taurus_chr1.trna1117-AspGTC (27905397-27905468) Asp (GTC) 72 bp Sc: 45.74
TCCTCATTAGTATAGTGGTTAGTATCCTTACCAGTCATGTGGGAGACTGGGGTTCAATTC
CCCAATGGGGAG
>Bos_taurus_chr18.trna3439-AspGTC (58709396-58709325) Asp (GTC) 72 bp Sc: 48.22
TCCCTGGTGGTCTAGTGTTTAGGATTTGGCACTGTCACTGCTATGGCCTGGGTTTGATCC
CTGGTCAGGGAA
>Bos_taurus_chr10.trna5117-AspGTC (83991178-83991106) Asp (GTC) 73 bp Sc: 48.65
TTCCTGGTGGTCCAGTGGCTAAGATTCTGTGCTGTCAATACAGGGGGCCCAGGTTCGATT
CTTGGTCAGGGAA

>Bos_taurus_chr15.trna176-AspGTC (6363853-6363924) Asp (GTC) 72 bp Sc: 49.80
TCCTCCTTAGTATAGTGGTGAATATCCCTGCTTGTCACACAGGAGACCAGGGTTTGATTC
CCTGACGGGGAG
>Bos_taurus_chr25.trna3977-AspGTC (20392081-20392008) Asp (GTC) 74 bp Sc: 51.62
TCCCTGGTGGTCCAGTGGCTAGGACTCGGCACTGTCACTGCCGTGGGCCTGGGGTTCAAT
CCCTGGTCAGGGAA
>Bos_taurus_chr12.trna415-AspGTC (13100475-13100546) Asp (GTC) 72 bp Sc: 52.27
TCCTTGTTAGTACAGTGGTGACTATCCCTGCCTGTCACGCAGGAGTCAGGGGTTTGATTC
CCCAATGAGGAG
>Bos_taurus_chr1.trna10753-AspGTC (18852260-18852189) Asp (GTC) 72 bp Sc: 52.78
TCCCTGTTAGTCTAGTGTTGAGTATCCCCGCCTGTCACTCGGGAGACCGGGGTTCAATTC
CCCGACGGGGAG
>Bos_taurus_chr3.trna2193-AspGTC (56144200-56144271) Asp (GTC) 72 bp Sc: 55.16
TCTTCGTTAGTATAGTGGTGAATATCCCCGCCTGTCACGCCAGAGACCGGGGTTCGATTC
CCCGACGGGGAG
>Bos_taurus_chr9.trna5880-AspGTC (62555482-62555411) Asp (GTC) 72 bp Sc: 56.44
TCCTCGTTAGTACAGTGGTGAGTATCCCTGCCTGTCACGTGGGAGACCTGGGTTCGACTC
CCTGACGGGGAG
>Bos_taurus_chr3.trna9347-AspGTC (3317708-3317637) Asp (GTC) 72 bp Sc: 56.75
TCCTCGTTAGTATAGTGGTGAATATCCCTGCCTGTCACGCGGGAGACCAGGATTCGATTC
CCTGACGGGGAG
>Bos_taurus_chr19.trna4403-AspGTC (46561809-46561737) Asp (GTC) 73 bp Sc: 57.52
TCCCTGGTGGTTCAGTGGTTAGGACTCTGTGCTGTCACTGCTGAGGTCCCAGGTTGAATC
CCTGGTCAGGGAA
>Bos_taurus_chr10.trna776-AspGTC (17931838-17931909) Asp (GTC) 72 bp Sc: 57.66
TCCTCGTTAATGTAGTGGTGAGTATCCCCGCCTGTCACGCGGGAGACCGAGGTTCGATTC
CCCGACGAGGAG
>Bos_taurus_chrX.trna5528-AspGTC (141749486-141749557) Asp (GTC) 72 bp Sc: 57.78
TCCCTGGTGGTCCAGTGGTTAGGACTTGGCACTGTCACTGCCATGGCCCAGGTTCAATCT
CTCGTCAGGAAA
>Bos_taurus_chr18.trna4817-AspGTC (36741388-36741317) Asp (GTC) 72 bp Sc: 57.79
TCCTTGTTAGTATAGTGGTGATTATCCCTGCCTGTCATGCAGGAGACCGGGGTTTGAGTC
CCTGATGGGGAG
>Bos_taurus_chr7.trna8324-AspGTC (15748198-15748126) Asp (GTC) 73 bp Sc: 58.06
TCCCTGACAGTCCAGTGGTTAAGACTCTGTACTGTCACTACTGAGGGCCCAGGTTCAATC
CCTGGTCAGGGAA
>Bos_taurus_chr3.trna6610-AspGTC (77509101-77509030) Asp (GTC) 72 bp Sc: 62.73
TCCTCTTTAGTATAGTGGTGAGTATCCCCACCTGTCACGTGGGAGACCAGGGTTCGATTC
CCTGATGGGGAG
>Bos_taurus_chr3.trna9195-AspGTC (8095726-8095655) Asp (GTC) 72 bp Sc: 71.60
TCCTCGTTAGTATAGTGGTAAGTATCCCCGCCTGTCACGCGGGAGACCGGGGTTCGATTC
CCCGACGGGGAG
>Bos_taurus_chr17.trna2133-AspGTC (53128220-53128291) Asp (GTC) 72 bp Sc: 72.92
TCCTCGTTAGTATAGTGGTGAGTATCCCCGCCTGTCACGCGGGAGACCGGGGTTCGATTC
CCCGACGGGGAG
>Bos_taurus_chr17.trna2135-AspGTC (53131191-53131262) Asp (GTC) 72 bp Sc: 72.92
TCCTCGTTAGTATAGTGGTGAGTATCCCCGCCTGTCACGCGGGAGACCGGGGTTCGATTC
CCCGACGGGGAG
>Bos_taurus_chr19.trna5451-AspGTC (28460466-28460395) Asp (GTC) 72 bp Sc: 72.92
TCCTCGTTAGTATAGTGGTGAGTATCCCCGCCTGTCACGCGGGAGACCGGGGTTCGATTC
CCCGACGGGGAG
>Bos_taurus_chr23.trna1431-AspGTC (30858999-30859070) Asp (GTC) 72 bp Sc: 72.92
TCCTCGTTAGTATAGTGGTGAGTATCCCCGCCTGTCACGCGGGAGACCGGGGTTCGATTC
CCCGACGGGGAG
>Bos_taurus_chr23.trna3486-AspGTC (30940535-30940464) Asp (GTC) 72 bp Sc: 72.92
TCCTCGTTAGTATAGTGGTGAGTATCCCCGCCTGTCACGCGGGAGACCGGGGTTCGATTC
CCCGACGGGGAG
>Bos_taurus_chr3.trna275-AspGTC (8114684-8114755) Asp (GTC) 72 bp Sc: 72.92
TCCTCGTTAGTATAGTGGTGAGTATCCCCGCCTGTCACGCGGGAGACCGGGGTTCGATTC
CCCGACGGGGAG
>Bos_taurus_chr3.trna9191-AspGTC (8111896-8111825) Asp (GTC) 72 bp Sc: 72.92
TCCTCGTTAGTATAGTGGTGAGTATCCCCGCCTGTCACGCGGGAGACCGGGGTTCGATTC
CCCGACGGGGAG
>Bos_taurus_chr3.trna9194-AspGTC (8102369-8102298) Asp (GTC) 72 bp Sc: 72.92
TCCTCGTTAGTATAGTGGTGAGTATCCCCGCCTGTCACGCGGGAGACCGGGGTTCGATTC
CCCGACGGGGAG
>Bos_taurus_chr3.trna9203-AspGTC (8020026-8019955) Asp (GTC) 72 bp Sc: 72.92

TCCTCGTTAGTATAGTGGTGAGTATCCCCGCCTGTCACGCGGGAGACCGGGGTTCGATTC
CCCGACGGGGAG
>Bos_taurus_chr5.trna2281-AspGTC (60678961-60679032) Asp (GTC) 72 bp Sc: 72.92
TCCTCGTTAGTATAGTGGTGAGTATCCCCGCCTGTCACGCGGGAGACCGGGGTTCGATTC
CCCGACGGGGAG
>Bos_taurus_chr5.trna2388-AspGTC (63016480-63016551) Asp (GTC) 72 bp Sc: 74.76
TCCTCGTTAGTATAGTGGTTAGTATCCCCGCCTGTCACGCGGGAGACCGGGGTTCAATTC
CCCGACGGGGAG
>Bos_taurus_chr20.trna4727-CysACA (28239721-28239645) Cys (ACA) 77 bp Sc: 31.68
CTTTTTAGGTTGGCTCAGATGGTAAAGCATCTGCCTACAGTGTGGGAGACCCAGGTTCAA
TCCCTGGGTTGGGAAGA
>Bos_taurus_chr29.trna343-CysACA (9096381-9096453) Cys (ACA) 73 bp Sc: 34.47
TCCCTGGTGGCTCAGACGGTAAAATATCTGCCCACAATGCAGGAGACCCAGGTTTGATCC
CTGGGTCAGGAAG
>Bos_taurus_chr9.trna3719-CysACA (98867770-98867841) Cys (ACA) 72 bp Sc: 36.74
TCCCTATTGGCTCAGACAGTAAAGCATCTGCCTACAGTGTGGGAGACCCAGGTTCAATCC
CTGGGTTGGGAA
>Bos_taurus_chr8.trna3338-CysACA (92059766-92059836) Cys (ACA) 71 bp Sc: 37.03
TCCCTGGTGGCTCAGATGGCAAAGCATCTGCCTACACTGCAGAAGACTGGGTTCGAGCCC
TGGGTAGGGAA
>Bos_taurus_chr11.trna1403-CysACA (30467220-30467291) Cys (ACA) 72 bp Sc: 37.12
TCCCTGGTGGCTCAGACAGTAAGGCATCTGCCTACAATGCAGGAAACCCAGGTTCAGTCC
CTGGGTAGGGAA
>Bos_taurus_chrX.trna6171-CysACA (147977128-147977058) Cys (ACA) 71 bp Sc: 37.59
TCCCTGGTGGCTCAGAGGGTAAAGCATCTGCCTACAATGCGGAAGACCGGGTTTGATCCC
TGGGTAGGGAA
>Bos_taurus_chr21.trna2247-CysACA (52818067-52818138) Cys (ACA) 72 bp Sc: 37.78
TCCCTGCTGGCTCAGACAGTAAAGCGTCTGCCTACAATGTGGGAAATCCAGGTTCGATCC
CTGGGTCGGGAA
>Bos_taurus_chr16.trna4133-CysACA (65721912-65721841) Cys (ACA) 72 bp Sc: 39.02
TCCCTGGTGGCTCAGACAGTAAAGCATCTGCCTACACTGGAGGTGACCCAGGTTCGATTC
CTGGGTGGGGAA
>Bos_taurus_chr3.trna2224-CysACA (57567747-57567818) Cys (ACA) 72 bp Sc: 40.63
TCCCTGGTGGCTCAGATGGTAAAGCATCTACCTACAGTGTGGGAGACCCAGGTTTGATCC
CTGGTCAGGAAG
>Bos_taurus_chr5.trna374-CysACA (11378569-11378640) Cys (ACA) 72 bp Sc: 40.83
TCCCTGGTGGCTCAGACCTTAAAGTGTCCACCTACAATGTGGGAGACCCAGGTTCAATCC
CTGGGTAGGGAA
>Bos_taurus_chr19.trna1890-CysACA (36644721-36644793) Cys (ACA) 73 bp Sc: 41.26
TCCCTGGTGGCTCAGATGGTAAAAGCATGTGCCTACAAAGCAGGAGACCCGGGTTTGGTC
CCTGGCTAGGGAA
>Bos_taurus_chr12.trna5350-CysACA (50401722-50401651) Cys (ACA) 72 bp Sc: 41.63
TCCCTGGTGGCTCATAGGGTAAAGTGTCTGCCTACAATGTGGGAGACTCAGGTTCGATCC
CTGGCTTGGGAA
>Bos_taurus_chr23.trna1942-CysACA (41198640-41198712) Cys (ACA) 73 bp Sc: 42.66
TCCCTGGTGGCTCAGACAGTAAAAGCATCTGCCTACAAGGCAGGAGACCCAGGTTCAATC
CCTGGTTTGGGAA
>Bos_taurus_chr28.trna1862-CysACA (39557252-39557181) Cys (ACA) 72 bp Sc: 42.70
TTCCTGGTAGCTCAGACAGTAAAGCGCCTGCCTACAATGTGGGAGACCCAGGTTCGATCC
CTGGTCGGAAAG
>Bos_taurus_chr13.trna7392-CysACA (14095858-14095788) Cys (ACA) 71 bp Sc: 42.87
TCCCTGGTGGCTCACATGGTAAAGCATCTGCCTACAATGCGGAAGACCAGGTTCGATCCC
TGGTCGGGAAG
>Bos_taurus_chr5.trna6745-CysACA (91685534-91685462) Cys (ACA) 73 bp Sc: 43.26
TCCCTGGTGGCTCAGATGGTAAAAGCATCTGCCTACAATGTGGGAGACCCAGGTTTGATC
CCTGGGCAGGGAA
>Bos_taurus_chr25.trna3533-CysACA (26863808-26863737) Cys (ACA) 72 bp Sc: 43.39
TCCCTGGTGGCTCAGACAGTAAAGTGTCTGCCTACAATGCAGGAAACTCAGGTTCAATCC
CTGGTTTGGGAA
>Bos_taurus_chr1.trna4663-CysACA (129173164-129173235) Cys (ACA) 72 bp Sc: 43.97
TCCCTGCTGGCTCAGACGGTAAAGTGTCTGTCTACAATGCAGGAGACCCGGGTTTGAGCC
CTGGGTTGGGAA
>Bos_taurus_chr3.trna6301-CysACA (85645905-85645834) Cys (ACA) 72 bp Sc: 44.16
TCCCTGGTAGCTCAGATGGTAAAGCGTCTGCCTACAATGAGGGATACCCAGGTTCAATCC
CTGGGTAGGGAA
>Bos_taurus_chr5.trna3822-CysACA (95172179-95172250) Cys (ACA) 72 bp Sc: 44.73
TCCCTGGTGGCTCAGACAGTAAAGTGTCTGCCTACAGTGCAGGAGACCCAGGTTTGATCC

CTGGGTGGGGAA
>Bos_taurus_chr29.trna263-CysACA (7465847-7465918) Cys (ACA) 72 bp Sc: 44.74
TCCCTGGTGGCTCAGACGGTAAAGTGTCTGTCTACAATGCAGGAGACCCAGGTTTGATCC
TTGGGTGGGGAA
>Bos_taurus_chr16.trna4197-CysACA (64550471-64550400) Cys (ACA) 72 bp Sc: 46.43
TCCCTGGTGGCTCAGACGGTAAAGCATCTGCCTACAATGTGGAAGACCCAGGTTTGATCC
CTGGGTGGGGAA
>Bos_taurus_chr12.trna4893-CysACA (63149874-63149803) Cys (ACA) 72 bp Sc: 46.52
TCCCTGGTGGCTCAGATGGTAAAGTGTCTGTCTACAATGCGGGAGACCCAGGTTCTATCC
CTGGGTAGGGAA
>Bos_taurus_chr13.trna2015-CysACA (46233789-46233860) Cys (ACA) 72 bp Sc: 47.14
TCCCTAGTGGCTCAGACGGTAAAGCATCTGCCTACAATGAGGGAGACCCAGGTTCAATCC
CTGGGTAGGGAA
>Bos_taurus_chr22.trna1677-CysACA (45997205-45997276) Cys (ACA) 72 bp Sc: 47.24
TCCCTGCTGGCTCAGACAGTAAAGCGTTTGCCTACAATGCAGAAGACCCGGGTTCAATCC
CTGGGTCGGGAA
>Bos_taurus_chr24.trna2143-CysACA (49645409-49645479) Cys (ACA) 71 bp Sc: 47.28
TCCCTGGTAGCTCAGAGGGTAAAGCGTCTGCCTACAATGCAGGAGACCAGGTTTGATCCC
TGGGTAGGGAA
>Bos_taurus_chr16.trna6587-CysACA (8022858-8022787) Cys (ACA) 72 bp Sc: 47.38
TCCCTGGTGGCTTAGACAGTAAAGCATCTGCCTACAATGCAGGAGACCCAGGTTTGATCC
CTGGGTAGGGAA
>Bos_taurus_chr1.trna3644-CysACA (102212092-102212163) Cys (ACA) 72 bp Sc: 48.40
TCCCTGGTGGCTCAGATGGTAAAGCATCTGTCTACAATGCAGAAGACCCGGGTTCCATCC
CTGGGTAGGGAA
>Bos_taurus_chr16.trna838-CysACA (26143613-26143685) Cys (ACA) 73 bp Sc: 49.35
TCCCGGGTGGCTCAGATGGGAAAAGTGTCTGCTTACAATGCGGGAGACCCAGGTTCAATC
CCTGGTTCGGGAA
>Bos_taurus_chr20.trna4016-CysACA (45065071-45065000) Cys (ACA) 72 bp Sc: 49.36
TCCCTGTTGGCTTAGACAGTAAAGCATCTGCCTACAATGCAGGAGGCCCAGGTTCAATCC
CTGGGTCGGGAA
>Bos_taurus_chr4.trna7695-CysACA (39289992-39289921) Cys (ACA) 72 bp Sc: 49.40
TCCCTGTTGGCTCAGATGGTAAAGCATCTGCCTACAATGCTGGAGACCCAAGTTCAATCC
CTGGGTAGGGAA
>Bos_taurus_chr28.trna1899-CysACA (38555692-38555621) Cys (ACA) 72 bp Sc: 49.68
TCCCAGGTGGCTCAGATGGTAAAGCGTCTGCGTACAATGCAGAAGACCTGGGTTCGATCC
CTGGGTTGGGAA
>Bos_taurus_chr6.trna4745-CysACA (113356662-113356591) Cys (ACA) 72 bp Sc: 49.83
TCCCAGATGGCTCACATGGTAAAGCATCTGACTACAATGCAGGAGACCCAGGTTCGATCC
CTGGGTTGGGAA
>Bos_taurus_chr9.trna691-CysACA (23034218-23034289) Cys (ACA) 72 bp Sc: 49.90
TCCCTGGTGGCTCAGAGGATAAAGTGTCTGTCTACAATGTAGGAGACCCAGGTTCAATCC
CTGGTTTGGGAA
>Bos_taurus_chr16.trna2892-CysACA (70052080-70052151) Cys (ACA) 72 bp Sc: 50.16
TCCTTGGTGGCTCAAACAGTAAAGCGTCTGCCTACAATGCAGGAGACCCAGGTTCAATCC
CTGGGTAGGGAA
>Bos_taurus_chr17.trna3962-CysACA (65409123-65409052) Cys (ACA) 72 bp Sc: 50.38
TCCCGAATAGCTCAGACAGTAAAGCGTCTGCCTACAATGCGGGAGACCCAGGTTCGATCC
CTGGGTCGGGAA
>Bos_taurus_chr6.trna6248-CysACA (82234385-82234314) Cys (ACA) 72 bp Sc: 50.51
TCCCTACTGGCTCAGATGGTAAAGCATCTGCCTACAATGTGGGAGACCCAGGTTCAATCC
CTGGGTTGGGAA
>Bos_taurus_chr20.trna2677-CysACA (65835376-65835447) Cys (ACA) 72 bp Sc: 50.66
TCCCTGGTGGCTCAGATGGTAAAGTGTCTGCCTACAATGCAGGAGACCCGGGTTCTATCC
CTGGGTAGGGAA
>Bos_taurus_chr21.trna656-CysACA (18003148-18003220) Cys (ACA) 73 bp Sc: 51.05
TTCCTGGTGGCTCAGATGGTAAAGTGTCTGCCTACAATGCAGGAGACCCGGGTTCAATCC
CTGGTCCGGGAAG
>Bos_taurus_chr3.trna6790-CysACA (71915253-71915182) Cys (ACA) 72 bp Sc: 51.56
TCCCTGGTGGCTCAGACGGTAAAGCATCTGCCTACAATGTGGGAGACCCAGGTTCGATCC
CTGGGTAGGGAA
>Bos_taurus_chr6.trna6548-CysACA (72485468-72485397) Cys (ACA) 72 bp Sc: 51.84
TTCCTGCTAGCTCAGACGGTAAAGCGTCTGCCTACAATGCAGGAGACCTGGGTTCGATCC
CTGGGTTGGAAA
>Bos_taurus_chr1.trna274-CysACA (3992733-3992804) Cys (ACA) 72 bp Sc: 51.86
TCCCTGGTGGCTCAGACAGTAAAGCGTCTGCCTACAATGCAGGAGACCCAGGTTCAATCC
CTGGTTCGGGAA

>Bos_taurus_chr1.trna9615-CysACA (53941450-53941379) Cys (ACA) 72 bp Sc: 52.51
TCCCTGGTGGCTCAGATGGTAAAGCGTCTACCTACAATGCGGGAGACCCAGGTTCGATCC
CTGGGTAGGGAA
>Bos_taurus_chr11.trna5162-CysACA (97004776-97004705) Cys (ACA) 72 bp Sc: 52.56
TCCCTGGTGGCTCAGACGGTAAAGCGTCTGCCTACAATGTAAGAGACCCAGGTTCAATCC
CTGGCTCGGGAA
>Bos_taurus_chr20.trna4088-CysACA (42539144-42539073) Cys (ACA) 72 bp Sc: 52.67
TCCCTAGTGGCTCAGATGGTAAAGCATCTGTCTACAACACAGGAGACCCGGGTTCAATCC
CTGGGTGGGGAA
>Bos_taurus_chr28.trna1256-CysACA (33385709-33385780) Cys (ACA) 72 bp Sc: 52.94
TCCCTGGTGGCTCAGACGGTAAAGCATCTGCCTACAATGTAGGAGACCCAGGTTTGATCC
CTGGCTTGGGAA
>Bos_taurus_chr6.trna8199-CysACA (24023907-24023836) Cys (ACA) 72 bp Sc: 53.09
TCCCTGGTGGCTCAGACGGTAAAGCATCTGCCTACAATGCGGGAGACCCAGGTTCAACTC
CTGGGTAGGGAA
>Bos_taurus_chr7.trna4199-CysACA (101619115-101619186) Cys (ACA) 72 bp Sc: 53.62
TCCCTGGTGGCTCAGAGGGTAAAGCATCTGCCTACAATGCAGGAGACCTAGGTTCAATCC
CTGGGTAGGGAA
>Bos_taurus_chr7.trna4074-CysACA (98747273-98747344) Cys (ACA) 72 bp Sc: 54.02
TCCCCGCTGGCTCAGATGGTAAAGTGTCTGCCTACAATGCGGGAGACCCAGGTTCAATCC
CTGGGTTGGGAA
>Bos_taurus_chr28.trna336-CysACA (8101244-8101315) Cys (ACA) 72 bp Sc: 54.36
TCCCTGGTGGCTCAGATGGTAAAGTGTCTGTCTACAATGTAGAAGACCCAGGTTCAATCC
CTGGTTTGGGAA
>Bos_taurus_chr2.trna4640-CysACA (126162698-126162769) Cys (ACA) 72 bp Sc: 54.72
TCCCTGGTGGCTCAGACGGTAAAGCATCTACCTACAATGCAGGAGACCCAGGTTCGATCC
CTGGGTGGGGAA
[truncated: 465,525 more chars]
